# Decoding Pecan’s Fungal Foe: A Genomic Insight into *Colletotrichum plurivorum* Isolate W-6

**DOI:** 10.3390/jof11030203

**Published:** 2025-03-05

**Authors:** Ke Deng, Ying Zhang, Saibin Lv, Chulong Zhang, Lihong Xiao

**Affiliations:** 1 College of Forestry and Biotechnology, Zhejiang A&F University, Hangzhou 311300, China; dengke@163.com (K.D.); 2019602042067@stu.zafu.edu.cn (Y.Z.); 2018802641010@stu.zafu.edu.cn (S.L.); 2 Zhejiang Key Laboratory of Biology and Ecological Regulation of Crop Pathogens and Insects, College of Agriculture and Biotechnology, Zhejiang University, Hangzhou 310058, China; clzhang@zju.edu.cn; 3 Ministry of Agriculture and Rural Affairs Key Laboratory of Molecular Biology of Crop Pathogens and Insect Pests, College of Agriculture and Biotechnology, Zhejiang University, Hangzhou 310058, China

**Keywords:** *Carya*, pathogenicity, virulence, fungal pathogen, anthracnose

## Abstract

Pecan (*Carya illinoinensis*) is a world-renowned nut crop that is highly favored by consumers for its high content of healthy nutrients. For a long time, anthracnose has severely threatened the yield and quality of pecan, causing significant economic losses to the global pecan industry. Here, we report the 54.57-Mb gapless chromosome-level assembly of the pathogenic ascomycetes *Colletotrichum plurivorum* isolate W-6 from pecan plantations in Southeast China. Six of 12 chromosomes contain, at least, telomeric repeats (CCCTAA)n or (TTAGGG)n at one end. A total of 14,343 protein-coding genes were predicted. Pathogenicity- and virulence-related annotations revealed 137 to 4558 genes associated with the TCDB, PHI, Cyt_P450, DFVF, effector, and secretome databases, respectively. A comparative analysis of isolate W-6, together with 51 other *Colletotrichum* strains, reveled 13 genes unique to the *Orchidearum* complex to which isolate W-6 belongs, highlighting the major facilitator superfamily transporters. The detailed analyses of MFS transporters associated with secondary metabolite gene clusters in isolate W-6 led to the identification and protein structure analyses of two key virulence factor candidates in DHA1 subclass, prlG and azaK, which were reported as efflux transporters of antibiotics in other pathogenic fungi. The assembly and further functional investigation of two pathogenic genes identified here potentially provide important resources for better understanding the biology and lifestyle of *Colletotrichum* and pave the way for designing more efficient strategies to control anthracnose in pecan plantations.

## 1. Introduction

*Carya illinoinensis* (Wangenh) K. Koch, commonly known as pecan, is one of the most economically important nut crops of the *Carya* genus (Juglandaceae), native to temperate to tropical regions of northeastern and central Mexico and the southern United States [1]. Pecan cultivars are nowadays planted worldwide for their edible and highly nutritious nut kernels, which are rich in components beneficial to human health, including protein and polyunsaturated fatty acids such as oleic, linoleic, and linolenic acids [2,3]. Regular consumption of pecan nut kernels and its derived foods can effectively prevent atherosclerosis, improve heart health, enhance brain function, reduce the effects of cholesterol, and more [4].

It has been reported that much of the global attention on pecan is due to increasing consumption in China, making it the largest importer of pecans worldwide, and meanwhile, China is also constantly striving to develop its own walnut production industry [1]. As of 2022, China has significantly expanded its pecan cultivation, reaching an estimated 85,000 hectares, but due to the relatively recent pecan tree plantings, production was only 4500 mt [5]. This growth is primarily concentrated in the Yangtze River Basin and southern regions, notably in provinces such as Anhui, Jiangsu, and Zhejiang, with Pawnee, Wichita, Stuart, etc. as the key cultivars [5]. Increasing consumer demand has driven the domestication, genetic improvement, and identification of varieties with outstanding performance in yield and taste-related traits, but they have poor resistance to environmental stresses, including fungal pathogens [6]. Among the fungal diseases affecting pecans, anthracnose caused by *Colletotrichum* species has become a significant biotic factor limiting nuts yield and quality in pecan plantations [7,8,9,10,11]. Therefore, when planting pecans, it is important to focus on controlling the early stages of the disease and address key periods for diseases management [12]. Repeated and costly chemical fungicides have been used for disease control on pecan, which may lead to the development of fungicide resistance in fungal pathogens [13].

*Colletotrichum* fungi are a group of plant pathogens with a wide range of hosts that cause anthracnose, which is considered one of the top 10 plant fungal diseases. These pathogens significantly reduce yields in many crops worldwide and significantly impact economic development, particularly in tropical and subtropical regions [14,15]. Typically, *Colletotrichum*-infected host plants exhibit symptoms of anthracnose, including a dark brown leaf, stem and fruit spots, fruit rot, and the wilting of leaves, which often result in dieback and a reduction in plant quality [10,16,17,18,19,20,21].

*Colletotrichum* fungi have been extensively studied as model pathological systems in plant–pathogen interactions [22,23]. Over 200 *Colletotrichum* species have been identified based on the characteristics exhibited, including 15 species complexes and 14 singleton species [24,25]. Many of these species are known to be widespread causes of anthracnose in crops and other plants worldwide, especially in the tropic and subtropic regions [26,27,28,29,30,31]. To date, genome assemblies of nearly 50 pathogens of *Colletotrichum* have been reported, though only a few candidate virulence genes have been identified, primarily in a few crop host species, such as *Ste12* in *C. fructicola* and *C. orbiculare* [32,33], *CfEC92* and *CfPMK1* in *C. fructicola* [34,35], and *CgMFS1* in *C. gloeosporioides* from *Hevea brasiliensis* [36]. However, these assemblies provide an important genetic basis for further exploration of the mechanisms of *Colletotrichum* pathogens.

Pecan anthracnose was first reported in 1914 in the United States, caused by the fungus *Glomerella cingulata* [37]. In recent years, pecan anthracnose caused by *Colletotrichum* species has been widely reported in the United States, Brazil, South Korea, and other countries, resulting in significant yield losses [8,38,39,40,41]. With the expansion of pecan planting in China in recent years, several *Colletotrichum* isolates have been identified as causal agents of pecan anthracnose, leading to up to 50% leaf shedding, nearly half or all fruit decay, and ultimately, a decrease in yield or even failure and poor-quality of pecan nut kernels [7,9,10,42]. Although *Colletotrichum* fungi cause severe damage to the pecan industry, studies are limited to the isolation and identification, morphological characteristics, and pathogenicity test, without exploring the molecular mechanisms behind their pathogenicity. The prevention and control of pecan anthracnose are also restricted to physical and chemical methods, which are costly, polluting, and poorly effective.

In this study, a high-quality gapless reference genome of *C. plurivorum* isolate W-6 was obtained, and prediction of the centromic region and telomeric repeats of each chromosome were performed using ONT technology combined with the Illumina short reads. Then, the protein-coding genes and their functions of the genome assembly were annotated based on general databases. To identify potential key genes associated with pathogenicity of isolate W-6, annotation was performed using specific fungal pathogenicity and virulence-related databases, followed by a comparative analysis of isolate W-6 with other 51 *Colletotrichum* strains to identify pathogenicity-related genes unique to the *Orchidearum* complex to which isolate W-6 belongs. These genes, related to secondary metabolite (SM) gene clusters in the isolate W-6 genome, resulted in the identification and protein structure characterization of two key virulence factor candidates: prlG and azaK. The results will provide valuable genomic resources for a better understanding of the biology and lifestyle of *Colletotrichum* species and pave the way for designing more efficient disease control strategies in *Carya* nut tree plantations in the future.

## 2. Materials and Methods

### 2.1. Culture Conditions of Pathogenic Fungus Isolate W-6

This study used the virulent *C. plurivorum* isolate W-6, which was first isolated from young fruits of the susceptible pecan cultivar Wichita collected from plantations in Anhui and Jiangsu Provinces, China in August 2019 [10]. Irregular dark brown or black spotted lesions first appear on the surface and interior of infected fruits and spread to all leaves. In almost all trees of the susceptible variety Wichita, these symptoms result in 30% to 50% leaf shedding and nearly 50% fruit decay [10]. To sequence the genome, the pure mycelium of isolate W-6 was routinely maintained on potato dextrose agar (PDA, 20 g potato, 20 g of dextrose, 18 g agar, and 1000 mL distilled water) medium plates and incubated at 28 °C in a dark incubator (model: ZQLY-180E, Shanghai Zhichu Instrument Co., Ltd., Shanghai, China). Conidia were induced by transferring 5 cm hyphae-containing plugs onto fresh PDA plates for 6 days at 25 °C with a 12 h light/dark cycle. Conidia were inoculated into 250 mL conical flasks containing 100 mL potato dextrose broth (PDB) medium for 6 days, and the cultures were collected for DNA and RNA extraction for genome assembly.

### 2.2. Genome Sequencing and Assembly

High-molecular-weight genomic DNA of the strain was extracted from frozen mycelial samples using the DNA quick Plant System and purified using QIAGEN Genomic-tips (QIAGEN China Shanghai Co., Ltd., Shanghai, China). The quality and quantity of the genomic DNA were determined following the method described by Xiao et al. (2021) [6]. Five micrograms of purified genomic DNA, with fragments longer than 2 kb, was used to prepare a whole genome sequencing library, which was sequenced on the PromethION platform according to the manufacturer’s procedures (Oxford Nanopore Technologies, Oxford, UK) at Biomarker Technologies, Beijing, China. Meanwhile, two micrograms of the purified genomic DNA was used to construct a short-insertion library (350 bp), which was sequenced on the Illumina NovaSeq system following the manufacturer’s procedures (Illumina Inc., San Diego, CA, USA).

The SOAP package was used for the base-calling and the quality control of the raw data generated from the Illumina NovaSeq system [43]. To detect the presence of the possible contamination from other fungal and bacterial sequences in the sample for sequencing, 10,000 single reads were randomly selected and BLAST was launched against the NT database by BLAST v. ncbi-blat+ 2.2.29 with parameters of num_descriptions 100-num_alignments 100-evalue 1 × 10^−0.5^ [44], followed by a genome survey to estimate the genome size, GC content, heterozygosity, and repeat rate.

The guppy v3.2.z4 software (https://nanoporetech.com/zh/software/other/guppy, accessed on 19 August 2019) was employed for the base-calling of the raw data generated from Oxford Nanopore Technology (ONT), and the reads of adapters only, low quality and short fragments (<2 kb) were removed using the Albacore program in the MinKNOW software package v.4.5.0 (https://github.com/nanoporetech/minknow_api, accessed on 15 January 2022). The isolate W-6 genome was assembled using the cleaned subreads by NECAT program with the default parameters [45] (https://github.com/xiaochuanle/NECAT, accessed on 3 August 2020). The initial assembly was then corrected using the clean subreads by Racon v. 1.4.3 software (https://github.com/isovic/racon/releases/tag/1.4.3, accessed on 30 July 2019). The initial genome assembly with higher accuracy was obtained after the improvement using Illumina short reads by Pilon v. 1.22 [46]. To generate a gapless isolate W-6 genome assembly, over 10 kb PromethION reads were filtered out and used for searching the telomeric repeat sequence “TTAGGG” or “CCCTAA” [47], followed by a gap-filling step using the Illumina short-reads-corrected ONT long reads. The centromere regions were predicted by QUARTET v1.2.1 [48].

The consistency of the isolate W-6 assembly was evaluated by blasting Illumina short reads against the genome assembly using BWA v. 0.7.12 program) [49]. The 758 conserved core genes in fungi_odb10 database [50] of sets of Benchmarking Universal Single-Copy Orthologs (BUSCO) were used for evaluating the completeness and accuracy of assembled isolate W-6 genome by BUSCO v. 5.4.5 [51].

### 2.3. RNA Extraction and Sequencing

To aid the annotation of protein-coding genes, the RNAprep Pure Plant Plus Kit (DP411, Tiangen, Beijing, China) was used to extract total RNA from mycelia of the isolate W-6. RNA integrity, quality, and quantity were assessed by 1% agarose gel electrophoresis (200 V, 15 min, D2000 DNA marker, Vazyme, Nanjing, China), Nanodrop2000 (ThermoFisher Scientific, Wilmington, DE, USA), and Agient2100 Bioanalyzer (LabChip GX, PerkinElmer, Hopkinton, MA, USA), respectively. A library for RNA paired-end sequencing (PE150) was constructed and sequenced on the Illumina NovaSeq6000 platform following the manufacturer’s procedures (Illumina Inc., San Diego, CA, USA).

### 2.4. Identification and Characterization of Repetitive Elements

A specific repetitive sequences database was first established based on the principles of structural prediction. The ab initio prediction was then performed using LTR_FINDER v. 1.05 [52], MITE-Hunter [53] RepeatScout v. 1.0.5 [54], and PILER-DF v. 2.4 [55]. Repetitive sequences in the database were classified with PASTEClassifier [56] before being combined with the RepBase database [57]. After these steps, a final repetitive sequence database was built, and repetitive sequences were obtained using RepeatMasker v. 4.0.6 [58].

### 2.5. Gene Prediction and Annotation

The repeat-masked isolate W-6 genome was used for protein-coding gene model prediction through a combination of de novo prediction, homologous proteins-based prediction, and transcript-based prediction. De novo prediction of protein-coding genes was carried out using Genscan v1.0 [59], Augustus v. 2.4 [60], GlimmerHMM v. 3.0.4 [61], GeneID v. 1.4 [62], and SNAP version 2006-07-28 [63]. GeMoMa v. 1.3.1 [64] was used for homologous protein-based annotation against the National Center for Biotechnology Information (NCBI) protein database. To aid gene model prediction, pair-end RNA sequencing reads were assembled based on the isolate W-6 genome assembly using Hisat2 v. 2.0.4 and Stringtie v. 1.2.3 [65]. The unigenes of the transcripts were obtained using TransDecoder v. 2.0 (https://github.com/TransDecoder/TransDecoder, accessed on 26 January 2015) and PASA v. 2.0.2 [66]. To obtain the final protein-coding gene set, gene models from these approaches were integrated using EVM v. 1.1.1 [67] and improved further by PASA v2.0.2 [66]. The quality of the protein-coding genes prediction was assessed by the support rate of genes in the final gene set for each prediction approach. Transfer RNAs (tRNAs) were annotated by using tRNAscan-SE v1.3.1 [68] and ribosomal RNAs (rRNAs) and other non-coding RNAs (ncRNAs) were predicted using the Rfam-based database [69] by Infernal v. 1.1 [70]. To obtain the pseudogene set, GenBlastA software v1.0.1 [71] was employed to align the predicted protein sequences of isolate W-6 with those protein sequences included in the SwissProtDatabase to find the possible candidates, and then, the software GeneWise v2.2.0 [72] was used for finding the mutations with pre-terminated codon and frameshift in the gene sequences. Gene clusters in the isolate W-6 genome assembly were predicted using antiSMASH v6.0.0 [73].

Functional annotation of protein-coding genes was performed by BLAST alignment (e-value: 1 × 10^−5^) [74] against databases of KOG [75], KEGG [76], Swiss-Prot [77], TrEMBL [77], and Nr [78]. The Blast2go program [79] was used for gene ontology (GO) [80] annotation and Hmmer [81] was for Pfam [82] annotation. Furthermore, the pathogenicity-associated proteins of the fungus were blasted against the Carbohydrate-active EnZYmes Database (CAZyme) [83] (http://www.cazy.org/, accessed on 26 July 2023), the Transporter Classification Database (TCDB) [84] (http://www.tcdb.org/, accessed on 1 October 2020), the Pathogen–Host Interactions Database (PHI-base) [85,86] (http://www.phi-base.org/, accessed on 1 May 2024), the Cytochrome P450 Engineering Database (CYPED) [87] (CYPED v. 6.0: https://cyped.biocatnet.de/, accessed on 29 May 2015), and the Fungal Virulence Factors Database (DFVF) [88] (http://sysbio.unl.edu/DFVF/, accessed on 22 October 2012). Secretory proteins were detected using SignalP 4.0 [89], and transmembrane proteins were filtered using TMHMM v2.0 [90]. The secreted proteins and effector proteins were further analyzed using EffectorP v3.0 [91].

### 2.6. Major Facilitator Superfamily Identification and Phylogenetic Tree

The MFS transporters in the isolate W-6 were identified using the InterPro website (https://www.ebi.ac.uk/interpro/, accessed on 30 May 2024). MFS proteins were further classified based on their functional domains and the annotation of their orthologs in the TCBD [84] database. A phylogenetic tree was constructed using the Q.pfam+R10 model of iqtree2 [92], based on multiple sequence alignments of all MFS proteins generated using MAFFT v7.505 [93].

### 2.7. Structural Analysis of Secondary Metabolic Gene Clusters and MFS Transporters

The SM gene clusters containing the MFS transporters were identified by antiSMASH [73] prediction, and the genes within the SM gene clusters containing the MFS transporters were annotated. Shutdown gene clusters were mined. Structural features were visualized using SnapGene software v6.0.2 (https://www.snapgene.com, accessed on 18 July 2017).

Transmembrane domains of prlG and azaK were predicted using TMHMM-2.0 (https://services.healthtech.dtu.dk/services/TMHMM-2.0/, accessed on 7 July 2000), and their secondary and tertiary protein structures were characterized using Protter (http://wlab.ethz.ch/protter/start/, accessed on 15 October 2024) and swissmodel (https://swissmodel.expasy.org, accessed on 28 October 2024), respectively.

## 3. Results and Discussion

### 3.1. High-Quality Gapless Assembly of Pathogenic Isolate W-6 from Pecan

**Figure 1:**
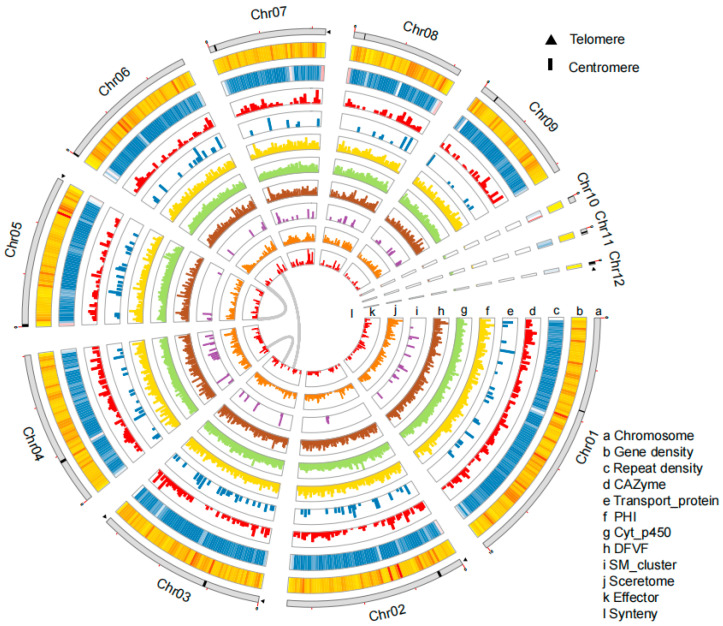
The features of isolate W-6 genome assembly.

The pathogenicity of *C. plurivorum* isolate W-6, which causes spots on leaves and young fruits of pecan, has been clearly identified and further confirmed based on Koch’s postulate in our previous report [10]. As one of the harmful pathogens in the main pecan production area of China, understanding its pathogenic mechanism is crucial for the effective prevention and control of the spot disease. However, the lack of genomic information about this pathogen hinders the identification of its pathogenic factors. Here, a genome survey of the isolate W-6 was first performed using paired-end short reads on the Illumina NovaSeq 6000 platform. This revealed a complex genome with a heterozygosity of 1.01% and repeat sequences 33.96% of the genome (Figure 1 and Figure S1; Table S2). The sequencing of isolate W-6 using the ONT PromethION system generated 11.21 Gb clean long single-molecule reads, resulting in an initial genome assembly of 54.60 Mb, with a contig-N50 length of 5.51 Mb and an average GC content of 55.59% across 12 contigs (Figure 1 and Figure S1; Table 1 and Tables S3–S5). Six of the assembled contigs contained at least one telomere, as evidenced by the presence of telomere repeats (TTAGGG)n or (CCCTAA)n, while Contig3 has both telomeres and, therefore, a full-length chromosome (Figure 1; Table 1 and Table S5). All contigs were predicted to have possible centromere sequences (Figure 1; Table 1 and Table S5). By integrating the results with previous reports on the chromosome number of *Colletotrichum* species (usually 10–13) [47], the 12-contig assembly of the isolate W-6 was considered a chromosome-level reference, including nine core chromosomes and three putative mini-chromosomes (Figure 1; Table 1 and Table S5). The accuracy of the assembly was evaluated by mapping Illumina NovaSeq data to the assembly, showing a high rate of correctly mapped reads (99.35%) and a 99.94% coverage of the isolate W-6 assembly (Table S6). The completeness of the assembly was assessed by searching the core fungal single-copy orthologs in the BUSCO obd10 database (758 genes) [51], which resulted in 98.6% of the genes in the isolate W-6 assembly being completely matched to those in the BUSCO database (Table S7).

The combination of homologous search and de novo prediction resulted in 8146 repetitive sequences with a total length of 5.79 Mb, representing 10.61% of the total isolate W-6 assembly (Table 1). Among the repetitive sequence categories, Class II/TIR was the most abundant, with a total length of 1.84 Mb, followed by the categories Class I/LTR/*Copia* (842.99 Kb) and Class I/LTR/*Gypsy* (586.72 Kb) (Table S8). Non-coding RNA prediction identified 619 genes in the isolate W-6 genome, containing 102 rRNAs, 472 tRNAs, and 45 other ncRNAs (Table S9). By integrating de novo prediction, homology search, and transcriptome assembly, a total of 14,343 protein-coding genes were annotated, with 75.73% (10,862) of the genes supported by transcriptome prediction and 99.8% BUSCO assessment in the isolate W-6 assembly (Table 1, Tables S10 and S11), suggesting a high credibility of the gene sets. The protein-coding genes were characterized by the average length of the gene (2.2 kb), exon (648.7 bp), CDS (514.6 bp), and intron (110.8 bp) (Table S12). Thirty-three of the predicted protein-coding genes were identified as pseudogenes by searching for early terminated codons and frameshifts (Table S13). In addition, 63 biosynthesis gene clusters, including 707 genes with a total length of 2.51 Mb, were identified in the isolate W-6 assembly (Table S14). These genes are mainly related to the biosynthesis of secondary metabolites (SMs), which likely function during the fungal infection of its host [93]. All the putative protein-coding genes were functionally annotated in seven general databases with the percentages of 24.16% (3467) to 97.44% (13,981) (Table S15).

Subcellular localization prediction for the protein-coding genes revealed 1916 genes encoding signal peptide-containing proteins, 3137 genes encoding proteins with transmembrane helix domains, 1451 secreted protein-encoding genes, and 478 effector protein-encoding genes (Figure 1; Tables S16 and S17). To facilitate further investigation into the pathogenicity of the fungal strain, the protein-coding genes were blasted against several specific databases, including Pathogen Host Interactions (PHI-base) [94], Virulence Factors in Fungal Pathogens (DFVF) [88], Carbohydrate-Active enZYmes (CAZymes) [83], Cytochrome P450 Engineering (CYPED) [87] and Transporter Classification (TCDB) [84]. The results showed that 4558 genes matched PHI-base, 3175 matched DFVF, 943 matched CAZymes, 1753 matched CYPED, and 137 matched TCDB (Figure 1; Table 2 and Tables S20–S25). The gap-free assembly and detailed annotation provides a high-quality reference for investigating the molecular mechanisms of pathogenicity in *Colletotrichum* pathogens and for developing efficient control strategies in the future.

### 3.2. Comparative Analyses of Colletotrichum Species and Identification of Potential Pathogenic Factors in Isolate W-6

**Figure 2:**
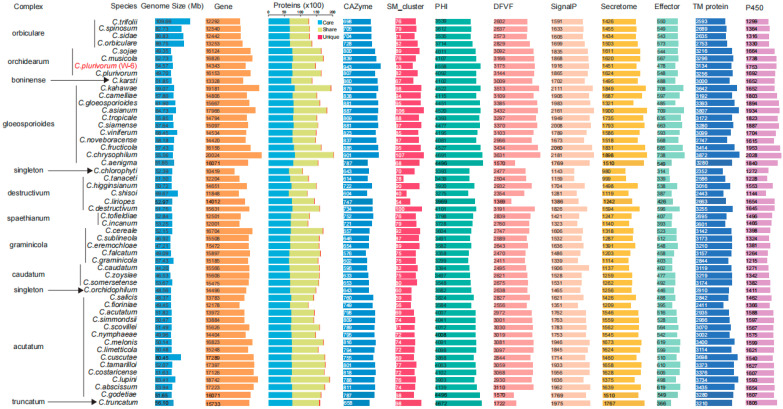
Comparative genomes of 52 *Colletotrichum* strains.

Our previous report showed that isolate W-6 belongs to the *Orchidearum* complex of *Colletotrichum* [10]. To better characterize the pathogenicity features among genomes of *Colletotrichum* species, we obtained sequences of an additional 51 genomes from NCBI and JGI genome database. In total, 52 genomes were used for comparison, including isolate W-6 and other 51 representative *Colletotrichum* strains of 10 complexes (49 species) and two singleton species. Comparative studies of the 52 *Colletotrichum* genomes revealed that the genome sizes of these species varied, with an average of 54.49 Mb (Table S26). The isolate W-6 genome was close to this average, while species in the Orbiculare complex had relatively larger genomes, ranging from 82.73 Mb to 109.66 Mb (Table S26). The isolate W-6 genome had a medium number of functionally annotated protein-coding genes in the general databases and specific databases for most virulence-related genes, but it contained the highest number of genes (943) encoding CAZymes and virulence-related genes in the PHI-base (Figure 2; Tables S20 and S26). CAZymes, produced by fungal plant pathogens, are vital for the degradation of plant polysaccharides, facilitating infection and/or nutrient acquisition during host colonization [95]. According to the similarity of ammino acid sequences in their protein domains, CAZymes are divided into six categories: glycoside hydrolases (GHs), glycosyl transferases (GTs), polysaccharide lyases (PLs), carbohydrate esters (CEs), auxiliary activities (AAs), and carbohydrate binding modules (CBMs). The isolate W-6 genome encoded a total of 151 subfamilies, belonging to eight PLs, 15 AAs, 11 CEs, 13 CBMs, 35 GTs, and 69 GHs (Tables S20 and S21). Among the *CAZyme* genes in the isolate W-6 genome, three genes (Chr04G0306.1, Chr05G0948.1 and Chr07G0081.1) belonging to CE, GT and GH subfamilies were unique to the pecan pathogen strain, likely indicating their important roles in the pathogenicity of isolate W-6. The expansion of genes with annotation in the PHI-base may also contribute to the pathogenicity of the fungal strain.

Plant fungal pathogens have complex and diverse pathogenic mechanisms, which are controlled by different genes and factors. The virulence factors of pathogenic fungi cover a wide range of components, including various effector proteins, secondary metabolites, and small RNAs, which participate in host penetration, the prevention or inhibition of host defense, and nutrient acquisition [96]. During the interaction between plants and pathogens, pathogens first cause damage to plant cells through virulence factors, thereby absorbing nutrients from plant cells to successfully colonize, develop, and reproduce. At the same time, they inactivate defense factors released by plants to increase the survival and growth of pathogens [22]. For example, fungalysin metalloprotease (Cgfl) is a conserved effector in the maize-host fungus *C. graminicola*, which enhances virulence in the maize anthracnose by degrading chitinases and participating in chitin signaling [97]. It has been reported that virulence-related genes involved in necrosis induction (*Hce2*), signal transduction (*CFEM*), protein oligosaccharide interaction (*CVNH*, *WSC*, *PAN*), and adherent development (*CAP22*, *CAS1*) are specifically present in *Colletotrichum* fungal pathogens and are distributed specifically within the genus [98].

To identify the potential pathogenic factors unique to the isolate W-6, we first found only four genes annotated in the PHI-base (one gene encoding protein kinase) and DFVF (three genes encoding Histone H3) databases among all 51 *Colletotrichum* species (Tables S26 and S27). However, these genes play a relatively small role on pathogenicity, according to research on their homologs in other pathogenic fungi [99,100,101]. We further searched for pathogenicity-related genes unique to the Orchidearum complex to which the isolate W-6 belongs, and identified a total of 154 genes, 46 of which were annotated in the InterPro database, including 13 genes annotated in the both the PHI-base and DFVF databases (Table 3 and Table S28). Among the 13 virulence-related genes, members of the MFS transporter family (four genes), G-protein coupled receptors (two genes), and ATP-binding cassette (ABC) transporters (two genes) represented relatively rich categories (Table 3 and Table S29). The homologous annotations of these genes in the databases indicated that three out of four MFS transporters were pathogenic, both G-protein coupled receptors were pathogenic, only one of the ABC transporters was pathogenic, and none of the two C2H2 transcription factors were pathogenic [102].

As is well-known, both ABC and MFS transporters are superfamilies and important members of multidrug resistance (MDR) transporters, which are essential for the full virulence of fungal pathogens [111]. The transporters enhance the multi-drug resistance of pathogens by increasing the efflux of fungicides [102], or by enhancing their pathogenicity through the efflux of toxins [112]. G-protein coupled receptors, as important components of signal transduction, transmit signals during pathogen–host interaction to promote fungal development and full virulence formation [113]. C2H2 transcription factors generally regulate the expression of various genes, such as those involved in spore formation, growth, development, and virulence-factor-encoding genes [114,115,116]. Therefore, the identification of virulence-related genes and the in-depth investigation of their functions will help clarify the mechanisms of pathogen virulence formation and their modes of action. This will also guide the future prevention and control strategies for anthracnose diseases in pecan and other plants.

### 3.3. Identification and Characterization of MFS Members in Isolate W-6

The MFS transporters represent the largest and most diverse superfamily of secondary metabolites transport proteins, found in essentially all organisms and currently including 105 subfamilies [117]. The main function of MFS proteins is to transport substrates across the cell membrane [118]. MFS transporters play an important role in the process of pathogen infection in plants, such as sporulation, sugar transport, stress response, spore penetration, and cell wall swelling pressure, and in the transport and formation of virulence factors. They also endow pathogenic fungi with resistance [119,120]. Our results showed that *MFS* genes represent the largest group among the virulence-related genes unique to species in the *Orchidearum* complex (Table 3 and Table S28), suggesting their potentially important roles in the pathogenicity of fungal isolates within this complex, including isolate W-6. Therefore, we assumed that MFS transporters play an important role in the virulence formation of isolate W-6, and some members of the family likely function as key factors in the pathogenicity of isolate W-6.

**Figure 3:**
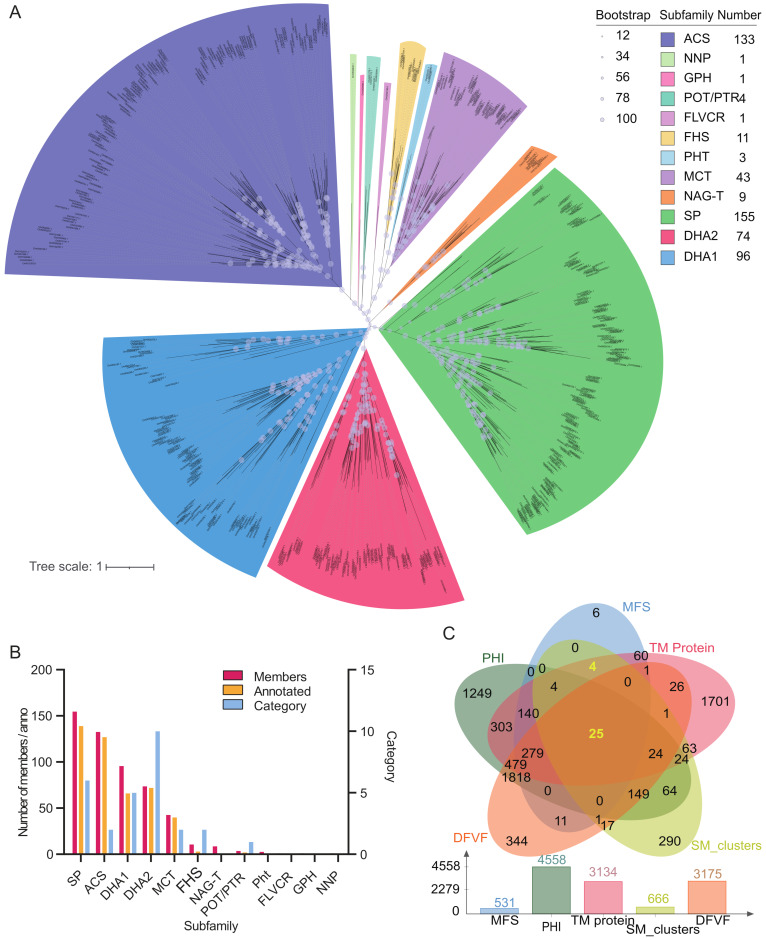
Phylogeny (**A**), classification (**B**), and potential virulence analyses (**C**) of MFSs in isolate W-6.

To identify the candidate MFSs responsible for pathogenicity in isolate W-6, we performed a genome-wide identification of MFS-encoding genes, which revealed 531 members (Figure 3; Table 4 and Table S29). Phylogenetic analysis revealed 12 subclasses that divided into two groups based on the members they contained: one includes five subclasses with over 40 members, namely, SP, ACS, DHA1, DHA2, and MCT, while the subclasses of NAG-T, POT/PTR, PHT, FLVCR, GPH, and NNP contain fewer than 10 members, with only one MFS found in the FLVCR, GPH, and NPP subclasses (Figure 3; Table 4, Tables S29 and S30).

As is well known, plants release defense factors during their interaction with pathogens to inhibit their growth and virulence formation. In turn, pathogens degrade, inactivate, or transport the defense factors outside the plant, indirectly affecting their virulence. MFS transporters also participate in the synthesis and transport of pathogenic virulence factors, directly affecting their function. Of the *MFSs* in the isolate W-6 assembly, 448 and 305 members were found to have virulence-related annotations in the PHI-base and DFVF database, respectively, accounting for 84.56% of the total *MFS* genes (Figure 3C; Table 4 and Table S26). In contrast, subclasses with more members also had a relatively higher proportion of virulence annotations (Table 4 and Table S30).

It has been reported that the DHA1 and DHA2 subclasses are the major members of multidrug resistance (MDR) in MSF transporters and play important roles in fungal pathogen resistance, not only pumping out antibiotics but also expelling pathogenic fungal toxins [121]. In contrast, members in the DHA2 subclass received the most significant number of virulence-related annotations in the databases, accounting for 97.3%, while those in the DHA1 subclass account only for 68.75% of the MFSs in the isolate W-6 (Figure 3C; Table 4 and Table S30). In other specific databases, the number of annotated DHA2 members is also the highest in the MFS subclasses, indicating their critical roles in various pathogenic processes in the isolate W-6. Members in the MCT subclass also received virulence-related annotations in up to 93.02% of cases (Table 4 and Table S26). It has been reported that MCTs affect the virulence of pathogens through nutritional or pH changes, thereby affecting the growth, development, and pathogenicity of fungi during infection [122]. However, MCTs are only annotated in two categories, are likely involved in a single biological process in the isolate W-6, and are crucial for the metabolism and homeostasis of pathogens [122].

MFS transporters in the SP subclass not only participate in sugar metabolism and the transportation of sugars within fungi, but also in the competition and transformation of sugars in host plants. They adjusted fungal metabolism through signal transduction to produce relevant virulence factors, thereby promoting plant cell necrosis [123,124,125,126]. Up to 89.68% of SP members received virulence annotations, second only to the DHA2 subclass in the isolate W-6 genome, which would greatly contribute to the successful infection in host tissues (Table 4 and Table S30). Among the MFSs, up to 95.40% of ACS members were annotated in the PHI-base and DFVF database (Table 4), reflecting their important roles in the isolate W-6 strain and plant host interaction. ACSs transporters play an important role in transporting various substances, such as vitamins and toxins, as reported in *Cercospora* species [127]. According to this study, during the transportation of fungal toxins, a large amount of reactive oxygen species (ROS) is released via photosensitization, which damages plant cells.

### 3.4. Homologs of prlG and azaK: The Key Candidates for Pathogenicity in Isolate W-6

From the perspective of plant-pathogen interactions, plant defense factors have, to some extent, promoted the virulence and reproductive capacity of plant pathogens. Multiple genes involved in the biosynthesis of small molecules such as toxins, antibiotics, and pigments in fungi could adaptively combine through vertical and horizontal transfer and cluster on chromosomes to form gene clusters [127,128,129,130]. A study on the causal agent of the rice “bakanae” disease pathogen *Fusarium fujikurio* revealed that the genes responsible for synthesizing mycotoxins were usually adjacent to each other in gene clusters, which typically include genes encoding polyketide synthases (PKSs), non-ribosomal peptide synthases (NRPSs), and chemical-modifying enzymes [131]. Similarly, MFS transporters were widely present in secondary metabolic gene clusters and participate in the transport of metabolites in fungal pathogens [129,132,133]. Recent studies revealed the three most-common conserved domains found near the type III PKS loci, corresponding to MFS transporters, fungal-specific transcription factors, and cytochrome P450 monooxygenases, respectively [132,133]. These reports suggest that MFS members are essential factors for the virulence of fungal pathogens, associated with the biosynthesis of mycotoxins in secondary metabolic gene clusters.

**Figure 4:**
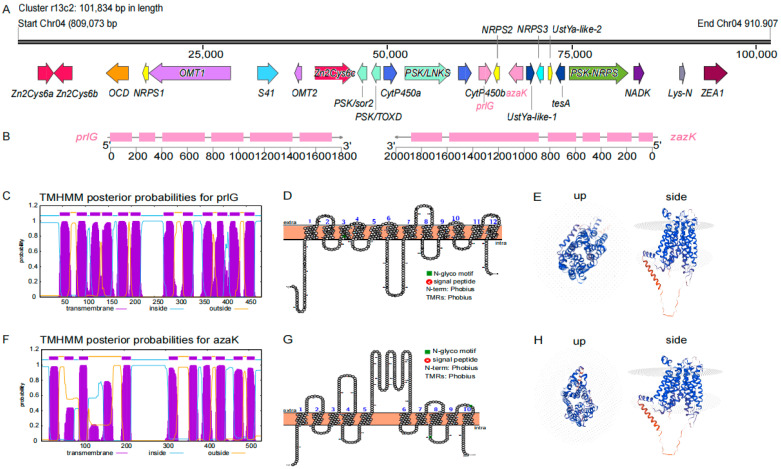
Details of SM cluster r13c2 and the structures of key candidates in MFSs. (**A**) Gene distribution on SM cluster r13c2. The total lenth of this cluster is 101,834 bp, located on chromosome 4 (Chr04), starting at 809,703 bp and ending at 910,907 bp. (**B**) The gene structures of *prlG* and *azaK*. The arrows represent the direction of the gene on the chromosome, and the number below represents the length of the genes, measured in bp. (**C**–**H**) Showing the predicted TM domains of protein, the 2D and 3D structures of prlG (**C**–**E**) and azaK (**F**–**H**).

In this study, we found 35 MFS-encoding genes from 20 SM gene clusters, accounting for 31.75% of the 63 SM gene clusters in the isolate W-6 genome (Table S31). Of the 35 MFSs, eight members belong to the DHA2 subclass and six to DHA1. Among the *MFS*-containing SM gene clusters, the most prominent one is Cluster r13c2, which consists of 24 well-annotated genes that center around functions in the biosynthesis and post-translational modification of multiple mycotoxins, including the ribosomally synthesized and post-translationally modified peptides (RiPPs) and non-ribosomally synthesized peptides (NRSPs) (Figure 4A; Table S32). Phomopsins are a group of hexapeptide mycotoxins belonging to the RiPPs, first reported to be produced by the pathogenic Ascomycetes *Aspergillus flavus* and *Ustilaginoidea virens* [134,135]. Most genes that cluster in r13c2 encode important enzymes associated with virulence and regulators, with 13 of them related to phomopsin biosynthesis and post-translational modification, including Zn2Cys6s, UstYa-likes, OCD, OMTs, and NRPS (Figure 4A; Table S32). Like an SM gene cluster in *Phomopsis leptostromiformis*, r13c2 also encodes enzymes and regulators for the phomopsin biosynthesis and multi-drug transporter proteins of MSFs [136].

Among the phomopsin-associated genes, homologs of Zn2Cys6s could regulate the expression of downstream oxidoyreducetase cellobiose dehydrogenase (OCD)-encoding genes [137] and are involved in regulating important virulence factors [138]. Additionally, some of the homologous Zn2Cys6s may function as ABC transporters participating in the regulation of gene expression related to resistance to azole compounds, the activation of gluconeogenesis enzymes, the regulation of drug response genes, and the control of secondary metabolite biosynthesis (such as ergot segments, fusaridine A, oryzanes, AAL toxins, and fumonisins) [139]. Three genes in the cluster encode NRPSs participate in the synthesis of phomopsin-associated proteins (Figure 4A; Table S32). Two *OMT genes* encoding SAM-dependent O-methyltransferasess have been reported to potentially participate in the methylation modification of phomopsin A to form phomospin E [140]. A homolog of the secondary metabolite-associated S41A peptidase plays a crucial role in the biosynthesis of various secondary metabolites, such as hexapeptide mycotoxins and anti-mitotic tetrapeptides [140]. Two homologs of UstYa were reported to participate in post-translational oxidative modification during the formation of cyclic peptides [134,140]. Meanwhile, *tesA*, which encodes thioesterase, functions by adding the last amino acid to peptide antibiotics in non-ribosomal peptide synthesis to form cyclic peptides [141,142].

To date, many reports have described the involvement of MFS transporters in the transport of various secondary metabolites in SM gene clusters [143,144,145,146,147], and there seems to be a certain degree of overlap in the substrates recognized by different MFS transporters [148]. As members of the DHA1 subclass in the MFS transporter family, both *prlG* and *azaK* have been reported to function in the efflux of antibiotics and secondary metabolites in SM gene clusters, leading to the development of drug resistance in pathogens [149,150,151]. However, the MFS transporters in SM gene clusters of pathogenic fungi of *Colletotrichum* species, including the isolate W-6, have not been functionally investigated. In this study, we identified the MFS homologs of prlG and azaK in the r13c2 cluster as candidates of isolate W-6 pathogenicity. We further characterized their gene features and putative protein structures. Both genes contain five introns and the gene length of *rplG* is shorter than that of *azaK*. They are distributed on two strands of DNA with only one *NRPS2* gene between them (Figure 4B). Predictions of transmembrane regions (TMRs) and two-dimensional structures showed 12 TMRs (Figure 4C,D) for prlG and 10 TMRs for azaK (Figure 4F,G), which resulted in the significant differences in their three-dimensional protein conformations (Figure 4E,H). These differences may lead to divergence in their functions related to pathogen virulence.

### 3.5. Conclusions and Perspectives

We obtained a nearly complete, ~55Mb gapless reference genome assembly of the pathogen *C. plurivorum* isolate W-6 from a cultivar ‘Wichita’ of pecan. This 12-chromosome assembly includes three mini-chromosomes and a total of 14,343 annotated protein models. Annotation using virulence-related specific databases, combined with the comparative genomic analysis of the isolate W-6 and 51 other genome-sequenced *Colletotrichum* strains, highlighted the MFS transporter members unique to the *Orchidearum* complex to which isolate W-6 belongs. Analysis of the MFS member-containing SM gene clusters further highlighted the r13c2 gene cluster containing a total of 24 virulence-related gene annotations in the PHI-base and DFVF databases, which have been reported to function in the synthesis and modification of RiPP mycotoxins (mainly hexapeptide phomopsins) and MFS transporters. prlG and azaK were finally identified as key candidates for the pathogenicity of the isolate W-6, and their protein structures were further characterized.

It is known that MFS transporters play an important role in the pathogenicity of pathogens and the defense signaling factors of low-immune hosts [36]. As in previous studies, MFS transporters are regulated by transcription factors at the beginning of SM gene clusters, and their expression can promote substrate transport and increase substrate yield and transport efficiency [152,153]. The findings here provide an important foundation for future studies on the pathogenicity of *Colletotrichum* species, which will facilitate the development of more efficient and environmentally friendly strategies to control anthracnose in pecan orchards.

## Figures and Tables

**Table 1:** Summary of the isolate W-6 genome assembly.

Sequencing Platform	ONT PromethION and Illunina NovaSeq 6000
Number of contigs	12
Assembly length (bp)	54,574,699
Contig N50 (bp)	6,029,146
T2T chromosome number	1
Maximum contig size(bp)	10,044,033
Minimum contig size (bp)	106,099
BUSCO completeness (%)	98.62
Repeat rate (%)	10.61
GC content	0.56
Predicted genes	14,343
SM_cluster	63
Signal P	1916
Secretome	1451
Transmembrone proteins	3137
Effector	478
PHI	4558
DFVF	3175
CAZyme	943
CYPED	1753
TCDB	137

**Table 2:** Distribution of virulence-related genes in isolate W-6 genome.

Chr ID	CAZyme	PHI-Base	Cyt_P450	DFVF	Effector	Secretome	SM_cluster	Gene
Chr01	217	911	353	607	115	335	13	2794
Chr02	37	632	228	434	51	161	2	1865
Chr03	108	533	206	396	55	158	4	1745
Chr04	122	546	242	394	53	195	11	1599
Chr05	71	412	162	288	48	118	4	1463
Chr06	76	449	163	314	36	130	4	1491
Chr07	195	388	147	261	56	143	8	1204
Chr08	63	325	116	231	32	108	7	1076
Chr09	54	357	134	246	31	101	7	1057
Chr10	0	1	1	1	1	1	0	20
Chr11	0	3	1	2	0	0	3	22
Chr12	0	1	0	1	0	0	0	7
Total	943	4558	1753	3175	478	1450	63	14,343

**Table 3:** Unique virulence-related genes of *Orchidearum* complex in PHI and DFVF databases.

GeneID	Family	Homology in Pathogen Species	PHI	DFVF	References
Chr01G2516.1	major facilitator superfamily (proton-linked monocarboxylate transporter)	*Magnaporthe oryzae*	PHI:812 *	Gene Symbol:MGG_10702	[103]
Chr02G0006.1	major facilitator superfamily (proton-linked monocarboxylate transporter)	*Magnaporthe oryzae*	PHI:812 *	Gene Symbol:MGG_10702	
Chr08G47.1	major facilitator superfamily (proton-linked monocarboxylate transporter)	*Magnaporthe oryzae*	PHI:812 *	Gene Symbol:MGG_10702	
Chr02G0666.1	major facilitator superfamily (siderophore transporter)	*Candida albicans*	PHI:513	N/A	[104]
Chr03G0348.1	aspartyl proteinase	*Candida albicans*	PHI:126 *	Gene Symbol:AFUA_3G01220	[105]
Chr04G0884.1	Aspergillus synthase	*Penicillium expansum*	PHI:3299	Gene Symbol:ACE1	[106]
Chr04G0877.1	ATP-binding cassette (ABC) transporter	*Zymoseptoria tritici*	PHI:1159 *	Gene Symbol:NULL	[107]
Chr03G0063.1	ferric reductases	*Cryptococcus neoformans*	PHI:3415	Gene Symbol:NOXA	[108]
Chr07G0020.1	G-protein coupled receptor	*Botrytis cinerea*	PHI:441 *	Gene Symbol:BTP1	[109]
Chr02G1777.1	G-protein coupled receptor	*Botrytis cinerea*	PHI:441 *	Gene Symbol:BTP1	
Chr05G0845.1	protein kinase	*Fusarium graminearum*	PHI:1226 *	Gene Symbol:SCH9	[110]
Chr01G1072.1	Tetratricopeptide-like helical domain superfamily	*Magnaporthe oryzae*	PHI:799 *	Gene Symbol:MGG_03530	[103]
Chr04G0933.1	C2H2transcription factor	*Fusarium graminearum*	PHI:1859	N/A	[110]

* Represents pathogenic/virulence factors reported in existing literature.

**Table 4:** Statistics of subfamilies in MFS family of the isolate W-6.

Subclass	Description	Number of Members	Annotation in PHI and DFVF Databases		Category
			Number	Percentage	
SP	The Sugar Porter (SP)	155	139	89.68	6
ACS	The Anion/Cation Symporter (ACS) Family	133	127	95.49	2
DHA1	The Drug/H+ Antiporter-1 (12 Spanner) (DHA1) Family	96	66	68.75	5
DHA2	The Drug/H+ Antiporter-2 (14 Spanner) (DHA2) Family	74	72	97.30	10
MCT	The Monocarboxylate Transporter (MCT) Family	43	40	93.02	2
FHS	The Fucose/H+ Symporter (FHS) Family	11	3	27.27	2
NAG-T	The N-Acetylglucosamine Transporter (NAG-T)	9	0	0.00	0
POT/PTR	The Proton-dependent Oligopeptide Transporter (POT/PTR) Family	4	2	50.00	1
PHT	The Proteobacterial Intraphagosomal Amino Acid Transporter (Pht) Family	3	0	0.00	0
FLVCR	The Feline Leukemia Virus Subgroup C Receptor (FLVCR)/Heme Importer Family	1	0	0.00	0
GPH	The Glycoside-Pentoside-Hexuronide (GPH)/Cation Symporter Family	1	0	0.00	0
NNP	The Nitrate/Nitrite Porter (NNP) family	1	0	0.00	0
Tatol	NA	531	449	84.56	27

## Data Availability

The authors confirm all supporting data, code, and protocols have been provided within the article or through supplementary data files. Genome sequencing data and assemblies of *C. plurivorum* isolate W-6 have been uploaded to NCBI BioProject PRJNA1170468, under BioSample numbers SAMN44359669.

## Supplementary Materials

The following supporting information can be downloaded at: https://www.mdpi.com/article/10.3390/jof11030203/s1. Figure S1. The genome survey of the isolate W-6. Table S1. Information on 51 genome-sequenced strains of *Colletotrichum* spp. and an outgroup speces. Table S2. Illumina NovaSeq data statistics for the isolate W-6 genome survey. Table S3. Oxford Nanopore data statistics for the isolate W-6 assembly. Table S4. Length distribution of clean reads of ONT data. Table S5. Detailed information of assembled contigs of the isolate W-6. Table S6. Assembly assessment of the isolate W-6 by Illumina NovaSeq data. Table S7. BUSCO assessment of assembly completeness of the isolate W-6 genome. Table S8. Predicted repeat elements in the isolate W-6 genome. Table S9. Prediction of non-coding RNAs. Table S10. Prediction of protein-coding genes in the isolate W-6 genome. Table S11. BUSCO assessment of protein-coding genes in the isolate W-6 genome. Table S12. Features of protein-coding genes in the isolate W-6 genome. Table S13. Statistics of pseudogenes in the isolate W-6 genome. Table S14. Information of predicted secondary metabolites cluster in isolate W-6 genome. Table S15. Function annotation of protein-coding gene based on general databases. Table S16. Signal peptide in the isolate W-6 genome. Table S17. List of transmembrane (TM) proteins in the isolate W-6 genome. Table S18. List of secreted protein in the isolate W-6 genome. Table S19. List of effectors in isolate W-6 genome. Table S20. Function annotation of protein-coding gene based on a specific database. Table S21. Prediction of CAZyme in isolate W-6 genome. Table S22. Information of TCDB transporters in the isolate W-6 genome. Table S23. Prediction of PHI proteins in the isolate W-6 genome. Table S24. Prediction of CYTP450 genes in the isolate W-6 genome. Table S25. Prediction of DFVF genes in the isolate W-6 genome. Table S26. Details of comparative analysis of 52 Colletotrichum species. Table S27. The isolate W-6 specific virulence-related genes in PHI and DFVF databases. Table S28. Pathogenicity-related genes unique to the Orchidearum complex. Table S29. Gene list of MFS superfamily. Table S30. Virulence-related annotation of MFS transporters. Table S31. Clusters containing MFSs. Table S32. Genes in Cluster r13c2.
