# Supplementary material for: Decoding Pecan’s Fungal Foe: A Genomic Insight into Colletotrichum plurivorum Isolate W-6
Source: J Fungi (Basel). 2025 Mar 5;11(3):203. doi: 10.3390/jof11030203 (PMC11943440; doi:10.3390/jof11030203)
Supplement: Supplementary file 1 [file jof-11-00203-s001.zip › Table S1.pdf]

Table S1. Information of 47 genome-sequenced strains of *Colletotrichum* spp. and an outgroup speces.

Complex	Taxon	Strain	GenBank accession	Host	Assembly	Genome size (bp)	Assembly level
	C.abscissum	IMI 504890	PRJNA350894	*Citrus sinensis*	CABS01	53,936,108	Scaffold
	C.acutatum	L51	PRJNA1060634	*Pyrus communis*	CACUL51	51,824,279	Scaffold
	C.costaricense	IMI 309622	PRJNA237763	*Coffea sp.*	CCOSS01	51,625,248	Scaffold
	C.cuscutae	IMI 304802	PRJNA238477	*Cuscuta*	CCUS01	80,453,496	Scaffold
	C.fioriniae	IMI 355084	PRJNA613862	*Quercus ilex*	ASM2794284 v1	49,445,812	Scaffold
	C.limetticola	KLA-Anders on	PRJNA504379	*Citrus x aurantiifolia*	CLIM01	50,483,870	Scaffold
acutatum	*C.lupini*	IMI 504893	PRJNA613862	*Lupinus albus*	ASM2327856 v1	63,407,421	Complete Genome
	C.melonis	Col 31	PRJNA928458	*Malus pumila*	CMEL01	50,135,825	Scaffold
	C.nymphaeae	SA-01	PRJNA564749	*Fragaria x ananassa*	CNYM01	49,956,273	Scaffold
	C.salicis	CBS 607.94	PRJNA476648	*Salix sp.*	CSAL01	48,373,413	Scaffold
	C.scovillei	Coll-524	PRJNA262440	*Capsicum annuum*	ASM1890767 v1	51,490,565	Scaffold
	C.simmondsii	CBS122122	PRJNA900520	*Carica papaya*	CSIM01	50,474,234	Scaffold
	C.tamarilloi	Tom-12	PRJNA506861	*Solanum betaceum*	CTAM01	52,071,711	Scaffold
boninense	*C.karsti*	CkLH20	PRJNA492188	*Camellia oleifera*	ASM1194739 v2	51,850,041	Scaffold
	C.caudatum	CBS 131602	PRJNA786750	*Sorghastrum nutans*	Colca1	44,202,764	Scaffold
caudatum	*C.somersetense*	CBS 131599	PRJNA577396	*Sorghastrum nutans*	Colso1	53,667,270	Scaffold
	C.zoysiae	MAFF 238573	PRJNA286731	*Zoysia tenuifolia*	Colzo1	46,533,813	Scaffold

destructivum	*C.destructivum*	CBS 520.97	PRJNA692809	*Medicago sativa*	ASM3444790v1	51,750,812	Complete Genome
	C.higginsianum	IMI 349063	PRJNA1029933	*Brassica rapa subsp. chinensis*	ASM167251v1	50,716,103	Chromosome
	C.shisoi	PG-2018a	PRJNA262442	*Perilla frutescens var. crispa*	ASM678308v1	69,667,657	Scaffold
	C.tanaceti	BRIP57315	PRJNA577394	*Tanacetum cinerariifolium*	ASM2622989v1	51,501,111	Scaffold
gloeosporioides	*C.asianum*	ICMP 18580	PRJNA350895	*Coffea sp.*	ASM980641v1	64,731,264	Scaffold
	C.camelliae	CcLH18	PRJNA350379	*Camellia oleifera*	ASM1194748v2	57,798,682	Scaffold
	C.chrysophilum	M932	PRJNA348398	*Malus pumila*	CCHY01	55,557,938	Scaffold
	C.fructicola	CfS4	PRJNA262368	*Fragaria × ananassa*	ASM1320187v1	57,426,314	Scaffold
	C.gloeosporioides	Lc1	PRJNA262441	*Liriodendron chinense*	NFU_CgLc1_1.0	61,904,035	Scaffold
	C.kahawae	CIFC_Que2	PRJNA431477	*Coffea arabica*	CKAH01	59,071,264	Scaffold
	C.noveboracense	Coll940	PRJNA577812	*Juglans nigra*	ASM2631915v1	58,178,553	Scaffold
	C.siamense	CAD5	PRJNA262221	*Manihot esculenta*	ASM1320175v1	57,642,425	Scaffold
	C.tropicale	CgS9275	PRJNA342923	*Morus alb*	ASM1320178v1	55,848,179	Scaffold
	C.viniferum	CGW01	PRJNA267650	*Vitis vinifera*	ASM1320176v1	68,452,207	Scaffold

	C.cereale	CBS 129662	PRJNA952538	*Bletilla ochracea*	Colce1	52,150,039	Scaffold
	C.eremochloae	CBS 129661	PRJNA239224	*Eremochloa ophiuroides*	Coler1	47,210,890	Scaffold
graminicola	*C.falcatum*	MAFF306170	PRJNA348678	*Saccharum officinarum*	Colfa1	49,085,377	Scaffold
	C.graminicola	M1.001	PRJNA262217	*Zea mays*	ASM2922662v1	57,426,633	Chromosome
	C.sublineola	S3.001	PRJNA445190	*Sorghum bicolor*	Colsu1	46,916,122	Scaffold
	C.orbiculare	104-T	PRJNA858866	*Cucumis sativus*	KSG_Corb_v2	89,748,316	Scaffold
orbiculare	*C.sidae*	CBS 518.97	PRJNA262370	*Sida spinosa*	ASM436793v1	86,827,816	Scaffold
	C.spinosum	CBS 515.97	PRJNA445190	*Xanthium spinosum*	ASM436682v1	82,734,851	Scaffold
	C.trifolii	543-2	PRJNA350752	*Medicago sativa*	KSG_Cotri_v1	109,659,959	Scaffold
	C.musicola	LFN0074	PRJNA476648	*Glycine max*	CMUS01	52,725,698	Scaffold
orchidearum	*C.plurivorum*	LFN00145	PRJNA476648	*Glycine max*	CPLU01	49,703,650	Scaffold
	C.sojae	LFN0009	PRJNA171217	*Glycine max*	CSOJ01	49,351,125	Scaffold
singleton	*C.chlorophyti*	NTL11	PRJNA360503	*Solanum lycopersicum*	ASM193710v1	52,387,045	Scaffold
	C.orchidophilum	IMI 309357	PRJNA476648	*Phalaenopsis sp.*	CORC01	48,556,462	Scaffold
spaethianum	*C.incanum*	MAFF238712	PRJNA47061	*Raphanus sativus* var. *longipinnatus*	ASM185523v1	53,254,579	Scaffold
	C.tofieldiae	861	PRJNA577398	*Arabidopsis thaliana*	Ct0861v1	52,836,184	Scaffold
Outgroup	*Verticillium alfalfae*	VaMs.102	PRJNA29511	*Medicago* spp.	ASM15082v1	32,833,495	Scaffold
