# Supplementary material for: Decoding Pecan’s Fungal Foe: A Genomic Insight into Colletotrichum plurivorum Isolate W-6
Source: J Fungi (Basel). 2025 Mar 5;11(3):203. doi: 10.3390/jof11030203 (PMC11943440; doi:10.3390/jof11030203)
Supplement: Supplementary file 1 [file jof-11-00203-s001.zip › Table S10.pdf]

Table S10. Prediction of protein-coding genes in isolate W-6 genome.

Method	Software	Reference species	Gene number
Ab initio-based prediction	Augustus		11,389
	Genscan		12,127
	Gene ID		20,754
	Glimmer HMM		13,137
	SNAP		13,649
RNA-seq-based prediction	PASA		22,878
	TransDecoder		24,212
Homology-based prediction	GeMoMa	*Colletotrichum fructicola*	13,358
		Colletotrichum gloeosporioides	12,391
		Glomerella graminicola	11,429
		Colletotrichum higginsianum	12,413
Integration	EVM		14,343
