# Supplementary material for: Decoding Pecan’s Fungal Foe: A Genomic Insight into Colletotrichum plurivorum Isolate W-6
Source: J Fungi (Basel). 2025 Mar 5;11(3):203. doi: 10.3390/jof11030203 (PMC11943440; doi:10.3390/jof11030203)
Supplement: Supplementary file 1 [file jof-11-00203-s001.zip › Table S11.pdf]

Table S11. BUSCO assessment of protein-coding genes in isolate W-6 genome.

Complete BUSCOs (C)	756 (99.8%)
Complete and single-copy BUSCOs (S)	754 (99.5%)
Complete and duplicated BUSCOs (D)	2 (0.3%)
Fragmented BUSCOs (F)	1 (0.1%)
Missing BUSCOs (M)	1 (0.1%)
Total conserved BUSCO genes (fungi obd10)	758
