# Supplementary material for: Decoding Pecan’s Fungal Foe: A Genomic Insight into Colletotrichum plurivorum Isolate W-6
Source: J Fungi (Basel). 2025 Mar 5;11(3):203. doi: 10.3390/jof11030203 (PMC11943440; doi:10.3390/jof11030203)
Supplement: Supplementary file 1 [file jof-11-00203-s001.zip › Table S12.pdf]

Table S12. Features of protein-coding genes in isolate W-6 genome.

Gene number	14,343
Total length of genes (bp)	31,576,597
Average length of genes (bp)	2,200.77
Total length of exons (bp)	28,327,651
Average length of exon (bp)	648.69
Exon unmber	43,669
Average number of exon per gene	3.04
Total length of CDSs (bp)	21,868,383
Average length of CDS (bp)	514.62
CDS number	42,494
Total length of introns (bp)	3,248,946
Average length of intron (bp)	110.81
Intron number	29,321
Average number of intron per gene	2.04
