# Supplementary material for: Decoding Pecan’s Fungal Foe: A Genomic Insight into Colletotrichum plurivorum Isolate W-6
Source: J Fungi (Basel). 2025 Mar 5;11(3):203. doi: 10.3390/jof11030203 (PMC11943440; doi:10.3390/jof11030203)
Supplement: Supplementary file 1 [file jof-11-00203-s001.zip › Table S13.pdf]

Table S13. Statistics of pesudogenes in isolate W-6 genome.

Pesudogene number	Pesudogene size (bp)	Average Pesudogene Length (bp)
33	31,768	962.66
