# Supplementary material for: Decoding Pecan’s Fungal Foe: A Genomic Insight into Colletotrichum plurivorum Isolate W-6
Source: J Fungi (Basel). 2025 Mar 5;11(3):203. doi: 10.3390/jof11030203 (PMC11943440; doi:10.3390/jof11030203)
Supplement: Supplementary file 1 [file jof-11-00203-s001.zip › Table S14.pdf]

Table S14. Information of predicted secondary metabolites cluster in isolate W-6 genome.

| cluster ID or Gene ID | contig ID | Start | End | Length(bp) |
|---|---|---|---|---|
| r1c1 | Chr01 | 1,071,857 | 1,084,725 | 12,869 |
| Chr01G0294.1 | Chr01 | 1,073,153 | 1,074,608 | 1,456 |
| Chr01G0295.1 | Chr01 | 1,075,996 | 1,077,873 | 1,878 |
| Chr01G0296.1 | Chr01 | 1,079,338 | 1,081,447 | 2,110 |
| Chr01G0297.1 | Chr01 | 1,082,962 | 1,084,724 | 1,763 |
| r1c2 | Chr01 | 2,005,504 | 2,061,191 | 55,688 |
| Chr01G0558.1 | Chr01 | 2,007,984 | 2,010,926 | 2,943 |
| Chr01G0559.1 | Chr01 | 2,011,074 | 2,014,673 | 3,600 |
| Chr01G0560.1 | Chr01 | 2,015,863 | 2,017,719 | 1,857 |
| Chr01G0561.1 | Chr01 | 2,019,610 | 2,022,260 | 2,651 |
| Chr01G0562.1 | Chr01 | 2,022,319 | 2,023,343 | 1,025 |
| Chr01G0563.1 | Chr01 | 2,023,716 | 2,027,959 | 4,244 |
| Chr01G0564.1 | Chr01 | 2,028,241 | 2,028,783 | 543 |
| Chr01G0565.1 | Chr01 | 2,030,123 | 2,032,021 | 1,899 |
| Chr01G0566.1 | Chr01 | 2,035,807 | 2,036,585 | 779 |
| Chr01G0567.1 | Chr01 | 2,040,747 | 2,042,870 | 2,124 |
| Chr01G0568.1 | Chr01 | 2,043,181 | 2,046,748 | 3,568 |
| Chr01G0569.1 | Chr01 | 2,048,347 | 2,052,347 | 4,001 |
| Chr01G0570.1 | Chr01 | 2,053,918 | 2,054,781 | 864 |
| Chr01G0571.1 | Chr01 | 2,055,635 | 2,056,647 | 1,013 |
| Chr01G0572.1 | Chr01 | 2,056,770 | 2,060,354 | 3,585 |
| Chr01G0573.1 | Chr01 | 2,060,780 | 2,061,190 | 411 |
| r1c3 | Chr01 | 2,212,654 | 2,229,560 | 16,907 |
| Chr01G0617.1 | Chr01 | 2,214,218 | 2,215,373 | 1,156 |
| Chr01G0618.1 | Chr01 | 2,215,939 | 2,218,198 | 2,260 |
| Chr01G0619.1 | Chr01 | 2,217,667 | 2,218,098 | 432 |
| Chr01G0620.1 | Chr01 | 2,222,062 | 2,223,955 | 1,894 |
| Chr01G0621.1 | Chr01 | 2,224,350 | 2,225,437 | 1,088 |
| Chr01G0622.1 | Chr01 | 2,225,832 | 2,228,076 | 2,245 |
| r1c4 | Chr01 | 3,045,221 | 3,089,299 | 44,079 |
| Chr01G0870.1 | Chr01 | 3,048,453 | 3,050,314 | 1,862 |
| Chr01G0871.1 | Chr01 | 3,050,854 | 3,052,878 | 2,025 |
| Chr01G0872.1 | Chr01 | 3,053,419 | 3,054,375 | 957 |
| Chr01G0873.1 | Chr01 | 3,054,767 | 3,056,594 | 1,828 |
| Chr01G0874.1 | Chr01 | 3,057,331 | 3,059,863 | 2,533 |
| Chr01G0875.1 | Chr01 | 3,060,284 | 3,062,961 | 2,678 |
| Chr01G0876.1 | Chr01 | 3,062,398 | 3,071,031 | 8,634 |
| Chr01G0877.1 | Chr01 | 3,072,056 | 3,072,823 | 768 |
| Chr01G0878.1 | Chr01 | 3,073,297 | 3,075,072 | 1,776 |
| Chr01G0879.1 | Chr01 | 3,076,893 | 3,078,482 | 1,590 |
| Chr01G0880.1 | Chr01 | 3,079,260 | 3,084,057 | 4,798 |
| r1c5 | Chr01 | 4,020,441 | 4,061,888 | 41,448 |

| | | | | |
|---|---|---|---|---|
| Chr01G1155.1 | Chr01 | 4,022,339 | 4,023,799 | 1,461 |
| Chr01G1156.1 | Chr01 | 4,024,588 | 4,025,957 | 1,370 |
| Chr01G1157.1 | Chr01 | 4,026,704 | 4,026,955 | 252 |
| Chr01G1158.1 | Chr01 | 4,027,011 | 4,027,495 | 485 |
| Chr01G1159.1 | Chr01 | 4,028,299 | 4,032,862 | 4,564 |
| Chr01G1160.1 | Chr01 | 4,032,840 | 4,036,138 | 3,299 |
| Chr01G1161.1 | Chr01 | 4,036,123 | 4,038,527 | 2,405 |
| Chr01G1162.1 | Chr01 | 4,038,338 | 4,038,730 | 393 |
| Chr01G1163.1 | Chr01 | 4,039,496 | 4,042,139 | 2,644 |
| Chr01G1164.1 | Chr01 | 4,043,486 | 4,045,727 | 2,242 |
| Chr01G1165.1 | Chr01 | 4,045,770 | 4,047,214 | 1,445 |
| Chr01G1166.1 | Chr01 | 4,046,490 | 4,048,369 | 1,880 |
| Chr01G1167.1 | Chr01 | 4,048,854 | 4,049,985 | 1,132 |
| Chr01G1168.1 | Chr01 | 4,050,337 | 4,051,332 | 996 |
| Chr01G1169.1 | Chr01 | 4,052,049 | 4,054,372 | 2,324 |
| Chr01G1170.1 | Chr01 | 4,054,737 | 4,059,255 | 4,519 |
| r1c6 | Chr01 | 5,370,974 | 5,419,397 | 48,424 |
| Chr01G1506.1 | Chr01 | 5,372,257 | 5,376,987 | 4,731 |
| Chr01G1507.1 | Chr01 | 5,372,617 | 5,372,979 | 363 |
| Chr01G1508.1 | Chr01 | 5,378,214 | 5,379,183 | 970 |
| Chr01G1509.1 | Chr01 | 5,380,648 | 5,382,873 | 2,226 |
| Chr01G1510.1 | Chr01 | 5381998 | 5385187 | 3190 |
| Chr01G1511.1 | Chr01 | 5387505 | 5390098 | 2594 |
| Chr01G1512.1 | Chr01 | 5390795 | 5399965 | 9171 |
| Chr01G1513.1 | Chr01 | 5407037 | 5409519 | 2483 |
| Chr01G1514.1 | Chr01 | 5410907 | 5412343 | 1437 |
| r1c7 | Chr01 | 5494742 | 5561984 | 67243 |
| Chr01G1531.1 | Chr01 | 5498016 | 5500893 | 2878 |
| Chr01G1532.1 | Chr01 | 5502541 | 5504681 | 2141 |
| Chr01G1533.1 | Chr01 | 5506066 | 5507645 | 1580 |
| Chr01G1534.1 | Chr01 | 5507938 | 5508708 | 771 |
| Chr01G1535.1 | Chr01 | 5509209 | 5510468 | 1260 |
| Chr01G1536.1 | Chr01 | 5510817 | 5512096 | 1280 |
| Chr01G1537.1 | Chr01 | 5512779 | 5513783 | 1005 |
| Chr01G1538.1 | Chr01 | 5514741 | 5521425 | 6685 |
| Chr01G1539.1 | Chr01 | 5522187 | 5522786 | 600 |
| Chr01G1540.1 | Chr01 | 5524967 | 5527766 | 2800 |
| Chr01G1541.1 | Chr01 | 5529842 | 5530063 | 222 |
| Chr01G1542.1 | Chr01 | 5530409 | 5533467 | 3059 |
| Chr01G1543.1 | Chr01 | 5534413 | 5541983 | 7571 |
| Chr01G1544.1 | Chr01 | 5543024 | 5544490 | 1467 |
| Chr01G1545.1 | Chr01 | 5549298 | 5552948 | 3651 |
| Chr01G1546.1 | Chr01 | 5554802 | 5557127 | 2326 |
| Chr01G1547.1 | Chr01 | 5557188 | 5561031 | 3844 |

| | | | | |
|---|---|---|---|---|
| r1c9 | Chr01 | 6524357 | 6539516 | 15160 |
| Chr01G1845.1 | Chr01 | 6526813 | 6529020 | 2208 |
| Chr01G1846.1 | Chr01 | 6529470 | 6531555 | 2086 |
| Chr01G1847.1 | Chr01 | 6532563 | 6534838 | 2276 |
| r1c10 | Chr01 | 6638419 | 6678246 | 39828 |
| Chr01G1876.1 | Chr01 | 6639387 | 6640651 | 1265 |
| Chr01G1877.1 | Chr01 | 6641797 | 6643032 | 1236 |
| Chr01G1878.1 | Chr01 | 6643207 | 6644192 | 986 |
| Chr01G1879.1 | Chr01 | 6643687 | 6644599 | 913 |
| Chr01G1880.1 | Chr01 | 6644749 | 6648647 | 3899 |
| Chr01G1881.1 | Chr01 | 6647625 | 6648474 | 850 |
| Chr01G1882.1 | Chr01 | 6648916 | 6654149 | 5234 |
| Chr01G1883.1 | Chr01 | 6649258 | 6651161 | 1904 |
| Chr01G1884.1 | Chr01 | 6654869 | 6659000 | 4132 |
| Chr01G1885.1 | Chr01 | 6660243 | 6662650 | 2408 |
| Chr01G1886.1 | Chr01 | 6663617 | 6666196 | 2580 |
| Chr01G1887.1 | Chr01 | 6667751 | 6669513 | 1763 |
| Chr01G1888.1 | Chr01 | 6669505 | 6674995 | 5491 |
| r1c11 | Chr01 | 6772053 | 6792944 | 20892 |
| Chr01G1922.1 | Chr01 | 6772424 | 6773251 | 828 |
| Chr01G1923.1 | Chr01 | 6774213 | 6775322 | 1110 |
| Chr01G1924.1 | Chr01 | 6775558 | 6776154 | 597 |
| Chr01G1925.1 | Chr01 | 6776721 | 6778808 | 2088 |
| Chr01G1926.1 | Chr01 | 6781952 | 6782943 | 992 |
| Chr01G1927.1 | Chr01 | 6784237 | 6785149 | 913 |
| Chr01G1928.1 | Chr01 | 6785445 | 6786470 | 1026 |
| r1c12 | Chr01 | 7327542 | 7349068 | 21527 |
| Chr01G2069.1 | Chr01 | 7328393 | 7329355 | 963 |
| Chr01G2070.1 | Chr01 | 7329445 | 7332236 | 2792 |
| Chr01G2071.1 | Chr01 | 7333493 | 7335806 | 2314 |
| Chr01G2072.1 | Chr01 | 7337541 | 7339067 | 1527 |
| Chr01G2073.1 | Chr01 | 7342443 | 7343693 | 1251 |
| Chr01G2074.1 | Chr01 | 7343802 | 7345699 | 1898 |
| r1c13 | Chr01 | 9249692 | 9293174 | 43483 |
| Chr01G2589.1 | Chr01 | 9252529 | 9253446 | 918 |
| Chr01G2590.1 | Chr01 | 9254907 | 9256910 | 2004 |
| Chr01G2591.1 | Chr01 | 9257418 | 9265980 | 8563 |
| Chr01G2592.1 | Chr01 | 9259351 | 9265980 | 6630 |
| Chr01G2593.1 | Chr01 | 9268349 | 9269134 | 786 |
| Chr01G2594.1 | Chr01 | 9269389 | 9273449 | 4061 |
| Chr01G2595.1 | Chr01 | 9274139 | 9276051 | 1913 |
| Chr01G2596.1 | Chr01 | 9274300 | 9280192 | 5893 |
| Chr01G2597.1 | Chr01 | 9280408 | 9282054 | 1647 |
| Chr01G2598.1 | Chr01 | 9282638 | 9283738 | 1101 |

| | | | | |
|---|---|---|---|---|
| Chr01G2599.1 | Chr01 | 9284245 | 9285914 | 1670 |
| Chr01G2600.1 | Chr01 | 9286223 | 9287864 | 1642 |
| Chr01G2601.1 | Chr01 | 9287957 | 9290918 | 2962 |
| r14c1 | Chr02 | 431027 | 448500 | 17474 |
| Chr02G0107.1 | Chr02 | 431027 | 431959 | 933 |
| Chr02G0108.1 | Chr02 | 432991 | 433629 | 639 |
| Chr02G0109.1 | Chr02 | 434288 | 436568 | 2281 |
| Chr02G0110.1 | Chr02 | 437707 | 439194 | 1488 |
| Chr02G0111.1 | Chr02 | 440183 | 442667 | 2485 |
| Chr02G0112.1 | Chr02 | 443162 | 446521 | 3360 |
| Chr02G0113.1 | Chr02 | 447070 | 448500 | 1431 |
| r14c2 | Chr02 | 1561067 | 1582447 | 21381 |
| Chr02G0420.1 | Chr02 | 1562707 | 1564016 | 1310 |
| Chr02G0421.1 | Chr02 | 1564689 | 1565661 | 973 |
| Chr02G0422.1 | Chr02 | 1566094 | 1566834 | 741 |
| Chr02G0423.1 | Chr02 | 1567880 | 1569808 | 1929 |
| Chr02G0424.1 | Chr02 | 1571067 | 1572447 | 1381 |
| Chr02G0425.1 | Chr02 | 1574225 | 1574908 | 684 |
| Chr02G0426.1 | Chr02 | 1575699 | 1576662 | 964 |
| Chr02G0427.1 | Chr02 | 1579521 | 1580585 | 1065 |
| r14c3 | Chr02 | 2428972 | 2474860 | 45889 |
| Chr02G0655.1 | Chr02 | 2429172 | 2431613 | 2442 |
| Chr02G0656.1 | Chr02 | 2432787 | 2438331 | 5545 |
| Chr02G0657.1 | Chr02 | 2436194 | 2438044 | 1851 |
| Chr02G0658.1 | Chr02 | 2438593 | 2441176 | 2584 |
| Chr02G0659.1 | Chr02 | 2441238 | 2443711 | 2474 |
| Chr02G0660.1 | Chr02 | 2443793 | 2446448 | 2656 |
| Chr02G0661.1 | Chr02 | 2446928 | 2448287 | 1360 |
| Chr02G0662.1 | Chr02 | 2448799 | 2455636 | 6838 |
| Chr02G0663.1 | Chr02 | 2457046 | 2457900 | 855 |
| Chr02G0664.1 | Chr02 | 2459264 | 2461456 | 2193 |
| Chr02G0665.1 | Chr02 | 2462341 | 2463261 | 921 |
| Chr02G0666.1 | Chr02 | 2463554 | 2465304 | 1751 |
| Chr02G0667.1 | Chr02 | 2467725 | 2469275 | 1551 |
| Chr02G0668.1 | Chr02 | 2469579 | 2471781 | 2203 |
| r4c1 | Chr02 | 4187368 | 4230766 | 43399 |
| Chr02G1109.1 | Chr02 | 4192861 | 4198705 | 5845 |
| Chr02G1110.1 | Chr02 | 4203945 | 4204970 | 1026 |
| Chr02G1111.1 | Chr02 | 4207368 | 4210766 | 3399 |
| Chr02G1112.1 | Chr02 | 4212067 | 4213404 | 1338 |
| Chr02G1113.1 | Chr02 | 4213605 | 4216086 | 2482 |
| Chr02G1114.1 | Chr02 | 4216105 | 4217778 | 1674 |
| Chr02G1115.1 | Chr02 | 4217212 | 4218530 | 1319 |
| Chr02G1116.1 | Chr02 | 4218961 | 4222740 | 3780 |

| | | | | |
|---|---|---|---|---|
| Chr02G1117.1 | Chr02 | 4223501 | 4226027 | 2527 |
| Chr02G1118.1 | Chr02 | 4226403 | 4228220 | 1818 |
| r4c2 | Chr02 | 4286899 | 4334813 | 47915 |
| Chr02G1130.1 | Chr02 | 4289584 | 4292310 | 2727 |
| Chr02G1131.1 | Chr02 | 4294158 | 4295597 | 1440 |
| Chr02G1132.1 | Chr02 | 4297583 | 4299394 | 1812 |
| Chr02G1133.1 | Chr02 | 4300433 | 4302228 | 1796 |
| Chr02G1134.1 | Chr02 | 4305194 | 4306143 | 950 |
| Chr02G1135.1 | Chr02 | 4306899 | 4314813 | 7915 |
| Chr02G1136.1 | Chr02 | 4316051 | 4317727 | 1677 |
| Chr02G1137.1 | Chr02 | 4318712 | 4320416 | 1705 |
| Chr02G1138.1 | Chr02 | 4321186 | 4322808 | 1623 |
| Chr02G1139.1 | Chr02 | 4321353 | 4326074 | 4722 |
| Chr02G1140.1 | Chr02 | 4326520 | 4327550 | 1031 |
| Chr02G1141.1 | Chr02 | 4327951 | 4329279 | 1329 |
| Chr02G1142.1 | Chr02 | 4329971 | 4334301 | 4331 |
| r7c1 | Chr03 | 1122781 | 1219060 | 96280 |
| Chr03G0290.1 | Chr03 | 1124265 | 1125583 | 1319 |
| Chr03G0291.1 | Chr03 | 1126122 | 1126677 | 556 |
| Chr03G0292.1 | Chr03 | 1127123 | 1128073 | 951 |
| Chr03G0293.1 | Chr03 | 1128507 | 1129491 | 985 |
| Chr03G0294.1 | Chr03 | 1130391 | 1131128 | 738 |
| Chr03G0295.1 | Chr03 | 1132781 | 1134085 | 1305 |
| Chr03G0296.1 | Chr03 | 1134576 | 1135951 | 1376 |
| Chr03G0297.1 | Chr03 | 1136398 | 1137824 | 1427 |
| Chr03G0298.1 | Chr03 | 1140193 | 1142125 | 1933 |
| Chr03G0299.1 | Chr03 | 1143146 | 1145624 | 2479 |
| Chr03G0300.1 | Chr03 | 1145690 | 1147083 | 1394 |
| Chr03G0301.1 | Chr03 | 1147819 | 1153471 | 5653 |
| Chr03G0302.1 | Chr03 | 1153674 | 1155898 | 2225 |
| Chr03G0303.1 | Chr03 | 1156899 | 1195714 | 38816 |
| Chr03G0304.1 | Chr03 | 1197169 | 1205879 | 8711 |
| Chr03G0305.1 | Chr03 | 1206636 | 1209808 | 3173 |
| Chr03G0306.1 | Chr03 | 1209940 | 1210799 | 860 |
| Chr03G0307.1 | Chr03 | 1211185 | 1212337 | 1153 |
| Chr03G0308.1 | Chr03 | 1212606 | 1214522 | 1917 |
| Chr03G0309.1 | Chr03 | 1214838 | 1215717 | 880 |
| r7c2 | Chr03 | 3989927 | 4058906 | 68980 |
| Chr03G1039.1 | Chr03 | 3991902 | 3993559 | 1658 |
| Chr03G1040.1 | Chr03 | 3994156 | 3996204 | 2049 |
| Chr03G1041.1 | Chr03 | 3997198 | 3998446 | 1249 |
| Chr03G1042.1 | Chr03 | 3998570 | 4003096 | 4527 |
| Chr03G1043.1 | Chr03 | 4009463 | 4017408 | 7946 |
| Chr03G1044.1 | Chr03 | 4017897 | 4018232 | 336 |

| | | | | |
|---|---|---|---|---|
| Chr03G1045.1 | Chr03 | 4021768 | 4023411 | 1644 |
| Chr03G1046.1 | Chr03 | 4023874 | 4024311 | 438 |
| Chr03G1047.1 | Chr03 | 4024422 | 4025135 | 714 |
| Chr03G1048.1 | Chr03 | 4026142 | 4027663 | 1522 |
| Chr03G1049.1 | Chr03 | 4028326 | 4029514 | 1189 |
| Chr03G1050.1 | Chr03 | 4032552 | 4034634 | 2083 |
| Chr03G1051.1 | Chr03 | 4035189 | 4036921 | 1733 |
| Chr03G1052.1 | Chr03 | 4037650 | 4044170 | 6521 |
| Chr03G1053.1 | Chr03 | 4044905 | 4046169 | 1265 |
| Chr03G1054.1 | Chr03 | 4046754 | 4049834 | 3081 |
| Chr03G1055.1 | Chr03 | 4051040 | 4053001 | 1962 |
| Chr03G1056.1 | Chr03 | 4054462 | 4055520 | 1059 |
| r7c3 | Chr03 | 5356320 | 5404443 | 48124 |
| Chr03G1385.1 | Chr03 | 5356863 | 5358615 | 1753 |
| Chr03G1386.1 | Chr03 | 5360598 | 5369816 | 9219 |
| Chr03G1387.1 | Chr03 | 5373472 | 5375201 | 1730 |
| Chr03G1388.1 | Chr03 | 5376320 | 5384443 | 8124 |
| Chr03G1389.1 | Chr03 | 5384918 | 5385955 | 1038 |
| Chr03G1390.1 | Chr03 | 5386452 | 5387483 | 1032 |
| Chr03G1391.1 | Chr03 | 5387771 | 5388748 | 978 |
| Chr03G1392.1 | Chr03 | 5388998 | 5390616 | 1619 |
| Chr03G1393.1 | Chr03 | 5391358 | 5392990 | 1633 |
| Chr03G1394.1 | Chr03 | 5393385 | 5394564 | 1180 |
| Chr03G1395.1 | Chr03 | 5396355 | 5400153 | 3799 |
| Chr03G1396.1 | Chr03 | 5400670 | 5404046 | 3377 |
| r7c4 | Chr03 | 6059069 | 6102524 | 43456 |
| Chr03G1589.1 | Chr03 | 6060900 | 6065727 | 4828 |
| Chr03G1590.1 | Chr03 | 6063887 | 6065416 | 1530 |
| Chr03G1591.1 | Chr03 | 6066255 | 6067523 | 1269 |
| Chr03G1592.1 | Chr03 | 6068041 | 6068940 | 900 |
| Chr03G1593.1 | Chr03 | 6072590 | 6074209 | 1620 |
| Chr03G1594.1 | Chr03 | 6077752 | 6078983 | 1232 |
| Chr03G1595.1 | Chr03 | 6079069 | 6082524 | 3456 |
| Chr03G1596.1 | Chr03 | 6083080 | 6084982 | 1903 |
| Chr03G1597.1 | Chr03 | 6085003 | 6087103 | 2101 |
| Chr03G1598.1 | Chr03 | 6087399 | 6089207 | 1809 |
| Chr03G1599.1 | Chr03 | 6089744 | 6091378 | 1635 |
| Chr03G1600.1 | Chr03 | 6091594 | 6094499 | 2906 |
| Chr03G1601.1 | Chr03 | 6097747 | 6100270 | 2524 |
| Chr03G1602.1 | Chr03 | 6100276 | 6101424 | 1149 |
| r13c1 | Chr04 | 166867 | 205768 | 38902 |
| Chr04G0024.1 | Chr04 | 166867 | 167794 | 928 |
| Chr04G0025.1 | Chr04 | 168916 | 169914 | 999 |
| Chr04G0026.1 | Chr04 | 177604 | 178243 | 640 |

| | | | | |
|---|---|---|---|---|
| Chr04G0027.1 | Chr04 | 179726 | 187082 | 7357 |
| Chr04G0028.1 | Chr04 | 188192 | 189265 | 1074 |
| Chr04G0029.1 | Chr04 | 189934 | 191772 | 1839 |
| Chr04G0030.1 | Chr04 | 192168 | 194066 | 1899 |
| Chr04G0031.1 | Chr04 | 197726 | 198656 | 931 |
| Chr04G0032.1 | Chr04 | 202608 | 203266 | 659 |
| Chr04G0033.1 | Chr04 | 203918 | 205768 | 1851 |
| r13c2 | Chr04 | 809073 | 910907 | 101835 |
| Chr04G0205.1 | Chr04 | 812423 | 814498 | 2076 |
| Chr04G0206.1 | Chr04 | 814521 | 816929 | 2409 |
| Chr04G0207.1 | Chr04 | 821535 | 824490 | 2956 |
| Chr04G0208.1 | Chr04 | 826382 | 827205 | 824 |
| Chr04G0209.1 | Chr04 | 827279 | 838141 | 10863 |
| Chr04G0210.1 | Chr04 | 841808 | 844591 | 2784 |
| Chr04G0211.1 | Chr04 | 846718 | 847698 | 981 |
| Chr04G0212.1 | Chr04 | 849463 | 854793 | 5331 |
| Chr04G0213.1 | Chr04 | 855147 | 856376 | 1230 |
| Chr04G0214.1 | Chr04 | 857021 | 858212 | 1192 |
| Chr04G0215.1 | Chr04 | 858631 | 860488 | 1858 |
| Chr04G0216.1 | Chr04 | 861466 | 867670 | 6205 |
| Chr04G0217.1 | Chr04 | 868648 | 870441 | 1794 |
| Chr04G0218.1 | Chr04 | 871375 | 873100 | 1726 |
| Chr04G0219.1 | Chr04 | 873322 | 874089 | 768 |
| Chr04G0220.1 | Chr04 | 875342 | 877227 | 1886 |
| Chr04G0221.1 | Chr04 | 877732 | 878796 | 1065 |
| Chr04G0222.1 | Chr04 | 879108 | 880028 | 921 |
| Chr04G0223.1 | Chr04 | 880609 | 881301 | 693 |
| Chr04G0224.1 | Chr04 | 881687 | 882824 | 1138 |
| Chr04G0225.1 | Chr04 | 883440 | 891411 | 7972 |
| Chr04G0226.1 | Chr04 | 892107 | 893516 | 1410 |
| Chr04G0227.1 | Chr04 | 894031 | 895648 | 1618 |
| Chr04G0228.1 | Chr04 | 896584 | 897682 | 1099 |
| Chr04G0229.1 | Chr04 | 898210 | 898974 | 765 |
| Chr04G0230.1 | Chr04 | 901550 | 904640 | 3091 |
| Chr04G0231.1 | Chr04 | 907400 | 909131 | 1732 |
| Chr04G0232.1 | Chr04 | 909981 | 910907 | 927 |
| r13c3 | Chr04 | 2981764 | 3038306 | 56543 |
| Chr04G0794.1 | Chr04 | 2981764 | 2983475 | 1712 |
| Chr04G0795.1 | Chr04 | 2984931 | 2985716 | 786 |
| Chr04G0796.1 | Chr04 | 2986719 | 2988073 | 1355 |
| Chr04G0797.1 | Chr04 | 2989479 | 2991666 | 2188 |
| Chr04G0798.1 | Chr04 | 2992219 | 2994144 | 1926 |
| Chr04G0799.1 | Chr04 | 2996235 | 2997818 | 1584 |
| Chr04G0800.1 | Chr04 | 2998529 | 3006542 | 8014 |

| | | | | |
|---|---|---|---|---|
| Chr04G0801.1 | Chr04 | 3007468 | 3009255 | 1788 |
| Chr04G0802.1 | Chr04 | 3010166 | 3011075 | 910 |
| Chr04G0803.1 | Chr04 | 3011657 | 3019053 | 7397 |
| Chr04G0804.1 | Chr04 | 3019130 | 3020267 | 1138 |
| Chr04G0805.1 | Chr04 | 3020791 | 3023330 | 2540 |
| Chr04G0806.1 | Chr04 | 3023709 | 3025703 | 1995 |
| Chr04G0807.1 | Chr04 | 3026070 | 3029137 | 3068 |
| Chr04G0808.1 | Chr04 | 3029742 | 3031437 | 1696 |
| Chr04G0809.1 | Chr04 | 3031616 | 3034131 | 2516 |
| Chr04G0810.1 | Chr04 | 3034313 | 3034924 | 612 |
| Chr04G0811.1 | Chr04 | 3037095 | 3038306 | 1212 |
| r13c4 | Chr04 | 3222661 | 3265804 | 43144 |
| Chr04G0878.1 | Chr04 | 3222661 | 3223050 | 390 |
| Chr04G0879.1 | Chr04 | 3223377 | 3225716 | 2340 |
| Chr04G0880.1 | Chr04 | 3231829 | 3232797 | 969 |
| Chr04G0881.1 | Chr04 | 3234100 | 3236170 | 2071 |
| Chr04G0882.1 | Chr04 | 3236318 | 3238282 | 1965 |
| Chr04G0883.1 | Chr04 | 3239065 | 3239928 | 864 |
| Chr04G0884.1 | Chr04 | 3240219 | 3246115 | 5897 |
| Chr04G0885.1 | Chr04 | 3246292 | 3247498 | 1207 |
| Chr04G0886.1 | Chr04 | 3253023 | 3254818 | 1796 |
| Chr04G0887.1 | Chr04 | 3257686 | 3263763 | 6078 |
| r13c5 | Chr04 | 3399659 | 3444848 | 45190 |
| Chr04G0918.1 | Chr04 | 3399659 | 3400673 | 1015 |
| Chr04G0919.1 | Chr04 | 3402437 | 3402873 | 437 |
| Chr04G0920.1 | Chr04 | 3403221 | 3404201 | 981 |
| Chr04G0921.1 | Chr04 | 3406049 | 3407137 | 1089 |
| Chr04G0922.1 | Chr04 | 3408302 | 3409278 | 977 |
| Chr04G0923.1 | Chr04 | 3411163 | 3412275 | 1113 |
| Chr04G0924.1 | Chr04 | 3413344 | 3415017 | 1674 |
| Chr04G0925.1 | Chr04 | 3416195 | 3418124 | 1930 |
| Chr04G0926.1 | Chr04 | 3418962 | 3428241 | 9280 |
| Chr04G0927.1 | Chr04 | 3428549 | 3429075 | 527 |
| Chr04G0928.1 | Chr04 | 3431156 | 3433112 | 1957 |
| Chr04G0929.1 | Chr04 | 3433943 | 3434519 | 577 |
| Chr04G0930.1 | Chr04 | 3434902 | 3438604 | 3703 |
| Chr04G0931.1 | Chr04 | 3441525 | 3443504 | 1980 |
| Chr04G0932.1 | Chr04 | 3444443 | 3444848 | 406 |
| r13c6 | Chr04 | 3587560 | 3658798 | 71239 |
| Chr04G0970.1 | Chr04 | 3598245 | 3599830 | 1586 |
| Chr04G0971.1 | Chr04 | 3600015 | 3602952 | 2938 |
| Chr04G0972.1 | Chr04 | 3604916 | 3606189 | 1274 |
| Chr04G0973.1 | Chr04 | 3606372 | 3614094 | 7723 |
| Chr04G0974.1 | Chr04 | 3615570 | 3617952 | 2383 |

| | | | | |
|---|---|---|---|---|
| Chr04G0975.1 | Chr04 | 3619232 | 3622554 | 3323 |
| Chr04G0976.1 | Chr04 | 3623400 | 3624517 | 1118 |
| Chr04G0977.1 | Chr04 | 3624839 | 3631569 | 6731 |
| Chr04G0978.1 | Chr04 | 3631694 | 3641484 | 9791 |
| Chr04G0979.1 | Chr04 | 3642244 | 3643472 | 1229 |
| Chr04G0980.1 | Chr04 | 3647059 | 3648145 | 1087 |
| Chr04G0981.1 | Chr04 | 3650651 | 3652492 | 1842 |
| Chr04G0982.1 | Chr04 | 3654750 | 3656075 | 1326 |
| Chr04G0983.1 | Chr04 | 3658262 | 3658798 | 537 |
| r13c7 | Chr04 | 4032266 | 4074579 | 42314 |
| Chr04G1074.1 | Chr04 | 4034008 | 4041835 | 7828 |
| Chr04G1075.1 | Chr04 | 4047723 | 4055850 | 8128 |
| Chr04G1076.1 | Chr04 | 4056791 | 4057522 | 732 |
| Chr04G1077.1 | Chr04 | 4058224 | 4059531 | 1308 |
| Chr04G1078.1 | Chr04 | 4060146 | 4061839 | 1694 |
| Chr04G1079.1 | Chr04 | 4061816 | 4062861 | 1046 |
| Chr04G1080.1 | Chr04 | 4063721 | 4065322 | 1602 |
| Chr04G1081.1 | Chr04 | 4066213 | 4068342 | 2130 |
| Chr04G1082.1 | Chr04 | 4068984 | 4070830 | 1847 |
| Chr04G1083.1 | Chr04 | 4071820 | 4074935 | 3116 |
| r13c8 | Chr04 | 4673811 | 4716875 | 43065 |
| Chr04G1259.1 | Chr04 | 4673811 | 4676117 | 2307 |
| Chr04G1260.1 | Chr04 | 4678445 | 4680173 | 1729 |
| Chr04G1261.1 | Chr04 | 4680639 | 4681981 | 1343 |
| Chr04G1262.1 | Chr04 | 4684269 | 4685599 | 1331 |
| Chr04G1263.1 | Chr04 | 4685990 | 4687862 | 1873 |
| Chr04G1264.1 | Chr04 | 4688682 | 4689874 | 1193 |
| Chr04G1265.1 | Chr04 | 4690360 | 4691053 | 694 |
| Chr04G1266.1 | Chr04 | 4693084 | 4700972 | 7889 |
| Chr04G1267.1 | Chr04 | 4702381 | 4703896 | 1516 |
| Chr04G1268.1 | Chr04 | 4704445 | 4705742 | 1298 |
| Chr04G1269.1 | Chr04 | 4705932 | 4706528 | 597 |
| Chr04G1270.1 | Chr04 | 4708324 | 4709382 | 1059 |
| Chr04G1271.1 | Chr04 | 4709926 | 4711650 | 1725 |
| Chr04G1272.1 | Chr04 | 4711637 | 4715088 | 3452 |
| r13c9 | Chr04 | 4731587 | 4782004 | 50418 |
| Chr04G1274.1 | Chr04 | 4734696 | 4738198 | 3503 |
| Chr04G1275.1 | Chr04 | 4738362 | 4742319 | 3958 |
| Chr04G1276.1 | Chr04 | 4744487 | 4746691 | 2205 |
| Chr04G1277.1 | Chr04 | 4748781 | 4751385 | 2605 |
| Chr04G1278.1 | Chr04 | 4751587 | 4762004 | 10418 |
| Chr04G1279.1 | Chr04 | 4763987 | 4765750 | 1764 |
| Chr04G1280.1 | Chr04 | 4766236 | 4767432 | 1197 |
| Chr04G1281.1 | Chr04 | 4769811 | 4772194 | 2384 |

| | | | | |
|---|---|---|---|---|
| Chr04G1282.1 | Chr04 | 4773814 | 4775315 | 1502 |
| Chr04G1283.1 | Chr04 | 4775647 | 4776215 | 569 |
| Chr04G1284.1 | Chr04 | 4776486 | 4777019 | 534 |
| Chr04G1285.1 | Chr04 | 4777702 | 4778481 | 780 |
| Chr04G1286.1 | Chr04 | 4778641 | 4781489 | 2849 |
| r13c10 | Chr04 | 5245012 | 5266363 | 21352 |
| Chr04G1391.1 | Chr04 | 5248223 | 5250693 | 2471 |
| Chr04G1392.1 | Chr04 | 5251244 | 5252268 | 1025 |
| Chr04G1393.1 | Chr04 | 5253004 | 5254352 | 1349 |
| Chr04G1394.1 | Chr04 | 5255012 | 5256363 | 1352 |
| Chr04G1395.1 | Chr04 | 5257696 | 5259406 | 1711 |
| Chr04G1396.1 | Chr04 | 5259659 | 5260888 | 1230 |
| Chr04G1397.1 | Chr04 | 5262327 | 5262871 | 545 |
| Chr04G1398.1 | Chr04 | 5263079 | 5265412 | 2334 |
| r13c11 | Chr04 | 5544231 | 5591605 | 47375 |
| Chr04G1488.1 | Chr04 | 5548835 | 5550812 | 1978 |
| Chr04G1489.1 | Chr04 | 5551015 | 5552067 | 1053 |
| Chr04G1490.1 | Chr04 | 5552515 | 5555394 | 2880 |
| Chr04G1491.1 | Chr04 | 5555730 | 5558109 | 2380 |
| Chr04G1492.1 | Chr04 | 5558691 | 5560052 | 1362 |
| Chr04G1493.1 | Chr04 | 5560322 | 5562482 | 2161 |
| Chr04G1494.1 | Chr04 | 5562576 | 5563598 | 1023 |
| Chr04G1495.1 | Chr04 | 5564100 | 5571760 | 7661 |
| Chr04G1496.1 | Chr04 | 5575377 | 5577338 | 1962 |
| Chr04G1497.1 | Chr04 | 5578454 | 5579258 | 805 |
| Chr04G1498.1 | Chr04 | 5580187 | 5581272 | 1086 |
| Chr04G1499.1 | Chr04 | 5581811 | 5583384 | 1574 |
| Chr04G1500.1 | Chr04 | 5588308 | 5589636 | 1329 |
| r2c3 | Chr05 | 180680 | 202109 | 21430 |
| Chr05G0020.1 | Chr05 | 180700 | 182516 | 1817 |
| Chr05G0021.1 | Chr05 | 183949 | 187525 | 3577 |
| Chr05G0022.1 | Chr05 | 188736 | 190405 | 1670 |
| Chr05G0023.1 | Chr05 | 190682 | 192111 | 1430 |
| Chr05G0024.1 | Chr05 | 192814 | 195711 | 2898 |
| Chr05G0025.1 | Chr05 | 196560 | 201719 | 5160 |
| r2c2 | Chr05 | 211640 | 232813 | 21174 |
| Chr05G0031.1 | Chr05 | 212546 | 215034 | 2489 |
| Chr05G0032.1 | Chr05 | 215662 | 216757 | 1096 |
| Chr05G0033.1 | Chr05 | 217094 | 218288 | 1195 |
| Chr05G0034.1 | Chr05 | 218635 | 220507 | 1873 |
| Chr05G0035.1 | Chr05 | 221642 | 222815 | 1174 |
| Chr05G0036.1 | Chr05 | 223694 | 225508 | 1815 |
| Chr05G0037.1 | Chr05 | 225709 | 227584 | 1876 |
| Chr05G0038.1 | Chr05 | 228570 | 230251 | 1682 |

| | | | | |
|---|---|---|---|---|
| r2c1 | Chr05 | 694065 | 753647 | 59583 |
| Chr05G0171.1 | Chr05 | 694450 | 696035 | 1586 |
| Chr05G0172.1 | Chr05 | 696899 | 698756 | 1858 |
| Chr05G0173.1 | Chr05 | 699771 | 701725 | 1955 |
| Chr05G0174.1 | Chr05 | 702121 | 704568 | 2448 |
| Chr05G0175.1 | Chr05 | 704998 | 707270 | 2273 |
| Chr05G0176.1 | Chr05 | 707459 | 708347 | 889 |
| Chr05G0177.1 | Chr05 | 710158 | 711165 | 1008 |
| Chr05G0178.1 | Chr05 | 714067 | 722322 | 8256 |
| Chr05G0179.1 | Chr05 | 722870 | 723931 | 1062 |
| Chr05G0180.1 | Chr05 | 724465 | 726174 | 1710 |
| Chr05G0181.1 | Chr05 | 726571 | 733649 | 7079 |
| Chr05G0182.1 | Chr05 | 734041 | 734926 | 886 |
| Chr05G0183.1 | Chr05 | 735398 | 737493 | 2096 |
| Chr05G0184.1 | Chr05 | 738208 | 738936 | 729 |
| Chr05G0185.1 | Chr05 | 739095 | 740881 | 1787 |
| Chr05G0186.1 | Chr05 | 741144 | 742800 | 1657 |
| Chr05G0187.1 | Chr05 | 748394 | 750563 | 2170 |
| Chr05G0188.1 | Chr05 | 750656 | 752331 | 1676 |
| r12c1 | Chr06 | 847933 | 892534 | 44602 |
| Chr06G0209.1 | Chr06 | 848301 | 849452 | 1152 |
| Chr06G0210.1 | Chr06 | 849932 | 852354 | 2423 |
| Chr06G0211.1 | Chr06 | 850914 | 852245 | 1332 |
| Chr06G0212.1 | Chr06 | 853083 | 854412 | 1330 |
| Chr06G0213.1 | Chr06 | 855292 | 856092 | 801 |
| Chr06G0214.1 | Chr06 | 857700 | 858975 | 1276 |
| Chr06G0215.1 | Chr06 | 859196 | 862740 | 3545 |
| Chr06G0216.1 | Chr06 | 863432 | 863734 | 303 |
| Chr06G0217.1 | Chr06 | 863940 | 867168 | 3229 |
| Chr06G0218.1 | Chr06 | 867933 | 872534 | 4602 |
| Chr06G0219.1 | Chr06 | 874354 | 875247 | 894 |
| Chr06G0220.1 | Chr06 | 877879 | 879865 | 1987 |
| Chr06G0221.1 | Chr06 | 882031 | 883634 | 1604 |
| Chr06G0222.1 | Chr06 | 886948 | 888666 | 1719 |
| Chr06G0223.1 | Chr06 | 888989 | 890044 | 1056 |
| r12c2 | Chr06 | 3819576 | 3863415 | 43840 |
| Chr06G1017.1 | Chr06 | 3820566 | 3821114 | 549 |
| Chr06G1018.1 | Chr06 | 3822078 | 3822511 | 434 |
| Chr06G1019.1 | Chr06 | 3826060 | 3829450 | 3391 |
| Chr06G1020.1 | Chr06 | 3831250 | 3832542 | 1293 |
| Chr06G1021.1 | Chr06 | 3832851 | 3834279 | 1429 |
| Chr06G1022.1 | Chr06 | 3834835 | 3836541 | 1707 |
| Chr06G1023.1 | Chr06 | 3837006 | 3838813 | 1808 |
| Chr06G1024.1 | Chr06 | 3839304 | 3843646 | 4343 |

| | | | | |
|---|---|---|---|---|
| Chr06G1025.1 | Chr06 | 3845097 | 3849603 | 4507 |
| Chr06G1026.1 | Chr06 | 3847711 | 3848119 | 409 |
| Chr06G1027.1 | Chr06 | 3849899 | 3850793 | 895 |
| Chr06G1028.1 | Chr06 | 3852221 | 3853045 | 825 |
| Chr06G1029.1 | Chr06 | 3853449 | 3857445 | 3997 |
| Chr06G1030.1 | Chr06 | 3860392 | 3862724 | 2333 |
| r12c3 | Chr06 | 5138183 | 5160708 | 22526 |
| Chr06G1397.1 | Chr06 | 5143247 | 5147226 | 3980 |
| Chr06G1398.1 | Chr06 | 5148183 | 5150708 | 2526 |
| Chr06G1399.1 | Chr06 | 5151277 | 5153556 | 2280 |
| Chr06G1400.1 | Chr06 | 5155874 | 5158099 | 2226 |
| Chr06G1401.1 | Chr06 | 5158615 | 5160384 | 1770 |
| r12c4 | Chr06 | 5352418 | 5409299 | 56882 |
| Chr06G1463.1 | Chr06 | 5354858 | 5355975 | 1118 |
| Chr06G1464.1 | Chr06 | 5357176 | 5357863 | 688 |
| Chr06G1465.1 | Chr06 | 5359070 | 5359836 | 767 |
| Chr06G1466.1 | Chr06 | 5361216 | 5365780 | 4565 |
| Chr06G1467.1 | Chr06 | 5366356 | 5367061 | 706 |
| Chr06G1468.1 | Chr06 | 5367132 | 5368893 | 1762 |
| Chr06G1469.1 | Chr06 | 5370100 | 5371245 | 1146 |
| Chr06G1470.1 | Chr06 | 5372418 | 5376374 | 3957 |
| Chr06G1471.1 | Chr06 | 5377631 | 5378716 | 1086 |
| Chr06G1472.1 | Chr06 | 5379338 | 5380312 | 975 |
| Chr06G1473.1 | Chr06 | 5380655 | 5389299 | 8645 |
| Chr06G1474.1 | Chr06 | 5389588 | 5390402 | 815 |
| Chr06G1475.1 | Chr06 | 5392324 | 5392747 | 424 |
| Chr06G1476.1 | Chr06 | 5398099 | 5398404 | 306 |
| Chr06G1477.1 | Chr06 | 5399252 | 5400758 | 1507 |
| Chr06G1478.1 | Chr06 | 5402532 | 5403410 | 879 |
| Chr06G1479.1 | Chr06 | 5403551 | 5406728 | 3178 |
| Chr06G1480.1 | Chr06 | 5406964 | 5408969 | 2006 |
| r3c1 | Chr07 | 390263 | 441421 | 51159 |
| Chr07G0086.1 | Chr07 | 395002 | 395870 | 869 |
| Chr07G0087.1 | Chr07 | 396080 | 397666 | 1587 |
| Chr07G0088.1 | Chr07 | 398181 | 400499 | 2319 |
| Chr07G0089.1 | Chr07 | 401015 | 403614 | 2600 |
| Chr07G0090.1 | Chr07 | 406156 | 407342 | 1187 |
| Chr07G0091.1 | Chr07 | 407783 | 422738 | 14956 |
| Chr07G0092.1 | Chr07 | 423471 | 425457 | 1987 |
| Chr07G0093.1 | Chr07 | 425914 | 426882 | 969 |
| Chr07G0094.1 | Chr07 | 427158 | 428493 | 1336 |
| Chr07G0095.1 | Chr07 | 428940 | 429900 | 961 |
| Chr07G0096.1 | Chr07 | 430177 | 435165 | 4989 |
| Chr07G0097.1 | Chr07 | 435617 | 439392 | 3776 |

| | | | | |
|---|---|---|---|---|
| Chr07G0098.1 | Chr07 | 439543 | 441421 | 1879 |
| r3c2 | Chr07 | 477792 | 539051 | 61260 |
| Chr07G0111.1 | Chr07 | 477792 | 479073 | 1282 |
| Chr07G0112.1 | Chr07 | 479803 | 481437 | 1635 |
| Chr07G0113.1 | Chr07 | 481812 | 483966 | 2155 |
| Chr07G0114.1 | Chr07 | 485551 | 487551 | 2001 |
| Chr07G0115.1 | Chr07 | 489472 | 490144 | 673 |
| Chr07G0116.1 | Chr07 | 493056 | 495079 | 2024 |
| Chr07G0117.1 | Chr07 | 495525 | 496523 | 999 |
| Chr07G0118.1 | Chr07 | 497745 | 505158 | 7414 |
| Chr07G0119.1 | Chr07 | 505975 | 506988 | 1014 |
| Chr07G0120.1 | Chr07 | 507265 | 508062 | 798 |
| Chr07G0121.1 | Chr07 | 508744 | 509526 | 783 |
| Chr07G0122.1 | Chr07 | 509834 | 511516 | 1683 |
| Chr07G0123.1 | Chr07 | 512760 | 519261 | 6502 |
| Chr07G0124.1 | Chr07 | 519851 | 520779 | 929 |
| Chr07G0125.1 | Chr07 | 521073 | 523522 | 2450 |
| Chr07G0126.1 | Chr07 | 523942 | 526291 | 2350 |
| Chr07G0127.1 | Chr07 | 528032 | 529728 | 1697 |
| Chr07G0128.1 | Chr07 | 530745 | 531738 | 994 |
| Chr07G0129.1 | Chr07 | 531741 | 535023 | 3283 |
| Chr07G0130.1 | Chr07 | 536950 | 537473 | 524 |
| Chr07G0131.1 | Chr07 | 537858 | 539051 | 1194 |
| r3c3 | Chr07 | 912408 | 928545 | 16138 |
| Chr07G0235.1 | Chr07 | 912408 | 913035 | 628 |
| Chr07G0236.1 | Chr07 | 916237 | 918066 | 1830 |
| Chr07G0237.1 | Chr07 | 918207 | 919816 | 1610 |
| Chr07G0238.1 | Chr07 | 920156 | 921585 | 1430 |
| Chr07G0239.1 | Chr07 | 922374 | 923935 | 1562 |
| r3c4 | Chr07 | 1290418 | 1311812 | 21395 |
| Chr07G0346.1 | Chr07 | 1292880 | 1294214 | 1335 |
| Chr07G0347.1 | Chr07 | 1295621 | 1296334 | 714 |
| Chr07G0348.1 | Chr07 | 1296570 | 1297243 | 674 |
| Chr07G0349.1 | Chr07 | 1299051 | 1299971 | 921 |
| Chr07G0350.1 | Chr07 | 1300418 | 1301812 | 1395 |
| Chr07G0351.1 | Chr07 | 1305131 | 1305755 | 625 |
| Chr07G0352.1 | Chr07 | 1306318 | 1307525 | 1208 |
| Chr07G0353.1 | Chr07 | 1307922 | 1309148 | 1227 |
| Chr07G0354.1 | Chr07 | 1309433 | 1311632 | 2200 |
| r3c5 | Chr07 | 1919022 | 1936797 | 17776 |
| Chr07G0542.1 | Chr07 | 1921094 | 1922697 | 1604 |
| Chr07G0543.1 | Chr07 | 1925251 | 1925981 | 731 |
| Chr07G0544.1 | Chr07 | 1927424 | 1928580 | 1157 |
| Chr07G0545.1 | Chr07 | 1934275 | 1936468 | 2194 |

| | | | | |
|---|---|---|---|---|
| r3c6 | Chr07 | 2420249 | 2442081 | 21833 |
| Chr07G0685.1 | Chr07 | 2420617 | 2422975 | 2359 |
| Chr07G0686.1 | Chr07 | 2427451 | 2428837 | 1387 |
| Chr07G0687.1 | Chr07 | 2430249 | 2432081 | 1833 |
| Chr07G0688.1 | Chr07 | 2432402 | 2433709 | 1308 |
| Chr07G0689.1 | Chr07 | 2434565 | 2435508 | 944 |
| r3c7 | Chr07 | 3638746 | 3690735 | 51990 |
| Chr07G0988.1 | Chr07 | 3640166 | 3643380 | 3215 |
| Chr07G0989.1 | Chr07 | 3643451 | 3645679 | 2229 |
| Chr07G0990.1 | Chr07 | 3646270 | 3648184 | 1915 |
| Chr07G0991.1 | Chr07 | 3653997 | 3656136 | 2140 |
| Chr07G0992.1 | Chr07 | 3656920 | 3658079 | 1160 |
| Chr07G0993.1 | Chr07 | 3658746 | 3670735 | 11990 |
| Chr07G0994.1 | Chr07 | 3671716 | 3674841 | 3126 |
| Chr07G0995.1 | Chr07 | 3675436 | 3681531 | 6096 |
| Chr07G0996.1 | Chr07 | 3675929 | 3677914 | 1986 |
| Chr07G0997.1 | Chr07 | 3678589 | 3679413 | 825 |
| Chr07G0998.1 | Chr07 | 3682551 | 3685966 | 3416 |
| Chr07G0999.1 | Chr07 | 3686025 | 3687677 | 1653 |
| r3c8 | Chr07 | 4094677 | 4138041 | 43365 |
| Chr07G1118.1 | Chr07 | 4097367 | 4098586 | 1220 |
| Chr07G1119.1 | Chr07 | 4099293 | 4100956 | 1664 |
| Chr07G1120.1 | Chr07 | 4101051 | 4102108 | 1058 |
| Chr07G1121.1 | Chr07 | 4102483 | 4104897 | 2415 |
| Chr07G1122.1 | Chr07 | 4105247 | 4106701 | 1455 |
| Chr07G1123.1 | Chr07 | 4107007 | 4108452 | 1446 |
| Chr07G1124.1 | Chr07 | 4108680 | 4110391 | 1712 |
| Chr07G1125.1 | Chr07 | 4110502 | 4112527 | 2026 |
| Chr07G1126.1 | Chr07 | 4113212 | 4114425 | 1214 |
| Chr07G1127.1 | Chr07 | 4114677 | 4118041 | 3365 |
| Chr07G1128.1 | Chr07 | 4121129 | 4122130 | 1002 |
| Chr07G1129.1 | Chr07 | 4122486 | 4124171 | 1686 |
| Chr07G1130.1 | Chr07 | 4124871 | 4127116 | 2246 |
| Chr07G1131.1 | Chr07 | 4128428 | 4131101 | 2674 |
| Chr07G1132.1 | Chr07 | 4134435 | 4136051 | 1617 |
| r5c1 | Chr08 | 428954 | 444803 | 15850 |
| Chr08G0109.1 | Chr08 | 428954 | 430643 | 1690 |
| Chr08G0110.1 | Chr08 | 430832 | 432750 | 1919 |
| Chr08G0111.1 | Chr08 | 433666 | 435041 | 1376 |
| Chr08G0112.1 | Chr08 | 436051 | 438412 | 2362 |
| Chr08G0113.1 | Chr08 | 439780 | 443923 | 4144 |
| Chr08G0114.1 | Chr08 | 441482 | 443288 | 1807 |
| r5c2 | Chr08 | 1382914 | 1397714 | 14801 |
| Chr08G0326.1 | Chr08 | 1382914 | 1384896 | 1983 |

| | | | | |
|---|---|---|---|---|
| Chr08G0327.1 | Chr08 | 1387451 | 1389550 | 2100 |
| Chr08G0328.1 | Chr08 | 1390818 | 1393312 | 2495 |
| Chr08G0329.1 | Chr08 | 1393451 | 1394925 | 1475 |
| r5c3 | Chr08 | 1529437 | 1550885 | 21449 |
| Chr08G0361.1 | Chr08 | 1534240 | 1537262 | 3023 |
| Chr08G0362.1 | Chr08 | 1539437 | 1540885 | 1449 |
| r5c4 | Chr08 | 1922679 | 1966326 | 43648 |
| Chr08G0436.1 | Chr08 | 1926861 | 1928516 | 1656 |
| Chr08G0437.1 | Chr08 | 1929161 | 1932745 | 3585 |
| Chr08G0438.1 | Chr08 | 1933638 | 1935485 | 1848 |
| Chr08G0439.1 | Chr08 | 1939954 | 1947778 | 7825 |
| Chr08G0440.1 | Chr08 | 1949144 | 1954073 | 4930 |
| Chr08G0441.1 | Chr08 | 1954215 | 1957343 | 3129 |
| Chr08G0442.1 | Chr08 | 1958599 | 1962357 | 3759 |
| Chr08G0443.1 | Chr08 | 1964614 | 1966326 | 1713 |
| r5c5 | Chr08 | 2031870 | 2075134 | 43265 |
| Chr08G0457.1 | Chr08 | 2035984 | 2040690 | 4707 |
| Chr08G0458.1 | Chr08 | 2041509 | 2042159 | 651 |
| Chr08G0459.1 | Chr08 | 2043812 | 2045516 | 1705 |
| Chr08G0460.1 | Chr08 | 2047641 | 2048971 | 1331 |
| Chr08G0461.1 | Chr08 | 2049318 | 2051544 | 2227 |
| Chr08G0462.1 | Chr08 | 2051817 | 2056213 | 4397 |
| Chr08G0463.1 | Chr08 | 2057914 | 2058792 | 879 |
| Chr08G0464.1 | Chr08 | 2060630 | 2063648 | 3019 |
| Chr08G0465.1 | Chr08 | 2063707 | 2065426 | 1720 |
| Chr08G0466.1 | Chr08 | 2065549 | 2067733 | 2185 |
| Chr08G0467.1 | Chr08 | 2068613 | 2070097 | 1485 |
| Chr08G0468.1 | Chr08 | 2071776 | 2074096 | 2321 |
| r5c6 | Chr08 | 2836795 | 2859218 | 22424 |
| Chr08G0656.1 | Chr08 | 2839664 | 2840927 | 1264 |
| Chr08G0657.1 | Chr08 | 2842151 | 2846078 | 3928 |
| Chr08G0658.1 | Chr08 | 2844175 | 2845773 | 1599 |
| Chr08G0659.1 | Chr08 | 2846122 | 2849674 | 3553 |
| Chr08G0660.1 | Chr08 | 2850242 | 2851782 | 1541 |
| Chr08G0661.1 | Chr08 | 2851858 | 2852630 | 773 |
| Chr08G0662.1 | Chr08 | 2854612 | 2855565 | 954 |
| Chr08G0663.1 | Chr08 | 2855803 | 2859052 | 3250 |
| r5c7 | Chr08 | 2955217 | 3004915 | 49699 |
| Chr08G0694.1 | Chr08 | 2955638 | 2956517 | 880 |
| Chr08G0695.1 | Chr08 | 2957544 | 2959977 | 2434 |
| Chr08G0696.1 | Chr08 | 2960345 | 2963684 | 3340 |
| Chr08G0697.1 | Chr08 | 2966087 | 2967193 | 1107 |
| Chr08G0698.1 | Chr08 | 2967396 | 2968055 | 660 |
| Chr08G0699.1 | Chr08 | 2968604 | 2969758 | 1155 |

| | | | | |
|---|---|---|---|---|
| Chr08G0700.1 | Chr08 | 2970334 | 2972035 | 1702 |
| Chr08G0701.1 | Chr08 | 2972693 | 2973043 | 351 |
| Chr08G0702.1 | Chr08 | 2973301 | 2974266 | 966 |
| Chr08G0703.1 | Chr08 | 2975217 | 2984915 | 9699 |
| Chr08G0704.1 | Chr08 | 2985474 | 2986659 | 1186 |
| Chr08G0705.1 | Chr08 | 2987015 | 2988990 | 1976 |
| Chr08G0706.1 | Chr08 | 2989122 | 2989907 | 786 |
| Chr08G0707.1 | Chr08 | 2992378 | 2994127 | 1750 |
| Chr08G0708.1 | Chr08 | 2995112 | 2996289 | 1178 |
| Chr08G0709.1 | Chr08 | 2997151 | 2997561 | 411 |
| Chr08G0710.1 | Chr08 | 2999116 | 3001480 | 2365 |
| Chr08G0711.1 | Chr08 | 3001694 | 3002571 | 878 |
| Chr08G0712.1 | Chr08 | 3003506 | 3004785 | 1280 |
| r8c1 | Chr09 | 689599 | 710912 | 21314 |
| Chr09G0167.1 | Chr09 | 697357 | 697803 | 447 |
| r8c2 | Chr09 | 975727 | 1018654 | 42928 |
| Chr09G0218.1 | Chr09 | 976138 | 977243 | 1106 |
| Chr09G0219.1 | Chr09 | 976665 | 979642 | 2978 |
| Chr09G0220.1 | Chr09 | 981117 | 984090 | 2974 |
| Chr09G0221.1 | Chr09 | 987874 | 990208 | 2335 |
| Chr09G0222.1 | Chr09 | 992233 | 992715 | 483 |
| Chr09G0223.1 | Chr09 | 994277 | 995754 | 1478 |
| Chr09G0224.1 | Chr09 | 997491 | 999534 | 2044 |
| Chr09G0225.1 | Chr09 | 1002835 | 1003876 | 1042 |
| Chr09G0226.1 | Chr09 | 1006861 | 1007874 | 1014 |
| Chr09G0227.1 | Chr09 | 1008035 | 1009059 | 1025 |
| Chr09G0228.1 | Chr09 | 1009789 | 1010517 | 729 |
| Chr09G0229.1 | Chr09 | 1012803 | 1014034 | 1232 |
| Chr09G0230.1 | Chr09 | 1015083 | 1016727 | 1645 |
| r8c3 | Chr09 | 2323855 | 2363787 | 39933 |
| Chr09G0557.1 | Chr09 | 2330763 | 2334033 | 3271 |
| Chr09G0558.1 | Chr09 | 2340044 | 2341235 | 1192 |
| Chr09G0559.1 | Chr09 | 2346403 | 2347154 | 752 |
| Chr09G0560.1 | Chr09 | 2349458 | 2355334 | 5877 |
| Chr09G0561.1 | Chr09 | 2356444 | 2357642 | 1199 |
| r8c4 | Chr09 | 2646375 | 2676613 | 30239 |
| Chr09G0629.1 | Chr09 | 2647321 | 2648217 | 897 |
| Chr09G0630.1 | Chr09 | 2648770 | 2651433 | 2664 |
| Chr09G0631.1 | Chr09 | 2652548 | 2653559 | 1012 |
| Chr09G0632.1 | Chr09 | 2653644 | 2654536 | 893 |
| Chr09G0633.1 | Chr09 | 2654959 | 2657017 | 2059 |
| Chr09G0634.1 | Chr09 | 2659087 | 2669892 | 10806 |
| r8c5 | Chr09 | 2735040 | 2779096 | 44057 |
| Chr09G0655.1 | Chr09 | 2746133 | 2750182 | 4050 |

| | | | | |
|---|---|---|---|---|
| Chr09G0656.1 | Chr09 | 2754497 | 2758588 | 4092 |
| Chr09G0657.1 | Chr09 | 2754497 | 2761682 | 7186 |
| Chr09G0658.1 | Chr09 | 2761993 | 2763747 | 1755 |
| Chr09G0659.1 | Chr09 | 2764392 | 2765687 | 1296 |
| Chr09G0660.1 | Chr09 | 2766923 | 2768537 | 1615 |
| Chr09G0661.1 | Chr09 | 2769559 | 2770359 | 801 |
| Chr09G0662.1 | Chr09 | 2777599 | 2778889 | 1291 |
| Chr09G0663.1 | Chr09 | 2779023 | 2787565 | 8543 |
| Chr09G0664.1 | Chr09 | 2788357 | 2789265 | 909 |
| r8c6 | Chr09 | 3959944 | 4014892 | 54949 |
| Chr09G1005.1 | Chr09 | 3963460 | 3968337 | 4878 |
| Chr09G1006.1 | Chr09 | 3970056 | 3972308 | 2253 |
| Chr09G1007.1 | Chr09 | 3972855 | 3974541 | 1687 |
| Chr09G1008.1 | Chr09 | 3980633 | 3981888 | 1256 |
| Chr09G1009.1 | Chr09 | 3983076 | 3984830 | 1755 |
| Chr09G1010.1 | Chr09 | 3985770 | 3989057 | 3288 |
| Chr09G1011.1 | Chr09 | 3992605 | 3995003 | 2399 |
| Chr09G1012.1 | Chr09 | 4003544 | 4005588 | 2045 |
| r8c7 | Chr09 | 4081459 | 4129958 | 48500 |
| Chr09G1031.1 | Chr09 | 4082928 | 4085211 | 2284 |
| Chr09G1032.1 | Chr09 | 4085739 | 4086851 | 1113 |
| Chr09G1033.1 | Chr09 | 4087301 | 4088886 | 1586 |
| Chr09G1034.1 | Chr09 | 4089366 | 4124909 | 35544 |
| Chr09G1035.1 | Chr09 | 4125944 | 4126977 | 1034 |
| Chr09G1036.1 | Chr09 | 4127303 | 4128379 | 1077 |
