# Supplementary material for: Decoding Pecan’s Fungal Foe: A Genomic Insight into Colletotrichum plurivorum Isolate W-6
Source: J Fungi (Basel). 2025 Mar 5;11(3):203. doi: 10.3390/jof11030203 (PMC11943440; doi:10.3390/jof11030203)
Supplement: Supplementary file 1 [file jof-11-00203-s001.zip › Table S15.pdf]

Table S15. Function annotation of protein-coding gene based on general databases.

Database	Number	Percentage (%)
GO_annotation	9,499	66.23
KEGG_annotation	3,467	24.17
KOG_class_annotation	5,882	41.01
Pfam_annotation	10,255	71.5
Swissprot_annotation	8,026	55.96
TrEMBL_annotation	13,981	97.48
nr_annotation	13,976	97.44
Anntation Gene	13,991	97.55
no-Annotation Gene	352	2.45
Total Gene	14,343	100.00
