# Supplementary material for: Decoding Pecan’s Fungal Foe: A Genomic Insight into Colletotrichum plurivorum Isolate W-6
Source: J Fungi (Basel). 2025 Mar 5;11(3):203. doi: 10.3390/jof11030203 (PMC11943440; doi:10.3390/jof11030203)
Supplement: Supplementary file 1 [file jof-11-00203-s001.zip › Table S16.pdf]

Table S16. Signal peptide in isolate W-6 genome.

Sequence ID	Start	End	Score
Chr01G0008.1	1	28	0.547
Chr01G0015.1	1	24	0.924
Chr01G0016.1	1	18	0.661
Chr01G0034.1	1	27	0.69
Chr01G0040.1	1	1	0.528
Chr01G0056.1	1	20	0.738
Chr01G0057.1	1	20	0.503
Chr01G0058.1	1	20	0.882
Chr01G0059.1	1	16	0.803
Chr01G0060.1	1	17	0.69
Chr01G0068.1	1	24	0.815
Chr01G0077.1	1	18	0.685
Chr01G0078.1	1	19	0.72
Chr01G0084.1	1	17	0.827
Chr01G0086.1	1	19	0.861
Chr01G0087.1	1	21	0.707
Chr01G0095.1	1	20	0.672
Chr01G0098.1	1	20	0.835
Chr01G0110.1	1	21	0.842
Chr01G0113.1	1	18	0.621
Chr01G0115.1	1	22	0.498
Chr01G0120.1	1	20	0.723
Chr01G0122.1	1	20	0.873
Chr01G0140.1	1	19	0.876
Chr01G0141.1	1	18	0.847
Chr01G0148.1	1	16	0.644
Chr01G0149.1	1	18	0.58
Chr01G0150.1	1	19	0.517
Chr01G0159.1	1	27	0.757
Chr01G0161.1	1	19	0.686
Chr01G0164.1	1	21	0.598
Chr01G0165.1	1	24	0.788
Chr01G0166.1	1	16	0.689
Chr01G0167.1	1	20	0.806
Chr01G0168.1	1	17	0.872
Chr01G0172.1	1	16	0.9
Chr01G0179.1	1	16	0.669
Chr01G0182.1	1	22	0.806
Chr01G0186.1	1	22	0.798
Chr01G0192.1	1	16	0.874
Chr01G0195.1	1	16	0.728
Chr01G0197.1	1	21	0.603

Chr01G0200.1	1	19	0.881
Chr01G0201.1	1	23	0.619
Chr01G0202.1	1	22	0.731
Chr01G0205.1	1	18	0.776
Chr01G0207.1	1	19	0.754
Chr01G0208.1	1	17	0.626
Chr01G0209.1	1	22	0.813
Chr01G0236.1	1	21	0.888
Chr01G0238.1	1	20	0.697
Chr01G0239.1	1	17	0.939
Chr01G0244.1	1	20	0.774
Chr01G0249.1	1	22	0.647
Chr01G0259.1	1	18	0.805
Chr01G0260.1	1	19	0.785
Chr01G0263.1	1	19	0.863
Chr01G0267.1	1	21	0.868
Chr01G0271.1	1	18	0.76
Chr01G0273.1	1	17	0.771
Chr01G0276.1	1	20	0.81
Chr01G0278.1	1	19	0.921
Chr01G0280.1	1	18	0.753
Chr01G0281.1	1	19	0.747
Chr01G0282.1	1	19	0.855
Chr01G0283.1	1	21	0.759
Chr01G0290.1	1	18	0.861
Chr01G0291.1	1	17	0.933
Chr01G0293.1	1	20	0.94
Chr01G0308.1	1	18	0.677
Chr01G0315.1	1	18	0.782
Chr01G0332.1	1	17	0.571
Chr01G0333.1	1	20	0.789
Chr01G0334.1	1	17	0.585
Chr01G0336.1	1	19	0.865
Chr01G0343.1	1	19	0.73
Chr01G0345.1	1	22	0.857
Chr01G0348.1	1	19	0.755
Chr01G0354.1	1	17	0.744
Chr01G0367.1	1	21	0.748
Chr01G0374.1	1	23	0.86
Chr01G0380.1	1	16	0.847
Chr01G0386.1	1	16	0.656
Chr01G0392.1	1	18	0.722
Chr01G0404.1	1	18	0.846
Chr01G0406.1	1	19	0.82

Chr01G0416.1	1	22	0.751
Chr01G0417.1	1	17	0.567
Chr01G0422.1	1	22	0.667
Chr01G0423.1	1	17	0.645
Chr01G0425.1	1	17	0.805
Chr01G0433.1	1	21	0.809
Chr01G0435.1	1	15	0.763
Chr01G0452.1	1	19	0.769
Chr01G0456.1	1	1	0.46
Chr01G0458.1	1	20	0.827
Chr01G0464.1	1	17	0.723
Chr01G0465.1	1	20	0.576
Chr01G0475.1	1	27	0.774
Chr01G0479.1	1	15	0.834
Chr01G0482.1	1	20	0.676
Chr01G0485.1	1	25	0.638
Chr01G0486.1	1	18	0.769
Chr01G0497.1	1	18	0.628
Chr01G0499.1	1	21	0.863
Chr01G0511.1	1	17	0.872
Chr01G0517.1	1	18	0.867
Chr01G0531.1	1	18	0.7
Chr01G0532.1	1	17	0.813
Chr01G0534.1	1	18	0.754
Chr01G0543.1	1	20	0.908
Chr01G0553.1	1	19	0.817
Chr01G0554.1	1	27	0.662
Chr01G0555.1	1	31	0.718
Chr01G0566.1	1	20	0.614
Chr01G0567.1	1	21	0.741
Chr01G0575.1	1	18	0.579
Chr01G0578.1	1	20	0.591
Chr01G0580.1	1	21	0.876
Chr01G0584.1	1	21	0.907
Chr01G0599.1	1	17	0.693
Chr01G0601.1	1	25	0.857
Chr01G0608.1	1	18	0.631
Chr01G0615.1	1	15	0.785
Chr01G0619.1	1	15	0.598
Chr01G0625.1	1	23	0.637
Chr01G0626.1	1	17	0.587
Chr01G0631.1	1	20	0.759
Chr01G0634.1	1	16	0.48
Chr01G0635.1	1	3	0.534

Chr01G0636.1	1	19	0.455
Chr01G0641.1	1	18	0.714
Chr01G0647.1	1	23	0.897
Chr01G0649.1	1	15	0.779
Chr01G0652.1	1	18	0.655
Chr01G0655.1	1	19	0.871
Chr01G0656.1	1	25	0.787
Chr01G0672.1	1	18	0.593
Chr01G0675.1	1	18	0.774
Chr01G0679.1	1	15	0.783
Chr01G0689.1	1	21	0.8
Chr01G0693.1	1	21	0.558
Chr01G0719.1	1	17	0.767
Chr01G0723.1	1	20	0.807
Chr01G0724.1	1	16	0.544
Chr01G0742.1	1	25	0.46
Chr01G0750.1	1	19	0.502
Chr01G0753.1	1	21	0.8
Chr01G0754.1	1	17	0.621
Chr01G0760.1	1	21	0.706
Chr01G0762.1	1	16	0.539
Chr01G0765.1	1	15	0.642
Chr01G0768.1	1	20	0.86
Chr01G0774.1	1	19	0.781
Chr01G0775.1	1	20	0.619
Chr01G0779.1	1	20	0.774
Chr01G0780.1	1	17	0.709
Chr01G0781.1	1	22	0.799
Chr01G0782.1	1	15	0.638
Chr01G0802.1	1	17	0.565
Chr01G0809.1	1	21	0.885
Chr01G0813.1	1	22	0.726
Chr01G0814.1	1	18	0.685
Chr01G0818.1	1	19	0.71
Chr01G0819.1	1	18	0.654
Chr01G0829.1	1	18	0.643
Chr01G0883.1	1	33	0.703
Chr01G0893.1	1	23	0.786
Chr01G0912.1	1	17	0.603
Chr01G0928.1	1	19	0.673
Chr01G0947.1	1	21	0.856
Chr01G0949.1	1	15	0.664
Chr01G0956.1	1	16	0.501
Chr01G0961.1	1	26	0.594

Chr01G0967.1	1	20	0.95
Chr01G0977.1	1	19	0.79
Chr01G0987.1	1	19	0.793
Chr01G0993.1	1	20	0.702
Chr01G0997.1	1	19	0.848
Chr01G1002.1	1	18	0.861
Chr01G1007.1	1	16	0.784
Chr01G1012.1	1	16	0.687
Chr01G1018.1	1	18	0.847
Chr01G1026.1	1	16	0.789
Chr01G1027.1	1	20	0.689
Chr01G1036.1	1	19	0.631
Chr01G1037.1	1	17	0.707
Chr01G1038.1	1	19	0.679
Chr01G1045.1	1	19	0.884
Chr01G1052.1	1	18	0.936
Chr01G1061.1	1	16	0.89
Chr01G1073.1	1	18	0.784
Chr01G1074.1	1	22	0.7
Chr01G1086.1	1	21	0.696
Chr01G1092.1	1	20	0.603
Chr01G1104.1	1	18	0.782
Chr01G1105.1	1	25	0.612
Chr01G1112.1	1	18	0.728
Chr01G1113.1	1	21	0.648
Chr01G1121.1	1	23	0.625
Chr01G1125.1	1	16	0.722
Chr01G1136.1	1	21	0.753
Chr01G1140.1	1	19	0.832
Chr01G1152.1	1	19	0.674
Chr01G1159.1	1	17	0.651
Chr01G1258.1	1	24	0.691
Chr01G1269.1	1	21	0.753
Chr01G1273.1	1	26	0.682
Chr01G1274.1	1	18	0.73
Chr01G1300.1	1	17	0.813
Chr01G1313.1	1	15	0.858
Chr01G1327.1	1	23	0.591
Chr01G1328.1	1	19	0.739
Chr01G1332.1	1	19	0.888
Chr01G1337.1	1	19	0.537
Chr01G1338.1	1	19	0.608
Chr01G1340.1	1	15	0.494
Chr01G1351.1	1	16	0.835

Chr01G1361.1	1	18	0.744
Chr01G1363.1	1	18	0.658
Chr01G1366.1	1	22	0.838
Chr01G1368.1	1	19	0.467
Chr01G1374.1	1	21	0.639
Chr01G1393.1	1	18	0.508
Chr01G1394.1	1	21	0.521
Chr01G1404.1	1	17	0.681
Chr01G1406.1	1	23	0.816
Chr01G1416.1	1	18	0.842
Chr01G1422.1	1	27	0.579
Chr01G1426.1	1	17	0.67
Chr01G1430.1	1	17	0.735
Chr01G1441.1	1	21	0.815
Chr01G1454.1	1	18	0.851
Chr01G1460.1	1	24	0.625
Chr01G1463.1	1	17	0.779
Chr01G1466.1	1	16	0.753
Chr01G1468.1	1	20	0.837
Chr01G1470.1	1	15	0.607
Chr01G1472.1	1	4	0.474
Chr01G1477.1	1	18	0.641
Chr01G1479.1	1	17	0.664
Chr01G1485.1	1	25	0.8
Chr01G1488.1	1	18	0.725
Chr01G1489.1	1	19	0.865
Chr01G1491.1	1	18	0.859
Chr01G1496.1	1	21	0.779
Chr01G1503.1	1	20	0.739
Chr01G1507.1	1	17	0.806
Chr01G1524.1	1	23	0.654
Chr01G1525.1	1	19	0.812
Chr01G1540.1	1	20	0.768
Chr01G1542.1	1	37	0.481
Chr01G1546.1	1	21	0.84
Chr01G1567.1	1	18	0.774
Chr01G1568.1	1	17	0.478
Chr01G1571.1	1	16	0.865
Chr01G1573.1	1	20	0.727
Chr01G1578.1	1	21	0.852
Chr01G1580.1	1	16	0.757
Chr01G1596.1	1	18	0.893
Chr01G1602.1	1	18	0.857
Chr01G1604.1	1	19	0.886

Chr01G1610.1	1	18	0.689
Chr01G1619.1	1	21	0.787
Chr01G1620.1	1	24	0.862
Chr01G1624.1	1	20	0.573
Chr01G1631.1	1	19	0.896
Chr01G1641.1	1	18	0.839
Chr01G1648.1	1	19	0.867
Chr01G1654.1	1	18	0.475
Chr01G1655.1	1	20	0.657
Chr01G1658.1	1	28	0.47
Chr01G1665.1	1	18	0.669
Chr01G1668.1	1	16	0.453
Chr01G1676.1	1	19	0.704
Chr01G1692.1	1	19	0.883
Chr01G1695.1	1	19	0.739
Chr01G1699.1	1	22	0.735
Chr01G1700.1	1	21	0.484
Chr01G1702.1	1	24	0.744
Chr01G1754.1	1	16	0.789
Chr01G1770.1	1	21	0.729
Chr01G1774.1	1	17	0.747
Chr01G1775.1	1	23	0.728
Chr01G1778.1	1	19	0.819
Chr01G1780.1	1	23	0.865
Chr01G1793.1	1	34	0.74
Chr01G1795.1	1	26	0.559
Chr01G1798.1	1	23	0.628
Chr01G1802.1	1	16	0.722
Chr01G1809.1	1	24	0.456
Chr01G1811.1	1	18	0.671
Chr01G1818.1	1	23	0.642
Chr01G1824.1	1	20	0.782
Chr01G1829.1	1	20	0.816
Chr01G1830.1	1	21	0.743
Chr01G1836.1	1	21	0.735
Chr01G1838.1	1	19	0.75
Chr01G1848.1	1	19	0.786
Chr01G1855.1	1	19	0.765
Chr01G1861.1	1	29	0.558
Chr01G1865.1	1	18	0.923
Chr01G1868.1	1	20	0.692
Chr01G1874.1	1	28	0.704
Chr01G1879.1	1	18	0.571
Chr01G1898.1	1	17	0.716

Chr01G1900.1	1	21	0.823
Chr01G1901.1	1	19	0.592
Chr01G1910.1	1	19	0.806
Chr01G1921.1	1	20	0.682
Chr01G1949.1	1	21	0.525
Chr01G1953.1	1	17	0.885
Chr01G1954.1	1	19	0.676
Chr01G1957.1	1	15	0.881
Chr01G1960.1	1	20	0.729
Chr01G1962.1	1	23	0.83
Chr01G1963.1	1	26	0.816
Chr01G1969.1	1	3	0.512
Chr01G1970.1	1	18	0.767
Chr01G1977.1	1	21	0.681
Chr01G1990.1	1	16	0.754
Chr01G1992.1	1	19	0.898
Chr01G1994.1	1	21	0.87
Chr01G1997.1	1	1	0.463
Chr01G2024.1	1	19	0.901
Chr01G2034.1	1	18	0.792
Chr01G2042.1	1	22	0.551
Chr01G2044.1	1	18	0.894
Chr01G2046.1	1	16	0.697
Chr01G2066.1	1	19	0.858
Chr01G2069.1	1	19	0.457
Chr01G2071.1	1	15	0.478
Chr01G2088.1	1	20	0.706
Chr01G2099.1	1	22	0.518
Chr01G2102.1	1	20	0.728
Chr01G2112.1	1	26	0.595
Chr01G2129.1	1	19	0.715
Chr01G2136.1	1	16	0.848
Chr01G2142.1	1	29	0.816
Chr01G2143.1	1	21	0.678
Chr01G2145.1	1	24	0.775
Chr01G2150.1	1	21	0.771
Chr01G2228.1	1	19	0.651
Chr01G2234.1	1	20	0.772
Chr01G2236.1	1	19	0.949
Chr01G2250.1	1	17	0.464
Chr01G2275.1	1	25	0.8
Chr01G2276.1	1	21	0.881
Chr01G2313.1	1	19	0.919
Chr01G2318.1	1	23	0.737

Chr01G2344.1	1	22	0.572
Chr01G2350.1	1	23	0.533
Chr01G2392.1	1	21	0.632
Chr01G2412.1	1	20	0.848
Chr01G2420.1	1	18	0.826
Chr01G2421.1	1	17	0.654
Chr01G2428.1	1	18	0.787
Chr01G2429.1	1	23	0.731
Chr01G2430.1	1	16	0.669
Chr01G2432.1	1	16	0.951
Chr01G2435.1	1	20	0.591
Chr01G2438.1	1	19	0.731
Chr01G2442.1	1	17	0.574
Chr01G2450.1	1	20	0.793
Chr01G2452.1	1	19	0.704
Chr01G2453.1	1	23	0.663
Chr01G2459.1	1	22	0.718
Chr01G2461.1	1	17	0.755
Chr01G2472.1	1	22	0.62
Chr01G2479.1	1	18	0.682
Chr01G2482.1	1	19	0.892
Chr01G2484.1	1	18	0.608
Chr01G2490.1	1	17	0.669
Chr01G2493.1	1	32	0.595
Chr01G2497.1	1	17	0.659
Chr01G2500.1	1	19	0.773
Chr01G2502.1	1	22	0.475
Chr01G2506.1	1	19	0.86
Chr01G2510.1	1	18	0.688
Chr01G2512.1	1	17	0.773
Chr01G2521.1	1	24	0.674
Chr01G2524.1	1	17	0.497
Chr01G2526.1	1	17	0.859
Chr01G2531.1	1	24	0.913
Chr01G2534.1	1	19	0.761
Chr01G2538.1	1	21	0.58
Chr01G2542.1	1	20	0.59
Chr01G2544.1	1	16	0.542
Chr01G2550.1	1	17	0.633
Chr01G2551.1	1	26	0.705
Chr01G2558.1	1	23	0.804
Chr01G2570.1	1	17	0.683
Chr01G2577.1	1	20	0.903
Chr01G2581.1	1	16	0.881

Chr01G2582.1	1	20	0.746
Chr01G2585.1	1	20	0.844
Chr01G2589.1	1	18	0.633
Chr01G2593.1	1	19	0.806
Chr01G2601.1	1	18	0.623
Chr01G2614.1	1	30	0.761
Chr01G2624.1	1	17	0.763
Chr01G2627.1	1	20	0.744
Chr01G2630.1	1	17	0.664
Chr01G2637.1	1	23	0.46
Chr01G2645.1	1	20	0.863
Chr01G2653.1	1	17	0.606
Chr01G2654.1	1	16	0.831
Chr01G2680.1	1	17	0.67
Chr01G2690.1	1	18	0.597
Chr01G2697.1	1	23	0.742
Chr01G2713.1	1	20	0.519
Chr01G2715.1	1	18	0.769
Chr01G2717.1	1	24	0.821
Chr01G2718.1	1	23	0.863
Chr01G2720.1	1	19	0.859
Chr01G2735.1	1	18	0.806
Chr01G2739.1	1	19	0.687
Chr01G2747.1	1	23	0.857
Chr01G2750.1	1	18	0.881
Chr01G2751.1	1	20	0.858
Chr01G2774.1	1	25	0.661
Chr01G2779.1	1	18	0.775
Chr01G2782.1	1	26	0.746
Chr05G0844.1	1	26	0.56
Chr05G0841.1	1	22	0.825
Chr05G0822.1	1	19	0.78
Chr05G0820.1	1	23	0.53
Chr05G0813.1	1	32	0.539
Chr05G0812.1	1	22	0.729
Chr05G0797.1	1	19	0.878
Chr05G0761.1	1	18	0.67
Chr05G0754.1	1	21	0.699
Chr05G0750.1	1	22	0.944
Chr05G0744.1	1	22	0.892
Chr05G0738.1	1	17	0.796
Chr05G0731.1	1	22	0.641
Chr05G0729.1	1	19	0.675
Chr05G0660.1	1	22	0.804

Chr05G0644.1	1	19	0.874
Chr05G0627.1	1	18	0.798
Chr05G0625.1	1	18	0.773
Chr05G0618.1	1	20	0.728
Chr05G0605.1	1	17	0.782
Chr05G0603.1	1	19	0.631
Chr05G0602.1	1	18	0.844
Chr05G0593.1	1	18	0.866
Chr05G0584.1	1	16	0.757
Chr05G0558.1	1	21	0.775
Chr05G0537.1	1	18	0.614
Chr05G0536.1	1	23	0.688
Chr05G0514.1	1	21	0.628
Chr05G0511.1	1	18	0.897
Chr05G0490.1	1	33	0.815
Chr05G0486.1	1	20	0.834
Chr05G0471.1	1	18	0.837
Chr05G0470.1	1	20	0.603
Chr05G0469.1	1	21	0.764
Chr05G0425.1	1	19	0.717
Chr05G0411.1	1	23	0.685
Chr05G0397.1	1	23	0.635
Chr05G0396.1	1	22	0.524
Chr05G0386.1	1	16	0.764
Chr05G0380.1	1	16	0.734
Chr05G0378.1	1	24	0.482
Chr05G0368.1	1	20	0.693
Chr05G0357.1	1	18	0.856
Chr05G0348.1	1	18	0.622
Chr05G0347.1	1	19	0.889
Chr05G0341.1	1	17	0.683
Chr05G0336.1	1	16	0.576
Chr05G0334.1	1	19	0.728
Chr05G0332.1	1	21	0.882
Chr05G0330.1	1	18	0.757
Chr05G0329.1	1	17	0.772
Chr05G0323.1	1	17	0.773
Chr05G0321.1	1	24	0.833
Chr05G0318.1	1	21	0.651
Chr05G0316.1	1	17	0.647
Chr05G0313.1	1	18	0.545
Chr05G0304.1	1	24	0.699
Chr05G0296.1	1	17	0.82
Chr05G0295.1	1	17	0.651

Chr05G0283.1	1	18	0.596
Chr05G0279.1	1	19	0.665
Chr05G0277.1	1	17	0.586
Chr05G0262.1	1	17	0.79
Chr05G0258.1	1	19	0.77
Chr05G0253.1	1	19	0.748
Chr05G0246.1	1	25	0.709
Chr05G0243.1	1	20	0.469
Chr05G0234.1	1	21	0.734
Chr05G0227.1	1	25	0.851
Chr05G0221.1	1	21	0.873
Chr05G0209.1	1	18	0.824
Chr05G0208.1	1	20	0.778
Chr05G0207.1	1	19	0.511
Chr05G0202.1	1	19	0.753
Chr05G0198.1	1	2	0.487
Chr05G0196.1	1	19	0.69
Chr05G0194.1	1	19	0.794
Chr05G0191.1	1	16	0.535
Chr05G0189.1	1	19	0.654
Chr05G0172.1	1	17	0.905
Chr05G0166.1	1	18	0.778
Chr05G0165.1	1	19	0.856
Chr05G0164.1	1	20	0.883
Chr05G0145.1	1	16	0.48
Chr05G0134.1	1	18	0.784
Chr05G0133.1	1	20	0.857
Chr05G0131.1	1	20	0.807
Chr05G0130.1	1	15	0.746
Chr05G0129.1	1	18	0.546
Chr05G0125.1	1	22	0.745
Chr05G0119.1	1	18	0.691
Chr05G0107.1	1	21	0.846
Chr05G0095.1	1	19	0.627
Chr05G0093.1	1	18	0.701
Chr05G0092.1	1	18	0.719
Chr05G0078.1	1	16	0.795
Chr05G0075.1	1	18	0.706
Chr05G0074.1	1	16	0.786
Chr05G0059.1	1	22	0.806
Chr05G0058.1	1	35	0.828
Chr05G0054.1	1	20	0.576
Chr05G0042.1	1	18	0.679
Chr05G0035.1	1	18	0.591

Chr07G0012.1	1	20	0.872
Chr07G0013.1	1	19	0.71
Chr07G0014.1	1	21	0.86
Chr07G0021.1	1	19	0.675
Chr07G0025.1	1	17	0.726
Chr07G0036.1	1	17	0.895
Chr07G0050.1	1	23	0.829
Chr07G0069.1	1	17	0.754
Chr07G0071.1	1	17	0.866
Chr07G0080.1	1	27	0.767
Chr07G0081.1	1	17	0.61
Chr07G0101.1	1	22	0.794
Chr07G0103.1	1	18	0.814
Chr07G0112.1	1	18	0.781
Chr07G0115.1	1	32	0.584
Chr07G0139.1	1	16	0.836
Chr07G0140.1	1	19	0.787
Chr07G0149.1	1	19	0.862
Chr07G0151.1	1	26	0.61
Chr07G0164.1	1	22	0.624
Chr07G0165.1	1	16	0.645
Chr07G0172.1	1	17	0.845
Chr07G0176.1	1	18	0.6
Chr07G0177.1	1	18	0.745
Chr07G0181.1	1	24	0.763
Chr07G0188.1	1	20	0.916
Chr07G0190.1	1	22	0.783
Chr07G0196.1	1	17	0.76
Chr07G0205.1	1	34	0.587
Chr07G0227.1	1	21	0.714
Chr07G0228.1	1	23	0.851
Chr07G0235.1	1	19	0.862
Chr07G0236.1	1	17	0.702
Chr07G0243.1	1	18	0.698
Chr07G0249.1	1	18	0.793
Chr07G0251.1	1	24	0.802
Chr07G0252.1	1	19	0.497
Chr07G0253.1	1	23	0.565
Chr07G0263.1	1	17	0.63
Chr07G0269.1	1	19	0.771
Chr07G0270.1	1	19	0.885
Chr07G0271.1	1	21	0.861
Chr07G0273.1	1	18	0.797
Chr07G0277.1	1	18	0.787

Chr07G0282.1	1	22	0.922
Chr07G0288.1	1	20	0.81
Chr07G0291.1	1	19	0.66
Chr07G0292.1	1	19	0.881
Chr07G0293.1	1	22	0.898
Chr07G0298.1	1	18	0.737
Chr07G0314.1	1	21	0.827
Chr07G0315.1	1	29	0.893
Chr07G0316.1	1	20	0.717
Chr07G0318.1	1	20	0.764
Chr07G0319.1	1	20	0.9
Chr07G0323.1	1	20	0.808
Chr07G0324.1	1	18	0.786
Chr07G0328.1	1	23	0.618
Chr07G0329.1	1	19	0.804
Chr07G0331.1	1	23	0.894
Chr07G0337.1	1	20	0.845
Chr07G0341.1	1	19	0.751
Chr07G0351.1	1	20	0.813
Chr07G0361.1	1	30	0.864
Chr07G0362.1	1	16	0.586
Chr07G0363.1	1	22	0.838
Chr07G0365.1	1	23	0.735
Chr07G0368.1	1	19	0.538
Chr07G0369.1	1	22	0.884
Chr07G0382.1	1	18	0.779
Chr07G0385.1	1	24	0.515
Chr07G0389.1	1	20	0.672
Chr07G0401.1	1	21	0.839
Chr07G0411.1	1	25	0.486
Chr07G0413.1	1	19	0.654
Chr07G0414.1	1	19	0.673
Chr07G0415.1	1	19	0.763
Chr07G0419.1	1	16	0.643
Chr07G0423.1	1	17	0.489
Chr07G0439.1	1	19	0.851
Chr07G0441.1	1	22	0.739
Chr07G0446.1	1	17	0.796
Chr07G0456.1	1	22	0.795
Chr07G0471.1	1	17	0.771
Chr07G0489.1	1	19	0.658
Chr07G0503.1	1	21	0.781
Chr07G0504.1	1	23	0.842
Chr07G0505.1	1	18	0.752

Chr07G0508.1	1	18	0.748
Chr07G0526.1	1	20	0.904
Chr07G0528.1	1	17	0.741
Chr07G0531.1	1	18	0.807
Chr07G0532.1	1	22	0.696
Chr07G0547.1	1	17	0.558
Chr07G0548.1	1	19	0.697
Chr07G0550.1	1	19	0.785
Chr07G0552.1	1	16	0.72
Chr07G0563.1	1	18	0.632
Chr07G0564.1	1	25	0.734
Chr07G0568.1	1	17	0.76
Chr07G0571.1	1	27	0.847
Chr07G0574.1	1	21	0.887
Chr07G0575.1	1	23	0.69
Chr07G0600.1	1	18	0.779
Chr07G0611.1	1	26	0.705
Chr07G0615.1	1	19	0.58
Chr07G0617.1	1	19	0.72
Chr07G0624.1	1	18	0.879
Chr07G0626.1	1	31	0.806
Chr07G0637.1	1	25	0.732
Chr07G0638.1	1	21	0.794
Chr07G0658.1	1	18	0.733
Chr07G0661.1	1	1	0.503
Chr07G0701.1	1	30	0.838
Chr07G0705.1	1	18	0.728
Chr07G0707.1	1	16	0.761
Chr07G0743.1	1	25	0.611
Chr07G0754.1	1	27	0.752
Chr07G0785.1	1	17	0.704
Chr07G0802.1	1	20	0.829
Chr07G0809.1	1	22	0.506
Chr07G0812.1	1	23	0.701
Chr07G0842.1	1	21	0.871
Chr07G0846.1	1	20	0.808
Chr07G0860.1	1	19	0.839
Chr07G0861.1	1	17	0.783
Chr07G0879.1	1	16	0.633
Chr07G0881.1	1	23	0.77
Chr07G0891.1	1	21	0.705
Chr07G0893.1	1	20	0.653
Chr07G0904.1	1	24	0.661
Chr07G0908.1	1	20	0.816

Chr07G0924.1	1	18	0.884
Chr07G0926.1	1	23	0.463
Chr07G0928.1	1	23	0.918
Chr07G0934.1	1	17	0.661
Chr07G0935.1	1	26	0.608
Chr07G0936.1	1	18	0.784
Chr07G0937.1	1	19	0.817
Chr07G0938.1	1	22	0.857
Chr07G0940.1	1	21	0.596
Chr07G0943.1	1	19	0.651
Chr07G0947.1	1	19	0.916
Chr07G0952.1	1	19	0.858
Chr07G0967.1	1	19	0.638
Chr07G0976.1	1	22	0.777
Chr07G0978.1	1	30	0.628
Chr07G0979.1	1	20	0.832
Chr07G0981.1	1	20	0.518
Chr07G0990.1	1	25	0.67
Chr07G0997.1	1	19	0.522
Chr07G1001.1	1	16	0.646
Chr07G1003.1	1	20	0.771
Chr07G1007.1	1	23	0.714
Chr07G1009.1	1	20	0.777
Chr07G1016.1	1	20	0.644
Chr07G1031.1	1	21	0.647
Chr07G1047.1	1	18	0.677
Chr07G1049.1	1	22	0.815
Chr07G1050.1	1	18	0.754
Chr07G1063.1	1	20	0.84
Chr07G1069.1	1	20	0.757
Chr07G1074.1	1	21	0.746
Chr07G1089.1	1	20	0.539
Chr07G1105.1	1	16	0.528
Chr07G1108.1	1	22	0.614
Chr07G1109.1	1	21	0.764
Chr07G1117.1	1	16	0.754
Chr07G1126.1	1	16	0.825
Chr07G1128.1	1	18	0.622
Chr07G1132.1	1	20	0.813
Chr07G1133.1	1	19	0.622
Chr07G1134.1	1	20	0.812
Chr07G1135.1	1	18	0.9
Chr07G1139.1	1	20	0.813
Chr07G1142.1	1	22	0.835

Chr07G1161.1	1	21	0.561
Chr07G1166.1	1	19	0.638
Chr07G1167.1	1	20	0.584
Chr07G1168.1	1	21	0.742
Chr07G1169.1	1	18	0.809
Chr07G1176.1	1	16	0.84
Chr07G1189.1	1	1	0.471
Chr02G0806.1	1	20	0.843
Chr02G0813.1	1	19	0.627
Chr02G0825.1	1	26	0.916
Chr02G0829.1	1	20	0.774
Chr02G0841.1	1	22	0.709
Chr02G0852.1	1	19	0.548
Chr02G0878.1	1	26	0.483
Chr02G0950.1	1	18	0.546
Chr02G0968.1	1	32	0.547
Chr02G0993.1	1	18	0.648
Chr02G1041.1	1	23	0.643
Chr02G1051.1	1	17	0.671
Chr02G1076.1	1	18	0.653
Chr02G1092.1	1	17	0.57
Chr02G1099.1	1	22	0.721
Chr02G1101.1	1	18	0.826
Chr02G1120.1	1	17	0.873
Chr02G1121.1	1	20	0.736
Chr02G1125.1	1	22	0.533
Chr02G1127.1	1	18	0.609
Chr02G1131.1	1	16	0.73
Chr02G1132.1	1	19	0.823
Chr02G1139.1	1	19	0.926
Chr02G1147.1	1	19	0.911
Chr02G1158.1	1	26	0.859
Chr02G1170.1	1	18	0.744
Chr02G1177.1	1	18	0.752
Chr02G1178.1	1	20	0.507
Chr02G1190.1	1	20	0.554
Chr02G1199.1	1	19	0.478
Chr02G1212.1	1	16	0.811
Chr02G1213.1	1	25	0.825
Chr02G1227.1	1	17	0.819
Chr02G1238.1	1	22	0.908
Chr02G1252.1	1	17	0.543
Chr02G1260.1	1	19	0.775
Chr02G1269.1	1	19	0.678

Chr02G1276.1	1	22	0.562
Chr02G1277.1	1	28	0.749
Chr02G1286.1	1	31	0.665
Chr02G1287.1	1	16	0.668
Chr02G1316.1	1	19	0.677
Chr02G1330.1	1	24	0.591
Chr02G1331.1	1	18	0.867
Chr02G1332.1	1	17	0.833
Chr02G1335.1	1	18	0.714
Chr02G1339.1	1	20	0.904
Chr02G1340.1	1	19	0.729
Chr02G1348.1	1	15	0.626
Chr02G1356.1	1	20	0.839
Chr02G1357.1	1	22	0.821
Chr02G1358.1	1	21	0.781
Chr02G1359.1	1	20	0.767
Chr02G1360.1	1	22	0.723
Chr02G1362.1	1	18	0.66
Chr02G1366.1	1	17	0.546
Chr02G1375.1	1	18	0.848
Chr02G1378.1	1	19	0.804
Chr02G1383.1	1	21	0.604
Chr02G1385.1	1	24	0.577
Chr02G1387.1	1	24	0.839
Chr02G1388.1	1	23	0.878
Chr02G1404.1	1	18	0.964
Chr02G1405.1	1	23	0.466
Chr02G1406.1	1	19	0.938
Chr02G1407.1	1	19	0.741
Chr02G1417.1	1	21	0.817
Chr02G1429.1	1	18	0.633
Chr02G1433.1	1	21	0.586
Chr02G1438.1	1	15	0.653
Chr02G1439.1	1	22	0.8
Chr02G1446.1	1	17	0.796
Chr02G1467.1	1	19	0.67
Chr02G1468.1	1	17	0.607
Chr02G1471.1	1	21	0.744
Chr02G1476.1	1	20	0.898
Chr02G1477.1	1	20	0.845
Chr02G1486.1	1	16	0.573
Chr02G1497.1	1	19	0.784
Chr02G1517.1	1	1	0.535
Chr02G1522.1	1	24	0.86

Chr02G1524.1	1	22	0.837
Chr02G1525.1	1	22	0.691
Chr02G1528.1	1	17	0.755
Chr02G1534.1	1	25	0.889
Chr02G1535.1	1	19	0.671
Chr02G1537.1	1	16	0.56
Chr02G1558.1	1	4	0.473
Chr02G1560.1	1	19	0.685
Chr02G1565.1	1	15	0.786
Chr02G1570.1	1	23	0.832
Chr02G1572.1	1	18	0.881
Chr02G1575.1	1	20	0.774
Chr02G1577.1	1	20	0.506
Chr02G1582.1	1	24	0.766
Chr02G1587.1	1	15	0.69
Chr02G1589.1	1	21	0.471
Chr02G1598.1	1	27	0.793
Chr02G1601.1	1	16	0.872
Chr02G1622.1	1	21	0.707
Chr02G1639.1	1	25	0.641
Chr02G1641.1	1	15	0.774
Chr02G1650.1	1	18	0.839
Chr02G1652.1	1	19	0.876
Chr02G1653.1	1	19	0.671
Chr02G1662.1	1	21	0.802
Chr02G1678.1	1	15	0.517
Chr02G1689.1	1	19	0.886
Chr02G1694.1	1	18	0.816
Chr02G1699.1	1	20	0.839
Chr02G1700.1	1	26	0.487
Chr02G1716.1	1	17	0.702
Chr02G1723.1	1	17	0.718
Chr02G1748.1	1	20	0.886
Chr02G1756.1	1	17	0.782
Chr02G1768.1	1	19	0.792
Chr02G1769.1	1	18	0.738
Chr02G1782.1	1	18	0.715
Chr02G1784.1	1	17	0.79
Chr02G1795.1	1	20	0.606
Chr02G1799.1	1	17	0.557
Chr02G1802.1	1	18	0.729
Chr02G1803.1	1	18	0.614
Chr02G1808.1	1	31	0.872
Chr02G1812.1	1	18	0.733

Chr02G1816.1	1	25	0.891
Chr02G1818.1	1	20	0.887
Chr02G1825.1	1	36	0.485
Chr02G1835.1	1	18	0.693
Chr02G1842.1	1	20	0.791
Chr02G1844.1	1	17	0.53
Chr02G1848.1	1	23	0.756
Chr02G1853.1	1	19	0.488
Chr02G1854.1	1	19	0.759
Chr08G0020.1	1	17	0.685
Chr08G0021.1	1	22	0.785
Chr08G0027.1	1	18	0.618
Chr08G0033.1	1	25	0.891
Chr08G0034.1	1	16	0.672
Chr08G0036.1	1	15	0.74
Chr08G0047.1	1	23	0.896
Chr08G0052.1	1	19	0.776
Chr08G0054.1	1	22	0.742
Chr08G0062.1	1	22	0.61
Chr08G0063.1	1	18	0.787
Chr08G0070.1	1	19	0.83
Chr08G0071.1	1	17	0.701
Chr08G0079.1	1	18	0.864
Chr08G0086.1	1	19	0.871
Chr08G0087.1	1	17	0.633
Chr08G0092.1	1	29	0.864
Chr08G0105.1	1	21	0.728
Chr08G0106.1	1	20	0.743
Chr08G0121.1	1	35	0.497
Chr08G0132.1	1	19	0.562
Chr08G0135.1	1	17	0.718
Chr08G0155.1	1	18	0.94
Chr08G0158.1	1	17	0.513
Chr08G0165.1	1	17	0.763
Chr08G0166.1	1	17	0.699
Chr08G0171.1	1	18	0.771
Chr08G0179.1	1	21	0.768
Chr08G0182.1	1	19	0.751
Chr08G0183.1	1	16	0.666
Chr08G0194.1	1	17	0.602
Chr08G0219.1	1	27	0.802
Chr08G0221.1	1	30	0.674
Chr08G0223.1	1	20	0.886
Chr08G0251.1	1	17	0.708

Chr08G0252.1	1	18	0.756
Chr08G0276.1	1	23	0.906
Chr08G0279.1	1	20	0.483
Chr08G0301.1	1	20	0.47
Chr08G0312.1	1	18	0.662
Chr08G0319.1	1	15	0.767
Chr08G0323.1	1	19	0.529
Chr08G0338.1	1	16	0.879
Chr08G0339.1	1	21	0.605
Chr08G0361.1	1	18	0.569
Chr08G0368.1	1	25	0.89
Chr08G0384.1	1	17	0.597
Chr08G0395.1	1	16	0.53
Chr08G0398.1	1	22	0.53
Chr08G0401.1	1	19	0.621
Chr08G0412.1	1	17	0.707
Chr08G0413.1	1	18	0.805
Chr08G0435.1	1	18	0.667
Chr08G0437.1	1	20	0.679
Chr08G0443.1	1	24	0.63
Chr08G0466.1	1	22	0.673
Chr08G0467.1	1	23	0.645
Chr08G0471.1	1	26	0.845
Chr08G0472.1	1	20	0.737
Chr08G0473.1	1	19	0.774
Chr08G0477.1	1	19	0.673
Chr08G0484.1	1	25	0.809
Chr08G0492.1	1	18	0.745
Chr08G0496.1	1	18	0.694
Chr08G0500.1	1	19	0.706
Chr08G0501.1	1	18	0.644
Chr08G0506.1	1	19	0.75
Chr08G0527.1	1	18	0.676
Chr08G0543.1	1	21	0.765
Chr08G0616.1	1	26	0.941
Chr08G0637.1	1	21	0.873
Chr08G0644.1	1	18	0.851
Chr08G0649.1	1	16	0.662
Chr08G0651.1	1	19	0.835
Chr08G0652.1	1	18	0.736
Chr08G0656.1	1	19	0.77
Chr08G0665.1	1	20	0.778
Chr08G0679.1	1	18	0.855
Chr08G0682.1	1	20	0.829

Chr08G0685.1	1	30	0.7
Chr08G0693.1	1	23	0.837
Chr08G0694.1	1	25	0.729
Chr08G0725.1	1	19	0.894
Chr08G0728.1	1	20	0.878
Chr08G0734.1	1	17	0.568
Chr08G0738.1	1	22	0.473
Chr08G0739.1	1	22	0.877
Chr08G0740.1	1	21	0.904
Chr08G0756.1	1	20	0.828
Chr08G0761.1	1	19	0.563
Chr08G0763.1	1	21	0.792
Chr08G0771.1	1	17	0.783
Chr08G0783.1	1	17	0.582
Chr08G0787.1	1	18	0.91
Chr08G0788.1	1	27	0.907
Chr08G0789.1	1	22	0.828
Chr08G0801.1	1	16	0.69
Chr08G0803.1	1	24	0.805
Chr08G0805.1	1	20	0.618
Chr08G0806.1	1	19	0.725
Chr08G0819.1	1	17	0.601
Chr08G0821.1	1	22	0.512
Chr08G0822.1	1	21	0.865
Chr08G0832.1	1	26	0.754
Chr08G0833.1	1	31	0.727
Chr08G0834.1	1	24	0.853
Chr08G0837.1	1	19	0.706
Chr08G0841.1	1	20	0.85
Chr08G0842.1	1	24	0.669
Chr08G0843.1	1	16	0.786
Chr08G0846.1	1	18	0.713
Chr08G0851.1	1	20	0.663
Chr08G0866.1	1	18	0.842
Chr08G0874.1	1	19	0.865
Chr08G0878.1	1	22	0.677
Chr08G0879.1	1	19	0.509
Chr08G0880.1	1	18	0.756
Chr08G0884.1	1	22	0.772
Chr08G0902.1	1	21	0.861
Chr08G0917.1	1	16	0.727
Chr08G0918.1	1	19	0.67
Chr08G0922.1	1	18	0.667
Chr08G0929.1	1	20	0.782

Chr08G0934.1	1	4	0.563
Chr08G0936.1	1	17	0.565
Chr08G0944.1	1	19	0.493
Chr08G0951.1	1	17	0.765
Chr08G0952.1	1	21	0.665
Chr08G0964.1	1	21	0.662
Chr08G0969.1	1	17	0.764
Chr08G0972.1	1	16	0.519
Chr08G0980.1	1	3	0.559
Chr08G0985.1	1	24	0.578
Chr08G0986.1	1	17	0.785
Chr08G0991.1	1	16	0.692
Chr08G0993.1	1	19	0.714
Chr08G0998.1	1	20	0.904
Chr08G1017.1	1	17	0.62
Chr08G1021.1	1	16	0.863
Chr08G1037.1	1	16	0.727
Chr08G1039.1	1	17	0.838
Chr08G1042.1	1	17	0.697
Chr08G1048.1	1	24	0.754
Chr08G1056.1	1	17	0.786
Chr05G0897.1	1	19	0.796
Chr05G0906.1	1	19	0.664
Chr05G0914.1	1	21	0.858
Chr05G0929.1	1	20	0.569
Chr05G0933.1	1	17	0.839
Chr05G0935.1	1	19	0.812
Chr05G0940.1	1	19	0.879
Chr05G0945.1	1	18	0.714
Chr05G0948.1	1	35	0.537
Chr05G0949.1	1	22	0.805
Chr05G0954.1	1	18	0.784
Chr05G0967.1	1	23	0.712
Chr05G0971.1	1	22	0.541
Chr05G0993.1	1	21	0.91
Chr05G1012.1	1	1	0.516
Chr05G1030.1	1	26	0.89
Chr05G1067.1	1	23	0.686
Chr05G1082.1	1	19	0.791
Chr05G1092.1	1	18	0.641
Chr05G1093.1	1	15	0.604
Chr05G1106.1	1	20	0.615
Chr05G1108.1	1	26	0.695
Chr05G1140.1	1	19	0.719

Chr05G1150.1	1	15	0.719
Chr05G1151.1	1	20	0.913
Chr05G1157.1	1	20	0.84
Chr05G1166.1	1	19	0.78
Chr05G1186.1	1	1	0.452
Chr05G1215.1	1	33	0.584
Chr05G1241.1	1	17	0.578
Chr05G1247.1	1	22	0.855
Chr05G1248.1	1	21	0.516
Chr05G1262.1	1	20	0.846
Chr05G1272.1	1	18	0.766
Chr05G1275.1	1	21	0.726
Chr05G1288.1	1	21	0.508
Chr05G1314.1	1	26	0.774
Chr05G1320.1	1	30	0.863
Chr05G1327.1	1	17	0.534
Chr05G1334.1	1	18	0.844
Chr05G1338.1	1	24	0.767
Chr05G1339.1	1	17	0.776
Chr05G1346.1	1	21	0.59
Chr05G1357.1	1	25	0.601
Chr05G1375.1	1	18	0.799
Chr05G1384.1	1	26	0.73
Chr05G1400.1	1	19	0.759
Chr05G1401.1	1	20	0.757
Chr05G1409.1	1	19	0.77
Chr05G1415.1	1	15	0.762
Chr05G1420.1	1	17	0.811
Chr05G1422.1	1	16	0.818
Chr05G1433.1	1	22	0.614
Chr03G0020.1	1	15	0.589
Chr03G0031.1	1	22	0.841
Chr03G0032.1	1	15	0.768
Chr03G0037.1	1	25	0.47
Chr03G0044.1	1	15	0.492
Chr03G0045.1	1	1	0.453
Chr03G0046.1	1	20	0.883
Chr03G0057.1	1	15	0.752
Chr03G0059.1	1	19	0.721
Chr03G0062.1	1	17	0.833
Chr03G0068.1	1	17	0.865
Chr03G0086.1	1	19	0.689
Chr03G0088.1	1	19	0.709
Chr03G0090.1	1	19	0.813

Chr03G0116.1	1	23	0.602
Chr03G0117.1	1	21	0.752
Chr03G0119.1	1	21	0.857
Chr03G0120.1	1	22	0.916
Chr03G0122.1	1	21	0.747
Chr03G0126.1	1	19	0.729
Chr03G0128.1	1	20	0.696
Chr03G0129.1	1	18	0.789
Chr03G0155.1	1	20	0.537
Chr03G0181.1	1	25	0.452
Chr03G0206.1	1	23	0.845
Chr03G0209.1	1	18	0.741
Chr03G0212.1	1	16	0.705
Chr03G0214.1	1	23	0.677
Chr03G0215.1	1	20	0.837
Chr03G0219.1	1	24	0.531
Chr03G0224.1	1	20	0.666
Chr03G0237.1	1	20	0.731
Chr03G0239.1	1	28	0.52
Chr03G0240.1	1	36	0.715
Chr03G0243.1	1	18	0.865
Chr03G0255.1	1	17	0.727
Chr03G0273.1	1	20	0.838
Chr03G0278.1	1	20	0.622
Chr03G0280.1	1	18	0.726
Chr03G0281.1	1	22	0.656
Chr03G0284.1	1	20	0.745
Chr03G0294.1	1	27	0.926
Chr03G0320.1	1	21	0.72
Chr03G0337.1	1	22	0.877
Chr03G0341.1	1	19	0.809
Chr03G0344.1	1	18	0.806
Chr03G0346.1	1	19	0.616
Chr03G0347.1	1	23	0.853
Chr03G0366.1	1	22	0.702
Chr03G0369.1	1	19	0.651
Chr03G0371.1	1	21	0.903
Chr03G0372.1	1	20	0.803
Chr03G0373.1	1	19	0.563
Chr03G0374.1	1	24	0.589
Chr03G0376.1	1	21	0.667
Chr03G0390.1	1	19	0.704
Chr03G0391.1	1	21	0.777
Chr03G0392.1	1	21	0.916

Chr03G0402.1	1	23	0.747
Chr03G0413.1	1	21	0.739
Chr03G0414.1	1	19	0.784
Chr03G0420.1	1	24	0.805
Chr03G0421.1	1	24	0.803
Chr03G0454.1	1	22	0.636
Chr03G0455.1	1	25	0.779
Chr03G0472.1	1	25	0.709
Chr03G0524.1	1	18	0.699
Chr03G0525.1	1	18	0.84
Chr03G0526.1	1	18	0.788
Chr03G0532.1	1	19	0.913
Chr03G0533.1	1	15	0.662
Chr03G0544.1	1	18	0.774
Chr03G0547.1	1	31	0.786
Chr03G0548.1	1	20	0.615
Chr03G0558.1	1	18	0.559
Chr03G0570.1	1	16	0.596
Chr03G0597.1	1	20	0.634
Chr03G0598.1	1	18	0.807
Chr03G0601.1	1	20	0.904
Chr03G0613.1	1	20	0.809
Chr03G0614.1	1	18	0.736
Chr03G0615.1	1	19	0.901
Chr03G0616.1	1	19	0.567
Chr03G0647.1	1	15	0.698
Chr03G0651.1	1	17	0.791
Chr03G0678.1	1	22	0.767
Chr03G0683.1	1	21	0.757
Chr03G0685.1	1	19	0.762
Chr03G0687.1	1	17	0.757
Chr03G0709.1	1	18	0.594
Chr03G0743.1	1	19	0.915
Chr03G0747.1	1	21	0.465
Chr03G0757.1	1	20	0.696
Chr03G0759.1	1	19	0.908
Chr03G0763.1	1	31	0.82
Chr03G0767.1	1	16	0.784
Chr03G0793.1	1	30	0.627
Chr03G0815.1	1	18	0.531
Chr03G0849.1	1	23	0.932
Chr03G0868.1	1	15	0.685
Chr03G0876.1	1	18	0.674
Chr03G0896.1	1	24	0.9

Chr03G0897.1	1	18	0.862
Chr03G0898.1	1	19	0.741
Chr03G0903.1	1	22	0.821
Chr03G0939.1	1	26	0.869
Chr03G0947.1	1	16	0.784
Chr03G0958.1	1	22	0.781
Chr03G0995.1	1	17	0.752
Chr03G1034.1	1	17	0.905
Chr03G1036.1	1	24	0.891
Chr03G1041.1	1	17	0.87
Chr03G1086.1	1	19	0.535
Chr03G1089.1	1	20	0.81
Chr03G1110.1	1	35	0.778
Chr03G1135.1	1	33	0.665
Chr03G1142.1	1	16	0.695
Chr03G1152.1	1	23	0.723
Chr03G1161.1	1	18	0.671
Chr03G1177.1	1	21	0.724
Chr03G1180.1	1	39	0.556
Chr03G1184.1	1	22	0.807
Chr03G1188.1	1	20	0.869
Chr03G1189.1	1	25	0.505
Chr03G1193.1	1	20	0.847
Chr03G1211.1	1	18	0.814
Chr03G1215.1	1	22	0.699
Chr03G1226.1	1	21	0.857
Chr03G1233.1	1	19	0.706
Chr03G1240.1	1	19	0.867
Chr03G1247.1	1	16	0.493
Chr03G1269.1	1	17	0.872
Chr03G1272.1	1	16	0.734
Chr03G1276.1	1	25	0.619
Chr03G1282.1	1	18	0.764
Chr03G1291.1	1	21	0.496
Chr03G1292.1	1	23	0.629
Chr03G1299.1	1	15	0.803
Chr03G1312.1	1	18	0.794
Chr03G1315.1	1	22	0.79
Chr03G1318.1	1	20	0.821
Chr03G1319.1	1	21	0.456
Chr03G1320.1	1	17	0.674
Chr03G1324.1	1	16	0.927
Chr03G1329.1	1	18	0.84
Chr03G1331.1	1	24	0.498

Chr03G1335.1	1	25	0.795
Chr03G1340.1	1	19	0.59
Chr03G1343.1	1	18	0.8
Chr03G1344.1	1	17	0.723
Chr03G1345.1	1	15	0.793
Chr03G1347.1	1	17	0.792
Chr03G1348.1	1	17	0.736
Chr03G1350.1	1	17	0.747
Chr03G1365.1	1	18	0.815
Chr03G1367.1	1	24	0.679
Chr03G1368.1	1	15	0.466
Chr03G1376.1	1	17	0.542
Chr03G1377.1	1	19	0.785
Chr03G1378.1	1	17	0.788
Chr03G1382.1	1	20	0.89
Chr03G1394.1	1	17	0.691
Chr03G1398.1	1	19	0.782
Chr03G1404.1	1	19	0.596
Chr03G1410.1	1	16	0.733
Chr03G1415.1	1	16	0.789
Chr03G1420.1	1	16	0.844
Chr03G1423.1	1	17	0.894
Chr03G1434.1	1	18	0.844
Chr03G1443.1	1	19	0.587
Chr03G1452.1	1	17	0.871
Chr03G1455.1	1	19	0.77
Chr03G1456.1	1	18	0.698
Chr03G1466.1	1	20	0.835
Chr03G1468.1	1	21	0.761
Chr03G1471.1	1	19	0.532
Chr03G1472.1	1	20	0.932
Chr03G1489.1	1	18	0.927
Chr03G1493.1	1	17	0.807
Chr03G1498.1	1	19	0.793
Chr03G1502.1	1	20	0.627
Chr03G1503.1	1	19	0.791
Chr03G1504.1	1	18	0.886
Chr03G1511.1	1	20	0.859
Chr03G1513.1	1	19	0.751
Chr03G1528.1	1	18	0.796
Chr03G1533.1	1	22	0.585
Chr03G1543.1	1	19	0.88
Chr03G1554.1	1	30	0.895
Chr03G1565.1	1	15	0.658

Chr03G1570.1	1	16	0.832
Chr03G1578.1	1	18	0.538
Chr03G1582.1	1	16	0.848
Chr03G1592.1	1	20	0.828
Chr03G1600.1	1	19	0.661
Chr03G1601.1	1	20	0.872
Chr03G1609.1	1	19	0.742
Chr03G1616.1	1	19	0.743
Chr03G1620.1	1	21	0.766
Chr03G1623.1	1	21	0.857
Chr03G1625.1	1	18	0.751
Chr03G1647.1	1	19	0.665
Chr03G1648.1	1	17	0.807
Chr03G1649.1	1	21	0.721
Chr03G1654.1	1	20	0.785
Chr03G1659.1	1	18	0.527
Chr03G1661.1	1	21	0.826
Chr03G1663.1	1	20	0.652
Chr03G1669.1	1	20	0.767
Chr03G1674.1	1	24	0.763
Chr03G1676.1	1	18	0.823
Chr03G1678.1	1	18	0.638
Chr03G1692.1	1	23	0.899
Chr03G1709.1	1	19	0.58
Chr03G1710.1	1	18	0.725
Chr03G1720.1	1	21	0.796
Chr03G1736.1	1	18	0.796
Chr03G1737.1	1	24	0.841
Chr03G1738.1	1	25	0.786
Chr09G0003.1	1	19	0.735
Chr09G0005.1	1	19	0.833
Chr09G0006.1	1	18	0.772
Chr09G0020.1	1	19	0.661
Chr09G0033.1	1	19	0.661
Chr09G0034.1	1	22	0.602
Chr09G0045.1	1	21	0.744
Chr09G0059.1	1	19	0.492
Chr09G0063.1	1	19	0.595
Chr09G0066.1	1	18	0.796
Chr09G0084.1	1	19	0.801
Chr09G0085.1	1	17	0.648
Chr09G0106.1	1	24	0.561
Chr09G0141.1	1	16	0.604
Chr09G0157.1	1	20	0.667

Chr09G0173.1	1	20	0.575
Chr09G0179.1	1	23	0.651
Chr09G0198.1	1	20	0.712
Chr09G0206.1	1	17	0.743
Chr09G0212.1	1	19	0.717
Chr09G0213.1	1	26	0.614
Chr09G0214.1	1	17	0.756
Chr09G0229.1	1	20	0.784
Chr09G0241.1	1	18	0.835
Chr09G0260.1	1	17	0.697
Chr09G0262.1	1	26	0.517
Chr09G0277.1	1	22	0.659
Chr09G0279.1	1	19	0.731
Chr09G0285.1	1	1	0.503
Chr09G0289.1	1	22	0.464
Chr09G0310.1	1	18	0.797
Chr09G0311.1	1	19	0.594
Chr09G0355.1	1	27	0.74
Chr09G0368.1	1	20	0.751
Chr09G0377.1	1	23	0.711
Chr09G0397.1	1	25	0.762
Chr09G0404.1	1	22	0.878
Chr09G0419.1	1	17	0.641
Chr09G0420.1	1	17	0.712
Chr09G0423.1	1	23	0.869
Chr09G0439.1	1	19	0.755
Chr09G0440.1	1	18	0.702
Chr09G0441.1	1	23	0.651
Chr09G0448.1	1	21	0.892
Chr09G0450.1	1	16	0.721
Chr09G0471.1	1	24	0.834
Chr09G0485.1	1	21	0.657
Chr09G0494.1	1	26	0.757
Chr09G0499.1	1	1	0.494
Chr09G0505.1	1	17	0.71
Chr09G0507.1	1	29	0.525
Chr09G0531.1	1	17	0.795
Chr09G0540.1	1	16	0.811
Chr09G0544.1	1	21	0.68
Chr09G0553.1	1	19	0.629
Chr09G0563.1	1	16	0.897
Chr09G0564.1	1	19	0.913
Chr09G0574.1	1	25	0.925
Chr09G0584.1	1	23	0.894

Chr09G0592.1	1	20	0.834
Chr09G0597.1	1	19	0.772
Chr09G0601.1	1	19	0.572
Chr09G0604.1	1	21	0.959
Chr09G0613.1	1	18	0.716
Chr09G0641.1	1	17	0.765
Chr09G0646.1	1	20	0.837
Chr09G0653.1	1	23	0.674
Chr09G0656.1	1	21	0.735
Chr09G0657.1	1	21	0.718
Chr09G0659.1	1	19	0.624
Chr09G0671.1	1	28	0.724
Chr09G0673.1	1	25	0.704
Chr09G0674.1	1	19	0.674
Chr09G0675.1	1	19	0.812
Chr09G0679.1	1	16	0.685
Chr09G0680.1	1	18	0.829
Chr09G0682.1	1	17	0.812
Chr09G0696.1	1	17	0.804
Chr09G0707.1	1	16	0.735
Chr09G0716.1	1	24	0.844
Chr09G0719.1	1	24	0.63
Chr09G0721.1	1	22	0.665
Chr09G0724.1	1	22	0.677
Chr09G0749.1	1	15	0.866
Chr09G0775.1	1	19	0.784
Chr09G0784.1	1	16	0.869
Chr09G0786.1	1	20	0.569
Chr09G0799.1	1	21	0.838
Chr09G0813.1	1	22	0.802
Chr09G0821.1	1	27	0.584
Chr09G0825.1	1	18	0.762
Chr09G0830.1	1	19	0.699
Chr09G0834.1	1	19	0.93
Chr09G0835.1	1	18	0.52
Chr09G0844.1	1	18	0.591
Chr09G0845.1	1	20	0.78
Chr09G0848.1	1	15	0.71
Chr09G0855.1	1	17	0.601
Chr09G0858.1	1	17	0.832
Chr09G0860.1	1	20	0.457
Chr09G0861.1	1	19	0.875
Chr09G0866.1	1	19	0.729
Chr09G0871.1	1	17	0.75

Chr09G0888.1	1	19	0.744
Chr09G0896.1	1	20	0.867
Chr09G0900.1	1	17	0.809
Chr09G0902.1	1	15	0.77
Chr09G0903.1	1	19	0.778
Chr09G0904.1	1	19	0.671
Chr09G0907.1	1	16	0.55
Chr09G0909.1	1	19	0.84
Chr09G0918.1	1	17	0.765
Chr09G0921.1	1	18	0.942
Chr09G0923.1	1	26	0.6
Chr09G0937.1	1	22	0.75
Chr09G0957.1	1	19	0.515
Chr09G0960.1	1	18	0.765
Chr09G0966.1	1	18	0.879
Chr09G0975.1	1	19	0.89
Chr09G0976.1	1	20	0.725
Chr09G0981.1	1	21	0.634
Chr09G0989.1	1	26	0.606
Chr09G0991.1	1	19	0.822
Chr09G0998.1	1	22	0.687
Chr09G1008.1	1	24	0.758
Chr09G1009.1	1	28	0.736
Chr09G1012.1	1	19	0.789
Chr09G1019.1	1	20	0.879
Chr09G1027.1	1	21	0.84
Chr09G1046.1	1	18	0.74
Chr09G1047.1	1	19	0.777
Chr09G1050.1	1	22	0.778
Chr06G0005.1	1	20	0.613
Chr06G0026.1	1	22	0.65
Chr06G0048.1	1	21	0.783
Chr06G0067.1	1	23	0.691
Chr06G0071.1	1	18	0.828
Chr06G0081.1	1	20	0.869
Chr06G0083.1	1	19	0.751
Chr06G0085.1	1	18	0.732
Chr06G0092.1	1	19	0.47
Chr06G0096.1	1	19	0.67
Chr06G0104.1	1	19	0.876
Chr06G0127.1	1	16	0.718
Chr06G0134.1	1	15	0.511
Chr06G0140.1	1	19	0.799
Chr06G0144.1	1	16	0.857

Chr06G0145.1	1	24	0.894
Chr06G0149.1	1	17	0.472
Chr06G0152.1	1	19	0.819
Chr06G0157.1	1	20	0.77
Chr06G0160.1	1	23	0.602
Chr06G0166.1	1	17	0.655
Chr06G0169.1	1	21	0.841
Chr06G0170.1	1	21	0.821
Chr06G0173.1	1	18	0.523
Chr06G0175.1	1	17	0.828
Chr06G0176.1	1	19	0.677
Chr06G0193.1	1	16	0.693
Chr06G0197.1	1	34	0.895
Chr06G0206.1	1	22	0.74
Chr06G0209.1	1	19	0.857
Chr06G0210.1	1	20	0.704
Chr06G0214.1	1	21	0.755
Chr06G0217.1	1	23	0.909
Chr06G0227.1	1	15	0.668
Chr06G0229.1	1	22	0.756
Chr06G0231.1	1	17	0.785
Chr06G0232.1	1	19	0.787
Chr06G0254.1	1	24	0.718
Chr06G0257.1	1	21	0.769
Chr06G0271.1	1	26	0.734
Chr06G0275.1	1	20	0.495
Chr06G0277.1	1	20	0.806
Chr06G0278.1	1	17	0.678
Chr06G0285.1	1	20	0.93
Chr06G0293.1	1	21	0.942
Chr06G0299.1	1	18	0.689
Chr06G0313.1	1	19	0.824
Chr06G0320.1	1	22	0.534
Chr06G0337.1	1	21	0.717
Chr06G0346.1	1	26	0.607
Chr06G0352.1	1	21	0.487
Chr06G0359.1	1	16	0.76
Chr06G0363.1	1	23	0.842
Chr06G0365.1	1	21	0.867
Chr06G0367.1	1	18	0.82
Chr06G0405.1	1	20	0.452
Chr06G0414.1	1	24	0.879
Chr06G0442.1	1	17	0.486
Chr06G0444.1	1	20	0.546

Chr06G0457.1	1	17	0.651
Chr06G0458.1	1	35	0.539
Chr06G0534.1	1	15	0.451
Chr06G0546.1	1	25	0.879
Chr06G0609.1	1	19	0.74
Chr06G0656.1	1	21	0.727
Chr06G0660.1	1	25	0.611
Chr06G0709.1	1	27	0.869
Chr06G0722.1	1	21	0.609
Chr06G0740.1	1	20	0.774
Chr06G0761.1	1	18	0.76
Chr06G0764.1	1	20	0.809
Chr06G0775.1	1	21	0.67
Chr06G0781.1	1	19	0.778
Chr06G0783.1	1	18	0.753
Chr06G0820.1	1	25	0.865
Chr06G0821.1	1	18	0.868
Chr06G0823.1	1	19	0.822
Chr06G0825.1	1	28	0.847
Chr06G0826.1	1	20	0.819
Chr06G0846.1	1	22	0.875
Chr06G0887.1	1	17	0.706
Chr06G0902.1	1	21	0.887
Chr06G0907.1	1	21	0.457
Chr06G0913.1	1	24	0.815
Chr06G0934.1	1	20	0.709
Chr06G0942.1	1	17	0.656
Chr06G0945.1	1	19	0.793
Chr06G0954.1	1	16	0.553
Chr06G0969.1	1	29	0.827
Chr06G0980.1	1	11	0.675
Chr06G0981.1	1	23	0.594
Chr06G0989.1	1	20	0.79
Chr06G0993.1	1	25	0.769
Chr06G0998.1	1	27	0.574
Chr06G1014.1	1	23	0.822
Chr06G1026.1	1	18	0.811
Chr06G1027.1	1	16	0.844
Chr06G1033.1	1	17	0.803
Chr06G1037.1	1	17	0.802
Chr06G1042.1	1	20	0.841
Chr06G1045.1	1	22	0.474
Chr06G1075.1	1	22	0.682
Chr06G1076.1	1	17	0.777

Chr06G1127.1	1	21	0.867
Chr06G1142.1	1	17	0.795
Chr06G1151.1	1	4	0.516
Chr06G1154.1	1	17	0.831
Chr06G1158.1	1	29	0.482
Chr06G1159.1	1	24	0.776
Chr06G1160.1	1	18	0.506
Chr06G1161.1	1	31	0.613
Chr06G1162.1	1	26	0.825
Chr06G1166.1	1	18	0.734
Chr06G1167.1	1	21	0.764
Chr06G1168.1	1	23	0.916
Chr06G1169.1	1	24	0.647
Chr06G1172.1	1	19	0.527
Chr06G1173.1	1	23	0.931
Chr06G1174.1	1	24	0.796
Chr06G1177.1	1	21	0.943
Chr06G1179.1	1	20	0.727
Chr06G1181.1	1	18	0.631
Chr06G1187.1	1	16	0.511
Chr06G1190.1	1	20	0.807
Chr06G1203.1	1	18	0.775
Chr06G1204.1	1	19	0.876
Chr06G1207.1	1	20	0.861
Chr06G1209.1	1	17	0.637
Chr06G1210.1	1	21	0.879
Chr06G1213.1	1	21	0.779
Chr06G1215.1	1	21	0.916
Chr06G1216.1	1	24	0.8
Chr06G1229.1	1	25	0.924
Chr06G1236.1	1	17	0.618
Chr06G1237.1	1	19	0.775
Chr06G1238.1	1	18	0.752
Chr06G1242.1	1	19	0.746
Chr06G1243.1	1	18	0.656
Chr06G1248.1	1	21	0.676
Chr06G1272.1	1	17	0.789
Chr06G1285.1	1	26	0.77
Chr06G1299.1	1	20	0.856
Chr06G1305.1	1	18	0.847
Chr06G1323.1	1	20	0.803
Chr06G1324.1	1	21	0.655
Chr06G1328.1	1	21	0.77
Chr06G1340.1	1	17	0.75

Chr06G1347.1	1	26	0.913
Chr06G1352.1	1	23	0.629
Chr06G1353.1	1	21	0.496
Chr06G1357.1	1	21	0.86
Chr06G1365.1	1	17	0.487
Chr06G1381.1	1	18	0.742
Chr06G1382.1	1	17	0.804
Chr06G1391.1	1	17	0.765
Chr06G1392.1	1	17	0.797
Chr06G1393.1	1	18	0.709
Chr06G1405.1	1	18	0.746
Chr06G1407.1	1	16	0.915
Chr06G1410.1	1	18	0.798
Chr06G1411.1	1	19	0.823
Chr06G1413.1	1	25	0.597
Chr06G1414.1	1	16	0.646
Chr06G1418.1	1	19	0.84
Chr06G1420.1	1	20	0.649
Chr06G1421.1	1	23	0.68
Chr06G1423.1	1	20	0.584
Chr06G1424.1	1	24	0.886
Chr06G1428.1	1	20	0.669
Chr06G1436.1	1	26	0.5
Chr06G1440.1	1	18	0.524
Chr06G1449.1	1	20	0.691
Chr06G1451.1	1	19	0.927
Chr06G1454.1	1	22	0.596
Chr06G1455.1	1	17	0.675
Chr06G1458.1	1	20	0.668
Chr06G1474.1	1	19	0.504
Chr06G1477.1	1	18	0.74
Chr06G1489.1	1	20	0.872
Chr04G0024.1	1	17	0.809
Chr04G0035.1	1	20	0.634
Chr04G0042.1	1	18	0.656
Chr04G0043.1	1	18	0.723
Chr04G0048.1	1	17	0.548
Chr04G0052.1	1	19	0.67
Chr04G0056.1	1	35	0.493
Chr04G0057.1	1	21	0.746
Chr04G0060.1	1	20	0.732
Chr04G0063.1	1	20	0.776
Chr04G0069.1	1	20	0.588
Chr04G0082.1	1	23	0.906

Chr04G0083.1	1	20	0.727
Chr04G0087.1	1	21	0.789
Chr04G0090.1	1	19	0.87
Chr04G0091.1	1	16	0.773
Chr04G0092.1	1	18	0.65
Chr04G0104.1	1	18	0.714
Chr04G0108.1	1	17	0.727
Chr04G0121.1	1	33	0.72
Chr04G0124.1	1	19	0.792
Chr04G0127.1	1	20	0.804
Chr04G0136.1	1	22	0.802
Chr04G0137.1	1	23	0.5
Chr04G0148.1	1	19	0.859
Chr04G0158.1	1	18	0.763
Chr04G0175.1	1	18	0.756
Chr04G0176.1	1	22	0.682
Chr04G0186.1	1	20	0.766
Chr04G0187.1	1	15	0.643
Chr04G0201.1	1	20	0.749
Chr04G0204.1	1	20	0.744
Chr04G0207.1	1	19	0.719
Chr04G0210.1	1	19	0.488
Chr04G0229.1	1	15	0.662
Chr04G0231.1	1	17	0.884
Chr04G0232.1	1	18	0.774
Chr04G0235.1	1	21	0.823
Chr04G0239.1	1	19	0.818
Chr04G0241.1	1	17	0.662
Chr04G0242.1	1	21	0.713
Chr04G0248.1	1	22	0.658
Chr04G0249.1	1	17	0.812
Chr04G0251.1	1	17	0.878
Chr04G0253.1	1	34	0.682
Chr04G0263.1	1	16	0.657
Chr04G0269.1	1	18	0.793
Chr04G0274.1	1	15	0.732
Chr04G0276.1	1	18	0.79
Chr04G0277.1	1	17	0.702
Chr04G0279.1	1	21	0.767
Chr04G0281.1	1	19	0.886
Chr04G0284.1	1	18	0.824
Chr04G0298.1	1	17	0.816
Chr04G0301.1	1	20	0.592
Chr04G0302.1	1	18	0.828

Chr04G0303.1	1	20	0.92
Chr04G0306.1	1	21	0.668
Chr04G0323.1	1	20	0.616
Chr04G0325.1	1	21	0.572
Chr04G0326.1	1	21	0.682
Chr04G0330.1	1	18	0.68
Chr04G0338.1	1	20	0.512
Chr04G0340.1	1	18	0.634
Chr04G0341.1	1	21	0.728
Chr04G0342.1	1	18	0.624
Chr04G0347.1	1	20	0.498
Chr04G0348.1	1	20	0.833
Chr04G0351.1	1	19	0.773
Chr04G0354.1	1	24	0.795
Chr04G0377.1	1	18	0.714
Chr04G0383.1	1	21	0.823
Chr04G0391.1	1	19	0.838
Chr04G0394.1	1	17	0.738
Chr04G0404.1	1	19	0.759
Chr04G0446.1	1	17	0.675
Chr04G0459.1	1	20	0.664
Chr04G0467.1	1	16	0.492
Chr04G0479.1	1	21	0.688
Chr04G0491.1	1	23	0.609
Chr04G0524.1	1	19	0.785
Chr04G0538.1	1	20	0.704
Chr04G0543.1	1	28	0.64
Chr04G0554.1	1	18	0.725
Chr04G0559.1	1	15	0.609
Chr04G0571.1	1	35	0.5
Chr04G0584.1	1	18	0.777
Chr04G0585.1	1	16	0.69
Chr04G0586.1	1	23	0.513
Chr04G0593.1	1	18	0.693
Chr04G0600.1	1	20	0.863
Chr04G0610.1	1	18	0.687
Chr04G0611.1	1	25	0.816
Chr04G0629.1	1	19	0.926
Chr04G0630.1	1	23	0.764
Chr04G0633.1	1	20	0.745
Chr04G0637.1	1	19	0.801
Chr04G0642.1	1	34	0.654
Chr04G0655.1	1	20	0.812
Chr04G0662.1	1	24	0.774

Chr04G0685.1	1	20	0.891
Chr04G0691.1	1	20	0.852
Chr04G0695.1	1	17	0.809
Chr04G0696.1	1	18	0.77
Chr04G0699.1	1	23	0.713
Chr04G0705.1	1	21	0.87
Chr04G0709.1	1	21	0.918
Chr04G0720.1	1	19	0.78
Chr04G0727.1	1	24	0.564
Chr04G0733.1	1	17	0.606
Chr04G0737.1	1	22	0.746
Chr04G0739.1	1	20	0.765
Chr04G0745.1	1	17	0.688
Chr04G0746.1	1	18	0.692
Chr04G0749.1	1	22	0.784
Chr04G0755.1	1	24	0.704
Chr04G0758.1	1	19	0.639
Chr04G0760.1	1	17	0.818
Chr04G0772.1	1	21	0.637
Chr04G0773.1	1	24	0.803
Chr04G0777.1	1	19	0.504
Chr04G0785.1	1	17	0.732
Chr04G0787.1	1	23	0.939
Chr04G0791.1	1	20	0.824
Chr04G0793.1	1	19	0.45
Chr04G0794.1	1	19	0.635
Chr04G0808.1	1	17	0.723
Chr04G0813.1	1	34	0.463
Chr04G0820.1	1	19	0.608
Chr04G0828.1	1	20	0.823
Chr04G0830.1	1	19	0.857
Chr04G0831.1	1	19	0.74
Chr04G0832.1	1	26	0.663
Chr04G0841.1	1	21	0.835
Chr04G0843.1	1	23	0.648
Chr04G0845.1	1	20	0.708
Chr04G0849.1	1	19	0.623
Chr04G0862.1	1	16	0.867
Chr04G0870.1	1	24	0.829
Chr04G0874.1	1	17	0.591
Chr04G0876.1	1	17	0.612
Chr04G0902.1	1	23	0.846
Chr04G0903.1	1	18	0.805
Chr04G0904.1	1	18	0.751

Chr04G0915.1	1	20	0.864
Chr04G0917.1	1	23	0.762
Chr04G0918.1	1	20	0.571
Chr04G0919.1	1	20	0.713
Chr04G0920.1	1	21	0.779
Chr04G0921.1	1	19	0.757
Chr04G0927.1	1	21	0.664
Chr04G0931.1	1	22	0.662
Chr04G0932.1	1	20	0.816
Chr04G0940.1	1	27	0.894
Chr04G0946.1	1	16	0.821
Chr04G0947.1	1	26	0.801
Chr04G0951.1	1	19	0.798
Chr04G0952.1	1	20	0.875
Chr04G0955.1	1	21	0.648
Chr04G0956.1	1	16	0.889
Chr04G0957.1	1	16	0.74
Chr04G0972.1	1	18	0.643
Chr04G0981.1	1	18	0.79
Chr04G0985.1	1	20	0.611
Chr04G0986.1	1	22	0.838
Chr04G0987.1	1	21	0.826
Chr04G0988.1	1	22	0.883
Chr04G0995.1	1	17	0.886
Chr04G0999.1	1	23	0.869
Chr04G1005.1	1	22	0.692
Chr04G1014.1	1	24	0.817
Chr04G1018.1	1	16	0.568
Chr04G1019.1	1	19	0.857
Chr04G1021.1	1	16	0.801
Chr04G1026.1	1	18	0.645
Chr04G1065.1	1	17	0.639
Chr04G1095.1	1	21	0.653
Chr04G1107.1	1	19	0.52
Chr04G1110.1	1	18	0.89
Chr04G1111.1	1	25	0.616
Chr04G1116.1	1	19	0.739
Chr04G1118.1	1	21	0.668
Chr04G1129.1	1	22	0.917
Chr04G1131.1	1	18	0.771
Chr04G1140.1	1	19	0.798
Chr04G1141.1	1	19	0.69
Chr04G1149.1	1	21	0.709
Chr04G1154.1	1	19	0.757

Chr04G1156.1	1	16	0.828
Chr04G1157.1	1	20	0.5
Chr04G1202.1	1	18	0.518
Chr04G1218.1	1	19	0.733
Chr04G1220.1	1	18	0.816
Chr04G1228.1	1	35	0.51
Chr04G1232.1	1	21	0.665
Chr04G1234.1	1	18	0.869
Chr04G1236.1	1	29	0.876
Chr04G1237.1	1	21	0.583
Chr04G1241.1	1	26	0.481
Chr04G1243.1	1	23	0.756
Chr04G1262.1	1	18	0.88
Chr04G1267.1	1	18	0.8
Chr04G1269.1	1	17	0.604
Chr04G1292.1	1	17	0.597
Chr04G1299.1	1	21	0.702
Chr04G1304.1	1	20	0.761
Chr04G1314.1	1	18	0.751
Chr04G1340.1	1	16	0.7
Chr04G1345.1	1	18	0.854
Chr04G1346.1	1	18	0.813
Chr04G1352.1	1	18	0.67
Chr04G1353.1	1	19	0.666
Chr04G1370.1	1	24	0.696
Chr04G1373.1	1	19	0.777
Chr04G1382.1	1	20	0.718
Chr04G1385.1	1	23	0.762
Chr04G1395.1	1	19	0.752
Chr04G1398.1	1	18	0.637
Chr04G1402.1	1	23	0.897
Chr04G1405.1	1	20	0.677
Chr04G1406.1	1	20	0.689
Chr04G1411.1	1	20	0.639
Chr04G1413.1	1	23	0.566
Chr04G1415.1	1	19	0.841
Chr04G1417.1	1	20	0.884
Chr04G1418.1	1	19	0.826
Chr04G1422.1	1	21	0.745
Chr04G1425.1	1	18	0.815
Chr04G1428.1	1	22	0.807
Chr04G1432.1	1	17	0.827
Chr04G1433.1	1	20	0.832
Chr04G1435.1	1	19	0.766

Chr04G1443.1	1	18	0.694
Chr04G1455.1	1	17	0.827
Chr04G1463.1	1	19	0.458
Chr04G1468.1	1	20	0.843
Chr04G1478.1	1	23	0.746
Chr04G1483.1	1	20	0.757
Chr04G1492.1	1	20	0.502
Chr04G1499.1	1	1	0.463
Chr04G1500.1	1	18	0.605
Chr04G1516.1	1	18	0.801
Chr04G1521.1	1	31	0.763
Chr04G1524.1	1	18	0.831
Chr04G1537.1	1	26	0.717
Chr04G1544.1	1	19	0.808
Chr04G1545.1	1	22	0.883
Chr04G1552.1	1	20	0.601
Chr04G1559.1	1	15	0.716
Chr04G1583.1	1	18	0.714
Chr04G1585.1	1	25	0.565
Chr04G1586.1	1	25	0.748
Chr04G1588.1	1	18	0.82
Chr04G1595.1	1	22	0.819
Chr02G0012.1	1	25	0.919
Chr02G0020.1	1	18	0.616
Chr02G0023.1	1	22	0.859
Chr02G0024.1	1	27	0.935
Chr02G0025.1	1	41	0.714
Chr02G0030.1	1	17	0.764
Chr02G0048.1	1	19	0.835
Chr02G0065.1	1	16	0.618
Chr02G0066.1	1	16	0.676
Chr02G0070.1	1	23	0.504
Chr02G0073.1	1	20	0.613
Chr02G0081.1	1	29	0.911
Chr02G0090.1	1	21	0.494
Chr02G0091.1	1	19	0.774
Chr02G0093.1	1	18	0.668
Chr02G0096.1	1	21	0.832
Chr02G0102.1	1	19	0.685
Chr02G0120.1	1	17	0.884
Chr02G0135.1	1	17	0.796
Chr02G0144.1	1	19	0.849
Chr02G0148.1	1	20	0.502
Chr02G0151.1	1	24	0.776

Chr02G0155.1	1	20	0.921
Chr02G0157.1	1	36	0.691
Chr02G0169.1	1	22	0.904
Chr02G0182.1	1	16	0.688
Chr02G0201.1	1	17	0.77
Chr02G0230.1	1	20	0.693
Chr02G0231.1	1	19	0.735
Chr02G0236.1	1	18	0.506
Chr02G0241.1	1	20	0.852
Chr02G0252.1	1	17	0.776
Chr02G0257.1	1	20	0.809
Chr02G0260.1	1	25	0.881
Chr02G0262.1	1	26	0.879
Chr02G0269.1	1	19	0.683
Chr02G0270.1	1	19	0.496
Chr02G0274.1	1	16	0.618
Chr02G0308.1	1	17	0.733
Chr02G0325.1	1	22	0.513
Chr02G0358.1	1	18	0.827
Chr02G0359.1	1	16	0.554
Chr02G0366.1	1	16	0.794
Chr02G0367.1	1	23	0.5
Chr02G0379.1	1	19	0.637
Chr02G0399.1	1	33	0.896
Chr02G0401.1	1	20	0.75
Chr02G0402.1	1	22	0.795
Chr02G0404.1	1	20	0.896
Chr02G0406.1	1	19	0.77
Chr02G0410.1	1	19	0.638
Chr02G0411.1	1	21	0.855
Chr02G0413.1	1	25	0.936
Chr02G0429.1	1	19	0.743
Chr02G0441.1	1	17	0.649
Chr02G0445.1	1	18	0.815
Chr02G0452.1	1	17	0.678
Chr02G0453.1	1	20	0.655
Chr02G0468.1	1	20	0.861
Chr02G0477.1	1	16	0.65
Chr02G0478.1	1	18	0.856
Chr02G0481.1	1	18	0.474
Chr02G0482.1	1	23	0.671
Chr02G0495.1	1	23	0.734
Chr02G0508.1	1	19	0.847
Chr02G0509.1	1	18	0.624

Chr02G0515.1	1	18	0.743
Chr02G0520.1	1	20	0.84
Chr02G0526.1	1	21	0.653
Chr02G0530.1	1	26	0.929
Chr02G0531.1	1	19	0.537
Chr02G0533.1	1	20	0.779
Chr02G0534.1	1	19	0.817
Chr02G0536.1	1	21	0.648
Chr02G0537.1	1	25	0.867
Chr02G0538.1	1	17	0.834
Chr02G0541.1	1	17	0.722
Chr02G0546.1	1	15	0.73
Chr02G0555.1	1	19	0.909
Chr02G0564.1	1	24	0.727
Chr02G0568.1	1	20	0.68
Chr02G0600.1	1	30	0.792
Chr02G0608.1	1	18	0.738
Chr02G0616.1	1	16	0.881
Chr02G0631.1	1	19	0.707
Chr02G0666.1	1	24	0.648
Chr02G0675.1	1	19	0.871
Chr02G0691.1	1	18	0.669
Chr02G0703.1	1	21	0.68
Chr02G0705.1	1	21	0.513
Chr02G0734.1	1	26	0.626
Chr10G0002.1	1	26	0.56
