# Supplementary material for: Decoding Pecan’s Fungal Foe: A Genomic Insight into Colletotrichum plurivorum Isolate W-6
Source: J Fungi (Basel). 2025 Mar 5;11(3):203. doi: 10.3390/jof11030203 (PMC11943440; doi:10.3390/jof11030203)
Supplement: Supplementary file 1 [file jof-11-00203-s001.zip › Table S17.pdf]

Table S17. List of transmembrane (TM) proteins in isolate W-6 genome.

TM protein ID	Number of predicted transmembrane_helix
Chr01G0002.1	3
Chr01G0011.1	4
Chr01G0012.1	1
Chr01G0015.1	8
Chr01G0022.1	12
Chr01G0029.1	11
Chr01G0032.1	12
Chr01G0041.1	3
Chr01G0043.1	2
Chr01G0053.1	11
Chr01G0054.1	1
Chr01G0069.1	1
Chr01G0081.1	4
Chr01G0094.1	7
Chr01G0100.1	12
Chr01G0111.1	12
Chr01G0116.1	1
Chr01G0117.1	5
Chr01G0126.1	12
Chr01G0127.1	1
Chr01G0128.1	3
Chr01G0130.1	2
Chr01G0132.1	14
Chr01G0133.1	6
Chr01G0134.1	3
Chr01G0135.1	12
Chr01G0137.1	11
Chr01G0140.1	1
Chr01G0143.1	13
Chr01G0152.1	14
Chr01G0159.1	1
Chr01G0163.1	1
Chr01G0165.1	1
Chr01G0170.1	1
Chr01G0172.1	7
Chr01G0173.1	1
Chr01G0175.1	10
Chr01G0176.1	12
Chr01G0184.1	12
Chr01G0189.1	7
Chr01G0197.1	1
Chr01G0199.1	10

Chr01G0202.1	1
Chr01G0210.1	5
Chr01G0216.1	14
Chr01G0219.1	12
Chr01G0221.1	1
Chr01G0226.1	4
Chr01G0227.1	12
Chr01G0228.1	5
Chr01G0231.1	8
Chr01G0236.1	1
Chr01G0237.1	6
Chr01G0245.1	10
Chr01G0252.1	14
Chr01G0256.1	8
Chr01G0257.1	11
Chr01G0260.1	7
Chr01G0261.1	1
Chr01G0264.1	12
Chr01G0267.1	7
Chr01G0270.1	10
Chr01G0272.1	5
Chr01G0279.1	12
Chr01G0285.1	1
Chr01G0288.1	7
Chr01G0291.1	1
Chr01G0292.1	1
Chr01G0293.1	2
Chr01G0294.1	7
Chr01G0295.1	1
Chr01G0297.1	1
Chr01G0298.1	2
Chr01G0306.1	1
Chr01G0310.1	7
Chr01G0313.1	11
Chr01G0326.1	12
Chr01G0343.1	1
Chr01G0344.1	6
Chr01G0348.1	1
Chr01G0349.1	3
Chr01G0350.1	9
Chr01G0351.1	8
Chr01G0353.1	11
Chr01G0354.1	3
Chr01G0371.1	13

Chr01G0372.1	8
Chr01G0376.1	11
Chr01G0377.1	2
Chr01G0382.1	9
Chr01G0383.1	9
Chr01G0384.1	4
Chr01G0387.1	4
Chr01G0388.1	1
Chr01G0389.1	1
Chr01G0395.1	10
Chr01G0407.1	2
Chr01G0409.1	10
Chr01G0413.1	7
Chr01G0418.1	1
Chr01G0422.1	1
Chr01G0423.1	1
Chr01G0433.1	1
Chr01G0443.1	4
Chr01G0456.1	2
Chr01G0457.1	6
Chr01G0463.1	7
Chr01G0468.1	11
Chr01G0470.1	8
Chr01G0477.1	8
Chr01G0479.1	1
Chr01G0484.1	9
Chr01G0485.1	1
Chr01G0489.1	7
Chr01G0491.1	1
Chr01G0501.1	2
Chr01G0503.1	1
Chr01G0504.1	12
Chr01G0509.1	12
Chr01G0520.1	12
Chr01G0526.1	12
Chr01G0530.1	1
Chr01G0542.1	5
Chr01G0547.1	1
Chr01G0550.1	10
Chr01G0559.1	10
Chr01G0560.1	12
Chr01G0575.1	1
Chr01G0576.1	1
Chr01G0578.1	1

Chr01G0582.1	1
Chr01G0584.1	1
Chr01G0587.1	12
Chr01G0592.1	8
Chr01G0604.1	1
Chr01G0608.1	1
Chr01G0613.1	2
Chr01G0614.1	1
Chr01G0625.1	1
Chr01G0627.1	7
Chr01G0629.1	2
Chr01G0635.1	5
Chr01G0640.1	6
Chr01G0642.1	1
Chr01G0644.1	6
Chr01G0647.1	3
Chr01G0648.1	1
Chr01G0650.1	14
Chr01G0651.1	3
Chr01G0653.1	11
Chr01G0661.1	2
Chr01G0662.1	9
Chr01G0665.1	14
Chr01G0670.1	1
Chr01G0671.1	12
Chr01G0674.1	11
Chr01G0680.1	3
Chr01G0682.1	7
Chr01G0687.1	3
Chr01G0695.1	4
Chr01G0696.1	1
Chr01G0703.1	10
Chr01G0707.1	1
Chr01G0710.1	1
Chr01G0711.1	4
Chr01G0714.1	2
Chr01G0717.1	2
Chr01G0718.1	7
Chr01G0720.1	1
Chr01G0727.1	6
Chr01G0729.1	6
Chr01G0732.1	3
Chr01G0735.1	6
Chr01G0739.1	13

Chr01G0740.1	4
Chr01G0742.1	1
Chr01G0745.1	3
Chr01G0747.1	11
Chr01G0750.1	11
Chr01G0752.1	1
Chr01G0759.1	1
Chr01G0766.1	7
Chr01G0769.1	7
Chr01G0771.1	1
Chr01G0772.1	11
Chr01G0776.1	12
Chr01G0806.1	10
Chr01G0817.1	7
Chr01G0821.1	12
Chr01G0826.1	5
Chr01G0828.1	6
Chr01G0833.1	1
Chr01G0834.1	3
Chr01G0836.1	1
Chr01G0837.1	1
Chr01G0838.1	7
Chr01G0840.1	1
Chr01G0849.1	11
Chr01G0853.1	1
Chr01G0860.1	2
Chr01G0862.1	1
Chr01G0866.1	5
Chr01G0867.1	1
Chr01G0871.1	12
Chr01G0878.1	1
Chr01G0879.1	10
Chr01G0880.1	14
Chr01G0883.1	1
Chr01G0904.1	1
Chr01G0906.1	4
Chr01G0908.1	7
Chr01G0910.1	1
Chr01G0914.1	10
Chr01G0917.1	11
Chr01G0918.1	2
Chr01G0923.1	7
Chr01G0941.1	14
Chr01G0947.1	2

Chr01G0948.1	10
Chr01G0950.1	4
Chr01G0951.1	11
Chr01G0955.1	7
Chr01G0959.1	3
Chr01G0963.1	2
Chr01G0967.1	2
Chr01G0971.1	1
Chr01G0978.1	6
Chr01G0979.1	1
Chr01G0989.1	10
Chr01G0994.1	2
Chr01G1000.1	14
Chr01G1001.1	13
Chr01G1003.1	12
Chr01G1005.1	1
Chr01G1009.1	2
Chr01G1010.1	2
Chr01G1011.1	5
Chr01G1015.1	3
Chr01G1022.1	11
Chr01G1023.1	1
Chr01G1025.1	11
Chr01G1039.1	4
Chr01G1043.1	1
Chr01G1050.1	8
Chr01G1051.1	3
Chr01G1055.1	12
Chr01G1060.1	5
Chr01G1063.1	2
Chr01G1064.1	6
Chr01G1069.1	3
Chr01G1071.1	12
Chr01G1075.1	4
Chr01G1078.1	7
Chr01G1081.1	11
Chr01G1082.1	11
Chr01G1101.1	10
Chr01G1102.1	1
Chr01G1106.1	12
Chr01G1107.1	1
Chr01G1111.1	3
Chr01G1114.1	7
Chr01G1128.1	11

Chr01G1135.1	1
Chr01G1140.1	11
Chr01G1148.1	9
Chr01G1149.1	7
Chr01G1162.1	2
Chr01G1171.1	1
Chr01G1172.1	1
Chr01G1182.1	2
Chr01G1183.1	1
Chr01G1188.1	15
Chr01G1189.1	6
Chr01G1198.1	1
Chr01G1208.1	1
Chr01G1219.1	6
Chr01G1223.1	2
Chr01G1226.1	1
Chr01G1227.1	1
Chr01G1240.1	4
Chr01G1244.1	5
Chr01G1245.1	2
Chr01G1250.1	1
Chr01G1255.1	5
Chr01G1258.1	2
Chr01G1273.1	12
Chr01G1280.1	9
Chr01G1284.1	1
Chr01G1288.1	1
Chr01G1290.1	4
Chr01G1301.1	1
Chr01G1316.1	1
Chr01G1318.1	1
Chr01G1324.1	12
Chr01G1328.1	8
Chr01G1329.1	2
Chr01G1330.1	11
Chr01G1339.1	2
Chr01G1348.1	9
Chr01G1374.1	1
Chr01G1377.1	1
Chr01G1382.1	1
Chr01G1383.1	3
Chr01G1385.1	1
Chr01G1391.1	1
Chr01G1396.1	10

Chr01G1398.1	1
Chr01G1399.1	3
Chr01G1400.1	12
Chr01G1410.1	11
Chr01G1415.1	12
Chr01G1418.1	1
Chr01G1427.1	4
Chr01G1434.1	2
Chr01G1441.1	1
Chr01G1442.1	11
Chr01G1444.1	1
Chr01G1450.1	1
Chr01G1451.1	1
Chr01G1455.1	1
Chr01G1456.1	4
Chr01G1467.1	1
Chr01G1481.1	8
Chr01G1490.1	6
Chr01G1493.1	12
Chr01G1494.1	12
Chr01G1498.1	4
Chr01G1503.1	1
Chr01G1504.1	7
Chr01G1510.1	1
Chr01G1520.1	11
Chr01G1525.1	1
Chr01G1526.1	5
Chr01G1531.1	14
Chr01G1533.1	1
Chr01G1537.1	7
Chr01G1550.1	5
Chr01G1553.1	3
Chr01G1554.1	1
Chr01G1555.1	12
Chr01G1556.1	12
Chr01G1558.1	2
Chr01G1560.1	3
Chr01G1562.1	2
Chr01G1564.1	10
Chr01G1574.1	2
Chr01G1581.1	7
Chr01G1587.1	2
Chr01G1588.1	3
Chr01G1591.1	12

Chr01G1592.1	6
Chr01G1594.1	12
Chr01G1596.1	9
Chr01G1597.1	12
Chr01G1598.1	10
Chr01G1600.1	5
Chr01G1606.1	2
Chr01G1611.1	14
Chr01G1612.1	11
Chr01G1616.1	10
Chr01G1617.1	7
Chr01G1623.1	9
Chr01G1625.1	3
Chr01G1645.1	1
Chr01G1650.1	1
Chr01G1661.1	6
Chr01G1662.1	3
Chr01G1664.1	1
Chr01G1669.1	9
Chr01G1682.1	1
Chr01G1693.1	10
Chr01G1696.1	1
Chr01G1697.1	4
Chr01G1701.1	4
Chr01G1706.1	1
Chr01G1709.1	11
Chr01G1712.1	11
Chr01G1714.1	12
Chr01G1721.1	12
Chr01G1722.1	8
Chr01G1724.1	1
Chr01G1727.1	2
Chr01G1731.1	5
Chr01G1738.1	1
Chr01G1739.1	1
Chr01G1740.1	1
Chr01G1750.1	3
Chr01G1761.1	12
Chr01G1763.1	9
Chr01G1764.1	1
Chr01G1765.1	12
Chr01G1769.1	4
Chr01G1771.1	11
Chr01G1773.1	8

Chr01G1776.1	12
Chr01G1780.1	7
Chr01G1788.1	1
Chr01G1790.1	11
Chr01G1791.1	1
Chr01G1793.1	1
Chr01G1794.1	6
Chr01G1795.1	1
Chr01G1796.1	10
Chr01G1800.1	1
Chr01G1805.1	3
Chr01G1812.1	15
Chr01G1815.1	12
Chr01G1817.1	7
Chr01G1821.1	9
Chr01G1826.1	3
Chr01G1835.1	6
Chr01G1840.1	9
Chr01G1842.1	12
Chr01G1847.1	1
Chr01G1856.1	2
Chr01G1857.1	1
Chr01G1862.1	4
Chr01G1864.1	11
Chr01G1866.1	7
Chr01G1867.1	9
Chr01G1869.1	1
Chr01G1870.1	7
Chr01G1874.1	8
Chr01G1875.1	1
Chr01G1876.1	1
Chr01G1885.1	3
Chr01G1886.1	4
Chr01G1887.1	1
Chr01G1890.1	1
Chr01G1902.1	9
Chr01G1912.1	7
Chr01G1918.1	14
Chr01G1925.1	1
Chr01G1931.1	10
Chr01G1932.1	6
Chr01G1933.1	11
Chr01G1936.1	3
Chr01G1945.1	2

Chr01G1947.1	1
Chr01G1948.1	3
Chr01G1951.1	1
Chr01G1955.1	5
Chr01G1958.1	1
Chr01G1959.1	5
Chr01G1967.1	14
Chr01G1969.1	3
Chr01G1974.1	10
Chr01G1982.1	11
Chr01G1985.1	1
Chr01G1991.1	6
Chr01G2000.1	1
Chr01G2009.1	2
Chr01G2014.1	2
Chr01G2016.1	2
Chr01G2017.1	1
Chr01G2025.1	2
Chr01G2026.1	5
Chr01G2027.1	1
Chr01G2028.1	13
Chr01G2029.1	1
Chr01G2030.1	1
Chr01G2034.1	1
Chr01G2051.1	5
Chr01G2055.1	4
Chr01G2056.1	9
Chr01G2061.1	5
Chr01G2062.1	2
Chr01G2065.1	13
Chr01G2068.1	2
Chr01G2070.1	7
Chr01G2077.1	3
Chr01G2080.1	8
Chr01G2085.1	2
Chr01G2087.1	7
Chr01G2091.1	1
Chr01G2092.1	1
Chr01G2093.1	10
Chr01G2099.1	1
Chr01G2100.1	4
Chr01G2109.1	14
Chr01G2110.1	3
Chr01G2113.1	1

Chr01G2114.1	8
Chr01G2123.1	1
Chr01G2131.1	5
Chr01G2132.1	1
Chr01G2134.1	1
Chr01G2136.1	1
Chr01G2140.1	11
Chr01G2143.1	2
Chr01G2151.1	8
Chr01G2154.1	5
Chr01G2156.1	3
Chr01G2159.1	1
Chr01G2160.1	1
Chr01G2161.1	1
Chr01G2162.1	1
Chr01G2164.1	7
Chr01G2171.1	1
Chr01G2172.1	10
Chr01G2178.1	1
Chr01G2181.1	1
Chr01G2182.1	1
Chr01G2186.1	1
Chr01G2192.1	4
Chr01G2196.1	14
Chr01G2202.1	2
Chr01G2205.1	3
Chr01G2216.1	1
Chr01G2228.1	1
Chr01G2233.1	10
Chr01G2234.1	2
Chr01G2247.1	1
Chr01G2253.1	3
Chr01G2262.1	10
Chr01G2263.1	2
Chr01G2277.1	1
Chr01G2280.1	1
Chr01G2284.1	1
Chr01G2289.1	2
Chr01G2291.1	12
Chr01G2293.1	1
Chr01G2299.1	2
Chr01G2304.1	1
Chr01G2311.1	5
Chr01G2312.1	7

Chr01G2315.1	1
Chr01G2324.1	1
Chr01G2335.1	3
Chr01G2345.1	12
Chr01G2353.1	3
Chr01G2357.1	4
Chr01G2361.1	8
Chr01G2373.1	11
Chr01G2375.1	9
Chr01G2378.1	4
Chr01G2391.1	4
Chr01G2395.1	4
Chr01G2399.1	9
Chr01G2406.1	2
Chr01G2407.1	2
Chr01G2409.1	1
Chr01G2411.1	12
Chr01G2412.1	7
Chr01G2415.1	11
Chr01G2416.1	14
Chr01G2419.1	4
Chr01G2423.1	4
Chr01G2434.1	7
Chr01G2437.1	1
Chr01G2445.1	9
Chr01G2451.1	4
Chr01G2455.1	9
Chr01G2458.1	11
Chr01G2459.1	1
Chr01G2462.1	7
Chr01G2464.1	4
Chr01G2468.1	8
Chr01G2469.1	2
Chr01G2474.1	1
Chr01G2494.1	12
Chr01G2500.1	1
Chr01G2501.1	1
Chr01G2504.1	6
Chr01G2508.1	4
Chr01G2511.1	8
Chr01G2515.1	12
Chr01G2516.1	12
Chr01G2517.1	1
Chr01G2518.1	4

Chr01G2528.1	11
Chr01G2529.1	1
Chr01G2530.1	1
Chr01G2531.1	1
Chr01G2533.1	2
Chr01G2535.1	2
Chr01G2537.1	1
Chr01G2539.1	1
Chr01G2546.1	10
Chr01G2550.1	5
Chr01G2560.1	10
Chr01G2561.1	14
Chr01G2566.1	7
Chr01G2569.1	3
Chr01G2571.1	12
Chr01G2573.1	1
Chr01G2586.1	7
Chr01G2588.1	6
Chr01G2596.1	1
Chr01G2606.1	11
Chr01G2607.1	9
Chr01G2609.1	10
Chr01G2612.1	1
Chr01G2629.1	8
Chr01G2632.1	7
Chr01G2633.1	12
Chr01G2636.1	8
Chr01G2643.1	1
Chr01G2644.1	2
Chr01G2651.1	6
Chr01G2657.1	8
Chr01G2658.1	1
Chr01G2666.1	4
Chr01G2672.1	1
Chr01G2681.1	1
Chr01G2682.1	3
Chr01G2687.1	2
Chr01G2689.1	1
Chr01G2694.1	12
Chr01G2698.1	10
Chr01G2704.1	1
Chr01G2707.1	11
Chr01G2716.1	5
Chr01G2718.1	2

Chr01G2719.1	1
Chr01G2735.1	1
Chr01G2736.1	4
Chr01G2738.1	10
Chr01G2740.1	4
Chr01G2742.1	1
Chr01G2744.1	1
Chr01G2745.1	12
Chr01G2746.1	12
Chr01G2748.1	4
Chr01G2760.1	4
Chr01G2761.1	2
Chr01G2770.1	11
Chr01G2773.1	7
Chr01G2775.1	12
Chr01G2780.1	12
Chr01G2782.1	1
Chr01G2785.1	6
Chr05G0872.1	1
Chr05G0859.1	10
Chr05G0858.1	3
Chr05G0854.1	2
Chr05G0850.1	1
Chr05G0849.1	1
Chr05G0848.1	7
Chr05G0844.1	8
Chr05G0828.1	2
Chr05G0827.1	4
Chr05G0826.1	6
Chr05G0823.1	10
Chr05G0814.1	4
Chr05G0809.1	12
Chr05G0804.1	5
Chr05G0801.1	2
Chr05G0797.1	1
Chr05G0794.1	3
Chr05G0789.1	13
Chr05G0779.1	5
Chr05G0769.1	4
Chr05G0763.1	6
Chr05G0752.1	3
Chr05G0750.1	2
Chr05G0744.1	1
Chr05G0740.1	1

Chr05G0729.1	1
Chr05G0724.1	6
Chr05G0719.1	3
Chr05G0716.1	4
Chr05G0709.1	1
Chr05G0705.1	1
Chr05G0698.1	5
Chr05G0688.1	1
Chr05G0683.1	2
Chr05G0679.1	1
Chr05G0662.1	4
Chr05G0645.1	12
Chr05G0637.1	2
Chr05G0636.1	3
Chr05G0623.1	1
Chr05G0620.1	12
Chr05G0618.1	5
Chr05G0611.1	4
Chr05G0608.1	4
Chr05G0596.1	10
Chr05G0584.1	1
Chr05G0581.1	4
Chr05G0578.1	4
Chr05G0577.1	2
Chr05G0576.1	1
Chr05G0568.1	1
Chr05G0567.1	1
Chr05G0565.1	10
Chr05G0560.1	1
Chr05G0559.1	1
Chr05G0550.1	1
Chr05G0544.1	1
Chr05G0535.1	1
Chr05G0534.1	1
Chr05G0531.1	1
Chr05G0525.1	10
Chr05G0523.1	11
Chr05G0518.1	1
Chr05G0512.1	1
Chr05G0505.1	2
Chr05G0501.1	12
Chr05G0490.1	1
Chr05G0486.1	1
Chr05G0485.1	6

Chr05G0471.1	1
Chr05G0469.1	1
Chr05G0465.1	2
Chr05G0458.1	3
Chr05G0433.1	1
Chr05G0431.1	2
Chr05G0426.1	4
Chr05G0419.1	1
Chr05G0416.1	10
Chr05G0407.1	10
Chr05G0402.1	9
Chr05G0399.1	1
Chr05G0397.1	9
Chr05G0396.1	1
Chr05G0394.1	2
Chr05G0391.1	3
Chr05G0389.1	1
Chr05G0384.1	1
Chr05G0381.1	1
Chr05G0379.1	12
Chr05G0377.1	7
Chr05G0362.1	2
Chr05G0358.1	1
Chr05G0355.1	1
Chr05G0354.1	2
Chr05G0349.1	7
Chr05G0340.1	1
Chr05G0339.1	7
Chr05G0338.1	1
Chr05G0336.1	1
Chr05G0335.1	1
Chr05G0331.1	10
Chr05G0328.1	10
Chr05G0324.1	2
Chr05G0321.1	4
Chr05G0320.1	3
Chr05G0319.1	3
Chr05G0315.1	1
Chr05G0311.1	12
Chr05G0307.1	2
Chr05G0299.1	12
Chr05G0298.1	2
Chr05G0296.1	8
Chr05G0293.1	4

Chr05G0292.1	11
Chr05G0291.1	12
Chr05G0281.1	1
Chr05G0273.1	5
Chr05G0272.1	10
Chr05G0271.1	1
Chr05G0270.1	12
Chr05G0269.1	2
Chr05G0263.1	12
Chr05G0259.1	14
Chr05G0252.1	1
Chr05G0243.1	1
Chr05G0241.1	4
Chr05G0225.1	6
Chr05G0223.1	4
Chr05G0221.1	6
Chr05G0220.1	12
Chr05G0216.1	2
Chr05G0214.1	4
Chr05G0215.1	1
Chr05G0211.1	12
Chr05G0202.1	1
Chr05G0199.1	13
Chr05G0198.1	14
Chr05G0188.1	11
Chr05G0185.1	1
Chr05G0183.1	1
Chr05G0173.1	12
Chr05G0171.1	7
Chr05G0162.1	10
Chr05G0158.1	11
Chr05G0156.1	12
Chr05G0154.1	11
Chr05G0151.1	2
Chr05G0137.1	12
Chr05G0136.1	9
Chr05G0135.1	11
Chr05G0127.1	14
Chr05G0125.1	1
Chr05G0124.1	1
Chr05G0122.1	6
Chr05G0118.1	6
Chr05G0114.1	10
Chr05G0108.1	7

Chr05G0099.1	4
Chr05G0094.1	2
Chr05G0090.1	13
Chr05G0089.1	9
Chr05G0088.1	10
Chr05G0085.1	11
Chr05G0081.1	1
Chr05G0077.1	2
Chr05G0076.1	10
Chr05G0073.1	1
Chr05G0070.1	1
Chr05G0069.1	1
Chr05G0068.1	13
Chr05G0067.1	9
Chr05G0066.1	4
Chr05G0062.1	7
Chr05G0061.1	2
Chr05G0050.1	1
Chr05G0049.1	12
Chr05G0045.1	4
Chr05G0037.1	1
Chr05G0031.1	9
Chr05G0029.1	10
Chr05G0025.1	12
Chr05G0022.1	1
Chr05G0007.1	6
Chr07G0004.1	8
Chr07G0008.1	4
Chr07G0012.1	1
Chr07G0017.1	4
Chr07G0020.1	7
Chr07G0023.1	1
Chr07G0031.1	4
Chr07G0037.1	6
Chr07G0041.1	3
Chr07G0052.1	1
Chr07G0054.1	5
Chr07G0059.1	12
Chr07G0064.1	1
Chr07G0071.1	5
Chr07G0079.1	10
Chr07G0085.1	10
Chr07G0087.1	1
Chr07G0088.1	12

Chr07G0092.1	14
Chr07G0097.1	1
Chr07G0098.1	1
Chr07G0099.1	1
Chr07G0100.1	1
Chr07G0101.1	1
Chr07G0102.1	4
Chr07G0103.1	1
Chr07G0105.1	9
Chr07G0108.1	4
Chr07G0109.1	10
Chr07G0110.1	1
Chr07G0113.1	1
Chr07G0114.1	11
Chr07G0116.1	14
Chr07G0122.1	1
Chr07G0127.1	1
Chr07G0129.1	7
Chr07G0131.1	5
Chr07G0137.1	3
Chr07G0151.1	1
Chr07G0158.1	1
Chr07G0159.1	3
Chr07G0162.1	12
Chr07G0164.1	7
Chr07G0166.1	1
Chr07G0168.1	6
Chr07G0175.1	5
Chr07G0185.1	7
Chr07G0188.1	1
Chr07G0193.1	9
Chr07G0200.1	10
Chr07G0201.1	2
Chr07G0204.1	14
Chr07G0212.1	4
Chr07G0215.1	7
Chr07G0216.1	11
Chr07G0217.1	1
Chr07G0226.1	10
Chr07G0227.1	7
Chr07G0229.1	2
Chr07G0230.1	1
Chr07G0234.1	12
Chr07G0242.1	1

Chr07G0264.1	1
Chr07G0265.1	2
Chr07G0270.1	7
Chr07G0272.1	5
Chr07G0276.1	12
Chr07G0285.1	9
Chr07G0287.1	1
Chr07G0289.1	12
Chr07G0294.1	7
Chr07G0302.1	4
Chr07G0305.1	1
Chr07G0308.1	1
Chr07G0313.1	11
Chr07G0317.1	3
Chr07G0322.1	7
Chr07G0327.1	12
Chr07G0331.1	1
Chr07G0332.1	6
Chr07G0333.1	1
Chr07G0336.1	13
Chr07G0338.1	9
Chr07G0339.1	6
Chr07G0343.1	3
Chr07G0346.1	1
Chr07G0359.1	1
Chr07G0366.1	10
Chr07G0367.1	11
Chr07G0375.1	1
Chr07G0377.1	1
Chr07G0380.1	4
Chr07G0381.1	7
Chr07G0383.1	14
Chr07G0387.1	8
Chr07G0389.1	1
Chr07G0400.1	2
Chr07G0402.1	2
Chr07G0404.1	10
Chr07G0409.1	4
Chr07G0416.1	11
Chr07G0420.1	7
Chr07G0422.1	1
Chr07G0429.1	1
Chr07G0430.1	1
Chr07G0432.1	1

Chr07G0435.1	3
Chr07G0438.1	12
Chr07G0440.1	4
Chr07G0445.1	12
Chr07G0449.1	1
Chr07G0453.1	7
Chr07G0466.1	6
Chr07G0483.1	11
Chr07G0488.1	1
Chr07G0492.1	12
Chr07G0501.1	10
Chr07G0511.1	1
Chr07G0513.1	1
Chr07G0514.1	1
Chr07G0515.1	12
Chr07G0517.1	11
Chr07G0524.1	1
Chr07G0530.1	10
Chr07G0534.1	1
Chr07G0537.1	7
Chr07G0546.1	1
Chr07G0556.1	9
Chr07G0561.1	1
Chr07G0566.1	11
Chr07G0572.1	7
Chr07G0573.1	9
Chr07G0579.1	5
Chr07G0581.1	7
Chr07G0584.1	4
Chr07G0585.1	10
Chr07G0587.1	9
Chr07G0602.1	7
Chr07G0608.1	1
Chr07G0616.1	1
Chr07G0620.1	10
Chr07G0626.1	1
Chr07G0637.1	1
Chr07G0638.1	9
Chr07G0649.1	3
Chr07G0650.1	2
Chr07G0655.1	2
Chr07G0660.1	1
Chr07G0668.1	12
Chr07G0672.1	2

Chr07G0673.1	1
Chr07G0674.1	5
Chr07G0686.1	1
Chr07G0690.1	1
Chr07G0698.1	1
Chr07G0701.1	6
Chr07G0703.1	14
Chr07G0705.1	2
Chr07G0706.1	6
Chr07G0711.1	12
Chr07G0716.1	1
Chr07G0718.1	2
Chr07G0722.1	1
Chr07G0724.1	11
Chr07G0733.1	11
Chr07G0743.1	1
Chr07G0750.1	1
Chr07G0767.1	4
Chr07G0773.1	9
Chr07G0777.1	11
Chr07G0788.1	4
Chr07G0801.1	6
Chr07G0806.1	23
Chr07G0811.1	2
Chr07G0812.1	1
Chr07G0815.1	8
Chr07G0818.1	7
Chr07G0841.1	5
Chr07G0845.1	4
Chr07G0848.1	16
Chr07G0855.1	12
Chr07G0860.1	10
Chr07G0870.1	5
Chr07G0872.1	7
Chr07G0876.1	4
Chr07G0877.1	2
Chr07G0884.1	1
Chr07G0887.1	12
Chr07G0890.1	7
Chr07G0892.1	4
Chr07G0894.1	10
Chr07G0897.1	12
Chr07G0899.1	1
Chr07G0906.1	12

Chr07G0907.1	10
Chr07G0912.1	12
Chr07G0916.1	1
Chr07G0919.1	12
Chr07G0929.1	9
Chr07G0931.1	1
Chr07G0938.1	1
Chr07G0945.1	1
Chr07G0953.1	5
Chr07G0960.1	1
Chr07G0961.1	6
Chr07G0967.1	3
Chr07G0975.1	10
Chr07G0976.1	7
Chr07G0977.1	12
Chr07G0980.1	10
Chr07G0982.1	1
Chr07G0985.1	12
Chr07G0991.1	1
Chr07G0998.1	4
Chr07G0999.1	1
Chr07G1002.1	1
Chr07G1005.1	11
Chr07G1010.1	5
Chr07G1013.1	8
Chr07G1019.1	10
Chr07G1037.1	11
Chr07G1039.1	3
Chr07G1040.1	7
Chr07G1041.1	10
Chr07G1042.1	1
Chr07G1052.1	1
Chr07G1053.1	19
Chr07G1057.1	1
Chr07G1064.1	3
Chr07G1067.1	12
Chr07G1074.1	1
Chr07G1076.1	2
Chr07G1080.1	10
Chr07G1087.1	3
Chr07G1101.1	12
Chr07G1102.1	1
Chr07G1104.1	1
Chr07G1111.1	9

Chr07G1116.1	7
Chr07G1119.1	1
Chr07G1130.1	1
Chr07G1137.1	11
Chr07G1140.1	11
Chr07G1141.1	12
Chr07G1142.1	1
Chr07G1143.1	7
Chr07G1147.1	3
Chr07G1163.1	4
Chr07G1170.1	7
Chr07G1172.1	11
Chr07G1175.1	8
Chr07G1180.1	8
Chr07G1181.1	12
Chr07G1185.1	1
Chr07G1188.1	11
Chr07G1190.1	2
Chr02G0784.1	1
Chr02G0798.1	1
Chr02G0802.1	1
Chr02G0803.1	6
Chr02G0806.1	1
Chr02G0824.1	4
Chr02G0825.1	1
Chr02G0828.1	7
Chr02G0829.1	2
Chr02G0830.1	2
Chr02G0839.1	10
Chr02G0841.1	2
Chr02G0844.1	5
Chr02G0858.1	14
Chr02G0860.1	1
Chr02G0869.1	12
Chr02G0873.1	2
Chr02G0874.1	5
Chr02G0875.1	1
Chr02G0876.1	3
Chr02G0877.1	1
Chr02G0885.1	2
Chr02G0891.1	1
Chr02G0892.1	3
Chr02G0897.1	10
Chr02G0915.1	3

Chr02G0925.1	1
Chr02G0927.1	1
Chr02G0931.1	1
Chr02G0942.1	1
Chr02G0944.1	1
Chr02G0947.1	4
Chr02G0962.1	1
Chr02G0979.1	1
Chr02G0988.1	2
Chr02G0997.1	1
Chr02G1004.1	2
Chr02G1008.1	1
Chr02G1009.1	9
Chr02G1015.1	6
Chr02G1016.1	9
Chr02G1022.1	2
Chr02G1030.1	2
Chr02G1041.1	1
Chr02G1044.1	6
Chr02G1050.1	3
Chr02G1051.1	1
Chr02G1053.1	12
Chr02G1054.1	12
Chr02G1056.1	2
Chr02G1060.1	5
Chr02G1071.1	12
Chr02G1072.1	4
Chr02G1088.1	1
Chr02G1095.1	1
Chr02G1103.1	1
Chr02G1121.1	6
Chr02G1124.1	2
Chr02G1128.1	1
Chr02G1129.1	1
Chr02G1130.1	7
Chr02G1133.1	1
Chr02G1137.1	1
Chr02G1138.1	1
Chr02G1139.1	1
Chr02G1158.1	2
Chr02G1159.1	1
Chr02G1163.1	1
Chr02G1165.1	1
Chr02G1168.1	2

Chr02G1170.1	1
Chr02G1172.1	2
Chr02G1175.1	11
Chr02G1180.1	6
Chr02G1186.1	1
Chr02G1188.1	1
Chr02G1197.1	5
Chr02G1204.1	4
Chr02G1213.1	1
Chr02G1215.1	1
Chr02G1218.1	10
Chr02G1222.1	7
Chr02G1225.1	3
Chr02G1231.1	1
Chr02G1234.1	1
Chr02G1243.1	1
Chr02G1247.1	6
Chr02G1248.1	11
Chr02G1254.1	3
Chr02G1256.1	12
Chr02G1259.1	2
Chr02G1265.1	12
Chr02G1266.1	1
Chr02G1270.1	1
Chr02G1271.1	1
Chr02G1272.1	5
Chr02G1273.1	11
Chr02G1277.1	6
Chr02G1279.1	1
Chr02G1284.1	12
Chr02G1288.1	1
Chr02G1289.1	1
Chr02G1291.1	6
Chr02G1304.1	1
Chr02G1306.1	11
Chr02G1308.1	1
Chr02G1310.1	11
Chr02G1317.1	1
Chr02G1321.1	10
Chr02G1322.1	2
Chr02G1326.1	6
Chr02G1332.1	6
Chr02G1333.1	7
Chr02G1336.1	12

Chr02G1337.1	12
Chr02G1346.1	2
Chr02G1353.1	1
Chr02G1355.1	7
Chr02G1361.1	7
Chr02G1363.1	1
Chr02G1367.1	2
Chr02G1369.1	5
Chr02G1373.1	2
Chr02G1374.1	1
Chr02G1376.1	6
Chr02G1377.1	5
Chr02G1380.1	12
Chr02G1381.1	1
Chr02G1382.1	7
Chr02G1386.1	11
Chr02G1387.1	1
Chr02G1388.1	1
Chr02G1395.1	8
Chr02G1397.1	10
Chr02G1407.1	1
Chr02G1408.1	6
Chr02G1411.1	6
Chr02G1420.1	7
Chr02G1423.1	15
Chr02G1425.1	16
Chr02G1427.1	7
Chr02G1428.1	4
Chr02G1436.1	11
Chr02G1437.1	12
Chr02G1441.1	12
Chr02G1445.1	10
Chr02G1455.1	1
Chr02G1464.1	8
Chr02G1469.1	8
Chr02G1471.1	2
Chr02G1472.1	4
Chr02G1475.1	12
Chr02G1476.1	1
Chr02G1480.1	6
Chr02G1485.1	16
Chr02G1494.1	12
Chr02G1498.1	11
Chr02G1500.1	12

Chr02G1501.1	7
Chr02G1502.1	5
Chr02G1504.1	14
Chr02G1506.1	5
Chr02G1508.1	1
Chr02G1509.1	3
Chr02G1510.1	1
Chr02G1512.1	4
Chr02G1515.1	1
Chr02G1517.1	2
Chr02G1519.1	8
Chr02G1532.1	5
Chr02G1533.1	1
Chr02G1534.1	1
Chr02G1540.1	6
Chr02G1544.1	10
Chr02G1546.1	11
Chr02G1547.1	6
Chr02G1549.1	3
Chr02G1568.1	1
Chr02G1570.1	1
Chr02G1576.1	7
Chr02G1591.1	12
Chr02G1596.1	14
Chr02G1597.1	7
Chr02G1606.1	2
Chr02G1609.1	12
Chr02G1611.1	1
Chr02G1613.1	10
Chr02G1615.1	6
Chr02G1623.1	7
Chr02G1624.1	12
Chr02G1626.1	8
Chr02G1631.1	14
Chr02G1632.1	5
Chr02G1637.1	1
Chr02G1639.1	2
Chr02G1642.1	1
Chr02G1644.1	12
Chr02G1652.1	1
Chr02G1662.1	1
Chr02G1664.1	1
Chr02G1672.1	1
Chr02G1674.1	2

Chr02G1676.1	14
Chr02G1679.1	6
Chr02G1682.1	1
Chr02G1683.1	1
Chr02G1685.1	1
Chr02G1698.1	2
Chr02G1702.1	14
Chr02G1708.1	7
Chr02G1723.1	1
Chr02G1727.1	12
Chr02G1734.1	1
Chr02G1741.1	8
Chr02G1747.1	4
Chr02G1760.1	10
Chr02G1762.1	4
Chr02G1770.1	12
Chr02G1771.1	12
Chr02G1773.1	7
Chr02G1777.1	4
Chr02G1779.1	10
Chr02G1781.1	6
Chr02G1790.1	10
Chr02G1796.1	10
Chr02G1800.1	10
Chr02G1806.1	6
Chr02G1808.1	1
Chr02G1811.1	11
Chr02G1816.1	2
Chr02G1817.1	4
Chr02G1820.1	1
Chr02G1824.1	1
Chr02G1829.1	7
Chr02G1834.1	12
Chr02G1836.1	10
Chr02G1838.1	1
Chr02G1844.1	7
Chr02G1847.1	1
Chr02G1851.1	1
Chr02G1852.1	12
Chr02G1855.1	12
Chr02G1860.1	1
Chr08G0007.1	3
Chr08G0008.1	8
Chr08G0013.1	1

Chr08G0017.1	3
Chr08G0021.1	3
Chr08G0025.1	1
Chr08G0028.1	5
Chr08G0029.1	6
Chr08G0032.1	1
Chr08G0040.1	1
Chr08G0044.1	11
Chr08G0047.1	1
Chr08G0049.1	7
Chr08G0050.1	10
Chr08G0057.1	4
Chr08G0065.1	1
Chr08G0078.1	7
Chr08G0084.1	1
Chr08G0091.1	1
Chr08G0092.1	1
Chr08G0093.1	1
Chr08G0094.1	1
Chr08G0104.1	8
Chr08G0110.1	2
Chr08G0112.1	1
Chr08G0114.1	9
Chr08G0118.1	1
Chr08G0119.1	12
Chr08G0121.1	1
Chr08G0138.1	8
Chr08G0145.1	12
Chr08G0150.1	1
Chr08G0155.1	2
Chr08G0156.1	1
Chr08G0160.1	1
Chr08G0176.1	10
Chr08G0184.1	1
Chr08G0185.1	1
Chr08G0187.1	9
Chr08G0189.1	1
Chr08G0192.1	7
Chr08G0199.1	1
Chr08G0203.1	1
Chr08G0205.1	1
Chr08G0219.1	4
Chr08G0230.1	11
Chr08G0231.1	2

Chr08G0233.1	11
Chr08G0238.1	1
Chr08G0241.1	2
Chr08G0243.1	10
Chr08G0267.1	1
Chr08G0273.1	1
Chr08G0274.1	1
Chr08G0299.1	9
Chr08G0306.1	1
Chr08G0307.1	7
Chr08G0312.1	1
Chr08G0313.1	5
Chr08G0318.1	10
Chr08G0320.1	7
Chr08G0321.1	9
Chr08G0322.1	1
Chr08G0327.1	1
Chr08G0328.1	6
Chr08G0332.1	7
Chr08G0340.1	1
Chr08G0359.1	1
Chr08G0360.1	1
Chr08G0361.1	1
Chr08G0364.1	8
Chr08G0368.1	4
Chr08G0369.1	1
Chr08G0372.1	4
Chr08G0378.1	1
Chr08G0382.1	1
Chr08G0386.1	11
Chr08G0388.1	12
Chr08G0407.1	10
Chr08G0410.1	8
Chr08G0411.1	4
Chr08G0415.1	10
Chr08G0418.1	2
Chr08G0432.1	7
Chr08G0435.1	1
Chr08G0438.1	13
Chr08G0443.1	1
Chr08G0447.1	12
Chr08G0449.1	4
Chr08G0453.1	2
Chr08G0460.1	10

Chr08G0462.1	1
Chr08G0463.1	4
Chr08G0468.1	13
Chr08G0470.1	7
Chr08G0471.1	1
Chr08G0472.1	1
Chr08G0474.1	11
Chr08G0498.1	1
Chr08G0507.1	3
Chr08G0508.1	1
Chr08G0513.1	2
Chr08G0518.1	9
Chr08G0519.1	11
Chr08G0524.1	1
Chr08G0526.1	5
Chr08G0528.1	1
Chr08G0539.1	2
Chr08G0541.1	12
Chr08G0543.1	12
Chr08G0549.1	4
Chr08G0566.1	9
Chr08G0577.1	1
Chr08G0582.1	1
Chr08G0589.1	4
Chr08G0600.1	1
Chr08G0613.1	14
Chr08G0616.1	10
Chr08G0626.1	1
Chr08G0636.1	1
Chr08G0637.1	5
Chr08G0640.1	1
Chr08G0642.1	1
Chr08G0643.1	12
Chr08G0651.1	1
Chr08G0654.1	1
Chr08G0666.1	4
Chr08G0667.1	1
Chr08G0668.1	6
Chr08G0670.1	1
Chr08G0671.1	10
Chr08G0677.1	10
Chr08G0679.1	1
Chr08G0684.1	5
Chr08G0685.1	1

Chr08G0686.1	1
Chr08G0689.1	11
Chr08G0693.1	1
Chr08G0700.1	1
Chr08G0715.1	10
Chr08G0718.1	4
Chr08G0719.1	1
Chr08G0721.1	1
Chr08G0723.1	6
Chr08G0728.1	1
Chr08G0729.1	7
Chr08G0730.1	7
Chr08G0731.1	4
Chr08G0732.1	11
Chr08G0739.1	1
Chr08G0740.1	6
Chr08G0741.1	7
Chr08G0742.1	1
Chr08G0745.1	4
Chr08G0746.1	10
Chr08G0752.1	1
Chr08G0754.1	1
Chr08G0764.1	1
Chr08G0778.1	1
Chr08G0780.1	6
Chr08G0790.1	5
Chr08G0796.1	8
Chr08G0804.1	5
Chr08G0811.1	1
Chr08G0816.1	7
Chr08G0823.1	11
Chr08G0825.1	12
Chr08G0826.1	14
Chr08G0827.1	1
Chr08G0832.1	1
Chr08G0834.1	1
Chr08G0838.1	1
Chr08G0839.1	1
Chr08G0840.1	1
Chr08G0846.1	1
Chr08G0850.1	12
Chr08G0852.1	1
Chr08G0856.1	5
Chr08G0862.1	1

Chr08G0865.1	10
Chr08G0870.1	10
Chr08G0875.1	13
Chr08G0876.1	1
Chr08G0878.1	1
Chr08G0883.1	7
Chr08G0885.1	14
Chr08G0886.1	7
Chr08G0891.1	9
Chr08G0893.1	3
Chr08G0899.1	6
Chr08G0902.1	1
Chr08G0905.1	10
Chr08G0913.1	6
Chr08G0916.1	1
Chr08G0927.1	9
Chr08G0942.1	12
Chr08G0943.1	1
Chr08G0950.1	2
Chr08G0956.1	10
Chr08G0957.1	1
Chr08G0959.1	10
Chr08G0963.1	2
Chr08G0965.1	10
Chr08G0967.1	7
Chr08G0969.1	1
Chr08G0970.1	9
Chr08G0973.1	1
Chr08G0975.1	6
Chr08G0978.1	1
Chr08G0987.1	1
Chr08G0995.1	13
Chr08G1003.1	12
Chr08G1004.1	1
Chr08G1006.1	12
Chr08G1007.1	1
Chr08G1011.1	10
Chr08G1012.1	1
Chr08G1014.1	11
Chr08G1018.1	6
Chr08G1022.1	1
Chr08G1024.1	8
Chr08G1027.1	4
Chr08G1032.1	1

Chr08G1036.1	1
Chr08G1043.1	1
Chr08G1044.1	12
Chr08G1045.1	8
Chr08G1049.1	10
Chr08G1057.1	12
Chr08G1066.1	1
Chr05G0878.1	1
Chr05G0881.1	8
Chr05G0882.1	4
Chr05G0889.1	4
Chr05G0890.1	2
Chr05G0895.1	1
Chr05G0904.1	1
Chr05G0907.1	7
Chr05G0909.1	3
Chr05G0912.1	1
Chr05G0916.1	2
Chr05G0917.1	1
Chr05G0919.1	1
Chr05G0921.1	2
Chr05G0924.1	11
Chr05G0925.1	6
Chr05G0934.1	11
Chr05G0939.1	7
Chr05G0941.1	7
Chr05G0944.1	1
Chr05G0948.1	1
Chr05G0953.1	12
Chr05G0954.1	1
Chr05G0966.1	7
Chr05G0973.1	1
Chr05G0975.1	1
Chr05G0987.1	2
Chr05G0993.1	4
Chr05G1006.1	4
Chr05G1009.1	1
Chr05G1012.1	5
Chr05G1013.1	12
Chr05G1018.1	1
Chr05G1025.1	9
Chr05G1027.1	1
Chr05G1028.1	7
Chr05G1030.1	2

Chr05G1034.1	5
Chr05G1038.1	6
Chr05G1041.1	12
Chr05G1043.1	1
Chr05G1049.1	2
Chr05G1053.1	2
Chr05G1056.1	1
Chr05G1057.1	7
Chr05G1059.1	10
Chr05G1062.1	1
Chr05G1065.1	1
Chr05G1069.1	6
Chr05G1070.1	2
Chr05G1072.1	11
Chr05G1075.1	7
Chr05G1081.1	5
Chr05G1085.1	1
Chr05G1097.1	9
Chr05G1100.1	4
Chr05G1105.1	2
Chr05G1108.1	7
Chr05G1111.1	2
Chr05G1118.1	2
Chr05G1120.1	11
Chr05G1121.1	2
Chr05G1126.1	1
Chr05G1130.1	6
Chr05G1141.1	11
Chr05G1149.1	5
Chr05G1151.1	1
Chr05G1155.1	1
Chr05G1159.1	1
Chr05G1186.1	1
Chr05G1201.1	1
Chr05G1206.1	4
Chr05G1210.1	13
Chr05G1212.1	1
Chr05G1219.1	1
Chr05G1223.1	1
Chr05G1229.1	3
Chr05G1234.1	2
Chr05G1249.1	9
Chr05G1251.1	2
Chr05G1256.1	3

Chr05G1257.1	1
Chr05G1258.1	7
Chr05G1261.1	1
Chr05G1270.1	1
Chr05G1271.1	4
Chr05G1274.1	1
Chr05G1281.1	7
Chr05G1284.1	6
Chr05G1287.1	6
Chr05G1288.1	4
Chr05G1289.1	2
Chr05G1296.1	8
Chr05G1304.1	10
Chr05G1305.1	2
Chr05G1307.1	11
Chr05G1315.1	10
Chr05G1337.1	3
Chr05G1341.1	12
Chr05G1347.1	1
Chr05G1349.1	2
Chr05G1362.1	1
Chr05G1377.1	9
Chr05G1384.1	1
Chr05G1385.1	4
Chr05G1413.1	2
Chr05G1419.1	6
Chr05G1429.1	11
Chr05G1432.1	1
Chr05G1441.1	7
Chr05G1449.1	3
Chr05G1450.1	4
Chr03G0005.1	5
Chr03G0007.1	12
Chr03G0008.1	1
Chr03G0012.1	9
Chr03G0013.1	10
Chr03G0022.1	8
Chr03G0024.1	7
Chr03G0025.1	1
Chr03G0051.1	1
Chr03G0052.1	1
Chr03G0058.1	5
Chr03G0062.1	5
Chr03G0063.1	6

Chr03G0064.1	7
Chr03G0067.1	11
Chr03G0071.1	4
Chr03G0072.1	13
Chr03G0077.1	11
Chr03G0078.1	4
Chr03G0079.1	5
Chr03G0080.1	7
Chr03G0087.1	11
Chr03G0090.1	1
Chr03G0093.1	4
Chr03G0095.1	1
Chr03G0096.1	1
Chr03G0110.1	7
Chr03G0114.1	1
Chr03G0116.1	1
Chr03G0118.1	12
Chr03G0119.1	2
Chr03G0120.1	1
Chr03G0123.1	7
Chr03G0128.1	1
Chr03G0131.1	1
Chr03G0137.1	1
Chr03G0138.1	3
Chr03G0139.1	1
Chr03G0144.1	1
Chr03G0145.1	10
Chr03G0146.1	3
Chr03G0152.1	6
Chr03G0155.1	1
Chr03G0169.1	12
Chr03G0172.1	1
Chr03G0190.1	1
Chr03G0195.1	3
Chr03G0200.1	1
Chr03G0206.1	6
Chr03G0210.1	2
Chr03G0211.1	1
Chr03G0215.1	7
Chr03G0216.1	10
Chr03G0220.1	5
Chr03G0223.1	6
Chr03G0228.1	11
Chr03G0232.1	7

Chr03G0241.1	14
Chr03G0246.1	24
Chr03G0247.1	12
Chr03G0250.1	13
Chr03G0252.1	1
Chr03G0253.1	1
Chr03G0259.1	8
Chr03G0260.1	1
Chr03G0272.1	13
Chr03G0275.1	7
Chr03G0278.1	1
Chr03G0282.1	4
Chr03G0283.1	12
Chr03G0286.1	4
Chr03G0290.1	1
Chr03G0291.1	1
Chr03G0296.1	1
Chr03G0301.1	12
Chr03G0302.1	7
Chr03G0307.1	3
Chr03G0322.1	4
Chr03G0323.1	1
Chr03G0335.1	5
Chr03G0338.1	3
Chr03G0339.1	2
Chr03G0340.1	2
Chr03G0343.1	7
Chr03G0350.1	10
Chr03G0354.1	9
Chr03G0357.1	2
Chr03G0362.1	1
Chr03G0367.1	1
Chr03G0368.1	11
Chr03G0372.1	1
Chr03G0378.1	1
Chr03G0381.1	1
Chr03G0382.1	3
Chr03G0385.1	13
Chr03G0387.1	1
Chr03G0389.1	4
Chr03G0400.1	7
Chr03G0401.1	1
Chr03G0404.1	6
Chr03G0407.1	3

Chr03G0410.1	1
Chr03G0433.1	7
Chr03G0434.1	12
Chr03G0441.1	2
Chr03G0444.1	2
Chr03G0445.1	5
Chr03G0449.1	10
Chr03G0450.1	6
Chr03G0451.1	10
Chr03G0472.1	1
Chr03G0477.1	6
Chr03G0493.1	1
Chr03G0496.1	6
Chr03G0498.1	2
Chr03G0500.1	5
Chr03G0502.1	4
Chr03G0512.1	6
Chr03G0516.1	1
Chr03G0518.1	1
Chr03G0527.1	6
Chr03G0528.1	10
Chr03G0529.1	12
Chr03G0531.1	4
Chr03G0545.1	7
Chr03G0547.1	1
Chr03G0552.1	10
Chr03G0557.1	4
Chr03G0558.1	1
Chr03G0569.1	1
Chr03G0570.1	1
Chr03G0579.1	7
Chr03G0587.1	4
Chr03G0588.1	7
Chr03G0589.1	10
Chr03G0597.1	1
Chr03G0602.1	1
Chr03G0603.1	1
Chr03G0604.1	2
Chr03G0607.1	1
Chr03G0610.1	3
Chr03G0613.1	1
Chr03G0614.1	1
Chr03G0615.1	1
Chr03G0616.1	1

Chr03G0631.1	12
Chr03G0644.1	4
Chr03G0643.1	12
Chr03G0651.1	4
Chr03G0652.1	1
Chr03G0658.1	1
Chr03G0662.1	1
Chr03G0667.1	14
Chr03G0668.1	10
Chr03G0673.1	6
Chr03G0676.1	2
Chr03G0678.1	1
Chr03G0681.1	1
Chr03G0684.1	1
Chr03G0688.1	14
Chr03G0691.1	5
Chr03G0698.1	1
Chr03G0701.1	1
Chr03G0704.1	10
Chr03G0712.1	8
Chr03G0715.1	7
Chr03G0716.1	4
Chr03G0728.1	12
Chr03G0731.1	5
Chr03G0747.1	3
Chr03G0751.1	1
Chr03G0757.1	1
Chr03G0759.1	7
Chr03G0771.1	3
Chr03G0781.1	6
Chr03G0782.1	9
Chr03G0784.1	4
Chr03G0789.1	2
Chr03G0801.1	1
Chr03G0803.1	4
Chr03G0806.1	8
Chr03G0807.1	7
Chr03G0808.1	2
Chr03G0809.1	6
Chr03G0810.1	1
Chr03G0812.1	5
Chr03G0819.1	2
Chr03G0820.1	2
Chr03G0826.1	10

Chr03G0829.1	2
Chr03G0830.1	1
Chr03G0836.1	11
Chr03G0839.1	8
Chr03G0848.1	10
Chr03G0849.1	7
Chr03G0850.1	3
Chr03G0852.1	6
Chr03G0868.1	7
Chr03G0880.1	11
Chr03G0882.1	4
Chr03G0888.1	7
Chr03G0896.1	1
Chr03G0899.1	8
Chr03G0900.1	9
Chr03G0903.1	1
Chr03G0905.1	2
Chr03G0912.1	1
Chr03G0919.1	7
Chr03G0921.1	1
Chr03G0922.1	8
Chr03G0923.1	5
Chr03G0924.1	3
Chr03G0928.1	1
Chr03G0937.1	4
Chr03G0939.1	1
Chr03G0940.1	8
Chr03G0943.1	1
Chr03G0948.1	9
Chr03G0955.1	12
Chr03G0965.1	2
Chr03G0972.1	15
Chr03G0978.1	1
Chr03G0982.1	9
Chr03G0987.1	1
Chr03G0992.1	13
Chr03G1012.1	8
Chr03G1029.1	6
Chr03G1030.1	13
Chr03G1039.1	8
Chr03G1044.1	1
Chr03G1045.1	2
Chr03G1048.1	12
Chr03G1051.1	1

Chr03G1054.1	12
Chr03G1060.1	4
Chr03G1061.1	4
Chr03G1065.1	1
Chr03G1070.1	6
Chr03G1076.1	2
Chr03G1094.1	7
Chr03G1110.1	1
Chr03G1118.1	11
Chr03G1119.1	1
Chr03G1121.1	3
Chr03G1127.1	8
Chr03G1128.1	4
Chr03G1139.1	3
Chr03G1146.1	1
Chr03G1150.1	2
Chr03G1152.1	1
Chr03G1153.1	7
Chr03G1156.1	4
Chr03G1157.1	1
Chr03G1158.1	1
Chr03G1164.1	1
Chr03G1166.1	2
Chr03G1167.1	11
Chr03G1170.1	6
Chr03G1174.1	3
Chr03G1181.1	12
Chr03G1184.1	1
Chr03G1187.1	4
Chr03G1191.1	10
Chr03G1193.1	8
Chr03G1196.1	1
Chr03G1197.1	1
Chr03G1202.1	1
Chr03G1207.1	9
Chr03G1215.1	1
Chr03G1217.1	7
Chr03G1225.1	6
Chr03G1231.1	1
Chr03G1232.1	1
Chr03G1246.1	7
Chr03G1251.1	2
Chr03G1255.1	2
Chr03G1256.1	3

Chr03G1262.1	2
Chr03G1264.1	6
Chr03G1265.1	7
Chr03G1268.1	11
Chr03G1274.1	7
Chr03G1285.1	12
Chr03G1286.1	1
Chr03G1287.1	12
Chr03G1288.1	2
Chr03G1301.1	11
Chr03G1303.1	1
Chr03G1304.1	1
Chr03G1308.1	2
Chr03G1313.1	1
Chr03G1314.1	7
Chr03G1316.1	4
Chr03G1321.1	1
Chr03G1323.1	14
Chr03G1324.1	1
Chr03G1326.1	1
Chr03G1328.1	6
Chr03G1329.1	1
Chr03G1335.1	1
Chr03G1340.1	6
Chr03G1341.1	4
Chr03G1357.1	10
Chr03G1372.1	2
Chr03G1375.1	2
Chr03G1383.1	1
Chr03G1385.1	1
Chr03G1387.1	12
Chr03G1395.1	10
Chr03G1399.1	6
Chr03G1400.1	10
Chr03G1406.1	5
Chr03G1408.1	3
Chr03G1409.1	1
Chr03G1413.1	1
Chr03G1418.1	1
Chr03G1422.1	3
Chr03G1426.1	4
Chr03G1428.1	1
Chr03G1430.1	10
Chr03G1435.1	5

Chr03G1437.1	4
Chr03G1454.1	6
Chr03G1459.1	12
Chr03G1467.1	7
Chr03G1470.1	12
Chr03G1475.1	6
Chr03G1480.1	12
Chr03G1484.1	1
Chr03G1486.1	1
Chr03G1489.1	1
Chr03G1491.1	15
Chr03G1497.1	14
Chr03G1500.1	1
Chr03G1501.1	5
Chr03G1505.1	1
Chr03G1516.1	1
Chr03G1517.1	11
Chr03G1522.1	12
Chr03G1524.1	1
Chr03G1526.1	11
Chr03G1527.1	12
Chr03G1530.1	3
Chr03G1533.1	4
Chr03G1534.1	3
Chr03G1535.1	6
Chr03G1538.1	1
Chr03G1539.1	6
Chr03G1541.1	1
Chr03G1549.1	12
Chr03G1556.1	11
Chr03G1560.1	4
Chr03G1566.1	7
Chr03G1567.1	12
Chr03G1568.1	7
Chr03G1573.1	7
Chr03G1576.1	11
Chr03G1578.1	1
Chr03G1580.1	1
Chr03G1583.1	6
Chr03G1584.1	13
Chr03G1590.1	9
Chr03G1593.1	1
Chr03G1596.1	12
Chr03G1603.1	1

Chr03G1605.1	4
Chr03G1607.1	12
Chr03G1608.1	2
Chr03G1611.1	7
Chr03G1612.1	7
Chr03G1616.1	1
Chr03G1618.1	1
Chr03G1619.1	1
Chr03G1624.1	3
Chr03G1625.1	1
Chr03G1632.1	11
Chr03G1636.1	11
Chr03G1637.1	12
Chr03G1638.1	12
Chr03G1646.1	2
Chr03G1654.1	4
Chr03G1655.1	7
Chr03G1657.1	7
Chr03G1660.1	7
Chr03G1661.1	2
Chr03G1663.1	11
Chr03G1665.1	1
Chr03G1666.1	6
Chr03G1671.1	1
Chr03G1681.1	12
Chr03G1682.1	12
Chr03G1690.1	12
Chr03G1696.1	7
Chr03G1699.1	9
Chr03G1701.1	9
Chr03G1706.1	2
Chr03G1707.1	1
Chr03G1710.1	3
Chr03G1719.1	11
Chr03G1722.1	12
Chr03G1732.1	1
Chr03G1735.1	12
Chr03G1738.1	1
Chr03G1741.1	1
Chr09G0009.1	10
Chr09G0014.1	9
Chr09G0018.1	10
Chr09G0027.1	6
Chr09G0033.1	4

Chr09G0045.1	1
Chr09G0047.1	1
Chr09G0054.1	2
Chr09G0062.1	7
Chr09G0063.1	1
Chr09G0070.1	2
Chr09G0074.1	6
Chr09G0080.1	11
Chr09G0088.1	11
Chr09G0091.1	5
Chr09G0092.1	1
Chr09G0094.1	1
Chr09G0095.1	11
Chr09G0097.1	1
Chr09G0103.1	8
Chr09G0115.1	7
Chr09G0116.1	5
Chr09G0117.1	5
Chr09G0120.1	6
Chr09G0129.1	2
Chr09G0149.1	2
Chr09G0153.1	1
Chr09G0156.1	2
Chr09G0158.1	1
Chr09G0169.1	3
Chr09G0173.1	1
Chr09G0174.1	5
Chr09G0179.1	1
Chr09G0180.1	9
Chr09G0187.1	1
Chr09G0190.1	7
Chr09G0199.1	1
Chr09G0207.1	1
Chr09G0210.1	2
Chr09G0219.1	10
Chr09G0224.1	2
Chr09G0230.1	1
Chr09G0233.1	12
Chr09G0240.1	10
Chr09G0243.1	3
Chr09G0249.1	6
Chr09G0253.1	5
Chr09G0256.1	1
Chr09G0257.1	4

Chr09G0258.1	3
Chr09G0262.1	4
Chr09G0271.1	1
Chr09G0276.1	9
Chr09G0277.1	5
Chr09G0281.1	2
Chr09G0286.1	5
Chr09G0302.1	8
Chr09G0304.1	3
Chr09G0306.1	9
Chr09G0307.1	6
Chr09G0309.1	5
Chr09G0313.1	6
Chr09G0327.1	1
Chr09G0333.1	1
Chr09G0334.1	5
Chr09G0335.1	1
Chr09G0340.1	1
Chr09G0351.1	8
Chr09G0356.1	1
Chr09G0358.1	1
Chr09G0369.1	1
Chr09G0370.1	1
Chr09G0371.1	2
Chr09G0374.1	3
Chr09G0378.1	3
Chr09G0382.1	1
Chr09G0384.1	14
Chr09G0392.1	15
Chr09G0395.1	3
Chr09G0396.1	9
Chr09G0397.1	7
Chr09G0403.1	4
Chr09G0411.1	12
Chr09G0418.1	4
Chr09G0423.1	1
Chr09G0428.1	1
Chr09G0432.1	3
Chr09G0437.1	4
Chr09G0448.1	4
Chr09G0451.1	1
Chr09G0459.1	1
Chr09G0467.1	1
Chr09G0471.1	7

Chr09G0475.1	2
Chr09G0478.1	1
Chr09G0489.1	10
Chr09G0492.1	1
Chr09G0501.1	4
Chr09G0507.1	2
Chr09G0514.1	1
Chr09G0527.1	2
Chr09G0529.1	1
Chr09G0534.1	2
Chr09G0535.1	8
Chr09G0536.1	3
Chr09G0539.1	1
Chr09G0550.1	1
Chr09G0568.1	5
Chr09G0570.1	2
Chr09G0575.1	5
Chr09G0577.1	1
Chr09G0579.1	9
Chr09G0584.1	1
Chr09G0591.1	9
Chr09G0602.1	12
Chr09G0609.1	1
Chr09G0615.1	3
Chr09G0620.1	4
Chr09G0629.1	1
Chr09G0630.1	2
Chr09G0636.1	2
Chr09G0637.1	7
Chr09G0640.1	12
Chr09G0657.1	5
Chr09G0665.1	4
Chr09G0667.1	11
Chr09G0668.1	2
Chr09G0670.1	1
Chr09G0672.1	5
Chr09G0675.1	1
Chr09G0676.1	1
Chr09G0678.1	7
Chr09G0686.1	7
Chr09G0692.1	4
Chr09G0697.1	10
Chr09G0700.1	12
Chr09G0705.1	10

Chr09G0709.1	6
Chr09G0710.1	7
Chr09G0724.1	1
Chr09G0730.1	14
Chr09G0731.1	12
Chr09G0734.1	3
Chr09G0740.1	4
Chr09G0741.1	9
Chr09G0743.1	9
Chr09G0757.1	1
Chr09G0762.1	4
Chr09G0771.1	3
Chr09G0773.1	1
Chr09G0782.1	1
Chr09G0783.1	6
Chr09G0786.1	9
Chr09G0799.1	5
Chr09G0806.1	4
Chr09G0808.1	7
Chr09G0828.1	3
Chr09G0830.1	1
Chr09G0834.1	1
Chr09G0839.1	12
Chr09G0845.1	2
Chr09G0847.1	3
Chr09G0861.1	8
Chr09G0866.1	1
Chr09G0883.1	1
Chr09G0884.1	12
Chr09G0887.1	1
Chr09G0896.1	1
Chr09G0901.1	1
Chr09G0905.1	1
Chr09G0910.1	8
Chr09G0911.1	12
Chr09G0913.1	10
Chr09G0919.1	12
Chr09G0921.1	1
Chr09G0925.1	1
Chr09G0929.1	8
Chr09G0936.1	6
Chr09G0944.1	3
Chr09G0947.1	10
Chr09G0951.1	1

Chr09G0952.1	1
Chr09G0979.1	9
Chr09G0981.1	2
Chr09G0984.1	10
Chr09G0992.1	14
Chr09G0997.1	7
Chr09G0999.1	7
Chr09G1000.1	5
Chr09G1002.1	1
Chr09G1011.1	4
Chr09G1018.1	12
Chr09G1019.1	6
Chr09G1021.1	6
Chr09G1031.1	13
Chr09G1033.1	1
Chr09G1034.1	1
Chr09G1035.1	4
Chr09G1038.1	1
Chr09G1040.1	1
Chr09G1042.1	4
Chr09G1043.1	1
Chr09G1051.1	2
Chr09G1055.1	1
Chr09G1057.1	2
Chr06G0025.1	1
Chr06G0027.1	1
Chr06G0045.1	3
Chr06G0054.1	5
Chr06G0055.1	1
Chr06G0057.1	12
Chr06G0060.1	7
Chr06G0061.1	6
Chr06G0072.1	2
Chr06G0082.1	12
Chr06G0091.1	1
Chr06G0092.1	1
Chr06G0102.1	10
Chr06G0108.1	7
Chr06G0110.1	7
Chr06G0114.1	3
Chr06G0116.1	4
Chr06G0121.1	11
Chr06G0128.1	1
Chr06G0132.1	6

Chr06G0139.1	1
Chr06G0141.1	14
Chr06G0143.1	6
Chr06G0151.1	14
Chr06G0155.1	1
Chr06G0156.1	1
Chr06G0159.1	12
Chr06G0163.1	12
Chr06G0165.1	4
Chr06G0168.1	12
Chr06G0173.1	1
Chr06G0181.1	12
Chr06G0197.1	7
Chr06G0199.1	7
Chr06G0221.1	8
Chr06G0222.1	1
Chr06G0223.1	1
Chr06G0228.1	12
Chr06G0230.1	6
Chr06G0236.1	12
Chr06G0240.1	1
Chr06G0248.1	4
Chr06G0249.1	6
Chr06G0251.1	8
Chr06G0255.1	8
Chr06G0256.1	10
Chr06G0258.1	1
Chr06G0261.1	5
Chr06G0265.1	6
Chr06G0273.1	10
Chr06G0282.1	1
Chr06G0285.1	3
Chr06G0286.1	10
Chr06G0288.1	14
Chr06G0301.1	2
Chr06G0325.1	2
Chr06G0329.1	5
Chr06G0331.1	4
Chr06G0333.1	11
Chr06G0337.1	1
Chr06G0338.1	6
Chr06G0341.1	1
Chr06G0343.1	10
Chr06G0353.1	2

Chr06G0361.1	8
Chr06G0362.1	12
Chr06G0363.1	9
Chr06G0365.1	6
Chr06G0369.1	11
Chr06G0374.1	1
Chr06G0378.1	7
Chr06G0395.1	10
Chr06G0396.1	1
Chr06G0400.1	12
Chr06G0401.1	1
Chr06G0406.1	12
Chr06G0408.1	1
Chr06G0409.1	1
Chr06G0410.1	10
Chr06G0419.1	1
Chr06G0420.1	1
Chr06G0426.1	1
Chr06G0438.1	6
Chr06G0447.1	4
Chr06G0451.1	10
Chr06G0457.1	1
Chr06G0463.1	7
Chr06G0465.1	1
Chr06G0467.1	2
Chr06G0469.1	6
Chr06G0477.1	1
Chr06G0478.1	4
Chr06G0483.1	1
Chr06G0491.1	3
Chr06G0500.1	8
Chr06G0501.1	1
Chr06G0518.1	4
Chr06G0536.1	1
Chr06G0538.1	1
Chr06G0543.1	9
Chr06G0546.1	1
Chr06G0551.1	3
Chr06G0565.1	5
Chr06G0572.1	4
Chr06G0575.1	9
Chr06G0579.1	5
Chr06G0585.1	2
Chr06G0586.1	2

Chr06G0589.1	14
Chr06G0591.1	1
Chr06G0593.1	11
Chr06G0594.1	10
Chr06G0595.1	8
Chr06G0597.1	4
Chr06G0624.1	8
Chr06G0625.1	1
Chr06G0626.1	1
Chr06G0629.1	1
Chr06G0633.1	2
Chr06G0634.1	6
Chr06G0638.1	5
Chr06G0642.1	1
Chr06G0651.1	7
Chr06G0653.1	11
Chr06G0656.1	1
Chr06G0664.1	8
Chr06G0666.1	1
Chr06G0669.1	3
Chr06G0687.1	4
Chr06G0688.1	1
Chr06G0690.1	1
Chr06G0693.1	10
Chr06G0698.1	6
Chr06G0707.1	1
Chr06G0709.1	1
Chr06G0711.1	1
Chr06G0717.1	5
Chr06G0722.1	1
Chr06G0726.1	2
Chr06G0727.1	9
Chr06G0730.1	1
Chr06G0741.1	4
Chr06G0747.1	5
Chr06G0751.1	7
Chr06G0752.1	1
Chr06G0753.1	7
Chr06G0754.1	4
Chr06G0763.1	7
Chr06G0772.1	1
Chr06G0773.1	1
Chr06G0776.1	7
Chr06G0788.1	1

Chr06G0790.1	2
Chr06G0791.1	11
Chr06G0796.1	3
Chr06G0799.1	2
Chr06G0801.1	14
Chr06G0805.1	1
Chr06G0810.1	4
Chr06G0819.1	4
Chr06G0820.1	1
Chr06G0823.1	1
Chr06G0826.1	1
Chr06G0848.1	1
Chr06G0849.1	12
Chr06G0852.1	1
Chr06G0873.1	4
Chr06G0877.1	10
Chr06G0878.1	11
Chr06G0883.1	1
Chr06G0887.1	1
Chr06G0895.1	5
Chr06G0899.1	7
Chr06G0910.1	4
Chr06G0913.1	1
Chr06G0916.1	3
Chr06G0919.1	5
Chr06G0921.1	1
Chr06G0928.1	10
Chr06G0934.1	1
Chr06G0936.1	12
Chr06G0943.1	6
Chr06G0948.1	1
Chr06G0964.1	1
Chr06G0965.1	9
Chr06G0969.1	1
Chr06G0970.1	9
Chr06G0976.1	1
Chr06G0981.1	2
Chr06G0987.1	5
Chr06G0989.1	2
Chr06G0990.1	1
Chr06G0992.1	2
Chr06G1001.1	1
Chr06G1009.1	2
Chr06G1025.1	7

Chr06G1038.1	11
Chr06G1039.1	1
Chr06G1041.1	12
Chr06G1047.1	1
Chr06G1050.1	7
Chr06G1054.1	7
Chr06G1071.1	1
Chr06G1072.1	3
Chr06G1079.1	4
Chr06G1088.1	1
Chr06G1099.1	1
Chr06G1101.1	11
Chr06G1102.1	1
Chr06G1104.1	1
Chr06G1113.1	2
Chr06G1116.1	4
Chr06G1126.1	1
Chr06G1129.1	10
Chr06G1132.1	1
Chr06G1133.1	7
Chr06G1134.1	16
Chr06G1135.1	9
Chr06G1139.1	14
Chr06G1140.1	1
Chr06G1141.1	1
Chr06G1148.1	1
Chr06G1153.1	4
Chr06G1161.1	8
Chr06G1162.1	1
Chr06G1163.1	5
Chr06G1164.1	1
Chr06G1170.1	4
Chr06G1182.1	1
Chr06G1183.1	1
Chr06G1184.1	11
Chr06G1185.1	4
Chr06G1186.1	1
Chr06G1187.1	1
Chr06G1189.1	10
Chr06G1192.1	2
Chr06G1198.1	12
Chr06G1201.1	1
Chr06G1204.1	7
Chr06G1208.1	1

Chr06G1210.1	1
Chr06G1212.1	2
Chr06G1216.1	1
Chr06G1219.1	7
Chr06G1220.1	1
Chr06G1225.1	12
Chr06G1228.1	1
Chr06G1237.1	1
Chr06G1241.1	6
Chr06G1247.1	2
Chr06G1250.1	4
Chr06G1254.1	13
Chr06G1256.1	5
Chr06G1266.1	1
Chr06G1271.1	3
Chr06G1273.1	1
Chr06G1274.1	5
Chr06G1275.1	12
Chr06G1278.1	2
Chr06G1285.1	2
Chr06G1289.1	1
Chr06G1299.1	10
Chr06G1304.1	1
Chr06G1318.1	2
Chr06G1322.1	6
Chr06G1326.1	9
Chr06G1331.1	1
Chr06G1343.1	5
Chr06G1344.1	2
Chr06G1347.1	5
Chr06G1363.1	1
Chr06G1364.1	1
Chr06G1367.1	1
Chr06G1377.1	1
Chr06G1380.1	4
Chr06G1394.1	11
Chr06G1396.1	11
Chr06G1399.1	10
Chr06G1400.1	12
Chr06G1404.1	12
Chr06G1411.1	1
Chr06G1415.1	1
Chr06G1416.1	9
Chr06G1427.1	12

Chr06G1431.1	12
Chr06G1434.1	1
Chr06G1436.1	4
Chr06G1437.1	10
Chr06G1443.1	5
Chr06G1444.1	1
Chr06G1449.1	1
Chr06G1450.1	1
Chr06G1454.1	7
Chr06G1462.1	1
Chr06G1464.1	4
Chr06G1469.1	1
Chr06G1474.1	1
Chr06G1482.1	6
Chr04G0028.1	9
Chr04G0031.1	2
Chr04G0033.1	14
Chr04G0037.1	7
Chr04G0040.1	17
Chr04G0042.1	4
Chr04G0050.1	11
Chr04G0056.1	10
Chr04G0058.1	12
Chr04G0060.1	1
Chr04G0079.1	5
Chr04G0080.1	10
Chr04G0081.1	11
Chr04G0082.1	1
Chr04G0089.1	5
Chr04G0094.1	10
Chr04G0095.1	3
Chr04G0097.1	5
Chr04G0101.1	12
Chr04G0111.1	6
Chr04G0116.1	1
Chr04G0121.1	5
Chr04G0125.1	3
Chr04G0128.1	6
Chr04G0129.1	1
Chr04G0130.1	1
Chr04G0134.1	10
Chr04G0138.1	12
Chr04G0139.1	12
Chr04G0141.1	1

Chr04G0142.1	8
Chr04G0145.1	10
Chr04G0149.1	7
Chr04G0153.1	9
Chr04G0164.1	12
Chr04G0165.1	10
Chr04G0166.1	11
Chr04G0168.1	14
Chr04G0169.1	7
Chr04G0171.1	14
Chr04G0173.1	11
Chr04G0189.1	3
Chr04G0201.1	8
Chr04G0203.1	6
Chr04G0208.1	1
Chr04G0212.1	7
Chr04G0215.1	1
Chr04G0217.1	1
Chr04G0218.1	12
Chr04G0219.1	1
Chr04G0220.1	10
Chr04G0221.1	1
Chr04G0223.1	1
Chr04G0224.1	1
Chr04G0234.1	3
Chr04G0239.1	1
Chr04G0242.1	1
Chr04G0243.1	3
Chr04G0245.1	11
Chr04G0247.1	1
Chr04G0252.1	8
Chr04G0253.1	2
Chr04G0255.1	3
Chr04G0259.1	4
Chr04G0260.1	4
Chr04G0267.1	1
Chr04G0272.1	13
Chr04G0283.1	1
Chr04G0285.1	1
Chr04G0289.1	6
Chr04G0293.1	12
Chr04G0297.1	7
Chr04G0299.1	7
Chr04G0304.1	12

Chr04G0309.1	12
Chr04G0310.1	7
Chr04G0311.1	1
Chr04G0320.1	2
Chr04G0328.1	6
Chr04G0331.1	11
Chr04G0333.1	10
Chr04G0335.1	12
Chr04G0336.1	11
Chr04G0344.1	1
Chr04G0350.1	1
Chr04G0353.1	1
Chr04G0357.1	12
Chr04G0358.1	5
Chr04G0360.1	1
Chr04G0363.1	1
Chr04G0373.1	6
Chr04G0375.1	6
Chr04G0382.1	12
Chr04G0385.1	12
Chr04G0387.1	2
Chr04G0388.1	9
Chr04G0389.1	12
Chr04G0391.1	1
Chr04G0392.1	7
Chr04G0396.1	4
Chr04G0399.1	10
Chr04G0412.1	9
Chr04G0414.1	1
Chr04G0423.1	1
Chr04G0425.1	1
Chr04G0434.1	1
Chr04G0439.1	9
Chr04G0441.1	7
Chr04G0447.1	1
Chr04G0452.1	5
Chr04G0454.1	6
Chr04G0457.1	1
Chr04G0459.1	2
Chr04G0473.1	6
Chr04G0480.1	3
Chr04G0491.1	1
Chr04G0497.1	1
Chr04G0500.1	2

Chr04G0505.1	1
Chr04G0510.1	14
Chr04G0513.1	1
Chr04G0523.1	1
Chr04G0526.1	14
Chr04G0532.1	6
Chr04G0533.1	6
Chr04G0538.1	1
Chr04G0544.1	9
Chr04G0557.1	1
Chr04G0558.1	6
Chr04G0569.1	12
Chr04G0572.1	1
Chr04G0579.1	1
Chr04G0580.1	8
Chr04G0586.1	1
Chr04G0592.1	3
Chr04G0600.1	1
Chr04G0605.1	4
Chr04G0612.1	2
Chr04G0613.1	4
Chr04G0614.1	16
Chr04G0620.1	1
Chr04G0625.1	2
Chr04G0628.1	1
Chr04G0632.1	1
Chr04G0634.1	7
Chr04G0636.1	2
Chr04G0639.1	10
Chr04G0641.1	16
Chr04G0642.1	1
Chr04G0646.1	11
Chr04G0648.1	1
Chr04G0649.1	1
Chr04G0658.1	10
Chr04G0659.1	12
Chr04G0662.1	1
Chr04G0671.1	10
Chr04G0677.1	1
Chr04G0679.1	10
Chr04G0697.1	1
Chr04G0700.1	14
Chr04G0702.1	8
Chr04G0705.1	7

Chr04G0708.1	10
Chr04G0709.1	1
Chr04G0714.1	6
Chr04G0715.1	2
Chr04G0719.1	1
Chr04G0728.1	1
Chr04G0731.1	7
Chr04G0740.1	6
Chr04G0741.1	8
Chr04G0750.1	5
Chr04G0753.1	7
Chr04G0756.1	10
Chr04G0761.1	1
Chr04G0768.1	3
Chr04G0776.1	1
Chr04G0782.1	12
Chr04G0785.1	1
Chr04G0786.1	4
Chr04G0787.1	1
Chr04G0788.1	4
Chr04G0789.1	1
Chr04G0790.1	2
Chr04G0792.1	4
Chr04G0798.1	1
Chr04G0801.1	12
Chr04G0804.1	6
Chr04G0805.1	1
Chr04G0807.1	2
Chr04G0812.1	6
Chr04G0814.1	2
Chr04G0816.1	8
Chr04G0818.1	11
Chr04G0838.1	1
Chr04G0844.1	4
Chr04G0847.1	4
Chr04G0848.1	1
Chr04G0849.1	2
Chr04G0865.1	1
Chr04G0866.1	6
Chr04G0867.1	3
Chr04G0877.1	8
Chr04G0878.1	1
Chr04G0886.1	9
Chr04G0888.1	6

Chr04G0902.1	1
Chr04G0904.1	1
Chr04G0905.1	1
Chr04G0908.1	3
Chr04G0911.1	4
Chr04G0916.1	6
Chr04G0917.1	6
Chr04G0918.1	1
Chr04G0924.1	1
Chr04G0928.1	14
Chr04G0929.1	4
Chr04G0936.1	12
Chr04G0939.1	6
Chr04G0940.1	2
Chr04G0941.1	1
Chr04G0944.1	11
Chr04G0947.1	1
Chr04G0949.1	11
Chr04G0952.1	8
Chr04G0954.1	10
Chr04G0959.1	12
Chr04G0960.1	1
Chr04G0961.1	4
Chr04G0969.1	7
Chr04G0980.1	2
Chr04G0992.1	2
Chr04G0996.1	4
Chr04G0997.1	7
Chr04G0998.1	1
Chr04G1001.1	9
Chr04G1003.1	12
Chr04G1007.1	8
Chr04G1013.1	5
Chr04G1016.1	10
Chr04G1024.1	6
Chr04G1027.1	8
Chr04G1028.1	3
Chr04G1030.1	8
Chr04G1033.1	12
Chr04G1046.1	1
Chr04G1047.1	2
Chr04G1050.1	1
Chr04G1051.1	14
Chr04G1053.1	4

Chr04G1054.1	1
Chr04G1055.1	12
Chr04G1057.1	4
Chr04G1059.1	1
Chr04G1065.1	1
Chr04G1069.1	2
Chr04G1070.1	1
Chr04G1077.1	2
Chr04G1078.1	1
Chr04G1080.1	12
Chr04G1087.1	1
Chr04G1088.1	2
Chr04G1106.1	4
Chr04G1113.1	1
Chr04G1122.1	9
Chr04G1123.1	2
Chr04G1127.1	6
Chr04G1129.1	1
Chr04G1134.1	6
Chr04G1155.1	1
Chr04G1158.1	10
Chr04G1161.1	1
Chr04G1162.1	6
Chr04G1163.1	1
Chr04G1164.1	1
Chr04G1165.1	2
Chr04G1169.1	7
Chr04G1179.1	1
Chr04G1181.1	4
Chr04G1182.1	4
Chr04G1192.1	12
Chr04G1197.1	1
Chr04G1198.1	4
Chr04G1199.1	4
Chr04G1203.1	8
Chr04G1206.1	4
Chr04G1205.1	11
Chr04G1209.1	2
Chr04G1212.1	1
Chr04G1216.1	1
Chr04G1217.1	11
Chr04G1224.1	1
Chr04G1226.1	1
Chr04G1228.1	1

Chr04G1229.1	9
Chr04G1236.1	1
Chr04G1239.1	2
Chr04G1243.1	10
Chr04G1246.1	10
Chr04G1248.1	8
Chr04G1249.1	11
Chr04G1260.1	12
Chr04G1263.1	13
Chr04G1276.1	14
Chr04G1279.1	11
Chr04G1284.1	3
Chr04G1285.1	1
Chr04G1294.1	1
Chr04G1302.1	7
Chr04G1305.1	1
Chr04G1310.1	8
Chr04G1326.1	1
Chr04G1328.1	1
Chr04G1333.1	14
Chr04G1335.1	2
Chr04G1336.1	1
Chr04G1340.1	1
Chr04G1348.1	13
Chr04G1349.1	12
Chr04G1355.1	1
Chr04G1360.1	4
Chr04G1361.1	1
Chr04G1366.1	6
Chr04G1388.1	13
Chr04G1389.1	1
Chr04G1392.1	1
Chr04G1395.1	1
Chr04G1397.1	3
Chr04G1401.1	11
Chr04G1402.1	7
Chr04G1404.1	3
Chr04G1405.1	1
Chr04G1407.1	1
Chr04G1409.1	1
Chr04G1410.1	2
Chr04G1412.1	7
Chr04G1417.1	1
Chr04G1421.1	12

Chr04G1424.1	11
Chr04G1427.1	1
Chr04G1428.1	1
Chr04G1429.1	1
Chr04G1434.1	12
Chr04G1440.1	1
Chr04G1444.1	1
Chr04G1446.1	12
Chr04G1447.1	1
Chr04G1449.1	6
Chr04G1454.1	6
Chr04G1455.1	1
Chr04G1458.1	1
Chr04G1459.1	1
Chr04G1460.1	2
Chr04G1469.1	15
Chr04G1475.1	7
Chr04G1476.1	4
Chr04G1478.1	1
Chr04G1482.1	7
Chr04G1484.1	6
Chr04G1498.1	1
Chr04G1503.1	12
Chr04G1509.1	1
Chr04G1510.1	4
Chr04G1513.1	11
Chr04G1518.1	3
Chr04G1519.1	12
Chr04G1521.1	1
Chr04G1527.1	9
Chr04G1529.1	8
Chr04G1533.1	1
Chr04G1535.1	7
Chr04G1536.1	1
Chr04G1539.1	8
Chr04G1542.1	4
Chr04G1545.1	1
Chr04G1546.1	6
Chr04G1555.1	11
Chr04G1567.1	3
Chr04G1568.1	7
Chr04G1571.1	11
Chr04G1572.1	4
Chr04G1577.1	1

Chr04G1580.1	1
Chr04G1584.1	1
Chr04G1588.1	1
Chr04G1594.1	4
Chr04G1595.1	1
Chr04G1596.1	1
Chr04G1597.1	2
Chr02G0003.1	1
Chr02G0004.1	1
Chr02G0006.1	11
Chr02G0011.1	11
Chr02G0012.1	1
Chr02G0014.1	1
Chr02G0016.1	3
Chr02G0019.1	1
Chr02G0020.1	1
Chr02G0026.1	1
Chr02G0029.1	1
Chr02G0031.1	10
Chr02G0032.1	5
Chr02G0033.1	10
Chr02G0035.1	14
Chr02G0036.1	7
Chr02G0038.1	12
Chr02G0040.1	12
Chr02G0044.1	1
Chr02G0045.1	2
Chr02G0049.1	11
Chr02G0052.1	10
Chr02G0054.1	8
Chr02G0056.1	7
Chr02G0059.1	12
Chr02G0062.1	12
Chr02G0064.1	8
Chr02G0071.1	1
Chr02G0073.1	1
Chr02G0075.1	3
Chr02G0081.1	1
Chr02G0083.1	11
Chr02G0091.1	1
Chr02G0094.1	6
Chr02G0095.1	9
Chr02G0098.1	14
Chr02G0100.1	12

Chr02G0103.1	1
Chr02G0117.1	2
Chr02G0118.1	4
Chr02G0121.1	9
Chr02G0122.1	4
Chr02G0126.1	9
Chr02G0128.1	10
Chr02G0134.1	3
Chr02G0140.1	3
Chr02G0142.1	1
Chr02G0145.1	12
Chr02G0150.1	12
Chr02G0153.1	12
Chr02G0155.1	1
Chr02G0156.1	8
Chr02G0158.1	7
Chr02G0165.1	1
Chr02G0171.1	1
Chr02G0180.1	12
Chr02G0186.1	2
Chr02G0187.1	4
Chr02G0191.1	3
Chr02G0199.1	12
Chr02G0202.1	3
Chr02G0203.1	6
Chr02G0205.1	3
Chr02G0209.1	4
Chr02G0210.1	2
Chr02G0218.1	11
Chr02G0219.1	5
Chr02G0223.1	5
Chr02G0229.1	13
Chr02G0235.1	3
Chr02G0240.1	1
Chr02G0248.1	8
Chr02G0257.1	14
Chr02G0258.1	1
Chr02G0260.1	2
Chr02G0270.1	1
Chr02G0272.1	1
Chr02G0273.1	9
Chr02G0288.1	1
Chr02G0291.1	1
Chr02G0294.1	12

Chr02G0306.1	5
Chr02G0307.1	7
Chr02G0317.1	1
Chr02G0323.1	12
Chr02G0325.1	1
Chr02G0330.1	1
Chr02G0340.1	1
Chr02G0352.1	1
Chr02G0355.1	5
Chr02G0357.1	12
Chr02G0367.1	1
Chr02G0371.1	8
Chr02G0376.1	8
Chr02G0378.1	11
Chr02G0393.1	5
Chr02G0399.1	1
Chr02G0400.1	1
Chr02G0401.1	1
Chr02G0402.1	13
Chr02G0427.1	6
Chr02G0432.1	11
Chr02G0444.1	1
Chr02G0447.1	1
Chr02G0455.1	11
Chr02G0460.1	4
Chr02G0470.1	4
Chr02G0473.1	7
Chr02G0475.1	7
Chr02G0477.1	6
Chr02G0481.1	1
Chr02G0483.1	1
Chr02G0485.1	7
Chr02G0488.1	3
Chr02G0491.1	2
Chr02G0492.1	6
Chr02G0499.1	9
Chr02G0503.1	11
Chr02G0505.1	12
Chr02G0511.1	1
Chr02G0513.1	1
Chr02G0521.1	7
Chr02G0523.1	1
Chr02G0524.1	3
Chr02G0525.1	10

Chr02G0527.1	6
Chr02G0530.1	1
Chr02G0537.1	1
Chr02G0539.1	8
Chr02G0540.1	1
Chr02G0543.1	1
Chr02G0544.1	2
Chr02G0550.1	7
Chr02G0553.1	1
Chr02G0554.1	1
Chr02G0561.1	5
Chr02G0564.1	7
Chr02G0567.1	1
Chr02G0574.1	2
Chr02G0576.1	1
Chr02G0581.1	1
Chr02G0584.1	7
Chr02G0585.1	2
Chr02G0586.1	11
Chr02G0589.1	1
Chr02G0592.1	2
Chr02G0593.1	11
Chr02G0594.1	21
Chr02G0600.1	1
Chr02G0605.1	9
Chr02G0614.1	4
Chr02G0616.1	2
Chr02G0618.1	9
Chr02G0619.1	9
Chr02G0626.1	11
Chr02G0635.1	1
Chr02G0641.1	10
Chr02G0658.1	9
Chr02G0666.1	10
Chr02G0678.1	9
Chr02G0684.1	9
Chr02G0685.1	1
Chr02G0688.1	7
Chr02G0693.1	1
Chr02G0703.1	1
Chr02G0704.1	6
Chr02G0707.1	2
Chr02G0710.1	4
Chr02G0725.1	1

Chr02G0734.1	1
Chr02G0737.1	1
Chr02G0741.1	3
Chr02G0752.1	14
Chr02G0767.1	7
Chr02G0771.1	1
Chr02G0773.1	11
Chr02G0779.1	6
Chr10G0003.1	1
Chr10G0004.1	4
Chr10G0007.1	2
Chr10G0010.1	8
