# Supplementary material for: Decoding Pecan’s Fungal Foe: A Genomic Insight into Colletotrichum plurivorum Isolate W-6
Source: J Fungi (Basel). 2025 Mar 5;11(3):203. doi: 10.3390/jof11030203 (PMC11943440; doi:10.3390/jof11030203)
Supplement: Supplementary file 1 [file jof-11-00203-s001.zip › Table S18.pdf]

Table S18. List of secreted protein in isolate W-6 genome.

Gene ID	Chromosome	Start	End
Chr01G0016.1	Chr01	124538	126462
Chr01G0034.1	Chr01	179115	182071
Chr01G0040.1	Chr01	190166	190912
Chr01G0056.1	Chr01	252171	253184
Chr01G0057.1	Chr01	256268	259433
Chr01G0058.1	Chr01	259640	260290
Chr01G0059.1	Chr01	263685	264242
Chr01G0060.1	Chr01	265004	265836
Chr01G0068.1	Chr01	294488	297101
Chr01G0077.1	Chr01	326046	327659
Chr01G0078.1	Chr01	329013	361942
Chr01G0084.1	Chr01	377346	379774
Chr01G0086.1	Chr01	386711	387386
Chr01G0087.1	Chr01	387746	388170
Chr01G0095.1	Chr01	405517	406692
Chr01G0098.1	Chr01	418034	419398
Chr01G0110.1	Chr01	446856	451032
Chr01G0113.1	Chr01	462608	466365
Chr01G0115.1	Chr01	468627	471083
Chr01G0120.1	Chr01	490198	492031
Chr01G0122.1	Chr01	494560	494901
Chr01G0141.1	Chr01	546546	547026
Chr01G0148.1	Chr01	573738	576160
Chr01G0149.1	Chr01	578610	579829
Chr01G0150.1	Chr01	580218	582381
Chr01G0161.1	Chr01	605851	609925
Chr01G0164.1	Chr01	622250	622882
Chr01G0166.1	Chr01	627706	628294
Chr01G0167.1	Chr01	628920	629603
Chr01G0168.1	Chr01	631669	633244
Chr01G0179.1	Chr01	663306	665432
Chr01G0182.1	Chr01	671142	672740
Chr01G0186.1	Chr01	697676	698385
Chr01G0192.1	Chr01	712874	714204
Chr01G0195.1	Chr01	725910	729286
Chr01G0200.1	Chr01	754098	755510
Chr01G0201.1	Chr01	759318	760886
Chr01G0205.1	Chr01	770632	771540
Chr01G0207.1	Chr01	775322	777282
Chr01G0208.1	Chr01	779540	782421
Chr01G0209.1	Chr01	786161	788123
Chr01G0238.1	Chr01	891227	892306

Chr01G0239.1	Chr01	893636	894725
Chr01G0244.1	Chr01	906276	907492
Chr01G0249.1	Chr01	923401	925888
Chr01G0259.1	Chr01	950444	951530
Chr01G0263.1	Chr01	967967	969043
Chr01G0271.1	Chr01	997523	999078
Chr01G0276.1	Chr01	1011443	1014747
Chr01G0278.1	Chr01	1019758	1020406
Chr01G0280.1	Chr01	1025128	1025969
Chr01G0281.1	Chr01	1028512	1029352
Chr01G0282.1	Chr01	1029749	1030389
Chr01G0283.1	Chr01	1032496	1033075
Chr01G0290.1	Chr01	1065215	1066840
Chr01G0308.1	Chr01	1123337	1124461
Chr01G0315.1	Chr01	1152238	1152757
Chr01G0332.1	Chr01	1194520	1195224
Chr01G0333.1	Chr01	1197090	1197563
Chr01G0334.1	Chr01	1199720	1200987
Chr01G0345.1	Chr01	1244694	1246437
Chr01G0367.1	Chr01	1331332	1333389
Chr01G0374.1	Chr01	1351392	1351604
Chr01G0386.1	Chr01	1392895	1394777
Chr01G0392.1	Chr01	1420861	1422464
Chr01G0404.1	Chr01	1457387	1457741
Chr01G0406.1	Chr01	1461248	1461591
Chr01G0416.1	Chr01	1497967	1500436
Chr01G0417.1	Chr01	1501882	1502823
Chr01G0425.1	Chr01	1541702	1542122
Chr01G0435.1	Chr01	1574786	1575391
Chr01G0452.1	Chr01	1656963	1658432
Chr01G0458.1	Chr01	1678631	1679532
Chr01G0464.1	Chr01	1702880	1703169
Chr01G0465.1	Chr01	1704299	1707337
Chr01G0475.1	Chr01	1742875	1743465
Chr01G0482.1	Chr01	1781188	1782896
Chr01G0486.1	Chr01	1791240	1793422
Chr01G0497.1	Chr01	1832490	1834870
Chr01G0499.1	Chr01	1840065	1841507
Chr01G0511.1	Chr01	1873036	1873956
Chr01G0517.1	Chr01	1885193	1886093
Chr01G0531.1	Chr01	1922595	1923529
Chr01G0532.1	Chr01	1924516	1925667
Chr01G0534.1	Chr01	1940560	1941666
Chr01G0543.1	Chr01	1961805	1963589

Chr01G0553.1	Chr01	1988439	1991860
Chr01G0554.1	Chr01	1994613	1999827
Chr01G0555.1	Chr01	2000841	2002303
Chr01G0566.1	Chr01	2035807	2036585
Chr01G0567.1	Chr01	2040747	2042870
Chr01G0580.1	Chr01	2101360	2103450
Chr01G0599.1	Chr01	2164105	2165680
Chr01G0601.1	Chr01	2170826	2171812
Chr01G0615.1	Chr01	2210794	2212047
Chr01G0619.1	Chr01	2217667	2218098
Chr01G0626.1	Chr01	2236533	2237336
Chr01G0631.1	Chr01	2252303	2254067
Chr01G0634.1	Chr01	2272743	2274112
Chr01G0636.1	Chr01	2276719	2278536
Chr01G0641.1	Chr01	2292674	2294233
Chr01G0649.1	Chr01	2314608	2314908
Chr01G0652.1	Chr01	2325943	2327731
Chr01G0655.1	Chr01	2336116	2337195
Chr01G0656.1	Chr01	2339076	2340483
Chr01G0672.1	Chr01	2384090	2385629
Chr01G0675.1	Chr01	2392704	2396228
Chr01G0679.1	Chr01	2402264	2403137
Chr01G0689.1	Chr01	2427411	2430027
Chr01G0693.1	Chr01	2449653	2451831
Chr01G0719.1	Chr01	2532095	2532280
Chr01G0723.1	Chr01	2543234	2545662
Chr01G0724.1	Chr01	2546187	2546975
Chr01G0753.1	Chr01	2667918	2670059
Chr01G0754.1	Chr01	2670269	2670766
Chr01G0760.1	Chr01	2684064	2685092
Chr01G0762.1	Chr01	2688683	2689330
Chr01G0765.1	Chr01	2703528	2704317
Chr01G0768.1	Chr01	2710325	2711053
Chr01G0774.1	Chr01	2726589	2727681
Chr01G0775.1	Chr01	2727957	2729868
Chr01G0779.1	Chr01	2741693	2742529
Chr01G0780.1	Chr01	2742795	2744064
Chr01G0781.1	Chr01	2745632	2747759
Chr01G0782.1	Chr01	2749809	2752525
Chr01G0802.1	Chr01	2809906	2811204
Chr01G0809.1	Chr01	2828318	2828903
Chr01G0813.1	Chr01	2837006	2839478
Chr01G0814.1	Chr01	2840298	2841594
Chr01G0819.1	Chr01	2864788	2865950

Chr01G0829.1	Chr01	2892059	2892906
Chr01G0893.1	Chr01	3116633	3118862
Chr01G0912.1	Chr01	3185021	3186372
Chr01G0928.1	Chr01	3241078	3242795
Chr01G0949.1	Chr01	3311619	3312833
Chr01G0956.1	Chr01	3339623	3341407
Chr01G0961.1	Chr01	3356080	3357222
Chr01G0977.1	Chr01	3404245	3405947
Chr01G0987.1	Chr01	3428631	3430089
Chr01G0993.1	Chr01	3442021	3442850
Chr01G0997.1	Chr01	3467355	3467754
Chr01G1002.1	Chr01	3483931	3484479
Chr01G1007.1	Chr01	3501092	3501720
Chr01G1012.1	Chr01	3518353	3519561
Chr01G1018.1	Chr01	3534229	3535377
Chr01G1026.1	Chr01	3552295	3554627
Chr01G1027.1	Chr01	3556020	3557342
Chr01G1036.1	Chr01	3581266	3581769
Chr01G1037.1	Chr01	3582918	3583394
Chr01G1038.1	Chr01	3585925	3586992
Chr01G1045.1	Chr01	3611157	3612596
Chr01G1061.1	Chr01	3691175	3693790
Chr01G1073.1	Chr01	3728294	3728742
Chr01G1074.1	Chr01	3730777	3732085
Chr01G1086.1	Chr01	3769242	3771149
Chr01G1092.1	Chr01	3786231	3787015
Chr01G1104.1	Chr01	3826820	3827077
Chr01G1112.1	Chr01	3851590	3853504
Chr01G1113.1	Chr01	3854994	3856178
Chr01G1121.1	Chr01	3883787	3887289
Chr01G1136.1	Chr01	3947014	3950028
Chr01G1152.1	Chr01	4011037	4013210
Chr01G1159.1	Chr01	4028299	4032862
Chr01G1269.1	Chr01	4492706	4495266
Chr01G1274.1	Chr01	4515345	4517781
Chr01G1300.1	Chr01	4620779	4622495
Chr01G1313.1	Chr01	4670040	4671426
Chr01G1327.1	Chr01	4760801	4761565
Chr01G1332.1	Chr01	4778997	4780433
Chr01G1337.1	Chr01	4792319	4793684
Chr01G1338.1	Chr01	4794379	4795530
Chr01G1340.1	Chr01	4800432	4800968
Chr01G1351.1	Chr01	4848463	4848845
Chr01G1361.1	Chr01	4880712	4882408

Chr01G1363.1	Chr01	4887540	4888020
Chr01G1366.1	Chr01	4897329	4898410
Chr01G1368.1	Chr01	4900646	4902039
Chr01G1393.1	Chr01	4993433	4995346
Chr01G1394.1	Chr01	5005365	5007353
Chr01G1404.1	Chr01	5046394	5047014
Chr01G1406.1	Chr01	5051682	5052101
Chr01G1416.1	Chr01	5088090	5088654
Chr01G1422.1	Chr01	5100935	5101763
Chr01G1426.1	Chr01	5113852	5114808
Chr01G1430.1	Chr01	5123096	5124180
Chr01G1454.1	Chr01	5195354	5195719
Chr01G1460.1	Chr01	5212754	5213588
Chr01G1463.1	Chr01	5225509	5225938
Chr01G1466.1	Chr01	5234781	5235743
Chr01G1468.1	Chr01	5238598	5239687
Chr01G1470.1	Chr01	5243049	5243924
Chr01G1472.1	Chr01	5250902	5251243
Chr01G1479.1	Chr01	5267314	5267874
Chr01G1485.1	Chr01	5287162	5288864
Chr01G1488.1	Chr01	5296990	5297800
Chr01G1489.1	Chr01	5297970	5299693
Chr01G1491.1	Chr01	5304416	5304808
Chr01G1496.1	Chr01	5325734	5327357
Chr01G1507.1	Chr01	5372617	5372979
Chr01G1524.1	Chr01	5467917	5469912
Chr01G1542.1	Chr01	5530409	5533467
Chr01G1546.1	Chr01	5554802	5557127
Chr01G1568.1	Chr01	5627474	5628585
Chr01G1571.1	Chr01	5658721	5659059
Chr01G1573.1	Chr01	5663969	5665395
Chr01G1578.1	Chr01	5679900	5680891
Chr01G1580.1	Chr01	5685989	5687226
Chr01G1604.1	Chr01	5789053	5789370
Chr01G1610.1	Chr01	5813817	5815365
Chr01G1620.1	Chr01	5863619	5864882
Chr01G1624.1	Chr01	5875716	5877162
Chr01G1641.1	Chr01	5914961	5916576
Chr01G1648.1	Chr01	5933993	5934244
Chr01G1655.1	Chr01	5949777	5950856
Chr01G1658.1	Chr01	5956039	5956977
Chr01G1665.1	Chr01	5970656	5971238
Chr01G1668.1	Chr01	5978750	5980602
Chr01G1676.1	Chr01	5996489	5997108

Chr01G1692.1	Chr01	6043997	6044227
Chr01G1695.1	Chr01	6052882	6053646
Chr01G1699.1	Chr01	6069481	6070773
Chr01G1702.1	Chr01	6076373	6078673
Chr01G1754.1	Chr01	6236466	6237349
Chr01G1770.1	Chr01	6283728	6285014
Chr01G1774.1	Chr01	6305125	6306601
Chr01G1775.1	Chr01	6308013	6309122
Chr01G1778.1	Chr01	6316648	6317665
Chr01G1798.1	Chr01	6381855	6383065
Chr01G1802.1	Chr01	6390181	6391797
Chr01G1811.1	Chr01	6421031	6422517
Chr01G1818.1	Chr01	6442435	6443529
Chr01G1824.1	Chr01	6462903	6463879
Chr01G1829.1	Chr01	6473987	6475217
Chr01G1830.1	Chr01	6475327	6477194
Chr01G1836.1	Chr01	6495149	6495364
Chr01G1838.1	Chr01	6497481	6499230
Chr01G1848.1	Chr01	6537413	6540137
Chr01G1855.1	Chr01	6570140	6571923
Chr01G1861.1	Chr01	6583720	6584981
Chr01G1865.1	Chr01	6595844	6596769
Chr01G1879.1	Chr01	6643687	6644599
Chr01G1898.1	Chr01	6707072	6707462
Chr01G1900.1	Chr01	6709542	6711422
Chr01G1901.1	Chr01	6712941	6714302
Chr01G1910.1	Chr01	6735124	6736737
Chr01G1949.1	Chr01	6882793	6883935
Chr01G1953.1	Chr01	6895040	6898227
Chr01G1954.1	Chr01	6900585	6902147
Chr01G1960.1	Chr01	6932986	6933742
Chr01G1962.1	Chr01	6938034	6940803
Chr01G1963.1	Chr01	6940983	6943002
Chr01G1970.1	Chr01	6966258	6967446
Chr01G1990.1	Chr01	7037020	7038706
Chr01G1992.1	Chr01	7047049	7049220
Chr01G1994.1	Chr01	7054538	7055112
Chr01G1997.1	Chr01	7060176	7062450
Chr01G2024.1	Chr01	7180191	7180553
Chr01G2042.1	Chr01	7233710	7235292
Chr01G2044.1	Chr01	7246680	7249921
Chr01G2046.1	Chr01	7256327	7258495
Chr01G2066.1	Chr01	7322242	7322973
Chr01G2069.1	Chr01	7328393	7329355

Chr01G2071.1	Chr01	7333493	7335612
Chr01G2088.1	Chr01	7388125	7394508
Chr01G2102.1	Chr01	7438561	7439847
Chr01G2112.1	Chr01	7469851	7470981
Chr01G2129.1	Chr01	7521734	7523421
Chr01G2142.1	Chr01	7570863	7572742
Chr01G2145.1	Chr01	7575230	7578191
Chr01G2150.1	Chr01	7594753	7598599
Chr01G2236.1	Chr01	7886384	7889995
Chr01G2250.1	Chr01	7936470	7937718
Chr01G2275.1	Chr01	8015555	8019345
Chr01G2276.1	Chr01	8020248	8021167
Chr01G2313.1	Chr01	8163351	8165449
Chr01G2318.1	Chr01	8188446	8189627
Chr01G2350.1	Chr01	8332840	8335715
Chr01G2392.1	Chr01	8516233	8517405
Chr01G2421.1	Chr01	8629121	8631985
Chr01G2428.1	Chr01	8661819	8662595
Chr01G2429.1	Chr01	8664052	8665524
Chr01G2430.1	Chr01	8666176	8667505
Chr01G2432.1	Chr01	8674878	8676530
Chr01G2435.1	Chr01	8685070	8686869
Chr01G2438.1	Chr01	8694365	8696254
Chr01G2442.1	Chr01	8706533	8707974
Chr01G2450.1	Chr01	8731128	8731667
Chr01G2452.1	Chr01	8736776	8738898
Chr01G2453.1	Chr01	8739811	8740924
Chr01G2472.1	Chr01	8819978	8820606
Chr01G2479.1	Chr01	8849376	8850636
Chr01G2482.1	Chr01	8855377	8856484
Chr01G2484.1	Chr01	8861879	8863042
Chr01G2490.1	Chr01	8881380	8882229
Chr01G2493.1	Chr01	8891362	8893371
Chr01G2497.1	Chr01	8903208	8903989
Chr01G2502.1	Chr01	8940841	8943023
Chr01G2506.1	Chr01	8955104	8955433
Chr01G2510.1	Chr01	8972654	8973643
Chr01G2512.1	Chr01	8978173	8980166
Chr01G2521.1	Chr01	9025162	9026924
Chr01G2524.1	Chr01	9033614	9035999
Chr01G2526.1	Chr01	9041773	9041964
Chr01G2534.1	Chr01	9073199	9074248
Chr01G2538.1	Chr01	9087058	9090567
Chr01G2542.1	Chr01	9098692	9101832

Chr01G2544.1	Chr01	9104069	9105114
Chr01G2551.1	Chr01	9124977	9127661
Chr01G2558.1	Chr01	9146564	9150739
Chr01G2570.1	Chr01	9188779	9190479
Chr01G2577.1	Chr01	9208656	9209366
Chr01G2585.1	Chr01	9237253	9237669
Chr01G2589.1	Chr01	9252529	9253446
Chr01G2593.1	Chr01	9268349	9269134
Chr01G2601.1	Chr01	9287957	9290918
Chr01G2614.1	Chr01	9322934	9324321
Chr01G2624.1	Chr01	9349212	9350276
Chr01G2627.1	Chr01	9357438	9359084
Chr01G2630.1	Chr01	9370207	9373159
Chr01G2637.1	Chr01	9393124	9394683
Chr01G2645.1	Chr01	9425536	9425871
Chr01G2653.1	Chr01	9449934	9451362
Chr01G2654.1	Chr01	9452141	9453955
Chr01G2680.1	Chr01	9558610	9560019
Chr01G2690.1	Chr01	9588572	9590983
Chr01G2697.1	Chr01	9616391	9617500
Chr01G2713.1	Chr01	9664298	9665353
Chr01G2715.1	Chr01	9670142	9671551
Chr01G2717.1	Chr01	9675907	9678880
Chr01G2720.1	Chr01	9686754	9688705
Chr01G2739.1	Chr01	9807070	9808093
Chr01G2747.1	Chr01	9832744	9833529
Chr01G2750.1	Chr01	9839826	9841001
Chr01G2751.1	Chr01	9841222	9842842
Chr01G2779.1	Chr01	9938001	9939955
Chr05G0841.1	Chr05	3280786	3282138
Chr05G0822.1	Chr05	3200098	3200442
Chr05G0820.1	Chr05	3195446	3196883
Chr05G0813.1	Chr05	3175399	3176250
Chr05G0812.1	Chr05	3174073	3174984
Chr05G0761.1	Chr05	2989319	2991004
Chr05G0754.1	Chr05	2966706	2967593
Chr05G0738.1	Chr05	2896085	2897166
Chr05G0731.1	Chr05	2879383	2881585
Chr05G0660.1	Chr05	2621881	2623962
Chr05G0644.1	Chr05	2542864	2544773
Chr05G0627.1	Chr05	2496862	2498538
Chr05G0625.1	Chr05	2492211	2494099
Chr05G0605.1	Chr05	2432313	2434041
Chr05G0602.1	Chr05	2418389	2420705

Chr05G0593.1	Chr05	2387104	2391290
Chr05G0558.1	Chr05	2267473	2269066
Chr05G0537.1	Chr05	2205821	2206801
Chr05G0536.1	Chr05	2202866	2203893
Chr05G0514.1	Chr05	2109314	2111049
Chr05G0511.1	Chr05	2098774	2100915
Chr05G0470.1	Chr05	1918484	1919102
Chr05G0425.1	Chr05	1754732	1755555
Chr05G0411.1	Chr05	1687486	1689031
Chr05G0386.1	Chr05	1595378	1596362
Chr05G0380.1	Chr05	1575260	1577203
Chr05G0378.1	Chr05	1567608	1569548
Chr05G0368.1	Chr05	1519632	1520892
Chr05G0357.1	Chr05	1466726	1467437
Chr05G0348.1	Chr05	1421117	1422011
Chr05G0347.1	Chr05	1416016	1419946
Chr05G0341.1	Chr05	1362205	1363441
Chr05G0334.1	Chr05	1339871	1340963
Chr05G0332.1	Chr05	1332639	1333331
Chr05G0330.1	Chr05	1326791	1329434
Chr05G0329.1	Chr05	1325439	1326016
Chr05G0323.1	Chr05	1304210	1304469
Chr05G0318.1	Chr05	1281801	1282262
Chr05G0316.1	Chr05	1274464	1277014
Chr05G0313.1	Chr05	1259864	1261943
Chr05G0304.1	Chr05	1196313	1197991
Chr05G0295.1	Chr05	1155153	1161426
Chr05G0283.1	Chr05	1114091	1115429
Chr05G0279.1	Chr05	1102114	1102839
Chr05G0277.1	Chr05	1090146	1091124
Chr05G0262.1	Chr05	1038570	1040099
Chr05G0258.1	Chr05	1014774	1016116
Chr05G0246.1	Chr05	974110	975857
Chr05G0234.1	Chr05	932349	932707
Chr05G0227.1	Chr05	907337	908924
Chr05G0209.1	Chr05	840505	841872
Chr05G0208.1	Chr05	838540	839976
Chr05G0207.1	Chr05	834305	836856
Chr05G0196.1	Chr05	774611	776790
Chr05G0194.1	Chr05	770190	770875
Chr05G0191.1	Chr05	763169	765081
Chr05G0189.1	Chr05	752723	754465
Chr05G0172.1	Chr05	696899	698756
Chr05G0166.1	Chr05	678247	678787

Chr05G0164.1	Chr05	671864	674647
Chr05G0145.1	Chr05	595134	602282
Chr05G0134.1	Chr05	549396	550816
Chr05G0133.1	Chr05	549052	549328
Chr05G0131.1	Chr05	538248	542087
Chr05G0130.1	Chr05	533035	534044
Chr05G0129.1	Chr05	531214	531951
Chr05G0119.1	Chr05	499522	500215
Chr05G0107.1	Chr05	460044	461966
Chr05G0095.1	Chr05	418046	419188
Chr05G0093.1	Chr05	413086	414607
Chr05G0092.1	Chr05	409271	410005
Chr05G0078.1	Chr05	367154	367553
Chr05G0075.1	Chr05	357748	358425
Chr05G0074.1	Chr05	355924	357473
Chr05G0059.1	Chr05	294758	297069
Chr05G0058.1	Chr05	285796	290962
Chr05G0054.1	Chr05	276990	278139
Chr05G0042.1	Chr05	241034	242341
Chr05G0035.1	Chr05	221642	222815
Chr07G0013.1	Chr07	101267	101889
Chr07G0021.1	Chr07	158572	160702
Chr07G0025.1	Chr07	171525	172449
Chr07G0036.1	Chr07	198034	198540
Chr07G0050.1	Chr07	247493	248708
Chr07G0069.1	Chr07	322432	324265
Chr07G0080.1	Chr07	367654	369247
Chr07G0081.1	Chr07	370936	371538
Chr07G0112.1	Chr07	479803	481437
Chr07G0115.1	Chr07	489472	490144
Chr07G0139.1	Chr07	557709	560344
Chr07G0140.1	Chr07	562392	564306
Chr07G0172.1	Chr07	671240	671714
Chr07G0176.1	Chr07	681283	682509
Chr07G0177.1	Chr07	682988	685234
Chr07G0181.1	Chr07	709370	710513
Chr07G0190.1	Chr07	745404	747238
Chr07G0196.1	Chr07	771848	774931
Chr07G0205.1	Chr07	813085	815117
Chr07G0228.1	Chr07	894637	895854
Chr07G0235.1	Chr07	912408	913035
Chr07G0243.1	Chr07	936468	937716
Chr07G0249.1	Chr07	955703	957325
Chr07G0252.1	Chr07	964299	966141

Chr07G0253.1	Chr07	969049	970293
Chr07G0263.1	Chr07	1001457	1001624
Chr07G0269.1	Chr07	1019006	1020430
Chr07G0271.1	Chr07	1028752	1033228
Chr07G0273.1	Chr07	1037691	1039830
Chr07G0277.1	Chr07	1049527	1051006
Chr07G0282.1	Chr07	1072836	1074610
Chr07G0288.1	Chr07	1103369	1104826
Chr07G0291.1	Chr07	1113182	1113481
Chr07G0292.1	Chr07	1114816	1115010
Chr07G0293.1	Chr07	1115365	1117637
Chr07G0298.1	Chr07	1132895	1133883
Chr07G0314.1	Chr07	1194836	1196974
Chr07G0315.1	Chr07	1198017	1199092
Chr07G0316.1	Chr07	1200592	1201731
Chr07G0318.1	Chr07	1205710	1207231
Chr07G0319.1	Chr07	1211136	1212868
Chr07G0324.1	Chr07	1228628	1229383
Chr07G0328.1	Chr07	1237598	1240456
Chr07G0329.1	Chr07	1241235	1241500
Chr07G0337.1	Chr07	1263496	1263814
Chr07G0341.1	Chr07	1274540	1275516
Chr07G0361.1	Chr07	1331043	1332002
Chr07G0362.1	Chr07	1333305	1335860
Chr07G0363.1	Chr07	1338323	1340247
Chr07G0365.1	Chr07	1350434	1350928
Chr07G0368.1	Chr07	1358980	1361297
Chr07G0382.1	Chr07	1400942	1403130
Chr07G0385.1	Chr07	1411701	1412643
Chr07G0401.1	Chr07	1476212	1478403
Chr07G0413.1	Chr07	1515966	1518378
Chr07G0414.1	Chr07	1518620	1520266
Chr07G0415.1	Chr07	1524844	1525927
Chr07G0419.1	Chr07	1539178	1540410
Chr07G0423.1	Chr07	1554288	1555994
Chr07G0439.1	Chr07	1604713	1605819
Chr07G0441.1	Chr07	1612182	1615575
Chr07G0446.1	Chr07	1624663	1626383
Chr07G0456.1	Chr07	1673827	1674681
Chr07G0471.1	Chr07	1709943	1710201
Chr07G0489.1	Chr07	1757954	1758277
Chr07G0503.1	Chr07	1801234	1802553
Chr07G0504.1	Chr07	1804799	1807300
Chr07G0508.1	Chr07	1826501	1827272

Chr07G0526.1	Chr07	1876747	1878315
Chr07G0531.1	Chr07	1888217	1888948
Chr07G0532.1	Chr07	1890142	1891269
Chr07G0547.1	Chr07	1937472	1939639
Chr07G0548.1	Chr07	1941880	1945255
Chr07G0550.1	Chr07	1949288	1951039
Chr07G0552.1	Chr07	1957673	1959132
Chr07G0563.1	Chr07	1988448	1988891
Chr07G0564.1	Chr07	1995256	1995848
Chr07G0568.1	Chr07	2008710	2009660
Chr07G0571.1	Chr07	2014409	2016342
Chr07G0574.1	Chr07	2030809	2031299
Chr07G0575.1	Chr07	2032058	2033499
Chr07G0600.1	Chr07	2124418	2126371
Chr07G0611.1	Chr07	2163988	2165133
Chr07G0615.1	Chr07	2173187	2174450
Chr07G0617.1	Chr07	2178281	2179457
Chr07G0658.1	Chr07	2317990	2318541
Chr07G0661.1	Chr07	2323071	2324752
Chr07G0707.1	Chr07	2506601	2507119
Chr07G0754.1	Chr07	2749024	2751759
Chr07G0785.1	Chr07	2877665	2878595
Chr07G0802.1	Chr07	2946098	2948009
Chr07G0809.1	Chr07	2989321	2989827
Chr07G0842.1	Chr07	3162343	3164585
Chr07G0846.1	Chr07	3175882	3177153
Chr07G0861.1	Chr07	3224664	3225156
Chr07G0879.1	Chr07	3285471	3286644
Chr07G0881.1	Chr07	3290379	3295178
Chr07G0891.1	Chr07	3347678	3348331
Chr07G0893.1	Chr07	3351515	3355166
Chr07G0904.1	Chr07	3387045	3389050
Chr07G0908.1	Chr07	3401872	3403081
Chr07G0924.1	Chr07	3458145	3458969
Chr07G0926.1	Chr07	3461007	3462052
Chr07G0928.1	Chr07	3469948	3472034
Chr07G0935.1	Chr07	3487066	3487806
Chr07G0936.1	Chr07	3488620	3489556
Chr07G0937.1	Chr07	3489679	3490314
Chr07G0940.1	Chr07	3496647	3497316
Chr07G0943.1	Chr07	3503938	3504567
Chr07G0947.1	Chr07	3514362	3516500
Chr07G0952.1	Chr07	3529125	3533038
Chr07G0978.1	Chr07	3602186	3603983

Chr07G0979.1	Chr07	3604881	3606612
Chr07G0981.1	Chr07	3611793	3612997
Chr07G0990.1	Chr07	3646270	3648271
Chr07G0997.1	Chr07	3678589	3679413
Chr07G1001.1	Chr07	3693987	3694811
Chr07G1003.1	Chr07	3699150	3699804
Chr07G1007.1	Chr07	3709069	3710160
Chr07G1009.1	Chr07	3713624	3714066
Chr07G1016.1	Chr07	3750640	3751563
Chr07G1031.1	Chr07	3808787	3810099
Chr07G1047.1	Chr07	3864870	3865850
Chr07G1049.1	Chr07	3870499	3874167
Chr07G1050.1	Chr07	3875019	3875429
Chr07G1089.1	Chr07	4002184	4002784
Chr07G1105.1	Chr07	4062705	4063832
Chr07G1108.1	Chr07	4072453	4072754
Chr07G1117.1	Chr07	4092401	4093558
Chr07G1126.1	Chr07	4113212	4114425
Chr07G1128.1	Chr07	4121129	4122130
Chr07G1132.1	Chr07	4134435	4136051
Chr07G1133.1	Chr07	4136455	4139292
Chr07G1134.1	Chr07	4142104	4142797
Chr07G1135.1	Chr07	4143174	4143683
Chr07G1139.1	Chr07	4151285	4151896
Chr07G1161.1	Chr07	4274487	4277370
Chr07G1166.1	Chr07	4287908	4289098
Chr07G1167.1	Chr07	4290397	4292410
Chr07G1168.1	Chr07	4292885	4293750
Chr07G1169.1	Chr07	4295992	4297685
Chr07G1176.1	Chr07	4321345	4322529
Chr07G1189.1	Chr07	4380312	4382997
Chr02G0813.1	Chr02	3153605	3156000
Chr02G0852.1	Chr02	3287354	3289156
Chr02G0878.1	Chr02	3385123	3387600
Chr02G0950.1	Chr02	3612610	3613454
Chr02G0968.1	Chr02	3661861	3665846
Chr02G0993.1	Chr02	3761509	3763019
Chr02G1076.1	Chr02	4032634	4033715
Chr02G1092.1	Chr02	4094129	4094805
Chr02G1099.1	Chr02	4146653	4147833
Chr02G1101.1	Chr02	4151237	4153970
Chr02G1120.1	Chr02	4237690	4238966
Chr02G1125.1	Chr02	4252381	4254988
Chr02G1127.1	Chr02	4262252	4263188

Chr02G1131.1	Chr02	4294158	4295597
Chr02G1132.1	Chr02	4297583	4299394
Chr02G1147.1	Chr02	4348097	4350944
Chr02G1177.1	Chr02	4464951	4466669
Chr02G1178.1	Chr02	4471140	4474008
Chr02G1190.1	Chr02	4516527	4518322
Chr02G1199.1	Chr02	4553976	4554501
Chr02G1227.1	Chr02	4663114	4665373
Chr02G1238.1	Chr02	4699791	4700525
Chr02G1252.1	Chr02	4731978	4732583
Chr02G1260.1	Chr02	4750242	4752035
Chr02G1269.1	Chr02	4785685	4786860
Chr02G1276.1	Chr02	4807183	4808716
Chr02G1286.1	Chr02	4840175	4844329
Chr02G1287.1	Chr02	4845109	4845787
Chr02G1316.1	Chr02	4942137	4944464
Chr02G1331.1	Chr02	4993964	4994245
Chr02G1335.1	Chr02	5020109	5020614
Chr02G1339.1	Chr02	5042577	5044370
Chr02G1340.1	Chr02	5045061	5045574
Chr02G1348.1	Chr02	5071012	5073102
Chr02G1356.1	Chr02	5092492	5093465
Chr02G1357.1	Chr02	5094081	5098250
Chr02G1358.1	Chr02	5098456	5100558
Chr02G1359.1	Chr02	5101883	5102156
Chr02G1360.1	Chr02	5104349	5105518
Chr02G1362.1	Chr02	5110382	5112563
Chr02G1366.1	Chr02	5121193	5123934
Chr02G1375.1	Chr02	5156716	5158116
Chr02G1383.1	Chr02	5181776	5184065
Chr02G1404.1	Chr02	5236659	5238137
Chr02G1405.1	Chr02	5238238	5239664
Chr02G1406.1	Chr02	5241307	5241841
Chr02G1417.1	Chr02	5283482	5285907
Chr02G1429.1	Chr02	5330561	5333831
Chr02G1433.1	Chr02	5344682	5345122
Chr02G1438.1	Chr02	5364262	5365406
Chr02G1446.1	Chr02	5383759	5384757
Chr02G1467.1	Chr02	5456333	5458313
Chr02G1468.1	Chr02	5460076	5460423
Chr02G1477.1	Chr02	5493050	5494227
Chr02G1486.1	Chr02	5527789	5529022
Chr02G1497.1	Chr02	5559553	5560104
Chr02G1522.1	Chr02	5632120	5633136

Chr02G1524.1	Chr02	5636127	5637890
Chr02G1525.1	Chr02	5641516	5643533
Chr02G1528.1	Chr02	5650595	5651983
Chr02G1535.1	Chr02	5685453	5685857
Chr02G1537.1	Chr02	5689467	5689874
Chr02G1558.1	Chr02	5769638	5769976
Chr02G1560.1	Chr02	5772408	5773124
Chr02G1565.1	Chr02	5817120	5817952
Chr02G1572.1	Chr02	5844776	5846081
Chr02G1575.1	Chr02	5852898	5853566
Chr02G1577.1	Chr02	5860526	5865102
Chr02G1582.1	Chr02	5881381	5882235
Chr02G1587.1	Chr02	5915469	5916301
Chr02G1598.1	Chr02	5940560	5942260
Chr02G1601.1	Chr02	5952256	5954343
Chr02G1622.1	Chr02	6037129	6038482
Chr02G1641.1	Chr02	6115942	6117322
Chr02G1650.1	Chr02	6141903	6142480
Chr02G1653.1	Chr02	6153435	6155353
Chr02G1678.1	Chr02	6261396	6262715
Chr02G1689.1	Chr02	6285893	6287862
Chr02G1694.1	Chr02	6311383	6312777
Chr02G1699.1	Chr02	6325545	6326414
Chr02G1700.1	Chr02	6327492	6332206
Chr02G1716.1	Chr02	6394989	6397066
Chr02G1748.1	Chr02	6495557	6497521
Chr02G1756.1	Chr02	6526867	6527415
Chr02G1768.1	Chr02	6570574	6572027
Chr02G1769.1	Chr02	6573016	6573378
Chr02G1782.1	Chr02	6626276	6627390
Chr02G1784.1	Chr02	6632137	6633549
Chr02G1795.1	Chr02	6668511	6670510
Chr02G1799.1	Chr02	6681099	6681921
Chr02G1802.1	Chr02	6687491	6688255
Chr02G1803.1	Chr02	6688735	6691646
Chr02G1812.1	Chr02	6714347	6715729
Chr02G1818.1	Chr02	6730447	6734081
Chr02G1825.1	Chr02	6755289	6756797
Chr02G1835.1	Chr02	6787267	6788607
Chr02G1842.1	Chr02	6812344	6815144
Chr02G1848.1	Chr02	6833820	6834667
Chr02G1853.1	Chr02	6844356	6845754
Chr02G1854.1	Chr02	6847306	6848445
Chr08G0020.1	Chr08	153048	153911

Chr08G0033.1	Chr08	188588	189527
Chr08G0036.1	Chr08	193866	197506
Chr08G0052.1	Chr08	241281	243054
Chr08G0054.1	Chr08	251756	257684
Chr08G0062.1	Chr08	270867	272672
Chr08G0063.1	Chr08	272953	273455
Chr08G0070.1	Chr08	291259	293326
Chr08G0071.1	Chr08	300808	301947
Chr08G0079.1	Chr08	324131	324611
Chr08G0086.1	Chr08	345439	347422
Chr08G0087.1	Chr08	348169	349997
Chr08G0105.1	Chr08	406804	407402
Chr08G0106.1	Chr08	409033	410666
Chr08G0132.1	Chr08	496129	497889
Chr08G0135.1	Chr08	505142	507140
Chr08G0158.1	Chr08	576833	578620
Chr08G0165.1	Chr08	593545	594664
Chr08G0166.1	Chr08	596284	598158
Chr08G0171.1	Chr08	616248	617108
Chr08G0179.1	Chr08	713934	714345
Chr08G0182.1	Chr08	720209	721010
Chr08G0183.1	Chr08	722634	724400
Chr08G0194.1	Chr08	756588	758861
Chr08G0221.1	Chr08	942059	944668
Chr08G0223.1	Chr08	952192	954198
Chr08G0252.1	Chr08	1086021	1087404
Chr08G0279.1	Chr08	1176781	1178679
Chr08G0301.1	Chr08	1269137	1274296
Chr08G0319.1	Chr08	1357445	1358130
Chr08G0323.1	Chr08	1371170	1374164
Chr08G0338.1	Chr08	1438165	1439039
Chr08G0339.1	Chr08	1440037	1440650
Chr08G0384.1	Chr08	1667194	1668347
Chr08G0395.1	Chr08	1700139	1702606
Chr08G0398.1	Chr08	1721403	1722407
Chr08G0401.1	Chr08	1727587	1728423
Chr08G0412.1	Chr08	1773012	1773772
Chr08G0413.1	Chr08	1774139	1778346
Chr08G0437.1	Chr08	1929161	1932745
Chr08G0466.1	Chr08	2065549	2067733
Chr08G0467.1	Chr08	2068613	2070097
Chr08G0473.1	Chr08	2087382	2088338
Chr08G0477.1	Chr08	2098294	2098999
Chr08G0484.1	Chr08	2116865	2122413

Chr08G0492.1	Chr08	2153975	2155780
Chr08G0496.1	Chr08	2181915	2184590
Chr08G0500.1	Chr08	2206830	2208727
Chr08G0501.1	Chr08	2212598	2213432
Chr08G0506.1	Chr08	2237815	2240949
Chr08G0527.1	Chr08	2332675	2333502
Chr08G0644.1	Chr08	2805372	2806546
Chr08G0649.1	Chr08	2819962	2821359
Chr08G0652.1	Chr08	2827672	2830022
Chr08G0656.1	Chr08	2839664	2840927
Chr08G0665.1	Chr08	2868524	2870560
Chr08G0682.1	Chr08	2915968	2917226
Chr08G0694.1	Chr08	2955638	2956517
Chr08G0725.1	Chr08	3036469	3040101
Chr08G0734.1	Chr08	3065797	3067090
Chr08G0738.1	Chr08	3081844	3083290
Chr08G0756.1	Chr08	3136387	3140216
Chr08G0761.1	Chr08	3150992	3152612
Chr08G0763.1	Chr08	3159013	3159869
Chr08G0771.1	Chr08	3191354	3196721
Chr08G0783.1	Chr08	3235473	3237497
Chr08G0787.1	Chr08	3251182	3252171
Chr08G0788.1	Chr08	3253715	3257236
Chr08G0789.1	Chr08	3261277	3263402
Chr08G0801.1	Chr08	3303261	3304046
Chr08G0803.1	Chr08	3306269	3308207
Chr08G0805.1	Chr08	3310968	3312122
Chr08G0806.1	Chr08	3314026	3315082
Chr08G0819.1	Chr08	3355532	3356427
Chr08G0821.1	Chr08	3360913	3361795
Chr08G0833.1	Chr08	3400363	3402784
Chr08G0837.1	Chr08	3414514	3415184
Chr08G0841.1	Chr08	3423140	3424590
Chr08G0842.1	Chr08	3424724	3426072
Chr08G0843.1	Chr08	3426795	3434429
Chr08G0866.1	Chr08	3507656	3508161
Chr08G0874.1	Chr08	3526911	3527551
Chr08G0879.1	Chr08	3541881	3545304
Chr08G0880.1	Chr08	3546141	3546871
Chr08G0884.1	Chr08	3554215	3555576
Chr08G0917.1	Chr08	3661593	3662709
Chr08G0918.1	Chr08	3666303	3667526
Chr08G0922.1	Chr08	3677548	3678484
Chr08G0929.1	Chr08	3695866	3696897

Chr08G0934.1	Chr08	3710091	3712544
Chr08G0936.1	Chr08	3723261	3726376
Chr08G0944.1	Chr08	3750987	3752198
Chr08G0951.1	Chr08	3781563	3783537
Chr08G0952.1	Chr08	3783678	3785702
Chr08G0964.1	Chr08	3830235	3831548
Chr08G0972.1	Chr08	3854843	3857334
Chr08G0980.1	Chr08	3876967	3878732
Chr08G0985.1	Chr08	3888574	3890714
Chr08G0986.1	Chr08	3891753	3892553
Chr08G0991.1	Chr08	3911884	3912805
Chr08G0993.1	Chr08	3919087	3920838
Chr08G1017.1	Chr08	3987824	3988516
Chr08G1021.1	Chr08	3999276	4000310
Chr08G1037.1	Chr08	4049755	4050247
Chr08G1039.1	Chr08	4052667	4053374
Chr08G1042.1	Chr08	4059671	4060579
Chr08G1048.1	Chr08	4075460	4076626
Chr08G1056.1	Chr08	4113015	4114833
Chr05G0897.1	Chr05	3513022	3514881
Chr05G0906.1	Chr05	3549101	3553645
Chr05G0914.1	Chr05	3571538	3572803
Chr05G0929.1	Chr05	3614666	3616370
Chr05G0933.1	Chr05	3642936	3643644
Chr05G0935.1	Chr05	3647758	3650949
Chr05G0940.1	Chr05	3660865	3662611
Chr05G0945.1	Chr05	3674888	3676269
Chr05G0949.1	Chr05	3684839	3687235
Chr05G0967.1	Chr05	3751083	3752860
Chr05G0971.1	Chr05	3760336	3765668
Chr05G1067.1	Chr05	4193632	4195013
Chr05G1082.1	Chr05	4267960	4268418
Chr05G1092.1	Chr05	4307903	4312803
Chr05G1093.1	Chr05	4313090	4314076
Chr05G1106.1	Chr05	4355962	4359633
Chr05G1140.1	Chr05	4570796	4573805
Chr05G1150.1	Chr05	4605202	4608739
Chr05G1157.1	Chr05	4629891	4633727
Chr05G1241.1	Chr05	4918243	4920216
Chr05G1247.1	Chr05	4937284	4940257
Chr05G1248.1	Chr05	4940265	4943161
Chr05G1262.1	Chr05	4968506	4971542
Chr05G1272.1	Chr05	5019768	5020025
Chr05G1275.1	Chr05	5024922	5026184

Chr05G1314.1	Chr05	5177546	5180666
Chr05G1320.1	Chr05	5210815	5211500
Chr05G1327.1	Chr05	5248755	5249502
Chr05G1338.1	Chr05	5293816	5295274
Chr05G1339.1	Chr05	5297951	5299075
Chr05G1346.1	Chr05	5312949	5314653
Chr05G1357.1	Chr05	5344861	5346893
Chr05G1375.1	Chr05	5385724	5387053
Chr05G1400.1	Chr05	5504631	5505692
Chr05G1401.1	Chr05	5507837	5508991
Chr05G1409.1	Chr05	5531791	5533329
Chr05G1415.1	Chr05	5557683	5558189
Chr05G1420.1	Chr05	5569740	5570201
Chr05G1422.1	Chr05	5574405	5574728
Chr03G0020.1	Chr03	91074	92306
Chr03G0031.1	Chr03	121568	122517
Chr03G0032.1	Chr03	122648	123205
Chr03G0037.1	Chr03	132946	135500
Chr03G0044.1	Chr03	148465	149790
Chr03G0045.1	Chr03	152495	153235
Chr03G0046.1	Chr03	154341	155840
Chr03G0057.1	Chr03	205873	213056
Chr03G0059.1	Chr03	225745	228900
Chr03G0068.1	Chr03	250823	251258
Chr03G0086.1	Chr03	321304	323210
Chr03G0088.1	Chr03	332330	333694
Chr03G0117.1	Chr03	450015	451685
Chr03G0126.1	Chr03	473059	474546
Chr03G0129.1	Chr03	482812	484197
Chr03G0181.1	Chr03	719857	722097
Chr03G0212.1	Chr03	857670	859284
Chr03G0214.1	Chr03	873110	875159
Chr03G0219.1	Chr03	884899	887114
Chr03G0224.1	Chr03	901901	904290
Chr03G0237.1	Chr03	952237	952573
Chr03G0239.1	Chr03	956236	959013
Chr03G0240.1	Chr03	959362	960525
Chr03G0243.1	Chr03	969445	971243
Chr03G0255.1	Chr03	1030612	1031542
Chr03G0273.1	Chr03	1079978	1080213
Chr03G0280.1	Chr03	1094746	1096578
Chr03G0281.1	Chr03	1098143	1101670
Chr03G0284.1	Chr03	1104560	1106962
Chr03G0294.1	Chr03	1130391	1131128

Chr03G0320.1	Chr03	1259604	1260926
Chr03G0337.1	Chr03	1315322	1315902
Chr03G0341.1	Chr03	1326530	1327594
Chr03G0344.1	Chr03	1336663	1337336
Chr03G0346.1	Chr03	1340482	1341296
Chr03G0347.1	Chr03	1342144	1344005
Chr03G0366.1	Chr03	1417237	1419615
Chr03G0369.1	Chr03	1429578	1430423
Chr03G0371.1	Chr03	1438239	1439887
Chr03G0373.1	Chr03	1442655	1443765
Chr03G0374.1	Chr03	1444692	1446720
Chr03G0376.1	Chr03	1450108	1451187
Chr03G0390.1	Chr03	1509408	1512191
Chr03G0391.1	Chr03	1512456	1514009
Chr03G0392.1	Chr03	1514799	1518139
Chr03G0402.1	Chr03	1543038	1544974
Chr03G0414.1	Chr03	1572645	1573088
Chr03G0420.1	Chr03	1591145	1591532
Chr03G0421.1	Chr03	1592690	1593205
Chr03G0454.1	Chr03	1698440	1700254
Chr03G0455.1	Chr03	1701437	1703347
Chr03G0524.1	Chr03	1965851	1967199
Chr03G0525.1	Chr03	1968461	1969990
Chr03G0532.1	Chr03	1996771	1997361
Chr03G0533.1	Chr03	1997836	1998751
Chr03G0544.1	Chr03	2049141	2050435
Chr03G0548.1	Chr03	2057648	2059633
Chr03G0598.1	Chr03	2274807	2276891
Chr03G0601.1	Chr03	2279969	2280433
Chr03G0647.1	Chr03	2429287	2430402
Chr03G0683.1	Chr03	2578984	2579639
Chr03G0685.1	Chr03	2589267	2590562
Chr03G0687.1	Chr03	2594803	2594988
Chr03G0709.1	Chr03	2703001	2705295
Chr03G0763.1	Chr03	2875298	2876973
Chr03G0767.1	Chr03	2899433	2901538
Chr03G0793.1	Chr03	3021132	3024080
Chr03G0815.1	Chr03	3080981	3081520
Chr03G0876.1	Chr03	3327432	3328851
Chr03G0897.1	Chr03	3402880	3403579
Chr03G0947.1	Chr03	3574874	3576450
Chr03G0958.1	Chr03	3629858	3631888
Chr03G0995.1	Chr03	3808977	3809494
Chr03G1034.1	Chr03	3980792	3980989

Chr03G1036.1	Chr03	3985934	3986999
Chr03G1041.1	Chr03	3997198	3998446
Chr03G1086.1	Chr03	4163194	4163597
Chr03G1089.1	Chr03	4166920	4169090
Chr03G1135.1	Chr03	4366964	4369147
Chr03G1142.1	Chr03	4393925	4395942
Chr03G1161.1	Chr03	4469800	4472371
Chr03G1177.1	Chr03	4530626	4532188
Chr03G1180.1	Chr03	4539673	4542120
Chr03G1188.1	Chr03	4582082	4585573
Chr03G1189.1	Chr03	4585580	4587212
Chr03G1211.1	Chr03	4656678	4659491
Chr03G1226.1	Chr03	4712598	4714376
Chr03G1233.1	Chr03	4736538	4741171
Chr03G1240.1	Chr03	4773781	4775527
Chr03G1247.1	Chr03	4814584	4815923
Chr03G1272.1	Chr03	4928355	4929562
Chr03G1276.1	Chr03	4938262	4939781
Chr03G1282.1	Chr03	4961263	4962145
Chr03G1291.1	Chr03	4990482	4991675
Chr03G1292.1	Chr03	4992874	4993889
Chr03G1299.1	Chr03	5019424	5021492
Chr03G1312.1	Chr03	5059505	5060498
Chr03G1315.1	Chr03	5066478	5069091
Chr03G1318.1	Chr03	5079526	5080112
Chr03G1319.1	Chr03	5082712	5083533
Chr03G1320.1	Chr03	5085115	5087675
Chr03G1331.1	Chr03	5114517	5116193
Chr03G1343.1	Chr03	5175987	5176954
Chr03G1345.1	Chr03	5183502	5184515
Chr03G1347.1	Chr03	5190214	5190657
Chr03G1348.1	Chr03	5193224	5197082
Chr03G1350.1	Chr03	5202366	5202782
Chr03G1365.1	Chr03	5295813	5296833
Chr03G1367.1	Chr03	5299493	5302790
Chr03G1368.1	Chr03	5312192	5313824
Chr03G1376.1	Chr03	5331468	5333349
Chr03G1377.1	Chr03	5334567	5336537
Chr03G1378.1	Chr03	5336895	5338384
Chr03G1382.1	Chr03	5348305	5348987
Chr03G1394.1	Chr03	5393385	5394564
Chr03G1398.1	Chr03	5408825	5410957
Chr03G1410.1	Chr03	5451658	5452202
Chr03G1415.1	Chr03	5462257	5463336

Chr03G1420.1	Chr03	5475838	5478447
Chr03G1423.1	Chr03	5484089	5484981
Chr03G1434.1	Chr03	5530849	5532427
Chr03G1443.1	Chr03	5552541	5553028
Chr03G1452.1	Chr03	5584661	5589750
Chr03G1455.1	Chr03	5596627	5597243
Chr03G1456.1	Chr03	5599649	5602908
Chr03G1466.1	Chr03	5631616	5632316
Chr03G1468.1	Chr03	5637388	5639290
Chr03G1471.1	Chr03	5644913	5645962
Chr03G1472.1	Chr03	5646302	5647618
Chr03G1493.1	Chr03	5738048	5739885
Chr03G1498.1	Chr03	5750202	5750549
Chr03G1502.1	Chr03	5767273	5771191
Chr03G1503.1	Chr03	5772867	5773563
Chr03G1504.1	Chr03	5774564	5774963
Chr03G1511.1	Chr03	5788023	5788914
Chr03G1513.1	Chr03	5791927	5792380
Chr03G1528.1	Chr03	5829621	5830420
Chr03G1543.1	Chr03	5875469	5875811
Chr03G1554.1	Chr03	5924135	5925602
Chr03G1565.1	Chr03	5960633	5961434
Chr03G1570.1	Chr03	5987418	5989005
Chr03G1582.1	Chr03	6026258	6027901
Chr03G1592.1	Chr03	6068041	6068940
Chr03G1600.1	Chr03	6091594	6094499
Chr03G1609.1	Chr03	6118888	6120353
Chr03G1620.1	Chr03	6148234	6149956
Chr03G1623.1	Chr03	6159007	6162224
Chr03G1647.1	Chr03	6250912	6251680
Chr03G1648.1	Chr03	6252996	6256979
Chr03G1649.1	Chr03	6258129	6259086
Chr03G1659.1	Chr03	6287124	6291925
Chr03G1676.1	Chr03	6346744	6347327
Chr03G1678.1	Chr03	6351512	6354210
Chr03G1692.1	Chr03	6387933	6390103
Chr03G1709.1	Chr03	6444672	6446398
Chr03G1720.1	Chr03	6475418	6476675
Chr03G1736.1	Chr03	6512957	6516110
Chr03G1737.1	Chr03	6526119	6526505
Chr09G0003.1	Chr09	39729	41101
Chr09G0005.1	Chr09	44527	46405
Chr09G0006.1	Chr09	47166	47876
Chr09G0020.1	Chr09	91742	94512

Chr09G0034.1	Chr09	136038	137344
Chr09G0059.1	Chr09	288525	288881
Chr09G0066.1	Chr09	310440	311350
Chr09G0084.1	Chr09	385756	387282
Chr09G0085.1	Chr09	387420	390284
Chr09G0106.1	Chr09	463181	464207
Chr09G0141.1	Chr09	568737	570491
Chr09G0157.1	Chr09	647409	648662
Chr09G0198.1	Chr09	888129	889811
Chr09G0212.1	Chr09	956586	957098
Chr09G0213.1	Chr09	959240	961566
Chr09G0214.1	Chr09	967919	969439
Chr09G0241.1	Chr09	1075230	1077307
Chr09G0260.1	Chr09	1148652	1150212
Chr09G0279.1	Chr09	1208288	1210005
Chr09G0285.1	Chr09	1227036	1229081
Chr09G0289.1	Chr09	1238283	1241154
Chr09G0310.1	Chr09	1346304	1346945
Chr09G0311.1	Chr09	1360057	1361318
Chr09G0355.1	Chr09	1513980	1516101
Chr09G0368.1	Chr09	1568805	1570300
Chr09G0377.1	Chr09	1603235	1604610
Chr09G0404.1	Chr09	1739687	1742245
Chr09G0419.1	Chr09	1784886	1785716
Chr09G0420.1	Chr09	1788222	1792565
Chr09G0439.1	Chr09	1847778	1848542
Chr09G0440.1	Chr09	1849655	1852460
Chr09G0441.1	Chr09	1852633	1854674
Chr09G0450.1	Chr09	1884523	1886141
Chr09G0485.1	Chr09	2036136	2038917
Chr09G0494.1	Chr09	2090249	2092788
Chr09G0499.1	Chr09	2110739	2112556
Chr09G0531.1	Chr09	2236551	2239550
Chr09G0540.1	Chr09	2267961	2270606
Chr09G0544.1	Chr09	2280254	2283371
Chr09G0553.1	Chr09	2306211	2307145
Chr09G0563.1	Chr09	2388726	2391126
Chr09G0564.1	Chr09	2400239	2400695
Chr09G0574.1	Chr09	2426613	2429683
Chr09G0592.1	Chr09	2494836	2497370
Chr09G0597.1	Chr09	2526697	2528320
Chr09G0601.1	Chr09	2535276	2535843
Chr09G0604.1	Chr09	2549076	2550396
Chr09G0613.1	Chr09	2596109	2598721

Chr09G0641.1	Chr09	2691486	2695288
Chr09G0646.1	Chr09	2706569	2708027
Chr09G0653.1	Chr09	2730600	2731970
Chr09G0656.1	Chr09	2754497	2758588
Chr09G0659.1	Chr09	2764392	2765687
Chr09G0671.1	Chr09	2820739	2823282
Chr09G0673.1	Chr09	2833557	2836690
Chr09G0674.1	Chr09	2837689	2838853
Chr09G0679.1	Chr09	2849679	2851407
Chr09G0680.1	Chr09	2853434	2854029
Chr09G0682.1	Chr09	2856878	2857385
Chr09G0696.1	Chr09	2900843	2902717
Chr09G0707.1	Chr09	2942953	2944070
Chr09G0716.1	Chr09	2983100	2984056
Chr09G0719.1	Chr09	2992788	2994018
Chr09G0721.1	Chr09	3002430	3003143
Chr09G0749.1	Chr09	3085386	3085802
Chr09G0775.1	Chr09	3165453	3166775
Chr09G0813.1	Chr09	3303574	3304857
Chr09G0821.1	Chr09	3321475	3323517
Chr09G0825.1	Chr09	3335910	3337088
Chr09G0835.1	Chr09	3376483	3379866
Chr09G0844.1	Chr09	3411823	3412474
Chr09G0848.1	Chr09	3421086	3423124
Chr09G0855.1	Chr09	3448927	3449470
Chr09G0858.1	Chr09	3458805	3460564
Chr09G0860.1	Chr09	3467282	3468930
Chr09G0871.1	Chr09	3497424	3498364
Chr09G0888.1	Chr09	3554087	3555755
Chr09G0900.1	Chr09	3595539	3597048
Chr09G0902.1	Chr09	3604265	3605705
Chr09G0903.1	Chr09	3608021	3610848
Chr09G0904.1	Chr09	3612108	3613224
Chr09G0907.1	Chr09	3621462	3622280
Chr09G0909.1	Chr09	3631227	3632831
Chr09G0918.1	Chr09	3662331	3666750
Chr09G0923.1	Chr09	3679367	3679708
Chr09G0937.1	Chr09	3718170	3718664
Chr09G0957.1	Chr09	3784930	3788914
Chr09G0960.1	Chr09	3799765	3800632
Chr09G0966.1	Chr09	3819453	3821733
Chr09G0975.1	Chr09	3844808	3845115
Chr09G0976.1	Chr09	3849656	3850710
Chr09G0989.1	Chr09	3900163	3904605

Chr09G0991.1	Chr09	3909510	3910505
Chr09G0998.1	Chr09	3934051	3935882
Chr09G1008.1	Chr09	3980633	3981888
Chr09G1009.1	Chr09	3983076	3984830
Chr09G1012.1	Chr09	4003544	4005588
Chr09G1027.1	Chr09	4064280	4065400
Chr09G1046.1	Chr09	4172295	4173636
Chr09G1047.1	Chr09	4177516	4179000
Chr09G1050.1	Chr09	4193667	4194022
Chr06G0026.1	Chr06	209552	214538
Chr06G0048.1	Chr06	342695	343492
Chr06G0067.1	Chr06	400657	402154
Chr06G0071.1	Chr06	408436	408769
Chr06G0081.1	Chr06	429258	430999
Chr06G0083.1	Chr06	439475	440756
Chr06G0085.1	Chr06	447863	452036
Chr06G0096.1	Chr06	473110	476607
Chr06G0104.1	Chr06	499618	500569
Chr06G0127.1	Chr06	563140	563826
Chr06G0134.1	Chr06	588187	591175
Chr06G0140.1	Chr06	608890	609692
Chr06G0144.1	Chr06	624397	625758
Chr06G0145.1	Chr06	626366	627805
Chr06G0149.1	Chr06	636900	638099
Chr06G0152.1	Chr06	646820	648674
Chr06G0157.1	Chr06	658758	659329
Chr06G0160.1	Chr06	666078	668125
Chr06G0169.1	Chr06	691726	693181
Chr06G0170.1	Chr06	694524	695500
Chr06G0175.1	Chr06	710486	711775
Chr06G0176.1	Chr06	715025	716470
Chr06G0206.1	Chr06	828610	830432
Chr06G0209.1	Chr06	848301	849452
Chr06G0210.1	Chr06	849932	852354
Chr06G0214.1	Chr06	857700	858975
Chr06G0217.1	Chr06	863940	867168
Chr06G0227.1	Chr06	902094	902582
Chr06G0229.1	Chr06	904881	906247
Chr06G0231.1	Chr06	910961	912852
Chr06G0232.1	Chr06	913007	914749
Chr06G0254.1	Chr06	989642	991328
Chr06G0257.1	Chr06	1000488	1001752
Chr06G0271.1	Chr06	1042960	1044268
Chr06G0275.1	Chr06	1057701	1059494

Chr06G0277.1	Chr06	1063559	1064388
Chr06G0293.1	Chr06	1115476	1117827
Chr06G0299.1	Chr06	1133157	1134776
Chr06G0313.1	Chr06	1194711	1196953
Chr06G0320.1	Chr06	1228650	1231633
Chr06G0346.1	Chr06	1302760	1305455
Chr06G0352.1	Chr06	1338297	1339665
Chr06G0359.1	Chr06	1360963	1363812
Chr06G0367.1	Chr06	1403115	1405069
Chr06G0405.1	Chr06	1547660	1549693
Chr06G0414.1	Chr06	1584156	1585797
Chr06G0442.1	Chr06	1676722	1678537
Chr06G0444.1	Chr06	1685401	1686910
Chr06G0458.1	Chr06	1734465	1738407
Chr06G0534.1	Chr06	2034489	2034962
Chr06G0609.1	Chr06	2285769	2288515
Chr06G0660.1	Chr06	2493356	2495886
Chr06G0740.1	Chr06	2762843	2766411
Chr06G0761.1	Chr06	2851381	2853573
Chr06G0764.1	Chr06	2860911	2862356
Chr06G0775.1	Chr06	2900184	2902224
Chr06G0781.1	Chr06	2922870	2924611
Chr06G0821.1	Chr06	3093916	3096505
Chr06G0825.1	Chr06	3108666	3110838
Chr06G0846.1	Chr06	3181914	3182972
Chr06G0902.1	Chr06	3396323	3399367
Chr06G0907.1	Chr06	3414911	3417015
Chr06G0942.1	Chr06	3535139	3538257
Chr06G0945.1	Chr06	3549048	3550578
Chr06G0954.1	Chr06	3586315	3589968
Chr06G0980.1	Chr06	3690365	3690715
Chr06G0993.1	Chr06	3758025	3759164
Chr06G0998.1	Chr06	3770188	3772212
Chr06G1014.1	Chr06	3811089	3811811
Chr06G1026.1	Chr06	3847711	3848119
Chr06G1027.1	Chr06	3849899	3850793
Chr06G1033.1	Chr06	3869136	3869877
Chr06G1037.1	Chr06	3879099	3881022
Chr06G1042.1	Chr06	3903259	3906316
Chr06G1045.1	Chr06	3913633	3914642
Chr06G1075.1	Chr06	4024612	4027454
Chr06G1127.1	Chr06	4243287	4245217
Chr06G1142.1	Chr06	4320476	4321042
Chr06G1154.1	Chr06	4346671	4347607

Chr06G1158.1	Chr06	4353451	4354140
Chr06G1159.1	Chr06	4360401	4361075
Chr06G1160.1	Chr06	4363294	4364019
Chr06G1166.1	Chr06	4384926	4386368
Chr06G1167.1	Chr06	4390514	4391755
Chr06G1168.1	Chr06	4392340	4393164
Chr06G1169.1	Chr06	4393448	4396252
Chr06G1172.1	Chr06	4402323	4404018
Chr06G1174.1	Chr06	4407412	4409113
Chr06G1177.1	Chr06	4418745	4420929
Chr06G1179.1	Chr06	4426049	4427965
Chr06G1181.1	Chr06	4432983	4433318
Chr06G1203.1	Chr06	4508637	4511070
Chr06G1207.1	Chr06	4526405	4527220
Chr06G1209.1	Chr06	4532518	4533021
Chr06G1213.1	Chr06	4544200	4545711
Chr06G1215.1	Chr06	4554995	4555890
Chr06G1229.1	Chr06	4608376	4609854
Chr06G1236.1	Chr06	4629125	4630990
Chr06G1238.1	Chr06	4634294	4634514
Chr06G1242.1	Chr06	4652377	4653424
Chr06G1243.1	Chr06	4659011	4660866
Chr06G1248.1	Chr06	4674140	4676063
Chr06G1305.1	Chr06	4841757	4843277
Chr06G1323.1	Chr06	4927343	4929678
Chr06G1324.1	Chr06	4930912	4932264
Chr06G1328.1	Chr06	4941083	4941486
Chr06G1340.1	Chr06	4978868	4979721
Chr06G1352.1	Chr06	5013333	5014348
Chr06G1353.1	Chr06	5015547	5016740
Chr06G1365.1	Chr06	5051133	5052632
Chr06G1381.1	Chr06	5098575	5099043
Chr06G1391.1	Chr06	5118250	5119046
Chr06G1392.1	Chr06	5120777	5122486
Chr06G1393.1	Chr06	5125367	5126071
Chr06G1405.1	Chr06	5172205	5173173
Chr06G1407.1	Chr06	5177770	5178018
Chr06G1410.1	Chr06	5187043	5187720
Chr06G1413.1	Chr06	5194466	5195967
Chr06G1414.1	Chr06	5197326	5198750
Chr06G1418.1	Chr06	5211067	5221152
Chr06G1420.1	Chr06	5227061	5228035
Chr06G1421.1	Chr06	5228454	5230313
Chr06G1423.1	Chr06	5235107	5236867

Chr06G1424.1	Chr06	5238653	5239649
Chr06G1428.1	Chr06	5246964	5248240
Chr06G1451.1	Chr06	5312569	5314074
Chr06G1455.1	Chr06	5325986	5328361
Chr06G1458.1	Chr06	5339486	5341092
Chr06G1477.1	Chr06	5399252	5400758
Chr06G1489.1	Chr06	5466124	5467265
Chr04G0024.1	Chr04	166867	167794
Chr04G0035.1	Chr04	210278	212176
Chr04G0043.1	Chr04	255469	256809
Chr04G0048.1	Chr04	271164	272448
Chr04G0052.1	Chr04	285967	287704
Chr04G0057.1	Chr04	297537	298109
Chr04G0063.1	Chr04	324959	326899
Chr04G0069.1	Chr04	343739	343981
Chr04G0083.1	Chr04	395856	399461
Chr04G0087.1	Chr04	407423	409494
Chr04G0090.1	Chr04	419018	421780
Chr04G0091.1	Chr04	421880	423960
Chr04G0092.1	Chr04	428541	429368
Chr04G0104.1	Chr04	463923	464348
Chr04G0108.1	Chr04	475714	476906
Chr04G0124.1	Chr04	539122	540246
Chr04G0137.1	Chr04	584397	585568
Chr04G0148.1	Chr04	616569	617223
Chr04G0158.1	Chr04	648337	649244
Chr04G0175.1	Chr04	708647	709149
Chr04G0176.1	Chr04	710654	711965
Chr04G0186.1	Chr04	738223	738889
Chr04G0187.1	Chr04	740124	740882
Chr04G0204.1	Chr04	808793	811103
Chr04G0207.1	Chr04	821535	824490
Chr04G0210.1	Chr04	841808	844591
Chr04G0229.1	Chr04	898210	898974
Chr04G0231.1	Chr04	907400	909131
Chr04G0232.1	Chr04	909981	910907
Chr04G0235.1	Chr04	922987	923937
Chr04G0241.1	Chr04	956190	957539
Chr04G0248.1	Chr04	974707	976910
Chr04G0249.1	Chr04	980743	981257
Chr04G0251.1	Chr04	987597	988391
Chr04G0263.1	Chr04	1030513	1031670
Chr04G0269.1	Chr04	1046843	1048996
Chr04G0274.1	Chr04	1069774	1072012

Chr04G0276.1	Chr04	1087633	1088409
Chr04G0277.1	Chr04	1088830	1089911
Chr04G0279.1	Chr04	1093028	1094675
Chr04G0281.1	Chr04	1101164	1101373
Chr04G0298.1	Chr04	1149842	1153132
Chr04G0301.1	Chr04	1160144	1161441
Chr04G0302.1	Chr04	1162439	1164313
Chr04G0303.1	Chr04	1166920	1167549
Chr04G0323.1	Chr04	1247633	1248520
Chr04G0325.1	Chr04	1251958	1253486
Chr04G0326.1	Chr04	1256663	1258398
Chr04G0330.1	Chr04	1269647	1270194
Chr04G0340.1	Chr04	1298481	1303149
Chr04G0341.1	Chr04	1304212	1305895
Chr04G0342.1	Chr04	1308856	1310713
Chr04G0347.1	Chr04	1331761	1334156
Chr04G0348.1	Chr04	1335424	1335819
Chr04G0351.1	Chr04	1345873	1346367
Chr04G0354.1	Chr04	1351964	1353398
Chr04G0377.1	Chr04	1441183	1441920
Chr04G0383.1	Chr04	1483988	1485906
Chr04G0394.1	Chr04	1524013	1525344
Chr04G0404.1	Chr04	1561367	1567293
Chr04G0446.1	Chr04	1734574	1736784
Chr04G0467.1	Chr04	1806543	1809208
Chr04G0479.1	Chr04	1844561	1846241
Chr04G0524.1	Chr04	2055456	2057841
Chr04G0543.1	Chr04	2136052	2137458
Chr04G0554.1	Chr04	2178959	2180995
Chr04G0559.1	Chr04	2206339	2208952
Chr04G0571.1	Chr04	2253328	2254271
Chr04G0584.1	Chr04	2310819	2311350
Chr04G0585.1	Chr04	2314120	2316082
Chr04G0593.1	Chr04	2335348	2336932
Chr04G0610.1	Chr04	2392780	2395652
Chr04G0611.1	Chr04	2395923	2399014
Chr04G0629.1	Chr04	2483667	2484186
Chr04G0630.1	Chr04	2486470	2487652
Chr04G0633.1	Chr04	2495839	2498938
Chr04G0637.1	Chr04	2510570	2511792
Chr04G0655.1	Chr04	2569716	2570422
Chr04G0691.1	Chr04	2671769	2674870
Chr04G0695.1	Chr04	2683348	2684665
Chr04G0696.1	Chr04	2685714	2687072

Chr04G0699.1	Chr04	2692666	2694704
Chr04G0720.1	Chr04	2758489	2759901
Chr04G0727.1	Chr04	2777709	2778746
Chr04G0733.1	Chr04	2796529	2797793
Chr04G0737.1	Chr04	2807168	2809506
Chr04G0739.1	Chr04	2813361	2814423
Chr04G0745.1	Chr04	2830156	2831824
Chr04G0746.1	Chr04	2832095	2833289
Chr04G0749.1	Chr04	2839150	2840926
Chr04G0755.1	Chr04	2859722	2862312
Chr04G0758.1	Chr04	2870764	2871705
Chr04G0760.1	Chr04	2877562	2878102
Chr04G0772.1	Chr04	2914996	2916186
Chr04G0773.1	Chr04	2917062	2918999
Chr04G0777.1	Chr04	2929769	2930460
Chr04G0791.1	Chr04	2972068	2974018
Chr04G0793.1	Chr04	2976430	2980404
Chr04G0794.1	Chr04	2981764	2983475
Chr04G0808.1	Chr04	3029742	3031437
Chr04G0813.1	Chr04	3043045	3045058
Chr04G0820.1	Chr04	3058883	3060631
Chr04G0828.1	Chr04	3080505	3081810
Chr04G0831.1	Chr04	3086597	3088485
Chr04G0832.1	Chr04	3089405	3090083
Chr04G0841.1	Chr04	3110525	3112072
Chr04G0843.1	Chr04	3123835	3125278
Chr04G0845.1	Chr04	3128384	3129419
Chr04G0862.1	Chr04	3168774	3170679
Chr04G0870.1	Chr04	3197966	3199274
Chr04G0874.1	Chr04	3207411	3208077
Chr04G0876.1	Chr04	3211491	3214525
Chr04G0903.1	Chr04	3330514	3334333
Chr04G0915.1	Chr04	3390395	3391191
Chr04G0919.1	Chr04	3402437	3402873
Chr04G0920.1	Chr04	3403221	3404201
Chr04G0921.1	Chr04	3406049	3407137
Chr04G0927.1	Chr04	3428549	3429075
Chr04G0931.1	Chr04	3441525	3443504
Chr04G0932.1	Chr04	3444443	3444848
Chr04G0946.1	Chr04	3509499	3510684
Chr04G0951.1	Chr04	3537864	3539093
Chr04G0955.1	Chr04	3548208	3550667
Chr04G0956.1	Chr04	3551237	3552058
Chr04G0957.1	Chr04	3553240	3554593

Chr04G0972.1	Chr04	3604916	3606189
Chr04G0981.1	Chr04	3650651	3652492
Chr04G0986.1	Chr04	3671457	3677953
Chr04G0987.1	Chr04	3684294	3688077
Chr04G0988.1	Chr04	3691308	3692484
Chr04G0995.1	Chr04	3714741	3716679
Chr04G0999.1	Chr04	3727187	3727906
Chr04G1005.1	Chr04	3747055	3748408
Chr04G1014.1	Chr04	3785091	3786004
Chr04G1018.1	Chr04	3836279	3841276
Chr04G1019.1	Chr04	3842047	3843458
Chr04G1021.1	Chr04	3848183	3848983
Chr04G1026.1	Chr04	3862798	3864397
Chr04G1095.1	Chr04	4112185	4113174
Chr04G1107.1	Chr04	4151538	4154229
Chr04G1110.1	Chr04	4169221	4171097
Chr04G1111.1	Chr04	4172236	4173812
Chr04G1116.1	Chr04	4187858	4190618
Chr04G1118.1	Chr04	4196051	4198040
Chr04G1131.1	Chr04	4230643	4231770
Chr04G1140.1	Chr04	4252474	4253941
Chr04G1141.1	Chr04	4254779	4258419
Chr04G1149.1	Chr04	4275401	4277756
Chr04G1154.1	Chr04	4291304	4292230
Chr04G1156.1	Chr04	4298215	4300495
Chr04G1157.1	Chr04	4302821	4304891
Chr04G1202.1	Chr04	4479700	4482061
Chr04G1218.1	Chr04	4525208	4525462
Chr04G1220.1	Chr04	4529083	4530326
Chr04G1232.1	Chr04	4587867	4590513
Chr04G1234.1	Chr04	4595571	4596025
Chr04G1237.1	Chr04	4601879	4602835
Chr04G1241.1	Chr04	4617226	4619392
Chr04G1267.1	Chr04	4702381	4703896
Chr04G1269.1	Chr04	4705932	4706528
Chr04G1292.1	Chr04	4812375	4814839
Chr04G1299.1	Chr04	4845743	4848260
Chr04G1304.1	Chr04	4860545	4862332
Chr04G1314.1	Chr04	4903088	4910146
Chr04G1346.1	Chr04	5034098	5034277
Chr04G1352.1	Chr04	5053999	5056126
Chr04G1353.1	Chr04	5056334	5057101
Chr04G1370.1	Chr04	5119433	5121815
Chr04G1382.1	Chr04	5191345	5192955

Chr04G1385.1	Chr04	5200884	5226418
Chr04G1398.1	Chr04	5263079	5265412
Chr04G1406.1	Chr04	5294041	5294634
Chr04G1411.1	Chr04	5304689	5306707
Chr04G1413.1	Chr04	5310581	5312326
Chr04G1415.1	Chr04	5315934	5316277
Chr04G1422.1	Chr04	5345327	5345644
Chr04G1425.1	Chr04	5353092	5356150
Chr04G1432.1	Chr04	5373468	5374323
Chr04G1433.1	Chr04	5377565	5378424
Chr04G1435.1	Chr04	5381487	5383082
Chr04G1443.1	Chr04	5396811	5398378
Chr04G1463.1	Chr04	5448367	5450954
Chr04G1468.1	Chr04	5467716	5469515
Chr04G1483.1	Chr04	5531186	5532861
Chr04G1492.1	Chr04	5558691	5560052
Chr04G1499.1	Chr04	5581811	5583384
Chr04G1500.1	Chr04	5588308	5589636
Chr04G1516.1	Chr04	5656954	5658684
Chr04G1524.1	Chr04	5683064	5685064
Chr04G1537.1	Chr04	5742945	5744795
Chr04G1544.1	Chr04	5773615	5774825
Chr04G1552.1	Chr04	5810487	5816012
Chr04G1559.1	Chr04	5826656	5827300
Chr04G1583.1	Chr04	5903687	5905161
Chr04G1585.1	Chr04	5911050	5913062
Chr02G0023.1	Chr02	126928	127458
Chr02G0024.1	Chr02	128590	129523
Chr02G0025.1	Chr02	130869	136698
Chr02G0030.1	Chr02	147314	149880
Chr02G0048.1	Chr02	212331	217888
Chr02G0066.1	Chr02	276901	277617
Chr02G0090.1	Chr02	344308	345369
Chr02G0093.1	Chr02	357668	359209
Chr02G0096.1	Chr02	367376	371130
Chr02G0102.1	Chr02	398937	401824
Chr02G0120.1	Chr02	465710	466952
Chr02G0135.1	Chr02	520802	521288
Chr02G0144.1	Chr02	551007	551595
Chr02G0148.1	Chr02	560233	561360
Chr02G0151.1	Chr02	569004	570987
Chr02G0157.1	Chr02	605480	607620
Chr02G0169.1	Chr02	650251	652662
Chr02G0182.1	Chr02	705916	708173

Chr02G0230.1	Chr02	870768	871464
Chr02G0231.1	Chr02	872663	873063
Chr02G0236.1	Chr02	886637	890225
Chr02G0241.1	Chr02	897812	899863
Chr02G0252.1	Chr02	929475	930267
Chr02G0274.1	Chr02	1016209	1017075
Chr02G0308.1	Chr02	1140794	1143942
Chr02G0358.1	Chr02	1314014	1318360
Chr02G0366.1	Chr02	1344509	1346564
Chr02G0379.1	Chr02	1406368	1409185
Chr02G0404.1	Chr02	1508748	1509791
Chr02G0406.1	Chr02	1511576	1513981
Chr02G0410.1	Chr02	1530348	1531207
Chr02G0411.1	Chr02	1531350	1531767
Chr02G0413.1	Chr02	1535535	1540171
Chr02G0429.1	Chr02	1584715	1587080
Chr02G0441.1	Chr02	1628509	1630396
Chr02G0445.1	Chr02	1643705	1644094
Chr02G0452.1	Chr02	1665745	1666920
Chr02G0453.1	Chr02	1667156	1668209
Chr02G0468.1	Chr02	1708142	1709435
Chr02G0478.1	Chr02	1750521	1751240
Chr02G0482.1	Chr02	1795136	1796654
Chr02G0495.1	Chr02	1832228	1832879
Chr02G0508.1	Chr02	1883762	1884345
Chr02G0509.1	Chr02	1885245	1885827
Chr02G0515.1	Chr02	1898921	1900492
Chr02G0520.1	Chr02	1927087	1930646
Chr02G0526.1	Chr02	1948041	1949924
Chr02G0531.1	Chr02	1963339	1964601
Chr02G0533.1	Chr02	1967925	1969289
Chr02G0534.1	Chr02	1971413	1972930
Chr02G0536.1	Chr02	1977061	1978725
Chr02G0538.1	Chr02	1984246	1984949
Chr02G0541.1	Chr02	1994306	1995195
Chr02G0546.1	Chr02	2039997	2040885
Chr02G0555.1	Chr02	2074087	2076468
Chr02G0568.1	Chr02	2126012	2127429
Chr02G0608.1	Chr02	2267826	2268359
Chr02G0631.1	Chr02	2351838	2355334
Chr02G0675.1	Chr02	2493242	2493616
Chr02G0691.1	Chr02	2569655	2570747
Chr02G0705.1	Chr02	2640322	2642644
Chr10G0002.1	Chr10	30118	30668
