# Supplementary material for: Decoding Pecan’s Fungal Foe: A Genomic Insight into Colletotrichum plurivorum Isolate W-6
Source: J Fungi (Basel). 2025 Mar 5;11(3):203. doi: 10.3390/jof11030203 (PMC11943440; doi:10.3390/jof11030203)
Supplement: Supplementary file 1 [file jof-11-00203-s001.zip › Table S19.pdf]

Table S19. List of effectors in isolate W-6 genome.

Gene ID	Cytoplasmic effector	Apoplastic effector	Prediction
Chr01G0056.1	-	Y (0.967)	Apoplastic effector
Chr01G0058.1	-	Y (0.697)	Apoplastic effector
Chr01G0059.1	Y (0.783)	-	Cytoplasmic effector
Chr01G0084.1	-	Y (0.849)	Apoplastic effector
Chr01G0086.1	Y (0.65)	Y (0.971)	Apoplastic/cytoplasmic effector
Chr01G0087.1	-	Y (0.696)	Apoplastic effector
Chr01G0122.1	Y (0.776)	Y (0.676)	Cytoplasmic/apoplastic effector
Chr01G0141.1	-	Y (0.995)	Apoplastic effector
Chr01G0164.1	Y (0.818)	Y (0.664)	Cytoplasmic/apoplastic effector
Chr01G0192.1	-	Y (0.512)	Apoplastic effector
Chr01G0263.1	-	Y (0.61)	Apoplastic effector
Chr01G0278.1	Y (0.862)	-	Cytoplasmic effector
Chr01G0281.1	Y (0.557)	Y (0.605)	Apoplastic/cytoplasmic effector
Chr01G0282.1	Y (0.845)	Y (0.502)	Cytoplasmic/apoplastic effector
Chr01G0283.1	-	Y (0.536)	Apoplastic effector
Chr01G0315.1	Y (0.94)	-	Cytoplasmic effector
Chr01G0332.1	-	Y (0.748)	Apoplastic effector
Chr01G0333.1	Y (0.82)	Y (0.78)	Cytoplasmic/apoplastic effector
Chr01G0334.1	-	Y (0.755)	Apoplastic effector
Chr01G0374.1	-	Y (0.9)	Apoplastic effector
Chr01G0404.1	Y (0.515)	Y (0.796)	Apoplastic/cytoplasmic effector
Chr01G0406.1	Y (0.78)	Y (0.876)	Apoplastic/cytoplasmic effector
Chr01G0425.1	-	Y (0.633)	Apoplastic effector
Chr01G0458.1	-	Y (0.936)	Apoplastic effector
Chr01G0464.1	Y (0.553)	Y (0.774)	Apoplastic/cytoplasmic effector
Chr01G0511.1	-	Y (0.631)	Apoplastic effector
Chr01G0517.1	-	Y (0.824)	Apoplastic effector
Chr01G0531.1	-	Y (0.781)	Apoplastic effector
Chr01G0532.1	-	Y (0.552)	Apoplastic effector
Chr01G0543.1	-	Y (0.75)	Apoplastic effector
Chr01G0555.1	-	Y (0.608)	Apoplastic effector
Chr01G0619.1	-	Y (0.751)	Apoplastic effector
Chr01G0626.1	-	Y (0.776)	Apoplastic effector
Chr01G0649.1	-	Y (0.798)	Apoplastic effector
Chr01G0655.1	Y (0.574)	-	Cytoplasmic effector
Chr01G0719.1	-	Y (0.866)	Apoplastic effector
Chr01G0754.1	-	Y (0.831)	Apoplastic effector
Chr01G0765.1	-	Y (0.736)	Apoplastic effector
Chr01G0774.1	-	Y (0.545)	Apoplastic effector
Chr01G0780.1	Y (0.602)	Y (0.818)	Apoplastic/cytoplasmic effector
Chr01G0819.1	-	Y (0.547)	Apoplastic effector
Chr01G0829.1	-	Y (0.523)	Apoplastic effector

Chr01G0912.1	-	Y (0.521)	Apoplastic effector
Chr01G0961.1	-	Y (0.593)	Apoplastic effector
Chr01G0993.1	-	Y (0.724)	Apoplastic effector
Chr01G0997.1	-	Y (0.816)	Apoplastic effector
Chr01G1007.1	Y (0.672)	-	Cytoplasmic effector
Chr01G1012.1	-	Y (0.681)	Apoplastic effector
Chr01G1026.1	-	Y (0.55)	Apoplastic effector
Chr01G1037.1	Y (0.506)	-	Cytoplasmic effector
Chr01G1061.1	-	Y (0.522)	Apoplastic effector
Chr01G1073.1	Y (0.617)	Y (0.991)	Apoplastic/cytoplasmic effector
Chr01G1074.1	-	Y (0.611)	Apoplastic effector
Chr01G1092.1	Y (0.582)	-	Cytoplasmic effector
Chr01G1104.1	Y (0.749)	-	Cytoplasmic effector
Chr01G1269.1	-	Y (0.579)	Apoplastic effector
Chr01G1327.1	Y (0.602)	Y (0.952)	Apoplastic/cytoplasmic effector
Chr01G1351.1	Y (0.63)	Y (0.839)	Apoplastic/cytoplasmic effector
Chr01G1361.1	-	Y (0.696)	Apoplastic effector
Chr01G1363.1	Y (0.723)	Y (0.925)	Apoplastic/cytoplasmic effector
Chr01G1406.1	-	Y (0.724)	Apoplastic effector
Chr01G1416.1	-	Y (0.996)	Apoplastic effector
Chr01G1430.1	Y (0.536)	Y (0.76)	Apoplastic/cytoplasmic effector
Chr01G1454.1	Y (0.573)	Y (0.764)	Apoplastic/cytoplasmic effector
Chr01G1460.1	-	Y (0.655)	Apoplastic effector
Chr01G1463.1	-	Y (0.806)	Apoplastic effector
Chr01G1470.1	Y (0.653)	-	Cytoplasmic effector
Chr01G1472.1	Y (0.822)	-	Cytoplasmic effector
Chr01G1479.1	Y (0.765)	-	Cytoplasmic effector
Chr01G1488.1	-	Y (0.54)	Apoplastic effector
Chr01G1489.1	-	Y (0.682)	Apoplastic effector
Chr01G1491.1	Y (0.623)	-	Cytoplasmic effector
Chr01G1507.1	Y (0.693)	-	Cytoplasmic effector
Chr01G1571.1	Y (0.813)	Y (0.842)	Apoplastic/cytoplasmic effector
Chr01G1604.1	-	Y (0.986)	Apoplastic effector
Chr01G1648.1	-	Y (0.897)	Apoplastic effector
Chr01G1665.1	Y (0.515)	-	Cytoplasmic effector
Chr01G1676.1	-	Y (0.707)	Apoplastic effector
Chr01G1692.1	-	Y (0.888)	Apoplastic effector
Chr01G1695.1	Y (0.644)	-	Cytoplasmic effector
Chr01G1699.1	-	Y (0.531)	Apoplastic effector
Chr01G1702.1	Y (0.569)	-	Cytoplasmic effector
Chr01G1754.1	-	Y (0.647)	Apoplastic effector
Chr01G1778.1	-	Y (0.568)	Apoplastic effector
Chr01G1798.1	Y (0.695)	-	Cytoplasmic effector
Chr01G1824.1	-	Y (0.636)	Apoplastic effector

Chr01G1829.1	Y (0.758)	-	Cytoplasmic effector
Chr01G1879.1	Y (0.693)	-	Cytoplasmic effector
Chr01G1898.1	-	Y (0.718)	Apoplastic effector
Chr01G1960.1	-	Y (0.759)	Apoplastic effector
Chr01G1970.1	-	Y (0.577)	Apoplastic effector
Chr01G1992.1	-	Y (0.533)	Apoplastic effector
Chr01G1994.1	-	Y (0.824)	Apoplastic effector
Chr01G2024.1	-	Y (0.658)	Apoplastic effector
Chr01G2102.1	-	Y (0.526)	Apoplastic effector
Chr01G2129.1	Y (0.61)	-	Cytoplasmic effector
Chr01G2150.1	-	Y (0.895)	Apoplastic effector
Chr01G2276.1	-	Y (0.647)	Apoplastic effector
Chr01G2392.1	-	Y (0.805)	Apoplastic effector
Chr01G2421.1	-	Y (0.562)	Apoplastic effector
Chr01G2428.1	-	Y (0.689)	Apoplastic effector
Chr01G2450.1	-	Y (0.619)	Apoplastic effector
Chr01G2484.1	-	Y (0.548)	Apoplastic effector
Chr01G2490.1	-	Y (0.758)	Apoplastic effector
Chr01G2497.1	-	Y (0.796)	Apoplastic effector
Chr01G2510.1	-	Y (0.567)	Apoplastic effector
Chr01G2526.1	-	Y (0.577)	Apoplastic effector
Chr01G2544.1	-	Y (0.592)	Apoplastic effector
Chr01G2551.1	Y (0.58)	-	Cytoplasmic effector
Chr01G2577.1	-	Y (0.61)	Apoplastic effector
Chr01G2585.1	Y (0.75)	-	Cytoplasmic effector
Chr01G2645.1	-	Y (0.806)	Apoplastic effector
Chr01G2713.1	-	Y (0.678)	Apoplastic effector
Chr01G2739.1	-	Y (0.624)	Apoplastic effector
Chr01G2779.1	-	Y (0.571)	Apoplastic effector
Chr05G0822.1	Y (0.605)	-	Cytoplasmic effector
Chr05G0813.1	-	Y (0.716)	Apoplastic effector
Chr05G0754.1	-	Y (0.545)	Apoplastic effector
Chr05G0738.1	-	Y (0.783)	Apoplastic effector
Chr05G0625.1	-	Y (0.65)	Apoplastic effector
Chr05G0558.1	Y (0.579)	-	Cytoplasmic effector
Chr05G0537.1	Y (0.893)	-	Cytoplasmic effector
Chr05G0536.1	-	Y (0.796)	Apoplastic effector
Chr05G0514.1	-	Y (0.505)	Apoplastic effector
Chr05G0425.1	-	Y (0.582)	Apoplastic effector
Chr05G0411.1	-	Y (0.649)	Apoplastic effector
Chr05G0380.1	Y (0.571)	-	Cytoplasmic effector
Chr05G0357.1	-	Y (0.607)	Apoplastic effector
Chr05G0341.1	-	Y (0.597)	Apoplastic effector
Chr05G0332.1	-	Y (0.705)	Apoplastic effector

Chr05G0330.1	Y (0.707)	Y (0.91)	Apoplastic/cytoplasmic effector
Chr05G0323.1	Y (0.799)	-	Cytoplasmic effector
Chr05G0318.1	-	Y (0.545)	Apoplastic effector
Chr05G0283.1	-	Y (0.852)	Apoplastic effector
Chr05G0277.1	-	Y (0.617)	Apoplastic effector
Chr05G0246.1	-	Y (0.647)	Apoplastic effector
Chr05G0234.1	Y (0.586)	Y (0.863)	Apoplastic/cytoplasmic effector
Chr05G0194.1	-	Y (0.835)	Apoplastic effector
Chr05G0191.1	-	Y (0.594)	Apoplastic effector
Chr05G0166.1	Y (0.666)	Y (0.962)	Apoplastic/cytoplasmic effector
Chr05G0133.1	-	Y (0.93)	Apoplastic effector
Chr05G0130.1	Y (0.871)	Y (0.758)	Cytoplasmic/apoplastic effector
Chr05G0129.1	Y (0.815)	-	Cytoplasmic effector
Chr05G0119.1	Y (0.561)	-	Cytoplasmic effector
Chr05G0107.1	-	Y (0.618)	Apoplastic effector
Chr05G0078.1	-	Y (0.764)	Apoplastic effector
Chr05G0075.1	-	Y (0.612)	Apoplastic effector
Chr07G0025.1	-	Y (0.867)	Apoplastic effector
Chr07G0036.1	Y (0.84)	-	Cytoplasmic effector
Chr07G0115.1	Y (0.858)	-	Cytoplasmic effector
Chr07G0139.1	-	Y (0.793)	Apoplastic effector
Chr07G0140.1	Y (0.526)	-	Cytoplasmic effector
Chr07G0181.1	-	Y (0.594)	Apoplastic effector
Chr07G0196.1	-	Y (0.67)	Apoplastic effector
Chr07G0235.1	-	Y (0.727)	Apoplastic effector
Chr07G0282.1	-	Y (0.577)	Apoplastic effector
Chr07G0291.1	Y (0.515)	Y (0.873)	Apoplastic/cytoplasmic effector
Chr07G0292.1	Y (0.518)	Y (0.827)	Apoplastic/cytoplasmic effector
Chr07G0316.1	-	Y (0.6)	Apoplastic effector
Chr07G0318.1	Y (0.504)	Y (0.839)	Apoplastic/cytoplasmic effector
Chr07G0324.1	Y (0.823)	-	Cytoplasmic effector
Chr07G0329.1	-	Y (0.859)	Apoplastic effector
Chr07G0337.1	Y (0.707)	Y (0.797)	Apoplastic/cytoplasmic effector
Chr07G0385.1	-	Y (0.582)	Apoplastic effector
Chr07G0415.1	-	Y (0.661)	Apoplastic effector
Chr07G0419.1	-	Y (0.745)	Apoplastic effector
Chr07G0471.1	-	Y (0.595)	Apoplastic effector
Chr07G0489.1	Y (0.648)	-	Cytoplasmic effector
Chr07G0508.1	-	Y (0.543)	Apoplastic effector
Chr07G0531.1	-	Y (0.511)	Apoplastic effector
Chr07G0563.1	-	Y (0.82)	Apoplastic effector
Chr07G0574.1	Y (0.853)	-	Cytoplasmic effector
Chr07G0600.1	-	Y (0.788)	Apoplastic effector
Chr07G0615.1	Y (0.56)	-	Cytoplasmic effector

Chr07G0617.1	Y (0.797)	-	Cytoplasmic effector
Chr07G0658.1	-	Y (0.647)	Apoplastic effector
Chr07G0707.1	-	Y (0.822)	Apoplastic effector
Chr07G0754.1	Y (0.688)	-	Cytoplasmic effector
Chr07G0802.1	-	Y (0.535)	Apoplastic effector
Chr07G0842.1	Y (0.637)	-	Cytoplasmic effector
Chr07G0861.1	-	Y (0.799)	Apoplastic effector
Chr07G0879.1	-	Y (0.636)	Apoplastic effector
Chr07G0891.1	Y (0.615)	Y (0.785)	Apoplastic/cytoplasmic effector
Chr07G0908.1	-	Y (0.767)	Apoplastic effector
Chr07G0924.1	-	Y (0.606)	Apoplastic effector
Chr07G0935.1	-	Y (0.74)	Apoplastic effector
Chr07G0936.1	-	Y (0.799)	Apoplastic effector
Chr07G0937.1	-	Y (0.861)	Apoplastic effector
Chr07G0940.1	-	Y (0.575)	Apoplastic effector
Chr07G0943.1	-	Y (0.796)	Apoplastic effector
Chr07G0979.1	Y (0.786)	Y (0.633)	Cytoplasmic/apoplastic effector
Chr07G1003.1	Y (0.536)	Y (0.731)	Apoplastic/cytoplasmic effector
Chr07G1009.1	-	Y (0.911)	Apoplastic effector
Chr07G1050.1	-	Y (0.664)	Apoplastic effector
Chr07G1108.1	Y (0.809)	Y (0.775)	Cytoplasmic/apoplastic effector
Chr07G1128.1	Y (0.599)	Y (0.57)	Cytoplasmic/apoplastic effector
Chr07G1133.1	-	Y (0.891)	Apoplastic effector
Chr07G1134.1	Y (0.824)	-	Cytoplasmic effector
Chr07G1135.1	Y (0.793)	-	Cytoplasmic effector
Chr07G1139.1	Y (0.688)	Y (0.687)	Cytoplasmic/apoplastic effector
Chr07G1166.1	-	Y (0.606)	Apoplastic effector
Chr07G1168.1	Y (0.66)	Y (0.64)	Cytoplasmic/apoplastic effector
Chr07G1169.1	-	Y (0.671)	Apoplastic effector
Chr02G1076.1	Y (0.791)	-	Cytoplasmic effector
Chr02G1092.1	-	Y (0.751)	Apoplastic effector
Chr02G1127.1	-	Y (0.837)	Apoplastic effector
Chr02G1177.1	-	Y (0.574)	Apoplastic effector
Chr02G1178.1	Y (0.655)	-	Cytoplasmic effector
Chr02G1238.1	Y (0.686)	Y (0.638)	Cytoplasmic/apoplastic effector
Chr02G1269.1	-	Y (0.754)	Apoplastic effector
Chr02G1287.1	Y (0.759)	-	Cytoplasmic effector
Chr02G1331.1	-	Y (0.864)	Apoplastic effector
Chr02G1335.1	Y (0.541)	Y (0.675)	Apoplastic/cytoplasmic effector
Chr02G1340.1	-	Y (0.748)	Apoplastic effector
Chr02G1356.1	-	Y (0.857)	Apoplastic effector
Chr02G1359.1	Y (0.629)	Y (0.768)	Apoplastic/cytoplasmic effector
Chr02G1375.1	-	Y (0.553)	Apoplastic effector
Chr02G1406.1	Y (0.729)	Y (0.652)	Cytoplasmic/apoplastic effector

Chr02G1433.1	Y (0.525)	Y (0.836)	Apoplastic/cytoplasmic effector
Chr02G1468.1	Y (0.793)	Y (0.594)	Cytoplasmic/apoplastic effector
Chr02G1486.1	-	Y (0.588)	Apoplastic effector
Chr02G1524.1	-	Y (0.525)	Apoplastic effector
Chr02G1535.1	-	Y (0.863)	Apoplastic effector
Chr02G1537.1	-	Y (0.848)	Apoplastic effector
Chr02G1558.1	Y (0.89)	-	Cytoplasmic effector
Chr02G1565.1	-	Y (0.527)	Apoplastic effector
Chr02G1575.1	-	Y (0.597)	Apoplastic effector
Chr02G1587.1	-	Y (0.615)	Apoplastic effector
Chr02G1641.1	-	Y (0.762)	Apoplastic effector
Chr02G1650.1	Y (0.773)	Y (0.93)	Apoplastic/cytoplasmic effector
Chr02G1689.1	-	Y (0.525)	Apoplastic effector
Chr02G1756.1	Y (0.964)	Y (0.959)	Cytoplasmic/apoplastic effector
Chr02G1769.1	-	Y (0.83)	Apoplastic effector
Chr02G1795.1	-	Y (0.589)	Apoplastic effector
Chr02G1799.1	-	Y (0.949)	Apoplastic effector
Chr02G1802.1	-	Y (0.867)	Apoplastic effector
Chr02G1818.1	Y (0.527)	-	Cytoplasmic effector
Chr08G0054.1	Y (0.659)	-	Cytoplasmic effector
Chr08G0063.1	-	Y (0.67)	Apoplastic effector
Chr08G0070.1	Y (0.511)	-	Cytoplasmic effector
Chr08G0079.1	Y (0.55)	Y (0.993)	Apoplastic/cytoplasmic effector
Chr08G0171.1	-	Y (0.785)	Apoplastic effector
Chr08G0179.1	Y (0.627)	Y (0.813)	Apoplastic/cytoplasmic effector
Chr08G0182.1	-	Y (0.924)	Apoplastic effector
Chr08G0194.1	-	Y (0.514)	Apoplastic effector
Chr08G0223.1	Y (0.604)	Y (0.592)	Cytoplasmic/apoplastic effector
Chr08G0338.1	Y (0.619)	Y (0.891)	Apoplastic/cytoplasmic effector
Chr08G0395.1	-	Y (0.637)	Apoplastic effector
Chr08G0398.1	Y (0.644)	-	Cytoplasmic effector
Chr08G0477.1	-	Y (0.973)	Apoplastic effector
Chr08G0500.1	Y (0.578)	-	Cytoplasmic effector
Chr08G0501.1	-	Y (0.58)	Apoplastic effector
Chr08G0649.1	-	Y (0.671)	Apoplastic effector
Chr08G0652.1	Y (0.599)	Y (0.822)	Apoplastic/cytoplasmic effector
Chr08G0656.1	Y (0.585)	-	Cytoplasmic effector
Chr08G0694.1	-	Y (0.511)	Apoplastic effector
Chr08G0801.1	-	Y (0.657)	Apoplastic effector
Chr08G0821.1	-	Y (0.601)	Apoplastic effector
Chr08G0837.1	Y (0.805)	-	Cytoplasmic effector
Chr08G0866.1	Y (0.667)	-	Cytoplasmic effector
Chr08G0874.1	Y (0.699)	-	Cytoplasmic effector
Chr08G0880.1	Y (0.781)	-	Cytoplasmic effector

Chr08G0884.1	-	Y (0.835)	Apoplastic effector
Chr08G0986.1	-	Y (0.641)	Apoplastic effector
Chr08G0991.1	-	Y (0.551)	Apoplastic effector
Chr08G1017.1	-	Y (0.568)	Apoplastic effector
Chr08G1021.1	Y (0.542)	-	Cytoplasmic effector
Chr08G1037.1	Y (0.764)	-	Cytoplasmic effector
Chr08G1039.1	-	Y (0.557)	Apoplastic effector
Chr05G0897.1	Y (0.747)	-	Cytoplasmic effector
Chr05G0933.1	-	Y (0.778)	Apoplastic effector
Chr05G0935.1	-	Y (0.595)	Apoplastic effector
Chr05G0971.1	Y (0.581)	-	Cytoplasmic effector
Chr05G1067.1	-	Y (0.523)	Apoplastic effector
Chr05G1093.1	-	Y (0.506)	Apoplastic effector
Chr05G1106.1	-	Y (0.547)	Apoplastic effector
Chr05G1140.1	-	Y (0.675)	Apoplastic effector
Chr05G1262.1	-	Y (0.71)	Apoplastic effector
Chr05G1272.1	Y (0.51)	-	Cytoplasmic effector
Chr05G1320.1	-	Y (0.734)	Apoplastic effector
Chr05G1375.1	-	Y (0.572)	Apoplastic effector
Chr05G1401.1	-	Y (0.724)	Apoplastic effector
Chr05G1409.1	-	Y (0.68)	Apoplastic effector
Chr05G1415.1	Y (0.51)	Y (0.865)	Apoplastic/cytoplasmic effector
Chr05G1422.1	-	Y (0.771)	Apoplastic effector
Chr03G0032.1	-	Y (0.503)	Apoplastic effector
Chr03G0057.1	-	Y (0.554)	Apoplastic effector
Chr03G0059.1	-	Y (0.525)	Apoplastic effector
Chr03G0068.1	-	Y (0.513)	Apoplastic effector
Chr03G0088.1	-	Y (0.732)	Apoplastic effector
Chr03G0237.1	-	Y (0.926)	Apoplastic effector
Chr03G0273.1	Y (0.833)	Y (0.84)	Apoplastic/cytoplasmic effector
Chr03G0294.1	-	Y (0.802)	Apoplastic effector
Chr03G0320.1	Y (0.517)	-	Cytoplasmic effector
Chr03G0337.1	-	Y (0.571)	Apoplastic effector
Chr03G0341.1	-	Y (0.56)	Apoplastic effector
Chr03G0344.1	Y (0.894)	-	Cytoplasmic effector
Chr03G0346.1	-	Y (0.841)	Apoplastic effector
Chr03G0366.1	Y (0.783)	-	Cytoplasmic effector
Chr03G0369.1	-	Y (0.678)	Apoplastic effector
Chr03G0371.1	Y (0.681)	-	Cytoplasmic effector
Chr03G0373.1	Y (0.689)	-	Cytoplasmic effector
Chr03G0376.1	-	Y (0.709)	Apoplastic effector
Chr03G0414.1	Y (0.798)	-	Cytoplasmic effector
Chr03G0524.1	-	Y (0.583)	Apoplastic effector
Chr03G0532.1	-	Y (0.671)	Apoplastic effector

Chr03G0687.1	Y (0.524)	Y (0.896)	Apoplastic/cytoplasmic effector
Chr03G0815.1	Y (0.556)	-	Cytoplasmic effector
Chr03G0958.1	-	Y (0.719)	Apoplastic effector
Chr03G1034.1	Y (0.67)	Y (0.861)	Apoplastic/cytoplasmic effector
Chr03G1036.1	Y (0.714)	-	Cytoplasmic effector
Chr03G1041.1	Y (0.51)	Y (0.89)	Apoplastic/cytoplasmic effector
Chr03G1086.1	Y (0.698)	Y (0.771)	Apoplastic/cytoplasmic effector
Chr03G1189.1	Y (0.722)	-	Cytoplasmic effector
Chr03G1240.1	Y (0.704)	-	Cytoplasmic effector
Chr03G1247.1	-	Y (0.531)	Apoplastic effector
Chr03G1291.1	Y (0.597)	-	Cytoplasmic effector
Chr03G1292.1	Y (0.69)	-	Cytoplasmic effector
Chr03G1312.1	-	Y (0.589)	Apoplastic effector
Chr03G1318.1	-	Y (0.711)	Apoplastic effector
Chr03G1347.1	Y (0.645)	Y (0.927)	Apoplastic/cytoplasmic effector
Chr03G1348.1	Y (0.645)	Y (0.926)	Apoplastic/cytoplasmic effector
Chr03G1365.1	Y (0.622)	-	Cytoplasmic effector
Chr03G1378.1	-	Y (0.781)	Apoplastic effector
Chr03G1423.1	-	Y (0.726)	Apoplastic effector
Chr03G1443.1	-	Y (0.784)	Apoplastic effector
Chr03G1455.1	Y (0.706)	-	Cytoplasmic effector
Chr03G1466.1	-	Y (0.82)	Apoplastic effector
Chr03G1471.1	Y (0.844)	-	Cytoplasmic effector
Chr03G1493.1	Y (0.739)	-	Cytoplasmic effector
Chr03G1498.1	Y (0.592)	-	Cytoplasmic effector
Chr03G1503.1	-	Y (0.556)	Apoplastic effector
Chr03G1504.1	Y (0.903)	-	Cytoplasmic effector
Chr03G1513.1	Y (0.804)	-	Cytoplasmic effector
Chr03G1543.1	Y (0.838)	Y (0.792)	Cytoplasmic/apoplastic effector
Chr03G1554.1	-	Y (0.608)	Apoplastic effector
Chr03G1565.1	-	Y (0.79)	Apoplastic effector
Chr03G1647.1	-	Y (0.848)	Apoplastic effector
Chr03G1649.1	Y (0.567)	-	Cytoplasmic effector
Chr03G1676.1	-	Y (0.89)	Apoplastic effector
Chr09G0006.1	-	Y (0.562)	Apoplastic effector
Chr09G0059.1	Y (0.766)	-	Cytoplasmic effector
Chr09G0106.1	Y (0.656)	-	Cytoplasmic effector
Chr09G0141.1	Y (0.587)	-	Cytoplasmic effector
Chr09G0198.1	-	Y (0.631)	Apoplastic effector
Chr09G0212.1	-	Y (0.677)	Apoplastic effector
Chr09G0214.1	-	Y (0.505)	Apoplastic effector
Chr09G0241.1	-	Y (0.561)	Apoplastic effector
Chr09G0310.1	-	Y (0.886)	Apoplastic effector
Chr09G0311.1	-	Y (0.542)	Apoplastic effector

Chr09G0419.1	-	Y (0.592)	Apoplastic effector
Chr09G0441.1	-	Y (0.592)	Apoplastic effector
Chr09G0450.1	-	Y (0.68)	Apoplastic effector
Chr09G0564.1	Y (0.518)	Y (0.824)	Apoplastic/cytoplasmic effector
Chr09G0601.1	Y (0.651)	-	Cytoplasmic effector
Chr09G0604.1	Y (0.597)	-	Cytoplasmic effector
Chr09G0646.1	-	Y (0.51)	Apoplastic effector
Chr09G0653.1	-	Y (0.623)	Apoplastic effector
Chr09G0659.1	-	Y (0.523)	Apoplastic effector
Chr09G0680.1	-	Y (0.871)	Apoplastic effector
Chr09G0707.1	-	Y (0.659)	Apoplastic effector
Chr09G0749.1	-	Y (0.837)	Apoplastic effector
Chr09G0858.1	-	Y (0.604)	Apoplastic effector
Chr09G0900.1	-	Y (0.688)	Apoplastic effector
Chr09G0904.1	-	Y (0.688)	Apoplastic effector
Chr09G0923.1	-	Y (0.911)	Apoplastic effector
Chr09G0960.1	-	Y (0.829)	Apoplastic effector
Chr09G0975.1	-	Y (0.956)	Apoplastic effector
Chr09G0991.1	-	Y (0.619)	Apoplastic effector
Chr09G1047.1	-	Y (0.586)	Apoplastic effector
Chr09G1050.1	Y (0.559)	-	Cytoplasmic effector
Chr06G0048.1	-	Y (0.68)	Apoplastic effector
Chr06G0067.1	Y (0.613)	-	Cytoplasmic effector
Chr06G0071.1	Y (0.797)	Y (0.561)	Cytoplasmic/apoplastic effector
Chr06G0104.1	-	Y (0.878)	Apoplastic effector
Chr06G0127.1	Y (0.839)	-	Cytoplasmic effector
Chr06G0144.1	-	Y (0.772)	Apoplastic effector
Chr06G0157.1	-	Y (0.666)	Apoplastic effector
Chr06G0160.1	Y (0.73)	-	Cytoplasmic effector
Chr06G0169.1	-	Y (0.538)	Apoplastic effector
Chr06G0210.1	Y (0.744)	Y (0.956)	Apoplastic/cytoplasmic effector
Chr06G0277.1	Y (0.809)	-	Cytoplasmic effector
Chr06G0761.1	Y (0.723)	-	Cytoplasmic effector
Chr06G0942.1	-	Y (0.645)	Apoplastic effector
Chr06G1014.1	Y (0.782)	-	Cytoplasmic effector
Chr06G1026.1	Y (0.816)	Y (0.722)	Cytoplasmic/apoplastic effector
Chr06G1027.1	Y (0.904)	-	Cytoplasmic effector
Chr06G1045.1	Y (0.848)	-	Cytoplasmic effector
Chr06G1142.1	-	Y (0.769)	Apoplastic effector
Chr06G1158.1	Y (0.58)	-	Cytoplasmic effector
Chr06G1159.1	-	Y (0.702)	Apoplastic effector
Chr06G1166.1	Y (0.846)	-	Cytoplasmic effector
Chr06G1168.1	Y (0.776)	-	Cytoplasmic effector
Chr06G1181.1	-	Y (0.773)	Apoplastic effector

Chr06G1209.1	Y (0.895)	-	Cytoplasmic effector
Chr06G1238.1	-	Y (0.711)	Apoplastic effector
Chr06G1243.1	-	Y (0.672)	Apoplastic effector
Chr06G1248.1	-	Y (0.656)	Apoplastic effector
Chr06G1352.1	Y (0.69)	-	Cytoplasmic effector
Chr06G1353.1	Y (0.597)	-	Cytoplasmic effector
Chr06G1381.1	Y (0.691)	Y (0.927)	Apoplastic/cytoplasmic effector
Chr06G1391.1	-	Y (0.566)	Apoplastic effector
Chr06G1393.1	-	Y (0.729)	Apoplastic effector
Chr06G1407.1	-	Y (0.795)	Apoplastic effector
Chr06G1410.1	Y (0.852)	Y (0.753)	Cytoplasmic/apoplastic effector
Chr06G1413.1	Y (0.501)	-	Cytoplasmic effector
Chr06G1489.1	-	Y (0.853)	Apoplastic effector
Chr04G0063.1	-	Y (0.649)	Apoplastic effector
Chr04G0069.1	Y (0.751)	Y (0.645)	Cytoplasmic/apoplastic effector
Chr04G0092.1	Y (0.722)	Y (0.616)	Cytoplasmic/apoplastic effector
Chr04G0104.1	-	Y (0.791)	Apoplastic effector
Chr04G0148.1	-	Y (0.562)	Apoplastic effector
Chr04G0175.1	Y (0.746)	-	Cytoplasmic effector
Chr04G0186.1	-	Y (0.856)	Apoplastic effector
Chr04G0187.1	-	Y (0.792)	Apoplastic effector
Chr04G0249.1	-	Y (0.829)	Apoplastic effector
Chr04G0251.1	-	Y (0.732)	Apoplastic effector
Chr04G0263.1	Y (0.575)	Y (0.713)	Apoplastic/cytoplasmic effector
Chr04G0276.1	-	Y (0.783)	Apoplastic effector
Chr04G0281.1	-	Y (0.712)	Apoplastic effector
Chr04G0301.1	Y (0.543)	Y (0.571)	Apoplastic/cytoplasmic effector
Chr04G0302.1	-	Y (0.761)	Apoplastic effector
Chr04G0303.1	Y (0.844)	-	Cytoplasmic effector
Chr04G0330.1	Y (0.5)	-	Cytoplasmic effector
Chr04G0341.1	-	Y (0.579)	Apoplastic effector
Chr04G0348.1	-	Y (0.643)	Apoplastic effector
Chr04G0351.1	Y (0.731)	-	Cytoplasmic effector
Chr04G0354.1	-	Y (0.506)	Apoplastic effector
Chr04G0584.1	Y (0.53)	Y (0.63)	Apoplastic/cytoplasmic effector
Chr04G0585.1	Y (0.803)	-	Cytoplasmic effector
Chr04G0593.1	Y (0.71)	-	Cytoplasmic effector
Chr04G0629.1	Y (0.583)	-	Cytoplasmic effector
Chr04G0633.1	-	Y (0.587)	Apoplastic effector
Chr04G0655.1	Y (0.806)	-	Cytoplasmic effector
Chr04G0695.1	Y (0.839)	Y (0.568)	Cytoplasmic/apoplastic effector
Chr04G0758.1	-	Y (0.682)	Apoplastic effector
Chr04G0760.1	Y (0.891)	-	Cytoplasmic effector
Chr04G0772.1	-	Y (0.526)	Apoplastic effector

Chr04G0832.1	-	Y (0.803)	Apoplastic effector
Chr04G0845.1	Y (0.508)	Y (0.962)	Apoplastic/cytoplasmic effector
Chr04G0874.1	Y (0.661)	Y (0.946)	Apoplastic/cytoplasmic effector
Chr04G0919.1	Y (0.831)	Y (0.918)	Apoplastic/cytoplasmic effector
Chr04G0927.1	Y (0.757)	Y (0.537)	Cytoplasmic/apoplastic effector
Chr04G0932.1	Y (0.771)	Y (0.997)	Apoplastic/cytoplasmic effector
Chr04G0951.1	Y (0.649)	Y (0.939)	Apoplastic/cytoplasmic effector
Chr04G0999.1	-	Y (0.693)	Apoplastic effector
Chr04G1014.1	-	Y (0.512)	Apoplastic effector
Chr04G1131.1	-	Y (0.679)	Apoplastic effector
Chr04G1149.1	-	Y (0.668)	Apoplastic effector
Chr04G1218.1	-	Y (0.892)	Apoplastic effector
Chr04G1234.1	Y (0.786)	Y (0.964)	Apoplastic/cytoplasmic effector
Chr04G1237.1	-	Y (0.788)	Apoplastic effector
Chr04G1346.1	-	Y (0.737)	Apoplastic effector
Chr04G1415.1	-	Y (0.772)	Apoplastic effector
Chr04G1432.1	Y (0.545)	-	Cytoplasmic effector
Chr04G1433.1	-	Y (0.582)	Apoplastic effector
Chr04G1443.1	-	Y (0.619)	Apoplastic effector
Chr04G1544.1	Y (0.737)	-	Cytoplasmic effector
Chr04G1559.1	-	Y (0.694)	Apoplastic effector
Chr04G1583.1	Y (0.674)	-	Cytoplasmic effector
Chr02G0023.1	-	Y (0.513)	Apoplastic effector
Chr02G0024.1	-	Y (0.576)	Apoplastic effector
Chr02G0135.1	-	Y (0.726)	Apoplastic effector
Chr02G0144.1	-	Y (0.631)	Apoplastic effector
Chr02G0231.1	Y (0.681)	Y (0.713)	Apoplastic/cytoplasmic effector
Chr02G0252.1	-	Y (0.854)	Apoplastic effector
Chr02G0358.1	Y (0.893)	-	Cytoplasmic effector
Chr02G0406.1	-	Y (0.829)	Apoplastic effector
Chr02G0410.1	-	Y (0.741)	Apoplastic effector
Chr02G0411.1	Y (0.704)	Y (0.9)	Apoplastic/cytoplasmic effector
Chr02G0429.1	-	Y (0.782)	Apoplastic effector
Chr02G0445.1	Y (0.72)	Y (0.677)	Cytoplasmic/apoplastic effector
Chr02G0495.1	-	Y (0.51)	Apoplastic effector
Chr02G0508.1	-	Y (0.736)	Apoplastic effector
Chr02G0546.1	Y (0.614)	-	Cytoplasmic effector
Chr02G0555.1	Y (0.755)	-	Cytoplasmic effector
Chr02G0675.1	Y (0.539)	-	Cytoplasmic effector
Chr10G0002.1	Y (0.787)	-	Cytoplasmic effector
