# Supplementary material for: Decoding Pecan’s Fungal Foe: A Genomic Insight into Colletotrichum plurivorum Isolate W-6
Source: J Fungi (Basel). 2025 Mar 5;11(3):203. doi: 10.3390/jof11030203 (PMC11943440; doi:10.3390/jof11030203)
Supplement: Supplementary file 1 [file jof-11-00203-s001.zip › Table S2.pdf]

Table S2. Illumina NovaSeq data statistics for the isolate W-6 genome survey.

Platform	Library	Read length	Raw data (Gb)	Depth (×)	Q20 (%)	Q30 (%)
Illumina NovaSeq 6000	350 bp	150 bp (PE)	3.16	62.4	97.63	93.85

Note: The average k-mer deepth (peak) is 53.
