# Supplementary material for: Decoding Pecan’s Fungal Foe: A Genomic Insight into Colletotrichum plurivorum Isolate W-6
Source: J Fungi (Basel). 2025 Mar 5;11(3):203. doi: 10.3390/jof11030203 (PMC11943440; doi:10.3390/jof11030203)
Supplement: Supplementary file 1 [file jof-11-00203-s001.zip › Table S20.pdf]

Table S20. Function annotation of protein-coding genes based on specific database.

Database		Number
CAZyme		943
CAZyme type	AA	191
	CBM	58
	CE	172
	GH	361
	GT	113
	PL	48
PHI		4,558
TCDB		137
P450		1753
DFVF		3,175
