# Supplementary material for: Decoding Pecan’s Fungal Foe: A Genomic Insight into Colletotrichum plurivorum Isolate W-6
Source: J Fungi (Basel). 2025 Mar 5;11(3):203. doi: 10.3390/jof11030203 (PMC11943440; doi:10.3390/jof11030203)
Supplement: Supplementary file 1 [file jof-11-00203-s001.zip › Table S21.pdf]

Table S21.Prediction of CAZyme in isolate W-6 genome.

protein_id	CAZyme_family	protein_length	protein_start	protien_end	hmm_length	hmm_start	hmm_end	e-value	coverage
Chr04G1393.1	GT1.hmm	429	31	420	382	11	381	2.60E-40	0.968586387
Chr05G0470.1	AA9.hmm	185	7	153	220	2	157	1.60E-06	0.704545455
Chr05G0054.1	GH131.hmm	377	22	264	255	1	255	3.90E-103	0.996078431
Chr08G0808.1	CE1.hmm	572	131	207	227	13	94	2.80E-05	0.356828194
Chr06G0359.1	AA9.hmm	185	7	153	220	2	157	1.60E-06	0.704545455
Chr05G0540.1	CE3.hmm	247	8	190	194	1	255	3.90E-103	0.996078431
Chr01G1892.1	CE1.hmm	572	131	207	227	13	94	2.80E-05	0.356828194
Chr07G0308.1	GH3.hmm	774	113	335	216	4	215	9.60E-64	0.976851852
Chr09G0416.1	CBM20.hmm	293	20	70	90	24	64	0.00059	0.444444444
Chr08G0437.1	GH35.hmm	1005	53	371	307	2	157	1.60E-06	0.704545455
Chr08G0728.1	AA1.hmm	602	36	375	943	450	792	2.30E-90	0.362672322
Chr07G1058.1	GH5.hmm	443	63	397	275	4	215	9.60E-64	0.976851852
Chr03G0847.1	GH1.hmm	476	2	469	429	4	215	9.60E-64	0.976851852
Chr01G2461.1	AA2.hmm	317	60	264	255	1	255	3.90E-103	0.996078431
Chr04G0978.1	CE10.hmm	2516	2171	2493	341	66	316	2.20E-24	0.73313783
Chr01G0057.1	GH43.hmm	1046	732	1013	248	2	157	1.60E-06	0.704545455
Chr05G0740.1	CE10.hmm	2516	2171	2493	341	66	316	2.20E-24	0.73313783
Chr07G0476.1	CE10.hmm	2516	2171	2493	341	66	316	2.20E-24	0.73313783
Chr06G0293.1	AA3.hmm	623	35	597	618	88	402	2.90E-89	0.508090615
Chr07G1215.1	GT4.hmm	478	213	348	160	6	143	2.30E-11	0.497797357
Chr07G0069.1	GH43.hmm	1046	732	1013	248	2	157	1.60E-06	0.704545455
Chr04G0381.1	GH38.hmm	1093	302	565	269	2	157	1.60E-06	0.704545455
Chr05G1043.1	GH47.hmm	709	183	676	446	1	255	3.90E-103	0.996078431
Chr08G0737.1	CE10.hmm	2516	2171	2493	341	66	316	2.20E-24	0.73313783

Chr07G0941.1	GH55.hmm	973	13	395	740	345	719	1.20E-120	0.505405405
Chr01G1863.1	GH127.hmm	640	9	550	524	1	255	3.90E-103	0.996078431
Chr01G1702.1	GH18.hmm	406	43	376	296	29	292	1.40E-54	0.888513514
Chr01G2452.1	GH28.hmm	443	63	397	275	4	215	9.60E-64	0.976851852
Chr07G1694.1	PL1.hmm	378	103	297	202	16	201	1.40E-45	0.915841584
Chr04G1203.1	GT58.hmm	430	32	396	364	2	157	1.60E-06	0.704545455
Chr09G0084.1	GH43.hmm	1046	732	1013	248	2	157	1.60E-06	0.704545455
Chr07G1271.1	GT8.hmm	386	98	333	257	29	292	1.40E-54	0.888513514
Chr01G2484.1	AA9.hmm	185	7	153	220	2	157	1.60E-06	0.704545455
Chr08G0252.1	CE8.hmm	408	52	363	288	5	264	1.10E-55	0.899305556
Chr07G1362.1	GH16.hmm	255	61	251	189	19	189	1.90E-24	0.899470899
Chr08G0043.1	CE10.hmm	2516	2171	2493	341	66	316	2.20E-24	0.73313783
Chr01G1860.1	CE10.hmm	2516	2171	2493	341	66	316	2.20E-24	0.73313783
Chr06G0117.1	GH2.hmm	852	5	227	220	2	157	1.60E-06	0.704545455
Chr04G1414.1	CE3.hmm	247	8	190	194	1	255	3.90E-103	0.996078431
Chr06G0182.1	CE10.hmm	2516	2171	2493	341	66	316	2.20E-24	0.73313783
Chr07G1002.1	GT31.hmm	446	118	302	255	1	255	3.90E-103	0.996078431
Chr09G0005.1	AA7.hmm	607	131	207	227	13	94	2.80E-05	0.356828194
Chr04G0987.1	GH16.hmm	255	61	251	189	19	189	1.90E-24	0.899470899
Chr03G0763.1	GH16.hmm	255	61	251	189	19	189	1.90E-24	0.899470899
Chr09G0529.1	GT90.hmm	456	146	320	250	2	157	1.60E-06	0.704545455
Chr06G1075.1	GH3.hmm	774	113	335	216	4	215	9.60E-64	0.976851852
Chr04G1141.1	GT1.hmm	429	31	420	382	11	381	2.60E-40	0.968586387
Chr03G1550.1	GH43.hmm	1046	732	1013	248	2	157	1.60E-06	0.704545455
Chr03G1680.1	GH109.hmm	376	10	128	126	1	255	3.90E-103	0.996078431
Chr01G0266.1	GT1.hmm	429	31	420	382	11	381	2.60E-40	0.968586387

Chr04G0907.1	GT8.hmm	386	98	333	257	29	292	1.40E-54	0.888513514
Chr07G0169.1	CE5.hmm	234	54	226	189	19	189	1.90E-24	0.899470899
Chr09G0716.1	GH43.hmm	1046	732	1013	248	2	157	1.60E-06	0.704545455
Chr04G1021.1	CE12.hmm	266	33	244	210	1	255	3.90E-103	0.996078431
Chr01G0742.1	GH76.hmm	493	30	399	358	12	343	3.00E-102	0.924581006
Chr01G1774.1	CBM1.hmm	432	21	49	29	1	255	3.90E-103	0.996078431
Chr07G1164.1	AA3.hmm	623	35	597	618	88	402	2.90E-89	0.508090615
Chr07G1364.1	AA13.hmm	384	19	250	232	1	255	3.90E-103	0.996078431
Chr01G0710.1	GH5.hmm	443	63	397	275	4	215	9.60E-64	0.976851852
Chr04G1045.1	AA3.hmm	623	35	597	618	88	402	2.90E-89	0.508090615
Chr08G0756.1	CE10.hmm	2516	2171	2493	341	66	316	2.20E-24	0.73313783
Chr04G0327.1	AA3.hmm	623	35	597	618	88	402	2.90E-89	0.508090615
Chr01G0161.1	AA12.hmm	463	25	416	401	4	215	9.60E-64	0.976851852
Chr07G0050.1	GH53.hmm	386	98	333	257	29	292	1.40E-54	0.888513514
Chr06G1169.1	GH92.hmm	791	277	776	491	2	157	1.60E-06	0.704545455
Chr01G0601.1	GH62.hmm	328	27	298	278	1	255	3.90E-103	0.996078431
Chr05G0386.1	GH131.hmm	377	22	264	255	1	255	3.90E-103	0.996078431
Chr05G1142.1	GH114.hmm	828	566	766	190	10	190	3.00E-26	0.340175953
Chr07G0522.1	AA7.hmm	607	131	207	227	13	94	2.80E-05	0.356828194
Chr04G0748.1	AA3.hmm	623	35	597	618	88	402	2.90E-89	0.508090615
Chr07G0484.1	GT2.hmm	238	7	153	220	2	157	1.60E-06	0.704545455
Chr04G0325.1	GH71.hmm	487	44	433	375	1	255	3.90E-103	0.996078431
Chr01G2236.1	GH47.hmm	709	183	676	446	1	255	3.90E-103	0.996078431
Chr06G0609.1	CE4.hmm	829	624	744	752	28	555	1.60E-97	0.700797872
Chr08G0166.1	CE10.hmm	2516	2171	2493	341	66	316	2.20E-24	0.73313783
Chr03G0929.1	CBM50.hmm	272	141	178	40	7	189	1.90E-24	0.899470899

Chr07G0149.1	GH7.hmm	746	26	399	358	12	343	3.00E-102	0.924581006
Chr04G1314.1	CE2.hmm	429	31	420	382	11	381	2.60E-40	0.968586387
Chr04G1111.1	CBM50.hmm	272	141	178	40	7	189	1.90E-24	0.899470899
Chr04G1277.1	AA3.hmm	623	35	597	618	88	402	2.90E-89	0.508090615
Chr01G0292.1	GH47.hmm	709	183	676	446	1	255	3.90E-103	0.996078431
Chr07G0619.1	GT39.hmm	738	63	397	275	4	215	9.60E-64	0.976851852
Chr04G0876.1	GH95.hmm	796	39	788	722	19	189	1.90E-24	0.899470899
Chr06G1237.1	GH17.hmm	289	29	285	189	19	189	1.90E-24	0.899470899
Chr06G0149.1	GH105.hmm	380	38	580	446	1	255	3.90E-103	0.996078431
Chr08G0553.1	GH43.hmm	1046	732	1013	248	2	157	1.60E-06	0.704545455
Chr06G0942.1	AA11.hmm	425	18	309	248	2	157	1.60E-06	0.704545455
Chr07G0311.1	CE10.hmm	2516	2171	2493	341	66	316	2.20E-24	0.73313783
Chr01G0553.1	AA1.hmm	602	36	375	943	450	792	2.30E-90	0.362672322
Chr01G1327.1	PL3.hmm	244	41	218	197	2	157	1.60E-06	0.704545455
Chr04G0269.1	GH43.hmm	1046	732	1013	248	2	157	1.60E-06	0.704545455
Chr01G1736.1	GH43.hmm	1046	732	1013	248	2	157	1.60E-06	0.704545455
Chr04G0323.1	CE10.hmm	2516	2171	2493	341	66	316	2.20E-24	0.73313783
Chr03G1317.1	GH99.hmm	266	33	244	210	1	255	3.90E-103	0.996078431
Chr04G1425.1	GH55.hmm	973	13	395	740	345	719	1.20E-120	0.505405405
Chr07G0310.1	GH3.hmm	774	113	335	216	4	215	9.60E-64	0.976851852
Chr05G0347.1	GH55.hmm	973	13	395	740	345	719	1.20E-120	0.505405405
Chr04G1334.1	AA7.hmm	607	131	207	227	13	94	2.80E-05	0.356828194
Chr01G1834.1	CBM35.hmm	408	52	363	288	5	264	1.10E-55	0.899305556
Chr07G1700.1	GH18.hmm	406	43	376	296	29	292	1.40E-54	0.888513514
Chr04G1533.1	GH3.hmm	774	113	335	216	4	215	9.60E-64	0.976851852
Chr07G0236.1	GH43.hmm	1046	732	1013	248	2	157	1.60E-06	0.704545455

Chr05G0725.1	AA2.hmm	317	60	264	255	1	255	3.90E-103	0.996078431
Chr07G0600.1	AA3.hmm	623	35	597	618	88	402	2.90E-89	0.508090615
Chr01G1242.1	GH47.hmm	709	183	676	446	1	255	3.90E-103	0.996078431
Chr01G2624.1	CE16.hmm	292	28	287	189	19	189	1.90E-24	0.899470899
Chr05G0152.1	GH109.hmm	376	10	128	126	1	255	3.90E-103	0.996078431
Chr05G0660.1	CE10.hmm	2516	2171	2493	341	66	316	2.20E-24	0.73313783
Chr08G0832.1	GH18.hmm	406	43	376	296	29	292	1.40E-54	0.888513514
Chr07G0735.1	CE10.hmm	2516	2171	2493	341	66	316	2.20E-24	0.73313783
Chr01G0308.1	AA9.hmm	185	7	153	220	2	157	1.60E-06	0.704545455
Chr04G0395.1	CE10.hmm	2516	2171	2493	341	66	316	2.20E-24	0.73313783
Chr04G0796.1	GH109.hmm	376	10	128	126	1	255	3.90E-103	0.996078431
Chr07G0536.1	GH128.hmm	320	45	279	224	5	264	1.10E-55	0.899305556
Chr03G0207.1	CE4.hmm	829	624	744	752	28	555	1.60E-97	0.700797872
Chr01G2393.1	GH79.hmm	567	65	359	341	66	316	2.20E-24	0.73313783
Chr05G0530.1	CBM63.hmm	308	221	266	40	7	189	1.90E-24	0.899470899
Chr09G0034.1	GH6.hmm	394	63	397	275	4	215	9.60E-64	0.976851852
Chr07G0439.1	GH53.hmm	386	98	333	257	29	292	1.40E-54	0.888513514
Chr06G0173.1	GH18.hmm	406	43	376	296	29	292	1.40E-54	0.888513514
Chr06G1179.1	AA7.hmm	607	131	207	227	13	94	2.80E-05	0.356828194
Chr07G0047.1	GT1.hmm	429	31	420	382	11	381	2.60E-40	0.968586387
Chr01G1754.1	AA9.hmm	185	7	153	220	2	157	1.60E-06	0.704545455
Chr01G0238.1	PL3.hmm	244	41	218	197	2	157	1.60E-06	0.704545455
Chr07G0720.1	GH95.hmm	796	39	788	722	19	189	1.90E-24	0.899470899
Chr05G0340.1	GT32.hmm	440	101	185	90	24	64	0.00059	0.444444444
Chr08G0471.1	AA1.hmm	602	36	375	943	450	792	2.30E-90	0.362672322
Chr01G0513.1	AA2.hmm	317	60	264	255	1	255	3.90E-103	0.996078431

Chr08G0065.1	GT8.hmm	386	98	333	257	29	292	1.40E-54	0.888513514
Chr07G1168.1	PL3.hmm	244	41	218	197	2	157	1.60E-06	0.704545455
Chr04G0279.1	CBM35.hmm	408	52	363	288	5	264	1.10E-55	0.899305556
Chr08G0925.1	GH2.hmm	852	5	227	220	2	157	1.60E-06	0.704545455
Chr08G0251.1	GH28.hmm	443	63	397	275	4	215	9.60E-64	0.976851852
Chr04G0532.1	GT2.hmm	238	7	153	220	2	157	1.60E-06	0.704545455
Chr04G1418.1	CBM18.hmm	1432	492	537	382	11	381	2.60E-40	0.968586387
Chr04G0284.1	GH6.hmm	394	63	397	275	4	215	9.60E-64	0.976851852
Chr01G2429.1	GH5.hmm	443	63	397	275	4	215	9.60E-64	0.976851852
Chr04G1262.1	GH131.hmm	377	22	264	255	1	255	3.90E-103	0.996078431
Chr07G0252.1	CE5.hmm	234	54	226	189	19	189	1.90E-24	0.899470899
Chr04G0354.1	CE16.hmm	292	28	287	189	19	189	1.90E-24	0.899470899
Chr01G1990.1	GH7.hmm	746	26	399	358	12	343	3.00E-102	0.924581006
Chr01G1602.1	GH7.hmm	746	26	399	358	12	343	3.00E-102	0.924581006
Chr03G1488.1	CBM21.hmm	767	324	428	107	6	143	2.30E-11	0.497797357
Chr06G1440.1	GH12.hmm	261	99	245	156	2	157	1.60E-06	0.704545455
Chr06G0502.1	GH16.hmm	255	61	251	189	19	189	1.90E-24	0.899470899
Chr04G0393.1	CE10.hmm	2516	2171	2493	341	66	316	2.20E-24	0.73313783
Chr03G1271.1	CE10.hmm	2516	2171	2493	341	66	316	2.20E-24	0.73313783
Chr05G1157.1	GH37.hmm	686	51	625	491	2	157	1.60E-06	0.704545455
Chr09G0918.1	GH74.hmm	885	74	182	233	7	189	1.90E-24	0.899470899
Chr08G0182.1	PL3.hmm	244	41	218	197	2	157	1.60E-06	0.704545455
Chr07G0179.1	AA9.hmm	185	7	153	220	2	157	1.60E-06	0.704545455
Chr04G0207.1	AA8.hmm	797	5	227	220	2	157	1.60E-06	0.704545455
Chr05G0676.1	GH38.hmm	1093	302	565	269	2	157	1.60E-06	0.704545455
Chr01G1832.1	AA4.hmm	572	131	207	227	13	94	2.80E-05	0.356828194

Chr09G0485.1	GH16.hmm	255	61	251	189	19	189	1.90E-24	0.899470899
Chr06G0274.1	AA2.hmm	317	60	264	255	1	255	3.90E-103	0.996078431
Chr04G1498.1	GT32.hmm	440	101	185	90	24	64	0.00059	0.444444444
Chr06G0241.1	CE5.hmm	234	54	226	189	19	189	1.90E-24	0.899470899
Chr09G0684.1	AA7.hmm	607	131	207	227	13	94	2.80E-05	0.356828194
Chr06G0934.1	GH76.hmm	493	30	399	358	12	343	3.00E-102	0.924581006
Chr01G2690.1	GH92.hmm	791	277	776	491	2	157	1.60E-06	0.704545455
Chr03G1206.1	CE1.hmm	572	131	207	227	13	94	2.80E-05	0.356828194
Chr03G1119.1	GT90.hmm	456	146	320	250	2	157	1.60E-06	0.704545455
Chr05G0247.1	CBM18.hmm	1432	492	537	382	11	381	2.60E-40	0.968586387
Chr01G0396.1	AA3.hmm	623	35	597	618	88	402	2.90E-89	0.508090615
Chr07G0103.1	AA7.hmm	607	131	207	227	13	94	2.80E-05	0.356828194
Chr01G2378.1	GT2.hmm	238	7	153	220	2	157	1.60E-06	0.704545455
Chr07G0829.1	AA1.hmm	602	36	375	943	450	792	2.30E-90	0.362672322
Chr01G1233.1	AA7.hmm	607	131	207	227	13	94	2.80E-05	0.356828194
Chr07G0206.1	AA3.hmm	623	35	597	618	88	402	2.90E-89	0.508090615
Chr07G0249.1	GH79.hmm	567	65	359	341	66	316	2.20E-24	0.73313783
Chr09G0553.1	AA9.hmm	185	7	153	220	2	157	1.60E-06	0.704545455
Chr05G0906.1	AA5.hmm	1217	709	1211	1281	559	1039	3.20E-152	0.37470726
Chr07G1375.1	PL1.hmm	378	103	297	202	16	201	1.40E-45	0.915841584
Chr08G0466.1	CE10.hmm	2516	2171	2493	341	66	316	2.20E-24	0.73313783
Chr03G1731.1	GH109.hmm	376	10	128	126	1	255	3.90E-103	0.996078431
Chr03G0598.1	CE10.hmm	2516	2171	2493	341	66	316	2.20E-24	0.73313783
Chr07G1572.1	CE5.hmm	234	54	226	189	19	189	1.90E-24	0.899470899
Chr01G0237.1	GT2.hmm	238	7	153	220	2	157	1.60E-06	0.704545455
Chr08G0952.1	AA3.hmm	623	35	597	618	88	402	2.90E-89	0.508090615

Chr06G0825.1	GH43.hmm	1046	732	1013	248	2	157	1.60E-06	0.704545455
Chr03G0916.1	CE1.hmm	572	131	207	227	13	94	2.80E-05	0.356828194
Chr08G0132.1	GH10.hmm	586	25	416	401	4	215	9.60E-64	0.976851852
Chr08G0048.1	GT1.hmm	429	31	420	382	11	381	2.60E-40	0.968586387
Chr05G1378.1	GH5.hmm	443	63	397	275	4	215	9.60E-64	0.976851852
Chr01G2438.1	GH3.hmm	774	113	335	216	4	215	9.60E-64	0.976851852
Chr01G2681.1	GT71.hmm	525	171	401	209	1	255	3.90E-103	0.996078431
Chr07G0500.1	GH3.hmm	774	113	335	216	4	215	9.60E-64	0.976851852
Chr04G0204.1	AA3.hmm	623	35	597	618	88	402	2.90E-89	0.508090615
Chr07G0401.1	GH13.hmm	539	59	365	342	1	255	3.90E-103	0.996078431
Chr08G1026.1	CE10.hmm	2516	2171	2493	341	66	316	2.20E-24	0.73313783
Chr07G0120.1	CE16.hmm	292	28	287	189	19	189	1.90E-24	0.899470899
Chr04G1251.1	GH55.hmm	973	13	395	740	345	719	1.20E-120	0.505405405
Chr07G1165.1	GH31.hmm	665	209	664	427	1	255	3.90E-103	0.996078431
Chr01G0071.1	GT1.hmm	429	31	420	382	11	381	2.60E-40	0.968586387
Chr01G0458.1	CBM63.hmm	308	221	266	40	7	189	1.90E-24	0.899470899
Chr05G0253.1	GH20.hmm	720	172	509	337	9	290	1.60E-88	0.955782313
Chr07G0341.1	CE12.hmm	266	33	244	210	1	255	3.90E-103	0.996078431
Chr01G0679.1	CE5.hmm	234	54	226	189	19	189	1.90E-24	0.899470899
Chr07G1398.1	GH33.hmm	379	4	123	126	1	255	3.90E-103	0.996078431
Chr01G1074.1	PL1.hmm	378	103	297	202	16	201	1.40E-45	0.915841584
Chr01G0016.1	AA7.hmm	607	131	207	227	13	94	2.80E-05	0.356828194
Chr01G1165.1	GH109.hmm	376	10	128	126	1	255	3.90E-103	0.996078431
Chr01G0765.1	AA9.hmm	185	7	153	220	2	157	1.60E-06	0.704545455
Chr08G0557.1	CE1.hmm	572	131	207	227	13	94	2.80E-05	0.356828194
Chr01G0869.1	GH2.hmm	852	5	227	220	2	157	1.60E-06	0.704545455

Chr08G0733.1	AA7.hmm	607	131	207	227	13	94	2.80E-05	0.356828194
Chr07G0266.1	GH10.hmm	586	25	416	401	4	215	9.60E-64	0.976851852
Chr07G1795.1	AA9.hmm	185	7	153	220	2	157	1.60E-06	0.704545455
Chr08G0459.1	AA7.hmm	607	131	207	227	13	94	2.80E-05	0.356828194
Chr04G0717.1	GH3.hmm	774	113	335	216	4	215	9.60E-64	0.976851852
Chr07G0129.1	GH109.hmm	376	10	128	126	1	255	3.90E-103	0.996078431
Chr01G0730.1	CE10.hmm	2516	2171	2493	341	66	316	2.20E-24	0.73313783
Chr06G0346.1	GH36.hmm	748	42	465	458	11	381	2.60E-40	0.968586387
Chr05G0416.1	GT57.hmm	599	75	305	233	7	189	1.90E-24	0.899470899
Chr03G1365.1	CE1.hmm	572	131	207	227	13	94	2.80E-05	0.356828194
Chr01G0576.1	GT4.hmm	478	213	348	160	6	143	2.30E-11	0.497797357
Chr03G0181.1	GH78.hmm	729	88	668	504	9	290	1.60E-88	0.955782313
Chr09G0726.1	CE10.hmm	2516	2171	2493	341	66	316	2.20E-24	0.73313783
Chr07G0547.1	AA3.hmm	623	35	597	618	88	402	2.90E-89	0.508090615
Chr07G0090.1	GH74.hmm	885	74	182	233	7	189	1.90E-24	0.899470899
Chr03G1177.1	GH13.hmm	539	59	365	342	1	255	3.90E-103	0.996078431
Chr07G0455.1	GH109.hmm	376	10	128	126	1	255	3.90E-103	0.996078431
Chr03G0208.1	GH18.hmm	406	43	376	296	29	292	1.40E-54	0.888513514
Chr07G0546.1	CE5.hmm	234	54	226	189	19	189	1.90E-24	0.899470899
Chr04G0533.1	GT2.hmm	238	7	153	220	2	157	1.60E-06	0.704545455
Chr07G0316.1	AA9.hmm	185	7	153	220	2	157	1.60E-06	0.704545455
Chr03G1312.1	PL3.hmm	244	41	218	197	2	157	1.60E-06	0.704545455
Chr01G1992.1	GH18.hmm	406	43	376	296	29	292	1.40E-54	0.888513514
Chr01G0192.1	PL9.hmm	427	16	410	373	1	255	3.90E-103	0.996078431
Chr07G0409.1	PL1.hmm	378	103	297	202	16	201	1.40E-45	0.915841584
Chr01G0893.1	GH125.hmm	540	112	271	257	29	292	1.40E-54	0.888513514

Chr05G0048.1	AA9.hmm	185	7	153	220	2	157	1.60E-06	0.704545455
Chr07G0705.1	GH128.hmm	320	45	279	224	5	264	1.10E-55	0.899305556
Chr07G0504.1	GH2.hmm	852	5	227	220	2	157	1.60E-06	0.704545455
Chr08G0086.1	AA7.hmm	607	131	207	227	13	94	2.80E-05	0.356828194
Chr04G0384.1	GH115.hmm	966	23	322	248	2	157	1.60E-06	0.704545455
Chr07G0423.1	AA4.hmm	572	131	207	227	13	94	2.80E-05	0.356828194
Chr01G1819.1	GH88.hmm	462	57	452	329	4	215	9.60E-64	0.976851852
Chr04G0981.1	AA3.hmm	623	35	597	618	88	402	2.90E-89	0.508090615
Chr07G0416.1	PL1.hmm	378	103	297	202	16	201	1.40E-45	0.915841584
Chr07G1689.1	PL1.hmm	378	103	297	202	16	201	1.40E-45	0.915841584
Chr09G0823.1	GH88.hmm	462	57	452	329	4	215	9.60E-64	0.976851852
Chr03G0046.1	CBM18.hmm	1432	492	537	382	11	381	2.60E-40	0.968586387
Chr07G1379.1	CE10.hmm	2516	2171	2493	341	66	316	2.20E-24	0.73313783
Chr05G0378.1	AA7.hmm	607	131	207	227	13	94	2.80E-05	0.356828194
Chr06G1297.1	CE10.hmm	2516	2171	2493	341	66	316	2.20E-24	0.73313783
Chr06G1349.1	CBM18.hmm	1432	492	537	382	11	381	2.60E-40	0.968586387
Chr03G0391.1	GH43.hmm	1046	732	1013	248	2	157	1.60E-06	0.704545455
Chr06G0984.1	GT3.hmm	714	26	399	358	12	343	3.00E-102	0.924581006
Chr09G0673.1	AA8.hmm	797	5	227	220	2	157	1.60E-06	0.704545455
Chr09G0127.1	GT28.hmm	231	44	433	375	1	255	3.90E-103	0.996078431
Chr07G0304.1	CE10.hmm	2516	2171	2493	341	66	316	2.20E-24	0.73313783
Chr03G1326.1	GT25.hmm	396	42	465	458	11	381	2.60E-40	0.968586387
Chr07G1601.1	AA3.hmm	623	35	597	618	88	402	2.90E-89	0.508090615
Chr01G0345.1	CE10.hmm	2516	2171	2493	341	66	316	2.20E-24	0.73313783
Chr06G0477.1	GT2.hmm	238	7	153	220	2	157	1.60E-06	0.704545455
Chr01G0418.1	GT32.hmm	440	101	185	90	24	64	0.00059	0.444444444

Chr08G0092.1	GH76.hmm	493	30	399	358	12	343	3.00E-102	0.924581006
Chr01G1557.1	GH32.hmm	678	77	220	341	66	316	2.20E-24	0.73313783
Chr01G2437.1	GT71.hmm	525	171	401	209	1	255	3.90E-103	0.996078431
Chr04G1232.1	AA3.hmm	623	35	597	618	88	402	2.90E-89	0.508090615
Chr06G0412.1	CE1.hmm	572	131	207	227	13	94	2.80E-05	0.356828194
Chr09G0491.1	GH37.hmm	686	51	625	491	2	157	1.60E-06	0.704545455
Chr03G1528.1	CE3.hmm	247	8	190	194	1	255	3.90E-103	0.996078431
Chr07G1486.1	AA9.hmm	185	7	153	220	2	157	1.60E-06	0.704545455
Chr08G0062.1	GH43.hmm	1046	732	1013	248	2	157	1.60E-06	0.704545455
Chr01G1026.1	GH131.hmm	377	22	264	255	1	255	3.90E-103	0.996078431
Chr04G1324.1	CE10.hmm	2516	2171	2493	341	66	316	2.20E-24	0.73313783
Chr09G0800.1	GT8.hmm	386	98	333	257	29	292	1.40E-54	0.888513514
Chr07G0228.1	CE3.hmm	247	8	190	194	1	255	3.90E-103	0.996078431
Chr07G1316.1	AA3.hmm	623	35	597	618	88	402	2.90E-89	0.508090615
Chr03G1718.1	CE4.hmm	829	624	744	752	28	555	1.60E-97	0.700797872
Chr04G0758.1	AA9.hmm	185	7	153	220	2	157	1.60E-06	0.704545455
Chr06G0773.1	GH16.hmm	255	61	251	189	19	189	1.90E-24	0.899470899
Chr07G1167.1	GH79.hmm	567	65	359	341	66	316	2.20E-24	0.73313783
Chr05G0334.1	AA9.hmm	185	7	153	220	2	157	1.60E-06	0.704545455
Chr01G2774.1	CE10.hmm	2516	2171	2493	341	66	316	2.20E-24	0.73313783
Chr09G1000.1	AA7.hmm	607	131	207	227	13	94	2.80E-05	0.356828194
Chr05G1339.1	AA2.hmm	317	60	264	255	1	255	3.90E-103	0.996078431
Chr03G0369.1	AA9.hmm	185	7	153	220	2	157	1.60E-06	0.704545455
Chr08G0846.1	AA11.hmm	425	18	309	248	2	157	1.60E-06	0.704545455
Chr01G0479.1	GH128.hmm	320	45	279	224	5	264	1.10E-55	0.899305556
Chr04G0485.1	GT41.hmm	1583	1289	1572	705	293	519	1.60E-52	0.320567376

Chr05G0246.1	CBM18.hmm	1432	492	537	382	11	381	2.60E-40	0.968586387
Chr03G0057.1	CBM18.hmm	1432	492	537	382	11	381	2.60E-40	0.968586387
Chr01G1366.1	GH43.hmm	1046	732	1013	248	2	157	1.60E-06	0.704545455
Chr07G1325.1	GH71.hmm	487	44	433	375	1	255	3.90E-103	0.996078431
Chr07G0066.1	CE3.hmm	247	8	190	194	1	255	3.90E-103	0.996078431
Chr01G1405.1	GH131.hmm	377	22	264	255	1	255	3.90E-103	0.996078431
Chr03G1468.1	CE10.hmm	2516	2171	2493	341	66	316	2.20E-24	0.73313783
Chr07G0021.1	AA3.hmm	623	35	597	618	88	402	2.90E-89	0.508090615
Chr05G0145.1	GT20.hmm	903	159	612	475	33	111	3.30E-08	0.780487805
Chr07G0482.1	GH18.hmm	406	43	376	296	29	292	1.40E-54	0.888513514
Chr01G0033.1	GH51.hmm	507	14	319	341	66	316	2.20E-24	0.73313783
Chr07G0419.1	PL3.hmm	244	41	218	197	2	157	1.60E-06	0.704545455
Chr05G0856.1	GT15.hmm	422	65	359	341	66	316	2.20E-24	0.73313783
Chr06G0175.1	GH6.hmm	394	63	397	275	4	215	9.60E-64	0.976851852
Chr06G0661.1	AA3.hmm	623	35	597	618	88	402	2.90E-89	0.508090615
Chr04G0341.1	GH28.hmm	443	63	397	275	4	215	9.60E-64	0.976851852
Chr07G0518.1	GH71.hmm	487	44	433	375	1	255	3.90E-103	0.996078431
Chr03G0058.1	GT2.hmm	238	7	153	220	2	157	1.60E-06	0.704545455
Chr04G0865.1	GT33.hmm	467	45	279	224	5	264	1.10E-55	0.899305556
Chr06G0610.1	CE1.hmm	572	131	207	227	13	94	2.80E-05	0.356828194
Chr03G0214.1	AA3.hmm	623	35	597	618	88	402	2.90E-89	0.508090615
Chr08G1005.1	GH2.hmm	852	5	227	220	2	157	1.60E-06	0.704545455
Chr07G0930.1	GH5.hmm	443	63	397	275	4	215	9.60E-64	0.976851852
Chr01G0923.1	GT2.hmm	238	7	153	220	2	157	1.60E-06	0.704545455
Chr01G1301.1	GH92.hmm	791	277	776	491	2	157	1.60E-06	0.704545455
Chr03G1604.1	GH1.hmm	476	2	469	429	4	215	9.60E-64	0.976851852

Chr08G0413.1	GH27.hmm	784	118	302	255	1	255	3.90E-103	0.996078431
Chr07G0497.1	CE3.hmm	247	8	190	194	1	255	3.90E-103	0.996078431
Chr01G0497.1	GH3.hmm	774	113	335	216	4	215	9.60E-64	0.976851852
Chr03G0088.1	GH28.hmm	443	63	397	275	4	215	9.60E-64	0.976851852
Chr04G0831.1	CE10.hmm	2516	2171	2493	341	66	316	2.20E-24	0.73313783
Chr07G0848.1	GT48.hmm	1944	869	1648	739	1	255	3.90E-103	0.996078431
Chr03G1548.1	GH95.hmm	796	39	788	722	19	189	1.90E-24	0.899470899
Chr01G0702.1	AA7.hmm	607	131	207	227	13	94	2.80E-05	0.356828194
Chr06G0099.1	GT32.hmm	440	101	185	90	24	64	0.00059	0.444444444
Chr06G0955.1	CBM18.hmm	1432	492	537	382	11	381	2.60E-40	0.968586387
Chr09G0699.1	AA9.hmm	185	7	153	220	2	157	1.60E-06	0.704545455
Chr09G0097.1	GT69.hmm	484	169	401	209	1	255	3.90E-103	0.996078431
Chr07G0044.1	GT69.hmm	484	169	401	209	1	255	3.90E-103	0.996078431
Chr01G0534.1	GH10.hmm	586	25	416	401	4	215	9.60E-64	0.976851852
Chr01G1641.1	GH28.hmm	443	63	397	275	4	215	9.60E-64	0.976851852
Chr01G0733.1	CE10.hmm	2516	2171	2493	341	66	316	2.20E-24	0.73313783
Chr09G0582.1	GH127.hmm	640	9	550	524	1	255	3.90E-103	0.996078431
Chr03G1283.1	GH31.hmm	665	209	664	427	1	255	3.90E-103	0.996078431
Chr06G0320.1	PL4.hmm	669	6	551	618	88	402	2.90E-89	0.508090615
Chr07G1016.1	AA9.hmm	185	7	153	220	2	157	1.60E-06	0.704545455
Chr07G1051.1	GH72.hmm	427	16	410	373	1	255	3.90E-103	0.996078431
Chr07G0936.1	PL3.hmm	244	41	218	197	2	157	1.60E-06	0.704545455
Chr06G0026.1	CE3.hmm	247	8	190	194	1	255	3.90E-103	0.996078431
Chr03G1174.1	GT32.hmm	440	101	185	90	24	64	0.00059	0.444444444
Chr06G0169.1	CBM50.hmm	272	141	178	40	7	189	1.90E-24	0.899470899
Chr04G1327.1	GT32.hmm	440	101	185	90	24	64	0.00059	0.444444444

Chr01G1337.1	AA9.hmm	185	7	153	220	2	157	1.60E-06	0.704545455
Chr03G1674.1	GH55.hmm	973	13	395	740	345	719	1.20E-120	0.505405405
Chr07G0025.1	GH55.hmm	973	13	395	740	345	719	1.20E-120	0.505405405
Chr04G0749.1	AA7.hmm	607	131	207	227	13	94	2.80E-05	0.356828194
Chr01G0720.1	GH47.hmm	709	183	676	446	1	255	3.90E-103	0.996078431
Chr07G0986.1	GH31.hmm	665	209	664	427	1	255	3.90E-103	0.996078431
Chr04G0820.1	GH43.hmm	1046	732	1013	248	2	157	1.60E-06	0.704545455
Chr01G1738.1	CBM50.hmm	272	141	178	40	7	189	1.90E-24	0.899470899
Chr07G1477.1	AA9.hmm	185	7	153	220	2	157	1.60E-06	0.704545455
Chr01G2442.1	GH105.hmm	380	38	580	446	1	255	3.90E-103	0.996078431
Chr04G0497.1	GT62.hmm	480	96	359	341	66	316	2.20E-24	0.73313783
Chr01G2717.1	GH76.hmm	493	30	399	358	12	343	3.00E-102	0.924581006
Chr06G1367.1	GT32.hmm	440	101	185	90	24	64	0.00059	0.444444444
Chr07G1590.1	CBM67.hmm	917	156	332	341	66	316	2.20E-24	0.73313783
Chr01G1685.1	CE10.hmm	2516	2171	2493	341	66	316	2.20E-24	0.73313783
Chr03G1442.1	GH106.hmm	903	159	612	475	33	111	3.30E-08	0.780487805
Chr09G0900.1	GH28.hmm	443	63	397	275	4	215	9.60E-64	0.976851852
Chr08G0527.1	AA9.hmm	185	7	153	220	2	157	1.60E-06	0.704545455
Chr04G0559.1	GH92.hmm	791	277	776	491	2	157	1.60E-06	0.704545455
Chr08G0375.1	AA4.hmm	572	131	207	227	13	94	2.80E-05	0.356828194
Chr01G0947.1	CE5.hmm	234	54	226	189	19	189	1.90E-24	0.899470899
Chr01G0068.1	CBM66.hmm	824	212	354	341	66	316	2.20E-24	0.73313783
Chr04G1188.1	CE10.hmm	2516	2171	2493	341	66	316	2.20E-24	0.73313783
Chr03G1398.1	AA3.hmm	623	35	597	618	88	402	2.90E-89	0.508090615
Chr03G0743.1	GH5.hmm	443	63	397	275	4	215	9.60E-64	0.976851852
Chr03G1320.1	GH5.hmm	443	63	397	275	4	215	9.60E-64	0.976851852

Chr01G1045.1	GH30.hmm	479	14	319	341	66	316	2.20E-24	0.73313783
Chr01G0193.1	AA7.hmm	607	131	207	227	13	94	2.80E-05	0.356828194
Chr01G2432.1	PL9.hmm	427	16	410	373	1	255	3.90E-103	0.996078431
Chr07G0686.1	CE4.hmm	829	624	744	752	28	555	1.60E-97	0.700797872
Chr01G2016.1	GT1.hmm	429	31	420	382	11	381	2.60E-40	0.968586387
Chr01G1522.1	CE1.hmm	572	131	207	227	13	94	2.80E-05	0.356828194
Chr07G0081.1	GT15.hmm	422	65	359	341	66	316	2.20E-24	0.73313783
Chr06G1418.1	GH16.hmm	255	61	251	189	19	189	1.90E-24	0.899470899
Chr05G0490.1	GT71.hmm	525	171	401	209	1	255	3.90E-103	0.996078431
Chr07G0960.1	GH18.hmm	406	43	376	296	29	292	1.40E-54	0.888513514
Chr05G1087.1	AA2.hmm	317	60	264	255	1	255	3.90E-103	0.996078431
Chr05G1024.1	GT69.hmm	484	169	401	209	1	255	3.90E-103	0.996078431
Chr09G0597.1	GH10.hmm	586	25	416	401	4	215	9.60E-64	0.976851852
Chr09G0358.1	GH47.hmm	709	183	676	446	1	255	3.90E-103	0.996078431
Chr01G2056.1	GT90.hmm	456	146	320	250	2	157	1.60E-06	0.704545455
Chr04G0274.1	GH20.hmm	720	172	509	337	9	290	1.60E-88	0.955782313
Chr03G1367.1	GH106.hmm	903	159	612	475	33	111	3.30E-08	0.780487805
Chr03G0212.1	CBM1.hmm	432	21	49	29	1	255	3.90E-103	0.996078431
Chr04G0035.1	AA3.hmm	623	35	597	618	88	402	2.90E-89	0.508090615
Chr09G0531.1	GH72.hmm	427	16	410	373	1	255	3.90E-103	0.996078431
Chr06G1421.1	GH127.hmm	640	9	550	524	1	255	3.90E-103	0.996078431
Chr09G0904.1	AA9.hmm	185	7	153	220	2	157	1.60E-06	0.704545455
Chr01G0334.1	GH28.hmm	443	63	397	275	4	215	9.60E-64	0.976851852
Chr07G0243.1	PL3.hmm	244	41	218	197	2	157	1.60E-06	0.704545455
Chr03G0209.1	CBM13.hmm	490	55	139	233	7	189	1.90E-24	0.899470899
Chr01G1619.1	GH55.hmm	973	13	395	740	345	719	1.20E-120	0.505405405

Chr03G0205.1	CE1.hmm	572	131	207	227	13	94	2.80E-05	0.356828194
Chr03G0456.1	GH43.hmm	1046	732	1013	248	2	157	1.60E-06	0.704545455
Chr09G0066.1	GH16.hmm	255	61	251	189	19	189	1.90E-24	0.899470899
Chr04G0151.1	GH43.hmm	1046	732	1013	248	2	157	1.60E-06	0.704545455
Chr01G1086.1	AA3.hmm	623	35	597	618	88	402	2.90E-89	0.508090615
Chr07G1525.1	AA1.hmm	602	36	375	943	450	792	2.30E-90	0.362672322
Chr03G1153.1	GT57.hmm	599	75	305	233	7	189	1.90E-24	0.899470899
Chr01G0924.1	AA7.hmm	607	131	207	227	13	94	2.80E-05	0.356828194
Chr09G0990.1	GH3.hmm	774	113	335	216	4	215	9.60E-64	0.976851852
Chr01G1699.1	GH43.hmm	1046	732	1013	248	2	157	1.60E-06	0.704545455
Chr04G1051.1	GT2.hmm	238	7	153	220	2	157	1.60E-06	0.704545455
Chr07G1237.1	CE5.hmm	234	54	226	189	19	189	1.90E-24	0.899470899
Chr04G0268.1	GH109.hmm	376	10	128	126	1	255	3.90E-103	0.996078431
Chr03G0334.1	AA7.hmm	607	131	207	227	13	94	2.80E-05	0.356828194
Chr06G0107.1	GT1.hmm	429	31	420	382	11	381	2.60E-40	0.968586387
Chr07G0099.1	AA7.hmm	607	131	207	227	13	94	2.80E-05	0.356828194
Chr07G1166.1	CE5.hmm	234	54	226	189	19	189	1.90E-24	0.899470899
Chr05G1140.1	GH132.hmm	435	127	298	278	1	255	3.90E-103	0.996078431
Chr04G0875.1	AA7.hmm	607	131	207	227	13	94	2.80E-05	0.356828194
Chr04G0524.1	GH15.hmm	611	47	353	455	2	157	1.60E-06	0.704545455
Chr09G0694.1	CBM67.hmm	917	156	332	341	66	316	2.20E-24	0.73313783
Chr06G0652.1	CE10.hmm	2516	2171	2493	341	66	316	2.20E-24	0.73313783
Chr07G1133.1	GH11.hmm	214	40	276	248	2	157	1.60E-06	0.704545455
Chr01G0699.1	GT1.hmm	429	31	420	382	11	381	2.60E-40	0.968586387
Chr01G1770.1	CE15.hmm	397	44	433	375	1	255	3.90E-103	0.996078431
Chr01G0303.1	CBM67.hmm	917	156	332	341	66	316	2.20E-24	0.73313783

Chr07G1358.1	GH93.hmm	379	4	123	126	1	255	3.90E-103	0.996078431
Chr07G0080.1	AA7.hmm	607	131	207	227	13	94	2.80E-05	0.356828194
Chr04G1352.1	GH28.hmm	443	63	397	275	4	215	9.60E-64	0.976851852
Chr04G0230.1	AA7.hmm	607	131	207	227	13	94	2.80E-05	0.356828194
Chr05G1030.1	GH74.hmm	885	74	182	233	7	189	1.90E-24	0.899470899
Chr07G1455.1	GT31.hmm	446	118	302	255	1	255	3.90E-103	0.996078431
Chr01G1528.1	AA7.hmm	607	131	207	227	13	94	2.80E-05	0.356828194
Chr07G0379.1	GH81.hmm	868	188	861	622	12	343	3.00E-102	0.924581006
Chr09G0724.1	GH76.hmm	493	30	399	358	12	343	3.00E-102	0.924581006
Chr05G0384.1	CE10.hmm	2516	2171	2493	341	66	316	2.20E-24	0.73313783
Chr01G0835.1	AA7.hmm	607	131	207	227	13	94	2.80E-05	0.356828194
Chr07G1812.1	PL1.hmm	378	103	297	202	16	201	1.40E-45	0.915841584
Chr04G0733.1	AA2.hmm	317	60	264	255	1	255	3.90E-103	0.996078431
Chr03G0605.1	GH13.hmm	539	59	365	342	1	255	3.90E-103	0.996078431
Chr07G0624.1	GH53.hmm	386	98	333	257	29	292	1.40E-54	0.888513514
Chr03G1290.1	CE10.hmm	2516	2171	2493	341	66	316	2.20E-24	0.73313783
Chr07G1125.1	CE10.hmm	2516	2171	2493	341	66	316	2.20E-24	0.73313783
Chr07G0517.1	CBM24.hmm	961	27	298	278	1	255	3.90E-103	0.996078431
Chr07G0877.1	GT4.hmm	478	213	348	160	6	143	2.30E-11	0.497797357
Chr05G0209.1	PL1.hmm	378	103	297	202	16	201	1.40E-45	0.915841584
Chr07G1175.1	GT90.hmm	456	146	320	250	2	157	1.60E-06	0.704545455
Chr05G0191.1	GH7.hmm	746	26	399	358	12	343	3.00E-102	0.924581006
Chr05G0603.1	GH13.hmm	539	59	365	342	1	255	3.90E-103	0.996078431
Chr01G1135.1	GT31.hmm	446	118	302	255	1	255	3.90E-103	0.996078431
Chr03G1620.1	GH28.hmm	443	63	397	275	4	215	9.60E-64	0.976851852
Chr01G2524.1	AA2.hmm	317	60	264	255	1	255	3.90E-103	0.996078431

Chr01G0626.1	AA9.hmm	185	7	153	220	2	157	1.60E-06	0.704545455
Chr07G1561.1	CE10.hmm	2516	2171	2493	341	66	316	2.20E-24	0.73313783
Chr01G0084.1	GH7.hmm	746	26	399	358	12	343	3.00E-102	0.924581006
Chr04G0610.1	GH76.hmm	493	30	399	358	12	343	3.00E-102	0.924581006
Chr01G0476.1	CE10.hmm	2516	2171	2493	341	66	316	2.20E-24	0.73313783
Chr04G1189.1	GH2.hmm	852	5	227	220	2	157	1.60E-06	0.704545455
Chr06G0443.1	GH132.hmm	435	127	298	278	1	255	3.90E-103	0.996078431
Chr04G1535.1	GT2.hmm	238	7	153	220	2	157	1.60E-06	0.704545455
Chr07G1417.1	GH3.hmm	774	113	335	216	4	215	9.60E-64	0.976851852
Chr01G1404.1	CE5.hmm	234	54	226	189	19	189	1.90E-24	0.899470899
Chr01G0344.1	GT2.hmm	238	7	153	220	2	157	1.60E-06	0.704545455
Chr03G0374.1	CE10.hmm	2516	2171	2493	341	66	316	2.20E-24	0.73313783
Chr03G0548.1	PL4.hmm	669	6	551	618	88	402	2.90E-89	0.508090615
Chr01G0079.1	GT1.hmm	429	31	420	382	11	381	2.60E-40	0.968586387
Chr05G0227.1	GH76.hmm	493	30	399	358	12	343	3.00E-102	0.924581006
Chr01G1496.1	GH28.hmm	443	63	397	275	4	215	9.60E-64	0.976851852
Chr04G0352.1	CE3.hmm	247	8	190	194	1	255	3.90E-103	0.996078431
Chr01G2454.1	CE10.hmm	2516	2171	2493	341	66	316	2.20E-24	0.73313783
Chr07G0920.1	GH43.hmm	1046	732	1013	248	2	157	1.60E-06	0.704545455
Chr05G0167.1	GH3.hmm	774	113	335	216	4	215	9.60E-64	0.976851852
Chr06G1327.1	GH78.hmm	729	88	668	504	9	290	1.60E-88	0.955782313
Chr05G0605.1	GH18.hmm	406	43	376	296	29	292	1.40E-54	0.888513514
Chr01G2145.1	GH76.hmm	493	30	399	358	12	343	3.00E-102	0.924581006
Chr01G1012.1	AA9.hmm	185	7	153	220	2	157	1.60E-06	0.704545455
Chr07G0802.1	CBM18.hmm	1432	492	537	382	11	381	2.60E-40	0.968586387
Chr01G1921.1	GH105.hmm	380	38	580	446	1	255	3.90E-103	0.996078431

Chr01G2157.1	GH3.hmm	774	113	335	216	4	215	9.60E-64	0.976851852
Chr01G1524.1	GH47.hmm	709	183	676	446	1	255	3.90E-103	0.996078431
Chr01G1578.1	GH131.hmm	377	22	264	255	1	255	3.90E-103	0.996078431
Chr07G1586.1	GH13.hmm	539	59	365	342	1	255	3.90E-103	0.996078431
Chr06G0883.1	GT15.hmm	422	65	359	341	66	316	2.20E-24	0.73313783
Chr09G0075.1	GH3.hmm	774	113	335	216	4	215	9.60E-64	0.976851852
Chr01G1426.1	CE1.hmm	572	131	207	227	13	94	2.80E-05	0.356828194
Chr09G0821.1	AA3.hmm	623	35	597	618	88	402	2.90E-89	0.508090615
Chr03G0376.1	PL3.hmm	244	41	218	197	2	157	1.60E-06	0.704545455
Chr01G0956.1	AA7.hmm	607	131	207	227	13	94	2.80E-05	0.356828194
Chr08G0496.1	CE10.hmm	2516	2171	2493	341	66	316	2.20E-24	0.73313783
Chr04G0608.1	CE16.hmm	292	28	287	189	19	189	1.90E-24	0.899470899
Chr04G1528.1	GH105.hmm	380	38	580	446	1	255	3.90E-103	0.996078431
Chr01G0263.1	GH11.hmm	214	40	276	248	2	157	1.60E-06	0.704545455
Chr08G0106.1	AA7.hmm	607	131	207	227	13	94	2.80E-05	0.356828194
Chr07G1782.1	GH43.hmm	1046	732	1013	248	2	157	1.60E-06	0.704545455
Chr07G0293.1	AA7.hmm	607	131	207	227	13	94	2.80E-05	0.356828194
Chr03G0243.1	CE10.hmm	2516	2171	2493	341	66	316	2.20E-24	0.73313783
Chr09G0751.1	GH109.hmm	376	10	128	126	1	255	3.90E-103	0.996078431
Chr08G0693.1	AA7.hmm	607	131	207	227	13	94	2.80E-05	0.356828194
Chr09G0991.1	PL1.hmm	378	103	297	202	16	201	1.40E-45	0.915841584
Chr07G0631.1	AA11.hmm	425	18	309	248	2	157	1.60E-06	0.704545455
Chr04G0951.1	PL3.hmm	244	41	218	197	2	157	1.60E-06	0.704545455
Chr01G1441.1	GT1.hmm	429	31	420	382	11	381	2.60E-40	0.968586387
Chr01G0209.1	AA7.hmm	607	131	207	227	13	94	2.80E-05	0.356828194
Chr04G1107.1	GH78.hmm	729	88	668	504	9	290	1.60E-88	0.955782313

Chr01G1468.1	CE16.hmm	292	28	287	189	19	189	1.90E-24	0.899470899
Chr01G0336.1	GH78.hmm	729	88	668	504	9	290	1.60E-88	0.955782313
Chr03G0059.1	CBM18.hmm	1432	492	537	382	11	381	2.60E-40	0.968586387
Chr08G0789.1	AA7.hmm	607	131	207	227	13	94	2.80E-05	0.356828194
Chr07G0298.1	CE12.hmm	266	33	244	210	1	255	3.90E-103	0.996078431
Chr07G0201.1	AA7.hmm	607	131	207	227	13	94	2.80E-05	0.356828194
Chr03G1233.1	CBM18.hmm	1432	492	537	382	11	381	2.60E-40	0.968586387
Chr07G1176.1	GH10.hmm	586	25	416	401	4	215	9.60E-64	0.976851852
Chr05G1355.1	GT20.hmm	903	159	612	475	33	111	3.30E-08	0.780487805
Chr03G1377.1	PL1.hmm	378	103	297	202	16	201	1.40E-45	0.915841584
Chr01G0555.1	CBM18.hmm	1432	492	537	382	11	381	2.60E-40	0.968586387
Chr07G0924.1	CE4.hmm	829	624	744	752	28	555	1.60E-97	0.700797872
Chr04G1516.1	GH43.hmm	1046	732	1013	248	2	157	1.60E-06	0.704545455
Chr09G0679.1	AA7.hmm	607	131	207	227	13	94	2.80E-05	0.356828194
Chr03G1571.1	CE8.hmm	408	52	363	288	5	264	1.10E-55	0.899305556
Chr09G0695.1	GH1.hmm	476	2	469	429	4	215	9.60E-64	0.976851852
Chr07G0891.1	GT34.hmm	313	68	493	458	11	381	2.60E-40	0.968586387
Chr07G0176.1	CE5.hmm	234	54	226	189	19	189	1.90E-24	0.899470899
Chr04G1304.1	AA7.hmm	607	131	207	227	13	94	2.80E-05	0.356828194
Chr07G1128.1	AA9.hmm	185	7	153	220	2	157	1.60E-06	0.704545455
Chr03G1498.1	CBM19.hmm	95	53	371	307	2	157	1.60E-06	0.704545455
Chr06G1392.1	AA1.hmm	602	36	375	943	450	792	2.30E-90	0.362672322
Chr03G1484.1	CE10.hmm	2516	2171	2493	341	66	316	2.20E-24	0.73313783
Chr03G1692.1	GH12.hmm	261	99	245	156	2	157	1.60E-06	0.704545455
Chr05G0074.1	AA7.hmm	607	131	207	227	13	94	2.80E-05	0.356828194
Chr04G0632.1	GT69.hmm	484	169	401	209	1	255	3.90E-103	0.996078431

Chr04G1267.1	GH28.hmm	443	63	397	275	4	215	9.60E-64	0.976851852
Chr06G1462.1	GT90.hmm	456	146	320	250	2	157	1.60E-06	0.704545455
Chr03G0031.1	CBM1.hmm	432	21	49	29	1	255	3.90E-103	0.996078431
Chr04G0295.1	GH3.hmm	774	113	335	216	4	215	9.60E-64	0.976851852
Chr03G1266.1	AA7.hmm	607	131	207	227	13	94	2.80E-05	0.356828194
Chr05G1314.1	GH32.hmm	678	77	220	341	66	316	2.20E-24	0.73313783
Chr01G0482.1	GH27.hmm	784	118	302	255	1	255	3.90E-103	0.996078431
Chr07G1716.1	GH72.hmm	427	16	410	373	1	255	3.90E-103	0.996078431
Chr01G2276.1	GH45.hmm	231	44	433	375	1	255	3.90E-103	0.996078431
Chr03G0651.1	GT22.hmm	516	6	551	618	88	402	2.90E-89	0.508090615
Chr01G1603.1	AA3.hmm	623	35	597	618	88	402	2.90E-89	0.508090615
Chr08G0724.1	GH18.hmm	406	43	376	296	29	292	1.40E-54	0.888513514
Chr07G0554.1	GT68.hmm	537	342	475	475	33	111	3.30E-08	0.780487805
Chr01G0208.1	GH74.hmm	885	74	182	233	7	189	1.90E-24	0.899470899
Chr05G1067.1	CBM50.hmm	272	141	178	40	7	189	1.90E-24	0.899470899
Chr03G1563.1	GT32.hmm	440	101	185	90	24	64	0.00059	0.444444444
Chr01G1061.1	PL9.hmm	427	16	410	373	1	255	3.90E-103	0.996078431
Chr08G0944.1	GH105.hmm	380	38	580	446	1	255	3.90E-103	0.996078431
Chr09G0198.1	CBM1.hmm	432	21	49	29	1	255	3.90E-103	0.996078431
Chr09G0085.1	GH31.hmm	665	209	664	427	1	255	3.90E-103	0.996078431
Chr01G2672.1	GH43.hmm	1046	732	1013	248	2	157	1.60E-06	0.704545455
Chr04G0735.1	GT55.hmm	441	8	190	194	1	255	3.90E-103	0.996078431
Chr03G0390.1	PL1.hmm	378	103	297	202	16	201	1.40E-45	0.915841584
Chr01G1538.1	CE10.hmm	2516	2171	2493	341	66	316	2.20E-24	0.73313783
Chr01G1021.1	CE10.hmm	2516	2171	2493	341	66	316	2.20E-24	0.73313783
Chr01G0182.1	GH43.hmm	1046	732	1013	248	2	157	1.60E-06	0.704545455

Chr01G1737.1	GT26.hmm	286	51	625	491	2	157	1.60E-06	0.704545455
Chr01G2583.1	CBM18.hmm	1432	492	537	382	11	381	2.60E-40	0.968586387
Chr06G1167.1	GH5.hmm	443	63	397	275	4	215	9.60E-64	0.976851852
Chr01G1234.1	CE10.hmm	2516	2171	2493	341	66	316	2.20E-24	0.73313783
Chr07G0165.1	AA3.hmm	623	35	597	618	88	402	2.90E-89	0.508090615
Chr05G0397.1	GT22.hmm	516	6	551	618	88	402	2.90E-89	0.508090615
Chr01G0486.1	GH131.hmm	377	22	264	255	1	255	3.90E-103	0.996078431
Chr03G1404.1	GH28.hmm	443	63	397	275	4	215	9.60E-64	0.976851852
Chr08G0876.1	CBM13.hmm	490	55	139	233	7	189	1.90E-24	0.899470899
Chr07G1630.1	CE10.hmm	2516	2171	2493	341	66	316	2.20E-24	0.73313783
Chr05G0841.1	CE3.hmm	247	8	190	194	1	255	3.90E-103	0.996078431
Chr06G0367.1	GH17.hmm	289	29	285	189	19	189	1.90E-24	0.899470899
Chr04G0106.1	AA3.hmm	623	35	597	618	88	402	2.90E-89	0.508090615
Chr01G1610.1	CE10.hmm	2516	2171	2493	341	66	316	2.20E-24	0.73313783
Chr08G0830.1	AA7.hmm	607	131	207	227	13	94	2.80E-05	0.356828194
Chr08G0985.1	AA3.hmm	623	35	597	618	88	402	2.90E-89	0.508090615
Chr06G0144.1	PL3.hmm	244	41	218	197	2	157	1.60E-06	0.704545455
Chr01G1492.1	CBM1.hmm	432	21	49	29	1	255	3.90E-103	0.996078431
Chr04G0306.1	CE5.hmm	234	54	226	189	19	189	1.90E-24	0.899470899
Chr07G1847.1	GT31.hmm	446	118	302	255	1	255	3.90E-103	0.996078431
Chr06G0239.1	CE1.hmm	572	131	207	227	13	94	2.80E-05	0.356828194
Chr08G0479.1	GH35.hmm	1005	53	371	307	2	157	1.60E-06	0.704545455
Chr03G0610.1	CE10.hmm	2516	2171	2493	341	66	316	2.20E-24	0.73313783
Chr07G0534.1	GH43.hmm	1046	732	1013	248	2	157	1.60E-06	0.704545455
Chr01G1269.1	GH16.hmm	255	61	251	189	19	189	1.90E-24	0.899470899
Chr07G1731.1	AA7.hmm	607	131	207	227	13	94	2.80E-05	0.356828194

Chr06G1449.1	GH76.hmm	493	30	399	358	12	343	3.00E-102	0.924581006
Chr07G1161.1	GH95.hmm	796	39	788	722	19	189	1.90E-24	0.899470899
Chr03G0443.1	GH13.hmm	539	59	365	342	1	255	3.90E-103	0.996078431
Chr01G1654.1	CE10.hmm	2516	2171	2493	341	66	316	2.20E-24	0.73313783
Chr06G0913.1	GH63.hmm	825	595	806	570	319	565	1.20E-167	0.989417989
Chr08G0047.1	PL4.hmm	669	6	551	618	88	402	2.90E-89	0.508090615
Chr01G1977.1	GH93.hmm	379	4	123	126	1	255	3.90E-103	0.996078431
Chr04G1488.1	AA1.hmm	602	36	375	943	450	792	2.30E-90	0.362672322
Chr09G0241.1	CE8.hmm	408	52	363	288	5	264	1.10E-55	0.899305556
Chr07G0946.1	CE10.hmm	2516	2171	2493	341	66	316	2.20E-24	0.73313783
Chr09G0980.1	GH13.hmm	539	59	365	342	1	255	3.90E-103	0.996078431
Chr06G0321.1	GH76.hmm	493	30	399	358	12	343	3.00E-102	0.924581006
Chr09G0773.1	GT62.hmm	480	96	359	341	66	316	2.20E-24	0.73313783
Chr06G1354.1	CE10.hmm	2516	2171	2493	341	66	316	2.20E-24	0.73313783
Chr01G0280.1	CE12.hmm	266	33	244	210	1	255	3.90E-103	0.996078431
Chr03G1558.1	AA7.hmm	607	131	207	227	13	94	2.80E-05	0.356828194
Chr07G0611.1	GH93.hmm	379	4	123	126	1	255	3.90E-103	0.996078431
Chr06G0497.1	AA5.hmm	1217	709	1211	1281	559	1039	3.20E-152	0.37470726
Chr05G0944.1	AA1.hmm	602	36	375	943	450	792	2.30E-90	0.362672322
Chr07G1788.1	CE4.hmm	829	624	744	752	28	555	1.60E-97	0.700797872
Chr05G1150.1	GH67.hmm	834	17	515	275	4	215	9.60E-64	0.976851852
Chr05G0536.1	GH16.hmm	255	61	251	189	19	189	1.90E-24	0.899470899
Chr07G0065.1	GH3.hmm	774	113	335	216	4	215	9.60E-64	0.976851852
Chr03G1431.1	GH109.hmm	376	10	128	126	1	255	3.90E-103	0.996078431
Chr08G0712.1	CE4.hmm	829	624	744	752	28	555	1.60E-97	0.700797872
Chr01G1343.1	GH109.hmm	376	10	128	126	1	255	3.90E-103	0.996078431

Chr07G0024.1	CBM18.hmm	1432	492	537	382	11	381	2.60E-40	0.968586387
Chr04G0263.1	GH28.hmm	443	63	397	275	4	215	9.60E-64	0.976851852
Chr01G1963.1	GH1.hmm	476	2	469	429	4	215	9.60E-64	0.976851852
Chr01G1983.1	GH109.hmm	376	10	128	126	1	255	3.90E-103	0.996078431
Chr01G2439.1	AA3.hmm	623	35	597	618	88	402	2.90E-89	0.508090615
Chr01G0168.1	CE16.hmm	292	28	287	189	19	189	1.90E-24	0.899470899
Chr07G0668.1	GT66.hmm	749	21	49	29	1	255	3.90E-103	0.996078431
Chr07G1151.1	CE10.hmm	2516	2171	2493	341	66	316	2.20E-24	0.73313783
Chr09G0020.1	AA9.hmm	185	7	153	220	2	157	1.60E-06	0.704545455
Chr03G1263.1	AA3.hmm	623	35	597	618	88	402	2.90E-89	0.508090615
Chr03G1683.1	GH109.hmm	376	10	128	126	1	255	3.90E-103	0.996078431
Chr07G1748.1	AA7.hmm	607	131	207	227	13	94	2.80E-05	0.356828194
Chr04G0720.1	CBM1.hmm	432	21	49	29	1	255	3.90E-103	0.996078431
Chr03G1664.1	CBM67.hmm	917	156	332	341	66	316	2.20E-24	0.73313783
Chr06G0206.1	AA7.hmm	607	131	207	227	13	94	2.80E-05	0.356828194
Chr05G0062.1	GT90.hmm	456	146	320	250	2	157	1.60E-06	0.704545455
Chr07G0323.1	GH5.hmm	443	63	397	275	4	215	9.60E-64	0.976851852
Chr05G1155.1	GT17.hmm	379	4	123	126	1	255	3.90E-103	0.996078431
Chr04G1110.1	CBM18.hmm	1432	492	537	382	11	381	2.60E-40	0.968586387
Chr07G1117.1	CE8.hmm	408	52	363	288	5	264	1.10E-55	0.899305556
Chr06G0660.1	GH78.hmm	729	88	668	504	9	290	1.60E-88	0.955782313
Chr07G0545.1	AA4.hmm	572	131	207	227	13	94	2.80E-05	0.356828194
Chr04G0696.1	PL1.hmm	378	103	297	202	16	201	1.40E-45	0.915841584
Chr03G0762.1	GH64.hmm	390	126	267	189	19	189	1.90E-24	0.899470899
Chr04G0946.1	CE10.hmm	2516	2171	2493	341	66	316	2.20E-24	0.73313783
Chr07G0182.1	AA3.hmm	623	35	597	618	88	402	2.90E-89	0.508090615

Chr06G1422.1	CE3.hmm	247	8	190	194	1	255	3.90E-103	0.996078431
Chr07G0929.1	GH15.hmm	611	47	353	455	2	157	1.60E-06	0.704545455
Chr04G1128.1	CE10.hmm	2516	2171	2493	341	66	316	2.20E-24	0.73313783
Chr04G0060.1	GH72.hmm	427	16	410	373	1	255	3.90E-103	0.996078431
Chr04G0745.1	AA7.hmm	607	131	207	227	13	94	2.80E-05	0.356828194
Chr06G1218.1	AA7.hmm	607	131	207	227	13	94	2.80E-05	0.356828194
Chr04G1419.1	CBM50.hmm	272	141	178	40	7	189	1.90E-24	0.899470899
Chr07G0101.1	GH43.hmm	1046	732	1013	248	2	157	1.60E-06	0.704545455
Chr07G1392.1	CE10.hmm	2516	2171	2493	341	66	316	2.20E-24	0.73313783
Chr04G1212.1	GT15.hmm	422	65	359	341	66	316	2.20E-24	0.73313783
Chr05G0967.1	CE10.hmm	2516	2171	2493	341	66	316	2.20E-24	0.73313783
Chr01G2529.1	GH125.hmm	540	112	271	257	29	292	1.40E-54	0.888513514
Chr07G1439.1	CE10.hmm	2516	2171	2493	341	66	316	2.20E-24	0.73313783
Chr08G0694.1	GH16.hmm	255	61	251	189	19	189	1.90E-24	0.899470899
Chr01G0115.1	GH3.hmm	774	113	335	216	4	215	9.60E-64	0.976851852
Chr07G0235.1	CBM50.hmm	272	141	178	40	7	189	1.90E-24	0.899470899
Chr01G2421.1	CE1.hmm	572	131	207	227	13	94	2.80E-05	0.356828194
Chr07G0868.1	GH78.hmm	729	88	668	504	9	290	1.60E-88	0.955782313
Chr04G1246.1	GT59.hmm	701	69	211	334	107	257	6.30E-06	0.449101796
Chr01G1300.1	GH125.hmm	540	112	271	257	29	292	1.40E-54	0.888513514
Chr03G0684.1	GH55.hmm	973	13	395	740	345	719	1.20E-120	0.505405405
Chr01G0056.1	PL3.hmm	244	41	218	197	2	157	1.60E-06	0.704545455
Chr05G1074.1	GH5.hmm	443	63	397	275	4	215	9.60E-64	0.976851852
Chr01G2497.1	PL3.hmm	244	41	218	197	2	157	1.60E-06	0.704545455
Chr01G1869.1	GH12.hmm	261	99	245	156	2	157	1.60E-06	0.704545455
Chr03G1709.1	AA9.hmm	185	7	153	220	2	157	1.60E-06	0.704545455

Chr07G0414.1	GH16.hmm	255	61	251	189	19	189	1.90E-24	0.899470899
Chr06G0231.1	AA3.hmm	623	35	597	618	88	402	2.90E-89	0.508090615
Chr04G0956.1	CE5.hmm	234	54	226	189	19	189	1.90E-24	0.899470899
Chr04G0127.1	CE1.hmm	572	131	207	227	13	94	2.80E-05	0.356828194
Chr05G0134.1	GH16.hmm	255	61	251	189	19	189	1.90E-24	0.899470899
Chr05G0709.1	GT62.hmm	480	96	359	341	66	316	2.20E-24	0.73313783
Chr07G1260.1	PL11.hmm	597	64	296	257	29	292	1.40E-54	0.888513514
Chr06G1102.1	GT17.hmm	379	4	123	126	1	255	3.90E-103	0.996078431
Chr04G1006.1	GH28.hmm	443	63	397	275	4	215	9.60E-64	0.976851852
Chr04G1585.1	AA3.hmm	623	35	597	618	88	402	2.90E-89	0.508090615
Chr08G0818.1	GH28.hmm	443	63	397	275	4	215	9.60E-64	0.976851852
Chr07G1595.1	CE1.hmm	572	131	207	227	13	94	2.80E-05	0.356828194
Chr08G0646.1	CE10.hmm	2516	2171	2493	341	66	316	2.20E-24	0.73313783
Chr09G0920.1	GH2.hmm	852	5	227	220	2	157	1.60E-06	0.704545455
Chr05G0274.1	AA7.hmm	607	131	207	227	13	94	2.80E-05	0.356828194
Chr03G1496.1	GH109.hmm	376	10	128	126	1	255	3.90E-103	0.996078431
Chr07G1314.1	AA7.hmm	607	131	207	227	13	94	2.80E-05	0.356828194
Chr03G1635.1	AA7.hmm	607	131	207	227	13	94	2.80E-05	0.356828194
Chr03G1188.1	GH35.hmm	1005	53	371	307	2	157	1.60E-06	0.704545455
Chr06G1451.1	GH29.hmm	501	25	416	401	4	215	9.60E-64	0.976851852
Chr03G1601.1	CE10.hmm	2516	2171	2493	341	66	316	2.20E-24	0.73313783
Chr03G1423.1	CE5.hmm	234	54	226	189	19	189	1.90E-24	0.899470899
Chr07G1385.1	GH92.hmm	791	277	776	491	2	157	1.60E-06	0.704545455
Chr03G1226.1	GH16.hmm	255	61	251	189	19	189	1.90E-24	0.899470899
Chr09G0239.1	AA3.hmm	623	35	597	618	88	402	2.90E-89	0.508090615
Chr01G0402.1	CE10.hmm	2516	2171	2493	341	66	316	2.20E-24	0.73313783

Chr01G1274.1	GH92.hmm	791	277	776	491	2	157	1.60E-06	0.704545455
Chr03G1565.1	CE5.hmm	234	54	226	189	19	189	1.90E-24	0.899470899
Chr04G0136.1	GH3.hmm	774	113	335	216	4	215	9.60E-64	0.976851852
Chr01G1027.1	AA11.hmm	425	18	309	248	2	157	1.60E-06	0.704545455
Chr01G1792.1	AA6.hmm	204	4	123	126	1	255	3.90E-103	0.996078431
Chr04G0292.1	CE10.hmm	2516	2171	2493	341	66	316	2.20E-24	0.73313783
Chr04G0773.1	CE10.hmm	2516	2171	2493	341	66	316	2.20E-24	0.73313783
Chr09G0707.1	CE8.hmm	408	52	363	288	5	264	1.10E-55	0.899305556
Chr04G0338.1	AA7.hmm	607	131	207	227	13	94	2.80E-05	0.356828194
Chr07G0406.1	GH7.hmm	746	26	399	358	12	343	3.00E-102	0.924581006
Chr01G0281.1	GH16.hmm	255	61	251	189	19	189	1.90E-24	0.899470899
Chr07G0270.1	AA3.hmm	623	35	597	618	88	402	2.90E-89	0.508090615
Chr06G1037.1	CE10.hmm	2516	2171	2493	341	66	316	2.20E-24	0.73313783
Chr01G2368.1	AA4.hmm	572	131	207	227	13	94	2.80E-05	0.356828194
Chr04G0231.1	AA7.hmm	607	131	207	227	13	94	2.80E-05	0.356828194
Chr07G1467.1	GH105.hmm	380	38	580	446	1	255	3.90E-103	0.996078431
Chr07G0367.1	AA3.hmm	623	35	597	618	88	402	2.90E-89	0.508090615
Chr01G0995.1	PL4.hmm	669	6	551	618	88	402	2.90E-89	0.508090615
Chr01G0631.1	CE10.hmm	2516	2171	2493	341	66	316	2.20E-24	0.73313783
Chr01G1495.1	GH78.hmm	729	88	668	504	9	290	1.60E-88	0.955782313
Chr06G1173.1	AA2.hmm	317	60	264	255	1	255	3.90E-103	0.996078431
Chr05G0341.1	PL1.hmm	378	103	297	202	16	201	1.40E-45	0.915841584
Chr01G0641.1	AA7.hmm	607	131	207	227	13	94	2.80E-05	0.356828194
Chr07G1577.1	AA7.hmm	607	131	207	227	13	94	2.80E-05	0.356828194
Chr01G0343.1	GH12.hmm	261	99	245	156	2	157	1.60E-06	0.704545455
Chr01G2680.1	AA2.hmm	317	60	264	255	1	255	3.90E-103	0.996078431

Chr06G0740.1	AA2.hmm	317	60	264	255	1	255	3.90E-103	0.996078431
Chr01G0452.1	GH28.hmm	443	63	397	275	4	215	9.60E-64	0.976851852
Chr01G1899.1	CE1.hmm	572	131	207	227	13	94	2.80E-05	0.356828194
Chr03G0244.1	GH109.hmm	376	10	128	126	1	255	3.90E-103	0.996078431
Chr03G0899.1	GT2.hmm	238	7	153	220	2	157	1.60E-06	0.704545455
Chr01G2247.1	GH47.hmm	709	183	676	446	1	255	3.90E-103	0.996078431
Chr05G1346.1	GH18.hmm	406	43	376	296	29	292	1.40E-54	0.888513514
Chr05G1384.1	GH47.hmm	709	183	676	446	1	255	3.90E-103	0.996078431
Chr04G0699.1	AA3.hmm	623	35	597	618	88	402	2.90E-89	0.508090615
Chr05G0245.1	GH16.hmm	255	61	251	189	19	189	1.90E-24	0.899470899
Chr01G0217.1	GH88.hmm	462	57	452	329	4	215	9.60E-64	0.976851852
Chr07G0441.1	GH35.hmm	1005	53	371	307	2	157	1.60E-06	0.704545455
Chr08G1047.1	CBM67.hmm	917	156	332	341	66	316	2.20E-24	0.73313783
Chr01G0251.1	CE4.hmm	829	624	744	752	28	555	1.60E-97	0.700797872
Chr01G1159.1	CE1.hmm	572	131	207	227	13	94	2.80E-05	0.356828194
Chr03G1345.1	CE5.hmm	234	54	226	189	19	189	1.90E-24	0.899470899
Chr07G1565.1	GH12.hmm	261	99	245	156	2	157	1.60E-06	0.704545455
Chr04G0137.1	PL1.hmm	378	103	297	202	16	201	1.40E-45	0.915841584
Chr08G0685.1	GH31.hmm	665	209	664	427	1	255	3.90E-103	0.996078431
Chr01G2739.1	GH43.hmm	1046	732	1013	248	2	157	1.60E-06	0.704545455
Chr03G0347.1	CE10.hmm	2516	2171	2493	341	66	316	2.20E-24	0.73313783
Chr01G2569.1	CE10.hmm	2516	2171	2493	341	66	316	2.20E-24	0.73313783
Chr01G0236.1	AA11.hmm	425	18	309	248	2	157	1.60E-06	0.704545455
Chr04G0794.1	AA8.hmm	797	5	227	220	2	157	1.60E-06	0.704545455
Chr01G2069.1	GH16.hmm	255	61	251	189	19	189	1.90E-24	0.899470899
Chr01G0543.1	PL1.hmm	378	103	297	202	16	201	1.40E-45	0.915841584

Chr01G0120.1	GH16.hmm	255	61	251	189	19	189	1.90E-24	0.899470899
Chr04G1536.1	GH16.hmm	255	61	251	189	19	189	1.90E-24	0.899470899
Chr07G0368.1	GH51.hmm	507	14	319	341	66	316	2.20E-24	0.73313783
Chr08G0833.1	CBM50.hmm	272	141	178	40	7	189	1.90E-24	0.899470899
Chr04G1119.1	GH127.hmm	640	9	550	524	1	255	3.90E-103	0.996078431
Chr06G0187.1	AA7.hmm	607	131	207	227	13	94	2.80E-05	0.356828194
Chr08G0799.1	CBM67.hmm	917	156	332	341	66	316	2.20E-24	0.73313783
Chr03G0051.1	GT15.hmm	422	65	359	341	66	316	2.20E-24	0.73313783
Chr07G1127.1	AA9.hmm	185	7	153	220	2	157	1.60E-06	0.704545455
Chr01G2517.1	CE10.hmm	2516	2171	2493	341	66	316	2.20E-24	0.73313783
Chr04G1282.1	CE10.hmm	2516	2171	2493	341	66	316	2.20E-24	0.73313783
Chr01G2014.1	GH31.hmm	665	209	664	427	1	255	3.90E-103	0.996078431
Chr07G1805.1	AA7.hmm	607	131	207	227	13	94	2.80E-05	0.356828194
Chr08G0194.1	CE4.hmm	829	624	744	752	28	555	1.60E-97	0.700797872
Chr09G0674.1	GH43.hmm	1046	732	1013	248	2	157	1.60E-06	0.704545455
Chr01G0026.1	CE10.hmm	2516	2171	2493	341	66	316	2.20E-24	0.73313783
Chr04G1370.1	PL4.hmm	669	6	551	618	88	402	2.90E-89	0.508090615
Chr05G1031.1	GH17.hmm	289	29	285	189	19	189	1.90E-24	0.899470899
Chr07G0096.1	AA2.hmm	317	60	264	255	1	255	3.90E-103	0.996078431
Chr01G1547.1	GT4.hmm	478	213	348	160	6	143	2.30E-11	0.497797357
Chr04G1586.1	GH54.hmm	482	221	266	40	7	189	1.90E-24	0.899470899
Chr01G2435.1	AA7.hmm	607	131	207	227	13	94	2.80E-05	0.356828194
Chr07G0232.1	GH35.hmm	1005	53	371	307	2	157	1.60E-06	0.704545455
Chr03G1495.1	GH109.hmm	376	10	128	126	1	255	3.90E-103	0.996078431
Chr09G0646.1	PL1.hmm	378	103	297	202	16	201	1.40E-45	0.915841584
Chr03G1472.1	GH10.hmm	586	25	416	401	4	215	9.60E-64	0.976851852

Chr07G0309.1	GH31.hmm	665	209	664	427	1	255	3.90E-103	0.996078431
Chr04G1411.1	AA3.hmm	623	35	597	618	88	402	2.90E-89	0.508090615
Chr01G1981.1	GH109.hmm	376	10	128	126	1	255	3.90E-103	0.996078431
Chr07G1190.1	GH10.hmm	586	25	416	401	4	215	9.60E-64	0.976851852
Chr01G2542.1	CE4.hmm	829	624	744	752	28	555	1.60E-97	0.700797872
Chr01G1401.1	CE10.hmm	2516	2171	2493	341	66	316	2.20E-24	0.73313783
Chr08G0036.1	AA12.hmm	463	25	416	401	4	215	9.60E-64	0.976851852
Chr01G2570.1	CE16.hmm	292	28	287	189	19	189	1.90E-24	0.899470899
Chr07G0291.1	GH5.hmm	443	63	397	275	4	215	9.60E-64	0.976851852
Chr03G0255.1	GH16.hmm	255	61	251	189	19	189	1.90E-24	0.899470899
Chr07G1518.1	GH3.hmm	774	113	335	216	4	215	9.60E-64	0.976851852
Chr06G1168.1	CE3.hmm	247	8	190	194	1	255	3.90E-103	0.996078431
Chr07G0382.1	AA7.hmm	607	131	207	227	13	94	2.80E-05	0.356828194
Chr05G0514.1	GH16.hmm	255	61	251	189	19	189	1.90E-24	0.899470899
Chr07G1746.1	GT1.hmm	429	31	420	382	11	381	2.60E-40	0.968586387
Chr07G0508.1	GH12.hmm	261	99	245	156	2	157	1.60E-06	0.704545455
Chr07G0568.1	CE16.hmm	292	28	287	189	19	189	1.90E-24	0.899470899
Chr01G2313.1	PL4.hmm	669	6	551	618	88	402	2.90E-89	0.508090615
Chr04G1351.1	AA7.hmm	607	131	207	227	13	94	2.80E-05	0.356828194
Chr03G0320.1	AA2.hmm	317	60	264	255	1	255	3.90E-103	0.996078431
Chr07G1275.1	GH5.hmm	443	63	397	275	4	215	9.60E-64	0.976851852
Chr01G0531.1	CE1.hmm	572	131	207	227	13	94	2.80E-05	0.356828194
Chr03G0235.1	GH95.hmm	796	39	788	722	19	189	1.90E-24	0.899470899
Chr06G0957.1	CBM18.hmm	1432	492	537	382	11	381	2.60E-40	0.968586387
Chr03G1378.1	PL1.hmm	378	103	297	202	16	201	1.40E-45	0.915841584
Chr07G1001.1	CE5.hmm	234	54	226	189	19	189	1.90E-24	0.899470899

Chr05G0600.1	CE10.hmm	2516	2171	2493	341	66	316	2.20E-24	0.73313783
Chr05G0095.1	CE5.hmm	234	54	226	189	19	189	1.90E-24	0.899470899
Chr01G1076.1	PL4.hmm	669	6	551	618	88	402	2.90E-89	0.508090615
Chr03G1553.1	GH55.hmm	973	13	395	740	345	719	1.20E-120	0.505405405
Chr08G0656.1	CE12.hmm	266	33	244	210	1	255	3.90E-103	0.996078431
Chr07G0947.1	CBM1.hmm	432	21	49	29	1	255	3.90E-103	0.996078431
Chr08G0566.1	GT39.hmm	738	63	397	275	4	215	9.60E-64	0.976851852
Chr01G1870.1	GT2.hmm	238	7	153	220	2	157	1.60E-06	0.704545455
Chr04G0824.1	CBM21.hmm	767	324	428	107	6	143	2.30E-11	0.497797357
Chr01G2627.1	CE10.hmm	2516	2171	2493	341	66	316	2.20E-24	0.73313783
Chr06G0049.1	PL17.hmm	204	4	123	126	1	255	3.90E-103	0.996078431
Chr08G0964.1	AA9.hmm	185	7	153	220	2	157	1.60E-06	0.704545455
Chr08G1048.1	GH5.hmm	443	63	397	275	4	215	9.60E-64	0.976851852
Chr09G0382.1	GT90.hmm	456	146	320	250	2	157	1.60E-06	0.704545455
Chr04G1398.1	GH81.hmm	868	188	861	622	12	343	3.00E-102	0.924581006
Chr03G1554.1	CBM18.hmm	1432	492	537	382	11	381	2.60E-40	0.968586387
Chr07G0995.1	GH12.hmm	261	99	245	156	2	157	1.60E-06	0.704545455
Chr07G0155.1	CBM50.hmm	272	141	178	40	7	189	1.90E-24	0.899470899
Chr01G0822.1	GH128.hmm	320	45	279	224	5	264	1.10E-55	0.899305556
Chr09G0540.1	GH55.hmm	973	13	395	740	345	719	1.20E-120	0.505405405
Chr07G1825.1	GH5.hmm	443	63	397	275	4	215	9.60E-64	0.976851852
Chr03G0126.1	GH10.hmm	586	25	416	401	4	215	9.60E-64	0.976851852
Chr09G0998.1	AA7.hmm	607	131	207	227	13	94	2.80E-05	0.356828194
Chr06G1174.1	AA5.hmm	1217	709	1211	1281	559	1039	3.20E-152	0.37470726
Chr07G1672.1	GT4.hmm	478	213	348	160	6	143	2.30E-11	0.497797357
Chr08G0654.1	GT21.hmm	538	128	376	296	29	292	1.40E-54	0.888513514

Chr03G0496.1	GT22.hmm	516	6	551	618	88	402	2.90E-89	0.508090615
Chr01G2102.1	PL9.hmm	427	16	410	373	1	255	3.90E-103	0.996078431
Chr05G0172.1	CE10.hmm	2516	2171	2493	341	66	316	2.20E-24	0.73313783
Chr09G0989.1	CE3.hmm	247	8	190	194	1	255	3.90E-103	0.996078431
Chr07G1652.1	GH71.hmm	487	44	433	375	1	255	3.90E-103	0.996078431
Chr09G0916.1	AA7.hmm	607	131	207	227	13	94	2.80E-05	0.356828194
Chr08G0843.1	GH55.hmm	973	13	395	740	345	719	1.20E-120	0.505405405
Chr01G1338.1	GH16.hmm	255	61	251	189	19	189	1.90E-24	0.899470899
Chr07G0048.1	AA1.hmm	602	36	375	943	450	792	2.30E-90	0.362672322
Chr01G1840.1	GT50.hmm	1311	133	344	458	11	381	2.60E-40	0.968586387
Chr07G1339.1	GH43.hmm	1046	732	1013	248	2	157	1.60E-06	0.704545455
Chr06G0217.1	AA7.hmm	607	131	207	227	13	94	2.80E-05	0.356828194
Chr04G1122.1	GT22.hmm	516	6	551	618	88	402	2.90E-89	0.508090615
Chr06G1213.1	AA7.hmm	607	131	207	227	13	94	2.80E-05	0.356828194
Chr05G0107.1	PL1.hmm	378	103	297	202	16	201	1.40E-45	0.915841584
Chr07G1497.1	CBM50.hmm	272	141	178	40	7	189	1.90E-24	0.899470899
Chr01G2544.1	AA9.hmm	185	7	153	220	2	157	1.60E-06	0.704545455
Chr03G1132.1	AA4.hmm	572	131	207	227	13	94	2.80E-05	0.356828194
Chr05G0364.1	GH133.hmm	1568	1090	1545	372	6	143	2.30E-11	0.497797357
Chr01G0630.1	AA3.hmm	623	35	597	618	88	402	2.90E-89	0.508090615
Chr03G0455.1	AA7.hmm	607	131	207	227	13	94	2.80E-05	0.356828194
Chr05G0948.1	GH16.hmm	255	61	251	189	19	189	1.90E-24	0.899470899
Chr07G0530.1	GH2.hmm	852	5	227	220	2	157	1.60E-06	0.704545455
Chr06G1455.1	AA8.hmm	797	5	227	220	2	157	1.60E-06	0.704545455
Chr04G0985.1	GH10.hmm	586	25	416	401	4	215	9.60E-64	0.976851852
Chr04G0453.1	CE1.hmm	572	131	207	227	13	94	2.80E-05	0.356828194

Chr08G0862.1	GT34.hmm	313	68	493	458	11	381	2.60E-40	0.968586387
Chr07G0187.1	GH29.hmm	501	25	416	401	4	215	9.60E-64	0.976851852
Chr01G2012.1	GH18.hmm	406	43	376	296	29	292	1.40E-54	0.888513514
Chr07G0881.1	GT24.hmm	1481	1165	1412	248	2	157	1.60E-06	0.704545455
Chr01G1140.1	GH13.hmm	539	59	365	342	1	255	3.90E-103	0.996078431
Chr04G0957.1	GH28.hmm	443	63	397	275	4	215	9.60E-64	0.976851852
Chr07G1136.1	AA7.hmm	607	131	207	227	13	94	2.80E-05	0.356828194
Chr07G1589.1	CE10.hmm	2516	2171	2493	341	66	316	2.20E-24	0.73313783
Chr07G0186.1	GT1.hmm	429	31	420	382	11	381	2.60E-40	0.968586387
Chr08G0087.1	GH5.hmm	443	63	397	275	4	215	9.60E-64	0.976851852
Chr09G0579.1	GT2.hmm	238	7	153	220	2	157	1.60E-06	0.704545455
Chr03G1331.1	CE10.hmm	2516	2171	2493	341	66	316	2.20E-24	0.73313783
Chr01G0386.1	GH16.hmm	255	61	251	189	19	189	1.90E-24	0.899470899
Chr05G0952.1	GH3.hmm	774	113	335	216	4	215	9.60E-64	0.976851852
Chr08G0742.1	GT1.hmm	429	31	420	382	11	381	2.60E-40	0.968586387
Chr07G1426.1	GH3.hmm	774	113	335	216	4	215	9.60E-64	0.976851852
Chr01G2530.1	GT71.hmm	525	171	401	209	1	255	3.90E-103	0.996078431
Chr07G0271.1	GH3.hmm	774	113	335	216	4	215	9.60E-64	0.976851852
Chr01G0296.1	PL10.hmm	684	413	536	455	2	157	1.60E-06	0.704545455
Chr07G1169.1	CBM18.hmm	1432	492	537	382	11	381	2.60E-40	0.968586387
Chr04G1023.1	CE10.hmm	2516	2171	2493	341	66	316	2.20E-24	0.73313783
Chr04G0342.1	GH43.hmm	1046	732	1013	248	2	157	1.60E-06	0.704545455
Chr06G0807.1	GH109.hmm	376	10	128	126	1	255	3.90E-103	0.996078431
Chr01G1052.1	CBM67.hmm	917	156	332	341	66	316	2.20E-24	0.73313783
Chr04G0863.1	GH3.hmm	774	113	335	216	4	215	9.60E-64	0.976851852
Chr07G0415.1	CE8.hmm	408	52	363	288	5	264	1.10E-55	0.899305556

Chr03G0122.1	GH106.hmm	903	159	612	475	33	111	3.30E-08	0.780487805
Chr06G0193.1	CBM1.hmm	432	21	49	29	1	255	3.90E-103	0.996078431
Chr01G0200.1	CE1.hmm	572	131	207	227	13	94	2.80E-05	0.356828194
Chr05G0125.1	GH1.hmm	476	2	469	429	4	215	9.60E-64	0.976851852
Chr07G0255.1	AA7.hmm	607	131	207	227	13	94	2.80E-05	0.356828194
Chr07G1261.1	CE10.hmm	2516	2171	2493	341	66	316	2.20E-24	0.73313783
Chr06G0214.1	CE3.hmm	247	8	190	194	1	255	3.90E-103	0.996078431
Chr01G0554.1	GH55.hmm	973	13	395	740	345	719	1.20E-120	0.505405405
Chr01G0273.1	GH18.hmm	406	43	376	296	29	292	1.40E-54	0.888513514
Chr01G1542.1	CBM1.hmm	432	21	49	29	1	255	3.90E-103	0.996078431
Chr07G0699.1	GH109.hmm	376	10	128	126	1	255	3.90E-103	0.996078431
Chr04G0248.1	AA3.hmm	623	35	597	618	88	402	2.90E-89	0.508090615
Chr01G0991.1	CE10.hmm	2516	2171	2493	341	66	316	2.20E-24	0.73313783
Chr01G2177.1	GT1.hmm	429	31	420	382	11	381	2.60E-40	0.968586387
Chr09G0194.1	CBM48.hmm	582	29	285	189	19	189	1.90E-24	0.899470899
Chr01G2582.1	CBM50.hmm	272	141	178	40	7	189	1.90E-24	0.899470899
Chr03G0533.1	GH131.hmm	377	22	264	255	1	255	3.90E-103	0.996078431
Chr03G0323.1	GT2.hmm	238	7	153	220	2	157	1.60E-06	0.704545455
Chr06G1364.1	GH17.hmm	289	29	285	189	19	189	1.90E-24	0.899470899
Chr04G0841.1	GH7.hmm	746	26	399	358	12	343	3.00E-102	0.924581006
Chr05G0598.1	GH15.hmm	611	47	353	455	2	157	1.60E-06	0.704545455
Chr09G0945.1	CE1.hmm	572	131	207	227	13	94	2.80E-05	0.356828194
Chr08G0161.1	CE1.hmm	572	131	207	227	13	94	2.80E-05	0.356828194
Chr05G0400.1	GH13.hmm	539	59	365	342	1	255	3.90E-103	0.996078431
Chr03G1678.1	GH43.hmm	1046	732	1013	248	2	157	1.60E-06	0.704545455
Chr04G0071.1	CBM1.hmm	432	21	49	29	1	255	3.90E-103	0.996078431

Chr08G0993.1	PL1.hmm	378	103	297	202	16	201	1.40E-45	0.915841584
Chr03G0523.1	GT1.hmm	429	31	420	382	11	381	2.60E-40	0.968586387
Chr03G0119.1	GH75.hmm	285	29	285	189	19	189	1.90E-24	0.899470899
Chr01G1444.1	CE10.hmm	2516	2171	2493	341	66	316	2.20E-24	0.73313783
Chr01G0207.1	AA3.hmm	623	35	597	618	88	402	2.90E-89	0.508090615
Chr07G0575.1	CE4.hmm	829	624	744	752	28	555	1.60E-97	0.700797872
Chr06G0775.1	AA7.hmm	607	131	207	227	13	94	2.80E-05	0.356828194
Chr07G0846.1	GH17.hmm	289	29	285	189	19	189	1.90E-24	0.899470899
Chr01G0981.1	CE3.hmm	247	8	190	194	1	255	3.90E-103	0.996078431
Chr04G1584.1	GT23.hmm	453	40	276	248	2	157	1.60E-06	0.704545455
Chr07G1842.1	CE10.hmm	2516	2171	2493	341	66	316	2.20E-24	0.73313783
Chr07G1404.1	GH18.hmm	406	43	376	296	29	292	1.40E-54	0.888513514
Chr03G0525.1	GH5.hmm	443	63	397	275	4	215	9.60E-64	0.976851852
Chr06G0808.1	GT8.hmm	386	98	333	257	29	292	1.40E-54	0.888513514
Chr07G0634.1	GT69.hmm	484	169	401	209	1	255	3.90E-103	0.996078431
Chr01G0034.1	GH47.hmm	572	102	564	446	2	446	3.10E-162	0.995515695
Chr01G0112.1	GT20.hmm	998	143	675	475	8	471	5.30E-152	0.974736842
Chr01G0179.1	PL27.hmm	709	57	697	610	2	610	2.90E-200	0.996721311
Chr01G0775.1	AA1.hmm	580	79	478	358	10	353	8.00E-21	0.958100559
Chr01G1821.1	GH154.hmm	1163	527	841	348	65	347	1.10E-109	0.810344828
Chr01G1954.1	GH43_24.hmm	450	46	279	246	2	245	3.80E-81	0.987804878
Chr01G2555.1	AA16.hmm	1186	1040	1180	167	25	167	1.80E-50	0.850299401
Chr01G2719.1	AA1.hmm	658	93	620	358	9	357	4.20E-48	0.972067039
Chr01G2720.1	AA1.hmm	592	39	554	358	7	356	2.70E-71	0.974860335
Chr02G0025.1	GH55_2.hmm	1821	583	1339	740	3	725	1.50E-280	0.975675676
Chr02G0044.1	GT69.hmm	528	121	399	239	1	238	1.80E-73	0.991631799

Chr02G0047.1	GT1.hmm	439	79	431	382	41	375	2.00E-28	0.87434555
Chr02G0048.1	AA1.hmm	1135	54	554	358	5	358	6.30E-86	0.98603352
Chr02G0054.1	GT76.hmm	440	16	439	407	4	407	8.10E-111	0.99017199
Chr02G0065.1	GH3.hmm	766	87	301	216	3	216	6.00E-56	0.986111111
Chr02G0066.1	CE3.hmm	239	32	225	194	1	194	1.50E-51	0.994845361
Chr02G0080.1	AA7.hmm	454	40	230	458	14	210	8.50E-48	0.427947598
Chr02G0081.1	GT15.hmm	548	40	352	273	3	270	1.90E-60	0.978021978
Chr02G0103.1	AA7.hmm	542	110	536	458	8	458	1.20E-72	0.982532751
Chr02G0120.1	CE16.hmm	360	35	325	267	2	263	2.50E-60	0.97752809
Chr02G0129.1	GH179.hmm	397	42	223	338	13	184	4.40E-17	0.50591716
Chr02G0151.1	AA1_3.hmm	598	62	377	312	6	305	2.90E-109	0.958333333
Chr02G0157.1	AA8.hmm	291	51	228	182	1	180	1.30E-41	0.983516484
Chr02G0182.1	AA3_2.hmm	592	27	589	568	2	567	7.30E-168	0.99471831
Chr02G0201.1	AA7.hmm	407	139	322	458	19	196	9.30E-39	0.386462882
Chr02G0206.1	AA3_2.hmm	582	5	579	568	2	567	3.50E-164	0.99471831
Chr02G0252.1	CE5.hmm	233	27	231	189	2	189	4.60E-41	0.989417989
Chr02G0255.1	AA7.hmm	496	38	471	458	10	450	3.00E-68	0.96069869
Chr02G0270.1	AA3_2.hmm	485	1	482	568	70	567	1.20E-110	0.875
Chr02G0291.1	GH5_9.hmm	713	346	669	303	1	303	2.20E-102	0.99669967
Chr02G0308.1	GH3.hmm	775	113	335	216	4	215	2.30E-63	0.976851852
Chr02G0309.1	GH31_3.hmm	764	256	696	429	1	429	1.10E-193	0.997668998
Chr02G0310.1	GH3.hmm	1003	158	382	216	10	215	1.00E-48	0.949074074
Chr02G0311.1	CE9.hmm	440	16	410	373	1	365	3.60E-116	0.975871314
Chr02G0367.1	AA3_2.hmm	608	45	605	568	2	561	4.20E-143	0.98415493
Chr02G0379.1	GH81.hmm	869	188	861	622	12	622	5.80E-220	0.980707395
Chr02G0401.1	GH13_1.hmm	540	58	365	306	1	306	6.30E-118	0.996732026

Chr02G0402.1	GH13_22.hmm	2342	101	503	400	1	400	2.20E-209	0.9975
Chr02G0406.1	GH7.hmm	462	21	453	415	1	414	1.30E-190	0.995180723
Chr02G0409.1	PL1.hmm	367	127	298	202	32	201	2.80E-48	0.836633663
Chr02G0423.1	AA4.hmm	564	22	269	522	2	217	4.10E-89	0.411877395
Chr02G0429.1	GH11.hmm	228	43	221	177	1	176	2.00E-70	0.988700565
Chr02G0446.1	GT61.hmm	283	84	205	119	4	117	1.10E-24	0.949579832
Chr02G0482.1	GH18.hmm	427	38	386	296	5	285	1.70E-79	0.945945946
Chr02G0497.1	CE3.hmm	1249	1	154	194	37	194	7.70E-35	0.809278351
Chr02G0500.1	GH3.hmm	879	73	284	216	6	216	4.90E-67	0.972222222
