# Supplementary material for: Decoding Pecan’s Fungal Foe: A Genomic Insight into Colletotrichum plurivorum Isolate W-6
Source: J Fungi (Basel). 2025 Mar 5;11(3):203. doi: 10.3390/jof11030203 (PMC11943440; doi:10.3390/jof11030203)
Supplement: Supplementary file 1 [file jof-11-00203-s001.zip › Table S22.pdf]

Table S22.Information of TCDB transportors in isolate W-6 genome.

Query	Best_Hit	Q.start	Q.end	S.start	S.end	Alignment _Length	Identity	Bit_Score	E-value	tcdb_ classification	Hit_Description
Chr01G0050.1	gnl|TC-DB|Q 12644 3.D.1.6.2	28	227	16	219	204	85.29%	358.607	4.87288e-128	3.D.1.6.2	NADH-ubiquinone oxidoreductase 23 kDa subunit, mitochondrial - Neurospora crassa.
Chr01G0051.1	gnl|TC-DB|P 11943 3.D.1.6.2	29	138	22	134	113	69.91%	151.369	6.82047e-49	3.D.1.6.2	Acyl carrier protein, mitochondrial - Neurospora crassa.
Chr01G0069.1	gnl|TC-DB|Q 7S1I2 3.D.1.6.2	1	114	1	115	115	73.04%	173.711	3.80988e-58	3.D.1.6.2	NADH:ubiquinone oxidoreductase 14kD subunit - Neurospora crassa.
Chr01G0117.1	gnl|TC-DB|P 40416 3.A.1.210.1	91	701	78	689	614	57.65%	714.531	0.0	3.A.1.210.1	Mitochondrial transporter ATM1 precursor - Saccharomyces cerevisiae (Baker's yeast).
Chr01G0376.1	gnl|TC-DB|Q 8J0U9 2.A.1.1.58	9	520	15	527	519	54.53%	566.614	0.0	2.A.1.1.58	Monosaccharide transporter - Aspergillus niger.
Chr01G0489.1	gnl|TC-DB|P 39986 3.A.3.10.1	26	1268	28	1209	1259	52.1%	1268.83	0.0	3.A.3.10.1	Probable cation-transporting ATPase 1 - Saccharomyces cerevisiae (Baker's yeast).
Chr01G0501.1	gnl|TC-DB|P 36046 3.A.8.1.1	147	219	276	348	73	75.34%	141.739	3.45743e-38	3.A.8.1.1	Intermembrane space import and assembly protein 40, mitochondrial - Saccharomyces cerevisiae (Baker's yeast).

Chr01G0714.1	gnl|TC-DB|P 39515 3.A.8.1.1	1	151	1	154	154	56.49%	171.4	4.46499e-56	3.A.8.1.1	Mitochondrial import inner membrane translocase subunit TIM17 (Mitochondrial protein import protein 2) (Mitochondrial inner membrane protein MIM17) - Saccharomyces cerevisiae (Baker's yeast).
Chr01G0901.1	gnl|TC-DB|P 25711 3.D.1.6.2	1	202	1	218	222	54.95%	225.713	6.52194e-76	3.D.1.6.2	NADH-ubiquinone oxidoreductase 21 kDa subunit, mitochondrial - Neurospora crassa.
Chr01G1175.1	gnl|TC-DB|Q 99297 2.A.29.2.5	2	296	5	304	302	58.61%	379.793	9.65912e-134	2.A.29.2.5	PUTATIVE MITOCHONDRIAL CARRIER YOR222W - Saccharomyces cerevisiae (Baker's yeast).
Chr01G1240.1	gnl|TC-DB|P 47818 2.A.89.1.1	78	269	96	288	196	52.55%	168.703	1.41872e-50	2.A.89.1.1	Protein CCC1 - Saccharomyces cerevisiae (Baker's yeast).
Chr01G1290.1	gnl|TC-DB|B 8N1Q6 1.A.55.4.1	1	149	1	149	149	58.39%	169.474	1.46414e-55	1.A.55.4.1	Clathrin-coated vesicle protein, putative OS=Aspergillus flavus (strain ATCC 200026 / FGSC A1120 / NRRL 3357 / JCM 12722 / SRRC 167) GN=AFLA_032100 PE=4 SV=1
Chr01G1348.1	gnl|TC-DB|P 15710	1	553	1	587	593	60.37%	736.102	0.0	2.A.20.2.1	PHOSPHATE-REPRESSIBLE PHOSPHATE PERMEASE -

	2.A.20.2.1										Neurospora crassa.
Chr01G1709.1	gnl|TC-DB|P 11636 2.A.1.1.7	1	519	1	523	523	71.13%	771.155	0.0	2.A.1.1.7	Quinate permease (Quinate transporter) - Neurospora crassa.
Chr01G1714.1	gnl|TC-DB|Q 07307 2.A.40.4.1	1	580	5	565	580	61.03%	697.967	0.0	2.A.40.4.1	URIC ACID-XANTHINE PERMEASE (UAPA TRANSPORTER) - Emericella nidulans (Aspergillus nidulans).
Chr01G1959.1	gnl|TC-DB|Q 5B2K4 2.A.96.1.3	47	299	50	298	254	55.12%	256.914	1.40582e-85	2.A.96.1.3	Putative uncharacterized protein (Acetate permease) OS=Emericella nidulans GN=acpA PE=4 SV=1
Chr01G2138.1	gnl|TC-DB|Q 9Y5S9 3.A.18.1.1	40	123	68	151	84	60.71%	114.775	1.44994e-33	3.A.18.1.1	RNA-binding protein 8A - Homo sapiens (Human).
Chr01G2154.1	gnl|TC-DB|P 32798 2.A.4.2.1	4	131	5	133	129	56.59%	146.747	3.44295e-39	2.A.4.2.1	COBALT UPTAKE PROTEIN COT1 - Saccharomyces cerevisiae (Baker's yeast).
Chr01G2217.1	gnl|TC-DB|P 08107 1.A.33.1.3	8	601	5	599	598	57.02%	675.626	0.0	1.A.33.1.3	Heat shock 70 kDa protein 1 (HSP70.1) (HSP70-1/HSP70-2) - Homo sapiens (Human).
Chr01G2345.1	gnl|TC-DB|B 1PXD0 2.A.39.2.4	9	516	8	508	509	57.76%	580.482	0.0	2.A.39.2.4	Cytosine-purine permease OS=Emericella nidulans GN=fcyB PE=2 SV=1
Chr01G2373.1	gnl|TC-DB|Q 99385	630	790	248	408	161	50.31%	181.03	1.27432e-50	2.A.19.2.2	CHROMOSOME IV READING FRAME ORF YDL128W -

<table>
<tr><th></th><th></th><th></th><th></th><th></th><th></th><th></th><th></th><th></th><th></th><th></th><th></th></tr>
<tr><td></td><td>2.A.19.2.2</td><td></td><td></td><td></td><td></td><td></td><td></td><td></td><td></td><td></td><td>Saccharomyces cerevisiae (Baker's yeast).</td></tr>
<tr><td>Chr05G0854.1</td><td>gnl|TC-DB|A9XIK8 1.A.56.1.10</td><td>34</td><td>183</td><td>33</td><td>182</td><td>150</td><td>65.33%</td><td>202.601</td><td>1.15919e-67</td><td>1.A.56.1.10</td><td>CTR2 long splice variant OS=Colletotrichum gloeosporioides f. sp. aeschynomenes GN=CTR2 PE=2 SV=1</td></tr>
<tr><td>Chr05G0804.1</td><td>gnl|TC-DB|Q9UUX9 3.A.3.2.6</td><td>12</td><td>1062</td><td>9</td><td>1025</td><td>1057</td><td>71.52%</td><td>1465.29</td><td>0.0</td><td>3.A.3.2.6</td><td>PUTATIVE CALCIUM P-TYPE ATPASE (FRAGMENT) - Neurospora crassa.</td></tr>
<tr><td>Chr05G0778.1</td><td>gnl|TC-DB|Q01356 2.A.29.9.1</td><td>5</td><td>331</td><td>15</td><td>362</td><td>348</td><td>64.37%</td><td>449.514</td><td>6.68384e-160</td><td>2.A.29.9.1</td><td>AMINO ACID TRANSPORTER ARG-13 - Neurospora crassa.</td></tr>
<tr><td>Chr05G0765.1</td><td>gnl|TC-DB|P07143 3.D.3.3.1</td><td>38</td><td>315</td><td>35</td><td>309</td><td>278</td><td>57.91%</td><td>343.199</td><td>4.97655e-119</td><td>3.D.3.3.1</td><td>Cytochrome C1, heme protein precursor - Saccharomyces cerevisiae (Baker's yeast).</td></tr>
<tr><td>Chr05G0666.1</td><td>gnl|TC-DB|P38919 3.A.18.1.1</td><td>13</td><td>396</td><td>25</td><td>411</td><td>387</td><td>67.18%</td><td>549.666</td><td>0.0</td><td>3.A.18.1.1</td><td>Eukaryotic initiation factor 4A-III - Homo sapiens (Human).</td></tr>
<tr><td>Chr05G0573.1</td><td>gnl|TC-DB|Q03015 3.D.1.6.2</td><td>1</td><td>101</td><td>1</td><td>101</td><td>101</td><td>63.37%</td><td>133.265</td><td>9.53835e-43</td><td>3.D.1.6.2</td><td>NADH-ubiquinone oxidoreductase 12 kDa subunit, mitochondrial - Neurospora crassa.</td></tr>
<tr><td>Chr05G0516.1</td><td>gnl|TC-DB|P38077 3.A.2.1.3</td><td>35</td><td>299</td><td>37</td><td>309</td><td>274</td><td>50.36%</td><td>266.929</td><td>3.04595e-89</td><td>3.A.2.1.3</td><td>ATP synthase gamma chain, mitochondrial precursor (EC 3.6.1.34) - Saccharomyces</td></tr>
</table>

											cerevisiae (Baker's yeast).
Chr05G0479.1	gnl|TC-DB|P07251 3.A.2.1.3	40	553	32	545	514	79.18%	854.358	0.0	3.A.2.1.3	ATP synthase alpha chain, mitochondrial precursor (EC 3.6.1.34) - Saccharomyces cerevisiae (Baker's yeast).
Chr05G0438.1	gnl|TC-DB|P22142 3.D.1.6.2	2	473	3	478	476	82.56%	819.305	0.0	3.D.1.6.2	NADH-ubiquinone oxidoreductase 49 kDa subunit, mitochondrial - Neurospora crassa.
Chr05G0433.1	gnl|TC-DB|P35724 1.A.35.2.2	577	852	653	967	316	51.27%	314.309	9.4355e-94	1.A.35.2.2	MANGANESE RESISTANCE PROTEIN - Saccharomyces cerevisiae (Baker's yeast).
Chr05G0399.1	gnl|TC-DB|P63144 8.A.5.1.3	7	341	67	389	335	50.45%	338.961	1.85692e-115	8.A.5.1.3	Voltage-gated potassium channel subunit beta-1 - Rattus norvegicus (Rat).
Chr05G0394.1	gnl|TC-DB|P40035 2.A.29.4.4	80	375	10	300	297	57.24%	339.732	8.97916e-117	2.A.29.4.4	Mitochondrial phosphate carrier protein 2 OS=Saccharomyces cerevisiae GN=PIC2 PE=1 SV=1
Chr05G0137.1	gnl|TC-DB|O74724 2.A.72.3.2	15	810	22	862	853	60.14%	1026.16	0.0	2.A.72.3.2	POTASSIUM TRANSPORTER - Neurospora crassa.
Chr05G0088.1	gnl|TC-DB|Q2U3D2 3.A.3.1.7	87	1097	40	1049	1021	61.31%	1299.26	0.0	3.A.3.1.7	Na+/K+ ATPase OS=Aspergillus oryzae GN=AO090038000088 PE=3 SV=1
Chr07G0146.1	gnl|TC-DB|P61326	8	154	5	146	147	68.71%	206.068	5.18225e-70	3.A.18.1.1	Protein mago nashi homolog - Homo sapiens (Human).

	3.A.18.1.1										
Chr07G0438.1	gnl|TC-DB|Q8J0V1 2.A.1.1.57	8	541	3	530	541	57.86%	615.15	0.0	2.A.1.1.57	Monosaccharide transporter - Aspergillus niger.
Chr07G0445.1	gnl|TC-DB|P43548 2.A.3.10.14	31	503	35	506	473	53.07%	515.383	1.76745e-179	2.A.3.10.14	General amino acid permease AGP3 - Saccharomyces cerevisiae (Baker's yeast).
Chr07G0733.1	gnl|TC-DB|Q8NKD5 1.A.11.3.3	36	464	22	450	429	63.64%	571.622	0.0	1.A.11.3.3	Ammonium transporter - Hebeloma cylindrosporum.
Chr07G0828.1	gnl|TC-DB|Q875B9 3.D.1.6.2	9	92	2	85	84	77.38%	134.806	9.35441e-43	3.D.1.6.2	Part of an hypothetical protein Pa5D0005 (Fragment) - Podospora anserina.
Chr07G0857.1	gnl|TC-DB|P25284 3.D.1.6.2	1	372	1	375	375	73.87%	590.497	0.0	3.D.1.6.2	NADH-ubiquinone oxidoreductase 40 kDa subunit, mitochondrial - Neurospora crassa.
Chr07G1181.1	gnl|TC-DB|Q8J0U9 2.A.1.1.58	12	510	15	524	510	50.59%	509.605	4.92658e-177	2.A.1.1.58	Monosaccharide transporter - Aspergillus niger.
Chr02G0828.1	gnl|TC-DB|Q9P8U8 9.A.10.1.3	16	321	15	318	306	50.33%	315.849	3.43584e-107	9.A.10.1.3	High-affinity iron permease CaFTR2 - Candida albicans (Yeast).
Chr02G0943.1	gnl|TC-DB|Q01519 3.D.4.8.1	15	86	11	82	72	63.89%	109.768	7.32575e-34	3.D.4.8.1	Cytochrome c oxidase polypeptide VIb (EC 1.9.3.1) (AED) - Saccharomyces

											cerevisiae (Baker's yeast).
Chr02G0957.1	gnl|TC-DB|Q541A5 3.A.16.1.1	20	212	13	203	194	51.03%	212.231	9.19803e-67	3.A.16.1.1	Ubiquitin fusion degradation 1-like - Homo sapiens (Human).
Chr02G0978.1	gnl|TC-DB|P24918 3.D.1.6.2	1	742	1	744	744	85.48%	1372.07	0.0	3.D.1.6.2	NADH-ubiquinone oxidoreductase 78 kDa subunit, mitochondrial - Neurospora crassa.
Chr02G1018.1	gnl|TC-DB|P08107 1.A.33.1.3	5	611	6	611	607	68.53%	875.544	0.0	1.A.33.1.3	Heat shock 70 kDa protein 1 (HSP70.1) (HSP70-1/HSP70-2) - Homo sapiens (Human).
Chr02G1113.1	gnl|TC-DB|P16140 3.A.2.2.3	9	503	20	517	504	80.95%	833.943	0.0	3.A.2.2.3	VACUOLAR ATP SYNTHASE SUBUNIT B (EC 3.6.1.34) (V-ATPASE B SUBUNIT) (VACUOLAR PROTON PUMP B SUBUNIT) (V-ATPASE 57 KDA SUBUNIT) - Saccharomyces cerevisiae (Baker's yeast).
Chr02G1163.1	gnl|TC-DB|P19516 3.D.4.8.1	70	257	79	259	188	56.91%	228.409	9.71673e-75	3.D.4.8.1	Cytochrome c oxidase assembly protein COX11, mitochondrial precursor - Saccharomyces cerevisiae (Baker's yeast).
Chr02G1225.1	gnl|TC-DB|P18239 2.A.29.1.7	15	311	23	318	297	75.76%	464.537	9.71369e-167	2.A.29.1.7	ADP,ATP carrier protein 2 - Saccharomyces cerevisiae (Baker's yeast).
Chr02G1386.1	gnl|TC-DB|P	2	783	5	812	822	55.96%	867.07	0.0	2.A.53.1.2	SULFATE PERMEASE II -

	23622										Neurospora crassa.
Chr02G1648.1	2.A.53.1.2 gnl|TC-DB|P 23641 2.A.29.4.3	34	322	17	306	290	60.0%	354.755	1.92745e-123	2.A.29.4.3	Mitochondrial phosphate carrier protein OS=Saccharomyces cerevisiae GN=MIR1 PE=1 SV=1
Chr02G1702.1	gnl|TC-DB|P 42839 2.A.19.7.1	925	1086	721	885	165	58.18%	198.749	7.5615e-53	2.A.19.7.1	Putative cation exchanger YNL321W - Saccharomyces cerevisiae (Baker's yeast).
Chr02G1753.1	gnl|TC-DB|P 32366 3.A.2.2.3	1	365	1	345	369	52.85%	403.29	1.9199e-141	3.A.2.2.3	VACUOLAR ATP SYNTHASE SUBUNIT D (EC 3.6.1.34) (V-ATPASE D SUBUNIT) (VACUOLAR PROTON PUMP D SUBUNIT) (V-ATPASE 39 KDA SUBUNIT) (V-ATPASE SUBUNIT M39) - Saccharomyces cerevisiae (Baker's yeast).
Chr08G0237.1	gnl|TC-DB|Q 13838 3.A.18.1.1	4	432	7	424	429	63.87%	577.4	0.0	3.A.18.1.1	Spliceosome RNA helicase BAT1 - Homo sapiens (Human).
Chr08G0449.1	gnl|TC-DB|P 32842 3.A.2.2.3	8	161	11	161	154	71.43%	215.698	2.31341e-73	3.A.2.2.3	PROTEOLIPID PROTEIN VMA11 - Saccharomyces cerevisiae (Baker's yeast).
Chr08G0539.1	gnl|TC-DB|Q 02854 3.D.1.6.2	3	189	1	188	188	63.83%	246.514	8.78999e-85	3.D.1.6.2	NADH-ubiquinone oxidoreductase 21 kDa subunit - Neurospora crassa.
Chr08G0543.1	gnl|TC-DB|Q	30	595	10	575	568	68.31%	796.193	0.0	2.A.1.40.1	Purine transporter - Emericella

	7Z8R3										nidulans (Aspergillus nidulans).
Chr08G0729.1	2.A.1.40.1 gnl|TC-DB|Q 9P8U8 9.A.10.1.3	16	329	15	324	316	50.32%	327.405	5.56737e-111	9.A.10.1.3	High-affinity iron permease CaFTR2 - Candida albicans (Yeast).
Chr08G0898.1	gnl|TC-DB|P 38919 3.A.18.1.1	1	401	7	411	405	77.78%	676.396	0.0	3.A.18.1.1	Eukaryotic initiation factor 4A-III - Homo sapiens (Human).
Chr08G0914.1	gnl|TC-DB|P 61011 3.A.5.9.1	1	419	1	423	423	58.87%	530.791	0.0	3.A.5.9.1	Signal recognition particle 54 kDa protein (SRP54) - Homo sapiens (Human).
Chr08G1002.1	gnl|TC-DB|O 47950 3.D.1.6.2	11	148	71	226	156	75.64%	246.514	5.53357e-85	3.D.1.6.2	NADH-ubiquinone oxidoreductase 19.3 kDa subunit, mitochondrial - Neurospora crassa.
Chr08G1011.1	gnl|TC-DB|B 5B9V9 3.A.3.9.5	42	1036	45	1054	1017	55.36%	1110.13	0.0	3.A.3.9.5	Na+ or K+ P-type ATPase OS=Ustilago maydis GN=ena1 PE=3 SV=1
Chr05G1041.1	gnl|TC-DB|P 19145 2.A.3.10.2	93	617	73	602	536	56.72%	560.451	0.0	2.A.3.10.2	General amino-acid permease GAP1 - Saccharomyces cerevisiae (Baker's yeast).
Chr05G1072.1	gnl|TC-DB|P 40260 1.A.11.3.1	32	455	14	436	425	52.0%	449.514	3.20738e-155	1.A.11.3.1	AMMONIUM TRANSPORTER MEP1 - Saccharomyces cerevisiae (Baker's yeast).
Chr05G1097.1	gnl|TC-DB|Q 9SY55	1	979	1	976	989	53.08%	986.482	0.0	3.A.3.2.19	3.A.3.2.19 Calcium-transporting ATPase 3, endoplasmic

	3.A.3.2.19										reticulum-type
Chr05G1171.1	gnl|TC-DB|P 32835 9.A.50.1.1	2	216	5	219	215	84.65%	376.326	1.37991e-134	9.A.50.1.1	GTP-binding nuclear protein GSP1/CNR1 OS=Saccharomyces cerevisiae GN=GSP1 PE=1 SV=1
Chr05G1307.1	gnl|TC-DB|Q 7RVX9 2.A.1.9.2	33	590	5	550	562	62.28%	709.909	0.0	2.A.1.9.2	Inorganic phosphate transporter PHO84 OS=Neurospora crassa GN=NCU08325 PE=4 SV=2
Chr03G0042.1	gnl|TC-DB|P 04037 3.D.4.8.1	142	259	31	147	118	55.93%	141.739	1.30217e-42	3.D.4.8.1	Cytochrome c oxidase polypeptide IV, mitochondrial precursor (EC 1.9.3.1) - Saccharomyces cerevisiae (Baker's yeast).
Chr03G0067.1	gnl|TC-DB|Q 6IT47 2.A.18.4.2	36	484	13	458	451	52.11%	445.277	2.55251e-154	2.A.18.4.2	Aromatic and neutral aliphatic amino acid permease - Penicillium chrysogenum (Penicillium notatum).
Chr03G0145.1	gnl|TC-DB|Q 99271 2.A.36.4.1	3	482	4	490	488	54.92%	560.451	0.0	2.A.36.4.1	NA(+)/H(+) ANTIPORTER - Saccharomyces cerevisiae (Baker's yeast).
Chr03G0228.1	gnl|TC-DB|P 11636 2.A.1.1.7	1	526	1	534	535	56.45%	595.504	0.0	2.A.1.1.7	Quinate permease (Quinate transporter) - Neurospora crassa.
Chr03G0307.1	gnl|TC-DB|P 39515 3.A.8.1.1	2	135	4	137	134	55.22%	150.984	5.80196e-48	3.A.8.1.1	Mitochondrial import inner membrane translocase subunit TIM17 (Mitochondrial protein

											import protein 2) (Mitochondrial inner membrane protein MIM17) - Saccharomyces cerevisiae (Baker's yeast).
Chr03G0434.1	gnl|TC-DB|Q8NJ22 2.A.1.1.33	73	536	82	555	477	51.57%	471.855	5.2944e-162	2.A.1.1.33	Hexose transporter (Similarity) - Kluyveromyces lactis (Yeast).
Chr03G0557.1	gnl|TC-DB|P25515 3.A.2.2.3	4	156	6	157	153	62.75%	181.8	4.29876e-60	3.A.2.2.3	VACUOLAR ATP SYNTHASE 16 KDA PROTEOLIPID SUBUNIT (EC 3.6.1.34) - Saccharomyces cerevisiae (Baker's yeast).
Chr03G0591.1	gnl|TC-DB|P25694 3.A.16.1.2	10	794	4	794	796	75.38%	1226.85	0.0	3.A.16.1.2	Cell division control protein 48 - Saccharomyces cerevisiae (Baker's yeast).
Chr03G0621.1	gnl|TC-DB|P42114 3.D.1.6.2	1	124	1	124	124	72.58%	202.216	2.97862e-69	3.D.1.6.2	NADH-ubiquinone oxidoreductase 14.8 kDa subunit - Neurospora crassa.
Chr03G0700.1	gnl|TC-DB|Q7RWU3 3.D.1.6.2	2	167	1	164	168	63.69%	216.853	8.28957e-74	3.D.1.6.2	NADH:ubiquinone oxidoreductase 18.4kD subunit - Neurospora crassa.
Chr03G0728.1	gnl|TC-DB|Q2VQZ4 2.A.3.10.22	57	580	16	536	525	64.0%	665.996	0.0	2.A.3.10.22	Putative amino acid permease OS=Glomus mosseae PE=2 SV=1
Chr03G0747.1	gnl|TC-DB|P32332 2.A.29.15.1	23	302	41	321	283	51.94%	303.523	2.3016e-103	2.A.29.15.1	MITOCHONDRIAL CARRIER PROTEIN PMT - Saccharomyces cerevisiae (Baker's yeast).

Chr03G0806.1	gnl|TC-DB|Q5A477 2.A.7.13.2	42	372	50	371	331	57.4%	353.214	5.11544e-121	2.A.7.13.2	2.A.7.13.2 GDP-mannose transporter
Chr03G0820.1	gnl|TC-DB|P35724 1.A.35.2.2	387	465	664	742	79	56.96%	100.523	1.75613e-22	1.A.35.2.2	MANGANESE RESISTANCE PROTEIN - Saccharomyces cerevisiae (Baker's yeast).
Chr03G0888.1	gnl|TC-DB|Q7RWT8 2.A.7.25.6	4	347	47	414	372	69.09%	488.419	3.08289e-174	2.A.7.25.6	Putative uncharacterized protein - Neurospora crassa.
Chr03G1065.1	gnl|TC-DB|Q07914 3.A.8.1.1	37	102	98	163	66	60.61%	96.6709	1.94512e-27	3.A.8.1.1	Chromosome XII reading frame ORF YLR008C - Saccharomyces cerevisiae (Baker's yeast).
Chr03G1121.1	gnl|TC-DB|O15258 9.B.82.1.2	44	206	27	184	164	60.98%	208.764	2.43087e-69	9.B.82.1.2	Protein RER1 - Homo sapiens (Human).
Chr03G1127.1	gnl|TC-DB|P28584 2.A.38.2.3	411	797	427	841	417	52.28%	426.402	9.47215e-136	2.A.38.2.3	Potassium transport protein, low-affinity - Saccharomyces cerevisiae (Baker's yeast).
Chr03G1181.1	gnl|TC-DB|B0Y3B6 3.A.1.201.11	65	1349	63	1348	1292	60.06%	1556.19	0.0	3.A.1.201.11	ABC multidrug transporter Mdr1 OS=Aspergillus fumigatus (strain CEA10 / CBS 144.89 / FGSC A1163) GN=AFUB_053630 PE=4 SV=1
Chr03G1207.1	gnl|TC-DB|P39524 3.A.3.8.2	240	1370	182	1311	1142	56.74%	1337.4	0.0	3.A.3.8.2	POTENTIAL PHOSPHOLIPID-TRANSPORTING ATPASE 1 (EC 3.6.3.13) -

											Saccharomyces cerevisiae (Baker's yeast).
Chr03G1252.1	gnl|TC-DB|Q12165 3.A.2.1.3	31	151	32	150	122	54.92%	131.724	4.5335e-36	3.A.2.1.3	ATP synthase delta chain, mitochondrial precursor (EC 3.6.1.34) - Saccharomyces cerevisiae (Baker's yeast).
Chr03G1491.1	gnl|TC-DB|P33413 2.A.21.6.1	5	533	6	532	529	51.8%	538.88	0.0	2.A.21.6.1	UREA ACTIVE TRANSPORTER - Saccharomyces cerevisiae (Baker's yeast).
Chr03G1637.1	gnl|TC-DB|P50276 2.A.3.8.4	3	536	12	555	546	50.18%	544.658	0.0	2.A.3.8.4	High affinity methionine permease - Saccharomyces cerevisiae (Baker's yeast).
Chr03G1712.1	gnl|TC-DB|P33303 2.A.29.13.1	16	310	11	305	298	56.71%	326.25	4.35864e-112	2.A.29.13.1	REGULATOR OF ACETYL-COA SYNTHETASE ACTIVITY - Saccharomyces cerevisiae (Baker's yeast).
Chr09G0009.1	gnl|TC-DB|P38680 2.A.18.4.1	13	475	6	469	470	53.19%	462.611	4.26482e-161	2.A.18.4.1	N amino acid transport system protein (Methyltryptophan resistance protein) - Neurospora crassa.
Chr09G0018.1	gnl|TC-DB|Q2MEV7 2.A.1.1.51	31	512	42	509	486	51.03%	468.003	1.15523e-161	2.A.1.1.51	Glucose/xylose symporter 1 - Candida intermedia.
Chr09G0381.1	gnl|TC-DB|O47950 3.D.1.6.2	1	222	1	226	226	77.43%	352.829	9.73408e-126	3.D.1.6.2	NADH-ubiquinone oxidoreductase 19.3 kDa subunit, mitochondrial - Neurospora

											crassa.
Chr09G0460.1	gnl|TC-DB|P 40915 3.D.1.6.2	1	261	1	263	268	70.52%	390.963	1.46245e-139	3.D.1.6.2	NADH-ubiquinone oxidoreductase 24 kDa subunit, mitochondrial - Neurospora crassa.
Chr09G0740.1	gnl|TC-DB|P 25515 3.A.2.2.3	3	161	2	160	159	69.81%	211.075	1.18376e-71	3.A.2.2.3	VACUOLAR ATP SYNTHASE 16 KDA PROTEOLIPID SUBUNIT (EC 3.6.1.34) - Saccharomyces cerevisiae (Baker's yeast).
Chr09G0828.1	gnl|TC-DB|P 39515 3.A.8.1.1	2	149	4	146	148	61.49%	182.185	2.46558e-60	3.A.8.1.1	Mitochondrial import inner membrane translocase subunit TIM17 (Mitochondrial protein import protein 2) (Mitochondrial inner membrane protein MIM17) - Saccharomyces cerevisiae (Baker's yeast).
Chr09G0892.1	gnl|TC-DB|Q 7RZ09 3.D.1.6.2	1	83	1	87	87	79.31%	137.887	3.87177e-45	3.D.1.6.2	Putative uncharacterized protein - Neurospora crassa.
Chr06G0086.1	gnl|TC-DB|P 23641 2.A.29.4.3	24	307	17	300	284	53.17%	296.975	1.48773e-100	2.A.29.4.3	Mitochondrial phosphate carrier protein OS=Saccharomyces cerevisiae GN=MIR1 PE=1 SV=1
Chr06G0132.1	gnl|TC-DB|P 04035 2.A.6.6.5	717	1137	455	872	424	56.37%	486.878	5.71699e-156	2.A.6.6.5	3-HYDROXY-3-METHYLGLUTA RYL-COENZYME A REDUCTASE (EC 1.1.1.34) (HMG-COA REDUCTASE) -

											Homo sapiens (Human).
Chr06G0329.1	gnl|TC-DB|P23968 3.A.2.2.3	16	198	31	213	183	62.3%	212.616	6.10867e-71	3.A.2.2.3	VACUOLAR ATP SYNTHASE 22 KDA PROTEOLIPID SUBUNIT (EC 3.6.1.34) - Saccharomyces cerevisiae (Baker's yeast).
Chr06G0333.1	gnl|TC-DB|A7LKG1 2.A.49.1.2	68	830	57	803	763	70.51%	1095.49	0.0	2.A.49.1.2	CLC voltage-gated chloride channel - Fusarium oxysporum f. sp. lycopersici.
Chr06G0639.1	gnl|TC-DB|Q12482 2.A.29.14.4	302	633	487	817	341	55.13%	363.999	1.79402e-114	2.A.29.14.4	Hypothetical protein YPR020C - Saccharomyces cerevisiae (Baker's yeast).
Chr06G0877.1	gnl|TC-DB|P36062 2.A.18.7.1	365	747	300	691	394	56.85%	403.29	5.14423e-131	2.A.18.7.1	Hypothetical 75.5 kDa protein in SDH1-CIM5/YTA3 intergenic region - Saccharomyces cerevisiae (Baker's yeast).
Chr06G0953.1	gnl|TC-DB|P38606 3.A.2.2.4	216	812	13	617	606	64.69%	817.379	0.0	3.A.2.2.4	Vacuolar ATP synthase catalytic subunit A - Homo sapiens (Human).
Chr06G1007.1	gnl|TC-DB|Q8X0V6 3.D.1.6.2	23	136	1	117	117	58.97%	137.117	1.94584e-43	3.D.1.6.2	Related to nadh-ubiquinone oxidoreductase subunit b17.2 - Neurospora crassa.
Chr06G1041.1	gnl|TC-DB|P11024 3.D.2.3.1	96	1106	50	1081	1038	52.5%	988.793	0.0	3.D.2.3.1	NAD(P) TRANSHYDROGENASE, MITOCHONDRIAL PRECURSOR (EC 1.6.1.2) (PYRIDINE NUCLEOTIDE

											TRANSHYDROGENASE) (NICOTINAMIDE NUCLEOTIDE TRANSHYDROGENASE) - Bos taurus (Bovine).
Chr06G1050.1	gnl|TC-DB|P39524 3.A.3.8.2	719	830	623	731	112	50.0%	116.701	1.24479e-26	3.A.3.8.2	POTENTIAL PHOSPHOLIPID-TRANSPORTING ATPASE 1 (EC 3.6.3.13) - Saccharomyces cerevisiae (Baker's yeast).
Chr06G1135.1	gnl|TC-DB|P61619 3.A.5.9.1	3	475	2	475	475	62.74%	606.29	0.0	3.A.5.9.1	Protein transport protein Sec61 alpha subunit isoform 1 (Sec61 alpha- 1) - Homo sapiens (Human).
Chr06G1290.1	gnl|TC-DB|Q07842 3.D.1.6.2	1	94	1	94	94	76.6%	159.844	2.04354e-53	3.D.1.6.2	NADH-ubiquinone oxidoreductase 10.5 kDa subunit - Neurospora crassa.
Chr06G1296.1	gnl|TC-DB|P30902 3.A.2.1.3	3	164	4	165	162	51.85%	179.489	8.25139e-58	3.A.2.1.3	ATP SYNTHASE D CHAIN, MITOCHONDRIAL (EC 3.6.1.34) - Saccharomyces cerevisiae (Baker's yeast).
Chr06G1437.1	gnl|TC-DB|B5B9V9 3.A.3.9.5	44	1033	48	1054	1013	56.27%	1143.64	0.0	3.A.3.9.5	Na+ or K+ P-type ATPase OS=Ustilago maydis GN=ena1 PE=3 SV=1
Chr04G0050.1	gnl|TC-DB|Q8NKD5 1.A.11.3.3	28	477	22	476	459	61.22%	573.933	0.0	1.A.11.3.3	Ammonium transporter - Hebeloma cylindrosporum.

Chr04G0079.1	gnl|TC-DB|P38360 3.A.3.5.14	316	1106	237	990	793	52.46%	799.275	0.0	3.A.3.5.14	Probable copper-transporting ATPase - Saccharomyces cerevisiae (Baker's yeast).
Chr04G0198.1	gnl|TC-DB|P25343 8.A.34.2.1	1	257	1	259	261	52.87%	283.878	3.38795e-97	8.A.34.2.1	Reduced viability upon starvation protein 161 - Saccharomyces cerevisiae (Baker's yeast).
Chr04G0439.1	gnl|TC-DB|Q7Z8R3 2.A.1.40.1	41	607	7	568	572	50.7%	566.614	0.0	2.A.1.40.1	Purine transporter - Emericella nidulans (Aspergillus nidulans).
Chr04G0464.1	gnl|TC-DB|Q9P6T9 3.D.1.6.2	1	103	1	104	104	61.54%	141.354	7.16058e-46	3.D.1.6.2	Putative uncharacterized protein 15E6.140 - Neurospora crassa.
Chr04G0494.1	gnl|TC-DB|P32610 3.A.2.2.3	1	255	1	256	258	67.83%	341.658	2.7798e-120	3.A.2.2.3	VACUOLAR ATP SYNTHASE SUBUNIT D (EC 3.6.1.34) (V-ATPASE D SUBUNIT) (VACUOLAR PROTON PUMP D SUBUNIT) - Saccharomyces cerevisiae (Baker's yeast).
Chr04G0551.1	gnl|TC-DB|P0A6Y8 1.A.33.1.2	46	646	2	600	606	56.93%	682.174	0.0	1.A.33.1.2	Chaperone protein dnaK - Escherichia coli.
Chr04G0654.1	gnl|TC-DB|P39111 3.A.2.2.3	10	120	5	118	114	64.04%	145.206	7.09512e-47	3.A.2.2.3	VACUOLAR ATP SYNTHASE SUBUNIT F (EC 3.6.1.34) (V-ATPASE F SUBUNIT) (VACUOLAR PROTON PUMP F SUBUNIT) (V-ATPASE 14 KDA

											SUBUNIT) - Saccharomyces cerevisiae (Baker's yeast).
Chr04G0807.1	gnl|TC-DB|P35724 1.A.35.2.2	422	559	810	947	138	52.17%	159.073	1.39369e-41	1.A.35.2.2	MANGANESE RESISTANCE PROTEIN - Saccharomyces cerevisiae (Baker's yeast).
Chr04G0886.1	gnl|TC-DB|Q99385 2.A.19.2.2	431	574	257	399	144	50.69%	158.303	1.92607e-43	2.A.19.2.2	CHROMOSOME IV READING FRAME ORF YDL128W - Saccharomyces cerevisiae (Baker's yeast).
Chr04G1150.1	gnl|TC-DB|P08067 1.A.24.1.1	44	235	24	215	193	63.73%	271.552	1.66637e-93	1.A.24.1.1	Ubiquinol-cytochrome C reductase iron-sulfur subunit, mitochondrial precursor (EC 1.10.2.2) (Rieske iron-sulfur protein) (RISP) - Saccharomyces cerevisiae (Baker's yeast).
Chr04G1229.1	gnl|TC-DB|Q5AZI1 2.A.7.25.7	21	646	15	567	627	52.79%	591.652	0.0	2.A.7.25.7	Putative uncharacterized protein OS=Emericella nidulans GN=AN6299.2 PE=4 SV=1
Chr04G1388.1	gnl|TC-DB|P78577 3.A.1.205.7	52	1426	53	1425	1379	68.96%	2014.96	0.0	3.A.1.205.7	AtrB - Aspergillus nidulans.
Chr04G1464.1	gnl|TC-DB|P21976 3.D.1.6.2	1	170	1	169	170	70.0%	252.292	2.20348e-87	3.D.1.6.2	NADH-ubiquinone oxidoreductase 20.8 kDa subunit - Neurospora crassa.
Chr04G1519.1	gnl|TC-DB|Q8J0V1	20	551	7	529	540	61.48%	664.455	0.0	2.A.1.1.57	Monosaccharide transporter - Aspergillus niger.

	2.A.1.1.57										
Chr02G0126.1	gnl|TC-DB|P15710 2.A.20.2.1	1	584	1	589	600	61.67%	743.806	0.0	2.A.20.2.1	PHOSPHATE-REPRESSIBLE PHOSPHATE PERMEASE - Neurospora crassa.
Chr02G0185.1	gnl|TC-DB|P24917 3.D.1.6.2	2	476	11	492	483	87.16%	878.241	0.0	3.D.1.6.2	NADH-ubiquinone oxidoreductase 51 kDa subunit, mitochondrial - Neurospora crassa.
Chr02G0244.1	gnl|TC-DB|P23710 3.D.1.6.2	22	272	24	270	252	77.38%	423.32	1.34552e-151	3.D.1.6.2	NADH-ubiquinone oxidoreductase 30.4 kDa subunit, mitochondrial - Neurospora crassa.
Chr02G0399.1	gnl|TC-DB|P08107 1.A.33.1.3	43	647	7	611	607	57.5%	727.628	0.0	1.A.33.1.3	Heat shock 70 kDa protein 1 (HSP70.1) (HSP70-1/HSP70-2) - Homo sapiens (Human).
Chr02G0605.1	gnl|TC-DB|P07038 3.A.3.3.1	3	922	4	920	921	80.46%	1465.29	0.0	3.A.3.3.1	PLASMA MEMBRANE ATPASE (EC 3.6.3.6) (PROTON PUMP) - Neurospora crassa.
Chr02G0609.1	gnl|TC-DB|Q04013 2.A.29.29.1	17	317	13	314	302	69.87%	436.032	1.25881e-155	2.A.29.29.1	Mitochondrial DNA replication protein YHM2 OS=Saccharomyces cerevisiae GN=YHM2 PE=1 SV=1
Chr02G0627.1	gnl|TC-DB|P38988 2.A.29.21.1	5	301	1	298	298	69.13%	427.557	1.06104e-152	2.A.29.21.1	Putative mitochondrial carrier protein YHM1/SHM1 - Saccharomyces cerevisiae (Baker's yeast).

Chr02G0645.1	gnl|TC-DB|P00830 3.A.2.1.3	47	499	44	496	453	84.11%	772.696	0.0	3.A.2.1.3	ATP SYNTHASE BETA CHAIN, MITOCHONDRIAL PRECURSOR (EC 3.6.1.34) - Saccharomyces cerevisiae (Baker's yeast).
Chr02G0669.1	gnl|TC-DB|O95166 1.A.9.5.2	1	116	1	116	116	59.48%	151.754	1.43625e-49	1.A.9.5.2	Gamma-aminobutyric acid receptor-associated protein - Homo sapiens (Human).
