# Supplementary material for: Decoding Pecan’s Fungal Foe: A Genomic Insight into Colletotrichum plurivorum Isolate W-6
Source: J Fungi (Basel). 2025 Mar 5;11(3):203. doi: 10.3390/jof11030203 (PMC11943440; doi:10.3390/jof11030203)
Supplement: Supplementary file 1 [file jof-11-00203-s001.zip › Table S23.pdf]

Table S23.Prediction of PHI proteins in isolate W-6 genome.

Query_id	Subject_id	E_value	Identity	Score	Subject_annotation
Chr01G0001.1	211	2.00E-28	73/188(38.83)	114	PHI:211|CaTUP1|AAB63195|5476|Candida_albicans|Reduced_virulence
Chr01G0008.1	1260	5.00E-26	80/235(34.04)	113	PHI:1260|FGSG_13944|I1RUC7|5518|Gibberella_zeae_(related:_Fusarium_graminearum)|Unaffected_pathogenicity
Chr01G0012.1	4194	9.00E-30	127/505(25.15)	119	AKT7|PHI:4194|V5XZS6|5599|Alternaria_alternata|increased_virulence_(Hyper virulence)
Chr01G0013.1	223	2.00E-49	96/261(36.78)	174	PHI:223|PEP1|AAK11166|140110|Nectria_haematococca_(related:_Fusarium_solani)|Reduced_virulence
Chr01G0014.1	1912	8.00E-102	232/672(34.52)	325	PHI:1912|GzZC227|I1RHF1|5518|Gibberella_zeae_(related:_Fusarium_graminearum)|Unaffected_pathogenicity
Chr01G0015.1	404	2.00E-33	112/442(25.34)	130	PHI:404|PTH11|AAD30436|318829|Magnaporthe_oryzae|Reduced_virulence
Chr01G0016.1	716	0	281/570(49.30)	580	PHI:716|ZEB1|ABB90284|5518|Fusarium_graminearum|Unaffected_pathogenicity
Chr01G0021.1	2171	0	485/698(69.48)	1051	PHI:2171|Peroxisomal_copper_amine_oxidase|MGG_02681|148305|Magnaporthe_oryzae_(related:_Magnaporthe_grisea)|Reduced_virulence
Chr01G0023.1	133	1.00E-117	214/570(37.54)	362	PHI:133|AKT1|BAA36588|5599|Alternaria_alternata|Loss_of_pathogenicity
Chr01G0024.1	881	3.00E-12	45/132(34.09)	63.9	PHI:881|MGG_04556|EDJ96020|318829|Magnaporthe_oryzae|Reduced_virulence
Chr01G0025.1	784	2.00E-160	219/284(77.11)	450	PHI:784|MGG_00056|EDK03390|318829|Magnaporthe_oryzae|Reduced_virulence
Chr01G0026.1	2032	5.00E-07	38/120(31.67)	48.5	PHI:2032|VTL1|G4NGA7|148305|Magnaporthe_oryzae_(related:_Magnaporthe_grisea)|Unaffected_pathogenicity
Chr01G0029.1	1662	1.00E-68	132/441(29.93)	239	PHI:1662|GzCCHC002|I1REJ1|5518|Gibberella_zeae_(related:_Fusarium_graminearum)|Unaffected_pathogenicity
Chr01G0030.1	2378	1.00E-08	94/383(24.54)	55.1	PHI:2378|DEP4|D2E9W9|29001|Alternaria_brassicicola|Mixed_outcome

Chr01G0031.1	2034	1.00E-09	64/249(25.70)	56.6	PHI:2034|MFP1|G4MZY1|148305|Magnaporthe_oryzae_(related:_Magnaporthe_grisea)|Reduced_virulence
Chr01G0032.1	2968	4.00E-44	131/472(27.75)	162	PHI:2968|Hxs1|J9VQA5|5207|Cryptococcus_neoformans|Reduced_virulence
Chr01G0034.1	2510	1.00E-62	159/499(31.86)	214	PHI:2510|msdS/AfmsdC|Q6PWQ1|746128|Aspergillus_fumigatus|Unaffected_pathogenicity
Chr01G0035.1	2247	1.00E-08	61/220(27.73)	52.4	PHI:2247|Sch1|EAT82552|13684|Phaeosphaeria_nodorum_(related:_Stagonospora_nodorum)|Unaffected_pathogenicity
Chr01G0044.1	876	2.00E-60	240/945(25.40)	225	PHI:876|MGG_11671|EDK03349|318829|Magnaporthe_oryzae|Reduced_virulence
Chr01G0053.1	3381	3.00E-73	168/472(35.59)	248	FVEG_12533|PHI:3381|W7N2B4|117187|Fusarium_verticillioides|unaffected_pathogenicity_
Chr01G0054.1	2393	1.00E-40	112/442(25.34)	151	PHI:2393|Related_to_O-methylsterigmatocystin_oxidoreductase|I1R980|5518|Gibberella_zeae_(related:_Fusarium_graminearum)|Increased_virulence_(Hypervirulence)
Chr01G0055.1	4194	4.00E-18	59/192(30.73)	84.7	AKT7|PHI:4194|V5XZS6|5599|Alternaria_alternata|increased_virulence_(Hypervirulence)
Chr01G0056.1	180	3.00E-93	137/233(58.80)	275	PHI:180|PELD|AAC49420|140110|Nectria_haematococca_(related:_Fusarium_solani)|Reduced_virulence
Chr01G0063.1	1867	6.00E-56	174/595(29.24)	201	PHI:1867|GzZC182|I1RL02|5518|Gibberella_zeae_(related:_Fusarium_graminearum)|Unaffected_pathogenicity
Chr01G0066.1	1233	0	324/491(65.99)	619	PHI:1233|FGSG_13944|I1RUG2|5518|Gibberella_zeae_(related:_Fusarium_graminearum)|Lethal
Chr01G0071.1	2087	0	947/1491(63.51)	1786	PHI:2087|Moatg26|MGG_03459|148305|Magnaporthe_oryzae_(related:_Magnaporthe_grisea)|Unaffected_pathogenicity
Chr01G0083.1	1807	0	509/809(62.92)	942	PHI:1807|GzZC122|I1RUM5|5518|Gibberella_zeae_(related:_Fusarium_graminearum)|Lethal

Chr01G0084.1	566	6.00E-68	156/453(34.44)	223	PHI:566|cel2|AAK19621|5017|Cochliobolus_carbonum|Unaffected_pathogenicity
Chr01G0085.1	1806	3.00E-23	101/409(24.69)	100	PHI:1806|GzZC121|I1RUM8|5518|Gibberella_zeae_(related:_Fusarium_graminearum)|Unaffected_pathogenicity
Chr01G0088.1	1884	1.00E-12	39/127(30.71)	63.5	PHI:1884|GzZC199|I1RJ58|5518|Gibberella_zeae_(related:_Fusarium_graminearum)|Unaffected_pathogenicity
Chr01G0090.1	1935	6.00E-10	33/93(35.48)	56.6	PHI:1935|GzZC250|I1RFP7|5518|Gibberella_zeae_(related:_Fusarium_graminearum)|Unaffected_pathogenicity
Chr01G0091.1	1690	4.00E-37	121/472(25.64)	142	PHI:1690|GzZC005|I1S463|5518|Gibberella_zeae_(related:_Fusarium_graminearum)|Unaffected_pathogenicity
Chr01G0092.1	2959	3.00E-91	162/324(50.00)	276	PHI:2959|mdh|C5BF98|67780|Edwardsiella_ictaluri|Reduced_virulence
Chr01G0093.1	504	2.00E-37	115/365(31.51)	137	PHI:504|LEU2|CAA42366|4932|Saccharomyces_cerevisiae|Reduced_virulence
Chr01G0094.1	441	6.00E-21	94/400(23.50)	92	PHI:441|BTP1|CAE55153|40559|Botrytis_cinerea|Reduced_virulence
Chr01G0096.1	1527	6.00E-37	100/357(28.01)	138	PHI:1527|GzHOMEL040|I1S9A1|5518|Gibberella_zeae_(related:_Fusarium_graminearum)|Lethal
Chr01G0097.1	2256	8.00E-31	117/382(30.63)	119	PHI:2256|Xdh1|Q0UA24|13684|Phaeosphaeria_nodorum_(related:_Stagonospora_nodorum)|Unaffected_pathogenicity
Chr01G0100.1	2968	2.00E-27	118/452(26.11)	112	PHI:2968|Hxs1|J9VQA5|5207|Cryptococcus_neoformans|Reduced_virulence
Chr01G0101.1	1456	2.00E-45	129/440(29.32)	167	PHI:1456|GzAra004|I1RN15|5518|Gibberella_zeae_(related:_Fusarium_graminearum)|Unaffected_pathogenicity
Chr01G0102.1	1981	1.00E-11	93/408(22.79)	64.3	PHI:1981|GzZC296|I1RIY9|5518|Gibberella_zeae_(related:_Fusarium_graminearum)|Unaffected_pathogenicity
Chr01G0105.1	2835	8.00E-14	56/173(32.37)	67.4	PHI:2835|RED2|C3JXE8|5016|Cochliobolus_heterostrophus|Reduced_virulence
Chr01G0106.1	1949	5.00E-10	77/340(22.65)	57.4	PHI:1949|GzZC264|I1RI60|5518|Gibberella_zeae_(related:_Fusarium_graminearum)|Lethal

Chr01G0107.1	2190	1.00E-12	68/225(30.22)	67.8	PHI:2190|MoCYP51A|G4MRP8|148305|Magnaporthe_oryzae_(related:_Magnaporthe_grisea)|Reduced_virulence
Chr01G0108.1	1902	8.00E-27	170/808(21.04)	114	PHI:1902|GzZC217|I1RJS7|5518|Gibberella_zeae_(related:_Fusarium_graminearum)|Unaffected_pathogenicity
Chr01G0111.1	2968	2.00E-53	140/467(29.98)	189	PHI:2968|Hxs1|J9VQA5|5207|Cryptococcus_neoformans|Reduced_virulence
Chr01G0112.1	242	0	395/858(46.04)	744	PHI:242|CaTPS2|CAC17748|5476|Candida_albicans|Reduced_virulence
Chr01G0113.1	2117	6.00E-15	76/262(29.01)	75.5	PHI:2117|SPM1|P58371|148305|Magnaporthe_oryzae_(related:_Magnaporthe_grisea)|Reduced_virulence
Chr01G0115.1	191	7.00E-152	306/814(37.59)	465	PHI:191|TOM1|AAB08446|39703|Septoria_lycopersici|Unaffected_pathogenicity
Chr01G0116.1	2376	3.00E-34	104/361(28.81)	132	PHI:2376|DEP2|D2E9W7|29001|Alternaria_brassicicola|Mixed_outcome
Chr01G0117.1	2042	4.00E-48	94/247(38.06)	181	PHI:2042|ABC3|Q3Y5V5|148305|Magnaporthe_oryzae_(related:_Magnaporthe_grisea)|Loss_of_pathogenicity
Chr01G0119.1	1047	2.00E-15	81/306(26.47)	74.3	PHI:1047|CTB6|ABK64183|29003|Cercospora_nicotianae|Reduced_virulence
Chr01G0123.1	1694	9.00E-12	61/225(27.11)	65.9	PHI:1694|GzZC009|I1S3X0|5518|Gibberella_zeae_(related:_Fusarium_graminearum)|Unaffected_pathogenicity
Chr01G0126.1	1555	1.00E-18	55/210(26.19)	86.3	PHI:1555|GzMyb019|I1RDG6|5518|Gibberella_zeae_(related:_Fusarium_graminearum)|Unaffected_pathogenicity
Chr01G0128.1	3237	1.00E-24	69/176(39.20)	94.7	ctrA2|PHI:3237|B0YDG4|746128|Aspergillus_fumigatus|mixed_outcome_
Chr01G0130.1	2240	5.00E-10	40/120(33.33)	57.8	PHI:2240|Srt1|Q4PBY9|5270|Ustilago_maydis|reduced_virulence
Chr01G0132.1	2976	7.00E-62	198/721(27.46)	221	PHI:2976|CgOPT1|C6ZRH8|29905|Colletotrichum_gloeosporioides_f._sp._aeschynomenes|Reduced_virulence
Chr01G0135.1	812	4.00E-14	93/373(24.93)	71.2	PHI:812|MGG_10702|EDJ94108|318829|Magnaporthe_oryzae|Reduced_virulence
Chr01G0137.1	812	7.00E-51	107/335(31.94)	178	PHI:812|MGG_10702|EDJ94108|318829|Magnaporthe_oryzae|Reduced_virulence

Chr01G0139.1	465	1.00E-34	114/346(32.95)	139	PHI:465|KIN2|AAB63337|5270|Ustilago_maydis|Reduced_virulence
Chr01G0143.1	544	2.00E-122	212/531(39.92)	376	PHI:544|BCMFS1|AAF64435|332648|Botrytis_cinerea|Unaffected_pathogenicity
Chr01G0144.1	1766	2.00E-12	100/444(22.52)	66.2	PHI:1766|GzZC081|I1S160|5518|Gibberella_zeae_(related:_Fusarium_graminearum)|Lethal
Chr01G0148.1	2807	4.00E-50	113/275(41.09)	177	PHI:2807|Ss-Sl2|A7EKS0|5180|Sclerotinia_sclerotiorum|Unaffected_pathogenicity
Chr01G0151.1	2802	1.00E-28	86/260(33.08)	108	PHI:2802|3hnr|B2ZRQ4|93612|Setosphaeria_turcica|Unaffected_pathogenicity
Chr01G0152.1	2704	3.00E-77	157/543(28.91)	255	PHI:2704|Tri12|F6MFB4|5518|Fusarium_graminearum|Reduced_virulence
Chr01G0153.1	2322	7.00E-91	132/260(50.77)	272	PHI:2322|SidH|Q4WF54|746128|Aspergillus_fumigatus|Reduced_virulence
Chr01G0154.1	2322	5.00E-69	110/267(41.20)	216	PHI:2322|SidH|Q4WF54|746128|Aspergillus_fumigatus|Reduced_virulence
Chr01G0155.1	2034	2.00E-40	98/287(34.15)	149	PHI:2034|MFP1|G4MZY1|148305|Magnaporthe_oryzae_(related:_Magnaporthe_grisea)|Reduced_virulence
Chr01G0157.1	508	2.00E-57	175/567(30.86)	201	PHI:508|AFT1|BAB69076|5599|Alternaria_alternata|Loss_of_pathogenicity
Chr01G0163.1	4194	6.00E-26	130/499(26.05)	108	AKT7|PHI:4194|V5XZS6|5599|Alternaria_alternata|increased_virulence_(Hypervirulence)
Chr01G0169.1	2844	2.00E-32	85/264(32.20)	119	PHI:2844|BRM2|O93802|5599|Alternaria_alternata|Unaffected_pathogenicity
Chr01G0170.1	2357	2.00E-19	128/507(25.25)	89.4	PHI:2357|CYP52X1|E2EAF6|475271|Beauveria_bassiana|Reduced_virulence
Chr01G0171.1	1893	4.00E-94	178/470(37.87)	296	PHI:1893|GzZC208|I1RNY0|5518|Gibberella_zeae_(related:_Fusarium_graminearum)|Unaffected_pathogenicity
Chr01G0173.1	1891	1.00E-34	119/462(25.76)	138	PHI:1891|GzZC206|I1RRM0|5518|Gibberella_zeae_(related:_Fusarium_graminearum)|Unaffected_pathogenicity
Chr01G0175.1	1662	8.00E-60	135/470(28.72)	213	PHI:1662|GzCCHC002|I1REJ1|5518|Gibberella_zeae_(related:_Fusarium_graminearum)|Unaffected_pathogenicity
Chr01G0176.1	26	3.00E-121	197/468(42.09)	371	PHI:26|CaMDR1|CAA37820|5476|Candida_albicans|Reduced_virulence
Chr01G0188.1	1792	1.00E-173	288/677(42.54)	516	PHI:1792|GzZC107|I1RVS7|5518|Gibberella_zeae_(related:_Fusarium_gramin

					earum)|Unaffected_pathogenicity
Chr01G0189.1	441	4.00E-27	82/336(24.40)	108	PHI:441|BTP1|CAE55153|40559|Botrytis_cinerea|Reduced_virulence
Chr01G0191.1	3387	2.00E-23	105/357(29.41)	99.8	FVEG_12523|PHI:3387|W7MT31|117187|Fusarium_verticillioides|unaffected_pathogenicity_
Chr01G0193.1	1046	5.00E-44	133/468(28.42)	160	PHI:1046|CTB5|ABK64182|29003|Cercospora_nicotianae|Reduced_virulence
Chr01G0194.1	3038	2.00E-06	52/220(23.64)	47.4	FgERG5B|PHI:3038|I1RIP4|5518|Fusarium_graminearum|reduced_virulence_
Chr01G0195.1	2654	2.00E-09	54/178(30.34)	58.5	PHI:2654|DUR1,2|Q59VF3|5476|Candida_albicans|Reduced_virulence
Chr01G0200.1	2451	2.00E-63	113/275(41.09)	205	PHI:2451|FAEB1|I1S3N8|5518|Gibberella_zeae_(related:_Fusarium_graminearum)|Unaffected_pathogenicity
Chr01G0207.1	922	4.00E-66	199/618(32.20)	229	PHI:922|um03615||5270|Ustilago_maydis|Unaffected_pathogenicity
Chr01G0209.1	1046	6.00E-33	116/460(25.22)	128	PHI:1046|CTB5|ABK64182|29003|Cercospora_nicotianae|Reduced_virulence
Chr01G0210.1	404	3.00E-11	51/197(25.89)	62	PHI:404|PTH11|AAD30436|318829|Magnaporthe_oryzae|Reduced_virulence
Chr01G0218.1	1821	5.00E-107	190/399(47.62)	336	PHI:1821|GzZC136|I1RWY8|5518|Gibberella_zeae_(related:_Fusarium_graminearum)|Unaffected_pathogenicity
Chr01G0219.1	1662	2.00E-18	97/461(21.04)	86.7	PHI:1662|GzCCHC002|I1REJ1|5518|Gibberella_zeae_(related:_Fusarium_graminearum)|Unaffected_pathogenicity
Chr01G0222.1	2315	6.00E-51	101/291(34.71)	172	PHI:2315|ChLae1|G4XKY9|5016|Cochliobolus_heterostrophus|Mixed_outcome
Chr01G0224.1	3268	4.00E-109	210/581(36.14)	345	YraS|PHI:3268|U3M7S2|29486|Yersinia_ruckeri|unaffected_pathogenicity_
Chr01G0227.1	1662	8.00E-68	156/538(29.00)	240	PHI:1662|GzCCHC002|I1REJ1|5518|Gibberella_zeae_(related:_Fusarium_graminearum)|Unaffected_pathogenicity
Chr01G0229.1	504	6.00E-43	124/379(32.72)	152	PHI:504|LEU2|CAA42366|4932|Saccharomyces_cerevisiae|Reduced_virulence
Chr01G0231.1	1662	2.00E-36	100/344(29.07)	142	PHI:1662|GzCCHC002|I1REJ1|5518|Gibberella_zeae_(related:_Fusarium_graminearum)|Unaffected_pathogenicity
Chr01G0232.1	1690	0	308/522(59.00)	592	PHI:1690|GzZC005|I1S463|5518|Gibberella_zeae_(related:_Fusarium_graminearum)|Unaffected_pathogenicity
Chr01G0233.1	2380	5.00E-11	29/67(43.28)	58.9	PHI:2380|DEP6|D2E9X1|29001|Alternaria_brassicicola|Mixed_outcome

Chr01G0237.1	3448	5.00E-20	72/275(26.18)	90.9	pgaC|PHI:3448|B5Y383|573|Klebsiella_pneumoniae|reduced_virulence_
Chr01G0238.1	4620	2.00E-43	98/252(38.89)	150	PcPL20|PHI:4620|A0A0D3LXG7|4784|Phytophthora_capsici|mixed_outcome
Chr01G0240.1	1269	1.00E-55	127/417(30.46)	193	PHI:1269|FGSG_02838|I1RFK9|5518|Gibberella_zeae_(related:_Fusarium_graminearum)|Unaffected_pathogenicity
Chr01G0242.1	2839	2.00E-62	121/350(34.57)	203	PHI:2839|RED1|Q8NJQ2|5016|Cochliobolus_heterostrophus|Reduced_virulence
Chr01G0245.1	2968	2.00E-29	134/524(25.57)	119	PHI:2968|Hxs1|J9VQA5|5207|Cryptococcus_neoformans|Reduced_virulence
Chr01G0246.1	1390	9.00E-27	61/167(36.53)	109	PHI:1390|GzC2H053|I1RU53|5518|Gibberella_zeae_(related:_Fusarium_graminearum)|Unaffected_pathogenicity
Chr01G0247.1	1390	8.00E-19	62/201(30.85)	87.8	PHI:1390|GzC2H053|I1RU53|5518|Gibberella_zeae_(related:_Fusarium_graminearum)|Unaffected_pathogenicity
Chr01G0250.1	1762	2.00E-39	147/565(26.02)	152	PHI:1762|GzZC077|I1RXJ4|5518|Gibberella_zeae_(related:_Fusarium_graminearum)|Unaffected_pathogenicity
Chr01G0252.1	1662	2.00E-60	162/558(29.03)	217	PHI:1662|GzCCHC002|I1REJ1|5518|Gibberella_zeae_(related:_Fusarium_graminearum)|Unaffected_pathogenicity
Chr01G0255.1	2034	5.00E-14	64/190(33.68)	69.7	PHI:2034|MFP1|G4MZY1|148305|Magnaporthe_oryzae_(related:_Magnaporthe_grisea)|Reduced_virulence
Chr01G0256.1	1662	2.00E-37	88/311(28.30)	144	PHI:1662|GzCCHC002|I1REJ1|5518|Gibberella_zeae_(related:_Fusarium_graminearum)|Unaffected_pathogenicity
Chr01G0258.1	2654	2.00E-13	73/234(31.20)	70.1	PHI:2654|DUR1,2|Q59VF3|5476|Candida_albicans|Reduced_virulence
Chr01G0260.1	2107	8.00E-21	62/202(30.69)	93.6	PHI:2107|Zinc-regulated_transporter_2|MGG_05905|148305|Magnaporthe_oryzae_(related:_Magnaporthe_grisea)|Mixed_outcome
Chr01G0262.1	3257	4.00E-26	90/287(31.36)	106	MoIlv1|PHI:3257|G4MU34|318829|Magnaporthe_oryzae|reduced_virulence_
Chr01G0263.1	572	8.00E-97	147/229(64.19)	285	PHI:572|XYL2|AAC62815|5017|Cochliobolus_carbonum|Unaffected_pathogenicity
Chr01G0264.1	538	5.00E-59	157/507(30.97)	206	PHI:538|FRT1|AAU87358|40559|Botrytis_cinerea|Unaffected_pathogenicity

Chr01G0266.1	243	0	756/917(82.44)	1541	PHI:243|CHIP6|AAD00894|5457|Colletotrichum_gloeosporioides|Reduced_virulence
Chr01G0267.1	3415	3.00E-14	84/380(22.11)	73.6	Fre2|PHI:3415|T2BNJ5|5207|Cryptococcus_neoformans|mixed_outcome_
Chr01G0270.1	2266	1.00E-85	147/427(34.43)	275	PHI:2266|Ptr2|0|13684|Phaeosphaeria_nodorum_(related:_Stagonospora_nodorum)|Unaffected_pathogenicity
Chr01G0272.1	404	3.00E-26	77/298(25.84)	107	PHI:404|PTH11|AAD30436|318829|Magnaporthe_oryzae|Reduced_virulence
Chr01G0273.1	144	1.00E-28	96/333(28.83)	113	PHI:144|CHT42|AAC05829|29875|Trichoderma_virens|Reduced_virulence
Chr01G0277.1	1260	8.00E-22	79/232(34.05)	99	PHI:1260|FGSG_13944|I1RUC7|5518|Gibberella_zeae_(related:_Fusarium_graminearum)|Unaffected_pathogenicity
Chr01G0279.1	510	5.00E-14	90/407(22.11)	71.6	PHI:510|CaNAG3|EAK93097|5476|Candida_albicans|Reduced_virulence
Chr01G0285.1	2357	1.00E-107	206/565(36.46)	333	PHI:2357|CYP52X1|E2EAF6|475271|Beauveria_bassiana|Reduced_virulence
Chr01G0289.1	1396	0	441/755(58.41)	704	PHI:1396|GzC2H059|I1RUM9|5518|Gibberella_zeae_(related:_Fusarium_graminearum)|Reduced_virulence
Chr01G0292.1	2488	1.00E-58	171/520(32.88)	204	PHI:2488|Man1|G4ND25|148305|Magnaporthe_oryzae_(related:_Magnaporthe_grisea)|Unaffected_pathogenicity
Chr01G0295.1	2535	1.00E-06	64/239(26.78)	48.1	PHI:2535|ERG11B|E9QY26|746128|Aspergillus_fumigatus|Mixed_outcome
Chr01G0297.1	4194	2.00E-15	67/243(27.57)	76.6	AKT7|PHI:4194|V5XZS6|5599|Alternaria_alternata|increased_virulence_(Hypervirulence)
Chr01G0298.1	4194	3.00E-13	62/245(25.31)	70.9	AKT7|PHI:4194|V5XZS6|5599|Alternaria_alternata|increased_virulence_(Hypervirulence)
Chr01G0299.1	2393	2.00E-08	44/170(25.88)	53.9	PHI:2393|Related_to_O-methylsterigmatocystin_oxidoreductase|I1R980|5518|Gibberella_zeae_(related:_Fusarium_graminearum)|Increased_virulence_(Hypervirulence)
Chr01G0302.1	1260	3.00E-07	29/88(32.95)	50.4	PHI:1260|FGSG_13944|I1RUC7|5518|Gibberella_zeae_(related:_Fusarium_graminearum)|Unaffected_pathogenicity
Chr01G0308.1	3216	2.00E-38	93/241(38.59)	138	MoCDIP4|PHI:3216|G4MVX4|318829|Magnaporthe_oryzae|mixed_outcome_

Chr01G0310.1	404	2.00E-37	105/368(28.53)	141	PHI:404|PTH11|AAD30436|318829|Magnaporthe_oryzae|Reduced_virulence
Chr01G0317.1	358	5.00E-07	114/506(22.53)	49.7	PHI:358|ILV2|AAR29084|5207|Cryptococcus_neoformans|Loss_of_pathogenicity
Chr01G0318.1	3028	6.00E-35	109/383(28.46)	134	Vatr2|PHI:3028|A5CVB7|28447|Clavibacter_michiganensis|reduced_virulence_
Chr01G0319.1	2147	2.00E-43	92/241(38.17)	154	PHI:2147|Erl1|G5EH97|148305|Magnaporthe_oryzae_(related:_Magnaporthe_grisea)|Reduced_virulence
Chr01G0323.1	2147	1.00E-09	38/105(36.19)	56.6	PHI:2147|Erl1|G5EH97|148305|Magnaporthe_oryzae_(related:_Magnaporthe_grisea)|Reduced_virulence
Chr01G0331.1	1693	0	290/554(52.35)	551	PHI:1693|GzZC008|I1RYA7|5518|Gibberella_zeae_(related:_Fusarium_graminearum)|Unaffected_pathogenicity
Chr01G0334.1	115	2.00E-69	146/428(34.11)	226	PHI:115|PGX1|AAC26146|5017|Cochliobolus_carbonum|Unaffected_pathogenicity
Chr01G0337.1	3388	2.00E-18	42/103(40.78)	75.1	FVEG_12522|PHI:3388|W7N2B2|117187|Fusarium_verticillioides|unaffected_pathogenicity_
Chr01G0338.1	1998	7.00E-67	127/345(36.81)	219	PHI:1998|GzZC313|I1R9B9|5518|Gibberella_zeae_(related:_Fusarium_graminearum)|Unaffected_pathogenicity
Chr01G0339.1	2034	3.00E-07	44/163(26.99)	49.3	PHI:2034|MFP1|G4MZY1|148305|Magnaporthe_oryzae_(related:_Magnaporthe_grisea)|Reduced_virulence
Chr01G0341.1	387	3.00E-139	217/450(48.22)	416	PHI:387|UGD1|AAK95561|5207|Cryptococcus_neoformans|Loss_of_pathogenicity
Chr01G0343.1	2978	2.00E-09	66/277(23.83)	54.7	PHI:2978|MoCel12A|G4N5V2|148305|Magnaporthe_oryzae|Unaffected_pathogenicity
Chr01G0344.1	3448	2.00E-06	56/257(21.79)	47.8	pgaC|PHI:3448|B5Y383|573|Klebsiella_pneumoniae|reduced_virulence_
Chr01G0345.1	2927	9.00E-57	156/486(32.10)	198	PHI:2927|lip5|J9N2Z8|59765|Fusarium_oxysporum_f._sp._Lycopersici|Unaffected_pathogenicity
Chr01G0350.1	3457	4.00E-22	125/503(24.85)	96.7	PHO84|PHI:3457|J9VMW8|5207|Cryptococcus_neoformans|mixed_outcome_

Chr01G0351.1	2042	2.00E-51	107/276(38.77)	196	PHI:2042|ABC3|Q3Y5V5|148305|Magnaporthe_oryzae_(related:_Magnaporthe_grisea)|Loss_of_pathogenicity
Chr01G0353.1	511	4.00E-08	47/173(27.17)	53.1	PHI:511|CaNAG4|EAK93098|5476|Candida_albicans|Reduced_virulence
Chr01G0357.1	1982	5.00E-146	260/689(37.74)	447	PHI:1982|GzZC297|I1RIT3|5518|Gibberella_zeae_(related:_Fusarium_graminearum)|Unaffected_pathogenicity
Chr01G0360.1	2654	2.00E-08	46/168(27.38)	55.1	PHI:2654|DUR1,2|Q59VF3|5476|Candida_albicans|Reduced_virulence
Chr01G0361.1	59	4.00E-08	42/131(32.06)	51.2	PHI:59|THR1|BAA18962|5462|Colletotrichum_lagenarium|Reduced_virulence
Chr01G0362.1	386	2.00E-78	108/192(56.25)	234	PHI:386|TSA1|AAP68994|5207|Cryptococcus_neoformans|Reduced_virulence
Chr01G0365.1	1974	4.00E-08	28/101(27.72)	53.1	PHI:1974|GzZC289|I1RQN6|5518|Gibberella_zeae_(related:_Fusarium_graminearum)|Unaffected_pathogenicity
Chr01G0370.1	1795	3.00E-16	119/468(25.43)	79.3	PHI:1795|GzZC110|I1RSX8|5518|Gibberella_zeae_(related:_Fusarium_graminearum)|Unaffected_pathogenicity
Chr01G0371.1	544	6.00E-111	205/560(36.61)	345	PHI:544|BCMFS1|AAF64435|332648|Botrytis_cinerea|Unaffected_pathogenicity
Chr01G0376.1	2968	9.00E-97	182/534(34.08)	305	PHI:2968|Hxs1|J9VQA5|5207|Cryptococcus_neoformans|Reduced_virulence
Chr01G0382.1	513	2.00E-38	127/492(25.81)	146	PHI:513|ARN1_(related:_SIT1)|EAK97011|5476|Candida_albicans|Reduced_virulence
Chr01G0383.1	812	3.00E-33	95/315(30.16)	126	PHI:812|MGG_10702|EDJ94108|318829|Magnaporthe_oryzae|Reduced_virulence
Chr01G0393.1	1956	2.00E-38	171/687(24.89)	149	PHI:1956|GzZC271|I1RLT2|5518|Gibberella_zeae_(related:_Fusarium_graminearum)|Unaffected_pathogenicity
Chr01G0395.1	1662	6.00E-37	114/431(26.45)	144	PHI:1662|GzCCHC002|I1REJ1|5518|Gibberella_zeae_(related:_Fusarium_graminearum)|Unaffected_pathogenicity
Chr01G0396.1	922	4.00E-40	159/616(25.81)	152	PHI:922|um03615||5270|Ustilago_maydis|Unaffected_pathogenicity
Chr01G0397.1	2393	2.00E-12	61/265(23.02)	68.2	PHI:2393|Related_to_O-methylsterigmatocystin_oxidoreductase|I1R980|5518|Gibberella_zeae_(related:_Fusarium_graminearum)|Increased_virulence_(Hyp

					ervirulence)
Chr01G0401.1	1572	0	502/775(64.77)	1074	PHI:1572|GzOB012|I1RE94|5518|Gibberella_zeae_(related:_Fusarium_graminearum)|Unaffected_pathogenicity
Chr01G0409.1	1555	4.00E-40	117/487(24.02)	150	PHI:1555|GzMyb019|I1RDG6|5518|Gibberella_zeae_(related:_Fusarium_graminearum)|Unaffected_pathogenicity
Chr01G0412.1	1424	9.00E-08	23/55(41.82)	47.4	PHI:1424|GzC2H092|I1S3J7|5518|Gibberella_zeae_(related:_Fusarium_graminearum)|Unaffected_pathogenicity
Chr01G0414.1	2322	2.00E-25	90/309(29.13)	101	PHI:2322|SidH|Q4WF54|746128|Aspergillus_fumigatus|Reduced_virulence
Chr01G0426.1	1378	0	455/860(52.91)	680	PHI:1378|GzC2H041|I1RQG9|5518|Gibberella_zeae_(related:_Fusarium_graminearum)|Unaffected_pathogenicity
Chr01G0436.1	2025	4.00E-06	32/127(25.20)	45.8	PHI:2025|HDL1|G4MQZ9|148305|Magnaporthe_oryzae_(related:_Magnaporthe_grisea)|Unaffected_pathogenicity
Chr01G0440.1	507	1.00E-28	67/188(35.64)	109	PHI:507|CaRAS1|AAF03566|5476|Candida_albicans|Reduced_virulence
Chr01G0445.1	2531	0	316/538(58.74)	627	PHI:2531|PRI1|Q4WXC5|746128|Aspergillus_fumigatus|Lethal
Chr01G0447.1	3109	4.00E-173	302/605(49.92)	506	FgSKN7|PHI:3109|I1RQL4|5518|Fusarium_graminearum|mixed_outcome_
Chr01G0449.1	1527	3.00E-21	87/384(22.66)	94.7	PHI:1527|GzHOMEL040|I1S9A1|5518|Gibberella_zeae_(related:_Fusarium_graminearum)|Lethal
Chr01G0450.1	2543	1.00E-175	296/719(41.17)	523	PHI:2543|GCD6|Q4WLS1|746128|Aspergillus_fumigatus|Mixed_outcome
Chr01G0452.1	181	4.00E-28	108/420(25.71)	112	PHI:181|PGX1|AAK81847|5507|Fusarium_oxysporum|Unaffected_pathogenicity
Chr01G0453.1	4194	3.00E-28	112/471(23.78)	115	AKT7|PHI:4194|V5XZS6|5599|Alternaria_alternata|increased_virulence_(Hypervirulence)
Chr01G0457.1	404	1.00E-07	44/191(23.04)	50.4	PHI:404|PTH11|AAD30436|318829|Magnaporthe_oryzae|Reduced_virulence
Chr01G0459.1	2315	2.00E-49	103/293(35.15)	168	PHI:2315|ChLae1|G4XKY9|5016|Cochliobolus_heterostrophus|Mixed_outcome
Chr01G0465.1	3662	3.00E-06	37/114(32.46)	48.1	PspB_(not_PD0218)|PHI:3662|Q87ET0|2371|Xylella_fastidiosa|Increased_virulence_(Hypervirulence)

Chr01G0468.1	1030	0	593/1383(42.88)	1129	PHI:1030|bcatrA|CAA93142|40559|Botrytis_cinerea|Unaffected_pathogenicity
Chr01G0470.1	2539	0	305/437(69.79)	628	PHI:2539|CDS1|Q4WJ71|746128|Aspergillus_fumigatus|Mixed_outcome
Chr01G0478.1	598	1.00E-91	164/359(45.68)	281	PHI:598|THIOL|ABB55459|5022|Leptosphaeria_maculans|Reduced_virulence
Chr01G0480.1	375	0	406/776(52.32)	640	PHI:375|REN1|BAC55015|5507|Fusarium_oxysporum|Unaffected_pathogenicit y
Chr01G0483.1	1823	1.00E-57	125/397(31.49)	198	PHI:1823|GzZC138|I1RXK4|5518|Gibberella_zeae_(related:_Fusarium_gramin earum)|Unaffected_pathogenicity
Chr01G0484.1	2968	8.00E-34	132/509(25.93)	131	PHI:2968|Hxs1|J9VQA5|5207|Cryptococcus_neoformans|Reduced_virulence
Chr01G0487.1	2008	2.00E-06	21/33(63.64)	49.3	PHI:2008|HST1|EDJ98541|148305|Magnaporthe_oryzae_(related:_Magnaporth e_grisea)|Unaffected_pathogenicity
Chr01G0489.1	2100	0	1034/1321(78.27)	2133	PHI:2100|Spf1|MGG_12005|148305|Magnaporthe_oryzae_(related:_Magnaport he_grisea)|Mixed_outcome
Chr01G0492.1	2959	4.00E-91	162/317(51.10)	276	PHI:2959|mdh|C5BF98|67780|Edwardsiella_ictaluri|Reduced_virulence
Chr01G0497.1	748	0	325/733(44.34)	600	PHI:748|um00446|Not_available|5270|Ustilago_maydis|Unaffected_pathogenici ty
Chr01G0499.1	697	5.00E-19	93/339(27.43)	86.3	PHI:697|ugt51E1|AAM81358|5022|Leptosphaeria_maculans|Unaffected_patho genicity
Chr01G0504.1	511	8.00E-33	111/440(25.23)	129	PHI:511|CaNAG4|EAK93098|5476|Candida_albicans|Reduced_virulence
Chr01G0506.1	2174	7.00E-69	120/312(38.46)	218	PHI:2174|NMR2|MGG_02860|148305|Magnaporthe_oryzae_(related:_Magnap orthe_grisea)|Reduced_virulence
Chr01G0507.1	1721	6.00E-104	176/443(39.73)	316	PHI:1721|GzZC036|I1S914|5518|Gibberella_zeae_(related:_Fusarium_gramine arum)|Unaffected_pathogenicity
Chr01G0514.1	2315	3.00E-60	108/291(37.11)	196	PHI:2315|ChLae1|G4XKY9|5016|Cochliobolus_heterostrophus|Mixed_outcome
Chr01G0516.1	2315	9.00E-52	109/308(35.39)	175	PHI:2315|ChLae1|G4XKY9|5016|Cochliobolus_heterostrophus|Mixed_outcome
Chr01G0520.1	510	4.00E-74	166/534(31.09)	247	PHI:510|CaNAG3|EAK93097|5476|Candida_albicans|Reduced_virulence
Chr01G0522.1	2644	3.00E-16	32/100(32.00)	68.9	PHI:2644|thioredoxin_1|P0AA28|90371|Salmonella_enterica_serovar_Typhimur

					ium|Reduced_virulence
Chr01G0526.1	1555	1.00E-38	120/510(23.53)	145	PHI:1555|GzMyb019|I1RDG6|5518|Gibberella_zeae_(related:_Fusarium_graminearum)|Unaffected_pathogenicity
Chr01G0530.1	1923	0	357/727(49.11)	662	PHI:1923|GzZC238|I1RHB3|5518|Gibberella_zeae_(related:_Fusarium_graminearum)|Unaffected_pathogenicity
Chr01G0531.1	2451	4.00E-81	133/290(45.86)	248	PHI:2451|FAEB1|I1S3N8|5518|Gibberella_zeae_(related:_Fusarium_graminearum)|Unaffected_pathogenicity
Chr01G0534.1	2204	4.00E-145	203/309(65.70)	414	PHI:2204|endo-1,4-beta-xylanase_[GH10_family]|MGG_01542|148305|Magnaporthe_oryzae_(related:_Magnaporthe_grisea)|Reduced_virulence
Chr01G0537.1	1683	0	447/690(64.78)	867	PHI:1683|GzRad001|I1RK33|5518|Gibberella_zeae_(related:_Fusarium_graminearum)|Lethal
Chr01G0538.1	1293	0	550/780(70.51)	1091	PHI:1293|GzAPSES001|I1RK32|5518|Gibberella_zeae_(related:_Fusarium_graminearum)|Reduced_virulence
Chr01G0543.1	3226	3.00E-92	165/337(48.96)	285	pnl1|PHI:3226|T2C7K6|36651|Penicillium_digitatum|reduced_virulence_
Chr01G0550.1	2968	9.00E-95	178/525(33.90)	300	PHI:2968|Hxs1|J9VQA5|5207|Cryptococcus_neoformans|Reduced_virulence
Chr01G0552.1	4194	2.00E-10	65/265(24.53)	60.8	AKT7|PHI:4194|V5XZS6|5599|Alternaria_alternata|increased_virulence_(Hypervirulence)
Chr01G0553.1	552	7.00E-132	236/609(38.75)	399	PHI:552|BcLCC2|AAK77953|40559|Botrytis_cinerea|Unaffected_pathogenicity
Chr01G0558.1	1992	6.00E-27	80/250(32.00)	113	PHI:1992|GzZC307|I1R983|5518|Gibberella_zeae_(related:_Fusarium_graminearum)|Unaffected_pathogenicity
Chr01G0559.1	2968	7.00E-31	131/527(24.86)	123	PHI:2968|Hxs1|J9VQA5|5207|Cryptococcus_neoformans|Reduced_virulence
Chr01G0560.1	1662	1.00E-28	108/487(22.18)	117	PHI:1662|GzCCHC002|I1REJ1|5518|Gibberella_zeae_(related:_Fusarium_graminearum)|Unaffected_pathogenicity
Chr01G0561.1	1923	0	320/729(43.90)	579	PHI:1923|GzZC238|I1RHB3|5518|Gibberella_zeae_(related:_Fusarium_graminearum)|Unaffected_pathogenicity
Chr01G0563.1	3384	3.00E-88	270/979(27.58)	308	FVEG_12530|PHI:3384|W7N2C1|117187|Fusarium_verticillioides|unaffected_p

					athogenicity_
Chr01G0566.1	256	2.00E-106	152/231(65.80)	309	PHI:256|GAS1|AAK52794|318829|Magnaporthe_oryzae|Reduced_virulence
Chr01G0568.1	3384	8.00E-152	331/1073(30.85)	481	FVEG_12530|PHI:3384|W7N2C1|117187|Fusarium_verticillioides|unaffected_pathogenicity_
Chr01G0569.1	438	2.00E-18	72/245(29.39)	87.8	PHI:438|BcBOT1_(related:_CND5)|AAQ16576|40559|Botrytis_cinerea|Reduced_virulence
Chr01G0571.1	509	9.00E-66	111/274(40.51)	209	PHI:509|AFT3|BAB69078|5599|Alternaria_alternata|Loss_of_pathogenicity
Chr01G0572.1	2930	2.00E-62	139/487(28.54)	223	PHI:2930|ctf2|J9MFF7|59765|Fusarium_oxysporum_f._sp._Lycopersici|Reduced_virulence
Chr01G0576.1	2441	1.00E-08	34/127(26.77)	54.3	PHI:2441|MgAlg2|F9XJV5|1047171|Mycosphaerella_graminicola_(related:_Zymoseptoria_triticii)|Loss_of_pathogenicity
Chr01G0577.1	387	3.00E-146	229/471(48.62)	435	PHI:387|UGD1|AAK95561|5207|Cryptococcus_neoformans|Loss_of_pathogenicity
Chr01G0583.1	112	2.00E-84	155/466(33.26)	269	PHI:112|MAK1|AAC49410|140110|Nectria_haematococca_(related:_Fusarium_solani)|Reduced_virulence
Chr01G0585.1	2802	2.00E-18	71/289(24.57)	80.5	PHI:2802|3hnr|B2ZRQ4|93612|Setosphaeria_turcica|Unaffected_pathogenicity
Chr01G0586.1	1707	3.00E-146	261/659(39.61)	450	PHI:1707|GzZC022|I1RJY6|5518|Gibberella_zeae_(related:_Fusarium_graminearum)|Unaffected_pathogenicity
Chr01G0587.1	812	2.00E-72	133/352(37.78)	233	PHI:812|MGG_10702|EDJ94108|318829|Magnaporthe_oryzae|Reduced_virulence
Chr01G0591.1	211	9.00E-44	98/242(40.50)	164	PHI:211|CaTUP1|AAB63195|5476|Candida_albicans|Reduced_virulence
Chr01G0592.1	1555	3.00E-51	126/445(28.31)	182	PHI:1555|GzMyb019|I1RDG6|5518|Gibberella_zeae_(related:_Fusarium_graminearum)|Unaffected_pathogenicity
Chr01G0602.1	439	1.00E-21	45/99(45.45)	89	PHI:439|TRI6|BAA83722|5518|Fusarium_graminearum|Reduced_virulence
Chr01G0616.1	1270	4.00E-57	110/288(38.19)	189	PHI:1270|FGSG_06420|I1RHA1|5518|Gibberella_zeae_(related:_Fusarium_graminearum)|Unaffected_pathogenicity

Chr01G0617.1	59	2.00E-06	50/212(23.58)	45.4	PHI:59|THR1|BAA18962|5462|Colletotrichum_lagenarium|Reduced_virulence
Chr01G0618.1	2022	8.00E-06	48/210(22.86)	43.9	PHI:2022|BUF1|MGG_02252|148305|Magnaporthe_oryzae_(related:_Magnaporthe_grisea)|Loss_of_pathogenicity
Chr01G0621.1	2802	2.00E-09	60/217(27.65)	54.7	PHI:2802|3hnr|B2ZRQ4|93612|Setosphaeria_turcica|Unaffected_pathogenicity
Chr01G0622.1	4194	2.00E-54	145/507(28.60)	191	AKT7|PHI:4194|V5XZS6|5599|Alternaria_alternata|increased_virulence_(Hypervirulence)
Chr01G0623.1	1935	3.00E-06	24/53(45.28)	47	PHI:1935|GzZC250|I1RFP7|5518|Gibberella_zeae_(related:_Fusarium_graminearum)|Unaffected_pathogenicity
Chr01G0626.1	1575	1.00E-27	82/242(33.88)	105	PHI:1575|GzOB015|I1RIQ3|5518|Gibberella_zeae_(related:_Fusarium_graminearum)|Unaffected_pathogenicity
Chr01G0627.1	441	3.00E-19	80/318(25.16)	86.7	PHI:441|BTP1|CAE55153|40559|Botrytis_cinerea|Reduced_virulence
Chr01G0631.1	541	1.00E-165	256/547(46.80)	484	PHI:541|LIP1|AAU87359|332648|Botrytis_cinerea|Unaffected_pathogenicity
Chr01G0632.1	1833	0	397/607(65.40)	760	PHI:1833|GzZC148|I1RK16|5518|Gibberella_zeae_(related:_Fusarium_graminearum)|Unaffected_pathogenicity
Chr01G0633.1	1795	3.00E-39	141/554(25.45)	150	PHI:1795|GzZC110|I1RSX8|5518|Gibberella_zeae_(related:_Fusarium_graminearum)|Unaffected_pathogenicity
Chr01G0640.1	441	1.00E-23	67/223(30.04)	97.8	PHI:441|BTP1|CAE55153|40559|Botrytis_cinerea|Reduced_virulence
Chr01G0641.1	716	2.00E-18	58/171(33.92)	85.5	PHI:716|ZEB1|ABB90284|5518|Fusarium_graminearum|Unaffected_pathogenicity
Chr01G0642.1	438	1.00E-33	109/440(24.77)	130	PHI:438|BcBOT1_(related:_CND5)|AAQ16576|40559|Botrytis_cinerea|Reduced_virulence
Chr01G0646.1	2294	5.00E-06	27/98(27.55)	46.2	PHI:2294|FgFRP1|I1RCK7|5518|Gibberella_zeae_(related:_Fusarium_graminearum)|Mixed_outcome
Chr01G0648.1	2393	5.00E-41	125/390(32.05)	152	PHI:2393|Related_to_O-methylsterigmatocystin_oxidoreductase|I1R980|5518|Gibberella_zeae_(related:_Fusarium_graminearum)|Increased_virulence_(Hypervirulence)

Chr01G0650.1	544	1.00E-133	225/566(39.75)	404	PHI:544|BCMFS1|AAF64435|332648|Botrytis_cinerea|Unaffected_pathogenicity
Chr01G0655.1	2898	8.00E-16	77/303(25.41)	74.3	PHI:2896|BEC1005|CCU82697|62688|Blumeria_graminis_f._sp._hordei|Effector_(plant_avirulence_determinant)
Chr01G0662.1	510	1.00E-18	90/393(22.90)	85.9	PHI:510|CaNAG3|EAK93097|5476|Candida_albicans|Reduced_virulence
Chr01G0664.1	1742	3.00E-35	154/631(24.41)	139	PHI:1742|GzZC057|I1S7H5|5518|Gibberella_zeae_(related:_Fusarium_graminearum)|Unaffected_pathogenicity
Chr01G0665.1	1161	6.00E-52	134/486(27.57)	186	PHI:1161|MgMfs1|A4ZGP3|54734|Mycosphaerella_graminicola_(related:_Zymoseptoria_triticii)|Chemistry_target
Chr01G0667.1	2269	8.00E-27	75/264(28.41)	103	PHI:2269|Mdh1|0|13684|Phaeosphaeria_nodorum_(related:_Stagonospora_nodorum)|Unaffected_pathogenicity
Chr01G0671.1	1662	4.00E-65	151/499(30.26)	229	PHI:1662|GzCCHC002|I1REJ1|5518|Gibberella_zeae_(related:_Fusarium_graminearum)|Unaffected_pathogenicity
Chr01G0673.1	59	4.00E-06	51/194(26.29)	44.3	PHI:59|THR1|BAA18962|5462|Colletotrichum_lagenarium|Reduced_virulence
Chr01G0674.1	3457	3.00E-08	47/193(24.35)	53.5	PHO84|PHI:3457|J9VMW8|5207|Cryptococcus_neoformans|mixed_outcome_
Chr01G0675.1	3662	2.00E-07	43/123(34.96)	52	PspB_(not_PD0218)|PHI:3662|Q87ET0|2371|Xylella_fastidiosa|Increased_virulence_(Hypervirulence)
Chr01G0678.1	1773	3.00E-16	61/234(26.07)	75.1	PHI:1773|GzZC088|I1S409|5518|Gibberella_zeae_(related:_Fusarium_graminearum)|Unaffected_pathogenicity
Chr01G0679.1	747	1.00E-18	58/196(29.59)	79.3	PHI:747|um00445|Not_available|5270|Ustilago_maydis|Unaffected_pathogenicity
Chr01G0681.1	2838	4.00E-22	73/254(28.74)	90.1	PHI:2838|TOX9|D2SZX8|5016|Cochliobolus_heterostrophus|Reduced_virulence
Chr01G0682.1	2094	0	417/630(66.19)	884	PHI:2094|Yvc1|MGG_09828|148305|Magnaporthe_oryzae_(related:_Magnaporthe_grisea)|Mixed_outcome
Chr01G0690.1	2067	1.00E-08	59/216(27.31)	56.6	PHI:2067|ABC4|MGG_00937|148305|Magnaporthe_oryzae_(related:_Magnapo

					rthe_grisea)|Loss_of_pathogenicity
Chr01G0702.1	1046	2.00E-53	148/474(31.22)	186	PHI:1046|CTB5|ABK64182|29003|Cercospora_nicotianae|Reduced_virulence
Chr01G0703.1	3381	1.00E-49	127/467(27.19)	179	FVEG_12533|PHI:3381|W7N2B4|117187|Fusarium_verticillioides|unaffected_pathogenicity_
Chr01G0706.1	1891	7.00E-13	85/360(23.61)	69.3	PHI:1891|GzZC206|I1RRM0|5518|Gibberella_zeae_(related:_Fusarium_graminearum)|Unaffected_pathogenicity
Chr01G0707.1	2908	5.00E-08	51/179(28.49)	52.4	PHI:2908|CYP51B|I1RBR4|5518|Fusarium_graminearum|Mixed_outcome
Chr01G0716.1	2521	2.00E-59	129/357(36.13)	211	PHI:2521|GUS1|Q4WEM7|746128|Aspergillus_fumigatus|Lethal
Chr01G0720.1	2510	1.00E-57	165/511(32.29)	201	PHI:2510|msdS/AfmsdC|Q6PWQ1|746128|Aspergillus_fumigatus|Unaffected_pathogenicity
Chr01G0727.1	441	6.00E-26	86/292(29.45)	104	PHI:441|BTP1|CAE55153|40559|Botrytis_cinerea|Reduced_virulence
Chr01G0728.1	1960	1.00E-40	135/550(24.55)	158	PHI:1960|GzZC275|I1RDE2|5518|Gibberella_zeae_(related:_Fusarium_graminearum)|Unaffected_pathogenicity
Chr01G0733.1	541	8.00E-33	98/287(34.15)	130	PHI:541|LIP1|AAU87359|332648|Botrytis_cinerea|Unaffected_pathogenicity
Chr01G0747.1	2107	5.00E-08	29/57(50.88)	52.8	PHI:2107|Zinc-regulated_transporter_2|MGG_05905|148305|Magnaporthe_oryzae_(related:_Magnaporthe_grisea)|Mixed_outcome
Chr01G0751.1	1773	6.00E-11	29/57(50.88)	61.6	PHI:1773|GzZC088|I1S409|5518|Gibberella_zeae_(related:_Fusarium_graminearum)|Unaffected_pathogenicity
Chr01G0757.1	2256	5.00E-44	107/341(31.38)	155	PHI:2256|Xdh1|Q0UA24|13684|Phaeosphaeria_nodorum_(related:_Stagonospora_nodorum)|Unaffected_pathogenicity
Chr01G0763.1	2625	5.00E-114	187/439(42.60)	342	PHI:2625|purA|P65882|90371|Salmonella_enterica_serovar_Typhimurium|Reduced_virulence
Chr01G0765.1	3378	5.00E-31	86/226(38.05)	114	Plegl1|PHI:3378|G9JLA8|285811|Pyrenochaeta_lycopersici|unaffected_pathogenicity_
Chr01G0767.1	2393	3.00E-09	48/200(24.00)	56.2	PHI:2393|Related_to_O-methylsterigmatocystin_oxidoreductase|I1R980|5518|Gibberella_zeae_(related:_Fusarium_graminearum)|Increased_virulence_(Hyp

					ervirulence)
Chr01G0768.1	2251	4.00E-06	52/199(26.13)	44.3	PHI:2251|Gox1|Q696X2|13684|Phaeosphaeria_nodorum_(related:_Stagonospora_nodorum)|Unaffected_pathogenicity
Chr01G0770.1	2171	5.00E-121	231/678(34.07)	379	PHI:2171|Peroxisomal_copper_amine_oxidase|MGG_02681|148305|Magnaporthe_oryzae_(related:_Magnaporthe_grisea)|Reduced_virulence
Chr01G0771.1	2991	1.00E-15	42/101(41.58)	75.9	PHI:2991|MGG_01426.6|Q52G60|148305|Magnaporthe_oryzae|Reduced_virulence
Chr01G0775.1	552	1.00E-07	59/246(23.98)	52	PHI:552|BcLCC2|AAK77953|40559|Botrytis_cinerea|Unaffected_pathogenicity
Chr01G0778.1	220	5.00E-64	150/438(34.25)	212	PHI:220|MAN1|AAG10203|5207|Cryptococcus_neoformans|Reduced_virulence
Chr01G0786.1	2358	4.00E-118	213/514(41.44)	386	PHI:2358|PLD|Q4WZL4|746128|Aspergillus_fumigatus|Reduced_virulence
Chr01G0789.1	2837	4.00E-43	85/227(37.44)	146	PHI:2837|OXI1|D2SZX7|5016|Cochliobolus_heterostrophus|Reduced_virulence
Chr01G0792.1	1901	2.00E-120	173/272(63.60)	380	PHI:1901|GzZC216|I1RK07|5518|Gibberella_zeae_(related:_Fusarium_graminearum)|Unaffected_pathogenicity
Chr01G0794.1	2257	1.00E-130	213/373(57.10)	381	PHI:2257|Pbl1|Q0UHM8|13684|Phaeosphaeria_nodorum_(related:_Stagonospora_nodorum)|Unaffected_pathogenicity
Chr01G0802.1	923	2.00E-11	94/397(23.68)	63.2	PHI:923|um03616||5270|Ustilago_maydis|Unaffected_pathogenicity
Chr01G0807.1	2895	2.00E-13	87/317(27.44)	68.9	PHI:2895|F-avi4330|B9JV05|373|Agrobacterium_vitis|Loss_of_pathogenicity
Chr01G0812.1	4194	1.00E-12	59/209(28.23)	67.4	AKT7|PHI:4194|V5XZS6|5599|Alternaria_alternata|increased_virulence_(Hypervirulence)
Chr01G0813.1	2171	2.00E-14	93/412(22.57)	73.9	PHI:2171|Peroxisomal_copper_amine_oxidase|MGG_02681|148305|Magnaporthe_oryzae_(related:_Magnaporthe_grisea)|Reduced_virulence
Chr01G0814.1	2611	2.00E-41	96/343(27.99)	149	PHI:2611|CMLE|H9C592|59765|Fusarium_oxysporum_f._sp._Lycopersici|Loss_of_pathogenicity
Chr01G0816.1	881	3.00E-44	119/360(33.06)	155	PHI:881|MGG_04556|EDJ96020|318829|Magnaporthe_oryzae|Reduced_virulence
Chr01G0821.1	2096	0	605/1044(57.95)	1241	PHI:2096|Calcium-transporting_ATPase_3|MGG_02074|148305|Magnaporthe_

					oryzae_(related:_Magnaporthe_grisea)|Loss_of_pathogenicity
Chr01G0830.1	1458	9.00E-55	135/456(29.61)	200	PHI:1458|GzAra006|Q4I7F9|5518|Gibberella_zeae_(related:_Fusarium_graminearum)|Unaffected_pathogenicity
Chr01G0835.1	1046	8.00E-31	114/467(24.41)	121	PHI:1046|CTB5|ABK64182|29003|Cercospora_nicotianae|Reduced_virulence
Chr01G0836.1	1828	0	294/642(45.79)	564	PHI:1828|GzZC143|I1S351|5518|Gibberella_zeae_(related:_Fusarium_graminearum)|Unaffected_pathogenicity
Chr01G0837.1	2376	1.00E-55	127/386(32.90)	193	PHI:2376|DEP2|D2E9W7|29001|Alternaria_brassicicola|Mixed_outcome
Chr01G0839.1	1391	1.00E-13	67/238(28.15)	72	PHI:1391|GzC2H054|I1RU55|5518|Gibberella_zeae_(related:_Fusarium_graminearum)|Unaffected_pathogenicity
Chr01G0842.1	1616	3.00E-47	121/404(29.95)	178	PHI:1616|GzssDB001|I1RE55|5518|Gibberella_zeae_(related:_Fusarium_graminearum)|Unaffected_pathogenicity
Chr01G0843.1	1751	6.00E-147	298/619(48.14)	440	PHI:1751|GzZC066|I1RZ96|5518|Gibberella_zeae_(related:_Fusarium_graminearum)|Unaffected_pathogenicity
Chr01G0845.1	2655	8.00E-165	317/819(38.71)	503	PHI:2655|lon|K1C7N5|287|Pseudomonas_aeruginosa|Reduced_virulence
Chr01G0846.1	1649	5.00E-128	189/287(65.85)	367	PHI:1649|GzWing021|I1RZ94|5518|Gibberella_zeae_(related:_Fusarium_graminearum)|Unaffected_pathogenicity
Chr01G0851.1	1228	6.00E-11	85/379(22.43)	61.2	PHI:1228|FGSG_07745|I1RR00|5518|Gibberella_zeae_(related:_Fusarium_graminearum)|Lethal
Chr01G0852.1	2544	3.00E-15	43/182(23.63)	75.9	PHI:2544|PAB1|Q4WK03|746128|Aspergillus_fumigatus|Mixed_outcome
Chr01G0858.1	2528	5.00E-15	93/430(21.63)	76.3	PHI:2528|SLY1|Q4WYU7|746128|Aspergillus_fumigatus|Lethal
Chr01G0865.1	2185	8.00E-29	105/252(41.67)	120	PHI:2185|PIC1|EHA54868|148305|Magnaporthe_oryzae_(related:_Magnaporthe_grisea)|Unaffected_pathogenicity
Chr01G0868.1	1699	7.00E-78	201/658(30.55)	264	PHI:1699|GzZC014|I1RL94|5518|Gibberella_zeae_(related:_Fusarium_graminearum)|Unaffected_pathogenicity
Chr01G0870.1	2570	4.00E-92	177/507(34.91)	293	PHI:2570|CYB2|Q6FM61|5478|Candida_glabrata|Reduced_virulence
Chr01G0871.1	2240	2.00E-21	112/480(23.33)	95.1	PHI:2240|Srt1|Q4PBY9|5270|Ustilago_maydis|reduced_virulence

Chr01G0875.1	2549	2.00E-37	157/532(29.51)	145	PHI:2549|MAK5|Q4WMS3|746128|Aspergillus_fumigatus|Mixed_outcome
Chr01G0876.1	55	0	866/2685(32.25)	1176	PHI:55|PKS1|AAB08104|5016|Cochliobolus_heterostrophus|Reduced_virulence
Chr01G0878.1	2357	3.00E-86	180/515(34.95)	277	PHI:2357|CYP52X1|E2EAF6|475271|Beauveria_bassiana|Reduced_virulence
Chr01G0879.1	2968	6.00E-29	135/510(26.47)	116	PHI:2968|Hxs1|J9VQA5|5207|Cryptococcus_neoformans|Reduced_virulence
Chr01G0880.1	267	5.00E-69	180/624(28.85)	254	PHI:267|MLT1|AAD51594|5476|Candida_albicans|Reduced_virulence
Chr01G0881.1	2315	3.00E-63	114/292(39.04)	204	PHI:2315|ChLae1|G4XKY9|5016|Cochliobolus_heterostrophus|Mixed_outcome
Chr01G0890.1	3332	1.00E-08	49/153(32.03)	52.8	snf7|PHI:3332|A0A095CFR8|552467|Cryptococcus_gattii|loss_of_pathogenicity_
Chr01G0891.1	1684	3.00E-65	107/204(52.45)	214	PHI:1684|GzRad002|I1RK66|5518|Gibberella_zeae_(related:_Fusarium_graminearum)|Unaffected_pathogenicity
Chr01G0893.1	785	0	385/527(73.06)	810	PHI:785|MGG_04128|EDJ99431|318829|Magnaporthe_oryzae|Reduced_virulence
Chr01G0894.1	3028	8.00E-27	123/462(26.62)	110	Vatr2|PHI:3028|A5CVB7|28447|Clavibacter_michiganensis|reduced_virulence_
Chr01G0895.1	1637	0	380/503(75.55)	779	PHI:1637|GzWing009|Q4IEV4|5518|Gibberella_zeae_(related:_Fusarium_graminearum)|Lethal
Chr01G0896.1	1554	2.00E-17	61/179(34.08)	80.1	PHI:1554|GzMyb018|I1RC64|5518|Gibberella_zeae_(related:_Fusarium_graminearum)|Unaffected_pathogenicity
Chr01G0897.1	2643	3.00E-39	110/374(29.41)	146	PHI:2643|CFAS|A4HTK3|5671|Leishmania_infantum|Reduced_virulence
Chr01G0899.1	1573	0	730/957(76.28)	1490	PHI:1573|GzOB013|I1RFJ2|5518|Gibberella_zeae_(related:_Fusarium_graminearum)|Lethal
Chr01G0902.1	806	2.00E-87	138/250(55.20)	262	PHI:806|MGG_04137|EDJ99440|318829|Magnaporthe_oryzae|Reduced_virulence
Chr01G0904.1	3629	1.00E-23	86/335(25.67)	102	Rv0392c|PHI:3629|P95200|1773|Mycobacterium_tuberculosis|unaffected_pathogenicity_
Chr01G0906.1	339	5.00E-34	62/157(39.49)	125	PHI:339|CLPT1|CAC41973|290576|Colletotrichum_lindemuthianum|Reduced_v

					irulence
Chr01G0908.1	404	1.00E-34	95/347(27.38)	132	PHI:404|PTH11|AAD30436|318829|Magnaporthe_oryzae|Reduced_virulence
Chr01G0910.1	4194	2.00E-10	61/228(26.75)	60.8	AKT7|PHI:4194|V5XZS6|5599|Alternaria_alternata|increased_virulence_(Hyper virulence)
Chr01G0911.1	1420	6.00E-15	116/474(24.47)	78.2	PHI:1420|GzC2H088|I1S172|5518|Gibberella_zeae_(related:_Fusarium_grami nearum)|Unaffected_pathogenicity
Chr01G0914.1	538	3.00E-20	118/483(24.43)	91.7	PHI:538|FRT1|AAU87358|40559|Botrytis_cinerea|Unaffected_pathogenicity
Chr01G0917.1	510	5.00E-20	124/548(22.63)	90.9	PHI:510|CaNAG3|EAK93097|5476|Candida_albicans|Reduced_virulence
Chr01G0922.1	1159	4.00E-07	37/99(37.37)	50.8	PHI:1159|MgAtr7|A5H456|54734|Mycosphaerella_graminicola_(related:_Zymos eptoria_tritici)|Chemistry_target
Chr01G0923.1	336	0	777/1052(73.86)	1533	PHI:336|CHS2|AAT77182|5507|Fusarium_oxysporum|Reduced_virulence
Chr01G0930.1	1458	2.00E-34	123/425(28.94)	137	PHI:1458|GzAra006|Q4I7F9|5518|Gibberella_zeae_(related:_Fusarium_gramin earum)|Unaffected_pathogenicity
Chr01G0931.1	1393	2.00E-09	52/236(22.03)	57	PHI:1393|GzC2H056|I1RU69|5518|Gibberella_zeae_(related:_Fusarium_grami nearum)|Unaffected_pathogenicity
Chr01G0941.1	2731	0	397/556(71.40)	781	PHI:2731|VdSge1|G2XD29|27337|Verticillium_dahliae|Loss_of_pathogenicity
Chr01G0944.1	439	2.00E-21	46/100(46.00)	87.8	PHI:439|TRI6|BAA83722|5518|Fusarium_graminearum|Reduced_virulence
Chr01G0947.1	407	7.00E-06	36/134(26.87)	43.5	PHI:407|PBC1|CAB40372|76659|Pyrenopeziza_brassicae|Loss_of_pathogenici ty
Chr01G0948.1	1555	3.00E-28	110/459(23.97)	114	PHI:1555|GzMyb019|I1RDG6|5518|Gibberella_zeae_(related:_Fusarium_grami nearum)|Unaffected_pathogenicity
Chr01G0955.1	441	2.00E-21	71/279(25.45)	92	PHI:441|BTP1|CAE55153|40559|Botrytis_cinerea|Reduced_virulence
Chr01G0956.1	716	0	326/539(60.48)	700	PHI:716|ZEB1|ABB90284|5518|Fusarium_graminearum|Unaffected_pathogenic ity
Chr01G0958.1	1754	1.00E-85	137/257(53.31)	269	PHI:1754|GzZC069|I1S1D6|5518|Gibberella_zeae_(related:_Fusarium_gramin earum)|Unaffected_pathogenicity

Chr01G0963.1	4194	6.00E-16	91/358(25.42)	78.2	AKT7|PHI:4194|V5XZS6|5599|Alternaria_alternata|increased_virulence_(Hypervirulence)
Chr01G0974.1	1974	7.00E-20	92/342(26.90)	90.5	PHI:1974|GzZC289|I1RQN6|5518|Gibberella_zeae_(related:_Fusarium_graminearum)|Unaffected_pathogenicity
Chr01G0975.1	1651	6.00E-49	117/321(36.45)	170	PHI:1651|GzWing023|I1S0U2|5518|Gibberella_zeae_(related:_Fusarium_graminearum)|Unaffected_pathogenicity
Chr01G0978.1	441	2.00E-09	77/304(25.33)	56.2	PHI:441|BTP1|CAE55153|40559|Botrytis_cinerea|Reduced_virulence
Chr01G0980.1	2544	1.00E-07	64/271(23.62)	50.4	PHI:2544|PAB1|Q4WK03|746128|Aspergillus_fumigatus|Mixed_outcome
Chr01G0989.1	1662	2.00E-35	121/492(24.59)	139	PHI:1662|GzCCHC002|I1REJ1|5518|Gibberella_zeae_(related:_Fusarium_graminearum)|Unaffected_pathogenicity
Chr01G0998.1	3386	6.00E-10	72/250(28.80)	57	FVEG_12528|PHI:3386|W7NCN7|117187|Fusarium_verticillioides|unaffected_pathogenicity_
Chr01G0999.1	1047	3.00E-11	41/115(35.65)	59.7	PHI:1047|CTB6|ABK64183|29003|Cercospora_nicotianae|Reduced_virulence
Chr01G1000.1	2976	9.00E-55	173/726(23.83)	199	PHI:2976|CgOPT1|C6ZRH8|29905|Colletotrichum_gloeosporioides_f._sp._aeschynomenes|Reduced_virulence
Chr01G1001.1	2976	2.00E-143	254/721(35.23)	441	PHI:2976|CgOPT1|C6ZRH8|29905|Colletotrichum_gloeosporioides_f._sp._aeschynomenes|Reduced_virulence
Chr01G1003.1	812	4.00E-45	111/363(30.58)	160	PHI:812|MGG_10702|EDJ94108|318829|Magnaporthe_oryzae|Reduced_virulence
Chr01G1011.1	2099	0	834/1290(64.65)	1591	PHI:2099|Pmc1|MGG_07971|148305|Magnaporthe_oryzae_(related:_Magnaporthe_grisea)|Mixed_outcome
Chr01G1012.1	3378	2.00E-59	117/246(47.56)	191	Plegl1|PHI:3378|G9JLA8|285811|Pyrenochaeta_lycopersici|unaffected_pathogenicity_
Chr01G1014.1	2639	0	378/542(69.74)	771	PHI:2639|Ilv3B|Q4WK65|746128|Aspergillus_fumigatus|Mixed_outcome
Chr01G1016.1	1816	5.00E-23	79/300(26.33)	101	PHI:1816|GzZC131|I1RRS3|5518|Gibberella_zeae_(related:_Fusarium_graminearum)|Unaffected_pathogenicity

Chr01G1018.1	479	9.00E-86	138/274(50.36)	259	PHI:479|MEP1|AAQ07436|199306|Coccidioides_posadasii|Reduced_virulence
Chr01G1019.1	1932	3.00E-34	118/452(26.11)	134	PHI:1932|GzZC247|I1REH9|5518|Gibberella_zeae_(related:_Fusarium_graminearum)|Unaffected_pathogenicity
Chr01G1022.1	2266	6.00E-144	224/535(41.87)	430	PHI:2266|Ptr2|0|13684|Phaeosphaeria_nodorum_(related:_Stagonospora_nodorum)|Unaffected_pathogenicity
Chr01G1028.1	3389	3.00E-107	201/540(37.22)	334	FVEG_12521|PHI:3389|W7MS01|117187|Fusarium_verticillioides|unaffected_pathogenicity_
Chr01G1029.1	2597	7.00E-122	208/470(44.26)	365	PHI:2597|Pir32|Q59PW0|746128|Aspergillus_fumigatus|Mixed_outcome
Chr01G1030.1	143	3.00E-30	87/327(26.61)	116	PHI:143|CHT|AAA33353|5563|Gloeocercospora_sorghi|Unaffected_pathogenicity
Chr01G1031.1	1921	4.00E-07	45/190(23.68)	50.4	PHI:1921|GzZC236|I1RCH8|5518|Gibberella_zeae_(related:_Fusarium_graminearum)|Reduced_virulence
Chr01G1032.1	3385	9.00E-156	216/423(51.06)	451	FVEG_12529|PHI:3385|W7MS09|117187|Fusarium_verticillioides|unaffected_pathogenicity_
Chr01G1039.1	3036	4.00E-135	189/323(58.51)	388	FgERG3B|PHI:3036|I1RM19|5518|Fusarium_graminearum|reduced_virulence_
Chr01G1044.1	2315	3.00E-58	109/291(37.46)	191	PHI:2315|ChLae1|G4XKY9|5016|Cochliobolus_heterostrophus|Mixed_outcome
Chr01G1048.1	1675	2.00E-06	57/210(27.14)	48.9	PHI:1675|GzDHHC003|Q4I8B6|5518|Gibberella_zeae_(related:_Fusarium_graminearum)|Unaffected_pathogenicity
Chr01G1053.1	211	2.00E-56	122/311(39.23)	203	PHI:211|CaTUP1|AAB63195|5476|Candida_albicans|Reduced_virulence
Chr01G1055.1	391	0	1060/1478(71.72)	2216	PHI:391|ABC2|BAC67162|318829|Magnaporthe_oryzae|Unaffected_pathogenicity
Chr01G1064.1	874	3.00E-103	212/481(44.07)	321	PHI:874|MGG_00435|EDK02952|318829|Magnaporthe_oryzae|Loss_of_pathogenicity
Chr01G1072.1	799	7.00E-48	144/434(33.18)	181	PHI:799|MGG_03530|EDJ94565|318829|Magnaporthe_oryzae|Reduced_virulence
Chr01G1074.1	3226	1.00E-133	202/372(54.30)	389	pnl1|PHI:3226|T2C7K6|36651|Penicillium_digitatum|reduced_virulence_

Chr01G1078.1	441	5.00E-15	88/351(25.07)	73.2	PHI:441|BTP1|CAE55153|40559|Botrytis_cinerea|Reduced_virulence
Chr01G1080.1	1956	6.00E-48	170/607(28.01)	177	PHI:1956|GzZC271|I1RLT2|5518|Gibberella_zeae_(related:_Fusarium_graminearum)|Unaffected_pathogenicity
Chr01G1081.1	1662	8.00E-32	114/478(23.85)	127	PHI:1662|GzCCHC002|I1REJ1|5518|Gibberella_zeae_(related:_Fusarium_graminearum)|Unaffected_pathogenicity
Chr01G1083.1	2315	5.00E-12	29/95(30.53)	58.9	PHI:2315|ChLae1|G4XKY9|5016|Cochliobolus_heterostrophus|Mixed_outcome
Chr01G1086.1	922	3.00E-15	97/381(25.46)	76.3	PHI:922|um03615||5270|Ustilago_maydis|Unaffected_pathogenicity
Chr01G1098.1	1890	4.00E-132	284/876(32.42)	416	PHI:1890|GzZC205|I1RTH7|5518|Gibberella_zeae_(related:_Fusarium_graminearum)|Unaffected_pathogenicity
Chr01G1099.1	2269	5.00E-33	84/264(31.82)	122	PHI:2269|Mdh1|0|13684|Phaeosphaeria_nodorum_(related:_Stagonospora_nodorum)|Unaffected_pathogenicity
Chr01G1101.1	2968	1.00E-75	149/496(30.04)	249	PHI:2968|Hxs1|J9VQA5|5207|Cryptococcus_neoformans|Reduced_virulence
Chr01G1106.1	812	1.00E-22	83/305(27.21)	97.8	PHI:812|MGG_10702|EDJ94108|318829|Magnaporthe_oryzae|Reduced_virulence
Chr01G1109.1	479	2.00E-45	96/253(37.94)	153	PHI:479|MEP1|AAQ07436|199306|Coccidioides_posadasii|Reduced_virulence
Chr01G1111.1	2197	2.00E-151	233/481(48.44)	441	PHI:2197|MoRgs6|XP_364773|148305|Magnaporthe_oryzae_(related:_Magnaporthe_grisea)|Unaffected_pathogenicity
Chr01G1114.1	441	3.00E-10	58/286(20.28)	58.9	PHI:441|BTP1|CAE55153|40559|Botrytis_cinerea|Reduced_virulence
Chr01G1117.1	1895	1.00E-11	26/45(57.78)	65.5	PHI:1895|GzZC210|I1RNU2|5518|Gibberella_zeae_(related:_Fusarium_graminearum)|Unaffected_pathogenicity
Chr01G1121.1	901	6.00E-56	141/473(29.81)	197	PHI:901|um01886||5270|Ustilago_maydis|Unaffected_pathogenicity
Chr01G1124.1	2908	2.00E-11	85/353(24.08)	63.5	PHI:2908|CYP51B|I1RBR4|5518|Fusarium_graminearum|Mixed_outcome
Chr01G1125.1	901	8.00E-84	155/441(35.15)	270	PHI:901|um01886||5270|Ustilago_maydis|Unaffected_pathogenicity
Chr01G1134.1	3301	6.00E-07	49/184(26.63)	46.6	patN|PHI:3301|A0A075TRB3|27334|Penicillium_expansum|unaffected_pathogenicity_
Chr01G1139.1	482	3.00E-39	93/292(31.85)	142	PHI:482|LAEA|AAR01218|5085|Aspergillus_fumigatus|Reduced_virulence

Chr01G1140.1	2503	0	1283/2474(51.86)	2528	PHI:2503|AGS1|Q96UQ6|746128|Aspergillus_fumigatus|Unaffected_pathogenicity
Chr01G1148.1	1555	0	401/510(78.63)	855	PHI:1555|GzMyb019|I1RDG6|5518|Gibberella_zeae_(related:_Fusarium_graminearum)|Unaffected_pathogenicity
Chr01G1153.1	1423	2.00E-21	57/181(31.49)	97.8	PHI:1423|GzC2H091|I1S2R1|5518|Gibberella_zeae_(related:_Fusarium_graminearum)|Unaffected_pathogenicity
Chr01G1159.1	2451	3.00E-102	162/306(52.94)	303	PHI:2451|FAEB1|I1S3N8|5518|Gibberella_zeae_(related:_Fusarium_graminearum)|Unaffected_pathogenicity
Chr01G1160.1	1790	0	484/807(59.98)	944	PHI:1790|GzZC105|I1RVT7|5518|Gibberella_zeae_(related:_Fusarium_graminearum)|Lethal
Chr01G1170.1	1554	8.00E-11	55/170(32.35)	63.5	PHI:1554|GzMyb018|I1RC64|5518|Gibberella_zeae_(related:_Fusarium_graminearum)|Unaffected_pathogenicity
Chr01G1171.1	2038	1.00E-06	69/274(25.18)	47	PHI:2038|Mir1|MGG_02370|148305|Magnaporthe_oryzae_(related:_Magnaporthe_grisea)|Unaffected_pathogenicity
Chr01G1172.1	2802	5.00E-15	73/279(26.16)	70.9	PHI:2802|3hnr|B2ZRQ4|93612|Setosphaeria_turcica|Unaffected_pathogenicity
Chr01G1175.1	2038	2.00E-13	60/221(27.15)	67	PHI:2038|Mir1|MGG_02370|148305|Magnaporthe_oryzae_(related:_Magnaporthe_grisea)|Unaffected_pathogenicity
Chr01G1177.1	2080	0	509/975(52.21)	798	PHI:2080|Moatg13|MGG_00454|148305|Magnaporthe_oryzae_(related:_Magnaporthe_grisea)|Reduced_virulence
Chr01G1179.1	2821	2.00E-27	81/256(31.64)	118	PHI:2821|SNF2|Q5ALP9|5476|Candida_albicans|Reduced_virulence
Chr01G1181.1	424	0	349/552(63.22)	716	PHI:424|PCK1|EAL19243|5207|Cryptococcus_neoformans|Reduced_virulence
Chr01G1183.1	2357	3.00E-25	127/509(24.95)	106	PHI:2357|CYP52X1|E2EAF6|475271|Beauveria_bassiana|Reduced_virulence
Chr01G1184.1	2205	6.00E-13	57/184(30.98)	67	PHI:2205|JmjC_domain-containing_protein_5_[GH10_family]|MGG_01543|148305|Magnaporthe_oryzae_(related:_Magnaporthe_grisea)|Reduced_virulence
Chr01G1188.1	2067	0	948/1667(56.87)	1810	PHI:2067|ABC4|MGG_00937|148305|Magnaporthe_oryzae_(related:_Magnaporthe_grisea)|Loss_of_pathogenicity

Chr01G1189.1	404	4.00E-27	59/211(27.96)	110	PHI:404|PTH11|AAD30436|318829|Magnaporthe_oryzae|Reduced_virulence
Chr01G1190.1	3161	6.00E-30	98/341(28.74)	127	Mohik5|PHI:3161|G4MKP6|318829|Magnaporthe_oryzae|loss_of_pathogenicity_
Chr01G1193.1	1789	0	337/780(43.21)	553	PHI:1789|GzZC104|I1RVU7|5518|Gibberella_zeae_(related:_Fusarium_graminearum)|Unaffected_pathogenicity
Chr01G1197.1	1646	0	496/995(49.85)	858	PHI:1646|GzWing018|I1RW28|5518|Gibberella_zeae_(related:_Fusarium_graminearum)|Reduced_virulence
Chr01G1201.1	673	7.00E-41	78/198(39.39)	140	PHI:673|Can1|AAZ30050|178876|Cryptococcus_neoformans|Unaffected_pathogenicity
Chr01G1204.1	2141	0	655/1103(59.38)	1127	PHI:2141|CDC15|XP_001406795|148305|Magnaporthe_oryzae_(related:_Magnaporthe_grisea)|Loss_of_pathogenicity
Chr01G1205.1	1439	0	1393/2205(63.17)	2712	PHI:1439|GzNot001|I1RW22|5518|Gibberella_zeae_(related:_Fusarium_graminearum)|Lethal
Chr01G1210.1	1527	1.00E-19	77/318(24.21)	87	PHI:1527|GzHOMEL040|I1S9A1|5518|Gibberella_zeae_(related:_Fusarium_graminearum)|Lethal
Chr01G1211.1	2020	2.00E-10	75/306(24.51)	59.3	PHI:2020|Tup1|XP_759427|5270|Ustilago_maydis|Mixed_outcome
Chr01G1213.1	3179	0	665/1025(64.88)	1252	MoKIN1|PHI:3179|G4MY59|318829|Magnaporthe_oryzae|reduced_virulence_
Chr01G1216.1	1321	9.00E-08	22/55(40.00)	49.7	PHI:1321|ZEB2|I1RFC6|5518|Gibberella_zeae_(related:_Fusarium_graminearum)|Unaffected_pathogenicity
Chr01G1222.1	2552	3.00E-06	46/182(25.27)	45.8	PHI:2552|NOP4|Q4WNM3|746128|Aspergillus_fumigatus|Mixed_outcome
Chr01G1223.1	2195	0	831/1255(66.22)	1592	PHI:2195|MoRgs4|XP_368254|148305|Magnaporthe_oryzae_(related:_Magnaporthe_grisea)|Mixed_outcome
Chr01G1230.1	2033	4.00E-10	54/184(29.35)	60.1	PHI:2033|MgPex6|G4NBI6|148305|Magnaporthe_oryzae_(related:_Magnaporthe_grisea)|Loss_of_pathogenicity
Chr01G1233.1	1046	1.00E-08	28/77(36.36)	53.9	PHI:1046|CTB5|ABK64182|29003|Cercospora_nicotianae|Reduced_virulence
Chr01G1234.1	2025	5.00E-08	41/137(29.93)	52.8	PHI:2025|HDL1|G4MQZ9|148305|Magnaporthe_oryzae_(related:_Magnaporthe

					_grisea)|Unaffected_pathogenicity
Chr01G1235.1	876	0	455/1045(43.54)	795	PHI:876|MGG_11671|EDK03349|318829|Magnaporthe_oryzae|Reduced_virulence
Chr01G1236.1	886	9.00E-12	61/257(23.74)	63.5	PHI:886|MGG_13052|EDK06087|318829|Magnaporthe_oryzae|Reduced_virulence
Chr01G1242.1	2488	0	320/497(64.39)	629	PHI:2488|Man1|G4ND25|148305|Magnaporthe_oryzae_(related:_Magnaporthe_grisea)|Unaffected_pathogenicity
Chr01G1247.1	2042	2.00E-09	65/285(22.81)	58.2	PHI:2042|ABC3|Q3Y5V5|148305|Magnaporthe_oryzae_(related:_Magnaporthe_grisea)|Loss_of_pathogenicity
Chr01G1248.1	820	0	426/446(95.52)	858	PHI:820|beta-tubulin|AAA34230|5025|Venturia_inaequalis|Chemistry_target
Chr01G1255.1	1676	9.00E-15	46/141(32.62)	73.6	PHI:1676|GzDHHC004|Q4I2M7|5518|Gibberella_zeae_(related:_Fusarium_graminearum)|Unaffected_pathogenicity
Chr01G1259.1	1313	1.00E-149	278/535(51.96)	440	PHI:1313|GzbHLH014|I1RY71|5518|Gibberella_zeae_(related:_Fusarium_graminearum)|Unaffected_pathogenicity
Chr01G1262.1	2079	2.00E-49	74/124(59.68)	157	PHI:2079|Moatg12|MGG_00598|148305|Magnaporthe_oryzae_(related:_Magnaporthe_grisea)|Loss_of_pathogenicity
Chr01G1266.1	1692	0	435/565(76.99)	884	PHI:1692|GzZC007|I1RYR6|5518|Gibberella_zeae_(related:_Fusarium_graminearum)|Unaffected_pathogenicity
Chr01G1267.1	2008	9.00E-11	45/146(30.82)	59.7	PHI:2008|HST1|EDJ98541|148305|Magnaporthe_oryzae_(related:_Magnaporthe_grisea)|Unaffected_pathogenicity
Chr01G1268.1	881	9.00E-32	103/332(31.02)	121	PHI:881|MGG_04556|EDJ96020|318829|Magnaporthe_oryzae|Reduced_virulence
Chr01G1276.1	2181	1.00E-50	88/148(59.46)	164	PHI:2181|MoDUO1|XP_365782|148305|Magnaporthe_oryzae_(related:_Magnaporthe_grisea)|Reduced_virulence
Chr01G1280.1	2099	0	512/1088(47.06)	947	PHI:2099|Pmc1|MGG_07971|148305|Magnaporthe_oryzae_(related:_Magnaporthe_grisea)|Mixed_outcome

Chr01G1281.1	2008	2.00E-178	273/535(51.03)	516	PHI:2008|HST1|EDJ98541|148305|Magnaporthe_oryzae_(related:_Magnaporthe_grisea)|Unaffected_pathogenicity
Chr01G1282.1	1246	0	660/1030(64.08)	1251	PHI:1246|FGSG_05549|I1RYQ5|5518|Gibberella_zeae_(related:_Fusarium_graminearum)|Unaffected_pathogenicity
Chr01G1283.1	157	2.00E-19	84/330(25.45)	86.7	PHI:157|TOXF|AAD45321|5017|Cochliobolus_carbonum|Loss_of_pathogenicity
Chr01G1285.1	1681	8.00E-12	93/362(25.69)	67.8	PHI:1681|GzNF001|I1REN7|5518|Gibberella_zeae_(related:_Fusarium_graminearum)|Unaffected_pathogenicity
Chr01G1288.1	438	1.00E-19	57/188(30.32)	89	PHI:438|BcBOT1_(related:_CND5)|AAQ16576|40559|Botrytis_cinerea|Reduced_virulence
Chr01G1292.1	598	8.00E-95	158/358(44.13)	290	PHI:598|THIOL|ABB55459|5022|Leptosphaeria_maculans|Reduced_virulence
Chr01G1298.1	2544	0	484/725(66.76)	837	PHI:2544|PAB1|Q4WK03|746128|Aspergillus_fumigatus|Mixed_outcome
Chr01G1300.1	785	2.00E-131	217/512(42.38)	394	PHI:785|MGG_04128|EDJ99431|318829|Magnaporthe_oryzae|Reduced_virulence
Chr01G1306.1	2339	7.00E-16	50/140(35.71)	69.3	PHI:2339|UhCnb1|I2G5N7|120017|Ustilago_hordei|Reduced_virulence
Chr01G1311.1	1401	0	307/454(67.62)	604	PHI:1401|GzC2H064|I1RVY6|5518|Gibberella_zeae_(related:_Fusarium_graminearum)|Unaffected_pathogenicity
Chr01G1316.1	2376	8.00E-42	127/411(30.90)	154	PHI:2376|DEP2|D2E9W7|29001|Alternaria_brassicicola|Mixed_outcome
Chr01G1317.1	1953	6.00E-106	181/423(42.79)	323	PHI:1953|GzZC268|I1RKB4|5518|Gibberella_zeae_(related:_Fusarium_graminearum)|Unaffected_pathogenicity
Chr01G1321.1	2256	2.00E-130	188/357(52.66)	380	PHI:2256|Xdh1|Q0UA24|13684|Phaeosphaeria_nodorum_(related:_Stagonospora_nodorum)|Unaffected_pathogenicity
Chr01G1323.1	2269	2.00E-87	134/253(52.96)	263	PHI:2269|Mdh1|0|13684|Phaeosphaeria_nodorum_(related:_Stagonospora_nodorum)|Unaffected_pathogenicity
Chr01G1324.1	2968	8.00E-31	117/463(25.27)	122	PHI:2968|Hxs1|J9VQA5|5207|Cryptococcus_neoformans|Reduced_virulence
Chr01G1325.1	1819	3.00E-70	119/253(47.04)	232	PHI:1819|GzZC134|I1S7J0|5518|Gibberella_zeae_(related:_Fusarium_graminearum)|Unaffected_pathogenicity

Chr01G1326.1	2269	2.00E-66	122/275(44.36)	209	PHI:2269|Mdh1|0|13684|Phaeosphaeria_nodorum_(related:_Stagonospora_nodorum)|Unaffected_pathogenicity
Chr01G1327.1	179	1.00E-63	116/254(45.67)	199	PHI:179|PELA|AAA33338|140110|Nectria_haematococca_(related:_Fusarium_solani)|Reduced_virulence
Chr01G1328.1	404	3.00E-42	101/345(29.28)	155	PHI:404|PTH11|AAD30436|318829|Magnaporthe_oryzae|Reduced_virulence
Chr01G1330.1	2968	8.00E-35	135/513(26.32)	134	PHI:2968|Hxs1|J9VQA5|5207|Cryptococcus_neoformans|Reduced_virulence
Chr01G1337.1	3378	1.00E-71	109/214(50.93)	223	Plegl1|PHI:3378|G9JLA8|285811|Pyrenochaeta_lycopersici|unaffected_pathogenicity_
Chr01G1342.1	1675	6.00E-08	57/248(22.98)	54.3	PHI:1675|GzDHHC003|Q4I8B6|5518|Gibberella_zeae_(related:_Fusarium_graminearum)|Unaffected_pathogenicity
Chr01G1347.1	1904	2.00E-51	168/622(27.01)	186	PHI:1904|GzZC219|I1RHR0|5518|Gibberella_zeae_(related:_Fusarium_graminearum)|Unaffected_pathogenicity
Chr01G1353.1	881	3.00E-31	78/240(32.50)	119	PHI:881|MGG_04556|EDJ96020|318829|Magnaporthe_oryzae|Reduced_virulence
Chr01G1356.1	885	1.00E-91	168/307(54.72)	318	PHI:885|MGG_02240|EDJ98842|318829|Magnaporthe_oryzae|Reduced_virulence
Chr01G1358.1	2357	4.00E-12	44/122(36.07)	65.5	PHI:2357|CYP52X1|E2EAF6|475271|Beauveria_bassiana|Reduced_virulence
Chr01G1359.1	1939	2.00E-19	76/278(27.34)	85.9	PHI:1939|GzZC254|I1RMX8|5518|Gibberella_zeae_(related:_Fusarium_graminearum)|Unaffected_pathogenicity
Chr01G1375.1	1863	4.00E-11	43/121(35.54)	62	PHI:1863|GzZC178|I1RL85|5518|Gibberella_zeae_(related:_Fusarium_graminearum)|Unaffected_pathogenicity
Chr01G1376.1	438	2.00E-20	64/192(33.33)	88.2	PHI:438|BcBOT1_(related:_CND5)|AAQ16576|40559|Botrytis_cinerea|Reduced_virulence
Chr01G1377.1	4194	6.00E-06	30/119(25.21)	43.9	AKT7|PHI:4194|V5XZS6|5599|Alternaria_alternata|increased_virulence_(Hypervirulence)
Chr01G1379.1	1851	5.00E-81	218/696(31.32)	270	PHI:1851|GzZC166|I1RXJ3|5518|Gibberella_zeae_(related:_Fusarium_gramin

					earum)|Lethal
Chr01G1384.1	2175	9.00E-67	136/325(41.85)	213	PHI:2175|NMR3|MGG_09705|148305|Magnaporthe_oryzae_(related:_Magnaporthe_grisea)|Reduced_virulence
Chr01G1391.1	2393	1.00E-26	102/443(23.02)	110	PHI:2393|Related_to_O-methylsterigmatocystin_oxidoreductase|I1R980|5518|Gibberella_zeae_(related:_Fusarium_graminearum)|Increased_virulence_(Hypervirulence)
Chr01G1395.1	319	8.00E-94	197/495(39.80)	328	PHI:319|SQL2|AAO19638|5270|Ustilago_maydis|Reduced_virulence
Chr01G1396.1	1555	2.00E-53	123/444(27.70)	188	PHI:1555|GzMyb019|I1RDG6|5518|Gibberella_zeae_(related:_Fusarium_graminearum)|Unaffected_pathogenicity
Chr01G1400.1	1662	4.00E-14	55/233(23.61)	72.8	PHI:1662|GzCCHC002|I1REJ1|5518|Gibberella_zeae_(related:_Fusarium_graminearum)|Unaffected_pathogenicity
Chr01G1402.1	1968	0	322/690(46.67)	595	PHI:1968|GzZC283|I1RB12|5518|Gibberella_zeae_(related:_Fusarium_graminearum)|Unaffected_pathogenicity
Chr01G1404.1	2383	1.00E-38	84/186(45.16)	132	PHI:2383|MfCUT1|Q2VF46|38448|Monilinia_fructicola|Increased_virulence_(hypervirulence)
Chr01G1415.1	1662	7.00E-16	82/361(22.71)	78.2	PHI:1662|GzCCHC002|I1REJ1|5518|Gibberella_zeae_(related:_Fusarium_graminearum)|Unaffected_pathogenicity
Chr01G1417.1	2227	6.00E-15	87/355(24.51)	74.3	PHI:2227|pao|D4III8|5270|Ustilago_maydis|Mixed_outcome
Chr01G1418.1	1852	3.00E-43	88/278(31.65)	157	PHI:1852|GzZC167|I1S170|5518|Gibberella_zeae_(related:_Fusarium_graminearum)|Unaffected_pathogenicity
Chr01G1424.1	1317	3.00E-10	32/102(31.37)	61.6	PHI:1317|GzBrom002|I1RQE9|5518|Gibberella_zeae_(related:_Fusarium_graminearum)|Reduced_virulence
Chr01G1426.1	2451	3.00E-66	112/278(40.29)	211	PHI:2451|FAEB1|I1S3N8|5518|Gibberella_zeae_(related:_Fusarium_graminearum)|Unaffected_pathogenicity
Chr01G1433.1	1279	5.00E-38	121/390(31.03)	147	PHI:1279|FGSG_12132|I1RU59|5518|Gibberella_zeae_(related:_Fusarium_graminearum)|Unaffected_pathogenicity

Chr01G1435.1	1685	0	402/575(69.91)	780	PHI:1685|GzRad003|I1S5Z3|5518|Gibberella_zeae_(related:_Fusarium_graminearum)|Unaffected_pathogenicity
Chr01G1442.1	1662	6.00E-76	144/434(33.18)	261	PHI:1662|GzCCHC002|I1REJ1|5518|Gibberella_zeae_(related:_Fusarium_graminearum)|Unaffected_pathogenicity
Chr01G1443.1	1792	0	321/690(46.52)	609	PHI:1792|GzZC107|I1RVS7|5518|Gibberella_zeae_(related:_Fusarium_graminearum)|Unaffected_pathogenicity
Chr01G1444.1	2032	8.00E-45	113/372(30.38)	165	PHI:2032|VTL1|G4NGA7|148305|Magnaporthe_oryzae_(related:_Magnaporthe_grisea)|Unaffected_pathogenicity
Chr01G1447.1	3383	1.00E-78	135/342(39.47)	248	FVEG_12531|PHI:3383|W7N2A8|117187|Fusarium_verticillioides|unaffected_pathogenicity_
Chr01G1450.1	2393	4.00E-08	39/172(22.67)	52.8	PHI:2393|Related_to_O-methylsterigmatocystin_oxidoreductase|I1R980|5518|Gibberella_zeae_(related:_Fusarium_graminearum)|Increased_virulence_(Hypervirulence)
Chr01G1453.1	2315	5.00E-54	110/293(37.54)	180	PHI:2315|ChLae1|G4XKY9|5016|Cochliobolus_heterostrophus|Mixed_outcome
Chr01G1455.1	4194	3.00E-31	118/498(23.69)	124	AKT7|PHI:4194|V5XZS6|5599|Alternaria_alternata|increased_virulence_(Hypervirulence)
Chr01G1457.1	3116	1.00E-06	79/335(23.58)	47.8	PSPTO_0371|PHI:3116|Q88AL8|317|Pseudomonas_syringae|mixed_outcome_
Chr01G1466.1	479	9.00E-52	109/296(36.82)	172	PHI:479|MEP1|AAQ07436|199306|Coccidioides_posadasii|Reduced_virulence
Chr01G1471.1	876	0	484/1227(39.45)	802	PHI:876|MGG_11671|EDK03349|318829|Magnaporthe_oryzae|Reduced_virulence
Chr01G1480.1	1872	7.00E-93	164/420(39.05)	290	PHI:1872|GzZC187|I1RTU2|5518|Gibberella_zeae_(related:_Fusarium_graminearum)|Lethal
Chr01G1481.1	3381	2.00E-07	46/161(28.57)	50.4	FVEG_12533|PHI:3381|W7N2B4|117187|Fusarium_verticillioides|unaffected_pathogenicity_
Chr01G1483.1	2654	3.00E-09	44/130(33.85)	57	PHI:2654|DUR1,2|Q59VF3|5476|Candida_albicans|Reduced_virulence
Chr01G1484.1	1392	8.00E-89	187/457(40.92)	288	PHI:1392|GzC2H055|I1RU67|5518|Gibberella_zeae_(related:_Fusarium_grami

					nearum)|Unaffected_pathogenicity
Chr01G1485.1	4194	9.00E-29	120/479(25.05)	116	AKT7|PHI:4194|V5XZS6|5599|Alternaria_alternata|increased_virulence_(Hyper virulence)
Chr01G1487.1	1260	1.00E-46	101/284(35.56)	172	PHI:1260|FGSG_13944|I1RUC7|5518|Gibberella_zeae_(related:_Fusarium_graminearum)|Unaffected_pathogenicity
Chr01G1492.1	569	2.00E-11	27/42(64.29)	62.4	PHI:569|XYL3|AAC06239|5507|Fusarium_oxysporum|Unaffected_pathogenicity
Chr01G1493.1	2747	9.00E-30	99/415(23.86)	119	PHI:2747|Cnt|A8YZD7|1280|Staphylococcus_aureus|Reduced_virulence
Chr01G1494.1	2240	1.00E-44	121/478(25.31)	164	PHI:2240|Srt1|Q4PBY9|5270|Ustilago_maydis|reduced_virulence
Chr01G1496.1	115	3.00E-18	96/411(23.36)	84.3	PHI:115|PGX1|AAC26146|5017|Cochliobolus_carbonum|Unaffected_pathogenicity
Chr01G1501.1	1520	3.00E-14	42/180(23.33)	67	PHI:1520|GzHOMEL024|I1RWS3|5518|Gibberella_zeae_(related:_Fusarium_graminearum)|Unaffected_pathogenicity
Chr01G1504.1	441	3.00E-28	85/309(27.51)	110	PHI:441|BTP1|CAE55153|40559|Botrytis_cinerea|Reduced_virulence
Chr01G1509.1	2020	2.00E-26	75/285(26.32)	110	PHI:2020|Tup1|XP_759427|5270|Ustilago_maydis|Mixed_outcome
Chr01G1512.1	2511	0	501/1697(29.52)	706	PHI:2511|Pes1|Q4WT66|746128|Aspergillus_fumigatus|Reduced_virulence
Chr01G1513.1	2025	9.00E-06	37/129(28.68)	45.1	PHI:2025|HDL1|G4MQZ9|148305|Magnaporthe_oryzae_(related:_Magnaporthe_grisea)|Unaffected_pathogenicity
Chr01G1516.1	1047	2.00E-13	54/207(26.09)	67.8	PHI:1047|CTB6|ABK64183|29003|Cercospora_nicotianae|Reduced_virulence
Chr01G1517.1	1402	9.00E-07	24/43(55.81)	49.7	PHI:1402|GzC2H065|I1RWB8|5518|Gibberella_zeae_(related:_Fusarium_graminearum)|Unaffected_pathogenicity
Chr01G1518.1	2570	5.00E-07	24/60(40.00)	51.6	PHI:2570|CYB2|Q6FM61|5478|Candida_glabrata|Reduced_virulence
Chr01G1519.1	1399	1.00E-12	45/169(26.63)	68.9	PHI:1399|GzC2H062|I1RV74|5518|Gibberella_zeae_(related:_Fusarium_graminearum)|Unaffected_pathogenicity
Chr01G1521.1	2248	3.00E-19	79/278(28.42)	89.7	PHI:2248|Als1|Q1L2E2|13684|Phaeosphaeria_nodorum_(related:_Stagonospora_nodorum)|Mixed_outcome
Chr01G1523.1	438	3.00E-15	98/432(22.69)	77.4	PHI:438|BcBOT1_(related:_CND5)|AAQ16576|40559|Botrytis_cinerea|Reduced

					_virulence
Chr01G1524.1	2488	7.00E-42	169/525(32.19)	155	PHI:2488|Man1|G4ND25|148305|Magnaporthe_oryzae_(related:_Magnaporthe_grisea)|Unaffected_pathogenicity
Chr01G1527.1	2156	1.00E-10	28/70(40.00)	60.1	PHI:2156|Moatf1|MGG_08212|148305|Magnaporthe_oryzae_(related:_Magnaporthe_grisea)|Reduced_virulence
Chr01G1528.1	716	3.00E-15	58/184(31.52)	75.5	PHI:716|ZEB1|ABB90284|5518|Fusarium_graminearum|Unaffected_pathogenicity
Chr01G1531.1	2976	6.00E-126	244/722(33.80)	398	PHI:2976|CgOPT1|C6ZRH8|29905|Colletotrichum_gloeosporioides_f._sp._aeschynomenes|Reduced_virulence
Chr01G1534.1	401	3.00E-66	109/225(48.44)	204	PHI:401|SOD2|AAB86583|5476|Candida_albicans|Unaffected_pathogenicity
Chr01G1538.1	713	8.00E-150	391/1405(27.83)	513	PHI:713|PKS13_(related:_ZEA2)|ABB90282|5518|Fusarium_graminearum|Unaffected_pathogenicity
Chr01G1542.1	569	4.00E-15	32/56(57.14)	73.6	PHI:569|XYL3|AAC06239|5507|Fusarium_oxysporum|Unaffected_pathogenicity
Chr01G1543.1	2290	0	948/2536(37.38)	1546	PHI:2290|BcBOA6|B1GVX7|40559|Botrytis_cinerea|Reduced_virulence
Chr01G1544.1	1926	7.00E-75	193/525(36.76)	243	PHI:1926|GzZC241|I1RKP6|5518|Gibberella_zeae_(related:_Fusarium_graminearum)|Unaffected_pathogenicity
Chr01G1545.1	2304	3.00E-137	207/421(49.17)	402	PHI:2304|BCFHG1|CAP74387|40559|Botrytis_cinerea|Unaffected_pathogenicity
Chr01G1548.1	2315	5.00E-49	99/291(34.02)	166	PHI:2315|ChLae1|G4XKY9|5016|Cochliobolus_heterostrophus|Mixed_outcome
Chr01G1550.1	441	2.00E-17	64/282(22.70)	80.9	PHI:441|BTP1|CAE55153|40559|Botrytis_cinerea|Reduced_virulence
Chr01G1552.1	1260	2.00E-06	41/152(26.97)	48.9	PHI:1260|FGSG_13944|I1RUC7|5518|Gibberella_zeae_(related:_Fusarium_graminearum)|Unaffected_pathogenicity
Chr01G1553.1	441	2.00E-09	45/177(25.42)	55.1	PHI:441|BTP1|CAE55153|40559|Botrytis_cinerea|Reduced_virulence
Chr01G1555.1	2968	2.00E-38	137/487(28.13)	145	PHI:2968|Hxs1|J9VQA5|5207|Cryptococcus_neoformans|Reduced_virulence
Chr01G1556.1	812	4.00E-80	144/359(40.11)	253	PHI:812|MGG_10702|EDJ94108|318829|Magnaporthe_oryzae|Reduced_virulence

Chr01G1561.1	2190	2.00E-09	61/253(24.11)	57	PHI:2190|MoCYP51A|G4MRP8|148305|Magnaporthe_oryzae_(related:_Magnaporthe_grisea)|Reduced_virulence
Chr01G1569.1	1422	0	359/650(55.23)	565	PHI:1422|GzC2H090|I1S1B7|5518|Gibberella_zeae_(related:_Fusarium_graminearum)|Reduced_virulence
Chr01G1570.1	491	5.00E-35	85/275(30.91)	132	PHI:491|YVH1|EAK96144|5476|Candida_albicans|Reduced_virulence
Chr01G1574.1	438	1.00E-20	110/475(23.16)	92.8	PHI:438|BcBOT1_(related:_CND5)|AAQ16576|40559|Botrytis_cinerea|Reduced_virulence
Chr01G1577.1	1503	4.00E-10	70/270(25.93)	59.3	PHI:1503|GzHMG035|I1S828|5518|Gibberella_zeae_(related:_Fusarium_graminearum)|Unaffected_pathogenicity
Chr01G1581.1	2094	1.00E-12	111/567(19.58)	68.6	PHI:2094|Yvc1|MGG_09828|148305|Magnaporthe_oryzae_(related:_Magnaporthe_grisea)|Mixed_outcome
Chr01G1582.1	211	1.00E-12	66/256(25.78)	68.9	PHI:211|CaTUP1|AAB63195|5476|Candida_albicans|Reduced_virulence
Chr01G1584.1	283	1.00E-51	139/493(28.19)	193	PHI:283|CDC24|AAO25556|5476|Candida_albicans|Loss_of_pathogenicity
Chr01G1587.1	4506	1.00E-06	34/96(35.42)	47.8	So_(soft)|PHI:4506|K9Y567|35717|Epichloe_festucae|effector_(plant_avirulence_determinant)
Chr01G1590.1	1925	2.00E-22	142/651(21.81)	99.4	PHI:1925|GzZC240|I1RH59|5518|Gibberella_zeae_(related:_Fusarium_graminearum)|Unaffected_pathogenicity
Chr01G1591.1	1555	2.00E-49	126/482(26.14)	178	PHI:1555|GzMyb019|I1RDG6|5518|Gibberella_zeae_(related:_Fusarium_graminearum)|Unaffected_pathogenicity
Chr01G1593.1	3364	8.00E-06	34/107(31.78)	43.5	MMAR_1663|PHI:3364|B2HHI1|1781|Mycobacterium_marinum|reduced_virulence_
Chr01G1594.1	3381	3.00E-70	157/450(34.89)	238	FVEG_12533|PHI:3381|W7N2B4|117187|Fusarium_verticillioides|unaffected_pathogenicity_
Chr01G1596.1	3415	2.00E-24	109/419(26.01)	106	Fre2|PHI:3415|T2BNJ5|5207|Cryptococcus_neoformans|mixed_outcome_
Chr01G1597.1	812	6.00E-77	136/362(37.57)	246	PHI:812|MGG_10702|EDJ94108|318829|Magnaporthe_oryzae|Reduced_virulence

Chr01G1602.1	566	6.00E-142	214/418(51.20)	413	PHI:566|cel2|AAK19621|5017|Cochliobolus_carbonum|Unaffected_pathogenicity
Chr01G1603.1	922	1.00E-45	181/614(29.48)	168	PHI:922|um03615||5270|Ustilago_maydis|Unaffected_pathogenicity
Chr01G1608.1	1047	1.00E-16	73/281(25.98)	77.4	PHI:1047|CTB6|ABK64183|29003|Cercospora_nicotianae|Reduced_virulence
Chr01G1611.1	1161	3.00E-16	88/375(23.47)	79	PHI:1161|MgMfs1|A4ZGP3|54734|Mycosphaerella_graminicola_(related:_Zymoseptoria_tritici)|Chemistry_target
Chr01G1612.1	310	0	933/1506(61.95)	1882	PHI:310|MgAtr4|AAK15314|54734|Mycosphaerella_graminicola|Reduced_virulence
Chr01G1613.1	1420	1.00E-12	96/428(22.43)	70.5	PHI:1420|GzC2H088|I1S172|5518|Gibberella_zeae_(related:_Fusarium_graminearum)|Unaffected_pathogenicity
Chr01G1615.1	2269	3.00E-32	87/264(32.95)	120	PHI:2269|Mdh1|0|13684|Phaeosphaeria_nodorum_(related:_Stagonospora_nodorum)|Unaffected_pathogenicity
Chr01G1616.1	538	5.00E-23	109/459(23.75)	100	PHI:538|FRT1|AAU87358|40559|Botrytis_cinerea|Unaffected_pathogenicity
Chr01G1617.1	404	9.00E-17	65/248(26.21)	79.3	PHI:404|PTH11|AAD30436|318829|Magnaporthe_oryzae|Reduced_virulence
Chr01G1622.1	1153	7.00E-06	55/247(22.27)	45.4	PHI:1153|cyp51/erg11|B6E223|54734|Mycosphaerella_graminicola_(related:_Zymoseptoria_tritici)|Chemistry_target
Chr01G1624.1	184	1.00E-09	45/142(31.69)	55.1	PHI:184|RBT4|AAG09789|5476|Candida_albicans|Reduced_virulence
Chr01G1641.1	115	1.00E-80	154/424(36.32)	258	PHI:115|PGX1|AAC26146|5017|Cochliobolus_carbonum|Unaffected_pathogenicity
Chr01G1650.1	438	2.00E-12	76/306(24.84)	66.6	PHI:438|BcBOT1_(related:_CND5)|AAQ16576|40559|Botrytis_cinerea|Reduced_virulence
Chr01G1651.1	2022	9.00E-23	81/262(30.92)	92.4	PHI:2022|BUF1|MGG_02252|148305|Magnaporthe_oryzae_(related:_Magnaporthe_grisea)|Loss_of_pathogenicity
Chr01G1654.1	2927	5.00E-102	206/520(39.62)	317	PHI:2927|lip5|J9N2Z8|59765|Fusarium_oxysporum_f._sp._Lycopersici|Unaffected_pathogenicity
Chr01G1656.1	3037	2.00E-12	50/186(26.88)	67	FgERG5A|PHI:3037|I1RE80|5518|Fusarium_graminearum|reduced_virulence_

Chr01G1659.1	881	6.00E-28	98/329(29.79)	109	PHI:881|MGG_04556|EDJ96020|318829|Magnaporthe_oryzae|Reduced_virulence
Chr01G1660.1	1893	2.00E-66	152/485(31.34)	224	PHI:1893|GzZC208|I1RNY0|5518|Gibberella_zeae_(related:_Fusarium_graminearum)|Unaffected_pathogenicity
Chr01G1661.1	404	5.00E-28	71/272(26.10)	112	PHI:404|PTH11|AAD30436|318829|Magnaporthe_oryzae|Reduced_virulence
Chr01G1667.1	2930	5.00E-90	156/472(33.05)	301	PHI:2930|ctf2|J9MFF7|59765|Fusarium_oxysporum_f._sp._Lycopersici|Reduced_virulence
Chr01G1669.1	1555	5.00E-41	118/482(24.48)	152	PHI:1555|GzMyb019|I1RDG6|5518|Gibberella_zeae_(related:_Fusarium_graminearum)|Unaffected_pathogenicity
Chr01G1678.1	2654	2.00E-13	110/419(26.25)	70.1	PHI:2654|DUR1,2|Q59VF3|5476|Candida_albicans|Reduced_virulence
Chr01G1682.1	1852	2.00E-59	111/263(42.21)	201	PHI:1852|GzZC167|I1S170|5518|Gibberella_zeae_(related:_Fusarium_graminearum)|Unaffected_pathogenicity
Chr01G1685.1	2927	1.00E-99	200/477(41.93)	311	PHI:2927|lip5|J9N2Z8|59765|Fusarium_oxysporum_f._sp._Lycopersici|Unaffected_pathogenicity
Chr01G1686.1	1976	8.00E-08	17/32(53.12)	51.6	PHI:1976|GzZC291|I1RLP8|5518|Gibberella_zeae_(related:_Fusarium_graminearum)|Lethal
Chr01G1691.1	1896	6.00E-120	230/613(37.52)	380	PHI:1896|GzZC211|I1RNR1|5518|Gibberella_zeae_(related:_Fusarium_graminearum)|Unaffected_pathogenicity
Chr01G1693.1	2101	9.00E-50	142/485(29.28)	177	PHI:2101|Vacuolar_calcium_ion_transporter|MGG_11454|148305|Magnaporthe_oryzae_(related:_Magnaporthe_grisea)|Unaffected_pathogenicity
Chr01G1702.1	144	6.00E-22	93/365(25.48)	94.4	PHI:144|CHT42|AAC05829|29875|Trichoderma_virens|Reduced_virulence
Chr01G1709.1	2968	6.00E-45	120/449(26.73)	164	PHI:2968|Hxs1|J9VQA5|5207|Cryptococcus_neoformans|Reduced_virulence
Chr01G1710.1	1883	2.00E-44	203/801(25.34)	168	PHI:1883|GzZC198|I1RJ71|5518|Gibberella_zeae_(related:_Fusarium_graminearum)|Unaffected_pathogenicity
Chr01G1717.1	2336	6.00E-47	141/428(32.94)	175	PHI:2336|NIA1|O00101|13684|Stagonospora_nodorum_(related:_Phaeosphaeria_nodorum)|Unaffected_pathogenicity

Chr01G1723.1	2385	5.00E-118	224/348(64.37)	345	PHI:2385|MYT2|I1RTN5|5518|Gibberella_zeae_(related:_Fusarium_graminearum)|Reduced_virulence
Chr01G1725.1	3243	2.00E-71	116/217(53.46)	237	lsfA|PHI:3243|Q02QT9|287|Pseudomonas_aeruginosa|reduced_virulence_
Chr01G1726.1	3351	2.00E-14	120/522(22.99)	74.3	mbtE|PHI:3351|I6Y0L1|1773|Mycobacterium_tuberculosis|reduced_virulence_
Chr01G1729.1	2030	0	328/720(45.56)	576	PHI:2030|TGL3-1|G4N492|148305|Magnaporthe_oryzae_(related:_Magnaporthe_grisea)|Unaffected_pathogenicity
Chr01G1732.1	2643	4.00E-08	31/105(29.52)	51.6	PHI:2643|CFAS|A4HTK3|5671|Leishmania_infantum|Reduced_virulence
Chr01G1734.1	2269	2.00E-34	88/258(34.11)	126	PHI:2269|Mdh1|0|13684|Phaeosphaeria_nodorum_(related:_Stagonospora_nodorum)|Unaffected_pathogenicity
Chr01G1735.1	1727	1.00E-10	45/163(27.61)	60.8	PHI:1727|GzZC042|I1S5Q2|5518|Gibberella_zeae_(related:_Fusarium_graminearum)|Unaffected_pathogenicity
Chr01G1737.1	2247	4.00E-26	86/282(30.50)	103	PHI:2247|Sch1|EAT82552|13684|Phaeosphaeria_nodorum_(related:_Stagonospora_nodorum)|Unaffected_pathogenicity
Chr01G1742.1	2321	7.00E-50	146/521(28.02)	180	PHI:2321|SidI|Q4WR83|746128|Aspergillus_fumigatus|Reduced_virulence
Chr01G1743.1	2022	2.00E-25	82/269(30.48)	100	PHI:2022|BUF1|MGG_02252|148305|Magnaporthe_oryzae_(related:_Magnaporthe_grisea)|Loss_of_pathogenicity
Chr01G1747.1	2378	1.00E-08	62/225(27.56)	55.1	PHI:2378|DEP4|D2E9W9|29001|Alternaria_brassicicola|Mixed_outcome
Chr01G1748.1	784	1.00E-25	81/268(30.22)	101	PHI:784|MGG_00056|EDK03390|318829|Magnaporthe_oryzae|Reduced_virulence
Chr01G1749.1	1919	0	514/999(51.45)	904	PHI:1919|GzZC234|I1RCQ4|5518|Gibberella_zeae_(related:_Fusarium_graminearum)|Unaffected_pathogenicity
Chr01G1750.1	1245	0	439/740(59.32)	771	PHI:1245|FGSG_01559|I1RTL1|5518|Gibberella_zeae_(related:_Fusarium_graminearum)|Unaffected_pathogenicity
Chr01G1753.1	3382	9.00E-07	22/49(44.90)	47.8	FVEG_12532|PHI:3382|W7MS18|117187|Fusarium_verticillioides|unaffected_pathogenicity_
Chr01G1754.1	3216	1.00E-31	81/219(36.99)	117	MoCDIP4|PHI:3216|G4MVX4|318829|Magnaporthe_oryzae|mixed_outcome_

Chr01G1756.1	55	2.00E-24	88/273(32.23)	103	PHI:55|PKS1|AAB08104|5016|Cochliobolus_heterostrophus|Reduced_virulence
Chr01G1757.1	2179	6.00E-06	56/193(29.02)	47.4	PHI:2179|Moplaa|F8SM03|148305|Magnaporthe_oryzae_(related:_Magnaporthe_grisea)|Reduced_virulence
Chr01G1758.1	2089	2.00E-114	256/679(37.70)	358	PHI:2089|Moatg28|MGG_08061|148305|Magnaporthe_oryzae_(related:_Magnaporthe_grisea)|Unaffected_pathogenicity
Chr01G1760.1	3134	8.00E-126	216/481(44.91)	378	pykF|PHI:3134|Q66A26|633|Yersinia_pseudotuberculosis|reduced_virulence_
Chr01G1761.1	3381	9.00E-93	180/465(38.71)	300	FVEG_12533|PHI:3381|W7N2B4|117187|Fusarium_verticillioides|unaffected_pathogenicity_
Chr01G1763.1	2042	8.00E-59	159/555(28.65)	220	PHI:2042|ABC3|Q3Y5V5|148305|Magnaporthe_oryzae_(related:_Magnaporthe_grisea)|Loss_of_pathogenicity
Chr01G1765.1	511	8.00E-52	142/535(26.54)	186	PHI:511|CaNAG4|EAK93098|5476|Candida_albicans|Reduced_virulence
Chr01G1771.1	1555	1.00E-49	132/501(26.35)	177	PHI:1555|GzMyb019|I1RDG6|5518|Gibberella_zeae_(related:_Fusarium_graminearum)|Unaffected_pathogenicity
Chr01G1773.1	538	6.00E-53	141/487(28.95)	191	PHI:538|FRT1|AAU87358|40559|Botrytis_cinerea|Unaffected_pathogenicity
Chr01G1774.1	2207	6.00E-14	42/95(44.21)	70.5	PHI:2207|endo-1,4-beta-xylanase_[GH10_family]|MGG_02245|148305|Magnaporthe_oryzae_(related:_Magnaporthe_grisea)|Reduced_virulence
Chr01G1780.1	2693	3.00E-46	111/295(37.63)	179	PHI:2693|GcABC-G1|F0XP73|226899|Grosmannia_clavigera|Reduced_virulence
Chr01G1782.1	3028	2.00E-15	83/360(23.06)	75.5	Vatr2|PHI:3028|A5CVB7|28447|Clavibacter_michiganensis|reduced_virulence_
Chr01G1784.1	504	2.00E-44	127/369(34.42)	156	PHI:504|LEU2|CAA42366|4932|Saccharomyces_cerevisiae|Reduced_virulence
Chr01G1790.1	812	6.00E-85	152/380(40.00)	266	PHI:812|MGG_10702|EDJ94108|318829|Magnaporthe_oryzae|Reduced_virulence
Chr01G1791.1	2279	1.00E-22	75/245(30.61)	92	PHI:2279|Conserved_hypothetical_protein|J9N0G7|5507|Fusarium_oxysporum|Unaffected_pathogenicity
Chr01G1799.1	1814	1.00E-123	229/556(41.19)	395	PHI:1814|GzZC129|I1RV04|5518|Gibberella_zeae_(related:_Fusarium_gramin

					earum)|Lethal
Chr01G1805.1	2020	8.00E-19	68/306(22.22)	88.2	PHI:2020|Tup1|XP_759427|5270|Ustilago_maydis|Mixed_outcome
Chr01G1808.1	1260	4.00E-06	41/138(29.71)	46.6	PHI:1260|FGSG_13944|I1RUC7|5518|Gibberella_zeae_(related:_Fusarium_graminearum)|Unaffected_pathogenicity
Chr01G1810.1	2269	5.00E-35	87/260(33.46)	127	PHI:2269|Mdh1|0|13684|Phaeosphaeria_nodorum_(related:_Stagonospora_nodorum)|Unaffected_pathogenicity
Chr01G1811.1	2570	7.00E-56	122/379(32.19)	193	PHI:2570|CYB2|Q6FM61|5478|Candida_glabrata|Reduced_virulence
Chr01G1812.1	2976	2.00E-86	195/626(31.15)	292	PHI:2976|CgOPT1|C6ZRH8|29905|Colletotrichum_gloeosporioides_f._sp._aeschynomenes|Reduced_virulence
Chr01G1814.1	784	1.00E-21	86/271(31.73)	94.7	PHI:784|MGG_00056|EDK03390|318829|Magnaporthe_oryzae|Reduced_virulence
Chr01G1815.1	1555	5.00E-31	120/462(25.97)	123	PHI:1555|GzMyb019|I1RDG6|5518|Gibberella_zeae_(related:_Fusarium_graminearum)|Unaffected_pathogenicity
Chr01G1820.1	1821	1.00E-164	246/386(63.73)	488	PHI:1821|GzZC136|I1RWY8|5518|Gibberella_zeae_(related:_Fusarium_graminearum)|Unaffected_pathogenicity
Chr01G1821.1	1662	1.00E-19	86/406(21.18)	92.8	PHI:1662|GzCCHC002|I1REJ1|5518|Gibberella_zeae_(related:_Fusarium_graminearum)|Unaffected_pathogenicity
Chr01G1822.1	2022	6.00E-08	54/196(27.55)	50.1	PHI:2022|BUF1|MGG_02252|148305|Magnaporthe_oryzae_(related:_Magnaporthe_grisea)|Loss_of_pathogenicity
Chr01G1829.1	2305	2.00E-22	48/102(47.06)	87	PHI:2305|BcFKBP12||40559|Botrytis_cinerea|Mixed_outcome
Chr01G1837.1	2247	2.00E-32	98/275(35.64)	120	PHI:2247|Sch1|EAT82552|13684|Phaeosphaeria_nodorum_(related:_Stagonospora_nodorum)|Unaffected_pathogenicity
Chr01G1838.1	2654	2.00E-16	81/257(31.52)	80.5	PHI:2654|DUR1,2|Q59VF3|5476|Candida_albicans|Reduced_virulence
Chr01G1842.1	2968	3.00E-29	113/433(26.10)	118	PHI:2968|Hxs1|J9VQA5|5207|Cryptococcus_neoformans|Reduced_virulence
Chr01G1844.1	2315	9.00E-52	104/290(35.86)	174	PHI:2315|ChLae1|G4XKY9|5016|Cochliobolus_heterostrophus|Mixed_outcome
Chr01G1845.1	1414	2.00E-12	33/70(47.14)	66.6	PHI:1414|GzC2H081|I1RZL0|5518|Gibberella_zeae_(related:_Fusarium_grami

					nearum)|Unaffected_pathogenicity
Chr01G1848.1	2117	4.00E-61	141/380(37.11)	207	PHI:2117|SPM1|P58371|148305|Magnaporthe_oryzae_(related:_Magnaporthe_grisea)|Reduced_virulence
Chr01G1852.1	2315	2.00E-48	104/279(37.28)	165	PHI:2315|ChLae1|G4XKY9|5016|Cochliobolus_heterostrophus|Mixed_outcome
Chr01G1855.1	903	2.00E-133	212/478(44.35)	402	PHI:903|um01888||5270|Ustilago_maydis|Unaffected_pathogenicity
Chr01G1856.1	2907	0	336/505(66.53)	712	PHI:2907|Cyp51A|I6YDU0|5518|Fusarium_graminearum|Mixed_outcome
Chr01G1857.1	4194	6.00E-55	144/490(29.39)	192	AKT7|PHI:4194|V5XZS6|5599|Alternaria_alternata|increased_virulence_(Hyper virulence)
Chr01G1858.1	2034	4.00E-13	62/205(30.24)	67	PHI:2034|MFP1|G4MZY1|148305|Magnaporthe_oryzae_(related:_Magnaporthe_grisea)|Reduced_virulence
Chr01G1864.1	538	7.00E-21	130/522(24.90)	93.6	PHI:538|FRT1|AAU87358|40559|Botrytis_cinerea|Unaffected_pathogenicity
Chr01G1871.1	1399	1.00E-10	40/121(33.06)	60.5	PHI:1399|GzC2H062|I1RV74|5518|Gibberella_zeae_(related:_Fusarium_graminearum)|Unaffected_pathogenicity
Chr01G1874.1	4211	7.00E-26	99/365(27.12)	110	FRE3|PHI:4211|J9VNH2|5207|Cryptococcus_neoformans|effector_(plant_avirulence_determinant)
Chr01G1880.1	3257	0	417/554(75.27)	853	MoIlv1|PHI:3257|G4MU34|318829|Magnaporthe_oryzae|reduced_virulence_
Chr01G1884.1	3019	0	739/1166(63.38)	1570	MoLys2|PHI:3019|G5EI34|318829|Magnaporthe_oryzae|reduced_virulence_
Chr01G1887.1	438	2.00E-23	123/520(23.65)	100	PHI:438|BcBOT1_(related:_CND5)|AAQ16576|40559|Botrytis_cinerea|Reduced_virulence
Chr01G1890.1	3354	3.00E-11	36/133(27.07)	63.2	rtxA1|PHI:3354|A0A023NA98|672|Vibrio_vulnificus|reduced_virulence_
Chr01G1891.1	19	6.00E-148	230/486(47.33)	437	PHI:19|NMT|AAA17547|5207|Cryptococcus_neoformans|Reduced_virulence
Chr01G1894.1	3301	2.00E-08	65/263(24.71)	51.2	patN|PHI:3301|A0A075TRB3|27334|Penicillium_expansum|unaffected_pathogenicity_
Chr01G1900.1	903	3.00E-115	201/517(38.88)	355	PHI:903|um01888||5270|Ustilago_maydis|Unaffected_pathogenicity
Chr01G1909.1	1342	4.00E-07	30/89(33.71)	44.7	PHI:1342|GzC2H002|I1RA24|5518|Gibberella_zeae_(related:_Fusarium_graminearum)|Unaffected_pathogenicity

Chr01G1911.1	1407	1.00E-11	80/348(22.99)	65.1	PHI:1407|GzC2H071|I1RX64|5518|Gibberella_zeae_(related:_Fusarium_graminearum)|Lethal
Chr01G1912.1	2199	0	387/611(63.34)	764	PHI:2199|MoRgs8|XP_001405673|148305|Magnaporthe_oryzae_(related:_Magnaporthe_grisea)|Unaffected_pathogenicity
Chr01G1917.1	112	7.00E-27	110/437(25.17)	110	PHI:112|MAK1|AAC49410|140110|Nectria_haematococca_(related:_Fusarium_solani)|Reduced_virulence
Chr01G1918.1	2067	0	822/1664(49.40)	1513	PHI:2067|ABC4|MGG_00937|148305|Magnaporthe_oryzae_(related:_Magnaporthe_grisea)|Loss_of_pathogenicity
Chr01G1919.1	2844	2.00E-29	85/279(30.47)	112	PHI:2844|BRM2|O93802|5599|Alternaria_alternata|Unaffected_pathogenicity
Chr01G1925.1	413	6.00E-92	166/432(38.43)	286	PHI:413|MPD1|AAT84078|13684|Stagonospora_nodorum|Unaffected_pathogenicity
Chr01G1929.1	1709	3.00E-29	125/532(23.50)	120	PHI:1709|GzZC024|I1RR13|5518|Gibberella_zeae_(related:_Fusarium_graminearum)|Unaffected_pathogenicity
Chr01G1930.1	2520	9.00E-39	132/477(27.67)	152	PHI:2520|LYS4|Q4WUL6|746128|Aspergillus_fumigatus|Lethal
Chr01G1931.1	1555	2.00E-29	113/463(24.41)	118	PHI:1555|GzMyb019|I1RDG6|5518|Gibberella_zeae_(related:_Fusarium_graminearum)|Unaffected_pathogenicity
Chr01G1933.1	1555	1.00E-40	119/471(25.27)	151	PHI:1555|GzMyb019|I1RDG6|5518|Gibberella_zeae_(related:_Fusarium_graminearum)|Unaffected_pathogenicity
Chr01G1940.1	1279	5.00E-44	100/307(32.57)	158	PHI:1279|FGSG_12132|I1RU59|5518|Gibberella_zeae_(related:_Fusarium_graminearum)|Unaffected_pathogenicity
Chr01G1943.1	1279	1.00E-23	108/440(24.55)	100	PHI:1279|FGSG_12132|I1RU59|5518|Gibberella_zeae_(related:_Fusarium_graminearum)|Unaffected_pathogenicity
Chr01G1944.1	3126	2.00E-44	129/448(28.79)	159	argD|PHI:3126|D4I307|552|Erwinia_amylovora|mixed_outcome_
Chr01G1945.1	4194	1.00E-52	149/495(30.10)	186	AKT7|PHI:4194|V5XZS6|5599|Alternaria_alternata|increased_virulence_(Hypervirulence)
Chr01G1952.1	1260	3.00E-08	34/108(31.48)	50.1	PHI:1260|FGSG_13944|I1RUC7|5518|Gibberella_zeae_(related:_Fusarium_gr

					aminearum)|Unaffected_pathogenicity
Chr01G1956.1	1420	1.00E-11	94/447(21.03)	66.6	PHI:1420|GzC2H088|I1S172|5518|Gibberella_zeae_(related:_Fusarium_graminearum)|Unaffected_pathogenicity
Chr01G1957.1	2117	4.00E-75	152/372(40.86)	244	PHI:2117|SPM1|P58371|148305|Magnaporthe_oryzae_(related:_Magnaporthe_grisea)|Reduced_virulence
Chr01G1958.1	2960	4.00E-18	105/356(29.49)	84.7	PHI:2960|frdA|C5BDL7|67780|Edwardsiella_ictaluri|Reduced_virulence
Chr01G1967.1	267	6.00E-125	391/1423(27.48)	426	PHI:267|MLT1|AAD51594|5476|Candida_albicans|Reduced_virulence
Chr01G1973.1	2834	9.00E-153	223/471(47.35)	449	PHI:2834|LAM1|C3PTB1|5016|Cochliobolus_heterostrophus|Reduced_virulence
Chr01G1974.1	1555	6.00E-18	64/277(23.10)	84	PHI:1555|GzMyb019|I1RDG6|5518|Gibberella_zeae_(related:_Fusarium_graminearum)|Unaffected_pathogenicity
Chr01G1975.1	2654	5.00E-14	100/389(25.71)	72.8	PHI:2654|DUR1,2|Q59VF3|5476|Candida_albicans|Reduced_virulence
Chr01G1980.1	1992	3.00E-07	86/394(21.83)	50.8	PHI:1992|GzZC307|I1R983|5518|Gibberella_zeae_(related:_Fusarium_graminearum)|Unaffected_pathogenicity
Chr01G1982.1	2968	9.00E-55	153/502(30.48)	193	PHI:2968|Hxs1|J9VQA5|5207|Cryptococcus_neoformans|Reduced_virulence
Chr01G1987.1	2020	5.00E-11	50/212(23.58)	63.9	PHI:2020|Tup1|XP_759427|5270|Ustilago_maydis|Mixed_outcome
Chr01G1990.1	566	5.00E-56	150/450(33.33)	191	PHI:566|cel2|AAK19621|5017|Cochliobolus_carbonum|Unaffected_pathogenicity
Chr01G1992.1	2388	5.00E-38	122/375(32.53)	142	PHI:2388|Chi2|0|5530|Metarhizium_anisopliae|Mixed_outcome
Chr01G1993.1	3387	6.00E-11	74/258(28.68)	61.6	FVEG_12523|PHI:3387|W7MT31|117187|Fusarium_verticillioides|unaffected_pathogenicity_
Chr01G2000.1	2395	8.00E-137	248/415(59.76)	400	PHI:2395|GzSYN1|I1RBM2|5518|Gibberella_zeae_(related:_Fusarium_graminearum)|Reduced_virulence
Chr01G2003.1	616	4.00E-43	94/249(37.75)	150	PHI:616|sseA|AAO68047|216597|Salmonella_enterica|Reduced_virulence
Chr01G2007.1	3024	1.00E-63	94/183(51.37)	211	Cln1|PHI:3024|J9VV14|5207|Cryptococcus_neoformans|unaffected_pathogenicity_

Chr01G2010.1	211	4.00E-54	119/318(37.42)	196	PHI:211|CaTUP1|AAB63195|5476|Candida_albicans|Reduced_virulence
Chr01G2012.1	144	2.00E-58	133/389(34.19)	196	PHI:144|CHT42|AAC05829|29875|Trichoderma_virens|Reduced_virulence
Chr01G2014.1	1071	1.00E-37	97/319(30.41)	150	PHI:1071|Gas1|CAF05793|5270|Ustilago_maydis|Loss_of_pathogenicity
Chr01G2015.1	3382	2.00E-09	22/46(47.83)	55.8	FVEG_12532|PHI:3382|W7MS18|117187|Fusarium_verticillioides|unaffected_pathogenicity_
Chr01G2017.1	4194	4.00E-42	129/474(27.22)	155	AKT7|PHI:4194|V5XZS6|5599|Alternaria_alternata|increased_virulence_(Hypervirulence)
Chr01G2019.1	2524	5.00E-85	127/192(66.15)	251	PHI:2524|nudC|Q70ZY8|746128|Aspergillus_fumigatus|Lethal
Chr01G2022.1	1605	9.00E-12	39/97(40.21)	65.1	PHI:1605|GzOB046|I1S216|5518|Gibberella_zeae_(related:_Fusarium_graminearum)|Unaffected_pathogenicity
Chr01G2025.1	4194	2.00E-22	112/444(25.23)	98.2	AKT7|PHI:4194|V5XZS6|5599|Alternaria_alternata|increased_virulence_(Hypervirulence)
Chr01G2028.1	544	2.00E-133	222/567(39.15)	412	PHI:544|BCMFS1|AAF64435|332648|Botrytis_cinerea|Unaffected_pathogenicity
Chr01G2033.1	2537	6.00E-171	233/315(73.97)	482	PHI:2537|TRR1|Q4WQJ0|746128|Aspergillus_fumigatus|Mixed_outcome
Chr01G2039.1	253	7.00E-61	157/469(33.48)	221	PHI:253|FOS1|AAK27436|5085|Aspergillus_fumigatus|Reduced_virulence
Chr01G2042.1	697	8.00E-26	91/330(27.58)	106	PHI:697|ugt51E1|AAM81358|5022|Leptosphaeria_maculans|Unaffected_pathogenicity
Chr01G2043.1	2130	5.00E-28	100/360(27.78)	117	PHI:2130|MoHox4|MGG_06285|148305|Magnaporthe_oryzae_(related:_Magnaporthe_grisea)|reduced_virulence
Chr01G2044.1	105	3.00E-144	243/568(42.78)	434	PHI:105|PLB1|AAC61890|5476|Candida_albicans|Reduced_virulence
Chr01G2045.1	1479	0	570/1206(47.26)	837	PHI:1479|GzHMG011|I1RD01|5518|Gibberella_zeae_(related:_Fusarium_graminearum)|Unaffected_pathogenicity
Chr01G2049.1	2654	1.00E-10	53/158(33.54)	61.6	PHI:2654|DUR1,2|Q59VF3|5476|Candida_albicans|Reduced_virulence
Chr01G2051.1	576	0	487/557(87.43)	1046	PHI:576|NoxA|BAE72680|35717|Epichloe_festucae|Enhanced_antagonism
Chr01G2054.1	1648	2.00E-117	252/723(34.85)	403	PHI:1648|GzWing020|I1RWP1|5518|Gibberella_zeae_(related:_Fusarium_gra

					minearum)|Reduced_virulence
Chr01G2055.1	1673	3.00E-168	240/455(52.75)	482	PHI:1673|GzDHHC001|Q4IMZ7|5518|Gibberella_zeae_(related:_Fusarium_graminearum)|Unaffected_pathogenicity
Chr01G2056.1	2822	2.00E-25	138/573(24.08)	110	PHI:2822|Cxt1p|Q5K8R6|5207|Cryptococcus_neoformans|Reduced_virulence
Chr01G2059.1	2037	3.00E-149	235/458(51.31)	438	PHI:2037|MoRic8|XP_001405357|148305|Magnaporthe_oryzae_(related:_Magnaporthe_grisea)|Loss_of_pathogenicity
Chr01G2061.1	1561	2.00E-143	212/292(72.60)	407	PHI:1561|GzOB001|I1R9W2|5518|Gibberella_zeae_(related:_Fusarium_graminearum)|Unaffected_pathogenicity
Chr01G2070.1	404	9.00E-30	68/259(26.25)	117	PHI:404|PTH11|AAD30436|318829|Magnaporthe_oryzae|Reduced_virulence
Chr01G2072.1	2394	1.00E-48	106/305(34.75)	169	PHI:2394|Conserved_hypothetical_protein|I1S104|5518|Gibberella_zeae_(related:_Fusarium_graminearum)|Increased_virulence_(Hypervirulence)
Chr01G2074.1	1251	0	382/539(70.87)	797	PHI:1251|FGSG_09150|I1RD19|5518|Gibberella_zeae_(related:_Fusarium_graminearum)|Unaffected_pathogenicity
Chr01G2077.1	2194	0	288/363(79.34)	580	PHI:2194|MoRgs3|XP_360603|148305|Magnaporthe_oryzae_(related:_Magnaporthe_grisea)|Reduced_virulence
Chr01G2079.1	2336	2.00E-30	69/221(31.22)	120	PHI:2336|NIA1|O00101|13684|Stagonospora_nodorum_(related:_Phaeosphaeria_nodorum)|Unaffected_pathogenicity
Chr01G2080.1	244	0	1082/1167(92.72)	2193	PHI:244|CLAP1|AAN62846|290576|Colletotrichum_lindemuthianum|Loss_of_pathogenicity
Chr01G2082.1	1531	1.00E-154	204/267(76.40)	433	PHI:1531|GzSsu72|Q4IPC8|5518|Gibberella_zeae_(related:_Fusarium_graminearum)|Unaffected_pathogenicity
Chr01G2083.1	3039	0	563/970(58.04)	957	MoSPA2|PHI:3039|U3MXJ8|318829|Magnaporthe_oryzae|mixed_outcome_
Chr01G2094.1	2474	0	340/546(62.27)	686	PHI:2474|imd1|E3P6S0|5207|Cryptococcus_neoformans|Reduced_virulence
Chr01G2095.1	3278	3.00E-11	68/290(23.45)	65.9	CoIra1|PHI:3278|N4V0R3|5465|Colletotrichum_orbiculare|reduced_virulence_
Chr01G2101.1	1713	0	342/652(52.45)	639	PHI:1713|GzZC028|I1RGG4|5518|Gibberella_zeae_(related:_Fusarium_graminearum)|Unaffected_pathogenicity

Chr01G2105.1	1414	4.00E-06	25/67(37.31)	47.4	PHI:1414|GzC2H081|I1RZL0|5518|Gibberella_zeae_(related:_Fusarium_graminearum)|Unaffected_pathogenicity
Chr01G2106.1	1998	4.00E-31	86/274(31.39)	122	PHI:1998|GzZC313|I1R9B9|5518|Gibberella_zeae_(related:_Fusarium_graminearum)|Unaffected_pathogenicity
Chr01G2108.1	2514	8.00E-10	40/156(25.64)	58.9	PHI:2514|PFS2|Q4X1Y0|746128|Aspergillus_fumigatus|Lethal
Chr01G2109.1	267	1.00E-169	458/1580(28.99)	555	PHI:267|MLT1|AAD51594|5476|Candida_albicans|Reduced_virulence
Chr01G2113.1	438	3.00E-22	62/211(29.38)	98.6	PHI:438|BcBOT1_(related:_CND5)|AAQ16576|40559|Botrytis_cinerea|Reduced_virulence
Chr01G2114.1	267	1.00E-114	322/1233(26.12)	390	PHI:267|MLT1|AAD51594|5476|Candida_albicans|Reduced_virulence
Chr01G2117.1	2074	0	318/496(64.11)	633	PHI:2074|Moatg6|MGG_03694|148305|Magnaporthe_oryzae_(related:_Magnaporthe_grisea)|Loss_of_pathogenicity
Chr01G2118.1	1218	0	325/341(95.31)	676	PHI:1218|FGSG_01559|I1RAY2|5518|Gibberella_zeae_(related:_Fusarium_graminearum)|Lethal
Chr01G2119.1	1471	1.00E-24	64/143(44.76)	108	PHI:1471|GzHMG003|I1RAY3|5518|Gibberella_zeae_(related:_Fusarium_graminearum)|Unaffected_pathogenicity
Chr01G2125.1	1563	1.00E-104	144/161(89.44)	298	PHI:1563|GzOB003|I1RAX7|5518|Gibberella_zeae_(related:_Fusarium_graminearum)|Unaffected_pathogenicity
Chr01G2131.1	796	8.00E-42	90/215(41.86)	145	PHI:796|MGG_02423|EDJ98620|318829|Magnaporthe_oryzae|Reduced_virulence
Chr01G2135.1	2086	0	307/497(61.77)	595	PHI:2086|Moatg24|MGG_03638|148305|Magnaporthe_oryzae_(related:_Magnaporthe_grisea)|Unaffected_pathogenicity
Chr01G2136.1	306	0	302/653(46.25)	578	PHI:306|KEX2|AAB80929|5476|Candida_albicans|Reduced_virulence
Chr01G2137.1	4506	3.00E-53	146/519(28.13)	202	So_(soft)|PHI:4506|K9Y567|35717|Epichloe_festucae|effector_(plant_avirulence_determinant)
Chr01G2138.1	2544	1.00E-07	21/72(29.17)	47.4	PHI:2544|PAB1|Q4WK03|746128|Aspergillus_fumigatus|Mixed_outcome
Chr01G2139.1	2175	5.00E-07	45/147(30.61)	47.8	PHI:2175|NMR3|MGG_09705|148305|Magnaporthe_oryzae_(related:_Magnap

					orthe_grisea)|Reduced_virulence
Chr01G2140.1	258	0	1069/1485(71.99)	2252	PHI:258|GPABC1|CAC40023|5128|Gibberella_pulicaris|Reduced_virulence
Chr01G2141.1	2247	5.00E-10	60/203(29.56)	57	PHI:2247|Sch1|EAT82552|13684|Phaeosphaeria_nodorum_(related:_Stagonospora_nodorum)|Unaffected_pathogenicity
Chr01G2149.1	2195	5.00E-07	25/82(30.49)	47.4	PHI:2195|MoRgs4|XP_368254|148305|Magnaporthe_oryzae_(related:_Magnaporthe_grisea)|Mixed_outcome
Chr01G2153.1	1597	0	754/1573(47.93)	1371	PHI:1597|GzOB037|I1RYF6|5518|Gibberella_zeae_(related:_Fusarium_graminearum)|Unaffected_pathogenicity
Chr01G2157.1	24	1.00E-44	131/408(32.11)	170	PHI:24|Avenacinase_gene|AAB09777|29850|Gaeumannomyces_graminis|Loss_of_pathogenicity
Chr01G2161.1	4194	4.00E-55	150/533(28.14)	193	AKT7|PHI:4194|V5XZS6|5599|Alternaria_alternata|increased_virulence_(Hypervirulence)
Chr01G2162.1	4194	1.00E-15	96/374(25.67)	77	AKT7|PHI:4194|V5XZS6|5599|Alternaria_alternata|increased_virulence_(Hypervirulence)
Chr01G2164.1	1555	1.00E-12	96/442(21.72)	67	PHI:1555|GzMyb019|I1RDG6|5518|Gibberella_zeae_(related:_Fusarium_graminearum)|Unaffected_pathogenicity
Chr01G2165.1	1974	7.00E-40	140/526(26.62)	149	PHI:1974|GzZC289|I1RQN6|5518|Gibberella_zeae_(related:_Fusarium_graminearum)|Unaffected_pathogenicity
Chr01G2167.1	1994	0	296/617(47.97)	568	PHI:1994|GzZC309|I1R9S1|5518|Gibberella_zeae_(related:_Fusarium_graminearum)|Unaffected_pathogenicity
Chr01G2172.1	511	7.00E-37	123/461(26.68)	142	PHI:511|CaNAG4|EAK93098|5476|Candida_albicans|Reduced_virulence
Chr01G2173.1	1816	5.00E-42	171/680(25.15)	162	PHI:1816|GzZC131|I1RRS3|5518|Gibberella_zeae_(related:_Fusarium_graminearum)|Unaffected_pathogenicity
Chr01G2177.1	243	0	395/942(41.93)	704	PHI:243|CHIP6|AAD00894|5457|Colletotrichum_gloeosporioides|Reduced_virulence
Chr01G2181.1	2042	2.00E-12	66/233(28.33)	68.6	PHI:2042|ABC3|Q3Y5V5|148305|Magnaporthe_oryzae_(related:_Magnaporthe

					_grisea)|Loss_of_pathogenicity
Chr01G2185.1	2616	1.00E-25	82/239(34.31)	106	PHI:2616|InlA|Q2L8G9|1639|Listeria_monocytogenes|Unaffected_pathogenicity
Chr01G2190.1	1876	2.00E-171	312/785(39.75)	513	PHI:1876|GzZC191|I1RD28|5518|Gibberella_zeae_(related:_Fusarium_graminearum)|Unaffected_pathogenicity
Chr01G2196.1	1662	3.00E-50	158/539(29.31)	186	PHI:1662|GzCCHC002|I1REJ1|5518|Gibberella_zeae_(related:_Fusarium_graminearum)|Unaffected_pathogenicity
Chr01G2197.1	1974	2.00E-83	167/557(29.98)	273	PHI:1974|GzZC289|I1RQN6|5518|Gibberella_zeae_(related:_Fusarium_graminearum)|Unaffected_pathogenicity
Chr01G2203.1	1317	3.00E-16	42/105(40.00)	78.2	PHI:1317|GzBrom002|I1RQE9|5518|Gibberella_zeae_(related:_Fusarium_graminearum)|Reduced_virulence
Chr01G2214.1	2321	8.00E-35	167/641(26.05)	137	PHI:2321|SidI|Q4WR83|746128|Aspergillus_fumigatus|Reduced_virulence
Chr01G2217.1	2058	1.00E-30	112/420(26.67)	125	PHI:2058|LHS1|MGG_06648.5|148305|Magnaporthe_oryzae_(related:_Magnaporthe_grisea)|Reduced_virulence
Chr01G2225.1	3234	0	352/485(72.58)	717	MoLYS20|PHI:3234|G4NCG1|318829|Magnaporthe_oryzae|reduced_virulence_
Chr01G2226.1	2821	4.00E-08	52/172(30.23)	53.9	PHI:2821|SNF2|Q5ALP9|5476|Candida_albicans|Reduced_virulence
Chr01G2236.1	2510	2.00E-24	138/567(24.34)	105	PHI:2510|msdS/AfmsdC|Q6PWQ1|746128|Aspergillus_fumigatus|Unaffected_pathogenicity
Chr01G2238.1	1967	0	548/747(73.36)	1126	PHI:1967|GzZC282|I1RB16|5518|Gibberella_zeae_(related:_Fusarium_graminearum)|Reduced_virulence
Chr01G2239.1	320	2.00E-06	64/242(26.45)	49.3	PHI:320|SSN6|AAL54912|5476|Candida_albicans|Reduced_virulence
Chr01G2245.1	1301	3.00E-51	79/110(71.82)	159	PHI:1301|GzbHLH002|I1RB45|5518|Gibberella_zeae_(related:_Fusarium_graminearum)|Unaffected_pathogenicity
Chr01G2247.1	2510	1.00E-60	153/500(30.60)	209	PHI:2510|msdS/AfmsdC|Q6PWQ1|746128|Aspergillus_fumigatus|Unaffected_pathogenicity
Chr01G2264.1	1966	8.00E-78	188/583(32.25)	259	PHI:1966|GzZC281|I1RB21|5518|Gibberella_zeae_(related:_Fusarium_gramin

					earum)|Unaffected_pathogenicity
Chr01G2266.1	3387	6.00E-24	76/228(33.33)	101	FVEG_12523|PHI:3387|W7MT31|117187|Fusarium_verticillioides|unaffected_pathogenicity_
Chr01G2273.1	194	0	456/729(62.55)	933	PHI:194|URE1|AAC62257|5207|Cryptococcus_neoformans|Reduced_virulence
Chr01G2274.1	1473	6.00E-86	178/325(54.77)	264	PHI:1473|GzHMG005|I1RB25|5518|Gibberella_zeae_(related:_Fusarium_graminearum)|Reduced_virulence
Chr01G2279.1	1627	6.00E-07	17/34(50.00)	45.1	PHI:1627|GzTF2S002|I1RW90|5518|Gibberella_zeae_(related:_Fusarium_graminearum)|Unaffected_pathogenicity
Chr01G2286.1	3356	0	405/700(57.86)	548	Mads2|PHI:3356|W7M2E5|117187|Fusarium_verticillioides|mixed_outcome_
Chr01G2290.1	1393	3.00E-11	57/246(23.17)	63.5	PHI:1393|GzC2H056|I1RU69|5518|Gibberella_zeae_(related:_Fusarium_graminearum)|Unaffected_pathogenicity
Chr01G2291.1	2968	3.00E-87	163/534(30.52)	280	PHI:2968|Hxs1|J9VQA5|5207|Cryptococcus_neoformans|Reduced_virulence
Chr01G2293.1	2960	2.00E-22	99/308(32.14)	99	PHI:2960|frdA|C5BDL7|67780|Edwardsiella_ictaluri|Reduced_virulence
Chr01G2295.1	2529	0	281/399(70.43)	585	PHI:2529|ERG10|Q4WCL5|746128|Aspergillus_fumigatus|Lethal
Chr01G2298.1	1943	0	583/907(64.28)	1168	PHI:1943|GzZC258|I1RY79|5518|Gibberella_zeae_(related:_Fusarium_graminearum)|Unaffected_pathogenicity
Chr01G2304.1	799	5.00E-06	31/90(34.44)	47	PHI:799|MGG_03530|EDJ94565|318829|Magnaporthe_oryzae|Reduced_virulence
Chr01G2309.1	2247	6.00E-11	57/192(29.69)	59.7	PHI:2247|Sch1|EAT82552|13684|Phaeosphaeria_nodorum_(related:_Stagonospora_nodorum)|Unaffected_pathogenicity
Chr01G2315.1	2401	7.00E-06	20/45(44.44)	44.3	PHI:2401|CaRING1|G0T3B3|456327|Xanthomonas_campestris_pv_vesicatoria|Mixed_outcome
Chr01G2317.1	213	2.00E-61	95/150(63.33)	189	PHI:213|CPA1|AAF69795|5207|Cryptococcus_neoformans|Reduced_virulence
Chr01G2319.1	190	2.00E-10	39/107(36.45)	62	PHI:190|TEC1|EAL04411|5476|Candida_albicans|Reduced_virulence
Chr01G2320.1	2552	2.00E-20	67/194(34.54)	94	PHI:2552|NOP4|Q4WNM3|746128|Aspergillus_fumigatus|Mixed_outcome
Chr01G2322.1	2017	3.00E-76	224/586(38.23)	256	PHI:2017|COM1|MGG_01215|148305|Magnaporthe_oryzae_(related:_Magnap

					orthe_grisea)|Reduced_virulence
Chr01G2332.1	1456	6.00E-128	218/530(41.13)	404	PHI:1456|GzAra004|I1RN15|5518|Gibberella_zeae_(related:_Fusarium_graminearum)|Unaffected_pathogenicity
Chr01G2336.1	1427	2.00E-130	213/320(66.56)	387	PHI:1427|GzC2H095|I1S4N1|5518|Gibberella_zeae_(related:_Fusarium_graminearum)|Unaffected_pathogenicity
Chr01G2338.1	346	7.00E-86	138/348(39.66)	280	PHI:346|CRU1|AAN10186|5270|Ustilago_maydis|Reduced_virulence
Chr01G2340.1	2315	1.00E-79	130/303(42.90)	244	PHI:2315|ChLae1|G4XKY9|5016|Cochliobolus_heterostrophus|Mixed_outcome
Chr01G2342.1	2255	0	268/369(72.63)	569	PHI:2255|Abd1|Q0U2A0|13684|Phaeosphaeria_nodorum_(related:_Stagonospora_nodorum)|Unaffected_pathogenicity
Chr01G2346.1	2825	7.00E-09	40/125(32.00)	57.4	PHI:2825|SET1|Q5ABG1|5476|Candida_albicans|Reduced_virulence
Chr01G2351.1	2174	1.00E-10	67/274(24.45)	59.3	PHI:2174|NMR2|MGG_02860|148305|Magnaporthe_oryzae_(related:_Magnaporthe_grisea)|Reduced_virulence
Chr01G2355.1	2382	2.00E-75	117/270(43.33)	241	PHI:2382|Upa2|Q6QIY0|5270|Ustilago_maydis|Unaffected_pathogenicity
Chr01G2356.1	1522	5.00E-07	63/308(20.45)	48.1	PHI:1522|GzHOMEL026|I1RXA5|5518|Gibberella_zeae_(related:_Fusarium_graminearum)|Lethal
Chr01G2358.1	211	6.00E-07	68/284(23.94)	50.4	PHI:211|CaTUP1|AAB63195|5476|Candida_albicans|Reduced_virulence
Chr01G2362.1	1564	4.00E-34	51/78(65.38)	113	PHI:1564|GzOB004|Q4IPZ1|5518|Gibberella_zeae_(related:_Fusarium_graminearum)|Unaffected_pathogenicity
Chr01G2363.1	1565	0	330/546(60.44)	662	PHI:1565|GzOB005|I1RBF6|5518|Gibberella_zeae_(related:_Fusarium_graminearum)|Unaffected_pathogenicity
Chr01G2367.1	1458	2.00E-29	124/480(25.83)	121	PHI:1458|GzAra006|Q4I7F9|5518|Gibberella_zeae_(related:_Fusarium_graminearum)|Unaffected_pathogenicity
Chr01G2370.1	2832	3.00E-88	136/260(52.31)	267	PHI:2832|AKT3-1|Q9P4U9|5599|Alternaria_alternata|Loss_of_pathogenicity
Chr01G2373.1	2103	7.00E-102	199/329(60.49)	333	PHI:2103|Vacuolar_calcium_ion_transporter|MGG_08710|148305|Magnaporthe_oryzae_(related:_Magnaporthe_grisea)|Unaffected_pathogenicity
Chr01G2374.1	2113	0	702/1271(55.23)	1133	PHI:2113|Kin4|MGG_01196|148305|Magnaporthe_oryzae_(related:_Magnaport

					he_grisea)|Mixed_outcome
Chr01G2383.1	2654	6.00E-23	114/435(26.21)	101	PHI:2654|DUR1,2|Q59VF3|5476|Candida_albicans|Reduced_virulence
Chr01G2385.1	1443	0	315/437(72.08)	606	PHI:1443|GzGATA001|I1RB11|5518|Gibberella_zeae_(related:_Fusarium_graminearum)|Unaffected_pathogenicity
Chr01G2390.1	3162	0	751/1370(54.82)	1479	Mohik8|PHI:3162|G4MXJ1|318829|Magnaporthe_oryzae|loss_of_pathogenicity_
Chr01G2400.1	2823	1.00E-140	258/637(40.50)	424	PHI:2823|PES1|Q59X38|5476|Candida_albicans|Reduced_virulence
Chr01G2401.1	1472	3.00E-122	256/650(39.38)	378	PHI:1472|GzHMG004|I1RAZ9|5518|Gibberella_zeae_(related:_Fusarium_graminearum)|Unaffected_pathogenicity
Chr01G2402.1	465	1.00E-67	150/378(39.68)	244	PHI:465|KIN2|AAB63337|5270|Ustilago_maydis|Reduced_virulence
Chr01G2408.1	339	5.00E-35	76/203(37.44)	123	PHI:339|CLPT1|CAC41973|290576|Colletotrichum_lindemuthianum|Reduced_virulence
Chr01G2411.1	1662	2.00E-137	240/559(42.93)	439	PHI:1662|GzCCHC002|I1REJ1|5518|Gibberella_zeae_(related:_Fusarium_graminearum)|Unaffected_pathogenicity
Chr01G2412.1	404	5.00E-42	113/382(29.58)	155	PHI:404|PTH11|AAD30436|318829|Magnaporthe_oryzae|Reduced_virulence
Chr01G2413.1	2034	2.00E-14	60/204(29.41)	71.6	PHI:2034|MFP1|G4MZY1|148305|Magnaporthe_oryzae_(related:_Magnaporthe_grisea)|Reduced_virulence
Chr01G2414.1	1701	3.00E-65	161/480(33.54)	224	PHI:1701|GzZC016|I1RKS9|5518|Gibberella_zeae_(related:_Fusarium_graminearum)|Unaffected_pathogenicity
Chr01G2415.1	812	1.00E-90	159/366(43.44)	280	PHI:812|MGG_10702|EDJ94108|318829|Magnaporthe_oryzae|Reduced_virulence
Chr01G2416.1	544	1.00E-07	100/459(21.79)	52	PHI:544|BCMFS1|AAF64435|332648|Botrytis_cinerea|Unaffected_pathogenicity
Chr01G2417.1	2147	2.00E-17	81/291(27.84)	81.3	PHI:2147|Erl1|G5EH97|148305|Magnaporthe_oryzae_(related:_Magnaporthe_grisea)|Reduced_virulence
Chr01G2418.1	2147	2.00E-45	96/250(38.40)	158	PHI:2147|Erl1|G5EH97|148305|Magnaporthe_oryzae_(related:_Magnaporthe_

					grisea)|Reduced_virulence
Chr01G2421.1	2451	5.00E-71	125/280(44.64)	224	PHI:2451|FAEB1|I1S3N8|5518|Gibberella_zeae_(related:_Fusarium_graminearum)|Unaffected_pathogenicity
Chr01G2425.1	1916	7.00E-54	158/586(26.96)	194	PHI:1916|GzZC231|I1RSG1|5518|Gibberella_zeae_(related:_Fusarium_graminearum)|Unaffected_pathogenicity
Chr01G2434.1	441	1.00E-27	98/393(24.94)	110	PHI:441|BTP1|CAE55153|40559|Botrytis_cinerea|Reduced_virulence
Chr01G2435.1	1046	1.00E-42	123/463(26.57)	156	PHI:1046|CTB5|ABK64182|29003|Cercospora_nicotianae|Reduced_virulence
Chr01G2436.1	1940	7.00E-26	107/421(25.42)	108	PHI:1940|GzZC255|I1RMT2|5518|Gibberella_zeae_(related:_Fusarium_graminearum)|Unaffected_pathogenicity
Chr01G2437.1	3794	4.00E-07	36/134(26.87)	50.1	MNN2|PHI:3794|Q6FQP8|5478|Candida_glabrata|mixed_outcome
Chr01G2438.1	748	7.00E-10	78/316(24.68)	59.3	PHI:748|um00446|Not_available|5270|Ustilago_maydis|Unaffected_pathogenicity
Chr01G2440.1	1260	8.00E-09	33/100(33.00)	53.1	PHI:1260|FGSG_13944|I1RUC7|5518|Gibberella_zeae_(related:_Fusarium_graminearum)|Unaffected_pathogenicity
Chr01G2445.1	1555	3.00E-30	98/418(23.44)	121	PHI:1555|GzMyb019|I1RDG6|5518|Gibberella_zeae_(related:_Fusarium_graminearum)|Unaffected_pathogenicity
Chr01G2452.1	115	6.00E-46	125/411(30.41)	164	PHI:115|PGX1|AAC26146|5017|Cochliobolus_carbonum|Unaffected_pathogenicity
Chr01G2454.1	2927	1.00E-85	189/552(34.24)	276	PHI:2927|lip5|J9N2Z8|59765|Fusarium_oxysporum_f._sp._Lycopersici|Unaffected_pathogenicity
Chr01G2457.1	1260	9.00E-16	62/169(36.69)	77.4	PHI:1260|FGSG_13944|I1RUC7|5518|Gibberella_zeae_(related:_Fusarium_graminearum)|Unaffected_pathogenicity
Chr01G2460.1	881	1.00E-31	74/220(33.64)	121	PHI:881|MGG_04556|EDJ96020|318829|Magnaporthe_oryzae|Reduced_virulence
Chr01G2463.1	1939	3.00E-69	120/263(45.63)	220	PHI:1939|GzZC254|I1RMX8|5518|Gibberella_zeae_(related:_Fusarium_graminearum)|Unaffected_pathogenicity

Chr01G2467.1	1249	0	459/618(74.27)	868	PHI:1249|FGSG_00132|I1RB79|5518|Gibberella_zeae_(related:_Fusarium_graminearum)|Unaffected_pathogenicity
Chr01G2473.1	2506	3.00E-19	61/201(30.35)	90.1	PHI:2506|Amr1|G3F820|29001|Alternaria_brassicicola|Increased_virulence_(Hypervirulence)
Chr01G2474.1	438	2.00E-50	140/477(29.35)	180	PHI:438|BcBOT1_(related:_CND5)|AAQ16576|40559|Botrytis_cinerea|Reduced_virulence
Chr01G2475.1	1051	8.00E-11	93/389(23.91)	61.2	PHI:1051|CTB3|ABC79591|29003|Cercospora_nicotianae|Reduced_virulence
Chr01G2476.1	1393	7.00E-107	237/778(30.46)	347	PHI:1393|GzC2H056|I1RU69|5518|Gibberella_zeae_(related:_Fusarium_graminearum)|Unaffected_pathogenicity
Chr01G2478.1	2836	8.00E-07	31/93(33.33)	46.2	PHI:2836|RED3|C3JXE9|5016|Cochliobolus_heterostrophus|Reduced_virulence
Chr01G2479.1	2117	7.00E-75	166/411(40.39)	243	PHI:2117|SPM1|P58371|148305|Magnaporthe_oryzae_(related:_Magnaporthe_grisea)|Reduced_virulence
Chr01G2480.1	112	1.00E-71	151/467(32.33)	236	PHI:112|MAK1|AAC49410|140110|Nectria_haematococca_(related:_Fusarium_solani)|Reduced_virulence
Chr01G2484.1	3216	5.00E-29	87/234(37.18)	109	MoCDIP4|PHI:3216|G4MVX4|318829|Magnaporthe_oryzae|mixed_outcome_
Chr01G2489.1	1263	0	384/650(59.08)	714	PHI:1263|FGSG_04770|I1RB84|5518|Gibberella_zeae_(related:_Fusarium_graminearum)|Unaffected_pathogenicity
Chr01G2491.1	1342	3.00E-06	32/111(28.83)	45.4	PHI:1342|GzC2H002|I1RA24|5518|Gibberella_zeae_(related:_Fusarium_graminearum)|Unaffected_pathogenicity
Chr01G2492.1	1399	2.00E-09	47/190(24.74)	57.4	PHI:1399|GzC2H062|I1RV74|5518|Gibberella_zeae_(related:_Fusarium_graminearum)|Unaffected_pathogenicity
Chr01G2494.1	511	4.00E-14	97/369(26.29)	72.4	PHI:511|CaNAG4|EAK93098|5476|Candida_albicans|Reduced_virulence
Chr01G2497.1	179	6.00E-60	105/217(48.39)	190	PHI:179|PELA|AAA33338|140110|Nectria_haematococca_(related:_Fusarium_solani)|Reduced_virulence
Chr01G2501.1	1816	2.00E-20	80/316(25.32)	91.7	PHI:1816|GzZC131|I1RRS3|5518|Gibberella_zeae_(related:_Fusarium_gramin

					earum)|Unaffected_pathogenicity
Chr01G2503.1	1393	8.00E-31	74/221(33.48)	122	PHI:1393|GzC2H056|I1RU69|5518|Gibberella_zeae_(related:_Fusarium_graminearum)|Unaffected_pathogenicity
Chr01G2507.1	2371	2.00E-07	30/115(26.09)	51.6	PHI:2371|bcpkaR|C0H5W5|40559|Botrytis_cinerea|Reduced_virulence
Chr01G2511.1	26	6.00E-44	127/460(27.61)	162	PHI:26|CaMDR1|CAA37820|5476|Candida_albicans|Reduced_virulence
Chr01G2516.1	812	3.00E-42	96/342(28.07)	152	PHI:812|MGG_10702|EDJ94108|318829|Magnaporthe_oryzae|Reduced_virulence
Chr01G2517.1	2032	2.00E-24	94/318(29.56)	103	PHI:2032|VTL1|G4NGA7|148305|Magnaporthe_oryzae_(related:_Magnaporthe_grisea)|Unaffected_pathogenicity
Chr01G2528.1	1555	8.00E-43	127/491(25.87)	158	PHI:1555|GzMyb019|I1RDG6|5518|Gibberella_zeae_(related:_Fusarium_graminearum)|Unaffected_pathogenicity
Chr01G2529.1	785	6.00E-108	200/529(37.81)	339	PHI:785|MGG_04128|EDJ99431|318829|Magnaporthe_oryzae|Reduced_virulence
Chr01G2532.1	1756	9.00E-48	93/197(47.21)	180	PHI:1756|GzZC071|I1S4Q3|5518|Gibberella_zeae_(related:_Fusarium_graminearum)|Unaffected_pathogenicity
Chr01G2535.1	3238	2.00E-57	94/192(48.96)	183	ctrC|PHI:3238|B0XUP5|746128|Aspergillus_fumigatus|mixed_outcome_
Chr01G2537.1	1260	2.00E-19	70/223(31.39)	92.4	PHI:1260|FGSG_13944|I1RUC7|5518|Gibberella_zeae_(related:_Fusarium_graminearum)|Unaffected_pathogenicity
Chr01G2539.1	438	2.00E-09	32/102(31.37)	52	PHI:438|BcBOT1_(related:_CND5)|AAQ16576|40559|Botrytis_cinerea|Reduced_virulence
Chr01G2544.1	3216	2.00E-43	126/308(40.91)	150	MoCDIP4|PHI:3216|G4MVX4|318829|Magnaporthe_oryzae|mixed_outcome_
Chr01G2546.1	2968	2.00E-36	125/489(25.56)	139	PHI:2968|Hxs1|J9VQA5|5207|Cryptococcus_neoformans|Reduced_virulence
Chr01G2547.1	1260	9.00E-08	60/204(29.41)	53.9	PHI:1260|FGSG_13944|I1RUC7|5518|Gibberella_zeae_(related:_Fusarium_graminearum)|Unaffected_pathogenicity
Chr01G2548.1	2378	7.00E-10	59/214(27.57)	58.5	PHI:2378|DEP4|D2E9W9|29001|Alternaria_brassicicola|Mixed_outcome
Chr01G2549.1	419	9.00E-30	91/322(28.26)	115	PHI:419|CSH1|AAP93915|5476|Candida_albicans|Reduced_virulence

Chr01G2550.1	404	2.00E-34	100/385(25.97)	134	PHI:404|PTH11|AAD30436|318829|Magnaporthe_oryzae|Reduced_virulence
Chr01G2558.1	3662	3.00E-07	40/114(35.09)	51.6	PspB_(not_PD0218)|PHI:3662|Q87ET0|2371|Xylella_fastidiosa|Increased_virulence_(Hypervirulence)
Chr01G2559.1	1974	1.00E-08	36/86(41.86)	55.5	PHI:1974|GzZC289|I1RQN6|5518|Gibberella_zeae_(related:_Fusarium_graminearum)|Unaffected_pathogenicity
Chr01G2560.1	2968	9.00E-34	120/453(26.49)	130	PHI:2968|Hxs1|J9VQA5|5207|Cryptococcus_neoformans|Reduced_virulence
Chr01G2561.1	267	2.00E-58	167/591(28.26)	219	PHI:267|MLT1|AAD51594|5476|Candida_albicans|Reduced_virulence
Chr01G2567.1	2315	3.00E-64	119/293(40.61)	207	PHI:2315|ChLae1|G4XKY9|5016|Cochliobolus_heterostrophus|Mixed_outcome
Chr01G2571.1	1662	2.00E-24	75/267(28.09)	105	PHI:1662|GzCCHC002|I1REJ1|5518|Gibberella_zeae_(related:_Fusarium_graminearum)|Unaffected_pathogenicity
Chr01G2574.1	2171	0	324/688(47.09)	612	PHI:2171|Peroxisomal_copper_amine_oxidase|MGG_02681|148305|Magnaporthe_oryzae_(related:_Magnaporthe_grisea)|Reduced_virulence
Chr01G2576.1	1399	6.00E-08	40/155(25.81)	49.3	PHI:1399|GzC2H062|I1RV74|5518|Gibberella_zeae_(related:_Fusarium_graminearum)|Unaffected_pathogenicity
Chr01G2577.1	3214	5.00E-28	63/162(38.89)	103	MoCDIP2|PHI:3214|G4MML4|318829|Magnaporthe_oryzae|mixed_outcome_
Chr01G2580.1	1909	2.00E-46	134/509(26.33)	169	PHI:1909|GzZC224|I1RHG6|5518|Gibberella_zeae_(related:_Fusarium_graminearum)|Unaffected_pathogenicity
Chr01G2583.1	144	3.00E-09	70/259(27.03)	58.2	PHI:144|CHT42|AAC05829|29875|Trichoderma_virens|Reduced_virulence
Chr01G2584.1	1707	7.00E-24	114/461(24.73)	102	PHI:1707|GzZC022|I1RJY6|5518|Gibberella_zeae_(related:_Fusarium_graminearum)|Unaffected_pathogenicity
Chr01G2586.1	404	1.00E-13	80/340(23.53)	69.7	PHI:404|PTH11|AAD30436|318829|Magnaporthe_oryzae|Reduced_virulence
Chr01G2587.1	1651	9.00E-104	172/397(43.32)	315	PHI:1651|GzWing023|I1S0U2|5518|Gibberella_zeae_(related:_Fusarium_graminearum)|Unaffected_pathogenicity
Chr01G2588.1	438	1.00E-52	146/490(29.80)	190	PHI:438|BcBOT1_(related:_CND5)|AAQ16576|40559|Botrytis_cinerea|Reduced_virulence
Chr01G2590.1	2315	1.00E-60	115/298(38.59)	197	PHI:2315|ChLae1|G4XKY9|5016|Cochliobolus_heterostrophus|Mixed_outcome

Chr01G2591.1	1651	2.00E-20	86/334(25.75)	90.1	PHI:1651|GzWing023|I1S0U2|5518|Gibberella_zeae_(related:_Fusarium_graminearum)|Unaffected_pathogenicity
Chr01G2594.1	3384	2.00E-82	277/1033(26.82)	289	FVEG_12530|PHI:3384|W7N2C1|117187|Fusarium_verticillioides|unaffected_pathogenicity_
Chr01G2596.1	1835	0	310/606(51.16)	625	PHI:1835|GzZC150|I1RIL1|5518|Gibberella_zeae_(related:_Fusarium_graminearum)|Unaffected_pathogenicity
Chr01G2604.1	1914	1.00E-11	25/34(73.53)	65.1	PHI:1914|GzZC229|I1RQ27|5518|Gibberella_zeae_(related:_Fusarium_graminearum)|Unaffected_pathogenicity
Chr01G2605.1	2269	8.00E-29	81/253(32.02)	110	PHI:2269|Mdh1|0|13684|Phaeosphaeria_nodorum_(related:_Stagonospora_nodorum)|Unaffected_pathogenicity
Chr01G2606.1	1555	8.00E-40	119/455(26.15)	149	PHI:1555|GzMyb019|I1RDG6|5518|Gibberella_zeae_(related:_Fusarium_graminearum)|Unaffected_pathogenicity
Chr01G2607.1	1662	2.00E-72	157/483(32.51)	250	PHI:1662|GzCCHC002|I1REJ1|5518|Gibberella_zeae_(related:_Fusarium_graminearum)|Unaffected_pathogenicity
Chr01G2612.1	3633	3.00E-19	50/156(32.05)	84.7	Rv0469|PHI:3633|Q6MX39|1773|Mycobacterium_tuberculosis|increased_virulence_
Chr01G2618.1	1260	1.00E-09	42/138(30.43)	58.5	PHI:1260|FGSG_13944|I1RUC7|5518|Gibberella_zeae_(related:_Fusarium_graminearum)|Unaffected_pathogenicity
Chr01G2627.1	2927	3.00E-111	190/487(39.01)	342	PHI:2927|lip5|J9N2Z8|59765|Fusarium_oxysporum_f._sp._Lycopersici|Unaffected_pathogenicity
Chr01G2629.1	2240	3.00E-33	131/504(25.99)	130	PHI:2240|Srt1|Q4PBY9|5270|Ustilago_maydis|reduced_virulence
Chr01G2632.1	441	1.00E-31	94/305(30.82)	122	PHI:441|BTP1|CAE55153|40559|Botrytis_cinerea|Reduced_virulence
Chr01G2633.1	812	6.00E-62	129/361(35.73)	206	PHI:812|MGG_10702|EDJ94108|318829|Magnaporthe_oryzae|Reduced_virulence
Chr01G2634.1	886	1.00E-06	46/183(25.14)	47	PHI:886|MGG_13052|EDK06087|318829|Magnaporthe_oryzae|Reduced_virulence

Chr01G2635.1	876	0	458/1162(39.41)	780	PHI:876|MGG_11671|EDK03349|318829|Magnaporthe_oryzae|Reduced_virulence
Chr01G2638.1	1269	1.00E-70	119/283(42.05)	225	PHI:1269|FGSG_02838|I1RFK9|5518|Gibberella_zeae_(related:_Fusarium_graminearum)|Unaffected_pathogenicity
Chr01G2640.1	241	1.00E-15	71/273(26.01)	79.7	PHI:241|CAC1|AAG60619|5207|Cryptococcus_neoformans|Loss_of_pathogenicity
Chr01G2641.1	1755	1.00E-162	254/604(42.05)	484	PHI:1755|GzZC070|I1S1A5|5518|Gibberella_zeae_(related:_Fusarium_graminearum)|Unaffected_pathogenicity
Chr01G2643.1	438	2.00E-39	124/471(26.33)	148	PHI:438|BcBOT1_(related:_CND5)|AAQ16576|40559|Botrytis_cinerea|Reduced_virulence
Chr01G2646.1	413	3.00E-26	114/453(25.17)	107	PHI:413|MPD1|AAT84078|13684|Stagonospora_nodorum|Unaffected_pathogenicity
Chr01G2650.1	1948	7.00E-50	150/545(27.52)	179	PHI:1948|GzZC263|I1RI81|5518|Gibberella_zeae_(related:_Fusarium_graminearum)|Unaffected_pathogenicity
Chr01G2652.1	438	5.00E-14	54/206(26.21)	71.6	PHI:438|BcBOT1_(related:_CND5)|AAQ16576|40559|Botrytis_cinerea|Reduced_virulence
Chr01G2653.1	72	2.00E-11	67/236(28.39)	62.4	PHI:72|SAP2|AAM21050|5476|Candida_albicans|Reduced_virulence
Chr01G2659.1	106	5.00E-167	253/483(52.38)	486	PHI:106|CAT1|AAC39448|5476|Candida_albicans|Reduced_virulence
Chr01G2667.1	1260	9.00E-10	51/149(34.23)	59.7	PHI:1260|FGSG_13944|I1RUC7|5518|Gibberella_zeae_(related:_Fusarium_graminearum)|Unaffected_pathogenicity
Chr01G2668.1	1527	8.00E-37	100/357(28.01)	138	PHI:1527|GzHOMEL040|I1S9A1|5518|Gibberella_zeae_(related:_Fusarium_graminearum)|Lethal
Chr01G2681.1	3794	2.00E-12	67/266(25.19)	67	MNN2|PHI:3794|Q6FQP8|5478|Candida_glabrata|mixed_outcome
Chr01G2682.1	3274	2.00E-14	123/555(22.16)	74.3	dhbF|PHI:3274|W2E906|147375|Paenibacillus_larvae|unaffected_pathogenicity_
Chr01G2684.1	273	2.00E-55	106/307(34.53)	182	PHI:273|SPT3|AAD33888|5476|Candida_albicans|Loss_of_pathogenicity

Chr01G2688.1	413	3.00E-32	111/417(26.62)	124	PHI:413|MPD1|AAT84078|13684|Stagonospora_nodorum|Unaffected_pathogenicity
Chr01G2689.1	4194	5.00E-64	147/518(28.38)	217	AKT7|PHI:4194|V5XZS6|5599|Alternaria_alternata|increased_virulence_(Hypervirulence)
Chr01G2691.1	800	4.00E-07	60/249(24.10)	50.8	PHI:800|MGG_13324|EDK00897|318829|Magnaporthe_oryzae|Reduced_virulence
Chr01G2692.1	1209	1.00E-24	66/209(31.58)	101	PHI:1209|FGSG_04770|I1RLH1|5518|Gibberella_zeae_(related:_Fusarium_graminearum)|Reduced_virulence
Chr01G2694.1	2693	0	1092/1539(70.96)	2233	PHI:2693|GcABC-G1|F0XP73|226899|Grosmannia_clavigera|Reduced_virulence
Chr01G2698.1	1555	2.00E-44	118/457(25.82)	162	PHI:1555|GzMyb019|I1RDG6|5518|Gibberella_zeae_(related:_Fusarium_graminearum)|Unaffected_pathogenicity
Chr01G2702.1	598	2.00E-129	199/370(53.78)	379	PHI:598|THIOL|ABB55459|5022|Leptosphaeria_maculans|Reduced_virulence
Chr01G2703.1	1260	7.00E-11	49/152(32.24)	62.8	PHI:1260|FGSG_13944|I1RUC7|5518|Gibberella_zeae_(related:_Fusarium_graminearum)|Unaffected_pathogenicity
Chr01G2705.1	521	3.00E-27	106/166(63.86)	102	PHI:521|CgDN24|AAB92223|5457|Colletotrichum_gloeosporioides|Unaffected_pathogenicity
Chr01G2707.1	1555	7.00E-44	118/431(27.38)	160	PHI:1555|GzMyb019|I1RDG6|5518|Gibberella_zeae_(related:_Fusarium_graminearum)|Unaffected_pathogenicity
Chr01G2710.1	254	7.00E-07	50/190(26.32)	47.4	PHI:254|FOW1|BAB85760|5507|Fusarium_oxysporum|Reduced_virulence
Chr01G2711.1	1711	2.00E-177	271/470(57.66)	519	PHI:1711|GzZC026|I1REQ5|5518|Gibberella_zeae_(related:_Fusarium_graminearum)|Unaffected_pathogenicity
Chr01G2712.1	1051	3.00E-11	98/387(25.32)	62.8	PHI:1051|CTB3|ABC79591|29003|Cercospora_nicotianae|Reduced_virulence
Chr01G2714.1	157	9.00E-17	86/335(25.67)	78.2	PHI:157|TOXF|AAD45321|5017|Cochliobolus_carbonum|Loss_of_pathogenicity
Chr01G2719.1	2700	2.00E-50	158/597(26.47)	183	PHI:2700|lac2|J7MF98|5465|Colletotrichum_orbiculare|Reduced_virulence
Chr01G2720.1	2700	4.00E-91	193/598(32.27)	293	PHI:2700|lac2|J7MF98|5465|Colletotrichum_orbiculare|Reduced_virulence

Chr01G2725.1	211	2.00E-58	121/311(38.91)	209	PHI:211|CaTUP1|AAB63195|5476|Candida_albicans|Reduced_virulence
Chr01G2726.1	876	2.00E-79	168/432(38.89)	266	PHI:876|MGG_11671|EDK03349|318829|Magnaporthe_oryzae|Reduced_virule nce
Chr01G2727.1	876	0	460/1162(39.59)	780	PHI:876|MGG_11671|EDK03349|318829|Magnaporthe_oryzae|Reduced_virule nce
Chr01G2728.1	886	1.00E-06	46/183(25.14)	47	PHI:886|MGG_13052|EDK06087|318829|Magnaporthe_oryzae|Reduced_virule nce
Chr01G2738.1	2968	8.00E-40	131/466(28.11)	149	PHI:2968|Hxs1|J9VQA5|5207|Cryptococcus_neoformans|Reduced_virulence
Chr01G2746.1	2968	1.00E-44	130/496(26.21)	164	PHI:2968|Hxs1|J9VQA5|5207|Cryptococcus_neoformans|Reduced_virulence
Chr01G2758.1	876	0	460/1162(39.59)	780	PHI:876|MGG_11671|EDK03349|318829|Magnaporthe_oryzae|Reduced_virule nce
Chr01G2759.1	886	1.00E-06	46/183(25.14)	47	PHI:886|MGG_13052|EDK06087|318829|Magnaporthe_oryzae|Reduced_virule nce
Chr01G2763.1	2643	8.00E-06	48/176(27.27)	43.9	PHI:2643|CFAS|A4HTK3|5671|Leishmania_infantum|Reduced_virulence
Chr01G2767.1	112	8.00E-33	110/415(26.51)	127	PHI:112|MAK1|AAC49410|140110|Nectria_haematococca_(related:_Fusarium_ solani)|Reduced_virulence
Chr01G2768.1	1999	2.00E-80	209/696(30.03)	271	PHI:1999|GzZC314|I1R9E5|5518|Gibberella_zeae_(related:_Fusarium_gramin earum)|Unaffected_pathogenicity
Chr01G2770.1	3381	2.00E-11	93/401(23.19)	63.5	FVEG_12533|PHI:3381|W7N2B4|117187|Fusarium_verticillioides|unaffected_p athogenicity_
Chr01G2773.1	441	2.00E-15	77/316(24.37)	73.9	PHI:441|BTP1|CAE55153|40559|Botrytis_cinerea|Reduced_virulence
Chr01G2774.1	2926	0	401/538(74.54)	847	PHI:2926|lip3|J9NE50|59765|Fusarium_oxysporum_f._sp._Lycopersici|Unaffect ed_pathogenicity
Chr01G2775.1	513	9.00E-52	150/577(26.00)	186	PHI:513|ARN1_(related:_SIT1)|EAK97011|5476|Candida_albicans|Reduced_vir ulence
Chr01G2780.1	544	1.00E-37	146/571(25.57)	144	PHI:544|BCMFS1|AAF64435|332648|Botrytis_cinerea|Unaffected_pathogenicit

					y
Chr01G2787.1	1260	2.00E-21	78/226(34.51)	97.8	PHI:1260|FGSG_13944|I1RUC7|5518|Gibberella_zeae_(related:_Fusarium_graminearum)|Unaffected_pathogenicity
Chr01G2789.1	1260	6.00E-23	77/224(34.38)	103	PHI:1260|FGSG_13944|I1RUC7|5518|Gibberella_zeae_(related:_Fusarium_graminearum)|Unaffected_pathogenicity
Chr01G2790.1	2020	6.00E-14	46/135(34.07)	72.8	PHI:2020|Tup1|XP_759427|5270|Ustilago_maydis|Mixed_outcome
Chr01G1131.1	2549	1.00E-07	30/112(26.79)	52	PHI:2549|MAK5|Q4WMS3|746128|Aspergillus_fumigatus|Mixed_outcome
Chr05G0871.1	1371	5.00E-59	104/279(37.28)	190	PHI:1371|GzC2H034|I1RM60|5518|Gibberella_zeae_(related:_Fusarium_graminearum)|Unaffected_pathogenicity
Chr05G0869.1	211	9.00E-56	119/310(38.39)	202	PHI:211|CaTUP1|AAB63195|5476|Candida_albicans|Reduced_virulence
Chr05G0868.1	2020	7.00E-48	109/317(34.38)	181	PHI:2020|Tup1|XP_759427|5270|Ustilago_maydis|Mixed_outcome
Chr05G0866.1	1522	1.00E-109	147/238(61.76)	320	PHI:1522|GzHOMEL026|I1RXA5|5518|Gibberella_zeae_(related:_Fusarium_graminearum)|Lethal
Chr05G0859.1	538	2.00E-14	113/469(24.09)	72.8	PHI:538|FRT1|AAU87358|40559|Botrytis_cinerea|Unaffected_pathogenicity
Chr05G0856.1	3097	2.00E-104	160/379(42.22)	320	Kre2/Mnt1|PHI:3097|J5JEX5|176275|Beauveria_bassiana|reduced_virulence_
Chr05G0854.1	3238	1.00E-13	51/193(26.42)	64.3	ctrC|PHI:3238|B0XUP5|746128|Aspergillus_fumigatus|mixed_outcome_
Chr05G0847.1	1557	6.00E-87	173/459(37.69)	275	PHI:1557|GzNH002|I1RBU9|5518|Gibberella_zeae_(related:_Fusarium_graminearum)|Unaffected_pathogenicity
Chr05G0845.1	1226	4.00E-27	69/220(31.36)	112	PHI:1226|FGSG_11614|I1RP88|5518|Gibberella_zeae_(related:_Fusarium_graminearum)|Lethal
Chr05G0840.1	2030	8.00E-77	180/533(33.77)	260	PHI:2030|TGL3-1|G4N492|148305|Magnaporthe_oryzae_(related:_Magnaporthe_grisea)|Unaffected_pathogenicity
Chr05G0836.1	1319	0	422/608(69.41)	835	PHI:1319|GzbZIP001|I1RAI5|5518|Gibberella_zeae_(related:_Fusarium_graminearum)|Reduced_virulence
Chr05G0831.1	1271	1.00E-38	74/197(37.56)	138	PHI:1271|FGSG_00792|I1R9J1|5518|Gibberella_zeae_(related:_Fusarium_graminearum)|Unaffected_pathogenicity

Chr05G0829.1	2315	1.00E-27	54/139(38.85)	105	PHI:2315|ChLae1|G4XKY9|5016|Cochliobolus_heterostrophus|Mixed_outcome
Chr05G0828.1	1424	6.00E-21	42/61(68.85)	96.3	PHI:1424|GzC2H092|I1S3J7|5518|Gibberella_zeae_(related:_Fusarium_graminearum)|Unaffected_pathogenicity
Chr05G0824.1	2315	5.00E-43	94/280(33.57)	150	PHI:2315|ChLae1|G4XKY9|5016|Cochliobolus_heterostrophus|Mixed_outcome
Chr05G0823.1	1662	6.00E-28	110/482(22.82)	115	PHI:1662|GzCCHC002|I1REJ1|5518|Gibberella_zeae_(related:_Fusarium_graminearum)|Unaffected_pathogenicity
Chr05G0819.1	1870	2.00E-66	170/592(28.72)	233	PHI:1870|GzZC185|I1RDS9|5518|Gibberella_zeae_(related:_Fusarium_graminearum)|Unaffected_pathogenicity
Chr05G0818.1	2393	7.00E-112	191/490(38.98)	342	PHI:2393|Related_to_O-methylsterigmatocystin_oxidoreductase|I1R980|5518|Gibberella_zeae_(related:_Fusarium_graminearum)|Increased_virulence_(Hypervirulence)
Chr05G0809.1	2968	7.00E-55	142/484(29.34)	192	PHI:2968|Hxs1|J9VQA5|5207|Cryptococcus_neoformans|Reduced_virulence
Chr05G0808.1	1562	0	765/1040(73.56)	1508	PHI:1562|GzOB002|I1RAH4|5518|Gibberella_zeae_(related:_Fusarium_graminearum)|Lethal
Chr05G0804.1	440	0	489/1014(48.22)	892	PHI:440|PMR1|CAB87245|5476|Candida_albicans|Reduced_virulence
Chr05G0801.1	1629	0	615/788(78.05)	1236	PHI:1629|GzWing001|I1RAH2|5518|Gibberella_zeae_(related:_Fusarium_graminearum)|Unaffected_pathogenicity
Chr05G0799.1	208	1.00E-20	116/493(23.53)	92.4	PHI:208|CaNAG5|BAB43816|5476|Candida_albicans|Reduced_virulence
Chr05G0792.1	4506	0	875/1303(67.15)	1641	So_(soft)|PHI:4506|K9Y567|35717|Epichloe_festucae|effector_(plant_avirulence_determinant)
Chr05G0787.1	1489	5.00E-115	223/449(49.67)	347	PHI:1489|GzHMG021|I1RSJ5|5518|Gibberella_zeae_(related:_Fusarium_graminearum)|Unaffected_pathogenicity
Chr05G0785.1	2323	0	1044/1310(79.69)	2077	PHI:2323|mhk1|E9F082|568076|Metarhizium_robertsii|Reduced_virulence
Chr05G0782.1	1257	0	487/692(70.38)	963	PHI:1257|FGSG_13509|I1RSK0|5518|Gibberella_zeae_(related:_Fusarium_graminearum)|Unaffected_pathogenicity
Chr05G0780.1	1348	0	662/892(74.22)	1195	PHI:1348|GzC2H008|I1RC07|5518|Gibberella_zeae_(related:_Fusarium_grami

					nearum)|Reduced_virulence
Chr05G0778.1	254	6.00E-07	28/122(22.95)	47.8	PHI:254|FOW1|BAB85760|5507|Fusarium_oxysporum|Reduced_virulence
Chr05G0777.1	1458	0	545/743(73.35)	1028	PHI:1458|GzAra006|Q4I7F9|5518|Gibberella_zeae_(related:_Fusarium_graminearum)|Unaffected_pathogenicity
Chr05G0774.1	3270	4.00E-168	238/284(83.80)	468	ARSEF_2860|PHI:3270|J5K8Q8|176275|Beauveria_bassiana|mixed_outcome_
Chr05G0767.1	1566	2.00E-101	144/307(46.91)	309	PHI:1566|GzOB006|I1RC95|5518|Gibberella_zeae_(related:_Fusarium_graminearum)|Lethal
Chr05G0762.1	213	1.00E-36	69/141(48.94)	136	PHI:213|CPA1|AAF69795|5207|Cryptococcus_neoformans|Reduced_virulence
Chr05G0760.1	3383	9.00E-47	112/326(34.36)	165	FVEG_12531|PHI:3383|W7N2A8|117187|Fusarium_verticillioides|unaffected_pathogenicity_
Chr05G0753.1	2835	2.00E-57	95/200(47.50)	189	PHI:2835|RED2|C3JXE8|5016|Cochliobolus_heterostrophus|Reduced_virulence
Chr05G0749.1	2748	1.00E-06	37/132(28.03)	49.7	PHI:2748|snf1|Q4PF20|5270|Ustilago_maydis|Reduced_virulence
Chr05G0747.1	2038	3.00E-06	60/208(28.85)	45.4	PHI:2038|Mir1|MGG_02370|148305|Magnaporthe_oryzae_(related:_Magnaporthe_grisea)|Unaffected_pathogenicity
Chr05G0745.1	3084	1.00E-15	39/129(30.23)	71.2	A1S_2343|PHI:3084|A3M771|470|Acinetobacter_baumannii|reduced_virulence_
Chr05G0743.1	1917	0	511/696(73.42)	1060	PHI:1917|GzZC232|I1RSE9|5518|Gibberella_zeae_(related:_Fusarium_graminearum)|Reduced_virulence
Chr05G0740.1	2032	3.00E-125	196/501(39.12)	381	PHI:2032|VTL1|G4NGA7|148305|Magnaporthe_oryzae_(related:_Magnaporthe_grisea)|Unaffected_pathogenicity
Chr05G0734.1	2540	0	240/311(77.17)	520	PHI:2540|IPP1|Q4WX65|746128|Aspergillus_fumigatus|Mixed_outcome
Chr05G0729.1	2114	0	442/575(76.87)	868	PHI:2114|Calnexin|MGG_01607|148305|Magnaporthe_oryzae_(related:_Magnaporthe_grisea)|Loss_of_pathogenicity
Chr05G0724.1	167	0	306/570(53.68)	569	PHI:167|CHIP3|AAF00024|5457|Colletotrichum_gloeosporioides|Unaffected_pathogenicity

Chr05G0722.1	1382	0	353/570(61.93)	594	PHI:1382|GzC2H045|I1RRX1|5518|Gibberella_zeae_(related:_Fusarium_graminearum)|Reduced_virulence
Chr05G0718.1	1681	4.00E-24	100/394(25.38)	105	PHI:1681|GzNF001|I1REN7|5518|Gibberella_zeae_(related:_Fusarium_graminearum)|Unaffected_pathogenicity
Chr05G0716.1	348	7.00E-22	63/189(33.33)	88.6	PHI:348|CSH3|EAK92385|5476|Candida_albicans|Reduced_virulence
Chr05G0711.1	2544	6.00E-07	24/111(21.62)	48.1	PHI:2544|PAB1|Q4WK03|746128|Aspergillus_fumigatus|Mixed_outcome
Chr05G0706.1	335	5.00E-45	76/117(64.96)	143	PHI:335|CGRA|AAG28884|5085|Aspergillus_fumigatus|Reduced_virulence
Chr05G0701.1	1658	3.00E-155	204/209(97.61)	430	PHI:1658|GzCCCH002|I1RRW3|5518|Gibberella_zeae_(related:_Fusarium_graminearum)|Unaffected_pathogenicity
Chr05G0693.1	1685	7.00E-14	63/224(28.12)	70.5	PHI:1685|GzRad003|I1S5Z3|5518|Gibberella_zeae_(related:_Fusarium_graminearum)|Unaffected_pathogenicity
Chr05G0686.1	1385	8.00E-07	69/321(21.50)	50.8	PHI:1385|GzC2H048|I1RSF7|5518|Gibberella_zeae_(related:_Fusarium_graminearum)|Unaffected_pathogenicity
Chr05G0677.1	1248	0	293/427(68.62)	573	PHI:1248|FGSG_03146|I1RRU2|5518|Gibberella_zeae_(related:_Fusarium_graminearum)|Unaffected_pathogenicity
Chr05G0671.1	1414	4.00E-14	37/81(45.68)	71.2	PHI:1414|GzC2H081|I1RZL0|5518|Gibberella_zeae_(related:_Fusarium_graminearum)|Unaffected_pathogenicity
Chr05G0670.1	822	3.00E-153	200/244(81.97)	431	PHI:822|Ip|AAB97419|54734|Mycosphaerella_graminicola|Chemistry_target
Chr05G0669.1	2844	1.00E-20	76/281(27.05)	87	PHI:2844|BRM2|O93802|5599|Alternaria_alternata|Unaffected_pathogenicity
Chr05G0667.1	501	2.00E-171	282/606(46.53)	501	PHI:501|ADE2|CAA99327|4932|Saccharomyces_cerevisiae|Reduced_virulence
Chr05G0666.1	423	5.00E-98	158/366(43.17)	305	PHI:423|VAD1|AAV41010|5207|Cryptococcus_neoformans|Reduced_virulence
Chr05G0665.1	1622	0	307/473(64.90)	654	PHI:1622|GzJUM004|I1RRU8|5518|Gibberella_zeae_(related:_Fusarium_graminearum)|Unaffected_pathogenicity
Chr05G0660.1	2032	2.00E-31	139/553(25.14)	125	PHI:2032|VTL1|G4NGA7|148305|Magnaporthe_oryzae_(related:_Magnaporthe_grisea)|Unaffected_pathogenicity
Chr05G0657.1	2453	0	875/1405(62.28)	1661	PHI:2453|Snt2|D9J222|61369|Fusarium_oxysporum_f.sp._melonis|Reduced_vi

					rulence
Chr05G0655.1	3060	0	779/1164(66.92)	1497	Molrg1|PHI:3060|G4MZD0|318829|Magnaporthe_oryzae|mixed_outcome_
Chr05G0654.1	1133	4.00E-09	65/282(23.05)	55.1	PHI:1133|Lmepi|B9DR51|5022|Leptosphaeria_maculans|Loss_of_pathogenicity
Chr05G0652.1	1476	2.00E-12	50/167(29.94)	63.5	PHI:1476|GzHMG008|I1RC98|5518|Gibberella_zeae_(related:_Fusarium_graminearum)|Unaffected_pathogenicity
Chr05G0650.1	2513	0	592/701(84.45)	1211	PHI:2513|GFA1|E9R5E2|746128|Aspergillus_fumigatus|Mixed_outcome
Chr05G0649.1	1566	0	369/391(94.37)	753	PHI:1566|GzOB006|I1RC95|5518|Gibberella_zeae_(related:_Fusarium_graminearum)|Lethal
Chr05G0646.1	1458	9.00E-08	39/136(28.68)	54.3	PHI:1458|GzAra006|Q4I7F9|5518|Gibberella_zeae_(related:_Fusarium_graminearum)|Unaffected_pathogenicity
Chr05G0632.1	1579	9.00E-130	257/750(34.27)	417	PHI:1579|GzOB019|I1RM25|5518|Gibberella_zeae_(related:_Fusarium_graminearum)|Unaffected_pathogenicity
Chr05G0627.1	350	2.00E-25	57/109(52.29)	97.8	PHI:350|EMP1|AAR06609|318829|Magnaporthe_oryzae|Reduced_virulence
Chr05G0622.1	2020	1.00E-25	79/262(30.15)	108	PHI:2020|Tup1|XP_759427|5270|Ustilago_maydis|Mixed_outcome
Chr05G0621.1	469	2.00E-123	184/353(52.12)	363	PHI:469|TOXG|AAD47837|5017|Cochliobolus_carbonum|Reduced_virulence
Chr05G0620.1	511	4.00E-40	123/476(25.84)	150	PHI:511|CaNAG4|EAK93098|5476|Candida_albicans|Reduced_virulence
Chr05G0619.1	1241	0	386/527(73.24)	759	PHI:1241|FGSG_07816|I1RAE2|5518|Gibberella_zeae_(related:_Fusarium_graminearum)|Unaffected_pathogenicity
Chr05G0616.1	3131	0	515/650(79.23)	1029	FgSCH9|PHI:3131|I1RAE5|5518|Fusarium_graminearum|mixed_outcome_
Chr05G0610.1	1343	1.00E-164	252/383(65.80)	468	PHI:1343|GzC2H003|I1RAF0|5518|Gibberella_zeae_(related:_Fusarium_graminearum)|Reduced_virulence
Chr05G0607.1	2960	2.00E-131	237/580(40.86)	400	PHI:2960|frdA|C5BDL7|67780|Edwardsiella_ictaluri|Reduced_virulence
Chr05G0605.1	144	8.00E-131	186/393(47.33)	387	PHI:144|CHT42|AAC05829|29875|Trichoderma_virens|Reduced_virulence
Chr05G0604.1	1920	0	576/827(69.65)	1129	PHI:1920|GzZC235|I1RCK0|5518|Gibberella_zeae_(related:_Fusarium_graminearum)|Unaffected_pathogenicity
Chr05G0603.1	2745	1.00E-06	30/87(34.48)	48.5	PHI:2745|treY|Q02LV5|287|Pseudomonas_aeruginosa|Mixed_outcome

Chr05G0599.1	1316	3.00E-70	125/278(44.96)	217	PHI:1316|GzBrom001|I1RBZ7|5518|Gibberella_zeae_(related:_Fusarium_graminearum)|Unaffected_pathogenicity
Chr05G0597.1	2544	6.00E-11	38/129(29.46)	61.2	PHI:2544|PAB1|Q4WK03|746128|Aspergillus_fumigatus|Mixed_outcome
Chr05G0591.1	2644	2.00E-06	21/74(28.38)	43.5	PHI:2644|thioredoxin_1|P0AA28|90371|Salmonella_enterica_serovar_Typhimurium|Reduced_virulence
Chr05G0590.1	789	5.00E-07	22/54(40.74)	50.4	PHI:789|MGG_04116|EDJ99418|318829|Magnaporthe_oryzae|Reduced_virulence
Chr05G0588.1	186	2.00E-42	158/654(24.16)	161	PHI:186|RIM8|AAD51715|5476|Candida_albicans|Reduced_virulence
Chr05G0587.1	1632	0	389/612(63.56)	771	PHI:1632|GzWing004|I1RBU0|5518|Gibberella_zeae_(related:_Fusarium_graminearum)|Unaffected_pathogenicity
Chr05G0580.1	1567	8.00E-115	150/170(88.24)	325	PHI:1567|GzOB007|I1RCB7|5518|Gibberella_zeae_(related:_Fusarium_graminearum)|Unaffected_pathogenicity
Chr05G0574.1	2552	6.00E-15	55/197(27.92)	74.7	PHI:2552|NOP4|Q4WNM3|746128|Aspergillus_fumigatus|Mixed_outcome
Chr05G0568.1	2908	0	421/525(80.19)	857	PHI:2908|CYP51B|I1RBR4|5518|Fusarium_graminearum|Mixed_outcome
Chr05G0566.1	464	7.00E-83	188/473(39.75)	291	PHI:464|KIN1|AAB63336|5270|Ustilago_maydis|Unaffected_pathogenicity
Chr05G0557.1	339	2.00E-07	24/84(28.57)	46.2	PHI:339|CLPT1|CAC41973|290576|Colletotrichum_lindemuthianum|Reduced_virulence
Chr05G0556.1	4559	1.00E-133	251/636(39.47)	421	T6SS2|PHI:4559|Q6TKU1|562|Escherichia_coli|effector_(plant_avirulence_determinant)
Chr05G0548.1	1347	2.00E-69	173/463(37.37)	233	PHI:1347|GzC2H007|I1RBT2|5518|Gibberella_zeae_(related:_Fusarium_graminearum)|Reduced_virulence
Chr05G0546.1	1458	2.00E-85	161/450(35.78)	292	PHI:1458|GzAra006|Q4I7F9|5518|Gibberella_zeae_(related:_Fusarium_graminearum)|Unaffected_pathogenicity
Chr05G0542.1	2570	4.00E-101	189/478(39.54)	317	PHI:2570|CYB2|Q6FM61|5478|Candida_glabrata|Reduced_virulence
Chr05G0538.1	1526	9.00E-12	36/125(28.80)	67	PHI:1526|GzHOMEL036|I1S0J3|5518|Gibberella_zeae_(related:_Fusarium_graminearum)|Unaffected_pathogenicity

Chr05G0529.1	2219	4.00E-10	26/67(38.81)	59.7	PHI:2219|don1|XP_758565|5270|Ustilago_maydis|Reduced_virulence
Chr05G0527.1	413	3.00E-28	107/401(26.68)	115	PHI:413|MPD1|AAT84078|13684|Stagonospora_nodorum|Unaffected_pathogenicity
Chr05G0526.1	59	4.00E-23	63/195(32.31)	93.6	PHI:59|THR1|BAA18962|5462|Colletotrichum_lagenarium|Reduced_virulence
Chr05G0525.1	3381	2.00E-15	100/413(24.21)	75.9	FVEG_12533|PHI:3381|W7N2B4|117187|Fusarium_verticillioides|unaffected_pathogenicity_
Chr05G0524.1	1723	0	468/933(50.16)	926	PHI:1723|GzZC038|I1S832|5518|Gibberella_zeae_(related:_Fusarium_graminearum)|Unaffected_pathogenicity
Chr05G0523.1	2042	0	604/1346(44.87)	1137	PHI:2042|ABC3|Q3Y5V5|148305|Magnaporthe_oryzae_(related:_Magnaporthe_grisea)|Loss_of_pathogenicity
Chr05G0522.1	1274	8.00E-11	56/189(29.63)	62.4	PHI:1274|FGSG_02488|I1RME6|5518|Gibberella_zeae_(related:_Fusarium_graminearum)|Unaffected_pathogenicity
Chr05G0520.1	2158	0	318/404(78.71)	667	PHI:2158|MoCMK1|EU_984498|148305|Magnaporthe_oryzae_(related:_Magnaporthe_grisea)|Reduced_virulence
Chr05G0518.1	2528	2.00E-07	74/313(23.64)	51.2	PHI:2528|SLY1|Q4WYU7|746128|Aspergillus_fumigatus|Lethal
Chr05G0517.1	1503	0	456/702(64.96)	951	PHI:1503|GzHMG035|I1S828|5518|Gibberella_zeae_(related:_Fusarium_graminearum)|Unaffected_pathogenicity
Chr05G0515.1	1291	0	622/936(66.45)	1135	PHI:1291|TOP1|I1RRX4|5518|Gibberella_zeae_(related:_Fusarium_graminearum)|Reduced_virulence
Chr05G0513.1	3028	2.00E-33	97/307(31.60)	130	Vatr2|PHI:3028|A5CVB7|28447|Clavibacter_michiganensis|reduced_virulence_
Chr05G0512.1	2357	5.00E-101	206/553(37.25)	316	PHI:2357|CYP52X1|E2EAF6|475271|Beauveria_bassiana|Reduced_virulence
Chr05G0504.1	2821	1.00E-84	184/568(32.39)	299	PHI:2821|SNF2|Q5ALP9|5476|Candida_albicans|Reduced_virulence
Chr05G0503.1	1260	3.00E-16	75/245(30.61)	79.3	PHI:1260|FGSG_13944|I1RUC7|5518|Gibberella_zeae_(related:_Fusarium_graminearum)|Unaffected_pathogenicity
Chr05G0499.1	2568	4.00E-87	141/303(46.53)	263	PHI:2568|CAP2|Q5AMP9|5476|Candida_albicans|Reduced_virulence
Chr05G0494.1	1768	1.00E-49	76/125(60.80)	159	PHI:1768|GzZC083|I1RAN6|5518|Gibberella_zeae_(related:_Fusarium_gramin

					earum)|Unaffected_pathogenicity
Chr05G0490.1	2884	6.00E-48	136/413(32.93)	174	PHI:2884|MNN21|Q59KJ7|5476|Candida_albicans|Reduced_virulence
Chr05G0485.1	404	1.00E-16	62/265(23.40)	80.5	PHI:404|PTH11|AAD30436|318829|Magnaporthe_oryzae|Reduced_virulence
Chr05G0482.1	1385	0	621/725(85.66)	1243	PHI:1385|GzC2H048|I1RSF7|5518|Gibberella_zeae_(related:_Fusarium_graminearum)|Unaffected_pathogenicity
Chr05G0479.1	3411	1.00E-37	103/345(29.86)	142	bscN|PHI:3411|O68539|518|Bordetella_bronchiseptica|reduced_virulence_
Chr05G0467.1	1843	0	438/921(47.56)	727	PHI:1843|GzZC158|I1S4X3|5518|Gibberella_zeae_(related:_Fusarium_graminearum)|Unaffected_pathogenicity
Chr05G0466.1	3066	0	781/1398(55.87)	1226	MoRga6|PHI:3066|G4MQB0|318829|Magnaporthe_oryzae|mixed_outcome_
Chr05G0460.1	2644	4.00E-12	24/80(30.00)	60.5	PHI:2644|thioredoxin_1|P0AA28|90371|Salmonella_enterica_serovar_Typhimurium|Reduced_virulence
Chr05G0450.1	3065	0	412/806(51.12)	670	MoRga5|PHI:3065|G4MQQ3|318829|Magnaporthe_oryzae|mixed_outcome_
Chr05G0448.1	1587	0	633/809(78.24)	1296	PHI:1587|GzOB027|I1RSI4|5518|Gibberella_zeae_(related:_Fusarium_graminearum)|Unaffected_pathogenicity
Chr05G0446.1	2177	0	768/1269(60.52)	1451	PHI:2177|PAS1|MGG_09299|148305|Magnaporthe_oryzae_(related:_Magnaporthe_grisea)|Unaffected_pathogenicity
Chr05G0442.1	2075	7.00E-06	28/84(33.33)	45.4	PHI:2075|Moatg7|MGG_07297|148305|Magnaporthe_oryzae_(related:_Magnaporthe_grisea)|Loss_of_pathogenicity
Chr05G0440.1	1205	0	536/891(60.16)	970	PHI:1205|(Sc_CDC15)|I1RA39|5518|Gibberella_zeae_(related:_Fusarium_graminearum)|Reduced_virulence
Chr05G0438.1	445	0	409/468(87.39)	844	PHI:445|NOS1||5518|Fusarium_graminearum|Reduced_virulence
Chr05G0436.1	1453	0	401/708(56.64)	712	PHI:1453|GzAra001|I1RA50|5518|Gibberella_zeae_(related:_Fusarium_graminearum)|Lethal
Chr05G0432.1	1399	4.00E-10	48/168(28.57)	60.8	PHI:1399|GzC2H062|I1RV74|5518|Gibberella_zeae_(related:_Fusarium_graminearum)|Unaffected_pathogenicity
Chr05G0430.1	2544	3.00E-10	27/72(37.50)	58.2	PHI:2544|PAB1|Q4WK03|746128|Aspergillus_fumigatus|Mixed_outcome

Chr05G0420.1	1384	8.00E-86	137/152(90.13)	258	PHI:1384|GzC2H047|I1RSD5|5518|Gibberella_zeae_(related:_Fusarium_graminearum)|Reduced_virulence
Chr05G0417.1	2020	3.00E-31	95/338(28.11)	129	PHI:2020|Tup1|XP_759427|5270|Ustilago_maydis|Mixed_outcome
Chr05G0413.1	1393	3.00E-09	47/206(22.82)	57.4	PHI:1393|GzC2H056|I1RU69|5518|Gibberella_zeae_(related:_Fusarium_graminearum)|Unaffected_pathogenicity
Chr05G0407.1	2968	2.00E-20	102/416(24.52)	92	PHI:2968|Hxs1|J9VQA5|5207|Cryptococcus_neoformans|Reduced_virulence
Chr05G0402.1	1555	5.00E-133	192/495(38.79)	399	PHI:1555|GzMyb019|I1RDG6|5518|Gibberella_zeae_(related:_Fusarium_graminearum)|Unaffected_pathogenicity
Chr05G0401.1	1720	2.00E-116	223/552(40.40)	362	PHI:1720|GzZC035|I1S956|5518|Gibberella_zeae_(related:_Fusarium_graminearum)|Unaffected_pathogenicity
Chr05G0400.1	2745	5.00E-06	27/89(30.34)	46.6	PHI:2745|treY|Q02LV5|287|Pseudomonas_aeruginosa|Mixed_outcome
Chr05G0399.1	419	2.00E-30	96/309(31.07)	117	PHI:419|CSH1|AAP93915|5476|Candida_albicans|Reduced_virulence
Chr05G0398.1	332	0	1789/2131(83.95)	3604	PHI:332|CAC1|BAD04045|5462|Colletotrichum_lagenarium|Loss_of_pathogenicity
Chr05G0394.1	2038	8.00E-58	119/291(40.89)	191	PHI:2038|Mir1|MGG_02370|148305|Magnaporthe_oryzae_(related:_Magnaporthe_grisea)|Unaffected_pathogenicity
Chr05G0392.1	1417	2.00E-29	60/177(33.90)	111	PHI:1417|GzC2H084|I1S0W0|5518|Gibberella_zeae_(related:_Fusarium_graminearum)|Unaffected_pathogenicity
Chr05G0378.1	716	0	279/538(51.86)	585	PHI:716|ZEB1|ABB90284|5518|Fusarium_graminearum|Unaffected_pathogenicity
Chr05G0377.1	441	1.00E-25	88/332(26.51)	103	PHI:441|BTP1|CAE55153|40559|Botrytis_cinerea|Reduced_virulence
Chr05G0375.1	876	0	436/1113(39.17)	720	PHI:876|MGG_11671|EDK03349|318829|Magnaporthe_oryzae|Reduced_virulence
Chr05G0374.1	1566	5.00E-09	63/230(27.39)	55.5	PHI:1566|GzOB006|I1RC95|5518|Gibberella_zeae_(related:_Fusarium_graminearum)|Lethal
Chr05G0373.1	2378	3.00E-71	180/567(31.75)	239	PHI:2378|DEP4|D2E9W9|29001|Alternaria_brassicicola|Mixed_outcome

Chr05G0372.1	1871	9.00E-166	306/742(41.24)	497	PHI:1871|GzZC186|I1RTX0|5518|Gibberella_zeae_(related:_Fusarium_graminearum)|Lethal
Chr05G0371.1	2269	2.00E-31	85/271(31.37)	117	PHI:2269|Mdh1|0|13684|Phaeosphaeria_nodorum_(related:_Stagonospora_nodorum)|Unaffected_pathogenicity
Chr05G0365.1	876	0	484/1227(39.45)	802	PHI:876|MGG_11671|EDK03349|318829|Magnaporthe_oryzae|Reduced_virulence
Chr05G0349.1	2199	3.00E-148	254/624(40.71)	442	PHI:2199|MoRgs8|XP_001405673|148305|Magnaporthe_oryzae_(related:_Magnaporthe_grisea)|Unaffected_pathogenicity
Chr05G0342.1	784	3.00E-07	26/100(26.00)	47.8	PHI:784|MGG_00056|EDK03390|318829|Magnaporthe_oryzae|Reduced_virulence
Chr05G0341.1	2476	2.00E-85	161/336(47.92)	261	PHI:2476|CcpelA|G8AA67|27358|Colletotrichum_coccodes|Mixed_outcome
Chr05G0339.1	167	2.00E-22	77/257(29.96)	98.2	PHI:167|CHIP3|AAF00024|5457|Colletotrichum_gloeosporioides|Unaffected_pathogenicity
Chr05G0335.1	2909	1.00E-09	73/278(26.26)	58.2	PHI:2909|CYP51C|I1S2M5|5518|Fusarium_graminearum|Mixed_outcome
Chr05G0334.1	1581	2.00E-93	135/257(52.53)	281	PHI:1581|GzOB021|I1RPX8|5518|Gibberella_zeae_(related:_Fusarium_graminearum)|Unaffected_pathogenicity
Chr05G0328.1	2099	0	513/1129(45.44)	909	PHI:2099|Pmc1|MGG_07971|148305|Magnaporthe_oryzae_(related:_Magnaporthe_grisea)|Mixed_outcome
Chr05G0325.1	2270	4.00E-16	62/224(27.68)	77.4	PHI:2270|Mpd1|Q0U6E8|13684|Phaeosphaeria_nodorum_(related:_Stagonospora_nodorum)|Unaffected_pathogenicity
Chr05G0322.1	1974	8.00E-59	146/545(26.79)	205	PHI:1974|GzZC289|I1RQN6|5518|Gibberella_zeae_(related:_Fusarium_graminearum)|Unaffected_pathogenicity
Chr05G0317.1	1443	6.00E-14	33/76(43.42)	68.2	PHI:1443|GzGATA001|I1RB11|5518|Gibberella_zeae_(related:_Fusarium_graminearum)|Unaffected_pathogenicity
Chr05G0311.1	267	2.00E-58	164/615(26.67)	219	PHI:267|MLT1|AAD51594|5476|Candida_albicans|Reduced_virulence
Chr05G0306.1	223	9.00E-34	65/156(41.67)	124	PHI:223|PEP1|AAK11166|140110|Nectria_haematococca_(related:_Fusarium_

					solani)|Reduced_virulence
Chr05G0302.1	886	1.00E-06	46/183(25.14)	47	PHI:886|MGG_13052|EDK06087|318829|Magnaporthe_oryzae|Reduced_virulence
Chr05G0301.1	876	0	459/1162(39.50)	781	PHI:876|MGG_11671|EDK03349|318829|Magnaporthe_oryzae|Reduced_virulence
Chr05G0299.1	812	3.00E-79	145/343(42.27)	252	PHI:812|MGG_10702|EDJ94108|318829|Magnaporthe_oryzae|Reduced_virulence
Chr05G0296.1	803	2.00E-52	203/705(28.79)	195	PHI:803|MGG_04629|EDJ95969|318829|Magnaporthe_oryzae|Reduced_virulence
Chr05G0292.1	3457	4.00E-21	115/443(25.96)	93.6	PHO84|PHI:3457|J9VMW8|5207|Cryptococcus_neoformans|mixed_outcome_
Chr05G0289.1	1754	7.00E-103	156/284(54.93)	315	PHI:1754|GzZC069|I1S1D6|5518|Gibberella_zeae_(related:_Fusarium_graminearum)|Unaffected_pathogenicity
Chr05G0278.1	2315	5.00E-11	43/167(25.75)	59.7	PHI:2315|ChLae1|G4XKY9|5016|Cochliobolus_heterostrophus|Mixed_outcome
Chr05G0274.1	1046	2.00E-49	137/473(28.96)	176	PHI:1046|CTB5|ABK64182|29003|Cercospora_nicotianae|Reduced_virulence
Chr05G0273.1	404	2.00E-23	69/242(28.51)	99.8	PHI:404|PTH11|AAD30436|318829|Magnaporthe_oryzae|Reduced_virulence
Chr05G0272.1	2968	1.00E-22	112/469(23.88)	98.2	PHI:2968|Hxs1|J9VQA5|5207|Cryptococcus_neoformans|Reduced_virulence
Chr05G0270.1	267	6.00E-64	173/581(29.78)	237	PHI:267|MLT1|AAD51594|5476|Candida_albicans|Reduced_virulence
Chr05G0263.1	2042	3.00E-164	414/1384(29.91)	529	PHI:2042|ABC3|Q3Y5V5|148305|Magnaporthe_oryzae_(related:_Magnaporthe_grisea)|Loss_of_pathogenicity
Chr05G0257.1	504	3.00E-22	90/364(24.73)	93.6	PHI:504|LEU2|CAA42366|4932|Saccharomyces_cerevisiae|Reduced_virulence
Chr05G0255.1	1260	6.00E-13	72/212(33.96)	70.1	PHI:1260|FGSG_13944|I1RUC7|5518|Gibberella_zeae_(related:_Fusarium_graminearum)|Unaffected_pathogenicity
Chr05G0252.1	3038	9.00E-19	58/173(33.53)	86.7	FgERG5B|PHI:3038|I1RIP4|5518|Fusarium_graminearum|reduced_virulence_
Chr05G0250.1	2839	2.00E-107	171/348(49.14)	320	PHI:2839|RED1|Q8NJQ2|5016|Cochliobolus_heterostrophus|Reduced_virulence
Chr05G0246.1	144	5.00E-07	37/130(28.46)	48.9	PHI:144|CHT42|AAC05829|29875|Trichoderma_virens|Reduced_virulence

Chr05G0232.1	2009	2.00E-06	26/59(44.07)	48.1	PHI:2009|HOS4|EDK06449|148305|Magnaporthe_oryzae_(related:_Magnaporthe_grisea)|Unaffected_pathogenicity
Chr05G0228.1	2033	5.00E-15	57/184(30.98)	76.3	PHI:2033|MgPex6|G4NBI6|148305|Magnaporthe_oryzae_(related:_Magnaporthe_grisea)|Loss_of_pathogenicity
Chr05G0225.1	2329	4.00E-45	103/291(35.40)	159	PHI:2329|CTB4|A0ST42|29003|Cercospora_nicotianae|Reduced_virulence
Chr05G0224.1	1975	2.00E-61	157/452(34.73)	210	PHI:1975|GzZC290|I1RQI0|5518|Gibberella_zeae_(related:_Fusarium_graminearum)|Unaffected_pathogenicity
Chr05G0221.1	404	4.00E-80	136/396(34.34)	262	PHI:404|PTH11|AAD30436|318829|Magnaporthe_oryzae|Reduced_virulence
Chr05G0218.1	1279	1.00E-34	166/605(27.44)	139	PHI:1279|FGSG_12132|I1RU59|5518|Gibberella_zeae_(related:_Fusarium_graminearum)|Unaffected_pathogenicity
Chr05G0211.1	2240	8.00E-40	120/478(25.10)	150	PHI:2240|Srt1|Q4PBY9|5270|Ustilago_maydis|reduced_virulence
Chr05G0210.1	2643	1.00E-06	37/143(25.87)	47.4	PHI:2643|CFAS|A4HTK3|5671|Leishmania_infantum|Reduced_virulence
Chr05G0209.1	222	3.00E-44	93/235(39.57)	154	PHI:222|PELB|AAD09857|5457|Colletotrichum_gloeosporioides|Reduced_virulence
Chr05G0206.1	2544	3.00E-15	69/324(21.30)	75.1	PHI:2544|PAB1|Q4WK03|746128|Aspergillus_fumigatus|Mixed_outcome
Chr05G0203.1	1181	0	506/709(71.37)	940	PHI:1181|(Sc_Kic1)|I1RNY7|5518|Gibberella_zeae_(related:_Fusarium_graminearum)|Reduced_virulence
Chr05G0200.1	1047	1.00E-10	40/130(30.77)	59.7	PHI:1047|CTB6|ABK64183|29003|Cercospora_nicotianae|Reduced_virulence
Chr05G0199.1	513	6.00E-44	131/511(25.64)	163	PHI:513|ARN1_(related:_SIT1)|EAK97011|5476|Candida_albicans|Reduced_virulence
Chr05G0198.1	2976	3.00E-129	234/692(33.82)	404	PHI:2976|CgOPT1|C6ZRH8|29905|Colletotrichum_gloeosporioides_f._sp._aeschynomenes|Reduced_virulence
Chr05G0197.1	55	8.00E-12	75/301(24.92)	64.3	PHI:55|PKS1|AAB08104|5016|Cochliobolus_heterostrophus|Reduced_virulence
Chr05G0191.1	566	2.00E-67	153/452(33.85)	222	PHI:566|cel2|AAK19621|5017|Cochliobolus_carbonum|Unaffected_pathogenicity

Chr05G0190.1	1856	2.00E-29	126/487(25.87)	117	PHI:1856|GzZC171|I1S377|5518|Gibberella_zeae_(related:_Fusarium_graminearum)|Unaffected_pathogenicity
Chr05G0188.1	812	3.00E-82	150/381(39.37)	261	PHI:812|MGG_10702|EDJ94108|318829|Magnaporthe_oryzae|Reduced_virulence
Chr05G0187.1	1051	3.00E-23	103/417(24.70)	99.8	PHI:1051|CTB3|ABC79591|29003|Cercospora_nicotianae|Reduced_virulence
Chr05G0186.1	419	2.00E-79	145/330(43.94)	247	PHI:419|CSH1|AAP93915|5476|Candida_albicans|Reduced_virulence
Chr05G0185.1	2393	1.00E-10	42/172(24.42)	61.2	PHI:2393|Related_to_O-methylsterigmatocystin_oxidoreductase|I1R980|5518|Gibberella_zeae_(related:_Fusarium_graminearum)|Increased_virulence_(Hypervirulence)
Chr05G0184.1	2838	1.00E-19	72/265(27.17)	83.2	PHI:2838|TOX9|D2SZX8|5016|Cochliobolus_heterostrophus|Reduced_virulence
Chr05G0183.1	438	1.00E-43	122/465(26.24)	160	PHI:438|BcBOT1_(related:_CND5)|AAQ16576|40559|Botrytis_cinerea|Reduced_virulence
Chr05G0182.1	2290	2.00E-85	127/212(59.91)	278	PHI:2290|BcBOA6|B1GVX7|40559|Botrytis_cinerea|Reduced_virulence
Chr05G0181.1	2290	0	535/1610(33.23)	713	PHI:2290|BcBOA6|B1GVX7|40559|Botrytis_cinerea|Reduced_virulence
Chr05G0180.1	4194	3.00E-48	135/503(26.84)	174	AKT7|PHI:4194|V5XZS6|5599|Alternaria_alternata|increased_virulence_(Hypervirulence)
Chr05G0178.1	3387	0	487/1289(37.78)	776	FVEG_12523|PHI:3387|W7MT31|117187|Fusarium_verticillioides|unaffected_pathogenicity_
Chr05G0175.1	3274	4.00E-24	68/205(33.17)	102	dhbF|PHI:3274|W2E906|147375|Paenibacillus_larvae|unaffected_pathogenicity_
Chr05G0174.1	438	8.00E-08	57/229(24.89)	52	PHI:438|BcBOT1_(related:_CND5)|AAQ16576|40559|Botrytis_cinerea|Reduced_virulence
Chr05G0173.1	1161	6.00E-101	184/522(35.25)	320	PHI:1161|MgMfs1|A4ZGP3|54734|Mycosphaerella_graminicola_(related:_Zymoseptoria_triticii)|Chemistry_target
Chr05G0172.1	541	0	312/560(55.71)	639	PHI:541|LIP1|AAU87359|332648|Botrytis_cinerea|Unaffected_pathogenicity

Chr05G0170.1	1881	0	256/457(56.02)	516	PHI:1881|GzZC196|I1RJA3|5518|Gibberella_zeae_(related:_Fusarium_graminearum)|Unaffected_pathogenicity
Chr05G0167.1	191	1.00E-42	133/428(31.07)	165	PHI:191|TOM1|AAB08446|39703|Septoria_lycopersici|Unaffected_pathogenicity
Chr05G0163.1	1878	5.00E-88	172/529(32.51)	294	PHI:1878|GzZC193|I1RPF6|5518|Gibberella_zeae_(related:_Fusarium_graminearum)|Unaffected_pathogenicity
Chr05G0162.1	2968	3.00E-58	140/486(28.81)	201	PHI:2968|Hxs1|J9VQA5|5207|Cryptococcus_neoformans|Reduced_virulence
Chr05G0160.1	1651	2.00E-46	118/403(29.28)	164	PHI:1651|GzWing023|I1S0U2|5518|Gibberella_zeae_(related:_Fusarium_graminearum)|Unaffected_pathogenicity
Chr05G0156.1	510	1.00E-180	268/474(56.54)	524	PHI:510|CaNAG3|EAK93097|5476|Candida_albicans|Reduced_virulence
Chr05G0154.1	2968	2.00E-66	161/508(31.69)	224	PHI:2968|Hxs1|J9VQA5|5207|Cryptococcus_neoformans|Reduced_virulence
Chr05G0153.1	1992	2.00E-24	117/421(27.79)	105	PHI:1992|GzZC307|I1R983|5518|Gibberella_zeae_(related:_Fusarium_graminearum)|Unaffected_pathogenicity
Chr05G0146.1	2020	8.00E-27	95/350(27.14)	112	PHI:2020|Tup1|XP_759427|5270|Ustilago_maydis|Mixed_outcome
Chr05G0145.1	242	1.00E-134	262/690(37.97)	426	PHI:242|CaTPS2|CAC17748|5476|Candida_albicans|Reduced_virulence
Chr05G0141.1	1887	1.00E-41	141/510(27.65)	158	PHI:1887|GzZC202|I1RHZ6|5518|Gibberella_zeae_(related:_Fusarium_graminearum)|Unaffected_pathogenicity
Chr05G0138.1	157	6.00E-103	164/364(45.05)	310	PHI:157|TOXF|AAD45321|5017|Cochliobolus_carbonum|Loss_of_pathogenicity
Chr05G0136.1	2968	5.00E-50	140/516(27.13)	179	PHI:2968|Hxs1|J9VQA5|5207|Cryptococcus_neoformans|Reduced_virulence
Chr05G0135.1	2968	2.00E-23	124/476(26.05)	101	PHI:2968|Hxs1|J9VQA5|5207|Cryptococcus_neoformans|Reduced_virulence
Chr05G0132.1	1689	5.00E-60	157/552(28.44)	216	PHI:1689|GzZC004|I1S018|5518|Gibberella_zeae_(related:_Fusarium_graminearum)|Unaffected_pathogenicity
Chr05G0131.1	3662	1.00E-06	36/120(30.00)	49.7	PspB_(not_PD0218)|PHI:3662|Q87ET0|2371|Xylella_fastidiosa|Increased_virulence_(Hypervirulence)
Chr05G0127.1	544	0	310/559(55.46)	643	PHI:544|BCMFS1|AAF64435|332648|Botrytis_cinerea|Unaffected_pathogenicity

Chr05G0126.1	55	6.00E-10	59/222(26.58)	58.2	PHI:55|PKS1|AAB08104|5016|Cochliobolus_heterostrophus|Reduced_virulence
Chr05G0123.1	2821	7.00E-71	137/318(43.08)	261	PHI:2821|SNF2|Q5ALP9|5476|Candida_albicans|Reduced_virulence
Chr05G0114.1	2096	0	490/1030(47.57)	884	PHI:2096|Calcium-transporting_ATPase_3|MGG_02074|148305|Magnaporthe_oryzae_(related:_Magnaporthe_grisea)|Loss_of_pathogenicity
Chr05G0115.1	2279	6.00E-20	65/260(25.00)	83.6	PHI:2279|Conserved_hypothetical_protein|J9N0G7|5507|Fusarium_oxysporum|Unaffected_pathogenicity
Chr05G0111.1	2190	6.00E-07	43/148(29.05)	45.4	PHI:2190|MoCYP51A|G4MRP8|148305|Magnaporthe_oryzae_(related:_Magnaporthe_grisea)|Reduced_virulence
Chr05G0100.1	1879	3.00E-37	161/659(24.43)	146	PHI:1879|GzZC194|I1RJB8|5518|Gibberella_zeae_(related:_Fusarium_graminearum)|Unaffected_pathogenicity
Chr05G0098.1	4194	2.00E-30	128/505(25.35)	121	AKT7|PHI:4194|V5XZS6|5599|Alternaria_alternata|increased_virulence_(Hypervirulence)
Chr05G0092.1	2341	4.00E-80	122/239(51.05)	242	PHI:2341|BeNEP1|Q0E7H5|278938|Botrytis_elliptica|Unaffected_pathogenicity
Chr05G0090.1	2976	1.00E-76	191/686(27.84)	263	PHI:2976|CgOPT1|C6ZRH8|29905|Colletotrichum_gloeosporioides_f._sp._aeschynomenes|Reduced_virulence
Chr05G0088.1	440	3.00E-86	272/1004(27.09)	298	PHI:440|PMR1|CAB87245|5476|Candida_albicans|Reduced_virulence
Chr05G0085.1	812	3.00E-20	99/360(27.50)	89	PHI:812|MGG_10702|EDJ94108|318829|Magnaporthe_oryzae|Reduced_virulence
Chr05G0076.1	2968	3.00E-50	144/512(28.12)	179	PHI:2968|Hxs1|J9VQA5|5207|Cryptococcus_neoformans|Reduced_virulence
Chr05G0074.1	716	3.00E-12	119/505(23.56)	65.9	PHI:716|ZEB1|ABB90284|5518|Fusarium_graminearum|Unaffected_pathogenicity
Chr05G0073.1	4194	2.00E-29	127/493(25.76)	118	AKT7|PHI:4194|V5XZS6|5599|Alternaria_alternata|increased_virulence_(Hypervirulence)
Chr05G0071.1	1816	2.00E-53	103/319(32.29)	192	PHI:1816|GzZC131|I1RRS3|5518|Gibberella_zeae_(related:_Fusarium_graminearum)|Unaffected_pathogenicity

Chr05G0068.1	2976	9.00E-125	245/730(33.56)	395	PHI:2976|CgOPT1|C6ZRH8|29905|Colletotrichum_gloeosporioides_f._sp._aeschynomenes|Reduced_virulence
Chr05G0062.1	2822	3.00E-29	132/554(23.83)	122	PHI:2822|Cxt1p|Q5K8R6|5207|Cryptococcus_neoformans|Reduced_virulence
Chr05G0059.1	201	4.00E-17	55/179(30.73)	77	PHI:201|AVR-Pita_(related:_AVR2-YAMO)|AAK00131|318829|Magnaporthe_oryzae|Effector_(plant_avirulence_determinant)
Chr05G0058.1	1225	0	733/1265(57.94)	1351	PHI:1225|FGSG_12132|I1RP25|5518|Gibberella_zeae_(related:_Fusarium_graminearum)|Lethal
Chr05G0057.1	1429	0	468/713(65.64)	874	PHI:1429|GzC2H097|I1S7I6|5518|Gibberella_zeae_(related:_Fusarium_graminearum)|Unaffected_pathogenicity
Chr05G0049.1	1454	3.00E-08	57/230(24.78)	53.5	PHI:1454|GzAra002|I1RCI0|5518|Gibberella_zeae_(related:_Fusarium_graminearum)|Unaffected_pathogenicity
Chr05G0048.1	1575	3.00E-31	74/190(38.95)	117	PHI:1575|GzOB015|I1RIQ3|5518|Gibberella_zeae_(related:_Fusarium_graminearum)|Unaffected_pathogenicity
Chr05G0043.1	1252	0	433/675(64.15)	723	PHI:1252|FGSG_12132|I1RP15|5518|Gibberella_zeae_(related:_Fusarium_graminearum)|Unaffected_pathogenicity
Chr05G0037.1	2909	7.00E-12	87/365(23.84)	64.7	PHI:2909|CYP51C|I1S2M5|5518|Fusarium_graminearum|Mixed_outcome
Chr05G0036.1	3385	8.00E-137	197/395(49.87)	402	FVEG_12529|PHI:3385|W7MS09|117187|Fusarium_verticillioides|unaffected_pathogenicity_
Chr05G0034.1	438	4.00E-42	121/439(27.56)	155	PHI:438|BcBOT1_(related:_CND5)|AAQ16576|40559|Botrytis_cinerea|Reduced_virulence
Chr05G0031.1	1161	7.00E-43	116/420(27.62)	157	PHI:1161|MgMfs1|A4ZGP3|54734|Mycosphaerella_graminicola_(related:_Zymoseptoria_tritici)|Chemistry_target
Chr05G0029.1	1555	8.00E-39	129/518(24.90)	146	PHI:1555|GzMyb019|I1RDG6|5518|Gibberella_zeae_(related:_Fusarium_graminearum)|Unaffected_pathogenicity
Chr05G0027.1	1527	5.00E-37	100/357(28.01)	139	PHI:1527|GzHOMEL040|I1S9A1|5518|Gibberella_zeae_(related:_Fusarium_graminearum)|Lethal

Chr05G0025.1	1030	0	531/1526(34.80)	836	PHI:1030|bcatrA|CAA93142|40559|Botrytis_cinerea|Unaffected_pathogenicity
Chr05G0024.1	1051	9.00E-18	95/373(25.47)	83.2	PHI:1051|CTB3|ABC79591|29003|Cercospora_nicotianae|Reduced_virulence
Chr05G0022.1	438	1.00E-46	135/485(27.84)	169	PHI:438|BcBOT1_(related:_CND5)|AAQ16576|40559|Botrytis_cinerea|Reduced_virulence
Chr05G0021.1	2570	6.00E-10	25/56(44.64)	60.5	PHI:2570|CYB2|Q6FM61|5478|Candida_glabrata|Reduced_virulence
Chr05G0019.1	1420	9.00E-12	85/346(24.57)	67.8	PHI:1420|GzC2H088|I1S172|5518|Gibberella_zeae_(related:_Fusarium_graminearum)|Unaffected_pathogenicity
Chr05G0018.1	1260	3.00E-38	116/353(32.86)	151	PHI:1260|FGSG_13944|I1RUC7|5518|Gibberella_zeae_(related:_Fusarium_graminearum)|Unaffected_pathogenicity
Chr05G0013.1	1522	4.00E-90	129/257(50.19)	271	PHI:1522|GzHOMEL026|I1RXA5|5518|Gibberella_zeae_(related:_Fusarium_graminearum)|Lethal
Chr05G0009.1	211	5.00E-59	126/310(40.65)	211	PHI:211|CaTUP1|AAB63195|5476|Candida_albicans|Reduced_virulence
Chr05G0007.1	1662	1.00E-33	84/298(28.19)	130	PHI:1662|GzCCHC002|I1REJ1|5518|Gibberella_zeae_(related:_Fusarium_graminearum)|Unaffected_pathogenicity
Chr07G0002.1	876	2.00E-62	137/360(38.06)	216	PHI:876|MGG_11671|EDK03349|318829|Magnaporthe_oryzae|Reduced_virulence
Chr07G0003.1	211	5.00E-28	74/188(39.36)	116	PHI:211|CaTUP1|AAB63195|5476|Candida_albicans|Reduced_virulence
Chr07G0005.1	1260	2.00E-32	82/235(34.89)	133	PHI:1260|FGSG_13944|I1RUC7|5518|Gibberella_zeae_(related:_Fusarium_graminearum)|Unaffected_pathogenicity
Chr07G0007.1	1443	2.00E-11	22/45(48.89)	61.2	PHI:1443|GzGATA001|I1RB11|5518|Gibberella_zeae_(related:_Fusarium_graminearum)|Unaffected_pathogenicity
Chr07G0010.1	223	9.00E-40	77/200(38.50)	145	PHI:223|PEP1|AAK11166|140110|Nectria_haematococca_(related:_Fusarium_solani)|Reduced_virulence
Chr07G0016.1	1675	5.00E-09	32/103(31.07)	56.2	PHI:1675|GzDHHC003|Q4I8B6|5518|Gibberella_zeae_(related:_Fusarium_graminearum)|Unaffected_pathogenicity
Chr07G0020.1	441	8.00E-19	89/355(25.07)	85.9	PHI:441|BTP1|CAE55153|40559|Botrytis_cinerea|Reduced_virulence

Chr07G0021.1	922	4.00E-57	195/640(30.47)	204	PHI:922|um03615||5270|Ustilago_maydis|Unaffected_pathogenicity
Chr07G0023.1	2834	2.00E-06	25/91(27.47)	46.6	PHI:2834|LAM1|C3PTB1|5016|Cochliobolus_heterostrophus|Reduced_virulence
Chr07G0026.1	1811	1.00E-83	128/228(56.14)	267	PHI:1811|GzZC126|I1RUD7|5518|Gibberella_zeae_(related:_Fusarium_graminearum)|Unaffected_pathogenicity
Chr07G0029.1	1458	6.00E-69	139/397(35.01)	233	PHI:1458|GzAra006|Q4I7F9|5518|Gibberella_zeae_(related:_Fusarium_graminearum)|Unaffected_pathogenicity
Chr07G0034.1	465	4.00E-18	65/199(32.66)	86.7	PHI:465|KIN2|AAB63337|5270|Ustilago_maydis|Reduced_virulence
Chr07G0048.1	1618	1.00E-18	52/197(26.40)	83.2	PHI:1618|GzFET5|Q4IQT8|5518|Gibberella_zeae_(related:_Fusarium_graminearum)|Unaffected_pathogenicity
Chr07G0051.1	1420	8.00E-11	94/434(21.66)	63.9	PHI:1420|GzC2H088|I1S172|5518|Gibberella_zeae_(related:_Fusarium_graminearum)|Unaffected_pathogenicity
Chr07G0052.1	1260	3.00E-16	76/238(31.93)	81.3	PHI:1260|FGSG_13944|I1RUC7|5518|Gibberella_zeae_(related:_Fusarium_graminearum)|Unaffected_pathogenicity
Chr07G0059.1	1662	7.00E-61	144/534(26.97)	217	PHI:1662|GzCCHC002|I1REJ1|5518|Gibberella_zeae_(related:_Fusarium_graminearum)|Unaffected_pathogenicity
Chr07G0070.1	1914	2.00E-06	24/43(55.81)	48.9	PHI:1914|GzZC229|I1RQ27|5518|Gibberella_zeae_(related:_Fusarium_graminearum)|Unaffected_pathogenicity
Chr07G0072.1	1260	2.00E-16	60/194(30.93)	80.9	PHI:1260|FGSG_13944|I1RUC7|5518|Gibberella_zeae_(related:_Fusarium_graminearum)|Unaffected_pathogenicity
Chr07G0084.1	413	7.00E-25	108/399(27.07)	103	PHI:413|MPD1|AAT84078|13684|Stagonospora_nodorum|Unaffected_pathogenicity
Chr07G0088.1	3381	3.00E-07	42/177(23.73)	50.4	FVEG_12533|PHI:3381|W7N2B4|117187|Fusarium_verticillioides|unaffected_pathogenicity_
Chr07G0089.1	1958	4.00E-44	168/668(25.15)	167	PHI:1958|GzZC273|I1RMD1|5518|Gibberella_zeae_(related:_Fusarium_graminearum)|Unaffected_pathogenicity

Chr07G0090.1	233	1.00E-21	55/150(36.67)	93.6	PHI:233|TOXE|AAD13811|5017|Cochliobolus_carbonum|Reduced_virulence
Chr07G0091.1	12	0	2196/5257(41.77)	3811	PHI:12|HTS1|AAA33023|5017|Cochliobolus_carbonum|Loss_of_pathogenicity
Chr07G0092.1	60	1.00E-164	248/514(48.25)	481	PHI:60|TOXA|AAB36607|5017|Cochliobolus_carbonum|Lethal
Chr07G0094.1	2247	4.00E-06	38/116(32.76)	44.3	PHI:2247|Sch1|EAT82552|13684|Phaeosphaeria_nodorum_(related:_Stagonospora_nodorum)|Unaffected_pathogenicity
Chr07G0095.1	784	1.00E-32	103/281(36.65)	120	PHI:784|MGG_00056|EDK03390|318829|Magnaporthe_oryzae|Reduced_virulence
Chr07G0096.1	96	0	555/1111(49.95)	1114	PHI:96|FAS2|AAA34345|5476|Candida_albicans|Loss_of_pathogenicity
Chr07G0097.1	157	8.00E-133	196/332(59.04)	402	PHI:157|TOXF|AAD45321|5017|Cochliobolus_carbonum|Loss_of_pathogenicity
Chr07G0098.1	4194	2.00E-141	219/520(42.12)	420	AKT7|PHI:4194|V5XZS6|5599|Alternaria_alternata|increased_virulence_(Hypervirulence)
Chr07G0099.1	4194	1.00E-145	220/505(43.56)	448	AKT7|PHI:4194|V5XZS6|5599|Alternaria_alternata|increased_virulence_(Hypervirulence)
Chr07G0100.1	4194	2.00E-39	122/473(25.79)	147	AKT7|PHI:4194|V5XZS6|5599|Alternaria_alternata|increased_virulence_(Hypervirulence)
Chr07G0105.1	1555	4.00E-27	96/444(21.62)	111	PHI:1555|GzMyb019|I1RDG6|5518|Gibberella_zeae_(related:_Fusarium_graminearum)|Unaffected_pathogenicity
Chr07G0106.1	2315	2.00E-45	102/292(34.93)	156	PHI:2315|ChLae1|G4XKY9|5016|Cochliobolus_heterostrophus|Mixed_outcome
Chr07G0112.1	1134	1.00E-11	88/318(27.67)	64.7	PHI:1134|ipa|B0LLU0|5022|Leptosphaeria_maculans|Increased_virulence_(Hypervirulence)
Chr07G0114.1	513	3.00E-66	152/518(29.34)	227	PHI:513|ARN1_(related:_SIT1)|EAK97011|5476|Candida_albicans|Reduced_virulence
Chr07G0116.1	2731	3.00E-123	200/548(36.50)	375	PHI:2731|VdSge1|G2XD29|27337|Verticillium_dahliae|Loss_of_pathogenicity
Chr07G0117.1	784	8.00E-11	55/217(25.35)	58.9	PHI:784|MGG_00056|EDK03390|318829|Magnaporthe_oryzae|Reduced_virulence
Chr07G0118.1	714	0	1450/2355(61.57)	2927	PHI:714|PKS4_(related:_ZEA1)|ABB90283|5518|Fusarium_graminearum|Unaff

					ected_pathogenicity
Chr07G0119.1	2844	3.00E-26	89/281(31.67)	102	PHI:2844|BRM2|O93802|5599|Alternaria_alternata|Unaffected_pathogenicity
Chr07G0122.1	4194	2.00E-55	142/490(28.98)	193	AKT7|PHI:4194|V5XZS6|5599|Alternaria_alternata|increased_virulence_(Hyper virulence)
Chr07G0123.1	713	0	1122/2111(53.15)	2244	PHI:713|PKS13_(related:_ZEA2)|ABB90282|5518|Fusarium_graminearum|Unaf fected_pathogenicity
Chr07G0124.1	2837	7.00E-17	49/178(27.53)	75.1	PHI:2837|OXI1|D2SZX7|5016|Cochliobolus_heterostrophus|Reduced_virulence
Chr07G0125.1	4194	5.00E-34	116/469(24.73)	132	AKT7|PHI:4194|V5XZS6|5599|Alternaria_alternata|increased_virulence_(Hyper virulence)
Chr07G0126.1	2964	1.00E-39	128/394(32.49)	151	PHI:2964|AsnB|G7TM01|129394|Xanthomonas_oryzae_pv._Oryzicola|Reduce d_virulence
Chr07G0127.1	2357	2.00E-120	183/435(42.07)	364	PHI:2357|CYP52X1|E2EAF6|475271|Beauveria_bassiana|Reduced_virulence
Chr07G0138.1	1566	3.00E-87	152/390(38.97)	273	PHI:1566|GzOB006|I1RC95|5518|Gibberella_zeae_(related:_Fusarium_gramin earum)|Lethal
Chr07G0139.1	487	3.00E-41	68/94(72.34)	132	PHI:487|MHP1|O94196|148305|Magnaporthe_grisea|Reduced_virulence
Chr07G0149.1	566	8.00E-123	195/396(49.24)	375	PHI:566|cel2|AAK19621|5017|Cochliobolus_carbonum|Unaffected_pathogenicit y
Chr07G0152.1	1260	5.00E-16	73/250(29.20)	79	PHI:1260|FGSG_13944|I1RUC7|5518|Gibberella_zeae_(related:_Fusarium_gr aminearum)|Unaffected_pathogenicity
Chr07G0153.1	1717	2.00E-128	223/477(46.75)	396	PHI:1717|GzZC032|I1R9K8|5518|Gibberella_zeae_(related:_Fusarium_gramin earum)|Unaffected_pathogenicity
Chr07G0159.1	4194	7.00E-32	115/465(24.73)	126	AKT7|PHI:4194|V5XZS6|5599|Alternaria_alternata|increased_virulence_(Hyper virulence)
Chr07G0162.1	544	4.00E-14	120/494(24.29)	72.4	PHI:544|BCMFS1|AAF64435|332648|Botrytis_cinerea|Unaffected_pathogenicit y
Chr07G0164.1	404	9.00E-29	102/418(24.40)	115	PHI:404|PTH11|AAD30436|318829|Magnaporthe_oryzae|Reduced_virulence

Chr07G0165.1	922	2.00E-22	159/683(23.28)	99.8	PHI:922|um03615||5270|Ustilago_maydis|Unaffected_pathogenicity
Chr07G0168.1	405	0	809/1489(54.33)	1550	PHI:405|PDE1|AAK07740|318829|Magnaporthe_oryzae|Reduced_virulence
Chr07G0169.1	2849	3.00E-45	96/237(40.51)	151	PHI:2849|cutA|Q99174|70790|Fusarium_solani_f._sp._cucurbitae|Unaffected_pathogenicity
Chr07G0174.1	2553	3.00E-30	72/193(37.31)	122	PHI:2553|VPS4|Q5AG40|5476|Candida_albicans|Loss_of_pathogenicity
Chr07G0178.1	3277	1.00E-37	115/372(30.91)	139	gnoA|PHI:3277|Q4WIQ3|746128|Aspergillus_fumigatus|unaffected_pathogenicity_
Chr07G0179.1	3216	2.00E-46	89/216(41.20)	156	MoCDIP4|PHI:3216|G4MVX4|318829|Magnaporthe_oryzae|mixed_outcome_
Chr07G0182.1	2534	7.00E-14	98/388(25.26)	71.2	PHI:2534|ERG11A|Q4WNT5|746128|Aspergillus_fumigatus|Mixed_outcome
Chr07G0186.1	243	5.00E-170	292/679(43.00)	518	PHI:243|CHIP6|AAD00894|5457|Colletotrichum_gloeosporioides|Reduced_virulence
Chr07G0189.1	1609	7.00E-27	118/499(23.65)	111	PHI:1609|GzP53L002|I1RJ69|5518|Gibberella_zeae_(related:_Fusarium_graminearum)|Unaffected_pathogenicity
Chr07G0193.1	2968	2.00E-34	111/424(26.18)	133	PHI:2968|Hxs1|J9VQA5|5207|Cryptococcus_neoformans|Reduced_virulence
Chr07G0194.1	169	0	344/453(75.94)	720	PHI:169|CLTA1|AAG25917|290576|Colletotrichum_lindemuthianum|Loss_of_pathogenicity
Chr07G0195.1	1987	0	547/954(57.34)	1019	PHI:1987|GzZC302|I1RAP2|5518|Gibberella_zeae_(related:_Fusarium_graminearum)|Reduced_virulence
Chr07G0196.1	566	2.00E-74	169/456(37.06)	242	PHI:566|cel2|AAK19621|5017|Cochliobolus_carbonum|Unaffected_pathogenicity
Chr07G0204.1	2377	1.00E-124	212/530(40.00)	379	PHI:2377|DEP3|D2E9W8|29001|Alternaria_brassicicola|Mixed_outcome
Chr07G0207.1	2964	5.00E-10	121/503(24.06)	59.3	PHI:2964|AsnB|G7TM01|129394|Xanthomonas_oryzae_pv._Oryzicola|Reduced_virulence
Chr07G0209.1	2834	9.00E-28	61/155(39.35)	109	PHI:2834|LAM1|C3PTB1|5016|Cochliobolus_heterostrophus|Reduced_virulence
Chr07G0224.1	393	0	528/665(79.40)	1045	PHI:393|CpCOT1|CAH04535|5111|Claviceps_purpurea|Loss_of_pathogenicity

Chr07G0233.1	1749	1.00E-12	108/444(24.32)	67.8	PHI:1749|GzZC064|I1S4A5|5518|Gibberella_zeae_(related:_Fusarium_graminearum)|Unaffected_pathogenicity
Chr07G0234.1	2968	1.00E-25	112/468(23.93)	108	PHI:2968|Hxs1|J9VQA5|5207|Cryptococcus_neoformans|Reduced_virulence
Chr07G0235.1	2404	4.00E-59	103/162(63.58)	182	PHI:2404|SLP_1|G4N906|148305|Magnaporthe_oryzae_(related:_Magnaporthe_grisea)|Reduced_virulence
Chr07G0237.1	4194	5.00E-08	38/127(29.92)	51.6	AKT7|PHI:4194|V5XZS6|5599|Alternaria_alternata|increased_virulence_(Hyper virulence)
Chr07G0243.1	179	7.00E-49	90/194(46.39)	163	PHI:179|PELA|AAA33338|140110|Nectria_haematococca_(related:_Fusarium_solani)|Reduced_virulence
Chr07G0244.1	1972	0	360/656(54.88)	701	PHI:1972|GzZC287|I1RF57|5518|Gibberella_zeae_(related:_Fusarium_graminearum)|Unaffected_pathogenicity
Chr07G0247.1	2269	5.00E-06	55/226(24.34)	44.7	PHI:2269|Mdh1|0|13684|Phaeosphaeria_nodorum_(related:_Stagonospora_nodorum)|Unaffected_pathogenicity
Chr07G0250.1	508	1.00E-39	136/481(28.27)	149	PHI:508|AFT1|BAB69076|5599|Alternaria_alternata|Loss_of_pathogenicity
Chr07G0260.1	2553	1.00E-06	42/150(28.00)	48.5	PHI:2553|VPS4|Q5AG40|5476|Candida_albicans|Loss_of_pathogenicity
Chr07G0264.1	3038	3.00E-10	51/193(26.42)	59.7	FgERG5B|PHI:3038|I1RIP4|5518|Fusarium_graminearum|reduced_virulence_
Chr07G0266.1	2208	5.00E-130	186/311(59.81)	384	PHI:2208|endo-1,4-beta-xylanase_[GH10_family]|MGG_05464|148305|Magnaporthe_oryzae_(related:_Magnaporthe_grisea)|Reduced_virulence
Chr07G0267.1	881	4.00E-31	94/323(29.10)	119	PHI:881|MGG_04556|EDJ96020|318829|Magnaporthe_oryzae|Reduced_virulence
Chr07G0270.1	404	4.00E-33	79/342(23.10)	134	PHI:404|PTH11|AAD30436|318829|Magnaporthe_oryzae|Reduced_virulence
Chr07G0271.1	24	0	433/777(55.73)	839	PHI:24|Avenacinase_gene|AAB09777|29850|Gaeumannomyces_graminis|Loss_of_pathogenicity
Chr07G0273.1	901	3.00E-35	135/478(28.24)	137	PHI:901|um01886||5270|Ustilago_maydis|Unaffected_pathogenicity
Chr07G0275.1	3386	1.00E-11	58/218(26.61)	62.4	FVEG_12528|PHI:3386|W7NCN7|117187|Fusarium_verticillioides|unaffected_pathogenicity_

Chr07G0280.1	2115	6.00E-152	220/336(65.48)	441	PHI:2115|Annexin_A7|MGG_06847|148305|Magnaporthe_oryzae_(related:_Magnaporthe_grisea)|Loss_of_pathogenicity
Chr07G0289.1	812	1.00E-32	97/298(32.55)	125	PHI:812|MGG_10702|EDJ94108|318829|Magnaporthe_oryzae|Reduced_virulence
Chr07G0290.1	2171	7.00E-128	248/663(37.41)	396	PHI:2171|Peroxisomal_copper_amine_oxidase|MGG_02681|148305|Magnaporthe_oryzae_(related:_Magnaporthe_grisea)|Reduced_virulence
Chr07G0293.1	716	1.00E-106	214/584(36.64)	335	PHI:716|ZEB1|ABB90284|5518|Fusarium_graminearum|Unaffected_pathogenicity
Chr07G0294.1	404	1.00E-14	70/261(26.82)	73.6	PHI:404|PTH11|AAD30436|318829|Magnaporthe_oryzae|Reduced_virulence
Chr07G0299.1	2340	3.00E-16	81/282(28.72)	73.9	PHI:2340|aiiA|Q9L8R8|337|Burkholderia_glumae|Mixed_outcome
Chr07G0300.1	881	1.00E-24	73/238(30.67)	100	PHI:881|MGG_04556|EDJ96020|318829|Magnaporthe_oryzae|Reduced_virulence
Chr07G0311.1	541	5.00E-26	59/124(47.58)	111	PHI:541|LIP1|AAU87359|332648|Botrytis_cinerea|Unaffected_pathogenicity
Chr07G0316.1	3378	4.00E-58	102/223(45.74)	187	Plegl1|PHI:3378|G9JLA8|285811|Pyrenochaeta_lycopersici|unaffected_pathogenicity_
Chr07G0318.1	180	5.00E-81	132/250(52.80)	244	PHI:180|PELD|AAC49420|140110|Nectria_haematococca_(related:_Fusarium_solani)|Reduced_virulence
Chr07G0321.1	1974	8.00E-09	72/312(23.08)	55.1	PHI:1974|GzZC289|I1RQN6|5518|Gibberella_zeae_(related:_Fusarium_graminearum)|Unaffected_pathogenicity
Chr07G0327.1	513	4.00E-139	216/523(41.30)	419	PHI:513|ARN1_(related:_SIT1)|EAK97011|5476|Candida_albicans|Reduced_virulence
Chr07G0335.1	1893	5.00E-74	166/508(32.68)	244	PHI:1893|GzZC208|I1RNY0|5518|Gibberella_zeae_(related:_Fusarium_graminearum)|Unaffected_pathogenicity
Chr07G0336.1	1030	0	801/1462(54.79)	1615	PHI:1030|bcatrA|CAA93142|40559|Botrytis_cinerea|Unaffected_pathogenicity
Chr07G0339.1	404	6.00E-20	64/278(23.02)	88.6	PHI:404|PTH11|AAD30436|318829|Magnaporthe_oryzae|Reduced_virulence
Chr07G0344.1	1455	7.00E-50	95/206(46.12)	167	PHI:1455|GzAra003|I1RKU6|5518|Gibberella_zeae_(related:_Fusarium_gramin

					earum)|Unaffected_pathogenicity
Chr07G0348.1	58	4.00E-133	178/188(94.68)	372	PHI:58|SCD1|BAA13009|5462|Colletotrichum_lagenarium|Reduced_virulence
Chr07G0349.1	2279	7.00E-25	76/251(30.28)	99.4	PHI:2279|Conserved_hypothetical_protein|J9N0G7|5507|Fusarium_oxysporum|Unaffected_pathogenicity
Chr07G0352.1	2269	3.00E-68	112/266(42.11)	213	PHI:2269|Mdh1|0|13684|Phaeosphaeria_nodorum_(related:_Stagonospora_nodorum)|Unaffected_pathogenicity
Chr07G0353.1	3629	2.00E-16	89/341(26.10)	78.2	Rv0392c|PHI:3629|P95200|1773|Mycobacterium_tuberculosis|unaffected_pathogenicity_
Chr07G0354.1	1908	2.00E-138	209/414(50.48)	412	PHI:1908|GzZC223|I1RHH3|5518|Gibberella_zeae_(related:_Fusarium_graminearum)|Unaffected_pathogenicity
Chr07G0355.1	784	8.00E-20	70/236(29.66)	84.7	PHI:784|MGG_00056|EDK03390|318829|Magnaporthe_oryzae|Reduced_virulence
Chr07G0364.1	3387	1.00E-22	73/241(30.29)	97.4	FVEG_12523|PHI:3387|W7MT31|117187|Fusarium_verticillioides|unaffected_pathogenicity_
Chr07G0366.1	1555	7.00E-21	112/492(22.76)	93.2	PHI:1555|GzMyb019|I1RDG6|5518|Gibberella_zeae_(related:_Fusarium_graminearum)|Unaffected_pathogenicity
Chr07G0371.1	55	2.00E-14	79/309(25.57)	71.6	PHI:55|PKS1|AAB08104|5016|Cochliobolus_heterostrophus|Reduced_virulence
Chr07G0372.1	1269	2.00E-99	172/434(39.63)	306	PHI:1269|FGSG_02838|I1RFK9|5518|Gibberella_zeae_(related:_Fusarium_graminearum)|Unaffected_pathogenicity
Chr07G0378.1	226	3.00E-10	50/167(29.94)	60.1	PHI:226|PEX6|AAK16738|5462|Colletotrichum_lagenarium|Loss_of_pathogenicity
Chr07G0381.1	404	2.00E-19	73/268(27.24)	87.4	PHI:404|PTH11|AAD30436|318829|Magnaporthe_oryzae|Reduced_virulence
Chr07G0382.1	716	5.00E-97	197/569(34.62)	310	PHI:716|ZEB1|ABB90284|5518|Fusarium_graminearum|Unaffected_pathogenicity
Chr07G0383.1	513	4.00E-52	153/608(25.16)	187	PHI:513|ARN1_(related:_SIT1)|EAK97011|5476|Candida_albicans|Reduced_vir

					ulence
Chr07G0388.1	358	0	380/672(56.55)	722	PHI:358|ILV2|AAR29084|5207|Cryptococcus_neoformans|Loss_of_pathogenicity
Chr07G0394.1	270	7.00E-81	119/191(62.30)	240	PHI:270|RHO1|BAA24262|5476|Candida_albicans|Loss_of_pathogenicity
Chr07G0396.1	1364	5.00E-79	199/555(35.86)	260	PHI:1364|GzC2H024|I1RJQ3|5518|Gibberella_zeae_(related:_Fusarium_graminearum)|Reduced_virulence
Chr07G0397.1	1365	6.00E-159	262/535(48.97)	466	PHI:1365|GzC2H025|I1RJQ4|5518|Gibberella_zeae_(related:_Fusarium_graminearum)|Unaffected_pathogenicity
Chr07G0404.1	2042	6.00E-53	108/290(37.24)	200	PHI:2042|ABC3|Q3Y5V5|148305|Magnaporthe_oryzae_(related:_Magnaporthe_grisea)|Loss_of_pathogenicity
Chr07G0415.1	1028	8.00E-70	142/333(42.64)	222	PHI:1028|bcpme1|CAC29255|40559|Botrytis_cinerea|Reduced_virulence
Chr07G0416.1	222	1.00E-21	99/300(33.00)	94.4	PHI:222|PELB|AAD09857|5457|Colletotrichum_gloeosporioides|Reduced_virulence
Chr07G0417.1	3126	7.00E-38	105/341(30.79)	141	argD|PHI:3126|D4I307|552|Erwinia_amylovora|mixed_outcome_
Chr07G0418.1	1737	0	330/688(47.97)	647	PHI:1737|GzZC052|I1S4E2|5518|Gibberella_zeae_(related:_Fusarium_graminearum)|Unaffected_pathogenicity
Chr07G0419.1	179	1.00E-113	162/231(70.13)	331	PHI:179|PELA|AAA33338|140110|Nectria_haematococca_(related:_Fusarium_solani)|Reduced_virulence
Chr07G0420.1	441	2.00E-24	78/292(26.71)	100	PHI:441|BTP1|CAE55153|40559|Botrytis_cinerea|Reduced_virulence
Chr07G0425.1	1918	7.00E-25	45/70(64.29)	107	PHI:1918|GzZC233|I1RT69|5518|Gibberella_zeae_(related:_Fusarium_graminearum)|Unaffected_pathogenicity
Chr07G0427.1	419	9.00E-69	130/322(40.37)	220	PHI:419|CSH1|AAP93915|5476|Candida_albicans|Reduced_virulence
Chr07G0429.1	4194	2.00E-50	140/479(29.23)	179	AKT7|PHI:4194|V5XZS6|5599|Alternaria_alternata|increased_virulence_(Hypervirulence)
Chr07G0431.1	4194	2.00E-40	132/485(27.22)	151	AKT7|PHI:4194|V5XZS6|5599|Alternaria_alternata|increased_virulence_(Hypervirulence)

Chr07G0432.1	2535	6.00E-06	50/209(23.92)	47	PHI:2535|ERG11B|E9QY26|746128|Aspergillus_fumigatus|Mixed_outcome
Chr07G0433.1	1695	3.00E-92	197/572(34.44)	300	PHI:1695|GzZC010|I1S2I3|5518|Gibberella_zeae_(related:_Fusarium_graminearum)|Unaffected_pathogenicity
Chr07G0435.1	4194	2.00E-55	141/479(29.44)	194	AKT7|PHI:4194|V5XZS6|5599|Alternaria_alternata|increased_virulence_(Hypervirulence)
Chr07G0436.1	881	7.00E-30	89/301(29.57)	114	PHI:881|MGG_04556|EDJ96020|318829|Magnaporthe_oryzae|Reduced_virulence
Chr07G0438.1	2968	7.00E-75	160/523(30.59)	247	PHI:2968|Hxs1|J9VQA5|5207|Cryptococcus_neoformans|Reduced_virulence
Chr07G0442.1	1835	0	290/593(48.90)	570	PHI:1835|GzZC150|I1RIL1|5518|Gibberella_zeae_(related:_Fusarium_graminearum)|Unaffected_pathogenicity
Chr07G0443.1	2844	8.00E-25	77/260(29.62)	98.2	PHI:2844|BRM2|O93802|5599|Alternaria_alternata|Unaffected_pathogenicity
Chr07G0448.1	1221	0	337/522(64.56)	621	PHI:1221|FGSG_03146|I1RJM9|5518|Gibberella_zeae_(related:_Fusarium_graminearum)|Lethal
Chr07G0450.1	1206	0	487/829(58.75)	778	PHI:1206|(Sp_Prp4)|I1RJM8|5518|Gibberella_zeae_(related:_Fusarium_graminearum)|Reduced_virulence
Chr07G0451.1	211	1.00E-57	123/311(39.55)	207	PHI:211|CaTUP1|AAB63195|5476|Candida_albicans|Reduced_virulence
Chr07G0466.1	441	3.00E-13	51/195(26.15)	68.2	PHI:441|BTP1|CAE55153|40559|Botrytis_cinerea|Reduced_virulence
Chr07G0469.1	1809	0	337/747(45.11)	619	PHI:1809|GzZC124|I1REW1|5518|Gibberella_zeae_(related:_Fusarium_graminearum)|Lethal
Chr07G0476.1	886	2.00E-06	41/157(26.11)	46.6	PHI:886|MGG_13052|EDK06087|318829|Magnaporthe_oryzae|Reduced_virulence
Chr07G0477.1	876	0	456/1164(39.18)	780	PHI:876|MGG_11671|EDK03349|318829|Magnaporthe_oryzae|Reduced_virulence
Chr07G0484.1	2656	4.00E-22	61/221(27.60)	90.9	PHI:2656|pmrF|D0ZPP5|90371|Salmonella_enterica_serovar_Typhimurium|Reduced_virulence
Chr07G0485.1	2034	3.00E-27	71/183(38.80)	110	PHI:2034|MFP1|G4MZY1|148305|Magnaporthe_oryzae_(related:_Magnaporthe

					_grisea)|Reduced_virulence
Chr07G0490.1	2315	2.00E-39	90/296(30.41)	142	PHI:2315|ChLae1|G4XKY9|5016|Cochliobolus_heterostrophus|Mixed_outcome
Chr07G0491.1	1725	7.00E-63	166/535(31.03)	217	PHI:1725|GzZC040|I1SAE7|5518|Gibberella_zeae_(related:_Fusarium_graminearum)|Unaffected_pathogenicity
Chr07G0492.1	1555	1.00E-42	119/476(25.00)	157	PHI:1555|GzMyb019|I1RDG6|5518|Gibberella_zeae_(related:_Fusarium_graminearum)|Unaffected_pathogenicity
Chr07G0493.1	1854	6.00E-27	138/589(23.43)	114	PHI:1854|GzZC169|I1S054|5518|Gibberella_zeae_(related:_Fusarium_graminearum)|Unaffected_pathogenicity
Chr07G0498.1	2895	4.00E-14	89/333(26.73)	71.2	PHI:2895|F-avi4330|B9JV05|373|Agrobacterium_vitis|Loss_of_pathogenicity
Chr07G0500.1	805	0	520/969(53.66)	606	PHI:805|MGG_00124|EDK03309|318829|Magnaporthe_oryzae|Reduced_virulence
Chr07G0501.1	1555	2.00E-30	107/465(23.01)	121	PHI:1555|GzMyb019|I1RDG6|5518|Gibberella_zeae_(related:_Fusarium_graminearum)|Unaffected_pathogenicity
Chr07G0508.1	2978	5.00E-36	90/248(36.29)	128	PHI:2978|MoCel12A|G4N5V2|148305|Magnaporthe_oryzae|Unaffected_pathogenicity
Chr07G0515.1	1555	3.00E-16	98/443(22.12)	78.6	PHI:1555|GzMyb019|I1RDG6|5518|Gibberella_zeae_(related:_Fusarium_graminearum)|Unaffected_pathogenicity
Chr07G0516.1	2378	8.00E-07	29/104(27.88)	49.3	PHI:2378|DEP4|D2E9W9|29001|Alternaria_brassicicola|Mixed_outcome
Chr07G0522.1	1260	2.00E-12	58/151(38.41)	67.4	PHI:1260|FGSG_13944|I1RUC7|5518|Gibberella_zeae_(related:_Fusarium_graminearum)|Unaffected_pathogenicity
Chr07G0523.1	2247	1.00E-39	98/273(35.90)	140	PHI:2247|Sch1|EAT82552|13684|Phaeosphaeria_nodorum_(related:_Stagonospora_nodorum)|Unaffected_pathogenicity
Chr07G0528.1	291	7.00E-06	22/67(32.84)	41.2	PHI:291|CPPH1|CAD10781|5111|Claviceps_purpurea|Unaffected_pathogenicity
Chr07G0531.1	2341	1.00E-86	125/239(52.30)	258	PHI:2341|BeNEP1|Q0E7H5|278938|Botrytis_elliptica|Unaffected_pathogenicity
Chr07G0535.1	2117	6.00E-06	60/209(28.71)	46.6	PHI:2117|SPM1|P58371|148305|Magnaporthe_oryzae_(related:_Magnaporthe

					_grisea)|Reduced_virulence
Chr07G0542.1	2336	2.00E-17	53/207(25.60)	80.1	PHI:2336|NIA1|O00101|13684|Stagonospora_nodorum_(related:_Phaeosphaeria_nodorum)|Unaffected_pathogenicity
Chr07G0548.1	2117	1.00E-07	55/199(27.64)	52.4	PHI:2117|SPM1|P58371|148305|Magnaporthe_oryzae_(related:_Magnaporthe_grisea)|Reduced_virulence
Chr07G0551.1	1782	2.00E-51	146/578(25.26)	186	PHI:1782|GzZC097|I1S3H4|5518|Gibberella_zeae_(related:_Fusarium_graminearum)|Lethal
Chr07G0552.1	201	9.00E-13	51/180(28.33)	64.3	PHI:201|AVR-Pita_(related:_AVR2-YAMO)|AAK00131|318829|Magnaporthe_oryzae|Effector_(plant_avirulence_determinant)
Chr07G0554.1	2315	7.00E-54	106/289(36.68)	179	PHI:2315|ChLae1|G4XKY9|5016|Cochliobolus_heterostrophus|Mixed_outcome
Chr07G0556.1	1662	1.00E-17	85/413(20.58)	84	PHI:1662|GzCCHC002|I1REJ1|5518|Gibberella_zeae_(related:_Fusarium_graminearum)|Unaffected_pathogenicity
Chr07G0559.1	489	1.00E-18	39/88(44.32)	75.1	PHI:489|TRX1|AAW46720|5207|Cryptococcus_neoformans|Reduced_virulence
Chr07G0560.1	1741	2.00E-42	152/614(24.76)	160	PHI:1741|GzZC056|I1S780|5518|Gibberella_zeae_(related:_Fusarium_graminearum)|Unaffected_pathogenicity
Chr07G0561.1	784	1.00E-10	79/287(27.53)	58.2	PHI:784|MGG_00056|EDK03390|318829|Magnaporthe_oryzae|Reduced_virulence
Chr07G0566.1	2968	8.00E-65	141/504(27.98)	221	PHI:2968|Hxs1|J9VQA5|5207|Cryptococcus_neoformans|Reduced_virulence
Chr07G0569.1	1047	4.00E-15	60/206(29.13)	73.2	PHI:1047|CTB6|ABK64183|29003|Cercospora_nicotianae|Reduced_virulence
Chr07G0578.1	419	6.00E-32	91/325(28.00)	121	PHI:419|CSH1|AAP93915|5476|Candida_albicans|Reduced_virulence
Chr07G0581.1	258	0	616/1063(57.95)	1285	PHI:258|GPABC1|CAC40023|5128|Gibberella_pulicaris|Reduced_virulence
Chr07G0583.1	1825	4.00E-83	136/384(35.42)	265	PHI:1825|GzZC140|I1RY88|5518|Gibberella_zeae_(related:_Fusarium_graminearum)|Unaffected_pathogenicity
Chr07G0585.1	1662	7.00E-34	130/476(27.31)	134	PHI:1662|GzCCHC002|I1REJ1|5518|Gibberella_zeae_(related:_Fusarium_graminearum)|Unaffected_pathogenicity
Chr07G0586.1	817	5.00E-53	90/159(56.60)	184	PHI:817|MGG_12252|EDK03444|318829|Magnaporthe_oryzae|Reduced_virule

					nce
Chr07G0589.1	2085	0	741/1371(54.05)	1364	PHI:2085|Moatg11|MGG_04486|148305|Magnaporthe_oryzae_(related:_Magnaporthe_grisea)|Unaffected_pathogenicity
Chr07G0591.1	1470	2.00E-54	80/87(91.95)	168	PHI:1470|GzHMG002|Q4IQX3|5518|Gibberella_zeae_(related:_Fusarium_graminearum)|Reduced_virulence
Chr07G0593.1	3325	2.00E-15	112/450(24.89)	76.3	PGM|PHI:3325|B5TQV9|1348|Streptococcus_parauberis|reduced_virulence_
Chr07G0602.1	404	9.00E-26	68/252(26.98)	106	PHI:404|PTH11|AAD30436|318829|Magnaporthe_oryzae|Reduced_virulence
Chr07G0604.1	3629	6.00E-29	110/429(25.64)	115	Rv0392c|PHI:3629|P95200|1773|Mycobacterium_tuberculosis|unaffected_pathogenicity_
Chr07G0605.1	2530	0	324/451(71.84)	677	PHI:2530|TUB1|Q4WKG5|746128|Aspergillus_fumigatus|Mixed_outcome
Chr07G0612.1	2895	3.00E-09	56/173(32.37)	55.8	PHI:2895|F-avi4330|B9JV05|373|Agrobacterium_vitis|Loss_of_pathogenicity
Chr07G0622.1	443	2.00E-73	141/367(38.42)	237	PHI:443|CBL1||5518|Fusarium_graminearum|Reduced_virulence
Chr07G0623.1	1933	2.00E-177	265/487(54.41)	509	PHI:1933|GzZC248|I1RC73|5518|Gibberella_zeae_(related:_Fusarium_graminearum)|Reduced_virulence
Chr07G0631.1	1554	0	449/662(67.82)	960	PHI:1554|GzMyb018|I1RC64|5518|Gibberella_zeae_(related:_Fusarium_graminearum)|Unaffected_pathogenicity
Chr07G0632.1	2714	0	482/660(73.03)	931	PHI:2714|PEX5|I1RC71|5518|Fusarium_graminearum|Reduced_virulence
Chr07G0634.1	455	1.00E-16	78/282(27.66)	79	PHI:455|CAP59|AAC13946|5207|Cryptococcus_neoformans|Loss_of_pathogenicity
Chr07G0639.1	2552	4.00E-06	25/88(28.41)	45.1	PHI:2552|NOP4|Q4WNM3|746128|Aspergillus_fumigatus|Mixed_outcome
Chr07G0640.1	1303	0	297/498(59.64)	520	PHI:1303|GzbHLH004|I1RC70|5518|Gibberella_zeae_(related:_Fusarium_graminearum)|Unaffected_pathogenicity
Chr07G0641.1	1934	7.00E-85	180/487(36.96)	269	PHI:1934|GzZC249|I1RC69|5518|Gibberella_zeae_(related:_Fusarium_graminearum)|Unaffected_pathogenicity
Chr07G0643.1	1219	0	581/896(64.84)	1022	PHI:1219|FGSG_05549|I1RC35|5518|Gibberella_zeae_(related:_Fusarium_graminearum)|Lethal

Chr07G0646.1	1486	3.00E-64	209/427(48.95)	211	PHI:1486|GzHMG018|I1RNM1|5518|Gibberella_zeae_(related:_Fusarium_graminearum)|Unaffected_pathogenicity
Chr07G0654.1	1618	8.00E-169	230/294(78.23)	472	PHI:1618|GzFET5|Q4IQT8|5518|Gibberella_zeae_(related:_Fusarium_graminearum)|Unaffected_pathogenicity
Chr07G0656.1	877	0	339/375(90.40)	689	PHI:877|MGG_00383|EDK03005|318829|Magnaporthe_oryzae|Reduced_virulence
Chr07G0663.1	2034	1.00E-36	99/296(33.45)	138	PHI:2034|MFP1|G4MZY1|148305|Magnaporthe_oryzae_(related:_Magnaporthe_grisea)|Reduced_virulence
Chr07G0664.1	3068	1.00E-05	67/241(27.80)	47	BbAC|PHI:3068|J4UTE1|176275|Beauveria_bassiana|reduced_virulence_
Chr07G0670.1	1256	0	639/1236(51.70)	1200	PHI:1256|FGSG_02153|I1RAA6|5518|Gibberella_zeae_(related:_Fusarium_graminearum)|Unaffected_pathogenicity
Chr07G0677.1	2544	5.00E-11	28/95(29.47)	58.2	PHI:2544|PAB1|Q4WK03|746128|Aspergillus_fumigatus|Mixed_outcome
Chr07G0678.1	2636	1.00E-08	49/152(32.24)	52.8	PHI:2636|TgCDPK3|Q8MYK0|5811|Toxoplasma_gondii|Reduced_virulence
Chr07G0683.1	1579	0	385/810(47.53)	779	PHI:1579|GzOB019|I1RM25|5518|Gibberella_zeae_(related:_Fusarium_graminearum)|Unaffected_pathogenicity
Chr07G0684.1	2527	2.00E-07	49/177(27.68)	51.6	PHI:2527|SEC31|Q4X0M4|746128|Aspergillus_fumigatus|Mixed_outcome
Chr07G0688.1	443	2.00E-27	105/404(25.99)	110	PHI:443|CBL1||5518|Fusarium_graminearum|Reduced_virulence
Chr07G0694.1	1260	1.00E-06	29/92(31.52)	50.4	PHI:1260|FGSG_13944|I1RUC7|5518|Gibberella_zeae_(related:_Fusarium_graminearum)|Unaffected_pathogenicity
Chr07G0695.1	4506	3.00E-44	171/662(25.83)	170	So_(soft)|PHI:4506|K9Y567|35717|Epichloe_festucae|effector_(plant_avirulence_determinant)
Chr07G0697.1	1302	9.00E-140	250/430(58.14)	406	PHI:1302|GzbHLH003|I1RC37|5518|Gibberella_zeae_(related:_Fusarium_graminearum)|Unaffected_pathogenicity
Chr07G0702.1	2315	6.00E-63	109/290(37.59)	209	PHI:2315|ChLae1|G4XKY9|5016|Cochliobolus_heterostrophus|Mixed_outcome
Chr07G0704.1	2844	4.00E-16	74/270(27.41)	74.3	PHI:2844|BRM2|O93802|5599|Alternaria_alternata|Unaffected_pathogenicity
Chr07G0708.1	1986	2.00E-119	250/734(34.06)	375	PHI:1986|GzZC301|I1RA77|5518|Gibberella_zeae_(related:_Fusarium_gramin

					earum)|Unaffected_pathogenicity
Chr07G0709.1	1718	4.00E-30	76/197(38.58)	117	PHI:1718|GzZC033|I1S9X1|5518|Gibberella_zeae_(related:_Fusarium_graminearum)|Unaffected_pathogenicity
Chr07G0711.1	511	1.00E-11	111/522(21.26)	64.7	PHI:511|CaNAG4|EAK93098|5476|Candida_albicans|Reduced_virulence
Chr07G0713.1	2895	2.00E-07	56/194(28.87)	50.1	PHI:2895|F-avi4330|B9JV05|373|Agrobacterium_vitis|Loss_of_pathogenicity
Chr07G0715.1	1353	0	480/720(66.67)	833	PHI:1353|GzC2H013|I1RCM1|5518|Gibberella_zeae_(related:_Fusarium_graminearum)|Reduced_virulence
Chr07G0716.1	2292	2.00E-125	178/311(57.23)	366	PHI:2292|aoxA|B0XVF7|746128|Aspergillus_fumigatus|Unaffected_pathogenicity
Chr07G0719.1	2609	1.00E-07	37/146(25.34)	48.9	PHI:2609|KRR1|AAB96910.2|746128|Aspergillus_fumigatus|Mixed_outcome
Chr07G0721.1	3111	1.00E-06	30/87(34.48)	47.4	FgAP1|PHI:3111|I1RWW4|5518|Fusarium_graminearum|mixed_outcome_
Chr07G0723.1	1212	0	1397/1944(71.86)	2588	PHI:1212|FGSG_12149|I1RCJ5|5518|Gibberella_zeae_(related:_Fusarium_graminearum)|Reduced_virulence
Chr07G0724.1	3457	5.00E-35	140/517(27.08)	137	PHO84|PHI:3457|J9VMW8|5207|Cryptococcus_neoformans|mixed_outcome_
Chr07G0725.1	1260	2.00E-06	74/251(29.48)	49.7	PHI:1260|FGSG_13944|I1RUC7|5518|Gibberella_zeae_(related:_Fusarium_graminearum)|Unaffected_pathogenicity
Chr07G0730.1	1721	2.00E-26	118/498(23.69)	108	PHI:1721|GzZC036|I1S914|5518|Gibberella_zeae_(related:_Fusarium_graminearum)|Unaffected_pathogenicity
Chr07G0731.1	2351	0	281/343(81.92)	603	PHI:2351|AMT1|XP_381310|5518|Gibberella_zeae_(related:_Fusarium_graminearum)|Reduced_virulence
Chr07G0733.1	2710	0	252/390(64.62)	534	PHI:2710|mepB|H8ZZ79|474922|Colletotrichum_gloeosporioides|Reduced_virulence
Chr07G0734.1	875	0	456/984(46.34)	872	PHI:875|MGG_04587|EDK05593|318829|Magnaporthe_oryzae|Loss_of_pathogenicity
Chr07G0735.1	2520	2.00E-40	133/468(28.42)	157	PHI:2520|LYS4|Q4WUL6|746128|Aspergillus_fumigatus|Lethal
Chr07G0736.1	2008	7.00E-14	56/171(32.75)	72	PHI:2008|HST1|EDJ98541|148305|Magnaporthe_oryzae_(related:_Magnaporth

					e_grisea)|Unaffected_pathogenicity
Chr07G0737.1	1552	1.00E-121	216/480(45.00)	410	PHI:1552|GzMyb016|I1S0N5|5518|Gibberella_zeae_(related:_Fusarium_graminearum)|Unaffected_pathogenicity
Chr07G0738.1	2067	1.00E-08	42/174(24.14)	55.5	PHI:2067|ABC4|MGG_00937|148305|Magnaporthe_oryzae_(related:_Magnaporthe_grisea)|Loss_of_pathogenicity
Chr07G0744.1	1462	3.00E-62	148/301(49.17)	200	PHI:1462|GzCCAAT002|I1RC79|5518|Gibberella_zeae_(related:_Fusarium_graminearum)|Reduced_virulence
Chr07G0746.1	862	5.00E-66	200/702(28.49)	237	PHI:862|ClaSSD1|BAE66713|5462|Colletotrichum_lagenarium|Loss_of_pathogenicity
Chr07G0749.1	2184	3.00E-60	216/639(33.80)	215	PHI:2184|HTF1|MGG_00184|148305|Magnaporthe_oryzae_(related:_Magnaporthe_grisea)|Unaffected_pathogenicity
Chr07G0750.1	541	7.00E-46	145/457(31.73)	167	PHI:541|LIP1|AAU87359|332648|Botrytis_cinerea|Unaffected_pathogenicity
Chr07G0752.1	1198	0	1013/1369(74.00)	2101	PHI:1198|(Sc_Ssk2/Ssk22)|I1RA81|5518|Gibberella_zeae_(related:_Fusarium_graminearum)|Reduced_virulence
Chr07G0757.1	443	4.00E-06	33/98(33.67)	45.8	PHI:443|CBL1||5518|Fusarium_graminearum|Reduced_virulence
Chr07G0762.1	1522	4.00E-06	28/93(30.11)	45.4	PHI:1522|GzHOMEL026|I1RXA5|5518|Gibberella_zeae_(related:_Fusarium_graminearum)|Lethal
Chr07G0764.1	801	2.00E-174	311/597(52.09)	512	PHI:801|MGG_04621|EDJ95977|318829|Magnaporthe_oryzae|Reduced_virulence
Chr07G0766.1	3287	1.00E-25	78/258(30.23)	112	CgVps34|PHI:3287|Q6FSR7|5478|Candida_glabrata|reduced_virulence_
Chr07G0771.1	1616	3.00E-29	78/231(33.77)	114	PHI:1616|GzssDB001|I1RE55|5518|Gibberella_zeae_(related:_Fusarium_graminearum)|Unaffected_pathogenicity
Chr07G0773.1	803	0	627/1164(53.87)	1109	PHI:803|MGG_04629|EDJ95969|318829|Magnaporthe_oryzae|Reduced_virulence
Chr07G0777.1	1454	0	461/668(69.01)	918	PHI:1454|GzAra002|I1RCI0|5518|Gibberella_zeae_(related:_Fusarium_graminearum)|Unaffected_pathogenicity

Chr07G0780.1	1350	1.00E-82	161/302(53.31)	252	PHI:1350|GzC2H010|I1RCI2|5518|Gibberella_zeae_(related:_Fusarium_graminearum)|Unaffected_pathogenicity
Chr07G0783.1	280	0	379/675(56.15)	728	PHI:280|CCN1|AAG36938|5207|Cryptococcus_neoformans|Reduced_virulence
Chr07G0789.1	1304	0	365/546(66.85)	648	PHI:1304|GzbHLH005|I1RCJ0|5518|Gibberella_zeae_(related:_Fusarium_graminearum)|Reduced_virulence
Chr07G0792.1	1351	0	458/1109(41.30)	743	PHI:1351|GzC2H011|I1RCJ3|5518|Gibberella_zeae_(related:_Fusarium_graminearum)|Unaffected_pathogenicity
Chr07G0798.1	1921	0	599/932(64.27)	1177	PHI:1921|GzZC236|I1RCH8|5518|Gibberella_zeae_(related:_Fusarium_graminearum)|Reduced_virulence
Chr07G0799.1	211	4.00E-11	54/214(25.23)	61.2	PHI:211|CaTUP1|AAB63195|5476|Candida_albicans|Reduced_virulence
Chr07G0801.1	2563	1.00E-27	71/209(33.97)	109	PHI:2563|DPP3|Q5AH74|5476|Candida_albicans|Reduced_virulence
Chr07G0803.1	338	1.00E-93	135/267(50.56)	297	PHI:338|CLB2|AAP94020|5270|Ustilago_maydis|Reduced_virulence
Chr07G0804.1	1569	0	274/369(74.25)	553	PHI:1569|GzOB009|I1RCH5|5518|Gibberella_zeae_(related:_Fusarium_graminearum)|Lethal
Chr07G0806.1	2092	0	1307/2129(61.39)	2480	PHI:2092|Cch1|MGG_05643|148305|Magnaporthe_oryzae_(related:_Magnaporthe_grisea)|Reduced_virulence
Chr07G0808.1	1478	4.00E-71	168/470(35.74)	237	PHI:1478|GzHMG010|I1RCP3|5518|Gibberella_zeae_(related:_Fusarium_graminearum)|Unaffected_pathogenicity
Chr07G0814.1	2915	0	392/417(94.00)	817	PHI:2915|Ppr1|W7LRW9|117187|Fusarium_verticillioides|Unaffected_pathogenicity
Chr07G0819.1	2038	1.00E-06	36/144(25.00)	48.1	PHI:2038|Mir1|MGG_02370|148305|Magnaporthe_oryzae_(related:_Magnaporthe_grisea)|Unaffected_pathogenicity
Chr07G0823.1	1632	1.00E-28	83/271(30.63)	119	PHI:1632|GzWing004|I1RBU0|5518|Gibberella_zeae_(related:_Fusarium_graminearum)|Unaffected_pathogenicity
Chr07G0824.1	2601	7.00E-22	74/305(24.26)	93.6	PHI:2601|Asc1|P83774|746128|Aspergillus_fumigatus|Mixed_outcome
Chr07G0825.1	1675	6.00E-15	63/197(31.98)	71.2	PHI:1675|GzDHHC003|Q4I8B6|5518|Gibberella_zeae_(related:_Fusarium_gra

					minearum)|Unaffected_pathogenicity
Chr07G0829.1	1468	1.00E-90	131/134(97.76)	260	PHI:1468|GzCCAAT008|Q4HTT1|5518|Gibberella_zeae_(related:_Fusarium_graminearum)|Unaffected_pathogenicity
Chr07G0832.1	2072	0	292/486(60.08)	585	PHI:2072|Moatg4|MGG_03580|148305|Magnaporthe_oryzae_(related:_Magnaporthe_grisea)|Loss_of_pathogenicity
Chr07G0833.1	1414	1.00E-170	312/554(56.32)	495	PHI:1414|GzC2H081|I1RZL0|5518|Gibberella_zeae_(related:_Fusarium_graminearum)|Unaffected_pathogenicity
Chr07G0835.1	443	3.00E-11	79/277(28.52)	63.2	PHI:443|CBL1||5518|Fusarium_graminearum|Reduced_virulence
Chr07G0838.1	2553	6.00E-77	150/396(37.88)	256	PHI:2553|VPS4|Q5AG40|5476|Candida_albicans|Loss_of_pathogenicity
Chr07G0839.1	1600	1.00E-55	84/122(68.85)	172	PHI:1600|GzOB041|I1RZK5|5518|Gibberella_zeae_(related:_Fusarium_graminearum)|Unaffected_pathogenicity
Chr07G0841.1	2077	0	414/813(50.92)	834	PHI:2077|Moatg9|MGG_09559|148305|Magnaporthe_oryzae_(related:_Magnaporthe_grisea)|Loss_of_pathogenicity
Chr07G0842.1	1414	8.00E-07	27/66(40.91)	48.9	PHI:1414|GzC2H081|I1RZL0|5518|Gibberella_zeae_(related:_Fusarium_graminearum)|Unaffected_pathogenicity
Chr07G0846.1	816	4.00E-41	94/270(34.81)	151	PHI:816|MGG_04582|EDJ95999|318829|Magnaporthe_oryzae|Reduced_virulence
Chr07G0847.1	2248	5.00E-16	83/342(24.27)	78.2	PHI:2248|Als1|Q1L2E2|13684|Phaeosphaeria_nodorum_(related:_Stagonospora_nodorum)|Mixed_outcome
Chr07G0848.1	2533	0	1453/1944(74.74)	2921	PHI:2533|FKS1|Q4WLT4|746128|Aspergillus_fumigatus|Lethal
Chr07G0849.1	1493	2.00E-134	218/395(55.19)	411	PHI:1493|GzHMG025|I1RUP7|5518|Gibberella_zeae_(related:_Fusarium_graminearum)|Unaffected_pathogenicity
Chr07G0852.1	2841	3.00E-10	62/251(24.70)	58.2	PHI:2841|CnSEC14-2|F6K8L6|5207|Cryptococcus_neoformans|Unaffected_pathogenicity
Chr07G0854.1	1397	5.00E-57	80/119(67.23)	174	PHI:1397|GzC2H060|I1RUQ2|5518|Gibberella_zeae_(related:_Fusarium_graminearum)|Unaffected_pathogenicity

Chr07G0860.1	1057	4.00E-11	68/254(26.77)	62.8	PHI:1057|MTP1|ABP98949|318829|Magnaporthe_oryzae|Unaffected_pathogenicity
Chr07G0861.1	3214	4.00E-09	33/93(35.48)	48.5	MoCDIP2|PHI:3214|G4MML4|318829|Magnaporthe_oryzae|mixed_outcome_
Chr07G0865.1	1447	0	614/940(65.32)	1150	PHI:1447|GzGATA005|I1RUP1|5518|Gibberella_zeae_(related:_Fusarium_graminearum)|Unaffected_pathogenicity
Chr07G0867.1	2821	5.00E-16	41/103(39.81)	80.9	PHI:2821|SNF2|Q5ALP9|5476|Candida_albicans|Reduced_virulence
Chr07G0880.1	1526	3.00E-69	180/574(31.36)	245	PHI:1526|GzHOMEL036|I1S0J3|5518|Gibberella_zeae_(related:_Fusarium_graminearum)|Unaffected_pathogenicity
Chr07G0881.1	361	6.00E-174	421/1480(28.45)	561	PHI:361|KRE5|EAL01265|5476|Candida_albicans|Reduced_virulence
Chr07G0882.1	211	3.00E-17	63/232(27.16)	83.6	PHI:211|CaTUP1|AAB63195|5476|Candida_albicans|Reduced_virulence
Chr07G0884.1	799	0	498/946(52.64)	801	PHI:799|MGG_03530|EDJ94565|318829|Magnaporthe_oryzae|Reduced_virulence
Chr07G0885.1	1047	3.00E-15	72/270(26.67)	73.6	PHI:1047|CTB6|ABK64183|29003|Cercospora_nicotianae|Reduced_virulence
Chr07G0887.1	812	6.00E-80	148/386(38.34)	255	PHI:812|MGG_10702|EDJ94108|318829|Magnaporthe_oryzae|Reduced_virulence
Chr07G0888.1	1660	3.00E-152	365/1156(31.57)	507	PHI:1660|GzCCCH004|I1S4C9|5518|Gibberella_zeae_(related:_Fusarium_graminearum)|Unaffected_pathogenicity
Chr07G0890.1	441	1.00E-11	64/274(23.36)	62.4	PHI:441|BTP1|CAE55153|40559|Botrytis_cinerea|Reduced_virulence
Chr07G0894.1	3457	1.00E-14	110/458(24.02)	73.6	PHO84|PHI:3457|J9VMW8|5207|Cryptococcus_neoformans|mixed_outcome_
Chr07G0897.1	2968	2.00E-34	134/487(27.52)	134	PHI:2968|Hxs1|J9VQA5|5207|Cryptococcus_neoformans|Reduced_virulence
Chr07G0899.1	2909	4.00E-14	79/329(24.01)	72	PHI:2909|CYP51C|I1S2M5|5518|Fusarium_graminearum|Mixed_outcome
Chr07G0906.1	1161	7.00E-38	127/536(23.69)	144	PHI:1161|MgMfs1|A4ZGP3|54734|Mycosphaerella_graminicola_(related:_Zymoseptoria_triticii)|Chemistry_target
Chr07G0907.1	1555	2.00E-30	121/511(23.68)	122	PHI:1555|GzMyb019|I1RDG6|5518|Gibberella_zeae_(related:_Fusarium_graminearum)|Unaffected_pathogenicity
Chr07G0909.1	2256	6.00E-28	90/335(26.87)	109	PHI:2256|Xdh1|Q0UA24|13684|Phaeosphaeria_nodorum_(related:_Stagonosp

					ora_nodorum)|Unaffected_pathogenicity
Chr07G0912.1	812	1.00E-33	93/302(30.79)	128	PHI:812|MGG_10702|EDJ94108|318829|Magnaporthe_oryzae|Reduced_virulence
Chr07G0916.1	4194	7.00E-51	145/511(28.38)	181	AKT7|PHI:4194|V5XZS6|5599|Alternaria_alternata|increased_virulence_(Hyper virulence)
Chr07G0919.1	2968	2.00E-39	131/500(26.20)	148	PHI:2968|Hxs1|J9VQA5|5207|Cryptococcus_neoformans|Reduced_virulence
Chr07G0921.1	2175	2.00E-55	123/326(37.73)	183	PHI:2175|NMR3|MGG_09705|148305|Magnaporthe_oryzae_(related:_Magnaporthe_grisea)|Reduced_virulence
Chr07G0929.1	2968	4.00E-24	109/426(25.59)	103	PHI:2968|Hxs1|J9VQA5|5207|Cryptococcus_neoformans|Reduced_virulence
Chr07G0930.1	323	4.00E-10	80/357(22.41)	59.3	PHI:323|VFGLU1|AAO63562|93591|Verticillium_fungicola|Reduced_virulence
Chr07G0931.1	1743	7.00E-174	283/580(48.79)	512	PHI:1743|GzZC058|I1S8J4|5518|Gibberella_zeae_(related:_Fusarium_graminearum)|Unaffected_pathogenicity
Chr07G0932.1	2315	1.00E-52	104/295(35.25)	176	PHI:2315|ChLae1|G4XKY9|5016|Cochliobolus_heterostrophus|Mixed_outcome
Chr07G0933.1	438	6.00E-37	125/488(25.61)	140	PHI:438|BcBOT1_(related:_CND5)|AAQ16576|40559|Botrytis_cinerea|Reduced_virulence
Chr07G0936.1	179	2.00E-65	114/225(50.67)	205	PHI:179|PELA|AAA33338|140110|Nectria_haematococca_(related:_Fusarium_solani)|Reduced_virulence
Chr07G0942.1	223	4.00E-26	44/84(52.38)	100	PHI:223|PEP1|AAK11166|140110|Nectria_haematococca_(related:_Fusarium_solani)|Reduced_virulence
Chr07G0944.1	1788	1.00E-61	195/694(28.10)	219	PHI:1788|GzZC103|I1S1N1|5518|Gibberella_zeae_(related:_Fusarium_graminearum)|Unaffected_pathogenicity
Chr07G0947.1	569	8.00E-13	30/53(56.60)	67.8	PHI:569|XYL3|AAC06239|5507|Fusarium_oxysporum|Unaffected_pathogenicity
Chr07G0949.1	1695	0	335/612(54.74)	633	PHI:1695|GzZC010|I1S2I3|5518|Gibberella_zeae_(related:_Fusarium_graminearum)|Unaffected_pathogenicity
Chr07G0951.1	1500	0	1082/1538(70.35)	2108	PHI:1500|GzHMG032|I1S2F2|5518|Gibberella_zeae_(related:_Fusarium_graminearum)|Unaffected_pathogenicity

Chr07G0955.1	419	1.00E-27	86/318(27.04)	108	PHI:419|CSH1|AAP93915|5476|Candida_albicans|Reduced_virulence
Chr07G0956.1	3258	4.00E-08	60/238(25.21)	52	C14DM|PHI:3258|Q4QGX0|5664|Leishmania_major|reduced_virulence_
Chr07G0960.1	144	9.00E-126	189/396(47.73)	373	PHI:144|CHT42|AAC05829|29875|Trichoderma_virens|Reduced_virulence
Chr07G0963.1	2553	0	274/439(62.41)	567	PHI:2553|VPS4|Q5AG40|5476|Candida_albicans|Loss_of_pathogenicity
Chr07G0971.1	1279	7.00E-20	46/133(34.59)	88.2	PHI:1279|FGSG_12132|I1RU59|5518|Gibberella_zeae_(related:_Fusarium_graminearum)|Unaffected_pathogenicity
Chr07G0975.1	510	9.00E-20	110/443(24.83)	89.7	PHI:510|CaNAG3|EAK93097|5476|Candida_albicans|Reduced_virulence
Chr07G0977.1	2968	8.00E-40	118/475(24.84)	149	PHI:2968|Hxs1|J9VQA5|5207|Cryptococcus_neoformans|Reduced_virulence
Chr07G0978.1	901	1.00E-65	144/429(33.57)	222	PHI:901|um01886||5270|Ustilago_maydis|Unaffected_pathogenicity
Chr07G0980.1	2968	5.00E-35	131/516(25.39)	135	PHI:2968|Hxs1|J9VQA5|5207|Cryptococcus_neoformans|Reduced_virulence
Chr07G0984.1	2378	6.00E-73	178/590(30.17)	244	PHI:2378|DEP4|D2E9W9|29001|Alternaria_brassicicola|Mixed_outcome
Chr07G0985.1	1662	1.00E-73	150/498(30.12)	256	PHI:1662|GzCCHC002|I1REJ1|5518|Gibberella_zeae_(related:_Fusarium_graminearum)|Unaffected_pathogenicity
Chr07G0986.1	1071	2.00E-28	146/605(24.13)	120	PHI:1071|Gas1|CAF05793|5270|Ustilago_maydis|Loss_of_pathogenicity
Chr07G0988.1	2988	5.00E-06	49/196(25.00)	47	PHI:2988|MoAPS2|G4NA99|148305|Magnaporthe_oryzae|Reduced_virulence
Chr07G0990.1	2207	8.00E-09	20/32(62.50)	54.3	PHI:2207|endo-1,4-beta-xylanase_[GH10_family]|MGG_02245|148305|Magnaporthe_oryzae_(related:_Magnaporthe_grisea)|Reduced_virulence
Chr07G0991.1	2357	3.00E-13	55/212(25.94)	69.3	PHI:2357|CYP52X1|E2EAF6|475271|Beauveria_bassiana|Reduced_virulence
Chr07G0993.1	2290	0	970/2508(38.68)	1675	PHI:2290|BcBOA6|B1GVX7|40559|Botrytis_cinerea|Reduced_virulence
Chr07G0994.1	2376	9.00E-13	87/375(23.20)	68.6	PHI:2376|DEP2|D2E9W7|29001|Alternaria_brassicicola|Mixed_outcome
Chr07G0995.1	2978	5.00E-86	138/270(51.11)	258	PHI:2978|MoCel12A|G4N5V2|148305|Magnaporthe_oryzae|Unaffected_pathogenicity
Chr07G1000.1	1842	0	385/646(59.60)	782	PHI:1842|GzZC157|I1RBA2|5518|Gibberella_zeae_(related:_Fusarium_graminearum)|Unaffected_pathogenicity
Chr07G1005.1	2968	1.00E-21	88/422(20.85)	95.9	PHI:2968|Hxs1|J9VQA5|5207|Cryptococcus_neoformans|Reduced_virulence
Chr07G1016.1	1581	1.00E-68	112/269(41.64)	216	PHI:1581|GzOB021|I1RPX8|5518|Gibberella_zeae_(related:_Fusarium_gramin

					earum)|Unaffected_pathogenicity
Chr07G1023.1	1332	1.00E-11	27/76(35.53)	60.8	PHI:1332|GzbZIP014|I1RXD5|5518|Gibberella_zeae_(related:_Fusarium_graminearum)|Unaffected_pathogenicity
Chr07G1024.1	1941	2.00E-18	43/100(43.00)	82.8	PHI:1941|GzZC256|I1RGD9|5518|Gibberella_zeae_(related:_Fusarium_graminearum)|Unaffected_pathogenicity
Chr07G1025.1	1941	2.00E-17	43/100(43.00)	82.8	PHI:1941|GzZC256|I1RGD9|5518|Gibberella_zeae_(related:_Fusarium_graminearum)|Unaffected_pathogenicity
Chr07G1027.1	1893	2.00E-64	152/480(31.67)	218	PHI:1893|GzZC208|I1RNY0|5518|Gibberella_zeae_(related:_Fusarium_graminearum)|Unaffected_pathogenicity
Chr07G1035.1	1978	3.00E-55	134/478(28.03)	196	PHI:1978|GzZC293|I1RLK0|5518|Gibberella_zeae_(related:_Fusarium_graminearum)|Lethal
Chr07G1039.1	4194	5.00E-54	137/486(28.19)	191	AKT7|PHI:4194|V5XZS6|5599|Alternaria_alternata|increased_virulence_(Hypervirulence)
Chr07G1041.1	3381	1.00E-10	52/243(21.40)	61.2	FVEG_12533|PHI:3381|W7N2B4|117187|Fusarium_verticillioides|unaffected_pathogenicity_
Chr07G1044.1	1742	2.00E-08	77/343(22.45)	53.1	PHI:1742|GzZC057|I1S7H5|5518|Gibberella_zeae_(related:_Fusarium_graminearum)|Unaffected_pathogenicity
Chr07G1046.1	1651	6.00E-21	86/332(25.90)	91.3	PHI:1651|GzWing023|I1S0U2|5518|Gibberella_zeae_(related:_Fusarium_graminearum)|Unaffected_pathogenicity
Chr07G1049.1	3662	5.00E-06	41/124(33.06)	48.1	PspB_(not_PD0218)|PHI:3662|Q87ET0|2371|Xylella_fastidiosa|Increased_virulence_(Hypervirulence)
Chr07G1056.1	1260	3.00E-15	60/157(38.22)	77.4	PHI:1260|FGSG_13944|I1RUC7|5518|Gibberella_zeae_(related:_Fusarium_graminearum)|Unaffected_pathogenicity
Chr07G1057.1	4194	2.00E-134	201/516(38.95)	401	AKT7|PHI:4194|V5XZS6|5599|Alternaria_alternata|increased_virulence_(Hypervirulence)
Chr07G1062.1	2990	2.00E-06	50/189(26.46)	48.1	PHI:2990|MGG_02474.6|G4MRY7|148305|Magnaporthe_oryzae|Reduced_virul

					ence
Chr07G1067.1	2240	2.00E-38	125/475(26.32)	145	PHI:2240|Srt1|Q4PBY9|5270|Ustilago_maydis|reduced_virulence
Chr07G1072.1	2570	8.00E-08	33/98(33.67)	52.4	PHI:2570|CYB2|Q6FM61|5478|Candida_glabrata|Reduced_virulence
Chr07G1073.1	800	1.00E-06	61/212(28.77)	48.9	PHI:800|MGG_13324|EDK00897|318829|Magnaporthe_oryzae|Reduced_virulence
Chr07G1079.1	2032	2.00E-40	140/495(28.28)	151	PHI:2032|VTL1|G4NGA7|148305|Magnaporthe_oryzae_(related:_Magnaporthe_grisea)|Unaffected_pathogenicity
Chr07G1081.1	1269	3.00E-89	148/361(41.00)	278	PHI:1269|FGSG_02838|I1RFK9|5518|Gibberella_zeae_(related:_Fusarium_graminearum)|Unaffected_pathogenicity
Chr07G1084.1	423	0	307/407(75.43)	650	PHI:423|VAD1|AAV41010|5207|Cryptococcus_neoformans|Reduced_virulence
Chr07G1091.1	2215	0	1123/2120(52.97)	2094	PHI:2215|BIM1|G4N983|148305|Magnaporthe_oryzae_(related:_Magnaporthe_grisea)|Reduced_virulence
Chr07G1093.1	1297	0	611/1142(53.50)	1004	PHI:1297|GzAT001|I1RPU1|5518|Gibberella_zeae_(related:_Fusarium_graminearum)|Reduced_virulence
Chr07G1096.1	1260	7.00E-06	52/204(25.49)	48.1	PHI:1260|FGSG_13944|I1RUC7|5518|Gibberella_zeae_(related:_Fusarium_graminearum)|Unaffected_pathogenicity
Chr07G1101.1	1662	1.00E-37	120/450(26.67)	145	PHI:1662|GzCCHC002|I1REJ1|5518|Gibberella_zeae_(related:_Fusarium_graminearum)|Unaffected_pathogenicity
Chr07G1103.1	2315	1.00E-46	101/290(34.83)	161	PHI:2315|ChLae1|G4XKY9|5016|Cochliobolus_heterostrophus|Mixed_outcome
Chr07G1105.1	201	2.00E-08	48/184(26.09)	51.6	PHI:201|AVR-Pita_(related:_AVR2-YAMO)|AAK00131|318829|Magnaporthe_oryzae|Effector_(plant_avirulence_determinant)
Chr07G1107.1	2315	7.00E-35	76/177(42.94)	127	PHI:2315|ChLae1|G4XKY9|5016|Cochliobolus_heterostrophus|Mixed_outcome
Chr07G1111.1	2968	7.00E-30	121/450(26.89)	120	PHI:2968|Hxs1|J9VQA5|5207|Cryptococcus_neoformans|Reduced_virulence
Chr07G1116.1	2094	5.00E-08	129/657(19.63)	53.5	PHI:2094|Yvc1|MGG_09828|148305|Magnaporthe_oryzae_(related:_Magnaporthe_grisea)|Mixed_outcome
Chr07G1117.1	1028	4.00E-103	171/352(48.58)	308	PHI:1028|bcpme1|CAC29255|40559|Botrytis_cinerea|Reduced_virulence

Chr07G1119.1	1051	2.00E-13	100/389(25.71)	69.7	PHI:1051|CTB3|ABC79591|29003|Cercospora_nicotianae|Reduced_virulence
Chr07G1120.1	1460	1.00E-102	160/317(50.47)	305	PHI:1460|GzAra008|I1S2F6|5518|Gibberella_zeae_(related:_Fusarium_graminearum)|Unaffected_pathogenicity
Chr07G1121.1	1994	1.00E-76	217/708(30.65)	261	PHI:1994|GzZC309|I1R9S1|5518|Gibberella_zeae_(related:_Fusarium_graminearum)|Unaffected_pathogenicity
Chr07G1126.1	3214	7.00E-08	32/111(28.83)	47	MoCDIP2|PHI:3214|G4MML4|318829|Magnaporthe_oryzae|mixed_outcome_
Chr07G1127.1	3384	2.00E-78	266/1031(25.80)	276	FVEG_12530|PHI:3384|W7N2C1|117187|Fusarium_verticillioides|unaffected_pathogenicity_
Chr07G1128.1	1581	1.00E-27	76/235(32.34)	105	PHI:1581|GzOB021|I1RPX8|5518|Gibberella_zeae_(related:_Fusarium_graminearum)|Unaffected_pathogenicity
Chr07G1133.1	572	1.00E-91	141/231(61.04)	270	PHI:572|XYL2|AAC62815|5017|Cochliobolus_carbonum|Unaffected_pathogenicity
Chr07G1137.1	2968	2.00E-54	144/510(28.24)	191	PHI:2968|Hxs1|J9VQA5|5207|Cryptococcus_neoformans|Reduced_virulence
Chr07G1138.1	1936	6.00E-81	222/763(29.10)	272	PHI:1936|GzZC251|I1RFG1|5518|Gibberella_zeae_(related:_Fusarium_graminearum)|Unaffected_pathogenicity
Chr07G1140.1	1662	2.00E-39	124/455(27.25)	152	PHI:1662|GzCCHC002|I1REJ1|5518|Gibberella_zeae_(related:_Fusarium_graminearum)|Unaffected_pathogenicity
Chr07G1141.1	1555	9.00E-48	129/482(26.76)	172	PHI:1555|GzMyb019|I1RDG6|5518|Gibberella_zeae_(related:_Fusarium_graminearum)|Unaffected_pathogenicity
Chr07G1143.1	577	0	500/576(86.81)	1073	PHI:577|NoxB|BAE72682|35717|Epichloe_festucae|Wild-type_mutualism
Chr07G1153.1	1605	0	284/524(54.20)	547	PHI:1605|GzOB046|I1S216|5518|Gibberella_zeae_(related:_Fusarium_graminearum)|Unaffected_pathogenicity
Chr07G1154.1	2400	2.00E-163	285/489(58.28)	471	PHI:2400|Fgp2|I1S215|5518|Gibberella_zeae_(related:_Fusarium_graminearum)|Unaffected_pathogenicity
Chr07G1158.1	1652	5.00E-33	57/72(79.17)	120	PHI:1652|GzWing024|I1S219|5518|Gibberella_zeae_(related:_Fusarium_graminearum)|Unaffected_pathogenicity

Chr07G1164.1	199	2.00E-53	184/652(28.22)	192	PHI:199|AOX1|AAF82788|5499|Cladosporium_fulvum|Reduced_virulence
Chr07G1165.1	1071	4.00E-23	109/453(24.06)	102	PHI:1071|Gas1|CAF05793|5270|Ustilago_maydis|Loss_of_pathogenicity
Chr07G1166.1	407	2.00E-06	35/124(28.23)	45.1	PHI:407|PBC1|CAB40372|76659|Pyrenopeziza_brassicae|Loss_of_pathogenicity
Chr07G1168.1	180	5.00E-71	125/227(55.07)	218	PHI:180|PELD|AAC49420|140110|Nectria_haematococca_(related:_Fusarium_solani)|Reduced_virulence
Chr07G1170.1	441	1.00E-11	63/255(24.71)	62.8	PHI:441|BTP1|CAE55153|40559|Botrytis_cinerea|Reduced_virulence
Chr07G1171.1	2837	2.00E-30	78/243(32.10)	114	PHI:2837|OXI1|D2SZX7|5016|Cochliobolus_heterostrophus|Reduced_virulence
Chr07G1172.1	2266	0	330/533(61.91)	684	PHI:2266|Ptr2|0|13684|Phaeosphaeria_nodorum_(related:_Stagonospora_nodorum)|Unaffected_pathogenicity
Chr07G1175.1	2822	2.00E-33	139/538(25.84)	135	PHI:2822|Cxt1p|Q5K8R6|5207|Cryptococcus_neoformans|Reduced_virulence
Chr07G1176.1	2204	4.00E-115	163/307(53.09)	338	PHI:2204|endo-1,4-beta-xylanase_[GH10_family]|MGG_01542|148305|Magnaporthe_oryzae_(related:_Magnaporthe_grisea)|Reduced_virulence
Chr07G1177.1	1503	2.00E-21	82/278(29.50)	94	PHI:1503|GzHMG035|I1S828|5518|Gibberella_zeae_(related:_Fusarium_graminearum)|Unaffected_pathogenicity
Chr07G1180.1	538	2.00E-53	146/517(28.24)	191	PHI:538|FRT1|AAU87358|40559|Botrytis_cinerea|Unaffected_pathogenicity
Chr07G1181.1	2968	8.00E-114	182/484(37.60)	348	PHI:2968|Hxs1|J9VQA5|5207|Cryptococcus_neoformans|Reduced_virulence
Chr07G1182.1	1528	7.00E-10	29/71(40.85)	57.4	PHI:1528|GzHOMEL041|I1SAN0|5518|Gibberella_zeae_(related:_Fusarium_graminearum)|Unaffected_pathogenicity
Chr07G1186.1	1785	0	300/520(57.69)	615	PHI:1785|GzZC100|I1S231|5518|Gibberella_zeae_(related:_Fusarium_graminearum)|Unaffected_pathogenicity
Chr07G1188.1	2309	2.00E-90	328/1327(24.72)	319	PHI:2309|BcatrB|Q9UW03|40559|Botrytis_cinerea|Reduced_virulence
Chr07G1193.1	223	5.00E-23	41/84(48.81)	92	PHI:223|PEP1|AAK11166|140110|Nectria_haematococca_(related:_Fusarium_solani)|Reduced_virulence
Chr07G1194.1	2020	1.00E-45	110/323(34.06)	174	PHI:2020|Tup1|XP_759427|5270|Ustilago_maydis|Mixed_outcome
Chr07G1196.1	1620	1.00E-17	45/127(35.43)	85.9	PHI:1620|GzJUM002|I1RD69|5518|Gibberella_zeae_(related:_Fusarium_grami

					nearum)|Unaffected_pathogenicity
Chr07G1204.1	2549	3.00E-07	24/87(27.59)	48.9	PHI:2549|MAK5|Q4WMS3|746128|Aspergillus_fumigatus|Mixed_outcome
Chr02G0784.1	2624	8.00E-77	184/430(42.79)	261	PHI:2624|aroA|P07637|90371|Salmonella_enterica_serovar_Typhimurium|Reduced_virulence
Chr02G0785.1	1367	3.00E-177	315/584(53.94)	520	PHI:1367|GzC2H028|I1RK94|5518|Gibberella_zeae_(related:_Fusarium_graminearum)|Unaffected_pathogenicity
Chr02G0790.1	1368	5.00E-78	162/339(47.79)	248	PHI:1368|GzC2H029|I1RK97|5518|Gibberella_zeae_(related:_Fusarium_graminearum)|Unaffected_pathogenicity
Chr02G0793.1	2229	1.00E-06	25/66(37.88)	48.9	PHI:2229|rok1|XP_759848|5270|Ustilago_maydis|Mixed_outcome
Chr02G0794.1	2084	0	363/475(76.42)	710	PHI:2084|Moatg18|MGG_03139|148305|Magnaporthe_oryzae_(related:_Magnaporthe_grisea)|Reduced_virulence
Chr02G0801.1	2432	2.00E-81	145/277(52.35)	258	PHI:2432|FgRgsA|I1RKA4|5518|Gibberella_zeae_(related:_Fusarium_gramineharum)|Reduced_virulence
Chr02G0802.1	3272	6.00E-33	137/512(26.76)	130	glpD|PHI:3272|Q8CWG4|992166|Yersinia_pestis|unaffected_pathogenicity_
Chr02G0804.1	2601	7.00E-08	54/217(24.88)	51.2	PHI:2601|Asc1|P83774|746128|Aspergillus_fumigatus|Mixed_outcome
Chr02G0807.1	2530	0	411/443(92.78)	871	PHI:2530|TUB1|Q4WKG5|746128|Aspergillus_fumigatus|Mixed_outcome
Chr02G0810.1	27	4.00E-112	150/183(81.97)	318	PHI:27|CAP20|AAA77678|5457|Colletotrichum_gloeosporioides|Loss_of_pathogenicity
Chr02G0812.1	1345	3.00E-09	19/33(57.58)	55.1	PHI:1345|GzC2H005|I1RAW2|5518|Gibberella_zeae_(related:_Fusarium_graminearum)|Unaffected_pathogenicity
Chr02G0815.1	3071	1.00E-13	55/190(28.95)	67.8	PsTatD4|PHI:3071|G4Z2B4|67593|Phytophthora_sojae|reduced_virulence_
Chr02G0817.1	1325	6.00E-82	141/259(54.44)	249	PHI:1325|GzbZIP007|I1RMI1|5518|Gibberella_zeae_(related:_Fusarium_graminearum)|Reduced_virulence
Chr02G0818.1	810	6.00E-26	53/93(56.99)	97.1	PHI:810|MGG_02436|EDJ98604|318829|Magnaporthe_oryzae|Reduced_virulence
Chr02G0823.1	2544	2.00E-15	49/172(28.49)	76.6	PHI:2544|PAB1|Q4WK03|746128|Aspergillus_fumigatus|Mixed_outcome

Chr02G0824.1	2570	8.00E-09	28/67(41.79)	54.7	PHI:2570|CYB2|Q6FM61|5478|Candida_glabrata|Reduced_virulence
Chr02G0826.1	2491	1.00E-18	57/155(36.77)	87	PHI:2491|FgPTC1|I1RJS9|5518|Gibberella_zeae_(related:_Fusarium_graminearum)|Reduced_virulence
Chr02G0828.1	485	4.00E-114	171/324(52.78)	337	PHI:485|FTR1|AAF69680|5476|Candida_albicans|Loss_of_pathogenicity
Chr02G0829.1	2920	0	464/603(76.95)	1000	PHI:2920|FET3-1|E3QRA4|31870|Colletotrichum_graminicola|Reduced_virulence
Chr02G0832.1	506	6.00E-35	70/140(50.00)	128	PHI:506|URA3|AAA34824|4932|Saccharomyces_cerevisiae|Reduced_virulence
Chr02G0833.1	1954	0	268/558(48.03)	531	PHI:1954|GzZC269|I1RMG7|5518|Gibberella_zeae_(related:_Fusarium_graminearum)|Unaffected_pathogenicity
Chr02G0837.1	1484	7.00E-160	318/703(45.23)	478	PHI:1484|GzHMG016|I1RMG1|5518|Gibberella_zeae_(related:_Fusarium_graminearum)|Unaffected_pathogenicity
Chr02G0846.1	2866	8.00E-09	28/69(40.58)	55.5	PHI:2866|ORF19.3625|Q59Y24|5476|Candida_albicans|Reduced_virulence
Chr02G0850.1	2545	1.00E-07	25/66(37.88)	50.1	PHI:2545|TIF35|Q4X1I3|746128|Aspergillus_fumigatus|Mixed_outcome
Chr02G0854.1	882	2.00E-54	103/335(30.75)	201	PHI:882|MGG_04985|EDJ95433|318829|Magnaporthe_oryzae|Reduced_virulence
Chr02G0858.1	1161	2.00E-35	138/527(26.19)	138	PHI:1161|MgMfs1|A4ZGP3|54734|Mycosphaerella_graminicola_(related:_Zymoseptoria_triticii)|Chemistry_target
Chr02G0859.1	2911	3.00E-63	174/573(30.37)	224	PHI:2911|Ss-pth2|A7F6V9|5180|Sclerotinia_sclerotiorum|Reduced_virulence
Chr02G0862.1	339	4.00E-86	122/201(60.70)	254	PHI:339|CLPT1|CAC41973|290576|Colletotrichum_lindemuthianum|Reduced_virulence
Chr02G0864.1	211	2.00E-15	81/319(25.39)	75.1	PHI:211|CaTUP1|AAB63195|5476|Candida_albicans|Reduced_virulence
Chr02G0865.1	2514	1.00E-07	58/266(21.80)	52.4	PHI:2514|PFS2|Q4X1Y0|746128|Aspergillus_fumigatus|Lethal
Chr02G0866.1	1452	0	384/571(67.25)	687	PHI:1452|GzHSF003|I1S282|5518|Gibberella_zeae_(related:_Fusarium_graminearum)|Unaffected_pathogenicity
Chr02G0869.1	2968	2.00E-40	123/471(26.11)	151	PHI:2968|Hxs1|J9VQA5|5207|Cryptococcus_neoformans|Reduced_virulence
Chr02G0871.1	2315	1.00E-56	110/292(37.67)	187	PHI:2315|ChLae1|G4XKY9|5016|Cochliobolus_heterostrophus|Mixed_outcome

Chr02G0872.1	482	3.00E-33	83/277(29.96)	123	PHI:482|LAEA|AAR01218|5085|Aspergillus_fumigatus|Reduced_virulence
Chr02G0877.1	2441	3.00E-08	102/468(21.79)	53.1	PHI:2441|MgAlg2|F9XJV5|1047171|Mycosphaerella_graminicola_(related:_Zymoseptoria_triticii)|Loss_of_pathogenicity
Chr02G0878.1	2411	2.00E-115	174/365(47.67)	353	PHI:2411|Ss-ggt1|A7F946|5180|Sclerotinia_sclerotiorum|Reduced_virulence
Chr02G0884.1	881	3.00E-37	92/344(26.74)	136	PHI:881|MGG_04556|EDJ96020|318829|Magnaporthe_oryzae|Reduced_virulence
Chr02G0885.1	2357	6.00E-90	183/517(35.40)	286	PHI:2357|CYP52X1|E2EAF6|475271|Beauveria_bassiana|Reduced_virulence
Chr02G0897.1	538	7.00E-52	149/588(25.34)	187	PHI:538|FRT1|AAU87358|40559|Botrytis_cinerea|Unaffected_pathogenicity
Chr02G0899.1	1289	0	372/442(84.16)	773	PHI:1289|FGSG_04416|I1RKK5|5518|Gibberella_zeae_(related:_Fusarium_graminearum)|Unaffected_pathogenicity
Chr02G0902.1	502	0	270/501(53.89)	531	PHI:502|ADE4|CAA89133|4932|Saccharomyces_cerevisiae|Reduced_virulence
Chr02G0903.1	1267	0	593/774(76.61)	1184	PHI:1267|FGSG_05586|I1RKK7|5518|Gibberella_zeae_(related:_Fusarium_graminearum)|Unaffected_pathogenicity
Chr02G0908.1	2428	8.00E-31	62/132(46.97)	110	PHI:2428|ypd1|F8UWJ7|5207|Cryptococcus_neoformans|Lethal
Chr02G0912.1	270	1.00E-108	148/178(83.15)	311	PHI:270|RHO1|BAA24262|5476|Candida_albicans|Loss_of_pathogenicity
Chr02G0914.1	2748	3.00E-51	140/495(28.28)	185	PHI:2748|snf1|Q4PF20|5270|Ustilago_maydis|Reduced_virulence
Chr02G0923.1	2970	0	247/351(70.37)	513	PHI:2970|argB|Q4WNW5|746128|Aspergillus_fumigatus|Unaffected_pathogenicity
Chr02G0926.1	1614	0	400/654(61.16)	662	PHI:1614|GzSART1|I1RQD8|5518|Gibberella_zeae_(related:_Fusarium_graminearum)|Unaffected_pathogenicity
Chr02G0931.1	2016	6.00E-98	164/246(66.67)	287	PHI:2016|PTH2|MGG_01099|148305|Magnaporthe_oryzae_(related:_Magnaporthe_grisea)|Loss_of_pathogenicity
Chr02G0932.1	2310	8.00E-06	31/115(26.96)	45.4	PHI:2310|VGB|I2DB61|27337|Verticillium_dahliae|Reduced_virulence
Chr02G0940.1	2256	2.00E-38	99/337(29.38)	140	PHI:2256|Xdh1|Q0UA24|13684|Phaeosphaeria_nodorum_(related:_Stagonospora_nodorum)|Unaffected_pathogenicity
Chr02G0946.1	2527	0	685/1340(51.12)	1202	PHI:2527|SEC31|Q4X0M4|746128|Aspergillus_fumigatus|Mixed_outcome

Chr02G0948.1	1310	2.00E-108	221/496(44.56)	331	PHI:1310|GzbHLH011|I1RQC1|5518|Gibberella_zeae_(related:_Fusarium_graminearum)|Unaffected_pathogenicity
Chr02G0951.1	1047	5.00E-21	91/360(25.28)	89.7	PHI:1047|CTB6|ABK64183|29003|Cercospora_nicotianae|Reduced_virulence
Chr02G0969.1	871	0	341/508(67.13)	707	PHI:871|MGG_12656|EDK01997|318829|Magnaporthe_oryzae|Reduced_virulence
Chr02G0973.1	2694	4.00E-31	100/315(31.75)	124	PHI:2694|rsmB|Q6D000|29471|Pectobacterium_atrosepticum|Mixed_outcome
Chr02G0976.1	419	8.00E-08	75/324(23.15)	50.4	PHI:419|CSH1|AAP93915|5476|Candida_albicans|Reduced_virulence
Chr02G0978.1	3343	1.00E-06	23/53(43.40)	49.3	nasA|PHI:3343|B5SDM5|305|Ralstonia_solanacearum|reduced_virulence_
Chr02G0982.1	882	5.00E-35	94/338(27.81)	140	PHI:882|MGG_04985|EDJ95433|318829|Magnaporthe_oryzae|Reduced_virulence
Chr02G0986.1	270	5.00E-18	54/197(27.41)	76.6	PHI:270|RHO1|BAA24262|5476|Candida_albicans|Loss_of_pathogenicity
Chr02G0998.1	1659	1.00E-172	254/336(75.60)	486	PHI:1659|GzCCCH003|I1S245|5518|Gibberella_zeae_(related:_Fusarium_graminearum)|Unaffected_pathogenicity
Chr02G0999.1	1992	6.00E-33	68/157(43.31)	125	PHI:1992|GzZC307|I1R983|5518|Gibberella_zeae_(related:_Fusarium_graminearum)|Unaffected_pathogenicity
Chr02G1000.1	1482	1.00E-47	129/330(39.09)	165	PHI:1482|GzHMG014|I1RKH7|5518|Gibberella_zeae_(related:_Fusarium_graminearum)|Unaffected_pathogenicity
Chr02G1001.1	1177	0	487/615(79.19)	971	PHI:1177|(Sc_Fpk1)|I1RKH9|5518|Gibberella_zeae_(related:_Fusarium_graminearum)|Reduced_virulence
Chr02G1002.1	2491	2.00E-10	45/145(31.03)	60.5	PHI:2491|FgPTC1|I1RJS9|5518|Gibberella_zeae_(related:_Fusarium_graminearum)|Reduced_virulence
Chr02G1013.1	1475	8.00E-146	281/658(42.71)	442	PHI:1475|GzHMG007|I1RBB7|5518|Gibberella_zeae_(related:_Fusarium_graminearum)|Unaffected_pathogenicity
Chr02G1016.1	538	7.00E-36	160/627(25.52)	140	PHI:538|FRT1|AAU87358|40559|Botrytis_cinerea|Unaffected_pathogenicity
Chr02G1018.1	2058	9.00E-33	85/303(28.05)	132	PHI:2058|LHS1|MGG_06648.5|148305|Magnaporthe_oryzae_(related:_Magnaporthe_grisea)|Reduced_virulence

Chr02G1020.1	1501	7.00E-83	184/401(45.89)	268	PHI:1501|GzHMG033|I1S4R6|5518|Gibberella_zeae_(related:_Fusarium_graminearum)|Unaffected_pathogenicity
Chr02G1022.1	512	6.00E-165	304/834(36.45)	501	PHI:512|CaNAG6|BAB43823|5476|Candida_albicans|Reduced_virulence
Chr02G1031.1	600	4.00E-13	51/155(32.90)	64.3	PHI:600|CBP1|AAF63734|5207|Cryptococcus_neoformans|Reduced_virulence
Chr02G1033.1	2038	1.00E-12	79/316(25.00)	65.5	PHI:2038|Mir1|MGG_02370|148305|Magnaporthe_oryzae_(related:_Magnaporthe_grisea)|Unaffected_pathogenicity
Chr02G1040.1	3274	6.00E-17	129/560(23.04)	82.8	dhbF|PHI:3274|W2E906|147375|Paenibacillus_larvae|unaffected_pathogenicity_
Chr02G1043.1	446	0	380/695(54.68)	700	PHI:446|TBL1||5518|Fusarium_graminearum|Reduced_virulence
Chr02G1045.1	320	2.00E-152	225/380(59.21)	478	PHI:320|SSN6|AAL54912|5476|Candida_albicans|Reduced_virulence
Chr02G1049.1	178	2.00E-20	68/272(25.00)	92.8	PHI:178|pabaA|AAD31929|5085|Aspergillus_fumigatus|Reduced_virulence
Chr02G1050.1	2197	1.00E-16	100/447(22.37)	79	PHI:2197|MoRgs6|XP_364773|148305|Magnaporthe_oryzae_(related:_Magnaporthe_grisea)|Unaffected_pathogenicity
Chr02G1051.1	33	8.00E-73	131/374(35.03)	239	PHI:33|PHR1|AAA68196|5476|Candida_albicans|Reduced_virulence
Chr02G1052.1	1198	8.00E-07	53/211(25.12)	49.7	PHI:1198|(Sc_Ssk2/Ssk22)|I1RA81|5518|Gibberella_zeae_(related:_Fusarium_graminearum)|Reduced_virulence
Chr02G1053.1	538	2.00E-43	148/510(29.02)	161	PHI:538|FRT1|AAU87358|40559|Botrytis_cinerea|Unaffected_pathogenicity
Chr02G1054.1	2329	3.00E-98	172/470(36.60)	311	PHI:2329|CTB4|A0ST42|29003|Cercospora_nicotianae|Reduced_virulence
Chr02G1056.1	2835	5.00E-26	64/180(35.56)	103	PHI:2835|RED2|C3JXE8|5016|Cochliobolus_heterostrophus|Reduced_virulence
Chr02G1057.1	2310	5.00E-19	69/264(26.14)	85.5	PHI:2310|VGB|I2DB61|27337|Verticillium_dahliae|Reduced_virulence
Chr02G1059.1	1469	5.00E-78	285/849(33.57)	282	PHI:1469|GzHMG001|I1R9Z0|5518|Gibberella_zeae_(related:_Fusarium_graminearum)|Unaffected_pathogenicity
Chr02G1063.1	413	2.00E-09	81/364(22.25)	56.6	PHI:413|MPD1|AAT84078|13684|Stagonospora_nodorum|Unaffected_pathogenicity
Chr02G1066.1	1538	0	448/854(52.46)	704	PHI:1538|GzMyb002|I1RA07|5518|Gibberella_zeae_(related:_Fusarium_grami

					nearum)|Reduced_virulence
Chr02G1068.1	277	9.00E-16	62/168(36.90)	74.7	PHI:277|BCP1|AAQ16572|40559|Botrytis_cinerea|Reduced_virulence
Chr02G1069.1	1631	0	394/809(48.70)	648	PHI:1631|GzWing003|I1RBL8|5518|Gibberella_zeae_(related:_Fusarium_graminearum)|Unaffected_pathogenicity
Chr02G1070.1	277	1.00E-27	61/140(43.57)	102	PHI:277|BCP1|AAQ16572|40559|Botrytis_cinerea|Reduced_virulence
Chr02G1071.1	26	7.00E-101	166/464(35.78)	318	PHI:26|CaMDR1|CAA37820|5476|Candida_albicans|Reduced_virulence
Chr02G1073.1	2269	2.00E-08	67/235(28.51)	52	PHI:2269|Mdh1|0|13684|Phaeosphaeria_nodorum_(related:_Stagonospora_nodorum)|Unaffected_pathogenicity
Chr02G1082.1	1260	1.00E-11	60/198(30.30)	66.2	PHI:1260|FGSG_13944|I1RUC7|5518|Gibberella_zeae_(related:_Fusarium_graminearum)|Unaffected_pathogenicity
Chr02G1086.1	378	6.00E-133	176/242(72.73)	399	PHI:378|SIT4|EAK91146|5476|Candida_albicans|Reduced_virulence
Chr02G1091.1	1992	1.00E-14	61/226(26.99)	75.1	PHI:1992|GzZC307|I1R983|5518|Gibberella_zeae_(related:_Fusarium_graminearum)|Unaffected_pathogenicity
Chr02G1094.1	1047	8.00E-24	99/358(27.65)	97.8	PHI:1047|CTB6|ABK64183|29003|Cercospora_nicotianae|Reduced_virulence
Chr02G1095.1	1566	5.00E-55	102/243(41.98)	193	PHI:1566|GzOB006|I1RC95|5518|Gibberella_zeae_(related:_Fusarium_graminearum)|Lethal
Chr02G1096.1	2237	9.00E-52	84/133(63.16)	179	PHI:2237|Rbf1|Q4P9P1|5270|Ustilago_maydis|Loss_of_pathogenicity
Chr02G1100.1	2269	1.00E-23	78/262(29.77)	95.9	PHI:2269|Mdh1|0|13684|Phaeosphaeria_nodorum_(related:_Stagonospora_nodorum)|Unaffected_pathogenicity
Chr02G1105.1	1540	6.00E-78	134/184(72.83)	273	PHI:1540|GzMyb004|I1RCX3|5518|Gibberella_zeae_(related:_Fusarium_graminearum)|Unaffected_pathogenicity
Chr02G1109.1	2020	8.00E-54	125/340(36.76)	200	PHI:2020|Tup1|XP_759427|5270|Ustilago_maydis|Mixed_outcome
Chr02G1111.1	3384	4.00E-54	234/918(25.49)	203	FVEG_12530|PHI:3384|W7N2C1|117187|Fusarium_verticillioides|unaffected_pathogenicity_
Chr02G1113.1	4613	1.00E-29	93/336(27.68)	117	esaN|PHI:4613|D0ZDL7|636|Edwardsiella_tarda|mixed_outcome
Chr02G1117.1	1423	2.00E-116	198/436(45.41)	365	PHI:1423|GzC2H091|I1S2R1|5518|Gibberella_zeae_(related:_Fusarium_grami

					nearum)|Unaffected_pathogenicity
Chr02G1119.1	1799	0	549/829(66.22)	1080	PHI:1799|GzZC114|I1RBN6|5518|Gibberella_zeae_(related:_Fusarium_graminearum)|Unaffected_pathogenicity
Chr02G1121.1	404	5.00E-39	119/452(26.33)	147	PHI:404|PTH11|AAD30436|318829|Magnaporthe_oryzae|Reduced_virulence
Chr02G1122.1	2835	1.00E-50	82/180(45.56)	169	PHI:2835|RED2|C3JXE8|5016|Cochliobolus_heterostrophus|Reduced_virulence
Chr02G1125.1	2927	3.00E-50	143/456(31.36)	180	PHI:2927|lip5|J9N2Z8|59765|Fusarium_oxysporum_f._sp._Lycopersici|Unaffected_pathogenicity
Chr02G1126.1	1399	2.00E-15	62/228(27.19)	76.3	PHI:1399|GzC2H062|I1RV74|5518|Gibberella_zeae_(related:_Fusarium_graminearum)|Unaffected_pathogenicity
Chr02G1127.1	1581	5.00E-73	107/238(44.96)	225	PHI:1581|GzOB021|I1RPX8|5518|Gibberella_zeae_(related:_Fusarium_graminearum)|Unaffected_pathogenicity
Chr02G1129.1	4194	1.00E-49	126/476(26.47)	177	AKT7|PHI:4194|V5XZS6|5599|Alternaria_alternata|increased_virulence_(Hypervirulence)
Chr02G1131.1	201	3.00E-12	52/210(24.76)	62.8	PHI:201|AVR-Pita_(related:_AVR2-YAMO)|AAK00131|318829|Magnaporthe_oryzae|Effector_(plant_avirulence_determinant)
Chr02G1133.1	2510	3.00E-64	161/501(32.14)	219	PHI:2510|msdS/AfmsdC|Q6PWQ1|746128|Aspergillus_fumigatus|Unaffected_pathogenicity
Chr02G1134.1	2279	5.00E-26	70/240(29.17)	102	PHI:2279|Conserved_hypothetical_protein|J9N0G7|5507|Fusarium_oxysporum|Unaffected_pathogenicity
Chr02G1135.1	55	0	906/2593(34.94)	1412	PHI:55|PKS1|AAB08104|5016|Cochliobolus_heterostrophus|Reduced_virulence
Chr02G1136.1	716	7.00E-11	59/215(27.44)	61.6	PHI:716|ZEB1|ABB90284|5518|Fusarium_graminearum|Unaffected_pathogenicity
Chr02G1137.1	4194	1.00E-51	129/463(27.86)	183	AKT7|PHI:4194|V5XZS6|5599|Alternaria_alternata|increased_virulence_(Hypervirulence)

Chr02G1138.1	2357	7.00E-117	206/522(39.46)	357	PHI:2357|CYP52X1|E2EAF6|475271|Beauveria_bassiana|Reduced_virulence
Chr02G1145.1	1499	2.00E-08	63/177(35.59)	52.4	PHI:1499|GzHMG031|I1S242|5518|Gibberella_zeae_(related:_Fusarium_graminearum)|Unaffected_pathogenicity
Chr02G1148.1	1579	0	357/753(47.41)	654	PHI:1579|GzOB019|I1RM25|5518|Gibberella_zeae_(related:_Fusarium_graminearum)|Unaffected_pathogenicity
Chr02G1149.1	2353	0	508/781(65.04)	1003	PHI:2353|AMT1|XP_381310|5518|Gibberella_zeae_(related:_Fusarium_graminearum)|Reduced_virulence
Chr02G1154.1	270	2.00E-69	101/180(56.11)	214	PHI:270|RHO1|BAA24262|5476|Candida_albicans|Loss_of_pathogenicity
Chr02G1169.1	2315	2.00E-41	96/290(33.10)	148	PHI:2315|ChLae1|G4XKY9|5016|Cochliobolus_heterostrophus|Mixed_outcome
Chr02G1174.1	2654	0	862/1840(46.85)	1645	PHI:2654|DUR1,2|Q59VF3|5476|Candida_albicans|Reduced_virulence
Chr02G1176.1	1783	0	328/639(51.33)	626	PHI:1783|GzZC098|I1S2C5|5518|Gibberella_zeae_(related:_Fusarium_graminearum)|Unaffected_pathogenicity
Chr02G1177.1	697	4.00E-28	100/372(26.88)	113	PHI:697|ugt51E1|AAM81358|5022|Leptosphaeria_maculans|Unaffected_pathogenicity
Chr02G1180.1	3370	0	418/853(49.00)	816	vph1|PHI:3370|C4YGV6|5476|Candida_albicans|reduced_virulence_
Chr02G1190.1	2209	0	250/364(68.68)	523	PHI:2209|endo-1,4-beta-xylanase_[GH10_family]|MGG_07868|148305|Magnaporthe_oryzae_(related:_Magnaporthe_grisea)|Reduced_virulence
Chr02G1197.1	404	9.00E-31	85/337(25.22)	121	PHI:404|PTH11|AAD30436|318829|Magnaporthe_oryzae|Reduced_virulence
Chr02G1200.1	1458	8.00E-08	32/108(29.63)	52	PHI:1458|GzAra006|Q4I7F9|5518|Gibberella_zeae_(related:_Fusarium_graminearum)|Unaffected_pathogenicity
Chr02G1201.1	22	9.00E-17	44/125(35.20)	83.2	PHI:22|UAC1|AAA57469|5270|Ustilago_maydis|Loss_of_pathogenicity
Chr02G1212.1	126	2.00E-06	53/201(26.37)	47	PHI:126|SAP5|CAA82923|5476|Candida_albicans|Reduced_virulence
Chr02G1213.1	2058	0	603/1027(58.71)	1122	PHI:2058|LHS1|MGG_06648.5|148305|Magnaporthe_oryzae_(related:_Magnaporthe_grisea)|Reduced_virulence
Chr02G1214.1	2837	1.00E-18	63/233(27.04)	80.1	PHI:2837|OXI1|D2SZX7|5016|Cochliobolus_heterostrophus|Reduced_virulence
Chr02G1216.1	1458	3.00E-12	38/123(30.89)	67.4	PHI:1458|GzAra006|Q4I7F9|5518|Gibberella_zeae_(related:_Fusarium_gramin

					earum)|Unaffected_pathogenicity
Chr02G1218.1	2099	0	526/1169(45.00)	924	PHI:2099|Pmc1|MGG_07971|148305|Magnaporthe_oryzae_(related:_Magnaporthe_grisea)|Mixed_outcome
Chr02G1219.1	1047	1.00E-18	94/357(26.33)	82.8	PHI:1047|CTB6|ABK64183|29003|Cercospora_nicotianae|Reduced_virulence
Chr02G1222.1	2375	4.00E-56	130/371(35.04)	190	PHI:2375|DEP1|D2E9W6|29001|Alternaria_brassicicola|Mixed_outcome
Chr02G1225.1	2038	2.00E-08	77/296(26.01)	52	PHI:2038|Mir1|MGG_02370|148305|Magnaporthe_oryzae_(related:_Magnaporthe_grisea)|Unaffected_pathogenicity
Chr02G1232.1	1857	2.00E-43	153/554(27.62)	166	PHI:1857|GzZC172|I1S3I1|5518|Gibberella_zeae_(related:_Fusarium_graminearum)|Unaffected_pathogenicity
Chr02G1234.1	413	2.00E-18	99/399(24.81)	84.7	PHI:413|MPD1|AAT84078|13684|Stagonospora_nodorum|Unaffected_pathogenicity
Chr02G1235.1	598	2.00E-08	45/146(30.82)	53.5	PHI:598|THIOL|ABB55459|5022|Leptosphaeria_maculans|Reduced_virulence
Chr02G1237.1	407	4.00E-46	87/185(47.03)	155	PHI:407|PBC1|CAB40372|76659|Pyrenopeziza_brassicae|Loss_of_pathogenicity
Chr02G1239.1	2315	7.00E-39	89/292(30.48)	140	PHI:2315|ChLae1|G4XKY9|5016|Cochliobolus_heterostrophus|Mixed_outcome
Chr02G1242.1	1889	7.00E-28	93/265(35.09)	112	PHI:1889|GzZC204|I1RHX4|5518|Gibberella_zeae_(related:_Fusarium_graminearum)|Unaffected_pathogenicity
Chr02G1247.1	167	4.00E-11	42/97(43.30)	63.2	PHI:167|CHIP3|AAF00024|5457|Colletotrichum_gloeosporioides|Unaffected_pathogenicity
Chr02G1250.1	1895	2.00E-49	183/707(25.88)	181	PHI:1895|GzZC210|I1RNU2|5518|Gibberella_zeae_(related:_Fusarium_graminearum)|Unaffected_pathogenicity
Chr02G1253.1	2106	2.00E-48	137/465(29.46)	177	PHI:2106|1-phosphatidylinositol-4,5-bisphosphate_phosphodiesterase_delta_1|MGG_05332|148305|Magnaporthe_oryzae_(related:_Magnaporthe_grisea)|Reduced_virulence
Chr02G1256.1	1555	1.00E-30	116/498(23.29)	122	PHI:1555|GzMyb019|I1RDG6|5518|Gibberella_zeae_(related:_Fusarium_graminearum)|Unaffected_pathogenicity

Chr02G1257.1	3028	7.00E-24	111/405(27.41)	100	Vatr2|PHI:3028|A5CVB7|28447|Clavibacter_michiganensis|reduced_virulence_
Chr02G1258.1	477	6.00E-99	175/525(33.33)	310	PHI:477|ICL1|EAK93039|5476|Candida_albicans|Reduced_virulence
Chr02G1259.1	2393	4.00E-43	130/438(29.68)	158	PHI:2393|Related_to_O-methylsterigmatocystin_oxidoreductase|I1R980|5518|Gibberella_zeae_(related:_Fusarium_graminearum)|Increased_virulence_(Hypervirulence)
Chr02G1261.1	2025	2.00E-07	45/128(35.16)	49.7	PHI:2025|HDL1|G4MQZ9|148305|Magnaporthe_oryzae_(related:_Magnaporthe_grisea)|Unaffected_pathogenicity
Chr02G1262.1	438	3.00E-24	108/443(24.38)	105	PHI:438|BcBOT1_(related:_CND5)|AAQ16576|40559|Botrytis_cinerea|Reduced_virulence
Chr02G1265.1	1555	8.00E-34	124/485(25.57)	131	PHI:1555|GzMyb019|I1RDG6|5518|Gibberella_zeae_(related:_Fusarium_graminearum)|Unaffected_pathogenicity
Chr02G1266.1	1759	9.00E-83	196/603(32.50)	275	PHI:1759|GzZC074|I1RXS2|5518|Gibberella_zeae_(related:_Fusarium_graminearum)|Unaffected_pathogenicity
Chr02G1268.1	1576	2.00E-30	107/400(26.75)	122	PHI:1576|GzOB016|I1RJY5|5518|Gibberella_zeae_(related:_Fusarium_graminearum)|Unaffected_pathogenicity
Chr02G1272.1	2954	6.00E-23	87/310(28.06)	96.3	PHI:2954|TgNST1|B6KLD2|5811|Toxoplasma_gondii|Reduced_virulence
Chr02G1273.1	2240	9.00E-40	119/421(28.27)	149	PHI:2240|Srt1|Q4PBY9|5270|Ustilago_maydis|reduced_virulence
Chr02G1274.1	1880	2.00E-163	276/643(42.92)	484	PHI:1880|GzZC195|I1RJB0|5518|Gibberella_zeae_(related:_Fusarium_graminearum)|Unaffected_pathogenicity
Chr02G1275.1	323	4.00E-07	66/282(23.40)	49.3	PHI:323|VFGLU1|AAO63562|93591|Verticillium_fungicola|Reduced_virulence
Chr02G1277.1	404	6.00E-91	139/347(40.06)	288	PHI:404|PTH11|AAD30436|318829|Magnaporthe_oryzae|Reduced_virulence
Chr02G1281.1	2378	6.00E-08	83/392(21.17)	53.1	PHI:2378|DEP4|D2E9W9|29001|Alternaria_brassicicola|Mixed_outcome
Chr02G1282.1	1687	1.00E-156	277/682(40.62)	468	PHI:1687|GzZC002|I1S584|5518|Gibberella_zeae_(related:_Fusarium_graminearum)|Unaffected_pathogenicity
Chr02G1283.1	226	7.00E-20	75/274(27.37)	91.3	PHI:226|PEX6|AAK16738|5462|Colletotrichum_lagenarium|Loss_of_pathogenicity

Chr02G1291.1	167	3.00E-18	70/228(30.70)	84.3	PHI:167|CHIP3|AAF00024|5457|Colletotrichum_gloeosporioides|Unaffected_pathogenicity
Chr02G1305.1	1832	1.00E-158	231/466(49.57)	469	PHI:1832|GzZC147|I1RH29|5518|Gibberella_zeae_(related:_Fusarium_graminearum)|Unaffected_pathogenicity
Chr02G1308.1	4194	3.00E-54	131/477(27.46)	190	AKT7|PHI:4194|V5XZS6|5599|Alternaria_alternata|increased_virulence_(Hypervirulence)
Chr02G1309.1	1763	1.00E-150	274/707(38.76)	476	PHI:1763|GzZC078|I1RXE8|5518|Gibberella_zeae_(related:_Fusarium_graminearum)|Unaffected_pathogenicity
Chr02G1310.1	543	0	829/1470(56.39)	1720	PHI:543|BCATRD|CAC41639|40559|Botrytis_cinerea|Unaffected_pathogenicity
Chr02G1314.1	1046	1.00E-09	30/77(38.96)	57.8	PHI:1046|CTB5|ABK64182|29003|Cercospora_nicotianae|Reduced_virulence
Chr02G1316.1	922	2.00E-77	195/580(33.62)	259	PHI:922|um03615||5270|Ustilago_maydis|Unaffected_pathogenicity
Chr02G1321.1	2329	3.00E-44	133/464(28.66)	161	PHI:2329|CTB4|A0ST42|29003|Cercospora_nicotianae|Reduced_virulence
Chr02G1322.1	2535	7.00E-19	91/383(23.76)	87	PHI:2535|ERG11B|E9QY26|746128|Aspergillus_fumigatus|Mixed_outcome
Chr02G1326.1	404	4.00E-13	63/248(25.40)	68.6	PHI:404|PTH11|AAD30436|318829|Magnaporthe_oryzae|Reduced_virulence
Chr02G1330.1	479	1.00E-55	115/307(37.46)	182	PHI:479|MEP1|AAQ07436|199306|Coccidioides_posadasii|Reduced_virulence
Chr02G1332.1	404	3.00E-27	92/393(23.41)	111	PHI:404|PTH11|AAD30436|318829|Magnaporthe_oryzae|Reduced_virulence
Chr02G1336.1	511	4.00E-25	124/508(24.41)	106	PHI:511|CaNAG4|EAK93098|5476|Candida_albicans|Reduced_virulence
Chr02G1337.1	1555	2.00E-36	121/468(25.85)	139	PHI:1555|GzMyb019|I1RDG6|5518|Gibberella_zeae_(related:_Fusarium_graminearum)|Unaffected_pathogenicity
Chr02G1338.1	1527	2.00E-37	100/357(28.01)	140	PHI:1527|GzHOMEL040|I1S9A1|5518|Gibberella_zeae_(related:_Fusarium_graminearum)|Lethal
Chr02G1341.1	886	2.00E-06	41/157(26.11)	46.6	PHI:886|MGG_13052|EDK06087|318829|Magnaporthe_oryzae|Reduced_virulence
Chr02G1342.1	876	0	455/1164(39.09)	778	PHI:876|MGG_11671|EDK03349|318829|Magnaporthe_oryzae|Reduced_virulence
Chr02G1346.1	2042	7.00E-13	70/245(28.57)	69.3	PHI:2042|ABC3|Q3Y5V5|148305|Magnaporthe_oryzae_(related:_Magnaporthe

					_grisea)|Loss_of_pathogenicity
Chr02G1354.1	1784	6.00E-77	160/449(35.63)	248	PHI:1784|GzZC099|I1S2A9|5518|Gibberella_zeae_(related:_Fusarium_graminearum)|Unaffected_pathogenicity
Chr02G1361.1	441	1.00E-12	70/311(22.51)	66.2	PHI:441|BTP1|CAE55153|40559|Botrytis_cinerea|Reduced_virulence
Chr02G1365.1	860	6.00E-08	58/205(28.29)	52.8	PHI:860|MSP1|AAX07670|318829|Magnaporthe_oryzae|Reduced_virulence
Chr02G1367.1	4194	2.00E-23	112/466(24.03)	100	AKT7|PHI:4194|V5XZS6|5599|Alternaria_alternata|increased_virulence_(Hypervirulence)
Chr02G1368.1	2835	2.00E-08	46/165(27.88)	52.8	PHI:2835|RED2|C3JXE8|5016|Cochliobolus_heterostrophus|Reduced_virulence
Chr02G1375.1	2476	1.00E-104	171/295(57.97)	311	PHI:2476|CcpelA|G8AA67|27358|Colletotrichum_coccodes|Mixed_outcome
Chr02G1380.1	1555	4.00E-18	74/324(22.84)	84.7	PHI:1555|GzMyb019|I1RDG6|5518|Gibberella_zeae_(related:_Fusarium_graminearum)|Unaffected_pathogenicity
Chr02G1382.1	1555	2.00E-168	238/512(46.48)	491	PHI:1555|GzMyb019|I1RDG6|5518|Gibberella_zeae_(related:_Fusarium_graminearum)|Unaffected_pathogenicity
Chr02G1391.1	3126	6.00E-38	105/353(29.75)	141	argD|PHI:3126|D4I307|552|Erwinia_amylovora|mixed_outcome_
Chr02G1393.1	1769	4.00E-11	113/520(21.73)	62.4	PHI:1769|GzZC084|I1RXB2|5518|Gibberella_zeae_(related:_Fusarium_graminearum)|Unaffected_pathogenicity
Chr02G1394.1	1956	2.00E-21	100/388(25.77)	95.5	PHI:1956|GzZC271|I1RLT2|5518|Gibberella_zeae_(related:_Fusarium_graminearum)|Unaffected_pathogenicity
Chr02G1395.1	1662	3.00E-10	109/507(21.50)	60.5	PHI:1662|GzCCHC002|I1REJ1|5518|Gibberella_zeae_(related:_Fusarium_graminearum)|Unaffected_pathogenicity
Chr02G1397.1	2968	1.00E-43	134/545(24.59)	160	PHI:2968|Hxs1|J9VQA5|5207|Cryptococcus_neoformans|Reduced_virulence
Chr02G1400.1	2570	2.00E-111	188/483(38.92)	344	PHI:2570|CYB2|Q6FM61|5478|Candida_glabrata|Reduced_virulence
Chr02G1402.1	419	5.00E-94	152/331(45.92)	284	PHI:419|CSH1|AAP93915|5476|Candida_albicans|Reduced_virulence
Chr02G1403.1	1759	0	339/639(53.05)	683	PHI:1759|GzZC074|I1RXS2|5518|Gibberella_zeae_(related:_Fusarium_graminearum)|Unaffected_pathogenicity

Chr02G1404.1	144	4.00E-110	170/391(43.48)	334	PHI:144|CHT42|AAC05829|29875|Trichoderma_virens|Reduced_virulence
Chr02G1412.1	465	2.00E-52	127/399(31.83)	198	PHI:465|KIN2|AAB63337|5270|Ustilago_maydis|Reduced_virulence
Chr02G1413.1	1634	1.00E-71	98/126(77.78)	212	PHI:1634|GzWing006|I1RDI9|5518|Gibberella_zeae_(related:_Fusarium_graminearum)|Unaffected_pathogenicity
Chr02G1417.1	24	3.00E-138	287/804(35.70)	429	PHI:24|Avenacinase_gene|AAB09777|29850|Gaeumannomyces_graminis|Loss_of_pathogenicity
Chr02G1419.1	1651	9.00E-114	178/399(44.61)	341	PHI:1651|GzWing023|I1S0U2|5518|Gibberella_zeae_(related:_Fusarium_graminearum)|Unaffected_pathogenicity
Chr02G1420.1	404	2.00E-12	67/291(23.02)	65.9	PHI:404|PTH11|AAD30436|318829|Magnaporthe_oryzae|Reduced_virulence
Chr02G1423.1	2976	0	305/696(43.82)	631	PHI:2976|CgOPT1|C6ZRH8|29905|Colletotrichum_gloeosporioides_f._sp._aeschynomenes|Reduced_virulence
Chr02G1424.1	2895	1.00E-11	63/204(30.88)	63.5	PHI:2895|F-avi4330|B9JV05|373|Agrobacterium_vitis|Loss_of_pathogenicity
Chr02G1425.1	267	2.00E-123	343/1352(25.37)	422	PHI:267|MLT1|AAD51594|5476|Candida_albicans|Reduced_virulence
Chr02G1426.1	191	8.00E-39	127/442(28.73)	152	PHI:191|TOM1|AAB08446|39703|Septoria_lycopersici|Unaffected_pathogenicity
Chr02G1427.1	404	8.00E-19	66/253(26.09)	86.7	PHI:404|PTH11|AAD30436|318829|Magnaporthe_oryzae|Reduced_virulence
Chr02G1431.1	1279	4.00E-29	127/478(26.57)	120	PHI:1279|FGSG_12132|I1RU59|5518|Gibberella_zeae_(related:_Fusarium_graminearum)|Unaffected_pathogenicity
Chr02G1432.1	1279	1.00E-44	160/514(31.13)	166	PHI:1279|FGSG_12132|I1RU59|5518|Gibberella_zeae_(related:_Fusarium_graminearum)|Unaffected_pathogenicity
Chr02G1437.1	267	1.00E-98	360/1453(24.78)	347	PHI:267|MLT1|AAD51594|5476|Candida_albicans|Reduced_virulence
Chr02G1439.1	541	3.00E-72	177/534(33.15)	241	PHI:541|LIP1|AAU87359|332648|Botrytis_cinerea|Unaffected_pathogenicity
Chr02G1440.1	494	9.00E-06	25/68(36.76)	47	PHI:494|PPOA|EAL89712|5085|Aspergillus_fumigatus|Increased_virulence_(Hypervirulence)
Chr02G1441.1	1555	7.00E-30	102/483(21.12)	120	PHI:1555|GzMyb019|I1RDG6|5518|Gibberella_zeae_(related:_Fusarium_graminearum)|Unaffected_pathogenicity

Chr02G1443.1	3387	5.00E-06	51/197(25.89)	45.8	FVEG_12523|PHI:3387|W7MT31|117187|Fusarium_verticillioides|unaffected_pathogenicity_
Chr02G1447.1	1279	2.00E-41	140/491(28.51)	161	PHI:1279|FGSG_12132|I1RU59|5518|Gibberella_zeae_(related:_Fusarium_graminearum)|Unaffected_pathogenicity
Chr02G1448.1	2895	2.00E-07	54/206(26.21)	50.1	PHI:2895|F-avi4330|B9JV05|373|Agrobacterium_vitis|Loss_of_pathogenicity
Chr02G1452.1	2838	7.00E-46	95/280(33.93)	155	PHI:2838|TOX9|D2SZX8|5016|Cochliobolus_heterostrophus|Reduced_virulence
Chr02G1459.1	2315	5.00E-24	55/163(33.74)	95.9	PHI:2315|ChLae1|G4XKY9|5016|Cochliobolus_heterostrophus|Mixed_outcome
Chr02G1463.1	2315	2.00E-51	104/290(35.86)	174	PHI:2315|ChLae1|G4XKY9|5016|Cochliobolus_heterostrophus|Mixed_outcome
Chr02G1464.1	2240	2.00E-31	114/500(22.80)	124	PHI:2240|Srt1|Q4PBY9|5270|Ustilago_maydis|reduced_virulence
Chr02G1466.1	2500	2.00E-18	97/388(25.00)	89	PHI:2500|rcsC|D4I7B7|552|Erwinia_amylovora|Loss_of_pathogenicity
Chr02G1469.1	1555	2.00E-157	225/505(44.55)	464	PHI:1555|GzMyb019|I1RDG6|5518|Gibberella_zeae_(related:_Fusarium_graminearum)|Unaffected_pathogenicity
Chr02G1477.1	3216	9.00E-39	93/226(41.15)	140	MoCDIP4|PHI:3216|G4MVX4|318829|Magnaporthe_oryzae|mixed_outcome_
Chr02G1485.1	2976	0	618/727(85.01)	1308	PHI:2976|CgOPT1|C6ZRH8|29905|Colletotrichum_gloeosporioides_f._sp._aeschynomenes|Reduced_virulence
Chr02G1486.1	3378	2.00E-25	78/232(33.62)	99.4	Plegl1|PHI:3378|G9JLA8|285811|Pyrenochaeta_lycopersici|unaffected_pathogenicity_
Chr02G1489.1	1761	0	337/761(44.28)	601	PHI:1761|GzZC076|I1RXN7|5518|Gibberella_zeae_(related:_Fusarium_graminearum)|Unaffected_pathogenicity
Chr02G1491.1	413	9.00E-141	214/433(49.42)	411	PHI:413|MPD1|AAT84078|13684|Stagonospora_nodorum|Unaffected_pathogenicity
Chr02G1492.1	1159	1.00E-99	167/334(50.00)	334	PHI:1159|MgAtr7|A5H456|54734|Mycosphaerella_graminicola_(related:_Zymoseptoria_tritici)|Chemistry_target
Chr02G1494.1	812	2.00E-77	143/339(42.18)	247	PHI:812|MGG_10702|EDJ94108|318829|Magnaporthe_oryzae|Reduced_virulence

Chr02G1495.1	1741	2.00E-67	214/737(29.04)	234	PHI:1741|GzZC056|I1S780|5518|Gibberella_zeae_(related:_Fusarium_graminearum)|Unaffected_pathogenicity
Chr02G1497.1	2404	1.00E-15	46/101(45.54)	69.3	PHI:2404|SLP_1|G4N906|148305|Magnaporthe_oryzae_(related:_Magnaporthe_grisea)|Reduced_virulence
Chr02G1500.1	3381	1.00E-85	171/452(37.83)	281	FVEG_12533|PHI:3381|W7N2B4|117187|Fusarium_verticillioides|unaffected_pathogenicity_
Chr02G1501.1	441	3.00E-31	106/363(29.20)	120	PHI:441|BTP1|CAE55153|40559|Botrytis_cinerea|Reduced_virulence
Chr02G1502.1	441	1.00E-26	58/176(32.95)	106	PHI:441|BTP1|CAE55153|40559|Botrytis_cinerea|Reduced_virulence
Chr02G1504.1	2976	9.00E-147	247/692(35.69)	449	PHI:2976|CgOPT1|C6ZRH8|29905|Colletotrichum_gloeosporioides_f._sp._aeschynomenes|Reduced_virulence
Chr02G1505.1	1810	1.00E-95	201/592(33.95)	304	PHI:1810|GzZC125|I1RUJ0|5518|Gibberella_zeae_(related:_Fusarium_graminearum)|Unaffected_pathogenicity
Chr02G1508.1	981	9.00E-09	27/65(41.54)	53.9	PHI:981|hopI1|AAL84247|59511|Pseudomonas_syringae|Effector_(plant_avirulence_determinant)
Chr02G1518.1	748	8.00E-46	134/409(32.76)	174	PHI:748|um00446|Not_available|5270|Ustilago_maydis|Unaffected_pathogenicity
Chr02G1519.1	2240	2.00E-39	135/478(28.24)	148	PHI:2240|Srt1|Q4PBY9|5270|Ustilago_maydis|reduced_virulence
Chr02G1523.1	419	1.00E-30	93/314(29.62)	117	PHI:419|CSH1|AAP93915|5476|Candida_albicans|Reduced_virulence
Chr02G1525.1	552	5.00E-12	118/482(24.48)	65.9	PHI:552|BcLCC2|AAK77953|40559|Botrytis_cinerea|Unaffected_pathogenicity
Chr02G1530.1	1907	0	508/775(65.55)	1028	PHI:1907|GzZC222|I1RHJ0|5518|Gibberella_zeae_(related:_Fusarium_graminearum)|Unaffected_pathogenicity
Chr02G1533.1	3272	5.00E-36	169/568(29.75)	140	glpD|PHI:3272|Q8CWG4|992166|Yersinia_pestis|unaffected_pathogenicity_
Chr02G1536.1	3028	3.00E-13	101/418(24.16)	68.6	Vatr2|PHI:3028|A5CVB7|28447|Clavibacter_michiganensis|reduced_virulence_
Chr02G1539.1	2279	6.00E-48	93/277(33.57)	162	PHI:2279|Conserved_hypothetical_protein|J9N0G7|5507|Fusarium_oxysporum|Unaffected_pathogenicity
Chr02G1540.1	441	7.00E-26	97/384(25.26)	105	PHI:441|BTP1|CAE55153|40559|Botrytis_cinerea|Reduced_virulence

Chr02G1541.1	2247	3.00E-24	91/280(32.50)	97.8	PHI:2247|Sch1|EAT82552|13684|Phaeosphaeria_nodorum_(related:_Stagonospora_nodorum)|Unaffected_pathogenicity
Chr02G1544.1	1555	3.00E-17	76/323(23.53)	82	PHI:1555|GzMyb019|I1RDG6|5518|Gibberella_zeae_(related:_Fusarium_graminearum)|Unaffected_pathogenicity
Chr02G1547.1	404	5.00E-18	90/383(23.50)	84.3	PHI:404|PTH11|AAD30436|318829|Magnaporthe_oryzae|Reduced_virulence
Chr02G1552.1	2562	2.00E-92	195/561(34.76)	310	PHI:2562|Cwt1|Q59M50|5476|Candida_albicans|Reduced_virulence
Chr02G1557.1	1047	3.00E-26	105/373(28.15)	105	PHI:1047|CTB6|ABK64183|29003|Cercospora_nicotianae|Reduced_virulence
Chr02G1561.1	2025	0	495/788(62.82)	972	PHI:2025|HDL1|G4MQZ9|148305|Magnaporthe_oryzae_(related:_Magnaporthe_grisea)|Unaffected_pathogenicity
Chr02G1563.1	1961	0	501/855(58.60)	915	PHI:1961|GzZC276|I1RD74|5518|Gibberella_zeae_(related:_Fusarium_graminearum)|Unaffected_pathogenicity
Chr02G1565.1	2978	6.00E-43	94/260(36.15)	146	PHI:2978|MoCel12A|G4N5V2|148305|Magnaporthe_oryzae|Unaffected_pathogenicity
Chr02G1566.1	1355	1.00E-17	121/445(27.19)	81.6	PHI:1355|GzC2H015|I1RD84|5518|Gibberella_zeae_(related:_Fusarium_graminearum)|Unaffected_pathogenicity
Chr02G1567.1	2543	2.00E-07	35/125(28.00)	51.2	PHI:2543|GCD6|Q4WLS1|746128|Aspergillus_fumigatus|Mixed_outcome
Chr02G1570.1	2088	6.00E-102	169/365(46.30)	306	PHI:2088|Moatg27|MGG_02386|148305|Magnaporthe_oryzae_(related:_Magnaporthe_grisea)|Unaffected_pathogenicity
Chr02G1572.1	69	2.00E-59	89/163(54.60)	186	PHI:69|CUTA|CAA93255|40559|Botrytis_cinerea|Unaffected_pathogenicity
Chr02G1574.1	3126	8.00E-95	154/395(38.99)	293	argD|PHI:3126|D4I307|552|Erwinia_amylovora|mixed_outcome_
Chr02G1577.1	716	8.00E-10	63/234(26.92)	58.2	PHI:716|ZEB1|ABB90284|5518|Fusarium_graminearum|Unaffected_pathogenicity
Chr02G1583.1	2020	5.00E-55	123/346(35.55)	203	PHI:2020|Tup1|XP_759427|5270|Ustilago_maydis|Mixed_outcome
Chr02G1589.1	2032	4.00E-37	134/511(26.22)	141	PHI:2032|VTL1|G4NGA7|148305|Magnaporthe_oryzae_(related:_Magnaporthe_grisea)|Unaffected_pathogenicity
Chr02G1591.1	2968	5.00E-42	119/456(26.10)	155	PHI:2968|Hxs1|J9VQA5|5207|Cryptococcus_neoformans|Reduced_virulence

Chr02G1596.1	544	2.00E-10	68/282(24.11)	60.1	PHI:544|BCMFS1|AAF64435|332648|Botrytis_cinerea|Unaffected_pathogenicity
Chr02G1597.1	2968	6.00E-47	121/405(29.88)	167	PHI:2968|Hxs1|J9VQA5|5207|Cryptococcus_neoformans|Reduced_virulence
Chr02G1601.1	922	1.00E-63	197/630(31.27)	222	PHI:922|um03615||5270|Ustilago_maydis|Unaffected_pathogenicity
Chr02G1602.1	1320	0	360/577(62.39)	600	PHI:1320|ZIF1|I1RD66|5518|Gibberella_zeae_(related:_Fusarium_graminearum)|Reduced_virulence
Chr02G1604.1	1420	8.00E-10	90/417(21.58)	61.2	PHI:1420|GzC2H088|I1S172|5518|Gibberella_zeae_(related:_Fusarium_graminearum)|Unaffected_pathogenicity
Chr02G1609.1	1662	7.00E-22	104/429(24.24)	97.1	PHI:1662|GzCCHC002|I1REJ1|5518|Gibberella_zeae_(related:_Fusarium_graminearum)|Unaffected_pathogenicity
Chr02G1611.1	2357	4.00E-12	47/140(33.57)	65.9	PHI:2357|CYP52X1|E2EAF6|475271|Beauveria_bassiana|Reduced_virulence
Chr02G1612.1	2022	4.00E-20	73/266(27.44)	84.7	PHI:2022|BUF1|MGG_02252|148305|Magnaporthe_oryzae_(related:_Magnaporthe_grisea)|Loss_of_pathogenicity
Chr02G1613.1	2968	4.00E-34	117/457(25.60)	133	PHI:2968|Hxs1|J9VQA5|5207|Cryptococcus_neoformans|Reduced_virulence
Chr02G1614.1	2247	6.00E-09	61/208(29.33)	53.5	PHI:2247|Sch1|EAT82552|13684|Phaeosphaeria_nodorum_(related:_Stagonospora_nodorum)|Unaffected_pathogenicity
Chr02G1616.1	2247	3.00E-21	77/200(38.50)	88.2	PHI:2247|Sch1|EAT82552|13684|Phaeosphaeria_nodorum_(related:_Stagonospora_nodorum)|Unaffected_pathogenicity
Chr02G1617.1	3364	9.00E-06	35/103(33.98)	42.4	MMAR_1663|PHI:3364|B2HHI1|1781|Mycobacterium_marinum|reduced_virulence_
Chr02G1622.1	2117	2.00E-52	133/421(31.59)	182	PHI:2117|SPM1|P58371|148305|Magnaporthe_oryzae_(related:_Magnaporthe_grisea)|Reduced_virulence
Chr02G1624.1	3381	5.00E-19	118/493(23.94)	87.8	FVEG_12533|PHI:3381|W7N2B4|117187|Fusarium_verticillioides|unaffected_pathogenicity_
Chr02G1625.1	2654	4.00E-06	43/125(34.40)	48.1	PHI:2654|DUR1,2|Q59VF3|5476|Candida_albicans|Reduced_virulence
Chr02G1626.1	1555	0	265/489(54.19)	561	PHI:1555|GzMyb019|I1RDG6|5518|Gibberella_zeae_(related:_Fusarium_grami

					nearum)|Unaffected_pathogenicity
Chr02G1631.1	2377	1.00E-75	161/530(30.38)	250	PHI:2377|DEP3|D2E9W8|29001|Alternaria_brassicicola|Mixed_outcome
Chr02G1632.1	3417	0	579/998(58.02)	1139	Ohmm|PHI:3417|W5ZQ93|176275|Beauveria_bassiana|increased_virulence_
Chr02G1636.1	1960	0	565/1017(55.56)	991	PHI:1960|GzZC275|I1RDE2|5518|Gibberella_zeae_(related:_Fusarium_graminearum)|Unaffected_pathogenicity
Chr02G1638.1	1735	2.00E-17	100/436(22.94)	82.8	PHI:1735|GzZC050|I1S6X4|5518|Gibberella_zeae_(related:_Fusarium_graminearum)|Unaffected_pathogenicity
Chr02G1644.1	2968	7.00E-35	122/464(26.29)	134	PHI:2968|Hxs1|J9VQA5|5207|Cryptococcus_neoformans|Reduced_virulence
Chr02G1646.1	1750	5.00E-155	265/562(47.15)	463	PHI:1750|GzZC065|I1S487|5518|Gibberella_zeae_(related:_Fusarium_graminearum)|Unaffected_pathogenicity
Chr02G1648.1	2038	1.00E-171	247/310(79.68)	481	PHI:2038|Mir1|MGG_02370|148305|Magnaporthe_oryzae_(related:_Magnaporthe_grisea)|Unaffected_pathogenicity
Chr02G1657.1	3176	0	599/1269(47.20)	908	MoSak1|PHI:3176|G4MNZ0|318829|Magnaporthe_oryzae|mixed_outcome_
Chr02G1658.1	872	1.00E-80	162/444(36.49)	284	PHI:872|MGG_03284|EDK00201|318829|Magnaporthe_oryzae|Reduced_virulence
Chr02G1659.1	143	3.00E-09	29/99(29.29)	55.8	PHI:143|CHT|AAA33353|5563|Gloeocercospora_sorghi|Unaffected_pathogenicity
Chr02G1669.1	332	1.00E-23	110/365(30.14)	107	PHI:332|CAC1|BAD04045|5462|Colletotrichum_lagenarium|Loss_of_pathogenicity
Chr02G1672.1	2441	1.00E-142	222/424(52.36)	419	PHI:2441|MgAlg2|F9XJV5|1047171|Mycosphaerella_graminicola_(related:_Zymoseptoria_triticii)|Loss_of_pathogenicity
Chr02G1675.1	413	1.00E-18	65/231(28.14)	85.5	PHI:413|MPD1|AAT84078|13684|Stagonospora_nodorum|Unaffected_pathogenicity
Chr02G1676.1	225	1.00E-58	106/241(43.98)	196	PHI:225|PEP5|AAK16922|140110|Nectria_haematococca_(related:_Fusarium_solani)|Reduced_virulence
Chr02G1679.1	404	2.00E-31	76/253(30.04)	122	PHI:404|PTH11|AAD30436|318829|Magnaporthe_oryzae|Reduced_virulence

Chr02G1680.1	1992	6.00E-10	46/174(26.44)	56.6	PHI:1992|GzZC307|I1R983|5518|Gibberella_zeae_(related:_Fusarium_graminearum)|Unaffected_pathogenicity
Chr02G1682.1	2802	3.00E-15	67/278(24.10)	74.3	PHI:2802|3hnr|B2ZRQ4|93612|Setosphaeria_turcica|Unaffected_pathogenicity
Chr02G1683.1	2357	5.00E-15	127/559(22.72)	75.1	PHI:2357|CYP52X1|E2EAF6|475271|Beauveria_bassiana|Reduced_virulence
Chr02G1688.1	3028	1.00E-12	90/364(24.73)	66.6	Vatr2|PHI:3028|A5CVB7|28447|Clavibacter_michiganensis|reduced_virulence_
Chr02G1689.1	3226	4.00E-123	184/331(55.59)	362	pnl1|PHI:3226|T2C7K6|36651|Penicillium_digitatum|reduced_virulence_
Chr02G1690.1	876	0	459/1162(39.50)	781	PHI:876|MGG_11671|EDK03349|318829|Magnaporthe_oryzae|Reduced_virulence
Chr02G1691.1	886	1.00E-06	46/183(25.14)	47	PHI:886|MGG_13052|EDK06087|318829|Magnaporthe_oryzae|Reduced_virulence
Chr02G1694.1	4618	1.00E-82	139/339(41.00)	258	PcPL15|PHI:4618|A0A0D3LX64|4784|Phytophthora_capsici|mixed_outcome
Chr02G1696.1	3126	5.00E-25	115/427(26.93)	103	argD|PHI:3126|D4I307|552|Erwinia_amylovora|mixed_outcome_
Chr02G1697.1	1891	0	453/787(57.56)	915	PHI:1891|GzZC206|I1RRM0|5518|Gibberella_zeae_(related:_Fusarium_graminearum)|Unaffected_pathogenicity
Chr02G1700.1	144	2.00E-17	65/257(25.29)	84	PHI:144|CHT42|AAC05829|29875|Trichoderma_virens|Reduced_virulence
Chr02G1702.1	2105	0	802/1176(68.20)	1433	PHI:2105|Calcium_permease|MGG_01381|148305|Magnaporthe_oryzae_(related:_Magnaporthe_grisea)|Mixed_outcome
Chr02G1703.1	1566	6.00E-108	171/372(45.97)	324	PHI:1566|GzOB006|I1RC95|5518|Gibberella_zeae_(related:_Fusarium_graminearum)|Lethal
Chr02G1704.1	3594	6.00E-56	113/275(41.09)	208	Ptp2|PHI:3594|H6VMG0|5207|Cryptococcus_neoformans|mixed_outcome_
Chr02G1705.1	3630	9.00E-55	130/384(33.85)	203	Rv2467|PHI:3630|L7N655|1773|Mycobacterium_tuberculosis|increased_virulence_
Chr02G1707.1	1620	0	781/1201(65.03)	1414	PHI:1620|GzJUM002|I1RD69|5518|Gibberella_zeae_(related:_Fusarium_graminearum)|Unaffected_pathogenicity
Chr02G1709.1	2844	8.00E-18	49/189(25.93)	78.2	PHI:2844|BRM2|O93802|5599|Alternaria_alternata|Unaffected_pathogenicity
Chr02G1713.1	211	1.00E-13	77/309(24.92)	71.2	PHI:211|CaTUP1|AAB63195|5476|Candida_albicans|Reduced_virulence

Chr02G1714.1	281	2.00E-147	184/311(59.16)	421	PHI:281|CDC10|CAA81090|5476|Candida_albicans|Reduced_virulence
Chr02G1716.1	434	9.00E-174	234/428(54.67)	499	PHI:434|GEL2|EAL88984|5085|Aspergillus_fumigatus|Reduced_virulence
Chr02G1717.1	2387	0	444/489(90.80)	920	PHI:2387|ACL2|I1RPR1|5518|Gibberella_zeae_(related:_Fusarium_graminearum)|Loss_of_pathogenicity
Chr02G1718.1	2386	0	365/410(89.02)	763	PHI:2386|ACL1|I1S7N4|5518|Gibberella_zeae_(related:_Fusarium_graminearum)|Loss_of_pathogenicity
Chr02G1724.1	1498	1.00E-64	178/507(35.11)	225	PHI:1498|GzHMG030|I1S1Y4|5518|Gibberella_zeae_(related:_Fusarium_graminearum)|Lethal
Chr02G1727.1	3381	1.00E-66	163/473(34.46)	229	FVEG_12533|PHI:3381|W7N2B4|117187|Fusarium_verticillioides|unaffected_pathogenicity_
Chr02G1731.1	716	0	267/545(48.99)	545	PHI:716|ZEB1|ABB90284|5518|Fusarium_graminearum|Unaffected_pathogenicity
Chr02G1732.1	112	3.00E-87	162/460(35.22)	276	PHI:112|MAK1|AAC49410|140110|Nectria_haematococca_(related:_Fusarium_solani)|Reduced_virulence
Chr02G1734.1	2802	9.00E-10	39/161(24.22)	53.1	PHI:2802|3hnr|B2ZRQ4|93612|Setosphaeria_turcica|Unaffected_pathogenicity
Chr02G1736.1	2983	3.00E-07	86/344(25.00)	50.8	PHI:2983|MGG_06279.6|G4N8E3|148305|Magnaporthe_oryzae|Reduced_virulence
Chr02G1737.1	1653	8.00E-62	85/119(71.43)	187	PHI:1653|GzWing026|I1S2A0|5518|Gibberella_zeae_(related:_Fusarium_graminearum)|Unaffected_pathogenicity
Chr02G1740.1	1458	7.00E-40	127/464(27.37)	152	PHI:1458|GzAra006|Q4I7F9|5518|Gibberella_zeae_(related:_Fusarium_graminearum)|Unaffected_pathogenicity
Chr02G1746.1	243	1.00E-153	253/588(43.03)	474	PHI:243|CHIP6|AAD00894|5457|Colletotrichum_gloeosporioides|Reduced_virulence
Chr02G1748.1	716	4.00E-81	193/603(32.01)	266	PHI:716|ZEB1|ABB90284|5518|Fusarium_graminearum|Unaffected_pathogenicity
Chr02G1750.1	1566	2.00E-37	91/244(37.30)	143	PHI:1566|GzOB006|I1RC95|5518|Gibberella_zeae_(related:_Fusarium_gramin

					earum)|Lethal
Chr02G1751.1	319	3.00E-11	58/227(25.55)	65.9	PHI:319|SQL2|AAO19638|5270|Ustilago_maydis|Reduced_virulence
Chr02G1760.1	812	2.00E-79	138/358(38.55)	252	PHI:812|MGG_10702|EDJ94108|318829|Magnaporthe_oryzae|Reduced_virulence
Chr02G1763.1	1468	1.00E-35	66/105(62.86)	120	PHI:1468|GzCCAAT008|Q4HTT1|5518|Gibberella_zeae_(related:_Fusarium_graminearum)|Unaffected_pathogenicity
Chr02G1767.1	479	2.00E-123	178/282(63.12)	357	PHI:479|MEP1|AAQ07436|199306|Coccidioides_posadasii|Reduced_virulence
Chr02G1771.1	3381	7.00E-47	129/444(29.05)	171	FVEG_12533|PHI:3381|W7N2B4|117187|Fusarium_verticillioides|unaffected_pathogenicity_
Chr02G1772.1	2378	2.00E-69	174/598(29.10)	236	PHI:2378|DEP4|D2E9W9|29001|Alternaria_brassicicola|Mixed_outcome
Chr02G1773.1	441	1.00E-10	63/266(23.68)	60.1	PHI:441|BTP1|CAE55153|40559|Botrytis_cinerea|Reduced_virulence
Chr02G1774.1	464	2.00E-23	100/349(28.65)	103	PHI:464|KIN1|AAB63336|5270|Ustilago_maydis|Unaffected_pathogenicity
Chr02G1777.1	441	3.00E-08	41/162(25.31)	49.3	PHI:441|BTP1|CAE55153|40559|Botrytis_cinerea|Reduced_virulence
Chr02G1779.1	2266	1.00E-86	180/609(29.56)	283	PHI:2266|Ptr2|0|13684|Phaeosphaeria_nodorum_(related:_Stagonospora_nodorum)|Unaffected_pathogenicity
Chr02G1780.1	2518	1.00E-06	26/98(26.53)	46.6	PHI:2518|MET16|Q4WWN9|746128|Aspergillus_fumigatus|Lethal
Chr02G1781.1	441	5.00E-25	73/251(29.08)	102	PHI:441|BTP1|CAE55153|40559|Botrytis_cinerea|Reduced_virulence
Chr02G1783.1	2022	1.00E-09	56/191(29.32)	55.5	PHI:2022|BUF1|MGG_02252|148305|Magnaporthe_oryzae_(related:_Magnaporthe_grisea)|Loss_of_pathogenicity
Chr02G1789.1	362	5.00E-52	181/665(27.22)	191	PHI:362|LYSF|CAC48042|5085|Aspergillus_fumigatus|Loss_of_pathogenicity
Chr02G1790.1	2240	5.00E-20	119/493(24.14)	90.5	PHI:2240|Srt1|Q4PBY9|5270|Ustilago_maydis|reduced_virulence
Chr02G1795.1	1581	1.00E-61	100/229(43.67)	198	PHI:1581|GzOB021|I1RPX8|5518|Gibberella_zeae_(related:_Fusarium_graminearum)|Unaffected_pathogenicity
Chr02G1800.1	2968	2.00E-50	128/484(26.45)	180	PHI:2968|Hxs1|J9VQA5|5207|Cryptococcus_neoformans|Reduced_virulence
Chr02G1805.1	716	3.00E-103	202/580(34.83)	327	PHI:716|ZEB1|ABB90284|5518|Fusarium_graminearum|Unaffected_pathogenicity

Chr02G1806.1	441	4.00E-11	69/269(25.65)	61.2	PHI:441|BTP1|CAE55153|40559|Botrytis_cinerea|Reduced_virulence
Chr02G1807.1	1974	5.00E-20	119/481(24.74)	91.7	PHI:1974|GzZC289|I1RQN6|5518|Gibberella_zeae_(related:_Fusarium_graminearum)|Unaffected_pathogenicity
Chr02G1809.1	1752	6.00E-39	138/553(24.95)	151	PHI:1752|GzZC067|I1RZR8|5518|Gibberella_zeae_(related:_Fusarium_graminearum)|Unaffected_pathogenicity
Chr02G1810.1	200	2.00E-11	75/349(21.49)	62.8	PHI:200|ARG1|BAB40769|5507|Fusarium_oxysporum|Reduced_virulence
Chr02G1811.1	1662	4.00E-44	122/438(27.85)	166	PHI:1662|GzCCHC002|I1REJ1|5518|Gibberella_zeae_(related:_Fusarium_graminearum)|Unaffected_pathogenicity
Chr02G1812.1	4618	1.00E-83	162/404(40.10)	262	PcPL15|PHI:4618|A0A0D3LX64|4784|Phytophthora_capsici|mixed_outcome
Chr02G1813.1	1260	2.00E-08	35/103(33.98)	54.7	PHI:1260|FGSG_13944|I1RUC7|5518|Gibberella_zeae_(related:_Fusarium_graminearum)|Unaffected_pathogenicity
Chr02G1817.1	796	5.00E-131	178/214(83.18)	369	PHI:796|MGG_02423|EDJ98620|318829|Magnaporthe_oryzae|Reduced_virulence
Chr02G1822.1	2345	0	601/851(70.62)	1174	PHI:2345|FSR1|Q2TJF8|117187|Gibberella_moniliformis_(related:_Fusarium_verticillioides)|Loss_of_pathogenicity
Chr02G1825.1	547	2.00E-109	153/310(49.35)	328	PHI:547|CEL5A|AAT40313|40559|Botrytis_cinerea|Unaffected_pathogenicity
Chr02G1829.1	1662	2.00E-39	99/322(30.75)	151	PHI:1662|GzCCHC002|I1REJ1|5518|Gibberella_zeae_(related:_Fusarium_graminearum)|Unaffected_pathogenicity
Chr02G1830.1	1902	4.00E-85	244/841(29.01)	291	PHI:1902|GzZC217|I1RJS7|5518|Gibberella_zeae_(related:_Fusarium_graminearum)|Unaffected_pathogenicity
Chr02G1831.1	3329	1.00E-43	95/278(34.17)	153	Ss-oah1|PHI:3329|A7ESB3|5180|Sclerotinia_sclerotiorum|mixed_outcome_
Chr02G1834.1	3663	1.00E-20	98/410(23.90)	91.3	PD0681|PHI:3663|Q87DK1|2371|Xylella_fastidiosa|increased_virulence_(Hypervirulence)
Chr02G1837.1	1975	2.00E-59	135/441(30.61)	204	PHI:1975|GzZC290|I1RQI0|5518|Gibberella_zeae_(related:_Fusarium_graminearum)|Unaffected_pathogenicity
Chr02G1842.1	541	0	307/529(58.03)	637	PHI:541|LIP1|AAU87359|332648|Botrytis_cinerea|Unaffected_pathogenicity

Chr02G1843.1	1974	7.00E-90	184/587(31.35)	290	PHI:1974|GzZC289|I1RQN6|5518|Gibberella_zeae_(related:_Fusarium_graminearum)|Unaffected_pathogenicity
Chr02G1846.1	1685	4.00E-11	64/214(29.91)	61.6	PHI:1685|GzRad003|I1S5Z3|5518|Gibberella_zeae_(related:_Fusarium_graminearum)|Unaffected_pathogenicity
Chr02G1852.1	3457	3.00E-19	113/447(25.28)	87.8	PHO84|PHI:3457|J9VMW8|5207|Cryptococcus_neoformans|mixed_outcome_
Chr02G1853.1	2117	2.00E-54	145/439(33.03)	189	PHI:2117|SPM1|P58371|148305|Magnaporthe_oryzae_(related:_Magnaporthe_grisea)|Reduced_virulence
Chr02G1855.1	2976	9.00E-57	170/714(23.81)	205	PHI:2976|CgOPT1|C6ZRH8|29905|Colletotrichum_gloeosporioides_f._sp._aeschynomenes|Reduced_virulence
Chr02G1858.1	1458	1.00E-07	31/92(33.70)	49.3	PHI:1458|GzAra006|Q4I7F9|5518|Gibberella_zeae_(related:_Fusarium_graminearum)|Unaffected_pathogenicity
Chr02G1860.1	4194	2.00E-29	130/520(25.00)	119	AKT7|PHI:4194|V5XZS6|5599|Alternaria_alternata|increased_virulence_(Hypervirulence)
Chr02G1861.1	886	2.00E-08	62/277(22.38)	54.3	PHI:886|MGG_13052|EDK06087|318829|Magnaporthe_oryzae|Reduced_virulence
Chr02G1862.1	876	3.00E-83	165/432(38.19)	277	PHI:876|MGG_11671|EDK03349|318829|Magnaporthe_oryzae|Reduced_virulence
Chr08G0004.1	211	2.00E-37	94/238(39.50)	145	PHI:211|CaTUP1|AAB63195|5476|Candida_albicans|Reduced_virulence
Chr08G0008.1	817	7.00E-19	55/158(34.81)	86.3	PHI:817|MGG_12252|EDK03444|318829|Magnaporthe_oryzae|Reduced_virulence
Chr08G0012.1	876	0	484/1227(39.45)	802	PHI:876|MGG_11671|EDK03349|318829|Magnaporthe_oryzae|Reduced_virulence
Chr08G0014.1	1260	3.00E-36	112/353(31.73)	144	PHI:1260|FGSG_13944|I1RUC7|5518|Gibberella_zeae_(related:_Fusarium_graminearum)|Unaffected_pathogenicity
Chr08G0017.1	267	6.00E-63	158/581(27.19)	223	PHI:267|MLT1|AAD51594|5476|Candida_albicans|Reduced_virulence
Chr08G0018.1	1260	2.00E-09	39/116(33.62)	53.1	PHI:1260|FGSG_13944|I1RUC7|5518|Gibberella_zeae_(related:_Fusarium_gr

					aminearum)|Unaffected_pathogenicity
Chr08G0025.1	1977	0	359/701(51.21)	648	PHI:1977|GzZC292|I1RLM4|5518|Gibberella_zeae_(related:_Fusarium_graminearum)|Unaffected_pathogenicity
Chr08G0026.1	1763	2.00E-177	285/640(44.53)	524	PHI:1763|GzZC078|I1RXE8|5518|Gibberella_zeae_(related:_Fusarium_graminearum)|Unaffected_pathogenicity
Chr08G0027.1	3236	5.00E-28	87/301(28.90)	114	Aph1|PHI:3236|J9VHR6|5207|Cryptococcus_neoformans|mixed_outcome_
Chr08G0028.1	404	9.00E-25	80/274(29.20)	103	PHI:404|PTH11|AAD30436|318829|Magnaporthe_oryzae|Reduced_virulence
Chr08G0037.1	1398	0	507/897(56.52)	714	PHI:1398|GzC2H061|I1RV65|5518|Gibberella_zeae_(related:_Fusarium_graminearum)|Unaffected_pathogenicity
Chr08G0042.1	2315	3.00E-36	89/267(33.33)	130	PHI:2315|ChLae1|G4XKY9|5016|Cochliobolus_heterostrophus|Mixed_outcome
Chr08G0044.1	2968	7.00E-33	123/528(23.30)	128	PHI:2968|Hxs1|J9VQA5|5207|Cryptococcus_neoformans|Reduced_virulence
Chr08G0045.1	1399	7.00E-132	278/701(39.66)	407	PHI:1399|GzC2H062|I1RV74|5518|Gibberella_zeae_(related:_Fusarium_graminearum)|Unaffected_pathogenicity
Chr08G0049.1	404	5.00E-14	66/261(25.29)	69.7	PHI:404|PTH11|AAD30436|318829|Magnaporthe_oryzae|Reduced_virulence
Chr08G0051.1	1812	4.00E-114	243/647(37.56)	357	PHI:1812|GzZC127|I1RV71|5518|Gibberella_zeae_(related:_Fusarium_graminearum)|Unaffected_pathogenicity
Chr08G0053.1	1234	0	1995/2439(81.80)	4123	PHI:1234|FGSG_07812|I1RV69|5518|Gibberella_zeae_(related:_Fusarium_graminearum)|Lethal
Chr08G0059.1	1048	6.00E-55	137/432(31.71)	189	PHI:1048|CTB7|ABK64184|29003|Cercospora_nicotianae|Reduced_virulence
Chr08G0064.1	1284	3.00E-21	73/272(26.84)	90.5	PHI:1284|FGSG_13509|I1RI73|5518|Gibberella_zeae_(related:_Fusarium_graminearum)|Unaffected_pathogenicity
Chr08G0071.1	257	4.00E-98	159/287(55.40)	292	PHI:257|GAS2|AAF74764|318829|Magnaporthe_oryzae|Reduced_virulence
Chr08G0072.1	1664	2.00E-09	39/94(41.49)	57.8	PHI:1664|GzCCHC004|I1RMT3|5518|Gibberella_zeae_(related:_Fusarium_graminearum)|Unaffected_pathogenicity
Chr08G0073.1	2382	3.00E-119	169/310(54.52)	347	PHI:2382|Upa2|Q6QIY0|5270|Ustilago_maydis|Unaffected_pathogenicity
Chr08G0075.1	1294	4.00E-141	243/436(55.73)	411	PHI:1294|GzAPSES002|I1RMT5|5518|Gibberella_zeae_(related:_Fusarium_gr

					aminearum)|Unaffected_pathogenicity
Chr08G0081.1	769	7.00E-91	127/154(82.47)	265	PHI:769|CPSOD1|CAC50073|5111|Claviceps_purpurea|Unaffected_pathogenicity
Chr08G0082.1	1530	0	649/888(73.09)	1343	PHI:1530|Gzsnd|I1RWP4|5518|Gibberella_zeae_(related:_Fusarium_graminearum)|Unaffected_pathogenicity
Chr08G0085.1	2147	2.00E-08	43/145(29.66)	53.5	PHI:2147|Erl1|G5EH97|148305|Magnaporthe_oryzae_(related:_Magnaporthe_grisea)|Reduced_virulence
Chr08G0086.1	716	1.00E-90	186/591(31.47)	291	PHI:716|ZEB1|ABB90284|5518|Fusarium_graminearum|Unaffected_pathogenicity
Chr08G0090.1	2969	0	602/914(65.86)	1201	PHI:2969|argEF|Q4WCZ1|746128|Aspergillus_fumigatus|Reduced_virulence
Chr08G0093.1	2357	1.00E-107	201/497(40.44)	334	PHI:2357|CYP52X1|E2EAF6|475271|Beauveria_bassiana|Reduced_virulence
Chr08G0095.1	2090	1.00E-112	234/443(52.82)	338	PHI:2090|Moatg29|MGG_02790|148305|Magnaporthe_oryzae_(related:_Magnaporthe_grisea)|Unaffected_pathogenicity
Chr08G0096.1	2393	1.00E-21	112/468(23.93)	95.1	PHI:2393|Related_to_O-methylsterigmatocystin_oxidoreductase|I1R980|5518|Gibberella_zeae_(related:_Fusarium_graminearum)|Increased_virulence_(Hypervirulence)
Chr08G0097.1	1790	6.00E-88	193/627(30.78)	297	PHI:1790|GzZC105|I1RVT7|5518|Gibberella_zeae_(related:_Fusarium_graminearum)|Lethal
Chr08G0098.1	1363	3.00E-46	97/284(34.15)	159	PHI:1363|TRI15|I1RJ74|5518|Gibberella_zeae_(related:_Fusarium_graminearum)|Unaffected_pathogenicity
Chr08G0099.1	1566	4.00E-06	54/195(27.69)	47	PHI:1566|GzOB006|I1RC95|5518|Gibberella_zeae_(related:_Fusarium_graminearum)|Lethal
Chr08G0100.1	2519	6.00E-156	225/449(50.11)	451	PHI:2519|LYS9|Q4WQ27|746128|Aspergillus_fumigatus|Lethal
Chr08G0104.1	2968	2.00E-29	120/489(24.54)	118	PHI:2968|Hxs1|J9VQA5|5207|Cryptococcus_neoformans|Reduced_virulence
Chr08G0106.1	716	7.00E-17	99/365(27.12)	80.9	PHI:716|ZEB1|ABB90284|5518|Fusarium_graminearum|Unaffected_pathogenicity

Chr08G0109.1	2227	9.00E-12	89/334(26.65)	64.7	PHI:2227|pao|D4III8|5270|Ustilago_maydis|Mixed_outcome
Chr08G0115.1	277	6.00E-37	70/138(50.72)	127	PHI:277|BCP1|AAQ16572|40559|Botrytis_cinerea|Reduced_virulence
Chr08G0118.1	208	2.00E-15	109/487(22.38)	77	PHI:208|CaNAG5|BAB43816|5476|Candida_albicans|Reduced_virulence
Chr08G0123.1	1627	3.00E-63	86/110(78.18)	188	PHI:1627|GzTF2S002|I1RW90|5518|Gibberella_zeae_(related:_Fusarium_graminearum)|Unaffected_pathogenicity
Chr08G0132.1	2206	1.00E-115	165/320(51.56)	351	PHI:2206|endo-1,4-beta-xylanase_precursor_[GH10_family]|MGG_14243|148305|Magnaporthe_oryzae_(related:_Magnaporthe_grisea)|Reduced_virulence
Chr08G0133.1	1278	2.00E-131	226/482(46.89)	417	PHI:1278|FGSG_09150|I1S453|5518|Gibberella_zeae_(related:_Fusarium_graminearum)|Unaffected_pathogenicity
Chr08G0138.1	3415	2.00E-34	104/395(26.33)	137	Fre2|PHI:3415|T2BNJ5|5207|Cryptococcus_neoformans|mixed_outcome_
Chr08G0141.1	2256	1.00E-25	104/362(28.73)	103	PHI:2256|Xdh1|Q0UA24|13684|Phaeosphaeria_nodorum_(related:_Stagonospora_nodorum)|Unaffected_pathogenicity
Chr08G0143.1	1681	2.00E-12	87/376(23.14)	68.2	PHI:1681|GzNF001|I1REN7|5518|Gibberella_zeae_(related:_Fusarium_graminearum)|Unaffected_pathogenicity
Chr08G0144.1	1741	3.00E-116	250/762(32.81)	368	PHI:1741|GzZC056|I1S780|5518|Gibberella_zeae_(related:_Fusarium_graminearum)|Unaffected_pathogenicity
Chr08G0145.1	2042	0	455/1349(33.73)	716	PHI:2042|ABC3|Q3Y5V5|148305|Magnaporthe_oryzae_(related:_Magnaporthe_grisea)|Loss_of_pathogenicity
Chr08G0158.1	2117	0	384/537(71.51)	757	PHI:2117|SPM1|P58371|148305|Magnaporthe_oryzae_(related:_Magnaporthe_grisea)|Reduced_virulence
Chr08G0166.1	541	6.00E-42	151/480(31.46)	156	PHI:541|LIP1|AAU87359|332648|Botrytis_cinerea|Unaffected_pathogenicity
Chr08G0173.1	355	2.00E-37	120/424(28.30)	142	PHI:355|GzmetE||5518|Fusarium_graminearum|Reduced_virulence
Chr08G0176.1	2008	3.00E-35	69/167(41.32)	139	PHI:2008|HST1|EDJ98541|148305|Magnaporthe_oryzae_(related:_Magnaporthe_grisea)|Unaffected_pathogenicity
Chr08G0180.1	2336	4.00E-31	111/373(29.76)	126	PHI:2336|NIA1|O00101|13684|Stagonospora_nodorum_(related:_Phaeosphaeria_nodorum)|Unaffected_pathogenicity

Chr08G0182.1	179	6.00E-55	101/227(44.49)	177	PHI:179|PELA|AAA33338|140110|Nectria_haematococca_(related:_Fusarium_solani)|Reduced_virulence
Chr08G0183.1	901	1.00E-44	137/489(28.02)	165	PHI:901|um01886||5270|Ustilago_maydis|Unaffected_pathogenicity
Chr08G0184.1	2376	9.00E-43	105/404(25.99)	158	PHI:2376|DEP2|D2E9W7|29001|Alternaria_brassicicola|Mixed_outcome
Chr08G0185.1	4194	1.00E-27	114/396(28.79)	114	AKT7|PHI:4194|V5XZS6|5599|Alternaria_alternata|increased_virulence_(Hypervirulence)
Chr08G0193.1	1964	1.00E-06	71/330(21.52)	47.8	PHI:1964|GzZC279|I1RQU2|5518|Gibberella_zeae_(related:_Fusarium_graminearum)|Unaffected_pathogenicity
Chr08G0196.1	1277	3.00E-45	141/480(29.38)	173	PHI:1277|FGSG_12887|I1S5L1|5518|Gibberella_zeae_(related:_Fusarium_graminearum)|Unaffected_pathogenicity
Chr08G0197.1	2654	4.00E-18	67/174(38.51)	85.5	PHI:2654|DUR1,2|Q59VF3|5476|Candida_albicans|Reduced_virulence
Chr08G0200.1	1467	2.00E-80	113/138(81.88)	235	PHI:1467|GzCCAAT007|I1RXZ0|5518|Gibberella_zeae_(related:_Fusarium_graminearum)|Unaffected_pathogenicity
Chr08G0201.1	2247	7.00E-12	56/188(29.79)	61.6	PHI:2247|Sch1|EAT82552|13684|Phaeosphaeria_nodorum_(related:_Stagonospora_nodorum)|Unaffected_pathogenicity
Chr08G0207.1	4203	2.00E-10	58/191(30.37)	61.2	SFI4|PHI:4203|D0NCC1|4787|Phytophthora_infestans|mixed_outcome
Chr08G0209.1	211	2.00E-40	84/218(38.53)	156	PHI:211|CaTUP1|AAB63195|5476|Candida_albicans|Reduced_virulence
Chr08G0210.1	1522	3.00E-97	127/238(53.36)	289	PHI:1522|GzHOMEL026|I1RXA5|5518|Gibberella_zeae_(related:_Fusarium_graminearum)|Lethal
Chr08G0212.1	211	7.00E-56	122/311(39.23)	200	PHI:211|CaTUP1|AAB63195|5476|Candida_albicans|Reduced_virulence
Chr08G0217.1	2315	8.00E-43	89/281(31.67)	150	PHI:2315|ChLae1|G4XKY9|5016|Cochliobolus_heterostrophus|Mixed_outcome
Chr08G0225.1	784	1.00E-13	71/219(32.42)	66.6	PHI:784|MGG_00056|EDK03390|318829|Magnaporthe_oryzae|Reduced_virulence
Chr08G0233.1	2976	9.00E-169	236/494(47.77)	499	PHI:2976|CgOPT1|C6ZRH8|29905|Colletotrichum_gloeosporioides_f._sp._aeschynomenes|Reduced_virulence
Chr08G0237.1	423	4.00E-77	152/420(36.19)	252	PHI:423|VAD1|AAV41010|5207|Cryptococcus_neoformans|Reduced_virulence

Chr08G0238.1	438	3.00E-11	63/235(26.81)	63.2	PHI:438|BcBOT1_(related:_CND5)|AAQ16576|40559|Botrytis_cinerea|Reduced_virulence
Chr08G0242.1	1393	1.00E-15	60/203(29.56)	78.2	PHI:1393|GzC2H056|I1RU69|5518|Gibberella_zeae_(related:_Fusarium_graminearum)|Unaffected_pathogenicity
Chr08G0251.1	181	2.00E-179	258/414(62.32)	511	PHI:181|PGX1|AAK81847|5507|Fusarium_oxysporum|Unaffected_pathogenicity
Chr08G0252.1	1029	8.00E-15	91/326(27.91)	72.4	PHI:1029|bcpme2|CAD21438|40559|Botrytis_cinerea|Unaffected_pathogenicity
Chr08G0255.1	1660	6.00E-21	140/558(25.09)	98.2	PHI:1660|GzCCCH004|I1S4C9|5518|Gibberella_zeae_(related:_Fusarium_graminearum)|Unaffected_pathogenicity
Chr08G0261.1	211	1.00E-51	115/290(39.66)	188	PHI:211|CaTUP1|AAB63195|5476|Candida_albicans|Reduced_virulence
Chr08G0263.1	1590	8.00E-80	121/159(76.10)	234	PHI:1590|GzOB030|I1RWI5|5518|Gibberella_zeae_(related:_Fusarium_graminearum)|Lethal
Chr08G0271.1	2058	8.00E-19	88/342(25.73)	87.8	PHI:2058|LHS1|MGG_06648.5|148305|Magnaporthe_oryzae_(related:_Magnaporthe_grisea)|Reduced_virulence
Chr08G0272.1	1589	3.00E-124	169/245(68.98)	354	PHI:1589|GzOB029|I1RWH5|5518|Gibberella_zeae_(related:_Fusarium_graminearum)|Unaffected_pathogenicity
Chr08G0273.1	1404	4.00E-49	95/170(55.88)	181	PHI:1404|GzC2H067|I1RWH6|5518|Gibberella_zeae_(related:_Fusarium_graminearum)|Unaffected_pathogenicity
Chr08G0275.1	1681	9.00E-11	81/371(21.83)	63.2	PHI:1681|GzNF001|I1REN7|5518|Gibberella_zeae_(related:_Fusarium_graminearum)|Unaffected_pathogenicity
Chr08G0277.1	508	1.00E-144	243/571(42.56)	432	PHI:508|AFT1|BAB69076|5599|Alternaria_alternata|Loss_of_pathogenicity
Chr08G0279.1	68	1.00E-40	128/398(32.16)	150	PHI:68|SAP1|AAA34368|5476|Candida_albicans|Reduced_virulence
Chr08G0282.1	211	4.00E-52	116/311(37.30)	191	PHI:211|CaTUP1|AAB63195|5476|Candida_albicans|Reduced_virulence
Chr08G0285.1	1578	0	363/588(61.73)	682	PHI:1578|GzOB018|I1RKY2|5518|Gibberella_zeae_(related:_Fusarium_graminearum)|Unaffected_pathogenicity
Chr08G0286.1	2632	5.00E-07	63/282(22.34)	49.3	PHI:2632|argH|Q2FIB5|1280|Staphylococcus_aureus|Reduced_virulence

Chr08G0292.1	2269	9.00E-50	103/265(38.87)	168	PHI:2269|Mdh1|0|13684|Phaeosphaeria_nodorum_(related:_Stagonospora_nodorum)|Unaffected_pathogenicity
Chr08G0297.1	2594	7.00E-07	24/66(36.36)	49.7	PHI:2594|SET3|Q59ZX1|746128|Aspergillus_fumigatus|Mixed_outcome
Chr08G0300.1	2019	4.00E-98	242/653(37.06)	328	PHI:2019|MoPLC3|MGG_08315|148305|Magnaporthe_oryzae_(related:_Magnaporthe_grisea)|Loss_of_pathogenicity
Chr08G0304.1	2055	2.00E-180	243/311(78.14)	503	PHI:2055|PPT1|Q1L5B8|31870|Colletotrichum_graminicola_(related:_Glomerella_graminicola)|Loss_of_pathogenicity
Chr08G0306.1	2027	0	405/612(66.18)	824	PHI:2027|TGL1-1|G4NJL1|148305|Magnaporthe_oryzae_(related:_Magnaporthe_grisea)|Unaffected_pathogenicity
Chr08G0309.1	1648	0	1835/2398(76.52)	3625	PHI:1648|GzWing020|I1RWP1|5518|Gibberella_zeae_(related:_Fusarium_graminearum)|Reduced_virulence
Chr08G0311.1	2115	2.00E-14	61/225(27.11)	73.2	PHI:2115|Annexin_A7|MGG_06847|148305|Magnaporthe_oryzae_(related:_Magnaporthe_grisea)|Loss_of_pathogenicity
Chr08G0317.1	211	3.00E-62	129/311(41.48)	221	PHI:211|CaTUP1|AAB63195|5476|Candida_albicans|Reduced_virulence
Chr08G0318.1	267	6.00E-73	181/567(31.92)	266	PHI:267|MLT1|AAD51594|5476|Candida_albicans|Reduced_virulence
Chr08G0319.1	3214	6.00E-08	29/95(30.53)	48.1	MoCDIP2|PHI:3214|G4MML4|318829|Magnaporthe_oryzae|mixed_outcome_
Chr08G0323.1	2388	1.00E-26	96/318(30.19)	111	PHI:2388|Chi2|0|5530|Metarhizium_anisopliae|Mixed_outcome
Chr08G0330.1	1052	3.00E-14	39/110(35.45)	73.6	PHI:1052|PHL1|ACB38886|135779|Cercospora_zeae-maydis|Unaffected_pathogenicity
Chr08G0331.1	2964	3.00E-33	130/404(32.18)	132	PHI:2964|AsnB|G7TM01|129394|Xanthomonas_oryzae_pv._Oryzicola|Reduced_virulence
Chr08G0332.1	441	2.00E-14	76/350(21.71)	71.6	PHI:441|BTP1|CAE55153|40559|Botrytis_cinerea|Reduced_virulence
Chr08G0335.1	1269	6.00E-81	154/431(35.73)	257	PHI:1269|FGSG_02838|I1RFK9|5518|Gibberella_zeae_(related:_Fusarium_graminearum)|Unaffected_pathogenicity
Chr08G0336.1	1701	1.00E-67	159/477(33.33)	233	PHI:1701|GzZC016|I1RKS9|5518|Gibberella_zeae_(related:_Fusarium_graminearum)|Unaffected_pathogenicity

Chr08G0354.1	1795	2.00E-19	136/573(23.73)	89.4	PHI:1795|GzZC110|I1RSX8|5518|Gibberella_zeae_(related:_Fusarium_graminearum)|Unaffected_pathogenicity
Chr08G0357.1	2844	5.00E-09	54/213(25.35)	53.9	PHI:2844|BRM2|O93802|5599|Alternaria_alternata|Unaffected_pathogenicity
Chr08G0358.1	62	1.00E-58	87/159(54.72)	186	PHI:62|caRSR1|AAB81286|5476|Candida_albicans|Reduced_virulence
Chr08G0363.1	1338	2.00E-111	193/326(59.20)	327	PHI:1338|GzbZIP020|I1S8Y4|5518|Gibberella_zeae_(related:_Fusarium_graminearum)|Unaffected_pathogenicity
Chr08G0368.1	2602	1.00E-10	73/280(26.07)	62	PHI:2602|Rim9|Q4PH32|5270|Ustilago_maydis|Unaffected_pathogenicity
Chr08G0370.1	2244	3.00E-26	84/359(23.40)	107	PHI:2244|37865|gi|46099354|5270|Ustilago_maydis|Unaffected_pathogenicity
Chr08G0373.1	1550	6.00E-163	283/553(51.18)	478	PHI:1550|GzMyb014|I1RWN5|5518|Gibberella_zeae_(related:_Fusarium_graminearum)|Unaffected_pathogenicity
Chr08G0381.1	1752	1.00E-07	24/55(43.64)	50.8	PHI:1752|GzZC067|I1RZR8|5518|Gibberella_zeae_(related:_Fusarium_graminearum)|Unaffected_pathogenicity
Chr08G0386.1	3283	3.00E-09	56/200(28.00)	56.6	ampG|PHI:3283|Q8P471|339|Xanthomonas_campestris|increased_virulence_
Chr08G0388.1	812	3.00E-61	127/367(34.60)	205	PHI:812|MGG_10702|EDJ94108|318829|Magnaporthe_oryzae|Reduced_virulence
Chr08G0389.1	1806	1.00E-16	48/120(40.00)	78.6	PHI:1806|GzZC121|I1RUM8|5518|Gibberella_zeae_(related:_Fusarium_graminearum)|Unaffected_pathogenicity
Chr08G0390.1	1322	1.00E-13	64/232(27.59)	68.2	PHI:1322|GzbZIP004|I1RG94|5518|Gibberella_zeae_(related:_Fusarium_graminearum)|Unaffected_pathogenicity
Chr08G0391.1	1806	1.00E-37	154/530(29.06)	144	PHI:1806|GzZC121|I1RUM8|5518|Gibberella_zeae_(related:_Fusarium_graminearum)|Unaffected_pathogenicity
Chr08G0394.1	1187	0	523/670(78.06)	1075	PHI:1187|(Sc_Dbf2/Dbf20)|I1RWG6|5518|Gibberella_zeae_(related:_Fusarium_graminearum)|Reduced_virulence
Chr08G0397.1	287	0	804/986(81.54)	1507	PHI:287|CLNR1|AAN65464|290576|Colletotrichum_lindemuthianum|Loss_of_pathogenicity
Chr08G0405.1	1458	2.00E-54	137/432(31.71)	200	PHI:1458|GzAra006|Q4I7F9|5518|Gibberella_zeae_(related:_Fusarium_gramin

					earum)|Unaffected_pathogenicity
Chr08G0407.1	2097	4.00E-89	286/1079(26.51)	315	PHI:2097|Neo1|MGG_04066|148305|Magnaporthe_oryzae_(related:_Magnaporthe_grisea)|Loss_of_pathogenicity
Chr08G0411.1	429	1.00E-152	198/224(88.39)	425	PHI:429|ClPLS1|CAD43407|290576|Colletotrichum_lindemuthianum|Loss_of_pathogenicity
Chr08G0414.1	1821	3.00E-64	134/340(39.41)	221	PHI:1821|GzZC136|I1RWY8|5518|Gibberella_zeae_(related:_Fusarium_graminearum)|Unaffected_pathogenicity
Chr08G0415.1	1662	2.00E-71	154/502(30.68)	249	PHI:1662|GzCCHC002|I1REJ1|5518|Gibberella_zeae_(related:_Fusarium_graminearum)|Unaffected_pathogenicity
Chr08G0417.1	1534	4.00E-123	180/223(80.72)	350	PHI:1534|GzMADS001|I1RWM0|5518|Gibberella_zeae_(related:_Fusarium_graminearum)|Unaffected_pathogenicity
Chr08G0418.1	3237	4.00E-47	79/158(50.00)	154	ctrA2|PHI:3237|B0YDG4|746128|Aspergillus_fumigatus|mixed_outcome_
Chr08G0419.1	1434	1.00E-76	172/372(46.24)	246	PHI:1434|GzC2H103|I1S8Y5|5518|Gibberella_zeae_(related:_Fusarium_graminearum)|Unaffected_pathogenicity
Chr08G0420.1	2267	0	438/541(80.96)	935	PHI:2267|Mls1|0|13684|Phaeosphaeria_nodorum_(related:_Stagonospora_nodorum)|Loss_of_pathogenicity
Chr08G0421.1	1184	0	429/969(44.27)	670	PHI:1184|(Sc_Gin4-like)|I1RWM5|5518|Gibberella_zeae_(related:_Fusarium_graminearum)|Reduced_virulence
Chr08G0422.1	1500	8.00E-07	35/134(26.12)	50.4	PHI:1500|GzHMG032|I1S2F2|5518|Gibberella_zeae_(related:_Fusarium_graminearum)|Unaffected_pathogenicity
Chr08G0425.1	2106	1.00E-127	260/670(38.81)	398	PHI:2106|1-phosphatidylinositol-4,5-bisphosphate_phosphodiesterase_delta_1|MGG_05332|148305|Magnaporthe_oryzae_(related:_Magnaporthe_grisea)|Reduced_virulence
Chr08G0427.1	1746	0	377/944(39.94)	629	PHI:1746|GzZC061|I1RWV5|5518|Gibberella_zeae_(related:_Fusarium_graminearum)|Unaffected_pathogenicity
Chr08G0431.1	3162	4.00E-135	298/742(40.16)	451	Mohik8|PHI:3162|G4MXJ1|318829|Magnaporthe_oryzae|loss_of_pathogenicity

					_
Chr08G0438.1	1161	5.00E-41	119/447(26.62)	154	PHI:1161|MgMfs1|A4ZGP3|54734|Mycosphaerella_graminicola_(related:_Zymoseptoria_triticii)|Chemistry_target
Chr08G0439.1	3387	0	779/2376(32.79)	1159	FVEG_12523|PHI:3387|W7MT31|117187|Fusarium_verticillioides|unaffected_pathogenicity_
Chr08G0440.1	2821	4.00E-22	53/148(35.81)	100	PHI:2821|SNF2|Q5ALP9|5476|Candida_albicans|Reduced_virulence
Chr08G0446.1	1182	0	1422/2492(57.06)	2832	PHI:1182|(Sc_Mec1)|I1S8Y9|5518|Gibberella_zeae_(related:_Fusarium_graminearum)|Reduced_virulence
Chr08G0447.1	2377	5.00E-20	99/389(25.45)	90.5	PHI:2377|DEP3|D2E9W8|29001|Alternaria_brassicicola|Mixed_outcome
Chr08G0450.1	1647	0	981/1256(78.11)	1868	PHI:1647|GzWing019|I1RWA9|5518|Gibberella_zeae_(related:_Fusarium_graminearum)|Reduced_virulence
Chr08G0455.1	3287	4.00E-179	340/982(34.62)	543	CgVps34|PHI:3287|Q6FSR7|5478|Candida_glabrata|reduced_virulence_
Chr08G0459.1	1046	7.00E-13	50/168(29.76)	67.4	PHI:1046|CTB5|ABK64182|29003|Cercospora_nicotianae|Reduced_virulence
Chr08G0460.1	3381	8.00E-65	141/406(34.73)	220	FVEG_12533|PHI:3381|W7N2B4|117187|Fusarium_verticillioides|unaffected_pathogenicity_
Chr08G0462.1	3384	3.00E-169	335/1069(31.34)	527	FVEG_12530|PHI:3384|W7N2C1|117187|Fusarium_verticillioides|unaffected_pathogenicity_
Chr08G0464.1	3126	6.00E-07	43/139(30.94)	49.7	argD|PHI:3126|D4I307|552|Erwinia_amylovora|mixed_outcome_
Chr08G0465.1	2248	5.00E-25	96/369(26.02)	105	PHI:2248|Als1|Q1L2E2|13684|Phaeosphaeria_nodorum_(related:_Stagonospora_nodorum)|Mixed_outcome
Chr08G0466.1	2032	2.00E-109	202/506(39.92)	343	PHI:2032|VTL1|G4NGA7|148305|Magnaporthe_oryzae_(related:_Magnaporthe_grisea)|Unaffected_pathogenicity
Chr08G0468.1	812	1.00E-65	122/337(36.20)	216	PHI:812|MGG_10702|EDJ94108|318829|Magnaporthe_oryzae|Reduced_virulence
Chr08G0469.1	1904	7.00E-17	62/232(26.72)	80.9	PHI:1904|GzZC219|I1RHR0|5518|Gibberella_zeae_(related:_Fusarium_graminearum)|Unaffected_pathogenicity

Chr08G0470.1	485	4.00E-114	166/334(49.70)	339	PHI:485|FTR1|AAF69680|5476|Candida_albicans|Loss_of_pathogenicity
Chr08G0471.1	2921	0	487/621(78.42)	1008	PHI:2921|FET3-2|E3Q7G1|31870|Colletotrichum_graminicola|Reduced_virulence
Chr08G0474.1	2968	1.00E-50	138/477(28.93)	181	PHI:2968|Hxs1|J9VQA5|5207|Cryptococcus_neoformans|Reduced_virulence
Chr08G0482.1	1051	1.00E-48	109/346(31.50)	177	PHI:1051|CTB3|ABC79591|29003|Cercospora_nicotianae|Reduced_virulence
Chr08G0483.1	2315	2.00E-54	106/292(36.30)	184	PHI:2315|ChLae1|G4XKY9|5016|Cochliobolus_heterostrophus|Mixed_outcome
Chr08G0487.1	1591	2.00E-116	225/492(45.73)	351	PHI:1591|GzOB031|I1RWQ7|5518|Gibberella_zeae_(related:_Fusarium_graminearum)|Reduced_virulence
Chr08G0488.1	1424	1.00E-81	176/480(36.67)	277	PHI:1424|GzC2H092|I1S3J7|5518|Gibberella_zeae_(related:_Fusarium_graminearum)|Unaffected_pathogenicity
Chr08G0496.1	541	5.00E-68	191/605(31.57)	231	PHI:541|LIP1|AAU87359|332648|Botrytis_cinerea|Unaffected_pathogenicity
Chr08G0498.1	1420	1.00E-08	96/421(22.80)	57.4	PHI:1420|GzC2H088|I1S172|5518|Gibberella_zeae_(related:_Fusarium_graminearum)|Unaffected_pathogenicity
Chr08G0502.1	881	2.00E-21	73/288(25.35)	90.1	PHI:881|MGG_04556|EDJ96020|318829|Magnaporthe_oryzae|Reduced_virulence
Chr08G0505.1	1406	0	420/616(68.18)	787	PHI:1406|GzC2H070|I1RX04|5518|Gibberella_zeae_(related:_Fusarium_graminearum)|Lethal
Chr08G0511.1	1193	0	455/512(88.87)	911	PHI:1193|(Sc_Sky1)|I1RGD6|5518|Gibberella_zeae_(related:_Fusarium_graminearum)|Reduced_virulence
Chr08G0514.1	1941	0	521/831(62.70)	989	PHI:1941|GzZC256|I1RGD9|5518|Gibberella_zeae_(related:_Fusarium_graminearum)|Unaffected_pathogenicity
Chr08G0517.1	1360	8.00E-139	248/458(54.15)	415	PHI:1360|GzC2H020|I1RGE3|5518|Gibberella_zeae_(related:_Fusarium_graminearum)|Unaffected_pathogenicity
Chr08G0518.1	1555	8.00E-41	120/494(24.29)	152	PHI:1555|GzMyb019|I1RDG6|5518|Gibberella_zeae_(related:_Fusarium_graminearum)|Unaffected_pathogenicity
Chr08G0519.1	2240	3.00E-41	137/509(26.92)	154	PHI:2240|Srt1|Q4PBY9|5270|Ustilago_maydis|reduced_virulence

Chr08G0521.1	1660	4.00E-38	109/374(29.14)	152	PHI:1660|GzCCCH004|I1S4C9|5518|Gibberella_zeae_(related:_Fusarium_graminearum)|Unaffected_pathogenicity
Chr08G0524.1	1838	0	291/633(45.97)	548	PHI:1838|GzZC153|I1RGC8|5518|Gibberella_zeae_(related:_Fusarium_graminearum)|Unaffected_pathogenicity
Chr08G0525.1	1359	1.00E-51	116/176(65.91)	164	PHI:1359|GzC2H019|I1RGC9|5518|Gibberella_zeae_(related:_Fusarium_graminearum)|Unaffected_pathogenicity
Chr08G0527.1	1581	5.00E-21	64/237(27.00)	87	PHI:1581|GzOB021|I1RPX8|5518|Gibberella_zeae_(related:_Fusarium_graminearum)|Unaffected_pathogenicity
Chr08G0529.1	1414	4.00E-08	55/210(26.19)	52.8	PHI:1414|GzC2H081|I1RZL0|5518|Gibberella_zeae_(related:_Fusarium_graminearum)|Unaffected_pathogenicity
Chr08G0537.1	1336	1.00E-66	145/289(50.17)	222	PHI:1336|GzbZIP018|I1S462|5518|Gibberella_zeae_(related:_Fusarium_graminearum)|Unaffected_pathogenicity
Chr08G0551.1	1164	0	371/564(65.78)	641	PHI:1164|RRG1|I1RX94|5518|Gibberella_zeae_(related:_Fusarium_graminearum)|Reduced_virulence
Chr08G0555.1	174	3.00E-46	75/145(51.72)	149	PHI:174|GNA1|BAA36496|5476|Candida_albicans|Reduced_virulence
Chr08G0558.1	2269	4.00E-31	83/263(31.56)	115	PHI:2269|Mdh1|0|13684|Phaeosphaeria_nodorum_(related:_Stagonospora_nodorum)|Unaffected_pathogenicity
Chr08G0559.1	2255	3.00E-69	134/341(39.30)	223	PHI:2255|Abd1|Q0U2A0|13684|Phaeosphaeria_nodorum_(related:_Stagonospora_nodorum)|Unaffected_pathogenicity
Chr08G0562.1	1522	4.00E-06	28/93(30.11)	45.4	PHI:1522|GzHOMEL026|I1RXA5|5518|Gibberella_zeae_(related:_Fusarium_graminearum)|Lethal
Chr08G0566.1	451	0	331/737(44.91)	625	PHI:451|PMT1|AAC31119|5476|Candida_albicans|Loss_of_pathogenicity
Chr08G0569.1	1770	2.00E-124	205/416(49.28)	377	PHI:1770|GzZC085|I1RX79|5518|Gibberella_zeae_(related:_Fusarium_graminearum)|Unaffected_pathogenicity
Chr08G0576.1	1771	2.00E-74	219/463(47.30)	242	PHI:1771|GzZC086|I1RX74|5518|Gibberella_zeae_(related:_Fusarium_graminearum)|Unaffected_pathogenicity

Chr08G0586.1	2842	4.00E-74	123/260(47.31)	236	PHI:2842|CnSFH5|F6K8L7|5207|Cryptococcus_neoformans|Mixed_outcome
Chr08G0587.1	2825	3.00E-22	56/136(41.18)	100	PHI:2825|SET1|Q5ABG1|5476|Candida_albicans|Reduced_virulence
Chr08G0589.1	1677	3.00E-128	183/406(45.07)	380	PHI:1677|GzDHHC005|Q4I1J3|5518|Gibberella_zeae_(related:_Fusarium_graminearum)|Unaffected_pathogenicity
Chr08G0590.1	1407	0	454/685(66.28)	917	PHI:1407|GzC2H071|I1RX64|5518|Gibberella_zeae_(related:_Fusarium_graminearum)|Lethal
Chr08G0596.1	1215	0	346/583(59.35)	647	PHI:1215|FGSG_02838|I1RX56|5518|Gibberella_zeae_(related:_Fusarium_graminearum)|Reduced_virulence
Chr08G0599.1	3014	0	1075/2032(52.90)	1791	MoAND1|PHI:3014|G5EHD3|318829|Magnaporthe_oryzae|reduced_virulence_
Chr08G0601.1	893	0	1039/1674(62.07)	1988	PHI:893|MGG_02986|EDK01543|318829|Magnaporthe_oryzae|Reduced_virulence
Chr08G0603.1	1666	0	346/448(77.23)	694	PHI:1666|GzCCHC006|I1RX47|5518|Gibberella_zeae_(related:_Fusarium_graminearum)|Unaffected_pathogenicity
Chr08G0607.1	1496	3.00E-19	55/173(31.79)	81.6	PHI:1496|MAT1-2-1|I1RX44|5518|Gibberella_zeae_(related:_Fusarium_graminearum)|Unaffected_pathogenicity
Chr08G0613.1	131	2.00E-75	163/563(28.95)	252	PHI:131|TRI12|AAD12756|5514|Fusarium_sporotrichioides|Unaffected_pathogenicity
Chr08G0615.1	265	0	377/581(64.89)	759	PHI:265|MET3|AAL92174|5207|Cryptococcus_neoformans|Loss_of_pathogenicity
Chr08G0620.1	3063	0	422/800(52.75)	736	MoRga3|PHI:3063|G4NKL4|318829|Magnaporthe_oryzae|mixed_outcome_
Chr08G0623.1	2054	1.00E-142	190/199(95.48)	397	PHI:2054|Rac1|A0SXQ7|148305|Magnaporthe_oryzae_(related:_Magnaporthe_grisea)|Loss_of_pathogenicity
Chr08G0624.1	1047	8.00E-58	134/359(37.33)	191	PHI:1047|CTB6|ABK64183|29003|Cercospora_nicotianae|Reduced_virulence
Chr08G0634.1	2154	5.00E-66	122/215(56.74)	215	PHI:2154|RBP35|MGG_02741|148305|Magnaporthe_oryzae_(related:_Magnaporthe_grisea)|Reduced_virulence
Chr08G0635.1	2020	3.00E-10	37/126(29.37)	60.5	PHI:2020|Tup1|XP_759427|5270|Ustilago_maydis|Mixed_outcome

Chr08G0643.1	812	2.00E-53	122/380(32.11)	183	PHI:812|MGG_10702|EDJ94108|318829|Magnaporthe_oryzae|Reduced_virulence
Chr08G0645.1	1867	2.00E-29	126/497(25.35)	120	PHI:1867|GzZC182|I1RL02|5518|Gibberella_zeae_(related:_Fusarium_graminearum)|Unaffected_pathogenicity
Chr08G0654.1	4493	0	287/511(56.16)	535	CGT|PHI:4493|G4MS28|318829|Magnaporthe_oryzae_(related:_Magnaporthe_grisea)|effector_(plant_avirulence_determinant)
Chr08G0658.1	792	5.00E-96	155/373(41.55)	304	PHI:792|MGG_09250|EDJ97946|318829|Magnaporthe_oryzae|Reduced_virulence
Chr08G0660.1	1640	2.00E-67	153/418(36.60)	220	PHI:1640|GzWing012|I1RPI0|5518|Gibberella_zeae_(related:_Fusarium_graminearum)|Unaffected_pathogenicity
Chr08G0663.1	1413	3.00E-123	306/952(32.14)	397	PHI:1413|GzC2H080|I1RZD4|5518|Gibberella_zeae_(related:_Fusarium_graminearum)|Unaffected_pathogenicity
Chr08G0670.1	2393	3.00E-36	132/489(26.99)	138	PHI:2393|Related_to_O-methylsterigmatocystin_oxidoreductase|I1R980|5518|Gibberella_zeae_(related:_Fusarium_graminearum)|Increased_virulence_(Hypervirulence)
Chr08G0671.1	1662	5.00E-67	139/436(31.88)	234	PHI:1662|GzCCHC002|I1REJ1|5518|Gibberella_zeae_(related:_Fusarium_graminearum)|Unaffected_pathogenicity
Chr08G0674.1	1958	4.00E-09	27/75(36.00)	54.3	PHI:1958|GzZC273|I1RMD1|5518|Gibberella_zeae_(related:_Fusarium_graminearum)|Unaffected_pathogenicity
Chr08G0675.1	1047	7.00E-15	73/266(27.44)	72	PHI:1047|CTB6|ABK64183|29003|Cercospora_nicotianae|Reduced_virulence
Chr08G0676.1	3028	3.00E-22	112/429(26.11)	96.3	Vatr2|PHI:3028|A5CVB7|28447|Clavibacter_michiganensis|reduced_virulence_
Chr08G0677.1	1662	7.00E-58	148/477(31.03)	207	PHI:1662|GzCCHC002|I1REJ1|5518|Gibberella_zeae_(related:_Fusarium_graminearum)|Unaffected_pathogenicity
Chr08G0685.1	1071	0	425/1085(39.17)	767	PHI:1071|Gas1|CAF05793|5270|Ustilago_maydis|Loss_of_pathogenicity
Chr08G0691.1	2490	5.00E-08	42/154(27.27)	52.4	PHI:2490|MCA|E9B636|5665|Leishmania_mexicana|Mixed_outcome
Chr08G0693.1	716	3.00E-95	196/572(34.27)	305	PHI:716|ZEB1|ABB90284|5518|Fusarium_graminearum|Unaffected_pathogenic

					ity
Chr08G0698.1	2838	3.00E-21	66/234(28.21)	87.4	PHI:2838|TOX9|D2SZX8|5016|Cochliobolus_heterostrophus|Reduced_virulence
Chr08G0700.1	4194	1.00E-52	143/486(29.42)	186	AKT7|PHI:4194|V5XZS6|5599|Alternaria_alternata|increased_virulence_(Hyper virulence)
Chr08G0702.1	482	7.00E-13	64/201(31.84)	65.5	PHI:482|LAEA|AAR01218|5085|Aspergillus_fumigatus|Reduced_virulence
Chr08G0703.1	325	0	1135/3075(36.91)	1840	PHI:325|ACE1|CAG28797|318829|Magnaporthe_oryzae|Effector_(plant_avirulence_determinant)
Chr08G0705.1	4194	9.00E-53	128/469(27.29)	186	AKT7|PHI:4194|V5XZS6|5599|Alternaria_alternata|increased_virulence_(Hyper virulence)
Chr08G0706.1	2269	7.00E-11	71/259(27.41)	58.5	PHI:2269|Mdh1|0|13684|Phaeosphaeria_nodorum_(related:_Stagonospora_nodorum)|Unaffected_pathogenicity
Chr08G0707.1	3225	1.00E-129	183/418(43.78)	381	Ndo1|PHI:3225|T2C913|36651|Penicillium_digitatum|unaffected_pathogenicity_
Chr08G0711.1	2269	2.00E-37	92/256(35.94)	132	PHI:2269|Mdh1|0|13684|Phaeosphaeria_nodorum_(related:_Stagonospora_nodorum)|Unaffected_pathogenicity
Chr08G0713.1	2654	8.00E-13	55/179(30.73)	68.6	PHI:2654|DUR1,2|Q59VF3|5476|Candida_albicans|Reduced_virulence
Chr08G0716.1	2907	5.00E-08	63/252(25.00)	52.8	PHI:2907|Cyp51A|I6YDU0|5518|Fusarium_graminearum|Mixed_outcome
Chr08G0719.1	112	2.00E-26	108/389(27.76)	108	PHI:112|MAK1|AAC49410|140110|Nectria_haematococca_(related:_Fusarium_solani)|Reduced_virulence
Chr08G0720.1	1413	4.00E-54	250/1020(24.51)	202	PHI:1413|GzC2H080|I1RZD4|5518|Gibberella_zeae_(related:_Fusarium_graminearum)|Unaffected_pathogenicity
Chr08G0724.1	409	3.00E-07	53/225(23.56)	48.5	PHI:409|BbCHIT1|AAN41259|176275|Beauveria_bassiana|Increased_virulence_(Hypervirulence)
Chr08G0728.1	2920	0	421/601(70.05)	891	PHI:2920|FET3-1|E3QRA4|31870|Colletotrichum_graminicola|Reduced_virulence
Chr08G0729.1	485	2.00E-117	173/331(52.27)	347	PHI:485|FTR1|AAF69680|5476|Candida_albicans|Loss_of_pathogenicity

Chr08G0730.1	3415	3.00E-40	137/552(24.82)	152	Fre2|PHI:3415|T2BNJ5|5207|Cryptococcus_neoformans|mixed_outcome_
Chr08G0731.1	2563	5.00E-16	56/195(28.72)	75.5	PHI:2563|DPP3|Q5AH74|5476|Candida_albicans|Reduced_virulence
Chr08G0732.1	2968	1.00E-24	109/461(23.64)	104	PHI:2968|Hxs1|J9VQA5|5207|Cryptococcus_neoformans|Reduced_virulence
Chr08G0733.1	716	2.00E-13	58/162(35.80)	69.3	PHI:716|ZEB1|ABB90284|5518|Fusarium_graminearum|Unaffected_pathogenicity
Chr08G0734.1	2898	4.00E-30	94/309(30.42)	114	PHI:2896|BEC1005|CCU82697|62688|Blumeria_graminis_f._sp._hordei|Effector_(plant_avirulence_determinant)
Chr08G0738.1	2540	8.00E-68	114/291(39.18)	219	PHI:2540|IPP1|Q4WX65|746128|Aspergillus_fumigatus|Mixed_outcome
Chr08G0743.1	2315	8.00E-63	115/292(39.38)	202	PHI:2315|ChLae1|G4XKY9|5016|Cochliobolus_heterostrophus|Mixed_outcome
Chr08G0744.1	2315	6.00E-61	115/291(39.52)	198	PHI:2315|ChLae1|G4XKY9|5016|Cochliobolus_heterostrophus|Mixed_outcome
Chr08G0745.1	2376	5.00E-54	130/443(29.35)	194	PHI:2376|DEP2|D2E9W7|29001|Alternaria_brassicicola|Mixed_outcome
Chr08G0746.1	544	2.00E-81	147/405(36.30)	262	PHI:544|BCMFS1|AAF64435|332648|Botrytis_cinerea|Unaffected_pathogenicity
Chr08G0760.1	2315	4.00E-60	113/293(38.57)	196	PHI:2315|ChLae1|G4XKY9|5016|Cochliobolus_heterostrophus|Mixed_outcome
Chr08G0764.1	2393	3.00E-45	132/483(27.33)	164	PHI:2393|Related_to_O-methylsterigmatocystin_oxidoreductase|I1R980|5518|Gibberella_zeae_(related:_Fusarium_graminearum)|Increased_virulence_(Hypervirulence)
Chr08G0775.1	482	3.00E-53	111/292(38.01)	180	PHI:482|LAEA|AAR01218|5085|Aspergillus_fumigatus|Reduced_virulence
Chr08G0778.1	4194	4.00E-31	122/498(24.50)	123	AKT7|PHI:4194|V5XZS6|5599|Alternaria_alternata|increased_virulence_(Hypervirulence)
Chr08G0786.1	2393	8.00E-108	181/486(37.24)	331	PHI:2393|Related_to_O-methylsterigmatocystin_oxidoreductase|I1R980|5518|Gibberella_zeae_(related:_Fusarium_graminearum)|Increased_virulence_(Hypervirulence)
Chr08G0789.1	716	4.00E-101	196/571(34.33)	321	PHI:716|ZEB1|ABB90284|5518|Fusarium_graminearum|Unaffected_pathogenicity
Chr08G0790.1	404	8.00E-14	54/207(26.09)	69.3	PHI:404|PTH11|AAD30436|318829|Magnaporthe_oryzae|Reduced_virulence

Chr08G0792.1	2255	6.00E-111	172/328(52.44)	331	PHI:2255|Abd1|Q0U2A0|13684|Phaeosphaeria_nodorum_(related:_Stagonospora_nodorum)|Unaffected_pathogenicity
Chr08G0794.1	1729	7.00E-11	26/58(44.83)	62.4	PHI:1729|GzZC044|I1S5H3|5518|Gibberella_zeae_(related:_Fusarium_graminearum)|Unaffected_pathogenicity
Chr08G0795.1	1901	1.00E-10	64/276(23.19)	61.2	PHI:1901|GzZC216|I1RK07|5518|Gibberella_zeae_(related:_Fusarium_graminearum)|Unaffected_pathogenicity
Chr08G0798.1	3234	6.00E-07	77/329(23.40)	49.3	MoLYS20|PHI:3234|G4NCG1|318829|Magnaporthe_oryzae|reduced_virulence_
Chr08G0806.1	479	5.00E-49	100/240(41.67)	164	PHI:479|MEP1|AAQ07436|199306|Coccidioides_posadasii|Reduced_virulence
Chr08G0811.1	1522	1.00E-08	63/279(22.58)	53.1	PHI:1522|GzHOMEL026|I1RXA5|5518|Gibberella_zeae_(related:_Fusarium_graminearum)|Lethal
Chr08G0815.1	1773	2.00E-59	127/418(30.38)	200	PHI:1773|GzZC088|I1S409|5518|Gibberella_zeae_(related:_Fusarium_graminearum)|Unaffected_pathogenicity
Chr08G0818.1	115	1.00E-46	131/393(33.33)	168	PHI:115|PGX1|AAC26146|5017|Cochliobolus_carbonum|Unaffected_pathogenicity
Chr08G0825.1	2240	9.00E-28	116/469(24.73)	114	PHI:2240|Srt1|Q4PBY9|5270|Ustilago_maydis|reduced_virulence
Chr08G0826.1	1662	8.00E-49	139/474(29.32)	182	PHI:1662|GzCCHC002|I1REJ1|5518|Gibberella_zeae_(related:_Fusarium_graminearum)|Unaffected_pathogenicity
Chr08G0827.1	1824	2.00E-167	271/559(48.48)	496	PHI:1824|GzZC139|I1RYC6|5518|Gibberella_zeae_(related:_Fusarium_graminearum)|Unaffected_pathogenicity
Chr08G0830.1	1046	1.00E-06	54/197(27.41)	48.1	PHI:1046|CTB5|ABK64182|29003|Cercospora_nicotianae|Reduced_virulence
Chr08G0832.1	144	3.00E-28	109/386(28.24)	117	PHI:144|CHT42|AAC05829|29875|Trichoderma_virens|Reduced_virulence
Chr08G0847.1	208	5.00E-10	86/391(21.99)	58.9	PHI:208|CaNAG5|BAB43816|5476|Candida_albicans|Reduced_virulence
Chr08G0848.1	1886	0	275/494(55.67)	542	PHI:1886|GzZC201|I1RU19|5518|Gibberella_zeae_(related:_Fusarium_graminearum)|Unaffected_pathogenicity
Chr08G0849.1	206	4.00E-79	129/246(52.44)	242	PHI:206|CaNAG1|BAB43821|5476|Candida_albicans|Reduced_virulence

Chr08G0853.1	1576	3.00E-60	129/370(34.86)	208	PHI:1576|GzOB016|I1RJY5|5518|Gibberella_zeae_(related:_Fusarium_graminearum)|Unaffected_pathogenicity
Chr08G0860.1	1399	8.00E-18	48/137(35.04)	85.5	PHI:1399|GzC2H062|I1RV74|5518|Gibberella_zeae_(related:_Fusarium_graminearum)|Unaffected_pathogenicity
Chr08G0865.1	2098	0	779/1090(71.47)	1592	PHI:2098|Calcium-transporting_ATPase_3|MGG_05078|148305|Magnaporthe_oryzae_(related:_Magnaporthe_grisea)|Loss_of_pathogenicity
Chr08G0870.1	2968	3.00E-64	160/516(31.01)	218	PHI:2968|Hxs1|J9VQA5|5207|Cryptococcus_neoformans|Reduced_virulence
Chr08G0875.1	131	9.00E-45	139/535(25.98)	166	PHI:131|TRI12|AAD12756|5514|Fusarium_sporotrichioides|Unaffected_pathogenicity
Chr08G0885.1	513	7.00E-40	136/572(23.78)	150	PHI:513|ARN1_(related:_SIT1)|EAK97011|5476|Candida_albicans|Reduced_virulence
Chr08G0886.1	3415	3.00E-28	111/498(22.29)	117	Fre2|PHI:3415|T2BNJ5|5207|Cryptococcus_neoformans|mixed_outcome_
Chr08G0887.1	1051	1.00E-34	111/411(27.01)	134	PHI:1051|CTB3|ABC79591|29003|Cercospora_nicotianae|Reduced_virulence
Chr08G0888.1	1193	7.00E-25	58/175(33.14)	103	PHI:1193|(Sc_Sky1)|I1RGD6|5518|Gibberella_zeae_(related:_Fusarium_graminearum)|Reduced_virulence
Chr08G0894.1	1621	0	1047/1551(67.50)	2081	PHI:1621|GzRUM1|I1RP98|5518|Gibberella_zeae_(related:_Fusarium_graminearum)|Unaffected_pathogenicity
Chr08G0896.1	2233	4.00E-17	43/141(30.50)	79.3	PHI:2233|Smu1|Q4P5N0|5270|Ustilago_maydis|reduced_virulence
Chr08G0897.1	1375	0	371/518(71.62)	759	PHI:1375|GzC2H038|I1RPA0|5518|Gibberella_zeae_(related:_Fusarium_graminearum)|Unaffected_pathogenicity
Chr08G0898.1	423	4.00E-89	153/366(41.80)	283	PHI:423|VAD1|AAV41010|5207|Cryptococcus_neoformans|Reduced_virulence
Chr08G0899.1	404	3.00E-24	78/316(24.68)	103	PHI:404|PTH11|AAD30436|318829|Magnaporthe_oryzae|Reduced_virulence
Chr08G0905.1	1555	3.00E-37	127/493(25.76)	142	PHI:1555|GzMyb019|I1RDG6|5518|Gibberella_zeae_(related:_Fusarium_graminearum)|Unaffected_pathogenicity
Chr08G0906.1	482	2.00E-49	99/278(35.61)	170	PHI:482|LAEA|AAR01218|5085|Aspergillus_fumigatus|Reduced_virulence
Chr08G0911.1	2248	3.00E-17	76/270(28.15)	83.2	PHI:2248|Als1|Q1L2E2|13684|Phaeosphaeria_nodorum_(related:_Stagonospor

					a_nodorum)|Mixed_outcome
Chr08G0913.1	2563	6.00E-19	70/265(26.42)	84.3	PHI:2563|DPP3|Q5AH74|5476|Candida_albicans|Reduced_virulence
Chr08G0916.1	4194	2.00E-32	131/527(24.86)	127	AKT7|PHI:4194|V5XZS6|5599|Alternaria_alternata|increased_virulence_(Hyper virulence)
Chr08G0920.1	1824	6.00E-73	176/548(32.12)	248	PHI:1824|GzZC139|I1RYC6|5518|Gibberella_zeae_(related:_Fusarium_gramin earum)|Unaffected_pathogenicity
Chr08G0921.1	2267	0	380/525(72.38)	813	PHI:2267|Mls1|0|13684|Phaeosphaeria_nodorum_(related:_Stagonospora_nod orum)|Loss_of_pathogenicity
Chr08G0930.1	2521	0	394/630(62.54)	784	PHI:2521|GUS1|Q4WEM7|746128|Aspergillus_fumigatus|Lethal
Chr08G0933.1	1326	1.00E-141	319/618(51.62)	427	PHI:1326|GzbZIP008|I1RPG1|5518|Gibberella_zeae_(related:_Fusarium_grami nearum)|Unaffected_pathogenicity
Chr08G0936.1	3236	2.00E-24	111/445(24.94)	103	Aph1|PHI:3236|J9VHR6|5207|Cryptococcus_neoformans|mixed_outcome_
Chr08G0937.1	2393	4.00E-87	136/299(45.48)	274	PHI:2393|Related_to_O-methylsterigmatocystin_oxidoreductase|I1R980|5518|Gibberella_zeae_(related:_Fusarium_graminearum)|Increased_virulence_(Hyp ervirulence)
Chr08G0939.1	714	4.00E-114	283/882(32.09)	394	PHI:714|PKS4_(related:_ZEA1)|ABB90283|5518|Fusarium_graminearum|Unaff ected_pathogenicity
Chr08G0940.1	419	5.00E-09	72/341(21.11)	53.9	PHI:419|CSH1|AAP93915|5476|Candida_albicans|Reduced_virulence
Chr08G0942.1	3381	8.00E-50	139/489(28.43)	180	FVEG_12533|PHI:3381|W7N2B4|117187|Fusarium_verticillioides|unaffected_p athogenicity_
Chr08G0948.1	1420	3.00E-07	81/366(22.13)	49.7	PHI:1420|GzC2H088|I1S172|5518|Gibberella_zeae_(related:_Fusarium_grami nearum)|Unaffected_pathogenicity
Chr08G0952.1	922	5.00E-54	178/613(29.04)	194	PHI:922|um03615||5270|Ustilago_maydis|Unaffected_pathogenicity
Chr08G0953.1	1458	3.00E-09	39/143(27.27)	57.4	PHI:1458|GzAra006|Q4I7F9|5518|Gibberella_zeae_(related:_Fusarium_gramin earum)|Unaffected_pathogenicity
Chr08G0954.1	1893	7.00E-46	129/434(29.72)	166	PHI:1893|GzZC208|I1RNY0|5518|Gibberella_zeae_(related:_Fusarium_gramin

					earum)|Unaffected_pathogenicity
Chr08G0955.1	1861	7.00E-09	55/258(21.32)	55.1	PHI:1861|GzZC176|I1RUV3|5518|Gibberella_zeae_(related:_Fusarium_graminearum)|Unaffected_pathogenicity
Chr08G0956.1	511	3.00E-09	56/253(22.13)	56.6	PHI:511|CaNAG4|EAK93098|5476|Candida_albicans|Reduced_virulence
Chr08G0957.1	438	5.00E-38	129/500(25.80)	143	PHI:438|BcBOT1_(related:_CND5)|AAQ16576|40559|Botrytis_cinerea|Reduced_virulence
Chr08G0958.1	1992	1.00E-14	61/192(31.77)	72.4	PHI:1992|GzZC307|I1R983|5518|Gibberella_zeae_(related:_Fusarium_graminearum)|Unaffected_pathogenicity
Chr08G0959.1	538	5.00E-46	146/523(27.92)	168	PHI:538|FRT1|AAU87358|40559|Botrytis_cinerea|Unaffected_pathogenicity
Chr08G0964.1	1581	5.00E-90	137/269(50.93)	275	PHI:1581|GzOB021|I1RPX8|5518|Gibberella_zeae_(related:_Fusarium_graminearum)|Unaffected_pathogenicity
Chr08G0965.1	1662	7.00E-34	108/432(25.00)	134	PHI:1662|GzCCHC002|I1REJ1|5518|Gibberella_zeae_(related:_Fusarium_graminearum)|Unaffected_pathogenicity
Chr08G0966.1	2315	8.00E-50	91/278(32.73)	169	PHI:2315|ChLae1|G4XKY9|5016|Cochliobolus_heterostrophus|Mixed_outcome
Chr08G0967.1	404	1.00E-28	86/338(25.44)	116	PHI:404|PTH11|AAD30436|318829|Magnaporthe_oryzae|Reduced_virulence
Chr08G0969.1	487	6.00E-33	55/100(55.00)	110	PHI:487|MHP1|O94196|148305|Magnaporthe_grisea|Reduced_virulence
Chr08G0985.1	199	1.00E-121	220/644(34.16)	377	PHI:199|AOX1|AAF82788|5499|Cladosporium_fulvum|Reduced_virulence
Chr08G0987.1	438	1.00E-42	131/484(27.07)	157	PHI:438|BcBOT1_(related:_CND5)|AAQ16576|40559|Botrytis_cinerea|Reduced_virulence
Chr08G0988.1	1625	4.00E-95	165/303(54.46)	303	PHI:1625|GzOpi|I1RXB9|5518|Gibberella_zeae_(related:_Fusarium_graminearum)|Unaffected_pathogenicity
Chr08G0993.1	3226	1.00E-71	145/347(41.79)	231	pnl1|PHI:3226|T2C7K6|36651|Penicillium_digitatum|reduced_virulence_
Chr08G0996.1	1435	8.00E-42	152/511(29.75)	160	PHI:1435|GzC2H104|I1S9G1|5518|Gibberella_zeae_(related:_Fusarium_graminearum)|Unaffected_pathogenicity
Chr08G1001.1	1765	8.00E-48	151/610(24.75)	176	PHI:1765|GzZC080|I1RY91|5518|Gibberella_zeae_(related:_Fusarium_graminearum)|Unaffected_pathogenicity

Chr08G1003.1	513	1.00E-47	152/587(25.89)	174	PHI:513|ARN1_(related:_SIT1)|EAK97011|5476|Candida_albicans|Reduced_virulence
Chr08G1006.1	2968	2.00E-31	119/498(23.90)	124	PHI:2968|Hxs1|J9VQA5|5207|Cryptococcus_neoformans|Reduced_virulence
Chr08G1007.1	1959	2.00E-56	171/603(28.36)	201	PHI:1959|GzZC274|I1RM85|5518|Gibberella_zeae_(related:_Fusarium_graminearum)|Unaffected_pathogenicity
Chr08G1010.1	447	9.00E-27	103/359(28.69)	110	PHI:447|MCSA|CAI61947|5085|Aspergillus_fumigatus|Reduced_virulence
Chr08G1011.1	2095	0	856/1080(79.26)	1760	PHI:2095|Calcium-transporting_ATPase_3|MGG_10730|148305|Magnaporthe_oryzae_(related:_Magnaporthe_grisea)|Reduced_virulence
Chr08G1023.1	2247	2.00E-07	43/125(34.40)	49.3	PHI:2247|Sch1|EAT82552|13684|Phaeosphaeria_nodorum_(related:_Stagonospora_nodorum)|Unaffected_pathogenicity
Chr08G1024.1	167	3.00E-37	156/537(29.05)	142	PHI:167|CHIP3|AAF00024|5457|Colletotrichum_gloeosporioides|Unaffected_pathogenicity
Chr08G1025.1	2148	0	564/1142(49.39)	1077	PHI:2148|7,8-LDS|MGG_13239|148305|Magnaporthe_oryzae_(related:_Magnaporthe_grisea)|Unaffected_pathogenicity
Chr08G1026.1	541	3.00E-38	147/492(29.88)	145	PHI:541|LIP1|AAU87359|332648|Botrytis_cinerea|Unaffected_pathogenicity
Chr08G1028.1	2522	0	292/531(54.99)	549	PHI:2522|SPE2|Q4WEN8|746128|Aspergillus_fumigatus|Lethal
Chr08G1032.1	4194	3.00E-41	122/485(25.15)	153	AKT7|PHI:4194|V5XZS6|5599|Alternaria_alternata|increased_virulence_(Hypervirulence)
Chr08G1033.1	1878	0	368/775(47.48)	670	PHI:1878|GzZC193|I1RPF6|5518|Gibberella_zeae_(related:_Fusarium_graminearum)|Unaffected_pathogenicity
Chr08G1034.1	2550	9.00E-114	203/438(46.35)	342	PHI:2550|NOB1|Q4WES1|746128|Aspergillus_fumigatus|Mixed_outcome
Chr08G1044.1	2968	8.00E-21	110/455(24.18)	93.2	PHI:2968|Hxs1|J9VQA5|5207|Cryptococcus_neoformans|Reduced_virulence
Chr08G1046.1	784	1.00E-07	41/137(29.93)	49.3	PHI:784|MGG_00056|EDK03390|318829|Magnaporthe_oryzae|Reduced_virulence
Chr08G1048.1	323	2.00E-14	86/377(22.81)	72	PHI:323|VFGLU1|AAO63562|93591|Verticillium_fungicola|Reduced_virulence
Chr08G1049.1	2968	5.00E-42	133/432(30.79)	155	PHI:2968|Hxs1|J9VQA5|5207|Cryptococcus_neoformans|Reduced_virulence

Chr08G1051.1	1818	1.00E-31	59/164(35.98)	123	PHI:1818|GzZC133|I1S7V2|5518|Gibberella_zeae_(related:_Fusarium_graminearum)|Unaffected_pathogenicity
Chr08G1057.1	812	6.00E-44	100/332(30.12)	157	PHI:812|MGG_10702|EDJ94108|318829|Magnaporthe_oryzae|Reduced_virulence
Chr08G1061.1	886	3.00E-06	40/176(22.73)	45.8	PHI:886|MGG_13052|EDK06087|318829|Magnaporthe_oryzae|Reduced_virulence
Chr08G1069.1	1260	7.00E-24	56/158(35.44)	105	PHI:1260|FGSG_13944|I1RUC7|5518|Gibberella_zeae_(related:_Fusarium_graminearum)|Unaffected_pathogenicity
Chr08G1073.1	1260	5.00E-09	55/175(31.43)	57.4	PHI:1260|FGSG_13944|I1RUC7|5518|Gibberella_zeae_(related:_Fusarium_graminearum)|Unaffected_pathogenicity
Chr05G0881.1	511	4.00E-17	53/192(27.60)	80.1	PHI:511|CaNAG4|EAK93098|5476|Candida_albicans|Reduced_virulence
Chr05G0884.1	2315	4.00E-26	61/136(44.85)	107	PHI:2315|ChLae1|G4XKY9|5016|Cochliobolus_heterostrophus|Mixed_outcome
Chr05G0886.1	1466	2.00E-91	147/203(72.41)	268	PHI:1466|GzCCAAT006|I1RSG9|5518|Gibberella_zeae_(related:_Fusarium_graminearum)|Unaffected_pathogenicity
Chr05G0897.1	2644	5.00E-11	29/101(28.71)	58.5	PHI:2644|thioredoxin_1|P0AA28|90371|Salmonella_enterica_serovar_Typhimurium|Reduced_virulence
Chr05G0903.1	223	9.00E-34	65/156(41.67)	124	PHI:223|PEP1|AAK11166|140110|Nectria_haematococca_(related:_Fusarium_solani)|Reduced_virulence
Chr05G0904.1	3354	3.00E-11	51/187(27.27)	61.6	rtxA1|PHI:3354|A0A023NA98|672|Vibrio_vulnificus|reduced_virulence_
Chr05G0905.1	1682	0	495/990(50.00)	946	PHI:1682|GzNF002|I1RSF8|5518|Gibberella_zeae_(related:_Fusarium_graminearum)|Unaffected_pathogenicity
Chr05G0906.1	352	1.00E-41	158/540(29.26)	162	PHI:352|GLO1|CAD79488|5270|Ustilago_maydis|Loss_of_pathogenicity
Chr05G0910.1	1260	1.00E-11	56/174(32.18)	64.7	PHI:1260|FGSG_13944|I1RUC7|5518|Gibberella_zeae_(related:_Fusarium_graminearum)|Unaffected_pathogenicity
Chr05G0918.1	3793	5.00E-105	161/334(48.20)	319	ANP1|PHI:3793|Q6FM27|5478|Candida_glabrata|effector_(plant_avirulence_determinant)

Chr05G0922.1	1273	0	791/1263(62.63)	1495	PHI:1273|FGSG_05549|I1RCM6|5518|Gibberella_zeae_(related:_Fusarium_graminearum)|Unaffected_pathogenicity
Chr05G0925.1	441	3.00E-24	91/325(28.00)	99.4	PHI:441|BTP1|CAE55153|40559|Botrytis_cinerea|Reduced_virulence
Chr05G0930.1	1461	1.00E-121	217/313(69.33)	352	PHI:1461|GzCCAAT001|I1RA31|5518|Gibberella_zeae_(related:_Fusarium_graminearum)|Unaffected_pathogenicity
Chr05G0934.1	1662	1.00E-147	261/607(43.00)	468	PHI:1662|GzCCHC002|I1REJ1|5518|Gibberella_zeae_(related:_Fusarium_graminearum)|Unaffected_pathogenicity
Chr05G0935.1	256	1.00E-15	62/201(30.85)	73.6	PHI:256|GAS1|AAK52794|318829|Magnaporthe_oryzae|Reduced_virulence
Chr05G0938.1	1048	3.00E-46	138/431(32.02)	165	PHI:1048|CTB7|ABK64184|29003|Cercospora_nicotianae|Reduced_virulence
Chr05G0941.1	404	5.00E-20	55/211(26.07)	89	PHI:404|PTH11|AAD30436|318829|Magnaporthe_oryzae|Reduced_virulence
Chr05G0944.1	397	8.00E-72	178/602(29.57)	245	PHI:397|LAC2|AAV64894|5207|Cryptococcus_neoformans|Unaffected_pathogenicity
Chr05G0951.1	1749	6.00E-103	212/591(35.87)	325	PHI:1749|GzZC064|I1S4A5|5518|Gibberella_zeae_(related:_Fusarium_graminearum)|Unaffected_pathogenicity
Chr05G0952.1	748	6.00E-52	124/361(34.35)	192	PHI:748|um00446|Not_available|5270|Ustilago_maydis|Unaffected_pathogenicity
Chr05G0953.1	2968	7.00E-23	100/395(25.32)	99.4	PHI:2968|Hxs1|J9VQA5|5207|Cryptococcus_neoformans|Reduced_virulence
Chr05G0964.1	2545	5.00E-08	28/72(38.89)	48.1	PHI:2545|TIF35|Q4X1I3|746128|Aspergillus_fumigatus|Mixed_outcome
Chr05G0965.1	800	3.00E-19	142/612(23.20)	89.7	PHI:800|MGG_13324|EDK00897|318829|Magnaporthe_oryzae|Reduced_virulence
Chr05G0967.1	2032	1.00E-52	166/548(30.29)	187	PHI:2032|VTL1|G4NGA7|148305|Magnaporthe_oryzae_(related:_Magnaporthe_grisea)|Unaffected_pathogenicity
Chr05G0969.1	442	0	599/770(77.79)	1245	PHI:442|MSY1||5518|Fusarium_graminearum|Reduced_virulence
Chr05G0975.1	4194	8.00E-54	134/475(28.21)	189	AKT7|PHI:4194|V5XZS6|5599|Alternaria_alternata|increased_virulence_(Hypervirulence)
Chr05G0983.1	1904	4.00E-49	148/516(28.68)	178	PHI:1904|GzZC219|I1RHR0|5518|Gibberella_zeae_(related:_Fusarium_gramin

					earum)|Unaffected_pathogenicity
Chr05G0993.1	404	4.00E-45	83/289(28.72)	160	PHI:404|PTH11|AAD30436|318829|Magnaporthe_oryzae|Reduced_virulence
Chr05G0999.1	1233	6.00E-06	34/129(26.36)	45.4	PHI:1233|FGSG_13944|I1RUG2|5518|Gibberella_zeae_(related:_Fusarium_graminearum)|Lethal
Chr05G1003.1	1678	3.00E-10	34/112(30.36)	58.2	PHI:1678|GzGRF|I1RMC1|5518|Gibberella_zeae_(related:_Fusarium_graminearum)|Unaffected_pathogenicity
Chr05G1013.1	812	2.00E-22	86/327(26.30)	95.1	PHI:812|MGG_10702|EDJ94108|318829|Magnaporthe_oryzae|Reduced_virulence
Chr05G1016.1	1568	5.00E-161	348/1089(31.96)	504	PHI:1568|GzOB008|I1RCE1|5518|Gibberella_zeae_(related:_Fusarium_graminearum)|Unaffected_pathogenicity
Chr05G1021.1	1426	2.00E-176	278/469(59.28)	514	PHI:1426|GzC2H094|I1S4M4|5518|Gibberella_zeae_(related:_Fusarium_graminearum)|Unaffected_pathogenicity
Chr05G1024.1	455	2.00E-26	91/302(30.13)	107	PHI:455|CAP59|AAC13946|5207|Cryptococcus_neoformans|Loss_of_pathogenicity
Chr05G1025.1	2607	0	385/790(48.73)	714	PHI:2607|Pmt4|Q4P380|5270|Ustilago_maydis|Reduced_virulence
Chr05G1028.1	2099	0	520/1145(45.41)	946	PHI:2099|Pmc1|MGG_07971|148305|Magnaporthe_oryzae_(related:_Magnaporthe_grisea)|Mixed_outcome
Chr05G1031.1	816	8.00E-125	267/561(47.59)	379	PHI:816|MGG_04582|EDJ95999|318829|Magnaporthe_oryzae|Reduced_virulence
Chr05G1034.1	3050	0	757/1146(66.06)	1497	CHSF|PHI:3050|Q4WC58|746128|Aspergillus_fumigatus|mixed_outcome_
Chr05G1035.1	799	4.00E-43	119/369(32.25)	165	PHI:799|MGG_03530|EDJ94565|318829|Magnaporthe_oryzae|Reduced_virulence
Chr05G1036.1	1260	6.00E-21	65/180(36.11)	95.9	PHI:1260|FGSG_13944|I1RUC7|5518|Gibberella_zeae_(related:_Fusarium_graminearum)|Unaffected_pathogenicity
Chr05G1038.1	404	2.00E-15	64/275(23.27)	76.3	PHI:404|PTH11|AAD30436|318829|Magnaporthe_oryzae|Reduced_virulence
Chr05G1039.1	784	8.00E-26	78/259(30.12)	102	PHI:784|MGG_00056|EDK03390|318829|Magnaporthe_oryzae|Reduced_virule

					nce
Chr05G1043.1	2510	9.00E-57	162/519(31.21)	200	PHI:2510|msdS/AfmsdC|Q6PWQ1|746128|Aspergillus_fumigatus|Unaffected_pathogenicity
Chr05G1044.1	2078	8.00E-98	140/234(59.83)	287	PHI:2078|Moatg10|MGG_14737|148305|Magnaporthe_oryzae_(related:_Magnaporthe_grisea)|Loss_of_pathogenicity
Chr05G1046.1	211	3.00E-06	35/107(32.71)	45.8	PHI:211|CaTUP1|AAB63195|5476|Candida_albicans|Reduced_virulence
Chr05G1048.1	211	4.00E-27	87/297(29.29)	112	PHI:211|CaTUP1|AAB63195|5476|Candida_albicans|Reduced_virulence
Chr05G1050.1	1243	2.00E-122	259/688(37.65)	385	PHI:1243|FGSG_06420|I1RBW7|5518|Gibberella_zeae_(related:_Fusarium_graminearum)|Unaffected_pathogenicity
Chr05G1051.1	2305	3.00E-18	52/116(44.83)	79.3	PHI:2305|BcFKBP12||40559|Botrytis_cinerea|Mixed_outcome
Chr05G1065.1	3236	1.00E-78	163/477(34.17)	260	Aph1|PHI:3236|J9VHR6|5207|Cryptococcus_neoformans|mixed_outcome_
Chr05G1072.1	2710	1.00E-141	209/396(52.78)	416	PHI:2710|mepB|H8ZZ79|474922|Colletotrichum_gloeosporioides|Reduced_virulence
Chr05G1076.1	1260	4.00E-11	67/224(29.91)	62	PHI:1260|FGSG_13944|I1RUC7|5518|Gibberella_zeae_(related:_Fusarium_graminearum)|Unaffected_pathogenicity
Chr05G1080.1	1300	0	370/680(54.41)	583	PHI:1300|GzbHLH001|I1RAL4|5518|Gibberella_zeae_(related:_Fusarium_graminearum)|Unaffected_pathogenicity
Chr05G1081.1	441	4.00E-08	38/171(22.22)	51.6	PHI:441|BTP1|CAE55153|40559|Botrytis_cinerea|Reduced_virulence
Chr05G1084.1	226	0	1255/1397(89.84)	2387	PHI:226|PEX6|AAK16738|5462|Colletotrichum_lagenarium|Loss_of_pathogenicity
Chr05G1085.1	2028	0	484/771(62.78)	980	PHI:2028|TGL1-2|G4NBI7|148305|Magnaporthe_oryzae_(related:_Magnaporthe_grisea)|Unaffected_pathogenicity
Chr05G1087.1	2183	7.00E-24	99/356(27.81)	100	PHI:2183|CPXB|EHA48040.1|148305|Magnaporthe_oryzae_(related:_Magnaporthe_grisea)|Unaffected_pathogenicity
Chr05G1091.1	3271	3.00E-150	200/218(91.74)	422	ARSEF_2860|PHI:3271|J4UI12|176275|Beauveria_bassiana|mixed_outcome_
Chr05G1092.1	2925	1.00E-88	129/210(61.43)	269	PHI:2925|lip2|J9MBA2|59765|Fusarium_oxysporum_f._sp._Lycopersici|Unaffec

					ted_pathogenicity
Chr05G1097.1	440	2.00E-158	339/990(34.24)	492	PHI:440|PMR1|CAB87245|5476|Candida_albicans|Reduced_virulence
Chr05G1103.1	1220	0	627/975(64.31)	1232	PHI:1220|FGSG_02488|I1RCF7|5518|Gibberella_zeae_(related:_Fusarium_graminearum)|Lethal
Chr05G1106.1	383	5.00E-26	55/145(37.93)	100	PHI:383|SOD5|EAL00626|5476|Candida_albicans|Loss_of_pathogenicity
Chr05G1109.1	1344	0	578/1193(48.45)	931	PHI:1344|GzC2H004|I1RAQ0|5518|Gibberella_zeae_(related:_Fusarium_graminearum)|Unaffected_pathogenicity
Chr05G1110.1	3314	3.00E-74	182/432(42.13)	240	cca1|PHI:3314|G4MNN4|318829|Magnaporthe_oryzae|loss_of_pathogenicity_
Chr05G1112.1	1539	1.00E-66	126/274(45.99)	209	PHI:1539|GzMyb003|I1RCG0|5518|Gibberella_zeae_(related:_Fusarium_graminearum)|Unaffected_pathogenicity
Chr05G1115.1	2109	2.00E-16	53/156(33.97)	70.9	PHI:2109|CNB|MGG_06933|148305|Magnaporthe_oryzae_(related:_Magnaporthe_grisea)|Mixed_outcome
Chr05G1116.1	2490	2.00E-51	108/295(36.61)	180	PHI:2490|MCA|E9B636|5665|Leishmania_mexicana|Mixed_outcome
Chr05G1126.1	2357	2.00E-23	124/477(26.00)	101	PHI:2357|CYP52X1|E2EAF6|475271|Beauveria_bassiana|Reduced_virulence
Chr05G1132.1	2219	7.00E-13	72/279(25.81)	71.6	PHI:2219|don1|XP_758565|5270|Ustilago_maydis|Reduced_virulence
Chr05G1133.1	2280	4.00E-55	88/138(63.77)	180	PHI:2280|Velvet_protein_family|J9MAX2|5507|Fusarium_oxysporum|Reduced_virulence
Chr05G1137.1	1938	2.00E-39	103/408(25.25)	155	PHI:1938|GzZC253|I1RMZ4|5518|Gibberella_zeae_(related:_Fusarium_graminearum)|Unaffected_pathogenicity
Chr05G1139.1	1354	0	325/591(54.99)	534	PHI:1354|GzC2H014|I1RCM9|5518|Gibberella_zeae_(related:_Fusarium_graminearum)|Reduced_virulence
Chr05G1142.1	1173	0	407/499(81.56)	849	PHI:1173|HDF1|I1RCN2|5518|Gibberella_zeae_(related:_Fusarium_graminearum)|Reduced_virulence
Chr05G1143.1	2866	1.00E-08	22/52(42.31)	53.5	PHI:2866|ORF19.3625|Q59Y24|5476|Candida_albicans|Reduced_virulence
Chr05G1147.1	3630	6.00E-56	131/338(38.76)	205	Rv2467|PHI:3630|L7N655|1773|Mycobacterium_tuberculosis|increased_virulence_

Chr05G1149.1	3207	2.00E-16	109/482(22.61)	80.5	Yvc1|PHI:3207|A1CLC0|746128|Aspergillus_fumigatus|reduced_virulence_
Chr05G1154.1	339	2.00E-11	49/178(27.53)	57.8	PHI:339|CLPT1|CAC41973|290576|Colletotrichum_lindemuthianum|Reduced_virulence
Chr05G1156.1	469	2.00E-101	161/334(48.20)	309	PHI:469|TOXG|AAD47837|5017|Cochliobolus_carbonum|Reduced_virulence
Chr05G1157.1	4575	6.00E-96	208/655(31.76)	311	NTH2|PHI:4575|Q5KEF4|5207|Cryptococcus_neoformans|mixed_outcome
Chr05G1162.1	1633	2.00E-108	167/333(50.15)	323	PHI:1633|GzWing005|I1RC15|5518|Gibberella_zeae_(related:_Fusarium_graminearum)|Unaffected_pathogenicity
Chr05G1170.1	1506	1.00E-141	274/574(47.74)	425	PHI:1506|GzHOME001|I1RC01|5518|Gibberella_zeae_(related:_Fusarium_graminearum)|Unaffected_pathogenicity
Chr05G1171.1	339	1.00E-21	55/213(25.82)	87.8	PHI:339|CLPT1|CAC41973|290576|Colletotrichum_lindemuthianum|Reduced_virulence
Chr05G1174.1	1352	1.00E-66	138/343(40.23)	228	PHI:1352|GzC2H012|I1RCK1|5518|Gibberella_zeae_(related:_Fusarium_graminearum)|Unaffected_pathogenicity
Chr05G1177.1	3060	8.00E-14	57/227(25.11)	72.8	Molrg1|PHI:3060|G4MZD0|318829|Magnaporthe_oryzae|mixed_outcome_
Chr05G1182.1	490	0	342/543(62.98)	662	PHI:490|FRP1|AAT85969|5507|Fusarium_oxysporum|Loss_of_pathogenicity
Chr05G1184.1	1477	6.00E-22	79/223(35.43)	96.7	PHI:1477|GzHMG009|I1RCK8|5518|Gibberella_zeae_(related:_Fusarium_graminearum)|Unaffected_pathogenicity
Chr05G1186.1	265	5.00E-20	63/188(33.51)	90.9	PHI:265|MET3|AAL92174|5207|Cryptococcus_neoformans|Loss_of_pathogenicity
Chr05G1193.1	1491	5.00E-09	30/161(18.63)	57.4	PHI:1491|GzHMG023|I1RST1|5518|Gibberella_zeae_(related:_Fusarium_graminearum)|Lethal
Chr05G1196.1	2544	9.00E-08	60/269(22.30)	51.6	PHI:2544|PAB1|Q4WK03|746128|Aspergillus_fumigatus|Mixed_outcome
Chr05G1199.1	1615	0	313/594(52.69)	585	PHI:1615|GzSGT1|I1RZJ1|5518|Gibberella_zeae_(related:_Fusarium_graminearum)|Unaffected_pathogenicity
Chr05G1201.1	358	2.00E-12	112/523(21.41)	68.2	PHI:358|ILV2|AAR29084|5207|Cryptococcus_neoformans|Loss_of_pathogenicity

Chr05G1216.1	2167	0	795/1285(61.87)	1630	PHI:2167|EXP5|G4N1A3|148305|Magnaporthe_oryzae_(related:_Magnaporthe_grisea)|Reduced_virulence
Chr05G1218.1	1974	0	394/589(66.89)	852	PHI:1974|GzZC289|I1RQN6|5518|Gibberella_zeae_(related:_Fusarium_graminearum)|Unaffected_pathogenicity
Chr05G1221.1	170	0	353/355(99.44)	736	PHI:170|CMK1|AAD50496|5462|Colletotrichum_lagenarium|Loss_of_pathogenicity
Chr05G1225.1	2490	2.00E-52	106/292(36.30)	182	PHI:2490|MCA|E9B636|5665|Leishmania_mexicana|Mixed_outcome
Chr05G1240.1	2962	7.00E-111	193/404(47.77)	336	PHI:2962|glyA|C5BEV2|67780|Edwardsiella_ictaluri|Reduced_virulence
Chr05G1241.1	697	0	284/381(74.54)	597	PHI:697|ugt51E1|AAM81358|5022|Leptosphaeria_maculans|Unaffected_pathogenicity
Chr05G1249.1	1555	3.00E-39	117/473(24.74)	147	PHI:1555|GzMyb019|I1RDG6|5518|Gibberella_zeae_(related:_Fusarium_graminearum)|Unaffected_pathogenicity
Chr05G1264.1	1420	6.00E-08	89/416(21.39)	55.1	PHI:1420|GzC2H088|I1S172|5518|Gibberella_zeae_(related:_Fusarium_graminearum)|Unaffected_pathogenicity
Chr05G1265.1	2315	1.00E-57	106/292(36.30)	190	PHI:2315|ChLae1|G4XKY9|5016|Cochliobolus_heterostrophus|Mixed_outcome
Chr05G1266.1	1552	5.00E-18	48/125(38.40)	86.7	PHI:1552|GzMyb016|I1S0N5|5518|Gibberella_zeae_(related:_Fusarium_graminearum)|Unaffected_pathogenicity
Chr05G1270.1	2393	6.00E-17	74/287(25.78)	80.9	PHI:2393|Related_to_O-methylsterigmatocystin_oxidoreductase|I1R980|5518|Gibberella_zeae_(related:_Fusarium_graminearum)|Increased_virulence_(Hypervirulence)
Chr05G1276.1	2315	9.00E-59	112/278(40.29)	192	PHI:2315|ChLae1|G4XKY9|5016|Cochliobolus_heterostrophus|Mixed_outcome
Chr05G1281.1	441	1.00E-21	87/367(23.71)	94	PHI:441|BTP1|CAE55153|40559|Botrytis_cinerea|Reduced_virulence
Chr05G1285.1	1948	7.00E-47	141/493(28.60)	169	PHI:1948|GzZC263|I1RI81|5518|Gibberella_zeae_(related:_Fusarium_graminearum)|Unaffected_pathogenicity
Chr05G1286.1	1317	0	824/1148(71.78)	1537	PHI:1317|GzBrom002|I1RQE9|5518|Gibberella_zeae_(related:_Fusarium_graminearum)|Reduced_virulence

Chr05G1287.1	3415	3.00E-24	115/485(23.71)	105	Fre2|PHI:3415|T2BNJ5|5207|Cryptococcus_neoformans|mixed_outcome_
Chr05G1290.1	1260	3.00E-17	67/176(38.07)	84.7	PHI:1260|FGSG_13944|I1RUC7|5518|Gibberella_zeae_(related:_Fusarium_graminearum)|Unaffected_pathogenicity
Chr05G1292.1	2544	7.00E-08	36/136(26.47)	53.1	PHI:2544|PAB1|Q4WK03|746128|Aspergillus_fumigatus|Mixed_outcome
Chr05G1294.1	1244	0	347/410(84.63)	722	PHI:1244|FGSG_00792|I1RA20|5518|Gibberella_zeae_(related:_Fusarium_graminearum)|Unaffected_pathogenicity
Chr05G1296.1	2538	7.00E-146	225/413(54.48)	425	PHI:2538|AUR1|Q4WXL8|746128|Aspergillus_fumigatus|Mixed_outcome
Chr05G1300.1	2020	4.00E-50	108/311(34.73)	189	PHI:2020|Tup1|XP_759427|5270|Ustilago_maydis|Mixed_outcome
Chr05G1304.1	1555	5.00E-19	63/283(22.26)	87.4	PHI:1555|GzMyb019|I1RDG6|5518|Gibberella_zeae_(related:_Fusarium_graminearum)|Unaffected_pathogenicity
Chr05G1307.1	3457	3.00E-180	276/535(51.59)	523	PHO84|PHI:3457|J9VMW8|5207|Cryptococcus_neoformans|mixed_outcome_
Chr05G1313.1	1342	3.00E-30	52/92(56.52)	109	PHI:1342|GzC2H002|I1RA24|5518|Gibberella_zeae_(related:_Fusarium_graminearum)|Unaffected_pathogenicity
Chr05G1315.1	2954	1.00E-14	65/239(27.20)	74.3	PHI:2954|TgNST1|B6KLD2|5811|Toxoplasma_gondii|Reduced_virulence
Chr05G1319.1	1608	4.00E-135	218/276(78.99)	385	PHI:1608|GzP53L001|Q4IR08|5518|Gibberella_zeae_(related:_Fusarium_graminearum)|Unaffected_pathogenicity
Chr05G1321.1	3278	1.00E-21	82/286(28.67)	98.6	CoIra1|PHI:3278|N4V0R3|5465|Colletotrichum_orbiculare|reduced_virulence_
Chr05G1323.1	1393	1.00E-13	55/209(26.32)	70.9	PHI:1393|GzC2H056|I1RU69|5518|Gibberella_zeae_(related:_Fusarium_graminearum)|Unaffected_pathogenicity
Chr05G1326.1	367	9.00E-35	82/246(33.33)	129	PHI:367|MRB1|AAT81148|5270|Ustilago_maydis|Reduced_virulence
Chr05G1327.1	184	7.00E-20	75/199(37.69)	84.3	PHI:184|RBT4|AAG09789|5476|Candida_albicans|Reduced_virulence
Chr05G1331.1	1451	0	570/804(70.90)	1097	PHI:1451|GzGH|I1RQL1|5518|Gibberella_zeae_(related:_Fusarium_graminearum)|Unaffected_pathogenicity
Chr05G1341.1	3381	1.00E-87	184/526(34.98)	288	FVEG_12533|PHI:3381|W7N2B4|117187|Fusarium_verticillioides|unaffected_pathogenicity_
Chr05G1346.1	144	2.00E-17	76/274(27.74)	81.3	PHI:144|CHT42|AAC05829|29875|Trichoderma_virens|Reduced_virulence

Chr05G1355.1	2172	0	430/524(82.06)	893	PHI:2172|TPS1|MGG_03860|148305|Magnaporthe_oryzae_(related:_Magnaporthe_grisea)|Loss_of_pathogenicity
Chr05G1361.1	219	2.00E-12	88/408(21.57)	68.2	PHI:219|LIG4|CAA64457|5476|Candida_albicans|Reduced_virulence
Chr05G1372.1	2022	2.00E-14	49/197(24.87)	68.9	PHI:2022|BUF1|MGG_02252|148305|Magnaporthe_oryzae_(related:_Magnaporthe_grisea)|Loss_of_pathogenicity
Chr05G1374.1	1727	5.00E-06	28/106(26.42)	45.8	PHI:1727|GzZC042|I1S5Q2|5518|Gibberella_zeae_(related:_Fusarium_graminearum)|Unaffected_pathogenicity
Chr05G1377.1	538	4.00E-30	118/498(23.69)	123	PHI:538|FRT1|AAU87358|40559|Botrytis_cinerea|Unaffected_pathogenicity
Chr05G1379.1	1195	0	1000/1699(58.86)	1606	PHI:1195|(Sc_Bck1)|I1RQI2|5518|Gibberella_zeae_(related:_Fusarium_graminearum)|Reduced_virulence
Chr05G1381.1	1260	3.00E-11	45/129(34.88)	62	PHI:1260|FGSG_13944|I1RUC7|5518|Gibberella_zeae_(related:_Fusarium_graminearum)|Unaffected_pathogenicity
Chr05G1384.1	2510	1.00E-27	73/197(37.06)	115	PHI:2510|msdS/AfmsdC|Q6PWQ1|746128|Aspergillus_fumigatus|Unaffected_pathogenicity
Chr05G1388.1	1284	8.00E-11	57/194(29.38)	61.6	PHI:1284|FGSG_13509|I1RI73|5518|Gibberella_zeae_(related:_Fusarium_graminearum)|Unaffected_pathogenicity
Chr05G1391.1	3594	2.00E-21	81/268(30.22)	95.5	Ptp2|PHI:3594|H6VMG0|5207|Cryptococcus_neoformans|mixed_outcome_
Chr05G1393.1	1566	2.00E-98	156/358(43.58)	301	PHI:1566|GzOB006|I1RC95|5518|Gibberella_zeae_(related:_Fusarium_graminearum)|Lethal
Chr05G1396.1	2841	1.00E-69	119/257(46.30)	219	PHI:2841|CnSEC14-2|F6K8L6|5207|Cryptococcus_neoformans|Unaffected_pathogenicity
Chr05G1398.1	1508	8.00E-40	116/378(30.69)	157	PHI:1508|GzHOME003|I1RNA9|5518|Gibberella_zeae_(related:_Fusarium_graminearum)|Unaffected_pathogenicity
Chr05G1401.1	256	7.00E-30	96/251(38.25)	111	PHI:256|GAS1|AAK52794|318829|Magnaporthe_oryzae|Reduced_virulence
Chr05G1402.1	1424	3.00E-17	94/374(25.13)	83.6	PHI:1424|GzC2H092|I1S3J7|5518|Gibberella_zeae_(related:_Fusarium_graminearum)|Unaffected_pathogenicity

Chr05G1412.1	1637	6.00E-37	85/259(32.82)	142	PHI:1637|GzWing009|Q4IEV4|5518|Gibberella_zeae_(related:_Fusarium_graminearum)|Lethal
Chr05G1421.1	1047	4.00E-18	87/299(29.10)	81.6	PHI:1047|CTB6|ABK64183|29003|Cercospora_nicotianae|Reduced_virulence
Chr05G1428.1	1697	8.00E-144	251/559(44.90)	434	PHI:1697|GzZC012|I1S3R7|5518|Gibberella_zeae_(related:_Fusarium_graminearum)|Unaffected_pathogenicity
Chr05G1435.1	2175	1.00E-11	63/170(37.06)	62.4	PHI:2175|NMR3|MGG_09705|148305|Magnaporthe_oryzae_(related:_Magnaporthe_grisea)|Reduced_virulence
Chr05G1436.1	1725	1.00E-18	64/219(29.22)	83.6	PHI:1725|GzZC040|I1SAE7|5518|Gibberella_zeae_(related:_Fusarium_graminearum)|Unaffected_pathogenicity
Chr05G1437.1	254	3.00E-105	156/271(57.56)	311	PHI:254|FOW1|BAB85760|5507|Fusarium_oxysporum|Reduced_virulence
Chr05G1440.1	2520	5.00E-35	125/458(27.29)	140	PHI:2520|LYS4|Q4WUL6|746128|Aspergillus_fumigatus|Lethal
Chr05G1441.1	2968	3.00E-19	97/370(26.22)	86.7	PHI:2968|Hxs1|J9VQA5|5207|Cryptococcus_neoformans|Reduced_virulence
Chr05G1442.1	1762	0	331/633(52.29)	649	PHI:1762|GzZC077|I1RXJ4|5518|Gibberella_zeae_(related:_Fusarium_graminearum)|Unaffected_pathogenicity
Chr05G1448.1	211	4.00E-53	115/289(39.79)	192	PHI:211|CaTUP1|AAB63195|5476|Candida_albicans|Reduced_virulence
Chr05G1455.1	1620	6.00E-16	42/127(33.07)	80.1	PHI:1620|GzJUM002|I1RD69|5518|Gibberella_zeae_(related:_Fusarium_graminearum)|Unaffected_pathogenicity
Chr05G1459.1	886	5.00E-10	48/193(24.87)	57.8	PHI:886|MGG_13052|EDK06087|318829|Magnaporthe_oryzae|Reduced_virulence
Chr05G1463.1	2549	3.00E-07	24/87(27.59)	48.9	PHI:2549|MAK5|Q4WMS3|746128|Aspergillus_fumigatus|Mixed_outcome
Chr03G0002.1	2315	3.00E-32	64/157(40.76)	118	PHI:2315|ChLae1|G4XKY9|5016|Cochliobolus_heterostrophus|Mixed_outcome
Chr03G0004.1	1902	9.00E-28	99/346(28.61)	113	PHI:1902|GzZC217|I1RJS7|5518|Gibberella_zeae_(related:_Fusarium_graminearum)|Unaffected_pathogenicity
Chr03G0005.1	1662	2.00E-20	50/187(26.74)	89	PHI:1662|GzCCHC002|I1REJ1|5518|Gibberella_zeae_(related:_Fusarium_graminearum)|Unaffected_pathogenicity
Chr03G0006.1	3329	1.00E-33	87/278(31.29)	125	Ss-oah1|PHI:3329|A7ESB3|5180|Sclerotinia_sclerotiorum|mixed_outcome_

Chr03G0007.1	2968	8.00E-47	144/505(28.51)	170	PHI:2968|Hxs1|J9VQA5|5207|Cryptococcus_neoformans|Reduced_virulence
Chr03G0008.1	1865	2.00E-173	281/667(42.13)	528	PHI:1865|GzZC180|I1S9C5|5518|Gibberella_zeae_(related:_Fusarium_graminearum)|Unaffected_pathogenicity
Chr03G0011.1	1726	4.00E-83	157/401(39.15)	270	PHI:1726|GzZC041|I1S9C7|5518|Gibberella_zeae_(related:_Fusarium_graminearum)|Unaffected_pathogenicity
Chr03G0012.1	1662	2.00E-39	113/436(25.92)	152	PHI:1662|GzCCHC002|I1REJ1|5518|Gibberella_zeae_(related:_Fusarium_graminearum)|Unaffected_pathogenicity
Chr03G0013.1	1555	4.00E-45	129/475(27.16)	165	PHI:1555|GzMyb019|I1RDG6|5518|Gibberella_zeae_(related:_Fusarium_graminearum)|Unaffected_pathogenicity
Chr03G0016.1	881	6.00E-07	54/220(24.55)	48.1	PHI:881|MGG_04556|EDJ96020|318829|Magnaporthe_oryzae|Reduced_virulence
Chr03G0022.1	1555	1.00E-55	139/462(30.09)	194	PHI:1555|GzMyb019|I1RDG6|5518|Gibberella_zeae_(related:_Fusarium_graminearum)|Unaffected_pathogenicity
Chr03G0024.1	441	2.00E-19	64/249(25.70)	85.9	PHI:441|BTP1|CAE55153|40559|Botrytis_cinerea|Reduced_virulence
Chr03G0025.1	438	4.00E-59	154/482(31.95)	204	PHI:438|BcBOT1_(related:_CND5)|AAQ16576|40559|Botrytis_cinerea|Reduced_virulence
Chr03G0028.1	2020	1.00E-19	81/299(27.09)	91.3	PHI:2020|Tup1|XP_759427|5270|Ustilago_maydis|Mixed_outcome
Chr03G0029.1	2644	4.00E-16	32/85(37.65)	69.3	PHI:2644|thioredoxin_1|P0AA28|90371|Salmonella_enterica_serovar_Typhimurium|Reduced_virulence
Chr03G0031.1	569	9.00E-12	27/53(50.94)	62	PHI:569|XYL3|AAC06239|5507|Fusarium_oxysporum|Unaffected_pathogenicity
Chr03G0033.1	346	2.00E-11	50/192(26.04)	63.5	PHI:346|CRU1|AAN10186|5270|Ustilago_maydis|Reduced_virulence
Chr03G0036.1	334	9.00E-10	71/299(23.75)	57.4	PHI:334|CGB1|AAO25585|5016|Cochliobolus_heterostrophus|Loss_of_pathogenicity
Chr03G0037.1	2227	2.00E-91	191/500(38.20)	290	PHI:2227|pao|D4III8|5270|Ustilago_maydis|Mixed_outcome
Chr03G0047.1	1544	8.00E-32	63/83(75.90)	117	PHI:1544|GzMyb008|I1RG68|5518|Gibberella_zeae_(related:_Fusarium_graminearum)|Unaffected_pathogenicity

Chr03G0048.1	1515	3.00E-123	192/343(55.98)	360	PHI:1515|GzHOMEL009|I1RG67|5518|Gibberella_zeae_(related:_Fusarium_graminearum)|Unaffected_pathogenicity
Chr03G0050.1	2639	0	390/543(71.82)	788	PHI:2639|Ilv3B|Q4WK65|746128|Aspergillus_fumigatus|Mixed_outcome
Chr03G0051.1	3097	0	379/507(74.75)	779	Kre2/Mnt1|PHI:3097|J5JEX5|176275|Beauveria_bassiana|reduced_virulence_
Chr03G0054.1	2086	4.00E-09	34/95(35.79)	57	PHI:2086|Moatg24|MGG_03638|148305|Magnaporthe_oryzae_(related:_Magnaporthe_grisea)|Unaffected_pathogenicity
Chr03G0056.1	387	7.00E-09	92/378(24.34)	55.1	PHI:387|UGD1|AAK95561|5207|Cryptococcus_neoformans|Loss_of_pathogenicity
Chr03G0058.1	2226	8.00E-35	123/513(23.98)	139	PHI:2226|Mcs1|XP_759351|5270|Ustilago_maydis|Reduced_virulence
Chr03G0062.1	4211	5.00E-12	76/334(22.75)	65.9	FRE3|PHI:4211|J9VNH2|5207|Cryptococcus_neoformans|effector_(plant_avirulence_determinant)
Chr03G0063.1	3415	3.00E-35	124/448(27.68)	138	Fre2|PHI:3415|T2BNJ5|5207|Cryptococcus_neoformans|mixed_outcome_
Chr03G0070.1	1527	4.00E-40	104/362(28.73)	148	PHI:1527|GzHOMEL040|I1S9A1|5518|Gibberella_zeae_(related:_Fusarium_graminearum)|Lethal
Chr03G0073.1	1442	2.00E-24	113/521(21.69)	103	PHI:1442|GzDNL|I1RG27|5518|Gibberella_zeae_(related:_Fusarium_graminearum)|Unaffected_pathogenicity
Chr03G0074.1	2171	1.00E-128	248/667(37.18)	398	PHI:2171|Peroxisomal_copper_amine_oxidase|MGG_02681|148305|Magnaporthe_oryzae_(related:_Magnaporthe_grisea)|Reduced_virulence
Chr03G0075.1	2315	2.00E-54	108/278(38.85)	178	PHI:2315|ChLae1|G4XKY9|5016|Cochliobolus_heterostrophus|Mixed_outcome
Chr03G0080.1	441	3.00E-32	92/347(26.51)	124	PHI:441|BTP1|CAE55153|40559|Botrytis_cinerea|Reduced_virulence
Chr03G0081.1	2844	8.00E-19	72/277(25.99)	81.6	PHI:2844|BRM2|O93802|5599|Alternaria_alternata|Unaffected_pathogenicity
Chr03G0082.1	1358	0	825/1159(71.18)	1557	PHI:1358|GzC2H018|I1RG87|5518|Gibberella_zeae_(related:_Fusarium_graminearum)|Unaffected_pathogenicity
Chr03G0083.1	1420	0	334/787(42.44)	582	PHI:1420|GzC2H088|I1S172|5518|Gibberella_zeae_(related:_Fusarium_graminearum)|Unaffected_pathogenicity
Chr03G0084.1	1552	1.00E-16	76/288(26.39)	82.8	PHI:1552|GzMyb016|I1S0N5|5518|Gibberella_zeae_(related:_Fusarium_grami

					nearum)|Unaffected_pathogenicity
Chr03G0086.1	2898	2.00E-25	90/310(29.03)	102	PHI:2896|BEC1005|CCU82697|62688|Blumeria_graminis_f._sp._hordei|Effector_(plant_avirulence_determinant)
Chr03G0087.1	812	8.00E-42	107/354(30.23)	152	PHI:812|MGG_10702|EDJ94108|318829|Magnaporthe_oryzae|Reduced_virulence
Chr03G0088.1	114	4.00E-165	235/348(67.53)	469	PHI:114|PG1|AAC05015|5507|Fusarium_oxysporum|Unaffected_pathogenicity
Chr03G0089.1	714	8.00E-22	103/361(28.53)	94.7	PHI:714|PKS4_(related:_ZEA1)|ABB90283|5518|Fusarium_graminearum|Unaffected_pathogenicity
Chr03G0090.1	73	2.00E-48	134/359(37.33)	172	PHI:73|SAP3|AAA34372|5476|Candida_albicans|Reduced_virulence
Chr03G0091.1	224	2.00E-34	77/213(36.15)	122	PHI:224|PEP2|AAK11167|140110|Nectria_haematococca_(related:_Fusarium_solani)|Reduced_virulence
Chr03G0092.1	112	0	264/447(59.06)	560	PHI:112|MAK1|AAC49410|140110|Nectria_haematococca_(related:_Fusarium_solani)|Reduced_virulence
Chr03G0098.1	1260	1.00E-08	50/163(30.67)	56.2	PHI:1260|FGSG_13944|I1RUC7|5518|Gibberella_zeae_(related:_Fusarium_graminearum)|Unaffected_pathogenicity
Chr03G0099.1	1552	3.00E-23	91/332(27.41)	104	PHI:1552|GzMyb016|I1S0N5|5518|Gibberella_zeae_(related:_Fusarium_graminearum)|Unaffected_pathogenicity
Chr03G0108.1	1194	1.00E-07	56/201(27.86)	50.1	PHI:1194|(Sc_Mkk1/Mkk2)|I1RT06|5518|Gibberella_zeae_(related:_Fusarium_graminearum)|Reduced_virulence
Chr03G0110.1	337	0	268/334(80.24)	573	PHI:337|CHS7|AAT77184|5507|Fusarium_oxysporum|Reduced_virulence
Chr03G0118.1	1662	3.00E-33	132/470(28.09)	132	PHI:1662|GzCCHC002|I1REJ1|5518|Gibberella_zeae_(related:_Fusarium_graminearum)|Unaffected_pathogenicity
Chr03G0119.1	2403	1.00E-112	162/263(61.60)	328	PHI:2403|CSN1|A9QUB2|169388|Fusarium_solani|Mixed_outcome
Chr03G0120.1	2802	5.00E-19	79/276(28.62)	84.7	PHI:2802|3hnr|B2ZRQ4|93612|Setosphaeria_turcica|Unaffected_pathogenicity
Chr03G0123.1	404	3.00E-15	67/249(26.91)	73.9	PHI:404|PTH11|AAD30436|318829|Magnaporthe_oryzae|Reduced_virulence
Chr03G0126.1	2206	1.00E-133	195/325(60.00)	394	PHI:2206|endo-1,4-beta-xylanase_precursor_[GH10_family]|MGG_14243|1483

					05|Magnaporthe_oryzae_(related:_Magnaporthe_grisea)|Reduced_virulence
Chr03G0131.1	413	2.00E-17	110/417(26.38)	81.6	PHI:413|MPD1|AAT84078|13684|Stagonospora_nodorum|Unaffected_pathogenicity
Chr03G0143.1	1773	1.00E-17	97/379(25.59)	82	PHI:1773|GzZC088|I1S409|5518|Gibberella_zeae_(related:_Fusarium_graminearum)|Unaffected_pathogenicity
Chr03G0147.1	2020	4.00E-20	52/125(41.60)	93.2	PHI:2020|Tup1|XP_759427|5270|Ustilago_maydis|Mixed_outcome
Chr03G0148.1	1423	7.00E-19	116/509(22.79)	87.8	PHI:1423|GzC2H091|I1S2R1|5518|Gibberella_zeae_(related:_Fusarium_graminearum)|Unaffected_pathogenicity
Chr03G0152.1	404	5.00E-10	47/191(24.61)	58.9	PHI:404|PTH11|AAD30436|318829|Magnaporthe_oryzae|Reduced_virulence
Chr03G0153.1	2378	8.00E-62	168/592(28.38)	214	PHI:2378|DEP4|D2E9W9|29001|Alternaria_brassicicola|Mixed_outcome
Chr03G0159.1	2491	0	378/595(63.53)	678	PHI:2491|FgPTC1|I1RJS9|5518|Gibberella_zeae_(related:_Fusarium_graminearum)|Reduced_virulence
Chr03G0160.1	1527	1.00E-40	104/343(30.32)	149	PHI:1527|GzHOMEL040|I1S9A1|5518|Gibberella_zeae_(related:_Fusarium_graminearum)|Lethal
Chr03G0161.1	1902	0	685/1086(63.08)	1289	PHI:1902|GzZC217|I1RJS7|5518|Gibberella_zeae_(related:_Fusarium_graminearum)|Unaffected_pathogenicity
Chr03G0165.1	1162	3.00E-127	189/294(64.29)	366	PHI:1162|BDM1|I1RJS3|5518|Gibberella_zeae_(related:_Fusarium_graminearum)|Mixed_outcome
Chr03G0166.1	2310	0	352/359(98.05)	740	PHI:2310|VGB|I2DB61|27337|Verticillium_dahliae|Reduced_virulence
Chr03G0170.1	3629	7.00E-36	125/458(27.29)	137	Rv0392c|PHI:3629|P95200|1773|Mycobacterium_tuberculosis|unaffected_pathogenicity_
Chr03G0175.1	2121	0	382/496(77.02)	733	PHI:2121|MST50p|MGG_05199|148305|Magnaporthe_oryzae_(related:_Magnaporthe_grisea)|Loss_of_pathogenicity
Chr03G0176.1	2248	2.00E-46	129/436(29.59)	171	PHI:2248|Als1|Q1L2E2|13684|Phaeosphaeria_nodorum_(related:_Stagonospora_nodorum)|Mixed_outcome
Chr03G0177.1	12	5.00E-06	108/480(22.50)	47	PHI:12|HTS1|AAA33023|5017|Cochliobolus_carbonum|Loss_of_pathogenicity

Chr03G0180.1	2180	0	461/884(52.15)	670	PHI:2180|MoTea4|G4N7A0|148305|Magnaporthe_oryzae_(related:_Magnaporthe_grisea)|Loss_of_pathogenicity
Chr03G0182.1	981	1.00E-12	39/92(42.39)	66.6	PHI:981|hopI1|AAL84247|59511|Pseudomonas_syringae|Effector_(plant_avirulence_determinant)
Chr03G0183.1	2521	1.00E-07	56/250(22.40)	52.4	PHI:2521|GUS1|Q4WEM7|746128|Aspergillus_fumigatus|Lethal
Chr03G0184.1	1025	0	386/585(65.98)	733	PHI:1025|bcnoxR|CAP12326|40559|Botrytis_cinerea|Reduced_virulence
Chr03G0190.1	1458	5.00E-60	143/441(32.43)	211	PHI:1458|GzAra006|Q4I7F9|5518|Gibberella_zeae_(related:_Fusarium_graminearum)|Unaffected_pathogenicity
Chr03G0191.1	35	9.00E-167	280/441(63.49)	478	PHI:35|CON7|AAB69694|318829|Magnaporthe_oryzae|Reduced_virulence
Chr03G0206.1	404	6.00E-35	104/433(24.02)	135	PHI:404|PTH11|AAD30436|318829|Magnaporthe_oryzae|Reduced_virulence
Chr03G0208.1	144	5.00E-44	110/365(30.14)	156	PHI:144|CHT42|AAC05829|29875|Trichoderma_virens|Reduced_virulence
Chr03G0212.1	547	2.00E-106	157/305(51.48)	325	PHI:547|CEL5A|AAT40313|40559|Botrytis_cinerea|Unaffected_pathogenicity
Chr03G0214.1	922	2.00E-68	194/650(29.85)	236	PHI:922|um03615||5270|Ustilago_maydis|Unaffected_pathogenicity
Chr03G0215.1	404	7.00E-29	89/359(24.79)	116	PHI:404|PTH11|AAD30436|318829|Magnaporthe_oryzae|Reduced_virulence
Chr03G0216.1	1555	5.00E-37	110/484(22.73)	141	PHI:1555|GzMyb019|I1RDG6|5518|Gibberella_zeae_(related:_Fusarium_graminearum)|Unaffected_pathogenicity
Chr03G0217.1	1455	3.00E-12	86/328(26.22)	65.1	PHI:1455|GzAra003|I1RKU6|5518|Gibberella_zeae_(related:_Fusarium_graminearum)|Unaffected_pathogenicity
Chr03G0220.1	404	9.00E-12	51/213(23.94)	63.5	PHI:404|PTH11|AAD30436|318829|Magnaporthe_oryzae|Reduced_virulence
Chr03G0221.1	2837	6.00E-23	77/248(31.05)	93.2	PHI:2837|OXI1|D2SZX7|5016|Cochliobolus_heterostrophus|Reduced_virulence
Chr03G0228.1	2968	3.00E-43	131/493(26.57)	159	PHI:2968|Hxs1|J9VQA5|5207|Cryptococcus_neoformans|Reduced_virulence
Chr03G0232.1	2107	2.00E-41	125/391(31.97)	152	PHI:2107|Zinc-regulated_transporter_2|MGG_05905|148305|Magnaporthe_oryzae_(related:_Magnaporthe_grisea)|Mixed_outcome
Chr03G0234.1	1798	2.00E-15	130/597(21.78)	77	PHI:1798|GzZC113|I1RSP9|5518|Gibberella_zeae_(related:_Fusarium_graminearum)|Unaffected_pathogenicity
Chr03G0241.1	1161	2.00E-57	144/452(31.86)	201	PHI:1161|MgMfs1|A4ZGP3|54734|Mycosphaerella_graminicola_(related:_Zymo

					septoria_triticii)|Chemistry_target
Chr03G0242.1	55	6.00E-25	80/242(33.06)	104	PHI:55|PKS1|AAB08104|5016|Cochliobolus_heterostrophus|Reduced_virulence
Chr03G0243.1	541	0	335/580(57.76)	674	PHI:541|LIP1|AAU87359|332648|Botrytis_cinerea|Unaffected_pathogenicity
Chr03G0245.1	860	6.00E-40	92/253(36.36)	150	PHI:860|MSP1|AAX07670|318829|Magnaporthe_oryzae|Reduced_virulence
Chr03G0246.1	2968	4.00E-50	143/522(27.39)	184	PHI:2968|Hxs1|J9VQA5|5207|Cryptococcus_neoformans|Reduced_virulence
Chr03G0250.1	2693	0	754/1451(51.96)	1514	PHI:2693|GcABC-G1|F0XP73|226899|Grosmannia_clavigera|Reduced_virulence
Chr03G0252.1	1816	1.00E-23	102/468(21.79)	103	PHI:1816|GzZC131|I1RRS3|5518|Gibberella_zeae_(related:_Fusarium_graminearum)|Unaffected_pathogenicity
Chr03G0259.1	1662	9.00E-14	59/252(23.41)	71.2	PHI:1662|GzCCHC002|I1REJ1|5518|Gibberella_zeae_(related:_Fusarium_graminearum)|Unaffected_pathogenicity
Chr03G0260.1	438	1.00E-42	120/461(26.03)	157	PHI:438|BcBOT1_(related:_CND5)|AAQ16576|40559|Botrytis_cinerea|Reduced_virulence
Chr03G0261.1	2256	2.00E-36	104/354(29.38)	135	PHI:2256|Xdh1|Q0UA24|13684|Phaeosphaeria_nodorum_(related:_Stagonospora_nodorum)|Unaffected_pathogenicity
Chr03G0277.1	2060	0	626/1010(61.98)	1205	PHI:2060|MgLig4|B6ZH51|148305|Magnaporthe_oryzae_(related:_Magnaporthe_grisea)|Unaffected_pathogenicity
Chr03G0280.1	903	2.00E-159	230/500(46.00)	469	PHI:903|um01888||5270|Ustilago_maydis|Unaffected_pathogenicity
Chr03G0290.1	413	6.00E-25	112/400(28.00)	103	PHI:413|MPD1|AAT84078|13684|Stagonospora_nodorum|Unaffected_pathogenicity
Chr03G0292.1	784	2.00E-14	78/280(27.86)	69.3	PHI:784|MGG_00056|EDK03390|318829|Magnaporthe_oryzae|Reduced_virulence
Chr03G0293.1	2175	1.00E-45	108/314(34.39)	157	PHI:2175|NMR3|MGG_09705|148305|Magnaporthe_oryzae_(related:_Magnaporthe_grisea)|Reduced_virulence
Chr03G0296.1	3025	1.00E-08	33/110(30.00)	53.5	RnPKS1|PHI:3025|X5IFG8|77044|Rosellinia_necatrix|unaffected_pathogenicity

_

Chr03G0298.1	1321	2.00E-07	53/196(27.04)	50.1	PHI:1321|ZEB2|I1RFC6|5518|Gibberella_zeae_(related:_Fusarium_graminearum)|Unaffected_pathogenicity
Chr03G0301.1	267	7.00E-117	375/1496(25.07)	403	PHI:267|MLT1|AAD51594|5476|Candida_albicans|Reduced_virulence
Chr03G0303.1	160	0	1207/3609(33.44)	1791	PHI:160|AMT|AAF01762|5599|Alternaria_alternata|Loss_of_pathogenicity
Chr03G0304.1	255	0	1147/2586(44.35)	2103	PHI:255|FUM1_(related:_FUM5)|AAD43562|5127|Gibberella_moniliformis|Unaffected_pathogenicity
Chr03G0305.1	508	4.00E-121	215/575(37.39)	372	PHI:508|AFT1|BAB69076|5599|Alternaria_alternata|Loss_of_pathogenicity
Chr03G0314.1	2082	5.00E-58	97/198(48.99)	182	PHI:2082|Moatg16|MGG_05255|148305|Magnaporthe_oryzae_(related:_Magnaporthe_grisea)|Loss_of_pathogenicity
Chr03G0319.1	280	9.00E-08	47/193(24.35)	54.3	PHI:280|CCN1|AAG36938|5207|Cryptococcus_neoformans|Reduced_virulence
Chr03G0328.1	1835	9.00E-32	116/425(27.29)	130	PHI:1835|GzZC150|I1RIL1|5518|Gibberella_zeae_(related:_Fusarium_graminearum)|Unaffected_pathogenicity
Chr03G0329.1	1812	2.00E-25	57/126(45.24)	108	PHI:1812|GzZC127|I1RV71|5518|Gibberella_zeae_(related:_Fusarium_graminearum)|Unaffected_pathogenicity
Chr03G0334.1	1046	1.00E-44	120/466(25.75)	162	PHI:1046|CTB5|ABK64182|29003|Cercospora_nicotianae|Reduced_virulence
Chr03G0335.1	404	2.00E-24	50/168(29.76)	102	PHI:404|PTH11|AAD30436|318829|Magnaporthe_oryzae|Reduced_virulence
Chr03G0341.1	3213	0	288/355(81.13)	555	MoCDIP1|PHI:3213|G4N8Y3|318829|Magnaporthe_oryzae|mixed_outcome_
Chr03G0343.1	441	2.00E-30	100/350(28.57)	118	PHI:441|BTP1|CAE55153|40559|Botrytis_cinerea|Reduced_virulence
Chr03G0347.1	2032	3.00E-103	200/524(38.17)	323	PHI:2032|VTL1|G4NGA7|148305|Magnaporthe_oryzae_(related:_Magnaporthe_grisea)|Unaffected_pathogenicity
Chr03G0348.1	126	9.00E-12	90/369(24.39)	63.2	PHI:126|SAP5|CAA82923|5476|Candida_albicans|Reduced_virulence
Chr03G0349.1	1812	1.00E-30	124/444(27.93)	124	PHI:1812|GzZC127|I1RV71|5518|Gibberella_zeae_(related:_Fusarium_graminearum)|Unaffected_pathogenicity
Chr03G0350.1	1662	3.00E-82	162/502(32.27)	279	PHI:1662|GzCCHC002|I1REJ1|5518|Gibberella_zeae_(related:_Fusarium_graminearum)|Unaffected_pathogenicity

Chr03G0359.1	2552	4.00E-06	24/85(28.24)	47	PHI:2552|NOP4|Q4WNM3|746128|Aspergillus_fumigatus|Mixed_outcome
Chr03G0368.1	1662	2.00E-40	105/412(25.49)	155	PHI:1662|GzCCHC002|I1REJ1|5518|Gibberella_zeae_(related:_Fusarium_graminearum)|Unaffected_pathogenicity
Chr03G0369.1	3216	7.00E-47	94/224(41.96)	159	MoCDIP4|PHI:3216|G4MVX4|318829|Magnaporthe_oryzae|mixed_outcome_
Chr03G0374.1	541	2.00E-41	144/389(37.02)	155	PHI:541|LIP1|AAU87359|332648|Botrytis_cinerea|Unaffected_pathogenicity
Chr03G0376.1	179	5.00E-58	109/211(51.66)	188	PHI:179|PELA|AAA33338|140110|Nectria_haematococca_(related:_Fusarium_solani)|Reduced_virulence
Chr03G0377.1	413	7.00E-32	118/434(27.19)	123	PHI:413|MPD1|AAT84078|13684|Stagonospora_nodorum|Unaffected_pathogenicity
Chr03G0378.1	438	4.00E-43	119/471(25.27)	158	PHI:438|BcBOT1_(related:_CND5)|AAQ16576|40559|Botrytis_cinerea|Reduced_virulence
Chr03G0379.1	1401	1.00E-07	34/150(22.67)	51.6	PHI:1401|GzC2H064|I1RVY6|5518|Gibberella_zeae_(related:_Fusarium_graminearum)|Unaffected_pathogenicity
Chr03G0385.1	2693	0	827/1442(57.35)	1704	PHI:2693|GcABC-G1|F0XP73|226899|Grosmannia_clavigera|Reduced_virulence
Chr03G0386.1	1825	1.00E-147	224/556(40.29)	441	PHI:1825|GzZC140|I1RY88|5518|Gibberella_zeae_(related:_Fusarium_graminearum)|Unaffected_pathogenicity
Chr03G0387.1	2393	2.00E-37	138/494(27.94)	141	PHI:2393|Related_to_O-methylsterigmatocystin_oxidoreductase|I1R980|5518|Gibberella_zeae_(related:_Fusarium_graminearum)|Increased_virulence_(Hypervirulence)
Chr03G0388.1	1741	3.00E-17	35/79(44.30)	75.5	PHI:1741|GzZC056|I1S780|5518|Gibberella_zeae_(related:_Fusarium_graminearum)|Unaffected_pathogenicity
Chr03G0394.1	488	7.00E-26	66/230(28.70)	110	PHI:488|IRS4|EAK93583|5476|Candida_albicans|Reduced_virulence
Chr03G0403.1	2022	7.00E-06	29/125(23.20)	44.3	PHI:2022|BUF1|MGG_02252|148305|Magnaporthe_oryzae_(related:_Magnaporthe_grisea)|Loss_of_pathogenicity
Chr03G0404.1	3415	2.00E-25	93/355(26.20)	108	Fre2|PHI:3415|T2BNJ5|5207|Cryptococcus_neoformans|mixed_outcome_

Chr03G0408.1	2908	9.00E-18	85/377(22.55)	83.6	PHI:2908|CYP51B|I1RBR4|5518|Fusarium_graminearum|Mixed_outcome
Chr03G0409.1	2336	8.00E-34	83/251(33.07)	133	PHI:2336|NIA1|O00101|13684|Stagonospora_nodorum_(related:_Phaeosphaeria_nodorum)|Unaffected_pathogenicity
Chr03G0411.1	1924	5.00E-59	181/624(29.01)	211	PHI:1924|GzZC239|I1RH93|5518|Gibberella_zeae_(related:_Fusarium_graminearum)|Unaffected_pathogenicity
Chr03G0415.1	1727	6.00E-06	31/106(29.25)	45.4	PHI:1727|GzZC042|I1S5Q2|5518|Gibberella_zeae_(related:_Fusarium_graminearum)|Unaffected_pathogenicity
Chr03G0423.1	358	4.00E-23	77/277(27.80)	100	PHI:358|ILV2|AAR29084|5207|Cryptococcus_neoformans|Loss_of_pathogenicity
Chr03G0429.1	2315	9.00E-60	110/289(38.06)	195	PHI:2315|ChLae1|G4XKY9|5016|Cochliobolus_heterostrophus|Mixed_outcome
Chr03G0434.1	538	0	349/575(60.70)	656	PHI:538|FRT1|AAU87358|40559|Botrytis_cinerea|Unaffected_pathogenicity
Chr03G0436.1	1260	3.00E-20	70/190(36.84)	94	PHI:1260|FGSG_13944|I1RUC7|5518|Gibberella_zeae_(related:_Fusarium_graminearum)|Unaffected_pathogenicity
Chr03G0445.1	2296	0	448/1080(41.48)	765	PHI:2296|tmpL|ABW87261.1|29001|Alternaria_brassicicola|Reduced_virulence
Chr03G0446.1	2322	2.00E-39	88/256(34.38)	138	PHI:2322|SidH|Q4WF54|746128|Aspergillus_fumigatus|Reduced_virulence
Chr03G0448.1	1260	7.00E-12	76/253(30.04)	67.8	PHI:1260|FGSG_13944|I1RUC7|5518|Gibberella_zeae_(related:_Fusarium_graminearum)|Unaffected_pathogenicity
Chr03G0449.1	2240	9.00E-24	123/502(24.50)	102	PHI:2240|Srt1|Q4PBY9|5270|Ustilago_maydis|reduced_virulence
Chr03G0450.1	404	2.00E-16	57/264(21.59)	79.3	PHI:404|PTH11|AAD30436|318829|Magnaporthe_oryzae|Reduced_virulence
Chr03G0453.1	1978	1.00E-74	154/483(31.88)	245	PHI:1978|GzZC293|I1RLK0|5518|Gibberella_zeae_(related:_Fusarium_graminearum)|Lethal
Chr03G0464.1	1543	3.00E-109	182/254(71.65)	318	PHI:1543|GzMyb007|I1RFQ3|5518|Gibberella_zeae_(related:_Fusarium_graminearum)|Unaffected_pathogenicity
Chr03G0466.1	2821	3.00E-81	170/485(35.05)	290	PHI:2821|SNF2|Q5ALP9|5476|Candida_albicans|Reduced_virulence
Chr03G0472.1	2247	3.00E-07	58/213(27.23)	48.5	PHI:2247|Sch1|EAT82552|13684|Phaeosphaeria_nodorum_(related:_Stagonospora_nodorum)|Unaffected_pathogenicity

Chr03G0474.1	509	3.00E-07	42/175(24.00)	48.1	PHI:509|AFT3|BAB69078|5599|Alternaria_alternata|Loss_of_pathogenicity
Chr03G0477.1	1555	3.00E-62	142/475(29.89)	212	PHI:1555|GzMyb019|I1RDG6|5518|Gibberella_zeae_(related:_Fusarium_graminearum)|Unaffected_pathogenicity
Chr03G0480.1	1827	9.00E-96	205/517(39.65)	305	PHI:1827|GzZC142|I1S2W8|5518|Gibberella_zeae_(related:_Fusarium_graminearum)|Unaffected_pathogenicity
Chr03G0487.1	251	0	352/353(99.72)	736	PHI:251|FGA1|BAB69488|5507|Fusarium_oxysporum|Reduced_virulence
Chr03G0491.1	178	6.00E-17	68/219(31.05)	82.8	PHI:178|pabaA|AAD31929|5085|Aspergillus_fumigatus|Reduced_virulence
Chr03G0495.1	3062	0	447/779(57.38)	742	MoRga2|PHI:3062|G4N7K5|318829|Magnaporthe_oryzae|mixed_outcome_
Chr03G0497.1	3126	9.00E-75	132/399(33.08)	240	argD|PHI:3126|D4I307|552|Erwinia_amylovora|mixed_outcome_
Chr03G0499.1	2069	0	546/896(60.94)	984	PHI:2069|Moatg1|MGG_06393|148305|Magnaporthe_oryzae_(related:_Magnaporthe_grisea)|Loss_of_pathogenicity
Chr03G0510.1	505	0	484/1079(44.86)	939	PHI:505|MIP1|AAA17543|4932|Saccharomyces_cerevisiae|Reduced_virulence
Chr03G0512.1	2309	2.00E-58	176/582(30.24)	212	PHI:2309|BcatrB|Q9UW03|40559|Botrytis_cinerea|Reduced_virulence
Chr03G0513.1	1862	2.00E-158	260/577(45.06)	470	PHI:1862|GzZC177|I1RL97|5518|Gibberella_zeae_(related:_Fusarium_graminearum)|Unaffected_pathogenicity
Chr03G0516.1	1675	6.00E-16	70/194(36.08)	80.5	PHI:1675|GzDHHC003|Q4I8B6|5518|Gibberella_zeae_(related:_Fusarium_graminearum)|Unaffected_pathogenicity
Chr03G0517.1	882	0	404/851(47.47)	635	PHI:882|MGG_04985|EDJ95433|318829|Magnaporthe_oryzae|Reduced_virulence
Chr03G0523.1	243	0	390/918(42.48)	649	PHI:243|CHIP6|AAD00894|5457|Colletotrichum_gloeosporioides|Reduced_virulence
Chr03G0525.1	323	2.00E-80	155/419(36.99)	254	PHI:323|VFGLU1|AAO63562|93591|Verticillium_fungicola|Reduced_virulence
Chr03G0530.1	1546	0	749/1606(46.64)	1104	PHI:1546|GzMyb010|Q4IB96|5518|Gibberella_zeae_(related:_Fusarium_graminearum)|Lethal
Chr03G0538.1	4559	2.00E-131	287/767(37.42)	419	T6SS2|PHI:4559|Q6TKU1|562|Escherichia_coli|effector_(plant_avirulence_determinant)

Chr03G0543.1	2119	0	703/926(75.92)	1369	PHI:2119|MST11p|MGG_14847|148305|Magnaporthe_oryzae_(related:_Magnaporthe_grisea)|Loss_of_pathogenicity
Chr03G0545.1	441	4.00E-23	103/372(27.69)	97.1	PHI:441|BTP1|CAE55153|40559|Botrytis_cinerea|Reduced_virulence
Chr03G0549.1	1773	1.00E-61	128/421(30.40)	206	PHI:1773|GzZC088|I1S409|5518|Gibberella_zeae_(related:_Fusarium_graminearum)|Unaffected_pathogenicity
Chr03G0550.1	3479	0	710/880(80.68)	1276	Vdcla4|PHI:3479|G2X6S4|27337|Verticillium_dahliae|unaffected_pathogenicity_
Chr03G0554.1	3281	9.00E-22	92/334(27.54)	92.8	AS87_04050|PHI:3281|A0A097IB07|34085|Riemerella_anatipestifer|reduced_virulence_
Chr03G0555.1	2315	5.00E-33	89/273(32.60)	125	PHI:2315|ChLae1|G4XKY9|5016|Cochliobolus_heterostrophus|Mixed_outcome
Chr03G0558.1	68	4.00E-62	153/411(37.23)	207	PHI:68|SAP1|AAA34368|5476|Candida_albicans|Reduced_virulence
Chr03G0564.1	1967	7.00E-36	95/376(25.27)	141	PHI:1967|GzZC282|I1RB16|5518|Gibberella_zeae_(related:_Fusarium_graminearum)|Reduced_virulence
Chr03G0565.1	1739	0	365/854(42.74)	672	PHI:1739|GzZC054|I1S7B9|5518|Gibberella_zeae_(related:_Fusarium_graminearum)|Unaffected_pathogenicity
Chr03G0566.1	1507	4.00E-137	238/449(53.01)	401	PHI:1507|GzHOME002|I1RNA7|5518|Gibberella_zeae_(related:_Fusarium_graminearum)|Unaffected_pathogenicity
Chr03G0568.1	1508	0	519/990(52.42)	920	PHI:1508|GzHOME003|I1RNA9|5518|Gibberella_zeae_(related:_Fusarium_graminearum)|Unaffected_pathogenicity
Chr03G0573.1	1224	0	1335/2274(58.71)	2645	PHI:1224|FGSG_09150|Q4IB89|5518|Gibberella_zeae_(related:_Fusarium_graminearum)|Lethal
Chr03G0579.1	441	2.00E-22	71/290(24.48)	96.7	PHI:441|BTP1|CAE55153|40559|Botrytis_cinerea|Reduced_virulence
Chr03G0580.1	1381	2.00E-129	243/550(44.18)	391	PHI:1381|GzC2H044|I1RRI0|5518|Gibberella_zeae_(related:_Fusarium_graminearum)|Unaffected_pathogenicity
Chr03G0582.1	1738	2.00E-18	66/259(25.48)	85.5	PHI:1738|GzZC053|I1S4B9|5518|Gibberella_zeae_(related:_Fusarium_graminearum)|Unaffected_pathogenicity
Chr03G0584.1	1892	0	364/789(46.13)	652	PHI:1892|GzZC207|I1RRH8|5518|Gibberella_zeae_(related:_Fusarium_gramin

					earum)|Unaffected_pathogenicity
Chr03G0589.1	2042	5.00E-50	134/449(29.84)	190	PHI:2042|ABC3|Q3Y5V5|148305|Magnaporthe_oryzae_(related:_Magnaporthe_grisea)|Loss_of_pathogenicity
Chr03G0591.1	226	1.00E-72	178/505(35.25)	258	PHI:226|PEX6|AAK16738|5462|Colletotrichum_lagenarium|Loss_of_pathogenicity
Chr03G0598.1	2032	8.00E-24	101/302(33.44)	103	PHI:2032|VTL1|G4NGA7|148305|Magnaporthe_oryzae_(related:_Magnaporthe_grisea)|Unaffected_pathogenicity
Chr03G0604.1	2643	4.00E-31	97/282(34.40)	123	PHI:2643|CFAS|A4HTK3|5671|Leishmania_infantum|Reduced_virulence
Chr03G0605.1	2745	8.00E-07	24/68(35.29)	49.3	PHI:2745|treY|Q02LV5|287|Pseudomonas_aeruginosa|Mixed_outcome
Chr03G0606.1	1529	0	888/1229(72.25)	1723	PHI:1529|Gzscp|I1RS41|5518|Gibberella_zeae_(related:_Fusarium_graminearum)|Reduced_virulence
Chr03G0608.1	2086	2.00E-07	83/355(23.38)	51.2	PHI:2086|Moatg24|MGG_03638|148305|Magnaporthe_oryzae_(related:_Magnaporthe_grisea)|Unaffected_pathogenicity
Chr03G0609.1	1585	0	246/378(65.08)	511	PHI:1585|GzOB025|I1RS43|5518|Gibberella_zeae_(related:_Fusarium_graminearum)|Unaffected_pathogenicity
Chr03G0610.1	2025	6.00E-07	71/306(23.20)	48.9	PHI:2025|HDL1|G4MQZ9|148305|Magnaporthe_oryzae_(related:_Magnaporthe_grisea)|Unaffected_pathogenicity
Chr03G0611.1	1465	4.00E-47	88/121(72.73)	164	PHI:1465|GzCCAAT005|I1RNC6|5518|Gibberella_zeae_(related:_Fusarium_graminearum)|Unaffected_pathogenicity
Chr03G0612.1	3287	2.00E-21	74/262(28.24)	97.8	CgVps34|PHI:3287|Q6FSR7|5478|Candida_glabrata|reduced_virulence_
Chr03G0617.1	1180	0	384/509(75.44)	765	PHI:1180|(Sc_Sat4)|I1RS32|5518|Gibberella_zeae_(related:_Fusarium_graminearum)|Reduced_virulence
Chr03G0623.1	1383	4.00E-99	154/274(56.20)	292	PHI:1383|GzC2H046|I1RS27|5518|Gibberella_zeae_(related:_Fusarium_graminearum)|Unaffected_pathogenicity
Chr03G0626.1	1584	1.00E-176	240/254(94.49)	488	PHI:1584|GzOB024|I1RS24|5518|Gibberella_zeae_(related:_Fusarium_graminearum)|Unaffected_pathogenicity

Chr03G0630.1	1920	2.00E-85	217/699(31.04)	289	PHI:1920|GzZC235|I1RCK0|5518|Gibberella_zeae_(related:_Fusarium_graminearum)|Unaffected_pathogenicity
Chr03G0631.1	26	4.00E-71	141/467(30.19)	237	PHI:26|CaMDR1|CAA37820|5476|Candida_albicans|Reduced_virulence
Chr03G0641.1	339	3.00E-07	49/194(25.26)	46.6	PHI:339|CLPT1|CAC41973|290576|Colletotrichum_lindemuthianum|Reduced_virulence
Chr03G0643.1	812	3.00E-18	99/392(25.26)	84	PHI:812|MGG_10702|EDJ94108|318829|Magnaporthe_oryzae|Reduced_virulence
Chr03G0646.1	106	0	283/482(58.71)	573	PHI:106|CAT1|AAC39448|5476|Candida_albicans|Reduced_virulence
Chr03G0654.1	339	8.00E-32	66/200(33.00)	115	PHI:339|CLPT1|CAC41973|290576|Colletotrichum_lindemuthianum|Reduced_virulence
Chr03G0656.1	2989	0	667/1073(62.16)	1253	PHI:2989|MGG_06243.6|G4N8I4|148305|Magnaporthe_oryzae|Reduced_virulence
Chr03G0660.1	1460	7.00E-23	83/282(29.43)	94	PHI:1460|GzAra008|I1S2F6|5518|Gibberella_zeae_(related:_Fusarium_graminearum)|Unaffected_pathogenicity
Chr03G0667.1	259	4.00E-46	125/346(36.13)	177	PHI:259|GPI7|AAL83897|5476|Candida_albicans|Reduced_virulence
Chr03G0668.1	2968	1.00E-23	109/413(26.39)	102	PHI:2968|Hxs1|J9VQA5|5207|Cryptococcus_neoformans|Reduced_virulence
Chr03G0670.1	2336	4.00E-30	77/246(31.30)	119	PHI:2336|NIA1|O00101|13684|Stagonospora_nodorum_(related:_Phaeosphaeria_nodorum)|Unaffected_pathogenicity
Chr03G0673.1	1925	5.00E-08	27/76(35.53)	53.5	PHI:1925|GzZC240|I1RH59|5518|Gibberella_zeae_(related:_Fusarium_graminearum)|Unaffected_pathogenicity
Chr03G0674.1	1643	0	415/784(52.93)	681	PHI:1643|GzWing015|I1RS37|5518|Gibberella_zeae_(related:_Fusarium_graminearum)|Reduced_virulence
Chr03G0679.1	3177	0	361/627(57.58)	692	MoTos3|PHI:3177|G4N7H1|318829|Magnaporthe_oryzae|mixed_outcome_
Chr03G0686.1	2315	1.00E-59	115/290(39.66)	194	PHI:2315|ChLae1|G4XKY9|5016|Cochliobolus_heterostrophus|Mixed_outcome
Chr03G0688.1	267	2.00E-104	375/1498(25.03)	364	PHI:267|MLT1|AAD51594|5476|Candida_albicans|Reduced_virulence
Chr03G0689.1	1683	2.00E-10	30/82(36.59)	58.9	PHI:1683|GzRad001|I1RK33|5518|Gibberella_zeae_(related:_Fusarium_grami

					nearum)|Lethal
Chr03G0704.1	1555	1.00E-24	63/248(25.40)	104	PHI:1555|GzMyb019|I1RDG6|5518|Gibberella_zeae_(related:_Fusarium_graminearum)|Unaffected_pathogenicity
Chr03G0706.1	1665	1.00E-42	115/281(40.93)	146	PHI:1665|GzCCHC005|I1RS06|5518|Gibberella_zeae_(related:_Fusarium_graminearum)|Unaffected_pathogenicity
Chr03G0708.1	2202	0	501/913(54.87)	953	PHI:2202|PdeH|D0ERY7|148305|Magnaporthe_oryzae_(related:_Magnaporthe_grisea)|Mixed_outcome
Chr03G0709.1	901	8.00E-114	206/482(42.74)	351	PHI:901|um01886||5270|Ustilago_maydis|Unaffected_pathogenicity
Chr03G0712.1	672	1.00E-72	241/886(27.20)	261	PHI:672|CAS1|AAL35099|192011|Cryptococcus_neoformans|Increased_virulence_(Hypervirulence)
Chr03G0722.1	438	2.00E-25	121/485(24.95)	106	PHI:438|BcBOT1_(related:_CND5)|AAQ16576|40559|Botrytis_cinerea|Reduced_virulence
Chr03G0729.1	1259	0	646/1084(59.59)	1104	PHI:1259|FGSG_03499|I1RNK3|5518|Gibberella_zeae_(related:_Fusarium_graminearum)|Unaffected_pathogenicity
Chr03G0735.1	2544	1.00E-30	80/333(24.02)	123	PHI:2544|PAB1|Q4WK03|746128|Aspergillus_fumigatus|Mixed_outcome
Chr03G0736.1	1214	0	726/918(79.08)	1342	PHI:1214|FGSG_07816|I1RN54|5518|Gibberella_zeae_(related:_Fusarium_graminearum)|Reduced_virulence
Chr03G0739.1	893	1.00E-25	106/420(25.24)	112	PHI:893|MGG_02986|EDK01543|318829|Magnaporthe_oryzae|Reduced_virulence
Chr03G0741.1	1681	8.00E-17	83/325(25.54)	84	PHI:1681|GzNF001|I1REN7|5518|Gibberella_zeae_(related:_Fusarium_graminearum)|Unaffected_pathogenicity
Chr03G0747.1	254	6.00E-07	40/126(31.75)	47.8	PHI:254|FOW1|BAB85760|5507|Fusarium_oxysporum|Reduced_virulence
Chr03G0748.1	211	4.00E-07	70/295(23.73)	48.9	PHI:211|CaTUP1|AAB63195|5476|Candida_albicans|Reduced_virulence
Chr03G0749.1	200	0	402/466(86.27)	851	PHI:200|ARG1|BAB40769|5507|Fusarium_oxysporum|Reduced_virulence
Chr03G0754.1	784	6.00E-20	81/283(28.62)	84.7	PHI:784|MGG_00056|EDK03390|318829|Magnaporthe_oryzae|Reduced_virulence

Chr03G0756.1	1268	0	340/470(72.34)	670	PHI:1268|FGSG_07816|I1RN43|5518|Gibberella_zeae_(related:_Fusarium_graminearum)|Unaffected_pathogenicity
Chr03G0760.1	213	6.00E-24	59/127(46.46)	97.4	PHI:213|CPA1|AAF69795|5207|Cryptococcus_neoformans|Reduced_virulence
Chr03G0761.1	1974	3.00E-31	117/418(27.99)	125	PHI:1974|GzZC289|I1RQN6|5518|Gibberella_zeae_(related:_Fusarium_graminearum)|Unaffected_pathogenicity
Chr03G0764.1	1374	0	655/812(80.67)	1358	PHI:1374|GzC2H037|I1RN37|5518|Gibberella_zeae_(related:_Fusarium_graminearum)|Unaffected_pathogenicity
Chr03G0765.1	319	2.00E-68	153/464(32.97)	250	PHI:319|SQL2|AAO19638|5270|Ustilago_maydis|Reduced_virulence
Chr03G0768.1	1846	1.00E-121	173/361(47.92)	365	PHI:1846|GzZC161|I1S7A6|5518|Gibberella_zeae_(related:_Fusarium_graminearum)|Unaffected_pathogenicity
Chr03G0773.1	1223	0	288/326(88.34)	595	PHI:1223|FGSG_12887|I1RN31|5518|Gibberella_zeae_(related:_Fusarium_graminearum)|Lethal
Chr03G0774.1	281	8.00E-08	53/226(23.45)	52	PHI:281|CDC10|CAA81090|5476|Candida_albicans|Reduced_virulence
Chr03G0778.1	1638	0	407/677(60.12)	721	PHI:1638|GzWing010|I1RN27|5518|Gibberella_zeae_(related:_Fusarium_graminearum)|Unaffected_pathogenicity
Chr03G0781.1	3415	1.00E-44	142/539(26.35)	166	Fre2|PHI:3415|T2BNJ5|5207|Cryptococcus_neoformans|mixed_outcome_
Chr03G0785.1	2256	6.00E-28	83/297(27.95)	110	PHI:2256|Xdh1|Q0UA24|13684|Phaeosphaeria_nodorum_(related:_Stagonospora_nodorum)|Unaffected_pathogenicity
Chr03G0787.1	1373	1.00E-98	196/392(50.00)	303	PHI:1373|GzC2H036|I1RN20|5518|Gibberella_zeae_(related:_Fusarium_graminearum)|Unaffected_pathogenicity
Chr03G0788.1	2604	1.00E-63	197/753(26.16)	229	PHI:2604|Rim13|Q4PCT8|5270|Ustilago_maydis|Unaffected_pathogenicity
Chr03G0796.1	3054	2.00E-135	202/276(73.19)	385	FvHAP5|PHI:3054|W7MQF2|117187|Fusarium_verticillioides|reduced_virulence_
Chr03G0805.1	2034	8.00E-10	40/148(27.03)	57.8	PHI:2034|MFP1|G4MZY1|148305|Magnaporthe_oryzae_(related:_Magnaporthe_grisea)|Reduced_virulence
Chr03G0811.1	2528	1.00E-20	120/488(24.59)	95.5	PHI:2528|SLY1|Q4WYU7|746128|Aspergillus_fumigatus|Lethal

Chr03G0813.1	2399	1.00E-73	143/290(49.31)	236	PHI:2399|Fgp1|I1S5P3|5518|Gibberella_zeae_(related:_Fusarium_graminearum)|Reduced_virulence
Chr03G0822.1	1564	3.00E-06	17/39(43.59)	39.3	PHI:1564|GzOB004|Q4IPZ1|5518|Gibberella_zeae_(related:_Fusarium_graminearum)|Unaffected_pathogenicity
Chr03G0825.1	1258	3.00E-141	193/243(79.42)	413	PHI:1258|FGSG_10591|I1S5M8|5518|Gibberella_zeae_(related:_Fusarium_graminearum)|Unaffected_pathogenicity
Chr03G0828.1	2020	2.00E-148	216/360(60.00)	447	PHI:2020|Tup1|XP_759427|5270|Ustilago_maydis|Mixed_outcome
Chr03G0829.1	3035	6.00E-156	207/348(59.48)	443	FgERG3A|PHI:3035|I1RFM2|5518|Fusarium_graminearum|reduced_virulence_
Chr03G0832.1	2959	4.00E-87	156/324(48.15)	265	PHI:2959|mdh|C5BF98|67780|Edwardsiella_ictaluri|Reduced_virulence
Chr03G0834.1	1680	3.00E-13	45/196(22.96)	70.1	PHI:1680|GzMIZ002|I1S004|5518|Gibberella_zeae_(related:_Fusarium_graminearum)|Unaffected_pathogenicity
Chr03G0836.1	259	3.00E-163	327/916(35.70)	500	PHI:259|GPI7|AAL83897|5476|Candida_albicans|Reduced_virulence
Chr03G0838.1	2552	0	357/752(47.47)	612	PHI:2552|NOP4|Q4WNM3|746128|Aspergillus_fumigatus|Mixed_outcome
Chr03G0842.1	1305	2.00E-38	97/195(49.74)	144	PHI:1305|GzbHLH006|I1RFN6|5518|Gibberella_zeae_(related:_Fusarium_graminearum)|Unaffected_pathogenicity
Chr03G0843.1	2321	1.00E-28	142/556(25.54)	117	PHI:2321|SidI|Q4WR83|746128|Aspergillus_fumigatus|Reduced_virulence
Chr03G0852.1	292	3.00E-08	57/230(24.78)	53.1	PHI:292|CPRa|AAK31936|5207|Cryptococcus_neoformans|Reduced_virulence
Chr03G0853.1	1588	1.00E-58	88/127(69.29)	179	PHI:1588|GzOB028|I1RSY2|5518|Gibberella_zeae_(related:_Fusarium_graminearum)|Lethal
Chr03G0855.1	1526	5.00E-48	160/573(27.92)	185	PHI:1526|GzHOMEL036|I1S0J3|5518|Gibberella_zeae_(related:_Fusarium_graminearum)|Unaffected_pathogenicity
Chr03G0856.1	1795	3.00E-143	244/658(37.08)	432	PHI:1795|GzZC110|I1RSX8|5518|Gibberella_zeae_(related:_Fusarium_graminearum)|Unaffected_pathogenicity
Chr03G0862.1	3629	3.00E-41	127/459(27.67)	153	Rv0392c|PHI:3629|P95200|1773|Mycobacterium_tuberculosis|unaffected_pathogenicity_
Chr03G0869.1	2020	2.00E-23	81/300(27.00)	103	PHI:2020|Tup1|XP_759427|5270|Ustilago_maydis|Mixed_outcome

Chr03G0870.1	3101	8.00E-30	96/331(29.00)	117	BCKDH|PHI:3101|Q8IEJ6|5833|Plasmodium_falciparum|reduced_virulence_
Chr03G0871.1	2060	7.00E-28	146/585(24.96)	118	PHI:2060|MgLig4|B6ZH51|148305|Magnaporthe_oryzae_(related:_Magnaporthe_grisea)|Unaffected_pathogenicity
Chr03G0874.1	2544	3.00E-08	67/332(20.18)	54.7	PHI:2544|PAB1|Q4WK03|746128|Aspergillus_fumigatus|Mixed_outcome
Chr03G0875.1	1595	0	332/577(57.54)	626	PHI:1595|GzOB035|I1RX22|5518|Gibberella_zeae_(related:_Fusarium_graminearum)|Unaffected_pathogenicity
Chr03G0877.1	2052	8.00E-140	187/194(96.39)	390	PHI:2052|Cdc42|Q9P8I4|148305|Magnaporthe_oryzae_(related:_Magnaporthe_grisea)|Reduced_virulence
Chr03G0880.1	286	3.00E-149	271/744(36.42)	464	PHI:286|CLC-A|AAO73005|5207|Cryptococcus_neoformans|Reduced_virulence
Chr03G0899.1	2699	1.00E-07	45/157(28.66)	52.8	PHI:2699|HrpM|Q8PPR7|611301|Xanthomonas_citri_ssp._Citri|Loss_of_pathogenicity
Chr03G0904.1	341	0	460/524(87.79)	884	PHI:341|CPK1|BAD04044|5462|Colletotrichum_lagenarium|Loss_of_pathogenicity
Chr03G0906.1	2106	0	368/710(51.83)	695	PHI:2106|1-phosphatidylinositol-4,5-bisphosphate_phosphodiesterase_delta_1|MGG_05332|148305|Magnaporthe_oryzae_(related:_Magnaporthe_grisea)|Reduced_virulence
Chr03G0908.1	1775	2.00E-51	120/384(31.25)	176	PHI:1775|GzZC090|I1S5X3|5518|Gibberella_zeae_(related:_Fusarium_graminearum)|Unaffected_pathogenicity
Chr03G0913.1	1414	1.00E-14	39/80(48.75)	74.3	PHI:1414|GzC2H081|I1RZL0|5518|Gibberella_zeae_(related:_Fusarium_graminearum)|Unaffected_pathogenicity
Chr03G0918.1	1380	1.00E-135	208/394(52.79)	405	PHI:1380|GzC2H043|I1RRG4|5518|Gibberella_zeae_(related:_Fusarium_graminearum)|Unaffected_pathogenicity
Chr03G0920.1	2514	0	286/512(55.86)	540	PHI:2514|PFS2|Q4X1Y0|746128|Aspergillus_fumigatus|Lethal
Chr03G0925.1	1772	3.00E-47	85/210(40.48)	164	PHI:1772|GzZC087|I1S053|5518|Gibberella_zeae_(related:_Fusarium_graminearum)|Reduced_virulence

Chr03G0927.1	2282	9.00E-146	269/422(63.74)	421	PHI:2282|MeaB|J9MHC1|5507|Fusarium_oxysporum|Increased_virulence_(Hypervirulence)
Chr03G0928.1	2908	2.00E-09	54/236(22.88)	57.4	PHI:2908|CYP51B|I1RBR4|5518|Fusarium_graminearum|Mixed_outcome
Chr03G0933.1	1519	1.00E-124	251/580(43.28)	379	PHI:1519|GzHOMEL018|I1RSV8|5518|Gibberella_zeae_(related:_Fusarium_graminearum)|Unaffected_pathogenicity
Chr03G0935.1	2009	0	565/1079(52.36)	858	PHI:2009|HOS4|EDK06449|148305|Magnaporthe_oryzae_(related:_Magnaporthe_grisea)|Unaffected_pathogenicity
Chr03G0948.1	2101	1.00E-103	182/421(43.23)	318	PHI:2101|Vacuolar_calcium_ion_transporter|MGG_11454|148305|Magnaporthe_oryzae_(related:_Magnaporthe_grisea)|Unaffected_pathogenicity
Chr03G0950.1	504	4.00E-168	232/364(63.74)	476	PHI:504|LEU2|CAA42366|4932|Saccharomyces_cerevisiae|Reduced_virulence
Chr03G0956.1	2216	1.00E-66	180/326(55.21)	212	PHI:2216|PemG1|A0N0D1|148305|Magnaporthe_oryzae_(related:_Magnaporthe_grisea)|Effector_(plant_avirulence_determinant)
Chr03G0962.1	2382	4.00E-95	133/274(48.54)	285	PHI:2382|Upa2|Q6QIY0|5270|Ustilago_maydis|Unaffected_pathogenicity
Chr03G0963.1	2453	3.00E-16	58/224(25.89)	82	PHI:2453|Snt2|D9J222|61369|Fusarium_oxysporum_f.sp._melonis|Reduced_virulence
Chr03G0964.1	204	2.00E-06	45/130(34.62)	48.9	PHI:204|CaCDC35|AAG18428|5476|Candida_albicans|Loss_of_pathogenicity
Chr03G0970.1	1730	5.00E-47	141/497(28.37)	174	PHI:1730|GzZC045|I1S6C7|5518|Gibberella_zeae_(related:_Fusarium_graminearum)|Unaffected_pathogenicity
Chr03G0972.1	2976	0	328/671(48.88)	666	PHI:2976|CgOPT1|C6ZRH8|29905|Colletotrichum_gloeosporioides_f._sp._aeschynomenes|Reduced_virulence
Chr03G0973.1	2895	2.00E-31	82/295(27.80)	130	PHI:2895|F-avi4330|B9JV05|373|Agrobacterium_vitis|Loss_of_pathogenicity
Chr03G0979.1	2825	7.00E-21	48/141(34.04)	95.9	PHI:2825|SET1|Q5ABG1|5476|Candida_albicans|Reduced_virulence
Chr03G0981.1	3126	5.00E-23	108/429(25.17)	98.6	argD|PHI:3126|D4I307|552|Erwinia_amylovora|mixed_outcome_
Chr03G0982.1	286	4.00E-101	215/606(35.48)	335	PHI:286|CLC-A|AAO73005|5207|Cryptococcus_neoformans|Reduced_virulence
Chr03G0989.1	806	8.00E-07	32/130(24.62)	47.4	PHI:806|MGG_04137|EDJ99440|318829|Magnaporthe_oryzae|Reduced_virule

					nce
Chr03G0990.1	1309	2.00E-114	200/333(60.06)	335	PHI:1309|GzbHLH010|I1RNI6|5518|Gibberella_zeae_(related:_Fusarium_graminearum)|Lethal
Chr03G0992.1	267	0	597/1627(36.69)	993	PHI:267|MLT1|AAD51594|5476|Candida_albicans|Reduced_virulence
Chr03G0993.1	1371	9.00E-71	115/268(42.91)	219	PHI:1371|GzC2H034|I1RM60|5518|Gibberella_zeae_(related:_Fusarium_graminearum)|Unaffected_pathogenicity
Chr03G1002.1	2600	0	359/705(50.92)	640	PHI:2600|GAT1|Q4WZG4|746128|Aspergillus_fumigatus|Mixed_outcome
Chr03G1006.1	1239	1.00E-139	210/354(59.32)	404	PHI:1239|FGSG_12149|I1RFC7|5518|Gibberella_zeae_(related:_Fusarium_graminearum)|Unaffected_pathogenicity
Chr03G1009.1	1458	3.00E-25	122/473(25.79)	108	PHI:1458|GzAra006|Q4I7F9|5518|Gibberella_zeae_(related:_Fusarium_graminearum)|Unaffected_pathogenicity
Chr03G1013.1	66	4.00E-20	55/206(26.70)	92	PHI:66|CPP1|AAC05307|5476|Candida_albicans|Reduced_virulence
Chr03G1020.1	1518	9.00E-18	43/120(35.83)	79	PHI:1518|GzHOMEL016|I1RS54|5518|Gibberella_zeae_(related:_Fusarium_graminearum)|Unaffected_pathogenicity
Chr03G1023.1	1253	0	797/1220(65.33)	1558	PHI:1253|FGSG_11614|I1RS57|5518|Gibberella_zeae_(related:_Fusarium_graminearum)|Unaffected_pathogenicity
Chr03G1030.1	544	2.00E-51	144/560(25.71)	185	PHI:544|BCMFS1|AAF64435|332648|Botrytis_cinerea|Unaffected_pathogenicity
Chr03G1037.1	1358	6.00E-11	66/275(24.00)	63.2	PHI:1358|GzC2H018|I1RG87|5518|Gibberella_zeae_(related:_Fusarium_graminearum)|Unaffected_pathogenicity
Chr03G1039.1	1662	3.00E-82	169/506(33.40)	279	PHI:1662|GzCCHC002|I1REJ1|5518|Gibberella_zeae_(related:_Fusarium_graminearum)|Unaffected_pathogenicity
Chr03G1042.1	315	0	398/601(66.22)	680	PHI:315|PacC|AAM95700|5507|Fusarium_oxysporum|Increased_virulence_(Hypervirulence)
Chr03G1043.1	255	0	965/2598(37.14)	1545	PHI:255|FUM1_(related:_FUM5)|AAD43562|5127|Gibberella_moniliformis|Unaffected_pathogenicity

Chr03G1048.1	3381	2.00E-65	145/430(33.72)	223	FVEG_12533|PHI:3381|W7N2B4|117187|Fusarium_verticillioides|unaffected_pathogenicity_
Chr03G1049.1	419	6.00E-06	24/101(23.76)	44.7	PHI:419|CSH1|AAP93915|5476|Candida_albicans|Reduced_virulence
Chr03G1051.1	2393	2.00E-13	43/178(24.16)	69.7	PHI:2393|Related_to_O-methylsterigmatocystin_oxidoreductase|I1R980|5518|Gibberella_zeae_(related:_Fusarium_graminearum)|Increased_virulence_(Hypervirulence)
Chr03G1052.1	255	0	472/1205(39.17)	823	PHI:255|FUM1_(related:_FUM5)|AAD43562|5127|Gibberella_moniliformis|Unaffected_pathogenicity
Chr03G1069.1	1432	5.00E-150	247/373(66.22)	433	PHI:1432|GzC2H100|I1S7Z8|5518|Gibberella_zeae_(related:_Fusarium_graminearum)|Unaffected_pathogenicity
Chr03G1072.1	2866	1.00E-06	23/56(41.07)	48.5	PHI:2866|ORF19.3625|Q59Y24|5476|Candida_albicans|Reduced_virulence
Chr03G1077.1	1491	0	593/906(65.45)	942	PHI:1491|GzHMG023|I1RST1|5518|Gibberella_zeae_(related:_Fusarium_graminearum)|Lethal
Chr03G1078.1	2315	2.00E-52	102/289(35.29)	176	PHI:2315|ChLae1|G4XKY9|5016|Cochliobolus_heterostrophus|Mixed_outcome
Chr03G1084.1	1566	9.00E-49	100/248(40.32)	179	PHI:1566|GzOB006|I1RC95|5518|Gibberella_zeae_(related:_Fusarium_graminearum)|Lethal
Chr03G1092.1	2821	4.00E-80	180/528(34.09)	291	PHI:2821|SNF2|Q5ALP9|5476|Candida_albicans|Reduced_virulence
Chr03G1093.1	1500	5.00E-10	33/98(33.67)	61.2	PHI:1500|GzHMG032|I1S2F2|5518|Gibberella_zeae_(related:_Fusarium_graminearum)|Unaffected_pathogenicity
Chr03G1094.1	440	2.00E-66	224/822(27.25)	238	PHI:440|PMR1|CAB87245|5476|Candida_albicans|Reduced_virulence
Chr03G1096.1	294	0	666/693(96.10)	1364	PHI:294|CST1|BAC11803|5462|Colletotrichum_lagenarium|Loss_of_pathogenicity
Chr03G1098.1	2020	5.00E-11	53/196(27.04)	62.8	PHI:2020|Tup1|XP_759427|5270|Ustilago_maydis|Mixed_outcome
Chr03G1099.1	124	2.00E-59	128/375(34.13)	223	PHI:124|INT1|AAA96019|5476|Candida_albicans|Reduced_virulence
Chr03G1102.1	2821	0	588/1237(47.53)	1041	PHI:2821|SNF2|Q5ALP9|5476|Candida_albicans|Reduced_virulence
Chr03G1108.1	59	5.00E-16	49/194(25.26)	73.6	PHI:59|THR1|BAA18962|5462|Colletotrichum_lagenarium|Reduced_virulence

Chr03G1119.1	2822	8.00E-54	165/568(29.05)	194	PHI:2822|Cxt1p|Q5K8R6|5207|Cryptococcus_neoformans|Reduced_virulence
Chr03G1122.1	2322	3.00E-33	81/258(31.40)	122	PHI:2322|SidH|Q4WF54|746128|Aspergillus_fumigatus|Reduced_virulence
Chr03G1135.1	1166	0	422/612(68.95)	788	PHI:1166|ATG15|I1RFN8|5518|Gibberella_zeae_(related:_Fusarium_graminearum)|Reduced_virulence
Chr03G1139.1	3035	3.00E-06	41/154(26.62)	46.2	FgERG3A|PHI:3035|I1RFM2|5518|Fusarium_graminearum|reduced_virulence_
Chr03G1141.1	1656	0	359/702(51.14)	568	PHI:1656|GzYL|I1RFN9|5518|Gibberella_zeae_(related:_Fusarium_graminearum)|Lethal
Chr03G1150.1	1935	0	552/876(63.01)	1098	PHI:1935|GzZC250|I1RFP7|5518|Gibberella_zeae_(related:_Fusarium_graminearum)|Unaffected_pathogenicity
Chr03G1153.1	1441	0	647/1029(62.88)	1150	PHI:1441|GzDDT|I1RFP4|5518|Gibberella_zeae_(related:_Fusarium_graminearum)|Reduced_virulence
Chr03G1155.1	1933	2.00E-17	65/250(26.00)	81.6	PHI:1933|GzZC248|I1RC73|5518|Gibberella_zeae_(related:_Fusarium_graminearum)|Reduced_virulence
Chr03G1157.1	923	9.00E-11	59/219(26.94)	60.8	PHI:923|um03616||5270|Ustilago_maydis|Unaffected_pathogenicity
Chr03G1162.1	2694	1.00E-09	74/266(27.82)	58.2	PHI:2694|rsmB|Q6D000|29471|Pectobacterium_atrosepticum|Mixed_outcome
Chr03G1167.1	3381	0	310/537(57.73)	636	FVEG_12533|PHI:3381|W7N2B4|117187|Fusarium_verticillioides|unaffected_pathogenicity_
Chr03G1170.1	2968	2.00E-16	117/475(24.63)	79.3	PHI:2968|Hxs1|J9VQA5|5207|Cryptococcus_neoformans|Reduced_virulence
Chr03G1172.1	781	2.00E-79	119/186(63.98)	257	PHI:781|MGG_00131|EDK03302|318829|Magnaporthe_oryzae|Reduced_virulence
Chr03G1173.1	1052	0	392/624(62.82)	805	PHI:1052|PHL1|ACB38886|135779|Cercospora_zeae-maydis|Unaffected_pathogenicity
Chr03G1174.1	364	5.00E-22	65/232(28.02)	94.4	PHI:364|MIT1|EAK95175|5476|Candida_albicans|Reduced_virulence
Chr03G1177.1	2745	2.00E-08	27/78(34.62)	53.9	PHI:2745|treY|Q02LV5|287|Pseudomonas_aeruginosa|Mixed_outcome
Chr03G1178.1	2315	1.00E-59	111/291(38.14)	194	PHI:2315|ChLae1|G4XKY9|5016|Cochliobolus_heterostrophus|Mixed_outcome
Chr03G1181.1	2042	0	473/1309(36.13)	766	PHI:2042|ABC3|Q3Y5V5|148305|Magnaporthe_oryzae_(related:_Magnaporthe

					_grisea)|Loss_of_pathogenicity
Chr03G1186.1	1547	3.00E-92	232/587(39.52)	298	PHI:1547|GzMyb011|I1RRN6|5518|Gibberella_zeae_(related:_Fusarium_graminearum)|Unaffected_pathogenicity
Chr03G1191.1	1555	2.00E-19	118/528(22.35)	88.6	PHI:1555|GzMyb019|I1RDG6|5518|Gibberella_zeae_(related:_Fusarium_graminearum)|Unaffected_pathogenicity
Chr03G1193.1	3415	9.00E-24	94/394(23.86)	103	Fre2|PHI:3415|T2BNJ5|5207|Cryptococcus_neoformans|mixed_outcome_
Chr03G1199.1	187	2.00E-06	19/62(30.65)	48.1	PHI:187|RUM1|AAG02418|5270|Ustilago_maydis|Loss_of_pathogenicity
Chr03G1204.1	887	2.00E-39	84/190(44.21)	141	PHI:887|MGG_05174|EDJ95219|318829|Magnaporthe_oryzae|Reduced_virulence
Chr03G1207.1	2097	2.00E-119	351/1226(28.63)	405	PHI:2097|Neo1|MGG_04066|148305|Magnaporthe_oryzae_(related:_Magnaporthe_grisea)|Loss_of_pathogenicity
Chr03G1211.1	1034	0	523/715(73.15)	1071	PHI:1034|Cpcat1|CAA04716|5111|Claviceps_purpurea|Unaffected_pathogenicity
Chr03G1213.1	981	6.00E-08	26/67(38.81)	50.4	PHI:981|hopI1|AAL84247|59511|Pseudomonas_syringae|Effector_(plant_avirulence_determinant)
Chr03G1218.1	423	2.00E-55	122/378(32.28)	196	PHI:423|VAD1|AAV41010|5207|Cryptococcus_neoformans|Reduced_virulence
Chr03G1219.1	4468	0	418/529(79.02)	842	mkkA|PHI:4468|A0A068BFA5|35717|Epichloe_festucae|effector_(plant_avirulence_determinant)
Chr03G1227.1	1867	3.00E-62	175/529(33.08)	217	PHI:1867|GzZC182|I1RL02|5518|Gibberella_zeae_(related:_Fusarium_graminearum)|Unaffected_pathogenicity
Chr03G1236.1	1328	6.00E-39	174/513(33.92)	147	PHI:1328|GzbZIP010|I1RRD2|5518|Gibberella_zeae_(related:_Fusarium_graminearum)|Reduced_virulence
Chr03G1244.1	2168	1.00E-132	497/1581(31.44)	447	PHI:2168|Hypothetical_protein|MGG_11346|148305|Magnaporthe_oryzae_(related:_Magnaporthe_grisea)|Reduced_virulence
Chr03G1251.1	3786	6.00E-17	58/201(28.86)	76.3	PA0005|PHI:3786|Q9I7C1|287|Pseudomonas_aeruginosa|mixed_outcome
Chr03G1254.1	173	2.00E-06	33/129(25.58)	44.7	PHI:173|CTG1|AAC03782|5466|Colletotrichum_trifolii|Loss_of_pathogenicity

Chr03G1255.1	2030	7.00E-49	147/524(28.05)	182	PHI:2030|TGL3-1|G4N492|148305|Magnaporthe_oryzae_(related:_Magnaporthe_grisea)|Unaffected_pathogenicity
Chr03G1258.1	339	2.00E-09	48/173(27.75)	52	PHI:339|CLPT1|CAC41973|290576|Colletotrichum_lindemuthianum|Reduced_virulence
Chr03G1261.1	1229	7.00E-166	303/536(56.53)	491	PHI:1229|FGSG_02153|I1RRB9|5518|Gibberella_zeae_(related:_Fusarium_graminearum)|Lethal
Chr03G1263.1	922	2.00E-36	163/612(26.63)	141	PHI:922|um03615||5270|Ustilago_maydis|Unaffected_pathogenicity
Chr03G1264.1	441	2.00E-12	63/292(21.58)	64.7	PHI:441|BTP1|CAE55153|40559|Botrytis_cinerea|Reduced_virulence
Chr03G1265.1	404	4.00E-15	77/262(29.39)	74.3	PHI:404|PTH11|AAD30436|318829|Magnaporthe_oryzae|Reduced_virulence
Chr03G1266.1	1046	3.00E-07	49/175(28.00)	49.7	PHI:1046|CTB5|ABK64182|29003|Cercospora_nicotianae|Reduced_virulence
Chr03G1268.1	2240	5.00E-30	119/459(25.93)	120	PHI:2240|Srt1|Q4PBY9|5270|Ustilago_maydis|reduced_virulence
Chr03G1270.1	2835	2.00E-54	94/213(44.13)	180	PHI:2835|RED2|C3JXE8|5016|Cochliobolus_heterostrophus|Reduced_virulence
Chr03G1272.1	2807	2.00E-30	109/367(29.70)	117	PHI:2807|Ss-Sl2|A7EKS0|5180|Sclerotinia_sclerotiorum|Unaffected_pathogenicity
Chr03G1274.1	404	3.00E-14	86/350(24.57)	71.6	PHI:404|PTH11|AAD30436|318829|Magnaporthe_oryzae|Reduced_virulence
Chr03G1278.1	2201	5.00E-164	278/581(47.85)	479	PHI:2201|PdeL|D0ERY8|148305|Magnaporthe_oryzae_(related:_Magnaporthe_grisea)|Mixed_outcome
Chr03G1281.1	304	0	1343/1698(79.09)	2687	PHI:304|GzCPS1|AAP12366|5518|Fusarium_graminearum|Reduced_virulence
Chr03G1283.1	1071	9.00E-27	156/672(23.21)	115	PHI:1071|Gas1|CAF05793|5270|Ustilago_maydis|Loss_of_pathogenicity
Chr03G1285.1	538	4.00E-31	121/476(25.42)	124	PHI:538|FRT1|AAU87358|40559|Botrytis_cinerea|Unaffected_pathogenicity
Chr03G1286.1	2393	1.00E-50	127/449(28.29)	179	PHI:2393|Related_to_O-methylsterigmatocystin_oxidoreductase|I1R980|5518|Gibberella_zeae_(related:_Fusarium_graminearum)|Increased_virulence_(Hypervirulence)
Chr03G1298.1	1277	7.00E-10	43/169(25.44)	58.9	PHI:1277|FGSG_12887|I1S5L1|5518|Gibberella_zeae_(related:_Fusarium_graminearum)|Unaffected_pathogenicity

Chr03G1299.1	2654	6.00E-07	45/150(30.00)	50.1	PHI:2654|DUR1,2|Q59VF3|5476|Candida_albicans|Reduced_virulence
Chr03G1302.1	3301	6.00E-20	72/253(28.46)	84	patN|PHI:3301|A0A075TRB3|27334|Penicillium_expansum|unaffected_pathogenicity_
Chr03G1303.1	2393	2.00E-08	65/340(19.12)	53.5	PHI:2393|Related_to_O-methylsterigmatocystin_oxidoreductase|I1R980|5518|Gibberella_zeae_(related:_Fusarium_graminearum)|Increased_virulence_(Hypervirulence)
Chr03G1304.1	2269	6.00E-13	66/283(23.32)	65.1	PHI:2269|Mdh1|0|13684|Phaeosphaeria_nodorum_(related:_Stagonospora_nodorum)|Unaffected_pathogenicity
Chr03G1305.1	2654	1.00E-09	66/209(31.58)	57.8	PHI:2654|DUR1,2|Q59VF3|5476|Candida_albicans|Reduced_virulence
Chr03G1307.1	1958	8.00E-32	176/746(23.59)	129	PHI:1958|GzZC273|I1RMD1|5518|Gibberella_zeae_(related:_Fusarium_graminearum)|Unaffected_pathogenicity
Chr03G1309.1	2140	1.00E-121	210/378(55.56)	358	PHI:2140|MoVam7|XP_001907727.1|148305|Magnaporthe_oryzae_(related:_Magnaporthe_grisea)|Loss_of_pathogenicity
Chr03G1311.1	1458	1.00E-58	136/400(34.00)	216	PHI:1458|GzAra006|Q4I7F9|5518|Gibberella_zeae_(related:_Fusarium_graminearum)|Unaffected_pathogenicity
Chr03G1312.1	179	3.00E-74	122/248(49.19)	229	PHI:179|PELA|AAA33338|140110|Nectria_haematococca_(related:_Fusarium_solani)|Reduced_virulence
Chr03G1317.1	2269	5.00E-144	188/266(70.68)	406	PHI:2269|Mdh1|0|13684|Phaeosphaeria_nodorum_(related:_Stagonospora_nodorum)|Unaffected_pathogenicity
Chr03G1320.1	547	9.00E-37	76/254(29.92)	138	PHI:547|CEL5A|AAT40313|40559|Botrytis_cinerea|Unaffected_pathogenicity
Chr03G1321.1	2147	2.00E-25	71/230(30.87)	103	PHI:2147|Erl1|G5EH97|148305|Magnaporthe_oryzae_(related:_Magnaporthe_grisea)|Reduced_virulence
Chr03G1323.1	513	3.00E-39	139/551(25.23)	149	PHI:513|ARN1_(related:_SIT1)|EAK97011|5476|Candida_albicans|Reduced_virulence
Chr03G1325.1	2315	2.00E-49	98/285(34.39)	167	PHI:2315|ChLae1|G4XKY9|5016|Cochliobolus_heterostrophus|Mixed_outcome
Chr03G1329.1	891	1.00E-12	70/284(24.65)	67.8	PHI:891|MGG_01748|EDK04531|318829|Magnaporthe_oryzae|Reduced_virule

					nce
Chr03G1331.1	2032	2.00E-46	130/513(25.34)	169	PHI:2032|VTL1|G4NGA7|148305|Magnaporthe_oryzae_(related:_Magnaporthe_grisea)|Unaffected_pathogenicity
Chr03G1334.1	673	1.00E-36	67/194(34.54)	130	PHI:673|Can1|AAZ30050|178876|Cryptococcus_neoformans|Unaffected_pathogenicity
Chr03G1345.1	2383	5.00E-53	94/169(55.62)	171	PHI:2383|MfCUT1|Q2VF46|38448|Monilinia_fructicola|Increased_virulence_(hypervirulence)
Chr03G1351.1	1796	3.00E-55	112/351(31.91)	196	PHI:1796|GzZC111|I1RSR7|5518|Gibberella_zeae_(related:_Fusarium_graminearum)|Unaffected_pathogenicity
Chr03G1353.1	494	0	381/1106(34.45)	636	PHI:494|PPOA|EAL89712|5085|Aspergillus_fumigatus|Increased_virulence_(Hypervirulence)
Chr03G1357.1	1555	1.00E-39	121/501(24.15)	149	PHI:1555|GzMyb019|I1RDG6|5518|Gibberella_zeae_(related:_Fusarium_graminearum)|Unaffected_pathogenicity
Chr03G1358.1	1784	2.00E-58	142/452(31.42)	198	PHI:1784|GzZC099|I1S2A9|5518|Gibberella_zeae_(related:_Fusarium_graminearum)|Unaffected_pathogenicity
Chr03G1360.1	3386	2.00E-12	72/272(26.47)	65.1	FVEG_12528|PHI:3386|W7NCN7|117187|Fusarium_verticillioides|unaffected_pathogenicity_
Chr03G1362.1	1527	5.00E-37	100/357(28.01)	139	PHI:1527|GzHOMEL040|I1S9A1|5518|Gibberella_zeae_(related:_Fusarium_graminearum)|Lethal
Chr03G1368.1	2570	2.00E-57	120/361(33.24)	196	PHI:2570|CYB2|Q6FM61|5478|Candida_glabrata|Reduced_virulence
Chr03G1369.1	2315	1.00E-35	85/284(29.93)	130	PHI:2315|ChLae1|G4XKY9|5016|Cochliobolus_heterostrophus|Mixed_outcome
Chr03G1370.1	2315	5.00E-37	86/290(29.66)	134	PHI:2315|ChLae1|G4XKY9|5016|Cochliobolus_heterostrophus|Mixed_outcome
Chr03G1371.1	482	9.00E-35	88/286(30.77)	129	PHI:482|LAEA|AAR01218|5085|Aspergillus_fumigatus|Reduced_virulence
Chr03G1377.1	4618	2.00E-67	154/386(39.90)	223	PcPL15|PHI:4618|A0A0D3LX64|4784|Phytophthora_capsici|mixed_outcome
Chr03G1378.1	2476	2.00E-118	181/300(60.33)	346	PHI:2476|CcpelA|G8AA67|27358|Colletotrichum_coccodes|Mixed_outcome
Chr03G1385.1	4194	2.00E-51	127/477(26.62)	182	AKT7|PHI:4194|V5XZS6|5599|Alternaria_alternata|increased_virulence_(Hyper

					virulence)
Chr03G1387.1	3381	1.00E-67	157/461(34.06)	230	FVEG_12533|PHI:3381|W7N2B4|117187|Fusarium_verticillioides|unaffected_pathogenicity_
Chr03G1388.1	2260	3.00E-138	471/1888(24.95)	481	PHI:2260|PKS1|G8DNT0|45130|Cochliobolus_sativus|Unaffected_pathogenicity
Chr03G1389.1	2247	3.00E-24	77/218(35.32)	97.8	PHI:2247|Sch1|EAT82552|13684|Phaeosphaeria_nodorum_(related:_Stagonospora_nodorum)|Unaffected_pathogenicity
Chr03G1390.1	3387	3.00E-07	61/193(31.61)	49.7	FVEG_12523|PHI:3387|W7MT31|117187|Fusarium_verticillioides|unaffected_pathogenicity_
Chr03G1393.1	413	1.00E-55	144/436(33.03)	191	PHI:413|MPD1|AAT84078|13684|Stagonospora_nodorum|Unaffected_pathogenicity
Chr03G1396.1	2083	1.00E-143	227/447(50.78)	425	PHI:2083|Moatg17|MGG_07667|148305|Magnaporthe_oryzae_(related:_Magnaporthe_grisea)|Loss_of_pathogenicity
Chr03G1398.1	922	2.00E-15	91/297(30.64)	77.4	PHI:922|um03615||5270|Ustilago_maydis|Unaffected_pathogenicity
Chr03G1399.1	404	3.00E-23	63/264(23.86)	99.4	PHI:404|PTH11|AAD30436|318829|Magnaporthe_oryzae|Reduced_virulence
Chr03G1400.1	1992	1.00E-30	94/287(32.75)	127	PHI:1992|GzZC307|I1R983|5518|Gibberella_zeae_(related:_Fusarium_graminearum)|Unaffected_pathogenicity
Chr03G1404.1	181	3.00E-141	218/418(52.15)	412	PHI:181|PGX1|AAK81847|5507|Fusarium_oxysporum|Unaffected_pathogenicity
Chr03G1411.1	2175	6.00E-07	71/258(27.52)	47.8	PHI:2175|NMR3|MGG_09705|148305|Magnaporthe_oryzae_(related:_Magnaporthe_grisea)|Reduced_virulence
Chr03G1413.1	2279	5.00E-28	83/282(29.43)	108	PHI:2279|Conserved_hypothetical_protein|J9N0G7|5507|Fusarium_oxysporum|Unaffected_pathogenicity
Chr03G1416.1	1228	0	379/550(68.91)	728	PHI:1228|FGSG_07745|I1RR00|5518|Gibberella_zeae_(related:_Fusarium_graminearum)|Lethal
Chr03G1423.1	407	4.00E-07	40/133(30.08)	46.6	PHI:407|PBC1|CAB40372|76659|Pyrenopeziza_brassicae|Loss_of_pathogenici

					ty
Chr03G1430.1	1662	2.00E-67	152/514(29.57)	237	PHI:1662|GzCCHC002|I1REJ1|5518|Gibberella_zeae_(related:_Fusarium_graminearum)|Unaffected_pathogenicity
Chr03G1435.1	441	9.00E-09	71/356(19.94)	53.9	PHI:441|BTP1|CAE55153|40559|Botrytis_cinerea|Reduced_virulence
Chr03G1436.1	1651	5.00E-98	167/401(41.65)	300	PHI:1651|GzWing023|I1S0U2|5518|Gibberella_zeae_(related:_Fusarium_graminearum)|Unaffected_pathogenicity
Chr03G1441.1	2174	2.00E-06	63/267(23.60)	45.8	PHI:2174|NMR2|MGG_02860|148305|Magnaporthe_oryzae_(related:_Magnaporthe_grisea)|Reduced_virulence
Chr03G1450.1	1371	8.00E-62	109/298(36.58)	198	PHI:1371|GzC2H034|I1RM60|5518|Gibberella_zeae_(related:_Fusarium_graminearum)|Unaffected_pathogenicity
Chr03G1453.1	1769	6.00E-23	102/455(22.42)	98.6	PHI:1769|GzZC084|I1RXB2|5518|Gibberella_zeae_(related:_Fusarium_graminearum)|Unaffected_pathogenicity
Chr03G1454.1	441	9.00E-11	76/344(22.09)	60.5	PHI:441|BTP1|CAE55153|40559|Botrytis_cinerea|Reduced_virulence
Chr03G1456.1	903	6.00E-149	222/486(45.68)	442	PHI:903|um01888||5270|Ustilago_maydis|Unaffected_pathogenicity
Chr03G1459.1	1662	2.00E-30	113/462(24.46)	123	PHI:1662|GzCCHC002|I1REJ1|5518|Gibberella_zeae_(related:_Fusarium_graminearum)|Unaffected_pathogenicity
Chr03G1467.1	404	1.00E-18	60/241(24.90)	84.3	PHI:404|PTH11|AAD30436|318829|Magnaporthe_oryzae|Reduced_virulence
Chr03G1468.1	541	1.00E-75	194/595(32.61)	251	PHI:541|LIP1|AAU87359|332648|Botrytis_cinerea|Unaffected_pathogenicity
Chr03G1469.1	2315	4.00E-52	102/290(35.17)	175	PHI:2315|ChLae1|G4XKY9|5016|Cochliobolus_heterostrophus|Mixed_outcome
Chr03G1472.1	2208	3.00E-140	190/312(60.90)	408	PHI:2208|endo-1,4-beta-xylanase_[GH10_family]|MGG_05464|148305|Magnaporthe_oryzae_(related:_Magnaporthe_grisea)|Reduced_virulence
Chr03G1473.1	2405	2.00E-17	53/150(35.33)	85.1	PHI:2405|MoSSK1|G5EH08|148305|Magnaporthe_oryzae_(related:_Magnaporthe_grisea)|Reduced_virulence
Chr03G1474.1	1047	8.00E-80	139/347(40.06)	249	PHI:1047|CTB6|ABK64183|29003|Cercospora_nicotianae|Reduced_virulence
Chr03G1475.1	441	1.00E-06	36/156(23.08)	47.4	PHI:441|BTP1|CAE55153|40559|Botrytis_cinerea|Reduced_virulence
Chr03G1478.1	1710	1.00E-159	293/706(41.50)	479	PHI:1710|GzZC025|I1RIH3|5518|Gibberella_zeae_(related:_Fusarium_gramine

					arum)|Unaffected_pathogenicity
Chr03G1480.1	2968	1.00E-30	119/493(24.14)	122	PHI:2968|Hxs1|J9VQA5|5207|Cryptococcus_neoformans|Reduced_virulence
Chr03G1481.1	1802	4.00E-39	118/408(28.92)	148	PHI:1802|GzZC117|I1RVB2|5518|Gibberella_zeae_(related:_Fusarium_graminearum)|Unaffected_pathogenicity
Chr03G1485.1	2835	7.00E-41	79/209(37.80)	145	PHI:2835|RED2|C3JXE8|5016|Cochliobolus_heterostrophus|Reduced_virulence
Chr03G1491.1	2558	6.00E-10	37/134(27.61)	59.3	PHI:2558|DUR31|Q59P00|5476|Candida_albicans|Reduced_virulence
Chr03G1494.1	1818	1.00E-59	101/233(43.35)	202	PHI:1818|GzZC133|I1S7V2|5518|Gibberella_zeae_(related:_Fusarium_graminearum)|Unaffected_pathogenicity
Chr03G1497.1	544	1.00E-61	159/547(29.07)	214	PHI:544|BCMFS1|AAF64435|332648|Botrytis_cinerea|Unaffected_pathogenicity
Chr03G1500.1	2908	3.00E-09	72/292(24.66)	56.2	PHI:2908|CYP51B|I1RBR4|5518|Fusarium_graminearum|Mixed_outcome
Chr03G1508.1	143	5.00E-58	114/318(35.85)	193	PHI:143|CHT|AAA33353|5563|Gloeocercospora_sorghi|Unaffected_pathogenicity
Chr03G1509.1	2835	1.00E-07	48/176(27.27)	48.9	PHI:2835|RED2|C3JXE8|5016|Cochliobolus_heterostrophus|Reduced_virulence
Chr03G1510.1	413	2.00E-22	114/440(25.91)	96.7	PHI:413|MPD1|AAT84078|13684|Stagonospora_nodorum|Unaffected_pathogenicity
Chr03G1517.1	1662	2.00E-59	142/482(29.46)	212	PHI:1662|GzCCHC002|I1REJ1|5518|Gibberella_zeae_(related:_Fusarium_graminearum)|Unaffected_pathogenicity
Chr03G1518.1	784	4.00E-80	134/266(50.38)	253	PHI:784|MGG_00056|EDK03390|318829|Magnaporthe_oryzae|Reduced_virulence
Chr03G1519.1	1870	2.00E-121	193/355(54.37)	374	PHI:1870|GzZC185|I1RDS9|5518|Gibberella_zeae_(related:_Fusarium_graminearum)|Unaffected_pathogenicity
Chr03G1520.1	2611	2.00E-08	41/138(29.71)	52.4	PHI:2611|CMLE|H9C592|59765|Fusarium_oxysporum_f._sp._Lycopersici|Loss_of_pathogenicity

Chr03G1522.1	2968	1.00E-34	123/511(24.07)	133	PHI:2968|Hxs1|J9VQA5|5207|Cryptococcus_neoformans|Reduced_virulence
Chr03G1524.1	1835	1.00E-149	245/602(40.70)	453	PHI:1835|GzZC150|I1RIL1|5518|Gibberella_zeae_(related:_Fusarium_graminearum)|Unaffected_pathogenicity
Chr03G1526.1	1555	2.00E-46	121/501(24.15)	168	PHI:1555|GzMyb019|I1RDG6|5518|Gibberella_zeae_(related:_Fusarium_graminearum)|Unaffected_pathogenicity
Chr03G1530.1	404	5.00E-14	45/142(31.69)	68.2	PHI:404|PTH11|AAD30436|318829|Magnaporthe_oryzae|Reduced_virulence
Chr03G1535.1	441	1.00E-19	90/380(23.68)	86.7	PHI:441|BTP1|CAE55153|40559|Botrytis_cinerea|Reduced_virulence
Chr03G1539.1	1675	0	515/703(73.26)	1105	PHI:1675|GzDHHC003|Q4I8B6|5518|Gibberella_zeae_(related:_Fusarium_graminearum)|Unaffected_pathogenicity
Chr03G1540.1	2654	2.00E-62	163/508(32.09)	234	PHI:2654|DUR1,2|Q59VF3|5476|Candida_albicans|Reduced_virulence
Chr03G1549.1	2968	2.00E-19	112/457(24.51)	88.6	PHI:2968|Hxs1|J9VQA5|5207|Cryptococcus_neoformans|Reduced_virulence
Chr03G1551.1	1820	3.00E-44	105/297(35.35)	158	PHI:1820|GzZC135|I1S8F7|5518|Gibberella_zeae_(related:_Fusarium_graminearum)|Unaffected_pathogenicity
Chr03G1556.1	812	2.00E-51	111/364(30.49)	178	PHI:812|MGG_10702|EDJ94108|318829|Magnaporthe_oryzae|Reduced_virulence
Chr03G1558.1	1046	1.00E-28	77/212(36.32)	115	PHI:1046|CTB5|ABK64182|29003|Cercospora_nicotianae|Reduced_virulence
Chr03G1559.1	1420	2.00E-08	74/343(21.57)	56.2	PHI:1420|GzC2H088|I1S172|5518|Gibberella_zeae_(related:_Fusarium_graminearum)|Unaffected_pathogenicity
Chr03G1561.1	1838	6.00E-60	140/464(30.17)	206	PHI:1838|GzZC153|I1RGC8|5518|Gibberella_zeae_(related:_Fusarium_graminearum)|Unaffected_pathogenicity
Chr03G1565.1	2849	5.00E-51	104/234(44.44)	166	PHI:2849|cutA|Q99174|70790|Fusarium_solani_f._sp._cucurbitae|Unaffected_pathogenicity
Chr03G1566.1	2376	1.00E-53	130/438(29.68)	192	PHI:2376|DEP2|D2E9W7|29001|Alternaria_brassicicola|Mixed_outcome
Chr03G1567.1	3381	1.00E-80	180/517(34.82)	266	FVEG_12533|PHI:3381|W7N2B4|117187|Fusarium_verticillioides|unaffected_pathogenicity_
Chr03G1568.1	2309	7.00E-41	152/562(27.05)	161	PHI:2309|BcatrB|Q9UW03|40559|Botrytis_cinerea|Reduced_virulence

Chr03G1569.1	2654	9.00E-18	68/184(36.96)	84.3	PHI:2654|DUR1,2|Q59VF3|5476|Candida_albicans|Reduced_virulence
Chr03G1570.1	2807	4.00E-34	103/282(36.52)	129	PHI:2807|Ss-Sl2|A7EKS0|5180|Sclerotinia_sclerotiorum|Unaffected_pathogenicity
Chr03G1571.1	1029	1.00E-27	59/125(47.20)	103	PHI:1029|bcpme2|CAD21438|40559|Botrytis_cinerea|Unaffected_pathogenicity
Chr03G1572.1	1393	2.00E-08	21/37(56.76)	53.5	PHI:1393|GzC2H056|I1RU69|5518|Gibberella_zeae_(related:_Fusarium_graminearum)|Unaffected_pathogenicity
Chr03G1577.1	2315	1.00E-48	103/292(35.27)	166	PHI:2315|ChLae1|G4XKY9|5016|Cochliobolus_heterostrophus|Mixed_outcome
Chr03G1580.1	2393	8.00E-41	127/479(26.51)	152	PHI:2393|Related_to_O-methylsterigmatocystin_oxidoreductase|I1R980|5518|Gibberella_zeae_(related:_Fusarium_graminearum)|Increased_virulence_(Hypervirulence)
Chr03G1583.1	3415	7.00E-42	132/491(26.88)	158	Fre2|PHI:3415|T2BNJ5|5207|Cryptococcus_neoformans|mixed_outcome_
Chr03G1586.1	800	0	649/1209(53.68)	1184	PHI:800|MGG_13324|EDK00897|318829|Magnaporthe_oryzae|Reduced_virulence
Chr03G1589.1	1034	3.00E-159	272/649(41.91)	480	PHI:1034|Cpcat1|CAA04716|5111|Claviceps_purpurea|Unaffected_pathogenicity
Chr03G1594.1	59	0	254/267(95.13)	518	PHI:59|THR1|BAA18962|5462|Colletotrichum_lagenarium|Reduced_virulence
Chr03G1595.1	3384	6.00E-64	266/1048(25.38)	233	FVEG_12530|PHI:3384|W7N2C1|117187|Fusarium_verticillioides|unaffected_pathogenicity_
Chr03G1597.1	1783	6.00E-59	191/662(28.85)	209	PHI:1783|GzZC098|I1S2C5|5518|Gibberella_zeae_(related:_Fusarium_graminearum)|Unaffected_pathogenicity
Chr03G1601.1	541	5.00E-64	177/570(31.05)	219	PHI:541|LIP1|AAU87359|332648|Botrytis_cinerea|Unaffected_pathogenicity
Chr03G1603.1	438	8.00E-25	104/442(23.53)	105	PHI:438|BcBOT1_(related:_CND5)|AAQ16576|40559|Botrytis_cinerea|Reduced_virulence
Chr03G1605.1	2103	1.00E-39	74/145(51.03)	140	PHI:2103|Vacuolar_calcium_ion_transporter|MGG_08710|148305|Magnaporthe_oryzae_(related:_Magnaporthe_grisea)|Unaffected_pathogenicity
Chr03G1607.1	2968	3.00E-27	102/415(24.58)	112	PHI:2968|Hxs1|J9VQA5|5207|Cryptococcus_neoformans|Reduced_virulence

Chr03G1611.1	441	3.00E-27	93/339(27.43)	108	PHI:441|BTP1|CAE55153|40559|Botrytis_cinerea|Reduced_virulence
Chr03G1612.1	404	2.00E-16	81/345(23.48)	77.8	PHI:404|PTH11|AAD30436|318829|Magnaporthe_oryzae|Reduced_virulence
Chr03G1617.1	2315	3.00E-57	113/294(38.44)	189	PHI:2315|ChLae1|G4XKY9|5016|Cochliobolus_heterostrophus|Mixed_outcome
Chr03G1619.1	438	1.00E-65	155/477(32.49)	222	PHI:438|BcBOT1_(related:_CND5)|AAQ16576|40559|Botrytis_cinerea|Reduced_virulence
Chr03G1620.1	181	0	299/434(68.89)	625	PHI:181|PGX1|AAK81847|5507|Fusarium_oxysporum|Unaffected_pathogenicity
Chr03G1623.1	2117	3.00E-06	26/57(45.61)	48.1	PHI:2117|SPM1|P58371|148305|Magnaporthe_oryzae_(related:_Magnaporthe_grisea)|Reduced_virulence
Chr03G1627.1	1391	1.00E-22	40/91(43.96)	96.7	PHI:1391|GzC2H054|I1RU55|5518|Gibberella_zeae_(related:_Fusarium_graminearum)|Unaffected_pathogenicity
Chr03G1631.1	1893	6.00E-76	162/487(33.26)	249	PHI:1893|GzZC208|I1RNY0|5518|Gibberella_zeae_(related:_Fusarium_graminearum)|Unaffected_pathogenicity
Chr03G1632.1	2042	0	420/1241(33.84)	687	PHI:2042|ABC3|Q3Y5V5|148305|Magnaporthe_oryzae_(related:_Magnaporthe_grisea)|Loss_of_pathogenicity
Chr03G1634.1	1828	0	319/675(47.26)	600	PHI:1828|GzZC143|I1S351|5518|Gibberella_zeae_(related:_Fusarium_graminearum)|Unaffected_pathogenicity
Chr03G1635.1	1046	6.00E-31	124/460(26.96)	122	PHI:1046|CTB5|ABK64182|29003|Cercospora_nicotianae|Reduced_virulence
Chr03G1636.1	812	7.00E-15	86/356(24.16)	73.2	PHI:812|MGG_10702|EDJ94108|318829|Magnaporthe_oryzae|Reduced_virulence
Chr03G1638.1	3381	1.00E-48	131/468(27.99)	177	FVEG_12533|PHI:3381|W7N2B4|117187|Fusarium_verticillioides|unaffected_pathogenicity_
Chr03G1645.1	1879	8.00E-29	108/420(25.71)	120	PHI:1879|GzZC194|I1RJB8|5518|Gibberella_zeae_(related:_Fusarium_graminearum)|Unaffected_pathogenicity
Chr03G1650.1	223	4.00E-47	88/255(34.51)	167	PHI:223|PEP1|AAK11166|140110|Nectria_haematococca_(related:_Fusarium_solani)|Reduced_virulence

Chr03G1652.1	1330	1.00E-16	41/67(61.19)	71.6	PHI:1330|GzbZIP012|I1RUH0|5518|Gibberella_zeae_(related:_Fusarium_graminearum)|Unaffected_pathogenicity
Chr03G1655.1	404	8.00E-13	64/280(22.86)	67	PHI:404|PTH11|AAD30436|318829|Magnaporthe_oryzae|Reduced_virulence
Chr03G1658.1	1963	0	305/465(65.59)	600	PHI:1963|GzZC278|I1RR01|5518|Gibberella_zeae_(related:_Fusarium_graminearum)|Unaffected_pathogenicity
Chr03G1659.1	748	0	317/667(47.53)	577	PHI:748|um00446|Not_available|5270|Ustilago_maydis|Unaffected_pathogenicity
Chr03G1660.1	2728	5.00E-32	132/492(26.83)	126	PHI:2728|FgERG4|I1RZZ3|5518|Fusarium_graminearum|Reduced_virulence
Chr03G1663.1	2240	4.00E-36	136/504(26.98)	138	PHI:2240|Srt1|Q4PBY9|5270|Ustilago_maydis|reduced_virulence
Chr03G1665.1	4194	5.00E-17	101/400(25.25)	80.1	AKT7|PHI:4194|V5XZS6|5599|Alternaria_alternata|increased_virulence_(Hypervirulence)
Chr03G1670.1	2020	4.00E-06	41/165(24.85)	46.2	PHI:2020|Tup1|XP_759427|5270|Ustilago_maydis|Mixed_outcome
Chr03G1671.1	881	2.00E-31	90/334(26.95)	121	PHI:881|MGG_04556|EDJ96020|318829|Magnaporthe_oryzae|Reduced_virulence
Chr03G1672.1	316	8.00E-18	70/298(23.49)	82.8	PHI:316|PiGPB1|AAP55639|4787|Phytophthora_infestans|Reduced_virulence
Chr03G1677.1	1806	7.00E-12	41/129(31.78)	60.8	PHI:1806|GzZC121|I1RUM8|5518|Gibberella_zeae_(related:_Fusarium_graminearum)|Unaffected_pathogenicity
Chr03G1681.1	2968	8.00E-33	128/485(26.39)	128	PHI:2968|Hxs1|J9VQA5|5207|Cryptococcus_neoformans|Reduced_virulence
Chr03G1682.1	812	3.00E-26	85/317(26.81)	106	PHI:812|MGG_10702|EDJ94108|318829|Magnaporthe_oryzae|Reduced_virulence
Chr03G1685.1	1802	3.00E-84	198/642(30.84)	285	PHI:1802|GzZC117|I1RVB2|5518|Gibberella_zeae_(related:_Fusarium_graminearum)|Unaffected_pathogenicity
Chr03G1692.1	2978	1.00E-10	62/250(24.80)	58.9	PHI:2978|MoCel12A|G4N5V2|148305|Magnaporthe_oryzae|Unaffected_pathogenicity
Chr03G1693.1	2553	4.00E-17	59/181(32.60)	82.4	PHI:2553|VPS4|Q5AG40|5476|Candida_albicans|Loss_of_pathogenicity
Chr03G1699.1	538	1.00E-46	154/540(28.52)	171	PHI:538|FRT1|AAU87358|40559|Botrytis_cinerea|Unaffected_pathogenicity

Chr03G1702.1	436	2.00E-51	83/174(47.70)	163	PHI:436|ESS1|AAN03477|5207|Cryptococcus_neoformans|Reduced_virulence
Chr03G1703.1	1627	9.00E-10	36/109(33.03)	50.4	PHI:1627|GzTF2S002|I1RW90|5518|Gibberella_zeae_(related:_Fusarium_graminearum)|Unaffected_pathogenicity
Chr03G1709.1	1575	9.00E-32	88/234(37.61)	120	PHI:1575|GzOB015|I1RIQ3|5518|Gibberella_zeae_(related:_Fusarium_graminearum)|Unaffected_pathogenicity
Chr03G1711.1	211	1.00E-22	68/199(34.17)	99.8	PHI:211|CaTUP1|AAB63195|5476|Candida_albicans|Reduced_virulence
Chr03G1712.1	2038	3.00E-17	80/284(28.17)	78.2	PHI:2038|Mir1|MGG_02370|148305|Magnaporthe_oryzae_(related:_Magnaporthe_grisea)|Unaffected_pathogenicity
Chr03G1714.1	1389	0	476/823(57.84)	897	PHI:1389|GzC2H052|I1RTJ6|5518|Gibberella_zeae_(related:_Fusarium_graminearum)|Lethal
Chr03G1715.1	784	8.00E-26	80/262(30.53)	101	PHI:784|MGG_00056|EDK03390|318829|Magnaporthe_oryzae|Reduced_virulence
Chr03G1716.1	2654	4.00E-12	110/485(22.68)	66.6	PHI:2654|DUR1,2|Q59VF3|5476|Candida_albicans|Reduced_virulence
Chr03G1717.1	881	5.00E-29	62/160(38.75)	112	PHI:881|MGG_04556|EDJ96020|318829|Magnaporthe_oryzae|Reduced_virulence
Chr03G1723.1	2269	5.00E-30	81/260(31.15)	113	PHI:2269|Mdh1|0|13684|Phaeosphaeria_nodorum_(related:_Stagonospora_nodorum)|Unaffected_pathogenicity
Chr03G1725.1	2256	3.00E-58	119/343(34.69)	194	PHI:2256|Xdh1|Q0UA24|13684|Phaeosphaeria_nodorum_(related:_Stagonospora_nodorum)|Unaffected_pathogenicity
Chr03G1728.1	1879	7.00E-28	97/370(26.22)	118	PHI:1879|GzZC194|I1RJB8|5518|Gibberella_zeae_(related:_Fusarium_graminearum)|Unaffected_pathogenicity
Chr03G1729.1	1695	1.00E-16	84/368(22.83)	80.1	PHI:1695|GzZC010|I1S2I3|5518|Gibberella_zeae_(related:_Fusarium_graminearum)|Unaffected_pathogenicity
Chr03G1733.1	1769	2.00E-11	97/387(25.06)	62	PHI:1769|GzZC084|I1RXB2|5518|Gibberella_zeae_(related:_Fusarium_graminearum)|Unaffected_pathogenicity
Chr03G1734.1	3126	4.00E-42	111/347(31.99)	153	argD|PHI:3126|D4I307|552|Erwinia_amylovora|mixed_outcome_

Chr03G1736.1	3662	2.00E-06	50/160(31.25)	48.9	PspB_(not_PD0218)|PHI:3662|Q87ET0|2371|Xylella_fastidiosa|Increased_virulence_(Hypervirulence)
Chr03G1745.1	2549	3.00E-07	24/87(27.59)	48.9	PHI:2549|MAK5|Q4WMS3|746128|Aspergillus_fumigatus|Mixed_outcome
Chr09G0001.1	1527	2.00E-22	88/361(24.38)	96.3	PHI:1527|GzHOMEL040|I1S9A1|5518|Gibberella_zeae_(related:_Fusarium_graminearum)|Lethal
Chr09G0004.1	2256	2.00E-18	74/279(26.52)	83.6	PHI:2256|Xdh1|Q0UA24|13684|Phaeosphaeria_nodorum_(related:_Stagonospora_nodorum)|Unaffected_pathogenicity
Chr09G0005.1	716	2.00E-69	173/581(29.78)	235	PHI:716|ZEB1|ABB90284|5518|Fusarium_graminearum|Unaffected_pathogenicity
Chr09G0007.1	1936	2.00E-158	282/721(39.11)	474	PHI:1936|GzZC251|I1RFG1|5518|Gibberella_zeae_(related:_Fusarium_graminearum)|Unaffected_pathogenicity
Chr09G0010.1	262	1.00E-31	65/175(37.14)	113	PHI:262|MAD2|EAK94586|5476|Candida_albicans|Reduced_virulence
Chr09G0014.1	3455	3.00E-42	157/528(29.73)	157	Apt1|PHI:3455|J9VIH5|5207|Cryptococcus_neoformans|reduced_virulence_
Chr09G0015.1	1458	3.00E-74	157/400(39.25)	252	PHI:1458|GzAra006|Q4I7F9|5518|Gibberella_zeae_(related:_Fusarium_graminearum)|Unaffected_pathogenicity
Chr09G0018.1	2968	2.00E-92	165/501(32.93)	293	PHI:2968|Hxs1|J9VQA5|5207|Cryptococcus_neoformans|Reduced_virulence
Chr09G0019.1	1527	2.00E-12	32/111(28.83)	63.5	PHI:1527|GzHOMEL040|I1S9A1|5518|Gibberella_zeae_(related:_Fusarium_graminearum)|Lethal
Chr09G0020.1	3216	2.00E-32	83/213(38.97)	123	MoCDIP4|PHI:3216|G4MVX4|318829|Magnaporthe_oryzae|mixed_outcome_
Chr09G0021.1	412	7.00E-09	27/72(37.50)	52.4	PHI:412|CHAP1|AAS64313|5016|Cochliobolus_heterostrophus|Unaffected_pathogenicity
Chr09G0029.1	2038	2.00E-09	69/283(24.38)	55.5	PHI:2038|Mir1|MGG_02370|148305|Magnaporthe_oryzae_(related:_Magnaporthe_grisea)|Unaffected_pathogenicity
Chr09G0032.1	1497	2.00E-81	223/631(35.34)	267	PHI:1497|GzHMG029|I1RZL9|5518|Gibberella_zeae_(related:_Fusarium_graminearum)|Reduced_virulence
Chr09G0035.1	2601	2.00E-150	205/317(64.67)	426	PHI:2601|Asc1|P83774|746128|Aspergillus_fumigatus|Mixed_outcome

Chr09G0036.1	1318	6.00E-102	155/306(50.65)	303	PHI:1318|GzBrom003|I1RZM2|5518|Gibberella_zeae_(related:_Fusarium_graminearum)|Unaffected_pathogenicity
Chr09G0037.1	2368	1.00E-07	37/133(27.82)	51.6	PHI:2368|bac|Q9P880|40559|Botrytis_cinerea|Reduced_virulence
Chr09G0039.1	2962	1.00E-92	173/372(46.51)	287	PHI:2962|glyA|C5BEV2|67780|Edwardsiella_ictaluri|Reduced_virulence
Chr09G0043.1	1579	3.00E-46	89/195(45.64)	179	PHI:1579|GzOB019|I1RM25|5518|Gibberella_zeae_(related:_Fusarium_graminearum)|Unaffected_pathogenicity
Chr09G0044.1	2544	3.00E-06	34/164(20.73)	48.5	PHI:2544|PAB1|Q4WK03|746128|Aspergillus_fumigatus|Mixed_outcome
Chr09G0051.1	2844	2.00E-33	87/275(31.64)	122	PHI:2844|BRM2|O93802|5599|Alternaria_alternata|Unaffected_pathogenicity
Chr09G0053.1	1753	0	607/839(72.35)	1204	PHI:1753|GzZC068|I1RZN4|5518|Gibberella_zeae_(related:_Fusarium_graminearum)|Unaffected_pathogenicity
Chr09G0054.1	3238	1.00E-38	70/175(40.00)	133	ctrC|PHI:3238|B0XUP5|746128|Aspergillus_fumigatus|mixed_outcome_
Chr09G0055.1	815	1.00E-23	93/250(37.20)	105	PHI:815|MGG_04685|EDJ95941|318829|Magnaporthe_oryzae|Reduced_virulence
Chr09G0067.1	2044	0	442/819(53.97)	561	PHI:2044|MoSOM1|XP_362263|148305|Magnaporthe_oryzae_(related:_Magnaporthe_grisea)|Loss_of_pathogenicity
Chr09G0069.1	2020	3.00E-25	73/295(24.75)	104	PHI:2020|Tup1|XP_759427|5270|Ustilago_maydis|Mixed_outcome
Chr09G0071.1	1663	3.00E-122	200/239(83.68)	348	PHI:1663|GzCCHC003|I1RFZ8|5518|Gibberella_zeae_(related:_Fusarium_graminearum)|Unaffected_pathogenicity
Chr09G0075.1	748	8.00E-47	120/358(33.52)	177	PHI:748|um00446|Not_available|5270|Ustilago_maydis|Unaffected_pathogenicity
Chr09G0081.1	1727	4.00E-30	61/175(34.86)	120	PHI:1727|GzZC042|I1S5Q2|5518|Gibberella_zeae_(related:_Fusarium_graminearum)|Unaffected_pathogenicity
Chr09G0083.1	1357	0	539/798(67.54)	1037	PHI:1357|GzC2H017|I1RFY9|5518|Gibberella_zeae_(related:_Fusarium_graminearum)|Unaffected_pathogenicity
Chr09G0085.1	1071	3.00E-45	109/338(32.25)	174	PHI:1071|Gas1|CAF05793|5270|Ustilago_maydis|Loss_of_pathogenicity
Chr09G0090.1	1574	0	700/868(80.65)	1374	PHI:1574|GzOB014|I1RFX8|5518|Gibberella_zeae_(related:_Fusarium_gramin

					earum)|Lethal
Chr09G0093.1	1795	6.00E-31	142/530(26.79)	125	PHI:1795|GzZC110|I1RSX8|5518|Gibberella_zeae_(related:_Fusarium_graminearum)|Unaffected_pathogenicity
Chr09G0096.1	1650	0	400/766(52.22)	567	PHI:1650|GzWing022|I1S006|5518|Gibberella_zeae_(related:_Fusarium_graminearum)|Lethal
Chr09G0097.1	455	4.00E-39	99/302(32.78)	146	PHI:455|CAP59|AAC13946|5207|Cryptococcus_neoformans|Loss_of_pathogenicity
Chr09G0112.1	1680	3.00E-81	158/372(42.47)	288	PHI:1680|GzMIZ002|I1S004|5518|Gibberella_zeae_(related:_Fusarium_graminearum)|Unaffected_pathogenicity
Chr09G0114.1	2029	5.00E-106	179/338(52.96)	317	PHI:2029|TGL2|G4NEK6|148305|Magnaporthe_oryzae_(related:_Magnaporthe_grisea)|Unaffected_pathogenicity
Chr09G0117.1	441	6.00E-16	76/319(23.82)	76.3	PHI:441|BTP1|CAE55153|40559|Botrytis_cinerea|Reduced_virulence
Chr09G0120.1	3415	7.00E-32	141/557(25.31)	128	Fre2|PHI:3415|T2BNJ5|5207|Cryptococcus_neoformans|mixed_outcome_
Chr09G0122.1	2020	2.00E-10	50/187(26.74)	58.9	PHI:2020|Tup1|XP_759427|5270|Ustilago_maydis|Mixed_outcome
Chr09G0124.1	1552	0	963/1120(85.98)	1890	PHI:1552|GzMyb016|I1S0N5|5518|Gibberella_zeae_(related:_Fusarium_graminearum)|Unaffected_pathogenicity
Chr09G0126.1	316	2.00E-12	83/309(26.86)	66.2	PHI:316|PiGPB1|AAP55639|4787|Phytophthora_infestans|Reduced_virulence
Chr09G0128.1	1260	3.00E-15	70/199(35.18)	78.6	PHI:1260|FGSG_13944|I1RUC7|5518|Gibberella_zeae_(related:_Fusarium_graminearum)|Unaffected_pathogenicity
Chr09G0142.1	1525	0	396/663(59.73)	670	PHI:1525|GzHOMEL035|Q4HY90|5518|Gibberella_zeae_(related:_Fusarium_graminearum)|Unaffected_pathogenicity
Chr09G0143.1	1772	0	423/693(61.04)	772	PHI:1772|GzZC087|I1S053|5518|Gibberella_zeae_(related:_Fusarium_graminearum)|Reduced_virulence
Chr09G0144.1	1203	0	412/456(90.35)	848	PHI:1203|(Sc_Yck1/2/3)|I1S050|5518|Gibberella_zeae_(related:_Fusarium_graminearum)|Reduced_virulence
Chr09G0148.1	433	1.00E-09	76/336(22.62)	59.7	PHI:433|CTB1|AAT69682|29003|Cercospora_nicotianae|Reduced_virulence

Chr09G0153.1	3374	0	770/1147(67.13)	1389	FgSln1|PHI:3374|I1S028|5518|Fusarium_graminearum|reduced_virulence_
Chr09G0161.1	1668	4.00E-135	219/455(48.13)	398	PHI:1668|GzCCHC008|I1S0C1|5518|Gibberella_zeae_(related:_Fusarium_graminearum)|Unaffected_pathogenicity
Chr09G0162.1	419	5.00E-29	92/328(28.05)	112	PHI:419|CSH1|AAP93915|5476|Candida_albicans|Reduced_virulence
Chr09G0168.1	1210	0	906/1424(63.62)	1587	PHI:1210|FGSG_01058|I1S077|5518|Gibberella_zeae_(related:_Fusarium_graminearum)|Reduced_virulence
Chr09G0174.1	167	3.00E-49	151/516(29.26)	177	PHI:167|CHIP3|AAF00024|5457|Colletotrichum_gloeosporioides|Unaffected_pathogenicity
Chr09G0175.1	1201	9.00E-114	164/270(60.74)	330	PHI:1201|(Bud32)|Q4HYC1|5518|Gibberella_zeae_(related:_Fusarium_graminearum)|Reduced_virulence
Chr09G0181.1	3257	6.00E-21	82/285(28.77)	92	MoIlv1|PHI:3257|G4MU34|318829|Magnaporthe_oryzae|reduced_virulence_
Chr09G0184.1	3014	3.00E-23	55/160(34.38)	105	MoAND1|PHI:3014|G5EHD3|318829|Magnaporthe_oryzae|reduced_virulence_
Chr09G0186.1	3279	1.00E-177	237/240(98.75)	489	CoRAS2|PHI:3279|N4UYN1|5465|Colletotrichum_orbiculare|reduced_virulence_
Chr09G0187.1	3023	9.00E-07	32/106(30.19)	48.1	opy2|PHI:3023|Q5A753|5476|Candida_albicans|reduced_virulence_
Chr09G0189.1	1619	7.00E-13	43/134(32.09)	69.3	PHI:1619|GzJUM001|I1RBK8|5518|Gibberella_zeae_(related:_Fusarium_graminearum)|Unaffected_pathogenicity
Chr09G0190.1	1055	0	838/915(91.58)	1744	PHI:1055|CgCHSIII|AAL23718|31870|Colletotrichum_graminicola|Unaffected_pathogenicity
Chr09G0194.1	3174	5.00E-07	29/97(29.90)	49.7	MoSip2|PHI:3174|G4MNE3|318829|Magnaporthe_oryzae|loss_of_pathogenicity_
Chr09G0195.1	1415	2.00E-115	305/908(33.59)	379	PHI:1415|GzC2H082|I1RZZ5|5518|Gibberella_zeae_(related:_Fusarium_graminearum)|Unaffected_pathogenicity
Chr09G0198.1	179	1.00E-125	170/239(71.13)	363	PHI:179|PELA|AAA33338|140110|Nectria_haematococca_(related:_Fusarium_solani)|Reduced_virulence
Chr09G0204.1	854	6.00E-16	68/232(29.31)	74.7	PHI:854|um01947|EAK82380|5270|Ustilago_maydis|Reduced_virulence

Chr09G0208.1	3060	3.00E-14	45/137(32.85)	73.9	Molrg1|PHI:3060|G4MZD0|318829|Magnaporthe_oryzae|mixed_outcome_
Chr09G0209.1	860	8.00E-06	34/126(26.98)	46.2	PHI:860|MSP1|AAX07670|318829|Magnaporthe_oryzae|Reduced_virulence
Chr09G0213.1	552	3.00E-141	237/594(39.90)	424	PHI:552|BcLCC2|AAK77953|40559|Botrytis_cinerea|Unaffected_pathogenicity
Chr09G0220.1	2075	0	452/712(63.48)	932	PHI:2075|Moatg7|MGG_07297|148305|Magnaporthe_oryzae_(related:_Magnaporthe_grisea)|Loss_of_pathogenicity
Chr09G0231.1	2425	0	741/2238(33.11)	1107	PHI:2425|PKS2|Q6RKG2|5016|Cochliobolus_heterostrophus|Reduced_virulence
Chr09G0232.1	2838	1.00E-06	41/168(24.40)	45.8	PHI:2838|TOX9|D2SZX8|5016|Cochliobolus_heterostrophus|Reduced_virulence
Chr09G0233.1	2042	0	818/1341(61.00)	1612	PHI:2042|ABC3|Q3Y5V5|148305|Magnaporthe_oryzae_(related:_Magnaporthe_grisea)|Loss_of_pathogenicity
Chr09G0234.1	2570	9.00E-07	21/45(46.67)	50.8	PHI:2570|CYB2|Q6FM61|5478|Candida_glabrata|Reduced_virulence
Chr09G0235.1	1420	7.00E-07	102/537(18.99)	49.7	PHI:1420|GzC2H088|I1S172|5518|Gibberella_zeae_(related:_Fusarium_graminearum)|Unaffected_pathogenicity
Chr09G0236.1	59	2.00E-06	55/224(24.55)	45.8	PHI:59|THR1|BAA18962|5462|Colletotrichum_lagenarium|Reduced_virulence
Chr09G0237.1	1816	5.00E-28	119/442(26.92)	117	PHI:1816|GzZC131|I1RRS3|5518|Gibberella_zeae_(related:_Fusarium_graminearum)|Unaffected_pathogenicity
Chr09G0239.1	922	6.00E-48	165/605(27.27)	175	PHI:922|um03615||5270|Ustilago_maydis|Unaffected_pathogenicity
Chr09G0241.1	1028	3.00E-78	153/343(44.61)	254	PHI:1028|bcpme1|CAC29255|40559|Botrytis_cinerea|Reduced_virulence
Chr09G0243.1	790	1.00E-131	239/496(48.19)	390	PHI:790|MGG_07259|EDK03989|318829|Magnaporthe_oryzae|Reduced_virulence
Chr09G0244.1	1691	2.00E-87	141/422(33.41)	278	PHI:1691|GzZC006|I1RZU0|5518|Gibberella_zeae_(related:_Fusarium_graminearum)|Unaffected_pathogenicity
Chr09G0246.1	591	3.00E-52	113/260(43.46)	190	PHI:591|ORP1|AAK51698|318829|Magnaporthe_oryzae|Loss_of_pathogenicity
Chr09G0247.1	243	1.00E-76	180/537(33.52)	265	PHI:243|CHIP6|AAD00894|5457|Colletotrichum_gloeosporioides|Reduced_virulence

Chr09G0253.1	576	7.00E-32	152/624(24.36)	128	PHI:576|NoxA|BAE72680|35717|Epichloe_festucae|Enhanced_antagonism
Chr09G0255.1	2651	5.00E-13	47/151(31.13)	60.5	PHI:2651|yedX|Q8ZQ52|90371|Salmonella_enterica_serovar_Typhimurium|Unaffected_pathogenicity
Chr09G0262.1	2602	3.00E-12	59/208(28.37)	62.8	PHI:2602|Rim9|Q4PH32|5270|Ustilago_maydis|Unaffected_pathogenicity
Chr09G0265.1	1667	0	339/476(71.22)	660	PHI:1667|GzCCHC007|I1RZP2|5518|Gibberella_zeae_(related:_Fusarium_graminearum)|Unaffected_pathogenicity
Chr09G0273.1	784	7.00E-21	78/260(30.00)	87	PHI:784|MGG_00056|EDK03390|318829|Magnaporthe_oryzae|Reduced_virulence
Chr09G0279.1	257	2.00E-143	198/282(70.21)	407	PHI:257|GAS2|AAF74764|318829|Magnaporthe_oryzae|Reduced_virulence
Chr09G0287.1	1048	1.00E-49	137/446(30.72)	174	PHI:1048|CTB7|ABK64184|29003|Cercospora_nicotianae|Reduced_virulence
Chr09G0288.1	1857	6.00E-85	219/767(28.55)	284	PHI:1857|GzZC172|I1S3I1|5518|Gibberella_zeae_(related:_Fusarium_graminearum)|Unaffected_pathogenicity
Chr09G0291.1	876	0	413/1038(39.79)	713	PHI:876|MGG_11671|EDK03349|318829|Magnaporthe_oryzae|Reduced_virulence
Chr09G0294.1	1556	1.00E-38	68/114(59.65)	139	PHI:1556|GzNH001|I1RZY2|5518|Gibberella_zeae_(related:_Fusarium_graminearum)|Reduced_virulence
Chr09G0296.1	1602	0	495/630(78.57)	996	PHI:1602|GzOB043|I1RZY1|5518|Gibberella_zeae_(related:_Fusarium_graminearum)|Unaffected_pathogenicity
Chr09G0297.1	2544	9.00E-22	62/208(29.81)	97.1	PHI:2544|PAB1|Q4WK03|746128|Aspergillus_fumigatus|Mixed_outcome
Chr09G0305.1	1514	0	426/1005(42.39)	728	PHI:1514|GzHOME012|I1RYV5|5518|Gibberella_zeae_(related:_Fusarium_graminearum)|Unaffected_pathogenicity
Chr09G0306.1	812	3.00E-32	105/358(29.33)	124	PHI:812|MGG_10702|EDJ94108|318829|Magnaporthe_oryzae|Reduced_virulence
Chr09G0307.1	441	5.00E-12	62/267(23.22)	64.3	PHI:441|BTP1|CAE55153|40559|Botrytis_cinerea|Reduced_virulence
Chr09G0308.1	1651	2.00E-51	128/402(31.84)	178	PHI:1651|GzWing023|I1S0U2|5518|Gibberella_zeae_(related:_Fusarium_graminearum)|Unaffected_pathogenicity

Chr09G0324.1	373	0	567/1553(36.51)	852	PHI:373|PLD1|EAK93902|5476|Candida_albicans|Reduced_virulence
Chr09G0325.1	823	9.00E-95	155/442(35.07)	294	PHI:823|beta-tubulin|CAA56936|38038|Rhynchosporium_secalis|Chemistry_target
Chr09G0328.1	1752	0	613/887(69.11)	1225	PHI:1752|GzZC067|I1RZR8|5518|Gibberella_zeae_(related:_Fusarium_graminearum)|Unaffected_pathogenicity
Chr09G0339.1	1135	2.00E-56	153/473(32.35)	194	PHI:1135|LmIFRD|C6KED4|5022|Leptosphaeria_maculans|Loss_of_pathogenicity
Chr09G0341.1	211	6.00E-19	66/290(22.76)	84.3	PHI:211|CaTUP1|AAB63195|5476|Candida_albicans|Reduced_virulence
Chr09G0342.1	1247	0	575/766(75.07)	1165	PHI:1247|FGSG_02488|I1S0G8|5518|Gibberella_zeae_(related:_Fusarium_graminearum)|Unaffected_pathogenicity
Chr09G0344.1	2520	7.00E-43	142/472(30.08)	164	PHI:2520|LYS4|Q4WUL6|746128|Aspergillus_fumigatus|Lethal
Chr09G0349.1	697	7.00E-23	96/332(28.92)	97.1	PHI:697|ugt51E1|AAM81358|5022|Leptosphaeria_maculans|Unaffected_pathogenicity
Chr09G0351.1	1555	6.00E-16	105/473(22.20)	77.4	PHI:1555|GzMyb019|I1RDG6|5518|Gibberella_zeae_(related:_Fusarium_graminearum)|Unaffected_pathogenicity
Chr09G0352.1	1958	1.00E-18	61/214(28.50)	86.3	PHI:1958|GzZC273|I1RMD1|5518|Gibberella_zeae_(related:_Fusarium_graminearum)|Unaffected_pathogenicity
Chr09G0356.1	2396	9.00E-130	189/319(59.25)	375	PHI:2396|GzSYN2|I1RZS5|5518|Gibberella_zeae_(related:_Fusarium_graminearum)|Reduced_virulence
Chr09G0357.1	1660	4.00E-32	130/505(25.74)	133	PHI:1660|GzCCCH004|I1S4C9|5518|Gibberella_zeae_(related:_Fusarium_graminearum)|Unaffected_pathogenicity
Chr09G0358.1	2488	6.00E-65	166/497(33.40)	222	PHI:2488|Man1|G4ND25|148305|Magnaporthe_oryzae_(related:_Magnaporthe_grisea)|Unaffected_pathogenicity
Chr09G0359.1	213	2.00E-66	99/154(64.29)	208	PHI:213|CPA1|AAF69795|5207|Cryptococcus_neoformans|Reduced_virulence
Chr09G0360.1	3278	9.00E-22	89/359(24.79)	100	Colra1|PHI:3278|N4V0R3|5465|Colletotrichum_orbiculare|reduced_virulence_
Chr09G0362.1	1440	0	333/498(66.87)	566	PHI:1440|GzNot002|I1SA65|5518|Gibberella_zeae_(related:_Fusarium_gramin

					earum)|Reduced_virulence
Chr09G0363.1	1417	2.00E-44	70/127(55.12)	151	PHI:1417|GzC2H084|I1S0W0|5518|Gibberella_zeae_(related:_Fusarium_graminearum)|Unaffected_pathogenicity
Chr09G0370.1	4194	6.00E-11	69/305(22.62)	62.4	AKT7|PHI:4194|V5XZS6|5599|Alternaria_alternata|increased_virulence_(Hypervirulence)
Chr09G0374.1	1629	9.00E-30	51/115(44.35)	112	PHI:1629|GzWing001|I1RAH2|5518|Gibberella_zeae_(related:_Fusarium_graminearum)|Unaffected_pathogenicity
Chr09G0382.1	2822	3.00E-37	147/586(25.09)	145	PHI:2822|Cxt1p|Q5K8R6|5207|Cryptococcus_neoformans|Reduced_virulence
Chr09G0384.1	1161	1.00E-22	121/510(23.73)	99.8	PHI:1161|MgMfs1|A4ZGP3|54734|Mycosphaerella_graminicola_(related:_Zymoseptoria_tritici)|Chemistry_target
Chr09G0387.1	1689	0	530/909(58.31)	952	PHI:1689|GzZC004|I1S018|5518|Gibberella_zeae_(related:_Fusarium_graminearum)|Unaffected_pathogenicity
Chr09G0389.1	1816	1.00E-69	150/409(36.67)	241	PHI:1816|GzZC131|I1RRS3|5518|Gibberella_zeae_(related:_Fusarium_graminearum)|Unaffected_pathogenicity
Chr09G0392.1	874	1.00E-22	91/303(30.03)	100	PHI:874|MGG_00435|EDK02952|318829|Magnaporthe_oryzae|Loss_of_pathogenicity
Chr09G0394.1	1420	1.00E-09	96/458(20.96)	59.3	PHI:1420|GzC2H088|I1S172|5518|Gibberella_zeae_(related:_Fusarium_graminearum)|Unaffected_pathogenicity
Chr09G0402.1	2022	4.00E-20	69/265(26.04)	85.1	PHI:2022|BUF1|MGG_02252|148305|Magnaporthe_oryzae_(related:_Magnaporthe_grisea)|Loss_of_pathogenicity
Chr09G0411.1	511	2.00E-35	114/444(25.68)	137	PHI:511|CaNAG4|EAK93098|5476|Candida_albicans|Reduced_virulence
Chr09G0425.1	3808	0	378/558(67.74)	658	Atf1|PHI:3808|I1S0C0|5518|Fusarium_graminearum_(related:_Gibberella_zeae)|effector_(plant_avirulence_determinant)
Chr09G0428.1	4194	9.00E-30	120/513(23.39)	120	AKT7|PHI:4194|V5XZS6|5599|Alternaria_alternata|increased_virulence_(Hypervirulence)
Chr09G0436.1	1533	5.00E-78	115/154(74.68)	229	PHI:1533|GzLam002|I1S0F5|5518|Gibberella_zeae_(related:_Fusarium_grami

					nearum)|Reduced_virulence
Chr09G0443.1	2821	6.00E-93	198/518(38.22)	333	PHI:2821|SNF2|Q5ALP9|5476|Candida_albicans|Reduced_virulence
Chr09G0444.1	3098	7.00E-104	173/359(48.19)	311	URA4|PHI:3098|J9VJS8|5207|Cryptococcus_neoformans|reduced_virulence_
Chr09G0446.1	1603	0	383/546(70.15)	816	PHI:1603|GzOB044|I1S0A1|5518|Gibberella_zeae_(related:_Fusarium_graminearum)|Unaffected_pathogenicity
Chr09G0448.1	404	6.00E-20	65/270(24.07)	88.2	PHI:404|PTH11|AAD30436|318829|Magnaporthe_oryzae|Reduced_virulence
Chr09G0449.1	255	3.00E-23	62/214(28.97)	99.4	PHI:255|FUM1_(related:_FUM5)|AAD43562|5127|Gibberella_moniliformis|Unaffected_pathogenicity
Chr09G0451.1	2321	1.00E-19	138/584(23.63)	90.5	PHI:2321|SidI|Q4WR83|746128|Aspergillus_fumigatus|Reduced_virulence
Chr09G0453.1	2065	0	370/638(57.99)	583	PHI:2065|MSTU1|Q4R1B9|148305|Magnaporthe_oryzae_(related:_Magnaporthe_grisea)|Loss_of_pathogenicity
Chr09G0456.1	1185	0	520/1101(47.23)	822	PHI:1185|(Sc_Swe1)|I1S0J9|5518|Gibberella_zeae_(related:_Fusarium_graminearum)|Reduced_virulence
Chr09G0459.1	133	7.00E-62	156/489(31.90)	214	PHI:133|AKT1|BAA36588|5599|Alternaria_alternata|Loss_of_pathogenicity
Chr09G0462.1	1416	2.00E-77	190/545(34.86)	257	PHI:1416|GzC2H083|I1S0D8|5518|Gibberella_zeae_(related:_Fusarium_graminearum)|Unaffected_pathogenicity
Chr09G0464.1	1436	3.00E-63	124/316(39.24)	216	PHI:1436|GzC2H105|I1SA30|5518|Gibberella_zeae_(related:_Fusarium_graminearum)|Reduced_virulence
Chr09G0468.1	412	2.00E-08	22/54(40.74)	51.6	PHI:412|CHAP1|AAS64313|5016|Cochliobolus_heterostrophus|Unaffected_pathogenicity
Chr09G0471.1	404	4.00E-39	107/411(26.03)	147	PHI:404|PTH11|AAD30436|318829|Magnaporthe_oryzae|Reduced_virulence
Chr09G0473.1	231	0	346/391(88.49)	726	PHI:231|RPK1|AAK31209|5462|Colletotrichum_lagenarium|Loss_of_pathogenicity
Chr09G0481.1	319	2.00E-08	24/59(40.68)	54.3	PHI:319|SQL2|AAO19638|5270|Ustilago_maydis|Reduced_virulence
Chr09G0483.1	2304	5.00E-07	66/258(25.58)	49.3	PHI:2304|BCFHG1|CAP74387|40559|Botrytis_cinerea|Unaffected_pathogenicity

Chr09G0486.1	1601	0	1043/1411(73.92)	2069	PHI:1601|GzOB042|Q4HYQ4|5518|Gibberella_zeae_(related:_Fusarium_graminearum)|Unaffected_pathogenicity
Chr09G0487.1	165	0	484/513(94.35)	933	PHI:165|CgMEK1_(related:_EMK1)|AAD55385|5457|Colletotrichum_gloeosporioides|Loss_of_pathogenicity
Chr09G0490.1	305	0	454/547(83.00)	949	PHI:305|ICL1|AAN28719|318829|Magnaporthe_oryzae|Reduced_virulence
Chr09G0491.1	794	0	595/739(80.51)	1272	PHI:794|MGG_09471|EDJ95622|318829|Magnaporthe_oryzae|Reduced_virulence
Chr09G0495.1	1197	0	551/735(74.97)	1086	PHI:1197|(Snf1)|I1RZP6|5518|Gibberella_zeae_(related:_Fusarium_graminearum)|Reduced_virulence
Chr09G0498.1	1424	3.00E-18	57/192(29.69)	84.7	PHI:1424|GzC2H092|I1S3J7|5518|Gibberella_zeae_(related:_Fusarium_graminearum)|Unaffected_pathogenicity
Chr09G0508.1	2491	3.00E-25	102/350(29.14)	106	PHI:2491|FgPTC1|I1RJS9|5518|Gibberella_zeae_(related:_Fusarium_graminearum)|Reduced_virulence
Chr09G0512.1	280	7.00E-13	132/595(22.18)	69.7	PHI:280|CCN1|AAG36938|5207|Cryptococcus_neoformans|Reduced_virulence
Chr09G0513.1	2735	7.00E-32	82/239(34.31)	120	PHI:2735|RsmA|D0KML5|55208|Pectobacterium_wasabiae|Increased_virulence_(hypervirulence)
Chr09G0515.1	489	3.00E-14	38/123(30.89)	63.2	PHI:489|TRX1|AAW46720|5207|Cryptococcus_neoformans|Reduced_virulence
Chr09G0522.1	1391	2.00E-22	64/194(32.99)	100	PHI:1391|GzC2H054|I1RU55|5518|Gibberella_zeae_(related:_Fusarium_graminearum)|Unaffected_pathogenicity
Chr09G0523.1	3630	8.00E-17	71/268(26.49)	81.6	Rv2467|PHI:3630|L7N655|1773|Mycobacterium_tuberculosis|increased_virulence_
Chr09G0525.1	465	3.00E-68	149/362(41.16)	248	PHI:465|KIN2|AAB63337|5270|Ustilago_maydis|Reduced_virulence
Chr09G0526.1	2570	2.00E-12	26/63(41.27)	60.1	PHI:2570|CYB2|Q6FM61|5478|Candida_glabrata|Reduced_virulence
Chr09G0528.1	2315	1.00E-56	101/288(35.07)	187	PHI:2315|ChLae1|G4XKY9|5016|Cochliobolus_heterostrophus|Mixed_outcome
Chr09G0530.1	419	5.00E-29	85/319(26.65)	113	PHI:419|CSH1|AAP93915|5476|Candida_albicans|Reduced_virulence
Chr09G0531.1	434	1.00E-83	146/349(41.83)	266	PHI:434|GEL2|EAL88984|5085|Aspergillus_fumigatus|Reduced_virulence

Chr09G0532.1	2141	3.00E-11	68/264(25.76)	64.3	PHI:2141|CDC15|XP_001406795|148305|Magnaporthe_oryzae_(related:_Magnaporthe_grisea)|Loss_of_pathogenicity
Chr09G0533.1	423	2.00E-64	132/404(32.67)	219	PHI:423|VAD1|AAV41010|5207|Cryptococcus_neoformans|Reduced_virulence
Chr09G0537.1	2322	1.00E-17	67/282(23.76)	77.8	PHI:2322|SidH|Q4WF54|746128|Aspergillus_fumigatus|Reduced_virulence
Chr09G0546.1	84	0	300/355(84.51)	626	PHI:84|MAGC|AAB65427|318829|Magnaporthe_oryzae|Unaffected_pathogenicity
Chr09G0547.1	2239	2.00E-141	194/293(66.21)	418	PHI:2239|Spe-Sdh|Q4P245|5270|Ustilago_maydis|reduced_virulence
Chr09G0553.1	1581	2.00E-80	123/254(48.43)	244	PHI:1581|GzOB021|I1RPX8|5518|Gibberella_zeae_(related:_Fusarium_graminearum)|Unaffected_pathogenicity
Chr09G0557.1	2506	0	416/1109(37.51)	673	PHI:2506|Amr1|G3F820|29001|Alternaria_brassicicola|Increased_virulence_(Hypervirulence)
Chr09G0558.1	2022	2.00E-76	119/252(47.22)	235	PHI:2022|BUF1|MGG_02252|148305|Magnaporthe_oryzae_(related:_Magnaporthe_grisea)|Loss_of_pathogenicity
Chr09G0560.1	2203	6.00E-158	246/462(53.25)	455	PHI:2203|Transcription_factor|G4MU26|148305|Magnaporthe_oryzae_(related:_Magnaporthe_grisea)|Reduced_virulence
Chr09G0561.1	1522	7.00E-06	68/305(22.30)	44.7	PHI:1522|GzHOMEL026|I1RXA5|5518|Gibberella_zeae_(related:_Fusarium_graminearum)|Lethal
Chr09G0562.1	40	0	1996/2169(92.02)	4087	PHI:40|PKS1|BAA18956|5462|Colletotrichum_lagenarium|Reduced_virulence
Chr09G0563.1	2920	5.00E-143	231/536(43.10)	428	PHI:2920|FET3-1|E3QRA4|31870|Colletotrichum_graminicola|Reduced_virulence
Chr09G0570.1	2038	1.00E-06	45/175(25.71)	46.6	PHI:2038|Mir1|MGG_02370|148305|Magnaporthe_oryzae_(related:_Magnaporthe_grisea)|Unaffected_pathogenicity
Chr09G0578.1	1617	1.00E-39	73/145(50.34)	131	PHI:1617|GzssDB002|I1S0G0|5518|Gibberella_zeae_(related:_Fusarium_graminearum)|Unaffected_pathogenicity
Chr09G0579.1	1054	0	822/903(91.03)	1714	PHI:1054|CgCHSI|AAL23717|31870|Colletotrichum_graminicola|Unaffected_pathogenicity

Chr09G0580.1	2528	0	471/727(64.79)	942	PHI:2528|SLY1|Q4WYU7|746128|Aspergillus_fumigatus|Lethal
Chr09G0584.1	901	3.00E-51	129/435(29.66)	185	PHI:901|um01886||5270|Ustilago_maydis|Unaffected_pathogenicity
Chr09G0590.1	3146	1.00E-36	139/564(24.65)	141	GGT|PHI:3146|O25743|210|Helicobacter_pylori|unaffected_pathogenicity_
Chr09G0591.1	1662	3.00E-28	99/390(25.38)	116	PHI:1662|GzCCHC002|I1REJ1|5518|Gibberella_zeae_(related:_Fusarium_graminearum)|Unaffected_pathogenicity
Chr09G0592.1	552	2.00E-174	259/583(44.43)	507	PHI:552|BcLCC2|AAK77953|40559|Botrytis_cinerea|Unaffected_pathogenicity
Chr09G0594.1	3332	5.00E-49	104/230(45.22)	160	snf7|PHI:3332|A0A095CFR8|552467|Cryptococcus_gattii|loss_of_pathogenicity_
Chr09G0597.1	2208	3.00E-125	175/316(55.38)	372	PHI:2208|endo-1,4-beta-xylanase_[GH10_family]|MGG_05464|148305|Magnaporthe_oryzae_(related:_Magnaporthe_grisea)|Reduced_virulence
Chr09G0598.1	3071	2.00E-11	51/176(28.98)	60.8	PsTatD4|PHI:3071|G4Z2B4|67593|Phytophthora_sojae|reduced_virulence_
Chr09G0600.1	881	2.00E-36	74/210(35.24)	134	PHI:881|MGG_04556|EDJ96020|318829|Magnaporthe_oryzae|Reduced_virulence
Chr09G0602.1	1161	6.00E-46	138/561(24.60)	169	PHI:1161|MgMfs1|A4ZGP3|54734|Mycosphaerella_graminicola_(related:_Zymoseptoria_triticii)|Chemistry_target
Chr09G0604.1	1414	1.00E-12	38/82(46.34)	66.2	PHI:1414|GzC2H081|I1RZL0|5518|Gibberella_zeae_(related:_Fusarium_graminearum)|Unaffected_pathogenicity
Chr09G0605.1	1579	0	351/650(54.00)	750	PHI:1579|GzOB019|I1RM25|5518|Gibberella_zeae_(related:_Fusarium_graminearum)|Unaffected_pathogenicity
Chr09G0610.1	316	6.00E-14	53/217(24.42)	70.5	PHI:316|PiGPB1|AAP55639|4787|Phytophthora_infestans|Reduced_virulence
Chr09G0627.1	2964	2.00E-170	286/592(48.31)	503	PHI:2964|AsnB|G7TM01|129394|Xanthomonas_oryzae_pv._Oryzicola|Reduced_virulence
Chr09G0629.1	981	8.00E-12	38/84(45.24)	62.4	PHI:981|hopI1|AAL84247|59511|Pseudomonas_syringae|Effector_(plant_avirulence_determinant)
Chr09G0630.1	4194	6.00E-28	134/518(25.87)	114	AKT7|PHI:4194|V5XZS6|5599|Alternaria_alternata|increased_virulence_(Hypervirulence)

Chr09G0633.1	1281	7.00E-62	177/548(32.30)	216	PHI:1281|FGSG_07742|I1REQ8|5518|Gibberella_zeae_(related:_Fusarium_graminearum)|Unaffected_pathogenicity
Chr09G0634.1	1279	6.00E-31	104/345(30.14)	129	PHI:1279|FGSG_12132|I1RU59|5518|Gibberella_zeae_(related:_Fusarium_graminearum)|Unaffected_pathogenicity
Chr09G0636.1	2393	1.00E-148	212/483(43.89)	436	PHI:2393|Related_to_O-methylsterigmatocystin_oxidoreductase|I1R980|5518|Gibberella_zeae_(related:_Fusarium_graminearum)|Increased_virulence_(Hypervirulence)
Chr09G0637.1	404	1.00E-15	54/224(24.11)	75.9	PHI:404|PTH11|AAD30436|318829|Magnaporthe_oryzae|Reduced_virulence
Chr09G0638.1	3274	4.00E-36	172/671(25.63)	146	dhbF|PHI:3274|W2E906|147375|Paenibacillus_larvae|unaffected_pathogenicity_
Chr09G0640.1	812	1.00E-60	118/330(35.76)	202	PHI:812|MGG_10702|EDJ94108|318829|Magnaporthe_oryzae|Reduced_virulence
Chr09G0641.1	1159	7.00E-41	99/322(30.75)	161	PHI:1159|MgAtr7|A5H456|54734|Mycosphaerella_graminicola_(related:_Zymoseptoria_triticii)|Chemistry_target
Chr09G0642.1	1159	5.00E-67	127/330(38.48)	232	PHI:1159|MgAtr7|A5H456|54734|Mycosphaerella_graminicola_(related:_Zymoseptoria_triticii)|Chemistry_target
Chr09G0646.1	4618	1.00E-76	159/377(42.18)	243	PcPL15|PHI:4618|A0A0D3LX64|4784|Phytophthora_capsici|mixed_outcome
Chr09G0651.1	1520	3.00E-12	31/80(38.75)	60.1	PHI:1520|GzHOMEL024|I1RWS3|5518|Gibberella_zeae_(related:_Fusarium_graminearum)|Unaffected_pathogenicity
Chr09G0655.1	1260	8.00E-13	61/197(30.96)	69.3	PHI:1260|FGSG_13944|I1RUC7|5518|Gibberella_zeae_(related:_Fusarium_graminearum)|Unaffected_pathogenicity
Chr09G0656.1	922	1.00E-31	179/672(26.64)	128	PHI:922|um03615||5270|Ustilago_maydis|Unaffected_pathogenicity
Chr09G0657.1	404	9.00E-85	139/345(40.29)	273	PHI:404|PTH11|AAD30436|318829|Magnaporthe_oryzae|Reduced_virulence
Chr09G0660.1	2315	5.00E-38	91/292(31.16)	137	PHI:2315|ChLae1|G4XKY9|5016|Cochliobolus_heterostrophus|Mixed_outcome
Chr09G0663.1	55	0	856/2628(32.57)	1134	PHI:55|PKS1|AAB08104|5016|Cochliobolus_heterostrophus|Reduced_virulence

Chr09G0664.1	3390	3.00E-11	42/130(32.31)	58.9	FVEG_12520|PHI:3390|W7MT28|117187|Fusarium_verticillioides|unaffected_pathogenicity_
Chr09G0665.1	441	2.00E-14	62/267(23.22)	70.9	PHI:441|BTP1|CAE55153|40559|Botrytis_cinerea|Reduced_virulence
Chr09G0670.1	438	7.00E-56	134/478(28.03)	194	PHI:438|BcBOT1_(related:_CND5)|AAQ16576|40559|Botrytis_cinerea|Reduced_virulence
Chr09G0671.1	2171	8.00E-20	149/672(22.17)	91.7	PHI:2171|Peroxisomal_copper_amine_oxidase|MGG_02681|148305|Magnaporthe_oryzae_(related:_Magnaporthe_grisea)|Reduced_virulence
Chr09G0672.1	2297	5.00E-100	211/558(37.81)	325	PHI:2297|tmpL|B0YAP6|746128|Aspergillus_fumigatus|Reduced_virulence
Chr09G0673.1	922	2.00E-08	103/393(26.21)	55.1	PHI:922|um03615||5270|Ustilago_maydis|Unaffected_pathogenicity
Chr09G0678.1	404	7.00E-17	67/281(23.84)	79.7	PHI:404|PTH11|AAD30436|318829|Magnaporthe_oryzae|Reduced_virulence
Chr09G0679.1	716	4.00E-16	56/172(32.56)	77.8	PHI:716|ZEB1|ABB90284|5518|Fusarium_graminearum|Unaffected_pathogenicity
Chr09G0684.1	1046	3.00E-31	72/192(37.50)	122	PHI:1046|CTB5|ABK64182|29003|Cercospora_nicotianae|Reduced_virulence
Chr09G0686.1	441	7.00E-15	67/279(24.01)	72.4	PHI:441|BTP1|CAE55153|40559|Botrytis_cinerea|Reduced_virulence
Chr09G0689.1	2844	8.00E-29	86/258(33.33)	109	PHI:2844|BRM2|O93802|5599|Alternaria_alternata|Unaffected_pathogenicity
Chr09G0690.1	211	8.00E-10	54/206(26.21)	58.5	PHI:211|CaTUP1|AAB63195|5476|Candida_albicans|Reduced_virulence
Chr09G0697.1	2240	5.00E-32	112/472(23.73)	126	PHI:2240|Srt1|Q4PBY9|5270|Ustilago_maydis|reduced_virulence
Chr09G0699.1	1581	4.00E-71	112/254(44.09)	223	PHI:1581|GzOB021|I1RPX8|5518|Gibberella_zeae_(related:_Fusarium_graminearum)|Unaffected_pathogenicity
Chr09G0700.1	3381	2.00E-22	121/532(22.74)	99	FVEG_12533|PHI:3381|W7N2B4|117187|Fusarium_verticillioides|unaffected_pathogenicity_
Chr09G0702.1	1893	4.00E-34	71/164(43.29)	129	PHI:1893|GzZC208|I1RNY0|5518|Gibberella_zeae_(related:_Fusarium_graminearum)|Unaffected_pathogenicity
Chr09G0704.1	1878	3.00E-42	127/517(24.56)	162	PHI:1878|GzZC193|I1RPF6|5518|Gibberella_zeae_(related:_Fusarium_graminearum)|Unaffected_pathogenicity
Chr09G0705.1	2968	3.00E-45	126/495(25.45)	165	PHI:2968|Hxs1|J9VQA5|5207|Cryptococcus_neoformans|Reduced_virulence

Chr09G0706.1	1823	8.00E-67	129/392(32.91)	223	PHI:1823|GzZC138|I1RXK4|5518|Gibberella_zeae_(related:_Fusarium_graminearum)|Unaffected_pathogenicity
Chr09G0707.1	1029	1.00E-95	158/336(47.02)	289	PHI:1029|bcpme2|CAD21438|40559|Botrytis_cinerea|Unaffected_pathogenicity
Chr09G0708.1	1393	1.00E-09	42/152(27.63)	56.6	PHI:1393|GzC2H056|I1RU69|5518|Gibberella_zeae_(related:_Fusarium_graminearum)|Unaffected_pathogenicity
Chr09G0711.1	2654	3.00E-07	53/180(29.44)	50.4	PHI:2654|DUR1,2|Q59VF3|5476|Candida_albicans|Reduced_virulence
Chr09G0712.1	1260	8.00E-06	34/114(29.82)	43.9	PHI:1260|FGSG_13944|I1RUC7|5518|Gibberella_zeae_(related:_Fusarium_graminearum)|Unaffected_pathogenicity
Chr09G0715.1	668	3.00E-47	124/393(31.55)	166	PHI:668|GNO1|AAP41027|178876|Cryptococcus_neoformans|Reduced_virulence
Chr09G0722.1	2839	8.00E-35	105/330(31.82)	130	PHI:2839|RED1|Q8NJQ2|5016|Cochliobolus_heterostrophus|Reduced_virulence
Chr09G0726.1	2025	2.00E-06	29/93(31.18)	47.4	PHI:2025|HDL1|G4MQZ9|148305|Magnaporthe_oryzae_(related:_Magnaporthe_grisea)|Unaffected_pathogenicity
Chr09G0730.1	1662	5.00E-105	203/505(40.20)	346	PHI:1662|GzCCHC002|I1REJ1|5518|Gibberella_zeae_(related:_Fusarium_graminearum)|Unaffected_pathogenicity
Chr09G0731.1	1555	1.00E-22	113/459(24.62)	98.6	PHI:1555|GzMyb019|I1RDG6|5518|Gibberella_zeae_(related:_Fusarium_graminearum)|Unaffected_pathogenicity
Chr09G0732.1	1418	9.00E-109	188/354(53.11)	325	PHI:1418|GzC2H085|I1S0W9|5518|Gibberella_zeae_(related:_Fusarium_graminearum)|Lethal
Chr09G0741.1	2728	0	391/604(64.74)	806	PHI:2728|FgERG4|I1RZZ3|5518|Fusarium_graminearum|Reduced_virulence
Chr09G0743.1	1662	3.00E-41	112/430(26.05)	157	PHI:1662|GzCCHC002|I1REJ1|5518|Gibberella_zeae_(related:_Fusarium_graminearum)|Unaffected_pathogenicity
Chr09G0744.1	2033	4.00E-09	56/174(32.18)	56.6	PHI:2033|MgPex6|G4NBI6|148305|Magnaporthe_oryzae_(related:_Magnaporthe_grisea)|Loss_of_pathogenicity
Chr09G0745.1	2038	4.00E-07	65/268(24.25)	48.1	PHI:2038|Mir1|MGG_02370|148305|Magnaporthe_oryzae_(related:_Magnaport

					he_grisea)|Unaffected_pathogenicity
Chr09G0746.1	1879	0	416/738(56.37)	786	PHI:1879|GzZC194|I1RJB8|5518|Gibberella_zeae_(related:_Fusarium_graminearum)|Unaffected_pathogenicity
Chr09G0754.1	3277	0	316/378(83.60)	667	gnoA|PHI:3277|Q4WIQ3|746128|Aspergillus_fumigatus|unaffected_pathogenicity_
Chr09G0758.1	1554	2.00E-24	69/189(36.51)	105	PHI:1554|GzMyb018|I1RC64|5518|Gibberella_zeae_(related:_Fusarium_graminearum)|Unaffected_pathogenicity
Chr09G0767.1	2022	4.00E-31	83/275(30.18)	116	PHI:2022|BUF1|MGG_02252|148305|Magnaporthe_oryzae_(related:_Magnaporthe_grisea)|Loss_of_pathogenicity
Chr09G0771.1	2038	3.00E-06	30/114(26.32)	46.2	PHI:2038|Mir1|MGG_02370|148305|Magnaporthe_oryzae_(related:_Magnaporthe_grisea)|Unaffected_pathogenicity
Chr09G0776.1	2169	4.00E-138	245/469(52.24)	408	PHI:2169|Conserved_hypothetical_protein|MGG_06188|148305|Magnaporthe_oryzae_(related:_Magnaporthe_grisea)|Reduced_virulence
Chr09G0777.1	2321	1.00E-49	148/518(28.57)	179	PHI:2321|SidI|Q4WR83|746128|Aspergillus_fumigatus|Reduced_virulence
Chr09G0781.1	1419	2.00E-136	212/315(67.30)	391	PHI:1419|GzC2H086|I1S0X7|5518|Gibberella_zeae_(related:_Fusarium_graminearum)|Unaffected_pathogenicity
Chr09G0785.1	2837	1.00E-17	64/230(27.83)	77	PHI:2837|OXI1|D2SZX7|5016|Cochliobolus_heterostrophus|Reduced_virulence
Chr09G0791.1	2161	0	1019/1432(71.16)	1930	PHI:2161|37135|MGG_04100|148305|Magnaporthe_oryzae_(related:_Magnaporthe_grisea)|Loss_of_pathogenicity
Chr09G0795.1	1670	5.00E-76	127/189(67.20)	227	PHI:1670|GzCCHC010|I1S0Q0|5518|Gibberella_zeae_(related:_Fusarium_graminearum)|Unaffected_pathogenicity
Chr09G0796.1	2570	3.00E-10	27/62(43.55)	60.1	PHI:2570|CYB2|Q6FM61|5478|Candida_glabrata|Reduced_virulence
Chr09G0797.1	2227	4.00E-14	115/491(23.42)	72.4	PHI:2227|pao|D4III8|5270|Ustilago_maydis|Mixed_outcome
Chr09G0799.1	1675	4.00E-07	53/176(30.11)	51.6	PHI:1675|GzDHHC003|Q4I8B6|5518|Gibberella_zeae_(related:_Fusarium_graminearum)|Unaffected_pathogenicity
Chr09G0801.1	211	2.00E-14	74/287(25.78)	72.8	PHI:211|CaTUP1|AAB63195|5476|Candida_albicans|Reduced_virulence

Chr09G0803.1	1163	0	477/1212(39.36)	630	PHI:1163|ERB1_(enhanced_branching_1)|I1S042|5518|Gibberella_zeae_(related:_Fusarium_graminearum)|Reduced_virulence
Chr09G0804.1	2091	2.00E-108	168/264(63.64)	317	PHI:2091|MgATG5|EF486491|148305|Magnaporthe_oryzae_(related:_Magnaporthe_grisea)|Loss_of_pathogenicity
Chr09G0808.1	441	2.00E-08	37/171(21.64)	53.1	PHI:441|BTP1|CAE55153|40559|Botrytis_cinerea|Reduced_virulence
Chr09G0809.1	1526	0	665/1066(62.38)	1287	PHI:1526|GzHOMEL036|I1S0J3|5518|Gibberella_zeae_(related:_Fusarium_graminearum)|Unaffected_pathogenicity
Chr09G0814.1	1709	0	321/569(56.41)	629	PHI:1709|GzZC024|I1RR13|5518|Gibberella_zeae_(related:_Fusarium_graminearum)|Unaffected_pathogenicity
Chr09G0816.1	2638	6.00E-71	180/578(31.14)	240	PHI:2638|Ilv3A|Q4X099|746128|Aspergillus_fumigatus|Reduced_virulence
Chr09G0817.1	784	8.00E-36	91/271(33.58)	129	PHI:784|MGG_00056|EDK03390|318829|Magnaporthe_oryzae|Reduced_virulence
Chr09G0821.1	922	1.00E-58	193/621(31.08)	208	PHI:922|um03615||5270|Ustilago_maydis|Unaffected_pathogenicity
Chr09G0822.1	500	2.00E-06	39/124(31.45)	47.4	PHI:500|YHB1|EAK91807|5476|Candida_albicans|Reduced_virulence
Chr09G0824.1	1821	7.00E-96	171/356(48.03)	315	PHI:1821|GzZC136|I1RWY8|5518|Gibberella_zeae_(related:_Fusarium_graminearum)|Unaffected_pathogenicity
Chr09G0829.1	2020	8.00E-09	72/329(21.88)	57.4	PHI:2020|Tup1|XP_759427|5270|Ustilago_maydis|Mixed_outcome
Chr09G0833.1	2526	2.00E-120	170/248(68.55)	347	PHI:2526|LUC7|Q4WMR3|746128|Aspergillus_fumigatus|Lethal
Chr09G0835.1	3662	1.00E-06	43/117(36.75)	49.3	PspB_(not_PD0218)|PHI:3662|Q87ET0|2371|Xylella_fastidiosa|Increased_virulence_(Hypervirulence)
Chr09G0836.1	1514	3.00E-174	326/791(41.21)	536	PHI:1514|GzHOME012|I1RYV5|5518|Gibberella_zeae_(related:_Fusarium_graminearum)|Unaffected_pathogenicity
Chr09G0838.1	413	4.00E-27	97/383(25.33)	109	PHI:413|MPD1|AAT84078|13684|Stagonospora_nodorum|Unaffected_pathogenicity
Chr09G0839.1	3381	5.00E-43	140/492(28.46)	159	FVEG_12533|PHI:3381|W7N2B4|117187|Fusarium_verticillioides|unaffected_pathogenicity_

Chr09G0846.1	223	8.00E-53	100/254(39.37)	183	PHI:223|PEP1|AAK11166|140110|Nectria_haematococca_(related:_Fusarium_solani)|Reduced_virulence
Chr09G0850.1	1420	5.00E-15	76/377(20.16)	76.6	PHI:1420|GzC2H088|I1S172|5518|Gibberella_zeae_(related:_Fusarium_graminearum)|Unaffected_pathogenicity
Chr09G0853.1	1322	2.00E-10	23/67(34.33)	55.8	PHI:1322|GzbZIP004|I1RG94|5518|Gibberella_zeae_(related:_Fusarium_graminearum)|Unaffected_pathogenicity
Chr09G0855.1	3214	6.00E-08	33/105(31.43)	47	MoCDIP2|PHI:3214|G4MML4|318829|Magnaporthe_oryzae|mixed_outcome_
Chr09G0857.1	263	0	405/418(96.89)	845	PHI:263|MAF1|AAL50116|5462|Colletotrichum_lagenarium|Reduced_virulence
Chr09G0860.1	480	6.00E-15	85/369(23.04)	74.7	PHI:480|SAP7|EAK94461|5476|Candida_albicans|Reduced_virulence
Chr09G0861.1	404	7.00E-24	88/346(25.43)	101	PHI:404|PTH11|AAD30436|318829|Magnaporthe_oryzae|Reduced_virulence
Chr09G0862.1	1260	1.00E-07	31/90(34.44)	53.1	PHI:1260|FGSG_13944|I1RUC7|5518|Gibberella_zeae_(related:_Fusarium_graminearum)|Unaffected_pathogenicity
Chr09G0870.1	2175	8.00E-07	43/119(36.13)	45.4	PHI:2175|NMR3|MGG_09705|148305|Magnaporthe_oryzae_(related:_Magnaporthe_grisea)|Reduced_virulence
Chr09G0872.1	339	2.00E-08	28/86(32.56)	49.7	PHI:339|CLPT1|CAC41973|290576|Colletotrichum_lindemuthianum|Reduced_virulence
Chr09G0874.1	220	5.00E-98	186/453(41.06)	302	PHI:220|MAN1|AAG10203|5207|Cryptococcus_neoformans|Reduced_virulence
Chr09G0875.1	1868	5.00E-23	50/92(54.35)	98.6	PHI:1868|GzZC183|I1RKR4|5518|Gibberella_zeae_(related:_Fusarium_graminearum)|Unaffected_pathogenicity
Chr09G0876.1	249	7.00E-11	49/158(31.01)	59.3	PHI:249|CYP1|AAG13968|318829|Magnaporthe_oryzae|Reduced_virulence
Chr09G0881.1	1175	0	352/441(79.82)	694	PHI:1175|(Sc_Srb10)|I1RKR8|5518|Gibberella_zeae_(related:_Fusarium_graminearum)|Reduced_virulence
Chr09G0884.1	2968	2.00E-23	105/422(24.88)	101	PHI:2968|Hxs1|J9VQA5|5207|Cryptococcus_neoformans|Reduced_virulence
Chr09G0894.1	2315	3.00E-14	42/150(28.00)	66.6	PHI:2315|ChLae1|G4XKY9|5016|Cochliobolus_heterostrophus|Mixed_outcome
Chr09G0897.1	1006	9.00E-43	128/361(35.46)	159	PHI:1006|HMR1|Not_Available|5518|Fusarium_graminearum|Reduced_virulence

Chr09G0899.1	2022	4.00E-08	58/209(27.75)	50.4	PHI:2022|BUF1|MGG_02252|148305|Magnaporthe_oryzae_(related:_Magnaporthe_grisea)|Loss_of_pathogenicity
Chr09G0900.1	3	7.00E-166	234/365(64.11)	470	PHI:3|PGN1|AAA79885|5017|Cochliobolus_carbonum|Unaffected_pathogenicity
Chr09G0901.1	397	2.00E-92	194/600(32.33)	300	PHI:397|LAC2|AAV64894|5207|Cryptococcus_neoformans|Unaffected_pathogenicity
Chr09G0904.1	1581	1.00E-35	83/244(34.02)	128	PHI:1581|GzOB021|I1RPX8|5518|Gibberella_zeae_(related:_Fusarium_graminearum)|Unaffected_pathogenicity
Chr09G0906.1	2553	2.00E-07	28/76(36.84)	51.2	PHI:2553|VPS4|Q5AG40|5476|Candida_albicans|Loss_of_pathogenicity
Chr09G0907.1	2034	6.00E-18	55/178(30.90)	81.3	PHI:2034|MFP1|G4MZY1|148305|Magnaporthe_oryzae_(related:_Magnaporthe_grisea)|Reduced_virulence
Chr09G0908.1	2393	4.00E-126	199/486(40.95)	378	PHI:2393|Related_to_O-methylsterigmatocystin_oxidoreductase|I1R980|5518|Gibberella_zeae_(related:_Fusarium_graminearum)|Increased_virulence_(Hypervirulence)
Chr09G0910.1	2728	2.00E-30	134/494(27.13)	121	PHI:2728|FgERG4|I1RZZ3|5518|Fusarium_graminearum|Reduced_virulence
Chr09G0912.1	1810	7.00E-11	78/345(22.61)	62	PHI:1810|GzZC125|I1RUJ0|5518|Gibberella_zeae_(related:_Fusarium_graminearum)|Unaffected_pathogenicity
Chr09G0913.1	3381	4.00E-58	160/510(31.37)	206	FVEG_12533|PHI:3381|W7N2B4|117187|Fusarium_verticillioides|unaffected_pathogenicity_
Chr09G0915.1	2357	2.00E-12	104/457(22.76)	66.6	PHI:2357|CYP52X1|E2EAF6|475271|Beauveria_bassiana|Reduced_virulence
Chr09G0916.1	197	3.00E-101	197/561(35.12)	318	PHI:197|ALO1|AAC98913|5476|Candida_albicans|Reduced_virulence
Chr09G0919.1	538	7.00E-26	128/539(23.75)	109	PHI:538|FRT1|AAU87358|40559|Botrytis_cinerea|Unaffected_pathogenicity
Chr09G0921.1	2899	6.00E-09	53/187(28.34)	51.2	PHI:2901|BEC1040|CCU82707|62688|Blumeria_graminis_f._sp._hordei|Effector_(plant_avirulence_determinant)
Chr09G0922.1	2038	2.00E-85	154/310(49.68)	261	PHI:2038|Mir1|MGG_02370|148305|Magnaporthe_oryzae_(related:_Magnaporthe_grisea)|Unaffected_pathogenicity

Chr09G0927.1	1566	4.00E-07	59/198(29.80)	50.1	PHI:1566|GzOB006|I1RC95|5518|Gibberella_zeae_(related:_Fusarium_graminearum)|Lethal
Chr09G0930.1	1914	2.00E-12	25/50(50.00)	67.8	PHI:1914|GzZC229|I1RQ27|5518|Gibberella_zeae_(related:_Fusarium_graminearum)|Unaffected_pathogenicity
Chr09G0931.1	157	9.00E-15	82/358(22.91)	72.4	PHI:157|TOXF|AAD45321|5017|Cochliobolus_carbonum|Loss_of_pathogenicity
Chr09G0932.1	2315	5.00E-56	112/324(34.57)	186	PHI:2315|ChLae1|G4XKY9|5016|Cochliobolus_heterostrophus|Mixed_outcome
Chr09G0934.1	3630	1.00E-12	85/364(23.35)	69.3	Rv2467|PHI:3630|L7N655|1773|Mycobacterium_tuberculosis|increased_virulence_
Chr09G0942.1	2315	2.00E-46	105/297(35.35)	160	PHI:2315|ChLae1|G4XKY9|5016|Cochliobolus_heterostrophus|Mixed_outcome
Chr09G0943.1	2654	2.00E-14	56/180(31.11)	74.7	PHI:2654|DUR1,2|Q59VF3|5476|Candida_albicans|Reduced_virulence
Chr09G0946.1	1269	4.00E-16	72/307(23.45)	75.9	PHI:1269|FGSG_02838|I1RFK9|5518|Gibberella_zeae_(related:_Fusarium_graminearum)|Unaffected_pathogenicity
Chr09G0948.1	419	5.00E-16	75/318(23.58)	75.1	PHI:419|CSH1|AAP93915|5476|Candida_albicans|Reduced_virulence
Chr09G0949.1	1296	0	434/681(63.73)	894	PHI:1296|GzAPSES004|I1S0Z2|5518|Gibberella_zeae_(related:_Fusarium_graminearum)|Reduced_virulence
Chr09G0955.1	2022	3.00E-07	53/231(22.94)	47.8	PHI:2022|BUF1|MGG_02252|148305|Magnaporthe_oryzae_(related:_Magnaporthe_grisea)|Loss_of_pathogenicity
Chr09G0957.1	2700	0	522/594(87.88)	1116	PHI:2700|lac2|J7MF98|5465|Colletotrichum_orbiculare|Reduced_virulence
Chr09G0960.1	2118	1.00E-75	111/138(80.43)	222	PHI:2118|MgSM1|MGG_05344|148305|Magnaporthe_oryzae_(related:_Magnaporthe_grisea)|Effector_(plant_avirulence_determinant)
Chr09G0962.1	612	6.00E-07	39/122(31.97)	48.1	PHI:612|orf408|AAL20306|216597|Salmonella_enterica|Reduced_virulence
Chr09G0964.1	2020	2.00E-12	69/283(24.38)	65.5	PHI:2020|Tup1|XP_759427|5270|Ustilago_maydis|Mixed_outcome
Chr09G0967.1	3147	3.00E-06	24/87(27.59)	46.6	rfaE|PHI:3147|B8F4X8|738|Haemophilus_parasuis|reduced_virulence_
Chr09G0968.1	157	4.00E-24	90/337(26.71)	99.8	PHI:157|TOXF|AAD45321|5017|Cochliobolus_carbonum|Loss_of_pathogenicity
Chr09G0969.1	1197	3.00E-09	37/118(31.36)	53.9	PHI:1197|(Snf1)|I1RZP6|5518|Gibberella_zeae_(related:_Fusarium_graminearum)|Reduced_virulence

Chr09G0970.1	1424	1.00E-16	109/441(24.72)	82	PHI:1424|GzC2H092|I1S3J7|5518|Gibberella_zeae_(related:_Fusarium_graminearum)|Unaffected_pathogenicity
Chr09G0973.1	2315	2.00E-51	100/290(34.48)	174	PHI:2315|ChLae1|G4XKY9|5016|Cochliobolus_heterostrophus|Mixed_outcome
Chr09G0978.1	1787	0	270/473(57.08)	530	PHI:1787|GzZC102|I1S1P9|5518|Gibberella_zeae_(related:_Fusarium_graminearum)|Unaffected_pathogenicity
Chr09G0979.1	2240	4.00E-39	119/472(25.21)	147	PHI:2240|Srt1|Q4PBY9|5270|Ustilago_maydis|reduced_virulence
Chr09G0980.1	2745	2.00E-07	30/99(30.30)	51.6	PHI:2745|treY|Q02LV5|287|Pseudomonas_aeruginosa|Mixed_outcome
Chr09G0981.1	1260	1.00E-09	44/124(35.48)	59.3	PHI:1260|FGSG_13944|I1RUC7|5518|Gibberella_zeae_(related:_Fusarium_graminearum)|Unaffected_pathogenicity
Chr09G0984.1	2968	7.00E-24	88/323(27.24)	102	PHI:2968|Hxs1|J9VQA5|5207|Cryptococcus_neoformans|Reduced_virulence
Chr09G0990.1	748	4.00E-26	159/639(24.88)	112	PHI:748|um00446|Not_available|5270|Ustilago_maydis|Unaffected_pathogenicity
Chr09G0991.1	222	1.00E-167	250/299(83.61)	471	PHI:222|PELB|AAD09857|5457|Colletotrichum_gloeosporioides|Reduced_virulence
Chr09G0992.1	2731	2.00E-15	75/291(25.77)	76.3	PHI:2731|VdSge1|G2XD29|27337|Verticillium_dahliae|Loss_of_pathogenicity
Chr09G0994.1	1442	3.00E-131	229/557(41.11)	395	PHI:1442|GzDNL|I1RG27|5518|Gibberella_zeae_(related:_Fusarium_graminearum)|Unaffected_pathogenicity
Chr09G0995.1	3057	2.00E-11	81/277(29.24)	63.5	VvpE|PHI:3057|C3VHN8|672|Vibrio_vulnificus|mixed_outcome_
Chr09G0997.1	2954	2.00E-34	67/145(46.21)	131	PHI:2954|TgNST1|B6KLD2|5811|Toxoplasma_gondii|Reduced_virulence
Chr09G0998.1	1046	1.00E-50	143/482(29.67)	180	PHI:1046|CTB5|ABK64182|29003|Cercospora_nicotianae|Reduced_virulence
Chr09G1000.1	1046	3.00E-11	55/186(29.57)	63.5	PHI:1046|CTB5|ABK64182|29003|Cercospora_nicotianae|Reduced_virulence
Chr09G1004.1	1262	8.00E-99	181/437(41.42)	315	PHI:1262|FGSG_04416|I1RQS1|5518|Gibberella_zeae_(related:_Fusarium_graminearum)|Unaffected_pathogenicity
Chr09G1005.1	1327	6.00E-109	246/629(39.11)	350	PHI:1327|GzbZIP009|I1RQS2|5518|Gibberella_zeae_(related:_Fusarium_graminearum)|Unaffected_pathogenicity
Chr09G1007.1	1856	2.00E-26	115/445(25.84)	108	PHI:1856|GzZC171|I1S377|5518|Gibberella_zeae_(related:_Fusarium_gramine

					arum)|Unaffected_pathogenicity
Chr09G1010.1	1393	1.00E-06	22/55(40.00)	49.7	PHI:1393|GzC2H056|I1RU69|5518|Gibberella_zeae_(related:_Fusarium_graminearum)|Unaffected_pathogenicity
Chr09G1012.1	3017	5.00E-128	250/584(42.81)	399	Mpr1|PHI:3017|J9VXZ9|5207|Cryptococcus_neoformans|reduced_virulence_
Chr09G1013.1	3607	0	732/2859(25.60)	679	sidN|PHI:3607|K7NCV2|35717|Epichloe_festucae|increased_virulence_
Chr09G1014.1	1010	0	315/547(57.59)	626	PHI:1010|SID1|XM_385547|5518|Fusarium_graminearum|Reduced_virulence
Chr09G1015.1	1937	6.00E-170	297/612(48.53)	510	PHI:1937|GzZC252|I1RN12|5518|Gibberella_zeae_(related:_Fusarium_graminearum)|Unaffected_pathogenicity
Chr09G1016.1	2654	4.00E-15	83/278(29.86)	75.9	PHI:2654|DUR1,2|Q59VF3|5476|Candida_albicans|Reduced_virulence
Chr09G1018.1	1555	9.00E-44	125/483(25.88)	161	PHI:1555|GzMyb019|I1RDG6|5518|Gibberella_zeae_(related:_Fusarium_graminearum)|Unaffected_pathogenicity
Chr09G1021.1	2563	2.00E-53	90/222(40.54)	177	PHI:2563|DPP3|Q5AH74|5476|Candida_albicans|Reduced_virulence
Chr09G1023.1	2594	2.00E-08	24/58(41.38)	55.5	PHI:2594|SET3|Q59ZX1|746128|Aspergillus_fumigatus|Mixed_outcome
Chr09G1026.1	1372	3.00E-78	180/333(54.05)	244	PHI:1372|GzC2H035|I1RN03|5518|Gibberella_zeae_(related:_Fusarium_graminearum)|Unaffected_pathogenicity
Chr09G1031.1	544	1.00E-156	237/543(43.65)	463	PHI:544|BCMFS1|AAF64435|332648|Botrytis_cinerea|Unaffected_pathogenicity
Chr09G1037.1	55	0	868/2666(32.56)	1127	PHI:55|PKS1|AAB08104|5016|Cochliobolus_heterostrophus|Reduced_virulence
Chr09G1041.1	211	3.00E-23	86/284(30.28)	102	PHI:211|CaTUP1|AAB63195|5476|Candida_albicans|Reduced_virulence
Chr09G1044.1	1721	1.00E-13	99/409(24.21)	69.7	PHI:1721|GzZC036|I1S914|5518|Gibberella_zeae_(related:_Fusarium_graminearum)|Unaffected_pathogenicity
Chr09G1045.1	3301	9.00E-12	59/196(30.10)	60.5	patN|PHI:3301|A0A075TRB3|27334|Penicillium_expansum|unaffected_pathogenicity_
Chr09G1047.1	479	2.00E-82	131/271(48.34)	250	PHI:479|MEP1|AAQ07436|199306|Coccidioides_posadasii|Reduced_virulence
Chr09G1048.1	2038	6.00E-07	64/264(24.24)	47.4	PHI:2038|Mir1|MGG_02370|148305|Magnaporthe_oryzae_(related:_Magnaport

					he_grisea)|Unaffected_pathogenicity
Chr09G1049.1	876	2.00E-84	174/475(36.63)	284	PHI:876|MGG_11671|EDK03349|318829|Magnaporthe_oryzae|Reduced_virulence
Chr09G1051.1	244	5.00E-29	67/151(44.37)	112	PHI:244|CLAP1|AAN62846|290576|Colletotrichum_lindemuthianum|Loss_of_pathogenicity
Chr09G1053.1	1279	7.00E-25	89/298(29.87)	108	PHI:1279|FGSG_12132|I1RU59|5518|Gibberella_zeae_(related:_Fusarium_graminearum)|Unaffected_pathogenicity
Chr09G1054.1	1279	1.00E-54	149/505(29.50)	199	PHI:1279|FGSG_12132|I1RU59|5518|Gibberella_zeae_(related:_Fusarium_graminearum)|Unaffected_pathogenicity
Chr06G0001.1	2549	3.00E-07	24/87(27.59)	48.9	PHI:2549|MAK5|Q4WMS3|746128|Aspergillus_fumigatus|Mixed_outcome
Chr06G0003.1	1423	3.00E-27	126/500(25.20)	114	PHI:1423|GzC2H091|I1S2R1|5518|Gibberella_zeae_(related:_Fusarium_graminearum)|Unaffected_pathogenicity
Chr06G0012.1	1522	9.00E-19	48/109(44.04)	76.6	PHI:1522|GzHOMEL026|I1RXA5|5518|Gibberella_zeae_(related:_Fusarium_graminearum)|Lethal
Chr06G0020.1	2020	9.00E-48	107/313(34.19)	181	PHI:2020|Tup1|XP_759427|5270|Ustilago_maydis|Mixed_outcome
Chr06G0022.1	1260	3.00E-19	68/182(37.36)	91.7	PHI:1260|FGSG_13944|I1RUC7|5518|Gibberella_zeae_(related:_Fusarium_graminearum)|Unaffected_pathogenicity
Chr06G0024.1	1522	7.00E-151	201/329(61.09)	429	PHI:1522|GzHOMEL026|I1RXA5|5518|Gibberella_zeae_(related:_Fusarium_graminearum)|Lethal
Chr06G0027.1	223	5.00E-07	24/85(28.24)	46.6	PHI:223|PEP1|AAK11166|140110|Nectria_haematococca_(related:_Fusarium_solani)|Reduced_virulence
Chr06G0030.1	1458	1.00E-06	31/91(34.07)	50.4	PHI:1458|GzAra006|Q4I7F9|5518|Gibberella_zeae_(related:_Fusarium_graminearum)|Unaffected_pathogenicity
Chr06G0036.1	1260	1.00E-09	52/180(28.89)	57	PHI:1260|FGSG_13944|I1RUC7|5518|Gibberella_zeae_(related:_Fusarium_graminearum)|Unaffected_pathogenicity
Chr06G0038.1	876	0	459/1195(38.41)	757	PHI:876|MGG_11671|EDK03349|318829|Magnaporthe_oryzae|Reduced_virule

					nce
Chr06G0041.1	2019	0	426/661(64.45)	868	PHI:2019|MoPLC3|MGG_08315|148305|Magnaporthe_oryzae_(related:_Magnaporthe_grisea)|Loss_of_pathogenicity
Chr06G0043.1	1527	2.00E-37	100/357(28.01)	140	PHI:1527|GzHOMEL040|I1S9A1|5518|Gibberella_zeae_(related:_Fusarium_graminearum)|Lethal
Chr06G0050.1	2175	7.00E-45	102/310(32.90)	155	PHI:2175|NMR3|MGG_09705|148305|Magnaporthe_oryzae_(related:_Magnaporthe_grisea)|Reduced_virulence
Chr06G0053.1	1741	3.00E-75	198/729(27.16)	258	PHI:1741|GzZC056|I1S780|5518|Gibberella_zeae_(related:_Fusarium_graminearum)|Unaffected_pathogenicity
Chr06G0055.1	3037	2.00E-07	46/201(22.89)	51.2	FgERG5A|PHI:3037|I1RE80|5518|Fusarium_graminearum|reduced_virulence_
Chr06G0057.1	2042	0	468/1316(35.56)	734	PHI:2042|ABC3|Q3Y5V5|148305|Magnaporthe_oryzae_(related:_Magnaporthe_grisea)|Loss_of_pathogenicity
Chr06G0060.1	404	8.00E-23	81/343(23.62)	97.4	PHI:404|PTH11|AAD30436|318829|Magnaporthe_oryzae|Reduced_virulence
Chr06G0061.1	404	2.00E-25	84/339(24.78)	105	PHI:404|PTH11|AAD30436|318829|Magnaporthe_oryzae|Reduced_virulence
Chr06G0064.1	881	2.00E-132	192/337(56.97)	383	PHI:881|MGG_04556|EDJ96020|318829|Magnaporthe_oryzae|Reduced_virulence
Chr06G0066.1	2022	1.00E-51	88/259(33.98)	169	PHI:2022|BUF1|MGG_02252|148305|Magnaporthe_oryzae_(related:_Magnaporthe_grisea)|Loss_of_pathogenicity
Chr06G0067.1	2644	2.00E-06	22/84(26.19)	44.7	PHI:2644|thioredoxin_1|P0AA28|90371|Salmonella_enterica_serovar_Typhimurium|Reduced_virulence
Chr06G0080.1	1957	0	494/908(54.41)	980	PHI:1957|GzZC272|I1RLS0|5518|Gibberella_zeae_(related:_Fusarium_graminearum)|Unaffected_pathogenicity
Chr06G0082.1	2968	3.00E-55	140/492(28.46)	194	PHI:2968|Hxs1|J9VQA5|5207|Cryptococcus_neoformans|Reduced_virulence
Chr06G0083.1	17	1.00E-27	97/333(29.13)	110	PHI:17|ACP|CAA43678|5482|Candida_tropicalis|Reduced_virulence
Chr06G0084.1	465	3.00E-62	134/325(41.23)	226	PHI:465|KIN2|AAB63337|5270|Ustilago_maydis|Reduced_virulence
Chr06G0086.1	2038	1.00E-150	221/281(78.65)	428	PHI:2038|Mir1|MGG_02370|148305|Magnaporthe_oryzae_(related:_Magnaport

					he_grisea)|Unaffected_pathogenicity
Chr06G0087.1	1369	6.00E-121	206/347(59.37)	352	PHI:1369|GzC2H031|I1RLS1|5518|Gibberella_zeae_(related:_Fusarium_graminearum)|Unaffected_pathogenicity
Chr06G0096.1	1503	2.00E-06	48/212(22.64)	48.1	PHI:1503|GzHMG035|I1S828|5518|Gibberella_zeae_(related:_Fusarium_graminearum)|Unaffected_pathogenicity
Chr06G0098.1	2304	6.00E-133	197/419(47.02)	390	PHI:2304|BCFHG1|CAP74387|40559|Botrytis_cinerea|Unaffected_pathogenicity
Chr06G0102.1	511	2.00E-56	164/502(32.67)	196	PHI:511|CaNAG4|EAK93098|5476|Candida_albicans|Reduced_virulence
Chr06G0103.1	1804	6.00E-123	218/574(37.98)	382	PHI:1804|GzZC119|I1RUX5|5518|Gibberella_zeae_(related:_Fusarium_graminearum)|Unaffected_pathogenicity
Chr06G0104.1	2450	5.00E-07	25/85(29.41)	41.2	PHI:2450|ToxB|Q8J0U6|45151|Pyrenophora_tritici-repentis|Reduced_virulence
Chr06G0106.1	1773	9.00E-55	121/415(29.16)	187	PHI:1773|GzZC088|I1S409|5518|Gibberella_zeae_(related:_Fusarium_graminearum)|Unaffected_pathogenicity
Chr06G0107.1	243	3.00E-166	305/839(36.35)	509	PHI:243|CHIP6|AAD00894|5457|Colletotrichum_gloeosporioides|Reduced_virulence
Chr06G0109.1	1856	8.00E-116	187/427(43.79)	349	PHI:1856|GzZC171|I1S377|5518|Gibberella_zeae_(related:_Fusarium_graminearum)|Unaffected_pathogenicity
Chr06G0113.1	1346	1.00E-09	32/93(34.41)	51.6	PHI:1346|GzC2H006|I1RB57|5518|Gibberella_zeae_(related:_Fusarium_graminearum)|Unaffected_pathogenicity
Chr06G0115.1	2256	1.00E-174	230/349(65.90)	493	PHI:2256|Xdh1|Q0UA24|13684|Phaeosphaeria_nodorum_(related:_Stagonospora_nodorum)|Unaffected_pathogenicity
Chr06G0118.1	339	9.00E-32	76/242(31.40)	117	PHI:339|CLPT1|CAC41973|290576|Colletotrichum_lindemuthianum|Reduced_virulence
Chr06G0119.1	2034	2.00E-09	52/237(21.94)	55.5	PHI:2034|MFP1|G4MZY1|148305|Magnaporthe_oryzae_(related:_Magnaporthe_grisea)|Reduced_virulence
Chr06G0121.1	2266	8.00E-129	220/589(37.35)	394	PHI:2266|Ptr2|0|13684|Phaeosphaeria_nodorum_(related:_Stagonospora_nod

					orum)|Unaffected_pathogenicity
Chr06G0126.1	2378	2.00E-06	79/377(20.95)	47.4	PHI:2378|DEP4|D2E9W9|29001|Alternaria_brassicicola|Mixed_outcome
Chr06G0128.1	4194	6.00E-26	116/470(24.68)	108	AKT7|PHI:4194|V5XZS6|5599|Alternaria_alternata|increased_virulence_(Hyper virulence)
Chr06G0129.1	1049	1.00E-34	117/444(26.35)	132	PHI:1049|CTB2|ABK64180|29003|Cercospora_nicotianae|Reduced_virulence
Chr06G0131.1	1566	9.00E-06	55/191(28.80)	45.8	PHI:1566|GzOB006|I1RC95|5518|Gibberella_zeae_(related:_Fusarium_gramin earum)|Lethal
Chr06G0132.1	1006	0	842/1158(72.71)	1706	PHI:1006|HMR1|Not_Available|5518|Fusarium_graminearum|Reduced_virulenc e
Chr06G0141.1	441	5.00E-14	54/189(28.57)	71.6	PHI:441|BTP1|CAE55153|40559|Botrytis_cinerea|Reduced_virulence
Chr06G0143.1	2563	5.00E-18	66/255(25.88)	81.3	PHI:2563|DPP3|Q5AH74|5476|Candida_albicans|Reduced_virulence
Chr06G0144.1	179	2.00E-55	106/222(47.75)	184	PHI:179|PELA|AAA33338|140110|Nectria_haematococca_(related:_Fusarium_ solani)|Reduced_virulence
Chr06G0148.1	2269	3.00E-21	45/117(38.46)	84.3	PHI:2269|Mdh1|0|13684|Phaeosphaeria_nodorum_(related:_Stagonospora_no dorum)|Unaffected_pathogenicity
Chr06G0151.1	513	5.00E-48	141/550(25.64)	175	PHI:513|ARN1_(related:_SIT1)|EAK97011|5476|Candida_albicans|Reduced_vir ulence
Chr06G0163.1	1555	9.00E-32	116/440(26.36)	125	PHI:1555|GzMyb019|I1RDG6|5518|Gibberella_zeae_(related:_Fusarium_grami nearum)|Unaffected_pathogenicity
Chr06G0168.1	513	2.00E-61	162/629(25.76)	215	PHI:513|ARN1_(related:_SIT1)|EAK97011|5476|Candida_albicans|Reduced_vir ulence
Chr06G0173.1	144	7.00E-17	76/282(26.95)	82	PHI:144|CHT42|AAC05829|29875|Trichoderma_virens|Reduced_virulence
Chr06G0179.1	1474	1.00E-62	234/674(34.72)	227	PHI:1474|GzHMG006|I1RB89|5518|Gibberella_zeae_(related:_Fusarium_grami nearum)|Unaffected_pathogenicity
Chr06G0181.1	1662	2.00E-77	157/466(33.69)	265	PHI:1662|GzCCHC002|I1REJ1|5518|Gibberella_zeae_(related:_Fusarium_gra minearum)|Unaffected_pathogenicity

Chr06G0182.1	2032	3.00E-49	122/400(30.50)	177	PHI:2032|VTL1|G4NGA7|148305|Magnaporthe_oryzae_(related:_Magnaporthe_grisea)|Unaffected_pathogenicity
Chr06G0187.1	716	7.00E-15	57/167(34.13)	74.3	PHI:716|ZEB1|ABB90284|5518|Fusarium_graminearum|Unaffected_pathogenicity
Chr06G0189.1	1654	2.00E-16	88/267(32.96)	78.2	PHI:1654|GzWing027|I1S4Q8|5518|Gibberella_zeae_(related:_Fusarium_graminearum)|Unaffected_pathogenicity
Chr06G0191.1	1052	5.00E-22	63/180(35.00)	98.2	PHI:1052|PHL1|ACB38886|135779|Cercospora_zeae-maydis|Unaffected_pathogenicity
Chr06G0193.1	547	2.00E-111	155/305(50.82)	334	PHI:547|CEL5A|AAT40313|40559|Botrytis_cinerea|Unaffected_pathogenicity
Chr06G0196.1	65	2.00E-07	44/171(25.73)	51.6	PHI:65|CPGB-1|AAC49838|5116|Cryphonectria_parasitica|Reduced_virulence
Chr06G0197.1	404	2.00E-101	150/352(42.61)	318	PHI:404|PTH11|AAD30436|318829|Magnaporthe_oryzae|Reduced_virulence
Chr06G0199.1	404	6.00E-22	65/271(23.99)	95.1	PHI:404|PTH11|AAD30436|318829|Magnaporthe_oryzae|Reduced_virulence
Chr06G0206.1	1046	2.00E-14	53/174(30.46)	72.4	PHI:1046|CTB5|ABK64182|29003|Cercospora_nicotianae|Reduced_virulence
Chr06G0208.1	1260	3.00E-16	80/244(32.79)	80.9	PHI:1260|FGSG_13944|I1RUC7|5518|Gibberella_zeae_(related:_Fusarium_graminearum)|Unaffected_pathogenicity
Chr06G0215.1	1424	6.00E-06	33/79(41.77)	47.4	PHI:1424|GzC2H092|I1S3J7|5518|Gibberella_zeae_(related:_Fusarium_graminearum)|Unaffected_pathogenicity
Chr06G0217.1	1046	1.00E-21	50/155(32.26)	94.7	PHI:1046|CTB5|ABK64182|29003|Cercospora_nicotianae|Reduced_virulence
Chr06G0218.1	2261	2.00E-108	317/1049(30.22)	378	PHI:2261|NPS6|G8DNS9|45130|Cochliobolus_sativus|Reduced_virulence
Chr06G0220.1	1153	5.00E-12	76/299(25.42)	65.1	PHI:1153|cyp51/erg11|B6E223|54734|Mycosphaerella_graminicola_(related:_Zymoseptoria_triticii)|Chemistry_target
Chr06G0221.1	2728	0	296/477(62.05)	618	PHI:2728|FgERG4|I1RZZ3|5518|Fusarium_graminearum|Reduced_virulence
Chr06G0222.1	438	9.00E-66	144/479(30.06)	221	PHI:438|BcBOT1_(related:_CND5)|AAQ16576|40559|Botrytis_cinerea|Reduced_virulence
Chr06G0225.1	1983	0	458/943(48.57)	809	PHI:1983|GzZC298|I1RB90|5518|Gibberella_zeae_(related:_Fusarium_graminearum)|Lethal

Chr06G0226.1	3119	2.00E-07	22/52(42.31)	44.7	PSPTO_2696|PHI:3119|Q882D2|317|Pseudomonas_syringae|mixed_outcome_
Chr06G0230.1	404	9.00E-14	60/261(22.99)	69.7	PHI:404|PTH11|AAD30436|318829|Magnaporthe_oryzae|Reduced_virulence
Chr06G0231.1	922	9.00E-88	201/609(33.00)	287	PHI:922|um03615||5270|Ustilago_maydis|Unaffected_pathogenicity
Chr06G0233.1	1279	3.00E-56	159/512(31.05)	199	PHI:1279|FGSG_12132|I1RU59|5518|Gibberella_zeae_(related:_Fusarium_graminearum)|Unaffected_pathogenicity
Chr06G0236.1	2968	2.00E-23	117/500(23.40)	101	PHI:2968|Hxs1|J9VQA5|5207|Cryptococcus_neoformans|Reduced_virulence
Chr06G0241.1	69	8.00E-09	43/143(30.07)	51.6	PHI:69|CUTA|CAA93255|40559|Botrytis_cinerea|Unaffected_pathogenicity
Chr06G0245.1	216	0	354/545(64.95)	708	PHI:216|HDC1|AAK35180|5017|Cochliobolus_carbonum|Reduced_virulence
Chr06G0249.1	404	4.00E-31	83/316(26.27)	124	PHI:404|PTH11|AAD30436|318829|Magnaporthe_oryzae|Reduced_virulence
Chr06G0251.1	1662	2.00E-34	88/334(26.35)	136	PHI:1662|GzCCHC002|I1REJ1|5518|Gibberella_zeae_(related:_Fusarium_graminearum)|Unaffected_pathogenicity
Chr06G0253.1	1742	0	392/729(53.77)	734	PHI:1742|GzZC057|I1S7H5|5518|Gibberella_zeae_(related:_Fusarium_graminearum)|Unaffected_pathogenicity
Chr06G0255.1	2107	9.00E-95	170/405(41.98)	299	PHI:2107|Zinc-regulated_transporter_2|MGG_05905|148305|Magnaporthe_oryzae_(related:_Magnaporthe_grisea)|Mixed_outcome
Chr06G0256.1	1662	1.00E-19	84/364(23.08)	90.5	PHI:1662|GzCCHC002|I1REJ1|5518|Gibberella_zeae_(related:_Fusarium_graminearum)|Unaffected_pathogenicity
Chr06G0258.1	3038	3.00E-11	49/165(29.70)	62.4	FgERG5B|PHI:3038|I1RIP4|5518|Fusarium_graminearum|reduced_virulence_
Chr06G0259.1	1984	3.00E-176	280/695(40.29)	519	PHI:1984|GzZC299|I1RB67|5518|Gibberella_zeae_(related:_Fusarium_graminearum)|Unaffected_pathogenicity
Chr06G0263.1	277	1.00E-109	157/211(74.41)	316	PHI:277|BCP1|AAQ16572|40559|Botrytis_cinerea|Reduced_virulence
Chr06G0264.1	1630	3.00E-167	231/404(57.18)	476	PHI:1630|GzWing002|I1RB71|5518|Gibberella_zeae_(related:_Fusarium_graminearum)|Unaffected_pathogenicity
Chr06G0268.1	784	5.00E-12	48/157(30.57)	62	PHI:784|MGG_00056|EDK03390|318829|Magnaporthe_oryzae|Reduced_virulence
Chr06G0272.1	3126	4.00E-30	106/352(30.11)	118	argD|PHI:3126|D4I307|552|Erwinia_amylovora|mixed_outcome_

Chr06G0273.1	511	1.00E-19	126/636(19.81)	90.5	PHI:511|CaNAG4|EAK93098|5476|Candida_albicans|Reduced_virulence
Chr06G0274.1	2183	0	483/805(60.00)	932	PHI:2183|CPXB|EHA48040.1|148305|Magnaporthe_oryzae_(related:_Magnaporthe_grisea)|Unaffected_pathogenicity
Chr06G0278.1	2393	9.00E-57	141/467(30.19)	196	PHI:2393|Related_to_O-methylsterigmatocystin_oxidoreductase|I1R980|5518|Gibberella_zeae_(related:_Fusarium_graminearum)|Increased_virulence_(Hypervirulence)
Chr06G0279.1	1424	7.00E-07	34/108(31.48)	45.4	PHI:1424|GzC2H092|I1S3J7|5518|Gibberella_zeae_(related:_Fusarium_graminearum)|Unaffected_pathogenicity
Chr06G0287.1	1551	0	573/780(73.46)	1141	PHI:1551|GzMyb015|I1RZG7|5518|Gibberella_zeae_(related:_Fusarium_graminearum)|Unaffected_pathogenicity
Chr06G0288.1	1161	1.00E-46	143/504(28.37)	172	PHI:1161|MgMfs1|A4ZGP3|54734|Mycosphaerella_graminicola_(related:_Zymoseptoria_triticii)|Chemistry_target
Chr06G0291.1	2532	0	316/426(74.18)	651	PHI:2532|HEM15|Q4WUA3|746128|Aspergillus_fumigatus|Lethal
Chr06G0292.1	3328	0	453/653(69.37)	914	MrKu70|PHI:3328|E9EMQ0|568076|Metarhizium_robertsii|unaffected_pathogenicity_
Chr06G0293.1	922	1.00E-83	213/608(35.03)	277	PHI:922|um03615||5270|Ustilago_maydis|Unaffected_pathogenicity
Chr06G0300.1	1896	2.00E-173	270/596(45.30)	520	PHI:1896|GzZC211|I1RNR1|5518|Gibberella_zeae_(related:_Fusarium_graminearum)|Unaffected_pathogenicity
Chr06G0306.1	789	0	682/1205(56.60)	924	PHI:789|MGG_04116|EDJ99418|318829|Magnaporthe_oryzae|Reduced_virulence
Chr06G0311.1	1174	0	616/1132(54.42)	1013	PHI:1174|HDF3|I1RNQ2|5518|Gibberella_zeae_(related:_Fusarium_graminearum)|Reduced_virulence
Chr06G0313.1	105	6.00E-69	190/595(31.93)	236	PHI:105|PLB1|AAC61890|5476|Candida_albicans|Reduced_virulence
Chr06G0315.1	1816	1.00E-43	121/449(26.95)	166	PHI:1816|GzZC131|I1RRS3|5518|Gibberella_zeae_(related:_Fusarium_graminearum)|Unaffected_pathogenicity
Chr06G0319.1	211	1.00E-07	36/106(33.96)	52.8	PHI:211|CaTUP1|AAB63195|5476|Candida_albicans|Reduced_virulence

Chr06G0326.1	1618	9.00E-40	89/275(32.36)	142	PHI:1618|GzFET5|Q4IQT8|5518|Gibberella_zeae_(related:_Fusarium_graminearum)|Unaffected_pathogenicity
Chr06G0333.1	286	0	396/777(50.97)	768	PHI:286|CLC-A|AAO73005|5207|Cryptococcus_neoformans|Reduced_virulence
Chr06G0334.1	1464	4.00E-63	104/183(56.83)	193	PHI:1464|GzCCAAT004|I1RMV4|5518|Gibberella_zeae_(related:_Fusarium_graminearum)|Reduced_virulence
Chr06G0336.1	1222	0	1047/1536(68.16)	2193	PHI:1222|FGSG_00132|I1RMV6|5518|Gibberella_zeae_(related:_Fusarium_graminearum)|Lethal
Chr06G0339.1	2382	0	266/303(87.79)	572	PHI:2382|Upa2|Q6QIY0|5270|Ustilago_maydis|Unaffected_pathogenicity
Chr06G0345.1	2821	2.00E-21	69/225(30.67)	98.2	PHI:2821|SNF2|Q5ALP9|5476|Candida_albicans|Reduced_virulence
Chr06G0357.1	1255	0	454/752(60.37)	832	PHI:1255|FGSG_07745|I1RQ67|5518|Gibberella_zeae_(related:_Fusarium_graminearum)|Unaffected_pathogenicity
Chr06G0359.1	3378	2.00E-28	82/231(35.50)	111	Plegl1|PHI:3378|G9JLA8|285811|Pyrenochaeta_lycopersici|unaffected_pathogenicity_
Chr06G0362.1	267	4.00E-121	365/1424(25.63)	416	PHI:267|MLT1|AAD51594|5476|Candida_albicans|Reduced_virulence
Chr06G0366.1	211	1.00E-57	132/339(38.94)	206	PHI:211|CaTUP1|AAB63195|5476|Candida_albicans|Reduced_virulence
Chr06G0367.1	2898	2.00E-41	102/304(33.55)	149	PHI:2896|BEC1005|CCU82697|62688|Blumeria_graminis_f._sp._hordei|Effector_(plant_avirulence_determinant)
Chr06G0368.1	1904	0	276/609(45.32)	535	PHI:1904|GzZC219|I1RHR0|5518|Gibberella_zeae_(related:_Fusarium_graminearum)|Unaffected_pathogenicity
Chr06G0369.1	2042	0	583/1329(43.87)	1103	PHI:2042|ABC3|Q3Y5V5|148305|Magnaporthe_oryzae_(related:_Magnaporthe_grisea)|Loss_of_pathogenicity
Chr06G0374.1	2038	6.00E-08	79/321(24.61)	50.8	PHI:2038|Mir1|MGG_02370|148305|Magnaporthe_oryzae_(related:_Magnaporthe_grisea)|Unaffected_pathogenicity
Chr06G0378.1	404	2.00E-06	45/222(20.27)	47.8	PHI:404|PTH11|AAD30436|318829|Magnaporthe_oryzae|Reduced_virulence
Chr06G0384.1	2694	1.00E-06	31/82(37.80)	48.9	PHI:2694|rsmB|Q6D000|29471|Pectobacterium_atrosepticum|Mixed_outcome

Chr06G0387.1	343	0	405/511(79.26)	845	PHI:343|CPRGS-1|AAT92283|5116|Cryphonectria_parasitica|Loss_of_pathogenicity
Chr06G0389.1	1487	0	373/558(66.85)	796	PHI:1487|GzHMG019|I1RQ92|5518|Gibberella_zeae_(related:_Fusarium_graminearum)|Unaffected_pathogenicity
Chr06G0396.1	413	3.00E-20	95/381(24.93)	89.7	PHI:413|MPD1|AAT84078|13684|Stagonospora_nodorum|Unaffected_pathogenicity
Chr06G0401.1	2393	6.00E-64	143/465(30.75)	216	PHI:2393|Related_to_O-methylsterigmatocystin_oxidoreductase|I1R980|5518|Gibberella_zeae_(related:_Fusarium_graminearum)|Increased_virulence_(Hypervirulence)
Chr06G0402.1	612	4.00E-28	86/276(31.16)	110	PHI:612|orf408|AAL20306|216597|Salmonella_enterica|Reduced_virulence
Chr06G0407.1	1958	0	629/987(63.73)	1203	PHI:1958|GzZC273|I1RMD1|5518|Gibberella_zeae_(related:_Fusarium_graminearum)|Unaffected_pathogenicity
Chr06G0411.1	2994	3.00E-08	88/348(25.29)	53.9	PHI:2994|MGG_06355.6|G4N7V2|148305|Magnaporthe_oryzae|Reduced_virulence
Chr06G0412.1	3354	6.00E-06	37/128(28.91)	45.1	rtxA1|PHI:3354|A0A023NA98|672|Vibrio_vulnificus|reduced_virulence_
Chr06G0416.1	1678	5.00E-180	252/368(68.48)	509	PHI:1678|GzGRF|I1RMC1|5518|Gibberella_zeae_(related:_Fusarium_graminearum)|Unaffected_pathogenicity
Chr06G0417.1	2155	0	257/345(74.49)	540	PHI:2155|MoPEX7|B8Q8Z5|148305|Magnaporthe_oryzae_(related:_Magnaporthe_grisea)|Loss_of_pathogenicity
Chr06G0418.1	2549	6.00E-37	137/464(29.53)	145	PHI:2549|MAK5|Q4WMS3|746128|Aspergillus_fumigatus|Mixed_outcome
Chr06G0424.1	2863	1.00E-11	25/70(35.71)	65.5	PHI:2863|Lys144|Q5A7T6|5476|Candida_albicans|Reduced_virulence
Chr06G0427.1	1500	7.00E-32	246/1023(24.05)	132	PHI:1500|GzHMG032|I1S2F2|5518|Gibberella_zeae_(related:_Fusarium_graminearum)|Unaffected_pathogenicity
Chr06G0429.1	1414	2.00E-09	27/66(40.91)	58.2	PHI:1414|GzC2H081|I1RZL0|5518|Gibberella_zeae_(related:_Fusarium_graminearum)|Unaffected_pathogenicity
Chr06G0430.1	2334	0	764/1013(75.42)	1587	PHI:2334|BcNma|E2GL14|40559|Botrytis_cinerea|Reduced_virulence

Chr06G0432.1	273	5.00E-98	162/311(52.09)	294	PHI:273|SPT3|AAD33888|5476|Candida_albicans|Loss_of_pathogenicity
Chr06G0435.1	1667	6.00E-06	19/32(59.38)	46.6	PHI:1667|GzCCHC007|I1RZP2|5518|Gibberella_zeae_(related:_Fusarium_graminearum)|Unaffected_pathogenicity
Chr06G0439.1	465	0	530/988(53.64)	966	PHI:465|KIN2|AAB63337|5270|Ustilago_maydis|Reduced_virulence
Chr06G0441.1	2529	2.00E-111	188/394(47.72)	335	PHI:2529|ERG10|Q4WCL5|746128|Aspergillus_fumigatus|Lethal
Chr06G0448.1	1449	9.00E-156	312/602(51.83)	460	PHI:1449|GzGATA007|I1RYV4|5518|Gibberella_zeae_(related:_Fusarium_graminearum)|Unaffected_pathogenicity
Chr06G0451.1	1662	1.00E-36	130/461(28.20)	143	PHI:1662|GzCCHC002|I1REJ1|5518|Gibberella_zeae_(related:_Fusarium_graminearum)|Unaffected_pathogenicity
Chr06G0455.1	2544	4.00E-08	29/90(32.22)	52.4	PHI:2544|PAB1|Q4WK03|746128|Aspergillus_fumigatus|Mixed_outcome
Chr06G0457.1	891	9.00E-171	352/934(37.69)	521	PHI:891|MGG_01748|EDK04531|318829|Magnaporthe_oryzae|Reduced_virulence
Chr06G0467.1	2038	3.00E-08	61/239(25.52)	52	PHI:2038|Mir1|MGG_02370|148305|Magnaporthe_oryzae_(related:_Magnaporthe_grisea)|Unaffected_pathogenicity
Chr06G0469.1	441	4.00E-34	98/317(30.91)	129	PHI:441|BTP1|CAE55153|40559|Botrytis_cinerea|Reduced_virulence
Chr06G0470.1	2844	4.00E-27	83/267(31.09)	105	PHI:2844|BRM2|O93802|5599|Alternaria_alternata|Unaffected_pathogenicity
Chr06G0471.1	1582	0	248/304(81.58)	521	PHI:1582|GzOB022|I1RQ81|5518|Gibberella_zeae_(related:_Fusarium_graminearum)|Unaffected_pathogenicity
Chr06G0472.1	2601	8.00E-06	45/166(27.11)	44.3	PHI:2601|Asc1|P83774|746128|Aspergillus_fumigatus|Mixed_outcome
Chr06G0473.1	419	2.00E-36	95/319(29.78)	134	PHI:419|CSH1|AAP93915|5476|Candida_albicans|Reduced_virulence
Chr06G0477.1	2656	7.00E-09	30/97(30.93)	54.3	PHI:2656|pmrF|D0ZPP5|90371|Salmonella_enterica_serovar_Typhimurium|Reduced_virulence
Chr06G0478.1	825	0	269/495(54.34)	525	PHI:825|SE|AAQ18216|5551|Trichophyton_rubrum|Chemistry_target
Chr06G0480.1	2177	6.00E-54	117/312(37.50)	199	PHI:2177|PAS1|MGG_09299|148305|Magnaporthe_oryzae_(related:_Magnaporthe_grisea)|Unaffected_pathogenicity
Chr06G0482.1	339	5.00E-147	198/202(98.02)	409	PHI:339|CLPT1|CAC41973|290576|Colletotrichum_lindemuthianum|Reduced_v

					irulence
Chr06G0488.1	1334	4.00E-48	141/289(48.79)	163	PHI:1334|GzbZIP016|I1RZI7|5518|Gibberella_zeae_(related:_Fusarium_graminearum)|Reduced_virulence
Chr06G0496.1	339	1.00E-22	46/118(38.98)	91.7	PHI:339|CLPT1|CAC41973|290576|Colletotrichum_lindemuthianum|Reduced_virulence
Chr06G0497.1	352	2.00E-10	120/553(21.70)	61.2	PHI:352|GLO1|CAD79488|5270|Ustilago_maydis|Loss_of_pathogenicity
Chr06G0498.1	2570	0	265/490(54.08)	530	PHI:2570|CYB2|Q6FM61|5478|Candida_glabrata|Reduced_virulence
Chr06G0506.1	1413	0	626/978(64.01)	1233	PHI:1413|GzC2H080|I1RZD4|5518|Gibberella_zeae_(related:_Fusarium_graminearum)|Unaffected_pathogenicity
Chr06G0508.1	1414	2.00E-11	34/86(39.53)	63.2	PHI:1414|GzC2H081|I1RZL0|5518|Gibberella_zeae_(related:_Fusarium_graminearum)|Unaffected_pathogenicity
Chr06G0511.1	2327	0	348/361(96.40)	725	PHI:2327|HOG1|P0C431|5518|Gibberella_zeae_(related:_Fusarium_graminearum)|Reduced_virulence
Chr06G0512.1	2062	0	734/886(82.84)	1554	PHI:2062|Gph1|XP_363893|148305|Magnaporthe_oryzae_(related:_Magnaporthe_grisea)|Mixed_outcome
Chr06G0513.1	82	0	322/355(90.70)	684	PHI:82|MAGA|AAB65425|318829|Magnaporthe_oryzae|Unaffected_pathogenicity
Chr06G0516.1	2655	5.00E-163	280/704(39.77)	504	PHI:2655|lon|K1C7N5|287|Pseudomonas_aeruginosa|Reduced_virulence
Chr06G0517.1	1275	0	356/573(62.13)	650	PHI:1275|FGSG_03146|I1S7R2|5518|Gibberella_zeae_(related:_Fusarium_graminearum)|Unaffected_pathogenicity
Chr06G0518.1	2605	0	347/454(76.43)	733	PHI:2605|hgc1|Q5ABE2|746128|Aspergillus_fumigatus|Mixed_outcome
Chr06G0519.1	1526	1.00E-19	48/129(37.21)	92.4	PHI:1526|GzHOMEL036|I1S0J3|5518|Gibberella_zeae_(related:_Fusarium_graminearum)|Unaffected_pathogenicity
Chr06G0522.1	482	6.00E-33	81/290(27.93)	123	PHI:482|LAEA|AAR01218|5085|Aspergillus_fumigatus|Reduced_virulence
Chr06G0526.1	1914	6.00E-142	269/628(42.83)	447	PHI:1914|GzZC229|I1RQ27|5518|Gibberella_zeae_(related:_Fusarium_graminearum)|Unaffected_pathogenicity

Chr06G0530.1	159	9.00E-88	129/237(54.43)	261	PHI:159|URA5|AAC62627|5037|Histoplasma_capsulatum|Loss_of_pathogenicity
Chr06G0535.1	1377	0	616/1005(61.29)	1135	PHI:1377|GzC2H040|I1RQ34|5518|Gibberella_zeae_(related:_Fusarium_graminearum)|Unaffected_pathogenicity
Chr06G0541.1	2256	6.00E-10	73/288(25.35)	57.4	PHI:2256|Xdh1|Q0UA24|13684|Phaeosphaeria_nodorum_(related:_Stagonospora_nodorum)|Unaffected_pathogenicity
Chr06G0547.1	2609	0	248/306(81.05)	530	PHI:2609|KRR1|AAB96910.2|746128|Aspergillus_fumigatus|Mixed_outcome
Chr06G0548.1	2176	2.00E-65	162/366(44.26)	217	PHI:2176|ASD4|MGG_06050|148305|Magnaporthe_oryzae_(related:_Magnaporthe_grisea)|Loss_of_pathogenicity
Chr06G0559.1	1524	2.00E-127	226/514(43.97)	386	PHI:1524|GzHOMEL033|I1RZF2|5518|Gibberella_zeae_(related:_Fusarium_graminearum)|Unaffected_pathogenicity
Chr06G0563.1	3037	0	394/531(74.20)	858	FgERG5A|PHI:3037|I1RE80|5518|Fusarium_graminearum|reduced_virulence_
Chr06G0567.1	1571	2.00E-97	132/160(82.50)	280	PHI:1571|GzOB011|I1RE76|5518|Gibberella_zeae_(related:_Fusarium_graminearum)|Unaffected_pathogenicity
Chr06G0571.1	2058	1.00E-35	125/495(25.25)	142	PHI:2058|LHS1|MGG_06648.5|148305|Magnaporthe_oryzae_(related:_Magnaporthe_grisea)|Reduced_virulence
Chr06G0572.1	2301	0	551/746(73.86)	1108	PHI:2301|CHS7|B5M4A8|148305|Magnaporthe_oryzae_(related:_Magnaporthe_grisea)|Reduced_virulence
Chr06G0574.1	2336	0	552/899(61.40)	1150	PHI:2336|NIA1|O00101|13684|Stagonospora_nodorum_(related:_Phaeosphaeria_nodorum)|Unaffected_pathogenicity
Chr06G0575.1	2101	7.00E-55	126/370(34.05)	191	PHI:2101|Vacuolar_calcium_ion_transporter|MGG_11454|148305|Magnaporthe_oryzae_(related:_Magnaporthe_grisea)|Unaffected_pathogenicity
Chr06G0581.1	2601	4.00E-07	36/156(23.08)	48.9	PHI:2601|Asc1|P83774|746128|Aspergillus_fumigatus|Mixed_outcome
Chr06G0587.1	2981	0	702/893(78.61)	1413	PHI:2981|Ctf1|A6N6J8|59765|Fusarium_oxysporum_f._sp._Lycopersici|Reduced_virulence
Chr06G0589.1	259	5.00E-11	55/198(27.78)	63.9	PHI:259|GPI7|AAL83897|5476|Candida_albicans|Reduced_virulence

Chr06G0590.1	1481	1.00E-78	194/453(42.83)	253	PHI:1481|GzHMG013|I1RE60|5518|Gibberella_zeae_(related:_Fusarium_graminearum)|Unaffected_pathogenicity
Chr06G0591.1	860	0	296/417(70.98)	594	PHI:860|MSP1|AAX07670|318829|Magnaporthe_oryzae|Reduced_virulence
Chr06G0592.1	443	0	366/437(83.75)	741	PHI:443|CBL1||5518|Fusarium_graminearum|Reduced_virulence
Chr06G0596.1	1616	5.00E-174	356/868(41.01)	531	PHI:1616|GzssDB001|I1RE55|5518|Gibberella_zeae_(related:_Fusarium_graminearum)|Unaffected_pathogenicity
Chr06G0600.1	1542	0	478/699(68.38)	930	PHI:1542|GzMyb006|I1RE51|5518|Gibberella_zeae_(related:_Fusarium_graminearum)|Unaffected_pathogenicity
Chr06G0601.1	2911	0	464/613(75.69)	1011	PHI:2911|Ss-pth2|A7F6V9|5180|Sclerotinia_sclerotiorum|Reduced_virulence
Chr06G0602.1	210	1.00E-92	198/550(36.00)	294	PHI:210|CAP1|AAD42978|5476|Candida_albicans|Loss_of_pathogenicity
Chr06G0604.1	1570	0	695/902(77.05)	1367	PHI:1570|GzOB010|I1RE46|5518|Gibberella_zeae_(related:_Fusarium_graminearum)|Unaffected_pathogenicity
Chr06G0605.1	3126	5.00E-48	125/350(35.71)	170	argD|PHI:3126|D4I307|552|Erwinia_amylovora|mixed_outcome_
Chr06G0617.1	317	1.00E-99	134/187(71.66)	287	PHI:317|RHBA|AAN17787|5085|Aspergillus_fumigatus|Reduced_virulence
Chr06G0621.1	1648	1.00E-144	321/842(38.12)	491	PHI:1648|GzWing020|I1RWP1|5518|Gibberella_zeae_(related:_Fusarium_graminearum)|Reduced_virulence
Chr06G0624.1	2100	6.00E-78	228/773(29.50)	281	PHI:2100|Spf1|MGG_12005|148305|Magnaporthe_oryzae_(related:_Magnaporthe_grisea)|Mixed_outcome
Chr06G0639.1	2038	9.00E-12	75/287(26.13)	63.9	PHI:2038|Mir1|MGG_02370|148305|Magnaporthe_oryzae_(related:_Magnaporthe_grisea)|Unaffected_pathogenicity
Chr06G0646.1	819	1.00E-127	256/695(36.83)	394	PHI:819|MGG_07061|EDK03750|318829|Magnaporthe_oryzae|Reduced_virulence
Chr06G0650.1	112	2.00E-21	98/402(24.38)	94	PHI:112|MAK1|AAC49410|140110|Nectria_haematococca_(related:_Fusarium_solani)|Reduced_virulence
Chr06G0651.1	404	6.00E-23	60/245(24.49)	98.6	PHI:404|PTH11|AAD30436|318829|Magnaporthe_oryzae|Reduced_virulence
Chr06G0653.1	812	4.00E-61	130/363(35.81)	204	PHI:812|MGG_10702|EDJ94108|318829|Magnaporthe_oryzae|Reduced_virule

					nce
Chr06G0654.1	1915	0	421/651(64.67)	749	PHI:1915|GzZC230|I1RSL0|5518|Gibberella_zeae_(related:_Fusarium_graminearum)|Reduced_virulence
Chr06G0655.1	338	2.00E-76	118/258(45.74)	256	PHI:338|CLB2|AAP94020|5270|Ustilago_maydis|Reduced_virulence
Chr06G0659.1	1579	5.00E-92	226/693(32.61)	322	PHI:1579|GzOB019|I1RM25|5518|Gibberella_zeae_(related:_Fusarium_graminearum)|Unaffected_pathogenicity
Chr06G0663.1	893	7.00E-67	189/660(28.64)	244	PHI:893|MGG_02986|EDK01543|318829|Magnaporthe_oryzae|Reduced_virulence
Chr06G0668.1	1492	0	339/477(71.07)	665	PHI:1492|GzHMG024|Q4I5U9|5518|Gibberella_zeae_(related:_Fusarium_graminearum)|Unaffected_pathogenicity
Chr06G0670.1	3174	3.00E-147	273/456(59.87)	431	MoSip2|PHI:3174|G4MNE3|318829|Magnaporthe_oryzae|loss_of_pathogenicity_
Chr06G0673.1	2109	7.00E-118	168/174(96.55)	332	PHI:2109|CNB|MGG_06933|148305|Magnaporthe_oryzae_(related:_Magnaporthe_grisea)|Mixed_outcome
Chr06G0681.1	2382	3.00E-60	116/291(39.86)	200	PHI:2382|Upa2|Q6QIY0|5270|Ustilago_maydis|Unaffected_pathogenicity
Chr06G0684.1	1388	1.00E-153	232/343(67.64)	436	PHI:1388|GzC2H051|I1RT91|5518|Gibberella_zeae_(related:_Fusarium_graminearum)|Unaffected_pathogenicity
Chr06G0685.1	3389	0	287/500(57.40)	577	FVEG_12521|PHI:3389|W7MS01|117187|Fusarium_verticillioides|unaffected_pathogenicity_
Chr06G0686.1	1644	0	455/741(61.40)	912	PHI:1644|GzRFX1|I1RTB9|5518|Gibberella_zeae_(related:_Fusarium_graminearum)|Unaffected_pathogenicity
Chr06G0688.1	2274	2.00E-148	281/680(41.32)	446	PHI:2274|Mid1|C6GZ42|5111|Claviceps_purpurea|Loss_of_pathogenicity
Chr06G0693.1	2968	2.00E-45	132/516(25.58)	165	PHI:2968|Hxs1|J9VQA5|5207|Cryptococcus_neoformans|Reduced_virulence
Chr06G0695.1	1552	6.00E-16	81/330(24.55)	80.9	PHI:1552|GzMyb016|I1S0N5|5518|Gibberella_zeae_(related:_Fusarium_graminearum)|Unaffected_pathogenicity
Chr06G0696.1	1232	0	315/411(76.64)	644	PHI:1232|FGSG_03499|I1RTC2|5518|Gibberella_zeae_(related:_Fusarium_gra

					minearum)|Lethal
Chr06G0703.1	1424	2.00E-23	40/56(71.43)	103	PHI:1424|GzC2H092|I1S3J7|5518|Gibberella_zeae_(related:_Fusarium_graminearum)|Unaffected_pathogenicity
Chr06G0706.1	1645	0	631/869(72.61)	1274	PHI:1645|GzWing017|I1RTD1|5518|Gibberella_zeae_(related:_Fusarium_graminearum)|Unaffected_pathogenicity
Chr06G0709.1	213	5.00E-70	107/160(66.88)	212	PHI:213|CPA1|AAF69795|5207|Cryptococcus_neoformans|Reduced_virulence
Chr06G0715.1	2825	3.00E-80	152/321(47.35)	283	PHI:2825|SET1|Q5ABG1|5476|Candida_albicans|Reduced_virulence
Chr06G0716.1	1623	4.00E-10	63/198(31.82)	58.2	PHI:1623|GzJUM005|I1S9T2|5518|Gibberella_zeae_(related:_Fusarium_graminearum)|Unaffected_pathogenicity
Chr06G0720.1	1548	2.00E-17	36/83(43.37)	82	PHI:1548|GzMyb012|I1RTE5|5518|Gibberella_zeae_(related:_Fusarium_graminearum)|Unaffected_pathogenicity
Chr06G0721.1	2570	2.00E-106	195/493(39.55)	338	PHI:2570|CYB2|Q6FM61|5478|Candida_glabrata|Reduced_virulence
Chr06G0725.1	2514	9.00E-07	25/95(26.32)	49.3	PHI:2514|PFS2|Q4X1Y0|746128|Aspergillus_fumigatus|Lethal
Chr06G0728.1	339	4.00E-38	69/174(39.66)	131	PHI:339|CLPT1|CAC41973|290576|Colletotrichum_lindemuthianum|Reduced_virulence
Chr06G0730.1	3459	5.00E-43	124/411(30.17)	157	NTPDase1|PHI:3459|Q4QFI1|5664|Leishmania_major|mixed_outcome_
Chr06G0731.1	2644	2.00E-07	25/70(35.71)	47.8	PHI:2644|thioredoxin_1|P0AA28|90371|Salmonella_enterica_serovar_Typhimurium|Reduced_virulence
Chr06G0732.1	465	6.00E-65	133/315(42.22)	228	PHI:465|KIN2|AAB63337|5270|Ustilago_maydis|Reduced_virulence
Chr06G0734.1	3485	6.00E-61	115/212(54.25)	190	MoSPC3|PHI:3485|G4MYT5|318829|Magnaporthe_oryzae|loss_of_pathogenicity_
Chr06G0737.1	2519	0	323/446(72.42)	688	PHI:2519|LYS9|Q4WQ27|746128|Aspergillus_fumigatus|Lethal
Chr06G0749.1	1260	6.00E-14	62/197(31.47)	72	PHI:1260|FGSG_13944|I1RUC7|5518|Gibberella_zeae_(related:_Fusarium_graminearum)|Unaffected_pathogenicity
Chr06G0751.1	441	3.00E-28	84/282(29.79)	111	PHI:441|BTP1|CAE55153|40559|Botrytis_cinerea|Reduced_virulence
Chr06G0755.1	1393	4.00E-08	34/118(28.81)	53.5	PHI:1393|GzC2H056|I1RU69|5518|Gibberella_zeae_(related:_Fusarium_grami

					nearum)|Unaffected_pathogenicity
Chr06G0757.1	1610	0	424/668(63.47)	822	PHI:1610|GzP53L003|I1RSN1|5518|Gibberella_zeae_(related:_Fusarium_graminearum)|Unaffected_pathogenicity
Chr06G0761.1	2644	1.00E-12	28/80(35.00)	62.4	PHI:2644|thioredoxin_1|P0AA28|90371|Salmonella_enterica_serovar_Typhimurium|Reduced_virulence
Chr06G0764.1	2117	9.00E-49	149/455(32.75)	174	PHI:2117|SPM1|P58371|148305|Magnaporthe_oryzae_(related:_Magnaporthe_grisea)|Reduced_virulence
Chr06G0765.1	1798	0	339/670(50.60)	634	PHI:1798|GzZC113|I1RSP9|5518|Gibberella_zeae_(related:_Fusarium_graminearum)|Unaffected_pathogenicity
Chr06G0768.1	2020	3.00E-28	72/295(24.41)	119	PHI:2020|Tup1|XP_759427|5270|Ustilago_maydis|Mixed_outcome
Chr06G0769.1	2244	1.00E-75	128/350(36.57)	239	PHI:2244|37865|gi|46099354|5270|Ustilago_maydis|Unaffected_pathogenicity
Chr06G0770.1	1287	0	343/457(75.05)	694	PHI:1287|FGSG_13944|I1RT82|5518|Gibberella_zeae_(related:_Fusarium_graminearum)|Unaffected_pathogenicity
Chr06G0775.1	716	5.00E-112	210/551(38.11)	349	PHI:716|ZEB1|ABB90284|5518|Fusarium_graminearum|Unaffected_pathogenicity
Chr06G0776.1	441	5.00E-23	95/336(28.27)	97.4	PHI:441|BTP1|CAE55153|40559|Botrytis_cinerea|Reduced_virulence
Chr06G0780.1	320	3.00E-06	26/114(22.81)	47.8	PHI:320|SSN6|AAL54912|5476|Candida_albicans|Reduced_virulence
Chr06G0785.1	1562	2.00E-105	205/528(38.83)	342	PHI:1562|GzOB002|I1RAH4|5518|Gibberella_zeae_(related:_Fusarium_graminearum)|Lethal
Chr06G0789.1	1816	0	651/912(71.38)	1239	PHI:1816|GzZC131|I1RRS3|5518|Gibberella_zeae_(related:_Fusarium_graminearum)|Unaffected_pathogenicity
Chr06G0790.1	1566	8.00E-50	100/250(40.00)	179	PHI:1566|GzOB006|I1RC95|5518|Gibberella_zeae_(related:_Fusarium_graminearum)|Lethal
Chr06G0792.1	2134	4.00E-176	349/680(51.32)	521	PHI:2134|MoHox5|MGG_07437|148305|Magnaporthe_oryzae_(related:_Magnaporthe_grisea)|reduced_virulence
Chr06G0794.1	2834	3.00E-11	48/158(30.38)	61.6	PHI:2834|LAM1|C3PTB1|5016|Cochliobolus_heterostrophus|Reduced_virulenc

					e
Chr06G0800.1	1457	0	391/570(68.60)	803	PHI:1457|GzAra005|I1RRR9|5518|Gibberella_zeae_(related:_Fusarium_graminearum)|Unaffected_pathogenicity
Chr06G0801.1	544	4.00E-46	147/552(26.63)	169	PHI:544|BCMFS1|AAF64435|332648|Botrytis_cinerea|Unaffected_pathogenicity
Chr06G0803.1	1872	5.00E-11	28/59(47.46)	62.8	PHI:1872|GzZC187|I1RTU2|5518|Gibberella_zeae_(related:_Fusarium_graminearum)|Lethal
Chr06G0804.1	2356	3.00E-104	138/166(83.13)	297	PHI:2356|MoHYR1|G4N178|148305|Magnaporthe_oryzae_(related:_Magnaporthe_grisea)|Reduced_virulence
Chr06G0813.1	1414	6.00E-15	42/91(46.15)	73.2	PHI:1414|GzC2H081|I1RZL0|5518|Gibberella_zeae_(related:_Fusarium_graminearum)|Unaffected_pathogenicity
Chr06G0816.1	1274	0	1075/1629(65.99)	2165	PHI:1274|FGSG_02488|I1RME6|5518|Gibberella_zeae_(related:_Fusarium_graminearum)|Unaffected_pathogenicity
Chr06G0818.1	121	1.00E-116	164/215(76.28)	333	PHI:121|PTH3|AAB88888|318829|Magnaporthe_oryzae|Loss_of_pathogenicity
Chr06G0830.1	1685	8.00E-10	57/204(27.94)	57.4	PHI:1685|GzRad003|I1S5Z3|5518|Gibberella_zeae_(related:_Fusarium_graminearum)|Unaffected_pathogenicity
Chr06G0842.1	2544	3.00E-28	58/170(34.12)	114	PHI:2544|PAB1|Q4WK03|746128|Aspergillus_fumigatus|Mixed_outcome
Chr06G0843.1	2038	1.00E-08	74/308(24.03)	52.8	PHI:2038|Mir1|MGG_02370|148305|Magnaporthe_oryzae_(related:_Magnaporthe_grisea)|Unaffected_pathogenicity
Chr06G0845.1	1433	8.00E-74	135/304(44.41)	236	PHI:1433|GzC2H102|I1S8E5|5518|Gibberella_zeae_(related:_Fusarium_graminearum)|Unaffected_pathogenicity
Chr06G0847.1	1159	3.00E-31	121/450(26.89)	127	PHI:1159|MgAtr7|A5H456|54734|Mycosphaerella_graminicola_(related:_Zymoseptoria_tritici)|Chemistry_target
Chr06G0848.1	2190	5.00E-13	48/190(25.26)	68.2	PHI:2190|MoCYP51A|G4MRP8|148305|Magnaporthe_oryzae_(related:_Magnaporthe_grisea)|Reduced_virulence
Chr06G0849.1	511	3.00E-26	106/432(24.54)	109	PHI:511|CaNAG4|EAK93098|5476|Candida_albicans|Reduced_virulence

Chr06G0851.1	211	6.00E-08	44/131(33.59)	53.1	PHI:211|CaTUP1|AAB63195|5476|Candida_albicans|Reduced_virulence
Chr06G0860.1	2525	1.00E-162	217/354(61.30)	461	PHI:2525|TOM40|Q4WDL0|746128|Aspergillus_fumigatus|Lethal
Chr06G0866.1	1216	0	478/652(73.31)	957	PHI:1216|FGSG_06420|I1RT48|5518|Gibberella_zeae_(related:_Fusarium_graminearum)|Reduced_virulence
Chr06G0869.1	211	2.00E-08	54/213(25.35)	53.5	PHI:211|CaTUP1|AAB63195|5476|Candida_albicans|Reduced_virulence
Chr06G0871.1	1200	0	362/394(91.88)	761	PHI:1200|(Gsk3)|I1RT36|5518|Gibberella_zeae_(related:_Fusarium_graminearum)|Reduced_virulence
Chr06G0881.1	3028	2.00E-08	79/340(23.24)	53.5	Vatr2|PHI:3028|A5CVB7|28447|Clavibacter_michiganensis|reduced_virulence_
Chr06G0882.1	2020	1.00E-11	73/307(23.78)	63.2	PHI:2020|Tup1|XP_759427|5270|Ustilago_maydis|Mixed_outcome
Chr06G0883.1	3096	0	291/394(73.86)	637	Ktr4|PHI:3096|J5K093|176275|Beauveria_bassiana|reduced_virulence_
Chr06G0888.1	3629	6.00E-06	96/405(23.70)	45.4	Rv0392c|PHI:3629|P95200|1773|Mycobacterium_tuberculosis|unaffected_pathogenicity_
Chr06G0893.1	2734	4.00E-28	85/304(27.96)	113	PHI:2734|metF|Q97S30|1313|Streptococcus_Pneumoniae|Mixed_outcome
Chr06G0897.1	1918	0	436/906(48.12)	781	PHI:1918|GzZC233|I1RT69|5518|Gibberella_zeae_(related:_Fusarium_graminearum)|Unaffected_pathogenicity
Chr06G0899.1	2097	0	987/1310(75.34)	1977	PHI:2097|Neo1|MGG_04066|148305|Magnaporthe_oryzae_(related:_Magnaporthe_grisea)|Loss_of_pathogenicity
Chr06G0915.1	178	2.00E-143	311/873(35.62)	445	PHI:178|pabaA|AAD31929|5085|Aspergillus_fumigatus|Reduced_virulence
Chr06G0925.1	2034	9.00E-10	39/120(32.50)	57.8	PHI:2034|MFP1|G4MZY1|148305|Magnaporthe_oryzae_(related:_Magnaporthe_grisea)|Reduced_virulence
Chr06G0928.1	2968	2.00E-48	150/516(29.07)	175	PHI:2968|Hxs1|J9VQA5|5207|Cryptococcus_neoformans|Reduced_virulence
Chr06G0930.1	1488	4.00E-96	262/631(41.52)	335	PHI:1488|GzHMG020|I1RSA6|5518|Gibberella_zeae_(related:_Fusarium_graminearum)|Unaffected_pathogenicity
Chr06G0931.1	2832	1.00E-10	43/149(28.86)	60.1	PHI:2832|AKT3-1|Q9P4U9|5599|Alternaria_alternata|Loss_of_pathogenicity
Chr06G0938.1	3028	4.00E-22	128/469(27.29)	96.7	Vatr2|PHI:3028|A5CVB7|28447|Clavibacter_michiganensis|reduced_virulence_
Chr06G0940.1	1227	0	2973/3902(76.19)	6188	PHI:1227|FGSG_07742|I1RPV7|5518|Gibberella_zeae_(related:_Fusarium_gra

					minearum)|Lethal
Chr06G0946.1	862	0	908/957(94.88)	1784	PHI:862|ClaSSD1|BAE66713|5462|Colletotrichum_lagenarium|Loss_of_pathogenicity
Chr06G0953.1	3411	8.00E-28	99/351(28.21)	115	bscN|PHI:3411|O68539|518|Bordetella_bronchiseptica|reduced_virulence_
Chr06G0955.1	144	7.00E-16	70/274(25.55)	79	PHI:144|CHT42|AAC05829|29875|Trichoderma_virens|Reduced_virulence
Chr06G0958.1	2060	4.00E-15	122/499(24.45)	77.4	PHI:2060|MgLig4|B6ZH51|148305|Magnaporthe_oryzae_(related:_Magnaporthe_grisea)|Unaffected_pathogenicity
Chr06G0963.1	1606	6.00E-109	158/214(73.83)	325	PHI:1606|GzOB047|I1S8E2|5518|Gibberella_zeae_(related:_Fusarium_graminearum)|Reduced_virulence
Chr06G0964.1	831	1.00E-116	156/215(72.56)	333	PHI:831|ERG2|CAA80454|318829|Magnaporthe_oryzae|chemistry_target
Chr06G0965.1	2266	7.00E-126	213/552(38.59)	387	PHI:2266|Ptr2|0|13684|Phaeosphaeria_nodorum_(related:_Stagonospora_nodorum)|Unaffected_pathogenicity
Chr06G0967.1	1796	0	397/761(52.17)	776	PHI:1796|GzZC111|I1RSR7|5518|Gibberella_zeae_(related:_Fusarium_graminearum)|Unaffected_pathogenicity
Chr06G0970.1	267	5.00E-111	229/625(36.64)	384	PHI:267|MLT1|AAD51594|5476|Candida_albicans|Reduced_virulence
Chr06G0972.1	1458	3.00E-77	160/458(34.93)	262	PHI:1458|GzAra006|Q4I7F9|5518|Gibberella_zeae_(related:_Fusarium_graminearum)|Unaffected_pathogenicity
Chr06G0974.1	2601	5.00E-15	53/190(27.89)	73.6	PHI:2601|Asc1|P83774|746128|Aspergillus_fumigatus|Mixed_outcome
Chr06G0977.1	1671	4.00E-114	267/759(35.18)	384	PHI:1671|GzCCHC011|Q4HWE2|5518|Gibberella_zeae_(related:_Fusarium_graminearum)|Reduced_virulence
Chr06G0978.1	2155	3.00E-16	71/281(25.27)	77	PHI:2155|MoPEX7|B8Q8Z5|148305|Magnaporthe_oryzae_(related:_Magnaporthe_grisea)|Loss_of_pathogenicity
Chr06G0983.1	1211	0	368/599(61.44)	642	PHI:1211|FGSG_06970|I1RRQ8|5518|Gibberella_zeae_(related:_Fusarium_graminearum)|Reduced_virulence
Chr06G0994.1	792	4.00E-36	109/396(27.53)	137	PHI:792|MGG_09250|EDJ97946|318829|Magnaporthe_oryzae|Reduced_virulence

Chr06G0995.1	2269	2.00E-81	129/277(46.57)	249	PHI:2269|Mdh1|0|13684|Phaeosphaeria_nodorum_(related:_Stagonospora_nodorum)|Unaffected_pathogenicity
Chr06G0996.1	2110	0	494/566(87.28)	1034	PHI:2110|Serine/threonine-protein_phosphatase_2B_catalytic_subunit|MGG_07456|148305|Magnaporthe_oryzae_(related:_Magnaporthe_grisea)|Unaffected_pathogenicity
Chr06G0999.1	1579	3.00E-52	112/282(39.72)	198	PHI:1579|GzOB019|I1RM25|5518|Gibberella_zeae_(related:_Fusarium_graminearum)|Unaffected_pathogenicity
Chr06G1000.1	2218	3.00E-18	112/452(24.78)	89.4	PHI:2218|drf1|XP_757288|5270|Ustilago_maydis|Reduced_virulence
Chr06G1006.1	2175	6.00E-74	137/311(44.05)	231	PHI:2175|NMR3|MGG_09705|148305|Magnaporthe_oryzae_(related:_Magnaporthe_grisea)|Reduced_virulence
Chr06G1015.1	3175	0	256/353(72.52)	516	MoSnf4|PHI:3175|G4NGW8|318829|Magnaporthe_oryzae|loss_of_pathogenicity_
Chr06G1016.1	3067	9.00E-127	238/460(51.74)	415	MoRga7|PHI:3067|G4NGW7|318829|Magnaporthe_oryzae|mixed_outcome_
Chr06G1018.1	1596	1.00E-37	61/87(70.11)	130	PHI:1596|GzOB036|I1RXD4|5518|Gibberella_zeae_(related:_Fusarium_graminearum)|Unaffected_pathogenicity
Chr06G1019.1	1332	9.00E-28	65/155(41.94)	108	PHI:1332|GzbZIP014|I1RXD5|5518|Gibberella_zeae_(related:_Fusarium_graminearum)|Unaffected_pathogenicity
Chr06G1021.1	1535	0	240/395(60.76)	517	PHI:1535|GzMADS002|I1RXD6|5518|Gibberella_zeae_(related:_Fusarium_graminearum)|Unaffected_pathogenicity
Chr06G1022.1	2315	1.00E-51	105/278(37.77)	174	PHI:2315|ChLae1|G4XKY9|5016|Cochliobolus_heterostrophus|Mixed_outcome
Chr06G1024.1	2259	1.00E-75	283/1066(26.55)	271	PHI:2259|AAR1|G8DNT1|45130|Cochliobolus_sativus|Reduced_virulence
Chr06G1029.1	2020	3.00E-11	79/356(22.19)	64.7	PHI:2020|Tup1|XP_759427|5270|Ustilago_maydis|Mixed_outcome
Chr06G1036.1	339	8.00E-18	51/184(27.72)	77.8	PHI:339|CLPT1|CAC41973|290576|Colletotrichum_lindemuthianum|Reduced_virulence
Chr06G1037.1	541	2.00E-55	160/491(32.59)	196	PHI:541|LIP1|AAU87359|332648|Botrytis_cinerea|Unaffected_pathogenicity
Chr06G1038.1	2042	0	460/1308(35.17)	760	PHI:2042|ABC3|Q3Y5V5|148305|Magnaporthe_oryzae_(related:_Magnaporthe

					_grisea)|Loss_of_pathogenicity
Chr06G1039.1	1828	0	281/584(48.12)	555	PHI:1828|GzZC143|I1S351|5518|Gibberella_zeae_(related:_Fusarium_graminearum)|Unaffected_pathogenicity
Chr06G1040.1	2020	1.00E-58	126/311(40.51)	213	PHI:2020|Tup1|XP_759427|5270|Ustilago_maydis|Mixed_outcome
Chr06G1049.1	1511	0	479/621(77.13)	895	PHI:1511|GzHOME009|I1RXF3|5518|Gibberella_zeae_(related:_Fusarium_graminearum)|Reduced_virulence
Chr06G1050.1	405	0	621/1397(44.45)	1177	PHI:405|PDE1|AAK07740|318829|Magnaporthe_oryzae|Reduced_virulence
Chr06G1053.1	55	8.00E-07	58/270(21.48)	48.5	PHI:55|PKS1|AAB08104|5016|Cochliobolus_heterostrophus|Reduced_virulence
Chr06G1061.1	2067	8.00E-11	60/209(28.71)	59.7	PHI:2067|ABC4|MGG_00937|148305|Magnaporthe_oryzae_(related:_Magnaporthe_grisea)|Loss_of_pathogenicity
Chr06G1064.1	3257	2.00E-12	83/277(29.96)	65.5	MoIlv1|PHI:3257|G4MU34|318829|Magnaporthe_oryzae|reduced_virulence_
Chr06G1068.1	2542	0	324/469(69.08)	663	PHI:2542|ERG12|Q4WP25|746128|Aspergillus_fumigatus|Mixed_outcome
Chr06G1071.1	807	6.00E-32	49/61(80.33)	114	PHI:807|MGG_06951|EDJ98115|318829|Magnaporthe_oryzae|Reduced_virulence
Chr06G1072.1	807	0	361/455(79.34)	737	PHI:807|MGG_06951|EDJ98115|318829|Magnaporthe_oryzae|Reduced_virulence
Chr06G1075.1	748	4.00E-138	257/648(39.66)	432	PHI:748|um00446|Not_available|5270|Ustilago_maydis|Unaffected_pathogenicity
Chr06G1076.1	2924	4.00E-120	176/342(51.46)	352	PHI:2924|lip1|J9MXG1|59765|Fusarium_oxysporum_f._sp._Lycopersici|Unaffected_pathogenicity
Chr06G1077.1	1552	9.00E-22	88/357(24.65)	99.4	PHI:1552|GzMyb016|I1S0N5|5518|Gibberella_zeae_(related:_Fusarium_graminearum)|Unaffected_pathogenicity
Chr06G1078.1	890	5.00E-160	258/599(43.07)	475	PHI:890|MGG_07015|EDJ98043|318829|Magnaporthe_oryzae|Reduced_virulence
Chr06G1079.1	1674	0	329/547(60.15)	616	PHI:1674|GzDHHC002|Q4IA62|5518|Gibberella_zeae_(related:_Fusarium_gra

					minearum)|Unaffected_pathogenicity
Chr06G1081.1	1026	0	520/1017(51.13)	980	PHI:1026|bcplc1|AAB39564|40559|Botrytis_cinerea|Reduced_virulence
Chr06G1082.1	873	4.00E-167	262/519(50.48)	506	PHI:873|MGG_02443|EDJ98595|318829|Magnaporthe_oryzae|Reduced_virulence
Chr06G1084.1	2715	1.00E-06	32/89(35.96)	48.9	PHI:2715|PEX6|I1RNL3|5518|Fusarium_graminearum|Reduced_virulence
Chr06G1086.1	2916	0	557/666(83.63)	1090	PHI:2916|Ppr2|W7M5P0|117187|Fusarium_verticillioides|Unaffected_pathogenicity
Chr06G1090.1	177	0	281/419(67.06)	582	PHI:177|ODC|CAB56523|13684|Stagonospora_nodorum|Reduced_virulence
Chr06G1096.1	1430	7.00E-30	62/140(44.29)	119	PHI:1430|GzC2H098|I1S7L4|5518|Gibberella_zeae_(related:_Fusarium_graminearum)|Unaffected_pathogenicity
Chr06G1107.1	2020	4.00E-18	92/401(22.94)	86.7	PHI:2020|Tup1|XP_759427|5270|Ustilago_maydis|Mixed_outcome
Chr06G1112.1	325	3.00E-06	85/373(22.79)	48.1	PHI:325|ACE1|CAG28797|318829|Magnaporthe_oryzae|Effector_(plant_avirulence_determinant)
Chr06G1117.1	1769	9.00E-116	210/508(41.34)	351	PHI:1769|GzZC084|I1RXB2|5518|Gibberella_zeae_(related:_Fusarium_graminearum)|Unaffected_pathogenicity
Chr06G1119.1	2315	6.00E-54	108/291(37.11)	179	PHI:2315|ChLae1|G4XKY9|5016|Cochliobolus_heterostrophus|Mixed_outcome
Chr06G1120.1	2930	0	484/660(73.33)	994	PHI:2930|ctf2|J9MFF7|59765|Fusarium_oxysporum_f._sp._Lycopersici|Reduced_virulence
Chr06G1133.1	1517	0	526/912(57.68)	886	PHI:1517|GzHOMEL013|I1RPZ1|5518|Gibberella_zeae_(related:_Fusarium_graminearum)|Lethal
Chr06G1134.1	267	2.00E-161	371/1226(30.26)	536	PHI:267|MLT1|AAD51594|5476|Candida_albicans|Reduced_virulence
Chr06G1136.1	1408	5.00E-127	201/444(45.27)	378	PHI:1408|GzC2H072|I1RXE9|5518|Gibberella_zeae_(related:_Fusarium_graminearum)|Unaffected_pathogenicity
Chr06G1137.1	2520	0	465/723(64.32)	946	PHI:2520|LYS4|Q4WUL6|746128|Aspergillus_fumigatus|Lethal
Chr06G1138.1	1763	0	384/708(54.24)	743	PHI:1763|GzZC078|I1RXE8|5518|Gibberella_zeae_(related:_Fusarium_graminearum)|Unaffected_pathogenicity

Chr06G1139.1	544	6.00E-177	264/549(48.09)	518	PHI:544|BCMFS1|AAF64435|332648|Botrytis_cinerea|Unaffected_pathogenicity
Chr06G1156.1	358	8.00E-49	147/562(26.16)	178	PHI:358|ILV2|AAR29084|5207|Cryptococcus_neoformans|Loss_of_pathogenicity
Chr06G1161.1	404	3.00E-28	76/315(24.13)	118	PHI:404|PTH11|AAD30436|318829|Magnaporthe_oryzae|Reduced_virulence
Chr06G1171.1	2315	1.00E-57	109/290(37.59)	189	PHI:2315|ChLae1|G4XKY9|5016|Cochliobolus_heterostrophus|Mixed_outcome
Chr06G1174.1	352	9.00E-08	67/267(25.09)	52	PHI:352|GLO1|CAD79488|5270|Ustilago_maydis|Loss_of_pathogenicity
Chr06G1176.1	2376	3.00E-50	120/408(29.41)	178	PHI:2376|DEP2|D2E9W7|29001|Alternaria_brassicicola|Mixed_outcome
Chr06G1177.1	184	1.00E-24	52/134(38.81)	97.4	PHI:184|RBT4|AAG09789|5476|Candida_albicans|Reduced_virulence
Chr06G1178.1	1269	1.00E-38	130/434(29.95)	143	PHI:1269|FGSG_02838|I1RFK9|5518|Gibberella_zeae_(related:_Fusarium_graminearum)|Unaffected_pathogenicity
Chr06G1179.1	716	3.00E-102	201/565(35.58)	322	PHI:716|ZEB1|ABB90284|5518|Fusarium_graminearum|Unaffected_pathogenicity
Chr06G1180.1	1913	3.00E-10	21/38(55.26)	60.8	PHI:1913|GzZC228|I1RP37|5518|Gibberella_zeae_(related:_Fusarium_graminearum)|Unaffected_pathogenicity
Chr06G1183.1	1895	1.00E-179	311/678(45.87)	543	PHI:1895|GzZC210|I1RNU2|5518|Gibberella_zeae_(related:_Fusarium_graminearum)|Unaffected_pathogenicity
Chr06G1184.1	2710	3.00E-109	182/408(44.61)	331	PHI:2710|mepB|H8ZZ79|474922|Colletotrichum_gloeosporioides|Reduced_virulence
Chr06G1191.1	853	8.00E-06	28/89(31.46)	44.3	PHI:853|YAP1|EAK83313|5270|Ustilago_maydis|Reduced_virulence
Chr06G1197.1	1781	0	343/731(46.92)	623	PHI:1781|GzZC096|I1S389|5518|Gibberella_zeae_(related:_Fusarium_graminearum)|Unaffected_pathogenicity
Chr06G1199.1	2378	1.00E-09	49/204(24.02)	58.2	PHI:2378|DEP4|D2E9W9|29001|Alternaria_brassicicola|Mixed_outcome
Chr06G1200.1	784	4.00E-24	64/200(32.00)	97.1	PHI:784|MGG_00056|EDK03390|318829|Magnaporthe_oryzae|Reduced_virulence
Chr06G1204.1	404	1.00E-29	99/401(24.69)	119	PHI:404|PTH11|AAD30436|318829|Magnaporthe_oryzae|Reduced_virulence

Chr06G1205.1	2315	1.00E-59	110/292(37.67)	195	PHI:2315|ChLae1|G4XKY9|5016|Cochliobolus_heterostrophus|Mixed_outcome
Chr06G1208.1	2357	3.00E-08	77/321(23.99)	53.9	PHI:2357|CYP52X1|E2EAF6|475271|Beauveria_bassiana|Reduced_virulence
Chr06G1211.1	1047	1.00E-20	100/361(27.70)	88.6	PHI:1047|CTB6|ABK64183|29003|Cercospora_nicotianae|Reduced_virulence
Chr06G1213.1	716	2.00E-10	72/268(26.87)	60.5	PHI:716|ZEB1|ABB90284|5518|Fusarium_graminearum|Unaffected_pathogenicity
Chr06G1214.1	1806	6.00E-21	106/490(21.63)	94.4	PHI:1806|GzZC121|I1RUM8|5518|Gibberella_zeae_(related:_Fusarium_graminearum)|Unaffected_pathogenicity
Chr06G1218.1	1046	5.00E-36	91/260(35.00)	137	PHI:1046|CTB5|ABK64182|29003|Cercospora_nicotianae|Reduced_virulence
Chr06G1219.1	441	1.00E-11	73/305(23.93)	63.2	PHI:441|BTP1|CAE55153|40559|Botrytis_cinerea|Reduced_virulence
Chr06G1220.1	4194	1.00E-25	120/476(25.21)	107	AKT7|PHI:4194|V5XZS6|5599|Alternaria_alternata|increased_virulence_(Hypervirulence)
Chr06G1222.1	886	1.00E-06	46/183(25.14)	47	PHI:886|MGG_13052|EDK06087|318829|Magnaporthe_oryzae|Reduced_virulence
Chr06G1223.1	876	0	459/1162(39.50)	781	PHI:876|MGG_11671|EDK03349|318829|Magnaporthe_oryzae|Reduced_virulence
Chr06G1234.1	2315	1.00E-45	99/292(33.90)	157	PHI:2315|ChLae1|G4XKY9|5016|Cochliobolus_heterostrophus|Mixed_outcome
Chr06G1236.1	72	2.00E-30	98/332(29.52)	119	PHI:72|SAP2|AAM21050|5476|Candida_albicans|Reduced_virulence
Chr06G1237.1	61	1.00E-63	110/306(35.95)	203	PHI:61|BGL2|AAA21151|5476|Candida_albicans|Reduced_virulence
Chr06G1238.1	3123	9.00E-30	48/54(88.89)	99.4	CoMC69|PHI:3123|H7CE70|5465|Colletotrichum_orbiculare|effector_(plant_avirulence_determinant)_
Chr06G1240.1	2020	3.00E-49	109/311(35.05)	186	PHI:2020|Tup1|XP_759427|5270|Ustilago_maydis|Mixed_outcome
Chr06G1254.1	2558	6.00E-160	242/474(51.05)	470	PHI:2558|DUR31|Q59P00|5476|Candida_albicans|Reduced_virulence
Chr06G1259.1	2315	3.00E-56	109/290(37.59)	185	PHI:2315|ChLae1|G4XKY9|5016|Cochliobolus_heterostrophus|Mixed_outcome
Chr06G1261.1	881	1.00E-17	62/201(30.85)	80.5	PHI:881|MGG_04556|EDJ96020|318829|Magnaporthe_oryzae|Reduced_virulence
Chr06G1265.1	799	6.00E-45	103/332(31.02)	173	PHI:799|MGG_03530|EDJ94565|318829|Magnaporthe_oryzae|Reduced_virule

					nce
Chr06G1271.1	2197	2.00E-29	109/461(23.64)	117	PHI:2197|MoRgs6|XP_364773|148305|Magnaporthe_oryzae_(related:_Magnaporthe_grisea)|Unaffected_pathogenicity
Chr06G1273.1	2393	6.00E-43	133/481(27.65)	157	PHI:2393|Related_to_O-methylsterigmatocystin_oxidoreductase|I1R980|5518|Gibberella_zeae_(related:_Fusarium_graminearum)|Increased_virulence_(Hypervirulence)
Chr06G1275.1	26	5.00E-17	52/233(22.32)	82	PHI:26|CaMDR1|CAA37820|5476|Candida_albicans|Reduced_virulence
Chr06G1276.1	2644	1.00E-06	28/95(29.47)	43.1	PHI:2644|thioredoxin_1|P0AA28|90371|Salmonella_enterica_serovar_Typhimurium|Reduced_virulence
Chr06G1279.1	2130	5.00E-07	54/204(26.47)	47.8	PHI:2130|MoHox4|MGG_06285|148305|Magnaporthe_oryzae_(related:_Magnaporthe_grisea)|reduced_virulence
Chr06G1287.1	1455	5.00E-13	76/308(24.68)	66.2	PHI:1455|GzAra003|I1RKU6|5518|Gibberella_zeae_(related:_Fusarium_graminearum)|Unaffected_pathogenicity
Chr06G1299.1	2968	1.00E-42	127/474(26.79)	157	PHI:2968|Hxs1|J9VQA5|5207|Cryptococcus_neoformans|Reduced_virulence
Chr06G1304.1	4194	9.00E-48	133/466(28.54)	171	AKT7|PHI:4194|V5XZS6|5599|Alternaria_alternata|increased_virulence_(Hypervirulence)
Chr06G1307.1	1260	3.00E-12	73/216(33.80)	67.8	PHI:1260|FGSG_13944|I1RUC7|5518|Gibberella_zeae_(related:_Fusarium_graminearum)|Unaffected_pathogenicity
Chr06G1315.1	2269	2.00E-21	81/289(28.03)	90.1	PHI:2269|Mdh1|0|13684|Phaeosphaeria_nodorum_(related:_Stagonospora_nodorum)|Unaffected_pathogenicity
Chr06G1320.1	1306	1.00E-57	150/331(45.32)	193	PHI:1306|GzbHLH007|I1RGF4|5518|Gibberella_zeae_(related:_Fusarium_graminearum)|Unaffected_pathogenicity
Chr06G1323.1	901	7.00E-41	130/482(26.97)	154	PHI:901|um01886||5270|Ustilago_maydis|Unaffected_pathogenicity
Chr06G1326.1	1662	6.00E-38	111/447(24.83)	147	PHI:1662|GzCCHC002|I1REJ1|5518|Gibberella_zeae_(related:_Fusarium_graminearum)|Unaffected_pathogenicity
Chr06G1336.1	1279	3.00E-53	142/434(32.72)	193	PHI:1279|FGSG_12132|I1RU59|5518|Gibberella_zeae_(related:_Fusarium_gra

					minearum)|Unaffected_pathogenicity
Chr06G1337.1	1279	7.00E-35	134/507(26.43)	141	PHI:1279|FGSG_12132|I1RU59|5518|Gibberella_zeae_(related:_Fusarium_graminearum)|Unaffected_pathogenicity
Chr06G1338.1	2290	4.00E-16	39/90(43.33)	73.9	PHI:2290|BcBOA6|B1GVX7|40559|Botrytis_cinerea|Reduced_virulence
Chr06G1341.1	2179	0	345/801(43.07)	640	PHI:2179|Moplaa|F8SM03|148305|Magnaporthe_oryzae_(related:_Magnaporthe_grisea)|Reduced_virulence
Chr06G1342.1	2601	2.00E-10	42/131(32.06)	58.5	PHI:2601|Asc1|P83774|746128|Aspergillus_fumigatus|Mixed_outcome
Chr06G1343.1	3415	3.00E-39	103/370(27.84)	150	Fre2|PHI:3415|T2BNJ5|5207|Cryptococcus_neoformans|mixed_outcome_
Chr06G1344.1	3238	9.00E-44	78/178(43.82)	146	ctrC|PHI:3238|B0XUP5|746128|Aspergillus_fumigatus|mixed_outcome_
Chr06G1358.1	1527	5.00E-40	104/341(30.50)	147	PHI:1527|GzHOMEL040|I1S9A1|5518|Gibberella_zeae_(related:_Fusarium_graminearum)|Lethal
Chr06G1360.1	1263	2.00E-37	88/239(36.82)	135	PHI:1263|FGSG_04770|I1RB84|5518|Gibberella_zeae_(related:_Fusarium_graminearum)|Unaffected_pathogenicity
Chr06G1362.1	2037	7.00E-34	67/155(43.23)	123	PHI:2037|MoRic8|XP_001405357|148305|Magnaporthe_oryzae_(related:_Magnaporthe_grisea)|Loss_of_pathogenicity
Chr06G1364.1	816	2.00E-11	55/188(29.26)	64.3	PHI:816|MGG_04582|EDJ95999|318829|Magnaporthe_oryzae|Reduced_virulence
Chr06G1368.1	1939	6.00E-49	99/278(35.61)	167	PHI:1939|GzZC254|I1RMX8|5518|Gibberella_zeae_(related:_Fusarium_graminearum)|Unaffected_pathogenicity
Chr06G1369.1	1330	1.00E-13	29/59(49.15)	65.1	PHI:1330|GzbZIP012|I1RUH0|5518|Gibberella_zeae_(related:_Fusarium_graminearum)|Unaffected_pathogenicity
Chr06G1370.1	1904	4.00E-124	214/613(34.91)	382	PHI:1904|GzZC219|I1RHR0|5518|Gibberella_zeae_(related:_Fusarium_graminearum)|Unaffected_pathogenicity
Chr06G1372.1	1974	3.00E-22	102/401(25.44)	97.1	PHI:1974|GzZC289|I1RQN6|5518|Gibberella_zeae_(related:_Fusarium_graminearum)|Unaffected_pathogenicity
Chr06G1373.1	1047	6.00E-29	104/352(29.55)	112	PHI:1047|CTB6|ABK64183|29003|Cercospora_nicotianae|Reduced_virulence

Chr06G1379.1	1566	2.00E-06	59/219(26.94)	48.1	PHI:1566|GzOB006|I1RC95|5518|Gibberella_zeae_(related:_Fusarium_graminearum)|Lethal
Chr06G1385.1	143	6.00E-36	95/343(27.70)	133	PHI:143|CHT|AAA33353|5563|Gloeocercospora_sorghi|Unaffected_pathogenicity
Chr06G1392.1	552	2.00E-176	272/571(47.64)	513	PHI:552|BcLCC2|AAK77953|40559|Botrytis_cinerea|Unaffected_pathogenicity
Chr06G1394.1	1555	4.00E-43	115/456(25.22)	159	PHI:1555|GzMyb019|I1RDG6|5518|Gibberella_zeae_(related:_Fusarium_graminearum)|Unaffected_pathogenicity
Chr06G1397.1	438	2.00E-20	118/501(23.55)	92	PHI:438|BcBOT1_(related:_CND5)|AAQ16576|40559|Botrytis_cinerea|Reduced_virulence
Chr06G1398.1	2394	2.00E-37	88/280(31.43)	136	PHI:2394|Conserved_hypothetical_protein|I1S104|5518|Gibberella_zeae_(related:_Fusarium_graminearum)|Increased_virulence_(Hypervirulence)
Chr06G1399.1	1662	6.00E-71	138/447(30.87)	246	PHI:1662|GzCCHC002|I1REJ1|5518|Gibberella_zeae_(related:_Fusarium_graminearum)|Unaffected_pathogenicity
Chr06G1400.1	2240	2.00E-40	128/492(26.02)	151	PHI:2240|Srt1|Q4PBY9|5270|Ustilago_maydis|reduced_virulence
Chr06G1402.1	1803	5.00E-29	136/558(24.37)	120	PHI:1803|GzZC118|I1RUX9|5518|Gibberella_zeae_(related:_Fusarium_graminearum)|Unaffected_pathogenicity
Chr06G1403.1	1942	7.00E-09	103/460(22.39)	56.6	PHI:1942|GzZC257|I1RG47|5518|Gibberella_zeae_(related:_Fusarium_graminearum)|Unaffected_pathogenicity
Chr06G1404.1	812	5.00E-45	110/335(32.84)	160	PHI:812|MGG_10702|EDJ94108|318829|Magnaporthe_oryzae|Reduced_virulence
Chr06G1406.1	2033	1.00E-18	62/166(37.35)	87	PHI:2033|MgPex6|G4NBI6|148305|Magnaporthe_oryzae_(related:_Magnaporthe_grisea)|Loss_of_pathogenicity
Chr06G1412.1	2315	3.00E-53	102/292(34.93)	179	PHI:2315|ChLae1|G4XKY9|5016|Cochliobolus_heterostrophus|Mixed_outcome
Chr06G1416.1	1662	2.00E-19	52/221(23.53)	89.4	PHI:1662|GzCCHC002|I1REJ1|5518|Gibberella_zeae_(related:_Fusarium_graminearum)|Unaffected_pathogenicity
Chr06G1417.1	2983	5.00E-10	73/338(21.60)	59.7	PHI:2983|MGG_06279.6|G4N8E3|148305|Magnaporthe_oryzae|Reduced_virul

					ence
Chr06G1427.1	812	9.00E-73	130/321(40.50)	234	PHI:812|MGG_10702|EDJ94108|318829|Magnaporthe_oryzae|Reduced_virulence
Chr06G1428.1	2117	3.00E-59	144/425(33.88)	201	PHI:2117|SPM1|P58371|148305|Magnaporthe_oryzae_(related:_Magnaporthe_grisea)|Reduced_virulence
Chr06G1431.1	812	5.00E-52	106/351(30.20)	179	PHI:812|MGG_10702|EDJ94108|318829|Magnaporthe_oryzae|Reduced_virulence
Chr06G1432.1	3225	0	233/409(56.97)	515	Ndo1|PHI:3225|T2C913|36651|Penicillium_digitatum|unaffected_pathogenicity_
Chr06G1434.1	1887	0	463/729(63.51)	900	PHI:1887|GzZC202|I1RHZ6|5518|Gibberella_zeae_(related:_Fusarium_graminearum)|Unaffected_pathogenicity
Chr06G1437.1	2095	0	729/1088(67.00)	1453	PHI:2095|Calcium-transporting_ATPase_3|MGG_10730|148305|Magnaporthe_oryzae_(related:_Magnaporthe_grisea)|Reduced_virulence
Chr06G1439.1	881	7.00E-17	58/211(27.49)	77.8	PHI:881|MGG_04556|EDJ96020|318829|Magnaporthe_oryzae|Reduced_virulence
Chr06G1440.1	2978	1.00E-12	61/200(30.50)	63.5	PHI:2978|MoCel12A|G4N5V2|148305|Magnaporthe_oryzae|Unaffected_pathogenicity
Chr06G1443.1	404	4.00E-06	75/356(21.07)	45.8	PHI:404|PTH11|AAD30436|318829|Magnaporthe_oryzae|Reduced_virulence
Chr06G1444.1	438	6.00E-48	131/463(28.29)	172	PHI:438|BcBOT1_(related:_CND5)|AAQ16576|40559|Botrytis_cinerea|Reduced_virulence
Chr06G1447.1	410	5.00E-69	106/197(53.81)	213	PHI:410|SOD2|AAW56834|5207|Cryptococcus_neoformans|Loss_of_pathogenicity
Chr06G1454.1	404	5.00E-33	128/536(23.88)	130	PHI:404|PTH11|AAD30436|318829|Magnaporthe_oryzae|Reduced_virulence
Chr06G1455.1	922	2.00E-14	146/624(23.40)	73.9	PHI:922|um03615||5270|Ustilago_maydis|Unaffected_pathogenicity
Chr06G1456.1	496	0	555/1093(50.78)	1126	PHI:496|PPOC|EAL92371|5085|Aspergillus_fumigatus|Increased_virulence_(Hypervirulence)
Chr06G1462.1	2822	9.00E-52	148/574(25.78)	188	PHI:2822|Cxt1p|Q5K8R6|5207|Cryptococcus_neoformans|Reduced_virulence

Chr06G1468.1	1456	8.00E-94	184/490(37.55)	303	PHI:1456|GzAra004|I1RN15|5518|Gibberella_zeae_(related:_Fusarium_graminearum)|Unaffected_pathogenicity
Chr06G1469.1	4194	3.00E-20	87/376(23.14)	89.7	AKT7|PHI:4194|V5XZS6|5599|Alternaria_alternata|increased_virulence_(Hypervirulence)
Chr06G1470.1	1049	5.00E-31	111/409(27.14)	125	PHI:1049|CTB2|ABK64180|29003|Cercospora_nicotianae|Reduced_virulence
Chr06G1473.1	255	0	1002/2573(38.94)	1656	PHI:255|FUM1_(related:_FUM5)|AAD43562|5127|Gibberella_moniliformis|Unaffected_pathogenicity
Chr06G1476.1	1453	3.00E-09	28/68(41.18)	51.2	PHI:1453|GzAra001|I1RA50|5518|Gibberella_zeae_(related:_Fusarium_graminearum)|Lethal
Chr06G1482.1	1662	7.00E-59	116/346(33.53)	206	PHI:1662|GzCCHC002|I1REJ1|5518|Gibberella_zeae_(related:_Fusarium_graminearum)|Unaffected_pathogenicity
Chr06G1483.1	876	0	454/1156(39.27)	764	PHI:876|MGG_11671|EDK03349|318829|Magnaporthe_oryzae|Reduced_virulence
Chr06G1484.1	886	1.00E-06	46/183(25.14)	47	PHI:886|MGG_13052|EDK06087|318829|Magnaporthe_oryzae|Reduced_virulence
Chr06G1488.1	1260	5.00E-12	50/154(32.47)	67	PHI:1260|FGSG_13944|I1RUC7|5518|Gibberella_zeae_(related:_Fusarium_graminearum)|Unaffected_pathogenicity
Chr06G1490.1	320	3.00E-07	72/269(26.77)	52.4	PHI:320|SSN6|AAL54912|5476|Candida_albicans|Reduced_virulence
Chr06G1491.1	1458	6.00E-07	39/126(30.95)	51.2	PHI:1458|GzAra006|Q4I7F9|5518|Gibberella_zeae_(related:_Fusarium_graminearum)|Unaffected_pathogenicity
Chr04G0003.1	1260	1.00E-27	84/217(38.71)	118	PHI:1260|FGSG_13944|I1RUC7|5518|Gibberella_zeae_(related:_Fusarium_graminearum)|Unaffected_pathogenicity
Chr04G0004.1	1527	9.00E-41	109/391(27.88)	150	PHI:1527|GzHOMEL040|I1S9A1|5518|Gibberella_zeae_(related:_Fusarium_graminearum)|Lethal
Chr04G0005.1	1458	7.00E-07	31/91(34.07)	50.8	PHI:1458|GzAra006|Q4I7F9|5518|Gibberella_zeae_(related:_Fusarium_graminearum)|Unaffected_pathogenicity

Chr04G0008.1	871	0	516/991(52.07)	1033	PHI:871|MGG_12656|EDK01997|318829|Magnaporthe_oryzae|Reduced_virulence
Chr04G0009.1	1620	5.00E-13	47/144(32.64)	70.9	PHI:1620|GzJUM002|I1RD69|5518|Gibberella_zeae_(related:_Fusarium_graminearum)|Unaffected_pathogenicity
Chr04G0015.1	1236	1.00E-15	66/215(30.70)	79	PHI:1236|FGSG_04770|I1RYF8|5518|Gibberella_zeae_(related:_Fusarium_graminearum)|Lethal
Chr04G0017.1	2020	4.00E-47	104/311(33.44)	179	PHI:2020|Tup1|XP_759427|5270|Ustilago_maydis|Mixed_outcome
Chr04G0018.1	1260	7.00E-33	92/232(39.66)	134	PHI:1260|FGSG_13944|I1RUC7|5518|Gibberella_zeae_(related:_Fusarium_graminearum)|Unaffected_pathogenicity
Chr04G0027.1	2379	0	1199/2412(49.71)	2211	PHI:2379|DEP5|D2E9X0|29001|Alternaria_brassicicola|Mixed_outcome
Chr04G0028.1	2375	2.00E-27	108/362(29.83)	108	PHI:2375|DEP1|D2E9W6|29001|Alternaria_brassicicola|Mixed_outcome
Chr04G0029.1	2378	4.00E-179	254/556(45.68)	519	PHI:2378|DEP4|D2E9W9|29001|Alternaria_brassicicola|Mixed_outcome
Chr04G0033.1	2377	2.00E-112	196/551(35.57)	347	PHI:2377|DEP3|D2E9W8|29001|Alternaria_brassicicola|Mixed_outcome
Chr04G0034.1	881	1.00E-131	184/339(54.28)	383	PHI:881|MGG_04556|EDJ96020|318829|Magnaporthe_oryzae|Reduced_virulence
Chr04G0035.1	922	2.00E-75	208/617(33.71)	255	PHI:922|um03615||5270|Ustilago_maydis|Unaffected_pathogenicity
Chr04G0036.1	2380	5.00E-35	153/595(25.71)	139	PHI:2380|DEP6|D2E9X1|29001|Alternaria_brassicicola|Mixed_outcome
Chr04G0038.1	800	2.00E-14	66/229(28.82)	74.3	PHI:800|MGG_13324|EDK00897|318829|Magnaporthe_oryzae|Reduced_virulence
Chr04G0042.1	404	1.00E-17	68/283(24.03)	84.3	PHI:404|PTH11|AAD30436|318829|Magnaporthe_oryzae|Reduced_virulence
Chr04G0047.1	2419	2.00E-07	50/228(21.93)	49.3	PHI:2419|CID1|I1RKF3|5518|Gibberella_zeae_(related:_Fusarium_graminearum)|Reduced_virulence
Chr04G0049.1	2837	2.00E-21	62/198(31.31)	88.2	PHI:2837|OXI1|D2SZX7|5016|Cochliobolus_heterostrophus|Reduced_virulence
Chr04G0050.1	2710	0	376/406(92.61)	763	PHI:2710|mepB|H8ZZ79|474922|Colletotrichum_gloeosporioides|Reduced_virulence
Chr04G0051.1	4194	3.00E-47	129/474(27.22)	170	AKT7|PHI:4194|V5XZS6|5599|Alternaria_alternata|increased_virulence_(Hyper

					virulence)
Chr04G0056.1	1057	0	300/478(62.76)	620	PHI:1057|MTP1|ABP98949|318829|Magnaporthe_oryzae|Unaffected_pathogenicity
Chr04G0058.1	1555	5.00E-39	133/522(25.48)	147	PHI:1555|GzMyb019|I1RDG6|5518|Gibberella_zeae_(related:_Fusarium_graminearum)|Unaffected_pathogenicity
Chr04G0060.1	2808	0	315/524(60.11)	636	PHI:2808|gas1|Q2KN79|59765|Fusarium_oxysporum_f._sp._Lycopersici|Reduced_virulence
Chr04G0062.1	2654	5.00E-19	59/169(34.91)	89.7	PHI:2654|DUR1,2|Q59VF3|5476|Candida_albicans|Reduced_virulence
Chr04G0071.1	2207	1.00E-10	30/71(42.25)	60.8	PHI:2207|endo-1,4-beta-xylanase_[GH10_family]|MGG_02245|148305|Magnaporthe_oryzae_(related:_Magnaporthe_grisea)|Reduced_virulence
Chr04G0074.1	552	6.00E-107	230/582(39.52)	338	PHI:552|BcLCC2|AAK77953|40559|Botrytis_cinerea|Unaffected_pathogenicity
Chr04G0079.1	244	3.00E-58	158/497(31.79)	217	PHI:244|CLAP1|AAN62846|290576|Colletotrichum_lindemuthianum|Loss_of_pathogenicity
Chr04G0080.1	2747	3.00E-24	92/382(24.08)	102	PHI:2747|Cnt|A8YZD7|1280|Staphylococcus_aureus|Reduced_virulence
Chr04G0082.1	2654	1.00E-18	110/438(25.11)	87.4	PHI:2654|DUR1,2|Q59VF3|5476|Candida_albicans|Reduced_virulence
Chr04G0086.1	410	2.00E-62	98/196(50.00)	195	PHI:410|SOD2|AAW56834|5207|Cryptococcus_neoformans|Loss_of_pathogenicity
Chr04G0090.1	479	3.00E-11	73/247(29.55)	62	PHI:479|MEP1|AAQ07436|199306|Coccidioides_posadasii|Reduced_virulence
Chr04G0091.1	3214	2.00E-06	25/77(32.47)	43.9	MoCDIP2|PHI:3214|G4MML4|318829|Magnaporthe_oryzae|mixed_outcome_
Chr04G0093.1	1904	6.00E-35	148/581(25.47)	136	PHI:1904|GzZC219|I1RHR0|5518|Gibberella_zeae_(related:_Fusarium_graminearum)|Unaffected_pathogenicity
Chr04G0094.1	1662	2.00E-38	90/323(27.86)	149	PHI:1662|GzCCHC002|I1REJ1|5518|Gibberella_zeae_(related:_Fusarium_graminearum)|Unaffected_pathogenicity
Chr04G0098.1	2315	9.00E-58	103/290(35.52)	190	PHI:2315|ChLae1|G4XKY9|5016|Cochliobolus_heterostrophus|Mixed_outcome
Chr04G0101.1	2968	4.00E-66	148/539(27.46)	224	PHI:2968|Hxs1|J9VQA5|5207|Cryptococcus_neoformans|Reduced_virulence
Chr04G0105.1	1904	5.00E-10	60/253(23.72)	58.5	PHI:1904|GzZC219|I1RHR0|5518|Gibberella_zeae_(related:_Fusarium_gramin

					earum)|Unaffected_pathogenicity
Chr04G0106.1	199	2.00E-137	241/648(37.19)	417	PHI:199|AOX1|AAF82788|5499|Cladosporium_fulvum|Reduced_virulence
Chr04G0107.1	1981	0	294/663(44.34)	547	PHI:1981|GzZC296|I1RIY9|5518|Gibberella_zeae_(related:_Fusarium_graminearum)|Unaffected_pathogenicity
Chr04G0109.1	1651	8.00E-20	88/355(24.79)	88.2	PHI:1651|GzWing023|I1S0U2|5518|Gibberella_zeae_(related:_Fusarium_graminearum)|Unaffected_pathogenicity
Chr04G0111.1	404	3.00E-28	68/275(24.73)	114	PHI:404|PTH11|AAD30436|318829|Magnaporthe_oryzae|Reduced_virulence
Chr04G0112.1	1681	0	1152/2285(50.42)	2189	PHI:1681|GzNF001|I1REN7|5518|Gibberella_zeae_(related:_Fusarium_graminearum)|Unaffected_pathogenicity
Chr04G0114.1	1554	2.00E-14	39/126(30.95)	72.4	PHI:1554|GzMyb018|I1RC64|5518|Gibberella_zeae_(related:_Fusarium_graminearum)|Unaffected_pathogenicity
Chr04G0115.1	890	2.00E-09	56/236(23.73)	57.8	PHI:890|MGG_07015|EDJ98043|318829|Magnaporthe_oryzae|Reduced_virulence
Chr04G0118.1	1423	3.00E-15	41/112(36.61)	73.6	PHI:1423|GzC2H091|I1S2R1|5518|Gibberella_zeae_(related:_Fusarium_graminearum)|Unaffected_pathogenicity
Chr04G0125.1	285	7.00E-23	90/344(26.16)	101	PHI:285|CHSV|AAO49384|5507|Fusarium_oxysporum|Increased_virulence_(Hypervirulence)
Chr04G0128.1	404	8.00E-37	86/291(29.55)	139	PHI:404|PTH11|AAD30436|318829|Magnaporthe_oryzae|Reduced_virulence
Chr04G0129.1	2393	5.00E-113	184/473(38.90)	345	PHI:2393|Related_to_O-methylsterigmatocystin_oxidoreductase|I1R980|5518|Gibberella_zeae_(related:_Fusarium_graminearum)|Increased_virulence_(Hypervirulence)
Chr04G0131.1	143	0	279/348(80.17)	589	PHI:143|CHT|AAA33353|5563|Gloeocercospora_sorghi|Unaffected_pathogenicity
Chr04G0132.1	1046	3.00E-07	35/119(29.41)	49.7	PHI:1046|CTB5|ABK64182|29003|Cercospora_nicotianae|Reduced_virulence
Chr04G0134.1	2968	2.00E-46	132/496(26.61)	168	PHI:2968|Hxs1|J9VQA5|5207|Cryptococcus_neoformans|Reduced_virulence
Chr04G0135.1	1750	9.00E-91	195/534(36.52)	308	PHI:1750|GzZC065|I1S487|5518|Gibberella_zeae_(related:_Fusarium_gramine

					arum)|Unaffected_pathogenicity
Chr04G0136.1	191	5.00E-29	189/729(25.93)	121	PHI:191|TOM1|AAB08446|39703|Septoria_lycopersici|Unaffected_pathogenicity
Chr04G0137.1	2476	0	275/326(84.36)	535	PHI:2476|CcpelA|G8AA67|27358|Colletotrichum_coccodes|Mixed_outcome
Chr04G0139.1	1662	1.00E-31	126/525(24.00)	127	PHI:1662|GzCCHC002|I1REJ1|5518|Gibberella_zeae_(related:_Fusarium_graminearum)|Unaffected_pathogenicity
Chr04G0142.1	510	9.00E-10	87/405(21.48)	58.5	PHI:510|CaNAG3|EAK93097|5476|Candida_albicans|Reduced_virulence
Chr04G0149.1	404	3.00E-13	61/225(27.11)	68.2	PHI:404|PTH11|AAD30436|318829|Magnaporthe_oryzae|Reduced_virulence
Chr04G0164.1	2240	4.00E-22	113/436(25.92)	97.4	PHI:2240|Srt1|Q4PBY9|5270|Ustilago_maydis|reduced_virulence
Chr04G0165.1	2968	3.00E-20	95/351(27.07)	91.3	PHI:2968|Hxs1|J9VQA5|5207|Cryptococcus_neoformans|Reduced_virulence
Chr04G0166.1	2968	9.00E-49	144/513(28.07)	181	PHI:2968|Hxs1|J9VQA5|5207|Cryptococcus_neoformans|Reduced_virulence
Chr04G0167.1	413	2.00E-20	98/410(23.90)	90.9	PHI:413|MPD1|AAT84078|13684|Stagonospora_nodorum|Unaffected_pathogenicity
Chr04G0168.1	225	3.00E-52	97/242(40.08)	179	PHI:225|PEP5|AAK16922|140110|Nectria_haematococca_(related:_Fusarium_solani)|Reduced_virulence
Chr04G0169.1	404	2.00E-22	74/280(26.43)	96.3	PHI:404|PTH11|AAD30436|318829|Magnaporthe_oryzae|Reduced_virulence
Chr04G0173.1	2976	3.00E-117	224/660(33.94)	374	PHI:2976|CgOPT1|C6ZRH8|29905|Colletotrichum_gloeosporioides_f._sp._aeschynomenes|Reduced_virulence
Chr04G0190.1	1856	1.00E-35	118/480(24.58)	135	PHI:1856|GzZC171|I1S377|5518|Gibberella_zeae_(related:_Fusarium_graminearum)|Unaffected_pathogenicity
Chr04G0192.1	1566	9.00E-99	147/323(45.51)	303	PHI:1566|GzOB006|I1RC95|5518|Gibberella_zeae_(related:_Fusarium_graminearum)|Lethal
Chr04G0195.1	3138	0	517/573(90.23)	1072	ELP3|PHI:3138|I1REF2|5518|Fusarium_graminearum|reduced_virulence_
Chr04G0201.1	404	2.00E-27	95/397(23.93)	112	PHI:404|PTH11|AAD30436|318829|Magnaporthe_oryzae|Reduced_virulence
Chr04G0202.1	823	0	324/447(72.48)	669	PHI:823|beta-tubulin|CAA56936|38038|Rhynchosporium_secalis|Chemistry_target

Chr04G0203.1	404	8.00E-14	63/265(23.77)	70.1	PHI:404|PTH11|AAD30436|318829|Magnaporthe_oryzae|Reduced_virulence
Chr04G0204.1	922	2.00E-92	221/629(35.14)	300	PHI:922|um03615||5270|Ustilago_maydis|Unaffected_pathogenicity
Chr04G0205.1	1861	1.00E-06	28/90(31.11)	48.1	PHI:1861|GzZC176|I1RUV3|5518|Gibberella_zeae_(related:_Fusarium_graminearum)|Unaffected_pathogenicity
Chr04G0206.1	1809	2.00E-70	199/765(26.01)	244	PHI:1809|GzZC124|I1REW1|5518|Gibberella_zeae_(related:_Fusarium_graminearum)|Lethal
Chr04G0207.1	199	3.00E-12	99/376(26.33)	67.4	PHI:199|AOX1|AAF82788|5499|Cladosporium_fulvum|Reduced_virulence
Chr04G0209.1	2511	0	694/2255(30.78)	1073	PHI:2511|Pes1|Q4WT66|746128|Aspergillus_fumigatus|Reduced_virulence
Chr04G0212.1	1942	3.00E-08	21/45(46.67)	55.1	PHI:1942|GzZC257|I1RG47|5518|Gibberella_zeae_(related:_Fusarium_graminearum)|Unaffected_pathogenicity
Chr04G0213.1	255	4.00E-20	52/162(32.10)	90.5	PHI:255|FUM1_(related:_FUM5)|AAD43562|5127|Gibberella_moniliformis|Unaffected_pathogenicity
Chr04G0214.1	3387	3.00E-06	58/192(30.21)	46.6	FVEG_12523|PHI:3387|W7MT31|117187|Fusarium_verticillioides|unaffected_pathogenicity_
Chr04G0215.1	2393	2.00E-09	55/236(23.31)	56.6	PHI:2393|Related_to_O-methylsterigmatocystin_oxidoreductase|I1R980|5518|Gibberella_zeae_(related:_Fusarium_graminearum)|Increased_virulence_(Hypervirulence)
Chr04G0216.1	255	0	504/1327(37.98)	868	PHI:255|FUM1_(related:_FUM5)|AAD43562|5127|Gibberella_moniliformis|Unaffected_pathogenicity
Chr04G0217.1	2393	5.00E-11	57/220(25.91)	62	PHI:2393|Related_to_O-methylsterigmatocystin_oxidoreductase|I1R980|5518|Gibberella_zeae_(related:_Fusarium_graminearum)|Increased_virulence_(Hypervirulence)
Chr04G0218.1	26	4.00E-79	152/471(32.27)	258	PHI:26|CaMDR1|CAA37820|5476|Candida_albicans|Reduced_virulence
Chr04G0222.1	2260	5.00E-12	80/293(27.30)	63.2	PHI:2260|PKS1|G8DNT0|45130|Cochliobolus_sativus|Unaffected_pathogenicity
Chr04G0225.1	255	0	1026/2609(39.33)	1742	PHI:255|FUM1_(related:_FUM5)|AAD43562|5127|Gibberella_moniliformis|Unaff

					ected_pathogenicity
Chr04G0230.1	716	1.00E-104	206/600(34.33)	330	PHI:716|ZEB1|ABB90284|5518|Fusarium_graminearum|Unaffected_pathogenicity
Chr04G0231.1	716	2.00E-14	111/471(23.57)	72.8	PHI:716|ZEB1|ABB90284|5518|Fusarium_graminearum|Unaffected_pathogenicity
Chr04G0242.1	2251	2.00E-09	41/131(31.30)	53.5	PHI:2251|Gox1|Q696X2|13684|Phaeosphaeria_nodorum_(related:_Stagonospora_nodorum)|Unaffected_pathogenicity
Chr04G0245.1	2968	3.00E-25	126/494(25.51)	106	PHI:2968|Hxs1|J9VQA5|5207|Cryptococcus_neoformans|Reduced_virulence
Chr04G0248.1	199	4.00E-16	116/476(24.37)	79.3	PHI:199|AOX1|AAF82788|5499|Cladosporium_fulvum|Reduced_virulence
Chr04G0252.1	2968	6.00E-29	114/391(29.16)	118	PHI:2968|Hxs1|J9VQA5|5207|Cryptococcus_neoformans|Reduced_virulence
Chr04G0256.1	714	6.00E-08	42/138(30.43)	50.8	PHI:714|PKS4_(related:_ZEA1)|ABB90283|5518|Fusarium_graminearum|Unaffected_pathogenicity
Chr04G0257.1	1552	2.00E-75	136/323(42.11)	273	PHI:1552|GzMyb016|I1S0N5|5518|Gibberella_zeae_(related:_Fusarium_graminearum)|Unaffected_pathogenicity
Chr04G0258.1	2042	2.00E-07	87/331(26.28)	51.6	PHI:2042|ABC3|Q3Y5V5|148305|Magnaporthe_oryzae_(related:_Magnaporthe_grisea)|Loss_of_pathogenicity
Chr04G0262.1	59	1.00E-06	33/122(27.05)	46.2	PHI:59|THR1|BAA18962|5462|Colletotrichum_lagenarium|Reduced_virulence
Chr04G0263.1	3	2.00E-156	216/357(60.50)	446	PHI:3|PGN1|AAA79885|5017|Cochliobolus_carbonum|Unaffected_pathogenicity
Chr04G0273.1	2482	4.00E-19	43/118(36.44)	91.3	PHI:2482|AlHK1|Q09JB7|160389|Alternaria_longipes|Increased_virulence_(Hypervirulence)
Chr04G0277.1	2022	4.00E-22	74/263(28.14)	91.7	PHI:2022|BUF1|MGG_02252|148305|Magnaporthe_oryzae_(related:_Magnaporthe_grisea)|Loss_of_pathogenicity
Chr04G0280.1	1681	4.00E-118	246/681(36.12)	401	PHI:1681|GzNF001|I1REN7|5518|Gibberella_zeae_(related:_Fusarium_graminearum)|Unaffected_pathogenicity
Chr04G0282.1	1888	8.00E-09	21/39(53.85)	55.1	PHI:1888|GzZC203|I1RHY2|5518|Gibberella_zeae_(related:_Fusarium_gramin

					earum)|Unaffected_pathogenicity
Chr04G0283.1	2190	2.00E-10	62/234(26.50)	60.8	PHI:2190|MoCYP51A|G4MRP8|148305|Magnaporthe_oryzae_(related:_Magnaporthe_grisea)|Reduced_virulence
Chr04G0285.1	438	1.00E-07	43/166(25.90)	51.2	PHI:438|BcBOT1_(related:_CND5)|AAQ16576|40559|Botrytis_cinerea|Reduced_virulence
Chr04G0289.1	441	8.00E-13	79/338(23.37)	66.6	PHI:441|BTP1|CAE55153|40559|Botrytis_cinerea|Reduced_virulence
Chr04G0293.1	2240	4.00E-24	119/508(23.43)	103	PHI:2240|Srt1|Q4PBY9|5270|Ustilago_maydis|reduced_virulence
Chr04G0294.1	1788	3.00E-118	226/633(35.70)	371	PHI:1788|GzZC103|I1S1N1|5518|Gibberella_zeae_(related:_Fusarium_graminearum)|Unaffected_pathogenicity
Chr04G0295.1	191	5.00E-47	136/431(31.55)	177	PHI:191|TOM1|AAB08446|39703|Septoria_lycopersici|Unaffected_pathogenicity
Chr04G0297.1	404	3.00E-22	63/261(24.14)	95.5	PHI:404|PTH11|AAD30436|318829|Magnaporthe_oryzae|Reduced_virulence
Chr04G0298.1	3662	3.00E-11	68/210(32.38)	64.7	PspB_(not_PD0218)|PHI:3662|Q87ET0|2371|Xylella_fastidiosa|Increased_virulence_(Hypervirulence)
Chr04G0299.1	441	4.00E-16	90/374(24.06)	76.3	PHI:441|BTP1|CAE55153|40559|Botrytis_cinerea|Reduced_virulence
Chr04G0304.1	812	8.00E-21	95/332(28.61)	90.9	PHI:812|MGG_10702|EDJ94108|318829|Magnaporthe_oryzae|Reduced_virulence
Chr04G0307.1	1767	9.00E-21	72/287(25.09)	93.2	PHI:1767|GzZC082|I1S134|5518|Gibberella_zeae_(related:_Fusarium_graminearum)|Unaffected_pathogenicity
Chr04G0309.1	1662	5.00E-55	133/453(29.36)	199	PHI:1662|GzCCHC002|I1REJ1|5518|Gibberella_zeae_(related:_Fusarium_graminearum)|Unaffected_pathogenicity
Chr04G0310.1	441	4.00E-20	61/232(26.29)	88.2	PHI:441|BTP1|CAE55153|40559|Botrytis_cinerea|Reduced_virulence
Chr04G0311.1	1051	2.00E-13	59/199(29.65)	68.9	PHI:1051|CTB3|ABC79591|29003|Cercospora_nicotianae|Reduced_virulence
Chr04G0315.1	3281	4.00E-12	93/356(26.12)	63.5	AS87_04050|PHI:3281|A0A097IB07|34085|Riemerella_anatipestifer|reduced_virulence_
Chr04G0316.1	3069	3.00E-09	39/101(38.61)	58.2	MrAC|PHI:3069|E9F4P0|568076|Metarhizium_robertsii|reduced_virulence_

Chr04G0324.1	2632	3.00E-29	96/442(21.72)	117	PHI:2632|argH|Q2FIB5|1280|Staphylococcus_aureus|Reduced_virulence
Chr04G0327.1	922	1.00E-46	179/617(29.01)	171	PHI:922|um03615||5270|Ustilago_maydis|Unaffected_pathogenicity
Chr04G0328.1	441	2.00E-25	95/383(24.80)	103	PHI:441|BTP1|CAE55153|40559|Botrytis_cinerea|Reduced_virulence
Chr04G0333.1	1662	3.00E-34	125/465(26.88)	135	PHI:1662|GzCCHC002|I1REJ1|5518|Gibberella_zeae_(related:_Fusarium_graminearum)|Unaffected_pathogenicity
Chr04G0335.1	2968	2.00E-35	117/462(25.32)	136	PHI:2968|Hxs1|J9VQA5|5207|Cryptococcus_neoformans|Reduced_virulence
Chr04G0338.1	1046	1.00E-45	133/463(28.73)	165	PHI:1046|CTB5|ABK64182|29003|Cercospora_nicotianae|Reduced_virulence
Chr04G0341.1	115	4.00E-65	138/395(34.94)	215	PHI:115|PGX1|AAC26146|5017|Cochliobolus_carbonum|Unaffected_pathogenicity
Chr04G0346.1	3310	0	385/792(48.61)	665	Mocod1|PHI:3310|G4MKH9|318829|Magnaporthe_oryzae|mixed_outcome_
Chr04G0347.1	811	2.00E-30	83/271(30.63)	115	PHI:811|MGG_10510|EDK06580|318829|Magnaporthe_oryzae|Reduced_virulence
Chr04G0353.1	1741	0	382/769(49.67)	708	PHI:1741|GzZC056|I1S780|5518|Gibberella_zeae_(related:_Fusarium_graminearum)|Unaffected_pathogenicity
Chr04G0355.1	3387	2.00E-14	90/332(27.11)	72	FVEG_12523|PHI:3387|W7MT31|117187|Fusarium_verticillioides|unaffected_pathogenicity_
Chr04G0357.1	513	9.00E-48	144/572(25.17)	174	PHI:513|ARN1_(related:_SIT1)|EAK97011|5476|Candida_albicans|Reduced_virulence
Chr04G0359.1	2608	1.00E-06	39/132(29.55)	47.4	PHI:2608|ACRTS2|F8R4Y0|5599|Alternaria_alternata|Reduced_virulence
Chr04G0360.1	2908	1.00E-15	66/289(22.84)	77	PHI:2908|CYP51B|I1RBR4|5518|Fusarium_graminearum|Mixed_outcome
Chr04G0362.1	465	8.00E-61	132/345(38.26)	223	PHI:465|KIN2|AAB63337|5270|Ustilago_maydis|Reduced_virulence
Chr04G0364.1	2638	0	448/610(73.44)	890	PHI:2638|Ilv3A|Q4X099|746128|Aspergillus_fumigatus|Reduced_virulence
Chr04G0365.1	3005	4.00E-66	97/195(49.74)	205	PHI:3005|ClpP|P63786|1280|Staphylococcus_aureus|Reduced_virulence
Chr04G0369.1	1414	3.00E-06	26/64(40.62)	47	PHI:1414|GzC2H081|I1RZL0|5518|Gibberella_zeae_(related:_Fusarium_graminearum)|Unaffected_pathogenicity
Chr04G0376.1	1424	2.00E-07	24/69(34.78)	50.8	PHI:1424|GzC2H092|I1S3J7|5518|Gibberella_zeae_(related:_Fusarium_gramin

					earum)|Unaffected_pathogenicity
Chr04G0382.1	1662	5.00E-65	136/434(31.34)	229	PHI:1662|GzCCHC002|I1REJ1|5518|Gibberella_zeae_(related:_Fusarium_graminearum)|Unaffected_pathogenicity
Chr04G0385.1	538	8.00E-51	148/526(28.14)	182	PHI:538|FRT1|AAU87358|40559|Botrytis_cinerea|Unaffected_pathogenicity
Chr04G0387.1	438	2.00E-41	125/483(25.88)	154	PHI:438|BcBOT1_(related:_CND5)|AAQ16576|40559|Botrytis_cinerea|Reduced_virulence
Chr04G0388.1	1555	0	255/497(51.31)	558	PHI:1555|GzMyb019|I1RDG6|5518|Gibberella_zeae_(related:_Fusarium_graminearum)|Unaffected_pathogenicity
Chr04G0389.1	2968	5.00E-27	124/490(25.31)	112	PHI:2968|Hxs1|J9VQA5|5207|Cryptococcus_neoformans|Reduced_virulence
Chr04G0390.1	1884	1.00E-18	54/162(33.33)	85.5	PHI:1884|GzZC199|I1RJ58|5518|Gibberella_zeae_(related:_Fusarium_graminearum)|Unaffected_pathogenicity
Chr04G0391.1	72	3.00E-16	99/374(26.47)	77	PHI:72|SAP2|AAM21050|5476|Candida_albicans|Reduced_virulence
Chr04G0392.1	2968	1.00E-15	76/315(24.13)	75.5	PHI:2968|Hxs1|J9VQA5|5207|Cryptococcus_neoformans|Reduced_virulence
Chr04G0393.1	541	7.00E-70	186/553(33.63)	236	PHI:541|LIP1|AAU87359|332648|Botrytis_cinerea|Unaffected_pathogenicity
Chr04G0394.1	2117	2.00E-54	149/430(34.65)	189	PHI:2117|SPM1|P58371|148305|Magnaporthe_oryzae_(related:_Magnaporthe_grisea)|Reduced_virulence
Chr04G0395.1	2032	2.00E-07	36/148(24.32)	49.7	PHI:2032|VTL1|G4NGA7|148305|Magnaporthe_oryzae_(related:_Magnaporthe_grisea)|Unaffected_pathogenicity
Chr04G0399.1	2968	3.00E-40	135/513(26.32)	150	PHI:2968|Hxs1|J9VQA5|5207|Cryptococcus_neoformans|Reduced_virulence
Chr04G0400.1	1902	6.00E-20	175/822(21.29)	93.2	PHI:1902|GzZC217|I1RJS7|5518|Gibberella_zeae_(related:_Fusarium_graminearum)|Unaffected_pathogenicity
Chr04G0401.1	1254	0	397/672(59.08)	701	PHI:1254|FGSG_07742|I1RDX3|5518|Gibberella_zeae_(related:_Fusarium_graminearum)|Unaffected_pathogenicity
Chr04G0405.1	463	0	515/709(72.64)	988	PHI:463|HSP90|AAA02743|4932|Saccharomyces_cerevisiae|Increased_virulence_(Hypervirulence)
Chr04G0409.1	2086	2.00E-14	53/174(30.46)	73.2	PHI:2086|Moatg24|MGG_03638|148305|Magnaporthe_oryzae_(related:_Magn

					aporthe_grisea)|Unaffected_pathogenicity
Chr04G0410.1	211	2.00E-22	77/284(27.11)	99.4	PHI:211|CaTUP1|AAB63195|5476|Candida_albicans|Reduced_virulence
Chr04G0412.1	1662	0	412/549(75.05)	792	PHI:1662|GzCCHC002|I1REJ1|5518|Gibberella_zeae_(related:_Fusarium_graminearum)|Unaffected_pathogenicity
Chr04G0414.1	923	7.00E-37	123/425(28.94)	142	PHI:923|um03616||5270|Ustilago_maydis|Unaffected_pathogenicity
Chr04G0421.1	1869	0	402/557(72.17)	823	PHI:1869|GzZC184|I1RDY4|5518|Gibberella_zeae_(related:_Fusarium_graminearum)|Unaffected_pathogenicity
Chr04G0429.1	2907	2.00E-14	54/197(27.41)	73.2	PHI:2907|Cyp51A|I6YDU0|5518|Fusarium_graminearum|Mixed_outcome
Chr04G0432.1	2844	6.00E-29	88/272(32.35)	110	PHI:2844|BRM2|O93802|5599|Alternaria_alternata|Unaffected_pathogenicity
Chr04G0450.1	1356	0	408/630(64.76)	731	PHI:1356|GzC2H016|I1RE06|5518|Gibberella_zeae_(related:_Fusarium_graminearum)|Unaffected_pathogenicity
Chr04G0452.1	2401	3.00E-06	25/80(31.25)	46.2	PHI:2401|CaRING1|G0T3B3|456327|Xanthomonas_campestris_pv_vesicatoria|Mixed_outcome
Chr04G0454.1	2042	5.00E-56	133/338(39.35)	207	PHI:2042|ABC3|Q3Y5V5|148305|Magnaporthe_oryzae_(related:_Magnaporthe_grisea)|Loss_of_pathogenicity
Chr04G0459.1	2395	2.00E-07	28/119(23.53)	49.7	PHI:2395|GzSYN1|I1RBM2|5518|Gibberella_zeae_(related:_Fusarium_graminearum)|Reduced_virulence
Chr04G0460.1	2640	9.00E-21	52/140(37.14)	82.8	PHI:2640|CNB1|C4XXN5|36911|Candida_lusitaniae|Reduced_virulence
Chr04G0466.1	2544	3.00E-09	29/83(34.94)	55.8	PHI:2544|PAB1|Q4WK03|746128|Aspergillus_fumigatus|Mixed_outcome
Chr04G0469.1	2802	4.00E-25	80/262(30.53)	99	PHI:2802|3hnr|B2ZRQ4|93612|Setosphaeria_turcica|Unaffected_pathogenicity
Chr04G0471.1	2196	6.00E-26	56/142(39.44)	111	PHI:2196|MoRgs5|XP_363151|148305|Magnaporthe_oryzae_(related:_Magnaporthe_grisea)|Unaffected_pathogenicity
Chr04G0472.1	346	7.00E-174	275/512(53.71)	509	PHI:346|CRU1|AAN10186|5270|Ustilago_maydis|Reduced_virulence
Chr04G0474.1	1500	2.00E-59	322/1342(23.99)	221	PHI:1500|GzHMG032|I1S2F2|5518|Gibberella_zeae_(related:_Fusarium_graminearum)|Unaffected_pathogenicity
Chr04G0484.1	1541	3.00E-63	153/324(47.22)	202	PHI:1541|GzFlbD|I1RE42|5518|Gibberella_zeae_(related:_Fusarium_graminea

					rum)|Unaffected_pathogenicity
Chr04G0487.1	1520	2.00E-17	49/205(23.90)	77.8	PHI:1520|GzHOMEL024|I1RWS3|5518|Gibberella_zeae_(related:_Fusarium_graminearum)|Unaffected_pathogenicity
Chr04G0491.1	256	2.00E-116	166/252(65.87)	335	PHI:256|GAS1|AAK52794|318829|Magnaporthe_oryzae|Reduced_virulence
Chr04G0492.1	280	4.00E-08	57/237(24.05)	53.9	PHI:280|CCN1|AAG36938|5207|Cryptococcus_neoformans|Reduced_virulence
Chr04G0502.1	1577	4.00E-76	110/146(75.34)	224	PHI:1577|GzOB017|I1RK79|5518|Gibberella_zeae_(related:_Fusarium_graminearum)|Unaffected_pathogenicity
Chr04G0505.1	3459	3.00E-24	103/414(24.88)	103	NTPDase1|PHI:3459|Q4QFI1|5664|Leishmania_major|mixed_outcome_
Chr04G0507.1	1699	7.00E-174	288/579(49.74)	516	PHI:1699|GzZC014|I1RL94|5518|Gibberella_zeae_(related:_Fusarium_graminearum)|Unaffected_pathogenicity
Chr04G0510.1	544	3.00E-45	130/447(29.08)	166	PHI:544|BCMFS1|AAF64435|332648|Botrytis_cinerea|Unaffected_pathogenicity
Chr04G0511.1	2844	3.00E-17	76/268(28.36)	76.3	PHI:2844|BRM2|O93802|5599|Alternaria_alternata|Unaffected_pathogenicity
Chr04G0512.1	2034	0	723/895(80.78)	1494	PHI:2034|MFP1|G4MZY1|148305|Magnaporthe_oryzae_(related:_Magnaporthe_grisea)|Reduced_virulence
Chr04G0518.1	2321	8.00E-12	106/480(22.08)	65.5	PHI:2321|SidI|Q4WR83|746128|Aspergillus_fumigatus|Reduced_virulence
Chr04G0519.1	2560	2.00E-21	64/188(34.04)	85.5	PHI:2560|CNB1|Q6FLU4|5478|Candida_glabrata|Reduced_virulence
Chr04G0522.1	2315	6.00E-56	100/289(34.60)	185	PHI:2315|ChLae1|G4XKY9|5016|Cochliobolus_heterostrophus|Mixed_outcome
Chr04G0523.1	4194	2.00E-30	132/481(27.44)	121	AKT7|PHI:4194|V5XZS6|5599|Alternaria_alternata|increased_virulence_(Hypervirulence)
Chr04G0525.1	1757	0	439/737(59.57)	862	PHI:1757|GzZC072|I1RYY1|5518|Gibberella_zeae_(related:_Fusarium_graminearum)|Unaffected_pathogenicity
Chr04G0526.1	544	7.00E-170	260/576(45.14)	499	PHI:544|BCMFS1|AAF64435|332648|Botrytis_cinerea|Unaffected_pathogenicity
Chr04G0527.1	1909	9.00E-32	126/497(25.35)	125	PHI:1909|GzZC224|I1RHG6|5518|Gibberella_zeae_(related:_Fusarium_graminearum)|Unaffected_pathogenicity

Chr04G0531.1	1288	0	318/419(75.89)	620	PHI:1288|FGSG_07812|I1RE83|5518|Gibberella_zeae_(related:_Fusarium_graminearum)|Unaffected_pathogenicity
Chr04G0532.1	2302	0	1304/1804(72.28)	2682	PHI:2302|CHS5|G4XVC0|148305|Magnaporthe_oryzae_(related:_Magnaporthe_grisea)|Loss_of_pathogenicity
Chr04G0533.1	1056	0	1705/1869(91.23)	3556	PHI:1056|CgCHSV|AAL23719|31870|Colletotrichum_graminicola|Reduced_virulence
Chr04G0536.1	2305	8.00E-51	81/117(69.23)	157	PHI:2305|BcFKBP12||40559|Botrytis_cinerea|Mixed_outcome
Chr04G0538.1	3208	1.00E-152	273/467(58.46)	479	VdMsb|PHI:3208|G2WS91|27337|Verticillium_dahliae|mixed_outcome_
Chr04G0541.1	1393	5.00E-11	46/181(25.41)	63.5	PHI:1393|GzC2H056|I1RU69|5518|Gibberella_zeae_(related:_Fusarium_graminearum)|Unaffected_pathogenicity
Chr04G0542.1	1959	0	501/609(82.27)	1060	PHI:1959|GzZC274|I1RM85|5518|Gibberella_zeae_(related:_Fusarium_graminearum)|Unaffected_pathogenicity
Chr04G0545.1	2545	3.00E-127	188/290(64.83)	366	PHI:2545|TIF35|Q4X1I3|746128|Aspergillus_fumigatus|Mixed_outcome
Chr04G0551.1	2058	9.00E-23	114/447(25.50)	100	PHI:2058|LHS1|MGG_06648.5|148305|Magnaporthe_oryzae_(related:_Magnaporthe_grisea)|Reduced_virulence
Chr04G0554.1	407	2.00E-45	88/205(42.93)	154	PHI:407|PBC1|CAB40372|76659|Pyrenopeziza_brassicae|Loss_of_pathogenicity
Chr04G0560.1	3312	1.00E-142	288/750(38.40)	437	gta1|PHI:3312|G4MT75|318829|Magnaporthe_oryzae|reduced_virulence_
Chr04G0566.1	3061	0	783/1212(64.60)	1412	MoRga1|PHI:3061|G4NF65|318829|Magnaporthe_oryzae|mixed_outcome_
Chr04G0569.1	1662	4.00E-26	101/456(22.15)	111	PHI:1662|GzCCHC002|I1REJ1|5518|Gibberella_zeae_(related:_Fusarium_graminearum)|Unaffected_pathogenicity
Chr04G0573.1	1514	0	718/1243(57.76)	1360	PHI:1514|GzHOME012|I1RYV5|5518|Gibberella_zeae_(related:_Fusarium_graminearum)|Unaffected_pathogenicity
Chr04G0575.1	2734	3.00E-40	104/307(33.88)	147	PHI:2734|metF|Q97S30|1313|Streptococcus_Pneumoniae|Mixed_outcome
Chr04G0580.1	2094	2.00E-19	77/327(23.55)	90.9	PHI:2094|Yvc1|MGG_09828|148305|Magnaporthe_oryzae_(related:_Magnaporthe_grisea)|Mixed_outcome

Chr04G0581.1	2804	2.00E-20	78/283(27.56)	93.2	PHI:2804|ecp6|Q2FDM5|93612|Setosphaeria_turcica|Reduced_virulence
Chr04G0587.1	2382	4.00E-68	99/153(64.71)	219	PHI:2382|Upa2|Q6QIY0|5270|Ustilago_maydis|Unaffected_pathogenicity
Chr04G0589.1	853	5.00E-06	38/127(29.92)	45.1	PHI:853|YAP1|EAK83313|5270|Ustilago_maydis|Reduced_virulence
Chr04G0600.1	2644	6.00E-11	28/81(34.57)	59.3	PHI:2644|thioredoxin_1|P0AA28|90371|Salmonella_enterica_serovar_Typhimurium|Reduced_virulence
Chr04G0601.1	2358	4.00E-173	276/488(56.56)	532	PHI:2358|PLD|Q4WZL4|746128|Aspergillus_fumigatus|Reduced_virulence
Chr04G0604.1	824	3.00E-19	87/350(24.86)	91.7	PHI:824|GyrA|BAC98434|337|Burkholderia_glumae|Chemistry_target
Chr04G0614.1	267	2.00E-122	347/1327(26.15)	419	PHI:267|MLT1|AAD51594|5476|Candida_albicans|Reduced_virulence
Chr04G0616.1	1612	2.00E-76	176/330(53.33)	248	PHI:1612|GzP53L005|I1RZ82|5518|Gibberella_zeae_(related:_Fusarium_graminearum)|Unaffected_pathogenicity
Chr04G0624.1	2455	3.00E-173	282/433(65.13)	493	PHI:2455|CRE1|J9MZK9|59765|Fusarium_oxysporum_f._sp._Lycopersici|Unaffected_pathogenicity
Chr04G0631.1	2042	1.00E-10	66/256(25.78)	62.4	PHI:2042|ABC3|Q3Y5V5|148305|Magnaporthe_oryzae_(related:_Magnaporthe_grisea)|Loss_of_pathogenicity
Chr04G0632.1	455	2.00E-38	95/302(31.46)	143	PHI:455|CAP59|AAC13946|5207|Cryptococcus_neoformans|Loss_of_pathogenicity
Chr04G0634.1	244	1.00E-71	255/919(27.75)	257	PHI:244|CLAP1|AAN62846|290576|Colletotrichum_lindemuthianum|Loss_of_pathogenicity
Chr04G0637.1	811	2.00E-114	174/327(53.21)	335	PHI:811|MGG_10510|EDK06580|318829|Magnaporthe_oryzae|Reduced_virulence
Chr04G0639.1	3663	1.00E-24	101/411(24.57)	103	PD0681|PHI:3663|Q87DK1|2371|Xylella_fastidiosa|increased_virulence_(Hypervirulence)
Chr04G0641.1	2976	3.00E-146	268/791(33.88)	456	PHI:2976|CgOPT1|C6ZRH8|29905|Colletotrichum_gloeosporioides_f._sp._aeschynomenes|Reduced_virulence
Chr04G0643.1	2008	5.00E-11	93/355(26.20)	60.8	PHI:2008|HST1|EDJ98541|148305|Magnaporthe_oryzae_(related:_Magnaporthe_grisea)|Unaffected_pathogenicity

Chr04G0649.1	3236	2.00E-59	142/445(31.91)	205	Aph1|PHI:3236|J9VHR6|5207|Cryptococcus_neoformans|mixed_outcome_
Chr04G0652.1	2520	2.00E-41	174/708(24.58)	159	PHI:2520|LYS4|Q4WUL6|746128|Aspergillus_fumigatus|Lethal
Chr04G0654.1	435	2.00E-47	71/115(61.74)	149	PHI:435|VMA7|EAL02110|5476|Candida_albicans|Loss_of_pathogenicity
Chr04G0656.1	504	2.00E-28	106/354(29.94)	111	PHI:504|LEU2|CAA42366|4932|Saccharomyces_cerevisiae|Reduced_virulence
Chr04G0657.1	2594	3.00E-09	31/118(26.27)	58.2	PHI:2594|SET3|Q59ZX1|746128|Aspergillus_fumigatus|Mixed_outcome
Chr04G0658.1	2515	0	302/458(65.94)	650	PHI:2515|ALG7|Q4X146|746128|Aspergillus_fumigatus|Lethal
Chr04G0659.1	3381	3.00E-63	167/532(31.39)	220	FVEG_12533|PHI:3381|W7N2B4|117187|Fusarium_verticillioides|unaffected_pathogenicity_
Chr04G0665.1	2255	4.00E-65	130/368(35.33)	213	PHI:2255|Abd1|Q0U2A0|13684|Phaeosphaeria_nodorum_(related:_Stagonospora_nodorum)|Unaffected_pathogenicity
Chr04G0666.1	911	5.00E-33	111/375(29.60)	125	PHI:911|um11451||5270|Ustilago_maydis|Reduced_virulence
Chr04G0670.1	1399	2.00E-13	56/162(34.57)	70.9	PHI:1399|GzC2H062|I1RV74|5518|Gibberella_zeae_(related:_Fusarium_graminearum)|Unaffected_pathogenicity
Chr04G0674.1	2020	7.00E-14	64/266(24.06)	72	PHI:2020|Tup1|XP_759427|5270|Ustilago_maydis|Mixed_outcome
Chr04G0675.1	2205	5.00E-120	203/482(42.12)	364	PHI:2205|JmjC_domain-containing_protein_5_[GH10_family]|MGG_01543|148305|Magnaporthe_oryzae_(related:_Magnaporthe_grisea)|Reduced_virulence
Chr04G0679.1	1555	3.00E-27	109/438(24.89)	111	PHI:1555|GzMyb019|I1RDG6|5518|Gibberella_zeae_(related:_Fusarium_graminearum)|Unaffected_pathogenicity
Chr04G0680.1	2269	2.00E-33	86/252(34.13)	122	PHI:2269|Mdh1|0|13684|Phaeosphaeria_nodorum_(related:_Stagonospora_nodorum)|Unaffected_pathogenicity
Chr04G0683.1	1775	5.00E-12	27/66(40.91)	65.5	PHI:1775|GzZC090|I1S5X3|5518|Gibberella_zeae_(related:_Fusarium_graminearum)|Unaffected_pathogenicity
Chr04G0688.1	1526	3.00E-16	38/127(29.92)	79.7	PHI:1526|GzHOMEL036|I1S0J3|5518|Gibberella_zeae_(related:_Fusarium_graminearum)|Unaffected_pathogenicity
Chr04G0696.1	4618	1.00E-74	151/382(39.53)	238	PcPL15|PHI:4618|A0A0D3LX64|4784|Phytophthora_capsici|mixed_outcome
Chr04G0697.1	4194	3.00E-31	115/479(24.01)	124	AKT7|PHI:4194|V5XZS6|5599|Alternaria_alternata|increased_virulence_(Hyper

					virulence)
Chr04G0698.1	482	2.00E-55	108/315(34.29)	184	PHI:482|LAEA|AAR01218|5085|Aspergillus_fumigatus|Reduced_virulence
Chr04G0699.1	922	1.00E-70	202/632(31.96)	241	PHI:922|um03615||5270|Ustilago_maydis|Unaffected_pathogenicity
Chr04G0700.1	513	2.00E-154	240/583(41.17)	459	PHI:513|ARN1_(related:_SIT1)|EAK97011|5476|Candida_albicans|Reduced_virulence
Chr04G0705.1	803	7.00E-09	63/270(23.33)	56.6	PHI:803|MGG_04629|EDJ95969|318829|Magnaporthe_oryzae|Reduced_virulence
Chr04G0706.1	1675	9.00E-09	47/147(31.97)	57	PHI:1675|GzDHHC003|Q4I8B6|5518|Gibberella_zeae_(related:_Fusarium_graminearum)|Unaffected_pathogenicity
Chr04G0707.1	2196	1.00E-70	164/554(29.60)	246	PHI:2196|MoRgs5|XP_363151|148305|Magnaporthe_oryzae_(related:_Magnaporthe_grisea)|Unaffected_pathogenicity
Chr04G0712.1	2279	8.00E-16	69/245(28.16)	72.8	PHI:2279|Conserved_hypothetical_protein|J9N0G7|5507|Fusarium_oxysporum|Unaffected_pathogenicity
Chr04G0716.1	419	2.00E-14	73/288(25.35)	70.9	PHI:419|CSH1|AAP93915|5476|Candida_albicans|Reduced_virulence
Chr04G0717.1	191	0	444/772(57.51)	899	PHI:191|TOM1|AAB08446|39703|Septoria_lycopersici|Unaffected_pathogenicity
Chr04G0720.1	2207	1.00E-08	28/55(50.91)	53.9	PHI:2207|endo-1,4-beta-xylanase_[GH10_family]|MGG_02245|148305|Magnaporthe_oryzae_(related:_Magnaporthe_grisea)|Reduced_virulence
Chr04G0721.1	143	8.00E-32	66/175(37.71)	122	PHI:143|CHT|AAA33353|5563|Gloeocercospora_sorghi|Unaffected_pathogenicity
Chr04G0722.1	1924	2.00E-151	262/653(40.12)	461	PHI:1924|GzZC239|I1RH93|5518|Gibberella_zeae_(related:_Fusarium_graminearum)|Unaffected_pathogenicity
Chr04G0724.1	2841	2.00E-20	80/277(28.88)	88.2	PHI:2841|CnSEC14-2|F6K8L6|5207|Cryptococcus_neoformans|Unaffected_pathogenicity
Chr04G0737.1	3662	4.00E-06	90/354(25.42)	47.8	PspB_(not_PD0218)|PHI:3662|Q87ET0|2371|Xylella_fastidiosa|Increased_virulence_(Hypervirulence)

Chr04G0740.1	4211	5.00E-28	107/465(23.01)	115	FRE3|PHI:4211|J9VNH2|5207|Cryptococcus_neoformans|effector_(plant_avirulence_determinant)
Chr04G0743.1	2654	2.00E-11	48/135(35.56)	64.7	PHI:2654|DUR1,2|Q59VF3|5476|Candida_albicans|Reduced_virulence
Chr04G0744.1	1741	5.00E-41	146/609(23.97)	156	PHI:1741|GzZC056|I1S780|5518|Gibberella_zeae_(related:_Fusarium_graminearum)|Unaffected_pathogenicity
Chr04G0745.1	1046	1.00E-38	135/469(28.78)	145	PHI:1046|CTB5|ABK64182|29003|Cercospora_nicotianae|Reduced_virulence
Chr04G0747.1	106	2.00E-156	234/486(48.15)	456	PHI:106|CAT1|AAC39448|5476|Candida_albicans|Reduced_virulence
Chr04G0748.1	922	6.00E-54	177/612(28.92)	193	PHI:922|um03615||5270|Ustilago_maydis|Unaffected_pathogenicity
Chr04G0749.1	1046	1.00E-28	123/485(25.36)	115	PHI:1046|CTB5|ABK64182|29003|Cercospora_nicotianae|Reduced_virulence
Chr04G0750.1	404	2.00E-06	47/209(22.49)	46.6	PHI:404|PTH11|AAD30436|318829|Magnaporthe_oryzae|Reduced_virulence
Chr04G0758.1	3378	7.00E-79	118/217(54.38)	238	Plegl1|PHI:3378|G9JLA8|285811|Pyrenochaeta_lycopersici|unaffected_pathogenicity_
Chr04G0767.1	2835	1.00E-09	54/195(27.69)	55.5	PHI:2835|RED2|C3JXE8|5016|Cochliobolus_heterostrophus|Reduced_virulence
Chr04G0770.1	1553	0	341/422(80.81)	667	PHI:1553|GzMyb017|I1S7F8|5518|Gibberella_zeae_(related:_Fusarium_graminearum)|Reduced_virulence
Chr04G0773.1	2032	7.00E-46	153/545(28.07)	168	PHI:2032|VTL1|G4NGA7|148305|Magnaporthe_oryzae_(related:_Magnaporthe_grisea)|Unaffected_pathogenicity
Chr04G0776.1	438	6.00E-35	121/475(25.47)	134	PHI:438|BcBOT1_(related:_CND5)|AAQ16576|40559|Botrytis_cinerea|Reduced_virulence
Chr04G0779.1	1795	2.00E-15	103/475(21.68)	76.6	PHI:1795|GzZC110|I1RSX8|5518|Gibberella_zeae_(related:_Fusarium_graminearum)|Unaffected_pathogenicity
Chr04G0780.1	3038	0	371/494(75.10)	766	FgERG5B|PHI:3038|I1RIP4|5518|Fusarium_graminearum|reduced_virulence_
Chr04G0781.1	1727	1.00E-06	30/108(27.78)	47.4	PHI:1727|GzZC042|I1S5Q2|5518|Gibberella_zeae_(related:_Fusarium_graminearum)|Unaffected_pathogenicity
Chr04G0782.1	2329	5.00E-20	113/467(24.20)	90.5	PHI:2329|CTB4|A0ST42|29003|Cercospora_nicotianae|Reduced_virulence

Chr04G0783.1	3301	4.00E-13	58/213(27.23)	65.1	patN|PHI:3301|A0A075TRB3|27334|Penicillium_expansum|unaffected_pathogenicity_
Chr04G0789.1	4194	1.00E-66	150/499(30.06)	224	AKT7|PHI:4194|V5XZS6|5599|Alternaria_alternata|increased_virulence_(Hypervirulence)
Chr04G0794.1	199	6.00E-07	84/356(23.60)	49.7	PHI:199|AOX1|AAF82788|5499|Cladosporium_fulvum|Reduced_virulence
Chr04G0797.1	1974	4.00E-22	66/210(31.43)	96.7	PHI:1974|GzZC289|I1RQN6|5518|Gibberella_zeae_(related:_Fusarium_graminearum)|Unaffected_pathogenicity
Chr04G0798.1	4194	2.00E-43	120/478(25.10)	160	AKT7|PHI:4194|V5XZS6|5599|Alternaria_alternata|increased_virulence_(Hypervirulence)
Chr04G0799.1	1952	5.00E-08	32/87(36.78)	50.8	PHI:1952|GzZC267|I1RDH3|5518|Gibberella_zeae_(related:_Fusarium_graminearum)|Unaffected_pathogenicity
Chr04G0800.1	55	0	925/2591(35.70)	1459	PHI:55|PKS1|AAB08104|5016|Cochliobolus_heterostrophus|Reduced_virulence
Chr04G0801.1	1161	0	263/505(52.08)	550	PHI:1161|MgMfs1|A4ZGP3|54734|Mycosphaerella_graminicola_(related:_Zymoseptoria_triticii)|Chemistry_target
Chr04G0802.1	3390	3.00E-24	81/266(30.45)	97.1	FVEG_12520|PHI:3390|W7MT28|117187|Fusarium_verticillioides|unaffected_pathogenicity_
Chr04G0803.1	714	0	749/2394(31.29)	989	PHI:714|PKS4_(related:_ZEA1)|ABB90283|5518|Fusarium_graminearum|Unaffected_pathogenicity
Chr04G0804.1	2375	6.00E-19	96/378(25.40)	84.3	PHI:2375|DEP1|D2E9W6|29001|Alternaria_brassicicola|Mixed_outcome
Chr04G0805.1	2535	2.00E-16	51/202(25.25)	79.3	PHI:2535|ERG11B|E9QY26|746128|Aspergillus_fumigatus|Mixed_outcome
Chr04G0809.1	2964	1.00E-36	139/435(31.95)	142	PHI:2964|AsnB|G7TM01|129394|Xanthomonas_oryzae_pv._Oryzicola|Reduced_virulence
Chr04G0812.1	441	2.00E-22	86/361(23.82)	94.7	PHI:441|BTP1|CAE55153|40559|Botrytis_cinerea|Reduced_virulence
Chr04G0813.1	1279	2.00E-71	192/579(33.16)	246	PHI:1279|FGSG_12132|I1RU59|5518|Gibberella_zeae_(related:_Fusarium_graminearum)|Unaffected_pathogenicity

Chr04G0816.1	1662	1.00E-16	57/244(23.36)	80.5	PHI:1662|GzCCHC002|I1REJ1|5518|Gibberella_zeae_(related:_Fusarium_graminearum)|Unaffected_pathogenicity
Chr04G0818.1	1662	0	313/500(62.60)	636	PHI:1662|GzCCHC002|I1REJ1|5518|Gibberella_zeae_(related:_Fusarium_graminearum)|Unaffected_pathogenicity
Chr04G0822.1	1772	3.00E-34	77/206(37.38)	127	PHI:1772|GzZC087|I1S053|5518|Gibberella_zeae_(related:_Fusarium_graminearum)|Reduced_virulence
Chr04G0825.1	1314	6.00E-11	43/106(40.57)	57	PHI:1314|GzbHLH015|I1S143|5518|Gibberella_zeae_(related:_Fusarium_graminearum)|Unaffected_pathogenicity
Chr04G0831.1	541	4.00E-75	201/552(36.41)	249	PHI:541|LIP1|AAU87359|332648|Botrytis_cinerea|Unaffected_pathogenicity
Chr04G0833.1	1893	7.00E-69	161/468(34.40)	238	PHI:1893|GzZC208|I1RNY0|5518|Gibberella_zeae_(related:_Fusarium_graminearum)|Unaffected_pathogenicity
Chr04G0836.1	2315	5.00E-64	112/289(38.75)	205	PHI:2315|ChLae1|G4XKY9|5016|Cochliobolus_heterostrophus|Mixed_outcome
Chr04G0841.1	566	6.00E-68	153/455(33.63)	223	PHI:566|cel2|AAK19621|5017|Cochliobolus_carbonum|Unaffected_pathogenicity
Chr04G0842.1	1747	2.00E-63	112/327(34.25)	206	PHI:1747|GzZC062|I1S6W1|5518|Gibberella_zeae_(related:_Fusarium_graminearum)|Lethal
Chr04G0851.1	1411	4.00E-91	165/238(69.33)	270	PHI:1411|GzC2H078|I1RZ11|5518|Gibberella_zeae_(related:_Fusarium_graminearum)|Lethal
Chr04G0859.1	2315	4.00E-54	108/292(36.99)	181	PHI:2315|ChLae1|G4XKY9|5016|Cochliobolus_heterostrophus|Mixed_outcome
Chr04G0860.1	2174	5.00E-08	36/112(32.14)	51.2	PHI:2174|NMR2|MGG_02860|148305|Magnaporthe_oryzae_(related:_Magnaporthe_grisea)|Reduced_virulence
Chr04G0861.1	1399	8.00E-17	64/203(31.53)	81.3	PHI:1399|GzC2H062|I1RV74|5518|Gibberella_zeae_(related:_Fusarium_graminearum)|Unaffected_pathogenicity
Chr04G0862.1	903	2.00E-159	236/547(43.14)	471	PHI:903|um01888||5270|Ustilago_maydis|Unaffected_pathogenicity
Chr04G0863.1	191	0	317/763(41.55)	541	PHI:191|TOM1|AAB08446|39703|Septoria_lycopersici|Unaffected_pathogenicity

Chr04G0875.1	1046	7.00E-23	66/179(36.87)	98.6	PHI:1046|CTB5|ABK64182|29003|Cercospora_nicotianae|Reduced_virulence
Chr04G0877.1	1159	0	375/1054(35.58)	637	PHI:1159|MgAtr7|A5H456|54734|Mycosphaerella_graminicola_(related:_Zymoseptoria_triticii)|Chemistry_target
Chr04G0878.1	391	7.00E-17	35/91(38.46)	74.3	PHI:391|ABC2|BAC67162|318829|Magnaporthe_oryzae|Unaffected_pathogenicity
Chr04G0879.1	3300	7.00E-68	204/714(28.57)	239	patL|PHI:3300|A0A075TXZ8|27334|Penicillium_expansum|unaffected_pathogenicity_
Chr04G0882.1	2393	2.00E-49	131/476(27.52)	176	PHI:2393|Related_to_O-methylsterigmatocystin_oxidoreductase|I1R980|5518|Gibberella_zeae_(related:_Fusarium_graminearum)|Increased_virulence_(Hypervirulence)
Chr04G0884.1	3299	0	1031/1819(56.68)	1923	patK|PHI:3299|A0A075TRC0|27334|Penicillium_expansum|unaffected_pathogenicity_
Chr04G0885.1	1047	6.00E-20	68/211(32.23)	86.7	PHI:1047|CTB6|ABK64183|29003|Cercospora_nicotianae|Reduced_virulence
Chr04G0886.1	2104	0	365/606(60.23)	618	PHI:2104|Vacuolar_calcium_ion_transporter|MGG_04159|148305|Magnaporthe_oryzae_(related:_Magnaporthe_grisea)|Unaffected_pathogenicity
Chr04G0887.1	1655	0	1141/1952(58.45)	2164	PHI:1655|GzWing028|I1S9V9|5518|Gibberella_zeae_(related:_Fusarium_graminearum)|Unaffected_pathogenicity
Chr04G0891.1	2068	0	702/1301(53.96)	1176	PHI:2068|DES1|XP_361689|148305|Magnaporthe_oryzae_(related:_Magnaporthe_grisea)|Reduced_virulence
Chr04G0892.1	1627	3.00E-12	42/119(35.29)	57.4	PHI:1627|GzTF2S002|I1RW90|5518|Gibberella_zeae_(related:_Fusarium_graminearum)|Unaffected_pathogenicity
Chr04G0897.1	3280	7.00E-158	213/214(99.53)	437	CoRAS1|PHI:3280|N4VQQ5|5465|Colletotrichum_orbiculare|
Chr04G0906.1	2315	9.00E-67	121/290(41.72)	212	PHI:2315|ChLae1|G4XKY9|5016|Cochliobolus_heterostrophus|Mixed_outcome
Chr04G0912.1	96	0	1212/1901(63.76)	2463	PHI:96|FAS2|AAA34345|5476|Candida_albicans|Loss_of_pathogenicity
Chr04G0913.1	97	0	1104/2065(53.46)	2285	PHI:97|TOXC|AAC62818|5017|Cochliobolus_carbonum|Reduced_virulence
Chr04G0916.1	3415	3.00E-29	120/479(25.05)	120	Fre2|PHI:3415|T2BNJ5|5207|Cryptococcus_neoformans|mixed_outcome_

Chr04G0917.1	3415	3.00E-16	90/404(22.28)	80.1	Fre2|PHI:3415|T2BNJ5|5207|Cryptococcus_neoformans|mixed_outcome_
Chr04G0924.1	2357	2.00E-13	66/268(24.63)	70.1	PHI:2357|CYP52X1|E2EAF6|475271|Beauveria_bassiana|Reduced_virulence
Chr04G0925.1	2321	1.00E-65	159/512(31.05)	223	PHI:2321|SidI|Q4WR83|746128|Aspergillus_fumigatus|Reduced_virulence
Chr04G0926.1	255	0	946/2589(36.54)	1481	PHI:255|FUM1_(related:_FUM5)|AAD43562|5127|Gibberella_moniliformis|Unaffected_pathogenicity
Chr04G0928.1	2731	4.00E-14	77/290(26.55)	72.8	PHI:2731|VdSge1|G2XD29|27337|Verticillium_dahliae|Loss_of_pathogenicity
Chr04G0933.1	1859	2.00E-06	27/65(41.54)	47.4	PHI:1859|GzZC174|I1S3H3|5518|Gibberella_zeae_(related:_Fusarium_graminearum)|Unaffected_pathogenicity
Chr04G0936.1	26	5.00E-114	203/605(33.55)	353	PHI:26|CaMDR1|CAA37820|5476|Candida_albicans|Reduced_virulence
Chr04G0943.1	355	0	397/475(83.58)	815	PHI:355|GzmetE||5518|Fusarium_graminearum|Reduced_virulence
Chr04G0944.1	3457	2.00E-149	249/542(45.94)	449	PHO84|PHI:3457|J9VMW8|5207|Cryptococcus_neoformans|mixed_outcome_
Chr04G0945.1	1964	4.00E-98	186/481(38.67)	304	PHI:1964|GzZC279|I1RQU2|5518|Gibberella_zeae_(related:_Fusarium_graminearum)|Unaffected_pathogenicity
Chr04G0950.1	1872	9.00E-06	14/33(42.42)	46.6	PHI:1872|GzZC187|I1RTU2|5518|Gibberella_zeae_(related:_Fusarium_graminearum)|Lethal
Chr04G0951.1	180	2.00E-90	143/239(59.83)	268	PHI:180|PELD|AAC49420|140110|Nectria_haematococca_(related:_Fusarium_solani)|Reduced_virulence
Chr04G0952.1	404	8.00E-29	97/349(27.79)	116	PHI:404|PTH11|AAD30436|318829|Magnaporthe_oryzae|Reduced_virulence
Chr04G0954.1	1555	2.00E-19	95/407(23.34)	87.8	PHI:1555|GzMyb019|I1RDG6|5518|Gibberella_zeae_(related:_Fusarium_graminearum)|Unaffected_pathogenicity
Chr04G0956.1	2849	2.00E-72	113/228(49.56)	221	PHI:2849|cutA|Q99174|70790|Fusarium_solani_f._sp._cucurbitae|Unaffected_pathogenicity
Chr04G0957.1	181	1.00E-18	108/423(25.53)	84.7	PHI:181|PGX1|AAK81847|5507|Fusarium_oxysporum|Unaffected_pathogenicity
Chr04G0960.1	438	9.00E-12	58/225(25.78)	64.3	PHI:438|BcBOT1_(related:_CND5)|AAQ16576|40559|Botrytis_cinerea|Reduced_virulence

Chr04G0962.1	419	6.00E-19	82/318(25.79)	83.6	PHI:419|CSH1|AAP93915|5476|Candida_albicans|Reduced_virulence
Chr04G0964.1	1552	8.00E-55	102/191(53.40)	201	PHI:1552|GzMyb016|I1S0N5|5518|Gibberella_zeae_(related:_Fusarium_graminearum)|Unaffected_pathogenicity
Chr04G0965.1	1580	3.00E-176	297/409(72.62)	500	PHI:1580|GzOB020|I1RM35|5518|Gibberella_zeae_(related:_Fusarium_graminearum)|Unaffected_pathogenicity
Chr04G0966.1	2547	4.00E-161	340/683(49.78)	484	PHI:2547|ESF1|Q4WHH1|746128|Aspergillus_fumigatus|Mixed_outcome
Chr04G0970.1	1526	8.00E-07	26/87(29.89)	48.5	PHI:1526|GzHOMEL036|I1S0J3|5518|Gibberella_zeae_(related:_Fusarium_graminearum)|Unaffected_pathogenicity
Chr04G0973.1	255	0	1142/2629(43.44)	2026	PHI:255|FUM1_(related:_FUM5)|AAD43562|5127|Gibberella_moniliformis|Unaffected_pathogenicity
Chr04G0974.1	2315	2.00E-57	107/296(36.15)	191	PHI:2315|ChLae1|G4XKY9|5016|Cochliobolus_heterostrophus|Mixed_outcome
Chr04G0975.1	1389	1.00E-93	232/830(27.95)	313	PHI:1389|GzC2H052|I1RTJ6|5518|Gibberella_zeae_(related:_Fusarium_graminearum)|Lethal
Chr04G0976.1	3387	3.00E-18	88/327(26.91)	83.6	FVEG_12523|PHI:3387|W7MT31|117187|Fusarium_verticillioides|unaffected_pathogenicity_
Chr04G0977.1	2700	2.00E-74	181/598(30.27)	259	PHI:2700|lac2|J7MF98|5465|Colletotrichum_orbiculare|Reduced_virulence
Chr04G0978.1	713	2.00E-139	382/1364(28.01)	483	PHI:713|PKS13_(related:_ZEA2)|ABB90282|5518|Fusarium_graminearum|Unaffected_pathogenicity
Chr04G0981.1	922	5.00E-52	187/627(29.82)	188	PHI:922|um03615||5270|Ustilago_maydis|Unaffected_pathogenicity
Chr04G0984.1	1856	1.00E-94	162/428(37.85)	294	PHI:1856|GzZC171|I1S377|5518|Gibberella_zeae_(related:_Fusarium_graminearum)|Unaffected_pathogenicity
Chr04G0985.1	2204	2.00E-47	102/304(33.55)	163	PHI:2204|endo-1,4-beta-xylanase_[GH10_family]|MGG_01542|148305|Magnaporthe_oryzae_(related:_Magnaporthe_grisea)|Reduced_virulence
Chr04G0989.1	1579	0	946/1217(77.73)	1865	PHI:1579|GzOB019|I1RM25|5518|Gibberella_zeae_(related:_Fusarium_graminearum)|Unaffected_pathogenicity
Chr04G0994.1	2315	2.00E-58	108/291(37.11)	191	PHI:2315|ChLae1|G4XKY9|5016|Cochliobolus_heterostrophus|Mixed_outcome

Chr04G0997.1	167	1.00E-41	159/551(28.86)	155	PHI:167|CHIP3|AAF00024|5457|Colletotrichum_gloeosporioides|Unaffected_pathogenicity
Chr04G0999.1	698	3.00E-35	82/219(37.44)	126	PHI:698|CtxA|AAF94614|243277|Vibrio_cholerae|Reduced_virulence
Chr04G1003.1	3381	3.00E-91	174/460(37.83)	294	FVEG_12533|PHI:3381|W7N2B4|117187|Fusarium_verticillioides|unaffected_pathogenicity_
Chr04G1007.1	2968	5.00E-38	133/500(26.60)	144	PHI:2968|Hxs1|J9VQA5|5207|Cryptococcus_neoformans|Reduced_virulence
Chr04G1008.1	423	2.00E-45	118/373(31.64)	170	PHI:423|VAD1|AAV41010|5207|Cryptococcus_neoformans|Reduced_virulence
Chr04G1010.1	1133	0	263/374(70.32)	535	PHI:1133|Lmepi|B9DR51|5022|Leptosphaeria_maculans|Loss_of_pathogenicity
Chr04G1011.1	55	6.00E-26	75/217(34.56)	108	PHI:55|PKS1|AAB08104|5016|Cochliobolus_heterostrophus|Reduced_virulence
Chr04G1013.1	404	2.00E-23	88/336(26.19)	99.4	PHI:404|PTH11|AAD30436|318829|Magnaporthe_oryzae|Reduced_virulence
Chr04G1027.1	1555	6.00E-30	116/477(24.32)	120	PHI:1555|GzMyb019|I1RDG6|5518|Gibberella_zeae_(related:_Fusarium_graminearum)|Unaffected_pathogenicity
Chr04G1030.1	1555	0	266/508(52.36)	558	PHI:1555|GzMyb019|I1RDG6|5518|Gibberella_zeae_(related:_Fusarium_graminearum)|Unaffected_pathogenicity
Chr04G1033.1	1662	2.00E-64	142/462(30.74)	227	PHI:1662|GzCCHC002|I1REJ1|5518|Gibberella_zeae_(related:_Fusarium_graminearum)|Unaffected_pathogenicity
Chr04G1038.1	2315	2.00E-60	112/294(38.10)	197	PHI:2315|ChLae1|G4XKY9|5016|Cochliobolus_heterostrophus|Mixed_outcome
Chr04G1041.1	1763	5.00E-29	139/598(23.24)	120	PHI:1763|GzZC078|I1RXE8|5518|Gibberella_zeae_(related:_Fusarium_graminearum)|Unaffected_pathogenicity
Chr04G1045.1	199	9.00E-51	183/647(28.28)	184	PHI:199|AOX1|AAF82788|5499|Cladosporium_fulvum|Reduced_virulence
Chr04G1048.1	2839	3.00E-120	177/346(51.16)	355	PHI:2839|RED1|Q8NJQ2|5016|Cochliobolus_heterostrophus|Reduced_virulence
Chr04G1049.1	3391	3.00E-07	39/149(26.17)	49.3	FVEG_12519|PHI:3391|W7N293|117187|Fusarium_verticillioides|unaffected_pathogenicity_
Chr04G1051.1	2968	2.00E-42	126/475(26.53)	162	PHI:2968|Hxs1|J9VQA5|5207|Cryptococcus_neoformans|Reduced_virulence

Chr04G1054.1	438	1.00E-42	130/448(29.02)	157	PHI:438|BcBOT1_(related:_CND5)|AAQ16576|40559|Botrytis_cinerea|Reduced_virulence
Chr04G1056.1	1904	5.00E-44	161/587(27.43)	163	PHI:1904|GzZC219|I1RHR0|5518|Gibberella_zeae_(related:_Fusarium_graminearum)|Unaffected_pathogenicity
Chr04G1058.1	438	5.00E-20	97/378(25.66)	92.4	PHI:438|BcBOT1_(related:_CND5)|AAQ16576|40559|Botrytis_cinerea|Reduced_virulence
Chr04G1061.1	217	6.00E-19	111/481(23.08)	87	PHI:217|HXK1|EAK93243|5476|Candida_albicans|Reduced_virulence
Chr04G1064.1	734	0	482/654(73.70)	956	PHI:734|FOW2|BAE98264|5507|Fusarium_oxysporum|Loss_of_pathogenicity
Chr04G1071.1	2401	3.00E-10	23/51(45.10)	56.6	PHI:2401|CaRING1|G0T3B3|456327|Xanthomonas_campestris_pv_vesicatoria|Mixed_outcome
Chr04G1072.1	1420	6.00E-09	100/429(23.31)	56.6	PHI:1420|GzC2H088|I1S172|5518|Gibberella_zeae_(related:_Fusarium_graminearum)|Unaffected_pathogenicity
Chr04G1073.1	1941	3.00E-70	150/462(32.47)	247	PHI:1941|GzZC256|I1RGD9|5518|Gibberella_zeae_(related:_Fusarium_graminearum)|Unaffected_pathogenicity
Chr04G1074.1	1022	0	624/956(65.27)	1162	PHI:1022|XLNR|ABN41464|5508|Fusarium_oxysporum|Unaffected_pathogenicity
Chr04G1075.1	55	0	916/2671(34.29)	1209	PHI:55|PKS1|AAB08104|5016|Cochliobolus_heterostrophus|Reduced_virulence
Chr04G1078.1	2357	1.00E-85	168/438(38.36)	276	PHI:2357|CYP52X1|E2EAF6|475271|Beauveria_bassiana|Reduced_virulence
Chr04G1080.1	3381	4.00E-77	175/520(33.65)	256	FVEG_12533|PHI:3381|W7N2B4|117187|Fusarium_verticillioides|unaffected_pathogenicity_
Chr04G1083.1	2244	7.00E-89	152/362(41.99)	276	PHI:2244|37865|gi|46099354|5270|Ustilago_maydis|Unaffected_pathogenicity
Chr04G1084.1	2378	4.00E-06	39/149(26.17)	43.9	PHI:2378|DEP4|D2E9W9|29001|Alternaria_brassicicola|Mixed_outcome
Chr04G1085.1	228	3.00E-15	105/433(24.25)	77	PHI:228|PLB1|AAF65220|5207|Cryptococcus_neoformans|Reduced_virulence
Chr04G1090.1	1384	3.00E-07	23/62(37.10)	43.9	PHI:1384|GzC2H047|I1RSD5|5518|Gibberella_zeae_(related:_Fusarium_graminearum)|Reduced_virulence

Chr04G1092.1	1404	7.00E-09	45/131(34.35)	54.7	PHI:1404|GzC2H067|I1RWH6|5518|Gibberella_zeae_(related:_Fusarium_graminearum)|Unaffected_pathogenicity
Chr04G1101.1	1192	6.00E-158	221/361(61.22)	451	PHI:1192|(Sc_Cak1)|I1RLX6|5518|Gibberella_zeae_(related:_Fusarium_graminearum)|Reduced_virulence
Chr04G1109.1	223	4.00E-48	95/262(36.26)	167	PHI:223|PEP1|AAK11166|140110|Nectria_haematococca_(related:_Fusarium_solani)|Reduced_virulence
Chr04G1110.1	144	3.00E-16	85/372(22.85)	78.6	PHI:144|CHT42|AAC05829|29875|Trichoderma_virens|Reduced_virulence
Chr04G1117.1	1832	4.00E-140	216/448(48.21)	421	PHI:1832|GzZC147|I1RH29|5518|Gibberella_zeae_(related:_Fusarium_graminearum)|Unaffected_pathogenicity
Chr04G1118.1	901	6.00E-46	136/472(28.81)	169	PHI:901|um01886||5270|Ustilago_maydis|Unaffected_pathogenicity
Chr04G1124.1	2502	0	363/569(63.80)	700	PHI:2502|FgTep1p|I1RM09|5518|Gibberella_zeae_(related:_Fusarium_graminearum)|Reduced_virulence
Chr04G1125.1	4194	1.00E-36	126/490(25.71)	139	AKT7|PHI:4194|V5XZS6|5599|Alternaria_alternata|increased_virulence_(Hypervirulence)
Chr04G1137.1	2109	9.00E-06	38/152(25.00)	42.7	PHI:2109|CNB|MGG_06933|148305|Magnaporthe_oryzae_(related:_Magnaporthe_grisea)|Mixed_outcome
Chr04G1139.1	1485	2.00E-131	279/628(44.43)	419	PHI:1485|GzHMG017|I1RMP0|5518|Gibberella_zeae_(related:_Fusarium_graminearum)|Unaffected_pathogenicity
Chr04G1140.1	2903	4.00E-72	121/327(37.00)	226	PHI:2897|BEC1019|KJ571201.1|62688|Blumeria_graminis_f._sp._hordei|Effector_(plant_avirulence_determinant)
Chr04G1141.1	243	4.00E-167	259/602(43.02)	509	PHI:243|CHIP6|AAD00894|5457|Colletotrichum_gloeosporioides|Reduced_virulence
Chr04G1142.1	1363	1.00E-40	108/359(30.08)	145	PHI:1363|TRI15|I1RJ74|5518|Gibberella_zeae_(related:_Fusarium_graminearum)|Unaffected_pathogenicity
Chr04G1146.1	1260	3.00E-15	63/173(36.42)	73.6	PHI:1260|FGSG_13944|I1RUC7|5518|Gibberella_zeae_(related:_Fusarium_graminearum)|Unaffected_pathogenicity

Chr04G1155.1	438	1.00E-29	133/477(27.88)	119	PHI:438|BcBOT1_(related:_CND5)|AAQ16576|40559|Botrytis_cinerea|Reduced_virulence
Chr04G1158.1	3457	6.00E-06	117/493(23.73)	46.2	PHO84|PHI:3457|J9VMW8|5207|Cryptococcus_neoformans|mixed_outcome_
Chr04G1159.1	1714	9.00E-154	281/667(42.13)	466	PHI:1714|GzZC029|I1RQU8|5518|Gibberella_zeae_(related:_Fusarium_graminearum)|Lethal
Chr04G1160.1	2393	2.00E-31	118/478(24.69)	126	PHI:2393|Related_to_O-methylsterigmatocystin_oxidoreductase|I1R980|5518|Gibberella_zeae_(related:_Fusarium_graminearum)|Increased_virulence_(Hypervirulence)
Chr04G1162.1	3207	1.00E-15	81/380(21.32)	77.8	Yvc1|PHI:3207|A1CLC0|746128|Aspergillus_fumigatus|reduced_virulence_
Chr04G1164.1	3633	4.00E-31	89/286(31.12)	119	Rv0469|PHI:3633|Q6MX39|1773|Mycobacterium_tuberculosis|increased_virulence_
Chr04G1165.1	1047	3.00E-33	96/303(31.68)	125	PHI:1047|CTB6|ABK64183|29003|Cercospora_nicotianae|Reduced_virulence
Chr04G1169.1	167	2.00E-60	148/431(34.34)	206	PHI:167|CHIP3|AAF00024|5457|Colletotrichum_gloeosporioides|Unaffected_pathogenicity
Chr04G1178.1	888	6.00E-145	202/301(67.11)	411	PHI:888|MGG_01707|EDK04578|318829|Magnaporthe_oryzae|Reduced_virulence
Chr04G1184.1	1516	4.00E-154	248/375(66.13)	448	PHI:1516|GzHOMEL012|I1RMR8|5518|Gibberella_zeae_(related:_Fusarium_graminearum)|Lethal
Chr04G1191.1	262	2.00E-12	30/109(27.52)	62	PHI:262|MAD2|EAK94586|5476|Candida_albicans|Reduced_virulence
Chr04G1192.1	1662	7.00E-21	62/232(26.72)	94.4	PHI:1662|GzCCHC002|I1REJ1|5518|Gibberella_zeae_(related:_Fusarium_graminearum)|Unaffected_pathogenicity
Chr04G1194.1	886	2.00E-06	41/157(26.11)	46.6	PHI:886|MGG_13052|EDK06087|318829|Magnaporthe_oryzae|Reduced_virulence
Chr04G1195.1	876	0	456/1164(39.18)	780	PHI:876|MGG_11671|EDK03349|318829|Magnaporthe_oryzae|Reduced_virulence
Chr04G1196.1	2315	2.00E-60	111/292(38.01)	197	PHI:2315|ChLae1|G4XKY9|5016|Cochliobolus_heterostrophus|Mixed_outcome

Chr04G1197.1	4194	1.00E-45	135/477(28.30)	166	AKT7|PHI:4194|V5XZS6|5599|Alternaria_alternata|increased_virulence_(Hypervirulence)
Chr04G1198.1	441	8.00E-13	73/274(26.64)	66.2	PHI:441|BTP1|CAE55153|40559|Botrytis_cinerea|Reduced_virulence
Chr04G1201.1	2644	1.00E-16	35/83(42.17)	70.1	PHI:2644|thioredoxin_1|P0AA28|90371|Salmonella_enterica_serovar_Typhimurium|Reduced_virulence
Chr04G1204.1	1598	1.00E-67	99/136(72.79)	202	PHI:1598|GzOB038|I1RZ35|5518|Gibberella_zeae_(related:_Fusarium_graminearum)|Reduced_virulence
Chr04G1208.1	1237	0	888/1210(73.39)	1672	PHI:1237|FGSG_01058|I1RZ37|5518|Gibberella_zeae_(related:_Fusarium_graminearum)|Lethal
Chr04G1211.1	1599	9.00E-80	123/182(67.58)	236	PHI:1599|GzOB039|I1RZ39|5518|Gibberella_zeae_(related:_Fusarium_graminearum)|Unaffected_pathogenicity
Chr04G1212.1	3095	0	261/435(60.00)	560	Ktr1|PHI:3095|J5JDI1|176275|Beauveria_bassiana|unaffected_pathogenicity_
Chr04G1217.1	2976	6.00E-50	172/719(23.92)	185	PHI:2976|CgOPT1|C6ZRH8|29905|Colletotrichum_gloeosporioides_f._sp._aeschynomenes|Reduced_virulence
Chr04G1221.1	876	0	482/1223(39.41)	803	PHI:876|MGG_11671|EDK03349|318829|Magnaporthe_oryzae|Reduced_virulence
Chr04G1222.1	1935	2.00E-10	56/228(24.56)	60.5	PHI:1935|GzZC250|I1RFP7|5518|Gibberella_zeae_(related:_Fusarium_graminearum)|Unaffected_pathogenicity
Chr04G1224.1	438	2.00E-41	118/456(25.88)	153	PHI:438|BcBOT1_(related:_CND5)|AAQ16576|40559|Botrytis_cinerea|Reduced_virulence
Chr04G1226.1	3301	7.00E-08	53/222(23.87)	49.7	patN|PHI:3301|A0A075TRB3|27334|Penicillium_expansum|unaffected_pathogenicity_
Chr04G1230.1	2490	6.00E-07	27/93(29.03)	48.9	PHI:2490|MCA|E9B636|5665|Leishmania_mexicana|Mixed_outcome
Chr04G1232.1	922	2.00E-87	213/627(33.97)	287	PHI:922|um03615||5270|Ustilago_maydis|Unaffected_pathogenicity
Chr04G1233.1	2032	3.00E-19	64/232(27.59)	87.4	PHI:2032|VTL1|G4NGA7|148305|Magnaporthe_oryzae_(related:_Magnaporthe_grisea)|Unaffected_pathogenicity

Chr04G1238.1	800	2.00E-07	61/231(26.41)	51.2	PHI:800|MGG_13324|EDK00897|318829|Magnaporthe_oryzae|Reduced_virulence
Chr04G1239.1	1209	8.00E-12	48/129(37.21)	65.9	PHI:1209|FGSG_04770|I1RLH1|5518|Gibberella_zeae_(related:_Fusarium_graminearum)|Reduced_virulence
Chr04G1241.1	3610	2.00E-07	59/249(23.69)	50.1	iucD|PHI:3610|Q6KD45|562|Escherichia_coli|mixed_outcome_
Chr04G1256.1	1423	6.00E-17	108/459(23.53)	83.2	PHI:1423|GzC2H091|I1S2R1|5518|Gibberella_zeae_(related:_Fusarium_graminearum)|Unaffected_pathogenicity
Chr04G1257.1	2269	4.00E-29	80/267(29.96)	111	PHI:2269|Mdh1|0|13684|Phaeosphaeria_nodorum_(related:_Stagonospora_nodorum)|Unaffected_pathogenicity
Chr04G1259.1	1901	1.00E-18	74/293(25.26)	87.4	PHI:1901|GzZC216|I1RK07|5518|Gibberella_zeae_(related:_Fusarium_graminearum)|Unaffected_pathogenicity
Chr04G1260.1	2968	2.00E-44	131/464(28.23)	163	PHI:2968|Hxs1|J9VQA5|5207|Cryptococcus_neoformans|Reduced_virulence
Chr04G1261.1	2322	2.00E-83	136/284(47.89)	253	PHI:2322|SidH|Q4WF54|746128|Aspergillus_fumigatus|Reduced_virulence
Chr04G1263.1	2377	2.00E-126	209/531(39.36)	383	PHI:2377|DEP3|D2E9W8|29001|Alternaria_brassicicola|Mixed_outcome
Chr04G1266.1	12	7.00E-180	703/2622(26.81)	619	PHI:12|HTS1|AAA33023|5017|Cochliobolus_carbonum|Loss_of_pathogenicity
Chr04G1267.1	115	3.00E-81	156/429(36.36)	259	PHI:115|PGX1|AAC26146|5017|Cochliobolus_carbonum|Unaffected_pathogenicity
Chr04G1270.1	2247	3.00E-27	84/267(31.46)	106	PHI:2247|Sch1|EAT82552|13684|Phaeosphaeria_nodorum_(related:_Stagonospora_nodorum)|Unaffected_pathogenicity
Chr04G1271.1	1949	4.00E-56	120/368(32.61)	190	PHI:1949|GzZC264|I1RI60|5518|Gibberella_zeae_(related:_Fusarium_graminearum)|Lethal
Chr04G1272.1	334	5.00E-20	74/301(24.58)	89.7	PHI:334|CGB1|AAO25585|5016|Cochliobolus_heterostrophus|Loss_of_pathogenicity
Chr04G1273.1	2270	3.00E-152	224/397(56.42)	437	PHI:2270|Mpd1|Q0U6E8|13684|Phaeosphaeria_nodorum_(related:_Stagonospora_nodorum)|Unaffected_pathogenicity
Chr04G1275.1	1226	0	562/639(87.95)	1162	PHI:1226|FGSG_11614|I1RP88|5518|Gibberella_zeae_(related:_Fusarium_gra

					minearum)|Lethal
Chr04G1276.1	544	6.00E-60	168/592(28.38)	209	PHI:544|BCMFS1|AAF64435|332648|Botrytis_cinerea|Unaffected_pathogenicity
Chr04G1277.1	199	0	523/665(78.65)	1109	PHI:199|AOX1|AAF82788|5499|Cladosporium_fulvum|Reduced_virulence
Chr04G1278.1	2511	0	597/1762(33.88)	1028	PHI:2511|Pes1|Q4WT66|746128|Aspergillus_fumigatus|Reduced_virulence
Chr04G1279.1	1161	1.00E-158	243/577(42.11)	467	PHI:1161|MgMfs1|A4ZGP3|54734|Mycosphaerella_graminicola_(related:_Zymoseptoria_triticii)|Chemistry_target
Chr04G1291.1	675	7.00E-69	222/871(25.49)	253	PHI:675|ROM2|AAW45289|192011|Cryptococcus_neoformans|Increased_virulence_(Hypervirulence)
Chr04G1295.1	1235	0	322/330(97.58)	672	PHI:1235|FGSG_04416|I1RWQ2|5518|Gibberella_zeae_(related:_Fusarium_graminearum)|Lethal
Chr04G1297.1	3088	0	279/392(71.17)	558	CPK2|PHI:3088|I1RWQ0|5518|Fusarium_graminearum|mixed_outcome_
Chr04G1302.1	441	3.00E-15	92/384(23.96)	73.9	PHI:441|BTP1|CAE55153|40559|Botrytis_cinerea|Reduced_virulence
Chr04G1304.1	197	3.00E-11	54/231(23.38)	62.8	PHI:197|ALO1|AAC98913|5476|Candida_albicans|Reduced_virulence
Chr04G1305.1	4194	2.00E-43	134/488(27.46)	159	AKT7|PHI:4194|V5XZS6|5599|Alternaria_alternata|increased_virulence_(Hypervirulence)
Chr04G1306.1	2022	5.00E-07	47/214(21.96)	47	PHI:2022|BUF1|MGG_02252|148305|Magnaporthe_oryzae_(related:_Magnaporthe_grisea)|Loss_of_pathogenicity
Chr04G1316.1	1410	2.00E-87	171/293(58.36)	265	PHI:1410|GzC2H076|I1RYG0|5518|Gibberella_zeae_(related:_Fusarium_graminearum)|Unaffected_pathogenicity
Chr04G1317.1	1236	0	509/739(68.88)	997	PHI:1236|FGSG_04770|I1RYF8|5518|Gibberella_zeae_(related:_Fusarium_graminearum)|Lethal
Chr04G1323.1	1402	0	419/848(49.41)	725	PHI:1402|GzC2H065|I1RWB8|5518|Gibberella_zeae_(related:_Fusarium_graminearum)|Unaffected_pathogenicity
Chr04G1324.1	2025	7.00E-08	35/103(33.98)	51.2	PHI:2025|HDL1|G4MQZ9|148305|Magnaporthe_oryzae_(related:_Magnaporthe_grisea)|Unaffected_pathogenicity

Chr04G1328.1	17	1.00E-42	126/404(31.19)	155	PHI:17|ACP|CAA43678|5482|Candida_tropicalis|Reduced_virulence
Chr04G1330.1	1399	4.00E-11	46/160(28.75)	63.5	PHI:1399|GzC2H062|I1RV74|5518|Gibberella_zeae_(related:_Fusarium_graminearum)|Unaffected_pathogenicity
Chr04G1333.1	267	6.00E-107	375/1516(24.74)	372	PHI:267|MLT1|AAD51594|5476|Candida_albicans|Reduced_virulence
Chr04G1334.1	197	2.00E-110	201/542(37.08)	342	PHI:197|ALO1|AAC98913|5476|Candida_albicans|Reduced_virulence
Chr04G1341.1	112	3.00E-17	84/330(25.45)	81.3	PHI:112|MAK1|AAC49410|140110|Nectria_haematococca_(related:_Fusarium_solani)|Reduced_virulence
Chr04G1348.1	1161	3.00E-124	206/556(37.05)	379	PHI:1161|MgMfs1|A4ZGP3|54734|Mycosphaerella_graminicola_(related:_Zymoseptoria_triticii)|Chemistry_target
Chr04G1349.1	1662	3.00E-37	103/405(25.43)	145	PHI:1662|GzCCHC002|I1REJ1|5518|Gibberella_zeae_(related:_Fusarium_graminearum)|Unaffected_pathogenicity
Chr04G1350.1	1559	6.00E-120	163/256(63.67)	344	PHI:1559|GzNEG001|I1RK21|5518|Gibberella_zeae_(related:_Fusarium_graminearum)|Unaffected_pathogenicity
Chr04G1351.1	1046	3.00E-46	144/486(29.63)	166	PHI:1046|CTB5|ABK64182|29003|Cercospora_nicotianae|Reduced_virulence
Chr04G1352.1	115	4.00E-77	140/380(36.84)	248	PHI:115|PGX1|AAC26146|5017|Cochliobolus_carbonum|Unaffected_pathogenicity
Chr04G1355.1	1916	9.00E-31	120/417(28.78)	126	PHI:1916|GzZC231|I1RSG1|5518|Gibberella_zeae_(related:_Fusarium_graminearum)|Unaffected_pathogenicity
Chr04G1360.1	1676	0	414/680(60.88)	799	PHI:1676|GzDHHC004|Q4I2M7|5518|Gibberella_zeae_(related:_Fusarium_graminearum)|Unaffected_pathogenicity
Chr04G1363.1	2518	5.00E-128	184/305(60.33)	370	PHI:2518|MET16|Q4WWN9|746128|Aspergillus_fumigatus|Lethal
Chr04G1366.1	4211	8.00E-36	121/505(23.96)	140	FRE3|PHI:4211|J9VNH2|5207|Cryptococcus_neoformans|effector_(plant_avirulence_determinant)
Chr04G1367.1	1260	3.00E-15	76/244(31.15)	77.8	PHI:1260|FGSG_13944|I1RUC7|5518|Gibberella_zeae_(related:_Fusarium_graminearum)|Unaffected_pathogenicity
Chr04G1369.1	3313	2.00E-18	106/456(23.25)	87.4	gpf1|PHI:3313|G4NII8|318829|Magnaporthe_oryzae|loss_of_pathogenicity_

Chr04G1374.1	2601	2.00E-08	68/275(24.73)	52.8	PHI:2601|Asc1|P83774|746128|Aspergillus_fumigatus|Mixed_outcome
Chr04G1379.1	1260	5.00E-22	75/224(33.48)	100	PHI:1260|FGSG_13944|I1RUC7|5518|Gibberella_zeae_(related:_Fusarium_graminearum)|Unaffected_pathogenicity
Chr04G1385.1	3418	1.00E-06	47/210(22.38)	52.8	ssp|PHI:3418|Q5DPX0|29385|Staphylococcus_saprophyticus|mixed_outcome_
Chr04G1388.1	2309	0	997/1405(70.96)	2086	PHI:2309|BcatrB|Q9UW03|40559|Botrytis_cinerea|Reduced_virulence
Chr04G1389.1	1688	5.00E-135	258/686(37.61)	413	PHI:1688|GzZC003|I1RWZ1|5518|Gibberella_zeae_(related:_Fusarium_graminearum)|Unaffected_pathogenicity
Chr04G1390.1	1735	3.00E-07	69/295(23.39)	50.1	PHI:1735|GzZC050|I1S6X4|5518|Gibberella_zeae_(related:_Fusarium_graminearum)|Unaffected_pathogenicity
Chr04G1391.1	1455	1.00E-11	70/291(24.05)	64.3	PHI:1455|GzAra003|I1RKU6|5518|Gibberella_zeae_(related:_Fusarium_graminearum)|Unaffected_pathogenicity
Chr04G1392.1	2279	1.00E-11	63/286(22.03)	61.2	PHI:2279|Conserved_hypothetical_protein|J9N0G7|5507|Fusarium_oxysporum|Unaffected_pathogenicity
Chr04G1395.1	4194	5.00E-52	141/477(29.56)	184	AKT7|PHI:4194|V5XZS6|5599|Alternaria_alternata|increased_virulence_(Hypervirulence)
Chr04G1396.1	1784	3.00E-60	149/463(32.18)	202	PHI:1784|GzZC099|I1S2A9|5518|Gibberella_zeae_(related:_Fusarium_graminearum)|Unaffected_pathogenicity
Chr04G1402.1	803	8.00E-27	130/479(27.14)	114	PHI:803|MGG_04629|EDJ95969|318829|Magnaporthe_oryzae|Reduced_virulence
Chr04G1411.1	922	3.00E-91	218/615(35.45)	297	PHI:922|um03615||5270|Ustilago_maydis|Unaffected_pathogenicity
Chr04G1412.1	1555	4.00E-23	106/475(22.32)	100	PHI:1555|GzMyb019|I1RDG6|5518|Gibberella_zeae_(related:_Fusarium_graminearum)|Unaffected_pathogenicity
Chr04G1416.1	2315	3.00E-55	102/290(35.17)	183	PHI:2315|ChLae1|G4XKY9|5016|Cochliobolus_heterostrophus|Mixed_outcome
Chr04G1418.1	144	1.00E-14	77/299(25.75)	75.5	PHI:144|CHT42|AAC05829|29875|Trichoderma_virens|Reduced_virulence
Chr04G1420.1	2570	3.00E-112	190/473(40.17)	345	PHI:2570|CYB2|Q6FM61|5478|Candida_glabrata|Reduced_virulence
Chr04G1421.1	1662	6.00E-35	117/459(25.49)	138	PHI:1662|GzCCHC002|I1REJ1|5518|Gibberella_zeae_(related:_Fusarium_gra

					minearum)|Unaffected_pathogenicity
Chr04G1424.1	513	2.00E-61	162/607(26.69)	215	PHI:513|ARN1_(related:_SIT1)|EAK97011|5476|Candida_albicans|Reduced_virulence
Chr04G1427.1	2644	3.00E-19	33/80(41.25)	79	PHI:2644|thioredoxin_1|P0AA28|90371|Salmonella_enterica_serovar_Typhimurium|Reduced_virulence
Chr04G1428.1	2644	5.00E-19	28/72(38.89)	77	PHI:2644|thioredoxin_1|P0AA28|90371|Salmonella_enterica_serovar_Typhimurium|Reduced_virulence
Chr04G1429.1	1260	1.00E-26	89/237(37.55)	114	PHI:1260|FGSG_13944|I1RUC7|5518|Gibberella_zeae_(related:_Fusarium_graminearum)|Unaffected_pathogenicity
Chr04G1434.1	538	2.00E-35	135/462(29.22)	137	PHI:538|FRT1|AAU87358|40559|Botrytis_cinerea|Unaffected_pathogenicity
Chr04G1436.1	1270	4.00E-49	96/286(33.57)	169	PHI:1270|FGSG_06420|I1RHA1|5518|Gibberella_zeae_(related:_Fusarium_graminearum)|Unaffected_pathogenicity
Chr04G1446.1	2968	7.00E-49	132/476(27.73)	176	PHI:2968|Hxs1|J9VQA5|5207|Cryptococcus_neoformans|Reduced_virulence
Chr04G1447.1	1879	8.00E-28	108/425(25.41)	117	PHI:1879|GzZC194|I1RJB8|5518|Gibberella_zeae_(related:_Fusarium_graminearum)|Unaffected_pathogenicity
Chr04G1449.1	441	2.00E-24	106/391(27.11)	100	PHI:441|BTP1|CAE55153|40559|Botrytis_cinerea|Reduced_virulence
Chr04G1457.1	2020	7.00E-07	59/230(25.65)	48.9	PHI:2020|Tup1|XP_759427|5270|Ustilago_maydis|Mixed_outcome
Chr04G1463.1	184	3.00E-18	71/238(29.83)	81.3	PHI:184|RBT4|AAG09789|5476|Candida_albicans|Reduced_virulence
Chr04G1467.1	2961	0	490/991(49.45)	919	PHI:2961|gcvP|C5BAT0|67780|Edwardsiella_ictaluri|Reduced_virulence
Chr04G1468.1	901	5.00E-85	159/474(33.54)	275	PHI:901|um01886||5270|Ustilago_maydis|Unaffected_pathogenicity
Chr04G1469.1	2558	2.00E-06	29/123(23.58)	48.5	PHI:2558|DUR31|Q59P00|5476|Candida_albicans|Reduced_virulence
Chr04G1473.1	2084	2.00E-30	90/306(29.41)	119	PHI:2084|Moatg18|MGG_03139|148305|Magnaporthe_oryzae_(related:_Magnaporthe_grisea)|Reduced_virulence
Chr04G1474.1	2244	0	258/356(72.47)	543	PHI:2244|37865|gi|46099354|5270|Ustilago_maydis|Unaffected_pathogenicity
Chr04G1477.1	2321	2.00E-109	215/604(35.60)	343	PHI:2321|SidI|Q4WR83|746128|Aspergillus_fumigatus|Reduced_virulence
Chr04G1479.1	2171	6.00E-106	204/559(36.49)	336	PHI:2171|Peroxisomal_copper_amine_oxidase|MGG_02681|148305|Magnapor

					the_oryzae_(related:_Magnaporthe_grisea)|Reduced_virulence
Chr04G1481.1	1877	1.00E-79	180/587(30.66)	268	PHI:1877|GzZC192|I1RPI7|5518|Gibberella_zeae_(related:_Fusarium_graminearum)|Unaffected_pathogenicity
Chr04G1482.1	404	2.00E-18	55/251(21.91)	85.1	PHI:404|PTH11|AAD30436|318829|Magnaporthe_oryzae|Reduced_virulence
Chr04G1484.1	874	1.00E-11	80/310(25.81)	63.5	PHI:874|MGG_00435|EDK02952|318829|Magnaporthe_oryzae|Loss_of_pathogenicity
Chr04G1488.1	552	8.00E-112	206/550(37.45)	347	PHI:552|BcLCC2|AAK77953|40559|Botrytis_cinerea|Unaffected_pathogenicity
Chr04G1491.1	1795	1.00E-24	109/425(25.65)	105	PHI:1795|GzZC110|I1RSX8|5518|Gibberella_zeae_(related:_Fusarium_graminearum)|Unaffected_pathogenicity
Chr04G1495.1	55	0	756/2458(30.76)	1003	PHI:55|PKS1|AAB08104|5016|Cochliobolus_heterostrophus|Reduced_virulence
Chr04G1496.1	482	3.00E-26	80/270(29.63)	105	PHI:482|LAEA|AAR01218|5085|Aspergillus_fumigatus|Reduced_virulence
Chr04G1499.1	1269	4.00E-77	154/423(36.41)	248	PHI:1269|FGSG_02838|I1RFK9|5518|Gibberella_zeae_(related:_Fusarium_graminearum)|Unaffected_pathogenicity
Chr04G1503.1	3381	1.00E-20	113/484(23.35)	93.2	FVEG_12533|PHI:3381|W7N2B4|117187|Fusarium_verticillioides|unaffected_pathogenicity_
Chr04G1508.1	1867	2.00E-28	65/159(40.88)	113	PHI:1867|GzZC182|I1RL02|5518|Gibberella_zeae_(related:_Fusarium_graminearum)|Unaffected_pathogenicity
Chr04G1510.1	2160	9.00E-158	225/320(70.31)	445	PHI:2160|MoSHO1|MGG_09125|148305|Magnaporthe_oryzae_(related:_Magnaporthe_grisea)|Reduced_virulence
Chr04G1515.1	482	7.00E-49	105/295(35.59)	170	PHI:482|LAEA|AAR01218|5085|Aspergillus_fumigatus|Reduced_virulence
Chr04G1519.1	2968	1.00E-80	163/509(32.02)	263	PHI:2968|Hxs1|J9VQA5|5207|Cryptococcus_neoformans|Reduced_virulence
Chr04G1520.1	1802	1.00E-65	176/634(27.76)	227	PHI:1802|GzZC117|I1RVB2|5518|Gibberella_zeae_(related:_Fusarium_graminearum)|Unaffected_pathogenicity
Chr04G1523.1	419	1.00E-70	127/321(39.56)	224	PHI:419|CSH1|AAP93915|5476|Candida_albicans|Reduced_virulence
Chr04G1524.1	3017	2.00E-71	179/574(31.18)	246	Mpr1|PHI:3017|J9VXZ9|5207|Cryptococcus_neoformans|reduced_virulence_

Chr04G1525.1	2269	4.00E-08	48/155(30.97)	51.6	PHI:2269|Mdh1|0|13684|Phaeosphaeria_nodorum_(related:_Stagonospora_nodorum)|Unaffected_pathogenicity
Chr04G1529.1	1662	3.00E-55	114/344(33.14)	195	PHI:1662|GzCCHC002|I1REJ1|5518|Gibberella_zeae_(related:_Fusarium_graminearum)|Unaffected_pathogenicity
Chr04G1533.1	748	0	313/683(45.83)	557	PHI:748|um00446|Not_available|5270|Ustilago_maydis|Unaffected_pathogenicity
Chr04G1535.1	2699	2.00E-06	43/156(27.56)	48.5	PHI:2699|HrpM|Q8PPR7|611301|Xanthomonas_citri_ssp._Citri|Loss_of_pathogenicity
Chr04G1541.1	1190	0	441/670(65.82)	833	PHI:1190|(Sc_Pbs2)|I1RWL6|5518|Gibberella_zeae_(related:_Fusarium_graminearum)|Reduced_virulence
Chr04G1542.1	2337	6.00E-08	59/220(26.82)	50.8	PHI:2337|SUR7|Q5A4M8|5476|Candida_albicans|Reduced_virulence
Chr04G1543.1	1861	2.00E-116	185/440(42.05)	351	PHI:1861|GzZC176|I1RUV3|5518|Gibberella_zeae_(related:_Fusarium_graminearum)|Unaffected_pathogenicity
Chr04G1548.1	1061	2.00E-134	186/208(89.42)	377	PHI:1061|MgRho3|ABK60346|318829|Magnaporthe_oryzae|Loss_of_pathogenicity
Chr04G1552.1	2654	2.00E-09	49/157(31.21)	58.2	PHI:2654|DUR1,2|Q59VF3|5476|Candida_albicans|Reduced_virulence
Chr04G1555.1	538	9.00E-51	140/491(28.51)	184	PHI:538|FRT1|AAU87358|40559|Botrytis_cinerea|Unaffected_pathogenicity
Chr04G1556.1	2255	3.00E-129	200/374(53.48)	377	PHI:2255|Abd1|Q0U2A0|13684|Phaeosphaeria_nodorum_(related:_Stagonospora_nodorum)|Unaffected_pathogenicity
Chr04G1557.1	2837	8.00E-53	100/227(44.05)	172	PHI:2837|OXI1|D2SZX7|5016|Cochliobolus_heterostrophus|Reduced_virulence
Chr04G1560.1	447	0	339/467(72.59)	698	PHI:447|MCSA|CAI61947|5085|Aspergillus_fumigatus|Reduced_virulence
Chr04G1561.1	261	6.00E-170	245/539(45.45)	496	PHI:261|ICL1|AAM89498|5022|Leptosphaeria_maculans|Reduced_virulence
Chr04G1562.1	784	2.00E-27	94/267(35.21)	107	PHI:784|MGG_00056|EDK03390|318829|Magnaporthe_oryzae|Reduced_virulence
Chr04G1564.1	2315	3.00E-30	90/309(29.13)	116	PHI:2315|ChLae1|G4XKY9|5016|Cochliobolus_heterostrophus|Mixed_outcome
Chr04G1566.1	2654	7.00E-114	205/531(38.61)	377	PHI:2654|DUR1,2|Q59VF3|5476|Candida_albicans|Reduced_virulence

Chr04G1567.1	3237	9.00E-28	66/156(42.31)	103	ctrA2|PHI:3237|B0YDG4|746128|Aspergillus_fumigatus|mixed_outcome_
Chr04G1568.1	4211	6.00E-22	108/463(23.33)	97.4	FRE3|PHI:4211|J9VNH2|5207|Cryptococcus_neoformans|effector_(plant_avirulence_determinant)
Chr04G1569.1	1675	2.00E-09	49/177(27.68)	58.5	PHI:1675|GzDHHC003|Q4I8B6|5518|Gibberella_zeae_(related:_Fusarium_graminearum)|Unaffected_pathogenicity
Chr04G1570.1	1867	3.00E-48	101/300(33.67)	174	PHI:1867|GzZC182|I1RL02|5518|Gibberella_zeae_(related:_Fusarium_graminearum)|Unaffected_pathogenicity
Chr04G1575.1	358	5.00E-31	132/533(24.77)	126	PHI:358|ILV2|AAR29084|5207|Cryptococcus_neoformans|Loss_of_pathogenicity
Chr04G1576.1	2117	5.00E-10	91/330(27.58)	59.7	PHI:2117|SPM1|P58371|148305|Magnaporthe_oryzae_(related:_Magnaporthe_grisea)|Reduced_virulence
Chr04G1585.1	922	3.00E-54	174/629(27.66)	195	PHI:922|um03615||5270|Ustilago_maydis|Unaffected_pathogenicity
Chr04G1590.1	552	3.00E-72	169/514(32.88)	240	PHI:552|BcLCC2|AAK77953|40559|Botrytis_cinerea|Unaffected_pathogenicity
Chr04G1592.1	1514	2.00E-71	120/246(48.78)	240	PHI:1514|GzHOME012|I1RYV5|5518|Gibberella_zeae_(related:_Fusarium_graminearum)|Unaffected_pathogenicity
Chr04G1597.1	1591	3.00E-71	115/199(57.79)	245	PHI:1591|GzOB031|I1RWQ7|5518|Gibberella_zeae_(related:_Fusarium_graminearum)|Reduced_virulence
Chr02G0004.1	4194	9.00E-30	127/505(25.15)	119	AKT7|PHI:4194|V5XZS6|5599|Alternaria_alternata|increased_virulence_(Hypervirulence)
Chr02G0006.1	812	3.00E-39	98/333(29.43)	143	PHI:812|MGG_10702|EDJ94108|318829|Magnaporthe_oryzae|Reduced_virulence
Chr02G0010.1	2839	2.00E-94	152/346(43.93)	285	PHI:2839|RED1|Q8NJQ2|5016|Cochliobolus_heterostrophus|Reduced_virulence
Chr02G0011.1	812	5.00E-87	149/379(39.31)	273	PHI:812|MGG_10702|EDJ94108|318829|Magnaporthe_oryzae|Reduced_virulence
Chr02G0031.1	1662	2.00E-82	149/425(35.06)	279	PHI:1662|GzCCHC002|I1REJ1|5518|Gibberella_zeae_(related:_Fusarium_gra

					minearum)|Unaffected_pathogenicity
Chr02G0033.1	2099	0	479/1100(43.55)	862	PHI:2099|Pmc1|MGG_07971|148305|Magnaporthe_oryzae_(related:_Magnaporthe_grisea)|Mixed_outcome
Chr02G0034.1	1872	1.00E-74	144/383(37.60)	240	PHI:1872|GzZC187|I1RTU2|5518|Gibberella_zeae_(related:_Fusarium_graminearum)|Lethal
Chr02G0035.1	2731	2.00E-12	72/311(23.15)	66.6	PHI:2731|VdSge1|G2XD29|27337|Verticillium_dahliae|Loss_of_pathogenicity
Chr02G0036.1	441	3.00E-15	81/334(24.25)	74.3	PHI:441|BTP1|CAE55153|40559|Botrytis_cinerea|Reduced_virulence
Chr02G0039.1	1456	0	305/598(51.00)	596	PHI:1456|GzAra004|I1RN15|5518|Gibberella_zeae_(related:_Fusarium_graminearum)|Unaffected_pathogenicity
Chr02G0041.1	1934	3.00E-10	28/52(53.85)	58.5	PHI:1934|GzZC249|I1RC69|5518|Gibberella_zeae_(related:_Fusarium_graminearum)|Unaffected_pathogenicity
Chr02G0044.1	455	7.00E-10	54/197(27.41)	58.5	PHI:455|CAP59|AAC13946|5207|Cryptococcus_neoformans|Loss_of_pathogenicity
Chr02G0046.1	1279	3.00E-37	164/599(27.38)	145	PHI:1279|FGSG_12132|I1RU59|5518|Gibberella_zeae_(related:_Fusarium_graminearum)|Unaffected_pathogenicity
Chr02G0048.1	4194	3.00E-48	139/474(29.32)	179	AKT7|PHI:4194|V5XZS6|5599|Alternaria_alternata|increased_virulence_(Hypervirulence)
Chr02G0049.1	1662	1.00E-40	116/430(26.98)	155	PHI:1662|GzCCHC002|I1REJ1|5518|Gibberella_zeae_(related:_Fusarium_graminearum)|Unaffected_pathogenicity
Chr02G0056.1	2042	7.00E-54	108/278(38.85)	200	PHI:2042|ABC3|Q3Y5V5|148305|Magnaporthe_oryzae_(related:_Magnaporthe_grisea)|Loss_of_pathogenicity
Chr02G0059.1	1662	5.00E-94	167/468(35.68)	311	PHI:1662|GzCCHC002|I1REJ1|5518|Gibberella_zeae_(related:_Fusarium_graminearum)|Unaffected_pathogenicity
Chr02G0060.1	1885	2.00E-21	138/571(24.17)	95.5	PHI:1885|GzZC200|I1RJ54|5518|Gibberella_zeae_(related:_Fusarium_graminearum)|Unaffected_pathogenicity
Chr02G0062.1	538	2.00E-16	100/440(22.73)	79.3	PHI:538|FRT1|AAU87358|40559|Botrytis_cinerea|Unaffected_pathogenicity

Chr02G0065.1	24	0	349/781(44.69)	608	PHI:24|Avenacinase_gene|AAB09777|29850|Gaeumannomyces_graminis|Loss_of_pathogenicity
Chr02G0068.1	255	8.00E-18	80/333(24.02)	82	PHI:255|FUM1_(related:_FUM5)|AAD43562|5127|Gibberella_moniliformis|Unaffected_pathogenicity
Chr02G0069.1	1769	9.00E-14	88/365(24.11)	70.1	PHI:1769|GzZC084|I1RXB2|5518|Gibberella_zeae_(related:_Fusarium_graminearum)|Unaffected_pathogenicity
Chr02G0071.1	4194	6.00E-12	55/195(28.21)	65.1	AKT7|PHI:4194|V5XZS6|5599|Alternaria_alternata|increased_virulence_(Hypervirulence)
Chr02G0076.1	2549	0	413/788(52.41)	707	PHI:2549|MAK5|Q4WMS3|746128|Aspergillus_fumigatus|Mixed_outcome
Chr02G0080.1	716	4.00E-13	62/228(27.19)	68.2	PHI:716|ZEB1|ABB90284|5518|Fusarium_graminearum|Unaffected_pathogenicity
Chr02G0081.1	3453	1.00E-34	86/335(25.67)	132	ktr3|PHI:3453|Q5KM27|5207|Cryptococcus_neoformans|reduced_virulence_
Chr02G0083.1	510	1.00E-18	99/459(21.57)	86.3	PHI:510|CaNAG3|EAK93097|5476|Candida_albicans|Reduced_virulence
Chr02G0084.1	1514	0	327/718(45.54)	573	PHI:1514|GzHOME012|I1RYV5|5518|Gibberella_zeae_(related:_Fusarium_graminearum)|Unaffected_pathogenicity
Chr02G0086.1	2322	4.00E-20	66/241(27.39)	85.1	PHI:2322|SidH|Q4WF54|746128|Aspergillus_fumigatus|Reduced_virulence
Chr02G0089.1	2909	1.00E-08	56/234(23.93)	54.7	PHI:2909|CYP51C|I1S2M5|5518|Fusarium_graminearum|Mixed_outcome
Chr02G0092.1	817	1.00E-09	35/119(29.41)	57.8	PHI:817|MGG_12252|EDK03444|318829|Magnaporthe_oryzae|Reduced_virulence
Chr02G0095.1	1662	6.00E-67	140/442(31.67)	235	PHI:1662|GzCCHC002|I1REJ1|5518|Gibberella_zeae_(related:_Fusarium_graminearum)|Unaffected_pathogenicity
Chr02G0096.1	2183	0	614/786(78.12)	1246	PHI:2183|CPXB|EHA48040.1|148305|Magnaporthe_oryzae_(related:_Magnaporthe_grisea)|Unaffected_pathogenicity
Chr02G0098.1	1662	8.00E-93	179/456(39.25)	310	PHI:1662|GzCCHC002|I1REJ1|5518|Gibberella_zeae_(related:_Fusarium_graminearum)|Unaffected_pathogenicity
Chr02G0099.1	1399	2.00E-06	34/126(26.98)	48.9	PHI:1399|GzC2H062|I1RV74|5518|Gibberella_zeae_(related:_Fusarium_grami

					nearum)|Unaffected_pathogenicity
Chr02G0100.1	812	3.00E-48	113/370(30.54)	169	PHI:812|MGG_10702|EDJ94108|318829|Magnaporthe_oryzae|Reduced_virulence
Chr02G0101.1	784	1.00E-18	70/231(30.30)	82	PHI:784|MGG_00056|EDK03390|318829|Magnaporthe_oryzae|Reduced_virulence
Chr02G0103.1	716	6.00E-14	56/187(29.95)	71.6	PHI:716|ZEB1|ABB90284|5518|Fusarium_graminearum|Unaffected_pathogenicity
Chr02G0105.1	781	4.00E-23	61/186(32.80)	97.8	PHI:781|MGG_00131|EDK03302|318829|Magnaporthe_oryzae|Reduced_virulence
Chr02G0111.1	2490	2.00E-09	46/153(30.07)	57.4	PHI:2490|MCA|E9B636|5665|Leishmania_mexicana|Mixed_outcome
Chr02G0113.1	2147	9.00E-27	81/257(31.52)	107	PHI:2147|Erl1|G5EH97|148305|Magnaporthe_oryzae_(related:_Magnaporthe_grisea)|Reduced_virulence
Chr02G0114.1	2147	1.00E-36	100/295(33.90)	136	PHI:2147|Erl1|G5EH97|148305|Magnaporthe_oryzae_(related:_Magnaporthe_grisea)|Reduced_virulence
Chr02G0121.1	2968	2.00E-31	110/445(24.72)	124	PHI:2968|Hxs1|J9VQA5|5207|Cryptococcus_neoformans|Reduced_virulence
Chr02G0123.1	2895	3.00E-09	59/191(30.89)	56.2	PHI:2895|F-avi4330|B9JV05|373|Agrobacterium_vitis|Loss_of_pathogenicity
Chr02G0125.1	784	4.00E-06	64/227(28.19)	45.1	PHI:784|MGG_00056|EDK03390|318829|Magnaporthe_oryzae|Reduced_virulence
Chr02G0133.1	3386	5.00E-09	56/201(27.86)	54.7	FVEG_12528|PHI:3386|W7NCN7|117187|Fusarium_verticillioides|unaffected_pathogenicity_
Chr02G0136.1	2174	1.00E-08	32/90(35.56)	52.8	PHI:2174|NMR2|MGG_02860|148305|Magnaporthe_oryzae_(related:_Magnaporthe_grisea)|Reduced_virulence
Chr02G0137.1	2020	7.00E-13	58/235(24.68)	70.1	PHI:2020|Tup1|XP_759427|5270|Ustilago_maydis|Mixed_outcome
Chr02G0138.1	1260	3.00E-07	51/195(26.15)	52.4	PHI:1260|FGSG_13944|I1RUC7|5518|Gibberella_zeae_(related:_Fusarium_graminearum)|Unaffected_pathogenicity
Chr02G0139.1	223	9.00E-34	65/156(41.67)	124	PHI:223|PEP1|AAK11166|140110|Nectria_haematococca_(related:_Fusarium_

					solani)|Reduced_virulence
Chr02G0142.1	2357	1.00E-15	97/392(24.74)	77	PHI:2357|CYP52X1|E2EAF6|475271|Beauveria_bassiana|Reduced_virulence
Chr02G0143.1	1713	0	368/557(66.07)	734	PHI:1713|GzZC028|I1RGG4|5518|Gibberella_zeae_(related:_Fusarium_graminearum)|Unaffected_pathogenicity
Chr02G0145.1	3381	5.00E-19	108/463(23.33)	87.4	FVEG_12533|PHI:3381|W7N2B4|117187|Fusarium_verticillioides|unaffected_pathogenicity_
Chr02G0149.1	1159	5.00E-57	116/325(35.69)	206	PHI:1159|MgAtr7|A5H456|54734|Mycosphaerella_graminicola_(related:_Zymoseptoria_tritici)|Chemistry_target
Chr02G0150.1	812	7.00E-49	104/369(28.18)	172	PHI:812|MGG_10702|EDJ94108|318829|Magnaporthe_oryzae|Reduced_virulence
Chr02G0151.1	552	1.00E-90	202/599(33.72)	292	PHI:552|BcLCC2|AAK77953|40559|Botrytis_cinerea|Unaffected_pathogenicity
Chr02G0153.1	511	2.00E-68	145/460(31.52)	232	PHI:511|CaNAG4|EAK93098|5476|Candida_albicans|Reduced_virulence
Chr02G0156.1	167	6.00E-17	73/257(28.40)	80.5	PHI:167|CHIP3|AAF00024|5457|Colletotrichum_gloeosporioides|Unaffected_pathogenicity
Chr02G0158.1	404	2.00E-12	81/341(23.75)	65.5	PHI:404|PTH11|AAD30436|318829|Magnaporthe_oryzae|Reduced_virulence
Chr02G0159.1	2269	1.00E-07	69/264(26.14)	49.3	PHI:2269|Mdh1|0|13684|Phaeosphaeria_nodorum_(related:_Stagonospora_nodorum)|Unaffected_pathogenicity
Chr02G0160.1	2315	3.00E-52	106/291(36.43)	175	PHI:2315|ChLae1|G4XKY9|5016|Cochliobolus_heterostrophus|Mixed_outcome
Chr02G0169.1	2411	4.00E-143	214/366(58.47)	424	PHI:2411|Ss-ggt1|A7F946|5180|Sclerotinia_sclerotiorum|Reduced_virulence
Chr02G0170.1	1635	1.00E-16	63/171(36.84)	81.3	PHI:1635|GzWing007|I1RFX1|5518|Gibberella_zeae_(related:_Fusarium_graminearum)|Unaffected_pathogenicity
Chr02G0173.1	1269	7.00E-45	128/420(30.48)	160	PHI:1269|FGSG_02838|I1RFK9|5518|Gibberella_zeae_(related:_Fusarium_graminearum)|Unaffected_pathogenicity
Chr02G0176.1	2269	2.00E-22	69/257(26.85)	91.7	PHI:2269|Mdh1|0|13684|Phaeosphaeria_nodorum_(related:_Stagonospora_nodorum)|Unaffected_pathogenicity
Chr02G0177.1	1820	5.00E-33	97/297(32.66)	126	PHI:1820|GzZC135|I1S8F7|5518|Gibberella_zeae_(related:_Fusarium_gramine

					arum)|Unaffected_pathogenicity
Chr02G0180.1	513	1.00E-46	149/576(25.87)	171	PHI:513|ARN1_(related:_SIT1)|EAK97011|5476|Candida_albicans|Reduced_virulence
Chr02G0182.1	922	3.00E-75	207/620(33.39)	253	PHI:922|um03615||5270|Ustilago_maydis|Unaffected_pathogenicity
Chr02G0190.1	2654	4.00E-14	58/182(31.87)	73.2	PHI:2654|DUR1,2|Q59VF3|5476|Candida_albicans|Reduced_virulence
Chr02G0201.1	716	7.00E-115	174/353(49.29)	348	PHI:716|ZEB1|ABB90284|5518|Fusarium_graminearum|Unaffected_pathogenicity
Chr02G0203.1	441	1.00E-11	80/333(24.02)	62.8	PHI:441|BTP1|CAE55153|40559|Botrytis_cinerea|Reduced_virulence
Chr02G0204.1	2247	5.00E-34	92/279(32.97)	125	PHI:2247|Sch1|EAT82552|13684|Phaeosphaeria_nodorum_(related:_Stagonospora_nodorum)|Unaffected_pathogenicity
Chr02G0205.1	4194	1.00E-33	124/472(26.27)	130	AKT7|PHI:4194|V5XZS6|5599|Alternaria_alternata|increased_virulence_(Hypervirulence)
Chr02G0206.1	922	5.00E-63	187/632(29.59)	219	PHI:922|um03615||5270|Ustilago_maydis|Unaffected_pathogenicity
Chr02G0213.1	2543	9.00E-13	92/428(21.50)	66.6	PHI:2543|GCD6|Q4WLS1|746128|Aspergillus_fumigatus|Mixed_outcome
Chr02G0216.1	1793	0	589/862(68.33)	1065	PHI:1793|GzZC108|I1RWT7|5518|Gibberella_zeae_(related:_Fusarium_graminearum)|Reduced_virulence
Chr02G0222.1	134	4.00E-29	86/282(30.50)	112	PHI:134|AKT2|BAA36589|5599|Alternaria_alternata|Loss_of_pathogenicity
Chr02G0223.1	1662	2.00E-25	72/288(25.00)	107	PHI:1662|GzCCHC002|I1REJ1|5518|Gibberella_zeae_(related:_Fusarium_graminearum)|Unaffected_pathogenicity
Chr02G0225.1	1783	5.00E-41	169/607(27.84)	156	PHI:1783|GzZC098|I1S2C5|5518|Gibberella_zeae_(related:_Fusarium_graminearum)|Unaffected_pathogenicity
Chr02G0226.1	1592	8.00E-86	168/373(45.04)	264	PHI:1592|GzOB032|I1RWS0|5518|Gibberella_zeae_(related:_Fusarium_graminearum)|Unaffected_pathogenicity
Chr02G0228.1	211	5.00E-52	107/259(41.31)	189	PHI:211|CaTUP1|AAB63195|5476|Candida_albicans|Reduced_virulence
Chr02G0229.1	544	2.00E-171	251/551(45.55)	500	PHI:544|BCMFS1|AAF64435|332648|Botrytis_cinerea|Unaffected_pathogenicity

Chr02G0237.1	1447	1.00E-46	73/145(50.34)	164	PHI:1447|GzGATA005|I1RUP1|5518|Gibberella_zeae_(related:_Fusarium_graminearum)|Unaffected_pathogenicity
Chr02G0241.1	901	5.00E-34	133/495(26.87)	134	PHI:901|um01886||5270|Ustilago_maydis|Unaffected_pathogenicity
Chr02G0245.1	368	9.00E-81	165/405(40.74)	269	PHI:368|NOT4|AAF66693|5476|Candida_albicans|Reduced_virulence
Chr02G0254.1	438	1.00E-38	131/493(26.57)	145	PHI:438|BcBOT1_(related:_CND5)|AAQ16576|40559|Botrytis_cinerea|Reduced_virulence
Chr02G0255.1	716	1.00E-09	54/186(29.03)	58.2	PHI:716|ZEB1|ABB90284|5518|Fusarium_graminearum|Unaffected_pathogenicity
Chr02G0257.1	1167	0	876/1270(68.98)	1821	PHI:1167|NPC1|I1RY25|5518|Gibberella_zeae_(related:_Fusarium_graminearum)|Reduced_virulence
Chr02G0261.1	1312	5.00E-152	244/432(56.48)	474	PHI:1312|GzbHLH013|I1RVV8|5518|Gibberella_zeae_(related:_Fusarium_graminearum)|Unaffected_pathogenicity
Chr02G0269.1	3662	1.00E-08	42/120(35.00)	55.8	PspB_(not_PD0218)|PHI:3662|Q87ET0|2371|Xylella_fastidiosa|Increased_virulence_(Hypervirulence)
Chr02G0270.1	922	1.00E-44	146/514(28.40)	164	PHI:922|um03615||5270|Ustilago_maydis|Unaffected_pathogenicity
Chr02G0271.1	1773	1.00E-34	115/434(26.50)	131	PHI:1773|GzZC088|I1S409|5518|Gibberella_zeae_(related:_Fusarium_graminearum)|Unaffected_pathogenicity
Chr02G0272.1	2534	2.00E-15	94/371(25.34)	75.9	PHI:2534|ERG11A|Q4WNT5|746128|Aspergillus_fumigatus|Mixed_outcome
Chr02G0273.1	267	1.00E-61	143/490(29.18)	229	PHI:267|MLT1|AAD51594|5476|Candida_albicans|Reduced_virulence
Chr02G0277.1	2196	8.00E-173	327/830(39.40)	528	PHI:2196|MoRgs5|XP_363151|148305|Magnaporthe_oryzae_(related:_Magnaporthe_grisea)|Unaffected_pathogenicity
Chr02G0282.1	1418	6.00E-07	21/53(39.62)	47.8	PHI:1418|GzC2H085|I1S0W9|5518|Gibberella_zeae_(related:_Fusarium_graminearum)|Lethal
Chr02G0285.1	2996	1.00E-143	282/493(57.20)	421	PHI:2996|MGG_06507.6|G4N6W9|148305|Magnaporthe_oryzae|Reduced_virulence
Chr02G0288.1	2336	3.00E-09	26/99(26.26)	52	PHI:2336|NIA1|O00101|13684|Stagonospora_nodorum_(related:_Phaeosphaer

					ia_nodorum)|Unaffected_pathogenicity
Chr02G0291.1	323	5.00E-10	42/158(26.58)	59.3	PHI:323|VFGLU1|AAO63562|93591|Verticillium_fungicola|Reduced_virulence
Chr02G0294.1	3381	4.00E-105	191/483(39.54)	332	FVEG_12533|PHI:3381|W7N2B4|117187|Fusarium_verticillioides|unaffected_pathogenicity_
Chr02G0295.1	2562	3.00E-17	64/267(23.97)	82.8	PHI:2562|Cwt1|Q59M50|5476|Candida_albicans|Reduced_virulence
Chr02G0298.1	2401	2.00E-06	47/159(29.56)	44.3	PHI:2401|CaRING1|G0T3B3|456327|Xanthomonas_campestris_pv_vesicatoria|Mixed_outcome
Chr02G0300.1	1242	0	317/442(71.72)	607	PHI:1242|FGSG_02838|I1RWG2|5518|Gibberella_zeae_(related:_Fusarium_graminearum)|Unaffected_pathogenicity
Chr02G0301.1	3111	0	358/584(61.30)	608	FgAP1|PHI:3111|I1RWW4|5518|Fusarium_graminearum|mixed_outcome_
Chr02G0304.1	2025	9.00E-06	34/132(25.76)	46.6	PHI:2025|HDL1|G4MQZ9|148305|Magnaporthe_oryzae_(related:_Magnaporthe_grisea)|Unaffected_pathogenicity
Chr02G0307.1	2107	1.00E-89	170/431(39.44)	284	PHI:2107|Zinc-regulated_transporter_2|MGG_05905|148305|Magnaporthe_oryzae_(related:_Magnaporthe_grisea)|Mixed_outcome
Chr02G0308.1	191	7.00E-48	196/729(26.89)	179	PHI:191|TOM1|AAB08446|39703|Septoria_lycopersici|Unaffected_pathogenicity
Chr02G0309.1	1071	1.00E-18	89/394(22.59)	88.6	PHI:1071|Gas1|CAF05793|5270|Ustilago_maydis|Loss_of_pathogenicity
Chr02G0310.1	748	2.00E-11	77/321(23.99)	65.1	PHI:748|um00446|Not_available|5270|Ustilago_maydis|Unaffected_pathogenicity
Chr02G0311.1	207	2.00E-92	169/430(39.30)	286	PHI:207|CaNAG2|BAB43813|5476|Candida_albicans|Reduced_virulence
Chr02G0313.1	1611	0	308/532(57.89)	574	PHI:1611|GzP53L004|I1RVV2|5518|Gibberella_zeae_(related:_Fusarium_graminearum)|Unaffected_pathogenicity
Chr02G0314.1	206	7.00E-71	118/246(47.97)	224	PHI:206|CaNAG1|BAB43821|5476|Candida_albicans|Reduced_virulence
Chr02G0315.1	208	8.00E-38	133/486(27.37)	143	PHI:208|CaNAG5|BAB43816|5476|Candida_albicans|Reduced_virulence
Chr02G0316.1	1679	1.00E-56	138/351(39.32)	191	PHI:1679|GzMIZ001|I1RW03|5518|Gibberella_zeae_(related:_Fusarium_graminearum)|Unaffected_pathogenicity

Chr02G0322.1	3101	8.00E-121	188/427(44.03)	361	BCKDH|PHI:3101|Q8IEJ6|5833|Plasmodium_falciparum|reduced_virulence_
Chr02G0323.1	1662	2.00E-63	130/458(28.38)	224	PHI:1662|GzCCHC002|I1REJ1|5518|Gibberella_zeae_(related:_Fusarium_graminearum)|Unaffected_pathogenicity
Chr02G0324.1	1742	8.00E-26	122/549(22.22)	111	PHI:1742|GzZC057|I1S7H5|5518|Gibberella_zeae_(related:_Fusarium_graminearum)|Unaffected_pathogenicity
Chr02G0329.1	2844	1.00E-50	105/267(39.33)	171	PHI:2844|BRM2|O93802|5599|Alternaria_alternata|Unaffected_pathogenicity
Chr02G0330.1	1036	1.00E-06	49/182(26.92)	47	PHI:1036|Rac|CAO82105|5111|Claviceps_purpurea|Loss_of_pathogenicity
Chr02G0331.1	334	7.00E-07	46/159(28.93)	48.9	PHI:334|CGB1|AAO25585|5016|Cochliobolus_heterostrophus|Loss_of_pathogenicity
Chr02G0356.1	2611	0	278/365(76.16)	601	PHI:2611|CMLE|H9C592|59765|Fusarium_oxysporum_f._sp._Lycopersici|Loss_of_pathogenicity
Chr02G0357.1	267	6.00E-75	181/645(28.06)	273	PHI:267|MLT1|AAD51594|5476|Candida_albicans|Reduced_virulence
Chr02G0359.1	3078	1.00E-60	182/608(29.93)	216	ssnA|PHI:3078|C5VVJ6|1307|Streptococcus_suis|reduced_virulence_
Chr02G0361.1	1379	0	354/570(62.11)	646	PHI:1379|GzC2H042|I1RQS8|5518|Gibberella_zeae_(related:_Fusarium_graminearum)|Reduced_virulence
Chr02G0367.1	922	3.00E-54	181/614(29.48)	195	PHI:922|um03615||5270|Ustilago_maydis|Unaffected_pathogenicity
Chr02G0369.1	1178	0	281/324(86.73)	587	PHI:1178|(Sc_Cdc28)|I1RW16|5518|Gibberella_zeae_(related:_Fusarium_graminearum)|Reduced_virulence
Chr02G0371.1	244	4.00E-112	315/1024(30.76)	379	PHI:244|CLAP1|AAN62846|290576|Colletotrichum_lindemuthianum|Loss_of_pathogenicity
Chr02G0375.1	1565	3.00E-79	168/508(33.07)	262	PHI:1565|GzOB005|I1RBF6|5518|Gibberella_zeae_(related:_Fusarium_graminearum)|Unaffected_pathogenicity
Chr02G0376.1	503	6.00E-130	213/453(47.02)	388	PHI:503|COX15|AAB64668|4932|Saccharomyces_cerevisiae|Reduced_virulence
Chr02G0378.1	2101	0	298/504(59.13)	553	PHI:2101|Vacuolar_calcium_ion_transporter|MGG_11454|148305|Magnaporthe_oryzae_(related:_Magnaporthe_grisea)|Unaffected_pathogenicity

Chr02G0380.1	1593	3.00E-75	129/287(44.95)	232	PHI:1593|GzOB033|I1RWS6|5518|Gibberella_zeae_(related:_Fusarium_graminearum)|Unaffected_pathogenicity
Chr02G0381.1	981	4.00E-11	34/71(47.89)	60.8	PHI:981|hopI1|AAL84247|59511|Pseudomonas_syringae|Effector_(plant_avirulence_determinant)
Chr02G0384.1	2251	2.00E-171	221/318(69.50)	481	PHI:2251|Gox1|Q696X2|13684|Phaeosphaeria_nodorum_(related:_Stagonospora_nodorum)|Unaffected_pathogenicity
Chr02G0388.1	1299	0	533/940(56.70)	1001	PHI:1299|GzAT003|I1RYN7|5518|Gibberella_zeae_(related:_Fusarium_graminearum)|Unaffected_pathogenicity
Chr02G0389.1	2269	9.00E-54	102/255(40.00)	179	PHI:2269|Mdh1|0|13684|Phaeosphaeria_nodorum_(related:_Stagonospora_nodorum)|Unaffected_pathogenicity
Chr02G0390.1	518	0	613/1021(60.04)	1037	PHI:518|MST20|AAP93639|318829|Magnaporthe_oryzae|Unaffected_pathogenicity
Chr02G0392.1	2321	2.00E-17	108/467(23.13)	83.6	PHI:2321|SidI|Q4WR83|746128|Aspergillus_fumigatus|Reduced_virulence
Chr02G0399.1	2058	1.00E-35	127/450(28.22)	141	PHI:2058|LHS1|MGG_06648.5|148305|Magnaporthe_oryzae_(related:_Magnaporthe_grisea)|Reduced_virulence
Chr02G0401.1	2504	7.00E-08	34/121(28.10)	52.8	PHI:2504|AGS2|Q96UR0|746128|Aspergillus_fumigatus|Unaffected_pathogenicity
Chr02G0402.1	2504	0	1002/1817(55.15)	1963	PHI:2504|AGS2|Q96UR0|746128|Aspergillus_fumigatus|Unaffected_pathogenicity
Chr02G0403.1	2393	4.00E-42	121/468(25.85)	155	PHI:2393|Related_to_O-methylsterigmatocystin_oxidoreductase|I1R980|5518|Gibberella_zeae_(related:_Fusarium_graminearum)|Increased_virulence_(Hypervirulence)
Chr02G0406.1	566	1.00E-76	165/460(35.87)	246	PHI:566|cel2|AAK19621|5017|Cochliobolus_carbonum|Unaffected_pathogenicity
Chr02G0407.1	256	1.00E-25	79/245(32.24)	101	PHI:256|GAS1|AAK52794|318829|Magnaporthe_oryzae|Reduced_virulence
Chr02G0409.1	222	2.00E-16	60/218(27.52)	76.6	PHI:222|PELB|AAD09857|5457|Colletotrichum_gloeosporioides|Reduced_virul

					ence
Chr02G0414.1	2020	2.00E-48	109/312(34.94)	184	PHI:2020|Tup1|XP_759427|5270|Ustilago_maydis|Mixed_outcome
Chr02G0415.1	1522	6.00E-118	155/238(65.13)	342	PHI:1522|GzHOMEL026|I1RXA5|5518|Gibberella_zeae_(related:_Fusarium_graminearum)|Lethal
Chr02G0417.1	1260	9.00E-25	81/224(36.16)	108	PHI:1260|FGSG_13944|I1RUC7|5518|Gibberella_zeae_(related:_Fusarium_graminearum)|Unaffected_pathogenicity
Chr02G0418.1	1921	4.00E-12	35/94(37.23)	67	PHI:1921|GzZC236|I1RCH8|5518|Gibberella_zeae_(related:_Fusarium_graminearum)|Reduced_virulence
Chr02G0419.1	143	6.00E-76	132/320(41.25)	239	PHI:143|CHT|AAA33353|5563|Gloeocercospora_sorghi|Unaffected_pathogenicity
Chr02G0420.1	2835	3.00E-07	42/165(25.45)	48.5	PHI:2835|RED2|C3JXE8|5016|Cochliobolus_heterostrophus|Reduced_virulence
Chr02G0422.1	2247	4.00E-08	44/147(29.93)	50.4	PHI:2247|Sch1|EAT82552|13684|Phaeosphaeria_nodorum_(related:_Stagonospora_nodorum)|Unaffected_pathogenicity
Chr02G0429.1	2213	8.00E-104	146/234(62.39)	301	PHI:2213|endo-1,4-beta-xylanase_I_[GH10_family]|MGG_07955|148305|Magnaporthe_oryzae_(related:_Magnaporthe_grisea)|Reduced_virulence
Chr02G0431.1	1714	2.00E-172	312/768(40.62)	513	PHI:1714|GzZC029|I1RQU8|5518|Gibberella_zeae_(related:_Fusarium_graminearum)|Lethal
Chr02G0433.1	2171	8.00E-109	192/478(40.17)	339	PHI:2171|Peroxisomal_copper_amine_oxidase|MGG_02681|148305|Magnaporthe_oryzae_(related:_Magnaporthe_grisea)|Reduced_virulence
Chr02G0436.1	1260	6.00E-09	42/114(36.84)	54.3	PHI:1260|FGSG_13944|I1RUC7|5518|Gibberella_zeae_(related:_Fusarium_graminearum)|Unaffected_pathogenicity
Chr02G0438.1	481	0	430/655(65.65)	749	PHI:481|KLAP1|AAX14039|27357|Colletotrichum_acutatum|Loss_of_pathogenicity
Chr02G0439.1	1423	4.00E-07	62/234(26.50)	51.2	PHI:1423|GzC2H091|I1S2R1|5518|Gibberella_zeae_(related:_Fusarium_graminearum)|Unaffected_pathogenicity

Chr02G0442.1	1882	2.00E-46	113/400(28.25)	171	PHI:1882|GzZC197|I1RJ84|5518|Gibberella_zeae_(related:_Fusarium_graminearum)|Unaffected_pathogenicity
Chr02G0443.1	1458	4.00E-33	119/406(29.31)	132	PHI:1458|GzAra006|Q4I7F9|5518|Gibberella_zeae_(related:_Fusarium_graminearum)|Unaffected_pathogenicity
Chr02G0444.1	2401	3.00E-06	21/46(45.65)	42.7	PHI:2401|CaRING1|G0T3B3|456327|Xanthomonas_campestris_pv_vesicatoria|Mixed_outcome
Chr02G0447.1	127	1.00E-07	65/234(27.78)	51.2	PHI:127|SAP6|CAA82924|5476|Candida_albicans|Reduced_virulence
Chr02G0448.1	59	6.00E-07	47/174(27.01)	47.4	PHI:59|THR1|BAA18962|5462|Colletotrichum_lagenarium|Reduced_virulence
Chr02G0449.1	1949	8.00E-56	128/380(33.68)	189	PHI:1949|GzZC264|I1RI60|5518|Gibberella_zeae_(related:_Fusarium_graminearum)|Lethal
Chr02G0454.1	1815	5.00E-104	238/774(30.75)	337	PHI:1815|GzZC130|I1RL60|5518|Gibberella_zeae_(related:_Fusarium_graminearum)|Unaffected_pathogenicity
Chr02G0468.1	2117	5.00E-51	141/437(32.27)	179	PHI:2117|SPM1|P58371|148305|Magnaporthe_oryzae_(related:_Magnaporthe_grisea)|Reduced_virulence
Chr02G0472.1	1992	6.00E-107	214/529(40.45)	340	PHI:1992|GzZC307|I1R983|5518|Gibberella_zeae_(related:_Fusarium_graminearum)|Unaffected_pathogenicity
Chr02G0473.1	2107	0	339/613(55.30)	614	PHI:2107|Zinc-regulated_transporter_2|MGG_05905|148305|Magnaporthe_oryzae_(related:_Magnaporthe_grisea)|Mixed_outcome
Chr02G0475.1	874	9.00E-08	66/290(22.76)	51.6	PHI:874|MGG_00435|EDK02952|318829|Magnaporthe_oryzae|Loss_of_pathogenicity
Chr02G0476.1	2025	3.00E-06	38/127(29.92)	46.2	PHI:2025|HDL1|G4MQZ9|148305|Magnaporthe_oryzae_(related:_Magnaporthe_grisea)|Unaffected_pathogenicity
Chr02G0479.1	438	2.00E-06	30/96(31.25)	46.6	PHI:438|BcBOT1_(related:_CND5)|AAQ16576|40559|Botrytis_cinerea|Reduced_virulence
Chr02G0481.1	17	3.00E-19	90/379(23.75)	87.8	PHI:17|ACP|CAA43678|5482|Candida_tropicalis|Reduced_virulence
Chr02G0482.1	144	1.00E-178	250/413(60.53)	507	PHI:144|CHT42|AAC05829|29875|Trichoderma_virens|Reduced_virulence

Chr02G0483.1	438	4.00E-172	242/497(48.69)	497	PHI:438|BcBOT1_(related:_CND5)|AAQ16576|40559|Botrytis_cinerea|Reduced_virulence
Chr02G0485.1	441	1.00E-35	95/314(30.25)	134	PHI:441|BTP1|CAE55153|40559|Botrytis_cinerea|Reduced_virulence
Chr02G0489.1	1527	1.00E-21	83/321(25.86)	93.2	PHI:1527|GzHOMEL040|I1S9A1|5518|Gibberella_zeae_(related:_Fusarium_graminearum)|Lethal
Chr02G0496.1	1047	1.00E-23	98/348(28.16)	97.1	PHI:1047|CTB6|ABK64183|29003|Cercospora_nicotianae|Reduced_virulence
Chr02G0500.1	191	3.00E-46	116/341(34.02)	175	PHI:191|TOM1|AAB08446|39703|Septoria_lycopersici|Unaffected_pathogenicity
Chr02G0503.1	2240	1.00E-31	119/517(23.02)	125	PHI:2240|Srt1|Q4PBY9|5270|Ustilago_maydis|reduced_virulence
Chr02G0505.1	267	2.00E-53	175/662(26.44)	203	PHI:267|MLT1|AAD51594|5476|Candida_albicans|Reduced_virulence
Chr02G0506.1	881	7.00E-19	55/154(35.71)	82.8	PHI:881|MGG_04556|EDJ96020|318829|Magnaporthe_oryzae|Reduced_virulence
Chr02G0507.1	1759	2.00E-61	148/490(30.20)	216	PHI:1759|GzZC074|I1RXS2|5518|Gibberella_zeae_(related:_Fusarium_graminearum)|Unaffected_pathogenicity
Chr02G0512.1	166	3.00E-73	154/374(41.18)	245	PHI:166|CHIP2|AAD53262|5457|Colletotrichum_gloeosporioides|Unaffected_pathogenicity
Chr02G0514.1	2256	4.00E-29	103/357(28.85)	113	PHI:2256|Xdh1|Q0UA24|13684|Phaeosphaeria_nodorum_(related:_Stagonospora_nodorum)|Unaffected_pathogenicity
Chr02G0521.1	441	3.00E-22	94/387(24.29)	94.7	PHI:441|BTP1|CAE55153|40559|Botrytis_cinerea|Reduced_virulence
Chr02G0522.1	1046	1.00E-49	145/474(30.59)	175	PHI:1046|CTB5|ABK64182|29003|Cercospora_nicotianae|Reduced_virulence
Chr02G0525.1	538	6.00E-18	86/332(25.90)	84.3	PHI:538|FRT1|AAU87358|40559|Botrytis_cinerea|Unaffected_pathogenicity
Chr02G0531.1	697	7.00E-13	88/345(25.51)	66.6	PHI:697|ugt51E1|AAM81358|5022|Leptosphaeria_maculans|Unaffected_pathogenicity
Chr02G0535.1	1420	1.00E-28	118/503(23.46)	118	PHI:1420|GzC2H088|I1S172|5518|Gibberella_zeae_(related:_Fusarium_graminearum)|Unaffected_pathogenicity
Chr02G0539.1	244	3.00E-103	312/1048(29.77)	353	PHI:244|CLAP1|AAN62846|290576|Colletotrichum_lindemuthianum|Loss_of_p

					athogenicity
Chr02G0546.1	2849	5.00E-47	97/232(41.81)	155	PHI:2849|cutA|Q99174|70790|Fusarium_solani_f._sp._cucurbitae|Unaffected_pathogenicity
Chr02G0547.1	922	3.00E-81	218/619(35.22)	270	PHI:922|um03615||5270|Ustilago_maydis|Unaffected_pathogenicity
Chr02G0548.1	1279	1.00E-35	136/481(28.27)	143	PHI:1279|FGSG_12132|I1RU59|5518|Gibberella_zeae_(related:_Fusarium_graminearum)|Unaffected_pathogenicity
Chr02G0549.1	1279	1.00E-42	118/385(30.65)	159	PHI:1279|FGSG_12132|I1RU59|5518|Gibberella_zeae_(related:_Fusarium_graminearum)|Unaffected_pathogenicity
Chr02G0551.1	1816	3.00E-28	124/520(23.85)	119	PHI:1816|GzZC131|I1RRS3|5518|Gibberella_zeae_(related:_Fusarium_graminearum)|Unaffected_pathogenicity
Chr02G0556.1	1260	1.00E-07	38/130(29.23)	53.5	PHI:1260|FGSG_13944|I1RUC7|5518|Gibberella_zeae_(related:_Fusarium_graminearum)|Unaffected_pathogenicity
Chr02G0560.1	2517	0	523/736(71.06)	1089	PHI:2517|TRPS|Q4X0J6|746128|Aspergillus_fumigatus|Lethal
Chr02G0564.1	3415	3.00E-20	66/235(28.09)	92.8	Fre2|PHI:3415|T2BNJ5|5207|Cryptococcus_neoformans|mixed_outcome_
Chr02G0568.1	697	4.00E-06	71/328(21.65)	45.8	PHI:697|ugt51E1|AAM81358|5022|Leptosphaeria_maculans|Unaffected_pathogenicity
Chr02G0570.1	1458	2.00E-45	116/396(29.29)	169	PHI:1458|GzAra006|Q4I7F9|5518|Gibberella_zeae_(related:_Fusarium_graminearum)|Unaffected_pathogenicity
Chr02G0573.1	2020	2.00E-14	58/238(24.37)	74.7	PHI:2020|Tup1|XP_759427|5270|Ustilago_maydis|Mixed_outcome
Chr02G0574.1	2038	3.00E-07	30/85(35.29)	48.5	PHI:2038|Mir1|MGG_02370|148305|Magnaporthe_oryzae_(related:_Magnaporthe_grisea)|Unaffected_pathogenicity
Chr02G0578.1	2419	5.00E-151	205/322(63.66)	428	PHI:2419|CID1|I1RKF3|5518|Gibberella_zeae_(related:_Fusarium_graminearum)|Reduced_virulence
Chr02G0579.1	1260	8.00E-20	62/151(41.06)	92	PHI:1260|FGSG_13944|I1RUC7|5518|Gibberella_zeae_(related:_Fusarium_graminearum)|Unaffected_pathogenicity
Chr02G0584.1	405	5.00E-155	284/612(46.41)	505	PHI:405|PDE1|AAK07740|318829|Magnaporthe_oryzae|Reduced_virulence

Chr02G0586.1	3381	6.00E-81	166/439(37.81)	264	FVEG_12533|PHI:3381|W7N2B4|117187|Fusarium_verticillioides|unaffected_pathogenicity_
Chr02G0587.1	4194	9.00E-60	137/508(26.97)	205	AKT7|PHI:4194|V5XZS6|5599|Alternaria_alternata|increased_virulence_(Hypervirulence)
Chr02G0590.1	346	6.00E-16	56/196(28.57)	78.2	PHI:346|CRU1|AAN10186|5270|Ustilago_maydis|Reduced_virulence
Chr02G0593.1	511	7.00E-09	111/506(21.94)	55.8	PHI:511|CaNAG4|EAK93098|5476|Candida_albicans|Reduced_virulence
Chr02G0594.1	3381	3.00E-42	120/402(29.85)	163	FVEG_12533|PHI:3381|W7N2B4|117187|Fusarium_verticillioides|unaffected_pathogenicity_
Chr02G0600.1	922	7.00E-29	164/648(25.31)	119	PHI:922|um03615||5270|Ustilago_maydis|Unaffected_pathogenicity
Chr02G0602.1	1884	1.00E-09	26/83(31.33)	58.2	PHI:1884|GzZC199|I1RJ58|5518|Gibberella_zeae_(related:_Fusarium_graminearum)|Unaffected_pathogenicity
Chr02G0605.1	440	3.00E-68	208/780(26.67)	243	PHI:440|PMR1|CAB87245|5476|Candida_albicans|Reduced_virulence
Chr02G0609.1	254	0	268/311(86.17)	507	PHI:254|FOW1|BAB85760|5507|Fusarium_oxysporum|Reduced_virulence
Chr02G0610.1	447	3.00E-164	250/459(54.47)	474	PHI:447|MCSA|CAI61947|5085|Aspergillus_fumigatus|Reduced_virulence
Chr02G0613.1	2321	5.00E-10	120/514(23.35)	59.7	PHI:2321|SidI|Q4WR83|746128|Aspergillus_fumigatus|Reduced_virulence
Chr02G0615.1	3385	0	315/433(72.75)	647	FVEG_12529|PHI:3385|W7MS09|117187|Fusarium_verticillioides|unaffected_pathogenicity_
Chr02G0619.1	3029	0	379/703(53.91)	761	PMT2|PHI:3029|Q5KAF1|5207|Cryptococcus_neoformans|increased_virulence_
Chr02G0620.1	339	4.00E-33	81/266(30.45)	120	PHI:339|CLPT1|CAC41973|290576|Colletotrichum_lindemuthianum|Reduced_virulence
Chr02G0625.1	2315	1.00E-45	94/281(33.45)	157	PHI:2315|ChLae1|G4XKY9|5016|Cochliobolus_heterostrophus|Mixed_outcome
Chr02G0626.1	2968	4.00E-51	141/478(29.50)	183	PHI:2968|Hxs1|J9VQA5|5207|Cryptococcus_neoformans|Reduced_virulence
Chr02G0633.1	860	3.00E-43	96/251(38.25)	162	PHI:860|MSP1|AAX07670|318829|Magnaporthe_oryzae|Reduced_virulence
Chr02G0636.1	2746	5.00E-19	83/299(27.76)	88.6	PHI:2746|treZ|Q02LV7|287|Pseudomonas_aeruginosa|Mixed_outcome
Chr02G0638.1	1913	4.00E-43	146/538(27.14)	158	PHI:1913|GzZC228|I1RP37|5518|Gibberella_zeae_(related:_Fusarium_gramin

					earum)|Unaffected_pathogenicity
Chr02G0642.1	3126	4.00E-28	86/329(26.14)	112	argD|PHI:3126|D4I307|552|Erwinia_amylovora|mixed_outcome_
Chr02G0644.1	3004	2.00E-67	138/363(38.02)	225	PHI:3004|ClpX|A6QHK8|1280|Staphylococcus_aureus|Reduced_virulence
Chr02G0645.1	3411	5.00E-44	125/413(30.27)	160	bscN|PHI:3411|O68539|518|Bordetella_bronchiseptica|reduced_virulence_
Chr02G0650.1	339	9.00E-54	86/211(40.76)	172	PHI:339|CLPT1|CAC41973|290576|Colletotrichum_lindemuthianum|Reduced_virulence
Chr02G0651.1	2546	2.00E-75	132/317(41.64)	236	PHI:2546|BRX1|Q4WKJ9|746128|Aspergillus_fumigatus|Mixed_outcome
Chr02G0653.1	1172	0	473/716(66.06)	1023	PHI:1172|HDF2|I1RKC4|5518|Gibberella_zeae_(related:_Fusarium_graminearum)|Reduced_virulence
Chr02G0660.1	1953	3.00E-151	232/470(49.36)	444	PHI:1953|GzZC268|I1RKB4|5518|Gibberella_zeae_(related:_Fusarium_graminearum)|Unaffected_pathogenicity
Chr02G0662.1	2261	0	919/1963(46.82)	1711	PHI:2261|NPS6|G8DNS9|45130|Cochliobolus_sativus|Reduced_virulence
Chr02G0664.1	2321	0	329/569(57.82)	695	PHI:2321|SidI|Q4WR83|746128|Aspergillus_fumigatus|Reduced_virulence
Chr02G0666.1	513	1.00E-07	114/521(21.88)	52	PHI:513|ARN1_(related:_SIT1)|EAK97011|5476|Candida_albicans|Reduced_virulence
Chr02G0669.1	2076	1.00E-78	112/118(94.92)	229	PHI:2076|Moatg8|MGG_01062|148305|Magnaporthe_oryzae_(related:_Magnaporthe_grisea)|Loss_of_pathogenicity
Chr02G0670.1	911	6.00E-21	76/291(26.12)	89.4	PHI:911|um11451||5270|Ustilago_maydis|Reduced_virulence
Chr02G0677.1	1604	1.00E-95	137/140(97.86)	274	PHI:1604|GzOB045|I1S1V9|5518|Gibberella_zeae_(related:_Fusarium_graminearum)|Unaffected_pathogenicity
Chr02G0679.1	1624	0	425/597(71.19)	867	PHI:1624|GzJUM006|I1SAP7|5518|Gibberella_zeae_(related:_Fusarium_graminearum)|Unaffected_pathogenicity
Chr02G0687.1	2858	3.00E-112	248/549(45.17)	345	PHI:2858|FvVE1|A0MAR2|117187|Fusarium_verticillioides|Loss_of_pathogenicity
Chr02G0690.1	1391	1.00E-07	36/140(25.71)	51.2	PHI:1391|GzC2H054|I1RU55|5518|Gibberella_zeae_(related:_Fusarium_graminearum)|Unaffected_pathogenicity

Chr02G0692.1	883	1.00E-08	58/127(45.67)	47	PHI:883|MGG_08560|EDJ97072|318829|Magnaporthe_oryzae|Reduced_virulence
Chr02G0701.1	1537	1.00E-116	211/329(64.13)	340	PHI:1537|MYT1|I1RA01|5518|Gibberella_zeae_(related:_Fusarium_graminearum)|Unaffected_pathogenicity
Chr02G0703.1	2401	3.00E-10	34/84(40.48)	57.4	PHI:2401|CaRING1|G0T3B3|456327|Xanthomonas_campestris_pv_vesicatoria|Mixed_outcome
Chr02G0706.1	1989	0	248/464(53.45)	535	PHI:1989|GzZC304|I1R9Z6|5518|Gibberella_zeae_(related:_Fusarium_graminearum)|Unaffected_pathogenicity
Chr02G0707.1	1629	7.00E-89	186/529(35.16)	298	PHI:1629|GzWing001|I1RAH2|5518|Gibberella_zeae_(related:_Fusarium_graminearum)|Unaffected_pathogenicity
Chr02G0713.1	3348	4.00E-11	34/78(43.59)	57	nfu|PHI:3348|Q2FID9|1280|Staphylococcus_aureus|reduced_virulence_
Chr02G0714.1	1345	9.00E-68	113/237(47.68)	213	PHI:1345|GzC2H005|I1RAW2|5518|Gibberella_zeae_(related:_Fusarium_graminearum)|Unaffected_pathogenicity
Chr02G0716.1	319	2.00E-12	52/255(20.39)	68.2	PHI:319|SQL2|AAO19638|5270|Ustilago_maydis|Reduced_virulence
Chr02G0726.1	2293	3.00E-51	73/97(75.26)	162	PHI:2293|cycA|B0XTA5|746128|Aspergillus_fumigatus|Reduced_virulence
Chr02G0727.1	226	1.00E-60	146/413(35.35)	221	PHI:226|PEX6|AAK16738|5462|Colletotrichum_lagenarium|Loss_of_pathogenicity
Chr02G0735.1	2032	3.00E-41	133/464(28.66)	154	PHI:2032|VTL1|G4NGA7|148305|Magnaporthe_oryzae_(related:_Magnaporthe_grisea)|Unaffected_pathogenicity
Chr02G0738.1	1579	0	307/672(45.68)	581	PHI:1579|GzOB019|I1RM25|5518|Gibberella_zeae_(related:_Fusarium_graminearum)|Unaffected_pathogenicity
Chr02G0740.1	1629	2.00E-09	64/232(27.59)	58.9	PHI:1629|GzWing001|I1RAH2|5518|Gibberella_zeae_(related:_Fusarium_graminearum)|Unaffected_pathogenicity
Chr02G0747.1	3331	5.00E-06	36/120(30.00)	43.1	snf7|PHI:3331|J9VU41|5207|Cryptococcus_neoformans|loss_of_pathogenicity_
Chr02G0749.1	3278	0	1270/1420(89.44)	2564	Colra1|PHI:3278|N4V0R3|5465|Colletotrichum_orbiculare|reduced_virulence_
Chr02G0751.1	2354	0	287/529(54.25)	558	PHI:2354|AMT2|XP_390894|5518|Gibberella_zeae_(related:_Fusarium_gramin

					earum)|Reduced_virulence
Chr02G0759.1	1238	0	513/797(64.37)	954	PHI:1238|FGSG_06970|I1S1V3|5518|Gibberella_zeae_(related:_Fusarium_graminearum)|Lethal
Chr02G0767.1	337	5.00E-25	76/284(26.76)	100	PHI:337|CHS7|AAT77184|5507|Fusarium_oxysporum|Reduced_virulence
Chr02G0768.1	1376	0	319/479(66.60)	660	PHI:1376|GzC2H039|I1RPM7|5518|Gibberella_zeae_(related:_Fusarium_graminearum)|Unaffected_pathogenicity
Chr02G0775.1	253	4.00E-66	157/429(36.60)	239	PHI:253|FOS1|AAK27436|5085|Aspergillus_fumigatus|Reduced_virulence
Chr02G0778.1	854	4.00E-84	161/358(44.97)	285	PHI:854|um01947|EAK82380|5270|Ustilago_maydis|Reduced_virulence
Chr02G0780.1	3161	0	1156/1962(58.92)	2104	Mohik5|PHI:3161|G4MKP6|318829|Magnaporthe_oryzae|loss_of_pathogenicity_
Chr11G0005.1	876	2.00E-54	123/356(34.55)	193	PHI:876|MGG_11671|EDK03349|318829|Magnaporthe_oryzae|Reduced_virulence
Chr11G0007.1	1263	7.00E-64	167/545(30.64)	221	PHI:1263|FGSG_04770|I1RB84|5518|Gibberella_zeae_(related:_Fusarium_graminearum)|Unaffected_pathogenicity
Chr11G0014.1	1420	2.00E-08	98/434(22.58)	54.3	PHI:1420|GzC2H088|I1S172|5518|Gibberella_zeae_(related:_Fusarium_graminearum)|Unaffected_pathogenicity
Chr10G0009.1	2553	8.00E-10	59/191(30.89)	58.9	PHI:2553|VPS4|Q5AG40|5476|Candida_albicans|Loss_of_pathogenicity
Chr12G0007.1	2549	3.00E-07	24/87(27.59)	48.9	PHI:2549|MAK5|Q4WMS3|746128|Aspergillus_fumigatus|Mixed_outcome
