# Supplementary material for: Decoding Pecan’s Fungal Foe: A Genomic Insight into Colletotrichum plurivorum Isolate W-6
Source: J Fungi (Basel). 2025 Mar 5;11(3):203. doi: 10.3390/jof11030203 (PMC11943440; doi:10.3390/jof11030203)
Supplement: Supplementary file 1 [file jof-11-00203-s001.zip › Table S24.pdf]

Table S24. Prediction of CYTP450 genes in isolate W-6 genome.

Query_id	Query_length	Identity	Subject_id	Subject_length	Query_start	Query_end	Subject_start	Subject_end	Positive	Gap	Align_length	Score	E_value	Query_annotation	Subject_annotation
Chr04G0720.1	471	76.3	genemark-scaffold15-processed-gene-0.52-mRNA-1[PseudohalonectrialignicolaM95]	905	21	58	536	573	0.69	0	37	73.6	4.0e-13	gene=Chr04G0720.1	genemark-scaffold15-processed-gene-0.52-mRNA-1[PseudohalonectrialignicolaM95]
Chr09G0805.1	374	50.9	Clame_scaffold9-5.100[Cladoniametacorallifera]	12517	275	327	2616	2660	0.54	1	52	48.9	8.3e-06	gene=Chr09G0805.1	Clame_scaffold9-5.100[Cladoniametacorallifera]
Chr03G0059.1	549	59.1	NOG_01398-R0[MagnaporthesalviniiM69]	425	500	542	304	347	0.22	1	42	54.3	2.9e-07	gene=Chr03G0059.1	NOG_01398-R0[MagnaporthesalviniiM69]
Chr01G0827.1	753	56.1	Clame_scaffold11-8.74[Cladoniametacora	8336	181	221	2034	2074	0.22	0	40	50.4	5.7e-06	gene=Chr01G0827.1	Clame_scaffold11-8.74[Cladoniametacorallifera]

			llifera]												
Chr03G0057.1	475	56.2	NOG_01398-R0[Magnaporthesalvinii M69]	425	370	417	297	341	0.22	1	47	57.4	3.0e-08	gene=Chr03G0057.1	NOG_01398-R0[MagnaporthesalviniiM69]
Chr06G1089.1	1206	55.1	Clama_scaffold_5-4.0[Cladoniamacilenta]	21962	1074	1121	15839	15885	0.23	2	47	57	9.8e-08	gene=Chr06G1089.1	Clama_scaffold_5-4.0[Cladoniamacilenta]
Chr04G0395.1	357	45.3	Genemark.6490_g[Ceriporiopsissubvermispora]	617	267	330	76	130	0.26	2	63	54.7	1.4e-07	gene=Chr04G0395.1	Genemark.6490_g[Ceriporiopsissubvermispora]
Chr05G0423.1	99	75.8	estExt_GeneWisePlus.C_110630[AspergillusnigerATCC1015]	2417	4	69	998	1063	0.27	0	65	102.4	1.7e-22	gene=Chr05G0423.1	estExt_GeneWisePlus.C_110630[AspergillusnigerATCC1015]
Chr03G1305.1	478	33.8	AB00197.1[Alternariabrassicicola]	1041	163	239	189	250	0.27	2	76	50.8	2.8e-06	gene=Chr03G1305.1	AB00197.1[Alternariabrassicicola]
Chr09G0560.1	420	52.9	EEA23504	1213	3	53	533	583	0.2	0	50	60.5	3.1e-09	gene=C	EEA23504.1[Penicilli

			.1[Penicilliummarneffei]						8					hr09G0560.1	ummarneffei]
Chr08G0043.1	360	36.7	Genemark.6490_g[Ceriporiopsissubvermispora]	617	249	308	62	120	0.30	1	59	51.6	1.2e-06	gene=Chr08G0043.1	Genemark.6490_g[Ceriporiopsissubvermispora]
Chr04G0970.1	432	41.4	PTRG_10301[Pyrenophoratritici-repentis]	1311	287	344	689	746	0.31	0	57	51.2	1.9e-06	gene=Chr04G0970.1	PTRG_10301[Pyrenophoratritici-repentis]
Chr06G1370.1	648	30.1	FVEG_04314T0[Fusariumverticillioides]	898	50	124	7	99	0.31	1	74	51.6	2.2e-06	gene=Chr06G1370.1	FVEG_04314T0[Fusariumverticillioides]
Chr03G1233.1	879	45.8	NOG_01398-R0[MagnaporthesalviniiM69]	425	809	876	274	341	0.31	2	67	55.1	2.7e-07	gene=Chr03G1233.1	NOG_01398-R0[MagnaporthesalviniiM69]
Chr05G1089.1	1312	49.2	Clama_scaffold_5-4.0[Cladoniamacilenta]	21962	848	906	15814	15874	0.31	2	58	55.8	2.4e-07	gene=Chr05G1089.1	Clama_scaffold_5-4.0[Cladoniamacilenta]

Chr04G0224.1	304	35.1	XP_007802185.1[EndocarponpusillumZ07020]	730	180	264	136	220	0.33	4	84	61.6	1.0e-09	gene=Chr04G0224.1	XP_007802185.1[EndocarponpusillumZ07020]
Chr07G0707.1	140	36.8	Endpu_scaffold7-6.70[EndocarponpusillumR61883]	936	35	102	22	89	0.35	0	67	57.4	8.7e-09	gene=Chr07G0707.1	Endpu_scaffold7-6.70[EndocarponpusillumR61883]
Chr04G0426.1	587	42	Clame_scaffold18-19.70[Cladoniametacorallifera]	7866	234	300	2176	2244	0.35	1	66	57	4.8e-08	gene=Chr04G0426.1	Clame_scaffold18-19.70[Cladoniametacorallifera]
Chr05G0229.1	254	38.2	Clama_scaffold_28-0.22[Cladoniamacilenta]	3845	68	135	3462	3529	0.35	0	67	63.2	2.9e-10	gene=Chr05G0229.1	Clama_scaffold_28-0.22[Cladoniamacilenta]
Chr11G0005.1	401	62.3	Pa_3_950[Podosporaanserina]	960	237	305	1	69	0.36	0	68	99.4	5.7e-21	gene=Chr11G0005.1	Pa_3_950[Podosporaanserina]
Chr08G0194.1	661	51.4	NOG_01398-R0[Magnaporthe	425	591	659	270	341	0.36	1	68	61.2	2.9e-09	gene=Chr08G0194.1	NOG_01398-R0[MagnaporthesalviniiM69]

			salviniiM69]												
Chr08G0088.1	843	48.6	estExt_Genemark1.C_250095[Dichomitussqualens]	176	632	700	105	176	0.36	1	68	69.3	1.3e-11	gene=Chr08G0088.1	estExt_Genemark1.C_250095[Dichomitussqualens]
Chr04G1448.1	250	41	Clama_scaffold_28-0.22[Cladoniamacilenta]	3845	62	135	3455	3532	0.38	2	73	59.7	3.1e-09	gene=Chr04G1448.1	Clama_scaffold_28-0.22[Cladoniamacilenta]
Chr09G0470.1	487	40.2	Clame_scaffold18-19.70[Cladoniametacorallifera]	7866	148	229	3032	3107	0.38	2	81	57.4	3.0e-08	gene=Chr09G0470.1	Clame_scaffold18-19.70[Cladoniametacorallifera]
Chr02G1221.1	218	48.6	EEA28139.1[Penicilliummarneffei]	2025	29	101	603	675	0.38	2	72	68.6	5.9e-12	gene=Chr02G1221.1	EEA28139.1[Penicilliummarneffei]
Chr03G0544.1	274	34.6	Clama_scaffold_28-0.22[Cladoniamacilenta]	3845	82	159	3455	3532	0.39	0	77	58.5	7.7e-09	gene=Chr03G0544.1	Clama_scaffold_28-0.22[Cladoniamacilenta]

Chr02G1250.1	681	37	EAA64957.1[AspergillusnidulansFGSCA4]	1745	10	88	570	649	0.39	2	78	53.5	6.1e-07	gene=Chr02G1250.1	EAA64957.1[AspergillusnidulansFGSCA4]
Chr01G0605.1	350	42.9	Hanno_00979[Heterobasidionannosum03012]	2421	102	191	2225	2306	0.40	3	89	67.8	1.6e-11	gene=Chr01G0605.1	Hanno_00979[Heterobasidionannosum03012]
Chr01G2692.1	390	30.2	fgenesh1_pg.8_#_209[PleurotusostreatusPC9]	1659	212	314	1486	1581	0.41	3	102	57.4	2.4e-08	gene=Chr01G2692.1	fgenesh1_pg.8_#_209[PleurotusostreatusPC9]
Chr03G0339.1	217	57.9	Endocarpon_02817[Endocarponpusillum]	1255	1	76	169	244	0.41	0	75	94.7	7.6e-20	gene=Chr03G0339.1	Endocarpon_02817[Endocarponpusillum]
Chr01G0651.1	235	45.3	Clame_scaffold9-5.100[Cladoniametacorallifera]	12517	131	205	1381	1455	0.41	0	74	78.2	8.0e-15	gene=Chr01G0651.1	Clame_scaffold9-5.100[Cladoniametacorallifera]
Chr06G0378.1	513	33.3	EAA61211.1[Aspergil	742	153	239	144	228	0.41	1	86	62.4	9.9e-10	gene=Chr06G03	EAA61211.1[AspergillusnidulansFGSCA4

			lusnidulansFGSCA4]											78.1	]
Chr03G1512.1	158	33.7	XP_007802185.1[EndocarponpusillumZ07020]	730	49	144	126	218	0.43	3	95	64.3	8.1e-11	gene=Chr03G1512.1	XP_007802185.1[EndocarponpusillumZ07020]
Chr01G2290.1	619	36.4	Schco2.Schco1.fgenesh2_pg.11_#_122[SchizophyllumcommuneH4-8]	499	199	300	244	356	0.43	3	101	90.9	3.1e-18	gene=Chr01G2290.1	Schco2.Schco1.fgenesh2_pg.11_#_122[SchizophyllumcommuneH4-8]
Chr09G0651.1	140	35.1	Endocarpon_04277[Endocarponpusillum]	1455	23	114	150	240	0.43	3	91	57	1.1e-08	gene=Chr09G0651.1	Endocarpon_04277[Endocarponpusillum]
Chr01G1612.1	1512	39.3	Clama_scaffold_5-4.0[Cladoniamacilenta]	21962	395	1459	5459	6542	0.84	11	1064	746.5	3.4e-215	gene=Chr01G1612.1	Clama_scaffold_5-4.0[Cladoniamacilenta]
Chr04G0359.1	318	38.6	fgenesh1_pg.24_&#	2158	23	154	1731	1833	0.44	3	131	89.7	3.6e-18	gene=Chr04G03	fgenesh1_pg.24_#_34[Phlebiabrevis

			35;_34[PhlebiabrevisporaHHB-7030SS6]											59.1	poraHHB-7030SS6]
Chr09G0777.1	518	39.6	Endpu_scaffold7-12.77[EndocarponpusillumR61883]	5051	351	437	1571	1661	0.44	1	86	63.9	3.5e-10	gene=Chr09G0777.1	Endpu_scaffold7-12.77[EndocarponpusillumR61883]
Chr01G2694.1	1522	39.4	Clama_scaffold_5-4.0[Cladoniamacilenta]	21962	150	251	5365	5472	0.23	2	101	68.9	3.2e-11	gene=Chr01G2694.1	Clama_scaffold_5-4.0[Cladoniamacilenta]
Chr09G0627.1	726	44.3	estExt_fgenesh3_pg.C_900031[Postiaplacenta]	756	40	132	45	150	0.45	3	92	95.1	2.0e-19	gene=Chr09G0627.1	estExt_fgenesh3_pg.C_900031[Postiaplacenta]
Chr04G0512.1	903	35.2	fgenesh1_pg.78_#_34[Sebacinavermiferaspp.bescii]	316	9	113	6	104	0.45	3	104	60.1	8.7e-09	gene=Chr04G0512.1	fgenesh1_pg.78_#_34[Sebacinavermiferaspp.bescii]
Chr05G0021.1	1170	34.9	fgenesh1_	1073	617	718	846	950	0.4	4	101	56.6	1.2e-07	gene=C	fgenesh1_kg.38_&#

			kg.38_#_38_#_Locus16506v1rpkm5.70[Exidiaglandulosa]						6					hr05G0021.1	35;_38_#_Locus16506v1rpkm5.70[Exidiaglandulosa]
Chr09G0082.1	618	32.8	XP_007803552.1[EndocarponpusillumZ07020]	1439	17	130	786	893	0.46	3	113	58.2	2.3e-08	gene=Chr09G0082.1	XP_007803552.1[EndocarponpusillumZ07020]
Chr01G2321.1	101	64.1	C_scaffold_20000017[Phytophthorasojae]	950	8	99	858	949	0.47	0	91	124.8	3.2e-29	gene=Chr01G2321.1	C_scaffold_20000017[Phytophthorasojae]
Chr01G0753.1	702	31.4	fgenesh1_pg.344_#_5[ColletotrichumsublineolaCBS131301]	968	567	671	863	963	0.47	3	104	50.4	5.4e-06	gene=Chr01G0753.1	fgenesh1_pg.344_#_5[ColletotrichumsublineolaCBS131301]
Chr01G1215.1	361	34	Endpu_scaffold5-16.60[Endoca	2171	255	352	1302	1404	0.47	1	97	68.2	1.3e-11	gene=Chr01G1215.1	Endpu_scaffold5-16.60[EndocarponpusillumR61883]

			rponpusillumR61883]												
Chr09G0391.1	361	34	Endpu_scaffold5-16.60[EndocarponpusillumR61883]	2171	255	352	1302	1404	0.47	1	97	66.2	4.8e-11	gene=Chr09G0391.1	Endpu_scaffold5-16.60[EndocarponpusillumR61883]
Chr06G0425.1	361	34	Endpu_scaffold5-16.60[EndocarponpusillumR61883]	2171	255	352	1302	1404	0.47	1	97	67	2.8e-11	gene=Chr06G0425.1	Endpu_scaffold5-16.60[EndocarponpusillumR61883]
Chr06G0479.1	361	34	Endpu_scaffold5-16.60[EndocarponpusillumR61883]	2171	255	352	1302	1404	0.47	1	97	67	2.8e-11	gene=Chr06G0479.1	Endpu_scaffold5-16.60[EndocarponpusillumR61883]
Chr06G1363.1	271	38.3	Lema_T073080.1[Leptosphaeriamaculans]	495	164	264	254	367	0.47	3	100	85.5	5.8e-17	gene=Chr06G1363.1	Lema_T073080.1[Leptosphaeriamaculans]

Chr05G0557.1	184	86	Hanno_04867[Heterobasidionannosum03012]	897	1	179	717	895	0.47	0	178	322.4	1.9e-88	gene=Chr05G0557.1	Hanno_04867[Heterobasidionannosum03012]
Chr01G2725.1	1160	39.1	gm1.11142_g[Spherobolusstellatus]	717	557	648	31	121	0.47	1	91	61.6	3.8e-09	gene=Chr01G2725.1	gm1.11142_g[Sphaerobolusstellatus]
Chr02G1583.1	1169	39.1	gm1.11142_g[Spherobolusstellatus]	717	582	673	31	121	0.47	1	91	61.6	3.9e-09	gene=Chr02G1583.1	gm1.11142_g[Sphaerobolusstellatus]
Chr03G0426.1	135	40.9	Lema_T073080.1[Leptosphaeriamaculans]	495	25	129	238	364	0.48	3	104	87.4	7.6e-18	gene=Chr03G0426.1	Lema_T073080.1[Leptosphaeriamaculans]
Chr01G0664.1	691	38.2	EAL85053.1[AspergillusfumigatusAf293]	720	323	419	256	356	0.48	3	96	55.5	1.6e-07	gene=Chr01G0664.1	EAL85053.1[AspergillusfumigatusAf293]
Chr02G1368.1	406	30.4	gw1.9.669.1[AspergillusnigerATCC1015]	322	78	186	156	264	0.48	3	108	55.1	1.3e-07	gene=Chr02G1368.1	gw1.9.669.1[AspergillusnigerATCC1015]

Chr02G1728.1	137	48.9	fgeneshMC_pg.6_#_470[Mucorcircinelloides]	514	11	98	413	500	0.48	0	87	78.2	4.7e-15	gene=Chr02G1728.1	fgeneshMC_pg.6_#_470[Mucorcircinelloides]
Chr01G1542.1	401	27.6	gm1.8135_g[PhlebiabrevisporaHHB-7030SS6]	869	230	350	590	705	0.49	4	120	49.7	5.2e-06	gene=Chr01G1542.1	gm1.8135_g[PhlebiabrevisporaHHB-7030SS6]
Chr01G2385.1	484	28.5	Clame_scaffold18-19.70[Cladoniametacorallifera]	7866	340	455	2377	2506	0.23	3	115	58.5	1.4e-08	gene=Chr01G2385.1	Clame_scaffold18-19.70[Cladoniametacorallifera]
Chr03G1134.1	751	36.6	g16550.t1[ArmillariamelleaDSM3731]	1160	306	406	777	875	0.23	1	100	63.2	8.5e-10	gene=Chr03G1134.1	g16550.t1[ArmillariamelleaDSM3731]
Chr02G1490.1	250	35	Clama_scaffold_28-0.22[Cladoniamacilenta]	3845	39	139	3431	3533	0.23	1	100	62	6.3e-10	gene=Chr02G1490.1	Clama_scaffold_28-0.22[Cladoniamacilenta]
Chr08G1055.1	371	30.4	Clame_scaffold11-8.	8336	66	204	6988	7159	0.23	8	138	69.7	4.5e-12	gene=Chr08G10	Clame_scaffold11-8.74[Cladoniametacor

			74[Cladoniametacorallifera]											55.1	allifera]
Chr01G2694.1	1522	37	Clama_scaffold_5-4.0[Cladoniamacilenta]	21962	373	1468	5414	6530	0.83	16	1095	721.8	9.0e-208	gene=Chr01G2694.1	Clama_scaffold_5-4.0[Cladoniamacilenta]
Chr01G2726.1	453	54.6	Pa_3_950[Podosporaanserina]	960	268	362	1	97	0.23	1	94	115.9	6.7e-26	gene=Chr01G2726.1	Pa_3_950[Podosporaanserina]
Chr05G0375.1	1578	54.6	Pa_3_950[Podosporaanserina]	960	1393	1487	1	97	0.23	1	94	116.3	1.8e-25	gene=Chr05G0375.1	Pa_3_950[Podosporaanserina]
Chr03G1389.1	321	31	fgenesh1_pg.78_#_34[Sebacinavermiferaspp.bescii]	316	40	152	5	113	0.23	2	112	51.6	1.1e-06	gene=Chr03G1389.1	fgenesh1_pg.78_#_34[Sebacinavermiferaspp.bescii]
Chr01G1985.1	1249	40.6	estExt_fgenesh1_pg.C_3530010[Exidiaglandulosa]	1270	900	995	1019	1114	0.23	0	95	86.7	1.2e-16	gene=Chr01G1985.1	estExt_fgenesh1_pg.C_3530010[Exidiaglandulosa]
Chr05G0139.1	579	27.6	Clame_scaffold11-8.	8336	154	295	6525	6651	0.23	4	141	55.8	1.1e-07	gene=Chr05G01	Clame_scaffold11-8.74[Cladoniametacor

			74[Cladoniametacorallifera]											39.1	allifera]
Chr07G0762.1	363	30.6	Endpu_scaffold5-16.60[EndocarponpusillumR61883]	2171	242	354	1284	1404	0.23	2	112	69.7	4.4e-12	gene=Chr07G0762.1	Endpu_scaffold5-16.60[EndocarponpusillumR61883]
Chr08G0562.1	391	30.6	Endpu_scaffold5-16.60[EndocarponpusillumR61883]	2171	270	382	1284	1404	0.23	2	112	69.7	4.7e-12	gene=Chr08G0562.1	Endpu_scaffold5-16.60[EndocarponpusillumR61883]
Chr08G0126.1	252	30.3	Clame_scaffold9-5.100[Cladoniametacorallifera]	12517	58	183	8686	8821	0.23	4	125	53.5	2.3e-07	gene=Chr08G0126.1	Clame_scaffold9-5.100[Cladoniametacorallifera]
Chr07G1183.1	671	71.3	EEA28139.1[Penicilliummarneffei]	2025	155	269	827	941	0.24	0	114	177.9	2.1e-44	gene=Chr07G1183.1	EEA28139.1[Penicilliummarneffei]
Chr07G1144.1	148	34.9	estExt_fgenesh3_pg.	756	6	118	17	145	0.24	3	112	93.6	1.2e-19	gene=Chr07G11	estExt_fgenesh3_pg.C_900031[Postiapla

			C_900031[Postiaplacenta]											44.1	centa]
Chr01G2573.1	208	30.5	XP_007802185.1[EndocarponpusillumZ07020]	730	73	205	80	221	0.24	6	132	67.4	1.3e-11	gene=Chr01G2573.1	XP_007802185.1[EndocarponpusillumZ07020]
Chr07G0119.1	282	34.5	fgenesh1_pg.78_#_34[Sebacinavermiferaspp.bescii]	316	12	130	4	113	0.24	3	118	52.8	4.3e-07	gene=Chr07G0119.1	fgenesh1_pg.78_#_34[Sebacinavermiferaspp.bescii]
Chr01G0591.1	907	37.8	gm1.11142_g[Sphaerobolusstellatus]	717	523	633	17	121	0.24	3	110	60.5	6.7e-09	gene=Chr01G0591.1	gm1.11142_g[Sphaerobolusstellatus]
Chr05G0442.1	444	31.5	fgenesh1_kg.20_#_67_#_Locus6460v1rpkm23.60[Sphaerobolusstellatus	458	55	253	22	165	0.24	4	198	99.4	6.4e-21	gene=Chr05G0442.1	fgenesh1_kg.20_#_67_#_Locus6460v1rpkm23.60[Sphaerobolusstellatus]

			]												
Chr02G1310.1	1479	37.7	Clama_scaffold_5-4.0[Cladoniamacilenta]	21962	364	1434	5459	6542	0.82	10	1070	713	4.0e-205	gene=Chr02G1310.1	Clama_scaffold_5-4.0[Cladoniamacilenta]
Chr07G0817.1	1139	32.8	g14592.t1[ArmillariamelleaDSM3731]	2505	8	126	1824	1936	0.24	2	118	67.4	6.9e-11	gene=Chr07G0817.1	g14592.t1[ArmillariamelleaDSM3731]
Chr01G0432.1	796	42.9	Clame_scaffold11-8.74[Cladoniametacorallifera]	8336	15	125	1958	2073	0.24	5	110	81.3	3.2e-15	gene=Chr01G0432.1	Clame_scaffold11-8.74[Cladoniametacorallifera]
Chr01G2539.1	130	66.1	estExt_Genewise1Plus.C_150001[Neurosporadiscreta]	480	7	120	285	398	0.24	2	113	147.1	7.8e-36	gene=Chr01G2539.1	estExt_Genewise1Plus.C_150001[Neurosporadiscreta]
Chr08G0225.1	269	34.2	fgenesh1_pg.78_#_34[Sebacinavermiferaspp.bescii]	316	1	112	1	113	0.25	2	111	61.2	1.2e-09	gene=Chr08G0225.1	fgenesh1_pg.78_#_34[Sebacinavermiferaspp.bescii]

Chr02G0422.1	247	35.7	augustus_masked-scaffold10-processed-gene-15.1-mRNA-1[Cladoniametacorallifera]	832	12	136	559	673	0.25	4	124	61.2	1.1e-09	gene=Chr02G0422.1	augustus_masked-scaffold10-processed-gene-15.1-mRNA-1[Cladoniametacorallifera]
Chr03G0825.1	644	28.4	ATEG_09350.1[Aspergillusterreus]	1972	96	229	1371	1513	0.25	4	133	79	1.3e-14	gene=Chr03G0825.1	ATEG_09350.1[Aspergillusterreus]
Chr02G0392.1	693	30.9	Clama_scaffold_5-4.0[Cladoniamacilenta]	21962	453	574	20034	20152	0.25	3	121	62.8	1.0e-09	gene=Chr02G0392.1	Clama_scaffold_5-4.0[Cladoniamacilenta]
Chr01G1900.1	543	44.4	estExt_Genemark1.C_130001[Punctulariastrigosozonata]	634	118	225	244	360	0.25	2	107	95.9	8.6e-20	gene=Chr01G1900.1	estExt_Genemark1.C_130001[Punctulariastrigosozonata]
Chr08G0458.1	151	35.1	estExt_fgenesh3_pg.C_900031	756	9	126	19	152	0.25	3	117	89.4	2.2e-18	gene=Chr08G0458.1	estExt_fgenesh3_pg.C_900031[Postiaplacenta]

			[Postiaplacenta]												
Chr09G0558.1	279	43.8	fgenesh1_pg.78_#_34[Sebacinavermiferaspp.bescii]	316	25	127	6	110	0.25	1	102	83.6	2.3e-16	gene=Chr09G0558.1	fgenesh1_pg.78_#_34[Sebacinavermiferaspp.bescii]
Chr01G2207.1	237	36.9	MUStwsD_GLEAN_10005208[Omphalotusolearius]	578	99	219	12	125	0.25	2	120	88.2	7.8e-18	gene=Chr01G2207.1	MUStwsD_GLEAN_10005208[Omphalotusolearius]
Chr04G1523.1	345	32.2	fgenesh1_pg.12_#_108[StereumhirsutumFP-91666SS1]	1485	82	207	1185	1329	0.25	3	125	62.4	6.7e-10	gene=Chr04G1523.1	fgenesh1_pg.12_#_108[StereumhirsutumFP-91666SS1]
Chr04G0616.1	649	37	RO3G_08358[Rhizopusoryzae]	569	261	418	90	221	0.25	6	157	89.7	7.4e-18	gene=Chr04G0616.1	RO3G_08358[Rhizopusoryzae]
Chr06G0803.1	856	25.7	Clame_scaffold9-5.1	12517	683	818	1014	1156	0.25	3	135	54.3	4.5e-07	gene=Chr06G08	Clame_scaffold9-5.100[Cladoniametacor

			00[Cladoniametacorallifera]											03.1	allifera]
Chr02G1862.1	470	48.6	Pa_3_950[Podosporaanserina]	960	284	386	1	105	0.25	1	102	110.2	3.8e-24	gene=Chr02G1862.1	Pa_3_950[Podosporaanserina]
Chr02G1561.1	729	33.8	ATEG_00556.1[Aspergillusterreus]	456	215	353	87	208	0.25	5	138	63.2	8.3e-10	gene=Chr02G1561.1	ATEG_00556.1[Aspergillusterreus]
Chr04G0222.1	271	42.3	fgenesh1_pg.24_#_34[PhlebiabrevisporaHHB-7030SS6]	2158	5	124	1720	1834	0.25	6	119	80.1	2.4e-15	gene=Chr04G0222.1	fgenesh1_pg.24_#_34[PhlebiabrevisporaHHB-7030SS6]
Chr06G0871.1	395	27.7	ATEG_09350.1[Aspergillusterreus]	1972	143	313	1404	1560	0.26	7	170	60.1	3.8e-09	gene=Chr06G0871.1	ATEG_09350.1[Aspergillusterreus]
Chr06G0440.1	152	39.3	estExt_fgenesh3_pg.C_900031[Postiaplacenta]	756	9	127	17	151	0.26	3	118	98.6	3.7e-21	gene=Chr06G0440.1	estExt_fgenesh3_pg.C_900031[Postiaplacenta]
Chr08G0544.1	476	31.4	fgenesh1_	251	166	433	3	173	0.2	5	267	103.2	4.7e-22	gene=C	fgenesh1_pm.41_&#

			pm.41_#_21[Saccharomycescerevisiae YB210]						6					hr08G0544.1	35;_21[SaccharomycescerevisiaeYB210]
Chr02G0329.1	369	47.8	fgenesh1_pg.78_#_34[Sebacinavermiferaspp.bescii]	316	117	227	3	113	0.26	2	110	83.2	3.9e-16	gene=Chr02G0329.1	fgenesh1_pg.78_#_34[Sebacinavermiferaspp.bescii]
Chr01G0035.1	258	31	estExt_Genemark1.C_150090[Dacryopinaxsp.DJM731SSP1]	827	6	138	29	166	0.26	5	132	57	2.1e-08	gene=Chr01G0035.1	estExt_Genemark1.C_150090[Dacryopinaxsp.DJM731SSP1]
Chr09G0294.1	482	41.2	Clame_scaffold18-19.70[Cladoniametacorallifera]	7866	326	458	2384	2506	0.26	4	132	90.5	3.2e-18	gene=Chr09G0294.1	Clame_scaffold18-19.70[Cladoniametacorallifera]
Chr09G0729.1	668	32	Clame_scaffold11-8.74[Cladoniametacora	8336	13	156	1958	2090	0.27	4	143	70.1	6.2e-12	gene=Chr09G0729.1	Clame_scaffold11-8.74[Cladoniametacorallifera]

			llifera]												
Chr01G1188.1	1633	29.7	Clama_scaffold_5-4.0[Cladoniamacilenta]	21962	1294	1439	9885	10020	0.27	4	145	56.2	2.3e-07	gene=Chr01G1188.1	Clama_scaffold_5-4.0[Cladoniamacilenta]
Chr03G1594.1	283	46.4	fgenesh1_pg.78_#_34[Sebacinavermiferaspp.bescii]	316	25	134	2	113	0.27	1	109	95.5	5.8e-20	gene=Chr03G1594.1	fgenesh1_pg.78_#_34[Sebacinavermiferaspp.bescii]
Chr01G0641.1	475	35.2	AFL2G_00556[Aspergillusflavus]	837	101	222	456	575	0.27	1	121	71.2	2.0e-12	gene=Chr01G0641.1	AFL2G_00556[Aspergillusflavus]
Chr02G0176.1	265	30.5	gw1.9.669.1[AspergillusnigerATCC1015]	322	7	145	156	319	0.27	4	138	58.9	5.7e-09	gene=Chr02G0176.1	gw1.9.669.1[AspergillusnigerATCC1015]
Chr04G1557.1	256	48.7	fgenesh1_pg.78_#_34[Sebacinavermiferaspp.bescii]	316	2	114	3	113	0.27	1	112	95.1	6.9e-20	gene=Chr04G1557.1	fgenesh1_pg.78_#_34[Sebacinavermiferaspp.bescii]
Chr08G0511.1	565	27.2	HCB0268	1240	87	242	262	407	0.2	6	155	59.7	7.1e-09	gene=C	HCB02684.1[Histopl

			4.1[Histoplasmacapsulatum]						7					hr08G0511.1	asmacapsulatum]
Chr07G0165.1	674	28.7	gm1.11182_g[Sphaerobolusstellatus]	620	525	666	476	614	0.28	2	141	59.7	8.5e-09	gene=Chr07G0165.1	gm1.11182_g[Sphaerobolusstellatus]
Chr03G1716.1	577	28.5	AB00197.1[Alternariabrassicicola]	1041	104	240	119	255	0.28	0	136	58.9	1.2e-08	gene=Chr03G1716.1	AB00197.1[Alternariabrassicicola]
Chr09G0462.1	608	39.4	GSTUMT00003726001[Tubermelanosporum]	553	342	516	327	466	0.28	4	174	127.9	2.3e-29	gene=Chr09G0462.1	GSTUMT00003726001[Tubermelanosporum]
Chr04G1037.1	341	34.2	maker-scaffold12.1-augustus-gene-11.200-mRNA-1[Raffaeleaquercus-mongolicae]	874	10	152	33	179	0.28	3	142	68.9	7.1e-12	gene=Chr04G1037.1	maker-scaffold12.1-augustus-gene-11.200-mRNA-1[Raffaeleaquercus-mongolicae]
Chr03G1554.1	433	44	SNOG_15414[Stago	723	59	232	13	154	0.28	3	173	151.8	1.1e-36	gene=Chr03G15	SNOG_15414[Stagonosporanodorum]

			nosporanodorum]											54.1	
Chr01G0555.1	367	39.7	SNOG_15414[Stagonosporanodorum]	723	57	229	16	156	0.28	3	172	140.2	2.7e-33	gene=Chr01G0555.1	SNOG_15414[Stagonosporanodorum]
Chr03G1237.1	360	30.1	Endpu_scaffold5-16.60[EndocarponpusillumR61883]	2171	202	349	1254	1402	0.28	7	147	60.8	2.0e-09	gene=Chr03G1237.1	Endpu_scaffold5-16.60[EndocarponpusillumR61883]
Chr09G0561.1	360	30.1	Endpu_scaffold5-16.60[EndocarponpusillumR61883]	2171	202	349	1254	1402	0.28	7	147	62	9.1e-10	gene=Chr09G0561.1	Endpu_scaffold5-16.60[EndocarponpusillumR61883]
Chr04G0688.1	616	34.1	PTRG_10301[Pyrenophoratritici-repentis]	1311	399	525	658	786	0.28	1	126	84	3.8e-16	gene=Chr04G0688.1	PTRG_10301[Pyrenophoratritici-repentis]
Chr01G2040.1	360	30.8	Endpu_scaffold5-16.60[Endoca	2171	202	349	1254	1402	0.29	7	147	62.4	7.0e-10	gene=Chr01G2040.1	Endpu_scaffold5-16.60[EndocarponpusillumR61883]

			rponpusillumR61883]												
Chr07G0447.1	360	30.8	Endpu_scaffold5-16.60[EndocarponpusillumR61883]	2171	202	349	1254	1402	0.29	7	147	63.2	4.1e-10	gene=Chr07G0447.1	Endpu_scaffold5-16.60[EndocarponpusillumR61883]
Chr09G0292.1	360	30.8	Endpu_scaffold5-16.60[EndocarponpusillumR61883]	2171	202	349	1254	1402	0.29	7	147	62.4	7.0e-10	gene=Chr09G0292.1	Endpu_scaffold5-16.60[EndocarponpusillumR61883]
Chr02G0955.1	360	30.8	Endpu_scaffold5-16.60[EndocarponpusillumR61883]	2171	202	349	1254	1402	0.29	7	147	62.4	7.0e-10	gene=Chr02G0955.1	Endpu_scaffold5-16.60[EndocarponpusillumR61883]
Chr09G1035.1	208	32.3	CH063_01019T0[Colletotrichumhigginsianum]	991	1	133	211	342	0.29	1	132	70.9	1.1e-12	gene=Chr09G1035.1	CH063_01019T0[Colletotrichumhigginsianum]

Chr07G0951.1	1550	27	MUStwsD_GLEAN_10001562[Omphalotusolearius]	2038	1355	1502	1837	1983	0.29	1	147	75.9	2.6e-13	gene=Chr07G0951.1	MUStwsD_GLEAN_10001562[Omphalotusolearius]
Chr03G0248.1	526	28.9	CLUG_01301.1[Candidalusitaniae]	563	331	487	340	523	0.29	6	156	73.9	3.4e-13	gene=Chr03G0248.1	CLUG_01301.1[Candidalusitaniae]
Chr03G1530.1	225	34.1	estExt_fgenesh1_pm.C_1380002[ColletotrichumsublineolaCBS131301]	890	6	136	668	802	0.29	1	130	83.6	1.8e-16	gene=Chr03G1530.1	estExt_fgenesh1_pm.C_1380002[ColletotrichumsublineolaCBS131301]
Chr07G0730.1	486	24	EEA19609.1[Penicilliummarneffei]	2642	134	354	2332	2563	0.29	9	220	69.7	5.9e-12	gene=Chr07G0730.1	EEA19609.1[Penicilliummarneffei]
Chr08G0331.1	773	37.2	fgenesh1_pg.737_#_2[Colletotrichumsublineol	439	637	769	295	439	0.29	1	132	110.5	4.8e-24	gene=Chr08G0331.1	fgenesh1_pg.737_#_2[ColletotrichumsublineolaCBS131301]

			aCBS131301]												
Chr02G1136.1	486	31.3	AFL2G_00556[Aspergillusflavus]	837	77	219	436	583	0.29	3	142	82	1.1e-15	gene=Chr02G1136.1	AFL2G_00556[Aspergillusflavus]
Chr01G0361.1	344	34.2	estExt_Genemark1.C_150090[Dacryopinaxsp.DJM731SSP1]	827	37	184	27	169	0.30	5	147	59.7	4.3e-09	gene=Chr01G0361.1	estExt_Genemark1.C_150090[Dacryopinaxsp.DJM731SSP1]
Chr04G0541.1	873	32	Schco2.Schco1.fgenesh2_pg.11_#_122[SchizophyllumcommuneH4-8]	499	400	567	162	358	0.30	10	167	98.6	2.1e-20	gene=Chr04G0541.1	Schco2.Schco1.fgenesh2_pg.11_#_122[SchizophyllumcommuneH4-8]
Chr04G0689.1	624	30.7	EEA23526.1[Penicilliummarneffei]	1874	419	587	405	566	0.30	5	168	78.6	1.6e-14	gene=Chr04G0689.1	EEA23526.1[Penicilliummarneffei]
Chr01G1141.1	465	57.5	EEA24033.1[Penicilli	344	1	127	1	125	0.30	1	126	152.5	6.6e-37	gene=Chr01G11	EEA24033.1[Penicilliummarneffei]

			ummarneffei]											41.1	
Chr02G0802.1	680	50.8	estExt_fgenesh1_pg.C_20253[Phytophthoracapsici]	353	343	468	1	126	0.30	2	125	144.1	3.4e-34	gene=Chr02G0802.1	estExt_fgenesh1_pg.C_20253[Phytophthoracapsici]
Chr03G1704.1	132	50	estExt_fgenesh1_pg.C_2340008[Calocera cornea]	682	1	128	543	678	0.30	4	127	119	2.3e-27	gene=Chr03G1704.1	estExt_fgenesh1_pg.C_2340008[Calocer acornea]
Chr01G0255.1	287	31.8	Hanno_06850[Heterobasidionannosum03012]	650	15	173	4	175	0.30	6	158	63.2	3.3e-10	gene=Chr01G0255.1	Hanno_06850[Heterobasidionannosum03012]
Chr02G1533.1	678	48.8	estExt_fgenesh1_pg.C_20253[Phytophthoracapsici]	353	335	461	1	127	0.30	2	126	131.3	2.3e-30	gene=Chr02G1533.1	estExt_fgenesh1_pg.C_20253[Phytophthoracapsici]
Chr03G1485.1	382	26.4	Hanno_06850[Heterobasidionannosum0	650	95	256	6	183	0.30	3	161	53.9	2.6e-07	gene=Chr03G1485.1	Hanno_06850[Heterobasidionannosum03012]

			3012]												
Chr03G1326.1	397	31.1	Clame_scaffold9-5.100[Cladoniametacorallifera]	12517	126	321	456	614	0.30	6	195	90.5	2.6e-18	gene=Chr03G1326.1	Clame_scaffold9-5.100[Cladoniametacorallifera]
Chr01G2383.1	609	30.3	AB00197.1[Alternariabrassicicola]	1041	168	318	100	247	0.31	3	150	60.1	5.9e-09	gene=Chr01G2383.1	AB00197.1[Alternariabrassicicola]
Chr06G0134.1	303	28.7	g19105.t1[ArmillariamelleaDSM3731]	888	13	187	19	201	0.31	8	174	77.8	1.4e-14	gene=Chr06G0134.1	g19105.t1[ArmillariamelleaDSM3731]
Chr04G0729.1	408	38.4	Clame_scaffold11-8.74[Cladoniametacorallifera]	8336	5	141	1943	2076	0.31	3	136	86.3	5.1e-17	gene=Chr04G0729.1	Clame_scaffold11-8.74[Cladoniametacorallifera]
Chr09G0653.1	457	36.6	estExt_fgenesh1_pg.C_3530010[Exidiaglandulosa]	1270	68	213	1038	1199	0.31	7	145	94	2.7e-19	gene=Chr09G0653.1	estExt_fgenesh1_pg.C_3530010[Exidiaglandulosa]
Chr05G0009.1	1220	54	EEA28139.1[Penicilli	2025	1081	1206	593	717	0.31	1	125	130.6	7.1e-30	gene=Chr05G00	EEA28139.1[Penicilliummarneffei]

			ummarneffei]											09.1	
Chr02G1657.1	1253	27.3	estExt_fgenesh1_pm.C_170119[Cenococcumgeophilum1.58]	1191	388	613	379	554	0.31	6	225	81.3	5.1e-15	gene=Chr02G1657.1	estExt_fgenesh1_pm.C_170119[Cenococcumgeophilum1.58]
Chr05G0863.1	475	59.7	TSTA_006840[Talaromycesstipitatus]	295	197	325	54	182	0.31	0	128	154.1	2.3e-37	gene=Chr05G0863.1	TSTA_006840[Talaromycesstipitatus]
Chr01G2356.1	351	26.1	Endpu_scaffold5-16.60[EndocarponpusillumR61883]	2171	172	340	1231	1402	0.31	5	168	73.6	3.0e-13	gene=Chr01G2356.1	Endpu_scaffold5-16.60[EndocarponpusillumR61883]
Chr05G0143.1	446	28	Clame_scaffold11-8.74[Cladoniametacorallifera]	8336	4	186	1988	2154	0.31	4	182	79.7	5.2e-15	gene=Chr05G0143.1	Clame_scaffold11-8.74[Cladoniametacorallifera]
Chr02G0103.1	542	26.6	CHG01242.1[Chaetomiumglo	1090	124	287	646	818	0.31	4	163	53.9	3.7e-07	gene=Chr02G0103.1	CHG01242.1[ChaetomiumglobosumCBS148.51]

			bosumCBS148.51]												
Chr03G1509.1	254	33.8	estExt_Genemark1.C_150090[Dacryopinaxsp.DJM731SSP1]	827	5	158	27	175	0.31	6	153	64.7	9.9e-11	gene=Chr03G1509.1	estExt_Genemark1.C_150090[Dacryopinaxsp.DJM731SSP1]
Chr06G0187.1	495	28.6	CHG01242.1[ChaetomiumglobosumCBS148.51]	1090	59	216	650	817	0.31	4	157	63.5	4.3e-10	gene=Chr06G0187.1	CHG01242.1[ChaetomiumglobosumCBS148.51]
Chr03G1261.1	714	25.4	HCB02684.1[Histoplasmacapsulatum]	1240	342	561	274	468	0.31	7	219	71.2	3.0e-12	gene=Chr03G1261.1	HCB02684.1[Histoplasmacapsulatum]
Chr07G0561.1	276	27.4	Clame_scaffold8-4.115[Cladoniametacorallifera]	16637	12	209	15230	15434	0.32	6	197	52.4	5.5e-07	gene=Chr07G0561.1	Clame_scaffold8-4.115[Cladoniametacorallifera]
Chr03G0152.1	435	25.3	EAA61211.1[AspergillusnidulansFGSCA4]	742	138	312	135	312	0.32	4	174	58.2	1.6e-08	gene=Chr03G0152.1	EAA61211.1[AspergillusnidulansFGSCA4]

Chr06G0815.1	229	44.1	estExt_fgenesh3_pg.C_900031[Postiaplacenta]	756	4	137	18	151	0.32	3	133	120.2	1.8e-27	gene=Chr06G0815.1	estExt_fgenesh3_pg.C_900031[Postiaplacenta]
Chr03G1307.1	674	26	EAL85053.1[AspergillusfumigatusAf293]	720	274	443	140	316	0.32	4	169	53.9	4.6e-07	gene=Chr03G1307.1	EAL85053.1[AspergillusfumigatusAf293]
Chr03G0328.1	1315	32	Clame_scaffold11-8.74[Cladoniametacorallifera]	8336	806	974	6746	6916	0.32	5	168	87.4	7.4e-17	gene=Chr03G0328.1	Clame_scaffold11-8.74[Cladoniametacorallifera]
Chr01G0931.1	455	34.8	Schco2.Schco1.fgenesh2_pg.11_#_122[SchizophyllumcommuneH4-8]	499	195	358	165	360	0.32	4	163	111.7	1.3e-24	gene=Chr01G0931.1	Schco2.Schco1.fgenesh2_pg.11_#_122[SchizophyllumcommuneH4-8]
Chr04G0596.1	398	36.9	g14592.t1[ArmillariamelleaDSM3731]	2505	2	149	837	991	0.32	5	147	72.4	7.5e-13	gene=Chr04G0596.1	g14592.t1[ArmillariamelleaDSM3731]

Chr08G0749.1	167	54.4	estExt_fgenesh3_pg.C_900031[Postiaplacenta]	756	10	143	17	152	0.33	1	133	160.2	1.1e-39	gene=Chr08G0749.1	estExt_fgenesh3_pg.C_900031[Postiaplacenta]
Chr04G1227.1	374	40	Clame_scaffold11-8.74[Cladoniametacorallifera]	8336	162	301	6757	6894	0.33	1	139	105.1	9.8e-23	gene=Chr04G1227.1	Clame_scaffold11-8.74[Cladoniametacorallifera]
Chr03G0214.1	644	34.9	gm1.11182_g[Sphaerobolusstellatus]	620	474	641	462	615	0.33	4	167	87.4	3.6e-17	gene=Chr03G0214.1	gm1.11182_g[Sphaerobolusstellatus]
Chr07G0503.1	392	30.4	AFL2G_07281[Aspergillusflavus]	701	24	179	1	158	0.33	2	155	80.5	2.7e-15	gene=Chr07G0503.1	AFL2G_07281[Aspergillusflavus]
Chr01G1402.1	822	26.5	EAL85053.1[AspergillusfumigatusAf293]	720	310	481	139	319	0.33	4	171	60.8	4.6e-09	gene=Chr01G1402.1	EAL85053.1[AspergillusfumigatusAf293]
Chr05G0835.1	384	37.4	estExt_fgenesh3_pg.C_130101[Postiapla	703	180	328	366	517	0.33	4	148	87.8	1.7e-17	gene=Chr05G0835.1	estExt_fgenesh3_pg.C_130101[Postiaplacenta]

			centa]												
Chr01G1584.1	1014	25.4	g14592.t1[ArmillariamelleaDSM3731]	2505	234	420	2204	2398	0.33	6	186	53.9	7.0e-07	gene=Chr01G1584.1	g14592.t1[ArmillariamelleaDSM3731]
Chr07G0127.1	490	89.3	XP_007598095.1[ColletotrichumfioriniaePJ7]	506	1	489	1	506	0.33	1	488	915.2	1.8e-266	gene=Chr07G0127.1	XP_007598095.1[ColletotrichumfioriniaePJ7]
Chr04G1317.1	716	28.5	ATEG_09350.1[Aspergillusterreus]	1972	82	298	1371	1571	0.33	9	216	82.4	1.3e-15	gene=Chr04G1317.1	ATEG_09350.1[Aspergillusterreus]
Chr06G0215.1	1011	43	Endocarpon_02817[Endocarponpusillum]	1255	1	152	95	245	0.33	4	151	122.1	2.1e-27	gene=Chr06G0215.1	Endocarpon_02817[Endocarponpusillum]
Chr07G0042.1	164	34.2	Clame_scaffold8-4.115[Cladoniametacorallifera]	16637	3	153	2700	2849	0.33	3	150	100.9	8.1e-22	gene=Chr07G0042.1	Clame_scaffold8-4.115[Cladoniametacorallifera]
Chr03G0499.1	862	31.6	OAT00923.1[Blasto	1246	34	230	361	539	0.33	3	196	104.8	2.9e-22	gene=Chr03G04	OAT00923.1[BlastomycesdermatitidisE

			mycesdermatitidisER-3]											99.1	R-3]
Chr02G0541.1	249	26.2	fgenesh1_pg.C_scaffold_63000067[Phytophthoraramorum]	793	33	231	593	780	0.33	5	198	67	2.0e-11	gene=Chr02G0541.1	fgenesh1_pg.C_scaffold_63000067[Phytophthoraramorum]
Chr04G0850.1	176	54.7	estExt_fgenesh3_pg.C_900031[Postiaplacenta]	756	3	147	8	152	0.33	5	144	152.9	1.9e-37	gene=Chr04G0850.1	estExt_fgenesh3_pg.C_900031[Postiaplacenta]
Chr05G0111.1	163	64.3	CH063_13135T0[Colletotrichumhigginsianum]	223	1	162	58	223	0.33	3	161	214.9	3.8e-56	gene=Chr05G0111.1	CH063_13135T0[Colletotrichumhigginsianum]
Chr02G0858.1	638	41.5	estExt_Genemark1.C_40179[Aspergilluscarbonarius]	665	88	233	522	664	0.34	3	145	91.7	1.9e-18	gene=Chr02G0858.1	estExt_Genemark1.C_40179[Aspergilluscarbonarius]
Chr01G1568.1	281	30.1	fgenesh1_	793	48	226	592	766	0.3	5	178	81.3	1.1e-15	gene=C	fgenesh1_pg.C_scaf

			pg.C_scaffold_63000067[Phytophthoraramorum]						4					hr01G1568.1	fold_63000067[Phytophthoraramorum]
Chr03G1254.1	190	34.1	Hanno_04867[Heterobasidionannosum03012]	897	19	186	732	890	0.34	2	167	114	1.1e-25	gene=Chr03G1254.1	Hanno_04867[Heterobasidionannosum03012]
Chr02G0630.1	907	41.2	fgenesh1_pg.C_scaffold_18000089[Phytophthoraramorum]	784	39	197	261	433	0.34	8	158	88.2	3.0e-17	gene=Chr02G0630.1	fgenesh1_pg.C_scaffold_18000089[Phytophthoraramorum]
Chr02G0190.1	653	32.7	AB00197.1[Alternariabrassicicola]	1041	201	361	99	256	0.34	3	160	74.7	2.5e-13	gene=Chr02G0190.1	AB00197.1[Alternariabrassicicola]
Chr02G0024.1	289	35.8	SNOG_15414[Stagonosporanodorum]	723	47	272	26	243	0.34	8	225	156.4	2.9e-38	gene=Chr02G0024.1	SNOG_15414[Stagonosporanodorum]
Chr02G1812.1	396	26.2	g8694.t1[Armillaria	1256	123	336	77	286	0.34	10	213	61.2	1.7e-09	gene=Chr02G18	g8694.t1[ArmillariamelleaDSM3731]

			melleaDSM3731]											12.1	
Chr04G0193.1	236	60.2	Clame_scaffold8-4.115[Cladoniametacorallifera]	16637	4	173	2698	2858	0.34	3	169	207.6	8.8e-54	gene=Chr04G0193.1	Clame_scaffold8-4.115[Cladoniametacorallifera]
Chr04G1325.1	169	54.5	HCEG_08413[HistoplasmacapsulatumH88]	423	12	153	264	408	0.34	1	141	160.6	8.8e-40	gene=Chr04G1325.1	HCEG_08413[HistoplasmacapsulatumH88]
Chr05G0123.1	1793	41.4	PTRG_10301[Pyrenophoratritici-repentis]	1311	1466	1608	643	787	0.34	1	142	118.2	5.3e-26	gene=Chr05G0123.1	PTRG_10301[Pyrenophoratritici-repentis]
Chr06G0470.1	266	28.4	Clame_scaffold8-4.115[Cladoniametacorallifera]	16637	4	190	15225	15430	0.35	5	186	65.5	6.1e-11	gene=Chr06G0470.1	Clame_scaffold8-4.115[Cladoniametacorallifera]
Chr06G1242.1	303	34.4	g19105.t1[ArmillariamelleaDSM3731]	888	13	178	15	195	0.35	7	165	91.7	9.0e-19	gene=Chr06G1242.1	g19105.t1[ArmillariamelleaDSM3731]

Chr08G0394.1	656	26.1	fgenesh1_pg.8_#_209[PleurotusostreatusPC9]	1659	253	499	1384	1584	0.35	4	246	97.4	3.6e-20	gene=Chr08G0394.1	fgenesh1_pg.8_#_209[PleurotusostreatusPC9]
Chr08G0255.1	2069	27.8	4127_t[AscocorynesarcoidesNRRL50072]	1621	1640	1836	1292	1491	0.35	9	196	68.9	4.3e-11	gene=Chr08G0255.1	4127_t[AscocorynesarcoidesNRRL50072]
Chr01G1473.1	394	30.8	maker-scaffold12.1-augustus-gene-11.200-mRNA-1[Raffaeleaquercus-mongolicae]	874	49	233	20	207	0.35	8	184	63.2	4.5e-10	gene=Chr01G1473.1	maker-scaffold12.1-augustus-gene-11.200-mRNA-1[Raffaeleaquercus-mongolicae]
Chr05G0925.1	345	25.7	maker-scaffold122-augustus-gene-0.90-mRNA-1[OphiocerasdolichostomumCB	1059	72	283	169	367	0.35	5	211	65.9	6.0e-11	gene=Chr05G0925.1	maker-scaffold122-augustus-gene-0.90-mRNA-1[OphiocerasdolichostomumCBS114926]

			S114926]												
Chr01G2309.1	291	30.4	XP_007602139.1[CollletotrichumfioriniaePJ7]	801	11	196	4	186	0.35	6	185	58.5	8.1e-09	gene=Chr01G2309.1	XP_007602139.1[CollletotrichumfioriniaePJ7]
Chr03G0250.1	1459	35.2	Clama_scaffold_5-4.0[Cladoniamacilenta]	21962	335	1414	5459	6542	1.00	17	1079	667.5	1.9e-191	gene=Chr03G0250.1	Clama_scaffold_5-4.0[Cladoniamacilenta]
Chr02G0903.1	791	25.1	estExt_fgenesh1_pm.C_170119[Cenococcumgeophilum1.58]	1191	15	246	271	473	0.35	6	231	68.9	1.6e-11	gene=Chr02G0903.1	estExt_fgenesh1_pm.C_170119[Cenococcumgeophilum1.58]
Chr06G1197.1	954	38.1	Clame_scaffold8-4.115[Cladoniametacorallifera]	16637	721	890	15230	15431	0.35	5	169	100.9	4.7e-21	gene=Chr06G1197.1	Clame_scaffold8-4.115[Cladoniametacorallifera]
Chr04G1101.1	390	27.3	HCB02684.1[Histoplasmacapsulatum]	1240	96	288	275	477	0.35	7	192	69.3	6.2e-12	gene=Chr04G1101.1	HCB02684.1[Histoplasmacapsulatum]
Chr08G0489.1	491	23.4	Clame_sc	8336	273	486	968	1174	0.3	4	213	58.5	1.4e-08	gene=C	Clame_scaffold11-8.

			affold11-8.74[Cladoniametacorallifera]						6					hr08G0489.1	74[Cladoniametacorallifera]
Chr04G1453.1	387	24.7	AMAG_10412T0[Allomycesmacrogynus]	687	90	296	279	483	0.36	4	206	73.9	2.5e-13	gene=Chr04G1453.1	AMAG_10412T0[Allomycesmacrogynus]
Chr08G0242.1	707	31.5	Schco2.Schco1.fgenesh2_pg.11_#_122[SchizophyllumcommuneH4-8]	499	211	406	138	363	0.36	10	195	105.1	1.8e-22	gene=Chr08G0242.1	Schco2.Schco1.fgenesh2_pg.11_#_122[SchizophyllumcommuneH4-8]
Chr04G0192.1	459	89	Clama_scaffold_5-4.0[Cladoniamacilenta]	21962	44	431	10050	10440	0.36	2	387	682.2	2.4e-196	gene=Chr04G0192.1	Clama_scaffold_5-4.0[Cladoniamacilenta]
Chr06G0907.1	262	39.6	Endocarpon_02817[Endocarponpusillum]	1255	1	159	95	247	0.36	3	158	110.9	1.2e-24	gene=Chr06G0907.1	Endocarpon_02817[Endocarponpusillum]
Chr01G0009.1	457	54.1	TSTA_006	295	163	307	37	182	0.3	1	144	153.7	2.9e-37	gene=C	TSTA_006840[Talar

			840[Talaromycesstipitatus]						6					hr01G0009.1	omycesstipitatus]
Chr04G1504.1	457	54.1	TSTA_006840[Talaromycesstipitatus]	295	163	307	37	182	0.36	1	144	153.7	2.9e-37	gene=Chr04G1504.1	TSTA_006840[Talaromycesstipitatus]
Chr07G0237.1	344	65.2	CH063_01447T0[Colletotrichumhigginsianum]	330	61	325	1	322	0.36	3	264	391	8.2e-109	gene=Chr07G0237.1	CH063_01447T0[Colletotrichumhigginsianum]
Chr08G1042.1	242	29.4	fgenesh1_pg.C_scaffold_63000067[Phytophthoraramorum]	793	37	230	593	780	0.36	5	193	66.6	2.5e-11	gene=Chr08G1042.1	fgenesh1_pg.C_scaffold_63000067[Phytophthoraramorum]
Chr08G0106.1	522	29.2	EAA63242.1[AspergillusnidulansFGSCA4]	1004	67	243	53	228	0.36	2	176	80.1	4.7e-15	gene=Chr08G0106.1	EAA63242.1[AspergillusnidulansFGSCA4]
Chr09G0529.1	457	24.9	KKY29248.1putative cytochromep450[Ph	766	166	428	41	258	0.36	6	262	82.8	6.3e-16	gene=Chr09G0529.1	KKY29248.1putative cytochromep450[Phaeomoniellachlamydospora][Phaeomonie

			aeomoniellachlamydospora][Phaeomoniellachlamydospora]												llachlamydospora]
Chr04G0628.1	515	30.4	EAA58861.1[AspergillusnidulansFGSCA4]	643	173	357	199	385	0.36	7	184	72.8	7.4e-13	gene=Chr04G0628.1	EAA58861.1[AspergillusnidulansFGSCA4]
Chr08G0197.1	513	28.5	AB00197.1[Alternariabrassicicola]	1041	81	293	154	351	0.36	8	212	62	1.3e-09	gene=Chr08G0197.1	AB00197.1[Alternariabrassicicola]
Chr02G1346.1	715	27.1	EfO2.075130.1[Epichloefestucae]	1182	467	671	919	1133	0.36	7	204	67.8	3.3e-11	gene=Chr02G1346.1	EfO2.075130.1[Epichloefestucae]
Chr03G1419.1	193	53	Pa_3_950[Podosporaanserina]	960	35	192	223	390	0.37	1	157	175.3	4.0e-44	gene=Chr03G1419.1	Pa_3_950[Podosporaanserina]
Chr05G1154.1	184	35.2	Hanno_04867[Heterobasidionannosum03012]	897	16	177	729	889	0.37	1	161	120.9	8.4e-28	gene=Chr05G1154.1	Hanno_04867[Heterobasidionannosum03012]

Chr04G0875.1	468	33.3	CHG01242.1[ChaetomiumglobosumCBS148.51]	1090	46	217	643	828	0.37	4	171	73.6	3.9e-13	gene=Chr04G0875.1	CHG01242.1[ChaetomiumglobosumCBS148.51]
Chr03G1304.1	297	58.2	gw1.9.669.1[AspergillusnigerATCC1015]	322	1	159	157	321	0.37	2	158	177.2	1.6e-44	gene=Chr03G1304.1	gw1.9.669.1[AspergillusnigerATCC1015]
Chr07G0594.1	226	57.3	SNOG_08385[Stagonosporanodorum]	473	39	188	1	150	0.37	0	149	183.7	1.3e-46	gene=Chr07G0594.1	SNOG_08385[Stagonosporanodorum]
Chr07G0450.1	822	28	HCB02684.1[Histoplasmacapsulatum]	1240	478	688	262	469	0.37	7	210	73.6	6.9e-13	gene=Chr07G0450.1	HCB02684.1[Histoplasmacapsulatum]
Chr01G2612.1	403	50.3	FGSG_08005T0[Fusariumgraminearum]	500	30	176	35	181	0.37	0	146	157.5	1.8e-38	gene=Chr01G2612.1	FGSG_08005T0[Fusariumgraminearum]
Chr01G1377.1	229	49.1	XP_007600826.1[ColletotrichumfioriniaePJ7]	423	16	174	12	182	0.37	1	158	163.7	1.4e-40	gene=Chr01G1377.1	XP_007600826.1[ColletotrichumfioriniaePJ7]

Chr04G0257.1	1894	43.4	PTRG_10301[Pyrenophoratritici-repentis]	1311	1600	1756	633	788	0.37	2	156	126.3	2.1e-28	gene=Chr04G0257.1	PTRG_10301[Pyrenophoratritici-repentis]
Chr04G1452.1	254	29.8	fgenesh1_pg.C_scaffold_63000067[Phytophthoraramorum]	793	41	242	593	780	0.37	5	201	75.5	5.6e-14	gene=Chr04G1452.1	fgenesh1_pg.C_scaffold_63000067[Phytophthoraramorum]
Chr03G1274.1	372	24	maker-scaffold122-augustus-gene-0.90-mRNA-1[OphiocerasdolichostomumCBS114926]	1059	60	298	143	383	0.37	9	238	54.3	2.0e-07	gene=Chr03G1274.1	maker-scaffold122-augustus-gene-0.90-mRNA-1[OphiocerasdolichostomumCBS114926]
Chr06G0010.1	316	52.1	TSTA_006840[Talaromycesstipitatus]	295	11	166	20	182	0.37	1	155	153.7	2.0e-37	gene=Chr06G0010.1	TSTA_006840[Talaromycesstipitatus]
Chr05G0008.1	316	52.1	TSTA_006840[Talaro	295	11	166	20	182	0.37	1	155	153.7	2.0e-37	gene=Chr05G00	TSTA_006840[Talaromycesstipitatus]

			mycesstipitatus]											08.1	
Chr09G0907.1	273	30.1	Hanno_06850[Heterobasidionannosum03012]	650	2	183	6	201	0.37	4	181	80.9	1.4e-15	gene=Chr09G0907.1	Hanno_06850[Heterobasidionannosum03012]
Chr04G0809.1	730	27.1	fgenesh1_pg.737_#_2[ColletotrichumsublineolaCBS131301]	439	143	727	142	439	0.37	7	584	203	6.7e-52	gene=Chr04G0809.1	fgenesh1_pg.737_#_2[ColletotrichumsublineolaCBS131301]
Chr02G1057.1	449	25.7	982_t[AscocorynesarcoidesNRRL50072]	273	105	315	12	224	0.37	11	210	52.8	6.9e-07	gene=Chr02G1057.1	982_t[AscocorynesarcoidesNRRL50072]
Chr07G0898.1	327	30.6	EAA58488.1[AspergillusnidulansFGSCA4]	1365	42	232	1066	1278	0.37	5	190	92	7.5e-19	gene=Chr07G0898.1	EAA58488.1[AspergillusnidulansFGSCA4]
Chr07G0224.1	643	26.7	HCB02684.1[Histoplasmacapsulatum]	1240	245	505	261	481	0.37	6	260	94.4	3.0e-19	gene=Chr07G0224.1	HCB02684.1[Histoplasmacapsulatum]

Chr04G0964.1	683	39.9	PTRG_10301[Pyrenophoratritici-repentis]	1311	404	575	607	777	0.38	4	171	119.4	9.1e-27	gene=Chr04G0964.1	PTRG_10301[Pyrenophoratritici-repentis]
Chr04G0421.1	652	26.6	Clame_scaffold9-5.100[Cladoniametacorallifera]	12517	311	529	9784	10010	0.38	9	218	62.8	9.7e-10	gene=Chr04G0421.1	Clame_scaffold9-5.100[Cladoniametacorallifera]
Chr06G0740.1	1126	31	estExt_fgenesh1_pg.C_3530010[Exidiaglandulosa]	1270	919	1104	1012	1212	0.38	6	185	89	2.2e-17	gene=Chr06G0740.1	estExt_fgenesh1_pg.C_3530010[Exidiaglandulosa]
Chr09G0661.1	267	34.1	Endocarpon_02817[Endocarponpusillum]	1255	1	201	1	184	0.38	5	200	107.5	1.4e-23	gene=Chr09G0661.1	Endocarpon_02817[Endocarponpusillum]
Chr09G0144.1	455	23.6	HCB02684.1[Histoplasmacapsulatum]	1240	13	248	275	511	0.38	10	235	52.4	9.1e-07	gene=Chr09G0144.1	HCB02684.1[Histoplasmacapsulatum]
Chr09G0728.1	427	28.9	augustus_masked-s	1265	4	201	834	1055	0.38	7	197	92	9.8e-19	gene=Chr09G07	augustus_masked-scaffold407-processe

			caffold407-processed-gene-0.5-mRNA-1[PseudohalonectrialignicolaM95]											28.1	d-gene-0.5-mRNA-1[PseudohalonectrialignicolaM95]
Chr03G1219.1	523	31.1	HCB02684.1[Histoplasmacapsulatum]	1240	303	500	349	541	0.38	7	197	75.9	8.9e-14	gene=Chr03G1219.1	HCB02684.1[Histoplasmacapsulatum]
Chr05G0852.1	242	32.9	XP_007599944.1[ColletotrichumfioriniaePJ7]	1113	21	213	82	286	0.38	9	192	96.7	2.2e-20	gene=Chr05G0852.1	XP_007599944.1[ColletotrichumfioriniaePJ7]
Chr04G1160.1	952	90.2	XP_007595104.1[ColletotrichumfioriniaePJ7]	532	428	948	1	531	0.38	1	520	959.1	2.1e-279	gene=Chr04G1160.1	XP_007595104.1[ColletotrichumfioriniaePJ7]
Chr07G0126.1	722	25.4	fgenesh1_pg.737_#_2[Colletotrichu	439	135	711	141	439	0.38	8	576	169.9	6.2e-42	gene=Chr07G0126.1	fgenesh1_pg.737_#_2[ColletotrichumsublineolaCBS131301]

			msublineolaCBS131301]												
Chr01G0652.1	370	51.6	maker-scaffold12.1-augustus-gene-11.200-mRNA-1[Raffaeleaquercus-mongolicae]	874	45	199	26	179	0.38	1	154	161	1.5e-39	gene=Chr01G0652.1	maker-scaffold12.1-augustus-gene-11.200-mRNA-1[Raffaeleaquercus-mongolicae]
Chr05G1169.1	641	36.8	Clame_scaffold8-4.115[Cladoniametacorallifera]	16637	347	547	2403	2651	0.38	6	200	134	3.4e-31	gene=Chr05G1169.1	Clame_scaffold8-4.115[Cladoniametacorallifera]
Chr05G0292.1	492	29	XP_007801092.1[EndocarponpusillumZ07020]	1039	34	235	42	247	0.38	6	201	72.4	9.2e-13	gene=Chr05G0292.1	XP_007801092.1[EndocarponpusillumZ07020]
Chr06G0957.1	713	33.3	SNOG_15414[Stagonosporanodorum]	723	35	282	15	253	0.38	8	247	144.4	2.8e-34	gene=Chr06G0957.1	SNOG_15414[Stagonosporanodorum]
Chr05G0455.1	353	28.4	ATEG_00	456	74	294	65	263	0.3	5	220	79.7	4.1e-15	gene=C	ATEG_00556.1[Asp

			556.1[Aspergillusterreus]						8					hr05G0455.1	ergillusterreus]
Chr01G1172.1	298	28	g15681.t1[Armillaria melleaDSM3731]	437	19	284	4	221	0.38	7	265	75.9	5.0e-14	gene=Chr01G1172.1	g15681.t1[Armillaria melleaDSM3731]
Chr01G0295.1	553	91.8	CH063_08303T0[Colletotrichumhigginsianum]	511	42	551	1	510	0.39	0	509	965.3	1.7e-281	gene=Chr01G0295.1	CH063_08303T0[Colletotrichumhigginsianum]
Chr03G1225.1	225	33	PGUG_03908.1[Candidaguilliermondii]	224	22	211	4	212	0.39	7	189	100.1	1.9e-21	gene=Chr03G1225.1	PGUG_03908.1[Candidaguilliermondii]
Chr07G1153.1	531	24.6	fgenesh1_pg.00058_#_49[PilodermacroceumF1598]	896	227	522	573	808	0.39	13	295	60.5	3.9e-09	gene=Chr07G1153.1	fgenesh1_pg.00058_#_49[PilodermacroceumF1598]
Chr09G0487.1	523	32.5	OAT00923.1[BlastomycesdermatitidisE	1246	176	373	382	583	0.39	11	197	70.1	4.9e-12	gene=Chr09G0487.1	OAT00923.1[BlastomycesdermatitidisER-3]

			R-3]												
Chr06G1315.1	369	26.7	g15681.t1[Armillaria melleaDSM3731]	437	87	359	4	221	0.39	4	272	102.1	8.1e-22	gene=Chr06G1315.1	g15681.t1[Armillaria melleaDSM3731]
Chr01G2258.1	368	88	FGSG_00863T0[Fusariumgraminearum]	475	1	368	108	475	0.39	0	367	651.4	3.6e-187	gene=Chr01G2258.1	FGSG_00863T0[Fusariumgraminearum]
Chr04G0082.1	548	28.9	AB00197.1[Alternariabrassicicola]	1041	34	249	52	263	0.39	6	215	61.2	2.4e-09	gene=Chr04G0082.1	AB00197.1[Alternariabrassicicola]
Chr02G0222.1	432	33.1	maker-scaffold7-augustus-gene-5.125-mRNA-1[Cladoniametacorallifera]	280	189	420	2	216	0.39	5	231	114	2.4e-25	gene=Chr02G0222.1	maker-scaffold7-augustus-gene-5.125-mRNA-1[Cladoniametacorallifera]
Chr01G0507.1	436	25.7	EEA19609.1[Penicilliummarneffei]	2642	198	427	2371	2638	0.39	6	229	64.3	2.2e-10	gene=Chr01G0507.1	EEA19609.1[Penicilliummarneffei]
Chr09G0642.1	439	31.3	CHG05266.1[Chaet	1130	89	316	685	924	0.39	8	227	106.3	5.1e-23	gene=Chr09G06	CHG05266.1[ChaetomiumglobosumCBS

			omiumglobosumCBS148.51]											42.1	148.51]
Chr01G0151.1	252	42.3	Clame_scaffold8-4.115[Cladoniametacorallifera]	16637	14	190	15230	15430	0.39	5	176	122.5	4.0e-28	gene=Chr01G0151.1	Clame_scaffold8-4.115[Cladoniametacorallifera]
Chr04G0047.1	370	31.7	Clama_scaffold_5-4.0[Cladoniamacilenta]	21962	10	240	10612	10856	0.39	9	230	122.9	4.5e-28	gene=Chr04G0047.1	Clama_scaffold_5-4.0[Cladoniamacilenta]
Chr01G1455.1	509	86.8	XP_007591783.1[ColletotrichumfioriniaePJ7]	486	1	508	1	486	0.39	1	507	918.3	2.2e-267	gene=Chr01G1455.1	XP_007591783.1[ColletotrichumfioriniaePJ7]
Chr09G0861.1	419	25.4	EAA61211.1[AspergillusnidulansFGSCA4]	742	132	351	53	267	0.39	6	219	67	3.3e-11	gene=Chr09G0861.1	EAA61211.1[AspergillusnidulansFGSCA4]
Chr01G1651.1	250	37.7	Clame_scaffold8-4.115[Cladoniametacorallifera]	16637	13	199	15230	15442	0.39	5	186	106.7	2.2e-23	gene=Chr01G1651.1	Clame_scaffold8-4.115[Cladoniametacorallifera]

Chr06G1148.1	277	30.2	Lema_T073080.1[Leptosphaeriamaculans]	495	33	271	56	364	0.39	9	238	142.1	5.3e-34	gene=Chr06G1148.1	Lema_T073080.1[Leptosphaeriamaculans]
Chr03G0317.1	576	28.7	FGSG_00863T0[Fusariumgraminearum]	475	312	519	230	436	0.39	6	207	78.2	2.0e-14	gene=Chr03G0317.1	FGSG_00863T0[Fusariumgraminearum]
Chr09G0881.1	469	27.9	estExt_fgenesh1_pm.C_170119[Cenococcumgeophilum1.58]	1191	51	267	280	487	0.39	8	216	82.8	6.5e-16	gene=Chr09G0881.1	estExt_fgenesh1_pm.C_170119[Cenococcumgeophilum1.58]
Chr03G0967.1	1085	40.6	Clame_scaffold8-4.115[Cladoniametacorallifera]	16637	658	888	4395	4613	0.39	5	230	164.1	5.1e-40	gene=Chr03G0967.1	Clame_scaffold8-4.115[Cladoniametacorallifera]
Chr07G0960.1	445	26.3	PLG_11783-R0[Pseudohalonectrialignicola M95]	427	55	318	12	252	0.39	8	263	101.7	1.3e-21	gene=Chr07G0960.1	PLG_11783-R0[Pseudohalonectrialignicola M95]
Chr05G0115.1	229	32.9	1430_t[As	481	1	197	55	260	0.3	4	196	100.5	1.5e-21	gene=C	1430_t[Ascocorynes

			cocorynesarcoidesNRRL50072]						9					hr05G0115.1	arcoidesNRRL50072]
Chr03G0385.1	1490	37.5	Clama_scaffold_5-4.0[Cladoniamacilenta]	21962	380	1447	5459	6539	0.82	16	1067	697.2	2.3e-200	gene=Chr03G0385.1	Clama_scaffold_5-4.0[Cladoniamacilenta]
Chr03G0617.1	491	34.7	HCB02684.1[Histoplasmacapsulatum]	1240	213	404	281	467	0.40	5	191	117.5	2.5e-26	gene=Chr03G0617.1	HCB02684.1[Histoplasmacapsulatum]
Chr05G0884.1	686	46	XP_007804354.1[EndocarponpusillumZ07020]	541	475	660	3	176	0.40	3	185	151.8	1.7e-36	gene=Chr05G0884.1	XP_007804354.1[EndocarponpusillumZ07020]
Chr04G0049.1	257	40.8	Clame_scaffold8-4.115[Cladoniametacorallifera]	16637	10	190	15230	15430	0.40	5	180	117.5	1.3e-26	gene=Chr04G0049.1	Clame_scaffold8-4.115[Cladoniametacorallifera]
Chr09G0872.1	187	64.6	Hanno_04867[Heterobasidionannosum0	897	1	178	717	894	0.41	0	177	254.2	6.5e-68	gene=Chr09G0872.1	Hanno_04867[Heterobasidionannosum03012]

			3012]												
Chr08G0550.1	272	27.9	estExt_fgenesh2_pg.C_10498[Schizophyllumcommune H4-8]	823	17	237	10	264	0.41	6	220	77.4	1.6e-14	gene=Chr08G0550.1	estExt_fgenesh2_pg.C_10498[SchizophyllumcommuneH4-8]
Chr04G0680.1	277	30.5	Endocarpon_03441[Endocarponpusillum]	1458	77	276	1260	1458	0.41	2	199	108.6	6.5e-24	gene=Chr04G0680.1	Endocarpon_03441[Endocarponpusillum]
Chr04G0401.1	699	31	HCB02684.1[Histoplasmacapsulatum]	1240	349	599	264	478	0.41	7	250	107.5	3.7e-23	gene=Chr04G0401.1	HCB02684.1[Histoplasmacapsulatum]
Chr02G1437.1	1494	24.8	EfO2.075130.1[Epichloefestucae]	1182	1186	1486	909	1155	0.41	10	300	72.4	2.8e-12	gene=Chr02G1437.1	EfO2.075130.1[Epichloefestucae]
Chr03G0005.1	223	35	Clame_scaffold8-4.115[Cladoniametacorallifera]	16637	26	206	2936	3118	0.41	2	180	112.8	2.8e-25	gene=Chr03G0005.1	Clame_scaffold8-4.115[Cladoniametacorallifera]
Chr06G0357.1	771	31.8	estExt_fge	1191	397	600	276	478	0.4	9	203	92.4	1.3e-18	gene=C	estExt_fgenesh1_p

			nesh1_pm.C_170119[Cenococcumgeophilum1.58]						1					hr06G0357.1	m.C_170119[Cenococcumgeophilum1.58]
Chr03G1258.1	182	44.6	Hanno_04867[Heterobasidionannosum03012]	897	13	178	732	897	0.41	0	165	159.8	1.6e-39	gene=Chr03G1258.1	Hanno_04867[Heterobasidionannosum03012]
Chr03G0002.1	223	45.7	XP_007804354.1[EndocarponpusillumZ07020]	541	7	178	26	195	0.41	2	171	158.7	4.4e-39	gene=Chr03G0002.1	XP_007804354.1[EndocarponpusillumZ07020]
Chr05G1177.1	810	29	MUStwsD_GLEAN_10005105[Omphalotusolearius]	1487	478	790	1174	1469	0.41	13	312	130.2	6.1e-30	gene=Chr05G1177.1	MUStwsD_GLEAN_10005105[Omphalotusolearius]
Chr09G0083.1	828	41.2	GSTUMT00003726001[Tubermelanosporum]	553	576	774	313	501	0.41	4	198	148.7	1.7e-35	gene=Chr09G0083.1	GSTUMT00003726001[Tubermelanosporum]

Chr01G1089.1	1176	49.1	fgenesh1_pg.81_#_33[Sebacinavermiferaspp.bescii]	630	982	1146	49	213	0.41	0	164	145.2	2.7e-34	gene=Chr01G1089.1	fgenesh1_pg.81_#_33[Sebacinavermiferaspp.bescii]
Chr04G1324.1	354	32	ATEG_00556.1[Aspergillusterreus]	456	90	307	65	280	0.41	8	217	102.4	6.0e-22	gene=Chr04G1324.1	ATEG_00556.1[Aspergillusterreus]
Chr07G0723.1	1889	31.8	HCB02684.1[Histoplasmacapsulatum]	1240	689	883	263	458	0.41	4	194	87.8	8.1e-17	gene=Chr07G0723.1	HCB02684.1[Histoplasmacapsulatum]
Chr02G1546.1	510	33.7	maker-scaffold4.1-snap-gene-7.120-mRNA-1[Raffaeleaquercus-mongolicae]	582	64	262	74	267	0.41	3	198	112.5	8.3e-25	gene=Chr02G1546.1	maker-scaffold4.1-snap-gene-7.120-mRNA-1[Raffaeleaquercus-mongolicae]
Chr05G0256.1	722	29.4	XP_007803552.1[EndocarponpusillumZ0	1439	263	527	783	1019	0.41	10	264	85.5	1.5e-16	gene=Chr05G0256.1	XP_007803552.1[EndocarponpusillumZ07020]

			7020]												
Chr02G0580.1	338	28.8	XP_007803552.1[EndocarponpusillumZ07020]	1439	4	227	787	1019	0.41	7	223	85.1	9.4e-17	gene=Chr02G0580.1	XP_007803552.1[EndocarponpusillumZ07020]
Chr02G0727.1	764	38.8	Clama_scaffold_5-4.0[Cladoniamacilenta]	21962	493	671	10207	10388	0.42	2	178	142.5	1.1e-33	gene=Chr02G0727.1	Clama_scaffold_5-4.0[Cladoniamacilenta]
Chr05G0058.1	1208	30.8	HCB02684.1[Histoplasmacapsulatum]	1240	812	1068	319	540	0.42	10	256	85.9	2.0e-16	gene=Chr05G0058.1	HCB02684.1[Histoplasmacapsulatum]
Chr02G1334.1	500	47.8	Endocarpon_02817[Endocarponpusillum]	1255	6	181	69	248	0.42	4	175	161.4	1.5e-39	gene=Chr02G1334.1	Endocarpon_02817[Endocarponpusillum]
Chr01G2670.1	318	33.6	Clame_scaffold8-4.115[Cladoniametacorallifera]	16637	21	230	16054	16257	0.42	7	209	113.6	2.3e-25	gene=Chr01G2670.1	Clame_scaffold8-4.115[Cladoniametacorallifera]
Chr03G1546.1	641	31.4	Clame_scaffold11-8.	8336	11	250	1945	2159	0.42	8	239	100.9	3.2e-21	gene=Chr03G15	Clame_scaffold11-8.74[Cladoniametacor

			74[Cladoniametacorallifera]											46.1	allifera]
Chr04G0067.1	207	51.8	Clama_scaffold_5-4.0[Cladoniamacilenta]	21962	14	202	18849	19036	0.42	3	188	193.7	1.2e-49	gene=Chr04G0067.1	Clama_scaffold_5-4.0[Cladoniamacilenta]
Chr01G2118.1	342	23.1	OAT00923.1[BlastomycesdermatitidisER-3]	1246	30	316	269	536	0.42	11	286	85.5	7.3e-17	gene=Chr01G2118.1	OAT00923.1[BlastomycesdermatitidisER-3]
Chr06G1488.1	987	28.1	XP_007803552.1[EndocarponpusillumZ07020]	1439	17	252	782	1019	0.42	10	235	68.9	2.0e-11	gene=Chr06G1488.1	XP_007803552.1[EndocarponpusillumZ07020]
Chr01G0041.1	513	24.2	estExt_Genemark1.C_370088[Galerina marginata]	959	51	309	45	391	0.42	9	258	88.6	1.3e-17	gene=Chr01G0041.1	estExt_Genemark1.C_370088[Galerina marginata]
Chr05G0669.1	305	25.4	XP_007804474.1[EndocarponpusillumZ0	1398	23	291	1150	1397	0.42	8	268	60.1	2.9e-09	gene=Chr05G0669.1	XP_007804474.1[EndocarponpusillumZ07020]

			7020]												
Chr03G1413.1	299	35.4	1430_t[AscocorynesarcoidesNRRL50072]	481	31	250	51	260	0.42	9	219	124.8	9.5e-29	gene=Chr03G1413.1	1430_t[AscocorynesarcoidesNRRL50072]
Chr09G0273.1	265	44.3	Clame_scaffold8-4.115[Cladoniametacorallifera]	16637	20	208	15230	15439	0.43	5	188	137.5	1.3e-32	gene=Chr09G0273.1	Clame_scaffold8-4.115[Cladoniametacorallifera]
Chr04G0636.1	381	59.9	Endpu_scaffold5-14.59[EndocarponpusillumR61883]	1473	118	315	332	543	0.43	3	197	254.6	1.0e-67	gene=Chr04G0636.1	Endpu_scaffold5-14.59[EndocarponpusillumR61883]
Chr01G1789.1	288	32	Lema_T073080.1[Leptosphaeriamaculans]	495	49	278	65	359	0.43	5	229	154.1	1.4e-37	gene=Chr01G1789.1	Lema_T073080.1[Leptosphaeriamaculans]
Chr01G0991.1	336	28.6	ATEG_00556.1[Aspergillusterreus]	456	57	295	24	280	0.43	8	238	84.7	1.2e-16	gene=Chr01G0991.1	ATEG_00556.1[Aspergillusterreus]

Chr07G0349.1	289	37	1430_t[AscocorynesarcoidesNRRL50072]	481	25	227	51	248	0.43	6	202	110.5	1.8e-24	gene=Chr07G0349.1	1430_t[AscocorynesarcoidesNRRL50072]
Chr03G0648.1	182	59.8	Hanno_04867[Heterobasidionannosum03012]	897	1	179	717	895	0.43	0	178	217.2	8.6e-57	gene=Chr03G0648.1	Hanno_04867[Heterobasidionannosum03012]
Chr05G0273.1	400	27.1	EAA61211.1[AspergillusnidulansFGSCA4]	742	94	318	75	308	0.43	5	224	82.8	5.5e-16	gene=Chr05G0273.1	EAA61211.1[AspergillusnidulansFGSCA4]
Chr02G0448.1	342	36.4	EAA58488.1[AspergillusnidulansFGSCA4]	1365	16	246	1067	1276	0.43	7	230	115.5	6.6e-26	gene=Chr02G0448.1	EAA58488.1[AspergillusnidulansFGSCA4]
Chr01G1838.1	560	30.1	AB00197.1[Alternariabrassicicola]	1041	34	250	52	263	0.43	4	216	76.3	7.3e-14	gene=Chr01G1838.1	AB00197.1[Alternariabrassicicola]
Chr02G0976.1	348	31.8	Clama_scaffold_5-4.0[Cladoniamacilenta]	21962	92	303	6709	6919	0.44	5	211	126.7	2.9e-29	gene=Chr02G0976.1	Clama_scaffold_5-4.0[Cladoniamacilenta]

Chr08G0862.1	364	30.9	EAA66095.1[AspergillusnidulansFGSCA4]	832	92	332	53	270	0.44	4	240	149.1	5.7e-36	gene=Chr08G0862.1	EAA66095.1[AspergillusnidulansFGSCA4]
Chr09G0689.1	257	28.7	XP_007602139.1[ColletotrichumfioriniaePJ7]	801	12	244	4	240	0.44	6	232	95.9	4.1e-20	gene=Chr09G0689.1	XP_007602139.1[ColletotrichumfioriniaePJ7]
Chr01G2519.1	353	41.3	Clame_scaffold8-4.115[Cladoniametacorallifera]	16637	131	347	12946	13142	0.44	6	216	162.2	6.3e-40	gene=Chr01G2519.1	Clame_scaffold8-4.115[Cladoniametacorallifera]
Chr03G0949.1	276	44.8	Endocarpon_02817[Endocarponpusillum]	1255	7	203	10	201	0.44	6	196	147.9	9.7e-36	gene=Chr03G0949.1	Endocarpon_02817[Endocarponpusillum]
Chr02G0653.1	748	28.2	estExt_fgenesh1_pg.C_450037[Phytophthorasojae]	872	188	434	639	865	0.44	7	246	93.6	5.9e-19	gene=Chr02G0653.1	estExt_fgenesh1_pg.C_450037[Phytophthorasojae]
Chr08G1066.1	1490	32.4	XP_007803552.1[En	1439	3	226	785	1019	0.44	8	223	106.3	1.7e-22	gene=Chr08G10	XP_007803552.1[EndocarponpusillumZ0

			docarponpusillumZ07020]											66.1	7020]
Chr04G1478.1	376	30.2	Clame_scaffold8-4.115[Cladoniametacorallifera]	16637	101	332	11992	12224	0.44	8	231	115.2	9.5e-26	gene=Chr04G1478.1	Clame_scaffold8-4.115[Cladoniametacorallifera]
Chr07G0902.1	335	30.7	EAA58488.1[AspergillusnidulansFGSCA4]	1365	37	252	1068	1289	0.44	3	215	89	6.5e-18	gene=Chr07G0902.1	EAA58488.1[AspergillusnidulansFGSCA4]
Chr05G0466.1	1634	27.9	CE479139_59193[PilodermacroceumF1598]	1380	1305	1579	678	987	0.44	12	274	68.9	3.4e-11	gene=Chr05G0466.1	CE479139_59193[PilodermacroceumF1598]
Chr02G0633.1	1053	34.4	Clama_scaffold_5-4.0[Cladoniamacilenta]	21962	761	963	10230	10436	0.44	4	202	114	5.9e-25	gene=Chr02G0633.1	Clama_scaffold_5-4.0[Cladoniamacilenta]
Chr05G0578.1	286	55	FGSG_09033T0[Fusariumgraminearum]	189	94	279	1	188	0.45	3	185	208.4	6.3e-54	gene=Chr05G0578.1	FGSG_09033T0[Fusariumgraminearum]
Chr02G0089.1	547	91	gm1.9153	560	1	546	1	546	0.4	0	545	1047.	2.6e-30	gene=C	gm1.9153_g[Colletot

			_g[Colletotrichumsub lineolaCBS131301]						5			7	6	hr02G0089.1	richumsublineolaCBS131301]
Chr03G0215.1	425	26.1	EAA63317.1[Aspergillusnidulan sFGSCA4]	1402	111	355	55	299	0.45	7	244	52	1.1e-06	gene=Chr03G0215.1	EAA63317.1[AspergillusnidulansFGSCA4]
Chr04G0763.1	194	61.2	EEA28139.1[Penicilliummarneffei]	2025	1	193	672	867	0.45	2	192	231.9	3.6e-61	gene=Chr04G0763.1	EEA28139.1[Penicilliummarneffei]
Chr01G0617.1	310	38	Clame_scaffold8-4.115[Cladoniametacorallifera]	16637	19	228	16054	16259	0.45	6	209	116.3	3.5e-26	gene=Chr01G0617.1	Clame_scaffold8-4.115[Cladoniametacorallifera]
Chr09G0392.1	979	23	EAA61211.1[Aspergillusnidulan sFGSCA4]	742	14	292	11	273	0.45	7	278	68.6	2.6e-11	gene=Chr09G0392.1	EAA61211.1[AspergillusnidulansFGSCA4]
Chr06G0743.1	604	34.6	Clame_scaffold8-4.115[Cladoniametacorallifera]	16637	324	534	2405	2630	0.45	4	210	126.3	6.6e-29	gene=Chr06G0743.1	Clame_scaffold8-4.115[Cladoniametacorallifera]

Chr08G0596.1	723	31.1	estExt_fgenesh1_pm.C_170119[Cenococcumgeophilum1.58]	1191	325	582	240	480	0.45	12	257	99.8	7.9e-21	gene=Chr08G0596.1	estExt_fgenesh1_pm.C_170119[Cenococcumgeophilum1.58]
Chr03G1460.1	522	42.6	Endocarpon_02817[Endocarponpusillum]	1255	23	242	25	248	0.45	5	219	176.8	3.7e-44	gene=Chr03G1460.1	Endocarpon_02817[Endocarponpusillum]
Chr03G0361.1	640	41.5	Endocarpon_02817[Endocarponpusillum]	1255	1	280	1	245	0.45	11	279	174.5	2.2e-43	gene=Chr03G0361.1	Endocarpon_02817[Endocarponpusillum]
Chr08G0134.1	230	48.2	snap_masked-scaffold9.1-processed-gene-6.111-mRNA-1[Raffaeleaquercus-mongolicae]	216	1	214	1	206	0.45	7	213	204.5	7.3e-53	gene=Chr08G0134.1	snap_masked-scaffold9.1-processed-gene-6.111-mRNA-1[Raffaeleaquercus-mongolicae]
Chr05G1430.1	573	41.2	Endocarp	1255	1	209	1	223	0.4	5	208	163.7	3.5e-40	gene=C	Endocarpon_02817[

			on_02817[Endocarponpusillum]						5					hr05G1430.1	Endocarponpusillum]
Chr01G2790.1	802	26.9	fgenesh1_pg.31_#_85[Fibulorhizoctoniasp.CBS109695]	1514	90	328	373	609	0.45	3	238	91.7	2.4e-18	gene=Chr01G2790.1	fgenesh1_pg.31_#_85[Fibulorhizoctoniasp.CBS109695]
Chr02G0002.1	1053	27	augustus_masked-scaffold407-processed-gene-0.5-mRNA-1[PseudohalonectrialignicolaM95]	1265	422	761	755	1049	0.46	14	339	94.7	3.7e-19	gene=Chr02G0002.1	augustus_masked-scaffold407-processed-gene-0.5-mRNA-1[PseudohalonectrialignicolaM95]
Chr01G2668.1	384	34.6	SS1G_01499.1[Sclerotiniasclerotiorum]	1043	2	212	656	864	0.46	6	210	126.7	3.2e-29	gene=Chr01G2668.1	SS1G_01499.1[Sclerotiniasclerotiorum]
Chr01G0096.1	384	34.6	SS1G_01499.1[Scle	1043	2	212	656	864	0.46	6	210	125.9	5.5e-29	gene=Chr01G00	SS1G_01499.1[Sclerotiniasclerotiorum]

			rotiniasclerotiorum]											96.1	
Chr03G1362.1	384	34.6	SS1G_01499.1[Sclerotiniasclerotiorum]	1043	2	212	656	864	0.46	6	210	125.9	5.5e-29	gene=Chr03G1362.1	SS1G_01499.1[Sclerotiniasclerotiorum]
Chr06G0043.1	384	34.6	SS1G_01499.1[Sclerotiniasclerotiorum]	1043	2	212	656	864	0.46	6	210	125.9	5.5e-29	gene=Chr06G0043.1	SS1G_01499.1[Sclerotiniasclerotiorum]
Chr05G0027.1	384	34.6	SS1G_01499.1[Sclerotiniasclerotiorum]	1043	2	212	656	864	0.46	6	210	125.9	5.5e-29	gene=Chr05G0027.1	SS1G_01499.1[Sclerotiniasclerotiorum]
Chr02G1338.1	384	34.6	SS1G_01499.1[Sclerotiniasclerotiorum]	1043	2	212	656	864	0.46	6	210	125.9	5.5e-29	gene=Chr02G1338.1	SS1G_01499.1[Sclerotiniasclerotiorum]
Chr02G1377.1	291	55.1	Clama_scaffold_5-4.0[Cladoniamacilenta]	21962	32	218	10441	10626	0.46	1	186	225.7	3.9e-59	gene=Chr02G1377.1	Clama_scaffold_5-4.0[Cladoniamacilenta]
Chr09G0851.1	382	30.5	Clame_scaffold11-8.74[Cladoniametacora	8336	89	327	3977	4199	0.46	4	238	132.1	7.6e-31	gene=Chr09G0851.1	Clame_scaffold11-8.74[Cladoniametacorallifera]

			llifera]												
Chr06G0054.1	308	30.8	MUStwsD_GLEAN_10000830[Omphalotusolearius]	927	33	284	574	801	0.46	4	251	125.9	4.4e-29	gene=Chr06G0054.1	MUStwsD_GLEAN_10000830[Omphalotusolearius]
Chr04G1200.1	1343	27.4	XP_007803552.1[EndocarponpusillumZ07020]	1439	7	266	786	1065	0.46	8	259	96.7	1.2e-19	gene=Chr04G1200.1	XP_007803552.1[EndocarponpusillumZ07020]
Chr06G0695.1	1452	27.9	EEA23526.1[Penicilliummarneffei]	1874	342	607	405	656	0.46	6	265	89.7	1.6e-17	gene=Chr06G0695.1	EEA23526.1[Penicilliummarneffei]
Chr04G0327.1	554	32.3	gm1.11182_g[Sphaerobolusstellatus]	620	250	549	362	614	0.46	14	299	106.3	6.5e-23	gene=Chr04G0327.1	gm1.11182_g[Sphaerobolusstellatus]
Chr02G0476.1	334	42.8	ATEG_00556.1[Aspergillusterreus]	456	92	284	85	276	0.46	2	192	139	5.4e-33	gene=Chr02G0476.1	ATEG_00556.1[Aspergillusterreus]
Chr03G0408.1	564	90.7	XP_007593930.1[Co	551	1	550	1	550	0.46	0	549	1058.5	1.5e-309	gene=Chr03G04	XP_007593930.1[ColletotrichumfioriniaeP

			lletotrichumfioriniaePJ7]											08.1	J7]
Chr01G1085.1	1194	42.9	Clame_scaffold8-4.115[Cladoniametacorallifera]	16637	646	855	4341	4538	0.46	2	209	177.2	6.4e-44	gene=Chr01G1085.1	Clame_scaffold8-4.115[Cladoniametacorallifera]
Chr03G0604.1	525	55.8	FGSG_08005T0[Fusariumgraminearum]	500	14	194	8	188	0.45	0	180	214.9	1.2e-55	gene=Chr03G0604.1	FGSG_08005T0[Fusariumgraminearum]
Chr09G0051.1	271	27.5	Endocarpon_03441[Endocarponpusillum]	1458	20	264	1216	1454	0.46	6	244	92.4	4.7e-19	gene=Chr09G0051.1	Endocarpon_03441[Endocarponpusillum]
Chr01G0196.1	213	44.3	EAA60936.1[AspergillusnidulansFGSCA4]	831	4	202	140	335	0.47	3	198	192.2	3.5e-49	gene=Chr01G0196.1	EAA60936.1[AspergillusnidulansFGSCA4]
Chr04G1564.1	378	29.3	XP_007804354.1[EndocarponpusillumZ07020]	541	81	337	50	286	0.47	7	256	126.3	4.1e-29	gene=Chr04G1564.1	XP_007804354.1[EndocarponpusillumZ07020]

Chr01G2509.1	629	44.4	Endocarpon_02817[Endocarponpusillum]	1255	18	252	22	265	0.47	8	234	181.4	1.8e-45	gene=Chr01G2509.1	Endocarpon_02817[Endocarponpusillum]
Chr07G0020.1	499	25.9	EAA61211.1[AspergillusnidulansFGSCA4]	742	108	370	16	272	0.47	5	262	68.6	1.3e-11	gene=Chr07G0020.1	EAA61211.1[AspergillusnidulansFGSCA4]
Chr01G2374.1	1166	26.7	estExt_Genewise1Plus.C_120159[Sistotremastrumsuecicum]	525	284	585	13	284	0.47	10	301	104	6.8e-22	gene=Chr01G2374.1	estExt_Genewise1Plus.C_120159[Sistotremastrumsuecicum]
Chr05G0371.1	278	29.8	XP_007804474.1[EndocarponpusillumZ07020]	1398	23	266	1150	1397	0.47	5	243	127.5	1.4e-29	gene=Chr05G0371.1	XP_007804474.1[EndocarponpusillumZ07020]
Chr02G1539.1	302	40.8	1430_t[AscocorynesarcoidesNRRL50072]	481	19	236	43	260	0.47	6	217	177.2	1.6e-44	gene=Chr02G1539.1	1430_t[AscocorynesarcoidesNRRL50072]
Chr01G0499.1	452	22.9	e_gw1.8.2	547	123	429	59	390	0.4	12	306	54.7	1.8e-07	gene=C	e_gw1.8.257.1[Paxill

			57.1[PaxillusinvolutusATCC200175]						7					hr01G0499.1	usinvolutusATCC200175]
Chr02G1750.1	763	31.3	Clama_scaffold_5-4.0[Cladoniamacilenta]	21962	217	461	10194	10429	0.47	6	244	127.1	4.9e-29	gene=Chr02G1750.1	Clama_scaffold_5-4.0[Cladoniamacilenta]
Chr08G0279.1	585	23.6	Clama_scaffold_28-0.22[Cladoniamacilenta]	3845	60	373	3531	3813	0.47	11	313	63.9	3.9e-10	gene=Chr08G0279.1	Clama_scaffold_28-0.22[Cladoniamacilenta]
Chr04G1198.1	330	28.5	estExt_fgenesh1_pm.C_1380002[ColletotrichumsublineolaCBS131301]	890	20	273	534	800	0.47	7	253	100.9	1.6e-21	gene=Chr04G1198.1	estExt_fgenesh1_pm.C_1380002[ColletotrichumsublineolaCBS131301]
Chr05G1126.1	567	90.5	XP_007599723.1[ColletotrichumfioriniaePJ7]	567	2	566	3	567	0.47	2	564	1044.6	2.2e-305	gene=Chr05G1126.1	XP_007599723.1[ColletotrichumfioriniaePJ7]
Chr09G0899.1	255	35.4	EAA65893	358	3	230	2	235	0.4	7	227	104	1.5e-22	gene=C	EAA65893.1[Aspergi

			.1[AspergillusnidulansFGSCA4]						7					hr09G0899.1	llusnidulansFGSCA4]
Chr02G0921.1	622	31.6	Clame_scaffold11-8.74[Cladoniametacorallifera]	8336	7	265	1946	2181	0.47	11	258	93.2	6.4e-19	gene=Chr02G0921.1	Clame_scaffold11-8.74[Cladoniametacorallifera]
Chr01G1189.1	395	23.8	EAA61211.1[AspergillusnidulansFGSCA4]	742	3	265	2	269	0.47	5	262	74.7	1.5e-13	gene=Chr01G1189.1	EAA61211.1[AspergillusnidulansFGSCA4]
Chr05G0616.1	715	32.1	HCB02684.1[Histoplasmacapsulatum]	1240	312	536	273	503	0.47	5	224	121.3	2.5e-27	gene=Chr05G0616.1	HCB02684.1[Histoplasmacapsulatum]
Chr01G1267.1	301	35.5	CE216775_210[SebacinavermiferaMAFF305830]	336	10	257	12	308	0.47	9	247	169.1	4.4e-42	gene=Chr01G1267.1	CE216775_210[SebacinavermiferaMAFF305830]
Chr08G0017.1	541	28.8	EfO2.075130.1[Epichloefestucae]	1182	265	528	906	1154	0.47	7	263	99	1.0e-20	gene=Chr08G0017.1	EfO2.075130.1[Epichloefestucae]
Chr07G0885.1	357	31.3	gm1.1009	821	15	267	3	244	0.4	10	252	85.1	1.0e-16	gene=C	gm1.10090_g[Hypho

			0_g[Hypholomasublateritium]						7					hr07G0885.1	lomasublateritium]
Chr06G0983.1	1114	29	fgenesh1_pg.8_#_209[PleurotusostreatusPC9]	1659	742	1026	1390	1641	0.47	8	284	113.6	8.2e-25	gene=Chr06G0983.1	fgenesh1_pg.8_#_209[PleurotusostreatusPC9]
Chr02G0227.1	402	31.1	Clame_scaffold18-19.70[Cladoniametacorallifera]	7866	1	238	3257	3503	0.47	7	237	124	2.2e-28	gene=Chr02G0227.1	Clame_scaffold18-19.70[Cladoniametacorallifera]
Chr03G0070.1	394	34.1	SS1G_01499.1[Sclerotiniasclerotiorum]	1043	3	222	647	864	0.47	6	219	132.5	6.0e-31	gene=Chr03G0070.1	SS1G_01499.1[Sclerotiniasclerotiorum]
Chr04G0004.1	394	34.1	SS1G_01499.1[Sclerotiniasclerotiorum]	1043	3	222	647	864	0.47	6	219	132.9	4.6e-31	gene=Chr04G0004.1	SS1G_01499.1[Sclerotiniasclerotiorum]
Chr03G1723.1	288	30.2	Endocarpon_03441[Endocarponpusillum]	1458	40	284	1214	1457	0.47	5	244	104.4	1.3e-22	gene=Chr03G1723.1	Endocarpon_03441[Endocarponpusillum]

Chr07G0046.1	301	33.2	Clame_scaffold8-4.115[Cladoniametacorallifera]	16637	14	276	16	264	0.47	8	262	136	4.2e-32	gene=Chr07G0046.1	Clame_scaffold8-4.115[Cladoniametacorallifera]
Chr09G0448.1	333	33.2	maker-scaffold122-augustus-gene-0.90-mRNA-1[OphiocerasdolichostomumCBS114926]	1059	72	313	184	409	0.47	5	241	118.6	7.6e-27	gene=Chr09G0448.1	maker-scaffold122-augustus-gene-0.90-mRNA-1[OphiocerasdolichostomumCBS114926]
Chr01G1501.1	197	57.9	SS1G_01499.1[Sclerotiniasclerotiorum]	1043	2	196	547	741	0.47	0	194	240.7	7.8e-64	gene=Chr01G1501.1	SS1G_01499.1[Sclerotiniasclerotiorum]
Chr01G0436.1	324	45.3	ATEG_00556.1[Aspergillusterreus]	456	64	282	65	277	0.48	5	218	171.4	9.6e-43	gene=Chr01G0436.1	ATEG_00556.1[Aspergillusterreus]
Chr07G0838.1	800	36.6	Clama_scaffold_5-4.0[Cladoniamacilenta]	21962	510	746	10211	10435	0.48	7	236	126.3	8.7e-29	gene=Chr07G0838.1	Clama_scaffold_5-4.0[Cladoniamacilenta]

Chr08G0440.1	1172	28	7575_t[AscocorynesarcoidesNRRL50072]	1484	446	745	386	666	0.48	11	299	106.7	1.1e-22	gene=Chr08G0440.1	7575_t[AscocorynesarcoidesNRRL50072]
Chr02G0613.1	706	29.6	Clama_scaffold_5-4.0[Cladoniamacilenta]	21962	254	569	19881	20154	0.48	14	315	103.2	7.0e-22	gene=Chr02G0613.1	Clama_scaffold_5-4.0[Cladoniamacilenta]
Chr04G0432.1	285	27.4	XP_007804474.1[EndocarponpusillumZ07020]	1398	22	271	1150	1394	0.48	5	249	89	5.5e-18	gene=Chr04G0432.1	XP_007804474.1[EndocarponpusillumZ07020]
Chr03G0770.1	357	34.6	g13734.t1[ArmillariamelleaDSM3731]	565	40	285	4	245	0.48	8	245	114.8	1.2e-25	gene=Chr03G0770.1	g13734.t1[ArmillariamelleaDSM3731]
Chr06G0563.1	534	90.1	gm1.7651_g[ColletotrichumsublineolaCBS131301]	533	1	533	1	533	0.48	0	532	1004.2	3.2e-293	gene=Chr06G0563.1	gm1.7651_g[ColletotrichumsublineolaCBS131301]
Chr03G1267.1	749	32.5	estExt_Genemark1.C_150090	827	451	706	25	274	0.48	9	255	101.7	2.2e-21	gene=Chr03G1267.1	estExt_Genemark1.C_150090[Dacryopinaxsp.DJM731SSP1

			[Dacryopinaxsp.DJM731SSP1]												]
Chr08G0939.1	1262	36.2	fgenesh1_pg.24_#_34[PhlebiabrevisporaHHB-7030SS6]	2158	1	222	353	582	0.48	6	221	130.2	9.6e-30	gene=Chr08G0939.1	fgenesh1_pg.24_#_34[PhlebiabrevisporaHHB-7030SS6]
Chr06G1240.1	1609	24.9	fgenesh1_pg.31_#_85[Fibulorhizoctoniasp.CBS109695]	1514	426	689	373	636	0.48	5	263	94.4	7.4e-19	gene=Chr06G1240.1	fgenesh1_pg.31_#_85[Fibulorhizoctoniasp.CBS109695]
Chr01G0722.1	326	29	Clame_scaffold8-4.115[Cladoniametacorallifera]	16637	1	281	1	262	0.48	13	280	97.1	2.3e-20	gene=Chr01G0722.1	Clame_scaffold8-4.115[Cladoniametacorallifera]
Chr05G0828.1	1291	25.2	Clame_scaffold9-5.100[Cladoniametacorallifera]	12517	1002	1280	11182	11450	0.48	8	278	66.2	1.7e-10	gene=Chr05G0828.1	Clame_scaffold9-5.100[Cladoniametacorallifera]
Chr06G1358.1	391	34.2	Endocarp	1455	28	281	323	557	0.4	7	253	136.7	3.2e-32	gene=C	Endocarpon_04277[

			on_04277[Endocarponpusillum]						8					hr06G1358.1	Endocarponpusillum]
Chr03G1223.1	592	44.4	Endocarpon_02817[Endocarponpusillum]	1255	1	216	1	240	0.48	7	215	196.4	5.1e-50	gene=Chr03G1223.1	Endocarpon_02817[Endocarponpusillum]
Chr02G1774.1	619	27.9	Hanno_03304[Heterobasidionannosum03012]	1417	106	383	760	1066	0.48	8	277	94	3.7e-19	gene=Chr02G1774.1	Hanno_03304[Heterobasidionannosum03012]
Chr08G0967.1	460	25	EAA61211.1[AspergillusnidulansFGSCA4]	742	87	350	11	273	0.48	6	263	82.4	8.3e-16	gene=Chr08G0967.1	EAA61211.1[AspergillusnidulansFGSCA4]
Chr04G0070.1	771	43.7	fgenesh1_kg.214_#_9_#_Locus8501v1rpkm7.50[Polyporusarcularius]	923	532	748	588	799	0.48	6	216	176	9.3e-44	gene=Chr04G0070.1	fgenesh1_kg.214_#_9_#_Locus8501v1rpkm7.50[Polyporusarcularius]

Chr03G0160.1	391	34.1	Endocarpon_04277[Endocarponpusillum]	1455	28	282	323	558	0.48	7	254	135.2	9.2e-32	gene=Chr03G0160.1	Endocarpon_04277[Endocarponpusillum]
Chr01G0272.1	371	22.2	EAA61211.1[AspergillusnidulansFGSCA4]	742	17	317	13	313	0.48	9	300	52.4	7.4e-07	gene=Chr01G0272.1	EAA61211.1[AspergillusnidulansFGSCA4]
Chr05G1246.1	1168	27.6	estExt_fgenesh1_pg.C_5_t10276[PhanerochaetecarnosaHHB-10118-Sp]	1237	640	901	731	1027	0.48	10	261	102.8	1.5e-21	gene=Chr05G1246.1	estExt_fgenesh1_pg.C_5_t10276[PhanerochaetecarnosaHHB-10118-Sp]
Chr04G1232.1	639	32.7	gm1.11182_g[Sphaerobolusstellatus]	620	302	617	362	615	0.48	7	315	124.4	2.7e-28	gene=Chr04G1232.1	gm1.11182_g[Sphaerobolusstellatus]
Chr09G0726.1	374	32.5	ATEG_00556.1[Aspergillusterreus]	456	85	318	48	280	0.48	5	233	102.1	8.3e-22	gene=Chr09G0726.1	ATEG_00556.1[Aspergillusterreus]
Chr05G0261.1	470	29.2	augustus_	1265	36	369	784	1062	0.4	15	333	125.9	6.7e-29	gene=C	augustus_masked-s

			masked-scaffold407-processed-gene-0.5-mRNA-1[PseudohalonectrialignicolaM95]						8					hr05G0261.1	caffold407-processed-gene-0.5-mRNA-1[PseudohalonectrialignicolaM95]
Chr01G1748.1	282	30.5	XP_007804474.1[EndocarponpusillumZ07020]	1398	26	279	1150	1396	0.48	6	253	115.5	5.4e-26	gene=Chr01G1748.1	XP_007804474.1[EndocarponpusillumZ07020]
Chr01G1814.1	929	28.2	XP_007804474.1[EndocarponpusillumZ07020]	1398	16	265	1150	1394	0.49	3	249	102.4	1.6e-21	gene=Chr01G1814.1	XP_007804474.1[EndocarponpusillumZ07020]
Chr04G0812.1	365	22.8	EAA61211.1[AspergillusnidulansFGSCA4]	742	19	320	25	328	0.49	11	301	61.6	1.2e-09	gene=Chr04G0812.1	EAA61211.1[AspergillusnidulansFGSCA4]
Chr01G1048.1	981	44.2	Endocarpon_02817[Endocarp	1255	8	254	11	245	0.49	7	246	194.5	3.2e-49	gene=Chr01G1048.1	Endocarpon_02817[Endocarponpusillum]

			onpusillum]												
Chr03G1715.1	263	33.1	XP_007804474.1[EndocarponpusillumZ07020]	1398	17	260	1156	1396	0.49	7	243	112.1	5.6e-25	gene=Chr03G1715.1	XP_007804474.1[EndocarponpusillumZ07020]
Chr04G0952.1	413	29.5	EAA61211.1[AspergillusnidulansFGSCA4]	742	100	346	18	264	0.49	7	246	84.3	2.0e-16	gene=Chr04G0952.1	EAA61211.1[AspergillusnidulansFGSCA4]
Chr06G1200.1	288	31.1	XP_007804474.1[EndocarponpusillumZ07020]	1398	9	285	1150	1396	0.49	7	276	105.5	5.7e-23	gene=Chr06G1200.1	XP_007804474.1[EndocarponpusillumZ07020]
Chr01G0339.1	341	39.7	EAA58488.1[AspergillusnidulansFGSCA4]	1365	39	246	1067	1280	0.49	3	207	119.4	4.6e-27	gene=Chr01G0339.1	EAA58488.1[AspergillusnidulansFGSCA4]
Chr03G0773.1	324	26.4	HCBG_09081[HistoplasmacapsulatumG186AR]	1260	2	293	265	540	0.49	10	291	110.2	2.6e-24	gene=Chr03G0773.1	HCBG_09081[HistoplasmacapsulatumG186AR]
Chr01G2569.1	1040	48	Endocarp	1255	410	620	23	249	0.4	3	210	209.5	1.0e-53	gene=C	Endocarpon_02817[

			on_02817[Endocarponpusillum]						9					hr01G2569.1	Endocarponpusillum]
Chr02G1028.1	795	39.5	Endocarpon_02817[Endocarponpusillum]	1255	22	249	23	250	0.49	6	227	163.7	4.9e-40	gene=Chr02G1028.1	Endocarpon_02817[Endocarponpusillum]
Chr05G0412.1	290	38	XP_007599944.1[ColletotrichumfioriniaePJ7]	1113	54	280	68	296	0.49	5	226	139	4.7e-33	gene=Chr05G0412.1	XP_007599944.1[ColletotrichumfioriniaePJ7]
Chr01G2605.1	303	29.9	Endocarpon_03441[Endocarponpusillum]	1458	55	302	1204	1458	0.49	7	247	110.2	2.5e-24	gene=Chr01G2605.1	Endocarpon_03441[Endocarponpusillum]
Chr09G0637.1	412	23.7	EAA61211.1[AspergillusnidulansFGSCA4]	742	14	326	7	292	0.49	7	312	75.5	9.1e-14	gene=Chr09G0637.1	EAA61211.1[AspergillusnidulansFGSCA4]
Chr01G1376.1	274	61.4	XP_007600826.1[Colletotrichu	423	2	211	214	422	0.49	1	209	268.5	4.9e-72	gene=Chr01G1376.1	XP_007600826.1[ColletotrichumfioriniaePJ7]

			mfioriniaePJ7]												
Chr03G1398.1	645	26.7	gm1.11182_g[Spherobolusstellatus]	620	317	639	336	614	0.49	10	322	85.1	1.8e-16	gene=Chr03G1398.1	gm1.11182_g[Sphaerobolusstellatus]
Chr04G0262.1	313	33.2	estExt_Genemark1.C_150090[Dacryopinaxsp.DJM731SSP1]	827	20	257	25	275	0.49	9	237	113.2	3.0e-25	gene=Chr04G0262.1	estExt_Genemark1.C_150090[Dacryopinaxsp.DJM731SSP1]
Chr04G1208.1	1171	28.6	fgenesh1_pg.8_#_209[Pleurotusostreatus PC9]	1659	848	1105	1387	1637	0.49	5	257	116.3	1.3e-25	gene=Chr04G1208.1	fgenesh1_pg.8_#_209[PleurotusostreatusPC9]
Chr02G1307.1	400	35.2	augustus_masked-scaffold407-processed-gene-0.5-mRNA-1[PseudohalonectrialignicolaM95	1265	1	232	814	1055	0.49	9	231	109	7.2e-24	gene=Chr02G1307.1	augustus_masked-scaffold407-processed-gene-0.5-mRNA-1[PseudohalonectrialignicolaM95]

			]												
Chr06G1455.1	792	24.9	maker-Caloplaca_flavorubescens_scaffold_19-augustus-gene-2.67.2-mRNA-1[Caloplacaflavorubescens]	1049	233	537	44	372	0.49	14	304	68.2	2.8e-11	gene=Chr06G1455.1	maker-Caloplaca_flavorubescens_scaffold_19-augustus-gene-2.67.2-mRNA-1[Caloplacaflavorubescens]
Chr09G0432.1	547	28.5	estExt_Genemark1.C_370088[Galerina marginata]	959	1	279	1	322	0.49	12	278	110.5	3.4e-24	gene=Chr09G0432.1	estExt_Genemark1.C_370088[Galerina marginata]
Chr05G0955.1	559	39.5	Endocarpon_02817[Endocarponpusillum]	1255	20	243	23	248	0.49	4	223	172.2	9.7e-43	gene=Chr05G0955.1	Endocarpon_02817[Endocarponpusillum]
Chr02G1526.1	543	27.6	augustus_masked-scaffold407-processe	1265	2	333	789	1065	0.49	10	331	120.2	4.3e-27	gene=Chr02G1526.1	augustus_masked-scaffold407-processed-gene-0.5-mRNA-1[Pseudohalonectrialig

			d-gene-0.5-mRNA-1[PseudohalonectrialignicolaM95]											nicolaM95]
Chr08G0759.1	443	31.9	Clame_scaffold18-19.70[Cladoniametacorallifera]	7866	76	329	3209	3498	0.49	6	253	156.4	4.4e-38	gene=Chr08G0759.1	Clame_scaffold18-19.70[Cladoniametacorallifera]
Chr07G0956.1	403	84	XP_007599910.1[ColletotrichumfioriniaePJ7]	398	3	402	4	398	0.50	1	399	685.6	1.9e-197	gene=Chr07G0956.1	XP_007599910.1[ColletotrichumfioriniaePJ7]
Chr01G2599.1	332	37.6	Clame_scaffold8-4.115[Cladoniametacorallifera]	16637	14	286	20	266	0.50	7	272	157.1	1.9e-38	gene=Chr01G2599.1	Clame_scaffold8-4.115[Cladoniametacorallifera]
Chr05G0446.1	1213	32.9	Clama_scaffold_5-4.0[Cladoniamacilenta]	21962	834	1067	10205	10436	0.50	2	233	148.3	3.3e-35	gene=Chr05G0446.1	Clama_scaffold_5-4.0[Cladoniamacilenta]
Chr03G1317.1	267	36.1	Endocarp	1458	37	264	1208	1456	0.5	5	227	151.8	6.5e-37	gene=C	Endocarpon_03441[

			on_03441[Endocarponpusillum]						0					hr03G1317.1	Endocarponpusillum]
Chr07G0297.1	333	30.2	EAA58488.1[AspergillusnidulansFGSCA4]	1365	36	285	1067	1322	0.50	5	249	97.1	2.4e-20	gene=Chr07G0297.1	EAA58488.1[AspergillusnidulansFGSCA4]
Chr09G1004.1	493	26.8	Clame_scaffold9-5.100[Cladoniametacorallifera]	12517	76	386	10282	10615	0.50	14	310	75.9	8.4e-14	gene=Chr09G1004.1	Clame_scaffold9-5.100[Cladoniametacorallifera]
Chr02G0759.1	1337	27.3	HCBG_09081[HistoplasmacapsulatumG186AR]	1260	271	560	263	542	0.50	5	289	135.6	2.4e-31	gene=Chr02G0759.1	HCBG_09081[HistoplasmacapsulatumG186AR]
Chr01G0418.1	341	30.1	Clame_scaffold8-4.115[Cladoniametacorallifera]	16637	52	306	11960	12220	0.50	4	254	147.1	2.0e-35	gene=Chr01G0418.1	Clame_scaffold8-4.115[Cladoniametacorallifera]
Chr01G0778.1	408	30.3	Clame_scaffold18-19.70[Clad	7866	133	391	4231	4523	0.50	9	258	136	5.6e-32	gene=Chr01G0778.1	Clame_scaffold18-19.70[Cladoniametacorallifera]

			oniametacorallifera]													
Chr02G1540.1	417	27.8	EAA61211.1[AspergillusnidulansFGSCA4]	742	21	288	4	292	0.50	10	267	82.4	7.5e-16	gene=Chr02G1540.1	EAA61211.1[AspergillusnidulansFGSCA4]
Chr01G0421.1	586	46.4	Endocarpon_02817[Endocarponpusillum]	1255	11	229	14	243	0.50	5	218	187.6	2.3e-47	gene=Chr01G0421.1	Endocarpon_02817[Endocarponpusillum]
Chr01G2679.1	1412	27.1	fgenesh1_pg.344_#_5[ColletotrichumsublineolaCBS131301]	968	373	680	238	559	0.50	11	307	110.9	6.7e-24	gene=Chr01G2679.1	fgenesh1_pg.344_#_5[ColletotrichumsublineolaCBS131301]
Chr08G0957.1	503	85	XP_007593864.1[ColletotrichumfioriniaePJ7]	521	1	501	1	520	0.50	1	500	927.2	4.6e-270	gene=Chr08G0957.1	XP_007593864.1[ColletotrichumfioriniaePJ7]
Chr05G1221.1	356	29	OAT00923.1[Blastomycesder	1246	21	311	269	537	0.50	11	290	115.9	5.3e-26	gene=Chr05G1221.1	OAT00923.1[BlastomycesdermatitidisER-3]

			matitidisER-3]												
Chr01G2312.1	500	36.1	SS1G_05772.1[Sclerotiniasclerotiorum]	770	1	499	441	770	0.50	3	498	308.9	5.9e-84	gene=Chr01G2312.1	SS1G_05772.1[Sclerotiniasclerotiorum]
Chr03G0679.1	644	32	HCB02684.1[Histoplasmacapsulatum]	1240	103	418	274	551	0.50	15	315	145.6	1.1e-34	gene=Chr03G0679.1	HCB02684.1[Histoplasmacapsulatum]
Chr03G1477.1	290	36.7	XP_007599944.1[ColletotrichumfioriniaePJ7]	1113	52	286	60	296	0.50	5	234	149.1	4.6e-36	gene=Chr03G1477.1	XP_007599944.1[ColletotrichumfioriniaePJ7]
Chr04G0754.1	659	49.3	Endocarpon_02817[Endocarponpusillum]	1255	21	233	25	243	0.50	6	212	195.7	9.7e-50	gene=Chr04G0754.1	Endocarpon_02817[Endocarponpusillum]
Chr03G1598.1	603	45.8	maker-scaffold12.1-augustus-gene-11.200-mRNA-1[Raffaelea	874	2	246	199	461	0.50	8	244	220.7	2.6e-57	gene=Chr03G1598.1	maker-scaffold12.1-augustus-gene-11.200-mRNA-1[Raffaeleaquercus-mongolicae]

			quercus-mongolicae]												
Chr04G1541.1	667	26.7	HCB02684.1[Histoplasmacapsulatum]	1240	334	610	291	567	0.50	8	276	84	4.1e-16	gene=Chr04G1541.1	HCB02684.1[Histoplasmacapsulatum]
Chr06G0591.1	426	35.8	Clama_scaffold_5-4.0[Cladoniamacilenta]	21962	131	360	10211	10436	0.50	5	229	134.8	1.3e-31	gene=Chr06G0591.1	Clama_scaffold_5-4.0[Cladoniamacilenta]
Chr03G0403.1	328	29.2	estExt_Genemark1.C_150090[Dacryopinaxsp.DJM731SSP1]	827	34	317	26	312	0.51	14	283	75.5	7.3e-14	gene=Chr03G0403.1	estExt_Genemark1.C_150090[Dacryopinaxsp.DJM731SSP1]
Chr07G0579.1	417	29.9	genemark-scaffold40-processed-gene-0.15-mRNA-1[PseudohalonectrialignicolaM95]	1158	51	327	2	258	0.51	9	276	103.2	4.1e-22	gene=Chr07G0579.1	genemark-scaffold40-processed-gene-0.15-mRNA-1[PseudohalonectrialignicolaM95]
Chr05G0182.1	238	41.1	fgenesh1_	2158	14	227	356	566	0.5	2	213	146.4	2.4e-35	gene=C	fgenesh1_pg.24_&#

			pg.24_#_34[PhlebiabrevisporaHHB-7030SS6]						1					hr05G0182.1	35;_34[PhlebiabrevisporaHHB-7030SS6]
Chr04G0429.1	513	88.7	CE325846_1339[ColletotrichumsublineolaCBS131301]	514	1	512	1	513	0.51	1	511	919.1	1.3e-267	gene=Chr04G0429.1	CE325846_1339[ColletotrichumsublineolaCBS131301]
Chr08G0711.1	255	32.3	Endocarpon_03441[Endocarponpusillum]	1458	16	254	1213	1458	0.51	6	238	120.9	1.2e-27	gene=Chr08G0711.1	Endocarpon_03441[Endocarponpusillum]
Chr09G0349.1	450	28.5	Clama_scaffold_28-0.22[Cladoniamacilenta]	3845	89	368	3518	3776	0.51	11	279	73.6	3.8e-13	gene=Chr09G0349.1	Clama_scaffold_28-0.22[Cladoniamacilenta]
Chr07G0955.1	380	41.6	fgenesh1_pg.12_#_108[StereumhirsutumFP-9	1485	59	326	1184	1449	0.51	8	267	206.5	3.2e-53	gene=Chr07G0955.1	fgenesh1_pg.12_#_108[StereumhirsutumFP-91666SS1]

			1666SS1]												
Chr07G1061.1	658	44.2	maker-scaffold7.1-augustus-gene-36.77-mRNA-1[Raffaelea quercus-mongolicae]	838	305	560	602	835	0.51	4	255	173.3	5.1e-43	gene=Chr07G1061.1	maker-scaffold7.1-augustus-gene-36.77-mRNA-1[Raffaeleaquercus-mongolicae]
Chr03G0756.1	471	25.7	fgenesh1_pg.8_#_209[Pleurotusostreatus PC9]	1659	74	404	1351	1637	0.51	11	330	102.8	6.1e-22	gene=Chr03G0756.1	fgenesh1_pg.8_#_209[PleurotusostreatusPC9]
Chr05G0512.1	536	89.3	XP_007601970.1[Colletotrichumfioriniae PJ7]	535	1	535	1	535	0.51	0	534	998.8	1.3e-291	gene=Chr05G0512.1	XP_007601970.1[ColletotrichumfioriniaePJ7]
Chr03G1471.1	325	28	Clama_scaffold_5-4.0[Cladonia macilenta]	21962	8	291	6676	6943	0.51	7	283	114	1.8e-25	gene=Chr03G1471.1	Clama_scaffold_5-4.0[Cladoniamacilenta]
Chr05G0097.1	905	41.8	Clame_scaffold8-4.115[Cladoni	16637	630	867	4331	4587	0.51	4	237	197.2	4.6e-50	gene=Chr05G0097.1	Clame_scaffold8-4.115[Cladoniametacorallifera]

			ametacorallifera]												
Chr04G0387.1	532	88	XP_007599475.1[ColletotrichumfioriniaePJ7]	492	41	530	1	491	0.51	1	489	915.2	1.9e-266	gene=Chr04G0387.1	XP_007599475.1[ColletotrichumfioriniaePJ7]
Chr09G0626.1	352	46	GSTUMT00003906001[Tubermelanosporum]	289	80	335	36	271	0.51	5	255	201.8	7.2e-52	gene=Chr09G0626.1	GSTUMT00003906001[Tubermelanosporum]
Chr04G1114.1	593	27.6	Clame_scaffold11-8.74[Cladoniametacorallifera]	8336	13	301	1948	2231	0.51	9	288	119	1.0e-26	gene=Chr04G1114.1	Clame_scaffold11-8.74[Cladoniametacorallifera]
Chr01G2586.1	370	24.2	EAA61211.1[AspergillusnidulansFGSCA4]	742	11	296	8	291	0.51	5	285	70.1	3.4e-12	gene=Chr01G2586.1	EAA61211.1[AspergillusnidulansFGSCA4]
Chr05G1056.1	1038	44.9	Clame_scaffold8-4.115[Cladoniametacorallifera]	16637	759	992	4330	4580	0.51	4	233	202.2	1.6e-51	gene=Chr05G1056.1	Clame_scaffold8-4.115[Cladoniametacorallifera]

Chr01G1518.1	1619	27.7	CE149827_15745[Bjerkanderaadusta]	1055	581	897	700	991	0.51	11	316	80.5	1.1e-14	gene=Chr01G1518.1	CE149827_15745[Bjerkanderaadusta]
Chr02G1134.1	300	35.1	1430_t[AscocorynesarcoidesNRRL50072]	481	15	277	55	322	0.51	13	262	127.1	1.9e-29	gene=Chr02G1134.1	1430_t[AscocorynesarcoidesNRRL50072]
Chr07G0963.1	435	35.6	Clama_scaffold_5-4.0[Cladoniamacilenta]	21962	123	352	10203	10436	0.51	5	229	136.3	4.6e-32	gene=Chr07G0963.1	Clama_scaffold_5-4.0[Cladoniamacilenta]
Chr06G0698.1	249	40.4	fgenesh1_pm.4_#_61[Dichomitussqualens]	282	5	226	7	227	0.51	4	221	152.5	3.5e-37	gene=Chr06G0698.1	fgenesh1_pm.4_#_61[Dichomitussqualens]
Chr06G0668.1	535	27.2	estExt_fgenesh1_pm.C_170119[Cenococcumgeophilum1.58]	1191	15	348	267	556	0.51	13	333	90.1	4.6e-18	gene=Chr06G0668.1	estExt_fgenesh1_pm.C_170119[Cenococcumgeophilum1.58]
Chr05G0014.1	919	26.1	fgenesh1_pg.31_&#	1514	409	682	378	657	0.51	5	273	103.6	7.0e-22	gene=Chr05G00	fgenesh1_pg.31_#_85[Fibulorhizoct

			35;_85[Fibulorhizoctoniasp.CBS109695]											14.1	oniasp.CBS109695]
Chr07G1057.1	524	88.7	XP_007590703.1[CollletotrichumfioriniaePJ7]	524	1	523	1	524	0.51	1	522	970.7	3.8e-283	gene=Chr07G1057.1	XP_007590703.1[CollletotrichumfioriniaePJ7]
Chr05G1300.1	1521	28	fgenesh1_pg.31_#_85[Fibulorhizoctoniasp.CBS109695]	1514	421	688	373	635	0.52	4	267	97.4	8.3e-20	gene=Chr05G1300.1	fgenesh1_pg.31_#_85[Fibulorhizoctoniasp.CBS109695]
Chr01G2114.1	1154	35.6	EfO2.075130.1[Epichloefestucae]	1182	914	1145	918	1154	0.52	5	231	127.9	4.3e-29	gene=Chr01G2114.1	EfO2.075130.1[Epichloefestucae]
Chr05G0120.1	569	47	Endocarpon_02817[Endocarponpusillum]	1255	17	238	20	245	0.52	6	221	188	1.7e-47	gene=Chr05G0120.1	Endocarpon_02817[Endocarponpusillum]
Chr01G0250.1	781	24.3	EAL85053.1[Aspergil	720	140	452	22	356	0.52	14	312	59.3	1.3e-08	gene=Chr01G02	EAL85053.1[AspergillusfumigatusAf293]

			lusfumigatusAf293]											50.1	
Chr02G0369.1	327	27.7	HCB02684.1[Histoplasmacapsulatum]	1240	2	325	273	559	0.52	12	323	99.8	3.6e-21	gene=Chr02G0369.1	HCB02684.1[Histoplasmacapsulatum]
Chr07G0752.1	1363	31.6	estExt_fgenesh1_pm.C_170119[Cenococcumgeophilum1.58]	1191	1064	1326	288	546	0.52	8	262	127.1	8.7e-29	gene=Chr07G0752.1	estExt_fgenesh1_pm.C_170119[Cenococcumgeophilum1.58]
Chr05G0947.1	794	36	Clame_scaffold8-4.115[Cladoniametacorallifera]	16637	436	729	4333	4586	0.52	4	293	176.4	7.3e-44	gene=Chr05G0947.1	Clame_scaffold8-4.115[Cladoniametacorallifera]
Chr01G1769.1	549	27.9	estExt_Genemark1.C_370088[Galerina marginata]	959	1	304	1	373	0.52	15	303	125.6	1.0e-28	gene=Chr01G1769.1	estExt_Genemark1.C_370088[Galerina marginata]
Chr01G2340.1	311	33.3	XP_007804354.1[EndocarponpusillumZ0	541	15	270	44	287	0.52	5	255	155.6	5.2e-38	gene=Chr01G2340.1	XP_007804354.1[EndocarponpusillumZ07020]

			7020]												
Chr05G0485.1	562	29.7	Lema_T013660.1[Leptosphaeriamaculans]	945	8	266	587	847	0.52	4	258	100.1	4.7e-21	gene=Chr05G0485.1	Lema_T013660.1[Leptosphaeriamaculans]
Chr01G2366.1	488	44.1	Endocarpon_02817[Endocarponpusillum]	1255	9	249	12	241	0.52	6	240	191.4	1.4e-48	gene=Chr01G2366.1	Endocarpon_02817[Endocarponpusillum]
Chr02G0201.1	407	28	M_BR29_EuGene_00112621[MagnaporthegriseaBR29]	952	44	347	426	746	0.52	10	303	141.7	1.0e-33	gene=Chr02G0201.1	M_BR29_EuGene_00112621[MagnaporthegriseaBR29]
Chr06G0696.1	405	29.6	OAT00923.1[BlastomycesdermatitidisER-3]	1246	62	353	268	536	0.52	11	291	97.1	2.9e-20	gene=Chr06G0696.1	OAT00923.1[BlastomycesdermatitidisER-3]
Chr01G1615.1	340	35.7	XP_007804474.1[EndocarponpusillumZ0	1398	86	339	1150	1398	0.52	5	253	154.1	1.7e-37	gene=Chr01G1615.1	XP_007804474.1[EndocarponpusillumZ07020]

			7020]												
Chr04G1271.1	461	28.8	EEA19609.1[Penicilliummarneffei]	2642	137	446	2299	2638	0.52	10	309	136.7	3.7e-32	gene=Chr04G1271.1	EEA19609.1[Penicilliummarneffei]
Chr01G2467.1	624	35	OAT00923.1[BlastomycesdermatitidisER-3]	1246	116	435	257	544	0.52	9	319	203.8	3.4e-52	gene=Chr01G2467.1	OAT00923.1[BlastomycesdermatitidisER-3]
Chr08G0084.1	429	26.1	fgenesh1_kg.6_#_2119_#_Locus1957v1rpkm87.08[SchizophyllumcommuneH4-8]	472	87	394	69	395	0.52	11	307	74.3	2.1e-13	gene=Chr08G0084.1	fgenesh1_kg.6_#_2119_#_Locus1957v1rpkm87.08[SchizophyllumcommuneH4-8]
Chr06G0376.1	569	67.8	Clama_scaffold_5-4.0[Cladoniamacilenta]	21962	78	490	17806	18162	0.52	1	412	566.2	2.4e-161	gene=Chr06G0376.1	Clama_scaffold_5-4.0[Cladoniamacilenta]
Chr06G1406.1	501	33.9	MUStwsD_GLEAN_10001533[	1200	215	482	773	1100	0.52	8	267	166	6.2e-41	gene=Chr06G1406.1	MUStwsD_GLEAN_10001533[Omphalotusolearius]

			Omphalotusolearius]												
Chr08G0415.1	563	24.4	Clame_scaffold8-4.115[Cladoniametacorallifera]	16637	85	369	2937	3227	0.52	2	284	114.4	2.4e-25	gene=Chr08G0415.1	Clame_scaffold8-4.115[Cladoniametacorallifera]
Chr03G0080.1	428	27.4	EAA61211.1[AspergillusnidulansFGSCA4]	742	27	297	14	282	0.52	6	270	94	2.6e-19	gene=Chr03G0080.1	EAA61211.1[AspergillusnidulansFGSCA4]
Chr02G1095.1	758	38.2	Clama_scaffold_5-4.0[Cladoniamacilenta]	21962	263	486	10208	10435	0.52	2	223	157.9	2.6e-38	gene=Chr02G1095.1	Clama_scaffold_5-4.0[Cladoniamacilenta]
Chr02G0449.1	439	27	EEA19609.1[Penicilliummarneffei]	2642	128	426	2293	2638	0.52	8	298	117.9	1.7e-26	gene=Chr02G0449.1	EEA19609.1[Penicilliummarneffei]
Chr09G0857.1	419	31.2	HCBG_09081[HistoplasmacapsulatumG186AR]	1260	21	314	271	539	0.52	10	293	121.3	1.5e-27	gene=Chr09G0857.1	HCBG_09081[HistoplasmacapsulatumG186AR]
Chr06G1233.1	300	36.4	MUStwsD	411	35	289	154	394	0.5	6	254	161.4	9.2e-40	gene=C	MUStwsD_GLEAN_

			_GLEAN_10007689[Omphalotusolearius]						2					hr06G1233.1	10007689[Omphalotusolearius]
Chr02G1430.1	448	29.3	Clame_scaffold11-8.74[Cladoniametacorallifera]	8336	31	321	6817	7159	0.52	8	290	145.2	1.0e-34	gene=Chr02G1430.1	Clame_scaffold11-8.74[Cladoniametacorallifera]
Chr09G0309.1	592	28.2	estExt_Genemark1.C_370088[Galerina marginata]	959	2	303	1	379	0.52	12	301	117.5	3.0e-26	gene=Chr09G0309.1	estExt_Genemark1.C_370088[Galerina marginata]
Chr05G0918.1	493	42	POb-2nd_00002412-RA[Polyporusbrumalis]	723	216	461	435	668	0.53	7	245	155.6	8.3e-38	gene=Chr05G0918.1	POb-2nd_00002412-RA[Polyporusbrumalis]
Chr02G1630.1	327	41.7	ATEG_00556.1[Aspergillusterreus]	456	49	284	47	276	0.53	4	235	169.5	3.7e-42	gene=Chr02G1630.1	ATEG_00556.1[Aspergillusterreus]
Chr05G1103.1	1002	34.8	OAT00923.1[Blasto	1246	71	333	271	538	0.53	10	262	140.6	5.6e-33	gene=Chr05G11	OAT00923.1[BlastomycesdermatitidisE

			mycesdermatitidisER-3]											03.1	R-3]
Chr02G1679.1	355	27.1	EAA61211.1[AspergillusnidulansFGSCA4]	742	6	275	12	287	0.53	6	269	87	2.6e-17	gene=Chr02G1679.1	EAA61211.1[AspergillusnidulansFGSCA4]
Chr06G0511.1	362	30.4	HCBG_09081[HistoplasmacapsulatumG186AR]	1260	13	300	266	540	0.53	9	287	114.4	1.6e-25	gene=Chr06G0511.1	HCBG_09081[HistoplasmacapsulatumG186AR]
Chr01G0548.1	621	48.7	Endocarpon_02817[Endocarponpusillum]	1255	19	246	15	245	0.53	5	227	204.9	1.5e-52	gene=Chr01G0548.1	Endocarpon_02817[Endocarponpusillum]
Chr04G0749.1	509	35.8	EAA63242.1[AspergillusnidulansFGSCA4]	1004	15	266	8	251	0.53	7	251	137.5	2.4e-32	gene=Chr04G0749.1	EAA63242.1[AspergillusnidulansFGSCA4]
Chr03G0845.1	291	44.6	Clama_scaffold_5-4.0[Cladoniamacilenta]	21962	8	287	6687	6952	0.53	3	279	256.9	1.6e-68	gene=Chr03G0845.1	Clama_scaffold_5-4.0[Cladoniamacilenta]
Chr08G0421.1	1204	37.9	HCB0268	1240	117	385	281	539	0.5	9	268	166.8	8.8e-41	gene=C	HCB02684.1[Histopl

			4.1[Histoplasmacapsulatum]						3					hr08G0421.1	asmacapsulatum]
Chr03G0543.1	902	31.6	Clama_scaffold_5-4.0[Cladoniamacilenta]	21962	627	897	8798	9089	0.53	7	270	146	1.2e-34	gene=Chr03G0543.1	Clama_scaffold_5-4.0[Cladoniamacilenta]
Chr04G0392.1	389	30	XP_007600712.1[ColletotrichumfioriniaePJ7]	959	30	305	85	404	0.53	8	275	124	2.1e-28	gene=Chr04G0392.1	XP_007600712.1[ColletotrichumfioriniaePJ7]
Chr02G1261.1	321	47.2	ATEG_00556.1[Aspergillusterreus]	456	61	278	63	277	0.53	3	217	191.4	8.9e-49	gene=Chr02G1261.1	ATEG_00556.1[Aspergillusterreus]
Chr03G1613.1	620	48.5	Endocarpon_02817[Endocarponpusillum]	1255	18	241	22	243	0.53	5	223	204.5	2.0e-52	gene=Chr03G1613.1	Endocarpon_02817[Endocarponpusillum]
Chr09G0499.1	571	33.5	Clame_scaffold11-8.74[Cladoniametacorallifera]	8336	322	567	920	1178	0.53	6	245	141.4	1.9e-33	gene=Chr09G0499.1	Clame_scaffold11-8.74[Cladoniametacorallifera]

Chr06G0362.1	1493	35.3	EfO2.075130.1[Epichloefestucae]	1182	1210	1474	905	1153	0.53	6	264	134.8	4.6e-31	gene=Chr06G0362.1	EfO2.075130.1[Epichloefestucae]
Chr09G0958.1	523	21.2	AFL2G_08515[Aspergillusflavus]	890	3	441	4	420	0.53	19	438	64.7	2.0e-10	gene=Chr09G0958.1	AFL2G_08515[Aspergillusflavus]
Chr02G0123.1	360	25.9	2325_t[AscocorynesarcoidesNRRL50072]	852	15	333	16	333	0.53	12	318	85.9	5.9e-17	gene=Chr02G0123.1	2325_t[AscocorynesarcoidesNRRL50072]
Chr07G0578.1	387	45.8	fgenesh1_pg.12_#_108[StereumhirsutumFP-91666SS1]	1485	65	350	1184	1449	0.53	4	285	255	7.9e-68	gene=Chr07G0578.1	fgenesh1_pg.12_#_108[StereumhirsutumFP-91666SS1]
Chr04G0974.1	439	35.9	XP_007804354.1[EndocarponpusillumZ07020]	541	138	385	27	274	0.53	4	247	190.3	2.7e-48	gene=Chr04G0974.1	XP_007804354.1[EndocarponpusillumZ07020]
Chr03G1084.1	1621	39.1	Clama_scaffold_5-4.	21962	580	817	10193	10435	0.53	4	237	179.9	1.4e-44	gene=Chr03G10	Clama_scaffold_5-4.0[Cladoniamacilenta]

			0[Cladonia macilenta]											84.1	
Chr06G1491.1	1519	25	gm1.898_g[TulasnellacalosporaAL13]	1440	862	1216	662	1019	0.53	13	354	93.2	1.6e-18	gene=Chr06G1491.1	gm1.898_g[TulasnellacalosporaAL13]
Chr02G0152.1	1308	24	Clame_scaffold11-8.74[Cladoniametacorallifera]	8336	296	604	1429	1760	0.53	8	308	72.4	2.4e-12	gene=Chr02G0152.1	Clame_scaffold11-8.74[Cladoniametacorallifera]
Chr05G1379.1	1782	33.1	fgenesh1_pg.8_#_209[PleurotusostreatusPC9]	1659	1496	1761	1390	1638	0.53	7	265	152.1	3.3e-36	gene=Chr05G1379.1	fgenesh1_pg.8_#_209[PleurotusostreatusPC9]
Chr06G0790.1	912	40.4	Clama_scaffold_5-4.0[Cladoniamacilenta]	21962	419	645	10211	10435	0.53	3	226	169.9	7.9e-42	gene=Chr06G0790.1	Clama_scaffold_5-4.0[Cladoniamacilenta]
Chr02G0414.1	1528	26.4	fgenesh1_pg.31_#_85[Fibulorhizoctoniasp.CBS109695]	1514	421	701	373	657	0.53	4	280	105.5	3.1e-22	gene=Chr02G0414.1	fgenesh1_pg.31_#_85[Fibulorhizoctoniasp.CBS109695]

Chr06G1388.1	608	26.3	Clame_scaffold18-19.70[Cladoniametacorallifera]	7866	163	463	3183	3540	0.53	9	300	120.2	4.8e-27	gene=Chr06G1388.1	Clame_scaffold18-19.70[Cladoniametacorallifera]
Chr05G0340.1	441	32.8	Clame_scaffold8-4.115[Cladoniametacorallifera]	16637	59	319	11974	12227	0.53	5	260	157.5	2.0e-38	gene=Chr05G0340.1	Clame_scaffold8-4.115[Cladoniametacorallifera]
Chr09G0634.1	1237	25.6	Clame_scaffold18-19.70[Cladoniametacorallifera]	7866	826	1151	3130	3503	0.54	12	325	107.5	6.5e-23	gene=Chr09G0634.1	Clame_scaffold18-19.70[Cladoniametacorallifera]
Chr06G0944.1	418	23.9	EAA58861.1[AspergillusnidulansFGSCA4]	643	8	341	9	380	0.54	13	333	59.3	6.9e-09	gene=Chr06G0944.1	EAA58861.1[AspergillusnidulansFGSCA4]
Chr05G0276.1	619	25.5	Clame_scaffold18-19.70[Cladoniametacorallifera]	7866	153	459	3183	3503	0.54	7	306	95.9	9.8e-20	gene=Chr05G0276.1	Clame_scaffold18-19.70[Cladoniametacorallifera]
Chr02G1465.1	619	48.3	Endocarpon_02817[	1255	1	249	1	246	0.54	7	248	214.9	1.4e-55	gene=Chr02G14	Endocarpon_02817[Endocarponpusillum]

			Endocarponpusillum]											65.1	
Chr08G1046.1	300	38.3	augustus_masked-scaffold10-processed-gene-15.1-mRNA-1[Cladoniametacorallifera]	832	7	248	555	802	0.54	5	241	153.7	1.9e-37	gene=Chr08G1046.1	augustus_masked-scaffold10-processed-gene-15.1-mRNA-1[Cladoniametacorallifera]
Chr01G1825.1	590	51.6	Endocarpon_02817[Endocarponpusillum]	1255	1	231	1	243	0.54	6	230	225.3	1.0e-58	gene=Chr01G1825.1	Endocarpon_02817[Endocarponpusillum]
Chr03G0707.1	1517	51.6	PTRG_11747[Pyrenophoratritici-repentis]	406	341	555	1	215	0.54	0	214	215.3	2.7e-55	gene=Chr03G0707.1	PTRG_11747[Pyrenophoratritici-repentis]
Chr09G0660.1	366	44.4	XP_007804354.1[EndocarponpusillumZ0	541	67	303	33	264	0.54	6	236	209.1	4.7e-54	gene=Chr09G0660.1	XP_007804354.1[EndocarponpusillumZ07020]

			7020]												
Chr01G1504.1	341	29.7	EAA61211.1[AspergillusnidulansFGSCA4]	742	32	298	25	292	0.54	6	266	94	2.0e-19	gene=Chr01G1504.1	EAA61211.1[AspergillusnidulansFGSCA4]
Chr04G0099.1	775	27.3	Clame_scaffold18-19.70[Cladoniametacorallifera]	7866	221	541	3180	3498	0.54	9	320	122.5	1.2e-27	gene=Chr04G0099.1	Clame_scaffold18-19.70[Cladoniametacorallifera]
Chr07G0670.1	1225	29.4	estExt_fgenesh1_pm.C_170119[Cenococcumgeophilum1.58]	1191	276	584	268	559	0.54	9	308	132.5	1.9e-30	gene=Chr07G0670.1	estExt_fgenesh1_pm.C_170119[Cenococcumgeophilum1.58]
Chr04G1297.1	398	33.3	HCB02684.1[Histoplasmacapsulatum]	1240	67	341	273	543	0.54	10	274	137.1	2.5e-32	gene=Chr04G1297.1	HCB02684.1[Histoplasmacapsulatum]
Chr01G2622.1	614	41.2	Clame_scaffold8-4.115[Cladoniametacorallifera]	16637	349	591	4330	4587	0.54	3	242	194.1	2.6e-49	gene=Chr01G2622.1	Clame_scaffold8-4.115[Cladoniametacorallifera]
Chr06G0480.1	756	37.5	Clama_sc	21962	471	701	10205	10436	0.5	1	230	163.3	6.1e-40	gene=C	Clama_scaffold_5-4.

			affold_5-4.0[Cladoniamacilenta]						4					hr06G0480.1	0[Cladoniamacilenta]
Chr01G1326.1	277	39.2	Endocarpon_03441[Endocarponpusillum]	1458	31	274	1215	1456	0.54	8	243	147.9	9.7e-36	gene=Chr01G1326.1	Endocarpon_03441[Endocarponpusillum]
Chr09G0678.1	386	27.6	EAA63317.1[AspergillusnidulansFGSCA4]	1402	8	315	17	320	0.54	14	307	73.6	3.2e-13	gene=Chr09G0678.1	EAA63317.1[AspergillusnidulansFGSCA4]
Chr08G0785.1	327	47.5	Endocarpon_02817[Endocarponpusillum]	1255	7	234	10	246	0.54	4	227	212.6	3.8e-55	gene=Chr08G0785.1	Endocarpon_02817[Endocarponpusillum]
Chr03G0075.1	274	36.1	XP_007804354.1[EndocarponpusillumZ07020]	541	3	241	60	298	0.54	3	238	186	3.2e-47	gene=Chr03G0075.1	XP_007804354.1[EndocarponpusillumZ07020]
Chr05G0908.1	532	38.7	Endocarpon_02817[Endocarponpusillum	1255	9	291	12	284	0.54	8	282	181	2.0e-45	gene=Chr05G0908.1	Endocarpon_02817[Endocarponpusillum]

			]												
Chr08G1032.1	509	87.4	CH063_13844T0[Colletotrichumhigginsianum]	506	1	508	1	506	0.54	1	507	921.4	2.6e-268	gene=Chr08G1032.1	CH063_13844T0[Colletotrichumhigginsianum]
Chr01G2549.1	383	48	fgenesh1_pg.12_#_108[StereumhirsutumFP-91666SS1]	1485	62	353	1184	1454	0.54	4	291	267.7	1.2e-71	gene=Chr01G2549.1	fgenesh1_pg.12_#_108[StereumhirsutumFP-91666SS1]
Chr07G0094.1	255	50.8	EAA65893.1[AspergillusnidulansFGSCA4]	358	1	230	1	236	0.54	5	229	226.1	2.6e-59	gene=Chr07G0094.1	EAA65893.1[AspergillusnidulansFGSCA4]
Chr01G1146.1	571	47.7	Endocarpon_02817[Endocarponpusillum]	1255	11	239	14	245	0.54	6	228	194.9	1.4e-49	gene=Chr01G1146.1	Endocarpon_02817[Endocarponpusillum]
Chr05G1084.1	1390	40.9	Clama_scaffold_5-4.0[Cladoniamacilenta]	21962	986	1219	10205	10434	0.54	5	233	164.9	3.9e-40	gene=Chr05G1084.1	Clama_scaffold_5-4.0[Cladoniamacilenta]
Chr03G0555.1	469	34	XP_00780	541	119	367	24	273	0.5	3	248	159.8	4.2e-39	gene=C	XP_007804354.1[En

			4354.1[Endocarponpusillum Z07020]						4					hr03G0555.1	docarponpusillumZ07020]
Chr03G0125.1	753	26.8	Clame_scaffold18-19.70[Cladoniametacorallifera]	7866	175	473	3181	3505	0.54	8	298	115.5	1.5e-25	gene=Chr03G0125.1	Clame_scaffold18-19.70[Cladoniametacorallifera]
Chr05G0225.1	306	36.3	Pa_7_5740[PodosporaanserinaSmat+]	576	38	291	77	321	0.54	5	253	108.2	9.4e-24	gene=Chr05G0225.1	Pa_7_5740[PodosporaanserinaSmat+]
Chr03G0904.1	511	34.1	estExt_fgenesh1_pm.C_170119[Cenococcumgeophilum1.58]	1191	199	456	282	549	0.55	7	257	170.2	3.4e-42	gene=Chr03G0904.1	estExt_fgenesh1_pm.C_170119[Cenococcumgeophilum1.58]
Chr06G0204.1	340	24.7	estExt_Genemark1.C_150090[Dacryopinaxsp.DJM731SSP1]	827	3	339	8	330	0.55	13	336	70.5	2.4e-12	gene=Chr06G0204.1	estExt_Genemark1.C_150090[Dacryopinaxsp.DJM731SSP1]
Chr01G0162.1	679	27.2	augustus_	1265	180	501	754	1058	0.5	12	321	114	3.8e-25	gene=C	augustus_masked-s

			masked-scaffold407-processed-gene-0.5-mRNA-1[PseudohalonectrialignicolaM95]						5					hr01G0162.1	caffold407-processed-gene-0.5-mRNA-1[PseudohalonectrialignicolaM95]
Chr01G2708.1	670	30.6	augustus_masked-scaffold407-processed-gene-0.5-mRNA-1[PseudohalonectrialignicolaM95]	1265	224	502	755	1055	0.55	9	278	134.4	2.7e-31	gene=Chr01G2708.1	augustus_masked-scaffold407-processed-gene-0.5-mRNA-1[PseudohalonectrialignicolaM95]
Chr01G2334.1	558	43.2	MUStwsD_GLEAN_10004907[Omphalotusolearius]	1368	228	460	25	253	0.55	2	232	74.7	2.1e-13	gene=Chr01G2334.1	MUStwsD_GLEAN_10004907[Omphalotusolearius]
Chr04G0111.1	440	24.4	EAA61211	742	26	350	24	346	0.5	10	324	81.6	1.4e-15	gene=C	EAA61211.1[Aspergi

			.1[AspergillusnidulansFGSCA4]						5					hr04G0111.1	llusnidulansFGSCA4]
Chr03G0591.1	820	45.8	Clama_scaffold_5-4.0[Cladoniamacilenta]	21962	222	445	10211	10437	0.55	1	223	208.8	1.4e-53	gene=Chr03G0591.1	Clama_scaffold_5-4.0[Cladoniamacilenta]
Chr08G0678.1	629	25.6	augustus_masked-scaffold407-processed-gene-0.5-mRNA-1[PseudohalonectrialignicolaM95]	1265	162	493	668	1055	0.55	15	331	101.3	2.4e-21	gene=Chr08G0678.1	augustus_masked-scaffold407-processed-gene-0.5-mRNA-1[PseudohalonectrialignicolaM95]
Chr08G0613.1	608	22.2	Clame_scaffold11-8.74[Cladoniametacorallifera]	8336	77	451	7950	8329	0.55	17	374	52.4	1.2e-06	gene=Chr08G0613.1	Clame_scaffold11-8.74[Cladoniametacorallifera]
Chr04G0288.1	701	40.8	Clame_scaffold8-4.115[Cladoniametacora	16637	251	527	4331	4585	0.55	5	276	199.1	9.3e-51	gene=Chr04G0288.1	Clame_scaffold8-4.115[Cladoniametacorallifera]

			llifera]												
Chr04G1250.1	249	45.9	Endocarpon_02817[Endocarponpusillum]	1255	7	247	10	247	0.55	6	240	191.8	5.3e-49	gene=Chr04G1250.1	Endocarpon_02817[Endocarponpusillum]
Chr05G1346.1	439	83.5	XP_007602398.1[ColletotrichumfioriniaePJ7]	445	1	436	1	443	0.55	2	435	758.8	1.9e-219	gene=Chr05G1346.1	XP_007602398.1[ColletotrichumfioriniaePJ7]
Chr05G0979.1	1206	53	EEA28139.1[Penicilliummarneffei]	2025	1	283	597	928	0.55	5	282	317.8	3.1e-86	gene=Chr05G0979.1	EEA28139.1[Penicilliummarneffei]
Chr05G1038.1	447	24.5	EAA61211.1[AspergillusnidulansFGSCA4]	742	16	332	10	328	0.55	10	316	73.9	2.9e-13	gene=Chr05G1038.1	EAA61211.1[AspergillusnidulansFGSCA4]
Chr01G2211.1	303	33.7	EAA58488.1[AspergillusnidulansFGSCA4]	1365	2	270	1055	1326	0.55	11	268	127.5	1.5e-29	gene=Chr01G2211.1	EAA58488.1[AspergillusnidulansFGSCA4]
Chr03G1006.1	398	33.7	OAT00923.1[Blastomycesder	1246	123	376	271	537	0.55	5	253	159.5	4.6e-39	gene=Chr03G1006.1	OAT00923.1[BlastomycesdermatitidisER-3]

			matitidisER-3]												
Chr04G1275.1	641	34.2	HCB02684.1[Histoplasmacapsulatum]	1240	293	551	273	540	0.55	7	258	144.8	1.9e-34	gene=Chr04G1275.1	HCB02684.1[Histoplasmacapsulatum]
Chr01G2074.1	548	30.8	estExt_fgenesh1_pm.C_170119[Cenococcumgeophilum1.58]	1191	25	309	284	560	0.55	7	284	142.1	1.1e-33	gene=Chr01G2074.1	estExt_fgenesh1_pm.C_170119[Cenococcumgeophilum1.58]
Chr01G0562.1	324	38.5	Clama_scaffold_5-4.0[Cladoniamacilenta]	21962	1	283	6684	6943	0.55	7	282	176.4	3.0e-44	gene=Chr01G0562.1	Clama_scaffold_5-4.0[Cladoniamacilenta]
Chr07G0767.1	578	30	PLG_01751-R0[PseudohalonectrialignicolaM95]	462	285	566	146	416	0.56	4	281	132.9	6.7e-31	gene=Chr07G0767.1	PLG_01751-R0[PseudohalonectrialignicolaM95]
Chr01G0379.1	776	22.7	AMAG_10412T0[Allomycesmacrogynus]	687	182	577	135	486	0.56	16	395	72.4	1.5e-12	gene=Chr01G0379.1	AMAG_10412T0[Allomycesmacrogynus]
Chr01G2025.1	540	88.3	CH063_06	538	2	539	1	538	0.5	0	537	1000	6.0e-29	gene=C	CH063_06487T0[Co

			487T0[Colletotrichumhigginsianum]						6				2	hr01G2025.1	lletotrichumhigginsianum]
Chr04G1257.1	298	35.9	XP_007804474.1[EndocarponpusillumZ07020]	1398	43	295	1148	1397	0.56	3	252	151.4	9.5e-37	gene=Chr04G1257.1	XP_007804474.1[EndocarponpusillumZ07020]
Chr05G1104.1	557	40.4	Endocarpon_02817[Endocarponpusillum]	1255	1	299	1	277	0.56	8	298	212.2	8.4e-55	gene=Chr05G1104.1	Endocarpon_02817[Endocarponpusillum]
Chr09G0170.1	438	36.7	augustus_masked-scaffold9-processed-gene-0.95-mRNA-1[MagnaporthesalviniiM69]	2316	150	404	538	818	0.56	2	254	198.4	9.9e-51	gene=Chr09G0170.1	augustus_masked-scaffold9-processed-gene-0.95-mRNA-1[MagnaporthesalviniiM69]
Chr08G0292.1	363	36	XP_007804474.1[Endocarponp	1398	109	360	1150	1396	0.56	3	251	160.2	2.5e-39	gene=Chr08G0292.1	XP_007804474.1[EndocarponpusillumZ07020]

			usillumZ07020]												
Chr07G0202.1	993	24.8	Clame_scaffold18-19.70[Cladoniametacorallifera]	7866	206	576	3126	3502	0.56	13	370	111.3	3.6e-24	gene=Chr07G0202.1	Clame_scaffold18-19.70[Cladoniametacorallifera]
Chr08G0450.1	1231	24	g14592.t1[ArmillariamelleaDSM3731]	2505	517	846	2185	2500	0.56	6	329	85.9	2.0e-16	gene=Chr08G0450.1	g14592.t1[ArmillariamelleaDSM3731]
Chr02G0389.1	359	36.8	XP_007804474.1[EndocarponpusillumZ07020]	1398	112	358	1148	1398	0.56	4	246	162.5	4.9e-40	gene=Chr02G0389.1	XP_007804474.1[EndocarponpusillumZ07020]
Chr03G0025.1	515	87.4	XP_007597811.1[ColletotrichumfioriniaePJ7]	515	1	513	1	514	0.56	1	512	944.1	3.8e-275	gene=Chr03G0025.1	XP_007597811.1[ColletotrichumfioriniaePJ7]
Chr04G1529.1	367	27	Clame_scaffold8-4.115[Cladoniametacorallifera]	16637	56	346	2944	3241	0.56	3	290	123.2	3.4e-28	gene=Chr04G1529.1	Clame_scaffold8-4.115[Cladoniametacorallifera]

Chr09G0233.1	1359	41.3	EfO2.075130.1[Epichloefestucae]	1182	1109	1356	916	1159	0.56	6	247	180.3	8.7e-45	gene=Chr09G0233.1	EfO2.075130.1[Epichloefestucae]
Chr05G0520.1	407	34.5	OAT00923.1[BlastomycesdermatitidisER-3]	1246	26	286	268	539	0.56	7	260	160.2	2.8e-39	gene=Chr05G0520.1	OAT00923.1[BlastomycesdermatitidisER-3]
Chr06G1329.1	816	24.9	Clame_scaffold18-19.70[Cladoniametacorallifera]	7866	87	451	3126	3502	0.56	14	364	105.1	2.1e-22	gene=Chr06G1329.1	Clame_scaffold18-19.70[Cladoniametacorallifera]
Chr03G0026.1	296	41.7	Clama_scaffold_5-4.0[Cladoniamacilenta]	21962	14	273	6688	6939	0.56	5	259	214.2	1.2e-55	gene=Chr03G0026.1	Clama_scaffold_5-4.0[Cladoniamacilenta]
Chr01G1407.1	777	38.3	Clame_scaffold8-4.115[Cladoniametacorallifera]	16637	452	717	4332	4580	0.56	3	265	188.7	1.4e-47	gene=Chr01G1407.1	Clame_scaffold8-4.115[Cladoniametacorallifera]
Chr01G1780.1	1081	41.1	Clama_scaffold_5-4.0[Cladonia	21962	371	621	5930	6186	0.56	11	250	165.6	1.8e-40	gene=Chr01G1780.1	Clama_scaffold_5-4.0[Cladoniamacilenta]

			macilenta]												
Chr07G0352.1	277	32	Endocarpon_03441[Endocarponpusillum]	1458	7	273	1172	1455	0.56	5	266	136	3.8e-32	gene=Chr07G0352.1	Endocarpon_03441[Endocarponpusillum]
Chr01G0169.1	278	45.8	XP_007602139.1[ColletotrichumfioriniaePJ7]	801	16	264	4	240	0.56	3	248	205.7	3.9e-53	gene=Chr01G0169.1	XP_007602139.1[ColletotrichumfioriniaePJ7]
Chr07G0819.1	698	24.5	MUStwsD_GLEAN_10006674[Omphalotusolearius]	1989	21	344	1346	1657	0.56	8	323	63.9	4.6e-10	gene=Chr07G0819.1	MUStwsD_GLEAN_10006674[Omphalotusolearius]
Chr05G0440.1	859	32.2	fgenesh1_pg.8_#_209[PleurotusostreatusPC9]	1659	22	325	1357	1650	0.56	12	303	149.4	1.0e-35	gene=Chr05G0440.1	fgenesh1_pg.8_#_209[PleurotusostreatusPC9]
Chr06G0469.1	398	30.3	EAA61211.1[AspergillusnidulansFGSCA4]	742	3	279	25	303	0.56	8	276	107.5	2.1e-23	gene=Chr06G0469.1	EAA61211.1[AspergillusnidulansFGSCA4]

Chr01G1161.1	573	32.6	fgenesh1_pg.4_#_248[Ceriporiopsis subvermispora]	994	260	539	141	452	0.56	8	279	116.3	6.5e-26	gene=Chr01G1161.1	fgenesh1_pg.4_#_248[Ceriporiopsis subvermispora]
Chr02G0505.1	1424	25.3	EfO2.075130.1[Epichloefestucae]	1182	459	775	818	1139	0.56	8	316	82	3.4e-15	gene=Chr02G0505.1	EfO2.075130.1[Epichloefestucae]
Chr04G0750.1	367	31	genemark-scaffold40-processed-gene-0.15-mRNA-1[PseudohalonectrialignicolaM95]	1158	12	304	5	282	0.56	7	292	105.9	5.6e-23	gene=Chr04G0750.1	genemark-scaffold40-processed-gene-0.15-mRNA-1[PseudohalonectrialignicolaM95]
Chr04G0362.1	1139	33	Hanno_03304[Heterobasidionannosum03012]	1417	850	1133	751	1064	0.56	10	283	141.4	3.7e-33	gene=Chr04G0362.1	Hanno_03304[Heterobasidionannosum03012]
Chr07G0702.1	589	37.2	XP_007804354.1[En	541	49	297	44	289	0.57	3	248	191.8	1.2e-48	gene=Chr07G07	XP_007804354.1[EndocarponpusillumZ0

			docarponpusillumZ07020]											02.1	7020]
Chr03G1467.1	363	31.1	genemark-scaffold40-processed-gene-0.15-mRNA-1[Pseudohalonectrialignicola M95]	1158	1	279	1	274	0.57	5	278	120.2	2.8e-27	gene=Chr03G1467.1	genemark-scaffold40-processed-gene-0.15-mRNA-1[PseudohalonectrialignicolaM95]
Chr01G0673.1	259	51.5	AB07286.1[Alternariabrassicicola]	540	29	256	311	539	0.57	1	227	229.6	2.4e-60	gene=Chr01G0673.1	AB07286.1[Alternariabrassicicola]
Chr06G1379.1	813	42.3	Clame_scaffold8-4.115[Cladoniametacorallifera]	16637	495	767	4330	4580	0.57	4	272	214.5	2.5e-55	gene=Chr06G1379.1	Clame_scaffold8-4.115[Cladoniametacorallifera]
Chr03G1611.1	352	37.3	maker-scaffold122-augustus-gene-0.90-mRNA-1[	1059	31	307	115	372	0.57	6	276	182.6	4.5e-46	gene=Chr03G1611.1	maker-scaffold122-augustus-gene-0.90-mRNA-1[OphiocerasdolichostomumCBS114926]

			OphiocerasdolichostomumCBS114926]												
Chr05G0824.1	353	44.7	XP_007804354.1[EndocarponpusillumZ07020]	541	76	310	57	288	0.57	3	234	207.6	1.3e-53	gene=Chr05G0824.1	XP_007804354.1[EndocarponpusillumZ07020]
Chr08G0217.1	339	41.2	XP_007804354.1[EndocarponpusillumZ07020]	541	46	304	47	295	0.57	4	258	209.1	4.3e-54	gene=Chr08G0217.1	XP_007804354.1[EndocarponpusillumZ07020]
Chr09G0973.1	369	35.1	XP_007804354.1[EndocarponpusillumZ07020]	541	74	333	36	295	0.57	3	259	179.1	5.2e-45	gene=Chr09G0973.1	XP_007804354.1[EndocarponpusillumZ07020]
Chr06G1300.1	535	26.5	EAA58861.1[AspergillusnidulansFGSCA4]	643	3	343	4	388	0.57	11	340	107.5	2.8e-23	gene=Chr06G1300.1	EAA58861.1[AspergillusnidulansFGSCA4]
Chr05G0180.1	531	83.9	CH063_00510T0[Colletotrichu	456	76	529	1	449	0.57	1	453	803.9	6.3e-233	gene=Chr05G0180.1	CH063_00510T0[Colletotrichumhigginsianum]

			mhigginsianum]												
Chr03G1617.1	382	45.3	XP_007804354.1[EndocarponpusillumZ07020]	541	87	336	36	286	0.57	6	249	223.4	2.5e-58	gene=Chr03G1617.1	XP_007804354.1[EndocarponpusillumZ07020]
Chr04G1287.1	462	39.4	Endocarpon_02817[Endocarponpusillum]	1255	17	289	21	292	0.57	8	272	179.9	3.8e-45	gene=Chr04G1287.1	Endocarpon_02817[Endocarponpusillum]
Chr08G0019.1	355	31.4	estExt_Genemark1.C_150090[Dacryopinaxsp.DJM731SSP1]	827	43	343	28	318	0.57	11	300	135.6	6.4e-32	gene=Chr08G0019.1	estExt_Genemark1.C_150090[Dacryopinaxsp.DJM731SSP1]
Chr03G1640.1	427	28.3	Clame_scaffold18-19.70[Cladoniametacorallifera]	7866	77	384	3184	3503	0.57	8	307	134	2.2e-31	gene=Chr03G1640.1	Clame_scaffold18-19.70[Cladoniametacorallifera]
Chr01G0210.1	375	31.8	genemark-scaffold40-processe	1158	31	333	3	306	0.57	9	302	126.3	4.1e-29	gene=Chr01G0210.1	genemark-scaffold40-processed-gene-0.15-mRNA-1[Pseudoh

			d-gene-0.15-mRNA-1[PseudohalonectrialignicolaM95]												alonectrialignicolaM95]
Chr01G1323.1	280	36.7	Endocarpon_03441[Endocarponpusillum]	1458	21	278	1191	1457	0.57	6	257	153.3	2.3e-37	gene=Chr01G1323.1	Endocarpon_03441[Endocarponpusillum]
Chr02G1073.1	339	37.2	Clame_scaffold8-4.115[Cladoniametacorallifera]	16637	14	286	16033	16311	0.57	8	272	164.9	9.4e-41	gene=Chr02G1073.1	Clame_scaffold8-4.115[Cladoniametacorallifera]
Chr04G0535.1	1039	24.7	fgenesh1_kg.20_#_67_#_Locus6460v1rpkm23.60[Sphaerobolusstellatus]	458	423	896	2	354	0.57	12	473	114.4	4.5e-25	gene=Chr04G0535.1	fgenesh1_kg.20_#_67_#_Locus6460v1rpkm23.60[Sphaerobolusstellatus]
Chr09G0525.1	1735	35	Hanno_03	1417	93	380	777	1084	0.5	9	287	144.4	6.7e-34	gene=C	Hanno_03304[Heter

			304[Heterobasidionannosum03012]						7					hr09G0525.1	obasidionannosum03012]
Chr05G1308.1	586	48.3	Endocarpon_02817[Endocarponpusillum]	1255	7	245	10	248	0.57	5	238	200.3	3.5e-51	gene=Chr05G1308.1	Endocarpon_02817[Endocarponpusillum]
Chr04G0805.1	565	87.1	CH063_05033T0[Colletotrichumhigginsianum]	555	47	563	33	550	0.57	1	516	966.8	5.9e-282	gene=Chr04G0805.1	CH063_05033T0[Colletotrichumhigginsianum]
Chr03G0123.1	331	36.4	genemark-scaffold40-processed-gene-0.15-mRNA-1[PseudohalonectrialignicolaM95]	1158	12	273	3	258	0.58	4	261	144.4	1.3e-34	gene=Chr03G0123.1	genemark-scaffold40-processed-gene-0.15-mRNA-1[PseudohalonectrialignicolaM95]
Chr02G1491.1	432	22.7	augustus_masked-scaffold9-pr	2316	4	369	1845	2240	0.58	15	365	60.8	2.4e-09	gene=Chr02G1491.1	augustus_masked-scaffold9-processed-gene-0.95-mRNA-1[M

			ocessed-gene-0.95-mRNA-1[MagnaporthesalviniiM69]												agnaporthesalviniiM69]
Chr02G0820.1	466	84.8	augustus_masked-scaffold125-processed-gene-0.37-mRNA-1[PseudohalonectrialignicolaM95]	990	3	462	526	985	0.58	2	459	818.9	1.6e-237	gene=Chr02G0820.1	augustus_masked-scaffold125-processed-gene-0.37-mRNA-1[PseudohalonectrialignicolaM95]
Chr04G0643.1	355	32.8	CE216775_210[SebacinavermiferaMAFF305830]	336	15	339	11	331	0.58	12	324	163.3	2.9e-40	gene=Chr04G0643.1	CE216775_210[SebacinavermiferaMAFF305830]
Chr03G1579.1	344	35	Clame_scaffold8-4.115[Cladoniametacorallifera]	16637	13	286	16035	16311	0.58	8	273	144.8	1.0e-34	gene=Chr03G1579.1	Clame_scaffold8-4.115[Cladoniametacorallifera]

Chr09G1033.1	488	82.5	snap_masked-scaffold95-processed-gene-0.92-mRNA-1[OphiocerasdolichostomumCBS114926]	455	51	487	10	454	0.58	4	436	731.5	3.6e-211	gene=Chr09G1033.1	snap_masked-scaffold95-processed-gene-0.92-mRNA-1[OphiocerasdolichostomumCBS114926]
Chr02G1239.1	410	41	XP_007804354.1[EndocarponpusillumZ07020]	541	113	361	41	286	0.58	5	248	202.6	4.9e-52	gene=Chr02G1239.1	XP_007804354.1[EndocarponpusillumZ07020]
Chr05G0805.1	645	24	CE892521_2050[Sistotremastrumsuecicum]	703	1	361	1	355	0.58	13	360	51.2	2.9e-06	gene=Chr05G0805.1	CE892521_2050[Sistotremastrumsuecicum]
Chr07G0448.1	514	32.9	estExt_fgenesh1_pm.C_170119[Cenococcumgeophilum1.58]	1191	35	316	262	543	0.58	8	281	144.8	1.5e-34	gene=Chr07G0448.1	estExt_fgenesh1_pm.C_170119[Cenococcumgeophilum1.58]

Chr01G1734.1	318	37.7	Endocarpon_03441[Endocarponpusillum]	1458	65	317	1204	1458	0.58	3	252	162.2	5.7e-40	gene=Chr01G1734.1	Endocarpon_03441[Endocarponpusillum]
Chr06G0197.1	476	23.8	maker-scaffold122-augustus-gene-0.90-mRNA-1[OphiocerasdolichostomumCBS114926]	1059	33	375	26	363	0.58	8	342	87	3.5e-17	gene=Chr06G0197.1	maker-scaffold122-augustus-gene-0.90-mRNA-1[OphiocerasdolichostomumCBS114926]
Chr09G0495.1	735	38.8	OAT00923.1[BlastomycesdermatitidisER-3]	1246	59	322	261	536	0.58	10	263	175.6	1.2e-43	gene=Chr09G0495.1	OAT00923.1[BlastomycesdermatitidisER-3]
Chr02G1850.1	799	29.1	augustus_masked-scaffold407-processed-gene-0.5-mRNA-1[Pseudoha	1265	244	554	751	1056	0.58	12	310	128.6	1.8e-29	gene=Chr02G1850.1	augustus_masked-scaffold407-processed-gene-0.5-mRNA-1[PseudohalonectrialignicolaM95]

			lonectrialignicolaM95]												
Chr01G0172.1	402	36.4	XP_007595452.1[ColletotrichumfioriniaePJ7]	902	87	362	27	345	0.58	5	275	197.6	1.6e-50	gene=Chr01G0172.1	XP_007595452.1[ColletotrichumfioriniaePJ7]
Chr01G2665.1	849	27.1	fgenesh1_pg.344_#_5[ColletotrichumsublineolaCBS131301]	968	263	621	225	559	0.58	14	358	109.8	9.0e-24	gene=Chr01G2665.1	fgenesh1_pg.344_#_5[ColletotrichumsublineolaCBS131301]
Chr05G0374.1	474	43.5	Clame_scaffold8-4.115[Cladoniametacorallifera]	16637	168	428	4331	4579	0.58	3	260	220.7	2.0e-57	gene=Chr05G0374.1	Clame_scaffold8-4.115[Cladoniametacorallifera]
Chr04G0328.1	395	26.3	EAA61211.1[AspergillusnidulansFGSCA4]	742	20	329	12	321	0.58	5	309	103.2	3.9e-22	gene=Chr04G0328.1	EAA61211.1[AspergillusnidulansFGSCA4]
Chr03G0210.1	627	25.4	estExt_Genemark1.	836	98	429	85	406	0.58	9	331	81.3	2.5e-15	gene=Chr03G02	estExt_Genemark1.C_2070038[Fibulorhi

			C_2070038[Fibulorhizoctoniasp.CBS109695]											10.1	zoctoniasp.CBS109695]
Chr07G0535.1	732	34.4	augustus_masked-scaffold418-processed-gene-0.20-mRNA-1[MagnaporthesalviniiM69]	1986	45	314	649	931	0.58	6	269	137.5	3.5e-32	gene=Chr07G0535.1	augustus_masked-scaffold418-processed-gene-0.20-mRNA-1[MagnaporthesalviniiM69]
Chr03G1583.1	637	26.6	estExt_Genewise1Plus.C_100483[PleurotusostreatusPC15]	623	111	449	157	479	0.59	11	338	106.7	5.7e-23	gene=Chr03G1583.1	estExt_Genewise1Plus.C_100483[PleurotusostreatusPC15]
Chr06G0177.1	616	23.8	AMAG_10412T0[Allomycesmacrogynus]	687	209	601	135	486	0.59	14	392	74.7	2.3e-13	gene=Chr06G0177.1	AMAG_10412T0[Allomycesmacrogynus]
Chr02G0254.1	561	87.4	GLRG_09581T0[Col	547	16	561	1	548	0.59	1	545	986.9	5.5e-288	gene=Chr02G02	GLRG_09581T0[Colletotrichumgraminicol

			letotrichumgraminicolaM1.001]											54.1	aM1.001]
Chr03G1371.1	313	36.4	XP_007804354.1[EndocarponpusillumZ07020]	541	3	267	11	288	0.59	4	264	185.3	6.2e-47	gene=Chr03G1371.1	XP_007804354.1[EndocarponpusillumZ07020]
Chr08G0746.1	415	31.4	AFL2G_07229[Aspergillusflavus]	640	74	394	197	485	0.59	2	320	187.2	2.2e-47	gene=Chr08G0746.1	AFL2G_07229[Aspergillusflavus]
Chr05G1280.1	415	23.5	e_gw1.9.611.1[Hebelomacylindrosporumh7]	1540	30	400	294	693	0.59	14	370	60.5	3.1e-09	gene=Chr05G1280.1	e_gw1.9.611.1[Hebelomacylindrosporumh7]
Chr02G1713.1	663	36.5	estExt_Genewise1.C_2_t60028[OidiodendronmaiusZn]	418	16	663	16	418	0.59	8	647	373.6	2.6e-103	gene=Chr02G1713.1	estExt_Genewise1.C_2_t60028[OidiodendronmaiusZn]
Chr09G1053.1	1184	25.8	Clame_scaffold11-8.	8336	705	1050	6757	7159	0.59	11	345	158.7	2.4e-38	gene=Chr09G10	Clame_scaffold11-8.74[Cladoniametacor

			74[Cladoniametacorallifera]											53.1	allifera]
Chr02G1040.1	671	22.6	ODG_10834-R0[OphiocerasdolichostomumCBS114926]	2287	274	644	238	599	0.59	10	370	51.6	2.3e-06	gene=Chr02G1040.1	ODG_10834-R0[OphiocerasdolichostomumCBS114926]
Chr05G0228.1	644	31.9	MUStwsD_GLEAN_10001533[Omphalotusolearius]	1200	241	532	775	1101	0.59	5	291	152.9	7.0e-37	gene=Chr05G0228.1	MUStwsD_GLEAN_10001533[Omphalotusolearius]
Chr03G0375.1	804	24	Clame_scaffold11-8.74[Cladoniametacorallifera]	8336	213	565	1944	2316	0.59	9	352	103.2	8.0e-22	gene=Chr03G0375.1	Clame_scaffold11-8.74[Cladoniametacorallifera]
Chr04G0675.1	496	39.1	maker-scaffold2.1-augustus-gene-26.101-mRNA-1[Raffaelea	682	75	387	378	681	0.59	9	312	197.2	2.5e-50	gene=Chr04G0675.1	maker-scaffold2.1-augustus-gene-26.101-mRNA-1[Raffaeleaquercus-mongolicae]

			quercus-mongolicae]												
Chr09G0209.1	810	39.4	Clame_scaffold8-4.115[Cladoniametacorallifera]	16637	471	739	4332	4587	0.59	4	268	203.4	5.7e-52	gene=Chr09G0209.1	Clame_scaffold8-4.115[Cladoniametacorallifera]
Chr07G0209.1	290	38.4	EAA59126.1[AspergillusnidulansFGSCA4]	1153	1	258	571	830	0.59	5	257	189.5	3.0e-48	gene=Chr07G0209.1	EAA59126.1[AspergillusnidulansFGSCA4]
Chr07G0932.1	349	39.7	XP_007804354.1[EndocarponpusillumZ07020]	541	65	315	47	296	0.59	3	250	201.8	7.1e-52	gene=Chr07G0932.1	XP_007804354.1[EndocarponpusillumZ07020]
Chr03G1078.1	369	42.7	XP_007804354.1[EndocarponpusillumZ07020]	541	69	323	33	287	0.59	4	254	226.1	3.7e-59	gene=Chr03G1078.1	XP_007804354.1[EndocarponpusillumZ07020]
Chr04G1395.1	492	83.9	XP_007603214.1[ColletotrichumfioriniaePJ7]	496	1	491	1	494	0.60	3	490	863.6	6.2e-251	gene=Chr04G1395.1	XP_007603214.1[ColletotrichumfioriniaePJ7]

Chr04G1429.1	1067	47.7	Endocarpon_02817[Endocarponpusillum]	1255	11	266	15	276	0.60	6	255	234.6	3.0e-61	gene=Chr04G1429.1	Endocarpon_02817[Endocarponpusillum]
Chr01G2422.1	700	27	Clame_scaffold18-19.70[Cladoniametacorallifera]	7866	166	525	3127	3499	0.60	10	359	138.3	1.9e-32	gene=Chr01G2422.1	Clame_scaffold18-19.70[Cladoniametacorallifera]
Chr04G1515.1	447	39.9	XP_007804354.1[EndocarponpusillumZ07020]	541	140	408	34	296	0.60	5	268	204.9	1.1e-52	gene=Chr04G1515.1	XP_007804354.1[EndocarponpusillumZ07020]
Chr09G0019.1	244	47.5	SS1G_01499.1[Sclerotiniasclerotiorum]	1043	1	242	765	1005	0.60	1	241	221.5	6.1e-58	gene=Chr09G0019.1	SS1G_01499.1[Sclerotiniasclerotiorum]
Chr08G0959.1	512	26.5	XP_007600712.1[ColletotrichumfioriniaePJ7]	959	47	389	20	380	0.60	9	342	86.7	4.9e-17	gene=Chr08G0959.1	XP_007600712.1[ColletotrichumfioriniaePJ7]
Chr03G1369.1	342	38.2	XP_007804354.1[En	541	14	289	16	287	0.60	6	275	186.8	2.3e-47	gene=Chr03G13	XP_007804354.1[EndocarponpusillumZ0

			docarponpusillumZ07020]											69.1	7020]
Chr04G0252.1	643	26	XP_007600712.1[Colletotrichumfioriniae PJ7]	959	99	448	7	342	0.60	13	349	85.5	1.4e-16	gene=Chr04G0252.1	XP_007600712.1[ColletotrichumfioriniaePJ7]
Chr01G2590.1	372	37.8	XP_007804354.1[EndocarponpusillumZ07020]	541	69	329	31	289	0.60	2	260	204.9	9.0e-53	gene=Chr01G2590.1	XP_007804354.1[EndocarponpusillumZ07020]
Chr01G1442.1	529	25.4	Clame_scaffold8-4.115[Cladoniametacorallifera]	16637	67	391	2938	3269	0.60	5	324	114.4	2.3e-25	gene=Chr01G1442.1	Clame_scaffold8-4.115[Cladoniametacorallifera]
Chr02G0223.1	417	34.8	Clame_scaffold8-4.115[Cladoniametacorallifera]	16637	147	416	2942	3216	0.60	3	269	156	5.4e-38	gene=Chr02G0223.1	Clame_scaffold8-4.115[Cladoniametacorallifera]
Chr07G0408.1	956	26.2	Clame_scaffold18-19.70[Clad	7866	272	613	3117	3496	0.60	12	341	91.7	2.9e-18	gene=Chr07G0408.1	Clame_scaffold18-19.70[Cladoniametacorallifera]

			oniametacorallifera]												
Chr01G0690.1	1056	27.8	Clama_scaffold_5-4.0[Cladoniamacilenta]	21962	443	774	9561	9973	0.60	7	331	161	4.2e-39	gene=Chr01G0690.1	Clama_scaffold_5-4.0[Cladoniamacilenta]
Chr03G0476.1	813	38.5	Clama_scaffold_5-4.0[Cladoniamacilenta]	21962	528	787	2218	2481	0.60	4	259	184.5	2.7e-46	gene=Chr03G0476.1	Clama_scaffold_5-4.0[Cladoniamacilenta]
Chr04G1030.1	559	30.1	Clame_scaffold8-4.115[Cladoniametacorallifera]	16637	94	396	2950	3245	0.61	4	302	148.7	1.1e-35	gene=Chr04G1030.1	Clame_scaffold8-4.115[Cladoniametacorallifera]
Chr02G1832.1	310	43.3	Clama_scaffold_5-4.0[Cladoniamacilenta]	21962	40	297	6688	6943	0.61	4	257	212.6	3.6e-55	gene=Chr02G1832.1	Clama_scaffold_5-4.0[Cladoniamacilenta]
Chr08G0875.1	620	22.8	Clame_scaffold11-8.74[Cladoniametacorallifera]	8336	38	425	7915	8331	0.61	12	387	67.8	2.9e-11	gene=Chr08G0875.1	Clame_scaffold11-8.74[Cladoniametacorallifera]
Chr06G1031.1	323	33.2	estExt_Genemark1.	827	18	315	23	320	0.61	9	297	132.9	3.8e-31	gene=Chr06G10	estExt_Genemark1.C_150090[Dacryopi

			C_150090[Dacryopinaxsp.DJM731SSP1]												31.1	naxsp.DJM731SSP1]
Chr02G1094.1	342	31	gm1.10090_g[Hypholomasublateritium]	821	2	320	4	311	0.61	13	318	115.9	5.1e-26	gene=Chr02G1094.1	gm1.10090_g[Hypholomasublateritium]
Chr06G1220.1	541	81.2	FVEG_14002T0[Fusariumverticillioides]	409	140	539	9	408	0.61	0	399	693.7	9.3e-200	gene=Chr06G1220.1	FVEG_14002T0[Fusariumverticillioides]
Chr08G0937.1	449	67	XP_007591849.1[CollletotrichumfioriniaePJ7]	528	5	445	3	524	0.61	1	440	685.6	2.1e-197	gene=Chr08G0937.1	XP_007591849.1[CollletotrichumfioriniaePJ7]
Chr02G0021.1	1045	24	Clame_scaffold18-19.70[Cladoniametacorallifera]	7866	340	753	3109	3496	0.61	19	413	75.9	1.8e-13	gene=Chr02G0021.1	Clame_scaffold18-19.70[Cladoniametacorallifera]
Chr01G1182.1	293	37.8	maker-Caloplaca_flavorubescens_scaffol	1930	1	286	1066	1364	0.61	7	285	190.7	1.4e-48	gene=Chr01G1182.1	maker-Caloplaca_flavorubescens_scaffold_19-augustus-gene-3.79-mRNA-1[Calop

			d_19-augustus-gene-3.79-mRNA-1[Caloplacaflavorubescens]											lacaflavorubescens]
Chr06G0249.1	453	29.7	EAA61211.1[AspergillusnidulansFGSCA4]	742	19	321	3	308	0.61	6	302	94	2.7e-19	gene=Chr06G0249.1	EAA61211.1[AspergillusnidulansFGSCA4]
Chr02G0625.1	334	41	XP_007804354.1[EndocarponpusillumZ07020]	541	23	286	28	288	0.62	3	263	218.4	7.1e-57	gene=Chr02G0625.1	XP_007804354.1[EndocarponpusillumZ07020]
Chr04G1525.1	381	36.4	estExt_Genemark1.C_150090[Dacryopinaxsp.DJM731SSP1]	827	78	363	28	311	0.62	9	285	151.4	1.2e-36	gene=Chr04G1525.1	estExt_Genemark1.C_150090[Dacryopinaxsp.DJM731SSP1]
Chr08G1023.1	326	57.5	Clame_scaffold8-4.115[Cladoniametacorallifera]	16637	14	276	16046	16308	0.62	3	262	298.9	4.0e-81	gene=Chr08G1023.1	Clame_scaffold8-4.115[Cladoniametacorallifera]

Chr03G0477.1	487	28.1	Clame_scaffold8-4.115[Cladoniametacorallifera]	16637	32	350	2942	3256	0.62	5	318	126.7	4.1e-29	gene=Chr03G0477.1	Clame_scaffold8-4.115[Cladoniametacorallifera]
Chr04G0098.1	358	43.8	XP_007804354.1[EndocarponpusillumZ07020]	541	57	315	33	288	0.62	3	258	214.5	1.1e-55	gene=Chr04G0098.1	XP_007804354.1[EndocarponpusillumZ07020]
Chr06G0076.1	692	30.3	augustus_masked-scaffold407-processed-gene-0.5-mRNA-1[PseudohalonectrialignicolaM95]	1265	56	404	755	1082	0.62	11	348	129	1.2e-29	gene=Chr06G0076.1	augustus_masked-scaffold407-processed-gene-0.5-mRNA-1[PseudohalonectrialignicolaM95]
Chr09G0591.1	480	29.2	Clame_scaffold8-4.115[Cladoniametacorallifera]	16637	75	422	2975	3325	0.62	10	347	131	2.1e-30	gene=Chr09G0591.1	Clame_scaffold8-4.115[Cladoniametacorallifera]
Chr01G1453.1	348	38	XP_00780	541	34	311	4	295	0.6	6	277	182.2	5.8e-46	gene=C	XP_007804354.1[En

			4354.1[EndocarponpusillumZ07020]						2					hr01G1453.1	docarponpusillumZ07020]
Chr01G1617.1	380	34.8	genemark-scaffold40-processed-gene-0.15-mRNA-1[PseudohalonectrialignicolaM95]	1158	5	290	3	285	0.62	5	285	148.7	7.8e-36	gene=Chr01G1617.1	genemark-scaffold40-processed-gene-0.15-mRNA-1[PseudohalonectrialignicolaM95]
Chr04G0428.1	624	25.5	BC1G_15490[Botrytiscinerea]	1059	206	610	678	1046	0.62	18	404	98.6	1.5e-20	gene=Chr04G0428.1	BC1G_15490[Botrytiscinerea]
Chr01G2214.1	651	21.9	EAA60443.1[AspergillusnidulansFGSCA4]	2180	125	585	278	661	0.62	11	460	81.3	2.6e-15	gene=Chr01G2214.1	EAA60443.1[AspergillusnidulansFGSCA4]
Chr06G1337.1	1120	27.7	Clame_scaffold11-8.74[Cladoniametacorallifera]	8336	641	986	6757	7159	0.62	9	345	167.9	3.7e-41	gene=Chr06G1337.1	Clame_scaffold11-8.74[Cladoniametacorallifera]
Chr04G0487.1	296	59.7	SS1G_01	1043	38	295	527	784	0.6	0	257	335.9	2.7e-92	gene=C	SS1G_01499.1[Scle

			499.1[Sclerotiniasclerotiorum]						2					hr04G0487.1	rotiniasclerotiorum]
Chr04G0860.1	343	63.4	ATEG_07093.1[Aspergillusterreus]	1384	66	340	1106	1384	0.62	3	274	375.2	4.6e-104	gene=Chr04G0860.1	ATEG_07093.1[Aspergillusterreus]
Chr01G0297.1	571	84	CH063_09762T0[Colletotrichumhigginsianum]	475	91	565	1	475	0.62	2	474	839.7	1.1e-243	gene=Chr01G0297.1	CH063_09762T0[Colletotrichumhigginsianum]
Chr03G0843.1	579	26.8	Clame_scaffold8-4.115[Cladoniametacorallifera]	16637	211	563	10642	10988	0.62	13	352	98.2	1.8e-20	gene=Chr03G0843.1	Clame_scaffold8-4.115[Cladoniametacorallifera]
Chr04G1333.1	1471	26.8	Clame_scaffold11-8.74[Cladoniametacorallifera]	8336	977	1311	7417	7748	0.62	3	334	131.7	3.8e-30	gene=Chr04G1333.1	Clame_scaffold11-8.74[Cladoniametacorallifera]
Chr06G1412.1	388	43.8	XP_007804354.1[EndocarponpusillumZ0	541	90	347	34	288	0.62	2	257	243	3.1e-64	gene=Chr06G1412.1	XP_007804354.1[EndocarponpusillumZ07020]

			7020]												
Chr06G0228.1	451	31.9	AFL2G_07147[Aspergillusflavus]	1440	51	354	60	372	0.62	3	303	165.6	7.3e-41	gene=Chr06G0228.1	AFL2G_07147[Aspergillusflavus]
Chr08G0670.1	546	86.4	XP_007595028.1[ColletotrichumfioriniaePJ7]	543	1	545	1	543	0.62	1	544	1022.3	1.1e-298	gene=Chr08G0670.1	XP_007595028.1[ColletotrichumfioriniaePJ7]
Chr05G0199.1	591	25.3	ATEG_04721.1[Aspergillusterreus]	994	135	499	532	877	0.62	10	364	80.9	3.1e-15	gene=Chr05G0199.1	ATEG_04721.1[Aspergillusterreus]
Chr09G0791.1	1454	36.4	fgenesh1_pg.8_#_209[PleurotusostreatusPC9]	1659	56	331	1382	1658	0.62	4	275	180.3	9.3e-45	gene=Chr09G0791.1	fgenesh1_pg.8_#_209[PleurotusostreatusPC9]
Chr08G0760.1	361	38.2	XP_007804354.1[EndocarponpusillumZ07020]	541	37	334	15	299	0.62	6	297	209.1	4.6e-54	gene=Chr08G0760.1	XP_007804354.1[EndocarponpusillumZ07020]
Chr05G0906.1	1218	32.3	estExt_fgenesh1_pg.	1270	363	666	902	1204	0.62	9	303	152.5	1.7e-36	gene=Chr05G09	estExt_fgenesh1_pg.C_3530010[ExidiagI

			C_3530010[Exidiaglandulosa]											06.1	andulosa]
Chr04G0994.1	332	42.7	XP_007804354.1[EndocarponpusillumZ07020]	541	18	291	16	288	0.62	6	273	218.4	7.0e-57	gene=Chr04G0994.1	XP_007804354.1[EndocarponpusillumZ07020]
Chr02G1277.1	404	24.2	maker-scaffold122-augustus-gene-0.90-mRNA-1[OphiocerasdolichostomumCBS114926]	1059	25	394	23	387	0.62	10	369	104	2.3e-22	gene=Chr02G1277.1	maker-scaffold122-augustus-gene-0.90-mRNA-1[OphiocerasdolichostomumCBS114926]
Chr09G0686.1	361	31.7	EAA61211.1[AspergillusnidulansFGSCA4]	742	10	313	2	309	0.62	7	303	132.1	7.2e-31	gene=Chr09G0686.1	EAA61211.1[AspergillusnidulansFGSCA4]
Chr08G0318.1	1421	26.2	Clame_scaffold11-8.74[Cladoniametacorallifera]	8336	928	1266	7387	7748	0.63	5	338	127.5	7.0e-29	gene=Chr08G0318.1	Clame_scaffold11-8.74[Cladoniametacorallifera]

Chr06G0159.1	460	28.1	AFL2G_07147[Aspergillusflavus]	1440	32	366	36	370	0.63	5	334	143.7	3.0e-34	gene=Chr06G0159.1	AFL2G_07147[Aspergillusflavus]
Chr07G0264.1	535	85.9	XP_007602547.1[ColletotrichumfioriniaePJ7]	541	1	516	1	516	0.63	0	515	941.8	1.9e-274	gene=Chr07G0264.1	XP_007602547.1[ColletotrichumfioriniaePJ7]
Chr03G1340.1	332	39.2	XP_007595452.1[ColletotrichumfioriniaePJ7]	902	5	305	4	345	0.63	10	300	203.8	1.8e-52	gene=Chr03G1340.1	XP_007595452.1[ColletotrichumfioriniaePJ7]
Chr03G0260.1	489	84.8	CH063_08618T0[Colletotrichumhigginsianum]	488	1	488	1	488	0.63	0	487	881.3	2.8e-256	gene=Chr03G0260.1	CH063_08618T0[Colletotrichumhigginsianum]
Chr06G1119.1	340	42.4	XP_007804354.1[EndocarponpusillumZ07020]	541	38	300	27	287	0.63	2	262	217.2	1.6e-56	gene=Chr06G1119.1	XP_007804354.1[EndocarponpusillumZ07020]
Chr07G0105.1	461	26.6	Clame_scaffold8-4.1	16637	40	385	2967	3322	0.63	6	345	125.6	8.6e-29	gene=Chr07G01	Clame_scaffold8-4.115[Cladoniametacor

			15[Cladoniametacorallifera]											05.1	allifera]
Chr05G0341.1	322	39.5	g8694.t1[ArmillariamelleaDSM3731]	1256	11	307	4	296	0.63	4	296	211.8	6.4e-55	gene=Chr05G0341.1	g8694.t1[ArmillariamelleaDSM3731]
Chr04G0698.1	338	39.4	XP_007804354.1[EndocarponpusillumZ07020]	541	3	299	5	288	0.63	8	296	211.1	1.1e-54	gene=Chr04G0698.1	XP_007804354.1[EndocarponpusillumZ07020]
Chr02G0871.1	352	40.7	XP_007804354.1[EndocarponpusillumZ07020]	541	41	307	24	288	0.63	3	266	214.5	1.1e-55	gene=Chr02G0871.1	XP_007804354.1[EndocarponpusillumZ07020]
Chr03G1550.1	534	36.7	XP_007598108.1[ColletotrichumfioriniaePJ7]	877	9	310	25	326	0.63	7	301	213.4	3.6e-55	gene=Chr03G1550.1	XP_007598108.1[ColletotrichumfioriniaePJ7]
Chr02G0548.1	1259	29.1	augustus_masked-scaffold407-processe	1265	791	1129	674	1057	0.63	11	338	162.9	1.3e-39	gene=Chr02G0548.1	augustus_masked-scaffold407-processed-gene-0.5-mRNA-1[Pseudohalonectrialig

			d-gene-0.5-mRNA-1[PseudohalonectrialignicolaM95]												nicolaM95]
Chr03G1325.1	358	37.6	XP_007804354.1[EndocarponpusillumZ07020]	541	1	312	1	288	0.63	6	311	222.2	5.2e-58	gene=Chr03G1325.1	XP_007804354.1[EndocarponpusillumZ07020]
Chr04G0916.1	601	27	estExt_Genewise1Plus.C_100483[PleurotusostreatusPC15]	623	93	457	145	498	0.63	12	364	120.2	4.7e-27	gene=Chr04G0916.1	estExt_Genewise1Plus.C_100483[PleurotusostreatusPC15]
Chr03G0672.1	1497	33.8	g14592.t1[ArmillariamelleaDSM3731]	2505	456	763	822	1116	0.63	9	307	159.5	1.7e-38	gene=Chr03G0672.1	g14592.t1[ArmillariamelleaDSM3731]
Chr06G0866.1	634	43.3	fgenesh1_pg.8_#_209[PleurotusostreatusPC9]	1659	17	280	1384	1649	0.63	4	263	213.4	4.3e-55	gene=Chr06G0866.1	fgenesh1_pg.8_#_209[PleurotusostreatusPC9]

Chr04G1369.1	856	31.8	F503_06550m.01[OphiostomapiceaeUAMH11346]	1224	38	513	5	365	0.63	9	475	202.2	1.3e-51	gene=Chr04G1369.1	F503_06550m.01[OphiostomapiceaeUAMH11346]
Chr03G0220.1	361	42.2	estExt_fgenesh1_pm.C_1380002[ColletotrichumsublineolaCBS131301]	890	22	300	560	838	0.63	4	278	192.2	5.9e-49	gene=Chr03G0220.1	estExt_fgenesh1_pm.C_1380002[ColletotrichumsublineolaCBS131301]
Chr01G0113.1	784	38.6	augustus_masked-scaffold418-processed-gene-0.20-mRNA-1[MagnaporthesalviniiM69]	1986	36	316	625	920	0.63	10	280	150.6	4.2e-36	gene=Chr01G0113.1	augustus_masked-scaffold418-processed-gene-0.20-mRNA-1[MagnaporthesalviniiM69]
Chr02G1375.1	334	44.3	g8694.t1[ArmillariamelleaDSM3731]	1256	32	320	27	297	0.63	5	288	207.6	1.2e-53	gene=Chr02G1375.1	g8694.t1[ArmillariamelleaDSM3731]
Chr06G0700.1	290	38.4	estExt_fge	823	8	278	17	292	0.6	1	270	194.1	1.2e-49	gene=C	estExt_fgenesh2_pg

			nesh2_pg.C_10498[SchizophyllumcommuneH4-8]						3					hr06G0700.1	.C_10498[SchizophyllumcommuneH4-8]
Chr06G0401.1	524	84.5	CH063_09276T0[Colletotrichumhigginsianum]	522	39	521	38	520	0.63	0	482	869.8	9.2e-253	gene=Chr06G0401.1	CH063_09276T0[Colletotrichumhigginsianum]
Chr05G1142.1	829	38.2	estExt_fgenesh1_pg.C_450037[Phytophthorasojae]	872	112	401	577	872	0.63	7	289	208.4	1.8e-53	gene=Chr05G1142.1	estExt_fgenesh1_pg.C_450037[Phytophthorasojae]
Chr02G1829.1	480	29.1	Clame_scaffold8-4.115[Cladoniametacorallifera]	16637	47	363	2945	3267	0.63	5	316	145.6	8.4e-35	gene=Chr02G1829.1	Clame_scaffold8-4.115[Cladoniametacorallifera]
Chr02G0626.1	578	21.4	XP_007600712.1[ColletotrichumfioriniaePJ7]	959	24	436	21	426	0.64	11	412	66.2	7.8e-11	gene=Chr02G0626.1	XP_007600712.1[ColletotrichumfioriniaePJ7]
Chr03G0743.1	419	26.6	estExt_Ge	1040	2	415	426	859	0.6	15	413	148.3	1.1e-35	gene=C	estExt_Genewise1.C

			newise1.C_690045[Auriculariasubglabra]						4					hr03G0743.1	_690045[Auriculariasubglabra]
Chr05G1265.1	365	39.8	XP_007804354.1[EndocarponpusillumZ07020]	541	49	328	22	298	0.64	5	279	205.7	5.2e-53	gene=Chr05G1265.1	XP_007804354.1[EndocarponpusillumZ07020]
Chr09G0748.1	641	26.8	Clame_scaffold11-8.74[Cladoniametacorallifera]	8336	16	394	1947	2312	0.64	8	378	111.3	2.3e-24	gene=Chr09G0748.1	Clame_scaffold11-8.74[Cladoniametacorallifera]
Chr02G0018.1	652	31.1	Clame_scaffold18-19.70[Cladoniametacorallifera]	7866	171	493	3132	3503	0.64	9	322	189.9	5.2e-48	gene=Chr02G0018.1	Clame_scaffold18-19.70[Cladoniametacorallifera]
Chr05G0203.1	702	40.3	fgenesh1_pg.8_#_209[PleurotusostreatusPC9]	1659	16	285	1379	1650	0.64	3	269	210.7	3.1e-54	gene=Chr05G0203.1	fgenesh1_pg.8_#_209[PleurotusostreatusPC9]
Chr02G1332.1	397	31.6	maker-scaffold122-a	1059	26	359	40	349	0.64	6	333	177.2	2.1e-44	gene=Chr02G13	maker-scaffold122-augustus-gene-0.90-

			ugustus-gene-0.90-mRNA-1[OphiocerasdolichostomumCBS114926]											32.1	mRNA-1[OphiocerasdolichostomumCBS114926]
Chr09G0602.1	568	24.5	AFL2G_07229[Aspergillusflavus]	640	94	500	205	572	0.64	6	406	120.2	4.4e-27	gene=Chr09G0602.1	AFL2G_07229[Aspergillusflavus]
Chr01G2478.1	303	54.4	augustus_masked-scaffold10-processed-gene-15.1-mRNA-1[Cladoniametacorallifera]	832	6	299	555	832	0.64	5	293	283.1	2.1e-76	gene=Chr01G2478.1	augustus_masked-scaffold10-processed-gene-15.1-mRNA-1[Cladoniametacorallifera]
Chr06G0473.1	379	44	fgenesh1_pg.12_#_108[StereumhirsutumFP-91666SS1]	1485	60	356	1183	1463	0.64	4	296	257.7	1.2e-68	gene=Chr06G0473.1	fgenesh1_pg.12_#_108[StereumhirsutumFP-91666SS1]

Chr03G1178.1	347	37.8	XP_007804354.1[EndocarponpusillumZ07020]	541	35	314	16	295	0.64	4	279	192.6	4.3e-49	gene=Chr03G1178.1	XP_007804354.1[EndocarponpusillumZ07020]
Chr08G0285.1	585	34.4	fgenesh1_pg.00058_#_49[PilodermacroceumF1598]	896	161	580	480	812	0.64	9	419	215.7	8.0e-56	gene=Chr08G0285.1	fgenesh1_pg.00058_#_49[PilodermacroceumF1598]
Chr01G1532.1	692	30.3	Clame_scaffold11-8.74[Cladoniametacorallifera]	8336	174	529	6767	7156	0.65	13	355	169.9	6.0e-42	gene=Chr01G1532.1	Clame_scaffold11-8.74[Cladoniametacorallifera]
Chr04G0322.1	1447	38.2	EEA28139.1[Penicilliummarneffei]	2025	9	306	603	911	0.65	6	297	201.1	5.1e-51	gene=Chr04G0322.1	EEA28139.1[Penicilliummarneffei]
Chr01G0516.1	388	36.9	XP_007804354.1[EndocarponpusillumZ07020]	541	40	350	2	295	0.65	7	310	197.6	1.5e-50	gene=Chr01G0516.1	XP_007804354.1[EndocarponpusillumZ07020]
Chr07G0985.1	597	28.2	Clame_sc	16637	93	419	2937	3270	0.6	5	326	149.8	5.5e-36	gene=C	Clame_scaffold8-4.1

			affold8-4.115[Cladoniametacorallifera]						5					hr07G0985.1	15[Cladoniametacorallifera]
Chr02G0306.1	497	73.4	PLG_01751-R0[Pseudohalonectrialignicola M95]	462	84	478	72	449	0.65	2	394	578.6	4.0e-165	gene=Chr02G0306.1	PLG_01751-R0[Pseudohalonectrialignicola M95]
Chr07G0554.1	358	34	XP_007804354.1[Endocarponpusillum Z07020]	541	29	323	4	300	0.65	5	294	175.6	5.6e-44	gene=Chr07G0554.1	XP_007804354.1[Endocarponpusillum Z07020]
Chr02G0205.1	543	83.9	CH063_03746T0[Colletotrichumhigginsianum]	552	8	542	7	552	0.65	1	534	965.3	1.7e-281	gene=Chr02G0205.1	CH063_03746T0[Colletotrichumhigginsianum]
Chr08G0483.1	492	39.4	XP_007804354.1[Endocarponpusillum Z07020]	541	174	451	15	289	0.65	3	277	213.4	3.3e-55	gene=Chr08G0483.1	XP_007804354.1[Endocarponpusillum Z07020]
Chr03G1370.1	335	39.9	XP_007804354.1[En	541	10	295	9	287	0.65	5	285	209.9	2.5e-54	gene=Chr03G13	XP_007804354.1[Endocarponpusillum Z0

			docarponpusillumZ07020]											70.1	7020]
Chr04G1038.1	365	40.7	XP_007804354.1[EndocarponpusillumZ07020]	541	49	326	22	296	0.65	4	277	208.8	6.1e-54	gene=Chr04G1038.1	XP_007804354.1[EndocarponpusillumZ07020]
Chr06G0128.1	515	84.1	CE167925_24560[ColletotrichumsublineolaCBS131301]	524	12	511	15	517	0.65	1	499	882.5	1.3e-256	gene=Chr06G0128.1	CE167925_24560[ColletotrichumsublineolaCBS131301]
Chr06G1022.1	346	38.1	XP_007804354.1[EndocarponpusillumZ07020]	541	32	323	12	295	0.65	5	291	195.3	6.6e-50	gene=Chr06G1022.1	XP_007804354.1[EndocarponpusillumZ07020]
Chr06G0439.1	930	36.4	Hanno_03304[Heterobasidionannosum03012]	1417	48	368	751	1094	0.65	11	320	170.2	6.1e-42	gene=Chr06G0439.1	Hanno_03304[Heterobasidionannosum03012]
Chr09G0528.1	367	33.6	XP_007804354.1[En	541	37	351	19	333	0.65	7	314	183.3	2.8e-46	gene=Chr09G05	XP_007804354.1[EndocarponpusillumZ0

			docarponpusillumZ07020]											28.1	7020]
Chr07G0280.1	453	30.9	HCB02885.1[Histoplasmacapsulatum]	895	133	448	551	893	0.65	8	315	144.1	2.3e-34	gene=Chr07G0280.1	HCB02885.1[Histoplasmacapsulatum]
Chr05G0939.1	305	51.5	EAA59870.1[AspergillusnidulansFGSCA4]	779	5	289	461	766	0.65	4	284	338.6	4.3e-93	gene=Chr05G0939.1	EAA59870.1[AspergillusnidulansFGSCA4]
Chr03G1469.1	360	35.1	XP_007804354.1[EndocarponpusillumZ07020]	541	33	345	6	304	0.65	6	312	194.5	1.2e-49	gene=Chr03G1469.1	XP_007804354.1[EndocarponpusillumZ07020]
Chr09G0991.1	332	50	g8694.t1[ArmillariamelleaDSM3731]	1256	33	318	22	297	0.65	5	285	218	9.2e-57	gene=Chr09G0991.1	g8694.t1[ArmillariamelleaDSM3731]
Chr04G0137.1	327	40.9	g8694.t1[ArmillariamelleaDSM3731]	1256	3	304	4	287	0.65	7	301	193	3.1e-49	gene=Chr04G0137.1	g8694.t1[ArmillariamelleaDSM3731]
Chr08G0710.1	755	29.3	Clame_scaffold11-8.	8336	116	507	6759	7153	0.65	10	391	164.5	2.7e-40	gene=Chr08G07	Clame_scaffold11-8.74[Cladoniametacor

			74[Cladoniametacorallifera]										10.1	allifera]
Chr09G0641.1	1005	29.6	HCB00741.1[Histoplasmacapsulatum]	1461	154	539	1097	1460	0.65	12	385	182.2	1.7e-45	gene=Chr09G0641.1	HCB00741.1[Histoplasmacapsulatum]
Chr05G1074.1	505	36.2	estExt_Genewise1.C_690045[Auriculariasubglabra]	1040	137	464	436	810	0.65	9	327	211.1	1.7e-54	gene=Chr05G1074.1	estExt_Genewise1.C_690045[Auriculariasubglabra]
Chr05G1276.1	360	40.8	XP_007804354.1[EndocarponpusillumZ07020]	541	44	328	12	300	0.65	5	284	226.9	2.1e-59	gene=Chr05G1276.1	XP_007804354.1[EndocarponpusillumZ07020]
Chr05G0311.1	1419	28.2	Clame_scaffold11-8.74[Cladoniametacorallifera]	8336	925	1253	7392	7717	0.65	4	328	121.7	3.8e-27	gene=Chr05G0311.1	Clame_scaffold11-8.74[Cladoniametacorallifera]
Chr03G0012.1	496	28.7	Clame_scaffold8-4.115[Cladoniametacora	16637	47	375	2940	3269	0.65	5	328	148.7	1.0e-35	gene=Chr03G0012.1	Clame_scaffold8-4.115[Cladoniametacorallifera]

			llifera]												
Chr07G0106.1	337	36	XP_007804354.1[EndocarponpusillumZ07020]	541	11	309	7	301	0.66	6	298	190.7	1.6e-48	gene=Chr07G0106.1	XP_007804354.1[EndocarponpusillumZ07020]
Chr04G1196.1	379	36.2	XP_007804354.1[EndocarponpusillumZ07020]	541	61	370	12	311	0.66	6	309	203	3.5e-52	gene=Chr04G1196.1	XP_007804354.1[EndocarponpusillumZ07020]
Chr09G0657.1	466	25.9	maker-scaffold122-augustus-gene-0.90-mRNA-1[OphiocerasdolichostomumCBS114926]	1059	30	398	29	384	0.66	8	368	118.2	1.4e-26	gene=Chr09G0657.1	maker-scaffold122-augustus-gene-0.90-mRNA-1[OphiocerasdolichostomumCBS114926]
Chr02G0885.1	519	85.1	XP_007593095.1[ColletotrichumfioriniaePJ7]	518	1	518	1	518	0.66	0	517	944.1	3.8e-275	gene=Chr02G0885.1	XP_007593095.1[ColletotrichumfioriniaePJ7]
Chr09G0131.1	532	29.4	CNAG_06	506	120	447	160	494	0.6	7	327	124.8	1.7e-28	gene=C	CNAG_06628T0[Cry

			628T0[Cryptococcusneoformansvar.grubiiH99]						6					hr09G0131.1	ptococcusneoformansvar.grubiiH99]
Chr03G1378.1	332	36.1	g8694.t1[ArmillariamelleaDSM3731]	1256	3	321	4	300	0.66	4	318	184.1	1.5e-46	gene=Chr03G1378.1	g8694.t1[ArmillariamelleaDSM3731]
Chr05G1241.1	400	59.2	Clama_scaffold_28-0.22[Cladoniamacilenta]	3845	85	361	3535	3809	0.66	1	276	342.8	3.0e-94	gene=Chr05G1241.1	Clama_scaffold_28-0.22[Cladoniamacilenta]
Chr07G0490.1	396	41.4	XP_007804354.1[EndocarponpusillumZ07020]	541	65	353	6	288	0.66	6	288	235.7	5.1e-62	gene=Chr07G0490.1	XP_007804354.1[EndocarponpusillumZ07020]
Chr07G0978.1	479	28.9	estExt_Genemark1.C_130001[Punctulariastrigosozonata]	634	106	463	244	621	0.66	11	357	143.7	3.2e-34	gene=Chr07G0978.1	estExt_Genemark1.C_130001[Punctulariastrigosozonata]
Chr04G0906.1	325	38.1	XP_00780	541	9	295	12	296	0.6	4	286	208	9.3e-54	gene=C	XP_007804354.1[En

			4354.1[EndocarponpusillumZ07020]						6					hr04G0906.1	docarponpusillumZ07020]
Chr06G0245.1	688	38.8	estExt_fgenesh1_pg.C_450037[Phytophthorasojae]	872	54	342	565	871	0.66	5	288	225.3	1.2e-58	gene=Chr06G0245.1	estExt_fgenesh1_pg.C_450037[Phytophthorasojae]
Chr04G0836.1	332	37.3	XP_007804354.1[EndocarponpusillumZ07020]	541	1	294	1	295	0.66	5	293	200.7	1.5e-51	gene=Chr04G0836.1	XP_007804354.1[EndocarponpusillumZ07020]
Chr02G1106.1	497	83.8	NCU03415T0[NeurosporacrassaOR74A]	495	5	491	7	493	0.66	0	486	855.1	2.2e-248	gene=Chr02G1106.1	NCU03415T0[NeurosporacrassaOR74A]
Chr01G1328.1	429	34.5	maker-scaffold122-augustus-gene-0.90-mRNA-1[OphiocerasdolichostomumCB	1059	50	383	60	381	0.66	8	333	202.6	5.1e-52	gene=Chr01G1328.1	maker-scaffold122-augustus-gene-0.90-mRNA-1[OphiocerasdolichostomumCBS114926]

			S114926]											
Chr07G0101.1	557	85	XP_007598108.1[ColletotrichumfioriniaePJ7]	877	1	521	1	521	0.66	0	520	954.9	2.3e-278	gene=Chr07G0101.1	XP_007598108.1[ColletotrichumfioriniaePJ7]
Chr04G0726.1	336	62	EAA57917.1[AspergillusnidulansFGSCA4]	864	4	313	6	327	0.66	6	309	406.4	1.8e-113	gene=Chr04G0726.1	EAA57917.1[AspergillusnidulansFGSCA4]
Chr05G0862.1	1289	53.1	EEA28139.1[Penicilliummarneffei]	2025	11	320	617	953	0.66	6	309	331.6	2.2e-90	gene=Chr05G0862.1	EEA28139.1[Penicilliummarneffei]
Chr08G0966.1	372	40	XP_007804354.1[EndocarponpusillumZ07020]	541	45	327	7	288	0.66	3	282	213	3.3e-55	gene=Chr08G0966.1	XP_007804354.1[EndocarponpusillumZ07020]
Chr04G0925.1	511	28.8	Clame_scaffold8-4.115[Cladoniametacorallifera]	16637	150	505	10628	10994	0.67	14	355	136	7.0e-32	gene=Chr04G0925.1	Clame_scaffold8-4.115[Cladoniametacorallifera]
Chr04G0258.1	620	85.3	Clama_scaffold_5-4.	21962	50	578	9518	10046	0.67	0	528	914.8	2.9e-266	gene=Chr04G02	Clama_scaffold_5-4.0[Cladoniamacilenta]

			0[Cladoniamacilenta]											58.1	
Chr08G0575.1	524	25.5	Clama_scaffold_5-4.0[Cladoniamacilenta]	21962	5	411	13705	14100	0.67	15	406	93.2	5.4e-19	gene=Chr08G0575.1	Clama_scaffold_5-4.0[Cladoniamacilenta]
Chr04G1552.1	648	30.4	HCB00741.1[Histoplasmacapsulatum]	1461	157	555	1100	1459	0.67	13	398	163.7	4.0e-40	gene=Chr04G1552.1	HCB00741.1[Histoplasmacapsulatum]
Chr09G0744.1	531	29.6	MUStwsD_GLEAN_10001533[Omphalotusolearius]	1200	77	458	643	1103	0.67	17	381	156.8	4.0e-38	gene=Chr09G0744.1	MUStwsD_GLEAN_10001533[Omphalotusolearius]
Chr08G0906.1	369	42.8	XP_007804354.1[EndocarponpusillumZ07020]	541	64	339	12	288	0.67	3	275	219.5	3.5e-57	gene=Chr08G0906.1	XP_007804354.1[EndocarponpusillumZ07020]
Chr04G1534.1	1237	26.3	Clama_scaffold_5-4.0[Cladoniamacilenta]	21962	604	1117	9561	10044	0.67	15	513	172.2	2.1e-42	gene=Chr04G1534.1	Clama_scaffold_5-4.0[Cladoniamacilenta]
Chr01G1931.1	483	26	Clame_sc	16637	21	391	2909	3293	0.6	8	370	118.6	1.1e-26	gene=C	Clame_scaffold8-4.1

			affold8-4.115[Cladoniametacorallifera]						7					hr01G1931.1	15[Cladoniametacorallifera]
Chr06G1428.1	388	41.3	M_BR29_EuGene_00068431[MagnaporthegriseaBR29]	989	98	386	100	400	0.67	8	288	184.1	1.7e-46	gene=Chr06G1428.1	M_BR29_EuGene_00068431[MagnaporthegriseaBR29]
Chr02G0479.1	340	54.9	CMQ_6543m.01[Grosmanniaclavigerakw1407]	569	1	289	146	449	0.67	3	288	320.5	1.3e-87	gene=Chr02G0479.1	CMQ_6543m.01[Grosmanniaclavigerakw1407]
Chr01G0184.1	534	31.9	MUStwsD_GLEAN_10000830[Omphalotusolearius]	927	14	353	554	874	0.67	4	339	179.5	5.8e-45	gene=Chr01G0184.1	MUStwsD_GLEAN_10000830[Omphalotusolearius]
Chr02G1169.1	404	34.2	XP_007804354.1[EndocarponpusillumZ07020]	541	69	384	3	310	0.67	6	315	198	1.2e-50	gene=Chr02G1169.1	XP_007804354.1[EndocarponpusillumZ07020]

Chr01G1299.1	1192	30.2	AFL2G_01904[Aspergillusflavus]	4755	800	1188	4402	4751	0.67	13	388	170.6	6.0e-42	gene=Chr01G1299.1	AFL2G_01904[Aspergillusflavus]
Chr01G0298.1	1037	81.8	CH063_05852T0[Colletotrichumhigginsianum]	454	583	1033	1	451	0.67	0	450	781.6	6.5e-226	gene=Chr01G0298.1	CH063_05852T0[Colletotrichumhigginsianum]
Chr06G0451.1	494	27.5	Clame_scaffold8-4.115[Cladoniametacorallifera]	16637	43	393	2940	3287	0.67	7	350	132.9	5.8e-31	gene=Chr06G0451.1	Clame_scaffold8-4.115[Cladoniametacorallifera]
Chr06G1122.1	472	23.8	AFL2G_08515[Aspergillusflavus]	890	8	409	6	421	0.67	10	401	105.5	9.4e-23	gene=Chr06G1122.1	AFL2G_08515[Aspergillusflavus]
Chr08G0775.1	373	37.8	XP_007804354.1[EndocarponpusillumZ07020]	541	36	330	6	296	0.67	3	294	211.5	9.6e-55	gene=Chr08G0775.1	XP_007804354.1[EndocarponpusillumZ07020]
Chr02G1121.1	488	27.5	maker-scaffold122-augustus-g	1059	29	397	39	392	0.67	9	368	150.6	2.6e-36	gene=Chr02G1121.1	maker-scaffold122-augustus-gene-0.90-mRNA-1[Ophioceras

			ene-0.90-mRNA-1[OphiocerasdolichostomumCBS114926]												dolichostomumCBS114926]
Chr02G1357.1	994	38.8	estExt_Genewise1.C_4080032[Exidiaglandulosa]	354	39	344	36	354	0.68	9	305	229.9	7.0e-60	gene=Chr02G1357.1	estExt_Genewise1.C_4080032[Exidiaglandulosa]
Chr04G1416.1	359	41.7	XP_007804354.1[EndocarponpusillumZ07020]	541	34	322	12	295	0.68	4	288	233.4	2.3e-61	gene=Chr04G1416.1	XP_007804354.1[EndocarponpusillumZ07020]
Chr02G1265.1	485	31	Clame_scaffold8-4.115[Cladoniametacorallifera]	16637	57	382	2947	3286	0.68	2	325	157.1	2.8e-38	gene=Chr02G1265.1	Clame_scaffold8-4.115[Cladoniametacorallifera]
Chr01G0881.1	348	38.2	XP_007804354.1[EndocarponpusillumZ07020]	541	16	309	4	295	0.68	4	293	204.1	1.4e-52	gene=Chr01G0881.1	XP_007804354.1[EndocarponpusillumZ07020]

Chr04G0201.1	471	38	maker-scaffold122-augustus-gene-0.90-mRNA-1[OphiocerasdolichostomumCBS114926]	1059	21	365	32	343	0.68	6	344	228.8	7.4e-60	gene=Chr04G0201.1	maker-scaffold122-augustus-gene-0.90-mRNA-1[OphiocerasdolichostomumCBS114926]
Chr01G0514.1	368	40.3	XP_007804354.1[EndocarponpusillumZ07020]	541	47	354	15	316	0.68	9	307	210.7	1.6e-54	gene=Chr01G0514.1	XP_007804354.1[EndocarponpusillumZ07020]
Chr08G0438.1	597	26.5	ATEG_04721.1[Aspergillusterreus]	994	120	500	528	889	0.68	9	380	114	3.4e-25	gene=Chr08G0438.1	ATEG_04721.1[Aspergillusterreus]
Chr02G1557.1	360	33.1	gm1.10090_g[Hypholomasublateritium]	821	5	355	6	333	0.68	15	350	127.9	1.4e-29	gene=Chr02G1557.1	gm1.10090_g[Hypholomasublateritium]
Chr01G1858.1	301	51.8	Endocarpon_04277[Endocarponpusillum	1455	24	300	1168	1449	0.68	2	276	281.6	6.1e-76	gene=Chr01G1858.1	Endocarpon_04277[Endocarponpusillum]

			]												
Chr04G0859.1	387	44.4	XP_007804354.1[EndocarponpusillumZ07020]	541	58	354	10	294	0.68	4	296	251.5	8.7e-67	gene=Chr04G0859.1	XP_007804354.1[EndocarponpusillumZ07020]
Chr01G0222.1	370	36.4	XP_007804354.1[EndocarponpusillumZ07020]	541	43	357	8	310	0.68	6	314	218	1.0e-56	gene=Chr01G0222.1	XP_007804354.1[EndocarponpusillumZ07020]
Chr02G1009.1	465	27.9	CE89146_1070[Neolentinuslepideus]	421	103	457	21	367	0.68	6	354	134	2.4e-31	gene=Chr02G1009.1	CE89146_1070[Neolentinuslepideus]
Chr03G1188.1	975	41.8	estExt_Genewise1.C_4080032[Exidiaglandulosa]	354	22	324	30	354	0.68	7	302	257.3	4.0e-68	gene=Chr03G1188.1	estExt_Genewise1.C_4080032[Exidiaglandulosa]
Chr02G1682.1	776	82.1	CH063_02473T0[Colletotrichumhigginsianum]	539	26	503	28	508	0.68	1	477	818.9	2.7e-237	gene=Chr02G1682.1	CH063_02473T0[Colletotrichumhigginsianum]
Chr02G1651.1	762	31	Clame_sc	7866	145	493	3120	3501	0.6	12	348	179.9	6.3e-45	gene=C	Clame_scaffold18-1

			affold18-19.70[Cladoniametacorallifera]						8					hr02G1651.1	9.70[Cladoniametacorallifera]
Chr03G1286.1	520	84.2	estExt_Genewise1Plus.C_5260005[ColletotrichumsublineolaCBS131301]	524	4	517	5	518	0.68	0	513	899	1.4e-261	gene=Chr03G1286.1	estExt_Genewise1Plus.C_5260005[ColletotrichumsublineolaCBS131301]
Chr02G0420.1	322	40.4	EAA58488.1[AspergillusnidulansFGSCA4]	1365	22	320	1051	1365	0.68	5	298	229.6	3.0e-60	gene=Chr02G0420.1	EAA58488.1[AspergillusnidulansFGSCA4]
Chr06G0811.1	459	39.6	Clama_scaffold_5-4.0[Cladoniamacilenta]	21962	112	413	4811	5111	0.69	5	301	204.9	1.1e-52	gene=Chr06G0811.1	Clama_scaffold_5-4.0[Cladoniamacilenta]
Chr07G0100.1	508	83.6	XP_007598110.1[ColletotrichumfioriniaePJ7]	506	1	505	1	504	0.69	1	504	912.5	1.2e-265	gene=Chr07G0100.1	XP_007598110.1[ColletotrichumfioriniaePJ7]
Chr04G0569.1	575	30.1	Clame_sc	16637	104	438	2939	3272	0.6	6	334	126.7	4.8e-29	gene=C	Clame_scaffold8-4.1

			affold8-4.115[Cladoniametacorallifera]						9					hr04G0569.1	15[Cladoniametacorallifera]
Chr06G0903.1	756	31.5	Clame_scaffold11-8.74[Cladoniametacorallifera]	8336	132	510	6754	7153	0.69	12	378	193.4	5.5e-49	gene=Chr06G0903.1	Clame_scaffold11-8.74[Cladoniametacorallifera]
Chr04G0523.1	504	83.2	XP_007600443.1[ColletotrichumfioriniaePJ7]	503	1	500	1	501	0.69	1	499	843.6	6.8e-245	gene=Chr04G0523.1	XP_007600443.1[ColletotrichumfioriniaePJ7]
Chr03G0118.1	502	29.9	Clame_scaffold8-4.115[Cladoniametacorallifera]	16637	23	359	2936	3272	0.69	4	336	172.6	6.7e-43	gene=Chr03G0118.1	Clame_scaffold8-4.115[Cladoniametacorallifera]
Chr09G1034.1	584	79.4	GLRG_01977T0[ColletotrichumgraminicolaM1.001]	567	1	565	1	561	0.69	5	564	812	2.5e-235	gene=Chr09G1034.1	GLRG_01977T0[ColletotrichumgraminicolaM1.001]
Chr07G0429.1	503	76.1	CH063_01	521	1	492	1	518	0.6	4	491	800.4	6.5e-23	gene=C	CH063_01906T0[Co

			906T0[Colletotrichumhigginsianum]						9				2	hr07G0429.1	lletotrichumhigginsianum]
Chr04G0679.1	457	34.8	Clame_scaffold8-4.115[Cladoniametacorallifera]	16637	35	353	2942	3270	0.69	6	318	172.9	4.7e-43	gene=Chr04G0679.1	Clame_scaffold8-4.115[Cladoniametacorallifera]
Chr09G0530.1	378	44.4	fgenesh1_pg.12_#_108[StereumhirsutumFP-91666SS1]	1485	19	338	1143	1459	0.69	6	319	259.2	4.1e-69	gene=Chr09G0530.1	fgenesh1_pg.12_#_108[StereumhirsutumFP-91666SS1]
Chr07G0359.1	885	28.4	1093_t[AscocorynesarcoidesNRRL50072]	1211	226	659	24	439	0.69	15	433	160.2	6.0e-39	gene=Chr07G0359.1	1093_t[AscocorynesarcoidesNRRL50072]
Chr06G0021.1	1171	45.3	EEA28139.1[Penicilliummarneffei]	2025	10	306	603	907	0.69	9	296	221.9	2.2e-57	gene=Chr06G0021.1	EEA28139.1[Penicilliummarneffei]
Chr06G0011.1	913	45.3	EEA28139.1[Penicilli	2025	10	306	603	907	0.69	9	296	223	7.8e-58	gene=Chr06G00	EEA28139.1[Penicilliummarneffei]

			ummarneffei]											11.1	
Chr01G0409.1	494	28.9	Clame_scaffold8-4.115[Cladoniametacorallifera]	16637	58	400	2945	3292	0.69	4	342	171.4	1.5e-42	gene=Chr01G0409.1	Clame_scaffold8-4.115[Cladoniametacorallifera]
Chr01G1844.1	354	40.6	XP_007804354.1[EndocarponpusillumZ07020]	541	23	314	6	296	0.69	3	291	231.5	8.5e-61	gene=Chr01G1844.1	XP_007804354.1[EndocarponpusillumZ07020]
Chr03G0063.1	604	26.2	estExt_Genewise1Plus.C_100483[PleurotusostreatusPC15]	623	162	604	185	623	0.69	19	442	110.9	2.9e-24	gene=Chr03G0063.1	estExt_Genewise1Plus.C_100483[PleurotusostreatusPC15]
Chr02G0266.1	757	32	Clame_scaffold11-8.74[Cladoniametacorallifera]	8336	197	589	6752	7156	0.69	10	392	208	2.2e-53	gene=Chr02G0266.1	Clame_scaffold11-8.74[Cladoniametacorallifera]
Chr05G0098.1	488	82.7	CH063_00006T0[Colletotrichu	488	1	485	1	485	0.69	0	484	843.6	6.5e-245	gene=Chr05G0098.1	CH063_00006T0[Colletotrichumhigginsianum]

			mhigginsianum]													
Chr08G0985.1	636	25	gm1.11182_g[Sphaerobolusstellatus]	620	35	622	169	614	0.70	24	587	106.3	7.4e-23	gene=Chr08G0985.1	gm1.11182_g[Sphaerobolusstellatus]
Chr04G0269.1	601	37.2	XP_007598108.1[CoIletotrichumfioriniaePJ7]	877	1	331	7	334	0.70	9	330	209.5	5.9e-54	gene=Chr04G0269.1	XP_007598108.1[CoIletotrichumfioriniaePJ7]
Chr03G1299.1	650	31.2	HCB00741.1[Histoplasmacapsulatum]	1461	140	551	1098	1459	0.70	10	411	194.5	2.1e-49	gene=Chr03G1299.1	HCB00741.1[Histoplasmacapsulatum]
Chr01G0195.1	868	34.3	HCB00741.1[Histoplasmacapsulatum]	1461	165	581	1098	1459	0.70	13	416	216.9	5.3e-56	gene=Chr01G0195.1	HCB00741.1[Histoplasmacapsulatum]
Chr01G0301.1	614	27.2	Clame_scaffold18-19.70[Cladoniametacorallifera]	7866	118	494	3129	3540	0.70	11	376	147.5	2.8e-35	gene=Chr01G0301.1	Clame_scaffold18-19.70[Cladoniametacorallifera]
Chr06G0084.1	1064	32.4	Hanno_03304[Heter	1417	95	445	750	1120	0.70	10	350	158.7	2.1e-38	gene=Chr06G00	Hanno_03304[Heterobasidionannosum0

			obasidionannosum03012]											84.1	3012]
Chr05G0076.1	522	22.4	XP_007600712.1[CollletotrichumfioriniaePJ7]	959	27	454	25	447	0.70	13	427	78.2	1.8e-14	gene=Chr05G0076.1	XP_007600712.1[CollletotrichumfioriniaePJ7]
Chr03G0206.1	475	37.5	maker-scaffold122-augustus-gene-0.90-mRNA-1[OphiocerasdolichostomumCBS114926]	1059	30	360	39	351	0.70	5	330	237.7	1.6e-62	gene=Chr03G0206.1	maker-scaffold122-augustus-gene-0.90-mRNA-1[OphiocerasdolichostomumCBS114926]
Chr04G1490.1	899	25.3	Clame_scaffold11-8.74[Cladoniametacorallifera]	8336	165	581	6760	7197	0.70	14	416	143.3	7.8e-34	gene=Chr04G1490.1	Clame_scaffold11-8.74[Cladoniametacorallifera]
Chr03G1735.1	552	33.9	ATEG_07313.1[Aspergillusterreus]	836	173	491	1	329	0.70	4	318	192.2	9.0e-49	gene=Chr03G1735.1	ATEG_07313.1[Aspergillusterreus]

Chr01G2516.1	427	27.3	MGYG_03812[Microsporumgypseum]	526	25	417	37	416	0.70	10	392	150.6	2.3e-36	gene=Chr01G2516.1	MGYG_03812[Microsporumgypseum]
Chr03G0212.1	506	63	genemark-scaffold15-processed-gene-0.52-mRNA-1[PseudohalonectrialignicolaM95]	905	203	504	602	904	0.70	1	301	406	3.6e-113	gene=Chr03G0212.1	genemark-scaffold15-processed-gene-0.52-mRNA-1[PseudohalonectrialignicolaM95]
Chr06G0181.1	519	28.1	Clame_scaffold8-4.115[Cladoniametacorallifera]	16637	56	415	2938	3300	0.70	7	359	138.3	1.4e-32	gene=Chr06G0181.1	Clame_scaffold8-4.115[Cladoniametacorallifera]
Chr02G1631.1	563	28	ATEG_04721.1[Aspergillusterreus]	994	127	497	537	899	0.70	9	370	124	3.1e-28	gene=Chr02G1631.1	ATEG_04721.1[Aspergillusterreus]
Chr05G0201.1	653	28.7	Clame_scaffold18-19.70[Cladoniametac	7866	115	537	3130	3560	0.70	15	422	158.7	1.3e-38	gene=Chr05G0201.1	Clame_scaffold18-19.70[Cladoniametacorallifera]

			orallifera]												
Chr07G1195.1	359	45.5	EEA28139.1[Penicilliummarneffei]	2025	11	310	603	913	0.70	8	299	252.7	3.6e-67	gene=Chr07G1195.1	EEA28139.1[Penicilliummarneffei]
Chr04G0866.1	543	45.8	HCB04253.1[Histoplasmacapsulatum]	902	179	527	508	897	0.70	3	348	271.6	1.1e-72	gene=Chr04G0866.1	HCB04253.1[Histoplasmacapsulatum]
Chr06G1259.1	344	39.6	XP_007804354.1[EndocarponpusillumZ07020]	541	1	297	1	296	0.70	3	296	222.6	3.9e-58	gene=Chr06G1259.1	XP_007804354.1[EndocarponpusillumZ07020]
Chr03G1385.1	505	81.6	XP_007591898.1[ColletotrichumfioriniaePJ7]	478	27	504	3	478	0.70	1	477	826.6	8.6e-240	gene=Chr03G1385.1	XP_007591898.1[ColletotrichumfioriniaePJ7]
Chr07G1188.1	1356	32.2	Clama_scaffold_5-4.0[Cladoniamacilenta]	21962	99	448	5944	6296	0.70	13	349	145.2	3.1e-34	gene=Chr07G1188.1	Clama_scaffold_5-4.0[Cladoniamacilenta]
Chr02G1425.1	1454	26.9	Clame_scaffold11-8.74[Cladoni	8336	939	1309	7387	7757	0.70	4	370	149.1	2.3e-35	gene=Chr02G1425.1	Clame_scaffold11-8.74[Cladoniametacorallifera]

			ametacorallifera]												
Chr03G1448.1	1011	43.9	EEA28139.1[Penicilliummarneffei]	2025	10	316	603	917	0.70	10	306	223.8	5.1e-58	gene=Chr03G1448.1	EEA28139.1[Penicilliummarneffei]
Chr02G1316.1	598	29.8	gm1.11182_g[Sphaerobolusstellatus]	620	61	592	214	614	0.70	17	531	160.2	4.1e-39	gene=Chr02G1316.1	gm1.11182_g[Sphaerobolusstellatus]
Chr02G0372.1	1159	29.2	PHYM_05353.1[Phymatotrichopsisomnivora]	1090	780	1149	709	1070	0.70	12	369	135.6	2.1e-31	gene=Chr02G0372.1	PHYM_05353.1[Phymatotrichopsisomnivora]
Chr01G0395.1	519	33.2	Clame_scaffold8-4.115[Cladoniametacorallifera]	16637	53	389	2932	3267	0.70	7	336	182.6	6.7e-46	gene=Chr01G0395.1	Clame_scaffold8-4.115[Cladoniametacorallifera]
Chr04G0161.1	427	39.6	augustus_masked-scaffold9-processed-gene-0.95-mRNA-1[	2316	113	416	532	849	0.70	4	303	231.5	1.0e-60	gene=Chr04G0161.1	augustus_masked-scaffold9-processed-gene-0.95-mRNA-1[Magnaporthesalviniii M69]

			Magnaporthesalvinii M69]												
Chr01G2729.1	902	62.4	EEA28139.1[Penicilliummarneffei]	2025	3	313	603	914	0.71	2	310	397.5	2.3e-110	gene=Chr01G2729.1	EEA28139.1[Penicilliummarneffei]
Chr05G0022.1	530	83	e_gw1.419.23.1[Colletotrichumsublineola CBS131301]	524	5	521	5	518	0.71	1	516	917.5	3.9e-267	gene=Chr05G0022.1	e_gw1.419.23.1[Colletotrichumsublineola CBS131301]
Chr04G0226.1	470	43.6	Clame_scaffold8-4.115[Cladoniametacorallifera]	16637	89	444	2346	2698	0.71	5	355	297.4	1.7e-80	gene=Chr04G0226.1	Clame_scaffold8-4.115[Cladoniametacorallifera]
Chr04G1276.1	587	28.6	ATEG_04721.1[Aspergillusterreus]	994	139	518	528	888	0.71	7	379	144.1	3.0e-34	gene=Chr04G1276.1	ATEG_04721.1[Aspergillusterreus]
Chr08G0953.1	751	31.7	gm1.898_g[Tulasnellacalospora AL13]	1440	63	425	670	1052	0.71	8	362	188.3	1.8e-47	gene=Chr08G0953.1	gm1.898_g[Tulasnellacalospora AL13]

Chr07G0636.1	1182	65	EEA28139.1[Penicilliummarneffei]	2025	1	317	597	916	0.71	2	316	417.5	2.8e-116	gene=Chr07G0636.1	EEA28139.1[Penicilliummarneffei]
Chr01G1210.1	350	52.1	SS1G_01499.1[Sclerotiniasclerotiorum]	1043	38	349	527	864	0.71	1	311	367.9	7.5e-102	gene=Chr01G1210.1	SS1G_01499.1[Sclerotiniasclerotiorum]
Chr06G1204.1	453	37.7	maker-scaffold122-augustus-gene-0.90-mRNA-1[OphiocerasdolichostomumCBS114926]	1059	24	375	39	374	0.71	10	351	239.6	4.0e-63	gene=Chr06G1204.1	maker-scaffold122-augustus-gene-0.90-mRNA-1[OphiocerasdolichostomumCBS114926]
Chr06G1171.1	357	37.7	XP_007804354.1[EndocarponpusillumZ07020]	541	28	349	11	322	0.71	6	321	200.7	1.6e-51	gene=Chr06G1171.1	XP_007804354.1[EndocarponpusillumZ07020]
Chr09G0384.1	599	24.2	ATEG_04721.1[Aspergillusterreus]	994	152	564	538	950	0.71	11	412	79.7	7.0e-15	gene=Chr09G0384.1	ATEG_04721.1[Aspergillusterreus]

Chr05G1429.1	536	33.8	ATEG_07313.1[Aspergillusterreus]	836	162	493	1	361	0.71	5	331	182.6	6.9e-46	gene=Chr05G1429.1	ATEG_07313.1[Aspergillusterreus]
Chr09G0306.1	429	27	MGYG_03812[Microsporumgypseum]	526	16	414	10	405	0.71	8	398	130.6	2.5e-30	gene=Chr09G0306.1	MGYG_03812[Microsporumgypseum]
Chr07G0098.1	530	80.7	estExt_Genewise1Plus.C_870069[ColletotrichumsublineolaCBS131301]	546	1	526	1	543	0.71	2	525	890.6	5.1e-259	gene=Chr07G0098.1	estExt_Genewise1Plus.C_870069[ColletotrichumsublineolaCBS131301]
Chr07G0585.1	527	28.3	Clame_scaffold8-4.115[Cladoniametacorallifera]	16637	64	431	2926	3293	0.71	8	367	134.8	1.6e-31	gene=Chr07G0585.1	Clame_scaffold8-4.115[Cladoniametacorallifera]
Chr04G0798.1	524	83.6	XP_007596789.1[ColletotrichumfioriniaePJ7]	522	1	518	1	518	0.71	0	517	904	4.4e-263	gene=Chr04G0798.1	XP_007596789.1[ColletotrichumfioriniaePJ7]

Chr05G0568.1	526	83.6	XP_007591427.1[CollletotrichumfioriniaePJ7]	526	1	524	1	525	0.71	1	523	916.4	8.6e-267	gene=Chr05G0568.1	XP_007591427.1[CollletotrichumfioriniaePJ7]
Chr01G1234.1	505	36.1	maker-Caloplaca_flavorubescens_scaffold_0-augustus-gene-25.63-mRNA-1[Caloplacaflavorubescens]	1014	176	491	1	322	0.71	5	315	209.5	5.0e-54	gene=Chr01G1234.1	maker-Caloplaca_flavorubescens_scaffold_0-augustus-gene-25.63-mRNA-1[Caloplacaflavorubescens]
Chr04G1197.1	501	80.9	XP_007601871.1[CollletotrichumfioriniaePJ7]	490	4	500	1	490	0.71	1	496	831.6	2.6e-241	gene=Chr04G1197.1	XP_007601871.1[CollletotrichumfioriniaePJ7]
Chr03G0301.1	1486	27.4	Clame_scaffold11-8.74[Cladoniametacorallifera]	8336	967	1332	7388	7751	0.71	5	365	144.1	7.5e-34	gene=Chr03G0301.1	Clame_scaffold11-8.74[Cladoniametacorallifera]
Chr01G0560.1	510	33.2	Clame_sc	16637	42	371	2928	3269	0.7	2	329	194.5	1.7e-49	gene=C	Clame_scaffold8-4.1

			affold8-4.115[Cladoniametacorallifera]						1					hr01G0560.1	15[Cladoniametacorallifera]
Chr09G0874.1	454	60.5	Clame_scaffold18-19.70[Cladoniametacorallifera]	7866	131	429	4230	4528	0.71	0	298	374.8	8.0e-104	gene=Chr09G0874.1	Clame_scaffold18-19.70[Cladoniametacorallifera]
Chr01G2412.1	460	35.2	maker-scaffold122-augustus-gene-0.90-mRNA-1[OphiocerasdolichostomumCBS114926]	1059	14	366	19	356	0.71	9	352	204.9	1.1e-52	gene=Chr01G2412.1	maker-scaffold122-augustus-gene-0.90-mRNA-1[OphiocerasdolichostomumCBS114926]
Chr01G2722.1	923	64.4	EEA28139.1[Penicilliummarneffei]	2025	1	317	597	916	0.71	2	316	416	6.4e-116	gene=Chr01G2722.1	EEA28139.1[Penicilliummarneffei]
Chr03G0690.1	1347	64.4	EEA28139.1[Penicilliummarneffei]	2025	1	317	597	916	0.71	2	316	416.4	7.1e-116	gene=Chr03G0690.1	EEA28139.1[Penicilliummarneffei]

Chr04G0614.1	1500	29.6	Clame_scaffold11-8.74[Cladoniametacorallifera]	8336	949	1342	7378	7751	0.72	8	393	139	2.4e-32	gene=Chr04G0614.1	Clame_scaffold11-8.74[Cladoniametacorallifera]
Chr06G0570.1	2409	23.5	AB02265.1[Alternariabrassicicola]	1503	1814	2342	976	1471	0.72	20	528	105.1	6.3e-22	gene=Chr06G0570.1	AB02265.1[Alternariabrassicicola]
Chr04G0033.1	543	30	EEA23504.1[Penicilliummarneffei]	1213	104	463	70	420	0.72	5	359	160.2	3.7e-39	gene=Chr04G0033.1	EEA23504.1[Penicilliummarneffei]
Chr05G0031.1	450	32.6	Clame_scaffold11-8.74[Cladoniametacorallifera]	8336	44	413	7899	8289	0.72	7	369	211.8	8.9e-55	gene=Chr05G0031.1	Clame_scaffold11-8.74[Cladoniametacorallifera]
Chr02G1336.1	496	21.1	Pa_7_5740[PodosporaanserinaSmat+]	576	14	487	64	520	0.72	11	473	71.2	2.1e-12	gene=Chr02G1336.1	Pa_7_5740[PodosporaanserinaSmat+]
Chr05G0121.1	339	46.6	EEA28139.1[Penicilliummarneffei]	2025	3	300	617	922	0.72	4	297	266.9	1.8e-71	gene=Chr05G0121.1	EEA28139.1[Penicilliummarneffei]

Chr07G1146.1	1105	64.1	EEA28139.1[Penicilliummarneffei]	2025	1	317	597	916	0.72	2	316	410.6	3.2e-114	gene=Chr07G1146.1	EEA28139.1[Penicilliummarneffei]
Chr06G0193.1	393	71.8	genemark-scaffold15-processed-gene-0.52-mRNA-1[PseudohalonectrialignicolaM95]	905	20	391	537	905	0.72	2	371	509.6	1.8e-144	gene=Chr06G0193.1	genemark-scaffold15-processed-gene-0.52-mRNA-1[PseudohalonectrialignicolaM95]
Chr08G0518.1	510	31.5	Clame_scaffold8-4.115[Cladoniametacorallifera]	16637	46	391	2940	3288	0.72	5	345	167.5	2.2e-41	gene=Chr08G0518.1	Clame_scaffold8-4.115[Cladoniametacorallifera]
Chr06G0732.1	622	31.1	Hanno_03304[Heterobasidionannosum03012]	1417	137	508	760	1141	0.72	10	371	156	8.0e-38	gene=Chr06G0732.1	Hanno_03304[Heterobasidionannosum03012]
Chr01G1712.1	698	24.4	fgenesh1_pg.12_#_108[S	1485	11	492	623	1056	0.72	14	481	120.9	3.2e-27	gene=Chr01G1712.1	fgenesh1_pg.12_#_108[StereumhirsutumFP-91666SS1]

			tereumhirsutumFP-91666SS1]												
Chr01G0453.1	529	83.7	XP_007602402.1[CollletotrichumfioriniaePJ7]	528	1	527	1	527	0.72	0	526	954.5	2.9e-278	gene=Chr01G0453.1	XP_007602402.1[CollletotrichumfioriniaePJ7]
Chr02G1412.1	1776	29.4	Hanno_03304[Heterobasidionannosum03012]	1417	53	476	709	1093	0.72	12	423	144.1	9.0e-34	gene=Chr02G1412.1	Hanno_03304[Heterobasidionannosum03012]
Chr03G0396.1	711	27.4	Clame_scaffold18-19.70[Cladoniametacorallifera]	7866	134	558	3129	3538	0.72	14	424	139.4	8.9e-33	gene=Chr03G0396.1	Clame_scaffold18-19.70[Cladoniametacorallifera]
Chr03G0216.1	535	31.2	Clame_scaffold8-4.115[Cladoniametacorallifera]	16637	65	417	2942	3298	0.72	6	352	160.2	3.7e-39	gene=Chr03G0216.1	Clame_scaffold8-4.115[Cladoniametacorallifera]
Chr04G1455.1	674	24.6	1093_t[AscocorynesarcoidesN	1211	48	507	20	464	0.72	17	459	122.1	1.4e-27	gene=Chr04G1455.1	1093_t[AscocorynesarcoidesNRRL50072]

			RRL50072]												
Chr08G0437.1	1006	36.6	estExt_Genewise1.C_4080032[Exidiaglandulosa]	354	3	338	1	354	0.72	9	335	219.9	7.3e-57	gene=Chr08G0437.1	estExt_Genewise1.C_4080032[Exidiaglandulosa]
Chr02G1464.1	513	24.3	XP_007600712.1[ColletotrichumfioriniaePJ7]	959	46	454	12	441	0.72	10	408	92	1.2e-18	gene=Chr02G1464.1	XP_007600712.1[ColletotrichumfioriniaePJ7]
Chr01G1971.1	754	33.9	Clame_scaffold11-8.74[Cladoniametacorallifera]	8336	138	503	6767	7158	0.72	12	365	207.2	3.7e-53	gene=Chr01G1971.1	Clame_scaffold11-8.74[Cladoniametacorallifera]
Chr07G1140.1	498	31.3	Clame_scaffold8-4.115[Cladoniametacorallifera]	16637	62	405	2945	3292	0.72	4	343	172.2	8.6e-43	gene=Chr07G1140.1	Clame_scaffold8-4.115[Cladoniametacorallifera]
Chr01G0908.1	407	33.5	maker-scaffold122-augustus-gene-0.90-	1059	2	365	29	382	0.72	9	363	203	3.7e-52	gene=Chr01G0908.1	maker-scaffold122-augustus-gene-0.90-mRNA-1[OphiocerasdolichostomumCBS1

			mRNA-1[OphiocerasdolichostomumCBS114926]												14926]
Chr02G1337.1	498	34.3	Clame_scaffold8-4.115[Cladoniametacorallifera]	16637	50	380	2941	3286	0.72	3	330	215.7	6.8e-56	gene=Chr02G1337.1	Clame_scaffold8-4.115[Cladoniametacorallifera]
Chr06G0671.1	387	67.7	CAB38507.1[Schizosaccharomycespombe972h-]	346	42	386	3	346	0.72	3	344	490.7	8.5e-139	gene=Chr06G0671.1	CAB38507.1[Schizosaccharomycespombe972h-]
Chr05G1041.1	618	29.5	ATEG_07313.1[Aspergillusterreus]	836	227	599	2	374	0.73	5	372	149.4	7.5e-36	gene=Chr05G1041.1	ATEG_07313.1[Aspergillusterreus]
Chr04G0106.1	613	24.3	maker-Caloplaca_flavorubescens_scaffold_19-augustus-gene-2.67.2-mR	1049	15	476	48	509	0.73	21	461	94.4	2.8e-19	gene=Chr04G0106.1	maker-Caloplaca_flavorubescens_scaffold_19-augustus-gene-2.67.2-mRNA-1[Caloplacaflavorubescens]

			NA-1[Caloplacaflavorubescens]											
Chr09G0584.1	621	30.2	estExt_Genemark1.C_130001[Punctulariastrigosozonata]	634	79	451	244	624	0.73	11	372	158.3	1.6e-38	gene=Chr09G0584.1	estExt_Genemark1.C_130001[Punctulariastrigosozonata]
Chr05G1294.1	414	35	estExt_fgenesh1_pm.C_170119[Cenococcumgeophilum1.58]	1191	15	362	281	653	0.73	12	347	191	1.5e-48	gene=Chr05G1294.1	estExt_fgenesh1_pm.C_170119[Cenococcumgeophilum1.58]
Chr01G0948.1	474	35.2	Clame_scaffold8-4.115[Cladoniametacorallifera]	16637	39	381	2942	3308	0.73	7	342	181	1.8e-45	gene=Chr01G0948.1	Clame_scaffold8-4.115[Cladoniametacorallifera]
Chr02G1825.1	359	56.5	genemark-scaffold15-processed-gene-0.52-mRNA-	905	49	358	602	905	0.73	3	309	370.9	9.1e-103	gene=Chr02G1825.1	genemark-scaffold15-processed-gene-0.52-mRNA-1[PseudohalonectrialignicolaM95]

			1[PseudohalonectrialignicolaM95]												
Chr08G0110.1	520	81.9	CH063_02044T0[Colletotrichumhigginsianum]	491	14	504	1	491	0.73	0	490	855.9	1.4e-248	gene=Chr08G0110.1	CH063_02044T0[Colletotrichumhigginsianum]
Chr05G0055.1	329	40.1	EAA58488.1[AspergillusnidulansFGSCA4]	1365	8	328	1047	1365	0.73	5	320	252.3	4.3e-67	gene=Chr05G0055.1	EAA58488.1[AspergillusnidulansFGSCA4]
Chr08G0778.1	498	82.1	fgenesh1_kg.249_#_25_#_Locus2833v1rpkm43.07[ColletotrichumsublineolaCBS131301]	497	1	497	1	497	0.73	0	496	858.2	2.6e-249	gene=Chr08G0778.1	fgenesh1_kg.249_#_25_#_Locus2833v1rpkm43.07[ColletotrichumsublineolaCBS131301]
Chr08G0714.1	643	28.8	Clama_scaffold_5-4.0[Cladonia	21962	33	430	12829	13301	0.73	11	397	205.7	9.1e-53	gene=Chr08G0714.1	Clama_scaffold_5-4.0[Cladoniamacilenta]

			macilenta]												
Chr06G0028.1	1168	61	EEA28139.1[Penicilliummarneffei]	2025	1	328	597	937	0.73	3	327	412.9	6.8e-115	gene=Chr06G0028.1	EEA28139.1[Penicilliummarneffei]
Chr06G0267.1	420	55.1	XP_007598583.1[ColletotrichumfioriniaePJ7]	516	1	419	1	516	0.73	9	418	498	5.8e-141	gene=Chr06G0267.1	XP_007598583.1[ColletotrichumfioriniaePJ7]
Chr07G0874.1	325	40.5	AFL2G_07281[Aspergillusflavus]	701	2	322	9	326	0.73	6	320	233.4	2.1e-61	gene=Chr07G0874.1	AFL2G_07281[Aspergillusflavus]
Chr03G0722.1	484	79.7	GLRG_08461T0[ColletotrichumgraminicolaM1.001]	491	1	484	1	492	0.73	2	483	817.8	3.8e-237	gene=Chr03G0722.1	GLRG_08461T0[ColletotrichumgraminicolaM1.001]
Chr01G0376.1	569	24.2	XP_007600712.1[ColletotrichumfioriniaePJ7]	959	48	463	21	426	0.73	5	415	137.5	2.7e-32	gene=Chr01G0376.1	XP_007600712.1[ColletotrichumfioriniaePJ7]
Chr04G1421.1	546	28	Clame_sc	16637	95	491	2941	3326	0.7	8	396	150.6	3.0e-36	gene=C	Clame_scaffold8-4.1

			affold8-4.115[Cladoniametacorallifera]						3					hr04G1421.1	15[Cladoniametacorallifera]
Chr02G0247.1	1102	63.8	EEA28139.1[Penicilliummarneffei]	2025	1	323	597	922	0.73	2	322	420.2	4.0e-117	gene=Chr02G0247.1	EEA28139.1[Penicilliummarneffei]
Chr01G2113.1	767	56.9	XP_007597153.1[ColletotrichumfioriniaePJ7]	325	1	324	1	315	0.73	3	323	380.6	2.5e-105	gene=Chr01G2113.1	XP_007597153.1[ColletotrichumfioriniaePJ7]
Chr02G1063.1	464	34	augustus_masked-scaffold9-processed-gene-0.95-mRNA-1[MagnaporthesalviniiM69]	2316	45	401	1851	2233	0.73	10	356	172.9	4.7e-43	gene=Chr02G1063.1	augustus_masked-scaffold9-processed-gene-0.95-mRNA-1[MagnaporthesalviniiM69]
Chr02G0938.1	1182	31	maker-scaffold21-snap-gene-16.251-mR	4329	778	1179	3943	4327	0.73	13	401	176	1.4e-43	gene=Chr02G0938.1	maker-scaffold21-snap-gene-16.251-mRNA-1[Cladoniametacorallifera]

			NA-1[Cladoniametacorallifera]												
Chr05G0239.1	401	46.8	Clame_scaffold8-4.115[Cladoniametacorallifera]	16637	39	360	16002	16313	0.73	7	321	260.8	1.5e-69	gene=Chr05G0239.1	Clame_scaffold8-4.115[Cladoniametacorallifera]
Chr01G0628.1	332	42.8	EAA58488.1[AspergillusnidulansFGSCA4]	1365	10	330	1047	1364	0.74	7	320	245.4	5.4e-65	gene=Chr01G0628.1	EAA58488.1[AspergillusnidulansFGSCA4]
Chr01G1790.1	461	30.2	MGYG_03812[Microsporumgypseum]	526	54	443	23	394	0.74	7	389	179.1	6.5e-45	gene=Chr01G1790.1	MGYG_03812[Microsporumgypseum]
Chr03G1143.1	648	31.1	Clame_scaffold18-19.70[Cladoniametacorallifera]	7866	135	522	3111	3536	0.74	12	387	208.8	1.1e-53	gene=Chr03G1143.1	Clame_scaffold18-19.70[Cladoniametacorallifera]
Chr06G1058.1	1110	63	EEA28139.1[Penicilliummarneffei]	2025	1	327	597	924	0.74	2	326	417.5	2.6e-116	gene=Chr06G1058.1	EEA28139.1[Penicilliummarneffei]
Chr01G1213.1	1087	24.5	estExt_Ge	525	210	656	19	472	0.7	16	446	99.8	1.2e-20	gene=C	estExt_Genewise1Pl

			newise1Plus.C_120159[Sistotremastrumsuecicum]						4					hr01G1213.1	us.C_120159[Sistotremastrumsuecicum]
Chr09G1032.1	340	44.7	AFL2G_07281[Aspergillusflavus]	701	17	334	5	317	0.74	5	317	256.5	2.4e-68	gene=Chr09G1032.1	AFL2G_07281[Aspergillusflavus]
Chr02G0270.1	485	28.5	maker-Caloplaca_flavorubescens_scaffold_19-augustus-gene-2.67.2-mRNA-1[Caloplacaflavorubescens]	1049	11	427	136	554	0.74	17	416	136	6.7e-32	gene=Chr02G0270.1	maker-Caloplaca_flavorubescens_scaffold_19-augustus-gene-2.67.2-mRNA-1[Caloplacaflavorubescens]
Chr09G0124.1	1120	28.2	PTRG_10301[Pyrenophoratritici-repentis]	1311	236	628	332	786	0.74	11	392	177.2	6.0e-44	gene=Chr09G0124.1	PTRG_10301[Pyrenophoratritici-repentis]
Chr08G0388.1	495	30.8	MGYG_03	526	96	468	32	391	0.7	8	372	166.4	4.7e-41	gene=C	MGYG_03812[Micro

			812[Microsporumgypseum]						4					hr08G0388.1	sporumgypseum]
Chr08G0751.1	507	36.6	estExt_fgenesh1_pg.C_3530010[Exidiaglandulosa]	1270	14	389	549	904	0.74	8	375	244.2	1.8e-64	gene=Chr08G0751.1	estExt_fgenesh1_pg.C_3530010[Exidiaglandulosa]
Chr01G2470.1	640	31.6	Clame_scaffold11-8.74[Cladoniametacorallifera]	8336	125	508	6767	7191	0.74	13	383	198	1.9e-50	gene=Chr01G2470.1	Clame_scaffold11-8.74[Cladoniametacorallifera]
Chr02G0049.1	516	31.2	Clame_scaffold8-4.115[Cladoniametacorallifera]	16637	67	429	2941	3306	0.74	7	362	176.8	3.6e-44	gene=Chr02G0049.1	Clame_scaffold8-4.115[Cladoniametacorallifera]
Chr01G2647.1	1342	25.6	CNAG_03466T0[Cryptococcusneoformansvar.grubiiH99]	1123	635	1111	512	966	0.74	18	476	102.4	2.3e-21	gene=Chr01G2647.1	CNAG_03466T0[Cryptococcusneoformansvar.grubiiH99]
Chr07G0170.1	805	45.7	g16550.t1[Armillaria	1160	332	702	616	980	0.74	8	370	306.2	6.2e-83	gene=Chr07G01	g16550.t1[ArmillariamelleaDSM3731]

			melleaDSM3731]											70.1	
Chr01G0506.1	310	44.6	PLG_05741-R0[Pseudohalonectrialignicola M95]	313	6	309	8	313	0.74	3	303	255.8	3.7e-68	gene=Chr01G0506.1	PLG_05741-R0[Pseudohalonectrialignicola M95]
Chr06G0278.1	540	78.8	XP_007601178.1[Colletotrichumfioriniae PJ7]	565	1	539	1	565	0.74	2	538	931.8	2.0e-271	gene=Chr06G0278.1	XP_007601178.1[ColletotrichumfioriniaePJ7]
Chr01G0206.1	727	31.8	Clame_scaffold11-8.74[Cladoniametacorallifera]	8336	165	553	6752	7155	0.74	10	388	205.3	1.3e-52	gene=Chr01G0206.1	Clame_scaffold11-8.74[Cladoniametacorallifera]
Chr02G0315.1	542	26	Clame_scaffold11-8.74[Cladoniametacorallifera]	8336	119	531	5281	5715	0.74	17	412	88.6	1.4e-17	gene=Chr02G0315.1	Clame_scaffold11-8.74[Cladoniametacorallifera]
Chr06G1404.1	427	33.1	MGYG_03812[Microsporumgypseum]	526	16	410	37	400	0.74	8	394	203.8	2.3e-52	gene=Chr06G1404.1	MGYG_03812[Microsporumgypseum]

Chr05G0718.1	689	24	4127_t[AscocorynesarcoidesNRRL50072]	1621	196	672	1015	1492	0.74	18	476	88.2	2.3e-17	gene=Chr05G0718.1	4127_t[AscocorynesarcoidesNRRL50072]
Chr02G1809.1	688	23.4	EAL85053.1[AspergillusfumigatusAf293]	720	152	592	54	510	0.74	14	440	94.4	3.2e-19	gene=Chr02G1809.1	EAL85053.1[AspergillusfumigatusAf293]
Chr01G0231.1	471	30.8	Clame_scaffold8-4.115[Cladoniametacorallifera]	16637	52	410	2945	3306	0.74	6	358	171.4	1.4e-42	gene=Chr01G0231.1	Clame_scaffold8-4.115[Cladoniametacorallifera]
Chr08G0905.1	525	29	Clame_scaffold8-4.115[Cladoniametacorallifera]	16637	51	419	2935	3301	0.74	5	368	157.5	2.3e-38	gene=Chr08G0905.1	Clame_scaffold8-4.115[Cladoniametacorallifera]
Chr05G0649.1	392	45	Clama_scaffold_5-4.0[Cladoniamacilenta]	21962	59	367	10136	10455	0.74	1	308	296.2	3.1e-80	gene=Chr05G0649.1	Clama_scaffold_5-4.0[Cladoniamacilenta]
Chr01G0015.1	446	34.7	maker-scaffold122-augustus-g	1059	4	361	7	355	0.74	7	357	204.1	1.8e-52	gene=Chr01G0015.1	maker-scaffold122-augustus-gene-0.90-mRNA-1[Ophioceras

			ene-0.90-mRNA-1[OphiocerasdolichostomumCBS114926]												dolichostomumCBS114926]
Chr08G0235.1	575	42.5	Clame_scaffold9-5.100[Cladoniametacorallifera]	12517	154	552	3067	3419	0.74	8	398	292.7	5.1e-79	gene=Chr08G0235.1	Clame_scaffold9-5.100[Cladoniametacorallifera]
Chr02G0125.1	342	43.5	EAA58488.1[AspergillusnidulansFGSCA4]	1365	12	334	1048	1360	0.74	6	322	246.1	3.2e-65	gene=Chr02G0125.1	EAA58488.1[AspergillusnidulansFGSCA4]
Chr05G0686.1	1169	60	Suibr1.gm1.7563_g[Suillusbrevipes]	866	147	534	133	492	0.74	6	387	458.4	1.4e-128	gene=Chr05G0686.1	Suibr1.gm1.7563_g[Suillusbrevipes]
Chr01G2479.1	403	39.8	M_BR29_EuGene_00068431[MagnaporthegriseaBR29]	989	71	400	45	400	0.74	10	329	201.1	1.4e-51	gene=Chr01G2479.1	M_BR29_EuGene_00068431[MagnaporthegriseaBR29]
Chr01G2528.1	500	33.1	Clame_sc	16637	46	398	2939	3288	0.7	4	352	183	4.9e-46	gene=C	Clame_scaffold8-4.1

			affold8-4.115[Cladoniametacorallifera]						4					hr01G2528.1	15[Cladoniametacorallifera]
Chr01G1702.1	407	39	NOG_01398-R0[MagnaporthesalviniiM69]	425	23	378	12	423	0.74	5	355	284.6	9.8e-77	gene=Chr01G1702.1	NOG_01398-R0[MagnaporthesalviniiM69]
Chr05G0037.1	525	80.2	XP_007591577.1[ColletotrichumfioriniaePJ7]	513	46	514	44	512	0.75	0	468	770	9.9e-223	gene=Chr05G0037.1	XP_007591577.1[ColletotrichumfioriniaePJ7]
Chr04G0525.1	784	30.7	AFL2G_01551[Aspergillusflavus]	1045	221	607	580	941	0.75	4	386	193.7	4.4e-49	gene=Chr04G0525.1	AFL2G_01551[Aspergillusflavus]
Chr03G1568.1	1009	28.5	Clama_scaffold_5-4.0[Cladoniamacilenta]	21962	420	830	5928	6323	0.75	15	410	136.7	8.2e-32	gene=Chr03G1568.1	Clama_scaffold_5-4.0[Cladoniamacilenta]
Chr02G1796.1	450	44.5	AFL2G_07147[Aspergillusflavus]	1440	23	332	52	370	0.75	3	309	298.1	9.4e-81	gene=Chr02G1796.1	AFL2G_07147[Aspergillusflavus]

Chr02G1478.1	624	30.9	Clame_scaffold18-19.70[Cladoniametacorallifera]	7866	62	440	3112	3499	0.75	8	378	190.7	2.9e-48	gene=Chr02G1478.1	Clame_scaffold18-19.70[Cladoniametacorallifera]
Chr07G0270.1	1083	33.4	maker-scaffold122-augustus-gene-0.90-mRNA-1[OphiocerasdolichostomumCBS114926]	1059	8	396	14	403	0.75	10	388	200.7	4.9e-51	gene=Chr07G0270.1	maker-scaffold122-augustus-gene-0.90-mRNA-1[OphiocerasdolichostomumCBS114926]
Chr07G0907.1	508	29.7	Clame_scaffold8-4.115[Cladoniametacorallifera]	16637	14	384	2918	3287	0.75	8	370	165.6	8.3e-41	gene=Chr07G0907.1	Clame_scaffold8-4.115[Cladoniametacorallifera]
Chr06G0899.1	1311	33.2	XP_007598052.1[ColletotrichumfioriniaePJ7]	1915	851	1197	848	1207	0.75	5	346	179.9	1.1e-44	gene=Chr06G0899.1	XP_007598052.1[ColletotrichumfioriniaePJ7]
Chr04G1263.1	542	27.1	AFL2G_07229[Aspe	640	80	534	204	626	0.75	10	454	152.5	7.7e-37	gene=Chr04G12	AFL2G_07229[Aspergillusflavus]

			rgillusflavus]											63.1	
Chr02G1622.1	401	35.6	M_BR29_EuGene_00068431[MagnaporthegriseaBR29]	989	34	398	19	401	0.75	17	364	168.3	1.0e-41	gene=Chr02G1622.1	M_BR29_EuGene_00068431[MagnaporthegriseaBR29]
Chr01G0152.1	575	24	ATEG_04721.1[Aspergillusterreus]	994	125	574	537	972	0.75	12	449	79	1.2e-14	gene=Chr01G0152.1	ATEG_04721.1[Aspergillusterreus]
Chr03G0013.1	512	39.8	Clame_scaffold8-4.115[Cladoniametacorallifera]	16637	47	372	2941	3263	0.75	4	325	241.1	1.6e-63	gene=Chr03G0013.1	Clame_scaffold8-4.115[Cladoniametacorallifera]
Chr06G0764.1	462	36.7	M_BR29_EuGene_00068431[MagnaporthegriseaBR29]	989	30	434	18	406	0.75	15	404	211.5	1.2e-54	gene=Chr06G0764.1	M_BR29_EuGene_00068431[MagnaporthegriseaBR29]
Chr09G0171.1	1246	63.8	EEA28139.1[Penicilliummarneff	2025	1	331	597	930	0.75	2	330	429.5	7.5e-120	gene=Chr09G0171.1	EEA28139.1[Penicilliummarneffei]

			ei]												
Chr04G0510.1	554	26.8	ATEG_04721.1[Aspergillusterreus]	994	47	451	479	874	0.75	6	404	127.9	2.1e-29	gene=Chr04G0510.1	ATEG_04721.1[Aspergillusterreus]
Chr06G0231.1	605	27.9	gm1.11182_g[Sphaerobolusstellatus]	620	34	598	177	614	0.75	14	564	166.4	5.8e-41	gene=Chr06G0231.1	gm1.11182_g[Sphaerobolusstellatus]
Chr01G2606.1	471	31	Clame_scaffold8-4.115[Cladoniametacorallifera]	16637	41	400	2942	3301	0.75	5	359	165.6	7.7e-41	gene=Chr01G2606.1	Clame_scaffold8-4.115[Cladoniametacorallifera]
Chr03G1303.1	552	83.7	BBA_02823m.01[BeauveriabassianaARSEF2860]	556	2	552	6	556	0.75	0	550	972.2	1.4e-283	gene=Chr03G1303.1	BBA_02823m.01[BeauveriabassianaARSEF2860]
Chr05G1393.1	422	55.4	Clama_scaffold_5-4.0[Cladoniamacilenta]	21962	86	392	10133	10438	0.75	1	306	345.9	3.7e-95	gene=Chr05G1393.1	Clama_scaffold_5-4.0[Cladoniamacilenta]
Chr02G1553.1	525	70.5	Clame_scaffold9-5.100[Cladoni	12517	142	516	3041	3420	0.75	1	374	554.3	8.5e-158	gene=Chr02G1553.1	Clame_scaffold9-5.100[Cladoniametacorallifera]

			ametacorallifera]												
Chr09G0371.1	347	46.9	estExt_fgenesh2_pm.C_21000[Thielaviaterrestris]	984	28	346	651	984	0.76	2	318	282	5.4e-76	gene=Chr09G0371.1	estExt_fgenesh2_pm.C_21000[Thielaviaterrestris]
Chr03G1580.1	612	84.9	CE66914_16640[ColletotrichumsublineolaCBS131301]	613	1	611	1	613	0.76	3	610	1112.8	0.0e+00	gene=Chr03G1580.1	CE66914_16640[ColletotrichumsublineolaCBS131301]
Chr09G0278.1	547	77.9	Clame_scaffold18-19.70[Cladoniametacorallifera]	7866	27	502	2557	3029	0.76	3	475	759.2	1.8e-219	gene=Chr09G0278.1	Clame_scaffold18-19.70[Cladoniametacorallifera]
Chr06G0163.1	495	29.8	Clame_scaffold8-4.115[Cladoniametacorallifera]	16637	17	388	2905	3282	0.76	7	371	172.2	8.6e-43	gene=Chr06G0163.1	Clame_scaffold8-4.115[Cladoniametacorallifera]
Chr02G1853.1	418	37.3	M_BR29_EuGene_00068431[	989	1	415	1	399	0.76	20	414	198	1.2e-50	gene=Chr02G1853.1	M_BR29_EuGene_00068431[MagnaporthegriseaBR29]

			MagnaporthegriseaBR29]												
Chr03G0986.1	515	29.6	Clame_scaffold8-4.115[Cladoniametacorallifera]	16637	108	502	6491	6872	0.76	11	394	112.5	8.4e-25	gene=Chr03G0986.1	Clame_scaffold8-4.115[Cladoniametacorallifera]
Chr01G1895.1	1125	29.3	Clame_scaffold8-4.115[Cladoniametacorallifera]	16637	386	800	4136	4531	0.76	7	414	170.6	5.7e-42	gene=Chr01G1895.1	Clame_scaffold8-4.115[Cladoniametacorallifera]
Chr05G0767.1	440	42.2	Clama_scaffold_5-4.0[Cladoniamacilenta]	21962	85	407	10120	10438	0.76	2	322	277.3	1.7e-74	gene=Chr05G0767.1	Clama_scaffold_5-4.0[Cladoniamacilenta]
Chr01G1003.1	449	32.2	MGYG_03812[Microsporumgypseum]	526	50	422	32	391	0.76	8	372	178.7	8.3e-45	gene=Chr01G1003.1	MGYG_03812[Microsporumgypseum]
Chr02G1703.1	390	50	Clama_scaffold_5-4.0[Cladoniamacilenta]	21962	56	359	10136	10439	0.76	0	303	307.8	1.0e-83	gene=Chr02G1703.1	Clama_scaffold_5-4.0[Cladoniamacilenta]
Chr05G0227.1	479	38.8	CHG0652	860	48	404	1	343	0.7	9	356	266.5	3.2e-71	gene=C	CHG06520.1[Chaeto

			0.1[ChaetomiumglobosumCBS148.51]						6					hr05G0227.1	miumglobosumCBS148.51]
Chr01G1544.1	470	24.7	maker-scaffold_1-exonerate_est2genome-gene-0.0-mRNA-1[Cladoniamacilenta]	916	1	446	1	468	0.76	11	445	96.7	4.4e-20	gene=Chr01G1544.1	maker-scaffold_1-exonerate_est2genome-gene-0.0-mRNA-1[Cladoniamacilenta]
Chr01G1857.1	494	76.9	e_gw1.16.51.1[Cryphonectriaparasitica]	509	22	490	23	503	0.76	1	468	787.7	4.3e-228	gene=Chr01G1857.1	e_gw1.16.51.1[Cryphonectriaparasitica]
Chr04G0293.1	521	25.3	XP_007600712.1[ColletotrichumfioriniaePJ7]	959	54	476	22	448	0.76	12	422	87.4	2.9e-17	gene=Chr04G0293.1	XP_007600712.1[ColletotrichumfioriniaePJ7]
Chr04G1349.1	513	30	Clame_scaffold8-4.115[Cladoniametacorallifera]	16637	17	394	2898	3284	0.76	6	377	187.2	2.7e-47	gene=Chr04G1349.1	Clame_scaffold8-4.115[Cladoniametacorallifera]

Chr07G0122.1	496	78.6	XP_007589868.1[CollletotrichumfioriniaePJ7]	487	1	495	1	486	0.76	1	494	809.7	1.1e-234	gene=Chr07G0122.1	XP_007589868.1[CollletotrichumfioriniaePJ7]
Chr09G0001.1	377	53.7	SS1G_01499.1[Sclerotiniasclerotiorum]	1043	38	376	527	864	0.76	2	338	387.1	1.3e-107	gene=Chr09G0001.1	SS1G_01499.1[Sclerotiniasclerotiorum]
Chr01G0648.1	554	61.6	XP_007602109.1[CollletotrichumfioriniaePJ7]	443	5	457	15	422	0.76	4	452	558.1	6.2e-159	gene=Chr01G0648.1	XP_007602109.1[CollletotrichumfioriniaePJ7]
Chr01G0880.1	1430	30.1	Clame_scaffold11-8.74[Cladoniametacorallifera]	8336	906	1278	7379	7752	0.76	6	372	152.5	2.0e-36	gene=Chr01G0880.1	Clame_scaffold11-8.74[Cladoniametacorallifera]
Chr04G0649.1	506	28.9	Clame_scaffold18-19.70[Cladoniametacorallifera]	7866	85	501	3675	4107	0.77	13	416	171.8	1.1e-42	gene=Chr04G0649.1	Clame_scaffold18-19.70[Cladoniametacorallifera]
Chr09G1018.1	534	34.5	Clame_scaffold8-4.1	16637	64	408	2938	3278	0.77	1	344	210.7	2.4e-54	gene=Chr09G10	Clame_scaffold8-4.115[Cladoniametacor

			15[Cladoniametacorallifera]										18.1	allifera]
Chr01G1833.1	772	28.8	Clame_scaffold18-19.70[Cladoniametacorallifera]	7866	218	623	3126	3548	0.77	12	405	189.1	1.1e-47	gene=Chr01G1833.1	Clame_scaffold18-19.70[Cladoniametacorallifera]
Chr01G2746.1	538	23.6	XP_007600712.1[ColletotrichumfioriniaePJ7]	959	50	489	10	450	0.77	10	439	99.8	5.9e-21	gene=Chr01G2746.1	XP_007600712.1[ColletotrichumfioriniaePJ7]
Chr09G0471.1	485	32.5	maker-scaffold122-augustus-gene-0.90-mRNA-1[OphiocerasdolichostomumCBS114926]	1059	9	409	12	401	0.77	11	400	191.4	1.3e-48	gene=Chr09G0471.1	maker-scaffold122-augustus-gene-0.90-mRNA-1[OphiocerasdolichostomumCBS114926]
Chr05G0726.1	667	32.4	Clama_scaffold_5-4.0[Cladoniamacilenta]	21962	46	440	12827	13297	0.77	14	394	225.3	1.2e-58	gene=Chr05G0726.1	Clama_scaffold_5-4.0[Cladoniamacilenta]

Chr08G1064.1	333	60.5	EEA28139.1[Penicilliummarneffei]	2025	1	331	597	928	0.77	2	330	412.5	2.5e-115	gene=Chr08G1064.1	EEA28139.1[Penicilliummarneffei]
Chr03G1526.1	490	36.6	Clame_scaffold8-4.115[Cladoniametacorallifera]	16637	33	369	2943	3285	0.77	2	336	229.6	4.5e-60	gene=Chr03G1526.1	Clame_scaffold8-4.115[Cladoniametacorallifera]
Chr05G0183.1	517	81	CH063_07049T0[Colletotrichumhigginsianum]	512	1	511	1	509	0.77	2	510	838.2	2.9e-243	gene=Chr05G0183.1	CH063_07049T0[Colletotrichumhigginsianum]
Chr06G0222.1	511	79.4	estExt_Genewise1.C_380012[Trichodermavirens]	504	5	510	6	504	0.77	4	505	829.7	1.0e-240	gene=Chr06G0222.1	estExt_Genewise1.C_380012[Trichodermavirens]
Chr02G1584.1	333	60.2	EEA28139.1[Penicilliummarneffei]	2025	1	331	597	928	0.77	2	330	410.6	9.7e-115	gene=Chr02G1584.1	EEA28139.1[Penicilliummarneffei]
Chr02G1494.1	451	30.5	MGYG_03812[Microsporumgy	526	55	450	33	419	0.77	9	395	172.9	4.6e-43	gene=Chr02G1494.1	MGYG_03812[Microsporumgypseum]

			pseum]												
Chr02G1453.1	341	52.2	augustus_masked-scaffold9-processed-gene-0.95-mRNA-1[MagnaporthesalviniiM69]	2316	2	340	1454	1819	0.77	3	338	354.8	6.4e-98	gene=Chr02G1453.1	augustus_masked-scaffold9-processed-gene-0.95-mRNA-1[MagnaporthesalviniiM69]
Chr01G1848.1	424	36	M_BR29_EuGene_00068431[MagnaporthegriseaBR29]	989	44	421	15	399	0.77	11	377	190.3	2.6e-48	gene=Chr01G1848.1	M_BR29_EuGene_00068431[MagnaporthegriseaBR29]
Chr01G2561.1	1487	24.2	EfO2.075130.1[Epichloefestucae]	1182	392	874	725	1182	0.77	15	482	95.9	2.4e-19	gene=Chr01G2561.1	EfO2.075130.1[Epichloefestucae]
Chr06G0192.1	1011	63	EEA28139.1[Penicilliummarneffei]	2025	1	338	597	937	0.77	2	337	432.2	9.4e-121	gene=Chr06G0192.1	EEA28139.1[Penicilliummarneffei]
Chr02G0300.1	542	40.8	estExt_Genewise1Pl	525	14	358	15	359	0.78	6	344	260.8	2.0e-69	gene=Chr02G03	estExt_Genewise1Plus.C_120159[Sistotr

			us.C_120159[Sistotremastrumsuecicum]											00.1	emastrumsuecicum]
Chr02G0011.1	489	32.2	MGYG_03812[Microsporumgypseum]	526	40	422	8	374	0.78	7	382	181.8	1.1e-45	gene=Chr02G0011.1	MGYG_03812[Microsporumgypseum]
Chr04G0737.1	747	39.8	augustus_masked-scaffold418-processed-gene-0.20-mRNA-1[MagnaporthesalviniiM69]	1986	31	390	613	960	0.78	9	359	229.6	6.8e-60	gene=Chr04G0737.1	augustus_masked-scaffold418-processed-gene-0.20-mRNA-1[MagnaporthesalviniiM69]
Chr01G2515.1	543	34.2	ATEG_07313.1[Aspergillusterreus]	836	117	507	3	368	0.78	3	390	225.7	7.2e-59	gene=Chr01G2515.1	ATEG_07313.1[Aspergillusterreus]
Chr02G0132.1	426	30.4	estExt_Genemark1.C_190104[Auriculariasubglabr	1623	1	414	1212	1615	0.78	14	413	178.3	1.0e-44	gene=Chr02G0132.1	estExt_Genemark1.C_190104[Auriculariasubglabra]

			a]												
Chr06G1196.1	620	27.7	Clame_scaffold18-19.70[Cladoniametacorallifera]	7866	100	528	3119	3574	0.78	12	428	191	2.2e-48	gene=Chr06G1196.1	Clame_scaffold18-19.70[Cladoniametacorallifera]
Chr01G0202.1	467	29.4	maker-scaffold_1-exonerate_est2genome-gene-4.3-mRNA-1[Cladoniamacilenta]	629	33	466	214	629	0.78	11	433	198.4	1.1e-50	gene=Chr01G0202.1	maker-scaffold_1-exonerate_est2genome-gene-4.3-mRNA-1[Cladoniamacilenta]
Chr03G0087.1	456	30.2	MGYG_03812[Microsporumgypseum]	526	36	444	22	405	0.78	8	408	184.1	2.0e-46	gene=Chr03G0087.1	MGYG_03812[Microsporumgypseum]
Chr01G0157.1	562	33.6	Clame_scaffold8-4.115[Cladoniametacorallifera]	16637	179	542	10628	10992	0.78	6	363	172.9	5.7e-43	gene=Chr01G0157.1	Clame_scaffold8-4.115[Cladoniametacorallifera]
Chr04G0102.1	469	65	Clama_scaffold_5-4.0[Cladonia	21962	109	464	16692	17049	0.78	2	355	481.5	6.3e-136	gene=Chr04G0102.1	Clama_scaffold_5-4.0[Cladoniamacilenta]

			macilenta]												
Chr01G1773.1	633	25.5	XP_007600712.1[ColletotrichumfioriniaePJ7]	959	110	544	8	453	0.78	12	434	106.3	7.4e-23	gene=Chr01G1773.1	XP_007600712.1[ColletotrichumfioriniaePJ7]
Chr04G1585.1	600	28.5	gm1.11182_g[Sphaerobolusstellatus]	620	43	595	173	615	0.78	18	552	155.2	1.3e-37	gene=Chr04G1585.1	gm1.11182_g[Sphaerobolusstellatus]
Chr03G1558.1	524	27.7	EAA63242.1[AspergillusnidulansFGSCA4]	1004	75	505	66	483	0.78	10	430	157.5	2.3e-38	gene=Chr03G1558.1	EAA63242.1[AspergillusnidulansFGSCA4]
Chr01G0665.1	565	33.9	Clame_scaffold11-8.74[Cladoniametacorallifera]	8336	17	398	7935	8336	0.78	4	381	229.6	5.2e-60	gene=Chr01G0665.1	Clame_scaffold11-8.74[Cladoniametacorallifera]
Chr08G0835.1	408	34.2	estExt_Genemark1.C_190104[Auriculariasubglabra]	1623	16	398	1227	1610	0.78	12	382	215.3	7.3e-56	gene=Chr08G0835.1	estExt_Genemark1.C_190104[Auriculariasubglabra]
Chr09G0236.1	329	44	EAA58488	1365	1	327	1043	1364	0.7	3	326	278.5	5.7e-75	gene=C	EAA58488.1[Aspergi

			.1[AspergillusnidulansFGSCA4]						8					hr09G0236.1	llusnidulansFGSCA4]
Chr06G1114.1	1384	61.2	EEA28139.1[Penicilliummarneffei]	2025	1	340	597	937	0.78	2	339	422.5	1.0e-117	gene=Chr06G1114.1	EEA28139.1[Penicilliummarneffei]
Chr07G0941.1	974	39.2	fgenesh1_pg.21_#_32[TulasnellacalosporaAL13]	710	22	390	323	693	0.78	8	368	280	5.8e-75	gene=Chr07G0941.1	fgenesh1_pg.21_#_32[TulasnellacalosporaAL13]
Chr03G1699.1	577	24.9	XP_007600712.1[ColletotrichumfioriniaePJ7]	959	54	496	8	451	0.78	13	442	97.1	4.1e-20	gene=Chr03G1699.1	XP_007600712.1[ColletotrichumfioriniaePJ7]
Chr01G2162.1	565	82.7	CH063_08890T0[Colletotrichumhigginsianum]	565	1	564	1	565	0.79	2	563	990.7	3.8e-289	gene=Chr01G2162.1	CH063_08890T0[Colletotrichumhigginsianum]
Chr06G1469.1	382	41.2	e_gw1.3.1373.1[Mycosphaerell	486	30	375	18	352	0.79	5	345	276.9	1.9e-74	gene=Chr06G1469.1	e_gw1.3.1373.1[Mycosphaerellagraminicola]

			agraminicola]												
Chr02G0070.1	477	28.1	maker-scaffold_1-exonerate_est2genome-gene-4.3-mRNA-1[Cladoniamacilenta]	629	37	476	214	629	0.79	13	439	170.2	3.1e-42	gene=Chr02G0070.1	maker-scaffold_1-exonerate_est2genome-gene-4.3-mRNA-1[Cladoniamacilenta]
Chr03G0368.1	500	35.1	Clame_scaffold8-4.115[Cladoniametacorallifera]	16637	20	385	2929	3299	0.79	5	365	233.4	3.2e-61	gene=Chr03G0368.1	Clame_scaffold8-4.115[Cladoniametacorallifera]
Chr04G0138.1	468	47.3	AFL2G_07147[Aspergillusflavus]	1440	53	377	38	371	0.79	2	324	312.4	5.0e-85	gene=Chr04G0138.1	AFL2G_07147[Aspergillusflavus]
Chr02G1365.1	694	44.1	Clame_scaffold8-4.115[Cladoniametacorallifera]	16637	81	682	3943	4601	0.79	11	601	520.4	1.8e-147	gene=Chr02G1365.1	Clame_scaffold8-4.115[Cladoniametacorallifera]
Chr01G1230.1	543	34.1	MUStwsD_GLEAN_	1200	55	453	701	1103	0.79	11	398	212.2	8.2e-55	gene=Chr01G12	MUStwsD_GLEAN_10001533[Omphalot

			10001533[Omphalotusolearius]											30.1	usolearius]
Chr02G0489.1	353	54.2	SS1G_01499.1[Sclerotiniasclerotiorum]	1043	33	351	526	844	0.79	0	318	372.9	2.4e-103	gene=Chr02G0489.1	SS1G_01499.1[Sclerotiniasclerotiorum]
Chr02G1138.1	541	80	GLRG_10319T0[ColletotrichumgraminicolaM1.001]	553	19	540	23	551	0.79	2	521	842	2.1e-244	gene=Chr02G1138.1	GLRG_10319T0[ColletotrichumgraminicolaM1.001]
Chr01G0091.1	675	25.8	EAL85053.1[AspergillusfumigatusAf293]	720	3	484	44	561	0.79	20	481	142.9	7.6e-34	gene=Chr01G0091.1	EAL85053.1[AspergillusfumigatusAf293]
Chr07G0441.1	1007	58.7	estExt_Genewise1.C_4080032[Exidiaglandulosa]	354	24	355	25	354	0.79	2	331	446	6.3e-125	gene=Chr07G0441.1	estExt_Genewise1.C_4080032[Exidiaglandulosa]
Chr03G0247.1	571	31.7	ATEG_07313.1[Aspergillusterr	836	189	558	1	378	0.79	3	369	182.2	9.6e-46	gene=Chr03G0247.1	ATEG_07313.1[Aspergillusterreus]

			eus]											
Chr07G0492.1	491	36.7	Clame_scaffold8-4.115[Cladoniametacorallifera]	16637	27	375	2935	3285	0.79	4	348	197.6	1.9e-50	gene=Chr07G0492.1	Clame_scaffold8-4.115[Cladoniametacorallifera]
Chr06G0102.1	481	33.2	Pa_7_5740[PodosporaanserinaSmat+]	576	99	476	154	527	0.79	5	377	181	1.8e-45	gene=Chr06G0102.1	Pa_7_5740[PodosporaanserinaSmat+]
Chr04G0156.1	419	34.1	Clame_scaffold8-4.115[Cladoniametacorallifera]	16637	1	410	6883	7354	0.79	16	409	213.4	2.8e-55	gene=Chr04G0156.1	Clame_scaffold8-4.115[Cladoniametacorallifera]
Chr01G1856.1	517	74.3	XP_007594880.1[CollletotrichumfioriniaePJ7]	548	10	515	11	547	0.80	2	505	829.7	1.0e-240	gene=Chr01G1856.1	XP_007594880.1[CollletotrichumfioriniaePJ7]
Chr07G0313.1	511	31.3	KKY27696.1putativecytochromep450alkane[Phaeomoniellach	965	84	466	27	413	0.80	5	382	198.4	1.2e-50	gene=Chr07G0313.1	KKY27696.1putativecytochromep450alkane[Phaeomoniellachlamydospora][Phaeomoniellachlamydospora]

			lamydospora][Phaeomoniellachlamydospora]												
Chr01G0810.1	413	38.8	estExt_Genemark1.C_190104[Auriculariasubglabra]	1623	7	377	1230	1610	0.80	8	370	277.7	1.2e-74	gene=Chr01G0810.1	estExt_Genemark1.C_190104[Auriculariasubglabra]
Chr01G0326.1	470	46.8	AFL2G_07147[Aspergillusflavus]	1440	59	385	42	373	0.80	2	326	318.9	5.4e-87	gene=Chr01G0326.1	AFL2G_07147[Aspergillusflavus]
Chr01G2402.1	1181	31.1	Hanno_03304[Heterobasidionannosum03012]	1417	88	511	704	1133	0.80	12	423	174.5	4.1e-43	gene=Chr01G2402.1	Hanno_03304[Heterobasidionannosum03012]
Chr06G1394.1	500	40.7	Clame_scaffold8-4.115[Cladoniametacorallifera]	16637	43	385	2942	3285	0.80	3	342	251.9	8.6e-67	gene=Chr06G1394.1	Clame_scaffold8-4.115[Cladoniametacorallifera]
Chr05G0291.1	549	36.1	ATEG_07	836	171	518	7	358	0.8	2	347	228.8	8.6e-60	gene=C	ATEG_07313.1[Asp

			313.1[Aspergillusterreus]						0					hr05G0291.1	ergillusterreus]
Chr08G0705.1	520	76.8	GLRG_11514T0[ColletotrichumgraminicolaM1.001]	516	1	507	12	508	0.80	6	506	811.2	3.8e-235	gene=Chr08G0705.1	GLRG_11514T0[ColletotrichumgraminicolaM1.001]
Chr03G0334.1	479	27.4	EAA63242.1[AspergillusnidulansFGSCA4]	1004	4	447	25	468	0.80	8	443	167.9	1.6e-41	gene=Chr03G0334.1	EAA63242.1[AspergillusnidulansFGSCA4]
Chr04G0230.1	651	23.9	M_BR29_EuGene_00112621[MagnaporthegriseaBR29]	952	93	641	425	945	0.80	18	548	129.8	6.4e-30	gene=Chr04G0230.1	M_BR29_EuGene_00112621[MagnaporthegriseaBR29]
Chr08G0138.1	853	25.1	estExt_Genewise1Plus.C_100483[PleurotusostreatusPC15]	623	41	528	20	498	0.80	21	487	110.2	6.9e-24	gene=Chr08G0138.1	estExt_Genewise1Plus.C_100483[PleurotusostreatusPC15]
Chr03G1102.1	1446	26	PTRG_10	1311	606	1065	332	868	0.8	13	459	177.2	7.8e-44	gene=C	PTRG_10301[Pyren

			301[Pyrenophoratritici-repentis]						0					hr03G1102.1	ophoratritici-repentis]
Chr06G0100.1	344	52.7	EAA60936.1[AspergillusnidulansFGSCA4]	831	3	330	5	332	0.80	4	327	360.1	1.5e-99	gene=Chr06G0100.1	EAA60936.1[AspergillusnidulansFGSCA4]
Chr01G1450.1	517	59.8	XP_007591776.1[ColletotrichumfioriniaePJ7]	452	1	514	1	448	0.80	3	513	607.8	6.4e-174	gene=Chr01G1450.1	XP_007591776.1[ColletotrichumfioriniaePJ7]
Chr04G1175.1	1218	58.5	EEA28139.1[Penicilliummarneffei]	2025	1	343	597	943	0.80	3	342	410.2	4.6e-114	gene=Chr04G1175.1	EEA28139.1[Penicilliummarneffei]
Chr01G2177.1	1310	34.4	e_gw1.9.856.1[Hebelomacylindrosporumh7]	1092	141	582	666	1066	0.80	12	441	241.9	2.3e-63	gene=Chr01G2177.1	e_gw1.9.856.1[Hebelomacylindrosporumh7]
Chr02G1440.1	952	64.2	CHG08480.1[ChaetomiumglobosumCB	372	19	367	22	370	0.80	0	348	487.6	1.8e-137	gene=Chr02G1440.1	CHG08480.1[ChaetomiumglobosumCBS148.51]

			S148.51]												
Chr03G1266.1	462	29.3	EEA27467.1[Penicilliummarneffei]	520	22	452	17	451	0.80	11	430	188	1.4e-47	gene=Chr03G1266.1	EEA27467.1[Penicilliummarneffei]
Chr01G1765.1	613	25.1	Pa_7_5740[PodosporaanserinaSmat+]	576	58	607	53	524	0.80	10	549	158.7	1.2e-38	gene=Chr01G1765.1	Pa_7_5740[PodosporaanserinaSmat+]
Chr01G0633.1	661	28.6	maker-scaffold4.1-augustus-gene-8.80-mRNA-1[Raffaeleaquercus-mongolicae]	1671	176	595	1117	1553	0.80	11	419	171.4	2.0e-42	gene=Chr01G0633.1	maker-scaffold4.1-augustus-gene-8.80-mRNA-1[Raffaeleaquercus-mongolicae]
Chr01G2109.1	1554	34.5	Clame_scaffold11-8.74[Cladoniametacorallifera]	8336	1009	1366	7387	7748	0.80	2	357	210.7	6.8e-54	gene=Chr01G2109.1	Clame_scaffold11-8.74[Cladoniametacorallifera]
Chr05G0566.1	976	40.4	Hanno_03304[Heterobasidionannosum0	1417	222	625	709	1097	0.80	12	403	256.5	6.8e-68	gene=Chr05G0566.1	Hanno_03304[Heterobasidionannosum03012]

			3012]												
Chr07G0138.1	464	41.7	Clama_scaffold_5-4.0[Cladoniamacilenta]	21962	82	433	10075	10435	0.80	4	351	292	7.0e-79	gene=Chr07G0138.1	Clama_scaffold_5-4.0[Cladoniamacilenta]
Chr01G2698.1	491	29.7	Clame_scaffold8-4.115[Cladoniametacorallifera]	16637	16	411	2904	3306	0.80	6	395	171.4	1.5e-42	gene=Chr01G2698.1	Clame_scaffold8-4.115[Cladoniametacorallifera]
Chr01G1771.1	492	39.7	Clame_scaffold8-4.115[Cladoniametacorallifera]	16637	32	374	2938	3286	0.81	2	342	256.1	4.5e-68	gene=Chr01G1771.1	Clame_scaffold8-4.115[Cladoniametacorallifera]
Chr09G0357.1	1087	28.5	4127_t[AscocorynesarcoidesNRRL50072]	1621	449	879	1028	1502	0.81	17	430	135.6	2.0e-31	gene=Chr09G0357.1	4127_t[AscocorynesarcoidesNRRL50072]
Chr06G0970.1	1467	33.5	Clame_scaffold11-8.74[Cladoniametacorallifera]	8336	910	1274	7383	7748	0.81	2	364	197.2	7.4e-50	gene=Chr06G0970.1	Clame_scaffold11-8.74[Cladoniametacorallifera]
Chr04G1055.1	544	36.7	ATEG_07	836	161	514	1	358	0.8	4	353	242.3	7.4e-64	gene=C	ATEG_07313.1[Asp

			313.1[Aspergillusterreus]						1					hr04G1055.1	ergillusterreus]
Chr05G0033.1	355	43.2	fgenesh1_pg.C_scaffold_1000303[Cochliobolusheterostrophus]	729	14	344	396	728	0.81	1	330	297.4	1.3e-80	gene=Chr05G0033.1	fgenesh1_pg.C_scaffold_1000303[Cochliobolusheterostrophus]
Chr04G0610.1	459	44.4	CHG06520.1[ChaetomiumglobosumCBS148.51]	860	47	392	1	343	0.81	7	345	305.4	6.0e-83	gene=Chr04G0610.1	CHG06520.1[ChaetomiumglobosumCBS148.51]
Chr06G0038.1	1314	32.6	RO3G_11402[Rhizopusoryzae]	1687	302	680	784	1165	0.81	7	378	197.2	6.6e-50	gene=Chr06G0038.1	RO3G_11402[Rhizopusoryzae]
Chr03G1665.1	401	70.4	XP_007595499.1[CollletotrichumfioriniaePJ7]	498	1	388	1	388	0.81	0	387	598.6	3.0e-171	gene=Chr03G1665.1	XP_007595499.1[CollletotrichumfioriniaePJ7]
Chr02G0662.1	1935	32.9	EAA60443.1[Aspergillusnidulan	2180	229	855	223	842	0.81	11	626	321.2	4.5e-87	gene=Chr02G0662.1	EAA60443.1[AspergillusnidulansFGSCA4]

			sFGSCA4]												
Chr04G0780.1	557	81	XP_007602552.1[ColletotrichumfioriniaePJ7]	542	25	555	1	532	0.81	1	530	910.6	5.0e-265	gene=Chr04G0780.1	XP_007602552.1[ColletotrichumfioriniaePJ7]
Chr05G0029.1	508	35.7	Clame_scaffold8-4.115[Cladoniametacorallifera]	16637	30	394	2907	3285	0.81	5	364	213.8	2.6e-55	gene=Chr05G0029.1	Clame_scaffold8-4.115[Cladoniametacorallifera]
Chr04G0664.1	612	70.5	Umbilicaria_05246[Umbilicariamuehlenbergii]	1265	156	610	72	507	0.81	3	454	618.6	4.3e-177	gene=Chr04G0664.1	Umbilicaria_05246[Umbilicariamuehlenbergii]
Chr04G0265.1	621	28.2	Clame_scaffold11-8.74[Cladoniametacorallifera]	8336	139	588	6757	7281	0.81	14	449	206.1	6.7e-53	gene=Chr04G0265.1	Clame_scaffold11-8.74[Cladoniametacorallifera]
Chr05G0523.1	1318	30.2	EfO2.075130.1[Epichloefestucae]	1182	905	1315	725	1159	0.81	12	410	190.7	6.2e-48	gene=Chr05G0523.1	EfO2.075130.1[Epichloefestucae]
Chr07G0062.1	1060	24.7	e_gw1.3.6	1054	558	1038	510	1024	0.8	18	480	104.4	4.7e-22	gene=C	e_gw1.3.610.1[Plicat

			10.1[Plicaturopsiscrispa]						1					hr07G0062.1	uropsiscrispa]
Chr01G1341.1	702	34.3	Clame_scaffold11-8.74[Cladoniametacorallifera]	8336	165	573	6757	7199	0.81	11	408	248.8	1.0e-65	gene=Chr01G1341.1	Clame_scaffold11-8.74[Cladoniametacorallifera]
Chr07G1101.1	495	35.1	Clame_scaffold8-4.115[Cladoniametacorallifera]	16637	12	383	2898	3288	0.81	6	371	220.3	2.8e-57	gene=Chr07G1101.1	Clame_scaffold8-4.115[Cladoniametacorallifera]
Chr03G1357.1	526	33.7	Clame_scaffold8-4.115[Cladoniametacorallifera]	16637	60	427	2943	3308	0.82	2	367	214.2	2.1e-55	gene=Chr03G1357.1	Clame_scaffold8-4.115[Cladoniametacorallifera]
Chr01G0137.1	509	38.3	MGYG_03812[Microsporumgypseum]	526	110	482	32	391	0.82	8	372	206.8	3.2e-53	gene=Chr01G0137.1	MGYG_03812[Microsporumgypseum]
Chr04G0806.1	512	77.7	XP_007596781.1[Colletotrichumfioriniae	970	27	511	489	970	0.82	2	484	784.6	3.8e-227	gene=Chr04G0806.1	XP_007596781.1[ColletotrichumfioriniaePJ7]

			PJ7]												
Chr01G0509.1	558	34.8	ATEG_07313.1[Aspergillusterreus]	836	174	541	1	374	0.82	6	367	206.1	6.1e-53	gene=Chr01G0509.1	ATEG_07313.1[Aspergillusterreus]
Chr02G0468.1	400	39.9	M_BR29_EuGene_00068431[MagnaporthegriseaBR29]	989	32	399	19	402	0.82	11	367	235.3	6.7e-62	gene=Chr02G0468.1	M_BR29_EuGene_00068431[MagnaporthegriseaBR29]
Chr02G1339.1	577	33	XP_007598108.1[ColletotrichumfioriniaePJ7]	877	29	445	25	444	0.82	17	416	198.4	1.3e-50	gene=Chr02G1339.1	XP_007598108.1[ColletotrichumfioriniaePJ7]
Chr07G0878.1	702	26.5	Clame_scaffold11-8.74[Cladoniametacorallifera]	8336	22	517	1949	2419	0.82	19	495	119.8	7.2e-27	gene=Chr07G0878.1	Clame_scaffold11-8.74[Cladoniametacorallifera]
Chr02G1321.1	471	31.4	Pa_7_5740[PodosporaanserinaSmat+]	576	56	467	78	524	0.82	10	411	196.1	5.3e-50	gene=Chr02G1321.1	Pa_7_5740[PodosporaanserinaSmat+]
Chr09G0656.1	662	24.6	maker-Cal	1049	10	621	13	571	0.8	28	611	100.1	5.6e-21	gene=C	maker-Caloplaca_fla

			oplaca_flavorubescens_scaffold_19-augustus-gene-2.67.2-mRNA-1[Caloplacaflavorubescens]						2					hr09G0656.1	vorubescens_scaffold_19-augustus-gene-2.67.2-mRNA-1[Caloplacaflavorubescens]
Chr02G1746.1	817	33	e_gw1.9.856.1[Hebelomacylindrosporumh7]	1092	56	487	666	1078	0.82	13	431	223.4	5.4e-58	gene=Chr02G1746.1	e_gw1.9.856.1[Hebelomacylindrosporumh7]
Chr01G1591.1	520	39.9	Clame_scaffold8-4.115[Cladoniametacorallifera]	16637	54	404	2942	3287	0.82	2	350	256.1	4.8e-68	gene=Chr01G1591.1	Clame_scaffold8-4.115[Cladoniametacorallifera]
Chr07G0204.1	575	27.6	AFL2G_07229[Aspergillusflavus]	640	73	559	191	626	0.82	8	486	172.9	5.9e-43	gene=Chr07G0204.1	AFL2G_07229[Aspergillusflavus]
Chr06G1397.1	557	76.9	GLRG_11698T0[Col	477	1	469	1	471	0.82	1	468	751.1	5.0e-217	gene=Chr06G13	GLRG_11698T0[Colletotrichumgraminicol

			lletotrichumgraminicolaM1.001]											97.1	aM1.001]
Chr01G2291.1	568	27.2	XP_007600712.1[CollletotrichumfioriniaePJ7]	959	28	468	21	452	0.82	6	440	159.5	6.6e-39	gene=Chr01G2291.1	XP_007600712.1[CollletotrichumfioriniaePJ7]
Chr01G0270.1	437	28.4	EEQ28888.1[Microsporumcanis]	609	4	427	157	588	0.83	6	423	187.2	2.3e-47	gene=Chr01G0270.1	EEQ28888.1[Microsporumcanis]
Chr05G0173.1	624	32.3	AFL2G_07229[Aspergillusflavus]	640	88	610	197	630	0.83	13	522	222.6	7.0e-58	gene=Chr05G0173.1	AFL2G_07229[Aspergillusflavus]
Chr02G1200.1	478	33.9	gm1.898_g[TulasnellacalosporaAL13]	1440	17	427	666	1071	0.83	8	410	224.6	1.4e-58	gene=Chr02G1200.1	gm1.898_g[TulasnellacalosporaAL13]
Chr01G1957.1	422	33.5	M_BR29_EuGene_00068431[MagnaporthegriseaB	989	28	419	12	428	0.83	13	391	159.5	4.9e-39	gene=Chr01G1957.1	M_BR29_EuGene_00068431[MagnaporthegriseaBR29]

			R29]												
Chr02G0040.1	561	34.2	ATEG_07313.1[Aspergillusterreus]	836	166	535	1	374	0.83	1	369	213.4	3.8e-55	gene=Chr02G0040.1	ATEG_07313.1[Aspergillusterreus]
Chr08G1030.1	1001	58.7	EEA28139.1[Penicilliummarneffei]	2025	1	360	597	953	0.83	3	359	418.7	1.1e-116	gene=Chr08G1030.1	EEA28139.1[Penicilliummarneffei]
Chr01G0720.1	592	27.5	MUStwsD_GLEAN_10001562[Omphalotusolearius]	2038	109	589	317	827	0.83	17	480	168.7	1.1e-41	gene=Chr01G0720.1	MUStwsD_GLEAN_10001562[Omphalotusolearius]
Chr01G1081.1	492	34.5	Clame_scaffold8-4.115[Cladoniametacorallifera]	16637	13	383	2912	3287	0.83	3	370	207.6	1.8e-53	gene=Chr01G1081.1	Clame_scaffold8-4.115[Cladoniametacorallifera]
Chr01G0493.1	1241	59.6	EEA28139.1[Penicilliummarneffei]	2025	1	356	597	952	0.83	3	355	427.6	2.8e-119	gene=Chr01G0493.1	EEA28139.1[Penicilliummarneffei]
Chr09G0443.1	2191	24.3	PTRG_10301[Pyren	1311	800	1290	215	779	0.83	16	490	143.7	1.4e-33	gene=Chr09G04	PTRG_10301[Pyrenophoratritici-repentis]

			ophoratritici-repentis]											43.1	
Chr01G2633.1	462	33.3	MGYG_03812[Microsporumgypseum]	526	40	441	10	397	0.83	8	401	213.8	2.4e-55	gene=Chr01G2633.1	MGYG_03812[Microsporumgypseum]
Chr04G0904.1	818	28.4	1093_t[AscocorynesarcoidesNRRL50072]	1211	3	475	2	449	0.83	20	472	129.8	8.1e-30	gene=Chr04G0904.1	1093_t[AscocorynesarcoidesNRRL50072]
Chr07G1141.1	502	37.2	Clame_scaffold8-4.115[Cladoniametacorallifera]	16637	33	398	2934	3299	0.83	5	365	233.4	3.2e-61	gene=Chr07G1141.1	Clame_scaffold8-4.115[Cladoniametacorallifera]
Chr02G1054.1	634	36.2	Pa_7_5740[PodosporaanserinaSmat+]	576	109	581	59	524	0.83	7	472	293.1	4.3e-79	gene=Chr02G1054.1	Pa_7_5740[PodosporaanserinaSmat+]
Chr02G0004.1	516	53.8	M_BR29_EuGene_00026651[MagnaporthegriseaB	528	6	513	4	524	0.83	4	507	580.1	1.4e-165	gene=Chr02G0004.1	M_BR29_EuGene_00026651[MagnaporthegriseaBR29]

			R29]												
Chr01G2689.1	497	78.4	XP_007591393.1[ColletotrichumfioriniaePJ7]	495	1	496	1	495	0.83	1	495	825.5	1.9e-239	gene=Chr01G2689.1	XP_007591393.1[ColletotrichumfioriniaePJ7]
Chr04G1519.1	558	27.8	XP_007600712.1[ColletotrichumfioriniaePJ7]	959	32	488	13	443	0.83	11	456	139	9.1e-33	gene=Chr04G1519.1	XP_007600712.1[ColletotrichumfioriniaePJ7]
Chr01G2571.1	504	36.3	Clame_scaffold8-4.115[Cladoniametacorallifera]	16637	37	405	2935	3308	0.83	4	368	212.6	5.8e-55	gene=Chr01G2571.1	Clame_scaffold8-4.115[Cladoniametacorallifera]
Chr01G1933.1	507	38.5	Clame_scaffold8-4.115[Cladoniametacorallifera]	16637	47	424	2942	3311	0.83	4	377	246.1	4.8e-65	gene=Chr01G1933.1	Clame_scaffold8-4.115[Cladoniametacorallifera]
Chr06G0107.1	881	35.7	e_gw1.9.856.1[Hebelomacylindrosporumh7]	1092	137	549	666	1067	0.83	11	412	253.4	5.2e-67	gene=Chr06G0107.1	e_gw1.9.856.1[Hebelomacylindrosporumh7]

Chr06G1179.1	591	24.6	M_BR29_EuGene_00112621[MagnaporthegriseaBR29]	952	40	583	428	947	0.83	18	543	140.2	4.3e-33	gene=Chr06G1179.1	M_BR29_EuGene_00112621[MagnaporthegriseaBR29]
Chr09G0283.1	1005	27.8	Clame_scaffold8-4.115[Cladoniametacorallifera]	16637	326	838	4092	4586	0.83	13	512	179.9	8.4e-45	gene=Chr09G0283.1	Clame_scaffold8-4.115[Cladoniametacorallifera]
Chr07G0116.1	544	29.1	AFL2G_07229[Aspergillusflavus]	640	69	520	191	610	0.84	2	451	232.3	7.7e-61	gene=Chr07G0116.1	AFL2G_07229[Aspergillusflavus]
Chr03G0305.1	610	36.8	Clama_scaffold_5-4.0[Cladoniamacilenta]	21962	180	560	19848	20232	0.84	13	380	196.4	5.2e-50	gene=Chr03G0305.1	Clama_scaffold_5-4.0[Cladoniamacilenta]
Chr01G0266.1	919	32.8	e_gw1.9.856.1[Hebelomacylindrosporumh7]	1092	88	540	654	1078	0.84	14	452	229.2	1.1e-59	gene=Chr01G0266.1	e_gw1.9.856.1[Hebelomacylindrosporumh7]
Chr04G0139.1	555	38.2	Clame_scaffold8-4.1	16637	34	438	2914	3295	0.84	7	404	253.1	4.3e-67	gene=Chr04G01	Clame_scaffold8-4.115[Cladoniametacor

			15[Cladoniametacorallifera]											39.1	allifera]
Chr08G0158.1	536	33.6	M_BR29_EuGene_00068431[MagnaporthegriseaBR29]	989	36	485	13	431	0.84	14	449	198.7	9.3e-51	gene=Chr08G0158.1	M_BR29_EuGene_00068431[MagnaporthegriseaBR29]
Chr08G0916.1	536	80.5	XP_007601544.1[ColletotrichumfioriniaePJ7]	537	1	533	1	533	0.84	0	532	889.8	8.7e-259	gene=Chr08G0916.1	XP_007601544.1[ColletotrichumfioriniaePJ7]
Chr07G0612.1	370	53.7	2325_t[AscocorynesarcoidesNRRL50072]	852	7	354	3	351	0.84	3	347	369.4	2.7e-102	gene=Chr07G0612.1	2325_t[AscocorynesarcoidesNRRL50072]
Chr01G2543.1	377	47.6	2325_t[AscocorynesarcoidesNRRL50072]	852	9	361	4	353	0.84	4	352	332	4.9e-91	gene=Chr01G2543.1	2325_t[AscocorynesarcoidesNRRL50072]
Chr02G0067.1	671	23.8	fgenesh1_pg.89_&#	737	79	611	41	562	0.84	16	532	155.2	1.5e-37	gene=Chr02G00	fgenesh1_pg.89_#_4[Hydnomeruliu

			35;_4[Hydnomeruliu spinastri]											67.1	spinastri]
Chr01G0526.1	516	28.9	Clame_scaffold8-4.115[Cladoniametacorallifera]	16637	1	416	2886	3307	0.84	6	415	179.9	4.3e-45	gene=Chr01G0526.1	Clame_scaffold8-4.115[Cladoniametacorallifera]
Chr04G0581.1	2233	53.4	Suibr1.gm1.7563_g[Suillusbrevipes]	866	503	911	133	515	0.84	9	408	396.4	1.3e-109	gene=Chr04G0581.1	Suibr1.gm1.7563_g[Suillusbrevipes]
Chr05G0877.1	1514	57.1	EEA28139.1[Penicilliummarneffei]	2025	4	348	597	943	0.84	1	344	403.3	7.0e-112	gene=Chr05G0877.1	EEA28139.1[Penicilliummarneffei]
Chr03G0775.1	661	54.9	Clame_scaffold9-5.100[Cladoniametacorallifera]	12517	129	494	1	354	0.84	2	365	409.1	5.6e-114	gene=Chr03G0775.1	Clame_scaffold9-5.100[Cladoniametacorallifera]
Chr05G0174.1	507	71.2	M_BR29_EuGene_00043971[MagnaporthegriseaB	429	71	504	1	427	0.84	2	433	641	6.7e-184	gene=Chr05G0174.1	M_BR29_EuGene_00043971[MagnaporthegriseaBR29]

			R29]												
Chr02G0188.1	1020	56.8	EEA28139.1[Penicilliummarneffei]	2025	1	345	597	943	0.84	1	344	401	2.3e-111	gene=Chr02G0188.1	EEA28139.1[Penicilliummarneffei]
Chr01G0355.1	590	24.6	FOXG_20661T0[Fusariumoxysporumf.sp.lycopersici]	1035	54	563	23	559	0.84	20	509	143.3	5.1e-34	gene=Chr01G0355.1	FOXG_20661T0[Fusariumoxysporumf.sp.lycopersici]
Chr04G1305.1	499	73.9	CH063_01141T0[Colletotrichumhigginsianum]	484	20	497	1	483	0.84	3	477	755.4	2.4e-218	gene=Chr04G1305.1	CH063_01141T0[Colletotrichumhigginsianum]
Chr09G0645.1	870	40.9	fgenesh1_pg.C_scaffold_9000455[Phanerochaetechrysosporium]	685	3	375	314	683	0.84	9	372	257.3	3.6e-68	gene=Chr09G0645.1	fgenesh1_pg.C_scaffold_9000455[Phanerochaetechrysosporium]
Chr01G2707.1	485	43	Clame_scaffold8-4.115[Cladoni	16637	27	375	2940	3285	0.84	2	348	255.4	7.6e-68	gene=Chr01G2707.1	Clame_scaffold8-4.115[Cladoniametacorallifera]

			ametacorallifera]												
Chr07G0378.1	498	30	MUStwsD_GLEAN_10001533[Omphalotusolearius]	1200	23	457	575	1036	0.84	9	434	179.1	7.1e-45	gene=Chr07G0378.1	MUStwsD_GLEAN_10001533[Omphalotusolearius]
Chr01G2049.1	562	29.8	AB00197.1[Alternariabrassicicola]	1041	33	546	43	490	0.84	8	513	226.1	5.7e-59	gene=Chr01G2049.1	AB00197.1[Alternariabrassicicola]
Chr03G0709.1	546	37	estExt_Genemark1.C_130001[Punctulariastrigosozonata]	634	145	542	221	631	0.84	11	397	263.5	3.1e-70	gene=Chr03G0709.1	estExt_Genemark1.C_130001[Punctulariastrigosozonata]
Chr04G0803.1	2340	38.6	fgenesh1_pg.24_#_34[PhlebiabrevisporaHHB-7030SS6]	2158	24	397	412	784	0.85	9	373	237.3	1.0e-61	gene=Chr04G0803.1	fgenesh1_pg.24_#_34[PhlebiabrevisporaHHB-7030SS6]
Chr06G0581.1	492	33.7	PCON_03529m.01[	459	37	484	49	458	0.85	13	447	248.4	9.4e-66	gene=Chr06G05	PCON_03529m.01[Pyronemaconfluens

			Pyronema confluens CBS100304]										81.1	CBS100304]
Chr04G0394.1	417	56.1	M_BR29_EuGene_00068431[MagnaporthegriseaBR29]	989	1	415	1	401	0.85	12	414	402.1	4.3e-112	gene=Chr04G0394.1	M_BR29_EuGene_00068431[MagnaporthegriseaBR29]
Chr03G0992.1	1544	42.2	Clame_scaffold11-8.74[Cladoniametacorallifera]	8336	1023	1384	7391	7748	0.85	6	361	258.1	3.7e-68	gene=Chr03G0992.1	Clame_scaffold11-8.74[Cladoniametacorallifera]
Chr01G2501.1	434	47.4	AFL2G_07147[Aspergillusflavus]	1440	19	425	402	792	0.85	5	406	386.7	1.9e-107	gene=Chr01G2501.1	AFL2G_07147[Aspergillusflavus]
Chr06G0775.1	660	25.9	M_BR29_EuGene_00112621[MagnaporthegriseaBR29]	952	104	639	426	942	0.85	19	535	164.9	1.8e-40	gene=Chr06G0775.1	M_BR29_EuGene_00112621[MagnaporthegriseaBR29]
Chr02G1308.1	504	65.6	XP_00759	527	7	503	4	523	0.8	2	496	704.9	3.8e-20	gene=C	XP_007597139.1[Co

			7139.1[Colletotrichumfioriniae PJ7]						5				3	hr02G1308.1	lletotrichumfioriniaePJ7]
Chr05G0929.1	476	34	maker-scaffold_1-exonerate_est2genome-gene-4.3-mRNA-1[Cladoniamacilenta]	629	33	473	214	629	0.85	11	440	223.8	2.4e-58	gene=Chr05G0929.1	maker-scaffold_1-exonerate_est2genome-gene-4.3-mRNA-1[Cladoniamacilenta]
Chr02G0592.1	548	52.8	SNOG_00663[Stagonosporanodorum]	823	75	547	410	823	0.85	7	472	480.7	1.3e-135	gene=Chr02G0592.1	SNOG_00663[Stagonosporanodorum]
Chr08G0026.1	664	23.9	AFL2G_01551[Aspergillusflavus]	1045	84	590	482	963	0.85	8	506	150.2	4.7e-36	gene=Chr08G0026.1	AFL2G_01551[Aspergillusflavus]
Chr06G1138.1	729	27.9	AFL2G_01551[Aspergillusflavus]	1045	136	599	509	949	0.85	9	463	169.1	1.1e-41	gene=Chr06G1138.1	AFL2G_01551[Aspergillusflavus]
Chr08G0092.1	590	64	CHG06520.1[Chaet	860	52	443	1	392	0.85	5	391	544.3	9.9e-155	gene=Chr08G00	CHG06520.1[ChaetomiumglobosumCBS

			omiumglobosumCBS148.51]											92.1	148.51]
Chr02G1811.1	482	31.2	Clame_scaffold8-4.115[Cladoniametacorallifera]	16637	24	436	2913	3344	0.85	6	412	191.4	1.3e-48	gene=Chr02G1811.1	Clame_scaffold8-4.115[Cladoniametacorallifera]
Chr07G0125.1	519	72.9	XP_007589871.1[ColletotrichumfioriniaePJ7]	449	73	517	4	446	0.85	2	444	699.1	2.1e-201	gene=Chr07G0125.1	XP_007589871.1[ColletotrichumfioriniaePJ7]
Chr09G0022.1	397	49.9	CPSG_08542[CoccidioidesposadasiiSilveira]	844	1	384	1	388	0.85	5	383	374.8	7.0e-104	gene=Chr09G0022.1	CPSG_08542[CoccidioidesposadasiiSilveira]
Chr01G0956.1	576	26.7	M_BR29_EuGene_00112621[MagnaporthegriseaBR29]	952	43	573	426	950	0.85	18	530	196.1	6.5e-50	gene=Chr01G0956.1	M_BR29_EuGene_00112621[MagnaporthegriseaBR29]
Chr04G0631.1	742	28.5	Clama_scaffold_5-4.	21962	59	677	9566	10044	0.86	16	618	203	6.8e-52	gene=Chr04G06	Clama_scaffold_5-4.0[Cladoniamacilenta]

			0[Cladoniamacilenta]											31.1	
Chr05G0162.1	510	29.1	XP_007600712.1[ColletotrichumfioriniaePJ7]	959	20	442	19	443	0.86	6	422	164.5	1.8e-40	gene=Chr05G0162.1	XP_007600712.1[ColletotrichumfioriniaePJ7]
Chr03G0512.1	644	29.3	Clama_scaffold_5-4.0[Cladoniamacilenta]	21962	65	505	5944	6389	0.86	15	440	154.1	3.2e-37	gene=Chr03G0512.1	Clama_scaffold_5-4.0[Cladoniamacilenta]
Chr05G0093.1	465	33.3	maker-scaffold_1-exonerate_est2genome-gene-4.3-mRNA-1[Cladoniamacilenta]	629	43	464	214	625	0.86	10	421	216.5	3.7e-56	gene=Chr05G0093.1	maker-scaffold_1-exonerate_est2genome-gene-4.3-mRNA-1[Cladoniamacilenta]
Chr09G0291.1	1348	33.7	RO3G_02234[Rhizopusoryzae]	1415	358	752	512	909	0.86	7	394	213.8	7.0e-55	gene=Chr09G0291.1	RO3G_02234[Rhizopusoryzae]
Chr06G0136.1	656	22.8	Clame_scaffold11-8.74[Cladoni	8336	48	619	1948	2499	0.86	21	571	131.3	2.2e-30	gene=Chr06G0136.1	Clame_scaffold11-8.74[Cladoniametacorallifera]

			ametacorallifera]												
Chr01G0252.1	582	36.5	Clame_scaffold11-8.74[Cladoniametacorallifera]	8336	13	432	7897	8336	0.86	5	419	276.2	5.0e-74	gene=Chr01G0252.1	Clame_scaffold11-8.74[Cladoniametacorallifera]
Chr08G0086.1	587	27.7	e_gw1.81.158.1[Trichodermavirens]	1087	111	586	631	1081	0.86	9	475	192.6	7.3e-49	gene=Chr08G0086.1	e_gw1.81.158.1[Trichodermavirens]
Chr09G0380.1	818	50.3	AFL2G_01904[Aspergillusflavus]	4755	464	814	4400	4754	0.86	4	350	356.3	5.3e-98	gene=Chr09G0380.1	AFL2G_01904[Aspergillusflavus]
Chr05G0366.1	625	24.4	Clame_scaffold11-8.74[Cladoniametacorallifera]	8336	15	589	1948	2500	0.86	23	574	107.5	3.3e-23	gene=Chr05G0366.1	Clame_scaffold11-8.74[Cladoniametacorallifera]
Chr05G0799.1	508	37	Clame_scaffold11-8.74[Cladoniametacorallifera]	8336	91	503	5281	5733	0.86	11	412	262.3	6.5e-70	gene=Chr05G0799.1	Clame_scaffold11-8.74[Cladoniametacorallifera]
Chr01G0876.1	2614	32.1	fgenesh1_	2158	7	438	359	784	0.8	9	431	217.2	1.2e-55	gene=C	fgenesh1_pg.24_&#

			pg.24_#_34[PhlebiabrevisporaHHB-7030SS6]						6					hr01G0876.1	35;_34[PhlebiabrevisporaHHB-7030SS6]
Chr02G0600.1	622	25.4	maker-Caloplaca_flavorubescens_scaffold_19-augustus-gene-2.67.2-mRNA-1[Caloplacaflavorubescens]	1049	39	578	47	571	0.86	22	539	105.5	1.2e-22	gene=Chr02G0600.1	maker-Caloplaca_flavorubescens_scaffold_19-augustus-gene-2.67.2-mRNA-1[Caloplacaflavorubescens]
Chr03G1619.1	530	75.8	CH063_09867T0[Colletotrichumhigginsianum]	520	22	529	1	520	0.86	3	507	811.6	3.0e-235	gene=Chr03G1619.1	CH063_09867T0[Colletotrichumhigginsianum]
Chr05G1016.1	1084	25.8	Hanno_03950[Heterobasidionannosum03012]	1497	349	923	299	828	0.86	28	574	123.2	1.0e-27	gene=Chr05G1016.1	Hanno_03950[Heterobasidionannosum03012]

Chr05G0728.1	572	29.2	estExt_Genewise1.C_20782[PhycomycesblakesleeanusNRRL1555]	495	75	504	19	446	0.86	8	429	182.2	9.6e-46	gene=Chr05G0728.1	estExt_Genewise1.C_20782[PhycomycesblakesleeanusNRRL1555]
Chr01G1178.1	698	42.8	g17951.t1[ArmillariamelleaDSM3731]	1190	1	392	1	444	0.86	6	391	285	1.3e-76	gene=Chr01G1178.1	g17951.t1[ArmillariamelleaDSM3731]
Chr05G0270.1	1446	26.3	EfO2.075130.1[Epichloefestucae]	1182	960	1441	662	1138	0.87	18	481	94	8.7e-19	gene=Chr05G0270.1	EfO2.075130.1[Epichloefestucae]
Chr06G1313.1	659	28.2	Clame_scaffold8-4.115[Cladoniametacorallifera]	16637	92	589	4040	4580	0.87	14	497	208.4	1.4e-53	gene=Chr06G1313.1	Clame_scaffold8-4.115[Cladoniametacorallifera]
Chr07G0293.1	659	26	M_BR29_EuGene_00112621[MagnaporthegriseaBR29]	952	102	639	426	941	0.87	15	537	175.6	1.0e-43	gene=Chr07G0293.1	M_BR29_EuGene_00112621[MagnaporthegriseaBR29]

Chr02G1475.1	567	44.9	ATEG_07313.1[Aspergillusterreus]	836	166	523	1	358	0.87	2	357	316.6	3.2e-86	gene=Chr02G1475.1	ATEG_07313.1[Aspergillusterreus]
Chr08G0479.1	1110	55.6	estExt_Genewise1.C_4080032[Exidiaglandulosa]	354	98	453	1	354	0.87	2	355	451.8	1.3e-126	gene=Chr08G0479.1	estExt_Genewise1.C_4080032[Exidiaglandulosa]
Chr04G0027.1	2372	36.2	fgenesh1_pg.24_#_34[PhlebiabrevisporaHHB-7030SS6]	2158	48	443	352	740	0.87	9	395	228.8	3.7e-59	gene=Chr04G0027.1	fgenesh1_pg.24_#_34[PhlebiabrevisporaHHB-7030SS6]
Chr07G0438.1	548	28.3	XP_007600712.1[ColletotrichumfioriniaePJ7]	959	24	499	13	468	0.87	12	475	151.4	1.7e-36	gene=Chr07G0438.1	XP_007600712.1[ColletotrichumfioriniaePJ7]
Chr09G0239.1	551	28.7	maker-Caloplaca_flavorubescens_scaffold_19-augustus-gene-	1049	14	506	47	574	0.87	22	492	173.3	4.3e-43	gene=Chr09G0239.1	maker-Caloplaca_flavorubescens_scaffold_19-augustus-gene-2.67.2-mRNA-1[Caloplacaflavorubescens]

			2.67.2-mRNA-1[Caloplacaflavorubescens]												
Chr03G1722.1	571	47.8	ATEG_07313.1[Aspergillusterreus]	836	177	529	1	358	0.87	2	352	332	7.5e-91	gene=Chr03G1722.1	ATEG_07313.1[Aspergillusterreus]
Chr09G0358.1	603	29.2	MUStwsD_GLEAN_10001562[Omphalotusolearius]	2038	115	586	317	827	0.87	18	471	186.8	4.1e-47	gene=Chr09G0358.1	MUStwsD_GLEAN_10001562[Omphalotusolearius]
Chr03G1048.1	460	29.6	Pa_7_5740[PodosporaanserinaSmat+]	576	2	453	84	520	0.87	7	451	195.3	8.8e-50	gene=Chr03G1048.1	Pa_7_5740[PodosporaanserinaSmat+]
Chr07G0097.1	858	79.2	MIX22873_6_31[ColletotrichumsublineolaCBS131301]	544	1	529	1	529	0.87	0	528	868.6	3.3e-252	gene=Chr07G0097.1	MIX22873_6_31[ColletotrichumsublineolaCBS131301]
Chr02G0457.1	588	24.3	FOXG_20	1035	42	563	10	557	0.8	16	521	173.3	4.6e-43	gene=C	FOXG_20661T0[Fus

			661T0[Fusariumoxysporumf.sp.lycopersici]						7					hr02G0457.1	ariumoxysporumf.sp.lycopersici]
Chr04G1516.1	577	33.3	XP_007598108.1[ColletotrichumfioriniaePJ7]	877	4	470	7	466	0.87	16	466	214.2	2.3e-55	gene=Chr04G1516.1	XP_007598108.1[ColletotrichumfioriniaePJ7]
Chr07G0210.1	629	26.2	Clame_scaffold11-8.74[Cladoniametacorallifera]	8336	45	587	1959	2499	0.87	18	542	129.8	6.2e-30	gene=Chr07G0210.1	Clame_scaffold11-8.74[Cladoniametacorallifera]
Chr03G0169.1	455	47.5	AFL2G_07147[Aspergillusflavus]	1440	1	370	1	373	0.87	6	369	328.6	6.6e-90	gene=Chr03G0169.1	AFL2G_07147[Aspergillusflavus]
Chr07G0244.1	653	27.7	AFL2G_01551[Aspergillusflavus]	1045	57	511	495	945	0.88	8	454	156.8	4.9e-38	gene=Chr07G0244.1	AFL2G_01551[Aspergillusflavus]
Chr06G1444.1	488	75.5	estExt_Genemark1.C_630000	490	5	486	5	489	0.88	1	481	750.4	7.5e-217	gene=Chr06G1444.1	estExt_Genemark1.C_6300002[ColletotrichumsublineolaCBS

			2[Colletotrichumsublineola CBS 131301]											131301]
Chr08G0153.1	498	36.8	Clame_scaffold11-8.74[Cladoniametacorallifera]	8336	29	458	2533	3032	0.88	16	429	234.2	1.9e-61	gene=Chr08G0153.1	Clame_scaffold11-8.74[Cladoniametacorallifera]
Chr01G1887.1	506	73	XP_007596979.1[ColletotrichumfioriniaePJ7]	520	1	505	1	520	0.88	3	504	768.5	2.8e-222	gene=Chr01G1887.1	XP_007596979.1[ColletotrichumfioriniaePJ7]
Chr04G0248.1	644	24.1	maker-Caloplaca_flavorubescens_scaffold_19-augustus-gene-2.67.2-mRNA-1[Caloplacaflavorubescens]	1049	26	641	47	619	0.88	22	615	106.7	5.8e-23	gene=Chr04G0248.1	maker-Caloplaca_flavorubescens_scaffold_19-augustus-gene-2.67.2-mRNA-1[Caloplacaflavorubescens]
Chr01G2190.1	804	29.3	AFL2G_0	1045	178	643	544	981	0.8	8	465	171.8	1.8e-42	gene=C	AFL2G_01551[Aspe

			1551[Aspergillusflavus]						8					hr01G2190.1	rgillusflavus]
Chr04G1077.1	403	56	estExt_Genewise1.C_9890004[Colletotrichumsublineolacbs131301]	573	25	400	25	393	0.88	3	375	455.7	3.2e-128	gene=Chr04G1077.1	estExt_Genewise1.C_9890004[ColletotrichumsublineolaCBS131301]
Chr03G1459.1	520	34	Clame_scaffold8-4.115[Cladoniametacorallifera]	16637	19	421	2897	3308	0.88	6	402	219.5	4.9e-57	gene=Chr03G1459.1	Clame_scaffold8-4.115[Cladoniametacorallifera]
Chr01G1956.1	1350	21.3	EEA28139.1[Penicilliummarneffei]	2025	203	804	915	1490	0.88	18	601	75.1	3.9e-13	gene=Chr01G1956.1	EEA28139.1[Penicilliummarneffei]
Chr09G0684.1	506	29.4	EAA63242.1[AspergillusnidulansFGSCA4]	1004	39	487	34	483	0.88	10	448	167.5	2.2e-41	gene=Chr09G0684.1	EAA63242.1[AspergillusnidulansFGSCA4]
Chr08G0826.1	589	29.5	ATEG_04721.1[Aspergillusterr	994	129	558	528	966	0.88	3	429	197.2	3.0e-50	gene=Chr08G0826.1	ATEG_04721.1[Aspergillusterreus]

			eus]												
Chr02G1710.1	688	37.9	fgenesh1_kg.20_#_67_#_Locus6460v1rpkm23.60[Sphaerobolusstellatus]	458	64	557	2	457	0.88	13	493	293.1	4.6e-79	gene=Chr02G1710.1	fgenesh1_kg.20_#_67_#_Locus6460v1rpkm23.60[Sphaerobolusstellatus]
Chr07G0880.1	801	29	Clama_scaffold_5-4.0[Cladoniamacilenta]	21962	211	708	1219	1755	0.88	20	497	212.2	1.2e-54	gene=Chr07G0880.1	Clama_scaffold_5-4.0[Cladoniamacilenta]
Chr09G0370.1	654	81.9	CH063_03591T0[Colletotrichumhigginsianum]	643	1	653	1	643	0.88	4	652	1087.4	0.0e+00	gene=Chr09G0370.1	CH063_03591T0[Colletotrichumhigginsianum]
Chr06G0322.1	516	44.9	Clama_scaffold_5-4.0[Cladoniamacilenta]	21962	6	434	18233	18652	0.88	10	428	335.1	8.0e-92	gene=Chr06G0322.1	Clama_scaffold_5-4.0[Cladoniamacilenta]
Chr01G2239.1	1155	38.5	EEA28139.1[Penicilliummarneff	2025	10	398	603	1001	0.89	11	388	232.3	1.6e-60	gene=Chr01G2239.1	EEA28139.1[Penicilliummarneffei]

			ei]												
Chr06G1490.1	1323	38.5	EEA28139.1[Penicilliummarneffei]	2025	10	398	603	1001	0.89	11	388	231.1	4.2e-60	gene=Chr06G1490.1	EEA28139.1[Penicilliummarneffei]
Chr01G0835.1	507	28.3	XP_007804728.1[EndocarponpusillumZ07020]	1059	45	504	595	1054	0.89	11	459	181	1.9e-45	gene=Chr01G0835.1	XP_007804728.1[EndocarponpusillumZ07020]
Chr05G0646.1	1700	40.3	gm1.898_g[TulasnellacalosporaAL13]	1440	783	1199	656	1099	0.89	10	416	299.3	1.6e-80	gene=Chr05G0646.1	gm1.898_g[TulasnellacalosporaAL13]
Chr05G0378.1	574	27.2	M_BR29_EuGene_00112621[MagnaporthegriseaBR29]	952	41	571	426	950	0.89	16	530	187.2	3.0e-47	gene=Chr05G0378.1	M_BR29_EuGene_00112621[MagnaporthegriseaBR29]
Chr07G0021.1	633	27.8	MUStwsD_GLEAN_10004418[Omphalotusolearius]	1665	113	620	1171	1656	0.89	14	507	171.8	1.4e-42	gene=Chr07G0021.1	MUStwsD_GLEAN_10004418[Omphalotusolearius]

Chr02G1632.1	1009	30.1	Endpu_scaffold7-12.77[EndocarponpusillumR61883]	5051	54	512	1265	1735	0.89	17	458	167.9	3.3e-41	gene=Chr02G1632.1	Endpu_scaffold7-12.77[EndocarponpusillumR61883]
Chr07G0920.1	519	32.9	XP_007598108.1[CollletotrichumfioriniaePJ7]	877	6	469	25	500	0.89	18	463	229.2	6.2e-60	gene=Chr07G0920.1	XP_007598108.1[CollletotrichumfioriniaePJ7]
Chr06G1077.1	1071	26	7575_t[AscocorynesarcoidesNRRL50072]	1484	466	1040	356	921	0.89	19	574	183.7	6.2e-46	gene=Chr06G1077.1	7575_t[AscocorynesarcoidesNRRL50072]
Chr09G0663.1	2570	31.1	fgenesh1_pg.24_#_34[PhlebiabrevisporaHHB-7030SS6]	2158	12	438	352	784	0.89	6	426	213.4	1.7e-54	gene=Chr09G0663.1	fgenesh1_pg.24_#_34[PhlebiabrevisporaHHB-7030SS6]
Chr08G0847.1	549	46.1	Clame_scaffold11-8.74[Cladoniametacora	8336	65	543	5281	5717	0.89	5	478	396	4.1e-110	gene=Chr08G0847.1	Clame_scaffold11-8.74[Cladoniametacorallifera]

			llifera]												
Chr03G0387.1	523	77.5	GLRG_00248T0[Colletotrichumgraminicola M1.001]	522	5	523	5	523	0.89	2	518	850.9	4.4e-247	gene=Chr03G0387.1	GLRG_00248T0[ColletotrichumgraminicolaM1.001]
Chr01G0193.1	510	31.3	XP_007804728.1[EndocarponpusillumZ07020]	1059	57	502	587	1054	0.89	8	445	229.2	6.1e-60	gene=Chr01G0193.1	XP_007804728.1[EndocarponpusillumZ07020]
Chr04G1141.1	816	33.8	e_gw1.9.856.1[Hebelomacylindrosporumh7]	1092	79	535	638	1082	0.89	13	456	245	1.7e-64	gene=Chr04G1141.1	e_gw1.9.856.1[Hebelomacylindrosporumh7]
Chr02G1748.1	607	27.5	M_BR29_EuGene_00112621[MagnaporthegriseaBR29]	952	26	602	425	950	0.89	17	576	193.4	4.4e-49	gene=Chr02G1748.1	M_BR29_EuGene_00112621[MagnaporthegriseaBR29]
Chr01G1656.1	562	79.1	CH063_00608T0[Colletotrichu	564	15	560	18	562	0.89	1	545	890.2	7.0e-259	gene=Chr01G1656.1	CH063_00608T0[Colletotrichumhigginsianum]

			mhigginsianum]												
Chr01G1483.1	559	32.9	AB00197.1[Alternariabrassicicola]	1041	7	546	5	484	0.89	15	539	271.9	9.0e-73	gene=Chr01G1483.1	AB00197.1[Alternariabrassicicola]
Chr01G2643.1	528	76.5	XP_007599609.1[ColletotrichumfioriniaePJ7]	531	1	527	1	531	0.90	3	526	825.5	2.0e-239	gene=Chr01G2643.1	XP_007599609.1[ColletotrichumfioriniaePJ7]
Chr01G0034.1	572	32.1	MUStwsD_GLEAN_10001562[Omphalotusolearius]	2038	94	564	317	827	0.90	20	470	208	1.6e-53	gene=Chr01G0034.1	MUStwsD_GLEAN_10001562[Omphalotusolearius]
Chr01G0742.1	494	46.9	CHG06520.1[ChaetomiumglobosumCBS148.51]	860	44	440	1	392	0.90	9	396	375.9	3.9e-104	gene=Chr01G0742.1	CHG06520.1[ChaetomiumglobosumCBS148.51]
Chr09G0711.1	560	30.9	AB00197.1[Alternariabrassicicola]	1041	46	548	26	485	0.90	12	502	211.1	1.9e-54	gene=Chr09G0711.1	AB00197.1[Alternariabrassicicola]

Chr01G0936.1	503	31.9	CNAG_06628T0[Cryptococcusneoformansvar.grubiiH99]	506	2	423	37	457	0.90	6	421	200.7	2.3e-51	gene=Chr01G0936.1	CNAG_06628T0[Cryptococcusneoformansvar.grubiiH99]
Chr02G0098.1	534	36.9	ATEG_04721.1[Aspergillusterreus]	994	131	533	543	965	0.90	5	402	291.6	1.0e-78	gene=Chr02G0098.1	ATEG_04721.1[Aspergillusterreus]
Chr06G1304.1	441	67	XP_007597624.1[ColletotrichumfioriniaePJ7]	519	2	439	13	463	0.90	4	437	607.1	9.3e-174	gene=Chr06G1304.1	XP_007597624.1[ColletotrichumfioriniaePJ7]
Chr06G0952.1	1292	53.8	EEA28139.1[Penicilliummarneffei]	2025	1	411	597	998	0.90	8	410	417.9	2.3e-116	gene=Chr06G0952.1	EEA28139.1[Penicilliummarneffei]
Chr01G0401.1	905	40.3	fgenesh1_pg.00058_#_49[PilodermacroceumF1598]	896	166	629	446	893	0.90	11	463	324.7	1.9e-88	gene=Chr01G0401.1	fgenesh1_pg.00058_#_49[PilodermacroceumF1598]
Chr07G0186.1	859	35	e_gw1.9.8	1092	68	525	632	1078	0.9	14	457	249.2	9.6e-66	gene=C	e_gw1.9.856.1[Hebe

			56.1[Hebelomacylindrosporumh7]						0					hr07G0186.1	lomacylindrosporumh7]
Chr06G0934.1	462	52	CHG06520.1[ChaetomiumglobosumCBS148.51]	860	44	441	1	392	0.90	8	397	427.2	1.4e-119	gene=Chr06G0934.1	CHG06520.1[ChaetomiumglobosumCBS148.51]
Chr03G0445.1	1065	30.2	ODG_10834-R0[OphiocerasdolichostomumCBS114926]	2287	36	518	79	587	0.90	21	482	173.7	6.4e-43	gene=Chr03G0445.1	ODG_10834-R0[OphiocerasdolichostomumCBS114926]
Chr05G1043.1	710	33.6	MUStwsD_GLEAN_10001562[Omphalotusolearius]	2038	176	691	317	840	0.90	14	515	292.7	6.3e-79	gene=Chr05G1043.1	MUStwsD_GLEAN_10001562[Omphalotusolearius]
Chr07G0159.1	566	68.8	XP_007598057.1[ColletotrichumfioriniaePJ7]	598	29	562	10	595	0.90	3	533	823.9	6.2e-239	gene=Chr07G0159.1	XP_007598057.1[ColletotrichumfioriniaePJ7]

Chr06G0926.1	1385	53.4	EEA28139.1[Penicilliummarneffei]	2025	1	376	597	969	0.90	2	375	408.7	1.5e-113	gene=Chr06G0926.1	EEA28139.1[Penicilliummarneffei]
Chr01G2717.1	469	47.5	CHG06520.1[ChaetomiumglobosumCBS148.51]	860	47	446	1	392	0.90	9	399	379	4.4e-105	gene=Chr01G2717.1	CHG06520.1[ChaetomiumglobosumCBS148.51]
Chr02G1731.1	647	27.6	M_BR29_EuGene_00112621[MagnaporthegriseaBR29]	952	107	644	426	950	0.90	17	537	213	5.7e-55	gene=Chr02G1731.1	M_BR29_EuGene_00112621[MagnaporthegriseaBR29]
Chr07G0464.1	1179	52.9	EEA28139.1[Penicilliummarneffei]	2025	1	376	597	969	0.90	2	375	404.4	2.5e-112	gene=Chr07G0464.1	EEA28139.1[Penicilliummarneffei]
Chr01G0411.1	954	21.5	EEA28139.1[Penicilliummarneffei]	2025	132	713	890	1452	0.91	18	581	130.2	7.2e-30	gene=Chr01G0411.1	EEA28139.1[Penicilliummarneffei]
Chr03G1576.1	572	44.2	ATEG_07313.1[Aspergillusterr	836	182	550	1	371	0.91	1	368	337.8	1.4e-92	gene=Chr03G1576.1	ATEG_07313.1[Aspergillusterreus]

			eus]												
Chr01G2652.1	502	72	CE249606_587[ColletotrichumsublineolaCBS131301]	464	38	497	2	460	0.91	3	459	701	5.4e-202	gene=Chr01G2652.1	CE249606_587[ColletotrichumsublineolaCBS131301]
Chr02G0071.1	509	75.1	XP_007594324.1[ColletotrichumfioriniaePJ7]	511	4	508	3	511	0.91	3	504	813.5	7.6e-236	gene=Chr02G0071.1	XP_007594324.1[ColletotrichumfioriniaePJ7]
Chr09G0005.1	608	24.5	e_gw1.81.158.1[Trichodermavirens]	1087	55	592	542	1065	0.91	15	537	151.4	1.9e-36	gene=Chr09G0005.1	e_gw1.81.158.1[Trichodermavirens]
Chr02G1805.1	667	25.9	e_gw1.81.158.1[Trichodermavirens]	1087	126	645	542	1068	0.91	15	519	167.9	2.2e-41	gene=Chr02G1805.1	e_gw1.81.158.1[Trichodermavirens]
Chr07G0182.1	529	77.8	XP_007598042.1[ColletotrichumfioriniaePJ7]	532	1	528	5	532	0.91	0	527	843.6	7.1e-245	gene=Chr07G0182.1	XP_007598042.1[ColletotrichumfioriniaePJ7]
Chr05G1248.1	495	32.4	fgenesh1_	1893	25	492	119	662	0.9	14	467	239.2	5.7e-63	gene=C	fgenesh1_pg.C_scaf

			pg.C_scaffold_1000882[Sporobolomycesroseus]						1					hr05G1248.1	fold_1000882[Sporobolomycesroseus]
Chr01G1183.1	505	74.7	GLRG_10597T0[Colletotrichumgraminicola M1.001]	508	1	497	1	500	0.91	3	496	773.9	6.6e-224	gene=Chr01G1183.1	GLRG_10597T0[ColletotrichumgraminicolaM1.001]
Chr06G1449.1	459	47.8	CHG06520.1[ChaetomiumglobosumCBS148.51]	860	46	438	4	392	0.91	7	392	392.9	2.9e-109	gene=Chr06G1449.1	CHG06520.1[ChaetomiumglobosumCBS148.51]
Chr01G0016.1	567	28.1	M_BR29_EuGene_00112621[MagnaporthegriseaBR29]	952	30	566	426	952	0.91	16	536	225.7	7.5e-59	gene=Chr01G0016.1	M_BR29_EuGene_00112621[MagnaporthegriseaBR29]
Chr06G0456.1	655	23.6	estExt_fgenesh1_pm.C_610136[Auricular	1088	21	627	499	1049	0.91	19	606	127.9	2.5e-29	gene=Chr06G0456.1	estExt_fgenesh1_pm.C_610136[Auriculariasubglabra]

			iasubglabra]												
Chr04G1539.1	570	52.5	Clame_scaffold9-5.100[Cladoniametacorallifera]	12517	146	539	8236	8651	0.91	4	393	346.7	2.9e-95	gene=Chr04G1539.1	Clame_scaffold9-5.100[Cladoniametacorallifera]
Chr06G1084.1	652	32.3	MUStwsD_GLEAN_10001533[Omphalotusolearius]	1200	102	634	635	1097	0.91	13	532	206.5	5.4e-53	gene=Chr06G1084.1	MUStwsD_GLEAN_10001533[Omphalotusolearius]
Chr06G0225.1	968	29.9	AFL2G_01551[Aspergillusflavus]	1045	220	713	512	975	0.91	10	493	216.9	6.0e-56	gene=Chr06G0225.1	AFL2G_01551[Aspergillusflavus]
Chr03G1263.1	550	27.4	maker-Caloplaca_flavorubescens_scaffold_19-augustus-gene-2.67.2-mRNA-1[Caloplacaflavo	1049	3	504	46	565	0.92	17	501	136.7	4.4e-32	gene=Chr03G1263.1	maker-Caloplaca_flavorubescens_scaffold_19-augustus-gene-2.67.2-mRNA-1[Caloplacaflavorubescens]

			rubescens]												
Chr09G0724.1	459	53	CHG06520.1[ChaetomiumglobosumCBS148.51]	860	44	436	1	392	0.92	7	392	429.9	2.1e-120	gene=Chr09G0724.1	CHG06520.1[ChaetomiumglobosumCBS148.51]
Chr03G0466.1	1159	30.7	PTRG_10301[Pyrenophoratritici-repentis]	1311	380	889	253	825	0.92	14	509	265	2.3e-70	gene=Chr03G0466.1	PTRG_10301[Pyrenophoratritici-repentis]
Chr04G1075.1	2578	35	fgenesh1_pg.24_#_34[PhlebiabrevisporaHHB-7030SS6]	2158	5	419	359	784	0.92	6	414	246.9	1.4e-64	gene=Chr04G1075.1	fgenesh1_pg.24_#_34[PhlebiabrevisporaHHB-7030SS6]
Chr08G0093.1	582	74.9	XP_007601013.1[ColletotrichumfioriniaePJ7]	532	78	581	24	532	0.92	2	503	803.1	1.2e-232	gene=Chr08G0093.1	XP_007601013.1[ColletotrichumfioriniaePJ7]
Chr07G0382.1	657	28.1	M_BR29_EuGene_00112621[	952	96	641	426	945	0.92	14	545	198.7	1.1e-50	gene=Chr07G0382.1	M_BR29_EuGene_00112621[MagnaporthegriseaBR29]

			MagnaporthegriseaBR29]												
Chr01G0023.1	570	35.6	Clame_scaffold8-4.115[Cladoniametacorallifera]	16637	119	555	10558	10989	0.92	12	436	256.1	5.2e-68	gene=Chr01G0023.1	Clame_scaffold8-4.115[Cladoniametacorallifera]
Chr02G1133.1	574	29.9	MUStwsD_GLEAN_10001562[Omphalotusolearius]	2038	69	568	291	824	0.92	19	499	189.1	7.9e-48	gene=Chr02G1133.1	MUStwsD_GLEAN_10001562[Omphalotusolearius]
Chr02G1137.1	513	75.2	XP_007595806.1[ColletotrichumfioriniaePJ7]	512	1	506	1	509	0.92	2	505	813.9	5.8e-236	gene=Chr02G1137.1	XP_007595806.1[ColletotrichumfioriniaePJ7]
Chr09G0915.1	575	63	XP_007594950.1[ColletotrichumfioriniaePJ7]	403	168	571	1	403	0.93	3	403	527.7	9.4e-150	gene=Chr09G0915.1	XP_007594950.1[ColletotrichumfioriniaePJ7]
Chr01G0117.1	712	79.2	EfO2.075130.1[Epich	1182	24	708	520	1180	0.93	2	684	1032.7	1.1e-301	gene=Chr01G01	EfO2.075130.1[Epichloefestucae]

			loefestucae]											17.1	
Chr02G1638.1	648	38.3	Clame_scaffold9-5.100[Cladoniametacorallifera]	12517	118	611	709	1156	0.93	13	493	287.7	1.8e-77	gene=Chr02G1638.1	Clame_scaffold9-5.100[Cladoniametacorallifera]
Chr07G1031.1	417	58.3	GLRG_09104T0[ColletotrichumgraminicolaM1.001]	407	1	403	1	394	0.93	4	402	447.2	1.2e-125	gene=Chr07G1031.1	GLRG_09104T0[ColletotrichumgraminicolaM1.001]
Chr08G0713.1	543	32.2	AB00197.1[Alternariabrassicicola]	1041	12	534	4	490	0.93	12	522	278.1	1.2e-74	gene=Chr08G0713.1	AB00197.1[Alternariabrassicicola]
Chr02G1611.1	528	75.4	CH063_01289T0[Colletotrichumhigginsianum]	524	29	527	26	524	0.93	0	498	790.8	5.4e-229	gene=Chr02G1611.1	CH063_01289T0[Colletotrichumhigginsianum]
Chr05G0188.1	530	49.6	MGYG_03812[Microsporumgypseum]	526	135	527	30	414	0.93	2	392	399.4	3.5e-111	gene=Chr05G0188.1	MGYG_03812[Microsporumgypseum]

Chr02G1500.1	571	36.1	Pa_7_5740[PodosporaanserinaSmat+]	576	123	565	66	520	0.93	11	442	232.3	8.1e-61	gene=Chr02G1500.1	Pa_7_5740[PodosporaanserinaSmat+]
Chr08G0112.1	595	78.7	CH063_02042T0[Colletotrichumhigginsianum]	594	25	594	23	594	0.93	3	569	911.4	3.1e-265	gene=Chr08G0112.1	CH063_02042T0[Colletotrichumhigginsianum]
Chr01G1179.1	1628	24.7	EEA23526.1[Penicilliummarneffei]	1874	829	1546	405	971	0.93	18	717	163.7	1.0e-39	gene=Chr01G1179.1	EEA23526.1[Penicilliummarneffei]
Chr08G0145.1	1279	28	EfO2.075130.1[Epichloefestucae]	1182	778	1274	676	1159	0.93	12	496	166.4	1.2e-40	gene=Chr08G0145.1	EfO2.075130.1[Epichloefestucae]
Chr01G2411.1	606	44.3	ATEG_04721.1[Aspergillusterreus]	994	109	521	479	891	0.93	0	412	385.2	7.9e-107	gene=Chr01G2411.1	ATEG_04721.1[Aspergillusterreus]
Chr09G0296.1	634	71	Clame_scaffold18-19.70[Cladoniametacorallifera]	7866	140	623	158	637	0.93	4	483	709.1	2.5e-204	gene=Chr09G0296.1	Clame_scaffold18-19.70[Cladoniametacorallifera]

Chr01G0292.1	597	27.6	MUStwsD_GLEAN_10001562[Omphalotusolearius]	2038	60	594	281	827	0.93	19	534	186.4	5.3e-47	gene=Chr01G0292.1	MUStwsD_GLEAN_10001562[Omphalotusolearius]
Chr01G0587.1	433	50.4	MGYG_03812[Microsporumgypseum]	526	38	430	32	414	0.93	1	392	404.8	6.9e-113	gene=Chr01G0587.1	MGYG_03812[Microsporumgypseum]
Chr04G0697.1	504	74	GLRG_07145T0[CollletotrichumgraminicolaM1.001]	496	12	502	1	493	0.93	1	490	762.7	1.5e-220	gene=Chr04G0697.1	GLRG_07145T0[CollletotrichumgraminicolaM1.001]
Chr05G0274.1	501	29.6	EAA63242.1[AspergillusnidulansFGSCA4]	1004	3	483	11	483	0.93	12	480	190.3	3.1e-48	gene=Chr05G0274.1	EAA63242.1[AspergillusnidulansFGSCA4]
Chr09G0219.1	591	34.8	KKY16879.1putative membranetransporter[Phaeomoniellachla	993	108	571	57	499	0.93	8	463	268.1	1.4e-71	gene=Chr09G0219.1	KKY16879.1putative membranetransporter[Phaeomoniellachlamydospora][Phaeomoniellachlamydospora]

			mydospora][Phaeomoniellachlamydospora]												
Chr01G1543.1	2448	35.1	fgenesh1_pg.24_#_34[PhlebiabrevisporaHHB-7030SS6]	2158	10	450	359	787	0.93	10	440	253.4	1.5e-66	gene=Chr01G1543.1	fgenesh1_pg.24_#_34[PhlebiabrevisporaHHB-7030SS6]
Chr03G1632.1	1182	29	EfO2.075130.1[Epichloefestucae]	1182	674	1177	666	1159	0.93	15	503	175.6	1.9e-43	gene=Chr03G1632.1	EfO2.075130.1[Epichloefestucae]
Chr05G0414.1	588	33	PCON_06884m.01[PyronemaconfluensCBS100304]	501	84	555	23	462	0.93	7	471	237.7	2.0e-62	gene=Chr05G0414.1	PCON_06884m.01[PyronemaconfluensCBS100304]
Chr09G0636.1	538	76.6	XP_007600337.1[ColletotrichumfioriniaePJ7]	535	8	537	6	535	0.93	2	529	840.5	6.1e-244	gene=Chr09G0636.1	XP_007600337.1[ColletotrichumfioriniaePJ7]

Chr03G0308.1	605	28.5	Clame_scaffold8-4.115[Cladoniametacorallifera]	16637	88	594	3933	4488	0.93	12	506	206.5	5.0e-53	gene=Chr03G0308.1	Clame_scaffold8-4.115[Cladoniametacorallifera]
Chr01G0397.1	938	56.7	fgenesh1_pg.C_scaffold_16000117[Cryphonectriaparasitica]	583	481	893	153	581	0.94	3	412	513.5	3.0e-145	gene=Chr01G0397.1	fgenesh1_pg.C_scaffold_16000117[Cryphonectriaparasitica]
Chr01G1594.1	505	31	Pa_7_5740[PodosporaanserinaSmat+]	576	45	495	76	518	0.94	5	450	204.9	1.2e-52	gene=Chr01G1594.1	Pa_7_5740[PodosporaanserinaSmat+]
Chr05G1307.1	601	35.8	XP_007801092.1[EndocarponpusillumZ07020]	1039	37	511	5	512	0.94	12	474	293.1	4.1e-79	gene=Chr05G1307.1	XP_007801092.1[EndocarponpusillumZ07020]
Chr05G0535.1	513	56.2	EEA23526.1[Penicilliummarneffei]	1874	77	509	1449	1863	0.94	4	432	464.9	6.7e-131	gene=Chr05G0535.1	EEA23526.1[Penicilliummarneffei]
Chr04G1279.1	561	37.7	AFL2G_07229[Aspe	640	96	558	197	636	0.94	5	462	320.5	2.2e-87	gene=Chr04G12	AFL2G_07229[Aspergillusflavus]

			rgillusflavus]										79.1	
Chr01G2196.1	561	30.7	ATEG_04721.1[Aspergillusterreus]	994	51	531	479	963	0.94	3	480	218	1.5e-56	gene=Chr01G2196.1	ATEG_04721.1[Aspergillusterreus]
Chr01G1524.1	565	33	MUStwsD_GLEAN_10001562[Omphalotusolearius]	2038	39	552	317	849	0.94	15	513	271.2	1.6e-72	gene=Chr01G1524.1	MUStwsD_GLEAN_10001562[Omphalotusolearius]
Chr05G0504.1	1056	29.6	PTRG_10301[Pyrenophoratritici-repentis]	1311	505	1011	261	780	0.94	19	506	192.2	1.7e-48	gene=Chr05G0504.1	PTRG_10301[Pyrenophoratritici-repentis]
Chr04G0146.1	407	55.1	EEA26647.1[Penicilliummarneffei]	744	10	397	8	395	0.94	2	387	444.5	7.4e-125	gene=Chr04G0146.1	EEA26647.1[Penicilliummarneffei]
Chr07G0203.1	582	29.2	Clama_scaffold_28-0.22[Cladoniamacilenta]	3845	53	568	2066	2580	0.94	22	515	191.8	1.2e-48	gene=Chr07G0203.1	Clama_scaffold_28-0.22[Cladoniamacilenta]

Chr06G0369.1	1301	29.3	EfO2.075130.1[Epichloefestucae]	1182	824	1298	686	1159	0.94	13	474	180.3	8.3e-45	gene=Chr06G0369.1	EfO2.075130.1[Epichloefestucae]
Chr01G0194.1	522	70.5	CH063_00326T0[Colletotrichumhigginsianum]	547	1	518	1	545	0.94	2	517	803.9	6.1e-233	gene=Chr01G0194.1	CH063_00326T0[Colletotrichumhigginsianum]
Chr01G0611.1	488	43.6	HCB01166.1[Histoplasmacapsulatum]	1012	49	464	556	1008	0.95	7	415	347.1	1.9e-95	gene=Chr01G0611.1	HCB01166.1[Histoplasmacapsulatum]
Chr06G0848.1	500	68.6	e_gw1.2.60.1[ColletotrichumsublineolaCBS131301]	1052	1	486	1	485	0.95	4	485	673.7	9.2e-194	gene=Chr06G0848.1	e_gw1.2.60.1[ColletotrichumsublineolaCBS131301]
Chr08G0693.1	660	27.3	e_gw1.81.158.1[Trichodermavirens]	1087	109	638	542	1071	0.95	17	529	191	2.4e-48	gene=Chr08G0693.1	e_gw1.81.158.1[Trichodermavirens]
Chr06G1038.1	1274	28.3	EfO2.075130.1[Epichloefestuca	1182	770	1272	666	1159	0.95	10	502	183.7	7.3e-46	gene=Chr06G1038.1	EfO2.075130.1[Epichloefestucae]

			e]											
Chr04G1348.1	570	35.3	AFL2G_07229[Aspergillusflavus]	640	75	546	195	631	0.95	4	471	280.8	2.0e-75	gene=Chr04G1348.1	AFL2G_07229[Aspergillusflavus]
Chr03G0928.1	499	71.4	e_gw1.126.9.1[ColletotrichumsublineolaCBS131301]	512	1	498	1	511	0.95	2	497	760.8	5.7e-220	gene=Chr03G0928.1	e_gw1.126.9.1[ColletotrichumsublineolaCBS131301]
Chr08G0715.1	551	33.7	KKY16879.1putativemembranetransporter[Phaeomoniellachlamydospora][Phaeomoniellachlamydospora]	993	62	531	57	499	0.95	7	469	283.1	3.8e-76	gene=Chr08G0715.1	KKY16879.1putativemembranetransporter[Phaeomoniellachlamydospora][Phaeomoniellachlamydospora]
Chr06G1072.1	455	45.7	g9457.t1[ArmillariamelleaDSM3731]	1459	34	430	1038	1447	0.95	4	396	342.8	3.4e-94	gene=Chr06G1072.1	g9457.t1[ArmillariamelleaDSM3731]

Chr02G1071.1	591	29.9	Pa_7_5740[PodosporaanserinaSmat+]	576	122	590	68	528	0.95	6	468	228.4	1.2e-59	gene=Chr02G1071.1	Pa_7_5740[PodosporaanserinaSmat+]
Chr02G1550.1	474	33.4	gm1.3475_g[Botryobasidiumbotryosum]	520	12	463	32	503	0.95	12	451	245.4	7.7e-65	gene=Chr02G1550.1	gm1.3475_g[Botryobasidiumbotryosum]
Chr08G0277.1	579	36.5	Clame_scaffold8-4.115[Cladoniametacorallifera]	16637	121	571	10558	10999	0.95	16	450	245.7	7.2e-65	gene=Chr08G0277.1	Clame_scaffold8-4.115[Cladoniametacorallifera]
Chr01G0396.1	567	25.5	MUStwsD_GLEAN_10004418[Omphalotusolearius]	1665	10	564	1099	1660	0.95	17	554	154.1	2.8e-37	gene=Chr01G0396.1	MUStwsD_GLEAN_10004418[Omphalotusolearius]
Chr04G0338.1	503	54.5	AFL2G_00556[Aspergillusflavus]	837	107	501	445	836	0.95	4	394	451.4	7.5e-127	gene=Chr04G0338.1	AFL2G_00556[Aspergillusflavus]
Chr01G1945.1	512	75.1	XP_007594288.1[Colletotrichu	514	2	511	5	514	0.95	0	509	807	7.1e-234	gene=Chr01G1945.1	XP_007594288.1[ColletotrichumfioriniaePJ7]

			mfioriniae PJ7]													
Chr02G1303.1	582	29.9	estExt_Genewise1.C_20782[Phycomycesblakesleeanus NRRL1555]	495	65	551	7	473	0.95	13	486	176	7.0e-44	gene=Chr02G1303.1	estExt_Genewise1.C_20782[PhycomycesblakesleeanusNRRL1555]
Chr07G0343.1	414	53	Clame_scaffold9-5.100[Cladoniametacorallifera]	12517	3	402	6995	7403	0.95	4	399	400.2	1.6e-111	gene=Chr07G0343.1	Clame_scaffold9-5.100[Cladoniametacorallifera]
Chr03G1387.1	492	36	Pa_7_5740[PodosporaanserinaSmat+]	576	50	485	83	523	0.95	8	435	220.7	2.1e-57	gene=Chr03G1387.1	Pa_7_5740[PodosporaanserinaSmat+]
Chr01G1603.1	549	27.5	MUStwsD_GLEAN_10004418[Omphalotusolearius]	1665	9	541	1099	1658	0.95	16	532	155.6	9.2e-38	gene=Chr01G1603.1	MUStwsD_GLEAN_10004418[Omphalotusolearius]
Chr02G1727.1	569	31.6	Pa_7_5740[Podospo	576	72	537	78	529	0.95	8	465	191.4	1.6e-48	gene=Chr02G17	Pa_7_5740[PodosporaanserinaSmat+]

			raanserinaSmat+]												27.1	
Chr04G0745.1	517	31	XP_007804728.1[EndocarponpusillumZ07020]	1059	39	511	586	1054	0.95	10	472	236.5	3.9e-62		gene=Chr04G0745.1	XP_007804728.1[EndocarponpusillumZ07020]
Chr05G0185.1	511	57.9	M_BR29_EuGene_00043991[MagnaporthegriseaBR29]	501	14	508	34	499	0.96	6	494	580.1	1.4e-165		gene=Chr05G0185.1	M_BR29_EuGene_00043991[MagnaporthegriseaBR29]
Chr09G0913.1	587	31.4	Pa_7_5740[PodosporaanserinaSmat+]	576	115	579	62	529	0.96	12	464	185.3	1.2e-46		gene=Chr09G0913.1	Pa_7_5740[PodosporaanserinaSmat+]
Chr04G1125.1	502	68	AB04557.1[Alternariabrassicicola]	516	17	500	25	511	0.96	2	483	748.8	2.3e-216		gene=Chr04G1125.1	AB04557.1[Alternariabrassicicola]
Chr05G0178.1	2589	36.9	fgenesh1_pg.24_#_34[PhlebiabrevisporaHHB-	2158	46	477	352	784	0.96	10	431	264.6	6.7e-70		gene=Chr05G0178.1	fgenesh1_pg.24_#_34[PhlebiabrevisporaHHB-7030SS6]

			7030SS6]												
Chr04G0051.1	495	71.6	XP_007596389.1[Colletotrichumfioriniae PJ7]	491	18	493	14	488	0.96	1	475	738	3.9e-213	gene=Chr04G0051.1	XP_007596389.1[ColletotrichumfioriniaePJ7]
Chr04G0218.1	484	32.1	Pa_7_5740[PodosporaanserinaSmat+]	576	27	478	77	520	0.96	3	451	228	1.3e-59	gene=Chr04G0218.1	Pa_7_5740[PodosporaanserinaSmat+]
Chr06G1218.1	533	31.8	XP_007804728.1[EndocarponpusillumZ07020]	1059	32	529	582	1052	0.96	13	497	218.4	1.1e-56	gene=Chr06G1218.1	XP_007804728.1[EndocarponpusillumZ07020]
Chr01G0650.1	580	36.8	AFL2G_07229[Aspergillusflavus]	640	98	572	197	640	0.96	5	474	302	8.4e-82	gene=Chr01G0650.1	AFL2G_07229[Aspergillusflavus]
Chr08G0789.1	654	27.3	CHG01242.1[ChaetomiumglobosumCBS148.51]	1090	95	636	528	1074	0.96	19	541	165.6	1.1e-40	gene=Chr08G0789.1	CHG01242.1[ChaetomiumglobosumCBS148.51]
Chr05G0263.1	1364	28.7	EfO2.075130.1[Epich	1182	840	1362	663	1160	0.96	14	522	169.1	2.0e-41	gene=Chr05G02	EfO2.075130.1[Epichloefestucae]

			loefestucae]											63.1	
Chr07G0737.1	1607	28.3	PTRG_10301[Pyrenophoratritici-repentis]	1311	409	935	217	814	0.96	20	526	201.4	4.3e-51	gene=Chr07G0737.1	PTRG_10301[Pyrenophoratritici-repentis]
Chr06G1395.1	421	54.1	EEA26647.1[Penicilliummarneffei]	744	1	411	1	408	0.96	2	410	451.1	8.2e-127	gene=Chr06G1395.1	EEA26647.1[Penicilliummarneffei]
Chr02G0080.1	454	33.3	EEA27467.1[Penicilliummarneffei]	520	4	451	6	455	0.97	9	447	222.6	5.1e-58	gene=Chr02G0080.1	EEA27467.1[Penicilliummarneffei]
Chr01G0371.1	585	36.2	AFL2G_07229[Aspergillusflavus]	640	85	562	197	640	0.97	7	477	266.9	3.0e-71	gene=Chr01G0371.1	AFL2G_07229[Aspergillusflavus]
Chr08G0889.1	1270	29.3	Clame_scaffold8-4.115[Cladoniametacorallifera]	16637	430	979	4064	4580	0.97	15	549	204.9	3.1e-52	gene=Chr08G0889.1	Clame_scaffold8-4.115[Cladoniametacorallifera]
Chr03G1181.1	1351	29.4	EfO2.075130.1[Epich	1182	843	1346	658	1159	0.97	14	503	205.7	1.9e-52	gene=Chr03G11	EfO2.075130.1[Epichloefestucae]

			loefestucae]											gene=C 81.1	
Chr08G0952.1	597	26.8	MUStwsD_GLEAN_10004418[Omphalotusolearius]	1665	48	594	1099	1659	0.97	16	546	160.6	3.1e-39	gene=Chr08G0952.1	MUStwsD_GLEAN_10004418[Omphalotusolearius]
Chr05G0034.1	458	60.1	XP_007602139.1[CollletotrichumfioriniaePJ7]	801	18	457	336	799	0.97	1	439	574.7	5.3e-164	gene=Chr05G0034.1	XP_007602139.1[CollletotrichumfioriniaePJ7]
Chr04G0936.1	596	28.9	Pa_7_5740[PodosporaanserinaSmat+]	576	101	591	43	524	0.97	6	490	169.1	8.8e-42	gene=Chr04G0936.1	Pa_7_5740[PodosporaanserinaSmat+]
Chr04G0022.1	889	41.8	EEA28139.1[Penicilliummarneffei]	2025	64	472	603	1019	0.97	7	408	310.5	3.6e-84	gene=Chr04G0022.1	EEA28139.1[Penicilliummarneffei]
Chr09G1031.1	583	36.6	AFL2G_07229[Aspergillusflavus]	640	80	556	197	637	0.97	4	476	290.4	2.6e-78	gene=Chr09G1031.1	AFL2G_07229[Aspergillusflavus]
Chr01G2161.1	525	67.1	XP_00759	528	7	521	1	528	0.9	3	514	742.3	2.2e-21	gene=C	XP_007597569.1[Co

			7569.1[Colletotrichumfioriniae PJ7]						7				4	hr01G2161.1	lletotrichumfioriniaePJ7]
Chr01G1763.1	1359	25.7	EfO2.075130.1[Epichloefestucae]	1182	80	613	643	1170	0.97	13	533	137.1	8.4e-32	gene=Chr01G1763.1	EfO2.075130.1[Epichloefestucae]
Chr04G1351.1	490	33.3	XP_007804728.1[EndocarponpusillumZ07020]	1059	15	489	586	1058	0.97	10	474	249.6	4.2e-66	gene=Chr04G1351.1	XP_007804728.1[EndocarponpusillumZ07020]
Chr03G0200.1	571	38.2	fgenesh1_pg.C_scaffold_1000882[Sporobolomycesroseus]	1893	45	538	93	642	0.97	18	493	310.5	2.3e-84	gene=Chr03G0200.1	fgenesh1_pg.C_scaffold_1000882[Sporobolomycesroseus]
Chr06G1134.1	1801	48.5	Clame_scaffold11-8.74[Cladoniametacorallifera]	8336	1071	1464	7357	7748	0.97	1	393	378.3	2.9e-104	gene=Chr06G1134.1	Clame_scaffold11-8.74[Cladoniametacorallifera]
Chr08G0700.1	511	61.7	estExt_Genemark1.	547	7	507	8	539	0.97	5	500	693.7	8.8e-200	gene=Chr08G07	estExt_Genemark1.C_100007[Colletotric

			C_100007[Colletotrichumsublineo laCBS131301]											00.1	humsublineolaCBS131301]
Chr06G0406.1	581	32.4	maker-scaffold4.1-snap-gene-7.120-mRNA-1[Raffaeleaquercus-mongolicae]	582	38	538	74	571	0.98	8	500	247.7	1.9e-65	gene=Chr06G0406.1	maker-scaffold4.1-snap-gene-7.120-mRNA-1[Raffaeleaquercus-mongolicae]
Chr09G0906.1	825	25	Clame_scaffold8-4.115[Cladoniametacorallifera]	16637	132	785	4003	4588	0.98	19	653	143.7	5.5e-34	gene=Chr09G0906.1	Clame_scaffold8-4.115[Cladoniametacorallifera]
Chr07G0435.1	556	75.2	estExt_Genewise1.C_7450006[Colletotrichumsublineo laCBS131301]	562	11	549	9	552	0.98	1	538	883.2	8.5e-257	gene=Chr07G0435.1	estExt_Genewise1.C_7450006[ColletotrichumsublineolaCBS131301]
Chr03G1635.1	506	28.6	XP_00780	1059	1	504	552	1054	0.9	11	503	193.7	2.8e-49	gene=C	XP_007804728.1[En

			4728.1[EndocarponpusillumZ07020]						8					hr03G1635.1	docarponpusillumZ07020]
Chr04G1080.1	513	30.5	Pa_7_5740[PodosporaanserinaSmat+]	576	22	510	47	524	0.98	10	488	219.9	3.7e-57	gene=Chr04G1080.1	Pa_7_5740[PodosporaanserinaSmat+]
Chr04G0699.1	606	26.4	MUStwsD_GLEAN_10004418[Omphalotusolearius]	1665	51	600	1102	1660	0.98	16	549	181.8	1.3e-45	gene=Chr04G0699.1	MUStwsD_GLEAN_10004418[Omphalotusolearius]
Chr01G0941.1	571	41.7	EEA23504.1[Penicilliummarneffei]	1213	139	558	71	483	0.98	4	419	308.5	8.9e-84	gene=Chr01G0941.1	EEA23504.1[Penicilliummarneffei]
Chr01G2435.1	499	29.7	XP_007804728.1[EndocarponpusillumZ07020]	1059	8	497	565	1054	0.98	9	489	233	4.1e-61	gene=Chr01G2435.1	XP_007804728.1[EndocarponpusillumZ07020]
Chr02G0142.1	556	62.9	XP_007594273.1[Colletotrichu	474	70	517	1	454	0.98	3	447	614.4	7.4e-176	gene=Chr02G0142.1	XP_007594273.1[ColletotrichumfioriniaePJ7]

			mfioriniae PJ7]												
Chr01G2028.1	827	37.6	AFL2G_07229[Aspergillusflavus]	640	348	821	192	637	0.98	3	473	293.5	4.3e-79	gene=Chr01G2028.1	AFL2G_07229[Aspergillusflavus]
Chr06G1024.1	1280	25.7	ODG_10834-R0[OphiocerasdolichostomumCBS114926]	2287	12	607	82	693	0.98	22	595	137.5	6.1e-32	gene=Chr06G1024.1	ODG_10834-R0[OphiocerasdolichostomumCBS114926]
Chr01G1288.1	509	64.2	CH063_10745T0[Colletotrichumhigginsianum]	553	1	508	1	553	0.98	7	507	702.6	1.9e-202	gene=Chr01G1288.1	CH063_10745T0[Colletotrichumhigginsianum]
Chr08G0118.1	692	62.6	Clame_scaffold11-8.74[Cladoniametacorallifera]	8336	227	661	5281	5719	0.98	4	434	539.7	2.9e-153	gene=Chr08G0118.1	Clame_scaffold11-8.74[Cladoniametacorallifera]
Chr09G0998.1	547	33.8	XP_007804728.1[EndocarponpusillumZ0	1059	87	533	605	1054	0.98	4	446	266.5	3.7e-71	gene=Chr09G0998.1	XP_007804728.1[EndocarponpusillumZ07020]

			7020]												
Chr01G0143.1	601	37	AFL2G_07229[Aspergillusflavus]	640	113	591	195	640	0.98	5	478	290	3.4e-78	gene=Chr01G0143.1	AFL2G_07229[Aspergillusflavus]
Chr04G1251.1	470	36.2	fgenesh1_pg.21_#_32[TulasnellacalosporaAL13]	710	2	468	59	540	0.98	12	466	310.1	2.5e-84	gene=Chr04G1251.1	fgenesh1_pg.21_#_32[TulasnellacalosporaAL13]
Chr01G2247.1	585	32	MUStwsD_GLEAN_10001562[Omphalotusolearius]	2038	83	576	296	827	0.99	16	493	215.3	1.0e-55	gene=Chr01G2247.1	MUStwsD_GLEAN_10001562[Omphalotusolearius]
Chr04G0960.1	507	72.5	XP_007598618.1[ColletotrichumfioriniaePJ7]	510	1	505	1	509	0.99	1	504	755.7	1.9e-218	gene=Chr04G0960.1	XP_007598618.1[ColletotrichumfioriniaePJ7]
Chr07G0092.1	548	38.1	EEA23504.1[Penicilliummarneffei]	1213	88	530	49	487	0.99	9	442	284.6	1.3e-76	gene=Chr07G0092.1	EEA23504.1[Penicilliummarneffei]

Chr07G0916.1	490	67.6	XP_007597624.1[ColletotrichumfioriniaePJ7]	519	2	488	13	514	0.99	5	486	686.4	1.3e-197	gene=Chr07G0916.1	XP_007597624.1[ColletotrichumfioriniaePJ7]
Chr03G1497.1	570	30	ATEG_04721.1[Aspergillusterreus]	994	71	554	479	964	0.99	7	483	213	5.1e-55	gene=Chr03G1497.1	ATEG_04721.1[Aspergillusterreus]
Chr08G0114.1	586	41.2	Clame_scaffold11-8.74[Cladoniametacorallifera]	8336	44	505	4284	4750	0.99	11	461	312.8	4.8e-85	gene=Chr08G0114.1	Clame_scaffold11-8.74[Cladoniametacorallifera]
Chr01G0911.1	2063	26.3	EEA28139.1[Penicilliummarneffei]	2025	289	853	952	1493	0.99	17	564	162.2	3.7e-39	gene=Chr01G0911.1	EEA28139.1[Penicilliummarneffei]
Chr04G1373.1	663	31	MUStwsD_GLEAN_10002689[Omphalotusolearius]	1056	75	655	550	1052	0.99	14	580	256.1	6.1e-68	gene=Chr04G1373.1	MUStwsD_GLEAN_10002689[Omphalotusolearius]
Chr09G0701.1	487	36.1	gm1.3475_g[Botryo	520	37	481	43	512	0.99	8	444	298.1	1.0e-80	gene=Chr09G07	gm1.3475_g[Botryobasidiumbotryosum]

			basidiumbotryosum]											01.1	
Chr04G0547.1	824	29.8	Endpu_scaffold5-14.59[EndocarponpusillumR61883]	1473	109	670	583	1105	0.99	18	561	156.8	6.2e-38	gene=Chr04G0547.1	Endpu_scaffold5-14.59[EndocarponpusillumR61883]
Chr01G2243.1	486	34.5	e_gw1.13.512.1[PaxillusinvolutusATCC200175]	501	14	466	19	476	0.99	10	452	236.5	3.6e-62	gene=Chr01G2243.1	e_gw1.13.512.1[PaxillusinvolutusATCC200175]
Chr04G0944.1	735	33.6	XP_007801092.1[EndocarponpusillumZ07020]	1039	42	555	16	548	0.99	12	513	268.9	1.0e-71	gene=Chr04G0944.1	XP_007801092.1[EndocarponpusillumZ07020]
Chr02G1281.1	645	25.1	fgenesh1_pg.89_#_4[Hydnomeruliuspinastri]	737	76	642	44	623	0.99	19	566	167.2	3.6e-41	gene=Chr02G1281.1	fgenesh1_pg.89_#_4[Hydnomeruliuspinastri]
Chr08G0096.1	518	74	XP_007601009.1[Colletotrichu	521	1	515	1	515	0.99	0	514	793.5	8.2e-230	gene=Chr08G0096.1	XP_007601009.1[ColletotrichumfioriniaePJ7]

			mfioriniaePJ7]												
Chr03G0241.1	549	28.5	ATEG_04721.1[Aspergillusterreus]	994	39	540	479	986	0.99	6	501	199.5	5.6e-51	gene=Chr03G0241.1	ATEG_04721.1[Aspergillusterreus]
Chr01G0209.1	521	31.3	EAA63242.1[AspergillusnidulansFGSCA4]	1004	18	493	6	483	0.99	8	475	240.4	2.7e-63	gene=Chr01G0209.1	EAA63242.1[AspergillusnidulansFGSCA4]
Chr09G0954.1	602	31.4	Clame_scaffold18-19.70[Cladoniametacorallifera]	7866	1	590	699	1241	0.99	15	589	174.1	2.8e-43	gene=Chr09G0954.1	Clame_scaffold18-19.70[Cladoniametacorallifera]
Chr04G0743.1	580	31.4	AB00197.1[Alternariabrassicicola]	1041	9	578	5	504	0.99	13	569	234.2	2.2e-61	gene=Chr04G0743.1	AB00197.1[Alternariabrassicicola]
Chr05G0410.1	1034	50.9	EEA28139.1[Penicilliummarneffei]	2025	1	440	597	1021	0.99	6	439	421.8	1.3e-117	gene=Chr05G0410.1	EEA28139.1[Penicilliummarneffei]
Chr04G0789.1	496	70.9	XP_007601303.1[Colletotrichu	498	9	492	12	496	0.99	1	483	738.4	3.0e-213	gene=Chr04G0789.1	XP_007601303.1[ColletotrichumfioriniaePJ7]

			mfioriniaePJ7]											
Chr04G0801.1	555	46.2	AFL2G_07229[Aspergillusflavus]	640	70	545	197	637	0.99	5	475	403.7	2.0e-112	gene=Chr04G0801.1	AFL2G_07229[Aspergillusflavus]
Chr06G0258.1	473	42.1	MAPG_10536T0[MagnaporthepoaeATCC64411]	537	36	465	66	526	0.99	2	429	362.8	3.3e-100	gene=Chr06G0258.1	MAPG_10536T0[MagnaporthepoaeATCC64411]
Chr02G0206.1	582	28.1	MUStwsD_GLEAN_10004418[Omphalotusolearius]	1665	11	579	1099	1658	0.80	16	568	183	5.7e-46	gene=Chr02G0206.1	MUStwsD_GLEAN_10004418[Omphalotusolearius]
Chr01G0071.1	1474	52.1	e_gw1.9.856.1[Hebelomacylindrosporumh7]	1092	963	1381	665	1075	0.80	3	418	452.2	1.3e-126	gene=Chr01G0071.1	e_gw1.9.856.1[Hebelomacylindrosporumh7]
Chr04G0360.1	515	69.6	XP_007601935.1[Colletotrichumfioriniae	528	1	513	1	527	0.80	4	512	755.4	2.5e-218	gene=Chr04G0360.1	XP_007601935.1[ColletotrichumfioriniaePJ7]

			PJ7]												
Chr05G1036.1	1039	29.3	EEA28139.1[Penicilliummarneffei]	2025	32	562	972	1490	0.80	15	530	210.7	4.6e-54	gene=Chr05G1036.1	EEA28139.1[Penicilliummarneffei]
Chr04G0321.1	633	49.6	XP_007786991.1[EndocarponpusillumZ07020]	1112	162	630	651	1109	0.80	8	468	422.9	3.6e-118	gene=Chr04G0321.1	XP_007786991.1[EndocarponpusillumZ07020]
Chr03G0456.1	540	33.3	XP_007598108.1[ColletotrichumfioriniaePJ7]	877	6	472	25	502	0.80	13	466	255	1.1e-67	gene=Chr03G0456.1	XP_007598108.1[ColletotrichumfioriniaePJ7]
Chr02G0367.1	608	27.7	MUStwsD_GLEAN_10004418[Omphalotusolearius]	1665	51	604	1099	1651	0.80	20	553	140.6	3.4e-33	gene=Chr02G0367.1	MUStwsD_GLEAN_10004418[Omphalotusolearius]
Chr03G0072.1	582	32.4	Hanno_08615[Heterobasidionannosum03012]	1190	26	511	8	517	0.80	6	485	263.5	3.3e-70	gene=Chr03G0072.1	Hanno_08615[Heterobasidionannosum03012]

Chr02G0229.1	558	40.7	AFL2G_07229[Aspergillusflavus]	640	70	547	197	638	0.80	3	477	367.1	2.1e-101	gene=Chr02G0229.1	AFL2G_07229[Aspergillusflavus]
Chr03G1567.1	510	35.4	Pa_7_5740[PodosporaanserinaSmat+]	576	45	500	69	518	0.80	8	455	228	1.4e-59	gene=Chr03G1567.1	Pa_7_5740[PodosporaanserinaSmat+]
Chr03G1103.1	592	65.2	Clame_scaffold8-4.115[Cladoniametacorallifera]	16637	99	560	11017	11486	0.80	3	461	582.8	2.5e-166	gene=Chr03G1103.1	Clame_scaffold8-4.115[Cladoniametacorallifera]
Chr09G1037.1	2616	36	fgenesh1_pg.24_#_34[PhlebiabrevisporaHHB-7030SS6]	2158	7	456	360	807	0.80	9	449	263.5	1.5e-69	gene=Chr09G1037.1	fgenesh1_pg.24_#_34[PhlebiabrevisporaHHB-7030SS6]
Chr06G1139.1	642	43.5	AFL2G_07229[Aspergillusflavus]	640	80	554	197	638	0.80	5	474	363.6	2.6e-100	gene=Chr06G1139.1	AFL2G_07229[Aspergillusflavus]
Chr09G0821.1	616	28	MUStwsD_GLEAN_10004418[	1665	48	612	1099	1657	0.81	19	564	189.1	8.5e-48	gene=Chr09G0821.1	MUStwsD_GLEAN_10004418[Omphalotusolearius]

			Omphalotusolearius]												
Chr05G0127.1	597	42.6	AFL2G_07229[Aspergillusflavus]	640	116	587	197	637	0.81	3	471	389.4	4.1e-108	gene=Chr05G0127.1	AFL2G_07229[Aspergillusflavus]
Chr05G1341.1	620	35.2	Pa_7_5740[PodosporaanserinaSmat+]	576	91	561	65	529	0.81	12	470	272.3	7.6e-73	gene=Chr05G1341.1	Pa_7_5740[PodosporaanserinaSmat+]
Chr02G0547.1	613	31.1	maker-Caloplaca_flavorubescens_scaffold_19-augustus-gene-2.67.2-mRNA-1[Caloplacaflavorubescens]	1049	12	566	44	571	0.81	21	554	213.8	3.2e-55	gene=Chr02G0547.1	maker-Caloplaca_flavorubescens_scaffold_19-augustus-gene-2.67.2-mRNA-1[Caloplacaflavorubescens]
Chr02G1625.1	1042	34.5	AB00197.1[Alternariabrassicicola]	1041	9	536	5	490	0.81	10	527	281.6	2.1e-75	gene=Chr02G1625.1	AB00197.1[Alternariabrassicicola]

Chr07G1019.1	536	33.5	fgenesh1_pg.12_#_108[StereumhirsutumFP-91666SS1]	1485	40	509	671	1139	0.81	7	469	262.3	6.8e-70	gene=Chr07G1019.1	fgenesh1_pg.12_#_108[StereumhirsutumFP-91666SS1]
Chr01G2672.1	598	36.4	XP_007598108.1[ColletotrichumfioriniaePJ7]	877	28	543	12	515	0.81	17	515	331.3	1.3e-90	gene=Chr01G2672.1	XP_007598108.1[ColletotrichumfioriniaePJ7]
Chr06G0057.1	1268	27.4	EfO2.075130.1[Epichloefestucae]	1182	713	1265	617	1159	0.81	13	552	184.1	5.6e-46	gene=Chr06G0057.1	EfO2.075130.1[Epichloefestucae]
Chr04G1586.1	483	60	estExt_Genewise1.C_390001[Exidiaglandulosa]	503	22	483	16	503	0.81	3	461	555.4	3.5e-158	gene=Chr04G1586.1	estExt_Genewise1.C_390001[Exidiaglandulosa]
Chr02G0904.1	638	34.9	Clama_scaffold_28-0.22[Cladoniamacilenta]	3845	117	636	2082	2579	0.81	14	519	269.6	5.1e-72	gene=Chr02G0904.1	Clama_scaffold_28-0.22[Cladoniamacilenta]
Chr04G0029.1	547	37.9	Clame_sc	16637	13	478	14421	14920	0.8	8	465	352.4	5.1e-97	gene=C	Clame_scaffold8-4.1

			affold8-4.115[Cladoniametacorallifera]						1					hr04G0029.1	15[Cladoniametacorallifera]
Chr02G0483.1	509	64.3	XP_007596926.1[Colletotrichumfioriniae PJ7]	454	15	456	12	453	0.81	0	441	624.8	5.0e-179	gene=Chr02G0483.1	XP_007596926.1[ColletotrichumfioriniaePJ7]
Chr03G0651.1	517	44	HCB07277.1[Histoplasmacapsulatum]	1063	1	504	1	604	0.81	11	503	486.1	2.8e-137	gene=Chr03G0651.1	HCB07277.1[Histoplasmacapsulatum]
Chr02G0522.1	477	34	XP_007804728.1[EndocarponpusillumZ07020]	1059	7	476	582	1057	0.81	11	469	262.3	6.1e-70	gene=Chr02G0522.1	XP_007804728.1[EndocarponpusillumZ07020]
Chr01G0707.1	533	73.7	XP_007598492.1[Colletotrichumfioriniae PJ7]	529	1	528	1	529	0.81	1	527	802.4	1.8e-232	gene=Chr01G0707.1	XP_007598492.1[ColletotrichumfioriniaePJ7]
Chr01G0176.1	577	35.1	Pa_7_5740[Podosporaanserina	576	110	574	71	526	0.81	7	464	265.8	6.7e-71	gene=Chr01G0176.1	Pa_7_5740[PodosporaanserinaSmat+]

			Smat+]													
Chr04G0526.1	634	42.6	AFL2G_07229[Aspergillusflavus]	640	147	621	197	640	0.81	2	474	370.9	1.6e-102	gene=Chr04G0526.1	AFL2G_07229[Aspergillusflavus]
Chr06G1232.1	479	46.1	augustus_masked-Caloplaca_flavorubescens_scaffold_16-abinit-gene-0.40-mRNA-1[Caloplacaflavorubescens]	1008	1	471	1	511	0.82	11	470	412.9	2.8e-115	gene=Chr06G1232.1	augustus_masked-Caloplaca_flavorubescens_scaffold_16-abinit-gene-0.40-mRNA-1[Caloplacaflavorubescens]
Chr04G1003.1	502	37.9	Pa_7_5740[PodosporaanserinaSmat+]	576	33	499	79	524	0.82	3	466	250	3.3e-66	gene=Chr04G1003.1	Pa_7_5740[PodosporaanserinaSmat+]
Chr07G0933.1	499	68.5	fgenesh2_pm.4_#_883[Thielaviaterrestris]	488	22	494	12	484	0.82	0	472	709.1	2.0e-204	gene=Chr07G0933.1	fgenesh2_pm.4_#_883[Thielaviaterrestris]
Chr07G0437.1	480	37.3	Paxru1.fg	500	29	475	42	494	0.8	8	446	297.7	1.3e-80	gene=C	Paxru1.fgenesh1_kg

			enesh1_kg.1494_#_5_#_Locus356v1rpkm400.81[PaxillusadelphusVe08.2h10]						2					hr07G0437.1	.1494_#_5_#_Locus356v1rpkm400.81[PaxillusadelphusVe08.2h10]
Chr04G1155.1	531	70.5	XP_007595098.1[CollletotrichumfioriniaePJ7]	512	19	526	1	508	0.82	2	507	741.5	3.8e-214	gene=Chr04G1155.1	XP_007595098.1[CollletotrichumfioriniaePJ7]
Chr04G0204.1	609	27.5	MUStwsD_GLEAN_10004418[Omphalotusolearius]	1665	39	605	1099	1659	0.82	14	566	187.2	3.2e-47	gene=Chr04G0204.1	MUStwsD_GLEAN_10004418[Omphalotusolearius]
Chr09G0063.1	464	47.4	estExt_fgenesh1_pg.C_3530010[Exidiaglandulosa]	1270	9	454	545	992	0.82	10	445	436.8	1.8e-122	gene=Chr09G0063.1	estExt_fgenesh1_pg.C_3530010[Exidiaglandulosa]
Chr07G1039.1	560	75.4	XP_00759	559	1	559	1	559	0.8	2	558	888.6	2.0e-25	gene=C	XP_007597438.1[Co

			7438.1[Colletotrichumfioriniae PJ7]						2				8	hr07G1039.1	lletotrichumfioriniaePJ7]
Chr08G0703.1	2996	35.5	fgenesh1_pg.24_#_34[Phlebiabrevis poraHHB-7030SS6]	2158	2	483	346	820	0.82	13	481	259.6	2.5e-68	gene=Chr08G0703.1	fgenesh1_pg.24_#_34[Phlebiabrevis poraHHB-7030SS6]
Chr08G0099.1	631	28.5	Clame_scaffold8-4.115[Cladoniametacorallifera]	16637	46	603	3980	4587	0.82	15	557	227.3	2.9e-59	gene=Chr08G0099.1	Clame_scaffold8-4.115[Cladoniametacorallifera]
Chr09G0670.1	500	62.6	M_BR29_EuGene_00124391[MagnaporthegriseaBR29]	510	20	496	8	507	0.82	3	476	668.7	3.0e-192	gene=Chr09G0670.1	M_BR29_EuGene_00124391[MagnaporthegriseaBR29]
Chr07G0239.1	481	36.3	NCU03415T0[NeurosporacrassaOR74A]	495	24	472	36	489	0.82	6	448	293.9	1.9e-79	gene=Chr07G0239.1	NCU03415T0[NeurosporacrassaOR74A]
Chr03G1499.1	501	35.8	RO3G_10	494	15	496	18	490	0.8	11	481	276.9	2.5e-74	gene=C	RO3G_10182[Rhizo

			182[Rhizopusoryzae]						2					hr03G1499.1	pusoryzae]
Chr05G0169.1	467	49.9	PCON_06884m.01[Pyronemaconfluens CBS100304]	501	5	463	4	492	0.82	5	458	465.7	3.5e-131	gene=Chr05G0169.1	PCON_06884m.01[Pyronemaconfluens CBS100304]
Chr03G1110.1	750	49.2	1093_t[AscocorynesarcoidesNRRL50072]	1211	25	488	7	497	0.82	10	463	470.3	2.3e-132	gene=Chr03G1110.1	1093_t[AscocorynesarcoidesNRRL50072]
Chr02G0182.1	592	29.3	maker-Caloplaca_flavorubescens_scaffold_19-augustus-gene-2.67.2-mRNA-1[Caloplacaflavorubescens]	1049	2	550	6	571	0.82	22	548	157.5	2.6e-38	gene=Chr02G0182.1	maker-Caloplaca_flavorubescens_scaffold_19-augustus-gene-2.67.2-mRNA-1[Caloplacaflavorubescens]
Chr02G0331.1	624	33.5	EGG0649	748	112	610	218	738	0.8	12	498	271.9	1.0e-72	gene=C	EGG06499.1[Melam

			9.1[Melampsoralarici-populina98AG31]						2					hr02G0331.1	psoralarici-populina98AG31]
Chr02G0232.1	672	37	EAA64957.1[AspergillusnidulansFGSCA4]	1745	132	671	1220	1745	0.82	23	539	218.4	1.4e-56	gene=Chr02G0232.1	EAA64957.1[AspergillusnidulansFGSCA4]
Chr07G0109.1	521	33.1	fgenesh1_pg.12_#_108[StereumhirsutumFP-91666SS1]	1485	41	510	671	1147	0.82	7	469	238.8	7.9e-63	gene=Chr07G0109.1	fgenesh1_pg.12_#_108[StereumhirsutumFP-91666SS1]
Chr03G1500.1	522	72.5	XP_007602828.1[ColletotrichumfioriniaePJ7]	523	1	521	1	523	0.82	1	520	766.1	1.4e-221	gene=Chr03G1500.1	XP_007602828.1[ColletotrichumfioriniaePJ7]
Chr01G2782.1	581	45	1093_t[AscocorynesarcoidesNRRL50072]	1211	21	491	12	510	0.82	13	470	413.7	2.0e-115	gene=Chr01G2782.1	1093_t[AscocorynesarcoidesNRRL50072]
Chr03G1603.1	510	71.1	GLRG_07981T0[Col	508	1	506	1	505	0.82	1	505	763.5	9.0e-221	gene=Chr03G16	GLRG_07981T0[Colletotrichumgraminicol

			letotrichumgraminicolaM1.001]											03.1	aM1.001]
Chr01G1124.1	507	68.3	GLRG_09802T0[ColletotrichumgraminicolaM1.001]	515	1	503	1	515	0.83	1	502	741.5	3.6e-214	gene=Chr01G1124.1	GLRG_09802T0[ColletotrichumgraminicolaM1.001]
Chr01G1975.1	538	37.1	AB00197.1[Alternariabrassicicola]	1041	11	533	5	490	0.83	9	522	351.7	8.6e-97	gene=Chr01G1975.1	AB00197.1[Alternariabrassicicola]
Chr03G1133.1	819	65.3	Clama_scaffold_5-4.0[Cladoniamacilenta]	21962	198	779	17166	17771	0.83	2	581	810.4	1.0e-234	gene=Chr03G1133.1	Clama_scaffold_5-4.0[Cladoniamacilenta]
Chr02G1793.1	933	32.9	CNAG_03466T0[Cryptococcusneoformansvar.grubiiH99]	1123	379	914	361	890	0.83	19	535	235.3	1.6e-61	gene=Chr02G1793.1	CNAG_03466T0[Cryptococcusneoformansvar.grubiiH99]
Chr01G0309.1	633	32.8	fgenesh1_pg.89_&#	737	63	585	41	565	0.83	16	522	239.2	7.3e-63	gene=Chr01G03	fgenesh1_pg.89_#_4[Hydnomeruliu

			35;_4[Hydnomeruliuspinastri]											09.1	spinastri]
Chr09G0428.1	555	60.5	FFUJ_08031m.01[FusariumfujikuroiIMI58289]	509	25	555	18	509	0.83	4	530	647.5	7.8e-186	gene=Chr09G0428.1	FFUJ_08031m.01[FusariumfujikuroiIMI58289]
Chr01G1736.1	560	36.3	XP_007598108.1[ColletotrichumfioriniaePJ7]	877	12	509	25	506	0.83	13	497	276.6	3.7e-74	gene=Chr01G1736.1	XP_007598108.1[ColletotrichumfioriniaePJ7]
Chr02G0587.1	508	68.1	XP_007601303.1[ColletotrichumfioriniaePJ7]	498	21	498	20	498	0.83	1	477	696	1.8e-200	gene=Chr02G0587.1	XP_007601303.1[ColletotrichumfioriniaePJ7]
Chr01G1884.1	1171	23.2	EAA60443.1[AspergillusnidulansFGSCA4]	2180	6	714	201	830	0.83	18	708	132.5	1.8e-30	gene=Chr01G1884.1	EAA60443.1[AspergillusnidulansFGSCA4]
Chr08G0696.1	1029	24.8	Clame_scaffold8-4.115[Cladoniametacora	16637	207	883	3962	4589	0.83	18	676	205.3	1.9e-52	gene=Chr08G0696.1	Clame_scaffold8-4.115[Cladoniametacorallifera]

			llifera]												
Chr08G0987.1	526	72.2	XP_007598685.1[Colletotrichumfioriniae PJ7]	527	1	524	1	524	0.83	2	523	770.8	5.8e-223	gene=Chr08G0987.1	XP_007598685.1[ColletotrichumfioriniaePJ7]
Chr01G1761.1	589	38.2	Pa_7_5740[Podosporaanserina Smat+]	576	109	582	64	520	0.83	5	473	281.6	1.2e-75	gene=Chr01G1761.1	Pa_7_5740[PodosporaanserinaSmat+]
Chr04G1158.1	556	35.6	KKY16879.1putative membrane transporter[Phaeomoniellachlamydospora][Phaeomoniellachlamydospora]	993	34	536	16	499	0.84	8	502	324.3	1.5e-88	gene=Chr04G1158.1	KKY16879.1putative membranetransporter[Phaeomoniellachlamydospora][Phaeomoniellachlamydospora]
Chr01G1523.1	1140	88.1	GLRG_11426T0[Colletotrichumgraminicola M1.001	1109	53	1140	32	1110	0.84	8	1087	1933.3	0.0e+00	gene=Chr01G1523.1	GLRG_11426T0[ColletotrichumgraminicolaM1.001]

			]												
Chr02G1563.1	864	35.1	Clame_scaffold9-5.100[Cladoniametacorallifera]	12517	254	792	1477	1991	0.84	9	538	323.2	5.3e-88	gene=Chr02G1563.1	Clame_scaffold9-5.100[Cladoniametacorallifera]
Chr02G1601.1	610	34.2	maker-Caloplaca_flavorubescens_scaffold_19-augustus-gene-2.67.2-mRNA-1[Caloplacaflavorubescens]	1049	31	568	47	574	0.84	24	537	245.4	9.8e-65	gene=Chr02G1601.1	maker-Caloplaca_flavorubescens_scaffold_19-augustus-gene-2.67.2-mRNA-1[Caloplacaflavorubescens]
Chr01G0399.1	521	38.8	CNAG_06628T0[Cryptococcusneoformansvar.grubiiH99]	506	20	496	37	497	0.84	7	476	288.9	6.6e-78	gene=Chr01G0399.1	CNAG_06628T0[Cryptococcusneoformansvar.grubiiH99]
Chr02G0272.1	527	71.9	CH063_01365T0[Colletotrichu	525	1	526	1	525	0.85	1	525	768.5	2.9e-222	gene=Chr02G0272.1	CH063_01365T0[Colletotrichumhigginsianum]

			mhigginsianum]												
Chr07G0154.1	492	36.2	PCON_06884m.01[Pyronema confluens CBS100304]	501	18	483	22	487	0.85	6	465	294.3	1.5e-79	gene=Chr07G0154.1	PCON_06884m.01[Pyronemaconfluens CBS100304]
Chr02G0643.1	627	30.5	fgenesh1_pg.89_#_4[Hydnomeruliu spinastri]	737	38	592	16	565	0.85	15	554	242.7	6.6e-64	gene=Chr02G0643.1	fgenesh1_pg.89_#_4[Hydnomeruliu spinastri]
Chr03G1092.1	1895	28.7	PTRG_10301[Pyrenophoratritici-repentis]	1311	1256	1805	206	795	0.85	13	549	216.5	1.5e-55	gene=Chr03G1092.1	PTRG_10301[Pyrenophoratritici-repentis]
Chr04G1411.1	611	27.9	MUStwsD_GLEAN_10004418[Omphalotusolearius]	1665	28	608	1099	1659	0.85	12	580	204.9	1.5e-52	gene=Chr04G1411.1	MUStwsD_GLEAN_10004418[Omphalotusolearius]
Chr04G0748.1	582	29	MUStwsD_GLEAN_	1665	17	576	1099	1660	0.85	17	559	181	2.2e-45	gene=Chr04G07	MUStwsD_GLEAN_10004418[Omphalot

			10004418[Omphalotusolearius]												48.1	usolearius]
Chr04G0741.1	549	41.3	maker-scaffold4.1-snap-gene-7.120-mRNA-1[Raffaeleaquercus-mongolicae]	582	36	532	59	557	0.85	7	496	365.2	7.7e-101	gene=Chr04G0741.1	maker-scaffold4.1-snap-gene-7.120-mRNA-1[Raffaeleaquercus-mongolicae]
Chr01G2017.1	502	62.1	XP_007597422.1[Colletotrichumfioriniae PJ7]	451	1	451	1	451	0.85	0	450	601.7	4.5e-172	gene=Chr01G2017.1	XP_007597422.1[ColletotrichumfioriniaePJ7]
Chr01G0767.1	535	51.6	VDAG_05420[VerticilliumdahliaeVdLs.17]	502	57	529	53	502	0.85	1	472	503.8	1.3e-142	gene=Chr01G0767.1	VDAG_05420[VerticilliumdahliaeVdLs.17]
Chr09G0238.1	501	39.1	CNAG_06628T0[Cryptococcus neoformansvar.grubii	506	25	490	40	499	0.85	7	465	311.6	9.2e-85	gene=Chr09G0238.1	CNAG_06628T0[Cryptococcusneoformansvar.grubiiH99]

			H99]													
Chr01G0642.1	500	66.9	CH063_03085T0[Colletotrichumhigginsianum]	481	17	496	1	480	0.85	0	479	657.5	6.8e-189	gene=Chr01G0642.1	CH063_03085T0[Colletotrichumhigginsianum]
Chr02G0551.1	830	34	AFL2G_07147[Aspergillusflavus]	1440	248	796	403	919	0.85	14	548	305.1	1.4e-82	gene=Chr02G0551.1	AFL2G_07147[Aspergillusflavus]
Chr05G0335.1	537	71.5	XP_007603232.1[ColletotrichumfioriniaePJ7]	539	1	535	1	535	0.85	2	534	814.3	4.7e-236	gene=Chr05G0335.1	XP_007603232.1[ColletotrichumfioriniaePJ7]
Chr02G1367.1	516	68.3	GLRG_06806T0[ColletotrichumgraminicolaM1.001]	513	1	512	1	511	0.85	4	511	734.6	4.5e-212	gene=Chr02G1367.1	GLRG_06806T0[ColletotrichumgraminicolaM1.001]
Chr07G0250.1	555	33.1	Clama_scaffold_5-4.0[Cladoniamacilenta]	21962	19	541	19656	20234	0.85	13	522	267.7	1.7e-71	gene=Chr07G0250.1	Clama_scaffold_5-4.0[Cladoniamacilenta]
Chr04G1054.1	520	58.9	CH063_05	500	1	518	1	499	0.8	6	517	592.4	2.8e-16	gene=C	CH063_05046T0[Co

			046T0[Colletotrichumhigginsianum]						5				9	hr04G1054.1	lletotrichumhigginsianum]
Chr09G0459.1	569	36.9	Clama_scaffold_5-4.0[Cladoniamacilenta]	21962	11	548	19659	20233	0.85	16	537	343.2	3.2e-94	gene=Chr09G0459.1	Clama_scaffold_5-4.0[Cladoniamacilenta]
Chr05G0252.1	535	70.7	XP_007596379.1[CollletotrichumfioriniaePJ7]	542	1	534	1	542	0.85	2	533	805.4	2.2e-233	gene=Chr05G0252.1	XP_007596379.1[CollletotrichumfioriniaePJ7]
Chr06G0847.1	573	70.4	e_gw1.2.60.1[ColletotrichumsublineolaCBS131301]	1052	1	569	487	1049	0.86	7	568	791.2	4.5e-229	gene=Chr06G0847.1	e_gw1.2.60.1[ColletotrichumsublineolaCBS131301]
Chr01G0812.1	522	37.2	FFUJ_01995m.01[FusariumfujikuroiIMI58289]	501	14	500	19	490	0.86	6	486	322.4	5.4e-88	gene=Chr01G0812.1	FFUJ_01995m.01[FusariumfujikuroiIMI58289]
Chr03G0283.1	523	39.4	KKY16879.1putative	993	39	503	45	499	0.86	2	464	360.1	2.4e-99	gene=Chr03G02	KKY16879.1putativemembranetransporte

			membrane transporter[Phaeomoniellachlamydospora][Phaeomoniellachlamydospora]											83.1	r[Phaeomoniellachlamydospora][Phaeomoniellachlamydospora]
Chr02G0294.1	554	38.6	Pa_7_5740[PodosporaanserinaSmat+]	576	75	551	61	525	0.86	6	476	291.6	1.1e-78	gene=Chr02G0294.1	Pa_7_5740[PodosporaanserinaSmat+]
Chr01G0054.1	529	57.1	e_gw1.87.249.1[Fistulinahepatica]	574	36	526	51	572	0.86	2	490	630.9	7.2e-181	gene=Chr01G0054.1	e_gw1.87.249.1[Fistulinahepatica]
Chr04G1045.1	576	28.8	MUStwsD_GLEAN_10004418[Omphalotusolearius]	1665	13	568	1102	1660	0.86	14	555	210.3	3.3e-54	gene=Chr04G1045.1	MUStwsD_GLEAN_10004418[Omphalotusolearius]
Chr09G0730.1	582	36.3	ATEG_04721.1[Aspergillusterr	994	75	556	479	964	0.86	5	481	297	2.7e-80	gene=Chr09G0730.1	ATEG_04721.1[Aspergillusterreus]

			eus]												
Chr02G0798.1	644	28.6	EAL87112.1[AspergillusfumigatusAf293]	671	17	611	8	644	0.86	19	594	242.3	8.8e-64	gene=Chr02G0798.1	EAL87112.1[AspergillusfumigatusAf293]
Chr02G1129.1	515	36	NFIA_089120[Neosartoryafischeri]	487	16	497	7	481	0.86	4	481	325.9	4.8e-89	gene=Chr02G1129.1	NFIA_089120[Neosartoryafischeri]
Chr04G0215.1	519	52.5	XP_007596512.1[ColletotrichumfioriniaePJ7]	496	3	514	4	493	0.87	11	511	513.8	1.3e-145	gene=Chr04G0215.1	XP_007596512.1[ColletotrichumfioriniaePJ7]
Chr01G2641.1	728	36.2	AFL2G_01551[Aspergillusflavus]	1045	161	647	497	975	0.87	6	486	302.8	6.2e-82	gene=Chr01G2641.1	AFL2G_01551[Aspergillusflavus]
Chr04G0386.1	712	29.8	estExt_Genewise1Plus.C_60940[Cochliobolusheterostrophus]	1066	83	710	493	1065	0.87	22	627	239.6	6.3e-63	gene=Chr04G0386.1	estExt_Genewise1Plus.C_60940[Cochliobolusheterostrophus]
Chr03G1727.1	499	37	estExt_Genewise1Pl	501	16	490	21	494	0.87	5	474	323.2	3.0e-88	gene=Chr03G17	estExt_Genewise1Plus.C_220314[Sphae

			us.C_220314[Sphaerobolusstellatus]										27.1	robolusstellatus]
Chr03G0776.1	516	43	XP_007801776.1[EndocarponpusillumZ07020]	703	25	515	233	702	0.87	10	490	375.2	7.0e-104	gene=Chr03G0776.1	XP_007801776.1[EndocarponpusillumZ07020]
Chr02G0037.1	638	58.5	augustus_masked-scaffold35-processed-gene-0.55-mRNA-1[PseudohalonectrialignicolaM95]	1257	1	491	551	1071	0.87	5	490	633.6	1.3e-181	gene=Chr02G0037.1	augustus_masked-scaffold35-processed-gene-0.55-mRNA-1[PseudohalonectrialignicolaM95]
Chr07G0777.1	630	35.5	XP_007801092.1[EndocarponpusillumZ07020]	1039	31	598	27	589	0.87	14	567	303.9	2.4e-82	gene=Chr07G0777.1	XP_007801092.1[EndocarponpusillumZ07020]
Chr05G0073.1	499	62.3	Lema_T074100.1[Le	529	24	491	57	526	0.87	1	467	636.7	1.2e-182	gene=Chr05G00	Lema_T074100.1[Leptosphaeriamaculan

			ptosphaeriamaculans]											73.1	s]
Chr01G0163.1	513	67.3	XP_007592925.1[ColletotrichumfioriniaePJ7]	508	1	512	1	508	0.87	4	511	719.5	1.5e-207	gene=Chr01G0163.1	XP_007592925.1[ColletotrichumfioriniaePJ7]
Chr01G0622.1	542	51.2	e_gw1.1.1192.1[Cochliobolusheterostrophus]	471	54	540	4	471	0.88	3	486	522.7	2.8e-148	gene=Chr01G0622.1	e_gw1.1.1192.1[Cochliobolusheterostrophus]
Chr05G1270.1	560	69.8	XP_007601432.1[ColletotrichumfioriniaePJ7]	567	1	555	1	565	0.88	3	554	833.2	1.0e-241	gene=Chr05G1270.1	XP_007601432.1[ColletotrichumfioriniaePJ7]
Chr09G0494.1	646	30.4	fgenesh1_pg.3_#_78[Botryobasidiumbotryosum]	1250	10	638	2	608	0.88	29	628	227.6	2.3e-59	gene=Chr09G0494.1	fgenesh1_pg.3_#_78[Botryobasidiumbotryosum]
Chr02G0170.1	1169	30.5	augustus_masked-s	1986	411	988	714	1317	0.88	17	577	220.7	5.0e-57	gene=Chr02G01	augustus_masked-scaffold418-processe

			caffold418-processed-gene-0.20-mRNA-1[Magnaporthesalvini iM69]											70.1	d-gene-0.20-mRNA-1[Magnaporthesalvin iiM69]
Chr01G1485.1	503	64.6	CH063_01282T0[Colletotrichumhigginsianum]	499	1	498	1	495	0.88	2	497	679.1	2.2e-195	gene=Chr01G1485.1	CH063_01282T0[Colletotrichumhigginsianum]
Chr06G0020.1	1558	35.9	EEA28139.1[Penicilliummarneffei]	2025	67	606	602	1132	0.88	14	539	305.1	2.7e-82	gene=Chr06G0020.1	EEA28139.1[Penicilliummarneffei]
Chr05G0975.1	501	65.4	XP_007595863.1[ColletotrichumfioriniaePJ7]	500	1	496	1	494	0.88	4	495	682.2	2.6e-196	gene=Chr05G0975.1	XP_007595863.1[ColletotrichumfioriniaePJ7]
Chr09G0544.1	470	48	XP_007593653.1[ColletotrichumfioriniaePJ7]	478	1	469	1	478	0.88	4	468	450.7	1.2e-126	gene=Chr09G0544.1	XP_007593653.1[ColletotrichumfioriniaePJ7]

Chr02G1292.1	1014	28.8	augustus_masked-scaffold418-processed-gene-0.20-mRNA-1[Magnaporthesalvinii M69]	1986	203	824	721	1312	0.88	21	621	206.8	6.5e-53	gene=Chr02G1292.1	augustus_masked-scaffold418-processed-gene-0.20-mRNA-1[Magnaporthesalvinii M69]
Chr02G1505.1	583	36.7	EAA64957.1[AspergillusnidulansFGSCA4]	1745	3	547	560	1082	0.88	14	544	327.4	1.9e-89	gene=Chr02G1505.1	EAA64957.1[AspergillusnidulansFGSCA4]
Chr01G0853.1	693	30.1	fgenesh1_kg.38_#_38_#_Locus16506v1rpkm5.70[Exidiaglandulosa]	1073	59	693	491	1073	0.88	22	634	242.3	9.5e-64	gene=Chr01G0853.1	fgenesh1_kg.38_#_38_#_Locus16506v1rpkm5.70[Exidiaglandulosa]
Chr04G0015.1	1175	31.7	EEA28139.1[Penicilliummarneffei]	2025	96	628	947	1479	0.88	13	532	234.6	3.3e-61	gene=Chr04G0015.1	EEA28139.1[Penicilliummarneffei]
Chr04G1078.1	546	70.3	GLRG_11	547	14	546	14	548	0.8	1	532	762.7	1.6e-22	gene=C	GLRG_11881T0[Coll

			881T0[ColletotrichumgraminicolaM1.001]						8				0	hr04G1078.1	etotrichumgraminicolaM1.001]
Chr03G1051.1	504	44.9	CH063_02967T0[Colletotrichumhigginsianum]	492	25	500	8	490	0.88	6	475	433	2.8e-121	gene=Chr03G1051.1	CH063_02967T0[Colletotrichumhigginsianum]
Chr02G0914.1	557	45.7	PLG_07201-R0[PseudohalonectrialignicolaM95]	618	21	556	6	610	0.88	11	535	500.7	1.2e-141	gene=Chr02G0914.1	PLG_07201-R0[PseudohalonectrialignicolaM95]
Chr01G1574.1	534	44.6	MGG_04345T0[Magnaportheoryzae70-15]	530	32	530	1	521	0.88	8	498	462.2	4.5e-130	gene=Chr01G1574.1	MGG_04345T0[Magnaportheoryzae70-15]
Chr02G1695.1	492	40.4	PCON_06884m.01[Pyronema confluens CBS100304]	501	19	486	22	487	0.89	5	467	315.8	4.8e-86	gene=Chr02G1695.1	PCON_06884m.01[Pyronemaconfluens CBS100304]

Chr05G1065.1	638	59.6	Clame_scaffold18-19.70[Cladoniametacorallifera]	7866	150	636	3631	4127	0.89	9	486	589.3	2.9e-168	gene=Chr05G1065.1	Clame_scaffold18-19.70[Cladoniametacorallifera]
Chr09G0927.1	763	28	Clame_scaffold8-4.115[Cladoniametacorallifera]	16637	94	726	3971	4587	0.89	15	632	236.9	4.4e-62	gene=Chr09G0927.1	Clame_scaffold8-4.115[Cladoniametacorallifera]
Chr07G1098.1	476	47.8	NCU03415T0[NeurosporacrassaOR74A]	495	9	466	20	489	0.89	4	457	431	9.9e-121	gene=Chr07G1098.1	NCU03415T0[NeurosporacrassaOR74A]
Chr01G1247.1	751	39.8	Clama_scaffold_5-4.0[Cladoniamacilenta]	21962	195	704	9552	10044	0.89	6	509	359.4	5.8e-99	gene=Chr01G1247.1	Clama_scaffold_5-4.0[Cladoniamacilenta]
Chr09G0276.1	607	60.2	maker-scaffold4.1-snap-gene-7.120-mRNA-1[Raffaeleaquercus-mongolicae]	582	68	602	60	582	0.89	8	534	597	1.3e-170	gene=Chr09G0276.1	maker-scaffold4.1-snap-gene-7.120-mRNA-1[Raffaeleaquercus-mongolicae]

Chr03G0099.1	1161	29.6	7575_t[AscocorynesarcoidesNRRL50072]	1484	445	1136	330	925	0.89	22	691	224.6	3.4e-58	gene=Chr03G0099.1	7575_t[AscocorynesarcoidesNRRL50072]
Chr01G2394.1	535	40.3	PCON_06884m.01[PyronemaconfluensCBS100304]	501	55	527	22	494	0.89	6	472	337.8	1.3e-92	gene=Chr01G2394.1	PCON_06884m.01[PyronemaconfluensCBS100304]
Chr07G0257.1	494	38.9	PCON_06884m.01[PyronemaconfluensCBS100304]	501	16	491	18	492	0.89	5	475	311.6	9.1e-85	gene=Chr07G0257.1	PCON_06884m.01[PyronemaconfluensCBS100304]
Chr01G1622.1	486	54.2	CH063_07836T0[Colletotrichumhigginsianum]	603	1	477	118	597	0.89	9	476	520	1.7e-147	gene=Chr01G1622.1	CH063_07836T0[Colletotrichumhigginsianum]
Chr01G1953.1	640	36.5	MUStwsD_GLEAN_10002689[Omphalot	1056	46	624	529	1051	0.89	15	578	323.2	3.9e-88	gene=Chr01G1953.1	MUStwsD_GLEAN_10002689[Omphalotusolearius]

			usolearius]												
Chr07G0069.1	558	38.3	XP_007598108.1[ColletotrichumfioriniaePJ7]	877	1	506	7	518	0.89	18	505	323.6	2.6e-88	gene=Chr07G0069.1	XP_007598108.1[ColletotrichumfioriniaePJ7]
Chr02G1683.1	555	69.2	XP_007590496.1[ColletotrichumfioriniaePJ7]	561	8	540	7	537	0.90	1	532	754.6	4.5e-218	gene=Chr02G1683.1	XP_007590496.1[ColletotrichumfioriniaePJ7]
Chr07G0432.1	882	55.8	GGTG_04203T0[Gaeumannomycesgraminisvar.triticiR3-111a-1]	514	18	499	21	507	0.90	7	481	519.6	3.9e-147	gene=Chr07G0432.1	GGTG_04203T0[Gaeumannomycesgraminisvar.triticiR3-111a-1]
Chr03G0898.1	609	36.2	MUStwsD_GLEAN_10002689[Omphalotusolearius]	1056	48	604	531	1054	0.90	12	556	340.9	1.7e-93	gene=Chr03G0898.1	MUStwsD_GLEAN_10002689[Omphalotusolearius]
Chr04G0776.1	514	62.2	XP_00758	534	1	513	1	534	0.9	2	512	723.8	8.0e-20	gene=C	XP_007589894.1[Co

			9894.1[ColletotrichumfioriniaePJ7]						0				9	hr04G0776.1	lletotrichumfioriniaePJ7]
Chr06G0345.1	901	28.8	EEA23526.1[Penicilliummarneffei]	1874	283	882	338	977	0.90	17	599	230.3	4.8e-60	gene=Chr06G0345.1	EEA23526.1[Penicilliummarneffei]
Chr03G0715.1	540	69.1	XP_007594162.1[ColletotrichumfioriniaePJ7]	537	1	539	1	537	0.90	3	538	724.9	3.8e-209	gene=Chr03G0715.1	XP_007594162.1[ColletotrichumfioriniaePJ7]
Chr04G1224.1	491	57.8	estExt_Genemark1.C_6300002[ColletotrichumsublineolaCBS131301]	490	13	487	14	489	0.90	4	474	558.5	4.2e-159	gene=Chr04G1224.1	estExt_Genemark1.C_6300002[ColletotrichumsublineolaCBS131301]
Chr08G0185.1	554	69.6	CH063_07627T0[Colletotrichumhigginsianum]	551	12	552	9	548	0.90	2	540	811.2	4.1e-235	gene=Chr08G0185.1	CH063_07627T0[Colletotrichumhigginsianum]
Chr01G1512.1	2756	29.3	EAA60443	2180	1313	1919	228	816	0.9	17	606	242.7	2.9e-63	gene=C	EAA60443.1[Aspergi

			.1[AspergillusnidulansFGSCA4]						0					hr01G1512.1	llusnidulansFGSCA4]
Chr02G1309.1	1416	33.7	AFL2G_07147[Aspergillusflavus]	1440	155	747	403	963	0.91	10	592	332	1.9e-90	gene=Chr02G1309.1	AFL2G_07147[Aspergillusflavus]
Chr05G0156.1	597	39.6	Pa_7_5740[PodosporaanserinaSmat+]	576	72	568	30	529	0.91	8	496	299.7	4.3e-81	gene=Chr05G0156.1	Pa_7_5740[PodosporaanserinaSmat+]
Chr05G0934.1	629	61.9	ATEG_04721.1[Aspergillusterreus]	994	101	592	479	974	0.92	2	491	594.7	6.8e-170	gene=Chr05G0934.1	ATEG_04721.1[Aspergillusterreus]
Chr07G1056.1	1017	31.9	EEA28139.1[Penicilliummarneffei]	2025	3	560	945	1494	0.92	11	557	252.7	1.0e-66	gene=Chr07G1056.1	EEA28139.1[Penicilliummarneffei]
Chr04G0040.1	1555	33	fgenesh1_pg.12_#_108[StereumhirsutumFP-91666SS1]	1485	4	545	590	1148	0.92	8	541	283.1	1.1e-75	gene=Chr04G0040.1	fgenesh1_pg.12_#_108[StereumhirsutumFP-91666SS1]
Chr07G0894.1	530	55.8	KKY16879	993	37	528	36	517	0.9	5	491	532.3	3.5e-15	gene=C	KKY16879.1putative

			.1putative membrane transporter[Phaeomoniellachlamydospora][Phaeomoniellachlamydospora]						2				1	hr07G0894.1	membranetransporter[Phaeomoniellachlamydospora][Phaeomoniellachlamydospora]
Chr09G0630.1	533	61.2	MGG_04345T0[Magnaportheoryzae70-15]	530	27	528	22	522	0.93	3	501	648.3	4.4e-186	gene=Chr09G0630.1	MGG_04345T0[Magnaportheoryzae70-15]
Chr09G0002.1	587	66.5	estExt_fgenesh1_pm.C_50001[Sporotrichumthermophile]	1067	48	582	4	536	0.93	4	534	725.7	2.4e-209	gene=Chr09G0002.1	estExt_fgenesh1_pm.C_50001[Sporotrichumthermophile]
Chr01G0285.1	540	64.7	XP_007601657.1[Colletotrichumfioriniae PJ7]	530	1	538	1	529	0.93	6	537	686.4	1.5e-197	gene=Chr01G0285.1	XP_007601657.1[ColletotrichumfioriniaePJ7]

Chr06G1273.1	531	63.3	XP_007597170.1[CollletotrichumfioriniaePJ7]	537	16	530	16	537	0.93	3	514	703.7	8.8e-203	gene=Chr06G1273.1	XP_007597170.1[CollletotrichumfioriniaePJ7]
Chr04G0285.1	513	62.7	GLRG_01104T0[CollletotrichumgraminicolaM1.001]	514	1	513	1	515	0.93	3	512	674.1	7.2e-194	gene=Chr04G0285.1	GLRG_01104T0[CollletotrichumgraminicolaM1.001]
Chr04G0728.1	641	33	fgenesh1_pg.3_#_78[Botryobasidiumbotryosum]	1250	51	629	30	608	0.93	19	578	248.1	1.6e-65	gene=Chr04G0728.1	fgenesh1_pg.3_#_78[Botryobasidiumbotryosum]
Chr01G1744.1	499	43.2	Lacam1.CE103718_1381[LaccariaamethystinaLaAM-08-1]	500	16	496	16	496	0.93	5	480	392.1	5.3e-109	gene=Chr01G1744.1	Lacam1.CE103718_1381[LaccariaamethystinaLaAM-08-1]
Chr05G1390.1	615	54.6	XP_007801210.1[Endocarponp	1151	95	595	73	560	0.93	6	500	552	5.0e-157	gene=Chr05G1390.1	XP_007801210.1[EndocarponpusillumZ07020]

			usillumZ07020]												
Chr06G0055.1	525	38.9	AO090011000412[Aspergillusoryzae]	548	4	521	36	544	0.93	8	517	357.8	1.2e-98	gene=Chr06G0055.1	AO090011000412[Aspergillusoryzae]
Chr06G1208.1	595	70.4	CE312466_2694[ColletotrichumsublineolaCBS131301]	615	1	591	1	604	0.93	4	590	856.3	1.2e-248	gene=Chr06G1208.1	CE312466_2694[ColletotrichumsublineolaCBS131301]
Chr02G1779.1	598	36.2	EEQ28888.1[Microsporumcanis]	609	53	568	52	581	0.94	6	515	336.3	4.2e-92	gene=Chr02G1779.1	EEQ28888.1[Microsporumcanis]
Chr07G1172.1	583	40.9	EEQ28888.1[Microsporumcanis]	609	47	562	52	593	0.94	10	515	387.9	1.2e-107	gene=Chr07G1172.1	EEQ28888.1[Microsporumcanis]
Chr01G0485.1	599	40.5	MUStwsD_GLEAN_10002689[Omphalotusolearius]	1056	68	590	542	1050	0.94	10	522	352.4	5.6e-97	gene=Chr01G0485.1	MUStwsD_GLEAN_10002689[Omphalotusolearius]

Chr07G0505.1	897	28	augustus_masked-scaffold418-processed-gene-0.20-mRNA-1[Magnaporthesalvinii M69]	1986	38	671	625	1269	0.94	17	633	206.1	9.7e-53	gene=Chr07G0505.1	augustus_masked-scaffold418-processed-gene-0.20-mRNA-1[Magnaporthesalvinii M69]
Chr04G0882.1	525	59.8	ACLA_093640[AspergillusclavatusNRRL1]	541	15	524	32	541	0.94	4	509	654.4	6.1e-188	gene=Chr04G0882.1	ACLA_093640[AspergillusclavatusNRRL1]
Chr01G0653.1	590	43.5	fgenesh1_pg.12_#_108[StereumhirsutumFP-91666SS1]	1485	55	542	659	1144	0.94	4	487	421	1.3e-117	gene=Chr01G0653.1	fgenesh1_pg.12_#_108[StereumhirsutumFP-91666SS1]
Chr03G0378.1	494	46.5	An13g01350[AspergillusnigerCBS513.88]	485	12	489	5	483	0.94	3	477	435.3	5.4e-122	gene=Chr03G0378.1	An13g01350[AspergillusnigerCBS513.88]
Chr03G0252.1	685	38.6	AFL2G_07147[Aspe	1440	110	665	398	930	0.94	6	555	365.5	7.3e-101	gene=Chr03G02	AFL2G_07147[Aspergillusflavus]

			rgillusflavus]											52.1	
Chr04G0924.1	558	58.8	XP_007598630.1[ColletotrichumfioriniaePJ7]	559	24	553	36	559	0.95	8	529	623.6	1.2e-178	gene=Chr04G0924.1	XP_007598630.1[ColletotrichumfioriniaePJ7]
Chr01G2474.1	521	43.8	AFL2G_02642[Aspergillusflavus]	507	26	516	15	502	0.95	4	490	422.5	3.8e-118	gene=Chr01G2474.1	AFL2G_02642[Aspergillusflavus]
Chr05G0347.1	1212	35.2	fgenesh1_pg.21_#_32[TulasnellacalosporaAL13]	710	143	838	62	684	0.95	18	695	388.3	1.9e-107	gene=Chr05G0347.1	fgenesh1_pg.21_#_32[TulasnellacalosporaAL13]
Chr02G1343.1	579	44.3	Clama_scaffold_28-0.22[Cladoniamacilenta]	3845	55	577	2064	2579	0.95	11	522	426	3.9e-119	gene=Chr02G1343.1	Clama_scaffold_28-0.22[Cladoniamacilenta]
Chr01G0012.1	516	53.8	M_BR29_EuGene_00026651[Magnaport	528	6	513	4	524	0.95	4	507	580.5	1.1e-165	gene=Chr01G0012.1	M_BR29_EuGene_00026651[MagnaporthegriseaBR29]

			hegriseaBR29]												
Chr02G1860.1	559	53.8	M_BR29_EuGene_00026651[MagnaporthegriseaBR29]	528	49	556	4	524	0.95	4	507	580.9	9.1e-166	gene=Chr02G1860.1	M_BR29_EuGene_00026651[MagnaporthegriseaBR29]
Chr01G0552.1	560	60.7	e_gw1.5.304.1[Thielaviaterrestris]	602	2	559	10	602	0.95	6	557	723.4	1.1e-208	gene=Chr01G0552.1	e_gw1.5.304.1[Thielaviaterrestris]
Chr04G0217.1	523	63	XP_007598718.1[CollletotrichumfioriniaePJ7]	523	1	520	1	519	0.95	4	519	691	5.8e-199	gene=Chr04G0217.1	XP_007598718.1[CollletotrichumfioriniaePJ7]
Chr01G1358.1	532	58.4	CH063_04672T0[Colletotrichumhigginsianum]	523	44	531	35	522	0.95	0	487	585.9	2.7e-167	gene=Chr01G1358.1	CH063_04672T0[Colletotrichumhigginsianum]
Chr03G1235.1	564	53.9	NCU03415T0[NeurosporacrassaOR74A]	495	70	560	8	493	0.95	5	490	537.3	1.2e-152	gene=Chr03G1235.1	NCU03415T0[NeurosporacrassaOR74A]

Chr01G0351.1	1235	32.5	EfO2.075130.1[Epichloefestucae]	1182	673	1224	582	1157	0.95	6	551	314.7	2.7e-85	gene=Chr01G0351.1	EfO2.075130.1[Epichloefestucae]
Chr04G0145.1	606	44.1	fgenesh1_pg.12_#_108[StereumhirsutumFP-91666SS1]	1485	58	545	659	1145	0.95	3	487	410.2	2.3e-114	gene=Chr04G0145.1	fgenesh1_pg.12_#_108[StereumhirsutumFP-91666SS1]
Chr04G0220.1	531	38.8	maker-scaffold4.1-snap-gene-7.120-mRNA-1[Raffaeleaquercus-mongolicae]	582	2	530	58	581	0.95	6	528	372.1	6.1e-103	gene=Chr04G0220.1	maker-scaffold4.1-snap-gene-7.120-mRNA-1[Raffaeleaquercus-mongolicae]
Chr06G1396.1	584	43.5	fgenesh1_pg.12_#_108[StereumhirsutumFP-91666SS1]	1485	52	540	660	1149	0.95	3	488	410.2	2.2e-114	gene=Chr06G1396.1	fgenesh1_pg.12_#_108[StereumhirsutumFP-91666SS1]
Chr03G1167.1	588	60.6	Pa_7_5740[Podospo	576	27	584	37	575	0.95	7	557	624.8	5.8e-179	gene=Chr03G11	Pa_7_5740[PodosporaanserinaSmat+]

			raanserinaSmat+]											67.1	
Chr03G1054.1	565	36.9	maker-scaffold4.1-snap-gene-7.120-mRNA-1[Raffaeleaquercus-mongolicae]	582	25	564	61	581	0.95	6	539	346.3	3.8e-95	gene=Chr03G1054.1	maker-scaffold4.1-snap-gene-7.120-mRNA-1[Raffaeleaquercus-mongolicae]
Chr05G0220.1	606	43.4	fgenesh1_pg.12_#_108[StereumhirsutumFP-91666SS1]	1485	49	539	660	1149	0.95	3	490	388.7	7.2e-108	gene=Chr05G0220.1	fgenesh1_pg.12_#_108[StereumhirsutumFP-91666SS1]
Chr02G1588.1	503	52.4	Suilu1.fgenesh1_kg.167_#_13_#_Locus272v1rpkm555.92[SuillusluteusUH-Slu-Lm8-n1]	500	15	502	17	500	0.95	3	487	493.4	1.7e-139	gene=Chr02G1588.1	Suilu1.fgenesh1_kg.167_#_13_#_Locus272v1rpkm555.92[SuillusluteusUH-Slu-Lm8-n1]

Chr02G0025.1	1821	32.9	fgenesh1_pg.21_#_32[TulasnellacalosporaAL13]	710	588	1338	43	701	0.96	18	750	407.1	5.8e-113	gene=Chr02G0025.1	fgenesh1_pg.21_#_32[TulasnellacalosporaAL13]
Chr05G0131.1	1093	28.8	augustus_masked-scaffold418-processed-gene-0.20-mRNA-1[MagnaporthesalviniiM69]	1986	84	735	654	1317	0.96	22	651	227.6	3.8e-59	gene=Chr05G0131.1	augustus_masked-scaffold418-processed-gene-0.20-mRNA-1[MagnaporthesalviniiM69]
Chr04G0438.1	1835	38.8	Clame_scaffold9-5.100[Cladoniametacorallifera]	12517	743	1321	3655	4290	0.96	20	578	347.4	5.5e-95	gene=Chr04G0438.1	Clame_scaffold9-5.100[Cladoniametacorallifera]
Chr01G1742.1	558	48.9	Clama_scaffold_5-4.0[Cladoniamacilenta]	21962	1	534	19648	20233	0.96	5	533	572.4	3.2e-163	gene=Chr01G1742.1	Clama_scaffold_5-4.0[Cladoniamacilenta]
Chr01G0299.1	514	46.1	GLRG_09802T0[Col	515	17	507	16	514	0.96	1	490	473	2.4e-133	gene=Chr01G02	GLRG_09802T0[Colletotrichumgraminicol

			letotrichumgraminicolaM1.001]											99.1	aM1.001]
Chr07G0738.1	608	70.8	MUStwsD_GLEAN_10005257[Omphalotusolearius]	1051	1	595	1	593	0.96	4	594	881.3	3.5e-256	gene=Chr07G0738.1	MUStwsD_GLEAN_10005257[Omphalotusolearius]
Chr06G0589.1	977	36.6	gm1.26033_g[Sphaerobolusstellatus]	943	328	928	3	574	0.96	13	600	311.2	2.3e-84	gene=Chr06G0589.1	gm1.26033_g[Sphaerobolusstellatus]
Chr03G1492.1	599	33.3	EAA58861.1[AspergillusnidulansFGSCA4]	643	1	592	1	643	0.97	14	591	359	6.0e-99	gene=Chr03G1492.1	EAA58861.1[AspergillusnidulansFGSCA4]
Chr08G0764.1	537	65.4	XP_007597170.1[ColletotrichumfioriniaePJ7]	537	1	536	1	537	0.97	1	535	767.7	5.0e-222	gene=Chr08G0764.1	XP_007597170.1[ColletotrichumfioriniaePJ7]
Chr08G0716.1	543	62.5	XP_007596494.1[Colletotrichu	545	1	539	1	542	0.97	5	538	709.1	2.1e-204	gene=Chr08G0716.1	XP_007596494.1[ColletotrichumfioriniaePJ7]

			mfioriniaePJ7]												
Chr01G0107.1	564	64	fgenesh1_kg.123_#_25_#_Locus3965v1rpkm28.11[ColletotrichumsublineolaCBS131301]	537	30	562	6	536	0.97	2	532	734.9	3.8e-212	gene=Chr01G0107.1	fgenesh1_kg.123_#_25_#_Locus3965v1rpkm28.11[ColletotrichumsublineolaCBS131301]
Chr07G0899.1	539	50.5	XP_007598042.1[ColletotrichumfioriniaePJ7]	532	18	519	36	531	0.97	3	501	507.3	1.2e-143	gene=Chr07G0899.1	XP_007598042.1[ColletotrichumfioriniaePJ7]
Chr01G1619.1	1622	34.8	fgenesh1_pg.21_#_32[TulasnellacalosporaAL13]	710	138	869	62	709	0.97	23	731	398.7	1.9e-110	gene=Chr01G1619.1	fgenesh1_pg.21_#_32[TulasnellacalosporaAL13]
Chr08G0209.1	1346	35.8	EEA28139.1[Penicilliummarneff	2025	89	688	603	1190	0.98	17	599	322.4	1.4e-87	gene=Chr08G0209.1	EEA28139.1[Penicilliummarneffei]

			ei]												
Chr08G0843.1	2162	40.7	fgenesh1_pg.21_#_32[TulasnellacalosporaAL13]	710	135	834	62	689	0.98	20	699	486.5	9.0e-137	gene=Chr08G0843.1	fgenesh1_pg.21_#_32[TulasnellacalosporaAL13]
Chr01G0207.1	629	41	maker-Caloplaca_flavorubescens_scaffold_19-augustus-gene-2.67.2-mRNA-1[Caloplacaflavorubescens]	1049	29	590	27	574	0.98	14	561	378.6	7.7e-105	gene=Chr01G0207.1	maker-Caloplaca_flavorubescens_scaffold_19-augustus-gene-2.67.2-mRNA-1[Caloplacaflavorubescens]
Chr01G0910.1	592	65.5	XP_007594063.1[ColletotrichumfioriniaePJ7]	618	1	591	4	618	0.99	2	590	853.6	7.7e-248	gene=Chr01G0910.1	XP_007594063.1[ColletotrichumfioriniaePJ7]
Chr04G0912.1	1855	28.2	CH063_03903T0[Colletotrichu	1339	1	755	645	1339	0.99	15	754	281.2	4.9e-75	gene=Chr04G0912.1	CH063_03903T0[Colletotrichumhigginsianum]

			mhigginsianum]													
Chr10G0009.1	797	25.9	Clame_scaffold8-4.115[Cladoniametacorallifera]	16637	29	758	3902	4585	0.99	20	729	199.1	1.1e-50	gene=Chr10G0009.1	Clame_scaffold8-4.115[Cladoniametacorallifera]
Chr05G0424.1	875	57	OAT02915.1[BlastomycesdermatitidisER-3]	1286	288	845	296	835	0.99	7	557	651	1.1e-186	gene=Chr05G0424.1	OAT02915.1[BlastomycesdermatitidisER-3]
Chr04G0035.1	615	39.7	maker-Caloplaca_flavorubescens_scaffold_19-augustus-gene-2.67.2-mRNA-1[Caloplacaflavorubescens]	1049	21	573	32	571	0.99	7	552	381.7	8.9e-106	gene=Chr04G0035.1	maker-Caloplaca_flavorubescens_scaffold_19-augustus-gene-2.67.2-mRNA-1[Caloplacaflavorubescens]
Chr04G0271.1	819	43.2	EEA28139.1[Penicilliummarneff	2025	1	567	597	1178	0.99	14	566	392.9	5.1e-109	gene=Chr04G0271.1	EEA28139.1[Penicilliummarneffei]

			ei]												
Chr01G0878.1	538	52.6	GLRG_11881T0[ColletotrichumgraminicolaM1.001]	547	16	535	21	545	0.99	8	519	558.9	3.5e-159	gene=Chr01G0878.1	GLRG_11881T0[ColletotrichumgraminicolaM1.001]
Chr05G1266.1	835	27	7575_t[AscocorynesarcoidesNRRL50072]	1484	1	798	171	924	0.99	30	797	238.4	1.6e-62	gene=Chr05G1266.1	7575_t[AscocorynesarcoidesNRRL50072]
Chr04G0454.1	796	30.3	EfO2.075130.1[Epichloefestucae]	1182	148	759	559	1174	0.99	13	611	217.6	2.9e-56	gene=Chr04G0454.1	EfO2.075130.1[Epichloefestucae]
Chr02G0403.1	540	61.2	estExt_Genemark1.C_40002[ColletotrichumsublineolaCBS131301]	536	9	538	8	536	0.99	2	529	711.8	3.3e-205	gene=Chr02G0403.1	estExt_Genemark1.C_40002[ColletotrichumsublineolaCBS131301]
Chr08G0406.1	604	44.9	gm1.6363_g[Tulasnellacalosp	616	82	604	88	616	0.99	6	522	464.5	1.0e-130	gene=Chr08G0406.1	gm1.6363_g[TulasnellacalosporaAL13]

			oraAL13]												
Chr06G0220.1	521	52.3	XP_007598042.1[ColletotrichumfioriniaePJ7]	532	9	519	20	532	1.00	5	510	509.2	3.1e-144	gene=Chr06G0220.1	XP_007598042.1[ColletotrichumfioriniaePJ7]
Chr06G0965.1	632	54.2	EEQ28888.1[Microsporumcanis]	609	57	599	42	593	1.00	8	542	573.9	1.3e-163	gene=Chr06G0965.1	EEQ28888.1[Microsporumcanis]
Chr06G0131.1	675	34	Clame_scaffold8-4.115[Cladoniametacorallifera]	16637	42	643	3936	4587	1.00	14	601	337	2.8e-92	gene=Chr06G0131.1	Clame_scaffold8-4.115[Cladoniametacorallifera]
Chr05G0818.1	532	61.2	estExt_Genemark1.C_1500014[ColletotrichumsublineolaCBS131301]	529	1	529	1	528	0.90	1	528	692.6	2.0e-199	gene=Chr05G0818.1	estExt_Genemark1.C_1500014[ColletotrichumsublineolaCBS131301]
Chr02G0030.1	629	52.3	CHG08027.1[ChaetomiumglobosumCB	1067	6	607	502	1064	0.90	12	601	600.1	1.6e-171	gene=Chr02G0030.1	CHG08027.1[ChaetomiumglobosumCBS148.51]

			S148.51]												
Chr01G1561.1	552	61.9	XP_007591045.1[ColletotrichumfioriniaePJ7]	544	6	551	2	544	0.90	2	545	699.9	1.3e-201	gene=Chr01G1561.1	XP_007591045.1[ColletotrichumfioriniaePJ7]
Chr04G0129.1	548	54.8	estExt_Genemark1.C_1500014[ColletotrichumsublineolaCBS131301]	529	21	539	5	522	0.90	4	518	594.7	5.9e-170	gene=Chr04G0129.1	estExt_Genemark1.C_1500014[ColletotrichumsublineolaCBS131301]
Chr09G0908.1	532	61.1	XP_007601226.1[ColletotrichumfioriniaePJ7]	531	1	529	1	529	0.91	0	528	715.7	2.2e-206	gene=Chr09G0908.1	XP_007601226.1[ColletotrichumfioriniaePJ7]
Chr09G0235.1	697	42	EEA28139.1[Penicilliummarneffei]	2025	137	668	922	1450	0.91	8	531	380.6	2.2e-105	gene=Chr09G0235.1	EEA28139.1[Penicilliummarneffei]
Chr06G0640.1	992	57.6	FOXG_20661T0[Fusariumoxysporumf.sp.	1035	16	587	10	570	0.91	10	571	664.1	1.4e-190	gene=Chr06G0640.1	FOXG_20661T0[Fusariumoxysporumf.sp.lycopersici]

			lycopersici]												
Chr03G0684.1	2189	37.6	fgenesh1_pg.21_#_32[TulasnellacalosporaAL13]	710	178	893	62	705	0.91	19	715	463.4	8.3e-130	gene=Chr03G0684.1	fgenesh1_pg.21_#_32[TulasnellacalosporaAL13]
Chr01G2116.1	796	26.3	Clame_scaffold8-4.115[Cladoniametacorallifera]	16637	13	742	3846	4586	0.91	18	729	233.4	5.1e-61	gene=Chr01G2116.1	Clame_scaffold8-4.115[Cladoniametacorallifera]
Chr04G0283.1	542	58.6	XP_007593287.1[ColletotrichumfioriniaePJ7]	550	11	541	13	550	0.91	2	530	681.4	4.8e-196	gene=Chr04G0283.1	XP_007593287.1[ColletotrichumfioriniaePJ7]
Chr05G1299.1	687	37.7	Clama_scaffold_5-4.0[Cladoniamacilenta]	21962	7	648	12826	13446	0.91	17	641	434.9	9.9e-122	gene=Chr05G1299.1	Clama_scaffold_5-4.0[Cladoniamacilenta]
Chr03G0955.1	582	48.4	Lema_T115650.1[Leptosphaeriamaculan	1350	8	581	752	1344	0.91	10	573	475.7	4.3e-134	gene=Chr03G0955.1	Lema_T115650.1[Leptosphaeriamaculans]

			s]												
Chr09G0835.1	935	28.7	augustus_masked-scaffold418-processed-gene-0.20-mRNA-1[MagnaporthesalviniiM69]	1986	9	724	606	1312	0.91	27	715	216.1	9.8e-56	gene=Chr09G0835.1	augustus_masked-scaffold418-processed-gene-0.20-mRNA-1[MagnaporthesalviniiM69]
Chr07G0567.1	772	32.7	Clame_scaffold8-4.115[Cladoniametacorallifera]	16637	41	750	3946	4588	0.91	11	709	302	1.1e-81	gene=Chr07G0567.1	Clame_scaffold8-4.115[Cladoniametacorallifera]
Chr04G1278.1	2360	34.5	EAA60443.1[AspergillusnidulansFGSCA4]	2180	1215	1808	138	726	0.91	9	593	338.6	3.3e-92	gene=Chr04G1278.1	EAA60443.1[AspergillusnidulansFGSCA4]
Chr03G1553.1	1677	33.4	fgenesh1_pg.21_#_32[TulasnellacalosporaAL13]	710	451	1201	9	701	0.92	21	750	393.7	6.2e-109	gene=Chr03G1553.1	fgenesh1_pg.21_#_32[TulasnellacalosporaAL13]
Chr01G0554.1	1712	33.9	fgenesh1_	710	547	1283	36	700	0.9	18	736	406.8	7.2e-11	gene=C	fgenesh1_pg.21_&#

			pg.21_#_32[TulasnellacalosporaAL13]						2				3	hr01G0554.1	35;_32[TulasnellacalosporaAL13]
Chr01G1552.1	1118	24.9	EEA28139.1[Penicilliummarneffei]	2025	3	801	617	1394	0.92	28	798	191	4.0e-48	gene=Chr01G1552.1	EEA28139.1[Penicilliummarneffei]
Chr01G0170.1	539	46.6	XP_007600811.1[CollletotrichumfioriniaePJ7]	560	1	533	1	556	0.92	5	532	524.2	9.7e-149	gene=Chr01G0170.1	XP_007600811.1[CollletotrichumfioriniaePJ7]
Chr02G0390.1	1012	43.4	fgenesh1_pg.8_#_209[PleurotusostreatusPC9]	1659	255	1000	1028	1654	0.92	14	745	431.8	1.2e-120	gene=Chr02G0390.1	fgenesh1_pg.8_#_209[PleurotusostreatusPC9]
Chr01G1391.1	538	55.2	XP_007603378.1[CollletotrichumfioriniaePJ7]	543	1	536	1	542	0.92	3	535	630.9	7.3e-181	gene=Chr01G1391.1	XP_007603378.1[CollletotrichumfioriniaePJ7]
Chr07G0991.1	577	60.5	CH063_05727T0[Col	573	13	576	21	573	0.92	5	563	688	5.4e-198	gene=Chr07G09	CH063_05727T0[Collletotrichumhigginsia

			letotrichumhigginsianum]											91.1	num]
Chr07G0328.1	914	27.6	augustus_masked-scaffold418-processed-gene-0.20-mRNA-1[Magnaporthesalvinii M69]	1986	20	768	617	1331	0.92	34	748	163.3	7.4e-40	gene=Chr07G0328.1	augustus_masked-scaffold418-processed-gene-0.20-mRNA-1[Magnaporthesalvinii M69]
Chr07G0260.1	709	32.6	Clame_scaffold8-4.115[Cladoniametacorallifera]	16637	47	678	3935	4587	0.92	12	631	329.7	4.6e-90	gene=Chr07G0260.1	Clame_scaffold8-4.115[Cladoniametacorallifera]
Chr05G0501.1	665	37.4	MUStwsD_GLEAN_10004571[Omphalotusolearius]	1115	23	614	24	618	0.93	11	591	355.1	9.6e-98	gene=Chr05G0501.1	MUStwsD_GLEAN_10004571[Omphalotusolearius]
Chr06G0348.1	657	34.9	Clame_scaffold8-4.115[Cladoni	16637	28	631	3948	4587	0.93	11	603	370.2	2.9e-102	gene=Chr06G0348.1	Clame_scaffold8-4.115[Cladoniametacorallifera]

			ametacorallifera]												
Chr06G0121.1	619	49.3	EEQ28888.1[Microsporumcanis]	609	63	601	52	605	0.93	6	538	524.6	8.5e-149	gene=Chr06G0121.1	EEQ28888.1[Microsporumcanis]
Chr07G0504.1	642	40.3	gm1.13924_g[Exidiaglandulosa]	902	28	620	6	576	0.93	15	592	418.7	6.8e-117	gene=Chr07G0504.1	gm1.13924_g[Exidiaglandulosa]
Chr04G0290.1	684	40.2	Clama_scaffold_5-4.0[Cladoniamacilenta]	21962	1	648	12820	13446	0.93	19	647	478.4	7.7e-135	gene=Chr04G0290.1	Clama_scaffold_5-4.0[Cladoniamacilenta]
Chr07G0404.1	1154	34.8	EfO2.075130.1[Epichloefestucae]	1182	256	840	582	1175	0.94	6	584	318.2	2.3e-86	gene=Chr07G0404.1	EfO2.075130.1[Epichloefestucae]
Chr08G0786.1	530	55.2	estExt_Genemark1.C_1500014[ColletotrichumsublineolaCBS131301]	529	1	527	1	527	0.94	0	526	616.3	1.8e-176	gene=Chr08G0786.1	estExt_Genemark1.C_1500014[ColletotrichumsublineolaCBS131301]
Chr01G0055.1	546	53.9	estExt_Ge	571	1	536	20	562	0.9	3	535	594.7	5.9e-17	gene=C	estExt_Genewise1.C

			newise1.C_21591[Cryphonectriaparasitica]						4				0	hr01G0055.1	_21591[Cryphonectriaparasitica]
Chr01G2630.1	877	25.5	augustus_masked-scaffold418-processed-gene-0.20-mRNA-1[MagnaporthesalviniiM69]	1986	52	859	636	1456	0.94	30	807	197.6	3.4e-50	gene=Chr01G2630.1	augustus_masked-scaffold418-processed-gene-0.20-mRNA-1[MagnaporthesalviniiM69]
Chr01G0737.1	959	28.7	augustus_masked-scaffold418-processed-gene-0.20-mRNA-1[MagnaporthesalviniiM69]	1986	53	753	636	1326	0.94	17	700	252.3	1.3e-66	gene=Chr01G0737.1	augustus_masked-scaffold418-processed-gene-0.20-mRNA-1[MagnaporthesalviniiM69]
Chr02G1259.1	561	55.1	CH063_00146T0[Colletotrichu	556	23	552	19	550	0.94	2	529	618.6	3.9e-177	gene=Chr02G1259.1	CH063_00146T0[Colletotrichumhigginsianum]

			mhigginsianum]												
Chr08G0238.1	586	55.6	XP_007601246.1[Colletotrichumfioriniae PJ7]	567	1	583	1	567	0.94	5	582	644.4	7.0e-185	gene=Chr08G0238.1	XP_007601246.1[ColletotrichumfioriniaePJ7]
Chr01G0963.1	594	53.8	VDAG_00797[VerticilliumdahliaeVdLs.17]	537	49	578	1	528	0.94	1	529	589.3	2.7e-168	gene=Chr01G0963.1	VDAG_00797[VerticilliumdahliaeVdLs.17]
Chr07G0118.1	2334	27.7	fgenesh1_pg.24_#_34[PhlebiabrevisporaHHB-7030SS6]	2158	11	796	360	1129	0.94	21	785	236.9	1.3e-61	gene=Chr07G0118.1	fgenesh1_pg.24_#_34[PhlebiabrevisporaHHB-7030SS6]
Chr03G0589.1	1008	36	EfO2.075130.1[Epichloefestucae]	1182	336	916	570	1157	0.95	5	580	360.9	2.7e-99	gene=Chr03G0589.1	EfO2.075130.1[Epichloefestucae]
Chr02G1322.1	554	50.9	GLRG_06846T0[ColletotrichumgraminicolaM1.001	537	1	528	1	529	0.95	1	527	589	3.3e-168	gene=Chr02G1322.1	GLRG_06846T0[ColletotrichumgraminicolaM1.001]

			]												
Chr04G0981.1	614	49.4	maker-Caloplaca_flavorubescens_scaffold_19-augustus-gene-2.67.2-mRNA-1[Caloplacaflavorubescens]	1049	2	575	8	574	0.95	13	573	513.5	2.0e-145	gene=Chr04G0981.1	maker-Caloplaca_flavorubescens_scaffold_19-augustus-gene-2.67.2-mRNA-1[Caloplacaflavorubescens]
Chr06G0731.1	581	39.7	AB04508.1[Alternariabrassicicola]	989	1	573	389	987	0.95	7	572	428.7	6.0e-120	gene=Chr06G0731.1	AB04508.1[Alternariabrassicicola]
Chr08G0186.1	586	56.4	XP_007599944.1[ColletotrichumfioriniaePJ7]	1113	19	573	22	584	0.96	6	554	620.5	1.1e-177	gene=Chr08G0186.1	XP_007599944.1[ColletotrichumfioriniaePJ7]
Chr03G1674.1	787	37.8	fgenesh1_pg.21_#_32[TulasnellacalosporaAL	710	39	776	34	708	0.96	19	737	481.1	1.4e-135	gene=Chr03G1674.1	fgenesh1_pg.21_#_32[TulasnellacalosporaAL13]

			13]												
Chr04G1425.1	773	35.8	fgenesh1_pg.21_#_32[TulasnellacalosporaAL13]	710	9	762	32	709	0.96	24	753	446.4	3.7e-125	gene=Chr04G1425.1	fgenesh1_pg.21_#_32[TulasnellacalosporaAL13]
Chr02G0056.1	839	36.4	EfO2.075130.1[Epichloefestucae]	1182	238	830	573	1171	0.96	9	592	347.1	3.3e-95	gene=Chr02G0056.1	EfO2.075130.1[Epichloefestucae]
Chr01G0232.1	766	42.2	EAL85053.1[AspergillusfumigatusAf293]	720	3	621	2	626	0.97	19	618	412.5	5.8e-115	gene=Chr01G0232.1	EAL85053.1[AspergillusfumigatusAf293]
Chr01G1650.1	589	60.9	XP_007596814.1[ColletotrichumfioriniaePJ7]	586	6	587	10	586	0.97	4	581	743.8	8.5e-215	gene=Chr01G1650.1	XP_007596814.1[ColletotrichumfioriniaePJ7]
Chr01G0675.1	902	31.1	augustus_masked-scaffold418-processed-gene-0.20-mRNA-	1986	36	723	619	1312	0.97	20	687	278.5	1.6e-74	gene=Chr01G0675.1	augustus_masked-scaffold418-processed-gene-0.20-mRNA-1[MagnaporthesalviniiM69]

			1[MagnaporthesalviniiM69]												
Chr09G0289.1	937	29.5	augustus_masked-scaffold418-processed-gene-0.20-mRNA-1[MagnaporthesalviniiM69]	1986	5	742	604	1314	0.98	28	737	249.6	8.0e-66	gene=Chr09G0289.1	augustus_masked-scaffold418-processed-gene-0.20-mRNA-1[MagnaporthesalviniiM69]
Chr01G1973.1	588	54.4	EAA59126.1[AspergillusnidulansFGSCA4]	1153	3	585	546	1152	0.98	8	582	642.9	2.0e-184	gene=Chr01G1973.1	EAA59126.1[AspergillusnidulansFGSCA4]
Chr03G1736.1	903	26.8	augustus_masked-scaffold418-processed-gene-0.20-mRNA-1[MagnaporthesalviniiM69]	1986	55	855	630	1452	0.99	27	800	242.3	1.2e-63	gene=Chr03G1736.1	augustus_masked-scaffold418-processed-gene-0.20-mRNA-1[MagnaporthesalviniiM69]
Chr07G1049.1	1223	27.8	augustus_	1986	43	855	622	1456	0.9	39	812	199.1	1.6e-50	gene=C	augustus_masked-s

			masked-scaffold418-processed-gene-0.20-mRNA-1[MagnaporthesalviniiM69]						9					hr07G1049.1	caffold418-processed-gene-0.20-mRNA-1[MagnaporthesalviniiM69]
Chr01G0465.1	977	30.3	augustus_masked-scaffold418-processed-gene-0.20-mRNA-1[MagnaporthesalviniiM69]	1986	82	866	660	1413	0.85	39	784	240.4	5.1e-63	gene=Chr01G0465.1	augustus_masked-scaffold418-processed-gene-0.20-mRNA-1[MagnaporthesalviniiM69]
Chr04G0858.1	771	33	4127_t[AscocorynesarcoidesNRRL50072]	1621	23	746	775	1528	0.86	25	723	349	8.0e-96	gene=Chr04G0858.1	4127_t[AscocorynesarcoidesNRRL50072]
Chr01G2588.1	924	74.2	estExt_fgenesh1_pm.C_1380002[Colletot	890	1	856	1	851	0.86	6	855	1282.3	0.0e+00	gene=Chr01G2588.1	estExt_fgenesh1_pm.C_1380002[ColletotrichumsublineolaCBS131301]

			richumsublineolaCBS131301]												
Chr01G0569.1	1123	83.8	XP_007592486.1[ColletotrichumfioriniaePJ7]	1070	48	1122	1	1070	0.87	4	1074	1835.1	0.0e+00	gene=Chr01G0569.1	XP_007592486.1[ColletotrichumfioriniaePJ7]
Chr07G0428.1	718	31.2	augustus_masked-scaffold9-processed-gene-0.95-mRNA-1[Magnaporthesalvinii M69]	2316	5	706	131	846	0.87	13	701	323.6	3.3e-88	gene=Chr07G0428.1	augustus_masked-scaffold9-processed-gene-0.95-mRNA-1[MagnaporthesalviniiM69]
Chr04G1469.1	685	42	Hanno_08615[Heterobasidionannosum03012]	1190	6	633	8	650	0.88	7	627	470.3	2.1e-132	gene=Chr04G1469.1	Hanno_08615[Heterobasidionannosum03012]
Chr07G0096.1	1605	37.1	CH063_03903T0[Colletotrichumhigginsia	1339	1	669	645	1339	0.88	17	668	415.6	1.4e-115	gene=Chr07G0096.1	CH063_03903T0[Colletotrichumhigginsianum]

			num]												
Chr04G0447.1	739	50.3	Clame_scaffold9-5.100[Cladoniametacorallifera]	12517	99	708	6179	6808	0.88	7	609	532.3	4.9e-151	gene=Chr04G0447.1	Clame_scaffold9-5.100[Cladoniametacorallifera]
Chr04G0225.1	2498	25.9	fgenesh1_pg.24_#_34[PhlebiabrevisporaHHB-7030SS6]	2158	24	911	359	1209	0.89	29	887	236.9	1.4e-61	gene=Chr04G0225.1	fgenesh1_pg.24_#_34[PhlebiabrevisporaHHB-7030SS6]
Chr05G0782.1	698	60.8	estExt_fgenesh1_pm.C_170119[Cenococcumgeophilum1.58]	1191	29	692	8	670	0.89	8	663	787.3	8.0e-228	gene=Chr05G0782.1	estExt_fgenesh1_pm.C_170119[Cenococcumgeophilum1.58]
Chr05G1235.1	1087	54.7	AB02265.1[Alternariabrassicicola]	1503	482	1086	889	1503	0.89	4	604	592	7.6e-169	gene=Chr05G1235.1	AB02265.1[Alternariabrassicicola]
Chr03G1491.1	680	39.7	Hanno_08615[Heterobasidionannosum0	1190	6	657	7	671	0.90	9	651	440.3	2.3e-123	gene=Chr03G1491.1	Hanno_08615[Heterobasidionannosum03012]

			3012]												
Chr01G2558.1	905	28.1	augustus_masked-scaffold418-processed-gene-0.20-mRNA-1[MagnaporthesalviniiM69]	1986	44	897	625	1458	0.91	35	853	251.1	2.7e-66	gene=Chr01G2558.1	augustus_masked-scaffold418-processed-gene-0.20-mRNA-1[MagnaporthesalviniiM69]
Chr02G0803.1	932	42.7	FVEG_03368T0[Fusariumverticillioides]	884	5	701	3	657	0.91	9	696	531.2	1.4e-150	gene=Chr02G0803.1	FVEG_03368T0[Fusariumverticillioides]
Chr03G0550.1	857	49.4	Clama_scaffold_5-4.0[Cladoniamacilenta]	21962	7	854	8305	9108	0.92	13	847	714.5	8.1e-206	gene=Chr03G0550.1	Clama_scaffold_5-4.0[Cladoniamacilenta]
Chr04G0216.1	1974	25.9	fgenesh1_pg.24_#_34[PhlebiabrevisporaHHB-7030SS6]	2158	16	922	357	1237	0.93	27	906	258.1	4.8e-68	gene=Chr04G0216.1	fgenesh1_pg.24_#_34[PhlebiabrevisporaHHB-7030SS6]
Chr02G0048.1	1135	76	XP_007594345.1[Co	1053	1	1064	1	1047	0.93	4	1063	1718.4	0.0e+00	gene=Chr02G00	XP_007594345.1[ColletotrichumfioriniaeP

			lletotrichumfioriniaePJ7]											48.1	J7]
Chr07G0548.1	908	29.1	augustus_masked-scaffold418-processed-gene-0.20-mRNA-1[MagnaporthesalviniiM69]	1986	31	896	617	1452	0.94	30	865	280.4	4.1e-75	gene=Chr07G0548.1	augustus_masked-scaffold418-processed-gene-0.20-mRNA-1[MagnaporthesalviniiM69]
Chr04G0298.1	923	28.5	augustus_masked-scaffold418-processed-gene-0.20-mRNA-1[MagnaporthesalviniiM69]	1986	38	909	625	1456	0.94	33	871	244.2	3.3e-64	gene=Chr04G0298.1	augustus_masked-scaffold418-processed-gene-0.20-mRNA-1[MagnaporthesalviniiM69]
Chr04G0926.1	2516	28.2	fgenesh1_pg.24_#_34[PhlebiabrevisporaHHB-	2158	84	964	344	1209	0.95	30	880	330.9	7.4e-90	gene=Chr04G0926.1	fgenesh1_pg.24_#_34[PhlebiabrevisporaHHB-7030SS6]

			7030SS6]												
Chr03G1353.1	1155	83.6	GLRG_01818T0[ColletotrichumgraminicolaM1.001]	1162	1	1153	1	1152	0.95	2	1152	1994.5	0.0e+00	gene=Chr03G1353.1	GLRG_01818T0[ColletotrichumgraminicolaM1.001]
Chr03G1623.1	902	29.3	augustus_masked-scaffold418-processed-gene-0.20-mRNA-1[MagnaporthesalviniiM69]	1986	58	887	630	1452	0.95	24	829	291.2	2.3e-78	gene=Chr03G1623.1	augustus_masked-scaffold418-processed-gene-0.20-mRNA-1[MagnaporthesalviniiM69]
Chr02G0269.1	942	29.7	augustus_masked-scaffold418-processed-gene-0.20-mRNA-1[MagnaporthesalviniiM69]	1986	43	918	625	1456	0.95	30	875	285	1.7e-76	gene=Chr02G0269.1	augustus_masked-scaffold418-processed-gene-0.20-mRNA-1[MagnaporthesalviniiM69]
Chr06G0519.1	1097	28.2	EEA23526	1874	163	1067	147	969	0.9	20	904	332.8	8.4e-91	gene=C	EEA23526.1[Penicilli

			.1[Penicilliummarneffei]						7					hr06G0519.1	ummarneffei]
Chr07G0993.1	3964	29.3	fgenesh1_pg.24_#_34[PhlebiabrevisporaHHB-7030SS6]	2158	2	928	359	1216	0.98	27	926	315.8	3.9e-85	gene=Chr07G0993.1	fgenesh1_pg.24_#_34[PhlebiabrevisporaHHB-7030SS6]
Chr02G1017.1	702	50.5	estExt_GeneWisePlus.C_50091[AspergillusnigerATCC1015]	1375	3	695	152	826	0.99	7	692	691.4	6.0e-199	gene=Chr02G1017.1	estExt_GeneWisePlus.C_50091[AspergillusnigerATCC1015]
Chr07G0793.1	1009	32.8	EAA62169.1[AspergillusnidulansFGSCA4]	2412	8	955	7	946	0.86	32	947	417.2	3.1e-116	gene=Chr07G0793.1	EAA62169.1[AspergillusnidulansFGSCA4]
Chr09G0023.1	1457	30	estExt_fgenesh2_pg.C_10473[SchizophyllumcommuneH4-8]	2747	261	1134	888	1797	0.86	25	873	385.6	1.5e-106	gene=Chr09G0023.1	estExt_fgenesh2_pg.C_10473[SchizophyllumcommuneH4-8]
Chr09G0104.1	1072	63.6	Clame_sc	7866	233	1005	1604	2382	0.8	9	772	982.2	2.6e-28	gene=C	Clame_scaffold18-1

			affold18-1 9.70[Cladoniametacorallifera]						7				6	hr09G0104.1	9.70[Cladoniametacorallifera]
Chr08G0376.1	1271	30.3	CNAG_03466T0[Cryptococcusneoformansvar.grubiiH99]	1123	289	1163	222	1098	0.88	32	874	323.6	5.9e-88	gene=Chr08G0376.1	CNAG_03466T0[Cryptococcusneoformansvar.grubiiH99]
Chr01G1506.1	989	64.1	genemark-scaffold80-processed-gene-0.29-mRNA-1[PseudohalonectrialignicolaM95]	1451	99	980	4	930	0.88	8	881	887.9	6.1e-258	gene=Chr01G1506.1	genemark-scaffold80-processed-gene-0.29-mRNA-1[PseudohalonectrialignicolaM95]
Chr05G1290.1	1379	29.4	EEA28139.1[Penicilliummarneffei]	2025	8	946	573	1489	0.88	36	938	334.3	3.6e-91	gene=Chr05G1290.1	EEA28139.1[Penicilliummarneffei]
Chr04G0793.1	928	34.1	augustus_masked-scaffold418	1986	48	910	613	1456	0.89	25	862	444.5	1.7e-124	gene=Chr04G0793.1	augustus_masked-scaffold418-processed-gene-0.20-mRNA-

			-processed-gene-0.20-mRNA-1[MagnaporthesalviniiM69]												1[MagnaporthesalviniiM69]
Chr02G0865.1	845	46.8	CIMG_02482.2[Coccidioidesimmitis]	1385	68	838	650	1375	0.89	7	770	701.4	7.0e-202	gene=Chr02G0865.1	CIMG_02482.2[Coccidioidesimmitis]
Chr07G0725.1	1492	33	EEA28139.1[Penicilliummarneffei]	2025	51	939	602	1486	0.89	33	888	399.8	7.6e-111	gene=Chr07G0725.1	EEA28139.1[Penicilliummarneffei]
Chr08G0407.1	1517	29.5	XP_007598052.1[ColletotrichumfioriniaePJ7]	1915	80	1218	62	1212	0.91	27	1138	462.6	9.8e-130	gene=Chr08G0407.1	XP_007598052.1[ColletotrichumfioriniaePJ7]
Chr02G1262.1	1126	78.7	XP_007590595.1[ColletotrichumfioriniaePJ7]	1116	1	1125	1	1116	0.91	6	1124	1800	0.0e+00	gene=Chr02G1262.1	XP_007590595.1[ColletotrichumfioriniaePJ7]
Chr01G1962.1	901	35	augustus_masked-s	1986	25	891	600	1456	0.92	23	866	450.7	2.3e-126	gene=Chr01G19	augustus_masked-scaffold418-processe

			caffold418-processed-gene-0.20-mRNA-1[Magnaporthesalviniii M69]												62.1	d-gene-0.20-mRNA-1[Magnaporthesalviniii M69]
Chr02G1022.1	848	68.1	EEA23218.1[Penicilliummarneffei]	1467	2	847	616	1467	0.93	4	845	1141.3	0.0e+00	gene=Chr02G1022.1	EEA23218.1[Penicilliummarneffei]
Chr01G0277.1	1236	37.9	EEA28139.1[Penicilliummarneffei]	2025	37	885	602	1490	0.93	23	848	541.6	1.3e-153	gene=Chr01G0277.1	EEA28139.1[Penicilliummarneffei]
Chr04G0003.1	1544	31.7	EEA28139.1[Penicilliummarneffei]	2025	63	970	603	1490	0.93	28	907	373.2	7.9e-103	gene=Chr04G0003.1	EEA28139.1[Penicilliummarneffei]
Chr09G0231.1	2290	26.2	fgenesh1_pg.24_#_34[PhlebiabrevisporaHHB-7030SS6]	2158	92	1197	360	1396	0.94	33	1105	312	3.2e-84	gene=Chr09G0231.1	fgenesh1_pg.24_#_34[PhlebiabrevisporaHHB-7030SS6]
Chr06G1418.1	1191	36.1	augustus_	1986	310	1181	607	1456	0.9	26	871	438.7	1.2e-12	gene=C	augustus_masked-s

			masked-scaffold418-processed-gene-0.20-mRNA-1[MagnaporthesalviniiM69]						5				2	hr06G1418.1	caffold418-processed-gene-0.20-mRNA-1[MagnaporthesalviniiM69]
Chr09G0850.1	770	46.8	EEA28139.1[Penicilliummarneffei]	2025	1	747	597	1358	0.95	6	746	698.4	5.4e-201	gene=Chr09G0850.1	EEA28139.1[Penicilliummarneffei]
Chr02G1135.1	2529	25.7	fgenesh1_pg.24_#_34[PhlebiabrevisporaHHB-7030SS6]	2158	8	1168	351	1433	0.95	40	1160	299.7	1.8e-80	gene=Chr02G1135.1	fgenesh1_pg.24_#_34[PhlebiabrevisporaHHB-7030SS6]
Chr03G1600.1	887	37.4	augustus_masked-scaffold418-processed-gene-0.20-mRNA-1[Magnaporthesalvi	1986	7	868	606	1456	0.95	24	861	466.5	4.0e-131	gene=Chr03G1600.1	augustus_masked-scaffold418-processed-gene-0.20-mRNA-1[MagnaporthesalviniiM69]

			niiM69]												
Chr04G1058.1	1097	75.4	XP_007590036.1[ColletotrichumfioriniaePJ7]	1091	1	1096	1	1091	0.96	7	1095	1679.1	0.0e+00	gene=Chr04G1058.1	XP_007590036.1[ColletotrichumfioriniaePJ7]
Chr06G0218.1	1417	28	ODG_10834-R0[OphiocerasdolichostomumCBS114926]	2287	152	1187	1143	2158	0.96	33	1035	285.4	2.0e-76	gene=Chr06G0218.1	ODG_10834-R0[OphiocerasdolichostomumCBS114926]
Chr04G0474.1	1260	25.9	MUStwsD_GLEAN_10001562[Omphalotusolearius]	2038	3	1236	827	1989	0.99	35	1233	74.7	4.8e-13	gene=Chr04G0474.1	MUStwsD_GLEAN_10001562[Omphalotusolearius]
Chr02G0417.1	1571	38.7	EEA28139.1[Penicilliummarneffei]	2025	1	874	597	1466	0.99	17	873	560.8	2.7e-159	gene=Chr02G0417.1	EEA28139.1[Penicilliummarneffei]
Chr06G0022.1	2225	38.8	EEA28139.1[Penicilliummarneffei]	2025	1	870	597	1466	1.00	15	869	546.6	7.5e-155	gene=Chr06G0022.1	EEA28139.1[Penicilliummarneffei]

Chr04G1379.1	1776	38.8	EEA28139.1[Penicilliummarneffei]	2025	1	870	597	1466	1.00	17	869	552	1.4e-156	gene=Chr04G1379.1	EEA28139.1[Penicilliummarneffei]
Chr03G1207.1	1374	30.8	XP_007598052.1[ColletotrichumfioriniaePJ7]	1915	229	1290	52	1328	0.83	32	1061	536.2	6.3e-152	gene=Chr03G1207.1	XP_007598052.1[ColletotrichumfioriniaePJ7]
Chr01G2787.1	1367	38.1	EEA28139.1[Penicilliummarneffei]	2025	1	870	597	1466	0.83	13	869	539.7	5.7e-153	gene=Chr01G2787.1	EEA28139.1[Penicilliummarneffei]
Chr01G0008.1	2207	40.8	EEA28139.1[Penicilliummarneffei]	2025	74	939	597	1464	0.84	19	865	608.2	2.1e-173	gene=Chr01G0008.1	EEA28139.1[Penicilliummarneffei]
Chr07G0099.1	1100	45.6	M_BR29_EuGene_00112621[MagnaporthegriseaBR29]	952	26	1075	5	950	0.85	20	1049	884.8	5.8e-257	gene=Chr07G0099.1	M_BR29_EuGene_00112621[MagnaporthegriseaBR29]
Chr03G0084.1	1064	42.8	7575_t[AscocorynesarcoidesN	1484	146	1051	81	928	0.85	11	905	689.1	4.5e-198	gene=Chr03G0084.1	7575_t[AscocorynesarcoidesNRRL50072]

			RRL50072]												
Chr09G0809.1	1072	54	PTRG_10301[Pyrenophoratritici-repentis]	1311	109	959	191	1059	0.85	11	850	926.4	1.7e-269	gene=Chr09G0809.1	PTRG_10301[Pyrenophoratritici-repentis]
Chr01G2789.1	1642	39.7	EEA28139.1[Penicilliummarneffei]	2025	1	889	597	1479	0.85	20	888	571.2	2.1e-162	gene=Chr01G2789.1	EEA28139.1[Penicilliummarneffei]
Chr07G0581.1	1067	37.2	Clama_scaffold_5-4.0[Cladoniamacilenta]	21962	167	1065	5467	6358	0.86	11	898	569.3	5.2e-162	gene=Chr07G0581.1	Clama_scaffold_5-4.0[Cladoniamacilenta]
Chr04G0978.1	2517	27.4	fgenesh1_pg.24_#_34[PhlebiabrevisporaHHB-7030SS6]	2158	123	1302	83	1228	0.87	37	1179	396.4	1.4e-109	gene=Chr04G0978.1	fgenesh1_pg.24_#_34[PhlebiabrevisporaHHB-7030SS6]
Chr07G0168.1	1396	80.4	XP_007598052.1[ColletotrichumfioriniaePJ7]	1915	1	1376	1	1377	0.89	12	1375	2209.5	0.0e+00	gene=Chr07G0168.1	XP_007598052.1[ColletotrichumfioriniaePJ7]

Chr03G1093.1	1200	25	MUStwsD_GLEAN_10001562[Omphalotusolearius]	2038	1	1181	826	2002	0.89	26	1180	139	2.0e-32	gene=Chr03G1093.1	MUStwsD_GLEAN_10001562[Omphalotusolearius]
Chr04G0877.1	1028	35.5	Clama_scaffold_5-4.0[Cladoniamacilenta]	21962	82	1023	5478	6433	0.90	13	941	565.1	9.5e-161	gene=Chr04G0877.1	Clama_scaffold_5-4.0[Cladoniamacilenta]
Chr08G1025.1	1128	73.7	GLRG_10013T0[ColletotrichumgraminicolaM1.001]	1128	5	1126	3	1127	0.90	6	1121	1739.5	0.0e+00	gene=Chr08G1025.1	GLRG_10013T0[ColletotrichumgraminicolaM1.001]
Chr06G1456.1	1125	72.3	MIX15449_41_76[ColletotrichumsublineolaCBS131301]	1112	3	1124	8	1111	0.91	4	1121	1702.2	0.0e+00	gene=Chr06G1456.1	MIX15449_41_76[ColletotrichumsublineolaCBS131301]
Chr04G0420.1	944	54.3	Clame_scaffold9-5.100[Cladoniametacora	12517	1	892	8835	9732	0.92	5	891	980.3	8.7e-286	gene=Chr04G0420.1	Clame_scaffold9-5.100[Cladoniametacorallifera]

			llifera]											
Chr09G0476.1	1032	62.9	HCB03452.1[Histoplasmacapsulatum]	1646	3	941	4	934	0.92	4	938	1176.8	0.0e+00	gene=Chr09G0476.1	HCB03452.1[Histoplasmacapsulatum]
Chr05G0025.1	1702	32.6	Clama_scaffold_5-4.0[Cladoniamacilenta]	21962	385	1529	5459	6533	0.94	16	1144	567.4	3.2e-161	gene=Chr05G0025.1	Clama_scaffold_5-4.0[Cladoniamacilenta]
Chr04G0200.1	1069	42.6	Clama_scaffold_5-4.0[Cladoniamacilenta]	21962	13	1054	15435	16434	0.96	20	1041	772.3	4.1e-223	gene=Chr04G0200.1	Clama_scaffold_5-4.0[Cladoniamacilenta]
Chr01G2140.1	1490	36.4	Clama_scaffold_5-4.0[Cladoniamacilenta]	21962	399	1450	5459	6540	0.97	16	1051	659.1	7.0e-189	gene=Chr01G2140.1	Clama_scaffold_5-4.0[Cladoniamacilenta]
Chr07G0336.1	1481	35.5	Clama_scaffold_5-4.0[Cladoniamacilenta]	21962	102	1411	5362	6541	0.98	23	1309	771.9	7.3e-223	gene=Chr07G0336.1	Clama_scaffold_5-4.0[Cladoniamacilenta]
Chr03G0855.1	1845	31.9	Clama_scaffold_5-4.0[Cladoniamacilenta]	21962	311	1498	574	1889	0.98	43	1187	481.9	1.9e-135	gene=Chr03G0855.1	Clama_scaffold_5-4.0[Cladoniamacilenta]
Chr04G1164.1	512	46	FGSG_08	500	8	194	2	188	0.4	0	186	185.3	1.0e-46	gene=C	FGSG_08005T0[Fus

			005T0[Fusariumgraminearum]						6					hr04G1164.1	ariumgraminearum]
Chr01G1055.1	1494	34.8	Clama_scaffold_5-4.0[Cladoniamacilenta]	21962	117	1452	5331	6534	0.82	21	1335	748.4	8.8e-216	gene=Chr01G1055.1	Clama_scaffold_5-4.0[Cladoniamacilenta]
Chr03G0364.1	1358	33.9	XP_007803552.1[EndocarponpusillumZ07020]	1439	59	1279	110	1332	0.82	38	1220	633.6	2.9e-181	gene=Chr03G0364.1	XP_007803552.1[EndocarponpusillumZ07020]
Chr04G1388.1	1427	37.8	Clama_scaffold_5-4.0[Cladoniamacilenta]	21962	60	1386	5351	6539	0.82	21	1326	867.5	1.2e-251	gene=Chr04G1388.1	Clama_scaffold_5-4.0[Cladoniamacilenta]
Chr05G1384.1	1015	31.5	MUStwsD_GLEAN_10001562[Omphalotusolearius]	2038	227	405	317	487	0.35	6	178	86.7	9.7e-17	gene=Chr05G1384.1	MUStwsD_GLEAN_10001562[Omphalotusolearius]
Chr01G2221.1	1752	26.4	estExt_fgenesh2_pg.C_10473[Schizophy	2747	15	1462	529	2225	0.83	46	1447	475.7	1.3e-133	gene=Chr01G2221.1	estExt_fgenesh2_pg.C_10473[SchizophyllumcommuneH4-8]

			llumcommuneH4-8]												
Chr07G0431.1	570	70.1	CH063_05878T0[Colletotrichumhigginsianum]	968	77	566	475	961	0.98	2	489	696.4	1.5e-200	gene=Chr07G0431.1	CH063_05878T0[Colletotrichumhigginsianum]
Chr01G0468.1	1588	53.9	Clama_scaffold_5-4.0[Cladoniamacilenta]	21962	137	1536	5289	6543	0.84	7	1399	1478.4	0.0e+00	gene=Chr01G0468.1	Clama_scaffold_5-4.0[Cladoniamacilenta]
Chr08G0641.1	1542	29.2	EEA19609.1[Penicilliummarneffei]	2642	213	1539	190	1561	0.86	30	1326	517.3	3.4e-146	gene=Chr08G0641.1	EEA19609.1[Penicilliummarneffei]
Chr01G1538.1	2086	26	fgenesh1_pg.24_#_34[PhlebiabrevisporaHHB-7030SS6]	2158	228	1728	214	1717	0.88	45	1500	440.7	5.5e-123	gene=Chr01G1538.1	fgenesh1_pg.24_#_34[PhlebiabrevisporaHHB-7030SS6]
Chr04G0209.1	3225	28.7	EAA60443.1[AspergillusnidulansFGSCA4]	2180	529	2033	180	1633	0.94	40	1504	560.8	5.6e-159	gene=Chr04G0209.1	EAA60443.1[AspergillusnidulansFGSCA4]
Chr02G0584.1	1273	45.6	XP_00759	1915	15	1272	50	1377	0.9	20	1257	1097.	0.0e+00	gene=C	XP_007598052.1[Co

			8052.1[ColletotrichumfioriniaePJ7]						5			8		hr02G0584.1	lletotrichumfioriniaePJ7]
Chr03G0362.1	2999	26.3	estExt_Genewise1.C_8_t20489[Fomitiporiamediterranea]	2825	890	2513	991	2526	0.97	29	1623	545.4	2.3e-154	gene=Chr03G0362.1	estExt_Genewise1.C_8_t20489[Fomitiporiamediterranea]
Chr06G0427.1	1180	46.9	MUStwsD_GLEAN_10001562[Omphalotusolearius]	2038	1	1174	826	2005	0.91	2	1173	884	1.1e-256	gene=Chr06G0427.1	MUStwsD_GLEAN_10001562[Omphalotusolearius]
Chr01G2314.1	1634	59.1	PLG_01589-R0[PseudohalonectrialignicolaM95]	1848	18	1632	6	1848	0.94	21	1614	1973.7	0.0e+00	gene=Chr01G2314.1	PLG_01589-R0[PseudohalonectrialignicolaM95]
Chr06G1050.1	1588	47.3	XP_007598052.1[ColletotrichumfioriniaePJ7]	1915	151	1444	22	1328	0.94	20	1293	1157.5	0.0e+00	gene=Chr06G1050.1	XP_007598052.1[ColletotrichumfioriniaePJ7]

Chr01G0907.1	1536	68.1	maker-scaffold_6-exonerate_est2genome-gene-2.14-mRNA-1[Cladoniamacilenta]	1905	25	1490	10	1469	0.97	7	1465	2045.4	0.0e+00	gene=Chr01G0907.1	maker-scaffold_6-exonerate_est2genome-gene-2.14-mRNA-1[Cladoniamacilenta]
Chr09G0562.1	2173	26.7	fgenesh1_pg.24_#_34[PhlebiabrevisporaHHB-7030SS6]	2158	116	2031	85	1835	0.98	49	1915	597.4	3.6e-170	gene=Chr09G0562.1	fgenesh1_pg.24_#_34[PhlebiabrevisporaHHB-7030SS6]
Chr06G0816.1	1611	45	ATEG_09350.1[Aspergillusterreus]	1972	12	1371	639	1969	0.98	22	1359	976.1	2.8e-284	gene=Chr06G0816.1	ATEG_09350.1[Aspergillusterreus]
Chr03G0303.1	12679	25	Endpu_scaffold7-12.77[EndocarponpusillumR61883]	5051	8372	10387	172	2162	0.93	60	2015	449.5	7.1e-125	gene=Chr03G0303.1	Endpu_scaffold7-12.77[EndocarponpusillumR61883]
Chr02G0789.1	1714	45	estExt_fgenesh2_pg.	2747	1	1694	520	2225	0.94	26	1693	1211.4	0.0e+00	gene=Chr02G07	estExt_fgenesh2_pg.C_10473[Schizophy

			C_10473[SchizophyllumcommuneH4-8]											89.1	llumcommuneH4-8]
Chr04G1266.1	2564	35.2	ODG_10834-R0[OphiocerasdolichostomumCBS114926]	2287	460	2561	80	2285	0.92	59	2101	1158.7	0.0e+00	gene=Chr04G1266.1	ODG_10834-R0[OphiocerasdolichostomumCBS114926]
Chr07G0091.1	4960	23	Endpu_scaffold7-12.77[EndocarponpusillumR61883]	5051	1768	4870	172	3815	0.95	102	3102	598.6	3.7e-170	gene=Chr07G0091.1	Endpu_scaffold7-12.77[EndocarponpusillumR61883]
Chr01G1499.1	3221	58.3	maker-Caloplaca_flavorubescens_scaffold_1-augustus-gene-23.48-mRNA-1[Caloplacaflavorubescens]	3615	1	3176	1	3158	0.93	28	3175	3704.1	0.0e+00	gene=Chr01G1499.1	maker-Caloplaca_flavorubescens_scaffold_1-augustus-gene-23.48-mRNA-1[Caloplacaflavorubescens]

Chr02G1194.1	4059	42.9	maker-scaffold_10-exonerate_est2genome-gene-13.14-mRNA-1[Cladoniamacilenta]	4285	39	4058	129	4285	0.94	91	4019	2246.9	0.0e+00	gene=Chr02G1194.1	maker-scaffold_10-exonerate_est2genome-gene-13.14-mRNA-1[Cladoniamacilenta]
Chr09G1013.1	4891	36.1	Endpu_scaffold7-12.77[EndocarponpusillumR61883]	5051	12	4885	158	5046	0.96	84	4873	2754.2	0.0e+00	gene=Chr09G1013.1	Endpu_scaffold7-12.77[EndocarponpusillumR61883]
