# Supplementary material for: Decoding Pecan’s Fungal Foe: A Genomic Insight into Colletotrichum plurivorum Isolate W-6
Source: J Fungi (Basel). 2025 Mar 5;11(3):203. doi: 10.3390/jof11030203 (PMC11943440; doi:10.3390/jof11030203)
Supplement: Supplementary file 1 [file jof-11-00203-s001.zip › Table S25.pdf]

Table S25. Prediction of DFVF genes in isolate W-6 genome.

Query_id	Query_length	Query_start	Query_end	Subject_id	Subject_length	Subject_start	Subject_end	Identity	Positive	Gap	Align_length	Score	E_value	Query_annotation	Subject_annotation
Chr01G0001.1	412	158	333	UniProt ID:TUP1_CANAL	514	258	438	73/188 (38.83)	0.52	0.1	188	114	1.00E-28	gene=Chr01G0001	Gene Symbol:TUP1|Host:Isolated from a wide variety of substrates including humans|Disease:invasive candidal disease|Description:FUNCTION: Represses transcription by RNA polymerase II. Represses genes responsible for initiating filamentous growth and this repression is lifted under inducing environmental conditions.
Chr01G0013.1	622	41	292	UniProt ID:Q9C441_FUSSO	330	61	303	96/261 (36.78)	0.55	0.1	261	174	9.00E-50	gene=Chr01G0013	Gene Symbol:PEP1|Host:Multiple plant families. Some strains may cause infections in humans|Disease:Saprobe, facultative

														pathogen|Description:Unknown
Chr01G0015.1	445	35	445	UniProt ID:Q9Y784_MAGGR	631	36	465	112/442(25.34)	0.45	0.1	442	130	9.00E-34	gene=Chr01G0015	Gene Symbol:PTH11|Host:Digitaria (Poaceae)|Disease:Leaf spot|Description:Unknown
Chr01G0016.1	566	1	566	UniProt ID:Q2VLJ1_GIBZA	565	1	563	281/570(49.30)	0.66	0.02	570	580	0	gene=Chr01G0016	Gene Symbol:ZEB1|Host:Principal hosts: Poaceae, including Zea mays (corn), Triticum aestivum (wheat), and Oryza sativa (rice). Additional hosts: various plant families|Disease:Seedling blight, pre- and post-emergence blight, root and foot rot, brown rot, culm decay, head or kernel blight (scab or ear scab) of cereals. Leaf|Description:Unknown
Chr01G0023.1	569	1	559	UniProt ID:O93800_AL	578	2	562	214/570(37.54)	0.56	0.04	570	362	7.00E-118	gene=Chr01G0023	Gene Symbol:AKT1|Host:Plant|Disease:Leaf spot,

			TAL											rots|Description:Unknown
Chr01G0 024.1	320	39	170	UniProt ID:A4Q VF8_M AGO7	339	69	196	45/132 (34.09)	0.52	0.03	132	63.9	2.00E-12	gene=Chr 01G0024	Gene Symbol:MGG_04556|Host :Poaceae, especially important on Oryzae|Disease:Rice blast|Description:COFACT OR: Zinc (By similarity).
Chr01G0 025.1	283	2	283	UniProt ID:A4R GG9_M AGO7	286	3	286	219/28 4(77.1 1)	0.88	0.01	284	450	9.00E-16 1	gene=Chr 01G0025	Gene Symbol:MGG_00056|Host :Poaceae, especially important on Oryzae|Disease:Rice blast|Description:SIMILAR ITY: Belongs to the short-chain dehydrogenases/reductas es (SDR) family.
Chr01G0 032.1	505	10	504	UniProt ID:Q5A NE1_C ANAL	748	37	541	139/52 3(26.5 8)	0.46	0.09	523	156	5.00E-42	gene=Chr 01G0032	Gene Symbol:SNF3|Host:Isolate d from a wide variety of substrates including humans|Disease:invasive candidal disease|Description:SIMIL ARITY: Belongs to the

															major facilitator superfamily. Sugar transporter (TC 2.A.1.1) family.
Chr01G0035.1	257	6	220	UniProt ID:A4RGG9_MAGO7	286	17	227	57/232 (24.57)	0.43	0.16	232	49.3	6.00E-08	gene=Chr01G0035	Gene Symbol:MGG_00056|Host:Poaceae, especially important on Oryzae|Disease:Rice blast|Description:SIMILARITY: Belongs to the short-chain dehydrogenases/reductases (SDR) family.
Chr01G0044.1	1538	550	1472	UniProt ID:A4RGC8_MAGO7	1158	244	1085	240/945(25.40)	0.4	0.13	945	225	1.00E-60	gene=Chr01G0044	Gene Symbol:MGG_11671|Host:Poaceae, especially important on Oryzae|Disease:Rice blast|Description:SIMILARITY: Contains 1 reverse transcriptase domain.
Chr01G0053.1	594	114	591	UniProt ID:Q5ABU7_CANAL	564	95	564	148/492(30.08)	0.51	0.07	492	232	1.00E-68	gene=Chr01G0053	Gene Symbol:MDR1|Host:Isolated from a wide variety of substrates including

														humans|Disease:invasive candidal disease|Description:Unknown
Chr01G0055.1	545	311	509	UniProt ID:A4ULJ2_MYCGR	515	300	514	54/223 (24.22)	0.43	0.14	223	46.6	2.00E-06	gene=Chr01G0055	Gene Symbol:CYP51|Host:Triticum and possibly a few other grasses|Disease:Leaf spot or speckled leaf blotch of wheat|Description:COFACTOR: Heme group (By similarity).
Chr01G0056.1	237	8	235	UniProt ID:Q00845_NECHA	233	6	233	137/233(58.80)	0.73	0.04	233	275	1.00E-93	gene=Chr01G0056	Gene Symbol:PELD|Host:Trees of various plant families|Disease:Fruit rot, stem rot|Description:Unknown
Chr01G0063.1	919	182	217	UniProt ID:A8QJI7_FUSOX	938	89	124	17/36(47.22)	0.67	0	36	50.4	3.00E-07	gene=Chr01G0063	Gene Symbol:XLNR|Host:Multiple genera in multiple families|Disease:Blights, wilts, rots of various sorts|Description:SIMILARITY: Contains 1 Zn(2)-C6

														fungal-type DNA-binding domain.
Chr01G0066.1	576	258	554	UniProt ID:Q59ZI9_CANAL	329	81	329	71/308(23.05)	0.39	0.23	308	88.2	4.00E-20	gene=Chr01G0066	Gene Symbol:CKA2|Host:Isolated from a wide variety of substrates including humans|Disease:invasive candidal disease|Description:SIMILARITY: Belongs to the protein kinase superfamily.
Chr01G0071.1	1473	964	1372	UniProt ID:O93841_9PEZI	914	94	523	144/445(32.36)	0.5	0.11	445	236	7.00E-65	gene=Chr01G0071	Gene Symbol:CHIP6|Host:Multiple genera in multiple families|Disease:'Anthracnose of stems and leaves, dieback, root rot, leaf spot, blossom rot, fruit rot (dieback and ripe rot), seedling blight.' (Mordue 1971)|Description:Unknown
Chr01G0078.1	10597	5787	6345	UniProt ID:B9WJ72_C	1913	425	935	211/582(36.25)	0.47	0.16	582	98.6	2.00E-20	gene=Chr01G0078	Gene Symbol:ALS9|Host:humans|Disease:leptomeningeal

				ANDC										disease,occasional invasive candidal disease|Description:Unknown
Chr01G0083.1	842	205	258	UniProt ID:A8QJI7_FUSOX	938	84	136	22/54(40.74)	0.56	0.02	54	51.6	1.00E-07	gene=Chr01G0083	Gene Symbol:XLNR|Host:Multiple genera in multiple families|Disease:Blights, wilts, rots of various sorts|Description:SIMILARITY: Contains 1 Zn(2)-C6 fungal-type DNA-binding domain.
Chr01G0084.1	443	4	441	UniProt ID:Q9C1F9_COCCA	423	2	410	156/453(34.44)	0.5	0.13	453	223	3.00E-68	gene=Chr01G0084	Gene Symbol:CEL2|Host:Corn, Zea mays, sometimes on Sorghum (Poaceae) and various other plant families|Disease:Northern corn leaf spot, ear and kernel rot|Description:Unknown
Chr01G0085.1	661	47	103	UniProt ID:A0ST46_CERNC	397	13	65	20/57(35.09)	0.54	0.07	57	45.4	6.00E-06	gene=Chr01G0085	Gene Symbol:CTB8|Host:Numerous taxa in Solanaceae|Disease:Leaf

															spot|Description:Unknown Gene Symbol:LEU2|Host:Isolated from a wide variety of substrates including humans|Disease:invasive candidal disease|Description:FUNCTION: Catalyzes the oxidation of 3-carboxy-2-hydroxy-4-methylpentanoate (3-isopropylmalate) to 3-carboxy-4-methyl-2-oxopentanoate. The product decarboxylates to 4-methyl-2 oxopentanoate (By similarity).
Chr01G0093.1	365	8	354	UniProt ID:Q5AFI8_CANAL	373	8	361	106/360(29.44)	0.46	0.05	360	125	2.00E-33	gene=Chr01G0093	
Chr01G0094.1	476	61	441	UniProt ID:Q6A2T2_BOTFU	391	13	381	94/400 (23.50)	0.46	0.13	400	92	3.00E-21	gene=Chr01G0094	Gene Symbol:BTP1|Host:Various plant families|Disease:Grey mould. Parasite or saprophyte|Description:Unknown
Chr01G0	385	14	259	UniProt	383	21	277	78/264	0.44	0.09	264	81.6	5.00E-18	gene=Chr	Gene

097.1				ID:Q6XVN4_CRYNV				(29.55)						01G0097	Symbol:GNO1|Host:humans|Disease:cryptococcosis|Description:COFACTOR: Zinc (By similarity).
Chr01G0100.1	504	35	467	UniProt ID:Q5ANE1_CANAL	748	51	492	116/452(25.66)	0.44	0.06	452	119	2.00E-29	gene=Chr01G0100	Gene Symbol:SNF3|Host:Isolated from a wide variety of substrates including humans|Disease:invasive candidal disease|Description:SIMILARITY: Belongs to the major facilitator superfamily. Sugar transporter (TC 2.A.1.1) family.
Chr01G0105.1	304	5	185	UniProt ID:Q75WR5_9PLEO	265	13	199	49/194 (25.26)	0.45	0.1	194	56.2	4.00E-10	gene=Chr01G0105	Gene Symbol:BRN1|Host:Belamcanda chinensis: Korea,Gladiolus ?gandavensis: Korea,Iris japonica: China,Iris missouriensis (Leaf spot.): Idaho; Montana; Oregon; Washington,Iris sp. (Leaf spot.): China; Texas;

															Washing|Disease:Leaf spot|Description:SIMILARITY: Belongs to the short-chain dehydrogenases/reductases (SDR) family.
Chr01G0106.1	362	3	43	UniProt ID:A0ST46_CERNC	397	15	55	17/41(41.46)	0.63	0	41	43.9	8.00E-06	gene=Chr01G0106	Gene Symbol:CTB8|Host:Numerous taxa in Solanaceae|Disease:Leaf spot|Description:Unknown
Chr01G0107.1	563	347	518	UniProt ID:A4ULI9_MYCGR	502	321	501	47/181(25.97)	0.41	0.05	181	54.7	6.00E-09	gene=Chr01G0107	Gene Symbol:CYP51|Host:Triticum and possibly a few other grasses|Disease:Leaf spot or speckled leaf blotch of wheat|Description:COFACTOR: Heme group (By similarity).
Chr01G0108.1	667	2	69	UniProt ID:A6N6J8_FUSOX	903	37	103	28/73(38.36)	0.47	0.15	73	45.4	1.00E-05	gene=Chr01G0108	Gene Symbol:CTF1|Host:Multiple genera in multiple families|Disease:Blights, wilts, rots of various sorts|Description:SIMILAR

														ITY: Contains 1 Zn(2)-C6 fungal-type DNA-binding domain.
Chr01G0111.1	535	28	494	UniProt ID:Q5ANE1_CANAL	748	39	496	141/474(29.75)	0.48	0.05	474	196	4.00E-55	gene=Chr01G0111	Gene Symbol:SNF3|Host:Isolated from a wide variety of substrates including humans|Disease:invasive candidal disease|Description:SIMILARITY: Belongs to the major facilitator superfamily. Sugar transporter (TC 2.A.1.1) family.
Chr01G0112.1	997	139	967	UniProt ID:Q5AI14_CANAL	888	22	831	395/858(46.04)	0.64	0.09	858	744	0	gene=Chr01G0112	Gene Symbol:TPS2|Host:Isolated from a wide variety of substrates including humans|Disease:invasive candidal disease|Description:Unknown
Chr01G0113.1	783	1	420	UniProt ID:ORYZ_ASP	403	2	403	119/450(26.44)	0.41	0.17	450	83.2	7.00E-18	gene=Chr01G0113	Gene Symbol:ALP1|Host:humans|Disease:infection|Descri

				FU											ption:FUNCTION: Secreted alkaline protease that allows assimilation of proteinaceous substrates. Acts as a significant virulence factor in invasive aspergillosis. Involved in immune evasion from the human and mice complement systems during infection. Efficiently cleaves important components of the complement cascade such as such as C3, C4, C5, and C1q, as well as IgG, which leads to down-regulation of complement activation at the hyphal surface.
Chr01G0115.1	799	48	792	UniProt ID:Q99324_SEPLY	803	46	795	306/814(37.59)	0.53	0.16	814	465	4.00E-152	gene=Chr01G0115	Gene Symbol:B2TOM|Host:Primarily tomato, Lycopersicon esculentum, also Solanum spp. and other

															Solanaceae|Disease:Leaf spot|Description:Unknown
Chr01G0117.1	711	445	686	UniProt ID:Q3Y5V5_MAGGR	1321	1074	1317	94/247 (38.06)	0.62	0.03	247	181	2.00E-48	gene=Chr01G0117	Gene Symbol:ABC3|Host:Digitaria (Poaceae)|Disease:Leaf spot|Description:SIMILARITY: Belongs to the ABC transporter superfamily.
Chr01G0119.1	362	13	298	UniProt ID:A0ST44_CERNC	357	4	291	81/306 (26.47)	0.43	0.12	306	74.3	1.00E-15	gene=Chr01G0119	Gene Symbol:CTB6|Host:Numerous taxa in Solanaceae|Disease:Leaf spot|Description:Unknown
Chr01G0123.1	940	223	282	UniProt ID:C5GNK0_AJEDR	415	21	79	25/60(41.67)	0.53	0.02	60	49.7	5.00E-07	gene=Chr01G0123	Gene Symbol:BDCG_06357|Host:humans|Disease:cutaneous Blastomyces dermatitidis infection|Description:Unknown
Chr01G0135.1	456	1	365	UniProt ID:A4UC81_MAGO7	376	13	372	93/373 (24.93)	0.42	0.06	373	71.2	2.00E-14	gene=Chr01G0135	Gene Symbol:MGG_10702|Host:Poaceae, especially important on Oryzae|Disease:Rice blast|Description:Unknow

														n
Chr01G0137.1	508	92	423	UniProt ID:A4UC81_MAGO7	376	41	372	107/335(31.94)	0.53	0.02	335	178	4.00E-51	gene=Chr01G0137	Gene Symbol:MGG_10702|Host:Poaceae, especially important on Oryzae|Disease:Rice blast|Description:Unknown
Chr01G0139.1	722	2	333	UniProt ID:P87199_USTMD	968	4	333	114/346(32.95)	0.49	0.09	346	139	7.00E-35	gene=Chr01G0139	Gene Symbol:KIN2|Host:Euchlaena spp., Zea spp. (Poaceae)|Disease:Smut. Corn smut|Description:SIMILARITY: Belongs to the kinesin-like protein family.
Chr01G0143.1	600	65	591	UniProt ID:Q9P8L8_BOTFU	598	67	592	212/531(39.92)	0.6	0.02	531	376	9.00E-123	gene=Chr01G0143	Gene Symbol:BCMFS1|Host:Various plant families|Disease:Grey mould. Parasite or saprophyte|Description:Unknown
Chr01G0151.1	251	7	247	UniProt ID:Q75WR5_9	265	9	262	85/260(32.69)	0.49	0.1	260	105	2.00E-27	gene=Chr01G0151	Gene Symbol:BRN1|Host:Belamcanda chinensis:

				PLEO											Korea,Gladiolus ?gandavensis: Korea,Iris japonica: China,Iris missouriensis (Leaf spot.): Idaho; Montana; Oregon; Washington,Iris sp. (Leaf spot.): China; Texas; Washing|Disease:Leaf spot|Description:SIMILARITY: Belongs to the short-chain dehydrogenases/reductases (SDR) family.
Chr01G0152.1	574	22	574	UniProt ID:O93842_FUSSP	598	11	573	159/565(28.14)	0.52	0.02	565	255	3.00E-77	gene=Chr01G0152	Gene Symbol:TRI12|Host:animals|Disease:trichothecene|Description:Unknown
Chr01G0153.1	278	26	274	UniProt ID:Q9P4U9_ALTAL	296	25	269	66/255(25.88)	0.43	0.06	255	76.3	4.00E-17	gene=Chr01G0153	Gene Symbol:AKT3-1|Host:Plant|Disease:Leaf spot, rots|Description:Unknown
Chr01G0154.1	283	33	278	UniProt ID:Q9P4U9_ALTAL	296	25	268	76/252(30.16)	0.46	0.06	252	85.1	2.00E-20	gene=Chr01G0154	Gene Symbol:AKT3-1|Host:Plant|Disease:Leaf spot, rots|Description:Unknown
Chr01G0	561	8	545	UniProt	578	13	564	175/56	0.46	0.08	567	201	1.00E-57	gene=Chr	Gene

157.1				ID:Q96 VB5_A LTAL				7(30.8 6)						01G0157	Symbol:AFT1-1|Host:Plant|Disease:Leaf spot, rots|Description:Unknown
Chr01G0 169.1	277	15	272	UniProt ID:O93 802_AL TAL	267	12	263	85/264 (32.20)	0.51	0.07	264	119	1.00E-32	gene=Chr 01G0169	Gene Symbol:BRM2|Host:Plant|Disease:Leaf spot, rots|Description:SIMILARITY: Belongs to the short-chain dehydrogenases/reductases (SDR) family.
Chr01G0 171.1	514	8	40	UniProt ID:Q5A 4F3_C ANAL	624	14	46	15/33(45.45)	0.7	0	33	50.1	2.00E-07	gene=Chr 01G0171	Gene Symbol:ZCF37|Host:Isolated from a wide variety of substrates including humans|Disease:invasive candidal disease|Description:Unknown
Chr01G0 173.1	724	169	359	UniProt ID:Q0 WXM3 _FUSO X	663	248	436	50/198 (25.25)	0.49	0.08	198	67.8	1.00E-12	gene=Chr 01G0173	Gene Symbol:FOW2|Host:Multiple genera in multiple families|Disease:Blights, wilts, rots of various sorts|Description:SIMILARITY: Contains 1 Zn(2)-C6

															fungal-type DNA-binding domain.
Chr01G0176.1	576	116	573	UniProt ID:Q5ABU7_CANAL	564	102	564	197/468(42.09)	0.6	0.03	468	371	1.00E-121	gene=Chr01G0176	Gene Symbol:MDR1|Host:Isolated from a wide variety of substrates including humans|Disease:invasive candidal disease|Description:Unknown
Chr01G0189.1	407	34	358	UniProt ID:Q6A2T2_BOTFU	391	37	371	82/336(24.40)	0.43	0.04	336	108	2.00E-27	gene=Chr01G0189	Gene Symbol:BTP1|Host:Various plant families|Disease:Grey mould. Parasite or saprophyte|Description:Unknown
Chr01G0191.1	357	33	352	UniProt ID:Q92217_COCHE	2528	1848	2138	81/333(24.32)	0.43	0.17	333	82	8.00E-18	gene=Chr01G0191	Gene Symbol:PKS1|Host:Zea mays|Disease:Southern leaf blight of maize|Description:Unknown
Chr01G0193.1	509	82	504	UniProt ID:A0ST43_C	459	3	458	133/468(28.42)	0.43	0.12	468	160	3.00E-44	gene=Chr01G0193	Gene Symbol:CTB5|Host:Numerous taxa in

				ERNC										Solanaceae|Disease:Leaf spot|Description:Unknown
Chr01G0195.1	867	217	391	UniProt ID:Q59VF3_CANAL	1813	63	215	54/178 (30.34)	0.45	0.16	178	58.5	1.00E-09	gene=Chr01G0195	Gene Symbol:"DUR1,2"|Host:Isolated from a wide variety of substrates including humans|Disease:invasive candidal disease|Description:CAUTION: The sequence shown here is derived from an EMBL/GenBank/DDBJ whole genome shotgun (WGS) entry which is preliminary data.
Chr01G0200.1	373	339	372	UniProt ID:O59937_FUSOX	384	19	52	22/34(64.71)	0.74	0	34	56.6	6.00E-10	gene=Chr01G0200	Gene Symbol:XYL3|Host:Multiple genera in multiple families|Disease:Blights, wilts, rots of various sorts|Description:SIMILARITY: Belongs to the glycosyl hydrolase 10 (cellulase F) family.
Chr01G0207.1	628	46	625	UniProt ID:Q4P	693	81	671	199/618(32.2	0.48	0.11	618	229	2.00E-66	gene=Chr01G0207	Gene Symbol:UM03615.1|Host:

				8E8_USTMA				0)							Euchlaena spp., Zea spp. (Poaceae)|Disease:Smut. Corn smut|Description:COFACTOR: FAD (By similarity).
Chr01G0209.1	520	69	508	UniProt ID:A0ST43_CERNC	459	12	456	116/460(25.22)	0.45	0.08	460	128	3.00E-33	gene=Chr01G0209	Gene Symbol:CTB5|Host:Numerous taxa in Solanaceae|Disease:Leaf spot|Description:Unknown
Chr01G0210.1	374	55	248	UniProt ID:Q9Y784_MAGGR	631	133	319	51/197 (25.89)	0.46	0.07	197	62	2.00E-11	gene=Chr01G0210	Gene Symbol:PTH11|Host:Digitaria (Poaceae)|Disease:Leaf spot|Description:Unknown
Chr01G0222.1	369	82	364	UniProt ID:Q6TFC7_ASPFM	349	63	346	99/288 (34.38)	0.48	0.03	288	144	5.00E-40	gene=Chr01G0222	Gene Symbol:NULL|Host:humans|Disease:infection|Description:Unknown
Chr01G0229.1	368	9	365	UniProt ID:Q5AFI8_CANAL	373	4	371	120/377(31.83)	0.48	0.08	377	149	7.00E-42	gene=Chr01G0229	Gene Symbol:LEU2|Host:Isolated from a wide variety of substrates including humans|Disease:invasive candidal disease|Description:FUNC

															TION: Catalyzes the oxidation of 3-carboxy-2-hydroxy-4-methylpentanoate (3-isopropylmalate) to 3-carboxy-4-methyl-2-oxopentanoate. The product decarboxylates to 4-methyl-2 oxopentanoate (By similarity).
Chr01G0232.1	765	216	322	UniProt ID:Q0WXM3_FUSOX	663	332	444	36/113 (31.86)	0.49	0.05	113	46.2	6.00E-06	gene=Chr01G0232	Gene Symbol:FOW2|Host:Multiple genera in multiple families|Disease:Blights, wilts, rots of various sorts|Description:SIMILARITY: Contains 1 Zn(2)-C6 fungal-type DNA-binding domain.
Chr01G0237.1	761	486	699	UniProt ID:D1MYV6_MAGGR	568	152	361	64/223 (28.70)	0.47	0.1	223	77.8	7.00E-16	gene=Chr01G0237	Gene Symbol:CBL1|Host:Digitaria (Poaceae)|Disease:Leaf spot|Description:SIMILARITY: Contains 3 chitin-binding type-1 domains.

Chr01G0238.1	293	74	255	UniProt ID:Q04701_FUSSO	242	42	233	88/193 (45.60)	0.56	0.06	193	148	2.00E-43	gene=Chr01G0238	Gene Symbol:PELA|Host:Multiple plant families. Some strains may cause infections in humans|Disease:Saprobe, facultative pathogen|Description:Unknown
Chr01G0242.1	348	131	344	UniProt ID:Q92217_COCHE	2528	1931	2145	56/222 (25.23)	0.44	0.07	222	56.6	1.00E-09	gene=Chr01G0242	Gene Symbol:PKS1|Host:Zea mays|Disease:Southern leaf blight of maize|Description:Unknown
Chr01G0245.1	574	26	498	UniProt ID:Q5ANE1_CANAL	748	39	496	130/483(26.92)	0.45	0.07	483	136	7.00E-35	gene=Chr01G0245	Gene Symbol:SNF3|Host:Isolated from a wide variety of substrates including humans|Disease:invasive candidal disease|Description:SIMILARITY: Belongs to the major facilitator superfamily. Sugar transporter (TC 2.A.1.1)

														family.
Chr01G0250.1	780	29	493	UniProt ID:Q9HG15_COLLN	746	2	433	126/493(25.56)	0.42	0.18	493	97.1	1.00E-21	gene=Chr01G0250	Gene Symbol:CLTA1|Host:Multiple genera of Fabaceae. Rare reports on other taxa|Disease:Leaf, stem and pod anthracnose|Description:SIMILARITY: Contains 1 Zn(2)-C6 fungal-type DNA-binding domain.
Chr01G0252.1	581	12	555	UniProt ID:Q9P8L8_BOTFU	598	31	594	156/576(27.08)	0.48	0.08	576	196	2.00E-55	gene=Chr01G0252	Gene Symbol:BCMFS1|Host:Various plant families|Disease:Grey mould. Parasite or saprophyte|Description:Unknown
Chr01G0255.1	286	14	196	UniProt ID:A4RGG9_MAGO7	286	14	208	65/205(31.71)	0.47	0.16	205	45.8	9.00E-07	gene=Chr01G0255	Gene Symbol:MGG_00056|Host:Poaceae, especially important on Oryzae|Disease:Rice blast|Description:SIMILARITY: Belongs to the short-chain

														dehydrogenases/reductases (SDR) family.
Chr01G0258.1	445	32	253	UniProt ID:Q59VF3_CANAL	1813	26	248	73/234 (31.20)	0.46	0.1	234	70.1	1.00E-13	gene=Chr01G0258	Gene Symbol:"DUR1,2"|Host:Isolated from a wide variety of substrates including humans|Disease:invasive candidal disease|Description:CAUTION: The sequence shown here is derived from an EMBL/GenBank/DDBJ whole genome shotgun (WGS) entry which is preliminary data.
Chr01G0263.1	278	1	216	UniProt ID:Q00350_COCCA	231	1	228	147/229(64.19)	0.72	0.06	229	285	5.00E-97	gene=Chr01G0263	Gene Symbol:XYL2|Host:Corn, Zea mays, sometimes on Sorghum (Poaceae) and various other plant families|Disease:Northern corn leaf spot, ear and kernel rot|Description:CATALYTIC ACTIVITY: Endohydrolysis of

															(1->4)-beta-D-xylosidic linkages in xylans.
Chr01G0264.1	535	19	519	UniProt ID:Q5XTQ5_BOTFU	615	72	561	157/507(30.97)	0.49	0.05	507	206	3.00E-59	gene=Chr01G0264	Gene Symbol:FRT1|Host:Various plant families|Disease:Grey mould. Parasite or saprophyte|Description:SIMILARITY: Belongs to the major facilitator superfamily. Sugar transporter (TC 2.A.1.1) family.
Chr01G0266.1	918	6	916	UniProt ID:O93841_9PEZI	914	4	911	756/917(82.44)	0.9	0.02	917	1541	0	gene=Chr01G0266	Gene Symbol:CHIP6|Host:Multiple genera in multiple families|Disease:'Anthracnose of stems and leaves, dieback, root rot, leaf spot, blossom rot, fruit rot (dieback and ripe rot), seedling blight.' (Mordue 1971)|Description:Unknown
Chr01G0270.1	436	4	427	UniProt ID:Q6Y	625	169	589	147/427(34.4	0.57	0.02	427	275	9.00E-86	gene=Chr01G0270	Gene Symbol:NULL|Host:Multipl

				392_PHAND				3)							e genera of Poaceae and Blysmus compressus (Cyperaceae)|Disease:Glume blotch of wheat and other grasses|Description:Unknown
Chr01G0272.1	370	9	295	UniProt ID:Q9Y784_MAGGR	631	103	395	77/298 (25.84)	0.5	0.05	298	107	2.00E-26	gene=Chr01G0272	Gene Symbol:PTH11|Host:Digitaria (Poaceae)|Disease:Leaf spot|Description:Unknown
Chr01G0273.1	413	91	412	UniProt ID:O59928_HYPVI	430	121	406	96/333 (28.83)	0.42	0.17	333	113	8.00E-29	gene=Chr01G0273	Gene Symbol:NULL|Host:humans|Disease:infection|Description:SIMILARITY: Belongs to the glycosyl hydrolase 18 family.
Chr01G0279.1	461	65	452	UniProt ID:Q59RG1_CANAL	561	148	549	90/407 (22.11)	0.4	0.06	407	71.6	3.00E-14	gene=Chr01G0279	Gene Symbol:NAG3|Host:Isolated from a wide variety of substrates including humans|Disease:invasive candidal disease|Description:CAUTION: The sequence shown

															here is derived from an EMBL/GenBank/DDBJ whole genome shotgun (WGS) entry which is preliminary data.
Chr01G0285.1	539	323	504	UniProt ID:A4ULI9_MYCGR	502	300	501	46/211 (21.80)	0.43	0.18	211	56.6	2.00E-09	gene=Chr01G0285	Gene Symbol:CYP51|Host:Triticum and possibly a few other grasses|Disease:Leaf spot or speckled leaf blotch of wheat|Description:COFACTOR: Heme group (By similarity).
Chr01G0289.1	723	1	722	UniProt ID:A4QRX6_MAGO7	702	1	701	375/758(49.47)	0.6	0.12	758	490	8.00E-164	gene=Chr01G0289	Gene Symbol:MGG_03451|Host:Poaceae, especially important on Oryzae|Disease:Rice blast|Description:Unknown
Chr01G0297.1	570	237	500	UniProt ID:A4ULJ0_MYCGR	518	231	505	68/295 (23.05)	0.43	0.17	295	55.1	5.00E-09	gene=Chr01G0297	Gene Symbol:CYP51|Host:Triticum and possibly a few other grasses|Disease:Leaf spot

															or speckled leaf blotch of wheat|Description:COFACTOR: Heme group (By similarity).
Chr01G0298.1	1036	742	1010	UniProt ID:A4ULI7_MYCGR	517	237	515	73/290(25.17)	0.46	0.11	290	63.2	4.00E-11	gene=Chr01G0298	Gene Symbol:CYP51|Host:Triticum and possibly a few other grasses|Disease:Leaf spot or speckled leaf blotch of wheat|Description:COFACTOR: Heme group (By similarity).
Chr01G0310.1	413	21	380	UniProt ID:Q9Y784_MAGGR	631	105	465	105/368(28.53)	0.47	0.04	368	141	1.00E-37	gene=Chr01G0310	Gene Symbol:PTH11|Host:Digitaria (Poaceae)|Disease:Leaf spot|Description:Unknown
Chr01G0334.1	404	23	398	UniProt ID:Q96VZ3_FUSOX	455	50	443	131/399(32.83)	0.51	0.07	399	198	6.00E-59	gene=Chr01G0334	Gene Symbol:PGX1|Host:Multiple genera in multiple families|Disease:Blights, wilts, rots of various sorts|Description:SIMILARITY: Belongs to the glycosyl hydrolase 28

															family.
Chr01G0339.1	340	37	157	UniProt ID:Q75WR5_9PLEO	265	7	130	36/125 (28.80)	0.5	0.04	125	45.1	2.00E-06	gene=Chr01G0339	Gene Symbol:BRN1|Host:Belamcanda chinensis: Korea,Gladiolus ?gandavensis: Korea,Iris japonica: China,Iris missouriensis (Leaf spot.): Idaho; Montana; Oregon; Washington,Iris sp. (Leaf spot.): China; Texas; Washing|Disease:Leaf spot|Description:SIMILARITY: Belongs to the short-chain dehydrogenases/reductases (SDR) family.
Chr01G0341.1	643	71	513	UniProt ID:Q96VU5_CRYNE	468	1	423	217/450(48.22)	0.65	0.08	450	416	2.00E-139	gene=Chr01G0341	Gene Symbol:UGD1|Host:humans|Disease:cryptococcosis|Description:Unknown
Chr01G0345.1	542	47	509	UniProt ID:Q5XTQ4_BOTFU	574	53	563	143/544(26.29)	0.42	0.21	544	125	8.00E-32	gene=Chr01G0345	Gene Symbol:LIP1|Host:Various plant families|Disease:Grey mould. Parasite or

															saprophyte|Description:Unknown
Chr01G0351.1	1234	960	1224	UniProt ID:Q3Y5V5_MAGGR	1321	1042	1317	107/276(38.77)	0.6	0.04	276	196	1.00E-51	gene=Chr01G0351	Gene Symbol:ABC3|Host:Digitaria (Poaceae)|Disease:Leaf spot|Description:SIMILARITY: Belongs to the ABC transporter superfamily.
Chr01G0353.1	559	5	170	UniProt ID:Q59RG0_CANAL	581	80	235	47/173(27.17)	0.46	0.14	173	53.1	2.00E-08	gene=Chr01G0353	Gene Symbol:NAG4|Host:Isolated from a wide variety of substrates including humans|Disease:invasive candidal disease|Description:CAUTION: The sequence shown here is derived from an EMBL/GenBank/DDBJ whole genome shotgun (WGS) entry which is preliminary data.
Chr01G0357.1	810	31	555	UniProt ID:A6N6J8_FUSOX	903	45	526	118/547(21.57)	0.37	0.16	547	72.4	6.00E-14	gene=Chr01G0357	Gene Symbol:CTF1|Host:Multiple genera in multiple families|Disease:Blights, wilts, rots of various

														sorts|Description:SIMILARITY: Contains 1 Zn(2)-C6 fungal-type DNA-binding domain.
Chr01G0360.1	673	231	391	UniProt ID:Q59VF3_CANAL	1813	59	215	46/168 (27.38)	0.45	0.11	168	55.1	1.00E-08	gene=Chr01G0360	Gene Symbol:"DUR1,2"|Host:Isolated from a wide variety of substrates including humans|Disease:invasive candidal disease|Description:CAUTION: The sequence shown here is derived from an EMBL/GenBank/DDBJ whole genome shotgun (WGS) entry which is preliminary data.
Chr05G0430.1	310	9	80	UniProt ID:PABP_COCIM	768	238	309	29/72(40.28)	0.63	0	72	65.1	1.00E-12	gene=Chr05G0430	Gene Symbol:PAB1|Host:humans|Disease:coccidioidomycosis|Description:FUNCTION: Binds the poly(A) tail of mRNA. Appears to be an important mediator of the multiple roles of the poly(A) tail in mRNA

														biogenesis, stability and translation. In the nucleus, involved in both mRNA cleavage and polyadenylation. Is also required for efficient mRNA export to the cytoplasm. Acts in concert with a poly(A)-specific nuclease (PAN) to affect poly(A) tail shortening, which may occur concomitantly with either nucleocytoplasmic mRNA transport or translational initiation. In the cytoplasm, stimulates translation initiation and regulates mRNA decay through translation termination-coupled poly(A) shortening, probably mediated by PAN (By similarity).
Chr05G0420.1	371	268	355	UniProt ID:Q5A	821	717	801	34/89(38.20)	0.57	0.06	89	74.7	2.00E-15	gene=Chr05G0420	Gene Symbol:CAS5|Host:Isolate

				MH6_CANAL										d from a wide variety of substrates including humans|Disease:invasive candidal disease|Description:CAUTION: The sequence shown here is derived from an EMBL/GenBank/DDBJ whole genome shotgun (WGS) entry which is preliminary data.
Chr05G0417.1	962	413	723	UniProt ID:TUP1_CANAL	514	185	509	95/349 (27.22)	0.44	0.18	349	123	2.00E-30	gene=Chr05G0417	Gene Symbol:TUP1|Host:Isolated from a wide variety of substrates including humans|Disease:invasive candidal disease|Description:FUNCTION: Represses transcription by RNA polymerase II. Represses genes responsible for initiating filamentous growth and this repression is lifted under inducing environmental conditions.

Chr05G0407.1	529	56	492	UniProt ID:Q5ANE1_CANAL	748	51	492	103/451(22.84)	0.42	0.05	451	102	6.00E-24	gene=Chr05G0407	Gene Symbol:SNF3|Host:Isolated from a wide variety of substrates including humans|Disease:invasive candidal disease|Description:SIMILARITY: Belongs to the major facilitator superfamily. Sugar transporter (TC 2.A.1.1) family.
Chr05G0401.1	692	151	316	UniProt ID:Q5ALS7_CANAL	1144	364	527	42/171(24.56)	0.46	0.07	171	47	3.00E-06	gene=Chr05G0401	Gene Symbol:CTF1|Host:Isolated from a wide variety of substrates including humans|Disease:invasive candidal disease|Description:SIMILARITY: Contains 1 Zn(2)-C6 fungal-type DNA-binding domain.
Chr05G0399.1	352	15	325	UniProt ID:Q59QH2_CANAL	337	4	313	102/331(30.82)	0.49	0.12	331	128	1.00E-34	gene=Chr05G0399	Gene Symbol:CSH1|Host:Isolated from a wide variety of substrates including

															humans|Disease:invasive candidal disease|Description:CAUTION: The sequence shown here is derived from an EMBL/GenBank/DDBJ whole genome shotgun (WGS) entry which is preliminary data.
Chr05G0398.1	2195	85	2195	UniProt ID:Q75T35_GLOLA	2143	31	2143	1789/2131(83.95)	0.91	0.02	2131	3604	0	gene=Chr05G0398	Gene Symbol:CAC1|Host:melons,cucumber|Disease:anthracnose fruit rot|Description:Unknown
Chr05G0387.1	286	2	151	UniProt ID:Q59WE5_CANAL	323	20	166	46/155 (29.68)	0.4	0.08	155	42.7	1.00E-05	gene=Chr05G0387	Gene Symbol:PHO100|Host:Isolated from a wide variety of substrates including humans|Disease:invasive candidal disease|Description:CAUTION: The sequence shown here is derived from an EMBL/GenBank/DDBJ whole genome shotgun (WGS) entry which is

															preliminary data.
Chr05G0384.1	934	249	902	UniProt ID:DPP4_ASPFU	765	101	744	276/665(41.50)	0.56	0.05	665	462	9.00E-150	gene=Chr05G0384	Gene Symbol:DPP4|Host:humans|Disease:infection|Description:FUNCTION: Extracellular dipeptidyl-peptidase which removes N- terminal dipeptides sequentially from polypeptides having unsubstituted N-termini provided that the penultimate residue is proline. Contributes to pathogenicity (By similarity).
Chr05G0378.1	573	37	573	UniProt ID:Q2VLJ1_GIBZA	565	27	563	279/538(51.86)	0.68	0	538	585	0	gene=Chr05G0378	Gene Symbol:ZEB1|Host:Principal hosts: Poaceae, including Zea mays (corn), Triticum aestivum (wheat), and Oryza sativa (rice). Additional hosts: various plant families|Disease:Seedling blight, pre- and

															post-emergence blight, root and foot rot, brown rot, culm decay, head or kernel blight (scab or ear scab) of cereals. Leaf|Description:Unknown
Chr05G0377.1	368	15	336	UniProt ID:Q6A2T2_BOTFU	391	30	343	88/332(26.51)	0.45	0.08	332	103	8.00E-26	gene=Chr05G0377	Gene Symbol:BTP1|Host:Various plant families|Disease:Grey mould. Parasite or saprophyte|Description:Unknown
Chr05G0375.1	1577	511	1528	UniProt ID:A4RGC8_MAGO7	1158	45	1146	436/1113(39.17)	0.55	0.1	1113	720	0	gene=Chr05G0375	Gene Symbol:MGG_11671|Host:Poaceae, especially important on Oryzae|Disease:Rice blast|Description:SIMILARITY: Contains 1 reverse transcriptase domain.
Chr05G0372.1	758	20	60	UniProt ID:A4R0W3_MAGO7	1226	274	316	18/43(41.86)	0.65	0.05	43	47.4	3.00E-06	gene=Chr05G0372	Gene Symbol:MGG_09263|Host:Poaceae, especially important on Oryzae|Disease:Rice

															blast|Description:Unknown
Chr05G0371.1	277	1	263	UniProt ID:Q32WF7_PHAND	266	5	262	85/271 (31.37)	0.51	0.08	271	117	9.00E-32	gene=Chr05G0371	Gene Symbol:MDH1|Host:Multiple genera of Poaceae and Blysmus compressus (Cyperaceae)|Disease:Glume blotch of wheat and other grasses|Description:Unknown
Chr05G0365.1	1312	55	1253	UniProt ID:A4RGC8_MAGO7	1158	10	1155	484/1227(39.45)	0.57	0.09	1227	802	0	gene=Chr05G0365	Gene Symbol:MGG_11671|Host:Poaceae, especially important on Oryzae|Disease:Rice blast|Description:SIMILARITY: Contains 1 reverse transcriptase domain.
Chr05G0346.1	1198	283	582	UniProt ID:ORYZ_ASPFU	403	118	369	71/307 (23.13)	0.38	0.2	307	47	4.00E-06	gene=Chr05G0346	Gene Symbol:ALP1|Host:humans|Disease:infection|Description:FUNCTION: Secreted alkaline protease that allows assimilation of

															proteinaceous substrates. Acts as a significant virulence factor in invasive aspergillosis. Involved in immune evasion from the human and mice complement systems during infection. Efficiently cleaves important components of the complement cascade such as such as C3, C4, C5, and C1q, as well as IgG, which leads to down-regulation of complement activation at the hyphal surface.
Chr05G0342.1	260	50	146	UniProt ID:A4RGG9_MAGO7	286	127	226	26/100 (26.00)	0.54	0.03	100	47.8	2.00E-07	gene=Chr05G0342	Gene Symbol:MGG_00056|Host:Poaceae, especially important on Oryzae|Disease:Rice blast|Description:SIMILARITY: Belongs to the short-chain dehydrogenases/reductas

															es (SDR) family.
Chr05G0 341.1	321	68	321	UniProt ID:PLYB_COLGL	331	68	331	135/268(50.37)	0.66	0.07	268	250	3.00E-81	gene=Chr05G0341	Gene Symbol:PLB|Host:Multiple genera in multiple families|Disease:'Anthracnose of stems and leaves, dieback, root rot, leaf spot, blossom rot, fruit rot (dieback and ripe rot), seedling blight.' (Mordue 1971)|Description:FUNCTION: Acts as a virulence factor active in plant tissue maceration.
Chr05G0 339.1	507	43	282	UniProt ID:Q9UUS8_COLGL	567	72	317	77/257 (29.96)	0.49	0.11	257	98.2	9.00E-23	gene=Chr05G0339	Gene Symbol:CHIP3|Host:Multiple genera in multiple families|Disease:'Anthracnose of stems and leaves, dieback, root rot, leaf spot, blossom rot, fruit rot (dieback and ripe rot), seedling blight.' (Mordue 1971)|Description:Unknown
Chr05G0	536	253	495	UniProt	518	256	500	64/266	0.43	0.17	266	45.8	4.00E-06	gene=Chr	Gene

335.1				ID:A4ULJ0_MYCGR				(24.06)						05G0335	Symbol:CYP51|Host:Triticum and possibly a few other grasses|Disease:Leaf spot or speckled leaf blotch of wheat|Description:COFACTOR: Heme group (By similarity).
Chr05G0334.1	341	308	341	UniProt ID:O59937_FUSOX	384	20	53	20/34(58.82)	0.74	0	34	53.1	7.00E-09	gene=Chr05G0334	Gene Symbol:XYL3|Host:Multiple genera in multiple families|Disease:Blights, wilts, rots of various sorts|Description:SIMILARITY: Belongs to the glycosyl hydrolase 10 (cellulase F) family.
Chr05G0328.1	1186	163	1060	UniProt ID:Q9P872_CANAL	917	37	865	264/909(29.04)	0.51	0.1	909	342	4.00E-101	gene=Chr05G0328	Gene Symbol:PMR1|Host:Isolated from a wide variety of substrates including humans|Disease:invasive candidal disease|Description:SIMILARITY: Belongs to the cation transport ATPase

															(P-type) family.
Chr05G0 322.1	536	9	90	UniProt ID:Q5A 4F3_C ANAL	624	17	106	30/90(33.33)	0.43	0.09	90	55.5	4.00E-09	gene=Chr 05G0322	Gene Symbol:ZCF37|Host:Isolat ed from a wide variety of substrates including humans|Disease:invasive candidal disease|Description:Unkn own
Chr05G0 317.1	251	160	233	UniProt ID:Q5E GQ1_C RYNE	392	322	380	31/74(41.89)	0.49	0.2	74	58.5	6.00E-11	gene=Chr 05G0317	Gene Symbol:BWC2|Host:huma ns|Disease:cryptococcosis |Description:Unknown
Chr05G0 311.1	1418	858	141 5	UniProt ID:Q9U W87_C ANAL	1606	997	1598	164/61 5(26.6 7)	0.47	0.11	615	219	1.00E-58	gene=Chr 05G0311	Gene Symbol:MLT1|Host:Isolate d from a wide variety of substrates including humans|Disease:invasive candidal disease|Description:SIMIL ARITY: Belongs to the ABC transporter superfamily.
Chr05G0 306.1	264	1	155	UniProt ID:Q9C 441_F	330	165	303	65/156 (41.67)	0.58	0.12	156	124	5.00E-34	gene=Chr 05G0306	Gene Symbol:PEP1|Host:Multipl e plant families. Some

				USSO										strains may cause infections in humans|Disease:Saprobe, facultative pathogen|Description:Unknown
Chr05G0302.1	333	25	201	UniProt ID:A4R3I5_MAGO7	400	104	274	46/183 (25.14)	0.45	0.1	183	47	7.00E-07	gene=Chr05G0302	Gene Symbol:"MGG_11993, MGG_12837, MGG_13052"|Host:Poaceae, especially important on Oryzae|Disease:Rice blast|Description:Unknown
Chr05G0301.1	1234	71	1201	UniProt ID:A4RGC8_MAGO7	1158	31	1132	459/1162(39.50)	0.56	0.08	1162	781	0	gene=Chr05G0301	Gene Symbol:MGG_11671|Host:Poaceae, especially important on Oryzae|Disease:Rice blast|Description:SIMILARITY: Contains 1 reverse transcriptase domain.
Chr05G0299.1	464	40	379	UniProt ID:A4UC81_MAGO7	376	33	371	145/343(42.27)	0.6	0.02	343	252	2.00E-79	gene=Chr05G0299	Gene Symbol:MGG_10702|Host:Poaceae, especially important on

															Oryzae|Disease:Rice blast|Description:Unknown
Chr05G0296.1	793	119	750	UniProt ID:A4QVA7_MAGO7	1340	370	1065	203/705(28.79)	0.44	0.12	705	195	1.00E-52	gene=Chr05G0296	Gene Symbol:MGG_04629|Host:Poaceae, especially important on Oryzae|Disease:Rice blast|Description:Unknown
Chr05G0289.1	571	4	357	UniProt ID:Q0WXM3_FUSOX	663	109	445	93/377 (24.67)	0.41	0.17	377	77.8	4.00E-16	gene=Chr05G0289	Gene Symbol:FOW2|Host:Multiple genera in multiple families|Disease:Blights, wilts, rots of various sorts|Description:SIMILARITY: Contains 1 Zn(2)-C6 fungal-type DNA-binding domain.
Chr05G0278.1	286	71	229	UniProt ID:Q6TFC7_ASPFM	349	114	268	42/173 (24.28)	0.44	0.18	173	44.3	4.00E-06	gene=Chr05G0278	Gene Symbol:NULL|Host:humans|Disease:infection|Description:Unknown
Chr05G0274.1	500	47	499	UniProt ID:A0ST43_C	459	1	457	137/473(28.96)	0.45	0.08	473	176	1.00E-49	gene=Chr05G0274	Gene Symbol:CTB5|Host:Numerous taxa in

				ERNC											Solanaceae|Disease:Leaf spot|Description:Unknown
Chr05G0273.1	399	94	319	UniProt ID:Q9Y784_MAGGR	631	170	407	69/242 (28.51)	0.48	0.08	242	99.8	1.00E-23	gene=Chr05G0273	Gene Symbol:PTH11|Host:Digitaria (Poaceae)|Disease:Leaf spot|Description:Unknown
Chr05G0272.1	511	68	509	UniProt ID:Q5ANE1_CANAL	748	48	496	115/461(24.95)	0.42	0.07	461	92.8	8.00E-21	gene=Chr05G0272	Gene Symbol:SNF3|Host:Isolated from a wide variety of substrates including humans|Disease:invasive candidal disease|Description:SIMILARITY: Belongs to the major facilitator superfamily. Sugar transporter (TC 2.A.1.1) family.
Chr05G0270.1	1445	896	1444	UniProt ID:Q5A762_CANAL	1606	1022	1585	173/581(29.78)	0.5	0.08	581	237	3.00E-64	gene=Chr05G0270	Gene Symbol:MLT1|Host:Isolated from a wide variety of substrates including humans|Disease:invasive candidal disease|Description:SIMIL

															ARITY: Belongs to the ABC transporter superfamily.
Chr05G0265.1	394	104	320	UniProt ID:Q56J91_CANGB	372	64	274	56/217 (25.81)	0.47	0.03	217	95.5	7.00E-23	gene=Chr05G0265	Gene Symbol:ERG6|Host:humans|Disease:Occasional invasive candidal disease|Description:Unknown
Chr05G0263.1	1363	44	1362	UniProt ID:Q3Y5V5_MAGGR	1321	26	1319	414/1384(29.91)	0.48	0.11	1384	529	2.00E-164	gene=Chr05G0263	Gene Symbol:ABC3|Host:Digitaria (Poaceae)|Disease:Leaf spot|Description:SIMILARITY: Belongs to the ABC transporter superfamily.
Chr05G0257.1	375	40	368	UniProt ID:Q5AFI8_CANAL	373	7	361	87/364 (23.90)	0.46	0.12	364	84.7	3.00E-19	gene=Chr05G0257	Gene Symbol:LEU2|Host:Isolated from a wide variety of substrates including humans|Disease:invasive candidal disease|Description:FUNCTION: Catalyzes the oxidation of 3-carboxy-2-hydroxy-4-methylpentanoate

															(3-isopropylmalate) to 3-carboxy-4-methyl-2-oxopentanoate. The product decarboxylates to 4-methyl-2 oxopentanoate (By similarity).
Chr05G0253.1	720	123	419	UniProt ID:Q59NY2_CANAL	562	110	418	72/326 (22.09)	0.4	0.14	326	76.3	2.00E-15	gene=Chr05G0253	Gene Symbol:HEX1|Host:Isolated from a wide variety of substrates including humans|Disease:invasive candidal disease|Description:CAUTION: The sequence shown here is derived from an EMBL/GenBank/DDBJ whole genome shotgun (WGS) entry which is preliminary data.
Chr05G0252.1	534	247	510	UniProt ID:A4ULJ0_MYCGR	518	221	515	69/312 (22.12)	0.41	0.21	312	60.1	1.00E-10	gene=Chr05G0252	Gene Symbol:CYP51|Host:Triticum and possibly a few other grasses|Disease:Leaf spot or speckled leaf blotch of wheat|Description:COFAC

															TOR: Heme group (By similarity).
Chr05G0250.1	349	137	343	UniProt ID:Q92217_COCHE	2528	1931	2138	50/212 (23.58)	0.45	0.04	212	62.8	1.00E-11	gene=Chr05G0250	Gene Symbol:PKS1|Host:Zea mays|Disease:Southern leaf blight of maize|Description:Unknown
Chr05G0247.1	133	22	63	UniProt ID:D1MYV6_MAGGR	568	523	563	25/42(59.52)	0.71	0.02	42	43.1	1.00E-06	gene=Chr05G0247	Gene Symbol:CBL1|Host:Digitaria (Poaceae)|Disease:Leaf spot|Description:SIMILARITY: Contains 3 chitin-binding type-1 domains.
Chr05G0246.1	393	278	393	UniProt ID:O59928_HYPVI	430	142	262	37/130 (28.46)	0.48	0.18	130	48.9	3.00E-07	gene=Chr05G0246	Gene Symbol:NULL|Host:humans|Disease:infection|Description:SIMILARITY: Belongs to the glycosyl hydrolase 18 family.
Chr05G0235.1	139	11	101	UniProt ID:D2JLS9_9HYPO	131	36	124	49/91(53.85)	0.7	0.02	91	99.8	5.00E-28	gene=Chr05G0235	Gene Symbol:HIS3|Host:plants|Disease:allergy|Description:SUBUNIT: The nucleosome is a histone

															octamer containing two molecules each of H2A, H2B, H3 and H4 assembled in one H3-H4 heterotetramer and two H2A-H2B heterodimers. The octamer wraps approximately 147 bp of DNA (By similarity).
Chr05G0228.1	643	268	437	UniProt ID:Q0PND8_MAGGR	1375	1007	1167	57/184 (30.98)	0.48	0.2	184	76.3	3.00E-15	gene=Chr05G0228	Gene Symbol:PEX6|Host:Digitaria (Poaceae)|Disease:Leaf spot|Description:SIMILARITY: Belongs to the AAA ATPase family.
Chr05G0225.1	305	3	291	UniProt ID:A0ST42_CERNC	512	25	307	103/291(35.40)	0.52	0.03	291	159	2.00E-45	gene=Chr05G0225	Gene Symbol:CTB4|Host:Numerous taxa in Solanaceae|Disease:Leaf spot|Description:Unknown
Chr05G0224.1	487	43	84	UniProt ID:Q59MD2_CANAL	843	757	798	17/42(40.48)	0.67	0	42	49.3	4.00E-07	gene=Chr05G0224	Gene Symbol:UME6|Host:Isolated from a wide variety of substrates including humans|Disease:invasive candidal

															disease|Description:CAUTION: The sequence shown here is derived from an EMBL/GenBank/DDBJ whole genome shotgun (WGS) entry which is preliminary data.
Chr05G0221.1	480	33	419	UniProt ID:Q9Y784_MAGGR	631	29	421	136/396(34.34)		0.59	0.03	396	262	2.00E-80	gene=Chr05G0221	Gene Symbol:PTH11|Host:Digitaria (Poaceae)|Disease:Leaf spot|Description:Unknown
Chr05G0211.1	551	66	520	UniProt ID:Q5ANE1_CANAL	748	51	507	110/465(23.66)		0.41	0.04	465	105	8.00E-25	gene=Chr05G0211	Gene Symbol:SNF3|Host:Isolated from a wide variety of substrates including humans|Disease:invasive candidal disease|Description:SIMILARITY: Belongs to the major facilitator superfamily. Sugar transporter (TC 2.A.1.1) family.
Chr05G0210.1	384	16	382	UniProt ID:C5G	380	14	379	228/367(62.1		0.74	0	367	474	3.00E-167	gene=Chr05G0210	Gene Symbol:BDCG_09435|Ho

			YF3_AJEDR				3)						st:humans|Disease:cutaneous Blastomyces dermatitidis infection|Description:Unknown
Chr05G0209.1	336	58	288	UniProt ID:PLYB_COLGL	331	67	295	93/235 (39.57)	0.6	0.04	235	154	2.00E-44	gene=Chr05G0209	Gene Symbol:PLB|Host:Multiple genera in multiple families|Disease:'Anthracnose of stems and leaves, dieback, root rot, leaf spot, blossom rot, fruit rot (dieback and ripe rot), seedling blight.' (Mordue 1971)|Description:FUNCTION: Acts as a virulence factor active in plant tissue maceration.
Chr05G0206.1	419	36	346	UniProt ID:F2QU09_PICP7	626	30	293	76/325 (23.38)	0.38	0.23	325	75.5	1.00E-15	gene=Chr05G0206	Gene Symbol:PAB1|Host:humans|Disease:occasional infection|Description:Unknown
Chr05G0203.1	701	16	289	UniProt ID:Q6IWN3_U	827	549	824	115/277(41.52)	0.61	0.01	277	234	4.00E-67	gene=Chr05G0203	Gene Symbol:NULL|Host:Euchlaena spp., Zea spp.

			STMD											(Poaceae)|Disease:Smut. Corn smut|Description:CATALYTIC ACTIVITY: ATP + a protein = ADP + a phosphoprotein.
Chr05G0200.1	368	13	134	UniProt ID:A0ST44_CERNC	357	4	133	40/130 (30.77)	0.48	0.06	130	59.7	7.00E-11	gene=Chr05G0200	Gene Symbol:CTB6|Host:Numerous taxa in Solanaceae|Disease:Leaf spot|Description:Unknown
Chr05G0199.1	590	134	578	UniProt ID:Q9P8L8_BOTFU	598	166	594	97/459 (21.13)	0.38	0.1	459	47.8	1.00E-06	gene=Chr05G0199	Gene Symbol:BCMFS1|Host:Various plant families|Disease:Grey mould. Parasite or saprophyte|Description:Unknown
Chr05G0197.1	381	90	372	UniProt ID:Q92217_COCHE	2528	1858	2138	75/301 (24.92)	0.41	0.13	301	64.3	5.00E-12	gene=Chr05G0197	Gene Symbol:PKS1|Host:Zea mays|Disease:Southern leaf blight of maize|Description:Unknown
Chr05G0191.1	446	4	444	UniProt ID:Q9C	423	5	411	153/452(33.8	0.49	0.12	452	222	1.00E-67	gene=Chr05G0191	Gene Symbol:CEL2|Host:Corn,

			1F9_COCCA				5)							Zea mays, sometimes on Sorghum (Poaceae) and various other plant families|Disease:Northern corn leaf spot, ear and kernel rot|Description:Unknown
Chr05G0188.1	529	71	446	UniProt ID:A4UC81_MAGO7	376	11	373	150/381(39.37)	0.56	0.06	381	261	2.00E-82	gene=Chr05G0188	Gene Symbol:MGG_10702|Host:Poaceae, especially important on Oryzae|Disease:Rice blast|Description:Unknown
Chr05G0187.1	403	10	398	UniProt ID:Q2I0M6_CERNC	871	35	422	103/417(24.70)	0.43	0.14	417	99.8	2.00E-23	gene=Chr05G0187	Gene Symbol:CTB3|Host:Numerous taxa in Solanaceae|Disease:Leaf spot|Description:Unknown
Chr05G0186.1	361	4	360	UniProt ID:Q75ZG3_ALTAL	366	7	365	201/368(54.62)	0.69	0.05	368	395	7.00E-137	gene=Chr05G0186	Gene Symbol:AFTS1|Host:Plant|Disease:Leaf spot, rots|Description:Unknown
Chr05G0183.1	516	127	488	UniProt ID:A4ULI5_MY	515	116	510	91/414(21.98)	0.38	0.17	414	51.2	7.00E-08	gene=Chr05G0183	Gene Symbol:CYP51|Host:Triticum and possibly a few

				CGR											other grasses|Disease:Leaf spot or speckled leaf blotch of wheat|Description:COFACTOR: Heme group (By similarity).
Chr05G0182.1	237	36	223	UniProt ID:Q6RKH1_GIBZA	384	1	187	110/188(58.51)	0.74	0.01	188	231	1.00E-74	gene=Chr05G0182	Gene Symbol:PKS9|Host:Principal hosts: Poaceae, including Zea mays (corn), Triticum aestivum (wheat), and Oryza sativa (rice). Additional hosts: various plant families|Disease:Seedling blight, pre- and post-emergence blight, root and foot rot, brown rot, culm decay, head or kernel blight (scab or ear scab) of cereals. Leaf|Description:Unknown
Chr05G0181.1	2334	1	1598	UniProt ID:Q6ZX14_MAGGR	4034	366	1813	504/1629(30.94)	0.46	0.13	1629	620	0	gene=Chr05G0181	Gene Symbol:ACE1|Host:Digitaria (Poaceae)|Disease:Leaf spot|Description:Unknown

Chr05G0180.1	530	282	494	UniProt ID:A4ULJ0_MYCGR	518	248	503	59/256 (23.05)	0.36	0.17	256	56.2	2.00E-09	gene=Chr05G0180	Gene Symbol:CYP51|Host:Triticum and possibly a few other grasses|Disease:Leaf spot or speckled leaf blotch of wheat|Description:COFACTOR: Heme group (By similarity).
Chr05G0178.1	2588	54	1302	UniProt ID:Q92217_COCHE	2528	13	1235	479/1275(37.57)	0.54	0.06	1275	738	0	gene=Chr05G0178	Gene Symbol:PKS1|Host:Zea mays|Disease:Southern leaf blight of maize|Description:Unknown
Chr05G0175.1	402	99	245	UniProt ID:O59897_ASPFM	2146	1829	1975	41/149 (27.52)	0.51	0.03	149	61.6	4.00E-11	gene=Chr05G0175	Gene Symbol:ALB1|Host:humans|Disease:infection|Description:Unknown
Chr05G0173.1	623	34	623	UniProt ID:Q9P8L8_BOTFU	598	67	589	194/593(32.72)	0.52	0.12	593	316	1.00E-99	gene=Chr05G0173	Gene Symbol:BCMFS1|Host:Various plant families|Disease:Grey mould. Parasite or saprophyte|Description:Unknown

Chr05G0172.1	580	19	578	UniProt ID:Q5XTQ4_BOTFU	574	28	572	312/560(55.71)	0.71	0.03	560	639	0	gene=Chr05G0172	Gene Symbol:LIP1|Host:Various plant families|Disease:Grey mould. Parasite or saprophyte|Description:Unknown
Chr05G0167.1	1095	281	631	UniProt ID:Q99324_SEPLY	803	59	480	133/428(31.07)	0.45	0.19	428	165	6.00E-43	gene=Chr05G0167	Gene Symbol:B2TOM|Host:Primarily tomato, Lycopersicon esculentum, also Solanum spp. and other Solanaceae|Disease:Leaf spot|Description:Unknown
Chr05G0163.1	756	74	180	UniProt ID:A0ST46_CERNC	397	17	122	32/110(29.09)	0.52	0.06	110	47.8	1.00E-06	gene=Chr05G0163	Gene Symbol:CTB8|Host:Numerous taxa in Solanaceae|Disease:Leaf spot|Description:Unknown
Chr05G0162.1	509	20	469	UniProt ID:Q5ANE1_CANAL	748	42	496	132/464(28.45)	0.48	0.05	464	177	6.00E-49	gene=Chr05G0162	Gene Symbol:SNF3|Host:Isolated from a wide variety of substrates including humans|Disease:invasive candidal disease|Description:SIMIL

															ARITY: Belongs to the major facilitator superfamily. Sugar transporter (TC 2.A.1.1) family.
Chr05G0160.1	407	184	390	UniProt ID:Q2I0M6_CERNC	871	192	411	59/227(25.99)	0.43	0.12	227	77	4.00E-16	gene=Chr05G0160	Gene Symbol:CTB3|Host:Numerous taxa in Solanaceae|Disease:Leaf spot|Description:Unknown
Chr05G0156.1	596	97	565	UniProt ID:Q59RG1_CANAL	561	85	557	268/474(56.54)	0.72	0.01	474	524	0	gene=Chr05G0156	Gene Symbol:NAG3|Host:Isolated from a wide variety of substrates including humans|Disease:invasive candidal disease|Description:CAUTION: The sequence shown here is derived from an EMBL/GenBank/DDBJ whole genome shotgun (WGS) entry which is preliminary data.
Chr05G0154.1	557	26	525	UniProt ID:Q5ANE1_C	748	13	496	173/512(33.79)	0.53	0.08	512	267	1.00E-80	gene=Chr05G0154	Gene Symbol:SNF3|Host:Isolated from a wide variety of

				ANAL										substrates including humans|Disease:invasive candidal disease|Description:SIMILARITY: Belongs to the major facilitator superfamily. Sugar transporter (TC 2.A.1.1) family.
Chr05G0146.1	516	138	471	UniProt ID:TUP1_CANAL	514	195	509	96/364 (26.37)	0.4	0.22	364	90.5	3.00E-20	gene=Chr05G0146	Gene Symbol:TUP1|Host:Isolated from a wide variety of substrates including humans|Disease:invasive candidal disease|Description:FUNCTION: Represses transcription by RNA polymerase II. Represses genes responsible for initiating filamentous growth and this repression is lifted under inducing environmental conditions.
Chr05G0145.1	903	228	888	UniProt ID:Q5A	888	146	817	262/690(37.9	0.55	0.07	690	426	8.00E-135	gene=Chr05G0145	Gene Symbol:TPS2|Host:Isolate

				I14_CANAL				7)							d from a wide variety of substrates including humans|Disease:invasive candidal disease|Description:Unknown
Chr05G0136.1	556	4	472	UniProt ID:Q5ANE1_CANAL	748	24	496	149/491(30.35)	0.48	0.08	491	186	2.00E-51	gene=Chr05G0136	Gene Symbol:SNF3|Host:Isolated from a wide variety of substrates including humans|Disease:invasive candidal disease|Description:SIMILARITY: Belongs to the major facilitator superfamily. Sugar transporter (TC 2.A.1.1) family.
Chr05G0135.1	518	39	496	UniProt ID:Q5XTQ5_BOTFU	615	96	547	124/487(25.46)	0.42	0.13	487	98.6	8.00E-23	gene=Chr05G0135	Gene Symbol:FRT1|Host:Various plant families|Disease:Grey mould. Parasite or saprophyte|Description:SIMILARITY: Belongs to the major facilitator

															superfamily. Sugar transporter (TC 2.A.1.1) family.
Chr05G0131.1	1092	158	307	UniProt ID:SUB6_ARTBE	412	158	295	55/162 (33.95)	0.46	0.22	162	71.2	1.00E-13	gene=Chr05G0131	Gene Symbol:SUB6|Host:hedgehogs|Disease:ringworm,Kerion Celsi Disease|Description:FUNCTION: Secreted subtilisin-like serine protease with keratinolytic activity that contributes to pathogenicity (By similarity).
Chr05G0127.1	596	36	592	UniProt ID:Q9P8L8_BOTFU	598	37	594	310/559(55.46)	0.76	0.01	559	643	0	gene=Chr05G0127	Gene Symbol:BCMFS1|Host:Various plant families|Disease:Grey mould. Parasite or saprophyte|Description:Unknown
Chr05G0126.1	348	25	240	UniProt ID:Q92217_COCHE	2528	1847	2055	59/222 (26.58)	0.43	0.09	222	58.2	3.00E-10	gene=Chr05G0126	Gene Symbol:PKS1|Host:Zea mays|Disease:Southern leaf blight of maize|Description:Unkno

															wn
Chr05G0123.1	1792	901	1205	UniProt ID:Q5AM49_CANAL	1690	775	1080	137/318(43.08)	0.61	0.08	318	261	4.00E-71	gene=Chr05G0123	Gene Symbol:SNF2|Host:Isolated from a wide variety of substrates including humans|Disease:invasive candidal disease|Description:Unknown
Chr05G0114.1	1037	56	996	UniProt ID:Q9P872_CANAL	917	37	879	295/965(30.57)	0.48	0.15	965	365	1.00E-110	gene=Chr05G0114	Gene Symbol:PMR1|Host:Isolated from a wide variety of substrates including humans|Disease:invasive candidal disease|Description:SIMILARITY: Belongs to the cation transport ATPase (P-type) family.
Chr05G0100.1	727	13	63	UniProt ID:A8QJI7_FUSOX	938	87	136	23/51(45.10)	0.59	0.02	51	50.8	2.00E-07	gene=Chr05G0100	Gene Symbol:XLNR|Host:Multiple genera in multiple families|Disease:Blights, wilts, rots of various sorts|Description:SIMILARITY: Contains 1 Zn(2)-C6

															fungal-type DNA-binding domain.
Chr05G0092.1	233	5	230	UniProt ID:Q079H2_9HELO	246	7	244	122/239(51.05)	0.66	0.06	239	241	3.00E-80	gene=Chr05G0092	Gene Symbol:NEP1|Host:liliaceous plants|Disease:Streaking of lily leaves|Description:Unknown
Chr05G0088.1	1097	101	1086	UniProt ID:Q9P872_CANAL	917	22	905	272/1004(27.09)	0.47	0.14	1004	298	2.00E-86	gene=Chr05G0088	Gene Symbol:PMR1|Host:Isolated from a wide variety of substrates including humans|Disease:invasive candidal disease|Description:SIMILARITY: Belongs to the cation transport ATPase (P-type) family.
Chr05G0085.1	409	27	372	UniProt ID:A4UC81_MAGO7	376	29	372	99/360 (27.50)	0.45	0.08	360	89	1.00E-20	gene=Chr05G0085	Gene Symbol:MGG_10702|Host:Poaceae, especially important on Oryzae|Disease:Rice blast|Description:Unknown

Chr05G0076.1	521	11	518	UniProt ID:Q5ANE1_CANAL	748	32	552	139/546(25.46)	0.46	0.12	546	180	7.00E-50	gene=Chr05G0076	Gene Symbol:SNF3|Host:Isolated from a wide variety of substrates including humans|Disease:invasive candidal disease|Description:SIMILARITY: Belongs to the major facilitator superfamily. Sugar transporter (TC 2.A.1.1) family.
Chr05G0074.1	482	45	472	UniProt ID:Q2VLJ1_GIBZA	565	103	541	119/505(23.56)	0.36	0.28	505	65.9	2.00E-12	gene=Chr05G0074	Gene Symbol:ZEB1|Host:Principal hosts: Poaceae, including Zea mays (corn), Triticum aestivum (wheat), and Oryza sativa (rice). Additional hosts: various plant families|Disease:Seedling blight, pre- and post-emergence blight, root and foot rot, brown rot, culm decay, head or kernel blight (scab or ear

															scab) of cereals.
Chr05G0073.1	498	272	457	UniProt ID:A4ULI5_MYCGR	515	282	502	54/222 (24.32)	0.4	0.17	222	55.5	4.00E-09	gene=Chr05G0073	Leaf|Description:Unknown Gene Symbol:CYP51|Host:Triticum and possibly a few other grasses|Disease:Leaf spot or speckled leaf blotch of wheat|Description:COFACTOR: Heme group (By similarity).
Chr05G0071.1	557	21	81	UniProt ID:Q9HG15_COLLN	746	20	80	29/63(46.03)	0.6	0.06	63	57.8	1.00E-09	gene=Chr05G0071	Gene Symbol:CLTA1|Host:Multiple genera of Fabaceae. Rare reports on other taxa|Disease:Leaf, stem and pod anthracnose|Description:SIMILARITY: Contains 1 Zn(2)-C6 fungal-type DNA-binding domain.
Chr05G0063.1	530	9	522	UniProt ID:C0S733_PARBP	560	13	542	155/539(28.76)	0.49	0.06	539	179	5.00E-50	gene=Chr05G0063	Gene Symbol:PABG_03488|Host:humans|Disease:Paracoccidioidomycosis|Description:SIMILARITY: Belongs

														to the TCP-1 chaperonin family.
Chr05G0062.1	953	422	948	UniProt ID:CXT1_CRYNJ	694	172	662	132/554(23.83)	0.4	0.16	554	122	2.00E-29	gene=Chr05G0062	Gene Symbol:CXT1|Host:humans|Disease:cryptococcosis|Description:FUNCTION: Beta-1,2-xylosyltransferase that plays a key role in capsule polysaccharide synthesis by transferring xylose to alpha-1,3-dimannoside in a beta-1,2-linkage. Also mediates glycosylation of glycosphingolipids; constitutes the unique xylosyltransferase responsible for adding xylose to glycosphingolipids.
Chr05G0059.1	362	35	359	UniProt ID:NPIIC_ARTGP	358	32	357	131/334(39.22)	0.54	0.05	334	213	4.00E-66	gene=Chr05G0059	Gene Symbol:MGYG_02351|Host:Human, Mouse, Rat, Chicken, Pig, Rabbit, Bovine, Dog, African clawed frog,

Chr05G0058.1	1207	827	1069	UniProt ID:Q5AG71_CANAL	1462	135	330	74/248 (29.84)	0.48	0.23	248	92.4	9.00E-20	gene=Chr05G0058	Zebrafish|Disease:tinea capitis, tinea corpus, ringworm, and other dermatophytoses|Description:FUNCTION: Secreted metalloproteinase that allows assimilation of proteinaceous substrates. Shows high activities on basic nuclear substrates such as histone and protamine. May be involved in virulence (By similarity). Gene Symbol:HSL1|Host:Isolated from a wide variety of substrates including humans|Disease:invasive candidal disease|Description:CAUTION: The sequence shown here is derived from an EMBL/GenBank/DDBJ whole genome shotgun (WGS) entry which is

															preliminary data.
Chr05G0043.1	688	1	126	UniProt ID:Q5K995_CRYNJ	1005	327	439	43/126 (34.13)	0.51	0.1	126	77.4	1.00E-15	gene=Chr05G0043	Gene Symbol:CNK02740|Host:humans|Disease:cryptococcosis|Description:Unknown
Chr05G0037.1	524	248	482	UniProt ID:A4ULJ0_MYCGR	518	242	504	69/267 (25.84)	0.47	0.13	267	62.4	3.00E-11	gene=Chr05G0037	Gene Symbol:CYP51|Host:Triticum and possibly a few other grasses|Disease:Leaf spot or speckled leaf blotch of wheat|Description:COFACTOR: Heme group (By similarity).
Chr05G0034.1	457	221	430	UniProt ID:A4ULI5_MYCGR	515	263	510	56/248 (22.58)	0.43	0.15	248	59.3	2.00E-10	gene=Chr05G0034	Gene Symbol:CYP51|Host:Triticum and possibly a few other grasses|Disease:Leaf spot or speckled leaf blotch of wheat|Description:COFACTOR: Heme group (By similarity).
Chr05G0031.1	449	16	409	UniProt ID:Q9P	598	49	422	110/401(27.4	0.48	0.08	401	149	3.00E-40	gene=Chr05G0031	Gene Symbol:BCMFS1|Host:Va

				8L8_BOTFU				3)							rious plant families|Disease:Grey mould. Parasite or saprophyte|Description:Unknown
Chr05G0025.1	1701	113	1608	UniProt ID:O60034_BOTFU	1562	148	1562	531/1526(34.80)	0.52	0.09	1526	836	0	gene=Chr05G0025	Gene Symbol:NULL|Host:Various plant families|Disease:Grey mould. Parasite or saprophyte|Description:SIMILARITY: Belongs to the ABC transporter superfamily.
Chr05G0024.1	419	11	373	UniProt ID:Q2I0M6_CERNC	871	8	365	95/373 (25.47)	0.42	0.07	373	83.2	5.00E-18	gene=Chr05G0024	Gene Symbol:CTB3|Host:Numerous taxa in Solanaceae|Disease:Leaf spot|Description:Unknown
Chr05G0009.1	1219	710	990	UniProt ID:TUP1_CANAL	514	201	509	126/310(40.65)	0.56	0.1	310	211	3.00E-59	gene=Chr05G0009	Gene Symbol:TUP1|Host:Isolated from a wide variety of substrates including humans|Disease:invasive candidal disease|Description:FUNC

															TION: Represses transcription by RNA polymerase II. Represses genes responsible for initiating filamentous growth and this repression is lifted under inducing environmental conditions.
Chr07G0002.1	376	1	348	UniProt ID:A4RGC8_MAGO7	1158	801	1150	137/360(38.06)	0.57	0.06	360	216	9.00E-63	gene=Chr07G0002	Gene Symbol:MGG_11671|Host:Poaceae, especially important on Oryzae|Disease:Rice blast|Description:SIMILARITY: Contains 1 reverse transcriptase domain.
Chr07G0003.1	798	577	752	UniProt ID:TUP1_CANAL	514	258	438	74/188(39.36)	0.53	0.1	188	116	3.00E-28	gene=Chr07G0003	Gene Symbol:TUP1|Host:Isolated from a wide variety of substrates including humans|Disease:invasive candidal disease|Description:FUNCTION: Represses transcription by RNA polymerase II. Represses

															genes responsible for initiating filamentous growth and this repression is lifted under inducing environmental conditions.
Chr07G0007.1	278	223	260	UniProt ID:Q5EGQ1_CRYNE	392	343	380	23/38(60.53)	0.68	0	38	57.4	2.00E-10	gene=Chr07G0007	Gene Symbol:BWC2|Host:humans|Disease:cryptococcosis|Description:Unknown
Chr07G0010.1	523	4	198	UniProt ID:Q9C441_FUSSO	330	120	303	77/200 (38.50)	0.56	0.11	200	145	5.00E-40	gene=Chr07G0010	Gene Symbol:PEP1|Host:Multiple plant families. Some strains may cause infections in humans|Disease:Saprobe, facultative pathogen|Description:Unknown
Chr07G0020.1	498	113	435	UniProt ID:Q6A2T2_BOTFU	391	39	368	89/355 (25.07)	0.41	0.16	355	85.9	5.00E-19	gene=Chr07G0020	Gene Symbol:BTP1|Host:Various plant families|Disease:Grey mould. Parasite or saprophyte|Description:Unknown
Chr07G0	632	24	620	UniProt	693	73	671	195/64	0.46	0.13	640	204	2.00E-57	gene=Chr	Gene

021.1				ID:Q4P8E8_USTMA				0(30.47)						07G0021	Symbol:UM03615.1|Host: Euchlaena spp., Zea spp. (Poaceae)|Disease:Smut. Corn smut|Description:COFACTOR: FAD (By similarity).
Chr07G0029.1	476	42	438	UniProt ID:DHH1_CRYNV	616	27	421	137/399(34.34)	0.54	0.02	399	231	7.00E-69	gene=Chr07G0029	Gene Symbol:VAD1|Host:humans|Disease:cryptococcosis|Description:FUNCTION: ATP-dependent RNA helicase involved in mRNA turnover, and more specifically in mRNA decapping. Is involved in G1/S DNA- damage checkpoint recovery, probably through the regulation of the translational status of a subset of mRNAs. May also have a role in translation and mRNA nuclear export (By similarity). Is involved in virulence.

Chr07G0034.1	811	297	493	UniProt ID:P87199_USTMD	968	115	294	65/199 (32.66)	0.49	0.11	199	86.7	2.00E-18	gene=Chr07G0034	Gene Symbol:KIN2|Host:Euchlaena spp., Zea spp. (Poaceae)|Disease:Smut. Corn smut|Description:SIMILARITY: Belongs to the kinesin-like protein family.
Chr07G0060.1	716	18	391	UniProt ID:Q9UVJ1_CANAL	761	11	386	96/413 (23.24)	0.42	0.18	413	76.3	3.00E-15	gene=Chr07G0060	Gene Symbol:CHS6|Host:Isolated from a wide variety of substrates including humans|Disease:invasive candidal disease|Description:Unknown
Chr07G0084.1	451	10	366	UniProt ID:Q5GFD3_PHAND	437	9	377	108/399(27.07)	0.4	0.18	399	103	4.00E-25	gene=Chr07G0084	Gene Symbol:NULL|Host:Multiple genera of Poaceae and Blysmus compressus (Cyperaceae)|Disease:Glume blotch of wheat and other grasses|Description:Unknown
Chr07G0	542	97	522	UniProt	561	163	549	95/438	0.37	0.14	438	47	2.00E-06	gene=Chr	Gene

<table>
<tr><td>088.1</td><td></td><td></td><td></td><td>ID:Q59RG1_CANAL</td><td></td><td></td><td></td><td>(21.69)</td><td></td><td></td><td></td><td></td><td></td><td>07G0088</td><td>Symbol:NAG3|Host:Isolated from a wide variety of substrates including humans|Disease:invasive candidal disease|Description:CAUTION: The sequence shown here is derived from an EMBL/GenBank/DDBJ whole genome shotgun (WGS) entry which is preliminary data.</td></tr>
<tr><td>Chr07G0089.1</td><td>630</td><td>24</td><td>379</td><td>UniProt ID:Q9HG15_COLLN</td><td>746</td><td>37</td><td>404</td><td>87/405 (21.48)</td><td>0.38</td><td>0.21</td><td>405</td><td>58.5</td><td>6.00E-10</td><td>gene=Chr07G0089</td><td>Gene Symbol:CLTA1|Host:Multiple genera of Fabaceae. Rare reports on other taxa|Disease:Leaf, stem and pod anthracnose|Description:SIMILARITY: Contains 1 Zn(2)-C6 fungal-type DNA-binding domain.</td></tr>
<tr><td>Chr07G0091.1</td><td>4959</td><td>1663</td><td>4952</td><td>UniProt ID:Q9UVN5_ALTAL</td><td>4360</td><td>171</td><td>3476</td><td>1062/3417(31.08)</td><td>0.5</td><td>0.07</td><td>3417</td><td>1516</td><td>0</td><td>gene=Chr07G0091</td><td>Gene Symbol:AMT|Host:Plant|Disease:Leaf spot, rots|Description:Unknown</td></tr>
</table>

Chr07G0092.1	547	1	530	UniProt ID:Q9P8L8_BOTFU	598	48	589	220/544(40.44)	0.6	0.03	544	412	2.00E-137	gene=Chr07G0092	Gene Symbol:BCMFS1|Host:Various plant families|Disease:Grey mould. Parasite or saprophyte|Description:Unknown
Chr07G0094.1	254	1	225	UniProt ID:Q75WR5_9PLEO	265	9	219	55/228(24.12)	0.37	0.09	228	44.3	3.00E-06	gene=Chr07G0094	Gene Symbol:BRN1|Host:Belamcanda chinensis: Korea,Gladiolus ?gandavensis: Korea,Iris japonica: China,Iris missouriensis (Leaf spot.): Idaho; Montana; Oregon; Washington,Iris sp. (Leaf spot.): China; Texas; Washing|Disease:Leaf spot|Description:SIMILARITY: Belongs to the short-chain dehydrogenases/reductases (SDR) family.
Chr07G0095.1	267	6	259	UniProt ID:A4RGG9_M	286	13	285	103/281(36.65)	0.52	0.12	281	120	7.00E-33	gene=Chr07G0095	Gene Symbol:MGG_00056|Host:Poaceae, especially

				AGO7											important on Oryzae|Disease:Rice blast|Description:SIMILARITY: Belongs to the short-chain dehydrogenases/reductases (SDR) family.
Chr07G0097.1	857	271	497	UniProt ID:A4ULJ0_MYCGR	518	261	505	58/253 (22.92)	0.4	0.13	253	53.9	3.00E-08	gene=Chr07G0097	Gene Symbol:CYP51|Host:Triticum and possibly a few other grasses|Disease:Leaf spot or speckled leaf blotch of wheat|Description:COFACTOR: Heme group (By similarity).
Chr07G0099.1	1099	511	1076	UniProt ID:Q2VLJ1_GIBZA	565	31	562	173/575(30.09)	0.46	0.09	575	242	6.00E-70	gene=Chr07G0099	Gene Symbol:ZEB1|Host:Principal hosts: Poaceae, including Zea mays (corn), Triticum aestivum (wheat), and Oryza sativa (rice). Additional hosts: various plant families|Disease:Seedling blight, pre- and

															post-emergence blight, root and foot rot, brown rot, culm decay, head or kernel blight (scab or ear scab) of cereals. Leaf|Description:Unknown
Chr07G0106.1	336	34	335	UniProt ID:Q6TFC7_ASPFM	349	38	348	92/316 (29.11)	0.46	0.06	316	117	7.00E-31	gene=Chr07G0106	Gene Symbol:NULL|Host:humans|Disease:infection|Description:Unknown
Chr07G0112.1	533	198	407	UniProt ID:A4RJR0_MAGO7	938	199	416	61/233 (26.18)	0.42	0.16	233	53.5	2.00E-08	gene=Chr07G0112	Gene Symbol:MGG_01748|Host:Poaceae, especially important on Oryzae|Disease:Rice blast|Description:Unknown
Chr07G0114.1	578	80	551	UniProt ID:O93886_9PEZI	607	87	550	105/483(21.74)	0.38	0.06	483	62.4	3.00E-11	gene=Chr07G0114	Gene Symbol:NULL|Host:Fabaceae|Disease:Leaf spot, seed stain, etc|Description:Unknown
Chr07G0116.1	543	18	542	UniProt ID:Q9P8L8_BOTFU	598	56	584	179/536(33.40)	0.55	0.03	536	315	4.00E-100	gene=Chr07G0116	Gene Symbol:BCMFS1|Host:Various plant families|Disease:Grey

															mould. Parasite or saprophyte|Description:Unknown
Chr07G0117.1	283	2	213	UniProt ID:A4RGG9_MAGO7	286	11	217	55/217 (25.35)	0.42	0.07	217	58.9	4.00E-11	gene=Chr07G0117	Gene Symbol:MGG_00056|Host:Poaceae, especially important on Oryzae|Disease:Rice blast|Description:SIMILARITY: Belongs to the short-chain dehydrogenases/reductases (SDR) family.
Chr07G0118.1	2333	11	1336	UniProt ID:Q92217_COCHE	2528	13	1389	490/1414(34.65)	0.51	0.09	1414	746	0	gene=Chr07G0118	Gene Symbol:PKS1|Host:Zea mays|Disease:Southern leaf blight of maize|Description:Unknown
Chr07G0119.1	281	6	275	UniProt ID:Q75WR5_9PLEO	265	3	262	89/281 (31.67)	0.47	0.11	281	102	2.00E-26	gene=Chr07G0119	Gene Symbol:BRN1|Host:Belamcanda chinensis: Korea,Gladiolus ?gandavensis: Korea,Iris japonica: China,Iris missouriensis (Leaf spot.): Idaho;

															Montana; Oregon; Washington,Iris sp. (Leaf spot.): China; Texas; Washing|Disease:Leaf spot|Description:SIMILARITY: Belongs to the short-chain dehydrogenases/reductases (SDR) family.
Chr07G0123.1	2088	1	1717	UniProt ID:O59897_ASPFM	2146	1	1714	546/1769(30.86)	0.5	0.06	1769	804	0	gene=Chr07G0123	Gene Symbol:ALB1|Host:humans|Disease:infection|Description:Unknown
Chr07G0124.1	290	15	284	UniProt ID:Q75WR5_9PLEO	265	9	262	71/271 (26.20)	0.45	0.07	271	74.7	9.00E-17	gene=Chr07G0124	Gene Symbol:BRN1|Host:Belamcanda chinensis: Korea,Gladiolus ?gandavensis: Korea,Iris japonica: China,Iris missouriensis (Leaf spot.): Idaho; Montana; Oregon; Washington,Iris sp. (Leaf spot.): China; Texas; Washing|Disease:Leaf spot|Description:SIMILARITY: Belongs to the

															short-chain dehydrogenases/reductases (SDR) family.
Chr07G0138.1	463	212	433	UniProt ID:Q5EMY3_MAGGR	424	117	342	90/233 (38.63)	0.58	0.08	233	147	7.00E-40	gene=Chr07G0138	Gene Symbol:NULL|Host:Digitaria (Poaceae)|Disease:Leaf spot|Description:SIMILARITY: Belongs to the AAA ATPase family.
Chr07G0139.1	96	2	94	UniProt ID:O94196_MAGGR	102	9	100	68/94(72.34)	0.8	0.03	94	132	1.00E-41	gene=Chr07G0139	Gene Symbol:NULL|Host:Digitaria (Poaceae)|Disease:Leaf spot|Description:Unknown
Chr07G0149.1	746	9	401	UniProt ID:Q9C1F9_COCCA	423	7	396	195/396(49.24)	0.63	0.02	396	375	4.00E-123	gene=Chr07G0149	Gene Symbol:CEL2|Host:Corn, Zea mays, sometimes on Sorghum (Poaceae) and various other plant families|Disease:Northern corn leaf spot, ear and kernel rot|Description:Unknown
Chr07G0162.1	580	12	479	UniProt ID:Q9P8L8_BOTFU	598	45	511	120/494(24.29)	0.4	0.11	494	72.4	3.00E-14	gene=Chr07G0162	Gene Symbol:BCMFS1|Host:Various plant families|Disease:Grey

															mould. Parasite or saprophyte|Description:Unknown
Chr07G0164.1	411	37	411	UniProt ID:Q9Y784_MAGGR	631	36	434	102/418(24.40)	0.45	0.15	418	115	5.00E-29	gene=Chr07G0164	Gene Symbol:PTH11|Host:Digitaria (Poaceae)|Disease:Leaf spot|Description:Unknown
Chr07G0165.1	673	17	668	UniProt ID:Q4P8E8_USTMA	693	64	673	159/683(23.28)	0.39	0.15	683	99.8	1.00E-22	gene=Chr07G0165	Gene Symbol:UM03615.1|Host: Euchlaena spp., Zea spp. (Poaceae)|Disease:Smut. Corn smut|Description:COFACTOR: FAD (By similarity).
Chr07G0168.1	1395	9	1385	UniProt ID:Q9C2Y4_MAGGR	1501	9	1489	809/1489(54.33)	0.69	0.08	1489	1550	0	gene=Chr07G0168	Gene Symbol:PDE1|Host:Digitaria (Poaceae)|Disease:Leaf spot|Description:CATALYTIC ACTIVITY: ATP + H(2)O + phospholipid(In) = ADP + phosphate + phospholipid(Out).
Chr07G0169.1	234	44	229	UniProt ID:CUTI_ALTB	209	16	209	93/194(47.94)	0.59	0.04	194	162	5.00E-50	gene=Chr07G0169	Gene Symbol:CUTAB1|Host:Brassicaceae, especially

				R											cauliflower and white cabbage|Disease:Dark brown to almost black, circular, zonate leaf spot. Seed-borne. More common and causing more severe disease than Alternaria brassicae (Ellis 196|Description:FUNCTION: Catalyzes the hydrolysis of cutin, a polyester that forms the structure of plant cuticle. Allows pathogenic fungi to penetrate through the cuticular barrier into the host plant during the initial stage of the fungal infection (By similarity).
Chr07G0174.1	931	658	847	UniProt ID:Q5AG40_CANAL	439	170	357	72/193 (37.31)	0.54	0.04	193	122	2.00E-30	gene=Chr07G0174	Gene Symbol:VPS4|Host:Isolated from a wide variety of substrates including humans|Disease:invasive candidal disease|Description:SIMIL

															ARITY: Belongs to the AAA ATPase family.
Chr07G0176.1	275	21	221	UniProt ID:CUTI1_COLGL	224	42	213	61/210 (29.05)	0.42	0.22	210	42.4	9.00E-06	gene=Chr07G0176	Gene Symbol:CUTA|Host:Multiple genera in multiple families|Disease:'Anthracnose of stems and leaves, dieback, root rot, leaf spot, blossom rot, fruit rot (dieback and ripe rot), seedling blight.' (Mordue 1971)|Description:FUNCTION: Catalyzes the hydrolysis of cutin, a polyester that forms the structure of plant cuticle. Allows pathogenic fungi to penetrate through the cuticular barrier into the host plant during the initial stage of the fungal infection.
Chr07G0178.1	375	20	372	UniProt ID:Q6XVN4_CRYNV	383	23	375	109/368(29.62)	0.47	0.08	368	127	6.00E-34	gene=Chr07G0178	Gene Symbol:GNO1|Host:humans|Disease:cryptococcosis|Description:COFACTOR:

															Zinc (By similarity).
Chr07G0182.1	528	231	494	UniProt ID:A4ULI9_MYCGR	502	236	501	68/276 (24.64)	0.45	0.08	276	58.2	6.00E-10	gene=Chr07G0182	Gene Symbol:CYP51|Host:Triticum and possibly a few other grasses|Disease:Leaf spot or speckled leaf blotch of wheat|Description:COFACTOR: Heme group (By similarity).
Chr07G0186.1	858	24	671	UniProt ID:O93841_9PEZI	914	20	658	292/679(43.00)	0.59	0.1	679	518	3.00E-170	gene=Chr07G0186	Gene Symbol:CHIP6|Host:Multiple genera in multiple families|Disease:'Anthracnose of stems and leaves, dieback, root rot, leaf spot, blossom rot, fruit rot (dieback and ripe rot), seedling blight.' (Mordue 1971)|Description:Unknown
Chr07G0193.1	500	13	458	UniProt ID:Q5ANE1_CANAL	748	39	496	121/471(25.69)	0.45	0.08	471	133	3.00E-34	gene=Chr07G0193	Gene Symbol:SNF3|Host:Isolated from a wide variety of substrates including humans|Disease:invasive

															candidal disease|Description:SIMILARITY: Belongs to the major facilitator superfamily. Sugar transporter (TC 2.A.1.1) family.
Chr07G0194.1	643	1	453	UniProt ID:Q9HG15_COLLN	746	153	604	344/453(75.94)	0.85	0	453	720	0	gene=Chr07G0194	Gene Symbol:CLTA1|Host:Multiple genera of Fabaceae. Rare reports on other taxa|Disease:Leaf, stem and pod anthracnose|Description:SIMILARITY: Contains 1 Zn(2)-C6 fungal-type DNA-binding domain.
Chr07G0195.1	947	53	527	UniProt ID:Q9HG15_COLLN	746	7	482	122/523(23.33)	0.38	0.18	523	102	2.00E-23	gene=Chr07G0195	Gene Symbol:CLTA1|Host:Multiple genera of Fabaceae. Rare reports on other taxa|Disease:Leaf, stem and pod anthracnose|Description:SIMILARITY: Contains 1 Zn(2)-C6 fungal-type

															DNA-binding domain.
Chr07G0196.1	529	6	455	UniProt ID:Q9C1F9_COCCA	423	9	411	169/456(37.06)	0.5	0.13	456	242	1.00E-74	gene=Chr07G0196	Gene Symbol:CEL2|Host:Corn, Zea mays, sometimes on Sorghum (Poaceae) and various other plant families|Disease:Northern corn leaf spot, ear and kernel rot|Description:Unknown
Chr07G0204.1	574	1	504	UniProt ID:Q9P8L8_BOTFU	598	37	531	158/512(30.86)	0.5	0.05	512	271	5.00E-83	gene=Chr07G0204	Gene Symbol:BCMFS1|Host:Various plant families|Disease:Grey mould. Parasite or saprophyte|Description:Unknown
Chr07G0224.1	642	1	642	UniProt ID:Q5K2R7_CLAPU	655	1	655	528/665(79.40)	0.84	0.05	665	1045	0	gene=Chr07G0224	Gene Symbol:COT1|Host:outcrossing species|Disease:ergotism|Description:CATALYTIC ACTIVITY: ATP + a protein = ADP + a phosphoprotein.
Chr07G0	634	26	263	UniProt	903	29	270	64/269	0.38	0.22	269	47.4	2.00E-06	gene=Chr	Gene

233.1				ID:A6N6J8_FUSOX				(23.79)						07G0233	Symbol:CTF1|Host:Multiple genera in multiple families|Disease:Blights, wilts, rots of various sorts|Description:SIMILARITY: Contains 1 Zn(2)-C6 fungal-type DNA-binding domain.
Chr07G0234.1	525	50	520	UniProt ID:Q5ANE1_CANAL	748	45	514	123/488(25.20)	0.43	0.07	488	129	1.00E-32	gene=Chr07G0234	Gene Symbol:SNF3|Host:Isolated from a wide variety of substrates including humans|Disease:invasive candidal disease|Description:SIMILARITY: Belongs to the major facilitator superfamily. Sugar transporter (TC 2.A.1.1) family.
Chr07G0235.1	160	1	160	UniProt ID:B3VBK9_CLAFU	228	1	163	68/164 (41.46)	0.59	0.03	164	115	8.00E-33	gene=Chr07G0235	Gene Symbol:NULL|Host:Lycopersicon esculentum (Solanaceae)|Disease:Leaf mold of tomato|Description:Unkno

															wn
Chr07G0 243.1	307	77	269	UniProt ID:Q04 701_F USSO	242	42	233	90/194 (46.39)	0.61	0.02	194	163	4.00E-49	gene=Chr 07G0243	Gene Symbol:PELA|Host:Multipl e plant families. Some strains may cause infections in humans|Disease:Saprobe, facultative pathogen|Description:Unk nown
Chr07G0 244.1	652	180	303	UniProt ID:Q0 WXM3 _FUSO X	663	303	424	37/129 (28.68)	0.46	0.09	129	49.3	5.00E-07	gene=Chr 07G0244	Gene Symbol:FOW2|Host:Multip le genera in multiple families|Disease:Blights, wilts, rots of various sorts|Description:SIMILAR ITY: Contains 1 Zn(2)-C6 fungal-type DNA-binding domain.
Chr07G0 247.1	334	22	242	UniProt ID:Q32 WF7_P HAND	266	16	211	55/226 (24.34)	0.42	0.15	226	44.7	3.00E-06	gene=Chr 07G0247	Gene Symbol:MDH1|Host:Multip le genera of Poaceae and Blysmus compressus (Cyperaceae)|Disease:Glu me blotch of wheat and other

															grasses|Description:Unknown
Chr07G0250.1	554	97	545	UniProt ID:Q96VB5_ALTAL	578	100	563	136/481(28.27)	0.45	0.1	481	149	7.00E-40	gene=Chr07G0250	Gene Symbol:AFT1-1|Host:Plant|Disease:Leaf spot, rots|Description:Unknown
Chr07G0260.1	708	492	631	UniProt ID:Q5AG40_CANAL	439	171	318	42/150 (28.00)	0.47	0.08	150	48.5	7.00E-07	gene=Chr07G0260	Gene Symbol:VPS4|Host:Isolated from a wide variety of substrates including humans|Disease:invasive candidal disease|Description:SIMILARITY: Belongs to the AAA ATPase family.
Chr07G0264.1	534	122	489	UniProt ID:A4ULI5_MYCGR	515	129	506	83/401 (20.70)	0.38	0.14	401	54.7	7.00E-09	gene=Chr07G0264	Gene Symbol:CYP51|Host:Triticum and possibly a few other grasses|Disease:Leaf spot or speckled leaf blotch of wheat|Description:COFACTOR: Heme group (By similarity).
Chr07G0266.1	465	79	387	UniProt ID:O59	384	92	384	139/314(44.2	0.59	0.08	314	214	4.00E-65	gene=Chr07G0266	Gene Symbol:XYL3|Host:Multipl

				937_FUSOX				7)							e genera in multiple families|Disease:Blights, wilts, rots of various sorts|Description:SIMILARITY: Belongs to the glycosyl hydrolase 10 (cellulase F) family.
Chr07G0267.1	362	35	355	UniProt ID:A4QVF8_MAGO7	339	33	337	94/323 (29.10)	0.46	0.06	323	119	2.00E-31	gene=Chr07G0267	Gene Symbol:MGG_04556|Host:Poaceae, especially important on Oryzae|Disease:Rice blast|Description:COFACTOR: Zinc (By similarity).
Chr07G0270.1	1082	34	369	UniProt ID:Q9Y784_MAGGR	631	36	373	79/342 (23.10)	0.49	0.03	342	134	2.00E-33	gene=Chr07G0270	Gene Symbol:PTH11|Host:Digitaria (Poaceae)|Disease:Leaf spot|Description:Unknown
Chr07G0271.1	817	55	809	UniProt ID:Q99324_SEPLY	803	33	797	414/779(53.15)	0.68	0.05	779	806	0	gene=Chr07G0271	Gene Symbol:B2TOM|Host:Primarily tomato, Lycopersicon esculentum, also Solanum spp. and other Solanaceae|Disease:Leaf spot|Description:Unknown

Chr07G0273.1	613	12	597	UniProt ID:SPCA_ARTOC	651	13	603	303/598(50.67)	0.67	0.03	598	583	0	gene=Chr07G0273	Gene Symbol:SCPA|Host:humans, reptiles|Disease:dermatophytoses|Description:FUNCTION: Extracellular serine carboxypeptidase that contributes to pathogenicity (By similarity).
Chr07G0275.1	347	3	220	UniProt ID:A4QVF8_MAGO7	339	2	232	65/244(26.64)	0.39	0.16	244	48.9	1.00E-07	gene=Chr07G0275	Gene Symbol:MGG_04556|Host:Poaceae, especially important on Oryzae|Disease:Rice blast|Description:COFACTOR: Zinc (By similarity).
Chr07G0288.1	373	11	369	UniProt ID:LAP1_ARTOC	373	9	370	169/377(44.83)	0.58	0.09	377	286	9.00E-94	gene=Chr07G0288	Gene Symbol:LAP1|Host:humans, reptiles|Disease:dermatophytoses|Description:FUNCTION: Extracellular aminopeptidase which contributes to pathogenicity (By

															similarity).
Chr07G0289.1	443	35	322	UniProt ID:A4UC81_MAGO7	376	54	339	97/298 (32.55)	0.5	0.07	298	125	8.00E-33	gene=Chr07G0289	Gene Symbol:MGG_10702|Host:Poaceae, especially important on Oryzae|Disease:Rice blast|Description:Unknown
Chr07G0293.1	658	90	657	UniProt ID:Q2VLJ1_GIBZA	565	19	562	214/584(36.64)	0.53	0.1	584	335	6.00E-107	gene=Chr07G0293	Gene Symbol:ZEB1|Host:Principal hosts: Poaceae, including Zea mays (corn), Triticum aestivum (wheat), and Oryza sativa (rice). Additional hosts: various plant families|Disease:Seedling blight, pre- and post-emergence blight, root and foot rot, brown rot, culm decay, head or kernel blight (scab or ear scab) of cereals. Leaf|Description:Unknown
Chr07G0294.1	424	46	302	UniProt ID:Q9Y	631	133	380	70/261 (26.82)	0.48	0.07	261	73.6	5.00E-15	gene=Chr07G0294	Gene Symbol:PTH11|Host:Digit

				784_MAGGR											aria (Poaceae)|Disease:Leaf spot|Description:Unknown
Chr07G0300.1	367	6	243	UniProt ID:A4QVF8_MAGO7	339	3	233	73/238 (30.67)	0.48	0.03	238	100	6.00E-25	gene=Chr07G0300	Gene Symbol:MGG_04556|Host:Poaceae, especially important on Oryzae|Disease:Rice blast|Description:COFACTOR: Zinc (By similarity).
Chr07G0308.1	912	524	646	UniProt ID:LAP2_ARTOC	495	235	344	33/125 (26.40)	0.46	0.14	125	46.2	7.00E-06	gene=Chr07G0308	Gene Symbol:LAP2|Host:humans, reptiles|Disease:dermatophytoses|Description:FUNCTION: Extracellular aminopeptidase that releases a wide variety of amino acids from natural peptides and contributes to pathogenicity.
Chr07G0311.1	1021	68	191	UniProt ID:Q5XTQ4_BOTFU	574	129	249	59/124 (47.58)	0.6	0.02	124	111	3.00E-26	gene=Chr07G0311	Gene Symbol:LIP1|Host:Various plant families|Disease:Grey mould. Parasite or

															saprophyte|Description:Unknown
Chr07G0318.1	254	5	254	UniProt ID:Q00845_NECHA	233	3	225	132/250(52.80)	0.63	0.11	250	244	3.00E-81	gene=Chr07G0318	Gene Symbol:PELD|Host:Trees of various plant families|Disease:Fruit rot, stem rot|Description:Unknown
Chr07G0335.1	530	8	40	UniProt ID:Q5A4F3_CANAL	624	14	46	15/33(45.45)	0.7	0	33	51.2	9.00E-08	gene=Chr07G0335	Gene Symbol:ZCF37|Host:Isolated from a wide variety of substrates including humans|Disease:invasive candidal disease|Description:Unknown
Chr07G0336.1	1480	8	1421	UniProt ID:O60034_BOTFU	1562	48	1503	801/1462(54.79)	0.71	0.04	1462	1615	0	gene=Chr07G0336	Gene Symbol:NULL|Host:Various plant families|Disease:Grey mould. Parasite or saprophyte|Description:SIMILARITY: Belongs to the ABC transporter superfamily.
Chr07G0	365	30	305	UniProt	631	128	402	64/278	0.46	0.02	278	88.6	3.00E-20	gene=Chr	Gene

339.1				ID:Q9Y784_MAGGR				(23.02)						07G0339	Symbol:PTH11|Host:Digitaria (Poaceae)|Disease:Leaf spot|Description:Unknown
Chr07G0352.1	276	15	275	UniProt ID:Q32WF7_PHAND	266	1	265	112/266(42.11)	0.62	0.02	266	213	2.00E-68	gene=Chr07G0352	Gene Symbol:MDH1|Host:Multiple genera of Poaceae and Blysmus compressus (Cyperaceae)|Disease:Glume blotch of wheat and other grasses|Description:Unknown
Chr07G0354.1	659	49	86	UniProt ID:Q5A4F3_CANAL	624	9	46	19/38(50.00)	0.66	0	38	52	6.00E-08	gene=Chr07G0354	Gene Symbol:ZCF37|Host:Isolated from a wide variety of substrates including humans|Disease:invasive candidal disease|Description:Unknown
Chr07G0355.1	316	3	231	UniProt ID:A4RGG9_MAGO7	286	11	216	70/236(29.66)	0.48	0.16	236	84.7	4.00E-20	gene=Chr07G0355	Gene Symbol:MGG_00056|Host:Poaceae, especially important on Oryzae|Disease:Rice

															blast|Description:SIMILARITY: Belongs to the short-chain dehydrogenases/reductases (SDR) family.
Chr07G0364.1	335	156	322	UniProt ID:Q92217_COCHE	2528	725	903	58/183 (31.69)	0.45	0.11	183	78.2	8.00E-17	gene=Chr07G0364	Gene Symbol:PKS1|Host:Zea mays|Disease:Southern leaf blight of maize|Description:Unknown
Chr07G0371.1	323	30	311	UniProt ID:Q92217_COCHE	2528	1848	2138	79/309 (25.57)	0.44	0.15	309	71.6	1.00E-14	gene=Chr07G0371	Gene Symbol:PKS1|Host:Zea mays|Disease:Southern leaf blight of maize|Description:Unknown
Chr07G0378.1	497	248	408	UniProt ID:Q0PND8_MAGGR	1375	1004	1173	49/173 (28.32)	0.5	0.09	173	57.8	9.00E-10	gene=Chr07G0378	Gene Symbol:PEX6|Host:Digitaria (Poaceae)|Disease:Leaf spot|Description:SIMILARITY: Belongs to the AAA ATPase family.
Chr07G0381.1	369	14	279	UniProt ID:Q9Y784_M	631	112	375	73/268 (27.24)	0.49	0.02	268	87.4	9.00E-20	gene=Chr07G0381	Gene Symbol:PTH11|Host:Digitaria

				AGGR										(Poaceae)|Disease:Leaf spot|Description:Unknown Gene Symbol:ZEB1|Host:Principal hosts: Poaceae, including Zea mays (corn), Triticum aestivum (wheat), and Oryza sativa (rice). Additional hosts: various plant families|Disease:Seedling blight, pre- and post-emergence blight, root and foot rot, brown rot, culm decay, head or kernel blight (scab or ear scab) of cereals.
Chr07G0382.1	656	92	654	UniProt ID:Q2VLJ1_GIBZA	565	27	561	197/569(34.62)	0.51	0.07	569	310	3.00E-97	gene=Chr07G0382
Chr07G0383.1	595	7	593	UniProt ID:O93886_9PEZI	607	3	587	135/612(22.06)	0.43	0.08	612	72.8	2.00E-14	gene=Chr07G0383	Leaf|Description:Unknown Gene Symbol:NULL|Host:Fabaceae|Disease:Leaf spot, seed stain, etc|Description:Unknown
Chr07G0394.1	200	1	185	UniProt ID:Q2KG56_M	199	1	182	98/185(52.97)	0.65	0.02	185	196	1.00E-63	gene=Chr07G0394	Gene Symbol:"MGCH7_CH7G479,

				AGO7											MGG_02731"|Host:Poaceae, especially important on Oryzae|Disease:Rice blast|Description:SIMILARITY: Belongs to the small GTPase superfamily. Rho family.
Chr07G0401.1	385	18	312	UniProt ID:Q5AJC0_CANAL	470	76	386	85/323 (26.32)	0.46	0.12	323	100	2.00E-24	gene=Chr07G0401	Gene Symbol:UTR2|Host:Isolated from a wide variety of substrates including humans|Disease:invasive candidal disease|Description:CAUTION: The sequence shown here is derived from an EMBL/GenBank/DDBJ whole genome shotgun (WGS) entry which is preliminary data.
Chr07G0404.1	1153	546	824	UniProt ID:Q3Y5V5_MAGGR	1321	1030	1319	108/290(37.24)	0.59	0.04	290	200	4.00E-53	gene=Chr07G0404	Gene Symbol:ABC3|Host:Digitaria (Poaceae)|Disease:Leaf spot|Description:SIMILARITY: Belongs to the ABC transporter superfamily.

Chr07G0415.1	327	6	323	UniProtID:Q9C2Y1_BOTFU	346	14	345	142/333(42.64)	0.59	0.05	333	222	5.00E-70	gene=Chr07G0415	Gene Symbol:BCPME1|Host:Various plant families|Disease:Grey mould. Parasite or saprophyte|Description:CATALYTIC ACTIVITY: Pectin + n H(2)O = n methanol + pectate.
Chr07G0416.1	754	465	747	UniProtID:PLYB_COLGL	331	58	326	99/300(33.00)	0.48	0.16	300	94.4	7.00E-22	gene=Chr07G0416	Gene Symbol:PLB|Host:Multiple genera in multiple families|Disease:'Anthracnose of stems and leaves, dieback, root rot, leaf spot, blossom rot, fruit rot (dieback and ripe rot), seedling blight.' (Mordue 1971)|Description:FUNCTION: Acts as a virulence factor active in plant tissue maceration.
Chr07G0419.1	330	102	330	UniProtID:Q04701_FUSSO	242	14	242	162/231(70.13)	0.8	0.02	231	331	6.00E-114	gene=Chr07G0419	Gene Symbol:PELA|Host:Multiple plant families. Some strains may cause

														infections in humans|Disease:Saprobe, facultative pathogen|Description:Unknown
Chr07G0420.1	343	9	285	UniProt ID:Q6A2T2_BOTFU	391	31	321	78/292 (26.71)	0.47	0.05	292	100	1.00E-24	gene=Chr07G0420	Gene Symbol:BTP1|Host:Various plant families|Disease:Grey mould. Parasite or saprophyte|Description:Unknown
Chr07G0427.1	382	7	381	UniProt ID:Q75ZG3_ALTAL	366	8	365	195/385(50.65)	0.66	0.1	385	364	2.00E-124	gene=Chr07G0427	Gene Symbol:AFTS1|Host:Plant|Disease:Leaf spot, rots|Description:Unknown
Chr07G0429.1	502	236	469	UniProt ID:A4ULI6_MYCGR	517	256	515	59/264 (22.35)	0.37	0.13	264	48.9	4.00E-07	gene=Chr07G0429	Gene Symbol:CYP51|Host:Triticum and possibly a few other grasses|Disease:Leaf spot or speckled leaf blotch of wheat|Description:COFACTOR: Heme group (By similarity).
Chr07G0	569	307	546	UniProt	515	225	510	63/287	0.39	0.17	287	52.4	4.00E-08	gene=Chr	Gene

431.1				ID:A4U LJ2_M YCGR				(21.95)						07G0431	Symbol:CYP51|Host:Triticum and possibly a few other grasses|Disease:Leaf spot or speckled leaf blotch of wheat|Description:COFACTOR: Heme group (By similarity).
Chr07G0 435.1	555	350	513	UniProt ID:A4U LJ2_M YCGR	515	308	501	45/195 (23.08)	0.45	0.16	195	62.8	2.00E-11	gene=Chr 07G0435	Gene Symbol:CYP51|Host:Triticum and possibly a few other grasses|Disease:Leaf spot or speckled leaf blotch of wheat|Description:COFACTOR: Heme group (By similarity).
Chr07G0 436.1	302	2	301	UniProt ID:A4Q VF8_M AGO7	339	53	338	89/301 (29.57)	0.5	0.05	301	114	4.00E-30	gene=Chr 07G0436	Gene Symbol:MGG_04556|Host:Poaceae, especially important on Oryzae|Disease:Rice blast|Description:COFACTOR: Zinc (By similarity).
Chr07G0 438.1	547	24	537	UniProt ID:Q5A	748	36	525	195/519(37.5	0.56	0.07	519	333	3.00E-105	gene=Chr 07G0438	Gene Symbol:SNF3|Host:Isolate

				NE1_CANAL				7)						d from a wide variety of substrates including humans|Disease:invasive candidal disease|Description:SIMILARITY: Belongs to the major facilitator superfamily. Sugar transporter (TC 2.A.1.1) family.
Chr07G0442.1	749	194	528	UniProt ID:Q0WXM3_FUSOX	663	222	551	79/351 (22.51)	0.43	0.11	351	72.4	5.00E-14	gene=Chr07G0442	Gene Symbol:FOW2|Host:Multiple genera in multiple families|Disease:Blights, wilts, rots of various sorts|Description:SIMILARITY: Contains 1 Zn(2)-C6 fungal-type DNA-binding domain.
Chr07G0443.1	254	3	252	UniProt ID:Q75WR5_9PLEO	265	8	263	77/260 (29.62)	0.45	0.05	260	98.2	4.00E-25	gene=Chr07G0443	Gene Symbol:BRN1|Host:Belamcanda chinensis: Korea,Gladiolus ?gandavensis: Korea,Iris japonica: China,Iris missouriensis (Leaf spot.): Idaho;

															Montana; Oregon; Washington,Iris sp. (Leaf spot.): China; Texas; Washing|Disease:Leaf spot|Description:SIMILARITY: Belongs to the short-chain dehydrogenases/reductases (SDR) family.
Chr07G0448.1	513	53	339	UniProt ID:Q5AG71_CANAL	1462	63	338	93/302 (30.79)	0.49	0.14	302	119	3.00E-29	gene=Chr07G0448	Gene Symbol:HSL1|Host:Isolated from a wide variety of substrates including humans|Disease:invasive candidal disease|Description:CAUTION: The sequence shown here is derived from an EMBL/GenBank/DDBJ whole genome shotgun (WGS) entry which is preliminary data.
Chr07G0450.1	821	463	814	UniProt ID:Q86ZC3_USTMD	533	167	485	100/361(27.70)	0.46	0.14	361	114	2.00E-27	gene=Chr07G0450	Gene Symbol:KPP6|Host:Euchlaena spp., Zea spp. (Poaceae)|Disease:Smut.

															Corn smut|Description:CATALYTIC ACTIVITY: ATP + a protein = ADP + a phosphoprotein.
Chr07G0451.1	1115	618	898	UniProt ID:TUP1_CANAL	514	201	509	123/311(39.55)	0.55	0.1	311	207	7.00E-58	gene=Chr07G0451	Gene Symbol:TUP1|Host:Isolated from a wide variety of substrates including humans|Disease:invasive candidal disease|Description:FUNCTION: Represses transcription by RNA polymerase II. Represses genes responsible for initiating filamentous growth and this repression is lifted under inducing environmental conditions.
Chr07G0466.1	409	101	290	UniProt ID:Q6A2T2_BOTFU	391	119	307	51/195(26.15)	0.51	0.06	195	68.2	2.00E-13	gene=Chr07G0466	Gene Symbol:BTP1|Host:Various plant families|Disease:Grey mould. Parasite or saprophyte|Description:Un

Chr07G0476.1	334	1	151	UniProt ID:A4R3I5_MAGO7	400	80	225	41/157 (26.11)	0.46	0.11	157	46.6	1.00E-06	gene=Chr07G0476	known Gene Symbol:"MGG_11993, MGG_12837, MGG_13052"|Host:Poaceae, especially important on Oryzae|Disease:Rice blast|Description:Unknown
Chr07G0477.1	1234	71	1201	UniProt ID:A4RGC8_MAGO7	1158	31	1132	456/1164(39.18)	0.57	0.08	1164	780	0	gene=Chr07G0477	Gene Symbol:MGG_11671|Host:Poaceae, especially important on Oryzae|Disease:Rice blast|Description:SIMILARITY: Contains 1 reverse transcriptase domain.
Chr07G0485.1	289	19	286	UniProt ID:Q32WF7_PHAND	266	17	262	84/278 (30.22)	0.46	0.15	278	75.5	5.00E-17	gene=Chr07G0485	Gene Symbol:MDH1|Host:Multiple genera of Poaceae and Blysmus compressus (Cyperaceae)|Disease:Glume blotch of wheat and other grasses|Description:Unknown

Chr07G0490.1	395	107	390	UniProt ID:Q6TFC7_ASPFM	349	64	346	92/291 (31.62)	0.51	0.05	291	133	5.00E-36	gene=Chr07G0490	Gene Symbol:NULL|Host:humans|Disease:infection|Description:Unknown
Chr07G0491.1	611	5	66	UniProt ID:A4R0W3_MAGO7	1226	278	334	25/62(40.32)	0.5	0.08	62	47.4	2.00E-06	gene=Chr07G0491	Gene Symbol:MGG_09263|Host:Poaceae, especially important on Oryzae|Disease:Rice blast|Description:Unknown
Chr07G0493.1	728	198	446	UniProt ID:Q0WXM3_FUSOX	663	237	475	64/258 (24.81)	0.45	0.11	258	67.4	2.00E-12	gene=Chr07G0493	Gene Symbol:FOW2|Host:Multiple genera in multiple families|Disease:Blights, wilts, rots of various sorts|Description:SIMILARITY: Contains 1 Zn(2)-C6 fungal-type DNA-binding domain.
Chr07G0500.1	955	34	955	UniProt ID:A4RG88_MAGO7	950	35	950	520/969(53.66)	0.62	0.1	969	606	0	gene=Chr07G0500	Gene Symbol:MGG_00124|Host:Poaceae, especially important on Oryzae|Disease:Rice blast|Description:Unknow

															n
Chr07G0503.1	391	37	170	UniProt ID:C7GMI1_YEAS2	319	8	147	41/143 (28.67)	0.43	0.08	143	44.3	5.00E-06	gene=Chr07G0503	Gene Symbol:TRR1|Host:humans|Disease:occasional infection|Description:CATALYTIC ACTIVITY: Thioredoxin + NADP(+) = thioredoxin disulfide + NADPH.
Chr07G0505.1	896	33	292	UniProt ID:SUB2A_COCP7	406	31	285	78/290 (26.90)	0.4	0.22	290	62.4	4.00E-11	gene=Chr07G0505	Gene Symbol:CPC735_023170|Host:humans|Disease:coccidiomycosis|Description: FUNCTION: Secreted subtilisin-like serine protease with keratinolytic activity that contributes to pathogenicity (By similarity).
Chr07G0523.1	289	20	223	UniProt ID:Q32WF7_PHAND	266	6	215	62/218 (28.44)	0.44	0.1	218	57	2.00E-10	gene=Chr07G0523	Gene Symbol:MDH1|Host:Multiple genera of Poaceae and Blysmus compressus (Cyperaceae)|Disease:Glume blotch of wheat and other

															grasses|Description:Unknown
Chr07G0528.1	124	1	119	UniProt ID:CEUL_OPHUL	100	1	98	40/120 (33.33)	0.46	0.19	120	45.4	2.00E-08	gene=Chr07G0528	Gene Symbol:CU|Host:Primarily Ulmus spp. (Ulmaceae)|Disease:Dutch elm diseae|Description:FUNCTION: Has been implicated in the pathogenicity of this fungus on ELM. Accumulates at, and plugs intercellular openings in the xylem, or interacts with host parenchyma cells, thereby enhancing respiration and electrolyte loss.
Chr07G0531.1	231	6	231	UniProt ID:Q079H2_9HELO	246	8	246	125/239(52.30)	0.66	0.05	239	258	8.00E-87	gene=Chr07G0531	Gene Symbol:NEP1|Host:liliaceous plants|Disease:Streaking of lily leaves|Description:Unknown

Chr07G0535.1	731	176	365	UniProt ID:SUB4A_COCP7	397	180	358	58/197 (29.44)	0.45	0.13	197	65.9	3.00E-12	gene=Chr07G0535	Gene Symbol:CPC735_066880|Host:humans|Disease:coccidiomycosis|Description: FUNCTION: Secreted subtilisin-like serine protease with keratinolytic activity that contributes to pathogenicity (By similarity).
Chr07G0544.1	338	29	314	UniProt ID:BOT2_BOTFU	399	99	374	69/309 (22.33)	0.4	0.18	309	57	5.00E-10	gene=Chr07G0544	Gene Symbol:BOT2|Host:Various plant families|Disease:Grey mould. Parasite or saprophyte|Description:FUNCTION: Presilphiperfolan-8-beta-ol synthase, which catalyzes the cyclization of farnesyl diphosphate (FPP) to presilphiperfolan-8-beta-ol (PSP), the committed step in the biosynthesis of the terpenoid virulence factor botrydial. Botrydial is

														necessary for colonization of plant tissue by the T4 strain. It is a strain-dependent virulence factor since highly aggressive strains like SAS56 or B05 still retain substantial virulence when botrydial synthesis is impaired.
Chr07G0548.1	907	142	332	UniProt ID:SUB1_ARTGP	481	149	322	57/199 (28.64)	0.46	0.17	199	57.8	1.00E-09	gene=Chr07G0548	Gene Symbol:SUB1|Host:Human, Mouse, Rat, Chicken, Pig, Rabbit, Bovine, Dog, African clawed frog, Zebrafish|Disease:tinea capitis, tinea corpus, ringworm, and other dermatophytoses|Description:FUNCTION: Secreted subtilisin-like serine protease with keratinolytic activity that contributes to pathogenicity (By similarity).
Chr07G0	499	9	459	UniProt	495	11	470	163/48	0.5	0.11	483	219	2.00E-65	gene=Chr	Gene

550.1				ID:LAP2_ARTOC				3(33.75)						07G0550	Symbol:LAP2|Host:humans, reptiles|Disease:dermatophytoses|Description:FUNCTION: Extracellular aminopeptidase that releases a wide variety of amino acids from natural peptides and contributes to pathogenicity.
Chr07G0551.1	765	126	166	UniProt ID:Q5A4F3_CANAL	624	3	46	22/44(50.00)	0.7	0.07	44	54.7	1.00E-08	gene=Chr07G0551	Gene Symbol:ZCF37|Host:Isolated from a wide variety of substrates including humans|Disease:invasive candidal disease|Description:Unknown
Chr07G0552.1	341	19	338	UniProt ID:MEP7_COCP7	363	25	354	125/331(37.76)	0.56	0.04	331	230	6.00E-73	gene=Chr07G0552	Gene Symbol:MEP7|Host:humans|Disease:coccidiomycosis|Description:FUNCTION: Secreted metalloproteinase that allows assimilation of proteinaceous substrates.

															Shows high activities on basic nuclear substrates such as histone and protamine. May be involved in virulence (By similarity).
Chr07G0554.1	357	70	348	UniProt ID:Q6TFC7_ASPFM	349	63	346	88/288 (30.56)	0.48	0.05	288	136	2.00E-37	gene=Chr07G0554	Gene Symbol:NULL|Host:humans|Disease:infection|Description:Unknown
Chr07G0559.1	130	39	126	UniProt ID:Q5K8C9_CRYNJ	119	35	118	39/88(44.32)	0.58	0.05	88	75.1	8.00E-19	gene=Chr07G0559	Gene Symbol:CNL06140|Host:humans|Disease:cryptococcosis|Description:Unknown
Chr07G0560.1	675	16	487	UniProt ID:A6N6J8_FUSOX	903	51	524	118/507(23.27)	0.37	0.13	507	57.8	2.00E-09	gene=Chr07G0560	Gene Symbol:CTF1|Host:Multiple genera in multiple families|Disease:Blights, wilts, rots of various sorts|Description:SIMILARITY: Contains 1 Zn(2)-C6 fungal-type DNA-binding domain.
Chr07G0561.1	275	5	273	UniProt ID:A4R	286	14	269	79/287 (27.53)	0.42	0.17	287	58.2	8.00E-11	gene=Chr07G0561	Gene Symbol:MGG_00056|Host

				GG9_M AGO7											:Poaceae, especially important on Oryzae|Disease:Rice blast|Description:SIMILARITY: Belongs to the short-chain dehydrogenases/reductases (SDR) family.
Chr07G0566.1	571	10	559	UniProt ID:Q5ANE1_CANAL	748	14	546	180/573(31.41)	0.51	0.11	573	251	2.00E-74	gene=Chr07G0566	Gene Symbol:SNF3|Host:Isolated from a wide variety of substrates including humans|Disease:invasive candidal disease|Description:SIMILARITY: Belongs to the major facilitator superfamily. Sugar transporter (TC 2.A.1.1) family.
Chr07G0569.1	344	8	198	UniProt ID:A0ST44_CERNC	357	7	206	60/206 (29.13)	0.5	0.1	206	73.2	2.00E-15	gene=Chr07G0569	Gene Symbol:CTB6|Host:Numerous taxa in Solanaceae|Disease:Leaf spot|Description:Unknown
Chr07G0	399	37	263	UniProt	568	130	360	102/23	0.64	0.03	232	217	5.00E-65	gene=Chr	Gene

575.1				ID:D1MYV6_MAGGR				2(43.97)						07G0575	Symbol:CBL1|Host:Digitaria (Poaceae)|Disease:Leaf spot|Description:SIMILARITY: Contains 3 chitin-binding type-1 domains.
Chr07G0578.1	386	28	340	UniProt ID:Q59QH2_CANAL	337	8	324	96/331(29.00)	0.5	0.1	331	127	4.00E-34	gene=Chr07G0578	Gene Symbol:CSH1|Host:Isolated from a wide variety of substrates including humans|Disease:invasive candidal disease|Description:CAUTION: The sequence shown here is derived from an EMBL/GenBank/DDBJ whole genome shotgun (WGS) entry which is preliminary data.
Chr07G0581.1	1066	1	1061	UniProt ID:Q96WW9_GIBPU	1491	230	1275	616/1063(57.95)	0.75	0.02	1063	1285	0	gene=Chr07G0581	Gene Symbol:ABC1|Host:Various plant families|Disease:Tree canker, rot of potatoes (Samuels et al. 2006). Root rot (Booth

															1973)|Description:SIMILARITY: Belongs to the ABC transporter superfamily.
Chr07G0586.1	712	553	708	UniProt ID:A4RGM3_MAGO7	283	125	281	90/159 (56.60)	0.75	0.03	159	184	3.00E-53	gene=Chr07G0586	Gene Symbol:MGG_12252|Host:Poaceae, especially important on Oryzae|Disease:Rice blast|Description:Unknown
Chr07G0591.1	168	66	168	UniProt ID:Q2Q466_MAGGR	101	1	101	83/103 (80.58)	0.88	0.02	103	166	7.00E-54	gene=Chr07G0591	Gene Symbol:MNH6|Host:Digitaria (Poaceae)|Disease:Leaf spot|Description:Unknown
Chr07G0602.1	402	43	293	UniProt ID:Q9Y784_MAGGR	631	126	369	68/252 (26.98)	0.48	0.04	252	106	5.00E-26	gene=Chr07G0602	Gene Symbol:PTH11|Host:Digitaria (Poaceae)|Disease:Leaf spot|Description:Unknown
Chr07G0605.1	454	1	448	UniProt ID:C5GVY6_AJEDR	451	1	444	397/448(88.62)	0.94	0.01	448	808	0	gene=Chr07G0605	Gene Symbol:BDCG_08660|Host:humans|Disease:cutaneous Blastomyces dermatitidis infection|Description:FUN

															CTION: Tubulin is the major constituent of microtubules. It binds two moles of GTP, one at an exchangeable site on the beta chain and one at a non-exchangeable site on the alpha-chain (By similarity).
Chr07G0612.1	369	64	254	UniProt ID:Q2XW08_COCHE	2144	1499	1670	54/197 (27.41)	0.44	0.16	197	47.4	1.00E-06	gene=Chr07G0612	Gene Symbol:PKS2|Host:Zea mays|Disease:Southern leaf blight of maize|Description:Unknown
Chr07G0616.1	283	170	256	UniProt ID:Q5ABB1_CANAL	119	28	111	33/87(37.93)	0.57	0.03	87	62.4	3.00E-13	gene=Chr07G0616	Gene Symbol:TTR1|Host:Isolated from a wide variety of substrates including humans|Disease:invasive candidal disease|Description:CAUTION: The sequence shown here is derived from an EMBL/GenBank/DDBJ whole genome shotgun

															(WGS) entry which is preliminary data.
Chr07G0623.1	480	4	43	UniProt ID:Q59MD2_CANAL	843	758	797	19/40(47.50)	0.65	0	40	50.1	2.00E-07	gene=Chr07G0623	Gene Symbol:UME6|Host:Isolated from a wide variety of substrates including humans|Disease:invasive candidal disease|Description:CAUTION: The sequence shown here is derived from an EMBL/GenBank/DDBJ whole genome shotgun (WGS) entry which is preliminary data.
Chr07G0634.1	428	82	352	UniProt ID:Q00523_CRYNE	458	122	358	78/282(27.66)	0.43	0.2	282	79	7.00E-17	gene=Chr07G0634	Gene Symbol:NULL|Host:humans|Disease:cryptococcosis|Description:Unknown
Chr07G0639.1	288	77	280	UniProt ID:C5GSW0_AJEDR	783	139	321	51/209(24.40)	0.41	0.15	209	60.5	2.00E-11	gene=Chr07G0639	Gene Symbol:BDCG_07503|Host:humans|Disease:cutaneous Blastomyces dermatitidis infection|Description:Unknown

Chr07G0641.1	496	21	227	UniProt ID:Q9P4U8_ALTAL	435	15	260	60/253 (23.72)	0.37	0.21	253	45.4	4.00E-06	gene=Chr07G0641	Gene Symbol:AKTR-2|Host:Plant|Disease:Leaf spot, rots|Description:Unknown
Chr07G0643.1	863	501	790	UniProt ID:Q5AP71_CANAL	405	82	330	79/293 (26.96)	0.46	0.16	293	83.6	6.00E-18	gene=Chr07G0643	Gene Symbol:TPK1|Host:Isolated from a wide variety of substrates including humans|Disease:invasive candidal disease|Description:CATALYTIC ACTIVITY: ATP + a protein = ADP + a phosphoprotein.
Chr07G0656.1	396	22	396	UniProt ID:A4RFD4_MAGO7	393	19	393	339/375(90.40)	0.97	0	375	689	0	gene=Chr07G0656	Gene Symbol:MGG_00383|Host:Poaceae, especially important on Oryzae|Disease:Rice blast|Description:FUNCTION: Catalyzes the formation of S-adenosylmethionine from methionine and ATP (By similarity).
Chr07G0	1224	278	572	UniProt	408	9	273	81/296	0.49	0.11	296	121	5.00E-30	gene=Chr	Gene

670.1				ID:Q00 LS5_P HAND				(27.36)						07G0670	Symbol:CPKA|Host:Multiple genera of Poaceae and Blysmus compressus (Cyperaceae)|Disease:Glume blotch of wheat and other grasses|Description:SIMILARITY: Contains 1 protein kinase domain.
Chr07G0 677.1	165	2	96	UniProt ID:PAB P_COC IM	768	236	330	29/95(30.53)	0.54	0	95	65.5	1.00E-13	gene=Chr 07G0677	Gene Symbol:PAB1|Host:humans|Disease:coccidioidomycosis|Description:FUNCTION: Binds the poly(A) tail of mRNA. Appears to be an important mediator of the multiple roles of the poly(A) tail in mRNA biogenesis, stability and translation. In the nucleus, involved in both mRNA cleavage and polyadenylation. Is also required for efficient mRNA export to the cytoplasm. Acts in concert

													with a poly(A)-specific nuclease (PAN) to affect poly(A) tail shortening, which may occur concomitantly with either nucleocytoplasmic mRNA transport or translational initiation. In the cytoplasm, stimulates translation initiation and regulates mRNA decay through translation termination-coupled poly(A) shortening, probably mediated by PAN (By similarity).
Chr07G0678.1	273	101	243	UniProt ID:CANB_CRYNJ	175	15	170	51/161 (31.68)	0.45	0.14	161	50.8	7.00E-09	gene=Chr07G0678	Gene Symbol:CNB1|Host:humans|Disease:cryptococcosis|Description:FUNCTION: Regulatory subunit of calcineurin, a calcium-dependent, calmodulin stimulated protein phosphatase. Confers calcium

														sensitivity. Plays a central role in virulence and antifungal drug action.
Chr07G0684.1	539	265	458	UniProt ID:A6ZXD7_YEAS7	1273	107	311	58/217 (26.73)	0.41	0.16	217	49.7	4.00E-07	gene=Chr07G0684	Gene Symbol:SEC31|Host:humans|Disease:occasional infection|Description:CAUTION: The sequence shown here is derived from an EMBL/GenBank/DDBJ whole genome shotgun (WGS) entry which is preliminary data.
Chr07G0686.1	267	71	266	UniProt ID:D1MYV6_MAGGR	568	150	356	46/212 (21.70)	0.42	0.1	212	43.1	9.00E-06	gene=Chr07G0686	Gene Symbol:CBL1|Host:Digitaria (Poaceae)|Disease:Leaf spot|Description:SIMILARITY: Contains 3 chitin-binding type-1 domains.
Chr07G0687.1	358	16	342	UniProt ID:BOT2_BOTFU	399	57	374	74/336 (22.02)	0.46	0.08	336	70.5	2.00E-14	gene=Chr07G0687	Gene Symbol:BOT2|Host:Various plant families|Disease:Grey mould. Parasite or

																saprophyte|Description:FUNCTION: Presilphiperfolan-8-beta-ol synthase, which catalyzes the cyclization of farnesyl diphosphate (FPP) to presilphiperfolan-8-beta-ol (PSP), the committed step in the biosynthesis of the terpenoid virulence factor botrydial. Botrydial is necessary for colonization of plant tissue by the T4 strain. It is a strain-dependent virulence factor since highly aggressive strains like SAS56 or B05 still retain substantial virulence when botrydial synthesis is impaired.
Chr07G0702.1	588	46	322	UniProt ID:Q6TFC7_ASPFM	349	60	335	93/280 (33.21)	0.51	0.03	280	171	1.00E-48	gene=Chr07G0702	Gene Symbol:NULL|Host:humans|Disease:infection|Description:Unknown
Chr07G0	317	20	277	UniProt	265	11	264	74/270	0.4	0.1	270	74.3	2.00E-16	gene=Chr	Gene

704.1				ID:Q75WR5_9PLEO				(27.41)						07G0704	Symbol:BRN1|Host:Belamcanda chinensis: Korea,Gladiolus ?gandavensis: Korea,Iris japonica: China,Iris missouriensis (Leaf spot.): Idaho; Montana; Oregon; Washington,Iris sp. (Leaf spot.): China; Texas; Washing|Disease:Leaf spot|Description:SIMILARITY: Belongs to the short-chain dehydrogenases/reductases (SDR) family.
Chr07G0708.1	737	20	95	UniProt ID:A4R0W3_MAGO7	1226	264	338	27/76(35.53)	0.49	0.01	76	47.8	2.00E-06	gene=Chr07G0708	Gene Symbol:MGG_09263|Host:Poaceae, especially important on Oryzae|Disease:Rice blast|Description:Unknown
Chr07G0711.1	560	41	547	UniProt ID:Q59RG0_CANAL	581	107	569	111/522(21.26)	0.39	0.14	522	64.7	6.00E-12	gene=Chr07G0711	Gene Symbol:NAG4|Host:Isolated from a wide variety of substrates including

														humans|Disease:invasive candidal disease|Description:CAUTION: The sequence shown here is derived from an EMBL/GenBank/DDBJ whole genome shotgun (WGS) entry which is preliminary data.
Chr07G0713.1	354	61	186	UniProt ID:Q92217_COCHE	2528	1875	1983	34/128 (26.56)	0.45	0.16	128	44.7	5.00E-06	gene=Chr07G0713	Gene Symbol:PKS1|Host:Zea mays|Disease:Southern leaf blight of maize|Description:Unknown
Chr07G0715.1	687	400	561	UniProt ID:Q59RR0_CANAL	783	594	731	56/167 (33.53)	0.46	0.2	167	84.3	8.00E-18	gene=Chr07G0715	Gene Symbol:ACE2|Host:Isolated from a wide variety of substrates including humans|Disease:invasive candidal disease|Description:CAUTION: The sequence shown here is derived from an EMBL/GenBank/DDBJ whole genome shotgun

															(WGS) entry which is preliminary data.
Chr07G0719.1	257	85	230	UniProt ID:Q4X132_ASPFU	358	45	186	37/146 (25.34)	0.47	0.03	146	48.9	8.00E-08	gene=Chr07G0719	Gene Symbol:AFUA_2G11380|Host:humans|Disease:infection|Description:CAUTION: The sequence shown here is derived from an EMBL/GenBank/DDBJ whole genome shotgun (WGS) entry which is preliminary data.
Chr07G0723.1	1888	700	847	UniProt ID:O59918_USTMD	608	208	354	70/148 (47.30)	0.69	0.01	148	155	6.00E-40	gene=Chr07G0723	Gene Symbol:UKC1|Host:Euchlaena spp., Zea spp. (Poaceae)|Disease:Smut. Corn smut|Description:CATALYTIC ACTIVITY: ATP + a protein = ADP + a phosphoprotein.
Chr07G0734.1	977	23	976	UniProt ID:A4RMS2_MAGO7	987	45	986	456/984(46.34)	0.66	0.07	984	872	0	gene=Chr07G0734	Gene Symbol:MGG_04587|Host:Poaceae, especially important on Oryzae|Disease:Rice

														blast|Description:Unknown
Chr07G0735.1	790	65	512	UniProt ID:A3LS85_PICST	677	14	449	126/459(27.45)	0.44	0.07	459	147	5.00E-38	gene=Chr07G0735	Gene Symbol:LYS4|Host:humans|Disease:occasional infection|Description:Unknown
Chr07G0737.1	1606	448	930	UniProt ID:Q5AM49_CANAL	1690	772	1265	206/506(40.71)	0.6	0.07	506	377	4.00E-108	gene=Chr07G0737	Gene Symbol:SNF2|Host:Isolated from a wide variety of substrates including humans|Disease:invasive candidal disease|Description:Unknown
Chr07G0746.1	985	294	921	UniProt ID:Q2V086_GLOLA	956	61	750	200/702(28.49)	0.46	0.12	702	237	3.00E-66	gene=Chr07G0746	Gene Symbol:CLASSD1|Host:melons,cucumber|Disease:anthracnose fruit rot|Description:Unknown
Chr07G0750.1	512	56	466	UniProt ID:Q5XTQ4_BOTFU	574	3	429	145/457(31.73)	0.46	0.17	457	167	4.00E-46	gene=Chr07G0750	Gene Symbol:LIP1|Host:Various plant families|Disease:Grey mould. Parasite or saprophyte|Description:Un

														known
Chr07G0752.1	1362	35	1312	UniProt ID:Q7Z9J3_9PEZI	1372	57	1360	690/1324(52.11)	0.68	0.05	1324	1294	0	gene=Chr07G0752	Gene Symbol:CZK3|Host:Zea mays (Poaceae)|Disease:Gray leaf spot of corn|Description:Unknown
Chr07G0764.1	635	33	600	UniProt ID:A4QVB5_MAGO7	609	13	569	311/597(52.09)	0.61	0.12	597	512	9.00E-175	gene=Chr07G0764	Gene Symbol:MGG_04621|Host:Poaceae, especially important on Oryzae|Disease:Rice blast|Description:Unknown
Chr07G0766.1	1935	1681	1929	UniProt ID:Q5AB06_CANAL	1020	712	1014	80/311(25.72)	0.43	0.23	311	109	8.00E-25	gene=Chr07G0766	Gene Symbol:VPS34|Host:Isolated from a wide variety of substrates including humans|Disease:invasive candidal disease|Description:SIMILARITY: Contains 1 PI3K/PI4K domain.
Chr07G0773.1	1147	25	1130	UniProt ID:A4QVA7_M	1340	216	1337	627/1164(53.87)	0.67	0.09	1164	1109	0	gene=Chr07G0773	Gene Symbol:MGG_04629|Host:Poaceae, especially

				AGO7										important on Oryzae|Disease:Rice blast|Description:Unknown
Chr07G0780.1	295	117	194	UniProt ID:Q5A0E5_CANAL	310	214	286	30/78(38.46)	0.62	0.06	78	64.7	6.00E-13	gene=Chr07G0780	Gene Symbol:NRG1|Host:Isolated from a wide variety of substrates including humans|Disease:invasive candidal disease|Description:Unknown
Chr07G0792.1	1151	767	885	UniProt ID:Q5D1P9_GLOAC	670	407	540	66/135(48.89)	0.64	0.13	135	138	1.00E-34	gene=Chr07G0792	Gene Symbol:NULL|Host:Multiple genera in multiple families|Disease:Anthracnose, canker, leaf spot, shepherd's crook, fruit rot, etc|Description:Unknown
Chr07G0798.1	891	208	408	UniProt ID:Q5ALS7_CANAL	1144	351	545	57/214(26.64)	0.46	0.15	214	62.4	9.00E-11	gene=Chr07G0798	Gene Symbol:CTF1|Host:Isolated from a wide variety of substrates including humans|Disease:invasive candidal disease|Description:SIMIL

															ARITY: Contains 1 Zn(2)-C6 fungal-type DNA-binding domain.
Chr07G0799.1	333	39	244	UniProt ID:TUP1_CANAL	514	260	464	54/214 (25.23)	0.42	0.08	214	61.2	2.00E-11	gene=Chr07G0799	Gene Symbol:TUP1|Host:Isolated from a wide variety of substrates including humans|Disease:invasive candidal disease|Description:FUNCTION: Represses transcription by RNA polymerase II. Represses genes responsible for initiating filamentous growth and this repression is lifted under inducing environmental conditions.
Chr07G0802.1	401	105	393	UniProt ID:D1MYV6_MAGGR	568	76	359	172/291(59.11)	0.71	0.03	291	347	2.00E-114	gene=Chr07G0802	Gene Symbol:CBL1|Host:Digitaria (Poaceae)|Disease:Leaf spot|Description:SIMILARITY: Contains 3 chitin-binding type-1 domains.
Chr07G0	496	199	463	UniProt	604	316	581	135/26	0.73	0.01	267	297	8.00E-94	gene=Chr	Gene

803.1				ID:Q6X269_USTMD				7(50.56)						07G0803	Symbol:CLB2|Host:Euchlaena spp., Zea spp. (Poaceae)|Disease:Smut. Corn smut|Description:SIMILARITY: Belongs to the cyclin family.
Chr07G0808.1	540	220	304	UniProt ID:Q9C1I3_CANAL	601	202	286	32/91(35.16)	0.55	0.13	91	47.4	1.00E-06	gene=Chr07G0808	Gene Symbol:RFG1|Host:Isolated from a wide variety of substrates including humans|Disease:invasive candidal disease|Description:Unknown
Chr07G0824.1	487	175	415	UniProt ID:TUP1_CANAL	514	255	511	74/259(28.57)	0.46	0.08	259	90.5	2.00E-20	gene=Chr07G0824	Gene Symbol:TUP1|Host:Isolated from a wide variety of substrates including humans|Disease:invasive candidal disease|Description:FUNCTION: Represses transcription by RNA polymerase II. Represses genes responsible for

															initiating filamentous growth and this repression is lifted under inducing environmental conditions.
Chr07G0838.1	799	437	798	UniProt ID:Q5AG40_CANAL	439	72	438	150/396(37.88)	0.56	0.16	396	256	4.00E-77	gene=Chr07G0838	Gene Symbol:VPS4|Host:Isolated from a wide variety of substrates including humans|Disease:invasive candidal disease|Description:SIMILARITY: Belongs to the AAA ATPase family.
Chr07G0845.1	584	24	572	UniProt ID:Q5AEK8_CANAL	584	5	577	263/573(45.90)	0.62	0.04	573	499	6.00E-171	gene=Chr07G0845	Gene Symbol:CAO19.260, ORF19.260|Host:Isolated from a wide variety of substrates including humans|Disease:invasive candidal disease|Description:SIMILARITY: Contains 1 cytochrome b5 heme-binding domain.
Chr07G0846.1	403	142	399	UniProt ID:A4Q	541	280	540	94/270(34.81)	0.49	0.08	270	151	2.00E-41	gene=Chr07G0846	Gene Symbol:MGG_04582|Host

				VD7_M AGO7											:Poaceae, especially important on Oryzae|Disease:Rice blast|Description:Unknown
Chr07G0 847.1	516	115	434	UniProt ID:Q1L 2E2_P HAND	619	174	500	83/342 (24.27)	0.41	0.11	342	78.2	3.00E-16	gene=Chr 07G0847	Gene Symbol:ALS1|Host:Multiple genera of Poaceae and Blysmus compressus (Cyperaceae)|Disease:Glume blotch of wheat and other grasses|Description:COFACTOR: Pyridoxal phosphate (By similarity).
Chr07G0 848.1	1944	1	194 4	UniProt ID:Q2L 7J5_FU SSO	1935	1	1935	1556/1 961(79 .35)	0.88	0.02	1961	3148	0	gene=Chr 07G0848	Gene Symbol:FKS1|Host:Multiple plant families. Some strains may cause infections in humans|Disease:Saprobe, facultative pathogen|Description:Unknown
Chr07G0 852.1	466	131	348	UniProt ID:B9W	301	55	269	64/240 (26.67)	0.43	0.2	240	75.1	5.00E-16	gene=Chr 07G0852	Gene Symbol:SEC14|Host:hum

				GZ2_CANDC										ans|Disease:leptomeningeal disease,occasional invasive candidal disease|Description:SIMILARITY: Contains 1 CRAL-TRIO domain.
Chr07G0860.1	600	301	549	UniProt ID:A5HF03_MAGGR	520	199	399	68/254 (26.77)	0.4	0.23	254	62.8	2.00E-11	gene=Chr07G0860	Gene Symbol:NULL|Host:Digitaria (Poaceae)|Disease:Leaf spot|Description:Unknown
Chr07G0865.1	1032	563	887	UniProt ID:Q5EGQ2_CRYNE	445	50	402	121/359(33.70)	0.48	0.11	359	174	2.00E-47	gene=Chr07G0865	Gene Symbol:BWC1|Host:humans|Disease:cryptococcosis|Description:Unknown
Chr07G0867.1	1099	320	422	UniProt ID:Q5AM49_CANAL	1690	1511	1613	41/103 (39.81)	0.6	0	103	80.9	3.00E-16	gene=Chr07G0867	Gene Symbol:SNF2|Host:Isolated from a wide variety of substrates including humans|Disease:invasive candidal disease|Description:Unknown
Chr07G0880.1	800	187	711	UniProt ID:Q5AM49_CANAL	1690	760	1255	162/535(30.28)	0.48	0.09	535	227	2.00E-62	gene=Chr07G0880	Gene Symbol:SNF2|Host:Isolated from a wide variety of substrates including

														humans|Disease:invasive candidal disease|Description:Unknown
Chr07G0881.1	1481	29	1432	UniProt ID:Q5AEW4_CANAL	1447	30	1406	421/1480(28.45)	0.48	0.12	1480	561	4.00E-174	gene=Chr07G0881	Gene Symbol:KRE5|Host:Isolated from a wide variety of substrates including humans|Disease:invasive candidal disease|Description:CAUTION: The sequence shown here is derived from an EMBL/GenBank/DDBJ whole genome shotgun (WGS) entry which is preliminary data.
Chr07G0882.1	1214	60	287	UniProt ID:TUP1_CANAL	514	260	464	63/232(27.16)	0.44	0.13	232	83.6	2.00E-17	gene=Chr07G0882	Gene Symbol:TUP1|Host:Isolated from a wide variety of substrates including humans|Disease:invasive candidal disease|Description:FUNCTION: Represses transcription by RNA

															polymerase II. Represses genes responsible for initiating filamentous growth and this repression is lifted under inducing environmental conditions.
Chr07G0884.1	990	111	990	UniProt ID:A4QRN5_MAGO7	1015	119	1015	498/946(52.64)	0.63	0.12	946	801	0	gene=Chr07G0884	Gene Symbol:MGG_03530|Host:Poaceae, especially important on Oryzae|Disease:Rice blast|Description:Unknown
Chr07G0885.1	356	15	267	UniProt ID:A0ST44_CERNC	357	3	257	72/270(26.67)	0.4	0.12	270	73.6	2.00E-15	gene=Chr07G0885	Gene Symbol:CTB6|Host:Numerous taxa in Solanaceae|Disease:Leaf spot|Description:Unknown
Chr07G0887.1	503	38	415	UniProt ID:A4UC81_MAGO7	376	3	373	148/386(38.34)	0.54	0.06	386	255	3.00E-80	gene=Chr07G0887	Gene Symbol:MGG_10702|Host:Poaceae, especially important on Oryzae|Disease:Rice blast|Description:Unknown
Chr07G0	341	9	266	UniProt	391	34	289	64/274	0.41	0.12	274	62.4	7.00E-12	gene=Chr	Gene

890.1				ID:Q6A 2T2_B OTFU				(23.36)						07G0890	Symbol:BTP1|Host:Various plant families|Disease:Grey mould. Parasite or saprophyte|Description:Unknown
Chr07G0 897.1	554	64	533	UniProt ID:Q5A NE1_C ANAL	748	51	523	113/49 0(23.0 6)	0.42	0.08	490	119	4.00E-29	gene=Chr 07G0897	Gene Symbol:SNF3|Host:Isolated from a wide variety of substrates including humans|Disease:invasive candidal disease|Description:SIMILARITY: Belongs to the major facilitator superfamily. Sugar transporter (TC 2.A.1.1) family.
Chr07G0 899.1	538	175	486	UniProt ID:A4U LJ0_M YCGR	518	197	503	76/338 (22.49)	0.43	0.17	338	56.2	2.00E-09	gene=Chr 07G0899	Gene Symbol:CYP51|Host:Triticum and possibly a few other grasses|Disease:Leaf spot or speckled leaf blotch of wheat|Description:COFACTOR: Heme group (By

															similarity).
Chr07G0906.1	567	1	463	UniProt ID:Q9P8L8_BOTFU	598	37	522	127/491(25.87)	0.45	0.07	491	143	1.00E-37	gene=Chr07G0906	Gene Symbol:BCMFS1|Host:Various plant families|Disease:Grey mould. Parasite or saprophyte|Description:Unknown
Chr07G0909.1	326	2	206	UniProt ID:A4QVF8_MAGO7	339	8	213	69/213 (32.39)	0.48	0.07	213	89.7	2.00E-21	gene=Chr07G0909	Gene Symbol:MGG_04556|Host:Poaceae, especially important on Oryzae|Disease:Rice blast|Description:COFACTOR: Zinc (By similarity).
Chr07G0912.1	436	4	301	UniProt ID:A4UC81_MAGO7	376	28	321	93/302 (30.79)	0.49	0.04	302	128	6.00E-34	gene=Chr07G0912	Gene Symbol:MGG_10702|Host:Poaceae, especially important on Oryzae|Disease:Rice blast|Description:Unknown
Chr07G0916.1	489	246	464	UniProt ID:A4ULJ1_MYCGR	517	263	515	51/257 (19.84)	0.42	0.16	257	51.6	5.00E-08	gene=Chr07G0916	Gene Symbol:CYP51|Host:Triticum and possibly a few other

															grasses|Disease:Leaf spot or speckled leaf blotch of wheat|Description:COFACTOR: Heme group (By similarity).
Chr07G0919.1	546	64	520	UniProt ID:Q5ANE1_CANAL	748	33	496	104/479(21.71)	0.41	0.08	479	102	5.00E-24	gene=Chr07G0919	Gene Symbol:SNF3|Host:Isolated from a wide variety of substrates including humans|Disease:invasive candidal disease|Description:SIMILARITY: Belongs to the major facilitator superfamily. Sugar transporter (TC 2.A.1.1) family.
Chr07G0924.1	254	32	237	UniProt ID:D1MYV6_MAGGR	568	139	353	79/216 (36.57)	0.5	0.05	216	124	2.00E-33	gene=Chr07G0924	Gene Symbol:CBL1|Host:Digitaria (Poaceae)|Disease:Leaf spot|Description:SIMILARITY: Contains 3 chitin-binding type-1 domains.
Chr07G0929.1	509	73	481	UniProt ID:Q5A	748	51	496	117/448(26.1	0.43	0.09	448	118	3.00E-29	gene=Chr07G0929	Gene Symbol:SNF3|Host:Isolate

				NE1_CANAL				2)							d from a wide variety of substrates including humans|Disease:invasive candidal disease|Description:SIMILARITY: Belongs to the major facilitator superfamily. Sugar transporter (TC 2.A.1.1) family.
Chr07G0930.1	524	22	336	UniProt ID:Q7ZA48_9HYPO	418	47	383	80/357 (22.41)	0.36	0.17	357	59.3	2.00E-10	gene=Chr07G0930	Gene Symbol:GLU1|Host:humans|Disease:Verticillium disease or dry bubble|Description:Unknown
Chr07G0931.1	635	259	520	UniProt ID:A4R0W3_MAGO7	1226	801	1013	66/268 (24.63)	0.4	0.23	268	58.5	8.00E-10	gene=Chr07G0931	Gene Symbol:MGG_09263|Host:Poaceae, especially important on Oryzae|Disease:Rice blast|Description:Unknown
Chr07G0932.1	348	62	344	UniProt ID:Q6TFC7_A	349	63	346	93/287 (32.40)	0.49	0.02	287	140	6.00E-39	gene=Chr07G0932	Gene Symbol:NULL|Host:humans|Disease:infection|Desc

				SPFM											ription:Unknown
Chr07G0936.1	274	49	270	UniProt ID:Q04701_FUSSO	242	18	237	114/225(50.67)	0.6	0.04	225	205	1.00E-65	gene=Chr07G0936	Gene Symbol:PELA|Host:Multiple plant families. Some strains may cause infections in humans|Disease:Saprobe, facultative pathogen|Description:Unknown
Chr07G0942.1	188	1	84	UniProt ID:Q9C441_FUSSO	330	221	303	44/84(52.38)	0.71	0.01	84	100	2.00E-26	gene=Chr07G0942	Gene Symbol:PEP1|Host:Multiple plant families. Some strains may cause infections in humans|Disease:Saprobe, facultative pathogen|Description:Unknown
Chr07G0944.1	801	10	71	UniProt ID:A6N6J8_FUSOX	903	26	82	28/65(43.08)	0.58	0.17	65	50.8	2.00E-07	gene=Chr07G0944	Gene Symbol:CTF1|Host:Multiple genera in multiple families|Disease:Blights, wilts, rots of various sorts|Description:SIMILARITY: Contains 1 Zn(2)-C6

															fungal-type DNA-binding domain.
Chr07G0947.1	642	7	58	UniProt ID:O59937_FUSOX	384	4	56	30/53(56.60)	0.72	0.02	53	67.8	4.00E-13	gene=Chr07G0947	Gene Symbol:XYL3|Host:Multiple genera in multiple families|Disease:Blights, wilts, rots of various sorts|Description:SIMILARITY: Belongs to the glycosyl hydrolase 10 (cellulase F) family.
Chr07G0955.1	379	27	317	UniProt ID:Q7Z8E8_CANDU	320	1	315	86/318(27.04)	0.46	0.09	318	108	8.00E-28	gene=Chr07G0955	Gene Symbol:CSH1|Host:humans|Disease:leptomeningeal disease,occasional invasive candidal disease|Description:Unknown
Chr07G0956.1	402	285	378	UniProt ID:Q4WPX2_ASPFU	1079	938	1032	35/98(35.71)	0.53	0.07	98	50.8	8.00E-08	gene=Chr07G0956	Gene Symbol:PPOA|Host:humans|Disease:infection|Description:Unknown
Chr07G0959.1	154	1	78	UniProt ID:Q5ADS0_CANAL	229	1	78	76/78(97.44)	0.99	0	78	155	3.00E-48	gene=Chr07G0959	Gene Symbol:UBI4|Host:Isolated from a wide variety of substrates including

														humans|Disease:invasive candidal disease|Description:Unknown
Chr07G0960.1	444	52	441	UniProt ID:O59928_HYPVI	430	38	429	189/396(47.73)	0.65	0.03	396	373	5.00E-126	gene=Chr07G0960	Gene Symbol:NULL|Host:humans|Disease:infection|Description:SIMILARITY: Belongs to the glycosyl hydrolase 18 family.
Chr07G0963.1	434	1	433	UniProt ID:Q5AG40_CANAL	439	1	438	274/439(62.41)	0.77	0.02	439	567	0	gene=Chr07G0963	Gene Symbol:VPS4|Host:Isolated from a wide variety of substrates including humans|Disease:invasive candidal disease|Description:SIMILARITY: Belongs to the AAA ATPase family.
Chr07G0975.1	511	55	483	UniProt ID:Q59RG1_CANAL	561	124	536	110/443(24.83)	0.4	0.1	443	89.7	5.00E-20	gene=Chr07G0975	Gene Symbol:NAG3|Host:Isolated from a wide variety of substrates including humans|Disease:invasive candidal disease|Description:CAUT

															ION: The sequence shown here is derived from an EMBL/GenBank/DDBJ whole genome shotgun (WGS) entry which is preliminary data.
Chr07G0977.1	530	6	474	UniProt ID:Q5ANE1_CANAL	748	19	496	128/485(26.39)	0.48	0.05	485	151	4.00E-40	gene=Chr07G0977	Gene Symbol:SNF3|Host:Isolated from a wide variety of substrates including humans|Disease:invasive candidal disease|Description:SIMILARITY: Belongs to the major facilitator superfamily. Sugar transporter (TC 2.A.1.1) family.
Chr07G0978.1	478	62	477	UniProt ID:SPCA_ARTOC	651	46	540	171/496(34.48)	0.5	0.16	496	269	5.00E-83	gene=Chr07G0978	Gene Symbol:SCPA|Host:humans, reptiles|Disease:dermatophytoses|Description:FUNCTION: Extracellular serine carboxypeptidase that contributes to

															pathogenicity (By similarity).
Chr07G0980.1	529	31	475	UniProt ID:Q5ANE1_CANAL	748	48	496	128/462(27.71)	0.47	0.06	462	162	1.00E-43	gene=Chr07G0980	Gene Symbol:SNF3|Host:Isolated from a wide variety of substrates including humans|Disease:invasive candidal disease|Description:SIMILARITY: Belongs to the major facilitator superfamily. Sugar transporter (TC 2.A.1.1) family.
Chr07G0986.1	873	151	725	UniProt ID:Q705V7_USTMD	1061	308	868	146/605(24.13)	0.38	0.12	605	120	1.00E-28	gene=Chr07G0986	Gene Symbol:GAS1|Host:Euchlaena spp., Zea spp. (Poaceae)|Disease:Smut. Corn smut|Description:Unknown
Chr07G0990.1	430	399	430	UniProt ID:O59937_FUSOX	384	22	53	20/32(62.50)	0.75	0	32	52.4	2.00E-08	gene=Chr07G0990	Gene Symbol:XYL3|Host:Multiple genera in multiple families|Disease:Blights, wilts, rots of various

															sorts|Description:SIMILARITY: Belongs to the glycosyl hydrolase 10 (cellulase F) family.
Chr07G0993.1	3963	2	1887	UniProt ID:Q6ZX14_MAGGR	4034	11	1904	761/1931(39.41)	0.55	0.04	1931	1248	0	gene=Chr07G0993	Gene Symbol:ACE1|Host:Digitaria (Poaceae)|Disease:Leaf spot|Description:Unknown
Chr07G0994.1	797	394	748	UniProt ID:Q5GFD3_PHAND	437	15	376	86/381(22.57)	0.42	0.12	381	64.7	7.00E-12	gene=Chr07G0994	Gene Symbol:NULL|Host:Multiple genera of Poaceae and Blysmus compressus (Cyperaceae)|Disease:Glume blotch of wheat and other grasses|Description:Unknown
Chr07G1000.1	681	12	451	UniProt ID:Q9HG15_COLLN	746	20	436	110/472(23.31)	0.36	0.18	472	60.1	2.00E-10	gene=Chr07G1000	Gene Symbol:CLTA1|Host:Multiple genera of Fabaceae. Rare reports on other taxa|Disease:Leaf, stem and pod anthracnose|Description:SIMILARITY: Contains 1 Zn(2)-C6 fungal-type

														DNA-binding domain.
Chr07G1003.1	131	15	129	UniProt ID:E9EJV1_METAR	659	315	418	33/118 (27.97)	0.45	0.14	118	47.4	5.00E-08	gene=Chr07G1003	Gene Symbol:MAA_01356|Host: insects|Disease:infection|Description:Unknown
Chr07G1005.1	528	58	485	UniProt ID:Q5ANE1_CANAL	748	73	496	111/436(25.46)	0.43	0.05	436	99.8	5.00E-23	gene=Chr07G1005	Gene Symbol:SNF3|Host:Isolated from a wide variety of substrates including humans|Disease:invasive candidal disease|Description:SIMILARITY: Belongs to the major facilitator superfamily. Sugar transporter (TC 2.A.1.1) family.
Chr07G1024.1	267	16	76	UniProt ID:A8QJI7_FUSOX	938	84	139	22/61(36.07)	0.51	0.08	61	48.1	2.00E-07	gene=Chr07G1024	Gene Symbol:XLNR|Host:Multiple genera in multiple families|Disease:Blights, wilts, rots of various sorts|Description:SIMILARITY: Contains 1 Zn(2)-C6 fungal-type DNA-binding domain.

Chr07G1025.1	514	18	76	UniProt ID:A8QJI7_FUSOX	938	86	139	21/59(35.59)	0.51	0.08	59	46.6	3.00E-06	gene=Chr07G1025	Gene Symbol:XLNR|Host:Multiple genera in multiple families|Disease:Blights, wilts, rots of various sorts|Description:SIMILARITY: Contains 1 Zn(2)-C6 fungal-type DNA-binding domain.
Chr07G1027.1	529	8	76	UniProt ID:Q5A4F3_CANAL	624	14	74	23/69(33.33)	0.48	0.12	69	49.7	2.00E-07	gene=Chr07G1027	Gene Symbol:ZCF37|Host:Isolated from a wide variety of substrates including humans|Disease:invasive candidal disease|Description:Unknown
Chr07G1039.1	559	357	529	UniProt ID:A4ULJ2_MYCGR	515	306	508	54/208(25.96)	0.42	0.19	208	53.5	2.00E-08	gene=Chr07G1039	Gene Symbol:CYP51|Host:Triticum and possibly a few other grasses|Disease:Leaf spot or speckled leaf blotch of wheat|Description:COFACTOR: Heme group (By similarity).

Chr07G1 041.1	560	60	241	UniProt ID:Q59 RG0_C ANAL	581	130	314	46/185 (24.86)	0.47	0.02	185	61.2	8.00E-11	gene=Chr 07G1041	Gene Symbol:NAG4|Host:Isolat ed from a wide variety of substrates including humans|Disease:invasive candidal disease|Description:CAUT ION: The sequence shown here is derived from an EMBL/GenBank/DDBJ whole genome shotgun (WGS) entry which is preliminary data.
Chr07G1 046.1	395	56	381	UniProt ID:Q2I0 M6_CE RNC	871	88	412	87/349 (24.93)	0.44	0.13	349	90.5	2.00E-20	gene=Chr 07G1046	Gene Symbol:CTB3|Host:Numer ous taxa in Solanaceae|Disease:Leaf spot|Description:Unknown
Chr07G1 049.1	1222	157	332	UniProt ID:SUB 7D_CO CP7	394	145	313	65/184 (35.33)	0.49	0.13	184	72	5.00E-14	gene=Chr 07G1049	Gene Symbol:CPC735_005570| Host:humans|Disease:coc cidiomycosis|Description: FUNCTION: Secreted subtilisin-like serine protease with keratinolytic activity that contributes to

															pathogenicity (By similarity).
Chr07G1057.1	523	5	476	UniProt ID:A4ULJ0_MYCGR	518	17	508	108/528(20.45)	0.37	0.17	528	47.4	1.00E-06	gene=Chr07G1057	Gene Symbol:CYP51|Host:Triticum and possibly a few other grasses|Disease:Leaf spot or speckled leaf blotch of wheat|Description:COFACTOR: Heme group (By similarity).
Chr07G1067.1	547	37	525	UniProt ID:Q5ANE1_CANAL	748	20	519	136/521(26.10)	0.44	0.1	521	120	2.00E-29	gene=Chr07G1067	Gene Symbol:SNF3|Host:Isolated from a wide variety of substrates including humans|Disease:invasive candidal disease|Description:SIMILARITY: Belongs to the major facilitator superfamily. Sugar transporter (TC 2.A.1.1) family.
Chr07G1073.1	530	321	513	UniProt ID:A4R9C7_M	1305	340	531	61/212(28.77)	0.43	0.18	212	48.9	6.00E-07	gene=Chr07G1073	Gene Symbol:MGG_13324|Host:Poaceae, especially

				AGO7											important on Oryzae|Disease:Rice blast|Description:Unknown
Chr07G1079.1	546	42	493	UniProt ID:Q5XTQ4_BOTFU	574	52	550	150/516(29.07)	0.42	0.16	516	131	1.00E-33	gene=Chr07G1079	Gene Symbol:LIP1|Host:Various plant families|Disease:Grey mould. Parasite or saprophyte|Description:Unknown
Chr07G1084.1	522	15	420	UniProt ID:DHH1_CRYNV	616	4	410	307/407(75.43)	0.87	0	407	650	0	gene=Chr07G1084	Gene Symbol:VAD1|Host:humans|Disease:cryptococcosis|Description:FUNCTION: ATP-dependent RNA helicase involved in mRNA turnover, and more specifically in mRNA decapping. Is involved in G1/S DNA- damage checkpoint recovery, probably through the regulation of the translational status of a subset of mRNAs. May

														also have a role in translation and mRNA nuclear export (By similarity). Is involved in virulence.
Chr07G1093.1	1089	243	982	UniProt ID:Q59UR3_CANAL	987	108	867	188/875(21.49)	0.35	0.29	875	79	1.00E-15	gene=Chr07G1093	Gene Symbol:SWI1|Host:Isolated from a wide variety of substrates including humans|Disease:invasive candidal disease|Description:SIMILARITY: Contains 1 ARID domain.
Chr07G1103.1	384	77	378	UniProt ID:Q6TFC7_ASPFM	349	38	347	102/315(32.38)	0.46	0.06	315	132	6.00E-36	gene=Chr07G1103	Gene Symbol:NULL|Host:humans|Disease:infection|Description:Unknown
Chr07G1105.1	355	1	350	UniProt ID:MEP8_COCP7	358	1	349	122/359(33.98)	0.53	0.05	359	192	5.00E-58	gene=Chr07G1105	Gene Symbol:MEP8|Host:humans|Disease:coccidiomycosis|Description:FUNCTION: Secreted metalloproteinase that allows assimilation of proteinaceous substrates.

													Shows high activities on basic nuclear substrates such as histone and protamine. May be involved in virulence (By similarity).
Chr07G1107.1	276	70	236	UniProt ID:Q6TFC7_ASPFM	349	61	223	65/181 (35.91)	0.52	0.18	181	105	5.00E-27	gene=Chr07G1107	Gene Symbol:NULL|Host:humans|Disease:infection|Description:Unknown
Chr07G1111.1	531	124	526	UniProt ID:Q5ANE1_CANAL	748	92	514	100/427(23.42)	0.42	0.07	427	96.7	5.00E-22	gene=Chr07G1111	Gene Symbol:SNF3|Host:Isolated from a wide variety of substrates including humans|Disease:invasive candidal disease|Description:SIMILARITY: Belongs to the major facilitator superfamily. Sugar transporter (TC 2.A.1.1) family.
Chr07G1117.1	343	1	343	UniProt ID:Q9C2Y1_BOTFU	346	1	346	171/352(48.58)	0.65	0.04	352	308	2.00E-103	gene=Chr07G1117	Gene Symbol:BCPME1|Host:Various plant families|Disease:Grey

															mould. Parasite or saprophyte|Description:CATALYTIC ACTIVITY: Pectin + n H(2)O = n methanol + pectate.
Chr07G1119.1	439	7	357	UniProt ID:Q2I0M6_CERNC	871	458	820	100/389(25.71)	0.41	0.16	389	69.7	1.00E-13	gene=Chr07G1119	Gene Symbol:CTB3|Host:Numerous taxa in Solanaceae|Disease:Leaf spot|Description:Unknown
Chr07G1121.1	692	6	593	UniProt ID:A6N6J8_FUSOX	903	44	635	151/619(24.39)	0.42	0.09	619	179	2.00E-48	gene=Chr07G1121	Gene Symbol:CTF1|Host:Multiple genera in multiple families|Disease:Blights, wilts, rots of various sorts|Description:SIMILARITY: Contains 1 Zn(2)-C6 fungal-type DNA-binding domain.
Chr07G1127.1	1048	86	971	UniProt ID:Q6ZX14_MAGGR	4034	3113	4000	213/963(22.12)	0.37	0.16	963	62.4	1.00E-10	gene=Chr07G1127	Gene Symbol:ACE1|Host:Digitaria (Poaceae)|Disease:Leaf spot|Description:Unknown
Chr07G1133.1	214	1	212	UniProt ID:Q00350_C	231	1	230	141/231(61.04)	0.71	0.09	231	270	8.00E-92	gene=Chr07G1133	Gene Symbol:XYL2|Host:Corn, Zea mays, sometimes on

				OCCA										Sorghum (Poaceae) and various other plant families|Disease:Northern corn leaf spot, ear and kernel rot|Description:CATALYTIC ACTIVITY: Endohydrolysis of (1->4)-beta-D-xylosidic linkages in xylans.
Chr07G1137.1	506	1	463	UniProt ID:Q5ANE1_CANAL	748	27	496	130/481(27.03)	0.47	0.06	481	160	3.00E-43	gene=Chr07G1137	Gene Symbol:SNF3|Host:Isolated from a wide variety of substrates including humans|Disease:invasive candidal disease|Description:SIMILARITY: Belongs to the major facilitator superfamily. Sugar transporter (TC 2.A.1.1) family.
Chr07G1138.1	771	191	381	UniProt ID:Q0WXM3_FUSO	663	250	434	48/195(24.62)	0.39	0.07	195	49.7	5.00E-07	gene=Chr07G1138	Gene Symbol:FOW2|Host:Multiple genera in multiple families|Disease:Blights,

				X											wilts, rots of various sorts|Description:SIMILARITY: Contains 1 Zn(2)-C6 fungal-type DNA-binding domain.
Chr07G1143.1	571	1	571	UniProt ID:Q2PEN8_9HYPO	575	1	575	500/576(86.81)	0.94	0.01	576	1073	0	gene=Chr07G1143	Gene Symbol:NOXB|Host:plants|Disease:cool-season grasses|Description:SIMILARITY: Contains 1 FAD-binding FR-type domain.
Chr07G1164.1	576	3	572	UniProt ID:Q9P304_CLAFU	665	2	637	184/652(28.22)	0.43	0.15	652	192	9.00E-54	gene=Chr07G1164	Gene Symbol:AOX1|Host:Lycopersicon esculentum (Solanaceae)|Disease:Leaf mold of tomato|Description:COFACTOR: FAD (By similarity).
Chr07G1165.1	665	207	624	UniProt ID:Q705V7_USTMD	1061	398	822	109/453(24.06)	0.41	0.14	453	102	2.00E-23	gene=Chr07G1165	Gene Symbol:GAS1|Host:Euchlaena spp., Zea spp. (Poaceae)|Disease:Smut. Corn smut|Description:Unknow

															n
Chr07G1166.1	335	31	147	UniProt ID:CUTI_PYRBR	203	31	148	35/124 (28.23)	0.48	0.1	124	45.1	1.00E-06	gene=Chr07G1166	Gene Symbol:NULL|Host:Brassica spp. (Brassicaceae)|Disease:Light leaf spot|Description:FUNCTION: Catalyzes the hydrolysis of cutin, a polyester that forms the structure of plant cuticle. Allows pathogenic fungi to penetrate through the cuticular barrier into the host plant during the initial stage of the fungal infection (By similarity).
Chr07G1168.1	230	8	230	UniProt ID:Q00845_NECHA	233	9	225	125/227(55.07)	0.66	0.06	227	218	3.00E-71	gene=Chr07G1168	Gene Symbol:PELD|Host:Trees of various plant families|Disease:Fruit rot, stem rot|Description:Unknown
Chr07G1169.1	367	21	179	UniProt ID:D1MYV6_M	568	398	568	63/178 (35.39)	0.48	0.15	178	68.6	1.00E-13	gene=Chr07G1169	Gene Symbol:CBL1|Host:Digitaria (Poaceae)|Disease:Leaf

				AGGR											spot|Description:SIMILARITY: Contains 3 chitin-binding type-1 domains.
Chr07G1170.1	371	82	315	UniProt ID:Q6A2T2_BOTFU	391	122	361	63/255 (24.71)	0.44	0.14	255	62.8	8.00E-12	gene=Chr07G1170	Gene Symbol:BTP1|Host:Various plant families|Disease:Grey mould. Parasite or saprophyte|Description:Unknown
Chr07G1171.1	284	9	278	UniProt ID:Q75WR5_9PLEO	265	11	262	81/273 (29.67)	0.47	0.09	273	97.4	1.00E-24	gene=Chr07G1171	Gene Symbol:BRN1|Host:Belamcanda chinensis: Korea,Gladiolus ?gandavensis: Korea,Iris japonica: China,Iris missouriensis (Leaf spot.): Idaho; Montana; Oregon; Washington,Iris sp. (Leaf spot.): China; Texas; Washing|Disease:Leaf spot|Description:SIMILARITY: Belongs to the short-chain dehydrogenases/reductas

															es (SDR) family.
Chr07G1172.1	582	35	565	UniProt ID:Q6Y392_PHAND	625	66	597	330/533(61.91)	0.75	0.01	533	684	0	gene=Chr07G1172	Gene Symbol:NULL|Host:Multiple genera of Poaceae and Blysmus compressus (Cyperaceae)|Disease:Glume blotch of wheat and other grasses|Description:Unknown
Chr07G1175.1	855	352	853	UniProt ID:CXT1_CRYNJ	694	152	661	139/538(25.84)	0.42	0.12	538	135	1.00E-33	gene=Chr07G1175	Gene Symbol:CXT1|Host:humans|Disease:cryptococcosis|Description:FUNCTION: Beta-1,2-xylosyltransferase that plays a key role in capsule polysaccharide synthesis by transferring xylose to alpha-1,3-dimannoside in a beta-1,2-linkage. Also mediates glycosylation of glycosphingolipids; constitutes the unique xylosyltransferase responsible for adding

															xylose to glycosphingolipids.
Chr07G1176.1	337	39	336	UniProtID:O59937_FUSOX	384	82	384	172/303(56.77)	0.69	0.02	303	318	9.00E-107	gene=Chr07G1176	Gene Symbol:XYL3|Host:Multiple genera in multiple families|Disease:Blights, wilts, rots of various sorts|Description:SIMILARITY: Belongs to the glycosyl hydrolase 10 (cellulase F) family.
Chr07G1180.1	595	71	570	UniProtID:Q5XTQ5_BOTFU	615	57	562	146/517(28.24)	0.48	0.05	517	191	1.00E-53	gene=Chr07G1180	Gene Symbol:FRT1|Host:Various plant families|Disease:Grey mould. Parasite or saprophyte|Description:SIMILARITY: Belongs to the major facilitator superfamily. Sugar transporter (TC 2.A.1.1) family.
Chr07G1181.1	534	32	510	UniProtID:Q5ANE1_CANAL	748	28	508	171/493(34.69)	0.54	0.05	493	270	1.00E-81	gene=Chr07G1181	Gene Symbol:SNF3|Host:Isolated from a wide variety of substrates including

															humans|Disease:invasive candidal disease|Description:SIMILARITY: Belongs to the major facilitator superfamily. Sugar transporter (TC 2.A.1.1) family.
Chr07G1186.1	746	179	217	UniProt ID:Q5A4F3_CANAL	624	8	46	18/39(46.15)	0.69	0	39	52	8.00E-08	gene=Chr07G1186	Gene Symbol:ZCF37|Host:Isolated from a wide variety of substrates including humans|Disease:invasive candidal disease|Description:Unknown
Chr07G1188.1	1355	85	1295	UniProt ID:Q9UW03_BOTFU	1439	111	1346	328/1327(24.72)	0.42	0.16	1327	319	1.00E-90	gene=Chr07G1188	Gene Symbol:ATRB|Host:Various plant families|Disease:Grey mould. Parasite or saprophyte|Description:SIMILARITY: Belongs to the ABC transporter superfamily.
Chr07G1	193	1	84	UniProt	330	221	303	41/84(	0.67	0.01	84	92	3.00E-23	gene=Chr	Gene

193.1				ID:Q9C441_FUSSO				48.81)						07G1193	Symbol:PEP1|Host:Multiple plant families. Some strains may cause infections in humans|Disease:Saprobe, facultative pathogen|Description:Unknown
Chr07G1194.1	1230	783	1074	UniProt ID:TUP1_CANAL	514	201	509	103/317(32.49)	0.51	0.1	317	164	3.00E-43	gene=Chr07G1194	Gene Symbol:TUP1|Host:Isolated from a wide variety of substrates including humans|Disease:invasive candidal disease|Description:FUNCTION: Represses transcription by RNA polymerase II. Represses genes responsible for initiating filamentous growth and this repression is lifted under inducing environmental conditions.
Chr07G1196.1	986	341	464	UniProt ID:Q9HFW4_U	2289	730	856	36/127 (28.35)	0.46	0.02	127	54.3	3.00E-08	gene=Chr07G1196	Gene Symbol:RUM1|Host:Euchlaena spp., Zea spp.

				STMD											(Poaceae)|Disease:Smut. Corn smut|Description:SIMILARITY: Contains 1 ARID domain.
Chr07G1204.1	322	1	111	UniProt ID:C4YII6_CANAW	768	492	599	36/115 (31.30)	0.5	0.1	115	52.4	2.00E-08	gene=Chr07G1204	Gene Symbol:CAWG_04261|Host:Isolated from a wide variety of substrates including humans|Disease:invasive candidal disease|Description:SIMILARITY: Belongs to the DEAD box helicase family.
Chr02G0787.1	136	6	136	UniProt ID:D2JLS9_9HYPO	131	1	131	131/131(100.00)	1	0	131	265	1.00E-92	gene=Chr02G0787	Gene Symbol:HIS3|Host:plants|Disease:allergy|Description:SUBUNIT: The nucleosome is a histone octamer containing two molecules each of H2A, H2B, H3 and H4 assembled in one H3-H4 heterotetramer and two H2A-H2B heterodimers.

															The octamer wraps approximately 147 bp of DNA (By similarity).
Chr02G0790.1	432	261	347	UniProt ID:Q59RR0_CANAL	783	646	726	40/87(45.98)	0.61	0.07	87	75.5	1.00E-15	gene=Chr02G0790	Gene Symbol:ACE2|Host:Isolated from a wide variety of substrates including humans|Disease:invasive candidal disease|Description:CAUTION: The sequence shown here is derived from an EMBL/GenBank/DDBJ whole genome shotgun (WGS) entry which is preliminary data.
Chr02G0807.1	1713	1	443	UniProt ID:C0SI36_PARBP	448	1	443	410/443(92.55)	0.97	0	443	877	0	gene=Chr02G0807	Gene Symbol:PABG_07341|Host:humans|Disease:Paracoccidioidomycosis|Description:FUNCTION: Tubulin is the major constituent of microtubules. It binds two moles of GTP, one at an exchangeable site on the beta chain and one at a

															non-exchangeable site on the alpha-chain (By similarity).
Chr02G0810.1	183	1	183	UniProt ID:Q00368_9PEZI	183	1	183	150/183(81.97)	0.91	0	183	318	2.00E-112	gene=Chr02G0810	Gene Symbol:CAP20|Host:Multiple genera in multiple families|Disease:'Anthracnose of stems and leaves, dieback, root rot, leaf spot, blossom rot, fruit rot (dieback and ripe rot), seedling blight.' (Mordue 1971)|Description:Unknown
Chr02G0817.1	277	163	246	UniProt ID:Q5AFM2_CANAL	173	78	159	41/84(48.81)	0.74	0.02	84	80.1	3.00E-19	gene=Chr02G0817	Gene Symbol:MET28|Host:Isolated from a wide variety of substrates including humans|Disease:invasive candidal disease|Description:SIMILARITY: Belongs to the bZIP family.
Chr02G0818.1	219	129	219	UniProt ID:A4R2T9_M	117	25	111	53/93(56.99)	0.68	0.09	93	97.1	3.00E-26	gene=Chr02G0818	Gene Symbol:MGG_02436|Host:Poaceae, especially

			AGO7											important on Oryzae|Disease:Rice blast|Description:Unknown
Chr02G0823.1	572	180	359	UniProt ID:F2QU09_PICP7	626	45	213	50/181 (27.62)	0.52	0.07	181	80.9	6.00E-17	gene=Chr02G0823	Gene Symbol:PAB1|Host:humans|Disease:occasional infection|Description:Unknown
Chr02G0824.1	400	1	124	UniProt ID:Q4WGR1_ASPFU	456	326	446	34/124 (27.42)	0.43	0.02	124	48.9	3.00E-07	gene=Chr02G0824	Gene Symbol:AFUA_7G05920|Host:humans|Disease:infection|Description:COFACTOR: Iron (By similarity).
Chr02G0826.1	648	436	613	UniProt ID:Q5APH9_CANAL	375	184	369	63/201 (31.34)	0.48	0.19	201	68.2	4.00E-13	gene=Chr02G0826	Gene Symbol:PTC1|Host:Isolated from a wide variety of substrates including humans|Disease:invasive candidal disease|Description:SIMILARITY: Belongs to the PP2C family.
Chr02G0828.1	321	3	321	UniProt ID:Q59ZX2_C	381	2	317	174/322(54.04)	0.73	0.03	322	363	2.00E-124	gene=Chr02G0828	Gene Symbol:FTR1|Host:Isolated from a wide variety of

			ANAL											substrates including humans|Disease:invasive candidal disease|Description:CAUTION: The sequence shown here is derived from an EMBL/GenBank/DDBJ whole genome shotgun (WGS) entry which is preliminary data.
Chr02G0829.1	620	19	513	UniProt ID:Q5RLJ7_CRYNV	594	58	578	163/543(30.02)	0.45	0.13	543	205	2.00E-58	gene=Chr02G0829	Gene Symbol:NULL|Host:humans|Disease:cryptococcosis|Description:Unknown
Chr02G0832.1	370	10	145	UniProt ID:PYRF_CANAL	270	7	139	64/136 (47.06)	0.63	0.02	136	127	1.00E-34	gene=Chr02G0832	Gene Symbol:URA3|Host:Isolated from a wide variety of substrates including humans|Disease:invasive candidal disease|Description:CATALYTIC ACTIVITY: Orotidine 5'-phosphate = UMP + CO(2).
Chr02G0837.1	679	87	193	UniProt ID:Q9C	601	203	299	45/109 (41.28)	0.58	0.13	109	97.1	6.00E-22	gene=Chr02G0837	Gene Symbol:RFG1|Host:Isolat

				1I3_CANAL											ed from a wide variety of substrates including humans|Disease:invasive candidal disease|Description:Unknown
Chr02G0846.1	720	635	689	UniProt ID:Q9HFW4_USTMD	2289	1664	1716	23/56(41.07)	0.48	0.07	56	51.2	2.00E-07	gene=Chr02G0846	Gene Symbol:RUM1|Host:Euchlaena spp., Zea spp. (Poaceae)|Disease:Smut. Corn smut|Description:SIMILARITY: Contains 1 ARID domain.
Chr02G0850.1	389	193	253	UniProt ID:A6REW8_AJECN	288	221	281	22/61(36.07)	0.59	0	61	48.1	3.00E-07	gene=Chr02G0850	Gene Symbol:HCAG_08183|Host:humans|Disease:Darling's disease|Description:FUNCTION: Component of the eukaryotic translation initiation factor 3 (eIF-3) complex (By similarity).
Chr02G0854.1	1018	582	909	UniProt ID:A4QTS1_M	847	464	782	103/335(30.75)	0.53	0.07	335	201	1.00E-54	gene=Chr02G0854	Gene Symbol:MGG_04985|Host:Poaceae, especially

				AGO7											important on Oryzae|Disease:Rice blast|Description:Unknown
Chr02G0858.1	637	87	597	UniProt ID:Q9P8L8_BOTFU	598	100	591	140/530(26.42)	0.43	0.11	530	129	2.00E-32	gene=Chr02G0858	Gene Symbol:BCMFS1|Host:Various plant families|Disease:Grey mould. Parasite or saprophyte|Description:Unknown
Chr02G0859.1	899	112	625	UniProt ID:O42620_MAGGR	614	29	513	167/532(31.39)	0.49	0.12	532	212	1.00E-59	gene=Chr02G0859	Gene Symbol:PTH2|Host:Digitaria (Poaceae)|Disease:Leaf spot|Description:SIMILARITY: Belongs to the carnitine/choline acetyltransferase family.
Chr02G0862.1	202	5	202	UniProt ID:A6R9F0_AJECN	204	8	204	185/198(93.43)	0.95	0.01	198	361	3.00E-128	gene=Chr02G0862	Gene Symbol:HCAG_06941|Host:humans|Disease:Darling's disease|Description:SIMILARITY: Belongs to the small GTPase superfamily. Rab family.

Chr02G0864.1	399	52	356	UniProt ID:TUP1_CANAL	514	225	514	81/319 (25.39)	0.43	0.13	319	75.1	1.00E-15	gene=Chr02G0864	Gene Symbol:TUP1|Host:Isolated from a wide variety of substrates including humans|Disease:invasive candidal disease|Description:FUNCTION: Represses transcription by RNA polymerase II. Represses genes responsible for initiating filamentous growth and this repression is lifted under inducing environmental conditions.
Chr02G0865.1	844	99	267	UniProt ID:TUP1_CANAL	514	258	422	40/181 (22.10)	0.4	0.15	181	51.2	1.00E-07	gene=Chr02G0865	Gene Symbol:TUP1|Host:Isolated from a wide variety of substrates including humans|Disease:invasive candidal disease|Description:FUNCTION: Represses transcription by RNA polymerase II. Represses genes responsible for

															initiating filamentous growth and this repression is lifted under inducing environmental conditions.
Chr02G0866.1	574	104	281	UniProt ID:Q5UB48_CRYNV	1040	206	388	62/196 (31.63)	0.46	0.16	196	85.9	2.00E-18	gene=Chr02G0866	Gene Symbol:SKN7|Host:humans|Disease:cryptococcosis|Description:Unknown
Chr02G0869.1	518	58	495	UniProt ID:Q5ANE1_CANAL	748	50	503	109/458(23.80)	0.45	0.05	458	120	8.00E-30	gene=Chr02G0869	Gene Symbol:SNF3|Host:Isolated from a wide variety of substrates including humans|Disease:invasive candidal disease|Description:SIMILARITY: Belongs to the major facilitator superfamily. Sugar transporter (TC 2.A.1.1) family.
Chr02G0871.1	351	61	344	UniProt ID:Q6TFC7_ASPFM	349	61	346	109/290(37.59)	0.56	0.03	290	181	5.00E-54	gene=Chr02G0871	Gene Symbol:NULL|Host:humans|Disease:infection|Description:Unknown
Chr02G0872.1	283	46	280	UniProt ID:Q6T	349	61	335	83/277 (29.96)	0.47	0.16	277	123	2.00E-33	gene=Chr02G0872	Gene Symbol:NULL|Host:huma

			FC7_A SPFM											ns|Disease:infection|Description:Unknown
Chr02G0875.1	268	18	265	UniProt ID:Q5ANM6_CANAL	286	23	284	63/262 (24.05)	0.46	0.05	262	71.6	1.00E-15	gene=Chr02G0875	Gene Symbol:PEP12|Host:Isolated from a wide variety of substrates including humans|Disease:invasive candidal disease|Description:CAUTION: The sequence shown here is derived from an EMBL/GenBank/DDBJ whole genome shotgun (WGS) entry which is preliminary data.
Chr02G0883.1	439	99	430	UniProt ID:Q5A415_CANAL	361	40	358	119/337(35.31)	0.58	0.07	337	206	2.00E-62	gene=Chr02G0883	Gene Symbol:ARP2|Host:Isolated from a wide variety of substrates including humans|Disease:invasive candidal disease|Description:SIMILARITY: Belongs to the actin family.
Chr02G0884.1	353	6	349	UniProt ID:A4Q	339	3	336	92/344 (26.74)	0.47	0.03	344	136	2.00E-37	gene=Chr02G0884	Gene Symbol:MGG_04556|Host

				VF8_M AGO7											:Poaceae, especially important on Oryzae|Disease:Rice blast|Description:COFACTOR: Zinc (By similarity).
Chr02G0 885.1	518	15	480	UniProt ID:A4U LJ2_M YCGR	515	23	505	105/51 3(20.4 7)	0.36	0.15	513	54.7	6.00E-09	gene=Chr 02G0885	Gene Symbol:CYP51|Host:Triticum and possibly a few other grasses|Disease:Leaf spot or speckled leaf blotch of wheat|Description:COFACTOR: Heme group (By similarity).
Chr02G0 897.1	606	17	560	UniProt ID:Q5X TQ5_B OTFU	615	11	560	149/58 8(25.3 4)	0.45	0.14	588	187	4.00E-52	gene=Chr 02G0897	Gene Symbol:FRT1|Host:Various plant families|Disease:Grey mould. Parasite or saprophyte|Description:SIMILARITY: Belongs to the major facilitator superfamily. Sugar transporter (TC 2.A.1.1) family.
Chr02G0	564	1	180	UniProt	696	1	183	45/190	0.45	0.09	190	55.5	6.00E-09	gene=Chr	Gene

902.1				ID:F2QSG4_PICP7				(23.68)						02G0902	Symbol:GFA1|Host:humans|Disease:occasional infection|Description:Unknown
Chr02G0903.1	790	26	378	UniProt ID:Q8NKE9_USTMD	1166	56	433	170/392(43.37)	0.58	0.14	392	304	3.00E-89	gene=Chr02G0903	Gene Symbol:CRK1|Host:Euchlaena spp., Zea spp. (Poaceae)|Disease:Smut. Corn smut|Description:Unknown
Chr02G0912.1	195	7	195	UniProt ID:Q2KG56_MAGO7	199	10	199	105/190(55.26)	0.67	0.01	190	211	8.00E-70	gene=Chr02G0912	Gene Symbol:"MGCH7_CH7G479, MGG_02731"|Host:Poaceae, especially important on Oryzae|Disease:Rice blast|Description:SIMILARITY: Belongs to the small GTPase superfamily. Rho family.
Chr02G0940.1	352	3	230	UniProt ID:A4QVF8_MAGO7	339	2	231	71/234(30.34)	0.48	0.04	234	100	6.00E-25	gene=Chr02G0940	Gene Symbol:MGG_04556|Host:Poaceae, especially important on Oryzae|Disease:Rice

															blast|Description:COFACTOR: Zinc (By similarity).
Chr02G0945.1	153	9	117	UniProt ID:Q59Z39_CANAL	259	17	124	24/111 (21.62)	0.5	0.05	111	46.2	1.00E-07	gene=Chr02G0945	Gene Symbol:SAP49|Host:Isolated from a wide variety of substrates including humans|Disease:invasive candidal disease|Description:CAUTION: The sequence shown here is derived from an EMBL/GenBank/DDBJ whole genome shotgun (WGS) entry which is preliminary data.
Chr02G0946.1	1278	1	1278	UniProt ID:SEC31_COCIM	1261	1	1261	658/1318(49.92)	0.65	0.07	1318	1179	0	gene=Chr02G0946	Gene Symbol:SEC31|Host:humans|Disease:coccidioidomycosis|Description:FUNCTION: Component of the coat protein complex II (COPII) which promotes the formation of transport vesicles from the endoplasmic reticulum (ER). The coat has two

															main functions, the physical deformation of the endoplasmic reticulum membrane into vesicles and the selection of cargo molecules (By similarity).
Chr02G0951.1	341	4	336	UniProt ID:A0ST44_CERNC	357	2	344	91/360 (25.28)	0.44	0.12	360	89.7	3.00E-21	gene=Chr02G0951	Gene Symbol:CTB6|Host:Numerous taxa in Solanaceae|Disease:Leaf spot|Description:Unknown
Chr02G0969.1	1072	497	1003	UniProt ID:A4RCH6_MAGO7	1810	791	1268	341/508(67.13)	0.78	0.06	508	707	0	gene=Chr02G0969	Gene Symbol:MGG_12656|Host:Poaceae, especially important on Oryzae|Disease:Rice blast|Description:SIMILARITY: Contains 1 reverse transcriptase domain.
Chr02G0976.1	347	74	325	UniProt ID:Q59QH2_CANAL	337	16	325	72/316 (22.78)	0.38	0.22	316	54.3	3.00E-09	gene=Chr02G0976	Gene Symbol:CSH1|Host:Isolated from a wide variety of substrates including humans|Disease:invasive candidal disease|Description:CAUT

															ION: The sequence shown here is derived from an EMBL/GenBank/DDBJ whole genome shotgun (WGS) entry which is preliminary data.
Chr02G0982.1	767	418	755	UniProt ID:A4QTS1_MAGO7	847	472	782	94/338 (27.81)	0.46	0.08	338	140	3.00E-35	gene=Chr02G0982	Gene Symbol:MGG_04985|Host:Poaceae, especially important on Oryzae|Disease:Rice blast|Description:Unknown
Chr02G0984.1	434	225	433	UniProt ID:A3LTI2_PICST	320	114	317	122/210(58.10)	0.77	0.03	210	249	2.00E-79	gene=Chr02G0984	Gene Symbol:PRS1|Host:humans|Disease:occasional infection|Description:CATALYTIC ACTIVITY: ATP + D-ribose 5-phosphate = AMP + 5-phospho-alpha-D-ribose 1-diphosphate.
Chr02G0986.1	187	4	187	UniProt ID:Q2KG56_MAGO7	199	7	199	47/200 (23.50)	0.45	0.12	200	68.9	1.00E-15	gene=Chr02G0986	Gene Symbol:"MGCH7_CH7G479, MGG_02731"|Host:Poace

														ae, especially important on Oryzae|Disease:Rice blast|Description:SIMILARITY: Belongs to the small GTPase superfamily. Rho family.
Chr02G0996.1	255	72	145	UniProt ID:A6RH34_AJECN	633	44	117	25/75(33.33)	0.55	0.03	75	48.1	2.00E-07	gene=Chr02G0996	Gene Symbol:HCAG_08951|Host:humans|Disease:Darling's disease|Description:Unknown
Chr02G1001.1	620	246	577	UniProt ID:O59918_USTMD	608	209	562	138/361(38.23)	0.56	0.1	361	233	2.00E-68	gene=Chr02G1001	Gene Symbol:UKC1|Host:Euchlaena spp., Zea spp. (Poaceae)|Disease:Smut. Corn smut|Description:CATALYTIC ACTIVITY: ATP + a protein = ADP + a phosphoprotein.
Chr02G1002.1	609	224	484	UniProt ID:Q5APH9_CANAL	375	111	344	70/280(25.00)	0.42	0.23	280	51.2	7.00E-08	gene=Chr02G1002	Gene Symbol:PTC1|Host:Isolated from a wide variety of substrates including humans|Disease:invasive

															candidal disease|Description:SIMILARITY: Belongs to the PP2C family.
Chr02G1016.1	666	91	663	UniProt ID:Q5XTQ5_BOTFU	615	29	606	160/627(25.52)	0.43	0.16	627	140	4.00E-36	gene=Chr02G1016	Gene Symbol:FRT1|Host:Various plant families|Disease:Grey mould. Parasite or saprophyte|Description:SIMILARITY: Belongs to the major facilitator superfamily. Sugar transporter (TC 2.A.1.1) family.
Chr02G1022.1	847	13	831	UniProt ID:SEY1_CANAL	790	21	790	303/834(36.33)	0.53	0.09	834	497	7.00E-164	gene=Chr02G1022	Gene Symbol:SEY1|Host:Isolated from a wide variety of substrates including humans|Disease:invasive candidal disease|Description:FUNCTION: Cooperates with the reticulon proteins and tubule- shaping DP1 family proteins to generate

															and maintain the structure of the tubular endoplasmic reticulum network. Has GTPase activity, which is required for its function in ER organization (By similarity). Required for virulence and resistance to cycloheximide.
Chr02G1031.1	252	39	173	UniProt ID:Q9P4A1_CRYNE	249	18	171	51/155 (32.90)	0.46	0.14	155	64.3	3.00E-13	gene=Chr02G1031	Gene Symbol:CBP1|Host:humans|Disease:cryptococcosis|Description:Unknown
Chr02G1033.1	346	91	251	UniProt ID:Q8TGD1_FUSOX	318	157	315	46/168 (27.38)	0.43	0.1	168	44.7	3.00E-06	gene=Chr02G1033	Gene Symbol:FOW1|Host:Multiple genera in multiple families|Disease:Blights, wilts, rots of various sorts|Description:SIMILARITY: Belongs to the mitochondrial carrier family.
Chr02G1040.1	670	272	649	UniProt ID:O93800_ALTAL	578	201	576	94/401 (23.44)	0.38	0.12	401	65.1	6.00E-12	gene=Chr02G1040	Gene Symbol:AKT1|Host:Plant|Disease:Leaf spot, rots|Description:Unknown

Chr02G1045.1	890	46	407	UniProt ID:Q5ADP3_CANAL	1080	99	475	224/377(59.42)	0.75	0.04	377	482	2.00E-154	gene=Chr02G1045	Gene Symbol:SSN6|Host:Isolated from a wide variety of substrates including humans|Disease:invasive candidal disease|Description:CAUTION: The sequence shown here is derived from an EMBL/GenBank/DDBJ whole genome shotgun (WGS) entry which is preliminary data.
Chr02G1047.1	391	1	388	UniProt ID:RL3_YEAST	387	1	387	286/388(73.71)	0.87	0	388	576	0	gene=Chr02G1047	Gene Symbol:YOR29-14|Host:humans|Disease:occasional infection|Description:SUBUNIT: Component of the large ribosomal subunit. Mature ribosomes consist of a small (40S) and a large (60S) subunit. The 40S subunit contains 32 different proteins (encoded by 56 genes)

															and 1 molecule of RNA (18S). The 60S subunit contains 46 different proteins (encoded by 81 genes) and 3 molecules of RNA (25S, 5.8S and 5S).
Chr02G1049.1	520	246	496	UniProt ID:Q9Y7F1_ASPFM	824	549	812	68/272 (25.00)	0.44	0.11	272	92.8	9.00E-21	gene=Chr02G1049	Gene Symbol:PABAA|Host:humans|Disease:infection|Description:Unknown
Chr02G1051.1	427	6	353	UniProt ID:Q5A661_CANAL	548	14	366	131/374(35.03)	0.5	0.13	374	239	4.00E-73	gene=Chr02G1051	Gene Symbol:PHR1|Host:Isolated from a wide variety of substrates including humans|Disease:invasive candidal disease|Description:CAUTION: The sequence shown here is derived from an EMBL/GenBank/DDBJ whole genome shotgun (WGS) entry which is preliminary data.
Chr02G1053.1	529	14	516	UniProt ID:Q5XTQ5_B	615	74	543	148/510(29.02)	0.48	0.09	510	161	1.00E-43	gene=Chr02G1053	Gene Symbol:FRT1|Host:Various plant

				OTFU											families|Disease:Grey mould. Parasite or saprophyte|Description:SIMILARITY: Belongs to the major facilitator superfamily. Sugar transporter (TC 2.A.1.1) family.
Chr02G1054.1	633	116	582	UniProt ID:A0ST42_CERNC	512	46	510	172/470(36.60)	0.57	0.02	470	311	2.00E-98	gene=Chr02G1054	Gene Symbol:CTB4|Host:Numerous taxa in Solanaceae|Disease:Leaf spot|Description:Unknown
Chr02G1056.1	358	88	272	UniProt ID:A4RGG9_MAGO7	286	16	217	60/205 (29.27)	0.44	0.11	205	61.6	9.00E-12	gene=Chr02G1056	Gene Symbol:MGG_00056|Host:Poaceae, especially important on Oryzae|Disease:Rice blast|Description:SIMILARITY: Belongs to the short-chain dehydrogenases/reductases (SDR) family.
Chr02G1057.1	448	108	364	UniProt ID:Q6XPX0_F	359	69	324	69/264 (26.14)	0.48	0.06	264	84.3	7.00E-19	gene=Chr02G1057	Gene Symbol:FGB1|Host:Multiple genera in multiple

				USOX										families|Disease:Blights, wilts, rots of various sorts|Description:Unknown
Chr02G1063.1	463	41	377	UniProt ID:Q5GFD3_PHAND	437	3	354	81/364 (22.25)	0.41	0.11	364	56.6	1.00E-09	gene=Chr02G1063	Gene Symbol:NULL|Host:Multiple genera of Poaceae and Blysmus compressus (Cyperaceae)|Disease:Glume blotch of wheat and other grasses|Description:Unknown
Chr02G1068.1	551	10	173	UniProt ID:Q6WP53_BOTFU	223	63	201	62/168 (36.90)	0.47	0.2	168	74.7	5.00E-16	gene=Chr02G1068	Gene Symbol:BCP1|Host:Various plant families|Disease:Grey mould. Parasite or saprophyte|Description:FUNCTION: PPIases accelerate the folding of proteins (By similarity).
Chr02G1069.1	763	175	237	UniProt ID:Q5UB48_CRYNV	1040	206	282	27/77(35.06)	0.56	0.18	77	64.3	1.00E-11	gene=Chr02G1069	Gene Symbol:SKN7|Host:humans|Disease:cryptococcosis|Description:Unknown

Chr02G1070.1	166	4	140	UniProt ID:Q6WP53_BOTFU	223	67	202	61/140 (43.57)	0.56	0.05	140	102	6.00E-28	gene=Chr02G1070	Gene Symbol:BCP1|Host:Various plant families|Disease:Grey mould. Parasite or saprophyte|Description:FUNCTION: PPIases accelerate the folding of proteins (By similarity).
Chr02G1071.1	590	131	587	UniProt ID:Q5ABU7_CANAL	564	102	564	166/464(35.78)	0.55	0.02	464	318	4.00E-101	gene=Chr02G1071	Gene Symbol:MDR1|Host:Isolated from a wide variety of substrates including humans|Disease:invasive candidal disease|Description:Unknown
Chr02G1073.1	338	25	250	UniProt ID:Q32WF7_PHAND	266	3	216	67/235 (28.51)	0.4	0.13	235	52	1.00E-08	gene=Chr02G1073	Gene Symbol:MDH1|Host:Multiple genera of Poaceae and Blysmus compressus (Cyperaceae)|Disease:Glume blotch of wheat and other grasses|Description:Unknown

Chr02G1086.1	795	556	795	UniProt ID:Q59KY8_CANAL	314	73	314	176/242(72.73)	0.84	0.01	242	399	3.00E-133	gene=Chr02G1086	Gene Symbol:SIT4|Host:Isolated from a wide variety of substrates including humans|Disease:invasive candidal disease|Description:CATALYTIC ACTIVITY: A phosphoprotein + H(2)O = a protein + phosphate.
Chr02G1091.1	762	28	59	UniProt ID:Q5A4F3_CANAL	624	21	52	17/32(53.12)	0.69	0	32	47.4	2.00E-06	gene=Chr02G1091	Gene Symbol:ZCF37|Host:Isolated from a wide variety of substrates including humans|Disease:invasive candidal disease|Description:Unknown
Chr02G1094.1	341	4	334	UniProt ID:A0ST44_CERNC	357	6	339	99/358(27.65)	0.44	0.14	358	97.8	4.00E-24	gene=Chr02G1094	Gene Symbol:CTB6|Host:Numerous taxa in Solanaceae|Disease:Leaf spot|Description:Unknown
Chr02G1095.1	757	259	486	UniProt ID:Q5AG40_C	439	127	354	83/234(35.47)	0.57	0.05	234	133	3.00E-34	gene=Chr02G1095	Gene Symbol:VPS4|Host:Isolated from a wide variety of

				ANAL											substrates including humans|Disease:invasive candidal disease|Description:SIMILARITY: Belongs to the AAA ATPase family.
Chr02G1096.1	425	14	132	UniProt ID:A3QX02_ASPFM	570	434	547	54/119 (45.38)	0.61	0.04	119	101	4.00E-24	gene=Chr02G1096	Gene Symbol:ZAFA|Host:humans|Disease:infection|Description:Unknown
Chr02G1100.1	304	34	293	UniProt ID:Q32WF7_PHAND	266	17	264	78/262 (29.77)	0.51	0.06	262	95.9	6.00E-24	gene=Chr02G1100	Gene Symbol:MDH1|Host:Multiple genera of Poaceae and Blysmus compressus (Cyperaceae)|Disease:Glume blotch of wheat and other grasses|Description:Unknown
Chr02G1109.1	1357	986	1260	UniProt ID:TUP1_CANAL	514	201	503	119/305(39.02)	0.54	0.1	305	192	8.00E-53	gene=Chr02G1109	Gene Symbol:TUP1|Host:Isolated from a wide variety of substrates including humans|Disease:invasive candidal disease|Description:FUNC

															TION: Represses transcription by RNA polymerase II. Represses genes responsible for initiating filamentous growth and this repression is lifted under inducing environmental conditions.
Chr02G1111.1	1096	714	922	UniProt ID:Q6ZX14_MAGGR	4034	3717	3902	65/211 (30.81)	0.41	0.13	211	54.3	4.00E-08	gene=Chr02G1111	Gene Symbol:ACE1|Host:Digitaria (Poaceae)|Disease:Leaf spot|Description:Unknown
Chr02G1117.1	798	497	582	UniProt ID:Q9HFW4_USTMD	2289	319	401	31/88(35.23)	0.55	0.08	88	51.2	2.00E-07	gene=Chr02G1117	Gene Symbol:RUM1|Host:Euchlaena spp., Zea spp. (Poaceae)|Disease:Smut. Corn smut|Description:SIMILARITY: Contains 1 ARID domain.
Chr02G1119.1	814	255	460	UniProt ID:Q0WXM3_FUSOX	663	249	450	52/209 (24.88)	0.44	0.05	209	67	2.00E-12	gene=Chr02G1119	Gene Symbol:FOW2|Host:Multiple genera in multiple families|Disease:Blights, wilts, rots of various sorts|Description:SIMILAR

															ITY: Contains 1 Zn(2)-C6 fungal-type DNA-binding domain.
Chr02G1121.1	487	9	445	UniProt ID:Q9Y784_MAGGR	631	15	435	119/452(26.33)	0.44	0.1	452	147	3.00E-39	gene=Chr02G1121	Gene Symbol:PTH11|Host:Digitaria (Poaceae)|Disease:Leaf spot|Description:Unknown
Chr02G1122.1	308	52	239	UniProt ID:Q32WF7_PHAND	266	18	214	48/202(23.76)	0.39	0.09	202	43.9	4.00E-06	gene=Chr02G1122	Gene Symbol:MDH1|Host:Multiple genera of Poaceae and Blysmus compressus (Cyperaceae)|Disease:Glume blotch of wheat and other grasses|Description:Unknown
Chr02G1125.1	521	7	378	UniProt ID:Q5XTQ4_BOTFU	574	20	393	121/396(30.56)	0.45	0.12	396	118	2.00E-29	gene=Chr02G1125	Gene Symbol:LIP1|Host:Various plant families|Disease:Grey mould. Parasite or saprophyte|Description:Unknown
Chr02G1129.1	514	135	470	UniProt ID:A4U	517	146	505	82/375(21.87)	0.36	0.14	375	58.2	5.00E-10	gene=Chr02G1129	Gene Symbol:CYP51|Host:Tritic

				LJ1_MYCGR											um and possibly a few other grasses|Disease:Leaf spot or speckled leaf blotch of wheat|Description:COFACTOR: Heme group (By similarity).
Chr02G1131.1	377	1	370	UniProt ID:MEP8_COCP7	358	1	347	135/378(35.71)	0.52	0.1	378	226	6.00E-71	gene=Chr02G1131	Gene Symbol:MEP8|Host:humans|Disease:coccidiomycosis|Description:FUNCTION: Secreted metalloproteinase that allows assimilation of proteinaceous substrates. Shows high activities on basic nuclear substrates such as histone and protamine. May be involved in virulence (By similarity).
Chr02G1135.1	2528	12	2523	UniProt ID:Q92217_COCHE	2528	8	2522	906/2593(34.94)	0.54	0.06	2593	1412	0	gene=Chr02G1135	Gene Symbol:PKS1|Host:Zea mays|Disease:Southern leaf blight of maize|Description:Unkno

															wn
Chr02G1136.1	485	22	213	UniProt ID:Q2VLJ1_GIBZA	565	83	292	59/215 (27.44)	0.44	0.13	215	61.6	4.00E-11	gene=Chr02G1136	Gene Symbol:ZEB1|Host:Principal hosts: Poaceae, including Zea mays (corn), Triticum aestivum (wheat), and Oryza sativa (rice). Additional hosts: various plant families|Disease:Seedling blight, pre- and post-emergence blight, root and foot rot, brown rot, culm decay, head or kernel blight (scab or ear scab) of cereals. Leaf|Description:Unknown
Chr02G1138.1	540	314	522	UniProt ID:A4ULI6_MYCGR	517	298	511	55/231 (23.81)	0.39	0.17	231	55.5	4.00E-09	gene=Chr02G1138	Gene Symbol:CYP51|Host:Triticum and possibly a few other grasses|Disease:Leaf spot or speckled leaf blotch of wheat|Description:COFACTOR: Heme group (By similarity).

Chr02G1154.1	289	72	242	UniProt ID:CDC42_CANAL	191	5	174	88/172 (51.16)	0.69	0.02	172	183	2.00E-57	gene=Chr02G1154	Gene Symbol:CDC42|Host:Isolated from a wide variety of substrates including humans|Disease:invasive candidal disease|Description:FUNCTION: Involved in hyphal formation, virulence, morphogenesis.
Chr02G1169.1	403	108	392	UniProt ID:Q6TFC7_ASPFM	349	63	347	91/292 (31.16)	0.45	0.05	292	127	7.00E-34	gene=Chr02G1169	Gene Symbol:NULL|Host:humans|Disease:infection|Description:Unknown
Chr02G1174.1	1821	12	1819	UniProt ID:Q59VF3_CANAL	1813	7	1813	862/1840(46.85)	0.64	0.04	1840	1645	0	gene=Chr02G1174	Gene Symbol:"DUR1,2"|Host:Isolated from a wide variety of substrates including humans|Disease:invasive candidal disease|Description:CAUTION: The sequence shown here is derived from an EMBL/GenBank/DDBJ whole genome shotgun (WGS) entry which is

															preliminary data.
Chr02G1176.1	669	32	543	UniProt ID:A6N6J8_FUSOX	903	23	524	131/556(23.56)	0.38	0.18	556	78.2	7.00E-16	gene=Chr02G1176	Gene Symbol:CTF1|Host:Multiple genera in multiple families|Disease:Blights, wilts, rots of various sorts|Description:SIMILARITY: Contains 1 Zn(2)-C6 fungal-type DNA-binding domain.
Chr02G1177.1	531	102	480	UniProt ID:CTSD_ASPFU	474	71	452	145/384(37.76)	0.55	0.02	384	260	1.00E-80	gene=Chr02G1177	Gene Symbol:CTSD|Host:humans|Disease:infection|Description:FUNCTION: Secreted aspartic-type endopeptidase which is secreted and contributes to virulence.
Chr02G1180.1	859	1	852	UniProt ID:Q96WM3_CRYNE	849	1	848	419/870(48.16)	0.66	0.05	870	802	0	gene=Chr02G1180	Gene Symbol:VPH1|Host:humans|Disease:cryptococcosis|Description:Unknown
Chr02G1190.1	367	65	344	UniProt ID:O59937_FUSOX	384	117	384	113/288(39.24)	0.51	0.1	288	167	2.00E-48	gene=Chr02G1190	Gene Symbol:XYL3|Host:Multiple genera in multiple families|Disease:Blights,

															wilts, rots of various sorts|Description:SIMILARITY: Belongs to the glycosyl hydrolase 10 (cellulase F) family.
Chr02G1193.1	316	5	313	UniProt ID:C1G3Y7_PARBD	320	3	317	230/315(73.02)	0.84	0.02	315	476	4.00E-170	gene=Chr02G1193	Gene Symbol:PADG_01653|Host:humans|Disease:Paracoccidioidomycosis|Description:CATALYTIC ACTIVITY: ATP + D-ribose 5-phosphate = AMP + 5-phospho-alpha-D-ribose 1-diphosphate.
Chr02G1197.1	393	3	335	UniProt ID:Q9Y784_MAGGR	631	105	424	85/337(25.22)	0.45	0.06	337	121	5.00E-31	gene=Chr02G1197	Gene Symbol:PTH11|Host:Digitaria (Poaceae)|Disease:Leaf spot|Description:Unknown
Chr02G1198.1	511	64	505	UniProt ID:Q59RQ6_CANAL	491	40	475	133/449(29.62)	0.48	0.04	449	166	6.00E-46	gene=Chr02G1198	Gene Symbol:LPD1|Host:Isolated from a wide variety of substrates including humans|Disease:invasive candidal

														disease|Description:CATALYTIC ACTIVITY: Protein N(6)-(dihydrolipoyl)lysine + NAD(+) = protein N(6)-(lipoyl)lysine + NADH.
Chr02G1200.1	477	272	377	UniProt ID:F2QYD1_PICP7	758	477	584	26/108 (24.07)	0.48	0.02	108	48.9	5.00E-07	gene=Chr02G1200	Gene Symbol:MAK5|Host:humans|Disease:occasional infection|Description:SIMILARITY: Belongs to the DEAD box helicase family.
Chr02G1201.1	979	112	236	UniProt ID:CYAA_USTMA	2493	1161	1284	44/125 (35.20)	0.61	0.01	125	83.2	5.00E-17	gene=Chr02G1201	Gene Symbol:UAC1|Host:Euchlaena spp., Zea spp. (Poaceae)|Disease:Smut. Corn smut|Description:FUNCTION: Plays essential roles in regulation of cellular metabolism by catalyzing the synthesis of a second messenger, cAMP.
Chr02G1212.1	438	41	413	UniProt ID:Y7403_AR	430	32	418	105/401(26.18)	0.45	0.1	401	123	4.00E-32	gene=Chr02G1212	Gene Symbol:ARB_07403|Host:hedgehogs|Disease:ringw

				TBC										orm,Kerion Celsi Disease|Description:FUNCTION: Probable secreted aspartic-type endopeptidase which contributes to virulence (By similarity).
Chr02G1214.1	262	8	244	UniProt ID:Q75WR5_9PLEO	265	9	245	71/255 (27.84)	0.45	0.14	255	68.2	1.00E-14	gene=Chr02G1214	Gene Symbol:BRN1|Host:Belamcanda chinensis: Korea,Gladiolus ?gandavensis: Korea,Iris japonica: China,Iris missouriensis (Leaf spot.): Idaho; Montana; Oregon; Washington,Iris sp. (Leaf spot.): China; Texas; Washing|Disease:Leaf spot|Description:SIMILARITY: Belongs to the short-chain dehydrogenases/reductases (SDR) family.
Chr02G1216.1	807	638	740	UniProt ID:C4YII6_CAN	768	475	577	45/107 (42.06)	0.62	0.07	107	82	5.00E-17	gene=Chr02G1216	Gene Symbol:CAWG_04261|Host:Isolated from a wide

				AW										variety of substrates including humans|Disease:invasive candidal disease|Description:SIMILARITY: Belongs to the DEAD box helicase family.
Chr02G1218.1	1281	208	1170	UniProt ID:Q9P872_CANAL	917	52	906	278/983(28.28)	0.47	0.15	983	307	6.00E-89	gene=Chr02G1218	Gene Symbol:PMR1|Host:Isolated from a wide variety of substrates including humans|Disease:invasive candidal disease|Description:SIMILARITY: Belongs to the cation transport ATPase (P-type) family.
Chr02G1219.1	347	6	341	UniProt ID:A0ST44_CERNC	357	5	344	94/357 (26.33)	0.43	0.11	357	82.8	9.00E-19	gene=Chr02G1219	Gene Symbol:CTB6|Host:Numerous taxa in Solanaceae|Disease:Leaf spot|Description:Unknown
Chr02G1232.1	859	325	503	UniProt ID:Q0WXM3_FUSO	663	254	433	56/187 (29.95)	0.49	0.08	187	80.9	1.00E-16	gene=Chr02G1232	Gene Symbol:FOW2|Host:Multiple genera in multiple families|Disease:Blights,

			X											wilts, rots of various sorts|Description:SIMILARITY: Contains 1 Zn(2)-C6 fungal-type DNA-binding domain.
Chr02G1234.1	420	7	372	UniProtID:Q5GFD3_PHAND	437	12	384	99/399 (24.81)	0.42	0.15	399	84.7	9.00E-19	gene=Chr02G1234	Gene Symbol:NULL|Host:Multiple genera of Poaceae and Blysmus compressus (Cyperaceae)|Disease:Glume blotch of wheat and other grasses|Description:Unknown
Chr02G1235.1	461	223	368	UniProtID:Q0QWD8_LEPMC	359	216	332	45/146 (30.82)	0.45	0.2	146	53.5	9.00E-09	gene=Chr02G1235	Gene Symbol:THIOL|Host:Brassica spp. and other Brassicaceae|Disease:Black leg, canker, dry rot, leaf spot|Description:SIMILARITY: Belongs to the thiolase family.
Chr02G1237.1	312	102	283	UniProtID:CUTI_PYRBR	203	10	189	87/185 (47.03)	0.63	0.04	185	155	2.00E-46	gene=Chr02G1237	Gene Symbol:NULL|Host:Brassica spp. (Brassicaceae)|Disease:Li

															ght leaf spot|Description:FUNCTION: Catalyzes the hydrolysis of cutin, a polyester that forms the structure of plant cuticle. Allows pathogenic fungi to penetrate through the cuticular barrier into the host plant during the initial stage of the fungal infection (By similarity).
Chr02G1239.1	409	52	402	UniProt ID:Q6TFC7_ASPFM	349	4	348	109/358(30.45)	0.46	0.06	358	137	2.00E-37	gene=Chr02G1239	Gene Symbol:NULL|Host:humans|Disease:infection|Description:Unknown
Chr02G1242.1	756	34	82	UniProt ID:A4R0W3_MAGO7	1226	275	323	23/49(46.94)	0.59	0	49	58.2	1.00E-09	gene=Chr02G1242	Gene Symbol:MGG_09263|Host:Poaceae, especially important on Oryzae|Disease:Rice blast|Description:Unknown
Chr02G1247.1	647	498	590	UniProt ID:Q9UUS8_C	567	411	503	42/97(43.30)	0.6	0.08	97	63.2	2.00E-11	gene=Chr02G1247	Gene Symbol:CHIP3|Host:Multiple genera in multiple

				OLGL										families|Disease:'Anthracnose of stems and leaves, dieback, root rot, leaf spot, blossom rot, fruit rot (dieback and ripe rot), seedling blight.' (Mordue 1971)|Description:Unknown
Chr02G1250.1	680	11	62	UniProt ID:A0ST46_CERNC	397	21	72	22/53(41.51)	0.64	0.04	53	50.1	2.00E-07	gene=Chr02G1250	Gene Symbol:CTB8|Host:Numerous taxa in Solanaceae|Disease:Leaf spot|Description:Unknown
Chr02G1253.1	575	63	226	UniProt ID:P78585_BOTFU	994	378	533	62/165(37.58)	0.53	0.06	165	101	2.00E-23	gene=Chr02G1253	Gene Symbol:BCPLC1|Host:Various plant families|Disease:Grey mould. Parasite or saprophyte|Description:SIMILARITY: Contains 1 C2 domain.
Chr02G1258.1	519	7	513	UniProt ID:Q59RB8_CANAL	550	7	527	175/525(33.33)	0.54	0.04	525	310	4.00E-99	gene=Chr02G1258	Gene Symbol:ICL1|Host:Isolated from a wide variety of substrates including humans|Disease:invasive

														candidal disease|Description:SIMILARITY: Belongs to the isocitrate lyase/PEP mutase superfamily. Isocitrate lyase family.
Chr02G1262.1	1125	59	479	UniProtID:A4ULJ0_MYCGR	518	60	505	106/466(22.75)	0.41	0.14	466	68.9	7.00E-13	gene=Chr02G1262	Gene Symbol:CYP51|Host:Triticum and possibly a few other grasses|Disease:Leaf spot or speckled leaf blotch of wheat|Description:COFACTOR: Heme group (By similarity).
Chr02G1273.1	512	54	500	UniProtID:Q5ANE1_CANAL	748	44	496	113/464(24.35)	0.42	0.06	464	117	7.00E-29	gene=Chr02G1273	Gene Symbol:SNF3|Host:Isolated from a wide variety of substrates including humans|Disease:invasive candidal disease|Description:SIMILARITY: Belongs to the major facilitator superfamily. Sugar transporter (TC 2.A.1.1)

														family.
Chr02G1275.1	482	92	345	UniProt ID:Q7ZA48_9HYPO	418	118	383	66/282 (23.40)	0.37	0.16	282	49.3	2.00E-07	gene=Chr02G1275	Gene Symbol:GLU1|Host:humans|Disease:Verticillium disease or dry bubble|Description:Unknown
Chr02G1277.1	403	26	371	UniProt ID:Q9Y784_MAGGR	631	20	364	139/347(40.06)	0.63	0.01	347	288	3.00E-91	gene=Chr02G1277	Gene Symbol:PTH11|Host:Digitaria (Poaceae)|Disease:Leaf spot|Description:Unknown
Chr02G1282.1	677	27	607	UniProt ID:A6N6J8_FUSOX	903	46	581	142/599(23.71)	0.37	0.14	599	85.1	4.00E-18	gene=Chr02G1282	Gene Symbol:CTF1|Host:Multiple genera in multiple families|Disease:Blights, wilts, rots of various sorts|Description:SIMILARITY: Contains 1 Zn(2)-C6 fungal-type DNA-binding domain.
Chr02G1283.1	521	241	410	UniProt ID:Q0PND8_MAGGR	1375	990	1174	60/196 (30.61)	0.48	0.19	196	87.8	4.00E-19	gene=Chr02G1283	Gene Symbol:PEX6|Host:Digitaria (Poaceae)|Disease:Leaf spot|Description:SIMILARITY: Belongs to the AAA

Chr02G1291.1	423	15	231	UniProt ID:Q9UUS8_COLGL	567	104	316	70/228 (30.70)	0.43	0.11	228	84.3	1.00E-18	gene=Chr02G1291	ATPase family. Gene Symbol:CHIP3|Host:Multiple genera in multiple families|Disease:'Anthracnose of stems and leaves, dieback, root rot, leaf spot, blossom rot, fruit rot (dieback and ripe rot), seedling blight.' (Mordue 1971)|Description:Unknown
Chr02G1292.1	1013	200	467	UniProt ID:SUB10_ARTBC	522	133	351	75/277 (27.08)	0.38	0.24	277	56.6	5.00E-09	gene=Chr02G1292	Gene Symbol:SUB10|Host:hedgehogs|Disease:ringworm, Kerion Celsi Disease|Description:FUNCTION: Secreted subtilisin-like serine protease with keratinolytic activity that contributes to pathogenicity (By similarity).
Chr02G1305.1	720	13	408	UniProt ID:Q9HG15_C	746	16	373	99/407 (24.32)	0.39	0.15	407	62	8.00E-11	gene=Chr02G1305	Gene Symbol:CLTA1|Host:Multiple genera of Fabaceae.

				OLLN											Rare reports on other taxa|Disease:Leaf, stem and pod anthracnose|Description:SIMILARITY: Contains 1 Zn(2)-C6 fungal-type DNA-binding domain.
Chr02G1308.1	503	264	477	UniProt ID:A4ULJ2_MYCGR	515	257	510	66/259 (25.48)	0.43	0.19	259	63.2	1.00E-11	gene=Chr02G1308	Gene Symbol:CYP51|Host:Triticum and possibly a few other grasses|Disease:Leaf spot or speckled leaf blotch of wheat|Description:COFACTOR: Heme group (By similarity).
Chr02G1309.1	1415	18	426	UniProt ID:A6N6J8_FUSOX	903	48	470	92/460 (20.00)	0.36	0.19	460	52.8	1.00E-07	gene=Chr02G1309	Gene Symbol:CTF1|Host:Multiple genera in multiple families|Disease:Blights, wilts, rots of various sorts|Description:SIMILARITY: Contains 1 Zn(2)-C6 fungal-type DNA-binding domain.
Chr02G1	1478	17	146	UniProt	1501	21	1477	829/14	0.73	0.02	1470	1720	0	gene=Chr	Gene

310.1			6	ID:Q96VL9_BOTFU				70(56.39)						02G1310	Symbol:BCATRD|Host:Various plant families|Disease:Grey mould. Parasite or saprophyte|Description:SIMILARITY: Belongs to the ABC transporter superfamily.
Chr02G1314.1	482	92	168	UniProt ID:A0ST43_CERNC	459	114	190	30/77(38.96)	0.53	0	77	57.8	6.00E-10	gene=Chr02G1314	Gene Symbol:CTB5|Host:Numerous taxa in Solanaceae|Disease:Leaf spot|Description:Unknown
Chr02G1316.1	597	51	594	UniProt ID:Q4P8E8_USTMA	693	123	673	195/580(33.62)	0.5	0.11	580	259	1.00E-77	gene=Chr02G1316	Gene Symbol:UM03615.1|Host: Euchlaena spp., Zea spp. (Poaceae)|Disease:Smut. Corn smut|Description:COFACTOR: FAD (By similarity).
Chr02G1321.1	470	50	467	UniProt ID:A0ST42_CERNC	512	52	509	133/464(28.66)	0.45	0.11	464	161	2.00E-44	gene=Chr02G1321	Gene Symbol:CTB4|Host:Numerous taxa in Solanaceae|Disease:Leaf spot|Description:Unknown
Chr02G1	553	138	500	UniProt	515	121	503	95/403	0.43	0.15	403	84.3	3.00E-18	gene=Chr	Gene

322.1				ID:A4ULI5_MYCGR				(23.57)						02G1322	Symbol:CYP51|Host:Triticum and possibly a few other grasses|Disease:Leaf spot or speckled leaf blotch of wheat|Description:COFACTOR: Heme group (By similarity).
Chr02G1326.1	426	60	304	UniProt ID:Q9Y784_MAGGR	631	131	369	63/248 (25.40)	0.46	0.05	248	68.6	2.00E-13	gene=Chr02G1326	Gene Symbol:PTH11|Host:Digitaria (Poaceae)|Disease:Leaf spot|Description:Unknown
Chr02G1330.1	308	1	302	UniProt ID:MEP1_COCP7	276	1	275	115/307(37.46)	0.53	0.12	307	182	7.00E-56	gene=Chr02G1330	Gene Symbol:MEP1|Host:humans|Disease:coccidiomycosis|Description:FUNCTION: Secreted metalloproteinase that allows assimilation of proteinaceous substrates. Pays a pivotal role as a pathogenicity determinant during infections and contributes to the ability of the pathogen to persist

<table>
<tr><td></td><td></td><td></td><td></td><td></td><td></td><td></td><td></td><td></td><td></td><td></td><td></td><td></td><td></td><td></td><td></td></tr>
<tr><td></td><td></td><td></td><td></td><td></td><td></td><td></td><td></td><td></td><td></td><td></td><td></td><td></td><td></td><td></td><td>within the mammalian host. Digests an immunodominant cell surface antigen (SOWgp) and prevents host recognition of endospores during the phase of development when these fungal cells are most vulnerable to phagocytic cell defenses.</td></tr>
<tr><td>Chr02G1332.1</td><td>396</td><td>7</td><td>377</td><td>UniProt ID:Q9Y784_MAGGR</td><td>631</td><td>14</td><td>398</td><td>92/393 (23.41)</td><td>0.43</td><td>0.08</td><td>393</td><td>111</td><td>2.00E-27</td><td>gene=Chr02G1332</td><td>Gene Symbol:PTH11|Host:Digitaria (Poaceae)|Disease:Leaf spot|Description:Unknown</td></tr>
<tr><td>Chr02G1336.1</td><td>495</td><td>3</td><td>485</td><td>UniProt ID:Q59RG0_CANAL</td><td>581</td><td>87</td><td>567</td><td>124/508(24.41)</td><td>0.41</td><td>0.1</td><td>508</td><td>106</td><td>2.00E-25</td><td>gene=Chr02G1336</td><td>Gene Symbol:NAG4|Host:Isolated from a wide variety of substrates including humans|Disease:invasive candidal disease|Description:CAUTION: The sequence shown here is derived from an EMBL/GenBank/DDBJ</td></tr>
</table>

														whole genome shotgun (WGS) entry which is preliminary data.
Chr02G1341.1	334	1	151	UniProt ID:A4R3I5_MAGO7	400	80	225	41/157 (26.11)	0.46	0.11	157	46.6	1.00E-06	gene=Chr02G1341	Gene Symbol:"MGG_11993, MGG_12837, MGG_13052"|Host:Poaceae, especially important on Oryzae|Disease:Rice blast|Description:Unknown
Chr02G1342.1	1234	71	1201	UniProt ID:A4RGC8_MAGO7	1158	31	1132	455/1164(39.09)	0.57	0.08	1164	778	0	gene=Chr02G1342	Gene Symbol:MGG_11671|Host:Poaceae, especially important on Oryzae|Disease:Rice blast|Description:SIMILARITY: Contains 1 reverse transcriptase domain.
Chr02G1346.1	714	451	675	UniProt ID:Q3Y5V5_MAGGR	1321	1065	1301	70/245 (28.57)	0.48	0.11	245	69.3	4.00E-13	gene=Chr02G1346	Gene Symbol:ABC3|Host:Digitaria (Poaceae)|Disease:Leaf spot|Description:SIMILARITY: Belongs to the ABC transporter superfamily.
Chr02G1	189	18	155	UniProt	159	2	126	62/138	0.66	0.09	138	125	7.00E-37	gene=Chr	Gene

351.1				ID:Q59 QC5_C ANAL				(44.93)						02G1351	Symbol:APS3|Host:Isolated from a wide variety of substrates including humans|Disease:invasive candidal disease|Description:CAUTION: The sequence shown here is derived from an EMBL/GenBank/DDBJ whole genome shotgun (WGS) entry which is preliminary data.
Chr02G1 354.1	447	41	73	UniProt ID:Q5A 4F3_C ANAL	624	14	46	16/33(48.48)	0.7	0	33	50.8	9.00E-08	gene=Chr 02G1354	Gene Symbol:ZCF37|Host:Isolated from a wide variety of substrates including humans|Disease:invasive candidal disease|Description:Unknown
Chr02G1 361.1	414	78	377	UniProt ID:Q6A 2T2_B OTFU	391	51	345	70/311 (22.51)	0.45	0.09	311	66.2	6.00E-13	gene=Chr 02G1361	Gene Symbol:BTP1|Host:Various plant families|Disease:Grey mould. Parasite or saprophyte|Description:Un

														known
Chr02G1 365.1	693	491	678	UniProt ID:Q5E MY3_M AGGR	424	154	347	58/205 (28.29)	0.44	0.14	205	52.8	3.00E-08	gene=Chr 02G1365	Gene Symbol:NULL|Host:Digitar ia (Poaceae)|Disease:Leaf spot|Description:SIMILARI TY: Belongs to the AAA ATPase family.
Chr02G1 366.1	714	329	453	UniProt ID:LAP 2_ART OC	495	223	340	39/126 (30.95)	0.49	0.07	126	51.6	8.00E-08	gene=Chr 02G1366	Gene Symbol:LAP2|Host:human s, reptiles|Disease:dermatop hytoses|Description:FUNC TION: Extracellular aminopeptidase that releases a wide variety of amino acids from natural peptides and contributes to pathogenicity.
Chr02G1 367.1	515	258	481	UniProt ID:A4U LI5_MY CGR	515	253	503	55/255 (21.57)	0.4	0.14	255	50.4	1.00E-07	gene=Chr 02G1367	Gene Symbol:CYP51|Host:Tritic um and possibly a few other grasses|Disease:Leaf spot or speckled leaf blotch of wheat|Description:COFAC TOR: Heme group (By

														similarity).
Chr02G1375.1	333	25	333	UniProt ID:PLYB_COLGL	331	30	331	179/310(57.74)	0.71	0.03	310	304	5.00E-102	gene=Chr02G1375	Gene Symbol:PLB|Host:Multiple genera in multiple families|Disease:'Anthracnose of stems and leaves, dieback, root rot, leaf spot, blossom rot, fruit rot (dieback and ripe rot), seedling blight.' (Mordue 1971)|Description:FUNCTION: Acts as a virulence factor active in plant tissue maceration.
Chr02G1394.1	521	12	45	UniProt ID:A4R0W3_MAGO7	1226	283	316	17/34(50.00)	0.59	0	34	45.4	7.00E-06	gene=Chr02G1394	Gene Symbol:MGG_09263|Host:Poaceae, especially important on Oryzae|Disease:Rice blast|Description:Unknown
Chr02G1397.1	546	28	496	UniProt ID:Q5ANE1_CANAL	748	39	498	126/490(25.71)	0.45	0.1	490	140	2.00E-36	gene=Chr02G1397	Gene Symbol:SNF3|Host:Isolated from a wide variety of substrates including humans|Disease:invasive

															candidal disease|Description:SIMILARITY: Belongs to the major facilitator superfamily. Sugar transporter (TC 2.A.1.1) family.
Chr02G1402.1	339	3	338	UniProt ID:Q75ZG3_ALTAL	366	24	364	179/347(51.59)	0.64	0.05	347	335	8.00E-114	gene=Chr02G1402	Gene Symbol:AFTS1|Host:Plant|Disease:Leaf spot, rots|Description:Unknown
Chr02G1404.1	472	62	447	UniProt ID:O59928_HYPVI	430	39	429	170/391(43.48)	0.64	0.01	391	334	2.00E-110	gene=Chr02G1404	Gene Symbol:NULL|Host:humans|Disease:infection|Description:SIMILARITY: Belongs to the glycosyl hydrolase 18 family.
Chr02G1412.1	1775	53	445	UniProt ID:P87199_USTMD	968	5	339	127/399(31.83)	0.52	0.18	399	198	1.00E-52	gene=Chr02G1412	Gene Symbol:KIN2|Host:Euchlaena spp., Zea spp. (Poaceae)|Disease:Smut. Corn smut|Description:SIMILARITY: Belongs to the kinesin-like protein family.
Chr02G1	790	43	783	UniProt	803	43	796	287/81	0.49	0.15	810	428	5.00E-13	gene=Chr	Gene

417.1				ID:Q99324_SEPLY				0(35.43)					8	02G1417	Symbol:B2TOM|Host:Primarily tomato, Lycopersicon esculentum, also Solanum spp. and other Solanaceae|Disease:Leaf spot|Description:Unknown
Chr02G1419.1	415	101	397	UniProt ID:Q2I0M6_CERNC	871	111	412	82/316(25.95)	0.42	0.1	316	91.7	9.00E-21	gene=Chr02G1419	Gene Symbol:CTB3|Host:Numerous taxa in Solanaceae|Disease:Leaf spot|Description:Unknown
Chr02G1420.1	378	3	292	UniProt ID:Q9Y784_MAGGR	631	105	390	67/291(23.02)	0.43	0.02	291	65.9	1.00E-12	gene=Chr02G1420	Gene Symbol:PTH11|Host:Digitaria (Poaceae)|Disease:Leaf spot|Description:Unknown
Chr02G1424.1	342	29	196	UniProt ID:Q2XW08_COCHE	2144	1479	1615	51/168(30.36)	0.46	0.18	168	52.4	2.00E-08	gene=Chr02G1424	Gene Symbol:PKS2|Host:Zea mays|Disease:Southern leaf blight of maize|Description:Unknown
Chr02G1425.1	1453	218	1441	UniProt ID:Q9UW87_CANAL	1606	257	1591	343/1352(25.37)	0.45	0.11	1352	422	9.00E-124	gene=Chr02G1425	Gene Symbol:MLT1|Host:Isolated from a wide variety of substrates including

															humans|Disease:invasive candidal disease|Description:SIMILARITY: Belongs to the ABC transporter superfamily.
Chr02G1426.1	842	14	391	UniProt ID:Q99324_SEPLY	803	54	476	127/442(28.73)	0.46	0.19	442	152	4.00E-39	gene=Chr02G1426	Gene Symbol:B2TOM|Host:Primarily tomato, Lycopersicon esculentum, also Solanum spp. and other Solanaceae|Disease:Leaf spot|Description:Unknown
Chr02G1427.1	494	30	278	UniProt ID:Q9Y784_MAGGR	631	116	364	66/253 (26.09)	0.47	0.03	253	86.7	5.00E-19	gene=Chr02G1427	Gene Symbol:PTH11|Host:Digitaria (Poaceae)|Disease:Leaf spot|Description:Unknown
Chr02G1431.1	652	275	473	UniProt ID:Q00LS5_PHAND	408	38	209	61/199 (30.65)	0.46	0.14	199	71.2	5.00E-14	gene=Chr02G1431	Gene Symbol:CPKA|Host:Multiple genera of Poaceae and Blysmus compressus (Cyperaceae)|Disease:Glume blotch of wheat and other grasses|Description:SIMIL

															ARITY: Contains 1 protein kinase domain.
Chr02G1432.1	523	232	375	UniProt ID:A9Z1V6_PHAND	394	82	214	48/144 (33.33)	0.46	0.08	144	57	1.00E-09	gene=Chr02G1432	Gene Symbol:CPKB|Host:Multiple genera of Poaceae and Blysmus compressus (Cyperaceae)|Disease:Glume blotch of wheat and other grasses|Description:Unknown
Chr02G1437.1	1493	198	1484	UniProt ID:Q9UW87_CANAL	1606	231	1598	360/1453(24.78)	0.42	0.17	1453	347	8.00E-99	gene=Chr02G1437	Gene Symbol:MLT1|Host:Isolated from a wide variety of substrates including humans|Disease:invasive candidal disease|Description:SIMILARITY: Belongs to the ABC transporter superfamily.
Chr02G1439.1	541	45	532	UniProt ID:Q5XTQ4_BOTFU	574	55	566	177/534(33.15)	0.49	0.13	534	241	2.00E-72	gene=Chr02G1439	Gene Symbol:LIP1|Host:Various plant families|Disease:Grey mould. Parasite or

															saprophyte|Description:Unknown
Chr02G1440.1	951	312	378	UniProt ID:Q4WPX2_ASPFU	1079	960	1025	25/68(36.76)	0.59	0.04	68	47	5.00E-06	gene=Chr02G1440	Gene Symbol:PPOA|Host:humans|Disease:infection|Description:Unknown
Chr02G1447.1	1064	273	428	UniProt ID:Q9Y880_COCCA	880	123	253	46/159(28.93)	0.42	0.19	159	57.8	3.00E-09	gene=Chr02G1447	Gene Symbol:SNF1|Host:Corn, Zea mays, sometimes on Sorghum (Poaceae) and various other plant families|Disease:Northern corn leaf spot, ear and kernel rot|Description:Unknown
Chr02G1456.1	879	17	749	UniProt ID:PALA_CANAL	785	3	737	239/760(31.45)	0.53	0.07	760	371	1.00E-115	gene=Chr02G1456	Gene Symbol:RIM20|Host:Isolated from a wide variety of substrates including humans|Disease:invasive candidal disease|Description:FUNCTION: Required for the proteolytic cleavage of the transcription factor RIM101 in response to

															alkaline ambient pH. May act as a scaffold protein that recruits the calpain-like protease RIM13 via SNF7 to its substrate RIM101 (By similarity). Required for filamentation.
Chr02G1459.1	234	42	183	UniProt ID:Q6TFC7_ASPFM	349	61	215	59/160 (36.88)	0.51	0.14	160	94.7	1.00E-23	gene=Chr02G1459	Gene Symbol:NULL|Host:humans|Disease:infection|Description:Unknown
Chr02G1463.1	371	66	367	UniProt ID:Q6TFC7_ASPFM	349	50	347	103/309(33.33)	0.51	0.06	309	153	2.00E-43	gene=Chr02G1463	Gene Symbol:NULL|Host:humans|Disease:infection|Description:Unknown
Chr02G1464.1	512	105	497	UniProt ID:Q5ANE1_CANAL	748	95	510	100/422(23.70)	0.43	0.08	422	91.3	2.00E-20	gene=Chr02G1464	Gene Symbol:SNF3|Host:Isolated from a wide variety of substrates including humans|Disease:invasive candidal disease|Description:SIMILARITY: Belongs to the major facilitator superfamily. Sugar

															transporter (TC 2.A.1.1) family.
Chr02G1466.1	1242	1107	1239	UniProt ID:Q1A1Y7_CRYNV	1383	1235	1361	55/139 (39.57)	0.56	0.13	139	89	1.00E-18	gene=Chr02G1466	Gene Symbol:TCO1|Host:humans|Disease:cryptococcosis|Description:SIMILARITY: Contains 1 histidine kinase domain.
Chr02G1473.1	356	5	346	UniProt ID:A3LUV9_PICST	365	10	364	108/361(29.92)	0.5	0.07	361	147	5.00E-41	gene=Chr02G1473	Gene Symbol:MET22|Host:humans|Disease:occasional infection|Description:Unknown
Chr02G1489.1	763	209	393	UniProt ID:Q0WXM3_FUSOX	663	249	440	44/192 (22.92)	0.44	0.04	192	64.3	1.00E-11	gene=Chr02G1489	Gene Symbol:FOW2|Host:Multiple genera in multiple families|Disease:Blights, wilts, rots of various sorts|Description:SIMILARITY: Contains 1 Zn(2)-C6 fungal-type DNA-binding domain.
Chr02G1491.1	431	13	426	UniProt ID:Q5GFD3_PHAND	437	9	436	214/433(49.42)	0.66	0.06	433	411	5.00E-141	gene=Chr02G1491	Gene Symbol:NULL|Host:Multiple genera of Poaceae and Blysmus compressus

															(Cyperaceae)|Disease:Glume blotch of wheat and other grasses|Description:Unknown
Chr02G1492.1	638	19	349	UniProt ID:A5H456_MYCGR	1811	1	333	167/334(50.00)	0.67	0.01	334	334	6.00E-100	gene=Chr02G1492	Gene Symbol:NULL|Host:Triticum and possibly a few other grasses|Disease:Leaf spot or speckled leaf blotch of wheat|Description:Unknown
Chr02G1494.1	450	30	365	UniProt ID:A4UC81_MAGO7	376	46	371	143/339(42.18)	0.6	0.05	339	247	1.00E-77	gene=Chr02G1494	Gene Symbol:MGG_10702|Host:Poaceae, especially important on Oryzae|Disease:Rice blast|Description:Unknown
Chr02G1495.1	680	252	423	UniProt ID:Q9HG15_COLLN	746	230	407	51/192(26.56)	0.42	0.18	192	48.1	1.00E-06	gene=Chr02G1495	Gene Symbol:CLTA1|Host:Multiple genera of Fabaceae. Rare reports on other taxa|Disease:Leaf, stem and pod anthracnose|Description:S

														IMILARITY: Contains 1 Zn(2)-C6 fungal-type DNA-binding domain.
Chr02G1497.1	183	75	153	UniProt ID:B3VBK9_CLAFU	228	42	114	37/79(46.84)	0.62	0.08	79	70.1	5.00E-16	gene=Chr02G1497	Gene Symbol:NULL|Host:Lycopersicon esculentum (Solanaceae)|Disease:Leaf mold of tomato|Description:Unknown
Chr02G1500.1	570	128	565	UniProt ID:Q5ABU7_CANAL	564	95	559	158/470(33.62)	0.54	0.08	470	267	7.00E-82	gene=Chr02G1500	Gene Symbol:MDR1|Host:Isolated from a wide variety of substrates including humans|Disease:invasive candidal disease|Description:Unknown
Chr02G1501.1	380	28	363	UniProt ID:Q6A2T2_BOTFU	391	22	374	106/363(29.20)	0.45	0.1	363	120	2.00E-31	gene=Chr02G1501	Gene Symbol:BTP1|Host:Various plant families|Disease:Grey mould. Parasite or saprophyte|Description:Unknown
Chr02G1	347	6	179	UniProt	391	121	296	58/176	0.52	0.01	176	106	8.00E-27	gene=Chr	Gene

502.1				ID:Q6A 2T2_B OTFU				(32.95)						02G1502	Symbol:BTP1|Host:Various plant families|Disease:Grey mould. Parasite or saprophyte|Description:Unknown
Chr02G1 518.1	850	14	376	UniProt ID:Q99 324_S EPLY	803	54	482	141/44 2(31.9 0)	0.45	0.21	442	168	3.00E-44	gene=Chr 02G1518	Gene Symbol:B2TOM|Host:Primarily tomato, Lycopersicon esculentum, also Solanum spp. and other Solanaceae|Disease:Leaf spot|Description:Unknown
Chr02G1 519.1	523	114	489	UniProt ID:Q5A NE1_C ANAL	748	96	496	102/40 5(25.1 9)	0.44	0.08	405	116	2.00E-28	gene=Chr 02G1519	Gene Symbol:SNF3|Host:Isolated from a wide variety of substrates including humans|Disease:invasive candidal disease|Description:SIMILARITY: Belongs to the major facilitator superfamily. Sugar transporter (TC 2.A.1.1) family.
Chr02G1	327	11	299	UniProt	320	1	309	93/314	0.5	0.1	314	117	7.00E-31	gene=Chr	Gene

523.1				ID:Q7Z8E8_CANDU				(29.62)						02G1523	Symbol:CSH1|Host:humans|Disease:leptomeningeal disease,occasional invasive candidal disease|Description:Unknown
Chr02G1525.1	636	93	325	UniProt ID:B2C6F1_CRYGA	614	79	329	62/267 (23.22)	0.39	0.19	267	50.1	3.00E-07	gene=Chr02G1525	Gene Symbol:LAC1|Host:humans|Disease:pulmonary cryptococcosis, basal meningitis, and cerebral cryptococcomas|Description:Unknown
Chr02G1530.1	783	40	95	UniProt ID:Q0WXM3_FUSOX	663	96	153	21/58(36.21)	0.47	0.03	58	47.8	2.00E-06	gene=Chr02G1530	Gene Symbol:FOW2|Host:Multiple genera in multiple families|Disease:Blights, wilts, rots of various sorts|Description:SIMILARITY: Contains 1 Zn(2)-C6 fungal-type DNA-binding domain.
Chr02G1540.1	416	11	361	UniProt ID:Q6A2T2_BOTFU	391	24	382	97/384 (25.26)	0.42	0.15	384	105	4.00E-26	gene=Chr02G1540	Gene Symbol:BTP1|Host:Various plant families|Disease:Grey

															mould. Parasite or saprophyte|Description:Unknown
Chr02G1541.1	299	26	226	UniProt ID:A4RGG9_MAGO7	286	9	206	52/209 (24.88)	0.43	0.09	209	50.1	4.00E-08	gene=Chr02G1541	Gene Symbol:MGG_00056|Host:Poaceae, especially important on Oryzae|Disease:Rice blast|Description:SIMILARITY: Belongs to the short-chain dehydrogenases/reductases (SDR) family.
Chr02G1547.1	501	68	432	UniProt ID:Q9Y784_MAGGR	631	112	492	90/383 (23.50)	0.43	0.05	383	84.3	3.00E-18	gene=Chr02G1547	Gene Symbol:PTH11|Host:Digitaria (Poaceae)|Disease:Leaf spot|Description:Unknown
Chr02G1552.1	1417	37	502	UniProt ID:Q59M50_CANAL	578	36	578	195/561(34.76)	0.51	0.2	561	310	1.00E-92	gene=Chr02G1552	Gene Symbol:CWT1|Host:Isolated from a wide variety of substrates including humans|Disease:invasive candidal disease|Description:CAUTION: The sequence shown

															here is derived from an EMBL/GenBank/DDBJ whole genome shotgun (WGS) entry which is preliminary data.
Chr02G1557.1	359	5	359	UniProt ID:A0ST44_CERNC	357	6	350	105/373(28.15)	0.44	0.12	373	105	2.00E-26	gene=Chr02G1557	Gene Symbol:CTB6|Host:Numerous taxa in Solanaceae|Disease:Leaf spot|Description:Unknown
Chr02G1563.1	863	81	489	UniProt ID:Q0WXM3_FUSOX	663	40	450	108/448(24.11)	0.37	0.17	448	79	5.00E-16	gene=Chr02G1563	Gene Symbol:FOW2|Host:Multiple genera in multiple families|Disease:Blights, wilts, rots of various sorts|Description:SIMILARITY: Contains 1 Zn(2)-C6 fungal-type DNA-binding domain.
Chr02G1566.1	453	331	418	UniProt ID:Q8NK75_GLOLA	697	548	633	31/91(34.07)	0.47	0.09	91	48.9	4.00E-07	gene=Chr02G1566	Gene Symbol:CST1|Host:melons,cucumber|Disease:anthracnose fruit rot|Description:Unknown
Chr02G1567.1	550	12	141	UniProt ID:C7G	712	27	150	37/131(28.24)	0.51	0.06	131	57.4	1.00E-09	gene=Chr02G1567	Gene Symbol:GCD6|Host:huma

				MI4_Y EAS2										ns|Disease:occasional infection|Description:CAUTION: The sequence shown here is derived from an EMBL/GenBank/DDBJ whole genome shotgun (WGS) entry which is preliminary data.
Chr02G1 572.1	226	39	201	UniProt ID:CUTI_BOTFU	202	26	188	89/163 (54.60)	0.74	0	163	186	1.00E-59	gene=Chr 02G1572	Gene Symbol:CUTA|Host:Various plant families|Disease:Grey mould. Parasite or saprophyte|Description:FUNCTION: Catalyzes the hydrolysis of cutin, a polyester that forms the structure of plant cuticle. Allows pathogenic fungi to penetrate through the cuticular barrier into the host plant during the initial stage of the fungal infection (By similarity).
Chr02G1	507	77	290	UniProt	565	119	347	63/234	0.42	0.11	234	58.2	5.00E-10	gene=Chr	Gene

577.1				ID:Q2VLJ1_GIBZA				(26.92)						02G1577	Symbol:ZEB1|Host:Principal hosts: Poaceae, including Zea mays (corn), Triticum aestivum (wheat), and Oryza sativa (rice). Additional hosts: various plant families|Disease:Seedling blight, pre- and post-emergence blight, root and foot rot, brown rot, culm decay, head or kernel blight (scab or ear scab) of cereals. Leaf|Description:Unknown Gene
Chr02G1583.1	1168	647	927	UniProt ID:TUP1_CANAL	514	201	509	119/311(38.26)	0.54	0.1	311	197	1.00E-54	gene=Chr02G1583	Symbol:TUP1|Host:Isolated from a wide variety of substrates including humans|Disease:invasive candidal disease|Description:FUNCTION: Represses transcription by RNA polymerase II. Represses genes responsible for

															initiating filamentous growth and this repression is lifted under inducing environmental conditions.
Chr02G1589.1	536	46	510	UniProt ID:Q5XTQ4_BOTFU	574	53	545	156/539(28.94)	0.4	0.22	539	115	4.00E-28	gene=Chr02G1589	Gene Symbol:LIP1|Host:Various plant families|Disease:Grey mould. Parasite or saprophyte|Description:Unknown
Chr02G1591.1	473	14	441	UniProt ID:Q5ANE1_CANAL	748	44	496	124/456(27.19)	0.49	0.07	456	168	3.00E-46	gene=Chr02G1591	Gene Symbol:SNF3|Host:Isolated from a wide variety of substrates including humans|Disease:invasive candidal disease|Description:SIMILARITY: Belongs to the major facilitator superfamily. Sugar transporter (TC 2.A.1.1) family.
Chr02G1596.1	503	77	339	UniProt ID:Q9P8L8_B	598	137	406	68/282(24.11)	0.44	0.11	282	60.1	1.00E-10	gene=Chr02G1596	Gene Symbol:BCMFS1|Host:Various plant

				OTFU										families|Disease:Grey mould. Parasite or saprophyte|Description:Unknown
Chr02G1597.1	398	2	397	UniProt ID:Q5ANE1_CANAL	748	154	538	105/406(25.86)	0.42	0.08	406	129	3.00E-33	gene=Chr02G1597	Gene Symbol:SNF3|Host:Isolated from a wide variety of substrates including humans|Disease:invasive candidal disease|Description:SIMILARITY: Belongs to the major facilitator superfamily. Sugar transporter (TC 2.A.1.1) family.
Chr02G1601.1	609	27	607	UniProt ID:Q4P8E8_USTMA	693	79	685	197/630(31.27)	0.46	0.11	630	222	7.00E-64	gene=Chr02G1601	Gene Symbol:UM03615.1|Host: Euchlaena spp., Zea spp. (Poaceae)|Disease:Smut. Corn smut|Description:COFACTOR: FAD (By similarity).
Chr02G1612.1	262	3	254	UniProt ID:Q75WR5_9	265	8	262	72/265(27.17)	0.44	0.09	265	82	2.00E-19	gene=Chr02G1612	Gene Symbol:BRN1|Host:Belamcanda chinensis:

				PLEO										Korea,Gladiolus ?gandavensis: Korea,Iris japonica: China,Iris missouriensis (Leaf spot.): Idaho; Montana; Oregon; Washington,Iris sp. (Leaf spot.): China; Texas; Washing|Disease:Leaf spot|Description:SIMILARITY: Belongs to the short-chain dehydrogenases/reductases (SDR) family.
Chr02G1613.1	595	179	582	UniProt ID:Q5ANE1_CANAL	748	91	503	102/417(24.46)	0.44	0.04	417	117	2.00E-28	gene=Chr02G1613	Gene Symbol:SNF3|Host:Isolated from a wide variety of substrates including humans|Disease:invasive candidal disease|Description:SIMILARITY: Belongs to the major facilitator superfamily. Sugar transporter (TC 2.A.1.1) family.
Chr02G1	281	8	197	UniProt	265	11	199	52/198	0.41	0.09	198	47.4	2.00E-07	gene=Chr	Gene

614.1				ID:Q75 WR5_9 PLEO				(26.26)						02G1614	Symbol:BRN1|Host:Belam canda chinensis: Korea,Gladiolus ?gandav ensis: Korea,Iris japonica: China,Iris missouriensis (Leaf spot.): Idaho; Montana; Oregon; Washington,Iris sp. (Leaf spot.): China; Texas; Washing|Disease:Leaf spot|Description:SIMILARI TY: Belongs to the short-chain dehydrogenases/reductas es (SDR) family.
Chr02G1 616.1	268	1	201	UniProt ID:Q75 WR5_9 PLEO	265	7	200	55/209 (26.32)	0.45	0.11	209	63.2	1.00E-12	gene=Chr 02G1616	Gene Symbol:BRN1|Host:Belam canda chinensis: Korea,Gladiolus ?gandav ensis: Korea,Iris japonica: China,Iris missouriensis (Leaf spot.): Idaho; Montana; Oregon; Washington,Iris sp. (Leaf spot.): China; Texas; Washing|Disease:Leaf

															spot|Description:SIMILARITY: Belongs to the short-chain dehydrogenases/reductases (SDR) family.
Chr02G1622.1	400	2	397	UniProt ID:ORYZ_ASPFU	403	1	402	167/413(40.44)	0.56	0.07	413	279	3.00E-90	gene=Chr02G1622	Gene Symbol:ALP1|Host:humans|Disease:infection|Description:FUNCTION: Secreted alkaline protease that allows assimilation of proteinaceous substrates. Acts as a significant virulence factor in invasive aspergillosis. Involved in immune evasion from the human and mice complement systems during infection. Efficiently cleaves important components of the complement cascade such as such as C3, C4, C5, and C1q, as well as IgG, which leads to down-

															regulation of complement activation at the hyphal surface.
Chr02G1624.1	540	48	533	UniProt ID:Q59RG0_CANAL	581	119	574	104/499(20.84)	0.4	0.11	499	77	7.00E-16	gene=Chr02G1624	Gene Symbol:NAG4|Host:Isolated from a wide variety of substrates including humans|Disease:invasive candidal disease|Description:CAUTION: The sequence shown here is derived from an EMBL/GenBank/DDBJ whole genome shotgun (WGS) entry which is preliminary data.
Chr02G1625.1	1041	128	252	UniProt ID:Q59VF3_CANAL	1813	69	192	43/125(34.40)	0.51	0.01	125	48.1	2.00E-06	gene=Chr02G1625	Gene Symbol:"DUR1,2"|Host:Isolated from a wide variety of substrates including humans|Disease:invasive candidal disease|Description:CAUTION: The sequence shown here is derived from an EMBL/GenBank/DDBJ

														whole genome shotgun (WGS) entry which is preliminary data.
Chr02G1631.1	562	11	475	UniProt ID:Q9P8L8_BOTFU	598	56	516	114/467(24.41)	0.44	0.02	467	147	4.00E-39	gene=Chr02G1631	Gene Symbol:BCMFS1|Host:Various plant families|Disease:Grey mould. Parasite or saprophyte|Description:Unknown
Chr02G1632.1	1008	62	544	UniProt ID:Q6ZX14_MAGGR	4034	3088	3605	150/539(27.83)	0.46	0.14	539	151	7.00E-38	gene=Chr02G1632	Gene Symbol:ACE1|Host:Digitaria (Poaceae)|Disease:Leaf spot|Description:Unknown
Chr02G1636.1	1016	281	582	UniProt ID:A6N6J8_FUSOX	903	239	522	73/324(22.53)	0.38	0.19	324	52	1.00E-07	gene=Chr02G1636	Gene Symbol:CTF1|Host:Multiple genera in multiple families|Disease:Blights, wilts, rots of various sorts|Description:SIMILARITY: Contains 1 Zn(2)-C6 fungal-type DNA-binding domain.
Chr02G1638.1	647	9	60	UniProt ID:Q59M50_C	578	36	91	26/57(45.61)	0.54	0.11	57	48.9	6.00E-07	gene=Chr02G1638	Gene Symbol:CWT1|Host:Isolated from a wide variety of

				ANAL										substrates including humans|Disease:invasive candidal disease|Description:CAUTION: The sequence shown here is derived from an EMBL/GenBank/DDBJ whole genome shotgun (WGS) entry which is preliminary data.
Chr02G1644.1	519	60	509	UniProt ID:Q5ANE1_CANAL	748	52	503	113/474(23.84)	0.41	0.1	474	113	2.00E-27	gene=Chr02G1644	Gene Symbol:SNF3|Host:Isolated from a wide variety of substrates including humans|Disease:invasive candidal disease|Description:SIMILARITY: Belongs to the major facilitator superfamily. Sugar transporter (TC 2.A.1.1) family.
Chr02G1657.1	1252	391	667	UniProt ID:Q9Y880_COCCA	880	162	371	75/277 (27.08)	0.42	0.24	277	90.1	4.00E-19	gene=Chr02G1657	Gene Symbol:SNF1|Host:Corn, Zea mays, sometimes on Sorghum (Poaceae) and

															various other plant families|Disease:Northern corn leaf spot, ear and kernel rot|Description:Unknown
Chr02G1658.1	1931	934	1923	UniProt ID:Q59QA6_CANAL	1100	3	1097	292/1130(25.84)	0.46	0.15	1130	367	2.00E-106	gene=Chr02G1658	Gene Symbol:VPS11|Host:Isolated from a wide variety of substrates including humans|Disease:invasive candidal disease|Description:CAUTION: The sequence shown here is derived from an EMBL/GenBank/DDBJ whole genome shotgun (WGS) entry which is preliminary data.
Chr02G1669.1	1900	1355	1707	UniProt ID:Q75T35_GLOLA	2143	738	1071	110/365(30.14)	0.46	0.12	365	107	6.00E-24	gene=Chr02G1669	Gene Symbol:CAC1|Host:melons,cucumber|Disease:anthracnose fruit rot|Description:Unknown
Chr02G1675.1	474	182	406	UniProt ID:Q5GFD3_P	437	167	385	65/231(28.14)	0.43	0.08	231	85.5	7.00E-19	gene=Chr02G1675	Gene Symbol:NULL|Host:Multiple genera of Poaceae and

			HAND												Blysmus compressus (Cyperaceae)|Disease:Glume blotch of wheat and other grasses|Description:Unknown
Chr02G1676.1	580	255	494	UniProt ID:Q9C0P7_NECHA	247	2	241	106/241(43.98)	0.61	0.01	241	196	6.00E-59	gene=Chr02G1676	Gene Symbol:PEP5|Host:Trees of various plant families|Disease:Fruit rot, stem rot|Description:Unknown
Chr02G1679.1	354	23	274	UniProt ID:Q9Y784_MAGGR	631	125	377	76/253 (30.04)	0.52	0	253	122	1.00E-31	gene=Chr02G1679	Gene Symbol:PTH11|Host:Digitaria (Poaceae)|Disease:Leaf spot|Description:Unknown
Chr02G1682.1	775	503	765	UniProt ID:O93802_ALTAL	267	9	263	66/278 (23.74)	0.41	0.14	278	71.2	1.00E-14	gene=Chr02G1682	Gene Symbol:BRM2|Host:Plant|Disease:Leaf spot, rots|Description:SIMILARITY: Belongs to the short-chain dehydrogenases/reductases (SDR) family.
Chr02G1	554	126	519	UniProt	517	129	516	76/417	0.38	0.12	417	52	5.00E-08	gene=Chr	Gene

683.1				ID:A4ULJ1_MYCGR				(18.23)						02G1683	Symbol:CYP51|Host:Triticum and possibly a few other grasses|Disease:Leaf spot or speckled leaf blotch of wheat|Description:COFACTOR: Heme group (By similarity).
Chr02G1689.1	379	126	303	UniProt ID:PLYB_COLGL	331	96	272	57/196 (29.08)	0.47	0.19	196	59.3	7.00E-11	gene=Chr02G1689	Gene Symbol:PLB|Host:Multiple genera in multiple families|Disease:'Anthracnose of stems and leaves, dieback, root rot, leaf spot, blossom rot, fruit rot (dieback and ripe rot), seedling blight.' (Mordue 1971)|Description:FUNCTION: Acts as a virulence factor active in plant tissue maceration.
Chr02G1690.1	1234	71	1201	UniProt ID:A4RGC8_MAGO7	1158	31	1132	459/1162(39.50)	0.57	0.08	1162	781	0	gene=Chr02G1690	Gene Symbol:MGG_11671|Host:Poaceae, especially important on Oryzae|Disease:Rice

Chr02G1691.1	333	25	201	UniProt ID:A4R3I5_MAGO7	400	104	274	46/183 (25.14)	0.45	0.1	183	47	7.00E-07	gene=Chr02G1691	blast|Description:SIMILARITY: Contains 1 reverse transcriptase domain. Gene Symbol:"MGG_11993, MGG_12837, MGG_13052"|Host:Poaceae, especially important on Oryzae|Disease:Rice blast|Description:Unknown
Chr02G1694.1	378	120	321	UniProt ID:PLYB_COLGL	331	94	295	60/223 (26.91)	0.44	0.19	223	52.8	9.00E-09	gene=Chr02G1694	Gene Symbol:PLB|Host:Multiple genera in multiple families|Disease:'Anthracnose of stems and leaves, dieback, root rot, leaf spot, blossom rot, fruit rot (dieback and ripe rot), seedling blight.' (Mordue 1971)|Description:FUNCTION: Acts as a virulence factor active in plant tissue maceration.
Chr02G1697.1	794	22	425	UniProt ID:A6N	903	46	419	93/429 (21.68)	0.37	0.19	429	68.2	1.00E-12	gene=Chr02G1697	Gene Symbol:CTF1|Host:Multipl

				6J8_FUSOX										e genera in multiple families|Disease:Blights, wilts, rots of various sorts|Description:SIMILARITY: Contains 1 Zn(2)-C6 fungal-type DNA-binding domain.
Chr02G1700.1	1367	534	754	UniProt ID:O59928_HYPVI	430	66	299	65/257 (25.29)	0.42	0.23	257	84	1.00E-17	gene=Chr02G1700	Gene Symbol:NULL|Host:humans|Disease:infection|Description:SIMILARITY: Belongs to the glycosyl hydrolase 18 family.
Chr02G1703.1	389	125	361	UniProt ID:Q0PND8_MAGGR	1375	989	1229	96/244 (39.34)	0.57	0.04	244	159	4.00E-43	gene=Chr02G1703	Gene Symbol:PEX6|Host:Digitaria (Poaceae)|Disease:Leaf spot|Description:SIMILARITY: Belongs to the AAA ATPase family.
Chr02G1707.1	1640	355	534	UniProt ID:Q9HFW4_USTMD	2289	660	859	75/201 (37.31)	0.51	0.11	201	134	2.00E-32	gene=Chr02G1707	Gene Symbol:RUM1|Host:Euchlaena spp., Zea spp. (Poaceae)|Disease:Smut. Corn smut|Description:SIMILARITY: Contains 1 ARID

															domain.
Chr02G1709.1	273	98	268	UniProt ID:Q75WR5_9PLEO	265	79	262	49/189 (25.93)	0.48	0.12	189	78.2	4.00E-18	gene=Chr02G1709	Gene Symbol:BRN1|Host:Belamcanda chinensis: Korea,Gladiolus ?gandavensis: Korea,Iris japonica: China,Iris missouriensis (Leaf spot.): Idaho; Montana; Oregon; Washington,Iris sp. (Leaf spot.): China; Texas; Washing|Disease:Leaf spot|Description:SIMILARITY: Belongs to the short-chain dehydrogenases/reductases (SDR) family.
Chr02G1713.1	662	382	661	UniProt ID:TUP1_CANAL	514	177	468	77/309 (24.92)	0.42	0.15	309	71.2	6.00E-14	gene=Chr02G1713	Gene Symbol:TUP1|Host:Isolated from a wide variety of substrates including humans|Disease:invasive candidal disease|Description:FUNCTION: Represses transcription by RNA

															polymerase II. Represses genes responsible for initiating filamentous growth and this repression is lifted under inducing environmental conditions.
Chr02G1714.1	335	1	308	UniProt ID:CDC10_CANAL	357	3	313	184/311(59.16)	0.81	0.01	311	421	1.00E-147	gene=Chr02G1714	Gene Symbol:CDC10|Host:Isolated from a wide variety of substrates including humans|Disease:invasive candidal disease|Description:FUNCTION: Plays a role in the cell cycle. Involved in the formation of the ring of filaments in the neck region at the mother-bud junction during mitosis.
Chr02G1716.1	483	3	387	UniProt ID:GEL1_ASPFU	452	6	379	160/395(40.51)	0.57	0.08	395	271	7.00E-86	gene=Chr02G1716	Gene Symbol:GEL1|Host:humans|Disease:infection|Description:FUNCTION: Splits internally a 1,3-beta-glucan molecule and transfers the newly

															generated reducing end (the donor) to the non-reducing end of another 1,3-beta-glucan molecule (the acceptor) forming a 1,3-beta linkage, resulting in the elongation of 1,3-beta-glucan chains in the cell wall. Involved in cell wall morphogenesis (By similarity).
Chr02G1727.1	568	62	531	UniProt ID:Q59RG1_CANAL	561	89	557	148/476(31.09)	0.51	0.03	476	203	2.00E-58	gene=Chr02G1727	Gene Symbol:NAG3|Host:Isolated from a wide variety of substrates including humans|Disease:invasive candidal disease|Description:CAUTION: The sequence shown here is derived from an EMBL/GenBank/DDBJ whole genome shotgun (WGS) entry which is preliminary data.
Chr02G1731.1	646	103	645	UniProt ID:Q2V	565	27	562	267/545(48.9	0.65	0.02	545	545	0	gene=Chr02G1731	Gene Symbol:ZEB1|Host:Princip

				LJ1_GIBZA				9)						al hosts: Poaceae, including Zea mays (corn), Triticum aestivum (wheat), and Oryza sativa (rice). Additional hosts: various plant families|Disease:Seedling blight, pre- and post-emergence blight, root and foot rot, brown rot, culm decay, head or kernel blight (scab or ear scab) of cereals. Leaf|Description:Unknown Gene
Chr02G1732.1	471	13	470	UniProt ID:Q01446_NECHA	459	18	458	162/460(35.22)	0.54	0.05	460	276	2.00E-87	gene=Chr02G1732	Symbol:MAK1|Host:Trees of various plant families|Disease:Fruit rot, stem rot|Description:Unknown Gene
Chr02G1734.1	168	3	157	UniProt ID:O93802_ALTAL	267	115	263	39/161 (24.22)	0.45	0.11	161	50.8	5.00E-09	gene=Chr02G1734	Symbol:BRM2|Host:Plant|Disease:Leaf spot, rots|Description:SIMILARITY: Belongs to the short-chain

														dehydrogenases/reductases (SDR) family.
Chr02G1736.1	591	151	328	UniProt ID:Q0WXM3_FUSOX	663	264	444	46/190(24.21)	0.41	0.11	190	45.4	7.00E-06	gene=Chr02G1736	Gene Symbol:FOW2|Host:Multiple genera in multiple families|Disease:Blights, wilts, rots of various sorts|Description:SIMILARITY: Contains 1 Zn(2)-C6 fungal-type DNA-binding domain.
Chr02G1740.1	633	15	404	UniProt ID:F2QYD1_PICP7	758	201	585	121/423(28.61)	0.49	0.17	423	162	4.00E-43	gene=Chr02G1740	Gene Symbol:MAK5|Host:humans|Disease:occasional infection|Description:SIMILARITY: Belongs to the DEAD box helicase family.
Chr02G1746.1	816	6	572	UniProt ID:O93841_9PEZI	914	49	618	253/588(43.03)	0.6	0.07	588	474	6.00E-154	gene=Chr02G1746	Gene Symbol:CHIP6|Host:Multiple genera in multiple families|Disease:'Anthracnose of stems and leaves, dieback, root rot, leaf spot, blossom rot, fruit rot (dieback and ripe rot), seedling blight.' (Mordue

														1971)|Description:Unknown
Chr02G1748.1	606	5	605	UniProt ID:Q2VLJ1_GIBZA	565	11	564	193/603(32.01)	0.49	0.08	603	266	2.00E-81	gene=Chr02G1748	Gene Symbol:ZEB1|Host:Principal hosts: Poaceae, including Zea mays (corn), Triticum aestivum (wheat), and Oryza sativa (rice). Additional hosts: various plant families|Disease:Seedling blight, pre- and post-emergence blight, root and foot rot, brown rot, culm decay, head or kernel blight (scab or ear scab) of cereals. Leaf|Description:Unknown
Chr02G1750.1	762	254	512	UniProt ID:Q0PND8_MAGGR	1375	1019	1286	98/287 (34.15)	0.51	0.16	287	125	3.00E-30	gene=Chr02G1750	Gene Symbol:PEX6|Host:Digitaria (Poaceae)|Disease:Leaf spot|Description:SIMILARITY: Belongs to the AAA ATPase family.
Chr02G1751.1	1832	1576	1794	UniProt ID:Q87	1441	1132	1340	58/227 (25.55)	0.44	0.11	227	65.9	2.00E-11	gene=Chr02G1751	Gene Symbol:SQL2|Host:Euchl

			5L6_USTMD												aena spp., Zea spp. (Poaceae)|Disease:Smut. Corn smut|Description:SIMILARITY: Contains 1 N-terminal Ras-GEF domain.
Chr02G1760.1	451	6	359	UniProt ID:A4UC81_MAGO7	376	19	373	138/358(38.55)	0.59	0.02	358	252	1.00E-79	gene=Chr02G1760	Gene Symbol:MGG_10702|Host:Poaceae, especially important on Oryzae|Disease:Rice blast|Description:Unknown
Chr02G1761.1	384	67	381	UniProt ID:Q5A415_CANAL	361	37	357	102/331(30.82)	0.53	0.08	331	140	1.00E-38	gene=Chr02G1761	Gene Symbol:ARP2|Host:Isolated from a wide variety of substrates including humans|Disease:invasive candidal disease|Description:SIMILARITY: Belongs to the actin family.
Chr02G1767.1	324	44	324	UniProt ID:MEP1_COCP7	276	1	274	178/282(63.12)	0.77	0.03	282	357	1.00E-123	gene=Chr02G1767	Gene Symbol:MEP1|Host:humans|Disease:coccidiomycosis|Description:FUNCTION:

Chr02G1768.1	376	1	372	UniProt ID:LAP1_ARTOC	373	1	370	163/390(41.79)	0.57	0.1	390	275	9.00E-90	gene=Chr02G1768	Secreted metalloproteinase that allows assimilation of proteinaceous substrates. Pays a pivotal role as a pathogenicity determinant during infections and contributes to the ability of the pathogen to persist within the mammalian host. Digests an immunodominant cell surface antigen (SOWgp) and prevents host recognition of endospores during the phase of development when these fungal cells are most vulnerable to phagocytic cell defenses. Gene Symbol:LAP1|Host:humans, reptiles|Disease:dermatophytoses|Description:FUNCTION: Extracellular

															aminopeptidase which contributes to pathogenicity (By similarity).
Chr02G1771.1	502	58	497	UniProt ID:Q5ABU7_CANAL	564	105	564	117/470(24.89)	0.42	0.09	470	152	5.00E-41	gene=Chr02G1771	Gene Symbol:MDR1|Host:Isolated from a wide variety of substrates including humans|Disease:invasive candidal disease|Description:Unknown
Chr02G1773.1	456	131	376	UniProt ID:Q6A2T2_BOTFU	391	130	385	63/266(23.68)	0.44	0.11	266	60.1	8.00E-11	gene=Chr02G1773	Gene Symbol:BTP1|Host:Various plant families|Disease:Grey mould. Parasite or saprophyte|Description:Unknown
Chr02G1774.1	618	89	380	UniProt ID:P87198_USTMD	1459	306	631	100/349(28.65)	0.45	0.23	349	103	9.00E-24	gene=Chr02G1774	Gene Symbol:KIN1|Host:Euchlaena spp., Zea spp. (Poaceae)|Disease:Smut. Corn smut|Description:SIMILARITY: Belongs to the

														kinesin-like protein family.
Chr02G1777.1	163	12	162	UniProt ID:Q6A2T2_BOTFU	391	113	273	41/162 (25.31)	0.46	0.07	162	49.3	2.00E-08	gene=Chr02G1777	Gene Symbol:BTP1|Host:Various plant families|Disease:Grey mould. Parasite or saprophyte|Description:Unknown
Chr02G1779.1	597	6	597	UniProt ID:Q6Y392_PHAND	625	36	613	180/609(29.56)	0.5	0.08	609	283	7.00E-87	gene=Chr02G1779	Gene Symbol:NULL|Host:Multiple genera of Poaceae and Blysmus compressus (Cyperaceae)|Disease:Glume blotch of wheat and other grasses|Description:Unknown
Chr02G1780.1	293	186	283	UniProt ID:Q4WWN9_ASPFU	308	179	271	26/98(26.53)	0.5	0.05	98	46.6	6.00E-07	gene=Chr02G1780	Gene Symbol:AFUA_3G06540|Host:humans|Disease:infection|Description:CAUTION: The sequence shown here is derived from an EMBL/GenBank/DDBJ whole genome shotgun (WGS) entry which is

															preliminary data.
Chr02G1781.1	421	35	273	UniProt ID:Q6A2T2_BOTFU	391	43	293	73/251 (29.08)	0.48	0.05	251	102	3.00E-25	gene=Chr02G1781	Gene Symbol:BTP1|Host:Various plant families|Disease:Grey mould. Parasite or saprophyte|Description:Unknown
Chr02G1789.1	809	122	723	UniProt ID:A3LS85_PICST	677	17	602	176/637(27.63)	0.45	0.14	637	187	5.00E-51	gene=Chr02G1789	Gene Symbol:LYS4|Host:humans|Disease:occasional infection|Description:Unknown
Chr02G1790.1	528	99	492	UniProt ID:Q5ANE1_CANAL	748	87	496	111/450(24.67)	0.42	0.21	450	89.7	7.00E-20	gene=Chr02G1790	Gene Symbol:SNF3|Host:Isolated from a wide variety of substrates including humans|Disease:invasive candidal disease|Description:SIMILARITY: Belongs to the major facilitator superfamily. Sugar transporter (TC 2.A.1.1) family.
Chr02G1	515	35	511	UniProt	748	51	527	141/48	0.49	0.05	489	187	2.00E-52	gene=Chr	Gene

800.1				ID:Q5ANE1_CANAL				9(28.83)						02G1800	Symbol:SNF3|Host:Isolated from a wide variety of substrates including humans|Disease:invasive candidal disease|Description:SIMILARITY: Belongs to the major facilitator superfamily. Sugar transporter (TC 2.A.1.1) family.
Chr02G1805.1	666	105	665	UniProt ID:Q2VLJ1_GIBZA	565	21	562	202/580(34.83)	0.53	0.1	580	327	2.00E-103	gene=Chr02G1805	Gene Symbol:ZEB1|Host:Principal hosts: Poaceae, including Zea mays (corn), Triticum aestivum (wheat), and Oryza sativa (rice). Additional hosts: various plant families|Disease:Seedling blight, pre- and post-emergence blight, root and foot rot, brown rot, culm decay, head or kernel blight (scab or ear scab) of cereals.

Chr02G1806.1	369	23	278	UniProt ID:Q6A2T2_BOTFU	391	42	290	69/269 (25.65)	0.42	0.12	269	61.2	2.00E-11	gene=Chr02G1806	Leaf|Description:Unknown Gene Symbol:BTP1|Host:Various plant families|Disease:Grey mould. Parasite or saprophyte|Description:Unknown
Chr02G1809.1	687	339	518	UniProt ID:Q5ALS7_CANAL	1144	472	648	44/186 (23.66)	0.38	0.08	186	49.7	4.00E-07	gene=Chr02G1809	Gene Symbol:CTF1|Host:Isolated from a wide variety of substrates including humans|Disease:invasive candidal disease|Description:SIMILARITY: Contains 1 Zn(2)-C6 fungal-type DNA-binding domain.
Chr02G1810.1	473	90	428	UniProt ID:Q9C1T0_FUSOX	467	101	433	75/349 (21.49)	0.43	0.07	349	62.8	1.00E-11	gene=Chr02G1810	Gene Symbol:ARG1|Host:Multiple genera in multiple families|Disease:Blights, wilts, rots of various sorts|Description:Unknown
Chr02G1	395	121	325	UniProt	331	92	289	52/223	0.4	0.19	223	47.4	6.00E-07	gene=Chr	Gene

812.1				ID:PLYB_COLGL				(23.32)						02G1812	Symbol:PLB|Host:Multiple genera in multiple families|Disease:'Anthracnose of stems and leaves, dieback, root rot, leaf spot, blossom rot, fruit rot (dieback and ripe rot), seedling blight.' (Mordue 1971)|Description:FUNCTION: Acts as a virulence factor active in plant tissue maceration.
Chr02G1817.1	214	1	214	UniProt ID:A4R2V5_MAGO7	214	1	214	178/214(83.18)	0.92	0	214	369	3.00E-131	gene=Chr02G1817	Gene Symbol:MGG_02423|Host:Poaceae, especially important on Oryzae|Disease:Rice blast|Description:FUNCTION: Required for the retention of luminal endoplasmic reticulum proteins. Determines the specificity of the luminal ER protein retention system. Also required for normal vesicular traffic

															through the Golgi (By similarity).
Chr02G1822.1	846	9	846	UniProt ID:Q2TJF8_GIBMO	823	1	823	601/851(70.62)	0.8	0.05	851	1174	0	gene=Chr02G1822	Gene Symbol:FSR1|Host:Chrysanthemum sp.: Brazil ,Gossypium sp.: China,Saccharum officinarum: Taiwan,Litchi chinensis: China,Musa nana: China,Phyllostachys sp.: China,Sansevieria trifasciata: Hong Kong|Disease:Disease Note: Bakanae disease of rice; fig endosepsis; ear rot of maize|Description:Unknown
Chr02G1825.1	358	50	358	UniProt ID:Q6IVV6_BOTFU	424	118	423	153/310(49.35)	0.67	0.02	310	328	1.00E-109	gene=Chr02G1825	Gene Symbol:CEL5A|Host:Various plant families|Disease:Grey mould. Parasite or saprophyte|Description:Unknown
Chr02G1	346	124	204	UniProt	550	168	250	35/83(	0.57	0.02	83	56.2	9.00E-10	gene=Chr	Gene

831.1				ID:Q59 RB8_C ANAL				42.17)						02G1831	Symbol:ICL1|Host:Isolated from a wide variety of substrates including humans|Disease:invasive candidal disease|Description:SIMILARITY: Belongs to the isocitrate lyase/PEP mutase superfamily. Isocitrate lyase family.
Chr02G1 842.1	582	41	566	UniProt ID:Q5X TQ4_B OTFU	574	44	572	307/52 9(58.0 3)	0.73	0.01	529	637	0	gene=Chr 02G1842	Gene Symbol:LIP1|Host:Various plant families|Disease:Grey mould. Parasite or saprophyte|Description:Unknown
Chr02G1 853.1	417	27	417	UniProt ID:ORY Z_ASP FU	403	33	403	173/40 4(42.8 2)	0.56	0.11	404	262	1.00E-83	gene=Chr 02G1853	Gene Symbol:ALP1|Host:humans|Disease:infection|Description:FUNCTION: Secreted alkaline protease that allows assimilation of proteinaceous substrates. Acts as a significant

														virulence factor in invasive aspergillosis. Involved in immune evasion from the human and mice complement systems during infection. Efficiently cleaves important components of the complement cascade such as such as C3, C4, C5, and C1q, as well as IgG, which leads to down-regulation of complement activation at the hyphal surface.
Chr02G1861.1	657	142	414	UniProt ID:A4R3I5_MAGO7	400	103	350	62/277 (22.38)	0.41	0.12	277	54.3	1.00E-08	gene=Chr02G1861	Gene Symbol:"MGG_11993, MGG_12837, MGG_13052"|Host:Poaceae, especially important on Oryzae|Disease:Rice blast|Description:Unknown
Chr02G1862.1	469	1	419	UniProt ID:A4RGC8_M	1158	723	1146	165/432(38.19)	0.56	0.05	432	277	2.00E-83	gene=Chr02G1862	Gene Symbol:MGG_11671|Host:Poaceae, especially

				AGO7											important on Oryzae|Disease:Rice blast|Description:SIMILARITY: Contains 1 reverse transcriptase domain.
Chr08G0004.1	891	598	823	UniProt ID:TUP1_CANAL	514	258	486	94/238 (39.50)	0.53	0.09	238	145	1.00E-37	gene=Chr08G0004	Gene Symbol:TUP1|Host:Isolated from a wide variety of substrates including humans|Disease:invasive candidal disease|Description:FUNCTION: Represses transcription by RNA polymerase II. Represses genes responsible for initiating filamentous growth and this repression is lifted under inducing environmental conditions.
Chr08G0008.1	1166	991	1144	UniProt ID:A4RGM3_MAGO7	283	129	273	55/158 (34.81)	0.52	0.11	158	86.3	4.00E-19	gene=Chr08G0008	Gene Symbol:MGG_12252|Host:Poaceae, especially important on Oryzae|Disease:Rice blast|Description:Unknow

														n
Chr08G0 012.1	1312	55	125 3	UniProt ID:A4R GC8_M AGO7	1158	10	1155	484/12 27(39. 45)	0.57	0.09	1227	802	0	gene=Chr 08G0012	Gene Symbol:MGG_11671|Host :Poaceae, especially important on Oryzae|Disease:Rice blast|Description:SIMILAR ITY: Contains 1 reverse transcriptase domain.
Chr08G0 016.1	139	11	101	UniProt ID:D2J LS9_9 HYPO	131	36	124	49/91(53.85)	0.7	0.02	91	100	5.00E-28	gene=Chr 08G0016	Gene Symbol:HIS3|Host:plants| Disease:allergy|Descriptio n:SUBUNIT: The nucleosome is a histone octamer containing two molecules each of H2A, H2B, H3 and H4 assembled in one H3-H4 heterotetramer and two H2A-H2B heterodimers. The octamer wraps approximately 147 bp of DNA (By similarity).
Chr08G0 017.1	540	5	532	UniProt ID:Q5A 762_C	1606	1043	1603	158/58 1(27.1 9)	0.5	0.13	581	224	3.00E-63	gene=Chr 08G0017	Gene Symbol:MLT1|Host:Isolate d from a wide variety of

			ANAL											substrates including humans|Disease:invasive candidal disease|Description:SIMILARITY: Belongs to the ABC transporter superfamily.
Chr08G0025.1	683	189	337	UniProt ID:Q0WXM3_FUSOX	663	282	444	42/170 (24.71)	0.45	0.16	170	58.2	9.00E-10	gene=Chr08G0025	Gene Symbol:FOW2|Host:Multiple genera in multiple families|Disease:Blights, wilts, rots of various sorts|Description:SIMILARITY: Contains 1 Zn(2)-C6 fungal-type DNA-binding domain.
Chr08G0026.1	663	270	364	UniProt ID:Q0WXM3_FUSOX	663	337	433	27/97(27.84)	0.52	0.02	97	53.9	2.00E-08	gene=Chr08G0026	Gene Symbol:FOW2|Host:Multiple genera in multiple families|Disease:Blights, wilts, rots of various sorts|Description:SIMILARITY: Contains 1 Zn(2)-C6 fungal-type DNA-binding domain.
Chr08G0	394	2	271	UniProt	631	109	373	80/274	0.47	0.05	274	103	5.00E-25	gene=Chr	Gene

<table>
<tr><td>028.1</td><td></td><td></td><td></td><td>ID:Q9Y784_MAGGR</td><td></td><td></td><td></td><td>(29.20)</td><td></td><td></td><td></td><td></td><td></td><td>08G0028</td><td>Symbol:PTH11|Host:Digitaria (Poaceae)|Disease:Leaf spot|Description:Unknown</td></tr>
<tr><td>Chr08G0032.1</td><td>448</td><td>54</td><td>433</td><td>UniProt ID:Q5A415_CANAL</td><td>361</td><td>23</td><td>351</td><td>109/395(27.59)</td><td>0.42</td><td>0.21</td><td>395</td><td>121</td><td>1.00E-31</td><td>gene=Chr08G0032</td><td>Gene Symbol:ARP2|Host:Isolated from a wide variety of substrates including humans|Disease:invasive candidal disease|Description:SIMILARITY: Belongs to the actin family.</td></tr>
<tr><td>Chr08G0038.1</td><td>393</td><td>36</td><td>265</td><td>UniProt ID:Q75ZG3_ALTAL</td><td>366</td><td>59</td><td>316</td><td>70/273 (25.64)</td><td>0.42</td><td>0.21</td><td>273</td><td>49.3</td><td>2.00E-07</td><td>gene=Chr08G0038</td><td>Gene Symbol:AFTS1|Host:Plant|Disease:Leaf spot, rots|Description:Unknown</td></tr>
<tr><td>Chr08G0042.1</td><td>266</td><td>2</td><td>263</td><td>UniProt ID:Q6TFC7_ASPFM</td><td>349</td><td>86</td><td>347</td><td>79/271 (29.15)</td><td>0.49</td><td>0.07</td><td>271</td><td>117</td><td>2.00E-31</td><td>gene=Chr08G0042</td><td>Gene Symbol:NULL|Host:humans|Disease:infection|Description:Unknown</td></tr>
<tr><td>Chr08G0044.1</td><td>515</td><td>28</td><td>476</td><td>UniProt ID:Q5ANE1_CANAL</td><td>748</td><td>44</td><td>496</td><td>130/468(27.78)</td><td>0.47</td><td>0.07</td><td>468</td><td>141</td><td>1.00E-36</td><td>gene=Chr08G0044</td><td>Gene Symbol:SNF3|Host:Isolated from a wide variety of substrates including humans|Disease:invasive</td></tr>
</table>

															candidal disease|Description:SIMILARITY: Belongs to the major facilitator superfamily. Sugar transporter (TC 2.A.1.1) family.
Chr08G0049.1	306	3	261	UniProt ID:Q9Y784_MAGGR	631	104	356	66/261 (25.29)	0.51	0.04	261	69.7	3.00E-14	gene=Chr08G0049	Gene Symbol:PTH11|Host:Digitaria (Poaceae)|Disease:Leaf spot|Description:Unknown
Chr08G0051.1	682	70	102	UniProt ID:Q5A4F3_CANAL	624	14	46	18/33(54.55)	0.73	0	33	54.3	1.00E-08	gene=Chr08G0051	Gene Symbol:ZCF37|Host:Isolated from a wide variety of substrates including humans|Disease:invasive candidal disease|Description:Unknown
Chr08G0059.1	428	2	388	UniProt ID:A0ST45_CERNC	450	4	422	137/432(31.71)	0.51	0.13	432	189	3.00E-55	gene=Chr08G0059	Gene Symbol:CTB7|Host:Numerous taxa in Solanaceae|Disease:Leaf spot|Description:Unknown
Chr08G0	295	3	288	UniProt	290	4	276	159/28	0.67	0.05	287	292	2.00E-98	gene=Chr	Gene

071.1			ID:Q9P470_MAGGR				7(55.40)						08G0071	Symbol:MAS1|Host:Digitaria (Poaceae)|Disease:Leaf spot|Description:Unknown
Chr08G0073.1	320	23	297	UniProt ID:Q59KY8_CANAL	314	19	297	154/280(55.00)	0.74	0.02	280	327	1.00E-111	gene=Chr08G0073	Gene Symbol:SIT4|Host:Isolated from a wide variety of substrates including humans|Disease:invasive candidal disease|Description:CATALYTIC ACTIVITY: A phosphoprotein + H(2)O = a protein + phosphate.
Chr08G0081.1	229	76	229	UniProt ID:SODC_CANAL	154	1	154	112/154(72.73)	0.82	0	154	233	1.00E-78	gene=Chr08G0081	Gene Symbol:SOD1|Host:Isolated from a wide variety of substrates including humans|Disease:invasive candidal disease|Description:FUNCTION: Destroys radicals which are normally produced within the cells and which are toxic to biological systems.

Chr08G0086.1	586	7	584	UniProt ID:Q2VLJ1_GIBZA	565	9	562	186/591(31.47)	0.5	0.08	591	291	8.00E-91	gene=Chr08G0086	Gene Symbol:ZEB1|Host:Principal hosts: Poaceae, including Zea mays (corn), Triticum aestivum (wheat), and Oryza sativa (rice). Additional hosts: various plant families|Disease:Seedling blight, pre- and post-emergence blight, root and foot rot, brown rot, culm decay, head or kernel blight (scab or ear scab) of cereals. Leaf|Description:Unknown
Chr08G0090.1	916	34	914	UniProt ID:A6R615_AJECN	898	4	896	586/904(64.82)	0.77	0.04	904	1181	0	gene=Chr08G0090	Gene Symbol:HCAG_05073|Host:humans|Disease:Darling's disease|Description:PATHWAY: Amino-acid biosynthesis; L-arginine biosynthesis; N(2)-acetyl-L-ornithine from L-glutamate.

Chr08G0093.1	581	341	550	UniProt ID:A4ULI5_MYCGR	515	257	502	53/254 (20.87)	0.41	0.2	254	60.5	1.00E-10	gene=Chr08G0093	Gene Symbol:CYP51|Host:Triticum and possibly a few other grasses|Disease:Leaf spot or speckled leaf blotch of wheat|Description:COFACTOR: Heme group (By similarity).
Chr08G0100.1	436	4	436	UniProt ID:A3LQ43_PICST	444	3	444	227/449(50.56)	0.69	0.05	449	453	3.00E-157	gene=Chr08G0100	Gene Symbol:LYS9|Host:humans|Disease:occasional infection|Description:Unknown
Chr08G0104.1	494	26	494	UniProt ID:Q5ANE1_CANAL	748	85	539	116/505(22.97)	0.43	0.17	505	124	4.00E-31	gene=Chr08G0104	Gene Symbol:SNF3|Host:Isolated from a wide variety of substrates including humans|Disease:invasive candidal disease|Description:SIMILARITY: Belongs to the major facilitator superfamily. Sugar transporter (TC 2.A.1.1) family.

Chr08G0106.1	521	65	416	UniProt ID:Q2VLJ1_GIBZA	565	104	457	99/365 (27.12)	0.38	0.07	365	80.9	4.00E-17	gene=Chr08G0106	Gene Symbol:ZEB1|Host:Principal hosts: Poaceae, including Zea mays (corn), Triticum aestivum (wheat), and Oryza sativa (rice). Additional hosts: various plant families|Disease:Seedling blight, pre- and post-emergence blight, root and foot rot, brown rot, culm decay, head or kernel blight (scab or ear scab) of cereals. Leaf|Description:Unknown
Chr08G0115.1	163	6	139	UniProt ID:Q6WP53_BOTFU	223	67	203	70/138 (50.72)	0.62	0.04	138	127	3.00E-37	gene=Chr08G0115	Gene Symbol:BCP1|Host:Various plant families|Disease:Grey mould. Parasite or saprophyte|Description:FUNCTION: PPIases accelerate the folding of proteins (By similarity).
Chr08G0	691	197	657	UniProt	493	52	487	109/48	0.43	0.16	487	77	1.00E-15	gene=Chr	Gene

118.1				ID:Q9C0M1_CANAL				7(22.38)						08G0118	Symbol:CANAG5|Host:Isolated from a wide variety of substrates including humans|Disease:invasive candidal disease|Description:Unknown
Chr08G0132.1	586	27	336	UniProt ID:O59937_FUSOX	384	90	384	126/316(39.87)	0.58	0.09	316	205	1.00E-60	gene=Chr08G0132	Gene Symbol:XYL3|Host:Multiple genera in multiple families|Disease:Blights, wilts, rots of various sorts|Description:SIMILARITY: Belongs to the glycosyl hydrolase 10 (cellulase F) family.
Chr08G0133.1	1470	43	345	UniProt ID:Q8X1F0_CRYNE	654	365	625	86/317 (27.13)	0.44	0.22	317	60.8	4.00E-10	gene=Chr08G0133	Gene Symbol:NULL|Host:humans|Disease:cryptococcosis|Description:CATALYTIC ACTIVITY: ATP + a protein = ADP + a phosphoprotein.
Chr08G0138.1	852	160	563	UniProt ID:Q2PEN8_9	575	57	491	97/441 (22.00)	0.38	0.1	441	66.6	3.00E-12	gene=Chr08G0138	Gene Symbol:NOXB|Host:plants|Disease:cool-season

				HYPO											grasses|Description:SIMILARITY: Contains 1 FAD-binding FR-type domain.
Chr08G0141.1	419	14	257	UniProt ID:Q6XVN4_CRYNV	383	13	274	76/271 (28.04)	0.41	0.13	271	69.7	5.00E-14	gene=Chr08G0141	Gene Symbol:GNO1|Host:humans|Disease:cryptococcosis|Description:COFACTOR: Zinc (By similarity).
Chr08G0145.1	1278	20	1276	UniProt ID:Q3Y5V5_MAGGR	1321	5	1321	455/1349(33.73)	0.53	0.09	1349	716	0	gene=Chr08G0145	Gene Symbol:ABC3|Host:Digitaria (Poaceae)|Disease:Leaf spot|Description:SIMILARITY: Belongs to the ABC transporter superfamily.
Chr08G0153.1	497	9	385	UniProt ID:C1G2S7_PARBD	468	8	372	86/400 (21.50)	0.4	0.15	400	81.6	2.00E-17	gene=Chr08G0153	Gene Symbol:PADG_01243|Host:humans|Disease:Paracoccidioidomycosis|Description:Unknown
Chr08G0158.1	535	23	472	UniProt ID:SUB8_COCP7	497	21	458	311/452(68.81)	0.8	0.04	452	640	0	gene=Chr08G0158	Gene Symbol:CPC735_031240|Host:humans|Disease:coccidiomycosis|Description: FUNCTION: Secreted subtilisin-like serine

															protease with keratinolytic activity that contributes to pathogenicity (By similarity).
Chr08G0 166.1	540	52	468	UniProt ID:Q5X TQ4_B OTFU	574	45	512	151/48 0(31.4 6)	0.43	0.16	480	156	3.00E-42	gene=Chr 08G0166	Gene Symbol:LIP1|Host:Various plant families|Disease:Grey mould. Parasite or saprophyte|Description:Un known
Chr08G0 173.1	520	66	520	UniProt ID:A3L RS6_PI CST	454	1	454	275/46 8(58.7 6)	0.71	0.06	468	543	0	gene=Chr 08G0173	Gene Symbol:MET23|Host:hum ans|Disease:occasional infection|Description:Unkn own
Chr08G0 182.1	240	1	225	UniProt ID:Q04 701_F USSO	242	1	224	101/22 7(44.4 9)	0.59	0.02	227	177	4.00E-55	gene=Chr 08G0182	Gene Symbol:PELA|Host:Multipl e plant families. Some strains may cause infections in humans|Disease:Saprobe, facultative pathogen|Description:Unk nown
Chr08G0	588	13	516	UniProt	662	18	565	185/55	0.49	0.1	554	261	1.00E-78	gene=Chr	Gene

183.1				ID:SCPB_TRIRU				4(33.39)						08G0183	Symbol:SCPB|Host:humans|Disease:infection|Description:FUNCTION: Extracellular serine carboxypeptidase that contributes to pathogenicity.
Chr08G0194.1	660	315	659	UniProt ID:D1MYV6_MAGGR	568	137	567	162/431(37.59)	0.51	0.2	431	238	3.00E-70	gene=Chr08G0194	Gene Symbol:CBL1|Host:Digitaria (Poaceae)|Disease:Leaf spot|Description:SIMILARITY: Contains 3 chitin-binding type-1 domains.
Chr08G0196.1	988	224	387	UniProt ID:Q7Z9J3_9PEZI	1372	1164	1309	52/172(30.23)	0.44	0.2	172	51.2	3.00E-07	gene=Chr08G0196	Gene Symbol:CZK3|Host:Zea mays (Poaceae)|Disease:Gray leaf spot of corn|Description:Unknown
Chr08G0197.1	512	53	223	UniProt ID:Q59VF3_CANAL	1813	75	240	67/174(38.51)	0.52	0.06	174	85.5	2.00E-18	gene=Chr08G0197	Gene Symbol:"DUR1,2"|Host:Isolated from a wide variety of substrates including humans|Disease:invasive candidal

															disease|Description:CAUTION: The sequence shown here is derived from an EMBL/GenBank/DDBJ whole genome shotgun (WGS) entry which is preliminary data.
Chr08G0209.1	1345	1038	1243	UniProt ID:TUP1_CANAL	514	260	476	84/218 (38.53)	0.59	0.06	218	156	1.00E-40	gene=Chr08G0209	Gene Symbol:TUP1|Host:Isolated from a wide variety of substrates including humans|Disease:invasive candidal disease|Description:FUNCTION: Represses transcription by RNA polymerase II. Represses genes responsible for initiating filamentous growth and this repression is lifted under inducing environmental conditions.
Chr08G0212.1	967	621	901	UniProt ID:TUP1_CANAL	514	201	509	122/311(39.23)	0.55	0.1	311	200	4.00E-56	gene=Chr08G0212	Gene Symbol:TUP1|Host:Isolated from a wide variety of substrates including

															humans|Disease:invasive candidal disease|Description:FUNCTION: Represses transcription by RNA polymerase II. Represses genes responsible for initiating filamentous growth and this repression is lifted under inducing environmental conditions.
Chr08G0217.1	338	52	334	UniProt ID:Q6TFC7_ASPFM	349	64	346	93/294 (31.63)	0.5	0.07	294	148	8.00E-42	gene=Chr08G0217	Gene Symbol:NULL|Host:humans|Disease:infection|Description:Unknown
Chr08G0225.1	268	6	213	UniProt ID:A4RGG9_MAGO7	286	16	223	71/219 (32.42)	0.47	0.1	219	66.6	7.00E-14	gene=Chr08G0225	Gene Symbol:MGG_00056|Host:Poaceae, especially important on Oryzae|Disease:Rice blast|Description:SIMILARITY: Belongs to the short-chain dehydrogenases/reductases (SDR) family.
Chr08G0	434	15	433	UniProt	616	1	411	152/42	0.53	0.02	420	252	2.00E-77	gene=Chr	Gene

237.1				ID:DHH1_CRYNV				0(36.19)						08G0237	Symbol:VAD1|Host:humans|Disease:cryptococcosis|Description:FUNCTION: ATP-dependent RNA helicase involved in mRNA turnover, and more specifically in mRNA decapping. Is involved in G1/S DNA- damage checkpoint recovery, probably through the regulation of the translational status of a subset of mRNAs. May also have a role in translation and mRNA nuclear export (By similarity). Is involved in virulence.
Chr08G0243.1	506	47	483	UniProt ID:A0ST42_CERNC	512	59	445	104/448(23.21)	0.39	0.16	448	45.1	7.00E-06	gene=Chr08G0243	Gene Symbol:CTB4|Host:Numerous taxa in Solanaceae|Disease:Leaf spot|Description:Unknown
Chr08G0251.1	436	22	429	UniProt ID:Q96	455	30	443	258/414(62.3	0.75	0.01	414	511	1.00E-179	gene=Chr08G0251	Gene Symbol:PGX1|Host:Multipl

				VZ3_F USOX				2)							e genera in multiple families|Disease:Blights, wilts, rots of various sorts|Description:SIMILARITY: Belongs to the glycosyl hydrolase 28 family.
Chr08G0252.1	408	43	363	UniProt ID:Q8X116_BOTFU	348	35	313	91/326 (27.91)	0.42	0.16	326	72.4	5.00E-15	gene=Chr08G0252	Gene Symbol:BCPME2|Host:Various plant families|Disease:Grey mould. Parasite or saprophyte|Description:CATALYTIC ACTIVITY: Pectin + n H(2)O = n methanol + pectate.
Chr08G0261.1	958	627	890	UniProt ID:TUP1_CANAL	514	201	486	115/290(39.66)	0.56	0.1	290	188	6.00E-52	gene=Chr08G0261	Gene Symbol:TUP1|Host:Isolated from a wide variety of substrates including humans|Disease:invasive candidal disease|Description:FUNCTION: Represses transcription by RNA polymerase II. Represses

															genes responsible for initiating filamentous growth and this repression is lifted under inducing environmental conditions.
Chr08G0264.1	552	475	552	UniProt ID:Q59NL7_CANAL	781	692	781	34/92(36.96)	0.57	0.17	92	51.6	8.00E-08	gene=Chr08G0264	Gene Symbol:WAL1|Host:Isolated from a wide variety of substrates including humans|Disease:invasive candidal disease|Description:CAUTION: The sequence shown here is derived from an EMBL/GenBank/DDBJ whole genome shotgun (WGS) entry which is preliminary data.
Chr08G0273.1	761	647	699	UniProt ID:C1G7T1_PARBD	534	89	143	24/55(43.64)	0.51	0.04	55	52.8	4.00E-08	gene=Chr08G0273	Gene Symbol:PADG_03236|Host:humans|Disease:Paracoccidioidomycosis|Description:Unknown
Chr08G0277.1	578	1	565	UniProt ID:Q96VB5_A	578	1	562	243/571(42.56)	0.61	0.03	571	432	6.00E-145	gene=Chr08G0277	Gene Symbol:AFT1-1|Host:Plant|Disease:Leaf spot,

				LTAL											rots|Description:Unknown
Chr08G0278.1	920	754	821	UniProt ID:Q00639_BLUGR	249	182	249	24/68(35.29)	0.49	0	68	45.1	7.00E-06	gene=Chr08G0278	Gene Symbol:GEGH7|Host:Poaceae|Disease:Powdery mildew|Description:Unknown
Chr08G0279.1	584	48	401	UniProt ID:CARP2_CANAL	398	54	385	123/373(32.98)	0.47	0.16	373	150	4.00E-41	gene=Chr08G0279	Gene Symbol:SAP2|Host:Isolated from a wide variety of substrates including humans|Disease:invasive candidal disease|Description:CATALYTIC ACTIVITY: Preferential cleavage at the carboxyl of hydrophobic amino acids, but fails to cleave 15-Leu-|-Tyr-16, 16-Tyr-|-Leu-17 and 24-Phe-|-Phe-25 of insulin B chain. Activates trypsinogen, and degrades keratin.
Chr08G0282.1	1189	626	906	UniProt ID:TUP	514	201	509	116/311(37.3	0.55	0.1	311	191	2.00E-52	gene=Chr08G0282	Gene Symbol:TUP1|Host:Isolate

				1_CANAL				0)							d from a wide variety of substrates including humans|Disease:invasive candidal disease|Description:FUNCTION: Represses transcription by RNA polymerase II. Represses genes responsible for initiating filamentous growth and this repression is lifted under inducing environmental conditions.
Chr08G0292.1	362	100	361	UniProt ID:Q32WF7_PHAND	266	15	265	103/265(38.87)	0.57	0.06	265	168	5.00E-50	gene=Chr08G0292	Gene Symbol:MDH1|Host:Multiple genera of Poaceae and Blysmus compressus (Cyperaceae)|Disease:Glume blotch of wheat and other grasses|Description:Unknown
Chr08G0297.1	617	115	179	UniProt ID:Q59ZX1_CANAL	1069	351	412	24/66(36.36)	0.47	0.08	66	49.7	4.00E-07	gene=Chr08G0297	Gene Symbol:SET3|Host:Isolated from a wide variety of substrates including

														humans|Disease:invasive candidal disease|Description:SIMILARITY: Contains 1 SET domain.
Chr08G0300.1	1360	793	1357	UniProt ID:P78585_BOTFU	994	339	951	201/669(30.04)	0.43	0.24	669	213	2.00E-57	gene=Chr08G0300	Gene Symbol:BCPLC1|Host:Various plant families|Disease:Grey mould. Parasite or saprophyte|Description:SIMILARITY: Contains 1 C2 domain.
Chr08G0309.1	2384	232	699	UniProt ID:Q873Z8_FUSOX	1863	83	572	123/514(23.93)	0.42	0.14	514	97.1	1.00E-20	gene=Chr08G0309	Gene Symbol:CHSV|Host:Multiple genera in multiple families|Disease:Blights, wilts, rots of various sorts|Description:Unknown
Chr08G0317.1	1459	1086	1366	UniProt ID:TUP1_CANAL	514	201	509	129/311(41.48)	0.57	0.1	311	221	2.00E-62	gene=Chr08G0317	Gene Symbol:TUP1|Host:Isolated from a wide variety of substrates including humans|Disease:invasive candidal

															disease|Description:FUNCTION: Represses transcription by RNA polymerase II. Represses genes responsible for initiating filamentous growth and this repression is lifted under inducing environmental conditions.
Chr08G0318.1	1420	913	1414	UniProt ID:Q9UW87_CANAL	1606	1047	1598	181/567(31.92)	0.51	0.14	567	266	4.00E-73	gene=Chr08G0318	Gene Symbol:MLT1|Host:Isolated from a wide variety of substrates including humans|Disease:invasive candidal disease|Description:SIMILARITY: Belongs to the ABC transporter superfamily.
Chr08G0330.1	699	408	516	UniProt ID:B2CG58_9PEZI	691	404	512	39/110 (35.45)	0.57	0.02	110	73.6	2.00E-14	gene=Chr08G0330	Gene Symbol:PHL1|Host:Zea mays (Poaceae)|Disease:Gray leaf spot of corn|Description:Unknown
Chr08G0	419	23	347	UniProt	391	11	357	76/350	0.43	0.08	350	71.6	1.00E-14	gene=Chr	Gene

<table>
<tr><td>332.1</td><td></td><td></td><td></td><td>ID:Q6A2T2_BOTFU</td><td></td><td></td><td></td><td>(21.71)</td><td></td><td></td><td></td><td></td><td></td><td>08G0332</td><td>Symbol:BTP1|Host:Various plant families|Disease:Grey mould. Parasite or saprophyte|Description:Unknown</td></tr>
<tr><td>Chr08G0335.1</td><td>447</td><td>45</td><td>188</td><td>UniProt ID:Q000T6_BOTFU</td><td>413</td><td>22</td><td>157</td><td>39/146 (26.71)</td><td>0.45</td><td>0.08</td><td>146</td><td>45.4</td><td>4.00E-06</td><td>gene=Chr08G0335</td><td>Gene Symbol:BMP3|Host:Various plant families|Disease:Grey mould. Parasite or saprophyte|Description:CATALYTIC ACTIVITY: ATP + a protein = ADP + a phosphoprotein.</td></tr>
<tr><td>Chr08G0357.1</td><td>348</td><td>30</td><td>236</td><td>UniProt ID:Q75WR5_9PLEO</td><td>265</td><td>8</td><td>192</td><td>54/213 (25.35)</td><td>0.4</td><td>0.16</td><td>213</td><td>53.9</td><td>2.00E-09</td><td>gene=Chr08G0357</td><td>Gene Symbol:BRN1|Host:Belamcanda chinensis: Korea,Gladiolus ?gandavensis: Korea,Iris japonica: China,Iris missouriensis (Leaf spot.): Idaho; Montana; Oregon; Washington,Iris sp. (Leaf spot.): China; Texas; Washing|Disease:Leaf</td></tr>
</table>

															spot|Description:SIMILARITY: Belongs to the short-chain dehydrogenases/reductases (SDR) family.
Chr08G0358.1	222	26	181	UniProt ID:Q59XU5_CANAL	291	17	172	70/156 (44.87)	0.69	0	156	159	6.00E-48	gene=Chr08G0358	Gene Symbol:RAS1|Host:Isolated from a wide variety of substrates including humans|Disease:invasive candidal disease|Description:CAUTION: The sequence shown here is derived from an EMBL/GenBank/DDBJ whole genome shotgun (WGS) entry which is preliminary data.
Chr08G0363.1	320	141	215	UniProt ID:Q5J7N6_COCHE	589	166	240	38/75(50.67)	0.67	0	75	72.4	4.00E-15	gene=Chr08G0363	Gene Symbol:CHAP1|Host:Zea mays|Disease:Southern leaf blight of maize|Description:SIMILARITY: Belongs to the bZIP family.
Chr08G0	515	136	506	UniProt	402	2	305	82/386	0.42	0.25	386	68.6	2.00E-13	gene=Chr	Gene

370.1				ID:Q8NJ85_CANAL				(21.24)						08G0370	Symbol:CDC11|Host:Isolated from a wide variety of substrates including humans|Disease:invasive candidal disease|Description:SIMILARITY: Belongs to the septin family.
Chr08G0381.1	362	46	90	UniProt ID:A8QJI7_FUSOX	938	78	122	18/45(40.00)	0.62	0	45	48.5	4.00E-07	gene=Chr08G0381	Gene Symbol:XLNR|Host:Multiple genera in multiple families|Disease:Blights, wilts, rots of various sorts|Description:SIMILARITY: Contains 1 Zn(2)-C6 fungal-type DNA-binding domain.
Chr08G0388.1	494	46	408	UniProt ID:A4UC81_MAGO7	376	10	371	127/367(34.60)	0.52	0.02	367	205	2.00E-61	gene=Chr08G0388	Gene Symbol:MGG_10702|Host:Poaceae, especially important on Oryzae|Disease:Rice blast|Description:Unknown
Chr08G0389.1	348	126	182	UniProt ID:Q9H	746	20	82	24/63(38.10)	0.59	0.1	63	47.4	7.00E-07	gene=Chr08G0389	Gene Symbol:CLTA1|Host:Multi

				G15_COLLN										ple genera of Fabaceae. Rare reports on other taxa|Disease:Leaf, stem and pod anthracnose|Description:SIMILARITY: Contains 1 Zn(2)-C6 fungal-type DNA-binding domain.
Chr08G0390.1	307	222	294	UniProt ID:Q8J0I5_CLAPU	550	436	508	26/73(35.62)	0.56	0	73	55.8	9.00E-10	gene=Chr08G0390	Gene Symbol:CPTF1|Host:outcrossing species|Disease:ergotism|Description:SIMILARITY: Belongs to the bZIP family.
Chr08G0394.1	655	187	638	UniProt ID:O59918_USTMD	608	140	589	155/465(33.33)	0.51	0.06	465	259	7.00E-78	gene=Chr08G0394	Gene Symbol:UKC1|Host:Euchlaena spp., Zea spp. (Poaceae)|Disease:Smut. Corn smut|Description:CATALYTIC ACTIVITY: ATP + a protein = ADP + a phosphoprotein.
Chr08G0397.1	962	1	962	UniProt ID:Q8J	971	1	971	804/986(81.5	0.88	0.04	986	1507	0	gene=Chr08G0397	Gene Symbol:CLNR1|Host:Multi

				1X6_C OLLN				4)							ple genera of Fabaceae. Rare reports on other taxa|Disease:Leaf, stem and pod anthracnose|Description:Unknown
Chr08G0405.1	1021	530	904	UniProt ID:DHH1_CRYNV	616	38	382	109/376(28.99)	0.48	0.09	376	166	1.00E-43	gene=Chr08G0405	Gene Symbol:VAD1|Host:humans|Disease:cryptococcosis|Description:FUNCTION: ATP-dependent RNA helicase involved in mRNA turnover, and more specifically in mRNA decapping. Is involved in G1/S DNA- damage checkpoint recovery, probably through the regulation of the translational status of a subset of mRNAs. May also have a role in translation and mRNA nuclear export (By similarity). Is involved in virulence.

Chr08G0407.1	1516	831	1224	UniProt ID:Q9C2Y4_MAGGR	1501	887	1278	160/411(38.93)	0.57	0.09	411	266	3.00E-73	gene=Chr08G0407	Gene Symbol:PDE1|Host:Digitaria (Poaceae)|Disease:Leaf spot|Description:CATALYTIC ACTIVITY: ATP + H(2)O + phospholipid(In) = ADP + phosphate + phospholipid(Out).
Chr08G0411.1	224	1	224	UniProt ID:Q8J0E2_COLLN	224	1	224	198/224(88.39)	0.96	0	224	425	7.00E-153	gene=Chr08G0411	Gene Symbol:PLS1|Host:Multiple genera of Fabaceae. Rare reports on other taxa|Disease:Leaf, stem and pod anthracnose|Description:Unknown
Chr08G0414.1	821	193	391	UniProt ID:Q5ALS7_CANAL	1144	350	519	52/203(25.62)	0.41	0.18	203	53.9	3.00E-08	gene=Chr08G0414	Gene Symbol:CTF1|Host:Isolated from a wide variety of substrates including humans|Disease:invasive candidal disease|Description:SIMILARITY: Contains 1 Zn(2)-C6 fungal-type DNA-binding domain.

Chr08G0417.1	223	54	119	UniProt ID:A9YDN6_MAGGR	702	3	68	28/66(42.42)	0.62	0	66	64.3	5.00E-13	gene=Chr08G0417	Gene Symbol:SIG1|Host:Digitaria (Poaceae)|Disease:Leaf spot|Description:SUBCELLULAR LOCATION: Nucleus (By similarity).
Chr08G0419.1	445	367	418	UniProt ID:Q9P3Z8_CYBJA	345	17	68	24/52(46.15)	0.58	0	52	60.1	7.00E-11	gene=Chr08G0419	Gene Symbol:MIG1|Host:humans|Disease:Chronic urinary tract infection|Description:Unknown
Chr08G0420.1	542	2	542	UniProt ID:Q5J4D6_PHAND	543	3	543	438/541(80.96)	0.89	0	541	935	0	gene=Chr08G0420	Gene Symbol:MLS1|Host:Multiple genera of Poaceae and Blysmus compressus (Cyperaceae)|Disease:Glume blotch of wheat and other grasses|Description:CATALYTIC ACTIVITY: Acetyl-CoA + H(2)O + glyoxylate = (S)-malate + CoA.
Chr08G0421.1	1203	78	466	UniProt ID:Q5A	1462	36	409	168/396(42.4	0.63	0.07	396	338	2.00E-97	gene=Chr08G0421	Gene Symbol:HSL1|Host:Isolate

				G71_C ANAL				2)							d from a wide variety of substrates including humans|Disease:invasive candidal disease|Description:CAUTION: The sequence shown here is derived from an EMBL/GenBank/DDBJ whole genome shotgun (WGS) entry which is preliminary data.
Chr08G0425.1	758	335	757	UniProt ID:P78585_BOTFU	994	469	956	152/498(30.52)	0.46	0.17	498	172	2.00E-45	gene=Chr08G0425	Gene Symbol:BCPLC1|Host:Various plant families|Disease:Grey mould. Parasite or saprophyte|Description:SIMILARITY: Contains 1 C2 domain.
Chr08G0427.1	904	42	119	UniProt ID:Q96UQ9_MAGGR	715	545	624	35/80(43.75)	0.6	0.03	80	79.3	4.00E-16	gene=Chr08G0427	Gene Symbol:NULL|Host:Digitaria (Poaceae)|Disease:Leaf spot|Description:Unknown
Chr08G0431.1	1362	799	1343	UniProt ID:Q9C1Q7_A	708	283	700	134/549(24.41)	0.41	0.25	549	122	2.00E-29	gene=Chr08G0431	Gene Symbol:NULL|Host:humans|Disease:infection|Desc

				SPFM											ription:SIMILARITY: Contains 1 histidine kinase domain.
Chr08G0438.1	596	56	490	UniProt ID:Q9P8L8_BOTFU	598	99	518	116/438(26.48)	0.47	0.05	438	149	1.00E-39	gene=Chr08G0438	Gene Symbol:BCMFS1|Host:Various plant families|Disease:Grey mould. Parasite or saprophyte|Description:Unknown
Chr08G0439.1	2273	4	1254	UniProt ID:Q92217_COCHE	2528	14	1310	500/1325(37.74)	0.55	0.08	1325	836	0	gene=Chr08G0439	Gene Symbol:PKS1|Host:Zea mays|Disease:Southern leaf blight of maize|Description:Unknown
Chr08G0440.1	1171	995	1141	UniProt ID:Q5AM49_CANAL	1690	1098	1243	53/148(35.81)	0.57	0.02	148	100	2.00E-22	gene=Chr08G0440	Gene Symbol:SNF2|Host:Isolated from a wide variety of substrates including humans|Disease:invasive candidal disease|Description:Unknown
Chr08G0447.1	466	5	411	UniProt ID:Q9P	598	34	424	96/425(22.59)	0.4	0.12	425	67.4	6.00E-13	gene=Chr08G0447	Gene Symbol:BCMFS1|Host:Va

				8L8_BOTFU										rious plant families|Disease:Grey mould. Parasite or saprophyte|Description:Unknown
Chr08G0450.1	1230	405	1199	UniProt ID:Q5KB00_CRYNJ	1296	342	1153	413/822(50.24)	0.67	0.05	822	794	0	gene=Chr08G0450	Gene Symbol:CNI04280|Host:humans|Disease:cryptococcosis|Description:SIMILARITY: Contains 1 DH (DBL-homology) domain.
Chr08G0455.1	914	5	913	UniProt ID:Q5AB06_CANAL	1020	19	1019	377/1075(35.07)	0.51	0.22	1075	529	2.00E-172	gene=Chr08G0455	Gene Symbol:VPS34|Host:Isolated from a wide variety of substrates including humans|Disease:invasive candidal disease|Description:SIMILARITY: Contains 1 PI3K/PI4K domain.
Chr08G0459.1	463	41	194	UniProt ID:A0ST43_CERNC	459	17	179	50/168(29.76)	0.48	0.11	168	67.4	4.00E-13	gene=Chr08G0459	Gene Symbol:CTB5|Host:Numerous taxa in Solanaceae|Disease:Leaf spot|Description:Unknown
Chr08G0	419	16	412	UniProt	581	174	567	128/40	0.5	0.02	400	191	4.00E-55	gene=Chr	Gene

460.1				ID:Q59RG0_CANAL				0(32.00)						08G0460	Symbol:NAG4|Host:Isolated from a wide variety of substrates including humans|Disease:invasive candidal disease|Description:CAUTION: The sequence shown here is derived from an EMBL/GenBank/DDBJ whole genome shotgun (WGS) entry which is preliminary data.
Chr08G0462.1	1051	689	879	UniProt ID:Q6ZX14_MAGGR	4034	3715	3902	57/205 (27.80)	0.43	0.15	205	62.8	1.00E-10	gene=Chr08G0462	Gene Symbol:ACE1|Host:Digitaria (Poaceae)|Disease:Leaf spot|Description:Unknown
Chr08G0465.1	465	90	438	UniProt ID:Q1L2E2_PHAND	619	216	544	96/369 (26.02)	0.42	0.16	369	105	3.00E-25	gene=Chr08G0465	Gene Symbol:ALS1|Host:Multiple genera of Poaceae and Blysmus compressus (Cyperaceae)|Disease:Glume blotch of wheat and other grasses|Description:COFACTOR: Pyridoxal phosphate (By similarity).

Chr08G0466.1	688	48	488	UniProt ID:Q5XTQ4_BOTFU	574	60	537	152/493(30.83)	0.46	0.14	493	153	1.00E-40	gene=Chr08G0466	Gene Symbol:LIP1|Host:Various plant families|Disease:Grey mould. Parasite or saprophyte|Description:Unknown
Chr08G0468.1	465	38	371	UniProt ID:A4UC81_MAGO7	376	48	367	122/337(36.20)	0.55	0.06	337	216	8.00E-66	gene=Chr08G0468	Gene Symbol:MGG_10702|Host:Poaceae, especially important on Oryzae|Disease:Rice blast|Description:Unknown
Chr08G0469.1	552	59	91	UniProt ID:Q5A4F3_CANAL	624	14	46	18/33(54.55)	0.7	0	33	51.2	9.00E-08	gene=Chr08G0469	Gene Symbol:ZCF37|Host:Isolated from a wide variety of substrates including humans|Disease:invasive candidal disease|Description:Unknown
Chr08G0470.1	371	1	330	UniProt ID:Q59ZX2_CANAL	381	1	328	168/333(50.45)	0.72	0.02	333	355	8.00E-121	gene=Chr08G0470	Gene Symbol:FTR1|Host:Isolated from a wide variety of substrates including

														humans|Disease:invasive candidal disease|Description:CAUTION: The sequence shown here is derived from an EMBL/GenBank/DDBJ whole genome shotgun (WGS) entry which is preliminary data.
Chr08G0471.1	601	25	517	UniProt ID:Q5RLJ7_CRYNV	594	58	578	163/539(30.24)	0.48	0.12	539	219	2.00E-63	gene=Chr08G0471	Gene Symbol:NULL|Host:humans|Disease:cryptococcosis|Description:Unknown
Chr08G0474.1	550	15	500	UniProt ID:Q5ANE1_CANAL	748	27	496	164/502(32.67)	0.48	0.1	502	222	3.00E-64	gene=Chr08G0474	Gene Symbol:SNF3|Host:Isolated from a wide variety of substrates including humans|Disease:invasive candidal disease|Description:SIMILARITY: Belongs to the major facilitator superfamily. Sugar transporter (TC 2.A.1.1) family.
Chr08G0	501	115	456	UniProt	871	63	404	109/34	0.51	0.02	346	177	7.00E-49	gene=Chr	Gene

482.1				ID:Q2I0M6_CERNC				6(31.50)						08G0482	Symbol:CTB3|Host:Numerous taxa in Solanaceae|Disease:Leaf spot|Description:Unknown
Chr08G0483.1	491	203	487	UniProt ID:Q6TFC7_ASPFM	349	63	346	90/289 (31.14)	0.52	0.03	289	151	6.00E-42	gene=Chr08G0483	Gene Symbol:NULL|Host:humans|Disease:infection|Description:Unknown
Chr08G0487.1	425	249	425	UniProt ID:Q9UW16_MAGGR	181	1	181	129/181(71.27)	0.81	0.02	181	247	9.00E-81	gene=Chr08G0487	Gene Symbol:VATP|Host:Digitaria (Poaceae)|Disease:Leaf spot|Description:Unknown
Chr08G0496.1	568	3	566	UniProt ID:Q5XTQ4_BOTFU	574	2	569	191/605(31.57)	0.46	0.13	605	231	3.00E-68	gene=Chr08G0496	Gene Symbol:LIP1|Host:Various plant families|Disease:Grey mould. Parasite or saprophyte|Description:Unknown
Chr08G0502.1	304	29	283	UniProt ID:A4QVF8_MAGO7	339	33	316	73/288 (25.35)	0.42	0.13	288	90.1	9.00E-22	gene=Chr08G0502	Gene Symbol:MGG_04556|Host:Poaceae, especially important on Oryzae|Disease:Rice blast|Description:COFACTOR: Zinc (By similarity).

Chr08G0511.1	564	99	243	UniProt ID:Q9Y880_COCCA	880	62	195	49/152 (32.24)	0.47	0.16	152	69.7	2.00E-13	gene=Chr08G0511	Gene Symbol:SNF1|Host:Corn, Zea mays, sometimes on Sorghum (Poaceae) and various other plant families|Disease:Northern corn leaf spot, ear and kernel rot|Description:Unknown
Chr08G0514.1	829	309	659	UniProt ID:A6N6J8_FUSOX	903	242	594	89/379 (23.48)	0.41	0.14	379	68.2	1.00E-12	gene=Chr08G0514	Gene Symbol:CTF1|Host:Multiple genera in multiple families|Disease:Blights, wilts, rots of various sorts|Description:SIMILARITY: Contains 1 Zn(2)-C6 fungal-type DNA-binding domain.
Chr08G0519.1	547	140	538	UniProt ID:Q5ANE1_CANAL	748	91	513	114/437(26.09)	0.44	0.12	437	120	2.00E-29	gene=Chr08G0519	Gene Symbol:SNF3|Host:Isolated from a wide variety of substrates including humans|Disease:invasive candidal disease|Description:SIMILARITY: Belongs to the

														major facilitator superfamily. Sugar transporter (TC 2.A.1.1) family.
Chr08G0522.1	430	341	420	UniProt ID:Q59WH0_CANAL	445	365	441	31/80(38.75)	0.59	0.04	80	55.1	3.00E-09	gene=Chr08G0522	Gene Symbol:ADA2|Host:Isolated from a wide variety of substrates including humans|Disease:invasive candidal disease|Description:CAUTION: The sequence shown here is derived from an EMBL/GenBank/DDBJ whole genome shotgun (WGS) entry which is preliminary data.
Chr08G0524.1	687	10	160	UniProt ID:A4R0W3_MAGO7	1226	279	458	45/180(25.00)	0.39	0.16	180	45.4	9.00E-06	gene=Chr08G0524	Gene Symbol:MGG_09263|Host:Poaceae, especially important on Oryzae|Disease:Rice blast|Description:Unknown
Chr08G0532.1	381	7	381	UniProt ID:C5G	380	4	378	272/375(72.5	0.84	0	375	584	0	gene=Chr08G0532	Gene Symbol:BDCG_09435|Ho

				YF3_AJEDR				3)							st:humans|Disease:cutaneous Blastomyces dermatitidis infection|Description:Unknown
Chr08G0547.1	474	38	474	UniProt ID:Q59LF3_CANAL	474	82	473	101/442(22.85)	0.4	0.12	442	88.6	8.00E-20	gene=Chr08G0547	Gene Symbol:RVS167|Host:Isolated from a wide variety of substrates including humans|Disease:invasive candidal disease|Description:SIMILARITY: Contains 1 SH3 domain.
Chr08G0551.1	917	692	880	UniProt ID:Q5AKU6_CANAL	674	484	665	105/191(54.97)	0.73	0.06	191	214	6.00E-60	gene=Chr08G0551	Gene Symbol:SSK1|Host:Isolated from a wide variety of substrates including humans|Disease:invasive candidal disease|Description:Unknown
Chr08G0555.1	174	3	167	UniProt ID:C5GAK9_AJEDR	216	49	213	111/166(66.87)	0.8	0.01	166	233	3.00E-78	gene=Chr08G0555	Gene Symbol:BDCG_02048|Host:humans|Disease:cutaneous Blastomyces

															dermatitidis infection|Description:Unknown
Chr08G0558.1	260	5	260	UniProt ID:Q32WF7_PHAND	266	14	266	83/263 (31.56)	0.48	0.06	263	115	2.00E-31	gene=Chr08G0558	Gene Symbol:MDH1|Host:Multiple genera of Poaceae and Blysmus compressus (Cyperaceae)|Disease:Glume blotch of wheat and other grasses|Description:Unknown
Chr08G0559.1	378	14	306	UniProt ID:Q6XVN4_CRYNV	383	19	315	77/319 (24.14)	0.38	0.15	319	60.5	4.00E-11	gene=Chr08G0559	Gene Symbol:GNO1|Host:humans|Disease:cryptococcosis|Description:COFACTOR: Zinc (By similarity).
Chr08G0566.1	945	50	780	UniProt ID:PMT1_CANAL	877	65	776	331/737(44.91)	0.58	0.04	737	625	0	gene=Chr08G0566	Gene Symbol:PMT1|Host:Isolated from a wide variety of substrates including humans|Disease:invasive candidal disease|Description:FUNCTION: Transfers mannose from Dol-P-mannose to

															Ser or Thr residues on proteins.
Chr08G0586.1	473	135	350	UniProt ID:C1GM90_PARBD	353	64	275	61/227 (26.87)	0.42	0.11	227	62.8	1.00E-11	gene=Chr08G0586	Gene Symbol:PADG_08176|Host:humans|Disease:Paracoccidioidomycosis|Description:SIMILARITY: Contains 1 CRAL-TRIO domain.
Chr08G0587.1	815	474	604	UniProt ID:SET1_CANAL	1040	908	1040	56/136 (41.18)	0.57	0.06	136	100	2.00E-22	gene=Chr08G0587	Gene Symbol:SET1|Host:Isolated from a wide variety of substrates including humans|Disease:invasive candidal disease|Description:FUNCTION: Catalytic component of the COMPASS (Set1C) complex that specifically mono-, di- and trimethylates histone H3 to form H3K4me1/2/3, which subsequently plays a role in telomere length maintenance, transcription

															elongation regulation and pathogenesis of invasive candidiasis.
Chr08G0596.1	722	357	581	UniProt ID:Q5AG71_CANAL	1462	59	272	73/238 (30.67)	0.48	0.16	238	103	2.00E-23	gene=Chr08G0596	Gene Symbol:HSL1|Host:Isolated from a wide variety of substrates including humans|Disease:invasive candidal disease|Description:CAUTION: The sequence shown here is derived from an EMBL/GenBank/DDBJ whole genome shotgun (WGS) entry which is preliminary data.
Chr08G0601.1	1715	1	1620	UniProt ID:A4RB72_MAGO7	1715	1	1633	1039/1674(62.07)	0.75	0.06	1674	1988	0	gene=Chr08G0601	Gene Symbol:MGG_02986|Host:Poaceae, especially important on Oryzae|Disease:Rice blast|Description:CATALYTIC ACTIVITY: Deoxynucleoside triphosphate + DNA(n) = diphosphate + DNA(n+1).

Chr08G0607.1	243	141	218	UniProt ID:Q9C1I3_CANAL	601	205	286	32/82(39.02)	0.55	0.05	82	47.8	2.00E-07	gene=Chr08G0607	Gene Symbol:RFG1|Host:Isolated from a wide variety of substrates including humans|Disease:invasive candidal disease|Description:Unknown
Chr08G0613.1	607	47	593	UniProt ID:O93842_FUSSP	598	18	570	163/563(28.95)	0.52	0.05	563	252	1.00E-75	gene=Chr08G0613	Gene Symbol:TRI12|Host:animals|Disease:trichothecene|Description:Unknown
Chr08G0620.1	811	613	810	UniProt ID:F2QQX0_PICP7	3007	2807	3003	61/202(30.20)	0.53	0.04	202	93.2	3.00E-20	gene=Chr08G0620	Gene Symbol:CHS1|Host:humans|Disease:occasional infection|Description:SIMILARITY: Contains 1 PH domain.
Chr08G0622.1	361	220	276	UniProt ID:C0S9K6_PARBP	730	314	370	21/57(36.84)	0.53	0	57	47	9.00E-07	gene=Chr08G0622	Gene Symbol:PABG_04193|Host:humans|Disease:Paracoccidioidomycosis|Description:Unknown
Chr08G0623.1	199	1	199	UniProt ID:Q2KG56_M	199	1	199	190/199(95.48)	0.97	0	199	397	6.00E-143	gene=Chr08G0623	Gene Symbol:"MGCH7_CH7G479,

				AGO7											MGG_02731"|Host:Poaceae, especially important on Oryzae|Disease:Rice blast|Description:SIMILARITY: Belongs to the small GTPase superfamily. Rho family.
Chr08G0624.1	344	1	341	UniProt ID:A0ST44_CERNC	357	1	346	134/359(37.33)	0.54	0.09	359	191	4.00E-58	gene=Chr08G0624	Gene Symbol:CTB6|Host:Numerous taxa in Solanaceae|Disease:Leaf spot|Description:Unknown
Chr08G0635.1	647	343	457	UniProt ID:TUP1_CANAL	514	260	368	34/118 (28.81)	0.54	0.1	118	56.2	3.00E-09	gene=Chr08G0635	Gene Symbol:TUP1|Host:Isolated from a wide variety of substrates including humans|Disease:invasive candidal disease|Description:FUNCTION: Represses transcription by RNA polymerase II. Represses genes responsible for initiating filamentous growth and this repression is lifted under inducing

														environmental conditions.
Chr08G0643.1	448	1	362	UniProt ID:A4UC81_MAGO7	376	1	371	122/380(32.11)	0.49	0.07	380	183	1.00E-53	gene=Chr08G0643	Gene Symbol:MGG_10702|Host:Poaceae, especially important on Oryzae|Disease:Rice blast|Description:Unknown
Chr08G0645.1	711	34	85	UniProt ID:A0ST46_CERNC	397	11	65	25/55(45.45)	0.55	0.05	55	45.1	8.00E-06	gene=Chr08G0645	Gene Symbol:CTB8|Host:Numerous taxa in Solanaceae|Disease:Leaf spot|Description:Unknown
Chr08G0646.1	645	417	641	UniProt ID:DPP5_ARTOT	726	469	695	57/241(23.65)	0.4	0.12	241	62.4	4.00E-11	gene=Chr08G0646	Gene Symbol:DPP5|Host:humans, reptiles|Disease:dermatophytoses|Description:FUNCTION: Extracellular dipeptidyl-peptidase which removes N- terminal dipeptides sequentially from polypeptides having unsubstituted N-termini. Contributes to pathogenicity (By

															similarity).
Chr08G0653.1	381	103	179	UniProt ID:Q5ADS0_CANAL	229	75	151	42/77(54.55)	0.71	0	77	87.4	8.00E-21	gene=Chr08G0653	Gene Symbol:UBI4|Host:Isolated from a wide variety of substrates including humans|Disease:invasive candidal disease|Description:Unknown
Chr08G0654.1	538	16	507	UniProt ID:Q5AMQ4_CANAL	544	19	544	175/547(31.99)	0.48	0.14	547	264	3.00E-81	gene=Chr08G0654	Gene Symbol:HSX11|Host:Isolated from a wide variety of substrates including humans|Disease:invasive candidal disease|Description:CAUTION: The sequence shown here is derived from an EMBL/GenBank/DDBJ whole genome shotgun (WGS) entry which is preliminary data.
Chr08G0658.1	532	131	495	UniProt ID:A4R0Y1_MAGO7	573	206	563	155/373(41.55)	0.58	0.06	373	304	3.00E-96	gene=Chr08G0658	Gene Symbol:MGG_09250|Host:Poaceae, especially important on

															Oryzae|Disease:Rice blast|Description:Unknown
Chr08G0663.1	853	7	84	UniProt ID:C1G7T1_PARBD	534	74	149	28/78(35.90)	0.54	0.03	78	58.9	7.00E-10	gene=Chr08G0663	Gene Symbol:PADG_03236|Host:humans|Disease:Paracoccidioidomycosis|Description:Unknown
Chr08G0674.1	257	14	93	UniProt ID:Q9HG15_COLLN	746	20	97	26/86(30.23)	0.53	0.16	86	45.8	1.00E-06	gene=Chr08G0674	Gene Symbol:CLTA1|Host:Multiple genera of Fabaceae. Rare reports on other taxa|Disease:Leaf, stem and pod anthracnose|Description:SIMILARITY: Contains 1 Zn(2)-C6 fungal-type DNA-binding domain.
Chr08G0675.1	338	11	255	UniProt ID:A0ST44_CERNC	357	4	257	73/266(27.44)	0.44	0.12	266	72	4.00E-15	gene=Chr08G0675	Gene Symbol:CTB6|Host:Numerous taxa in Solanaceae|Disease:Leaf spot|Description:Unknown
Chr08G0685.1	985	1	984	UniProt ID:Q705V7_U	1061	1	1059	425/1085(39.17)	0.57	0.12	1085	767	0	gene=Chr08G0685	Gene Symbol:GAS1|Host:Euchlaena spp., Zea spp.

				STMD										(Poaceae)|Disease:Smut. Corn smut|Description:Unknown
Chr08G0693.1	659	94	655	UniProt ID:Q2VLJ1_GIBZA	565	27	562	196/572(34.27)	0.53	0.08	572	305	2.00E-95	gene=Chr08G0693	Gene Symbol:ZEB1|Host:Principal hosts: Poaceae, including Zea mays (corn), Triticum aestivum (wheat), and Oryza sativa (rice). Additional hosts: various plant families|Disease:Seedling blight, pre- and post-emergence blight, root and foot rot, brown rot, culm decay, head or kernel blight (scab or ear scab) of cereals. Leaf|Description:Unknown
Chr08G0700.1	510	347	476	UniProt ID:A4ULI9_MYCGR	502	348	501	42/158 (26.58)	0.41	0.2	158	53.1	2.00E-08	gene=Chr08G0700	Gene Symbol:CYP51|Host:Triticum and possibly a few other grasses|Disease:Leaf spot or speckled leaf blotch of

															wheat|Description:COFACTOR: Heme group (By similarity).
Chr08G0702.1	300	8	205	UniProt ID:Q6TFC7_ASPFM	349	65	250	64/201 (31.84)	0.44	0.09	201	65.5	4.00E-13	gene=Chr08G0702	Gene Symbol:NULL|Host:humans|Disease:infection|Description:Unknown
Chr08G0703.1	2995	12	2974	UniProt ID:Q6ZX14_MAGGR	4034	11	3025	1135/3075(36.91)	0.54	0.06	3075	1840	0	gene=Chr08G0703	Gene Symbol:ACE1|Host:Digitaria (Poaceae)|Disease:Leaf spot|Description:Unknown
Chr08G0705.1	519	270	484	UniProt ID:A4ULI8_MYCGR	517	269	513	47/245 (19.18)	0.4	0.12	245	56.6	2.00E-09	gene=Chr08G0705	Gene Symbol:CYP51|Host:Triticum and possibly a few other grasses|Disease:Leaf spot or speckled leaf blotch of wheat|Description:COFACTOR: Heme group (By similarity).
Chr08G0706.1	261	11	257	UniProt ID:Q32WF7_PHAND	266	17	262	71/259 (27.41)	0.43	0.1	259	58.5	4.00E-11	gene=Chr08G0706	Gene Symbol:MDH1|Host:Multiple genera of Poaceae and Blysmus compressus (Cyperaceae)|Disease:Glume blotch of wheat and

<table>
<tr><td></td><td></td><td></td><td></td><td></td><td></td><td></td><td></td><td></td><td></td><td></td><td></td><td></td><td></td><td></td><td>other grasses|Description:Unknown</td></tr>
<tr><td>Chr08G0711.1</td><td>254</td><td>4</td><td>252</td><td>UniProt ID:Q32WF7_PHAND</td><td>266</td><td>15</td><td>264</td><td>92/256 (35.94)</td><td>0.52</td><td>0.05</td><td>256</td><td>132</td><td>9.00E-38</td><td>gene=Chr08G0711</td><td>Gene Symbol:MDH1|Host:Multiple genera of Poaceae and Blysmus compressus (Cyperaceae)|Disease:Glume blotch of wheat and other grasses|Description:Unknown</td></tr>
<tr><td>Chr08G0713.1</td><td>542</td><td>116</td><td>294</td><td>UniProt ID:Q59VF3_CANAL</td><td>1813</td><td>69</td><td>242</td><td>55/179 (30.73)</td><td>0.5</td><td>0.03</td><td>179</td><td>68.6</td><td>5.00E-13</td><td>gene=Chr08G0713</td><td>Gene Symbol:"DUR1,2"|Host:Isolated from a wide variety of substrates including humans|Disease:invasive candidal disease|Description:CAUTION: The sequence shown here is derived from an EMBL/GenBank/DDBJ whole genome shotgun (WGS) entry which is preliminary data.</td></tr>
<tr><td>Chr08G0</td><td>475</td><td>15</td><td>394</td><td>UniProt</td><td>459</td><td>16</td><td>380</td><td>108/38</td><td>0.43</td><td>0.08</td><td>389</td><td>108</td><td>1.00E-26</td><td>gene=Chr</td><td>Gene</td></tr>
</table>

719.1				ID:Q01 446_N ECHA				9(27.7 6)						08G0719	Symbol:MAK1|Host:Trees of various plant families|Disease:Fruit rot, stem rot|Description:Unknown
Chr08G0 720.1	1010	30	85	UniProt ID:Q9C 1R5_C RYNE	606	392	448	22/57(38.60)	0.51	0.02	57	47.8	2.00E-06	gene=Chr 08G0720	Gene Symbol:STE12A|Host:humans|Disease:cryptococcosis|Description:Unknown
Chr08G0 724.1	347	75	264	UniProt ID:Q8J 1Y3_B EABA	348	108	326	53/225 (23.56)	0.4	0.18	225	48.5	2.00E-07	gene=Chr 08G0724	Gene Symbol:CHIT1|Host:various arthropod species|Disease:white muscardine disease|Description:SIMILARITY: Belongs to the glycosyl hydrolase 18 family.
Chr08G0 728.1	602	22	501	UniProt ID:Q5R LJ7_C RYNV	594	61	578	161/53 2(30.2 6)	0.46	0.12	532	197	1.00E-55	gene=Chr 08G0728	Gene Symbol:NULL|Host:humans|Disease:cryptococcosis|Description:Unknown
Chr08G0 729.1	364	3	329	UniProt ID:Q59 ZX2_C ANAL	381	2	323	178/32 9(54.1 0)	0.73	0.03	329	375	9.00E-12 9	gene=Chr 08G0729	Gene Symbol:FTR1|Host:Isolated from a wide variety of substrates including

															humans|Disease:invasive candidal disease|Description:CAUTION: The sequence shown here is derived from an EMBL/GenBank/DDBJ whole genome shotgun (WGS) entry which is preliminary data.
Chr08G0730.1	561	155	428	UniProt ID:B0BER9_CLAPU	557	100	431	81/340 (23.82)	0.37	0.22	340	61.2	7.00E-11	gene=Chr08G0730	Gene Symbol:NOX1|Host:outcrossing species|Disease:ergotism|Description:SIMILARITY: Contains 1 FAD-binding FR-type domain.
Chr08G0732.1	507	54	488	UniProt ID:Q5ANE1_CANAL	748	51	505	112/468(23.93)	0.42	0.1	468	102	8.00E-24	gene=Chr08G0732	Gene Symbol:SNF3|Host:Isolated from a wide variety of substrates including humans|Disease:invasive candidal disease|Description:SIMILARITY: Belongs to the major facilitator superfamily. Sugar

													transporter (TC 2.A.1.1) family.
Chr08G0733.1	502	82	231	UniProt ID:Q2VLJ1_GIBZA	565	127	283	58/162 (35.80)	0.47	0.1	162	69.3	1.00E-13	gene=Chr08G0733	Gene Symbol:ZEB1|Host:Principal hosts: Poaceae, including Zea mays (corn), Triticum aestivum (wheat), and Oryza sativa (rice). Additional hosts: various plant families|Disease:Seedling blight, pre- and post-emergence blight, root and foot rot, brown rot, culm decay, head or kernel blight (scab or ear scab) of cereals. Leaf|Description:Unknown
Chr08G0734.1	310	3	271	UniProt ID:Q5AMT2_CANAL	308	5	259	77/278 (27.70)	0.43	0.12	278	78.2	1.00E-17	gene=Chr08G0734	Gene Symbol:BGL2|Host:Isolated from a wide variety of substrates including humans|Disease:invasive candidal disease|Description:SIMILARITY: Belongs to the

															glycosyl hydrolase 17 family.
Chr08G0738.1	342	34	323	UniProt ID:F2QLB5_PICP7	286	2	285	119/292(40.75)	0.62	0.03	292	233	5.00E-75	gene=Chr08G0738	Gene Symbol:IPP1|Host:humans|Disease:occasional infection|Description:Unknown
Chr08G0743.1	335	40	326	UniProt ID:Q6TFC7_ASPFM	349	61	346	100/292(34.25)	0.52	0.04	292	160	1.00E-46	gene=Chr08G0743	Gene Symbol:NULL|Host:humans|Disease:infection|Description:Unknown
Chr08G0744.1	358	60	346	UniProt ID:Q6TFC7_ASPFM	349	61	346	104/291(35.74)	0.53	0.03	291	172	2.00E-50	gene=Chr08G0744	Gene Symbol:NULL|Host:humans|Disease:infection|Description:Unknown
Chr08G0746.1	414	5	384	UniProt ID:Q9P8L8_BOTFU	598	28	430	147/405(36.30)	0.56	0.07	405	262	1.00E-81	gene=Chr08G0746	Gene Symbol:BCMFS1|Host:Various plant families|Disease:Grey mould. Parasite or saprophyte|Description:Unknown
Chr08G0756.1	772	13	769	UniProt ID:DPP4_ASPFC	765	7	763	464/758(61.21)	0.77	0	758	967	0	gene=Chr08G0756	Gene Symbol:DPP4|Host:humans|Disease:infection|Description:FUNCTION:

															Extracellular dipeptidyl-peptidase which removes N- terminal dipeptides sequentially from polypeptides having unsubstituted N-termini provided that the penultimate residue is proline. Contributes to pathogenicity.
Chr08G0760.1	360	42	351	UniProt ID:Q6TFC7_ASPFM	349	40	346	105/314(33.44)	0.52	0.04	314	162	4.00E-47	gene=Chr08G0760	Gene Symbol:NULL|Host:humans|Disease:infection|Description:Unknown
Chr08G0775.1	372	77	360	UniProt ID:Q6TFC7_ASPFM	349	61	347	111/292(38.01)	0.54	0.04	292	180	2.00E-53	gene=Chr08G0775	Gene Symbol:NULL|Host:humans|Disease:infection|Description:Unknown
Chr08G0789.1	653	91	652	UniProt ID:Q2VLJ1_GIBZA	565	23	562	196/571(34.33)	0.52	0.07	571	321	2.00E-101	gene=Chr08G0789	Gene Symbol:ZEB1|Host:Principal hosts: Poaceae, including Zea mays (corn), Triticum aestivum (wheat), and Oryza sativa (rice). Additional hosts: various plant

															families|Disease:Seedling blight, pre- and post-emergence blight, root and foot rot, brown rot, culm decay, head or kernel blight (scab or ear scab) of cereals. Leaf|Description:Unknown
Chr08G0790.1	327	38	240	UniProt ID:Q9Y784_MAGGR	631	192	387	54/207 (26.09)	0.47	0.07	207	69.3	5.00E-14	gene=Chr08G0790	Gene Symbol:PTH11|Host:Digitaria (Poaceae)|Disease:Leaf spot|Description:Unknown
Chr08G0792.1	400	54	250	UniProt ID:Q6XVN4_CRYNV	383	22	236	65/221 (29.41)	0.43	0.14	221	69.7	5.00E-14	gene=Chr08G0792	Gene Symbol:GNO1|Host:humans|Disease:cryptococcosis|Description:COFACTOR: Zinc (By similarity).
Chr08G0794.1	681	31	77	UniProt ID:Q5A4F3_CANAL	624	8	54	22/47(46.81)	0.6	0	47	55.5	6.00E-09	gene=Chr08G0794	Gene Symbol:ZCF37|Host:Isolated from a wide variety of substrates including humans|Disease:invasive candidal disease|Description:Unknown

Chr08G0806.1	288	51	282	UniProt ID:MEP7_TRIVH	294	57	270	93/234 (39.74)	0.56	0.09	234	165	3.00E-49	gene=Chr08G0806	Gene Symbol:TRV_07111|Host: humans|Disease:infection|Description:FUNCTION: Secreted metalloproteinase that allows assimilation of proteinaceous substrates. Plays a pivotal role as a pathogenicity determinant during infections and contributes to the ability of the pathogen to persist within the mammalian host (By similarity).
Chr08G0815.1	429	19	51	UniProt ID:Q5A4F3_CANAL	624	14	46	15/33(45.45)	0.67	0	33	45.1	5.00E-06	gene=Chr08G0815	Gene Symbol:ZCF37|Host:Isolated from a wide variety of substrates including humans|Disease:invasive candidal disease|Description:Unknown
Chr08G0818.1	514	116	478	UniProt ID:Q96VZ3_F	455	58	421	108/377(28.65)	0.47	0.07	377	131	2.00E-34	gene=Chr08G0818	Gene Symbol:PGX1|Host:Multiple genera in multiple

				USOX										families|Disease:Blights, wilts, rots of various sorts|Description:SIMILARITY: Belongs to the glycosyl hydrolase 28 family.
Chr08G0825.1	539	114	507	UniProt ID:Q5XTQ5_BOTFU	615	155	548	96/410 (23.41)	0.43	0.08	410	73.6	9.00E-15	gene=Chr08G0825	Gene Symbol:FRT1|Host:Various plant families|Disease:Grey mould. Parasite or saprophyte|Description:SIMILARITY: Belongs to the major facilitator superfamily. Sugar transporter (TC 2.A.1.1) family.
Chr08G0826.1	588	30	485	UniProt ID:Q9P8L8_BOTFU	598	65	522	127/475(26.74)	0.49	0.08	475	166	5.00E-45	gene=Chr08G0826	Gene Symbol:BCMFS1|Host:Various plant families|Disease:Grey mould. Parasite or saprophyte|Description:Unknown
Chr08G0830.1	561	116	296	UniProt ID:A0S	459	3	183	54/197 (27.41)	0.41	0.16	197	48.1	7.00E-07	gene=Chr08G0830	Gene Symbol:CTB5|Host:Numer

				T43_CERNC											ous taxa in Solanaceae|Disease:Leaf spot|Description:Unknown
Chr08G0832.1	1221	213	559	UniProt ID:O59928_HYPVI	430	57	412	109/386(28.24)	0.44	0.18	386	117	2.00E-28	gene=Chr08G0832	Gene Symbol:NULL|Host:humans|Disease:infection|Description:SIMILARITY: Belongs to the glycosyl hydrolase 18 family.
Chr08G0843.1	2161	1481	1800	UniProt ID:SUB2_ARTOC	423	154	418	97/322 (30.12)	0.43	0.18	322	93.6	1.00E-20	gene=Chr08G0843	Gene Symbol:SUB2|Host:humans, reptiles|Disease:dermatophytoses|Description:FUNCTION: Secreted subtilisin-like serine protease with keratinolytic activity that contributes to pathogenicity (By similarity).
Chr08G0847.1	548	47	411	UniProt ID:Q9C0M1_CANAL	493	63	422	86/391 (21.99)	0.41	0.15	391	58.9	3.00E-10	gene=Chr08G0847	Gene Symbol:CANAG5|Host:Isolated from a wide variety of substrates including humans|Disease:invasive candidal

															disease|Description:Unknown
Chr08G0849.1	318	1	244	UniProt ID:Q9C1S8_CANAL	248	1	242	129/246(52.44)	0.67	0.02	246	242	2.00E-79	gene=Chr08G0849	Gene Symbol:CANAG1|Host:Isolated from a wide variety of substrates including humans|Disease:invasive candidal disease|Description:Unknown
Chr08G0865.1	1138	13	1055	UniProt ID:Q9P872_CANAL	917	28	904	308/1061(29.03)	0.46	0.19	1061	352	3.00E-105	gene=Chr08G0865	Gene Symbol:PMR1|Host:Isolated from a wide variety of substrates including humans|Disease:invasive candidal disease|Description:SIMILARITY: Belongs to the cation transport ATPase (P-type) family.
Chr08G0870.1	524	31	485	UniProt ID:Q5ANE1_CANAL	748	45	496	141/475(29.68)	0.48	0.09	475	189	7.00E-53	gene=Chr08G0870	Gene Symbol:SNF3|Host:Isolated from a wide variety of substrates including humans|Disease:invasive candidal

															disease|Description:SIMILARITY: Belongs to the major facilitator superfamily. Sugar transporter (TC 2.A.1.1) family.
Chr08G0875.1	619	74	603	UniProt ID:O93842_FUSSP	598	62	590	139/535(25.98)	0.46	0.02	535	166	5.00E-45	gene=Chr08G0875	Gene Symbol:TRI12|Host:animals|Disease:trichothecene|Description:Unknown
Chr01G0362.1	215	6	197	UniProt ID:Q7Z9J6_CRYNV	197	9	197	108/192(56.25)	0.75	0.02	192	234	1.00E-78	gene=Chr01G0362	Gene Symbol:TSA1|Host:humans|Disease:cryptococcosis|Description:Unknown
Chr01G0371.1	584	9	562	UniProt ID:Q9P8L8_BOTFU	598	40	592	205/560(36.61)	0.55	0.02	560	345	3.00E-111	gene=Chr01G0371	Gene Symbol:BCMFS1|Host:Various plant families|Disease:Grey mould. Parasite or saprophyte|Description:Unknown
Chr01G0376.1	568	34	518	UniProt ID:Q5ANE1_CANAL	748	30	509	166/494(33.60)	0.55	0.05	494	265	1.00E-79	gene=Chr01G0376	Gene Symbol:SNF3|Host:Isolated from a wide variety of substrates including humans|Disease:invasive

															candidal disease|Description:SIMILARITY: Belongs to the major facilitator superfamily. Sugar transporter (TC 2.A.1.1) family.
Chr01G0383.1	404	32	312	UniProt ID:A4UC81_MAGO7	376	59	371	95/315 (30.16)	0.47	0.11	315	126	2.00E-33	gene=Chr01G0383	Gene Symbol:MGG_10702|Host:Poaceae, especially important on Oryzae|Disease:Rice blast|Description:Unknown
Chr01G0386.1	409	91	244	UniProt ID:Q5AJC0_CANAL	470	132	296	63/166 (37.95)	0.58	0.08	166	107	1.00E-26	gene=Chr01G0386	Gene Symbol:UTR2|Host:Isolated from a wide variety of substrates including humans|Disease:invasive candidal disease|Description:CAUTION: The sequence shown here is derived from an EMBL/GenBank/DDBJ whole genome shotgun (WGS) entry which is

														preliminary data.
Chr01G0396.1	566	5	560	UniProt ID:Q4P8E8_USTMA	693	84	671	159/616(25.81)	0.44	0.14	616	152	2.00E-40	gene=Chr01G0396	Gene Symbol:UM03615.1|Host: Euchlaena spp., Zea spp. (Poaceae)|Disease:Smut. Corn smut|Description:COFACTOR: FAD (By similarity).
Chr01G0397.1	937	250	458	UniProt ID:A4ULI8_MYCGR	517	291	511	58/223 (26.01)	0.44	0.07	223	55.8	6.00E-09	gene=Chr01G0397	Gene Symbol:CYP51|Host:Triticum and possibly a few other grasses|Disease:Leaf spot or speckled leaf blotch of wheat|Description:COFACTOR: Heme group (By similarity).
Chr01G0409.1	493	42	281	UniProt ID:A0ST42_CERNC	512	52	285	55/251 (21.91)	0.41	0.11	251	44.7	8.00E-06	gene=Chr01G0409	Gene Symbol:CTB4|Host:Numerous taxa in Solanaceae|Disease:Leaf spot|Description:Unknown
Chr01G0414.1	338	45	335	UniProt ID:Q9P4U9_ALTAL	296	16	268	80/297 (26.94)	0.4	0.17	297	68.9	3.00E-14	gene=Chr01G0414	Gene Symbol:AKT3-1|Host:Plant|Disease:Leaf spot, rots|Description:Unknown

Chr01G0426.1	823	477	583	UniProt ID:Q59RR0_CANAL	783	639	744	52/107 (48.60)	0.64	0.01	107	123	9.00E-30	gene=Chr01G0426	Gene Symbol:ACE2|Host:Isolated from a wide variety of substrates including humans|Disease:invasive candidal disease|Description:CAUTION: The sequence shown here is derived from an EMBL/GenBank/DDBJ whole genome shotgun (WGS) entry which is preliminary data.
Chr01G0431.1	557	8	536	UniProt ID:C0S733_PARBP	560	9	553	159/567(28.04)	0.51	0.11	567	228	1.00E-67	gene=Chr01G0431	Gene Symbol:PABG_03488|Host:humans|Disease:Paracoccidioidomycosis|Description:SIMILARITY: Belongs to the TCP-1 chaperonin family.
Chr01G0440.1	266	23	209	UniProt ID:Q59XU5_CANAL	291	17	174	67/188 (35.64)	0.49	0.16	188	109	6.00E-29	gene=Chr01G0440	Gene Symbol:RAS1|Host:Isolated from a wide variety of substrates including humans|Disease:invasive candidal

															disease|Description:CAUTION: The sequence shown here is derived from an EMBL/GenBank/DDBJ whole genome shotgun (WGS) entry which is preliminary data.
Chr01G0445.1	517	1	517	UniProt ID:Q4WXC5_ASPFU	522	1	522	316/538(58.74)	0.72	0.07	538	627	0	gene=Chr01G0445	Gene Symbol:AFUA_3G09020|Host:humans|Disease:infection|Description:SIMILARITY: Belongs to the eukaryotic-type primase small subunit family.
Chr01G0447.1	602	4	195	UniProt ID:Q5A4X5_CANAL	559	10	222	86/213(40.38)	0.62	0.1	213	159	5.00E-43	gene=Chr01G0447	Gene Symbol:SKN7|Host:Isolated from a wide variety of substrates including humans|Disease:invasive candidal disease|Description:CAUTION: The sequence shown here is derived from an EMBL/GenBank/DDBJ whole genome shotgun (WGS) entry which is

															preliminary data.
Chr01G0450.1	727	3	714	UniProt ID:Q4WLS1_ASPFU	767	63	749	296/719(41.17)	0.61	0.05	719	523	7.00E-176	gene=Chr01G0450	Gene Symbol:AFUA_6G12530|Host:humans|Disease:infection|Description:CAUTION: The sequence shown here is derived from an EMBL/GenBank/DDBJ whole genome shotgun (WGS) entry which is preliminary data.
Chr01G0452.1	431	12	409	UniProt ID:Q96VZ3_FUSOX	455	43	433	108/420(25.71)	0.44	0.12	420	112	2.00E-28	gene=Chr01G0452	Gene Symbol:PGX1|Host:Multiple genera in multiple families|Disease:Blights, wilts, rots of various sorts|Description:SIMILARITY: Belongs to the glycosyl hydrolase 28 family.
Chr01G0457.1	346	92	277	UniProt ID:Q9Y784_MAGGR	631	182	360	44/191(23.04)	0.48	0.09	191	50.4	6.00E-08	gene=Chr01G0457	Gene Symbol:PTH11|Host:Digitaria (Poaceae)|Disease:Leaf spot|Description:Unknown
Chr01G0	345	44	334	UniProt	349	34	346	98/324	0.48	0.14	324	132	4.00E-36	gene=Chr	Gene

459.1				ID:Q6TFC7_ASPFM				(30.25)						01G0459	Symbol:NULL|Host:humans|Disease:infection|Description:Unknown
Chr01G0465.1	976	175	354	UniProt ID:SUB6_ARTBE	412	158	314	56/188 (29.79)	0.44	0.21	188	53.9	3.00E-08	gene=Chr01G0465	Gene Symbol:SUB6|Host:hedgehogs|Disease:ringworm,Kerion Celsi Disease|Description:FUNCTION: Secreted subtilisin-like serine protease with keratinolytic activity that contributes to pathogenicity (By similarity).
Chr01G0468.1	1587	213	1556	UniProt ID:O60034_BOTFU	1562	158	1514	593/1383(42.88)	0.62	0.05	1383	1129	0	gene=Chr01G0468	Gene Symbol:NULL|Host:Various plant families|Disease:Grey mould. Parasite or saprophyte|Description:SIMILARITY: Belongs to the ABC transporter superfamily.
Chr01G0470.1	461	25	460	UniProt ID:Q4WJ71_	432	4	432	305/437(69.79)	0.81	0.02	437	628	0	gene=Chr01G0470	Gene Symbol:AFUA_1G07010|Host:humans|Disease:infe

				ASPFU											ction|Description:CATALYTIC ACTIVITY: CTP + phosphatidate = diphosphate + CDP-diacylglycerol.
Chr01G0478.1	416	61	414	UniProt ID:Q0QWD8_LEPMC	359	1	358	164/359(45.68)	0.62	0.02	359	281	8.00E-92	gene=Chr01G0478	Gene Symbol:THIOL|Host:Brassica spp. and other Brassicaceae|Disease:Black leg, canker, dry rot, leaf spot|Description:SIMILARITY: Belongs to the thiolase family.
Chr01G0480.1	726	1	726	UniProt ID:Q876Z6_FUSOX	724	1	724	406/776(52.32)	0.63	0.13	776	640	0	gene=Chr01G0480	Gene Symbol:RENSA|Host:Multiple genera in multiple families|Disease:Blights, wilts, rots of various sorts|Description:Unknown
Chr01G0483.1	565	102	278	UniProt ID:Q5ALS7_CANAL	1144	351	539	47/196 (23.98)	0.42	0.13	196	46.6	3.00E-06	gene=Chr01G0483	Gene Symbol:CTF1|Host:Isolated from a wide variety of substrates including humans|Disease:invasive candidal

															disease|Description:SIMILARITY: Contains 1 Zn(2)-C6 fungal-type DNA-binding domain.
Chr01G0484.1	546	60	513	UniProt ID:Q5ANE1_CANAL	748	49	503	123/469(26.23)	0.44	0.06	469	130	5.00E-33	gene=Chr01G0484	Gene Symbol:SNF3|Host:Isolated from a wide variety of substrates including humans|Disease:invasive candidal disease|Description:SIMILARITY: Belongs to the major facilitator superfamily. Sugar transporter (TC 2.A.1.1) family.
Chr01G0485.1	598	31	586	UniProt ID:SED3_ASPFU	596	31	580	223/566(39.40)	0.55	0.05	566	364	2.00E-118	gene=Chr01G0485	Gene Symbol:SED3|Host:humans|Disease:infection|Description:FUNCTION: Secreted tripeptidyl-peptidase which degrades proteins at acidic pHs and is involved in virulence.
Chr01G0	1320	257	877	UniProt	917	136	671	144/64	0.38	0.2	644	80.1	5.00E-16	gene=Chr	Gene

489.1				ID:Q9P872_CANAL				4(22.36)						01G0489	Symbol:PMR1|Host:Isolated from a wide variety of substrates including humans|Disease:invasive candidal disease|Description:SIMILARITY: Belongs to the cation transport ATPase (P-type) family.
Chr01G0497.1	734	3	726	UniProt ID:Q99324_SEPLY	803	5	795	326/803(40.60)	0.56	0.11	803	541	0	gene=Chr01G0497	Gene Symbol:B2TOM|Host:Primarily tomato, Lycopersicon esculentum, also Solanum spp. and other Solanaceae|Disease:Leaf spot|Description:Unknown
Chr01G0499.1	451	33	433	UniProt ID:PEPA_COCP7	443	46	442	180/411(43.80)	0.62	0.06	411	326	2.00E-107	gene=Chr01G0499	Gene Symbol:PEP3|Host:humans|Disease:coccidiomycosis|Description:FUNCTION: Secreted aspartic endopeptidase that allows assimilation of proteinaceous substrates. Can catalyze hydrolysis of the major structural

															proteins of basement membrane, elastin, collagen, and laminin. Thought to play a significant role in virulence (By similarity).
Chr01G0504.1	526	79	516	UniProt ID:Q59RG0_CANAL	581	160	571	111/440(25.23)	0.43	0.07	440	129	5.00E-33	gene=Chr01G0504	Gene Symbol:NAG4|Host:Isolated from a wide variety of substrates including humans|Disease:invasive candidal disease|Description:CAUTION: The sequence shown here is derived from an EMBL/GenBank/DDBJ whole genome shotgun (WGS) entry which is preliminary data.
Chr01G0514.1	367	73	362	UniProt ID:Q6TFC7_ASPFM	349	61	348	101/292(34.59)	0.56	0.02	292	184	5.00E-55	gene=Chr01G0514	Gene Symbol:NULL|Host:humans|Disease:infection|Description:Unknown
Chr01G0516.1	387	78	380	UniProt ID:Q6TFC7_A	349	61	346	104/312(33.33)	0.5	0.11	312	154	9.00E-44	gene=Chr01G0516	Gene Symbol:NULL|Host:humans|Disease:infection|Desc

				SPFM											ription:Unknown
Chr01G0520.1	594	75	591	UniProt ID:Q59RG1_CANAL	561	25	554	166/534(31.09)	0.49	0.04	534	247	3.00E-74	gene=Chr01G0520	Gene Symbol:NAG3|Host:Isolated from a wide variety of substrates including humans|Disease:invasive candidal disease|Description:CAUTION: The sequence shown here is derived from an EMBL/GenBank/DDBJ whole genome shotgun (WGS) entry which is preliminary data.
Chr01G0530.1	919	384	700	UniProt ID:Q9HG15_COLLN	746	179	482	72/334(21.56)	0.38	0.14	334	55.8	7.00E-09	gene=Chr01G0530	Gene Symbol:CLTA1|Host:Multiple genera of Fabaceae. Rare reports on other taxa|Disease:Leaf, stem and pod anthracnose|Description:SIMILARITY: Contains 1 Zn(2)-C6 fungal-type DNA-binding domain.
Chr01G0534.1	316	30	315	UniProt ID:O59	384	84	384	189/301(62.7	0.73	0.05	301	347	2.00E-118	gene=Chr01G0534	Gene Symbol:XYL3|Host:Multipl

				937_FUSOX				9)							e genera in multiple families|Disease:Blights, wilts, rots of various sorts|Description:SIMILARITY: Belongs to the glycosyl hydrolase 10 (cellulase F) family.
Chr01G0537.1	674	176	658	UniProt ID:Q59UQ8_CANAL	445	35	441	133/507(26.23)	0.42	0.24	507	156	2.00E-42	gene=Chr01G0537	Gene Symbol:PEP7|Host:Isolated from a wide variety of substrates including humans|Disease:invasive candidal disease|Description:CAUTION: The sequence shown here is derived from an EMBL/GenBank/DDBJ whole genome shotgun (WGS) entry which is preliminary data.
Chr01G0538.1	849	78	183	UniProt ID:A4RED5_MAGO7	618	105	209	32/108 (29.63)	0.53	0.05	108	62	8.00E-11	gene=Chr01G0538	Gene Symbol:MGG_00692|Host:Poaceae, especially important on Oryzae|Disease:Rice blast|Description:Unknow

															n
Chr01G0543.1	455	414	455	UniProt ID:O59937_FUSOX	384	11	53	25/43(58.14)	0.72	0.02	43	57.4	6.00E-10	gene=Chr01G0543	Gene Symbol:XYL3|Host:Multiple genera in multiple families|Disease:Blights, wilts, rots of various sorts|Description:SIMILARITY: Belongs to the glycosyl hydrolase 10 (cellulase F) family.
Chr01G0550.1	559	17	483	UniProt ID:Q5ANE1_CANAL	748	34	496	177/474(37.34)	0.57	0.04	474	288	3.00E-88	gene=Chr01G0550	Gene Symbol:SNF3|Host:Isolated from a wide variety of substrates including humans|Disease:invasive candidal disease|Description:SIMILARITY: Belongs to the major facilitator superfamily. Sugar transporter (TC 2.A.1.1) family.
Chr01G0552.1	559	236	525	UniProt ID:A4ULJ2_MYCGR	515	230	501	66/294(22.45)	0.37	0.09	294	46.6	2.00E-06	gene=Chr01G0552	Gene Symbol:CYP51|Host:Triticum and possibly a few other

															grasses|Disease:Leaf spot or speckled leaf blotch of wheat|Description:COFACTOR: Heme group (By similarity).
Chr01G0553.1	596	63	548	UniProt ID:Q5RLJ7_CRYNV	594	54	553	145/517(28.05)	0.43	0.09	517	184	2.00E-51	gene=Chr01G0553	Gene Symbol:NULL|Host:humans|Disease:cryptococcosis|Description:Unknown
Chr01G0559.1	567	77	547	UniProt ID:Q5ANE1_CANAL	748	51	521	120/491(24.44)	0.43	0.08	491	129	2.00E-32	gene=Chr01G0559	Gene Symbol:SNF3|Host:Isolated from a wide variety of substrates including humans|Disease:invasive candidal disease|Description:SIMILARITY: Belongs to the major facilitator superfamily. Sugar transporter (TC 2.A.1.1) family.
Chr01G0561.1	822	75	123	UniProt ID:A4R0W3_MAGO7	1226	268	316	21/49(42.86)	0.55	0	49	51.2	2.00E-07	gene=Chr01G0561	Gene Symbol:MGG_09263|Host:Poaceae, especially important on Oryzae|Disease:Rice

															blast|Description:Unknown
Chr01G0563.1	1231	52	355	UniProt ID:Q96VB5_ALTAL	578	34	358	82/336 (24.40)	0.42	0.13	336	58.2	2.00E-09	gene=Chr01G0563	Gene Symbol:AFT1-1|Host:Plant|Disease:Leaf spot, rots|Description:Unknown
Chr01G0566.1	240	1	231	UniProt ID:Q96TN6_MAGGR	251	1	229	152/231(65.80)	0.77	0.01	231	309	1.00E-106	gene=Chr01G0566	Gene Symbol:MAS3|Host:Digitaria (Poaceae)|Disease:Leaf spot|Description:Unknown
Chr01G0568.1	1076	696	942	UniProt ID:Q6ZX14_MAGGR	4034	3715	3947	77/255 (30.20)	0.45	0.12	255	65.5	2.00E-11	gene=Chr01G0568	Gene Symbol:ACE1|Host:Digitaria (Poaceae)|Disease:Leaf spot|Description:Unknown
Chr01G0569.1	1122	261	479	UniProt ID:A4ULJ0_MYCGR	518	258	505	59/249 (23.69)	0.43	0.12	249	65.1	1.00E-11	gene=Chr01G0569	Gene Symbol:CYP51|Host:Triticum and possibly a few other grasses|Disease:Leaf spot or speckled leaf blotch of wheat|Description:COFACTOR: Heme group (By similarity).
Chr01G0571.1	316	12	285	UniProt ID:Q9P	296	6	268	110/274(40.1	0.6	0.04	274	210	4.00E-66	gene=Chr01G0571	Gene Symbol:AKT3-2|Host:Plan

				4U7_A LTAL				5)						t|Disease:Leaf spot, rots|Description:Unknown
Chr01G0 572.1	747	266	680	UniProt ID:A6N 6J8_FU SOX	903	226	633	124/42 9(28.9 0)	0.5	0.08	429	167	3.00E-44	gene=Chr 01G0572	Gene Symbol:CTF1|Host:Multipl e genera in multiple families|Disease:Blights, wilts, rots of various sorts|Description:SIMILAR ITY: Contains 1 Zn(2)-C6 fungal-type DNA-binding domain.
Chr01G0 577.1	658	78	546	UniProt ID:Q96 VU5_C RYNE	468	6	424	229/47 1(48.6 2)	0.64	0.11	471	435	1.00E-14 6	gene=Chr 01G0577	Gene Symbol:UGD1|Host:huma ns|Disease:cryptococcosis |Description:Unknown
Chr01G0 583.1	496	23	486	UniProt ID:Q01 446_N ECHA	459	16	454	155/46 6(33.2 6)	0.54	0.06	466	269	1.00E-84	gene=Chr 01G0583	Gene Symbol:MAK1|Host:Trees of various plant families|Disease:Fruit rot, stem rot|Description:Unknown
Chr01G0 585.1	296	11	286	UniProt ID:Q75 WR5_9 PLEO	265	11	262	71/289 (24.57)	0.42	0.17	289	77.4	1.00E-17	gene=Chr 01G0585	Gene Symbol:BRN1|Host:Belam canda chinensis: Korea,Gladiolus ?gandav ensis: Korea,Iris japonica:

														China,Iris missouriensis (Leaf spot.): Idaho; Montana; Oregon; Washington,Iris sp. (Leaf spot.): China; Texas; Washing|Disease:Leaf spot|Description:SIMILARITY: Belongs to the short-chain dehydrogenases/reductases (SDR) family.
Chr01G0586.1	800	291	490	UniProt ID:Q0WXM3_FUSOX	663	256	460	57/209 (27.27)	0.44	0.06	209	68.6	6.00E-13	gene=Chr01G0586	Gene Symbol:FOW2|Host:Multiple genera in multiple families|Disease:Blights, wilts, rots of various sorts|Description:SIMILARITY: Contains 1 Zn(2)-C6 fungal-type DNA-binding domain.
Chr01G0587.1	432	3	349	UniProt ID:A4UC81_MAGO7	376	24	373	133/352(37.78)	0.57	0.02	352	233	1.00E-72	gene=Chr01G0587	Gene Symbol:MGG_10702|Host:Poaceae, especially important on Oryzae|Disease:Rice blast|Description:Unknow

															n
Chr01G0591.1	906	611	840	UniProt ID:TUP1_CANAL	514	260	486	98/242 (40.50)	0.55	0.11	242	164	5.00E-44	gene=Chr01G0591	Gene Symbol:TUP1|Host:Isolated from a wide variety of substrates including humans|Disease:invasive candidal disease|Description:FUNCTION: Represses transcription by RNA polymerase II. Represses genes responsible for initiating filamentous growth and this repression is lifted under inducing environmental conditions.
Chr01G0602.1	295	151	236	UniProt ID:Q9UWE7_GIBZA	218	118	216	45/99(45.45)	0.59	0.13	99	89	8.00E-22	gene=Chr01G0602	Gene Symbol:TRI6|Host:Principal hosts: Poaceae, including Zea mays (corn), Triticum aestivum (wheat), and Oryza sativa (rice). Additional hosts: various plant families|Disease:Seedling blight, pre- and

															post-emergence blight, root and foot rot, brown rot, culm decay, head or kernel blight (scab or ear scab) of cereals. Leaf|Description:Unknown
Chr01G0605.1	349	1	337	UniProt ID:Q5A5C1_CANAL	344	1	279	94/340 (27.65)	0.46	0.19	340	127	2.00E-34	gene=Chr01G0605	Gene Symbol:YNL191|Host:Isolated from a wide variety of substrates including humans|Disease:invasive candidal disease|Description:Unknown
Chr01G0616.1	401	52	400	UniProt ID:HOG1_CRYPA	358	19	304	83/357 (23.25)	0.39	0.22	357	72.8	5.00E-15	gene=Chr01G0616	Gene Symbol:HOG1|Host:Castanea spp., Fagus sylvatica, Quercus spp. (Fagaceae)|Disease:Chestnut blight. Cankers|Description:FUNCTION: Mitogen-activated protein kinase involved in a signal transduction pathway that is activated by changes in the

Chr01G0 617.1	309	19	234	UniProt ID:Q75 WR5_9 PLEO	265	9	207	52/220 (23.64)	0.4	0.11	220	45.4	2.00E-06	gene=Chr 01G0617	osmolarity of the extracellular environment. Controls osmotic regulation of transcription of target genes (By similarity). Involved in the virulence and conidia formation. Mediates tannic acid-induced laccase expression and cryparin expression. Gene Symbol:BRN1|Host:Belam canda chinensis: Korea,Gladiolus ?gandav ensis: Korea,Iris japonica: China,Iris missouriensis (Leaf spot.): Idaho; Montana; Oregon; Washington,Iris sp. (Leaf spot.): China; Texas; Washing|Disease:Leaf spot|Description:SIMILARI TY: Belongs to the short-chain dehydrogenases/reductas

															es (SDR) family.<br>Gene Symbol:BOT2|Host:Various plant families|Disease:Grey mould. Parasite or saprophyte|Description:FUNCTION: Presilphiperfolan-8-beta-ol synthase, which catalyzes the cyclization of farnesyl diphosphate (FPP) to presilphiperfolan-8-beta-ol (PSP), the committed step in the biosynthesis of the terpenoid virulence factor botrydial. Botrydial is necessary for colonization of plant tissue by the T4 strain. It is a strain-dependent virulence factor since highly aggressive strains like SAS56 or B05 still retain substantial virulence when botrydial synthesis is
Chr01G0620.1	386	16	376	UniProt ID:BOT2_BOTFU	399	28	392	251/365(68.77)	0.84	0.01	365	555	0	gene=Chr01G0620	

															impaired.
Chr01G0621.1	317	56	186	UniProt ID:A4RGG9_MAGO7	286	69	193	40/132 (30.30)	0.51	0.06	132	48.5	1.00E-07	gene=Chr01G0621	Gene Symbol:MGG_00056|Host:Poaceae, especially important on Oryzae|Disease:Rice blast|Description:SIMILARITY: Belongs to the short-chain dehydrogenases/reductases (SDR) family.
Chr01G0627.1	421	12	316	UniProt ID:Q6A2T2_BOTFU	391	11	324	80/318 (25.16)	0.45	0.05	318	86.7	2.00E-19	gene=Chr01G0627	Gene Symbol:BTP1|Host:Various plant families|Disease:Grey mould. Parasite or saprophyte|Description:Unknown
Chr01G0631.1	548	29	546	UniProt ID:Q5XTQ4_BOTFU	574	39	572	256/547(46.80)	0.63	0.08	547	484	8.00E-166	gene=Chr01G0631	Gene Symbol:LIP1|Host:Various plant families|Disease:Grey mould. Parasite or saprophyte|Description:Unknown
Chr01G0	369	6	364	UniProt	373	3	372	174/37	0.63	0.05	374	328	3.00E-11	gene=Chr	Gene

634.1				ID:LAP 1_ART BC				4(46.5 2)					0	01G0634	Symbol:LAP1|Host:hedgehogs|Disease:ringworm,Kerion Celsi Disease|Description:FUNCTION: Extracellular aminopeptidase which contributes to pathogenicity (By similarity).
Chr01G0 640.1	354	88	306	UniProt ID:Q6A 2T2_B OTFU	391	116	332	67/223 (30.04)	0.47	0.04	223	97.8	7.00E-24	gene=Chr 01G0640	Gene Symbol:BTP1|Host:Various plant families|Disease:Grey mould. Parasite or saprophyte|Description:Unknown
Chr01G0 641.1	474	56	220	UniProt ID:Q2V LJ1_GI BZA	565	120	290	58/171 (33.92)	0.53	0.04	171	85.5	1.00E-18	gene=Chr 01G0641	Gene Symbol:ZEB1|Host:Principal hosts: Poaceae, including Zea mays (corn), Triticum aestivum (wheat), and Oryza sativa (rice). Additional hosts: various plant families|Disease:Seedling blight, pre- and

															post-emergence blight, root and foot rot, brown rot, culm decay, head or kernel blight (scab or ear scab) of cereals. Leaf|Description:Unknown
Chr01G0642.1	499	266	457	UniProt ID:A4ULI9_MYCGR	502	269	495	56/232 (24.14)	0.45	0.19	232	49.3	3.00E-07	gene=Chr01G0642	Gene Symbol:CYP51|Host:Triticum and possibly a few other grasses|Disease:Leaf spot or speckled leaf blotch of wheat|Description:COFACTOR: Heme group (By similarity).
Chr01G0650.1	579	32	572	UniProt ID:Q9P8L8_BOTFU	598	28	592	225/566(39.75)	0.6	0.05	566	404	7.00E-134	gene=Chr01G0650	Gene Symbol:BCMFS1|Host:Various plant families|Disease:Grey mould. Parasite or saprophyte|Description:Unknown
Chr01G0655.1	359	101	292	UniProt ID:Q5AMT2_CANAL	308	79	273	52/199 (26.13)	0.42	0.06	199	60.5	2.00E-11	gene=Chr01G0655	Gene Symbol:BGL2|Host:Isolated from a wide variety of substrates including

<table>
<tr><td></td><td></td><td></td><td></td><td></td><td></td><td></td><td></td><td></td><td></td><td></td><td></td><td></td><td></td><td></td><td>humans|Disease:invasive candidal disease|Description:SIMILARITY: Belongs to the glycosyl hydrolase 17 family.</td></tr>
<tr><td>Chr01G0662.1</td><td>509</td><td>61</td><td>434</td><td>UniProt ID:Q59RG1_CANAL</td><td>561</td><td>113</td><td>502</td><td>90/393 (22.90)</td><td>0.43</td><td>0.06</td><td>393</td><td>85.9</td><td>8.00E-19</td><td>gene=Chr01G0662</td><td>Gene Symbol:NAG3|Host:Isolated from a wide variety of substrates including humans|Disease:invasive candidal disease|Description:CAUTION: The sequence shown here is derived from an EMBL/GenBank/DDBJ whole genome shotgun (WGS) entry which is preliminary data.</td></tr>
<tr><td>Chr01G0664.1</td><td>690</td><td>26</td><td>427</td><td>UniProt ID:Q9HG15_COLLN</td><td>746</td><td>16</td><td>407</td><td>101/420(24.05)</td><td>0.43</td><td>0.11</td><td>420</td><td>110</td><td>5.00E-26</td><td>gene=Chr01G0664</td><td>Gene Symbol:CLTA1|Host:Multiple genera of Fabaceae. Rare reports on other taxa|Disease:Leaf, stem and pod anthracnose|Description:S</td></tr>
</table>

															IMILARITY: Contains 1 Zn(2)-C6 fungal-type DNA-binding domain.
Chr01G0665.1	564	25	507	UniProt ID:Q9P8L8_BOTFU	598	96	589	130/497(26.16)	0.48	0.03	497	176	2.00E-48	gene=Chr01G0665	Gene Symbol:BCMFS1|Host:Various plant families|Disease:Grey mould. Parasite or saprophyte|Description:Unknown
Chr01G0667.1	261	5	261	UniProt ID:Q32WF7_PHAND	266	11	266	75/264 (28.41)	0.46	0.06	264	103	5.00E-27	gene=Chr01G0667	Gene Symbol:MDH1|Host:Multiple genera of Poaceae and Blysmus compressus (Cyperaceae)|Disease:Glume blotch of wheat and other grasses|Description:Unknown
Chr01G0674.1	530	105	355	UniProt ID:Q59RG0_CANAL	581	175	433	58/264 (21.97)	0.41	0.07	264	45.1	7.00E-06	gene=Chr01G0674	Gene Symbol:NAG4|Host:Isolated from a wide variety of substrates including humans|Disease:invasive candidal disease|Description:CAUT

Chr01G0675.1	901	141	331	UniProt ID:ORYZ_ASPFU	403	153	323	59/198 (29.80)	0.41	0.17	198	50.1	3.00E-07	gene=Chr01G0675	ION: The sequence shown here is derived from an EMBL/GenBank/DDBJ whole genome shotgun (WGS) entry which is preliminary data. Gene Symbol:ALP1|Host:humans|Disease:infection|Description:FUNCTION: Secreted alkaline protease that allows assimilation of proteinaceous substrates. Acts as a significant virulence factor in invasive aspergillosis. Involved in immune evasion from the human and mice complement systems during infection. Efficiently cleaves important components of the complement cascade such as such as C3, C4, C5, and C1q, as well as IgG,

														which leads to down-regulation of complement activation at the hyphal surface.
Chr01G0690.1	1055	428	609	UniProt ID:Q3Y5V5_MAGGR	1321	1067	1275	60/214 (28.04)	0.44	0.17	214	52	2.00E-07	gene=Chr01G0690	Gene Symbol:ABC3|Host:Digitaria (Poaceae)|Disease:Leaf spot|Description:SIMILARITY: Belongs to the ABC transporter superfamily.
Chr01G0692.1	1447	997	1068	UniProt ID:C1G7S0_PARBD	466	358	422	26/72(36.11)	0.49	0.1	72	47.4	4.00E-06	gene=Chr01G0692	Gene Symbol:PADG_03225|Host:humans|Disease:Paracoccidioidomycosis|Description:COFACTOR: Iron (By similarity).
Chr01G0702.1	505	53	505	UniProt ID:A0ST43_CERNC	459	4	456	148/474(31.22)	0.47	0.09	474	186	1.00E-53	gene=Chr01G0702	Gene Symbol:CTB5|Host:Numerous taxa in Solanaceae|Disease:Leaf spot|Description:Unknown
Chr01G0703.1	511	58	509	UniProt ID:Q59RG0_CANAL	581	119	573	122/460(26.52)	0.48	0.03	460	160	1.00E-43	gene=Chr01G0703	Gene Symbol:NAG4|Host:Isolated from a wide variety of substrates including humans|Disease:invasive

														candidal disease|Description:CAUTION: The sequence shown here is derived from an EMBL/GenBank/DDBJ whole genome shotgun (WGS) entry which is preliminary data.
Chr01G0706.1	682	82	293	UniProt ID:Q0WXM3_FUSOX	663	246	445	46/216 (21.30)	0.4	0.09	216	59.7	3.00E-10	gene=Chr01G0706	Gene Symbol:FOW2|Host:Multiple genera in multiple families|Disease:Blights, wilts, rots of various sorts|Description:SIMILARITY: Contains 1 Zn(2)-C6 fungal-type DNA-binding domain.
Chr01G0716.1	628	103	520	UniProt ID:C5G7L2_AJEDR	708	215	622	150/424(35.38)	0.54	0.05	424	237	6.00E-69	gene=Chr01G0716	Gene Symbol:BDCG_00882|Host:humans|Disease:cutaneous Blastomyces dermatitidis infection|Description:SIMILARITY: Belongs to the class-I aminoacyl-tRNA synthetase family.

Chr08G0887.1	404	19	396	UniProt ID:Q2I0M6_CERNC	871	456	861	111/411(27.01)	0.45	0.09	411	134	6.00E-35	gene=Chr08G0887	Gene Symbol:CTB3|Host:Numerous taxa in Solanaceae|Disease:Leaf spot|Description:Unknown
Chr08G0888.1	418	104	309	UniProt ID:A6RGA6_AJECN	483	116	320	56/222(25.23)	0.45	0.15	222	52.8	2.00E-08	gene=Chr08G0888	Gene Symbol:HCAG_08672|Host:humans|Disease:Darling's disease|Description:Unknown
Chr08G0894.1	1738	474	1429	UniProt ID:Q9HFW4_USTMD	2289	540	1528	374/1046(35.76)	0.5	0.14	1046	591	1.00E-177	gene=Chr08G0894	Gene Symbol:RUM1|Host:Euchlaena spp., Zea spp. (Poaceae)|Disease:Smut. Corn smut|Description:SIMILARITY: Contains 1 ARID domain.
Chr08G0896.1	314	89	224	UniProt ID:STE20_USTMA	746	544	673	43/141(30.50)	0.5	0.11	141	79.3	2.00E-17	gene=Chr08G0896	Gene Symbol:SMU1|Host:Euchlaena spp., Zea spp. (Poaceae)|Disease:Smut. Corn smut|Description:FUNCTION: MAP4K component of

Chr08G0898.1	402	30	394	UniProt ID:DHH1_CRYNV	616	38	401	153/366(41.80)	0.58	0.01	366	283	2.00E-89	gene=Chr08G0898	the MAPK pathway required for the mating pheromone response and the regulation of cell polarity and cell cycle. Phosphorylates histone H2B to form H2BS10ph (By similarity). Gene Symbol:VAD1|Host:humans|Disease:cryptococcosis|Description:FUNCTION: ATP-dependent RNA helicase involved in mRNA turnover, and more specifically in mRNA decapping. Is involved in G1/S DNA- damage checkpoint recovery, probably through the regulation of the translational status of a subset of mRNAs. May also have a role in translation and mRNA nuclear export (By

														similarity). Is involved in virulence.
Chr08G0899.1	488	42	356	UniProt ID:Q9Y784_MAGGR	631	98	407	78/316 (24.68)	0.45	0.02	316	103	2.00E-24	gene=Chr08G0899	Gene Symbol:PTH11|Host:Digitaria (Poaceae)|Disease:Leaf spot|Description:Unknown
Chr08G0906.1	368	97	365	UniProt ID:Q6TFC7_ASPFM	349	63	335	99/278 (35.61)	0.54	0.05	278	170	9.00E-50	gene=Chr08G0906	Gene Symbol:NULL|Host:humans|Disease:infection|Description:Unknown
Chr08G0911.1	783	230	487	UniProt ID:Q1L2E2_PHAND	619	296	551	76/270 (28.15)	0.45	0.1	270	83.2	2.00E-17	gene=Chr08G0911	Gene Symbol:ALS1|Host:Multiple genera of Poaceae and Blysmus compressus (Cyperaceae)|Disease:Glume blotch of wheat and other grasses|Description:COFACTOR: Pyridoxal phosphate (By similarity).
Chr08G0920.1	764	56	108	UniProt ID:A8QJI7_FUSOX	938	87	139	19/53(35.85)	0.6	0	53	49.7	5.00E-07	gene=Chr08G0920	Gene Symbol:XLNR|Host:Multiple genera in multiple families|Disease:Blights, wilts, rots of various

															sorts|Description:SIMILARITY: Contains 1 Zn(2)-C6 fungal-type DNA-binding domain.
Chr08G0921.1	537	9	533	UniProt ID:Q5J4D6_PHAND	543	12	536	380/525(72.38)	0.82	0	525	813	0	gene=Chr08G0921	Gene Symbol:MLS1|Host:Multiple genera of Poaceae and Blysmus compressus (Cyperaceae)|Disease:Glume blotch of wheat and other grasses|Description:CATALYTIC ACTIVITY: Acetyl-CoA + H(2)O + glyoxylate = (S)-malate + CoA.
Chr08G0930.1	628	1	626	UniProt ID:Q4WEM7_ASPFU	715	88	712	394/630(62.54)	0.75	0.01	630	784	0	gene=Chr08G0930	Gene Symbol:AFUA_5G03560|Host:humans|Disease:infection|Description:SIMILARITY: Belongs to the class-I aminoacyl-tRNA synthetase family.
Chr08G0933.1	623	123	618	UniProt ID:Q59W43_C	634	60	631	149/593(25.13)	0.41	0.2	593	114	2.00E-27	gene=Chr08G0933	Gene Symbol:HAP43|Host:Isolated from a wide variety of

				ANAL											substrates including humans|Disease:invasive candidal disease|Description:SIMILARITY: Belongs to the bZIP family.
Chr08G0939.1	1261	293	1184	UniProt ID:Q92217_COCHE	2528	1411	2321	298/934(31.91)	0.49	0.07	934	356	9.00E-102	gene=Chr08G0939	Gene Symbol:PKS1|Host:Zea mays|Disease:Southern leaf blight of maize|Description:Unknown
Chr08G0940.1	322	11	321	UniProt ID:Q59QH2_CANAL	337	10	328	75/343(21.87)	0.41	0.16	343	57.8	2.00E-10	gene=Chr08G0940	Gene Symbol:CSH1|Host:Isolated from a wide variety of substrates including humans|Disease:invasive candidal disease|Description:CAUTION: The sequence shown here is derived from an EMBL/GenBank/DDBJ whole genome shotgun (WGS) entry which is preliminary data.
Chr08G0	523	26	518	UniProt	512	41	505	137/49	0.47	0.08	498	164	3.00E-45	gene=Chr	Gene

942.1				ID:A0ST42_CERNC				8(27.51)						08G0942	Symbol:CTB4|Host:Numerous taxa in Solanaceae|Disease:Leaf spot|Description:Unknown
Chr08G0952.1	596	43	593	UniProt ID:Q4P8E8_USTMA	693	84	673	178/613(29.04)	0.46	0.14	613	194	3.00E-54	gene=Chr08G0952	Gene Symbol:UM03615.1|Host: Euchlaena spp., Zea spp. (Poaceae)|Disease:Smut. Corn smut|Description:COFACTOR: FAD (By similarity).
Chr08G0953.1	750	278	391	UniProt ID:DHH1_CRYNV	616	256	369	35/114 (30.70)	0.46	0	114	55.5	8.00E-09	gene=Chr08G0953	Gene Symbol:VAD1|Host:humans|Disease:cryptococcosis|Description:FUNCTION: ATP-dependent RNA helicase involved in mRNA turnover, and more specifically in mRNA decapping. Is involved in G1/S DNA- damage checkpoint recovery, probably through the regulation of the translational status of a subset of mRNAs. May

															also have a role in translation and mRNA nuclear export (By similarity). Is involved in virulence.
Chr08G0954.1	474	8	40	UniProt ID:Q5A4F3_CANAL	624	14	46	17/33(51.52)	0.7	0	33	50.1	2.00E-07	gene=Chr08G0954	Gene Symbol:ZCF37|Host:Isolated from a wide variety of substrates including humans|Disease:invasive candidal disease|Description:Unknown
Chr08G0956.1	528	4	218	UniProt ID:Q59RG0_CANAL	581	74	308	56/253(22.13)	0.4	0.22	253	56.6	2.00E-09	gene=Chr08G0956	Gene Symbol:NAG4|Host:Isolated from a wide variety of substrates including humans|Disease:invasive candidal disease|Description:CAUTION: The sequence shown here is derived from an EMBL/GenBank/DDBJ whole genome shotgun (WGS) entry which is preliminary data.

Chr08G0957.1	502	255	467	UniProt ID:A4ULI5_MYCGR	515	261	503	51/243(20.99)	0.37	0.12	243	46.6	2.00E-06	gene=Chr08G0957	Gene Symbol:CYP51|Host:Triticum and possibly a few other grasses|Disease:Leaf spot or speckled leaf blotch of wheat|Description:COFACTOR: Heme group (By similarity).
Chr08G0959.1	511	5	494	UniProt ID:Q5XTQ5_BOTFU	615	65	564	146/523(27.92)	0.47	0.11	523	168	3.00E-46	gene=Chr08G0959	Gene Symbol:FRT1|Host:Various plant families|Disease:Grey mould. Parasite or saprophyte|Description:SIMILARITY: Belongs to the major facilitator superfamily. Sugar transporter (TC 2.A.1.1) family.
Chr08G0964.1	437	384	437	UniProt ID:O59937_FUSOX	384	1	54	22/54(40.74)	0.59	0	54	56.2	1.00E-09	gene=Chr08G0964	Gene Symbol:XYL3|Host:Multiple genera in multiple families|Disease:Blights, wilts, rots of various sorts|Description:SIMILAR

															ITY: Belongs to the glycosyl hydrolase 10 (cellulase F) family.
Chr08G0966.1	371	85	367	UniProt ID:Q6TFC7_ASPFM	349	65	349	93/290 (32.07)	0.51	0.04	290	144	3.00E-40	gene=Chr08G0966	Gene Symbol:NULL|Host:humans|Disease:infection|Description:Unknown
Chr08G0967.1	459	17	344	UniProt ID:Q9Y784_MAGGR	631	30	360	86/338 (25.44)	0.45	0.05	338	116	6.00E-29	gene=Chr08G0967	Gene Symbol:PTH11|Host:Digitaria (Poaceae)|Disease:Leaf spot|Description:Unknown
Chr08G0969.1	100	1	98	UniProt ID:O94196_MAGGR	102	1	100	55/100 (55.00)	0.68	0.02	100	110	4.00E-33	gene=Chr08G0969	Gene Symbol:NULL|Host:Digitaria (Poaceae)|Disease:Leaf spot|Description:Unknown
Chr08G0978.1	628	128	328	UniProt ID:Q5AAJ8_CANAL	359	146	306	56/201 (27.86)	0.44	0.2	201	71.6	2.00E-14	gene=Chr08G0978	Gene Symbol:RTT109|Host:Isolated from a wide variety of substrates including humans|Disease:invasive candidal disease|Description:CAUTION: The sequence shown here is derived from an EMBL/GenBank/DDBJ

															whole genome shotgun (WGS) entry which is preliminary data.
Chr08G0985.1	635	35	626	UniProt ID:Q9P304_CLAFU	665	6	639	220/644(34.16)	0.53	0.1	644	377	8.00E-122	gene=Chr08G0985	Gene Symbol:AOX1|Host:Lycopersicon esculentum (Solanaceae)|Disease:Leaf mold of tomato|Description:COFACTOR: FAD (By similarity).
Chr08G0987.1	525	251	490	UniProt ID:A4ULJ0_MYCGR	518	254	513	57/270 (21.11)	0.41	0.15	270	46.6	2.00E-06	gene=Chr08G0987	Gene Symbol:CYP51|Host:Triticum and possibly a few other grasses|Disease:Leaf spot or speckled leaf blotch of wheat|Description:COFACTOR: Heme group (By similarity).
Chr08G0993.1	446	122	301	UniProt ID:PLYB_COLGL	331	94	272	46/190 (24.21)	0.46	0.11	190	50.4	7.00E-08	gene=Chr08G0993	Gene Symbol:PLB|Host:Multiple genera in multiple families|Disease:'Anthracnose of stems and leaves, dieback, root rot, leaf spot,

														blossom rot, fruit rot (dieback and ripe rot), seedling blight.' (Mordue 1971)|Description:FUNCTION: Acts as a virulence factor active in plant tissue maceration.
Chr08G1001.1	637	15	57	UniProt ID:Q96UQ9_MAGGR	715	570	612	21/43(48.84)	0.67	0	43	53.1	3.00E-08	gene=Chr08G1001	Gene Symbol:NULL|Host:Digitaria (Poaceae)|Disease:Leaf spot|Description:Unknown
Chr08G1003.1	588	9	586	UniProt ID:O93886_9PEZI	607	17	573	134/590(22.71)	0.42	0.08	590	54.7	8.00E-09	gene=Chr08G1003	Gene Symbol:NULL|Host:Fabaceae|Disease:Leaf spot, seed stain, etc|Description:Unknown
Chr08G1006.1	529	48	509	UniProt ID:Q5XTQ5_BOTFU	615	105	561	120/474(25.32)	0.44	0.06	474	120	7.00E-30	gene=Chr08G1006	Gene Symbol:FRT1|Host:Various plant families|Disease:Grey mould. Parasite or saprophyte|Description:SIMILARITY: Belongs to the major facilitator superfamily. Sugar transporter (TC 2.A.1.1)

														family.
Chr08G1007.1	655	329	567	UniProt ID:A4R0W3_MAGO7	1226	818	1052	63/249 (25.30)	0.45	0.1	249	52	8.00E-08	gene=Chr08G1007	Gene Symbol:MGG_09263|Host:Poaceae, especially important on Oryzae|Disease:Rice blast|Description:Unknown
Chr08G1010.1	509	113	451	UniProt ID:Q50I20_ASPFM	465	122	458	103/359(28.69)	0.45	0.12	359	110	5.00E-27	gene=Chr08G1010	Gene Symbol:MCSA|Host:humans|Disease:infection|Description:SIMILARITY: Belongs to the citrate synthase family.
Chr08G1011.1	1095	31	1031	UniProt ID:Q9P872_CANAL	917	28	904	298/1016(29.33)	0.48	0.15	1016	382	1.00E-116	gene=Chr08G1011	Gene Symbol:PMR1|Host:Isolated from a wide variety of substrates including humans|Disease:invasive candidal disease|Description:SIMILARITY: Belongs to the cation transport ATPase (P-type) family.
Chr08G1024.1	511	25	504	UniProt ID:Q9U	567	46	559	156/537(29.0	0.44	0.15	537	142	2.00E-37	gene=Chr08G1024	Gene Symbol:CHIP3|Host:Multi

			US8_COLGL				5)							ple genera in multiple families|Disease:'Anthracnose of stems and leaves, dieback, root rot, leaf spot, blossom rot, fruit rot (dieback and ripe rot), seedling blight.' (Mordue 1971)|Description:Unknown
Chr08G1025.1	1127	16	1127	UniProt ID:Q4WPX2_ASPFU	1079	12	1072	516/1115(46.28)	0.65	0.05	1115	1044	0	gene=Chr08G1025	Gene Symbol:PPOA|Host:humans|Disease:infection|Description:Unknown
Chr08G1026.1	531	21	453	UniProt ID:Q5XTQ4_BOTFU	574	36	512	147/492(29.88)	0.45	0.15	492	145	2.00E-38	gene=Chr08G1026	Gene Symbol:LIP1|Host:Various plant families|Disease:Grey mould. Parasite or saprophyte|Description:Unknown
Chr08G1028.1	523	1	521	UniProt ID:C1GJS5_PARBD	520	1	517	317/548(57.85)	0.69	0.11	548	593	0	gene=Chr08G1028	Gene Symbol:PADG_07511|Host:humans|Disease:Paracoccidioidomycosis|Description:Unknown
Chr08G1	508	244	480	UniProt	515	241	502	53/269	0.4	0.14	269	45.4	4.00E-06	gene=Chr	Gene

032.1				ID:A4U LI5_MY CGR				(19.70)						08G1032	Symbol:CYP51|Host:Triticum and possibly a few other grasses|Disease:Leaf spot or speckled leaf blotch of wheat|Description:COFACTOR: Heme group (By similarity).
Chr08G1 033.1	795	140	248	UniProt ID:A0S T46_C ERNC	397	19	124	35/113 (30.97)	0.51	0.1	113	50.4	2.00E-07	gene=Chr 08G1033	Gene Symbol:CTB8|Host:Numerous taxa in Solanaceae|Disease:Leaf spot|Description:Unknown
Chr08G1 034.1	442	21	442	UniProt ID:C5G HR8_A JEDR	435	9	435	207/433(47.81)	0.64	0.04	433	366	3.00E-123	gene=Chr 08G1034	Gene Symbol:BDCG_04304|Host:humans|Disease:cutaneous Blastomyces dermatitidis infection|Description:Unknown
Chr08G1 044.1	536	59	513	UniProt ID:Q5A NE1_C ANAL	748	50	511	103/476(21.64)	0.39	0.07	476	75.5	2.00E-15	gene=Chr 08G1044	Gene Symbol:SNF3|Host:Isolated from a wide variety of substrates including humans|Disease:invasive candidal

													disease|Description:SIMILARITY: Belongs to the major facilitator superfamily. Sugar transporter (TC 2.A.1.1) family.
Chr08G1046.1	299	54	176	UniProt ID:A4RGG9_MAGO7	286	73	203	41/137 (29.93)	0.53	0.15	137	49.3	9.00E-08	gene=Chr08G1046	Gene Symbol:MGG_00056|Host:Poaceae, especially important on Oryzae|Disease:Rice blast|Description:SIMILARITY: Belongs to the short-chain dehydrogenases/reductases (SDR) family.
Chr08G1048.1	388	32	383	UniProt ID:Q7ZA48_9HYPO	418	47	407	86/377 (22.81)	0.35	0.11	377	72	1.00E-14	gene=Chr08G1048	Gene Symbol:GLU1|Host:humans|Disease:Verticillium disease or dry bubble|Description:Unknown
Chr08G1049.1	498	101	496	UniProt ID:Q5ANE1_CANAL	748	88	503	105/427(24.59)	0.47	0.1	427	130	2.00E-33	gene=Chr08G1049	Gene Symbol:SNF3|Host:Isolated from a wide variety of substrates including

														humans|Disease:invasive candidal disease|Description:SIMILARITY: Belongs to the major facilitator superfamily. Sugar transporter (TC 2.A.1.1) family.
Chr08G1051.1	719	217	593	UniProt ID:A6N6J8_FUSOX	903	247	617	92/394 (23.35)	0.43	0.1	394	68.2	8.00E-13	gene=Chr08G1051	Gene Symbol:CTF1|Host:Multiple genera in multiple families|Disease:Blights, wilts, rots of various sorts|Description:SIMILARITY: Contains 1 Zn(2)-C6 fungal-type DNA-binding domain.
Chr08G1052.1	417	49	233	UniProt ID:A4QVF8_MAGO7	339	1	179	46/189 (24.34)	0.43	0.07	189	48.1	4.00E-07	gene=Chr08G1052	Gene Symbol:MGG_04556|Host:Poaceae, especially important on Oryzae|Disease:Rice blast|Description:COFACTOR: Zinc (By similarity).
Chr08G1057.1	413	2	321	UniProt ID:A4U	376	36	367	100/332(30.1	0.48	0.04	332	157	3.00E-44	gene=Chr08G1057	Gene Symbol:MGG_10702|Host

				C81_MAGO7				2)						:Poaceae, especially important on Oryzae|Disease:Rice blast|Description:Unknown
Chr08G1061.1	376	142	315	UniProt ID:A4R3I5_MAGO7	400	103	265	40/176 (22.73)	0.44	0.09	176	45.8	2.00E-06	gene=Chr08G1061	Gene Symbol:"MGG_11993, MGG_12837, MGG_13052"|Host:Poaceae, especially important on Oryzae|Disease:Rice blast|Description:Unknown
Chr08G1070.1	932	655	868	UniProt ID:SUB4A_COCP7	397	151	357	66/223 (29.60)	0.44	0.11	223	55.1	8.00E-09	gene=Chr08G1070	Gene Symbol:CPC735_066880|Host:humans|Disease:coccidiomycosis|Description: FUNCTION: Secreted subtilisin-like serine protease with keratinolytic activity that contributes to pathogenicity (By similarity).
Chr05G0881.1	374	26	216	UniProt ID:Q59RG0_C	581	127	317	53/192 (27.60)	0.51	0.01	192	80.1	2.00E-17	gene=Chr05G0881	Gene Symbol:NAG4|Host:Isolated from a wide variety of

				ANAL										substrates including humans|Disease:invasive candidal disease|Description:CAUTION: The sequence shown here is derived from an EMBL/GenBank/DDBJ whole genome shotgun (WGS) entry which is preliminary data.
Chr05G0884.1	685	526	653	UniProt ID:Q6TFC7_ASPFM	349	63	189	55/130 (42.31)	0.55	0.04	130	95.1	4.00E-22	gene=Chr05G0884	Gene Symbol:NULL|Host:humans|Disease:infection|Description:Unknown
Chr05G0902.1	368	314	358	UniProt ID:RAD18_CANAL	378	27	71	16/45(35.56)	0.56	0	45	44.3	6.00E-06	gene=Chr05G0902	Gene Symbol:RAD18|Host:Isolated from a wide variety of substrates including humans|Disease:invasive candidal disease|Description:FUNCTION: E3 RING-finger protein, member of the UBC2/RAD6 epistasis group. Associates to the E2 ubiquitin conjugating

															enzyme UBC2/RAD6 to form the UBC2-RAD18 ubiquitin ligase complex involved in postreplicative repair (PRR) of damaged DNA (By similarity).
Chr05G0903.1	264	1	155	UniProt ID:Q9C441_FUSSO	330	165	303	65/156 (41.67)	0.58	0.12	156	124	5.00E-34	gene=Chr05G0903	Gene Symbol:PEP1|Host:Multiple plant families. Some strains may cause infections in humans|Disease:Saprobe, facultative pathogen|Description:Unknown
Chr05G0906.1	1217	772	1207	UniProt ID:Q7Z868_USTMD	862	99	629	158/540(29.26)	0.42	0.21	540	162	8.00E-42	gene=Chr05G0906	Gene Symbol:GLO1|Host:Euchlaena spp., Zea spp. (Poaceae)|Disease:Smut. Corn smut|Description:Unknown
Chr05G0922.1	1200	878	1014	UniProt ID:Q5K2R7_CLAPU	655	269	407	37/144 (25.69)	0.44	0.08	144	46.6	7.00E-06	gene=Chr05G0922	Gene Symbol:COT1|Host:outcrossing species|Disease:ergotism|

															Description:CATALYTIC ACTIVITY: ATP + a protein = ADP + a phosphoprotein.
Chr05G0925.1	344	33	316	UniProt ID:Q6A2T2_BOTFU	391	38	356	91/325 (28.00)	0.46	0.14	325	99.4	2.00E-24	gene=Chr05G0925	Gene Symbol:BTP1|Host:Various plant families|Disease:Grey mould. Parasite or saprophyte|Description:Unknown
Chr05G0934.1	628	32	581	UniProt ID:Q9P8L8_BOTFU	598	46	592	156/557(28.01)	0.49	0.03	557	226	7.00E-66	gene=Chr05G0934	Gene Symbol:BCMFS1|Host:Various plant families|Disease:Grey mould. Parasite or saprophyte|Description:Unknown
Chr05G0935.1	382	4	182	UniProt ID:Q96TN6_MAGGR	251	2	198	62/201 (30.85)	0.49	0.13	201	73.6	6.00E-16	gene=Chr05G0935	Gene Symbol:MAS3|Host:Digitaria (Poaceae)|Disease:Leaf spot|Description:Unknown
Chr05G0938.1	425	2	387	UniProt ID:A0ST45_C	450	7	425	138/431(32.02)	0.47	0.13	431	165	2.00E-46	gene=Chr05G0938	Gene Symbol:CTB7|Host:Numerous taxa in

				ERNC											Solanaceae|Disease:Leaf spot|Description:Unknown
Chr05G0941.1	389	92	302	UniProt ID:Q9Y784_MAGGR	631	162	367	55/211 (26.07)	0.47	0.02	211	89	3.00E-20	gene=Chr05G0941	Gene Symbol:PTH11|Host:Digitaria (Poaceae)|Disease:Leaf spot|Description:Unknown
Chr05G0944.1	751	147	737	UniProt ID:B2C6F1_CRYGA	614	36	586	180/623(28.89)	0.45	0.17	623	253	2.00E-74	gene=Chr05G0944	Gene Symbol:LAC1|Host:humans|Disease:pulmonary cryptococcosis, basal meningitis, and cerebral cryptococcomas|Description:Unknown
Chr05G0948.1	488	47	321	UniProt ID:Q5AJC0_CANAL	470	68	396	76/331 (22.96)	0.41	0.18	331	62.8	2.00E-11	gene=Chr05G0948	Gene Symbol:UTR2|Host:Isolated from a wide variety of substrates including humans|Disease:invasive candidal disease|Description:CAUTION: The sequence shown here is derived from an EMBL/GenBank/DDBJ whole genome shotgun (WGS) entry which is

														preliminary data.
Chr05G0952.1	843	7	369	UniProt ID:Q99324_SEPLY	803	54	486	132/441(29.93)	0.46	0.2	441	188	6.00E-51	gene=Chr05G0952	Gene Symbol:B2TOM|Host:Primarily tomato, Lycopersicon esculentum, also Solanum spp. and other Solanaceae|Disease:Leaf spot|Description:Unknown
Chr05G0953.1	517	42	497	UniProt ID:Q5ANE1_CANAL	748	51	514	126/475(26.53)	0.44	0.06	475	121	5.00E-30	gene=Chr05G0953	Gene Symbol:SNF3|Host:Isolated from a wide variety of substrates including humans|Disease:invasive candidal disease|Description:SIMILARITY: Belongs to the major facilitator superfamily. Sugar transporter (TC 2.A.1.1) family.
Chr05G0956.1	561	331	556	UniProt ID:C0S9K6_PARBP	730	22	238	63/231(27.27)	0.44	0.08	231	52.4	4.00E-08	gene=Chr05G0956	Gene Symbol:PABG_04193|Host:humans|Disease:Paracoccidioidomycosis|Description:Unknown
Chr05G0	166	66	134	UniProt	288	210	279	26/72(	0.61	0.07	72	48.9	2.00E-08	gene=Chr	Gene

964.1				ID:C1G0P8_PARBD				36.11)						05G0964	Symbol:PADG_00438|Host:humans|Disease:Paracoccidioidomycosis|Description:FUNCTION: Component of the eukaryotic translation initiation factor 3 (eIF-3) complex (By similarity).
Chr05G0965.1	607	7	603	UniProt ID:A4R9C7_MAGO7	1305	7	532	142/612(23.20)	0.4	0.17	612	89.7	2.00E-19	gene=Chr05G0965	Gene Symbol:MGG_13324|Host:Poaceae, especially important on Oryzae|Disease:Rice blast|Description:Unknown
Chr05G0967.1	573	57	535	UniProt ID:Q5XTQ4_BOTFU	574	58	538	157/520(30.19)	0.44	0.15	520	172	2.00E-47	gene=Chr05G0967	Gene Symbol:LIP1|Host:Various plant families|Disease:Grey mould. Parasite or saprophyte|Description:Unknown
Chr05G0983.1	556	28	62	UniProt ID:Q5A4F3_CANAL	624	12	46	17/35(48.57)	0.66	0	35	50.4	1.00E-07	gene=Chr05G0983	Gene Symbol:ZCF37|Host:Isolated from a wide variety of substrates including

															humans|Disease:invasive candidal disease|Description:Unknown
Chr05G0993.1	305	19	303	UniProt ID:Q9Y784_MAGGR	631	22	309	83/289 (28.72)	0.55	0.02	289	160	2.00E-45	gene=Chr05G0993	Gene Symbol:PTH11|Host:Digitaria (Poaceae)|Disease:Leaf spot|Description:Unknown
Chr05G1013.1	416	24	337	UniProt ID:A4UC81_MAGO7	376	55	371	86/327 (26.30)	0.45	0.07	327	95.1	1.00E-22	gene=Chr05G1013	Gene Symbol:MGG_10702|Host:Poaceae, especially important on Oryzae|Disease:Rice blast|Description:Unknown
Chr05G1021.1	704	344	408	UniProt ID:Q700F1_CANGB	703	549	607	22/65(33.85)	0.49	0.09	65	45.4	8.00E-06	gene=Chr05G1021	Gene Symbol:ACE2|Host:humans|Disease:Occasional invasive candidal disease|Description:Unknown
Chr05G1024.1	419	55	347	UniProt ID:Q00523_CRYNE	458	91	359	91/302 (30.13)	0.47	0.14	302	107	1.00E-26	gene=Chr05G1024	Gene Symbol:NULL|Host:humans|Disease:cryptococcosis|Description:Unknown

Chr05G1025.1	770	13	770	UniProt ID:Q59X23_CANAL	755	8	755	373/778(47.94)	0.64	0.06	778	711	0	gene=Chr05G1025	Gene Symbol:PMT4|Host:Isolated from a wide variety of substrates including humans|Disease:invasive candidal disease|Description:CAUTION: The sequence shown here is derived from an EMBL/GenBank/DDBJ whole genome shotgun (WGS) entry which is preliminary data.
Chr05G1028.1	1196	181	1088	UniProt ID:Q9P872_CANAL	917	65	881	269/919(29.27)	0.5	0.12	919	344	6.00E-102	gene=Chr05G1028	Gene Symbol:PMR1|Host:Isolated from a wide variety of substrates including humans|Disease:invasive candidal disease|Description:SIMILARITY: Belongs to the cation transport ATPase (P-type) family.
Chr05G1031.1	570	14	569	UniProt ID:A4QVD7_M	541	16	539	267/561(47.59)	0.6	0.07	561	379	4.00E-125	gene=Chr05G1031	Gene Symbol:MGG_04582|Host:Poaceae, especially

				AGO7											important on Oryzae|Disease:Rice blast|Description:Unknown
Chr05G1034.1	1229	83	1184	UniProt ID:CHS3_CANAL	1213	13	1153	624/1168(53.42)	0.67	0.08	1168	1202	0	gene=Chr05G1034	Gene Symbol:CHS3|Host:Isolated from a wide variety of substrates including humans|Disease:invasive candidal disease|Description:FUNCTION: Formation and repair of the disk-shaped septum in yeast and the cross walls of the hyphal phase.
Chr05G1035.1	750	359	716	UniProt ID:A4QRN5_MAGO7	1015	219	577	119/369(32.25)	0.51	0.06	369	165	2.00E-43	gene=Chr05G1035	Gene Symbol:MGG_03530|Host:Poaceae, especially important on Oryzae|Disease:Rice blast|Description:Unknown
Chr05G1038.1	446	13	282	UniProt ID:Q9Y784_M	631	110	366	64/275(23.27)	0.47	0.08	275	76.3	9.00E-16	gene=Chr05G1038	Gene Symbol:PTH11|Host:Digitaria

				AGGR										(Poaceae)|Disease:Leaf spot|Description:Unknown
Chr05G1 039.1	298	52	293	UniProt ID:A4RGG9_MAGO7	286	13	269	78/259 (30.12)	0.5	0.07	259	102	4.00E-26	gene=Chr05G1039	Gene Symbol:MGG_00056|Host:Poaceae, especially important on Oryzae|Disease:Rice blast|Description:SIMILARITY: Belongs to the short-chain dehydrogenases/reductases (SDR) family.
Chr05G1 046.1	363	19	124	UniProt ID:TUP1_CANAL	514	265	366	35/107 (32.71)	0.5	0.06	107	45.8	2.00E-06	gene=Chr05G1046	Gene Symbol:TUP1|Host:Isolated from a wide variety of substrates including humans|Disease:invasive candidal disease|Description:FUNCTION: Represses transcription by RNA polymerase II. Represses genes responsible for initiating filamentous growth and this repression is lifted under inducing

														environmental conditions.
Chr05G1048.1	555	184	453	UniProt ID:TUP1_CANAL	514	204	464	87/297 (29.29)	0.45	0.21	297	112	2.00E-27	gene=Chr05G1048	Gene Symbol:TUP1|Host:Isolated from a wide variety of substrates including humans|Disease:invasive candidal disease|Description:FUNCTION: Represses transcription by RNA polymerase II. Represses genes responsible for initiating filamentous growth and this repression is lifted under inducing environmental conditions.
Chr05G1050.1	696	68	544	UniProt ID:O59918_USTMD	608	132	547	163/500(32.60)	0.48	0.21	500	223	3.00E-64	gene=Chr05G1050	Gene Symbol:UKC1|Host:Euchlaena spp., Zea spp. (Poaceae)|Disease:Smut. Corn smut|Description:CATALYTIC ACTIVITY: ATP + a protein = ADP + a phosphoprotein.
Chr05G1	468	364	467	UniProt	117	1	116	52/116	0.55	0.1	116	79.3	1.00E-18	gene=Chr	Gene

051.1				ID:Q2LK92_BOTFU				(44.83)						05G1051	Symbol:PIC5|Host:Various plant families|Disease:Grey mould. Parasite or saprophyte|Description:SIMILARITY: Belongs to the FKBP-type PPIase family.
Chr05G1081.1	363	180	342	UniProt ID:Q6A2T2_BOTFU	391	204	374	38/171 (22.22)	0.47	0.05	171	51.6	3.00E-08	gene=Chr05G1081	Gene Symbol:BTP1|Host:Various plant families|Disease:Grey mould. Parasite or saprophyte|Description:Unknown
Chr05G1084.1	1389	23	1389	UniProt ID:Q0PND8_MAGGR	1375	15	1374	912/1390(65.61)	0.78	0.04	1390	1721	0	gene=Chr05G1084	Gene Symbol:PEX6|Host:Digitaria (Poaceae)|Disease:Leaf spot|Description:SIMILARITY: Belongs to the AAA ATPase family.
Chr05G1087.1	360	86	339	UniProt ID:KATG_PENMA	748	61	416	97/357 (27.17)	0.37	0.29	357	90.5	1.00E-20	gene=Chr05G1087	Gene Symbol:KATG|Host:humans|Disease:lethal systemic infection|Description:FUNCTION: Bifunctional enzyme with both catalase

															and broad- spectrum peroxidase activity (By similarity). May be involved in protection from the host during host infection.
Chr05G1089.1	1311	21	1041	UniProt ID:Q59YF3_CANAL	986	2	976	364/1049(34.70)	0.54	0.1	1049	568	0	gene=Chr05G1089	Gene Symbol:INP51|Host:Isolated from a wide variety of substrates including humans|Disease:invasive candidal disease|Description:CAUTION: The sequence shown here is derived from an EMBL/GenBank/DDBJ whole genome shotgun (WGS) entry which is preliminary data.
Chr05G1091.1	250	1	244	UniProt ID:1433_CANAL	264	24	258	170/244(69.67)	0.81	0.04	244	347	6.00E-121	gene=Chr05G1091	Gene Symbol:BMH1|Host:Isolated from a wide variety of substrates including humans|Disease:invasive candidal disease|Description:SIMIL

															ARITY: Belongs to the 14-3-3 family.
Chr05G1092.1	423	114	331	UniProt ID:Q6WER3_GIBZA	351	87	275	73/225 (32.44)	0.48	0.19	225	87.8	4.00E-20	gene=Chr05G1092	Gene Symbol:FGL1|Host:Principal hosts: Poaceae, including Zea mays (corn), Triticum aestivum (wheat), and Oryza sativa (rice). Additional hosts: various plant families|Disease:Seedling blight, pre- and post-emergence blight, root and foot rot, brown rot, culm decay, head or kernel blight (scab or ear scab) of cereals. Leaf|Description:Unknown
Chr05G1097.1	996	10	990	UniProt ID:Q9P872_CANAL	917	30	911	339/990(34.24)	0.52	0.12	990	492	1.00E-158	gene=Chr05G1097	Gene Symbol:PMR1|Host:Isolated from a wide variety of substrates including humans|Disease:invasive candidal disease|Description:SIMILARITY: Belongs to the

															cation transport ATPase (P-type) family.
Chr05G1103.1	1001	71	314	UniProt ID:Q01143_MAGGR	539	228	459	77/247 (31.17)	0.52	0.07	247	132	4.00E-33	gene=Chr05G1103	Gene Symbol:CPKA|Host:Digitaria (Poaceae)|Disease:Leaf spot|Description:CATALYTIC ACTIVITY: ATP + a protein = ADP + a phosphoprotein.
Chr05G1106.1	251	58	202	UniProt ID:Q5AD07_CANAL	228	41	178	55/145 (37.93)	0.54	0.05	145	100	3.00E-26	gene=Chr05G1106	Gene Symbol:SOD5|Host:Isolated from a wide variety of substrates including humans|Disease:invasive candidal disease|Description:FUNCTION: Destroys radicals which are normally produced within the cells and which are toxic to biological systems (By similarity).
Chr05G1115.1	158	5	147	UniProt ID:Q5AK12_C	201	44	183	46/148 (31.08)	0.54	0.09	148	70.1	3.00E-16	gene=Chr05G1115	Gene Symbol:CNB1|Host:Isolated from a wide variety of

				ANAL											substrates including humans|Disease:invasive candidal disease|Description:CAUTION: The sequence shown here is derived from an EMBL/GenBank/DDBJ whole genome shotgun (WGS) entry which is preliminary data.
Chr05G1119.1	362	15	362	UniProt ID:C0SJH4_PARBP	302	7	300	210/348(60.34)	0.7	0.16	348	379	3.00E-131	gene=Chr05G1119	Gene Symbol:PABG_07828|Host:humans|Disease:Paracoccidioidomycosis|Description:Unknown
Chr05G1126.1	566	288	529	UniProt ID:A4ULI8_MYCGR	517	245	505	67/266(25.19)	0.44	0.11	266	74.7	4.00E-15	gene=Chr05G1126	Gene Symbol:CYP51|Host:Triticum and possibly a few other grasses|Disease:Leaf spot or speckled leaf blotch of wheat|Description:COFACTOR: Heme group (By similarity).
Chr05G1132.1	1972	1222	1466	UniProt ID:Q5K	1296	471	686	55/264(20.83)	0.41	0.25	264	50.8	8.00E-07	gene=Chr05G1132	Gene Symbol:CNI04280|Host:h

				B00_CRYNJ											umans|Disease:cryptococcosis|Description:SIMILARITY: Contains 1 DH (DBL-homology) domain.
Chr05G1133.1	430	305	413	UniProt ID:A4RG81_MAGO7	558	413	513	43/109 (39.45)	0.52	0.07	109	75.9	9.00E-16	gene=Chr05G1133	Gene Symbol:MGG_00131|Host:Poaceae, especially important on Oryzae|Disease:Rice blast|Description:Unknown
Chr05G1137.1	964	384	556	UniProt ID:Q0WXM3_FUSOX	663	253	424	47/175 (26.86)	0.42	0.03	175	68.9	6.00E-13	gene=Chr05G1137	Gene Symbol:FOW2|Host:Multiple genera in multiple families|Disease:Blights, wilts, rots of various sorts|Description:SIMILARITY: Contains 1 Zn(2)-C6 fungal-type DNA-binding domain.
Chr05G1139.1	638	280	365	UniProt ID:Q59RR0_CANAL	783	647	732	37/88(42.05)	0.59	0.05	88	77.8	6.00E-16	gene=Chr05G1139	Gene Symbol:ACE2|Host:Isolated from a wide variety of substrates including humans|Disease:invasive candidal

														disease|Description:CAUTION: The sequence shown here is derived from an EMBL/GenBank/DDBJ whole genome shotgun (WGS) entry which is preliminary data.
Chr05G1140.1	435	161	426	UniProt ID:Q59NP5_CANAL	418	160	410	112/271(41.33)	0.55	0.09	271	190	8.00E-56	gene=Chr05G1140	Gene Symbol:SUN41|Host:Isolated from a wide variety of substrates including humans|Disease:invasive candidal disease|Description:CAUTION: The sequence shown here is derived from an EMBL/GenBank/DDBJ whole genome shotgun (WGS) entry which is preliminary data.
Chr05G1142.1	828	62	492	UniProt ID:Q5A839_CANAL	454	35	453	223/433(51.50)	0.68	0.04	433	434	3.00E-144	gene=Chr05G1142	Gene Symbol:HOS2|Host:Isolated from a wide variety of substrates including humans|Disease:invasive candidal

Chr05G1143.1	433	380	431	UniProt ID:Q9HFW4_USTMD	2289	1671	1722	23/55(41.82)	0.49	0.11	55	48.9	4.00E-07	gene=Chr05G1143	disease|Description:CATALYTIC ACTIVITY: Hydrolysis of an N(6)-acetyl-lysine residue of a histone to yield a deacetylated histone. Gene Symbol:RUM1|Host:Euchlaena spp., Zea spp. (Poaceae)|Disease:Smut. Corn smut|Description:SIMILARITY: Contains 1 ARID domain.
Chr05G1154.1	183	9	149	UniProt ID:F2QX13_PICP7	203	5	145	49/150(32.67)	0.48	0.12	150	72.4	7.00E-17	gene=Chr05G1154	Gene Symbol:YPT1|Host:humans|Disease:occasional infection|Description:SIMILARITY: Belongs to the small GTPase superfamily. Rab family.
Chr05G1157.1	686	163	633	UniProt ID:Q156F4_BOTFU	727	274	710	121/500(24.20)	0.36	0.18	500	87.4	7.00E-19	gene=Chr05G1157	Gene Symbol:NULL|Host:Various plant families|Disease:Grey mould. Parasite or

															saprophyte|Description:Unknown
Chr05G1160.1	83	1	79	UniProt ID:Q5ADS0_CANAL	229	1	79	40/79(50.63)	0.75	0	79	87.4	2.00E-23	gene=Chr05G1160	Gene Symbol:UBI4|Host:Isolated from a wide variety of substrates including humans|Disease:invasive candidal disease|Description:Unknown
Chr05G1171.1	246	4	216	UniProt ID:Q59P43_CANAL	214	2	214	184/213(86.38)	0.94	0	213	397	1.00E-141	gene=Chr05G1171	Gene Symbol:GSP1|Host:Isolated from a wide variety of substrates including humans|Disease:invasive candidal disease|Description:CAUTION: The sequence shown here is derived from an EMBL/GenBank/DDBJ whole genome shotgun (WGS) entry which is preliminary data.
Chr05G1182.1	544	1	539	UniProt ID:Q6B957_F	526	11	524	342/543(62.98)	0.74	0.06	543	662	0	gene=Chr05G1182	Gene Symbol:FRP1|Host:Multiple genera in multiple

				USOX											families|Disease:Blights, wilts, rots of various sorts|Description:SIMILARITY: Contains 1 F-box domain.
Chr05G1192.1	509	22	509	UniProt ID:Q59RQ6_CANAL	491	5	491	316/490(64.49)	0.8	0.01	490	595	0	gene=Chr05G1192	Gene Symbol:LPD1|Host:Isolated from a wide variety of substrates including humans|Disease:invasive candidal disease|Description:CATALYTIC ACTIVITY: Protein N(6)-(dihydrolipoyl)lysine + NAD(+) = protein N(6)-(lipoyl)lysine + NADH.
Chr05G1196.1	467	102	291	UniProt ID:F2QU09_PICP7	626	142	305	51/195(26.15)	0.42	0.18	195	62.4	2.00E-11	gene=Chr05G1196	Gene Symbol:PAB1|Host:humans|Disease:occasional infection|Description:Unknown
Chr05G1218.1	607	10	38	UniProt ID:Q5A4F3_CANAL	624	18	46	17/29(58.62)	0.66	0	29	48.5	7.00E-07	gene=Chr05G1218	Gene Symbol:ZCF37|Host:Isolated from a wide variety of substrates including

															humans|Disease:invasive candidal disease|Description:Unknown
Chr05G1221.1	355	1	355	UniProt ID:Q9UW09_GLOLA	355	1	355	353/355(99.44)	0.99	0	355	736	0	gene=Chr05G1221	Gene Symbol:CMK1|Host:melons,cucumber|Disease:anthracnose fruit rot|Description:CATALYTIC ACTIVITY: ATP + a protein = ADP + a phosphoprotein.
Chr05G1237.1	640	37	169	UniProt ID:VPS27_CANAL	841	35	178	43/152 (28.29)	0.53	0.18	152	49.7	4.00E-07	gene=Chr05G1237	Gene Symbol:VPS27|Host:Isolated from a wide variety of substrates including humans|Disease:invasive candidal disease|Description:FUNCTION: Component of the ESCRT-0 complex which is the sorting receptor for ubiquitinated cargo proteins at the multivesicular body (MVB) and recruits ESCRT-I to

														the MVB outer membrane (By similarity).
Chr05G1241.1	399	1	398	UniProt ID:CARP_ASPFU	398	1	398	293/399(73.43)	0.84	0.01	399	613	0	gene=Chr05G1241	Gene Symbol:PEP2|Host:humans|Disease:infection|Description:FUNCTION: Vacuolar aspartic endopeptidase which is probably also secreted and contributes to virulence.
Chr05G1265.1	364	54	355	UniProt ID:Q6TFC7_ASPFM	349	36	346	111/320(34.69)	0.53	0.08	320	176	5.00E-52	gene=Chr05G1265	Gene Symbol:NULL|Host:humans|Disease:infection|Description:Unknown
Chr05G1266.1	834	229	391	UniProt ID:Q5AM49_CANAL	1690	801	945	53/171(30.99)	0.48	0.2	171	74.3	2.00E-14	gene=Chr05G1266	Gene Symbol:SNF2|Host:Isolated from a wide variety of substrates including humans|Disease:invasive candidal disease|Description:Unknown
Chr05G1276.1	359	70	354	UniProt ID:Q6TFC7_A	349	61	347	105/288(36.46)	0.53	0.01	288	179	3.00E-53	gene=Chr05G1276	Gene Symbol:NULL|Host:humans|Disease:infection|Desc

			SPFM												ription:Unknown
Chr05G1281.1	436	10	359	UniProt ID:Q6A2T2_BOTFU	391	6	345	87/367 (23.71)	0.45	0.12	367	94	5.00E-22	gene=Chr05G1281	Gene Symbol:BTP1|Host:Various plant families|Disease:Grey mould. Parasite or saprophyte|Description:Unknown
Chr05G1286.1	1146	331	404	UniProt ID:Q5AM49_CANAL	1690	1532	1596	26/74(35.14)	0.57	0.12	74	49.7	1.00E-06	gene=Chr05G1286	Gene Symbol:SNF2|Host:Isolated from a wide variety of substrates including humans|Disease:invasive candidal disease|Description:Unknown
Chr05G1292.1	850	365	458	UniProt ID:C5GSW0_AJEDR	783	224	323	32/103 (31.07)	0.52	0.12	103	50.4	3.00E-07	gene=Chr05G1292	Gene Symbol:BDCG_07503|Host:humans|Disease:cutaneous Blastomyces dermatitidis infection|Description:Unknown
Chr05G1294.1	413	1	407	UniProt ID:A9Z1V6_P	394	1	392	308/416(74.04)	0.82	0.08	416	605	0	gene=Chr05G1294	Gene Symbol:CPKB|Host:Multiple genera of Poaceae and

				HAND										Blysmus compressus (Cyperaceae)|Disease:Glume blotch of wheat and other grasses|Description:Unknown
Chr05G1296.1	456	30	431	UniProt ID:Q4WXL8_ASPFU	436	32	434	225/413(54.48)	0.7	0.05	413	425	4.00E-146	gene=Chr05G1296	Gene Symbol:AFUA_3G09960|Host:humans|Disease:infection|Description:CAUTION: The sequence shown here is derived from an EMBL/GenBank/DDBJ whole genome shotgun (WGS) entry which is preliminary data.
Chr05G1300.1	1520	1086	1366	UniProt ID:TUP1_CANAL	514	201	509	111/311(35.69)	0.52	0.1	311	176	6.00E-47	gene=Chr05G1300	Gene Symbol:TUP1|Host:Isolated from a wide variety of substrates including humans|Disease:invasive candidal disease|Description:FUNCTION: Represses transcription by RNA polymerase II. Represses

															genes responsible for initiating filamentous growth and this repression is lifted under inducing environmental conditions.
Chr05G1 313.1	170	62	114	UniProt ID:Q70 0F1_C ANGB	703	523	577	27/55(49.09)	0.62	0.04	55	58.9	2.00E-11	gene=Chr 05G1313	Gene Symbol:ACE2|Host:huma ns|Disease:Occasional invasive candidal disease|Description:Unkn own
Chr05G1 321.1	779	208	474	UniProt ID:Q5A 506_C ANAL	1237	658	947	71/293 (24.23)	0.43	0.1	293	67.4	2.00E-12	gene=Chr 05G1321	Gene Symbol:BUD2|Host:Isolat ed from a wide variety of substrates including humans|Disease:invasive candidal disease|Description:SIMIL ARITY: Contains 1 C2 domain.
Chr05G1 326.1	410	171	410	UniProt ID:Q69 7D5_U STMD	274	54	274	82/246 (33.33)	0.5	0.13	246	129	5.00E-35	gene=Chr 05G1326	Gene Symbol:MRB1|Host:Euchl aena spp., Zea spp. (Poaceae)|Disease:Smut. Corn smut|Description:Unknow

														n
Chr05G1327.1	229	38	228	UniProt ID:Q9HFZ2_CANAL	358	153	343	75/199 (37.69)	0.52	0.08	199	84.3	4.00E-20	gene=Chr05G1327	Gene Symbol:RBT4|Host:Isolated from a wide variety of substrates including humans|Disease:invasive candidal disease|Description:Unknown
Chr05G1341.1	619	1	553	UniProt ID:Q59RG0_CANAL	581	1	570	179/576(31.08)	0.49	0.05	576	244	8.00E-73	gene=Chr05G1341	Gene Symbol:NAG4|Host:Isolated from a wide variety of substrates including humans|Disease:invasive candidal disease|Description:CAUTION: The sequence shown here is derived from an EMBL/GenBank/DDBJ whole genome shotgun (WGS) entry which is preliminary data.
Chr05G1346.1	438	35	264	UniProt ID:O59928_HYPVI	430	51	303	76/274 (27.74)	0.42	0.24	274	81.3	1.00E-17	gene=Chr05G1346	Gene Symbol:NULL|Host:humans|Disease:infection|Description:SIMILARITY:

Chr05G1355.1	530	1	511	UniProt ID:Q875L7_MAGGR	529	1	524	430/524(82.06)	0.89	0.02	524	893	0	gene=Chr05G1355	Belongs to the glycosyl hydrolase 18 family. Gene Symbol:TPS1|Host:Digitaria (Poaceae)|Disease:Leaf spot|Description:Unknown
Chr05G1358.1	544	23	535	UniProt ID:Q5AB74_CANAL	540	31	525	125/518(24.13)	0.49	0.05	518	160	2.00E-43	gene=Chr05G1358	Gene Symbol:CCT8|Host:Isolated from a wide variety of substrates including humans|Disease:invasive candidal disease|Description:SIMILARITY: Belongs to the TCP-1 chaperonin family.
Chr05G1361.1	870	454	840	UniProt ID:DNLI4_CANAL	928	298	630	87/408(21.32)	0.38	0.24	408	65.9	7.00E-12	gene=Chr05G1361	Gene Symbol:LIG4|Host:Isolated from a wide variety of substrates including humans|Disease:invasive candidal disease|Description:FUNCTION: Involved in ds DNA break (DSB) repair. Has a role in non-homologous integration (NHI) pathways

															where it is required in the final step of non-homologus end-joining (NHEJ). Not required for the repair of DSBs induced by ionizing radiation or UV light. Has a important role in morphogenesis, positively affecting the capacity to form hyphae.
Chr05G1372.1	281	1	238	UniProt ID:O93802_ALTAL	267	10	251	59/255 (23.14)	0.42	0.12	255	58.5	5.00E-11	gene=Chr05G1372	Gene Symbol:BRM2|Host:Plant|Disease:Leaf spot, rots|Description:SIMILARITY: Belongs to the short-chain dehydrogenases/reductases (SDR) family.
Chr05G1377.1	681	120	611	UniProt ID:Q5XTQ5_BOTFU	615	103	549	118/498(23.69)	0.45	0.11	498	123	2.00E-30	gene=Chr05G1377	Gene Symbol:FRT1|Host:Various plant families|Disease:Grey mould. Parasite or saprophyte|Description:SIMILARITY: Belongs to the

															major facilitator superfamily. Sugar transporter (TC 2.A.1.1) family.
Chr05G1379.1	1781	1144	1778	UniProt ID:A4RDS9_MAGO7	1514	864	1512	465/680(68.38)	0.77	0.11	680	855	0	gene=Chr05G1379	Gene Symbol:MGG_00883|Host:Poaceae, especially important on Oryzae|Disease:Rice blast|Description:Unknown
Chr05G1393.1	421	154	395	UniProt ID:Q0PND8_MAGGR	1375	985	1229	99/248(39.92)	0.57	0.04	248	161	2.00E-43	gene=Chr05G1393	Gene Symbol:PEX6|Host:Digitaria (Poaceae)|Disease:Leaf spot|Description:SIMILARITY: Belongs to the AAA ATPase family.
Chr05G1396.1	341	6	296	UniProt ID:C5GFB0_AJEDR	363	25	315	220/291(75.60)	0.85	0	291	478	1.00E-169	gene=Chr05G1396	Gene Symbol:BDCG_03063|Host:humans|Disease:cutaneous Blastomyces dermatitidis infection|Description:SIMILARITY: Contains 1 CRAL-TRIO domain.
Chr05G1	249	1	243	UniProt	251	1	218	96/251	0.48	0.16	251	111	4.00E-30	gene=Chr	Gene

401.1				ID:Q96 TN6_M AGGR				(38.25)						05G1401	Symbol:MAS3|Host:Digitaria (Poaceae)|Disease:Leaf spot|Description:Unknown
Chr05G1 403.1	643	362	578	UniProt ID:D1M GZ7_B EABA	379	136	343	62/231 (26.84)	0.45	0.16	231	47.8	1.00E-06	gene=Chr 05G1403	Gene Symbol:PR1|Host:various arthropod species|Disease:white muscardine disease|Description:SIMILARITY: Belongs to the peptidase S8 family.
Chr05G1 421.1	346	13	296	UniProt ID:A0S T44_C ERNC	357	4	292	87/299 (29.10)	0.44	0.08	299	81.6	2.00E-18	gene=Chr 05G1421	Gene Symbol:CTB6|Host:Numerous taxa in Solanaceae|Disease:Leaf spot|Description:Unknown
Chr05G1 428.1	722	15	62	UniProt ID:A6N 6J8_FU SOX	903	40	90	20/51(39.22)	0.51	0.06	51	45.4	9.00E-06	gene=Chr 05G1428	Gene Symbol:CTF1|Host:Multiple genera in multiple families|Disease:Blights, wilts, rots of various sorts|Description:SIMILARITY: Contains 1 Zn(2)-C6 fungal-type DNA-binding domain.

Chr05G1437.1	315	39	309	UniProt ID:Q8TGD1_FUSOX	318	37	306	156/271(57.56)	0.73	0	271	311	2.00E-105	gene=Chr05G1437	Gene Symbol:FOW1|Host:Multiple genera in multiple families|Disease:Blights, wilts, rots of various sorts|Description:SIMILARITY: Belongs to the mitochondrial carrier family.
Chr05G1440.1	795	59	502	UniProt ID:A3LS85_PICST	677	13	442	134/458(29.26)	0.45	0.09	458	151	4.00E-39	gene=Chr05G1440	Gene Symbol:LYS4|Host:humans|Disease:occasional infection|Description:Unknown
Chr05G1441.1	384	4	337	UniProt ID:Q5ANE1_CANAL	748	160	496	88/353(24.93)	0.42	0.1	353	55.1	3.00E-09	gene=Chr05G1441	Gene Symbol:SNF3|Host:Isolated from a wide variety of substrates including humans|Disease:invasive candidal disease|Description:SIMILARITY: Belongs to the major facilitator superfamily. Sugar transporter (TC 2.A.1.1) family.

Chr05G1442.1	650	34	427	UniProt ID:Q9HG15_COLLN	746	14	411	88/417 (21.10)	0.38	0.1	417	90.9	6.00E-20	gene=Chr05G1442	Gene Symbol:CLTA1|Host:Multiple genera of Fabaceae. Rare reports on other taxa|Disease:Leaf, stem and pod anthracnose|Description:SIMILARITY: Contains 1 Zn(2)-C6 fungal-type DNA-binding domain.
Chr05G1448.1	956	625	888	UniProt ID:TUP1_CANAL	514	201	486	115/289(39.79)	0.56	0.1	289	192	2.00E-53	gene=Chr05G1448	Gene Symbol:TUP1|Host:Isolated from a wide variety of substrates including humans|Disease:invasive candidal disease|Description:FUNCTION: Represses transcription by RNA polymerase II. Represses genes responsible for initiating filamentous growth and this repression is lifted under inducing environmental conditions.
Chr05G1	981	332	465	UniProt	2289	719	856	40/138	0.49	0.03	138	70.1	5.00E-13	gene=Chr	Gene

<table>
<tr><td>455.1</td><td></td><td></td><td></td><td>ID:Q9HFW4_USTMD</td><td></td><td></td><td></td><td>(28.99)</td><td></td><td></td><td></td><td></td><td></td><td>05G1455</td><td>Symbol:RUM1|Host:Euchlaena spp., Zea spp. (Poaceae)|Disease:Smut. Corn smut|Description:SIMILARITY: Contains 1 ARID domain.</td></tr>
<tr><td>Chr05G1459.1</td><td>365</td><td>104</td><td>288</td><td>UniProt ID:A4R3I5_MAGO7</td><td>400</td><td>86</td><td>265</td><td>48/193 (24.87)</td><td>0.44</td><td>0.11</td><td>193</td><td>57.8</td><td>3.00E-10</td><td>gene=Chr05G1459</td><td>Gene Symbol:"MGG_11993, MGG_12837, MGG_13052"|Host:Poaceae, especially important on Oryzae|Disease:Rice blast|Description:Unknown</td></tr>
<tr><td>Chr05G1463.1</td><td>322</td><td>1</td><td>111</td><td>UniProt ID:C4YII6_CANAW</td><td>768</td><td>492</td><td>599</td><td>36/115 (31.30)</td><td>0.5</td><td>0.1</td><td>115</td><td>52.4</td><td>2.00E-08</td><td>gene=Chr05G1463</td><td>Gene Symbol:CAWG_04261|Host:Isolated from a wide variety of substrates including humans|Disease:invasive candidal disease|Description:SIMILARITY: Belongs to the DEAD box helicase family.</td></tr>
<tr><td>Chr03G0</td><td>222</td><td>23</td><td>178</td><td>UniProt</td><td>349</td><td>61</td><td>215</td><td>65/159</td><td>0.58</td><td>0.04</td><td>159</td><td>117</td><td>8.00E-32</td><td>gene=Chr</td><td>Gene</td></tr>
</table>

002.1				ID:Q6TFC7_ASPFM				(40.88)						03G0002	Symbol:NULL|Host:humans|Disease:infection|Description:Unknown
Chr03G0006.1	324	126	187	UniProt ID:Q59RB8_CANAL	550	168	230	27/64(42.19)	0.58	0.05	64	49.7	1.00E-07	gene=Chr03G0006	Gene Symbol:ICL1|Host:Isolated from a wide variety of substrates including humans|Disease:invasive candidal disease|Description:SIMILARITY: Belongs to the isocitrate lyase/PEP mutase superfamily. Isocitrate lyase family.
Chr03G0007.1	528	24	488	UniProt ID:Q5ANE1_CANAL	748	37	496	121/482(25.10)	0.45	0.08	482	118	4.00E-29	gene=Chr03G0007	Gene Symbol:SNF3|Host:Isolated from a wide variety of substrates including humans|Disease:invasive candidal disease|Description:SIMILARITY: Belongs to the major facilitator superfamily. Sugar transporter (TC 2.A.1.1) family.

Chr03G0008.1	1190	607	833	UniProt ID:Q9HG15_COLLN	746	162	395	60/239 (25.10)	0.4	0.07	239	60.5	4.00E-10	gene=Chr03G0008	Gene Symbol:CLTA1|Host:Multiple genera of Fabaceae. Rare reports on other taxa|Disease:Leaf, stem and pod anthracnose|Description:SIMILARITY: Contains 1 Zn(2)-C6 fungal-type DNA-binding domain.
Chr03G0011.1	471	30	400	UniProt ID:Q9HG15_COLLN	746	20	367	87/378 (23.02)	0.4	0.1	378	91.7	1.00E-20	gene=Chr03G0011	Gene Symbol:CLTA1|Host:Multiple genera of Fabaceae. Rare reports on other taxa|Disease:Leaf, stem and pod anthracnose|Description:SIMILARITY: Contains 1 Zn(2)-C6 fungal-type DNA-binding domain.
Chr03G0016.1	378	10	223	UniProt ID:A4QVF8_MAGO7	339	8	203	54/220 (24.55)	0.41	0.14	220	48.1	3.00E-07	gene=Chr03G0016	Gene Symbol:MGG_04556|Host:Poaceae, especially important on Oryzae|Disease:Rice blast|Description:COFACT

														OR: Zinc (By similarity).
Chr03G0024.1	363	107	340	UniProt ID:Q6A2T2_BOTFU	391	116	362	64/249 (25.70)	0.47	0.07	249	85.9	1.00E-19	gene=Chr03G0024	Gene Symbol:BTP1|Host:Various plant families|Disease:Grey mould. Parasite or saprophyte|Description:Unknown
Chr03G0028.1	998	219	606	UniProt ID:TUP1_CANAL	514	152	474	87/394 (22.08)	0.4	0.2	394	68.9	6.00E-13	gene=Chr03G0028	Gene Symbol:TUP1|Host:Isolated from a wide variety of substrates including humans|Disease:invasive candidal disease|Description:FUNCTION: Represses transcription by RNA polymerase II. Represses genes responsible for initiating filamentous growth and this repression is lifted under inducing environmental conditions.
Chr03G0031.1	264	11	63	UniProt ID:O59937_F	384	4	56	27/53(50.94)	0.62	0	53	62	5.00E-12	gene=Chr03G0031	Gene Symbol:XYL3|Host:Multiple genera in multiple

			USOX											families|Disease:Blights, wilts, rots of various sorts|Description:SIMILARITY: Belongs to the glycosyl hydrolase 10 (cellulase F) family.
Chr03G0033.1	495	244	436	UniProt ID:A6ZXD7_YEAS7	1273	48	242	55/208 (26.44)	0.52	0.13	208	77.8	5.00E-16	gene=Chr03G0033	Gene Symbol:SEC31|Host:humans|Disease:occasional infection|Description:CAUTION: The sequence shown here is derived from an EMBL/GenBank/DDBJ whole genome shotgun (WGS) entry which is preliminary data.
Chr03G0036.1	477	90	375	UniProt ID:Q6XSF5_COCHE	351	23	298	71/299 (23.75)	0.44	0.12	299	57.4	5.00E-10	gene=Chr03G0036	Gene Symbol:NULL|Host:Zea mays|Disease:Southern leaf blight of maize|Description:Unknown
Chr03G0046.1	464	4	363	UniProt ID:Q5AJC0_C	470	9	376	164/370(44.32)	0.64	0.03	370	315	2.00E-102	gene=Chr03G0046	Gene Symbol:UTR2|Host:Isolated from a wide variety of

				ANAL										substrates including humans|Disease:invasive candidal disease|Description:CAUTION: The sequence shown here is derived from an EMBL/GenBank/DDBJ whole genome shotgun (WGS) entry which is preliminary data.
Chr03G0051.1	506	120	436	UniProt ID:A3LNY8_PICST	414	92	374	139/317(43.85)	0.6	0.11	317	279	8.00E-89	gene=Chr03G0051	Gene Symbol:KRE2|Host:humans|Disease:occasional infection|Description:Unknown
Chr03G0056.1	457	74	406	UniProt ID:Q96VU5_CRYNE	468	9	378	92/378(24.34)	0.37	0.14	378	55.1	4.00E-09	gene=Chr03G0056	Gene Symbol:UGD1|Host:humans|Disease:cryptococcosis|Description:Unknown
Chr03G0057.1	474	9	414	UniProt ID:D1MYV6_MAGGR	568	59	441	183/408(44.85)	0.61	0.07	408	347	8.00E-114	gene=Chr03G0057	Gene Symbol:CBL1|Host:Digitaria (Poaceae)|Disease:Leaf spot|Description:SIMILARITY: Contains 3 chitin-binding type-1 domains.

Chr03G0058.1	653	183	643	UniProt ID:Q8TFN4_COLGR	1866	1225	1691	122/487(25.05)	0.44	0.09	487	139	6.00E-35	gene=Chr03G0058	Gene Symbol:CHSV|Host:Poaceae especially Zea mays|Disease:Leaf spot, stalk rot, etc|Description:Unknown
Chr03G0059.1	548	1	537	UniProt ID:D1MYV6_MAGGR	568	1	567	256/579(44.21)	0.59	0.09	579	430	6.00E-145	gene=Chr03G0059	Gene Symbol:CBL1|Host:Digitaria (Poaceae)|Disease:Leaf spot|Description:SIMILARITY: Contains 3 chitin-binding type-1 domains.
Chr03G0063.1	603	317	588	UniProt ID:Q2PEP0_9HYPO	557	258	549	77/309 (24.92)	0.4	0.17	309	63.9	1.00E-11	gene=Chr03G0063	Gene Symbol:NOXA|Host:plants|Disease:cool-season grasses|Description:SIMILARITY: Contains 1 FAD-binding FR-type domain.
Chr03G0075.1	273	1	268	UniProt ID:Q6TFC7_ASPFM	349	77	346	94/272 (34.56)	0.49	0.02	272	157	6.00E-46	gene=Chr03G0075	Gene Symbol:NULL|Host:humans|Disease:infection|Description:Unknown
Chr03G0080.1	427	9	334	UniProt ID:Q6A	391	11	353	92/347 (26.51)	0.46	0.07	347	124	1.00E-32	gene=Chr03G0080	Gene Symbol:BTP1|Host:Variou

				2T2_BOTFU										s plant families|Disease:Grey mould. Parasite or saprophyte|Description:Unknown
Chr03G0081.1	319	51	319	UniProt ID:O93802_ALTAL	267	1	260	72/277 (25.99)	0.42	0.09	277	81.6	4.00E-19	gene=Chr03G0081	Gene Symbol:BRM2|Host:Plant|Disease:Leaf spot, rots|Description:SIMILARITY: Belongs to the short-chain dehydrogenases/reductases (SDR) family.
Chr03G0082.1	1182	60	136	UniProt ID:Q9P3Z8_CYBJA	345	14	83	37/77(48.05)	0.6	0.09	77	72.4	3.00E-14	gene=Chr03G0082	Gene Symbol:MIG1|Host:humans|Disease:Chronic urinary tract infection|Description:Unknown
Chr03G0083.1	1028	923	1023	UniProt ID:A3QX02_ASPFM	570	403	486	37/101 (36.63)	0.47	0.17	101	61.2	2.00E-10	gene=Chr03G0083	Gene Symbol:ZAFA|Host:humans|Disease:infection|Description:Unknown
Chr03G0084.1	1063	480	771	UniProt ID:Q5AM49_C	1690	801	1080	83/313 (26.52)	0.44	0.17	313	78.6	1.00E-15	gene=Chr03G0084	Gene Symbol:SNF2|Host:Isolated from a wide variety of

				ANAL											substrates including humans|Disease:invasive candidal disease|Description:Unknown
Chr03G0086.1	415	7	272	UniProt ID:Q5AMT2_CANAL	308	6	257	69/275 (25.09)	0.43	0.12	275	77.8	6.00E-17	gene=Chr03G0086	Gene Symbol:BGL2|Host:Isolated from a wide variety of substrates including humans|Disease:invasive candidal disease|Description:SIMILARITY: Belongs to the glycosyl hydrolase 17 family.
Chr03G0087.1	455	25	369	UniProt ID:A4UC81_MAGO7	376	29	371	107/354(30.23)	0.48	0.06	354	152	4.00E-42	gene=Chr03G0087	Gene Symbol:MGG_10702|Host:Poaceae, especially important on Oryzae|Disease:Rice blast|Description:Unknown
Chr03G0088.1	375	30	375	UniProt ID:O00094_FUSOX	370	24	370	235/348(67.53)	0.8	0.01	348	469	2.00E-165	gene=Chr03G0088	Gene Symbol:PG1|Host:Multiple genera in multiple families|Disease:Blights,

															wilts, rots of various sorts|Description:SIMILARITY: Belongs to the glycosyl hydrolase 28 family.
Chr03G0089.1	353	21	220	UniProt ID:A4QVF8_MAGO7	339	17	218	55/211 (26.07)	0.48	0.09	211	75.9	2.00E-16	gene=Chr03G0089	Gene Symbol:MGG_04556|Host:Poaceae, especially important on Oryzae|Disease:Rice blast|Description:COFACTOR: Zinc (By similarity).
Chr03G0090.1	577	138	480	UniProt ID:CARP3_CANAL	398	51	385	134/359(37.33)	0.52	0.11	359	172	1.00E-48	gene=Chr03G0090	Gene Symbol:SAP3|Host:Isolated from a wide variety of substrates including humans|Disease:invasive candidal disease|Description:CATALYTIC ACTIVITY: Preferential cleavage at the carboxyl of hydrophobic amino acids, but fails to cleave 15-Leu-|-Tyr-16, 16-Tyr-|-Leu-17 and

															24-Phe-|-Phe-25 of insulin B chain. Activates trypsinogen, and degrades keratin.
Chr03G0092.1	463	16	462	UniProt ID:Q01446_NECHA	459	16	458	264/447(59.06)	0.76	0.01	447	560	0	gene=Chr03G0092	Gene Symbol:MAK1|Host:Trees of various plant families|Disease:Fruit rot, stem rot|Description:Unknown
Chr03G0093.1	214	18	212	UniProt ID:Q9UVJ0_CANAL	200	7	200	79/203 (38.92)	0.57	0.08	203	128	3.00E-37	gene=Chr03G0093	Gene Symbol:CAO19.12623, CAO19.5156, ORF19.5156|Host:Isolated from a wide variety of substrates including humans|Disease:invasive candidal disease|Description:Unknown
Chr03G0099.1	1160	519	880	UniProt ID:Q5AM49_CANAL	1690	798	1117	92/375 (24.53)	0.43	0.18	375	96.3	6.00E-21	gene=Chr03G0099	Gene Symbol:SNF2|Host:Isolated from a wide variety of substrates including humans|Disease:invasive candidal

															disease|Description:Unknown
Chr03G0110.1	334	4	308	UniProt ID:CHS7_CANAL	310	3	309	150/307(48.86)	0.66	0.01	307	285	6.00E-95	gene=Chr03G0110	Gene Symbol:CHS7|Host:Isolated from a wide variety of substrates including humans|Disease:invasive candidal disease|Description:FUNCTION: Chaperone required for the export of the chitin synthase CHS3 from the endoplasmic reticulum (By similarity).
Chr03G0120.1	516	268	516	UniProt ID:O93802_ALTAL	267	5	267	79/271 (29.15)	0.46	0.11	271	83.2	9.00E-19	gene=Chr03G0120	Gene Symbol:BRM2|Host:Plant|Disease:Leaf spot, rots|Description:SIMILARITY: Belongs to the short-chain dehydrogenases/reductases (SDR) family.
Chr03G0123.1	330	35	277	UniProt ID:Q9Y784_MAGGR	631	128	368	67/249 (26.91)	0.44	0.06	249	73.9	2.00E-15	gene=Chr03G0123	Gene Symbol:PTH11|Host:Digitaria (Poaceae)|Disease:Leaf

														spot|Description:Unknown
Chr03G0126.1	495	27	337	UniProt ID:O59937_FUSOX	384	90	384	131/315(41.59)	0.58	0.08	315	221	3.00E-67	gene=Chr03G0126	Gene Symbol:XYL3|Host:Multiple genera in multiple families|Disease:Blights, wilts, rots of various sorts|Description:SIMILARITY: Belongs to the glycosyl hydrolase 10 (cellulase F) family.
Chr03G0131.1	446	2	384	UniProt ID:Q5GFD3_PHAND	437	6	393	110/417(26.38)	0.43	0.15	417	81.6	9.00E-18	gene=Chr03G0131	Gene Symbol:NULL|Host:Multiple genera of Poaceae and Blysmus compressus (Cyperaceae)|Disease:Glume blotch of wheat and other grasses|Description:Unknown
Chr03G0145.1	976	1	733	UniProt ID:Q59KS4_CANAL	800	1	700	322/749(42.99)	0.59	0.09	749	600	0	gene=Chr03G0145	Gene Symbol:CNH1|Host:Isolated from a wide variety of substrates including humans|Disease:invasive candidal disease|Description:CAUT

															ION: The sequence shown here is derived from an EMBL/GenBank/DDBJ whole genome shotgun (WGS) entry which is preliminary data.
Chr03G0147.1	915	706	847	UniProt ID:TUP1_CANAL	514	258	397	52/147 (35.37)	0.54	0.08	147	88.6	4.00E-19	gene=Chr03G0147	Gene Symbol:TUP1|Host:Isolated from a wide variety of substrates including humans|Disease:invasive candidal disease|Description:FUNCTION: Represses transcription by RNA polymerase II. Represses genes responsible for initiating filamentous growth and this repression is lifted under inducing environmental conditions.
Chr03G0149.1	907	632	849	UniProt ID:ORYZ_ASPFU	403	160	375	62/235 (26.38)	0.42	0.15	235	69.7	2.00E-13	gene=Chr03G0149	Gene Symbol:ALP1|Host:humans|Disease:infection|Description:FUNCTION: Secreted alkaline

															protease that allows assimilation of proteinaceous substrates. Acts as a significant virulence factor in invasive aspergillosis. Involved in immune evasion from the human and mice complement systems during infection. Efficiently cleaves important components of the complement cascade such as such as C3, C4, C5, and C1q, as well as IgG, which leads to down-regulation of complement activation at the hyphal surface.
Chr03G0 152.1	434	112	286	UniProt ID:Q9Y 784_M AGGR	631	203	377	47/191 (24.61)	0.46	0.17	191	58.9	3.00E-10	gene=Chr 03G0152	Gene Symbol:PTH11|Host:Digit aria (Poaceae)|Disease:Leaf spot|Description:Unknown
Chr03G0 157.1	914	30	908	UniProt ID:Q2H	879	20	875	520/90 7(57.3	0.72	0.09	907	956	0	gene=Chr 03G0157	Gene Symbol:CHGG_03112|Ho

				9J2_CHAGB				3)							st:Multiple genera in multiple families|Disease:Saprobe|Description:Unknown
Chr03G0159.1	585	129	467	UniProt ID:Q5APH9_CANAL	375	74	370	149/355(41.97)	0.53	0.21	355	252	3.00E-78	gene=Chr03G0159	Gene Symbol:PTC1|Host:Isolated from a wide variety of substrates including humans|Disease:invasive candidal disease|Description:SIMILARITY: Belongs to the PP2C family.
Chr03G0166.1	359	1	359	UniProt ID:Q6XPX0_FUSOX	359	1	359	348/359(96.94)	0.99	0	359	732	0	gene=Chr03G0166	Gene Symbol:FGB1|Host:Multiple genera in multiple families|Disease:Blights, wilts, rots of various sorts|Description:Unknown
Chr03G0175.1	483	10	469	UniProt ID:C6K2F1_MYCGR	558	15	523	210/520(40.38)	0.54	0.14	520	308	8.00E-99	gene=Chr03G0175	Gene Symbol:STE50|Host:Triticum and possibly a few other grasses|Disease:Leaf spot or speckled leaf blotch of

															wheat|Description:SIMILARITY: Contains 1 SAM (sterile alpha motif) domain.
Chr03G0176.1	671	194	614	UniProt ID:Q1L2E2_PHAND	619	142	550	129/436(29.59)	0.48	0.1	436	171	1.00E-46	gene=Chr03G0176	Gene Symbol:ALS1|Host:Multiple genera of Poaceae and Blysmus compressus (Cyperaceae)|Disease:Glume blotch of wheat and other grasses|Description:COFACTOR: Pyridoxal phosphate (By similarity).
Chr03G0183.1	625	64	273	UniProt ID:A6ZTU6_YEAS7	708	204	404	52/216(24.07)	0.44	0.1	216	62.8	3.00E-11	gene=Chr03G0183	Gene Symbol:GUS1|Host:humans|Disease:occasional infection|Description:SIMILARITY: Belongs to the class-I aminoacyl-tRNA synthetase family.
Chr03G0184.1	562	1	559	UniProt ID:B0BER8_BOTFU	540	1	537	386/585(65.98)	0.75	0.13	585	733	0	gene=Chr03G0184	Gene Symbol:NOXR|Host:Various plant families|Disease:Grey mould. Parasite or

															saprophyte|Description:Unknown
Chr03G0190.1	576	128	437	UniProt ID:DHH1_CRYNV	616	38	338	90/311 (28.94)	0.49	0.04	311	140	2.00E-36	gene=Chr03G0190	Gene Symbol:VAD1|Host:humans|Disease:cryptococcosis|Description:FUNCTION: ATP-dependent RNA helicase involved in mRNA turnover, and more specifically in mRNA decapping. Is involved in G1/S DNA- damage checkpoint recovery, probably through the regulation of the translational status of a subset of mRNAs. May also have a role in translation and mRNA nuclear export (By similarity). Is involved in virulence.
Chr03G0191.1	427	7	417	UniProt ID:O13337_MAGGR	441	9	439	280/441(63.49)	0.72	0.09	441	478	5.00E-167	gene=Chr03G0191	Gene Symbol:CON7|Host:Digitaria (Poaceae)|Disease:Leaf

Chr03G0206.1	474	1	425	UniProt ID:Q9Y784_MAGGR	631	2	425	104/433(24.02)	0.44	0.04	433	135	3.00E-35	gene=Chr03G0206	spot|Description:Unknown Gene Symbol:PTH11|Host:Digitaria (Poaceae)|Disease:Leaf spot|Description:Unknown
Chr03G0207.1	248	28	247	UniProt ID:D1MYV6_MAGGR	568	139	359	97/222(43.69)	0.61	0.01	222	192	7.00E-58	gene=Chr03G0207	Gene Symbol:CBL1|Host:Digitaria (Poaceae)|Disease:Leaf spot|Description:SIMILARITY: Contains 3 chitin-binding type-1 domains.
Chr03G0208.1	345	17	339	UniProt ID:O59928_HYPVI	430	41	405	110/365(30.14)	0.46	0.12	365	156	3.00E-44	gene=Chr03G0208	Gene Symbol:NULL|Host:humans|Disease:infection|Description:SIMILARITY: Belongs to the glycosyl hydrolase 18 family.
Chr03G0212.1	505	204	504	UniProt ID:Q6IVV6_BOTFU	424	118	422	157/305(51.48)	0.68	0.01	305	325	1.00E-106	gene=Chr03G0212	Gene Symbol:CEL5A|Host:Various plant families|Disease:Grey mould. Parasite or saprophyte|Description:Unknown

Chr03G0214.1	643	28	641	UniProt ID:Q4P8E8_USTMA	693	70	672	194/650(29.85)	0.47	0.13	650	236	1.00E-68	gene=Chr03G0214	Gene Symbol:UM03615.1|Host: Euchlaena spp., Zea spp. (Poaceae)|Disease:Smut. Corn smut|Description:COFACTOR: FAD (By similarity).
Chr03G0215.1	424	5	348	UniProt ID:Q9Y784_MAGGR	631	6	358	89/359 (24.79)	0.46	0.06	359	116	4.00E-29	gene=Chr03G0215	Gene Symbol:PTH11|Host:Digitaria (Poaceae)|Disease:Leaf spot|Description:Unknown
Chr03G0220.1	360	70	275	UniProt ID:Q9Y784_MAGGR	631	185	382	51/213 (23.94)	0.47	0.1	213	63.5	5.00E-12	gene=Chr03G0220	Gene Symbol:PTH11|Host:Digitaria (Poaceae)|Disease:Leaf spot|Description:Unknown
Chr03G0221.1	296	21	294	UniProt ID:O93802_ALTAL	267	12	267	76/278 (27.34)	0.44	0.09	278	84.7	4.00E-20	gene=Chr03G0221	Gene Symbol:BRM2|Host:Plant|Disease:Leaf spot, rots|Description:SIMILARITY: Belongs to the short-chain dehydrogenases/reductases (SDR) family.
Chr03G0	537	27	491	UniProt	748	42	496	132/47	0.49	0.06	474	171	6.00E-47	gene=Chr	Gene

228.1				ID:Q5ANE1_CANAL				4(27.85)						03G0228	Symbol:SNF3|Host:Isolated from a wide variety of substrates including humans|Disease:invasive candidal disease|Description:SIMILARITY: Belongs to the major facilitator superfamily. Sugar transporter (TC 2.A.1.1) family.
Chr03G0241.1	548	19	454	UniProt ID:Q9P8L8_BOTFU	598	94	539	137/452(30.31)	0.48	0.05	452	177	3.00E-49	gene=Chr03G0241	Gene Symbol:BCMFS1|Host:Various plant families|Disease:Grey mould. Parasite or saprophyte|Description:Unknown
Chr03G0242.1	359	12	244	UniProt ID:Q92217_COCHE	2528	2066	2300	80/242(33.06)	0.52	0.07	242	104	4.00E-25	gene=Chr03G0242	Gene Symbol:PKS1|Host:Zea mays|Disease:Southern leaf blight of maize|Description:Unknown
Chr03G0243.1	578	1	578	UniProt ID:Q5X	574	1	574	335/580(57.7	0.71	0.01	580	674	0	gene=Chr03G0243	Gene Symbol:LIP1|Host:Various

			TQ4_BOTFU				6)							plant families|Disease:Grey mould. Parasite or saprophyte|Description:Unknown
Chr03G0245.1	764	461	701	UniProt ID:Q5EMY3_MAGGR	424	93	339	92/253 (36.36)	0.55	0.07	253	150	3.00E-40	gene=Chr03G0245	Gene Symbol:NULL|Host:Digitaria (Poaceae)|Disease:Leaf spot|Description:SIMILARITY: Belongs to the AAA ATPase family.
Chr03G0246.1	1068	551	1021	UniProt ID:Q5ANE1_CANAL	748	39	498	137/483(28.36)	0.48	0.07	483	192	1.00E-51	gene=Chr03G0246	Gene Symbol:SNF3|Host:Isolated from a wide variety of substrates including humans|Disease:invasive candidal disease|Description:SIMILARITY: Belongs to the major facilitator superfamily. Sugar transporter (TC 2.A.1.1) family.
Chr03G0250.1	1458	4	1445	UniProt ID:Q96WW9_	1491	53	1481	724/1463(49.49)	0.67	0.04	1463	1467	0	gene=Chr03G0250	Gene Symbol:ABC1|Host:Various plant

				GIBPU										families|Disease:Tree canker, rot of potatoes (Samuels et al. 2006). Root rot (Booth 1973)|Description:SIMILARITY: Belongs to the ABC transporter superfamily.
Chr03G0252.1	684	183	455	UniProt ID:Q5ALS7_CANAL	1144	361	647	65/306 (21.24)	0.42	0.17	306	47	3.00E-06	gene=Chr03G0252	Gene Symbol:CTF1|Host:Isolated from a wide variety of substrates including humans|Disease:invasive candidal disease|Description:SIMILARITY: Contains 1 Zn(2)-C6 fungal-type DNA-binding domain.
Chr03G0260.1	488	217	451	UniProt ID:A4ULI5_MYCGR	515	233	501	58/277 (20.94)	0.39	0.18	277	62.8	2.00E-11	gene=Chr03G0260	Gene Symbol:CYP51|Host:Triticum and possibly a few other grasses|Disease:Leaf spot or speckled leaf blotch of wheat|Description:COFACTOR: Heme group (By similarity).

Chr03G0261.1	394	70	376	UniProt ID:Q6XVN4_CRYNV	383	28	363	94/340 (27.65)	0.41	0.11	340	78.2	8.00E-17	gene=Chr03G0261	Gene Symbol:GNO1|Host:humans|Disease:cryptococcosis|Description:COFACTOR: Zinc (By similarity).
Chr03G0277.1	1003	41	853	UniProt ID:DNLI4_CANAL	928	11	789	257/846(30.38)	0.52	0.12	846	374	4.00E-114	gene=Chr03G0277	Gene Symbol:LIG4|Host:Isolated from a wide variety of substrates including humans|Disease:invasive candidal disease|Description:FUNCTION: Involved in ds DNA break (DSB) repair. Has a role in non-homologous integration (NHI) pathways where it is required in the final step of non-homologus end-joining (NHEJ). Not required for the repair of DSBs induced by ionizing radiation or UV light. Has a important role in morphogenesis, positively affecting the capacity to

														form hyphae.
Chr03G0280.1	548	33	531	UniProt ID:Q4PDC5_USTMA	583	39	534	230/500(46.00)	0.63	0.01	500	469	8.00E-160	gene=Chr03G0280	Gene Symbol:UM01888.1|Host:Euchlaena spp., Zea spp.(Poaceae)|Disease:Smut. Corn smut|Description:CAUTION: The sequence shown here is derived from an EMBL/GenBank/DDBJ whole genome shotgun (WGS) entry which is preliminary data.
Chr03G0284.1	538	1	536	UniProt ID:MCPB_TRIEQ	538	1	537	306/540(56.67)	0.73	0.01	540	637	0	gene=Chr03G0284	Gene Symbol:MCPB|Host:humans|Disease:Malabar itch|Description:FUNCTION: Extracellular metalloprotease that contributes to pathogenicity (By similarity).
Chr03G0290.1	408	15	376	UniProt ID:Q5GFD3_PHAND	437	8	378	112/400(28.00)	0.42	0.17	400	103	4.00E-25	gene=Chr03G0290	Gene Symbol:NULL|Host:Multiple genera of Poaceae and Blysmus compressus

															(Cyperaceae)|Disease:Glume blotch of wheat and other grasses|Description:Unknown
Chr03G0292.1	277	8	275	UniProt ID:A4RGG9_MAGO7	286	17	271	78/280 (27.86)	0.44	0.13	280	69.3	9.00E-15	gene=Chr03G0292	Gene Symbol:MGG_00056|Host:Poaceae, especially important on Oryzae|Disease:Rice blast|Description:SIMILARITY: Belongs to the short-chain dehydrogenases/reductases (SDR) family.
Chr03G0296.1	326	15	122	UniProt ID:P79068_GLOLA	2187	1932	2038	30/110 (27.27)	0.47	0.05	110	50.1	9.00E-08	gene=Chr03G0296	Gene Symbol:PKS1|Host:melons,cucumber|Disease:anthracnose fruit rot|Description:Unknown
Chr03G0301.1	1485	141	1476	UniProt ID:Q9UW87_CANAL	1606	145	1598	375/1496(25.07)	0.44	0.14	1496	403	4.00E-117	gene=Chr03G0301	Gene Symbol:MLT1|Host:Isolated from a wide variety of substrates including humans|Disease:invasive candidal

															disease|Description:SIMILARITY: Belongs to the ABC transporter superfamily.
Chr03G0303.1	12678	4849	8333	UniProt ID:Q9UVN5_ALTAL	4360	63	3484	1207/3609(33.44)	0.51	0.09	3609	1791	0	gene=Chr03G0303	Gene Symbol:AMT|Host:Plant|Disease:Leaf spot, rots|Description:Unknown
Chr03G0304.1	2521	69	2504	UniProt ID:Q92217_COCHE	2528	11	2512	828/2603(31.81)	0.48	0.1	2603	1068	0	gene=Chr03G0304	Gene Symbol:PKS1|Host:Zea mays|Disease:Southern leaf blight of maize|Description:Unknown
Chr03G0305.1	609	1	569	UniProt ID:Q96VB5_ALTAL	578	1	566	215/575(37.39)	0.57	0.03	575	372	2.00E-121	gene=Chr03G0305	Gene Symbol:AFT1-1|Host:Plant|Disease:Leaf spot, rots|Description:Unknown
Chr03G0328.1	1314	14	425	UniProt ID:Q0WXM3_FUSOX	663	110	514	100/431(23.20)	0.38	0.1	431	67	4.00E-12	gene=Chr03G0328	Gene Symbol:FOW2|Host:Multiple genera in multiple families|Disease:Blights, wilts, rots of various sorts|Description:SIMILARITY: Contains 1 Zn(2)-C6 fungal-type DNA-binding

															domain.
Chr03G0329.1	622	8	40	UniProt ID:Q5A4F3_CANAL	624	14	46	16/33(48.48)	0.7	0	33	52	7.00E-08	gene=Chr03G0329	Gene Symbol:ZCF37|Host:Isolated from a wide variety of substrates including humans|Disease:invasive candidal disease|Description:Unknown
Chr03G0334.1	478	34	477	UniProt ID:A0ST43_CERNC	459	1	457	120/466(25.75)	0.44	0.07	466	162	7.00E-45	gene=Chr03G0334	Gene Symbol:CTB5|Host:Numerous taxa in Solanaceae|Disease:Leaf spot|Description:Unknown
Chr03G0335.1	388	125	292	UniProt ID:Q9Y784_MAGGR	631	194	360	50/168(29.76)	0.58	0.01	168	102	1.00E-24	gene=Chr03G0335	Gene Symbol:PTH11|Host:Digitaria (Poaceae)|Disease:Leaf spot|Description:Unknown
Chr03G0343.1	358	16	356	UniProt ID:Q6A2T2_BOTFU	391	22	350	100/350(28.57)	0.48	0.09	350	118	9.00E-31	gene=Chr03G0343	Gene Symbol:BTP1|Host:Various plant families|Disease:Grey mould. Parasite or saprophyte|Description:Unknown

Chr03G0347.1	571	45	487	UniProt ID:Q5XTQ4_BOTFU	574	55	533	152/503(30.22)	0.41	0.17	503	165	5.00E-45	gene=Chr03G0347	Gene Symbol:LIP1|Host:Various plant families|Disease:Grey mould. Parasite or saprophyte|Description:Unknown
Chr03G0348.1	380	5	361	UniProt ID:Y1220_ASPFU	439	92	437	131/368(35.60)	0.52	0.09	368	184	4.00E-54	gene=Chr03G0348	Gene Symbol:AFUA_3G01220|Host:humans|Disease:infection|Description:FUNCTION: Probable aspartic-type endopeptidase which contributes to virulence (By similarity).
Chr03G0349.1	550	17	49	UniProt ID:Q5A4F3_CANAL	624	14	46	18/33(54.55)	0.7	0	33	52.8	3.00E-08	gene=Chr03G0349	Gene Symbol:ZCF37|Host:Isolated from a wide variety of substrates including humans|Disease:invasive candidal disease|Description:Unknown
Chr03G0359.1	564	80	162	UniProt ID:Q4	740	42	126	24/85(28.24)	0.52	0.02	85	47	2.00E-06	gene=Chr03G0359	Gene Symbol:AFUA_4G06250|

				WNM3_ASPFU										Host:humans|Disease:infection|Description:CAUTION: The sequence shown here is derived from an EMBL/GenBank/DDBJ whole genome shotgun (WGS) entry which is preliminary data.
Chr03G0374.1	540	13	349	UniProt ID:Q5XTQ4_BOTFU	574	11	380	144/389(37.02)	0.47	0.18	389	155	1.00E-41	gene=Chr03G0374	Gene Symbol:LIP1|Host:Various plant families|Disease:Grey mould. Parasite or saprophyte|Description:Unknown
Chr03G0376.1	335	113	321	UniProt ID:Q04701_FUSSO	242	23	228	109/211(51.66)	0.63	0.03	211	188	3.00E-58	gene=Chr03G0376	Gene Symbol:PELA|Host:Multiple plant families. Some strains may cause infections in humans|Disease:Saprobe, facultative pathogen|Description:Unknown
Chr03G0377.1	400	2	394	UniProt ID:Q5G	437	6	418	118/434(27.1	0.44	0.14	434	123	4.00E-32	gene=Chr03G0377	Gene Symbol:NULL|Host:Multipl

				FD3_PHAND				9)							e genera of Poaceae and Blysmus compressus (Cyperaceae)|Disease:Glume blotch of wheat and other grasses|Description:Unknown
Chr03G0378.1	493	250	458	UniProt ID:A4ULJ1_MYCGR	517	252	504	58/257 (22.57)	0.39	0.2	257	63.9	8.00E-12	gene=Chr03G0378	Gene Symbol:CYP51|Host:Triticum and possibly a few other grasses|Disease:Leaf spot or speckled leaf blotch of wheat|Description:COFACTOR: Heme group (By similarity).
Chr03G0385.1	1489	45	1481	UniProt ID:Q96WW9_GIBPU	1491	46	1481	794/1444(54.99)	0.71	0.01	1444	1687	0	gene=Chr03G0385	Gene Symbol:ABC1|Host:Various plant families|Disease:Tree canker, rot of potatoes (Samuels et al. 2006). Root rot (Booth 1973)|Description:SIMILARITY: Belongs to the ABC transporter superfamily.

Chr03G0394.1	731	544	724	UniProt ID:IRS4_CANAL	638	412	630	66/230 (28.70)	0.46	0.26	230	110	4.00E-26	gene=Chr03G0394	Gene Symbol:IRS4|Host:Isolated from a wide variety of substrates including humans|Disease:invasive candidal disease|Description:FUNCTION: Positive regulator of phosphatidylinositol 4,5-bisphosphate turnover and negatively regulates signaling through the cell integrity pathway. Involved in rDNA silencing (By similarity).
Chr03G0402.1	439	69	432	UniProt ID:LAP4_ARTOC	372	20	367	126/370(34.05)	0.51	0.08	370	184	3.00E-54	gene=Chr03G0402	Gene Symbol:MCYG_03459|Host:humans, reptiles|Disease:dermatophytoses|Description:FUNCTION: Probable extracellular aminopeptidase which contributes to pathogenicity (By similarity).

Chr03G0404.1	595	169	583	UniProt ID:Q2PEN8_9HYPO	575	127	565	101/465(21.72)	0.36	0.16	465	53.9	1.00E-08	gene=Chr03G0404	Gene Symbol:NOXB|Host:plants|Disease:cool-season grasses|Description:SIMILARITY: Contains 1 FAD-binding FR-type domain.
Chr03G0408.1	563	319	513	UniProt ID:A4ULJ2_MYCGR	515	279	503	58/225(25.78)	0.44	0.13	225	77.4	5.00E-16	gene=Chr03G0408	Gene Symbol:CYP51|Host:Triticum and possibly a few other grasses|Disease:Leaf spot or speckled leaf blotch of wheat|Description:COFACTOR: Heme group (By similarity).
Chr03G0409.1	481	184	385	UniProt ID:Q59MV9_CANAL	398	122	304	56/210(26.67)	0.44	0.17	210	63.9	5.00E-12	gene=Chr03G0409	Gene Symbol:YHB1|Host:Isolated from a wide variety of substrates including humans|Disease:invasive candidal disease|Description:SIMILARITY: Belongs to the globin family.
Chr03G0	709	209	381	UniProt	663	254	434	54/185	0.49	0.09	185	84	9.00E-18	gene=Chr	Gene

411.1				ID:Q0 WXM3 _FUSO X				(29.19)						03G0411	Symbol:FOW2|Host:Multiple genera in multiple families|Disease:Blights, wilts, rots of various sorts|Description:SIMILARITY: Contains 1 Zn(2)-C6 fungal-type DNA-binding domain.
Chr03G0 429.1	349	44	331	UniProt ID:Q6T FC7_A SPFM	349	61	347	104/29 3(35.4 9)	0.54	0.04	293	171	2.00E-50	gene=Chr 03G0429	Gene Symbol:NULL|Host:humans|Disease:infection|Description:Unknown
Chr03G0 434.1	570	9	568	UniProt ID:Q5X TQ5_B OTFU	615	30	596	349/57 5(60.7 0)	0.75	0.04	575	656	0	gene=Chr 03G0434	Gene Symbol:FRT1|Host:Various plant families|Disease:Grey mould. Parasite or saprophyte|Description:SIMILARITY: Belongs to the major facilitator superfamily. Sugar transporter (TC 2.A.1.1) family.
Chr03G0 445.1	1064	45	533	UniProt ID:Q9U VN5_A	4360	1369	1874	156/53 7(29.0 5)	0.44	0.15	537	153	2.00E-38	gene=Chr 03G0445	Gene Symbol:AMT|Host:Plant|Disease:Leaf spot,

				LTAL											rots|Description:Unknown
Chr03G0446.1	262	12	262	UniProt ID:Q9P4U9_ALTAL	296	21	271	85/256 (33.20)	0.52	0.04	256	103	6.00E-27	gene=Chr03G0446	Gene Symbol:AKT3-1|Host:Plant|Disease:Leaf spot, rots|Description:Unknown
Chr03G0449.1	515	50	480	UniProt ID:Q5ANE1_CANAL	748	51	496	113/456(24.78)	0.43	0.08	456	90.9	3.00E-20	gene=Chr03G0449	Gene Symbol:SNF3|Host:Isolated from a wide variety of substrates including humans|Disease:invasive candidal disease|Description:SIMILARITY: Belongs to the major facilitator superfamily. Sugar transporter (TC 2.A.1.1) family.
Chr03G0450.1	525	9	272	UniProt ID:Q9Y784_MAGGR	631	106	364	57/264 (21.59)	0.45	0.02	264	79.3	1.00E-16	gene=Chr03G0450	Gene Symbol:PTH11|Host:Digitaria (Poaceae)|Disease:Leaf spot|Description:Unknown
Chr03G0453.1	541	83	256	UniProt ID:Q5ALS7_CANAL	1144	351	519	38/178 (21.35)	0.43	0.07	178	49.7	3.00E-07	gene=Chr03G0453	Gene Symbol:CTF1|Host:Isolated from a wide variety of substrates including

															humans|Disease:invasive candidal disease|Description:SIMILARITY: Contains 1 Zn(2)-C6 fungal-type DNA-binding domain.
Chr03G0462.1	863	1	863	UniProt ID:A6RGD8_AJECN	869	1	869	534/873(61.17)	0.77	0.02	873	1014	0	gene=Chr03G0462	Gene Symbol:HCAG_08704|Host:humans|Disease:Darling's disease|Description:SIMILARITY: Contains 1 PCI domain.
Chr03G0466.1	1158	387	857	UniProt ID:Q5AM49_CANAL	1690	780	1239	170/485(35.05)	0.54	0.08	485	290	1.00E-81	gene=Chr03G0466	Gene Symbol:SNF2|Host:Isolated from a wide variety of substrates including humans|Disease:invasive candidal disease|Description:Unknown
Chr03G0474.1	275	17	190	UniProt ID:Q96VB3_ALTAL	296	25	193	42/175(24.00)	0.4	0.04	175	48.1	1.00E-07	gene=Chr03G0474	Gene Symbol:AFT3-1|Host:Plant|Disease:Leaf spot, rots|Description:Unknown
Chr03G0	626	276	436	UniProt	746	270	433	54/168	0.49	0.07	168	82	3.00E-17	gene=Chr	Gene

480.1				ID:Q9HG15_COLLN				(32.14)						03G0480	Symbol:CLTA1|Host:Multiple genera of Fabaceae. Rare reports on other taxa|Disease:Leaf, stem and pod anthracnose|Description:SIMILARITY: Contains 1 Zn(2)-C6 fungal-type DNA-binding domain.
Chr03G0487.1	353	1	353	UniProt ID:Q96VA7_FUSOX	353	1	353	352/353(99.72)	1	0	353	736	0	gene=Chr03G0487	Gene Symbol:FGA1|Host:Multiple genera in multiple families|Disease:Blights, wilts, rots of various sorts|Description:Unknown
Chr03G0491.1	763	15	205	UniProt ID:Q9Y7F1_ASPFM	824	19	233	68/219 (31.05)	0.46	0.15	219	82.8	3.00E-17	gene=Chr03G0491	Gene Symbol:PABAA|Host:humans|Disease:infection|Description:Unknown
Chr03G0495.1	837	58	279	UniProt ID:F2QQX0_PICP7	3007	2807	2998	51/224 (22.77)	0.39	0.15	224	52.4	1.00E-07	gene=Chr03G0495	Gene Symbol:CHS1|Host:humans|Disease:occasional infection|Description:SIMILARITY: Contains 1 PH domain.

Chr03G0 499.1	861	44	265	UniProt ID:Q99 079_U STMD	405	191	400	72/242 (29.75)	0.47	0.21	242	99.4	5.00E-23	gene=Chr 03G0499	Gene Symbol:NULL|Host:Euchl aena spp., Zea spp. (Poaceae)|Disease:Smut. Corn smut|Description:CATALY TIC ACTIVITY: ATP + a protein = ADP + a phosphoprotein.
Chr03G0 503.1	828	504	807	UniProt ID:Q8T GW9_ CANAL	1085	101	431	69/352 (19.60)	0.36	0.2	352	46.2	7.00E-06	gene=Chr 03G0503	Gene Symbol:SSN6|Host:Isolate d from a wide variety of substrates including humans|Disease:invasive candidal disease|Description:Unkn own
Chr03G0 507.1	176	52	117	UniProt ID:Q9P 849_C ANAL	257	16	81	26/66(39.39)	0.56	0	66	47.8	5.00E-08	gene=Chr 03G0507	Gene Symbol:SAP45|Host:Isolat ed from a wide variety of substrates including humans|Disease:invasive candidal disease|Description:Unkn own
Chr03G0	643	27	587	UniProt	1439	791	1343	176/58	0.48	0.09	582	212	1.00E-58	gene=Chr	Gene

512.1				ID:Q9UW03_BOTFU				2(30.24)						03G0512	Symbol:ATRB|Host:Various plant families|Disease:Grey mould. Parasite or saprophyte|Description:SIMILARITY: Belongs to the ABC transporter superfamily.
Chr03G0517.1	836	39	836	UniProt ID:A4QTS1_MAGO7	847	26	847	404/851(47.47)	0.6	0.1	851	635	0	gene=Chr03G0517	Gene Symbol:MGG_04985|Host:Poaceae, especially important on Oryzae|Disease:Rice blast|Description:Unknown
Chr03G0523.1	1235	209	1097	UniProt ID:O93841_9PEZI	914	23	909	390/918(42.48)	0.57	0.07	918	649	0	gene=Chr03G0523	Gene Symbol:CHIP6|Host:Multiple genera in multiple families|Disease:'Anthracnose of stems and leaves, dieback, root rot, leaf spot, blossom rot, fruit rot (dieback and ripe rot), seedling blight.' (Mordue 1971)|Description:Unknown

Chr03G0525.1	422	7	409	UniProt ID:Q7ZA48_9HYPO	418	5	403	155/419(36.99)	0.53	0.09	419	254	1.00E-80	gene=Chr03G0525	Gene Symbol:GLU1|Host:humans|Disease:Verticillium disease or dry bubble|Description:Unknown
Chr03G0537.1	1169	451	527	UniProt ID:Q4WNM3_ASPFU	740	46	126	29/84(34.52)	0.54	0.12	84	47.4	4.00E-06	gene=Chr03G0537	Gene Symbol:AFUA_4G06250|Host:humans|Disease:infection|Description:CAUTION: The sequence shown here is derived from an EMBL/GenBank/DDBJ whole genome shotgun (WGS) entry which is preliminary data.
Chr03G0543.1	901	44	901	UniProt ID:C6KEF4_MYCGR	927	30	927	439/911(48.19)	0.64	0.07	911	756	0	gene=Chr03G0543	Gene Symbol:STE11|Host:Triticum and possibly a few other grasses|Disease:Leaf spot or speckled leaf blotch of wheat|Description:SIMILARITY: Contains 1 SAM (sterile alpha motif) domain.

Chr03G0545.1	393	5	354	UniProt ID:Q6A2T2_BOTFU	391	19	374	103/372(27.69)	0.46	0.1	372	97.1	2.00E-23	gene=Chr03G0545	Gene Symbol:BTP1|Host:Various plant families|Disease:Grey mould. Parasite or saprophyte|Description:Unknown
Chr03G0549.1	452	67	137	UniProt ID:Q5A4F3_CANAL	624	14	86	25/77(32.47)	0.49	0.13	77	49.7	2.00E-07	gene=Chr03G0549	Gene Symbol:ZCF37|Host:Isolated from a wide variety of substrates including humans|Disease:invasive candidal disease|Description:Unknown
Chr03G0550.1	856	14	856	UniProt ID:Q96UH9_MAGGR	856	1	856	674/867(77.74)	0.83	0.04	867	1245	0	gene=Chr03G0550	Gene Symbol:CHM1|Host:Digitaria (Poaceae)|Disease:Leaf spot|Description:CATALYTIC ACTIVITY: ATP + a protein = ADP + a phosphoprotein.
Chr03G0554.1	425	38	363	UniProt ID:Q7LJU0_C	410	80	401	88/341(25.81)	0.41	0.1	341	85.9	3.00E-19	gene=Chr03G0554	Gene Symbol:UXS1|Host:humans|Disease:cryptococcosis

				RYNE										|Description:Unknown
Chr03G0555.1	468	139	393	UniProt ID:Q6TFC7_ASPFM	349	63	326	81/270 (30.00)	0.47	0.08	270	109	2.00E-27	gene=Chr03G0555	Gene Symbol:NULL|Host:humans|Disease:infection|Description:Unknown
Chr03G0558.1	465	1	455	UniProt ID:OPSB_ASPFU	485	1	459	235/464(50.65)	0.65	0.03	464	414	6.00E-141	gene=Chr03G0558	Gene Symbol:OPSB|Host:humans|Disease:infection|Description:FUNCTION: Probable GPI-anchored aspartic-type endopeptidase which contributes to virulence (By similarity).
Chr03G0565.1	872	163	559	UniProt ID:A6N6J8_FUSOX	903	238	619	85/418 (20.33)	0.38	0.14	418	51.6	1.00E-07	gene=Chr03G0565	Gene Symbol:CTF1|Host:Multiple genera in multiple families|Disease:Blights, wilts, rots of various sorts|Description:SIMILARITY: Contains 1 Zn(2)-C6 fungal-type DNA-binding domain.
Chr03G0566.1	410	298	397	UniProt ID:Q99067_U	469	121	250	38/130 (29.23)	0.42	0.23	130	44.3	7.00E-06	gene=Chr03G0566	Gene Symbol:UHBE1|Host:Poaceae|Disease:Smut|Descri

				STHO										ption:SUBCELLULAR LOCATION: Nucleus (By similarity).
Chr03G0573.1	2913	2719	2819	UniProt ID:Q5AB06_CANAL	1020	858	956	33/104 (31.73)	0.54	0.08	104	54.7	8.00E-08	gene=Chr03G0573	Gene Symbol:VPS34|Host:Isolated from a wide variety of substrates including humans|Disease:invasive candidal disease|Description:SIMILARITY: Contains 1 PI3K/PI4K domain.
Chr03G0576.1	413	102	189	UniProt ID:Q59KZ0_CANAL	741	33	111	27/88(30.68)	0.48	0.1	88	46.6	2.00E-06	gene=Chr03G0576	Gene Symbol:NOP4|Host:Isolated from a wide variety of substrates including humans|Disease:invasive candidal disease|Description:CAUTION: The sequence shown here is derived from an EMBL/GenBank/DDBJ whole genome shotgun (WGS) entry which is preliminary data.
Chr03G0	506	5	288	UniProt	391	16	296	71/290	0.48	0.05	290	96.7	9.00E-23	gene=Chr	Gene

579.1				ID:Q6A2T2_BOTFU				(24.48)						03G0579	Symbol:BTP1|Host:Various plant families|Disease:Grey mould. Parasite or saprophyte|Description:Unknown
Chr03G0582.1	490	14	46	UniProt ID:Q5A4F3_CANAL	624	14	46	15/33(45.45)	0.82	0	33	52	5.00E-08	gene=Chr03G0582	Gene Symbol:ZCF37|Host:Isolated from a wide variety of substrates including humans|Disease:invasive candidal disease|Description:Unknown
Chr03G0587.1	742	62	388	UniProt ID:Q5AHB5_CANAL	571	76	374	76/342(22.22)	0.44	0.17	342	53.9	2.00E-08	gene=Chr03G0587	Gene Symbol:DFG16|Host:Isolated from a wide variety of substrates including humans|Disease:invasive candidal disease|Description:CAUTION: The sequence shown here is derived from an EMBL/GenBank/DDBJ whole genome shotgun (WGS) entry which is

															preliminary data.
Chr03G0589.1	1007	494	916	UniProt ID:Q3Y5V5_MAGGR	1321	871	1317	134/449(29.84)	0.5	0.06	449	190	3.00E-50	gene=Chr03G0589	Gene Symbol:ABC3|Host:Digitaria (Poaceae)|Disease:Leaf spot|Description:SIMILARITY: Belongs to the ABC transporter superfamily.
Chr03G0591.1	819	269	735	UniProt ID:Q0PND8_MAGGR	1375	763	1238	176/498(35.34)	0.53	0.11	498	248	2.00E-69	gene=Chr03G0591	Gene Symbol:PEX6|Host:Digitaria (Poaceae)|Disease:Leaf spot|Description:SIMILARITY: Belongs to the AAA ATPase family.
Chr03G0598.1	672	193	450	UniProt ID:Q5XTQ4_BOTFU	574	111	386	84/292(28.77)	0.45	0.17	292	94	4.00E-21	gene=Chr03G0598	Gene Symbol:LIP1|Host:Various plant families|Disease:Grey mould. Parasite or saprophyte|Description:Unknown
Chr03G0604.1	524	16	513	UniProt ID:Q5APD4_CANAL	513	7	507	306/506(60.47)	0.73	0.03	506	639	0	gene=Chr03G0604	Gene Symbol:CFA1|Host:Isolated from a wide variety of substrates including humans|Disease:invasive candidal

															disease|Description:CAUTION: The sequence shown here is derived from an EMBL/GenBank/DDBJ whole genome shotgun (WGS) entry which is preliminary data.
Chr03G0612.1	998	829	992	UniProt ID:Q5AB06_CANAL	1020	855	1017	49/172 (28.49)	0.53	0.1	172	80.9	2.00E-16	gene=Chr03G0612	Gene Symbol:VPS34|Host:Isolated from a wide variety of substrates including humans|Disease:invasive candidal disease|Description:SIMILARITY: Contains 1 PI3K/PI4K domain.
Chr03G0617.1	490	208	472	UniProt ID:Q5AG71_CANAL	1462	66	325	87/277 (31.41)	0.49	0.1	277	135	2.00E-34	gene=Chr03G0617	Gene Symbol:HSL1|Host:Isolated from a wide variety of substrates including humans|Disease:invasive candidal disease|Description:CAUTION: The sequence shown here is derived from an EMBL/GenBank/DDBJ

														whole genome shotgun (WGS) entry which is preliminary data.
Chr03G0630.1	765	258	440	UniProt ID:Q0WXM3_FUSOX	663	250	434	40/189 (21.16)	0.42	0.05	189	56.6	4.00E-09	gene=Chr03G0630	Gene Symbol:FOW2|Host:Multiple genera in multiple families|Disease:Blights, wilts, rots of various sorts|Description:SIMILARITY: Contains 1 Zn(2)-C6 fungal-type DNA-binding domain.
Chr03G0631.1	498	38	494	UniProt ID:Q5ABU7_CANAL	564	95	560	141/467(30.19)	0.52	0.02	467	237	2.00E-71	gene=Chr03G0631	Gene Symbol:MDR1|Host:Isolated from a wide variety of substrates including humans|Disease:invasive candidal disease|Description:Unknown
Chr03G0641.1	212	49	192	UniProt ID:C1GLV1_PARBD	161	6	121	44/147 (29.93)	0.46	0.23	147	56.2	3.00E-11	gene=Chr03G0641	Gene Symbol:PADG_08342|Host:humans|Disease:Paracoccidioidomycosis|Description:SIMILARITY: Belongs to the small GTPase

														superfamily. Rab family.
Chr03G0643.1	486	22	396	UniProt ID:A4UC81_MAGO7	376	4	376	99/392 (25.26)	0.41	0.09	392	84	2.00E-18	gene=Chr03G0643	Gene Symbol:MGG_10702|Host:Poaceae, especially important on Oryzae|Disease:Rice blast|Description:Unknown
Chr03G0646.1	504	17	495	UniProt ID:CATA_CANAL	487	3	484	283/482(58.71)	0.73	0.01	482	573	0	gene=Chr03G0646	Gene Symbol:CTA1|Host:Isolated from a wide variety of substrates including humans|Disease:invasive candidal disease|Description:FUNCTION: Occurs in almost all aerobically respiring organisms and serves to protect cells from the toxic effects of hydrogen peroxide. Required for hyphal growth.
Chr03G0648.1	181	18	143	UniProt ID:A7A233_YEAS7	215	21	150	40/131 (30.53)	0.44	0.05	131	52.8	6.00E-10	gene=Chr03G0648	Gene Symbol:SEC4|Host:humans|Disease:occasional infection|Description:SIMI

														LARITY: Belongs to the small GTPase superfamily. Rab family.
Chr03G0654.1	239	8	239	UniProt ID:Q59X89_CANAL	216	5	215	140/232(60.34)	0.69	0.09	232	271	3.00E-92	gene=Chr03G0654	Gene Symbol:"CAO19.13721, CAO19.589, CAO19.6364, CAO19.8221, O|Host:Isolated from a wide variety of substrates including humans|Disease:invasive candidal disease|Description:SIMILARITY: Belongs to the small GTPase superfamily. Rab family.
Chr03G0655.1	671	37	648	UniProt ID:C1G3C3_PARBD	650	37	633	305/633(48.18)	0.64	0.09	633	426	2.00E-140	gene=Chr03G0655	Gene Symbol:PADG_01439|Host:humans|Disease:Paracoccidioidomycosis|Description:Unknown
Chr03G0656.1	1094	341	761	UniProt ID:Q5ALS7_CANAL	1144	343	740	91/442(20.59)	0.4	0.15	442	55.5	1.00E-08	gene=Chr03G0656	Gene Symbol:CTF1|Host:Isolated from a wide variety of substrates including

															humans|Disease:invasive candidal disease|Description:SIMILARITY: Contains 1 Zn(2)-C6 fungal-type DNA-binding domain.
Chr03G0668.1	587	137	579	UniProt ID:Q5ANE1_CANAL	748	75	503	98/468 (20.94)	0.4	0.14	468	84.7	4.00E-18	gene=Chr03G0668	Gene Symbol:SNF3|Host:Isolated from a wide variety of substrates including humans|Disease:invasive candidal disease|Description:SIMILARITY: Belongs to the major facilitator superfamily. Sugar transporter (TC 2.A.1.1) family.
Chr03G0670.1	309	85	216	UniProt ID:Q59MV9_CANAL	398	180	306	44/136 (32.35)	0.51	0.1	136	62	9.00E-12	gene=Chr03G0670	Gene Symbol:YHB1|Host:Isolated from a wide variety of substrates including humans|Disease:invasive candidal disease|Description:SIMILARITY: Belongs to the

														globin family.
Chr03G0674.1	742	204	289	UniProt ID:Q5A7S7_CANAL	526	252	337	47/86(54.65)	0.77	0	86	107	3.00E-25	gene=Chr03G0674	Gene Symbol:FKH2|Host:Isolated from a wide variety of substrates including humans|Disease:invasive candidal disease|Description:SIMILARITY: Contains 1 fork-head DNA-binding domain.
Chr03G0679.1	643	90	409	UniProt ID:Q9Y880_COCCA	880	50	316	95/321(29.60)	0.46	0.17	321	140	7.00E-36	gene=Chr03G0679	Gene Symbol:SNF1|Host:Corn, Zea mays, sometimes on Sorghum (Poaceae) and various other plant families|Disease:Northern corn leaf spot, ear and kernel rot|Description:Unknown
Chr03G0684.1	2188	1697	1830	UniProt ID:SUB4A_COCP7	397	244	355	46/137(33.58)	0.42	0.2	137	47.4	6.00E-06	gene=Chr03G0684	Gene Symbol:CPC735_066880|Host:humans|Disease:coccidiomycosis|Description: FUNCTION: Secreted subtilisin-like serine

															protease with keratinolytic activity that contributes to pathogenicity (By similarity).
Chr03G0686.1	353	71	353	UniProt ID:Q6TFC7_ASPFM	349	63	346	102/288(35.42)	0.51	0.03	288	162	5.00E-47	gene=Chr03G0686	Gene Symbol:NULL|Host:humans|Disease:infection|Description:Unknown
Chr03G0688.1	1472	127	1463	UniProt ID:Q5A762_CANAL	1606	149	1598	375/1498(25.03)	0.42	0.14	1498	365	8.00E-105	gene=Chr03G0688	Gene Symbol:MLT1|Host:Isolated from a wide variety of substrates including humans|Disease:invasive candidal disease|Description:SIMILARITY: Belongs to the ABC transporter superfamily.
Chr03G0689.1	285	154	234	UniProt ID:VPS27_CANAL	841	210	283	32/83(38.55)	0.48	0.13	83	54.7	2.00E-09	gene=Chr03G0689	Gene Symbol:VPS27|Host:Isolated from a wide variety of substrates including humans|Disease:invasive candidal disease|Description:FUNCTION: Component of the

															ESCRT-0 complex which is the sorting receptor for ubiquitinated cargo proteins at the multivesicular body (MVB) and recruits ESCRT-I to the MVB outer membrane (By similarity).
Chr03G0697.1	2082	495	2053	UniProt ID:LAA1_YEAST	2014	458	1993	363/1638(22.16)	0.44	0.11	1638	298	4.00E-82	gene=Chr03G0697	Gene Symbol:LAA1|Host:humans|Disease:occasional infection|Description:FUNCTION: Involved in the trans-Golgi network (TGN)-endosome transport. Important for the correct localization of the adapter protein complex AP-1.
Chr03G0708.1	877	281	620	UniProt ID:Q5AI81_CANAL	571	204	570	141/381(37.01)	0.56	0.14	381	223	1.00E-63	gene=Chr03G0708	Gene Symbol:PDE2|Host:Isolated from a wide variety of substrates including humans|Disease:invasive candidal disease|Description:CAUT

															ION: The sequence shown here is derived from an EMBL/GenBank/DDBJ whole genome shotgun (WGS) entry which is preliminary data.
Chr03G0709.1	545	87	542	UniProt ID:Q4PDC7_USTMA	589	115	586	206/482(42.74)	0.58	0.07	482	351	4.00E-114	gene=Chr03G0709	Gene Symbol:UM01886.1|Host: Euchlaena spp., Zea spp. (Poaceae)|Disease:Smut. Corn smut|Description:CAUTION: The sequence shown here is derived from an EMBL/GenBank/DDBJ whole genome shotgun (WGS) entry which is preliminary data.
Chr03G0712.1	1368	44	852	UniProt ID:CAS1_CRYNJ	960	47	852	241/886(27.20)	0.44	0.18	886	261	7.00E-73	gene=Chr03G0712	Gene Symbol:CAS1|Host:humans|Disease:cryptococcosis|Description:FUNCTION: Probable O-acetyltransferase required for the O-acetylation of the capsular

															glucoronoxylmannans (GXM) involved in virulence.
Chr03G0729.1	1065	89	332	UniProt ID:Q5K995_CRYNJ	1005	196	418	65/253 (25.69)	0.45	0.15	253	85.1	1.00E-17	gene=Chr03G0729	Gene Symbol:CNK02740|Host:humans|Disease:cryptococcosis|Description:Unknown
Chr03G0734.1	560	208	272	UniProt ID:RAD18_CANAL	378	8	81	24/74(32.43)	0.43	0.12	74	44.7	7.00E-06	gene=Chr03G0734	Gene Symbol:RAD18|Host:Isolated from a wide variety of substrates including humans|Disease:invasive candidal disease|Description:FUNCTION: E3 RING-finger protein, member of the UBC2/RAD6 epistasis group. Associates to the E2 ubiquitin conjugating enzyme UBC2/RAD6 to form the UBC2-RAD18 ubiquitin ligase complex involved in postreplicative repair (PRR) of damaged DNA (By similarity).

Chr03G0735.1	480	84	376	UniProt ID:PABP_COCIM	768	52	315	77/300 (25.67)	0.44	0.14	300	120	5.00E-30	gene=Chr03G0735	Gene Symbol:PAB1|Host:humans|Disease:coccidioidomycosis|Description:FUNCTION: Binds the poly(A) tail of mRNA. Appears to be an important mediator of the multiple roles of the poly(A) tail in mRNA biogenesis, stability and translation. In the nucleus, involved in both mRNA cleavage and polyadenylation. Is also required for efficient mRNA export to the cytoplasm. Acts in concert with a poly(A)-specific nuclease (PAN) to affect poly(A) tail shortening, which may occur concomitantly with either nucleocytoplasmic mRNA transport or translational initiation. In the cytoplasm, stimulates translation

															initiation and regulates mRNA decay through translation termination-coupled poly(A) shortening, probably mediated by PAN (By similarity).
Chr03G0736.1	898	326	554	UniProt ID:HOG1_CANAL	377	21	242	83/232 (35.78)	0.56	0.06	232	120	4.00E-30	gene=Chr03G0736	Gene Symbol:HOG1|Host:Isolated from a wide variety of substrates including humans|Disease:invasive candidal disease|Description:FUNCTION: Mitogen-activated protein kinase involved in a signal transduction pathway that is activated by changes in the osmolarity of the extracellular environment. Controls osmotic regulation of transcription of target genes. Regulates stress- induced production and accumulation of

															glycerol and D-arabitol. HOG1 is also involved in virulence, morphogenesis and oxidative stress response especially through its role in chlamydospore formation, an oxygen-dependent morphogenetic program.
Chr03G0739.1	1472	852	1230	UniProtID:A4RB72_MAGO7	1715	1160	1568	106/420(25.24)	0.45	0.12	420	112	8.00E-26	gene=Chr03G0739	Gene Symbol:MGG_02986|Host:Poaceae, especially important on Oryzae|Disease:Rice blast|Description:CATALYTIC ACTIVITY: Deoxynucleoside triphosphate + DNA(n) = diphosphate + DNA(n+1).
Chr03G0747.1	310	58	175	UniProtID:Q8TGD1_FUSOX	318	167	281	40/126(31.75)	0.44	0.15	126	47.8	3.00E-07	gene=Chr03G0747	Gene Symbol:FOW1|Host:Multiple genera in multiple families|Disease:Blights, wilts, rots of various sorts|Description:SIMILARITY: Belongs to the

															mitochondrial carrier family.
Chr03G0748.1	389	62	337	UniProt ID:TUP1_CANAL	514	205	464	70/295 (23.73)	0.39	0.18	295	48.9	2.00E-07	gene=Chr03G0748	Gene Symbol:TUP1|Host:Isolated from a wide variety of substrates including humans|Disease:invasive candidal disease|Description:FUNCTION: Represses transcription by RNA polymerase II. Represses genes responsible for initiating filamentous growth and this repression is lifted under inducing environmental conditions.
Chr03G0749.1	467	1	466	UniProt ID:Q9C1T0_FUSOX	467	1	466	402/466(86.27)	0.93	0	466	851	0	gene=Chr03G0749	Gene Symbol:ARG1|Host:Multiple genera in multiple families|Disease:Blights, wilts, rots of various sorts|Description:Unknown
Chr03G0754.1	279	23	273	UniProt ID:A4R	286	16	267	81/283 (28.62)	0.43	0.22	283	84.7	4.00E-20	gene=Chr03G0754	Gene Symbol:MGG_00056|Host

				GG9_MAGO7											:Poaceae, especially important on Oryzae|Disease:Rice blast|Description:SIMILARITY: Belongs to the short-chain dehydrogenases/reductases (SDR) family.
Chr03G0756.1	470	1	469	UniProt ID:C5GS89_AJEDR	481	1	480	273/490(55.71)	0.71	0.06	490	483	7.00E-168	gene=Chr03G0756	Gene Symbol:BDCG_07142|Host:humans|Disease:cutaneous Blastomyces dermatitidis infection|Description:Unknown
Chr03G0760.1	570	328	450	UniProt ID:Q9P8W9_CRYNE	162	16	141	59/127 (46.46)	0.57	0.04	127	97.4	3.00E-24	gene=Chr03G0760	Gene Symbol:CPA1|Host:humans|Disease:cryptococcosis|Description:FUNCTION: PPIases accelerate the folding of proteins (By similarity).
Chr03G0764.1	805	26	78	UniProt ID:Q9P3Z8_CYBJA	345	15	67	28/53(52.83)	0.62	0	53	67.4	7.00E-13	gene=Chr03G0764	Gene Symbol:MIG1|Host:humans|Disease:Chronic urinary tract

															infection|Description:Unknown
Chr03G0765.1	1231	802	1204	UniProt ID:Q875L6_USTMD	1441	925	1385	153/464(32.97)	0.52	0.14	464	250	1.00E-68	gene=Chr03G0765	Gene Symbol:SQL2|Host:Euchlaena spp., Zea spp. (Poaceae)|Disease:Smut. Corn smut|Description:SIMILARITY: Contains 1 N-terminal Ras-GEF domain.
Chr03G0773.1	323	8	294	UniProt ID:A3LXZ6_PICST	310	5	294	161/290(55.52)	0.75	0.01	290	341	4.00E-117	gene=Chr03G0773	Gene Symbol:CDC28|Host:humans|Disease:occasional infection|Description:Unknown
Chr03G0774.1	677	214	429	UniProt ID:CDC10_CANAL	357	37	211	53/226(23.45)	0.41	0.27	226	52	4.00E-08	gene=Chr03G0774	Gene Symbol:CDC10|Host:Isolated from a wide variety of substrates including humans|Disease:invasive candidal disease|Description:FUNCTION: Plays a role in the cell cycle. Involved in the formation of the ring of filaments in the neck

															region at the mother-bud junction during mitosis.
Chr03G0778.1	667	62	436	UniProt ID:Q5A7S7_CANAL	526	22	365	145/386(37.56)	0.52	0.14	386	218	2.00E-63	gene=Chr03G0778	Gene Symbol:FKH2|Host:Isolated from a wide variety of substrates including humans|Disease:invasive candidal disease|Description:SIMILARITY: Contains 1 fork-head DNA-binding domain.
Chr03G0781.1	585	167	585	UniProt ID:Q2PEN8_9HYPO	575	127	568	106/469(22.60)	0.37	0.16	469	61.6	6.00E-11	gene=Chr03G0781	Gene Symbol:NOXB|Host:plants|Disease:cool-season grasses|Description:SIMILARITY: Contains 1 FAD-binding FR-type domain.
Chr03G0785.1	370	31	301	UniProt ID:Q6XVN4_CRYNV	383	34	315	81/302(26.82)	0.42	0.17	302	79.3	2.00E-17	gene=Chr03G0785	Gene Symbol:GNO1|Host:humans|Disease:cryptococcosis|Description:COFACTOR: Zinc (By similarity).
Chr03G0787.1	502	375	470	UniProt ID:Q70	703	519	603	26/96(27.08)	0.44	0.11	96	45.4	5.00E-06	gene=Chr03G0787	Gene Symbol:ACE2|Host:huma

				0F1_CANGB										ns|Disease:Occasional invasive candidal disease|Description:Unknown
Chr03G0788.1	865	184	701	UniProt ID:PALB_CANAL	717	147	573	131/526(24.90)	0.42	0.2	526	140	4.00E-35	gene=Chr03G0788	Gene Symbol:RIM13|Host:Isolated from a wide variety of substrates including humans|Disease:invasive candidal disease|Description:FUNCTION: Required for the proteolytic cleavage of the transcription factor RIM101 in response to alkaline ambient pH (By similarity).
Chr03G0802.1	135	34	129	UniProt ID:D2JLS9_9HYPO	131	36	128	67/96(69.79)	0.83	0.03	96	139	3.00E-43	gene=Chr03G0802	Gene Symbol:HIS3|Host:plants|Disease:allergy|Description:SUBUNIT: The nucleosome is a histone octamer containing two molecules each of H2A, H2B, H3 and H4 assembled in one H3-H4

														heterotetramer and two H2A-H2B heterodimers. The octamer wraps approximately 147 bp of DNA (By similarity).
Chr03G0805.1	360	107	256	UniProt ID:Q75WR5_9PLEO	265	80	228	38/151 (25.17)	0.4	0.02	151	49.7	7.00E-08	gene=Chr03G0805	Gene Symbol:BRN1|Host:Belamcanda chinensis: Korea,Gladiolus ?gandavensis: Korea,Iris japonica: China,Iris missouriensis (Leaf spot.): Idaho; Montana; Oregon; Washington,Iris sp. (Leaf spot.): China; Texas; Washing|Disease:Leaf spot|Description:SIMILARITY: Belongs to the short-chain dehydrogenases/reductases (SDR) family.
Chr03G0811.1	1224	30	567	UniProt ID:F2QZT0_PICP7	645	42	637	142/624(22.76)	0.43	0.18	624	108	4.00E-25	gene=Chr03G0811	Gene Symbol:SLY1|Host:humans|Disease:occasional infection|Description:Unknown

Chr03G0825.1	643	16	222	UniProt ID:Q9Y880_COCCA	880	62	261	72/211 (34.12)	0.53	0.07	211	124	2.00E-30	gene=Chr03G0825	Gene Symbol:SNF1|Host:Corn, Zea mays, sometimes on Sorghum (Poaceae) and various other plant families|Disease:Northern corn leaf spot, ear and kernel rot|Description:Unknown
Chr03G0828.1	597	252	593	UniProt ID:TUP1_CANAL	514	160	511	201/358(56.15)	0.71	0.06	358	413	3.00E-138	gene=Chr03G0828	Gene Symbol:TUP1|Host:Isolated from a wide variety of substrates including humans|Disease:invasive candidal disease|Description:FUNCTION: Represses transcription by RNA polymerase II. Represses genes responsible for initiating filamentous growth and this repression is lifted under inducing environmental conditions.
Chr03G0829.1	346	1	336	UniProt ID:Q59	386	1	383	186/383(48.5	0.63	0.12	383	352	5.00E-120	gene=Chr03G0829	Gene Symbol:ERG3|Host:Isolat

				VG6_C ANAL				6)							ed from a wide variety of substrates including humans|Disease:invasive candidal disease|Description:CAUTION: The sequence shown here is derived from an EMBL/GenBank/DDBJ whole genome shotgun (WGS) entry which is preliminary data.
Chr03G0834.1	513	201	394	UniProt ID:Q5A8Y9_CANAL	1545	202	392	59/194 (30.41)	0.52	0.02	194	122	3.00E-30	gene=Chr03G0834	Gene Symbol:SIZ1|Host:Isolated from a wide variety of substrates including humans|Disease:invasive candidal disease|Description:CAUTION: The sequence shown here is derived from an EMBL/GenBank/DDBJ whole genome shotgun (WGS) entry which is preliminary data.
Chr03G0838.1	758	27	655	UniProt ID:A6R	633	15	592	329/649(50.6	0.67	0.14	649	615	0	gene=Chr03G0838	Gene Symbol:HCAG_08951|Ho

				H34_AJECN				9)							st:humans|Disease:Darling's disease|Description:Unknown
Chr03G0843.1	578	211	577	UniProt ID:O93800_ALTAL	578	207	571	98/392 (25.00)	0.4	0.13	392	63.9	9.00E-12	gene=Chr03G0843	Gene Symbol:AKT1|Host:Plant|Disease:Leaf spot, rots|Description:Unknown
Chr03G0852.1	480	47	273	UniProt ID:Q9C1Q9_CRYNE	385	26	248	57/230 (24.78)	0.43	0.04	230	53.1	1.00E-08	gene=Chr03G0852	Gene Symbol:CPRA1|Host:humans|Disease:cryptococcosis|Description:Unknown
Chr03G0855.1	1844	839	1360	UniProt ID:Q5AM49_CANAL	1690	777	1251	144/542(26.57)	0.44	0.16	542	162	5.00E-41	gene=Chr03G0855	Gene Symbol:SNF2|Host:Isolated from a wide variety of substrates including humans|Disease:invasive candidal disease|Description:Unknown
Chr03G0868.1	396	156	345	UniProt ID:A6R119_AJECN	609	349	574	65/231 (28.14)	0.44	0.2	231	49.3	2.00E-07	gene=Chr03G0868	Gene Symbol:HCAG_03326|Host:humans|Disease:Darling's disease|Description:Unknown

Chr03G0869.1	674	331	552	UniProt ID:Q6XPX0_FUSOX	359	120	357	71/239 (29.71)	0.43	0.08	239	94.7	6.00E-22	gene=Chr03G0869	Gene Symbol:FGB1|Host:Multiple genera in multiple families|Disease:Blights, wilts, rots of various sorts|Description:Unknown
Chr03G0871.1	931	552	930	UniProt ID:DNLI4_CANAL	928	299	665	100/411(24.33)	0.41	0.18	411	84	2.00E-17	gene=Chr03G0871	Gene Symbol:LIG4|Host:Isolated from a wide variety of substrates including humans|Disease:invasive candidal disease|Description:FUNCTION: Involved in ds DNA break (DSB) repair. Has a role in non-homologous integration (NHI) pathways where it is required in the final step of non-homologus end-joining (NHEJ). Not required for the repair of DSBs induced by ionizing radiation or UV light. Has a important role in

														morphogenesis, positively affecting the capacity to form hyphae.
Chr03G0874.1	791	291	611	UniProt ID:PABP_COCIM	768	54	335	71/333 (21.32)	0.41	0.19	333	63.9	2.00E-11	gene=Chr03G0874 Gene Symbol:PAB1|Host:humans|Disease:coccidioidomycosis|Description:FUNCTION: Binds the poly(A) tail of mRNA. Appears to be an important mediator of the multiple roles of the poly(A) tail in mRNA biogenesis, stability and translation. In the nucleus, involved in both mRNA cleavage and polyadenylation. Is also required for efficient mRNA export to the cytoplasm. Acts in concert with a poly(A)-specific nuclease (PAN) to affect poly(A) tail shortening, which may occur concomitantly with either nucleocytoplasmic mRNA

															transport or translational initiation. In the cytoplasm, stimulates translation initiation and regulates mRNA decay through translation termination-coupled poly(A) shortening, probably mediated by PAN (By similarity).
Chr03G0877.1	194	2	194	UniProt ID:Q2PBY8_CLAPU	195	3	195	180/193(93.26)	0.99	0	193	379	1.00E-135	gene=Chr03G0877	Gene Symbol:CDC42|Host:outcrossing species|Disease:ergotism|Description:SIMILARITY: Belongs to the small GTPase superfamily. Rho family.
Chr03G0880.1	906	157	848	UniProt ID:Q874K8_CRYNV	864	46	769	271/744(36.42)	0.57	0.1	744	464	2.00E-149	gene=Chr03G0880	Gene Symbol:CLC-A|Host:humans|Disease:cryptococcosis|Description:Unknown
Chr03G0898.1	608	7	606	UniProt ID:SED4_ASPFU	594	11	592	287/601(47.75)	0.66	0.03	601	582	0	gene=Chr03G0898	Gene Symbol:SED4|Host:humans|Disease:infection|Description:FUNCTION:

Chr03G0904.1	510	1	510	UniProt ID:Q75T36_GLOLA	518	1	518	460/524(87.79)	0.9	0.04	524	884	0	gene=Chr03G0904	Secreted tripeptidyl-peptidase which degrades proteins at acidic pHs and is involved in virulence. Gene Symbol:CPK1|Host:melons,cucumber|Disease:anthracnose fruit rot|Description:CATALYTIC ACTIVITY: ATP + a protein = ADP + a phosphoprotein.
Chr03G0906.1	673	259	668	UniProt ID:P78585_BOTFU	994	469	949	150/485(30.93)	0.45	0.16	485	189	1.00E-51	gene=Chr03G0906	Gene Symbol:BCPLC1|Host:Various plant families|Disease:Grey mould. Parasite or saprophyte|Description:SIMILARITY: Contains 1 C2 domain.
Chr03G0918.1	545	70	192	UniProt ID:A3QX02_ASPFM	570	431	546	42/123(34.15)	0.55	0.06	123	71.6	3.00E-14	gene=Chr03G0918	Gene Symbol:ZAFA|Host:humans|Disease:infection|Description:Unknown
Chr03G0	629	82	300	UniProt	359	53	279	55/229	0.42	0.05	229	68.6	3.00E-13	gene=Chr	Gene

920.1				ID:GBB_CRYPA				(24.02)						03G0920	Symbol:GB-1|Host:Castanea spp., Fagus sylvatica, Quercus spp. (Fagaceae)|Disease:Chestnut blight. Cankers|Description:FUNCTION: Guanine nucleotide-binding proteins (G proteins) are involved as a modulator or transducer in various transmembrane signaling systems. The beta and gamma chains are required for the GTPase activity, for replacement of GDP by GTP, and for G protein- effector interaction.
Chr03G0930.1	1212	7	1212	UniProt ID:RSE1_CANAL	1219	40	1219	430/1255(34.26)	0.55	0.1	1255	660	0	gene=Chr03G0930	Gene Symbol:RSE1|Host:Isolated from a wide variety of substrates including humans|Disease:invasive candidal disease|Description:FUNC

														TION: Involved in pre-mRNA splicing and cell cycle control (By similarity).
Chr03G0950.1	366	3	365	UniProt ID:Q5AFI8_CANAL	373	5	370	221/368(60.05)	0.77	0.02	368	453	2.00E-159	gene=Chr03G0950	Gene Symbol:LEU2|Host:Isolated from a wide variety of substrates including humans|Disease:invasive candidal disease|Description:FUNCTION: Catalyzes the oxidation of 3-carboxy-2-hydroxy-4-methylpentanoate (3-isopropylmalate) to 3-carboxy-4-methyl-2-oxopentanoate. The product decarboxylates to 4-methyl-2 oxopentanoate (By similarity).
Chr03G0962.1	308	28	300	UniProt ID:Q59KY8_CANAL	314	17	294	118/279(42.29)	0.65	0.03	279	243	7.00E-79	gene=Chr03G0962	Gene Symbol:SIT4|Host:Isolated from a wide variety of substrates including humans|Disease:invasive

															candidal disease|Description:CATALYTIC ACTIVITY: A phosphoprotein + H(2)O = a protein + phosphate.
Chr03G0964.1	853	3	282	UniProt ID:B9WMV3_CANDC	767	10	241	91/284 (32.04)	0.52	0.2	284	155	3.00E-40	gene=Chr03G0964	Gene Symbol:CD36_34700|Host:humans|Disease:leptomeningeal disease,occasional disease,occasional invasive candidal disease|Description:Unknown
Chr03G0970.1	674	7	309	UniProt ID:A6N6J8_FUSOX	903	179	479	86/326 (26.38)	0.43	0.15	326	75.1	7.00E-15	gene=Chr03G0970	Gene Symbol:CTF1|Host:Multiple genera in multiple families|Disease:Blights, wilts, rots of various sorts|Description:SIMILARITY: Contains 1 Zn(2)-C6 fungal-type DNA-binding domain.
Chr03G0973.1	787	102	443	UniProt ID:Q92217_COCHE	2528	109	456	98/366 (26.78)	0.42	0.11	366	122	2.00E-29	gene=Chr03G0973	Gene Symbol:PKS1|Host:Zea mays|Disease:Southern leaf blight of

														maize|Description:Unknown
Chr03G0979.1	917	179	317	UniProt ID:SET1_CANAL	1040	900	1040	48/141 (34.04)	0.51	0.01	141	95.9	4.00E-21	gene=Chr03G0979	Gene Symbol:SET1|Host:Isolated from a wide variety of substrates including humans|Disease:invasive candidal disease|Description:FUNCTION: Catalytic component of the COMPASS (Set1C) complex that specifically mono-, di- and trimethylates histone H3 to form H3K4me1/2/3, which subsequently plays a role in telomere length maintenance, transcription elongation regulation and pathogenesis of invasive candidiasis.
Chr03G0982.1	879	314	877	UniProt ID:Q874K8_CRYNV	864	194	778	215/606(35.48)	0.56	0.1	606	335	2.00E-101	gene=Chr03G0982	Gene Symbol:CLC-A|Host:humans|Disease:cryptococcosis|Description:Unknown

Chr03G0988.1	502	123	418	UniProt ID:Q5A8L5_CANAL	492	154	441	97/313 (30.99)	0.49	0.13	313	165	8.00E-46	gene=Chr03G0988	Gene Symbol:POP2|Host:Isolated from a wide variety of substrates including humans|Disease:invasive candidal disease|Description:CAUTION: The sequence shown here is derived from an EMBL/GenBank/DDBJ whole genome shotgun (WGS) entry which is preliminary data.
Chr03G0989.1	413	126	252	UniProt ID:A4R575_MAGO7	249	26	155	32/130 (24.62)	0.45	0.02	130	47.4	5.00E-07	gene=Chr03G0989	Gene Symbol:MGG_04137|Host:Poaceae, especially important on Oryzae|Disease:Rice blast|Description:SIMILARITY: Contains 1 CTLH domain.
Chr03G0992.1	1543	6	1539	UniProt ID:Q9UW87_CANAL	1606	20	1603	597/1627(36.69)	0.55	0.08	1627	993	0	gene=Chr03G0992	Gene Symbol:MLT1|Host:Isolated from a wide variety of substrates including humans|Disease:invasive

														candidal disease|Description:SIMILARITY: Belongs to the ABC transporter superfamily.
Chr03G1002.1	696	33	696	UniProt ID:C5GA89_AJEDR	656	2	655	373/680(54.85)	0.71	0.06	680	681	0	gene=Chr03G1002 Gene Symbol:BDCG_01185|Host:humans|Disease:cutaneous Blastomyces dermatitidis infection|Description:Unknown
Chr03G1006.1	397	118	376	UniProt ID:O42793_COLTR	530	214	474	102/266(38.35)	0.61	0.05	266	203	2.00E-60	gene=Chr03G1006 Gene Symbol:PKAC|Host:Fabaceae|Disease:Southern anthracnose of clover and other legumes|Description:CATALYTIC ACTIVITY: ATP + a protein = ADP + a phosphoprotein.
Chr03G1009.1	604	171	581	UniProt ID:F2QYD1_PICP7	758	217	583	117/442(26.47)	0.41	0.24	442	97.8	3.00E-22	gene=Chr03G1009 Gene Symbol:MAK5|Host:humans|Disease:occasional infection|Description:SIMILARITY: Belongs to the

Chr03G1013.1	784	323	519	UniProtID:Q5APU2_CANAL	597	424	581	55/206(26.70)	0.46	0.28	206	92.8	2.00E-20	gene=Chr03G1013	DEAD box helicase family. Gene Symbol:CPP1|Host:Isolated from a wide variety of substrates including humans|Disease:invasive candidal disease|Description:CAUTION: The sequence shown here is derived from an EMBL/GenBank/DDBJ whole genome shotgun (WGS) entry which is preliminary data.
Chr03G1023.1	1206	891	1198	UniProtID:Q9Y880_COCCA	880	2	314	102/329(31.00)	0.52	0.11	329	156	9.00E-40	gene=Chr03G1023	Gene Symbol:SNF1|Host:Corn, Zea mays, sometimes on Sorghum (Poaceae) and various other plant families|Disease:Northern corn leaf spot, ear and kernel rot|Description:Unknown
Chr03G1030.1	549	10	547	UniProtID:Q9P8L8_B	598	41	588	144/560(25.71)	0.44	0.06	560	185	1.00E-51	gene=Chr03G1030	Gene Symbol:BCMFS1|Host:Various plant

				OTFU											families|Disease:Grey mould. Parasite or saprophyte|Description:Unknown
Chr03G1042.1	587	12	586	UniProt ID:PACC_FUSOX	609	30	607	398/601(66.22)	0.75	0.08	601	680	0	gene=Chr03G1042	Gene Symbol:PACC|Host:Multiple genera in multiple families|Disease:Blights, wilts, rots of various sorts|Description:FUNCTION: Transcription factor that mediates regulation of both acid- and alkaline-expressed genes in response to ambient pH. At alkaline ambient pH, activates transcription of alkaline-expressed genes (including pacC itself) and represses transcription of acid-expressed genes. Specifically recognizes and binds the consensus sequence 5'-GCCARG-3'. May act as a negative

														regulator of virulence to plants.
Chr03G1043.1	2509	21	2497	UniProt ID:Q92217_COCHE	2528	13	2518	824/2630(31.33)	0.48	0.11	2630	965	0	gene=Chr03G1043	Gene Symbol:PKS1|Host:Zea mays|Disease:Southern leaf blight of maize|Description:Unknown
Chr03G1048.1	459	2	455	UniProt ID:A0ST42_CERNC	512	63	507	132/455(29.01)	0.5	0.02	455	191	2.00E-55	gene=Chr03G1048	Gene Symbol:CTB4|Host:Numerous taxa in Solanaceae|Disease:Leaf spot|Description:Unknown
Chr03G1049.1	362	79	284	UniProt ID:Q75ZG3_ALTAL	366	146	336	45/206(21.84)	0.43	0.07	206	52.4	1.00E-08	gene=Chr03G1049	Gene Symbol:AFTS1|Host:Plant|Disease:Leaf spot, rots|Description:Unknown
Chr03G1052.1	1990	2	1141	UniProt ID:Q92217_COCHE	2528	10	1217	414/1225(33.80)	0.48	0.08	1225	579	3.00E-171	gene=Chr03G1052	Gene Symbol:PKS1|Host:Zea mays|Disease:Southern leaf blight of maize|Description:Unknown
Chr03G1078.1	368	78	364	UniProt ID:Q6TFC7_A	349	61	349	98/291(33.68)	0.52	0.02	291	168	6.00E-49	gene=Chr03G1078	Gene Symbol:NULL|Host:humans|Disease:infection|Desc

				SPFM											ription:Unknown
Chr03G1084.1	1620	589	818	UniProt ID:Q0PND8_MAGGR	1375	992	1224	102/239(42.68)	0.61	0.06	239	176	2.00E-45	gene=Chr03G1084	Gene Symbol:PEX6|Host:Digitaria (Poaceae)|Disease:Leaf spot|Description:SIMILARITY: Belongs to the AAA ATPase family.
Chr03G1092.1	1894	1293	1804	UniProt ID:Q5AM49_CANAL	1690	772	1251	180/528(34.09)	0.52	0.12	528	291	3.00E-80	gene=Chr03G1092	Gene Symbol:SNF2|Host:Isolated from a wide variety of substrates including humans|Disease:invasive candidal disease|Description:Unknown
Chr03G1094.1	1018	114	852	UniProt ID:Q9P872_CANAL	917	36	798	224/822(27.25)	0.45	0.17	822	238	1.00E-66	gene=Chr03G1094	Gene Symbol:PMR1|Host:Isolated from a wide variety of substrates including humans|Disease:invasive candidal disease|Description:SIMILARITY: Belongs to the cation transport ATPase (P-type) family.
Chr03G1	719	1	692	UniProt	697	1	684	666/69	0.97	0.01	693	1364	0	gene=Chr	Gene

096.1				ID:Q8NK75_GLOLA				3(96.10)						03G1096	Symbol:CST1|Host:melons,cucumber|Disease:anthracnose fruit rot|Description:Unknown
Chr03G1098.1	605	97	300	UniProt ID:Q8J214_USTMD	592	337	526	57/211(27.01)	0.41	0.13	211	60.1	2.00E-10	gene=Chr03G1098	Gene Symbol:CRU1|Host:Euchlaena spp., Zea spp. (Poaceae)|Disease:Smut. Corn smut|Description:Unknown
Chr03G1099.1	1471	1052	1426	UniProt ID:BUD4_CANAL	1711	1360	1687	128/377(33.95)	0.51	0.14	377	223	1.00E-59	gene=Chr03G1099	Gene Symbol:BUD4|Host:Isolated from a wide variety of substrates including humans|Disease:invasive candidal disease|Description:FUNCTION: Required for establishment of the axial budding pattern in yeast cells. May be involved in the selection of future sites of septation in hyphal cells. Contributes to morphogenesis and is

															important for induction of hyphal growth. Also plays a role in epithelial adherence, and is involved in intestinal colonization and systemic infection. The role in adhesion is probably minor compared with its role in morphogenesis.
Chr03G1102.1	1445	262	1417	UniProt ID:Q5AM49_CANAL	1690	504	1688	588/1237(47.53)	0.62	0.11	1237	1042	0	gene=Chr03G1102	Gene Symbol:SNF2|Host:Isolated from a wide variety of substrates including humans|Disease:invasive candidal disease|Description:Unknown
Chr03G1108.1	309	2	195	UniProt ID:O93802_ALTAL	267	9	187	48/197 (24.37)	0.45	0.11	197	60.8	1.00E-11	gene=Chr03G1108	Gene Symbol:BRM2|Host:Plant|Disease:Leaf spot, rots|Description:SIMILARITY: Belongs to the short-chain dehydrogenases/reductases (SDR) family.

Chr03G1119.1	620	77	608	UniProt ID:CXT1_CRYNJ	694	151	662	165/568(29.05)	0.44	0.16	568	194	5.00E-54	gene=Chr03G1119	Gene Symbol:CXT1|Host:humans|Disease:cryptococcosis|Description:FUNCTION: Beta-1,2-xylosyltransferase that plays a key role in capsule polysaccharide synthesis by transferring xylose to alpha-1,3-dimannoside in a beta-1,2-linkage. Also mediates glycosylation of glycosphingolipids; constitutes the unique xylosyltransferase responsible for adding xylose to glycosphingolipids.
Chr03G1122.1	288	26	285	UniProt ID:Q96VB3_ALTAL	296	7	268	65/263(24.71)	0.46	0.02	263	90.1	6.00E-22	gene=Chr03G1122	Gene Symbol:AFT3-1|Host:Plant|Disease:Leaf spot, rots|Description:Unknown
Chr03G1139.1	373	201	373	UniProt ID:Q59VG6_CANAL	386	205	373	53/186(28.49)	0.41	0.16	186	48.5	2.00E-07	gene=Chr03G1139	Gene Symbol:ERG3|Host:Isolated from a wide variety of substrates including

														humans|Disease:invasive candidal disease|Description:CAUTION: The sequence shown here is derived from an EMBL/GenBank/DDBJ whole genome shotgun (WGS) entry which is preliminary data.
Chr03G1142.1	435	23	432	UniProt ID:MCPAL_ARTBC	416	16	413	163/419(38.90)	0.58	0.07	419	295	4.00E-96	gene=Chr03G1142	Gene Symbol:ARB_03789|Host:hedgehogs|Disease:ringworm,Kerion Celsi Disease|Description:FUNCTION: Extracellular metalloprotease that contributes to pathogenicity (By similarity).
Chr03G1150.1	966	269	449	UniProt ID:Q0WXM3_FUSOX	663	255	434	48/183(26.23)	0.5	0.03	183	74.3	2.00E-14	gene=Chr03G1150	Gene Symbol:FOW2|Host:Multiple genera in multiple families|Disease:Blights, wilts, rots of various sorts|Description:SIMILARITY: Contains 1 Zn(2)-C6

															fungal-type DNA-binding domain.
Chr03G1155.1	428	32	71	UniProt ID:A4R0W3_MAGO7	1226	276	315	15/40(37.50)	0.6	0	40	45.1	6.00E-06	gene=Chr03G1155	Gene Symbol:MGG_09263|Host:Poaceae, especially important on Oryzae|Disease:Rice blast|Description:Unknown
Chr03G1157.1	410	52	248	UniProt ID:Q4P8E7_USTMA	703	280	488	59/219(26.94)	0.43	0.15	219	60.8	5.00E-11	gene=Chr03G1157	Gene Symbol:UM03616.1|Host: Euchlaena spp., Zea spp. (Poaceae)|Disease:Smut. Corn smut|Description:CAUTION: The sequence shown here is derived from an EMBL/GenBank/DDBJ whole genome shotgun (WGS) entry which is preliminary data.
Chr03G1167.1	587	49	519	UniProt ID:Q59RG0_CANAL	581	101	573	196/479(40.92)	0.58	0.03	479	319	2.00E-101	gene=Chr03G1167	Gene Symbol:NAG4|Host:Isolated from a wide variety of substrates including humans|Disease:invasive

															candidal disease|Description:CAUTION: The sequence shown here is derived from an EMBL/GenBank/DDBJ whole genome shotgun (WGS) entry which is preliminary data.
Chr03G1170.1	525	59	477	UniProt ID:Q5ANE1_CANAL	748	51	492	104/450(23.11)	0.37	0.09	450	88.6	2.00E-19	gene=Chr03G1170	Gene Symbol:SNF3|Host:Isolated from a wide variety of substrates including humans|Disease:invasive candidal disease|Description:SIMILARITY: Belongs to the major facilitator superfamily. Sugar transporter (TC 2.A.1.1) family.
Chr03G1172.1	440	223	406	UniProt ID:A4RG81_MAGO7	558	333	517	119/186(63.98)	0.79	0.02	186	257	1.00E-79	gene=Chr03G1172	Gene Symbol:MGG_00131|Host:Poaceae, especially important on Oryzae|Disease:Rice blast|Description:Unknow

														n
Chr03G1173.1	627	1	594	UniProt ID:B2CG58_9PEZI	691	1	623	392/624(62.82)	0.76	0.05	624	805	0	gene=Chr03G1173	Gene Symbol:PHL1|Host:Zea mays (Poaceae)|Disease:Gray leaf spot of corn|Description:Unknown
Chr03G1174.1	332	60	286	UniProt ID:Q59X54_CANAL	544	51	270	65/232(28.02)	0.45	0.07	232	94.4	3.00E-22	gene=Chr03G1174	Gene Symbol:MIT1|Host:Isolated from a wide variety of substrates including humans|Disease:invasive candidal disease|Description:CAUTION: The sequence shown here is derived from an EMBL/GenBank/DDBJ whole genome shotgun (WGS) entry which is preliminary data.
Chr03G1178.1	346	61	346	UniProt ID:Q6TFC7_ASPFM	349	61	348	91/290(31.38)	0.5	0.02	290	154	6.00E-44	gene=Chr03G1178	Gene Symbol:NULL|Host:humans|Disease:infection|Description:Unknown
Chr03G1181.1	1350	103	1348	UniProt ID:Q3Y	1321	32	1321	473/1309(36.	0.56	0.06	1309	766	0	gene=Chr03G1181	Gene Symbol:ABC3|Host:Digitar

				5V5_MAGGR				13)								ia (Poaceae)|Disease:Leaf spot|Description:SIMILARITY: Belongs to the ABC transporter superfamily.
Chr03G1194.1	547	1	547	UniProt ID:C0S733_PARBP	560	1	560	439/560(78.39)	0.91	0.02	560	918	0	gene=Chr03G1194	Gene Symbol:PABG_03488|Host:humans|Disease:Paracoccidioidomycosis|Description:SIMILARITY: Belongs to the TCP-1 chaperonin family.
Chr03G1199.1	673	132	191	UniProt ID:Q9HFW4_USTMD	2289	526	586	19/62(30.65)	0.56	0.05	62	48.1	1.00E-06	gene=Chr03G1199	Gene Symbol:RUM1|Host:Euchlaena spp., Zea spp. (Poaceae)|Disease:Smut. Corn smut|Description:SIMILARITY: Contains 1 ARID domain.
Chr03G1204.1	300	85	263	UniProt ID:A4QT57_MAGO7	350	148	333	84/190(44.21)	0.63	0.08	190	141	1.00E-39	gene=Chr03G1204	Gene Symbol:MGG_05174|Host:Poaceae, especially important on Oryzae|Disease:Rice blast|Description:Unknown

Chr03G1207.1	1373	793	1290	UniProt ID:Q9C2Y4_MAGGR	1501	875	1388	172/525(32.76)	0.53	0.07	525	276	1.00E-76	gene=Chr03G1207	Gene Symbol:PDE1|Host:Digitaria (Poaceae)|Disease:Leaf spot|Description:CATALYTIC ACTIVITY: ATP + H(2)O + phospholipid(In) = ADP + phosphate + phospholipid(Out).
Chr03G1208.1	133	59	127	UniProt ID:A6ZPY5_YEAS7	170	98	166	25/73(34.25)	0.56	0.11	73	40.4	5.00E-06	gene=Chr03G1208	Gene Symbol:ESS1|Host:humans|Disease:occasional infection|Description:SIMILARITY: Contains 1 PpiC domain.
Chr03G1211.1	712	1	712	UniProt ID:O60038_CLAPU	716	2	714	523/715(73.15)	0.81	0.01	715	1071	0	gene=Chr03G1211	Gene Symbol:CAT1|Host:outcrossing species|Disease:ergotism|Description:FUNCTION: Occurs in almost all aerobically respiring organisms and serves to protect cells from the toxic effects of hydrogen peroxide (By similarity).
Chr03G1	252	101	190	UniProt	1462	640	729	22/90(	0.59	0	90	45.1	2.00E-06	gene=Chr	Gene

213.1				ID:Q5AG71_CANAL				24.44)						03G1213	Symbol:HSL1|Host:Isolated from a wide variety of substrates including humans|Disease:invasive candidal disease|Description:CAUTION: The sequence shown here is derived from an EMBL/GenBank/DDBJ whole genome shotgun (WGS) entry which is preliminary data.
Chr03G1218.1	544	116	492	UniProt ID:DHH1_CRYNV	616	38	397	122/378(32.28)	0.53	0.05	378	196	1.00E-55	gene=Chr03G1218	Gene Symbol:VAD1|Host:humans|Disease:cryptococcosis|Description:FUNCTION: ATP-dependent RNA helicase involved in mRNA turnover, and more specifically in mRNA decapping. Is involved in G1/S DNA- damage checkpoint recovery, probably through the regulation of the translational status of a

															subset of mRNAs. May also have a role in translation and mRNA nuclear export (By similarity). Is involved in virulence.
Chr03G1219.1	522	189	513	UniProt ID:C5MK57_MYCGR	458	32	345	129/335(38.51)	0.55	0.09	335	210	3.00E-62	gene=Chr03G1219	Gene Symbol:STE7|Host:Triticum and possibly a few other grasses|Disease:Leaf spot or speckled leaf blotch of wheat|Description:Unknown
Chr03G1225.1	224	15	212	UniProt ID:Q9UVJ0_CANAL	200	2	196	93/200 (46.50)	0.65	0.04	200	157	3.00E-48	gene=Chr03G1225	Gene Symbol:CAO19.12623, CAO19.5156, ORF19.5156|Host:Isolated from a wide variety of substrates including humans|Disease:invasive candidal disease|Description:Unknown
Chr03G1232.1	501	355	476	UniProt ID:Q5AG71_C	1462	641	771	36/131 (27.48)	0.53	0.07	131	45.1	8.00E-06	gene=Chr03G1232	Gene Symbol:HSL1|Host:Isolated from a wide variety of

				ANAL											substrates including humans|Disease:invasive candidal disease|Description:CAUTION: The sequence shown here is derived from an EMBL/GenBank/DDBJ whole genome shotgun (WGS) entry which is preliminary data.
Chr03G1233.1	878	1	601	UniProt ID:D1MYV6_MAGGR	568	1	567	260/604(43.05)	0.58	0.07	604	435	2.00E-142	gene=Chr03G1233	Gene Symbol:CBL1|Host:Digitaria (Poaceae)|Disease:Leaf spot|Description:SIMILARITY: Contains 3 chitin-binding type-1 domains.
Chr03G1236.1	571	310	371	UniProt ID:Q5AFM2_CANAL	173	82	137	27/63(42.86)	0.65	0.13	63	48.9	8.00E-08	gene=Chr03G1236	Gene Symbol:MET28|Host:Isolated from a wide variety of substrates including humans|Disease:invasive candidal disease|Description:SIMILARITY: Belongs to the bZIP family.

Chr03G1254.1	189	17	133	UniProt ID:Q875J4_HYPVI	353	148	273	33/129 (25.58)	0.41	0.12	129	44.7	9.00E-07	gene=Chr03G1254	Gene Symbol:NULL|Host:humans|Disease:infection|Description:Unknown
Chr03G1258.1	181	10	178	UniProt ID:A6R9F0_AJECN	204	7	176	45/175 (25.71)	0.47	0.06	175	53.1	4.00E-10	gene=Chr03G1258	Gene Symbol:HCAG_06941|Host:humans|Disease:Darling's disease|Description:SIMILARITY: Belongs to the small GTPase superfamily. Rab family.
Chr03G1261.1	713	338	690	UniProt ID:HOG1_CRYPA	358	15	307	108/360(30.00)	0.43	0.21	360	121	9.00E-31	gene=Chr03G1261	Gene Symbol:HOG1|Host:Castanea spp., Fagus sylvatica, Quercus spp. (Fagaceae)|Disease:Chestnut blight. Cankers|Description:FUNCTION: Mitogen-activated protein kinase involved in a signal transduction pathway that is activated by changes in the osmolarity of the extracellular environment.

															Controls osmotic regulation of transcription of target genes (By similarity). Involved in the virulence and conidia formation. Mediates tannic acid-induced laccase expression and cryparin expression.
Chr03G1263.1	549	3	539	UniProt ID:Q4P8E8_USTMA	693	83	669	163/612(26.63)	0.45	0.16	612	141	1.00E-36	gene=Chr03G1263	Gene Symbol:UM03615.1|Host: Euchlaena spp., Zea spp. (Poaceae)|Disease:Smut. Corn smut|Description:COFACTOR: FAD (By similarity).
Chr03G1264.1	345	6	285	UniProt ID:Q6A2T2_BOTFU	391	65	350	63/292 (21.58)	0.42	0.06	292	64.7	1.00E-12	gene=Chr03G1264	Gene Symbol:BTP1|Host:Various plant families|Disease:Grey mould. Parasite or saprophyte|Description:Unknown
Chr03G1265.1	398	26	281	UniProt ID:Q9Y784_M	631	126	380	77/262 (29.39)	0.47	0.05	262	74.3	3.00E-15	gene=Chr03G1265	Gene Symbol:PTH11|Host:Digitaria

				AGGR											(Poaceae)|Disease:Leaf spot|Description:Unknown
Chr03G1266.1	461	50	212	UniProt ID:A0ST43_CERNC	459	12	185	49/175 (28.00)	0.47	0.07	175	49.7	2.00E-07	gene=Chr03G1266	Gene Symbol:CTB5|Host:Numerous taxa in Solanaceae|Disease:Leaf spot|Description:Unknown
Chr03G1268.1	512	110	506	UniProt ID:Q5ANE1_CANAL	748	97	521	106/440(24.09)	0.43	0.13	440	79.3	1.00E-16	gene=Chr03G1268	Gene Symbol:SNF3|Host:Isolated from a wide variety of substrates including humans|Disease:invasive candidal disease|Description:SIMILARITY: Belongs to the major facilitator superfamily. Sugar transporter (TC 2.A.1.1) family.
Chr03G1269.1	152	13	152	UniProt ID:C5FHM9_ARTOC	159	31	159	50/142 (35.21)	0.55	0.11	142	77.4	4.00E-19	gene=Chr03G1269	Gene Symbol:MCYG_01588|Host:humans, reptiles|Disease:dermatophytoses|Description:Unknown
Chr03G1	362	103	296	UniProt	266	12	214	50/210	0.41	0.11	210	51.6	2.00E-08	gene=Chr	Gene

270.1				ID:Q32WF7_PHAND				(23.81)						03G1270	Symbol:MDH1|Host:Multiple genera of Poaceae and Blysmus compressus (Cyperaceae)|Disease:Glume blotch of wheat and other grasses|Description:Unknown
Chr03G1274.1	371	27	351	UniProt ID:Q9Y784_MAGGR	631	105	441	86/350 (24.57)	0.43	0.11	350	71.6	2.00E-14	gene=Chr03G1274	Gene Symbol:PTH11|Host:Digitaria (Poaceae)|Disease:Leaf spot|Description:Unknown
Chr03G1278.1	520	15	384	UniProt ID:Q5AGE4_CANAL	426	2	373	107/402(26.62)	0.44	0.15	402	116	3.00E-29	gene=Chr03G1278	Gene Symbol:PDE1|Host:Isolated from a wide variety of substrates including humans|Disease:invasive candidal disease|Description:CAUTION: The sequence shown here is derived from an EMBL/GenBank/DDBJ whole genome shotgun (WGS) entry which is preliminary data.

Chr03G1281.1	1817	170	1810	UniProt ID:Q86ZP4_GIBZA	1692	1	1688	1343/1698(79.09)	0.86	0.04	1698	2687	0	gene=Chr03G1281	Gene Symbol:CPS1|Host:Principal hosts: Poaceae, including Zea mays (corn), Triticum aestivum (wheat), and Oryza sativa (rice). Additional hosts: various plant families|Disease:Seedling blight, pre- and post-emergence blight, root and foot rot, brown rot, culm decay, head or kernel blight (scab or ear scab) of cereals. Leaf|Description:Unknown
Chr03G1283.1	1077	335	979	UniProt ID:Q705V7_USTMD	1061	415	960	156/672(23.21)	0.39	0.23	672	115	5.00E-27	gene=Chr03G1283	Gene Symbol:GAS1|Host:Euchlaena spp., Zea spp. (Poaceae)|Disease:Smut. Corn smut|Description:Unknown
Chr03G1285.1	496	12	489	UniProt ID:Q5ANE1_C	748	36	519	122/499(24.45)	0.45	0.07	499	137	2.00E-35	gene=Chr03G1285	Gene Symbol:SNF3|Host:Isolated from a wide variety of

				ANAL											substrates including humans|Disease:invasive candidal disease|Description:SIMILARITY: Belongs to the major facilitator superfamily. Sugar transporter (TC 2.A.1.1) family.
Chr03G1298.1	506	101	185	UniProt ID:Q7Z9J3_9PEZI	1372	1226	1312	34/92(36.96)	0.49	0.13	92	47	2.00E-06	gene=Chr03G1298	Gene Symbol:CZK3|Host:Zea mays (Poaceae)|Disease:Gray leaf spot of corn|Description:Unknown
Chr03G1299.1	649	198	346	UniProt ID:Q59VF3_CANAL	1813	69	210	45/150(30.00)	0.48	0.06	150	50.1	4.00E-07	gene=Chr03G1299	Gene Symbol:"DUR1,2"|Host:Isolated from a wide variety of substrates including humans|Disease:invasive candidal disease|Description:CAUTION: The sequence shown here is derived from an EMBL/GenBank/DDBJ whole genome shotgun

															(WGS) entry which is preliminary data.
Chr03G1302.1	249	7	246	UniProt ID:A4RGG9_MAGO7	286	20	269	78/256 (30.47)	0.46	0.09	256	75.1	4.00E-17	gene=Chr03G1302	Gene Symbol:MGG_00056|Host:Poaceae, especially important on Oryzae|Disease:Rice blast|Description:SIMILARITY: Belongs to the short-chain dehydrogenases/reductases (SDR) family.
Chr03G1304.1	296	7	287	UniProt ID:Q32WF7_PHAND	266	12	262	66/283 (23.32)	0.41	0.12	283	65.1	3.00E-13	gene=Chr03G1304	Gene Symbol:MDH1|Host:Multiple genera of Poaceae and Blysmus compressus (Cyperaceae)|Disease:Glume blotch of wheat and other grasses|Description:Unknown
Chr03G1305.1	477	90	285	UniProt ID:Q59VF3_CANAL	1813	54	246	66/209 (31.58)	0.45	0.14	209	57.8	8.00E-10	gene=Chr03G1305	Gene Symbol:"DUR1,2"|Host:Isolated from a wide variety of substrates including humans|Disease:invasive

															candidal disease|Description:CAUTION: The sequence shown here is derived from an EMBL/GenBank/DDBJ whole genome shotgun (WGS) entry which is preliminary data.
Chr03G1307.1	673	265	574	UniProt ID:Q9HG15_COLLN	746	183	477	78/316 (24.68)	0.46	0.09	316	85.5	3.00E-18	gene=Chr03G1307	Gene Symbol:CLTA1|Host:Multiple genera of Fabaceae. Rare reports on other taxa|Disease:Leaf, stem and pod anthracnose|Description:SIMILARITY: Contains 1 Zn(2)-C6 fungal-type DNA-binding domain.
Chr03G1311.1	1775	238	612	UniProt ID:DHH1_CRYNV	616	13	383	122/380(32.11)	0.52	0.04	380	209	2.00E-57	gene=Chr03G1311	Gene Symbol:VAD1|Host:humans|Disease:cryptococcosis|Description:FUNCTION: ATP-dependent RNA helicase involved in mRNA turnover, and more specifically in mRNA

														decapping. Is involved in G1/S DNA- damage checkpoint recovery, probably through the regulation of the translational status of a subset of mRNAs. May also have a role in translation and mRNA nuclear export (By similarity). Is involved in virulence.
Chr03G1312.1	309	62	307	UniProt ID:Q04701_FUSSO	242	13	241	122/248(49.19)	0.63	0.08	248	229	2.00E-74	gene=Chr03G1312	Gene Symbol:PELA|Host:Multiple plant families. Some strains may cause infections in humans|Disease:Saprobe, facultative pathogen|Description:Unknown
Chr03G1317.1	266	1	266	UniProt ID:Q32WF7_PHAND	266	1	266	188/266(70.68)	0.83	0	266	406	3.00E-144	gene=Chr03G1317	Gene Symbol:MDH1|Host:Multiple genera of Poaceae and Blysmus compressus (Cyperaceae)|Disease:Glu

														me blotch of wheat and other grasses|Description:Unknown
Chr03G1320.1	470	69	319	UniProt ID:Q6IVV6_BOTFU	424	160	406	76/254 (29.92)	0.52	0.04	254	138	5.00E-37	gene=Chr03G1320	Gene Symbol:CEL5A|Host:Various plant families|Disease:Grey mould. Parasite or saprophyte|Description:Unknown
Chr03G1325.1	357	47	349	UniProt ID:Q6TFC7_ASPFM	349	43	346	101/314(32.17)	0.48	0.07	314	151	6.00E-43	gene=Chr03G1325	Gene Symbol:NULL|Host:humans|Disease:infection|Description:Unknown
Chr03G1329.1	589	176	440	UniProt ID:A4RJR0_MAGO7	938	151	421	70/284 (24.65)	0.44	0.11	284	67.8	8.00E-13	gene=Chr03G1329	Gene Symbol:MGG_01748|Host:Poaceae, especially important on Oryzae|Disease:Rice blast|Description:Unknown
Chr03G1331.1	558	52	510	UniProt ID:Q5XTQ4_BOTFU	574	53	537	142/505(28.12)	0.39	0.13	505	127	3.00E-32	gene=Chr03G1331	Gene Symbol:LIP1|Host:Various plant families|Disease:Grey

															mould. Parasite or saprophyte|Description:Unknown
Chr03G1334.1	280	74	262	UniProt ID:Q30E79_CRYNV	221	13	204	67/194 (34.54)	0.57	0.04	194	130	6.00E-37	gene=Chr03G1334	Gene Symbol:CAN1|Host:humans|Disease:cryptococcosis|Description:Unknown
Chr03G1345.1	255	82	250	UniProt ID:CUTI_ERYGR	236	65	232	87/171 (50.88)	0.68	0.03	171	176	2.00E-54	gene=Chr03G1345	Gene Symbol:CUT1|Host:Poaceae|Disease:Powdery mildew|Description:FUNCTION: Catalyzes the hydrolysis of cutin, a polyester that forms the structure of plant cuticle. Allows pathogenic fungi to penetrate through the cuticular barrier into the host plant during the initial stage of the fungal infection (By similarity).
Chr03G1351.1	488	46	169	UniProt ID:Q0WXM3_FUSOX	663	323	446	36/126 (28.57)	0.47	0.03	126	59.3	2.00E-10	gene=Chr03G1351	Gene Symbol:FOW2|Host:Multiple genera in multiple families|Disease:Blights, wilts, rots of various

															sorts|Description:SIMILARITY: Contains 1 Zn(2)-C6 fungal-type DNA-binding domain.
Chr03G1353.1	1154	29	1118	UniProt ID:Q4WPX2_ASPFU	1079	44	1072	381/1106(34.45)	0.53	0.08	1106	636	0	gene=Chr03G1353	Gene Symbol:PPOA|Host:humans|Disease:infection|Description:Unknown
Chr03G1358.1	408	6	47	UniProt ID:Q5A4F3_CANAL	624	14	54	18/42(42.86)	0.62	0.02	42	49.3	2.00E-07	gene=Chr03G1358	Gene Symbol:ZCF37|Host:Isolated from a wide variety of substrates including humans|Disease:invasive candidal disease|Description:Unknown
Chr03G1360.1	344	37	238	UniProt ID:A4QVF8_MAGO7	339	31	246	59/227(25.99)	0.41	0.16	227	56.2	6.00E-10	gene=Chr03G1360	Gene Symbol:MGG_04556|Host:Poaceae, especially important on Oryzae|Disease:Rice blast|Description:COFACTOR: Zinc (By similarity).
Chr03G1369.1	341	46	327	UniProt ID:Q6TFC7_A	349	64	346	85/287(29.62)	0.48	0.03	287	123	1.00E-32	gene=Chr03G1369	Gene Symbol:NULL|Host:humans|Disease:infection|Desc

				SPFM											ription:Unknown
Chr03G1370.1	334	52	334	UniProt ID:Q6TFC7_ASPFM	349	64	347	90/289 (31.14)	0.48	0.04	289	134	9.00E-37	gene=Chr03G1370	Gene Symbol:NULL|Host:humans|Disease:infection|Description:Unknown
Chr03G1371.1	312	37	305	UniProt ID:Q6TFC7_ASPFM	349	64	347	88/286 (30.77)	0.48	0.07	286	129	5.00E-35	gene=Chr03G1371	Gene Symbol:NULL|Host:humans|Disease:infection|Description:Unknown
Chr03G1377.1	499	451	485	UniProt ID:O59937_FUSOX	384	19	53	21/35(60.00)	0.66	0	35	52.4	3.00E-08	gene=Chr03G1377	Gene Symbol:XYL3|Host:Multiple genera in multiple families|Disease:Blights, wilts, rots of various sorts|Description:SIMILARITY: Belongs to the glycosyl hydrolase 10 (cellulase F) family.
Chr03G1378.1	331	31	331	UniProt ID:PLYB_COLGL	331	35	331	180/303(59.41)	0.75	0.03	303	313	2.00E-105	gene=Chr03G1378	Gene Symbol:PLB|Host:Multiple genera in multiple families|Disease:'Anthracnose of stems and leaves, dieback, root rot, leaf spot, blossom rot, fruit rot (dieback and ripe rot),

															seedling blight.' (Mordue 1971)|Description:FUNCTION: Acts as a virulence factor active in plant tissue maceration.
Chr03G1385.1	504	268	477	UniProt ID:A4ULJ2_MYCGR	515	269	509	56/246 (22.76)	0.41	0.17	246	54.3	7.00E-09	gene=Chr03G1385	Gene Symbol:CYP51|Host:Triticum and possibly a few other grasses|Disease:Leaf spot or speckled leaf blotch of wheat|Description:COFACTOR: Heme group (By similarity).
Chr03G1387.1	491	12	485	UniProt ID:Q59RG0_CANAL	581	84	572	155/493(31.44)	0.49	0.05	493	209	3.00E-61	gene=Chr03G1387	Gene Symbol:NAG4|Host:Isolated from a wide variety of substrates including humans|Disease:invasive candidal disease|Description:CAUTION: The sequence shown here is derived from an EMBL/GenBank/DDBJ whole genome shotgun (WGS) entry which is

															preliminary data.
Chr03G1388.1	2638	93	1805	UniProt ID:O59897_ASPFM	2146	113	1853	462/1845(25.04)	0.41	0.13	1845	419	3.00E-119	gene=Chr03G1388	Gene Symbol:ALB1|Host:humans|Disease:infection|Description:Unknown
Chr03G1389.1	320	34	234	UniProt ID:O93802_ALTAL	267	5	200	55/202(27.23)	0.45	0.03	202	72	2.00E-15	gene=Chr03G1389	Gene Symbol:BRM2|Host:Plant|Disease:Leaf spot, rots|Description:SIMILARITY: Belongs to the short-chain dehydrogenases/reductases (SDR) family.
Chr03G1390.1	343	3	218	UniProt ID:A4QVF8_MAGO7	339	4	223	60/240(25.00)	0.4	0.18	240	45.1	3.00E-06	gene=Chr03G1390	Gene Symbol:MGG_04556|Host:Poaceae, especially important on Oryzae|Disease:Rice blast|Description:COFACTOR: Zinc (By similarity).
Chr03G1393.1	440	9	431	UniProt ID:Q5GFD3_PHAND	437	11	425	144/436(33.03)	0.5	0.08	436	191	6.00E-56	gene=Chr03G1393	Gene Symbol:NULL|Host:Multiple genera of Poaceae and Blysmus compressus (Cyperaceae)|Disease:Glume blotch of wheat and

															other grasses|Description:Unknown
Chr03G1394.1	353	21	348	UniProt ID:NPIIC_ARTGP	358	30	357	105/343(30.61)	0.49	0.09	343	123	1.00E-32	gene=Chr03G1394	Gene Symbol:MGYG_02351|Host:Human, Mouse, Rat, Chicken, Pig, Rabbit, Bovine, Dog, African clawed frog, Zebrafish|Disease:tinea capitis, tinea corpus, ringworm, and other dermatophytoses|Description:FUNCTION: Secreted metalloproteinase that allows assimilation of proteinaceous substrates. Shows high activities on basic nuclear substrates such as histone and protamine. May be involved in virulence (By similarity).
Chr03G1398.1	644	127	403	UniProt ID:Q4P8E8_U	693	166	417	91/297 (30.64)	0.43	0.22	297	77.4	9.00E-16	gene=Chr03G1398	Gene Symbol:UM03615.1|Host: Euchlaena spp., Zea spp.

				STMA											(Poaceae)|Disease:Smut. Corn smut|Description:COFACTOR: FAD (By similarity).
Chr03G1399.1	421	5	267	UniProt ID:Q9Y784_MAGGR	631	104	364	63/264(23.86)	0.48	0.02	264	99.4	2.00E-23	gene=Chr03G1399	Gene Symbol:PTH11|Host:Digitaria (Poaceae)|Disease:Leaf spot|Description:Unknown
Chr03G1400.1	1505	3	522	UniProt ID:Q5ANE1_CANAL	748	31	581	128/569(22.50)	0.42	0.12	569	139	1.00E-34	gene=Chr03G1400	Gene Symbol:SNF3|Host:Isolated from a wide variety of substrates including humans|Disease:invasive candidal disease|Description:SIMILARITY: Belongs to the major facilitator superfamily. Sugar transporter (TC 2.A.1.1) family.
Chr03G1404.1	413	22	412	UniProt ID:Q96VZ3_FUSOX	455	29	443	218/418(52.15)	0.64	0.07	418	412	2.00E-141	gene=Chr03G1404	Gene Symbol:PGX1|Host:Multiple genera in multiple families|Disease:Blights, wilts, rots of various

															sorts|Description:SIMILARITY: Belongs to the glycosyl hydrolase 28 family.
Chr03G1420.1	427	7	427	UniProt ID:MCPAL_ARTOC	416	5	415	193/427(45.20)	0.62	0.05	427	379	7.00E-129	gene=Chr03G1420	Gene Symbol:MCYG_01475|Host:humans, reptiles|Disease:dermatophytoses|Description:FUNCTION: Extracellular metalloprotease that contributes to pathogenicity (By similarity).
Chr03G1423.1	224	78	206	UniProt ID:CUTI_ERYGR	236	128	224	39/129(30.23)	0.41	0.25	129	52.8	2.00E-09	gene=Chr03G1423	Gene Symbol:CUT1|Host:Poaceae|Disease:Powdery mildew|Description:FUNCTION: Catalyzes the hydrolysis of cutin, a polyester that forms the structure of plant cuticle. Allows pathogenic fungi to penetrate through the cuticular barrier into the host plant during the initial

															stage of the fungal infection (By similarity).
Chr03G1435.1	364	9	339	UniProt ID:Q6A2T2_BOTFU	391	44	390	71/356 (19.94)	0.39	0.1	356	53.9	5.00E-09	gene=Chr03G1435	Gene Symbol:BTP1|Host:Various plant families|Disease:Grey mould. Parasite or saprophyte|Description:Unknown
Chr03G1436.1	405	190	400	UniProt ID:Q2I0M6_CERNC	871	195	422	64/239 (26.78)	0.44	0.16	239	67	6.00E-13	gene=Chr03G1436	Gene Symbol:CTB3|Host:Numerous taxa in Solanaceae|Disease:Leaf spot|Description:Unknown
Chr03G1453.1	471	4	46	UniProt ID:Q5A4F3_CANAL	624	13	54	19/43(44.19)	0.65	0.02	43	51.2	7.00E-08	gene=Chr03G1453	Gene Symbol:ZCF37|Host:Isolated from a wide variety of substrates including humans|Disease:invasive candidal disease|Description:Unknown
Chr03G1454.1	463	15	344	UniProt ID:Q6A2T2_BOTFU	391	30	332	76/344 (22.09)	0.4	0.16	344	60.5	5.00E-11	gene=Chr03G1454	Gene Symbol:BTP1|Host:Various plant families|Disease:Grey

															mould. Parasite or saprophyte|Description:Unknown
Chr03G1456.1	546	49	530	UniProt ID:Q4PDC5_USTMA	583	58	533	222/486(45.68)	0.62	0.03	486	442	3.00E-149	gene=Chr03G1456	Gene Symbol:UM01888.1|Host: Euchlaena spp., Zea spp. (Poaceae)|Disease:Smut. Corn smut|Description:CAUTION: The sequence shown here is derived from an EMBL/GenBank/DDBJ whole genome shotgun (WGS) entry which is preliminary data.
Chr03G1467.1	362	25	264	UniProt ID:Q9Y784_MAGGR	631	125	362	60/241(24.90)	0.46	0.02	241	84.3	8.00E-19	gene=Chr03G1467	Gene Symbol:PTH11|Host:Digitaria (Poaceae)|Disease:Leaf spot|Description:Unknown
Chr03G1468.1	553	1	552	UniProt ID:Q5XTQ4_BOTFU	574	1	569	194/595(32.61)	0.48	0.12	595	251	8.00E-76	gene=Chr03G1468	Gene Symbol:LIP1|Host:Various plant families|Disease:Grey mould. Parasite or saprophyte|Description:Un

															known
Chr03G1469.1	359	74	358	UniProt ID:Q6TFC7_ASPFM	349	63	349	93/293 (31.74)	0.51	0.05	293	158	2.00E-45	gene=Chr03G1469	Gene Symbol:NULL|Host:humans|Disease:infection|Description:Unknown
Chr03G1472.1	414	31	338	UniProt ID:O59937_FUSOX	384	92	384	125/312(40.06)	0.55	0.07	312	206	2.00E-62	gene=Chr03G1472	Gene Symbol:XYL3|Host:Multiple genera in multiple families|Disease:Blights, wilts, rots of various sorts|Description:SIMILARITY: Belongs to the glycosyl hydrolase 10 (cellulase F) family.
Chr03G1473.1	1285	1163	1279	UniProt ID:Q66WN9_ALTBR	1328	1120	1234	41/117 (35.04)	0.56	0.02	117	82.4	1.00E-16	gene=Chr03G1473	Gene Symbol:NIK1|Host:Brassicaceae, especially cauliflower and white cabbage|Disease:Dark brown to almost black, circular, zonate leaf spot. Seed-borne. More common and causing more severe disease than Alternaria brassicae (Ellis 196|Description:SIMILARI

														TY: Contains 1 histidine kinase domain.
Chr03G1474.1	371	5	343	UniProt ID:A0ST44_CERNC	357	1	344	139/347(40.06)	0.6	0.03	347	249	5.00E-80	gene=Chr03G1474	Gene Symbol:CTB6|Host:Numerous taxa in Solanaceae|Disease:Leaf spot|Description:Unknown
Chr03G1475.1	413	77	231	UniProt ID:Q6A2T2_BOTFU	391	143	295	36/156 (23.08)	0.4	0.03	156	47.4	8.00E-07	gene=Chr03G1475	Gene Symbol:BTP1|Host:Various plant families|Disease:Grey mould. Parasite or saprophyte|Description:Unknown
Chr03G1478.1	716	223	403	UniProt ID:Q0WXM3_FUSOX	663	254	446	54/196 (27.55)	0.47	0.09	196	79	4.00E-16	gene=Chr03G1478	Gene Symbol:FOW2|Host:Multiple genera in multiple families|Disease:Blights, wilts, rots of various sorts|Description:SIMILARITY: Contains 1 Zn(2)-C6 fungal-type DNA-binding domain.
Chr03G1480.1	528	53	497	UniProt ID:Q5ANE1_C	748	51	496	111/464(23.92)	0.44	0.08	464	116	2.00E-28	gene=Chr03G1480	Gene Symbol:SNF3|Host:Isolated from a wide variety of

				ANAL										substrates including humans|Disease:invasive candidal disease|Description:SIMILARITY: Belongs to the major facilitator superfamily. Sugar transporter (TC 2.A.1.1) family.
Chr03G1485.1	381	88	294	UniProt ID:Q32WF7_PHAND	266	13	228	52/221 (23.53)	0.4	0.09	221	50.4	5.00E-08	gene=Chr03G1485	Gene Symbol:MDH1|Host:Multiple genera of Poaceae and Blysmus compressus (Cyperaceae)|Disease:Glume blotch of wheat and other grasses|Description:Unknown
Chr03G1494.1	706	186	593	UniProt ID:Q5ALS7_CANAL	1144	355	740	87/416 (20.91)	0.39	0.09	416	65.5	6.00E-12	gene=Chr03G1494	Gene Symbol:CTF1|Host:Isolated from a wide variety of substrates including humans|Disease:invasive candidal disease|Description:SIMILARITY: Contains 1

Chr03G1497.1	569	8	545	UniProt ID:Q9P8L8_BOTFU	598	53	584	159/547(29.07)	0.49	0.04	547	214	6.00E-62	gene=Chr03G1497	Zn(2)-C6 fungal-type DNA-binding domain. Gene Symbol:BCMFS1|Host:Various plant families|Disease:Grey mould. Parasite or saprophyte|Description:Unknown
Chr03G1509.1	253	7	98	UniProt ID:A4RGG9_MAGO7	286	17	110	33/95(34.74)	0.53	0.04	95	47	3.00E-07	gene=Chr03G1509	Gene Symbol:MGG_00056|Host:Poaceae, especially important on Oryzae|Disease:Rice blast|Description:SIMILARITY: Belongs to the short-chain dehydrogenases/reductases (SDR) family.
Chr03G1510.1	427	2	407	UniProt ID:Q5GFD3_PHAND	437	4	420	114/440(25.91)	0.45	0.13	440	96.7	9.00E-23	gene=Chr03G1510	Gene Symbol:NULL|Host:Multiple genera of Poaceae and Blysmus compressus (Cyperaceae)|Disease:Glume blotch of wheat and other

														grasses|Description:Unknown
Chr03G1518.1	542	262	520	UniProt ID:A4RGG9_MAGO7	286	10	272	134/266(50.38)	0.68	0.04	266	253	3.00E-80	gene=Chr03G1518	Gene Symbol:MGG_00056|Host:Poaceae, especially important on Oryzae|Disease:Rice blast|Description:SIMILARITY: Belongs to the short-chain dehydrogenases/reductases (SDR) family.
Chr03G1522.1	506	23	490	UniProt ID:Q5XTQ5_BOTFU	615	110	566	128/489(26.18)	0.44	0.11	489	100	2.00E-23	gene=Chr03G1522	Gene Symbol:FRT1|Host:Various plant families|Disease:Grey mould. Parasite or saprophyte|Description:SIMILARITY: Belongs to the major facilitator superfamily. Sugar transporter (TC 2.A.1.1) family.
Chr03G1524.1	762	228	425	UniProt ID:Q0WXM3	663	252	445	44/202(21.78)	0.44	0.06	202	52	8.00E-08	gene=Chr03G1524	Gene Symbol:FOW2|Host:Multiple genera in multiple

			_FUSOX												families|Disease:Blights, wilts, rots of various sorts|Description:SIMILARITY: Contains 1 Zn(2)-C6 fungal-type DNA-binding domain.
Chr03G1530.1	224	6	142	UniProt ID:Q9Y784_MAGGR	631	235	369	45/142 (31.69)	0.54	0.08	142	68.2	3.00E-14	gene=Chr03G1530	Gene Symbol:PTH11|Host:Digitaria (Poaceae)|Disease:Leaf spot|Description:Unknown
Chr03G1535.1	366	1	351	UniProt ID:Q6A2T2_BOTFU	391	1	363	90/380 (23.68)	0.44	0.12	380	86.7	7.00E-20	gene=Chr03G1535	Gene Symbol:BTP1|Host:Various plant families|Disease:Grey mould. Parasite or saprophyte|Description:Unknown
Chr03G1540.1	2280	60	563	UniProt ID:Q59VF3_CANAL	1813	615	1052	163/508(32.09)	0.49	0.15	508	234	1.00E-62	gene=Chr03G1540	Gene Symbol:"DUR1,2"|Host:Isolated from a wide variety of substrates including humans|Disease:invasive candidal disease|Description:CAUTION: The sequence shown

														here is derived from an EMBL/GenBank/DDBJ whole genome shotgun (WGS) entry which is preliminary data.
Chr03G1549.1	546	33	511	UniProtID:Q5ANE1_CANAL	748	11	496	125/515(24.27)	0.42	0.13	515	77	8.00E-16	gene=Chr03G1549	Gene Symbol:SNF3|Host:Isolated from a wide variety of substrates including humans|Disease:invasive candidal disease|Description:SIMILARITY: Belongs to the major facilitator superfamily. Sugar transporter (TC 2.A.1.1) family.
Chr03G1556.1	437	19	375	UniProtID:A4UC81_MAGO7	376	13	372	111/364(30.49)	0.51	0.03	364	178	9.00E-52	gene=Chr03G1556	Gene Symbol:MGG_10702|Host:Poaceae, especially important on Oryzae|Disease:Rice blast|Description:Unknown
Chr03G1558.1	523	63	271	UniProtID:A0S	459	3	196	77/212(36.32)	0.5	0.1	212	115	7.00E-29	gene=Chr03G1558	Gene Symbol:CTB5|Host:Numer

				T43_CERNC										ous taxa in Solanaceae|Disease:Leaf spot|Description:Unknown
Chr03G1565.1	226	1	226	UniProt ID:CUTI3_FUSSO	231	1	230	103/237(43.46)	0.62	0.08	237	166	2.00E-51	gene=Chr03G1565	Gene Symbol:CUT3|Host:Multiple plant families. Some strains may cause infections in humans|Disease:Saprobe, facultative pathogen|Description:FUNCTION: Catalyzes the hydrolysis of cutin, a polyester that forms the structure of plant cuticle. Allows pathogenic fungi to penetrate through the cuticular barrier into the host plant during the initial stage of the fungal infection (By similarity).
Chr03G1566.1	811	11	354	UniProt ID:Q2I0M6_CERNC	871	446	821	99/392(25.26)	0.4	0.16	392	63.5	3.00E-11	gene=Chr03G1566	Gene Symbol:CTB3|Host:Numerous taxa in Solanaceae|Disease:Leaf spot|Description:Unknown

Chr03G1567.1	509	47	501	UniProt ID:Q5ABU7_CANAL	564	95	558	150/468(32.05)	0.5	0.04	468	238	1.00E-71	gene=Chr03G1567	Gene Symbol:MDR1|Host:Isolated from a wide variety of substrates including humans|Disease:invasive candidal disease|Description:Unknown
Chr03G1568.1	1008	420	945	UniProt ID:Q9UW03_BOTFU	1439	810	1326	152/562(27.05)	0.47	0.14	562	161	4.00E-41	gene=Chr03G1568	Gene Symbol:ATRB|Host:Various plant families|Disease:Grey mould. Parasite or saprophyte|Description:SIMILARITY: Belongs to the ABC transporter superfamily.
Chr03G1569.1	497	41	217	UniProt ID:Q59VF3_CANAL	1813	69	238	68/184(36.96)	0.53	0.11	184	84.3	5.00E-18	gene=Chr03G1569	Gene Symbol:"DUR1,2"|Host:Isolated from a wide variety of substrates including humans|Disease:invasive candidal disease|Description:CAUTION: The sequence shown here is derived from an

														EMBL/GenBank/DDBJ whole genome shotgun (WGS) entry which is preliminary data.
Chr03G1571.1	145	24	145	UniProt ID:Q8X116_BOTFU	348	225	348	59/125 (47.20)	0.58	0.03	125	103	7.00E-28	gene=Chr03G1571	Gene Symbol:BCPME2|Host:Various plant families|Disease:Grey mould. Parasite or saprophyte|Description:CATALYTIC ACTIVITY: Pectin + n H(2)O = n methanol + pectate.
Chr03G1577.1	375	86	370	UniProt ID:Q6TFC7_ASPFM	349	63	347	98/288 (34.03)	0.49	0.02	288	152	3.00E-43	gene=Chr03G1577	Gene Symbol:NULL|Host:humans|Disease:infection|Description:Unknown
Chr03G1583.1	636	181	574	UniProt ID:B0BER9_CLAPU	557	100	529	100/452(22.12)	0.39	0.18	452	51.2	1.00E-07	gene=Chr03G1583	Gene Symbol:NOX1|Host:outcrossing species|Disease:ergotism|Description:SIMILARITY: Contains 1 FAD-binding FR-type domain.
Chr03G1586.1	1233	1	1142	UniProt ID:A4R	1305	1	1137	649/1209(53.	0.67	0.11	1209	1184	0	gene=Chr03G1586	Gene Symbol:MGG_13324|Host

				9C7_MAGO7				68)							:Poaceae, especially important on Oryzae|Disease:Rice blast|Description:Unknown
Chr03G1589.1	741	5	741	UniProt ID:Q9C476_CLAFU	745	6	745	494/742(66.58)	0.8	0.01	742	1049	0	gene=Chr03G1589	Gene Symbol:CAT-2|Host:Lycopersicon esculentum (Solanaceae)|Disease:Leaf mold of tomato|Description:FUNCTION: Occurs in almost all aerobically respiring organisms and serves to protect cells from the toxic effects of hydrogen peroxide (By similarity).
Chr03G1594.1	282	26	282	UniProt ID:O93802_ALTAL	267	9	267	193/259(74.52)	0.85	0.01	259	397	4.00E-140	gene=Chr03G1594	Gene Symbol:BRM2|Host:Plant|Disease:Leaf spot, rots|Description:SIMILARITY: Belongs to the short-chain dehydrogenases/reductases (SDR) family.
Chr03G1	1094	222	925	UniProt	4034	3210	3902	175/76	0.38	0.17	762	64.3	4.00E-11	gene=Chr	Gene

595.1				ID:Q6ZX14_MAGGR				2(22.97)						03G1595	Symbol:ACE1|Host:Digitaria (Poaceae)|Disease:Leaf spot|Description:Unknown
Chr03G1597.1	645	14	482	UniProt ID:A6N6J8_FUSOX	903	47	495	108/485(22.27)	0.38	0.11	485	67	2.00E-12	gene=Chr03G1597	Gene Symbol:CTF1|Host:Multiple genera in multiple families|Disease:Blights, wilts, rots of various sorts|Description:SIMILARITY: Contains 1 Zn(2)-C6 fungal-type DNA-binding domain.
Chr03G1600.1	886	142	297	UniProt ID:SUB6_TRISH	405	152	293	56/168(33.33)	0.43	0.23	168	70.9	9.00E-14	gene=Chr03G1600	Gene Symbol:SUB6|Host:humans|Disease:infection|Description:FUNCTION: Secreted subtilisin-like serine protease with keratinolytic activity that contributes to pathogenicity (By similarity).
Chr03G1601.1	553	18	542	UniProt ID:Q5XTQ4_BOTFU	574	38	565	177/570(31.05)	0.44	0.15	570	219	3.00E-64	gene=Chr03G1601	Gene Symbol:LIP1|Host:Various plant families|Disease:Grey

															mould. Parasite or saprophyte|Description:Unknown
Chr03G1603.1	509	267	475	UniProt ID:A4ULI5_MYCGR	515	266	503	49/240 (20.42)	0.38	0.14	240	46.6	2.00E-06	gene=Chr03G1603	Gene Symbol:CYP51|Host:Triticum and possibly a few other grasses|Disease:Leaf spot or speckled leaf blotch of wheat|Description:COFACTOR: Heme group (By similarity).
Chr03G1607.1	530	55	530	UniProt ID:Q5ANE1_CANAL	748	51	540	135/500(27.00)	0.44	0.07	500	117	1.00E-28	gene=Chr03G1607	Gene Symbol:SNF3|Host:Isolated from a wide variety of substrates including humans|Disease:invasive candidal disease|Description:SIMILARITY: Belongs to the major facilitator superfamily. Sugar transporter (TC 2.A.1.1) family.
Chr03G1611.1	351	13	336	UniProt ID:Q6A	391	20	350	93/339 (27.43)	0.47	0.07	339	108	2.00E-27	gene=Chr03G1611	Gene Symbol:BTP1|Host:Variou

				2T2_BOTFU										s plant families|Disease:Grey mould. Parasite or saprophyte|Description:Unknown
Chr03G1612.1	364	20	338	UniProt ID:Q9Y784_MAGGR	631	121	461	81/345 (23.48)	0.46	0.09	345	77.8	1.00E-16	gene=Chr03G1612	Gene Symbol:PTH11|Host:Digitaria (Poaceae)|Disease:Leaf spot|Description:Unknown
Chr03G1617.1	381	95	375	UniProt ID:Q6TFC7_ASPFM	349	63	346	97/288 (33.68)	0.52	0.04	288	166	6.00E-48	gene=Chr03G1617	Gene Symbol:NULL|Host:humans|Disease:infection|Description:Unknown
Chr03G1619.1	529	279	488	UniProt ID:A4ULJ2_MYCGR	515	265	503	63/244 (25.82)	0.41	0.16	244	55.1	4.00E-09	gene=Chr03G1619	Gene Symbol:CYP51|Host:Triticum and possibly a few other grasses|Disease:Leaf spot or speckled leaf blotch of wheat|Description:COFACTOR: Heme group (By similarity).
Chr03G1620.1	458	25	458	UniProt ID:Q96VZ3_F	455	23	455	299/434(68.89)	0.83	0	434	625	0	gene=Chr03G1620	Gene Symbol:PGX1|Host:Multiple genera in multiple

				USOX											families|Disease:Blights, wilts, rots of various sorts|Description:SIMILARITY: Belongs to the glycosyl hydrolase 28 family.
Chr03G1623.1	901	158	307	UniProt ID:SUB7_TRIEQ	401	152	284	55/156(35.26)	0.4	0.19	156	60.1	2.00E-10	gene=Chr03G1623	Gene Symbol:SUB7|Host:humans|Disease:Malabar itch|Description:FUNCTION: Secreted subtilisin-like serine protease with keratinolytic activity that contributes to pathogenicity (By similarity).
Chr03G1631.1	523	8	40	UniProt ID:Q5A4F3_CANAL	624	14	46	16/33(48.48)	0.7	0	33	51.2	9.00E-08	gene=Chr03G1631	Gene Symbol:ZCF37|Host:Isolated from a wide variety of substrates including humans|Disease:invasive candidal disease|Description:Unknown
Chr03G1632.1	1181	4	1177	UniProt ID:Q3Y	1321	88	1319	420/1241(33.	0.54	0.06	1241	687	0	gene=Chr03G1632	Gene Symbol:ABC3|Host:Digitar

			5V5_MAGGR				84)							ia (Poaceae)|Disease:Leaf spot|Description:SIMILARITY: Belongs to the ABC transporter superfamily.
Chr03G1634.1	733	21	93	UniProt ID:A4R0W3_MAGO7	1226	281	352	25/75(33.33)	0.52	0.07	75	46.6	4.00E-06	gene=Chr03G1634	Gene Symbol:MGG_09263|Host:Poaceae, especially important on Oryzae|Disease:Rice blast|Description:Unknown
Chr03G1635.1	505	71	504	UniProt ID:A0ST43_CERNC	459	12	456	124/460(26.96)	0.43	0.09	460	122	3.00E-31	gene=Chr03G1635	Gene Symbol:CTB5|Host:Numerous taxa in Solanaceae|Disease:Leaf spot|Description:Unknown
Chr03G1636.1	437	2	351	UniProt ID:A4UC81_MAGO7	376	33	371	86/356(24.16)	0.42	0.06	356	73.2	4.00E-15	gene=Chr03G1636	Gene Symbol:MGG_10702|Host:Poaceae, especially important on Oryzae|Disease:Rice blast|Description:Unknown
Chr03G1638.1	585	99	579	UniProt ID:Q5ABU7_C	564	101	561	127/495(25.66)	0.44	0.1	495	159	4.00E-43	gene=Chr03G1638	Gene Symbol:MDR1|Host:Isolated from a wide variety of

				ANAL										substrates including humans|Disease:invasive candidal disease|Description:Unknown
Chr03G1645.1	749	315	632	UniProt ID:A6N6J8_FUSOX	903	309	626	79/334 (23.65)	0.43	0.1	334	67	2.00E-12	gene=Chr03G1645	Gene Symbol:CTF1|Host:Multiple genera in multiple families|Disease:Blights, wilts, rots of various sorts|Description:SIMILARITY: Contains 1 Zn(2)-C6 fungal-type DNA-binding domain.
Chr03G1650.1	620	53	298	UniProt ID:Q9C441_FUSSO	330	74	311	88/255 (34.51)	0.55	0.1	255	167	2.00E-47	gene=Chr03G1650	Gene Symbol:PEP1|Host:Multiple plant families. Some strains may cause infections in humans|Disease:Saprobe, facultative pathogen|Description:Unknown
Chr03G1655.1	364	18	294	UniProt ID:Q9Y784_M	631	120	384	64/280 (22.86)	0.46	0.06	280	67	5.00E-13	gene=Chr03G1655	Gene Symbol:PTH11|Host:Digitaria

				AGGR											(Poaceae)|Disease:Leaf spot|Description:Unknown
Chr03G1658.1	826	330	462	UniProt ID:Q0WXM3_FUSOX	663	305	435	42/135 (31.11)	0.52	0.04	135	72	7.00E-14	gene=Chr03G1658	Gene Symbol:FOW2|Host:Multiple genera in multiple families|Disease:Blights, wilts, rots of various sorts|Description:SIMILARITY: Contains 1 Zn(2)-C6 fungal-type DNA-binding domain.
Chr03G1659.1	893	45	703	UniProt ID:Q99324_SEPLY	803	31	678	299/680(43.97)	0.59	0.08	680	492	4.00E-161	gene=Chr03G1659	Gene Symbol:B2TOM|Host:Primarily tomato, Lycopersicon esculentum, also Solanum spp. and other Solanaceae|Disease:Leaf spot|Description:Unknown
Chr03G1660.1	489	16	489	UniProt ID:Q59LS4_CANAL	448	14	448	182/493(36.92)	0.53	0.16	493	305	1.00E-98	gene=Chr03G1660	Gene Symbol:ERG24|Host:Isolated from a wide variety of substrates including humans|Disease:invasive candidal disease|Description:CAUTION: The sequence shown

														here is derived from an EMBL/GenBank/DDBJ whole genome shotgun (WGS) entry which is preliminary data.
Chr03G1663.1	501	8	463	UniProt ID:Q5ANE1_CANAL	748	39	496	132/477(27.67)	0.46	0.08	477	142	3.00E-37	gene=Chr03G1663	Gene Symbol:SNF3|Host:Isolated from a wide variety of substrates including humans|Disease:invasive candidal disease|Description:SIMILARITY: Belongs to the major facilitator superfamily. Sugar transporter (TC 2.A.1.1) family.
Chr03G1671.1	517	176	509	UniProt ID:A4QVF8_MAGO7	339	17	337	90/334 (26.95)	0.47	0.04	334	121	1.00E-31	gene=Chr03G1671	Gene Symbol:MGG_04556|Host:Poaceae, especially important on Oryzae|Disease:Rice blast|Description:COFACTOR: Zinc (By similarity).
Chr03G1672.1	711	369	645	UniProt ID:GBB	359	67	357	70/293 (23.89)	0.46	0.06	293	80.1	4.00E-17	gene=Chr03G1672	Gene Symbol:GB-1|Host:Castan

				_CRYPA											ea spp., Fagus sylvatica, Quercus spp. (Fagaceae)|Disease:Chestnut blight. Cankers|Description:FUNCTION: Guanine nucleotide-binding proteins (G proteins) are involved as a modulator or transducer in various transmembrane signaling systems. The beta and gamma chains are required for the GTPase activity, for replacement of GDP by GTP, and for G protein- effector interaction.
Chr03G1677.1	196	46	83	UniProt ID:A0ST46_CERNC	397	19	56	17/38(44.74)	0.66	0	38	44.3	1.00E-06	gene=Chr03G1677	Gene Symbol:CTB8|Host:Numerous taxa in Solanaceae|Disease:Leaf spot|Description:Unknown
Chr03G1681.1	523	53	491	UniProt ID:Q5ANE1_C	748	51	496	117/460(25.43)	0.43	0.08	460	107	1.00E-25	gene=Chr03G1681	Gene Symbol:SNF3|Host:Isolated from a wide variety of

				ANAL											substrates including humans|Disease:invasive candidal disease|Description:SIMILARITY: Belongs to the major facilitator superfamily. Sugar transporter (TC 2.A.1.1) family.
Chr03G1682.1	446	42	352	UniProt ID:A4UC81_MAGO7	376	66	369	85/317 (26.81)	0.43	0.06	317	106	2.00E-26	gene=Chr03G1682	Gene Symbol:MGG_10702|Host:Poaceae, especially important on Oryzae|Disease:Rice blast|Description:Unknown
Chr03G1693.1	953	707	874	UniProt ID:Q5AG40_CANAL	439	172	350	59/181 (32.60)	0.5	0.08	181	82.4	2.00E-17	gene=Chr03G1693	Gene Symbol:VPS4|Host:Isolated from a wide variety of substrates including humans|Disease:invasive candidal disease|Description:SIMILARITY: Belongs to the AAA ATPase family.
Chr03G1	576	37	538	UniProt	615	64	575	154/54	0.46	0.12	540	171	8.00E-47	gene=Chr	Gene

699.1				ID:Q5XTQ5_BOTFU				0(28.52)						03G1699	Symbol:FRT1|Host:Various plant families|Disease:Grey mould. Parasite or saprophyte|Description:SIMILARITY: Belongs to the major facilitator superfamily. Sugar transporter (TC 2.A.1.1) family.
Chr03G1702.1	177	2	177	UniProt ID:C1GD67_PARBD	190	5	190	125/186(67.20)	0.81	0.05	186	262	4.00E-90	gene=Chr03G1702	Gene Symbol:PADG_05203|Host:humans|Disease:Paracoccidioidomycosis|Description:SIMILARITY: Contains 1 PpiC domain.
Chr03G1711.1	766	418	597	UniProt ID:TUP1_CANAL	514	263	454	68/199(34.17)	0.53	0.13	199	99.8	6.00E-23	gene=Chr03G1711	Gene Symbol:TUP1|Host:Isolated from a wide variety of substrates including humans|Disease:invasive candidal disease|Description:FUNCTION: Represses transcription by RNA polymerase II. Represses

															genes responsible for initiating filamentous growth and this repression is lifted under inducing environmental conditions.
Chr03G1714.1	845	48	105	UniProt ID:Q96UQ9_MAGGR	715	563	623	34/61(55.74)	0.69	0.05	61	73.9	2.00E-14	gene=Chr03G1714	Gene Symbol:NULL|Host:Digitaria (Poaceae)|Disease:Leaf spot|Description:Unknown
Chr03G1715.1	262	7	260	UniProt ID:A4RGG9_MAGO7	286	18	271	80/262(30.53)	0.5	0.06	262	101	5.00E-26	gene=Chr03G1715	Gene Symbol:MGG_00056|Host:Poaceae, especially important on Oryzae|Disease:Rice blast|Description:SIMILARITY: Belongs to the short-chain dehydrogenases/reductases (SDR) family.
Chr03G1716.1	576	59	524	UniProt ID:Q59VF3_CANAL	1813	29	447	110/485(22.68)	0.37	0.18	485	66.6	2.00E-12	gene=Chr03G1716	Gene Symbol:"DUR1,2"|Host:Isolated from a wide variety of substrates including humans|Disease:invasive candidal disease|Description:CAUT

														ION: The sequence shown here is derived from an EMBL/GenBank/DDBJ whole genome shotgun (WGS) entry which is preliminary data.
Chr03G1717.1	299	2	157	UniProt ID:A4QVF8_MAGO7	339	62	211	62/160 (38.75)	0.57	0.09	160	112	3.00E-29	gene=Chr03G1717	Gene Symbol:MGG_04556|Host:Poaceae, especially important on Oryzae|Disease:Rice blast|Description:COFACTOR: Zinc (By similarity).
Chr03G1721.1	315	21	80	UniProt ID:Q5J7N6_COCHE	589	167	226	25/61(40.98)	0.66	0.03	61	43.5	9.00E-06	gene=Chr03G1721	Gene Symbol:CHAP1|Host:Zea mays|Disease:Southern leaf blight of maize|Description:SIMILARITY: Belongs to the bZIP family.
Chr03G1723.1	287	26	281	UniProt ID:Q32WF7_PHAND	266	14	262	81/260 (31.15)	0.5	0.06	260	113	3.00E-30	gene=Chr03G1723	Gene Symbol:MDH1|Host:Multiple genera of Poaceae and Blysmus compressus (Cyperaceae)|Disease:Glume blotch of wheat and

														other grasses|Description:Unknown
Chr03G1725.1	403	20	309	UniProt ID:Q6XVN4_CRYNV	383	17	297	82/316 (25.95)	0.39	0.19	316	71.2	1.00E-14	gene=Chr03G1725	Gene Symbol:GNO1|Host:humans|Disease:cryptococcosis|Description:COFACTOR: Zinc (By similarity).
Chr03G1728.1	848	232	772	UniProt ID:A6N6J8_FUSOX	903	121	665	137/584(23.46)	0.38	0.14	584	80.1	2.00E-16	gene=Chr03G1728	Gene Symbol:CTF1|Host:Multiple genera in multiple families|Disease:Blights, wilts, rots of various sorts|Description:SIMILARITY: Contains 1 Zn(2)-C6 fungal-type DNA-binding domain.
Chr03G1736.1	902	165	310	UniProt ID:SUB6_TRITO	412	158	289	47/160 (29.38)	0.42	0.26	160	48.1	1.00E-06	gene=Chr03G1736	Gene Symbol:SUB6|Host:humans|Disease:infection|Description:FUNCTION: Secreted subtilisin-like serine protease with keratinolytic activity that contributes to pathogenicity (By

															similarity).
Chr03G1745.1	322	1	111	UniProt ID:C4YII6_CANAW	768	492	599	36/115 (31.30)	0.5	0.1	115	52.4	2.00E-08	gene=Chr03G1745	Gene Symbol:CAWG_04261|Host:Isolated from a wide variety of substrates including humans|Disease:invasive candidal disease|Description:SIMILARITY: Belongs to the DEAD box helicase family.
Chr09G0004.1	451	40	286	UniProt ID:Q6XVN4_CRYNV	383	13	252	68/255 (26.67)	0.42	0.09	255	70.5	4.00E-14	gene=Chr09G0004	Gene Symbol:GNO1|Host:humans|Disease:cryptococcosis|Description:COFACTOR: Zinc (By similarity).
Chr09G0005.1	607	34	607	UniProt ID:Q2VLJ1_GIBZA	565	21	563	173/581(29.78)	0.46	0.08	581	235	1.00E-69	gene=Chr09G0005	Gene Symbol:ZEB1|Host:Principal hosts: Poaceae, including Zea mays (corn), Triticum aestivum (wheat), and Oryza sativa (rice). Additional hosts: various plant families|Disease:Seedling blight, pre- and

															post-emergence blight, root and foot rot, brown rot, culm decay, head or kernel blight (scab or ear scab) of cereals.
Chr09G0010.1	172	1	169	UniProt ID:Q59VQ3_CANAL	214	53	212	66/175 (37.71)	0.57	0.12	175	116	6.00E-33	gene=Chr09G0010	Leaf|Description:Unknown Gene Symbol:MAD2|Host:Isolated from a wide variety of substrates including humans|Disease:invasive candidal disease|Description:CAUTION: The sequence shown here is derived from an EMBL/GenBank/DDBJ whole genome shotgun (WGS) entry which is preliminary data.
Chr09G0015.1	603	202	547	UniProt ID:DHH1_CRYNV	616	56	383	115/350(32.86)	0.53	0.07	350	187	3.00E-52	gene=Chr09G0015	Gene Symbol:VAD1|Host:humans|Disease:cryptococcosis|Description:FUNCTION: ATP-dependent RNA helicase involved in mRNA turnover, and more

															specifically in mRNA decapping. Is involved in G1/S DNA- damage checkpoint recovery, probably through the regulation of the translational status of a subset of mRNAs. May also have a role in translation and mRNA nuclear export (By similarity). Is involved in virulence.
Chr09G0018.1	531	8	497	UniProt ID:Q5ANE1_CANAL	748	29	508	209/498(41.97)	0.62	0.05	498	412	8.00E-136	gene=Chr09G0018	Gene Symbol:SNF3|Host:Isolated from a wide variety of substrates including humans|Disease:invasive candidal disease|Description:SIMILARITY: Belongs to the major facilitator superfamily. Sugar transporter (TC 2.A.1.1) family.
Chr09G0	221	108	179	UniProt	589	166	235	27/72(	0.64	0.03	72	52.4	4.00E-09	gene=Chr	Gene

021.1				ID:Q5J 7N6_C OCHE				37.50)						09G0021	Symbol:CHAP1|Host:Zea mays|Disease:Southern leaf blight of maize|Description:SIMILARITY: Belongs to the bZIP family.
Chr09G0 035.1	316	56	313	UniProt ID:Q6X SF5_C OCHE	351	58	311	71/266 (26.69)	0.47	0.08	266	86.7	2.00E-20	gene=Chr 09G0035	Gene Symbol:NULL|Host:Zea mays|Disease:Southern leaf blight of maize|Description:Unknown
Chr09G0 037.1	439	108	186	UniProt ID:CCR 4_CAN AL	787	316	394	29/79(36.71)	0.51	0	79	52.8	3.00E-08	gene=Chr 09G0037	Gene Symbol:CCR4|Host:Isolated from a wide variety of substrates including humans|Disease:invasive candidal disease|Description:FUNCTION: Acts as catalytic component of the CCR4-NOT core complex, which in the nucleus seems to be a general transcription factor, and in the cytoplasm the major

															mRNA deadenylase involved in mRNA turnover. Ccr4 has 3'-5' RNase activity with a strong preference for polyadenylated substrates and also low exonuclease activity towards single stranded DNA. Discovered because of its role in the control of ADH2 gene expression. It is required for the expression of genes involved in non-fermentative growth (By similarity).
Chr09G0044.1	1079	583	847	UniProt ID:F2QU09_PICP7	626	133	424	60/298 (20.13)	0.42	0.13	298	56.2	7.00E-09	gene=Chr09G0044	Gene Symbol:PAB1|Host:humans|Disease:occasional infection|Description:Unknown
Chr09G0051.1	270	1	263	UniProt ID:Q75WR5_9PLEO	265	3	262	87/275 (31.64)	0.48	0.1	275	122	1.00E-33	gene=Chr09G0051	Gene Symbol:BRN1|Host:Belamcanda chinensis: Korea,Gladiolus ?gandavensis: Korea,Iris japonica:

														China,Iris missouriensis (Leaf spot.): Idaho; Montana; Oregon; Washington,Iris sp. (Leaf spot.): China; Texas; Washing|Disease:Leaf spot|Description:SIMILARITY: Belongs to the short-chain dehydrogenases/reductases (SDR) family.
Chr09G0053.1	860	72	480	UniProt ID:Q9HG15_COLLN	746	11	367	89/425 (20.94)	0.37	0.2	425	49.7	6.00E-07	gene=Chr09G0053	Gene Symbol:CLTA1|Host:Multiple genera of Fabaceae. Rare reports on other taxa|Disease:Leaf, stem and pod anthracnose|Description:SIMILARITY: Contains 1 Zn(2)-C6 fungal-type DNA-binding domain.
Chr09G0055.1	1148	4	223	UniProt ID:A4QV79_MAGO7	1446	306	547	93/250 (37.20)	0.5	0.15	250	105	8.00E-24	gene=Chr09G0055	Gene Symbol:MGG_04685|Host:Poaceae, especially important on Oryzae|Disease:Rice

														blast|Description:Unknown
Chr09G0069.1	354	41	345	UniProt ID:TUP1_CANAL	514	181	503	81/327 (24.77)	0.44	0.08	327	92.4	1.00E-21	gene=Chr09G0069	Gene Symbol:TUP1|Host:Isolated from a wide variety of substrates including humans|Disease:invasive candidal disease|Description:FUNCTION: Represses transcription by RNA polymerase II. Represses genes responsible for initiating filamentous growth and this repression is lifted under inducing environmental conditions.
Chr09G0075.1	833	9	371	UniProt ID:Q99324_SEPLY	803	52	485	128/440(29.09)	0.45	0.19	440	175	1.00E-46	gene=Chr09G0075	Gene Symbol:B2TOM|Host:Primarily tomato, Lycopersicon esculentum, also Solanum spp. and other Solanaceae|Disease:Leaf spot|Description:Unknown
Chr09G0085.1	919	555	867	UniProt ID:Q70	1061	598	924	109/338(32.2	0.51	0.11	338	174	2.00E-45	gene=Chr09G0085	Gene Symbol:GAS1|Host:Euchl

				5V7_USTMD				5)							aena spp., Zea spp. (Poaceae)|Disease:Smut. Corn smut|Description:Unknown
Chr09G0096.1	1133	637	708	UniProt ID:Q5A7S7_CANAL	526	241	312	25/75(33.33)	0.6	0.08	75	46.2	8.00E-06	gene=Chr09G0096	Gene Symbol:FKH2|Host:Isolated from a wide variety of substrates including humans|Disease:invasive candidal disease|Description:SIMILARITY: Contains 1 fork-head DNA-binding domain.
Chr09G0097.1	484	154	439	UniProt ID:Q00523_CRYNE	458	79	370	99/302(32.78)	0.49	0.09	302	146	2.00E-39	gene=Chr09G0097	Gene Symbol:NULL|Host:humans|Disease:cryptococcosis|Description:Unknown
Chr09G0117.1	405	23	321	UniProt ID:Q6A2T2_BOTFU	391	25	342	76/319(23.82)	0.44	0.07	319	76.3	3.00E-16	gene=Chr09G0117	Gene Symbol:BTP1|Host:Various plant families|Disease:Grey mould. Parasite or saprophyte|Description:Unknown

Chr09G0120.1	601	164	449	UniProt ID:B0BER9_CLAPU	557	101	441	69/344 (20.06)	0.35	0.18	344	51.6	7.00E-08	gene=Chr09G0120	Gene Symbol:NOX1|Host:outcrossing species|Disease:ergotism|Description:SIMILARITY: Contains 1 FAD-binding FR-type domain.
Chr09G0122.1	338	6	217	UniProt ID:Q6XPX0_FUSOX	359	156	356	47/214 (21.96)	0.44	0.07	214	54.7	2.00E-09	gene=Chr09G0122	Gene Symbol:FGB1|Host:Multiple genera in multiple families|Disease:Blights, wilts, rots of various sorts|Description:Unknown
Chr09G0124.1	1119	169	672	UniProt ID:Q5AM49_CANAL	1690	767	1285	228/522(43.68)	0.65	0.04	522	450	1.00E-135	gene=Chr09G0124	Gene Symbol:SNF2|Host:Isolated from a wide variety of substrates including humans|Disease:invasive candidal disease|Description:Unknown
Chr09G0126.1	608	458	602	UniProt ID:Q8J214_USTMD	592	291	432	37/147 (25.17)	0.49	0.05	147	54.3	1.00E-08	gene=Chr09G0126	Gene Symbol:CRU1|Host:Euchlaena spp., Zea spp. (Poaceae)|Disease:Smut.

															Corn smut|Description:Unknown
Chr09G0143.1	687	227	259	UniProt ID:Q5A4F3_CANAL	624	14	46	18/33(54.55)	0.7	0	33	49.3	5.00E-07	gene=Chr09G0143	Gene Symbol:ZCF37|Host:Isolated from a wide variety of substrates including humans|Disease:invasive candidal disease|Description:Unknown
Chr09G0144.1	454	3	222	UniProt ID:Q7Z9J3_9PEZI	1372	1094	1320	74/239(30.96)	0.48	0.13	239	76.3	1.00E-15	gene=Chr09G0144	Gene Symbol:CZK3|Host:Zea mays (Poaceae)|Disease:Gray leaf spot of corn|Description:Unknown
Chr09G0148.1	915	544	875	UniProt ID:Q6DQW3_CERNC	2196	917	1210	76/336(22.62)	0.4	0.14	336	59.7	6.00E-10	gene=Chr09G0148	Gene Symbol:NULL|Host:Numerous taxa in Solanaceae|Disease:Leaf spot|Description:Unknown
Chr09G0152.1	484	19	141	UniProt ID:Q59QC5_CANAL	159	2	131	34/130(26.15)	0.57	0.05	130	70.1	6.00E-15	gene=Chr09G0152	Gene Symbol:APS3|Host:Isolated from a wide variety of substrates including

														humans|Disease:invasive candidal disease|Description:CAUTION: The sequence shown here is derived from an EMBL/GenBank/DDBJ whole genome shotgun (WGS) entry which is preliminary data.
Chr09G0153.1	1181	3	742	UniProt ID:O42695_CANAL	1377	4	660	221/758(29.16)	0.46	0.16	758	229	5.00E-62	gene=Chr09G0153
														Gene Symbol:CASLN1|Host:Isolated from a wide variety of substrates including humans|Disease:invasive candidal disease|Description:SIMILARITY: Contains 1 histidine kinase domain.
Chr09G0162.1	347	12	321	UniProt ID:Q75ZG3_ALTAL	366	24	344	110/343(32.07)	0.48	0.16	343	129	1.00E-34	gene=Chr09G0162	Gene Symbol:AFTS1|Host:Plant|Disease:Leaf spot, rots|Description:Unknown
Chr09G0168.1	1387	962	1261	UniProt ID:Q86ZC3_USTMD	533	190	485	97/316(30.70)	0.52	0.11	316	140	2.00E-35	gene=Chr09G0168	Gene Symbol:KPP6|Host:Euchlaena spp., Zea spp. (Poaceae)|Disease:Smut.

															Corn smut|Description:CATALYTIC ACTIVITY: ATP + a protein = ADP + a phosphoprotein.
Chr09G0174.1	520	58	514	UniProt ID:Q9UUS8_COLGL	567	66	561	151/516(29.26)	0.46	0.15	516	177	2.00E-49	gene=Chr09G0174	Gene Symbol:CHIP3|Host:Multiple genera in multiple families|Disease:'Anthracnose of stems and leaves, dieback, root rot, leaf spot, blossom rot, fruit rot (dieback and ripe rot), seedling blight.' (Mordue 1971)|Description:Unknown
Chr09G0186.1	240	1	193	UniProt ID:Q875L5_USTMD	192	1	177	132/193(68.39)	0.8	0.08	193	273	2.00E-93	gene=Chr09G0186	Gene Symbol:RAS2|Host:Euchlaena spp., Zea spp. (Poaceae)|Disease:Smut. Corn smut|Description:Unknown
Chr09G0189.1	937	295	428	UniProt ID:Q9HFW4_U	2289	719	856	38/138 (27.54)	0.43	0.03	138	53.9	4.00E-08	gene=Chr09G0189	Gene Symbol:RUM1|Host:Euchlaena spp., Zea spp.

				STMD											(Poaceae)|Disease:Smut. Corn smut|Description:SIMILARITY: Contains 1 ARID domain.
Chr09G0190.1	913	1	913	UniProt ID:Q8TFN5_COLGR	912	1	912	838/915(91.58)	0.95	0.01	915	1744	0	gene=Chr09G0190	Gene Symbol:CHSIII|Host:Poaceae especially Zea mays|Disease:Leaf spot, stalk rot, etc|Description:Unknown
Chr09G0195.1	1016	138	209	UniProt ID:Q5AMH6_CANAL	821	724	799	26/78(33.33)	0.54	0.1	78	52.8	9.00E-08	gene=Chr09G0195	Gene Symbol:CAS5|Host:Isolated from a wide variety of substrates including humans|Disease:invasive candidal disease|Description:CAUTION: The sequence shown here is derived from an EMBL/GenBank/DDBJ whole genome shotgun (WGS) entry which is preliminary data.
Chr09G0196.1	478	76	363	UniProt ID:Q59	457	36	318	143/293(48.8	0.68	0.05	293	305	6.00E-99	gene=Chr09G0196	Gene Symbol:PCT1|Host:Isolate

				SI6_CANAL				1)								d from a wide variety of substrates including humans|Disease:invasive candidal disease|Description:CAUTION: The sequence shown here is derived from an EMBL/GenBank/DDBJ whole genome shotgun (WGS) entry which is preliminary data.
Chr09G0198.1	372	134	372	UniProt ID:Q04701_FUSSO	242	4	242	170/239(71.13)	0.82	0	239	363	5.00E-126	gene=Chr09G0198	Gene Symbol:PELA|Host:Multiple plant families. Some strains may cause infections in humans|Disease:Saprobe, facultative pathogen|Description:Unknown
Chr09G0204.1	262	13	240	UniProt ID:Q4PD87_USTMA	594	420	594	68/232(29.31)	0.41	0.26	232	74.7	3.00E-16	gene=Chr09G0204	Gene Symbol:UM01926.1|Host: Euchlaena spp., Zea spp. (Poaceae)|Disease:Smut. Corn smut|Description:CAUTIO

															N: The sequence shown here is derived from an EMBL/GenBank/DDBJ whole genome shotgun (WGS) entry which is preliminary data.
Chr09G0209.1	809	552	664	UniProt ID:Q5EMY3_MAGGR	424	155	280	34/126 (26.98)	0.48	0.1	126	46.2	4.00E-06	gene=Chr09G0209	Gene Symbol:NULL|Host:Digitaria (Poaceae)|Disease:Leaf spot|Description:SIMILARITY: Belongs to the AAA ATPase family.
Chr09G0213.1	590	48	546	UniProt ID:B2C6F1_CRYGA	614	36	544	155/535(28.97)	0.46	0.12	535	201	6.00E-57	gene=Chr09G0213	Gene Symbol:LAC1|Host:humans|Disease:pulmonary cryptococcosis, basal meningitis, and cerebral cryptococcomas|Description:Unknown
Chr09G0231.1	2289	91	2278	UniProt ID:Q2XW08_COCHE	2144	11	2135	746/2238(33.33)	0.53	0.07	2238	1126	0	gene=Chr09G0231	Gene Symbol:PKS2|Host:Zea mays|Disease:Southern leaf blight of maize|Description:Unknown
Chr09G0	1358	41	135	UniProt	1321	3	1321	818/13	0.76	0.03	1341	1612	0	gene=Chr	Gene

233.1			8	ID:Q3Y5V5_MAGGR				41(61.00)						09G0233	Symbol:ABC3|Host:Digitaria (Poaceae)|Disease:Leaf spot|Description:SIMILARITY: Belongs to the ABC transporter superfamily.
Chr09G0234.1	1652	1009	1072	UniProt ID:A5HEH7_9PEZI	479	367	430	22/64(34.38)	0.55	0	64	48.5	2.00E-06	gene=Chr09G0234	Gene Symbol:OLE1|Host:Multiple genera in multiple families|Disease:Cankers, dieback, and several other diseases. Also, a human pathogen|Description:COFACTOR: Iron (By similarity).
Chr09G0239.1	550	14	540	UniProt ID:Q4P8E8_USTMA	693	84	671	165/605(27.27)	0.43	0.16	605	175	3.00E-48	gene=Chr09G0239	Gene Symbol:UM03615.1|Host: Euchlaena spp., Zea spp. (Poaceae)|Disease:Smut. Corn smut|Description:COFACTOR: FAD (By similarity).
Chr09G0241.1	654	5	316	UniProt ID:Q9C2Y1_BOTFU	346	3	345	153/343(44.61)	0.58	0.09	343	254	2.00E-78	gene=Chr09G0241	Gene Symbol:BCPME1|Host:Various plant families|Disease:Grey mould. Parasite or

															saprophyte|Description:CATALYTIC ACTIVITY: Pectin + n H(2)O = n methanol + pectate.
Chr09G0243.1	455	1	455	UniProt ID:A4RI68_MAGO7	477	1	475	239/496(48.19)	0.63	0.13	496	390	8.00E-132	gene=Chr09G0243	Gene Symbol:MGG_07259|Host:Poaceae, especially important on Oryzae|Disease:Rice blast|Description:Unknown
Chr09G0246.1	865	336	563	UniProt ID:Q96V66_MAGGR	573	93	352	113/260(43.46)	0.57	0.12	260	190	2.00E-52	gene=Chr09G0246	Gene Symbol:NULL|Host:Digitaria (Poaceae)|Disease:Leaf spot|Description:Unknown
Chr09G0247.1	747	61	577	UniProt ID:O93841_9PEZI	914	91	612	180/537(33.52)	0.52	0.07	537	265	7.00E-77	gene=Chr09G0247	Gene Symbol:CHIP6|Host:Multiple genera in multiple families|Disease:'Anthracnose of stems and leaves, dieback, root rot, leaf spot, blossom rot, fruit rot (dieback and ripe rot), seedling blight.' (Mordue 1971)|Description:Unknown

Chr09G0253.1	753	171	735	UniProt ID:Q2PEP0_9HYPO	557	33	546	152/624(24.36)	0.38	0.27	624	128	4.00E-32	gene=Chr09G0253	Gene Symbol:NOXA|Host:plants|Disease:cool-season grasses|Description:SIMILARITY: Contains 1 FAD-binding FR-type domain.
Chr09G0273.1	264	12	261	UniProt ID:A4RGG9_MAGO7	286	13	269	78/260(30.00)	0.49	0.05	260	87	4.00E-21	gene=Chr09G0273	Gene Symbol:MGG_00056|Host:Poaceae, especially important on Oryzae|Disease:Rice blast|Description:SIMILARITY: Belongs to the short-chain dehydrogenases/reductases (SDR) family.
Chr09G0279.1	284	1	282	UniProt ID:Q9P470_MAGGR	290	1	282	198/282(70.21)	0.83	0	282	407	1.00E-143	gene=Chr09G0279	Gene Symbol:MAS1|Host:Digitaria (Poaceae)|Disease:Leaf spot|Description:Unknown
Chr09G0287.1	422	5	405	UniProt ID:A0ST45_CERNC	450	7	439	137/446(30.72)	0.48	0.13	446	174	6.00E-50	gene=Chr09G0287	Gene Symbol:CTB7|Host:Numerous taxa in Solanaceae|Disease:Leaf

Chr09G0288.1	743	247	446	UniProt ID:Q0WXM3_FUSOX	663	254	461	50/212 (23.58)	0.42	0.08	212	57.8	1.00E-09	gene=Chr09G0288	spot|Description:Unknown Gene Symbol:FOW2|Host:Multiple genera in multiple families|Disease:Blights, wilts, rots of various sorts|Description:SIMILARITY: Contains 1 Zn(2)-C6 fungal-type DNA-binding domain.
Chr09G0289.1	936	159	324	UniProt ID:SUB7A_COCP7	398	150	281	56/173 (32.37)	0.45	0.28	173	50.1	3.00E-07	gene=Chr09G0289	Gene Symbol:CPC735_050320|Host:humans|Disease:coccidiomycosis|Description: FUNCTION: Secreted subtilisin-like serine protease with keratinolytic activity that contributes to pathogenicity (By similarity).
Chr09G0291.1	1347	234	1268	UniProt ID:A4RGC8_MAGO7	1158	133	1088	413/1038(39.79)	0.56	0.08	1038	713	0	gene=Chr09G0291	Gene Symbol:MGG_11671|Host:Poaceae, especially important on Oryzae|Disease:Rice blast|Description:SIMILAR

														ITY: Contains 1 reverse transcriptase domain.
Chr09G0294.1	481	429	460	UniProt ID:Q5EGQ1_CRYNE	392	348	379	21/32(65.62)	0.81	0	32	57.8	5.00E-10	gene=Chr09G0294	Gene Symbol:BWC2|Host:humans|Disease:cryptococcosis|Description:Unknown
Chr09G0297.1	565	125	297	UniProt ID:PABP_COCIM	768	143	330	62/191(32.46)	0.51	0.11	191	100	5.00E-23	gene=Chr09G0297	Gene Symbol:PAB1|Host:humans|Disease:coccidioidomycosis|Description:FUNCTION: Binds the poly(A) tail of mRNA. Appears to be an important mediator of the multiple roles of the poly(A) tail in mRNA biogenesis, stability and translation. In the nucleus, involved in both mRNA cleavage and polyadenylation. Is also required for efficient mRNA export to the cytoplasm. Acts in concert with a poly(A)-specific nuclease (PAN) to affect poly(A) tail shortening,

														which may occur concomitantly with either nucleocytoplasmic mRNA transport or translational initiation. In the cytoplasm, stimulates translation initiation and regulates mRNA decay through translation termination-coupled poly(A) shortening, probably mediated by PAN (By similarity).
Chr09G0306.1	428	5	358	UniProt ID:A4UC81_MAGO7	376	25	371	105/358(29.33)	0.45	0.04	358	124	2.00E-32	gene=Chr09G0306	Gene Symbol:MGG_10702|Host:Poaceae, especially important on Oryzae|Disease:Rice blast|Description:Unknown
Chr09G0307.1	393	13	271	UniProt ID:Q6A2T2_BOTFU	391	40	297	62/267(23.22)	0.41	0.06	267	64.3	3.00E-12	gene=Chr09G0307	Gene Symbol:BTP1|Host:Various plant families|Disease:Grey mould. Parasite or saprophyte|Description:Un

															known
Chr09G0308.1	407	182	384	UniProt ID:Q2I0M6_CERNC	871	193	408	68/224 (30.36)	0.46	0.13	224	85.5	8.00E-19	gene=Chr09G0308	Gene Symbol:CTB3|Host:Numerous taxa in Solanaceae|Disease:Leaf spot|Description:Unknown
Chr09G0324.1	1805	377	1805	UniProt ID:Q59TT3_CANAL	1710	247	1710	567/1553(36.51)	0.5	0.14	1553	852	0	gene=Chr09G0324	Gene Symbol:PLD1|Host:Isolated from a wide variety of substrates including humans|Disease:invasive candidal disease|Description:CAUTION: The sequence shown here is derived from an EMBL/GenBank/DDBJ whole genome shotgun (WGS) entry which is preliminary data.
Chr09G0325.1	462	3	408	UniProt ID:Q7Z7T9_MONFR	398	2	398	143/407(35.14)	0.59	0.03	407	281	1.00E-90	gene=Chr09G0325	Gene Symbol:TUB2|Host:Rosaceae, primarily Prunus spp., also Pomoideae including apple (Malus) and pear (Pyrus). Reported on Vitis vinifera

															(Vitaceae)|Disease:Brown fruit rot, wilt, blight, canker|Description:FUNCTION: Tubulin is the major constituent of microtubules. It binds two moles of GTP, one at an exchangeable site on the beta chain and one at a non-exchangeable site on the alpha-chain (By similarity).
Chr09G0328.1	894	213	509	UniProt ID:A6N6J8_FUSOX	903	238	532	61/304(20.07)	0.41	0.05	304	54.7	2.00E-08	gene=Chr09G0328	Gene Symbol:CTF1|Host:Multiple genera in multiple families|Disease:Blights, wilts, rots of various sorts|Description:SIMILARITY: Contains 1 Zn(2)-C6 fungal-type DNA-binding domain.
Chr09G0332.1	379	61	370	UniProt ID:Q5A415_CANAL	361	31	350	138/322(42.86)	0.64	0.04	322	281	7.00E-92	gene=Chr09G0332	Gene Symbol:ARP2|Host:Isolated from a wide variety of substrates including humans|Disease:invasive

															candidal disease|Description:SIMILARITY: Belongs to the actin family.
Chr09G0341.1	318	40	311	UniProt ID:TUP1_CANAL	514	210	487	66/290 (22.76)	0.43	0.1	290	84.3	3.00E-19	gene=Chr09G0341	Gene Symbol:TUP1|Host:Isolated from a wide variety of substrates including humans|Disease:invasive candidal disease|Description:FUNCTION: Represses transcription by RNA polymerase II. Represses genes responsible for initiating filamentous growth and this repression is lifted under inducing environmental conditions.
Chr09G0344.1	818	175	732	UniProt ID:A3LS85_PICST	677	94	589	158/573(27.57)	0.43	0.16	573	156	1.00E-40	gene=Chr09G0344	Gene Symbol:LYS4|Host:humans|Disease:occasional infection|Description:Unknown
Chr09G0349.1	449	50	435	UniProt ID:PEP	395	20	394	159/399(39.8	0.51	0.09	399	222	3.00E-68	gene=Chr09G0349	Gene Symbol:PEP1|Host:huma

				A_ASPFU				5)						ns|Disease:infection|Description:FUNCTION: Secreted aspartic endopeptidase that allows assimilation of proteinaceous substrates. Can catalyze hydrolysis of the major structural proteins of basement membrane, elastin, collagen, and laminin. Thought to play a significant role in virulence (By similarity).
Chr09G0352.1	444	52	305	UniProt ID:Q9HG15_COLLN	746	173	407	60/261 (22.99)	0.38	0.13	261	50.4	1.00E-07	gene=Chr09G0352	Gene Symbol:CLTA1|Host:Multiple genera of Fabaceae. Rare reports on other taxa|Disease:Leaf, stem and pod anthracnose|Description:SIMILARITY: Contains 1 Zn(2)-C6 fungal-type DNA-binding domain.
Chr09G0356.1	335	240	322	UniProt ID:Q5A	286	199	279	25/83(30.12)	0.61	0.02	83	45.8	1.00E-06	gene=Chr09G0356	Gene Symbol:PEP12|Host:Isolat

				NM6_C ANAL											ed from a wide variety of substrates including humans|Disease:invasive candidal disease|Description:CAUTION: The sequence shown here is derived from an EMBL/GenBank/DDBJ whole genome shotgun (WGS) entry which is preliminary data.
Chr09G0 359.1	372	11	164	UniProt ID:Q9P 8W9_C RYNE	162	2	148	99/154 (64.29)	0.79	0.05	154	208	1.00E-66	gene=Chr 09G0359	Gene Symbol:CPA1|Host:humans|Disease:cryptococcosis|Description:FUNCTION: PPIases accelerate the folding of proteins (By similarity).
Chr09G0 360.1	1274	519	109 3	UniProt ID:Q5A 506_C ANAL	1237	348	965	166/65 5(25.3 4)	0.42	0.18	655	157	1.00E-39	gene=Chr 09G0360	Gene Symbol:BUD2|Host:Isolated from a wide variety of substrates including humans|Disease:invasive candidal disease|Description:SIMILARITY: Contains 1 C2

															domain.
Chr09G0362.1	540	1	142	UniProt ID:Q5A755_CANAL	662	88	232	45/149 (30.20)	0.58	0.07	149	75.5	3.00E-15	gene=Chr09G0362	Gene Symbol:NOT5|Host:Isolated from a wide variety of substrates including humans|Disease:invasive candidal disease|Description:CAUTION: The sequence shown here is derived from an EMBL/GenBank/DDBJ whole genome shotgun (WGS) entry which is preliminary data.
Chr09G0370.1	653	358	582	UniProt ID:A4ULI8_MYCGR	517	300	509	55/232 (23.71)	0.43	0.13	232	50.8	1.00E-07	gene=Chr09G0370	Gene Symbol:CYP51|Host:Triticum and possibly a few other grasses|Disease:Leaf spot or speckled leaf blotch of wheat|Description:COFACTOR: Heme group (By similarity).
Chr09G0379.1	420	273	367	UniProt ID:A6ZWK4_Y	685	272	380	36/112 (32.14)	0.49	0.18	112	50.8	8.00E-08	gene=Chr09G0379	Gene Symbol:NOP4|Host:humans|Disease:occasional

				EAS7											infection|Description:CAUTION: The sequence shown here is derived from an EMBL/GenBank/DDBJ whole genome shotgun (WGS) entry which is preliminary data.
Chr09G0382.1	644	71	636	UniProt ID:CXT1_CRYNJ	694	152	662	147/586(25.09)	0.39	0.16	586	145	2.00E-37	gene=Chr09G0382	Gene Symbol:CXT1|Host:humans|Disease:cryptococcosis|Description:FUNCTION: Beta-1,2-xylosyltransferase that plays a key role in capsule polysaccharide synthesis by transferring xylose to alpha-1,3-dimannoside in a beta-1,2-linkage. Also mediates glycosylation of glycosphingolipids; constitutes the unique xylosyltransferase responsible for adding xylose to glycosphingolipids.

Chr09G0384.1	598	12	577	UniProt ID:Q9P8L8_BOTFU	598	22	592	137/611(22.42)	0.39	0.14	611	87.4	5.00E-19	gene=Chr09G0384	Gene Symbol:BCMFS1|Host:Various plant families|Disease:Grey mould. Parasite or saprophyte|Description:Unknown
Chr09G0389.1	602	171	467	UniProt ID:Q5ALS7_CANAL	1144	339	644	76/317(23.97)	0.44	0.1	317	78.2	6.00E-16	gene=Chr09G0389	Gene Symbol:CTF1|Host:Isolated from a wide variety of substrates including humans|Disease:invasive candidal disease|Description:SIMILARITY: Contains 1 Zn(2)-C6 fungal-type DNA-binding domain.
Chr09G0392.1	978	13	294	UniProt ID:A4RF81_MAGO7	542	16	311	91/303(30.03)	0.45	0.09	303	100	8.00E-23	gene=Chr09G0392	Gene Symbol:MGG_00435|Host:Poaceae, especially important on Oryzae|Disease:Rice blast|Description:Unknown
Chr09G0402.1	263	12	255	UniProt ID:Q75	265	12	262	70/259(27.03)	0.46	0.09	259	82.8	1.00E-19	gene=Chr09G0402	Gene Symbol:BRN1|Host:Belam

				WR5_9 PLEO												canda chinensis: Korea,Gladiolus ?gandavensis: Korea,Iris japonica: China,Iris missouriensis (Leaf spot.): Idaho; Montana; Oregon; Washington,Iris sp. (Leaf spot.): China; Texas; Washing|Disease:Leaf spot|Description:SIMILARITY: Belongs to the short-chain dehydrogenases/reductases (SDR) family.
Chr09G0411.1	573	105	545	UniProt ID:Q59RG0_CANAL	581	121	549	114/444(25.68)	0.45	0.04	444	137	1.00E-35	gene=Chr09G0411		Gene Symbol:NAG4|Host:Isolated from a wide variety of substrates including humans|Disease:invasive candidal disease|Description:CAUTION: The sequence shown here is derived from an EMBL/GenBank/DDBJ whole genome shotgun (WGS) entry which is

															preliminary data.
Chr09G0425.1	541	1	541	UniProt ID:Q8J0I5_CLAPU	550	1	550	359/588(61.05)	0.68	0.14	588	560	0	gene=Chr09G0425	Gene Symbol:CPTF1|Host:outcrossing species|Disease:ergotism|Description:SIMILARITY: Belongs to the bZIP family.
Chr09G0443.1	2190	833	1322	UniProt ID:Q5AM49_CANAL	1690	771	1270	196/514(38.13)	0.58	0.07	514	333	3.00E-93	gene=Chr09G0443	Gene Symbol:SNF2|Host:Isolated from a wide variety of substrates including humans|Disease:invasive candidal disease|Description:Unknown
Chr09G0448.1	332	73	329	UniProt ID:Q9Y784_MAGGR	631	187	452	65/270 (24.07)	0.45	0.06	270	88.2	3.00E-20	gene=Chr09G0448	Gene Symbol:PTH11|Host:Digitaria (Poaceae)|Disease:Leaf spot|Description:Unknown
Chr09G0449.1	335	33	247	UniProt ID:Q92217_COCHE	2528	1846	2054	62/223 (27.80)	0.5	0.1	223	88.6	4.00E-20	gene=Chr09G0449	Gene Symbol:PKS1|Host:Zea mays|Disease:Southern leaf blight of maize|Description:Unkno

															wn
Chr09G0451.1	699	250	647	UniProt ID:O93800_ALTAL	578	201	563	96/407 (23.59)	0.4	0.13	407	80.5	8.00E-17	gene=Chr09G0451	Gene Symbol:AKT1|Host:Plant|Disease:Leaf spot, rots|Description:Unknown
Chr09G0453.1	704	90	704	UniProt ID:A4RED5_MAGO7	618	6	617	370/638(57.99)	0.68	0.08	638	583	0	gene=Chr09G0453	Gene Symbol:MGG_00692|Host:Poaceae, especially important on Oryzae|Disease:Rice blast|Description:Unknown
Chr09G0456.1	1211	806	1056	UniProt ID:Q5AP97_CANAL	1178	781	1024	105/270(38.89)	0.57	0.17	270	209	8.00E-56	gene=Chr09G0456	Gene Symbol:SWE1|Host:Isolated from a wide variety of substrates including humans|Disease:invasive candidal disease|Description:SIMILARITY: Belongs to the protein kinase superfamily.
Chr09G0459.1	568	74	554	UniProt ID:O93800_ALTAL	578	89	564	156/489(31.90)	0.5	0.04	489	214	4.00E-62	gene=Chr09G0459	Gene Symbol:AKT1|Host:Plant|Disease:Leaf spot, rots|Description:Unknown

Chr09G0464.1	841	561	611	UniProt ID:Q700F1_CANGB	703	553	600	21/51(41.18)	0.61	0.06	51	48.9	9.00E-07	gene=Chr09G0464	Gene Symbol:ACE2|Host:humans|Disease:Occasional invasive candidal disease|Description:Unknown
Chr09G0468.1	228	115	168	UniProt ID:Q5J7N6_COCHE	589	166	219	22/54(40.74)	0.72	0	54	51.6	1.00E-08	gene=Chr09G0468	Gene Symbol:CHAP1|Host:Zea mays|Disease:Southern leaf blight of maize|Description:SIMILARITY: Belongs to the bZIP family.
Chr09G0471.1	484	5	406	UniProt ID:Q9Y784_MAGGR	631	9	404	107/411(26.03)	0.47	0.06	411	147	2.00E-39	gene=Chr09G0471	Gene Symbol:PTH11|Host:Digitaria (Poaceae)|Disease:Leaf spot|Description:Unknown
Chr09G0473.1	392	56	374	UniProt ID:Q9HFV9_CRYNE	482	132	468	172/349(49.28)	0.64	0.12	349	294	3.00E-95	gene=Chr09G0473	Gene Symbol:PKR1|Host:humans|Disease:cryptococcosis|Description:Unknown
Chr09G0481.1	433	1	431	UniProt ID:Q59LF3_CANAL	474	35	474	222/447(49.66)	0.69	0.05	447	446	5.00E-154	gene=Chr09G0481	Gene Symbol:RVS167|Host:Isolated from a wide variety of substrates including

															humans|Disease:invasive candidal disease|Description:SIMILARITY: Contains 1 SH3 domain.
Chr09G0483.1	547	231	409	UniProt ID:Q59MV9_CANAL	398	146	325	53/188 (28.19)	0.43	0.09	188	48.9	4.00E-07	gene=Chr09G0483	Gene Symbol:YHB1|Host:Isolated from a wide variety of substrates including humans|Disease:invasive candidal disease|Description:SIMILARITY: Belongs to the globin family.
Chr09G0487.1	522	10	522	UniProt ID:Q9UQZ1_9PEZI	512	1	512	484/513(94.35)	0.96	0	513	933	0	gene=Chr09G0487	Gene Symbol:EMK1|Host:Multiple genera in multiple families|Disease:'Anthracnose of stems and leaves, dieback, root rot, leaf spot, blossom rot, fruit rot (dieback and ripe rot), seedling blight.' (Mordue 1971)|Description:Unknown
Chr09G0	546	11	545	UniProt	550	6	543	354/53	0.78	0.01	538	745	0	gene=Chr	Gene

490.1				ID:Q59RB8_CANAL				8(65.80)						09G0490	Symbol:ICL1|Host:Isolated from a wide variety of substrates including humans|Disease:invasive candidal disease|Description:SIMILARITY: Belongs to the isocitrate lyase/PEP mutase superfamily. Isocitrate lyase family.
Chr09G0491.1	740	3	740	UniProt ID:TREB_MAGO7	736	2	736	595/739(80.51)	0.91	0.01	739	1272	0	gene=Chr09G0491	Gene Symbol:NTH1|Host:Poaceae, especially important on Oryzae|Disease:Rice blast|Description:FUNCTION: Plays a role in pathogenicity, specifically in proliferation of invasive hyphae in rice blast disease.
Chr09G0495.1	734	4	734	UniProt ID:Q8J2N0_FUSOX	706	5	706	546/748(72.99)	0.81	0.08	748	1065	0	gene=Chr09G0495	Gene Symbol:SNF1|Host:Multiple genera in multiple families|Disease:Blights, wilts, rots of various sorts|Description:Unknow

															n
Chr09G0497.1	798	73	798	UniProt ID:C5GDC1_AJEDR	747	25	747	360/755(47.68)	0.65	0.08	755	658	0	gene=Chr09G0497	Gene Symbol:BDCG_02855|Host:humans|Disease:cutaneous Blastomyces dermatitidis infection|Description:Unknown
Chr09G0508.1	450	32	287	UniProt ID:Q5APH9_CANAL	375	92	371	86/301 (28.57)	0.44	0.22	301	105	4.00E-26	gene=Chr09G0508	Gene Symbol:PTC1|Host:Isolated from a wide variety of substrates including humans|Disease:invasive candidal disease|Description:SIMILARITY: Belongs to the PP2C family.
Chr09G0515.1	124	1	122	UniProt ID:Q5K8C9_CRYNJ	119	1	118	38/123 (30.89)	0.54	0.05	123	63.2	2.00E-14	gene=Chr09G0515	Gene Symbol:CNL06140|Host:humans|Disease:cryptococcosis|Description:Unknown
Chr09G0525.1	1734	8	365	UniProt ID:P87199_USTMD	968	4	340	149/362(41.16)	0.58	0.08	362	248	2.00E-68	gene=Chr09G0525	Gene Symbol:KIN2|Host:Euchlaena spp., Zea spp. (Poaceae)|Disease:Smut.

Chr09G0526.1	83	3	77	UniProtID:Q5AEK8_CANAL	584	8	81	21/75(28.00)	0.56	0.01	75	43.5	4.00E-07	gene=Chr09G0526	Corn smut|Description:SIMILARITY: Belongs to the kinesin-like protein family. Gene Symbol:CAO19.260, ORF19.260|Host:Isolated from a wide variety of substrates including humans|Disease:invasive candidal disease|Description:SIMILARITY: Contains 1 cytochrome b5 heme-binding domain.
Chr09G0528.1	366	66	348	UniProtID:Q6TFC7_ASPFM	349	65	346	96/289(33.22)	0.51	0.04	289	148	1.00E-41	gene=Chr09G0528	Gene Symbol:NULL|Host:humans|Disease:infection|Description:Unknown
Chr09G0530.1	377	8	321	UniProtID:Q75ZG3_ALTAL	366	9	357	103/352(29.26)	0.45	0.12	352	125	2.00E-33	gene=Chr09G0530	Gene Symbol:AFTS1|Host:Plant|Disease:Leaf spot, rots|Description:Unknown
Chr09G0531.1	454	5	454	UniProtID:GEL1_ASP	452	8	450	246/453(54.30)	0.7	0.03	453	466	1.00E-161	gene=Chr09G0531	Gene Symbol:GEL1|Host:humans|Disease:infection|Desc

				FU											ription:FUNCTION: Splits internally a 1,3-beta-glucan molecule and transfers the newly generated reducing end (the donor) to the non-reducing end of another 1,3-beta-glucan molecule (the acceptor) forming a 1,3-beta linkage, resulting in the elongation of 1,3-beta-glucan chains in the cell wall. Involved in cell wall morphogenesis (By similarity).
Chr09G0533.1	485	44	439	UniProt ID:DHH1_CRYNV	616	10	391	132/404(32.67)	0.56	0.07	404	219	1.00E-64	gene=Chr09G0533	Gene Symbol:VAD1|Host:humans|Disease:cryptococcosis|Description:FUNCTION: ATP-dependent RNA helicase involved in mRNA turnover, and more specifically in mRNA decapping. Is involved in G1/S DNA- damage checkpoint recovery,

															probably through the regulation of the translational status of a subset of mRNAs. May also have a role in translation and mRNA nuclear export (By similarity). Is involved in virulence.
Chr09G0537.1	283	18	281	UniProt ID:Q96VB3_ALTAL	296	22	269	67/267(25.09)	0.45	0.08	267	72.8	6.00E-16	gene=Chr09G0537	Gene Symbol:AFT3-1|Host:Plant|Disease:Leaf spot, rots|Description:Unknown
Chr09G0546.1	354	1	354	UniProt ID:O13316_MAGGR	355	1	355	300/355(84.51)	0.9	0	355	626	0	gene=Chr09G0546	Gene Symbol:MAGC|Host:Digitaria (Poaceae)|Disease:Leaf spot|Description:Unknown
Chr09G0547.1	291	5	291	UniProt ID:Q6RXX2_CRYNV	750	6	286	182/287(63.41)	0.79	0.02	287	379	7.00E-127	gene=Chr09G0547	Gene Symbol:NULL|Host:humans|Disease:cryptococcosis|Description:SIMILARITY: Belongs to the spermidine/spermine synthase family.
Chr09G0	973	7	57	UniProt	841	514	563	24/51(	0.67	0.02	51	56.2	7.00E-09	gene=Chr	Gene

557.1				ID:Q9Y 8E5_C RYNV				47.06)						09G0557	Symbol:NULL|Host:humans|Disease:cryptococcosis|Description:Unknown
Chr09G0 558.1	278	23	275	UniProt ID:Q75 WR5_9 PLEO	265	9	262	118/25 5(46.2 7)	0.63	0.01	255	226	3.00E-73	gene=Chr 09G0558	Gene Symbol:BRN1|Host:Belamcanda chinensis: Korea,Gladiolus ?gandavensis: Korea,Iris japonica: China,Iris missouriensis (Leaf spot.): Idaho; Montana; Oregon; Washington,Iris sp. (Leaf spot.): China; Texas; Washing|Disease:Leaf spot|Description:SIMILARITY: Belongs to the short-chain dehydrogenases/reductases (SDR) family.
Chr09G0 560.1	419	8	107	UniProt ID:A0S T46_C ERNC	397	18	125	32/109 (29.36)	0.47	0.09	109	53.1	1.00E-08	gene=Chr 09G0560	Gene Symbol:CTB8|Host:Numerous taxa in Solanaceae|Disease:Leaf spot|Description:Unknown
Chr09G0 562.1	2172	1	215 8	UniProt ID:P79	2187	1	2169	1996/2 169(92	0.95	0.01	2169	4087	0	gene=Chr 09G0562	Gene Symbol:PKS1|Host:melon

				068_G LOLA				.02)							s,cucumber|Disease:anthracnose fruit rot|Description:Unknown
Chr09G0563.1	563	18	510	UniProt ID:Q5RLJ7_CRYNV	594	61	578	157/546(28.75)	0.46	0.15	546	208	6.00E-60	gene=Chr09G0563	Gene Symbol:NULL|Host:humans|Disease:cryptococcosis|Description:Unknown
Chr09G0566.1	564	21	95	UniProt ID:Q59W09_CANAL	379	14	88	32/75(42.67)	0.6	0	75	78.2	1.00E-16	gene=Chr09G0566	Gene Symbol:GCS1|Host:Isolated from a wide variety of substrates including humans|Disease:invasive candidal disease|Description:CAUTION: The sequence shown here is derived from an EMBL/GenBank/DDBJ whole genome shotgun (WGS) entry which is preliminary data.
Chr09G0579.1	910	16	910	UniProt ID:Q8TFN6_COLGR	899	1	899	822/903(91.03)	0.95	0.01	903	1714	0	gene=Chr09G0579	Gene Symbol:CHSI|Host:Poaceae especially Zea mays|Disease:Leaf spot, stalk rot, etc|Description:Unknown

Chr09G0580.1	712	1	709	UniProt ID:Q4WYU7_ASPFU	712	5	711	471/727(64.79)	0.78	0.05	727	942	0	gene=Chr09G0580	Gene Symbol:AFUA_3G14320|Host:humans|Disease:infection|Description:CAUTION: The sequence shown here is derived from an EMBL/GenBank/DDBJ whole genome shotgun (WGS) entry which is preliminary data.
Chr09G0584.1	620	43	455	UniProt ID:Q4PDC7_USTMA	589	162	583	129/435(29.66)	0.5	0.08	435	185	2.00E-51	gene=Chr09G0584	Gene Symbol:UM01886.1|Host: Euchlaena spp., Zea spp. (Poaceae)|Disease:Smut. Corn smut|Description:CAUTION: The sequence shown here is derived from an EMBL/GenBank/DDBJ whole genome shotgun (WGS) entry which is preliminary data.
Chr09G0592.1	561	66	544	UniProt ID:Q5RLJ7_CRYNV	594	62	580	164/526(31.18)	0.48	0.1	526	228	2.00E-67	gene=Chr09G0592	Gene Symbol:NULL|Host:humans|Disease:cryptococcosis|Description:Unknown

Chr09G0594.1	225	4	150	UniProt ID:SNF7_CANAL	226	1	146	68/148 (45.95)	0.72	0.02	148	115	4.00E-32	gene=Chr09G0594	Gene Symbol:SNF7|Host:Isolated from a wide variety of substrates including humans|Disease:invasive candidal disease|Description:FUNCTION: Required for the sorting and concentration of proteins resulting in the entry of these proteins into the invaginating vesicles of the multivesicular body (MVB). Also required for the proteolytic cleavage of the transcription factor RIM101 in response to alkaline ambient pH.
Chr09G0597.1	484	27	333	UniProt ID:O59937_FUSOX	384	90	382	139/312(44.55)	0.57	0.08	312	220	5.00E-67	gene=Chr09G0597	Gene Symbol:XYL3|Host:Multiple genera in multiple families|Disease:Blights, wilts, rots of various sorts|Description:SIMILARITY: Belongs to the glycosyl hydrolase 10

															(cellulase F) family.
Chr09G0600.1	382	5	210	UniProt ID:A4QVF8_MAGO7	339	2	211	74/210 (35.24)	0.53	0.02	210	134	1.00E-36	gene=Chr09G0600	Gene Symbol:MGG_04556|Host:Poaceae, especially important on Oryzae|Disease:Rice blast|Description:COFACTOR: Zinc (By similarity).
Chr09G0602.1	567	8	563	UniProt ID:Q9P8L8_BOTFU	598	34	589	131/565(23.19)	0.42	0.03	565	154	3.00E-41	gene=Chr09G0602	Gene Symbol:BCMFS1|Host:Various plant families|Disease:Grey mould. Parasite or saprophyte|Description:Unknown
Chr09G0610.1	541	92	290	UniProt ID:Q6XPX0_FUSOX	359	170	357	47/200 (23.50)	0.42	0.07	200	62.8	1.00E-11	gene=Chr09G0610	Gene Symbol:FGB1|Host:Multiple genera in multiple families|Disease:Blights, wilts, rots of various sorts|Description:Unknown
Chr09G0633.1	558	358	450	UniProt ID:BUR1_CANAL	746	173	261	41/102 (40.20)	0.48	0.22	102	50.1	2.00E-07	gene=Chr09G0633	Gene Symbol:CRK1|Host:Isolated from a wide variety of substrates including

															humans|Disease:invasive candidal disease|Description:FUNCTION: Serine/threonine-protein kinase involved in transcription regulation. Phosphorylates the UBC2/RAD6 ubiquitin-conjugating enzyme (E2), leading to monoubiquitination of histone H2B and the silencing of telomeric-associated genes. Also required for histone H3 methylation. Necessary for the recovery from pheromone-induced growth arrest in the cell cycle G1 phase (By similarity). Required for pseudohyphal growth and virulence in mice.
Chr09G0	1236	227	435	UniProt	408	42	209	56/211	0.43	0.21	211	59.3	6.00E-10	gene=Chr	Gene

634.1				ID:Q00LS5_PHAND				(26.54)						09G0634	Symbol:CPKA|Host:Multiple genera of Poaceae and Blysmus compressus (Cyperaceae)|Disease:Glume blotch of wheat and other grasses|Description:SIMILARITY: Contains 1 protein kinase domain.
Chr09G0637.1	411	23	246	UniProt ID:Q9Y784_MAGGR	631	109	331	54/224 (24.11)	0.49	0	224	75.9	8.00E-16	gene=Chr09G0637	Gene Symbol:PTH11|Host:Digitaria (Poaceae)|Disease:Leaf spot|Description:Unknown
Chr09G0638.1	969	614	806	UniProt ID:O59897_ASPFM	2146	1775	1992	60/222 (27.03)	0.45	0.15	222	67.4	3.00E-12	gene=Chr09G0638	Gene Symbol:ALB1|Host:humans|Disease:infection|Description:Unknown
Chr09G0640.1	419	10	335	UniProt ID:A4UC81_MAGO7	376	42	369	118/330(35.76)	0.55	0.02	330	202	6.00E-61	gene=Chr09G0640	Gene Symbol:MGG_10702|Host:Poaceae, especially important on Oryzae|Disease:Rice blast|Description:Unknown
Chr09G0	1004	627	947	UniProt	1811	17	335	99/322	0.5	0.01	322	161	4.00E-41	gene=Chr	Gene

641.1				ID:A5H456_MYCGR				(30.75)						09G0641	Symbol:NULL|Host:Triticum and possibly a few other grasses|Disease:Leaf spot or speckled leaf blotch of wheat|Description:Unknown
Chr09G0642.1	438	55	379	UniProt ID:A5H456_MYCGR	1811	5	333	127/330(38.48)	0.56	0.02	330	232	3.00E-67	gene=Chr09G0642	Gene Symbol:NULL|Host:Triticum and possibly a few other grasses|Disease:Leaf spot or speckled leaf blotch of wheat|Description:Unknown
Chr09G0646.1	386	120	317	UniProt ID:PLYB_COLGL	331	92	289	56/212 (26.42)	0.46	0.13	212	58.2	2.00E-10	gene=Chr09G0646	Gene Symbol:PLB|Host:Multiple genera in multiple families|Disease:'Anthracnose of stems and leaves, dieback, root rot, leaf spot, blossom rot, fruit rot (dieback and ripe rot), seedling blight.' (Mordue 1971)|Description:FUNCTION: Acts as a virulence factor active in plant tissue maceration.

Chr09G0648.1	511	1	222	UniProt ID:Q59NG2_CANAL	564	42	259	101/222(45.50)	0.64	0.02	222	216	1.00E-63	gene=Chr09G0648	Gene Symbol:RAD52|Host:Isolated from a wide variety of substrates including humans|Disease:invasive candidal disease|Description:CAUTION: The sequence shown here is derived from an EMBL/GenBank/DDBJ whole genome shotgun (WGS) entry which is preliminary data.
Chr09G0650.1	487	262	323	UniProt ID:PABP_COCIM	768	236	297	22/62(35.48)	0.6	0	62	45.8	4.00E-06	gene=Chr09G0650	Gene Symbol:PAB1|Host:humans|Disease:coccidioidomycosis|Description:FUNCTION: Binds the poly(A) tail of mRNA. Appears to be an important mediator of the multiple roles of the poly(A) tail in mRNA biogenesis, stability and translation. In the nucleus, involved in both mRNA cleavage and

															polyadenylation. Is also required for efficient mRNA export to the cytoplasm. Acts in concert with a poly(A)-specific nuclease (PAN) to affect poly(A) tail shortening, which may occur concomitantly with either nucleocytoplasmic mRNA transport or translational initiation. In the cytoplasm, stimulates translation initiation and regulates mRNA decay through translation termination-coupled poly(A) shortening, probably mediated by PAN (By similarity).
Chr09G0656.1	661	38	658	UniProt ID:Q4P8E8_USTMA	693	84	671	179/672(26.64)	0.43	0.2	672	128	7.00E-32	gene=Chr09G0656	Gene Symbol:UM03615.1|Host: Euchlaena spp., Zea spp. (Poaceae)|Disease:Smut. Corn smut|Description:COFAC

															TOR: FAD (By similarity).
Chr09G0657.1	465	36	373	UniProtID:Q9Y784_MAGGR	631	31	373	139/345(40.29)	0.61	0.03	345	273	5.00E-85	gene=Chr09G0657	Gene Symbol:PTH11|Host:Digitaria (Poaceae)|Disease:Leaf spot|Description:Unknown
Chr09G0660.1	365	79	353	UniProtID:Q6TFC7_ASPFM	349	64	347	92/291(31.62)	0.49	0.08	291	129	8.00E-35	gene=Chr09G0660	Gene Symbol:NULL|Host:humans|Disease:infection|Description:Unknown
Chr09G0663.1	2569	15	2568	UniProtID:Q92217_COCHE	2528	8	2521	856/2628(32.57)	0.5	0.07	2628	1134	0	gene=Chr09G0663	Gene Symbol:PKS1|Host:Zea mays|Disease:Southern leaf blight of maize|Description:Unknown
Chr09G0665.1	325	11	267	UniProtID:Q6A2T2_BOTFU	391	94	347	62/267(23.22)	0.4	0.09	267	70.9	1.00E-14	gene=Chr09G0665	Gene Symbol:BTP1|Host:Various plant families|Disease:Grey mould. Parasite or saprophyte|Description:Unknown
Chr09G0673.1	844	463	803	UniProtID:Q4P8E8_U	693	308	673	103/393(26.21)	0.38	0.2	393	55.1	1.00E-08	gene=Chr09G0673	Gene Symbol:UM03615.1|Host: Euchlaena spp., Zea spp.

				STMA										(Poaceae)|Disease:Smut. Corn smut|Description:COFACTOR: FAD (By similarity).
Chr09G0678.1	385	26	299	UniProt ID:Q9Y784_MAGGR	631	106	373	67/281 (23.84)	0.46	0.07	281	79.7	4.00E-17	gene=Chr09G0678	Gene Symbol:PTH11|Host:Digitaria (Poaceae)|Disease:Leaf spot|Description:Unknown
Chr09G0679.1	468	57	221	UniProt ID:Q2VLJ1_GIBZA	565	123	294	56/172 (32.56)	0.49	0.04	172	77.8	2.00E-16	gene=Chr09G0679	Gene Symbol:ZEB1|Host:Principal hosts: Poaceae, including Zea mays (corn), Triticum aestivum (wheat), and Oryza sativa (rice). Additional hosts: various plant families|Disease:Seedling blight, pre- and post-emergence blight, root and foot rot, brown rot, culm decay, head or kernel blight (scab or ear scab) of cereals. Leaf|Description:Unknown
Chr09G0	505	60	246	UniProt	459	3	193	72/192	0.57	0.03	192	122	2.00E-31	gene=Chr	Gene

684.1				ID:A0ST43_CERNC				(37.50)						09G0684	Symbol:CTB5|Host:Numerous taxa in Solanaceae|Disease:Leaf spot|Description:Unknown
Chr09G0686.1	360	26	290	UniProt ID:Q6A2T2_BOTFU	391	33	301	67/279 (24.01)	0.44	0.09	279	72.4	4.00E-15	gene=Chr09G0686	Gene Symbol:BTP1|Host:Various plant families|Disease:Grey mould. Parasite or saprophyte|Description:Unknown
Chr09G0689.1	256	9	252	UniProt ID:O93802_ALTAL	267	10	263	86/258 (33.33)	0.51	0.07	258	109	5.00E-29	gene=Chr09G0689	Gene Symbol:BRM2|Host:Plant|Disease:Leaf spot, rots|Description:SIMILARITY: Belongs to the short-chain dehydrogenases/reductases (SDR) family.
Chr09G0690.1	613	407	591	UniProt ID:TUP1_CANAL	514	260	454	54/206 (26.21)	0.45	0.16	206	58.5	5.00E-10	gene=Chr09G0690	Gene Symbol:TUP1|Host:Isolated from a wide variety of substrates including humans|Disease:invasive candidal disease|Description:FUNC

															TION: Represses transcription by RNA polymerase II. Represses genes responsible for initiating filamentous growth and this repression is lifted under inducing environmental conditions.
Chr09G0697.1	527	52	481	UniProt ID:Q5ANE1_CANAL	748	30	464	114/442(25.79)	0.43	0.04	442	118	5.00E-29	gene=Chr09G0697	Gene Symbol:SNF3|Host:Isolated from a wide variety of substrates including humans|Disease:invasive candidal disease|Description:SIMILARITY: Belongs to the major facilitator superfamily. Sugar transporter (TC 2.A.1.1) family.
Chr09G0700.1	529	43	237	UniProt ID:Q59RG0_CANAL	581	116	313	48/198(24.24)	0.47	0.02	198	63.9	9.00E-12	gene=Chr09G0700	Gene Symbol:NAG4|Host:Isolated from a wide variety of substrates including humans|Disease:invasive candidal

														disease|Description:CAUTION: The sequence shown here is derived from an EMBL/GenBank/DDBJ whole genome shotgun (WGS) entry which is preliminary data.
Chr09G0702.1	355	8	123	UniProt ID:Q5A4F3_CANAL	624	14	112	35/119 (29.41)	0.42	0.19	119	51.6	3.00E-08	gene=Chr09G0702	Gene Symbol:ZCF37|Host:Isolated from a wide variety of substrates including humans|Disease:invasive candidal disease|Description:Unknown
Chr09G0704.1	777	77	195	UniProt ID:A0ST46_CERNC	397	18	142	37/134 (27.61)	0.45	0.18	134	45.1	9.00E-06	gene=Chr09G0704	Gene Symbol:CTB8|Host:Numerous taxa in Solanaceae|Disease:Leaf spot|Description:Unknown
Chr09G0705.1	516	22	469	UniProt ID:Q5ANE1_CANAL	748	44	496	139/463(30.02)	0.5	0.05	463	196	2.00E-55	gene=Chr09G0705	Gene Symbol:SNF3|Host:Isolated from a wide variety of substrates including humans|Disease:invasive candidal

															disease|Description:SIMILARITY: Belongs to the major facilitator superfamily. Sugar transporter (TC 2.A.1.1) family.
Chr09G0707.1	330	3	330	UniProt ID:Q8X116_BOTFU	348	16	348	158/336(47.02)	0.65	0.03	336	289	7.00E-96	gene=Chr09G0707	Gene Symbol:BCPME2|Host:Various plant families|Disease:Grey mould. Parasite or saprophyte|Description:CATALYTIC ACTIVITY: Pectin + n H(2)O = n methanol + pectate.
Chr09G0711.1	559	133	310	UniProt ID:Q59VF3_CANAL	1813	62	234	53/180 (29.44)	0.44	0.05	180	50.4	2.00E-07	gene=Chr09G0711	Gene Symbol:"DUR1,2"|Host:Isolated from a wide variety of substrates including humans|Disease:invasive candidal disease|Description:CAUTION: The sequence shown here is derived from an EMBL/GenBank/DDBJ whole genome shotgun

																(WGS) entry which is preliminary data.
Chr09G0715.1	432	53	432	UniProt ID:Q6XVN4_CRYNV	383	3	378	124/393(31.55)	0.5	0.08	393	166	2.00E-47	gene=Chr09G0715		Gene Symbol:GNO1|Host:humans|Disease:cryptococcosis|Description:COFACTOR: Zinc (By similarity).
Chr09G0722.1	350	137	258	UniProt ID:Q92217_COCHE	2528	1931	2054	31/125(24.80)	0.52	0.03	125	44.7	6.00E-06	gene=Chr09G0722		Gene Symbol:PKS1|Host:Zea mays|Disease:Southern leaf blight of maize|Description:Unknown
Chr09G0730.1	581	1	527	UniProt ID:Q9P8L8_BOTFU	598	37	564	162/538(30.11)	0.49	0.04	538	225	8.00E-66	gene=Chr09G0730		Gene Symbol:BCMFS1|Host:Various plant families|Disease:Grey mould. Parasite or saprophyte|Description:Unknown
Chr09G0732.1	414	198	316	UniProt ID:Q700F1_CANGB	703	512	612	38/121(31.40)	0.47	0.18	121	52.4	3.00E-08	gene=Chr09G0732		Gene Symbol:ACE2|Host:humans|Disease:Occasional invasive candidal disease|Description:Unknown

Chr09G0737.1	946	370	643	UniProt ID:Q59YF3_CANAL	986	249	539	68/306 (22.22)	0.46	0.15	306	72.4	7.00E-14	gene=Chr09G0737	Gene Symbol:INP51|Host:Isolated from a wide variety of substrates including humans|Disease:invasive candidal disease|Description:CAUTION: The sequence shown here is derived from an EMBL/GenBank/DDBJ whole genome shotgun (WGS) entry which is preliminary data.
Chr09G0741.1	594	160	593	UniProt ID:Q59LS4_CANAL	448	14	447	140/474(29.54)	0.45	0.17	474	139	7.00E-37	gene=Chr09G0741	Gene Symbol:ERG24|Host:Isolated from a wide variety of substrates including humans|Disease:invasive candidal disease|Description:CAUTION: The sequence shown here is derived from an EMBL/GenBank/DDBJ whole genome shotgun (WGS) entry which is preliminary data.

Chr09G0744.1	530	223	370	UniProt ID:Q0PND8_MAGGR	1375	1004	1170	56/174 (32.18)	0.48	0.19	174	56.6	2.00E-09	gene=Chr09G0744	Gene Symbol:PEX6|Host:Digitaria (Poaceae)|Disease:Leaf spot|Description:SIMILARITY: Belongs to the AAA ATPase family.
Chr09G0746.1	775	286	624	UniProt ID:A6N6J8_FUSOX	903	309	631	82/346 (23.70)	0.4	0.09	346	75.1	7.00E-15	gene=Chr09G0746	Gene Symbol:CTF1|Host:Multiple genera in multiple families|Disease:Blights, wilts, rots of various sorts|Description:SIMILARITY: Contains 1 Zn(2)-C6 fungal-type DNA-binding domain.
Chr09G0754.1	399	3	378	UniProt ID:Q6XVN4_CRYNV	383	2	377	270/377(71.62)	0.83	0.01	377	562	0	gene=Chr09G0754	Gene Symbol:GNO1|Host:humans|Disease:cryptococcosis|Description:COFACTOR: Zinc (By similarity).
Chr09G0767.1	282	4	275	UniProt ID:Q75WR5_9PLEO	265	8	262	82/275 (29.82)	0.49	0.08	275	113	4.00E-30	gene=Chr09G0767	Gene Symbol:BRN1|Host:Belamcanda chinensis: Korea,Gladiolus ?gandavensis: Korea,Iris japonica: China,Iris missouriensis

															(Leaf spot.): Idaho; Montana; Oregon; Washington,Iris sp. (Leaf spot.): China; Texas; Washing|Disease:Leaf spot|Description:SIMILARITY: Belongs to the short-chain dehydrogenases/reductases (SDR) family.
Chr09G0773.1	404	59	396	UniProt ID:A6R120_AJECN	376	27	375	128/353(36.26)	0.57	0.05	353	231	5.00E-72	gene=Chr09G0773	Gene Symbol:HCAG_03327|Host:humans|Disease:Darling's disease|Description:Unknown
Chr09G0777.1	517	167	501	UniProt ID:O93800_ALTAL	578	205	558	95/361(26.32)	0.48	0.09	361	104	9.00E-25	gene=Chr09G0777	Gene Symbol:AKT1|Host:Plant|Disease:Leaf spot, rots|Description:Unknown
Chr09G0785.1	282	1	276	UniProt ID:O93802_ALTAL	267	1	263	69/287(24.04)	0.43	0.12	287	57.8	9.00E-11	gene=Chr09G0785	Gene Symbol:BRM2|Host:Plant|Disease:Leaf spot, rots|Description:SIMILARITY: Belongs to the short-chain

															dehydrogenases/reductases (SDR) family.
Chr09G0791.1	1453	61	328	UniProt ID:Q9HFT9_CRYNE	1230	939	1212	115/279(41.22)	0.6	0.06	279	216	7.00E-58	gene=Chr09G0791	Gene Symbol:STE11ALPHA|Host:humans|Disease:cryptococcosis|Description:SIMILARITY: Contains 1 SAM (sterile alpha motif) domain.
Chr09G0801.1	481	72	351	UniProt ID:TUP1_CANAL	514	268	497	74/287(25.78)	0.44	0.22	287	72.8	9.00E-15	gene=Chr09G0801	Gene Symbol:TUP1|Host:Isolated from a wide variety of substrates including humans|Disease:invasive candidal disease|Description:FUNCTION: Represses transcription by RNA polymerase II. Represses genes responsible for initiating filamentous growth and this repression is lifted under inducing environmental conditions.
Chr09G0803.1	1192	1	1107	UniProt ID:A4R	1226	1	1139	428/1217(35.	0.47	0.15	1217	491	7.00E-153	gene=Chr09G0803	Gene Symbol:MGG_09263|Host

			0W3_MAGO7				17)								:Poaceae, especially important on Oryzae|Disease:Rice blast|Description:Unknown
Chr09G0808.1	397	118	276	UniProt ID:Q6A2T2_BOTFU	391	130	296	37/171 (21.64)	0.44	0.09	171	53.1	1.00E-08	gene=Chr09G0808	Gene Symbol:BTP1|Host:Various plant families|Disease:Grey mould. Parasite or saprophyte|Description:Unknown
Chr09G0809.1	1071	189	703	UniProt ID:Q5AM49_CANAL	1690	781	1239	154/529(29.11)	0.48	0.16	529	235	3.00E-64	gene=Chr09G0809	Gene Symbol:SNF2|Host:Isolated from a wide variety of substrates including humans|Disease:invasive candidal disease|Description:Unknown
Chr09G0814.1	775	50	612	UniProt ID:Q9HG15_COLLN	746	13	496	133/582(22.85)	0.39	0.2	582	94.7	6.00E-21	gene=Chr09G0814	Gene Symbol:CLTA1|Host:Multiple genera of Fabaceae. Rare reports on other taxa|Disease:Leaf, stem and pod

														anthracnose|Description:SIMILARITY: Contains 1 Zn(2)-C6 fungal-type DNA-binding domain.
Chr09G0817.1	269	3	266	UniProt ID:A4RGG9_MAGO7	286	13	274	91/271 (33.58)	0.51	0.06	271	129	4.00E-36	gene=Chr09G0817	Gene Symbol:MGG_00056|Host:Poaceae, especially important on Oryzae|Disease:Rice blast|Description:SIMILARITY: Belongs to the short-chain dehydrogenases/reductases (SDR) family.
Chr09G0821.1	615	43	612	UniProt ID:Q4P8E8_USTMA	693	84	672	193/621(31.08)	0.46	0.13	621	208	7.00E-59	gene=Chr09G0821	Gene Symbol:UM03615.1|Host: Euchlaena spp., Zea spp. (Poaceae)|Disease:Smut. Corn smut|Description:COFACTOR: FAD (By similarity).
Chr09G0822.1	564	284	403	UniProt ID:Q59MV9_CANAL	398	205	319	39/124 (31.45)	0.48	0.1	124	47.4	1.00E-06	gene=Chr09G0822	Gene Symbol:YHB1|Host:Isolated from a wide variety of substrates including humans|Disease:invasive

														candidal disease|Description:SIMILARITY: Belongs to the globin family.
Chr09G0824.1	1455	819	1037	UniProt ID:Q5ALS7_CANAL	1144	347	550	60/226 (26.55)	0.46	0.13	226	55.1	2.00E-08	gene=Chr09G0824	Gene Symbol:CTF1|Host:Isolated from a wide variety of substrates including humans|Disease:invasive candidal disease|Description:SIMILARITY: Contains 1 Zn(2)-C6 fungal-type DNA-binding domain.
Chr09G0829.1	1366	80	330	UniProt ID:TUP1_CANAL	514	257	498	57/257 (22.18)	0.41	0.08	257	52.4	1.00E-07	gene=Chr09G0829	Gene Symbol:TUP1|Host:Isolated from a wide variety of substrates including humans|Disease:invasive candidal disease|Description:FUNCTION: Represses transcription by RNA polymerase II. Represses genes responsible for initiating filamentous

															growth and this repression is lifted under inducing environmental conditions.
Chr09G0833.1	272	1	244	UniProt ID:C1GN68_PARBD	295	1	246	179/249(71.89)	0.82	0.03	249	363	9.00E-127	gene=Chr09G0833	Gene Symbol:PADG_08704|Host:humans|Disease:Paracoccidioidomycosis|Description:Unknown
Chr09G0835.1	934	140	344	UniProt ID:SUB1_ARTGP	481	149	317	59/212(27.83)	0.44	0.24	212	61.6	1.00E-10	gene=Chr09G0835	Gene Symbol:SUB1|Host:Human, Mouse, Rat, Chicken, Pig, Rabbit, Bovine, Dog, African clawed frog, Zebrafish|Disease:tinea capitis, tinea corpus, ringworm, and other dermatophytoses|Description:FUNCTION: Secreted subtilisin-like serine protease with keratinolytic activity that contributes to pathogenicity (By similarity).
Chr09G0838.1	437	1	350	UniProt ID:Q5GFD3_P	437	11	379	97/383(25.33)	0.44	0.12	383	109	2.00E-27	gene=Chr09G0838	Gene Symbol:NULL|Host:Multiple genera of Poaceae and

			HAND											Blysmus compressus (Cyperaceae)|Disease:Glume blotch of wheat and other grasses|Description:Unknown
Chr09G0839.1	486	21	482	UniProt ID:A0ST42_CERNC	512	51	508	129/472(27.33)	0.45	0.05	472	127	5.00E-33	gene=Chr09G0839	Gene Symbol:CTB4|Host:Numerous taxa in Solanaceae|Disease:Leaf spot|Description:Unknown
Chr09G0846.1	582	46	288	UniProt ID:Q9C441_FUSSO	330	70	303	100/254(39.37)	0.57	0.12	254	183	5.00E-53	gene=Chr09G0846	Gene Symbol:PEP1|Host:Multiple plant families. Some strains may cause infections in humans|Disease:Saprobe, facultative pathogen|Description:Unknown
Chr09G0853.1	170	92	149	UniProt ID:Q8J0I5_CLAPU	550	443	500	21/58(36.21)	0.6	0	58	46.2	2.00E-07	gene=Chr09G0853	Gene Symbol:CPTF1|Host:outcrossing species|Disease:ergotism|Description:SIMILARITY: Belongs to the bZIP

															family.
Chr09G0857.1	418	1	418	UniProt ID:Q8X125_GLOLA	418	1	418	405/418(96.89)	0.98	0	418	845	0	gene=Chr09G0857	Gene Symbol:MAF1|Host:melons,cucumber|Disease:anthracnose fruit rot|Description:CATALYTIC ACTIVITY: ATP + a protein = ADP + a phosphoprotein.
Chr09G0860.1	483	5	480	UniProt ID:Y1220_ASPFU	439	12	437	157/484(32.44)	0.51	0.14	484	218	7.00E-66	gene=Chr09G0860	Gene Symbol:AFUA_3G01220|Host:humans|Disease:infection|Description:FUNCTION: Probable aspartic-type endopeptidase which contributes to virulence (By similarity).
Chr09G0861.1	418	26	358	UniProt ID:Q9Y784_MAGGR	631	34	367	88/346(25.43)	0.45	0.07	346	101	4.00E-24	gene=Chr09G0861	Gene Symbol:PTH11|Host:Digitaria (Poaceae)|Disease:Leaf spot|Description:Unknown
Chr09G0872.1	186	18	143	UniProt ID:A7A233_Y	215	21	150	36/131(27.48)	0.48	0.05	131	58.9	4.00E-12	gene=Chr09G0872	Gene Symbol:SEC4|Host:humans|Disease:occasional

				EAS7										infection|Description:SIMILARITY: Belongs to the small GTPase superfamily. Rab family.
Chr09G0876.1	501	14	167	UniProt ID:Q9HFU3_MAGGR	215	71	209	49/158 (31.01)	0.45	0.15	158	59.3	4.00E-11	gene=Chr09G0876	Gene Symbol:CYP1|Host:Digitaria (Poaceae)|Disease:Leaf spot|Description:FUNCTION: PPIases accelerate the folding of proteins (By similarity).
Chr09G0881.1	468	59	421	UniProt ID:A3LXZ6_PICST	310	13	303	117/364(32.14)	0.51	0.2	364	192	2.00E-57	gene=Chr09G0881	Gene Symbol:CDC28|Host:humans|Disease:occasional infection|Description:Unknown
Chr09G0882.1	391	33	391	UniProt ID:Q5A415_CANAL	361	1	361	290/361(80.33)	0.91	0.01	361	617	0	gene=Chr09G0882	Gene Symbol:ARP2|Host:Isolated from a wide variety of substrates including humans|Disease:invasive candidal disease|Description:SIMILARITY: Belongs to the actin family.
Chr09G0	509	89	494	UniProt	748	87	486	111/42	0.45	0.12	428	105	4.00E-25	gene=Chr	Gene

884.1				ID:Q5ANE1_CANAL				8(25.93)						09G0884	Symbol:SNF3|Host:Isolated from a wide variety of substrates including humans|Disease:invasive candidal disease|Description:SIMILARITY: Belongs to the major facilitator superfamily. Sugar transporter (TC 2.A.1.1) family.
Chr09G0888.1	536	9	532	UniProt ID:MCPB_ARTOC	539	7	538	244/533(45.78)	0.63	0.02	533	487	1.00E-167	gene=Chr09G0888	Gene Symbol:MCPB|Host:humans, reptiles|Disease:dermatophytoses|Description:FUNCTION: Extracellular metalloprotease that contributes to pathogenicity (By similarity).
Chr09G0894.1	185	35	180	UniProt ID:Q6TFC7_ASPFM	349	189	336	38/152(25.00)	0.47	0.07	152	64.3	2.00E-13	gene=Chr09G0894	Gene Symbol:NULL|Host:humans|Disease:infection|Description:Unknown
Chr09G0	365	1	360	UniProt	370	1	367	204/36	0.71	0.02	367	402	3.00E-13	gene=Chr	Gene

900.1				ID:P78608_CLAPU				7(55.59)					9	09G0900	Symbol:PG2|Host:outcrossing species|Disease:ergotism|Description:SIMILARITY: Belongs to the glycosyl hydrolase 28 family.
Chr09G0901.1	699	127	678	UniProt ID:B2C6F1_CRYGA	614	32	569	192/565(33.98)	0.49	0.07	565	305	2.00E-94	gene=Chr09G0901	Gene Symbol:LAC1|Host:humans|Disease:pulmonary cryptococcosis, basal meningitis, and cerebral cryptococcomas|Description:Unknown
Chr09G0906.1	824	591	664	UniProt ID:Q5AG40_CANAL	439	171	246	28/76(36.84)	0.58	0.03	76	51.2	1.00E-07	gene=Chr09G0906	Gene Symbol:VPS4|Host:Isolated from a wide variety of substrates including humans|Disease:invasive candidal disease|Description:SIMILARITY: Belongs to the AAA ATPase family.
Chr09G0907.1	272	2	184	UniProt ID:O93802_ALTAL	267	13	199	57/192(29.69)	0.47	0.07	192	64.7	3.00E-13	gene=Chr09G0907	Gene Symbol:BRM2|Host:Plant|Disease:Leaf spot, rots|Description:SIMILARI

															TY: Belongs to the short-chain dehydrogenases/reductases (SDR) family.
Chr09G0909.1	489	1	450	UniProt ID:LAP2_ARTOC	495	1	471	173/494(35.02)	0.5	0.14	494	227	1.00E-68	gene=Chr09G0909	Gene Symbol:LAP2|Host:humans, reptiles|Disease:dermatophytoses|Description:FUNCTION: Extracellular aminopeptidase that releases a wide variety of amino acids from natural peptides and contributes to pathogenicity.
Chr09G0910.1	478	1	478	UniProt ID:Q59LS4_CANAL	448	6	448	188/494(38.06)	0.55	0.14	494	310	1.00E-100	gene=Chr09G0910	Gene Symbol:ERG24|Host:Isolated from a wide variety of substrates including humans|Disease:invasive candidal disease|Description:CAUTION: The sequence shown here is derived from an EMBL/GenBank/DDBJ whole genome shotgun

															(WGS) entry which is preliminary data.
Chr09G0912.1	539	8	64	UniProt ID:Q5A4F3_CANAL	624	14	69	22/58(37.93)	0.66	0.05	58	58.9	3.00E-10	gene=Chr09G0912	Gene Symbol:ZCF37|Host:Isolated from a wide variety of substrates including humans|Disease:invasive candidal disease|Description:Unknown
Chr09G0913.1	586	101	582	UniProt ID:Q59RG0_CANAL	581	79	581	146/507(28.80)	0.49	0.06	507	200	5.00E-57	gene=Chr09G0913	Gene Symbol:NAG4|Host:Isolated from a wide variety of substrates including humans|Disease:invasive candidal disease|Description:CAUTION: The sequence shown here is derived from an EMBL/GenBank/DDBJ whole genome shotgun (WGS) entry which is preliminary data.
Chr09G0915.1	574	340	531	UniProt ID:A4ULJ1_M	517	292	505	52/220(23.64)	0.44	0.15	220	50.8	1.00E-07	gene=Chr09G0915	Gene Symbol:CYP51|Host:Triticum and possibly a few

				YCGR											other grasses|Disease:Leaf spot or speckled leaf blotch of wheat|Description:COFACTOR: Heme group (By similarity).
Chr09G0916.1	544	24	497	UniProt ID:ALO_CANAL	557	10	543	197/561(35.12)	0.52	0.2	561	318	2.00E-101	gene=Chr09G0916	Gene Symbol:ALO1|Host:Isolated from a wide variety of substrates including humans|Disease:invasive candidal disease|Description:CATALYTIC ACTIVITY: D-arabinono-1,4-lactone + O(2) = dehydro-D-arabinono-1,4-lactone + H(2)O(2).
Chr09G0917.1	419	45	196	UniProt ID:Q75ZG3_ALTAL	366	62	233	57/181(31.49)	0.46	0.21	181	61.6	2.00E-11	gene=Chr09G0917	Gene Symbol:AFTS1|Host:Plant|Disease:Leaf spot, rots|Description:Unknown
Chr09G0919.1	545	24	531	UniProt ID:Q5XTQ5_BOTFU	615	59	569	128/539(23.75)	0.4	0.11	539	109	4.00E-26	gene=Chr09G0919	Gene Symbol:FRT1|Host:Various plant families|Disease:Grey

															mould. Parasite or saprophyte|Description:SIMILARITY: Belongs to the major facilitator superfamily. Sugar transporter (TC 2.A.1.1) family.
Chr09G0927.1	762	539	740	UniProt ID:Q5AG40_CANAL	439	171	371	54/209(25.84)	0.45	0.07	209	47.4	2.00E-06	gene=Chr09G0927	Gene Symbol:VPS4|Host:Isolated from a wide variety of substrates including humans|Disease:invasive candidal disease|Description:SIMILARITY: Belongs to the AAA ATPase family.
Chr09G0930.1	696	13	46	UniProt ID:Q5A4F3_CANAL	624	13	46	19/34(55.88)	0.74	0	34	53.5	3.00E-08	gene=Chr09G0930	Gene Symbol:ZCF37|Host:Isolated from a wide variety of substrates including humans|Disease:invasive candidal disease|Description:Unknown
Chr09G0932.1	384	44	383	UniProt ID:Q6T	349	44	346	110/342(32.1	0.48	0.12	342	179	6.00E-53	gene=Chr09G0932	Gene Symbol:NULL|Host:huma

			FC7_ASPFM				6)						ns|Disease:infection|Description:Unknown
Chr09G0942.1	368	67	367	UniProt ID:Q6TFC7_ASPFM	349	39	347	101/313(32.27)	0.46	0.05	313	136	2.00E-37	gene=Chr09G0942	Gene Symbol:NULL|Host:humans|Disease:infection|Description:Unknown
Chr09G0943.1	686	82	260	UniProt ID:Q59VF3_CANAL	1813	70	242	56/180(31.11)	0.5	0.04	180	74.7	1.00E-14	gene=Chr09G0943	Gene Symbol:"DUR1,2"|Host:Isolated from a wide variety of substrates including humans|Disease:invasive candidal disease|Description:CAUTION: The sequence shown here is derived from an EMBL/GenBank/DDBJ whole genome shotgun (WGS) entry which is preliminary data.
Chr09G0946.1	327	130	221	UniProt ID:HOG1_CANAL	377	159	241	31/92(33.70)	0.5	0.1	92	47	6.00E-07	gene=Chr09G0946	Gene Symbol:HOG1|Host:Isolated from a wide variety of substrates including humans|Disease:invasive candidal disease|Description:FUNC

															TION: Mitogen-activated protein kinase involved in a signal transduction pathway that is activated by changes in the osmolarity of the extracellular environment. Controls osmotic regulation of transcription of target genes. Regulates stress- induced production and accumulation of glycerol and D-arabitol. HOG1 is also involved in virulence, morphogenesis and oxidative stress response especially through its role in chlamydospore formation, an oxygen-dependent morphogenetic program.
Chr09G0948.1	350	3	300	UniProt ID:Q75ZG3_ALTAL	366	32	339	83/330 (25.15)	0.4	0.16	330	78.6	4.00E-17	gene=Chr09G0948	Gene Symbol:AFTS1|Host:Plant |Disease:Leaf spot, rots|Description:Unknown
Chr09G0	690	33	108	UniProt	618	133	205	26/76(	0.51	0.04	76	45.4	8.00E-06	gene=Chr	Gene

949.1				ID:A4RED5_MAGO7				34.21)						09G0949	Symbol:MGG_00692|Host:Poaceae, especially important on Oryzae|Disease:Rice blast|Description:Unknown
Chr09G0957.1	595	18	211	UniProt ID:B2C6F1_CRYGA	614	47	232	68/200 (34.00)	0.54	0.1	200	118	8.00E-29	gene=Chr09G0957	Gene Symbol:LAC1|Host:humans|Disease:pulmonary cryptococcosis, basal meningitis, and cerebral cryptococcomas|Description:Unknown
Chr09G0960.1	138	19	138	UniProt ID:Q8J0U4_LEPMC	136	20	136	67/120 (55.83)	0.73	0.03	120	138	5.00E-43	gene=Chr09G0960	Gene Symbol:SP1|Host:Brassica spp. and other Brassicaceae|Disease:Black leg, canker, dry rot, leaf spot|Description:Unknown
Chr09G0964.1	358	44	189	UniProt ID:TUP1_CANAL	514	222	368	41/149 (27.52)	0.54	0.03	149	65.1	1.00E-12	gene=Chr09G0964	Gene Symbol:TUP1|Host:Isolated from a wide variety of substrates including humans|Disease:invasive candidal disease|Description:FUNC

															TION: Represses transcription by RNA polymerase II. Represses genes responsible for initiating filamentous growth and this repression is lifted under inducing environmental conditions.
Chr09G0967.1	448	15	188	UniProt ID:Q59SI6_CANAL	457	116	283	53/176 (30.11)	0.49	0.06	176	96.3	1.00E-22	gene=Chr09G0967	Gene Symbol:PCT1|Host:Isolated from a wide variety of substrates including humans|Disease:invasive candidal disease|Description:CAUTION: The sequence shown here is derived from an EMBL/GenBank/DDBJ whole genome shotgun (WGS) entry which is preliminary data.
Chr09G0969.1	227	62	164	UniProt ID:Q8J2N0_FUSOX	706	168	272	37/118 (31.36)	0.47	0.24	118	53.5	2.00E-09	gene=Chr09G0969	Gene Symbol:SNF1|Host:Multiple genera in multiple families|Disease:Blights, wilts, rots of various

															sorts|Description:Unknown
Chr09G0973.1	368	82	365	UniProt ID:Q6TFC7_ASPFM	349	63	348	89/290 (30.69)	0.51	0.03	290	156	1.00E-44	gene=Chr09G0973	Gene Symbol:NULL|Host:humans|Disease:infection|Description:Unknown
Chr09G0978.1	530	53	92	UniProt ID:A8QJI7_FUSOX	938	82	121	18/40(45.00)	0.63	0	40	47	2.00E-06	gene=Chr09G0978	Gene Symbol:XLNR|Host:Multiple genera in multiple families|Disease:Blights, wilts, rots of various sorts|Description:SIMILARITY: Contains 1 Zn(2)-C6 fungal-type DNA-binding domain.
Chr09G0979.1	551	78	535	UniProt ID:Q5ANE1_CANAL	748	51	514	116/477(24.32)	0.43	0.07	477	130	5.00E-33	gene=Chr09G0979	Gene Symbol:SNF3|Host:Isolated from a wide variety of substrates including humans|Disease:invasive candidal disease|Description:SIMILARITY: Belongs to the major facilitator superfamily. Sugar transporter (TC 2.A.1.1)

															family.
Chr09G0982.1	444	18	444	UniProt ID:Q5AL34_CANAL	458	4	458	160/475(33.68)	0.54	0.14	475	235	2.00E-72	gene=Chr09G0982	Gene Symbol:PSA2|Host:Isolated from a wide variety of substrates including humans|Disease:invasive candidal disease|Description:CAUTION: The sequence shown here is derived from an EMBL/GenBank/DDBJ whole genome shotgun (WGS) entry which is preliminary data.
Chr09G0984.1	536	54	497	UniProt ID:Q5ANE1_CANAL	748	51	496	116/463(25.05)	0.42	0.08	463	107	1.00E-25	gene=Chr09G0984	Gene Symbol:SNF3|Host:Isolated from a wide variety of substrates including humans|Disease:invasive candidal disease|Description:SIMILARITY: Belongs to the major facilitator superfamily. Sugar transporter (TC 2.A.1.1) family.

Chr09G0990.1	912	237	845	UniProt ID:Q99324_SEPLY	803	105	754	174/695(25.04)	0.38	0.19	695	95.5	5.00E-21	gene=Chr09G0990	Gene Symbol:B2TOM|Host:Primarily tomato, Lycopersicon esculentum, also Solanum spp. and other Solanaceae|Disease:Leaf spot|Description:Unknown
Chr09G0991.1	331	32	329	UniProt ID:PLYB_COLGL	331	31	329	250/299(83.61)	0.91	0	299	471	7.00E-168	gene=Chr09G0991	Gene Symbol:PLB|Host:Multiple genera in multiple families|Disease:'Anthracnose of stems and leaves, dieback, root rot, leaf spot, blossom rot, fruit rot (dieback and ripe rot), seedling blight.' (Mordue 1971)|Description:FUNCTION: Acts as a virulence factor active in plant tissue maceration.
Chr09G0992.1	550	79	421	UniProt ID:O93886_9PEZI	607	111	461	82/360(22.78)	0.44	0.07	360	71.2	5.00E-14	gene=Chr09G0992	Gene Symbol:NULL|Host:Fabaceae|Disease:Leaf spot, seed stain, etc|Description:Unknown
Chr09G0	532	8	41	UniProt	663	118	151	16/34(	0.53	0	34	45.1	8.00E-06	gene=Chr	Gene

994.1				ID:Q0 WXM3 _FUSO X				47.06)						09G0994	Symbol:FOW2|Host:Multiple genera in multiple families|Disease:Blights, wilts, rots of various sorts|Description:SIMILARITY: Contains 1 Zn(2)-C6 fungal-type DNA-binding domain.
Chr09G0 998.1	546	92	535	UniProt ID:A0S T43_C ERNC	459	1	458	143/48 2(29.6 7)	0.44	0.13	482	180	6.00E-51	gene=Chr 09G0998	Gene Symbol:CTB5|Host:Numerous taxa in Solanaceae|Disease:Leaf spot|Description:Unknown
Chr09G1 000.1	826	54	226	UniProt ID:A0S T43_C ERNC	459	13	193	55/186 (29.57)	0.45	0.1	186	63.5	2.00E-11	gene=Chr 09G1000	Gene Symbol:CTB5|Host:Numerous taxa in Solanaceae|Disease:Leaf spot|Description:Unknown
Chr09G1 004.1	492	54	282	UniProt ID:Q1H GK2_M AGGR	803	112	316	69/238 (28.99)	0.47	0.18	238	74.3	5.00E-15	gene=Chr 09G1004	Gene Symbol:NULL|Host:Digitaria (Poaceae)|Disease:Leaf spot|Description:Unknown
Chr09G1 012.1	636	15	636	UniProt ID:MEP _NEOF I	634	14	634	428/62 5(68.4 8)	0.8	0.01	625	876	0	gene=Chr 09G1012	Gene Symbol:MEP|Host:human s|Disease:infection|Description:FUNCTION:

															Secreted metalloproteinase that allows assimilation of proteinaceous substrates and probably acts as a virulence factor (By similarity).
Chr09G1013.1	4890	44	2170	UniProt ID:Q9UVN5_ALTAL	4360	286	2431	618/2268(27.25)	0.43	0.12	2268	520	2.00E-148	gene=Chr09G1013	Gene Symbol:AMT|Host:Plant|Disease:Leaf spot, rots|Description:Unknown
Chr09G1014.1	546	41	535	UniProt ID:Q5SE95_ASPFM	501	14	483	243/502(48.41)	0.65	0.08	502	462	3.00E-158	gene=Chr09G1014	Gene Symbol:SIDA|Host:humans|Disease:infection|Description:Unknown
Chr09G1015.1	725	393	517	UniProt ID:Q5ALS7_CANAL	1144	501	642	39/142(27.46)	0.43	0.12	142	48.9	9.00E-07	gene=Chr09G1015	Gene Symbol:CTF1|Host:Isolated from a wide variety of substrates including humans|Disease:invasive candidal disease|Description:SIMILARITY: Contains 1 Zn(2)-C6 fungal-type DNA-binding domain.
Chr09G1	512	52	323	UniProt	1813	41	288	83/278	0.47	0.13	278	75.9	2.00E-15	gene=Chr	Gene

016.1				ID:Q59 VF3_C ANAL				(29.86)						09G1016	Symbol:"DUR1,2"|Host:Isolated from a wide variety of substrates including humans|Disease:invasive candidal disease|Description:CAUTION: The sequence shown here is derived from an EMBL/GenBank/DDBJ whole genome shotgun (WGS) entry which is preliminary data.
Chr09G1 023.1	822	43	97	UniProt ID:Q59 ZX1_C ANAL	1069	361	414	24/58(41.38)	0.55	0.12	58	55.5	1.00E-08	gene=Chr 09G1023	Gene Symbol:SET3|Host:Isolated from a wide variety of substrates including humans|Disease:invasive candidal disease|Description:SIMILARITY: Contains 1 SET domain.
Chr09G1 031.1	582	21	558	UniProt ID:Q9P 8L8_B OTFU	598	53	591	237/54 3(43.6 5)	0.64	0.02	543	463	8.00E-15 7	gene=Chr 09G1031	Gene Symbol:BCMFS1|Host:Various plant families|Disease:Grey mould. Parasite or

															saprophyte|Description:Unknown
Chr09G1032.1	339	121	324	UniProt ID:A3LVL9_PICST	319	111	308	59/215 (27.44)	0.41	0.13	215	49.7	9.00E-08	gene=Chr09G1032	Gene Symbol:TRR1|Host:humans|Disease:occasional infection|Description:CATALYTIC ACTIVITY: Thioredoxin + NADP(+) = thioredoxin disulfide + NADPH.
Chr09G1037.1	2615	7	2606	UniProt ID:Q92217_COCHE	2528	13	2518	868/2666(32.56)	0.49	0.08	2666	1127	0	gene=Chr09G1037	Gene Symbol:PKS1|Host:Zea mays|Disease:Southern leaf blight of maize|Description:Unknown
Chr09G1041.1	1030	656	915	UniProt ID:TUP1_CANAL	514	263	509	86/284 (30.28)	0.44	0.21	284	102	2.00E-23	gene=Chr09G1041	Gene Symbol:TUP1|Host:Isolated from a wide variety of substrates including humans|Disease:invasive candidal disease|Description:FUNCTION: Represses transcription by RNA polymerase II. Represses

															genes responsible for initiating filamentous growth and this repression is lifted under inducing environmental conditions.
Chr09G1045.1	238	3	201	UniProt ID:Q32WF7_PHAND	266	21	214	51/203 (25.12)	0.43	0.06	203	50.1	2.00E-08	gene=Chr09G1045	Gene Symbol:MDH1|Host:Multiple genera of Poaceae and Blysmus compressus (Cyperaceae)|Disease:Glume blotch of wheat and other grasses|Description:Unknown
Chr09G1047.1	278	6	275	UniProt ID:MEP1_COCP7	276	10	274	131/271(48.34)	0.61	0.03	271	250	1.00E-82	gene=Chr09G1047	Gene Symbol:MEP1|Host:humans|Disease:coccidiomycosis|Description:FUNCTION: Secreted metalloproteinase that allows assimilation of proteinaceous substrates. Pays a pivotal role as a pathogenicity determinant during infections and contributes to the ability of

															the pathogen to persist within the mammalian host. Digests an immunodominant cell surface antigen (SOWgp) and prevents host recognition of endospores during the phase of development when these fungal cells are most vulnerable to phagocytic cell defenses.
Chr09G1049.1	565	41	506	UniProt ID:A4RGC8_MAGO7	1158	696	1155	174/475(36.63)	0.55	0.05	475	284	1.00E-84	gene=Chr09G1049	Gene Symbol:MGG_11671|Host:Poaceae, especially important on Oryzae|Disease:Rice blast|Description:SIMILARITY: Contains 1 reverse transcriptase domain.
Chr09G1051.1	179	3	152	UniProt ID:Q8J286_COLLN	1167	970	1099	67/151(44.37)	0.57	0.15	151	112	3.00E-29	gene=Chr09G1051	Gene Symbol:CLAP1|Host:Multiple genera of Fabaceae. Rare reports on other taxa|Disease:Leaf, stem and pod

														anthracnose|Description:SIMILARITY: Belongs to the cation transport ATPase (P-type) family.
Chr09G1053.1	1183	252	402	UniProt ID:Q5AG71_CANAL	1462	144	270	45/152 (29.61)	0.43	0.17	152	54.3	3.00E-08	gene=Chr09G1053	Gene Symbol:HSL1|Host:Isolated from a wide variety of substrates including humans|Disease:invasive candidal disease|Description:CAUTION: The sequence shown here is derived from an EMBL/GenBank/DDBJ whole genome shotgun (WGS) entry which is preliminary data.
Chr09G1054.1	785	370	577	UniProt ID:C4YM45_CANAW	317	22	203	61/215 (28.37)	0.41	0.19	215	56.6	2.00E-09	gene=Chr09G1054	Gene Symbol:CAWG_01920|Host:Isolated from a wide variety of substrates including humans|Disease:invasive candidal disease|Description:Unknown

Chr06G0001.1	322	1	111	UniProt ID:C4YII6_CANAW	768	492	599	35/115 (30.43)	0.48	0.1	115	50.8	6.00E-08	gene=Chr06G0001	Gene Symbol:CAWG_04261|Host:Isolated from a wide variety of substrates including humans|Disease:invasive candidal disease|Description:SIMILARITY: Belongs to the DEAD box helicase family.
Chr06G0004.1	989	670	904	UniProt ID:SUB8_COCP7	497	155	394	61/254 (24.02)	0.42	0.13	254	57	3.00E-09	gene=Chr06G0004	Gene Symbol:CPC735_031240|Host:humans|Disease:coccidiomycosis|Description: FUNCTION: Secreted subtilisin-like serine protease with keratinolytic activity that contributes to pathogenicity (By similarity).
Chr06G0005.1	151	6	141	UniProt ID:Q2UKQ5_ASPOR	146	9	136	62/136 (45.59)	0.55	0.06	136	95.9	3.00E-26	gene=Chr06G0005	Gene Symbol:AO090003000715|Host:Isolated from varied species in numerous families as a saprophyte|Disease:Rots

															of various foods|Description:Unknown
Chr01G0722.1	325	16	281	UniProt ID:Q7Z8E8_CANDU	320	3	314	76/331 (22.96)	0.39	0.25	331	50.1	6.00E-08	gene=Chr01G0722	Gene Symbol:CSH1|Host:humans|Disease:leptomeningeal disease,occasional invasive candidal disease|Description:Unknown
Chr01G0727.1	368	5	283	UniProt ID:Q6A2T2_BOTFU	391	14	293	86/292 (29.45)	0.46	0.09	292	104	3.00E-26	gene=Chr01G0727	Gene Symbol:BTP1|Host:Various plant families|Disease:Grey mould. Parasite or saprophyte|Description:Unknown
Chr01G0733.1	586	83	356	UniProt ID:Q5XTQ4_BOTFU	574	58	335	98/287 (34.15)	0.47	0.08	287	130	5.00E-33	gene=Chr01G0733	Gene Symbol:LIP1|Host:Various plant families|Disease:Grey mould. Parasite or saprophyte|Description:Unknown
Chr01G0737.1	958	151	317	UniProt ID:SUB	522	140	285	52/174 (29.89)	0.44	0.2	174	57.8	2.00E-09	gene=Chr01G0737	Gene Symbol:SUB10|Host:hum

				10_TRIVH											ans|Disease:infection|Description:FUNCTION: Secreted subtilisin-like serine protease with keratinolytic activity that contributes to pathogenicity (By similarity).
Chr01G0751.1	528	10	49	UniProt ID:Q59MD2_CANAL	843	763	802	18/40(45.00)	0.6	0	40	45.4	6.00E-06	gene=Chr01G0751	Gene Symbol:UME6|Host:Isolated from a wide variety of substrates including humans|Disease:invasive candidal disease|Description:CAUTION: The sequence shown here is derived from an EMBL/GenBank/DDBJ whole genome shotgun (WGS) entry which is preliminary data.
Chr01G0757.1	358	12	332	UniProt ID:Q6XVN4_CRYNV	383	22	358	86/356(24.16)	0.39	0.15	356	72	6.00E-15	gene=Chr01G0757	Gene Symbol:GNO1|Host:humans|Disease:cryptococcosis|Description:COFACTOR: Zinc (By similarity).

Chr01G0761.1	588	46	586	UniProt ID:MCPB_TRITO	538	5	537	274/542(50.55)	0.65	0.02	542	548	0	gene=Chr01G0761	Gene Symbol:MCPB|Host:humans|Disease:infection|Description:FUNCTION: Extracellular metalloprotease that contributes to pathogenicity (By similarity).
Chr01G0768.1	242	53	241	UniProt ID:Q696X2_PHAND	321	6	194	52/199 (26.13)	0.43	0.1	199	44.3	2.00E-06	gene=Chr01G0768	Gene Symbol:GLO1|Host:Multiple genera of Poaceae and Blysmus compressus (Cyperaceae)|Disease:Glume blotch of wheat and other grasses|Description:Unknown
Chr01G0784.1	205	10	200	UniProt ID:Q59L90_CANAL	197	3	190	100/191(52.36)	0.66	0.02	191	194	7.00E-63	gene=Chr01G0784	Gene Symbol:HET1|Host:Isolated from a wide variety of substrates including humans|Disease:invasive candidal disease|Description:CAUTION: The sequence shown

<table>
<tr><td></td><td></td><td></td><td></td><td></td><td></td><td></td><td></td><td></td><td></td><td></td><td></td><td></td><td></td><td></td><td>here is derived from an EMBL/GenBank/DDBJ whole genome shotgun (WGS) entry which is preliminary data.</td></tr>
<tr><td>Chr01G0786.1</td><td>859</td><td>78</td><td>532</td><td>UniProt ID:Q59TT3_CANAL</td><td>1710</td><td>625</td><td>1061</td><td>191/473(40.38)</td><td>0.59</td><td>0.11</td><td>473</td><td>357</td><td>9.00E-106</td><td>gene=Chr01G0786</td><td>Gene Symbol:PLD1|Host:Isolated from a wide variety of substrates including humans|Disease:invasive candidal disease|Description:CAUTION: The sequence shown here is derived from an EMBL/GenBank/DDBJ whole genome shotgun (WGS) entry which is preliminary data.</td></tr>
<tr><td>Chr01G0789.1</td><td>255</td><td>4</td><td>254</td><td>UniProt ID:O93802_ALTAL</td><td>267</td><td>10</td><td>267</td><td>79/263 (30.04)</td><td>0.51</td><td>0.06</td><td>263</td><td>139</td><td>2.00E-40</td><td>gene=Chr01G0789</td><td>Gene Symbol:BRM2|Host:Plant|Disease:Leaf spot, rots|Description:SIMILARITY: Belongs to the short-chain dehydrogenases/reductases (SDR) family.</td></tr>
</table>

Chr01G0792.1	912	161	221	UniProt ID:Q5A4F3_CANAL	624	2	62	31/61(50.82)	0.64	0	61	79.3	4.00E-16	gene=Chr01G0792	Gene Symbol:ZCF37|Host:Isolated from a wide variety of substrates including humans|Disease:invasive candidal disease|Description:Unknown
Chr01G0802.1	406	31	348	UniProt ID:Q4P8E7_USTMA	703	287	656	94/397(23.68)	0.38	0.27	397	63.2	1.00E-11	gene=Chr01G0802	Gene Symbol:UM03616.1|Host:Euchlaena spp., Zea spp. (Poaceae)|Disease:Smut. Corn smut|Description:CAUTION: The sequence shown here is derived from an EMBL/GenBank/DDBJ whole genome shotgun (WGS) entry which is preliminary data.
Chr01G0807.1	344	26	226	UniProt ID:Q92217_COCHE	2528	1837	2034	57/209(27.27)	0.46	0.09	209	49.7	1.00E-07	gene=Chr01G0807	Gene Symbol:PKS1|Host:Zea mays|Disease:Southern leaf blight of maize|Description:Unknown

Chr01G0816.1	360	2	358	UniProt ID:A4QVF8_MAGO7	339	5	338	119/360(33.06)	0.5	0.08	360	155	1.00E-44	gene=Chr01G0816	Gene Symbol:MGG_04556|Host:Poaceae, especially important on Oryzae|Disease:Rice blast|Description:COFACTOR: Zinc (By similarity).
Chr01G0821.1	1066	17	1046	UniProt ID:Q9P872_CANAL	917	5	904	311/1049(29.65)	0.46	0.16	1049	346	2.00E-103	gene=Chr01G0821	Gene Symbol:PMR1|Host:Isolated from a wide variety of substrates including humans|Disease:invasive candidal disease|Description:SIMILARITY: Belongs to the cation transport ATPase (P-type) family.
Chr01G0830.1	900	83	464	UniProt ID:DHH1_CRYNV	616	31	378	116/383(30.29)	0.49	0.09	383	180	1.00E-48	gene=Chr01G0830	Gene Symbol:VAD1|Host:humans|Disease:cryptococcosis|Description:FUNCTION: ATP-dependent RNA helicase involved in mRNA turnover, and more specifically in mRNA decapping. Is involved in

															G1/S DNA- damage checkpoint recovery, probably through the regulation of the translational status of a subset of mRNAs. May also have a role in translation and mRNA nuclear export (By similarity). Is involved in virulence.
Chr01G0835.1	506	70	504	UniProt ID:A0ST43_CERNC	459	12	456	114/467(24.41)	0.42	0.12	467	121	4.00E-31	gene=Chr01G0835	Gene Symbol:CTB5|Host:Numerous taxa in Solanaceae|Disease:Leaf spot|Description:Unknown
Chr01G0836.1	659	15	67	UniProt ID:A8QJI7_FUSOX	938	84	131	21/53(39.62)	0.57	0.09	53	49.3	6.00E-07	gene=Chr01G0836	Gene Symbol:XLNR|Host:Multiple genera in multiple families|Disease:Blights, wilts, rots of various sorts|Description:SIMILARITY: Contains 1 Zn(2)-C6 fungal-type DNA-binding domain.
Chr01G0	484	5	337	UniProt	871	458	820	88/383	0.37	0.18	383	51.2	8.00E-08	gene=Chr	Gene

837.1				ID:Q2I0M6_CERNC				(22.98)					01G0837	Symbol:CTB3|Host:Numerous taxa in Solanaceae|Disease:Leaf spot|Description:Unknown
Chr01G0843.1	624	209	262	UniProt ID:Q59M50_CANAL	578	38	91	20/54(37.04)	0.52	0	54	49.3	4.00E-07	gene=Chr01G0843	Gene Symbol:CWT1|Host:Isolated from a wide variety of substrates including humans|Disease:invasive candidal disease|Description:CAUTION: The sequence shown here is derived from an EMBL/GenBank/DDBJ whole genome shotgun (WGS) entry which is preliminary data.
Chr01G0852.1	566	266	446	UniProt ID:PABP_COCIM	768	52	233	43/182(23.63)	0.46	0.01	182	74.3	6.00E-15	gene=Chr01G0852	Gene Symbol:PAB1|Host:humans|Disease:coccidioidomycosis|Description:FUNCTION: Binds the poly(A) tail of mRNA. Appears to be an important mediator of the multiple roles of the poly(A) tail in mRNA

														biogenesis, stability and translation. In the nucleus, involved in both mRNA cleavage and polyadenylation. Is also required for efficient mRNA export to the cytoplasm. Acts in concert with a poly(A)-specific nuclease (PAN) to affect poly(A) tail shortening, which may occur concomitantly with either nucleocytoplasmic mRNA transport or translational initiation. In the cytoplasm, stimulates translation initiation and regulates mRNA decay through translation termination-coupled poly(A) shortening, probably mediated by PAN (By similarity).
Chr01G0858.1	725	13	603	UniProt ID:A6Z	666	41	656	144/658(21.8	0.41	0.17	658	95.1	3.00E-21	gene=Chr01G0858	Gene Symbol:SLY1|Host:human

				YE8_YEAS7				8)						s|Disease:occasional infection|Description:CAUTION: The sequence shown here is derived from an EMBL/GenBank/DDBJ whole genome shotgun (WGS) entry which is preliminary data.
Chr01G0868.1	774	267	444	UniProt ID:Q0WXM3_FUSOX	663	268	448	50/195 (25.64)	0.45	0.16	195	55.5	8.00E-09	gene=Chr01G0868	Gene Symbol:FOW2|Host:Multiple genera in multiple families|Disease:Blights, wilts, rots of various sorts|Description:SIMILARITY: Contains 1 Zn(2)-C6 fungal-type DNA-binding domain.
Chr01G0870.1	482	5	57	UniProt ID:F2QZS1_PICP7	494	368	423	24/56(42.86)	0.63	0.05	56	46.6	2.00E-06	gene=Chr01G0870	Gene Symbol:OLE1|Host:humans|Disease:occasional infection|Description:COFACTOR: Iron (By similarity).
Chr01G0871.1	530	60	500	UniProt ID:Q5A	748	51	493	119/457(26.0	0.44	0.07	457	113	1.00E-27	gene=Chr01G0871	Gene Symbol:SNF3|Host:Isolate

				NE1_CANAL				4)							d from a wide variety of substrates including humans|Disease:invasive candidal disease|Description:SIMILARITY: Belongs to the major facilitator superfamily. Sugar transporter (TC 2.A.1.1) family.
Chr01G0875.1	604	28	432	UniProt ID:F2QYD1_PICP7	758	193	587	118/445(26.52)	0.44	0.2	445	166	1.00E-44	gene=Chr01G0875	Gene Symbol:MAK5|Host:humans|Disease:occasional infection|Description:SIMILARITY: Belongs to the DEAD box helicase family.
Chr01G0876.1	2613	6	2607	UniProt ID:Q92217_COCHE	2528	11	2518	866/2685(32.25)	0.5	0.1	2685	1176	0	gene=Chr01G0876	Gene Symbol:PKS1|Host:Zea mays|Disease:Southern leaf blight of maize|Description:Unknown
Chr01G0878.1	537	12	485	UniProt ID:A4ULI5_MYCGR	515	20	507	110/525(20.95)	0.37	0.17	525	68.2	4.00E-13	gene=Chr01G0878	Gene Symbol:CYP51|Host:Triticum and possibly a few other

														grasses|Disease:Leaf spot or speckled leaf blotch of wheat|Description:COFACTOR: Heme group (By similarity).
Chr01G0879.1	475	22	452	UniProt ID:Q5ANE1_CANAL	748	49	497	114/468(24.36)	0.42	0.12	468	96.3	4.00E-22	gene=Chr01G0879	Gene Symbol:SNF3|Host:Isolated from a wide variety of substrates including humans|Disease:invasive candidal disease|Description:SIMILARITY: Belongs to the major facilitator superfamily. Sugar transporter (TC 2.A.1.1) family.
Chr01G0880.1	1429	850	1421	UniProt ID:Q5A762_CANAL	1606	992	1598	180/624(28.85)	0.5	0.11	624	254	2.00E-69	gene=Chr01G0880	Gene Symbol:MLT1|Host:Isolated from a wide variety of substrates including humans|Disease:invasive candidal disease|Description:SIMILARITY: Belongs to the ABC transporter

															superfamily.
Chr01G0881.1	347	38	339	UniProt ID:Q6TFC7_ASPFM	349	36	346	105/316(33.23)	0.47	0.06	316	162	3.00E-47	gene=Chr01G0881	Gene Symbol:NULL|Host:humans|Disease:infection|Description:Unknown
Chr01G0885.1	490	1	198	UniProt ID:Q5A4X7_CANAL	404	1	198	130/198(65.66)	0.82	0	198	283	9.00E-91	gene=Chr01G0885	Gene Symbol:PRS11|Host:Isolated from a wide variety of substrates including humans|Disease:invasive candidal disease|Description:CATALYTIC ACTIVITY: ATP + D-ribose 5-phosphate = AMP + 5-phospho-alpha-D-ribose 1-diphosphate.
Chr01G0891.1	449	5	353	UniProt ID:RAD18_CANAL	378	2	376	105/395(26.58)	0.42	0.17	395	145	6.00E-40	gene=Chr01G0891	Gene Symbol:RAD18|Host:Isolated from a wide variety of substrates including humans|Disease:invasive candidal disease|Description:FUNCTION: E3 RING-finger protein, member of the

															UBC2/RAD6 epistasis group. Associates to the E2 ubiquitin conjugating enzyme UBC2/RAD6 to form the UBC2-RAD18 ubiquitin ligase complex involved in postreplicative repair (PRR) of damaged DNA (By similarity).
Chr01G0893.1	540	23	540	UniProt ID:A4R566_MAGO7	550	24	550	385/527(73.06)	0.84	0.02	527	810	0	gene=Chr01G0893	Gene Symbol:MGG_04128|Host:Poaceae, especially important on Oryzae|Disease:Rice blast|Description:Unknown
Chr01G0897.1	479	175	420	UniProt ID:Q5APD4_CANAL	513	200	442	85/251 (33.86)	0.47	0.05	251	123	1.00E-31	gene=Chr01G0897	Gene Symbol:CFA1|Host:Isolated from a wide variety of substrates including humans|Disease:invasive candidal disease|Description:CAUTION: The sequence shown here is derived from an EMBL/GenBank/DDBJ

														whole genome shotgun (WGS) entry which is preliminary data.
Chr01G0902.1	273	32	272	UniProt ID:A4R575_MAGO7	249	1	247	138/250(55.20)	0.68	0.05	250	262	1.00E-87	gene=Chr01G0902	Gene Symbol:MGG_04137|Host:Poaceae, especially important on Oryzae|Disease:Rice blast|Description:SIMILARITY: Contains 1 CTLH domain.
Chr01G0906.1	451	8	206	UniProt ID:F2QZW6_PICP7	218	2	214	81/215(37.67)	0.57	0.08	215	149	8.00E-43	gene=Chr01G0906	Gene Symbol:YPT1|Host:humans|Disease:occasional infection|Description:SIMILARITY: Belongs to the small GTPase superfamily. Rab family.
Chr01G0908.1	406	7	347	UniProt ID:Q9Y784_MAGGR	631	30	370	95/347(27.38)	0.48	0.03	347	132	9.00E-35	gene=Chr01G0908	Gene Symbol:PTH11|Host:Digitaria (Poaceae)|Disease:Leaf spot|Description:Unknown
Chr01G0910.1	591	508	559	UniProt ID:A4ULJ0_M	518	454	505	20/52(38.46)	0.62	0	52	46.6	3.00E-06	gene=Chr01G0910	Gene Symbol:CYP51|Host:Triticum and possibly a few

				YCGR											other grasses|Disease:Leaf spot or speckled leaf blotch of wheat|Description:COFACTOR: Heme group (By similarity).
Chr01G0914.1	539	53	494	UniProt ID:Q5ANE1_CANAL	748	51	496	109/459(23.75)	0.43	0.07	459	114	2.00E-27	gene=Chr01G0914	Gene Symbol:SNF3|Host:Isolated from a wide variety of substrates including humans|Disease:invasive candidal disease|Description:SIMILARITY: Belongs to the major facilitator superfamily. Sugar transporter (TC 2.A.1.1) family.
Chr01G0917.1	580	25	566	UniProt ID:Q59RG1_CANAL	561	57	543	124/548(22.63)	0.38	0.12	548	90.9	3.00E-20	gene=Chr01G0917	Gene Symbol:NAG3|Host:Isolated from a wide variety of substrates including humans|Disease:invasive candidal disease|Description:CAUTION: The sequence shown

														here is derived from an EMBL/GenBank/DDBJ whole genome shotgun (WGS) entry which is preliminary data.
Chr01G0922.1	658	174	267	UniProt ID:A5H456_MYCGR	1811	1291	1389	37/99(37.37)	0.52	0.05	99	50.8	2.00E-07	gene=Chr01G0922	Gene Symbol:NULL|Host:Triticum and possibly a few other grasses|Disease:Leaf spot or speckled leaf blotch of wheat|Description:Unknown
Chr01G0923.1	1229	185	1216	UniProt ID:Q5YCX0_FUSOX	1041	18	1041	777/1052(73.86)	0.82	0.05	1052	1533	0	gene=Chr01G0923	Gene Symbol:CHS2|Host:Multiple genera in multiple families|Disease:Blights, wilts, rots of various sorts|Description:Unknown
Chr01G0928.1	492	27	475	UniProt ID:LAP2_TRIVH	495	30	475	171/469(36.46)	0.53	0.09	469	228	1.00E-68	gene=Chr01G0928	Gene Symbol:LAP2|Host:humans|Disease:infection|Description:FUNCTION: Extracellular aminopeptidase that releases a wide variety of

															amino acids from natural peptides and contributes to pathogenicity (By similarity).
Chr01G0930.1	762	126	572	UniProt ID:C1GMG4_PARBD	772	174	605	130/489(26.58)	0.44	0.2	489	131	1.00E-32	gene=Chr01G0930	Gene Symbol:PADG_08450|Host:humans|Disease:Paracoccidioidomycosis|Description:SIMILARITY: Belongs to the DEAD box helicase family.
Chr01G0941.1	570	12	570	UniProt ID:Q9P8L8_BOTFU	598	34	597	234/565(41.42)	0.63	0.01	565	459	2.00E-155	gene=Chr01G0941	Gene Symbol:BCMFS1|Host:Various plant families|Disease:Grey mould. Parasite or saprophyte|Description:Unknown
Chr01G0944.1	263	141	227	UniProt ID:Q9UWE7_GIBZA	218	118	217	46/100 (46.00)	0.58	0.13	100	87.8	1.00E-21	gene=Chr01G0944	Gene Symbol:TRI6|Host:Principal hosts: Poaceae, including Zea mays (corn), Triticum aestivum (wheat), and Oryza sativa (rice). Additional hosts: various plant

															families|Disease:Seedling blight, pre- and post-emergence blight, root and foot rot, brown rot, culm decay, head or kernel blight (scab or ear scab) of cereals.
Chr01G0947.1	337	35	163	UniProt ID:CUTI_PYRBR	203	32	147	36/134 (26.87)	0.43	0.17	134	43.5	4.00E-06	gene=Chr01G0947	Leaf|Description:Unknown Gene Symbol:NULL|Host:Brassica spp. (Brassicaceae)|Disease:Light leaf spot|Description:FUNCTION: Catalyzes the hydrolysis of cutin, a polyester that forms the structure of plant cuticle. Allows pathogenic fungi to penetrate through the cuticular barrier into the host plant during the initial stage of the fungal infection (By similarity).
Chr01G0955.1	371	10	259	UniProt ID:Q6A	391	16	293	71/279 (25.45)	0.46	0.11	279	92	1.00E-21	gene=Chr01G0955	Gene Symbol:BTP1|Host:Variou

				2T2_BOTFU											s plant families|Disease:Grey mould. Parasite or saprophyte|Description:Unknown
Chr01G0956.1	575	36	574	UniProt ID:Q2VLJ1_GIBZA	565	24	562	326/539(60.48)	0.73	0	539	700	0	gene=Chr01G0956	Gene Symbol:ZEB1|Host:Principal hosts: Poaceae, including Zea mays (corn), Triticum aestivum (wheat), and Oryza sativa (rice). Additional hosts: various plant families|Disease:Seedling blight, pre- and post-emergence blight, root and foot rot, brown rot, culm decay, head or kernel blight (scab or ear scab) of cereals. Leaf|Description:Unknown
Chr01G0958.1	535	156	326	UniProt ID:Q0WXM3_FUSOX	663	265	445	41/185(22.16)	0.48	0.1	185	55.5	4.00E-09	gene=Chr01G0958	Gene Symbol:FOW2|Host:Multiple genera in multiple families|Disease:Blights, wilts, rots of various

															sorts|Description:SIMILARITY: Contains 1 Zn(2)-C6 fungal-type DNA-binding domain.
Chr01G0975.1	369	166	353	UniProt ID:Q2I0M6_CERNC	871	203	411	58/218 (26.61)	0.45	0.18	218	68.9	1.00E-13	gene=Chr01G0975	Gene Symbol:CTB3|Host:Numerous taxa in Solanaceae|Disease:Leaf spot|Description:Unknown
Chr01G0978.1	365	11	296	UniProt ID:Q6A2T2_BOTFU	391	39	330	77/304 (25.33)	0.43	0.1	304	56.2	1.00E-09	gene=Chr01G0978	Gene Symbol:BTP1|Host:Various plant families|Disease:Grey mould. Parasite or saprophyte|Description:Unknown
Chr01G0980.1	319	16	244	UniProt ID:PABP_COCIM	768	57	309	64/270 (23.70)	0.42	0.21	270	55.1	2.00E-09	gene=Chr01G0980	Gene Symbol:PAB1|Host:humans|Disease:coccidioidomycosis|Description:FUNCTION: Binds the poly(A) tail of mRNA. Appears to be an important mediator of the multiple roles of the poly(A) tail in mRNA biogenesis, stability and

													translation. In the nucleus, involved in both mRNA cleavage and polyadenylation. Is also required for efficient mRNA export to the cytoplasm. Acts in concert with a poly(A)-specific nuclease (PAN) to affect poly(A) tail shortening, which may occur concomitantly with either nucleocytoplasmic mRNA transport or translational initiation. In the cytoplasm, stimulates translation initiation and regulates mRNA decay through translation termination-coupled poly(A) shortening, probably mediated by PAN (By similarity).
Chr01G0998.1	345	18	209	UniProt ID:A4QVF8_M	339	21	230	60/219 (27.40)	0.42	0.16	219	47.4	4.00E-07	gene=Chr01G0998	Gene Symbol:MGG_04556|Host:Poaceae, especially

				AGO7										important on Oryzae|Disease:Rice blast|Description:COFACTOR: Zinc (By similarity).
Chr01G0999.1	240	15	117	UniProt ID:A0ST44_CERNC	357	9	122	41/115 (35.65)	0.51	0.11	115	59.7	2.00E-11	gene=Chr01G0999	Gene Symbol:CTB6|Host:Numerous taxa in Solanaceae|Disease:Leaf spot|Description:Unknown
Chr01G1003.1	448	15	365	UniProt ID:A4UC81_MAGO7	376	15	374	111/363(30.58)	0.49	0.04	363	160	2.00E-45	gene=Chr01G1003	Gene Symbol:MGG_10702|Host:Poaceae, especially important on Oryzae|Disease:Rice blast|Description:Unknown
Chr01G1011.1	1229	128	1030	UniProt ID:Q9P872_CANAL	917	36	864	259/911(28.43)	0.48	0.1	911	327	6.00E-96	gene=Chr01G1011	Gene Symbol:PMR1|Host:Isolated from a wide variety of substrates including humans|Disease:invasive candidal disease|Description:SIMILARITY: Belongs to the cation transport ATPase (P-type) family.

Chr01G1018.1	277	6	274	UniProt ID:MEP1_COCP7	276	7	274	138/274(50.36)	0.64	0.04	274	259	5.00E-86	gene=Chr01G1018	Gene Symbol:MEP1|Host:humans|Disease:coccidiomycosis|Description:FUNCTION: Secreted metalloproteinase that allows assimilation of proteinaceous substrates. Pays a pivotal role as a pathogenicity determinant during infections and contributes to the ability of the pathogen to persist within the mammalian host. Digests an immunodominant cell surface antigen (SOWgp) and prevents host recognition of endospores during the phase of development when these fungal cells are most vulnerable to phagocytic cell defenses.
Chr01G1022.1	534	2	515	UniProt ID:Q6Y	625	56	582	224/535(41.8	0.6	0.05	535	430	4.00E-144	gene=Chr01G1022	Gene Symbol:NULL|Host:Multipl

				392_PHAND				7)						e genera of Poaceae and Blysmus compressus (Cyperaceae)|Disease:Glume blotch of wheat and other grasses|Description:Unknown
Chr01G1029.1	426	16	424	UniProt ID:Q4WU51_ASPFU	491	19	484	208/470(44.26)	0.58	0.14	470	365	4.00E-122	gene=Chr01G1029	Gene Symbol:AFUA_5G07210|Host:humans|Disease:infection|Description:CAUTION: The sequence shown here is derived from an EMBL/GenBank/DDBJ whole genome shotgun (WGS) entry which is preliminary data.
Chr01G1039.1	316	1	316	UniProt ID:Q59VG6_CANAL	386	1	373	142/379(37.47)	0.54	0.18	379	234	2.00E-74	gene=Chr01G1039	Gene Symbol:ERG3|Host:Isolated from a wide variety of substrates including humans|Disease:invasive candidal disease|Description:CAUTION: The sequence shown here is derived from an

Chr01G1044.1	365	49	358	UniProt ID:Q6TFC7_ASPFM	349	40	348	106/316(33.54)	0.51	0.04	316	161	1.00E-46	gene=Chr01G1044	EMBL/GenBank/DDBJ whole genome shotgun (WGS) entry which is preliminary data. Gene Symbol:NULL|Host:humans|Disease:infection|Description:Unknown
Chr01G1053.1	1077	578	858	UniProt ID:TUP1_CANAL	514	201	509	122/311(39.23)	0.55	0.1	311	203	1.00E-56	gene=Chr01G1053	Gene Symbol:TUP1|Host:Isolated from a wide variety of substrates including humans|Disease:invasive candidal disease|Description:FUNCTION: Represses transcription by RNA polymerase II. Represses genes responsible for initiating filamentous growth and this repression is lifted under inducing environmental conditions.
Chr01G1055.1	1493	29	1493	UniProt ID:Q874F3_M	1484	13	1484	1060/1478(71.72)	0.84	0.01	1478	2216	0	gene=Chr01G1055	Gene Symbol:ABC2|Host:Digitaria (Poaceae)|Disease:Leaf

				AGGR											spot|Description:SIMILARITY: Belongs to the ABC transporter superfamily.
Chr01G1064.1	514	22	415	UniProt ID:A4RF81_MAGO7	542	8	481	212/481(44.07)	0.55	0.2	481	321	2.00E-103	gene=Chr01G1064	Gene Symbol:MGG_00435|Host:Poaceae, especially important on Oryzae|Disease:Rice blast|Description:Unknown
Chr01G1072.1	797	7	424	UniProt ID:A4QRN5_MAGO7	1015	173	573	144/434(33.18)	0.52	0.11	434	181	4.00E-48	gene=Chr01G1072	Gene Symbol:MGG_03530|Host:Poaceae, especially important on Oryzae|Disease:Rice blast|Description:Unknown
Chr01G1074.1	375	120	302	UniProt ID:PLYB_COLGL	331	90	272	59/200(29.50)	0.44	0.17	200	58.9	1.00E-10	gene=Chr01G1074	Gene Symbol:PLB|Host:Multiple genera in multiple families|Disease:'Anthracnose of stems and leaves, dieback, root rot, leaf spot, blossom rot, fruit rot (dieback and ripe rot), seedling blight.' (Mordue

														1971)|Description:FUNCTION: Acts as a virulence factor active in plant tissue maceration.
Chr01G1078.1	368	24	355	UniProt ID:Q6A2T2_BOTFU	391	25	354	88/351 (25.07)	0.44	0.11	351	73.2	3.00E-15	gene=Chr01G1078	Gene Symbol:BTP1|Host:Various plant families|Disease:Grey mould. Parasite or saprophyte|Description:Unknown
Chr01G1080.1	723	14	443	UniProt ID:Q9HG15_COLLN	746	15	436	109/467(23.34)	0.37	0.18	467	70.1	2.00E-13	gene=Chr01G1080	Gene Symbol:CLTA1|Host:Multiple genera of Fabaceae. Rare reports on other taxa|Disease:Leaf, stem and pod anthracnose|Description:SIMILARITY: Contains 1 Zn(2)-C6 fungal-type DNA-binding domain.
Chr06G0020.1	1557	1022	1302	UniProt ID:TUP1_CANAL	514	201	509	106/311(34.08)	0.53	0.1	311	175	9.00E-47	gene=Chr06G0020	Gene Symbol:TUP1|Host:Isolated from a wide variety of substrates including humans|Disease:invasive

Chr06G0027.1	215	2	78	UniProt ID:Q9C441_FUSSO	330	81	165	24/85(28.24)	0.48	0.09	85	46.6	3.00E-07	gene=Chr06G0027	candidal disease|Description:FUNCTION: Represses transcription by RNA polymerase II. Represses genes responsible for initiating filamentous growth and this repression is lifted under inducing environmental conditions. Gene Symbol:PEP1|Host:Multiple plant families. Some strains may cause infections in humans|Disease:Saprobe, facultative pathogen|Description:Unknown
Chr06G0038.1	1313	95	1254	UniProt ID:A4RGC8_MAGO7	1158	38	1150	459/1195(38.41)	0.55	0.1	1195	757	0	gene=Chr06G0038	Gene Symbol:MGG_11671|Host:Poaceae, especially important on Oryzae|Disease:Rice blast|Description:SIMILARITY: Contains 1 reverse

															transcriptase domain.
Chr06G0041.1	612	105	593	UniProt ID:P78585_BOTFU	994	415	937	188/572(32.87)	0.47	0.23	572	247	1.00E-71	gene=Chr06G0041	Gene Symbol:BCPLC1|Host:Various plant families|Disease:Grey mould. Parasite or saprophyte|Description:SIMILARITY: Contains 1 C2 domain.
Chr06G0057.1	1267	29	1265	UniProt ID:Q3Y5V5_MAGGR	1321	25	1319	468/1316(35.56)	0.54	0.08	1316	734	0	gene=Chr06G0057	Gene Symbol:ABC3|Host:Digitaria (Poaceae)|Disease:Leaf spot|Description:SIMILARITY: Belongs to the ABC transporter superfamily.
Chr06G0060.1	359	4	332	UniProt ID:Q9Y784_MAGGR	631	100	433	81/343 (23.62)	0.43	0.07	343	97.4	4.00E-23	gene=Chr06G0060	Gene Symbol:PTH11|Host:Digitaria (Poaceae)|Disease:Leaf spot|Description:Unknown
Chr06G0061.1	401	4	334	UniProt ID:Q9Y784_MAGGR	631	104	430	84/339 (24.78)	0.46	0.06	339	105	1.00E-25	gene=Chr06G0061	Gene Symbol:PTH11|Host:Digitaria (Poaceae)|Disease:Leaf spot|Description:Unknown
Chr06G0	340	3	339	UniProt	339	2	338	192/33	0.73	0	337	383	1.00E-13	gene=Chr	Gene

064.1				ID:A4QVF8_MAGO7				7(56.97)					2	06G0064	Symbol:MGG_04556|Host:Poaceae, especially important on Oryzae|Disease:Rice blast|Description:COFACTOR: Zinc (By similarity).
Chr06G0066.1	245	1	242	UniProt ID:Q75WR5_9PLEO	265	7	262	87/258 (33.72)	0.58	0.07	258	168	2.00E-51	gene=Chr06G0066	Gene Symbol:BRN1|Host:Belamcanda chinensis: Korea,Gladiolus ?gandavensis: Korea,Iris japonica: China,Iris missouriensis (Leaf spot.): Idaho; Montana; Oregon; Washington,Iris sp. (Leaf spot.): China; Texas; Washing|Disease:Leaf spot|Description:SIMILARITY: Belongs to the short-chain dehydrogenases/reductases (SDR) family.
Chr06G0080.1	895	263	624	UniProt ID:Q9HG15_COLLN	746	170	515	70/374 (18.72)	0.4	0.11	374	46.6	6.00E-06	gene=Chr06G0080	Gene Symbol:CLTA1|Host:Multiple genera of Fabaceae. Rare reports on other

															taxa|Disease:Leaf, stem and pod anthracnose|Description:SIMILARITY: Contains 1 Zn(2)-C6 fungal-type DNA-binding domain.
Chr06G0082.1	540	30	503	UniProt ID:Q5ANE1_CANAL	748	37	500	145/480(30.21)	0.48	0.05	480	183	9.00E-51	gene=Chr06G0082	Gene Symbol:SNF3|Host:Isolated from a wide variety of substrates including humans|Disease:invasive candidal disease|Description:SIMILARITY: Belongs to the major facilitator superfamily. Sugar transporter (TC 2.A.1.1) family.
Chr06G0083.1	407	4	404	UniProt ID:PEPA_ASPFU	395	7	394	197/406(48.52)	0.66	0.06	406	366	3.00E-124	gene=Chr06G0083	Gene Symbol:PEP1|Host:humans|Disease:infection|Description:FUNCTION: Secreted aspartic endopeptidase that allows assimilation of proteinaceous substrates.

															Can catalyze hydrolysis of the major structural proteins of basement membrane, elastin, collagen, and laminin. Thought to play a significant role in virulence (By similarity).
Chr06G0084.1	1063	67	386	UniProt ID:P87199_USTMD	968	25	340	134/325(41.23)	0.57	0.04	325	226	2.00E-62	gene=Chr06G0084	Gene Symbol:KIN2|Host:Euchlaena spp., Zea spp. (Poaceae)|Disease:Smut. Corn smut|Description:SIMILARITY: Belongs to the kinesin-like protein family.
Chr06G0098.1	421	3	416	UniProt ID:Q6XX21_CRYNV	504	81	502	177/428(41.36)	0.62	0.05	428	323	6.00E-106	gene=Chr06G0098	Gene Symbol:FHB1|Host:humans|Disease:cryptococcosis|Description:SIMILARITY: Belongs to the globin family.
Chr06G0102.1	480	16	473	UniProt ID:Q59RG0_CANAL	581	86	573	164/502(32.67)	0.49	0.12	502	196	1.00E-56	gene=Chr06G0102	Gene Symbol:NAG4|Host:Isolated from a wide variety of substrates including

															humans|Disease:invasive candidal disease|Description:CAUTION: The sequence shown here is derived from an EMBL/GenBank/DDBJ whole genome shotgun (WGS) entry which is preliminary data.
Chr06G0103.1	712	6	568	UniProt ID:A6N6J8_FUSOX	903	45	628	176/614(28.66)	0.44	0.13	614	207	1.00E-57	gene=Chr06G0103	Gene Symbol:CTF1|Host:Multiple genera in multiple families|Disease:Blights, wilts, rots of various sorts|Description:SIMILARITY: Contains 1 Zn(2)-C6 fungal-type DNA-binding domain.
Chr06G0104.1	87	9	87	UniProt ID:Q9C173_9PLEO	87	9	87	27/81(33.33)	0.51	0.05	81	38.9	2.00E-06	gene=Chr06G0104	Gene Symbol:TOXB|Host:Poaceae|Disease:Yellow leaf spot of cereals and grasses|Description:Unknown
Chr06G0106.1	418	34	80	UniProt ID:Q5A	624	3	46	19/47(40.43)	0.62	0.06	47	49.7	2.00E-07	gene=Chr06G0106	Gene Symbol:ZCF37|Host:Isolat

				4F3_CANAL											ed from a wide variety of substrates including humans|Disease:invasive candidal disease|Description:Unknown
Chr06G0107.1	880	125	878	UniProt ID:O93841_9PEZI	914	79	910	305/839(36.35)	0.55	0.11	839	509	2.00E-166	gene=Chr06G0107	Gene Symbol:CHIP6|Host:Multiple genera in multiple families|Disease:'Anthracnose of stems and leaves, dieback, root rot, leaf spot, blossom rot, fruit rot (dieback and ripe rot), seedling blight.' (Mordue 1971)|Description:Unknown
Chr06G0109.1	447	19	78	UniProt ID:Q0WXM3_FUSOX	663	110	169	18/60(30.00)	0.48	0	60	47.4	1.00E-06	gene=Chr06G0109	Gene Symbol:FOW2|Host:Multiple genera in multiple families|Disease:Blights, wilts, rots of various sorts|Description:SIMILARITY: Contains 1 Zn(2)-C6 fungal-type DNA-binding domain.

Chr06G0115.1	381	7	270	UniProt ID:Q6XVN4_CRYNV	383	11	288	75/288 (26.04)	0.42	0.12	288	65.5	1.00E-12	gene=Chr06G0115	Gene Symbol:GNO1|Host:humans|Disease:cryptococcosis|Description:COFACTOR: Zinc (By similarity).
Chr06G0118.1	330	23	245	UniProt ID:F2QX13_PICP7	203	1	173	79/223 (35.43)	0.52	0.22	223	133	1.00E-37	gene=Chr06G0118	Gene Symbol:YPT1|Host:humans|Disease:occasional infection|Description:SIMILARITY: Belongs to the small GTPase superfamily. Rab family.
Chr06G0119.1	262	3	213	UniProt ID:A4RGG9_MAGO7	286	14	226	62/226 (27.43)	0.45	0.12	226	48.5	1.00E-07	gene=Chr06G0119	Gene Symbol:MGG_00056|Host:Poaceae, especially important on Oryzae|Disease:Rice blast|Description:SIMILARITY: Belongs to the short-chain dehydrogenases/reductases (SDR) family.
Chr06G0121.1	618	15	591	UniProt ID:Q6Y392_PHAND	625	34	596	220/589(37.35)	0.56	0.06	589	394	5.00E-129	gene=Chr06G0121	Gene Symbol:NULL|Host:Multiple genera of Poaceae and Blysmus compressus

															(Cyperaceae)|Disease:Glume blotch of wheat and other grasses|Description:Unknown
Chr06G0129.1	453	11	436	UniProt ID:A0ST41_CERNC	461	12	421	117/444(26.35)	0.45	0.12	444	132	6.00E-35	gene=Chr06G0129	Gene Symbol:CTB2|Host:Numerous taxa in Solanaceae|Disease:Leaf spot|Description:Unknown
Chr06G0141.1	620	92	269	UniProt ID:Q6A2T2_BOTFU	391	116	301	54/189 (28.57)	0.48	0.07	189	71.6	3.00E-14	gene=Chr06G0141	Gene Symbol:BTP1|Host:Various plant families|Disease:Grey mould. Parasite or saprophyte|Description:Unknown
Chr06G0144.1	435	208	420	UniProt ID:Q04701_FUSSO	242	13	229	106/222(47.75)	0.57	0.06	222	184	1.00E-55	gene=Chr06G0144	Gene Symbol:PELA|Host:Multiple plant families. Some strains may cause infections in humans|Disease:Saprobe, facultative pathogen|Description:Unknown

Chr06G0148.1	120	1	117	UniProt ID:Q32WF7_PHAND	266	154	263	45/117 (38.46)	0.61	0.06	117	84.3	2.00E-21	gene=Chr06G0148	Gene Symbol:MDH1|Host:Multiple genera of Poaceae and Blysmus compressus (Cyperaceae)|Disease:Glume blotch of wheat and other grasses|Description:Unknown
Chr06G0151.1	585	141	578	UniProt ID:Q9P8L8_BOTFU	598	175	594	106/462(22.94)	0.41	0.14	462	47.8	1.00E-06	gene=Chr06G0151	Gene Symbol:BCMFS1|Host:Various plant families|Disease:Grey mould. Parasite or saprophyte|Description:Unknown
Chr06G0173.1	1281	557	801	UniProt ID:O59928_HYPVI	430	44	299	76/282 (26.95)	0.4	0.22	282	82	4.00E-17	gene=Chr06G0173	Gene Symbol:NULL|Host:humans|Disease:infection|Description:SIMILARITY: Belongs to the glycosyl hydrolase 18 family.
Chr06G0182.1	547	31	497	UniProt ID:Q5XTQ4_BOTFU	574	55	536	148/503(29.42)	0.45	0.11	503	168	4.00E-46	gene=Chr06G0182	Gene Symbol:LIP1|Host:Various plant families|Disease:Grey

															mould. Parasite or saprophyte|Description:Unknown
Chr06G0187.1	494	56	216	UniProt ID:Q2VLJ1_GIBZA	565	127	292	57/167 (34.13)	0.5	0.04	167	74.3	4.00E-15	gene=Chr06G0187	Gene Symbol:ZEB1|Host:Principal hosts: Poaceae, including Zea mays (corn), Triticum aestivum (wheat), and Oryza sativa (rice). Additional hosts: various plant families|Disease:Seedling blight, pre- and post-emergence blight, root and foot rot, brown rot, culm decay, head or kernel blight (scab or ear scab) of cereals. Leaf|Description:Unknown
Chr06G0189.1	570	268	324	UniProt ID:Q5A7S7_CANAL	526	257	312	24/57(42.11)	0.61	0.02	57	50.4	2.00E-07	gene=Chr06G0189	Gene Symbol:FKH2|Host:Isolated from a wide variety of substrates including humans|Disease:invasive candidal disease|Description:SIMIL

															ARITY: Contains 1 fork-head DNA-binding domain.
Chr06G0191.1	638	480	637	UniProt ID:B2CG58_9PEZI	691	407	585	63/180 (35.00)	0.51	0.13	180	98.2	3.00E-22	gene=Chr06G0191	Gene Symbol:PHL1|Host:Zea mays (Poaceae)|Disease:Gray leaf spot of corn|Description:Unknown
Chr06G0193.1	392	92	392	UniProt ID:Q6IVV6_BOTFU	424	120	424	155/305(50.82)	0.7	0.01	305	334	1.00E-111	gene=Chr06G0193	Gene Symbol:CEL5A|Host:Various plant families|Disease:Grey mould. Parasite or saprophyte|Description:Unknown
Chr06G0196.1	1233	123	288	UniProt ID:GBB_CRYPA	359	170	319	44/171 (25.73)	0.44	0.15	171	51.6	1.00E-07	gene=Chr06G0196	Gene Symbol:GB-1|Host:Castanea spp., Fagus sylvatica, Quercus spp. (Fagaceae)|Disease:Chestnut blight. Cankers|Description:FUNCTION: Guanine nucleotide-binding proteins (G proteins) are

															involved as a modulator or transducer in various transmembrane signaling systems. The beta and gamma chains are required for the GTPase activity, for replacement of GDP by GTP, and for G protein- effector interaction.
Chr06G0197.1	475	32	381	UniProt ID:Q9Y784_MAGGR	631	23	373	150/352(42.61)	0.68	0.01	352	318	1.00E-101	gene=Chr06G0197	Gene Symbol:PTH11|Host:Digitaria (Poaceae)|Disease:Leaf spot|Description:Unknown
Chr06G0199.1	390	33	302	UniProt ID:Q9Y784_MAGGR	631	105	369	65/271 (23.99)	0.48	0.03	271	95.1	4.00E-22	gene=Chr06G0199	Gene Symbol:PTH11|Host:Digitaria (Poaceae)|Disease:Leaf spot|Description:Unknown
Chr06G0206.1	507	74	241	UniProt ID:A0ST43_CERNC	459	14	186	53/174 (30.46)	0.45	0.04	174	72.4	1.00E-14	gene=Chr06G0206	Gene Symbol:CTB5|Host:Numerous taxa in Solanaceae|Disease:Leaf spot|Description:Unknown
Chr06G0	477	52	200	UniProt	459	2	156	50/155	0.54	0.04	155	94.7	8.00E-22	gene=Chr	Gene

217.1				ID:A0ST43_CERNC				(32.26)						06G0217	Symbol:CTB5|Host:Numerous taxa in Solanaceae|Disease:Leaf spot|Description:Unknown
Chr06G0218.1	1416	183	1166	UniProt ID:Q09MP5_COCMI	1761	27	984	303/1006(30.12)	0.47	0.07	1006	371	8.00E-107	gene=Chr06G0218	Gene Symbol:NPS6|Host:Principal hosts: cultivated Oryza sativa (rice) and Zizania aquatica (wild rice). Less common on non-cultivated Oryza spp. and other Poaceae under natural conditions. Able to infect a wide ra|Disease:Brown spot and seedling blight|Description:Unknown
Chr06G0220.1	520	211	485	UniProt ID:A4ULJ1_MYCGR	517	225	503	76/299(25.42)	0.43	0.15	299	65.1	4.00E-12	gene=Chr06G0220	Gene Symbol:CYP51|Host:Triticum and possibly a few other grasses|Disease:Leaf spot or speckled leaf blotch of wheat|Description:COFACTOR: Heme group (By similarity).

Chr06G0221.1	476	43	475	UniProtID:Q59LS4_CANAL	448	14	447	136/464(29.31)	0.46	0.13	464	167	6.00E-47	gene=Chr06G0221	Gene Symbol:ERG24|Host:Isolated from a wide variety of substrates including humans|Disease:invasive candidal disease|Description:CAUTION: The sequence shown here is derived from an EMBL/GenBank/DDBJ whole genome shotgun (WGS) entry which is preliminary data.
Chr06G0225.1	967	380	465	UniProtID:Q0WXM3_FUSOX	663	337	424	27/88(30.68)	0.51	0.02	88	51.6	2.00E-07	gene=Chr06G0225	Gene Symbol:FOW2|Host:Multiple genera in multiple families|Disease:Blights, wilts, rots of various sorts|Description:SIMILARITY: Contains 1 Zn(2)-C6 fungal-type DNA-binding domain.
Chr06G0230.1	350	23	247	UniProtID:Q9Y784_MAGGR	631	103	362	60/261(22.99)	0.45	0.14	261	69.7	5.00E-14	gene=Chr06G0230	Gene Symbol:PTH11|Host:Digitaria (Poaceae)|Disease:Leaf

														spot|Description:Unknown
Chr06G0231.1	604	25	598	UniProt ID:Q4P8E8_USTMA	693	82	671	201/609(33.00)	0.51	0.09	609	287	5.00E-88	gene=Chr06G0231 Gene Symbol:UM03615.1|Host: Euchlaena spp., Zea spp. (Poaceae)|Disease:Smut. Corn smut|Description:COFACTOR: FAD (By similarity).
Chr06G0236.1	548	69	537	UniProt ID:Q5ANE1_CANAL	748	51	514	122/501(24.35)	0.44	0.14	501	102	8.00E-24	gene=Chr06G0236 Gene Symbol:SNF3|Host:Isolated from a wide variety of substrates including humans|Disease:invasive candidal disease|Description:SIMILARITY: Belongs to the major facilitator superfamily. Sugar transporter (TC 2.A.1.1) family.
Chr06G0241.1	242	100	226	UniProt ID:CUTI_ERYGR	236	142	236	40/127(31.50)	0.44	0.25	127	53.1	2.00E-09	gene=Chr06G0241 Gene Symbol:CUT1|Host:Poaceae|Disease:Powdery mildew|Description:FUNCTION: Catalyzes the hydrolysis of cutin, a

															polyester that forms the structure of plant cuticle. Allows pathogenic fungi to penetrate through the cuticular barrier into the host plant during the initial stage of the fungal infection (By similarity).
Chr06G0245.1	687	3	537	UniProt ID:Q9C1C6_COCCA	648	10	531	354/545(64.95)	0.78	0.06	545	708	0	gene=Chr06G0245	Gene Symbol:HDC2|Host:Corn, Zea mays, sometimes on Sorghum (Poaceae) and various other plant families|Disease:Northern corn leaf spot, ear and kernel rot|Description:Unknown
Chr06G0249.1	452	23	335	UniProt ID:Q9Y784_MAGGR	631	103	406	83/316(26.27)	0.48	0.05	316	124	2.00E-31	gene=Chr06G0249	Gene Symbol:PTH11|Host:Digitaria (Poaceae)|Disease:Leaf spot|Description:Unknown
Chr06G0253.1	721	2	375	UniProt ID:Q9HG15_COLLN	746	10	367	103/389(26.48)	0.41	0.12	389	92.4	2.00E-20	gene=Chr06G0253	Gene Symbol:CLTA1|Host:Multiple genera of Fabaceae. Rare reports on other

															taxa|Disease:Leaf, stem and pod anthracnose|Description:SIMILARITY: Contains 1 Zn(2)-C6 fungal-type DNA-binding domain.
Chr06G0254.1	523	37	518	UniProt ID:LAP2_TRIEQ	495	2	485	209/503(41.55)	0.58	0.08	503	362	2.00E-119	gene=Chr06G0254	Gene Symbol:LAP2|Host:humans|Disease:Malabar itch|Description:FUNCTION: Extracellular aminopeptidase that releases a wide variety of amino acids from natural peptides and contributes to pathogenicity.
Chr06G0259.1	711	33	103	UniProt ID:Q59MD2_CANAL	843	763	834	29/72(40.28)	0.57	0.01	72	66.2	4.00E-12	gene=Chr06G0259	Gene Symbol:UME6|Host:Isolated from a wide variety of substrates including humans|Disease:invasive candidal disease|Description:CAUTION: The sequence shown here is derived from an EMBL/GenBank/DDBJ

														whole genome shotgun (WGS) entry which is preliminary data.
Chr06G0263.1	239	29	239	UniProt ID:Q6WP53_BOTFU	223	13	223	157/211(74.41)	0.82	0	211	316	8.00E-110	gene=Chr06G0263	Gene Symbol:BCP1|Host:Various plant families|Disease:Grey mould. Parasite or saprophyte|Description:FUNCTION: PPIases accelerate the folding of proteins (By similarity).
Chr06G0268.1	253	47	197	UniProt ID:A4RGG9_MAGO7	286	67	215	48/157(30.57)	0.5	0.09	157	62	3.00E-12	gene=Chr06G0268	Gene Symbol:MGG_00056|Host:Poaceae, especially important on Oryzae|Disease:Rice blast|Description:SIMILARITY: Belongs to the short-chain dehydrogenases/reductases (SDR) family.
Chr06G0273.1	623	13	618	UniProt ID:Q59RG0_CANAL	581	3	581	126/636(19.81)	0.38	0.14	636	90.5	6.00E-20	gene=Chr06G0273	Gene Symbol:NAG4|Host:Isolated from a wide variety of substrates including

															humans|Disease:invasive candidal disease|Description:CAUTION: The sequence shown here is derived from an EMBL/GenBank/DDBJ whole genome shotgun (WGS) entry which is preliminary data.
Chr06G0274.1	843	87	835	UniProt ID:KATG_PENMA	748	1	739	554/752(73.67)	0.83	0.02	752	1138	0	gene=Chr06G0274	Gene Symbol:KATG|Host:humans|Disease:lethal systemic infection|Description:FUNCTION: Bifunctional enzyme with both catalase and broad- spectrum peroxidase activity (By similarity). May be involved in protection from the host during host infection.
Chr06G0288.1	630	143	630	UniProt ID:Q9P8L8_BOTFU	598	106	592	135/505(26.73)	0.46	0.07	505	164	3.00E-44	gene=Chr06G0288	Gene Symbol:BCMFS1|Host:Various plant families|Disease:Grey mould. Parasite or

														saprophyte|Description:Unknown
Chr06G0291.1	429	1	425	UniProt ID:Q4WUA3_ASPFU	428	1	422	316/426(74.18)	0.83	0.01	426	651	0	gene=Chr06G0291	Gene Symbol:AFUA_5G07750|Host:humans|Disease:infection|Description:FUNCTION: Catalyzes the ferrous insertion into protoporphyrin IX (By similarity).
Chr06G0292.1	657	24	657	UniProt ID:A3KLI8_CLAPU	616	15	616	418/634(65.93)	0.81	0.05	634	840	0	gene=Chr06G0292	Gene Symbol:KU70|Host:outcrossing species|Disease:ergotism|Description:Unknown
Chr06G0293.1	623	37	604	UniProt ID:Q4P8E8_USTMA	693	84	676	213/608(35.03)	0.5	0.09	608	277	6.00E-84	gene=Chr06G0293	Gene Symbol:UM03615.1|Host: Euchlaena spp., Zea spp. (Poaceae)|Disease:Smut. Corn smut|Description:COFACTOR: FAD (By similarity).
Chr06G0306.1	1149	6	1145	UniProt ID:A4R553_MAGO7	1283	99	1279	682/1205(56.60)	0.67	0.07	1205	924	0	gene=Chr06G0306	Gene Symbol:MGG_04116|Host:Poaceae, especially important on

														Oryzae|Disease:Rice blast|Description:SIMILARITY: Contains 1 SH3 domain.
Chr06G0311.1	1197	354	407	UniProt ID:Q5A839_CANAL	454	158	211	23/56(41.07)	0.57	0.07	56	46.6	6.00E-06	gene=Chr06G0311	Gene Symbol:HOS2|Host:Isolated from a wide variety of substrates including humans|Disease:invasive candidal disease|Description:CATALYTIC ACTIVITY: Hydrolysis of an N(6)-acetyl-lysine residue of a histone to yield a deacetylated histone.
Chr06G0313.1	673	28	588	UniProt ID:PLB1_CANAL	605	24	562	190/595(31.93)	0.48	0.15	595	236	3.00E-69	gene=Chr06G0313	Gene Symbol:PLB1|Host:Isolated from a wide variety of substrates including humans|Disease:invasive candidal disease|Description:FUNCTION: Catalyzes the release of fatty acids from lysophospholipids.

															Phospholipase B may well contribute to pathogenicity by abetting the fungus in damaging and traversing host cell membranes, processes which likely increase the rapidity of disseminated infection.
Chr06G0319.1	1047	683	786	UniProt ID:TUP1_CANAL	514	271	373	36/106 (33.96)	0.52	0.05	106	52.8	7.00E-08	gene=Chr06G0319	Gene Symbol:TUP1|Host:Isolated from a wide variety of substrates including humans|Disease:invasive candidal disease|Description:FUNCTION: Represses transcription by RNA polymerase II. Represses genes responsible for initiating filamentous growth and this repression is lifted under inducing environmental conditions.
Chr06G0325.1	1534	53	1512	UniProt ID:NTE1_CAN	1386	27	1362	535/1519(35.22)	0.52	0.16	1519	833	0	gene=Chr06G0325	Gene Symbol:NTE1|Host:Isolated from a wide variety of

				AL										substrates including humans|Disease:invasive candidal disease|Description:FUNCTION: Intracellular phospholipase B that catalyzes the double deacylation of phosphatidylcholine (PC) to glycerophosphocholine (GroPCho). Plays an important role in membrane lipid homeostasis. Responsible for the rapid PC turnover in response to inositol, elevated temperatures, or when choline is present in the growth medium (By similarity).
Chr06G0333.1	851	49	791	UniProt ID:Q874K8_CRYNV	864	25	801	396/777(50.97)	0.7	0.04	777	768	0	gene=Chr06G0333	Gene Symbol:CLC-A|Host:humans|Disease:cryptococcosis|Description:Unknown
Chr06G0336.1	1529	97	291	UniProt ID:O13	415	101	319	57/230 (24.78)	0.4	0.2	230	63.9	3.00E-11	gene=Chr06G0336	Gene Symbol:MPS1|Host:Digita

				352_MAGGR										ria (Poaceae)|Disease:Leaf spot|Description:CATALYTIC ACTIVITY: ATP + a protein = ADP + a phosphoprotein.
Chr06G0339.1	328	30	328	UniProt ID:Q59KY8_CANAL	314	7	314	179/309(57.93)	0.75	0.04	309	382	7.00E-133	gene=Chr06G0339	Gene Symbol:SIT4|Host:Isolated from a wide variety of substrates including humans|Disease:invasive candidal disease|Description:CATALYTIC ACTIVITY: A phosphoprotein + H(2)O = a protein + phosphate.
Chr06G0345.1	900	679	899	UniProt ID:Q5AM49_CANAL	1690	1058	1273	69/225 (30.67)	0.52	0.06	225	98.2	1.00E-21	gene=Chr06G0345	Gene Symbol:SNF2|Host:Isolated from a wide variety of substrates including humans|Disease:invasive candidal disease|Description:Unknown
Chr06G0357.1	770	405	607	UniProt ID:Q5A	1462	66	276	69/218 (31.65)	0.46	0.1	218	104	6.00E-24	gene=Chr06G0357	Gene Symbol:HSL1|Host:Isolate

			G71_CANAL											d from a wide variety of substrates including humans|Disease:invasive candidal disease|Description:CAUTION: The sequence shown here is derived from an EMBL/GenBank/DDBJ whole genome shotgun (WGS) entry which is preliminary data.
Chr06G0359.1	508	412	495	UniProt ID:D1MYV6_MAGGR	568	488	567	39/84(46.43)	0.51	0.05	84	64.3	6.00E-12	gene=Chr06G0359	Gene Symbol:CBL1|Host:Digitaria (Poaceae)|Disease:Leaf spot|Description:SIMILARITY: Contains 3 chitin-binding type-1 domains.
Chr06G0362.1	1492	224	1479	UniProt ID:Q5A762_CANAL	1606	238	1603	365/1424(25.63)	0.45	0.16	1424	416	2.00E-121	gene=Chr06G0362	Gene Symbol:MLT1|Host:Isolated from a wide variety of substrates including humans|Disease:invasive candidal disease|Description:SIMILARITY: Belongs to the

Chr06G0365.1	447	38	354	UniProt ID:A6R119_AJECN	609	227	550	220/325(67.69)	0.75	0.03	325	418	4.00E-141	gene=Chr06G0365	ABC transporter superfamily. Gene Symbol:HCAG_03326|Host:humans|Disease:Darling's disease|Description:Unknown
Chr06G0366.1	1101	646	968	UniProt ID:TUP1_CANAL	514	201	509	132/339(38.94)	0.55	0.14	339	206	7.00E-58	gene=Chr06G0366	Gene Symbol:TUP1|Host:Isolated from a wide variety of substrates including humans|Disease:invasive candidal disease|Description:FUNCTION: Represses transcription by RNA polymerase II. Represses genes responsible for initiating filamentous growth and this repression is lifted under inducing environmental conditions.
Chr06G0367.1	459	5	272	UniProt ID:Q5AMT2_C	308	8	259	77/275(28.00)	0.46	0.11	275	71.6	7.00E-15	gene=Chr06G0367	Gene Symbol:BGL2|Host:Isolated from a wide variety of

				ANAL											substrates including humans|Disease:invasive candidal disease|Description:SIMILARITY: Belongs to the glycosyl hydrolase 17 family.
Chr06G0368.1	602	24	66	UniProt ID:Q5A4F3_CANAL	624	3	46	20/44(45.45)	0.61	0.02	44	50.4	2.00E-07	gene=Chr06G0368	Gene Symbol:ZCF37|Host:Isolated from a wide variety of substrates including humans|Disease:invasive candidal disease|Description:Unknown
Chr06G0369.1	1300	31	1300	UniProt ID:Q3Y5V5_MAGGR	1321	22	1321	583/1329(43.87)	0.64	0.07	1329	1103	0	gene=Chr06G0369	Gene Symbol:ABC3|Host:Digitaria (Poaceae)|Disease:Leaf spot|Description:SIMILARITY: Belongs to the ABC transporter superfamily.
Chr06G0378.1	512	33	244	UniProt ID:Q9Y784_MAGGR	631	125	325	45/222(20.27)	0.45	0.14	222	47.8	1.00E-06	gene=Chr06G0378	Gene Symbol:PTH11|Host:Digitaria (Poaceae)|Disease:Leaf spot|Description:Unknown

Chr06G0387.1	732	224	732	UniProt ID:Q6B971_CRYPA	510	1	510	405/511(79.26)	0.89	0.01	511	845	0	gene=Chr06G0387	Gene Symbol:CPRGS-1|Host:Castanea spp., Fagus sylvatica, Quercus spp. (Fagaceae)|Disease:Chestnut blight. Cankers|Description:SIMILARITY: Contains 1 RGS domain.
Chr06G0389.1	901	380	433	UniProt ID:Q9HFW4_USTMD	2289	534	587	20/56(35.71)	0.61	0.07	56	56.6	6.00E-09	gene=Chr06G0389	Gene Symbol:RUM1|Host:Euchlaena spp., Zea spp. (Poaceae)|Disease:Smut. Corn smut|Description:SIMILARITY: Contains 1 ARID domain.
Chr06G0396.1	443	5	368	UniProt ID:Q5GFD3_PHAND	437	9	372	95/381(24.93)	0.41	0.09	381	89.7	2.00E-20	gene=Chr06G0396	Gene Symbol:NULL|Host:Multiple genera of Poaceae and Blysmus compressus (Cyperaceae)|Disease:Glume blotch of wheat and other grasses|Description:Unknown

Chr06G0407.1	1001	66	546	UniProt ID:Q9HG15_COLLN	746	16	403	123/488(25.20)	0.39	0.22	488	102	4.00E-23	gene=Chr06G0407	Gene Symbol:CLTA1|Host:Multiple genera of Fabaceae. Rare reports on other taxa|Disease:Leaf, stem and pod anthracnose|Description:SIMILARITY: Contains 1 Zn(2)-C6 fungal-type DNA-binding domain.
Chr06G0417.1	346	2	342	UniProt ID:A4RKI4_MAGO7	323	3	322	242/345(70.14)	0.8	0.08	345	494	1.00E-176	gene=Chr06G0417	Gene Symbol:MGG_01481|Host:Poaceae, especially important on Oryzae|Disease:Rice blast|Description:Unknown
Chr06G0418.1	655	98	478	UniProt ID:C4YII6_CANAW	768	201	593	132/411(32.12)	0.48	0.12	411	142	2.00E-36	gene=Chr06G0418	Gene Symbol:CAWG_04261|Host:Isolated from a wide variety of substrates including humans|Disease:invasive candidal disease|Description:SIMILARITY: Belongs to the

Chr06G0424.1	708	81	117	UniProtID:Q5A4F3_CANAL	624	10	46	21/37(56.76)	0.73	0	37	61.2	1.00E-10	gene=Chr06G0424	DEAD box helicase family. Gene Symbol:ZCF37|Host:Isolated from a wide variety of substrates including humans|Disease:invasive candidal disease|Description:Unknown
Chr06G0432.1	321	7	305	UniProtID:Q9Y7E5_CANAL	328	4	312	162/311(52.09)	0.66	0.05	311	294	3.00E-98	gene=Chr06G0432	Gene Symbol:SPT3|Host:Isolated from a wide variety of substrates including humans|Disease:invasive candidal disease|Description:Unknown
Chr06G0434.1	143	34	128	UniProtID:Q59QC5_CANAL	159	18	118	37/102(36.27)	0.59	0.08	102	70.1	2.00E-16	gene=Chr06G0434	Gene Symbol:APS3|Host:Isolated from a wide variety of substrates including humans|Disease:invasive candidal disease|Description:CAUTION: The sequence shown here is derived from an

														EMBL/GenBank/DDBJ whole genome shotgun (WGS) entry which is preliminary data.
Chr06G0439.1	929	4	928	UniProt ID:P87199_USTMD	968	2	968	530/988(53.64)	0.69	0.09	988	966	0	gene=Chr06G0439	Gene Symbol:KIN2|Host:Euchlaena spp., Zea spp. (Poaceae)|Disease:Smut. Corn smut|Description:SIMILARITY: Belongs to the kinesin-like protein family.
Chr06G0441.1	428	3	428	UniProt ID:C0SHF9_PARBP	421	5	420	275/436(63.07)	0.76	0.07	436	541	0	gene=Chr06G0441	Gene Symbol:PABG_07114|Host:humans|Disease:Paracoccidioidomycosis|Description:SIMILARITY: Belongs to the thiolase family.
Chr06G0443.1	468	91	248	UniProt ID:Q59NP5_CANAL	418	202	364	62/167(37.13)	0.52	0.08	167	89	4.00E-20	gene=Chr06G0443	Gene Symbol:SUN41|Host:Isolated from a wide variety of substrates including humans|Disease:invasive candidal disease|Description:CAUTION: The sequence shown

														here is derived from an EMBL/GenBank/DDBJ whole genome shotgun (WGS) entry which is preliminary data.
Chr06G0448.1	595	286	342	UniProt ID:Q09RL3_CRYNE	963	302	358	41/57(71.93)	0.84	0	57	99.4	1.00E-22	gene=Chr06G0448	Gene Symbol:CIR1|Host:humans|Disease:cryptococcosis|Description:Unknown
Chr06G0454.1	373	9	310	UniProt ID:Q59W09_CANAL	379	3	315	136/327(41.59)	0.57	0.12	327	254	2.00E-81	gene=Chr06G0454	Gene Symbol:GCS1|Host:Isolated from a wide variety of substrates including humans|Disease:invasive candidal disease|Description:CAUTION: The sequence shown here is derived from an EMBL/GenBank/DDBJ whole genome shotgun (WGS) entry which is preliminary data.
Chr06G0455.1	378	49	302	UniProt ID:F2QU09_PICP7	626	54	305	69/274(25.18)	0.42	0.15	274	64.7	3.00E-12	gene=Chr06G0455	Gene Symbol:PAB1|Host:humans|Disease:occasional infection|Description:Unkn

															own
Chr06G0457.1	861	21	861	UniProt ID:A4RJR0_MAGO7	938	23	935	352/934(37.69)	0.52	0.12	934	521	5.00E-171	gene=Chr06G0457	Gene Symbol:MGG_01748|Host:Poaceae, especially important on Oryzae|Disease:Rice blast|Description:Unknown
Chr06G0469.1	397	2	299	UniProt ID:Q6A2T2_BOTFU	391	42	354	98/317 (30.91)	0.48	0.07	317	129	2.00E-34	gene=Chr06G0469	Gene Symbol:BTP1|Host:Various plant families|Disease:Grey mould. Parasite or saprophyte|Description:Unknown
Chr06G0470.1	265	5	255	UniProt ID:Q75WR5_9PLEO	265	7	262	83/267 (31.09)	0.48	0.1	267	105	2.00E-27	gene=Chr06G0470	Gene Symbol:BRN1|Host:Belamcanda chinensis: Korea,Gladiolus ?gandavensis: Korea,Iris japonica: China,Iris missouriensis (Leaf spot.): Idaho; Montana; Oregon; Washington,Iris sp. (Leaf spot.): China; Texas; Washing|Disease:Leaf

															spot|Description:SIMILARITY: Belongs to the short-chain dehydrogenases/reductases (SDR) family.
Chr06G0473.1	378	27	327	UniProt ID:Q7Z8E8_CANDU	320	1	314	95/319 (29.78)	0.5	0.07	319	134	1.00E-36	gene=Chr06G0473	Gene Symbol:CSH1|Host:humans|Disease:leptomeningeal disease,occasional invasive candidal disease|Description:Unknown
Chr06G0478.1	499	5	499	UniProt ID:Q4JEX9_TRIRU	489	22	488	269/495(54.34)	0.68	0.06	495	525	0	gene=Chr06G0478	Gene Symbol:NULL|Host:humans|Disease:infection|Description:Unknown
Chr06G0480.1	755	448	694	UniProt ID:Q0PND8_MAGGR	1375	965	1217	103/255(40.39)	0.6	0.04	255	177	7.00E-47	gene=Chr06G0480	Gene Symbol:PEX6|Host:Digitaria (Poaceae)|Disease:Leaf spot|Description:SIMILARITY: Belongs to the AAA ATPase family.
Chr06G0482.1	202	1	202	UniProt ID:Q96VL3_COLLN	202	1	202	198/202(98.02)	0.99	0	202	409	3.00E-147	gene=Chr06G0482	Gene Symbol:PT1|Host:Multiple genera of Fabaceae. Rare reports on other

															taxa|Disease:Leaf, stem and pod anthracnose|Description:SIMILARITY: Belongs to the small GTPase superfamily. Rab family.
Chr06G0488.1	328	156	229	UniProt ID:Q4PCH2_USTMA	758	125	206	28/82(34.15)	0.59	0.1	82	53.9	6.00E-09	gene=Chr06G0488	Gene Symbol:YAP1|Host:Euchlaena spp., Zea spp. (Poaceae)|Disease:Smut. Corn smut|Description:SIMILARITY: Belongs to the bZIP family.
Chr06G0489.1	753	107	273	UniProt ID:Q5A415_CANAL	361	32	195	45/170 (26.47)	0.46	0.05	170	70.9	5.00E-14	gene=Chr06G0489	Gene Symbol:ARP2|Host:Isolated from a wide variety of substrates including humans|Disease:invasive candidal disease|Description:SIMILARITY: Belongs to the actin family.
Chr06G0496.1	317	7	165	UniProt ID:Q59ZB7_C	217	12	169	56/163 (34.36)	0.56	0.06	163	107	3.00E-28	gene=Chr06G0496	Gene Symbol:YPT7|Host:Isolated from a wide variety of

				ANAL											substrates including humans|Disease:invasive candidal disease|Description:SIMILARITY: Belongs to the small GTPase superfamily. Rab family.
Chr06G0497.1	712	279	710	UniProt ID:Q7Z868_USTMD	862	88	618	120/553(21.70)	0.36	0.26	553	61.2	1.00E-10	gene=Chr06G0497	Gene Symbol:GLO1|Host:Euchlaena spp., Zea spp. (Poaceae)|Disease:Smut. Corn smut|Description:Unknown
Chr06G0498.1	497	6	55	UniProt ID:A5HEH7_9PEZI	479	361	410	21/50(42.00)	0.6	0	50	46.6	2.00E-06	gene=Chr06G0498	Gene Symbol:OLE1|Host:Multiple genera in multiple families|Disease:Cankers, dieback, and several other diseases. Also, a human pathogen|Description:COFACTOR: Iron (By similarity).
Chr06G0506.1	1028	78	150	UniProt ID:A6ZUW0_Y	504	15	91	29/77(37.66)	0.52	0.05	77	56.2	6.00E-09	gene=Chr06G0506	Gene Symbol:MIG1|Host:humans|Disease:occasional

				EAS7											infection|Description:CAUTION: The sequence shown here is derived from an EMBL/GenBank/DDBJ whole genome shotgun (WGS) entry which is preliminary data.
Chr06G0511.1	361	1	361	UniProt ID:HOG1_CRYPA	358	1	358	336/361(93.07)	0.96	0.01	361	707	0	gene=Chr06G0511	Gene Symbol:HOG1|Host:Castanea spp., Fagus sylvatica, Quercus spp. (Fagaceae)|Disease:Chestnut blight. Cankers|Description:FUNCTION: Mitogen-activated protein kinase involved in a signal transduction pathway that is activated by changes in the osmolarity of the extracellular environment. Controls osmotic regulation of transcription of target genes (By similarity). Involved in the

															virulence and conidia formation. Mediates tannic acid-induced laccase expression and cryparin expression.
Chr06G0513.1	355	1	355	UniProt ID:O13314_MAGGR	356	2	356	322/355(90.70)	0.96	0	355	684	0	gene=Chr06G0513	Gene Symbol:MAGA|Host:Digitaria (Poaceae)|Disease:Leaf spot|Description:Unknown
Chr06G0516.1	1110	551	678	UniProt ID:Q0PND8_MAGGR	1375	982	1109	40/137 (29.20)	0.46	0.13	137	46.6	7.00E-06	gene=Chr06G0516	Gene Symbol:PEX6|Host:Digitaria (Poaceae)|Disease:Leaf spot|Description:SIMILARITY: Belongs to the AAA ATPase family.
Chr06G0517.1	978	347	621	UniProt ID:Q9Y880_COCCA	880	90	359	74/300 (24.67)	0.39	0.18	300	58.2	2.00E-09	gene=Chr06G0517	Gene Symbol:SNF1|Host:Corn, Zea mays, sometimes on Sorghum (Poaceae) and various other plant families|Disease:Northern corn leaf spot, ear and kernel rot|Description:Unknown
Chr06G0	477	36	477	UniProt	473	32	473	341/44	0.89	0	442	743	0	gene=Chr	Gene

518.1				ID:C5GNL4_AJEDR				2(77.15)						06G0518	Symbol:BDCG_06371|Host:humans|Disease:cutaneous Blastomyces dermatitidis infection|Description:COFACTOR: Iron (By similarity).
Chr06G0519.1	1096	940	1096	UniProt ID:Q5AM49_CANAL	1690	1119	1274	58/161 (36.02)	0.53	0.06	161	90.1	4.00E-19	gene=Chr06G0519	Gene Symbol:SNF2|Host:Isolated from a wide variety of substrates including humans|Disease:invasive candidal disease|Description:Unknown
Chr06G0522.1	309	22	307	UniProt ID:Q6TFC7_ASPFM	349	65	346	81/290 (27.93)	0.47	0.04	290	123	4.00E-33	gene=Chr06G0522	Gene Symbol:NULL|Host:humans|Disease:infection|Description:Unknown
Chr06G0526.1	1011	31	71	UniProt ID:Q5A4F3_CANAL	624	3	46	25/44(56.82)	0.7	0.07	44	62.8	5.00E-11	gene=Chr06G0526	Gene Symbol:ZCF37|Host:Isolated from a wide variety of substrates including humans|Disease:invasive candidal disease|Description:Unkn

															own
Chr06G0530.1	234	2	234	UniProt ID:O74686_AJECA	244	8	244	129/237(54.43)	0.73	0.02	237	261	5.00E-88	gene=Chr06G0530	Gene Symbol:URA5|Host:humans|Disease:Darling's disease|Description:Unknown
Chr06G0532.1	464	1	464	UniProt ID:C5GWH6_AJEDR	468	1	468	342/468(73.08)	0.86	0.01	468	733	0	gene=Chr06G0532	Gene Symbol:BDCG_08802|Host:humans|Disease:cutaneous Blastomyces dermatitidis infection|Description:Unknown
Chr06G0535.1	1003	221	280	UniProt ID:C5J0G7_MYCGR	726	596	649	27/60(45.00)	0.58	0.1	60	59.3	8.00E-10	gene=Chr06G0535	Gene Symbol:NULL|Host:Triticum and possibly a few other grasses|Disease:Leaf spot or speckled leaf blotch of wheat|Description:Unknown
Chr06G0541.1	363	21	246	UniProt ID:A4QVF8_MAGO7	339	19	247	55/241(22.82)	0.41	0.11	241	43.5	8.00E-06	gene=Chr06G0541	Gene Symbol:MGG_04556|Host:Poaceae, especially important on Oryzae|Disease:Rice blast|Description:COFACT

Chr06G0547.1	359	37	342	UniProt ID:Q4X132_ASPFU	358	1	306	248/306(81.05)	0.92	0	306	530	0	gene=Chr06G0547	OR: Zinc (By similarity). Gene Symbol:AFUA_2G11380|Host:humans|Disease:infection|Description:CAUTION: The sequence shown here is derived from an EMBL/GenBank/DDBJ whole genome shotgun (WGS) entry which is preliminary data.
Chr06G0548.1	397	110	162	UniProt ID:Q8J1X6_COLLN	971	695	747	40/53(75.47)	0.79	0	53	94	1.00E-21	gene=Chr06G0548	Gene Symbol:CLNR1|Host:Multiple genera of Fabaceae. Rare reports on other taxa|Disease:Leaf, stem and pod anthracnose|Description:Unknown
Chr06G0563.1	533	325	512	UniProt ID:A4ULI5_MYCGR	515	298	512	60/223(26.91)	0.42	0.19	223	72.8	1.00E-14	gene=Chr06G0563	Gene Symbol:CYP51|Host:Triticum and possibly a few other grasses|Disease:Leaf spot or speckled leaf blotch of wheat|Description:COFAC

															TOR: Heme group (By similarity).
Chr06G0567.1	162	18	150	UniProt ID:Q9UW16_MAGGR	181	34	168	35/141 (24.82)	0.5	0.1	141	45.8	1.00E-07	gene=Chr06G0567	Gene Symbol:VATP|Host:Digitaria (Poaceae)|Disease:Leaf spot|Description:Unknown
Chr06G0572.1	727	180	476	UniProt ID:CHS3_CANAL	1213	840	1143	75/311 (24.12)	0.41	0.07	311	84	1.00E-17	gene=Chr06G0572	Gene Symbol:CHS3|Host:Isolated from a wide variety of substrates including humans|Disease:invasive candidal disease|Description:FUNCTION: Formation and repair of the disk-shaped septum in yeast and the cross walls of the hyphal phase.
Chr06G0577.1	1132	352	507	UniProt ID:MAK5_COCIM	783	188	360	42/175 (24.00)	0.48	0.12	175	48.9	1.00E-06	gene=Chr06G0577	Gene Symbol:MAK5|Host:humans|Disease:coccidioidomycosis|Description:FUNCTION: ATP-binding RNA helicase involved in the biogenesis of 60S ribosomal subunits and is

															required for the normal formation of 25S and 5.8S rRNAs (By similarity).
Chr06G0581.1	491	106	273	UniProt ID:TUP1_CANAL	514	270	424	48/171 (28.07)	0.42	0.11	171	47	2.00E-06	gene=Chr06G0581	Gene Symbol:TUP1|Host:Isolated from a wide variety of substrates including humans|Disease:invasive candidal disease|Description:FUNCTION: Represses transcription by RNA polymerase II. Represses genes responsible for initiating filamentous growth and this repression is lifted under inducing environmental conditions.
Chr06G0587.1	998	106	992	UniProt ID:A6N6J8_FUSOX	903	44	900	702/893(78.61)	0.86	0.05	893	1413	0	gene=Chr06G0587	Gene Symbol:CTF1|Host:Multiple genera in multiple families|Disease:Blights, wilts, rots of various sorts|Description:SIMILARITY: Contains 1 Zn(2)-C6 fungal-type DNA-binding

															domain.
Chr06G0591.1	425	18	423	UniProt ID:Q5EMY3_MAGGR	424	1	416	296/417(70.98)	0.83	0.03	417	594	0	gene=Chr06G0591	Gene Symbol:NULL|Host:Digitaria (Poaceae)|Disease:Leaf spot|Description:SIMILARITY: Belongs to the AAA ATPase family.
Chr06G0596.1	849	770	827	UniProt ID:A4R7D1_MAGO7	778	709	766	21/58(36.21)	0.57	0	58	49.3	7.00E-07	gene=Chr06G0596	Gene Symbol:MGG_03284|Host:Poaceae, especially important on Oryzae|Disease:Rice blast|Description:Unknown
Chr06G0600.1	691	336	448	UniProt ID:Q59WH0_CANAL	445	8	115	36/116(31.03)	0.47	0.09	116	55.5	6.00E-09	gene=Chr06G0600	Gene Symbol:ADA2|Host:Isolated from a wide variety of substrates including humans|Disease:invasive candidal disease|Description:CAUTION: The sequence shown here is derived from an EMBL/GenBank/DDBJ whole genome shotgun (WGS) entry which is

														preliminary data.
Chr06G0601.1	641	43	641	UniProt ID:O42620_MAGGR	614	12	614	480/603(79.60)	0.9	0.01	603	991	0	gene=Chr06G0601 Gene Symbol:PTH2|Host:Digitaria (Poaceae)|Disease:Leaf spot|Description:SIMILARITY: Belongs to the carnitine/choline acetyltransferase family.
Chr06G0602.1	528	7	528	UniProt ID:Q5A6P9_CANAL	545	13	545	198/550(36.00)	0.55	0.08	550	294	8.00E-93	gene=Chr06G0602 Gene Symbol:SRV2|Host:Isolated from a wide variety of substrates including humans|Disease:invasive candidal disease|Description:SIMILARITY: Belongs to the CAP family.
Chr06G0609.1	829	616	826	UniProt ID:D1MYV6_MAGGR	568	142	355	60/226(26.55)	0.46	0.12	226	80.1	2.00E-16	gene=Chr06G0609 Gene Symbol:CBL1|Host:Digitaria (Poaceae)|Disease:Leaf spot|Description:SIMILARITY: Contains 3 chitin-binding type-1 domains.
Chr06G0617.1	186	1	186	UniProt ID:Q8J	187	1	187	134/187(71.6	0.88	0.01	187	287	6.00E-100	gene=Chr06G0617 Gene Symbol:RHEB|Host:huma

			2P6_ASPFM				6)							ns|Disease:infection|Description:Unknown
Chr06G0621.1	1583	147	719	UniProt ID:Q8TFN4_COLGR	1866	84	729	153/670(22.84)	0.43	0.18	670	140	2.00E-34	gene=Chr06G0621	Gene Symbol:CHSV|Host:Poaceae especially Zea mays|Disease:Leaf spot, stalk rot, etc|Description:Unknown
Chr06G0624.1	1602	369	1132	UniProt ID:Q9P872_CANAL	917	103	751	167/792(21.09)	0.37	0.22	792	91.3	2.00E-19	gene=Chr06G0624	Gene Symbol:PMR1|Host:Isolated from a wide variety of substrates including humans|Disease:invasive candidal disease|Description:SIMILARITY: Belongs to the cation transport ATPase (P-type) family.
Chr06G0636.1	490	1	357	UniProt ID:Q5A5C1_CANAL	344	1	315	162/358(45.25)	0.63	0.12	358	311	1.00E-102	gene=Chr06G0636	Gene Symbol:YNL191|Host:Isolated from a wide variety of substrates including humans|Disease:invasive candidal disease|Description:Unknown

Chr06G0646.1	684	13	683	UniProt ID:A4RHH9_MAGO7	667	17	667	256/695(36.83)	0.55	0.1	695	394	8.00E-128	gene=Chr06G0646	Gene Symbol:MGG_07061|Host:Poaceae, especially important on Oryzae|Disease:Rice blast|Description:Unknown
Chr06G0650.1	444	8	404	UniProt ID:Q01446_NECHA	459	19	394	98/402(24.38)	0.41	0.08	402	94	9.00E-22	gene=Chr06G0650	Gene Symbol:MAK1|Host:Trees of various plant families|Disease:Fruit rot, stem rot|Description:Unknown
Chr06G0651.1	426	29	273	UniProt ID:Q9Y784_MAGGR	631	126	366	60/245(24.49)	0.49	0.02	245	98.6	3.00E-23	gene=Chr06G0651	Gene Symbol:PTH11|Host:Digitaria (Poaceae)|Disease:Leaf spot|Description:Unknown
Chr06G0653.1	444	1	357	UniProt ID:A4UC81_MAGO7	376	17	371	130/363(35.81)	0.53	0.04	363	204	2.00E-61	gene=Chr06G0653	Gene Symbol:MGG_10702|Host:Poaceae, especially important on Oryzae|Disease:Rice blast|Description:Unknown
Chr06G0	651	354	610	UniProt	604	322	578	118/25	0.7	0.01	258	256	9.00E-77	gene=Chr	Gene

655.1				ID:Q6X269_USTMD				8(45.74)						06G0655	Symbol:CLB2|Host:Euchlaena spp., Zea spp. (Poaceae)|Disease:Smut. Corn smut|Description:SIMILARITY: Belongs to the cyclin family.
Chr06G0663.1	1104	359	983	UniProt ID:A4RB72_MAGO7	1715	935	1572	189/660(28.64)	0.47	0.09	660	244	4.00E-67	gene=Chr06G0663	Gene Symbol:MGG_02986|Host:Poaceae, especially important on Oryzae|Disease:Rice blast|Description:CATALYTIC ACTIVITY: Deoxynucleoside triphosphate + DNA(n) = diphosphate + DNA(n+1).
Chr06G0668.1	534	30	367	UniProt ID:BUR1_CANAL	746	39	379	176/344(51.16)	0.67	0.03	344	352	7.00E-113	gene=Chr06G0668	Gene Symbol:CRK1|Host:Isolated from a wide variety of substrates including humans|Disease:invasive candidal disease|Description:FUNCTION: Serine/threonine-protein

															kinase involved in transcription regulation. Phosphorylates the UBC2/RAD6 ubiquitin-conjugating enzyme (E2), leading to monoubiquitination of histone H2B and the silencing of telomeric-associated genes. Also required for histone H3 methylation. Necessary for the recovery from pheromone-induced growth arrest in the cell cycle G1 phase (By similarity). Required for pseudohyphal growth and virulence in mice.
Chr06G0673.1	174	1	172	UniProtID:CANB_CRYNJ	175	1	172	122/172(70.93)	0.86	0	172	251	4.00E-86	gene=Chr06G0673	Gene Symbol:CNB1|Host:humans|Disease:cryptococcosis|Description:FUNCTION: Regulatory subunit of calcineurin, a

Chr06G0681.1	478	172	461	UniProt ID:Q5ABP4_CANAL	609	95	389	107/304(35.20)	0.53	0.08	304	186	9.00E-53	gene=Chr06G0681	calcium-dependent, calmodulin stimulated protein phosphatase. Confers calcium sensitivity. Plays a central role in virulence and antifungal drug action. Gene Symbol:CMP1|Host:Isolated from a wide variety of substrates including humans|Disease:invasive candidal disease|Description:CATALYTIC ACTIVITY: A phosphoprotein + H(2)O = a protein + phosphate.
Chr06G0686.1	803	219	273	UniProt ID:Q5AMQ6_CANAL	1111	466	534	21/69(30.43)	0.52	0.2	69	47.8	2.00E-06	gene=Chr06G0686	Gene Symbol:CAO19.12059, CAO19.4590, ORF19.4590|Host:Isolated from a wide variety of substrates including humans|Disease:invasive candidal disease|Description:CAUT

														ION: The sequence shown here is derived from an EMBL/GenBank/DDBJ whole genome shotgun (WGS) entry which is preliminary data.
Chr06G0693.1	516	12	512	UniProt ID:Q5ANE1_CANAL	748	32	541	133/527(25.24)	0.46	0.08	527	158	2.00E-42	gene=Chr06G0693	Gene Symbol:SNF3|Host:Isolated from a wide variety of substrates including humans|Disease:invasive candidal disease|Description:SIMILARITY: Belongs to the major facilitator superfamily. Sugar transporter (TC 2.A.1.1) family.
Chr06G0695.1	1451	342	532	UniProt ID:Q5AM49_CANAL	1690	801	955	47/199(23.62)	0.45	0.26	199	60.5	6.00E-10	gene=Chr06G0695	Gene Symbol:SNF2|Host:Isolated from a wide variety of substrates including humans|Disease:invasive candidal disease|Description:Unknown

Chr06G0696.1	404	61	357	UniProt ID:A3LXZ6_PICST	310	3	295	118/297(39.73)	0.59	0.01	297	225	7.00E-71	gene=Chr06G0696	Gene Symbol:CDC28|Host:humans|Disease:occasional infection|Description:Unknown
Chr06G0706.1	856	470	672	UniProt ID:F2QQX0_PICP7	3007	2807	3003	48/207(23.19)	0.42	0.07	207	46.6	6.00E-06	gene=Chr06G0706	Gene Symbol:CHS1|Host:humans|Disease:occasional infection|Description:SIMILARITY: Contains 1 PH domain.
Chr06G0709.1	207	37	196	UniProt ID:Q9P8W9_CRYNE	162	4	162	107/160(66.88)	0.76	0.01	160	212	3.00E-70	gene=Chr06G0709	Gene Symbol:CPA1|Host:humans|Disease:cryptococcosis|Description:FUNCTION: PPIases accelerate the folding of proteins (By similarity).
Chr06G0715.1	1258	941	1258	UniProt ID:SET1_CANAL	1040	736	1040	152/321(47.35)	0.63	0.06	321	283	2.00E-80	gene=Chr06G0715	Gene Symbol:SET1|Host:Isolated from a wide variety of substrates including humans|Disease:invasive candidal disease|Description:FUNCTION: Catalytic

															component of the COMPASS (Set1C) complex that specifically mono-, di- and trimethylates histone H3 to form H3K4me1/2/3, which subsequently plays a role in telomere length maintenance, transcription elongation regulation and pathogenesis of invasive candidiasis.
Chr06G0728.1	205	2	205	UniProt ID:Q59ZB7_CANAL	217	4	217	129/214(60.28)	0.76	0.05	214	262	2.00E-89	gene=Chr06G0728	Gene Symbol:YPT7|Host:Isolated from a wide variety of substrates including humans|Disease:invasive candidal disease|Description:SIMILARITY: Belongs to the small GTPase superfamily. Rab family.
Chr06G0732.1	621	120	428	UniProt ID:P87199_USTMD	968	52	340	133/315(42.22)	0.58	0.1	315	228	4.00E-65	gene=Chr06G0732	Gene Symbol:KIN2|Host:Euchlaena spp., Zea spp. (Poaceae)|Disease:Smut.

Chr06G0737.1	447	4	446	UniProt ID:C1G452_PARBD	450	8	450	322/443(72.69)	0.87	0	443	693	0	gene=Chr06G0737	Corn smut|Description:SIMILARITY: Belongs to the kinesin-like protein family. Gene Symbol:PADG_01718|Host:humans|Disease:Paracoccidioidomycosis|Description:Unknown
Chr06G0751.1	337	22	281	UniProt ID:Q6A2T2_BOTFU	391	22	296	84/282(29.79)	0.49	0.1	282	111	2.00E-28	gene=Chr06G0751	Gene Symbol:BTP1|Host:Various plant families|Disease:Grey mould. Parasite or saprophyte|Description:Unknown
Chr06G0764.1	461	1	427	UniProt ID:ORYZ_ASPFU	403	1	401	187/437(42.79)	0.56	0.11	437	307	2.00E-100	gene=Chr06G0764	Gene Symbol:ALP1|Host:humans|Disease:infection|Description:FUNCTION: Secreted alkaline protease that allows assimilation of proteinaceous substrates. Acts as a significant virulence factor in invasive

															aspergillosis. Involved in immune evasion from the human and mice complement systems during infection. Efficiently cleaves important components of the complement cascade such as such as C3, C4, C5, and C1q, as well as IgG, which leads to down-regulation of complement activation at the hyphal surface.
Chr06G0765.1	711	51	125	UniProt ID:A0ST46_CERNC	397	19	94	23/76(30.26)	0.53	0.01	76	46.2	4.00E-06	gene=Chr06G0765	Gene Symbol:CTB8|Host:Numerous taxa in Solanaceae|Disease:Leaf spot|Description:Unknown
Chr06G0768.1	894	288	555	UniProt ID:TUP1_CANAL	514	192	479	83/296(28.04)	0.49	0.12	296	109	6.00E-26	gene=Chr06G0768	Gene Symbol:TUP1|Host:Isolated from a wide variety of substrates including humans|Disease:invasive candidal disease|Description:FUNC

															TION: Represses transcription by RNA polymerase II. Represses genes responsible for initiating filamentous growth and this repression is lifted under inducing environmental conditions.
Chr06G0769.1	386	3	311	UniProt ID:CDC10_CANAL	357	6	316	113/314(35.99)	0.58	0.03	314	220	2.00E-68	gene=Chr06G0769	Gene Symbol:CDC10|Host:Isolated from a wide variety of substrates including humans|Disease:invasive candidal disease|Description:FUNCTION: Plays a role in the cell cycle. Involved in the formation of the ring of filaments in the neck region at the mother-bud junction during mitosis.
Chr06G0775.1	659	100	642	UniProt ID:Q2VLJ1_GIBZA	565	27	556	210/551(38.11)	0.55	0.05	551	349	3.00E-112	gene=Chr06G0775	Gene Symbol:ZEB1|Host:Principal hosts: Poaceae, including Zea mays (corn), Triticum aestivum (wheat),

															and Oryza sativa (rice). Additional hosts: various plant families|Disease:Seedling blight, pre- and post-emergence blight, root and foot rot, brown rot, culm decay, head or kernel blight (scab or ear scab) of cereals. Leaf|Description:Unknown
Chr06G0776.1	450	19	335	UniProt ID:Q6A2T2_BOTFU	391	9	329	95/336 (28.27)	0.43	0.1	336	97.4	3.00E-23	gene=Chr06G0776	Gene Symbol:BTP1|Host:Various plant families|Disease:Grey mould. Parasite or saprophyte|Description:Unknown
Chr06G0780.1	686	412	521	UniProt ID:Q8TGW9_CANAL	1085	347	460	26/114 (22.81)	0.53	0.04	114	47.8	2.00E-06	gene=Chr06G0780	Gene Symbol:SSN6|Host:Isolated from a wide variety of substrates including humans|Disease:invasive candidal disease|Description:Unknown

Chr06G0789.1	898	61	746	UniProt ID:A6N6J8_FUSOX	903	45	674	146/715(20.42)	0.37	0.16	715	69.3	5.00E-13	gene=Chr06G0789	Gene Symbol:CTF1|Host:Multiple genera in multiple families|Disease:Blights, wilts, rots of various sorts|Description:SIMILARITY: Contains 1 Zn(2)-C6 fungal-type DNA-binding domain.
Chr06G0790.1	911	417	665	UniProt ID:Q5AG40_CANAL	439	132	371	95/255(37.25)	0.56	0.08	255	139	6.00E-36	gene=Chr06G0790	Gene Symbol:VPS4|Host:Isolated from a wide variety of substrates including humans|Disease:invasive candidal disease|Description:SIMILARITY: Belongs to the AAA ATPase family.
Chr06G0801.1	547	1	530	UniProt ID:Q9P8L8_BOTFU	598	37	578	147/552(26.63)	0.45	0.06	552	169	3.00E-46	gene=Chr06G0801	Gene Symbol:BCMFS1|Host:Various plant families|Disease:Grey mould. Parasite or saprophyte|Description:Unknown
Chr06G0	1610	770	955	UniProt	1178	873	1036	63/190	0.49	0.16	190	81.3	3.00E-16	gene=Chr	Gene

816.1				ID:Q5AP97_CANAL				(33.16)						06G0816	Symbol:SWE1|Host:Isolated from a wide variety of substrates including humans|Disease:invasive candidal disease|Description:SIMILARITY: Belongs to the protein kinase superfamily.
Chr06G0818.1	229	9	228	UniProt ID:Q59LL5_CANAL	222	3	222	148/227(65.20)	0.78	0.06	227	298	9.00E-103	gene=Chr06G0818	Gene Symbol:HIS3|Host:Isolated from a wide variety of substrates including humans|Disease:invasive candidal disease|Description:CATALYTIC ACTIVITY: D-erythro-1-(imidazol-4-yl)glycerol 3- phosphate = 3-(imidazol-4-yl)-2-oxopropyl phosphate + H(2)O.
Chr06G0837.1	460	16	460	UniProt ID:ARGJ_AJECN	471	24	471	270/457(59.08)	0.73	0.05	457	524	0	gene=Chr06G0837	Gene Symbol:HCAG_02997|Host:humans|Disease:Darling's disease|Description:FUNC

														TION: Catalyzes two activities which are involved in the cyclic version of arginine biosynthesis: the synthesis of acetylglutamate from glutamate and acetyl-CoA, and of ornithine by transacetylation between acetylornithine and glutamate (By similarity).
Chr06G0842.1	368	8	205	UniProt ID:Q59Z39_CANAL	259	12	234	89/223 (39.91)	0.64	0.11	223	202	3.00E-63	gene=Chr06G0842	Gene Symbol:SAP49|Host:Isolated from a wide variety of substrates including humans|Disease:invasive candidal disease|Description:CAUTION: The sequence shown here is derived from an EMBL/GenBank/DDBJ whole genome shotgun (WGS) entry which is preliminary data.
Chr06G0	488	226	342	UniProt	570	434	547	49/119	0.53	0.06	119	82	1.00E-17	gene=Chr	Gene

845.1				ID:A3QX02_ASPFM				(41.18)						06G0845	Symbol:ZAFA|Host:humans|Disease:infection|Description:Unknown
Chr06G0847.1	572	126	534	UniProt ID:A5H456_MYCGR	1811	1	399	121/450(26.89)	0.42	0.2	450	127	2.00E-31	gene=Chr06G0847	Gene Symbol:NULL|Host:Triticum and possibly a few other grasses|Disease:Leaf spot or speckled leaf blotch of wheat|Description:Unknown
Chr06G0848.1	499	280	461	UniProt ID:A4ULI5_MYCGR	515	298	503	48/207(23.19)	0.43	0.13	207	60.1	1.00E-10	gene=Chr06G0848	Gene Symbol:CYP51|Host:Triticum and possibly a few other grasses|Disease:Leaf spot or speckled leaf blotch of wheat|Description:COFACTOR: Heme group (By similarity).
Chr06G0849.1	513	62	479	UniProt ID:Q59RG0_CANAL	581	130	550	106/432(24.54)	0.44	0.06	432	109	2.00E-26	gene=Chr06G0849	Gene Symbol:NAG4|Host:Isolated from a wide variety of substrates including humans|Disease:invasive candidal disease|Description:CAUT

															ION: The sequence shown here is derived from an EMBL/GenBank/DDBJ whole genome shotgun (WGS) entry which is preliminary data.
Chr06G0851.1	831	24	152	UniProt ID:TUP1_CANAL	514	265	377	44/131 (33.59)	0.56	0.15	131	53.1	3.00E-08	gene=Chr06G0851	Gene Symbol:TUP1|Host:Isolated from a wide variety of substrates including humans|Disease:invasive candidal disease|Description:FUNCTION: Represses transcription by RNA polymerase II. Represses genes responsible for initiating filamentous growth and this repression is lifted under inducing environmental conditions.
Chr06G0854.1	375	50	372	UniProt ID:Q5A415_CANAL	361	23	357	157/335(46.87)	0.69	0.04	335	340	3.00E-115	gene=Chr06G0854	Gene Symbol:ARP2|Host:Isolated from a wide variety of substrates including humans|Disease:invasive

															candidal disease|Description:SIMILARITY: Belongs to the actin family.
Chr06G0860.1	353	3	353	UniProt ID:Q4WDL0_ASPFU	355	2	355	217/354(61.30)	0.77	0.01	354	461	5.00E-163	gene=Chr06G0860	Gene Symbol:AFUA_6G05110|Host:humans|Disease:infection|Description:CAUTION: The sequence shown here is derived from an EMBL/GenBank/DDBJ whole genome shotgun (WGS) entry which is preliminary data.
Chr06G0866.1	633	17	280	UniProt ID:Q6IWN3_USTMD	827	554	819	118/267(44.19)	0.63	0.01	267	233	8.00E-67	gene=Chr06G0866	Gene Symbol:NULL|Host:Euchlaena spp., Zea spp. (Poaceae)|Disease:Smut. Corn smut|Description:CATALYTIC ACTIVITY: ATP + a protein = ADP + a phosphoprotein.
Chr06G0869.1	481	211	414	UniProt ID:TUP1_CAN	514	295	470	54/213(25.35)	0.4	0.22	213	53.5	1.00E-08	gene=Chr06G0869	Gene Symbol:TUP1|Host:Isolated from a wide variety of

			AL												substrates including humans|Disease:invasive candidal disease|Description:FUNCTION: Represses transcription by RNA polymerase II. Represses genes responsible for initiating filamentous growth and this repression is lifted under inducing environmental conditions.
Chr06G0871.1	394	14	325	UniProt ID:HOG1_CRYPA	358	1	306	105/324(32.41)	0.57	0.09	324	186	3.00E-55	gene=Chr06G0871	Gene Symbol:HOG1|Host:Castanea spp., Fagus sylvatica, Quercus spp. (Fagaceae)|Disease:Chestnut blight. Cankers|Description:FUNCTION: Mitogen-activated protein kinase involved in a signal transduction pathway that is activated by changes in the osmolarity of the extracellular environment.

															Controls osmotic regulation of transcription of target genes (By similarity). Involved in the virulence and conidia formation. Mediates tannic acid-induced laccase expression and cryparin expression.
Chr06G0882.1	362	18	235	UniProt ID:Q6XPX0_FUSOX	359	116	356	56/247 (22.67)	0.41	0.14	247	50.4	5.00E-08	gene=Chr06G0882	Gene Symbol:FGB1|Host:Multiple genera in multiple families|Disease:Blights, wilts, rots of various sorts|Description:Unknown
Chr06G0883.1	392	70	360	UniProt ID:Q5AJC8_CANAL	461	137	419	144/291(49.48)	0.67	0.03	291	308	4.00E-101	gene=Chr06G0883	Gene Symbol:MNT2|Host:Isolated from a wide variety of substrates including humans|Disease:invasive candidal disease|Description:CAUTION: The sequence shown here is derived from an EMBL/GenBank/DDBJ

														whole genome shotgun (WGS) entry which is preliminary data.
Chr06G0897.1	968	327	361	UniProt ID:Q5A4F3_CANAL	624	12	46	17/35(48.57)	0.6	0	35	46.2	7.00E-06	gene=Chr06G0897	Gene Symbol:ZCF37|Host:Isolated from a wide variety of substrates including humans|Disease:invasive candidal disease|Description:Unknown
Chr06G0899.1	1310	851	1255	UniProt ID:Q9C2Y4_MAGGR	1501	891	1329	133/443(30.02)	0.47	0.09	443	184	5.00E-48	gene=Chr06G0899	Gene Symbol:PDE1|Host:Digitaria (Poaceae)|Disease:Leaf spot|Description:CATALYTIC ACTIVITY: ATP + H(2)O + phospholipid(In) = ADP + phosphate + phospholipid(Out).
Chr06G0915.1	834	3	832	UniProt ID:Q9Y7F1_ASPFM	824	27	820	311/873(35.62)	0.5	0.14	873	445	1.00E-143	gene=Chr06G0915	Gene Symbol:PABAA|Host:humans|Disease:infection|Description:Unknown
Chr06G0928.1	567	55	538	UniProt ID:Q5ANE1_C	748	43	496	157/489(32.11)	0.49	0.08	489	213	1.00E-60	gene=Chr06G0928	Gene Symbol:SNF3|Host:Isolated from a wide variety of

				ANAL										substrates including humans|Disease:invasive candidal disease|Description:SIMILARITY: Belongs to the major facilitator superfamily. Sugar transporter (TC 2.A.1.1) family.
Chr06G0931.1	511	73	221	UniProt ID:Q9P4U9_ALTAL	296	24	165	43/149 (28.86)	0.45	0.05	149	60.1	6.00E-11	gene=Chr06G0931	Gene Symbol:AKT3-1|Host:Plant|Disease:Leaf spot, rots|Description:Unknown
Chr06G0946.1	1347	393	1347	UniProt ID:Q2V086_GLOLA	956	1	956	908/957(94.88)	0.98	0	957	1784	0	gene=Chr06G0946	Gene Symbol:CLASSD1|Host:melons,cucumber|Disease:anthracnose fruit rot|Description:Unknown
Chr06G0955.1	1259	454	692	UniProt ID:O59928_HYPVI	430	44	298	70/274 (25.55)	0.4	0.2	274	79	4.00E-16	gene=Chr06G0955	Gene Symbol:NULL|Host:humans|Disease:infection|Description:SIMILARITY: Belongs to the glycosyl hydrolase 18 family.
Chr06G0957.1	712	292	410	UniProt ID:D1M	568	394	568	59/175 (33.71)	0.47	0.32	175	79.3	2.00E-16	gene=Chr06G0957	Gene Symbol:CBL1|Host:Digitar

				YV6_MAGGR											ia (Poaceae)|Disease:Leaf spot|Description:SIMILARITY: Contains 3 chitin-binding type-1 domains.
Chr06G0958.1	1004	271	497	UniProt ID:DNLI4_CANAL	928	297	511	59/235 (25.11)	0.43	0.12	235	56.6	5.00E-09	gene=Chr06G0958	Gene Symbol:LIG4|Host:Isolated from a wide variety of substrates including humans|Disease:invasive candidal disease|Description:FUNCTION: Involved in ds DNA break (DSB) repair. Has a role in non-homologous integration (NHI) pathways where it is required in the final step of non-homologus end-joining (NHEJ). Not required for the repair of DSBs induced by ionizing radiation or UV light. Has a important role in morphogenesis, positively affecting the capacity to

															form hyphae.
Chr06G0963.1	530	1	214	UniProt ID:A4QSA8_MAGO7	213	1	212	135/220(61.36)	0.75	0.06	220	261	3.00E-84	gene=Chr06G0963	Gene Symbol:MGG_08628|Host:Poaceae, especially important on Oryzae|Disease:Rice blast|Description:Unknown
Chr06G0964.1	217	24	217	UniProt ID:ERG2_USTMA	241	40	241	118/202(58.42)	0.7	0.04	202	230	4.00E-76	gene=Chr06G0964	Gene Symbol:ERG2|Host:Euchlaena spp., Zea spp. (Poaceae)|Disease:Smut. Corn smut|Description:FUNCTION: Catalyzes the reaction which results in unsaturation at C-7 in the B ring of sterols.
Chr06G0965.1	631	68	599	UniProt ID:Q6Y392_PHAND	625	77	594	213/552(38.59)	0.57	0.1	552	387	4.00E-126	gene=Chr06G0965	Gene Symbol:NULL|Host:Multiple genera of Poaceae and Blysmus compressus (Cyperaceae)|Disease:Glume blotch of wheat and other grasses|Description:Unkn

															own
Chr06G0967.1	785	201	407	UniProt ID:Q0WXM3_FUSOX	663	250	450	55/213 (25.82)	0.47	0.08	213	81.6	5.00E-17	gene=Chr06G0967	Gene Symbol:FOW2|Host:Multiple genera in multiple families|Disease:Blights, wilts, rots of various sorts|Description:SIMILARITY: Contains 1 Zn(2)-C6 fungal-type DNA-binding domain.
Chr06G0970.1	1466	835	1438	UniProt ID:Q9UW87_CANAL	1606	985	1603	229/625(36.64)	0.56	0.04	625	384	3.00E-111	gene=Chr06G0970	Gene Symbol:MLT1|Host:Isolated from a wide variety of substrates including humans|Disease:invasive candidal disease|Description:SIMILARITY: Belongs to the ABC transporter superfamily.
Chr06G0972.1	694	216	558	UniProt ID:C4YII6_CANAW	768	203	580	137/395(34.68)	0.5	0.17	395	187	2.00E-51	gene=Chr06G0972	Gene Symbol:CAWG_04261|Host:Isolated from a wide variety of substrates including humans|Disease:invasive

															candidal disease|Description:SIMILARITY: Belongs to the DEAD box helicase family.
Chr06G0974.1	595	257	557	UniProt ID:TUP1_CANAL	514	260	511	66/304(21.71)	0.4	0.18	304	63.9	9.00E-12	gene=Chr06G0974	Gene Symbol:TUP1|Host:Isolated from a wide variety of substrates including humans|Disease:invasive candidal disease|Description:FUNCTION: Represses transcription by RNA polymerase II. Represses genes responsible for initiating filamentous growth and this repression is lifted under inducing environmental conditions.
Chr06G0977.1	1424	98	1255	UniProt ID:Q5AL09_CANAL	1422	1	1174	493/1201(41.05)	0.6	0.06	1201	872	0	gene=Chr06G0977	Gene Symbol:KEM1|Host:Isolated from a wide variety of substrates including humans|Disease:invasive candidal disease|Description:CAUT

															ION: The sequence shown here is derived from an EMBL/GenBank/DDBJ whole genome shotgun (WGS) entry which is preliminary data.
Chr06G0978.1	438	187	418	UniProt ID:TUP1_CANAL	514	295	510	77/248 (31.05)	0.45	0.19	248	74.3	3.00E-15	gene=Chr06G0978	Gene Symbol:TUP1|Host:Isolated from a wide variety of substrates including humans|Disease:invasive candidal disease|Description:FUNCTION: Represses transcription by RNA polymerase II. Represses genes responsible for initiating filamentous growth and this repression is lifted under inducing environmental conditions.
Chr06G0983.1	1113	742	1025	UniProt ID:A3LXZ6_PICST	310	13	296	115/288(39.93)	0.59	0.03	288	202	1.00E-58	gene=Chr06G0983	Gene Symbol:CDC28|Host:humans|Disease:occasional infection|Description:Unknown

Chr06G0994.1	438	55	425	UniProt ID:A4R0Y1_MAGO7	573	196	566	109/396(27.53)	0.43	0.13	396	137	2.00E-36	gene=Chr06G0994	Gene Symbol:MGG_09250|Host:Poaceae, especially important on Oryzae|Disease:Rice blast|Description:Unknown
Chr06G0995.1	334	60	333	UniProt ID:Q32WF7_PHAND	266	6	265	129/277(46.57)	0.65	0.07	277	249	1.00E-81	gene=Chr06G0995	Gene Symbol:MDH1|Host:Multiple genera of Poaceae and Blysmus compressus (Cyperaceae)|Disease:Glume blotch of wheat and other grasses|Description:Unknown
Chr06G0996.1	565	12	557	UniProt ID:Q5ABP4_CANAL	609	7	607	331/603(54.89)	0.71	0.1	603	681	0	gene=Chr06G0996	Gene Symbol:CMP1|Host:Isolated from a wide variety of substrates including humans|Disease:invasive candidal disease|Description:CATALYTIC ACTIVITY: A phosphoprotein + H(2)O = a protein + phosphate.

Chr06G1000.1	1722	1113	1579	UniProt ID:Q5AL52_CANAL	1732	1133	1597	182/473(38.48)	0.58	0.03	473	336	1.00E-94	gene=Chr06G1000	Gene Symbol:BNI1|Host:Isolated from a wide variety of substrates including humans|Disease:invasive candidal disease|Description:SIMILARITY: Contains 1 FH2 (formin homology 2) domain.
Chr06G1019.1	307	150	217	UniProt ID:Q8J0I5_CLAPU	550	433	500	31/68(45.59)	0.68	0	68	71.2	7.00E-15	gene=Chr06G1019	Gene Symbol:CPTF1|Host:outcrossing species|Disease:ergotism|Description:SIMILARITY: Belongs to the bZIP family.
Chr06G1022.1	345	68	342	UniProt ID:Q6TFC7_ASPFM	349	61	335	97/283(34.28)	0.51	0.06	283	154	3.00E-44	gene=Chr06G1022	Gene Symbol:NULL|Host:humans|Disease:infection|Description:Unknown
Chr06G1024.1	1279	34	890	UniProt ID:Q6ZX14_MAGGR	4034	3088	3938	250/916(27.29)	0.43	0.14	916	213	1.00E-56	gene=Chr06G1024	Gene Symbol:ACE1|Host:Digitaria (Poaceae)|Disease:Leaf spot|Description:Unknown
Chr06G1	1054	282	566	UniProt	514	252	508	70/295	0.43	0.16	295	61.6	1.00E-10	gene=Chr	Gene

029.1				ID:TUP1_CANAL				(23.73)						06G1029	Symbol:TUP1|Host:Isolated from a wide variety of substrates including humans|Disease:invasive candidal disease|Description:FUNCTION: Represses transcription by RNA polymerase II. Represses genes responsible for initiating filamentous growth and this repression is lifted under inducing environmental conditions.
Chr06G1034.1	345	8	330	UniProt ID:C5GU56_AJEDR	347	21	340	174/324(53.70)	0.71	0.02	324	333	6.00E-113	gene=Chr06G1034	Gene Symbol:BDCG_08122|Host:humans|Disease:cutaneous Blastomyces dermatitidis infection|Description:Unknown
Chr06G1036.1	308	110	278	UniProt ID:A7A233_YEAS7	215	9	173	50/171(29.24)	0.55	0.05	171	90.5	2.00E-22	gene=Chr06G1036	Gene Symbol:SEC4|Host:humans|Disease:occasional infection|Description:SIMILARITY: Belongs to the

Chr06G1037.1	591	46	487	UniProt ID:Q5XTQ4_BOTFU	574	56	516	160/491(32.59)	0.46	0.16	491	196	1.00E-55	gene=Chr06G1037	small GTPase superfamily. Rab family. Gene Symbol:LIP1|Host:Various plant families|Disease:Grey mould. Parasite or saprophyte|Description:Unknown
Chr06G1038.1	1273	33	1273	UniProt ID:Q3Y5V5_MAGGR	1321	22	1320	460/1308(35.17)	0.55	0.06	1308	760	0	gene=Chr06G1038	Gene Symbol:ABC3|Host:Digitaria (Poaceae)|Disease:Leaf spot|Description:SIMILARITY: Belongs to the ABC transporter superfamily.
Chr06G1040.1	1049	671	951	UniProt ID:TUP1_CANAL	514	201	509	124/311(39.87)	0.56	0.1	311	205	1.00E-57	gene=Chr06G1040	Gene Symbol:TUP1|Host:Isolated from a wide variety of substrates including humans|Disease:invasive candidal disease|Description:FUNCTION: Represses transcription by RNA polymerase II. Represses genes responsible for

														initiating filamentous growth and this repression is lifted under inducing environmental conditions.
Chr06G1050.1	1587	138	1444	UniProt ID:Q9C2Y4_MAGGR	1501	12	1388	621/1397(44.45)	0.63	0.08	1397	1177	0	gene=Chr06G1050	Gene Symbol:PDE1|Host:Digitaria (Poaceae)|Disease:Leaf spot|Description:CATALYTIC ACTIVITY: ATP + H(2)O + phospholipid(In) = ADP + phosphate + phospholipid(Out).
Chr06G1053.1	383	116	377	UniProt ID:Q92217_COCHE	2528	1896	2138	58/270(21.48)	0.43	0.13	270	48.5	4.00E-07	gene=Chr06G1053	Gene Symbol:PKS1|Host:Zea mays|Disease:Southern leaf blight of maize|Description:Unknown
Chr06G1061.1	280	25	187	UniProt ID:Q874F3_MAGGR	1484	875	1054	53/180(29.44)	0.47	0.09	180	47	6.00E-07	gene=Chr06G1061	Gene Symbol:ABC2|Host:Digitaria (Poaceae)|Disease:Leaf spot|Description:SIMILARITY: Belongs to the ABC transporter superfamily.
Chr06G1068.1	497	34	493	UniProt ID:C0S	490	26	483	322/460(70.0	0.82	0	460	667	0	gene=Chr06G1068	Gene Symbol:PABG_01505|Hos

				1P0_PARBP				0)							t:humans|Disease:Paracoccidioidomycosis|Description:SIMILARITY: Belongs to the GHMP kinase family.
Chr06G1071.1	87	1	61	UniProt ID:A4R1F0_MAGO7	514	1	61	49/61(80.33)	0.89	0	61	114	3.00E-32	gene=Chr06G1071	Gene Symbol:MGG_06951|Host:Poaceae, especially important on Oryzae|Disease:Rice blast|Description:Unknown
Chr06G1072.1	454	6	454	UniProt ID:A4R1F0_MAGO7	514	61	514	361/455(79.34)	0.89	0.02	455	737	0	gene=Chr06G1072	Gene Symbol:MGG_06951|Host:Poaceae, especially important on Oryzae|Disease:Rice blast|Description:Unknown
Chr06G1075.1	864	24	689	UniProt ID:Q99324_SEPLY	803	13	703	263/719(36.58)	0.51	0.11	719	391	4.00E-123	gene=Chr06G1075	Gene Symbol:B2TOM|Host:Primarily tomato, Lycopersicon esculentum, also Solanum spp. and other Solanaceae|Disease:Leaf spot|Description:Unknown

Chr06G1076.1	339	17	335	UniProt ID:Q6WER3_GIBZA	351	15	342	167/329(50.76)	0.66	0.03	329	347	2.00E-118	gene=Chr06G1076	Gene Symbol:FGL1|Host:Principal hosts: Poaceae, including Zea mays (corn), Triticum aestivum (wheat), and Oryza sativa (rice). Additional hosts: various plant families|Disease:Seedling blight, pre- and post-emergence blight, root and foot rot, brown rot, culm decay, head or kernel blight (scab or ear scab) of cereals. Leaf|Description:Unknown
Chr06G1077.1	1070	894	1050	UniProt ID:Q5AM49_CANAL	1690	1098	1253	56/159(35.22)	0.51	0.03	159	95.9	7.00E-21	gene=Chr06G1077	Gene Symbol:SNF2|Host:Isolated from a wide variety of substrates including humans|Disease:invasive candidal disease|Description:Unknown
Chr06G1078.1	657	77	655	UniProt ID:A4R	610	18	608	258/599(43.0	0.61	0.05	599	475	3.00E-160	gene=Chr06G1078	Gene Symbol:MGG_07015|Host

				178_MAGO7				7)							:Poaceae, especially important on Oryzae|Disease:Rice blast|Description:Unknown
Chr06G1081.1	1135	139	1126	UniProt ID:P78585_BOTFU	994	8	990	520/1017(51.13)	0.69	0.06	1017	980	0	gene=Chr06G1081	Gene Symbol:BCPLC1|Host:Various plant families|Disease:Grey mould. Parasite or saprophyte|Description:SIMILARITY: Contains 1 C2 domain.
Chr06G1082.1	866	370	864	UniProt ID:A4R2T0_MAGO7	774	278	772	262/519(50.48)	0.68	0.09	519	506	2.00E-167	gene=Chr06G1082	Gene Symbol:MGG_02443|Host:Poaceae, especially important on Oryzae|Disease:Rice blast|Description:Unknown
Chr06G1084.1	651	310	392	UniProt ID:Q0PND8_MAGGR	1375	1019	1107	32/92(34.78)	0.54	0.13	92	48.1	1.00E-06	gene=Chr06G1084	Gene Symbol:PEX6|Host:Digitaria (Poaceae)|Disease:Leaf spot|Description:SIMILARITY: Belongs to the AAA ATPase family.

Chr06G1089.1	1205	905	1144	UniProt ID:Q59YF3_CANAL	986	660	882	88/242 (36.36)	0.5	0.09	242	142	2.00E-35	gene=Chr06G1089	Gene Symbol:INP51|Host:Isolated from a wide variety of substrates including humans|Disease:invasive candidal disease|Description:CAUTION: The sequence shown here is derived from an EMBL/GenBank/DDBJ whole genome shotgun (WGS) entry which is preliminary data.
Chr06G1090.1	456	40	456	UniProt ID:Q9UVG2_PHAND	445	32	445	281/419(67.06)	0.79	0.02	419	582	0	gene=Chr06G1090	Gene Symbol:ODC1|Host:Multiple genera of Poaceae and Blysmus compressus (Cyperaceae)|Disease:Glume blotch of wheat and other grasses|Description:CATALYTIC ACTIVITY: L-ornithine = putrescine + CO(2).
Chr06G1107.1	903	533	706	UniProt ID:TUP	514	260	416	52/182 (28.57)	0.48	0.18	182	75.1	7.00E-15	gene=Chr06G1107	Gene Symbol:TUP1|Host:Isolate

				1_CANAL											d from a wide variety of substrates including humans|Disease:invasive candidal disease|Description:FUNCTION: Represses transcription by RNA polymerase II. Represses genes responsible for initiating filamentous growth and this repression is lifted under inducing environmental conditions.
Chr06G1112.1	711	259	613	UniProt ID:Q6ZX14_MAGGR	4034	3175	3518	85/373 (22.79)	0.37	0.13	373	48.1	2.00E-06	gene=Chr06G1112	Gene Symbol:ACE1|Host:Digitaria (Poaceae)|Disease:Leaf spot|Description:Unknown
Chr06G1117.1	508	2	39	UniProt ID:Q5A4F3_CANAL	624	9	46	19/38(50.00)	0.61	0	38	45.4	5.00E-06	gene=Chr06G1117	Gene Symbol:ZCF37|Host:Isolated from a wide variety of substrates including humans|Disease:invasive candidal disease|Description:Unknown
Chr06G1	339	29	338	UniProt	349	36	346	108/32	0.51	0.06	320	159	4.00E-46	gene=Chr	Gene

119.1				ID:Q6TFC7_ASPFM				0(33.75)						06G1119	Symbol:NULL|Host:humans|Disease:infection|Description:Unknown
Chr06G1120.1	675	23	527	UniProt ID:A6N6J8_FUSOX	903	204	687	144/530(27.17)	0.43	0.13	530	184	7.00E-50	gene=Chr06G1120	Gene Symbol:CTF1|Host:Multiple genera in multiple families|Disease:Blights, wilts, rots of various sorts|Description:SIMILARITY: Contains 1 Zn(2)-C6 fungal-type DNA-binding domain.
Chr06G1134.1	1800	494	1657	UniProt ID:Q5A762_CANAL	1606	451	1603	375/1227(30.56)	0.5	0.11	1227	538	2.00E-162	gene=Chr06G1134	Gene Symbol:MLT1|Host:Isolated from a wide variety of substrates including humans|Disease:invasive candidal disease|Description:SIMILARITY: Belongs to the ABC transporter superfamily.
Chr06G1137.1	778	27	777	UniProt ID:C5GXJ3_AJEDR	804	22	802	506/788(64.21)	0.77	0.06	788	1020	0	gene=Chr06G1137	Gene Symbol:BDCG_09181|Host:humans|Disease:cutaneous Blastomyces

														dermatitidis infection|Description:Unknown
Chr06G1138.1	728	204	373	UniProt ID:Q0WXM3_FUSOX	663	254	425	46/181 (25.41)	0.46	0.11	181	62.8	4.00E-11	gene=Chr06G1138	Gene Symbol:FOW2|Host:Multiple genera in multiple families|Disease:Blights, wilts, rots of various sorts|Description:SIMILARITY: Contains 1 Zn(2)-C6 fungal-type DNA-binding domain.
Chr06G1139.1	641	17	562	UniProt ID:Q9P8L8_BOTFU	598	60	598	264/549(48.09)	0.67	0.02	549	518	3.00E-177	gene=Chr06G1139	Gene Symbol:BCMFS1|Host:Various plant families|Disease:Grey mould. Parasite or saprophyte|Description:Unknown
Chr06G1145.1	339	92	284	UniProt ID:A6R757_AJECN	306	66	279	65/215 (30.23)	0.47	0.11	215	79.7	5.00E-18	gene=Chr06G1145	Gene Symbol:HCAG_05465|Host:humans|Disease:Darling's disease|Description:SIMILARITY: Contains 1 CRAL-TRIO domain.

Chr06G1161.1	732	68	356	UniProt ID:Q9Y784_MAGGR	631	63	373	76/315 (24.13)	0.49	0.1	315	118	2.00E-28	gene=Chr06G1161	Gene Symbol:PTH11|Host:Digitaria (Poaceae)|Disease:Leaf spot|Description:Unknown
Chr06G1171.1	356	59	346	UniProt ID:Q6TFC7_ASPFM	349	61	347	105/292(35.96)	0.54	0.03	292	168	3.00E-49	gene=Chr06G1171	Gene Symbol:NULL|Host:humans|Disease:infection|Description:Unknown
Chr06G1174.1	494	276	490	UniProt ID:Q7Z868_USTMD	862	360	603	67/267 (25.09)	0.38	0.28	267	52	5.00E-08	gene=Chr06G1174	Gene Symbol:GLO1|Host:Euchlaena spp., Zea spp. (Poaceae)|Disease:Smut. Corn smut|Description:Unknown
Chr06G1176.1	460	4	340	UniProt ID:Q5GFD3_PHAND	437	9	357	93/381 (24.41)	0.4	0.2	381	57	8.00E-10	gene=Chr06G1176	Gene Symbol:NULL|Host:Multiple genera of Poaceae and Blysmus compressus (Cyperaceae)|Disease:Glume blotch of wheat and other grasses|Description:Unknown
Chr06G1	210	45	174	UniProt	358	207	340	52/134	0.56	0.03	134	97.4	6.00E-25	gene=Chr	Gene

177.1				ID:Q9H FZ2_C ANAL				(38.81)						06G1177	Symbol:RBT4|Host:Isolated from a wide variety of substrates including humans|Disease:invasive candidal disease|Description:Unknown
Chr06G1 179.1	590	34	589	UniProt ID:Q2V LJ1_GI BZA	565	27	563	201/565(35.58)	0.51	0.07	565	322	2.00E-102	gene=Chr 06G1179	Gene Symbol:ZEB1|Host:Principal hosts: Poaceae, including Zea mays (corn), Triticum aestivum (wheat), and Oryza sativa (rice). Additional hosts: various plant families|Disease:Seedling blight, pre- and post-emergence blight, root and foot rot, brown rot, culm decay, head or kernel blight (scab or ear scab) of cereals. Leaf|Description:Unknown
Chr06G1 180.1	853	2	38	UniProt ID:Q5A 4F3_C	624	10	46	19/37(51.35)	0.65	0	37	53.9	2.00E-08	gene=Chr 06G1180	Gene Symbol:ZCF37|Host:Isolated from a wide variety of

				ANAL											substrates including humans|Disease:invasive candidal disease|Description:Unknown
Chr06G1183.1	1140	490	555	UniProt ID:Q5A4F3_CANAL	624	14	68	26/66(39.39)	0.58	0.17	66	61.2	2.00E-10	gene=Chr06G1183	Gene Symbol:ZCF37|Host:Isolated from a wide variety of substrates including humans|Disease:invasive candidal disease|Description:Unknown
Chr06G1191.1	307	17	104	UniProt ID:Q4PCH2_USTMA	758	121	209	28/89(31.46)	0.56	0.01	89	44.3	5.00E-06	gene=Chr06G1191	Gene Symbol:YAP1|Host:Euchlaena spp., Zea spp. (Poaceae)|Disease:Smut. Corn smut|Description:SIMILARITY: Belongs to the bZIP family.
Chr06G1197.1	953	700	950	UniProt ID:O93802_ALTAL	267	1	264	75/271(27.68)	0.49	0.1	271	106	9.00E-26	gene=Chr06G1197	Gene Symbol:BRM2|Host:Plant|Disease:Leaf spot, rots|Description:SIMILARITY: Belongs to the

															short-chain dehydrogenases/reductases (SDR) family.
Chr06G1200.1	287	86	285	UniProt ID:A4RGG9_MAGO7	286	81	271	64/200 (32.00)	0.52	0.05	200	97.1	3.00E-24	gene=Chr06G1200	Gene Symbol:MGG_00056|Host:Poaceae, especially important on Oryzae|Disease:Rice blast|Description:SIMILARITY: Belongs to the short-chain dehydrogenases/reductases (SDR) family.
Chr06G1204.1	452	25	418	UniProt ID:Q9Y784_MAGGR	631	36	422	99/401 (24.69)	0.44	0.05	401	119	7.00E-30	gene=Chr06G1204	Gene Symbol:PTH11|Host:Digitaria (Poaceae)|Disease:Leaf spot|Description:Unknown
Chr06G1205.1	364	67	353	UniProt ID:Q6TFC7_ASPFM	349	61	346	107/295(36.27)	0.55	0.06	295	181	4.00E-54	gene=Chr06G1205	Gene Symbol:NULL|Host:humans|Disease:infection|Description:Unknown
Chr06G1208.1	594	361	561	UniProt ID:A4ULJ2_MYCGR	515	305	503	47/211 (22.27)	0.41	0.1	211	47.8	1.00E-06	gene=Chr06G1208	Gene Symbol:CYP51|Host:Triticum and possibly a few other

															grasses|Disease:Leaf spot or speckled leaf blotch of wheat|Description:COFACTOR: Heme group (By similarity).
Chr06G1211.1	351	4	347	UniProt ID:A0ST44_CERNC	357	2	341	100/361(27.70)	0.45	0.11	361	88.6	7.00E-21	gene=Chr06G1211	Gene Symbol:CTB6|Host:Numerous taxa in Solanaceae|Disease:Leaf spot|Description:Unknown
Chr06G1213.1	503	51	301	UniProt ID:Q2VLJ1_GIBZA	565	106	369	72/268(26.87)	0.41	0.08	268	60.5	9.00E-11	gene=Chr06G1213	Gene Symbol:ZEB1|Host:Principal hosts: Poaceae, including Zea mays (corn), Triticum aestivum (wheat), and Oryza sativa (rice). Additional hosts: various plant families|Disease:Seedling blight, pre- and post-emergence blight, root and foot rot, brown rot, culm decay, head or kernel blight (scab or ear scab) of cereals. Leaf|Description:Unknown

Chr06G1214.1	692	12	107	UniProt ID:Q0WXM3_FUSOX	663	48	153	36/106 (33.96)	0.43	0.09	106	57.8	1.00E-09	gene=Chr06G1214	Gene Symbol:FOW2|Host:Multiple genera in multiple families|Disease:Blights, wilts, rots of various sorts|Description:SIMILARITY: Contains 1 Zn(2)-C6 fungal-type DNA-binding domain.
Chr06G1218.1	532	63	316	UniProt ID:A0ST43_CERNC	459	3	260	91/260 (35.00)	0.51	0.03	260	137	3.00E-36	gene=Chr06G1218	Gene Symbol:CTB5|Host:Numerous taxa in Solanaceae|Disease:Leaf spot|Description:Unknown
Chr06G1219.1	380	17	311	UniProt ID:Q6A2T2_BOTFU	391	40	335	73/305 (23.93)	0.43	0.06	305	63.2	6.00E-12	gene=Chr06G1219	Gene Symbol:BTP1|Host:Various plant families|Disease:Grey mould. Parasite or saprophyte|Description:Unknown
Chr06G1222.1	333	25	201	UniProt ID:A4R3I5_MAGO7	400	104	274	46/183 (25.14)	0.45	0.1	183	47	7.00E-07	gene=Chr06G1222	Gene Symbol:"MGG_11993, MGG_12837, MGG_13052"|Host:Poaceae, especially important

															on Oryzae|Disease:Rice blast|Description:Unknown
Chr06G1223.1	1234	71	1201	UniProt ID:A4RGC8_MAGO7	1158	31	1132	459/1162(39.50)	0.57	0.08	1162	781	0	gene=Chr06G1223	Gene Symbol:MGG_11671|Host:Poaceae, especially important on Oryzae|Disease:Rice blast|Description:SIMILARITY: Contains 1 reverse transcriptase domain.
Chr06G1234.1	347	61	344	UniProt ID:Q6TFC7_ASPFM	349	63	346	89/287(31.01)	0.46	0.02	287	135	5.00E-37	gene=Chr06G1234	Gene Symbol:NULL|Host:humans|Disease:infection|Description:Unknown
Chr06G1236.1	459	67	377	UniProt ID:OPSB_ASPFU	485	67	388	106/330(32.12)	0.5	0.08	330	138	7.00E-37	gene=Chr06G1236	Gene Symbol:OPSB|Host:humans|Disease:infection|Description:FUNCTION: Probable GPI-anchored aspartic-type endopeptidase which contributes to virulence (By similarity).
Chr06G1237.1	289	7	287	UniProt ID:Q5A	308	6	305	111/306(36.2	0.54	0.1	306	206	5.00E-65	gene=Chr06G1237	Gene Symbol:BGL2|Host:Isolate

				MT2_CANAL				7)							d from a wide variety of substrates including humans|Disease:invasive candidal disease|Description:SIMILARITY: Belongs to the glycosyl hydrolase 17 family.
Chr06G1240.1	1608	1228	1506	UniProt ID:TUP1_CANAL	514	201	509	116/311(37.30)	0.54	0.11	311	183	2.00E-49	gene=Chr06G1240	Gene Symbol:TUP1|Host:Isolated from a wide variety of substrates including humans|Disease:invasive candidal disease|Description:FUNCTION: Represses transcription by RNA polymerase II. Represses genes responsible for initiating filamentous growth and this repression is lifted under inducing environmental conditions.
Chr06G1259.1	343	9	329	UniProt ID:Q6TFC7_A	349	17	349	106/337(31.45)	0.48	0.06	337	156	7.00E-45	gene=Chr06G1259	Gene Symbol:NULL|Host:humans|Disease:infection|Desc

				SPFM											ription:Unknown
Chr06G1261.1	376	82	281	UniProt ID:A4QVF8_MAGO7	339	57	249	62/201 (30.85)	0.49	0.04	201	80.5	7.00E-18	gene=Chr06G1261	Gene Symbol:MGG_04556|Host:Poaceae, especially important on Oryzae|Disease:Rice blast|Description:COFACTOR: Zinc (By similarity).
Chr06G1265.1	919	586	912	UniProt ID:A4QRN5_MAGO7	1015	280	599	103/332(31.02)	0.52	0.05	332	173	3.00E-45	gene=Chr06G1265	Gene Symbol:MGG_03530|Host:Poaceae, especially important on Oryzae|Disease:Rice blast|Description:Unknown
Chr06G1275.1	698	112	342	UniProt ID:Q5ABU7_CANAL	564	94	325	52/233 (22.32)	0.45	0.01	233	82	3.00E-17	gene=Chr06G1275	Gene Symbol:MDR1|Host:Isolated from a wide variety of substrates including humans|Disease:invasive candidal disease|Description:Unknown
Chr06G1299.1	540	13	478	UniProt ID:Q5ANE1_C	748	37	504	137/480(28.54)	0.47	0.05	480	174	6.00E-48	gene=Chr06G1299	Gene Symbol:SNF3|Host:Isolated from a wide variety of

				ANAL											substrates including humans|Disease:invasive candidal disease|Description:SIMILARITY: Belongs to the major facilitator superfamily. Sugar transporter (TC 2.A.1.1) family.
Chr06G1310.1	209	4	208	UniProt ID:D8QLT4_SCHCM	201	5	200	106/205(51.71)	0.66	0.04	205	193	2.00E-62	gene=Chr06G1310	Gene Symbol:SCHCODRAFT_258971|Host:Usually saprotrophic on wood, but sometimes parasitic on various woody and herbaceous hosts|Disease:wood rot|Description:Unknown
Chr06G1315.1	368	87	364	UniProt ID:Q32WF7_PHAND	266	14	265	81/289(28.03)	0.46	0.17	289	90.1	1.00E-21	gene=Chr06G1315	Gene Symbol:MDH1|Host:Multiple genera of Poaceae and Blysmus compressus (Cyperaceae)|Disease:Glume blotch of wheat and other grasses|Description:Unkn

															own
Chr06G1323.1	621	21	601	UniProt ID:SCPB_ARTOC	655	22	610	373/589(63.33)	0.79	0.01	589	808	0	gene=Chr06G1323	Gene Symbol:SCPB|Host:humans, reptiles|Disease:dermatophytoses|Description:FUNCTION: Extracellular serine carboxypeptidase that contributes to pathogenicity (By similarity).
Chr06G1336.1	645	351	539	UniProt ID:Q59Z23_CANAL	444	172	331	51/200(25.50)	0.44	0.26	200	50.8	1.00E-07	gene=Chr06G1336	Gene Symbol:TPK2|Host:Isolated from a wide variety of substrates including humans|Disease:invasive candidal disease|Description:CATALYTIC ACTIVITY: ATP + a protein = ADP + a phosphoprotein.
Chr06G1337.1	1119	189	408	UniProt ID:Q7Z9J3_9PEZI	1372	1113	1310	57/225(25.33)	0.43	0.14	225	55.5	2.00E-08	gene=Chr06G1337	Gene Symbol:CZK3|Host:Zea mays (Poaceae)|Disease:Gray leaf spot of

Chr06G1338.1	169	1	83	UniProt ID:Q6ZX14_MAGGR	4034	2162	2244	31/83(37.35)	0.58	0	83	71.6	2.00E-15	gene=Chr06G1338	corn|Description:Unknown Gene Symbol:ACE1|Host:Digitaria (Poaceae)|Disease:Leaf spot|Description:Unknown
Chr06G1341.1	766	67	307	UniProt ID:TUP1_CANAL	514	259	509	69/266 (25.94)	0.45	0.15	266	74.3	8.00E-15	gene=Chr06G1341	Gene Symbol:TUP1|Host:Isolated from a wide variety of substrates including humans|Disease:invasive candidal disease|Description:FUNCTION: Represses transcription by RNA polymerase II. Represses genes responsible for initiating filamentous growth and this repression is lifted under inducing environmental conditions.
Chr06G1342.1	363	28	282	UniProt ID:TUP1_CANAL	514	260	491	58/257 (22.57)	0.4	0.11	257	52.4	2.00E-08	gene=Chr06G1342	Gene Symbol:TUP1|Host:Isolated from a wide variety of substrates including humans|Disease:invasive candidal

															disease|Description:FUNCTION: Represses transcription by RNA polymerase II. Represses genes responsible for initiating filamentous growth and this repression is lifted under inducing environmental conditions.
Chr06G1343.1	664	387	632	UniProt ID:A6ZIB8_MAGGR	582	305	561	62/274 (22.63)	0.37	0.16	274	57.8	1.00E-09	gene=Chr06G1343	Gene Symbol:NULL|Host:Digitaria (Poaceae)|Disease:Leaf spot|Description:SIMILARITY: Contains 1 FAD-binding FR-type domain.
Chr06G1360.1	202	9	138	UniProt ID:Q00LS5_PHAND	408	66	207	47/143 (32.87)	0.5	0.1	143	64.7	2.00E-13	gene=Chr06G1360	Gene Symbol:CPKA|Host:Multiple genera of Poaceae and Blysmus compressus (Cyperaceae)|Disease:Glume blotch of wheat and other grasses|Description:SIMILARITY: Contains 1 protein kinase domain.

Chr06G1364.1	738	478	649	UniProt ID:A4QVD7_MAGO7	541	311	483	55/188(29.26)	0.44	0.16	188	64.3	1.00E-11	gene=Chr06G1364	Gene Symbol:MGG_04582|Host:Poaceae, especially important on Oryzae|Disease:Rice blast|Description:Unknown
Chr06G1370.1	647	48	80	UniProt ID:Q5A4F3_CANAL	624	14	46	19/33(57.58)	0.7	0	33	52	8.00E-08	gene=Chr06G1370	Gene Symbol:ZCF37|Host:Isolated from a wide variety of substrates including humans|Disease:invasive candidal disease|Description:Unknown
Chr06G1373.1	340	4	340	UniProt ID:A0ST44_CERNC	357	6	346	104/352(29.55)	0.47	0.07	352	112	3.00E-29	gene=Chr06G1373	Gene Symbol:CTB6|Host:Numerous taxa in Solanaceae|Disease:Leaf spot|Description:Unknown
Chr06G1392.1	569	48	552	UniProt ID:B2C6F1_CRYGA	614	36	571	155/544(28.49)	0.46	0.09	544	217	5.00E-63	gene=Chr06G1392	Gene Symbol:LAC1|Host:humans|Disease:pulmonary cryptococcosis, basal meningitis, and cerebral cryptococcomas|Descripti

														on:Unknown
Chr06G1397.1	556	333	486	UniProt ID:A4ULJ2_MYCGR	515	338	512	49/180 (27.22)	0.43	0.17	180	51.6	6.00E-08	gene=Chr06G1397	Gene Symbol:CYP51|Host:Triticum and possibly a few other grasses|Disease:Leaf spot or speckled leaf blotch of wheat|Description:COFACTOR: Heme group (By similarity).
Chr06G1400.1	524	33	474	UniProt ID:Q5ANE1_CANAL	748	48	495	121/458(26.42)	0.45	0.06	458	133	4.00E-34	gene=Chr06G1400	Gene Symbol:SNF3|Host:Isolated from a wide variety of substrates including humans|Disease:invasive candidal disease|Description:SIMILARITY: Belongs to the major facilitator superfamily. Sugar transporter (TC 2.A.1.1) family.
Chr06G1403.1	752	4	83	UniProt ID:A6N6J8_FUSOX	903	34	126	31/93(33.33)	0.51	0.14	93	50.1	4.00E-07	gene=Chr06G1403	Gene Symbol:CTF1|Host:Multiple genera in multiple families|Disease:Blights,

															wilts, rots of various sorts|Description:SIMILARITY: Contains 1 Zn(2)-C6 fungal-type DNA-binding domain.
Chr06G1404.1	426	16	345	UniProt ID:A4UC81_MAGO7	376	64	373	110/335(32.84)		0.51	0.09	335	160	3.00E-45	gene=Chr06G1404	Gene Symbol:MGG_10702|Host:Poaceae, especially important on Oryzae|Disease:Rice blast|Description:Unknown
Chr06G1406.1	500	260	411	UniProt ID:Q0PND8_MAGGR	1375	1023	1173	62/166(37.35)		0.54	0.17	166	87	7.00E-19	gene=Chr06G1406	Gene Symbol:PEX6|Host:Digitaria (Poaceae)|Disease:Leaf spot|Description:SIMILARITY: Belongs to the AAA ATPase family.
Chr06G1412.1	387	101	385	UniProt ID:Q6TFC7_ASPFM	349	64	347	92/287(32.06)		0.5	0.02	287	155	5.00E-44	gene=Chr06G1412	Gene Symbol:NULL|Host:humans|Disease:infection|Description:Unknown
Chr06G1414.1	425	1	414	UniProt ID:MCPAL_ARTOC	416	1	415	160/433(36.95)		0.58	0.09	433	283	2.00E-91	gene=Chr06G1414	Gene Symbol:MCYG_01475|Host:humans, reptiles|Disease:dermatop

															hytoses|Description:FUNCTION: Extracellular metalloprotease that contributes to pathogenicity (By similarity).
Chr06G1418.1	1190	307	661	UniProt ID:SUB6_ARTBE	412	11	349	93/388(23.97)	0.36	0.21	388	49.3	8.00E-07	gene=Chr06G1418	Gene Symbol:SUB6|Host:hedgehogs|Disease:ringworm,Kerion Celsi Disease|Description:FUNCTION: Secreted subtilisin-like serine protease with keratinolytic activity that contributes to pathogenicity (By similarity).
Chr06G1427.1	458	55	368	UniProt ID:A4UC81_MAGO7	376	56	373	130/321(40.50)	0.56	0.03	321	234	5.00E-73	gene=Chr06G1427	Gene Symbol:MGG_10702|Host:Poaceae, especially important on Oryzae|Disease:Rice blast|Description:Unknown
Chr06G1428.1	387	1	385	UniProt ID:ORY	403	1	401	227/403(56.3	0.7	0.05	403	425	1.00E-147	gene=Chr06G1428	Gene Symbol:ALP1|Host:human

				Z_ASPFU				3)							s|Disease:infection|Description:FUNCTION: Secreted alkaline protease that allows assimilation of proteinaceous substrates. Acts as a significant virulence factor in invasive aspergillosis. Involved in immune evasion from the human and mice complement systems during infection. Efficiently cleaves important components of the complement cascade such as such as C3, C4, C5, and C1q, as well as IgG, which leads to down-regulation of complement activation at the hyphal surface.
Chr06G1431.1	439	4	350	UniProt ID:A4UC81_MAGO7	376	22	369	106/351(30.20)	0.51	0.02	351	179	3.00E-52	gene=Chr06G1431	Gene Symbol:MGG_10702|Host:Poaceae, especially important on

															Oryzae|Disease:Rice blast|Description:Unknown
Chr06G1437.1	1082	28	1028	UniProt ID:Q9P872_CANAL	917	21	904	307/1014(30.28)	0.48	0.14	1014	409	2.00E-126	gene=Chr06G1437	Gene Symbol:PMR1|Host:Isolated from a wide variety of substrates including humans|Disease:invasive candidal disease|Description:SIMILARITY: Belongs to the cation transport ATPase (P-type) family.
Chr06G1439.1	353	26	225	UniProt ID:A4QVF8_MAGO7	339	22	224	58/211 (27.49)	0.48	0.09	211	77.8	4.00E-17	gene=Chr06G1439	Gene Symbol:MGG_04556|Host:Poaceae, especially important on Oryzae|Disease:Rice blast|Description:COFACTOR: Zinc (By similarity).
Chr06G1443.1	362	25	344	UniProt ID:Q9Y784_MAGGR	631	105	455	75/356 (21.07)	0.37	0.12	356	45.8	2.00E-06	gene=Chr06G1443	Gene Symbol:PTH11|Host:Digitaria (Poaceae)|Disease:Leaf spot|Description:Unknown
Chr06G1	487	260	454	UniProt	515	277	502	56/227	0.43	0.15	227	60.1	1.00E-10	gene=Chr	Gene

<table>
<tr><td>444.1</td><td></td><td></td><td></td><td>ID:A4ULJ2_MYCGR</td><td></td><td></td><td></td><td>(24.67)</td><td></td><td></td><td></td><td></td><td></td><td>06G1444</td><td>Symbol:CYP51|Host:Triticum and possibly a few other grasses|Disease:Leaf spot or speckled leaf blotch of wheat|Description:COFACTOR: Heme group (By similarity).</td></tr>
<tr><td>Chr06G1447.1</td><td>250</td><td>40</td><td>236</td><td>UniProt ID:Q5IFQ6_CRYNV</td><td>234</td><td>30</td><td>222</td><td>106/197(53.81)</td><td>0.65</td><td>0.02</td><td>197</td><td>213</td><td>3.00E-69</td><td>gene=Chr06G1447</td><td>Gene Symbol:SOD2|Host:humans|Disease:cryptococcosis|Description:FUNCTION: Destroys radicals which are normally produced within the cells and which are toxic to biological systems (By similarity).</td></tr>
<tr><td>Chr06G1454.1</td><td>540</td><td>31</td><td>507</td><td>UniProt ID:Q9Y784_MAGGR</td><td>631</td><td>33</td><td>552</td><td>128/536(23.88)</td><td>0.43</td><td>0.14</td><td>536</td><td>130</td><td>3.00E-33</td><td>gene=Chr06G1454</td><td>Gene Symbol:PTH11|Host:Digitaria (Poaceae)|Disease:Leaf spot|Description:Unknown</td></tr>
<tr><td>Chr06G1455.1</td><td>791</td><td>229</td><td>745</td><td>UniProt ID:Q4P8E8_USTMA</td><td>693</td><td>77</td><td>671</td><td>146/624(23.40)</td><td>0.37</td><td>0.22</td><td>624</td><td>73.9</td><td>1.00E-14</td><td>gene=Chr06G1455</td><td>Gene Symbol:UM03615.1|Host: Euchlaena spp., Zea spp. (Poaceae)|Disease:Smut.</td></tr>
</table>

														Corn smut|Description:COFACTOR: FAD (By similarity).
Chr06G1456.1	1124	42	1123	UniProt ID:Q4WPX2_ASPFU	1079	11	1071	510/1087(46.92)	0.64	0.03	1087	1032	0	gene=Chr06G1456	Gene Symbol:PPOA|Host:humans|Disease:infection|Description:Unknown
Chr06G1462.1	675	118	666	UniProt ID:CXT1_CRYNJ	694	152	669	148/574(25.78)	0.44	0.14	574	188	5.00E-52	gene=Chr06G1462	Gene Symbol:CXT1|Host:humans|Disease:cryptococcosis|Description:FUNCTION: Beta-1,2-xylosyltransferase that plays a key role in capsule polysaccharide synthesis by transferring xylose to alpha-1,3-dimannoside in a beta-1,2-linkage. Also mediates glycosylation of glycosphingolipids; constitutes the unique xylosyltransferase responsible for adding xylose to glycosphingolipids.
Chr06G1	928	542	927	UniProt	461	36	436	111/40	0.48	0.08	409	125	3.00E-31	gene=Chr	Gene

470.1				ID:A0ST41_CERNC				9(27.14)						06G1470	Symbol:CTB2|Host:Numerous taxa in Solanaceae|Disease:Leaf spot|Description:Unknown
Chr06G1473.1	2522	46	2507	UniProt ID:Q92217_COCHE	2528	5	2512	765/2614(29.27)	0.46	0.1	2614	946	0	gene=Chr06G1473	Gene Symbol:PKS1|Host:Zea mays|Disease:Southern leaf blight of maize|Description:Unknown
Chr06G1477.1	479	166	423	UniProt ID:MCPA_ARTOC	422	162	373	76/266(28.57)	0.43	0.23	266	72.4	1.00E-14	gene=Chr06G1477	Gene Symbol:MCPA|Host:humans, reptiles|Disease:dermatophytoses|Description:FUNCTION: Extracellular metalloprotease that contributes to pathogenicity (By similarity).
Chr06G1483.1	1215	71	1182	UniProt ID:A4RGC8_MAGO7	1158	31	1132	454/1156(39.27)	0.56	0.08	1156	764	0	gene=Chr06G1483	Gene Symbol:MGG_11671|Host:Poaceae, especially important on Oryzae|Disease:Rice blast|Description:SIMILAR

															ITY: Contains 1 reverse transcriptase domain.
Chr06G1484.1	333	25	201	UniProt ID:A4R3I5_MAGO7	400	104	274	46/183 (25.14)	0.45	0.1	183	47	7.00E-07	gene=Chr06G1484	Gene Symbol:"MGG_11993, MGG_12837, MGG_13052"|Host:Poaceae, especially important on Oryzae|Disease:Rice blast|Description:Unknown
Chr06G1490.1	1322	896	1160	UniProt ID:Q5ADP3_CANAL	1080	96	320	71/266 (26.69)	0.4	0.16	266	56.2	1.00E-08	gene=Chr06G1490	Gene Symbol:SSN6|Host:Isolated from a wide variety of substrates including humans|Disease:invasive candidal disease|Description:CAUTION: The sequence shown here is derived from an EMBL/GenBank/DDBJ whole genome shotgun (WGS) entry which is preliminary data.
Chr06G1491.1	1518	1122	1247	UniProt ID:C4YII6_CAN	768	474	599	40/133 (30.08)	0.52	0.11	133	60.1	7.00E-10	gene=Chr06G1491	Gene Symbol:CAWG_04261|Host:Isolated from a wide

				AW											variety of substrates including humans|Disease:invasive candidal disease|Description:SIMILARITY: Belongs to the DEAD box helicase family.
Chr04G0008.1	1357	374	1357	UniProt ID:A4RCH6_MAGO7	1810	483	1422	516/991(52.07)	0.69	0.06	991	1033	0	gene=Chr04G0008	Gene Symbol:MGG_12656|Host:Poaceae, especially important on Oryzae|Disease:Rice blast|Description:SIMILARITY: Contains 1 reverse transcriptase domain.
Chr04G0009.1	984	335	470	UniProt ID:Q9HFW4_USTMD	2289	717	856	40/141 (28.37)	0.45	0.04	141	55.8	1.00E-08	gene=Chr04G0009	Gene Symbol:RUM1|Host:Euchlaena spp., Zea spp. (Poaceae)|Disease:Smut. Corn smut|Description:SIMILARITY: Contains 1 ARID domain.
Chr04G0015.1	1174	743	959	UniProt ID:FUZ7_UST	435	118	294	55/217 (25.35)	0.48	0.18	217	63.5	3.00E-11	gene=Chr04G0015	Gene Symbol:FUZ7|Host:Euchlaena spp., Zea spp.

				MA											(Poaceae)|Disease:Smut. Corn smut|Description:FUNCTION: Protein kinase that is necessary for a-locus-dependent processes, such as conjugation tube formation, filament formation, and maintenance of filamentous growth, and for a-locus-independent processes, such as tumor induction and teliospore germination.
Chr04G0017.1	1525	1088	1368	UniProt ID:TUP1_CANAL	514	201	509	105/311(33.76)	0.54	0.1	311	175	7.00E-47	gene=Chr04G0017	Gene Symbol:TUP1|Host:Isolated from a wide variety of substrates including humans|Disease:invasive candidal disease|Description:FUNCTION: Represses transcription by RNA polymerase II. Represses

															genes responsible for initiating filamentous growth and this repression is lifted under inducing environmental conditions.
Chr04G0027.1	2371	53	1329	UniProt ID:Q92217_COCHE	2528	11	1310	453/1340(33.81)	0.52	0.08	1340	692	0	gene=Chr04G0027	Gene Symbol:PKS1|Host:Zea mays|Disease:Southern leaf blight of maize|Description:Unknown
Chr04G0033.1	542	3	541	UniProt ID:Q9P8L8_BOTFU	598	66	597	147/549(26.78)	0.48	0.05	549	209	2.00E-60	gene=Chr04G0033	Gene Symbol:BCMFS1|Host:Various plant families|Disease:Grey mould. Parasite or saprophyte|Description:Unknown
Chr04G0034.1	393	55	393	UniProt ID:A4QVF8_MAGO7	339	2	338	184/339(54.28)	0.75	0.01	339	383	7.00E-132	gene=Chr04G0034	Gene Symbol:MGG_04556|Host:Poaceae, especially important on Oryzae|Disease:Rice blast|Description:COFACTOR: Zinc (By similarity).
Chr04G0	614	33	614	UniProt	693	81	674	208/61	0.49	0.09	617	255	9.00E-76	gene=Chr	Gene

<table>
<tr><td>035.1</td><td></td><td></td><td></td><td>ID:Q4P8E8_USTMA</td><td></td><td></td><td></td><td>7(33.71)</td><td></td><td></td><td></td><td></td><td></td><td>04G0035</td><td>Symbol:UM03615.1|Host: Euchlaena spp., Zea spp. (Poaceae)|Disease:Smut. Corn smut|Description:COFACTOR: FAD (By similarity).</td></tr>
<tr><td>Chr04G0036.1</td><td>721</td><td>14</td><td>86</td><td>UniProt ID:Q0WXM3_FUSOX</td><td>663</td><td>76</td><td>154</td><td>26/79(32.91)</td><td>0.51</td><td>0.08</td><td>79</td><td>48.9</td><td>7.00E-07</td><td>gene=Chr04G0036</td><td>Gene Symbol:FOW2|Host:Multiple genera in multiple families|Disease:Blights, wilts, rots of various sorts|Description:SIMILARITY: Contains 1 Zn(2)-C6 fungal-type DNA-binding domain.</td></tr>
<tr><td>Chr04G0038.1</td><td>551</td><td>271</td><td>476</td><td>UniProt ID:A4R9C7_MAGO7</td><td>1305</td><td>250</td><td>470</td><td>66/229 (28.82)</td><td>0.46</td><td>0.14</td><td>229</td><td>74.3</td><td>9.00E-15</td><td>gene=Chr04G0038</td><td>Gene Symbol:MGG_13324|Host:Poaceae, especially important on Oryzae|Disease:Rice blast|Description:Unknown</td></tr>
<tr><td>Chr04G0042.1</td><td>739</td><td>38</td><td>287</td><td>UniProt ID:Q9Y784_MAGGR</td><td>631</td><td>36</td><td>316</td><td>68/283 (24.03)</td><td>0.45</td><td>0.12</td><td>283</td><td>84.3</td><td>7.00E-18</td><td>gene=Chr04G0042</td><td>Gene Symbol:PTH11|Host:Digitaria (Poaceae)|Disease:Leaf</td></tr>
</table>

														spot|Description:Unknown
Chr04G0043.1	446	99	293	UniProt ID:MCPAL_ARTOC	416	148	316	59/199 (29.65)	0.45	0.17	199	69.3	9.00E-14	gene=Chr04G0043	Gene Symbol:MCYG_01475|Host:humans, reptiles|Disease:dermatophytoses|Description:FUNCTION: Extracellular metalloprotease that contributes to pathogenicity (By similarity).
Chr04G0049.1	256	2	253	UniProt ID:A4RGG9_MAGO7	286	13	269	76/261 (29.12)	0.44	0.05	261	84	5.00E-20	gene=Chr04G0049	Gene Symbol:MGG_00056|Host:Poaceae, especially important on Oryzae|Disease:Rice blast|Description:SIMILARITY: Belongs to the short-chain dehydrogenases/reductases (SDR) family.
Chr04G0052.1	373	1	369	UniProt ID:LAP1_ARTOC	373	1	370	152/389(39.07)	0.54	0.1	389	236	8.00E-75	gene=Chr04G0052	Gene Symbol:LAP1|Host:humans, reptiles|Disease:dermatophytoses|Description:FUNC

															TION: Extracellular aminopeptidase which contributes to pathogenicity (By similarity).
Chr04G0056.1	487	23	486	UniProt ID:A5HF03_MAGGR	520	44	520	300/478(62.76)	0.78	0.03	478	620	0	gene=Chr04G0056	Gene Symbol:NULL|Host:Digitaria (Poaceae)|Disease:Leaf spot|Description:Unknown
Chr04G0060.1	537	21	537	UniProt ID:Q2KN79_FUSOX	539	19	539	315/524(60.11)	0.72	0.02	524	636	0	gene=Chr04G0060	Gene Symbol:GAS1|Host:Multiple genera in multiple families|Disease:Blights, wilts, rots of various sorts|Description:Unknown
Chr04G0062.1	682	78	245	UniProt ID:Q59VF3_CANAL	1813	69	232	59/169(34.91)	0.53	0.04	169	89.7	3.00E-19	gene=Chr04G0062	Gene Symbol:"DUR1,2"|Host:Isolated from a wide variety of substrates including humans|Disease:invasive candidal disease|Description:CAUTION: The sequence shown here is derived from an EMBL/GenBank/DDBJ

															whole genome shotgun (WGS) entry which is preliminary data.
Chr04G0071.1	507	63	97	UniProt ID:O59937_FUSOX	384	22	56	20/35(57.14)	0.66	0	35	53.9	8.00E-09	gene=Chr04G0071	Gene Symbol:XYL3|Host:Multiple genera in multiple families|Disease:Blights, wilts, rots of various sorts|Description:SIMILARITY: Belongs to the glycosyl hydrolase 10 (cellulase F) family.
Chr04G0074.1	698	96	665	UniProt ID:B2C6F1_CRYGA	614	36	584	186/596(31.21)	0.45	0.12	596	214	4.00E-61	gene=Chr04G0074	Gene Symbol:LAC1|Host:humans|Disease:pulmonary cryptococcosis, basal meningitis, and cerebral cryptococcomas|Description:Unknown
Chr04G0079.1	1129	683	1105	UniProt ID:Q8J286_COLLN	1167	388	879	158/497(31.79)	0.5	0.16	497	217	1.00E-58	gene=Chr04G0079	Gene Symbol:CLAP1|Host:Multiple genera of Fabaceae. Rare reports on other taxa|Disease:Leaf, stem and pod anthracnose|Description:S

															IMILARITY: Belongs to the cation transport ATPase (P-type) family.
Chr04G0082.1	547	100	529	UniProt ID:Q59VF3_CANAL	1813	69	453	110/438(25.11)	0.43	0.14	438	87.4	6.00E-19	gene=Chr04G0082	Gene Symbol:"DUR1,2"|Host:Isolated from a wide variety of substrates including humans|Disease:invasive candidal disease|Description:CAUTION: The sequence shown here is derived from an EMBL/GenBank/DDBJ whole genome shotgun (WGS) entry which is preliminary data.
Chr04G0086.1	208	2	192	UniProt ID:Q5IFQ6_CRYNV	234	25	220	98/196 (50.00)	0.64	0.03	196	195	1.00E-62	gene=Chr04G0086	Gene Symbol:SOD2|Host:humans|Disease:cryptococcosis|Description:FUNCTION: Destroys radicals which are normally produced within the cells and which are toxic to biological systems (By similarity).
Chr04G0	675	379	605	UniProt	276	56	275	73/247	0.4	0.19	247	62	1.00E-11	gene=Chr	Gene

090.1				ID:MEP1_COCP7				(29.55)						04G0090	Symbol:MEP1|Host:humans|Disease:coccidiomycosis|Description:FUNCTION: Secreted metalloproteinase that allows assimilation of proteinaceous substrates. Pays a pivotal role as a pathogenicity determinant during infections and contributes to the ability of the pathogen to persist within the mammalian host. Digests an immunodominant cell surface antigen (SOWgp) and prevents host recognition of endospores during the phase of development when these fungal cells are most vulnerable to phagocytic cell defenses.
Chr04G0093.1	552	34	72	UniProt ID:Q5A4F3_C	624	8	46	20/39(51.28)	0.72	0	39	56.2	3.00E-09	gene=Chr04G0093	Gene Symbol:ZCF37|Host:Isolated from a wide variety of

				ANAL											substrates including humans|Disease:invasive candidal disease|Description:Unknown
Chr04G0094.1	489	6	246	UniProt ID:A0ST42_CERNC	512	21	252	59/254 (23.23)	0.43	0.14	254	50.4	1.00E-07	gene=Chr04G0094	Gene Symbol:CTB4|Host:Numerous taxa in Solanaceae|Disease:Leaf spot|Description:Unknown
Chr04G0098.1	357	66	353	UniProt ID:Q6TFC7_ASPFM	349	61	347	97/292 (33.22)	0.52	0.03	292	165	5.00E-48	gene=Chr04G0098	Gene Symbol:NULL|Host:humans|Disease:infection|Description:Unknown
Chr04G0101.1	556	10	535	UniProt ID:Q5ANE1_CANAL	748	16	520	162/539(30.06)	0.49	0.09	539	221	1.00E-63	gene=Chr04G0101	Gene Symbol:SNF3|Host:Isolated from a wide variety of substrates including humans|Disease:invasive candidal disease|Description:SIMILARITY: Belongs to the major facilitator superfamily. Sugar transporter (TC 2.A.1.1) family.

Chr04G0106.1	612	12	605	UniProt ID:Q9P304_CLAFU	665	5	640	241/648(37.19)	0.56	0.1	648	417	1.00E-137	gene=Chr04G0106	Gene Symbol:AOX1|Host:Lycopersicon esculentum (Solanaceae)|Disease:Leaf mold of tomato|Description:COFACTOR: FAD (By similarity).
Chr04G0109.1	394	59	379	UniProt ID:Q2I0M6_CERNC	871	85	411	77/339 (22.71)	0.45	0.09	339	85.9	5.00E-19	gene=Chr04G0109	Gene Symbol:CTB3|Host:Numerous taxa in Solanaceae|Disease:Leaf spot|Description:Unknown
Chr04G0111.1	439	9	280	UniProt ID:Q9Y784_MAGGR	631	103	377	68/275 (24.73)	0.48	0.01	275	114	2.00E-28	gene=Chr04G0111	Gene Symbol:PTH11|Host:Digitaria (Poaceae)|Disease:Leaf spot|Description:Unknown
Chr04G0115.1	796	148	372	UniProt ID:A4R178_MAGO7	610	372	589	56/236 (23.73)	0.47	0.12	236	57.8	1.00E-09	gene=Chr04G0115	Gene Symbol:MGG_07015|Host:Poaceae, especially important on Oryzae|Disease:Rice blast|Description:Unknown
Chr04G0	123	2	118	UniProt	119	4	118	53/117	0.7	0.02	117	115	2.00E-34	gene=Chr	Gene

117.1				ID:Q5ABB1_CANAL				(45.30)						04G0117	Symbol:TTR1|Host:Isolated from a wide variety of substrates including humans|Disease:invasive candidal disease|Description:CAUTION: The sequence shown here is derived from an EMBL/GenBank/DDBJ whole genome shotgun (WGS) entry which is preliminary data.
Chr04G0125.1	667	200	507	UniProt ID:Q873Z8_FUSOX	1863	1236	1571	90/344 (26.16)	0.45	0.13	344	101	4.00E-23	gene=Chr04G0125	Gene Symbol:CHSV|Host:Multiple genera in multiple families|Disease:Blights, wilts, rots of various sorts|Description:Unknown
Chr04G0128.1	384	7	296	UniProt ID:Q9Y784_MAGGR	631	103	389	86/291 (29.55)	0.5	0.02	291	139	5.00E-37	gene=Chr04G0128	Gene Symbol:PTH11|Host:Digitaria (Poaceae)|Disease:Leaf spot|Description:Unknown
Chr04G0132.1	506	50	168	UniProt ID:A0S	459	67	183	35/119 (29.41)	0.49	0.02	119	49.7	2.00E-07	gene=Chr04G0132	Gene Symbol:CTB5|Host:Numer

				T43_CERNC										ous taxa in Solanaceae|Disease:Leaf spot|Description:Unknown
Chr04G0134.1	501	16	468	UniProt ID:Q5ANE1_CANAL	748	42	496	134/471(28.45)	0.5	0.07	471	172	2.00E-47	gene=Chr04G0134	Gene Symbol:SNF3|Host:Isolated from a wide variety of substrates including humans|Disease:invasive candidal disease|Description:SIMILARITY: Belongs to the major facilitator superfamily. Sugar transporter (TC 2.A.1.1) family.
Chr04G0136.1	786	139	774	UniProt ID:Q99324_SEPLY	803	106	792	189/729(25.93)	0.4	0.19	729	121	3.00E-29	gene=Chr04G0136	Gene Symbol:B2TOM|Host:Primarily tomato, Lycopersicon esculentum, also Solanum spp. and other Solanaceae|Disease:Leaf spot|Description:Unknown
Chr04G0137.1	326	63	326	UniProt ID:PLYB_COLGL	331	69	329	162/265(61.13)	0.72	0.02	265	314	4.00E-106	gene=Chr04G0137	Gene Symbol:PLB|Host:Multiple genera in multiple families|Disease:'Anthracn

														ose of stems and leaves, dieback, root rot, leaf spot, blossom rot, fruit rot (dieback and ripe rot), seedling blight.' (Mordue 1971)|Description:FUNCTION: Acts as a virulence factor active in plant tissue maceration.
Chr04G0142.1	616	116	492	UniProt ID:Q59RG1_CANAL	561	83	474	87/405 (21.48)	0.37	0.1	405	58.5	5.00E-10	gene=Chr04G0142	Gene Symbol:NAG3|Host:Isolated from a wide variety of substrates including humans|Disease:invasive candidal disease|Description:CAUTION: The sequence shown here is derived from an EMBL/GenBank/DDBJ whole genome shotgun (WGS) entry which is preliminary data.
Chr04G0149.1	353	22	235	UniProt ID:Q9Y784_MAGGR	631	113	319	61/225 (27.11)	0.48	0.13	225	68.2	2.00E-13	gene=Chr04G0149	Gene Symbol:PTH11|Host:Digitaria (Poaceae)|Disease:Leaf

															spot|Description:Unknown
Chr04G0155.1	306	146	304	UniProt ID:Q59VG6_CANAL	386	210	358	44/159 (27.67)	0.43	0.06	159	49.3	1.00E-07	gene=Chr04G0155	Gene Symbol:ERG3|Host:Isolated from a wide variety of substrates including humans|Disease:invasive candidal disease|Description:CAUTION: The sequence shown here is derived from an EMBL/GenBank/DDBJ whole genome shotgun (WGS) entry which is preliminary data.
Chr04G0164.1	565	137	546	UniProt ID:Q5ANE1_CANAL	748	83	496	97/426 (22.77)	0.42	0.07	426	77.8	5.00E-16	gene=Chr04G0164	Gene Symbol:SNF3|Host:Isolated from a wide variety of substrates including humans|Disease:invasive candidal disease|Description:SIMILARITY: Belongs to the major facilitator superfamily. Sugar transporter (TC 2.A.1.1) family.

Chr04G0165.1	544	56	492	UniProt ID:Q5ANE1_CANAL	748	51	492	106/449(23.61)	0.4	0.04	449	95.9	1.00E-21	gene=Chr04G0165	Gene Symbol:SNF3|Host:Isolated from a wide variety of substrates including humans|Disease:invasive candidal disease|Description:SIMILARITY: Belongs to the major facilitator superfamily. Sugar transporter (TC 2.A.1.1) family.
Chr04G0166.1	1172	51	532	UniProt ID:Q5ANE1_CANAL	748	39	513	139/498(27.91)	0.47	0.08	498	204	2.00E-55	gene=Chr04G0166	Gene Symbol:SNF3|Host:Isolated from a wide variety of substrates including humans|Disease:invasive candidal disease|Description:SIMILARITY: Belongs to the major facilitator superfamily. Sugar transporter (TC 2.A.1.1) family.
Chr04G0167.1	462	2	369	UniProt ID:Q5G	437	11	385	98/410(23.90)	0.38	0.19	410	90.9	1.00E-20	gene=Chr04G0167	Gene Symbol:NULL|Host:Multipl

				FD3_PHAND											e genera of Poaceae and Blysmus compressus (Cyperaceae)|Disease:Glume blotch of wheat and other grasses|Description:Unknown
Chr04G0168.1	572	245	485	UniProt ID:Q9C0P7_NECHA	247	1	241	97/242 (40.08)	0.59	0.01	242	179	2.00E-52	gene=Chr04G0168	Gene Symbol:PEP5|Host:Trees of various plant families|Disease:Fruit rot, stem rot|Description:Unknown
Chr04G0169.1	376	6	282	UniProt ID:Q9Y784_MAGGR	631	101	378	74/280 (26.43)	0.48	0.02	280	96.3	1.00E-22	gene=Chr04G0169	Gene Symbol:PTH11|Host:Digitaria (Poaceae)|Disease:Leaf spot|Description:Unknown
Chr04G0190.1	505	29	73	UniProt ID:Q5A4F3_CANAL	624	1	46	17/46(36.96)	0.59	0.02	46	46.2	3.00E-06	gene=Chr04G0190	Gene Symbol:ZCF37|Host:Isolated from a wide variety of substrates including humans|Disease:invasive candidal disease|Description:Unknown

Chr04G0192.1	458	196	432	UniProt ID:Q0PND8_MAGGR	1375	989	1229	96/245 (39.18)	0.58	0.05	245	151	7.00E-40	gene=Chr04G0192	Gene Symbol:PEX6|Host:Digitaria (Poaceae)|Disease:Leaf spot|Description:SIMILARITY: Belongs to the AAA ATPase family.
Chr04G0198.1	261	1	258	UniProt ID:Q5AFE4_CANAL	293	30	289	143/262(54.58)	0.75	0.02	262	300	5.00E-102	gene=Chr04G0198	Gene Symbol:RVS161|Host:Isolated from a wide variety of substrates including humans|Disease:invasive candidal disease|Description:CAUTION: The sequence shown here is derived from an EMBL/GenBank/DDBJ whole genome shotgun (WGS) entry which is preliminary data.
Chr04G0200.1	1068	160	361	UniProt ID:Q59YF3_CANAL	986	619	805	53/209 (25.36)	0.45	0.14	209	69.7	6.00E-13	gene=Chr04G0200	Gene Symbol:INP51|Host:Isolated from a wide variety of substrates including humans|Disease:invasive candidal disease|Description:CAUT

															ION: The sequence shown here is derived from an EMBL/GenBank/DDBJ whole genome shotgun (WGS) entry which is preliminary data.
Chr04G0201.1	470	25	403	UniProt ID:Q9Y784_MAGGR	631	32	408	95/397 (23.93)	0.43	0.1	397	112	1.00E-27	gene=Chr04G0201	Gene Symbol:PTH11|Host:Digitaria (Poaceae)|Disease:Leaf spot|Description:Unknown
Chr04G0202.1	463	1	443	UniProt ID:A0ZT55_GLOAC	445	1	426	354/443(79.91)	0.89	0.04	443	726	0	gene=Chr04G0202	Gene Symbol:CATUB1|Host:Multiple genera in multiple families|Disease:Anthracnose, canker, leaf spot, shepherd's crook, fruit rot, etc|Description:FUNCTION: Tubulin is the major constituent of microtubules. It binds two moles of GTP, one at an exchangeable site on the beta chain and one at a non-exchangeable site on the alpha-chain (By

															similarity).
Chr04G0 203.1	369	12	269	UniProt ID:Q9Y 784_M AGGR	631	103	362	63/265 (23.77)	0.44	0.05	265	70.1	5.00E-14	gene=Chr 04G0203	Gene Symbol:PTH11|Host:Digit aria (Poaceae)|Disease:Leaf spot|Description:Unknown
Chr04G0 204.1	608	19	604	UniProt ID:Q4P 8E8_U STMA	693	69	673	221/62 9(35.1 4)	0.5	0.11	629	300	1.00E-92	gene=Chr 04G0204	Gene Symbol:UM03615.1|Host: Euchlaena spp., Zea spp. (Poaceae)|Disease:Smut. Corn smut|Description:COFAC TOR: FAD (By similarity).
Chr04G0 207.1	797	242	581	UniProt ID:Q9P 304_CL AFU	665	8	355	99/376 (26.33)	0.41	0.17	376	67.4	2.00E-12	gene=Chr 04G0207	Gene Symbol:AOX1|Host:Lycop ersicon esculentum (Solanaceae)|Disease:Lea f mold of tomato|Description:COFA CTOR: FAD (By similarity).
Chr04G0 209.1	3224	1670	313 5	UniProt ID:Q9U VN5_A LTAL	4360	907	2319	434/15 04(28. 86)	0.46	0.09	1504	544	3.00E-15 6	gene=Chr 04G0209	Gene Symbol:AMT|Host:Plant|D isease:Leaf spot, rots|Description:Unknown
Chr04G0	409	141	384	UniProt	4034	1351	1588	70/254	0.5	0.1	254	89.7	4.00E-20	gene=Chr	Gene

213.1				ID:Q6ZX14_MAGGR				(27.56)						04G0213	Symbol:ACE1|Host:Digitaria (Poaceae)|Disease:Leaf spot|Description:Unknown
Chr04G0216.1	1973	15	1246	UniProt ID:Q92217_COCHE	2528	9	1332	432/1339(32.26)	0.48	0.09	1339	574	1.00E-169	gene=Chr04G0216	Gene Symbol:PKS1|Host:Zea mays|Disease:Southern leaf blight of maize|Description:Unknown
Chr04G0218.1	483	20	478	UniProt ID:Q5ABU7_CANAL	564	93	559	152/471(32.27)	0.54	0.03	471	258	2.00E-79	gene=Chr04G0218	Gene Symbol:MDR1|Host:Isolated from a wide variety of substrates including humans|Disease:invasive candidal disease|Description:Unknown
Chr04G0222.1	270	4	258	UniProt ID:P79068_GLOLA	2187	1924	2174	72/273(26.37)	0.42	0.15	273	61.6	1.00E-11	gene=Chr04G0222	Gene Symbol:PKS1|Host:melons,cucumber|Disease:anthracnose fruit rot|Description:Unknown
Chr04G0225.1	2497	25	2490	UniProt ID:Q92217_COCHE	2528	13	2518	821/2606(31.50)	0.48	0.09	2606	1047	0	gene=Chr04G0225	Gene Symbol:PKS1|Host:Zea mays|Disease:Southern leaf blight of

															maize|Description:Unknown
Chr04G0229.1	187	112	179	UniProt ID:NPIID_ARTOC	353	283	349	30/73(41.10)	0.49	0.15	73	43.1	3.00E-06	gene=Chr04G0229	Gene Symbol:MCYG_07155|Host:humans, reptiles|Disease:dermatophytoses|Description:FUNCTION: Probable secreted metalloprotease that shows high activities on basic nuclear substrates such as histone and protamine (By similarity). May be involved in virulence.
Chr04G0230.1	650	61	648	UniProt ID:Q2VLJ1_GIBZA	565	11	562	206/600(34.33)	0.5	0.1	600	330	6.00E-105	gene=Chr04G0230	Gene Symbol:ZEB1|Host:Principal hosts: Poaceae, including Zea mays (corn), Triticum aestivum (wheat), and Oryza sativa (rice). Additional hosts: various plant families|Disease:Seedling blight, pre- and post-emergence blight,

															root and foot rot, brown rot, culm decay, head or kernel blight (scab or ear scab) of cereals. Leaf|Description:Unknown Gene Symbol:ZEB1|Host:Principal hosts: Poaceae, including Zea mays (corn), Triticum aestivum (wheat), and Oryza sativa (rice). Additional hosts: various plant families|Disease:Seedling blight, pre- and post-emergence blight, root and foot rot, brown rot, culm decay, head or kernel blight (scab or ear scab) of cereals. Leaf|Description:Unknown Gene
Chr04G0231.1	490	51	485	UniProt ID:Q2VLJ1_GIBZA	565	105	547	111/471(23.57)	0.4	0.14	471	72.8	1.00E-14	gene=Chr04G0231	
Chr04G0242.1	225	44	167	UniProt ID:Q696X2_PHAND	321	159	282	41/131(31.30)	0.45	0.11	131	53.5	1.00E-09	gene=Chr04G0242	Symbol:GLO1|Host:Multiple genera of Poaceae and Blysmus compressus (Cyperaceae)|Disease:Glu

															me blotch of wheat and other grasses|Description:Unknown
Chr04G0245.1	515	49	482	UniProt ID:Q5ANE1_CANAL	748	46	496	106/466(22.75)	0.42	0.1	466	89	1.00E-19	gene=Chr04G0245	Gene Symbol:SNF3|Host:Isolated from a wide variety of substrates including humans|Disease:invasive candidal disease|Description:SIMILARITY: Belongs to the major facilitator superfamily. Sugar transporter (TC 2.A.1.1) family.
Chr04G0248.1	643	25	495	UniProt ID:Q9P304_CLAFU	665	6	402	116/476(24.37)	0.39	0.18	476	79.3	2.00E-16	gene=Chr04G0248	Gene Symbol:AOX1|Host:Lycopersicon esculentum (Solanaceae)|Disease:Leaf mold of tomato|Description:COFACTOR: FAD (By similarity).
Chr04G0252.1	642	104	465	UniProt ID:Q5X	615	98	436	110/378(29.1	0.48	0.15	378	117	1.00E-28	gene=Chr04G0252	Gene Symbol:FRT1|Host:Variou

				TQ5_B OTFU				0)							s plant families|Disease:Grey mould. Parasite or saprophyte|Description:SIMILARITY: Belongs to the major facilitator superfamily. Sugar transporter (TC 2.A.1.1) family.
Chr04G0256.1	253	83	224	UniProt ID:Q92217_COCHE	2528	1875	1992	42/142 (29.58)	0.44	0.17	142	43.1	9.00E-06	gene=Chr04G0256	Gene Symbol:PKS1|Host:Zea mays|Disease:Southern leaf blight of maize|Description:Unknown
Chr04G0257.1	1893	1028	1339	UniProt ID:Q5AM49_CANAL	1690	776	1084	127/321(39.56)	0.61	0.07	321	257	8.00E-70	gene=Chr04G0257	Gene Symbol:SNF2|Host:Isolated from a wide variety of substrates including humans|Disease:invasive candidal disease|Description:Unknown
Chr04G0258.1	619	276	575	UniProt ID:Q3Y5V5_M	1321	973	1275	87/331 (26.28)	0.41	0.18	331	51.6	1.00E-07	gene=Chr04G0258	Gene Symbol:ABC3|Host:Digitaria (Poaceae)|Disease:Leaf

				AGGR											spot|Description:SIMILARITY: Belongs to the ABC transporter superfamily.
Chr04G0263.1	362	1	358	UniProt ID:Q4G495_BOTFU	374	8	372	197/368(53.53)	0.68	0.04	368	374	3.00E-128	gene=Chr04G0263	Gene Symbol:PG2|Host:Various plant families|Disease:Grey mould. Parasite or saprophyte|Description:SIMILARITY: Belongs to the glycosyl hydrolase 28 family.
Chr04G0273.1	1277	1143	1260	UniProt ID:Q66WN9_ALTBR	1328	1119	1234	43/118 (36.44)	0.63	0.02	118	91.3	2.00E-19	gene=Chr04G0273	Gene Symbol:NIK1|Host:Brassicaceae, especially cauliflower and white cabbage|Disease:Dark brown to almost black, circular, zonate leaf spot. Seed-borne. More common and causing more severe disease than Alternaria brassicae (Ellis 196|Description:SIMILARITY: Contains 1 histidine kinase domain.

Chr04G0274.1	708	54	409	UniProt ID:Q59NY2_CANAL	562	50	413	91/396 (22.98)	0.39	0.18	396	74.3	7.00E-15	gene=Chr04G0274	Gene Symbol:HEX1|Host:Isolated from a wide variety of substrates including humans|Disease:invasive candidal disease|Description:CAUTION: The sequence shown here is derived from an EMBL/GenBank/DDBJ whole genome shotgun (WGS) entry which is preliminary data.
Chr04G0277.1	322	67	317	UniProt ID:Q75WR5_9PLEO	265	9	263	77/267 (28.84)	0.47	0.1	267	91.3	2.00E-22	gene=Chr04G0277	Gene Symbol:BRN1|Host:Belamcanda chinensis: Korea,Gladiolus ?gandavensis: Korea,Iris japonica: China,Iris missouriensis (Leaf spot.): Idaho; Montana; Oregon; Washington,Iris sp. (Leaf spot.): China; Texas; Washing|Disease:Leaf spot|Description:SIMILARITY: Belongs to the

														short-chain dehydrogenases/reductases (SDR) family.
Chr04G0283.1	541	211	504	UniProt ID:A4ULI5_MYCGR	515	208	509	66/312 (21.15)	0.36	0.09	312	46.2	3.00E-06	gene=Chr04G0283	Gene Symbol:CYP51|Host:Triticum and possibly a few other grasses|Disease:Leaf spot or speckled leaf blotch of wheat|Description:COFACTOR: Heme group (By similarity).
Chr04G0289.1	416	38	361	UniProt ID:Q6A2T2_BOTFU	391	60	385	79/338 (23.37)	0.41	0.08	338	66.6	5.00E-13	gene=Chr04G0289	Gene Symbol:BTP1|Host:Various plant families|Disease:Grey mould. Parasite or saprophyte|Description:Unknown
Chr04G0293.1	520	18	505	UniProt ID:Q5ANE1_CANAL	748	16	503	108/509(21.22)	0.42	0.08	509	96.3	6.00E-22	gene=Chr04G0293	Gene Symbol:SNF3|Host:Isolated from a wide variety of substrates including humans|Disease:invasive candidal disease|Description:SIMIL

														ARITY: Belongs to the major facilitator superfamily. Sugar transporter (TC 2.A.1.1) family.
Chr04G0294.1	705	224	546	UniProt ID:Q0WXM3_FUSOX	663	260	575	74/336 (22.02)	0.4	0.1	336	62	6.00E-11	gene=Chr04G0294	Gene Symbol:FOW2|Host:Multiple genera in multiple families|Disease:Blights, wilts, rots of various sorts|Description:SIMILARITY: Contains 1 Zn(2)-C6 fungal-type DNA-binding domain.
Chr04G0295.1	831	17	371	UniProt ID:Q99324_SEPLY	803	57	481	136/431(31.55)	0.46	0.19	431	177	3.00E-47	gene=Chr04G0295	Gene Symbol:B2TOM|Host:Primarily tomato, Lycopersicon esculentum, also Solanum spp. and other Solanaceae|Disease:Leaf spot|Description:Unknown
Chr04G0297.1	376	19	278	UniProt ID:Q9Y784_MAGGR	631	111	370	63/261 (24.14)	0.5	0.01	261	95.5	2.00E-22	gene=Chr04G0297	Gene Symbol:PTH11|Host:Digitaria (Poaceae)|Disease:Leaf spot|Description:Unknown

Chr04G0298.1	922	153	316	UniProt ID:SU11B_COCP7	400	151	298	54/171 (31.58)	0.46	0.18	171	64.3	1.00E-11	gene=Chr04G0298	Gene Symbol:CPC735_047380|Host:humans|Disease:coccidiomycosis|Description: FUNCTION: Secreted subtilisin-like serine protease with keratinolytic activity that contributes to pathogenicity (By similarity).
Chr04G0299.1	362	14	354	UniProt ID:Q6A2T2_BOTFU	391	42	391	90/374 (24.06)	0.44	0.15	374	76.3	2.00E-16	gene=Chr04G0299	Gene Symbol:BTP1|Host:Various plant families|Disease:Grey mould. Parasite or saprophyte|Description:Unknown
Chr04G0304.1	434	27	347	UniProt ID:A4UC81_MAGO7	376	54	372	95/332 (28.61)	0.46	0.07	332	90.9	5.00E-21	gene=Chr04G0304	Gene Symbol:MGG_10702|Host:Poaceae, especially important on Oryzae|Disease:Rice blast|Description:Unknown
Chr04G0307.1	506	2	60	UniProt ID:Q70	703	552	607	25/59(42.37)	0.58	0.05	59	55.1	5.00E-09	gene=Chr04G0307	Gene Symbol:ACE2|Host:huma

				0F1_CANGB										ns|Disease:Occasional invasive candidal disease|Description:Unknown
Chr04G0310.1	370	97	308	UniProt ID:Q6A2T2_BOTFU	391	116	344	61/232 (26.29)	0.48	0.1	232	88.2	2.00E-20	gene=Chr04G0310	Gene Symbol:BTP1|Host:Various plant families|Disease:Grey mould. Parasite or saprophyte|Description:Unknown
Chr04G0311.1	351	16	206	UniProt ID:Q2I0M6_CERNC	871	457	650	59/199 (29.65)	0.46	0.07	199	68.9	1.00E-13	gene=Chr04G0311	Gene Symbol:CTB3|Host:Numerous taxa in Solanaceae|Disease:Leaf spot|Description:Unknown
Chr04G0316.1	768	160	754	UniProt ID:CCR4_CANAL	787	181	787	234/650(36.00)	0.55	0.15	650	426	1.00E-137	gene=Chr04G0316	Gene Symbol:CCR4|Host:Isolated from a wide variety of substrates including humans|Disease:invasive candidal disease|Description:FUNCTION: Acts as catalytic component of the CCR4-NOT core complex,

															which in the nucleus seems to be a general transcription factor, and in the cytoplasm the major mRNA deadenylase involved in mRNA turnover. Ccr4 has 3'-5' RNase activity with a strong preference for polyadenylated substrates and also low exonuclease activity towards single stranded DNA. Discovered because of its role in the control of ADH2 gene expression. It is required for the expression of genes involved in non-fermentative growth (By similarity).
Chr04G0324.1	487	86	378	UniProt ID:Q9C1T0_FUSOX	467	114	402	78/309 (25.24)	0.45	0.12	309	91.7	8.00E-21	gene=Chr04G0324	Gene Symbol:ARG1|Host:Multiple genera in multiple families|Disease:Blights, wilts, rots of various sorts|Description:Unknow

															n
Chr04G0327.1	553	5	549	UniProt ID:Q4P8E8_USTMA	693	83	671	179/617(29.01)	0.45	0.16	617	171	8.00E-47	gene=Chr04G0327	Gene Symbol:UM03615.1|Host: Euchlaena spp., Zea spp. (Poaceae)|Disease:Smut. Corn smut|Description:COFACTOR: FAD (By similarity).
Chr04G0328.1	394	22	388	UniProt ID:Q6A2T2_BOTFU	391	32	379	95/383(24.80)	0.46	0.13	383	103	1.00E-25	gene=Chr04G0328	Gene Symbol:BTP1|Host:Various plant families|Disease:Grey mould. Parasite or saprophyte|Description:Unknown
Chr04G0335.1	583	87	554	UniProt ID:Q5ANE1_CANAL	748	32	505	113/482(23.44)	0.43	0.05	482	118	1.00E-28	gene=Chr04G0335	Gene Symbol:SNF3|Host:Isolated from a wide variety of substrates including humans|Disease:invasive candidal disease|Description:SIMILARITY: Belongs to the major facilitator superfamily. Sugar transporter (TC 2.A.1.1)

														family.
Chr04G0338.1	502	67	500	UniProt ID:A0ST43_CERNC	459	8	456	133/463(28.73)	0.46	0.09	463	165	7.00E-46	gene=Chr04G0338	Gene Symbol:CTB5|Host:Numerous taxa in Solanaceae|Disease:Leaf spot|Description:Unknown
Chr04G0341.1	422	40	415	UniProt ID:Q96VZ3_FUSOX	455	60	435	117/384(30.47)	0.49	0.04	384	164	2.00E-46	gene=Chr04G0341	Gene Symbol:PGX1|Host:Multiple genera in multiple families|Disease:Blights, wilts, rots of various sorts|Description:SIMILARITY: Belongs to the glycosyl hydrolase 28 family.
Chr04G0346.1	740	1	53	UniProt ID:A8QJI7_FUSOX	938	88	139	20/53(37.74)	0.62	0.02	53	48.1	1.00E-06	gene=Chr04G0346	Gene Symbol:XLNR|Host:Multiple genera in multiple families|Disease:Blights, wilts, rots of various sorts|Description:SIMILARITY: Contains 1 Zn(2)-C6 fungal-type DNA-binding domain.
Chr04G0347.1	294	50	273	UniProt ID:A4Q	306	40	285	83/271(30.63)	0.4	0.27	271	115	1.00E-30	gene=Chr04G0347	Gene Symbol:MGG_10510|Host

				PV0_MAGO7											:Poaceae, especially important on Oryzae|Disease:Rice blast|Description:SIMILARITY: Belongs to the RNase T2 family.
Chr04G0355.1	338	6	260	UniProt ID:Q92217_COCHE	2528	1806	2054	63/266 (23.68)	0.47	0.11	266	56.6	9.00E-10	gene=Chr04G0355	Gene Symbol:PKS1|Host:Zea mays|Disease:Southern leaf blight of maize|Description:Unknown
Chr04G0357.1	589	11	582	UniProt ID:Q9P8L8_BOTFU	598	62	594	138/591(23.35)	0.41	0.13	591	79.7	1.00E-16	gene=Chr04G0357	Gene Symbol:BCMFS1|Host:Various plant families|Disease:Grey mould. Parasite or saprophyte|Description:Unknown
Chr04G0360.1	514	145	478	UniProt ID:A4ULJ2_MYCGR	515	155	507	81/369 (21.95)	0.38	0.14	369	68.9	2.00E-13	gene=Chr04G0360	Gene Symbol:CYP51|Host:Triticum and possibly a few other grasses|Disease:Leaf spot or speckled leaf blotch of wheat|Description:COFAC

															TOR: Heme group (By similarity).
Chr04G0362.1	1138	794	1123	UniProt ID:P87199_USTMD	968	1	329	132/345(38.26)	0.56	0.09	345	223	5.00E-61	gene=Chr04G0362	Gene Symbol:KIN2|Host:Euchlaena spp., Zea spp. (Poaceae)|Disease:Smut. Corn smut|Description:SIMILARITY: Belongs to the kinesin-like protein family.
Chr04G0385.1	532	2	511	UniProt ID:Q5XTQ5_BOTFU	615	41	550	148/526(28.14)	0.47	0.06	526	182	4.00E-51	gene=Chr04G0385	Gene Symbol:FRT1|Host:Various plant families|Disease:Grey mould. Parasite or saprophyte|Description:SIMILARITY: Belongs to the major facilitator superfamily. Sugar transporter (TC 2.A.1.1) family.
Chr04G0387.1	531	289	495	UniProt ID:A4ULI5_MYCGR	515	267	503	58/242(23.97)	0.41	0.17	242	57.4	1.00E-09	gene=Chr04G0387	Gene Symbol:CYP51|Host:Triticum and possibly a few other grasses|Disease:Leaf spot

															or speckled leaf blotch of wheat|Description:COFACTOR: Heme group (By similarity).
Chr04G0389.1	550	65	536	UniProt ID:Q5XTQ5_BOTFU	615	105	565	126/493(25.56)	0.44	0.11	493	112	4.00E-27	gene=Chr04G0389	Gene Symbol:FRT1|Host:Various plant families|Disease:Grey mould. Parasite or saprophyte|Description:SIMILARITY: Belongs to the major facilitator superfamily. Sugar transporter (TC 2.A.1.1) family.
Chr04G0391.1	381	1	347	UniProt ID:Q59MV8_CANAL	398	1	343	100/377(26.53)	0.42	0.17	377	77.8	1.00E-16	gene=Chr04G0391	Gene Symbol:SAP2|Host:Isolated from a wide variety of substrates including humans|Disease:invasive candidal disease|Description:SIMILARITY: Belongs to the peptidase A1 family.
Chr04G0392.1	388	29	314	UniProt ID:Q5A	748	102	435	79/343(23.03)	0.39	0.19	343	68.9	1.00E-13	gene=Chr04G0392	Gene Symbol:SNF3|Host:Isolate

				NE1_CANAL											d from a wide variety of substrates including humans|Disease:invasive candidal disease|Description:SIMILARITY: Belongs to the major facilitator superfamily. Sugar transporter (TC 2.A.1.1) family.
Chr04G0393.1	567	20	530	UniProt ID:Q5XTQ4_BOTFU	574	32	538	186/553(33.63)	0.48	0.16	553	236	4.00E-70	gene=Chr04G0393	Gene Symbol:LIP1|Host:Various plant families|Disease:Grey mould. Parasite or saprophyte|Description:Unknown
Chr04G0394.1	416	1	414	UniProt ID:ORYZ_ASPFU	403	4	402	200/424(47.17)	0.6	0.08	424	326	2.00E-108	gene=Chr04G0394	Gene Symbol:ALP1|Host:humans|Disease:infection|Description:FUNCTION: Secreted alkaline protease that allows assimilation of proteinaceous substrates. Acts as a significant

															virulence factor in invasive aspergillosis. Involved in immune evasion from the human and mice complement systems during infection. Efficiently cleaves important components of the complement cascade such as such as C3, C4, C5, and C1q, as well as IgG, which leads to down-regulation of complement activation at the hyphal surface.
Chr04G0399.1	489	11	463	UniProt ID:Q5ANE1_CANAL	748	37	496	141/469(30.06)	0.46	0.05	469	171	4.00E-47	gene=Chr04G0399	Gene Symbol:SNF3|Host:Isolated from a wide variety of substrates including humans|Disease:invasive candidal disease|Description:SIMILARITY: Belongs to the major facilitator superfamily. Sugar transporter (TC 2.A.1.1)

														family.
Chr04G0400.1	1075	15	207	UniProt ID:O93802_ALTAL	267	12	196	55/194 (28.35)	0.51	0.05	194	77.4	2.00E-16	gene=Chr04G0400	Gene Symbol:BRM2|Host:Plant|Disease:Leaf spot, rots|Description:SIMILARITY: Belongs to the short-chain dehydrogenases/reductases (SDR) family.
Chr04G0401.1	698	319	600	UniProt ID:Q9Y880_COCCA	880	32	262	95/282 (33.69)	0.48	0.18	282	146	2.00E-37	gene=Chr04G0401	Gene Symbol:SNF1|Host:Corn, Zea mays, sometimes on Sorghum (Poaceae) and various other plant families|Disease:Northern corn leaf spot, ear and kernel rot|Description:Unknown
Chr04G0405.1	704	5	704	UniProt ID:HSP90_CANAL	707	7	707	528/706(74.79)	0.86	0.02	706	997	0	gene=Chr04G0405	Gene Symbol:HSP90|Host:Isolated from a wide variety of substrates including humans|Disease:invasive candidal disease|Description:FUNCTION: Molecular

															chaperone that promotes the maturation, structural maintenance and proper regulation of specific target proteins involved for instance in cell cycle control and signal transduction. Undergoes a functional cycle that is linked to its ATPase activity. This cycle probably induces conformational changes in the client proteins, thereby causing their activation. Interacts dynamically with various co-chaperones that modulate its substrate recognition, ATPase cycle and chaperone function (By similarity).
Chr04G0410.1	776	407	674	UniProt ID:TUP1_CANAL	514	265	509	77/284 (27.11)	0.42	0.19	284	99.4	1.00E-22	gene=Chr04G0410	Gene Symbol:TUP1|Host:Isolated from a wide variety of substrates including humans|Disease:invasive

															candidal disease|Description:FUNCTION: Represses transcription by RNA polymerase II. Represses genes responsible for initiating filamentous growth and this repression is lifted under inducing environmental conditions.
Chr04G0414.1	547	194	533	UniProt ID:Q4P8E7_USTMA	703	274	695	123/425(28.94)	0.42	0.21	425	142	4.00E-37	gene=Chr04G0414	Gene Symbol:UM03616.1|Host: Euchlaena spp., Zea spp. (Poaceae)|Disease:Smut. Corn smut|Description:CAUTION: The sequence shown here is derived from an EMBL/GenBank/DDBJ whole genome shotgun (WGS) entry which is preliminary data.
Chr04G0421.1	651	14	47	UniProt ID:Q5A4F3_CANAL	624	13	46	17/34(50.00)	0.71	0	34	51.6	8.00E-08	gene=Chr04G0421	Gene Symbol:ZCF37|Host:Isolated from a wide variety of substrates including

															humans|Disease:invasive candidal disease|Description:Unknown
Chr04G0429.1	512	297	486	UniProt ID:A4ULJ2_MYCGR	515	302	511	54/210 (25.71)	0.46	0.1	210	61.6	4.00E-11	gene=Chr04G0429	Gene Symbol:CYP51|Host:Triticum and possibly a few other grasses|Disease:Leaf spot or speckled leaf blotch of wheat|Description:COFACTOR: Heme group (By similarity).
Chr04G0432.1	284	13	273	UniProt ID:O93802_ALTAL	267	8	266	88/272 (32.35)	0.49	0.09	272	110	3.00E-29	gene=Chr04G0432	Gene Symbol:BRM2|Host:Plant|Disease:Leaf spot, rots|Description:SIMILARITY: Belongs to the short-chain dehydrogenases/reductases (SDR) family.
Chr04G0450.1	608	268	356	UniProt ID:A3QX02_ASPFM	570	468	551	33/92(35.87)	0.47	0.12	92	50.1	2.00E-07	gene=Chr04G0450	Gene Symbol:ZAFA|Host:humans|Disease:infection|Description:Unknown
Chr04G0	795	483	787	UniProt	1321	351	681	133/33	0.56	0.12	338	207	3.00E-56	gene=Chr	Gene

454.1				ID:Q3Y5V5_MAGGR				8(39.35)						04G0454	Symbol:ABC3|Host:Digitaria (Poaceae)|Disease:Leaf spot|Description:SIMILARITY: Belongs to the ABC transporter superfamily.
Chr04G0459.1	371	276	362	UniProt ID:Q5ANM6_CANAL	286	198	284	32/87(36.78)	0.63	0	87	62.8	5.00E-12	gene=Chr04G0459	Gene Symbol:PEP12|Host:Isolated from a wide variety of substrates including humans|Disease:invasive candidal disease|Description:CAUTION: The sequence shown here is derived from an EMBL/GenBank/DDBJ whole genome shotgun (WGS) entry which is preliminary data.
Chr04G0460.1	149	8	142	UniProt ID:Q5AK12_CANAL	201	48	183	50/140(35.71)	0.59	0.06	140	82.4	1.00E-20	gene=Chr04G0460	Gene Symbol:CNB1|Host:Isolated from a wide variety of substrates including humans|Disease:invasive candidal disease|Description:CAUTION: The sequence shown

														here is derived from an EMBL/GenBank/DDBJ whole genome shotgun (WGS) entry which is preliminary data.
Chr04G0466.1	343	76	158	UniProt ID:C1GL98_PARBD	761	235	317	33/83(39.76)	0.59	0	83	66.2	7.00E-13	gene=Chr04G0466	Gene Symbol:PADG_07884|Host:humans|Disease:Paracoccidioidomycosis|Description:Unknown
Chr04G0469.1	252	3	250	UniProt ID:Q75WR5_9PLEO	265	8	263	78/262(29.77)	0.47	0.08	262	97.8	6.00E-25	gene=Chr04G0469	Gene Symbol:BRN1|Host:Belamcanda chinensis: Korea,Gladiolus ?gandavensis: Korea,Iris japonica: China,Iris missouriensis (Leaf spot.): Idaho; Montana; Oregon; Washington,Iris sp. (Leaf spot.): China; Texas; Washing|Disease:Leaf spot|Description:SIMILARITY: Belongs to the short-chain dehydrogenases/reductases (SDR) family.

Chr04G0472.1	614	115	591	UniProt ID:Q8J214_USTMD	592	65	568	275/512(53.71)	0.68	0.08	512	509	4.00E-174	gene=Chr04G0472	Gene Symbol:CRU1|Host:Euchlaena spp., Zea spp. (Poaceae)|Disease:Smut. Corn smut|Description:Unknown
Chr04G0479.1	542	46	526	UniProt ID:ECM14_ARTOC	593	57	553	239/506(47.23)	0.63	0.07	506	437	2.00E-147	gene=Chr04G0479	Gene Symbol:ECM14|Host:humans, reptiles|Disease:dermatophytoses|Description:FUNCTION: Probable carboxypeptidase that may be involved in cell wall organization and biogenesis (By similarity).
Chr04G0488.1	969	245	562	UniProt ID:Q59YF3_CANAL	986	162	479	75/344 (21.80)	0.4	0.15	344	63.9	3.00E-11	gene=Chr04G0488	Gene Symbol:INP51|Host:Isolated from a wide variety of substrates including humans|Disease:invasive candidal disease|Description:CAUTION: The sequence shown here is derived from an

														EMBL/GenBank/DDBJ whole genome shotgun (WGS) entry which is preliminary data.
Chr04G0491.1	256	3	254	UniProt ID:Q96TN6_MAGGR	251	2	249	166/252(65.87)	0.78	0.02	252	335	1.00E-116	gene=Chr04G0491	Gene Symbol:MAS3|Host:Digitaria (Poaceae)|Disease:Leaf spot|Description:Unknown
Chr04G0497.1	480	98	393	UniProt ID:A3F9N0_HANAN	368	71	367	120/301(39.87)	0.58	0.03	301	222	4.00E-68	gene=Chr04G0497	Gene Symbol:MNN9|Host:humans|Disease:infection|Description:Unknown
Chr04G0507.1	750	274	552	UniProt ID:Q5ALS7_CANAL	1144	360	657	73/303(24.09)	0.42	0.1	303	74.3	1.00E-14	gene=Chr04G0507	Gene Symbol:CTF1|Host:Isolated from a wide variety of substrates including humans|Disease:invasive candidal disease|Description:SIMILARITY: Contains 1 Zn(2)-C6 fungal-type DNA-binding domain.
Chr04G0510.1	553	6	443	UniProt ID:Q9P8L8_B	598	72	505	130/447(29.08)	0.48	0.05	447	166	2.00E-45	gene=Chr04G0510	Gene Symbol:BCMFS1|Host:Various plant

				OTFU											families|Disease:Grey mould. Parasite or saprophyte|Description:Unknown
Chr04G0511.1	271	1	250	UniProt ID:O93802_ALTAL	267	1	254	76/268 (28.36)	0.47	0.12	268	76.3	2.00E-17	gene=Chr04G0511	Gene Symbol:BRM2|Host:Plant|Disease:Leaf spot, rots|Description:SIMILARITY: Belongs to the short-chain dehydrogenases/reductases (SDR) family.
Chr04G0512.1	902	5	104	UniProt ID:Q59JX6_CANAL	103	4	103	55/100 (55.00)	0.8	0	100	112	1.00E-29	gene=Chr04G0512	Gene Symbol:FOX2|Host:Isolated from a wide variety of substrates including humans|Disease:invasive candidal disease|Description:CAUTION: The sequence shown here is derived from an EMBL/GenBank/DDBJ whole genome shotgun (WGS) entry which is preliminary data.
Chr04G0	700	268	632	UniProt	578	207	531	83/379	0.4	0.18	379	58.9	5.00E-10	gene=Chr	Gene

518.1				ID:Q96 VB5_A LTAL				(21.90)						04G0518	Symbol:AFT1-1|Host:Plant|Disease:Leaf spot, rots|Description:Unknown
Chr04G0 519.1	190	1	171	UniProt ID:CAN B_CRY NJ	175	1	158	57/174 (32.76)	0.56	0.11	174	75.5	5.00E-18	gene=Chr 04G0519	Gene Symbol:CNB1|Host:humans|Disease:cryptococcosis |Description:FUNCTION: Regulatory subunit of calcineurin, a calcium-dependent, calmodulin stimulated protein phosphatase. Confers calcium sensitivity. Plays a central role in virulence and antifungal drug action.
Chr04G0 522.1	358	75	358	UniProt ID:Q6T FC7_A SPFM	349	60	346	96/288 (33.33)	0.51	0.02	288	169	1.00E-49	gene=Chr 04G0522	Gene Symbol:NULL|Host:humans|Disease:infection|Description:Unknown
Chr04G0 525.1	783	32	72	UniProt ID:A6N 6J8_FU SOX	903	52	93	19/44(43.18)	0.59	0.11	44	45.4	1.00E-05	gene=Chr 04G0525	Gene Symbol:CTF1|Host:Multiple genera in multiple families|Disease:Blights, wilts, rots of various sorts|Description:SIMILAR

															ITY: Contains 1 Zn(2)-C6 fungal-type DNA-binding domain.
Chr04G0526.1	633	58	621	UniProt ID:Q9P8L8_BOTFU	598	22	592	260/576(45.14)	0.65	0.03	576	499	4.00E-170	gene=Chr04G0526	Gene Symbol:BCMFS1|Host:Various plant families|Disease:Grey mould. Parasite or saprophyte|Description:Unknown
Chr04G0527.1	550	21	63	UniProt ID:Q5A4F3_CANAL	624	1	46	20/46(43.48)	0.63	0.07	46	50.8	1.00E-07	gene=Chr04G0527	Gene Symbol:ZCF37|Host:Isolated from a wide variety of substrates including humans|Disease:invasive candidal disease|Description:Unknown
Chr04G0530.1	71	3	66	UniProt ID:SUB10_TRIVH	522	36	112	24/77(31.17)	0.49	0.17	77	40	4.00E-06	gene=Chr04G0530	Gene Symbol:SUB10|Host:humans|Disease:infection|Description:FUNCTION: Secreted subtilisin-like serine protease with keratinolytic activity that contributes to

															pathogenicity (By similarity).
Chr04G0532.1	1782	735	1569	UniProt ID:CHS6_USTMA	1180	61	890	490/839(58.40)	0.75	0.02	839	1012	0	gene=Chr04G0532	Gene Symbol:CHS6|Host:Euchlaena spp., Zea spp. (Poaceae)|Disease:Smut. Corn smut|Description:FUNCTION: Plays a major role in cell wall biogenesis. Involved in mating tube and dikaryotic hyphae formation. Essential for pathogenicity.
Chr04G0533.1	1866	1	1866	UniProt ID:Q8TFN4_COLGR	1866	1	1866	1705/1869(91.23)	0.96	0	1869	3556	0	gene=Chr04G0533	Gene Symbol:CHSV|Host:Poaceae especially Zea mays|Disease:Leaf spot, stalk rot, etc|Description:Unknown
Chr04G0536.1	113	1	113	UniProt ID:Q2LK92_BOTFU	117	1	117	81/117(69.23)	0.79	0.03	117	157	5.00E-51	gene=Chr04G0536	Gene Symbol:PIC5|Host:Various plant families|Disease:Grey mould. Parasite or saprophyte|Description:SI

															MILARITY: Belongs to the FKBP-type PPIase family.
Chr04G0542.1	695	346	577	UniProt ID:A4R0W3_MAGO7	1226	768	1012	64/248(25.81)	0.44	0.08	248	62.8	5.00E-11	gene=Chr04G0542	Gene Symbol:MGG_09263|Host:Poaceae, especially important on Oryzae|Disease:Rice blast|Description:Unknown
Chr04G0545.1	298	1	298	UniProt ID:C1G0P8_PARBD	288	1	288	200/299(66.89)	0.78	0.04	299	385	4.00E-135	gene=Chr04G0545	Gene Symbol:PADG_00438|Host:humans|Disease:Paracoccidioidomycosis|Description:FUNCTION: Component of the eukaryotic translation initiation factor 3 (eIF-3) complex (By similarity).
Chr04G0554.1	327	78	282	UniProt ID:CUTI_PYRBR	203	17	189	88/205(42.93)	0.56	0.16	205	154	1.00E-45	gene=Chr04G0554	Gene Symbol:NULL|Host:Brassica spp. (Brassicaceae)|Disease:Light leaf spot|Description:FUNCTION: Catalyzes the hydrolysis of cutin, a

															polyester that forms the structure of plant cuticle. Allows pathogenic fungi to penetrate through the cuticular barrier into the host plant during the initial stage of the fungal infection (By similarity).
Chr04G0566.1	1175	981	1173	UniProt ID:F2QQX0_PICP7	3007	2805	3001	53/197 (26.90)	0.47	0.02	197	77.4	4.00E-15	gene=Chr04G0566	Gene Symbol:CHS1|Host:humans|Disease:occasional infection|Description:SIMILARITY: Contains 1 PH domain.
Chr04G0581.1	2232	1011	1292	UniProt ID:Q59VF3_CANAL	1813	627	923	65/303 (21.45)	0.44	0.09	303	62.8	2.00E-10	gene=Chr04G0581	Gene Symbol:"DUR1,2"|Host:Isolated from a wide variety of substrates including humans|Disease:invasive candidal disease|Description:CAUTION: The sequence shown here is derived from an EMBL/GenBank/DDBJ whole genome shotgun (WGS) entry which is

															preliminary data.
Chr04G0587.1	414	5	155	UniProt ID:Q59KY8_CANAL	314	7	161	91/155 (58.71)	0.79	0.03	155	212	2.00E-65	gene=Chr04G0587	Gene Symbol:SIT4|Host:Isolated from a wide variety of substrates including humans|Disease:invasive candidal disease|Description:CATALYTIC ACTIVITY: A phosphoprotein + H(2)O = a protein + phosphate.
Chr04G0589.1	323	5	122	UniProt ID:Q4PCH2_USTMA	758	136	252	38/127 (29.92)	0.5	0.15	127	45.1	3.00E-06	gene=Chr04G0589	Gene Symbol:YAP1|Host:Euchlaena spp., Zea spp. (Poaceae)|Disease:Smut. Corn smut|Description:SIMILARITY: Belongs to the bZIP family.
Chr04G0593.1	403	1	384	UniProt ID:LAP1_ARTOC	373	1	371	198/385(51.43)	0.69	0.04	385	407	1.00E-140	gene=Chr04G0593	Gene Symbol:LAP1|Host:humans, reptiles|Disease:dermatophytoses|Description:FUNCTION: Extracellular aminopeptidase which

															contributes to pathogenicity (By similarity).
Chr04G0601.1	861	42	538	UniProt ID:Q59TT3_CANAL	1710	629	1051	180/515(34.95)	0.5	0.21	515	295	1.00E-84	gene=Chr04G0601	Gene Symbol:PLD1|Host:Isolated from a wide variety of substrates including humans|Disease:invasive candidal disease|Description:CAUTION: The sequence shown here is derived from an EMBL/GenBank/DDBJ whole genome shotgun (WGS) entry which is preliminary data.
Chr04G0605.1	328	36	326	UniProt ID:B8LZ89_TALSN	328	13	307	135/296(45.61)	0.67	0.02	296	270	7.00E-89	gene=Chr04G0605	Gene Symbol:TSTA_083650|Host:humans|Disease:occasional infection|Description:Unknown
Chr04G0614.1	1499	294	1499	UniProt ID:Q5A762_CANAL	1606	333	1606	347/1327(26.15)	0.45	0.13	1327	419	1.00E-122	gene=Chr04G0614	Gene Symbol:MLT1|Host:Isolated from a wide variety of substrates including

															humans|Disease:invasive candidal disease|Description:SIMILARITY: Belongs to the ABC transporter superfamily.
Chr04G0616.1	648	231	472	UniProt ID:Q5ACU9_CANAL	504	193	488	77/309 (24.92)	0.37	0.26	309	64.7	6.00E-12	gene=Chr04G0616	Gene Symbol:NDT80|Host:Isolated from a wide variety of substrates including humans|Disease:invasive candidal disease|Description:CAUTION: The sequence shown here is derived from an EMBL/GenBank/DDBJ whole genome shotgun (WGS) entry which is preliminary data.
Chr04G0619.1	477	23	121	UniProt ID:Q59W09_CANAL	379	20	115	48/99(48.48)	0.64	0.03	99	104	2.00E-25	gene=Chr04G0619	Gene Symbol:GCS1|Host:Isolated from a wide variety of substrates including humans|Disease:invasive candidal disease|Description:CAUT

														ION: The sequence shown here is derived from an EMBL/GenBank/DDBJ whole genome shotgun (WGS) entry which is preliminary data.
Chr04G0624.1	411	54	410	UniProt ID:C5GNK0_AJEDR	415	19	414	216/415(52.05)	0.62	0.19	415	308	3.00E-101	gene=Chr04G0624	Gene Symbol:BDCG_06357|Host:humans|Disease:cutaneous Blastomyces dermatitidis infection|Description:Unknown
Chr04G0631.1	741	485	712	UniProt ID:Q3Y5V5_MAGGR	1321	1075	1313	66/256(25.78)	0.45	0.18	256	62.4	7.00E-11	gene=Chr04G0631	Gene Symbol:ABC3|Host:Digitaria (Poaceae)|Disease:Leaf spot|Description:SIMILARITY: Belongs to the ABC transporter superfamily.
Chr04G0632.1	440	103	394	UniProt ID:Q00523_CRYNE	458	80	361	95/302(31.46)	0.49	0.1	302	143	1.00E-38	gene=Chr04G0632	Gene Symbol:NULL|Host:humans|Disease:cryptococcosis|Description:Unknown
Chr04G0634.1	1096	276	1070	UniProt ID:Q8J286_C	1167	196	1094	255/919(27.75)	0.46	0.16	919	257	6.00E-72	gene=Chr04G0634	Gene Symbol:CLAP1|Host:Multiple genera of Fabaceae.

				OLLN											Rare reports on other taxa|Disease:Leaf, stem and pod anthracnose|Description:SIMILARITY: Belongs to the cation transport ATPase (P-type) family.
Chr04G0637.1	323	1	323	UniProt ID:A4QPV0_MAGO7	306	1	302	174/327(53.21)	0.64	0.09	327	335	1.00E-114	gene=Chr04G0637	Gene Symbol:MGG_10510|Host:Poaceae, especially important on Oryzae|Disease:Rice blast|Description:SIMILARITY: Belongs to the RNase T2 family.
Chr04G0652.1	796	108	700	UniProt ID:C4YH28_CANAW	684	90	626	164/606(27.06)	0.46	0.14	606	195	7.00E-54	gene=Chr04G0652	Gene Symbol:CAWG_03369|Host:Isolated from a wide variety of substrates including humans|Disease:invasive candidal disease|Description:Unknown
Chr04G0654.1	120	10	120	UniProt ID:Q5A	122	8	122	71/115(61.74)	0.81	0.03	115	149	9.00E-48	gene=Chr04G0654	Gene Symbol:VMA7|Host:Isolat

				HJ0_CANAL										ed from a wide variety of substrates including humans|Disease:invasive candidal disease|Description:FUNCTION: Subunit of the peripheral V1 complex of vacuolar ATPase essential for assembly or catalytic function. V-ATPase is responsible for acidifying a variety of intracellular compartments in eukaryotic cells (By similarity).
Chr04G0656.1	381	53	377	UniProt ID:Q5AFI8_CANAL	373	8	361	96/358 (26.82)	0.45	0.1	358	99.4	3.00E-24	gene=Chr04G0656	Gene Symbol:LEU2|Host:Isolated from a wide variety of substrates including humans|Disease:invasive candidal disease|Description:FUNCTION: Catalyzes the oxidation of 3-carboxy-2-hydroxy-4-methylpentanoate

														(3-isopropylmalate) to 3-carboxy-4-methyl-2-oxopentanoate. The product decarboxylates to 4-methyl-2 oxopentanoate (By similarity).
Chr04G0657.1	934	312	413	UniProt ID:Q59ZX1_CANAL	1069	653	770	31/118 (26.27)	0.53	0.14	118	58.2	2.00E-09	gene=Chr04G0657	Gene Symbol:SET3|Host:Isolated from a wide variety of substrates including humans|Disease:invasive candidal disease|Description:SIMILARITY: Contains 1 SET domain.
Chr04G0658.1	466	3	458	UniProt ID:Q4X146_ASPFU	468	6	461	302/458(65.94)	0.83	0.01	458	650	0	gene=Chr04G0658	Gene Symbol:AFUA_2G11240|Host:humans|Disease:infection|Description:CAUTION: The sequence shown here is derived from an EMBL/GenBank/DDBJ whole genome shotgun (WGS) entry which is preliminary data.
Chr04G0	577	124	570	UniProt	564	95	557	131/46	0.48	0.05	467	206	4.00E-59	gene=Chr	Gene

659.1				ID:Q5ABU7_CANAL				7(28.05)						04G0659	Symbol:MDR1|Host:Isolated from a wide variety of substrates including humans|Disease:invasive candidal disease|Description:Unknown
Chr04G0665.1	428	19	222	UniProt ID:A4QVF8_MAGO7	339	2	199	64/210 (30.48)	0.46	0.09	210	67.4	3.00E-13	gene=Chr04G0665	Gene Symbol:MGG_04556|Host:Poaceae, especially important on Oryzae|Disease:Rice blast|Description:COFACTOR: Zinc (By similarity).
Chr04G0674.1	655	308	614	UniProt ID:Q6XPX0_FUSOX	359	63	316	68/320 (21.25)	0.38	0.25	320	64.3	6.00E-12	gene=Chr04G0674	Gene Symbol:FGB1|Host:Multiple genera in multiple families|Disease:Blights, wilts, rots of various sorts|Description:Unknown
Chr04G0680.1	276	26	271	UniProt ID:Q32WF7_PHAND	266	16	261	86/252 (34.13)	0.51	0.05	252	122	9.00E-34	gene=Chr04G0680	Gene Symbol:MDH1|Host:Multiple genera of Poaceae and Blysmus compressus (Cyperaceae)|Disease:Glu

															me blotch of wheat and other grasses|Description:Unknown
Chr04G0683.1	632	12	56	UniProt ID:Q5A4F3_CANAL	624	3	46	24/45(53.33)	0.71	0.02	45	60.8	1.00E-10	gene=Chr04G0683	Gene Symbol:ZCF37|Host:Isolated from a wide variety of substrates including humans|Disease:invasive candidal disease|Description:Unknown
Chr04G0688.1	615	403	559	UniProt ID:Q5AM49_CANAL	1690	1120	1276	52/158(32.91)	0.51	0.01	158	79	4.00E-16	gene=Chr04G0688	Gene Symbol:SNF2|Host:Isolated from a wide variety of substrates including humans|Disease:invasive candidal disease|Description:Unknown
Chr04G0696.1	383	116	302	UniProt ID:PLYB_COLGL	331	90	273	57/199(28.64)	0.46	0.14	199	63.5	4.00E-12	gene=Chr04G0696	Gene Symbol:PLB|Host:Multiple genera in multiple families|Disease:'Anthracnose of stems and leaves, dieback, root rot, leaf spot,

															blossom rot, fruit rot (dieback and ripe rot), seedling blight.' (Mordue 1971)|Description:FUNCTION: Acts as a virulence factor active in plant tissue maceration.
Chr04G0697.1	503	287	474	UniProt ID:A4ULI5_MYCGR	515	294	507	51/218 (23.39)	0.39	0.16	218	55.1	5.00E-09	gene=Chr04G0697	Gene Symbol:CYP51|Host:Triticum and possibly a few other grasses|Disease:Leaf spot or speckled leaf blotch of wheat|Description:COFACTOR: Heme group (By similarity).
Chr04G0698.1	337	31	336	UniProt ID:Q6TFC7_ASPFM	349	36	346	108/315(34.29)	0.52	0.04	315	184	1.00E-55	gene=Chr04G0698	Gene Symbol:NULL|Host:humans|Disease:infection|Description:Unknown
Chr04G0699.1	605	27	597	UniProt ID:Q4P8E8_USTMA	693	68	672	202/632(31.96)	0.48	0.14	632	241	7.00E-71	gene=Chr04G0699	Gene Symbol:UM03615.1|Host: Euchlaena spp., Zea spp. (Poaceae)|Disease:Smut. Corn smut|Description:COFAC

															TOR: FAD (By similarity).
Chr04G0700.1	611	19	454	UniProt ID:O93842_FUSSP	598	6	416	109/457(23.85)	0.39	0.15	457	54.3	1.00E-08	gene=Chr04G0700	Gene Symbol:TRI12|Host:animals|Disease:trichothecene|Description:Unknown
Chr04G0705.1	717	298	560	UniProt ID:A4QVA7_MAGO7	1340	523	781	63/270(23.33)	0.42	0.07	270	56.6	4.00E-09	gene=Chr04G0705	Gene Symbol:MGG_04629|Host:Poaceae, especially important on Oryzae|Disease:Rice blast|Description:Unknown
Chr04G0716.1	400	127	381	UniProt ID:Q59QH2_CANAL	337	44	320	79/282(28.01)	0.49	0.11	282	86.3	7.00E-20	gene=Chr04G0716	Gene Symbol:CSH1|Host:Isolated from a wide variety of substrates including humans|Disease:invasive candidal disease|Description:CAUTION: The sequence shown here is derived from an EMBL/GenBank/DDBJ whole genome shotgun (WGS) entry which is preliminary data.
Chr04G0	904	139	894	UniProt	803	31	796	444/77	0.72	0.03	772	899	0	gene=Chr	Gene

717.1				ID:Q99324_SEPLY				2(57.51)						04G0717	Symbol:B2TOM|Host:Primarily tomato, Lycopersicon esculentum, also Solanum spp. and other Solanaceae|Disease:Leaf spot|Description:Unknown
Chr04G0720.1	470	15	58	UniProt ID:O59937_FUSOX	384	13	56	25/44(56.82)	0.61	0	44	53.5	9.00E-09	gene=Chr04G0720	Gene Symbol:XYL3|Host:Multiple genera in multiple families|Disease:Blights, wilts, rots of various sorts|Description:SIMILARITY: Belongs to the glycosyl hydrolase 10 (cellulase F) family.
Chr04G0722.1	812	252	456	UniProt ID:Q0WXM3_FUSOX	663	255	450	55/212(25.94)	0.45	0.11	212	81.6	7.00E-17	gene=Chr04G0722	Gene Symbol:FOW2|Host:Multiple genera in multiple families|Disease:Blights, wilts, rots of various sorts|Description:SIMILARITY: Contains 1 Zn(2)-C6 fungal-type DNA-binding domain.
Chr04G0724.1	434	20	293	UniProt ID:A6Z	304	24	271	76/274(27.74)	0.49	0.09	274	120	1.00E-31	gene=Chr04G0724	Gene Symbol:SEC14|Host:hum

				MD8_YEAS7											ans|Disease:occasional infection|Description:SIMILARITY: Contains 1 CRAL-TRIO domain.
Chr04G0727.1	301	1	272	UniProt ID:Q59WE5_CANAL	323	1	300	97/308 (31.49)	0.46	0.14	308	107	1.00E-27	gene=Chr04G0727	Gene Symbol:PHO100|Host:Isolated from a wide variety of substrates including humans|Disease:invasive candidal disease|Description:CAUTION: The sequence shown here is derived from an EMBL/GenBank/DDBJ whole genome shotgun (WGS) entry which is preliminary data.
Chr04G0737.1	746	29	401	UniProt ID:SUB6_TRIEQ	412	25	372	110/408(26.96)	0.4	0.23	408	68.2	5.00E-13	gene=Chr04G0737	Gene Symbol:SUB6|Host:humans|Disease:Malabar itch|Description:FUNCTION: Secreted subtilisin-like serine protease with keratinolytic activity that contributes to pathogenicity (By

														similarity).
Chr04G0743.1	579	133	267	UniProt ID:Q59VF3_CANAL	1813	69	202	48/135 (35.56)	0.52	0.01	135	64.7	9.00E-12	gene=Chr04G0743	Gene Symbol:"DUR1,2"|Host:Isolated from a wide variety of substrates including humans|Disease:invasive candidal disease|Description:CAUTION: The sequence shown here is derived from an EMBL/GenBank/DDBJ whole genome shotgun (WGS) entry which is preliminary data.
Chr04G0744.1	667	10	49	UniProt ID:A8QJI7_FUSOX	938	95	131	18/40(45.00)	0.6	0.08	40	45.8	7.00E-06	gene=Chr04G0744	Gene Symbol:XLNR|Host:Multiple genera in multiple families|Disease:Blights, wilts, rots of various sorts|Description:SIMILARITY: Contains 1 Zn(2)-C6 fungal-type DNA-binding domain.
Chr04G0745.1	516	69	514	UniProt ID:A0ST43_C	459	8	459	135/469(28.78)	0.43	0.09	469	145	7.00E-39	gene=Chr04G0745	Gene Symbol:CTB5|Host:Numerous taxa in

				ERNC											Solanaceae|Disease:Leaf spot|Description:Unknown
Chr04G0747.1	491	4	487	UniProt ID:CATA_CANAL	487	3	480	234/486(48.15)	0.65	0.02	486	456	1.00E-156	gene=Chr04G0747	Gene Symbol:CTA1|Host:Isolated from a wide variety of substrates including humans|Disease:invasive candidal disease|Description:FUNCTION: Occurs in almost all aerobically respiring organisms and serves to protect cells from the toxic effects of hydrogen peroxide. Required for hyphal growth.
Chr04G0748.1	581	12	574	UniProt ID:Q4P8E8_USTMA	693	84	673	177/612(28.92)	0.44	0.12	612	193	3.00E-54	gene=Chr04G0748	Gene Symbol:UM03615.1|Host: Euchlaena spp., Zea spp. (Poaceae)|Disease:Smut. Corn smut|Description:COFACTOR: FAD (By similarity).
Chr04G0749.1	508	63	506	UniProt ID:A0ST43_C	459	1	459	123/485(25.36)	0.41	0.14	485	115	5.00E-29	gene=Chr04G0749	Gene Symbol:CTB5|Host:Numerous taxa in

				ERNC											Solanaceae|Disease:Leaf spot|Description:Unknown
Chr04G0750.1	366	30	233	UniProt ID:Q9Y784_MAGGR	631	120	319	47/209 (22.49)	0.41	0.07	209	46.6	1.00E-06	gene=Chr04G0750	Gene Symbol:PTH11|Host:Digitaria (Poaceae)|Disease:Leaf spot|Description:Unknown
Chr04G0767.1	333	56	247	UniProt ID:A4RGG9_MAGO7	286	16	215	56/207 (27.05)	0.43	0.11	207	47	5.00E-07	gene=Chr04G0767	Gene Symbol:MGG_00056|Host:Poaceae, especially important on Oryzae|Disease:Rice blast|Description:SIMILARITY: Belongs to the short-chain dehydrogenases/reductases (SDR) family.
Chr04G0770.1	533	19	533	UniProt ID:Q59WH0_CANAL	445	8	445	219/515(42.52)	0.58	0.15	515	397	8.00E-134	gene=Chr04G0770	Gene Symbol:ADA2|Host:Isolated from a wide variety of substrates including humans|Disease:invasive candidal disease|Description:CAUTION: The sequence shown here is derived from an

															EMBL/GenBank/DDBJ whole genome shotgun (WGS) entry which is preliminary data.
Chr04G0773.1	562	14	521	UniProt ID:Q5XTQ4_BOTFU	574	5	538	164/551(29.76)	0.44	0.11	551	166	2.00E-45	gene=Chr04G0773	Gene Symbol:LIP1|Host:Various plant families|Disease:Grey mould. Parasite or saprophyte|Description:Unknown
Chr04G0780.1	556	328	507	UniProt ID:A4ULJ2_MYCGR	515	269	485	54/217(24.88)	0.38	0.17	217	57.8	8.00E-10	gene=Chr04G0780	Gene Symbol:CYP51|Host:Triticum and possibly a few other grasses|Disease:Leaf spot or speckled leaf blotch of wheat|Description:COFACTOR: Heme group (By similarity).
Chr04G0781.1	489	3	51	UniProt ID:Q9P4U8_ALTAL	435	10	58	20/49(40.82)	0.55	0	49	45.8	3.00E-06	gene=Chr04G0781	Gene Symbol:AKTR-2|Host:Plant|Disease:Leaf spot, rots|Description:Unknown
Chr04G0782.1	526	17	471	UniProt ID:A0S	512	24	458	113/467(24.2	0.39	0.09	467	90.5	3.00E-20	gene=Chr04G0782	Gene Symbol:CTB4|Host:Numer

			T42_C ERNC				0)							ous taxa in Solanaceae|Disease:Leaf spot|Description:Unknown
Chr04G0 783.1	282	80	264	UniProt ID:Q32 WF7_P HAND	266	99	262	54/200 (27.00)	0.41	0.26	200	55.5	6.00E-10	gene=Chr 04G0783	Gene Symbol:MDH1|Host:Multiple genera of Poaceae and Blysmus compressus (Cyperaceae)|Disease:Glume blotch of wheat and other grasses|Description:Unknown
Chr04G0 789.1	495	269	472	UniProt ID:A4U LJ0_M YCGR	518	280	516	54/240 (22.50)	0.4	0.16	240	47.8	8.00E-07	gene=Chr 04G0789	Gene Symbol:CYP51|Host:Triticum and possibly a few other grasses|Disease:Leaf spot or speckled leaf blotch of wheat|Description:COFACTOR: Heme group (By similarity).
Chr04G0 793.1	927	186	342	UniProt ID:SUB 6_ART BE	412	158	296	49/170 (28.82)	0.43	0.26	170	54.3	2.00E-08	gene=Chr 04G0793	Gene Symbol:SUB6|Host:hedgehogs|Disease:ringworm,Kerion Celsi Disease|Description:FUN

															CTION: Secreted subtilisin-like serine protease with keratinolytic activity that contributes to pathogenicity (By similarity).
Chr04G0794.1	549	36	366	UniProt ID:Q9P304_CLAFU	665	8	332	84/356(23.60)	0.39	0.16	356	49.7	3.00E-07	gene=Chr04G0794	Gene Symbol:AOX1|Host:Lycopersicon esculentum (Solanaceae)|Disease:Leaf mold of tomato|Description:COFACTOR: FAD (By similarity).
Chr04G0797.1	477	3	38	UniProt ID:Q5A4F3_CANAL	624	11	46	16/36(44.44)	0.72	0	36	49.7	2.00E-07	gene=Chr04G0797	Gene Symbol:ZCF37|Host:Isolated from a wide variety of substrates including humans|Disease:invasive candidal disease|Description:Unknown
Chr04G0800.1	2551	31	2546	UniProt ID:Q92217_COCHE	2528	13	2524	925/2591(35.70)	0.54	0.06	2591	1459	0	gene=Chr04G0800	Gene Symbol:PKS1|Host:Zea mays|Disease:Southern leaf blight of

															maize|Description:Unknown
Chr04G0801.1	554	22	545	UniProt ID:Q9P8L8_BOTFU	598	69	589	272/526(51.71)	0.7	0.01	526	548	0	gene=Chr04G0801	Gene Symbol:BCMFS1|Host:Various plant families|Disease:Grey mould. Parasite or saprophyte|Description:Unknown
Chr04G0803.1	2339	23	1251	UniProt ID:Q92217_COCHE	2528	63	1300	485/1261(38.46)	0.54	0.04	1261	802	0	gene=Chr04G0803	Gene Symbol:PKS1|Host:Zea mays|Disease:Southern leaf blight of maize|Description:Unknown
Chr04G0805.1	564	339	535	UniProt ID:A4ULJ2_MYCGR	515	285	513	50/229(21.83)	0.43	0.14	229	69.3	2.00E-13	gene=Chr04G0805	Gene Symbol:CYP51|Host:Triticum and possibly a few other grasses|Disease:Leaf spot or speckled leaf blotch of wheat|Description:COFACTOR: Heme group (By similarity).
Chr04G0808.1	284	27	283	UniProt ID:MCP	422	24	283	118/265(44.5	0.62	0.05	265	236	3.00E-75	gene=Chr04G0808	Gene Symbol:MCPA|Host:huma

				A_TRIRU				3)							ns|Disease:infection|Description:FUNCTION: Extracellular metalloprotease that contributes to pathogenicity.
Chr04G0812.1	364	18	347	UniProtID:Q6A2T2_BOTFU	391	42	386	86/361(23.82)	0.43	0.13	361	94.7	9.00E-23	gene=Chr04G0812	Gene Symbol:BTP1|Host:Various plant families|Disease:Grey mould. Parasite or saprophyte|Description:Unknown
Chr04G0813.1	612	249	484	UniProtID:C4YM45_CANAW	317	5	204	67/240(27.92)	0.42	0.18	240	70.5	4.00E-14	gene=Chr04G0813	Gene Symbol:CAWG_01920|Host:Isolated from a wide variety of substrates including humans|Disease:invasive candidal disease|Description:Unknown
Chr04G0831.1	567	57	564	UniProtID:Q5XTQ4_BOTFU	574	58	571	201/552(36.41)	0.47	0.15	552	249	2.00E-75	gene=Chr04G0831	Gene Symbol:LIP1|Host:Various plant families|Disease:Grey

															mould. Parasite or saprophyte|Description:Unknown
Chr04G0833.1	1058	9	41	UniProt ID:Q5A4F3_CANAL	624	14	46	15/33(45.45)	0.67	0	33	47.8	2.00E-06	gene=Chr04G0833	Gene Symbol:ZCF37|Host:Isolated from a wide variety of substrates including humans|Disease:invasive candidal disease|Description:Unknown
Chr04G0836.1	331	41	325	UniProt ID:Q6TFC7_ASPFM	349	61	347	104/288(36.11)	0.56	0.01	288	195	8.00E-60	gene=Chr04G0836	Gene Symbol:NULL|Host:humans|Disease:infection|Description:Unknown
Chr04G0841.1	432	5	432	UniProt ID:Q9C1F9_COCCA	423	3	411	153/455(33.63)	0.49	0.16	455	223	4.00E-68	gene=Chr04G0841	Gene Symbol:CEL2|Host:Corn, Zea mays, sometimes on Sorghum (Poaceae) and various other plant families|Disease:Northern corn leaf spot, ear and kernel rot|Description:Unknown
Chr04G0842.1	361	12	44	UniProt ID:Q5A	624	14	46	19/33(57.58)	0.73	0	33	53.1	1.00E-08	gene=Chr04G0842	Gene Symbol:ZCF37|Host:Isolat

			4F3_CANAL											ed from a wide variety of substrates including humans|Disease:invasive candidal disease|Description:Unknown
Chr04G0859.1	386	97	386	UniProt ID:Q6TFC7_ASPFM	349	63	347	101/294(34.35)	0.54	0.04	294	163	4.00E-47	gene=Chr04G0859	Gene Symbol:NULL|Host:humans|Disease:infection|Description:Unknown
Chr04G0862.1	597	1	533	UniProt ID:Q4PDC5_USTMA	583	1	538	236/547(43.14)	0.61	0.04	547	471	9.00E-160	gene=Chr04G0862	Gene Symbol:UM01888.1|Host: Euchlaena spp., Zea spp. (Poaceae)|Disease:Smut. Corn smut|Description:CAUTION: The sequence shown here is derived from an EMBL/GenBank/DDBJ whole genome shotgun (WGS) entry which is preliminary data.
Chr04G0863.1	708	1	698	UniProt ID:Q99324_SEPLY	803	57	794	317/763(41.55)	0.56	0.12	763	541	0	gene=Chr04G0863	Gene Symbol:B2TOM|Host:Primarily tomato, Lycopersicon esculentum, also Solanum

																spp. and other Solanaceae|Disease:Leaf spot|Description:Unknown
Chr04G0869.1	232	60	166	UniProt ID:Q56J91_CANGB	372	114	220	36/112 (32.14)	0.48	0.09	112	54.7	7.00E-10	gene=Chr04G0869		Gene Symbol:ERG6|Host:humans|Disease:Occasional invasive candidal disease|Description:Unknown
Chr04G0875.1	467	43	213	UniProt ID:A0ST43_CERNC	459	15	193	66/179 (36.87)	0.54	0.04	179	98.6	4.00E-23	gene=Chr04G0875		Gene Symbol:CTB5|Host:Numerous taxa in Solanaceae|Disease:Leaf spot|Description:Unknown
Chr04G0877.1	1027	7	1026	UniProt ID:A5H456_MYCGR	1811	649	1685	375/1054(35.58)	0.53	0.05	1054	637	0	gene=Chr04G0877		Gene Symbol:NULL|Host:Triticum and possibly a few other grasses|Disease:Leaf spot or speckled leaf blotch of wheat|Description:Unknown
Chr04G0878.1	108	2	91	UniProt ID:Q874F3_MAGGR	1484	1384	1472	35/91(38.46)	0.57	0.03	91	74.3	4.00E-17	gene=Chr04G0878		Gene Symbol:ABC2|Host:Digitaria (Poaceae)|Disease:Leaf spot|Description:SIMILARITY: Belongs to the ABC

															transporter superfamily.
Chr04G0879.1	779	22	144	UniProt ID:A4R0W3_MAGO7	1226	278	419	39/143 (27.27)	0.38	0.15	143	50.1	5.00E-07	gene=Chr04G0879	Gene Symbol:MGG_09263|Host:Poaceae, especially important on Oryzae|Disease:Rice blast|Description:Unknown
Chr04G0884.1	1822	35	992	UniProt ID:Q6ZX14_MAGGR	4034	13	999	323/1007(32.08)	0.47	0.07	1007	395	2.00E-112	gene=Chr04G0884	Gene Symbol:ACE1|Host:Digitaria (Poaceae)|Disease:Leaf spot|Description:Unknown
Chr04G0885.1	347	13	211	UniProt ID:A0ST44_CERNC	357	4	206	68/211 (32.23)	0.44	0.09	211	86.7	3.00E-20	gene=Chr04G0885	Gene Symbol:CTB6|Host:Numerous taxa in Solanaceae|Disease:Leaf spot|Description:Unknown
Chr04G0891.1	1217	1	1217	UniProt ID:A4R599_MAGO7	1287	1	1287	702/1301(53.96)	0.67	0.08	1301	1174	0	gene=Chr04G0891	Gene Symbol:MGG_04163|Host:Poaceae, especially important on Oryzae|Disease:Rice blast|Description:Unknown
Chr04G0897.1	214	3	214	UniProt ID:Q9U	210	4	210	149/212(70.2	0.78	0.02	212	279	8.00E-96	gene=Chr04G0897	Gene Symbol:RAS1|Host:huma

				VQ4_C RYNE				8)							ns|Disease:cryptococcosis |Description:Unknown
Chr04G0 906.1	324	38	324	UniProt ID:Q6T FC7_A SPFM	349	61	346	98/293 (33.45)	0.5	0.04	293	159	6.00E-46	gene=Chr 04G0906	Gene Symbol:NULL|Host:huma ns|Disease:infection|Desc ription:Unknown
Chr04G0 924.1	557	278	525	UniProt ID:A4U LJ2_M YCGR	515	252	503	70/262 (26.72)	0.4	0.09	262	60.5	1.00E-10	gene=Chr 04G0924	Gene Symbol:CYP51|Host:Tritic um and possibly a few other grasses|Disease:Leaf spot or speckled leaf blotch of wheat|Description:COFAC TOR: Heme group (By similarity).
Chr04G0 925.1	510	150	502	UniProt ID:O93 800_AL TAL	578	191	560	114/37 5(30.4 0)	0.45	0.07	375	121	2.00E-30	gene=Chr 04G0925	Gene Symbol:AKT1|Host:Plant| Disease:Leaf spot, rots|Description:Unknown
Chr04G0 926.1	2515	90	128 6	UniProt ID:Q92 217_C OCHE	2528	7	1306	438/13 14(33. 33)	0.51	0.1	1314	605	8.00E-17 8	gene=Chr 04G0926	Gene Symbol:PKS1|Host:Zea mays|Disease:Southern leaf blight of maize|Description:Unkno wn
Chr04G0	622	189	471	UniProt	598	125	415	79/294	0.43	0.05	294	70.5	1.00E-13	gene=Chr	Gene

928.1				ID:Q9P8L8_BOTFU				(26.87)						04G0928	Symbol:BCMFS1|Host:Various plant families|Disease:Grey mould. Parasite or saprophyte|Description:Unknown
Chr04G0936.1	595	3	591	UniProt ID:Q5ABU7_CANAL	564	4	563	204/605(33.72)	0.52	0.1	605	354	7.00E-115	gene=Chr04G0936	Gene Symbol:MDR1|Host:Isolated from a wide variety of substrates including humans|Disease:invasive candidal disease|Description:Unknown
Chr04G0943.1	491	6	491	UniProt ID:Q4WU51_ASPFU	491	4	491	337/495(68.08)	0.8	0.03	495	692	0	gene=Chr04G0943	Gene Symbol:AFUA_5G07210|Host:humans|Disease:infection|Description:CAUTION: The sequence shown here is derived from an EMBL/GenBank/DDBJ whole genome shotgun (WGS) entry which is preliminary data.
Chr04G0944.1	734	74	311	UniProt ID:Q5A	748	41	241	59/238 (24.79)	0.41	0.16	238	47	3.00E-06	gene=Chr04G0944	Gene Symbol:SNF3|Host:Isolate

				NE1_CANAL											d from a wide variety of substrates including humans|Disease:invasive candidal disease|Description:SIMILARITY: Belongs to the major facilitator superfamily. Sugar transporter (TC 2.A.1.1) family.
Chr04G0951.1	242	8	240	UniProt ID:Q00845_NECHA	233	6	233	143/239(59.83)	0.7	0.07	239	268	9.00E-91	gene=Chr04G0951	Gene Symbol:PELD|Host:Trees of various plant families|Disease:Fruit rot, stem rot|Description:Unknown
Chr04G0952.1	412	6	329	UniProt ID:Q9Y784_MAGGR	631	1	345	97/349 (27.79)	0.47	0.08	349	116	4.00E-29	gene=Chr04G0952	Gene Symbol:PTH11|Host:Digitaria (Poaceae)|Disease:Leaf spot|Description:Unknown
Chr04G0956.1	227	1	227	UniProt ID:CUTI_COLCA	228	1	228	190/228(83.33)	0.89	0	228	385	2.00E-137	gene=Chr04G0956	Gene Symbol:CUTA|Host:Multiple genera in multiple families|Disease:Anthracnose, blight, dieback, leaf,

														fruit, and stem rots|Description:FUNCTION: Catalyzes the hydrolysis of cutin, a polyester that forms the structure of plant cuticle. Allows pathogenic fungi to penetrate through the cuticular barrier into the host plant during the initial stage of the fungal infection.
Chr04G0957.1	414	19	411	UniProt ID:Q96VZ3_FUSOX	455	46	447	108/423(25.53)	0.41	0.12	423	84.7	8.00E-19	gene=Chr04G0957	Gene Symbol:PGX1|Host:Multiple genera in multiple families|Disease:Blights, wilts, rots of various sorts|Description:SIMILARITY: Belongs to the glycosyl hydrolase 28 family.
Chr04G0960.1	506	294	478	UniProt ID:A4ULI4_MYCGR	517	304	510	47/208(22.60)	0.45	0.12	208	47.8	1.00E-06	gene=Chr04G0960	Gene Symbol:CYP51|Host:Triticum and possibly a few other grasses|Disease:Leaf spot

														or speckled leaf blotch of wheat|Description:COFACTOR: Heme group (By similarity).
Chr04G0962.1	348	11	316	UniProt ID:Q59QH2_CANAL	337	4	319	88/325 (27.08)	0.49	0.09	325	97.4	6.00E-24	gene=Chr04G0962	Gene Symbol:CSH1|Host:Isolated from a wide variety of substrates including humans|Disease:invasive candidal disease|Description:CAUTION: The sequence shown here is derived from an EMBL/GenBank/DDBJ whole genome shotgun (WGS) entry which is preliminary data.
Chr04G0964.1	682	401	597	UniProt ID:Q5AM49_CANAL	1690	1058	1256	90/204 (44.12)	0.59	0.06	204	169	1.00E-44	gene=Chr04G0964	Gene Symbol:SNF2|Host:Isolated from a wide variety of substrates including humans|Disease:invasive candidal disease|Description:Unknown
Chr04G0	727	4	726	UniProt	739	13	736	383/74	0.66	0.07	748	519	1.00E-17	gene=Chr	Gene

966.1				ID:C0SAS5_PARBP				8(51.20)					4	04G0966	Symbol:PABG_04780|Host:humans|Disease:Paracoccidioidomycosis|Description:Unknown
Chr04G0973.1	2516	30	2480	UniProt ID:Q92217_COCHE	2528	13	2512	821/2599(31.59)	0.49	0.1	2599	1026	0	gene=Chr04G0973	Gene Symbol:PKS1|Host:Zea mays|Disease:Southern leaf blight of maize|Description:Unknown
Chr04G0974.1	438	155	435	UniProt ID:Q6TFC7_ASPFM	349	63	346	98/293 (33.45)	0.52	0.07	293	156	4.00E-44	gene=Chr04G0974	Gene Symbol:NULL|Host:humans|Disease:infection|Description:Unknown
Chr04G0975.1	845	15	115	UniProt ID:A3LVN8_PICST	579	23	112	40/106 (37.74)	0.48	0.2	106	64.3	1.00E-11	gene=Chr04G0975	Gene Symbol:MIG1|Host:humans|Disease:occasional infection|Description:Unknown
Chr04G0976.1	347	17	227	UniProt ID:A4QVF8_MAGO7	339	17	231	52/222 (23.42)	0.44	0.08	222	62	1.00E-11	gene=Chr04G0976	Gene Symbol:MGG_04556|Host:Poaceae, especially important on Oryzae|Disease:Rice blast|Description:COFACTOR: Zinc (By similarity).

Chr04G0977.1	1394	808	1363	UniProt ID:Q5RLJ7_CRYNV	594	45	559	144/578(24.91)	0.39	0.15	578	144	2.00E-36	gene=Chr04G0977	Gene Symbol:NULL|Host:humans|Disease:cryptococcosis|Description:Unknown
Chr04G0978.1	2516	123	1725	UniProt ID:O59897_ASPFM	2146	111	1717	456/1709(26.68)	0.44	0.12	1709	470	3.00E-135	gene=Chr04G0978	Gene Symbol:ALB1|Host:humans|Disease:infection|Description:Unknown
Chr04G0981.1	613	34	610	UniProt ID:Q4P8E8_USTMA	693	81	671	187/627(29.82)	0.49	0.14	627	188	3.00E-52	gene=Chr04G0981	Gene Symbol:UM03615.1|Host: Euchlaena spp., Zea spp. (Poaceae)|Disease:Smut. Corn smut|Description:COFACTOR: FAD (By similarity).
Chr04G0984.1	447	26	61	UniProt ID:Q5A4F3_CANAL	624	14	48	16/36(44.44)	0.67	0.03	36	47.4	1.00E-06	gene=Chr04G0984	Gene Symbol:ZCF37|Host:Isolated from a wide variety of substrates including humans|Disease:invasive candidal disease|Description:Unknown
Chr04G0985.1	339	34	331	UniProt ID:O59937_F	384	90	384	98/304(32.24)	0.53	0.05	304	130	2.00E-35	gene=Chr04G0985	Gene Symbol:XYL3|Host:Multiple genera in multiple

				USOX											families|Disease:Blights, wilts, rots of various sorts|Description:SIMILARITY: Belongs to the glycosyl hydrolase 10 (cellulase F) family.
Chr04G0994.1	331	43	331	UniProt ID:Q6TFC7_ASPFM	349	61	349	94/294 (31.97)	0.49	0.03	294	152	2.00E-43	gene=Chr04G0994	Gene Symbol:NULL|Host:humans|Disease:infection|Description:Unknown
Chr04G0997.1	559	25	558	UniProt ID:Q9UUS8_COLGL	567	89	565	159/551(28.86)	0.41	0.17	551	155	7.00E-42	gene=Chr04G0997	Gene Symbol:CHIP3|Host:Multiple genera in multiple families|Disease:'Anthracnose of stems and leaves, dieback, root rot, leaf spot, blossom rot, fruit rot (dieback and ripe rot), seedling blight.' (Mordue 1971)|Description:Unknown
Chr04G0999.1	239	26	199	UniProt ID:E3R143_COLGM	229	34	223	82/192 (42.71)	0.54	0.1	192	134	5.00E-39	gene=Chr04G0999	Gene Symbol:GLRG_11978|Host:Poaceae especially Zea mays|Disease:Leaf spot, stalk rot,

															etc|Description:Unknown
Chr04G1003.1	501	2	499	UniProt ID:Q59RG0_CANAL	581	80	573	171/510(33.53)	0.53	0.05	510	262	9.00E-81	gene=Chr04G1003	Gene Symbol:NAG4|Host:Isolated from a wide variety of substrates including humans|Disease:invasive candidal disease|Description:CAUTION: The sequence shown here is derived from an EMBL/GenBank/DDBJ whole genome shotgun (WGS) entry which is preliminary data.
Chr04G1007.1	541	93	533	UniProt ID:Q5ANE1_CANAL	748	72	521	113/464(24.35)	0.42	0.08	464	83.6	7.00E-18	gene=Chr04G1007	Gene Symbol:SNF3|Host:Isolated from a wide variety of substrates including humans|Disease:invasive candidal disease|Description:SIMILARITY: Belongs to the major facilitator superfamily. Sugar transporter (TC 2.A.1.1) family.

Chr04G1008.1	860	108	474	UniProt ID:DHH1_CRYNV	616	33	392	118/373(31.64)	0.52	0.05	373	170	1.00E-45	gene=Chr04G1008	Gene Symbol:VAD1|Host:humans|Disease:cryptococcosis|Description:FUNCTION: ATP-dependent RNA helicase involved in mRNA turnover, and more specifically in mRNA decapping. Is involved in G1/S DNA- damage checkpoint recovery, probably through the regulation of the translational status of a subset of mRNAs. May also have a role in translation and mRNA nuclear export (By similarity). Is involved in virulence.
Chr04G1010.1	372	6	349	UniProt ID:Q7LJU0_CRYNE	410	90	402	78/365 (21.37)	0.39	0.2	365	57.4	4.00E-10	gene=Chr04G1010	Gene Symbol:UXS1|Host:humans|Disease:cryptococcosis|Description:Unknown
Chr04G1011.1	394	96	309	UniProt ID:Q92	2528	1843	2055	75/217 (34.56)	0.51	0.03	217	108	3.00E-26	gene=Chr04G1011	Gene Symbol:PKS1|Host:Zea

				217_COCHE										mays|Disease:Southern leaf blight of maize|Description:Unknown
Chr04G1013.1	388	5	326	UniProt ID:Q9Y784_MAGGR	631	94	409	88/336 (26.19)	0.46	0.1	336	99.4	1.00E-23	gene=Chr04G1013	Gene Symbol:PTH11|Host:Digitaria (Poaceae)|Disease:Leaf spot|Description:Unknown
Chr04G1026.1	492	53	466	UniProt ID:LAP2_ASPFU	501	61	483	150/440(34.09)	0.5	0.1	440	209	8.00E-62	gene=Chr04G1026	Gene Symbol:LAP2|Host:humans|Disease:infection|Description:FUNCTION: Extracellular aminopeptidase that releases a wide variety of amino acids from natural peptides and contributes to pathogenicity (By similarity).
Chr04G1038.1	364	54	355	UniProt ID:Q6TFC7_ASPFM	349	36	346	114/320(35.62)	0.54	0.08	320	192	3.00E-58	gene=Chr04G1038	Gene Symbol:NULL|Host:humans|Disease:infection|Description:Unknown
Chr04G1045.1	575	1	569	UniProt ID:Q9P	665	3	640	183/647(28.2	0.43	0.13	647	184	5.00E-51	gene=Chr04G1045	Gene Symbol:AOX1|Host:Lycop

			304_CLAFU				8)								ersicon esculentum (Solanaceae)|Disease:Leaf mold of tomato|Description:COFACTOR: FAD (By similarity).
Chr04G1048.1	408	185	310	UniProt ID:Q92217_COCHE	2528	1922	2049	35/129 (27.13)	0.48	0.03	129	56.2	2.00E-09	gene=Chr04G1048	Gene Symbol:PKS1|Host:Zea mays|Disease:Southern leaf blight of maize|Description:Unknown
Chr04G1049.1	360	14	168	UniProt ID:B9A5A2_CYBJA	329	56	232	49/177 (27.68)	0.45	0.12	177	62	1.00E-11	gene=Chr04G1049	Gene Symbol:CUMET2|Host:humans|Disease:Chronic urinary tract infection|Description:Unknown
Chr04G1051.1	1116	67	602	UniProt ID:Q5ANE1_CANAL	748	37	561	150/566(26.50)	0.45	0.13	566	192	9.00E-52	gene=Chr04G1051	Gene Symbol:SNF3|Host:Isolated from a wide variety of substrates including humans|Disease:invasive candidal disease|Description:SIMILARITY: Belongs to the

															major facilitator superfamily. Sugar transporter (TC 2.A.1.1) family.
Chr04G1054.1	519	323	486	UniProt ID:A4ULI5_MYCGR	515	307	502	50/196 (25.51)	0.44	0.16	196	55.1	4.00E-09	gene=Chr04G1054	Gene Symbol:CYP51|Host:Triticum and possibly a few other grasses|Disease:Leaf spot or speckled leaf blotch of wheat|Description:COFACTOR: Heme group (By similarity).
Chr04G1058.1	1096	219	441	UniProt ID:A4ULI5_MYCGR	515	258	509	61/256 (23.83)	0.41	0.14	256	55.8	7.00E-09	gene=Chr04G1058	Gene Symbol:CYP51|Host:Triticum and possibly a few other grasses|Disease:Leaf spot or speckled leaf blotch of wheat|Description:COFACTOR: Heme group (By similarity).
Chr04G1061.1	527	61	525	UniProt ID:Q59RW5_CANAL	493	83	493	111/481(23.08)	0.41	0.18	481	87	4.00E-19	gene=Chr04G1061	Gene Symbol:HXK1|Host:Isolated from a wide variety of substrates including

															humans|Disease:invasive candidal disease|Description:Unknown
Chr04G1064.1	668	28	668	UniProt ID:Q0WXM3_FUSOX	663	33	662	482/654(73.70)	0.83	0.06	654	956	0	gene=Chr04G1064	Gene Symbol:FOW2|Host:Multiple genera in multiple families|Disease:Blights, wilts, rots of various sorts|Description:SIMILARITY: Contains 1 Zn(2)-C6 fungal-type DNA-binding domain.
Chr04G1073.1	885	230	471	UniProt ID:Q5ALS7_CANAL	1144	353	576	67/247(27.13)	0.4	0.11	247	57.8	2.00E-09	gene=Chr04G1073	Gene Symbol:CTF1|Host:Isolated from a wide variety of substrates including humans|Disease:invasive candidal disease|Description:SIMILARITY: Contains 1 Zn(2)-C6 fungal-type DNA-binding domain.
Chr04G1074.1	947	1	947	UniProt ID:A8QJI7_FU	938	1	938	624/956(65.27)	0.75	0.03	956	1162	0	gene=Chr04G1074	Gene Symbol:XLNR|Host:Multiple genera in multiple

				SOX											families|Disease:Blights, wilts, rots of various sorts|Description:SIMILARITY: Contains 1 Zn(2)-C6 fungal-type DNA-binding domain.
Chr04G1075.1	2577	6	2577	UniProt ID:Q92217_COCHE	2528	13	2527	916/2671(34.29)	0.49	0.1	2671	1209	0	gene=Chr04G1075	Gene Symbol:PKS1|Host:Zea mays|Disease:Southern leaf blight of maize|Description:Unknown
Chr04G1078.1	545	313	500	UniProt ID:A4ULI5_MYCGR	515	305	511	50/226 (22.12)	0.42	0.25	226	61.6	5.00E-11	gene=Chr04G1078	Gene Symbol:CYP51|Host:Triticum and possibly a few other grasses|Disease:Leaf spot or speckled leaf blotch of wheat|Description:COFACTOR: Heme group (By similarity).
Chr04G1080.1	512	10	509	UniProt ID:A0ST42_CERNC	512	18	508	150/510(29.41)	0.49	0.06	510	212	2.00E-62	gene=Chr04G1080	Gene Symbol:CTB4|Host:Numerous taxa in Solanaceae|Disease:Leaf spot|Description:Unknown

Chr04G1083.1	452	59	351	UniProt ID:CDC10_CANAL	357	17	307	117/294(39.80)	0.62	0.01	294	233	1.00E-72	gene=Chr04G1083	Gene Symbol:CDC10|Host:Isolated from a wide variety of substrates including humans|Disease:invasive candidal disease|Description:FUNCTION: Plays a role in the cell cycle. Involved in the formation of the ring of filaments in the neck region at the mother-bud junction during mitosis.
Chr04G1085.1	827	197	586	UniProt ID:PLB1_CRYNV	637	61	426	105/433(24.25)	0.39	0.25	433	77	1.00E-15	gene=Chr04G1085	Gene Symbol:PLB1|Host:humans|Disease:cryptococcosis|Description:FUNCTION: Exhibits phospholipase B (PLB), lysophospholipase (LPL) and lysophospholipase/transacylase (LPTA) activities.
Chr04G1091.1	418	351	415	UniProt ID:Q5AMH6_CANAL	821	746	810	22/65(33.85)	0.49	0	65	44.3	8.00E-06	gene=Chr04G1091	Gene Symbol:CAS5|Host:Isolated from a wide variety of substrates including

															humans|Disease:invasive candidal disease|Description:CAUTION: The sequence shown here is derived from an EMBL/GenBank/DDBJ whole genome shotgun (WGS) entry which is preliminary data.
Chr04G1101.1	389	129	386	UniProt ID:A3LXZ6_PICST	310	49	297	79/265 (29.81)	0.47	0.09	265	115	6.00E-30	gene=Chr04G1101	Gene Symbol:CDC28|Host:humans|Disease:occasional infection|Description:Unknown
Chr04G1104.1	517	114	487	UniProt ID:Q5AGE7_CANAL	469	141	464	119/383(31.07)	0.48	0.18	383	154	6.00E-42	gene=Chr04G1104	Gene Symbol:PTH1|Host:Isolated from a wide variety of substrates including humans|Disease:invasive candidal disease|Description:CAUTION: The sequence shown here is derived from an EMBL/GenBank/DDBJ whole genome shotgun (WGS) entry which is

															preliminary data.
Chr04G1109.1	405	41	293	UniProt ID:Q9C441_FUSSO	330	61	303	95/262 (36.26)	0.54	0.11	262	167	3.00E-48	gene=Chr04G1109	Gene Symbol:PEP1|Host:Multiple plant families. Some strains may cause infections in humans|Disease:Saprobe, facultative pathogen|Description:Unknown
Chr04G1110.1	566	157	493	UniProt ID:O59928_HYPVI	430	44	392	85/372 (22.85)	0.41	0.16	372	78.6	2.00E-16	gene=Chr04G1110	Gene Symbol:NULL|Host:humans|Disease:infection|Description:SIMILARITY: Belongs to the glycosyl hydrolase 18 family.
Chr04G1117.1	705	3	404	UniProt ID:Q9HG15_COLLN	746	7	371	104/417(24.94)	0.4	0.16	417	65.5	5.00E-12	gene=Chr04G1117	Gene Symbol:CLTA1|Host:Multiple genera of Fabaceae. Rare reports on other taxa|Disease:Leaf, stem and pod anthracnose|Description:SIMILARITY: Contains 1 Zn(2)-C6 fungal-type DNA-binding domain.

Chr04G1118.1	552	7	550	UniProt ID:SPCA_ARTOC	651	3	573	206/582(35.40)	0.53	0.08	582	332	1.00E-105	gene=Chr04G1118	Gene Symbol:SCPA|Host:humans, reptiles|Disease:dermatophytoses|Description:FUNCTION: Extracellular serine carboxypeptidase that contributes to pathogenicity (By similarity).
Chr04G1125.1	501	266	475	UniProt ID:A4ULJ1_MYCGR	517	264	516	62/259(23.94)	0.41	0.21	259	47	2.00E-06	gene=Chr04G1125	Gene Symbol:CYP51|Host:Triticum and possibly a few other grasses|Disease:Leaf spot or speckled leaf blotch of wheat|Description:COFACTOR: Heme group (By similarity).
Chr04G1141.1	815	55	617	UniProt ID:O93841_9PEZI	914	22	615	259/602(43.02)	0.62	0.08	602	509	2.00E-167	gene=Chr04G1141	Gene Symbol:CHIP6|Host:Multiple genera in multiple families|Disease:'Anthracnose of stems and leaves, dieback, root rot, leaf spot, blossom rot, fruit rot

															(dieback and ripe rot), seedling blight.' (Mordue 1971)|Description:Unknown
Chr04G1144.1	567	1	551	UniProt ID:A3GFA7_PICST	549	1	526	155/561(27.63)	0.5	0.08	561	181	2.00E-50	gene=Chr04G1144	Gene Symbol:CCT8|Host:humans|Disease:occasional infection|Description:SIMILARITY: Belongs to the TCP-1 chaperonin family.
Chr04G1155.1	530	271	496	UniProt ID:A4ULJ0_MYCGR	518	257	505	57/251 (22.71)	0.39	0.11	251	47	2.00E-06	gene=Chr04G1155	Gene Symbol:CYP51|Host:Triticum and possibly a few other grasses|Disease:Leaf spot or speckled leaf blotch of wheat|Description:COFACTOR: Heme group (By similarity).
Chr04G1159.1	760	243	493	UniProt ID:Q9HG15_COLLN	746	184	436	59/268 (22.01)	0.4	0.12	268	53.5	3.00E-08	gene=Chr04G1159	Gene Symbol:CLTA1|Host:Multiple genera of Fabaceae. Rare reports on other taxa|Disease:Leaf, stem and pod anthracnose|Description:S

															IMILARITY: Contains 1 Zn(2)-C6 fungal-type DNA-binding domain.
Chr04G1164.1	511	13	498	UniProt ID:Q5APD4_CANAL	513	4	498	276/496(55.65)	0.73	0.02	496	596	0	gene=Chr04G1164	Gene Symbol:CFA1|Host:Isolated from a wide variety of substrates including humans|Disease:invasive candidal disease|Description:CAUTION: The sequence shown here is derived from an EMBL/GenBank/DDBJ whole genome shotgun (WGS) entry which is preliminary data.
Chr04G1165.1	351	1	291	UniProt ID:A0ST44_CERNC	357	1	293	96/303(31.68)	0.46	0.07	303	125	2.00E-33	gene=Chr04G1165	Gene Symbol:CTB6|Host:Numerous taxa in Solanaceae|Disease:Leaf spot|Description:Unknown
Chr04G1169.1	417	19	415	UniProt ID:Q9UUS8_COLGL	567	144	565	148/431(34.34)	0.52	0.1	431	206	1.00E-60	gene=Chr04G1169	Gene Symbol:CHIP3|Host:Multiple genera in multiple families|Disease:'Anthracnose of stems and leaves,

															dieback, root rot, leaf spot, blossom rot, fruit rot (dieback and ripe rot), seedling blight.' (Mordue 1971)|Description:Unknown
Chr04G1178.1	293	1	289	UniProt ID:A4RJV7_MAGO7	296	1	295	202/301(67.11)	0.78	0.06	301	411	4.00E-145	gene=Chr04G1178	Gene Symbol:MGG_01707|Host:Poaceae, especially important on Oryzae|Disease:Rice blast|Description:Unknown
Chr04G1183.1	610	30	579	UniProt ID:Q5AMK1_CANAL	632	42	577	174/605(28.76)	0.43	0.2	605	215	7.00E-62	gene=Chr04G1183	Gene Symbol:BEM1|Host:Isolated from a wide variety of substrates including humans|Disease:invasive candidal disease|Description:SIMILARITY: Contains 2 SH3 domains.
Chr04G1188.1	698	7	691	UniProt ID:Q4KRH1_ARTBE	726	20	697	233/707(32.96)	0.52	0.07	707	352	4.00E-111	gene=Chr04G1188	Gene Symbol:NULL|Host:hedgehogs|Disease:ringworm,Kerion Celsi

															Disease|Description:Unknown
Chr04G1191.1	260	33	141	UniProt ID:Q59VU9_CANAL	212	23	131	30/109 (27.52)	0.54	0	109	62	1.00E-12	gene=Chr04G1191	Gene Symbol:MAD2|Host:Isolated from a wide variety of substrates including humans|Disease:invasive candidal disease|Description:CAUTION: The sequence shown here is derived from an EMBL/GenBank/DDBJ whole genome shotgun (WGS) entry which is preliminary data.
Chr04G1194.1	334	1	151	UniProt ID:A4R3I5_MAGO7	400	80	225	41/157 (26.11)	0.46	0.11	157	46.6	1.00E-06	gene=Chr04G1194	Gene Symbol:"MGG_11993, MGG_12837, MGG_13052"|Host:Poaceae, especially important on Oryzae|Disease:Rice blast|Description:Unknown
Chr04G1195.1	1234	71	1201	UniProt ID:A4RGC8_M	1158	31	1132	456/1164(39.18)	0.57	0.08	1164	780	0	gene=Chr04G1195	Gene Symbol:MGG_11671|Host:Poaceae, especially

				AGO7										important on Oryzae|Disease:Rice blast|Description:SIMILARITY: Contains 1 reverse transcriptase domain.
Chr04G1196.1	378	77	375	UniProt ID:Q6TFC7_ASPFM	349	48	345	96/307 (31.27)	0.52	0.06	307	173	8.00E-51	gene=Chr04G1196	Gene Symbol:NULL|Host:humans|Disease:infection|Description:Unknown
Chr04G1198.1	329	21	271	UniProt ID:Q6A2T2_BOTFU	391	25	290	73/274 (26.64)	0.43	0.11	274	66.2	4.00E-13	gene=Chr04G1198	Gene Symbol:BTP1|Host:Various plant families|Disease:Grey mould. Parasite or saprophyte|Description:Unknown
Chr04G1208.1	1170	808	1168	UniProt ID:Q5AHG6_CANAL	787	352	722	146/376(38.83)	0.59	0.05	376	268	6.00E-77	gene=Chr04G1208	Gene Symbol:SCH9|Host:Isolated from a wide variety of substrates including humans|Disease:invasive candidal disease|Description:CATALYTIC ACTIVITY: ATP + a protein = ADP + a phosphoprotein.

Chr04G1212.1	424	1	424	UniProt ID:C1GDU0_PARBD	411	1	411	257/424(60.61)	0.76	0.03	424	551	0	gene=Chr04G1212	Gene Symbol:PADG_05426|Host:humans|Disease:Paracoccidioidomycosis|Description:Unknown
Chr04G1221.1	1309	52	1250	UniProt ID:A4RGC8_MAGO7	1158	10	1155	482/1223(39.41)	0.58	0.08	1223	803	0	gene=Chr04G1221	Gene Symbol:MGG_11671|Host:Poaceae, especially important on Oryzae|Disease:Rice blast|Description:SIMILARITY: Contains 1 reverse transcriptase domain.
Chr04G1222.1	469	37	138	UniProt ID:Q5ALS7_CANAL	1144	431	533	33/105(31.43)	0.5	0.05	105	45.8	4.00E-06	gene=Chr04G1222	Gene Symbol:CTF1|Host:Isolated from a wide variety of substrates including humans|Disease:invasive candidal disease|Description:SIMILARITY: Contains 1 Zn(2)-C6 fungal-type DNA-binding domain.
Chr04G1224.1	490	252	452	UniProt ID:A4ULJ2_M	515	266	499	59/236(25.00)	0.44	0.16	236	66.2	1.00E-12	gene=Chr04G1224	Gene Symbol:CYP51|Host:Triticum and possibly a few

				YCGR											other grasses|Disease:Leaf spot or speckled leaf blotch of wheat|Description:COFACTOR: Heme group (By similarity).
Chr04G1226.1	283	13	271	UniProt ID:Q32WF7_PHAND	266	29	264	66/274 (24.09)	0.37	0.19	274	47.4	2.00E-07	gene=Chr04G1226	Gene Symbol:MDH1|Host:Multiple genera of Poaceae and Blysmus compressus (Cyperaceae)|Disease:Glume blotch of wheat and other grasses|Description:Unknown
Chr04G1232.1	638	24	617	UniProt ID:Q4P8E8_USTMA	693	66	672	213/627(33.97)	0.51	0.08	627	287	1.00E-87	gene=Chr04G1232	Gene Symbol:UM03615.1|Host: Euchlaena spp., Zea spp. (Poaceae)|Disease:Smut. Corn smut|Description:COFACTOR: FAD (By similarity).
Chr04G1233.1	435	10	400	UniProt ID:Q5XTQ4_BOTFU	574	185	573	100/432(23.15)	0.34	0.19	432	74.7	2.00E-15	gene=Chr04G1233	Gene Symbol:LIP1|Host:Various plant families|Disease:Grey

															mould. Parasite or saprophyte|Description:Unknown
Chr04G1238.1	589	375	586	UniProt ID:A4R9C7_MAGO7	1305	336	535	61/231 (26.41)	0.38	0.22	231	51.2	1.00E-07	gene=Chr04G1238	Gene Symbol:MGG_13324|Host:Poaceae, especially important on Oryzae|Disease:Rice blast|Description:Unknown
Chr04G1239.1	1047	271	407	UniProt ID:O59918_USTMD	608	299	455	48/165 (29.09)	0.45	0.22	165	55.5	1.00E-08	gene=Chr04G1239	Gene Symbol:UKC1|Host:Euchlaena spp., Zea spp. (Poaceae)|Disease:Smut. Corn smut|Description:CATALYTIC ACTIVITY: ATP + a protein = ADP + a phosphoprotein.
Chr04G1257.1	297	29	293	UniProt ID:Q32WF7_PHAND	266	8	263	80/267 (29.96)	0.5	0.05	267	111	2.00E-29	gene=Chr04G1257	Gene Symbol:MDH1|Host:Multiple genera of Poaceae and Blysmus compressus (Cyperaceae)|Disease:Glume blotch of wheat and other

															grasses|Description:Unknown
Chr04G1259.1	768	41	84	UniProt ID:Q5A4F3_CANAL	624	11	54	20/44(45.45)	0.66	0	44	58.9	7.00E-10	gene=Chr04G1259	Gene Symbol:ZCF37|Host:Isolated from a wide variety of substrates including humans|Disease:invasive candidal disease|Description:Unknown
Chr04G1260.1	515	11	471	UniProt ID:Q5ANE1_CANAL	748	37	496	146/474(30.80)	0.49	0.06	474	185	1.00E-51	gene=Chr04G1260	Gene Symbol:SNF3|Host:Isolated from a wide variety of substrates including humans|Disease:invasive candidal disease|Description:SIMILARITY: Belongs to the major facilitator superfamily. Sugar transporter (TC 2.A.1.1) family.
Chr04G1261.1	294	29	292	UniProt ID:Q9P4U9_ALTAL	296	28	271	80/271(29.52)	0.45	0.13	271	88.6	2.00E-21	gene=Chr04G1261	Gene Symbol:AKT3-1|Host:Plant|Disease:Leaf spot, rots|Description:Unknown

Chr04G1263.1	541	22	469	UniProt ID:Q9P8L8_BOTFU	598	44	512	153/482(31.74)	0.5	0.1	482	224	9.00E-66	gene=Chr04G1263	Gene Symbol:BCMFS1|Host:Various plant families|Disease:Grey mould. Parasite or saprophyte|Description:Unknown
Chr04G1266.1	2563	135	2563	UniProt ID:Q9UVN5_ALTAL	4360	1038	3496	667/2591(25.74)	0.43	0.11	2591	595	8.00E-173	gene=Chr04G1266	Gene Symbol:AMT|Host:Plant|Disease:Leaf spot, rots|Description:Unknown
Chr04G1267.1	465	63	424	UniProt ID:Q96VZ3_FUSOX	455	69	434	133/377(35.28)	0.53	0.07	377	239	1.00E-73	gene=Chr04G1267	Gene Symbol:PGX1|Host:Multiple genera in multiple families|Disease:Blights, wilts, rots of various sorts|Description:SIMILARITY: Belongs to the glycosyl hydrolase 28 family.
Chr04G1271.1	460	3	98	UniProt ID:A0ST46_CERNC	397	19	126	37/108(34.26)	0.51	0.11	108	51.6	4.00E-08	gene=Chr04G1271	Gene Symbol:CTB8|Host:Numerous taxa in Solanaceae|Disease:Leaf spot|Description:Unknown
Chr04G1	852	1	255	UniProt	351	53	350	74/301	0.38	0.16	301	89.7	3.00E-20	gene=Chr	Gene

272.1				ID:Q6XSF5_COCHE				(24.58)						04G1272	Symbol:NULL|Host:Zea mays|Disease:Southern leaf blight of maize|Description:Unknown
Chr04G1275.1	640	176	614	UniProt ID:Q5AHG6_CANAL	787	302	719	207/452(45.80)	0.6	0.1	452	359	1.00E-113	gene=Chr04G1275	Gene Symbol:SCH9|Host:Isolated from a wide variety of substrates including humans|Disease:invasive candidal disease|Description:CATALYTIC ACTIVITY: ATP + a protein = ADP + a phosphoprotein.
Chr04G1276.1	586	8	584	UniProt ID:Q9P8L8_BOTFU	598	53	596	168/592(28.38)	0.47	0.11	592	209	3.00E-60	gene=Chr04G1276	Gene Symbol:BCMFS1|Host:Various plant families|Disease:Grey mould. Parasite or saprophyte|Description:Unknown
Chr04G1277.1	669	5	669	UniProt ID:Q9P304_CLAFU	665	3	665	523/665(78.65)	0.88	0	665	1109	0	gene=Chr04G1277	Gene Symbol:AOX1|Host:Lycopersicon esculentum (Solanaceae)|Disease:Lea

															f mold of tomato|Description:COFACTOR: FAD (By similarity).
Chr04G1278.1	2359	192	1809	UniProt ID:Q9UVN5_ALTAL	4360	250	1863	597/1667(35.81)	0.53	0.06	1667	931	0	gene=Chr04G1278	Gene Symbol:AMT|Host:Plant|Disease:Leaf spot, rots|Description:Unknown
Chr04G1279.1	560	5	560	UniProt ID:Q9P8L8_BOTFU	598	23	590	241/570(42.28)	0.63	0.03	570	462	9.00E-157	gene=Chr04G1279	Gene Symbol:BCMFS1|Host:Various plant families|Disease:Grey mould. Parasite or saprophyte|Description:Unknown
Chr04G1291.1	1702	804	1652	UniProt ID:Q5KB00_CRYNJ	1296	392	1160	222/871(25.49)	0.45	0.14	871	253	4.00E-69	gene=Chr04G1291	Gene Symbol:CNI04280|Host:humans|Disease:cryptococcosis|Description:SIMILARITY: Contains 1 DH (DBL-homology) domain.
Chr04G1295.1	334	10	239	UniProt ID:Q5AP71_CANAL	405	91	308	62/239(25.94)	0.46	0.13	239	66.6	3.00E-13	gene=Chr04G1295	Gene Symbol:TPK1|Host:Isolated from a wide variety of substrates including humans|Disease:invasive

															candidal disease|Description:CATALYTIC ACTIVITY: ATP + a protein = ADP + a phosphoprotein.
Chr04G1296.1	511	124	430	UniProt ID:Q59YF3_CANAL	986	659	879	69/311 (22.19)	0.38	0.3	311	72.4	3.00E-14	gene=Chr04G1296	Gene Symbol:INP51|Host:Isolated from a wide variety of substrates including humans|Disease:invasive candidal disease|Description:CAUTION: The sequence shown here is derived from an EMBL/GenBank/DDBJ whole genome shotgun (WGS) entry which is preliminary data.
Chr04G1297.1	397	48	397	UniProt ID:O42793_COLTR	530	195	530	168/355(47.32)	0.65	0.07	355	330	1.00E-108	gene=Chr04G1297	Gene Symbol:PKAC|Host:Fabaceae|Disease:Southern anthracnose of clover and other legumes|Description:CATALYTIC ACTIVITY: ATP + a protein = ADP + a

														phosphoprotein.
Chr04G1302.1	372	14	363	UniProt ID:Q6A2T2_BOTFU	391	25	391	92/384 (23.96)	0.42	0.13	384	73.9	2.00E-15	gene=Chr04G1302	Gene Symbol:BTP1|Host:Various plant families|Disease:Grey mould. Parasite or saprophyte|Description:Unknown
Chr04G1304.1	507	39	251	UniProt ID:ALO_CANAL	557	31	253	54/231 (23.38)	0.45	0.11	231	62.8	2.00E-11	gene=Chr04G1304	Gene Symbol:ALO1|Host:Isolated from a wide variety of substrates including humans|Disease:invasive candidal disease|Description:CATALYTIC ACTIVITY: D-arabinono-1,4-lactone + O(2) = dehydro-D-arabinono-1,4-lactone + H(2)O(2).
Chr04G1305.1	498	112	457	UniProt ID:A4ULI6_MYCGR	517	131	497	71/380 (18.68)	0.38	0.12	380	49.3	3.00E-07	gene=Chr04G1305	Gene Symbol:CYP51|Host:Triticum and possibly a few other grasses|Disease:Leaf spot or speckled leaf blotch of

															wheat|Description:COFACTOR: Heme group (By similarity).
Chr04G1316.1	313	248	307	UniProt ID:Q5AMH6_CANAL	821	747	806	22/60(36.67)	0.53	0	60	55.1	2.00E-09	gene=Chr04G1316	Gene Symbol:CAS5|Host:Isolated from a wide variety of substrates including humans|Disease:invasive candidal disease|Description:CAUTION: The sequence shown here is derived from an EMBL/GenBank/DDBJ whole genome shotgun (WGS) entry which is preliminary data.
Chr04G1317.1	715	9	288	UniProt ID:Q9UQZ1_9PEZI	512	59	317	91/299(30.43)	0.46	0.2	299	122	2.00E-30	gene=Chr04G1317	Gene Symbol:EMK1|Host:Multiple genera in multiple families|Disease:'Anthracnose of stems and leaves, dieback, root rot, leaf spot, blossom rot, fruit rot (dieback and ripe rot), seedling blight.' (Mordue 1971)|Description:Unknow

														n
Chr04G1328.1	494	57	488	UniProt ID:OPSB_ASPFU	485	69	484	154/443(34.76)	0.52	0.09	443	221	2.00E-66	gene=Chr04G1328	Gene Symbol:OPSB|Host:humans|Disease:infection|Description:FUNCTION: Probable GPI-anchored aspartic-type endopeptidase which contributes to virulence (By similarity).
Chr04G1333.1	1470	99	1469	UniProt ID:Q9UW87_CANAL	1606	137	1603	375/1516(24.74)	0.42	0.13	1516	372	3.00E-107	gene=Chr04G1333	Gene Symbol:MLT1|Host:Isolated from a wide variety of substrates including humans|Disease:invasive candidal disease|Description:SIMILARITY: Belongs to the ABC transporter superfamily.
Chr04G1334.1	565	26	476	UniProt ID:ALO_CANAL	557	17	543	201/542(37.08)	0.54	0.2	542	342	1.00E-110	gene=Chr04G1334	Gene Symbol:ALO1|Host:Isolated from a wide variety of substrates including humans|Disease:invasive candidal

															disease|Description:CATALYTIC ACTIVITY: D-arabinono-1,4-lactone + O(2) = dehydro-D-arabinono-1,4-lactone + H(2)O(2).
Chr04G1341.1	426	1	318	UniProt ID:Q01446_NECHA	459	22	329	84/330(25.45)	0.44	0.1	330	81.3	1.00E-17	gene=Chr04G1341	Gene Symbol:MAK1|Host:Trees of various plant families|Disease:Fruit rot, stem rot|Description:Unknown
Chr04G1348.1	569	4	532	UniProt ID:Q9P8L8_BOTFU	598	51	569	193/529(36.48)	0.59	0.02	529	363	3.00E-118	gene=Chr04G1348	Gene Symbol:BCMFS1|Host:Various plant families|Disease:Grey mould. Parasite or saprophyte|Description:Unknown
Chr04G1351.1	489	39	488	UniProt ID:A0ST43_CERNC	459	1	459	144/486(29.63)	0.45	0.13	486	166	2.00E-46	gene=Chr04G1351	Gene Symbol:CTB5|Host:Numerous taxa in Solanaceae|Disease:Leaf spot|Description:Unknown
Chr04G1352.1	462	62	434	UniProt ID:Q96	455	69	443	138/389(35.4	0.52	0.08	389	226	5.00E-69	gene=Chr04G1352	Gene Symbol:PGX1|Host:Multipl

				VZ3_F USOX				8)							e genera in multiple families|Disease:Blights, wilts, rots of various sorts|Description:SIMILARITY: Belongs to the glycosyl hydrolase 28 family.
Chr04G1355.1	868	271	763	UniProt ID:A6N6J8_FUSOX	903	179	662	115/523(21.99)	0.38	0.13	523	73.9	2.00E-14	gene=Chr04G1355	Gene Symbol:CTF1|Host:Multiple genera in multiple families|Disease:Blights, wilts, rots of various sorts|Description:SIMILARITY: Contains 1 Zn(2)-C6 fungal-type DNA-binding domain.
Chr04G1363.1	342	7	311	UniProt ID:Q4WWN9_ASPFU	308	2	294	184/305(60.33)	0.75	0.04	305	370	3.00E-128	gene=Chr04G1363	Gene Symbol:AFUA_3G06540|Host:humans|Disease:infection|Description:CAUTION: The sequence shown here is derived from an EMBL/GenBank/DDBJ whole genome shotgun (WGS) entry which is preliminary data.

Chr04G1366.1	627	328	625	UniProt ID:A6ZIB7_MAGGR	553	229	553	81/345 (23.48)	0.4	0.19	345	63.2	2.00E-11	gene=Chr04G1366	Gene Symbol:NULL|Host:Digitaria (Poaceae)|Disease:Leaf spot|Description:SIMILARITY: Contains 1 FAD-binding FR-type domain.
Chr04G1373.1	662	1	662	UniProt ID:SED1_ASPFU	644	1	644	277/691(40.09)	0.54	0.11	691	454	2.00E-151	gene=Chr04G1373	Gene Symbol:SED1|Host:humans|Disease:infection|Description:FUNCTION: Secreted tripeptidyl-peptidase which degrades proteins at acidic pHs and is involved in virulence.
Chr04G1388.1	1426	34	1426	UniProt ID:Q9UW03_BOTFU	1439	42	1439	997/1405(70.96)	0.83	0.01	1405	2086	0	gene=Chr04G1388	Gene Symbol:ATRB|Host:Various plant families|Disease:Grey mould. Parasite or saprophyte|Description:SIMILARITY: Belongs to the ABC transporter superfamily.
Chr04G1	491	252	456	UniProt	515	266	501	54/236	0.41	0.13	236	52.8	2.00E-08	gene=Chr	Gene

395.1				ID:A4ULJ2_MYCGR				(22.88)						04G1395	Symbol:CYP51|Host:Triticum and possibly a few other grasses|Disease:Leaf spot or speckled leaf blotch of wheat|Description:COFACTOR: Heme group (By similarity).
Chr04G1396.1	409	9	41	UniProt ID:Q5A4F3_CANAL	624	14	46	16/33(48.48)	0.7	0	33	49.7	2.00E-07	gene=Chr04G1396	Gene Symbol:ZCF37|Host:Isolated from a wide variety of substrates including humans|Disease:invasive candidal disease|Description:Unknown
Chr04G1402.1	707	146	590	UniProt ID:A4QVA7_MAGO7	1340	391	830	130/479(27.14)	0.42	0.15	479	114	4.00E-27	gene=Chr04G1402	Gene Symbol:MGG_04629|Host:Poaceae, especially important on Oryzae|Disease:Rice blast|Description:Unknown
Chr04G1411.1	610	21	606	UniProt ID:Q4P8E8_U	693	82	672	218/615(35.45)	0.52	0.09	615	297	2.00E-91	gene=Chr04G1411	Gene Symbol:UM03615.1|Host: Euchlaena spp., Zea spp.

				STMA											(Poaceae)|Disease:Smut. Corn smut|Description:COFACTOR: FAD (By similarity).
Chr04G1416.1	358	70	354	UniProt ID:Q6TFC7_ASPFM	349	63	348	99/290 (34.14)	0.49	0.03	290	155	2.00E-44	gene=Chr04G1416	Gene Symbol:NULL|Host:humans|Disease:infection|Description:Unknown
Chr04G1418.1	1432	608	894	UniProt ID:O59928_HYPVI	430	117	385	77/299 (25.75)	0.41	0.14	299	75.5	6.00E-15	gene=Chr04G1418	Gene Symbol:NULL|Host:humans|Disease:infection|Description:SIMILARITY: Belongs to the glycosyl hydrolase 18 family.
Chr04G1434.1	506	18	457	UniProt ID:Q5XTQ5_BOTFU	615	110	551	135/462(29.22)	0.47	0.09	462	137	1.00E-35	gene=Chr04G1434	Gene Symbol:FRT1|Host:Various plant families|Disease:Grey mould. Parasite or saprophyte|Description:SIMILARITY: Belongs to the major facilitator superfamily. Sugar transporter (TC 2.A.1.1) family.
Chr04G1	434	178	323	UniProt	509	155	276	40/149	0.45	0.2	149	46.2	2.00E-06	gene=Chr	Gene

436.1				ID:Q5AAG6_CANAL				(26.85)						04G1436	Symbol:MKC1|Host:Isolated from a wide variety of substrates including humans|Disease:invasive candidal disease|Description:CATALYTIC ACTIVITY: ATP + a protein = ADP + a phosphoprotein.
Chr04G1446.1	547	32	514	UniProt ID:Q5ANE1_CANAL	748	20	496	155/498(31.12)	0.51	0.07	498	230	3.00E-67	gene=Chr04G1446	Gene Symbol:SNF3|Host:Isolated from a wide variety of substrates including humans|Disease:invasive candidal disease|Description:SIMILARITY: Belongs to the major facilitator superfamily. Sugar transporter (TC 2.A.1.1) family.
Chr04G1447.1	798	288	702	UniProt ID:Q5ALS7_CANAL	1144	355	742	98/439 (22.32)	0.41	0.17	439	56.2	6.00E-09	gene=Chr04G1447	Gene Symbol:CTF1|Host:Isolated from a wide variety of substrates including humans|Disease:invasive

														candidal disease|Description:SIMILARITY: Contains 1 Zn(2)-C6 fungal-type DNA-binding domain.
Chr04G1449.1	390	7	384	UniProt ID:Q6A2T2_BOTFU	391	16	374	106/391(27.11)	0.43	0.12	391	100	1.00E-24	gene=Chr04G1449	Gene Symbol:BTP1|Host:Various plant families|Disease:Grey mould. Parasite or saprophyte|Description:Unknown
Chr04G1463.1	311	86	301	UniProt ID:Q9HFZ2_CANAL	358	148	357	71/238 (29.83)	0.42	0.21	238	81.3	2.00E-18	gene=Chr04G1463	Gene Symbol:RBT4|Host:Isolated from a wide variety of substrates including humans|Disease:invasive candidal disease|Description:Unknown
Chr04G1468.1	514	31	488	UniProt ID:Q4PDC7_USTMA	589	134	583	159/474(33.54)	0.49	0.08	474	275	3.00E-85	gene=Chr04G1468	Gene Symbol:UM01886.1|Host: Euchlaena spp., Zea spp. (Poaceae)|Disease:Smut. Corn smut|Description:CAUTIO

															N: The sequence shown here is derived from an EMBL/GenBank/DDBJ whole genome shotgun (WGS) entry which is preliminary data.
Chr04G1472.1	336	53	106	UniProt ID:F2QU09_PICP7	626	160	212	21/54(38.89)	0.59	0.02	54	45.8	2.00E-06	gene=Chr04G1472	Gene Symbol:PAB1|Host:humans|Disease:occasional infection|Description:Unknown
Chr04G1474.1	379	5	375	UniProt ID:Q8NJ85_CANAL	402	13	385	157/387(40.57)	0.63	0.08	387	293	3.00E-96	gene=Chr04G1474	Gene Symbol:CDC11|Host:Isolated from a wide variety of substrates including humans|Disease:invasive candidal disease|Description:SIMILARITY: Belongs to the septin family.
Chr04G1477.1	638	124	629	UniProt ID:Q96VB5_ALTAL	578	93	564	133/517(25.73)	0.42	0.11	517	105	1.00E-24	gene=Chr04G1477	Gene Symbol:AFT1-1|Host:Plant|Disease:Leaf spot, rots|Description:Unknown
Chr04G1482.1	476	32	281	UniProt ID:Q9Y	631	110	358	55/251(21.91)	0.48	0.01	251	85.1	1.00E-18	gene=Chr04G1482	Gene Symbol:PTH11|Host:Digit

			784_MAGGR												aria (Poaceae)|Disease:Leaf spot|Description:Unknown
Chr04G1484.1	383	5	275	UniProt ID:A4RF81_MAGO7	542	10	306	80/310 (25.81)	0.43	0.17	310	63.5	6.00E-12	gene=Chr04G1484	Gene Symbol:MGG_00435|Host:Poaceae, especially important on Oryzae|Disease:Rice blast|Description:Unknown
Chr04G1488.1	594	76	577	UniProt ID:Q5RLJ7_CRYNV	594	61	580	160/528(30.30)	0.49	0.06	528	242	5.00E-72	gene=Chr04G1488	Gene Symbol:NULL|Host:humans|Disease:cryptococcosis|Description:Unknown
Chr04G1490.1	898	705	889	UniProt ID:Q50I20_ASPFM	465	265	458	53/200 (26.50)	0.42	0.11	200	46.6	4.00E-06	gene=Chr04G1490	Gene Symbol:MCSA|Host:humans|Disease:infection|Description:SIMILARITY: Belongs to the citrate synthase family.
Chr04G1495.1	2399	1	2391	UniProt ID:Q92217_COCHE	2528	221	2521	756/2458(30.76)	0.49	0.09	2458	1003	0	gene=Chr04G1495	Gene Symbol:PKS1|Host:Zea mays|Disease:Southern leaf blight of maize|Description:Unknown

Chr04G1496.1	405	148	405	UniProt ID:Q6TFC7_ASPFM	349	70	338	80/270 (29.63)	0.47	0.05	270	105	2.00E-26	gene=Chr04G1496	Gene Symbol:NULL|Host:humans|Disease:infection|Description:Unknown
Chr04G1499.1	490	105	244	UniProt ID:F2Z6H2_CANGB	676	394	547	42/161 (26.09)	0.42	0.17	161	48.5	6.00E-07	gene=Chr04G1499	Gene Symbol:STE11|Host:humans|Disease:Occasional invasive candidal disease|Description:SIMILARITY: Contains 1 SAM (sterile alpha motif) domain.
Chr04G1503.1	521	3	488	UniProt ID:Q59RG1_CANAL	561	40	528	99/504 (19.64)	0.39	0.07	504	80.1	6.00E-17	gene=Chr04G1503	Gene Symbol:NAG3|Host:Isolated from a wide variety of substrates including humans|Disease:invasive candidal disease|Description:CAUTION: The sequence shown here is derived from an EMBL/GenBank/DDBJ whole genome shotgun (WGS) entry which is preliminary data.
Chr04G1	446	148	438	UniProt	349	61	347	105/29	0.52	0.04	295	170	4.00E-49	gene=Chr	Gene

515.1				ID:Q6TFC7_ASPFM				5(35.59)						04G1515	Symbol:NULL|Host:humans|Disease:infection|Description:Unknown
Chr04G1519.1	557	32	526	UniProt ID:Q5ANE1_CANAL	748	36	508	191/499(38.28)	0.57	0.06	499	328	2.00E-103	gene=Chr04G1519	Gene Symbol:SNF3|Host:Isolated from a wide variety of substrates including humans|Disease:invasive candidal disease|Description:SIMILARITY: Belongs to the major facilitator superfamily. Sugar transporter (TC 2.A.1.1) family.
Chr04G1520.1	664	317	653	UniProt ID:A4R0W3_MAGO7	1226	794	1111	74/362(20.44)	0.36	0.19	362	48.5	1.00E-06	gene=Chr04G1520	Gene Symbol:MGG_09263|Host:Poaceae, especially important on Oryzae|Disease:Rice blast|Description:Unknown
Chr04G1523.1	344	28	343	UniProt ID:Q75ZG3_ALTAL	366	13	365	168/356(47.19)	0.61	0.12	356	312	9.00E-105	gene=Chr04G1523	Gene Symbol:AFTS1|Host:Plant|Disease:Leaf spot, rots|Description:Unknown

Chr04G1524.1	631	17	629	UniProt ID:MEP1_ASPFN	635	18	632	279/623(44.78)	0.62	0.03	623	528	1.00E-180	gene=Chr04G1524	Gene Symbol:MEP|Host:Multiple genera in multiple families|Disease:Secondary pathogen of some plants. Rots of fruit and food where it produces highly carcinogenic chemicals|Description:FUNCTION: Secreted metalloproteinase that allows assimilation of proteinaceous substrates (By similarity).
Chr04G1525.1	380	75	221	UniProt ID:Q32WF7_PHAND	266	16	159	48/155(30.97)	0.47	0.12	155	51.6	2.00E-08	gene=Chr04G1525	Gene Symbol:MDH1|Host:Multiple genera of Poaceae and Blysmus compressus (Cyperaceae)|Disease:Glume blotch of wheat and other grasses|Description:Unknown
Chr04G1533.1	956	79	955	UniProt ID:Q99324_S	803	2	799	344/909(37.84)	0.53	0.16	909	513	3.00E-168	gene=Chr04G1533	Gene Symbol:B2TOM|Host:Primarily tomato, Lycopersicon

				EPLY											esculentum, also Solanum spp. and other Solanaceae|Disease:Leaf spot|Description:Unknown
Chr04G1541.1	666	259	579	UniProt ID:Q9UQZ1_9PEZI	512	46	323	118/335(35.22)	0.53	0.21	335	202	7.00E-58	gene=Chr04G1541	Gene Symbol:EMK1|Host:Multiple genera in multiple families|Disease:'Anthracnose of stems and leaves, dieback, root rot, leaf spot, blossom rot, fruit rot (dieback and ripe rot), seedling blight.' (Mordue 1971)|Description:Unknown
Chr04G1543.1	498	3	80	UniProt ID:Q59MD2_CANAL	843	759	838	29/80(36.25)	0.55	0.03	80	57.8	7.00E-10	gene=Chr04G1543	Gene Symbol:UME6|Host:Isolated from a wide variety of substrates including humans|Disease:invasive candidal disease|Description:CAUTION: The sequence shown here is derived from an EMBL/GenBank/DDBJ whole genome shotgun

														(WGS) entry which is preliminary data.
Chr04G1545.1	513	70	226	UniProt ID:Q5AJC0_CANAL	470	116	276	54/162 (33.33)	0.54	0.04	162	103	1.00E-24	gene=Chr04G1545	Gene Symbol:UTR2|Host:Isolated from a wide variety of substrates including humans|Disease:invasive candidal disease|Description:CAUTION: The sequence shown here is derived from an EMBL/GenBank/DDBJ whole genome shotgun (WGS) entry which is preliminary data.
Chr04G1548.1	203	1	203	UniProt ID:A0SXQ6_MAGGR	210	3	210	186/208(89.42)	0.93	0.02	208	377	1.00E-134	gene=Chr04G1548	Gene Symbol:RHO3|Host:Digitaria (Poaceae)|Disease:Leaf spot|Description:SIMILARITY: Belongs to the small GTPase superfamily. Rho family.
Chr04G1552.1	647	213	365	UniProt ID:Q59VF3_C	1813	69	215	49/157 (31.21)	0.45	0.09	157	58.2	1.00E-09	gene=Chr04G1552	Gene Symbol:"DUR1,2"|Host:Isolated from a wide variety

				ANAL											of substrates including humans|Disease:invasive candidal disease|Description:CAUTION: The sequence shown here is derived from an EMBL/GenBank/DDBJ whole genome shotgun (WGS) entry which is preliminary data.
Chr04G1555.1	663	140	626	UniProt ID:Q5XTQ5_BOTFU	615	95	561	140/491(28.51)	0.48	0.06	491	184	5.00E-51	gene=Chr04G1555	Gene Symbol:FRT1|Host:Various plant families|Disease:Grey mould. Parasite or saprophyte|Description:SIMILARITY: Belongs to the major facilitator superfamily. Sugar transporter (TC 2.A.1.1) family.
Chr04G1556.1	364	27	346	UniProt ID:Q6XVN4_CRYNV	383	22	362	87/356 (24.44)	0.39	0.14	356	58.2	2.00E-10	gene=Chr04G1556	Gene Symbol:GNO1|Host:humans|Disease:cryptococcosis|Description:COFACTOR: Zinc (By similarity).

Chr04G1557.1	255	2	250	UniProt ID:Q75WR5_9PLEO	265	8	262	88/260 (33.85)	0.54	0.06	260	137	2.00E-39	gene=Chr04G1557	Gene Symbol:BRN1|Host:Belamcanda chinensis: Korea,Gladiolus ?gandavensis: Korea,Iris japonica: China,Iris missouriensis (Leaf spot.): Idaho; Montana; Oregon; Washington,Iris sp. (Leaf spot.): China; Texas; Washing|Disease:Leaf spot|Description:SIMILARITY: Belongs to the short-chain dehydrogenases/reductases (SDR) family.
Chr04G1560.1	471	1	466	UniProt ID:Q50I20_ASPFM	465	1	465	339/467(72.59)	0.83	0.01	467	698	0	gene=Chr04G1560	Gene Symbol:MCSA|Host:humans|Disease:infection|Description:SIMILARITY: Belongs to the citrate synthase family.
Chr04G1561.1	598	66	597	UniProt ID:Q59RB8_CANAL	550	11	543	241/534(45.13)	0.63	0.01	534	491	3.00E-168	gene=Chr04G1561	Gene Symbol:ICL1|Host:Isolated from a wide variety of substrates including

														humans|Disease:invasive candidal disease|Description:SIMILARITY: Belongs to the isocitrate lyase/PEP mutase superfamily. Isocitrate lyase family.
Chr04G1562.1	287	12	265	UniProt ID:A4RGG9_MAGO7	286	14	273	94/267 (35.21)	0.53	0.07	267	107	9.00E-28	gene=Chr04G1562	Gene Symbol:MGG_00056|Host:Poaceae, especially important on Oryzae|Disease:Rice blast|Description:SIMILARITY: Belongs to the short-chain dehydrogenases/reductases (SDR) family.
Chr04G1564.1	377	80	376	UniProt ID:Q6TFC7_ASPFM	349	65	347	84/318 (26.42)	0.42	0.18	318	102	2.00E-25	gene=Chr04G1564	Gene Symbol:NULL|Host:humans|Disease:infection|Description:Unknown
Chr04G1566.1	724	34	560	UniProt ID:Q59VF3_CANAL	1813	614	1123	205/531(38.61)	0.61	0.05	531	377	4.00E-114	gene=Chr04G1566	Gene Symbol:"DUR1,2"|Host:Isolated from a wide variety of substrates including humans|Disease:invasive

															candidal disease|Description:CAUTION: The sequence shown here is derived from an EMBL/GenBank/DDBJ whole genome shotgun (WGS) entry which is preliminary data.
Chr04G1568.1	595	172	561	UniProt ID:Q2PEN8_9HYPO	575	131	550	101/447(22.60)	0.38	0.19	447	57.8	9.00E-10	gene=Chr04G1568	Gene Symbol:NOXB|Host:plants|Disease:cool-season grasses|Description:SIMILARITY: Contains 1 FAD-binding FR-type domain.
Chr04G1576.1	671	214	501	UniProt ID:SUB2_TRIRU	421	150	413	87/296(29.39)	0.44	0.14	296	72.4	2.00E-14	gene=Chr04G1576	Gene Symbol:SUB2|Host:humans|Disease:infection|Description:FUNCTION: Secreted subtilisin-like serine protease with keratinolytic activity that contributes to pathogenicity (By similarity).
Chr04G1	599	25	596	UniProt	693	69	673	174/62	0.45	0.13	629	195	2.00E-54	gene=Chr	Gene

585.1				ID:Q4P8E8_USTMA				9(27.66)						04G1585	Symbol:UM03615.1|Host:Euchlaena spp., Zea spp. (Poaceae)|Disease:Smut. Corn smut|Description:COFACTOR: FAD (By similarity).
Chr04G1590.1	491	2	486	UniProt ID:B2C6F1_CRYGA	614	63	569	161/532(30.26)	0.44	0.14	532	199	3.00E-57	gene=Chr04G1590	Gene Symbol:LAC1|Host:humans|Disease:pulmonary cryptococcosis, basal meningitis, and cerebral cryptococcomas|Description:Unknown
Chr04G1597.1	1044	1	199	UniProt ID:Q9UW16_MAGGR	181	1	181	110/199(55.28)	0.7	0.09	199	207	3.00E-62	gene=Chr04G1597	Gene Symbol:VATP|Host:Digitaria (Poaceae)|Disease:Leaf spot|Description:Unknown
Chr02G0006.1	393	2	301	UniProt ID:A4UC81_MAGO7	376	36	367	98/333(29.43)	0.46	0.1	333	143	2.00E-39	gene=Chr02G0006	Gene Symbol:MGG_10702|Host:Poaceae, especially important on Oryzae|Disease:Rice blast|Description:Unknown
Chr02G0010.1	309	104	211	UniProt ID:Q92	2528	1922	2049	34/129(26.36)	0.47	0.17	129	53.9	6.00E-09	gene=Chr02G0010	Gene Symbol:PKS1|Host:Zea

			217_COCHE												mays|Disease:Southern leaf blight of maize|Description:Unknown
Chr02G0011.1	488	5	381	UniProt ID:A4UC81_MAGO7	376	14	373	149/379(39.31)	0.6	0.06	379	273	3.00E-87	gene=Chr02G0011	Gene Symbol:MGG_10702|Host:Poaceae, especially important on Oryzae|Disease:Rice blast|Description:Unknown
Chr02G0024.1	288	227	272	UniProt ID:D1MYV6_MAGGR	568	521	567	23/47(48.94)	0.6	0.02	47	43.1	9.00E-06	gene=Chr02G0024	Gene Symbol:CBL1|Host:Digitaria (Poaceae)|Disease:Leaf spot|Description:SIMILARITY: Contains 3 chitin-binding type-1 domains.
Chr02G0033.1	1174	190	1059	UniProt ID:Q9P872_CANAL	917	52	850	249/885(28.14)	0.5	0.11	885	325	3.00E-95	gene=Chr02G0033	Gene Symbol:PMR1|Host:Isolated from a wide variety of substrates including humans|Disease:invasive candidal disease|Description:SIMILARITY: Belongs to the

															cation transport ATPase (P-type) family.
Chr02G0035.1	523	82	357	UniProt ID:Q9P8L8_BOTFU	598	137	418	68/288 (23.61)	0.44	0.06	288	63.2	1.00E-11	gene=Chr02G0035	Gene Symbol:BCMFS1|Host:Various plant families|Disease:Grey mould. Parasite or saprophyte|Description:Unknown
Chr02G0036.1	406	27	343	UniProt ID:Q6A2T2_BOTFU	391	32	349	81/334 (24.25)	0.43	0.1	334	74.3	2.00E-15	gene=Chr02G0036	Gene Symbol:BTP1|Host:Various plant families|Disease:Grey mould. Parasite or saprophyte|Description:Unknown
Chr02G0044.1	527	150	339	UniProt ID:Q00523_CRYNE	458	121	296	54/197 (27.41)	0.44	0.14	197	58.5	4.00E-10	gene=Chr02G0044	Gene Symbol:NULL|Host:humans|Disease:cryptococcosis|Description:Unknown
Chr02G0048.1	1134	63	559	UniProt ID:B2C6F1_CRYGA	614	69	551	140/523(26.77)	0.42	0.13	523	145	4.00E-37	gene=Chr02G0048	Gene Symbol:LAC1|Host:humans|Disease:pulmonary cryptococcosis, basal meningitis, and cerebral cryptococcomas|Descripti

															on:Unknown
Chr02G0056.1	838	548	814	UniProt ID:Q3Y5V5_MAGGR	1321	1042	1317	108/278(38.85)	0.59	0.05	278	200	4.00E-54	gene=Chr02G0056	Gene Symbol:ABC3|Host:Digitaria (Poaceae)|Disease:Leaf spot|Description:SIMILARITY: Belongs to the ABC transporter superfamily.
Chr02G0060.1	657	84	120	UniProt ID:Q59MD2_CANAL	843	762	798	20/37(54.05)	0.65	0	37	56.6	3.00E-09	gene=Chr02G0060	Gene Symbol:UME6|Host:Isolated from a wide variety of substrates including humans|Disease:invasive candidal disease|Description:CAUTION: The sequence shown here is derived from an EMBL/GenBank/DDBJ whole genome shotgun (WGS) entry which is preliminary data.
Chr02G0062.1	525	53	501	UniProt ID:Q5ANE1_CANAL	748	54	505	115/464(24.78)	0.41	0.06	464	94	4.00E-21	gene=Chr02G0062	Gene Symbol:SNF3|Host:Isolated from a wide variety of substrates including humans|Disease:invasive candidal

															disease|Description:SIMILARITY: Belongs to the major facilitator superfamily. Sugar transporter (TC 2.A.1.1) family.
Chr02G0065.1	765	36	756	UniProt ID:Q99324_SEPLY	803	31	794	336/781(43.02)	0.58	0.1	781	599	0	gene=Chr02G0065	Gene Symbol:B2TOM|Host:Primarily tomato, Lycopersicon esculentum, also Solanum spp. and other Solanaceae|Disease:Leaf spot|Description:Unknown
Chr02G0068.1	340	27	336	UniProt ID:Q92217_COCHE	2528	1841	2138	78/327 (23.85)	0.44	0.14	327	76.6	4.00E-16	gene=Chr02G0068	Gene Symbol:PKS1|Host:Zea mays|Disease:Southern leaf blight of maize|Description:Unknown
Chr02G0071.1	508	289	473	UniProt ID:A4ULJ2_MYCGR	515	298	503	51/212 (24.06)	0.45	0.16	212	44.7	9.00E-06	gene=Chr02G0071	Gene Symbol:CYP51|Host:Triticum and possibly a few other grasses|Disease:Leaf spot or speckled leaf blotch of wheat|Description:COFAC

															TOR: Heme group (By similarity).
Chr02G0076.1	756	7	753	UniProt ID:C5GH04_AJEDR	772	3	772	417/788(52.92)	0.67	0.07	788	723	0	gene=Chr02G0076	Gene Symbol:BDCG_03519|Host:humans|Disease:cutaneous Blastomyces dermatitidis infection|Description:SIMILARITY: Belongs to the DEAD box helicase family.
Chr02G0080.1	453	42	252	UniProt ID:Q2VLJ1_GIBZA	565	120	347	62/228(27.19)	0.46	0.07	228	68.2	3.00E-13	gene=Chr02G0080	Gene Symbol:ZEB1|Host:Principal hosts: Poaceae, including Zea mays (corn), Triticum aestivum (wheat), and Oryza sativa (rice). Additional hosts: various plant families|Disease:Seedling blight, pre- and post-emergence blight, root and foot rot, brown rot, culm decay, head or kernel blight (scab or ear scab) of cereals. Leaf|Description:Unknown

Chr02G0081.1	547	40	220	UniProt ID:A3LNY8_PICST	414	94	289	67/196 (34.18)	0.55	0.08	196	134	2.00E-35	gene=Chr02G0081	Gene Symbol:KRE2|Host:humans|Disease:occasional infection|Description:Unknown
Chr02G0083.1	486	52	478	UniProt ID:Q59RG1_CANAL	561	107	561	99/459 (21.57)	0.41	0.08	459	86.3	5.00E-19	gene=Chr02G0083	Gene Symbol:NAG3|Host:Isolated from a wide variety of substrates including humans|Disease:invasive candidal disease|Description:CAUTION: The sequence shown here is derived from an EMBL/GenBank/DDBJ whole genome shotgun (WGS) entry which is preliminary data.
Chr02G0086.1	281	16	215	UniProt ID:Q9P4U9_ALTAL	296	23	210	65/204 (31.86)	0.45	0.1	204	69.7	8.00E-15	gene=Chr02G0086	Gene Symbol:AKT3-1|Host:Plant|Disease:Leaf spot, rots|Description:Unknown
Chr02G0092.1	982	576	692	UniProt ID:A4RGM3_MAGO	283	173	282	35/119 (29.41)	0.51	0.09	119	57.8	7.00E-10	gene=Chr02G0092	Gene Symbol:MGG_12252|Host:Poaceae, especially important on

				7											Oryzae|Disease:Rice blast|Description:Unknown
Chr02G0096.1	780	55	780	UniProt ID:KATG_PENMA	748	15	741	488/733(66.58)	0.76	0.02	733	952	0	gene=Chr02G0096	Gene Symbol:KATG|Host:humans|Disease:lethal systemic infection|Description:FUNCTION: Bifunctional enzyme with both catalase and broad- spectrum peroxidase activity (By similarity). May be involved in protection from the host during host infection.
Chr02G0098.1	533	4	468	UniProt ID:Q9P8L8_BOTFU	598	37	518	146/489(29.86)	0.47	0.06	489	196	8.00E-56	gene=Chr02G0098	Gene Symbol:BCMFS1|Host:Various plant families|Disease:Grey mould. Parasite or saprophyte|Description:Unknown
Chr02G0100.1	464	14	377	UniProt ID:A4UC81_MAGO7	376	11	371	113/370(30.54)	0.5	0.04	370	169	2.00E-48	gene=Chr02G0100	Gene Symbol:MGG_10702|Host:Poaceae, especially important on

															Oryzae|Disease:Rice blast|Description:Unknown
Chr02G0101.1	357	37	259	UniProt ID:A4RGG9_MAGO7	286	9	216	70/231 (30.30)	0.47	0.13	231	82	7.00E-19	gene=Chr02G0101	Gene Symbol:MGG_00056|Host:Poaceae, especially important on Oryzae|Disease:Rice blast|Description:SIMILARITY: Belongs to the short-chain dehydrogenases/reductases (SDR) family.
Chr02G0102.1	901	170	342	UniProt ID:SUB3_ARTGP	397	149	315	57/182 (31.32)	0.44	0.13	182	47.4	2.00E-06	gene=Chr02G0102	Gene Symbol:SUB3|Host:Human, Mouse, Rat, Chicken, Pig, Rabbit, Bovine, Dog, African clawed frog, Zebrafish|Disease:tinea capitis, tinea corpus, ringworm, and other dermatophytoses|Description:FUNCTION: Secreted subtilisin-like serine protease with keratinolytic activity that contributes to

															pathogenicity (By similarity).
Chr02G0103.1	541	106	286	UniProt ID:Q2VLJ1_GIBZA	565	110	292	56/187 (29.95)	0.5	0.05	187	71.6	4.00E-14	gene=Chr02G0103	Gene Symbol:ZEB1|Host:Principal hosts: Poaceae, including Zea mays (corn), Triticum aestivum (wheat), and Oryza sativa (rice). Additional hosts: various plant families|Disease:Seedling blight, pre- and post-emergence blight, root and foot rot, brown rot, culm decay, head or kernel blight (scab or ear scab) of cereals. Leaf|Description:Unknown
Chr02G0105.1	370	27	196	UniProt ID:A4RG81_MAGO7	558	336	513	61/186 (32.80)	0.5	0.13	186	97.8	3.00E-23	gene=Chr02G0105	Gene Symbol:MGG_00131|Host:Poaceae, especially important on Oryzae|Disease:Rice blast|Description:Unknown
Chr02G0	478	21	432	UniProt	748	56	496	109/44	0.43	0.08	445	125	1.00E-31	gene=Chr	Gene

121.1				ID:Q5ANE1_CANAL				5(24.49)						02G0121	Symbol:SNF3|Host:Isolated from a wide variety of substrates including humans|Disease:invasive candidal disease|Description:SIMILARITY: Belongs to the major facilitator superfamily. Sugar transporter (TC 2.A.1.1) family.
Chr02G0125.1	341	25	245	UniProt ID:A4RGG9_MAGO7	286	7	213	64/227 (28.19)	0.39	0.11	227	45.1	2.00E-06	gene=Chr02G0125	Gene Symbol:MGG_00056|Host:Poaceae, especially important on Oryzae|Disease:Rice blast|Description:SIMILARITY: Belongs to the short-chain dehydrogenases/reductases (SDR) family.
Chr02G0133.1	350	58	186	UniProt ID:Q92217_COCHE	2528	1866	1983	36/131 (27.48)	0.48	0.11	131	45.8	2.00E-06	gene=Chr02G0133	Gene Symbol:PKS1|Host:Zea mays|Disease:Southern leaf blight of maize|Description:Unkno

															wn
Chr02G0 137.1	1036	74	203	UniProt ID:TUP 1_CAN AL	514	260	370	39/130 (30.00)	0.46	0.15	130	53.1	5.00E-08	gene=Chr 02G0137	Gene Symbol:TUP1|Host:Isolated from a wide variety of substrates including humans|Disease:invasive candidal disease|Description:FUNCTION: Represses transcription by RNA polymerase II. Represses genes responsible for initiating filamentous growth and this repression is lifted under inducing environmental conditions.
Chr02G0 139.1	264	1	155	UniProt ID:Q9C 441_F USSO	330	165	303	65/156 (41.67)	0.58	0.12	156	124	5.00E-34	gene=Chr 02G0139	Gene Symbol:PEP1|Host:Multiple plant families. Some strains may cause infections in humans|Disease:Saprobe, facultative pathogen|Description:Unknown
Chr02G0	555	194	495	UniProt	518	193	505	76/345	0.39	0.22	345	48.1	7.00E-07	gene=Chr	Gene

142.1				ID:A4ULJ0_MYCGR				(22.03)						02G0142	Symbol:CYP51|Host:Triticum and possibly a few other grasses|Disease:Leaf spot or speckled leaf blotch of wheat|Description:COFACTOR: Heme group (By similarity).
Chr02G0143.1	560	64	307	UniProt ID:Q5ALS7_CANAL	1144	360	614	60/267 (22.47)	0.41	0.13	267	53.9	2.00E-08	gene=Chr02G0143	Gene Symbol:CTF1|Host:Isolated from a wide variety of substrates including humans|Disease:invasive candidal disease|Description:SIMILARITY: Contains 1 Zn(2)-C6 fungal-type DNA-binding domain.
Chr02G0145.1	488	50	459	UniProt ID:A0ST42_CERNC	512	65	486	119/440(27.05)	0.42	0.11	440	74.3	4.00E-15	gene=Chr02G0145	Gene Symbol:CTB4|Host:Numerous taxa in Solanaceae|Disease:Leaf spot|Description:Unknown
Chr02G0149.1	551	168	489	UniProt ID:A5H456_M	1811	16	333	116/325(35.69)	0.57	0.03	325	206	3.00E-57	gene=Chr02G0149	Gene Symbol:NULL|Host:Triticum and possibly a few other

				YCGR											grasses|Disease:Leaf spot or speckled leaf blotch of wheat|Description:Unknown
Chr02G0150.1	480	30	392	UniProt ID:A4UC81_MAGO7	376	8	374	104/369(28.18)	0.48	0.02	369	172	4.00E-49	gene=Chr02G0150	Gene Symbol:MGG_10702|Host:Poaceae, especially important on Oryzae|Disease:Rice blast|Description:Unknown
Chr02G0151.1	597	62	575	UniProt ID:B2C6F1_CRYGA	614	43	569	166/551(30.13)	0.45	0.11	551	210	2.00E-60	gene=Chr02G0151	Gene Symbol:LAC1|Host:humans|Disease:pulmonary cryptococcosis, basal meningitis, and cerebral cryptococcomas|Description:Unknown
Chr02G0153.1	563	105	562	UniProt ID:Q59RG0_CANAL	581	119	577	145/460(31.52)	0.53	0.01	460	232	1.00E-68	gene=Chr02G0153	Gene Symbol:NAG4|Host:Isolated from a wide variety of substrates including humans|Disease:invasive candidal disease|Description:CAUTION: The sequence shown

															here is derived from an EMBL/GenBank/DDBJ whole genome shotgun (WGS) entry which is preliminary data.
Chr02G0156.1	487	53	294	UniProt ID:Q9UUS8_COLGL	567	64	314	73/257 (28.40)	0.43	0.08	257	80.5	4.00E-17	gene=Chr02G0156	Gene Symbol:CHIP3|Host:Multiple genera in multiple families|Disease:'Anthracnose of stems and leaves, dieback, root rot, leaf spot, blossom rot, fruit rot (dieback and ripe rot), seedling blight.' (Mordue 1971)|Description:Unknown
Chr02G0158.1	392	19	355	UniProt ID:Q9Y784_MAGGR	631	103	416	81/341 (23.75)	0.41	0.09	341	65.5	1.00E-12	gene=Chr02G0158	Gene Symbol:PTH11|Host:Digitaria (Poaceae)|Disease:Leaf spot|Description:Unknown
Chr02G0159.1	266	9	266	UniProt ID:Q32WF7_PHAND	266	17	266	69/264 (26.14)	0.41	0.08	264	49.3	6.00E-08	gene=Chr02G0159	Gene Symbol:MDH1|Host:Multiple genera of Poaceae and Blysmus compressus (Cyperaceae)|Disease:Glu

														me blotch of wheat and other grasses|Description:Unknown
Chr02G0160.1	351	63	346	UniProt ID:Q6TFC7_ASPFM	349	63	346	97/288 (33.68)	0.5	0.03	288	146	5.00E-41	gene=Chr02G0160	Gene Symbol:NULL|Host:humans|Disease:infection|Description:Unknown
Chr02G0170.1	1168	167	315	UniProt ID:Q2I0M6_CERNC	871	253	412	45/163 (27.61)	0.49	0.1	163	64.7	2.00E-11	gene=Chr02G0170	Gene Symbol:CTB3|Host:Numerous taxa in Solanaceae|Disease:Leaf spot|Description:Unknown
Chr02G0176.1	264	12	262	UniProt ID:Q32WF7_PHAND	266	11	264	69/257 (26.85)	0.44	0.04	257	91.7	1.00E-22	gene=Chr02G0176	Gene Symbol:MDH1|Host:Multiple genera of Poaceae and Blysmus compressus (Cyperaceae)|Disease:Glume blotch of wheat and other grasses|Description:Unknown
Chr02G0177.1	571	2	92	UniProt ID:A6N6J8_FUSOX	903	31	133	36/109 (33.03)	0.45	0.22	109	46.2	4.00E-06	gene=Chr02G0177	Gene Symbol:CTF1|Host:Multiple genera in multiple families|Disease:Blights,

															wilts, rots of various sorts|Description:SIMILARITY: Contains 1 Zn(2)-C6 fungal-type DNA-binding domain.
Chr02G0180.1	594	8	520	UniProt ID:Q9P8L8_BOTFU	598	29	528	117/527(22.20)	0.4	0.08	527	86.7	7.00E-19	gene=Chr02G0180	Gene Symbol:BCMFS1|Host:Various plant families|Disease:Grey mould. Parasite or saprophyte|Description:Unknown
Chr02G0182.1	591	13	588	UniProt ID:Q4P8E8_USTMA	693	69	672	207/620(33.39)	0.5	0.1	620	253	1.00E-75	gene=Chr02G0182	Gene Symbol:UM03615.1|Host: Euchlaena spp., Zea spp. (Poaceae)|Disease:Smut. Corn smut|Description:COFACTOR: FAD (By similarity).
Chr02G0190.1	652	222	396	UniProt ID:Q59VF3_CANAL	1813	70	242	58/182(31.87)	0.47	0.09	182	73.2	3.00E-14	gene=Chr02G0190	Gene Symbol:"DUR1,2"|Host:Isolated from a wide variety of substrates including humans|Disease:invasive candidal disease|Description:CAUT

														ION: The sequence shown here is derived from an EMBL/GenBank/DDBJ whole genome shotgun (WGS) entry which is preliminary data.
Chr02G0201.1	406	40	392	UniProt ID:Q2VLJ1_GIBZA	565	27	376	174/353(49.29)	0.66	0.01	353	348	4.00E-115	gene=Chr02G0201	Gene Symbol:ZEB1|Host:Principal hosts: Poaceae, including Zea mays (corn), Triticum aestivum (wheat), and Oryza sativa (rice). Additional hosts: various plant families|Disease:Seedling blight, pre- and post-emergence blight, root and foot rot, brown rot, culm decay, head or kernel blight (scab or ear scab) of cereals. Leaf|Description:Unknown
Chr02G0203.1	366	25	324	UniProt ID:Q6A2T2_BOTFU	391	42	360	80/333(24.02)	0.41	0.14	333	62.8	6.00E-12	gene=Chr02G0203	Gene Symbol:BTP1|Host:Various plant families|Disease:Grey

															mould. Parasite or saprophyte|Description:Unknown
Chr02G0205.1	542	349	509	UniProt ID:A4ULI5_MYCGR	515	309	503	46/196(23.47)	0.41	0.18	196	49.7	3.00E-07	gene=Chr02G0205	Gene Symbol:CYP51|Host:Triticum and possibly a few other grasses|Disease:Leaf spot or speckled leaf blotch of wheat|Description:COFACTOR: Heme group (By similarity).
Chr02G0206.1	581	6	579	UniProt ID:Q4P8E8_USTMA	693	84	673	187/632(29.59)	0.45	0.16	632	219	3.00E-63	gene=Chr02G0206	Gene Symbol:UM03615.1|Host: Euchlaena spp., Zea spp. (Poaceae)|Disease:Smut. Corn smut|Description:COFACTOR: FAD (By similarity).
Chr02G0213.1	364	1	364	UniProt ID:C5GX01_AJEDR	364	1	364	303/364(83.24)	0.91	0	364	634	0	gene=Chr02G0213	Gene Symbol:BDCG_08925|Host:humans|Disease:cutaneous Blastomyces dermatitidis infection|Description:Unknown

Chr02G0217.1	903	1	215	UniProt ID:Q5ADS0_CANAL	229	1	228	215/228(94.30)	0.94	0.06	228	424	9.00E-143	gene=Chr02G0217	Gene Symbol:UBI4|Host:Isolated from a wide variety of substrates including humans|Disease:invasive candidal disease|Description:Unknown
Chr02G0222.1	431	135	414	UniProt ID:O93801_ALTAL	262	3	258	86/282(30.50)	0.43	0.1	282	112	3.00E-29	gene=Chr02G0222	Gene Symbol:AKT2|Host:Plant|Disease:Leaf spot, rots|Description:Unknown
Chr02G0225.1	661	257	566	UniProt ID:Q5ALS7_CANAL	1144	351	658	76/322(23.60)	0.42	0.08	322	64.7	1.00E-11	gene=Chr02G0225	Gene Symbol:CTF1|Host:Isolated from a wide variety of substrates including humans|Disease:invasive candidal disease|Description:SIMILARITY: Contains 1 Zn(2)-C6 fungal-type DNA-binding domain.
Chr02G0228.1	975	633	882	UniProt ID:TUP1_CANAL	514	259	509	107/259(41.31)	0.58	0.07	259	189	3.00E-52	gene=Chr02G0228	Gene Symbol:TUP1|Host:Isolated from a wide variety of substrates including

															humans|Disease:invasive candidal disease|Description:FUNCTION: Represses transcription by RNA polymerase II. Represses genes responsible for initiating filamentous growth and this repression is lifted under inducing environmental conditions.
Chr02G0229.1	557	15	546	UniProt ID:Q9P8L8_BOTFU	598	43	589	251/551(45.55)	0.66	0.04	551	500	1.00E-171	gene=Chr02G0229	Gene Symbol:BCMFS1|Host:Various plant families|Disease:Grey mould. Parasite or saprophyte|Description:Unknown
Chr02G0241.1	668	9	604	UniProt ID:SCPB_TRIRU	662	10	613	260/620(41.94)	0.57	0.06	620	442	2.00E-146	gene=Chr02G0241	Gene Symbol:SCPB|Host:humans|Disease:infection|Description:FUNCTION: Extracellular serine carboxypeptidase that contributes to pathogenicity.

Chr02G0245.1	744	5	397	UniProt ID:Q9P927_CANAL	576	6	365	165/405(40.74)	0.57	0.14	405	269	5.00E-81	gene=Chr02G0245	Gene Symbol:NOT4|Host:Isolated from a wide variety of substrates including humans|Disease:invasive candidal disease|Description:SIMILARITY: Contains 1 RING-type zinc finger.
Chr02G0255.1	495	47	231	UniProt ID:Q2VLJ1_GIBZA	565	123	294	54/186 (29.03)	0.44	0.08	186	58.2	6.00E-10	gene=Chr02G0255	Gene Symbol:ZEB1|Host:Principal hosts: Poaceae, including Zea mays (corn), Triticum aestivum (wheat), and Oryza sativa (rice). Additional hosts: various plant families|Disease:Seedling blight, pre- and post-emergence blight, root and foot rot, brown rot, culm decay, head or kernel blight (scab or ear scab) of cereals. Leaf|Description:Unknown Gene
Chr02G0	941	145	341	UniProt	481	149	345	61/221	0.43	0.22	221	52	9.00E-08	gene=Chr	

269.1				ID:SUB 1_ART GP				(27.60)						02G0269	Symbol:SUB1|Host:Human, Mouse, Rat, Chicken, Pig, Rabbit, Bovine, Dog, African clawed frog, Zebrafish|Disease:tinea capitis, tinea corpus, ringworm, and other dermatophytoses|Description:FUNCTION: Secreted subtilisin-like serine protease with keratinolytic activity that contributes to pathogenicity (By similarity).
Chr02G0 270.1	484	11	482	UniProt ID:Q4P 8E8_U STMA	693	169	673	146/51 4(28.4 0)	0.46	0.1	514	164	6.00E-45	gene=Chr 02G0270	Gene Symbol:UM03615.1|Host: Euchlaena spp., Zea spp. (Poaceae)|Disease:Smut. Corn smut|Description:COFACTOR: FAD (By similarity).
Chr02G0 272.1	526	242	499	UniProt ID:A4U LJ2_M YCGR	515	245	508	60/275 (21.82)	0.44	0.1	275	58.5	4.00E-10	gene=Chr 02G0272	Gene Symbol:CYP51|Host:Triticum and possibly a few other grasses|Disease:Leaf spot

															or speckled leaf blotch of wheat|Description:COFACTOR: Heme group (By similarity).
Chr02G0273.1	1460	1006	1453	UniProt ID:Q9UW87_CANAL	1606	1099	1580	143/490(29.18)	0.49	0.1	490	229	8.00E-62	gene=Chr02G0273	Gene Symbol:MLT1|Host:Isolated from a wide variety of substrates including humans|Disease:invasive candidal disease|Description:SIMILARITY: Belongs to the ABC transporter superfamily.
Chr02G0285.1	455	133	241	UniProt ID:A3QX02_ASPFM	570	434	541	54/111(48.65)	0.59	0.05	111	97.4	1.00E-22	gene=Chr02G0285	Gene Symbol:ZAFA|Host:humans|Disease:infection|Description:Unknown
Chr02G0288.1	135	4	78	UniProt ID:A3LQC4_PICST	484	384	462	26/79(32.91)	0.52	0.05	79	43.1	1.00E-06	gene=Chr02G0288	Gene Symbol:OLE1|Host:humans|Disease:occasional infection|Description:COFACTOR: Iron (By similarity).
Chr02G0291.1	712	274	419	UniProt ID:Q7Z	418	33	177	42/158(26.58)	0.46	0.16	158	59.3	3.00E-10	gene=Chr02G0291	Gene Symbol:GLU1|Host:huma

				A48_9HYPO											ns|Disease:Verticillium disease or dry bubble|Description:Unknown
Chr02G0294.1	553	13	552	UniProt ID:Q59RG0_CANAL	581	39	575	183/546(33.52)	0.56	0.03	546	305	3.00E-96	gene=Chr02G0294	Gene Symbol:NAG4|Host:Isolated from a wide variety of substrates including humans|Disease:invasive candidal disease|Description:CAUTION: The sequence shown here is derived from an EMBL/GenBank/DDBJ whole genome shotgun (WGS) entry which is preliminary data.
Chr02G0295.1	688	409	675	UniProt ID:Q59M50_CANAL	578	398	575	64/267(23.97)	0.39	0.33	267	82.8	2.00E-17	gene=Chr02G0295	Gene Symbol:CWT1|Host:Isolated from a wide variety of substrates including humans|Disease:invasive candidal disease|Description:CAUTION: The sequence shown here is derived from an

															EMBL/GenBank/DDBJ whole genome shotgun (WGS) entry which is preliminary data.
Chr02G0300.1	541	13	302	UniProt ID:Q75T36_GLOLA	518	200	494	113/301(37.54)	0.57	0.06	301	197	6.00E-57	gene=Chr02G0300	Gene Symbol:CPK1|Host:melons,cucumber|Disease:anthracnose fruit rot|Description:CATALYTIC ACTIVITY: ATP + a protein = ADP + a phosphoprotein.
Chr02G0301.1	568	1	553	UniProt ID:Q5J7N6_COCHE	589	1	581	238/617(38.57)	0.52	0.16	617	313	4.00E-99	gene=Chr02G0301	Gene Symbol:CHAP1|Host:Zea mays|Disease:Southern leaf blight of maize|Description:SIMILARITY: Belongs to the bZIP family.
Chr02G0308.1	774	135	766	UniProt ID:Q99324_SEPLY	803	105	795	196/729(26.89)	0.41	0.19	729	179	4.00E-48	gene=Chr02G0308	Gene Symbol:B2TOM|Host:Primarily tomato, Lycopersicon esculentum, also Solanum spp. and other Solanaceae|Disease:Leaf spot|Description:Unknown

Chr02G0309.1	763	315	673	UniProt ID:Q705V7_USTMD	1061	458	839	89/394 (22.59)	0.43	0.12	394	88.6	6.00E-19	gene=Chr02G0309	Gene Symbol:GAS1|Host:Euchlaena spp., Zea spp. (Poaceae)|Disease:Smut. Corn smut|Description:Unknown
Chr02G0310.1	1002	177	382	UniProt ID:Q99324_SEPLY	803	103	299	57/218 (26.15)	0.46	0.15	218	61.6	1.00E-10	gene=Chr02G0310	Gene Symbol:B2TOM|Host:Primarily tomato, Lycopersicon esculentum, also Solanum spp. and other Solanaceae|Disease:Leaf spot|Description:Unknown
Chr02G0311.1	439	13	432	UniProt ID:Q59RW2_CANAL	413	3	399	169/430(39.30)	0.58	0.1	430	288	3.00E-93	gene=Chr02G0311	Gene Symbol:DAC1|Host:Isolated from a wide variety of substrates including humans|Disease:invasive candidal disease|Description:CAUTION: The sequence shown here is derived from an EMBL/GenBank/DDBJ whole genome shotgun (WGS) entry which is

															preliminary data.
Chr02G0314.1	421	1	244	UniProt ID:Q9C1S8_CANAL	248	1	242	118/246(47.97)	0.65	0.02	246	224	4.00E-71	gene=Chr02G0314	Gene Symbol:CANAG1|Host:Isolated from a wide variety of substrates including humans|Disease:invasive candidal disease|Description:Unknown
Chr02G0315.1	541	78	534	UniProt ID:Q9C0M1_CANAL	493	47	490	133/486(27.37)	0.45	0.15	486	143	5.00E-38	gene=Chr02G0315	Gene Symbol:CANAG5|Host:Isolated from a wide variety of substrates including humans|Disease:invasive candidal disease|Description:Unknown
Chr02G0324.1	1005	16	454	UniProt ID:Q9HG15_COLLN	746	20	456	98/488(20.08)	0.38	0.2	488	67.4	2.00E-12	gene=Chr02G0324	Gene Symbol:CLTA1|Host:Multiple genera of Fabaceae. Rare reports on other taxa|Disease:Leaf, stem and pod anthracnose|Description:SIMILARITY: Contains 1 Zn(2)-C6 fungal-type

														DNA-binding domain.
Chr02G0329.1	368	117	367	UniProt ID:O93802_ALTAL	267	9	267	105/267(39.33)	0.58	0.09	267	171	6.00E-51	gene=Chr02G0329	Gene Symbol:BRM2|Host:Plant|Disease:Leaf spot, rots|Description:SIMILARITY: Belongs to the short-chain dehydrogenases/reductases (SDR) family.
Chr02G0330.1	627	4	171	UniProt ID:A7A233_YEAS7	215	21	184	44/170(25.88)	0.44	0.05	170	52	2.00E-08	gene=Chr02G0330	Gene Symbol:SEC4|Host:humans|Disease:occasional infection|Description:SIMILARITY: Belongs to the small GTPase superfamily. Rab family.
Chr02G0331.1	623	386	533	UniProt ID:Q6XSF5_COCHE	351	67	209	46/159(28.93)	0.44	0.17	159	48.9	4.00E-07	gene=Chr02G0331	Gene Symbol:NULL|Host:Zea mays|Disease:Southern leaf blight of maize|Description:Unknown
Chr02G0351.1	415	360	413	UniProt ID:Q59LF3_CANAL	474	421	474	22/54(40.74)	0.61	0	54	54.3	5.00E-09	gene=Chr02G0351	Gene Symbol:RVS167|Host:Isolated from a wide variety of substrates including

															humans|Disease:invasive candidal disease|Description:SIMILARITY: Contains 1 SH3 domain.
Chr02G0353.1	599	15	122	UniProt ID:Q59W09_CANAL	379	6	112	36/108(33.33)	0.56	0.01	108	81.6	1.00E-17	gene=Chr02G0353	Gene Symbol:GCS1|Host:Isolated from a wide variety of substrates including humans|Disease:invasive candidal disease|Description:CAUTION: The sequence shown here is derived from an EMBL/GenBank/DDBJ whole genome shotgun (WGS) entry which is preliminary data.
Chr02G0357.1	1458	843	1447	UniProt ID:Q5A762_CANAL	1606	969	1598	181/645(28.06)	0.51	0.09	645	273	3.00E-75	gene=Chr02G0357	Gene Symbol:MLT1|Host:Isolated from a wide variety of substrates including humans|Disease:invasive candidal disease|Description:SIMILARITY: Belongs to the

															ABC transporter superfamily.
Chr02G0367.1	607	46	605	UniProt ID:Q4P8E8_USTMA	693	84	667	181/614(29.48)	0.44	0.14	614	195	2.00E-54	gene=Chr02G0367	Gene Symbol:UM03615.1|Host: Euchlaena spp., Zea spp. (Poaceae)|Disease:Smut. Corn smut|Description:COFACTOR: FAD (By similarity).
Chr02G0369.1	326	1	309	UniProt ID:C1G8Q1_PARBD	333	1	309	255/309(82.52)	0.9	0	309	532	0	gene=Chr02G0369	Gene Symbol:PADG_03637|Host:humans|Disease:Paracoccidioidomycosis|Description:Unknown
Chr02G0371.1	1211	204	1183	UniProt ID:Q8J286_COLLN	1167	125	1098	315/1024(30.76)	0.48	0.09	1024	379	2.00E-112	gene=Chr02G0371	Gene Symbol:CLAP1|Host:Multiple genera of Fabaceae. Rare reports on other taxa|Disease:Leaf, stem and pod anthracnose|Description:SIMILARITY: Belongs to the cation transport ATPase (P-type) family.
Chr02G0384.1	345	35	344	UniProt ID:Q69	321	3	320	221/318(69.5	0.81	0.03	318	481	1.00E-171	gene=Chr02G0384	Gene Symbol:GLO1|Host:Multipl

				6X2_P HAND				0)						e genera of Poaceae and Blysmus compressus (Cyperaceae)|Disease:Glume blotch of wheat and other grasses|Description:Unknown
Chr02G0389.1	358	105	356	UniProt ID:Q32WF7_PHAND	266	15	264	102/255(40.00)	0.59	0.03	255	179	5.00E-54	gene=Chr02G0389	Gene Symbol:MDH1|Host:Multiple genera of Poaceae and Blysmus compressus (Cyperaceae)|Disease:Glume blotch of wheat and other grasses|Description:Unknown
Chr02G0390.1	1011	337	1010	UniProt ID:C6KEF5_MYCGR	833	176	832	393/698(56.30)	0.67	0.09	698	663	0	gene=Chr02G0390	Gene Symbol:STE20|Host:Triticum and possibly a few other grasses|Disease:Leaf spot or speckled leaf blotch of wheat|Description:CATALYTIC ACTIVITY: ATP + a protein = ADP + a phosphoprotein.

Chr02G0392.1	692	142	639	UniProt ID:Q96VB5_ALTAL	578	89	554	120/533(22.51)	0.39	0.19	533	68.9	4.00E-13	gene=Chr02G0392	Gene Symbol:AFT1-1|Host:Plant|Disease:Leaf spot, rots|Description:Unknown
Chr02G0395.1	714	2	458	UniProt ID:VPS27_CANAL	841	1	549	178/555(32.07)	0.49	0.19	555	246	6.00E-71	gene=Chr02G0395	Gene Symbol:VPS27|Host:Isolated from a wide variety of substrates including humans|Disease:invasive candidal disease|Description:FUNCTION: Component of the ESCRT-0 complex which is the sorting receptor for ubiquitinated cargo proteins at the multivesicular body (MVB) and recruits ESCRT-I to the MVB outer membrane (By similarity).
Chr02G0397.1	551	27	544	UniProt ID:C0S733_PARBP	560	16	542	156/530(29.43)	0.52	0.03	530	211	2.00E-61	gene=Chr02G0397	Gene Symbol:PABG_03488|Host:humans|Disease:Paracoccidioidomycosis|Description:SIMILARITY: Belongs to the TCP-1 chaperonin

															family.
Chr02G0406.1	461	8	460	UniProt ID:Q9C1F9_COCCA	423	5	418	165/460(35.87)	0.5	0.12	460	246	8.00E-77	gene=Chr02G0406	Gene Symbol:CEL2|Host:Corn, Zea mays, sometimes on Sorghum (Poaceae) and various other plant families|Disease:Northern corn leaf spot, ear and kernel rot|Description:Unknown
Chr02G0407.1	359	2	236	UniProt ID:Q96TN6_MAGGR	251	1	236	79/245 (32.24)	0.51	0.08	245	101	7.00E-26	gene=Chr02G0407	Gene Symbol:MAS3|Host:Digitaria (Poaceae)|Disease:Leaf spot|Description:Unknown
Chr02G0409.1	366	99	302	UniProt ID:PLYB_COLGL	331	68	272	60/218 (27.52)	0.47	0.12	218	76.6	1.00E-16	gene=Chr02G0409	Gene Symbol:PLB|Host:Multiple genera in multiple families|Disease:'Anthracnose of stems and leaves, dieback, root rot, leaf spot, blossom rot, fruit rot (dieback and ripe rot), seedling blight.' (Mordue 1971)|Description:FUNCTION: Acts as a virulence

														factor active in plant tissue maceration.
Chr02G0413.1	1366	818	1077	UniProt ID:SU11A_COCP7	403	136	334	68/268 (25.37)	0.38	0.29	268	52	1.00E-07	gene=Chr02G0413	Gene Symbol:CPC735_035780|Host:humans|Disease:coccidiomycosis|Description: FUNCTION: Secreted subtilisin-like serine protease with keratinolytic activity that contributes to pathogenicity (By similarity).
Chr02G0414.1	1527	1048	1328	UniProt ID:TUP1_CANAL	514	201	509	106/311(34.08)	0.54	0.1	311	179	4.00E-48	gene=Chr02G0414	Gene Symbol:TUP1|Host:Isolated from a wide variety of substrates including humans|Disease:invasive candidal disease|Description:FUNCTION: Represses transcription by RNA polymerase II. Represses genes responsible for initiating filamentous growth and this repression is lifted under inducing

															environmental conditions.
Chr02G0418.1	715	4	65	UniProt ID:Q9HG15_COLLN	746	16	76	24/62(38.71)	0.55	0.02	62	47.8	2.00E-06	gene=Chr02G0418	Gene Symbol:CLTA1|Host:Multiple genera of Fabaceae. Rare reports on other taxa|Disease:Leaf, stem and pod anthracnose|Description:SIMILARITY: Contains 1 Zn(2)-C6 fungal-type DNA-binding domain.
Chr02G0420.1	321	30	240	UniProt ID:A4RGG9_MAGO7	286	2	213	55/229(24.02)	0.39	0.15	229	45.4	2.00E-06	gene=Chr02G0420	Gene Symbol:MGG_00056|Host:Poaceae, especially important on Oryzae|Disease:Rice blast|Description:SIMILARITY: Belongs to the short-chain dehydrogenases/reductases (SDR) family.
Chr02G0429.1	227	19	223	UniProt ID:Q2LMP0_BOTFU	227	23	226	122/206(59.22)	0.75	0.01	206	242	5.00E-81	gene=Chr02G0429	Gene Symbol:XYN11A|Host:Various plant families|Disease:Grey mould. Parasite or

															saprophyte|Description:CATALYTIC ACTIVITY: Endohydrolysis of (1->4)-beta-D-xylosidic linkages in xylans.
Chr02G0431.1	755	224	425	UniProt ID:Q9HG15_COLLN	746	184	370	47/204 (23.04)	0.41	0.09	204	57	3.00E-09	gene=Chr02G0431	Gene Symbol:CLTA1|Host:Multiple genera of Fabaceae. Rare reports on other taxa|Disease:Leaf, stem and pod anthracnose|Description:SIMILARITY: Contains 1 Zn(2)-C6 fungal-type DNA-binding domain.
Chr02G0438.1	688	59	688	UniProt ID:Q5D1P9_GLOAC	670	35	670	430/655(65.65)	0.75	0.07	655	749	0	gene=Chr02G0438	Gene Symbol:NULL|Host:Multiple genera in multiple families|Disease:Anthracnose, canker, leaf spot, shepherd's crook, fruit rot, etc|Description:Unknown
Chr02G0440.1	990	704	924	UniProt ID:SUB1_ARTGP	481	146	364	71/232 (30.60)	0.45	0.1	232	74.3	1.00E-14	gene=Chr02G0440	Gene Symbol:SUB1|Host:Human, Mouse, Rat, Chicken, Pig, Rabbit, Bovine, Dog,

															African clawed frog, Zebrafish|Disease:tinea capitis, tinea corpus, ringworm, and other dermatophytoses|Description:FUNCTION: Secreted subtilisin-like serine protease with keratinolytic activity that contributes to pathogenicity (By similarity).
Chr02G0442.1	899	29	93	UniProt ID:A8QJI7_FUSOX	938	57	132	24/76(31.58)	0.46	0.14	76	47.4	4.00E-06	gene=Chr02G0442	Gene Symbol:XLNR|Host:Multiple genera in multiple families|Disease:Blights, wilts, rots of various sorts|Description:SIMILARITY: Contains 1 Zn(2)-C6 fungal-type DNA-binding domain.
Chr02G0443.1	606	76	460	UniProt ID:C5GH04_AJEDR	772	175	606	127/445(28.54)	0.46	0.16	445	145	2.00E-37	gene=Chr02G0443	Gene Symbol:BDCG_03519|Host:humans|Disease:cutaneous Blastomyces dermatitidis infection|Description:SIMI

															LARITY: Belongs to the DEAD box helicase family.
Chr02G0447.1	629	108	325	UniProt ID:CARP6_CANAL	418	81	303	65/234 (27.78)	0.4	0.12	234	51.2	8.00E-08	gene=Chr02G0447	Gene Symbol:SAP6|Host:Isolated from a wide variety of substrates including humans|Disease:invasive candidal disease|Description:CATALYTIC ACTIVITY: Preferential cleavage at the carboxyl of hydrophobic amino acids, but fails to cleave 15-Leu-|-Tyr-16, 16-Tyr-|-Leu-17 and 24-Phe-|-Phe-25 of insulin B chain. Activates trypsinogen, and degrades keratin.
Chr02G0454.1	785	10	70	UniProt ID:Q0WXM3_FUSOX	663	88	151	23/64(35.94)	0.5	0.05	64	48.9	8.00E-07	gene=Chr02G0454	Gene Symbol:FOW2|Host:Multiple genera in multiple families|Disease:Blights, wilts, rots of various sorts|Description:SIMILAR

Chr02G0468.1	399	1	397	UniProt ID:ORYZ_ASPFU	403	1	402	217/419(51.79)	0.67	0.09	419	395	8.00E-136	gene=Chr02G0468	ITY: Contains 1 Zn(2)-C6 fungal-type DNA-binding domain. Gene Symbol:ALP1|Host:humans|Disease:infection|Description:FUNCTION: Secreted alkaline protease that allows assimilation of proteinaceous substrates. Acts as a significant virulence factor in invasive aspergillosis. Involved in immune evasion from the human and mice complement systems during infection. Efficiently cleaves important components of the complement cascade such as such as C3, C4, C5, and C1q, as well as IgG, which leads to down-regulation of complement activation at the hyphal

															surface.
Chr02G0475.1	457	20	275	UniProt ID:A4RF81_MAGO7	542	29	311	66/290 (22.76)	0.4	0.14	290	51.6	5.00E-08	gene=Chr02G0475	Gene Symbol:MGG_00435|Host:Poaceae, especially important on Oryzae|Disease:Rice blast|Description:Unknown
Chr02G0481.1	584	57	450	UniProt ID:OPSB_TRIVH	495	73	435	122/406(30.05)	0.49	0.14	406	138	3.00E-36	gene=Chr02G0481	Gene Symbol:OPSB|Host:humans|Disease:infection|Description:FUNCTION: Probable GPI-anchored aspartic-type endopeptidase which contributes to virulence (By similarity).
Chr02G0482.1	426	21	423	UniProt ID:O59928_HYPVI	430	20	429	250/413(60.53)	0.76	0.03	413	507	7.00E-179	gene=Chr02G0482	Gene Symbol:NULL|Host:humans|Disease:infection|Description:SIMILARITY: Belongs to the glycosyl hydrolase 18 family.
Chr02G0485.1	406	35	333	UniProt ID:Q6A2T2_B	391	43	356	95/314 (30.25)	0.51	0.05	314	134	6.00E-36	gene=Chr02G0485	Gene Symbol:BTP1|Host:Various plant

				OTFU										families|Disease:Grey mould. Parasite or saprophyte|Description:Unknown
Chr02G0496.1	335	4	329	UniProt ID:A0ST44_CERNC	357	6	344	98/348 (28.16)	0.45	0.09	348	97.1	7.00E-24	gene=Chr02G0496	Gene Symbol:CTB6|Host:Numerous taxa in Solanaceae|Disease:Leaf spot|Description:Unknown
Chr02G0500.1	878	84	373	UniProt ID:Q99324_SEPLY	803	94	428	116/341(34.02)	0.49	0.17	341	175	2.00E-46	gene=Chr02G0500	Gene Symbol:B2TOM|Host:Primarily tomato, Lycopersicon esculentum, also Solanum spp. and other Solanaceae|Disease:Leaf spot|Description:Unknown
Chr02G0503.1	523	171	499	UniProt ID:Q5XTQ5_BOTFU	615	223	550	79/339 (23.30)	0.43	0.06	339	75.1	3.00E-15	gene=Chr02G0503	Gene Symbol:FRT1|Host:Various plant families|Disease:Grey mould. Parasite or saprophyte|Description:SIMILARITY: Belongs to the major facilitator superfamily. Sugar transporter (TC 2.A.1.1)

														family.
Chr02G0505.1	1423	792	1416	UniProt ID:Q5A762_CANAL	1606	978	1588	176/662(26.59)	0.47	0.13	662	205	3.00E-54	gene=Chr02G0505	Gene Symbol:MLT1|Host:Isolated from a wide variety of substrates including humans|Disease:invasive candidal disease|Description:SIMILARITY: Belongs to the ABC transporter superfamily.
Chr02G0506.1	314	19	172	UniProt ID:A4QVF8_MAGO7	339	65	213	55/154(35.71)	0.53	0.03	154	82.8	4.00E-19	gene=Chr02G0506	Gene Symbol:MGG_04556|Host:Poaceae, especially important on Oryzae|Disease:Rice blast|Description:COFACTOR: Zinc (By similarity).
Chr02G0512.1	593	179	528	UniProt ID:Q9UUS7_9PEZI	567	93	462	154/374(41.18)	0.6	0.07	374	245	2.00E-73	gene=Chr02G0512	Gene Symbol:CHIP2|Host:Multiple genera in multiple families|Disease:Anthracnose of stems and leaves, dieback, root rot, leaf spot, blossom rot, fruit rot (dieback and ripe rot),

														seedling blight.' (Mordue 1971)|Description:Unknown
Chr02G0521.1	395	14	384	UniProt ID:Q6A2T2_BOTFU	391	14	384	94/387 (24.29)	0.44	0.08	387	94.7	2.00E-22	gene=Chr02G0521	Gene Symbol:BTP1|Host:Various plant families|Disease:Grey mould. Parasite or saprophyte|Description:Unknown
Chr02G0522.1	476	36	476	UniProt ID:A0ST43_CERNC	459	3	459	145/474(30.59)	0.45	0.11	474	175	8.00E-50	gene=Chr02G0522	Gene Symbol:CTB5|Host:Numerous taxa in Solanaceae|Disease:Leaf spot|Description:Unknown
Chr02G0525.1	535	59	374	UniProt ID:Q5ANE1_CANAL	748	51	370	88/326 (26.99)	0.45	0.05	326	92	1.00E-20	gene=Chr02G0525	Gene Symbol:SNF3|Host:Isolated from a wide variety of substrates including humans|Disease:invasive candidal disease|Description:SIMILARITY: Belongs to the major facilitator superfamily. Sugar transporter (TC 2.A.1.1)

															family.
Chr01G1083.1	119	23	113	UniProt ID:Q6TFC7_ASPFM	349	256	346	28/91(30.77)	0.45	0	91	53.1	3.00E-10	gene=Chr01G1083	Gene Symbol:NULL|Host:humans|Disease:infection|Description:Unknown
Chr01G1086.1	558	209	547	UniProt ID:Q4P8E8_USTMA	693	318	671	97/381(25.46)	0.38	0.18	381	76.3	1.00E-15	gene=Chr01G1086	Gene Symbol:UM03615.1|Host:Euchlaena spp., Zea spp. (Poaceae)|Disease:Smut. Corn smut|Description:COFACTOR: FAD (By similarity).
Chr01G1099.1	299	38	298	UniProt ID:Q32WF7_PHAND	266	8	265	84/264(31.82)	0.48	0.03	264	122	3.00E-33	gene=Chr01G1099	Gene Symbol:MDH1|Host:Multiple genera of Poaceae and Blysmus compressus (Cyperaceae)|Disease:Glume blotch of wheat and other grasses|Description:Unknown
Chr01G1101.1	533	14	478	UniProt ID:Q5ANE1_CANAL	748	43	493	159/482(32.99)	0.53	0.1	482	247	2.00E-73	gene=Chr01G1101	Gene Symbol:SNF3|Host:Isolated from a wide variety of substrates including humans|Disease:invasive

															candidal disease|Description:SIMILARITY: Belongs to the major facilitator superfamily. Sugar transporter (TC 2.A.1.1) family.
Chr01G1106.1	584	157	454	UniProt ID:A4UC81_MAGO7	376	73	369	83/305 (27.21)	0.47	0.05	305	97.8	6.00E-23	gene=Chr01G1106	Gene Symbol:MGG_10702|Host:Poaceae, especially important on Oryzae|Disease:Rice blast|Description:Unknown
Chr01G1109.1	243	18	243	UniProt ID:MEP1_COCP7	276	41	276	96/253 (37.94)	0.53	0.17	253	153	1.00E-45	gene=Chr01G1109	Gene Symbol:MEP1|Host:humans|Disease:coccidiomycosis|Description:FUNCTION: Secreted metalloproteinase that allows assimilation of proteinaceous substrates. Pays a pivotal role as a pathogenicity determinant during infections and contributes to the ability of

																the pathogen to persist within the mammalian host. Digests an immunodominant cell surface antigen (SOWgp) and prevents host recognition of endospores during the phase of development when these fungal cells are most vulnerable to phagocytic cell defenses.
Chr01G1 114.1	392	18	295	UniProt ID:Q6A 2T2_B OTFU	391	16	293	58/286 (20.28)		0.43	0.06	286	58.9	1.00E-10	gene=Chr 01G1114	Gene Symbol:BTP1|Host:Variou s plant families|Disease:Grey mould. Parasite or saprophyte|Description:Un known
Chr01G1 121.1	542	39	498	UniProt ID:Q4P DC7_U STMA	589	143	583	141/47 3(29.8 1)		0.47	0.1	473	197	3.00E-56	gene=Chr 01G1121	Gene Symbol:UM01886.1|Host: Euchlaena spp., Zea spp. (Poaceae)|Disease:Smut. Corn smut|Description:CAUTIO N: The sequence shown

															here is derived from an EMBL/GenBank/DDBJ whole genome shotgun (WGS) entry which is preliminary data.
Chr01G1125.1	450	18	445	UniProt ID:Q4PDC7_USTMA	589	147	583	155/441(35.15)	0.53	0.04	441	270	4.00E-84	gene=Chr01G1125	Gene Symbol:UM01886.1|Host: Euchlaena spp., Zea spp. (Poaceae)|Disease:Smut. Corn smut|Description:CAUTION: The sequence shown here is derived from an EMBL/GenBank/DDBJ whole genome shotgun (WGS) entry which is preliminary data.
Chr01G1139.1	377	92	375	UniProt ID:Q6TFC7_ASPFM	349	68	347	93/292 (31.85)	0.45	0.07	292	142	2.00E-39	gene=Chr01G1139	Gene Symbol:NULL|Host:humans|Disease:infection|Description:Unknown
Chr01G1160.1	792	21	130	UniProt ID:Q0WXM3_FUSOX	663	28	154	38/128 (29.69)	0.45	0.15	128	45.8	7.00E-06	gene=Chr01G1160	Gene Symbol:FOW2|Host:Multiple genera in multiple families|Disease:Blights, wilts, rots of various

															sorts|Description:SIMILARITY: Contains 1 Zn(2)-C6 fungal-type DNA-binding domain.
Chr01G1172.1	297	24	285	UniProt ID:Q75WR5_9PLEO	265	11	262	72/279 (25.81)	0.39	0.16	279	68.6	2.00E-14	gene=Chr01G1172	Gene Symbol:BRN1|Host:Belamcanda chinensis: Korea,Gladiolus ?gandavensis: Korea,Iris japonica: China,Iris missouriensis (Leaf spot.): Idaho; Montana; Oregon; Washington,Iris sp. (Leaf spot.): China; Texas; Washing|Disease:Leaf spot|Description:SIMILARITY: Belongs to the short-chain dehydrogenases/reductases (SDR) family.
Chr01G1178.1	697	230	278	UniProt ID:Q59LF3_CANAL	474	423	473	21/51(41.18)	0.57	0.04	51	47.8	1.00E-06	gene=Chr01G1178	Gene Symbol:RVS167|Host:Isolated from a wide variety of substrates including humans|Disease:invasive candidal

														disease|Description:SIMILARITY: Contains 1 SH3 domain.
Chr01G1179.1	1627	805	1047	UniProt ID:Q5AM49_CANAL	1690	784	1010	81/256 (31.64)	0.5	0.16	256	119	1.00E-27	gene=Chr01G1179	Gene Symbol:SNF2|Host:Isolated from a wide variety of substrates including humans|Disease:invasive candidal disease|Description:Unknown
Chr01G1181.1	591	51	590	UniProt ID:F5HAA8_CRYNB	551	1	549	349/552(63.22)	0.76	0.03	552	716	0	gene=Chr01G1181	Gene Symbol:CNBH3420|Host: humans|Disease:cryptococcosis|Description:CAUTION: The sequence shown here is derived from an EMBL/GenBank/DDBJ whole genome shotgun (WGS) entry which is preliminary data.
Chr01G1188.1	1632	1300	1466	UniProt ID:Q3Y5V5_MAGGR	1321	1102	1277	53/183 (28.96)	0.54	0.13	183	75.9	1.00E-14	gene=Chr01G1188	Gene Symbol:ABC3|Host:Digitaria (Poaceae)|Disease:Leaf spot|Description:SIMILARITY: Belongs to the ABC

															transporter superfamily.
Chr01G1189.1	394	73	280	UniProt ID:Q9Y784_MAGGR	631	167	377	59/211 (27.96)	0.49	0.01	211	110	2.00E-27	gene=Chr01G1189	Gene Symbol:PTH11|Host:Digitaria (Poaceae)|Disease:Leaf spot|Description:Unknown
Chr01G1190.1	1500	820	1062	UniProt ID:Q1A1Y7_CRYNV	1383	818	1051	82/250 (32.80)	0.51	0.09	250	126	4.00E-30	gene=Chr01G1190	Gene Symbol:TCO1|Host:humans|Disease:cryptococcosis|Description:SIMILARITY: Contains 1 histidine kinase domain.
Chr01G1193.1	782	116	248	UniProt ID:A8QJI7_FUSOX	938	11	139	41/139 (29.50)	0.46	0.12	139	54.7	2.00E-08	gene=Chr01G1193	Gene Symbol:XLNR|Host:Multiple genera in multiple families|Disease:Blights, wilts, rots of various sorts|Description:SIMILARITY: Contains 1 Zn(2)-C6 fungal-type DNA-binding domain.
Chr01G1201.1	241	15	207	UniProt ID:Q30E79_CRYNV	221	15	210	78/198 (39.39)	0.56	0.04	198	140	4.00E-41	gene=Chr01G1201	Gene Symbol:CAN1|Host:humans|Disease:cryptococcosis|Description:Unknown
Chr01G1	1061	50	106	UniProt	1040	27	1040	621/10	0.67	0.13	1084	1034	0	gene=Chr	Gene

204.1			1	ID:A4R2P0_MAGO7				84(57.29)						01G1204	Symbol:MGG_11899|Host:Poaceae, especially important on Oryzae|Disease:Rice blast|Description:SIMILARITY: Contains 1 SH3 domain.
Chr01G1211.1	303	25	288	UniProt ID:TUP1_CANAL	514	269	510	68/268(25.37)	0.4	0.11	268	55.8	8.00E-10	gene=Chr01G1211	Gene Symbol:TUP1|Host:Isolated from a wide variety of substrates including humans|Disease:invasive candidal disease|Description:FUNCTION: Represses transcription by RNA polymerase II. Represses genes responsible for initiating filamentous growth and this repression is lifted under inducing environmental conditions.
Chr01G1213.1	1086	187	608	UniProt ID:Q5K995_CRYNJ	1005	120	550	246/440(55.91)	0.68	0.06	440	480	5.00E-152	gene=Chr01G1213	Gene Symbol:CNK02740|Host:humans|Disease:cryptococcosis|Description:Unknow

															n
Chr01G1222.1	315	9	170	UniProt ID:C1GC36_PARBD	691	50	225	47/177 (26.55)	0.46	0.09	177	53.9	5.00E-09	gene=Chr01G1222	Gene Symbol:PADG_04558|Host:humans|Disease:Paracoccidioidomycosis|Description:Unknown
Chr01G1230.1	542	178	350	UniProt ID:Q0PND8_MAGGR	1375	1015	1170	54/184 (29.35)	0.46	0.21	184	60.1	2.00E-10	gene=Chr01G1230	Gene Symbol:PEX6|Host:Digitaria (Poaceae)|Disease:Leaf spot|Description:SIMILARITY: Belongs to the AAA ATPase family.
Chr01G1233.1	425	92	168	UniProt ID:A0ST43_CERNC	459	114	190	28/77(36.36)	0.49	0	77	53.9	8.00E-09	gene=Chr01G1233	Gene Symbol:CTB5|Host:Numerous taxa in Solanaceae|Disease:Leaf spot|Description:Unknown
Chr01G1235.1	1229	169	1201	UniProt ID:A4RGC8_MAGO7	1158	142	1150	455/1045(43.54)	0.61	0.05	1045	795	0	gene=Chr01G1235	Gene Symbol:MGG_11671|Host:Poaceae, especially important on Oryzae|Disease:Rice blast|Description:SIMILARITY: Contains 1 reverse transcriptase domain.
Chr01G1	402	95	349	UniProt	400	104	346	61/257	0.4	0.06	257	63.5	5.00E-12	gene=Chr	Gene

236.1				ID:A4R3I5_MAGO7				(23.74)						01G1236	Symbol:"MGG_11993, MGG_12837, MGG_13052"|Host:Poaceae, especially important on Oryzae|Disease:Rice blast|Description:Unknown
Chr01G1247.1	750	483	725	UniProt ID:Q3Y5V5_MAGGR	1321	1031	1297	65/285 (22.81)	0.44	0.21	285	58.2	1.00E-09	gene=Chr01G1247	Gene Symbol:ABC3|Host:Digitaria (Poaceae)|Disease:Leaf spot|Description:SIMILARITY: Belongs to the ABC transporter superfamily.
Chr01G1248.1	447	1	398	UniProt ID:Q7Z7T9_MONFR	398	1	398	386/398(96.98)	0.99	0	398	818	0	gene=Chr01G1248	Gene Symbol:TUB2|Host:Rosaceae, primarily Prunus spp., also Pomoideae including apple (Malus) and pear (Pyrus). Reported on Vitis vinifera (Vitaceae)|Disease:Brown fruit rot, wilt, blight, canker|Description:FUNCTION: Tubulin is the major constituent of microtubules. It binds two

															moles of GTP, one at an exchangeable site on the beta chain and one at a non-exchangeable site on the alpha-chain (By similarity).
Chr01G1250.1	429	71	424	UniProt ID:A3LUV9_PICST	365	4	364	163/369(44.17)	0.62	0.06	369	284	2.00E-92	gene=Chr01G1250	Gene Symbol:MET22|Host:humans|Disease:occasional infection|Description:Unknown
Chr01G1266.1	732	56	526	UniProt ID:A6N6J8_FUSOX	903	48	523	119/505(23.56)	0.39	0.12	505	72.4	5.00E-14	gene=Chr01G1266	Gene Symbol:CTF1|Host:Multiple genera in multiple families|Disease:Blights, wilts, rots of various sorts|Description:SIMILARITY: Contains 1 Zn(2)-C6 fungal-type DNA-binding domain.
Chr01G1268.1	358	29	356	UniProt ID:A4QVF8_MAGO7	339	22	336	103/332(31.02)	0.48	0.06	332	121	5.00E-32	gene=Chr01G1268	Gene Symbol:MGG_04556|Host:Poaceae, especially important on Oryzae|Disease:Rice blast|Description:COFACT

															OR: Zinc (By similarity).
Chr01G1269.1	378	65	304	UniProt ID:Q5AJC0_CANAL	470	112	384	81/274 (29.56)	0.49	0.13	274	108	3.00E-27	gene=Chr01G1269	Gene Symbol:UTR2|Host:Isolated from a wide variety of substrates including humans|Disease:invasive candidal disease|Description:CAUTION: The sequence shown here is derived from an EMBL/GenBank/DDBJ whole genome shotgun (WGS) entry which is preliminary data.
Chr01G1279.1	788	2	154	UniProt ID:Q59WE5_CANAL	323	20	166	51/164 (31.10)	0.45	0.17	164	50.8	1.00E-07	gene=Chr01G1279	Gene Symbol:PHO100|Host:Isolated from a wide variety of substrates including humans|Disease:invasive candidal disease|Description:CAUTION: The sequence shown here is derived from an EMBL/GenBank/DDBJ whole genome shotgun (WGS) entry which is

															preliminary data.
Chr01G1280.1	1396	281	1208	UniProtID:Q9P872_CANAL	917	52	886	277/940(29.47)	0.5	0.12	940	340	6.00E-100	gene=Chr01G1280	Gene Symbol:PMR1|Host:Isolated from a wide variety of substrates including humans|Disease:invasive candidal disease|Description:SIMILARITY: Belongs to the cation transport ATPase (P-type) family.
Chr01G1282.1	997	151	276	UniProtID:Q5AP97_CANAL	1178	891	996	37/128(28.91)	0.46	0.19	128	50.1	5.00E-07	gene=Chr01G1282	Gene Symbol:SWE1|Host:Isolated from a wide variety of substrates including humans|Disease:invasive candidal disease|Description:SIMILARITY: Belongs to the protein kinase superfamily.
Chr01G1288.1	508	289	482	UniProtID:A4ULJ0_MYCGR	518	298	509	56/218(25.69)	0.47	0.14	218	70.1	9.00E-14	gene=Chr01G1288	Gene Symbol:CYP51|Host:Triticum and possibly a few other grasses|Disease:Leaf spot

																or speckled leaf blotch of wheat|Description:COFACTOR: Heme group (By similarity).
Chr01G1292.1	416	62	416	UniProt ID:Q0QWD8_LEPMC	359	1	358	158/358(44.13)	0.6	0.01	358	290	4.00E-95	gene=Chr01G1292	Gene Symbol:THIOL|Host:Brassica spp. and other Brassicaceae|Disease:Black leg, canker, dry rot, leaf spot|Description:SIMILARITY: Belongs to the thiolase family.
Chr01G1298.1	760	27	749	UniProt ID:C5GSW0_AJEDR	783	20	759	495/764(64.79)	0.74	0.09	764	892	0	gene=Chr01G1298	Gene Symbol:BDCG_07503|Host:humans|Disease:cutaneous Blastomyces dermatitidis infection|Description:Unknown
Chr01G1300.1	512	23	507	UniProt ID:A4R566_MAGO7	550	37	541	217/512(42.38)	0.58	0.07	512	394	1.00E-131	gene=Chr01G1300	Gene Symbol:MGG_04128|Host:Poaceae, especially important on Oryzae|Disease:Rice blast|Description:Unknown

Chr01G1306.1	151	14	148	UniProt ID:CANB_CRYNJ	175	25	160	48/140 (34.29)	0.55	0.06	140	68.6	6.00E-16	gene=Chr01G1306	Gene Symbol:CNB1|Host:humans|Disease:cryptococcosis|Description:FUNCTION: Regulatory subunit of calcineurin, a calcium-dependent, calmodulin stimulated protein phosphatase. Confers calcium sensitivity. Plays a central role in virulence and antifungal drug action.
Chr01G1311.1	907	11	96	UniProt ID:C1G7T1_PARBD	534	73	157	36/86(41.86)	0.55	0.01	86	72	5.00E-14	gene=Chr01G1311	Gene Symbol:PADG_03236|Host:humans|Disease:Paracoccidioidomycosis|Description:Unknown
Chr01G1316.1	435	9	344	UniProt ID:Q2I0M6_CERNC	871	459	820	95/376 (25.27)	0.4	0.14	376	55.5	3.00E-09	gene=Chr01G1316	Gene Symbol:CTB3|Host:Numerous taxa in Solanaceae|Disease:Leaf spot|Description:Unknown
Chr01G1317.1	415	13	57	UniProt ID:Q0WXM3	663	111	157	23/47(48.94)	0.62	0.04	47	54.7	5.00E-09	gene=Chr01G1317	Gene Symbol:FOW2|Host:Multiple genera in multiple

				_FUSOX										families|Disease:Blights, wilts, rots of various sorts|Description:SIMILARITY: Contains 1 Zn(2)-C6 fungal-type DNA-binding domain.
Chr01G1321.1	364	7	345	UniProt ID:Q6XVN4_CRYNV	383	13	363	94/368 (25.54)	0.38	0.13	368	76.3	2.00E-16	gene=Chr01G1321	Gene Symbol:GNO1|Host:humans|Disease:cryptococcosis|Description:COFACTOR: Zinc (By similarity).
Chr01G1323.1	279	29	279	UniProt ID:Q32WF7_PHAND	266	15	266	134/253(52.96)	0.68	0.01	253	263	1.00E-87	gene=Chr01G1323	Gene Symbol:MDH1|Host:Multiple genera of Poaceae and Blysmus compressus (Cyperaceae)|Disease:Glume blotch of wheat and other grasses|Description:Unknown
Chr01G1324.1	521	72	489	UniProt ID:Q5XTQ5_BOTFU	615	140	551	102/442(23.08)	0.42	0.12	442	100	3.00E-23	gene=Chr01G1324	Gene Symbol:FRT1|Host:Various plant families|Disease:Grey mould. Parasite or saprophyte|Description:SI

															MILARITY: Belongs to the major facilitator superfamily. Sugar transporter (TC 2.A.1.1) family.
Chr01G1325.1	823	328	617	UniProt ID:Q5ALS7_CANAL	1144	352	651	75/308 (24.35)	0.42	0.08	308	77.8	1.00E-15	gene=Chr01G1325	Gene Symbol:CTF1|Host:Isolated from a wide variety of substrates including humans|Disease:invasive candidal disease|Description:SIMILARITY: Contains 1 Zn(2)-C6 fungal-type DNA-binding domain.
Chr01G1326.1	276	3	275	UniProt ID:Q32WF7_PHAND	266	1	265	122/275(44.36)	0.61	0.04	275	209	1.00E-66	gene=Chr01G1326	Gene Symbol:MDH1|Host:Multiple genera of Poaceae and Blysmus compressus (Cyperaceae)|Disease:Glume blotch of wheat and other grasses|Description:Unknown
Chr01G1327.1	244	1	241	UniProt ID:Q04	242	1	235	116/254(45.6	0.59	0.13	254	199	7.00E-64	gene=Chr01G1327	Gene Symbol:PELA|Host:Multipl

				701_F USSO				7)							e plant families. Some strains may cause infections in humans|Disease:Saprobe, facultative pathogen|Description:Unknown
Chr01G1328.1	428	24	361	UniProt ID:Q9Y784_MAGGR	631	30	370	101/345(29.28)	0.52	0.03	345	155	2.00E-42	gene=Chr01G1328	Gene Symbol:PTH11|Host:Digitaria (Poaceae)|Disease:Leaf spot|Description:Unknown
Chr01G1330.1	556	44	511	UniProt ID:Q5ANE1_CANAL	748	51	496	115/480(23.96)	0.43	0.1	480	113	2.00E-27	gene=Chr01G1330	Gene Symbol:SNF3|Host:Isolated from a wide variety of substrates including humans|Disease:invasive candidal disease|Description:SIMILARITY: Belongs to the major facilitator superfamily. Sugar transporter (TC 2.A.1.1) family.
Chr01G1347.1	651	73	104	UniProt ID:Q5A	624	13	44	17/32(53.12)	0.75	0	32	49.3	5.00E-07	gene=Chr01G1347	Gene Symbol:ZCF37|Host:Isolat

				4F3_CANAL										ed from a wide variety of substrates including humans|Disease:invasive candidal disease|Description:Unknown
Chr01G1353.1	360	1	238	UniProt ID:A4QVF8_MAGO7	339	1	228	78/240 (32.50)	0.49	0.06	240	119	2.00E-31	gene=Chr01G1353	Gene Symbol:MGG_04556|Host:Poaceae, especially important on Oryzae|Disease:Rice blast|Description:COFACTOR: Zinc (By similarity).
Chr01G1356.1	1152	775	1060	UniProt ID:A4R3H7_MAGO7	1108	756	1054	168/307(54.72)	0.68	0.09	307	318	8.00E-92	gene=Chr01G1356	Gene Symbol:MGG_02240|Host:Poaceae, especially important on Oryzae|Disease:Rice blast|Description:Unknown
Chr01G1379.1	701	11	359	UniProt ID:A6N6J8_FUSOX	903	46	408	87/388 (22.42)	0.38	0.16	388	47	3.00E-06	gene=Chr01G1379	Gene Symbol:CTF1|Host:Multiple genera in multiple families|Disease:Blights, wilts, rots of various sorts|Description:SIMILAR

															ITY: Contains 1 Zn(2)-C6 fungal-type DNA-binding domain.
Chr01G1391.1	537	286	479	UniProt ID:A4ULI9_MYCGR	502	291	501	53/218 (24.31)	0.4	0.14	218	45.8	4.00E-06	gene=Chr01G1391	Gene Symbol:CYP51|Host:Triticum and possibly a few other grasses|Disease:Leaf spot or speckled leaf blotch of wheat|Description:COFACTOR: Heme group (By similarity).
Chr01G1393.1	495	19	490	UniProt ID:LAP2_ASPFU	501	26	493	251/476(52.73)	0.68	0.03	476	464	7.00E-160	gene=Chr01G1393	Gene Symbol:LAP2|Host:humans|Disease:infection|Description:FUNCTION: Extracellular aminopeptidase that releases a wide variety of amino acids from natural peptides and contributes to pathogenicity (By similarity).
Chr01G1395.1	1236	745	1193	UniProt ID:Q875L6_U	1441	903	1385	197/495(39.80)	0.59	0.12	495	328	4.00E-94	gene=Chr01G1395	Gene Symbol:SQL2|Host:Euchlaena spp., Zea spp.

				STMD											(Poaceae)|Disease:Smut. Corn smut|Description:SIMILARITY: Contains 1 N-terminal Ras-GEF domain.
Chr01G1402.1	821	298	586	UniProt ID:Q5ALS7_CANAL	1144	351	643	76/321 (23.68)	0.44	0.19	321	63.9	3.00E-11	gene=Chr01G1402	Gene Symbol:CTF1|Host:Isolated from a wide variety of substrates including humans|Disease:invasive candidal disease|Description:SIMILARITY: Contains 1 Zn(2)-C6 fungal-type DNA-binding domain.
Chr01G1404.1	206	20	202	UniProt ID:CUTI_MONFR	201	26	196	84/186 (45.16)	0.58	0.1	186	132	7.00E-39	gene=Chr01G1404	Gene Symbol:CUT1|Host:Rosaceae, primarily Prunus spp., also Pomoideae including apple (Malus) and pear (Pyrus). Reported on Vitis vinifera (Vitaceae)|Disease:Brown fruit rot, wilt, blight, canker|Description:FUNCTION: Catalyzes the

														hydrolysis of cutin, a polyester that forms the structure of plant cuticle. Allows pathogenic fungi to penetrate through the cuticular barrier into the host plant during the initial stage of the fungal infection (By similarity).
Chr01G1433.1	580	271	441	UniProt ID:Q00LS5_PHAND	408	64	209	45/171 (26.32)	0.44	0.15	171	58.5	4.00E-10	gene=Chr01G1433	Gene Symbol:CPKA|Host:Multiple genera of Poaceae and Blysmus compressus (Cyperaceae)|Disease:Glume blotch of wheat and other grasses|Description:SIMILARITY: Contains 1 protein kinase domain.
Chr02G0531.1	384	25	360	UniProt ID:CARP_ARTBC	400	68	390	85/360 (23.61)	0.41	0.17	360	73.6	3.00E-15	gene=Chr02G0531	Gene Symbol:PEP2|Host:hedgehogs|Disease:ringworm,Kerion Celsi Disease|Description:FUNCTION: Vacuolar aspartic endopeptidase which is

														probably also secreted and contributes to virulence (By similarity).
Chr02G0539.1	1185	165	1156	UniProt ID:Q8J286_COLLN	1167	121	1098	312/1048(29.77)	0.46	0.12	1048	353	2.00E-103	gene=Chr02G0539	Gene Symbol:CLAP1|Host:Multiple genera of Fabaceae. Rare reports on other taxa|Disease:Leaf, stem and pod anthracnose|Description:SIMILARITY: Belongs to the cation transport ATPase (P-type) family.
Chr02G0546.1	226	1	224	UniProt ID:CUTI1_COLGL	224	1	224	104/231(45.02)	0.57	0.06	231	172	8.00E-54	gene=Chr02G0546	Gene Symbol:CUTA|Host:Multiple genera in multiple families|Disease:'Anthracnose of stems and leaves, dieback, root rot, leaf spot, blossom rot, fruit rot (dieback and ripe rot), seedling blight.' (Mordue 1971)|Description:FUNCTION: Catalyzes the hydrolysis of cutin, a polyester that forms the

															structure of plant cuticle. Allows pathogenic fungi to penetrate through the cuticular barrier into the host plant during the initial stage of the fungal infection.
Chr02G0547.1	612	12	604	UniProt ID:Q4P8E8_USTMA	693	81	672	218/619(35.22)	0.47	0.09	619	270	2.00E-81	gene=Chr02G0547	Gene Symbol:UM03615.1|Host: Euchlaena spp., Zea spp. (Poaceae)|Disease:Smut. Corn smut|Description:COFACTOR: FAD (By similarity).
Chr02G0548.1	1258	289	473	UniProt ID:F2Z6H2_CANGB	676	440	610	46/185 (24.86)	0.47	0.08	185	67.8	2.00E-12	gene=Chr02G0548	Gene Symbol:STE11|Host:humans|Disease:Occasional invasive candidal disease|Description:SIMILARITY: Contains 1 SAM (sterile alpha motif) domain.
Chr02G0560.1	709	1	709	UniProt ID:C5GT68_AJEDR	724	1	724	521/724(71.96)	0.84	0.02	724	1098	0	gene=Chr02G0560	Gene Symbol:BDCG_07611|Host:humans|Disease:cutaneous Blastomyces

<table>
<tr><td></td><td></td><td></td><td></td><td></td><td></td><td></td><td></td><td></td><td></td><td></td><td></td><td></td><td></td><td></td><td>dermatitidis infection|Description:Unknown</td></tr>
<tr><td>Chr02G0568.1</td><td>456</td><td>100</td><td>441</td><td>UniProt ID:Y1220_ASPFU</td><td>439</td><td>92</td><td>435</td><td>121/354(34.18)</td><td>0.52</td><td>0.06</td><td>354</td><td>186</td><td>5.00E-54</td><td>gene=Chr02G0568</td><td>Gene Symbol:AFUA_3G01220|Host:humans|Disease:infection|Description:FUNCTION: Probable aspartic-type endopeptidase which contributes to virulence (By similarity).</td></tr>
<tr><td>Chr02G0570.1</td><td>611</td><td>28</td><td>534</td><td>UniProt ID:C1GMG4_PARBD</td><td>772</td><td>81</td><td>678</td><td>166/606(27.39)</td><td>0.46</td><td>0.18</td><td>606</td><td>177</td><td>2.00E-48</td><td>gene=Chr02G0570</td><td>Gene Symbol:PADG_08450|Host:humans|Disease:Paracoccidioidomycosis|Description:SIMILARITY: Belongs to the DEAD box helicase family.</td></tr>
<tr><td>Chr02G0573.1</td><td>992</td><td>84</td><td>315</td><td>UniProt ID:TUP1_CANAL</td><td>514</td><td>259</td><td>500</td><td>61/252(24.21)</td><td>0.42</td><td>0.12</td><td>252</td><td>61.2</td><td>1.00E-10</td><td>gene=Chr02G0573</td><td>Gene Symbol:TUP1|Host:Isolated from a wide variety of substrates including humans|Disease:invasive candidal disease|Description:FUNC</td></tr>
</table>

																TION: Represses transcription by RNA polymerase II. Represses genes responsible for initiating filamentous growth and this repression is lifted under inducing environmental conditions.
Chr02G0574.1	297	29	294	UniProt ID:Q8TGD1_FUSOX	318	45	311	65/288 (22.57)		0.41	0.15	288	43.9	4.00E-06	gene=Chr02G0574	Gene Symbol:FOW1|Host:Multiple genera in multiple families|Disease:Blights, wilts, rots of various sorts|Description:SIMILARITY: Belongs to the mitochondrial carrier family.
Chr02G0584.1	1272	685	1261	UniProt ID:Q9C2Y4_MAGGR	1501	859	1466	284/612(46.41)		0.61	0.06	612	505	3.00E-155	gene=Chr02G0584	Gene Symbol:PDE1|Host:Digitaria (Poaceae)|Disease:Leaf spot|Description:CATALYTIC ACTIVITY: ATP + H(2)O + phospholipid(In) = ADP + phosphate + phospholipid(Out).
Chr02G0	436	10	429	UniProt	581	147	567	137/42		0.52	0.02	424	209	1.00E-61	gene=Chr	Gene

586.1				ID:Q59 RG0_C ANAL				4(32.3 1)					02G0586	Symbol:NAG4|Host:Isolated from a wide variety of substrates including humans|Disease:invasive candidal disease|Description:CAUTION: The sequence shown here is derived from an EMBL/GenBank/DDBJ whole genome shotgun (WGS) entry which is preliminary data.
Chr02G0 590.1	620	337	531	UniProt ID:Q8J 214_U STMD	592	337	513	56/196 (28.57)	0.49	0.1	196	78.2	4.00E-16	gene=Chr 02G0590	Gene Symbol:CRU1|Host:Euchlaena spp., Zea spp. (Poaceae)|Disease:Smut. Corn smut|Description:Unknown
Chr02G0 593.1	582	85	575	UniProt ID:Q59 RG0_C ANAL	581	122	568	111/50 6(21.9 4)	0.38	0.15	506	55.8	4.00E-09	gene=Chr 02G0593	Gene Symbol:NAG4|Host:Isolated from a wide variety of substrates including humans|Disease:invasive candidal disease|Description:CAUT

															ION: The sequence shown here is derived from an EMBL/GenBank/DDBJ whole genome shotgun (WGS) entry which is preliminary data.
Chr02G0594.1	1443	97	511	UniProt ID:Q5ABU7_CANAL	564	103	523	101/424(23.82)	0.46	0.03	424	126	8.00E-31	gene=Chr02G0594	Gene Symbol:MDR1|Host:Isolated from a wide variety of substrates including humans|Disease:invasive candidal disease|Description:Unknown
Chr02G0600.1	621	29	615	UniProt ID:Q4P8E8_USTMA	693	73	671	164/648(25.31)	0.39	0.17	648	119	4.00E-29	gene=Chr02G0600	Gene Symbol:UM03615.1|Host: Euchlaena spp., Zea spp. (Poaceae)|Disease:Smut. Corn smut|Description:COFACTOR: FAD (By similarity).
Chr02G0602.1	565	70	156	UniProt ID:Q5J7N6_COCHE	589	138	216	30/91(32.97)	0.49	0.18	91	45.4	7.00E-06	gene=Chr02G0602	Gene Symbol:CHAP1|Host:Zea mays|Disease:Southern leaf blight of maize|Description:SIMILA

															RITY: Belongs to the bZIP family.
Chr02G0605.1	922	80	779	UniProt ID:Q9P872_CANAL	917	36	797	208/780(26.67)	0.47	0.13	780	243	2.00E-68	gene=Chr02G0605	Gene Symbol:PMR1|Host:Isolated from a wide variety of substrates including humans|Disease:invasive candidal disease|Description:SIMILARITY: Belongs to the cation transport ATPase (P-type) family.
Chr02G0609.1	317	7	317	UniProt ID:Q8TGD1_FUSOX	318	9	318	268/311(86.17)	0.93	0	311	507	0	gene=Chr02G0609	Gene Symbol:FOW1|Host:Multiple genera in multiple families|Disease:Blights, wilts, rots of various sorts|Description:SIMILARITY: Belongs to the mitochondrial carrier family.
Chr02G0610.1	472	12	461	UniProt ID:Q50I20_ASPFM	465	5	459	250/459(54.47)	0.68	0.03	459	474	2.00E-164	gene=Chr02G0610	Gene Symbol:MCSA|Host:humans|Disease:infection|Description:SIMILARITY: Belongs to the citrate

															synthase family.
Chr02G0619.1	738	33	738	UniProt ID:Q5ADM9_CANAL	769	37	765	373/730(51.10)	0.68	0.03	730	749	0	gene=Chr02G0619	Gene Symbol:PMT2|Host:Isolated from a wide variety of substrates including humans|Disease:invasive candidal disease|Description:CAUTION: The sequence shown here is derived from an EMBL/GenBank/DDBJ whole genome shotgun (WGS) entry which is preliminary data.
Chr02G0620.1	267	5	267	UniProt ID:Q8J031_PICAN	203	4	203	76/263(28.90)	0.49	0.24	263	127	4.00E-36	gene=Chr02G0620	Gene Symbol:YPT1|Host:humans|Disease:occasional infection|Description:SIMILARITY: Belongs to the small GTPase superfamily. Rab family.
Chr02G0625.1	333	40	323	UniProt ID:Q6TFC7_ASPFM	349	64	346	93/292(31.85)	0.5	0.06	292	138	3.00E-38	gene=Chr02G0625	Gene Symbol:NULL|Host:humans|Disease:infection|Description:Unknown
Chr02G0	577	24	478	UniProt	748	44	496	131/47	0.47	0.1	478	164	3.00E-44	gene=Chr	Gene

626.1				ID:Q5ANE1_CANAL				8(27.41)						02G0626	Symbol:SNF3|Host:Isolated from a wide variety of substrates including humans|Disease:invasive candidal disease|Description:SIMILARITY: Belongs to the major facilitator superfamily. Sugar transporter (TC 2.A.1.1) family.
Chr02G0633.1	1052	722	963	UniProt ID:Q5EMY3_MAGGR	424	94	343	96/251 (38.25)	0.56	0.04	251	162	2.00E-43	gene=Chr02G0633	Gene Symbol:NULL|Host:Digitaria (Poaceae)|Disease:Leaf spot|Description:SIMILARITY: Belongs to the AAA ATPase family.
Chr02G0638.1	489	15	58	UniProt ID:Q59MD2_CANAL	843	763	806	19/44(43.18)	0.61	0	44	50.4	1.00E-07	gene=Chr02G0638	Gene Symbol:UME6|Host:Isolated from a wide variety of substrates including humans|Disease:invasive candidal disease|Description:CAUTION: The sequence shown here is derived from an

															EMBL/GenBank/DDBJ whole genome shotgun (WGS) entry which is preliminary data.
Chr02G0650.1	212	3	170	UniProt ID:F2QX13_PICP7	203	2	169	91/168 (54.17)	0.73	0	168	191	2.00E-61	gene=Chr02G0650	Gene Symbol:YPT1|Host:humans|Disease:occasional infection|Description:SIMILARITY: Belongs to the small GTPase superfamily. Rab family.
Chr02G0651.1	304	1	303	UniProt ID:A6ZND4_YEAS7	291	1	290	156/304(51.32)	0.69	0.05	304	315	1.00E-107	gene=Chr02G0651	Gene Symbol:BRX1|Host:humans|Disease:occasional infection|Description:CAUTION: The sequence shown here is derived from an EMBL/GenBank/DDBJ whole genome shotgun (WGS) entry which is preliminary data.
Chr02G0653.1	747	143	378	UniProt ID:Q5A839_CANAL	454	83	302	69/239 (28.87)	0.52	0.09	239	103	3.00E-24	gene=Chr02G0653	Gene Symbol:HOS2|Host:Isolated from a wide variety of substrates including

															humans|Disease:invasive candidal disease|Description:CATALYTIC ACTIVITY: Hydrolysis of an N(6)-acetyl-lysine residue of a histone to yield a deacetylated histone.
Chr02G0660.1	519	22	67	UniProt ID:Q0WXM3_FUSOX	663	108	153	18/46(39.13)	0.5	0	46	47.8	1.00E-06	gene=Chr02G0660	Gene Symbol:FOW2|Host:Multiple genera in multiple families|Disease:Blights, wilts, rots of various sorts|Description:SIMILARITY: Contains 1 Zn(2)-C6 fungal-type DNA-binding domain.
Chr02G0662.1	1934	179	1931	UniProt ID:Q5D6D3_COCHE	1761	1	1761	876/1782(49.16)	0.66	0.03	1782	1670	0	gene=Chr02G0662	Gene Symbol:NPS6|Host:Zea mays|Disease:Southern leaf blight of maize|Description:Unknown
Chr02G0664.1	574	7	574	UniProt ID:Q96VB5_A	578	4	578	152/605(25.12)	0.41	0.11	605	127	2.00E-32	gene=Chr02G0664	Gene Symbol:AFT1-1|Host:Plant|Disease:Leaf spot,

				LTAL											rots|Description:Unknown
Chr02G0684.1	580	10	455	UniProt ID:Q59KS4_CANAL	800	12	450	161/456(35.31)	0.57	0.06	456	284	4.00E-86	gene=Chr02G0684	Gene Symbol:CNH1|Host:Isolated from a wide variety of substrates including humans|Disease:invasive candidal disease|Description:CAUTION: The sequence shown here is derived from an EMBL/GenBank/DDBJ whole genome shotgun (WGS) entry which is preliminary data.
Chr02G0687.1	550	35	226	UniProt ID:A4RG81_MAGO7	558	334	513	58/194 (29.90)	0.46	0.08	194	78.2	3.00E-16	gene=Chr02G0687	Gene Symbol:MGG_00131|Host:Poaceae, especially important on Oryzae|Disease:Rice blast|Description:Unknown
Chr02G0696.1	2112	1754	2088	UniProt ID:C1G3N5_PARBD	362	12	330	86/367 (23.43)	0.38	0.22	367	54.3	4.00E-08	gene=Chr02G0696	Gene Symbol:PADG_01551|Host:humans|Disease:Paracoccidioidomycosis|Description:CATALYTIC

Chr02G0706.1	470	73	120	UniProt ID:Q59MD2_CANAL	843	752	799	20/48(41.67)	0.65	0	48	50.8	1.00E-07	gene=Chr02G0706	ACTIVITY: Thioredoxin + NADP(+) = thioredoxin disulfide + NADPH. Gene Symbol:UME6|Host:Isolated from a wide variety of substrates including humans|Disease:invasive candidal disease|Description:CAUTION: The sequence shown here is derived from an EMBL/GenBank/DDBJ whole genome shotgun (WGS) entry which is preliminary data.
Chr02G0716.1	802	543	788	UniProt ID:Q875L6_USTMD	1441	1134	1388	52/255(20.39)	0.45	0.04	255	68.2	1.00E-12	gene=Chr02G0716	Gene Symbol:SQL2|Host:Euchlaena spp., Zea spp. (Poaceae)|Disease:Smut. Corn smut|Description:SIMILARITY: Contains 1 N-terminal Ras-GEF domain.
Chr02G0727.1	763	330	722	UniProt ID:Q0P	1375	847	1224	140/412(33.9	0.52	0.13	412	212	5.00E-58	gene=Chr02G0727	Gene Symbol:PEX6|Host:Digitar

				ND8_MAGGR				8)							ia (Poaceae)|Disease:Leaf spot|Description:SIMILARITY: Belongs to the AAA ATPase family.
Chr02G0735.1	577	35	490	UniProt ID:Q5XTQ4_BOTFU	574	55	536	153/508(30.12)	0.42	0.15	508	145	2.00E-38	gene=Chr02G0735	Gene Symbol:LIP1|Host:Various plant families|Disease:Grey mould. Parasite or saprophyte|Description:Unknown
Chr02G0747.1	207	1	147	UniProt ID:SNF7_CANAL	226	6	152	42/148 (28.38)	0.51	0.01	148	52.4	2.00E-09	gene=Chr02G0747	Gene Symbol:SNF7|Host:Isolated from a wide variety of substrates including humans|Disease:invasive candidal disease|Description:FUNCTION: Required for the sorting and concentration of proteins resulting in the entry of these proteins into the invaginating vesicles of the multivesicular body (MVB). Also required for the proteolytic cleavage of

															the transcription factor RIM101 in response to alkaline ambient pH.
Chr02G0749.1	2593	1349	1448	UniProt ID:Q5A506_CANAL	1237	824	929	34/106 (32.08)	0.53	0.06	106	59.7	2.00E-09	gene=Chr02G0749	Gene Symbol:BUD2|Host:Isolated from a wide variety of substrates including humans|Disease:invasive candidal disease|Description:SIMILARITY: Contains 1 C2 domain.
Chr02G0759.1	1336	275	566	UniProt ID:Q9P932_CANAL	411	95	364	107/297(36.03)	0.55	0.11	297	195	1.00E-54	gene=Chr02G0759	Gene Symbol:TPK2|Host:Isolated from a wide variety of substrates including humans|Disease:invasive candidal disease|Description:CATALYTIC ACTIVITY: ATP + a protein = ADP + a phosphoprotein.
Chr02G0767.1	295	5	292	UniProt ID:CHS7_CANAL	310	2	288	75/293 (25.60)	0.43	0.04	293	96.7	4.00E-24	gene=Chr02G0767	Gene Symbol:CHS7|Host:Isolated from a wide variety of substrates including

															humans|Disease:invasive candidal disease|Description:FUNCTION: Chaperone required for the export of the chitin synthase CHS3 from the endoplasmic reticulum (By similarity).
Chr02G0775.1	2483	1924	2346	UniProt ID:Q9C1Q7_ASPFM	708	308	702	157/429(36.60)	0.54	0.09	429	239	2.00E-66	gene=Chr02G0775	Gene Symbol:NULL|Host:humans|Disease:infection|Description:SIMILARITY: Contains 1 histidine kinase domain.
Chr02G0778.1	1140	15	352	UniProt ID:Q4PD87_USTMA	594	2	354	161/358(44.97)	0.6	0.07	358	285	2.00E-84	gene=Chr02G0778	Gene Symbol:UM01926.1|Host: Euchlaena spp., Zea spp. (Poaceae)|Disease:Smut. Corn smut|Description:CAUTION: The sequence shown here is derived from an EMBL/GenBank/DDBJ whole genome shotgun (WGS) entry which is preliminary data.

Chr02G0780.1	2053	1387	1618	UniProt ID:Q1A1Y7_CRYNV	1383	826	1054	101/232(43.53)	0.64	0.01	232	192	3.00E-50	gene=Chr02G0780	Gene Symbol:TCO1|Host:humans|Disease:cryptococcosis|Description:SIMILARITY: Contains 1 histidine kinase domain.
Chr02G0781.1	538	21	527	UniProt ID:C0S733_PARBP	560	27	545	148/527(28.08)	0.51	0.05	527	207	6.00E-60	gene=Chr02G0781	Gene Symbol:PABG_03488|Host:humans|Disease:Paracoccidioidomycosis|Description:SIMILARITY: Belongs to the TCP-1 chaperonin family.
Chr11G0005.1	400	1	305	UniProt ID:A4RGC8_MAGO7	1158	723	1072	123/356(34.55)	0.5	0.16	356	193	9.00E-55	gene=Chr11G0005	Gene Symbol:MGG_11671|Host:Poaceae, especially important on Oryzae|Disease:Rice blast|Description:SIMILARITY: Contains 1 reverse transcriptase domain.
Chr11G0007.1	615	396	580	UniProt ID:Q00LS5_PHAND	408	58	251	59/200(29.50)	0.47	0.11	200	75.9	1.00E-15	gene=Chr11G0007	Gene Symbol:CPKA|Host:Multiple genera of Poaceae and Blysmus compressus (Cyperaceae)|Disease:Glu

														me blotch of wheat and other grasses|Description:SIMILARITY: Contains 1 protein kinase domain.
Chr10G0009.1	796	526	691	UniProt ID:Q5AG40_CANAL	439	111	300	59/191 (30.89)	0.46	0.14	191	58.9	5.00E-10	gene=Chr10G0009	Gene Symbol:VPS4|Host:Isolated from a wide variety of substrates including humans|Disease:invasive candidal disease|Description:SIMILARITY: Belongs to the AAA ATPase family.
Chr12G0007.1	322	1	111	UniProt ID:C4YII6_CANAW	768	492	599	36/115 (31.30)	0.5	0.1	115	52.4	2.00E-08	gene=Chr12G0007	Gene Symbol:CAWG_04261|Host:Isolated from a wide variety of substrates including humans|Disease:invasive candidal disease|Description:SIMILARITY: Belongs to the DEAD box helicase family.
Chr01G1443.1	666	240	454	UniProt ID:Q5A	1144	360	585	63/233 (27.04)	0.44	0.11	233	56.6	3.00E-09	gene=Chr01G1443	Gene Symbol:CTF1|Host:Isolate

				LS7_CANAL										d from a wide variety of substrates including humans|Disease:invasive candidal disease|Description:SIMILARITY: Contains 1 Zn(2)-C6 fungal-type DNA-binding domain.
Chr01G1444.1	581	73	444	UniProt ID:Q5XTQ4_BOTFU	574	55	447	127/402(31.59)	0.46	0.1	402	157	4.00E-42	gene=Chr01G1444	Gene Symbol:LIP1|Host:Various plant families|Disease:Grey mould. Parasite or saprophyte|Description:Unknown
Chr01G1453.1	347	48	342	UniProt ID:Q6TFC7_ASPFM	349	38	347	97/315 (30.79)	0.48	0.08	315	153	1.00E-43	gene=Chr01G1453	Gene Symbol:NULL|Host:humans|Disease:infection|Description:Unknown
Chr01G1455.1	508	261	482	UniProt ID:A4ULJ2_MYCGR	515	264	508	62/256 (24.22)	0.43	0.18	256	79.7	6.00E-17	gene=Chr01G1455	Gene Symbol:CYP51|Host:Triticum and possibly a few other grasses|Disease:Leaf spot or speckled leaf blotch of wheat|Description:COFAC

Chr01G1466.1	300	5	298	UniProt ID:MEP1_COCP7	276	7	273	109/296(36.82)	0.51	0.1	296	172	5.00E-52	gene=Chr01G1466	TOR: Heme group (By similarity). Gene Symbol:MEP1|Host:humans|Disease:coccidiomycosis|Description:FUNCTION: Secreted metalloproteinase that allows assimilation of proteinaceous substrates. Pays a pivotal role as a pathogenicity determinant during infections and contributes to the ability of the pathogen to persist within the mammalian host. Digests an immunodominant cell surface antigen (SOWgp) and prevents host recognition of endospores during the phase of development when these fungal cells are most vulnerable to phagocytic cell defenses.

Chr01G1471.1	1312	55	1253	UniProt ID:A4RGC8_MAGO7	1158	10	1155	484/1227(39.45)	0.57	0.09	1227	802	0	gene=Chr01G1471	Gene Symbol:MGG_11671|Host:Poaceae, especially important on Oryzae|Disease:Rice blast|Description:SIMILARITY: Contains 1 reverse transcriptase domain.
Chr01G1477.1	428	1	418	UniProt ID:MCPAL_ARTOC	416	1	415	178/430(41.40)	0.61	0.06	430	340	2.00E-113	gene=Chr01G1477	Gene Symbol:MCYG_01475|Host:humans, reptiles|Disease:dermatophytoses|Description:FUNCTION: Extracellular metalloprotease that contributes to pathogenicity (By similarity).
Chr01G1483.1	558	135	260	UniProt ID:Q59VF3_CANAL	1813	69	192	44/130(33.85)	0.5	0.08	130	57	2.00E-09	gene=Chr01G1483	Gene Symbol:"DUR1,2"|Host:Isolated from a wide variety of substrates including humans|Disease:invasive candidal disease|Description:CAUTION: The sequence shown

															here is derived from an EMBL/GenBank/DDBJ whole genome shotgun (WGS) entry which is preliminary data.
Chr01G1487.1	621	39	315	UniProt ID:Q00LS5_PHAND	408	12	281	74/283 (26.15)	0.44	0.07	283	95.9	4.00E-22	gene=Chr01G1487	Gene Symbol:CPKA|Host:Multiple genera of Poaceae and Blysmus compressus (Cyperaceae)|Disease:Glume blotch of wheat and other grasses|Description:SIMILARITY: Contains 1 protein kinase domain.
Chr01G1492.1	398	354	395	UniProt ID:O59937_FUSOX	384	11	52	27/42(64.29)	0.67	0	42	62.4	9.00E-12	gene=Chr01G1492	Gene Symbol:XYL3|Host:Multiple genera in multiple families|Disease:Blights, wilts, rots of various sorts|Description:SIMILARITY: Belongs to the glycosyl hydrolase 10 (cellulase F) family.
Chr01G1494.1	535	116	514	UniProt ID:Q5A	748	95	503	100/415(24.1	0.45	0.05	415	110	1.00E-26	gene=Chr01G1494	Gene Symbol:SNF3|Host:Isolate

				NE1_CANAL				0)						d from a wide variety of substrates including humans|Disease:invasive candidal disease|Description:SIMILARITY: Belongs to the major facilitator superfamily. Sugar transporter (TC 2.A.1.1) family.
Chr01G1496.1	445	121	376	UniProt ID:Q96VZ3_FUSOX	455	146	433	63/288 (21.88)	0.43	0.11	288	62.4	1.00E-11	gene=Chr01G1496	Gene Symbol:PGX1|Host:Multiple genera in multiple families|Disease:Blights, wilts, rots of various sorts|Description:SIMILARITY: Belongs to the glycosyl hydrolase 28 family.
Chr01G1504.1	340	4	300	UniProt ID:Q6A2T2_BOTFU	391	16	320	85/309 (27.51)	0.47	0.05	309	110	2.00E-28	gene=Chr01G1504	Gene Symbol:BTP1|Host:Various plant families|Disease:Grey mould. Parasite or saprophyte|Description:Unknown

Chr01G1509.1	462	173	388	UniProt ID:TUP1_CANAL	514	271	466	68/222 (30.63)	0.5	0.14	222	94.4	1.00E-21	gene=Chr01G1509	Gene Symbol:TUP1|Host:Isolated from a wide variety of substrates including humans|Disease:invasive candidal disease|Description:FUNCTION: Represses transcription by RNA polymerase II. Represses genes responsible for initiating filamentous growth and this repression is lifted under inducing environmental conditions.
Chr01G1512.1	2755	1234	2292	UniProt ID:Q9UVN5_ALTAL	4360	193	1239	311/1117(27.84)	0.45	0.11	1117	324	2.00E-89	gene=Chr01G1512	Gene Symbol:AMT|Host:Plant|Disease:Leaf spot, rots|Description:Unknown
Chr01G1515.1	1218	2	421	UniProt ID:Q9UVI6_CANAL	856	5	490	180/498(36.14)	0.54	0.18	498	282	3.00E-81	gene=Chr01G1515	Gene Symbol:SAP185|Host:Isolated from a wide variety of substrates including humans|Disease:invasive candidal disease|Description:Unkn

														own
Chr01G1516.1	357	11	204	UniProt ID:A0ST44_CERNC	357	4	202	54/207 (26.09)	0.44	0.1	207	67.8	1.00E-13	gene=Chr01G1516	Gene Symbol:CTB6|Host:Numerous taxa in Solanaceae|Disease:Leaf spot|Description:Unknown
Chr01G1518.1	1618	857	1068	UniProt ID:A5HEH7_9PEZI	479	221	430	52/223 (23.32)	0.4	0.11	223	48.9	2.00E-06	gene=Chr01G1518	Gene Symbol:OLE1|Host:Multiple genera in multiple families|Disease:Cankers, dieback, and several other diseases. Also, a human pathogen|Description:COFACTOR: Iron (By similarity).
Chr01G1521.1	798	209	467	UniProt ID:Q1L2E2_PHAND	619	260	522	79/278 (28.42)	0.43	0.12	278	89.7	2.00E-19	gene=Chr01G1521	Gene Symbol:ALS1|Host:Multiple genera of Poaceae and Blysmus compressus (Cyperaceae)|Disease:Glume blotch of wheat and other grasses|Description:COFACTOR: Pyridoxal phosphate (By similarity).
Chr01G1	1139	237	476	UniProt	515	236	503	65/276	0.41	0.16	276	56.6	5.00E-09	gene=Chr	Gene

523.1				ID:A4ULJ2_MYCGR				(23.55)						01G1523	Symbol:CYP51|Host:Triticum and possibly a few other grasses|Disease:Leaf spot or speckled leaf blotch of wheat|Description:COFACTOR: Heme group (By similarity).
Chr01G1527.1	369	204	270	UniProt ID:Q8J0I5_CLAPU	550	434	500	27/67(40.30)	0.64	0	67	59.7	1.00E-10	gene=Chr01G1527	Gene Symbol:CPTF1|Host:outcrossing species|Disease:ergotism|Description:SIMILARITY: Belongs to the bZIP family.
Chr01G1528.1	478	69	244	UniProt ID:Q2VLJ1_GIBZA	565	123	306	58/184(31.52)	0.51	0.04	184	75.5	1.00E-15	gene=Chr01G1528	Gene Symbol:ZEB1|Host:Principal hosts: Poaceae, including Zea mays (corn), Triticum aestivum (wheat), and Oryza sativa (rice). Additional hosts: various plant families|Disease:Seedling blight, pre- and post-emergence blight,

															root and foot rot, brown rot, culm decay, head or kernel blight (scab or ear scab) of cereals. Leaf|Description:Unknown
Chr01G1534.1	208	11	207	UniProt ID:Q5IFQ6_CRYNV	234	5	222	100/222(45.05)	0.62	0.13	222	204	4.00E-66	gene=Chr01G1534	Gene Symbol:SOD2|Host:humans|Disease:cryptococcosis|Description:FUNCTION: Destroys radicals which are normally produced within the cells and which are toxic to biological systems (By similarity).
Chr01G1538.1	2085	31	1684	UniProt ID:O59897_ASPFM	2146	40	1701	461/1753(26.30)	0.44	0.11	1753	512	1.00E-149	gene=Chr01G1538	Gene Symbol:ALB1|Host:humans|Disease:infection|Description:Unknown
Chr01G1542.1	400	21	76	UniProt ID:O59937_FUSOX	384	1	56	32/56(57.14)	0.68	0	56	73.6	3.00E-15	gene=Chr01G1542	Gene Symbol:XYL3|Host:Multiple genera in multiple families|Disease:Blights, wilts, rots of various sorts|Description:SIMILARITY: Belongs to the glycosyl hydrolase 10

															(cellulase F) family.
Chr01G1543.1	2447	9	2441	UniProt ID:Q6ZX14_MAGGR	4034	10	2505	887/2551(34.77)	0.5	0.07	2551	1334	0	gene=Chr01G1543	Gene Symbol:ACE1|Host:Digitaria (Poaceae)|Disease:Leaf spot|Description:Unknown
Chr01G1545.1	454	35	454	UniProt ID:Q6XX21_CRYNV	504	81	502	207/426(48.59)	0.65	0.02	426	369	2.00E-123	gene=Chr01G1545	Gene Symbol:FHB1|Host:humans|Disease:cryptococcosis|Description:SIMILARITY: Belongs to the globin family.
Chr01G1548.1	339	40	339	UniProt ID:Q6TFC7_ASPFM	349	45	347	99/317(31.23)	0.49	0.1	317	153	8.00E-44	gene=Chr01G1548	Gene Symbol:NULL|Host:humans|Disease:infection|Description:Unknown
Chr01G1550.1	400	8	257	UniProt ID:Q6A2T2_BOTFU	391	10	290	64/282(22.70)	0.45	0.12	282	80.9	1.00E-17	gene=Chr01G1550	Gene Symbol:BTP1|Host:Various plant families|Disease:Grey mould. Parasite or saprophyte|Description:Unknown
Chr01G1553.1	305	5	180	UniProt ID:Q6A2T2_BOTFU	391	128	296	45/177(25.42)	0.45	0.05	177	55.1	1.00E-09	gene=Chr01G1553	Gene Symbol:BTP1|Host:Various plant families|Disease:Grey

															mould. Parasite or saprophyte|Description:Unknown
Chr01G1555.1	532	27	529	UniProt ID:Q5ANE1_CANAL	748	42	528	122/515(23.69)	0.44	0.08	515	125	2.00E-31	gene=Chr01G1555	Gene Symbol:SNF3|Host:Isolated from a wide variety of substrates including humans|Disease:invasive candidal disease|Description:SIMILARITY: Belongs to the major facilitator superfamily. Sugar transporter (TC 2.A.1.1) family.
Chr01G1556.1	439	2	355	UniProt ID:A4UC81_MAGO7	376	26	371	144/359(40.11)	0.59	0.05	359	253	2.00E-80	gene=Chr01G1556	Gene Symbol:MGG_10702|Host:Poaceae, especially important on Oryzae|Disease:Rice blast|Description:Unknown
Chr01G1570.1	514	238	491	UniProt ID:Q59ZY7_CANAL	322	90	319	85/275(30.91)	0.47	0.24	275	132	3.00E-35	gene=Chr01G1570	Gene Symbol:YVH1|Host:Isolated from a wide variety of substrates including

														humans|Disease:invasive candidal disease|Description:CAUTION: The sequence shown here is derived from an EMBL/GenBank/DDBJ whole genome shotgun (WGS) entry which is preliminary data.
Chr01G1582.1	1024	348	589	UniProt ID:TUP1_CANAL	514	268	484	66/256 (25.78)	0.41	0.21	256	68.9	6.00E-13	gene=Chr01G1582	Gene Symbol:TUP1|Host:Isolated from a wide variety of substrates including humans|Disease:invasive candidal disease|Description:FUNCTION: Represses transcription by RNA polymerase II. Represses genes responsible for initiating filamentous growth and this repression is lifted under inducing environmental conditions.
Chr01G1583.1	212	68	150	UniProt ID:SNF	226	65	147	22/83(26.51)	0.61	0	83	42.7	4.00E-06	gene=Chr01G1583	Gene Symbol:SNF7|Host:Isolate

				7_CANAL											d from a wide variety of substrates including humans|Disease:invasive candidal disease|Description:FUNCTION: Required for the sorting and concentration of proteins resulting in the entry of these proteins into the invaginating vesicles of the multivesicular body (MVB). Also required for the proteolytic cleavage of the transcription factor RIM101 in response to alkaline ambient pH.
Chr01G1584.1	1013	113	563	UniProt ID:Q59YK6_CANAL	844	166	637	139/493(28.19)	0.47	0.13	493	193	7.00E-52	gene=Chr01G1584	Gene Symbol:CDC24|Host:Isolated from a wide variety of substrates including humans|Disease:invasive candidal disease|Description:SIMILARITY: Contains 1 DH (DBL-homology) domain.
Chr01G1	645	3	51	UniProt	624	7	54	19/49(	0.61	0.02	49	45.4	7.00E-06	gene=Chr	Gene

590.1				ID:Q5A4F3_CANAL				38.78)						01G1590	Symbol:ZCF37|Host:Isolated from a wide variety of substrates including humans|Disease:invasive candidal disease|Description:Unknown
Chr01G1593.1	258	23	183	UniProt ID:B9WDI5_CANDC	376	113	272	50/168 (29.76)	0.43	0.09	168	53.9	2.00E-09	gene=Chr01G1593	Gene Symbol:ERG6|Host:humans|Disease:leptomeningeal disease,occasional invasive candidal disease|Description:Unknown
Chr01G1594.1	504	37	496	UniProt ID:Q5ABU7_CANAL	564	93	558	146/472(30.93)	0.5	0.04	472	227	1.00E-67	gene=Chr01G1594	Gene Symbol:MDR1|Host:Isolated from a wide variety of substrates including humans|Disease:invasive candidal disease|Description:Unknown
Chr01G1597.1	460	16	373	UniProt ID:A4UC81_MAGO7	376	11	366	136/362(37.57)	0.59	0.03	362	246	3.00E-77	gene=Chr01G1597	Gene Symbol:MGG_10702|Host:Poaceae, especially important on

														Oryzae|Disease:Rice blast|Description:Unknown
Chr01G1600.1	590	1	580	UniProt ID:Q5AEK8_CANAL	584	3	578	259/591(43.82)	0.59	0.04	591	468	9.00E-159	gene=Chr01G1600 Gene Symbol:CAO19.260, ORF19.260|Host:Isolated from a wide variety of substrates including humans|Disease:invasive candidal disease|Description:SIMILARITY: Contains 1 cytochrome b5 heme-binding domain.
Chr01G1602.1	419	1	414	UniProt ID:Q9C1F9_COCCA	423	1	410	214/418(51.20)	0.66	0.03	418	413	4.00E-142	gene=Chr01G1602 Gene Symbol:CEL2|Host:Corn, Zea mays, sometimes on Sorghum (Poaceae) and various other plant families|Disease:Northern corn leaf spot, ear and kernel rot|Description:Unknown
Chr01G1603.1	548	3	541	UniProt ID:Q4P8E8_U	693	83	673	181/614(29.48)	0.45	0.16	614	168	7.00E-46	gene=Chr01G1603 Gene Symbol:UM03615.1|Host: Euchlaena spp., Zea spp.

				STMA										(Poaceae)|Disease:Smut. Corn smut|Description:COFACTOR: FAD (By similarity).
Chr01G1608.1	347	12	282	UniProt ID:A0ST44_CERNC	357	4	275	73/281 (25.98)	0.42	0.07	281	77.4	7.00E-17	gene=Chr01G1608	Gene Symbol:CTB6|Host:Numerous taxa in Solanaceae|Disease:Leaf spot|Description:Unknown
Chr01G1611.1	543	47	409	UniProt ID:Q9P8L8_BOTFU	598	94	454	97/370 (26.22)	0.43	0.04	370	68.6	3.00E-13	gene=Chr01G1611	Gene Symbol:BCMFS1|Host:Various plant families|Disease:Grey mould. Parasite or saprophyte|Description:Unknown
Chr01G1612.1	1511	1	1490	UniProt ID:Q9C1I7_MYCGR	1635	36	1525	933/1506(61.95)	0.75	0.02	1506	1882	0	gene=Chr01G1612	Gene Symbol:ATR4|Host:Triticum and possibly a few other grasses|Disease:Leaf spot or speckled leaf blotch of wheat|Description:SIMILARITY: Belongs to the ABC transporter superfamily.
Chr01G1615.1	339	77	337	UniProt ID:Q32	266	15	264	87/264 (32.95)	0.5	0.06	264	120	2.00E-32	gene=Chr01G1615	Gene Symbol:MDH1|Host:Multip

				WF7_PHAND										le genera of Poaceae and Blysmus compressus (Cyperaceae)|Disease:Glume blotch of wheat and other grasses|Description:Unknown
Chr01G1616.1	531	51	488	UniProt ID:Q5ANE1_CANAL	748	51	496	108/455(23.74)	0.43	0.06	455	105	6.00E-25	gene=Chr01G1616	Gene Symbol:SNF3|Host:Isolated from a wide variety of substrates including humans|Disease:invasive candidal disease|Description:SIMILARITY: Belongs to the major facilitator superfamily. Sugar transporter (TC 2.A.1.1) family.
Chr01G1617.1	379	28	265	UniProt ID:Q9Y784_MAGGR	631	128	363	65/248(26.21)	0.46	0.09	248	79.3	5.00E-17	gene=Chr01G1617	Gene Symbol:PTH11|Host:Digitaria (Poaceae)|Disease:Leaf spot|Description:Unknown
Chr01G1622.1	485	204	443	UniProt ID:A4U	517	282	509	55/247(22.27)	0.36	0.11	247	45.4	5.00E-06	gene=Chr01G1622	Gene Symbol:CYP51|Host:Tritic

				LJ1_MYCGR											um and possibly a few other grasses|Disease:Leaf spot or speckled leaf blotch of wheat|Description:COFACTOR: Heme group (By similarity).
Chr01G1624.1	263	123	262	UniProt ID:Q9HFZ2_CANAL	358	213	343	45/142 (31.69)	0.42	0.09	142	55.1	8.00E-10	gene=Chr01G1624	Gene Symbol:RBT4|Host:Isolated from a wide variety of substrates including humans|Disease:invasive candidal disease|Description:Unknown
Chr01G1641.1	463	35	427	UniProt ID:Q96VZ3_FUSOX	455	50	443	148/411(36.01)	0.52	0.09	411	235	2.00E-72	gene=Chr01G1641	Gene Symbol:PGX1|Host:Multiple genera in multiple families|Disease:Blights, wilts, rots of various sorts|Description:SIMILARITY: Belongs to the glycosyl hydrolase 28 family.
Chr01G1644.1	394	54	374	UniProt ID:BOT	399	61	385	113/329(34.3	0.53	0.04	329	199	4.00E-60	gene=Chr01G1644	Gene Symbol:BOT2|Host:Variou

				2_BOT FU				5)							s plant families|Disease:Grey mould. Parasite or saprophyte|Description:FUNCTION: Presilphiperfolan-8-beta-ol synthase, which catalyzes the cyclization of farnesyl diphosphate (FPP) to presilphiperfolan-8-beta-ol (PSP), the committed step in the biosynthesis of the terpenoid virulence factor botrydial. Botrydial is necessary for colonization of plant tissue by the T4 strain. It is a strain-dependent virulence factor since highly aggressive strains like SAS56 or B05 still retain substantial virulence when botrydial synthesis is impaired.
Chr01G1651.1	249	5	245	UniProt ID:O93	267	9	263	79/259 (30.50)	0.46	0.08	259	92	6.00E-23	gene=Chr01G1651	Gene Symbol:BRM2|Host:Plant|

			802_ALTAL											Disease:Leaf spot, rots|Description:SIMILARITY: Belongs to the short-chain dehydrogenases/reductases (SDR) family.
Chr01G1654.1	494	1	344	UniProt ID:Q5XTQ4_BOTFU	574	1	377	135/389(34.70)	0.48	0.15	389	153	3.00E-41	gene=Chr01G1654	Gene Symbol:LIP1|Host:Various plant families|Disease:Grey mould. Parasite or saprophyte|Description:Unknown
Chr01G1659.1	351	28	345	UniProt ID:A4QVF8_MAGO7	339	28	336	98/329(29.79)	0.45	0.09	329	109	4.00E-28	gene=Chr01G1659	Gene Symbol:MGG_04556|Host:Poaceae, especially important on Oryzae|Disease:Rice blast|Description:COFACTOR: Zinc (By similarity).
Chr01G1660.1	555	8	40	UniProt ID:Q5A4F3_CANAL	624	14	46	16/33(48.48)	0.7	0	33	49.7	3.00E-07	gene=Chr01G1660	Gene Symbol:ZCF37|Host:Isolated from a wide variety of substrates including humans|Disease:invasive candidal

															disease|Description:Unknown
Chr01G1661.1	356	10	275	UniProt ID:Q9Y784_MAGGR	631	100	365	71/272 (26.10)	0.53	0.04	272	112	3.00E-28	gene=Chr01G1661	Gene Symbol:PTH11|Host:Digitaria (Poaceae)|Disease:Leaf spot|Description:Unknown
Chr01G1667.1	721	29	598	UniProt ID:A6N6J8_FUSOX	903	45	631	154/625(24.64)	0.41	0.15	625	139	5.00E-35	gene=Chr01G1667	Gene Symbol:CTF1|Host:Multiple genera in multiple families|Disease:Blights, wilts, rots of various sorts|Description:SIMILARITY: Contains 1 Zn(2)-C6 fungal-type DNA-binding domain.
Chr01G1678.1	434	45	424	UniProt ID:Q59VF3_CANAL	1813	42	444	110/419(26.25)	0.43	0.13	419	70.1	9.00E-14	gene=Chr01G1678	Gene Symbol:"DUR1,2"|Host:Isolated from a wide variety of substrates including humans|Disease:invasive candidal disease|Description:CAUTION: The sequence shown here is derived from an EMBL/GenBank/DDBJ

														whole genome shotgun (WGS) entry which is preliminary data.
Chr01G1685.1	494	20	340	UniProt ID:Q5XTQ4_BOTFU	574	30	376	132/357(36.97)	0.48	0.13	357	157	1.00E-42	gene=Chr01G1685	Gene Symbol:LIP1|Host:Various plant families|Disease:Grey mould. Parasite or saprophyte|Description:Unknown
Chr01G1702.1	406	41	364	UniProt ID:O59928_HYPVI	430	57	390	93/365(25.48)	0.41	0.2	365	94.4	3.00E-22	gene=Chr01G1702	Gene Symbol:NULL|Host:humans|Disease:infection|Description:SIMILARITY: Belongs to the glycosyl hydrolase 18 family.
Chr01G1709.1	537	22	490	UniProt ID:Q5ANE1_CANAL	748	37	496	134/477(28.09)	0.48	0.05	477	165	1.00E-44	gene=Chr01G1709	Gene Symbol:SNF3|Host:Isolated from a wide variety of substrates including humans|Disease:invasive candidal disease|Description:SIMILARITY: Belongs to the major facilitator superfamily. Sugar

															transporter (TC 2.A.1.1) family.
Chr01G1710.1	738	4	68	UniProt ID:A8QJI7_FUSOX	938	69	132	29/65(44.62)	0.51	0.02	65	61.2	2.00E-10	gene=Chr01G1710	Gene Symbol:XLNR|Host:Multiple genera in multiple families|Disease:Blights, wilts, rots of various sorts|Description:SIMILARITY: Contains 1 Zn(2)-C6 fungal-type DNA-binding domain.
Chr01G1725.1	1300	4	191	UniProt ID:Q7Z9J6_CRYNV	197	10	185	58/190(30.53)	0.48	0.08	190	85.1	6.00E-19	gene=Chr01G1725	Gene Symbol:TSA1|Host:humans|Disease:cryptococcosis|Description:Unknown
Chr01G1726.1	630	51	590	UniProt ID:O93800_ALTAL	578	22	561	129/581(22.20)	0.36	0.14	581	52.8	4.00E-08	gene=Chr01G1726	Gene Symbol:AKT1|Host:Plant|Disease:Leaf spot, rots|Description:Unknown
Chr01G1732.1	336	24	259	UniProt ID:F2QWL8_PICP7	383	71	282	70/240(29.17)	0.41	0.13	240	84.3	2.00E-19	gene=Chr01G1732	Gene Symbol:ERG6|Host:humans|Disease:occasional infection|Description:Unknown
Chr01G1734.1	317	61	314	UniProt ID:Q32	266	15	263	88/258(34.11)	0.55	0.05	258	126	1.00E-34	gene=Chr01G1734	Gene Symbol:MDH1|Host:Multip

				WF7_P HAND											le genera of Poaceae and Blysmus compressus (Cyperaceae)|Disease:Glume blotch of wheat and other grasses|Description:Unknown
Chr01G1737.1	286	21	275	UniProt ID:Q32WF7_PHAND	266	11	260	67/266 (25.19)	0.43	0.1	266	48.1	1.00E-07	gene=Chr01G1737	Gene Symbol:MDH1|Host:Multiple genera of Poaceae and Blysmus compressus (Cyperaceae)|Disease:Glume blotch of wheat and other grasses|Description:Unknown
Chr01G1742.1	557	92	540	UniProt ID:Q96VB5_ALTAL	578	108	564	144/466(30.90)	0.47	0.06	466	180	5.00E-50	gene=Chr01G1742	Gene Symbol:AFT1-1|Host:Plant|Disease:Leaf spot, rots|Description:Unknown
Chr01G1743.1	267	6	259	UniProt ID:O93802_ALTAL	267	3	267	80/276 (28.99)	0.45	0.12	276	100	1.00E-25	gene=Chr01G1743	Gene Symbol:BRM2|Host:Plant|Disease:Leaf spot, rots|Description:SIMILARITY: Belongs to the short-chain

															dehydrogenases/reductases (SDR) family.
Chr01G1748.1	281	20	279	UniProt ID:A4RGG9_MAGO7	286	14	271	81/268 (30.22)	0.5	0.07	268	101	6.00E-26	gene=Chr01G1748	Gene Symbol:MGG_00056|Host:Poaceae, especially important on Oryzae|Disease:Rice blast|Description:SIMILARITY: Belongs to the short-chain dehydrogenases/reductases (SDR) family.
Chr01G1749.1	1008	40	573	UniProt ID:A6N6J8_FUSOX	903	46	523	116/567(20.46)	0.37	0.22	567	59.3	9.00E-10	gene=Chr01G1749	Gene Symbol:CTF1|Host:Multiple genera in multiple families|Disease:Blights, wilts, rots of various sorts|Description:SIMILARITY: Contains 1 Zn(2)-C6 fungal-type DNA-binding domain.
Chr01G1750.1	717	147	454	UniProt ID:Q00LS5_PHAND	408	22	268	79/313 (25.24)	0.42	0.23	313	80.5	6.00E-17	gene=Chr01G1750	Gene Symbol:CPKA|Host:Multiple genera of Poaceae and Blysmus compressus (Cyperaceae)|Disease:Glu

															me blotch of wheat and other grasses|Description:SIMILARITY: Contains 1 protein kinase domain.
Chr01G1756.1	341	30	296	UniProt ID:Q92217_COCHE	2528	1848	2108	88/273 (32.23)	0.49	0.07	273	103	1.00E-24	gene=Chr01G1756	Gene Symbol:PKS1|Host:Zea mays|Disease:Southern leaf blight of maize|Description:Unknown
Chr01G1757.1	940	685	871	UniProt ID:A6ZXD7_YEAS7	1273	65	246	50/199 (25.13)	0.43	0.15	199	49.3	1.00E-06	gene=Chr01G1757	Gene Symbol:SEC31|Host:humans|Disease:occasional infection|Description:CAUTION: The sequence shown here is derived from an EMBL/GenBank/DDBJ whole genome shotgun (WGS) entry which is preliminary data.
Chr01G1761.1	588	117	582	UniProt ID:Q5ABU7_CANAL	564	95	559	161/479(33.61)	0.52	0.06	479	267	7.00E-82	gene=Chr01G1761	Gene Symbol:MDR1|Host:Isolated from a wide variety of substrates including

														humans|Disease:invasive candidal disease|Description:Unknown
Chr01G1763.1	1358	797	1338	UniProt ID:Q3Y5V5_MAGGR	1321	763	1298	159/555(28.65)	0.48	0.06	555	220	4.00E-59	gene=Chr01G1763	Gene Symbol:ABC3|Host:Digitaria (Poaceae)|Disease:Leaf spot|Description:SIMILARITY: Belongs to the ABC transporter superfamily.
Chr01G1765.1	612	79	607	UniProt ID:Q59RG0_CANAL	581	121	573	142/535(26.54)	0.43	0.16	535	186	5.00E-52	gene=Chr01G1765	Gene Symbol:NAG4|Host:Isolated from a wide variety of substrates including humans|Disease:invasive candidal disease|Description:CAUTION: The sequence shown here is derived from an EMBL/GenBank/DDBJ whole genome shotgun (WGS) entry which is preliminary data.
Chr01G1773.1	632	100	568	UniProt ID:Q5XTQ5_B	615	70	549	141/487(28.95)	0.47	0.05	487	191	4.00E-53	gene=Chr01G1773	Gene Symbol:FRT1|Host:Various plant

				OTFU											families|Disease:Grey mould. Parasite or saprophyte|Description:SIMILARITY: Belongs to the major facilitator superfamily. Sugar transporter (TC 2.A.1.1) family.
Chr01G1774.1	432	8	65	UniProt ID:O59937_FUSOX	384	7	65	29/59(49.15)	0.64	0.02	59	61.2	3.00E-11	gene=Chr01G1774	Gene Symbol:XYL3|Host:Multiple genera in multiple families|Disease:Blights, wilts, rots of various sorts|Description:SIMILARITY: Belongs to the glycosyl hydrolase 10 (cellulase F) family.
Chr01G1780.1	1080	351	697	UniProt ID:Q9UW03_BOTFU	1439	797	1133	120/361(33.24)	0.53	0.11	361	171	5.00E-44	gene=Chr01G1780	Gene Symbol:ATRB|Host:Various plant families|Disease:Grey mould. Parasite or saprophyte|Description:SIMILARITY: Belongs to the ABC transporter superfamily.

Chr01G1784.1	356	1	348	UniProt ID:Q5AFI8_CANAL	373	1	357	125/378(33.07)	0.52	0.13	378	157	8.00E-45	gene=Chr01G1784	Gene Symbol:LEU2|Host:Isolated from a wide variety of substrates including humans|Disease:invasive candidal disease|Description:FUNCTION: Catalyzes the oxidation of 3-carboxy-2-hydroxy-4-methylpentanoate (3-isopropylmalate) to 3-carboxy-4-methyl-2-oxopentanoate. The product decarboxylates to 4-methyl-2 oxopentanoate (By similarity).
Chr01G1790.1	460	4	374	UniProt ID:A4UC81_MAGO7	376	5	373	152/380(40.00)	0.59	0.05	380	266	3.00E-85	gene=Chr01G1790	Gene Symbol:MGG_10702|Host:Poaceae, especially important on Oryzae|Disease:Rice blast|Description:Unknown
Chr01G1799.1	928	20	659	UniProt ID:A6N	903	46	611	156/660(23.6	0.4	0.17	660	113	1.00E-26	gene=Chr01G1799	Gene Symbol:CTF1|Host:Multipl

				6J8_FUSOX				4)							e genera in multiple families|Disease:Blights, wilts, rots of various sorts|Description:SIMILARITY: Contains 1 Zn(2)-C6 fungal-type DNA-binding domain.
Chr01G1805.1	672	372	628	UniProt ID:Q6XPX0_FUSOX	359	105	357	74/267 (27.72)	0.45	0.09	267	84.7	1.00E-18	gene=Chr01G1805	Gene Symbol:FGB1|Host:Multiple genera in multiple families|Disease:Blights, wilts, rots of various sorts|Description:Unknown
Chr01G1810.1	289	16	269	UniProt ID:Q32WF7_PHAND	266	8	261	87/260 (33.46)	0.52	0.05	260	127	3.00E-35	gene=Chr01G1810	Gene Symbol:MDH1|Host:Multiple genera of Poaceae and Blysmus compressus (Cyperaceae)|Disease:Glume blotch of wheat and other grasses|Description:Unknown
Chr01G1814.1	928	6	265	UniProt ID:A4RGG9_M	286	11	269	86/271 (31.73)	0.46	0.08	271	94.7	6.00E-22	gene=Chr01G1814	Gene Symbol:MGG_00056|Host:Poaceae, especially

				AGO7											important on Oryzae|Disease:Rice blast|Description:SIMILARITY: Belongs to the short-chain dehydrogenases/reductases (SDR) family.
Chr01G1820.1	864	217	395	UniProt ID:Q5ALS7_CANAL	1144	351	519	49/188 (26.06)	0.45	0.15	188	58.2	2.00E-09	gene=Chr01G1820	Gene Symbol:CTF1|Host:Isolated from a wide variety of substrates including humans|Disease:invasive candidal disease|Description:SIMILARITY: Contains 1 Zn(2)-C6 fungal-type DNA-binding domain.
Chr01G1822.1	265	10	201	UniProt ID:Q75WR5_9PLEO	265	8	200	53/203 (26.11)	0.43	0.1	203	46.6	4.00E-07	gene=Chr01G1822	Gene Symbol:BRN1|Host:Belamcanda chinensis: Korea,Gladiolus ?gandavensis: Korea,Iris japonica: China,Iris missouriensis (Leaf spot.): Idaho; Montana; Oregon; Washington,Iris sp. (Leaf

															spot.): China; Texas; Washing|Disease:Leaf spot|Description:SIMILARITY: Belongs to the short-chain dehydrogenases/reductases (SDR) family.
Chr01G1829.1	186	38	128	UniProt ID:Q2LK92_BOTFU	117	17	117	48/102 (47.06)	0.61	0.12	102	87	1.00E-22	gene=Chr01G1829	Gene Symbol:PIC5|Host:Various plant families|Disease:Grey mould. Parasite or saprophyte|Description:SIMILARITY: Belongs to the FKBP-type PPIase family.
Chr01G1837.1	307	46	275	UniProt ID:A4RGG9_MAGO7	286	16	261	65/250 (26.00)	0.46	0.1	250	76.3	5.00E-17	gene=Chr01G1837	Gene Symbol:MGG_00056|Host:Poaceae, especially important on Oryzae|Disease:Rice blast|Description:SIMILARITY: Belongs to the short-chain dehydrogenases/reductases (SDR) family.
Chr01G1	559	101	355	UniProt	1813	69	299	81/257	0.48	0.11	257	80.5	1.00E-16	gene=Chr	Gene

838.1				ID:Q59 VF3_C ANAL				(31.52)						01G1838	Symbol:"DUR1,2"|Host:Isolated from a wide variety of substrates including humans|Disease:invasive candidal disease|Description:CAUTION: The sequence shown here is derived from an EMBL/GenBank/DDBJ whole genome shotgun (WGS) entry which is preliminary data.
Chr01G1 842.1	559	70	539	UniProt ID:Q5A NE1_C ANAL	748	51	514	127/49 9(25.4 5)	0.42	0.13	499	115	4.00E-28	gene=Chr 01G1842	Gene Symbol:SNF3|Host:Isolated from a wide variety of substrates including humans|Disease:invasive candidal disease|Description:SIMILARITY: Belongs to the major facilitator superfamily. Sugar transporter (TC 2.A.1.1) family.
Chr01G1 844.1	353	44	343	UniProt ID:Q6T	349	47	346	105/30 5(34.4	0.5	0.03	305	159	1.00E-45	gene=Chr 01G1844	Gene Symbol:NULL|Host:huma

				FC7_A SPFM				3)							ns|Disease:infection|Description:Unknown
Chr01G1848.1	423	35	421	UniProt ID:ORYZ_ASPFU	403	19	401	177/389(45.50)	0.61	0.02	389	307	5.00E-101	gene=Chr01G1848	Gene Symbol:ALP1|Host:humans|Disease:infection|Description:FUNCTION: Secreted alkaline protease that allows assimilation of proteinaceous substrates. Acts as a significant virulence factor in invasive aspergillosis. Involved in immune evasion from the human and mice complement systems during infection. Efficiently cleaves important components of the complement cascade such as such as C3, C4, C5, and C1q, as well as IgG, which leads to down-regulation of complement activation at the hyphal surface.

Chr01G1852.1	353	80	348	UniProt ID:Q6TFC7_ASPFM	349	63	333	92/276 (33.33)	0.47	0.04	276	129	8.00E-35	gene=Chr01G1852	Gene Symbol:NULL|Host:humans|Disease:infection|Description:Unknown
Chr01G1855.1	537	59	526	UniProt ID:Q4PDC5_USTMA	583	67	534	212/478(44.35)	0.61	0.04	478	402	9.00E-134	gene=Chr01G1855	Gene Symbol:UM01888.1|Host: Euchlaena spp., Zea spp. (Poaceae)|Disease:Smut. Corn smut|Description:CAUTION: The sequence shown here is derived from an EMBL/GenBank/DDBJ whole genome shotgun (WGS) entry which is preliminary data.
Chr01G1856.1	516	5	490	UniProt ID:A4ULI4_MYCGR	517	13	515	275/507(54.24)	0.69	0.05	507	554	0	gene=Chr01G1856	Gene Symbol:CYP51|Host:Triticum and possibly a few other grasses|Disease:Leaf spot or speckled leaf blotch of wheat|Description:COFACTOR: Heme group (By similarity).
Chr01G1	300	13	200	UniProt	267	9	203	67/212	0.44	0.19	212	57.8	1.00E-10	gene=Chr	Gene

858.1				ID:O93802_ALTAL				(31.60)						01G1858	Symbol:BRM2|Host:Plant|Disease:Leaf spot, rots|Description:SIMILARITY: Belongs to the short-chain dehydrogenases/reductases (SDR) family.
Chr01G1864.1	528	79	500	UniProt ID:Q5ANE1_CANAL	748	74	496	112/442(25.34)	0.45	0.09	442	112	5.00E-27	gene=Chr01G1864	Gene Symbol:SNF3|Host:Isolated from a wide variety of substrates including humans|Disease:invasive candidal disease|Description:SIMILARITY: Belongs to the major facilitator superfamily. Sugar transporter (TC 2.A.1.1) family.
Chr01G1884.1	1170	51	986	UniProt ID:Q6ZX14_MAGGR	4034	3088	3925	247/964(25.62)	0.41	0.16	964	162	5.00E-41	gene=Chr01G1884	Gene Symbol:ACE1|Host:Digitaria (Poaceae)|Disease:Leaf spot|Description:Unknown
Chr01G1888.1	1091	491	788	UniProt ID:SUB2_TRIV	419	151	372	82/301(27.24)	0.4	0.27	301	68.6	7.00E-13	gene=Chr01G1888	Gene Symbol:SUB2|Host:humans|Disease:infection|Desc

				C											ription:FUNCTION: Secreted subtilisin-like serine protease with keratinolytic activity that contributes to pathogenicity (By similarity).
Chr01G1891.1	563	37	563	UniProt ID:A6RBC1_AJECN	529	8	529	272/530(51.32)	0.67	0.02	530	541	0	gene=Chr01G1891	Gene Symbol:HCAG_06259|Host:humans|Disease:Darling's disease|Description:FUNCTION: Adds a myristoyl group to the N-terminal glycine residue of certain cellular proteins (By similarity).
Chr01G1894.1	263	9	258	UniProt ID:Q32WF7_PHAND	266	17	262	54/271(19.93)	0.39	0.17	271	44.3	2.00E-06	gene=Chr01G1894	Gene Symbol:MDH1|Host:Multiple genera of Poaceae and Blysmus compressus (Cyperaceae)|Disease:Glume blotch of wheat and other grasses|Description:Unknown

Chr01G1900.1	542	31	525	UniProt ID:Q4PDC5_USTMA	583	36	534	201/517(38.88)	0.59	0.08	517	355	2.00E-115	gene=Chr01G1900	Gene Symbol:UM01888.1|Host: Euchlaena spp., Zea spp. (Poaceae)|Disease:Smut. Corn smut|Description:CAUTION: The sequence shown here is derived from an EMBL/GenBank/DDBJ whole genome shotgun (WGS) entry which is preliminary data.
Chr01G1917.1	472	46	471	UniProt ID:Q01446_NECHA	459	22	427	110/437(25.17)	0.41	0.1	437	110	4.00E-27	gene=Chr01G1917	Gene Symbol:MAK1|Host:Trees of various plant families|Disease:Fruit rot, stem rot|Description:Unknown
Chr01G1918.1	1609	483	699	UniProt ID:Q3Y5V5_MAGGR	1321	1084	1320	62/241(25.73)	0.45	0.12	241	70.1	9.00E-13	gene=Chr01G1918	Gene Symbol:ABC3|Host:Digitaria (Poaceae)|Disease:Leaf spot|Description:SIMILARITY: Belongs to the ABC transporter superfamily.
Chr01G1919.1	279	5	271	UniProt ID:O93	267	3	265	85/279(30.47)	0.51	0.1	279	112	9.00E-30	gene=Chr01G1919	Gene Symbol:BRM2|Host:Plant|

				802_ALTAL											Disease:Leaf spot, rots|Description:SIMILARITY: Belongs to the short-chain dehydrogenases/reductases (SDR) family.
Chr01G1925.1	436	3	416	UniProt ID:Q5GFD3_PHAND	437	7	432	166/432(38.43)	0.57	0.06	432	286	3.00E-92	gene=Chr01G1925	Gene Symbol:NULL|Host:Multiple genera of Poaceae and Blysmus compressus (Cyperaceae)|Disease:Glume blotch of wheat and other grasses|Description:Unknown
Chr01G1929.1	794	209	581	UniProt ID:Q9HG15_COLLN	746	179	493	85/377 (22.55)	0.41	0.18	377	71.2	1.00E-13	gene=Chr01G1929	Gene Symbol:CLTA1|Host:Multiple genera of Fabaceae. Rare reports on other taxa|Disease:Leaf, stem and pod anthracnose|Description:SIMILARITY: Contains 1 Zn(2)-C6 fungal-type DNA-binding domain.
Chr01G1	811	68	522	UniProt	677	17	449	136/47	0.45	0.11	470	150	1.00E-38	gene=Chr	Gene

930.1				ID:A3LS85_PICST				0(28.94)						01G1930	Symbol:LYS4|Host:humans|Disease:occasional infection|Description:Unknown
Chr01G1937.1	344	1	339	UniProt ID:O94101_BOTFU	342	1	339	272/339(80.24)	0.89	0	339	582	0	gene=Chr01G1937	Gene Symbol:BCNMT1|Host:Various plant families|Disease:Grey mould. Parasite or saprophyte|Description:Unknown
Chr01G1940.1	298	44	281	UniProt ID:C1G8Q1_PARBD	333	4	219	69/251 (27.49)	0.41	0.19	251	57.4	2.00E-10	gene=Chr01G1940	Gene Symbol:PADG_03637|Host:humans|Disease:Paracoccidioidomycosis|Description:Unknown
Chr01G1943.1	394	91	312	UniProt ID:Q7Z9J3_9PEZI	1372	1113	1310	64/240 (26.67)	0.41	0.25	240	53.9	1.00E-08	gene=Chr01G1943	Gene Symbol:CZK3|Host:Zea mays (Poaceae)|Disease:Gray leaf spot of corn|Description:Unknown
Chr01G1945.1	511	243	471	UniProt ID:A4ULJ2_MYCGR	515	255	501	55/255 (21.57)	0.42	0.13	255	50.8	1.00E-07	gene=Chr01G1945	Gene Symbol:CYP51|Host:Triticum and possibly a few other

															grasses|Disease:Leaf spot or speckled leaf blotch of wheat|Description:COFACTOR: Heme group (By similarity).
Chr01G1953.1	639	20	625	UniProt ID:SED3_ASPFU	596	26	586	228/610(37.38)	0.55	0.09	610	402	3.00E-132	gene=Chr01G1953	Gene Symbol:SED3|Host:humans|Disease:infection|Description:FUNCTION: Secreted tripeptidyl-peptidase which degrades proteins at acidic pHs and is involved in virulence.
Chr01G1957.1	421	23	389	UniProt ID:D1MGZ7_BEABA	379	21	377	192/367(52.32)	0.66	0.03	367	352	5.00E-119	gene=Chr01G1957	Gene Symbol:PR1|Host:various arthropod species|Disease:white muscardine disease|Description:SIMILARITY: Belongs to the peptidase S8 family.
Chr01G1962.1	900	134	344	UniProt ID:SUB6_TRIVH	412	115	314	67/230(29.13)	0.43	0.21	230	57	2.00E-09	gene=Chr01G1962	Gene Symbol:SUB6|Host:humans|Disease:infection|Description:FUNCTION:

															Secreted subtilisin-like serine protease with keratinolytic activity that contributes to pathogenicity (By similarity).
Chr01G1967.1	1446	195	1436	UniProt ID:Q5A762_CANAL	1606	219	1600	391/1423(27.48)	0.45	0.16	1423	426	3.00E-125	gene=Chr01G1967	Gene Symbol:MLT1|Host:Isolated from a wide variety of substrates including humans|Disease:invasive candidal disease|Description:SIMILARITY: Belongs to the ABC transporter superfamily.
Chr01G1975.1	537	122	478	UniProt ID:Q59VF3_CANAL	1813	61	420	100/389(25.71)	0.43	0.16	389	72.8	3.00E-14	gene=Chr01G1975	Gene Symbol:"DUR1,2"|Host:Isolated from a wide variety of substrates including humans|Disease:invasive candidal disease|Description:CAUTION: The sequence shown here is derived from an EMBL/GenBank/DDBJ

													whole genome shotgun (WGS) entry which is preliminary data.
Chr01G1980.1	648	8	51	UniProt ID:Q5A4F3_CANAL	624	19	61	20/44(45.45)	0.59	0.02	44	48.9	7.00E-07	gene=Chr01G1980	Gene Symbol:ZCF37|Host:Isolated from a wide variety of substrates including humans|Disease:invasive candidal disease|Description:Unknown
Chr01G1982.1	559	65	524	UniProt ID:Q5ANE1_CANAL	748	41	496	153/475(32.21)	0.51	0.07	475	229	2.00E-66	gene=Chr01G1982	Gene Symbol:SNF3|Host:Isolated from a wide variety of substrates including humans|Disease:invasive candidal disease|Description:SIMILARITY: Belongs to the major facilitator superfamily. Sugar transporter (TC 2.A.1.1) family.
Chr01G1987.1	870	109	299	UniProt ID:TUP1_CAN	514	284	468	51/199(25.63)	0.46	0.11	199	48.9	8.00E-07	gene=Chr01G1987	Gene Symbol:TUP1|Host:Isolated from a wide variety of

				AL										substrates including humans|Disease:invasive candidal disease|Description:FUNCTION: Represses transcription by RNA polymerase II. Represses genes responsible for initiating filamentous growth and this repression is lifted under inducing environmental conditions.
Chr01G1990.1	446	5	443	UniProt ID:Q9C1F9_COCCA	423	6	410	150/450(33.33)	0.48	0.12	450	191	3.00E-56	gene=Chr01G1990	Gene Symbol:CEL2|Host:Corn, Zea mays, sometimes on Sorghum (Poaceae) and various other plant families|Disease:Northern corn leaf spot, ear and kernel rot|Description:Unknown
Chr01G1992.1	548	399	487	UniProt ID:D1MYV6_MAGGR	568	418	567	46/150 (30.67)	0.37	0.41	150	49.3	4.00E-07	gene=Chr01G1992	Gene Symbol:CBL1|Host:Digitaria (Poaceae)|Disease:Leaf spot|Description:SIMILARITY: Contains 3

															chitin-binding type-1 domains.
Chr01G1993.1	369	22	240	UniProt ID:Q92217_COCHE	2528	1833	2031	68/225 (30.22)	0.47	0.14	225	60.5	7.00E-11	gene=Chr01G1993	Gene Symbol:PKS1|Host:Zea mays|Disease:Southern leaf blight of maize|Description:Unknown
Chr01G2007.1	431	95	341	UniProt ID:Q4U320_USTMD	520	130	377	105/251(41.83)	0.63	0.03	251	209	3.00E-62	gene=Chr01G2007	Gene Symbol:CLN1|Host:Euchlaena spp., Zea spp. (Poaceae)|Disease:Smut. Corn smut|Description:SIMILARITY: Belongs to the cyclin family.
Chr01G2010.1	1016	629	923	UniProt ID:TUP1_CANAL	514	201	509	119/318(37.42)	0.53	0.1	318	196	2.00E-54	gene=Chr01G2010	Gene Symbol:TUP1|Host:Isolated from a wide variety of substrates including humans|Disease:invasive candidal disease|Description:FUNCTION: Represses transcription by RNA polymerase II. Represses

															genes responsible for initiating filamentous growth and this repression is lifted under inducing environmental conditions.
Chr01G2012.1	365	8	364	UniProt ID:O59928_HYPVI	430	20	407	133/389(34.19)	0.49	0.08	389	196	1.00E-58	gene=Chr01G2012	Gene Symbol:NULL|Host:humans|Disease:infection|Description:SIMILARITY: Belongs to the glycosyl hydrolase 18 family.
Chr01G2014.1	1071	686	980	UniProt ID:Q705V7_USTMD	1061	602	917	97/319 (30.41)	0.5	0.08	319	150	6.00E-38	gene=Chr01G2014	Gene Symbol:GAS1|Host:Euchlaena spp., Zea spp. (Poaceae)|Disease:Smut. Corn smut|Description:Unknown
Chr01G2015.1	368	12	70	UniProt ID:Q96VB4_ALTAL	445	13	76	24/68(35.29)	0.53	0.19	68	43.9	8.00E-06	gene=Chr01G2015	Gene Symbol:AFTR-1|Host:Plant|Disease:Leaf spot, rots|Description:Unknown
Chr01G2017.1	501	142	481	UniProt ID:A4ULJ2_MYCGR	515	147	513	87/377 (23.08)	0.43	0.12	377	77	5.00E-16	gene=Chr01G2017	Gene Symbol:CYP51|Host:Triticum and possibly a few other

															grasses|Disease:Leaf spot or speckled leaf blotch of wheat|Description:COFACTOR: Heme group (By similarity).
Chr01G2028.1	826	268	824	UniProt ID:Q9P8L8_BOTFU	598	34	592	222/567(39.15)	0.59	0.03	567	412	9.00E-134	gene=Chr01G2028	Gene Symbol:BCMFS1|Host:Various plant families|Disease:Grey mould. Parasite or saprophyte|Description:Unknown
Chr01G2033.1	327	5	321	UniProt ID:C1G3N5_PARBD	362	10	332	241/323(74.61)	0.87	0.02	323	496	3.00E-177	gene=Chr01G2033	Gene Symbol:PADG_01551|Host:humans|Disease:Paracoccidioidomycosis|Description:CATALYTIC ACTIVITY: Thioredoxin + NADP(+) = thioredoxin disulfide + NADPH.
Chr01G2039.1	1401	783	1240	UniProt ID:Q9C1Q7_ASPFM	708	309	706	157/469(33.48)	0.53	0.17	469	221	4.00E-61	gene=Chr01G2039	Gene Symbol:NULL|Host:humans|Disease:infection|Description:SIMILARITY: Contains 1 histidine kinase domain.

Chr01G2042.1	491	144	487	UniProt ID:PEPA_ASPFU	395	59	392	167/344(48.55)	0.67	0.03	344	311	8.00E-102	gene=Chr01G2042	Gene Symbol:PEP1|Host:humans|Disease:infection|Description:FUNCTION: Secreted aspartic endopeptidase that allows assimilation of proteinaceous substrates. Can catalyze hydrolysis of the major structural proteins of basement membrane, elastin, collagen, and laminin. Thought to play a significant role in virulence (By similarity).
Chr01G2044.1	648	42	603	UniProt ID:PLB1_CANAL	605	19	564	243/568(42.78)	0.59	0.05	568	434	2.00E-144	gene=Chr01G2044	Gene Symbol:PLB1|Host:Isolated from a wide variety of substrates including humans|Disease:invasive candidal disease|Description:FUNCTION: Catalyzes the release of fatty acids from lysophospholipids.

															Phospholipase B may well contribute to pathogenicity by abetting the fungus in damaging and traversing host cell membranes, processes which likely increase the rapidity of disseminated infection.
Chr01G2 049.1	561	87	240	UniProt ID:Q59 VF3_C ANAL	1813	52	208	53/158 (33.54)	0.51	0.03	158	61.6	7.00E-11	gene=Chr 01G2049	Gene Symbol:"DUR1,2"|Host:Is olated from a wide variety of substrates including humans|Disease:invasive candidal disease|Description:CAUT ION: The sequence shown here is derived from an EMBL/GenBank/DDBJ whole genome shotgun (WGS) entry which is preliminary data.
Chr01G2 051.1	557	1	557	UniProt ID:Q2P EP0_9 HYPO	557	1	557	487/55 7(87.4 3)	0.94	0	557	1046	0	gene=Chr 01G2051	Gene Symbol:NOXA|Host:plants |Disease:cool-season grasses|Description:SIMIL ARITY: Contains 1

Chr01G2054.1	1228	114	566	UniProt ID:Q8TFN4_COLGR	1866	84	570	132/503(26.24)	0.45	0.13	503	147	2.00E-36	gene=Chr01G2054	FAD-binding FR-type domain. Gene Symbol:CHSV|Host:Poaceae especially Zea mays|Disease:Leaf spot, stalk rot, etc|Description:Unknown
Chr01G2056.1	978	415	970	UniProt ID:CXT1_CRYNJ	694	173	662	138/573(24.08)	0.39	0.17	573	110	1.00E-25	gene=Chr01G2056	Gene Symbol:CXT1|Host:humans|Disease:cryptococcosis|Description:FUNCTION: Beta-1,2-xylosyltransferase that plays a key role in capsule polysaccharide synthesis by transferring xylose to alpha-1,3-dimannoside in a beta-1,2-linkage. Also mediates glycosylation of glycosphingolipids; constitutes the unique xylosyltransferase responsible for adding xylose to glycosphingolipids.

Chr01G2070.1	339	13	267	UniProt ID:Q9Y784_MAGGR	631	105	362	68/259 (26.25)	0.53	0.02	259	117	5.00E-30	gene=Chr01G2070	Gene Symbol:PTH11|Host:Digitaria (Poaceae)|Disease:Leaf spot|Description:Unknown
Chr01G2072.1	452	220	348	UniProt ID:Q8NJV0_GIBZA	375	177	310	34/134 (25.37)	0.47	0.04	134	49.7	1.00E-07	gene=Chr01G2072	Gene Symbol:TRI5|Host:Principal hosts: Poaceae, including Zea mays (corn), Triticum aestivum (wheat), and Oryza sativa (rice). Additional hosts: various plant families|Disease:Seedling blight, pre- and post-emergence blight, root and foot rot, brown rot, culm decay, head or kernel blight (scab or ear scab) of cereals. Leaf|Description:Unknown
Chr01G2074.1	547	25	303	UniProt ID:Q1HGK2_MAGGR	803	132	386	95/282 (33.69)	0.53	0.11	282	153	2.00E-40	gene=Chr01G2074	Gene Symbol:NULL|Host:Digitaria (Poaceae)|Disease:Leaf spot|Description:Unknown
Chr01G2	1167	1	116	UniProt	1167	1	1165	1082/1	0.97	0	1167	2193	0	gene=Chr	Gene

080.1			7	ID:Q8J 286_C OLLN				167(92 .72)						01G2080	Symbol:CLAP1|Host:Multiple genera of Fabaceae. Rare reports on other taxa|Disease:Leaf, stem and pod anthracnose|Description:SIMILARITY: Belongs to the cation transport ATPase (P-type) family.
Chr01G2 083.1	931	95	208	UniProt ID:Q59 PD2_C ANAL	1466	2	113	58/120 (48.33)	0.6	0.12	120	105	6.00E-24	gene=Chr 01G2083	Gene Symbol:SPA2|Host:Isolated from a wide variety of substrates including humans|Disease:invasive candidal disease|Description:CAUTION: The sequence shown here is derived from an EMBL/GenBank/DDBJ whole genome shotgun (WGS) entry which is preliminary data.
Chr01G2 092.1	803	385	507	UniProt ID:LAP 2_TRIE Q	495	229	341	40/124 (32.26)	0.51	0.1	124	49.7	5.00E-07	gene=Chr 01G2092	Gene Symbol:LAP2|Host:humans|Disease:Malabar itch|Description:FUNCTIO

															N: Extracellular aminopeptidase that releases a wide variety of amino acids from natural peptides and contributes to pathogenicity.
Chr01G2101.1	709	15	486	UniProt ID:Q9HG15_COLLN	746	3	457	112/497(22.54)	0.41	0.13	497	67.8	1.00E-12	gene=Chr01G2101	Gene Symbol:CLTA1|Host:Multiple genera of Fabaceae. Rare reports on other taxa|Disease:Leaf, stem and pod anthracnose|Description:SIMILARITY: Contains 1 Zn(2)-C6 fungal-type DNA-binding domain.
Chr01G2106.1	603	178	372	UniProt ID:Q0WXM3_FUSOX	663	250	451	54/213 (25.35)	0.43	0.14	213	56.6	2.00E-09	gene=Chr01G2106	Gene Symbol:FOW2|Host:Multiple genera in multiple families|Disease:Blights, wilts, rots of various sorts|Description:SIMILARITY: Contains 1 Zn(2)-C6 fungal-type DNA-binding domain.
Chr01G2	723	17	195	UniProt	514	260	421	49/188	0.44	0.19	188	54.3	1.00E-08	gene=Chr	Gene

108.1				ID:TUP1_CANAL				(26.06)						01G2108	Symbol:TUP1|Host:Isolated from a wide variety of substrates including humans|Disease:invasive candidal disease|Description:FUNCTION: Represses transcription by RNA polymerase II. Represses genes responsible for initiating filamentous growth and this repression is lifted under inducing environmental conditions.
Chr01G2109.1	1553	68	1542	UniProt ID:Q5A762_CANAL	1606	112	1603	457/1580(28.92)	0.47	0.12	1580	555	3.00E-170	gene=Chr01G2109	Gene Symbol:MLT1|Host:Isolated from a wide variety of substrates including humans|Disease:invasive candidal disease|Description:SIMILARITY: Belongs to the ABC transporter superfamily.
Chr01G2114.1	1153	50	1144	UniProt ID:Q9U	1606	400	1598	322/1233(26.	0.45	0.14	1233	390	7.00E-115	gene=Chr01G2114	Gene Symbol:MLT1|Host:Isolate

				W87_CANAL				12)							d from a wide variety of substrates including humans|Disease:invasive candidal disease|Description:SIMILARITY: Belongs to the ABC transporter superfamily.
Chr01G2118.1	341	1	317	UniProt ID:Q59ZI9_CANAL	329	11	327	206/317(64.98)	0.82	0	317	437	4.00E-154	gene=Chr01G2118	Gene Symbol:CKA2|Host:Isolated from a wide variety of substrates including humans|Disease:invasive candidal disease|Description:SIMILARITY: Belongs to the protein kinase superfamily.
Chr01G2131.1	346	5	215	UniProt ID:A4R2V5_MAGO7	214	3	212	90/215 (41.86)	0.57	0.04	215	145	4.00E-42	gene=Chr01G2131	Gene Symbol:MGG_02423|Host:Poaceae, especially important on Oryzae|Disease:Rice blast|Description:FUNCTION: Required for the retention of luminal

															endoplasmic reticulum proteins. Determines the specificity of the luminal ER protein retention system. Also required for normal vesicular traffic through the Golgi (By similarity).
Chr01G2136.1	806	1	686	UniProt ID:Q5APK9_CANAL	936	4	731	315/736(42.80)	0.61	0.08	736	579	0	gene=Chr01G2136	Gene Symbol:KEX2|Host:Isolated from a wide variety of substrates including humans|Disease:invasive candidal disease|Description:CAUTION: The sequence shown here is derived from an EMBL/GenBank/DDBJ whole genome shotgun (WGS) entry which is preliminary data.
Chr01G2138.1	165	47	121	UniProt ID:C5GSC7_AJEDR	288	210	284	25/75(33.33)	0.51	0	75	47.8	5.00E-08	gene=Chr01G2138	Gene Symbol:BDCG_07180|Host:humans|Disease:cutaneous Blastomyces dermatitidis

															infection|Description:FUNCTION: Component of the eukaryotic translation initiation factor 3 (eIF-3) complex (By similarity).
Chr01G2140.1	1489	19	1483	UniProt ID:Q96WW9_GIBPU	1491	12	1481	1069/1485(71.99)	0.83	0.02	1485	2252	0	gene=Chr01G2140	Gene Symbol:ABC1|Host:Various plant families|Disease:Tree canker, rot of potatoes (Samuels et al. 2006). Root rot (Booth 1973)|Description:SIMILARITY: Belongs to the ABC transporter superfamily.
Chr01G2157.1	822	22	360	UniProt ID:Q99324_SEPLY	803	82	485	134/412(32.52)	0.44	0.2	412	169	9.00E-45	gene=Chr01G2157	Gene Symbol:B2TOM|Host:Primarily tomato, Lycopersicon esculentum, also Solanum spp. and other Solanaceae|Disease:Leaf spot|Description:Unknown
Chr01G2161.1	524	268	495	UniProt ID:A4ULI6_MYCGR	517	255	513	52/261(19.92)	0.41	0.13	261	46.6	2.00E-06	gene=Chr01G2161	Gene Symbol:CYP51|Host:Triticum and possibly a few other

															grasses|Disease:Leaf spot or speckled leaf blotch of wheat|Description:COFACTOR: Heme group (By similarity).
Chr01G2166.1	302	24	165	UniProt ID:C0S8M8_PARBP	377	116	255	40/147 (27.21)	0.4	0.08	147	45.1	2.00E-06	gene=Chr01G2166	Gene Symbol:PABG_03630|Host:humans|Disease:Paracoccidioidomycosis|Description:Unknown
Chr01G2167.1	718	35	626	UniProt ID:A6N6J8_FUSOX	903	48	631	182/621(29.31)	0.44	0.11	621	201	2.00E-55	gene=Chr01G2167	Gene Symbol:CTF1|Host:Multiple genera in multiple families|Disease:Blights, wilts, rots of various sorts|Description:SIMILARITY: Contains 1 Zn(2)-C6 fungal-type DNA-binding domain.
Chr01G2172.1	597	77	528	UniProt ID:Q59RG0_CANAL	581	127	573	123/461(26.68)	0.49	0.05	461	142	4.00E-37	gene=Chr01G2172	Gene Symbol:NAG4|Host:Isolated from a wide variety of substrates including humans|Disease:invasive candidal disease|Description:CAUT

															ION: The sequence shown here is derived from an EMBL/GenBank/DDBJ whole genome shotgun (WGS) entry which is preliminary data.
Chr01G2173.1	795	365	513	UniProt ID:A6N6J8_FUSOX	903	367	520	39/165 (23.64)	0.45	0.16	165	55.5	8.00E-09	gene=Chr01G2173	Gene Symbol:CTF1|Host:Multiple genera in multiple families|Disease:Blights, wilts, rots of various sorts|Description:SIMILARITY: Contains 1 Zn(2)-C6 fungal-type DNA-binding domain.
Chr01G2177.1	1309	64	983	UniProt ID:O93841_9PEZI	914	11	909	395/942(41.93)	0.59	0.07	942	704	0	gene=Chr01G2177	Gene Symbol:CHIP6|Host:Multiple genera in multiple families|Disease:'Anthracnose of stems and leaves, dieback, root rot, leaf spot, blossom rot, fruit rot (dieback and ripe rot), seedling blight.' (Mordue 1971)|Description:Unknown

Chr01G2181.1	822	540	756	UniProt ID:Q3Y5V5_MAGGR	1321	1087	1315	66/233 (28.33)	0.46	0.09	233	68.6	1.00E-12	gene=Chr01G2181	Gene Symbol:ABC3|Host:Digitaria (Poaceae)|Disease:Leaf spot|Description:SIMILARITY: Belongs to the ABC transporter superfamily.
Chr01G2185.1	382	138	306	UniProt ID:O13328_MAGGR	2160	826	999	54/176 (30.68)	0.49	0.05	176	55.5	3.00E-09	gene=Chr01G2185	Gene Symbol:MAC1|Host:Digitaria (Poaceae)|Disease:Leaf spot|Description:Unknown
Chr01G2190.1	803	225	380	UniProt ID:Q0WXM3_FUSOX	663	268	424	46/158 (29.11)	0.51	0.02	158	79	4.00E-16	gene=Chr01G2190	Gene Symbol:FOW2|Host:Multiple genera in multiple families|Disease:Blights, wilts, rots of various sorts|Description:SIMILARITY: Contains 1 Zn(2)-C6 fungal-type DNA-binding domain.
Chr01G2196.1	560	10	456	UniProt ID:Q9P8L8_BOTFU	598	72	518	123/457(26.91)	0.46	0.04	457	149	1.00E-39	gene=Chr01G2196	Gene Symbol:BCMFS1|Host:Various plant families|Disease:Grey mould. Parasite or saprophyte|Description:Un

														known
Chr01G2197.1	566	9	38	UniProtID:Q5A4F3_CANAL	624	17	46	17/30(56.67)	0.67	0	30	48.1	8.00E-07	gene=Chr01G2197	Gene Symbol:ZCF37|Host:Isolated from a wide variety of substrates including humans|Disease:invasive candidal disease|Description:Unknown
Chr01G2207.1	236	32	235	UniProtID:Q59SD1_CANAL	248	33	247	75/223(33.63)	0.56	0.12	223	113	6.00E-31	gene=Chr01G2207	Gene Symbol:VPS28|Host:Isolated from a wide variety of substrates including humans|Disease:invasive candidal disease|Description:CAUTION: The sequence shown here is derived from an EMBL/GenBank/DDBJ whole genome shotgun (WGS) entry which is preliminary data.
Chr01G2209.1	2507	389	501	UniProtID:VPS27_CANAL	841	170	284	34/118(28.81)	0.45	0.07	118	68.2	5.00E-12	gene=Chr01G2209	Gene Symbol:VPS27|Host:Isolated from a wide variety of substrates including

															humans|Disease:invasive candidal disease|Description:FUNCTION: Component of the ESCRT-0 complex which is the sorting receptor for ubiquitinated cargo proteins at the multivesicular body (MVB) and recruits ESCRT-I to the MVB outer membrane (By similarity).
Chr01G2214.1	650	227	636	UniProt ID:Q96VB5_ALTAL	578	207	560	110/421(26.13)	0.41	0.19	421	74.3	6.00E-15	gene=Chr01G2214	Gene Symbol:AFT1-1|Host:Plant|Disease:Leaf spot, rots|Description:Unknown
Chr01G2222.1	475	17	102	UniProt ID:TUP1_CANAL	514	242	329	30/90(33.33)	0.5	0.07	90	45.1	6.00E-06	gene=Chr01G2222	Gene Symbol:TUP1|Host:Isolated from a wide variety of substrates including humans|Disease:invasive candidal disease|Description:FUNCTION: Represses transcription by RNA polymerase II. Represses

															genes responsible for initiating filamentous growth and this repression is lifted under inducing environmental conditions.
Chr01G2226.1	715	307	465	UniProt ID:Q5AM49_CANAL	1690	1528	1684	52/172 (30.23)	0.45	0.16	172	53.9	2.00E-08	gene=Chr01G2226	Gene Symbol:SNF2|Host:Isolated from a wide variety of substrates including humans|Disease:invasive candidal disease|Description:Unknown
Chr01G2231.1	1434	779	1428	UniProt ID:CFT1_CRYNB	1431	782	1430	168/669(25.11)	0.43	0.06	669	175	5.00E-45	gene=Chr01G2231	Gene Symbol:CFT1|Host:humans|Disease:cryptococcosis|Description:FUNCTION: RNA-binding component of the cleavage and polyadenylation factor (CPF) complex, which plays a key role in polyadenylation-dependent pre-mRNA 3'-end formation and cooperates with cleavage factors

															including the CFIA complex and NAB4/CFIB. Involved in poly(A) site recognition. May be involved in coupling transcription termination and mRNA 3'-end formation (By similarity).
Chr01G2239.1	1154	812	1052	UniProt ID:Q8TGW9_CANAL	1085	95	303	64/242 (26.45)	0.42	0.14	242	49.3	1.00E-06	gene=Chr01G2239	Gene Symbol:SSN6|Host:Isolated from a wide variety of substrates including humans|Disease:invasive candidal disease|Description:Unknown
Chr01G2264.1	603	19	74	UniProt ID:Q5A4F3_CANAL	624	3	51	19/56(33.93)	0.52	0.13	56	48.1	1.00E-06	gene=Chr01G2264	Gene Symbol:ZCF37|Host:Isolated from a wide variety of substrates including humans|Disease:invasive candidal disease|Description:Unknown
Chr01G2266.1	335	34	295	UniProt ID:Q92	2528	1851	2111	76/267 (28.46)	0.46	0.04	267	93.6	1.00E-21	gene=Chr01G2266	Gene Symbol:PKS1|Host:Zea

				217_C OCHE											mays|Disease:Southern leaf blight of maize|Description:Unknown
Chr01G2269.1	624	65	537	UniProt ID:C7GJ52_YEAS2	492	55	491	176/482(36.51)	0.55	0.11	482	290	5.00E-91	gene=Chr01G2269	Gene Symbol:MSY1|Host:humans|Disease:occasional infection|Description:SIMILARITY: Belongs to the class-I aminoacyl-tRNA synthetase family.
Chr01G2286.1	654	1	276	UniProt ID:A9YDN6_MAGGR	702	1	285	182/302(60.26)	0.68	0.14	302	284	4.00E-86	gene=Chr01G2286	Gene Symbol:SIG1|Host:Digitaria (Poaceae)|Disease:Leaf spot|Description:SUBCELLULAR LOCATION: Nucleus (By similarity).
Chr01G2288.1	474	149	471	UniProt ID:Q5A415_CANAL	361	72	357	74/335(22.09)	0.42	0.18	335	82.4	4.00E-18	gene=Chr01G2288	Gene Symbol:ARP2|Host:Isolated from a wide variety of substrates including humans|Disease:invasive candidal disease|Description:SIMILARITY: Belongs to the actin family.

Chr01G2 291.1	567	16	541	UniProt ID:Q5A NE1_C ANAL	748	32	550	215/53 2(40.4 1)	0.61	0.04	532	401	6.00E-13 1	gene=Chr 01G2291	Gene Symbol:SNF3|Host:Isolate d from a wide variety of substrates including humans|Disease:invasive candidal disease|Description:SIMIL ARITY: Belongs to the major facilitator superfamily. Sugar transporter (TC 2.A.1.1) family.
Chr01G2 293.1	625	536	618	UniProt ID:Q5A EK8_C ANAL	584	2	83	26/83(31.33)	0.57	0.01	83	51.6	9.00E-08	gene=Chr 01G2293	Gene Symbol:CAO19.260, ORF19.260|Host:Isolated from a wide variety of substrates including humans|Disease:invasive candidal disease|Description:SIMIL ARITY: Contains 1 cytochrome b5 heme-binding domain.
Chr01G2 295.1	400	1	400	UniProt ID:C1G 6E6_P	399	1	399	282/40 0(70.5 0)	0.86	0	400	588	0	gene=Chr 01G2295	Gene Symbol:PADG_02751|Ho st:humans|Disease:Parac

				ARBD											occidioidomycosis|Description:SIMILARITY: Belongs to the thiolase family.
Chr01G2298.1	895	46	662	UniProt ID:A6N6J8_FUSOX	903	45	617	138/649(21.26)	0.38	0.17	649	86.7	3.00E-18	gene=Chr01G2298	Gene Symbol:CTF1|Host:Multiple genera in multiple families|Disease:Blights, wilts, rots of various sorts|Description:SIMILARITY: Contains 1 Zn(2)-C6 fungal-type DNA-binding domain.
Chr01G2303.1	333	14	187	UniProt ID:C5GEJ4_AJEDR	1259	18	201	48/195 (24.62)	0.41	0.16	195	45.8	2.00E-06	gene=Chr01G2303	Gene Symbol:BDCG_02713|Host:humans|Disease:cutaneous Blastomyces dermatitidis infection|Description:Unknown
Chr01G2304.1	749	60	148	UniProt ID:A4QRN5_MAGO7	1015	474	563	31/90(34.44)	0.47	0.01	90	47	3.00E-06	gene=Chr01G2304	Gene Symbol:MGG_03530|Host:Poaceae, especially important on Oryzae|Disease:Rice blast|Description:Unknown

Chr01G2309.1	290	83	193	UniProt ID:Q32WF7_PHAND	266	99	208	37/113 (32.74)	0.48	0.04	113	49.3	7.00E-08	gene=Chr01G2309	Gene Symbol:MDH1|Host:Multiple genera of Poaceae and Blysmus compressus (Cyperaceae)|Disease:Glume blotch of wheat and other grasses|Description:Unknown
Chr01G2317.1	182	14	163	UniProt ID:Q9P8W9_CRYNE	162	4	146	95/150 (63.33)	0.77	0.05	150	189	1.00E-61	gene=Chr01G2317	Gene Symbol:CPA1|Host:humans|Disease:cryptococcosis|Description:FUNCTION: PPIases accelerate the folding of proteins (By similarity).
Chr01G2319.1	921	100	206	UniProt ID:Q5ANJ4_CANAL	743	161	251	39/107 (36.45)	0.51	0.15	107	62	1.00E-10	gene=Chr01G2319	Gene Symbol:TEC1|Host:Isolated from a wide variety of substrates including humans|Disease:invasive candidal disease|Description:CAUTION: The sequence shown here is derived from an EMBL/GenBank/DDBJ

															whole genome shotgun (WGS) entry which is preliminary data.
Chr01G2320.1	864	636	815	UniProt ID:Q4WNM3_ASPFU	740	49	236	67/194 (34.54)	0.52	0.1	194	94	1.00E-20	gene=Chr01G2320	Gene Symbol:AFUA_4G06250|Host:humans|Disease:infection|Description:CAUTION: The sequence shown here is derived from an EMBL/GenBank/DDBJ whole genome shotgun (WGS) entry which is preliminary data.
Chr01G2338.1	601	231	572	UniProt ID:Q8J214_USTMD	592	236	568	138/348(39.66)	0.59	0.06	348	280	4.00E-86	gene=Chr01G2338	Gene Symbol:CRU1|Host:Euchlaena spp., Zea spp. (Poaceae)|Disease:Smut. Corn smut|Description:Unknown
Chr01G2340.1	310	15	308	UniProt ID:Q6TFC7_ASPFM	349	63	346	110/297(37.04)	0.56	0.05	297	208	5.00E-65	gene=Chr01G2340	Gene Symbol:NULL|Host:humans|Disease:infection|Description:Unknown
Chr01G2346.1	1276	767	891	UniProt ID:SET	1040	898	1014	40/125 (32.00)	0.47	0.06	125	57.4	4.00E-09	gene=Chr01G2346	Gene Symbol:SET1|Host:Isolate

				1_CANAL											d from a wide variety of substrates including humans|Disease:invasive candidal disease|Description:FUNCTION: Catalytic component of the COMPASS (Set1C) complex that specifically mono-, di- and trimethylates histone H3 to form H3K4me1/2/3, which subsequently plays a role in telomere length maintenance, transcription elongation regulation and pathogenesis of invasive candidiasis.
Chr01G2355.1	525	214	510	UniProt ID:Q59KY8_CANAL	314	9	308	116/303(38.28)	0.6	0.03	303	231	1.00E-71	gene=Chr01G2355	Gene Symbol:SIT4|Host:Isolated from a wide variety of substrates including humans|Disease:invasive candidal disease|Description:CATALYTIC ACTIVITY: A

														phosphoprotein + H(2)O = a protein + phosphate.
Chr01G2358.1	1125	797	1075	UniProt ID:TUP1_CANAL	514	260	510	68/284 (23.94)	0.42	0.13	284	50.4	4.00E-07	gene=Chr01G2358	Gene Symbol:TUP1|Host:Isolated from a wide variety of substrates including humans|Disease:invasive candidal disease|Description:FUNCTION: Represses transcription by RNA polymerase II. Represses genes responsible for initiating filamentous growth and this repression is lifted under inducing environmental conditions.
Chr01G2367.1	704	173	595	UniProt ID:F2QYD1_PICP7	758	202	584	123/456(26.97)	0.42	0.23	456	106	7.00E-25	gene=Chr01G2367	Gene Symbol:MAK5|Host:humans|Disease:occasional infection|Description:SIMILARITY: Belongs to the DEAD box helicase family.
Chr01G2370.1	299	26	283	UniProt ID:Q9P4U9_A	296	20	276	136/260(52.31)	0.67	0.02	260	267	1.00E-88	gene=Chr01G2370	Gene Symbol:AKT3-1|Host:Plant|Disease:Leaf spot,

				LTAL											rots|Description:Unknown
Chr01G2374.1	1165	246	587	UniProt ID:Q9Y880_COCCA	880	26	316	133/344(38.66)	0.54	0.16	344	238	3.00E-66	gene=Chr01G2374	Gene Symbol:SNF1|Host:Corn, Zea mays, sometimes on Sorghum (Poaceae) and various other plant families|Disease:Northern corn leaf spot, ear and kernel rot|Description:Unknown
Chr01G2383.1	608	126	542	UniProt ID:Q59VF3_CANAL	1813	17	417	114/435(26.21)	0.43	0.12	435	101	3.00E-23	gene=Chr01G2383	Gene Symbol:"DUR1,2"|Host:Isolated from a wide variety of substrates including humans|Disease:invasive candidal disease|Description:CAUTION: The sequence shown here is derived from an EMBL/GenBank/DDBJ whole genome shotgun (WGS) entry which is preliminary data.
Chr01G2385.1	483	424	465	UniProt ID:Q5EGQ1_C	392	346	392	31/47(65.96)	0.74	0.11	47	72.8	7.00E-15	gene=Chr01G2385	Gene Symbol:BWC2|Host:humans|Disease:cryptococcosis

				RYNE										|Description:Unknown
Chr01G2390.1	1556	1053	1534	UniProt ID:Q9C1Q7_ASPFM	708	306	699	132/486(27.16)	0.45	0.2	486	129	2.00E-31	gene=Chr01G2390	Gene Symbol:NULL|Host:humans|Disease:infection|Description:SIMILARITY: Contains 1 histidine kinase domain.
Chr01G2400.1	655	1	631	UniProt ID:PESC_CANAL	587	1	562	258/637(40.50)	0.59	0.13	637	424	7.00E-141	gene=Chr01G2400	Gene Symbol:NOP7|Host:Isolated from a wide variety of substrates including humans|Disease:invasive candidal disease|Description:FUNCTION: Component of the NOP7 complex, which is required for maturation of the 25S and 5.8S ribosomal RNAs and formation of the 60S ribosome (By similarity). Required for the transition from hyphal to yeast growth.
Chr01G2402.1	1180	92	461	UniProt ID:P87	968	4	367	150/378(39.6	0.6	0.06	378	244	7.00E-68	gene=Chr01G2402	Gene Symbol:KIN2|Host:Euchla

				199_USTMD				8)							ena spp., Zea spp. (Poaceae)|Disease:Smut. Corn smut|Description:SIMILARITY: Belongs to the kinesin-like protein family.
Chr01G2407.1	699	118	483	UniProt ID:Q59YF3_CANAL	986	154	479	87/380 (22.89)	0.41	0.18	380	82.4	3.00E-17	gene=Chr01G2407	Gene Symbol:INP51|Host:Isolated from a wide variety of substrates including humans|Disease:invasive candidal disease|Description:CAUTION: The sequence shown here is derived from an EMBL/GenBank/DDBJ whole genome shotgun (WGS) entry which is preliminary data.
Chr01G2408.1	221	22	187	UniProt ID:Q59X89_CANAL	216	12	181	104/171(60.82)	0.73	0.04	171	204	1.00E-66	gene=Chr01G2408	Gene Symbol:"CAO19.13721, CAO19.589, CAO19.6364, CAO19.8221, O|Host:Isolated from a wide variety of substrates

															including humans|Disease:invasive candidal disease|Description:SIMILARITY: Belongs to the small GTPase superfamily. Rab family.
Chr01G2411.1	605	43	591	UniProt ID:Q9P8L8_BOTFU	598	23	594	178/578(30.80)	0.5	0.06	578	273	2.00E-83	gene=Chr01G2411	Gene Symbol:BCMFS1|Host:Various plant families|Disease:Grey mould. Parasite or saprophyte|Description:Unknown
Chr01G2412.1	459	3	366	UniProt ID:Q9Y784_MAGGR	631	4	370	113/382(29.58)	0.5	0.09	382	155	3.00E-42	gene=Chr01G2412	Gene Symbol:PTH11|Host:Digitaria (Poaceae)|Disease:Leaf spot|Description:Unknown
Chr01G2413.1	336	39	267	UniProt ID:Q32WF7_PHAND	266	23	244	69/238(28.99)	0.42	0.11	238	54.3	2.00E-09	gene=Chr01G2413	Gene Symbol:MDH1|Host:Multiple genera of Poaceae and Blysmus compressus (Cyperaceae)|Disease:Glume blotch of wheat and other

																grasses|Description:Unknown
Chr01G2415.1	431	12	348	UniProt ID:A4UC81_MAGO7	376	16	374	159/366(43.44)	0.61	0.1	366	280	7.00E-91	gene=Chr01G2415		Gene Symbol:MGG_10702|Host:Poaceae, especially important on Oryzae|Disease:Rice blast|Description:Unknown
Chr01G2416.1	571	7	447	UniProt ID:Q9P8L8_BOTFU	598	28	457	100/459(21.79)	0.4	0.1	459	52	6.00E-08	gene=Chr01G2416		Gene Symbol:BCMFS1|Host:Various plant families|Disease:Grey mould. Parasite or saprophyte|Description:Unknown
Chr01G2425.1	692	2	347	UniProt ID:Q0WXM3_FUSOX	663	115	441	72/364(19.78)	0.37	0.15	364	68.6	5.00E-13	gene=Chr01G2425		Gene Symbol:FOW2|Host:Multiple genera in multiple families|Disease:Blights, wilts, rots of various sorts|Description:SIMILARITY: Contains 1 Zn(2)-C6 fungal-type DNA-binding domain.
Chr01G2	412	1	412	UniProt	416	1	416	208/42	0.66	0.05	425	414	1.00E-14	gene=Chr		Gene

430.1				ID:MCP AL_AR TOC				5(48.9 4)					2	01G2430	Symbol:MCYG_01475|Ho st:humans, reptiles|Disease:dermatop hytoses|Description:FUNC TION: Extracellular metalloprotease that contributes to pathogenicity (By similarity).
Chr01G2 434.1	386	7	385	UniProt ID:Q6A 2T2_B OTFU	391	5	375	98/393 (24.94)	0.44	0.09	393	110	6.00E-28	gene=Chr 01G2434	Gene Symbol:BTP1|Host:Variou s plant families|Disease:Grey mould. Parasite or saprophyte|Description:Un known
Chr01G2 435.1	498	55	497	UniProt ID:A0S T43_C ERNC	459	1	456	123/46 3(26.5 7)	0.43	0.06	463	156	6.00E-43	gene=Chr 01G2435	Gene Symbol:CTB5|Host:Numer ous taxa in Solanaceae|Disease:Leaf spot|Description:Unknown
Chr01G2 436.1	485	24	76	UniProt ID:Q59 MD2_C ANAL	843	768	819	22/53(41.51)	0.55	0.02	53	47	2.00E-06	gene=Chr 01G2436	Gene Symbol:UME6|Host:Isolat ed from a wide variety of substrates including humans|Disease:invasive

															candidal disease|Description:CAUTION: The sequence shown here is derived from an EMBL/GenBank/DDBJ whole genome shotgun (WGS) entry which is preliminary data.
Chr01G2438.1	629	161	425	UniProt ID:Q99324_SEPLY	803	108	361	71/276 (25.72)	0.38	0.12	276	54.7	1.00E-08	gene=Chr01G2438	Gene Symbol:B2TOM|Host:Primarily tomato, Lycopersicon esculentum, also Solanum spp. and other Solanaceae|Disease:Leaf spot|Description:Unknown
Chr01G2452.1	443	36	425	UniProt ID:Q96VZ3_FUSOX	455	46	434	116/396(29.29)	0.45	0.03	396	147	3.00E-40	gene=Chr01G2452	Gene Symbol:PGX1|Host:Multiple genera in multiple families|Disease:Blights, wilts, rots of various sorts|Description:SIMILARITY: Belongs to the glycosyl hydrolase 28 family.
Chr01G2454.1	538	54	364	UniProt ID:Q5X	574	61	370	109/329(33.1	0.47	0.11	329	143	6.00E-38	gene=Chr01G2454	Gene Symbol:LIP1|Host:Various

			TQ4_BOTFU				3)							plant families|Disease:Grey mould. Parasite or saprophyte|Description:Unknown
Chr01G2455.1	608	10	467	UniProt ID:Q59KS4_CANAL	800	12	437	155/466(33.26)	0.52	0.1	466	261	2.00E-77	gene=Chr01G2455	Gene Symbol:CNH1|Host:Isolated from a wide variety of substrates including humans|Disease:invasive candidal disease|Description:CAUTION: The sequence shown here is derived from an EMBL/GenBank/DDBJ whole genome shotgun (WGS) entry which is preliminary data.
Chr01G2460.1	363	5	223	UniProt ID:A4QVF8_MAGO7	339	2	214	74/220(33.64)	0.54	0.04	220	121	6.00E-32	gene=Chr01G2460	Gene Symbol:MGG_04556|Host:Poaceae, especially important on Oryzae|Disease:Rice blast|Description:COFACTOR: Zinc (By similarity).
Chr01G2	623	85	483	UniProt	589	107	503	216/41	0.66	0.1	419	395	4.00E-13	gene=Chr	Gene

467.1				ID:Q59<br>Z94_C<br>ANAL				9(51.5<br>5)					0	01G2467	Symbol:RCK2|Host:Isolat<br>ed from a wide variety of<br>substrates including<br>humans|Disease:invasive<br>candidal<br>disease|Description:SIMIL<br>ARITY: Contains 1 protein<br>kinase domain.
Chr01G2<br>473.1	794	11	60	UniProt<br>ID:Q9P<br>3Z8_C<br>YBJA	345	20	69	23/50(<br>46.00)	0.58	0	50	60.8	1.00E-10	gene=Chr<br>01G2473	Gene<br>Symbol:MIG1|Host:human<br>s|Disease:Chronic urinary<br>tract<br>infection|Description:Unkn<br>own
Chr01G2<br>474.1	520	312	490	UniProt<br>ID:A4U<br>LJ2_M<br>YCGR	515	299	503	54/210<br>(25.71)	0.4	0.17	210	48.1	7.00E-07	gene=Chr<br>01G2474	Gene<br>Symbol:CYP51|Host:Tritic<br>um and possibly a few<br>other<br>grasses|Disease:Leaf spot<br>or speckled leaf blotch of<br>wheat|Description:COFAC<br>TOR: Heme group (By<br>similarity).
Chr01G2<br>475.1	440	6	359	UniProt<br>ID:Q2I0<br>M6_CE	871	458	820	93/389<br>(23.91)	0.38	0.16	389	61.2	5.00E-11	gene=Chr<br>01G2475	Gene<br>Symbol:CTB3|Host:Numer<br>ous taxa in

				RNC											Solanaceae|Disease:Leaf spot|Description:Unknown
Chr01G2478.1	302	22	183	UniProt ID:A4RGG9_MAGO7	286	50	203	44/163 (26.99)	0.42	0.06	163	43.1	9.00E-06	gene=Chr01G2478	Gene Symbol:MGG_00056|Host:Poaceae, especially important on Oryzae|Disease:Rice blast|Description:SIMILARITY: Belongs to the short-chain dehydrogenases/reductases (SDR) family.
Chr01G2479.1	402	1	399	UniProt ID:ORYZ_ASPFU	403	1	401	228/407(56.02)	0.7	0.03	407	434	4.00E-151	gene=Chr01G2479	Gene Symbol:ALP1|Host:humans|Disease:infection|Description:FUNCTION: Secreted alkaline protease that allows assimilation of proteinaceous substrates. Acts as a significant virulence factor in invasive aspergillosis. Involved in immune evasion from the human and mice complement systems

															during infection. Efficiently cleaves important components of the complement cascade such as such as C3, C4, C5, and C1q, as well as IgG, which leads to down-regulation of complement activation at the hyphal surface.
Chr01G2480.1	505	37	495	UniProt ID:Q01446_NECHA	459	16	454	151/467(32.33)	0.52	0.08	467	236	6.00E-72	gene=Chr01G2480	Gene Symbol:MAK1|Host:Trees of various plant families|Disease:Fruit rot, stem rot|Description:Unknown
Chr01G2489.1	644	317	542	UniProt ID:C6KEF4_MYCGR	927	643	865	61/243 (25.10)	0.45	0.15	243	83.2	2.00E-17	gene=Chr01G2489	Gene Symbol:STE11|Host:Triticum and possibly a few other grasses|Disease:Leaf spot or speckled leaf blotch of wheat|Description:SIMILARITY: Contains 1 SAM (sterile alpha motif) domain.

Chr01G2494.1	579	166	520	UniProt ID:Q59RG0_CANAL	581	168	520	97/369 (26.29)	0.44	0.08	369	72.4	2.00E-14	gene=Chr01G2494	Gene Symbol:NAG4|Host:Isolated from a wide variety of substrates including humans|Disease:invasive candidal disease|Description:CAUTION: The sequence shown here is derived from an EMBL/GenBank/DDBJ whole genome shotgun (WGS) entry which is preliminary data.
Chr01G2497.1	239	3	217	UniProt ID:Q04701_FUSSO	242	4	218	105/217(48.39)	0.63	0.02	217	190	3.00E-60	gene=Chr01G2497	Gene Symbol:PELA|Host:Multiple plant families. Some strains may cause infections in humans|Disease:Saprobe, facultative pathogen|Description:Unknown
Chr01G2507.1	973	266	366	UniProt ID:Q9HFV9_CRYNE	482	348	438	36/101 (35.64)	0.5	0.1	101	48.5	1.00E-06	gene=Chr01G2507	Gene Symbol:PKR1|Host:humans|Disease:cryptococcosis|Description:Unknown

Chr01G2511.1	581	104	554	UniProt ID:Q5ABU7_CANAL	564	109	564	127/460(27.61)	0.47	0.03	460	163	3.00E-44	gene=Chr01G2511	Gene Symbol:MDR1|Host:Isolated from a wide variety of substrates including humans|Disease:invasive candidal disease|Description:Unknown
Chr01G2516.1	426	2	334	UniProt ID:A4UC81_MAGO7	376	36	367	96/342(28.07)	0.49	0.06	342	152	2.00E-42	gene=Chr01G2516	Gene Symbol:MGG_10702|Host:Poaceae, especially important on Oryzae|Disease:Rice blast|Description:Unknown
Chr01G2517.1	452	3	403	UniProt ID:Q5XTQ4_BOTFU	574	34	469	124/455(27.25)	0.41	0.16	455	95.9	3.00E-22	gene=Chr01G2517	Gene Symbol:LIP1|Host:Various plant families|Disease:Grey mould. Parasite or saprophyte|Description:Unknown
Chr01G2529.1	686	146	658	UniProt ID:A4R566_MAGO7	550	37	533	200/529(37.81)	0.54	0.09	529	339	4.00E-108	gene=Chr01G2529	Gene Symbol:MGG_04128|Host:Poaceae, especially important on

															Oryzae|Disease:Rice blast|Description:Unknown
Chr01G2530.1	496	91	287	UniProt ID:Q59WF4_CANAL	597	118	310	49/198 (24.75)	0.46	0.03	198	52.8	3.00E-08	gene=Chr01G2530	Gene Symbol:MNN2|Host:Isolated from a wide variety of substrates including humans|Disease:invasive candidal disease|Description:CAUTION: The sequence shown here is derived from an EMBL/GenBank/DDBJ whole genome shotgun (WGS) entry which is preliminary data.
Chr01G2542.1	761	548	758	UniProt ID:D1MYV6_MAGGR	568	142	355	53/219 (24.20)	0.43	0.06	219	72	5.00E-14	gene=Chr01G2542	Gene Symbol:CBL1|Host:Digitaria (Poaceae)|Disease:Leaf spot|Description:SIMILARITY: Contains 3 chitin-binding type-1 domains.
Chr01G2544.1	305	271	305	UniProt ID:O59937_F	384	19	54	20/36(55.56)	0.69	0.03	36	47	6.00E-07	gene=Chr01G2544	Gene Symbol:XYL3|Host:Multiple genera in multiple

				USOX											families|Disease:Blights, wilts, rots of various sorts|Description:SIMILARITY: Belongs to the glycosyl hydrolase 10 (cellulase F) family.
Chr01G2546.1	521	105	493	UniProt ID:Q5ANE1_CANAL	748	95	503	102/413(24.70)	0.46	0.07	413	108	6.00E-26	gene=Chr01G2546	Gene Symbol:SNF3|Host:Isolated from a wide variety of substrates including humans|Disease:invasive candidal disease|Description:SIMILARITY: Belongs to the major facilitator superfamily. Sugar transporter (TC 2.A.1.1) family.
Chr01G2549.1	382	24	339	UniProt ID:Q75ZG3_ALTAL	366	29	355	93/334(27.84)	0.49	0.07	334	119	5.00E-31	gene=Chr01G2549	Gene Symbol:AFTS1|Host:Plant|Disease:Leaf spot, rots|Description:Unknown
Chr01G2550.1	511	52	416	UniProt ID:Q9Y784_MAGGR	631	38	416	100/385(25.97)	0.47	0.07	385	134	1.00E-34	gene=Chr01G2550	Gene Symbol:PTH11|Host:Digitaria (Poaceae)|Disease:Leaf

Chr01G2558.1	904	58	294	UniProt ID:SU11A_COCP7	403	66	282	73/244 (29.92)	0.41	0.14	244	68.6	4.00E-13	gene=Chr01G2558	spot|Description:Unknown Gene Symbol:CPC735_035780|Host:humans|Disease:coccidiomycosis|Description: FUNCTION: Secreted subtilisin-like serine protease with keratinolytic activity that contributes to pathogenicity (By similarity).
Chr01G2559.1	663	2	34	UniProt ID:Q5A4F3_CANAL	624	14	46	17/33(51.52)	0.7	0	33	51.2	1.00E-07	gene=Chr01G2559	Gene Symbol:ZCF37|Host:Isolated from a wide variety of substrates including humans|Disease:invasive candidal disease|Description:Unknown
Chr01G2560.1	499	47	480	UniProt ID:Q5ANE1_CANAL	748	49	495	135/465(29.03)	0.46	0.11	465	146	1.00E-38	gene=Chr01G2560	Gene Symbol:SNF3|Host:Isolated from a wide variety of substrates including humans|Disease:invasive candidal disease|Description:SIMIL

															ARITY: Belongs to the major facilitator superfamily. Sugar transporter (TC 2.A.1.1) family.
Chr01G2561.1	1486	925	1474	UniProt ID:Q5A762_CANAL	1606	1044	1598	167/591(28.26)	0.48	0.13	591	219	1.00E-58	gene=Chr01G2561	Gene Symbol:MLT1|Host:Isolated from a wide variety of substrates including humans|Disease:invasive candidal disease|Description:SIMILARITY: Belongs to the ABC transporter superfamily.
Chr01G2567.1	360	1	351	UniProt ID:Q6TFC7_ASPFM	349	1	346	119/356(33.43)	0.47	0.04	356	166	2.00E-48	gene=Chr01G2567	Gene Symbol:NULL|Host:humans|Disease:infection|Description:Unknown
Chr01G2580.1	570	20	63	UniProt ID:Q5A4F3_CANAL	624	3	46	20/44(45.45)	0.66	0	44	56.6	2.00E-09	gene=Chr01G2580	Gene Symbol:ZCF37|Host:Isolated from a wide variety of substrates including humans|Disease:invasive candidal disease|Description:Unkn

														own
Chr01G2583.1	1340	559	758	UniProt ID:O59928_HYPVI	430	59	299	70/259 (27.03)	0.39	0.3	259	58.2	2.00E-09	gene=Chr01G2583	Gene Symbol:NULL|Host:humans|Disease:infection|Description:SIMILARITY: Belongs to the glycosyl hydrolase 18 family.
Chr01G2586.1	369	22	353	UniProt ID:Q9Y784_MAGGR	631	125	432	80/340 (23.53)	0.42	0.12	340	69.7	6.00E-14	gene=Chr01G2586	Gene Symbol:PTH11|Host:Digitaria (Poaceae)|Disease:Leaf spot|Description:Unknown
Chr01G2587.1	413	246	397	UniProt ID:Q2I0M6_CERNC	871	247	411	49/168 (29.17)	0.49	0.11	168	82	1.00E-17	gene=Chr01G2587	Gene Symbol:CTB3|Host:Numerous taxa in Solanaceae|Disease:Leaf spot|Description:Unknown
Chr01G2590.1	371	65	368	UniProt ID:Q6TFC7_ASPFM	349	49	349	99/308 (32.14)	0.5	0.04	308	164	1.00E-47	gene=Chr01G2590	Gene Symbol:NULL|Host:humans|Disease:infection|Description:Unknown
Chr01G2591.1	418	75	399	UniProt ID:Q2I0M6_CERNC	871	86	412	90/346 (26.01)	0.42	0.12	346	80.9	3.00E-17	gene=Chr01G2591	Gene Symbol:CTB3|Host:Numerous taxa in Solanaceae|Disease:Leaf spot|Description:Unknown

Chr01G2594.1	1062	682	915	UniProt ID:Q6ZX14_MAGGR	4034	3717	3930	66/240 (27.50)	0.41	0.13	240	56.6	7.00E-09	gene=Chr01G2594	Gene Symbol:ACE1|Host:Digitaria (Poaceae)|Disease:Leaf spot|Description:Unknown
Chr01G2596.1	914	377	568	UniProt ID:Q0WXM3_FUSOX	663	254	445	54/197 (27.41)	0.47	0.05	197	71.2	1.00E-13	gene=Chr01G2596	Gene Symbol:FOW2|Host:Multiple genera in multiple families|Disease:Blights, wilts, rots of various sorts|Description:SIMILARITY: Contains 1 Zn(2)-C6 fungal-type DNA-binding domain.
Chr01G2604.1	718	49	81	UniProt ID:Q5A4F3_CANAL	624	14	46	20/33(60.61)	0.76	0	33	55.5	7.00E-09	gene=Chr01G2604	Gene Symbol:ZCF37|Host:Isolated from a wide variety of substrates including humans|Disease:invasive candidal disease|Description:Unknown
Chr01G2605.1	302	51	297	UniProt ID:Q32WF7_PHAND	266	15	261	81/253 (32.02)	0.48	0.05	253	110	4.00E-29	gene=Chr01G2605	Gene Symbol:MDH1|Host:Multiple genera of Poaceae and Blysmus compressus (Cyperaceae)|Disease:Glu

														me blotch of wheat and other grasses|Description:Unknown
Chr01G2612.1	402	5	400	UniProt ID:Q5APD4_CANAL	513	6	412	179/410(43.66)	0.61	0.04	410	349	5.00E-116	gene=Chr01G2612	Gene Symbol:CFA1|Host:Isolated from a wide variety of substrates including humans|Disease:invasive candidal disease|Description:CAUTION: The sequence shown here is derived from an EMBL/GenBank/DDBJ whole genome shotgun (WGS) entry which is preliminary data.
Chr01G2627.1	514	43	372	UniProt ID:Q5XTQ4_BOTFU	574	55	386	116/355(32.68)	0.47	0.14	355	146	6.00E-39	gene=Chr01G2627	Gene Symbol:LIP1|Host:Various plant families|Disease:Grey mould. Parasite or saprophyte|Description:Unknown
Chr01G2629.1	566	79	558	UniProt ID:Q5A	748	51	531	120/499(24.0	0.43	0.07	499	112	6.00E-27	gene=Chr01G2629	Gene Symbol:SNF3|Host:Isolate

				NE1_CANAL				5)							d from a wide variety of substrates including humans|Disease:invasive candidal disease|Description:SIMILARITY: Belongs to the major facilitator superfamily. Sugar transporter (TC 2.A.1.1) family.
Chr01G2630.1	876	451	494	UniProt ID:SUB10_TRIVH	522	324	367	23/44(52.27)	0.73	0	44	47.8	2.00E-06	gene=Chr01G2630	Gene Symbol:SUB10|Host:humans|Disease:infection|Description:FUNCTION: Secreted subtilisin-like serine protease with keratinolytic activity that contributes to pathogenicity (By similarity).
Chr01G2632.1	380	10	302	UniProt ID:Q6A2T2_BOTFU	391	10	306	94/305(30.82)	0.47	0.07	305	122	6.00E-32	gene=Chr01G2632	Gene Symbol:BTP1|Host:Various plant families|Disease:Grey mould. Parasite or saprophyte|Description:Un

															known
Chr01G2633.1	461	31	376	UniProt ID:A4UC81_MAGO7	376	16	371	129/361(35.73)	0.54	0.06	361	206	3.00E-62	gene=Chr01G2633	Gene Symbol:MGG_10702|Host:Poaceae, especially important on Oryzae|Disease:Rice blast|Description:Unknown
Chr01G2634.1	333	25	201	UniProt ID:A4R3I5_MAGO7	400	104	274	46/183(25.14)	0.45	0.1	183	47	7.00E-07	gene=Chr01G2634	Gene Symbol:"MGG_11993, MGG_12837, MGG_13052"|Host:Poaceae, especially important on Oryzae|Disease:Rice blast|Description:Unknown
Chr01G2635.1	1234	71	1201	UniProt ID:A4RGC8_MAGO7	1158	31	1132	458/1162(39.41)	0.57	0.08	1162	780	0	gene=Chr01G2635	Gene Symbol:MGG_11671|Host:Poaceae, especially important on Oryzae|Disease:Rice blast|Description:SIMILARITY: Contains 1 reverse transcriptase domain.
Chr01G2638.1	285	139	279	UniProt ID:Q8J	416	197	321	41/154(26.62)	0.37	0.27	154	44.7	3.00E-06	gene=Chr01G2638	Gene Symbol:MK2|Host:outcros

				0E1_C LAPU											sing species|Disease:ergotism|Description:CATALYTIC ACTIVITY: ATP + a protein = ADP + a phosphoprotein.
Chr01G2 640.1	1191	584	850	UniProt ID:Q9C 443_C RYNE	2271	1033	1297	71/273 (26.01)	0.45	0.05	273	79.7	7.00E-16	gene=Chr 01G2640	Gene Symbol:CAC1|Host:humans|Disease:cryptococcosis|Description:Unknown
Chr01G2 641.1	727	29	87	UniProt ID:A8Q JI7_FU SOX	938	70	124	22/59(37.29)	0.56	0.07	59	50.1	4.00E-07	gene=Chr 01G2641	Gene Symbol:XLNR|Host:Multiple genera in multiple families|Disease:Blights, wilts, rots of various sorts|Description:SIMILARITY: Contains 1 Zn(2)-C6 fungal-type DNA-binding domain.
Chr01G2 643.1	527	288	495	UniProt ID:A4U LJ0_M YCGR	518	273	505	49/234 (20.94)	0.36	0.12	234	58.5	4.00E-10	gene=Chr 01G2643	Gene Symbol:CYP51|Host:Triticum and possibly a few other grasses|Disease:Leaf spot or speckled leaf blotch of wheat|Description:COFAC

														TOR: Heme group (By similarity).
Chr01G2646.1	445	7	428	UniProt ID:Q5GFD3_PHAND	437	11	434	114/453(25.17)	0.44	0.13	453	107	2.00E-26	gene=Chr01G2646	Gene Symbol:NULL|Host:Multiple genera of Poaceae and Blysmus compressus (Cyperaceae)|Disease:Glume blotch of wheat and other grasses|Description:Unknown
Chr01G2652.1	501	336	464	UniProt ID:A4ULI6_MYCGR	517	355	505	36/156 (23.08)	0.44	0.21	156	45.1	6.00E-06	gene=Chr01G2652	Gene Symbol:CYP51|Host:Triticum and possibly a few other grasses|Disease:Leaf spot or speckled leaf blotch of wheat|Description:COFACTOR: Heme group (By similarity).
Chr01G2653.1	414	165	393	UniProt ID:Q59MV8_CANAL	398	176	385	67/236 (28.39)	0.46	0.14	236	62.4	1.00E-11	gene=Chr01G2653	Gene Symbol:SAP2|Host:Isolated from a wide variety of substrates including humans|Disease:invasive candidal

																disease|Description:SIMILARITY: Belongs to the peptidase A1 family.
Chr01G2654.1	584	19	580	UniProt ID:SED2_ASPFU	602	20	596	236/593(39.80)	0.53	0.08	593	370	2.00E-120	gene=Chr01G2654	Gene Symbol:SED2|Host:humans|Disease:infection|Description:FUNCTION: Secreted tripeptidyl-peptidase which degrades proteins at acidic pHs and is involved in virulence.
Chr01G2659.1	587	27	565	UniProt ID:Q9C477_CLAFU	566	4	547	400/545(73.39)	0.83	0.01	545	845	0	gene=Chr01G2659	Gene Symbol:CAT-1|Host:Lycopersicon esculentum (Solanaceae)|Disease:Leaf mold of tomato|Description:FUNCTION: Occurs in almost all aerobically respiring organisms and serves to protect cells from the toxic effects of hydrogen peroxide (By similarity).
Chr01G2681.1	525	157	503	UniProt ID:Q59	597	171	560	90/417(21.58)	0.37	0.23	417	58.5	4.00E-10	gene=Chr01G2681	Gene Symbol:MNN2|Host:Isolat

				WF4_C ANAL										ed from a wide variety of substrates including humans|Disease:invasive candidal disease|Description:CAUTION: The sequence shown here is derived from an EMBL/GenBank/DDBJ whole genome shotgun (WGS) entry which is preliminary data.
Chr01G2684.1	292	4	272	UniProt ID:Q9Y7E5_CANAL	328	8	312	106/307(34.53)	0.52	0.13	307	182	1.00E-55	gene=Chr01G2684	Gene Symbol:SPT3|Host:Isolated from a wide variety of substrates including humans|Disease:invasive candidal disease|Description:Unknown
Chr01G2688.1	434	2	386	UniProt ID:Q5GFD3_PHAND	437	11	417	111/417(26.62)	0.41	0.1	417	124	2.00E-32	gene=Chr01G2688	Gene Symbol:NULL|Host:Multiple genera of Poaceae and Blysmus compressus (Cyperaceae)|Disease:Glume blotch of wheat and other

															grasses|Description:Unknown
Chr01G2689.1	496	238	474	UniProt ID:A4ULJ0_MYCGR	518	243	516	62/274 (22.63)	0.42	0.14	274	60.5	8.00E-11	gene=Chr01G2689	Gene Symbol:CYP51|Host:Triticum and possibly a few other grasses|Disease:Leaf spot or speckled leaf blotch of wheat|Description:COFACTOR: Heme group (By similarity).
Chr01G2691.1	645	374	610	UniProt ID:A4R9C7_MAGO7	1305	298	510	60/249 (24.10)	0.42	0.19	249	50.8	2.00E-07	gene=Chr01G2691	Gene Symbol:MGG_13324|Host:Poaceae, especially important on Oryzae|Disease:Rice blast|Description:Unknown
Chr01G2692.1	389	179	316	UniProt ID:Q5APR8_CANAL	976	759	887	43/140 (30.71)	0.49	0.09	140	65.1	3.00E-12	gene=Chr01G2692	Gene Symbol:CLA4|Host:Isolated from a wide variety of substrates including humans|Disease:invasive candidal disease|Description:CATALYTIC ACTIVITY: ATP + a

															protein = ADP + a phosphoprotein.
Chr01G2694.1	1521	28	1511	UniProt ID:O13407_MAGGR	1619	33	1542	1098/1516(72.43)	0.82	0.03	1516	2232	0	gene=Chr01G2694	Gene Symbol:ABC1|Host:Digitaria (Poaceae)|Disease:Leaf spot|Description:SIMILARITY: Belongs to the ABC transporter superfamily.
Chr01G2702.1	418	49	418	UniProt ID:Q0QWD8_LEPMC	359	1	358	199/370(53.78)	0.67	0.03	370	379	1.00E-129	gene=Chr01G2702	Gene Symbol:THIOL|Host:Brassica spp. and other Brassicaceae|Disease:Black leg, canker, dry rot, leaf spot|Description:SIMILARITY: Belongs to the thiolase family.
Chr01G2705.1	205	1	148	UniProt ID:O43117_COLGL	215	1	162	106/166(63.86)	0.65	0.13	166	102	1.00E-27	gene=Chr01G2705	Gene Symbol:NULL|Host:Multiple genera in multiple families|Disease:'Anthracnose of stems and leaves, dieback, root rot, leaf spot, blossom rot, fruit rot (dieback and ripe rot), seedling blight.' (Mordue 1971)|Description:Unknow

															n
Chr01G2710.1	298	15	189	UniProt ID:Q8TGD1_FUSOX	318	130	306	50/190 (26.32)	0.42	0.15	190	47.4	4.00E-07	gene=Chr01G2710	Gene Symbol:FOW1|Host:Multiple genera in multiple families|Disease:Blights, wilts, rots of various sorts|Description:SIMILARITY: Belongs to the mitochondrial carrier family.
Chr01G2712.1	408	9	348	UniProt ID:Q2I0M6_CERNC	871	447	822	98/387 (25.32)	0.39	0.15	387	62.8	2.00E-11	gene=Chr01G2712	Gene Symbol:CTB3|Host:Numerous taxa in Solanaceae|Disease:Leaf spot|Description:Unknown
Chr01G2719.1	657	74	395	UniProt ID:Q5RLJ7_CRYNV	594	62	355	81/330 (24.55)	0.41	0.13	330	74.3	6.00E-15	gene=Chr01G2719	Gene Symbol:NULL|Host:humans|Disease:cryptococcosis|Description:Unknown
Chr01G2720.1	591	22	569	UniProt ID:Q5RLJ7_CRYNV	594	61	568	154/566(27.21)	0.44	0.13	566	171	7.00E-47	gene=Chr01G2720	Gene Symbol:NULL|Host:humans|Disease:cryptococcosis|Description:Unknown
Chr01G2725.1	1159	748	1028	UniProt ID:TUP1_CAN	514	201	509	121/311(38.91)	0.57	0.1	311	209	1.00E-58	gene=Chr01G2725	Gene Symbol:TUP1|Host:Isolated from a wide variety of

				AL											substrates including humans|Disease:invasive candidal disease|Description:FUNCTION: Represses transcription by RNA polymerase II. Represses genes responsible for initiating filamentous growth and this repression is lifted under inducing environmental conditions.
Chr01G2726.1	452	1	403	UniProt ID:A4RGC8_MAGO7	1158	723	1146	168/432(38.89)	0.55	0.09	432	266	1.00E-79	gene=Chr01G2726	Gene Symbol:MGG_11671|Host:Poaceae, especially important on Oryzae|Disease:Rice blast|Description:SIMILARITY: Contains 1 reverse transcriptase domain.
Chr01G2727.1	1234	71	1201	UniProt ID:A4RGC8_MAGO7	1158	31	1132	460/1162(39.59)	0.57	0.08	1162	780	0	gene=Chr01G2727	Gene Symbol:MGG_11671|Host:Poaceae, especially important on Oryzae|Disease:Rice blast|Description:SIMILAR

															ITY: Contains 1 reverse transcriptase domain.
Chr01G2728.1	333	25	201	UniProt ID:A4R3I5_MAGO7	400	104	274	46/183 (25.14)	0.45	0.1	183	47	7.00E-07	gene=Chr01G2728	Gene Symbol:"MGG_11993, MGG_12837, MGG_13052"|Host:Poaceae, especially important on Oryzae|Disease:Rice blast|Description:Unknown
Chr01G2738.1	506	14	498	UniProt ID:Q5ANE1_CANAL	748	44	543	141/508(27.76)	0.49	0.06	508	181	2.00E-50	gene=Chr01G2738	Gene Symbol:SNF3|Host:Isolated from a wide variety of substrates including humans|Disease:invasive candidal disease|Description:SIMILARITY: Belongs to the major facilitator superfamily. Sugar transporter (TC 2.A.1.1) family.
Chr01G2746.1	537	67	515	UniProt ID:Q5ANE1_CANAL	748	50	503	120/460(26.09)	0.46	0.04	460	140	2.00E-36	gene=Chr01G2746	Gene Symbol:SNF3|Host:Isolated d from a wide variety of substrates including

														humans|Disease:invasive candidal disease|Description:SIMILARITY: Belongs to the major facilitator superfamily. Sugar transporter (TC 2.A.1.1) family.
Chr01G2758.1	1234	71	1201	UniProt ID:A4RGC8_MAGO7	1158	31	1132	460/1162(39.59)	0.57	0.08	1162	780	0	gene=Chr01G2758	Gene Symbol:MGG_11671|Host:Poaceae, especially important on Oryzae|Disease:Rice blast|Description:SIMILARITY: Contains 1 reverse transcriptase domain.
Chr01G2759.1	333	25	201	UniProt ID:A4R3I5_MAGO7	400	104	274	46/183(25.14)	0.45	0.1	183	47	7.00E-07	gene=Chr01G2759	Gene Symbol:"MGG_11993, MGG_12837, MGG_13052"|Host:Poaceae, especially important on Oryzae|Disease:Rice blast|Description:Unknown
Chr01G2767.1	509	82	484	UniProt ID:Q01	459	22	413	110/415(26.5	0.46	0.08	415	127	5.00E-33	gene=Chr01G2767	Gene Symbol:MAK1|Host:Trees

			446_NECHA				1)							of various plant families|Disease:Fruit rot, stem rot|Description:Unknown
Chr01G2768.1	752	228	454	UniProt ID:Q0WXM3_FUSOX	663	255	479	57/233 (24.46)	0.44	0.06	233	68.6	7.00E-13	gene=Chr01G2768	Gene Symbol:FOW2|Host:Multiple genera in multiple families|Disease:Blights, wilts, rots of various sorts|Description:SIMILARITY: Contains 1 Zn(2)-C6 fungal-type DNA-binding domain.
Chr01G2770.1	488	56	485	UniProt ID:Q59RG0_CANAL	581	166	570	99/443 (22.35)	0.38	0.12	443	62.4	2.00E-11	gene=Chr01G2770	Gene Symbol:NAG4|Host:Isolated from a wide variety of substrates including humans|Disease:invasive candidal disease|Description:CAUTION: The sequence shown here is derived from an EMBL/GenBank/DDBJ whole genome shotgun (WGS) entry which is preliminary data.

Chr01G2773.1	358	8	305	UniProt ID:Q6A2T2_BOTFU	391	34	333	77/316(24.37)	0.44	0.11	316	73.9	1.00E-15	gene=Chr01G2773	Gene Symbol:BTP1|Host:Various plant families|Disease:Grey mould. Parasite or saprophyte|Description:Unknown
Chr01G2774.1	540	1	339	UniProt ID:Q5XTQ4_BOTFU	574	3	382	110/391(28.13)	0.41	0.16	391	97.1	3.00E-22	gene=Chr01G2774	Gene Symbol:LIP1|Host:Various plant families|Disease:Grey mould. Parasite or saprophyte|Description:Unknown
Chr01G2775.1	582	147	524	UniProt ID:Q9P8L8_BOTFU	598	178	543	100/394(25.38)	0.4	0.11	394	71.2	5.00E-14	gene=Chr01G2775	Gene Symbol:BCMFS1|Host:Various plant families|Disease:Grey mould. Parasite or saprophyte|Description:Unknown
Chr01G2780.1	577	1	564	UniProt ID:Q9P8L8_BOTFU	598	32	567	146/571(25.57)	0.42	0.07	571	144	6.00E-38	gene=Chr01G2780	Gene Symbol:BCMFS1|Host:Various plant families|Disease:Grey mould. Parasite or

															saprophyte|Description:Unknown
Chr01G2790.1	801	621	742	UniProt ID:TUP1_CANAL	514	258	372	44/126 (34.92)	0.51	0.12	126	69.7	2.00E-13	gene=Chr01G2790	Gene Symbol:TUP1|Host:Isolated from a wide variety of substrates including humans|Disease:invasive candidal disease|Description:FUNCTION: Represses transcription by RNA polymerase II. Represses genes responsible for initiating filamentous growth and this repression is lifted under inducing environmental conditions.
Chr01G1131.1	550	214	339	UniProt ID:C4YII6_CANAW	768	474	599	41/133 (30.83)	0.53	0.11	133	60.8	9.00E-11	gene=Chr01G1131	Gene Symbol:CAWG_04261|Host:Isolated from a wide variety of substrates including humans|Disease:invasive candidal disease|Description:SIMILARITY: Belongs to the

															DEAD box helicase family.
Chr05G0869.1	1511	1055	1334	UniProt ID:TUP1_CANAL	514	201	509	119/310(38.39)	0.55	0.1	310	202	5.00E-56	gene=Chr05G0869	Gene Symbol:TUP1|Host:Isolated from a wide variety of substrates including humans|Disease:invasive candidal disease|Description:FUNCTION: Represses transcription by RNA polymerase II. Represses genes responsible for initiating filamentous growth and this repression is lifted under inducing environmental conditions.
Chr05G0868.1	1312	917	1197	UniProt ID:TUP1_CANAL	514	201	509	106/314(33.76)	0.53	0.12	314	172	5.00E-46	gene=Chr05G0868	Gene Symbol:TUP1|Host:Isolated from a wide variety of substrates including humans|Disease:invasive candidal disease|Description:FUNCTION: Represses transcription by RNA polymerase II. Represses

															genes responsible for initiating filamentous growth and this repression is lifted under inducing environmental conditions.
Chr05G0859.1	506	17	442	UniProt ID:Q5ANE1_CANAL	748	44	493	108/474(22.78)	0.41	0.15	474	87.8	3.00E-19	gene=Chr05G0859	Gene Symbol:SNF3|Host:Isolated from a wide variety of substrates including humans|Disease:invasive candidal disease|Description:SIMILARITY: Belongs to the major facilitator superfamily. Sugar transporter (TC 2.A.1.1) family.
Chr05G0856.1	422	64	360	UniProt ID:Q5AJC8_CANAL	461	138	417	115/297(38.72)	0.59	0.06	297	241	6.00E-75	gene=Chr05G0856	Gene Symbol:MNT2|Host:Isolated from a wide variety of substrates including humans|Disease:invasive candidal disease|Description:CAUTION: The sequence shown here is derived from an

														EMBL/GenBank/DDBJ whole genome shotgun (WGS) entry which is preliminary data.
Chr05G0847.1	494	20	126	UniProt ID:A0ST46_CERNC	397	25	139	33/115 (28.70)	0.43	0.07	115	47.8	8.00E-07	gene=Chr05G0847	Gene Symbol:CTB8|Host:Numerous taxa in Solanaceae|Disease:Leaf spot|Description:Unknown
Chr05G0846.1	765	416	485	UniProt ID:TUP1_CANAL	514	294	359	25/70(35.71)	0.56	0.06	70	45.8	6.00E-06	gene=Chr05G0846	Gene Symbol:TUP1|Host:Isolated from a wide variety of substrates including humans|Disease:invasive candidal disease|Description:FUNCTION: Represses transcription by RNA polymerase II. Represses genes responsible for initiating filamentous growth and this repression is lifted under inducing environmental conditions.
Chr05G0845.1	518	50	228	UniProt ID:Q8N	690	297	519	62/223 (27.80)	0.41	0.2	223	77	8.00E-16	gene=Chr05G0845	Gene Symbol:SCH9|Host:huma

				KF8_CRYNV										ns|Disease:cryptococcosis|Description:CATALYTIC ACTIVITY: ATP + a protein = ADP + a phosphoprotein.
Chr05G0839.1	404	63	341	UniProt ID:D4AUT8_ARTBC	734	366	664	106/306(34.64)	0.49	0.11	306	124	2.00E-31	gene=Chr05G0839	Gene Symbol:ARB_08005|Host:hedgehogs|Disease:ringworm,Kerion Celsi Disease|Description:CAUTION: The sequence shown here is derived from an EMBL/GenBank/DDBJ whole genome shotgun (WGS) entry which is preliminary data.
Chr05G0831.1	348	183	333	UniProt ID:HOG1_CANAL	377	158	298	47/153(30.72)	0.48	0.09	153	67	3.00E-13	gene=Chr05G0831	Gene Symbol:HOG1|Host:Isolated from a wide variety of substrates including humans|Disease:invasive candidal disease|Description:FUNCTION: Mitogen-activated protein kinase involved in

													a signal transduction pathway that is activated by changes in the osmolarity of the extracellular environment. Controls osmotic regulation of transcription of target genes. Regulates stress- induced production and accumulation of glycerol and D-arabitol. HOG1 is also involved in virulence, morphogenesis and oxidative stress response especially through its role in chlamydospore formation, an oxygen-dependent morphogenetic program.
Chr05G0829.1	198	39	197	UniProt ID:Q6TFC7_ASPFM	349	40	197	59/162 (36.42)	0.52	0.04	162	95.5	2.00E-24	gene=Chr05G0829	Gene Symbol:NULL|Host:humans|Disease:infection|Description:Unknown
Chr05G0824.1	352	67	347	UniProt ID:Q6TFC7_A	349	65	346	93/290 (32.07)	0.48	0.06	290	141	3.00E-39	gene=Chr05G0824	Gene Symbol:NULL|Host:humans|Disease:infection|Desc

				SPFM											ription:Unknown
Chr05G0809.1	514	23	474	UniProt ID:Q5ANE1_CANAL	748	39	496	133/468(28.42)	0.51	0.06	468	189	4.00E-53	gene=Chr05G0809	Gene Symbol:SNF3|Host:Isolated from a wide variety of substrates including humans|Disease:invasive candidal disease|Description:SIMILARITY: Belongs to the major facilitator superfamily. Sugar transporter (TC 2.A.1.1) family.
Chr05G0804.1	1062	59	1062	UniProt ID:Q9P872_CANAL	917	23	917	489/1014(48.22)	0.62	0.13	1014	892	0	gene=Chr05G0804	Gene Symbol:PMR1|Host:Isolated from a wide variety of substrates including humans|Disease:invasive candidal disease|Description:SIMILARITY: Belongs to the cation transport ATPase (P-type) family.
Chr05G0799.1	507	31	486	UniProt ID:Q9C0M1_C	493	20	490	116/493(23.53)	0.43	0.12	493	92.4	5.00E-21	gene=Chr05G0799	Gene Symbol:CANAG5|Host:Isolated from a wide variety

				ANAL											of substrates including humans|Disease:invasive candidal disease|Description:Unknown
Chr05G0787.1	456	106	181	UniProt ID:Q2Q466_MAGGR	101	14	91	27/78(34.62)	0.63	0.03	78	60.8	3.00E-12	gene=Chr05G0787	Gene Symbol:MNH6|Host:Digitaria (Poaceae)|Disease:Leaf spot|Description:Unknown
Chr05G0785.1	1302	13	1300	UniProt ID:Q9C1U1_MAGGR	1307	13	1307	1017/1300(78.23)	0.87	0.01	1300	2031	0	gene=Chr05G0785	Gene Symbol:HIK1|Host:Digitaria (Poaceae)|Disease:Leaf spot|Description:SIMILARITY: Contains 1 histidine kinase domain.
Chr05G0783.1	1106	827	903	UniProt ID:Q59W09_CANAL	379	10	81	31/77(40.26)	0.57	0.06	77	64.3	1.00E-11	gene=Chr05G0783	Gene Symbol:GCS1|Host:Isolated from a wide variety of substrates including humans|Disease:invasive candidal disease|Description:CAUTION: The sequence shown here is derived from an EMBL/GenBank/DDBJ

															whole genome shotgun (WGS) entry which is preliminary data.
Chr05G0782.1	697	311	579	UniProt ID:Q59Z94_CANAL	589	143	436	123/305(40.33)	0.55	0.15	305	213	1.00E-60	gene=Chr05G0782	Gene Symbol:RCK2|Host:Isolated from a wide variety of substrates including humans|Disease:invasive candidal disease|Description:SIMILARITY: Contains 1 protein kinase domain.
Chr05G0778.1	332	72	193	UniProt ID:Q8TGD1_FUSOX	318	161	280	28/122(22.95)	0.48	0.02	122	47.8	3.00E-07	gene=Chr05G0778	Gene Symbol:FOW1|Host:Multiple genera in multiple families|Disease:Blights, wilts, rots of various sorts|Description:SIMILARITY: Belongs to the mitochondrial carrier family.
Chr05G0777.1	742	281	705	UniProt ID:DHH1_CRYNV	616	21	401	132/426(30.99)	0.49	0.11	426	186	2.00E-51	gene=Chr05G0777	Gene Symbol:VAD1|Host:humans|Disease:cryptococcosis|Description:FUNCTION: ATP-dependent RNA

															helicase involved in mRNA turnover, and more specifically in mRNA decapping. Is involved in G1/S DNA- damage checkpoint recovery, probably through the regulation of the translational status of a subset of mRNAs. May also have a role in translation and mRNA nuclear export (By similarity). Is involved in virulence.
Chr05G0774.1	266	2	261	UniProt ID:1433_CANAL	264	4	258	198/260(76.15)	0.89	0.02	260	410	1.00E-145	gene=Chr05G0774	Gene Symbol:BMH1|Host:Isolated from a wide variety of substrates including humans|Disease:invasive candidal disease|Description:SIMILARITY: Belongs to the 14-3-3 family.
Chr05G0768.1	535	1	535	UniProt ID:C0S	560	1	543	160/555(28.8	0.53	0.06	555	227	2.00E-67	gene=Chr05G0768	Gene Symbol:PABG_03488|Hos

				733_P ARBP				3)							t:humans|Disease:Paracoccidioidomycosis|Description:SIMILARITY: Belongs to the TCP-1 chaperonin family.
Chr05G0767.1	439	162	420	UniProt ID:Q5AG40_CANAL	439	116	370	95/264 (35.98)	0.55	0.05	264	150	4.00E-41	gene=Chr05G0767	Gene Symbol:VPS4|Host:Isolated from a wide variety of substrates including humans|Disease:invasive candidal disease|Description:SIMILARITY: Belongs to the AAA ATPase family.
Chr05G0762.1	1407	478	614	UniProt ID:Q9P8W9_CRYNE	162	16	156	69/141 (48.94)	0.65	0.03	141	136	8.00E-37	gene=Chr05G0762	Gene Symbol:CPA1|Host:humans|Disease:cryptococcosis|Description:FUNCTION: PPIases accelerate the folding of proteins (By similarity).
Chr05G0753.1	382	90	275	UniProt ID:O93802_ALTAL	267	8	201	49/202 (24.26)	0.41	0.12	202	45.4	2.00E-06	gene=Chr05G0753	Gene Symbol:BRM2|Host:Plant|Disease:Leaf spot, rots|Description:SIMILARITY: Belongs to the

																short-chain dehydrogenases/reductases (SDR) family.
Chr05G0743.1	693	53	120	UniProt ID:Q59MD2_CANAL	843	763	832	25/70(35.71)	0.6	0.03	70	62.4	5.00E-11	gene=Chr05G0743	Gene Symbol:UME6|Host:Isolated from a wide variety of substrates including humans|Disease:invasive candidal disease|Description:CAUTION: The sequence shown here is derived from an EMBL/GenBank/DDBJ whole genome shotgun (WGS) entry which is preliminary data.
Chr05G0740.1	594	58	563	UniProt ID:Q5XTQ4_BOTFU	574	8	543	162/557(29.08)	0.43	0.13	557	177	5.00E-49	gene=Chr05G0740	Gene Symbol:LIP1|Host:Various plant families|Disease:Grey mould. Parasite or saprophyte|Description:Unknown
Chr05G0734.1	423	136	423	UniProt ID:C5GHC7_A	295	8	295	238/288(82.64)	0.91	0	288	524	0	gene=Chr05G0734	Gene Symbol:BDCG_03640|Host:humans|Disease:cutane

			JEDR												ous Blastomyces dermatitidis infection|Description:Unknown
Chr05G0724.1	553	6	552	UniProt ID:Q9UUS8_COLGL	567	6	566	306/570(53.68)	0.66	0.06	570	569	0	gene=Chr05G0724	Gene Symbol:CHIP3|Host:Multiple genera in multiple families|Disease:'Anthracnose of stems and leaves, dieback, root rot, leaf spot, blossom rot, fruit rot (dieback and ripe rot), seedling blight.' (Mordue 1971)|Description:Unknown
Chr05G0722.1	557	436	501	UniProt ID:Q9P3Z8_CYBJA	345	13	77	33/66(50.00)	0.68	0.02	66	79.3	5.00E-17	gene=Chr05G0722	Gene Symbol:MIG1|Host:humans|Disease:Chronic urinary tract infection|Description:Unknown
Chr05G0716.1	214	16	204	UniProt ID:Q59PJ5_CANAL	236	4	169	63/189(33.33)	0.52	0.12	189	88.6	4.00E-22	gene=Chr05G0716	Gene Symbol:CSH3|Host:Isolated from a wide variety of substrates including humans|Disease:invasive

															candidal disease|Description:CAUTION: The sequence shown here is derived from an EMBL/GenBank/DDBJ whole genome shotgun (WGS) entry which is preliminary data.
Chr05G0711.1	315	74	161	UniProt ID:A6ZRC1_YEAS7	577	203	291	31/89(34.83)	0.58	0.01	89	61.2	2.00E-11	gene=Chr05G0711	Gene Symbol:PAB1|Host:humans|Disease:occasional infection|Description:CAUTION: The sequence shown here is derived from an EMBL/GenBank/DDBJ whole genome shotgun (WGS) entry which is preliminary data.
Chr05G0709.1	367	10	367	UniProt ID:A6R120_AJECN	376	7	376	229/370(61.89)	0.77	0.03	370	471	3.00E-166	gene=Chr05G0709	Gene Symbol:HCAG_03327|Host:humans|Disease:Darling's disease|Description:Unknown
Chr05G0	2204	529	684	UniProt	772	220	386	48/169	0.47	0.09	169	48.9	3.00E-06	gene=Chr	Gene

692.1				ID:C1GMG4_PARBD				(28.40)						05G0692	Symbol:PADG_08450|Host:humans|Disease:Paracoccidioidomycosis|Description:SIMILARITY: Belongs to the DEAD box helicase family.
Chr05G0686.1	1168	118	400	UniProt ID:Q59VF3_CANAL	1813	631	921	71/304 (23.36)	0.41	0.11	304	47.8	4.00E-06	gene=Chr05G0686	Gene Symbol:"DUR1,2"|Host:Isolated from a wide variety of substrates including humans|Disease:invasive candidal disease|Description:CAUTION: The sequence shown here is derived from an EMBL/GenBank/DDBJ whole genome shotgun (WGS) entry which is preliminary data.
Chr05G0677.1	423	235	408	UniProt ID:Q9UQZ1_9PEZI	512	179	317	49/174 (28.16)	0.38	0.2	174	52.4	2.00E-08	gene=Chr05G0677	Gene Symbol:EMK1|Host:Multiple genera in multiple families|Disease:'Anthracnose of stems and leaves, dieback, root rot, leaf spot, blossom rot, fruit rot

															(dieback and ripe rot), seedling blight.' (Mordue 1971)|Description:Unknown
Chr05G0669.1	304	16	289	UniProt ID:Q75WR5_9PLEO	265	9	264	76/281 (27.05)	0.44	0.11	281	87	5.00E-21	gene=Chr05G0669	Gene Symbol:BRN1|Host:Belamcanda chinensis: Korea,Gladiolus ?gandavensis: Korea,Iris japonica: China,Iris missouriensis (Leaf spot.): Idaho; Montana; Oregon; Washington,Iris sp. (Leaf spot.): China; Texas; Washing|Disease:Leaf spot|Description:SIMILARITY: Belongs to the short-chain dehydrogenases/reductases (SDR) family.
Chr05G0667.1	604	6	603	UniProt ID:Q5ANJ5_CANAL	568	5	565	272/600(45.33)	0.63	0.07	600	477	2.00E-162	gene=Chr05G0667	Gene Symbol:ADE2|Host:Isolated from a wide variety of substrates including humans|Disease:invasive candidal

Chr05G0666.1	396	25	389	UniProt ID:DHH1_CRYNV	616	38	401	158/366(43.17)	0.62	0.01	366	305	3.00E-98	gene=Chr05G0666	disease|Description:CAUTION: The sequence shown here is derived from an EMBL/GenBank/DDBJ whole genome shotgun (WGS) entry which is preliminary data. Gene Symbol:VAD1|Host:humans|Disease:cryptococcosis|Description:FUNCTION: ATP-dependent RNA helicase involved in mRNA turnover, and more specifically in mRNA decapping. Is involved in G1/S DNA- damage checkpoint recovery, probably through the regulation of the translational status of a subset of mRNAs. May also have a role in translation and mRNA nuclear export (By similarity). Is involved in

														virulence.
Chr05G0660.1	548	4	340	UniProt ID:Q5XTQ4_BOTFU	574	1	362	108/378(28.57)	0.44	0.15	378	122	1.00E-30	gene=Chr05G0660	Gene Symbol:LIP1|Host:Various plant families|Disease:Grey mould. Parasite or saprophyte|Description:Unknown
Chr05G0655.1	1173	816	1004	UniProt ID:F2QQX0_PICP7	3007	2807	2981	46/194(23.71)	0.47	0.12	194	55.5	2.00E-08	gene=Chr05G0655	Gene Symbol:CHS1|Host:humans|Disease:occasional infection|Description:SIMILARITY: Contains 1 PH domain.
Chr05G0654.1	378	15	374	UniProt ID:Q7LJU0_CRYNE	410	89	410	81/372(21.77)	0.39	0.17	372	45.1	4.00E-06	gene=Chr05G0654	Gene Symbol:UXS1|Host:humans|Disease:cryptococcosis|Description:Unknown
Chr05G0650.1	701	1	701	UniProt ID:D3YIG6_SPOSC	708	1	708	632/708(89.27)	0.95	0.01	708	1293	0	gene=Chr05G0650	Gene Symbol:GFA1|Host:plants|Disease:Rose-picker's Disease|Description:Unknown
Chr05G0649.1	391	126	365	UniProt ID:Q5AG40_C	439	126	361	93/242(38.43)	0.61	0.03	242	155	2.00E-43	gene=Chr05G0649	Gene Symbol:VPS4|Host:Isolated from a wide variety of

				ANAL											substrates including humans|Disease:invasive candidal disease|Description:SIMILARITY: Belongs to the AAA ATPase family.
Chr05G0646.1	1699	804	1154	UniProt ID:C4YII6_CANAW	768	208	606	95/422 (22.51)	0.42	0.22	422	65.9	1.00E-11	gene=Chr05G0646	Gene Symbol:CAWG_04261|Host:Isolated from a wide variety of substrates including humans|Disease:invasive candidal disease|Description:SIMILARITY: Belongs to the DEAD box helicase family.
Chr05G0627.1	210	1	105	UniProt ID:Q6T7C1_MAGGR	207	1	109	57/109 (52.29)	0.65	0.04	109	97.8	1.00E-25	gene=Chr05G0627	Gene Symbol:EMP1|Host:Digitaria (Poaceae)|Disease:Leaf spot|Description:Unknown
Chr05G0622.1	516	289	508	UniProt ID:TUP1_CANAL	514	201	416	80/246 (32.52)	0.48	0.23	246	100	1.00E-23	gene=Chr05G0622	Gene Symbol:TUP1|Host:Isolated from a wide variety of substrates including humans|Disease:invasive

															candidal disease|Description:FUNCTION: Represses transcription by RNA polymerase II. Represses genes responsible for initiating filamentous growth and this repression is lifted under inducing environmental conditions.
Chr05G0620.1	515	42	509	UniProt ID:Q59RG0_CANAL	581	112	581	123/476(25.84)	0.48	0.03	476	150	2.00E-40	gene=Chr05G0620	Gene Symbol:NAG4|Host:Isolated from a wide variety of substrates including humans|Disease:invasive candidal disease|Description:CAUTION: The sequence shown here is derived from an EMBL/GenBank/DDBJ whole genome shotgun (WGS) entry which is preliminary data.
Chr05G0619.1	526	166	465	UniProt ID:Q5AP71_C	405	78	369	128/300(42.67)	0.64	0.03	300	251	5.00E-78	gene=Chr05G0619	Gene Symbol:TPK1|Host:Isolated from a wide variety of

				ANAL											substrates including humans|Disease:invasive candidal disease|Description:CATALYTIC ACTIVITY: ATP + a protein = ADP + a phosphoprotein.
Chr05G0616.1	714	128	659	UniProt ID:Q5AHG6_CANAL	787	198	729	326/557(58.53)	0.71	0.09	557	608	0	gene=Chr05G0616	Gene Symbol:SCH9|Host:Isolated from a wide variety of substrates including humans|Disease:invasive candidal disease|Description:CATALYTIC ACTIVITY: ATP + a protein = ADP + a phosphoprotein.
Chr05G0610.1	386	131	196	UniProt ID:C5J0G7_MYCGR	726	590	649	31/68(45.59)	0.65	0.15	68	57.8	5.00E-10	gene=Chr05G0610	Gene Symbol:NULL|Host:Triticum and possibly a few other grasses|Disease:Leaf spot or speckled leaf blotch of wheat|Description:Unknown
Chr05G0605.1	474	90	474	UniProt ID:O59	430	38	430	186/393(47.3	0.67	0.02	393	387	4.00E-131	gene=Chr05G0605	Gene Symbol:NULL|Host:huma

				928_HYPVI				3)						ns|Disease:infection|Description:SIMILARITY: Belongs to the glycosyl hydrolase 18 family.
Chr05G0604.1	836	237	422	UniProt ID:Q0WXM3_FUSOX	663	250	434	52/192 (27.08)	0.43	0.07	192	63.2	4.00E-11	gene=Chr05G0604	Gene Symbol:FOW2|Host:Multiple genera in multiple families|Disease:Blights, wilts, rots of various sorts|Description:SIMILARITY: Contains 1 Zn(2)-C6 fungal-type DNA-binding domain.
Chr05G0600.1	700	9	698	UniProt ID:DPP5_TRIEQ	726	20	707	298/705(42.27)	0.62	0.05	705	566	0	gene=Chr05G0600	Gene Symbol:DPP5|Host:humans|Disease:Malabar itch|Description:FUNCTION: Extracellular dipeptidyl-peptidase which removes N- terminal dipeptides sequentially from polypeptides having unsubstituted N-termini. Contributes to pathogenicity (By similarity).

Chr05G0597.1	383	210	339	UniProt ID:C1GL98_PARBD	761	209	338	36/130 (27.69)	0.45	0	130	59.7	1.00E-10	gene=Chr05G0597	Gene Symbol:PADG_07884|Host:humans|Disease:Paracoccidioidomycosis|Description:Unknown
Chr05G0590.1	787	715	785	UniProt ID:Q59LF3_CANAL	474	398	474	32/77(41.56)	0.56	0.08	77	57.8	1.00E-09	gene=Chr05G0590	Gene Symbol:RVS167|Host:Isolated from a wide variety of substrates including humans|Disease:invasive candidal disease|Description:SIMILARITY: Contains 1 SH3 domain.
Chr05G0588.1	773	19	742	UniProt ID:C5GWL2_AJEDR	776	18	760	319/797(40.03)	0.55	0.16	797	451	2.00E-147	gene=Chr05G0588	Gene Symbol:BDCG_08831|Host:humans|Disease:cutaneous Blastomyces dermatitidis infection|Description:Unknown
Chr05G0574.1	482	228	397	UniProt ID:F2QU09_PICP7	626	139	294	54/170 (31.76)	0.51	0.08	170	81.3	2.00E-17	gene=Chr05G0574	Gene Symbol:PAB1|Host:humans|Disease:occasional infection|Description:Unknown

Chr05G0568.1	525	1	503	UniProt ID:A4ULI4_MYCGR	517	1	517	310/519(59.73)	0.73	0.03	519	617	0	gene=Chr05G0568	Gene Symbol:CYP51|Host:Triticum and possibly a few other grasses|Disease:Leaf spot or speckled leaf blotch of wheat|Description:COFACTOR: Heme group (By similarity).
Chr05G0566.1	975	263	702	UniProt ID:P87198_USTMD	1459	313	750	188/473(39.75)	0.56	0.14	473	291	4.00E-83	gene=Chr05G0566	Gene Symbol:KIN1|Host:Euchlaena spp., Zea spp. (Poaceae)|Disease:Smut. Corn smut|Description:SIMILARITY: Belongs to the kinesin-like protein family.
Chr05G0557.1	183	18	138	UniProt ID:A7A233_YEAS7	215	21	145	36/126(28.57)	0.5	0.05	126	56.2	4.00E-11	gene=Chr05G0557	Gene Symbol:SEC4|Host:humans|Disease:occasional infection|Description:SIMILARITY: Belongs to the small GTPase superfamily. Rab family.
Chr05G0548.1	465	220	462	UniProt ID:A3Q	570	301	546	96/252(38.10)	0.52	0.06	252	153	1.00E-41	gene=Chr05G0548	Gene Symbol:ZAFA|Host:huma

				X02_ASPFM										ns|Disease:infection|Description:Unknown
Chr05G0546.1	1207	588	942	UniProt ID:DHH1_CRYNV	616	42	385	117/357(32.77)	0.54	0.04	357	206	1.00E-56	gene=Chr05G0546	Gene Symbol:VAD1|Host:humans|Disease:cryptococcosis|Description:FUNCTION: ATP-dependent RNA helicase involved in mRNA turnover, and more specifically in mRNA decapping. Is involved in G1/S DNA- damage checkpoint recovery, probably through the regulation of the translational status of a subset of mRNAs. May also have a role in translation and mRNA nuclear export (By similarity). Is involved in virulence.
Chr05G0542.1	495	9	102	UniProt ID:F2QZS1_PICP7	494	375	464	30/94(31.91)	0.49	0.04	94	47.4	1.00E-06	gene=Chr05G0542	Gene Symbol:OLE1|Host:humans|Disease:occasional infection|Description:COF

															ACTOR: Iron (By similarity).
Chr05G0538.1	1180	904	1030	UniProt ID:Q5AM49_CANAL	1690	1120	1247	35/131 (26.72)	0.48	0.05	131	55.8	1.00E-08	gene=Chr05G0538	Gene Symbol:SNF2|Host:Isolated from a wide variety of substrates including humans|Disease:invasive candidal disease|Description:Unknown
Chr05G0529.1	448	277	314	UniProt ID:Q59UQ8_CANAL	445	165	200	17/38(44.74)	0.68	0.05	38	50.4	1.00E-07	gene=Chr05G0529	Gene Symbol:PEP7|Host:Isolated from a wide variety of substrates including humans|Disease:invasive candidal disease|Description:CAUTION: The sequence shown here is derived from an EMBL/GenBank/DDBJ whole genome shotgun (WGS) entry which is preliminary data.
Chr05G0527.1	713	24	390	UniProt ID:Q5GFD3_P	437	9	386	107/401(26.68)	0.43	0.14	401	115	2.00E-28	gene=Chr05G0527	Gene Symbol:NULL|Host:Multiple genera of Poaceae and

				HAND											Blysmus compressus (Cyperaceae)|Disease:Glume blotch of wheat and other grasses|Description:Unknown
Chr05G0526.1	252	7	252	UniProt ID:O93802_ALTAL	267	15	267	81/263 (30.80)	0.47	0.1	263	89.4	5.00E-22	gene=Chr05G0526	Gene Symbol:BRM2|Host:Plant|Disease:Leaf spot, rots|Description:SIMILARITY: Belongs to the short-chain dehydrogenases/reductases (SDR) family.
Chr05G0525.1	421	1	399	UniProt ID:Q59RG0_CANAL	581	192	567	93/404 (23.02)	0.39	0.08	404	62.4	2.00E-11	gene=Chr05G0525	Gene Symbol:NAG4|Host:Isolated from a wide variety of substrates including humans|Disease:invasive candidal disease|Description:CAUTION: The sequence shown here is derived from an EMBL/GenBank/DDBJ whole genome shotgun (WGS) entry which is

															preliminary data.
Chr05G0524.1	1339	68	115	UniProt ID:Q5AMH6_CANAL	821	748	797	23/50(46.00)	0.7	0.04	50	61.2	3.00E-10	gene=Chr05G0524	Gene Symbol:CAS5|Host:Isolated from a wide variety of substrates including humans|Disease:invasive candidal disease|Description:CAUTION: The sequence shown here is derived from an EMBL/GenBank/DDBJ whole genome shotgun (WGS) entry which is preliminary data.
Chr05G0523.1	1317	39	1317	UniProt ID:Q3Y5V5_MAGGR	1321	14	1321	604/1346(44.87)	0.65	0.08	1346	1137	0	gene=Chr05G0523	Gene Symbol:ABC3|Host:Digitaria (Poaceae)|Disease:Leaf spot|Description:SIMILARITY: Belongs to the ABC transporter superfamily.
Chr05G0520.1	406	12	386	UniProt ID:Q00LS5_PHAND	408	1	391	291/391(74.42)	0.86	0.04	391	602	0	gene=Chr05G0520	Gene Symbol:CPKA|Host:Multiple genera of Poaceae and Blysmus compressus (Cyperaceae)|Disease:Glume blotch of wheat and

														other grasses|Description:SIMILARITY: Contains 1 protein kinase domain.
Chr05G0518.1	660	139	421	UniProt ID:Q4WYU7_ASPFU	712	167	463	74/313 (23.64)	0.42	0.15	313	51.2	1.00E-07	gene=Chr05G0518	Gene Symbol:AFUA_3G14320|Host:humans|Disease:infection|Description:CAUTION: The sequence shown here is derived from an EMBL/GenBank/DDBJ whole genome shotgun (WGS) entry which is preliminary data.
Chr05G0515.1	918	104	918	UniProt ID:Q59XR0_CANAL	780	2	780	405/818(49.51)	0.66	0.05	818	729	0	gene=Chr05G0515	Gene Symbol:TOP1|Host:Isolated from a wide variety of substrates including humans|Disease:invasive candidal disease|Description:SIMILARITY: Belongs to the eukaryotic type I topoisomerase family.
Chr05G0514.1	367	88	282	UniProt ID:Q5A	470	137	339	69/206 (33.50)	0.53	0.07	206	108	3.00E-27	gene=Chr05G0514	Gene Symbol:UTR2|Host:Isolate

				JC0_CANAL											d from a wide variety of substrates including humans|Disease:invasive candidal disease|Description:CAUTION: The sequence shown here is derived from an EMBL/GenBank/DDBJ whole genome shotgun (WGS) entry which is preliminary data.
Chr05G0512.1	535	313	497	UniProt ID:A4ULI9_MYCGR	502	299	502	50/215 (23.26)	0.42	0.19	215	56.2	2.00E-09	gene=Chr05G0512	Gene Symbol:CYP51|Host:Triticum and possibly a few other grasses|Disease:Leaf spot or speckled leaf blotch of wheat|Description:COFACTOR: Heme group (By similarity).
Chr05G0511.1	607	125	595	UniProt ID:Q59NY2_CANAL	562	81	546	156/485(32.16)	0.5	0.07	485	230	6.00E-68	gene=Chr05G0511	Gene Symbol:HEX1|Host:Isolated from a wide variety of substrates including humans|Disease:invasive candidal

<table>
<tr><td></td><td></td><td></td><td></td><td></td><td></td><td></td><td></td><td></td><td></td><td></td><td></td><td></td><td></td><td></td><td>disease|Description:CAUTION: The sequence shown here is derived from an EMBL/GenBank/DDBJ whole genome shotgun (WGS) entry which is preliminary data.</td></tr>
<tr><td>Chr05G0504.1</td><td>1055</td><td>492</td><td>1038</td><td>UniProt ID:Q5AM49_CANAL</td><td>1690</td><td>769</td><td>1264</td><td>184/568(32.39)</td><td>0.52</td><td>0.16</td><td>568</td><td>299</td><td>8.00E-85</td><td>gene=Chr05G0504</td><td>Gene Symbol:SNF2|Host:Isolated from a wide variety of substrates including humans|Disease:invasive candidal disease|Description:Unknown</td></tr>
<tr><td>Chr05G0490.1</td><td>500</td><td>106</td><td>377</td><td>UniProt ID:Q59WF4_CANAL</td><td>597</td><td>136</td><td>464</td><td>97/329 (29.48)</td><td>0.47</td><td>0.17</td><td>329</td><td>160</td><td>1.00E-43</td><td>gene=Chr05G0490</td><td>Gene Symbol:MNN2|Host:Isolated from a wide variety of substrates including humans|Disease:invasive candidal disease|Description:CAUTION: The sequence shown here is derived from an EMBL/GenBank/DDBJ whole genome shotgun</td></tr>
</table>

															(WGS) entry which is preliminary data.
Chr05G0485.1	561	1	264	UniProt ID:Q9Y784_MAGGR	631	104	362	62/265 (23.40)	0.45	0.03	265	80.5	7.00E-17	gene=Chr05G0485	Gene Symbol:PTH11|Host:Digitaria (Poaceae)|Disease:Leaf spot|Description:Unknown
Chr05G0482.1	1191	5	486	UniProt ID:Q59VF3_CANAL	1813	589	1052	178/483(36.85)	0.58	0.04	483	312	2.00E-88	gene=Chr05G0482	Gene Symbol:"DUR1,2"|Host:Isolated from a wide variety of substrates including humans|Disease:invasive candidal disease|Description:CAUTION: The sequence shown here is derived from an EMBL/GenBank/DDBJ whole genome shotgun (WGS) entry which is preliminary data.
Chr05G0466.1	1633	1284	1475	UniProt ID:F2QQX0_PICP7	3007	2807	3006	58/209 (27.75)	0.46	0.12	209	62.4	2.00E-10	gene=Chr05G0466	Gene Symbol:CHS1|Host:humans|Disease:occasional infection|Description:SIMILARITY: Contains 1 PH domain.

Chr05G0450.1	718	441	586	UniProt ID:F2QQX0_PICP7	3007	2834	2991	43/159 (27.04)	0.45	0.09	159	55.8	6.00E-09	gene=Chr05G0450	Gene Symbol:CHS1|Host:humans|Disease:occasional infection|Description:SIMILARITY: Contains 1 PH domain.
Chr05G0446.1	1212	760	1065	UniProt ID:Q0PND8_MAGGR	1375	892	1222	131/337(38.87)	0.59	0.11	337	207	2.00E-55	gene=Chr05G0446	Gene Symbol:PEX6|Host:Digitaria (Poaceae)|Disease:Leaf spot|Description:SIMILARITY: Belongs to the AAA ATPase family.
Chr05G0440.1	858	48	329	UniProt ID:C6KEF5_MYCGR	833	559	822	89/285 (31.23)	0.55	0.08	285	150	3.00E-38	gene=Chr05G0440	Gene Symbol:STE20|Host:Triticum and possibly a few other grasses|Disease:Leaf spot or speckled leaf blotch of wheat|Description:CATALYTIC ACTIVITY: ATP + a protein = ADP + a phosphoprotein.
