# Supplementary material for: Decoding Pecan’s Fungal Foe: A Genomic Insight into Colletotrichum plurivorum Isolate W-6
Source: J Fungi (Basel). 2025 Mar 5;11(3):203. doi: 10.3390/jof11030203 (PMC11943440; doi:10.3390/jof11030203)
Supplement: Supplementary file 1 [file jof-11-00203-s001.zip › Table S26.pdf]

Table S26. Details of comparative analysis of 52 *Colletotrichum* strains.

Complex	Species	Strain	Host	Genome Size	Gene	Core Gene	Share Gene	Unique Gene	CAZyme	SM_cluster	PHI	DFVF	SignalP	Secretome	Effector	TM protein	P450
acutatum	*C.abscissum*	IMI 504890	*Citrus sinensis*	53,936,108	17,223	7,123	10,083	17	811	74	4,139	3,110	1,962	1,639	619	3,435	1,654
	C.acutatum	L51	*Pyrus communis*	51,824,279	13,972	6,886	7,085	1	798	69	4,007	2,972	1,762	1,546	516	2,935	1,588
	C.costaricense	IMI 309622	*Coffea sp,*	51,625,248	17,126	7,039	10,087	0	818	72	4,102	3,068	1,956	1,628	606	3,376	1,607
	C.cuscutae	IMI 304802	*Cuscuta*	80,453,496	17,289	6,937	10,352	0	755	69	3,868	2,844	1,714	1,460	510	3,698	1,540
	C.fioriniae	IMI 355084	*Quercus ilex*	49,445,812	12,178	6,492	5,686	0	749	56	3,584	2,556	1,351	1,209	395	2,411	1,366
	C.limetticola	KLA-Anderson	*Citrus x aurantiifolia*	50,483,870	15,248	7,096	8,146	6	794	72	4,098	3,097	1,845	1,624	599	3,114	1,621
	C.lupini	IMI 504893	*Lupinus albus*	63,407,421	18,742	6,715	12,016	11	788	76	3,903	2,930	1,636	1,375	498	3,734	1,593
	C.melonis	Col 31	*Malus pumila*	50,135,825	16,823	7,032	9,786	5	816	74	4,091	3,081	1,946	1,673	619	3,400	1,599
	C.nymphaeae	SA-01	*Fragaria x ananassa*	49,956,273	14,404	6,967	7,435	2	795	72	4,008	3,019	1,753	1,545	542	3,002	1,575
	C.salicis	CBS 607.94	*Salix sp,*	48,373,413	13,783	6,869	6,912	2	760	59	3,824	2,827	1,621	1,426	488	2,842	1,462
	C.scovillei	Coll-524	*Capsicum*	51,490,	15,626	6,770	8,850	6	789	71	4,0	3,030	1,78	1,562	564	3,07	1,56

			annuum	565							12		3			0	7
	C.simmondsii	CBS122122	*Carica papaya*	50,474,234	13,884	6,907	6,975	2	809	74	4,041	3,001	1,763	1,559	528	2,966	1,597
	C.tamarilloi	Tom-12	*Solanum betaceum*	52,071,711	17,397	7,028	10,359	10	801	77	4,063	3,059	1,933	1,658	610	3,373	1,627
	C.godetiae	CBS 193.32	*Canarium spp.*	51,652,851	16,071	7,137	8,901	33	787	68	4,496	1,570	1,769	1,510	549	3,280	1,607
	C.karsti	CkLH20	*Camellia oleifera*	51,850,041	13,328	6,889	6,437	2	860	67	4,102	3,009	1,702	1,495	488	3,000	1,652
	C.caudatum	CBS 131602	*Sorghastrum nutans*	44,202,764	15,566	6,332	9,218	16	596	82	3,394	2,495	1,906	1,137	402	3,119	1,271
caudatum	*C.somersetense*	CBS 131599	*Sorghastrum nutans*	53,667,270	15,475	6,536	8,929	10	653	80	3,548	2,675	1,531	1,262	492	3,174	1,382
	C.zoysiae	MAFF 238573	*Zoysia tenuifolia*	46,533,813	15,606	6,451	9,135	20	633	75	3,497	2,621	1,528	1,259	477	3,219	1,342
	C.destructivum	CBS 520.97	*Medicago sativa*	51,750,812	15,631	7,215	8,408	8	734	100	4,101	3,191	1,826	1,594	596	3,255	1,645
destructivum	*C.higginsianum*	IMI 349063	*Brassica rapa subsp, chinensis*	50,716,103	14,651	6,967	7,676	8	722	90	3,920	2,932	1,704	1,498	538	3,016	1,553
	C.shisoi	PG-2018a	*Perilla frutescens var, crispa*	69,667,657	11,848	6,072	5,776	0	604	40	3,275	2,354	1,281	1,119	387	2,443	1,144
	C.tanaceti	BRIP57315	*Tanacetum*	51,501,111	12,204	6,543	5,630	31	614	28	3,439	2,504	1,159	999	339	2,586	1,228

			cinerariifolium														
	C.asianum	ICMP 18580	*Coffea sp,*	64,731,264	17,966	7,587	10,309	70	887	106	4,520	3,432	2,161	1,900	709	3,807	1,934
	C.camelliae	CcLH18	*Camellia oleifera*	57,798,682	14,806	7,304	7,498	4	838	94	4,115	3,109	1,906	1,687	597	3,192	1,803
	C.chrysophilum	M932	*Malus pumila*	55,557,938	20,024	7,988	12,028	8	901	107	4,691	3,631	2,181	1,898	738	3,872	2,028
	C.fructicola	CfS4	*Fragaria × ananassa*	57,426,314	16,156	7,723	8,413	20	886	95	4,527	3,434	2,060	1,831	685	3,414	1,953
	C.gloeosporioides	Lc1	*Liriodendron chinense*	61,904,035	15,667	7,576	8,088	3	881	95	4,451	3,385	1,983	1,321	485	3,393	1,894
gloeosporioides	*C.kahawae*	CIFC_Que2	*Coffea arabica*	59,071,264	19,181	7,938	11,190	53	879	98	4,522	3,513	2,111	1,849	708	3,642	1,652
	C.noveboracense	Coll940	*Juglans nigra*	58,178,553	14,420	7,229	7,191	0	837	87	4,081	2,966	1,673	1,518	568	2,747	1,615
	C.siamense	CAD5	*Manihot esculenta*	57,642,425	15,097	7,598	7,499	0	881	97	4,477	3,370	2,008	1,793	663	3,280	1,887
	C.tropicale	CgS9275	*Morus alb*	55,848,179	14,794	7,465	7,329	0	869	88	4,393	3,297	1,949	1,735	635	3,172	1,823
	C.viniferum	CGW01	*Vitis vinifera*	68,452,207	14,534	7,264	7,261	9	823	85	4,195	3,103	1,789	1,586	593	3,099	1,704
	C.aenigma	Cg56	*strawberry*	59,189,926	15,190	7,510	7,680	0	609	91	4,779	1,672	1,950	1,748	382	3,200	1,840
graminicol	*C.cereale*	CBS	*Bletilla*	52,150,	16,704	6,742	9,928	34	657	92	3,6	2,747	1,60	1,318	523	3,14	1,39

a		129662	*ochracea*	039							04		6			2	8
	C.eremochloae	CBS 129661	*Eremochloa ophiuroides*	47,210,890	15,472	6,569	8,891	12	654	89	3,582	2,643	1,636	1,391	548	3,210	1,381
	C.falcatum	MAFF306170	*Saccharum officinarum*	49,085,377	15,897	6,259	9,615	23	576	75	3,358	2,470	1,486	1,203	458	3,157	1,264
	C.graminicola	M1.001	*Zea mays*	57,426,633	15,185	6,184	8,997	4	602	75	3,299	2,411	1,339	1,114	403	2,844	1,215
	C.sublineola	S3.001	*Sorghum bicolor*	46,916,122	15,508	6,549	8,937	22	646	87	3,491	2,589	1,532	1,287	512	3,173	1,324
	C.orbiculare	104-T	*Cucumis sativus*	89,748,316	13,253	6,464	6,787	2	728	82	3,714	2,829	1,699	1,503	573	2,753	1,330
orbiculare	*C.sidae*	CBS 518.97	*Sida spinosa*	86,827,816	12,442	6,398	6,042	2	704	71	3,535	2,573	1,606	1,434	543	2,635	1,316
	C.spinosum	CBS 515.97	*Xanthium spinosum*	82,734,851	12,540	6,425	6,115	0	705	79	3,612	2,637	1,633	1,455	549	2,689	1,364
	C.trifolii	543-2	*Medicago sativa*	109,659,959	12,292	6,337	5,955	0	698	76	3,539	2,602	1,591	1,426	550	2,593	1,299
orchidearum	*C.plurivorum* (W-6)	W-6	*Carya illinoinensis*	54,574,699	14,343	7,093	7,243	7	943	63	4,558	3,175	1,916	1,451	478	3,134	1,753
	C.plurivorum	LFN00145	*Glycine max*	49,703,650	16,153	7,221	8,925	7	807	82	4,092	3,144	1,865	1,624	548	3,256	1,692

singleton	*C.musicola*	LFN0074	*Glycine max*	52,725,698	16,826	7,354	9,462	10	839	76	4,107	3,166	1,868	1,620	567	3,296	1,738
	C.sojae	LFN0009	*Glycine max*	49,351,125	16,124	7,196	8,923	5	800	89	4,011	3,092	1,835	1,611	544	3,216	1,664
	C.chlorophyti	NTL11	*Solanum lycopersicum*	52,387,045	10,419	6,166	4,251	2	643	70	3,393	2,477	1,143	980	314	2,352	1,272
	C.orchidophilum	IMI 309357	*Phalaenopsis sp,*	48,556,462	14,496	6,514	7,978	4	643	60	3,582	2,638	1,465	1,256	446	2,910	1,411
spaethianum	*C.incanum*	MAFF238712	*Raphanus sativus var, longipinnatus*	53,254,579	12,001	6,708	5,289	4	721	79	3,728	2,760	1,323	1,140	393	2,601	1,466
	C.tofieldiae	861	*Arabidopsis thaliana*	52,836,184	12,501	6,779	5,720	2	752	76	3,798	2,839	1,421	1,247	407	2,695	1,496
	C.liriopes	MAFF 242679	*Rohdea japonica*	52,972,150	14,012	7,080	6,900	32	747	54	3,969	1,369	1,386	1,242	426	2,663	1,654
truncatum	*C.truncatum*	CMES1059	*Glycine max*	56,102,516	15,733	7,202	8,334	197	658	98	4,672	1,722	1,975	1,767	366	3,210	1,806
Average				54,487,268.17	13,815.22	6,931.25	8,128.08	13.96	645.78	78.10	3,959.75	2,821.92	1,721.67	1,475.23	413.42	3,092.21	1,737
