# Supplementary material for: Decoding Pecan’s Fungal Foe: A Genomic Insight into Colletotrichum plurivorum Isolate W-6
Source: J Fungi (Basel). 2025 Mar 5;11(3):203. doi: 10.3390/jof11030203 (PMC11943440; doi:10.3390/jof11030203)
Supplement: Supplementary file 1 [file jof-11-00203-s001.zip › Table S27.pdf]

Table S27. Isolate W-6 specific virulance-related genes in PHI and DFVF databases.

DATA base	GeneID	Protein family	Homologous protein derived species	Reference
DFVF	Chr05G0235.1	Histone H3	*Magnaporthe oryzae*	Kwon *et al.*, 2018
	Chr08G0016.1			
	Chr06G0365.1	Imidazoleglycerol-phosphate dehydratase	*Ajellomyces capsulatus*	Sharpton *et al.*, 2009
PHI	Chr06G1488.1	protein kinase	*Fusarium graminearum*	Wang *et al.*, 2011

Kwon, S., Lee, J., Jeon, J., Kim, S., Park, S.-Y., Jeon, J.*, et al.* (2018) Role of the Histone Acetyltransferase Rtt109 in Development and Pathogenicity of the Rice Blast Fungus. *Molecular Plant-Microbe Interactions®,* **31,** 1200-1210.

Sharpton, T. J., Stajich, J. E., Rounsley, S. D., Gardner, M. J., Wortman, J. R., Jordar, V. S., et al. (2009) Comparative genomic analyses of the human fungal pathogens *Coccidioide*s and their relatives. Genome Research, **19**, 1722-1731.

Wang, C., Zhang, S., Hou, R., Zhao, Z., Zheng, Q., Xu, Q.*, et al.* (2011) Functional Analysis of the Kinome of the Wheat Scab Fungus *Fusarium graminearum*. *PLoS Pathogens,* **7**.
