# Supplementary material for: Decoding Pecan’s Fungal Foe: A Genomic Insight into Colletotrichum plurivorum Isolate W-6
Source: J Fungi (Basel). 2025 Mar 5;11(3):203. doi: 10.3390/jof11030203 (PMC11943440; doi:10.3390/jof11030203)
Supplement: Supplementary file 1 [file jof-11-00203-s001.zip › Table S28.pdf]

Table S28. Pathogenicity-related genes unique to the *Orchidearum* complex.

Orthogroup	*C.musicola*	*C.plurivorum*	*C.sojae*	*W-6.genome*
OG0010336	KAF6790309.1, KAF6804555.1, KAF6810210.1, KAF6816917.1, KAF6816918.1, KAF6822012.1, KAF6822013.1	KAF6802912.1, KAF6805529.1, KAF6805530.1, KAF6808064.1, KAF6821740.1, KAF6821741.1, KAF6824277.1, KAF6829476.1	KAF6800360.1	Chr07G0019.1
OG0011308	KAF6820299.1, KAF6820300.1, KAF6820301.1	KAF6834437.1, KAF6834438.1, KAF6834439.1, KAF6834440.1	KAF6806169.1, KAF6806170.1	Chr03G0256.1, Chr03G0257.1
OG0011584	KAF6783481.1, KAF6790665.1	KAF6802740.1, KAF6802858.1, KAF6803183.1, KAF6805329.1, KAF6819898.1, KAF6835992.1	KAF6781399.1	Chr04G0986.1
OG0012867	KAF6788194.1, KAF6807278.1, KAF6830074.1	KAF6812362.1	KAF6784752.1, KAF6797600.1	Chr07G0163.1
OG0012868	KAF6793703.1, KAF6807594.1	KAF6817560.1	KAF6814822.1	Chr01G2516.1, Chr02G0006.1, Chr08G1057.1
OG0012870	KAF6800958.1	KAF6828930.1, KAF6828931.1	KAF6804444.1, KAF6804445.1	Chr05G1332.1, Chr05G1333.1
OG0	KAF6803354.1, KAF6803355.1	KAF6834448.1, KAF6834449.1	KAF6799390.	Chr03G0265.1,

012871			1	Chr03G0266.1
OG0012875	KAF6839501.1	KAF6832676.1, KAF6832677.1	KAF6801123.1, KAF6801124.1	Chr01G1500.1, Chr01G1502.1
OG0013762	KAF6784009.1, KAF6803977.1	KAF6802600.1, KAF6803888.1	KAF6788777.1	Chr01G2785.1
OG0013765	KAF6812385.1, KAF6828672.1	KAF6810376.1, KAF6816731.1	KAF6795835.1	Chr01G2447.1
OG0013771	KAF6834011.1	KAF6827055.1	KAF6804818.1, KAF6811957.1	Chr06G1260.1, Chr06G1263.1
OG0013772	KAF6842491.1, KAF6842492.1	KAF6825311.1, KAF6825312.1	KAF6797608.1	Chr08G0492.1
OG0013773	KAF6843829.1	KAF6826914.1, KAF6839764.1	KAF6809074.1	Chr03G0559.1, Chr06G0079.1
OG0014841	KAF6783605.1	KAF6822659.1, KAF6823268.1	KAF6805934.1	Chr04G0738.1
OG0	KAF6796430.1, KAF6827638.1	KAF6832371.1	KAF6788743.	Chr03G1463.1

014845			1	
OG0014846	KAF6798280.1	KAF6806870.1	KAF6788134.1	Chr05G1460.1, Chr08G1074.1
OG0014849	KAF6802299.1, KAF6843605.1	KAF6825169.1	KAF6803690.1	Chr01G2718.1
OG0014851	KAF6803356.1, KAF6810201.1	KAF6834447.1	KAF6799389.1	Chr03G0264.1
OG0014855	KAF6807883.1, KAF6814536.1	KAF6810075.1	KAF6788785.1	Chr08G0009.1
OG0014856	KAF6809941.1	KAF6830357.1	KAF6806512.1, KAF6806513.1	Chr07G1029.1
OG0014864	KAF6826425.1	KAF6819477.1	KAF6803668.1	Chr04G1417.1, Chr07G0333.1
OG0014870	KAF6839002.1, KAF6839003.1	KAF6827637.1	KAF6797649.1	Chr01G0434.1
OG00160	KAF6782059.1	KAF6819189.1	KAF6806751.1	Chr04G1020.1

75				
OG0016076	KAF6784597.1	KAF6820885.1	KAF6793053.1	Chr06G0137.1
OG0016077	KAF6784601.1	KAF6824800.1	KAF6809827.1	Chr08G1004.1
OG0016079	KAF6786812.1	KAF6838869.1	KAF6807810.1	Chr08G0398.1
OG0016080	KAF6787095.1	KAF6829248.1	KAF6816371.1	Chr01G1072.1
OG0016083	KAF6790385.1	KAF6821739.1	KAF6786402.1	Chr07G0020.1
OG0016086	KAF6795044.1	KAF6807192.1	KAF6796960.1	Chr07G0013.1
OG0016087	KAF6796514.1	KAF6823072.1	KAF6798855.1	Chr04G0884.1
OG0016088	KAF6800353.1	KAF6822370.1	KAF6797642.1	Chr01G0427.1
OG0	KAF6802093.1	KAF6833103.1	KAF6816094.	Chr04G1554.1

0160 90			1	
OG0 0160 92	KAF6802412.1	KAF6822230.1	KAF6802210. 1	Chr05G0279.1
OG0 0160 93	KAF6802541.1	KAF6818376.1	KAF6791369. 1	Chr01G0961.1
OG0 0160 94	KAF6802974.1	KAF6828415.1	KAF6790356. 1	Chr01G0582.1
OG0 0160 95	KAF6803215.1	KAF6811925.1	KAF6813079. 1	Chr04G0933.1
OG0 0160 96	KAF6803274.1	KAF6837453.1	KAF6806126. 1	Chr08G0131.1
OG0 0160 97	KAF6803353.1	KAF6834450.1	KAF6799391. 1	Chr03G0267.1
OG0 0161 00	KAF6804843.1	KAF6823417.1	KAF6808534. 1	Chr01G2648.1
OG0 0161 02	KAF6806568.1	KAF6810536.1	KAF6802276. 1	Chr03G1382.1

OG0016103	KAF6807148.1	KAF6828754.1	KAF6800303.1	Chr02G1777.1
OG0016105	KAF6808100.1	KAF6809651.1	KAF6808546.1	Chr01G2636.1
OG0016106	KAF6808320.1	KAF6834228.1	KAF6807769.1	Chr08G0191.1
OG0016107	KAF6808558.1	KAF6816441.1	KAF6811020.1	Chr02G0179.1
OG0016108	KAF6808972.1	KAF6816490.1	KAF6808318.1	Chr03G1649.1
OG0016109	KAF6809030.1	KAF6832199.1	KAF6807446.1	Chr03G0348.1
OG0016110	KAF6809338.1	KAF6837130.1	KAF6787927.1	Chr08G0736.1
OG0016111	KAF6809952.1	KAF6816706.1	KAF6793014.1	Chr05G1305.1
OG00161	KAF6810514.1	KAF6810132.1	KAF6808808.1	Chr02G0469.1

12				
OG0016113	KAF6810687.1	KAF6832915.1	KAF6814863.1	Chr01G2477.1
OG0016114	KAF6811577.1	KAF6833555.1	KAF6819296.1	Chr02G0275.1
OG0016115	KAF6812011.1	KAF6823157.1	KAF6804508.1	Chr02G0464.1
OG0016116	KAF6812678.1	KAF6829868.1	KAF6810305.1	Chr05G0917.1
OG0016117	KAF6812679.1	KAF6829867.1	KAF6810306.1	Chr05G0916.1
OG0016118	KAF6813065.1	KAF6826666.1	KAF6790338.1	Chr04G0177.1
OG0016119	KAF6813277.1	KAF6826512.1	KAF6798726.1	Chr04G0306.1
OG0016120	KAF6813382.1	KAF6835587.1	KAF6816343.1	Chr01G1047.1
OG0	KAF6813383.1	KAF6835586.1	KAF6816342.	Chr01G1046.1

016121			1	
OG0016123	KAF6813677.1	KAF6825152.1	KAF6799413.1	Chr01G2699.1
OG0016124	KAF6814432.1	KAF6822816.1	KAF6808878.1	Chr06G1149.1
OG0016125	KAF6814618.1	KAF6838421.1	KAF6809146.1	Chr05G0129.1
OG0016126	KAF6815139.1	KAF6822559.1	KAF6813647.1	Chr05G0977.1
OG0016127	KAF6815408.1	KAF6840079.1	KAF6818627.1	Chr05G1129.1
OG0016128	KAF6816331.1	KAF6821795.1	KAF6796963.1	Chr01G1843.1
OG0016129	KAF6817107.1	KAF6828699.1	KAF6808602.1	Chr07G1089.1
OG0016130	KAF6817547.1	KAF6828844.1	KAF6795902.1	Chr04G0133.1

OG0016131	KAF6817646.1	KAF6816775.1	KAF6806964.1	Chr07G0177.1
OG0016132	KAF6818095.1	KAF6837886.1	KAF6818945.1	Chr03G0995.1
OG0016133	KAF6818839.1	KAF6825405.1	KAF6789412.1	Chr02G1685.1
OG0016134	KAF6819063.1	KAF6818086.1	KAF6797633.1	Chr01G1817.1
OG0016135	KAF6819198.1	KAF6840262.1	KAF6819322.1	Chr02G0386.1
OG0016137	KAF6821124.1	KAF6829290.1	KAF6816410.1	Chr01G1108.1
OG0016138	KAF6821444.1	KAF6816885.1	KAF6796501.1	Chr04G1406.1
OG0016139	KAF6821745.1	KAF6814518.1	KAF6807391.1	Chr05G0217.1
OG00161	KAF6822592.1	KAF6821960.1	KAF6813549.1	Chr02G1662.1

41				
OG0016142	KAF6822708.1	KAF6814193.1	KAF6802701.1	Chr02G0042.1
OG0016143	KAF6822888.1	KAF6808797.1	KAF6787865.1	Chr06G1478.1
OG0016144	KAF6823386.1	KAF6818512.1	KAF6787300.1	Chr05G0336.1
OG0016145	KAF6823688.1	KAF6826133.1	KAF6798801.1	Chr01G1674.1
OG0016146	KAF6823944.1	KAF6827726.1	KAF6794962.1	Chr04G0998.1
OG0016147	KAF6823995.1	KAF6813971.1	KAF6801029.1	Chr04G0823.1
OG0016149	KAF6824133.1	KAF6833312.1	KAF6791645.1	Chr03G0063.1
OG0016150	KAF6824763.1	KAF6835093.1	KAF6791734.1	Chr04G0092.1
OG0	KAF6825428.1	KAF6839719.1	KAF6812153.	Chr03G0601.1

016152			1	
OG0016153	KAF6825450.1	KAF6823067.1	KAF6786807.1	Chr04G0877.1
OG0016155	KAF6825721.1	KAF6832930.1	KAF6802929.1	Chr01G2466.1
OG0016156	KAF6825934.1	KAF6832406.1	KAF6789380.1	Chr03G1493.1
OG0016157	KAF6827095.1	KAF6819831.1	KAF6802722.1	Chr05G0233.1
OG0016158	KAF6827468.1	KAF6836856.1	KAF6812724.1	Chr05G1033.1
OG0016159	KAF6827592.1	KAF6836964.1	KAF6818335.1	Chr08G0344.1
OG0016160	KAF6828227.1	KAF6838531.1	KAF6818114.1	Chr01G2008.1
OG0016161	KAF6828531.1	KAF6828124.1	KAF6807365.1	Chr08G1021.1

OG0016162	KAF6828666.1	KAF6810370.1	KAF6795841.1	Chr01G2441.1
OG0016163	KAF6828668.1	KAF6810372.1	KAF6795839.1	Chr01G2443.1
OG0016164	KAF6828676.1	KAF6832949.1	KAF6795831.1	Chr01G2450.1
OG0016165	KAF6829024.1	KAF6836412.1	KAF6814077.1	Chr07G0081.1
OG0016167	KAF6829070.1	KAF6833964.1	KAF6807980.1	Chr09G0295.1
OG0016168	KAF6829382.1	KAF6837435.1	KAF6806887.1	Chr08G0146.1
OG0016169	KAF6829816.1	KAF6836275.1	KAF6808377.1	Chr06G0756.1
OG0016171	KAF6830028.1	KAF6804199.1	KAF6806396.1	Chr03G0331.1
OG00161	KAF6830095.1	KAF6823143.1	KAF6804496.1	Chr02G0450.1

72				
OG0016173	KAF6830315.1	KAF6815031.1	KAF6807913.1	Chr06G0242.1
OG0016175	KAF6830845.1	KAF6836697.1	KAF6808288.1	Chr03G1679.1
OG0016176	KAF6831087.1	KAF6838232.1	KAF6803967.1	Chr03G0133.1
OG0016177	KAF6831338.1	KAF6837801.1	KAF6818708.1	Chr01G2372.1
OG0016178	KAF6831980.1	KAF6833198.1	KAF6806785.1	Chr06G1436.1
OG0016179	KAF6832050.1	KAF6839704.1	KAF6812168.1	Chr03G0615.1
OG0016180	KAF6832051.1	KAF6839703.1	KAF6812169.1	Chr03G0616.1
OG0016181	KAF6832714.1	KAF6820080.1	KAF6811555.1	Chr03G1410.1
OG0	KAF6832876.1	KAF6826411.1	KAF6811458.	Chr04G1456.1

016182			1	
OG0016183	KAF6833540.1	KAF6837934.1	KAF6818992.1	Chr03G1035.1
OG0016184	KAF6833622.1	KAF6828924.1	KAF6804452.1	Chr05G1330.1
OG0016185	KAF6833776.1	KAF6820403.1	KAF6803114.1	Chr01G0186.1
OG0016186	KAF6835268.1	KAF6834265.1	KAF6786825.1	Chr08G0232.1
OG0016187	KAF6835383.1	KAF6835429.1	KAF6817582.1	Chr09G0206.1
OG0016189	KAF6835969.1	KAF6833556.1	KAF6819297.1	Chr02G0276.1
OG0016190	KAF6835983.1	KAF6824997.1	KAF6790055.1	Chr02G0106.1
OG0016191	KAF6836492.1	KAF6825725.1	KAF6793777.1	Chr01G2193.1

OG0016192	KAF6837241.1	KAF6822602.1	KAF6804310.1	Chr05G0357.1
OG0016194	KAF6838143.1	KAF6829413.1	KAF6816984.1	Chr04G0662.1
OG0016195	KAF6838493.1	KAF6816714.1	KAF6804526.1	Chr05G1301.1
OG0016197	KAF6839141.1	KAF6832117.1	KAF6821335.1	Chr02G0666.1
OG0016198	KAF6839535.1	KAF6835276.1	KAF6806943.1	Chr02G1573.1
OG0016199	KAF6839538.1	KAF6835279.1	KAF6806946.1	Chr02G1576.1
OG0016201	KAF6840335.1	KAF6830568.1	KAF6810096.1	Chr01G0724.1
OG0016202	KAF6840374.1	KAF6819053.1	KAF6803777.1	Chr05G0948.1
OG00162	KAF6840978.1	KAF6814912.1	KAF6785548.1	Chr04G0237.1

04				
OG0016205	KAF6841044.1	KAF6822050.1	KAF6811974.1	Chr06G1274.1
OG0016206	KAF6841074.1	KAF6832769.1	KAF6802747.1	Chr05G0171.1
OG0016208	KAF6841855.1	KAF6835177.1	KAF6798755.1	Chr06G0305.1
OG0016209	KAF6841861.1	KAF6822459.1	KAF6798751.1	Chr06G0301.1
OG0016210	KAF6842648.1	KAF6841420.1	KAF6806204.1	Chr05G0488.1
OG0016211	KAF6842725.1	KAF6808858.1	KAF6804938.1	Chr05G0845.1
OG0016212	KAF6842764.1	KAF6831636.1	KAF6803322.1	Chr02G1479.1
OG0016213	KAF6842975.1	KAF6818559.1	KAF6808694.1	Chr02G0243.1
OG0	KAF6843226.1	KAF6827182.1	KAF6804967.	Chr09G0859.1

016215			1	
OG0016216	KAF6843440.1	KAF6839119.1	KAF6819599.1	Chr09G0157.1
OG0016217	KAF6843903.1	KAF6826288.1	KAF6792065.1	Chr01G1129.1
OG0016218	KAF6844003.1	KAF6835783.1	KAF6790350.1	Chr04G0424.1
OG0016219	KAF6844276.1	KAF6840666.1	KAF6806069.1	Chr08G0593.1
OG0016220	KAF6844310.1	KAF6830996.1	KAF6811799.1	Chr01G1803.1
OG0016221	KAF6844356.1	KAF6830951.1	KAF6804096.1	Chr01G1764.1
OG0016222	KAF6844506.1	KAF6835921.1	KAF6818278.1	Chr08G0291.1
OG0016224	KAF6845460.1	KAF6827367.1	KAF6794559.1	Chr03G0494.1
