# Supplementary material for: Decoding Pecan’s Fungal Foe: A Genomic Insight into Colletotrichum plurivorum Isolate W-6
Source: J Fungi (Basel). 2025 Mar 5;11(3):203. doi: 10.3390/jof11030203 (PMC11943440; doi:10.3390/jof11030203)
Supplement: Supplementary file 1 [file jof-11-00203-s001.zip › Table S29.pdf]

Table S29. Gene list of MFS superfamily.

Gene ID	Chromosome ID	Start	End	Subclass
Chr01G0029.1	Chr01	160,710	162,798	The Anion:Cation Symporter (ACS) Family
Chr01G0032.1	Chr01	172,490	174,858	The Sugar Porter (SP)
Chr01G0053.1	Chr01	239,939	242,032	The Drug:H+ Antiporter-1 (12 Spanner) (DHA1) Family
Chr01G0100.1	Chr01	423,385	425,120	The Sugar Porter (SP)
Chr01G0111.1	Chr01	451,912	454,209	The Sugar Porter (SP)
Chr01G0126.1	Chr01	504,062	505,767	The Anion:Cation Symporter (ACS) Family
Chr01G0135.1	Chr01	533,369	534,838	The Monocarboxylate Transporter (MCT) Family
Chr01G0137.1	Chr01	536,229	538,952	The Monocarboxylate Transporter (MCT) Family
Chr01G0143.1	Chr01	551,547	553,767	The Drug:H+ Antiporter-2 (14 Spanner) (DHA2) Family
Chr01G0152.1	Chr01	588,580	590,516	The Drug:H+ Antiporter-2 (14 Spanner) (DHA2) Family
Chr01G0175.1	Chr01	652,231	654,182	The Anion:Cation Symporter (ACS) Family
Chr01G0176.1	Chr01	654,369	656,261	The Drug:H+ Antiporter-1 (12 Spanner) (DHA1) Family
Chr01G0219.1	Chr01	824,913	826,573	The Anion:Cation Symporter (ACS) Family
Chr01G0227.1	Chr01	860,647	863,859	The Anion:Cation Symporter (ACS) Family
Chr01G0231.1	Chr01	875,124	877,051	The Anion:Cation Symporter (ACS) Family
Chr01G0245.1	Chr01	909,609	912,421	The Sugar Porter (SP)
Chr01G0252.1	Chr01	933,141	934,945	The Drug:H+ Antiporter-2 (14 Spanner) (DHA2) Family
Chr01G0256.1	Chr01	941,483	943,824	The Anion:Cation Symporter (ACS) Family
Chr01G0264.1	Chr01	972,355	974,019	The Sugar Porter (SP)
Chr01G0279.1	Chr01	1,023,318	1,024,911	The Drug:H+ Antiporter-1 (12 Spanner) (DHA1) Family
Chr01G0326.1	Chr01	1,174,823	1,176,991	The Fucose: H+ Symporter (FHS) Family
Chr01G0350.1	Chr01	1,257,356	1,259,111	The Sugar Porter (SP)
Chr01G0353.1	Chr01	1,282,753	1,284,651	The Drug:H+ Antiporter-1 (12 Spanner) (DHA1) Family
Chr01G0371.1	Chr01	1,343,122	1,345,424	The Drug:H+ Antiporter-2 (14 Spanner) (DHA2) Family

Chr01G0376.1	Chr01	1,356,160	1,358,783	The Sugar Porter (SP)
Chr01G0382.1	Chr01	1,381,959	1,383,765	The Drug:H+ Antiporter-2 (14 Spanner) (DHA2) Family
Chr01G0383.1	Chr01	1,384,402	1,386,651	The Monocarboxylate Transporter (MCT) Family
Chr01G0395.1	Chr01	1,428,472	1,430,503	The Anion:Cation Symporter (ACS) Family
Chr01G0409.1	Chr01	1,475,307	1,477,334	The Anion:Cation Symporter (ACS) Family
Chr01G0484.1	Chr01	1,786,458	1,788,260	The Sugar Porter (SP)
Chr01G0504.1	Chr01	1,856,538	1,858,118	The Drug:H+ Antiporter-1 (12 Spanner) (DHA1) Family
Chr01G0520.1	Chr01	1,893,529	1,895,696	The Drug:H+ Antiporter-1 (12 Spanner) (DHA1) Family
Chr01G0526.1	Chr01	1,904,742	1,906,559	The Anion:Cation Symporter (ACS) Family
Chr01G0550.1	Chr01	1,976,875	1,979,543	The Sugar Porter (SP)
Chr01G0559.1	Chr01	2,011,074	2,014,673	The Sugar Porter (SP)
Chr01G0560.1	Chr01	2,015,863	2,017,719	The Anion:Cation Symporter (ACS) Family
Chr01G0587.1	Chr01	2,125,124	2,126,473	The Monocarboxylate Transporter (MCT) Family
Chr01G0592.1	Chr01	2,142,935	2,145,675	The Anion:Cation Symporter (ACS) Family
Chr01G0650.1	Chr01	2,322,507	2,324,554	The Drug:H+ Antiporter-2 (14 Spanner) (DHA2) Family
Chr01G0662.1	Chr01	2,360,305	2,362,444	The Drug:H+ Antiporter-1 (12 Spanner) (DHA1) Family
Chr01G0665.1	Chr01	2,368,334	2,370,253	The Drug:H+ Antiporter-2 (14 Spanner) (DHA2) Family
Chr01G0671.1	Chr01	2,382,308	2,383,977	The Anion:Cation Symporter (ACS) Family
Chr01G0674.1	Chr01	2,388,745	2,391,390	The Sugar Porter (SP)
Chr01G0703.1	Chr01	2,480,547	2,482,197	The Drug:H+ Antiporter-1 (12 Spanner) (DHA1) Family
Chr01G0871.1	Chr01	3,050,854	3,052,878	The Sugar Porter (SP)
Chr01G0879.1	Chr01	3,076,893	3,078,482	The Sugar Porter (SP)
Chr01G0914.1	Chr01	3,189,280	3,191,259	The Sugar Porter (SP)
Chr01G0917.1	Chr01	3,201,373	3,203,115	The Drug:H+ Antiporter-1 (12 Spanner) (DHA1) Family
Chr01G0941.1	Chr01	3,285,347	3,289,851	The Drug:H+ Antiporter-2 (14 Spanner) (DHA2) Family
Chr01G0948.1	Chr01	3,309,774	3,311,416	The Anion:Cation Symporter (ACS) Family

Chr01G0989.1	Chr01	3,433,845	3,436,681	The Anion:Cation Symporter (ACS) Family
Chr01G1003.1	Chr01	3,491,024	3,492,683	The Monocarboxylate Transporter (MCT) Family
Chr01G1025.1	Chr01	3,550,340	3,552,172	The N-Acetylglucosamine Transporter (NAG-T)
Chr01G1081.1	Chr01	3,754,546	3,756,668	The Anion:Cation Symporter (ACS) Family
Chr01G1101.1	Chr01	3,816,656	3,822,256	The Sugar Porter (SP)
Chr01G1106.1	Chr01	3,831,737	3,834,986	The Monocarboxylate Transporter (MCT) Family
Chr01G1148.1	Chr01	4,004,205	4,006,247	The Anion:Cation Symporter (ACS) Family
Chr01G1273.1	Chr01	4,512,933	4,514,385	The Drug:H+ Antiporter-1 (12 Spanner) (DHA1) Family
Chr01G1324.1	Chr01	4,745,936	4,747,660	The Sugar Porter (SP)
Chr01G1330.1	Chr01	4,771,990	4,774,134	The Sugar Porter (SP)
Chr01G1396.1	Chr01	5,025,055	5,026,971	The Anion:Cation Symporter (ACS) Family
Chr01G1400.1	Chr01	5,035,365	5,037,061	The Anion:Cation Symporter (ACS) Family
Chr01G1410.1	Chr01	5,070,076	5,072,644	The N-Acetylglucosamine Transporter (NAG-T)
Chr01G1415.1	Chr01	5,085,647	5,087,586	The Anion:Cation Symporter (ACS) Family
Chr01G1442.1	Chr01	5,170,969	5,172,949	The Anion:Cation Symporter (ACS) Family
Chr01G1481.1	Chr01	5,279,155	5,280,648	The Drug:H+ Antiporter-1 (12 Spanner) (DHA1) Family
Chr01G1494.1	Chr01	5,319,249	5,322,765	The Sugar Porter (SP)
Chr01G1555.1	Chr01	5,584,515	5,587,117	The Sugar Porter (SP)
Chr01G1556.1	Chr01	5,587,612	5,589,430	The Monocarboxylate Transporter (MCT) Family
Chr01G1591.1	Chr01	5,746,428	5,748,784	The Anion:Cation Symporter (ACS) Family
Chr01G1594.1	Chr01	5,753,398	5,755,025	The Drug:H+ Antiporter-1 (12 Spanner) (DHA1) Family
Chr01G1597.1	Chr01	5,760,881	5,762,504	The Monocarboxylate Transporter (MCT) Family
Chr01G1611.1	Chr01	5,817,012	5,819,867	The Drug:H+ Antiporter-2 (14 Spanner) (DHA2) Family
Chr01G1616.1	Chr01	5,837,705	5,840,595	The Sugar Porter (SP)
Chr01G1669.1	Chr01	5,981,200	5,982,833	The Anion:Cation Symporter (ACS) Family
Chr01G1709.1	Chr01	6,090,246	6,092,391	The Sugar Porter (SP)

Chr01G1761.1	Chr01	6,256,038	6,258,917	The Drug:H+ Antiporter-1 (12 Spanner) (DHA1) Family
Chr01G1765.1	Chr01	6,271,614	6,275,976	The Drug:H+ Antiporter-1 (12 Spanner) (DHA1) Family
Chr01G1771.1	Chr01	6,285,978	6,288,423	The Anion:Cation Symporter (ACS) Family
Chr01G1773.1	Chr01	6,300,263	6,302,645	The Sugar Porter (SP)
Chr01G1790.1	Chr01	6,354,121	6,355,503	The Monocarboxylate Transporter (MCT) Family
Chr01G1796.1	Chr01	6,366,950	6,368,625	The Drug:H+ Antiporter-1 (12 Spanner) (DHA1) Family
Chr01G1815.1	Chr01	6,436,350	6,438,109	The Anion:Cation Symporter (ACS) Family
Chr01G1821.1	Chr01	6,450,571	6,455,263	The Anion:Cation Symporter (ACS) Family
Chr01G1842.1	Chr01	6,514,781	6,517,817	The Sugar Porter (SP)
Chr01G1864.1	Chr01	6,593,752	6,595,646	The Sugar Porter (SP)
Chr01G1931.1	Chr01	6,797,191	6,798,865	The Anion:Cation Symporter (ACS) Family
Chr01G1933.1	Chr01	6,802,774	6,805,264	The Anion:Cation Symporter (ACS) Family
Chr01G1974.1	Chr01	6,974,877	6,977,556	The Anion:Cation Symporter (ACS) Family
Chr01G1982.1	Chr01	7,002,804	7,005,640	The Sugar Porter (SP)
Chr01G2028.1	Chr01	7,189,551	7,192,190	The Drug:H+ Antiporter-2 (14 Spanner) (DHA2) Family
Chr01G2093.1	Chr01	7,403,988	7,407,334	The Drug:H+ Antiporter-1 (12 Spanner) (DHA1) Family
Chr01G2164.1	Chr01	7,643,408	7,645,078	The Anion:Cation Symporter (ACS) Family
Chr01G2172.1	Chr01	7,667,941	7,669,852	The Drug:H+ Antiporter-1 (12 Spanner) (DHA1) Family
Chr01G2196.1	Chr01	7,746,028	7,747,771	The Drug:H+ Antiporter-2 (14 Spanner) (DHA2) Family
Chr01G2291.1	Chr01	8,066,603	8,068,494	The Sugar Porter (SP)
Chr01G2411.1	Chr01	8,596,565	8,599,427	The Drug:H+ Antiporter-2 (14 Spanner) (DHA2) Family
Chr01G2415.1	Chr01	8,613,804	8,615,199	The Monocarboxylate Transporter (MCT) Family
Chr01G2416.1	Chr01	8,615,323	8,617,509	The Drug:H+ Antiporter-2 (14 Spanner) (DHA2) Family
Chr01G2445.1	Chr01	8,713,543	8,715,421	The Anion:Cation Symporter (ACS) Family
Chr01G2494.1	Chr01	8,895,068	8,896,913	The Drug:H+ Antiporter-1 (12 Spanner) (DHA1) Family
Chr01G2511.1	Chr01	8,975,061	8,976,998	The Drug:H+ Antiporter-1 (12 Spanner) (DHA1) Family

Chr01G2516.1	Chr01	9,008,177	9,009,506	The Monocarboxylate Transporter (MCT) Family
Chr01G2528.1	Chr01	9,046,840	9,048,566	The Anion:Cation Symporter (ACS) Family
Chr01G2546.1	Chr01	9,109,485	9,111,222	The Sugar Porter (SP)
Chr01G2560.1	Chr01	9,154,878	9,157,611	The Sugar Porter (SP)
Chr01G2571.1	Chr01	9,190,840	9,200,126	The Anion:Cation Symporter (ACS) Family
Chr01G2606.1	Chr01	9,300,624	9,302,438	The Anion:Cation Symporter (ACS) Family
Chr01G2607.1	Chr01	9,302,945	9,304,861	The Anion:Cation Symporter (ACS) Family
Chr01G2609.1	Chr01	9,309,184	9,311,145	The N-Acetylglucosamine Transporter (NAG-T)
Chr01G2629.1	Chr01	9,367,049	9,370,084	The Sugar Porter (SP)
Chr01G2633.1	Chr01	9,382,539	9,384,052	The Monocarboxylate Transporter (MCT) Family
Chr01G2657.1	Chr01	9,458,550	9,461,085	The Drug:H+ Antiporter-1 (12 Spanner) (DHA1) Family
Chr01G2698.1	Chr01	9,617,730	9,620,094	The Anion:Cation Symporter (ACS) Family
Chr01G2707.1	Chr01	9,649,279	9,651,567	The Anion:Cation Symporter (ACS) Family
Chr01G2738.1	Chr01	9,803,821	9,805,395	The Sugar Porter (SP)
Chr01G2745.1	Chr01	9,826,203	9,828,221	The Drug:H+ Antiporter-1 (12 Spanner) (DHA1) Family
Chr01G2746.1	Chr01	9,830,076	9,832,113	The Sugar Porter (SP)
Chr01G2770.1	Chr01	9,915,337	9,916,953	The Drug:H+ Antiporter-1 (12 Spanner) (DHA1) Family
Chr01G2775.1	Chr01	9,928,842	9,930,834	The Drug:H+ Antiporter-2 (14 Spanner) (DHA2) Family
Chr01G2780.1	Chr01	9,943,119	9,945,216	The Drug:H+ Antiporter-2 (14 Spanner) (DHA2) Family
Chr02G0006.1	Chr02	51,465	52,778	The Monocarboxylate Transporter (MCT) Family
Chr02G0011.1	Chr02	78,270	79,736	The Monocarboxylate Transporter (MCT) Family
Chr02G0031.1	Chr02	150,434	152,068	The Anion:Cation Symporter (ACS) Family
Chr02G0035.1	Chr02	162,915	164,561	The Drug:H+ Antiporter-2 (14 Spanner) (DHA2) Family
Chr02G0038.1	Chr02	173,682	175,468	The Drug:H+ Antiporter-1 (12 Spanner) (DHA1) Family
Chr02G0049.1	Chr02	217,993	219,699	The Anion:Cation Symporter (ACS) Family
Chr02G0059.1	Chr02	256,903	258,611	The Anion:Cation Symporter (ACS) Family

Chr02G0062.1	Chr02	263,705	265,627	The Sugar Porter (SP)
Chr02G0064.1	Chr02	268,439	270,125	The Proteobacterial Intraphagosomal Amino Acid Transporter (Pht) Family
Chr02G0083.1	Chr02	327,457	329,086	The Drug:H+ Antiporter-1 (12 Spanner) (DHA1) Family
Chr02G0095.1	Chr02	363,924	365,513	The Anion:Cation Symporter (ACS) Family
Chr02G0098.1	Chr02	378,751	380,399	The Drug:H+ Antiporter-2 (14 Spanner) (DHA2) Family
Chr02G0100.1	Chr02	386,142	388,197	The Monocarboxylate Transporter (MCT) Family
Chr02G0121.1	Chr02	480,694	482,451	The Sugar Porter (SP)
Chr02G0145.1	Chr02	554,565	556,660	The Drug:H+ Antiporter-1 (12 Spanner) (DHA1) Family
Chr02G0150.1	Chr02	565,562	567,631	The Monocarboxylate Transporter (MCT) Family
Chr02G0153.1	Chr02	592,994	594,743	The Drug:H+ Antiporter-1 (12 Spanner) (DHA1) Family
Chr02G0180.1	Chr02	702,000	703,972	The Drug:H+ Antiporter-2 (14 Spanner) (DHA2) Family
Chr02G0199.1	Chr02	758,637	760,666	The Feline Leukemia Virus Subgroup C Receptor (FLVCR)/Heme Importer Family
Chr02G0223.1	Chr02	851,235	854,535	The Anion:Cation Symporter (ACS) Family
Chr02G0229.1	Chr02	867,458	869,224	The Drug:H+ Antiporter-2 (14 Spanner) (DHA2) Family
Chr02G0294.1	Chr02	1,087,787	1,090,163	The Drug:H+ Antiporter-1 (12 Spanner) (DHA1) Family
Chr02G0323.1	Chr02	1,198,711	1,200,401	The Anion:Cation Symporter (ACS) Family
Chr02G0455.1	Chr02	1,672,794	1,674,633	The Drug:H+ Antiporter-1 (12 Spanner) (DHA1) Family
Chr02G0503.1	Chr02	1,866,256	1,868,186	The Sugar Porter (SP)
Chr02G0525.1	Chr02	1,945,259	1,946,977	The Sugar Porter (SP)
Chr02G0586.1	Chr02	2,186,871	2,188,398	The Drug:H+ Antiporter-1 (12 Spanner) (DHA1) Family
Chr02G0593.1	Chr02	2,206,588	2,209,343	The Drug:H+ Antiporter-1 (12 Spanner) (DHA1) Family
Chr02G0594.1	Chr02	2,210,226	2,216,133	The Drug:H+ Antiporter-1 (12 Spanner) (DHA1) Family
Chr02G0626.1	Chr02	2,331,307	2,336,243	The Sugar Porter (SP)
Chr02G0666.1	Chr02	2,463,554	2,465,304	The Drug:H+ Antiporter-2 (14 Spanner) (DHA2) Family
Chr02G0858.1	Chr02	3,312,354	3,315,189	The Drug:H+ Antiporter-2 (14 Spanner) (DHA2) Family
Chr02G0869.1	Chr02	3,361,142	3,362,926	The Sugar Porter (SP)

Chr02G0897.1	Chr02	3,445,508	3,447,614	The Sugar Porter (SP)
Chr02G1016.1	Chr02	3,825,588	3,828,268	The Sugar Porter (SP)
Chr02G1053.1	Chr02	3,958,369	3,961,950	The Sugar Porter (SP)
Chr02G1054.1	Chr02	3,963,544	3,966,491	The Drug:H+ Antiporter-1 (12 Spanner) (DHA1) Family
Chr02G1071.1	Chr02	4,019,182	4,021,372	The Drug:H+ Antiporter-1 (12 Spanner) (DHA1) Family
Chr02G1256.1	Chr02	4,740,232	4,742,709	The Anion:Cation Symporter (ACS) Family
Chr02G1265.1	Chr02	4,772,703	4,774,836	The Anion:Cation Symporter (ACS) Family
Chr02G1273.1	Chr02	4,799,153	4,801,137	The Sugar Porter (SP)
Chr02G1321.1	Chr02	4,958,770	4,960,606	The Drug:H+ Antiporter-1 (12 Spanner) (DHA1) Family
Chr02G1336.1	Chr02	5,021,302	5,023,148	The Drug:H+ Antiporter-1 (12 Spanner) (DHA1) Family
Chr02G1337.1	Chr02	5,030,126	5,031,899	The Anion:Cation Symporter (ACS) Family
Chr02G1380.1	Chr02	5,173,296	5,176,843	The Anion:Cation Symporter (ACS) Family
Chr02G1382.1	Chr02	5,178,355	5,180,622	The Anion:Cation Symporter (ACS) Family
Chr02G1397.1	Chr02	5,221,214	5,223,260	The Sugar Porter (SP)
Chr02G1436.1	Chr02	5,355,799	5,357,735	The N-Acetylglucosamine Transporter (NAG-T)
Chr02G1441.1	Chr02	5,373,510	5,375,178	The Anion:Cation Symporter (ACS) Family
Chr02G1445.1	Chr02	5,381,612	5,383,420	The Proton-dependent Oligopeptide Transporter (POT/PTR) Family
Chr02G1464.1	Chr02	5,441,147	5,442,929	The Sugar Porter (SP)
Chr02G1469.1	Chr02	5,461,764	5,464,632	The Anion:Cation Symporter (ACS) Family
Chr02G1494.1	Chr02	5,550,542	5,552,218	The Monocarboxylate Transporter (MCT) Family
Chr02G1500.1	Chr02	5,567,631	5,570,636	The Drug:H+ Antiporter-1 (12 Spanner) (DHA1) Family
Chr02G1519.1	Chr02	5,622,605	5,624,530	The Sugar Porter (SP)
Chr02G1544.1	Chr02	5,710,978	5,712,972	The Anion:Cation Symporter (ACS) Family
Chr02G1546.1	Chr02	5,715,279	5,716,808	The Drug:H+ Antiporter-1 (12 Spanner) (DHA1) Family
Chr02G1591.1	Chr02	5,925,174	5,926,702	The Sugar Porter (SP)
Chr02G1596.1	Chr02	5,932,853	5,934,594	The Drug:H+ Antiporter-2 (14 Spanner) (DHA2) Family

Chr02G1597.1	Chr02	5,935,034	5,936,385	The Sugar Porter (SP)
Chr02G1609.1	Chr02	6,001,662	6,003,750	The Anion:Cation Symporter (ACS) Family
Chr02G1613.1	Chr02	6,011,618	6,013,925	The Sugar Porter (SP)
Chr02G1624.1	Chr02	6,041,414	6,043,424	The Drug:H+ Antiporter-1 (12 Spanner) (DHA1) Family
Chr02G1626.1	Chr02	6,048,110	6,051,245	The Anion:Cation Symporter (ACS) Family
Chr02G1631.1	Chr02	6,069,069	6,070,947	The Drug:H+ Antiporter-2 (14 Spanner) (DHA2) Family
Chr02G1644.1	Chr02	6,123,449	6,125,200	The Sugar Porter (SP)
Chr02G1676.1	Chr02	6,255,575	6,257,929	The Drug:H+ Antiporter-2 (14 Spanner) (DHA2) Family
Chr02G1727.1	Chr02	6,434,383	6,436,251	The Drug:H+ Antiporter-1 (12 Spanner) (DHA1) Family
Chr02G1760.1	Chr02	6,535,316	6,536,846	The Monocarboxylate Transporter (MCT) Family
Chr02G1770.1	Chr02	6,574,617	6,576,527	The Sugar Porter (SP)
Chr02G1771.1	Chr02	6,576,978	6,578,669	The Drug:H+ Antiporter-1 (12 Spanner) (DHA1) Family
Chr02G1779.1	Chr02	6,618,942	6,620,965	The Proton-dependent Oligopeptide Transporter (POT/PTR) Family
Chr02G1790.1	Chr02	6,651,106	6,653,065	The Sugar Porter (SP)
Chr02G1796.1	Chr02	6,671,211	6,673,206	The Fucose: H+ Symporter (FHS) Family
Chr02G1800.1	Chr02	6,682,255	6,684,102	The Sugar Porter (SP)
Chr02G1811.1	Chr02	6,710,506	6,712,376	The Anion:Cation Symporter (ACS) Family
Chr02G1829.1	Chr02	6,765,542	6,767,145	The Anion:Cation Symporter (ACS) Family
Chr02G1834.1	Chr02	6,784,086	6,786,362	The Fucose: H+ Symporter (FHS) Family
Chr02G1852.1	Chr02	6,842,103	6,843,798	The Sugar Porter (SP)
Chr03G0005.1	Chr03	59,427	60,242	The Anion:Cation Symporter (ACS) Family
Chr03G0007.1	Chr03	62,214	63,965	The Sugar Porter (SP)
Chr03G0012.1	Chr03	76,705	78,417	The Anion:Cation Symporter (ACS) Family
Chr03G0013.1	Chr03	79,279	81,153	The Anion:Cation Symporter (ACS) Family
Chr03G0022.1	Chr03	96,899	99,018	The Anion:Cation Symporter (ACS) Family
Chr03G0087.1	Chr03	326,296	331,580	The Monocarboxylate Transporter (MCT) Family

Chr03G0118.1	Chr03	452,103	454,402	The Anion:Cation Symporter (ACS) Family
Chr03G0169.1	Chr03	648,337	650,419	The Fucose: H+ Symporter (FHS) Family
Chr03G0216.1	Chr03	878,202	880,088	The Anion:Cation Symporter (ACS) Family
Chr03G0228.1	Chr03	916,782	918,650	The Sugar Porter (SP)
Chr03G0241.1	Chr03	961,157	962,833	The Drug:H+ Antiporter-2 (14 Spanner) (DHA2) Family
Chr03G0246.1	Chr03	977,343	994,415	The Sugar Porter (SP)
Chr03G0259.1	Chr03	1,042,271	1,043,895	The Anion:Cation Symporter (ACS) Family
Chr03G0283.1	Chr03	1,101,936	1,104,541	The Sugar Porter (SP)
Chr03G0350.1	Chr03	1,348,297	1,350,215	The Anion:Cation Symporter (ACS) Family
Chr03G0368.1	Chr03	1,423,587	1,425,198	The Anion:Cation Symporter (ACS) Family
Chr03G0434.1	Chr03	1,629,030	1,631,080	The Sugar Porter (SP)
Chr03G0449.1	Chr03	1,682,984	1,684,792	The Sugar Porter (SP)
Chr03G0477.1	Chr03	1,787,477	1,789,073	The Anion:Cation Symporter (ACS) Family
Chr03G0528.1	Chr03	1,978,833	1,980,866	The Sugar Porter (SP)
Chr03G0631.1	Chr03	2,371,164	2,373,888	The Drug:H+ Antiporter-1 (12 Spanner) (DHA1) Family
Chr03G0643.1	Chr03	2,416,502	2,419,774	The Monocarboxylate Transporter (MCT) Family
Chr03G0668.1	Chr03	2,516,014	2,517,810	The Sugar Porter (SP)
Chr03G0704.1	Chr03	2,662,810	2,665,049	The Anion:Cation Symporter (ACS) Family
Chr03G0955.1	Chr03	3,618,581	3,620,776	The Glycoside-Pentoside-Hexuronide (GPH):Cation Symporter Family
Chr03G1030.1	Chr03	3,961,367	3,966,482	The Drug:H+ Antiporter-2 (14 Spanner) (DHA2) Family
Chr03G1039.1	Chr03	3,991,902	3,993,559	The Anion:Cation Symporter (ACS) Family
Chr03G1048.1	Chr03	4,026,142	4,027,663	The Drug:H+ Antiporter-1 (12 Spanner) (DHA1) Family
Chr03G1054.1	Chr03	4,046,754	4,049,834	The Drug:H+ Antiporter-1 (12 Spanner) (DHA1) Family
Chr03G1167.1	Chr03	4,484,796	4,488,618	The Drug:H+ Antiporter-1 (12 Spanner) (DHA1) Family
Chr03G1170.1	Chr03	4,497,897	4,501,331	The Sugar Porter (SP)
Chr03G1191.1	Chr03	4,589,644	4,591,213	The Anion:Cation Symporter (ACS) Family

Chr03G1268.1	Chr03	4,910,601	4,912,416	The Sugar Porter (SP)
Chr03G1285.1	Chr03	4,971,463	4,973,463	The Sugar Porter (SP)
Chr03G1287.1	Chr03	4,976,751	4,978,406	The Monocarboxylate Transporter (MCT) Family
Chr03G1323.1	Chr03	5,094,270	5,096,172	The Drug:H+ Antiporter-2 (14 Spanner) (DHA2) Family
Chr03G1357.1	Chr03	5,219,678	5,221,608	The Anion:Cation Symporter (ACS) Family
Chr03G1387.1	Chr03	5,373,472	5,375,201	The Drug:H+ Antiporter-1 (12 Spanner) (DHA1) Family
Chr03G1400.1	Chr03	5,414,659	5,425,654	The Sugar Porter (SP)
Chr03G1430.1	Chr03	5,516,321	5,520,785	The Anion:Cation Symporter (ACS) Family
Chr03G1459.1	Chr03	5,612,566	5,616,435	The Anion:Cation Symporter (ACS) Family
Chr03G1480.1	Chr03	5,674,491	5,676,331	The Sugar Porter (SP)
Chr03G1497.1	Chr03	5,747,704	5,749,413	The Drug:H+ Antiporter-2 (14 Spanner) (DHA2) Family
Chr03G1517.1	Chr03	5,799,061	5,801,323	The Anion:Cation Symporter (ACS) Family
Chr03G1522.1	Chr03	5,812,424	5,814,060	The Sugar Porter (SP)
Chr03G1526.1	Chr03	5,821,846	5,823,625	The Anion:Cation Symporter (ACS) Family
Chr03G1549.1	Chr03	5,896,219	5,898,721	The Sugar Porter (SP)
Chr03G1556.1	Chr03	5,928,176	5,929,624	The Monocarboxylate Transporter (MCT) Family
Chr03G1567.1	Chr03	5,976,568	5,978,216	The Drug:H+ Antiporter-1 (12 Spanner) (DHA1) Family
Chr03G1590.1	Chr03	6,063,887	6,065,416	The Fucose: H+ Symporter (FHS) Family
Chr03G1607.1	Chr03	6,112,882	6,116,126	The Sugar Porter (SP)
Chr03G1636.1	Chr03	6,199,777	6,201,142	The Monocarboxylate Transporter (MCT) Family
Chr03G1638.1	Chr03	6,206,269	6,208,153	The Drug:H+ Antiporter-1 (12 Spanner) (DHA1) Family
Chr03G1663.1	Chr03	6,299,564	6,301,431	The Sugar Porter (SP)
Chr03G1681.1	Chr03	6,358,619	6,360,763	The Sugar Porter (SP)
Chr03G1682.1	Chr03	6,361,385	6,362,921	The Monocarboxylate Transporter (MCT) Family
Chr03G1690.1	Chr03	6,384,007	6,385,679	The Drug:H+ Antiporter-1 (12 Spanner) (DHA1) Family
Chr03G1699.1	Chr03	6,411,589	6,418,091	The Sugar Porter (SP)

Chr04G0033.1	Chr04	203,918	205,768	The Drug:H+ Antiporter-2 (14 Spanner) (DHA2) Family
Chr04G0058.1	Chr04	300,288	302,958	The Anion:Cation Symporter (ACS) Family
Chr04G0094.1	Chr04	431,922	435,460	The Anion:Cation Symporter (ACS) Family
Chr04G0101.1	Chr04	452,543	456,689	The Sugar Porter (SP)
Chr04G0134.1	Chr04	569,798	571,646	The Sugar Porter (SP)
Chr04G0138.1	Chr04	585,882	587,907	The Fucose: H+ Symporter (FHS) Family
Chr04G0139.1	Chr04	589,701	592,123	The Anion:Cation Symporter (ACS) Family
Chr04G0142.1	Chr04	595,739	597,852	The Drug:H+ Antiporter-1 (12 Spanner) (DHA1) Family
Chr04G0164.1	Chr04	660,288	661,985	The Sugar Porter (SP)
Chr04G0165.1	Chr04	663,321	667,284	The Sugar Porter (SP)
Chr04G0166.1	Chr04	665,953	671,082	The Sugar Porter (SP)
Chr04G0168.1	Chr04	676,335	678,171	The Drug:H+ Antiporter-2 (14 Spanner) (DHA2) Family
Chr04G0218.1	Chr04	871,375	873,100	The Drug:H+ Antiporter-1 (12 Spanner) (DHA1) Family
Chr04G0220.1	Chr04	875,342	877,227	The Drug:H+ Antiporter-1 (12 Spanner) (DHA1) Family
Chr04G0245.1	Chr04	967,183	969,060	The Sugar Porter (SP)
Chr04G0252.1	Chr04	988,663	992,033	The Sugar Porter (SP)
Chr04G0293.1	Chr04	1,133,145	1,134,901	The Sugar Porter (SP)
Chr04G0304.1	Chr04	1,172,995	1,174,367	The Monocarboxylate Transporter (MCT) Family
Chr04G0309.1	Chr04	1,187,130	1,190,123	The Anion:Cation Symporter (ACS) Family
Chr04G0331.1	Chr04	1,272,480	1,274,192	The Proteobacterial Intraphagosomal Amino Acid Transporter (Pht) Family
Chr04G0333.1	Chr04	1,279,745	1,281,603	The Anion:Cation Symporter (ACS) Family
Chr04G0335.1	Chr04	1,284,079	1,287,069	The Sugar Porter (SP)
Chr04G0336.1	Chr04	1,289,394	1,291,452	The Drug:H+ Antiporter-1 (12 Spanner) (DHA1) Family
Chr04G0357.1	Chr04	1,360,434	1,362,542	The Drug:H+ Antiporter-2 (14 Spanner) (DHA2) Family
Chr04G0382.1	Chr04	1,481,485	1,483,601	The Anion:Cation Symporter (ACS) Family
Chr04G0385.1	Chr04	1,489,930	1,491,848	The Sugar Porter (SP)

Chr04G0388.1	Chr04	1,500,755	1,502,702	The Anion:Cation Symporter (ACS) Family
Chr04G0389.1	Chr04	1,503,601	1,505,529	The Sugar Porter (SP)
Chr04G0399.1	Chr04	1,535,112	1,536,977	The Sugar Porter (SP)
Chr04G0510.1	Chr04	2,011,387	2,013,297	The Drug:H+ Antiporter-2 (14 Spanner) (DHA2) Family
Chr04G0526.1	Chr04	2,061,527	2,064,297	The Drug:H+ Antiporter-2 (14 Spanner) (DHA2) Family
Chr04G0569.1	Chr04	2,247,916	2,249,743	The Anion:Cation Symporter (ACS) Family
Chr04G0639.1	Chr04	2,517,313	2,518,825	The Fucose: H+ Symporter (FHS) Family
Chr04G0646.1	Chr04	2,538,560	2,541,236	The Drug:H+ Antiporter-1 (12 Spanner) (DHA1) Family
Chr04G0659.1	Chr04	2,586,033	2,588,007	The Drug:H+ Antiporter-1 (12 Spanner) (DHA1) Family
Chr04G0671.1	Chr04	2,620,324	2,621,922	The N-Acetylglucosamine Transporter (NAG-T)
Chr04G0679.1	Chr04	2,645,809	2,647,416	The Anion:Cation Symporter (ACS) Family
Chr04G0700.1	Chr04	2,695,370	2,697,205	The Drug:H+ Antiporter-2 (14 Spanner) (DHA2) Family
Chr04G0741.1	Chr04	2,817,610	2,819,310	The Drug:H+ Antiporter-1 (12 Spanner) (DHA1) Family
Chr04G0756.1	Chr04	2,863,406	2,865,290	The Drug:H+ Antiporter-1 (12 Spanner) (DHA1) Family
Chr04G0782.1	Chr04	2,944,785	2,946,702	The Drug:H+ Antiporter-1 (12 Spanner) (DHA1) Family
Chr04G0801.1	Chr04	3,007,468	3,009,255	The Drug:H+ Antiporter-2 (14 Spanner) (DHA2) Family
Chr04G0816.1	Chr04	3,050,812	3,052,335	The Anion:Cation Symporter (ACS) Family
Chr04G0818.1	Chr04	3,053,806	3,055,400	The Anion:Cation Symporter (ACS) Family
Chr04G0928.1	Chr04	3,431,156	3,433,112	The Drug:H+ Antiporter-2 (14 Spanner) (DHA2) Family
Chr04G0936.1	Chr04	3,459,010	3,461,118	The Drug:H+ Antiporter-1 (12 Spanner) (DHA1) Family
Chr04G0944.1	Chr04	3,503,810	3,507,343	The Sugar Porter (SP)
Chr04G0954.1	Chr04	3,546,065	3,547,486	The Anion:Cation Symporter (ACS) Family
Chr04G1003.1	Chr04	3,740,327	3,742,587	The Drug:H+ Antiporter-1 (12 Spanner) (DHA1) Family
Chr04G1007.1	Chr04	3,751,088	3,753,158	The Sugar Porter (SP)
Chr04G1016.1	Chr04	3,804,044	3,805,435	The Drug:H+ Antiporter-1 (12 Spanner) (DHA1) Family
Chr04G1027.1	Chr04	3,866,217	3,868,136	The Anion:Cation Symporter (ACS) Family

Chr04G1030.1	Chr04	3,872,976	3,875,512	The Anion:Cation Symporter (ACS) Family
Chr04G1033.1	Chr04	3,880,066	3,881,908	The Anion:Cation Symporter (ACS) Family
Chr04G1051.1	Chr04	3,947,672	3,952,319	The Sugar Porter (SP)
Chr04G1080.1	Chr04	4,063,721	4,065,322	The Drug:H+ Antiporter-1 (12 Spanner) (DHA1) Family
Chr04G1158.1	Chr04	4,306,574	4,309,484	The Sugar Porter (SP)
Chr04G1192.1	Chr04	4,425,233	4,427,426	The Anion:Cation Symporter (ACS) Family
Chr04G1205.1	Chr04	4,485,308	4,489,736	The Fucose: H+ Symporter (FHS) Family
Chr04G1260.1	Chr04	4,678,445	4,680,173	The Sugar Porter (SP)
Chr04G1263.1	Chr04	4,685,990	4,687,862	The Drug:H+ Antiporter-2 (14 Spanner) (DHA2) Family
Chr04G1276.1	Chr04	4,744,487	4,746,691	The Drug:H+ Antiporter-2 (14 Spanner) (DHA2) Family
Chr04G1279.1	Chr04	4,763,987	4,765,750	The Drug:H+ Antiporter-2 (14 Spanner) (DHA2) Family
Chr04G1348.1	Chr04	5,036,156	5,038,275	The Drug:H+ Antiporter-2 (14 Spanner) (DHA2) Family
Chr04G1349.1	Chr04	5,039,041	5,041,537	The Anion:Cation Symporter (ACS) Family
Chr04G1412.1	Chr04	5,307,766	5,309,495	The Anion:Cation Symporter (ACS) Family
Chr04G1421.1	Chr04	5,336,201	5,338,542	The Anion:Cation Symporter (ACS) Family
Chr04G1424.1	Chr04	5,348,987	5,350,930	The Drug:H+ Antiporter-2 (14 Spanner) (DHA2) Family
Chr04G1434.1	Chr04	5,378,950	5,380,590	The Sugar Porter (SP)
Chr04G1446.1	Chr04	5,403,354	5,405,061	The Sugar Porter (SP)
Chr04G1503.1	Chr04	5,599,353	5,601,172	The Drug:H+ Antiporter-1 (12 Spanner) (DHA1) Family
Chr04G1519.1	Chr04	5,665,079	5,667,443	The Sugar Porter (SP)
Chr04G1527.1	Chr04	5,689,532	5,692,365	The Drug:H+ Antiporter-1 (12 Spanner) (DHA1) Family
Chr04G1529.1	Chr04	5,697,221	5,700,810	The Anion:Cation Symporter (ACS) Family
Chr04G1555.1	Chr04	5,819,182	5,821,479	The Sugar Porter (SP)
Chr05G0007.1	Chr05	74,377	75,389	The Anion:Cation Symporter (ACS) Family
Chr05G0029.1	Chr05	208,376	211,925	The Anion:Cation Symporter (ACS) Family
Chr05G0031.1	Chr05	212,546	215,034	The Drug:H+ Antiporter-2 (14 Spanner) (DHA2) Family

Chr05G0049.1	Chr05	260,266	263,033	The Sugar Porter (SP)
Chr05G0076.1	Chr05	359,715	361,338	The Sugar Porter (SP)
Chr05G0085.1	Chr05	387,080	390,161	The Monocarboxylate Transporter (MCT) Family
Chr05G0127.1	Chr05	523,589	525,498	The Drug:H+ Antiporter-2 (14 Spanner) (DHA2) Family
Chr05G0135.1	Chr05	555,289	557,707	The Sugar Porter (SP)
Chr05G0136.1	Chr05	558,280	560,742	The Sugar Porter (SP)
Chr05G0154.1	Chr05	636,988	638,997	The Sugar Porter (SP)
Chr05G0156.1	Chr05	649,760	651,608	The Drug:H+ Antiporter-1 (12 Spanner) (DHA1) Family
Chr05G0162.1	Chr05	666,851	668,598	The Sugar Porter (SP)
Chr05G0173.1	Chr05	699,771	701,725	The Drug:H+ Antiporter-2 (14 Spanner) (DHA2) Family
Chr05G0188.1	Chr05	750,656	752,331	The Monocarboxylate Transporter (MCT) Family
Chr05G0199.1	Chr05	788,574	790,623	The Drug:H+ Antiporter-2 (14 Spanner) (DHA2) Family
Chr05G0211.1	Chr05	847,913	850,828	The Sugar Porter (SP)
Chr05G0225.1	Chr05	899,968	900,918	The Drug:H+ Antiporter-1 (12 Spanner) (DHA1) Family
Chr05G0272.1	Chr05	1,070,924	1,072,692	The Sugar Porter (SP)
Chr05G0292.1	Chr05	1,145,983	1,147,842	The Sugar Porter (SP)
Chr05G0299.1	Chr05	1,175,576	1,177,662	The Monocarboxylate Transporter (MCT) Family
Chr05G0379.1	Chr05	1,569,592	1,572,203	The Drug:H+ Antiporter-1 (12 Spanner) (DHA1) Family
Chr05G0402.1	Chr05	1,660,400	1,662,126	The Anion:Cation Symporter (ACS) Family
Chr05G0407.1	Chr05	1,675,382	1,677,279	The Sugar Porter (SP)
Chr05G0525.1	Chr05	2,160,576	2,162,580	The Drug:H+ Antiporter-1 (12 Spanner) (DHA1) Family
Chr05G0620.1	Chr05	2,479,795	2,481,605	The Drug:H+ Antiporter-1 (12 Spanner) (DHA1) Family
Chr05G0645.1	Chr05	2,552,633	2,554,838	The Nitrate/Nitrite Porter (NNP) family
Chr05G0809.1	Chr05	3,162,898	3,164,673	The Sugar Porter (SP)
Chr05G0823.1	Chr05	3,201,111	3,202,814	The Anion:Cation Symporter (ACS) Family
Chr05G0859.1	Chr05	3,339,415	3,341,135	The Sugar Porter (SP)

Chr05G0881.1	Chr05	3,458,992	3,460,291	The Drug:H+ Antiporter-1 (12 Spanner) (DHA1) Family
Chr05G0924.1	Chr05	3,598,651	3,600,358	The N-Acetylglucosamine Transporter (NAG-T)
Chr05G0934.1	Chr05	3,644,480	3,647,112	The Drug:H+ Antiporter-2 (14 Spanner) (DHA2) Family
Chr05G0953.1	Chr05	3,702,380	3,704,157	The Sugar Porter (SP)
Chr05G1013.1	Chr05	3,952,782	3,954,428	The Monocarboxylate Transporter (MCT) Family
Chr05G1120.1	Chr05	4,424,812	4,430,293	The Sugar Porter (SP)
Chr05G1141.1	Chr05	4,575,459	4,577,225	The Sugar Porter (SP)
Chr05G1210.1	Chr05	4,803,191	4,805,242	The Drug:H+ Antiporter-2 (14 Spanner) (DHA2) Family
Chr05G1249.1	Chr05	4,943,227	4,945,056	The Anion:Cation Symporter (ACS) Family
Chr05G1304.1	Chr05	5,143,699	5,145,999	The Anion:Cation Symporter (ACS) Family
Chr05G1307.1	Chr05	5,157,270	5,159,269	The Sugar Porter (SP)
Chr05G1341.1	Chr05	5,300,893	5,303,975	The Drug:H+ Antiporter-1 (12 Spanner) (DHA1) Family
Chr05G1377.1	Chr05	5,399,159	5,402,725	The Sugar Porter (SP)
Chr05G1441.1	Chr05	5,634,435	5,635,622	The Sugar Porter (SP)
Chr06G0082.1	Chr06	431,929	433,872	The Sugar Porter (SP)
Chr06G0102.1	Chr06	494,650	496,494	The Drug:H+ Antiporter-1 (12 Spanner) (DHA1) Family
Chr06G0151.1	Chr06	644,004	646,508	The Drug:H+ Antiporter-2 (14 Spanner) (DHA2) Family
Chr06G0159.1	Chr06	662,784	664,422	The Fucose: H+ Symporter (FHS) Family
Chr06G0163.1	Chr06	677,352	679,353	The Anion:Cation Symporter (ACS) Family
Chr06G0168.1	Chr06	688,158	690,166	The Drug:H+ Antiporter-2 (14 Spanner) (DHA2) Family
Chr06G0181.1	Chr06	736,652	738,993	The Anion:Cation Symporter (ACS) Family
Chr06G0228.1	Chr06	903,034	905,173	The Fucose: H+ Symporter (FHS) Family
Chr06G0236.1	Chr06	937,911	941,782	The Sugar Porter (SP)
Chr06G0251.1	Chr06	982,430	984,469	The Anion:Cation Symporter (ACS) Family
Chr06G0256.1	Chr06	996,959	999,322	The Anion:Cation Symporter (ACS) Family
Chr06G0273.1	Chr06	1,046,843	1,049,222	The Drug:H+ Antiporter-1 (12 Spanner) (DHA1) Family

Chr06G0286.1	Chr06	1,088,750	1,091,853	The Drug:H+ Antiporter-1 (12 Spanner) (DHA1) Family
Chr06G0288.1	Chr06	1,096,547	1,098,687	The Drug:H+ Antiporter-2 (14 Spanner) (DHA2) Family
Chr06G0406.1	Chr06	1,557,146	1,560,977	The Drug:H+ Antiporter-1 (12 Spanner) (DHA1) Family
Chr06G0451.1	Chr06	1,709,616	1,711,335	The Anion:Cation Symporter (ACS) Family
Chr06G0594.1	Chr06	2,231,486	2,238,042	The Proton-dependent Oligopeptide Transporter (POT/PTR) Family
Chr06G0653.1	Chr06	2,462,780	2,464,169	The Monocarboxylate Transporter (MCT) Family
Chr06G0693.1	Chr06	2,598,139	2,599,743	The Sugar Porter (SP)
Chr06G0801.1	Chr06	3,020,943	3,023,038	The Drug:H+ Antiporter-2 (14 Spanner) (DHA2) Family
Chr06G0849.1	Chr06	3,190,253	3,192,348	The Drug:H+ Antiporter-1 (12 Spanner) (DHA1) Family
Chr06G0928.1	Chr06	3,472,574	3,474,877	The Sugar Porter (SP)
Chr06G1139.1	Chr06	4,298,453	4,301,323	The Drug:H+ Antiporter-2 (14 Spanner) (DHA2) Family
Chr06G1225.1	Chr06	4,590,328	4,592,373	The Drug:H+ Antiporter-1 (12 Spanner) (DHA1) Family
Chr06G1275.1	Chr06	4,760,561	4,763,684	The Drug:H+ Antiporter-1 (12 Spanner) (DHA1) Family
Chr06G1299.1	Chr06	4,817,926	4,819,833	The Sugar Porter (SP)
Chr06G1326.1	Chr06	4,932,758	4,936,767	The Anion:Cation Symporter (ACS) Family
Chr06G1394.1	Chr06	5,127,374	5,129,186	The Anion:Cation Symporter (ACS) Family
Chr06G1399.1	Chr06	5,151,277	5,153,556	The Anion:Cation Symporter (ACS) Family
Chr06G1400.1	Chr06	5,155,874	5,158,099	The Sugar Porter (SP)
Chr06G1404.1	Chr06	5,168,395	5,169,928	The Monocarboxylate Transporter (MCT) Family
Chr06G1416.1	Chr06	5,204,076	5,205,756	The Anion:Cation Symporter (ACS) Family
Chr06G1427.1	Chr06	5,243,822	5,245,862	The Monocarboxylate Transporter (MCT) Family
Chr06G1431.1	Chr06	5,256,313	5,257,756	The Monocarboxylate Transporter (MCT) Family
Chr06G1482.1	Chr06	5,413,196	5,414,379	The Anion:Cation Symporter (ACS) Family
Chr07G0059.1	Chr07	293,436	295,312	The Anion:Cation Symporter (ACS) Family
Chr07G0088.1	Chr07	398,181	400,499	The Drug:H+ Antiporter-1 (12 Spanner) (DHA1) Family
Chr07G0092.1	Chr07	423,471	425,457	The Drug:H+ Antiporter-2 (14 Spanner) (DHA2) Family

Chr07G0105.1	Chr07	462,221	464,001	The Anion:Cation Symporter (ACS) Family
Chr07G0114.1	Chr07	485,551	487,551	The Drug:H+ Antiporter-2 (14 Spanner) (DHA2) Family
Chr07G0116.1	Chr07	493,056	495,079	The Drug:H+ Antiporter-2 (14 Spanner) (DHA2) Family
Chr07G0162.1	Chr07	636,520	638,511	The Drug:H+ Antiporter-2 (14 Spanner) (DHA2) Family
Chr07G0193.1	Chr07	756,303	757,989	The Sugar Porter (SP)
Chr07G0200.1	Chr07	788,112	790,570	The Drug:H+ Antiporter-1 (12 Spanner) (DHA1) Family
Chr07G0204.1	Chr07	810,824	813,067	The Drug:H+ Antiporter-2 (14 Spanner) (DHA2) Family
Chr07G0226.1	Chr07	889,070	890,934	The Drug:H+ Antiporter-1 (12 Spanner) (DHA1) Family
Chr07G0234.1	Chr07	909,004	911,375	The Sugar Porter (SP)
Chr07G0289.1	Chr07	1,105,406	1,107,710	The Monocarboxylate Transporter (MCT) Family
Chr07G0313.1	Chr07	1,186,506	1,188,097	The Sugar Porter (SP)
Chr07G0327.1	Chr07	1,235,279	1,237,440	The Drug:H+ Antiporter-2 (14 Spanner) (DHA2) Family
Chr07G0366.1	Chr07	1,351,891	1,354,070	The Anion:Cation Symporter (ACS) Family
Chr07G0383.1	Chr07	1,403,567	1,405,588	The Drug:H+ Antiporter-2 (14 Spanner) (DHA2) Family
Chr07G0416.1	Chr07	1,526,945	1,534,281	The Monocarboxylate Transporter (MCT) Family
Chr07G0438.1	Chr07	1,601,600	1,603,370	The Sugar Porter (SP)
Chr07G0492.1	Chr07	1,764,385	1,766,466	The Anion:Cation Symporter (ACS) Family
Chr07G0501.1	Chr07	1,790,890	1,796,601	The Anion:Cation Symporter (ACS) Family
Chr07G0515.1	Chr07	1,847,502	1,849,171	The Anion:Cation Symporter (ACS) Family
Chr07G0530.1	Chr07	1,886,193	1,887,854	The N-Acetylglucosamine Transporter (NAG-T)
Chr07G0556.1	Chr07	1,966,494	1,968,335	The Anion:Cation Symporter (ACS) Family
Chr07G0566.1	Chr07	2,002,856	2,005,361	The Sugar Porter (SP)
Chr07G0585.1	Chr07	2,070,406	2,072,809	The Anion:Cation Symporter (ACS) Family
Chr07G0703.1	Chr07	2,480,320	2,481,834	The Drug:H+ Antiporter-2 (14 Spanner) (DHA2) Family
Chr07G0711.1	Chr07	2,532,679	2,534,998	The Drug:H+ Antiporter-1 (12 Spanner) (DHA1) Family
Chr07G0724.1	Chr07	2,596,579	2,599,194	The Sugar Porter (SP)

Chr07G0777.1	Chr07	2,845,848	2,848,733	The Sugar Porter (SP)
Chr07G0887.1	Chr07	3,315,699	3,317,955	The Monocarboxylate Transporter (MCT) Family
Chr07G0894.1	Chr07	3,355,999	3,358,402	The Sugar Porter (SP)
Chr07G0897.1	Chr07	3,369,276	3,371,803	The Sugar Porter (SP)
Chr07G0906.1	Chr07	3,392,661	3,395,340	The Drug:H+ Antiporter-2 (14 Spanner) (DHA2) Family
Chr07G0907.1	Chr07	3,396,996	3,399,213	The Anion:Cation Symporter (ACS) Family
Chr07G0912.1	Chr07	3,413,635	3,414,945	The Monocarboxylate Transporter (MCT) Family
Chr07G0919.1	Chr07	3,431,237	3,433,085	The Sugar Porter (SP)
Chr07G0929.1	Chr07	3,472,557	3,474,355	The Sugar Porter (SP)
Chr07G0975.1	Chr07	3,588,167	3,589,801	The Drug:H+ Antiporter-1 (12 Spanner) (DHA1) Family
Chr07G0977.1	Chr07	3,598,992	3,600,853	The Sugar Porter (SP)
Chr07G0980.1	Chr07	3,607,592	3,609,875	The Sugar Porter (SP)
Chr07G0985.1	Chr07	3,627,501	3,630,277	The Anion:Cation Symporter (ACS) Family
Chr07G1005.1	Chr07	3,704,143	3,706,670	The Sugar Porter (SP)
Chr07G1041.1	Chr07	3,843,539	3,845,760	The Drug:H+ Antiporter-1 (12 Spanner) (DHA1) Family
Chr07G1067.1	Chr07	3,919,118	3,921,287	The Sugar Porter (SP)
Chr07G1101.1	Chr07	4,053,289	4,056,057	The Anion:Cation Symporter (ACS) Family
Chr07G1111.1	Chr07	4,078,169	4,079,970	The Sugar Porter (SP)
Chr07G1137.1	Chr07	4,145,674	4,147,755	The Sugar Porter (SP)
Chr07G1140.1	Chr07	4,152,242	4,154,190	The Anion:Cation Symporter (ACS) Family
Chr07G1141.1	Chr07	4,155,818	4,157,580	The Anion:Cation Symporter (ACS) Family
Chr07G1172.1	Chr07	4,302,827	4,305,364	The Proton-dependent Oligopeptide Transporter (POT/PTR) Family
Chr07G1180.1	Chr07	4,330,503	4,332,565	The Sugar Porter (SP)
Chr07G1181.1	Chr07	4,332,788	4,336,400	The Sugar Porter (SP)
Chr08G0044.1	Chr08	218,793	221,119	The Sugar Porter (SP)
Chr08G0050.1	Chr08	234,547	236,749	The N-Acetylglucosamine Transporter (NAG-T)

Chr08G0104.1	Chr08	401,948	403,609	The Sugar Porter (SP)
Chr08G0119.1	Chr08	458,366	459,872	The Drug:H+ Antiporter-1 (12 Spanner) (DHA1) Family
Chr08G0187.1	Chr08	733,941	735,560	The Drug:H+ Antiporter-1 (12 Spanner) (DHA1) Family
Chr08G0230.1	Chr08	992,266	994,578	The Drug:H+ Antiporter-1 (12 Spanner) (DHA1) Family
Chr08G0243.1	Chr08	1,058,078	1,060,875	The Drug:H+ Antiporter-1 (12 Spanner) (DHA1) Family
Chr08G0388.1	Chr08	1,675,139	1,678,087	The Monocarboxylate Transporter (MCT) Family
Chr08G0415.1	Chr08	1,781,692	1,784,259	The Anion:Cation Symporter (ACS) Family
Chr08G0438.1	Chr08	1,933,638	1,935,485	The Drug:H+ Antiporter-2 (14 Spanner) (DHA2) Family
Chr08G0447.1	Chr08	1,985,180	1,988,304	The Drug:H+ Antiporter-2 (14 Spanner) (DHA2) Family
Chr08G0460.1	Chr08	2,047,641	2,048,971	The Drug:H+ Antiporter-1 (12 Spanner) (DHA1) Family
Chr08G0468.1	Chr08	2,071,776	2,074,096	The Monocarboxylate Transporter (MCT) Family
Chr08G0474.1	Chr08	2,089,023	2,091,388	The Sugar Porter (SP)
Chr08G0518.1	Chr08	2,292,566	2,295,654	The Anion:Cation Symporter (ACS) Family
Chr08G0519.1	Chr08	2,297,446	2,299,469	The Sugar Porter (SP)
Chr08G0613.1	Chr08	2,705,468	2,708,119	The Drug:H+ Antiporter-2 (14 Spanner) (DHA2) Family
Chr08G0643.1	Chr08	2,798,346	2,799,913	The Monocarboxylate Transporter (MCT) Family
Chr08G0671.1	Chr08	2,888,964	2,890,855	The Anion:Cation Symporter (ACS) Family
Chr08G0677.1	Chr08	2,899,546	2,901,265	The Anion:Cation Symporter (ACS) Family
Chr08G0715.1	Chr08	3,011,649	3,013,490	The Sugar Porter (SP)
Chr08G0732.1	Chr08	3,060,978	3,062,768	The Sugar Porter (SP)
Chr08G0746.1	Chr08	3,105,344	3,106,655	The Drug:H+ Antiporter-2 (14 Spanner) (DHA2) Family
Chr08G0825.1	Chr08	3,370,834	3,372,575	The Sugar Porter (SP)
Chr08G0826.1	Chr08	3,374,660	3,376,590	The Drug:H+ Antiporter-2 (14 Spanner) (DHA2) Family
Chr08G0870.1	Chr08	3,514,812	3,517,249	The Sugar Porter (SP)
Chr08G0875.1	Chr08	3,528,964	3,531,356	The Drug:H+ Antiporter-2 (14 Spanner) (DHA2) Family
Chr08G0885.1	Chr08	3,562,570	3,564,344	The Drug:H+ Antiporter-2 (14 Spanner) (DHA2) Family

Chr08G0905.1	Chr08	3,628,476	3,630,097	The Anion:Cation Symporter (ACS) Family
Chr08G0942.1	Chr08	3,744,390	3,745,961	The Drug:H+ Antiporter-1 (12 Spanner) (DHA1) Family
Chr08G0956.1	Chr08	3,807,102	3,808,842	The Drug:H+ Antiporter-1 (12 Spanner) (DHA1) Family
Chr08G0959.1	Chr08	3,817,284	3,819,000	The Sugar Porter (SP)
Chr08G0965.1	Chr08	3,831,847	3,833,543	The Anion:Cation Symporter (ACS) Family
Chr08G0970.1	Chr08	3,844,175	3,849,048	The N-Acetylglucosamine Transporter (NAG-T)
Chr08G1003.1	Chr08	3,941,208	3,943,025	The Drug:H+ Antiporter-2 (14 Spanner) (DHA2) Family
Chr08G1006.1	Chr08	3,950,020	3,952,627	The Sugar Porter (SP)
Chr08G1044.1	Chr08	4,064,069	4,066,228	The Sugar Porter (SP)
Chr08G1045.1	Chr08	4,066,543	4,067,979	The Drug:H+ Antiporter-1 (12 Spanner) (DHA1) Family
Chr08G1049.1	Chr08	4,077,346	4,079,189	The Sugar Porter (SP)
Chr08G1057.1	Chr08	4,117,126	4,119,402	The Monocarboxylate Transporter (MCT) Family
Chr09G0018.1	Chr09	82,701	84,633	The Sugar Porter (SP)
Chr09G0219.1	Chr09	976,665	979,642	The Sugar Porter (SP)
Chr09G0240.1	Chr09	1,071,687	1,073,374	The Proteobacterial Intraphagosomal Amino Acid Transporter (Pht) Family
Chr09G0276.1	Chr09	1,197,181	1,199,398	The Drug:H+ Antiporter-1 (12 Spanner) (DHA1) Family
Chr09G0306.1	Chr09	1,332,917	1,334,813	The Monocarboxylate Transporter (MCT) Family
Chr09G0351.1	Chr09	1,503,752	1,505,464	The Anion:Cation Symporter (ACS) Family
Chr09G0384.1	Chr09	1,626,889	1,629,369	The Drug:H+ Antiporter-2 (14 Spanner) (DHA2) Family
Chr09G0392.1	Chr09	1,668,299	1,675,055	The Fucose: H+ Symporter (FHS) Family
Chr09G0411.1	Chr09	1,765,111	1,767,066	The Drug:H+ Antiporter-1 (12 Spanner) (DHA1) Family
Chr09G0591.1	Chr09	2,491,201	2,492,949	The Anion:Cation Symporter (ACS) Family
Chr09G0602.1	Chr09	2,536,028	2,538,544	The Drug:H+ Antiporter-2 (14 Spanner) (DHA2) Family
Chr09G0640.1	Chr09	2,689,480	2,690,855	The Monocarboxylate Transporter (MCT) Family
Chr09G0697.1	Chr09	2,904,084	2,905,820	The Sugar Porter (SP)
Chr09G0700.1	Chr09	2,915,321	2,917,111	The Drug:H+ Antiporter-1 (12 Spanner) (DHA1) Family

Chr09G0705.1	Chr09	2,933,055	2,934,941	The Sugar Porter (SP)
Chr09G0730.1	Chr09	3,028,338	3,030,409	The Drug:H+ Antiporter-2 (14 Spanner) (DHA2) Family
Chr09G0731.1	Chr09	3,031,851	3,034,564	The Anion:Cation Symporter (ACS) Family
Chr09G0743.1	Chr09	3,070,351	3,072,147	The Anion:Cation Symporter (ACS) Family
Chr09G0839.1	Chr09	3,391,741	3,393,720	The Drug:H+ Antiporter-1 (12 Spanner) (DHA1) Family
Chr09G0884.1	Chr09	3,543,400	3,545,487	The Sugar Porter (SP)
Chr09G0913.1	Chr09	3,643,802	3,645,654	The Drug:H+ Antiporter-1 (12 Spanner) (DHA1) Family
Chr09G0919.1	Chr09	3,668,079	3,670,056	The Sugar Porter (SP)
Chr09G0979.1	Chr09	3,855,015	3,857,086	The Sugar Porter (SP)
Chr09G0984.1	Chr09	3,874,760	3,878,297	The Sugar Porter (SP)
Chr09G0992.1	Chr09	3,914,609	3,916,309	The Drug:H+ Antiporter-2 (14 Spanner) (DHA2) Family
Chr09G1018.1	Chr09	4,041,132	4,043,366	The Anion:Cation Symporter (ACS) Family
Chr09G1031.1	Chr09	4,082,928	4,085,211	The Drug:H+ Antiporter-2 (14 Spanner) (DHA2) Family
