# Supplementary material for: Decoding Pecan’s Fungal Foe: A Genomic Insight into Colletotrichum plurivorum Isolate W-6
Source: J Fungi (Basel). 2025 Mar 5;11(3):203. doi: 10.3390/jof11030203 (PMC11943440; doi:10.3390/jof11030203)
Supplement: Supplementary file 1 [file jof-11-00203-s001.zip › Table S3.pdf]

Table S3. Oxford Nanopore data statistics for isolate W-6 assembly.

Data type	Total reads	Total base (bp)	N50 read length (bp)	N90 read length (bp)	Average read length (bp)	Maximum read length (bp)	Mean read quality	Depth (×)
Raw data	2,630,207	12,878,112,392	9,294	2,114	4,896	141,347	9.51	235.65
Clean data	1,616,038	11,212,610,042	10,120	3,090	6,938	89,503	9.77	205.17
