# Supplementary material for: Decoding Pecan’s Fungal Foe: A Genomic Insight into Colletotrichum plurivorum Isolate W-6
Source: J Fungi (Basel). 2025 Mar 5;11(3):203. doi: 10.3390/jof11030203 (PMC11943440; doi:10.3390/jof11030203)
Supplement: Supplementary file 1 [file jof-11-00203-s001.zip › Table S30.pdf]

Table S30. Virulent-related annotation of MFS transporters.

Subfamily	Number of members	Number of virulence annotations*	Percentage **	Category* **
The Sugar Porter (SP)	155	139	89.68	6
The Anion:Cation Symporter (ACS) Family	133	127	95.49	2
The Drug:H+ Antiporter-1 (12 Spanner) (DHA1) Family	96	66	68.75	5
The Drug:H+ Antiporter-2 (14 Spanner) (DHA2) Family	74	72	97.30	10
The Monocarboxylate Transporter (MCT) Family	43	40	93.02	2
The Fucose: H+ Symporter (FHS) Family	11	3	27.27	2
The N-Acetylglucosamine Transporter (NAG-T)	9	0	0	0
The Proton-dependent Oligopeptide Transporter (POT/PTR) Family	4	2	50	1
The Proteobacterial Intraphagosomal Amino Acid Transporter (Pht) Family	3	0	0	0
The Feline Leukemia Virus Subgroup C Receptor (FLVCR)/Heme Importer Family	1	0	0	0
The Glycoside-Pentoside-Hexuronide (GPH):Cation Symporter Family	1	0	0	0
The Nitrate/Nitrite Porter (NNP) family	1	0	0	0
Total	531	449	84.56	27

*, The number of virulence annotations is obtained based on the number of annotations obtained from DFVF and PHI

**, The number of virulence annotations as a percentage of the number of members

***, The number of annotated IDs based on DFVF and PHI databases
