# Supplementary material for: Decoding Pecan’s Fungal Foe: A Genomic Insight into Colletotrichum plurivorum Isolate W-6
Source: J Fungi (Basel). 2025 Mar 5;11(3):203. doi: 10.3390/jof11030203 (PMC11943440; doi:10.3390/jof11030203)
Supplement: Supplementary file 1 [file jof-11-00203-s001.zip › Table S31.pdf]

Table S31. Clusters containing MFSs.

Gene_cluster ID	Gene ID	Chromosome ID	Start	End	Length(bp)	Family
r1c2	Chr01G0559.1	Chr01	2011074	2014673	3600	The Sugar Porter (SP)
	Chr01G0560.1	Chr01	2015863	2017719	1857	The Anion:Cation Symporter (ACS) Family
r1c4	Chr01G0871.1	Chr01	3050854	3052878	2025	The Sugar Porter (SP)
	Chr01G0879.1	Chr01	3076893	3078482	1590	The Sugar Porter (SP)
r14c3	Chr02G0666.1	Chr02	2463554	2465304	1751	The Drug:H+ Antiporter-2 (14 Spanner) (DHA2) Family
r7c2	Chr03G1039.1	Chr03	3991902	3993559	1658	The Anion:Cation Symporter (ACS) Family
	Chr03G1048.1	Chr03	4026142	4027663	1522	The Drug:H+ Antiporter-1 (12 Spanner) (DHA1) Family
	Chr03G1054.1	Chr03	4046754	4049834	3081	The Drug:H+ Antiporter-1 (12 Spanner) (DHA1) Family
r7c3	Chr03G1387.1	Chr03	5373472	5375201	1730	The Drug:H+ Antiporter-1 (12 Spanner) (DHA1) Family
r7c4	Chr03G1590.1	Chr03	6063887	6065416	1530	The Fucose: H+ Symporter (FHS) Family
r13c1	Chr04G0033.1	Chr04	203918	205768	1851	The Drug:H+ Antiporter-2 (14 Spanner) (DHA2) Family
r13c2	Chr04G0218.1	Chr04	871375	873100	1726	The Drug:H+ Antiporter-1 (12 Spanner) (DHA1) Family
	Chr04G0220.1	Chr04	875342	877227	1886	The Drug:H+ Antiporter-1 (12 Spanner) (DHA1) Family
r13c3	Chr04G0801.1	Chr04	3007468	3009255	1788	The Drug:H+ Antiporter-2 (14 Spanner) (DHA2) Family
r13c5	Chr04G0928.1	Chr04	3431156	3433112	1957	The Drug:H+ Antiporter-2 (14 Spanner) (DHA2) Family
r13c7	Chr04G1080.1	Chr04	4063721	4065322	1602	The Drug:H+ Antiporter-1 (12 Spanner) (DHA1) Family
r13c8	Chr04G1260.1	Chr04	4678445	4680173	1729	The Sugar Porter (SP)
	Chr04G1263.1	Chr04	4685990	4687862	1873	The Drug:H+ Antiporter-2 (14 Spanner) (DHA2) Family
r13c9	Chr04G1276.1	Chr04	4744487	4746691	2205	The Drug:H+ Antiporter-2 (14 Spanner) (DHA2) Family
	Chr04G1279.1	Chr04	4763987	4765750	1764	The Drug:H+ Antiporter-2 (14 Spanner) (DHA2) Family
r2c2	Chr05G0031.1	Chr05	212546	215034	2489	The Drug:H+ Antiporter-2 (14 Spanner) (DHA2) Family
r2c1	Chr05G0173.1	Chr05	699771	701725	1955	The Drug:H+ Antiporter-2 (14 Spanner) (DHA2) Family
	Chr05G0188.1	Chr05	750656	752331	1676	The Monocarboxylate Transporter (MCT) Family
r12c3	Chr06G1399.1	Chr06	5151277	5153556	2280	The Anion:Cation Symporter (ACS) Family

	Chr06G1400.1	Chr06	5155874	5158099	2226	The Sugar Porter (SP)
r3c1	Chr07G0088.1	Chr07	398181	400499	2319	The Drug:H+ Antiporter-1 (12 Spanner) (DHA1) Family
	Chr07G0092.1	Chr07	423471	425457	1987	The Drug:H+ Antiporter-2 (14 Spanner) (DHA2) Family
r3c2	Chr07G0114.1	Chr07	485551	487551	2001	The Drug:H+ Antiporter-2 (14 Spanner) (DHA2) Family
	Chr07G0116.1	Chr07	493056	495079	2024	The Drug:H+ Antiporter-2 (14 Spanner) (DHA2) Family
r5c4	Chr08G0438.1	Chr08	1933638	1935485	1848	The Drug:H+ Antiporter-2 (14 Spanner) (DHA2) Family
r5c5	Chr08G0460.1	Chr08	2047641	2048971	1331	The Drug:H+ Antiporter-1 (12 Spanner) (DHA1) Family
	Chr08G0468.1	Chr08	2071776	2074096	2321	The Monocarboxylate Transporter (MCT) Family
r8c2	Chr09G0219.1	Chr09	976665	979642	2978	The Sugar Porter (SP)
r8c7	Chr09G1031.1	Chr09	4082928	4085211	2284	The Drug:H+ Antiporter-2 (14 Spanner) (DHA2) Family
