# Supplementary material for: Decoding Pecan’s Fungal Foe: A Genomic Insight into Colletotrichum plurivorum Isolate W-6
Source: J Fungi (Basel). 2025 Mar 5;11(3):203. doi: 10.3390/jof11030203 (PMC11943440; doi:10.3390/jof11030203)
Supplement: Supplementary file 1 [file jof-11-00203-s001.zip › Table S32.pdf]

Table S32. Genes in Cluster r13c2.

Gene Name	Gene ID	Description	Functional description
Zn2Cys6a	Chr04G0205.1	Zn2Cys6	Regulators of virulence factors
Zn2Cys6b	Chr04G0206.1	Zn2Cys6	Regulators of virulence factors
OCD	Chr04G0207.1	GMC Oxidoreductase Cellobiose Dehydrogenase	GMC Oxidoreductase Cellobiose Dehydrogenase is involved in the degradation of plant cell wall components
NRPS1	Chr04G0208.1	Phomopsin biosynthesis-associated protein	It is part of a gene cluster that mediates the biosynthesis of phomopsins
OMT1	Chr04G0209.1	SAM-dependent O-methyltransferase	SAM-dependent O-methyltransferase is involved in the methylation of Phomopsin A to phomopsin E
S41	Chr04G0210.1	Secondary metabolite-associated S41A peptidase	They play a crucial role in the biosynthesis of various secondary metabolites, such as hexapeptide mycotoxins and anti-mitotic tetrapeptides.
OMT2	Chr04G0211.1	SAM-dependent O-methyltransferase	SAM-dependent O-methyltransferase is involved in the methylation of Phomopsin A to phomopsin E
Zn2Cys6c	Chr04G0212.1	Zn2Cys6	ABC transporter regulating transcription factor
PSK/sor2	Chr04G0213.1	Non-reducing polyketide synthase sor2	It is a key enzyme in the sorbicillin cyclization step
PSK/TOXD	Chr04G0214.1	Trans-enoyl reductase *TOXD*	It is involved in the HC-toxin synthesis process
CytP450a	Chr04G0215.1	Cytochrome P450 monooygenase	Cytochrome P450 monooxygenase is essential for the synthesis of the echinocandins family hexapeptides.
PSK/L	Chr04G	Lovastatin nonaketide synthase	Lovastatin nonaketide synthase is the basis for the synthesis of Lovastatin and is involved in the

NKS	0216.1		synthesis of the precursor dihydromonacolin L.
CytP450b	Chr04G 0217.1	Cytochrome P450 monooygenase	Cytochrome P450 monooxygenase is essential for the synthesis of the echinocandins family hexapeptides.
prlG	Chr04G 0218.1	MFS(DHA1) prlG	Efflux pump that might be required for efficient secretion of pyrrolocin or other secondary metabolies produced by the pyrrolocin gene cluster
NRPS2	Chr04G 0219.1	Phomopsin biosynthesis-associated protein	It is part of a gene cluster that mediates the biosynthesis of phomopsins
azaK	Chr04G 0220.1	MFS(DHA1) azaK	Efflux pump that might be required for efficient secretion of azaphilones
UstYa-like-1	Chr04G 0221.1	Mycotoxin biosynthesis protein UstYa-like	It is involved in the post-translational oxidative modification during the formation of cyclic peptides.
tesA	Chr04G 0222.1	Thioesterase	Thioesterase adds the final amino acid to the peptide antibiotic during non-ribosomal synthesis, thereby forming a cyclic peptide.
NRPS3	Chr04G 0223.1	Phomopsin biosynthesis-associated protein	It is part of a gene cluster that mediates the biosynthesis of phomopsins
UstYa-like-2	Chr04G 0224.1	Mycotoxin biosynthesis protein UstYa-like	It is involved in the post-translational oxidative modification during the formation of cyclic peptides.
PKS-NRPS	Chr04G 0225.1	Polyketide synthase-Nonribosomal peptide synthetase (PKS-NRPS)	PKS-NRPS is a complex natural enzyme that is involved in the biosynthesis of polyketides and peptide toxins produced by fungi, and it is a large multi-modular enzyme.
NADK	Chr04G 0226.1	NAD kinase	It can maintain the pathogenic mechanism of pathogens by supporting virulence factors and protective enzymes.
Lys-N	Chr04G 0229.1	peptidyl-lys metalloendopeptidase	It is a metalloproteinase of the M35 family, capable of cleaving peptide chains and peptide bonds linked to Lys, which can trigger rapid accumulation of H2O2, inducing cell death.
ZEA1	Chr04G 0230.1	Non-reducing polyketide synthase ZEA1	It is a part of the gene cluster that mediates the biosynthesis of zearalenone (ZEA)
