# Supplementary material for: Decoding Pecan’s Fungal Foe: A Genomic Insight into Colletotrichum plurivorum Isolate W-6
Source: J Fungi (Basel). 2025 Mar 5;11(3):203. doi: 10.3390/jof11030203 (PMC11943440; doi:10.3390/jof11030203)
Supplement: Supplementary file 1 [file jof-11-00203-s001.zip › Table S4.pdf]

Table S4. Length distribution of clean reads of ONT data.

Reads length (kb)	Number of Reads	Length (bp)	Percentage (%)	Average read length (bp)
2~5	817,686	2,551,674,620	22.75	3,120.60
5~10	409,275	2,972,692,032	26.51	7,263.31
10~20	348,477	4,691,530,594	41.84	13,462.95
20~30	35,916	832,723,285	7.42	23,185.30
30~40	4,050	135,278,076	1.2	33,401.99
40~50	547	23,786,923	0.21	43,486.14
50~60	69	3,685,555	0.03	53,413.84
60~70	12	766,025	0	63,835.41
70~80	4	294,965	0	73,741.25
>=80	2	177,967	0	88,983.50
