# Supplementary material for: Decoding Pecan’s Fungal Foe: A Genomic Insight into Colletotrichum plurivorum Isolate W-6
Source: J Fungi (Basel). 2025 Mar 5;11(3):203. doi: 10.3390/jof11030203 (PMC11943440; doi:10.3390/jof11030203)
Supplement: Supplementary file 1 [file jof-11-00203-s001.zip › Table S5.pdf]

Table S5. Detailed information of assembled contigs of isolate W-6.

Contig ID	Chromsome ID	Length (bp)	Telomere				Centromere	
			Repeat element	Start	End	Length (bp)	Start	End
Contig01	Chr01	10,044,033	NA	NA	NA	NA	3,640,000	3,645,000
Contig02	Chr02	6,944,546	CCCTAA	20	85	66	750,000	1,180,000
Contig03	Chr03	6,591,936	CCCTAA/TTAGGG	5/6,591,841	83/6,591,936	78/96	1,940,000	2,330,000
Contig04	Chr04	6,029,146	NA	NA	NA	NA	1,600,000	1,800,000
Contig05	Chr05	5,841,298	TTAGGG	5,841,221	5,841,298	78	1,000	120,000
Contig06	Chr06	5,509,059	NA	NA	NA	NA	1,000	60,000
Contig07	Chr07	4,509,887	TTAGGG	4,509,780	4,509,887	108	255,000	264,000
Contig08	Chr08	4,302,628	NA	NA	NA	NA	4,160,000	430,000
Contig09	Chr09	4,258,435	NA	NA	NA	NA	646,000	651,000
Contig10	Chr10	237,091	NA	NA	NA	NA	18,000	23,000

Contig11	Chr11	200,541	NA	NA	NA	NA	152,000	157,000
Contig12	Chr12	106,099	TTAGGG	106,051	106,098	48	1,000	105,000
