# Supplementary material for: Decoding Pecan’s Fungal Foe: A Genomic Insight into Colletotrichum plurivorum Isolate W-6
Source: J Fungi (Basel). 2025 Mar 5;11(3):203. doi: 10.3390/jof11030203 (PMC11943440; doi:10.3390/jof11030203)
Supplement: Supplementary file 1 [file jof-11-00203-s001.zip › Table S6.pdf]

Table S6. Assembly assessment of isolate W-6 by Illumina NovaSeq data.

Library	Mapped (%)	Properly mapped (%)	Coverage (%)	Depth (X)
350bp (PE)	99.35	97.68	99.94	57.15
