# Supplementary material for: Decoding Pecan’s Fungal Foe: A Genomic Insight into Colletotrichum plurivorum Isolate W-6
Source: J Fungi (Basel). 2025 Mar 5;11(3):203. doi: 10.3390/jof11030203 (PMC11943440; doi:10.3390/jof11030203)
Supplement: Supplementary file 1 [file jof-11-00203-s001.zip › Table S7.pdf]

Table S7. BUSCO assessment of assembly completeness of isolate W-6 genome.

| | |
|---|---|
| Complete BUSCOs (C) | 747 (98.6%) |
| Complete and single-copy BUSCOs (S) | 744 (98.2%) |
| Complete and duplicated BUSCOs (D) | 3 (0.4%) |
| Fragmented BUSCOs (F) | 2 (0.3%) |
| Missing BUSCOs (M) | 9 (1.1%) |
| Total conserved BUSCO genes (fungi obd10) | 758 |
