# Supplementary material for: Decoding Pecan’s Fungal Foe: A Genomic Insight into Colletotrichum plurivorum Isolate W-6
Source: J Fungi (Basel). 2025 Mar 5;11(3):203. doi: 10.3390/jof11030203 (PMC11943440; doi:10.3390/jof11030203)
Supplement: Supplementary file 1 [file jof-11-00203-s001.zip › Table S8.pdf]

Table S8. Predicted repeat elements in isolate W-6 genome.

| Type | Number | Length (bp) | Percentage (%) |
|---|---|---|---|
| ClassI | 2,227 | 2,435,773 | 4.46 |
| ClassI/DIRS | 98 | 36,560 | 0.07 |
| ClassI/LINE | 499 | 448,480 | 0.82 |
| ClassI/LTR | 70 | 57,804 | 0.11 |
| ClassI/LTR/Copia | 545 | 842,991 | 1.54 |
| ClassI/LTR/Gypsy | 499 | 586,718 | 1.08 |
| ClassI/PLE\|LARD | 417 | 296,035 | 0.54 |
| ClassI/TRIM | 66 | 75,843 | 0.14 |
| ClassI/Unknown | 24 | 40,558 | 0.07 |
| ClassII | 3,102 | 2,323,736 | 4.26 |
| ClassII/Helitron | 201 | 295,964 | 0.54 |
| ClassII/MITE | 254 | 68,072 | 0.12 |
| ClassII/TIR | 2,549 | 1,842,095 | 3.38 |
| ClassII/Unknown | 98 | 117,605 | 0.22 |
| PotentialHostGene | 185 | 178,416 | 0.33 |
| SSR | 128 | 20,865 | 0.04 |
| Unknown | 2,504 | 834,177 | 1.53 |
| Total | 8,146 | 5,792,967 | 10.61 |
