# Supplementary material for: Decoding Pecan’s Fungal Foe: A Genomic Insight into Colletotrichum plurivorum Isolate W-6
Source: J Fungi (Basel). 2025 Mar 5;11(3):203. doi: 10.3390/jof11030203 (PMC11943440; doi:10.3390/jof11030203)
Supplement: Supplementary file 1 [file jof-11-00203-s001.zip › Table S9.pdf]

Table S9. Prediction of non-coding RNAs.

RNA classify	Number	Family number/Rfam_id
rRNA	102	4
5S_rRNA	102	5S_rRNA
18S_rRNA	0	SSU_rRNA_eukarya
5.8S_rRNA	0	5_8S_rRNA
28S_rRNA	0	LSU_rRNA_eukarya
tRNA	472	52
other ncRNA	45	30
