# Supplementary figures and images for: Decoding Pecan’s Fungal Foe: A Genomic Insight into Colletotrichum plurivorum Isolate W-6
Source: J Fungi (Basel). 2025 Mar 5;11(3):203. doi: 10.3390/jof11030203 (PMC11943440; doi:10.3390/jof11030203)

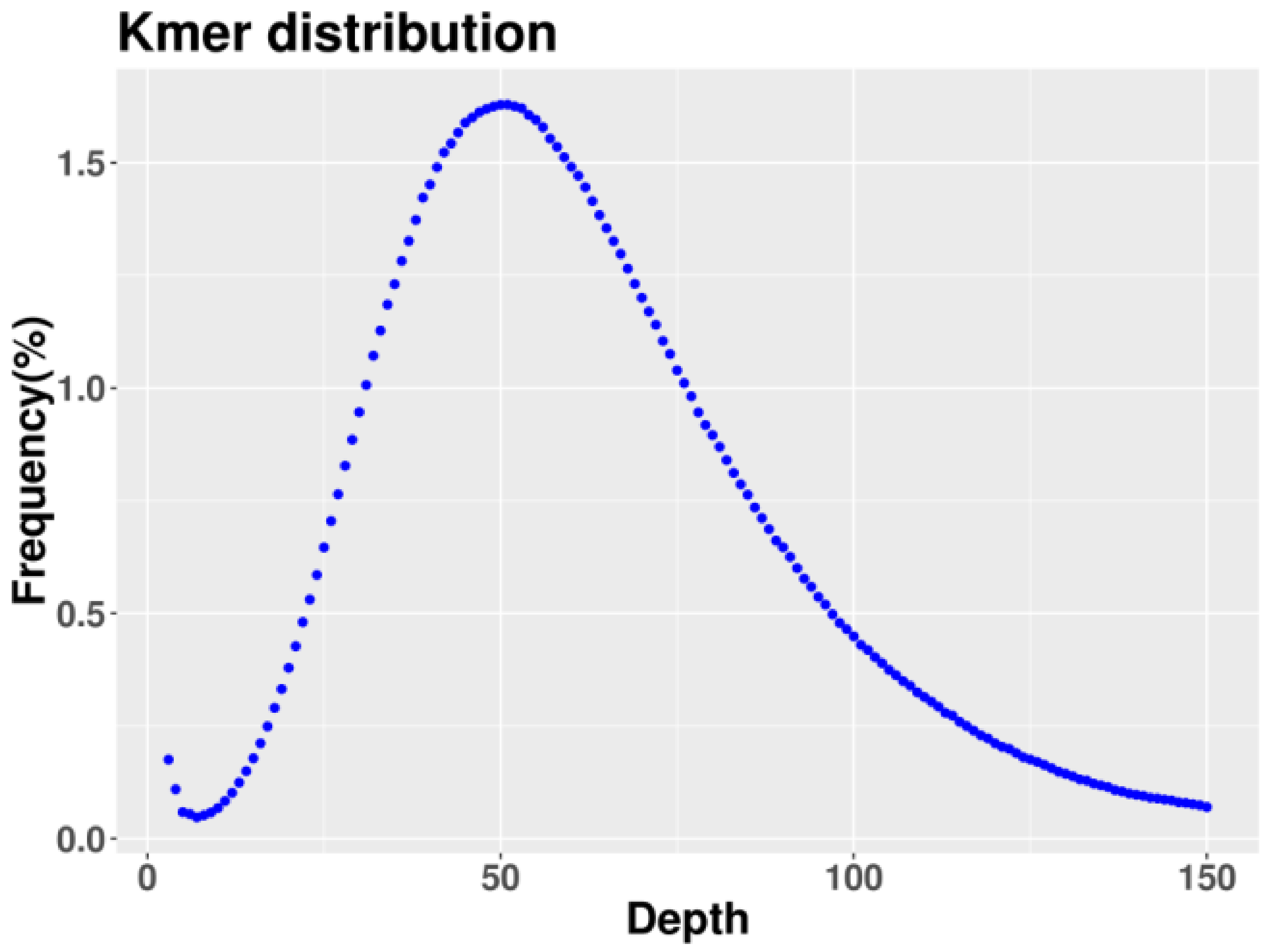

Supplement: Supplementary file 1 [file jof-11-00203-s001.zip › Figure S1.png]
